# Microsatellite Interruptions Stabilize Primate Genomes and Exist as Population-Specific Single Nucleotide Polymorphisms within Individual Human Genomes

**DOI:** 10.1371/journal.pgen.1004498

**Published:** 2014-07-17

**Authors:** Guruprasad Ananda, Suzanne E. Hile, Amanda Breski, Yanli Wang, Yogeshwar Kelkar, Kateryna D. Makova, Kristin A. Eckert

**Affiliations:** 1 Department of Biology, Penn State University, University Park, Pennsylvania, United States of America; 2 Department of Pathology, Gittlen Cancer Research Foundation, The Pennsylvania State University College of Medicine, Hershey, Pennsylvania, United States of America; 3 Center for Medical Genomics, Penn State University, University Park, Pennsylvania, United States of America; National Institute of Genetics, Japan

## Abstract

Interruptions of microsatellite sequences impact genome evolution and can alter disease manifestation. However, human polymorphism levels at interrupted microsatellites (iMSs) are not known at a genome-wide scale, and the pathways for gaining interruptions are poorly understood. Using the 1000 Genomes Phase-1 variant call set, we interrogated mono-, di-, tri-, and tetranucleotide repeats up to 10 units in length. We detected ∼26,000–40,000 iMSs within each of four human population groups (African, European, East Asian, and American). We identified population-specific iMSs within exonic regions, and discovered that known disease-associated iMSs contain alleles present at differing frequencies among the populations. By analyzing longer microsatellites in primate genomes, we demonstrate that single interruptions result in a genome-wide average two- to six-fold reduction in microsatellite mutability, as compared with perfect microsatellites. Centrally located interruptions lowered mutability dramatically, by two to three orders of magnitude. Using a biochemical approach, we tested directly whether the mutability of a specific iMS is lower because of decreased DNA polymerase strand slippage errors. Modeling the adenomatous polyposis coli tumor suppressor gene sequence, we observed that a single base substitution interruption reduced strand slippage error rates five- to 50-fold, relative to a perfect repeat, during synthesis by DNA polymerases α, β, or η. Computationally, we demonstrate that iMSs arise primarily by base substitution mutations within individual human genomes. Our biochemical survey of human DNA polymerase α, β, δ, κ, and η error rates within certain microsatellites suggests that interruptions are created most frequently by low fidelity polymerases. Our combined computational and biochemical results demonstrate that iMSs are abundant in human genomes and are sources of population-specific genetic variation that may affect genome stability. The genome-wide identification of iMSs in human populations presented here has important implications for current models describing the impact of microsatellite polymorphisms on gene expression.

## Author Summary

Microsatellites are short tandem repeat DNA sequences located throughout the human genome that display a high degree of inter-individual variation. This characteristic makes microsatellites an attractive tool for population genetics and forensics research. Some microsatellites affect gene expression, and mutations within such microsatellites can cause disease. Interruption mutations disrupt the perfect repeated array and are frequently associated with altered disease risk, but they have not been thoroughly studied in human genomes. We identified interrupted mono-, di-, tri- and tetranucleotide MSs (iMS) within individual genomes from African, European, Asian and American population groups. We show that many iMSs, including some within disease-associated genes, are unique to a single population group. By measuring the conservation of microsatellites between human and chimpanzee genomes, we demonstrate that interruptions decrease the probability of microsatellite mutations throughout the genome. We demonstrate that iMSs arise in the human genome by single base changes within the DNA, and provide biochemical data suggesting that these stabilizing changes may be created by error-prone DNA polymerases. Our genome-wide study supports the model in which iMSs act to stabilize individual genomes, and suggests that population-specific differences in microsatellite architecture may be an avenue by which genetic ancestry impacts individual disease risk.

## Introduction

Over 3% of the human genome consists of microsatellites, defined as short tandem repeats of 1–6 bases per motif unit, interspersed throughout the genome [1]. Strand slippage during DNA synthesis is facilitated by the presence of tandem repeats, and has been proposed to be the dominant mutational mechanism for microsatellites [2], [3]. Perfect microsatellites contain repeats of a single motif sequence, whereas interrupted microsatellites (iMSs) include tandem repeats of a single motif interrupted by other bases. Many microsatellites are located within coding and regulatory sequences [4], and can be important modifiers of gene expression, affecting transcription rate, RNA stability, splicing efficiency, and RNA-protein interactions [5]–[7]. Because microsatellite alleles are highly polymorphic, they may provide a large pool of heritable, phenotypic variants for subsequent selection [8]–[10]. Length variation at certain microsatellites contributes to natural variation in brain development and behavioral traits [11], and may modulate neurodegenerative disease risk [12].

Microsatellite interruptions also are known to have important consequences for human health and disease. For instance, germline interruptions of disease-causing microsatellite alleles act as a disease modifier for spinocerebellar ataxia type 10 [13], and alter the age of onset of spinocerebellar ataxia type 1 [14]. Importantly, the presence of interrupted alleles at the *FMR* gene (Fragile X syndrome) microsatellite diminishes the likelihood of repeat-expansion to disease length alleles in the next generation [15], [16]. Similarly, the presence of multiple interruptions at the *DM-1* gene microsatellite decreases the probability of both germline and somatic expansions [17], [18]. Furthermore, a population-specific, single nucleotide polymorphism within the *APC* gene coding region converts an iMS (AAATAAAA) to a perfect microsatellite (A)_8_, leading to an increased risk of somatic *APC* mutation and colorectal cancer in Ashkenazi Jews [19]. Biomedical interest in microsatellite interruptions has been renewed recently by the demonstration that iMSs within the *ATXN2* (*SCA2*) gene are associated with a different disease presentation than perfect expanded alleles [20]. These studies demonstrate that a complex relationship exists between microsatellites and disease, that involves not only length but also sequence polymorphisms. Importantly, iMSs might represent a reservoir of mutable alleles that can expand in subsequent generations, as was shown for *SCA2* [21] and myotonic dystrophy type 2 [22].

Microsatellite interruptions are major contributors to the microsatellite life cycle. According to the life cycle hypothesis, a microsatellite locus undergoes stages of birth, adulthood and death during its evolution [23]. Microsatellites are “born” from short tandem repeats (proto-microsatellites) when they reach a threshold length that alters their mutational behavior [24], [25]. Microsatellites display a characteristically high frequency of motif-based insertion/deletion (indel) mutations that drive high germline microsatellite mutation rates; this is in contrast to proto-microsatellites that have lower indel mutation frequencies than microsatellites [25], [26]. Microsatellites “die” when the length of the tandem repeat falls below the threshold, and interruptions are the major cause of microsatellite death [27], [28]. Some interruptions can persist for millions of years (MYs), e.g., for 19–35 MYs at one locus studied in artiodactyls [29]. These features can serve as an advantage when using iMSs as markers in population genetics, since interrupted repeats exhibit lower homoplasy than uninterrupted MSs. Indeed, for iMSs, the probability of acquiring an interruption by two independent events (i.e. the probability of a homoplasy) is much lower than the probability of inheriting this interruption from a common ancestor. Because of this, iMSs might be more appropriate markers than perfect microsatellites for studying population differentiation [30]. Interrupted microsatellites are more stable genetically (less mutable, but still polymorphic) than perfect repeats in natural chicken populations [31], and interruptions can reduce the mutability of specific microsatellite sequences [32]–[34]. However, the quantitative effects of interruptions on decreasing human microsatellite mutability have never been evaluated previously in a genome-wide study.

The significant role of iMSs in modifying the clinical manifestations of disease and their important contributions to genome evolution warrant a detailed understanding of iMSs. Specifically, the architecture of human genomes with regard to iMSs has not been previously investigated, and the mechanism by which interruptions arise has not been extensively studied. We used a multi-disciplinary approach combining computational and biochemical methods to address three biologically important questions regarding microsatellite interruptions. *First*, what is the quantitative effect of microsatellite interruptions on microsatellite mutability genome-wide? *Second*, how common are microsatellite interruptions within the human genome, where do they occur, and how often are human populations polymorphic for the presence/absence of interruptions? *Third*, what are the possible biochemical pathways giving rise to microsatellite interruptions? Our results reveal the highly dynamic nature of microsatellite mutagenesis in the human genome, one that includes a robust level of interruption variation, and demonstrate that iMSs provide a source of population-specific genetic modifiers potentially affecting the stability of individual human genomes.

## Results

### Reduction in microsatellite mutability due to interruptions

To understand the impact of microsatellite interruptions on human genome stability, we first set out to determine the genome-wide magnitude of microsatellite mutability reduction due to the presence of interruptions. For this analysis, we studied high-quality primate genome alignments using a comparative genomics approach. Mono-, di-, tri- and tetranucleotide microsatellites above the threshold repeat number were identified in human, chimpanzee, orangutan, macaque, and marmoset reference genomes (Table S1; penta- and hexanucleotide microsatellites were omitted due to their lower abundance and algorithmic difficulties in specifying all possible interruptions). iMSs were identified as microsatellites in which at least one perfect repeat stretch extended beyond the threshold repeat number. An interruption was required to be shorter than or equal to the microsatellite's motif size. For each of the five primate genomes examined, iMSs were more abundant than perfect microsatellites (Table S1). When only orthologous iMSs with one or two interruptions were considered (see Materials and Methods for details), iMSs numbered from 6,000–38,000, while perfect microsatellites numbered from 8,000–48,000, depending on the primate genome analyzed.

**Figure 1:**
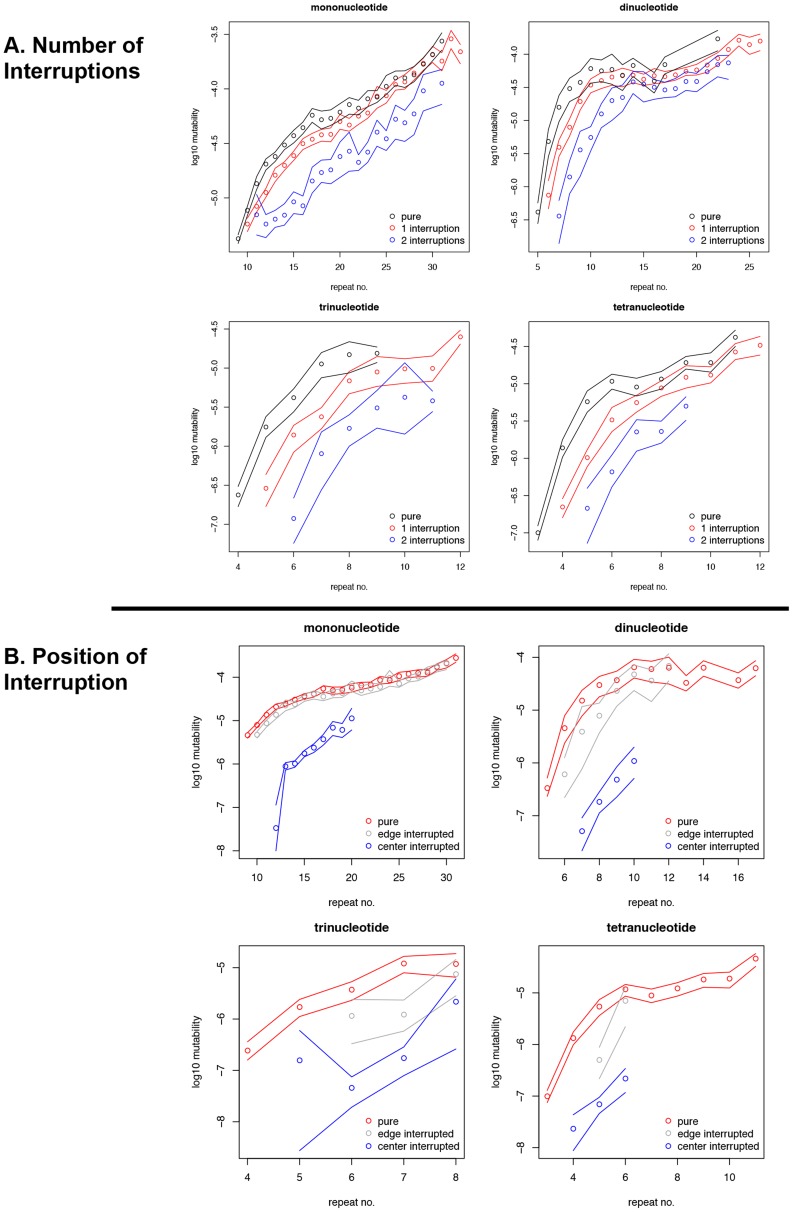
Effect of interruptions on microsatellite mutability in primate genomes. (A) Mutability of perfect (pure) microsatellites and that of microsatellites with one or two interruptions. (B) Mutability of perfect (pure) microsatellites and that of microsatellites with single interruptions that were located within the middle 25%, or in the fringe 25% (at either 5′ or 3′ end) of the microsatellite length. The number of repeats of a microsatellite was calculated by dividing the total length of the microsatellite, excepting the interrupting nucleotides, by the size of its repeating motif. At each repeat number the lines designate the 2.5th and 97.5th percentiles of empirical distributions that were obtained through bootstrap resampling. The repeats are binned based on their repeat number in the human genome (the reciprocal operation, when binning was based on repeat number in chimpanzee, did not change the results).

The mutability, or the average squared difference in repeat number (allele length) between two species [35], was contrasted for all perfect versus interrupted microsatellites present in human-chimpanzee genomic alignments. Namely, we performed a genome-wide comparison of the mutability of microsatellites with the same repeated motif that were perfect in both human and chimpanzee to that of microsatellites that were interrupted (with the same interruption(s)) in both of these species. For microsatellites of all motif sizes examined, short microsatellites with one interruption were less mutable than perfect microsatellites with the same overall repeat number (Figure 1A). The average, genome-wide mutability difference for mononucleotides was ∼two-fold at 12 repeat units, and up to ∼six-fold for di-, tri-, and tetranucleotide microsatellites with 6, 5, and 4 units, respectively. Microsatellites with two interruptions were, on average, one to two orders of magnitude more stable than uninterrupted microsatellites with the same repeat number (Figure 1A). The mutability difference between perfect and iMS loci was highest at shorter repeat numbers for all motifs. Thus, the quantitative effect of a single interruption on an individual microsatellite locus can be substantial. For example, more centrally located interruptions have a strong effect on mutability, dramatically lowering microsatellite mutability up to two to three orders of magnitude, whereas interruptions located on the microsatellite fringes have only a marginal effect (Figure 1B). The identity of the interrupting base has a non-significant effect on mononucleotide microsatellite mutability (Figure S1).

### Microsatellite interruptions in human populations

**Figure 2:**
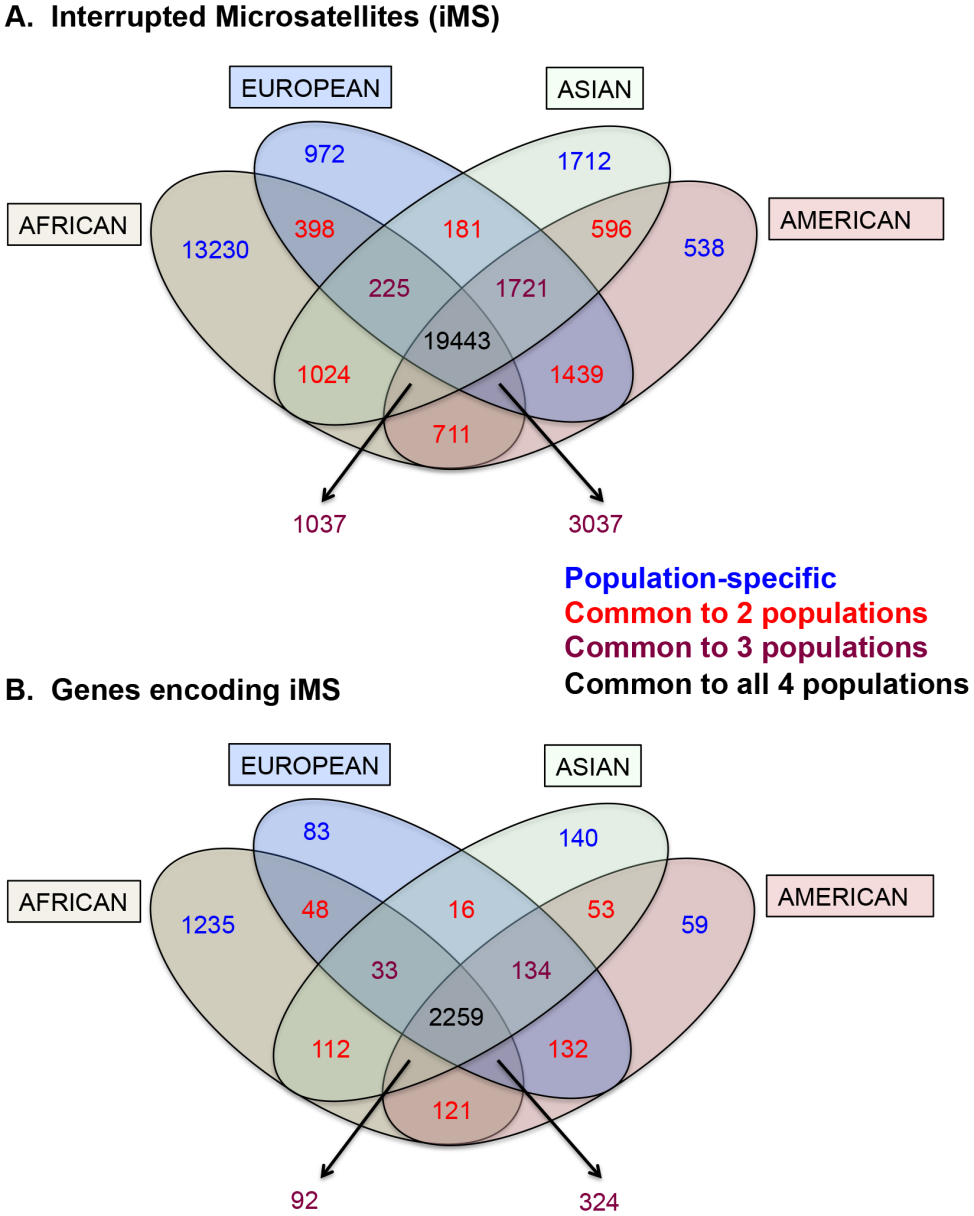
Distribution of interrupted microsatellites in human 1000 genomes populations. Venn diagram depicting (A) numbers of interrupted microsatellites (iMSs) across the four populations genome-wide, and (B) numbers of genes with iMSs in exons. Tan, blue, green, and red ellipses represent African, European, Asian, and American populations, respectively. Numbers in blue, red, maroon, and black represent counts of population-specific iMSs (absent in the other three), iMSs shared between two populations (and absent in the other two), iMSs shared between three populations (and absent in the fourth), and iMSs common to all populations, respectively.

Armed with the knowledge that interruptions significantly stabilize microsatellites genome-wide, we next examined individual human genome microsatellites for the presence of interruption polymorphisms. We found such polymorphisms to be highly abundant and informative for predicting population-specific microsatellite stabilization. In this analysis, we identified 1,814,151 perfect mono-, di-, tri-, and tetra-nucleotide microsatellites above the threshold length within the reference human genome (UCSC build hg19) [25]. Here, we imposed an upper limit on the microsatellite lengths analyzed (10, 9, 8, and 7 units for mono-, di-, tri- and tetranucleotide repeats, respectively), because we found next generation sequencing data at longer repeats to be biased due to sequencing errors and/or read-length limitations [25]. For microsatellites that are perfect in the reference genome, we analyzed the frequency of iMSs within four human population groups (African, European, East Asian, and American), using the 1000 Genomes Phase-1 variant call set [36]. Interruptions were defined as single nucleotide polymorphisms (SNPs) or indels leading to a sequence within the microsatellite that differs from the full motif unit. All indel and SNP variants (with allele frequency ≥0.05) were identified, and considered to be interruptions if they were located within a microsatellite but not at the starting/ending repeat unit. In this manner, we identified ∼26,000–40,000 polymorphic iMSs, depending on the population group (Table 1, Figure 2A; Datasets S1, S2, S3, S4, S5). A substantial number of interrupted alleles were present in all four population groups with different allele frequencies, corresponding to a fixation index (F_ST_) of 0.061 (range: 0.000–0.590; sd: 0.062; median: 0.041), which falls well within the range of SNP F_ST_ values (0.052–0.083) derived from pair-wise population comparisons of the 1000 Genomes Phase-1 project [36] (Dataset S6). Despite such low observed average level of population differentiation, numerous interruptions were shared by two or three population groups, or unique to a single population group (referred to as ‘population-specific’ interruptions henceforth)(Figure 2A). The greater number of interruptions within Africans compared to other population groups is likely due to a higher number of the 1000 Genomes variants in Africans, reflecting their high diversity [36], [37]. We also identified genes that encode polymorphic exonic iMSs. Among the four population groups studied, ∼3,000–4,000 genes contained polymorphic interruptions within exonic microsatellites (Table 2). Several genes encoding exonic iMS alleles are specific to only one population, or are shared by two or three populations (Figure 2B; Dataset S1, S2, S3, S4, S5). These data demonstrate that iMSs can provide an abundant source of population-specific alleles potentially stabilizing individual genomes by lowering microsatellite mutation rates.

**Table 1:** The number, types, and consequences of polymorphic iMS loci genome-wide in the 1000 genomes Phase-1 dataset, by population group.

Population	Number of Tandem Repeats				
	Total loci^a^	SNP Interruption		Indel Interruption	
		Death^b^	Degeneration^c^	Death	Degeneration
**African**	39,105	25,746	3,971	7,304	2,906
**European**	27,416	17,707	2,786	5,327	2,116
**Asian**	25,939	16,617	2,582	5,227	2,034
**American**	28,522	18,386	2,912	5,584	2,203

a The number of loci is less than the total number of SNP and Indel interruptions because some loci contain multiple interruptions.

b Interruptions resulting in iMS below the threshold length.

c Interruptions resulting in iMS at/above the threshold length.

**Table 2:** The number and types of exonic iMS in the 1000 Genomes Phase-1 dataset, by population.

Population	Total number of affected genes^a^	Mechanism^b^	
		SNP	Indel
**African**	4,224	3,478	1,213
**European**	3,029	2,323	879
**Asian**	2,839	2,158	819
**American**	3,174	2,427	908

a The number of genes is less than the total number of iMS because some genes contain multiple interruptions.

b Number of iMSs.

### Functional consequences of exonic iMS alleles

We performed more in-depth analyses of the polymorphic exonic iMSs identified above in the four human population groups to determine the potential functional impact of iMS presence on genome function. Only a few of the iMSs identified are predicted to cause frameshifts or nonsynonymous mutations (Figure S2, Table S2); the vast majority of population-specific interruptions are not expected to alter protein sequence. Thus, the primary effect of iMS may be to modulate the mutation rate of the underlying microsatellite. To gain further insight into the potential biological relevance of the iMSs, we performed Gene Ontology (GO) analyses for each set of genes encoding population-specific iMS alleles. The significantly (*p*<0.01) enriched GO terms are distinct for each population. For example, the GO terms enriched in the African-specific iMS genes included several neurological and organ development terms (Table S3), while those for the European-specific iMS genes were predominantly immunological terms (Table S4). Since the GO vocabularies are structured such that they can be queried at different levels, we examined the smallest sized GO terms, identified the associated genes containing the iMS, and queried these genes for clinical associations using Online Mendelian Inheritance in Man (www.omim.org). Several genes that we identified in this manner are associated with familial disease or disease susceptibility (Table 3). For example, we discovered three, African-specific interrupted mononucleotide microsatellites within the *HTT* (Huntington's) gene, which correspond to perfect microsatellites in European, Asian and American populations (Table 3). It is important to bear in mind that although the genes identified by this analysis are implicated in disease, the associated microsatellites have not been shown to play a causal role. Therefore, these iMSs will have to be studied further for their potential role in modulating disease risk.

**Table 3:** Examples of disease-associated genes corresponding to Gene Ontology terms significantly enriched (p<0.01) in population-specific iMS alleles.

Gene (Chr.)	GO terms (GOID)	Disease Association^a^	iMS Location^b^	MS Motif^c^
**AFR-specific Interruptions (perfect in EUR, AMR, ASN)**				
APOB (Chr. 2)	Positive regulation of cholesterol storage (GO:0010886);Lipoprotein catabolic process (GO:0042159)	Familial hypobetalipoproteinemia	21254729	ACC
ATM (Chr. 11)	Lipoprotein catabolic process (GO:0042159); Histone mRNA catabolic process (GO:0071044)	Ataxia telangiectasia; breast cancer susceptibility	108170038	TC
ENPP1 (Chr. 6)	Inorganic diphosphate transport (GO: 0030505)	Susceptibility to Type II diabetes, obesity	132169558	C
			132194964	AT
HTT (Chr. 4)	Neural plate development (GO:0001840);Neural plate formation (GO:0021990);Citrulline metabolic process (GO:0000052)	Huntington's disease	3129695	A
			3150527	A
			3177754	T
MSR1 (Chr. 8)	Positive regulator of cholesterol storage (GO:0010886)	Hereditary prostate cancer	16021086	TA
MYH9 (Chr. 22)	Establishment of T cell polarity (GO:0001768)	May-Hegglin/Fechtner syndrome	36676990	T
			36683802	A
POLG (Chr. 15)/	Gamma DNA polymerase complex (GO:0005760)	Progressive external opthalmoplegia; Mitochondrial depletion syndrome	89867478	ACG
DNA2 (Chr. 10)			70181580	T
SCN2A (Chr. 2)	Sodium channel complex (GO: 0034706)	Autism spectrum disorder; infantile epilepsy	166242996	A
SCN5A (Chr. 3)	Sodium channel complex (GO: 0034706)	Long QT syndrome, Brugada syndrome	38691860	AC
SCNN1B (Chr. 16)	Sodium channel complex (GO: 0034706)	Liddle syndrome	23312745	GA
SPTB (Chr. 14)	Long term strengthening of neuromuscular junction (GO:0042062)	Hereditary spherocytosis; elliptocytosis	65232615	TG
**EUR-specific Interruptions (perfect in AFR, AMR, ASN)**				
SDHA (Chr.5)	Mitochondrial respiratory chain complex II; Succinate dehydrogenase complex (GO: 0005749; 0045281)	Leigh syndrome	229004	A
SDHAF2 (Chr. 11)	Mitochondrial electron transport; protein-FAD linkage (GO:0006121; 0018293)	Paraganglioma 2	61205342	T
PCCA (Chr. 13)	Propionyl-CoA carboxylase activity (GO:0004658)	Propionic acidemia	101019895	T
**ASN-specific Interruptions (perfect in AFR, AMR, EUR)**				
POMC (Chr. 2)	Types 1, 3 and 4 melanocortin receptor binding (GO: 0070996, 0031781; 0031782)	Propiomelanocortin deficiency	25384471	GCT
**AMR-specific Interruptions (perfect in AFR, ASN, EUR)**				
SAG (Chr. 2)	Rhodopsin mediated signaling pathway; rhodopsin mediated phototransduction; opsin binding (GO:0016056; 0009586; 0002046)	Oguchi disease	234248618	T

a Genes with non-microsatellite variants known or suspected of being associated with disease/disease susceptibility [Online Mendelian Inheritance in Man (www.omim.org)].

b Exact chromosomal position (in basepair) of the interrupton(s) in the hg19 reference genome and the indicated chromosome.

c Sequence motif of the perfect microsatellite repeat(s).

We also examined polymorphisms in 15 genes containing exonic (coding and UTR) iMS alleles that are well known to be associated with microsatellite expansion diseases [38]. Eight loci (*ARX*, *CBFA1*, *FMR1*, *FMR2*, *HOXA13*, *OPMD*, *SCA3*, and *ZIC2*) contained no differences in microsatellite sequence from the reference genome in any of the four population groups studied. Four genes (*AIB1*, *SCA2*/*ATXN2*, *SCA17*, and *HOXD13*) contained iMS alleles that differed from the reference genome sequence, and the variants were present in all four population groups at differing allele frequencies (Table 4). For some loci/populations, the reference genome sequence is not the major allele (e.g., *SCA17*). The genetic consequences of the iMS variants include both sequences that are expected to increase mutability, and sequences expected to decrease mutability. For example, the *HOXD13* variant iMS allele is expected to have lower mutability than the reference genome iMS due to the presence of a third interruption that decreases the perfect tandem (GCG)_5_ repeat to a length below the mutability threshold (four units for trinucleotide repeats [25]). The frequency of this triply-interrupted allele varies from 0.76 in the African population to 0.26 in European and American populations. The *AIB1* locus contains four alternative iMS alleles present at varying frequencies among the populations, one of which is a doubly interrupted allele, leading to greater stabilization of the repeat due to disruption of the (CAG)_6_ array. For three loci (*DRPLA*, *SCA1*, and *FOXL2*), we observed instances of population-specific iMS alleles. *DRPLA* contained variant alleles in only two of the four population groups studied (African and American), both of which decrease the number of interrupting bases, relative to the reference genome, potentially increasing mutability of the repeat. Finally, we noted an increased number of interruptions within polyglutamine repeats compared to polyalanine repeats, consistent with previous observations about the high propensity of polyglutamine repeats to acquire length and nucleotide polymorphisms [39].

**Table 4:** Population allele frequencies for iMS in expanded microsatellite disease loci.

Locus^a^	hg19 sequence^b^/Variant allele sequence(s)	Allele Frequency			
		AFR	EUR	ASN	AMR
**Polyglutamine repeats**					
**AIB1**	(CAG)_6_ CAA (CAG)_9_	0.1464	0.3426	0.5699	0.4365
	a. (CAG)_6_ CAA( **CAG** )**_8_**	0.3455	0.3955	0.2115	0.3674
	b. (CAG)_3_ **CAA** (CAG)_2_ CAA(CAG)_9_	0.2012	0.1839	0.1329	0.1243
	c. (CAG)_6_ CAA **(** **CAG** **)_10_**	0.3069	0.0780	0.0857	0.0718
**DRPLA**	CAGCAACAGCAA (CAG)_15_	0.7032	1.0	1.0	0.8232
	a. CAGCAA (CAG)_15_	0.1748	0	0	0.0939
	b. CAGCAA **(** **CAG** **)_16_**	0.1220	0	0	0.0829
**SCA1/ATXN1**	(GCA)_12_ CAT (CAG) CAT (CAG)_14_	0.7094	0.8650	0.3794	0.7072
	a. (GCA)_12_ CAT (CAG) CAT (CAG) **CAT** (CAG)_12_	0.0772	0.0622	0.1556	0.1298
	b. (GCA)_12_ CAT **(** **CAG** **)_16_**	0.2134	0.0728	0.2622	0.1630
	c. (GCA)_12_ CAT (CAG) CAT (CAG)_3_ **CAT** (CAG)_10_	0	0	0.2028	0
**SCA2/ATXN2**	(CAG)_13_ CAA (CAG)_9_	0.5224	0.3135	0.5385	0.3785
	(CAG)_8_ **CAA** (CAG)_4_ CAA (CAG)_9_	0.4776	0.6865	0.4615	0.6215
**SCA17/TBP**	(CAG)_3_(CAA)_3_(CAG)_8_ CAA (CAG) CAA (CAG)_19_	0.1704	0.1667	0.0892	0.1105
	(CAG)_3_(CAA)_3_ **(** **CAG** **)_9_** CAA (CAG) CAA (CAG)_19_	0.8296	0.8333	0.9108	0.8895
**Polyalanine repeats**					
**HOXD13**	(GCG)_4_ GCA (GCG)_2_ GCT (GCG)_5_	0.2398	0.7407	0.6801	0.7431
	(GCG)_4_ GCA (GCG)_2_ GCT (GCG)_3_ **GCA**GCG c	0.7602	0.2593	0.3199	0.2569
**FOXL2** d	(CGG)(CCC)(CGG)(CGC)C(CGC)(CA)(CGC)_2_ (ACC)(CGC)(CTG)(CGG)(CGC)(CTC)(CGG)	0.5630	1.0	0.9301	1.0
	(CGG)(CCC)(CGG)(CGC)C**CCC** (CA)(CGC)_2_ (ACC)(CGC)(CTG)(CGG)(CGC)(CTC)(CGG)	0.4370	0	0.0699	0

a Chromosomal locations for iMS examined are as follows: AIB1: Chr. 20, 46279815-899; DRPLA: Chr. 12, 7045879-936; SCA1: Chr. 6, 16327866-954; SCA2: Chr. 12, 112036754-823; SCA17, Chr.6: 170870995-1103; HOXD13: Chr. 2, 176957786-825; FOXL2, Chr. 3, 138664861-903. The following loci were examined, but no differences from the reference genome were observed in any of the four populations: SCA3; CBFA1; ZIC2; OPMD; HOXA13; ARX; FMR1; FMR2.

b Underline indicates the position of the interruption within the hg19 reference sequence. Bold font indicates the variant sequence (relative to the reference sequence) identified within the four populations examined.

c This allele corresponds to the non-diseased sequence reported in reference [60].

d There is another microsatellite present at this locus [(CGC)(GGC)(TGC)(AGC)(CGC)(AGC)(TGC)2(AGC)(CGC)(TGC)(GGC)(TGC)(CGC)]; however it showed no differences from the reference genome in any of the four populations.

### Interrupted alleles: Heterozygosity and linkage disequilibrium

Low indel mutation rates of iMSs (Figure 1) also are expected to be reflected in their low indel polymorphism levels. To test this, we investigated the levels of heterozygosity and the presence of linkage disequilibrium (LD) between interrupted microsatellite alleles caused by indels and neighboring, population-matched SNPs from the 1000 Genomes Phase-1 data. Approximately 30–40% of iMSs display low levels of heterozygosity (below 0.2; Figures S3A–D). In fact, we observed a skew towards lower heterozygosity for iMSs as compared to that for perfect microsatellites (*p* = 0.028 for Asians; *p* = 0.066, *p* = 0.057, and *p* = 0.072 for Africans, Americans, and Europeans, respectively; Kolmogorov-Smirnov test).

In each of the four populations studied, 4,400 to 5,000 interruption-causing alleles (36–49% of the alleles investigated) were found to be in moderate LD (R^2^>0.80) with SNPs (Figure S4, Table S5), and 686 to 990 alleles (6–10%) were in perfect LD (R^2^ = 1) with SNPs. Interestingly, certain interruption alleles displayed perfect LD in some, but not all, populations (Table S5). Generally, iMS alleles in the African population displayed lower levels of LD compared to the other three populations (Figure S3), likely due to the abundance of low-frequency variants in Africans compared to non-African populations [36].

The exonic iMSs in perfect LD with neighboring SNPs were examined in more detail. Within each population, 6 to 11 of such alleles were identified (Table S6). For each allele, we examined the phenotype and disease relationships of the linked SNPs using SNPnexus web browser [40]–[42], and found associations with cancer, neurological, immune, cardiovascular, and metabolic disorders (Table S6). These associations reiterate a potential for iMSs to modulate disease risk in a population-specific manner.

### A case example: Mutability of an exonic iMS associated with colorectal cancer

We sought to directly verify the quantitative effect on mutability of a single base substitution interruption within an exonic microsatellite encoded within a human disease gene. We chose the well-established biological model of a population-specific iMS encoded within the *APC* tumor suppressor gene. In 6% of the Ashkenazi Jewish population, a centrally located iMS (AAATAAAA) within an exon of the APC gene is present in the germline as a perfect A_8_ microsatellite (AAAAAAAA); this nonsynonymous SNP leads to an I1307K variant, but has no effect on APC protein function [19]. Nevertheless, this population has a greater chance of producing an inactive *APC* gene in somatic tissues, which increases the risk of colorectal cancer [43]. The proposed mechanism accounting for this observation is the enhanced somatic mutability of the perfect A_8_ sequence, relative to the interrupted sequence [19], [44]. We modeled the germline sequences of the perfect and interrupted *APC* microsatellites, and measured DNA polymerase strand slippage error rates using our established *in vitro* assay. Briefly, in this analysis, defined tandem repeat sequences are inserted in-frame within a reporter gene. Vectors containing these reporter cassettes are used as templates for *in vitro* DNA synthesis reactions, and DNA polymerase errors that result in gene inactivation (frameshift, nonsense or missense mutations) are scored by genetic selection in *E. coli* [45], [46]. To determine the specificity of polymerase errors, independent mutants are isolated, and the DNA sequence changes within the reporter region are determined by dideoxy DNA sequence analysis of purified vector DNA [47].

**Figure 3:**
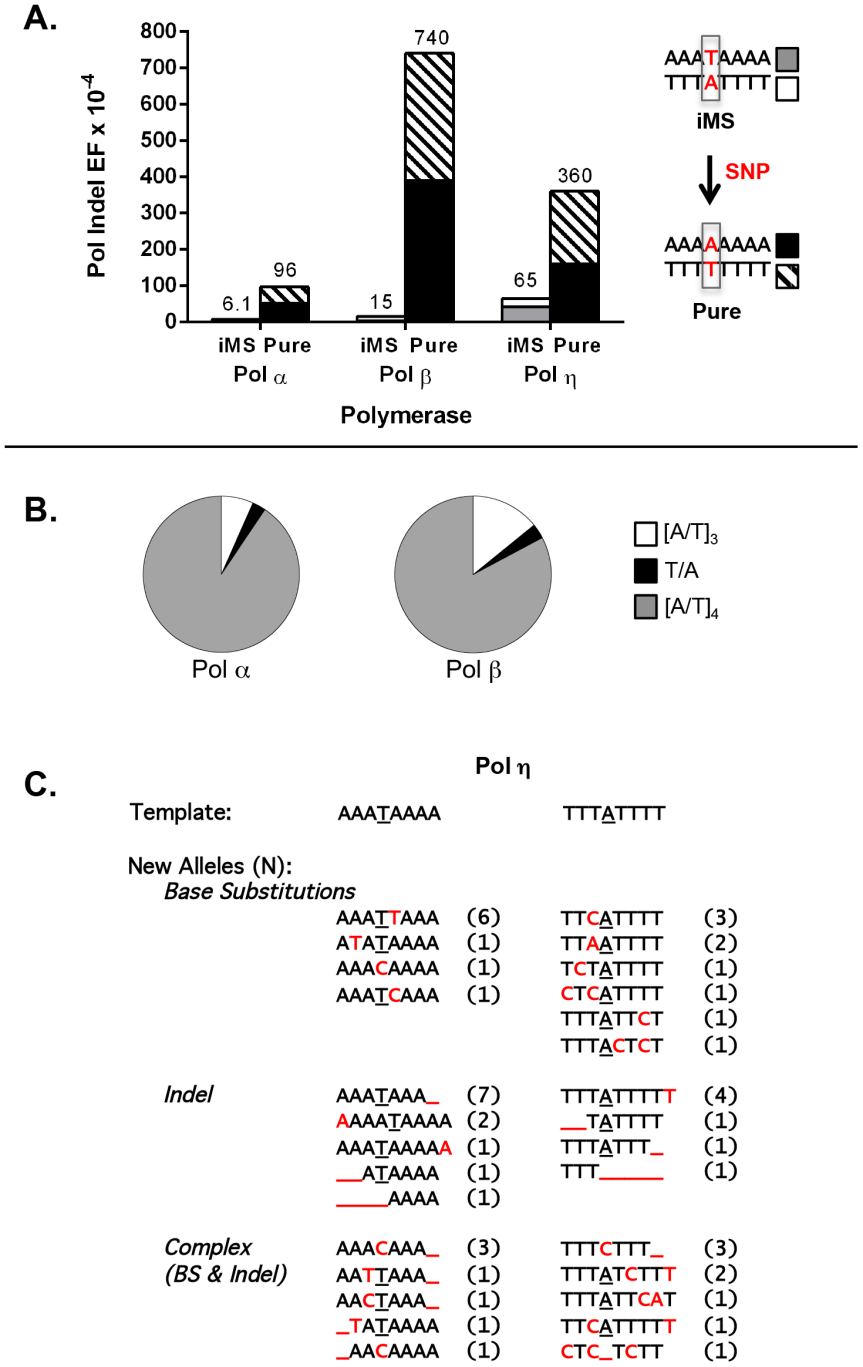
DNA polymerase error rates at interrupted microsatellites corresponding to sequences within the *APC* gene. (A). DNA polymerase indel error frequency. The Pol EF for each of the four alleles was determined separately from two independent polymerase reactions per single-stranded template (Table S5). Indel Pol EFs were calculated by multiplying the proportion of unit-based indel mutational events (as examples, [A]_8_→[A]_7_ for a perfect allele or A_3_TA_4_→A_3_TA_3_ for an iMS allele) by the microsatellite Pol EF. Numbers on the top of each column were obtained by adding the Indel Pol EFs of the complementary alleles in order to compare the difference in polymerase fidelity upon introduction of a single nucleotide polymorphism (SNP) that converts the double-stranded iMS sequence to a double-stranded perfect (pure) sequence. (B) Specificity of Pol α and Pol β mutational events within the iMS alleles. Proportions of mutational events found within the three-unit tandem repeat (open sectors), the interrupting base (black sectors), and the four-unit tandem repeat (gray sectors). Total mutational events for pols α and β were 74 and 35, respectively and all were indel events. Two pol α events generated the loss of the interrupting T within the A_3_TA_4_ iMS sequence (A_3_TA_4_→[A]_7_ and A_3_TA_4_→[A]_6_). One similar event occurred for pol β at the T_3_AT_4_ iMS sequence (T_3_AT_4_→[T]_4_). (C). Pol η mutational events within the iMS alleles generate sequence diversity. Events (71 total) are categorized according to the mutational mechanism that most likely created them. Red indicates individual mutational events. Underline indicates a missing base or bases. Number in parentheses shows the number of mutants carrying the new sequence.

For these experiments, we examined three DNA polymerases, representing distinct polymerase families and postulated to be required for distinct genome maintenance functions: Pol α, DNA replication; Pol β, DNA repair; and Pol η, translesion synthesis. The accuracy of each polymerase was measured on four DNA templates, representing the complementary strands of the perfect (A_8_ and T_8_) and iMS (A_3_ TA_4_ and T_3_ AT_4_) alleles in *APC* (Figure 3A). For the perfect allele templates, the polymerases created +1 A/T insertions, −1 A/T deletions, and A:T to T:A tranversions that lead to TAA nonsense codons (data not shown), which also are the types of inactivating APC somatic mutations observed within tumors from I1307K carriers [44]. For the iMS allele templates, the polymerase indel error frequency was five- to 50-fold lower than that for the perfect allele, depending on the polymerase, demonstrating strand slippage stabilization by this single interruption (Figure 3A; Table S7). We observed that the interrupting base is rarely removed by these polymerases; the predominant errors (>95%) are indels within the remaining perfect tandem repeat tracts (Figure 3B). The frequency of deleting the interrupting base to create a perfect allele was very low (9.2×10^−6^ and 2.2×10^−5^ for Pol α and Pol β, respectively), relative to other types of polymerase errors (Table S7). Moreover, the polymerase error frequencies at the residual repeats within the iMS alleles were similar to the error frequencies at similar short tandem repeats located elsewhere within the HSV-tk gene coding sequence (data not shown). These analyses strongly suggest that the single nucleotide interruption within the *APC* gene leads to the mutational death of the microsatellite.

DNA sequence analyses of Pol η errors produced on the interrupted templates emphasized the novel mutational signature of this enzyme within this specific microsatellite motif (Figure 3C). Intriguingly, Pol η has the unique ability to litter this iMS with additional errors, often creating a DNA synthesis product that is more random in sequence than the starting iMS template sequence. Despite this ability, the original interrupting base is maintained in the majority (79%) of Pol η synthesis products.

### Pathways of gaining interruptions

Despite the clear biological significance of iMSs on human genome stability and disease risk, very little is known regarding the biochemical pathways by which interruptions arise in microsatellites. Mutational events to create interrupted alleles could be produced during several cellular mutagenesis pathways, including cytosine deamination events, the creation of abasic sites, endogenous DNA damage-induced mutations and DNA polymerase errors, among others. We used two complementary approaches to gain insight into the potential pathways underlying the production of iMS in the human genome. *First*, the abundance of polymorphic interruptions and the short evolutionary time since divergence of the four 1000 Genomes population groups allowed us to examine the types of mutations leading to population-specific microsatellite interruptions in detail. (We observed a high degree of interruption gain/loss event saturation along primate phylogenetic branches, precluding us from deciphering interruption pathways in this data set. For instance, the resulting numbers of interruptions along the human or chimpanzee lineages since their ∼6 MY split were similar to that along the orangutan lineage since its ∼12 MY split from the human lineage (Figure S5)). *Second*, the fact that DNA polymerases can create interruption errors during *in vitro* synthesis of microsatellite-containing templates [45], [46], [48] afforded us the opportunity to examine one biochemical pathway- namely, polymerase errors during DNA synthesis.

#### Population genomics approach

**Figure 4:**
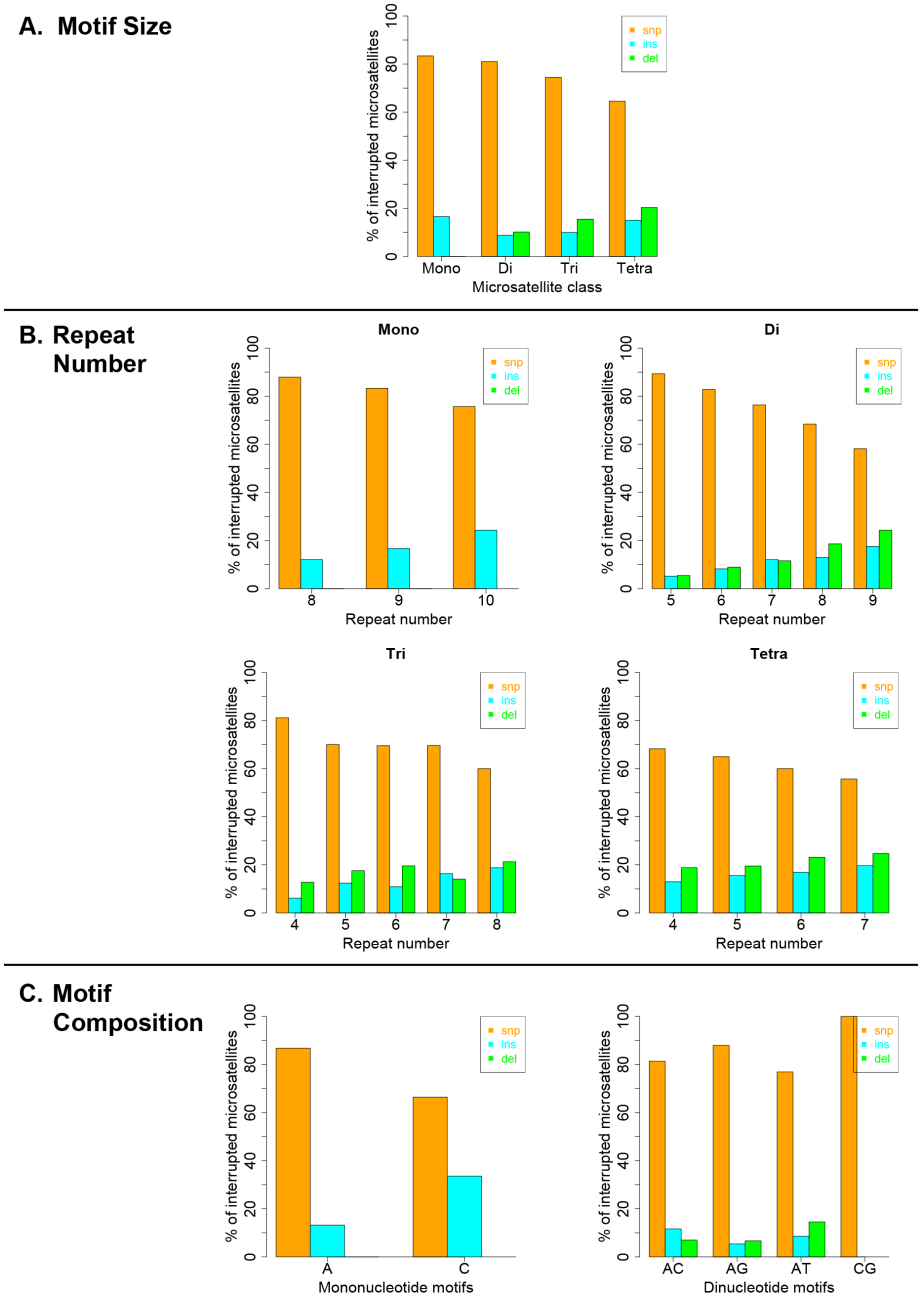
Pathways (substitutions, insertions, and deletions) driving the African population-specific interruptions. Repeats separated by (A) motif size, (B) repeat number, and (C) motif sequence for mono- and dinucleotides microsatellites.

Interruption variants identified in the 1000 genomes datasets were classified as either base substitutions (SNP variants) or indels (insertion/deletion variants that did not include a whole-motif insertion/deletion). The intrinsic properties of microsatellites (motif size, repeat number and motif composition) are known to be the primary factors dictating motif-based indel mutations within microsatellites [49], [50]. Therefore, we examined the effect of intrinsic sequence properties on the production of population-specific iMS alleles (Figure 4 presents results for the African population; the results for the other three populations are very similar; Figures S6, S7, S8). Base substitutions are the primary mutation type leading to iMSs in all four population groups, all motif classes and repeat numbers examined (Figures 4, S6, S7, S8). The relative proportion of substitution-based interruptions is lower in tetranucleotides compared to the other three motif classes (Figures 4A, S6). This may reflect the fact that numerous tetranucleotide motifs contain proto-microsatellites of two or three tandem repeats (i.e., TTCC or TTTC), which would be expected to increase the likelihood of indel interruption mutations. For all motif sizes, with increasing repeat number, the proportion of substitution-driven interruptions decreases, while indel-based interruptions increases (Figures 4B, S7). For mono- and dinucleotide microsatellites, we observed some differences in the proportion of iMS alleles based on motif composition (Figures 4C, S8). In particular, [G/C]_n_ alleles were found to have more insertion interruptions compared to [A/T]_n_ alleles. Interestingly, these trends reflect the order of slippage-driven polymorphism incidence ([G/C]>[A/T] for mononucleotides), as observed in the 1000 Genomes Pilot-1 dataset for length polymorphisms [25].

#### Biochemical approach

**Figure 5:**
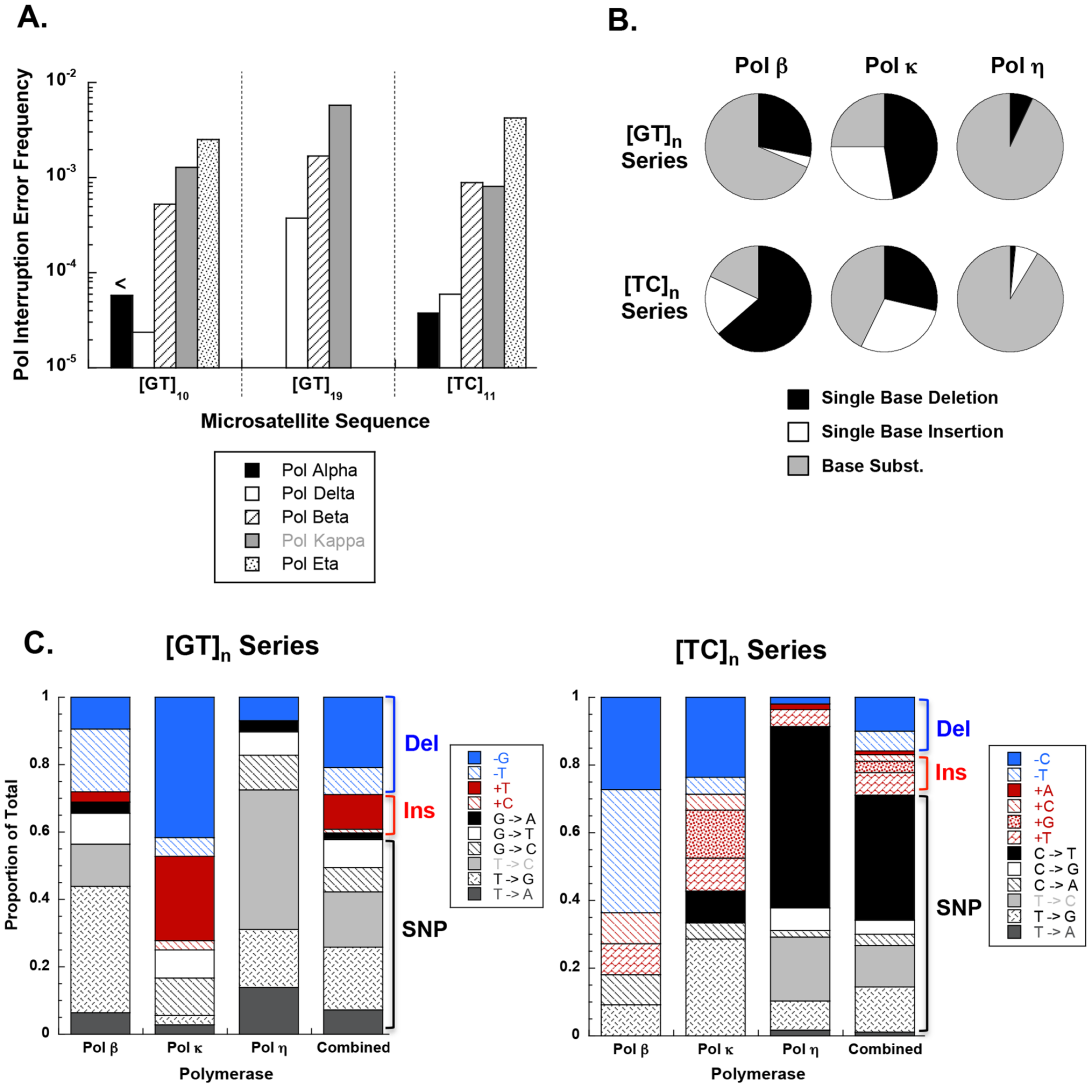
DNA polymerase interruption mutagenesis within [GT]_n_ and [TC]_n_ dinucleotide microsatellite sequences. (A) Interruption Pol EFs at the [GT]_10_, [GT]_19_, and [TC]_11_ alleles for B-family (pols α and δ), X-family (pol β) and Y-family (pols κ and η) DNA polymerases. Interruption Pol EFs were calculated from unpublished and published [24], [26], [45], [46], [48], [94] data by multiplying the proportion of interruption mutational events at each allele by the microsatellite Pol EF. Only detectable interruptions (ie, interruptions that produce a frameshift or a stop codon) were included in this analysis given that an event must be detectable to contribute toward the Pol EF. Less than symbol (<) indicates that no interruption events were found for pol α at the [GT]_10_ allele; the interruption Pol EF is estimated to be <5.7×10^−5^. The Pol EF was not determined for Pol α or Pol η using the GT_19_ template. (B) DNA polymerases utilize signature interruption mechanisms. Pie charts depict the proportion of mutational events generated by each possible interruption mechanism at [GT]_n_ and [TC]_n_ alleles. Graphs include both detectable and undetectable interruptions. Data used in the [GT]_n_ chart is a compilation of interruption events from pol β (N = 32) at [GT]_10_, [GT]_13_, and [GT]_19_, pol κ (N = 36) at [GT]_10_, [GT]_13_, and [GT]_19_, and pol η (N = 29) at [GT]_10_. The [TC]_n_ chart includes events from pol β (N = 11) at [TC]_11_ and [TC]_14_, pol κ (N = 21) at [TC]_11_ and [TC]_14_, and pol η (N = 58) at [TC]_11_. See Supplementary Figures S7 and S8 for complete representation of interruption mutations. (C) Detailed specificity of interruption events at [GT]_n_ and [TC]_n_ microsatellites. Columns in blue indicate the proportion of total interruptions that are single base deletions. Columns in red indicate the proportion that are single base insertions and columns in black/gray indicate the proportion that are base substitutions. Data used for this analysis is the same as that used in (B) for pols β, κ, and η. Data in combined column indicates the specificity obtained upon combining data from all three polymerases.

DNA synthesis errors by polymerases can arise during the biochemical pathways of DNA replication, recombination, repair, and translesion synthesis. The human DNA polymerases associated with these four pathways constitute distinct enzymatic families and have differing inherent accuracies [51]. To gain insight as to which DNA polymerases potentially may produce iMSs in the genome, we surveyed the *in vitro* frequency of detectable iMS errors (see below) that are created by polymerases involved in replication (Pols α and δ), recombination (Pols δ and η), repair (Pols β, δ, and κ), and translesion synthesis (Pols κ and η). Detectable interruption errors within dinucleotide microsatellites can be produced in our in-frame genetic reporter assay by single base indel errors or by base substitution errors that create a nonsense codon and inactivate the HSV-tk protein. For the dinucleotide motifs examined, we observed that genome-stabilizing iMSs are created most frequently by error-prone polymerases. The replicative human DNA polymerases α (Pol α) and δ (Pol δ) create interruption errors within [GT]_10_ and [TC]_11_ alleles at a very low frequency (∼10^−5^; Figure 5A). These results are similar to our previous report for yeast replicative Pol δ and Pol ε holoenzymes [52]. Conversely, the specialized translesion synthesis polymerases, Pol κ and Pol η, produce a relatively high frequency (∼10^−3^) of interruption errors within the same alleles (Figure 5A). The repair polymerase, Pol β, has an intermediate interruption error frequency that ranges from 10^−4^ to 10^−3^, depending on the allele sequence. For the polymerases examined, the interruption error frequency increases with allele length, up to 10^−2^ within the [GT]_19_ allele. Thus, iMS alleles within the specific microsatellites examined are readily created by human DNA polymerases.

We undertook an in-depth analysis of the interruption errors produced by polymerases within these templates to further understand the potential biochemical pathways by which iMS may arise in the human genome through DNA polymerase errors. Unique iMS mutational signatures are created by each DNA polymerase within the [GT]_n_ and [TC]_n_ alleles (Figure 5B; Table S8; Figures S9 and S10). Pol η produced a characteristically high proportion of base substitution errors on both templates, while Pol κ displayed a propensity for creating single base insertion errors within the dinucleotide alleles. The very few interruption errors produced by Pol δ were primarily (∼70–75%) base substitutions. We note that the interruption error specificity of Pols β and κ were somewhat influenced by the motif sequence. Pol η produced a unique error profile when synthesizing perfect mono- and dinucleotide templates, in that the synthesis products are characterized by a high degree of sequence diversity (Table 5). A full 20% of Pol η iMS DNA products contained two or more interruptions (14/71); in comparison, the related Y family Pol κ produced 2 or more interruptions in only 3% (2/55) of cases.

**Table 5:** Sequence diversity created *in vitro* by human DNA polymerase η base substitution errors within perfect microsatellites.

Starting Microsatellite Template Sequence			
[A]_8_	[T]_8_	[GT]_10_	[TC]_11_
iMS Mutational Events			
A **T** [A]_6_ a	**A** [T]_7_	G**C** [GT]_8_ G**A**	TC **C**C [TC]_4_ T**T** [TC]_4_
[A]_4_ **T** [A]_3_	[T]_2_ **C** [T]_5_	GT **C**T [GT]_2_ G**G** [GT]_4_ b	TC **C**C [TC]_6_ **CT** [TC]_2_
[A]_5_ **G** A b	[T]_5_ **C** [T]_2_	[GT]_2_ G**C** [GT]_3_ G**C**G**C** d	TC T**T** [TC]_4_ **G**C [TC]_4_
	[T]_6_ **C** T	[GT]_3_ G**G C**T [GT]_4_ b	[TC]_2_ **C**C TC T**T** [TC]_7_ c
	[T]_6_ **C** [T]_2_	[GT]_6_ G**G** **T**T GT b	[TC]_2_ T**T** [TC]_3_ T**T** [TC]_5_ c
			[TC]_3_ **CT** [TC]_8_ c
			[TC]_3_ **G**C [TC]_2_ T**T** [TC]_5_ c
			[TC]_3_ T**T** [TC]_5_ T**T** [TC]_2_ c
			[TC]_5_ T**T** [TC]_5_ T**T** c

Bold, interrupting base(s).

a Three independent occurrences.

b A substitution occurred with a 1 unit deletion.

c A substitution occurred with a 1 unit insertion.

d A substitution occurred with a 2 unit deletion.

We pooled all interruption errors created by the three polymerases most frequently producing iMS errors (Pols β, κ, η) using five dinucleotide templates, and calculated the proportion of interruption errors via base substitution, single base deletion, and insertion events (Figure 5C). For the [GT]_10–19_ dinucleotide motif, the majority of iMSs arose by base substitution errors (60%), followed by single base deletion errors (29%), and then by single base insertion errors (11%). A similar trend was observed for errors produced within the [TC]_11–14_ templates (71% base substitutions). These polymerase data for the types of iMS errors produced within dinucleotide alleles *in vitro* are in concordance with the human genome data for the types of interrupted dinucleotide microsatellites observed genome-wide (Figure 4C).

## Discussion

In this study, we answered three biologically significant questions regarding mono-, di- tri- and tetranucleotide microsatellite interruptions in the human genome. *First*, using primate genome alignments, we quantified the genome-wide effect of interruptions on decreasing microsatellite mutability, and found it can be significant and strong – from several fold to several orders of magnitude, compared with perfect repeats. *Second*, utilizing the 1000 Genomes Phase-1 dataset, we found iMS polymorphisms to be highly abundant and informative for predicting population-specific microsatellite stabilization, especially for exonic loci. The vast majority of the population-specific, exonic iMSs we identified are not expected to alter protein sequence; thus, the primary effect of interruptions may be to modulate the mutation rate of the underlying microsatellite. *Third*, we discovered that base substitutions are the primary type of interruption among MSs in all population groups, and for the four microsatellite classes examined. We surveyed five mammalian DNA polymerases involved in DNA replication, repair, and specialized functions, and found that, for the mono- and dinucleotide microsatellite sequences analyzed, iMSs are created most frequently by error-prone polymerases. Pol η is notable among the enzymes examined in that the microsatellite DNA synthesis products are characterized by a high degree of sequence diversity.

### Contribution of interruptions to human genome stabilization

Early studies of microsatellite interruptions demonstrated reduced mutation rates at a few iMS loci, as compared with perfect alleles of the same repeat number [28]–[30], [44]. A higher mutability of microsatellites was observed for interruptions closest to the repeat tract ends, as compared with centrally located interruptions [31], [33], [53], [54]. Such studies suggested that interruptions might effectively divide microsatellites into shorter repeat runs. Within the interrupted repeat itself, the mutation rates of the individual arms depend on the lengths of perfect tracts remaining within the iMS allele [55].

Here, we provide a detailed, genome-wide analysis of the mutability of perfect and interrupted MSs in completely sequenced primate genomes. For the four motif sizes examined, interruptions significantly reduced mutability when present (a) within shorter microsatellites, (b) in multiple numbers (i.e., two interruptions per microsatellite), or (c) near the center of the microsatellite (Figure 1) – all of which give rise to a shorter perfect repeat tract. Importantly, the magnitude of the effect of interruptions on microsatellite allele length variation ranged from a few-fold to several orders of magnitude for loci across the genome.

We also report here that the perfect microsatellites in the human reference genome analyzed here (≤10 units in length) are frequently found as iMS polymorphisms within the genomes of individuals from four population groups. Although the majority of iMS alleles were shared among all groups, many of the iMS alleles we detected were specific to only one population group, or shared between subsets of population groups (Figure 2). Our quantitative results for the stabilizing effects of interruptions in short microsatellites are biologically relevant here, as the vast majority of iMSs we identified in human genomes are within short microsatellites, just above the length threshold. Therefore, interruptions are expected to have a strong effect on stabilizing such microsatellites. Thus, iMSs are a likely source of population-specific genetic variants that can affect the stability of individual genomes by reducing the mutability of microsatellites. To the best of our knowledge, this is the first report of iMSs as an abundant source of population-specific genetic modifiers in the human genome. The full abundance of iMSs within the human genome must await future studies, when improvements in sequencing technology read length and accuracy will allow the interrogation of all microsatellite motif sizes, lengths, and sequences that are present within individual genomes.

### Impact of interruptions on genome function

The *APC* tumor suppressor gene illustrates a provocative example in which a single, population-specific, germline SNP can affect disease risk by altering the mutagenic potential of a microsatellite sequence. Our data directly support the previous model that the perfect [A_8_/T_8_] allele creates a hypermutable region within the *APC* gene, leading to cancer predisposition [19]. We measured DNA polymerase strand slippage error rates that are up to 50-fold lower during replication of the iMS sequences [A_3_ TA_4_/T_3_ AT_4_], compared to the perfect sequences [A_8_/T_8_] (Figure 3). Previous biochemical studies of trinucleotide microsatellites have shown that interruptions decrease slipped strand formation [56] and decrease the thermostability of secondary structures formed by repetitive sequences [57]. Our results advance these studies by demonstrating that the mechanism of reduced mutability by an interruption within a mononucleotide A/T allele is lowered polymerase strand slippage errors during DNA synthesis.

Expanding on the *APC* gene observation that SNPs can create perfect microsatellites and hypermutable sequences in disease states, we identified ∼3,000–4,000 genes (depending on the population group) that are perfect in the reference genome, but contain iMS within exonic regions (Figure 2). The exonic iMS alleles that are specific to only one or two populations likely represent a pool of genes that are at a risk for increased mutation in the other population groups. Madsen and colleagues reported that short tandem repeats/microsatellites in exons are overrepresented among human genes associated with cancer and immune system diseases [58]. We observed that while European-specific iMSs are enriched in genes associated with immunological function, African-specific iMSs are enriched in genes associated with neurological function. Thus, population-specific differences in microsatellite architecture (perfect *versus* interrupted) may be a widespread mechanism by which genetic ancestry impacts individual disease risk. While our focus has been on comparing population groups, our F_ST_ analysis indicated that many iMS alleles are not fixed within population groups, thus potentially providing a rich source of individual genetic variability.

Perfect microsatellites are at a higher risk for microsatellite expansion mutations that are causative for numerous neurological/neurodegenerative diseases [3], and the presence of interrupted alleles has been well documented to decrease disease risk. We investigated several genes previously described as harboring disease-associated, coding iMS alleles [38]. The genetic consequences of the iMS variants we identified include both sequences that are expected to increase mutability, and sequences that are expected to decrease mutability. Various *AIB1* iMS alleles have been noted previously in a survey of European DNA samples [59], consistent with the allelic distribution we observed for the 1000 Genomes European population group. One of the iMS variants we identified within *AIB1* occurs at a much higher allele frequency in the African population, and is expected to display higher mutability than the reference sequence, due to an increased perfect tandem repeat tract length. The two *HOXD13* iMS alleles we identified were observed previously in a pedigree analysis of 16 synpolydactyly families [60]. Importantly, repeat expansions in these families segregated with the disease phenotype; however, the iMSs were retained in all of the expanded alleles. Recently, amyotophic lateral sclerosis patients have been described as having moderately expanded *SCA2* iMS alleles that retain at least one of the interruptions [20], [61]. Both microsatellite length and purity (interruption) *SCA1* and *SCA2* polymorphisms have been described among unaffected individuals [62], [63], consistent with the iMS variant alleles we detected in this study.

### Pathways leading to microsatellite interruption

The pathways by which iMSs arise in genomes have not been extensively studied. Several cellular mechanisms could account for the production of iMS alleles in genomes, including (but not limited to) endogenous DNA damage-induced mutations and DNA synthesis errors during DNA replication, repair and/or recombination. The types of iMS ultimately observed in human genomes will be further shaped by DNA repair pathways and selection, which will serve to reduce the number of and narrow the types of mutational events within microsatellites. We demonstrate here that base substitutions are the primary type of iMS present in individual human genomes. We also used our established biochemical assay to determine the potential contribution of errors created by three distinct DNA polymerase families to the formation of iMS alleles. For the microsatellite templates and types of detectable errors examined, we observed that genome stabilizing microsatellite interruptions are created most frequently *in vitro* by error-prone, specialized Pols η and κ, while replicative Pols α and δ rarely created interruptions (Figure 5). The generality of our observations for all microsatellite sequences and human polymerases is not known, and must await future experimental analyses. Nevertheless, we observed that DNA Pol η is very efficient at making interruptions within perfect microsatellites and creates multiple errors within a single DNA synthetic event. Pol η also creates base substitution errors within the tandem repeat tracts of iMS templates, with the net result being a more random sequence. DNA Pol η serves several important functions in human genome stability. Germline mutations leading to loss of Pol η activity causes the cancer predisposition syndrome, xeroderma pigmentosum-variant [64], and enhanced cellular UV sensitivity [65]. Pol η has been well- characterized biochemically, and is capable of accurate translesion synthesis across UV photoproducts and other DNA lesions [64], [66]. Human Pol η also is required for the maintenance of common fragile sites and prevention of chromosomal rearrangments [67], [68]. On the other hand, Pol η performs a key role in targeted mutagenesis during somatic hypermutation of immunoglobulin genes, primarily targeting mutations to A:T basepairs [69]–[71]. Here, we show *in vitro* that Pol η litters mononucleotide A/T microsatellites with many base substitution errors (Figure 3C and Table 5), an error characteristic that is highly reminiscent of somatic hypermutation.

### Interruptions – The result of an interplay of replication, repair, and recombination

Previous studies of primate MSs reported that point mutations occur more frequently than expected within microsatellites, based on the overall genome divergence [72], and that there is a two-fold higher rate of base substitutions within coding microsatellites relative to other coding sequences [73]. In a study of microsatellite births and deaths, we observed that substitutions were the leading cause of death, and that the density of births/deaths is non-random throughout the genome [27]. Although interruptions can be removed from microsatellites, restoring long perfect repeat stretches and high mutability of microsatellites [27], our *in vitro* results suggest that this may be a rare event during DNA synthesis based on the small number of microsatellites examined.

Our discovery that interruptions are created more frequently by low fidelity repair and specialized polymerases than by high fidelity replicative polymerases suggests one potential mechanistic explanation for these observations. Based on our data to date, we would predict that the frequency of interruptions among microsatellites in the genome (of the same motif and number) will depend upon the relative activities of replication, repair and recombination DNA synthesis pathways, such that more iMSs are expected in genomic regions where either repair or specialized polymerases, such as Pols η, κ and β, are more frequently engaged. DNA synthesis by these polymerases would have the consequence of speeding up microsatellite death and impeding microsatellite resurrection [74]. For example, specialized polymerases may be engaged at the replication fork more often during synthesis of highly repetitive microsatellite sequences than of coding sequences, because replicative polymerases are inhibited [46], [68], [75]. Indeed, Pol κ was recently implicated in the synthesis of DNA at stalled replication forks in unstressed human cells [76]. Alternatively, an increased level of DNA damage within microsatellites, relative to coding sequences, would necessarily engage repair and specialized polymerases during the downstream pathways of gap-filling or translesion synthesis, respectively. A noncanonical pathway of mismatch repair that is activated by DNA lesions was shown to recruit Pol η to chromatin in a replication-independent manner [77]. Finally, Pol η activity may be targeted to specific genomic sequences, such as the highly mutable hotspots identified for somatic hypermutation of immunoglobulin genes.

### Perspective

Microsatellites present within regulatory regions of the genome can affect gene expression, and allele length polymorphisms are increasingly recognized as contributing to phenotypic variation and disease risk [5], [10], [12]. Indeed, it has been previously proposed that polymorphic microsatellite alleles present within candidate genes associated with a disease or trait should be considered as contributing to the trait [11]. Genomic microsatellites display genetic variation that includes both allele length and sequence polymorphisms. The genetic architecture of microsatellites can include stabilizing, interrupted alleles. Our study advances our understanding of the impact of microsatellite sequence variation by illuminating the sheer abundance of iMS alleles within individual human genomes and the magnitude of the genome stabilization effects. We have identified genes encoding exonic microsatellites that are present as protective, interrupted alleles in only one of four human population groups. These population-specific, iMS-containing genes are enriched in distinct functional pathways, suggesting that microsatellite sequence variation may contribute to the effects of genetic ancestry on disease risk. Importantly, our analyses demonstrate that many iMS alleles are not fixed within population groups, suggesting that microsatellite interruptions could be a source of genetic variability impacting individual phenotypic variation.

## Materials and Methods

### Identification of orthologous microsatellites in primate genomes

We identified perfect as well as interrupted microsatellites in human (hg18), chimpanzee (panTro2), orangutan (ponAbe2), macaque (rheMac2) and marmoset (calJac1) genomes using Sputnik [78] and a computational pipeline that we developed for proper extraction of iMSs (see below). In this approach, Sputnik is utilized to perform a genome-wide search for microsatellite ‘seeds’ (see Table S1 for search parameters) i.e., stretches of perfect mono-, di-, tri- and tetra-nucleotide repeats at or above the threshold repeat lengths of 9, 5, 4 and 3 units, respectively (following [24], [79]). Each seed's (e.g. [AC]_6_) flanking sequences are examined for the presence of (a) any additional seeds of any motif, or (b) additional instances of the repeat motif (e.g. [AC]_2_) with the intervening non-repeat nucleotides extending to not more than the length of the repeat motif itself (here, 2 bp). If additional complete repeats of the repeating motif or seeds composed of the same repeat motif are identified in the neighborhood of the seed, then the focal seed and the discovered extensions are merged into a single microsatellite. To complete the above example, if the focal seed [AC]_6_ exists such that (a) on its 3′ end, following a dinucleotide GT, there was discovered another seed [AC]_7_, and (b) on its 5′ end an immediately adjacent instance of [CA]_2_ is found, then the resultant focal seed is extended to include these additional repeats such that the final repeat becomes [AC]_7_GT[AC]_6_[CA]_2_. This extension process is continued iteratively into the flanking regions until no more additional instances of the focal motif are identified, or if the terminal additions to the microsatellites are composed of repeat instances that are smaller than two repeats long. After the extension process is terminated, each repeat is classified as an iMS if the above microsatellite extension process was possible, and as a perfect microsatellite if the extension was not possible. Compound microsatellites, created when adjacent seeds were composed of different motifs, are discarded.

We then identified orthologous microsatellites using the publicly available multiZ alignments of primate genomes [80]. From the identified set of orthologous microsatellites, we removed those that (1) were located within 25 bp of each other; (2) possessed at least one nucleotide of low sequence quality (namely, with PHRED score below 20); (3) had low-complexity flanking (20 bp upstream and 20 bp downstream) sequences; (4) had flanking sequence identity below 85% between any species pair; (5) differed in nucleotide sequence of the repeating motif, (6) had more than two interruptions in any species; (7) were interrupted microsatellites but differed in the sequence of the interrupting nucleotide(s) between species; (8) were interrupted microsatellite loci that differed in the context of the interruption (i.e., the repeat nucleotides immediately flanking the interruption) between species (Table S1). Our final set of microsatellite loci consisted of 30,715 perfect orthologous microsatellite loci and 46,356 orthologous microsatellites with one or two interruptions in the studied species.

The size of each iMS was measured in terms of repeat numbers and was calculated by dividing the total length of microsatellite-native sequence (i.e., all sequence other than the interrupting nucleotides) by the size of the repeating motif. Mutability values and their respective 95% confidence intervals (CI) were measured at multiple repeat numbers for microsatellites with 0, 1 and 2 interruptions separately, using methods previously implemented in [50].

### Identification of interruptions using the 1000 genomes Phase-1 dataset

We obtained variant calls (SNPs and indels) from the 1000 Genomes Phase-1 Project [36] for four population groups – Africans, Europeans, Asians and Americans. These calls were intersected with perfect microsatellites (mono-, di-, tri-, and tetra-nucleotide repeats of length ranges 8–10, 10–18, 12–24, and 16–28 bp respectively) identified from the human reference genome (UCSC build hg19) – the lower bounds of the chosen length ranges represent microsatellite thresholds and the upper bounds represent the length up to which indel calls generated from short-reads are reliable (see [25] for details). All indel and SNP variants present at an allele frequency ≥0.05 were identified separately for each population group. These variants were considered to be interruptions if they were located within a microsatellite but not at the starting/ending repeat unit. Additionally, for indels, only those indels that did not include a whole-motif insertion/deletion were considered to be interruptions. We next compared the list of iMS loci across populations to identify microsatellites interrupted in all populations and in subsets of populations. Population-specific interruptions were defined as those that are interrupted in one population, but remain perfect in the other three. We obtained coordinates of disease-associated loci [38] from the UCSC Genome Browser [81], [82], and intersected the 1000 Genomes Phase-1 Project variant calls to identify interruptions at these loci across the four population groups. Again, we used the allele frequency cut-off of 0.05 and the aforementioned filters to identify interruptions.

### F_ST_ estimation

For interruptions present in all four population groups, the frequencies of the interruption variant alleles (*p*) were extracted for each of the four population groups. For each interruption, heterozygozity (*H* = 2*pq*) values were computed separately for each population group, where *q* = 1-*p* denotes the frequency of the reference allele. The average of these population heterozygozities was computed as *H_S_*. Next, the average allele frequencies for the total population (*P*, *Q*) were computed by averaging the allele frequencies (*p* and *q*) over the four populations. Next, total heterozygosity was estimated as *H_T_* = 2*PQ*. *F_ST_* was then estimated as *F_ST_* = (*H_T_*−*H_S_*)/*H_T_* [83].

### Heterozygosity estimation and significance testing

Population allele frequencies for the variant iMSs as well as perfect microsatellites (those without interrupting variants) were obtained from the VCF files, and heterozygosity was estimated as *2pq*, where, *p* = allele frequency of the variant and *q* = 1-*p*. Frequencies of iMSs and perfect microsatellites were estimated at different heterozygosity bins (ranging from 0 to 0.5, with bin-size equal to 0.02), and the distributions of these frequencies were compared against each other using two-sample bootstrap Kolmogorov-Smirnov test with 10,000 iterations from the R “Matching” package [84].

### LD estimation and phenotype association

Pairwise correlation coefficient, R^2^ (proxy for LD), was calculated between interruption-causing indels and neighboring (located within a 1-Mb window around the indel), population-matched SNPs from the 1000 Genomes Phase-1 dataset using PLINK v1.07 (http://pngu.mgh.harvard.edu/purcell/plink/) [85]. For each indel, SNPs with the maximum R^2^ values were chosen for subsequent analysis. Indel-SNP pairs that showed a perfect LD (R^2^ = 1) were selected and intersected with a list of exon coordinates to identify exonic indel-SNP pairs in perfect LD using Galaxy. The SNPs from such perfect LD pairs were submitted to SNPnexus to obtain phenotype and disease associations.

### Gene Ontology analyses

iMS loci were intersected with exon coordinates obtained from the UCSC Genome Browser [81], [82] using Galaxy [86], [87], [88] and HUGO gene names [89] were obtained for exonic iMS. Using functions from the R package “GOstats” [90], we compared the exonic iMS-containing genes with all other genes in the genome to determine an over/underrepresentation of GO molecular functions, biochemical processes and cellular components in the selected gene set.

### *In vitro* polymerase assay

Purified calf thymus pol α-primase complex (pol α) was kindly supplied by Dr. Fred Perrino or the human complex was purchased from Chimerx (Madison, WI). Recombinant DNA pol β was purified as described [91]. The 4-subunit recombinant human Pol δ4 was purified as described [92] and was a generous gift of Dr. Marietta Lee. Purified full-length human pol κ and pol η were purchased from Enzymax (Lexington, KY). [GT]_n_ and [TC]_n_ microsatellite-containing herpes simplex virus type 1 thymidine kinase (HSV-tk) vectors have been previously described [26], [45]. Dinucleotide microsatellites were inserted in-frame between positions 111 and 112 of the HSV-tk sense strand. Additional vectors were constructed with in-frame inserts in the same position as above and the final sequences of [T]_8_, [A]_8_, [T]_3_ A [T]_4_ and [A]_3_ T [A]_4_. These sequences model the perfect and interrupted (iMS) alleles found within the *APC* gene (positions 3917–3924) of the Ashkenazi Jewish and non-Ashkenazi populations, respectively [19].

Linear DNA fragments and ssDNA were used to construct MluI (position 83) to StuI (position 180) gapped duplex (GD) molecules, as described [47], [93]. *In vitro* polymerase reactions for pol α [94], pol β [45], and pols δ, κ, and η [46] at dinucleotide microsatellite templates were previously described. For the *APC* gene model templates, polymerase reactions contained 1 pmol of oligonucleotide-primed ssDNA at 20 nM concentration. Reaction conditions were the same as in the references above except 20 units of Chimerx human pol α, 15 pmol of pol β, and 1–2 pmol of pol η were used. To sample reaction products for mutations, small fragments were prepared by MluI and StuI digestion and hybridized to the corresponding GD molecule as described [45]. Successful hybridization was verified by agarose gel analysis as described [52]. An aliquot of DNA from the final hybridization was used to transform *E.coli* strain FT334 for mutant frequency determination on VBA selective media [47]. The presence of 50 µg/mL chloramphenicol (Cm) selects for progeny of the polymerase-synthesized strand and the presence of 40 µM 5-fluoro-2′-deoxyuridine (FUdR) selects for bacteria carrying HSV-tk mutant plasmids. The observed HSV-tk mutant frequency (MF) is the number of FUdR^R^Cm^R^ colonies divided by the number of Cm^R^ colonies. To control for pre-existing mutations, we also determined the HSV-tk MF for each ssDNA used to construct the GD molecules. Independent mutants for DNA sequence analyses were isolated as described [47] from two polymerase reactions per template. The DNA sequence of the HSV-tk gene in the MluI-StuI region of each mutant was determined by dideoxy DNA sequence analysis of plasmid DNA as described [45].

### *In vitro* polymerase mutational specificity calculations

Pol η and Pol κ produce multiple mutational events per target sequence. In order to properly compare polymerase error frequencies (Pol EFs) among polymerases, we identified those mutational events that were detectable as single mutational events, and adjusted the observed HSV-tk MF to reflect multiple errors per target. First, Pol EFs were determined by the following equation: Pol EF = (Observed MF) − (ssDNA background MF) − (Outside target MF), where outside target MF is the frequency of errors occurring outside the gap target. Next, each mutational event was scored as detectable or undetectable. All frameshifts and those base substitutions that caused an amino acid change or a stop codon within coding sequences were considered detectable. Base substitutions within microsatellite sequences were only considered detectable when a stop codon was produced. Only detectable events were used for determining Pol EF_est_. Each mutational event was also scored as tandem or nontandem. Tandem events were those adjacent to one another, whereas nontandem were errors >1 nt apart. Pol EFs were then corrected for the existence of multiple nontandem mutations as described [46]. The Pol EF_est_ obtained is the overall Pol EF_est_ and includes mutational events within the microsatellite sequence and within the adjacent HSV-tk coding sequence (see Table S5 and accompanying footnotes). The Pol EF_est_ of a specific type of mutational event was calculated from the proportion of the specific mutational event (among the total analyzed) multiplied by Pol EF_est_. For analyses presented herein, we further subdivided the microsatellite Pol EF_est_ into unit-based indel Pol EF_est_ or interruption Pol EF_est_. A unit-based indel is an error that occurs when an entire microsatellite unit or units are inserted or deleted (i.e., [GT]_10_→[GT]_9_). An interruption is an indel or base substitution that disrupts the repetitive nature of the microsatellite sequence (i.e., [GT]_10_→[GT]_5_T[GT]_5_).

## Supporting Information

Dataset S1 All interrupted microsatellites in the four 1000 genomes populations, with functional effects.
(PDF)

Dataset S2 African (AFR) population-specific, exonic interrupted microsatellites.
(PDF)

Dataset S3 American (AMR) population-specific, exonic interrupted microsatellites.
(PDF)

Dataset S4 Asian (ASN) population-specific, exonic interrupted microsatellites.
(PDF)

Dataset S5 European (EUR) population-specific, exonic interrupted microsatellites.
(PDF)

Dataset S6 Fixation index values of interrupted microsatellites in four 1000 genomes populations.
(XLS)

Figure S1 Effect of interruption identity on microsatellite mutability. Mutability of singly-interrupted poly-A microsatellites binned according to their interruption ([A]_n_T[A]_n_, [A]_n_C[A]_n_ and [A]_n_G[A]_n_). Number of repeats of a microsatellite was calculated by dividing the total length of the microsatellite, excepting the interrupting nucleotides, by the size of its repeating motif. At each repeat number the lines designate the 2.5th and 97.5^th^ percentiles of empirical distributions that were obtained through resampling.
(PDF)

Figure S2 The effect of microsatellite interruptions on protein-coding sequences. (A). Interruptions present in more than one population group. (B). Interruptions present in individual population groups.
(PDF)

Figure S3 Proportion of iMS and perfect MS alleles at different levels of heterozygosity. (A). African population; (B). Asian population; (C). European population; (D). American population. The density of iMSs with heterozygosity below 10% is likely an underestimate since our data did not include variants with frequency below 5%.
(PDF)

Figure S4 Proportion of iMS alleles at different levels of linkage disequilibrium with neighboring, population-matched SNPs.
(PDF)

Figure S5 Microsatellite loci are saturated by gain/loss events. Numbers in blue and red indicate the number of interruptions gained and lost in the respective branch of the five-species primate tree.
(PDF)

Figure S6 Effect of motif size on population-specific interruptions in 1000 genomes datasets. (A). American population; (B). Asian population; (C). European population.
(PDF)

Figure S7 Effect of repeat number on population-specific interruptions in 1000 genomes datasets. (A). American population; (B). Asian population; (C). European population. Individual panels are data for mono-, di-, tri-, and tetranucleotide microsatellites within each population.
(PDF)

Figure S8 Effect of motif composition on population-specific interruptions in 1000 genomes datasets. (A). American population; (B). Asian population; (C). European population. Individual panels are data for mono- and dinucleotide microsatellites within each population.
(PDF)

Figure S9 Unique mutational signatures of polymerase interruption errors within [GT] dinucleotide microsatellites. (A). GT10 template; (B). GT13 template; (C). GT19 template. DNA synthesis proceeds from right to left. The middle line of sequence is the in-frame wild-type HSV-tk gene and subscripts indicate each dinucleotide unit of the microsatellite. Indels are shown above the sequence and base substitutions are shown below. Each symbol represents one mutational event: (**▵**) one base deletion; (**♦**) two base deletion; (**▴**) one base insertion with identity of the inserted base above the symbol. Symbols with subscripts indicate that the interruption event occurred with a 1 dinucleotide unit deletion (−1); 2 dinucleotide unit deletion (−2); 1 dinucleotide unit insertion (+1); or 2 dinucleotide unit insertion (+2). Interruption events for pol δ are gray, pol β are blue, pol κ are red, and pol η are black.
(PDF)

Figure S10 Unique mutational signatures of polymerase interruption errors within [TC] dinucleotide microsatellites. (A). TC11 template; (B). TC14 template. Symbols and subscripts are the same as that indicated in Figure S9.
(PDF)

Table S1 Numbers of perfect (pure) and interrupted orthologous microsatellites in primate genomes.
(DOCX)

Table S2 Functional effects of exonic iMS SNP/InDel polymorphisms in four populations.
(DOCX)

Table S3 Gene Ontology functions significantly overrepresented (p<0.01) in genes containing African population-specific iMSs.
(DOCX)

Table S4 Gene Ontology functions significantly overrepresented (p<0.01) in genes containing European population-specific iMSs.
(DOCX)

Table S5 Numbers of interruption-causing indels in linkage disequilibrium with SNPs.
(DOCX)

Table S6 Phenotype and disease associations of perfect linkage disequilibrium indel-SNP pairs.
(DOCX)

Table S7 Polymerase error frequencies within perfect and interrupted APC gene model templates.
(DOCX)

Table S8 Specificity of microsatellite interruptions created by DNA polymerases δ, β and κ.
(DOCX)
