# Supplementary material for: Microsatellite Interruptions Stabilize Primate Genomes and Exist as Population-Specific Single Nucleotide Polymorphisms within Individual Human Genomes
Source: PLoS Genet. 2014 Jul 17;10(7):e1004498. doi: 10.1371/journal.pgen.1004498 (PMC4102424; doi:10.1371/journal.pgen.1004498)
Supplement: Dataset S2 — African (AFR) population-specific, exonic interrupted microsatellites. (PDF) [file pgen.1004498.s002.pdf]

Dataset S2. African (AFR) population-specific, exonic interrupted microsatellites.

chr	start	end	motif	interruption_event	interruption_nt	interruption_pos	gene
10	10986558	10986567	T	snp	C	10986563	LOC254312
10	11904828	11904836	A	snp	C	11904833	C10orf47
10	11904828	11904836	A	snp	C	11904833	LOC219731
10	14595390	14595400	A	snp	G	14595391	FAM107B
10	15090297	15090305	C	snp	A	15090303	ACBD7
10	15090297	15090305	C	snp	A	15090303	OLAH
10	18826215	18826223	T	snp	C	18826218	CACNB2
10	18826215	18826223	T	snp	C	18826218	U80764
10	21861864	21861872	T	snp	C	21861868	MLLT10
10	24722200	24722209	T	snp	C	24722204	KIAA1217
10	24809616	24809625	A	snp	C	24809617	KIAA1217
10	28341712	28341721	T	snp	C	28341713	MPP7
10	29163826	29163834	G	snp	T	29163827	5S_rRNA
10	29163826	29163834	G	snp	T	29163829	5S_rRNA
10	29785105	29785113	A	snp	C	29785111	SVIL
10	30748780	30748789	T	snp	G	30748785	MAP3K8
10	32097845	32097853	A	snp	G	32097850	ARHGAP12
10	35478105	35478114	T	snp	G	35478110	CREM
10	35898086	35898096	A	snp	C	35898091	GJD4
10	37441066	37441075	T	snp	G	37441071	ANKRD30A
10	46049329	46049337	A	snp	C	46049335	MARCH8
10	51735343	51735352	G	snp	T	51735344	BC035067
10	51735343	51735352	G	snp	T	51735344	TIMM23
10	51735343	51735352	G	snp	T	51735344	TIMM23B
10	51810867	51810875	T	snp	C	51810873	FLJ31813
10	60477694	60477704	A	snp	C	60477701	BICC1
10	60477694	60477704	A	snp	C	60477701	LOC728640
10	61714347	61714355	T	snp	G	61714352	C10orf40
10	61788616	61788625	T	snp	G	61788622	ANK3
10	63982248	63982256	A	snp	C	63982253	RTKN2

10	65383738	65383746	T	snp	G	65383742	REEP3
10	69752752	69752760	A	snp	G	69752758	HERC4
10	69756745	69756755	A	snp	G	69756750	HERC4
10	70051808	70051816	A	snp	G	70051809	PBLD
10	70748013	70748021	A	snp	C	70748019	KIAA1279
10	70930184	70930194	A	snp	G	70930187	VPS26A
10	71017441	71017450	G	snp	A	71017444	HKDC1
10	71031619	71031628	T	snp	C	71031620	HK1
10	71154973	71154981	T	snp	G	71154979	HK1
10	73973322	73973332	C	snp	T	73973329	ANAPC16
10	73973322	73973332	C	snp	T	73973329	ASCC1
10	75202800	75202808	A	snp	C	75202801	PPP3CB
10	75884745	75884755	T	snp	C	75884751	AP3M1
10	75898977	75898986	A	snp	C	75898978	AP3M1
10	75898977	75898986	A	snp	C	75898980	AP3M1
10	78843596	78843604	A	snp	C	78843600	KCNMA1
10	80829903	80829912	G	snp	A	80829905	ZMIZ1
10	82184768	82184776	T	snp	C	82184769	C10orf58
10	90033586	90033595	A	snp	G	90033592	RNLS
10	90579132	90579140	A	snp	G	90579136	ANKRD22
10	90579132	90579140	A	snp	G	90579136	LIPM
10	91178464	91178474	T	snp	G	91178467	IFIT5
10	91399728	91399736	T	snp	C	91399731	PANK1
10	93611984	93611992	A	snp	C	93611990	TNKS2
10	93840517	93840525	T	snp	G	93840523	CPEB3
10	95274525	95274533	T	snp	C	95274527	CEP55
10	95349360	95349370	G	snp	T	95349364	O3FAR1
10	97425033	97425043	T	snp	G	97425041	TCTN3
10	97442990	97442999	A	snp	G	97442994	TCTN3
10	97974310	97974318	A	snp	G	97974311	BLNK
10	98127804	98127812	A	snp	C	98127808	TLL2
10	99019602	99019610	T	snp	G	99019606	ARHGAP19
10	101718910	101718919	T	snp	G	101718915	DNMBP

10	101718910	101718919	T	snp	G	101718915	DNMBP-AS1
10	101815106	101815116	C	snp	T	101815113	CPN1
10	101915049	101915058	C	snp	A	101915050	ERLIN1
10	104160643	104160651	G	snp	T	104160649	NFKB2
10	104449091	104449100	A	snp	G	104449092	ARL3
10	112658018	112658028	T	snp	C	112658026	BBIP1
10	112658018	112658028	T	snp	C	112658026	MIR4680
10	112658018	112658028	T	snp	C	112658026	PDCD4
10	114205635	114205643	T	snp	G	114205637	ZDHHC6
10	115334304	115334312	T	snp	C	115334305	HABP2
10	115355191	115355199	A	snp	G	115355194	NRAP
10	115962358	115962366	T	snp	G	115962360	TDRD1
10	117855993	117856003	T	snp	C	117855995	GFRA1
10	118390022	118390031	T	snp	C	118390024	PNLIPRP2
10	124035405	124035414	A	snp	C	124035410	BTBD16
10	124248088	124248096	A	snp	G	124248090	HTRA1
10	126848279	126848287	C	snp	T	126848284	CTBP2
10	127568225	127568233	T	snp	C	127568230	DHX32
10	127680207	127680215	T	snp	C	127680211	FANK1
10	128789801	128789810	T	snp	G	128789804	DOCK1
10	128909140	128909148	T	snp	C	128909144	DOCK1
10	134149079	134149087	G	snp	A	134149083	LRRC27
10	135051671	135051679	G	snp	A	135051677	VENTX
10	135075427	135075435	G	snp	A	135075431	ADAM8
10	135088633	135088643	C	snp	A	135088641	ADAM8
10	135368146	135368156	T	snp	G	135368152	CYP2E1
10	135368146	135368156	T	snp	G	135368152	CYP2E1
10	135368146	135368156	T	snp	G	135368152	SYCE1
10	135368146	135368156	T	snp	G	135368152	SYCE1
10	135368146	135368156	T	snp	G	135368152	SYCE1
11	441799	441807	C	snp	A	441805	ANO9
11	640341	640350	G	snp	A	640348	DRD4
11	993679	993688	C	snp	A	993683	AP2A2

11	1248192	1248201	G	snp	T	1248196	MUC5B
11	3667293	3667303	T	snp	G	3667296	ART1
11	3745507	3745515	A	snp	G	3745512	NUP98
11	4202734	4202742	T	snp	C	4202735	RRM1
11	4719274	4719282	T	snp	G	4719276	OR51E2
11	6949266	6949275	T	snp	C	6949268	ZNF215
11	7614341	7614349	T	snp	C	7614347	PPFIBP2
11	8246319	8246327	G	snp	A	8246325	LMO1
11	8721611	8721620	G	snp	T	8721612	ST5
11	8721611	8721620	G	snp	A	8721615	ST5
11	8941194	8941203	T	snp	G	8941196	AKIP1
11	8941194	8941203	T	snp	G	8941196	C11orf16
11	9537042	9537051	A	snp	C	9537043	DM376719
11	9537042	9537051	A	snp	C	9537043	ZNF143
11	10522976	10522984	G	snp	T	10522977	AMPD3
11	10522976	10522984	G	snp	T	10522982	AMPD3
11	10779128	10779137	A	snp	G	10779134	CTR9
11	11644219	11644229	C	snp	A	11644223	GALNTL4
11	15104588	15104597	A	snp	G	15104592	CALCB
11	16424307	16424315	A	snp	G	16424313	SOX6
11	18044643	18044651	A	snp	G	18044647	TPH1
11	18370295	18370303	A	snp	G	18370301	GTF2H1
11	18587437	18587446	T	snp	C	18587442	UEVLD
11	20419998	20420007	T	snp	G	20420004	PRMT3
11	27401922	27401930	T	snp	G	27401926	LGR4
11	27719733	27719741	T	snp	C	27719736	BDNF
11	27719733	27719741	T	snp	C	27719736	BDNF-AS1
11	27719733	27719741	T	snp	C	27719738	BDNF
11	27719733	27719741	T	snp	C	27719738	BDNF-AS1
11	30886296	30886306	T	snp	C	30886299	DCDC5
11	30899834	30899842	A	snp	C	30899835	DCDC5
11	32461940	32461948	T	snp	G	32461942	WT1
11	32461940	32461948	T	snp	G	32461942	WT1-AS

11	33078639	33078649	T	snp	C	33078642	TCP11L1
11	34979843	34979851	G	snp	A	34979849	PDHX
11	43589965	43589975	T	snp	G	43589968	BC031305
11	46624211	46624221	T	snp	G	46624217	HARBI1
11	46640295	46640305	T	snp	G	46640302	ATG13
11	46883312	46883321	A	snp	C	46883317	LOC100507401
11	46883312	46883321	A	snp	C	46883317	LRP4
11	46883312	46883321	A	snp	C	46883317	LRP4
11	57147920	57147929	A	snp	C	57147922	PRG3
11	57253804	57253813	T	snp	C	57253810	SLC43A1
11	57996404	57996412	T	snp	G	57996407	OR10Q1
11	58701100	58701110	T	snp	G	58701104	GLYATL1
11	58701100	58701110	T	snp	G	58701104	LOC283194
11	59733444	59733452	T	snp	G	59733449	AB231702
11	60049400	60049410	A	snp	G	60049406	MS4A4A
11	61608995	61609004	C	snp	T	61609001	FADS2
11	62495237	62495246	A	snp	C	62495238	HNRNPUL2
11	62495237	62495246	A	snp	C	62495238	TTC9C
11	64814811	64814820	T	snp	G	64814812	NAALADL1
11	65120386	65120396	T	snp	C	65120390	DPF2
11	65730942	65730952	T	snp	G	65730944	SART1
11	65825466	65825476	A	snp	G	65825469	SF3B2
11	70819983	70819992	C	snp	T	70819989	SHANK2
11	71203785	71203793	A	snp	C	71203789	NADSYN1
11	72290195	72290203	G	snp	A	72290198	PDE2A
11	72975317	72975326	C	snp	A	72975324	P2RY6
11	74035531	74035539	T	snp	C	74035534	BC048427
11	74057351	74057360	A	snp	C	74057354	PGM2L1
11	74690358	74690368	A	snp	G	74690365	SPCS2
11	75599500	75599509	A	snp	G	75599503	UVRAG
11	82613427	82613435	C	snp	A	82613433	C11orf82
11	83179890	83179898	A	snp	G	83179893	DLG2
11	83190417	83190426	G	snp	A	83190418	DLG2

11	94301877	94301885	T	snp	C	94301880	PIWIL4
11	94319811	94319819	C	snp	A	94319817	PIWIL4
11	95521369	95521378	T	snp	G	95521374	FAM76B
11	103033016	103033025	A	snp	G	103033020	DYNC2H1
11	103182317	103182327	A	snp	G	103182319	DYNC2H1
11	103817639	103817647	A	snp	G	103817644	PDGFD
11	104756323	104756332	A	snp	C	104756326	CASP12
11	104758111	104758119	A	snp	C	104758117	CASP12
11	104774008	104774016	A	snp	G	104774013	LOC643733
11	107382225	107382233	T	snp	C	107382229	ALKBH8
11	110207311	110207320	T	snp	C	110207318	AK124179
11	111383110	111383120	C	snp	T	111383114	BC021736
11	111383110	111383120	C	snp	T	111383114	BC021736
11	111383110	111383120	C	snp	T	111383114	BTG4
11	111383110	111383120	C	snp	T	111383114	MIR34B
11	111383110	111383120	C	snp	T	111383114	MIR34C
11	111943613	111943623	A	snp	C	111943617	PIH1D2
11	112095113	112095122	T	snp	G	112095117	BCO2
11	112098608	112098616	A	snp	C	112098612	PTS
11	114261515	114261523	A	snp	C	114261520	C11orf71
11	114280098	114280108	T	snp	C	114280105	RBM7
11	117023481	117023491	A	snp	C	117023486	PAFAH1B2
11	118478396	118478406	C	snp	A	118478403	PHLDB1
11	120348592	120348600	A	snp	G	120348596	ARHGEF12
11	121903703	121903711	T	snp	G	121903708	BC089451
11	122757290	122757298	T	snp	C	122757291	C11orf63
11	124951303	124951312	T	snp	C	124951310	SLC37A2
11	128425524	128425534	A	snp	C	128425527	ETS1
11	128992697	128992705	T	snp	C	128992702	ARHGAP32
11	129875576	129875586	A	snp	G	129875577	LINC00167
11	129875576	129875586	A	snp	G	129875577	PRDM10
11	130714222	130714231	T	snp	C	130714225	BC031979
11	134231044	134231054	A	snp	C	134231047	GLB1L2

12	970296	970306	A	snp	G	970297	WNK1
12	2055261	2055270	T	snp	C	2055265	DCP1B
12	3105016	3105024	A	snp	G	3105018	TEAD4
12	4382313	4382321	G	snp	T	4382319	CCND2
12	4459957	4459966	A	snp	G	4459959	C12orf5
12	4870783	4870793	T	snp	G	4870785	GALNT8
12	6473618	6473626	A	snp	C	6473619	SCNN1A
12	6690186	6690194	A	snp	C	6690189	AK096395
12	6690186	6690194	A	snp	C	6690189	AK096395
12	6690186	6690194	A	snp	C	6690189	AK096395
12	6690186	6690194	A	snp	C	6690189	AK096395
12	6690186	6690194	A	snp	C	6690189	CHD4
12	6690186	6690194	A	snp	C	6690189	CHD4
12	6690186	6690194	A	snp	C	6690189	CHD4
12	6690186	6690194	A	snp	C	6690189	CHD4
12	6690186	6690194	A	snp	C	6690189	SCARNA11
12	7044050	7044059	T	snp	G	7044055	ATN1
12	7864969	7864977	A	snp	G	7864975	DPPA3
12	8024578	8024587	A	snp	G	8024583	AY455283
12	8024578	8024587	A	snp	G	8024583	SLC2A14
12	8024578	8024587	A	snp	G	8024583	SLC2A14
12	8194273	8194281	T	snp	G	8194275	FOXJ2
12	8285506	8285516	T	snp	G	8285510	CLEC4A
12	8285506	8285516	T	snp	G	8285510	POU5F1P3
12	8976926	8976934	T	snp	C	8976929	A2ML1
12	9094546	9094554	A	snp	G	9094547	M6PR
12	9094546	9094554	A	snp	G	9094547	M6PR
12	9094546	9094554	A	snp	G	9094547	PHC1
12	9228021	9228029	T	snp	G	9228023	A2M
12	9385608	9385617	G	snp	T	9385611	A2MP1
12	9555620	9555629	G	snp	T	9555623	DQ599803
12	10281842	10281852	A	snp	G	10281846	CLEC7A
12	12046811	12046820	A	snp	G	12046812	ETV6

12	14660587	14660596	T	snp	C	14660589	PLBD1
12	14780705	14780714	A	snp	C	14780708	GUCY2C
12	15773728	15773737	T	snp	C	15773733	EPS8
12	18801880	18801888	A	snp	C	18801885	PIK3C2G
12	21391816	21391824	T	snp	C	21391817	SLCO1B1
12	21451025	21451034	T	snp	C	21451027	SLCO1A2
12	21524937	21524946	A	snp	G	21524942	IAPP
12	21524937	21524946	A	snp	G	21524942	SLCO1A2
12	22208346	22208355	T	snp	C	22208353	CMAS
12	23888220	23888229	T	snp	G	23888222	SOX5
12	25033390	25033400	A	snp	G	25033397	BCAT1
12	25307002	25307011	T	snp	G	25307007	CASC1
12	25385903	25385912	T	snp	C	25385909	KRAS
12	26100419	26100427	A	snp	G	26100425	LOC100506451
12	27064490	27064499	A	snp	G	27064497	ASUN
12	27522232	27522240	G	snp	T	27522234	ARNTL2
12	27950067	27950077	A	snp	C	27950075	KLHDC5
12	28125848	28125857	C	snp	A	28125851	PTHLH
12	29498482	29498490	A	snp	C	29498488	ERGIC2
12	29632748	29632756	T	snp	C	29632751	OVCH1
12	29725489	29725497	T	snp	C	29725493	TMTC1
12	31545378	31545386	A	snp	G	31545380	DENND5B
12	32903144	32903153	T	snp	G	32903147	YARS2
12	39740617	39740625	T	snp	C	39740622	KIF21A
12	40625398	40625407	A	snp	G	40625402	LRRK2
12	40713869	40713877	A	snp	G	40713872	LRRK2
12	44154690	44154698	T	snp	C	44154696	IRAK4
12	44167275	44167283	T	snp	C	44167278	IRAK4
12	48115078	48115087	T	snp	G	48115085	AL831948
12	48115078	48115087	T	snp	G	48115085	ENDOU
12	49254487	49254495	T	snp	G	49254493	RND1
12	49921053	49921063	T	snp	C	49921054	SPATS2
12	50229500	50229510	T	snp	G	50229508	BCDIN3D

12	50229500	50229510	T	snp	G	50229508	LOC100286844
12	50291547	50291556	C	snp	A	50291550	FAIM2
12	50506805	50506815	T	snp	G	50506812	C12orf62
12	50576141	50576150	G	snp	T	50576145	LIMA1
12	51632448	51632457	G	snp	T	51632454	DAZAP2
12	51772666	51772676	C	snp	T	51772673	GALNT6
12	53648257	53648267	T	snp	C	53648261	MFSD5
12	53804300	53804310	T	snp	G	53804306	SP1
12	53825863	53825871	T	snp	G	53825869	AMHR2
12	54511887	54511896	A	snp	C	54511889	FLJ12825
12	54694552	54694560	C	snp	A	54694558	NFE2
12	57318798	57318808	T	snp	G	57318806	SDR9C7
12	57393268	57393277	A	snp	G	57393274	ZBTB39
12	57422572	57422580	T	snp	G	57422575	MYO1A
12	57823582	57823590	A	snp	C	57823584	KIAA1002
12	57823582	57823590	A	snp	C	57823584	R3HDM2
12	59002629	59002639	A	snp	G	59002633	AK093124
12	59273245	59273255	T	snp	G	59273248	LRIG3
12	64058116	64058124	A	snp	C	64058120	DPY19L2
12	65217996	65218005	T	snp	C	65218003	TBC1D30
12	65369246	65369255	T	snp	C	65369251	FLJ41278
12	65859891	65859899	A	snp	G	65859892	MSRB3
12	66232376	66232384	T	snp	C	66232377	HMGA2
12	70071565	70071574	A	snp	G	70071569	BEST3
12	70749630	70749639	A	snp	G	70749631	CNOT2
12	71972516	71972524	T	snp	C	71972519	LGR5
12	72005150	72005160	A	snp	C	72005152	ZFC3H1
12	75441507	75441516	A	snp	G	75441511	KCNC2
12	75891675	75891684	A	snp	G	75891677	GLIPR1
12	75891675	75891684	A	snp	G	75891677	KRR1
12	76843741	76843749	C	snp	A	76843747	OSBPL8
12	77457432	77457442	A	snp	G	77457438	E2F7
12	85269882	85269890	A	snp	G	85269885	SLC6A15

12	91540100	91540108	T	snp	C	91540102	DCN
12	93196418	93196427	T	snp	G	93196421	EEA1
12	94646220	94646228	T	snp	G	94646221	PLXNC1
12	95870285	95870293	A	snp	G	95870289	METAP2
12	96412335	96412345	A	snp	C	96412337	LTA4H
12	96896997	96897005	T	snp	C	96897002	C12orf55
12	97132948	97132957	T	snp	C	97132954	C12orf63
12	100434484	100434493	A	snp	C	100434486	UHRF1BP1L
12	101015652	101015661	G	snp	A	101015659	GAS2L3
12	102040416	102040425	A	snp	G	102040417	MYBPC1
12	102054877	102054886	T	snp	C	102054878	MYBPC1
12	102549060	102549069	A	snp	C	102549062	PARPBP
12	103248607	103248616	A	snp	C	103248611	PAH
12	104286590	104286598	A	snp	G	104286591	GNN
12	104496721	104496730	C	snp	T	104496723	HCFC2
12	105593585	105593594	T	snp	G	105593591	APPL2
12	106458571	106458580	C	snp	A	106458578	NUAK1
12	106976381	106976389	C	snp	A	106976386	LOC100287944
12	106976381	106976389	C	snp	A	106976386	RFX4
12	107415069	107415079	A	snp	G	107415072	CRY1
12	109886818	109886826	A	snp	C	109886821	KCTD10
12	109886818	109886826	A	snp	C	109886821	MYO1H
12	110835012	110835021	A	snp	C	110835014	ANAPC7
12	113742711	113742720	A	snp	C	113742715	SLC24A6
12	114353836	114353844	T	snp	C	114353837	RBM19
12	117203926	117203935	T	snp	G	117203929	RNFT2
12	117205371	117205381	T	snp	G	117205372	RNFT2
12	117205371	117205381	T	snp	G	117205373	RNFT2
12	117684915	117684923	T	snp	G	117684916	NOS1
12	117902289	117902298	T	snp	C	117902296	KSR2
12	119865785	119865793	A	snp	C	119865791	AF086288
12	119865785	119865793	A	snp	C	119865791	CCDC60
12	121015909	121015917	C	snp	A	121015914	POP5

12	121015909	121015917	C	snp	A	121015914	RNF10
12	121443112	121443120	A	snp	G	121443115	C12orf43
12	121466420	121466428	C	snp	A	121466421	OASL
12	121647751	121647759	C	snp	T	121647752	P2RX4
12	122389160	122389169	A	snp	G	122389166	WDR66
12	122764620	122764628	T	snp	C	122764625	CLIP1
12	124095451	124095460	T	snp	G	124095454	DDX55
12	124395884	124395892	G	snp	A	124395887	DNAH10
12	124396125	124396133	G	snp	A	124396128	DNAH10
12	124396125	124396133	G	snp	T	124396130	DNAH10
12	124396688	124396696	G	snp	T	124396693	DNAH10
12	125400562	125400570	A	snp	G	125400566	UBB
12	125400562	125400570	A	snp	G	125400566	UBC
12	133330349	133330358	A	snp	C	133330353	ANKLE2
12	133763761	133763770	T	snp	G	133763763	ZNF268
13	19432718	19432726	T	snp	G	19432724	ANKRD20A9P
13	19998384	19998393	A	snp	C	19998390	TPTE2
13	20578403	20578412	T	snp	G	20578407	ZMYM2
13	21547830	21547839	A	snp	G	21547831	LATS2
13	21636037	21636047	A	snp	G	21636041	LATS2
13	21987146	21987155	A	snp	C	21987148	ZDHHC20
13	22112553	22112561	A	snp	C	22112559	EFHA1
13	25356487	25356497	T	snp	G	25356494	RNF17
13	25902336	25902345	A	snp	G	25902337	NUPL1
13	31715453	31715461	A	snp	C	31715454	HSPH1
13	31803948	31803957	T	snp	G	31803953	B3GALTL
13	31803948	31803957	T	snp	G	31803955	B3GALTL
13	32366295	32366304	T	snp	C	32366298	RXFP2
13	36939380	36939389	A	snp	G	36939384	SPG20
13	36939380	36939389	A	snp	G	36939384	SPG20
13	36939380	36939389	A	snp	G	36939384	SPG20OS
13	36939380	36939389	A	snp	G	36939384	SPG20OS
13	37007654	37007663	T	snp	C	37007658	CCNA1

13	37422192	37422200	T	snp	C	37422197	SMAD9
13	41486861	41486871	A	snp	G	41486865	LOC100616668
13	41486861	41486871	A	snp	G	41486865	SUGT1P3
13	45955845	45955854	C	snp	T	45955850	LOC100190939
13	46626455	46626464	G	snp	T	46626460	AK095119
13	46626455	46626464	G	snp	T	46626460	AK095119
13	46626455	46626464	G	snp	T	46626460	AK124928
13	46626455	46626464	G	snp	T	46626460	AK124928
13	46626455	46626464	G	snp	T	46626460	CPB2
13	46626455	46626464	G	snp	T	46626460	ZC3H13
13	47326994	47327002	T	snp	G	47326997	LRCH1
13	50266571	50266581	A	snp	C	50266573	EBPL
13	64320164	64320173	A	snp	C	64320166	LOC647264
13	70371199	70371207	T	snp	G	70371200	KLHL1
13	74766303	74766312	A	snp	C	74766310	Mir_340
13	75910942	75910952	T	snp	G	75910949	TBC1D4
13	75935820	75935829	T	snp	C	75935823	TBC1D4
13	76391053	76391061	T	snp	G	76391056	LMO7
13	99047025	99047033	A	snp	G	99047027	FARP1
13	107197114	107197122	A	snp	C	107197118	ARGLU1
13	111091149	111091158	C	snp	A	111091156	COL4A2
13	111941379	111941389	T	snp	G	111941384	ARHGEF7
13	114503068	114503077	G	snp	T	114503069	FAM70B
13	114507420	114507428	C	snp	T	114507425	FAM70B
13	114765431	114765440	G	snp	A	114765438	RASA3
13	114843800	114843808	T	snp	C	114843805	RASA3
13	114898557	114898566	C	snp	T	114898560	RASA3
13	114898685	114898693	C	snp	T	114898690	RASA3
14	20853517	20853526	T	snp	C	20853524	TEP1
14	21082318	21082326	C	snp	A	21082324	TRNA_Pro
14	21082318	21082326	C	snp	A	21082324	TRNA_Thr
14	21869705	21869715	T	snp	C	21869707	CHD8
14	21926844	21926854	A	snp	C	21926846	RAB2B

14	22309293	22309301	T	snp	C	22309294	TCRA
14	22309293	22309301	T	snp	C	22309294	TCRA
14	22309293	22309301	T	snp	C	22309294	TRA
14	22309293	22309301	T	snp	C	22309294	TRA
14	22309293	22309301	T	snp	C	22309294	TRAV12-1
14	22309293	22309301	T	snp	C	22309294	TRAV12-1
14	22888638	22888646	T	snp	G	22888643	AK093552
14	22888638	22888646	T	snp	G	22888643	AK125397
14	22888638	22888646	T	snp	G	22888643	AV4S1
14	22888638	22888646	T	snp	G	22888643	hADV29S1
14	22888638	22888646	T	snp	G	22888643	hADV36S1
14	22888638	22888646	T	snp	G	22888643	hADV38S2
14	22888638	22888646	T	snp	G	22888643	T-Cell
14	22888638	22888646	T	snp	G	22888643	TCRA
14	22888638	22888646	T	snp	G	22888643	TCRA
14	22888638	22888646	T	snp	G	22888643	TCRA
14	22888638	22888646	T	snp	G	22888643	TCRA
14	22888638	22888646	T	snp	G	22888643	TCRA
14	22888638	22888646	T	snp	G	22888643	TCR-alpha
14	22888638	22888646	T	snp	G	22888643	TCR-alpha
14	22888638	22888646	T	snp	G	22888643	TRA
14	22888638	22888646	T	snp	G	22888643	TRA
14	22888638	22888646	T	snp	G	22888643	TRA
14	22888638	22888646	T	snp	G	22888643	TRA@
14	22888638	22888646	T	snp	G	22888643	TRAC
14	22888638	22888646	T	snp	G	22888643	TRAC
14	22888638	22888646	T	snp	G	22888643	TRD
14	22946226	22946235	A	snp	G	22946230	AK093552
14	22946226	22946235	A	snp	G	22946230	AV4S1
14	22946226	22946235	A	snp	G	22946230	hADV29S1
14	22946226	22946235	A	snp	G	22946230	hADV36S1
14	22946226	22946235	A	snp	G	22946230	hADV38S2
14	22946226	22946235	A	snp	G	22946230	T-Cell

14	22946226	22946235	A	snp	G	22946230	TCRA
14	22946226	22946235	A	snp	G	22946230	TCRA
14	22946226	22946235	A	snp	G	22946230	TCRA
14	22946226	22946235	A	snp	G	22946230	TCRA
14	22946226	22946235	A	snp	G	22946230	TCRA
14	22946226	22946235	A	snp	G	22946230	TCRA
14	22946226	22946235	A	snp	G	22946230	TCR-alpha
14	22946226	22946235	A	snp	G	22946230	TCR-alpha
14	22946226	22946235	A	snp	G	22946230	TRA
14	22946226	22946235	A	snp	G	22946230	TRA
14	22946226	22946235	A	snp	G	22946230	TRA
14	22946226	22946235	A	snp	G	22946230	TRA@
14	22946226	22946235	A	snp	G	22946230	TRA@
14	22946226	22946235	A	snp	G	22946230	TRAC
14	22946226	22946235	A	snp	G	22946230	TRAC
14	22946226	22946235	A	snp	G	22946230	TRD
14	22946226	22946235	A	snp	G	22946230	X61074
14	23289191	23289199	C	snp	A	23289193	SLC7A7
14	23377239	23377247	T	snp	C	23377245	RBM23
14	23417663	23417673	G	snp	A	23417668	HAUS4
14	24739363	24739371	G	snp	T	24739365	HP08474
14	24739363	24739371	G	snp	T	24739365	RABGGTA
14	25042173	25042182	T	snp	C	25042180	CTSG
14	31399219	31399227	T	snp	C	31399225	STRN3
14	31593483	31593492	T	snp	C	31593484	HECTD1
14	31779357	31779366	T	snp	G	31779364	HEATR5A
14	37148384	37148392	T	snp	C	37148385	SLC25A21
14	38511499	38511508	T	snp	C	38511503	C14orf25
14	39533679	39533687	T	snp	G	39533681	SEC23A
14	39789307	39789316	T	snp	C	39789310	CTAGE5
14	49409831	49409841	T	snp	C	49409832	SNORD112
14	50578438	50578446	A	snp	C	50578440	METTL21D
14	50671131	50671139	A	snp	G	50671132	SOS2

14	52417704	52417714	A	snp	C	52417706	GNG2
14	53002759	53002769	A	snp	C	53002764	TXNDC16
14	53112187	53112195	T	snp	G	53112193	ERO1L
14	53525166	53525174	A	snp	G	53525172	DDHD1
14	55203701	55203709	G	snp	T	55203702	SAMD4A
14	55509650	55509658	T	snp	C	55509653	SOCS4
14	55625761	55625771	C	snp	T	55625763	DLGAP5
14	56137398	56137408	T	snp	C	56137406	KTN1
14	58926037	58926046	A	snp	C	58926038	KIAA0586
14	61450503	61450512	T	snp	G	61450505	SLC38A6
14	61857392	61857402	A	snp	C	61857393	PRKCH
14	62598134	62598142	G	snp	T	62598140	FLJ43390
14	64885193	64885201	A	snp	G	64885197	MTHFD1
14	71275978	71275987	C	snp	A	71275985	MAP3K9
14	71570897	71570905	T	snp	C	71570903	PCNX
14	73536922	73536931	T	snp	G	73536927	RBM25
14	73945830	73945838	T	snp	G	73945833	AK055876
14	73945830	73945838	T	snp	G	73945833	HEATR4
14	74386989	74386998	A	snp	G	74386990	ZNF410
14	74432423	74432431	A	snp	G	74432429	ENTPD5
14	74764267	74764275	T	snp	C	74764271	ABCD4
14	75229179	75229188	C	snp	A	75229186	YLPM1
14	75763088	75763096	A	snp	G	75763090	LOC731223
14	76087768	76087778	A	snp	G	76087776	FLVCR2
14	77579044	77579053	T	snp	G	77579050	KIAA1737
14	78022655	78022664	A	snp	G	78022662	SPTLC2
14	78138704	78138712	A	snp	G	78138707	ALKBH1
14	82458072	82458080	A	snp	C	82458077	Mir_633
14	88657946	88657955	T	snp	G	88657951	KCNK10
14	89312670	89312679	A	snp	G	89312674	TTC8
14	92257934	92257944	A	snp	G	92257936	TC2N
14	92526234	92526243	A	snp	C	92526239	ATXN3
14	93044595	93044603	A	snp	G	93044599	RIN3

14	93108456	93108466	T	snp	C	93108463	RIN3
14	94373600	94373609	A	snp	G	94373601	FAM181A-AS1
14	94547060	94547069	A	snp	C	94547063	DDX24
14	94547060	94547069	A	snp	C	94547063	IFI27L1
14	96120989	96120999	A	snp	C	96120996	TCL6
14	96991188	96991196	A	snp	G	96991190	PAPOLA
14	97017995	97018004	A	snp	G	97017996	PAPOLA
14	100764835	100764843	T	snp	C	100764838	SLC25A29
14	102358731	102358739	G	snp	A	102358734	PPP2R5C
14	102844042	102844050	A	snp	G	102844046	TECPR2
14	106137078	106137088	T	snp	G	106137080	abParts
14	106137078	106137088	T	snp	G	106137080	abParts
14	106137078	106137088	T	snp	G	106137080	DKFZp686O16217
14	106137078	106137088	T	snp	G	106137080	DKFZp686O16217
14	106137078	106137088	T	snp	G	106137080	IGH@
14	106137078	106137088	T	snp	G	106137080	IGH@
14	106137078	106137088	T	snp	G	106137080	IGHE
14	106137078	106137088	T	snp	G	106137080	IGHE
14	106137078	106137088	T	snp	G	106137080	IGHE
14	106137078	106137088	T	snp	G	106137080	IGHE
14	106137078	106137088	T	snp	G	106137080	IGHG1
14	106137078	106137088	T	snp	G	106137080	IGHG1
14	106476029	106476038	T	snp	G	106476030	abParts
14	106479109	106479117	C	snp	A	106479111	abParts
14	106491904	106491913	T	snp	G	106491907	abParts
14	106709250	106709258	T	snp	G	106709252	abParts
14	106846004	106846013	A	snp	C	106846011	abParts
14	107282166	107282176	A	snp	C	107282172	abParts
15	22839778	22839786	A	snp	G	22839782	TUBGCP5
15	25426117	25426125	T	snp	C	25426120	SNORD115-6
15	25426117	25426125	T	snp	C	25426120	SNURF-SNRPN
15	25426117	25426125	T	snp	C	25426120	SNURF-SNRPN
15	28499434	28499442	A	snp	C	28499435	HERC2

15	28986430	28986440	T	snp	G	28986431	WHAMMP2
15	29000470	29000479	T	snp	G	29000472	WHAMMP2
15	29083886	29083894	A	snp	G	29083892	LOC646278
15	31040511	31040519	A	snp	G	31040514	LOC100288637
15	31243170	31243179	A	snp	C	31243172	MTMR10
15	33068280	33068289	A	snp	G	33068283	FMN1
15	33193355	33193364	A	snp	G	33193359	FMN1
15	33877654	33877662	A	snp	G	33877658	RYR3
15	34065548	34065558	T	snp	C	34065556	RYR3
15	36218742	36218750	A	snp	G	36218744	MIR4510
15	36218742	36218750	A	snp	C	36218747	MIR4510
15	40270942	40270951	T	snp	G	40270943	EIF2AK4
15	40493488	40493497	A	snp	G	40493491	BUB1B
15	40846802	40846812	T	snp	G	40846810	C15orf57
15	41098407	41098416	T	snp	C	41098411	DNAJC17
15	41098407	41098416	T	snp	C	41098411	ZFYVE19
15	41589663	41589672	A	snp	C	41589666	OIP5-AS1
15	41798793	41798803	T	snp	C	41798795	LTK
15	42484985	42484994	T	snp	G	42484989	VPS39
15	42641027	42641035	T	snp	C	42641029	CAPN3
15	42641027	42641035	T	snp	C	42641029	CAPN3
15	42641027	42641035	T	snp	C	42641029	GANC
15	42641027	42641035	T	snp	C	42641029	GANC
15	42712787	42712796	A	snp	G	42712789	ZFP106
15	44625134	44625143	T	snp	G	44625141	CASC4
15	44861383	44861393	A	snp	G	44861385	SPG11
15	45176982	45176992	A	snp	G	45176984	U1
15	49799924	49799934	A	snp	C	49799932	C15orf33
15	49913097	49913107	C	snp	A	49913103	C15orf33
15	49913097	49913107	C	snp	A	49913103	DTWD1
15	49918365	49918374	A	snp	G	49918370	DTWD1
15	50555230	50555239	A	snp	G	50555235	HDC
15	50787867	50787875	T	snp	G	50787870	AX746640

15	50787867	50787875	T	snp	G	50787870	USP8
15	50873112	50873122	A	snp	C	50873120	TRPM7
15	50883255	50883264	T	snp	C	50883262	TRPM7
15	51029657	51029667	T	snp	C	51029659	SPPL2A
15	51569212	51569221	A	snp	G	51569219	CYP19A1
15	51569212	51569221	A	snp	G	51569219	DQ595419
15	51766474	51766482	A	snp	G	51766478	DMXL2
15	52698839	52698849	T	snp	G	52698843	MYO5A
15	52844604	52844613	A	snp	C	52844606	ARPP19
15	59517573	59517581	T	snp	C	59517577	MYO1E
15	59529297	59529306	T	snp	G	59529300	MYO1E
15	59806278	59806286	G	snp	T	59806283	FAM81A
15	59931125	59931133	A	snp	G	59931130	GTF2A2
15	63357848	63357856	T	snp	G	63357851	TPM1
15	65294896	65294905	A	snp	G	65294902	MTFMT
15	65352639	65352649	T	snp	C	65352640	RASL12
15	65477364	65477374	C	snp	T	65477369	CLPX
15	65822172	65822182	A	snp	C	65822174	PTPLAD1
15	65865625	65865634	T	snp	G	65865631	PTPLAD1
15	66044497	66044505	A	snp	G	66044498	DENND4A
15	66776477	66776486	C	snp	A	66776479	MAP2K1
15	72903112	72903121	A	snp	C	72903114	DQ582071
15	73022775	73022783	A	snp	G	73022777	BBS4
15	74288047	74288056	T	snp	G	74288053	PML
15	75012114	75012122	C	snp	A	75012116	CYP1A1
15	77545587	77545597	G	snp	T	77545591	PEAK1
15	78474776	78474784	C	snp	A	78474780	ACSBG1
15	78474776	78474784	C	snp	A	78474781	ACSBG1
15	79042733	79042741	T	snp	C	79042735	DQ596823
15	79501864	79501872	T	snp	G	79501866	LOC729911
15	79501864	79501872	T	snp	G	79501866	MIR184
15	83456472	83456482	A	snp	G	83456474	FSD2
15	83523157	83523166	A	snp	G	83523162	HOMER2

15	85470033	85470042	G	snp	T	85470039	SLC28A1
15	88726243	88726252	C	snp	T	88726244	NTRK3
15	89171975	89171984	A	snp	C	89171976	AEN
15	90152711	90152719	T	snp	C	90152717	C15orf42
15	90198730	90198738	C	snp	T	90198733	KIF7
15	90450618	90450626	A	snp	C	90450622	C15orf38
15	90450618	90450626	A	snp	C	90450622	C15orf38-AP3S2
15	91325617	91325626	A	snp	C	91325621	BLM
15	99927265	99927274	T	snp	G	99927271	LRRC28
16	460793	460802	C	snp	A	460795	DECR2
16	779924	779932	C	snp	T	779929	HAGHL
16	779924	779932	C	snp	T	779929	NARFL
16	1734744	1734752	A	snp	G	1734750	CRAMP1L
16	1734744	1734752	A	snp	G	1734750	HN1L
16	2013620	2013628	A	snp	C	2013625	RPS2
16	2013620	2013628	A	snp	C	2013625	RPS2
16	2013620	2013628	A	snp	C	2013625	RPS2
16	2013620	2013628	A	snp	C	2013625	RPS2
16	2013620	2013628	A	snp	C	2013625	SNORA64
16	2013620	2013628	A	snp	C	2013625	TCRBV20S1
16	2013620	2013628	A	snp	C	2013625	TCRBV20S1
16	2013620	2013628	A	snp	C	2013625	TCRBV20S1
16	2013620	2013628	A	snp	C	2013625	TCRBV20S1
16	2050937	2050946	A	snp	C	2050940	TCRBV20S1
16	2050937	2050946	A	snp	C	2050940	TCRBV20S1
16	2050937	2050946	A	snp	C	2050940	TCRBV20S1
16	2050937	2050946	A	snp	C	2050940	TCRBV20S1
16	2050937	2050946	A	snp	C	2050940	ZNF598
16	2050937	2050946	A	snp	C	2050940	ZNF598
16	2050937	2050946	A	snp	C	2050940	ZNF598
16	2050937	2050946	A	snp	C	2050940	ZNF598
16	2087452	2087461	G	snp	T	2087459	SLC9A3R2
16	2087452	2087461	G	snp	T	2087459	SLC9A3R2

16	2087452	2087461	G	snp	T	2087459	SLC9A3R2
16	2087452	2087461	G	snp	T	2087459	SLC9A3R2
16	2087452	2087461	G	snp	T	2087459	SLC9A3R2
16	2087452	2087461	G	snp	T	2087459	TCRBV20S1
16	2087452	2087461	G	snp	T	2087459	TCRBV20S1
16	2087452	2087461	G	snp	T	2087459	TCRBV20S1
16	2087452	2087461	G	snp	T	2087459	TCRBV20S1
16	2087452	2087461	G	snp	T	2087459	TCRBV20S1
16	2214127	2214136	G	snp	T	2214128	TRAF7
16	3101221	3101229	G	snp	T	3101222	BC045731
16	3101221	3101229	G	snp	T	3101222	MMP25
16	3101221	3101229	G	snp	T	3101222	MMP25
16	3294923	3294932	A	snp	C	3294930	MEFV
16	3303505	3303514	T	snp	C	3303509	MEFV
16	4038599	4038607	G	snp	T	4038605	ADCY9
16	4740203	4740211	T	snp	G	4740207	MGRN1
16	4788055	4788065	T	snp	G	4788058	C16orf71
16	4811694	4811704	A	snp	C	4811696	ZNF500
16	8719196	8719204	A	snp	G	8719202	METTL22
16	11055970	11055980	T	snp	C	11055971	CLEC16A
16	11914267	11914277	T	snp	C	11914272	BCAR4
16	11922056	11922064	A	snp	G	11922062	BCAR4
16	14698482	14698492	T	snp	C	14698485	PARN
16	15814589	15814598	A	snp	G	15814594	AX747846
16	15814589	15814598	A	snp	G	15814594	MYH11
16	15814589	15814598	A	snp	G	15814594	MYH11
16	15814589	15814598	A	snp	G	15814594	MYH11
16	15814589	15814598	A	snp	G	15814594	MYH11
16	15814589	15814598	A	snp	G	15814594	MYH11
16	15814589	15814598	A	snp	G	15814594	MYH11
16	15814589	15814598	A	snp	G	15814594	NDE1
16	15814589	15814598	A	snp	G	15814594	NDE1
16	15814589	15814598	A	snp	G	15814594	NDE1

16	15814589	15814598	A	snp	G	15814594	NDE1
16	15819019	15819027	A	snp	C	15819020	AX747846
16	15819019	15819027	A	snp	C	15819020	MYH11
16	15819019	15819027	A	snp	C	15819020	MYH11
16	15819019	15819027	A	snp	C	15819020	NDE1
16	15853919	15853927	C	snp	A	15853923	MYH11
16	16278975	16278983	C	snp	A	16278980	ABCC6
16	19085867	19085876	A	snp	G	19085872	COQ7
16	19428865	19428875	A	snp	G	19428873	TMC5
16	19694192	19694200	A	snp	C	19694194	C16orf62
16	19895405	19895413	C	snp	T	19895411	GPRC5B
16	20493482	20493491	A	snp	G	20493485	ACSM2A
16	20870892	20870902	A	snp	C	20870900	DCUN1D3
16	20870892	20870902	A	snp	C	20870900	ERI2
16	21129301	21129310	T	snp	G	21129305	DNAH3
16	23654507	23654517	T	snp	G	23654508	DCTN5
16	24232587	24232595	G	snp	T	24232588	PRKCB
16	24673814	24673824	A	snp	C	24673816	AK127191
16	24874159	24874168	A	snp	C	24874161	SLC5A11
16	24881800	24881810	A	snp	C	24881801	SLC5A11
16	27715443	27715452	C	snp	A	27715445	KIAA0556
16	29128985	29128994	A	snp	G	29128989	NPIPL1
16	29128985	29128994	A	snp	G	29128989	RRN3P2
16	29705850	29705858	C	snp	T	29705853	BOLA2
16	29705850	29705858	C	snp	T	29705853	QPRT
16	30510972	30510980	T	snp	G	30510977	ITGAL
16	30773598	30773608	G	snp	T	30773604	C16orf93
16	30773598	30773608	G	snp	T	30773604	RNF40
16	31238688	31238696	T	snp	G	31238689	TRIM72
16	46865342	46865350	C	snp	A	46865348	C16orf87
16	48268283	48268293	T	snp	G	48268286	ABCC11
16	48311579	48311588	T	snp	C	48311580	LONP2
16	48311579	48311588	T	snp	C	48311580	MIR548AE2

16	50323103	50323113	T	snp	G	50323109	ADCY7
16	50347346	50347356	A	snp	C	50347348	ADCY7
16	55360200	55360210	G	snp	T	55360207	IRX6
16	55564404	55564414	G	snp	A	55564407	LPCAT2
16	56678864	56678872	T	snp	C	56678869	MT1DP
16	56838974	56838982	T	snp	G	56838977	NUP93
16	57017792	57017801	G	snp	A	57017795	CETP
16	57987792	57987802	A	snp	G	57987795	CNGB1
16	58313561	58313569	T	snp	G	58313565	CCDC113
16	58313561	58313569	T	snp	G	58313565	PRSS54
16	66792646	66792656	A	snp	G	66792654	CCDC79
16	69338885	69338895	A	snp	G	69338893	SNTB2
16	69365121	69365129	G	snp	T	69365122	COG8
16	69365121	69365129	G	snp	T	69365122	PDF
16	70051037	70051046	T	snp	C	70051042	CLEC18A
16	70051037	70051046	T	snp	C	70051042	PDXDC2P
16	71513659	71513668	T	snp	C	71513664	ZNF19
16	71513659	71513668	T	snp	C	71513664	ZNF19
16	71513659	71513668	T	snp	C	71513664	ZNF23
16	72000503	72000511	T	snp	G	72000504	PKD1L3
16	74371831	74371841	T	snp	G	74371836	LOC283922
16	74655601	74655609	T	snp	G	74655604	RFWD3
16	74730527	74730535	A	snp	G	74730528	MLKL
16	74908790	74908799	T	snp	G	74908796	WDR59
16	75137900	75137908	G	snp	A	75137901	ZNRF1
16	77771371	77771381	T	snp	G	77771378	NUDT7
16	81173911	81173919	A	snp	G	81173915	PKD1L2
16	81176086	81176094	T	snp	C	81176092	PKD1L2
16	81412852	81412860	T	snp	G	81412858	GAN
16	81712240	81712248	C	snp	T	81712244	CMIP
16	81929180	81929189	A	snp	G	81929184	PLCG2
16	84808078	84808088	T	snp	C	84808080	USP10
16	87938693	87938702	C	snp	A	87938698	CA5A

16	88781784	88781794	A	snp	C	88781790	CTU2
16	88781784	88781794	A	snp	C	88781790	MIR4722
16	88781784	88781794	A	snp	C	88781790	PIEZO1
16	88781784	88781794	A	snp	C	88781790	PIEZO1
16	88900867	88900875	C	snp	T	88900868	GALNS
16	89518906	89518915	T	snp	C	89518907	AK097694
16	89518906	89518915	T	snp	C	89518907	ANKRD11
16	89848619	89848627	A	snp	C	89848624	FANCA
17	1201528	1201536	T	snp	C	1201530	TUSC5
17	1489986	1489996	A	snp	C	1489988	SLC43A2
17	1606876	1606884	T	snp	G	1606882	TLCD2
17	1609024	1609033	T	snp	G	1609028	TLCD2
17	1786457	1786466	T	snp	G	1786460	RPA1
17	2299192	2299200	G	snp	A	2299194	MNT
17	2888249	2888259	T	snp	C	2888257	RAP1GAP2
17	3617388	3617396	T	snp	G	3617394	ITGAE
17	3901499	3901508	T	snp	G	3901501	AB062083
17	3967357	3967367	G	snp	A	3967358	ZZEF1
17	3967357	3967367	G	snp	A	3967359	ZZEF1
17	4350101	4350109	G	snp	T	4350103	SPNS3
17	4699264	4699274	G	snp	T	4699272	PSMB6
17	4801279	4801287	C	snp	T	4801285	CHRNE
17	4801279	4801287	C	snp	T	4801285	MINK1
17	4805179	4805188	C	snp	A	4805185	C17orf107
17	4805179	4805188	C	snp	A	4805185	CHRNE
17	5239072	5239081	T	snp	C	5239073	RABEP1
17	5346440	5346448	C	snp	T	5346445	DHX33
17	6603519	6603527	T	snp	C	6603523	SLC13A5
17	6977169	6977178	C	snp	T	6977170	CLEC10A
17	7384038	7384048	A	snp	C	7384045	SLC35G6
17	7384038	7384048	A	snp	C	7384045	ZBTB4
17	7788420	7788428	C	snp	A	7788426	CHD3
17	8124957	8124966	C	snp	T	8124963	LINC00324

17	8124957	8124966	C	snp	T	8124963	LINC00324
17	8124957	8124966	C	snp	T	8124963	TRNA_Gly
17	9808693	9808702	G	snp	T	9808694	RCVRN
17	9923929	9923939	A	snp	C	9923930	GAS7
17	10446505	10446513	T	snp	G	10446507	AK097500
17	10446505	10446513	T	snp	G	10446507	AK097500
17	10446505	10446513	T	snp	G	10446507	AK097500
17	10446505	10446513	T	snp	G	10446507	AK097500
17	10446505	10446513	T	snp	G	10446507	AK097500
17	10446505	10446513	T	snp	G	10446507	MYH2
17	10446505	10446513	T	snp	G	10446507	MYH2
17	10446505	10446513	T	snp	G	10446507	MYH2
17	10446505	10446513	T	snp	G	10446507	MYH2
17	10446505	10446513	T	snp	G	10446507	MYH2
17	10532881	10532890	G	snp	A	10532883	MYH3
17	10558978	10558988	T	snp	C	10558983	MYH3
17	11829382	11829390	G	snp	T	11829384	DNAH9
17	13695078	13695086	A	snp	G	13695082	AK123263
17	16594920	16594930	A	snp	C	16594926	CCDC144A
17	18178975	18178985	T	snp	C	18178983	TOP3A
17	18188095	18188105	A	snp	G	18188096	TOP3A
17	18314136	18314144	C	snp	A	18314141	AX748015
17	19194917	19194926	T	snp	G	19194924	AX748411
17	19194917	19194926	T	snp	G	19194924	EPN2
17	20946556	20946564	G	snp	A	20946558	USP22
17	21091124	21091132	T	snp	G	21091126	DHRS7B
17	25929141	25929151	T	snp	C	25929148	KSR1
17	27384001	27384011	A	snp	C	27384006	PIPOX
17	27861104	27861114	T	snp	C	27861112	TAOK1
17	27967938	27967948	T	snp	G	27967946	SSH2
17	27967938	27967948	T	snp	G	27967946	U6
17	28846159	28846167	C	snp	T	28846161	GOSR1
17	29111121	29111130	T	snp	G	29111124	CRLF3

17	29661149	29661157	A	snp	C	29661153	NF1
17	29844224	29844232	G	snp	A	29844225	RAB11FIP4
17	29845846	29845854	T	snp	G	29845851	RAB11FIP4
17	30358361	30358370	T	snp	C	30358365	LRRC37B
17	30679328	30679338	T	snp	G	30679329	ZNF207
17	33814490	33814498	C	snp	T	33814495	SLFN12L
17	35721921	35721931	T	snp	C	35721929	ACACA
17	36000483	36000492	T	snp	C	36000488	DDX52
17	36689850	36689858	G	snp	T	36689851	SRCIN1
17	36893086	36893094	A	snp	C	36893089	PCGF2
17	37212920	37212929	A	snp	G	37212923	LOC100131347
17	37312311	37312320	T	snp	G	37312316	ARL5C
17	37791481	37791490	A	snp	C	37791486	PPP1R1B
17	38031861	38031869	T	snp	G	38031864	ZPBP2
17	38097004	38097012	T	snp	C	38097007	LRRC3C
17	39121003	39121011	T	snp	G	39121008	KRT39
17	39122301	39122310	T	snp	G	39122308	KRT39
17	40272592	40272601	C	snp	A	40272593	KAT2A
17	40703785	40703793	A	snp	G	40703786	BC043620
17	40703785	40703793	A	snp	G	40703786	HSD17B1
17	40703785	40703793	A	snp	G	40703786	HSD17B1
17	40705710	40705718	G	snp	A	40705714	BC043620
17	40705710	40705718	G	snp	A	40705714	HSD17B1
17	40705710	40705718	G	snp	A	40705714	HSD17B1
17	41231216	41231224	A	snp	C	41231220	BRCA1
17	41247598	41247607	A	snp	C	41247603	BRCA1
17	46137541	46137549	G	snp	T	46137545	NFE2L1
17	46152330	46152338	A	snp	G	46152332	CBX1
17	46474600	46474608	G	snp	T	46474601	SKAP1
17	46655142	46655151	C	snp	A	46655143	HOXB3
17	46655142	46655151	C	snp	A	46655143	HOXB3
17	46655142	46655151	C	snp	A	46655143	HOXB4
17	46655142	46655151	C	snp	A	46655143	HOXB4

17	46689004	46689013	A	snp	G	46689007	HOXB7
17	46689004	46689013	A	snp	G	46689007	HOXB8
17	47038038	47038046	T	snp	C	47038043	GIP
17	47233251	47233260	T	snp	G	47233258	B4GALNT2
17	47295004	47295013	C	snp	A	47295007	ABI3
17	47578434	47578442	T	snp	C	47578437	NGFR
17	48183468	48183476	G	snp	A	48183470	PDK2
17	48260625	48260633	A	snp	G	48260629	COL1A1
17	48676254	48676262	T	snp	G	48676260	CACNA1G
17	49232438	49232447	A	snp	C	49232445	NME1
17	49232438	49232447	A	snp	C	49232445	NME1
17	49232438	49232447	A	snp	C	49232445	NME1-NME2
17	49232438	49232447	A	snp	C	49232445	NME1-NME2
17	49232438	49232447	A	snp	C	49232445	NME2
17	49232438	49232447	A	snp	C	49232445	NME2
17	56235810	56235820	C	snp	A	56235818	MSX2P1
17	56691029	56691039	T	snp	C	56691033	TEX14
17	57247947	57247955	A	snp	C	57247952	PRR11
17	58349161	58349169	T	snp	C	58349166	USP32
17	61959897	61959907	G	snp	A	61959902	GH2
17	62120040	62120049	A	snp	G	62120044	DQ572107
17	62120040	62120049	A	snp	G	62120044	ERN1
17	62495664	62495674	A	snp	G	62495666	DDX5
17	62495664	62495674	A	snp	G	62495666	MIR3064
17	66247807	66247817	A	snp	C	66247812	AMZ2
17	66597743	66597752	G	snp	T	66597750	FAM20A
17	66984484	66984493	T	snp	C	66984487	ABCA9
17	67252575	67252584	T	snp	C	67252580	ABCA5
17	70594330	70594339	A	snp	G	70594334	LINC00511
17	71398046	71398055	G	snp	A	71398050	SDK2
17	73942957	73942967	T	snp	G	73942962	ACOX1
17	73981401	73981409	T	snp	C	73981407	CDK3
17	76063421	76063429	A	snp	G	76063423	TNRC6C

17	76112961	76112971	A	snp	G	76112964	TMC6
17	76162387	76162395	A	snp	C	76162393	C17orf99
17	76449937	76449945	T	snp	G	76449941	DNAH17
17	76573717	76573726	C	snp	A	76573723	DNAH17
17	77768647	77768655	C	snp	A	77768653	CBX8
17	78223718	78223726	A	snp	G	78223722	SLC26A11
17	78246082	78246090	A	snp	G	78246088	RNF213
17	79090442	79090451	C	snp	A	79090447	AATK
17	79090442	79090451	C	snp	A	79090447	AATK
17	79090442	79090451	C	snp	A	79090447	BAIAP2
17	79090442	79090451	C	snp	A	79090447	BAIAP2
17	79288330	79288338	C	snp	T	79288335	TMEM105
17	80091256	80091266	A	snp	G	80091264	CCDC57
17	80160333	80160343	A	snp	C	80160336	CCDC57
17	80191883	80191891	T	snp	G	80191889	SLC16A3
17	80444216	80444224	G	snp	T	80444218	NARF
17	80623102	80623112	A	snp	C	80623105	RAB40B
17	80786380	80786388	C	snp	T	80786382	TBCD
17	80786380	80786388	C	snp	T	80786382	ZNF750
18	645636	645644	A	snp	G	645642	CLUL1
18	721558	721568	A	snp	C	721562	YES1
18	2732008	2732016	A	snp	G	2732009	SMCHD1
18	3163447	3163457	T	snp	G	3163454	MYOM1
18	5420014	5420024	A	snp	G	5420020	EPB41L3
18	6590774	6590783	T	snp	G	6590780	LOC100130480
18	6788457	6788467	T	snp	G	6788460	ARHGAP28
18	11657633	11657643	C	snp	T	11657635	DQ582047
18	11657633	11657643	C	snp	T	11657635	DQ583379
18	11657633	11657643	C	snp	T	11657635	DQ596967
18	12493882	12493890	T	snp	G	12493888	SPIRE1
18	12657425	12657433	G	snp	A	12657430	AK095621
18	12657425	12657433	G	snp	A	12657430	SPIRE1
18	12678942	12678951	A	snp	G	12678948	CEP76

18	12678942	12678951	A	snp	G	12678948	PSMG2
18	12784750	12784758	T	snp	G	12784756	PTPN2
18	13099601	13099611	T	snp	G	13099602	CEP192
18	14533925	14533934	T	snp	C	14533927	POTEC
18	18571600	18571610	T	snp	C	18571604	ROCK1
18	21464349	21464358	T	snp	G	21464351	LAMA3
18	22033993	22034001	A	snp	G	22033999	IMPACT
18	28722636	28722645	T	snp	C	28722643	DSC1
18	28936389	28936398	T	snp	G	28936396	DSG1
18	29426549	29426557	A	snp	C	29426551	TRAPPC8
18	29692644	29692653	A	snp	G	29692650	RNF138
18	43591268	43591278	A	snp	G	43591272	PSTPIP2
18	45458514	45458523	T	snp	C	45458518	SMAD2
18	46903929	46903937	A	snp	G	46903934	DYM
18	47565711	47565719	T	snp	G	47565715	MYO5B
18	53303614	53303622	A	snp	G	53303615	TCF4
18	55213052	55213060	A	snp	C	55213057	FECH
18	55272861	55272870	A	snp	G	55272864	NARS
18	56000375	56000383	G	snp	T	56000377	NEDD4L
18	56368000	56368010	T	snp	G	56368003	MALT1
18	56650343	56650352	A	snp	G	56650346	ZNF532
18	56650831	56650839	C	snp	T	56650833	ZNF532
18	77724859	77724867	G	snp	T	77724862	HSBP1L1
19	291663	291671	G	snp	A	291665	PPAP2C
19	336851	336860	T	snp	C	336853	MIER2
19	404896	404905	G	snp	A	404897	C2CD4C
19	404896	404905	G	snp	A	404898	C2CD4C
19	632536	632546	G	snp	T	632544	POLRMT
19	709911	709921	G	snp	T	709912	PALM
19	873769	873778	C	snp	A	873773	MED16
19	1118879	1118889	A	snp	C	1118881	SBNO2
19	1118879	1118889	A	snp	C	1118882	SBNO2
19	1120677	1120686	T	snp	G	1120678	SBNO2

19	1396966	1396974	G	snp	A	1396969	AK126693
19	1396966	1396974	G	snp	A	1396969	GAMT
19	1396966	1396974	G	snp	A	1396969	NDUFS7
19	1527033	1527041	G	snp	A	1527035	PLK5
19	1812200	1812208	G	snp	A	1812203	ATP8B3
19	2084581	2084591	T	snp	C	2084585	MOB3A
19	2399719	2399727	T	snp	G	2399723	TMPRSS9
19	2512369	2512379	T	snp	G	2512370	GNG7
19	2821094	2821104	T	snp	C	2821102	ZNF554
19	2822214	2822222	T	snp	G	2822218	ZNF554
19	3115783	3115792	G	snp	A	3115789	GNA11
19	3744153	3744161	C	snp	T	3744158	TJP3
19	4307360	4307368	T	snp	C	4307363	FSD1
19	4544963	4544971	T	snp	G	4544967	SEMA6B
19	4771799	4771808	A	snp	G	4771805	MIR7-3HG
19	4903494	4903503	T	snp	C	4903495	ARRDC5
19	5152120	5152130	G	snp	T	5152124	KDM4B
19	5207404	5207412	T	snp	C	5207409	PTPRS
19	5756954	5756962	A	snp	C	5756960	TMEM146
19	5828414	5828422	G	snp	T	5828415	NRTN
19	6378584	6378592	G	snp	T	6378586	GTF2F1
19	6445125	6445135	T	snp	G	6445131	SLC25A23
19	6470591	6470599	T	snp	G	6470596	DENND1C
19	6477250	6477259	G	snp	T	6477255	DENND1C
19	7163592	7163602	A	snp	G	7163598	INSR
19	7511625	7511633	A	snp	G	7511631	ARHGEF18
19	7516473	7516483	T	snp	C	7516479	ARHGEF18
19	7614068	7614076	T	snp	C	7614071	PNPLA6
19	7691236	7691244	C	snp	T	7691242	XAB2
19	8548015	8548024	T	snp	G	8548018	HNRNPM
19	8932094	8932102	G	snp	T	8932095	ZNF558
19	9082114	9082122	A	snp	C	9082117	MUC16
19	10071663	10071671	C	snp	T	10071668	COL5A3

19	10171487	10171495	A	snp	G	10171490	C3P1
19	10342010	10342018	G	snp	T	10342012	MIR4322
19	10342010	10342018	G	snp	T	10342012	S1PR2
19	10416441	10416449	G	snp	A	10416443	FDX1L
19	10416441	10416449	G	snp	A	10416443	FDX1L
19	10416441	10416449	G	snp	A	10416443	ZGLP1
19	10416441	10416449	G	snp	A	10416443	ZGLP1
19	11274872	11274882	A	snp	C	11274875	KANK2
19	12625187	12625196	A	snp	C	12625193	ZNF709
19	12756654	12756663	T	snp	G	12756658	MAN2B1
19	12813201	12813210	T	snp	G	12813205	TNPO2
19	12821703	12821712	T	snp	G	12821707	TNPO2
19	14267378	14267386	G	snp	T	14267379	LOC100507373
19	14267378	14267386	G	snp	T	14267379	LOC100507373
19	14267378	14267386	G	snp	T	14267379	LOC100507373
19	14267378	14267386	G	snp	T	14267379	LOC100507373
19	14267378	14267386	G	snp	T	14267379	LPHN1
19	14267378	14267386	G	snp	T	14267379	LPHN1
19	14267378	14267386	G	snp	T	14267379	LPHN1
19	14267378	14267386	G	snp	T	14267379	LPHN1
19	15589163	15589172	A	snp	G	15589164	PGLYRP2
19	16211628	16211637	A	snp	G	16211629	AK095546
19	16211628	16211637	A	snp	G	16211629	TPM4
19	16275653	16275661	C	snp	T	16275654	CIB3
19	16278052	16278060	A	snp	C	16278053	CIB3
19	17183457	17183466	T	snp	G	17183461	HAUS8
19	17355544	17355552	G	snp	A	17355548	NR2F6
19	17572178	17572187	A	snp	G	17572183	NXNL1
19	17694244	17694254	G	snp	T	17694251	GLT25D1
19	18311106	18311115	A	snp	G	18311110	RAB3A
19	18379172	18379180	G	snp	A	18379178	KIAA1683
19	18701202	18701210	T	snp	G	18701204	C19orf60
19	19294384	19294392	T	snp	C	19294388	MEF2B

19	19294384	19294392	T	snp	C	19294388	MEF2BNB
19	19294384	19294392	T	snp	C	19294388	MEF2BNB-MEF2B
19	19294384	19294392	T	snp	C	19294388	MEF2BNB-MEF2B
19	19842878	19842887	A	snp	C	19842879	ZNF14
19	21674350	21674358	A	snp	G	21674351	LOC400680
19	21765377	21765385	A	snp	G	21765383	AX748435
19	23316138	23316148	T	snp	G	23316142	ZNF730
19	23556412	23556422	A	snp	C	23556420	ZNF91
19	23992201	23992210	A	snp	G	23992207	RPSA
19	24115133	24115142	T	snp	G	24115137	AK125686
19	24115133	24115142	T	snp	G	24115137	ZNF726
19	28285245	28285255	G	snp	T	28285253	BC024732
19	28285245	28285255	G	snp	T	28285253	LOC148189
19	33405536	33405545	A	snp	G	33405538	CEP89
19	38042735	38042743	G	snp	T	38042736	LOC100507433
19	38042735	38042743	G	snp	T	38042736	ZNF540
19	38975445	38975455	T	snp	C	38975448	RYR1
19	39370448	39370458	T	snp	G	39370455	SIRT2
19	39690127	39690137	T	snp	C	39690133	NCCRP1
19	39992837	39992847	A	snp	C	39992840	DLL3
19	39992837	39992847	A	snp	C	39992845	DLL3
19	40194417	40194426	A	snp	G	40194423	LGALS14
19	40574781	40574790	A	snp	G	40574784	ZNF780A
19	41315706	41315716	A	snp	C	41315713	AK097370
19	42072326	42072334	G	snp	A	42072330	CEACAM21
19	42342537	42342545	C	snp	T	42342539	LYPD4
19	43519089	43519097	A	snp	C	43519094	PSG11
19	43519089	43519097	A	snp	C	43519094	PSG6
19	44008368	44008377	C	snp	T	44008369	PHLDB3
19	45542629	45542639	A	snp	C	45542631	CLASRP
19	45862016	45862024	T	snp	C	45862021	ERCC2
19	46173211	46173220	G	snp	T	46173212	GIPR
19	46205487	46205495	G	snp	A	46205488	QPCTL

19	47543996	47544006	G	snp	T	47543997	NPAS1
19	47921171	47921181	C	snp	A	47921177	MEIS3
19	47921171	47921181	C	snp	A	47921179	MEIS3
19	48248179	48248188	A	snp	G	48248181	GLTSCR2
19	48741053	48741063	A	snp	G	48741060	CARD8
19	49168177	49168187	T	snp	C	49168181	NTN5
19	49168177	49168187	T	snp	C	49168181	NTN5
19	49168177	49168187	T	snp	C	49168181	SEC1
19	49168177	49168187	T	snp	C	49168181	SEC1
19	49215845	49215854	T	snp	G	49215850	MAMSTR
19	49215845	49215854	T	snp	G	49215852	MAMSTR
19	49218105	49218113	G	snp	T	49218110	MAMSTR
19	49337234	49337242	A	snp	G	49337236	HSD17B14
19	49364108	49364117	A	snp	G	49364109	PLEKHA4
19	49442535	49442545	A	snp	C	49442542	DHDH
19	49448594	49448604	A	snp	G	49448597	DHDH
19	49537160	49537168	C	snp	T	49537166	CGB2
19	49957277	49957287	T	snp	G	49957285	ALDH16A1
19	49965125	49965133	G	snp	A	49965130	ALDH16A1
19	50123756	50123766	G	snp	T	50123760	PRR12
19	50451299	50451309	A	snp	G	50451302	SIGLEC11
19	50965853	50965862	T	snp	C	50965857	MYBPC2
19	51326350	51326359	C	snp	A	51326357	KLK1
19	51747102	51747111	A	snp	G	51747103	CD33
19	52471663	52471672	T	snp	G	52471667	BC014606
19	52471663	52471672	T	snp	G	52471667	BC014606
19	52471663	52471672	T	snp	G	52471667	ZNF350
19	52471663	52471672	T	snp	G	52471667	ZNF350
19	52875440	52875449	A	snp	C	52875444	ZNF880
19	53848944	53848952	T	snp	C	53848945	ZNF845
19	54697599	54697607	G	snp	A	54697601	TSEN34
19	55537073	55537082	A	snp	C	55537080	GP6
19	56499766	56499776	A	snp	G	56499768	NLRP8

19	57029834	57029842	T	snp	G	57029836	ZNF471
19	57930251	57930261	T	snp	G	57930255	ZNF17
19	58070632	58070641	G	snp	A	58070639	ZNF550
19	58903697	58903707	A	snp	G	58903698	RPS5
19	59079184	59079193	T	snp	C	59079185	LOC100131691
19	59079184	59079193	T	snp	C	59079185	MZF1
1	870311	870319	G	snp	A	870316	SAMD11
1	1193427	1193437	T	snp	G	1193431	UBE2J2
1	3650311	3650319	C	snp	T	3650315	TP73
1	3650311	3650319	C	snp	T	3650315	TP73-AS1
1	6196074	6196083	A	snp	C	6196080	CHD5
1	6473522	6473531	T	snp	G	6473529	HES2
1	7725774	7725783	A	snp	G	7725775	CAMTA1
1	7805585	7805593	A	snp	G	7805587	CAMTA1
1	7854551	7854559	A	snp	G	7854554	PER3
1	8029500	8029510	G	snp	A	8029508	PARK7
1	8937769	8937779	A	snp	C	8937771	ENO1
1	8937769	8937779	A	snp	C	8937771	ENO1
1	8937769	8937779	A	snp	C	8937771	ENO1-AS1
1	9307011	9307019	C	snp	T	9307016	H6PD
1	10385044	10385053	A	snp	G	10385046	KIF1B
1	10857307	10857317	G	snp	A	10857311	CASZ1
1	12184229	12184238	T	snp	C	12184230	TNFRSF8
1	12347382	12347390	T	snp	C	12347386	VPS13D
1	12347382	12347390	T	snp	C	12347387	VPS13D
1	12857497	12857505	C	snp	T	12857503	PRAMEF1
1	13943890	13943898	T	snp	G	13943896	PDPN
1	15772049	15772058	G	snp	T	15772056	CTRC
1	17713953	17713963	G	snp	A	17713955	PADI6
1	21014537	21014547	T	snp	G	21014540	KIF17
1	21904199	21904207	C	snp	T	21904205	ALPL
1	22304227	22304237	T	snp	C	22304228	CELA3B
1	22311438	22311448	A	snp	C	22311439	CELA3B

1	24104825	24104833	C	snp	T	24104828	LOC100506963
1	24104825	24104833	C	snp	T	24104828	PITHD1
1	24405699	24405707	T	snp	C	24405700	MYOM3
1	24829679	24829687	G	snp	T	24829684	RCAN3
1	24829679	24829687	G	snp	T	24829684	RCAN3AS
1	25171669	25171679	A	snp	C	25171672	CLIC4
1	25171669	25171679	A	snp	C	25171672	Z24749
1	26510357	26510365	C	snp	T	26510362	CNKSR1
1	26612291	26612299	C	snp	A	26612296	UBXN11
1	28150600	28150608	A	snp	G	28150605	STX12
1	31191436	31191446	C	snp	A	31191437	LOC100129196
1	31191436	31191446	C	snp	A	31191437	MATN1
1	33503441	33503449	C	snp	T	33503447	AK2
1	34039109	34039118	A	snp	G	34039111	CSMD2
1	34039109	34039118	A	snp	C	34039114	CSMD2
1	34117524	34117532	G	snp	T	34117525	CSMD2
1	34631520	34631528	G	snp	A	34631523	CSMD2
1	35457392	35457401	A	snp	C	35457395	ZMYM6
1	35855052	35855060	A	snp	G	35855054	ZMYM4
1	36563156	36563164	C	snp	A	36563157	COL8A2
1	36703415	36703423	T	snp	G	36703421	THRAP3
1	39803449	39803459	T	snp	G	39803450	MACF1
1	40138109	40138118	T	snp	C	40138113	NT5C1A
1	40533953	40533961	T	snp	C	40533957	CAP1
1	40778843	40778853	T	snp	C	40778848	COL9A2
1	42999892	42999901	G	snp	A	42999898	CCDC30
1	43226329	43226337	T	snp	G	43226335	LEPRE1
1	46180465	46180474	T	snp	C	46180469	IPP
1	47281773	47281782	C	snp	A	47281779	CYP4B1
1	47716490	47716500	A	snp	G	47716491	STIL
1	47835242	47835252	T	snp	C	47835243	CMPK1
1	49056762	49056771	C	snp	A	49056769	AGBL4
1	61927732	61927741	T	snp	G	61927735	NFIA

1	62229671	62229680	T	snp	G	62229678	INADL
1	62263878	62263886	T	snp	G	62263879	INADL
1	62380192	62380200	A	snp	G	62380194	INADL
1	62904568	62904576	T	snp	G	62904574	USP1
1	64017377	64017387	T	snp	G	64017383	EFCAB7
1	65247390	65247398	T	snp	C	65247393	RAVER2
1	65614843	65614851	C	snp	T	65614848	AK4
1	67132862	67132871	C	snp	A	67132869	AK298300
1	67132862	67132871	C	snp	A	67132869	AK298300
1	67132862	67132871	C	snp	A	67132869	SGIP1
1	67132862	67132871	C	snp	A	67132869	SGIP1
1	67307044	67307052	A	snp	G	67307045	WDR78
1	71250281	71250290	T	snp	G	71250288	BC041441
1	76388585	76388594	A	snp	C	76388588	ASB17
1	78308227	78308236	T	snp	G	78308233	FAM73A
1	84465067	84465077	C	snp	A	84465072	TTLL7
1	84809555	84809563	A	snp	G	84809561	SAMD13
1	84878636	84878646	C	snp	A	84878641	DNASE2B
1	87182124	87182133	T	snp	G	87182131	SH3GLB1
1	93791094	93791104	T	snp	C	93791097	LOC100131564
1	93826600	93826608	G	snp	T	93826601	DR1
1	95322293	95322302	T	snp	C	95322300	SLC44A3
1	98387374	98387384	C	snp	A	98387378	DPYD
1	100128778	100128786	A	snp	G	100128782	PALMD
1	100587243	100587251	A	snp	C	100587249	SASS6
1	100638795	100638805	A	snp	C	100638800	LRRC39
1	109241121	109241129	T	snp	C	109241127	PRPF38B
1	109398647	109398657	T	snp	G	109398655	AKNAD1
1	113371008	113371017	A	snp	G	113371011	AX748125
1	117660126	117660134	A	snp	G	117660130	TRIM45
1	118549720	118549728	T	snp	G	118549721	SPAG17
1	146739066	146739075	T	snp	C	146739067	CHD1L
1	149872771	149872779	T	snp	G	149872774	BOLA1

1	149907175	149907184	C	snp	A	149907182	MTMR11
1	150302230	150302238	T	snp	G	150302236	PRPF3
1	150777704	150777714	G	snp	A	150777706	CTSK
1	150918928	150918937	T	snp	G	150918935	SETDB1
1	151010861	151010871	A	snp	G	151010863	BNIPL
1	151022040	151022048	T	snp	C	151022042	C1orf56
1	151022040	151022048	T	snp	C	151022042	CDC42SE1
1	151492610	151492620	T	snp	C	151492613	CGN
1	151816213	151816223	A	snp	G	151816219	LOC100132111
1	154113312	154113320	A	snp	C	154113314	NUP210L
1	155347813	155347823	A	snp	C	155347818	ASH1L
1	155706209	155706218	T	snp	G	155706216	DAP3
1	155706209	155706218	T	snp	G	155706216	YY1AP1
1	156017772	156017781	A	snp	G	156017779	UBQLN4
1	156494544	156494552	G	snp	T	156494546	IQGAP3
1	156694022	156694030	G	snp	T	156694023	ISG20L2
1	160186889	160186897	G	snp	A	160186892	DCAF8
1	160302354	160302364	A	snp	G	160302355	COPA
1	161751220	161751228	A	snp	G	161751225	ATF6
1	164743401	164743411	A	snp	G	164743403	LOC100505795
1	164743401	164743411	A	snp	G	164743403	PBX1
1	165599802	165599810	A	snp	G	165599803	MGST3
1	165623956	165623964	T	snp	C	165623961	MGST3
1	168663765	168663773	T	snp	G	168663771	DPT
1	169890931	169890939	A	snp	G	169890932	KIFAP3
1	170933762	170933770	A	snp	C	170933765	C1orf129
1	173867243	173867252	T	snp	G	173867250	DQ593451
1	174605509	174605517	A	snp	C	174605510	RABGAP1L
1	176105061	176105070	A	snp	G	176105065	RFWD2
1	178694076	178694085	C	snp	T	178694083	RALGPS2
1	178819640	178819649	T	snp	C	178819643	ANGPTL1
1	178819640	178819649	T	snp	C	178819643	RALGPS2
1	180144134	180144142	A	snp	C	180144135	QSOX1

1	180164131	180164140	G	snp	T	180164133	QSOX1
1	180164131	180164140	G	snp	T	180164135	QSOX1
1	180946380	180946388	T	snp	G	180946381	AK056657
1	180946380	180946388	T	snp	G	180946381	STX6
1	184728083	184728093	A	snp	C	184728087	AX747662
1	186925628	186925638	T	snp	G	186925632	PLA2G4A
1	186925628	186925638	T	snp	G	186925635	PLA2G4A
1	200905885	200905893	A	snp	G	200905886	C1orf81
1	201861902	201861911	G	snp	T	201861905	SHISA4
1	201952890	201952898	T	snp	G	201952893	RNPEP
1	201980845	201980854	A	snp	G	201980852	ELF3
1	202573399	202573407	C	snp	T	202573405	SYT2
1	202827393	202827403	A	snp	G	202827397	BC040684
1	202827393	202827403	A	snp	G	202827397	BC040684
1	202827393	202827403	A	snp	G	202827397	BC049825
1	202827393	202827403	A	snp	G	202827397	BC049825
1	202897424	202897432	A	snp	G	202897430	KLHL12
1	204371720	204371729	T	snp	G	204371721	PPP1R15B
1	204524988	204524996	A	snp	G	204524990	MDM4
1	205684222	205684230	A	snp	G	205684227	NUCKS1
1	207285446	207285456	A	snp	G	207285453	C4BPA
1	210408391	210408401	T	snp	C	210408396	C1orf133
1	210408391	210408401	T	snp	C	210408396	SERTAD4
1	212501671	212501680	A	snp	G	212501674	PPP2R5A
1	212617389	212617398	A	snp	C	212617395	NENF
1	212957348	212957357	A	snp	G	212957353	NSL1
1	212977389	212977398	A	snp	G	212977396	TATDN3
1	213057640	213057650	A	snp	G	213057648	FLVCR1
1	215812319	215812328	A	snp	C	215812325	USH2A
1	215972001	215972009	T	snp	G	215972002	USH2A
1	216495186	216495196	T	snp	G	216495190	USH2A
1	217792147	217792155	A	snp	G	217792148	GPATCH2
1	222887924	222887934	T	snp	G	222887926	BROX

1	223168205	223168215	T	snp	C	223168212	DISP1
1	223286557	223286565	T	snp	G	223286560	TLR5
1	223969507	223969516	A	snp	C	223969512	TP53BP2
1	224563198	224563207	T	snp	G	224563202	CNIH4
1	225677293	225677302	A	snp	G	225677300	ENAH
1	225940632	225940640	T	snp	G	225940636	AK124056
1	226352490	226352499	T	snp	G	226352497	ACBD3
1	227098195	227098203	T	snp	C	227098196	ADCK3
1	227922140	227922149	A	snp	C	227922141	JMJD4
1	227922140	227922149	A	snp	C	227922141	JMJD4
1	227922140	227922149	A	snp	C	227922141	JMJD4
1	227922140	227922149	A	snp	C	227922141	LOC100130093
1	227922140	227922149	A	snp	C	227922141	SNAP47
1	227922140	227922149	A	snp	C	227922141	SNAP47
1	227922140	227922149	A	snp	C	227922141	SNAP47
1	229586482	229586490	T	snp	G	229586485	NUP133
1	229735795	229735803	T	snp	C	229735801	TAF5L
1	233518877	233518886	T	snp	C	233518884	KIAA1804
1	234606022	234606031	A	snp	C	234606024	TARBP1
1	235633220	235633228	T	snp	G	235633223	B3GALNT2
1	237731675	237731684	A	snp	C	237731676	RYR2
1	241236715	241236724	A	snp	G	241236720	RGS7
1	241265888	241265896	T	snp	C	241265893	RGS7
1	242253556	242253565	A	snp	C	242253558	PLD5
1	243293174	243293183	A	snp	G	243293175	CEP170
1	244746525	244746533	A	snp	G	244746527	C1orf101
1	246704820	246704830	A	snp	G	246704828	TFB2M
1	247752620	247752629	A	snp	C	247752626	OR2G2
1	248032205	248032213	T	snp	G	248032211	OR2W3
1	248032205	248032213	T	snp	G	248032211	TRIM58
1	248085911	248085919	T	snp	C	248085912	OR2T8
20	869816	869824	C	snp	A	869821	ANGPT4
20	2096541	2096549	C	snp	A	2096547	STK35

20	2126561	2126569	T	snp	C	2126566	STK35
20	3839336	3839344	A	snp	C	3839341	MAVS
20	3856672	3856680	T	snp	C	3856677	MAVS
20	7895767	7895776	A	snp	C	7895773	HAO1
20	11248227	11248236	T	snp	C	11248231	LOC339593
20	17477747	17477755	A	snp	G	17477750	BFSP1
20	18039048	18039058	C	snp	T	18039050	OVOL2
20	18464104	18464113	T	snp	C	18464109	POLR3F
20	18470642	18470651	A	snp	C	18470645	RBBP9
20	18515840	18515849	C	snp	A	18515847	SEC23B
20	20372830	20372839	A	snp	C	20372836	RALGAPA2
20	22543597	22543605	A	snp	G	22543598	LINC00261
20	23808189	23808199	T	snp	G	23808196	CST2
20	25207766	25207774	C	snp	A	25207772	ENTPD6
20	25229530	25229538	T	snp	G	25229536	PYGB
20	25251793	25251801	C	snp	T	25251795	PYGB
20	31218829	31218838	A	snp	C	31218835	C20orf203
20	31961550	31961559	A	snp	C	31961557	CDK5RAP1
20	32210236	32210244	T	snp	G	32210237	CBFA2T2
20	34581973	34581982	T	snp	G	34581978	C20orf152
20	42263808	42263816	A	snp	C	42263809	IFT52
20	42844409	42844417	T	snp	C	42844412	LOC100505783
20	43037364	43037372	A	snp	C	43037365	HNF4A
20	43037364	43037372	A	snp	C	43037365	MIR3646
20	43159447	43159455	A	snp	G	43159448	PKIG
20	43737274	43737283	T	snp	G	43737277	WFDC5
20	44466395	44466404	T	snp	C	44466396	SNX21
20	45979213	45979222	T	snp	G	45979214	AK098067
20	45979213	45979222	T	snp	G	45979214	BC047609
20	45979213	45979222	T	snp	G	45979214	ZMYND8
20	45979213	45979222	T	snp	G	45979214	ZMYND8
20	47906287	47906297	T	snp	C	47906294	ZNFX1-AS1
20	48251979	48251988	G	snp	A	48251983	B4GALT5

20	50776519	50776528	A	snp	C	50776521	ZFP64
20	55046623	55046632	A	snp	C	55046627	C20orf43
20	55802854	55802862	T	snp	G	55802857	BMP7
20	61539335	61539345	T	snp	C	61539343	DIDO1
20	62167507	62167516	C	snp	T	62167513	PTK6
20	62378734	62378742	G	snp	A	62378739	SLC2A4RG
20	62378734	62378742	G	snp	A	62378739	ZBTB46
20	62612996	62613006	C	snp	T	62613004	PRPF6
21	10990693	10990702	C	snp	A	10990696	TPTE
21	15537721	15537731	A	snp	G	15537726	LIPI
21	19274501	19274511	T	snp	G	19274505	CHODL
21	27079048	27079057	T	snp	G	27079053	JAM2
21	30391234	30391243	T	snp	G	30391237	RWDD2B
21	31580609	31580618	A	snp	C	31580614	LINC00307
21	32126534	32126542	T	snp	G	32126540	KRTAP21-1
21	32597488	32597496	T	snp	G	32597494	TIAM1
21	34724908	34724916	A	snp	C	34724911	IFNAR1
21	34899984	34899992	A	snp	G	34899985	GART
21	35741851	35741861	A	snp	G	35741855	KCNE2
21	38130054	38130062	A	snp	G	38130060	HLCS
21	40759617	40759627	C	snp	A	40759624	WRB
21	42748315	42748323	A	snp	C	42748316	MX2
21	42748315	42748323	A	snp	C	42748318	MX2
21	43322301	43322311	A	snp	C	43322303	C2CD2
21	43443106	43443114	A	snp	C	43443112	ZNF295-AS1
21	45063848	45063858	A	snp	G	45063853	HSF2BP
21	45092648	45092656	A	snp	G	45092652	RRP1B
21	47406610	47406618	C	snp	A	47406611	COL6A1
21	47832012	47832021	T	snp	C	47832018	PCNT
22	18382588	18382598	A	snp	G	18382589	MICAL3
22	18574100	18574108	A	snp	G	18574103	PEX26
22	21304678	21304687	T	snp	G	21304682	BC033281
22	21304678	21304687	T	snp	G	21304682	CRKL

22	23094195	23094204	A	snp	C	23094200	abParts
22	23094195	23094204	A	snp	C	23094200	DKFZp667J0810
22	23805125	23805134	A	snp	C	23805129	LOC388882
22	23830152	23830161	T	snp	C	23830154	LOC388882
22	24868797	24868805	A	snp	C	24868803	C22orf45
22	24868797	24868805	A	snp	C	24868803	UPB1
22	24898870	24898879	T	snp	C	24898873	UPB1
22	24940235	24940243	G	snp	T	24940241	C22orf13
22	25250782	25250790	A	snp	G	25250786	SGSM1
22	25505934	25505943	A	snp	C	25505935	KIAA1671
22	25505934	25505943	A	snp	C	25505935	KIAA1671
22	25505934	25505943	A	snp	C	25505935	LOC100128531
22	25505934	25505943	A	snp	C	25505935	LOC100128531
22	26040122	26040132	T	snp	G	26040125	ADRBK2
22	26240231	26240239	A	snp	G	26240233	MYO18B
22	27026784	27026792	T	snp	C	27026787	CRYBA4
22	29120395	29120404	A	snp	C	29120399	CHEK2
22	29125913	29125923	T	snp	C	29125914	CHEK2
22	29835545	29835553	G	snp	T	29835551	RFPL1
22	29835545	29835553	G	snp	T	29835551	RFPL1-AS1
22	30426627	30426637	G	snp	T	30426635	MTMR3
22	30892548	30892557	C	snp	A	30892554	SEC14L4
22	30927417	30927427	A	snp	G	30927421	SEC14L6
22	31603272	31603280	A	snp	G	31603275	BC069815
22	31603272	31603280	A	snp	G	31603275	RNF185
22	31984821	31984829	A	snp	G	31984826	SFI1
22	32081927	32081935	T	snp	C	32081928	PRR14L
22	32617723	32617733	T	snp	G	32617729	SLC5A4
22	32743893	32743903	A	snp	C	32743894	JA536278
22	36676987	36676995	T	snp	G	36676990	MYH9
22	36683799	36683808	A	snp	C	36683802	MYH9
22	37532506	37532515	C	snp	T	37532513	IL2RB
22	38613195	38613205	A	snp	C	38613197	MAFF

22	39639850	39639858	G	snp	T	39639852	PDGFB
22	40697582	40697590	T	snp	G	40697588	TNRC6B
22	41210827	41210835	T	snp	G	41210830	MIR4766
22	41210827	41210835	T	snp	G	41210830	SLC25A17
22	41526533	41526541	A	snp	C	41526536	EP300
22	41739176	41739184	A	snp	C	41739182	ZC3H7B
22	42488911	42488919	A	snp	G	42488914	LOC100132273
22	43467095	43467103	T	snp	G	43467098	TTLL1
22	44259072	44259081	A	snp	C	44259074	SULT4A1
22	45794719	45794728	A	snp	C	45794722	SMC1B
22	46439734	46439744	A	snp	C	46439736	LOC100271722
22	46449889	46449897	G	snp	A	46449890	C22orf26
22	46449889	46449897	G	snp	A	46449890	LOC150381
22	46449889	46449897	G	snp	A	46449890	MIRLET7BHG
22	46632156	46632166	A	snp	C	46632159	PPARA
22	47769563	47769573	G	snp	A	47769569	LOC339685
22	50198510	50198520	A	snp	G	50198516	BRD1
22	51112354	51112363	G	snp	T	51112358	SHANK3
22	51112354	51112363	G	snp	A	51112360	SHANK3
2	1521712	1521721	C	snp	A	1521718	TPO
2	7080643	7080651	T	snp	C	7080645	RNF144A
2	11265650	11265658	T	snp	C	11265653	FLJ33534
2	11853719	11853728	T	snp	C	11853721	LPIN1
2	17964335	17964344	T	snp	G	17964338	GEN1
2	17964335	17964344	T	snp	G	17964338	SMC6
2	26067474	26067483	A	snp	G	26067480	ASXL2
2	26718821	26718829	C	snp	A	26718827	OTOF
2	27655441	27655450	T	snp	G	27655446	NRBP1
2	29134504	29134514	T	snp	C	29134512	WDR43
2	29431779	29431789	A	snp	G	29431781	ALK
2	31557739	31557748	T	snp	C	31557742	XDH
2	31597403	31597411	A	snp	G	31597409	XDH
2	33050630	33050638	G	snp	A	33050635	LINC00486

2	33162851	33162859	A	snp	G	33162853	LINC00486
2	33162851	33162859	A	snp	G	33162853	LOC100271832
2	36923896	36923905	G	snp	T	36923897	VIT
2	36923896	36923905	G	snp	T	36923899	VIT
2	37064354	37064362	T	snp	C	37064358	STRN
2	37519654	37519662	T	snp	G	37519655	PRKD3
2	38208970	38208980	A	snp	C	38208978	FAM82A1
2	39102669	39102677	C	snp	T	39102670	DHX57
2	39102669	39102677	C	snp	T	39102670	MORN2
2	43965700	43965709	T	snp	C	43965704	PLEKHH2
2	46583272	46583282	C	snp	T	46583280	EPAS1
2	46657041	46657051	A	snp	C	46657049	BC031304
2	47347883	47347891	T	snp	G	47347888	C2orf61
2	47629890	47629900	T	snp	G	47629897	MSH2
2	47713846	47713855	T	snp	C	47713848	AK056077
2	47713846	47713855	T	snp	C	47713848	MSH2
2	48031272	48031282	T	snp	G	48031279	MSH6
2	48731793	48731802	A	snp	C	48731796	PPP1R21
2	49004452	49004460	T	snp	C	49004458	STON1-GTF2A1L
2	54571902	54571910	A	snp	C	54571908	C2orf73
2	54895849	54895857	G	snp	T	54895850	SPTBN1
2	56598522	56598530	A	snp	C	56598524	CCDC85A
2	61002842	61002852	T	snp	C	61002845	PAPOLG
2	61307871	61307880	A	snp	C	61307873	KIAA1841
2	61709732	61709741	T	snp	G	61709735	XPO1
2	62372821	62372829	T	snp	G	62372823	BC071802
2	62448648	62448656	A	snp	G	62448654	B3GNT2
2	64416873	64416883	T	snp	C	64416879	LINC00309
2	65305841	65305849	T	snp	G	65305847	CEP68
2	66243054	66243062	A	snp	C	66243059	FLJ16124
2	69373508	69373516	T	snp	G	69373511	ANTXR1
2	69693391	69693399	G	snp	T	69693396	AAK1
2	71061922	71061931	A	snp	C	71061923	CD207

2	71338077	71338085	A	snp	C	71338083	MCEE
2	85107119	85107127	C	snp	T	85107120	C2orf89
2	85649288	85649298	T	snp	C	85649291	SH2D6
2	85659508	85659516	T	snp	G	85659512	SH2D6
2	85659532	85659542	T	snp	G	85659536	SH2D6
2	86075962	86075970	T	snp	C	86075966	ST3GAL5
2	86915641	86915649	T	snp	C	86915647	RNF103-CHMP3
2	87111275	87111284	T	snp	C	87111282	LOC100286979
2	87111275	87111284	T	snp	C	87111282	RMND5A
2	87114860	87114869	T	snp	G	87114863	LOC100286979
2	87114860	87114869	T	snp	G	87114863	RMND5A
2	89326076	89326084	T	snp	C	89326082	abParts
2	99009057	99009066	A	snp	G	99009061	CNGA3
2	101098490	101098498	G	snp	T	101098493	NMS
2	103035368	103035376	T	snp	C	103035374	IL18RAP
2	108619310	108619319	A	snp	G	108619315	SLC5A7
2	109098378	109098386	C	snp	A	109098383	GCC2
2	111417958	111417968	A	snp	C	111417963	BUB1
2	112550822	112550830	T	snp	G	112550824	ANAPC1
2	113531085	113531094	T	snp	C	113531092	IL1A
2	114004988	114004997	A	snp	C	114004993	LOC654433
2	114004988	114004997	A	snp	C	114004993	PAX8
2	114013023	114013033	A	snp	C	114013028	LOC654433
2	114013023	114013033	A	snp	C	114013028	PAX8
2	114020843	114020851	T	snp	G	114020847	LOC654433
2	114020843	114020851	T	snp	G	114020847	PAX8
2	115919055	115919065	C	snp	A	115919060	DPP10
2	115919055	115919065	C	snp	A	115919060	LOC389023
2	115919055	115919065	C	snp	A	115919061	DPP10
2	115919055	115919065	C	snp	A	115919061	LOC389023
2	128048667	128048677	A	snp	C	128048673	ERCC3
2	128239018	128239027	G	snp	A	128239019	IWS1
2	131805979	131805988	G	snp	T	131805983	FAM168B

2	132259417	132259425	A	snp	G	132259420	LOC150776
2	132263229	132263239	T	snp	G	132263237	LOC150776
2	136625594	136625603	A	snp	G	136625601	MCM6
2	145222132	145222140	T	snp	C	145222135	ZEB2
2	149817810	149817818	G	snp	T	149817816	KIF5C
2	153399490	153399498	A	snp	C	153399496	FMNL2
2	157470260	157470270	T	snp	C	157470261	GPD2
2	160526488	160526496	T	snp	C	160526492	BAZ2B
2	160571869	160571877	G	snp	A	160571875	MARCH7
2	160605853	160605862	T	snp	G	160605859	MARCH7
2	165586010	165586019	A	snp	C	165586014	COBLL1
2	166242990	166242998	A	snp	G	166242996	SCN2A
2	168922550	168922558	A	snp	C	168922552	STK39
2	170072509	170072518	A	snp	C	170072514	LRP2
2	174227477	174227486	T	snp	G	174227479	CDCA7
2	175265663	175265671	A	snp	G	175265665	SCRN3
2	176789404	176789413	A	snp	C	176789408	KIAA1715
2	178403473	178403483	T	snp	G	178403474	AGPS
2	182374176	182374184	T	snp	C	182374178	ITGA4
2	186625767	186625775	A	snp	G	186625769	FSIP2
2	186629183	186629192	T	snp	G	186629190	FSIP2
2	187692280	187692289	T	snp	G	187692284	ZSWIM2
2	190429174	190429182	T	snp	C	190429180	SLC40A1
2	191843823	191843831	A	snp	G	191843829	STAT1
2	191863407	191863417	A	snp	G	191863408	STAT1
2	196663434	196663443	T	snp	G	196663436	DNAH7
2	196821415	196821423	T	snp	C	196821416	DNAH7
2	198051196	198051206	T	snp	C	198051204	ANKRD44
2	198355147	198355157	T	snp	G	198355152	HSPD1
2	200512634	200512643	T	snp	G	200512640	BC035629
2	202131584	202131592	T	snp	G	202131586	CASP8
2	207610904	207610913	A	snp	C	207610908	MDH1B
2	208592249	208592258	T	snp	G	208592256	CCNYL1

2	208629376	208629385	A	snp	C	208629380	FZD5
2	209107474	209107482	T	snp	C	209107479	IDH1
2	210372977	210372985	T	snp	C	210372983	MAP2
2	211158847	211158856	T	snp	C	211158854	MYL1
2	212523105	212523115	A	snp	C	212523113	ERBB4
2	213869915	213869924	G	snp	A	213869918	IKZF2
2	214012403	214012412	A	snp	C	214012404	IKZF2
2	214012403	214012412	A	snp	C	214012406	IKZF2
2	215631927	215631935	T	snp	C	215631931	BARD1
2	216246205	216246214	T	snp	G	216246209	FN1
2	216256768	216256776	A	snp	G	216256774	FN1
2	218843803	218843811	G	snp	A	218843804	TNS1
2	219203684	219203692	T	snp	G	219203690	PNKD
2	219271200	219271208	C	snp	T	219271202	CTDSP1
2	219322793	219322803	A	snp	G	219322797	USP37
2	219424719	219424729	A	snp	C	219424722	USP37
2	219869552	219869562	G	snp	T	219869555	CCDC108
2	220130651	220130660	A	snp	G	220130652	TUBA4B
2	223496862	223496872	A	snp	G	223496869	FARSB
2	224749908	224749917	G	snp	T	224749909	WDFY1
2	224832410	224832418	T	snp	G	224832413	MRPL44
2	226518756	226518765	A	snp	G	226518760	NYAP2
2	227924351	227924360	A	snp	C	227924353	COL4A4
2	228120429	228120439	T	snp	G	228120430	AK056332
2	228120429	228120439	T	snp	G	228120430	AK056332
2	228120429	228120439	T	snp	G	228120430	AK056332
2	228120429	228120439	T	snp	G	228120430	BC035052
2	228120429	228120439	T	snp	G	228120430	BC035052
2	228120429	228120439	T	snp	G	228120430	BC035052
2	228120429	228120439	T	snp	G	228120430	COL4A3
2	228120429	228120439	T	snp	G	228120430	COL4A3
2	228120429	228120439	T	snp	G	228120430	COL4A3
2	228492859	228492868	T	snp	G	228492860	C2orf83

2	228572453	228572461	T	snp	C	228572459	AX746677
2	228572453	228572461	T	snp	C	228572459	SLC19A3
2	231685193	231685202	A	snp	G	231685197	CAB39
2	234873228	234873236	A	snp	C	234873230	TRPM8
2	238427730	238427739	T	snp	G	238427733	MLPH
2	239073512	239073521	A	snp	C	239073514	FAM132B
2	241808307	241808315	C	snp	T	241808313	AGXT
3	367138	367146	A	snp	G	367142	CHL1
3	1425348	1425357	A	snp	C	1425353	CNTN6
3	3885253	3885262	C	snp	A	3885258	LRRN1
3	3885253	3885262	C	snp	A	3885258	SUMF1
3	8722847	8722855	G	snp	T	8722851	C3orf32
3	9031326	9031334	A	snp	C	9031331	SRGAP3
3	10148979	10148989	T	snp	C	10148982	C3orf24
3	14485477	14485486	C	snp	T	14485479	SLC6A6
3	14485477	14485486	C	snp	A	14485483	SLC6A6
3	15113898	15113906	A	snp	G	15113899	ZFYVE20
3	15221876	15221885	T	snp	C	15221881	COL6A4P1
3	15248805	15248815	A	snp	G	15248811	CAPN7
3	15282163	15282172	A	snp	G	15282164	CAPN7
3	15315755	15315765	A	snp	G	15315761	SH3BP5
3	15605481	15605490	A	snp	C	15605483	HACL1
3	15684128	15684136	T	snp	G	15684134	BTD
3	15804554	15804562	T	snp	G	15804557	ANKRD28
3	15804554	15804562	T	snp	G	15804557	BC041363
3	17417165	17417175	A	snp	C	17417171	TBC1D5
3	18457302	18457312	G	snp	T	18457303	SATB1
3	29940516	29940524	A	snp	G	29940520	RBMS3
3	32187877	32187885	A	snp	G	32187879	GPD1L
3	32408488	32408496	T	snp	C	32408492	CMTM8
3	33421173	33421183	A	snp	C	33421177	FBXL2
3	33442888	33442896	A	snp	G	33442889	FBXL2
3	33442888	33442896	A	snp	G	33442889	UBP1

3	36888166	36888174	T	snp	G	36888171	TRANK1
3	37876661	37876670	G	snp	T	37876668	BC040563
3	38034921	38034930	A	snp	G	38034927	VILL
3	38527209	38527217	T	snp	C	38527214	ACVR2B
3	38830111	38830120	G	snp	A	38830116	SCN10A
3	39119880	39119889	T	snp	G	39119885	WDR48
3	40428601	40428609	G	snp	T	40428602	ENTPD3
3	46742521	46742531	C	snp	A	46742522	TMIE
3	47604270	47604280	G	snp	T	47604271	AK094639
3	47604270	47604280	G	snp	T	47604271	CSPG5
3	48503076	48503085	T	snp	G	48503082	ATRIP
3	48503076	48503085	T	snp	G	48503082	ATRIP
3	48503076	48503085	T	snp	G	48503082	TREX1
3	48503076	48503085	T	snp	G	48503082	TREX1
3	49448579	49448589	T	snp	C	49448582	RHOA
3	49508971	49508979	T	snp	C	49508975	DAG1
3	51975569	51975579	C	snp	T	51975576	PARP3
3	51975569	51975579	C	snp	T	51975576	RRP9
3	53219274	53219282	G	snp	T	53219277	PRKCD
3	56655211	56655221	A	snp	C	56655213	CCDC66
3	56655211	56655221	A	snp	C	56655213	CCDC66
3	56655211	56655221	A	snp	C	56655213	FAM208A
3	57400933	57400943	A	snp	G	57400935	DNAH12
3	58518090	58518099	T	snp	C	58518091	ACOX2
3	58630499	58630509	A	snp	G	58630500	FAM3D
3	66397442	66397452	A	snp	C	66397446	SLC25A26
3	69590940	69590948	C	snp	A	69590946	FRMD4B
3	75788826	75788835	T	snp	C	75788833	MIR4273
3	75788826	75788835	T	snp	C	75788833	ZNF717
3	86997101	86997111	T	snp	C	86997105	VGLL3
3	93612364	93612372	T	snp	C	93612368	PROS1
3	98503989	98503999	T	snp	G	98503992	ST3GAL6
3	98515111	98515120	A	snp	C	98515115	DCBLD2

3	98601263	98601273	T	snp	C	98601269	DCBLD2
3	100013093	100013102	T	snp	G	100013100	TBC1D23
3	101219388	101219397	A	snp	C	101219391	SENP7
3	101475599	101475607	A	snp	G	101475603	CEP97
3	108638335	108638343	T	snp	G	108638341	GUCA1C
3	108698023	108698031	A	snp	C	108698027	MORC1
3	111767190	111767198	T	snp	C	111767193	TMPRSS7
3	111785622	111785632	T	snp	C	111785628	TMPRSS7
3	112191199	112191207	T	snp	G	112191201	BTLA
3	113284379	113284388	A	snp	C	113284384	SIDT1
3	113287473	113287481	A	snp	G	113287477	SIDT1
3	113302488	113302496	A	snp	G	113302492	SIDT1
3	113848189	113848197	A	snp	C	113848192	DRD3
3	119248319	119248328	A	snp	G	119248320	CD80
3	119423024	119423033	T	snp	C	119423031	C3orf15
3	120133850	120133860	A	snp	G	120133856	FSTL1
3	121616524	121616533	A	snp	G	121616527	SLC15A2
3	124453108	124453118	A	snp	G	124453113	UMPS
3	124641387	124641395	T	snp	C	124641390	MUC13
3	124806627	124806635	T	snp	G	124806633	SLC12A8
3	125651236	125651246	A	snp	C	125651238	ALG1L
3	126181066	126181075	A	snp	C	126181070	ZXDC
3	128723211	128723219	C	snp	A	128723214	CCDC48
3	130369129	130369137	G	snp	T	130369130	COL6A6
3	130369129	130369137	G	snp	T	130369131	COL6A6
3	138402350	138402359	A	snp	G	138402353	PIK3CB
3	142466184	142466193	A	snp	G	142466187	TRPC1
3	142540748	142540757	T	snp	G	142540754	PCOLCE2
3	150345688	150345698	T	snp	G	150345689	SELT
3	150402579	150402589	T	snp	C	150402582	FAM194A
3	156259169	156259179	T	snp	C	156259177	SSR3
3	160220151	160220161	T	snp	C	160220157	KPNA4
3	169578000	169578008	T	snp	C	169578003	LRRC31

3	170110900	170110909	T	snp	G	170110902	SKIL
3	172064821	172064829	A	snp	C	172064827	FNDC3B
3	172080401	172080411	T	snp	G	172080402	FNDC3B
3	172312817	172312827	T	snp	C	172312825	AK127557
3	172477398	172477407	T	snp	G	172477401	ECT2
3	186015278	186015286	A	snp	G	186015282	DGKG
3	186562896	186562905	G	snp	T	186562897	ADIPOQ
3	194024547	194024556	A	snp	C	194024552	LOC100131551
3	194429509	194429517	T	snp	C	194429512	LOC100507391
3	195965315	195965324	G	snp	A	195965317	AF088041
3	195965315	195965324	G	snp	A	195965317	PCYT1A
3	196553346	196553355	T	snp	G	196553350	PAK2
4	467311	467319	T	snp	C	467316	ABCA11P
4	467311	467319	T	snp	C	467316	ABCA11P
4	467311	467319	T	snp	C	467316	ZNF721
4	467311	467319	T	snp	C	467316	ZNF721
4	532606	532614	G	snp	T	532607	PIGG
4	2990907	2990916	A	snp	G	2990912	GRK4
4	3129690	3129698	A	snp	C	3129695	HTT
4	3150524	3150534	A	snp	G	3150527	HTT
4	7434412	7434420	G	snp	T	7434414	PSAPL1
4	7434412	7434420	G	snp	T	7434414	SORCS2
4	7801978	7801986	A	snp	G	7801984	AFAP1
4	8021414	8021422	A	snp	G	8021416	ABLIM2
4	8078463	8078471	G	snp	A	8078468	ABLIM2
4	15447544	15447554	A	snp	G	15447547	C1QTNF7
4	15724869	15724878	T	snp	C	15724876	BST1
4	17183836	17183845	T	snp	G	17183842	BC029598
4	18023886	18023894	T	snp	G	18023890	LCORL
4	25154683	25154691	T	snp	C	25154687	SEPSECS
4	36162828	36162837	A	snp	C	36162832	ARAP2
4	37585225	37585234	T	snp	G	37585232	C4orf19
4	37864570	37864578	T	snp	G	37864571	PGM2

4	39267998	39268008	A	snp	C	39268004	WDR19
4	39268012	39268020	A	snp	G	39268015	WDR19
4	42415728	42415737	T	snp	C	42415735	AK027252
4	42415728	42415737	T	snp	C	42415735	ATP8A1
4	44713228	44713238	A	snp	G	44713234	GNPDA2
4	48173067	48173077	A	snp	C	48173071	TEC
4	54243137	54243145	A	snp	G	54243140	FIP1L1
4	54243137	54243145	A	snp	G	54243140	PDGFRA
4	55969946	55969956	A	snp	C	55969948	KDR
4	68929898	68929906	T	snp	C	68929901	BC041902
4	68929898	68929906	T	snp	C	68929901	BC041902
4	68929898	68929906	T	snp	C	68929901	LOC550112
4	68929898	68929906	T	snp	C	68929901	LOC550112
4	68929898	68929906	T	snp	C	68929901	SYT14L
4	68929898	68929906	T	snp	C	68929901	SYT14L
4	68929898	68929906	T	snp	C	68929901	TMPRSS11F
4	68929898	68929906	T	snp	C	68929901	TMPRSS11F
4	70346054	70346062	T	snp	G	70346056	UGT2B4
4	70799755	70799764	T	snp	C	70799758	CSN1S1
4	70936637	70936646	A	snp	G	70936641	CSN1S2AP
4	74007866	74007875	A	snp	G	74007869	ANKRD17
4	76282690	76282698	A	snp	G	76282696	LOC441025
4	76581768	76581776	A	snp	C	76581774	G3BP2
4	77022906	77022916	A	snp	G	77022910	ART3
4	79441560	79441568	A	snp	C	79441564	FRAS1
4	81106117	81106125	T	snp	C	81106123	PRDM8
4	81124892	81124901	C	snp	A	81124899	PRDM8
4	83801336	83801346	A	snp	G	83801342	SEC31A
4	84349504	84349512	A	snp	G	84349510	HELQ
4	85853399	85853409	A	snp	C	85853401	WDFY3
4	87141502	87141512	A	snp	C	87141510	BC038746
4	87141502	87141512	A	snp	C	87141510	MAPK10
4	88226473	88226482	A	snp	C	88226479	HSD17B13

4	88728178	88728187	A	snp	C	88728180	IBSP
4	89053656	89053664	A	snp	C	89053657	ABCG2
4	90167248	90167256	T	snp	C	90167252	GPRIN3
4	95500621	95500630	A	snp	G	95500627	PDLIM5
4	95588562	95588570	T	snp	G	95588564	PDLIM5
4	96012421	96012431	T	snp	G	96012426	BMPR1B
4	100263712	100263720	A	snp	G	100263714	ADH1C
4	100339590	100339598	A	snp	G	100339595	ADH7
4	100459863	100459871	A	snp	C	100459865	C4orf17
4	101950530	101950538	A	snp	C	101950535	PPP3CA
4	103553957	103553965	T	snp	C	103553959	MANBA
4	108603451	108603461	T	snp	G	108603456	PAPSS1
4	109677993	109678002	A	snp	C	109677995	AGXT2L1
4	109779967	109779975	A	snp	G	109779969	COL25A1
4	120058057	120058067	T	snp	C	120058065	MYOZ2
4	120414685	120414694	A	snp	C	120414692	LOC645513
4	120414685	120414694	A	snp	C	120414692	PDE5A
4	122721708	122721718	T	snp	G	122721713	EXOSC9
4	123662332	123662341	T	snp	G	123662338	BBS12
4	129778071	129778079	T	snp	C	129778075	PHF17
4	138949556	138949564	T	snp	G	138949559	LOC641365
4	139102738	139102747	A	snp	G	139102744	SLC7A11
4	142640634	142640642	A	snp	G	142640636	IL15
4	143043164	143043172	A	snp	C	143043168	INPP4B
4	145792599	145792609	A	snp	C	145792601	BC044611
4	151356126	151356134	A	snp	G	151356129	LRBA
4	151771128	151771137	A	snp	C	151771133	LRBA
4	151829655	151829663	T	snp	C	151829658	LRBA
4	152330289	152330297	C	snp	T	152330293	FAM160A1
4	153875839	153875849	A	snp	G	153875845	FHDC1
4	154266314	154266322	C	snp	A	154266318	MND1
4	154515387	154515395	A	snp	G	154515392	KIAA0922
4	156290514	156290524	A	snp	C	156290516	MAP9

4	156765478	156765486	A	snp	C	156765481	ACCN5
4	156838133	156838141	A	snp	C	156838135	TDO2
4	158281519	158281529	T	snp	G	158281522	GRIA2
4	165031823	165031831	T	snp	G	165031829	MARCH1
4	166220099	166220107	C	snp	A	166220102	KLHL2
4	166262191	166262201	T	snp	C	166262192	MSMO1
4	170662484	170662494	T	snp	G	170662488	C4orf27
4	174087950	174087958	A	snp	C	174087951	BC040577
4	175838811	175838820	A	snp	G	175838815	ADAM29
4	178283754	178283763	T	snp	C	178283756	NEIL3
4	183810587	183810595	T	snp	C	183810588	DCTD
4	184619257	184619266	T	snp	C	184619259	TRAPPC11
4	186272015	186272024	A	snp	C	186272020	SNX25
4	187073865	187073873	T	snp	G	187073871	FAM149A
4	187130932	187130940	G	snp	T	187130938	CYP4V2
4	187529532	187529540	T	snp	G	187529537	FAT1
5	345056	345064	G	snp	T	345057	AHRR
5	1112986	1112996	C	snp	A	1112993	SLC12A7
5	1308860	1308868	T	snp	C	1308864	MIR4457
5	1494529	1494539	G	snp	T	1494531	LPCAT1
5	1501535	1501544	C	snp	A	1501541	LPCAT1
5	1627264	1627272	T	snp	G	1627266	LOC728613
5	5321096	5321105	T	snp	C	5321097	ADAMTS16
5	11383882	11383890	A	snp	C	11383883	CTNND2
5	13701161	13701170	A	snp	C	13701166	DNAH5
5	13876600	13876608	T	snp	C	13876604	DNAH5
5	14280134	14280142	T	snp	G	14280138	TRIO
5	21475326	21475334	T	snp	G	21475332	GUSBP1
5	32254949	32254957	A	snp	G	32254952	MTMR12
5	34939832	34939842	T	snp	G	34939833	DNAJC21
5	35036998	35037007	T	snp	C	35037005	AGXT2
5	35710659	35710667	A	snp	G	35710665	SPEF2
5	37184001	37184010	A	snp	G	37184005	C5orf42

5	37244793	37244803	A	snp	C	37244794	C5orf42
5	37479850	37479859	T	snp	G	37479852	WDR70
5	38483483	38483491	T	snp	C	38483488	LIFR
5	38923696	38923705	T	snp	C	38923697	OSMR
5	52224234	52224243	A	snp	C	52224240	ITGA1
5	61689740	61689748	T	snp	C	61689742	DIMT1
5	63985693	63985703	T	snp	C	63985699	FAM159B
5	64947473	64947481	G	snp	T	64947478	C5orf44
5	64961018	64961027	A	snp	G	64961019	C5orf44
5	64961018	64961027	A	snp	G	64961019	SGTB
5	67097161	67097171	A	snp	C	67097162	BC042046
5	70845810	70845818	T	snp	C	70845812	BDP1
5	72868631	72868640	T	snp	G	72868632	UTP15
5	76758544	76758552	A	snp	G	76758549	WDR41
5	77754702	77754710	A	snp	G	77754707	SCAMP1
5	78250337	78250345	T	snp	C	78250338	ARSB
5	79929168	79929177	T	snp	G	79929173	DHFR
5	80689730	80689738	C	snp	A	80689735	ACOT12
5	80689730	80689738	C	snp	A	80689735	RNU5E-1
5	82806967	82806977	T	snp	C	82806971	VCAN
5	82948212	82948221	A	snp	G	82948215	HAPLN1
5	90606009	90606018	A	snp	C	90606013	AK091866
5	96078043	96078053	T	snp	G	96078050	CAST
5	96120904	96120913	A	snp	G	96120909	ERAP1
5	118466048	118466057	T	snp	G	118466051	DMXL1
5	118573843	118573851	T	snp	C	118573845	DMXL1
5	122165207	122165216	T	snp	G	122165208	SNX2
5	122426943	122426952	A	snp	G	122426948	PRDM6
5	127855318	127855328	T	snp	C	127855319	FBN2
5	132035886	132035895	T	snp	G	132035890	KIF3A
5	132425554	132425562	A	snp	C	132425556	HSPA4
5	134788831	134788839	T	snp	C	134788834	TIFAB
5	137029309	137029317	A	snp	G	137029314	KLHL3

5	137029309	137029317	A	snp	G	137029314	MYOT
5	137620251	137620259	T	snp	C	137620253	CDC25C
5	139003270	139003280	T	snp	G	139003276	UBE2D2
5	140177430	140177440	T	snp	G	140177436	PCDHA1
5	140177430	140177440	T	snp	G	140177436	PCDHA2
5	140177430	140177440	T	snp	G	140177436	PCDHA2
5	146753692	146753701	A	snp	G	146753697	STK32A
5	146755559	146755567	T	snp	G	146755564	STK32A
5	149199057	149199066	T	snp	G	149199060	PPARGC1B
5	149389570	149389579	A	snp	G	149389572	HMGXB3
5	156679025	156679033	A	snp	G	156679028	ITK
5	157099564	157099573	A	snp	C	157099566	C5orf52
5	159842906	159842916	T	snp	C	159842911	SLU7
5	167379580	167379589	T	snp	C	167379587	ODZ2
5	176037576	176037584	C	snp	T	176037581	GPRIN1
5	176830622	176830630	G	snp	A	176830626	F12
5	177379531	177379540	C	snp	A	177379535	AK126616
5	179269516	179269526	A	snp	C	179269523	C5orf45
5	179269533	179269543	A	snp	C	179269535	C5orf45
6	2668340	2668350	T	snp	C	2668347	MYLK4
6	2769643	2769652	A	snp	G	2769648	WRNIP1
6	8041888	8041897	T	snp	C	8041891	EEF1E1
6	8041888	8041897	T	snp	C	8041891	MUTED
6	8041888	8041897	T	snp	C	8041891	TXNDC5
6	8064685	8064693	T	snp	G	8064691	EEF1E1
6	8064685	8064693	T	snp	G	8064691	MUTED
6	8064685	8064693	T	snp	G	8064691	TXNDC5
6	10628569	10628577	T	snp	G	10628572	GCNT2
6	20152618	20152626	A	snp	G	20152622	MBOAT1
6	21743285	21743295	T	snp	C	21743290	LINC00340
6	22190873	22190881	T	snp	G	22190875	LINC00340
6	24686761	24686769	T	snp	C	24686767	ACOT13
6	24701143	24701153	T	snp	C	24701145	ACOT13

6	24701143	24701153	T	snp	C	24701145	C6orf62
6	24810990	24811000	T	snp	C	24810992	FAM65B
6	25086043	25086052	A	snp	G	25086045	CMAHP
6	26424033	26424041	T	snp	C	26424039	BTN2A3P
6	26856598	26856606	A	snp	C	26856599	GUSBP2
6	27878735	27878745	T	snp	G	27878737	OR2B2
6	30628501	30628509	A	snp	C	30628505	DHX16
6	31677035	31677044	T	snp	G	31677036	ABHD16A
6	31677035	31677044	T	snp	G	31677036	LY6G6F
6	31690753	31690761	T	snp	G	31690758	C6orf25
6	32267217	32267225	A	snp	G	32267223	C6orf10
6	32604870	32604879	A	snp	G	32604875	HLA-DQA1
6	32605979	32605987	T	snp	G	32605981	HLA-DQA1
6	32630299	32630307	A	snp	C	32630302	HLA-DQB1
6	32630340	32630348	A	snp	C	32630343	HLA-DQB1
6	33219138	33219148	A	snp	C	33219141	HCG25
6	33219138	33219148	A	snp	C	33219141	HCG25
6	33219138	33219148	A	snp	C	33219141	HCG25
6	33219138	33219148	A	snp	C	33219141	VPS52
6	33219138	33219148	A	snp	C	33219141	VPS52
6	33219138	33219148	A	snp	C	33219141	VPS52
6	33233651	33233661	T	snp	G	33233653	VPS52
6	34204282	34204292	G	snp	A	34204284	HMGA1
6	34665437	34665445	G	snp	A	34665441	C6orf106
6	39854995	39855003	A	snp	C	39854999	AX747174
6	39854995	39855003	A	snp	C	39854999	DAAM2
6	39855016	39855024	A	snp	C	39855018	AX747174
6	39855016	39855024	A	snp	C	39855018	DAAM2
6	41105716	41105725	A	snp	C	41105720	LOC221442
6	41304929	41304938	A	snp	C	41304934	NCR2
6	41746363	41746371	C	snp	T	41746368	FRS3
6	42109822	42109830	G	snp	T	42109823	C6orf132
6	42985966	42985974	T	snp	C	42985972	KLHDC3

6	43529119	43529129	T	snp	C	43529123	XPO5
6	43591126	43591134	A	snp	G	43591130	GTPBP2
6	43973227	43973237	G	snp	T	43973231	AK024736
6	43973227	43973237	G	snp	T	43973231	C6orf223
6	44124010	44124019	A	snp	G	44124016	TMEM63B
6	46702599	46702607	A	snp	G	46702600	PLA2G7
6	53279095	53279105	T	snp	G	53279101	7SK
6	54187131	54187141	A	snp	C	54187134	TINAG
6	55216701	55216711	T	snp	G	55216708	GFRAL
6	58246662	58246671	A	snp	C	58246668	GUSBP4
6	58288064	58288072	T	snp	G	58288068	GUSBP4
6	69684540	69684549	A	snp	G	69684541	BAI3
6	70386647	70386657	A	snp	G	70386652	LMBRD1
6	71664618	71664627	G	snp	A	71664620	B3GAT2
6	73103271	73103279	A	snp	C	73103275	RIMS1
6	75899297	75899305	T	snp	G	75899299	COL12A1
6	83727187	83727196	A	snp	C	83727188	UBE2CBP
6	86349424	86349433	A	snp	G	86349431	SYNCRIP
6	88343956	88343966	T	snp	G	88343964	ORC3
6	93949362	93949371	A	snp	C	93949369	EPHA7
6	97346570	97346580	A	snp	C	97346576	NDUFAF4
6	99978984	99978992	C	snp	A	99978986	LOC100130890
6	101163428	101163436	A	snp	C	101163432	ASCC3
6	105574101	105574110	A	snp	C	105574105	BVES
6	105594389	105594397	A	snp	C	105594390	C6orf112
6	107017268	107017278	T	snp	G	107017269	AIM1
6	109312269	109312278	A	snp	C	109312275	SESN1
6	111898615	111898623	T	snp	C	111898619	TRAF3IP2
6	111898615	111898623	T	snp	C	111898619	TRAF3IP2-AS1
6	112114243	112114252	A	snp	G	112114248	FYN
6	116978187	116978196	A	snp	C	116978188	ZUFSP
6	117084152	117084162	A	snp	G	117084160	FAM162B
6	121460157	121460165	T	snp	C	121460160	C6orf170

6	122757806	122757815	A	snp	G	122757810	BC022047
6	123819070	123819078	A	snp	C	123819073	TRDN
6	126070724	126070732	G	snp	A	126070726	BC036196
6	126070724	126070732	G	snp	A	126070726	HEY2
6	128564416	128564425	A	snp	C	128564422	PTPRK
6	129898178	129898187	A	snp	G	129898181	ARHGAP18
6	129960364	129960372	A	snp	G	129960367	ARHGAP18
6	131276466	131276475	A	snp	G	131276472	EPB41L2
6	132169552	132169560	C	snp	A	132169558	ENPP1
6	132784346	132784355	A	snp	C	132784352	STX7
6	133109508	133109516	T	snp	G	133109509	C6orf192
6	136592896	136592904	T	snp	C	136592901	BCLAF1
6	137194083	137194091	T	snp	G	137194087	PEX7
6	137326801	137326809	T	snp	G	137326803	IL20RA
6	138644772	138644782	A	snp	C	138644774	KIAA1244
6	138644772	138644782	A	snp	C	138644777	KIAA1244
6	141005283	141005293	A	snp	C	141005284	MIR4465
6	141939628	141939636	C	snp	T	141939633	AK097143
6	142715977	142715985	C	snp	A	142715978	GPR126
6	146267869	146267877	A	snp	G	146267872	SHPRH
6	148662830	148662838	A	snp	C	148662833	SASH1
6	149722186	149722195	A	snp	C	149722188	SUMO4
6	149722186	149722195	A	snp	C	149722188	TAB2
6	150209823	150209831	A	snp	G	150209827	LOC100652739
6	150209823	150209831	A	snp	G	150209827	LOC100652739
6	150209823	150209831	A	snp	G	150209827	RAET1E
6	150209823	150209831	A	snp	G	150209827	RAET1E
6	150209823	150209831	A	snp	G	150209827	RAET1E
6	150383206	150383215	T	snp	C	150383212	ULBP3
6	151675302	151675310	A	snp	C	151675305	AKAP12
6	151870041	151870049	T	snp	G	151870044	C6orf97
6	152264525	152264534	A	snp	C	152264528	ESR1
6	152645129	152645138	A	snp	G	152645133	SYNE1

6	152688900	152688910	A	snp	G	152688906	SYNE1
6	155729279	155729287	T	snp	G	155729285	NOX3
6	155775874	155775882	T	snp	C	155775876	NOX3
6	158360176	158360186	A	snp	G	158360183	SNX9
6	159186212	159186221	G	snp	T	159186214	EZR
6	159186212	159186221	G	snp	T	159186214	MIR3918
6	159186212	159186221	G	snp	T	159186214	SYTL3
6	159210275	159210284	T	snp	C	159210282	EZR
6	160101530	160101538	T	snp	G	160101531	BC016015
6	160101530	160101538	T	snp	G	160101531	SOD2
6	160173383	160173392	T	snp	G	160173385	WTAP
6	160465464	160465474	T	snp	G	160465465	IGF2R
6	166843612	166843622	T	snp	G	166843619	RPS6KA2
6	167413536	167413544	T	snp	C	167413538	CCR6
6	167413536	167413544	T	snp	C	167413538	CCR6
6	167413536	167413544	T	snp	C	167413538	FGFR1OP
6	167413536	167413544	T	snp	C	167413538	FGFR1OP
6	167730023	167730032	C	snp	A	167730029	UNC93A
6	167738157	167738165	T	snp	G	167738158	TTLL2
6	167795017	167795026	T	snp	C	167795018	TCP10
6	170064772	170064782	A	snp	G	170064775	WDR27
7	1203624	1203633	T	snp	G	1203630	AK090593
7	1476518	1476526	C	snp	A	1476519	MICALL2
7	3083177	3083185	C	snp	A	3083182	CARD11
7	4006313	4006323	T	snp	G	4006317	SDK1
7	4780954	4780964	T	snp	G	4780960	FOXK1
7	5633992	5634001	G	snp	A	5633998	FSCN1
7	7457170	7457180	G	snp	A	7457178	COL28A1
7	7559634	7559643	A	snp	G	7559639	COL28A1
7	7571927	7571936	T	snp	G	7571928	COL28A1
7	7635162	7635171	T	snp	C	7635163	MIOS
7	8100233	8100241	T	snp	C	8100235	GLCCI1
7	11872524	11872534	A	snp	C	11872529	THSD7A

7	20437587	20437597	T	snp	G	20437590	ITGB8
7	21631676	21631684	A	snp	C	21631682	DNAH11
7	23234974	23234982	T	snp	G	23234980	NUPL2
7	26679247	26679255	C	snp	A	26679253	C7orf71
7	28220455	28220463	G	snp	T	28220456	JAZF1
7	28220455	28220463	G	snp	T	28220456	JAZF1-AS1
7	29440022	29440030	A	snp	G	29440023	CHN2
7	29551797	29551807	A	snp	C	29551802	BC038570
7	29551797	29551807	A	snp	C	29551802	CHN2
7	29551797	29551807	A	snp	C	29551802	CHN2
7	29720690	29720698	T	snp	C	29720695	LOC646762
7	29720690	29720698	T	snp	C	29720695	MIR550A3
7	32662406	32662414	A	snp	C	32662408	AVL9
7	32662406	32662414	A	snp	C	32662408	DPY19L1P1
7	35352491	35352500	A	snp	C	35352496	LOC401324
7	37137213	37137222	A	snp	G	37137218	ELMO1
7	37874424	37874433	T	snp	C	37874428	BC043356
7	38385747	38385755	G	snp	T	38385753	LOC100506776
7	43480196	43480204	A	snp	G	43480198	HECW1
7	48231587	48231596	T	snp	G	48231592	ABCA13
7	48451933	48451941	T	snp	C	48451938	ABCA13
7	50473604	50473612	T	snp	C	50473609	IKZF1
7	55233857	55233867	A	snp	C	55233862	EGFR
7	56086051	56086061	A	snp	C	56086053	PSPH
7	56150284	56150294	A	snp	G	56150288	PHKG1
7	56150284	56150294	A	snp	G	56150288	PHKG1
7	56150284	56150294	A	snp	G	56150288	PHKG1
7	56150284	56150294	A	snp	G	56150288	PHKG1
7	56150284	56150294	A	snp	G	56150288	PSPH
7	56150284	56150294	A	snp	G	56150288	PSPH
7	56150284	56150294	A	snp	G	56150288	PSPH
7	56150284	56150294	A	snp	G	56150288	PSPH
7	56496593	56496601	T	snp	G	56496595	LOC650226

7	57242222	57242230	T	snp	C	57242224	GUSBP10
7	57242222	57242230	T	snp	C	57242224	GUSBP10
7	57242222	57242230	T	snp	C	57242224	MtDNA_ssA
7	57242222	57242230	T	snp	C	57242224	TRNA
7	63983310	63983320	A	snp	G	63983314	ZNF680
7	66461205	66461213	A	snp	C	66461211	SBDS
7	66461205	66461213	A	snp	C	66461211	TYW1
7	66475136	66475144	T	snp	G	66475142	TYW1
7	70162546	70162556	C	snp	T	70162552	AUTS2
7	70228800	70228810	T	snp	C	70228804	AUTS2
7	73038472	73038482	C	snp	A	73038474	MLXIPL
7	73254462	73254471	G	snp	T	73254463	WBSCR27
7	73479395	73479404	C	snp	A	73479401	ELN
7	75102964	75102972	T	snp	G	75102966	POM121C
7	75141148	75141157	A	snp	C	75141151	PMS2P3
7	75186655	75186664	A	snp	C	75186659	HIP1
7	75620710	75620718	T	snp	G	75620713	TMEM120A
7	75988727	75988736	G	snp	T	75988730	YWHAG
7	76032664	76032672	T	snp	C	76032665	SRCRB4D
7	76032664	76032672	T	snp	C	76032665	ZP3
7	76610355	76610363	C	snp	A	76610361	DTX2P1-UPK3BP1-PMS2P11
7	76903784	76903792	T	snp	G	76903785	CCDC146
7	76910834	76910842	T	snp	C	76910839	CCDC146
7	77033934	77033942	A	snp	G	77033936	PION
7	80267903	80267912	G	snp	T	80267906	CD36
7	80394874	80394882	T	snp	G	80394877	SEMA3C
7	81659639	81659647	A	snp	G	81659640	AK055932
7	81659639	81659647	A	snp	G	81659640	CACNA2D1
7	81963998	81964006	A	snp	C	81964003	CACNA2D1
7	83024566	83024574	A	snp	C	83024569	SEMA3E
7	87021439	87021447	T	snp	C	87021444	CROT
7	87445746	87445754	G	snp	T	87445747	RUNDC3B
7	87445746	87445754	G	snp	T	87445752	RUNDC3B

7	87761451	87761461	T	snp	G	87761457	ADAM22
7	89866026	89866034	T	snp	C	89866030	STEAP2
7	90192427	90192435	T	snp	C	90192428	CDK14
7	90707921	90707931	A	snp	C	90707929	CDK14
7	94056143	94056152	A	snp	G	94056144	COL1A2
7	95219250	95219260	T	snp	G	95219251	PDK4
7	97940858	97940866	A	snp	G	97940861	BAIAP2L1
7	98784916	98784924	A	snp	C	98784917	KPNA7
7	98791957	98791966	T	snp	G	98791963	KPNA7
7	99009010	99009019	T	snp	G	99009013	BUD31
7	99720988	99720997	A	snp	C	99720993	CNPY4
7	100273498	100273506	C	snp	T	100273499	GNB2
7	100399234	100399242	A	snp	C	100399235	EPHB4
7	101958616	101958625	T	snp	C	101958621	SH2B2
7	102075973	102075981	C	snp	T	102075975	ORAI2
7	103160505	103160513	T	snp	G	103160510	RELN
7	103161590	103161599	T	snp	C	103161597	RELN
7	104438472	104438480	G	snp	T	104438473	LHFPL3
7	104438472	104438480	G	snp	T	104438473	LOC645591
7	105146864	105146874	T	snp	G	105146866	PUS7
7	105672823	105672832	T	snp	G	105672825	CDHR3
7	107414306	107414316	T	snp	G	107414313	SLC26A3
7	107577448	107577458	T	snp	C	107577455	LAMB1
7	115894369	115894377	T	snp	C	115894375	BD495725
7	115894369	115894377	T	snp	C	115894375	TES
7	117398600	117398608	T	snp	G	117398603	CTTNBP2
7	123611497	123611506	T	snp	C	123611498	SPAM1
7	124569378	124569387	T	snp	C	124569381	AX746567
7	124569378	124569387	T	snp	C	124569381	BC142949
7	124569378	124569387	T	snp	C	124569381	BX648695
7	124569378	124569387	T	snp	C	124569381	POT1
7	126891389	126891398	T	snp	G	126891391	GRM8
7	128411333	128411343	T	snp	G	128411339	CALU

7	128411333	128411343	T	snp	G	128411339	OPN1SW
7	128504835	128504845	T	snp	G	128504843	ATP6V1F
7	128504835	128504845	T	snp	G	128504843	ATP6V1F
7	128504835	128504845	T	snp	G	128504843	ATP6V1F
7	128504835	128504845	T	snp	G	128504843	KCP
7	128504835	128504845	T	snp	G	128504843	KCP
7	128504835	128504845	T	snp	G	128504843	KCP
7	128680726	128680735	T	snp	C	128680729	TNPO3
7	128697691	128697700	A	snp	C	128697696	TPI1P2
7	129906353	129906361	T	snp	G	129906359	CPA2
7	134853043	134853053	C	snp	A	134853045	C7orf49
7	135121947	135121955	T	snp	C	135121951	CNOT4
7	137559791	137559800	T	snp	C	137559798	CREB3L2
7	137585558	137585568	A	snp	G	137585561	CREB3L2
7	137790647	137790656	A	snp	C	137790649	AKR1D1
7	137790647	137790656	A	snp	C	137790652	AKR1D1
7	138767621	138767630	T	snp	G	138767626	ZC3HAV1
7	139026462	139026471	G	snp	T	139026464	C7orf55
7	139026462	139026471	G	snp	T	139026464	LUC7L2
7	139026462	139026471	G	snp	T	139026464	LUC7L2
7	139026462	139026471	G	snp	T	139026464	TRNA
7	139026462	139026471	G	snp	T	139026464	TRNA_Arg
7	140049338	140049348	G	snp	T	140049341	SLC37A3
7	140101019	140101028	T	snp	C	140101023	AK131347
7	140174237	140174245	G	snp	A	140174243	MKRN1
7	141765743	141765752	T	snp	G	141765744	MGAM
7	147074228	147074236	C	snp	A	147074234	CNTNAP2
7	147074228	147074236	C	snp	A	147074234	MIR548F4
7	147074228	147074236	C	snp	A	147074234	MIR548I4
7	148937473	148937483	C	snp	A	148937480	ZNF212
7	153755561	153755570	G	snp	T	153755564	AK127966
7	153755561	153755570	G	snp	T	153755564	DPP6
7	154737178	154737187	A	snp	G	154737179	LOC100132707

7	154737178	154737187	A	snp	G	154737179	LOC100132707
7	154737178	154737187	A	snp	G	154737179	PAXIP1
7	154737178	154737187	A	snp	G	154737179	PAXIP1
8	443956	443965	T	snp	C	443958	C8orf42
8	614174	614182	A	snp	G	614175	ERICH1
8	1771615	1771624	G	snp	A	1771616	ARHGEF10
8	1819518	1819527	A	snp	C	1819521	ARHGEF10
8	1819518	1819527	A	snp	C	1819521	BC047307
8	2793300	2793308	T	snp	C	2793301	CSMD1
8	6260751	6260759	T	snp	G	6260752	LOC100287015
8	6370833	6370843	T	snp	C	6370837	ANGPT2
8	6370833	6370843	T	snp	C	6370837	MCPH1
8	6379875	6379884	A	snp	G	6379877	ANGPT2
8	6379875	6379884	A	snp	G	6379877	MCPH1
8	6390158	6390167	T	snp	C	6390161	ANGPT2
8	6390158	6390167	T	snp	C	6390161	MCPH1
8	6692706	6692714	G	snp	T	6692707	LOC100652791
8	6692706	6692714	G	snp	T	6692707	LOC100652791
8	6692706	6692714	G	snp	T	6692707	XKR5
8	8654518	8654526	A	snp	C	8654520	MFHAS1
8	11929620	11929629	T	snp	G	11929624	LOC100133267
8	12176189	12176198	T	snp	G	12176193	LOC100133267
8	12176189	12176198	T	snp	G	12176193	LOC100506990
8	17271631	17271641	T	snp	G	17271633	MTMR7
8	17486422	17486431	T	snp	G	17486424	PDGFRL
8	17532373	17532382	A	snp	G	17532376	MTUS1
8	19688181	19688191	T	snp	C	19688187	INTS10
8	22292005	22292014	A	snp	C	22292012	SLC39A14
8	23712167	23712176	T	snp	G	23712173	STC1
8	24770524	24770533	T	snp	G	24770528	AK308605
8	24770524	24770533	T	snp	G	24770528	NEFM
8	25324671	25324681	T	snp	C	25324677	CDCA2
8	25324671	25324681	T	snp	C	25324677	PPP2R2A

8	26264688	26264697	T	snp	G	26264692	BNIP3L
8	27400523	27400532	C	snp	A	27400529	EPHX2
8	28970581	28970591	A	snp	G	28970585	AF086219
8	28970581	28970591	A	snp	G	28970585	KIF13B
8	35092779	35092788	G	snp	T	35092781	UNC5D
8	38033474	38033483	C	snp	A	38033475	BAG4
8	38033474	38033483	C	snp	A	38033475	LSM1
8	38676312	38676322	T	snp	C	38676314	TACC1
8	41572381	41572389	A	snp	G	41572384	ANK1
8	41572381	41572389	A	snp	G	41572384	ANK1
8	41572381	41572389	A	snp	G	41572384	ANK1
8	41572381	41572389	A	snp	G	41572384	NKX6-3
8	41572381	41572389	A	snp	G	41572384	NKX6-3
8	41572381	41572389	A	snp	G	41572384	NKX6-3
8	42402110	42402119	A	snp	G	42402111	C8orf40
8	56878345	56878353	T	snp	G	56878347	LYN
8	57131807	57131816	A	snp	C	57131808	CHCHD7
8	59170348	59170356	T	snp	C	59170352	BC032030
8	59328609	59328617	T	snp	G	59328612	UBXN2B
8	66514092	66514101	A	snp	G	66514096	ARMC1
8	67041079	67041088	A	snp	C	67041085	TRIM55
8	68964325	68964333	A	snp	C	68964326	PREX2
8	68985343	68985351	A	snp	C	68985347	PREX2
8	70414915	70414924	T	snp	C	70414918	SULF1
8	70593828	70593836	A	snp	G	70593834	SLCO5A1
8	71581553	71581561	G	snp	T	71581558	LACTB2
8	71581553	71581561	G	snp	T	71581558	XKR9
8	72932593	72932602	A	snp	G	72932596	LOC100132891
8	72932593	72932602	A	snp	G	72932596	TRPA1
8	73958739	73958748	T	snp	G	73958744	TERF1
8	76190005	76190014	A	snp	G	76190007	BC062758
8	77595635	77595643	C	snp	A	77595641	LOC100192378
8	77595635	77595643	C	snp	A	77595641	ZFHX4

8	79649697	79649706	A	snp	G	79649700	IL7
8	82440305	82440313	T	snp	G	82440310	FABP12
8	82572356	82572364	A	snp	C	82572362	IMPA1
8	92970148	92970156	A	snp	G	92970152	RUNX1T1
8	95182984	95182994	T	snp	C	95182985	CDH17
8	95471549	95471558	T	snp	G	95471550	RAD54B
8	96060472	96060481	T	snp	C	96060479	C8orf38
8	96064849	96064858	T	snp	C	96064856	C8orf38
8	100588605	100588614	T	snp	G	100588609	VPS13B
8	101206173	101206183	T	snp	G	101206174	SPAG1
8	102385840	102385848	T	snp	C	102385841	AK291701
8	104389630	104389638	A	snp	C	104389635	CTHRC1
8	104479421	104479429	T	snp	C	104479422	BX641143
8	104898497	104898505	T	snp	G	104898499	RIMS2
8	110566177	110566185	T	snp	G	110566183	EBAG9
8	113811440	113811449	A	snp	C	113811441	CSMD3
8	113959816	113959825	A	snp	G	113959819	CSMD3
8	117659334	117659342	T	snp	C	117659338	EIF3H
8	118326032	118326041	A	snp	C	118326037	SNORA31
8	120258403	120258411	A	snp	C	120258404	MAL2
8	127571348	127571356	T	snp	G	127571354	FAM84B
8	131455461	131455470	C	snp	A	131455468	ASAP1
8	131811739	131811748	T	snp	C	131811741	ADCY8
8	133492917	133492926	C	snp	A	133492920	KCNQ3
8	133765016	133765026	A	snp	G	133765024	TMEM71
8	133960424	133960432	C	snp	T	133960429	TG
8	142443021	142443029	G	snp	T	142443022	FLJ43860
8	142490160	142490168	C	snp	T	142490165	FLJ43860
8	143425266	143425274	C	snp	A	143425270	TSNARE1
8	143620141	143620149	C	snp	T	143620145	BAI1
8	143621143	143621152	C	snp	A	143621149	BAI1
8	144406041	144406049	C	snp	A	144406042	TOP1MT
8	144669503	144669513	T	snp	G	144669511	EEF1D

8	145026033	145026041	C	snp	A	145026039	PLEC
8	145602159	145602167	C	snp	T	145602165	ADCK5
8	145690955	145690963	G	snp	T	145690959	CYHR1
8	145690955	145690963	G	snp	T	145690959	KIFC2
8	145735724	145735732	C	snp	T	145735725	MFSD3
8	145735724	145735732	C	snp	T	145735725	RECQL4
8	146004106	146004114	T	snp	C	146004110	ZNF34
9	504960	504969	G	snp	A	504966	KANK1
9	686580	686589	A	snp	C	686581	KANK1
9	732052	732061	G	snp	A	732054	KANK1
9	2109787	2109795	T	snp	C	2109791	SMARCA2
9	4834291	4834301	T	snp	G	4834298	RCL1
9	6556450	6556459	A	snp	G	6556454	GLDC
9	14113901	14113911	A	snp	C	14113902	NFIB
9	14119892	14119900	A	snp	G	14119893	NFIB
9	14773643	14773652	A	snp	G	14773647	FREM1
9	14788035	14788045	T	snp	C	14788039	FREM1
9	15579739	15579747	T	snp	G	15579743	C9orf93
9	18904697	18904705	A	snp	C	18904698	ADAMTSL1
9	21207298	21207306	T	snp	G	21207299	IFNA10
9	21207298	21207306	T	snp	G	21207299	IFNA14
9	26115557	26115566	T	snp	C	26115559	LOC100506422
9	27283246	27283255	T	snp	C	27283248	LINC00032
9	32494613	32494623	A	snp	C	32494617	DDX58
9	36665333	36665342	T	snp	C	36665334	MELK
9	37523381	37523390	A	snp	C	37523382	FBXO10
9	37791273	37791281	A	snp	C	37791277	AK098263
9	37915860	37915870	A	snp	G	37915866	SHB
9	68742275	68742283	A	snp	G	68742276	LOC100132352
9	69649333	69649341	A	snp	C	69649337	BC070322
9	74331368	74331377	A	snp	C	74331375	TMEM2
9	74968284	74968294	A	snp	C	74968287	ZFAND5
9	75538274	75538282	T	snp	C	75538277	ALDH1A1

9	78639193	78639201	T	snp	C	78639195	PCSK5
9	88692059	88692069	A	snp	C	88692063	GOLM1
9	93640290	93640298	T	snp	C	93640293	SYK
9	94710845	94710854	C	snp	A	94710846	ROR2
9	94710845	94710854	C	snp	A	94710849	ROR2
9	95100597	95100607	A	snp	C	95100598	CENPP
9	100263829	100263838	G	snp	A	100263834	TMOD1
9	101894355	101894363	T	snp	G	101894360	TGFBR1
9	101985597	101985605	A	snp	C	101985601	SEC61B
9	103003069	103003078	T	snp	C	103003075	INVS
9	104240202	104240210	G	snp	A	104240203	C9orf125
9	111691066	111691075	A	snp	G	111691069	IKBKAP
9	112010204	112010212	T	snp	G	112010207	EPB41L4B
9	112779168	112779177	T	snp	C	112779169	AKAP2
9	112779168	112779177	T	snp	C	112779169	PALM2-AKAP2
9	113546864	113546874	A	snp	G	113546869	MUSK
9	114178400	114178409	T	snp	G	114178401	KIAA0368
9	114431140	114431150	T	snp	C	114431147	DNAJC25-GNG10
9	114431140	114431150	T	snp	C	114431147	GNG10
9	115448191	115448199	A	snp	C	115448194	C9orf80
9	115955547	115955555	T	snp	C	115955552	FKBP15
9	115973613	115973621	T	snp	G	115973619	FKBP15
9	116169299	116169307	A	snp	C	116169302	POLE3
9	116818031	116818039	A	snp	C	116818032	ZNF618
9	117880145	117880154	A	snp	G	117880148	TNC
9	119801876	119801885	A	snp	G	119801878	ASTN2
9	125016348	125016356	A	snp	C	125016349	RBM18
9	125590010	125590019	A	snp	G	125590014	PDCL
9	129565990	129565999	A	snp	C	129565994	AX747444
9	129565990	129565999	A	snp	C	129565994	ZBTB43
9	130187860	130187869	T	snp	G	130187865	ZNF79
9	130634621	130634629	G	snp	A	130634623	AK1
9	130700955	130700963	T	snp	C	130700959	DPM2

9	131456518	131456527	T	snp	G	131456519	SET
9	131456518	131456527	T	snp	G	131456519	SET
9	131456518	131456527	T	snp	G	131456519	Y16709
9	131456518	131456527	T	snp	G	131456520	SET
9	131456518	131456527	T	snp	G	131456520	SET
9	131456518	131456527	T	snp	G	131456520	Y16709
9	131621645	131621655	A	snp	C	131621647	CCBL1
9	132576658	132576666	C	snp	A	132576664	TOR1A
9	133541150	133541158	C	snp	A	133541155	PRDM12
9	134006474	134006483	T	snp	G	134006480	NUP214
9	135157440	135157448	A	snp	G	135157442	SETX
9	136659826	136659834	G	snp	A	136659832	VAV2
9	137775825	137775833	G	snp	T	137775828	FCN2
9	137966925	137966935	C	snp	T	137966930	OLFM1
9	138456317	138456326	T	snp	C	138456322	PAEP
9	139397312	139397321	T	snp	C	139397313	NOTCH1
9	140290119	140290128	G	snp	T	140290120	EXD3
9	140499265	140499273	G	snp	T	140499266	ARRDC1
9	140632645	140632653	A	snp	C	140632650	EHMT1
X	1315929	1315938	A	snp	C	1315935	CRLF2
X	2699898	2699907	T	snp	G	2699905	XG
X	14868798	14868808	A	snp	G	14868805	FANCB
X	18277874	18277882	A	snp	G	18277879	SCML2
X	18607051	18607061	A	snp	C	18607053	CDKL5
X	19014006	19014015	T	snp	G	19014010	GPR64
X	19500369	19500379	A	snp	C	19500377	MAP3K15
X	24231005	24231014	T	snp	G	24231008	ZFX
X	27480403	27480411	A	snp	C	27480408	SMEK3P
X	38660108	38660117	A	snp	C	38660112	MID1IP1
X	41073729	41073739	A	snp	G	41073733	USP9X
X	41079310	41079318	T	snp	C	41079315	USP9X
X	43638034	43638043	A	snp	G	43638037	MAOB
X	47342912	47342921	C	snp	A	47342919	ZNF41

X	48435396	48435404	T	snp	C	48435401	RBM3
X	48565500	48565509	C	snp	A	48565507	SUV39H1
X	49452829	49452837	A	snp	C	49452832	PAGE1
X	53675473	53675482	A	snp	C	53675477	HUWE1
X	53675483	53675492	A	snp	C	53675487	HUWE1
X	55246034	55246044	T	snp	G	55246040	PAGE5
X	67263222	67263232	A	snp	G	67263228	OPHN1
X	69642813	69642822	C	snp	A	69642820	GDPD2
X	70837776	70837785	C	snp	T	70837783	BCYRN1
X	70837776	70837785	C	snp	T	70837783	BCYRN1
X	70837776	70837785	C	snp	T	70837783	CXCR3
X	70837776	70837785	C	snp	T	70837783	CXCR3
X	70838048	70838056	C	snp	T	70838053	BCYRN1
X	70838048	70838056	C	snp	T	70838053	BCYRN1
X	70838048	70838056	C	snp	T	70838053	CXCR3
X	70838048	70838056	C	snp	T	70838053	CXCR3
X	74743316	74743325	C	snp	A	74743323	ZDHHC15
X	84534381	84534391	A	snp	C	84534382	POF1B
X	100534956	100534966	A	snp	C	100534958	TAF7L
X	100534956	100534966	A	snp	C	100534961	TAF7L
X	100764432	100764441	T	snp	G	100764438	ARMCX4
X	101093565	101093574	G	snp	T	101093566	NXF5
X	107315483	107315491	T	snp	G	107315489	VSIG1
X	109601253	109601261	A	snp	C	109601259	AMMECR1
X	109601253	109601261	A	snp	C	109601259	RGAG1
X	110763286	110763294	A	snp	G	110763292	DKFZp686D0853
X	115571357	115571366	A	snp	G	115571361	SLC6A14
X	117750513	117750523	T	snp	G	117750521	DOCK11
X	119065169	119065179	A	snp	C	119065172	NKAP
X	119067903	119067911	A	snp	C	119067906	NKAP
X	119248491	119248500	A	snp	G	119248496	AK123976
X	119248491	119248500	A	snp	G	119248496	RHOXF1
X	122846843	122846852	A	snp	C	122846849	THOC2

X	125683148	125683157	A	snp	G	125683155	DCAF12L1
X	129272439	129272448	A	snp	G	129272440	AIFM1
X	129649093	129649102	A	snp	C	129649094	BC043223
X	135049991	135050001	T	snp	G	135049998	MMGT1
X	135382745	135382755	T	snp	C	135382748	GPR112
X	138285669	138285679	G	snp	A	138285674	FGF13
X	148045421	148045429	A	snp	C	148045427	AFF2
X	149826092	149826102	A	snp	G	149826100	MTM1
X	151122554	151122562	C	snp	A	151122555	GABRE
X	153137316	153137324	C	snp	T	153137321	L1CAM
X	153714027	153714037	G	snp	T	153714029	UBL4A
10	854664	854680	GT	snp	A	854674	LARP4B
10	1206093	1206107	AT	snp	C	1206101	LINC00200
10	5090560	5090570	TG	snp	C	5090566	AKR1C3
10	5978790	5978800	AG	snp	G	5978794	FBXO18
10	6879582	6879592	TA	snp	C	6879584	LOC100507127
10	12257683	12257693	TA	snp	A	12257685	CDC123
10	15889065	15889075	AT	snp	C	15889071	FAM188A
10	16873849	16873861	TG	snp	G	16873853	CUBN
10	20357543	20357555	TA	snp	G	20357548	PLXDC2
10	23322117	23322129	AT	snp	G	23322125	ARMC3
10	24755891	24755905	AT	snp	G	24755897	KIAA1217
10	32750680	32750692	AT	snp	G	32750688	CCDC7
10	44356823	44356833	GT	snp	A	44356827	LOC100506835
10	46245305	46245319	TA	snp	T	46245310	FAM21C
10	49609021	49609033	TC	snp	G	49609028	MAPK8
10	49930702	49930712	CA	snp	T	49930708	WDFY4
10	55944220	55944232	TG	snp	A	55944223	PCDH15
10	72135329	72135347	AC	snp	G	72135332	LRRC20
10	72300041	72300055	TG	snp	T	72300050	KIAA1274
10	72433020	72433038	GT	snp	A	72433024	ADAMTS14
10	73574402	73574418	AC	snp	G	73574404	CDH23
10	75203008	75203026	AG	snp	A	75203023	PPP3CB

10	76853489	76853503	AC	snp	A	76853496	DUSP13
10	81449164	81449174	AC	snp	T	81449168	LOC650623
10	88718828	88718838	CA	snp	A	88718832	SNCG
10	90500373	90500387	TG	snp	A	90500380	LIPK
10	90674434	90674452	AT	snp	A	90674439	STAMBPL1
10	91179688	91179698	TG	snp	A	91179693	IFIT5
10	99477906	99477916	GT	snp	C	99477912	MARVELD1
10	99477906	99477916	GT	snp	C	99477912	NR_026754
10	102036079	102036089	CT	snp	C	102036084	BLOC1S2
10	104639651	104639667	TA	snp	T	104639664	AS3MT
10	108380890	108380900	GT	snp	T	108380894	SORCS1
10	112679142	112679152	GA	snp	G	112679149	BBIP1
10	112679142	112679152	GA	snp	G	112679149	SHOC2
10	115423329	115423339	CA	snp	G	115423334	NRAP
10	116083292	116083302	CA	snp	G	116083295	AFAP1L2
10	123892371	123892383	CA	snp	T	123892375	TACC2
10	124321182	124321194	TC	snp	G	124321191	DMBT1
10	129241942	129241952	TG	snp	C	129241946	DOCK1
10	135037482	135037498	GT	snp	T	135037494	KNDC1
10	135151344	135151356	AC	snp	G	135151348	CALY
10	135151344	135151356	AC	snp	G	135151348	ZNF511
11	320389	320403	CA	snp	T	320393	BC040735
11	320389	320403	CA	snp	T	320393	BC040735
11	320389	320403	CA	snp	T	320393	IFITM3
11	320389	320403	CA	snp	T	320393	IFITM3
11	614364	614374	TG	snp	C	614366	IRF7
11	1781783	1781795	TG	snp	C	1781789	CTSD
11	1781783	1781795	TG	snp	C	1781789	CTSD
11	1781783	1781795	TG	snp	C	1781789	MOB2
11	1781783	1781795	TG	snp	C	1781789	MOB2
11	8647114	8647128	AC	snp	T	8647123	TRIM66
11	15502911	15502921	TG	snp	C	15502913	SnoMBII_202
11	17125164	17125178	TG	snp	A	17125167	PIK3C2A

11	34668638	34668652	CA	snp	C	34668641	EHF
11	45727008	45727018	TA	snp	G	45727011	DQ585129
11	45727008	45727018	TA	snp	G	45727011	DQ594272
11	45727008	45727018	TA	snp	G	45727011	DQ600737
11	47361828	47361840	CA	snp	G	47361837	MYBPC3
11	57154521	57154535	GA	snp	C	57154529	PRG2
11	57822447	57822457	TG	snp	C	57822449	OR9Q1
11	58909399	58909415	AG	snp	A	58909412	BC028022
11	58909399	58909415	AG	snp	A	58909412	FAM111A
11	60292162	60292174	TC	snp	G	60292171	MS4A13
11	61039773	61039785	AC	snp	T	61039782	VWCE
11	61285525	61285535	CA	snp	A	61285527	SYT7
11	62429577	62429591	AT	snp	A	62429580	C11orf48
11	63232757	63232771	AG	snp	G	63232763	HRASLS5
11	66279474	66279490	AT	snp	A	66279477	BBS1
11	66279535	66279547	TA	snp	C	66279543	BBS1
11	72535841	72535851	TG	snp	C	72535846	ATG16L2
11	73141364	73141376	GT	snp	G	73141369	FAM168A
11	74061964	74061982	AG	snp	A	74061979	PGM2L1
11	77376800	77376814	AC	snp	A	77376805	RSF1
11	83166708	83166722	GT	snp	G	83166719	DLG2
11	87845961	87845979	AT	snp	C	87845974	RAB38
11	88910344	88910362	GA	snp	G	88910349	TYR
11	89185478	89185488	TC	snp	T	89185481	NOX4
11	99828623	99828633	TA	snp	G	99828630	CNTN5
11	104869429	104869439	TG	snp	A	104869432	CASP5
11	107926341	107926357	AT	snp	A	107926344	CUL5
11	110481750	110481760	TA	snp	G	110481755	ARHGAP20
11	112109177	112109195	TA	snp	C	112109187	PTS
11	117887072	117887088	AG	snp	T	117887083	LOC100526771
11	123066024	123066034	CT	snp	G	123066031	CLMP
11	125480643	125480657	CA	snp	A	125480653	STT3A
11	126327281	126327291	GT	snp	T	126327287	KIRREL3

11	128992824	128992834	AT	snp	C	128992827	ARHGAP32
11	130340014	130340026	AT	snp	C	130340019	ADAMTS15
12	3429767	3429777	CA	snp	T	3429771	AK095365
12	3579361	3579371	TG	snp	A	3579368	DQ590289
12	3579361	3579371	TG	snp	A	3579368	DQ591331
12	3579361	3579371	TG	snp	A	3579368	DQ599425
12	3579361	3579371	TG	snp	A	3579368	PRMT8
12	3579361	3579371	TG	snp	A	3579368	PRMT8
12	3579361	3579371	TG	snp	A	3579368	PRMT8
12	3737212	3737224	CT	snp	C	3737215	EFCAB4B
12	6629357	6629373	AT	snp	A	6629360	NCAPD2
12	7034263	7034273	TG	snp	C	7034267	ATN1
12	7047139	7047149	GC	snp	A	7047142	ATN1
12	7970335	7970349	AT	snp	G	7970342	SLC2A14
12	8627922	8627936	TA	snp	C	8627925	CLEC6A
12	9021018	9021032	TG	snp	C	9021020	A2ML1
12	9310553	9310569	TC	snp	T	9310564	PZP
12	10168551	10168565	AC	snp	G	10168562	CLEC12B
12	10780601	10780611	TG	snp	T	10780608	STYK1
12	10871664	10871674	AC	snp	G	10871666	CSDA
12	11508875	11508885	TG	snp	C	11508878	PRB1
12	11548851	11548861	TG	snp	C	11548854	PRB2
12	15806751	15806761	TG	snp	A	15806756	EPS8
12	20889651	20889665	AC	snp	T	20889659	SLCO1C1
12	31299114	31299126	TA	snp	A	31299120	OVOS2
12	31299114	31299126	TA	snp	G	31299123	OVOS2
12	40692719	40692729	TA	snp	G	40692722	LRRK2
12	40940479	40940491	GT	snp	G	40940486	MUC19
12	49165397	49165409	TC	snp	T	49165404	ADCY6
12	49165397	49165409	TC	snp	T	49165404	MIR4701
12	75897576	75897588	AT	snp	C	75897581	KRR1
12	78583327	78583339	AC	snp	G	78583335	NAV3
12	82780233	82780243	CT	snp	T	82780235	C12orf26

12	86272995	86273005	TA	snp	G	86273000	NTS
12	91574163	91574179	AG	snp	A	91574174	DCN
12	93209721	93209733	AG	snp	C	93209726	EEA1
12	96398656	96398666	TC	snp	C	96398660	LTA4H
12	96896906	96896916	TC	snp	A	96896913	C12orf55
12	98896617	98896633	CA	snp	C	98896620	LOC643770
12	98896617	98896633	CA	snp	C	98896620	LOC643770
12	98896617	98896633	CA	snp	C	98896620	TRNA_Asp
12	101699389	101699407	TA	snp	G	101699404	UTP20
12	102148288	102148300	GA	snp	A	102148290	GNPTAB
12	103696899	103696909	CA	snp	T	103696906	C12orf42
12	104300927	104300943	AG	snp	A	104300930	GNN
12	104396750	104396766	AG	snp	C	104396763	GLT8D2
12	113716734	113716752	GT	snp	G	113716737	TPCN1
12	114385062	114385078	AC	snp	G	114385065	RBM19
12	116998057	116998069	TA	snp	G	116998060	MAP1LC3B2
12	117702303	117702317	TC	snp	T	117702306	NOS1
12	120590465	120590477	AT	snp	C	120590472	GCN1L1
12	121449268	121449278	TA	snp	C	121449270	C12orf43
12	122293618	122293634	AC	snp	G	122293622	HPD
12	123958075	123958087	AT	snp	G	123958077	RILPL1
12	123958075	123958087	AT	snp	G	123958077	SNRNP35
12	124978766	124978780	AC	snp	G	124978772	NCOR2
13	22068415	22068429	TC	snp	C	22068425	EFHA1
13	23870300	23870310	AT	snp	G	23870302	SGCG
13	23945531	23945541	AT	snp	C	23945534	SACS
13	24241207	24241225	TG	snp	C	24241215	TNFRSF19
13	25072734	25072744	TA	snp	C	25072740	PARP4
13	32798034	32798046	TG	snp	A	32798041	FRY
13	36920501	36920511	CG	snp	T	36920503	SPG20
13	36920501	36920511	CG	snp	T	36920503	SPG20
13	36920501	36920511	CG	snp	T	36920503	SPG20OS
13	36920501	36920511	CG	snp	T	36920503	SPG20OS

13	46155324	46155334	TA	snp	C	46155328	FAM194B
13	49070799	49070809	CA	snp	T	49070803	RCBTB2
13	49934041	49934051	AT	snp	C	49934044	CAB39L
13	51077089	51077099	TA	snp	G	51077094	BCMS
13	51077089	51077099	TA	snp	G	51077094	BCMS
13	51077089	51077099	TA	snp	G	51077094	DLEU1
13	51077089	51077099	TA	snp	G	51077096	BCMS
13	51077089	51077099	TA	snp	G	51077096	BCMS
13	51077089	51077099	TA	snp	G	51077096	DLEU1
13	76445400	76445418	TC	snp	G	76445412	AX747676
13	79933924	79933942	AT	snp	C	79933931	RBM26
13	88332062	88332072	TA	snp	C	88332066	SLITRK5
13	91150752	91150762	TC	snp	T	91150755	BC038529
13	96416975	96416987	AT	snp	C	96416978	DNAJC3
13	101753609	101753619	AT	snp	C	101753614	NALCN
13	110858224	110858236	TA	snp	C	110858230	COL4A1
13	111566723	111566737	CG	snp	G	111566731	ANKRD10
13	111953796	111953806	TG	snp	A	111953801	ARHGEF7
13	114289142	114289156	TG	snp	C	114289148	TFDP1
13	114757356	114757366	CA	snp	T	114757360	RASA3
14	21092781	21092795	AG	snp	C	21092786	TRNA_Leu
14	21791945	21791963	GT	snp	A	21791947	RPGRIP1
14	23535331	23535343	GA	snp	A	23535335	ACIN1
14	34931276	34931286	CG	snp	A	34931281	SPTSSA
14	36075603	36075613	TG	snp	A	36075610	RALGAPA1
14	39562966	39562976	AT	snp	C	39562971	SEC23A
14	47426124	47426136	CT	snp	A	47426129	MDGA2
14	55641412	55641424	AT	snp	G	55641420	DLGAP5
14	60074632	60074644	AG	snp	T	60074635	RTN1
14	63849647	63849657	TA	snp	C	63849649	PPP2R5E
14	65232611	65232625	TG	snp	G	65232615	SPTB
14	65556073	65556083	TC	snp	C	65556075	LOC100506321
14	65556073	65556083	TC	snp	C	65556075	MAX

14	69922028	69922040	TG	snp	T	69922035	SLC39A9
14	70420729	70420743	GT	snp	A	70420736	SMOC1
14	70988983	70988999	AG	snp	C	70988992	ADAM20
14	76421822	76421832	CT	snp	G	76421827	TTLL5
14	78325270	78325282	GT	snp	T	78325276	ADCK1
14	81728001	81728011	AT	snp	G	81728007	STON2
14	87386833	87386843	TC	snp	G	87386840	LOC283585
14	88634030	88634040	TG	snp	A	88634033	DQ599616
14	88858643	88858653	AT	snp	G	88858649	SPATA7
14	93360097	93360109	CT	snp	G	93360099	AK093301
14	94674047	94674059	TA	snp	C	94674050	PPP4R4
14	100408310	100408320	TA	snp	T	100408313	EML1
14	100604328	100604338	GA	snp	G	100604333	EVL
14	101378067	101378081	TA	snp	G	101378078	Mir_370
14	101528240	101528256	AG	snp	T	101528248	MIR377
14	102030523	102030533	AG	snp	C	102030526	DIO3
14	102817131	102817145	TC	snp	G	102817134	CINP
14	104095763	104095773	CG	snp	T	104095768	KLC1
14	106688389	106688399	TA	snp	G	106688396	abParts
14	106913829	106913845	TC	snp	T	106913842	abParts
14	107165970	107165984	TC	snp	T	107165981	abParts
15	22709777	22709787	TC	snp	C	22709781	abParts
15	22709777	22709787	TC	snp	C	22709781	abParts
15	22709777	22709787	TC	snp	C	22709781	abParts
15	22709777	22709787	TC	snp	C	22709781	abParts
15	22709777	22709787	TC	snp	C	22709781	GOLGA8DP
15	22709777	22709787	TC	snp	C	22709781	GOLGA8DP
15	22709777	22709787	TC	snp	C	22709781	GOLGA8DP
15	22709777	22709787	TC	snp	C	22709781	GOLGA8DP
15	22856080	22856098	AT	snp	C	22856093	TUBGCP5
15	31352099	31352111	CA	snp	T	31352105	TRPM1
15	38231093	38231107	GA	snp	A	38231101	TMCO5A
15	48417488	48417502	TA	snp	C	48417495	SLC24A5

15	48739862	48739876	GA	snp	T	48739867	FBN1
15	50932362	50932376	AC	snp	T	50932373	TRPM7
15	52028909	52028919	AC	snp	G	52028913	LYSMD2
15	57973962	57973972	AG	snp	A	57973965	GCOM1
15	57973962	57973972	AG	snp	A	57973965	GCOM1
15	57973962	57973972	AG	snp	A	57973965	MYZAP
15	57973962	57973972	AG	snp	A	57973965	MYZAP
15	57973962	57973972	AG	snp	A	57973965	MYZAP
15	57973962	57973972	AG	snp	A	57973965	POLR2M
15	63028182	63028192	AT	snp	G	63028188	TLN2
15	63030391	63030401	TC	snp	C	63030397	TLN2
15	63341745	63341757	TG	snp	T	63341748	AK310237
15	63341745	63341757	TG	snp	T	63341748	AX747619
15	63341745	63341757	TG	snp	T	63341748	TPM1
15	63341745	63341757	TG	snp	T	63341748	TPM1
15	72874925	72874941	AT	snp	T	72874937	ARIH1
15	75978614	75978630	CA	snp	G	75978619	CSPG4
15	76023634	76023646	AC	snp	G	76023636	DNM1P35
15	76023634	76023646	AC	snp	G	76023636	DNM1P35
15	76023634	76023646	AC	snp	G	76023636	ODF3L1
15	78450790	78450806	AT	snp	C	78450793	IDH3A
15	79031198	79031208	TA	snp	G	79031203	DQ586415
15	80036498	80036516	GA	snp	A	80036500	TRNA_Cys
15	91841486	91841498	TC	snp	T	91841493	SV2B
16	420899	420909	AT	snp	C	420906	MRPL28
16	420899	420909	AT	snp	C	420906	TMEM8A
16	928876	928886	GA	snp	C	928878	LMF1
16	1114742	1114754	AG	snp	G	1114750	LOC146336
16	1389744	1389756	CA	snp	G	1389750	BAIAP3
16	1390533	1390547	CA	snp	C	1390544	BAIAP3
16	1656927	1656939	CA	snp	T	1656935	IFT140
16	1657004	1657016	AC	snp	T	1657011	IFT140
16	3101198	3101212	TG	snp	T	3101207	BC045731

16	3101198	3101212	TG	snp	T	3101207	MMP25
16	3101198	3101212	TG	snp	T	3101207	MMP25
16	3209542	3209560	TC	snp	T	3209545	TRNA_Pro
16	3215741	3215753	GA	snp	C	3215749	TRNA_Lys
16	3529763	3529781	TG	snp	A	3529778	NAA60
16	3576556	3576568	AT	snp	G	3576559	CLUAP1
16	3576556	3576568	AT	snp	G	3576561	CLUAP1
16	4737522	4737532	AC	snp	C	4737528	MGRN1
16	4828806	4828818	AT	snp	C	4828809	SEPT12
16	4882393	4882403	AT	snp	C	4882396	GLYR1
16	8799116	8799132	GA	snp	C	8799120	ABAT
16	8799116	8799132	GA	snp	C	8799120	U7
16	11072519	11072529	GT	snp	C	11072524	CLEC16A
16	11272568	11272578	TG	snp	A	11272571	CLEC16A
16	11585534	11585544	CA	snp	T	11585538	AK126539
16	11815958	11815970	TC	snp	T	11815963	TXNDC11
16	18839166	18839176	TA	snp	A	18839170	SMG1
16	18862714	18862730	AC	snp	G	18862726	SMG1
16	19091993	19092003	AT	snp	C	19091996	COQ7
16	20374770	20374782	GT	snp	C	20374779	PDILT
16	20482746	20482756	AC	snp	G	20482750	ACSM2A
16	23312735	23312751	GA	snp	A	23312745	SCNN1B
16	23521096	23521108	GT	snp	G	23521101	GGA2
16	27899918	27899930	TC	snp	T	27899925	GSG1L
16	48386254	48386266	TA	snp	G	48386263	LONP2
16	48386254	48386266	TA	snp	G	48386263	MIR548AE2
16	50702259	50702269	TC	snp	G	50702264	AF143871
16	50702259	50702269	TC	snp	G	50702264	SNX20
16	56602109	56602127	AG	snp	A	56602120	MT4
16	56602109	56602127	AG	snp	A	56602124	MT4
16	57691510	57691520	TG	snp	C	57691515	GPR56
16	57830658	57830672	CA	snp	T	57830665	KIFC3
16	58429225	58429241	TA	snp	T	58429238	GINS3

16	58622173	58622183	GA	snp	A	58622177	CNOT1
16	68054784	68054798	TA	snp	G	68054787	DDX28
16	68054784	68054798	TA	snp	G	68054787	DUS2L
16	72197609	72197619	TA	snp	C	72197615	PMFBP1
16	72818443	72818459	AT	snp	G	72818455	AK021563
16	72818443	72818459	AT	snp	G	72818455	ZFHX3
16	74566091	74566109	AT	snp	G	74566106	GLG1
16	81410312	81410328	AC	snp	T	81410315	GAN
16	89705261	89705275	CA	snp	T	89705266	DPEP1
16	89705359	89705371	CA	snp	G	89705362	DPEP1
16	89980313	89980327	TG	snp	C	89980315	BC160930
17	6356444	6356462	CT	snp	T	6356454	PITPNM3
17	6558634	6558648	GT	snp	A	6558644	MIR4520A
17	6558634	6558648	GT	snp	A	6558644	MIR4520B
17	7644546	7644556	TC	snp	T	7644553	DNAH2
17	8300905	8300917	TC	snp	C	8300913	RNF222
17	8366124	8366136	TG	snp	C	8366128	NDEL1
17	8743395	8743405	AT	snp	T	8743399	PIK3R6
17	9569130	9569140	AG	snp	G	9569136	USP43
17	10435819	10435831	AT	snp	C	10435822	AK097500
17	10435819	10435831	AT	snp	C	10435822	AK097500
17	10435819	10435831	AT	snp	C	10435822	AK097500
17	10435819	10435831	AT	snp	C	10435822	MYH2
17	10435819	10435831	AT	snp	C	10435822	MYH2
17	10435819	10435831	AT	snp	C	10435822	MYH2
17	15587339	15587349	CA	snp	G	15587344	TRIM16
17	21477314	21477326	CA	snp	T	21477323	C17orf51
17	27401054	27401064	CA	snp	T	27401060	MYO18A
17	27401054	27401064	CA	snp	T	27401060	TIAF1
17	28943054	28943066	AT	snp	G	28943063	LRRC37BP1
17	28950530	28950540	TA	snp	C	28950534	LRRC37BP1
17	28950530	28950540	TA	snp	C	28950534	SH3GL1P2
17	29206419	29206437	TG	snp	A	29206432	ATAD5

17	32596519	32596533	AG	snp	C	32596530	CCL7
17	33761368	33761382	AG	snp	A	33761371	SLFN13
17	34341995	34342011	TA	snp	G	34341998	CCL23
17	36627165	36627175	AT	snp	C	36627168	ARHGAP23
17	36669310	36669320	TA	snp	A	36669312	ARHGAP23
17	38506450	38506464	TG	snp	C	38506456	RARA
17	38922718	38922732	TC	snp	T	38922729	KRT26
17	39020880	39020892	CA	snp	G	39020886	KRT12
17	39136306	39136316	AG	snp	A	39136311	KRT40
17	39929039	39929051	CT	snp	C	39929042	JUP
17	40553660	40553674	GA	snp	A	40553670	PTRF
17	40557682	40557696	TC	snp	A	40557690	PTRF
17	40557682	40557696	TC	snp	G	40557692	PTRF
17	41225765	41225783	TA	snp	T	41225780	BRCA1
17	41862429	41862443	AG	snp	A	41862432	C17orf105
17	45534640	45534650	AT	snp	C	45534645	MRPL45P2
17	45906662	45906680	AG	snp	A	45906665	MRPL10
17	48128034	48128044	AC	snp	G	48128036	LOC284080
17	48349383	48349395	AT	snp	G	48349391	TMEM92
17	53801050	53801060	AT	snp	C	53801053	TMEM100
17	60469440	60469452	AT	snp	A	60469449	EFCAB3
17	60882162	60882180	AC	snp	T	60882171	MARCH10
17	63154037	63154053	TG	snp	C	63154048	RGS9
17	66132777	66132793	AT	snp	G	66132790	LOC100499466
17	66930192	66930202	TA	snp	G	66930195	ABCA8
17	67160182	67160192	AT	snp	C	67160185	ABCA10
17	67214799	67214809	AT	snp	C	67214804	ABCA10
17	76793740	76793750	CA	snp	T	76793746	USP36
17	76919458	76919468	GT	snp	A	76919464	TIMP2
17	77705883	77705897	AT	snp	G	77705893	ENPP7
17	78145100	78145114	TC	snp	A	78145106	CARD14
17	78298596	78298606	TG	snp	T	78298599	RNF213
17	78316533	78316543	TA	snp	G	78316536	RNF213

18	157911	157929	TA	snp	A	157921	USP14
18	3253617	3253635	GT	snp	A	3253627	MYL12A
18	7042093	7042105	CA	snp	G	7042098	LAMA1
18	12007723	12007735	TC	snp	T	12007726	IMPA2
18	14852496	14852510	AT	snp	C	14852501	ANKRD30B
18	21375681	21375691	AT	snp	T	21375687	LAMA3
18	28917679	28917695	AG	snp	A	28917692	DSG1
18	29782401	29782413	CA	snp	T	29782403	MEP1B
18	32918631	32918641	AG	snp	C	32918638	ZNF24
18	43515636	43515646	CA	snp	G	43515638	EPG5
18	45376875	45376885	TA	snp	G	45376880	SMAD2
18	51880585	51880599	AT	snp	A	51880588	STARD6
18	56415612	56415624	TA	snp	G	56415618	MALT1
18	57569712	57569726	GT	snp	T	57569716	PMAIP1
18	64172785	64172795	AT	snp	C	64172788	CDH19
19	864042	864060	CA	snp	G	864057	CFD
19	1085060	1085070	AG	snp	T	1085066	HMHA1
19	1085060	1085070	AG	snp	T	1085066	HMHA1
19	1085060	1085070	AG	snp	T	1085066	POLR2E
19	1085448	1085458	TC	snp	C	1085454	HMHA1
19	1085448	1085458	TC	snp	C	1085454	POLR2E
19	2350453	2350463	TC	snp	G	2350457	SPPL2B
19	2645225	2645241	TC	snp	C	2645229	GNG7
19	3055064	3055074	GT	snp	C	3055069	AES
19	4230914	4230924	TC	snp	C	4230920	EBI3
19	4254531	4254543	GT	snp	G	4254536	CCDC94
19	4326042	4326052	AC	snp	G	4326049	STAP2
19	4655695	4655707	AT	snp	C	4655702	TNFAIP8L1
19	5743678	5743692	CT	snp	G	5743687	TMEM146
19	6429071	6429085	TA	snp	C	6429073	SLC25A41
19	6589104	6589114	CT	snp	G	6589107	CD70
19	6663506	6663516	TG	snp	A	6663511	TNFSF14
19	6710559	6710575	GA	snp	A	6710571	C3

19	7566745	7566763	AG	snp	G	7566751	C19orf45
19	7767885	7767899	AG	snp	A	7767894	FCER2
19	8199859	8199875	AT	snp	T	8199871	FBN3
19	8278946	8278960	CA	snp	G	8278955	CERS4
19	10183392	10183404	AG	snp	C	10183399	C3P1
19	11409054	11409064	CA	snp	T	11409060	TSPAN16
19	11469387	11469397	CT	snp	T	11469393	LPPR2
19	12428304	12428314	TG	snp	G	12428306	ZNF563
19	13250334	13250348	GT	snp	A	13250339	NACC1
19	14847477	14847487	AT	snp	G	14847482	EMR2
19	15635511	15635521	AG	snp	A	15635518	CYP4F22
19	17006695	17006711	GC	snp	T	17006699	CPAMD8
19	17017110	17017120	CT	snp	C	17017113	CPAMD8
19	17056987	17057001	TG	snp	A	17056998	CPAMD8
19	19307207	19307219	AT	snp	G	19307215	RFXANK
19	20295140	20295156	TG	snp	A	20295145	ZNF486
19	20808511	20808521	AT	snp	A	20808514	ZNF626
19	21202563	21202573	AT	snp	A	21202566	ZNF430
19	21509767	21509777	TA	snp	C	21509773	ZNF708
19	21579139	21579151	TC	snp	T	21579142	ZNF493
19	29881999	29882011	AG	snp	A	29882002	LOC284395
19	29881999	29882011	AG	snp	A	29882006	LOC284395
19	29886872	29886884	AC	snp	G	29886874	LOC284395
19	33111141	33111153	CA	snp	G	33111147	ANKRD27
19	33610965	33610977	TA	snp	T	33610974	GPATCH1
19	35629218	35629230	GT	snp	G	35629225	FXYD1
19	35629218	35629230	GT	snp	G	35629225	LGI4
19	36367793	36367803	TC	snp	G	36367798	APLP1
19	37289521	37289531	AT	snp	G	37289527	LOC284408
19	38826182	38826200	AT	snp	A	38826185	CATSPERG
19	41187573	41187587	AT	snp	G	41187583	NUMBL
19	42127484	42127494	CT	snp	A	42127487	CEACAM4
19	44841451	44841463	AT	snp	C	44841460	ZFP112

19	45032851	45032869	AC	snp	G	45032866	CEACAM20
19	45568153	45568163	TC	snp	T	45568160	CLASRP
19	47923778	47923792	AG	snp	C	47923787	MEIS3
19	49206105	49206115	CA	snp	G	49206107	FUT2
19	50118386	50118396	AG	snp	C	50118389	PRR12
19	50733514	50733532	AG	snp	T	50733527	MYH14
19	50764144	50764156	TG	snp	C	50764146	MYH14
19	51826528	51826542	TG	snp	C	51826533	IGLON5
19	52223262	52223272	CT	snp	C	52223265	HAS1
19	52870808	52870822	GT	snp	A	52870814	ZNF610
19	53238759	53238769	TC	snp	C	53238763	ZNF611
19	53356602	53356612	AT	snp	C	53356609	ZNF468
19	54190794	54190804	AG	snp	T	54190797	MIR1283-1
19	54190794	54190804	AG	snp	T	54190797	MIR519C
19	54562173	54562183	AT	snp	C	54562176	VSTM1
19	55287689	55287707	TA	snp	C	55287693	KIR2DL1
19	55287689	55287707	TA	snp	C	55287693	KIR2DL2
19	55287689	55287707	TA	snp	C	55287693	KIR2DL3
19	55287689	55287707	TA	snp	C	55287693	KIR2DL3
19	55287689	55287707	TA	snp	C	55287693	KIR2DS4
19	55332902	55332918	GA	snp	A	55332906	KIR2DS4
19	55332902	55332918	GA	snp	A	55332906	KIR3DL1
19	55332902	55332918	GA	snp	A	55332906	KIR3DL1
19	55902251	55902261	GA	snp	G	55902256	RPL28
19	56006583	56006601	AT	snp	G	56006593	SSC5D
19	56187091	56187101	TC	snp	T	56187096	EPN1
19	58546470	58546482	CA	snp	T	58546474	ZSCAN1
1	4001785	4001797	TC	snp	G	4001794	LOC728716
1	4002094	4002108	TC	snp	A	4002097	LOC728716
1	4475209	4475219	TC	snp	A	4475213	LOC284661
1	6604572	6604588	AC	snp	G	6604584	NOL9
1	10065181	10065193	AC	snp	A	10065190	RBP7
1	12027697	12027715	TA	snp	G	12027706	PLOD1

1	12027697	12027715	TA	snp	T	12027712	PLOD1
1	16045965	16045979	AG	snp	T	16045968	PLEKHM2
1	18435092	18435102	GT	snp	A	18435094	IGSF21
1	18619378	18619388	CA	snp	T	18619382	IGSF21
1	20666016	20666026	AG	snp	C	20666022	VWA5B1
1	22303589	22303599	TG	snp	C	22303592	CELA3B
1	27433853	27433865	AC	snp	T	27433858	SLC9A1
1	28056138	28056148	TC	snp	A	28056145	FAM76A
1	34083627	34083641	AG	snp	C	34083632	CSMD2
1	36180968	36180980	AC	snp	T	36180971	C1orf216
1	38185403	38185419	AC	snp	G	38185408	EPHA10
1	38221000	38221016	CA	snp	G	38221005	EPHA10
1	39491559	39491569	TC	snp	T	39491564	NDUFS5
1	43031410	43031426	TG	snp	G	43031412	CCDC30
1	45364028	45364040	TC	snp	T	45364033	EIF2B3
1	46093678	46093694	TG	snp	A	46093691	GPBP1L1
1	46158829	46158839	AT	snp	C	46158832	IPP
1	46158829	46158839	AT	snp	C	46158832	TMEM69
1	48648844	48648854	AT	snp	C	48648849	SKINTL
1	51933629	51933641	TA	snp	C	51933635	EPS15
1	53109550	53109566	AG	snp	C	53109553	FAM159A
1	53503870	53503880	TA	snp	G	53503873	SCP2
1	54509636	54509646	AT	snp	C	54509643	TMEM59
1	55189432	55189442	CA	snp	T	55189434	HEATR8-TTC4
1	55189432	55189442	CA	snp	T	55189434	TTC4
1	60223711	60223721	AC	snp	T	60223715	FGGY
1	62732164	62732174	CA	snp	T	62732169	KANK4
1	62738005	62738015	AC	snp	T	62738012	KANK4
1	64560231	64560243	TG	snp	A	64560234	BC040909
1	64560231	64560243	TG	snp	A	64560234	ROR1
1	65099457	65099467	TG	snp	A	65099460	CACHD1
1	67358736	67358748	AG	snp	A	67358739	WDR78
1	67392604	67392616	TA	snp	G	67392613	MIER1

1	67558756	67558766	AT	snp	G	67558762	C1orf141
1	76081086	76081100	CT	snp	A	76081088	SLC44A5
1	76350326	76350336	AC	snp	A	76350331	MSH4
1	85562141	85562155	TA	snp	G	85562151	WDR63
1	86847116	86847132	AC	snp	G	86847120	ODF2L
1	89293354	89293368	CT	snp	A	89293362	PKN2
1	89293354	89293368	CT	snp	A	89293364	PKN2
1	89301240	89301252	AT	snp	T	89301242	PKN2
1	92735353	92735363	AT	snp	C	92735358	GLMN
1	92788632	92788648	TA	snp	T	92788643	RPAP2
1	94219918	94219928	AG	snp	A	94219925	BCAR3
1	94219918	94219928	AG	snp	A	94219925	MIG7
1	94317477	94317493	GA	snp	G	94317490	AX746627
1	94549026	94549036	AT	snp	G	94549028	ABCA4
1	94696132	94696142	TA	snp	C	94696138	ARHGAP29
1	95293457	95293469	AG	snp	T	95293466	SLC44A3
1	98166769	98166779	AT	snp	C	98166771	DPYD
1	100617762	100617772	CT	snp	C	100617765	LRRC39
1	107682153	107682163	TA	snp	C	107682159	NTNG1
1	111059812	111059830	GA	snp	A	111059824	KCNA10
1	113554894	113554906	GA	snp	A	113554898	BC037540
1	113554894	113554906	GA	snp	T	113554901	BC037540
1	114339126	114339138	AT	snp	C	114339132	RSBN1
1	115089079	115089093	AT	snp	G	115089090	DENND2C
1	116311366	116311382	CA	snp	G	116311379	CASQ2
1	117213888	117213898	AG	snp	A	117213895	MIR320B1
1	117213912	117213930	AG	snp	A	117213915	MIR320B1
1	145375100	145375112	TA	snp	C	145375102	AX747132
1	150319751	150319765	AC	snp	G	150319761	PRPF3
1	152850902	152850912	TG	snp	A	152850905	SMCP
1	152958830	152958842	AC	snp	G	152958832	SPRR1A
1	152958830	152958842	AC	snp	T	152958839	SPRR1A
1	152976909	152976921	AG	snp	A	152976918	SPRR3

1	155268950	155268960	TC	snp	T	155268957	PKLR
1	156642738	156642748	AG	snp	C	156642743	NES
1	156842024	156842034	AT	snp	C	156842029	NTRK1
1	157096246	157096258	AT	snp	G	157096252	ETV3
1	157665675	157665693	TC	snp	C	157665679	FCRL3
1	158747500	158747512	TC	snp	G	158747505	OR6N2
1	159775908	159775918	AG	snp	C	159775915	FCRL6
1	160156023	160156033	TC	snp	G	160156026	ATP1A4
1	160605138	160605150	GC	snp	T	160605143	SLAMF1
1	161590688	161590698	TA	snp	C	161590692	TRNA_Asn
1	161640883	161640893	GA	snp	C	161640885	FCGR2B
1	161761054	161761064	AT	snp	G	161761061	ATF6
1	162767063	162767077	AC	snp	G	162767066	HSD17B7
1	164558504	164558514	CA	snp	T	164558511	PBX1
1	175300235	175300245	TC	snp	T	175300238	TNR
1	177251785	177251795	GA	snp	A	177251791	FAM5B
1	183875612	183875628	AT	snp	G	183875616	RGL1
1	185151789	185151799	CT	snp	G	185151795	SWT1
1	185926575	185926585	TA	snp	G	185926578	HMCN1
1	186863693	186863711	AG	snp	G	186863701	PLA2G4A
1	196747661	196747673	AT	snp	C	196747668	CFHR1
1	196747661	196747673	AT	snp	C	196747668	CFHR3
1	196747661	196747673	AT	snp	C	196747668	CFHR4
1	197575394	197575408	CA	snp	C	197575397	DENND1B
1	198665296	198665314	AT	snp	G	198665311	PTPRC
1	200310898	200310910	GT	snp	C	200310901	C1orf98
1	202575901	202575913	GA	snp	C	202575907	SYT2
1	204081658	204081668	TC	snp	T	204081665	SOX13
1	204966740	204966752	AC	snp	G	204966748	NFASC
1	212980263	212980275	AT	snp	T	212980271	TATDN3
1	213125697	213125713	TG	snp	T	213125700	VASH2
1	215748144	215748154	TA	snp	G	215748147	KCTD3
1	215748144	215748154	TA	snp	G	215748151	KCTD3

1	216404575	216404591	AT	snp	G	216404581	USH2A
1	226818407	226818419	AG	snp	G	226818413	ITPKB
1	227770485	227770499	TG	snp	A	227770496	ZNF678
1	229437651	229437661	TG	snp	A	229437658	RAB4A
1	232172767	232172777	GA	snp	C	232172773	DISC1
1	233353498	233353512	TA	snp	G	233353507	PCNXL2
1	236576713	236576723	TC	snp	T	236576716	EDARADD
1	237063497	237063507	AT	snp	G	237063503	MTR
1	246929093	246929103	CT	snp	G	246929099	SCCPDH
1	247693340	247693358	GT	snp	A	247693350	C1orf150
1	247693340	247693358	GT	snp	A	247693350	LOC148824
1	247693340	247693358	GT	snp	A	247693350	OR2C3
20	415586	415596	GC	snp	T	415591	DQ588114
20	415586	415596	GC	snp	T	415591	TBC1D20
20	8866154	8866164	GA	snp	C	8866161	PLCB1
20	13140426	13140436	CA	snp	T	13140430	SPTLC3
20	15001930	15001940	TG	snp	A	15001937	MACROD2
20	15001930	15001940	TG	snp	A	15001937	U6
20	15967530	15967544	GT	snp	A	15967532	MACROD2
20	19791508	19791522	TG	snp	T	19791511	BC090059
20	21346724	21346734	TA	snp	G	21346731	XRN2
20	23470913	23470925	TA	snp	A	23470917	CST8
20	30620662	30620672	TG	snp	C	30620668	C20orf160
20	32000344	32000354	AG	snp	G	32000350	SNTA1
20	36530676	36530686	CA	snp	G	36530682	VSTM2L
20	37230372	37230384	GT	snp	T	37230378	ARHGAP40
20	37277265	37277275	TC	snp	T	37277268	ARHGAP40
20	37277265	37277275	TC	snp	T	37277270	ARHGAP40
20	41933333	41933345	AG	snp	A	41933336	SCARNA15
20	44429014	44429024	AT	snp	C	44429017	DNTTIP1
20	44563832	44563850	GC	snp	T	44563843	PCIF1
20	44692166	44692176	CT	snp	G	44692169	NCOA5
20	44846067	44846079	CA	snp	T	44846071	CDH22

20	50809374	50809384	CT	snp	T	50809380	ZFP64
20	55917248	55917266	GT	snp	A	55917251	MIR5095
20	55917248	55917266	GT	snp	A	55917251	SPO11
20	57264849	57264859	CA	snp	G	57264853	NPEPL1
20	61524125	61524135	AC	snp	T	61524132	DIDO1
20	62163783	62163797	GT	snp	C	62163792	PTK6
21	17554887	17554903	TC	snp	T	17554900	LINC00478
21	23108905	23108917	AC	snp	G	23108911	LINC00317
21	31962181	31962197	TC	snp	T	31962194	KRTAP22-2
21	34168783	34168797	AG	snp	A	34168786	C21orf49
21	34168783	34168797	AG	snp	A	34168786	C21orf62
21	35883738	35883752	TC	snp	A	35883742	KCNE1
21	35883738	35883752	TC	snp	G	35883743	KCNE1
21	40603839	40603849	AT	snp	C	40603844	BRWD1
21	42709734	42709748	GT	snp	C	42709736	FAM3B
21	43483216	43483226	TC	snp	T	43483223	UMODL1
21	46066315	46066325	CA	snp	T	46066318	KRTAP10-11
21	46066315	46066325	CA	snp	T	46066318	TSPEAR
21	46116859	46116869	AC	snp	G	46116863	KRTAP10-12
21	46116859	46116869	AC	snp	G	46116863	TSPEAR
21	46281994	46282008	GT	snp	A	46281997	PTTG1IP
22	17150673	17150689	CT	snp	T	17150675	ANKRD62P1-PARP4P3
22	17150673	17150689	CT	snp	T	17150675	ANKRD62P1-PARP4P3
22	17150673	17150689	CT	snp	T	17150675	TPTEP1
22	17150673	17150689	CT	snp	T	17150675	TPTEP1
22	17663801	17663811	AC	snp	G	17663803	CECR1
22	20818275	20818287	TC	snp	T	20818278	KLHL22
22	24096939	24096957	CA	snp	G	24096942	VPREB3
22	24407619	24407631	CG	snp	A	24407624	CABIN1
22	25587924	25587934	TA	snp	G	25587929	KIAA1671
22	29075763	29075773	GC	snp	T	29075768	TTC28
22	29709352	29709362	GC	snp	A	29709358	GAS2L1
22	29709352	29709362	GC	snp	A	29709358	RASL10A

22	30151315	30151329	GA	snp	G	30151326	ZMAT5
22	30187694	30187710	AC	snp	T	30187701	ASCC2
22	30407378	30407390	TC	snp	G	30407387	MTMR3
22	32080942	32080954	CA	snp	A	32080948	PRR14L
22	32500480	32500490	CA	snp	T	32500484	SLC5A1
22	32545637	32545655	AT	snp	A	32545640	C22orf42
22	37710562	37710572	GA	snp	T	37710568	CYTH4
22	44554942	44554954	GA	snp	C	44554951	PARVB
22	45128756	45128766	CA	snp	T	45128762	ARHGAP8
22	45128756	45128766	CA	snp	T	45128762	ARHGAP8
22	45128756	45128766	CA	snp	T	45128762	PRR5
22	45128756	45128766	CA	snp	T	45128762	PRR5
22	45128756	45128766	CA	snp	T	45128762	PRR5-ARHGAP8
22	45128756	45128766	CA	snp	T	45128762	PRR5-ARHGAP8
22	45920985	45921001	AG	snp	A	45920998	FBLN1
22	47117187	47117201	AT	snp	T	47117197	CERK
22	48938527	48938537	GT	snp	C	48938533	FAM19A5
22	48938527	48938537	GT	snp	C	48938533	LOC284933
22	51010833	51010843	TC	snp	A	51010837	BC048192
22	51010833	51010843	TC	snp	A	51010837	CHKB
22	51010833	51010843	TC	snp	A	51010837	CHKB
22	51010833	51010843	TC	snp	A	51010837	CHKB-CPT1B
22	51010833	51010843	TC	snp	A	51010837	CHKB-CPT1B
22	51010833	51010843	TC	snp	A	51010837	CPT1B
22	51010833	51010843	TC	snp	A	51010837	CPT1B
2	10138727	10138737	AT	snp	C	10138732	GRHL1
2	10919554	10919568	AT	snp	C	10919557	ATP6V1C2
2	11810711	11810723	TG	snp	C	11810715	NTSR2
2	20823851	20823861	CT	snp	T	20823857	HS1BP3
2	20900827	20900837	TG	snp	C	20900831	C2orf43
2	26784890	26784900	CA	snp	G	26784893	C2orf70
2	27527129	27527145	AT	snp	G	27527142	TRIM54
2	31604836	31604846	GT	snp	C	31604843	XDH

2	31638586	31638600	AC	snp	G	31638594	Mir_584
2	31638586	31638600	AC	snp	G	31638594	XDH
2	33505582	33505594	AC	snp	G	33505588	LTBP1
2	33621252	33621266	GT	snp	A	33621262	LTBP1
2	37264288	37264302	AT	snp	G	37264293	HEATR5B
2	37264288	37264302	AT	snp	G	37264296	HEATR5B
2	39054699	39054713	AC	snp	T	39054702	DHX57
2	39412502	39412512	AT	snp	G	39412506	CDKL4
2	39412556	39412566	AT	snp	G	39412560	CDKL4
2	44161454	44161464	AT	snp	G	44161456	LRPPRC
2	44540540	44540550	TG	snp	A	44540547	SLC3A1
2	47302450	47302460	TA	snp	G	47302455	C2orf61
2	47302450	47302460	TA	snp	G	47302455	TTC7A
2	47302450	47302460	TA	snp	G	47302455	TTC7A
2	54082255	54082269	CT	snp	C	54082260	GPR75
2	54082255	54082269	CT	snp	C	54082260	GPR75-ASB3
2	55407296	55407312	AT	snp	G	55407306	C2orf63
2	55516585	55516597	GA	snp	C	55516587	CCDC88A
2	61721951	61721965	TA	snp	G	61721961	XPO1
2	66667181	66667199	CG	snp	T	66667185	MEIS1
2	73913158	73913172	CT	snp	G	73913168	ALMS1P
2	87114260	87114270	TA	snp	G	87114263	LOC100286979
2	87114260	87114270	TA	snp	G	87114263	LOC100286979
2	87114260	87114270	TA	snp	G	87114263	RMND5A
2	87114260	87114270	TA	snp	G	87114263	RMND5A
2	89373790	89373800	CT	snp	G	89373792	abParts
2	95511833	95511847	TA	snp	C	95511843	ANKRD20A8P
2	95976923	95976933	CA	snp	T	95976927	KCNIP3
2	99775861	99775879	TC	snp	G	99775864	LIPT1
2	99775861	99775879	TC	snp	G	99775864	MRPL30
2	102809526	102809540	CA	snp	G	102809533	IL1RL2
2	108945271	108945281	TG	snp	C	108945273	SULT1C2P1
2	109092543	109092555	TC	snp	G	109092545	GCC2

2	111471333	111471345	CA	snp	T	111471339	DQ579470
2	113978280	113978290	GT	snp	A	113978287	PAX8
2	119912803	119912819	AC	snp	G	119912813	C1QL2
2	120019946	120019956	TA	snp	T	120019949	STEAP3
2	120442827	120442841	AC	snp	G	120442832	TMEM177
2	120442827	120442841	AC	snp	G	120442837	TMEM177
2	121732417	121732427	AC	snp	T	121732424	GLI2
2	132266731	132266741	CT	snp	A	132266737	LOC150776
2	135625399	135625415	TA	snp	C	135625407	ACMSD
2	135625399	135625415	TA	snp	C	135625407	LOC100129961
2	150425404	150425422	AT	snp	C	150425419	MMADHC
2	153546795	153546809	AT	snp	C	153546798	PRPF40A
2	160731224	160731234	AT	snp	C	160731231	LY75
2	160731224	160731234	AT	snp	C	160731231	LY75
2	160731224	160731234	AT	snp	C	160731231	LY75-CD302
2	166768165	166768179	AT	snp	T	166768175	TTC21B
2	166868772	166868782	AT	snp	G	166868774	SCN1A
2	169745273	169745285	TA	snp	C	169745277	SPC25
2	169853319	169853329	AT	snp	A	169853322	ABCB11
2	171692992	171693002	TA	snp	C	171692994	GAD1
2	179192180	179192190	AG	snp	T	179192187	OSBPL6
2	183829231	183829241	TA	snp	C	183829235	NCKAP1
2	187501641	187501651	AT	snp	G	187501647	ITGAV
2	187702356	187702366	TA	snp	G	187702363	ZSWIM2
2	192290990	192291000	TG	snp	T	192290997	MYO1B
2	197586077	197586093	AT	snp	C	197586084	CCDC150
2	197862145	197862159	TA	snp	C	197862153	ANKRD44
2	198669102	198669114	CG	snp	A	198669109	PLCL1
2	201347489	201347505	TG	snp	T	201347492	SPATS2L
2	202213377	202213387	CA	snp	A	202213381	ALS2CR12
2	207946712	207946726	TG	snp	T	207946721	KLF7
2	208443361	208443375	TG	snp	A	208443371	CREB1
2	220502040	220502050	TG	snp	C	220502042	SLC4A3

2	233470171	233470187	AG	snp	G	233470183	EFHD1
2	233673654	233673666	AT	snp	C	233673657	GIGYF2
3	8681721	8681731	CA	snp	G	8681723	C3orf32
3	12232973	12232989	GT	snp	A	12232983	SYN2
3	12944789	12944799	CA	snp	A	12944791	IQSEC1
3	13611478	13611490	TG	snp	C	13611486	FBLN2
3	13759382	13759398	CT	snp	G	13759385	LOC285375
3	15778812	15778822	GT	snp	T	15778818	ANKRD28
3	20141251	20141267	AG	snp	C	20141264	KAT2B
3	20160263	20160273	AT	snp	T	20160265	KAT2B
3	30052613	30052629	AT	snp	G	30052616	RBMS3
3	32030099	32030113	CT	snp	C	32030110	ZNF860
3	32579429	32579439	AT	snp	T	32579431	DYNC1LI1
3	36934343	36934361	AT	snp	G	36934352	TRANK1
3	36934343	36934361	AT	snp	C	36934353	TRANK1
3	38173808	38173820	TC	snp	C	38173812	ACAA1
3	38691858	38691868	AC	snp	G	38691860	SCN5A
3	39168034	39168050	CA	snp	T	39168046	TTC21A
3	39168034	39168050	CA	snp	G	39168047	TTC21A
3	42601672	42601682	AT	snp	C	42601677	SEC22C
3	42741498	42741516	TG	snp	C	42741511	HHATL
3	50306629	50306639	CA	snp	T	50306631	SEMA3B
3	52131251	52131261	GT	snp	C	52131255	POC1A
3	52273418	52273428	GC	snp	A	52273420	BC039681
3	52273418	52273428	GC	snp	A	52273420	TWF2
3	53125466	53125476	GA	snp	A	53125468	RFT1
3	58620097	58620107	AG	snp	A	58620104	FAM3D
3	65479523	65479533	GA	snp	C	65479529	MAGI1
3	69236719	69236731	AT	snp	C	69236724	FRMD4B
3	82513668	82513684	TA	snp	A	82513678	BC031255
3	82513740	82513750	TA	snp	A	82513744	BC031255
3	100977407	100977421	CT	snp	T	100977409	IMPG2
3	108116832	108116844	CA	snp	T	108116834	MYH15

3	108288015	108288029	TA	snp	C	108288025	KIAA1524
3	111637039	111637049	TG	snp	A	111637044	PHLDB2
3	113898519	113898529	TC	snp	G	113898524	DRD3
3	122843199	122843211	GT	snp	A	122843203	PDIA5
3	124117163	124117175	TG	snp	A	124117165	KALRN
3	124483081	124483091	TC	snp	G	124483088	ITGB5
3	138007032	138007044	TG	snp	A	138007037	ARMC8
3	138007032	138007044	TG	snp	A	138007037	NME9
3	141688074	141688088	AT	snp	G	141688084	TFDP2
3	142053553	142053563	TA	snp	G	142053560	XRN1
3	142150734	142150744	AT	snp	G	142150736	XRN1
3	145820681	145820693	GT	snp	A	145820683	PLOD2
3	162920228	162920240	CA	snp	G	162920237	BC073807
3	162920228	162920240	CA	snp	G	162920237	LOC647107
3	167251342	167251352	GT	snp	C	167251347	WDR49
3	167507447	167507457	AG	snp	G	167507451	SERPINI1
3	168865503	168865521	GT	snp	A	168865509	MECOM
3	169830167	169830183	TA	snp	C	169830173	PHC3
3	171323297	171323307	AT	snp	C	171323304	PLD1
3	182584708	182584720	AT	snp	C	182584715	ATP11B
4	653361	653371	GT	snp	A	653363	PDE6B
4	763998	764010	CA	snp	G	764007	DKFZp547K2416
4	763998	764010	CA	snp	G	764007	PCGF3
4	1190899	1190909	TC	snp	C	1190903	AX747178
4	1190899	1190909	TC	snp	C	1190903	LOC100130872
4	1190899	1190909	TC	snp	C	1190903	SPON2
4	1221130	1221140	GT	snp	C	1221135	CTBP1
4	2657174	2657192	TG	snp	C	2657187	FAM193A
4	5838189	5838199	CA	snp	T	5838191	CRMP1
4	6272300	6272310	CT	snp	C	6272305	WFS1
4	8039021	8039031	CA	snp	G	8039028	ABLIM2
4	12249079	12249089	TG	snp	C	12249084	BC042433
4	14473175	14473185	GT	snp	A	14473179	BC070495

4	14473175	14473185	GT	snp	A	14473179	MGC4836
4	14979385	14979395	AT	snp	G	14979391	LOC441009
4	15706189	15706199	TG	snp	A	15706192	BST1
4	16257401	16257413	GA	snp	C	16257405	FLJ39653
4	17632830	17632844	AT	snp	C	17632833	CR936688
4	17632830	17632844	AT	snp	C	17632833	FAM184B
4	22458063	22458073	CT	snp	C	22458068	GPR125
4	38994368	38994382	TA	snp	T	38994379	TMEM156
4	42456678	42456696	GT	snp	A	42456680	ATP8A1
4	48106576	48106588	AT	snp	T	48106578	TXK
4	48135774	48135786	TA	snp	G	48135782	TXK
4	48169242	48169252	AT	snp	G	48169248	TEC
4	53728367	53728377	CG	snp	A	53728373	RASL11B
4	55163821	55163837	TG	snp	G	55163825	PDGFRA
4	68356609	68356619	AT	snp	G	68356614	CENPC1
4	71256793	71256803	AT	snp	G	71256799	SMR3B
4	77677558	77677570	TC	snp	G	77677567	SHROOM3
4	82025815	82025827	TA	snp	G	82025824	PRKG2
4	82367117	82367127	AG	snp	C	82367124	RASGEF1B
4	83787302	83787314	AG	snp	G	83787306	SEC31A
4	88402966	88402976	AC	snp	G	88402968	SPARCL1
4	89238363	89238373	TG	snp	C	89238368	BC027846
4	91839789	91839805	AT	snp	A	91839792	FAM190A
4	100760505	100760517	AT	snp	C	100760513	DAPP1
4	104120033	104120043	TC	snp	T	104120040	CENPE
4	105416876	105416886	TC	snp	C	105416880	AK094561
4	105416876	105416886	TC	snp	C	105416880	CXXC4
4	106511311	106511327	AG	snp	A	106511314	ARHGEF38
4	110611352	110611368	AC	snp	T	110611354	CASP6
4	113349284	113349294	TA	snp	G	113349287	ALPK1
4	122052333	122052347	TA	snp	G	122052340	TNIP3
4	123161611	123161621	GA	snp	G	123161618	KIAA1109
4	128814957	128814967	GA	snp	T	128814963	PLK4

4	128922032	128922042	TG	snp	A	128922037	C4orf29
4	132649186	132649196	TG	snp	C	132649192	BC131768
4	134062199	134062209	AT	snp	C	134062203	BC040219
4	139027189	139027201	AT	snp	C	139027192	LOC641364
4	139027189	139027201	AT	snp	C	139027192	LOC641365
4	141075643	141075653	GT	snp	A	141075647	MAML3
4	144107341	144107351	CA	snp	T	144107343	USP38
4	146081090	146081100	AC	snp	T	146081095	OTUD4
4	151770174	151770186	TA	snp	C	151770178	LRBA
4	164054969	164054983	TG	snp	C	164054972	NAF1
4	166419900	166419918	GT	snp	A	166419907	CPE
4	177040245	177040255	TC	snp	G	177040250	WDR17
4	177189807	177189817	AT	snp	G	177189809	ASB5
4	185696726	185696736	GA	snp	A	185696732	ACSL1
4	187195788	187195802	CT	snp	C	187195795	F11
4	189030305	189030319	AC	snp	T	189030310	TRIML2
4	189063800	189063814	AG	snp	A	189063805	TRIML1
4	190879289	190879301	TC	snp	A	190879292	FRG1
5	462731	462745	CA	snp	T	462733	EXOC3
5	640701	640711	CA	snp	T	640703	CEP72
5	640701	640711	CA	snp	G	640704	CEP72
5	7788815	7788825	TG	snp	A	7788822	ADCY2
5	7835065	7835081	AC	snp	T	7835072	C5orf49
5	13862962	13862972	AT	snp	G	13862968	DNAH5
5	17812200	17812210	AT	snp	C	17812203	BC028204
5	17812212	17812222	AT	snp	C	17812215	BC028204
5	17812224	17812234	AT	snp	C	17812227	BC028204
5	17812236	17812246	AT	snp	C	17812239	BC028204
5	17812248	17812258	AT	snp	C	17812251	BC028204
5	17812260	17812270	AT	snp	C	17812263	BC028204
5	17812272	17812282	AT	snp	G	17812274	BC028204
5	17812272	17812282	AT	snp	C	17812275	BC028204
5	17812284	17812294	AT	snp	C	17812287	BC028204

5	17812296	17812306	AT	snp	C	17812299	BC028204
5	17812460	17812472	AT	snp	G	17812464	BC028204
5	21624494	21624506	AT	snp	G	21624502	BC038535
5	24505513	24505523	CA	snp	T	24505519	CDH10
5	38500303	38500313	TA	snp	C	38500309	LIFR
5	40936899	40936911	GA	snp	C	40936907	C7
5	41260370	41260384	CA	snp	A	41260376	C6
5	56527830	56527840	TG	snp	A	56527833	GPBP1
5	68264236	68264250	GA	snp	A	68264240	AK128486
5	72200627	72200637	CA	snp	G	72200630	TNPO1
5	72800589	72800599	AT	snp	C	72800596	BTF3
5	75998482	75998492	CT	snp	G	75998487	IQGAP2
5	76371806	76371816	TA	snp	G	76371811	ZBED3
5	76371826	76371836	TA	snp	G	76371829	ZBED3
5	76371826	76371836	TA	snp	G	76371831	ZBED3
5	76371846	76371856	TA	snp	G	76371849	ZBED3
5	76749691	76749701	CA	snp	T	76749693	WDR41
5	78595808	78595824	AT	snp	G	78595816	JMY
5	85588329	85588339	TA	snp	G	85588334	NBPF22P
5	86634613	86634623	AT	snp	G	86634617	RASA1
5	94289873	94289885	AT	snp	G	94289875	MCTP1
5	94826193	94826209	AT	snp	T	94826205	TTC37
5	96209831	96209841	AG	snp	A	96209838	AK094985
5	101607313	101607325	AT	snp	C	101607316	SLCO4C1
5	109757117	109757133	AT	snp	A	109757120	TMEM232
5	112353508	112353518	AT	snp	G	112353510	DCP2
5	112353508	112353518	AT	snp	G	112353510	MCC
5	118605129	118605145	TG	snp	A	118605138	TNFAIP8
5	121977815	121977825	TA	snp	C	121977819	BC043373
5	122067217	122067231	AT	snp	C	122067224	BC043373
5	127301428	127301438	TA	snp	G	127301431	FLJ33630
5	128798292	128798310	TG	snp	G	128798306	ADAMTS19
5	128994060	128994070	AG	snp	A	128994063	ADAMTS19

5	132545085	132545103	GA	snp	C	132545097	FSTL4
5	133509745	133509755	AG	snp	T	133509751	SKP1
5	137224889	137224899	GA	snp	A	137224891	PKD2L2
5	140264852	140264862	AT	snp	G	140264854	PCDHA1
5	140264852	140264862	AT	snp	G	140264854	PCDHA10
5	140264852	140264862	AT	snp	G	140264854	PCDHA11
5	140264852	140264862	AT	snp	G	140264854	PCDHA12
5	140264852	140264862	AT	snp	G	140264854	PCDHA13
5	140264852	140264862	AT	snp	G	140264854	PCDHA13
5	140264852	140264862	AT	snp	G	140264854	PCDHA2
5	140264852	140264862	AT	snp	G	140264854	PCDHA3
5	140264852	140264862	AT	snp	G	140264854	PCDHA4
5	140264852	140264862	AT	snp	G	140264854	PCDHA5
5	140264852	140264862	AT	snp	G	140264854	PCDHA6
5	140264852	140264862	AT	snp	G	140264854	PCDHA7
5	140264852	140264862	AT	snp	G	140264854	PCDHA8
5	140264852	140264862	AT	snp	G	140264854	PCDHA9
5	147502947	147502961	TA	snp	C	147502949	SPINK5
5	147502947	147502961	TA	snp	G	147502957	SPINK5
5	147506887	147506897	TG	snp	A	147506890	SPINK5
5	147869706	147869716	TA	snp	G	147869711	HTR4
5	149441811	149441825	AT	snp	C	149441818	CSF1R
5	150845256	150845266	AG	snp	A	150845259	SLC36A1
5	167833971	167833987	AG	snp	A	167833974	WWC1
5	168728671	168728687	AC	snp	T	168728680	SLIT3
5	169507130	169507148	TG	snp	G	169507132	DOCK2
5	175386580	175386592	TA	snp	G	175386585	THOC3
5	177165017	177165027	GT	snp	C	177165022	FAM153A
6	3191924	3191934	TA	snp	G	3191931	LOC100507194
6	4041976	4041990	CA	snp	G	4041987	PRPF4B
6	7573393	7573403	TG	snp	T	7573396	DSP
6	8652020	8652030	GA	snp	C	8652024	HULC
6	8652020	8652030	GA	snp	C	8652024	LOC100506207

6	13231261	13231275	AG	snp	A	13231272	PHACTR1
6	17986440	17986450	AT	snp	G	17986446	KIF13A
6	18666915	18666925	TA	snp	C	18666921	AK098665
6	22111009	22111023	AG	snp	A	22111020	LINC00340
6	25137415	25137425	CA	snp	T	25137417	CMAHP
6	32290647	32290659	TC	snp	C	32290649	C6orf10
6	32485533	32485543	AG	snp	C	32485538	HLA-DRB5
6	32485533	32485543	AG	snp	C	32485540	HLA-DRB5
6	32974393	32974405	TG	snp	T	32974400	HLA-DOA
6	36285782	36285792	GA	snp	C	36285788	C6orf222
6	38975723	38975739	CA	snp	C	38975726	DNAH8
6	42608187	42608199	AG	snp	T	42608190	UBR2
6	43151475	43151487	CT	snp	T	43151483	CUL9
6	46792630	46792640	TA	snp	T	46792637	MEP1A
6	51936843	51936853	TG	snp	C	51936845	PKHD1
6	52271950	52271962	TC	snp	G	52271956	PAQR8
6	55407646	55407658	AT	snp	C	55407651	HMGCLL1
6	66052936	66052948	AT	snp	G	66052938	EYS
6	84799293	84799303	GA	snp	A	84799299	MRAP2
6	88378003	88378013	TG	snp	C	88378009	ORC3
6	105175387	105175397	AC	snp	T	105175390	HACE1
6	105175435	105175445	AC	snp	T	105175438	HACE1
6	105175483	105175493	AC	snp	T	105175486	HACE1
6	111693077	111693087	AG	snp	C	111693082	REV3L
6	117013439	117013449	TG	snp	A	117013446	KPNA5
6	122772921	122772939	AT	snp	A	122772924	SERINC1
6	127781132	127781150	AC	snp	A	127781137	C6orf174
6	127781132	127781150	AC	snp	A	127781137	KIAA0408
6	129854347	129854357	GA	snp	C	129854354	BC035400
6	132194958	132194972	AT	snp	G	132194964	ENPP1
6	134796976	134796988	TA	snp	G	134796979	AJ606328
6	134796976	134796988	TA	snp	G	134796979	AJ606328
6	134796976	134796988	TA	snp	G	134796979	AJ606330

6	134796976	134796988	TA	snp	G	134796979	AJ606330
6	134796976	134796988	TA	snp	G	134796979	BC040308
6	134796976	134796988	TA	snp	G	134796979	BC040308
6	134796976	134796988	TA	snp	G	134796979	LOC154092
6	134796976	134796988	TA	snp	G	134796979	LOC154092
6	136962648	136962660	AT	snp	C	136962651	MAP3K5
6	137320597	137320607	TA	snp	G	137320602	IL20RA
6	138413823	138413833	AT	snp	C	138413828	PERP
6	143083496	143083506	TA	snp	C	143083502	HIVEP2
6	149783539	149783553	CA	snp	T	149783543	ZC3H12D
6	150046730	150046740	AT	snp	C	150046733	NUP43
6	152127701	152127711	TG	snp	G	152127707	ESR1
6	152731556	152731574	AC	snp	A	152731559	SYNE1
6	154610915	154610927	AG	snp	A	154610920	CNKSR3
6	154610915	154610927	AG	snp	A	154610920	IPCEF1
6	154610915	154610927	AG	snp	A	154610920	IPCEF1
6	159689581	159689591	TC	snp	A	159689588	FNDC1
6	166822306	166822320	CA	snp	C	166822309	RPS6KA2
7	5518323	5518333	CA	snp	G	5518330	FBXL18
7	5889172	5889182	AT	snp	G	5889174	ZNF815
7	6049605	6049623	AT	snp	G	6049619	AIMP2
7	6049605	6049623	AT	snp	G	6049619	PMS2
7	12372608	12372622	GA	snp	C	12372614	VWDE
7	16842375	16842385	AT	snp	C	16842380	AGR2
7	22856799	22856809	TA	snp	C	22856803	TOMM7
7	27221324	27221338	AT	snp	G	27221330	HOXA11
7	29729590	29729604	AC	snp	T	29729601	DPY19L2P3
7	31116855	31116865	GT	snp	G	31116862	ADCYAP1R1
7	35671888	35671898	AT	snp	T	35671892	HERPUD2
7	36436540	36436552	CA	snp	T	36436546	ANLN
7	39744767	39744779	AC	snp	C	39744775	RALA
7	47314373	47314383	GT	snp	A	47314375	AK310472
7	47314373	47314383	GT	snp	A	47314375	TNS3

7	47866629	47866639	TG	snp	G	47866635	C7orf69
7	47866629	47866639	TG	snp	G	47866635	PKD1L1
7	55214870	55214880	CA	snp	T	55214872	EGFR
7	55270785	55270795	AC	snp	T	55270787	EGFR
7	66290853	66290863	CT	snp	G	66290859	LOC729156
7	66479589	66479601	AC	snp	G	66479596	TYW1
7	66761541	66761551	TA	snp	T	66761546	PMS2P4
7	72510854	72510864	AT	snp	C	72510861	FKBP6
7	72510854	72510864	AT	snp	C	72510861	FKBP6
7	72510854	72510864	AT	snp	C	72510861	FKBP6
7	72510854	72510864	AT	snp	C	72510861	PMS2L2
7	72510854	72510864	AT	snp	C	72510861	PMS2L2
7	72510854	72510864	AT	snp	C	72510861	PMS2L2
7	72510854	72510864	AT	snp	C	72510861	PMS2L2
7	73276432	73276442	TC	snp	C	73276434	WBSCR28
7	73802657	73802673	CA	snp	A	73802669	CLIP2
7	73974911	73974925	AG	snp	T	73974914	GTF2IRD1
7	74298311	74298329	GT	snp	C	74298326	STAG3L2
7	74925971	74925981	TA	snp	G	74925978	PMS2L14
7	74925971	74925981	TA	snp	G	74925978	PMS2L14
7	74925971	74925981	TA	snp	G	74925978	PMS2L14
7	74925971	74925981	TA	snp	G	74925978	PMS2L14
7	74925971	74925981	TA	snp	G	74925978	PMS2L14
7	74925971	74925981	TA	snp	G	74925978	PMS2L14
7	74925971	74925981	TA	snp	G	74925978	PMS2L14
7	74925971	74925981	TA	snp	G	74925978	PMS2L14
7	74925971	74925981	TA	snp	G	74925978	PMS2L14
7	74925971	74925981	TA	snp	G	74925978	PMS2L2
7	74925971	74925981	TA	snp	G	74925978	PMS2L2
7	74925971	74925981	TA	snp	G	74925978	PMS2L2
7	74925971	74925981	TA	snp	G	74925978	PMS2L2
7	74925971	74925981	TA	snp	G	74925978	PMS2L2
7	74925971	74925981	TA	snp	G	74925978	PMS2L2

7	74925971	74925981	TA	snp	G	74925978	PMS2P5
7	74925971	74925981	TA	snp	G	74925978	PMS2P5
7	74925971	74925981	TA	snp	G	74925978	PMS2P5
7	74982030	74982040	TA	snp	G	74982033	PMS2L2
7	74982050	74982060	TA	snp	G	74982057	PMS2L2
7	74982114	74982124	TA	snp	C	74982118	PMS2L2
7	86978344	86978354	CT	snp	A	86978346	CROT
7	87214273	87214289	CA	snp	C	87214276	ABCB1
7	96649515	96649529	CA	snp	A	96649521	DLX5
7	100772882	100772898	AG	snp	A	100772885	SERPINE1
7	101259809	101259819	AT	snp	C	101259814	MYL10
7	111366519	111366529	TA	snp	G	111366526	DOCK4
7	111387986	111387998	AT	snp	G	111387990	DOCK4
7	111639401	111639411	AT	snp	C	111639406	DOCK4
7	115893893	115893903	GT	snp	C	115893896	BD495725
7	115893893	115893903	GT	snp	C	115893896	TES
7	122055871	122055881	AC	snp	G	122055873	CADPS2
7	122130967	122130977	GA	snp	C	122130973	CADPS2
7	124469780	124469792	TA	snp	C	124469785	POT1
7	135613041	135613053	AC	snp	T	135613048	LUZP6
7	135613041	135613053	AC	snp	T	135613048	MTPN
7	137601570	137601584	AG	snp	C	137601581	CREB3L2
7	141431584	141431594	CT	snp	G	141431591	FLJ40852
7	141431584	141431594	CT	snp	G	141431591	WEE2
7	141431632	141431642	CT	snp	G	141431639	FLJ40852
7	141431632	141431642	CT	snp	G	141431639	WEE2
7	142345171	142345185	CT	snp	T	142345181	TCRBV10S1P
7	142345171	142345185	CT	snp	T	142345181	TCRBV10S1P
7	142345171	142345185	CT	snp	T	142345181	TCRBV2S1
7	142345171	142345185	CT	snp	T	142345181	TCRBV2S1
7	142345171	142345185	CT	snp	T	142345181	TCRBV5S1A1T
7	142345171	142345185	CT	snp	T	142345181	TCRBV5S1A1T
7	142345171	142345185	CT	snp	T	142345181	TCRVB

7	142345171	142345185	CT	snp	T	142345181	TCRVB
7	142373903	142373913	CA	snp	G	142373906	MTRNR2L6
7	142373903	142373913	CA	snp	G	142373906	TCRBV19S1P
7	142373903	142373913	CA	snp	G	142373906	TCRBV2S1
7	142373903	142373913	CA	snp	G	142373906	TCRBV5S1A1T
7	142373903	142373913	CA	snp	G	142373906	TCRVB
7	142621400	142621414	AT	snp	C	142621411	TRPV5
7	142621472	142621486	AT	snp	C	142621483	TRPV5
7	144346593	144346605	TA	snp	C	144346595	TPK1
7	146113368	146113378	TA	snp	G	146113373	CNTNAP2
7	146113368	146113378	TA	snp	G	146113373	Metazoa_SRP
7	149072136	149072146	AC	snp	C	149072138	TRNA_Cys
7	149148379	149148391	GT	snp	G	149148384	ZNF777
7	155189224	155189236	CA	snp	G	155189231	BC150495
7	155531072	155531084	CA	snp	G	155531079	RBM33
8	2953988	2953998	TC	snp	C	2953990	CSMD1
8	3045610	3045622	TA	snp	C	3045618	CSMD1
8	3165467	3165477	TC	snp	A	3165470	CSMD1
8	3224463	3224479	AC	snp	C	3224475	CSMD1
8	7841922	7841940	GT	snp	C	7841926	FAM66E
8	16021083	16021093	TA	snp	G	16021086	MSR1
8	16977994	16978004	TA	snp	G	16977999	EFHA2
8	17103681	17103691	CT	snp	C	17103686	CNOT7
8	17103681	17103691	CT	snp	C	17103686	VPS37A
8	17731168	17731180	AT	snp	C	17731177	FGL1
8	20040422	20040432	AC	snp	G	20040426	SLC18A1
8	20040422	20040432	AC	snp	A	20040427	SLC18A1
8	20040705	20040721	TG	snp	C	20040711	SLC18A1
8	24253182	24253198	AC	snp	G	24253194	ADAMDEC1
8	29605635	29605645	GA	snp	A	29605639	BC015784
8	29605635	29605645	GA	snp	A	29605639	BC082237
8	29605635	29605645	GA	snp	A	29605639	C8orf75
8	37598111	37598125	AT	snp	G	37598117	ERLIN2

8	42692647	42692657	TA	snp	C	42692649	THAP1
8	62467822	62467832	TA	snp	C	62467828	ASPH
8	66964142	66964152	AG	snp	C	66964148	DNAJC5B
8	82395812	82395826	TG	snp	G	82395822	FABP4
8	82597940	82597952	TA	snp	C	82597942	IMPA1
8	87680828	87680838	TG	snp	C	87680832	CNGB3
8	93728091	93728103	AG	snp	A	93728096	AK128161
8	95678307	95678317	TA	snp	C	95678311	ESRP1
8	110488511	110488525	GA	snp	A	110488515	PKHD1L1
8	114448523	114448533	AT	snp	C	114448528	CSMD3
8	120258392	120258404	CA	snp	T	120258396	MAL2
8	120576923	120576941	AT	snp	A	120576926	ENPP2
8	133758698	133758708	AT	snp	C	133758703	TMEM71
8	135847441	135847453	TG	snp	A	135847446	Mir_652
8	139207902	139207916	AC	snp	T	139207909	FAM135B
8	140999515	140999531	TG	snp	C	140999527	TRAPPC9
8	142431193	142431203	GT	snp	T	142431195	AK311257
8	142431193	142431203	GT	snp	T	142431195	PTP4A3
9	313761	313773	CA	snp	G	313766	DOCK8
9	386543	386555	AC	snp	C	386551	DOCK8
9	2524897	2524911	AG	snp	C	2524902	FLJ35024
9	2525978	2525988	AC	snp	T	2525982	FLJ35024
9	3271553	3271563	TC	snp	T	3271560	RFX3
9	15268506	15268516	CA	snp	G	15268513	TTC39B
9	16665486	16665498	AG	snp	A	16665489	BNC2
9	18721912	18721922	TG	snp	T	18721917	ADAMTSL1
9	27331466	27331482	TG	snp	C	27331476	MOB3B
9	36608414	36608430	TA	snp	G	36608419	MELK
9	36608414	36608430	TA	snp	G	36608425	MELK
9	38425030	38425040	AC	snp	G	38425037	IGFBPL1
9	39118360	39118370	AG	snp	C	39118364	CNTNAP3
9	71662252	71662264	AC	snp	T	71662259	FXN
9	71819652	71819666	GT	snp	T	71819662	TJP2

9	72374929	72374947	GC	snp	A	72374940	PTAR1
9	77743351	77743365	AT	snp	C	77743360	OSTF1
9	78784457	78784469	TA	snp	G	78784466	PCSK5
9	90298425	90298439	GA	snp	A	90298431	DAPK1
9	94973631	94973647	TC	snp	A	94973643	AK127087
9	94973631	94973647	TC	snp	A	94973643	IARS
9	95178254	95178264	TA	snp	C	95178256	CENPP
9	95178254	95178264	TA	snp	C	95178256	CENPP
9	95178254	95178264	TA	snp	C	95178256	OMD
9	95178254	95178264	TA	snp	C	95178256	OMD
9	106877546	106877556	TA	snp	C	106877548	SMC2
9	113697146	113697156	GA	snp	A	113697148	LPAR1
9	113697146	113697156	GA	snp	A	113697148	Y_RNA
9	114821599	114821609	TG	snp	C	114821601	MIR3134
9	114821599	114821609	TG	snp	C	114821601	SUSD1
9	114996229	114996243	AG	snp	C	114996240	MIR3134
9	114996229	114996243	AG	snp	C	114996240	PTBP3
9	117693496	117693506	GT	snp	C	117693499	TNFSF8
9	118165174	118165184	TA	snp	C	118165180	DEC1
9	123164276	123164286	TA	snp	T	123164283	CDK5RAP2
9	123209265	123209277	AC	snp	T	123209268	CDK5RAP2
9	124920996	124921008	AT	snp	C	124921001	NDUFA8
9	125153665	125153675	AT	snp	G	125153669	PTGS1
9	130628518	130628530	GT	snp	A	130628523	AK1
9	130988153	130988165	TC	snp	T	130988160	DNM1
9	131133976	131133986	CT	snp	A	131133982	URM1
9	132979128	132979138	CT	snp	G	132979130	NCS1
9	138902786	138902798	CA	snp	G	138902789	NACC2
X	217129	217145	TG	snp	A	217136	PLCXD1
X	1425057	1425067	GA	snp	C	1425063	CRLF2
X	1425057	1425067	GA	snp	C	1425063	CRLF2
X	1425057	1425067	GA	snp	C	1425063	CSF2RA
X	1425057	1425067	GA	snp	C	1425063	CSF2RA

X	1471624	1471638	AG	snp	T	1471632	CRLF2
X	1471624	1471638	AG	snp	T	1471632	CRLF2
X	1471624	1471638	AG	snp	T	1471632	IL3RA
X	1471624	1471638	AG	snp	T	1471632	IL3RA
X	1762343	1762355	TC	snp	T	1762352	ASMT
X	2650603	2650615	CT	snp	T	2650611	CD99
X	15444211	15444221	TA	snp	T	15444216	PIR
X	15444211	15444221	TA	snp	T	15444216	PIR-FIGF
X	37961217	37961233	GT	snp	C	37961230	SYTL5
X	38032146	38032160	GA	snp	A	38032152	SRPX
X	48369585	48369595	CT	snp	C	48369592	PORCN
X	49959473	49959485	TG	snp	T	49959482	AKAP4
X	53406702	53406712	CA	snp	A	53406708	SMC1A
X	55289271	55289281	AT	snp	C	55289274	PAGE3
X	55511947	55511965	AT	snp	A	55511950	USP51
X	69672455	69672465	GA	snp	C	69672461	DLG3
X	70117066	70117076	AT	snp	T	70117072	TEX11
X	72666209	72666221	TC	snp	T	72666216	CDX4
X	85236210	85236224	TA	snp	T	85236213	CHM
X	109932289	109932299	AT	snp	C	109932294	CHRDL1
X	111325620	111325632	GA	snp	A	111325626	TRPC5
X	111325620	111325632	GA	snp	A	111325626	ZCCHC16
X	114397900	114397910	CA	snp	T	114397904	LRCH2
X	114796415	114796425	GA	snp	G	114796420	AK127380
X	114796415	114796425	GA	snp	G	114796420	AK127380
X	114796415	114796425	GA	snp	G	114796420	AK127380
X	114796415	114796425	GA	snp	G	114796420	PLS3
X	114796415	114796425	GA	snp	G	114796420	PLS3
X	114796415	114796425	GA	snp	G	114796420	PLS3
X	117580383	117580393	AT	snp	T	117580385	WDR44
X	118228725	118228735	AG	snp	G	118228727	KIAA1210
X	130433073	130433085	AG	snp	G	130433081	IGSF1
X	132217057	132217067	TA	snp	C	132217059	USP26

X	135721657	135721675	AT	snp	A	135721660	LOC100128420
X	147002979	147002995	TA	snp	G	147002991	FMR1
X	147002979	147002995	TA	snp	G	147002991	FMR1-AS1
X	150911397	150911413	AT	snp	C	150911408	CNGA2
X	152811078	152811090	AC	snp	T	152811085	ATP2B3
10	14868193	14868217	AGA	snp	T	14868212	CDNF
10	17148303	17148318	ATA	snp	G	17148312	CUBN
10	17631267	17631288	ATC	snp	G	17631275	PTPLA
10	26436282	26436294	TGA	snp	T	26436286	MYO3A
10	26994404	26994416	ATT	snp	C	26994411	PDSS1
10	70588446	70588458	TTA	snp	C	70588449	STOX1
10	70670150	70670162	GTT	snp	C	70670157	DDX50
10	79397498	79397516	GCC	snp	T	79397503	KCNMA1
10	95352569	95352587	ATT	snp	T	95352581	RBP4
10	96960372	96960387	AGA	snp	G	96960377	C10orf129
10	98393326	98393338	TAT	snp	C	98393331	PIK3AP1
10	98393326	98393338	TAT	snp	G	98393333	PIK3AP1
10	102049297	102049312	AAT	snp	A	102049302	PKD2L1
10	105239328	105239343	CAA	snp	G	105239339	CALHM3
10	106165855	106165867	AAC	snp	G	106165858	CCDC147
10	118459731	118459743	CAT	snp	T	118459734	HSPA12A
10	120789542	120789554	CCT	snp	A	120789547	NANOS1
10	121302234	121302252	GAG	snp	A	121302243	RGS10
10	124035025	124035037	CAC	snp	G	124035029	BTBD16
10	124321346	124321358	CCT	snp	C	124321354	DMBT1
10	129351577	129351589	AAC	snp	T	129351582	NPS
11	428481	428493	ATG	snp	C	428488	ANO9
11	535289	535313	CCG	snp	T	535305	HRAS
11	1593694	1593706	CGC	snp	T	1593697	DUSP8
11	1593694	1593706	CGC	snp	T	1593697	LOC338651
11	1593694	1593706	CGC	snp	T	1593697	LOC338651
11	1593694	1593706	CGC	snp	T	1593697	MOB2
11	1593694	1593706	CGC	snp	T	1593697	MOB2

11	1593694	1593706	CGC	snp	T	1593697	MOB2
11	8893185	8893197	GCA	snp	T	8893192	ST5
11	16817340	16817352	AAT	snp	A	16817348	PLEKHA7
11	36632366	36632387	TGC	snp	A	36632382	C11orf74
11	60691973	60691997	GGC	snp	T	60691984	TMEM132A
11	67034122	67034146	GCG	snp	A	67034127	ADRBK1
11	67888779	67888794	GGC	snp	T	67888785	CHKA
11	75062773	75062788	GCC	snp	A	75062782	ARRB1
11	83771364	83771379	AAT	snp	G	83771368	DLG2
11	93063678	93063690	GCC	snp	T	93063685	CCDC67
11	100998099	100998111	GCC	snp	T	100998104	FJ515873
11	100998099	100998111	GCC	snp	T	100998104	PGR
11	124413240	124413252	AGA	snp	A	124413247	OR8B12
11	125035042	125035063	CCG	snp	G	125035058	PKNOX2
11	125619928	125619940	AAT	snp	C	125619934	PATE1
11	128641987	128641999	AGG	snp	A	128641994	FLI1
11	134122245	134122257	TCC	snp	T	134122249	THYN1
12	323599	323614	CCT	snp	G	323608	SLC6A12
12	1100455	1100467	GCA	snp	G	1100460	ERC1
12	2038970	2038985	GGA	snp	A	2038977	LOC100271702
12	4553741	4553756	TTC	snp	A	4553746	FGF6
12	4553741	4553756	TTC	snp	A	4553749	FGF6
12	6797707	6797719	GGA	snp	A	6797713	ZNF384
12	12484644	12484656	GGA	snp	A	12484648	MANSC1
12	15103596	15103608	TCA	snp	G	15103604	ARHGDIB
12	21428238	21428253	AAT	snp	C	21428245	SLCO1A2
12	21428238	21428253	AAT	snp	G	21428246	SLCO1A2
12	25386049	25386067	ACC	snp	A	25386056	KRAS
12	25386049	25386067	ACC	snp	A	25386062	KRAS
12	26593150	26593174	AAC	snp	G	26593163	ITPR2
12	29604099	29604120	ATA	snp	C	29604109	OVCH1
12	49580271	49580283	GGA	snp	C	49580278	TUBA1A
12	49721570	49721591	AAT	snp	A	49721575	TROAP

12	53436064	53436076	CTC	snp	T	53436072	EIF4B
12	53436064	53436076	CTC	snp	T	53436072	LOC283335
12	71897912	71897927	TTG	snp	T	71897923	LGR5
12	75824887	75824902	GAG	snp	A	75824892	GLIPR1L2
12	93192111	93192129	ATT	snp	T	93192123	EEA1
12	100166426	100166447	ACA	snp	T	100166434	ANKS1B
12	100440498	100440513	ATA	snp	A	100440508	UHRF1BP1L
12	110819136	110819154	TTG	snp	T	110819141	ANAPC7
12	123466181	123466193	CTC	snp	T	123466189	ARL6IP4
12	123466292	123466307	GAA	snp	G	123466303	ARL6IP4
12	124299599	124299617	CAA	snp	A	124299608	DNAH10
12	129441257	129441272	TCC	snp	T	129441268	GLT1D1
12	132547155	132547167	GAC	snp	A	132547161	EP400
13	29233415	29233430	GGC	snp	A	29233421	POMP
13	43137699	43137711	GAA	snp	C	43137703	TNFSF11
13	45151551	45151563	GGA	snp	A	45151558	LOC641467
13	45151551	45151563	GGA	snp	A	45151558	LOC641467
13	45151551	45151563	GGA	snp	A	45151558	TSC22D1
13	75935523	75935535	TTG	snp	C	75935529	TBC1D4
13	77460393	77460411	CCG	snp	A	77460407	KCTD12
13	110853725	110853737	GAG	snp	C	110853730	COL4A1
13	114156520	114156541	TTG	snp	T	114156531	TMCO3
14	24511653	24511671	TTG	snp	C	24511662	DHRS4L1
14	24511653	24511671	TTG	snp	C	24511662	DHRS4L2
14	29235532	29235544	ACC	snp	G	29235536	FOXG1
14	35245742	35245754	TCA	snp	G	35245747	BAZ1A
14	50705219	50705237	TAA	snp	C	50705222	L2HGDH
14	63862167	63862182	AGG	snp	A	63862174	PPP2R5E
14	69619702	69619717	CCG	snp	C	69619710	DCAF5
14	77579051	77579066	TTG	snp	T	77579056	KIAA1737
14	93185689	93185701	CCA	snp	G	93185694	LGMN
14	94126068	94126083	ATC	snp	G	94126077	UNC79
14	99637285	99637297	GGT	snp	G	99637293	BCL11B

14	101328249	101328264	CTC	snp	T	101328260	MEG3
14	103429046	103429058	GGT	snp	A	103429052	CDC42BPB
14	106993938	106993953	TAC	snp	C	106993944	abParts
15	23086364	23086388	GCC	snp	C	23086382	NIPA1
15	34816952	34816964	ATC	snp	C	34816959	GOLGA8B
15	35530026	35530044	GGA	snp	T	35530029	ANP32AP1
15	40650455	40650479	CCG	snp	A	40650460	DISP2
15	41989922	41989934	AGT	snp	G	41989925	MGA
15	69388921	69388933	AGG	snp	A	69388925	LINC00277
15	69388921	69388933	AGG	snp	A	69388925	MIR548H4
15	72523678	72523693	GCG	snp	T	72523688	PKM2
15	78203749	78203761	ATC	snp	T	78203757	DQ586415
15	78369948	78369963	CGC	snp	C	78369958	TBC1D2B
15	89867474	89867486	AGC	snp	A	89867478	POLG
15	90768314	90768326	TGC	snp	T	90768319	SEMA4B
15	101395410	101395425	TTC	snp	T	101395415	LOC145757
15	101817624	101817642	CCG	snp	T	101817629	SELS
16	284550	284562	GAG	snp	C	284555	ITFG3
16	284550	284562	GAG	snp	C	284555	LUC7L
16	838632	838644	CGG	snp	T	838636	CHTF18
16	838632	838644	CGG	snp	T	838636	RPUSD1
16	1031782	1031803	CGG	snp	T	1031797	LMF1
16	1031782	1031803	CGG	snp	T	1031797	SOX8
16	2390589	2390601	GGC	snp	T	2390595	ABCA17P
16	2390589	2390601	GGC	snp	T	2390595	ABCA17P
16	2390589	2390601	GGC	snp	T	2390595	ABCA3
16	2770513	2770531	ATT	snp	T	2770525	PRSS27
16	3111152	3111164	AAT	snp	G	3111158	BC045731
16	3111152	3111164	AAT	snp	G	3111158	MMP25
16	5121685	5121697	CTA	snp	A	5121691	ALG1
16	8901726	8901738	TAA	snp	C	8901731	PMM2
16	15471570	15471585	GGA	snp	A	15471580	NPIP
16	19503767	19503782	ATT	snp	G	19503772	TMC5

16	23646141	23646165	TCA	snp	C	23646153	PALB2
16	56459348	56459363	GCC	snp	T	56459353	AMFR
16	57126476	57126497	GCC	snp	T	57126483	CPNE2
16	57789552	57789567	CAT	snp	G	57789559	KATNB1
16	78142894	78142909	ACA	snp	G	78142897	WWOX
16	88496785	88496797	GGC	snp	A	88496790	ZNF469
16	88780081	88780093	GTG	snp	A	88780089	CTU2
17	260292	260304	GAG	snp	A	260298	C17orf97
17	1482442	1482463	AAT	snp	A	1482447	SLC43A2
17	3444939	3444957	ATG	snp	C	3444951	TRPV3
17	5185671	5185689	GGC	snp	A	5185679	RABEP1
17	7827186	7827204	CAC	snp	T	7827197	KCNAB3
17	18908061	18908073	GCG	snp	T	18908065	FAM83G
17	18908061	18908073	GCG	snp	T	18908065	FAM83G
17	18908061	18908073	GCG	snp	T	18908065	SLC5A10
17	18908061	18908073	GCG	snp	T	18908065	SLC5A10
17	18908061	18908073	GCG	snp	A	18908069	FAM83G
17	18908061	18908073	GCG	snp	A	18908069	FAM83G
17	18908061	18908073	GCG	snp	A	18908069	SLC5A10
17	18908061	18908073	GCG	snp	A	18908069	SLC5A10
17	21156728	21156740	CGG	snp	A	21156732	C17orf103
17	34942586	34942598	AAG	snp	A	34942594	GGNBP2
17	39189355	39189373	TTA	snp	C	39189359	KRTAP1-3
17	39742849	39742867	GCT	snp	A	39742855	JUP
17	39742849	39742867	GCT	snp	A	39742855	KRT14
17	44048490	44048505	AGG	snp	A	44048501	MAPT
17	44160044	44160056	CAG	snp	A	44160047	KIAA1267
17	48134824	48134836	AGG	snp	A	48134828	ITGA3
17	49337862	49337874	CGG	snp	T	49337866	MBTD1
17	49337862	49337874	CGG	snp	T	49337866	UTP18
17	61785560	61785578	TTG	snp	C	61785563	STRADA
17	65899971	65899983	GAA	snp	G	65899979	BPTF
17	66596762	66596774	CAG	snp	T	66596768	FAM20A

17	78181357	78181375	AAC	snp	A	78181371	CARD14
18	14763091	14763103	TTA	snp	C	14763094	ANKRD30B
18	19749009	19749021	GCG	snp	A	19749017	GATA6
18	20953712	20953724	AGG	snp	A	20953719	TMEM241
18	25081558	25081570	AAC	snp	G	25081561	AK127888
18	40038869	40038881	GAT	snp	A	40038874	LOC284260
18	72011110	72011122	TTA	snp	A	72011116	C18orf63
18	76740725	76740737	CGG	snp	T	76740731	SALL3
19	520504	520516	AAC	snp	A	520512	TPGS1
19	857094	857109	ATA	snp	C	857104	ELANE
19	1009185	1009203	AGC	snp	G	1009188	C19orf6
19	1009185	1009203	AGC	snp	G	1009188	FLJ00277
19	1009185	1009203	AGC	snp	G	1009188	GRIN3B
19	1395487	1395505	CGC	snp	A	1395499	AK126693
19	1395487	1395505	CGC	snp	A	1395499	NDUFS7
19	2859698	2859716	CCA	snp	G	2859706	ZNF555
19	3908622	3908637	TCC	snp	T	3908629	ATCAY
19	4211639	4211651	CAA	snp	G	4211644	ANKRD24
19	8001990	8002011	AAC	snp	G	8001998	TIMM44
19	8151331	8151352	TTA	snp	T	8151348	FBN3
19	8642947	8642959	AAC	snp	A	8642955	MYO1F
19	9004550	9004571	CAC	snp	A	9004562	MUC16
19	9010296	9010320	ATC	snp	T	9010316	MUC16
19	9016410	9016425	ACC	snp	T	9016418	MUC16
19	11307560	11307572	GGT	snp	A	11307563	KANK2
19	11536507	11536531	AAC	snp	T	11536516	CCDC151
19	11536507	11536531	AAC	snp	G	11536518	CCDC151
19	13318994	13319015	GGA	snp	G	13319005	CACNA1A
19	17721853	17721868	AAC	snp	T	17721858	UNC13A
19	17932851	17932863	TTA	snp	G	17932854	INSL3
19	35626615	35626630	GAT	snp	G	35626623	LGI4
19	36211354	36211366	AAG	snp	G	36211358	MLL4
19	36585458	36585479	AAT	snp	G	36585462	WDR62

19	38011746	38011758	TGG	snp	T	38011751	ZNF793
19	38634012	38634024	TCC	snp	T	38634019	SIPA1L3
19	39957684	39957699	AAT	snp	G	39957687	SUPT5H
19	41173874	41173895	TGC	snp	T	41173879	NUMBL
19	41889414	41889429	TTA	snp	T	41889422	BCKDHA
19	41889414	41889429	TTA	snp	T	41889422	TMEM91
19	44454816	44454840	AAT	snp	C	44454831	ZNF221
19	44454816	44454840	AAT	snp	C	44454834	ZNF221
19	46996180	46996192	CCT	snp	C	46996185	BC132841
19	46996180	46996192	CCT	snp	C	46996185	LOC100506012
19	46996180	46996192	CCT	snp	C	46996185	PNMAL2
19	47420971	47420983	TTG	snp	G	47420977	ARHGAP35
19	48494882	48494897	CCT	snp	T	48494886	BSPH1
19	48494882	48494897	CCT	snp	A	48494890	BSPH1
19	48800716	48800728	GCG	snp	A	48800724	CCDC114
19	49114405	49114420	TTG	snp	C	49114408	FAM83E
19	50832212	50832224	GCT	snp	A	50832216	KCNC3
19	50832212	50832224	GCT	snp	A	50832216	NR1H2
19	54238752	54238773	TAT	snp	A	54238763	MIR518D
19	55399308	55399320	AAG	snp	T	55399313	FCAR
19	55693895	55693916	AAC	snp	G	55693907	PTPRH
19	56114231	56114246	GAG	snp	A	56114236	FIZ1
19	56114231	56114246	GAG	snp	A	56114236	ZNF524
19	56224235	56224247	GGA	snp	A	56224241	NLRP9
1	3645985	3645997	GCA	snp	A	3645988	TP73
1	6529182	6529206	TCC	snp	T	6529187	PLEKHG5
1	9810275	9810287	AAT	snp	C	9810283	CLSTN1
1	11711167	11711179	TAT	snp	G	11711175	FBXO2
1	19567777	19567789	TTG	snp	A	19567784	KIAA0090
1	23107827	23107839	AAG	snp	G	23107831	EPHB2
1	26658997	26659015	TTG	snp	T	26659002	AIM1L
1	26799163	26799175	CCG	snp	T	26799167	HMGN2
1	29508342	29508354	GCG	snp	T	29508349	SRSF4

1	31466479	31466494	CAC	snp	G	31466487	PUM1
1	31653725	31653740	GTG	snp	C	31653732	NKAIN1
1	33146735	33146747	TTG	snp	A	33146743	RBBP4
1	33146735	33146747	TTG	snp	A	33146743	SYNC
1	35904440	35904458	AAC	snp	T	35904454	KIAA0319L
1	39848005	39848017	TAT	snp	C	39848013	MACF1
1	40930011	40930023	ATT	snp	C	40930018	ZNF643
1	47282620	47282632	GTG	snp	A	47282625	CYP4B1
1	63153860	63153875	GGC	snp	G	63153865	DOCK7
1	67593323	67593335	GTT	snp	C	67593327	C1orf141
1	68566989	68567010	TAT	snp	C	68566992	LOC100289178
1	68566989	68567010	TAT	snp	C	68566992	WLS
1	84944985	84944997	AGC	snp	G	84944988	RPF1
1	85593831	85593846	AAG	snp	G	85593840	WDR63
1	85930746	85930761	GGC	snp	C	85930749	DDAH1
1	89352593	89352611	ATT	snp	T	89352605	GTF2B
1	89665514	89665526	AAG	snp	A	89665522	GBP4
1	109102744	109102765	GGC	snp	T	109102761	FAM102B
1	112162406	112162418	CGC	snp	T	112162409	RAP1A
1	113120589	113120601	ATC	snp	G	113120592	ST7L
1	146697781	146697793	AGT	snp	C	146697789	FMO5
1	150251112	150251124	AGG	snp	A	150251119	C1orf54
1	151859907	151859925	AAT	snp	A	151859912	THEM4
1	154301252	154301267	GGC	snp	A	154301260	ATP8B2
1	156101066	156101084	AAT	snp	A	156101071	LMNA
1	156566450	156566462	GCT	snp	T	156566457	APOA1BP
1	156566450	156566462	GCT	snp	T	156566457	APOA1BP
1	156566450	156566462	GCT	snp	T	156566457	APOA1BP
1	156566450	156566462	GCT	snp	T	156566457	APOA1BP
1	156566450	156566462	GCT	snp	T	156566457	GPATCH4
1	156566450	156566462	GCT	snp	T	156566457	GPATCH4
1	156566450	156566462	GCT	snp	T	156566457	GPATCH4
1	156566450	156566462	GCT	snp	T	156566457	GPATCH4

1	156721555	156721567	CTC	snp	T	156721561	HDGF
1	156898316	156898331	TCT	snp	C	156898321	LRRC71
1	174245273	174245285	ATT	snp	C	174245277	RABGAP1L
1	186086569	186086584	TTG	snp	A	186086577	HMCN1
1	186086569	186086584	TTG	snp	A	186086577	HMCN1
1	186086569	186086584	TTG	snp	A	186086577	MIR548F1
1	186086569	186086584	TTG	snp	A	186086577	MIR548F1
1	203667527	203667542	CCA	snp	G	203667535	ATP2B4
1	210561169	210561181	GGT	snp	C	210561173	HHAT
1	216693168	216693180	AAG	snp	A	216693173	ESRRG
1	228590238	228590250	CTC	snp	C	228590245	TRIM11
1	237754389	237754413	CTC	snp	C	237754402	RYR2
1	237754433	237754445	CCT	snp	T	237754436	RYR2
20	260846	260861	AAC	snp	T	260851	C20orf96
20	1115672	1115687	GCC	snp	T	1115680	PSMF1
20	2297211	2297226	AAT	snp	G	2297221	TGM3
20	4765996	4766014	AAC	snp	G	4766009	RASSF2
20	23731647	23731659	CTC	snp	T	23731652	CST1
20	30556073	30556088	GGC	snp	T	30556081	XKR7
20	33735087	33735099	GCA	snp	T	33735091	EDEM2
20	35137985	35138000	AAT	snp	A	35137990	BC039668
20	35137985	35138000	AAT	snp	A	35137990	DLGAP4
20	37193512	37193524	CCT	snp	T	37193519	RALGAPB
20	37501268	37501283	TGC	snp	T	37501272	Metazoa_SRP
20	37501268	37501283	TGC	snp	T	37501272	PPP1R16B
20	44182832	44182844	TTG	snp	T	44182840	WFDC8
20	45984319	45984334	ACA	snp	A	45984329	AK098067
20	45984319	45984334	ACA	snp	A	45984329	BC047609
20	45984319	45984334	ACA	snp	A	45984329	ZMYND8
20	48099617	48099629	TTC	snp	T	48099622	KCNB1
20	49547656	49547668	GGC	snp	T	49547664	ADNP
21	22129886	22129898	TGA	snp	C	22129891	LINC00320
21	30494969	30494981	GCT	snp	T	30494975	C21orf7

21	32554054	32554075	CTT	snp	G	32554057	TIAM1
22	18050630	18050642	CCT	snp	G	18050635	SLC25A18
22	19166259	19166271	GGC	snp	A	19166262	CLTCL1
22	19166259	19166271	GGC	snp	A	19166262	SLC25A1
22	21318545	21318557	GGT	snp	T	21318551	AIFM3
22	21318545	21318557	GGT	snp	T	21318551	BC127858
22	25567693	25567711	AAT	snp	A	25567698	KIAA1671
22	38031633	38031645	TTG	snp	C	38031640	SH3BP1
22	38453185	38453197	GGC	snp	T	38453190	PICK1
22	48885281	48885299	GCG	snp	A	48885286	FAM19A5
2	21254724	21254736	AAC	snp	T	21254729	APOB
2	27608109	27608121	CTC	snp	T	27608114	PPM1G
2	29320627	29320639	CCA	snp	T	29320633	CLIP4
2	39005199	39005217	ATT	snp	C	39005204	GEMIN6
2	42274848	42274860	GCC	snp	A	42274851	PKDCC
2	45620528	45620540	TTG	snp	C	45620534	SRBD1
2	48589159	48589171	ATT	snp	C	48589165	FOXN2
2	55184127	55184139	TAC	snp	T	55184132	EML6
2	71221962	71221980	CCG	snp	C	71221967	TEX261
2	71662817	71662835	AAC	snp	A	71662825	ZNF638
2	79601264	79601276	TCA	snp	T	79601272	CTNNA2
2	89235790	89235802	TTG	snp	C	89235794	abParts
2	106810757	106810772	GCG	snp	T	106810766	UXS1
2	152491239	152491257	TTA	snp	C	152491244	NEB
2	160605860	160605881	TTG	snp	T	160605877	MARCH7
2	169727859	169727871	ATA	snp	G	169727862	SPC25
2	174129767	174129782	ACA	snp	T	174129771	MLK7-AS1
2	174129767	174129782	ACA	snp	T	174129771	ZAK
2	176957810	176957825	GCG	snp	A	176957821	HOXD13
2	178129390	178129405	GGC	snp	T	178129398	NFE2L2
2	204305087	204305099	GGT	snp	C	204305092	RAPH1
2	209054064	209054076	CCA	snp	G	209054072	C2orf80
2	217498281	217498293	GCC	snp	T	217498289	IGFBP2

2	219330667	219330679	TTA	snp	C	219330673	USP37
2	225449893	225449917	GGC	snp	G	225449898	CUL3
2	232659466	232659484	TTG	snp	A	232659480	COPS7B
2	233411049	233411061	TGT	snp	A	233411056	CHRNG
3	14106092	14106110	CAG	snp	T	14106102	TPRXL
3	14106092	14106110	CAG	snp	C	14106103	TPRXL
3	14106326	14106338	CAG	snp	C	14106331	TPRXL
3	16555218	16555233	CCG	snp	T	16555222	RFTN1
3	18466368	18466380	TGT	snp	T	18466375	SATB1
3	24379563	24379575	TTG	snp	C	24379566	THRB
3	25706340	25706358	GCC	snp	A	25706349	MIR4442
3	25706340	25706358	GCC	snp	A	25706349	TOP2B
3	25706340	25706358	GCC	snp	A	25706349	TOP2B
3	39229896	39229908	TGC	snp	C	39229899	XIRP1
3	42596467	42596488	AAC	snp	A	42596484	SEC22C
3	42740777	42740789	TAA	snp	A	42740780	HHATL
3	45267303	45267321	CGC	snp	T	45267309	TMEM158
3	45718597	45718609	TGC	snp	A	45718601	LIMD1
3	45718597	45718609	TGC	snp	A	45718601	LOC644714
3	46414019	46414040	ACA	snp	G	46414034	CCR5
3	51743026	51743038	CTG	snp	C	51743033	GRM2
3	62938253	62938265	TTG	snp	C	62938258	LOC285401
3	74348881	74348893	CAG	snp	T	74348887	CNTN3
3	128709955	128709967	ACA	snp	A	128709962	KIAA1257
3	133660840	133660852	ATT	snp	C	133660847	SLCO2A1
3	142443441	142443453	CCT	snp	A	142443449	TRPC1
3	154801359	154801371	AGT	snp	C	154801364	MME
3	171756904	171756919	AAC	snp	G	171756908	FNDC3B
3	178866320	178866335	CGC	snp	T	178866326	BC032034
3	178866320	178866335	CGC	snp	T	178866326	PIK3CA
3	182511419	182511431	GGC	snp	G	182511427	ATP11B
3	190123384	190123396	CCA	snp	T	190123391	CLDN16
4	4037258	4037273	TTC	snp	T	4037269	BC042823

4	7716927	7716939	CTC	snp	C	7716931	SORCS2
4	20396582	20396594	TGG	snp	A	20396590	SLIT2
4	20396582	20396594	TGG	snp	A	20396590	SLIT2-IT1
4	25824277	25824292	AGG	snp	A	25824282	SEL1L3
4	37455627	37455651	CCG	snp	T	37455640	C4orf19
4	38666560	38666572	CCA	snp	C	38666565	FLJ13197
4	38666560	38666572	CCA	snp	C	38666565	FLJ13197
4	38666560	38666572	CCA	snp	C	38666565	KLF3
4	38666560	38666572	CCA	snp	C	38666565	KLF3
4	69097705	69097717	AAT	snp	G	69097709	TMPRSS11B
4	70919454	70919475	TCC	snp	T	70919465	HTN1
4	71571506	71571521	CGC	snp	T	71571515	RUFY3
4	73182409	73182433	AAT	snp	C	73182413	ADAMTS3
4	76792609	76792621	TTC	snp	C	76792615	PPEF2
4	76957165	76957177	TGC	snp	T	76957170	ART3
4	76957165	76957177	TGC	snp	T	76957170	ART3
4	76957165	76957177	TGC	snp	T	76957170	ART3
4	76957165	76957177	TGC	snp	T	76957170	CXCL11
4	76957165	76957177	TGC	snp	T	76957170	CXCL11
4	76957165	76957177	TGC	snp	T	76957170	CXCL11
4	79755378	79755393	ATC	snp	C	79755388	BMP2K
4	84366728	84366740	TAT	snp	C	84366736	HELQ
4	86937092	86937110	ATT	snp	C	86937095	MAPK10
4	86937092	86937110	ATT	snp	C	86937098	MAPK10
4	126338119	126338131	AAT	snp	G	126338123	FAT4
4	126399435	126399447	TTA	snp	G	126399439	FAT4
4	153457150	153457165	GCG	snp	A	153457161	DKFZP434I0714
4	153457150	153457165	GCG	snp	A	153457161	FBXW7
4	169108193	169108205	AAT	snp	G	169108200	ANXA10
4	185973185	185973209	TTG	snp	C	185973188	BC043280
4	186298674	186298692	AAC	snp	G	186298688	BC128459
4	186298674	186298692	AAC	snp	G	186298688	LRP2BP
4	187071513	187071531	TTG	snp	T	187071518	FAM149A

4	187542975	187542990	AGT	snp	C	187542985	FAT1
5	1443970	1443985	TTG	snp	T	1443975	SLC6A3
5	10244641	10244653	AAC	snp	C	10244648	FAM173B
5	60193330	60193342	TAT	snp	C	60193338	ERCC8
5	64558465	64558477	GAA	snp	A	64558471	ADAMTS6
5	76114959	76114971	CGG	snp	T	76114962	F2RL1
5	78329777	78329789	CAA	snp	T	78329783	DMGDH
5	121798278	121798296	GAT	snp	C	121798286	BC029465
5	121798278	121798296	GAT	snp	C	121798286	SNCAIP
5	127419931	127419952	GCG	snp	A	127419948	FLJ33630
5	127419931	127419952	GCG	snp	A	127419948	SLC12A2
5	133484435	133484447	ATT	snp	C	133484440	TCF7
5	172350075	172350096	AGG	snp	T	172350092	ERGIC1
5	174951424	174951436	TTG	snp	C	174951430	SFXN1
5	176523855	176523867	TCC	snp	T	176523860	FGFR4
5	176524110	176524122	TCC	snp	G	176524117	FGFR4
5	177614360	177614372	GCC	snp	T	177614365	GMCL1P1
5	178772620	178772635	GCC	snp	G	178772630	ADAMTS2
5	180030319	180030331	GCC	snp	T	180030324	FLT4
6	3457325	3457349	CTC	snp	T	3457339	AK096549
6	3457325	3457349	CTC	snp	T	3457339	SLC22A23
6	26578017	26578041	GTT	snp	T	26578020	TRNA_Tyr
6	26682080	26682092	TTG	snp	C	26682086	TRNA_Ala
6	29759439	29759451	AAC	snp	T	29759444	HCG4
6	29759439	29759451	AAC	snp	T	29759444	LOC554223
6	30647000	30647012	GCA	snp	G	30647004	PPP1R18
6	31588701	31588716	GGC	snp	T	31588706	PRRC2A
6	32407460	32407475	TTG	snp	C	32407467	HLA-DRA
6	35197026	35197038	AAT	snp	A	35197034	SCUBE3
6	38566699	38566717	AAT	snp	A	38566704	BTBD9
6	44219918	44219930	AGA	snp	A	44219925	HSP90AB1
6	70926925	70926946	TGA	snp	C	70926932	COL9A1
6	90366299	90366311	CAA	snp	C	90366306	MDN1

6	91006512	91006536	GCT	snp	C	91006520	BACH2
6	121526444	121526465	AAG	snp	T	121526458	C6orf170
6	139176118	139176130	TTG	snp	C	139176121	ECT2L
6	151186857	151186881	CCG	snp	C	151186865	MTHFD1L
6	153311966	153311978	AAC	snp	T	153311969	MTRF1L
6	158505845	158505857	AAC	snp	T	158505853	SYNJ2
6	160859032	160859044	GGA	snp	C	160859036	SLC22A3
6	163955711	163955723	ATG	snp	A	163955718	QKI
7	565840	565852	GTG	snp	C	565843	FLJ44511
7	1191681	1191693	TGC	snp	A	1191688	ZFAND2A
7	5112026	5112044	TGC	snp	T	5112034	LOC389458
7	5112026	5112044	TGC	snp	T	5112034	LOC389458
7	5112026	5112044	TGC	snp	T	5112034	LOC389458
7	5112026	5112044	TGC	snp	T	5112034	RBAK-LOC389458
7	5112026	5112044	TGC	snp	T	5112034	RBAK-LOC389458
7	5112026	5112044	TGC	snp	T	5112034	RBAK-LOC389458
7	21743366	21743381	AAC	snp	A	21743377	DNAH11
7	32768518	32768533	GGC	snp	A	32768525	AK057321
7	32768518	32768533	GGC	snp	A	32768525	AVL9
7	32768518	32768533	GGC	snp	A	32768525	ZNRF2P1
7	53834846	53834858	CCA	snp	T	53834849	FLJ45974
7	92986680	92986692	CAG	snp	A	92986683	CCDC132
7	94036655	94036673	ACT	snp	G	94036661	COL1A2
7	100336487	100336499	TCT	snp	C	100336492	ZAN
7	100336487	100336499	TCT	snp	C	100336493	ZAN
7	112579811	112579832	GCC	snp	T	112579814	C7orf60
7	128423333	128423345	GGA	snp	C	128423340	TRNA
7	128423333	128423345	GGA	snp	C	128423340	TRNA_Pro
7	150864905	150864917	CCG	snp	T	150864912	GBX1
7	155727501	155727513	CTC	snp	T	155727509	Mir_598
7	155727609	155727621	TCC	snp	T	155727617	Mir_598
8	2087921	2087933	CAC	snp	T	2087926	MYOM2
8	11707580	11707592	TGG	snp	A	11707588	CTSB

8	21977119	21977131	CAA	snp	G	21977123	HR
8	22861392	22861404	CCA	snp	T	22861396	RHOBTB2
8	25271890	25271902	TCC	snp	T	25271894	DKFZp451J181
8	25271890	25271902	TCC	snp	T	25271894	PPP2R2A
8	37757465	37757480	AAT	snp	G	37757470	RAB11FIP1
8	38324421	38324433	CCA	snp	A	38324425	FGFR1
8	41548426	41548444	AAC	snp	T	41548434	ANK1
8	41548426	41548444	AAC	snp	T	41548434	ANK1
8	41548426	41548444	AAC	snp	T	41548434	ANK1
8	41548426	41548444	AAC	snp	T	41548434	NKX6-3
8	41548426	41548444	AAC	snp	T	41548434	NKX6-3
8	101076165	101076177	TCT	snp	G	101076173	RGS22
8	110408432	110408444	TAA	snp	C	110408438	PKHD1L1
8	117950724	117950745	GGC	snp	A	117950737	AL832163
8	117950724	117950745	GGC	snp	A	117950737	C8orf85
8	120767960	120767972	AAT	snp	G	120767964	TAF2
8	121824054	121824072	GCC	snp	A	121824062	SNTB1
8	124392110	124392131	TAT	snp	C	124392119	ATAD2
8	130365020	130365032	CCT	snp	T	130365024	CCDC26
8	139705222	139705234	TGC	snp	T	139705227	COL22A1
8	140743192	140743204	GGA	snp	A	140743199	TRAPPC9
8	142195932	142195947	AAT	snp	G	142195935	DENND3
8	142449575	142449587	TGC	snp	A	142449580	FLJ43860
8	143425132	143425156	GAG	snp	G	143425151	TSNARE1
8	144091554	144091575	AAT	snp	A	144091559	LOC100133669
8	144091554	144091575	AAT	snp	A	144091571	LOC100133669
8	144357341	144357353	CCT	snp	G	144357344	GLI4
8	144357341	144357353	CCT	snp	G	144357344	GLI4
8	144357341	144357353	CCT	snp	G	144357344	ZFP41
8	144357341	144357353	CCT	snp	G	144357344	ZFP41
9	12775885	12775897	AGC	snp	G	12775888	C9orf150
9	12775885	12775897	AGC	snp	T	12775893	C9orf150
9	27573206	27573230	CGC	snp	T	27573212	C9orf72

9	27941600	27941612	CCT	snp	C	27941605	BX538226
9	34016243	34016255	GAG	snp	A	34016248	UBAP2
9	35906583	35906598	CCA	snp	C	35906594	HRCT1
9	75315433	75315445	AGA	snp	A	75315437	TMC1
9	79464935	79464950	TGT	snp	A	79464939	PRUNE2
9	88461677	88461695	ATT	snp	G	88461680	AK124523
9	89561422	89561434	GCG	snp	A	89561430	GAS1
9	92112892	92112913	GGC	snp	A	92112901	SEMA4D
9	100851198	100851216	TTG	snp	T	100851203	TRIM14
9	108060616	108060637	TTG	snp	T	108060621	SLC44A1
9	108071738	108071753	TAT	snp	G	108071748	SLC44A1
9	117373813	117373837	GGC	snp	C	117373829	C9orf91
9	131187328	131187349	TTG	snp	T	131187333	CERCAM
9	131566503	131566518	GCT	snp	A	131566512	TBC1D13
9	134758087	134758099	CTC	snp	T	134758095	MED27
9	140647810	140647822	CAG	snp	T	140647816	EHMT1
X	1715371	1715389	TCC	snp	T	1715384	AKAP17A
X	1715371	1715389	TCC	snp	T	1715384	ASMT
X	18668097	18668109	CCG	snp	A	18668105	CDKL5
X	18668097	18668109	CCG	snp	A	18668105	RS1
X	20134976	20134988	GCC	snp	A	20134979	MAP7D2
X	21274118	21274136	TTA	snp	C	21274130	Mir_544
X	53239525	53239537	CCA	snp	T	53239532	KDM5C
X	69944498	69944510	TAA	snp	C	69944504	TEX11
X	92928108	92928126	GCT	snp	C	92928113	FAM133A
X	92928108	92928126	GCT	snp	C	92928113	NAP1L3
X	134476990	134477011	ACA	snp	G	134477005	BC029787
X	134476990	134477011	ACA	snp	G	134477005	DKFZp451F083
X	134476990	134477011	ACA	snp	G	134477005	ZNF449
X	140993905	140993917	CCT	snp	T	140993911	MAGEC1
X	144901395	144901407	AGA	snp	A	144901399	SLITRK2
10	14969613	14969633	GAGG	snp	A	14969620	DCLRE1C
10	26592373	26592397	AGGG	snp	A	26592388	GAD2

10	26592373	26592397	AGGG	snp	A	26592392	GAD2
10	34409356	34409372	AAAT	snp	T	34409366	PARD3
10	55954482	55954502	TCTA	snp	A	55954487	PCDH15
10	57360638	57360662	ATTT	snp	A	57360645	MTRNR2L5
10	57360638	57360662	ATTT	snp	A	57360645	PCDH15
10	59957061	59957089	AAAT	snp	C	59957070	IPMK
10	68934890	68934918	AGGG	snp	A	68934897	CTNNA3
10	71103904	71103928	ATCC	snp	T	71103922	HK1
10	71695825	71695845	AGGG	snp	A	71695838	COL13A1
10	81838055	81838075	TCAT	snp	C	81838070	FAM213A
10	81838055	81838075	TCAT	snp	C	81838070	FAM213A
10	81838055	81838075	TCAT	snp	C	81838070	LOC219347
10	97081357	97081373	ATGA	snp	A	97081363	SORBS1
10	97604496	97604516	GATG	snp	C	97604502	ENTPD1
10	97604496	97604516	GATG	snp	C	97604502	ENTPD1
10	97604496	97604516	GATG	snp	C	97604502	ENTPD1
10	97604496	97604516	GATG	snp	C	97604502	LOC728558
10	97604496	97604516	GATG	snp	C	97604502	LOC728558
10	116228028	116228052	TTTG	snp	T	116228047	ABLIM1
10	121435391	121435411	CCTT	snp	C	121435406	BAG3
11	22284765	22284781	TTTA	snp	A	22284770	ANO5
11	30944565	30944581	AGGG	snp	A	30944571	DCDC5
11	31494405	31494421	TTTA	snp	T	31494416	IMMP1L
11	55656687	55656707	AAAT	snp	G	55656693	SPRYD5
11	61044640	61044660	GGAG	snp	A	61044655	VWCE
11	62443394	62443422	AATA	snp	C	62443414	UBXN1
11	66242189	66242213	ATTT	snp	T	66242205	PELI3
11	73821127	73821147	AAAT	snp	A	73821134	C2CD3
11	126325704	126325724	AAAC	snp	C	126325716	KIRREL3
11	131408995	131409011	TTTG	snp	G	131409000	AK128059
11	131408995	131409011	TTTG	snp	G	131409000	AK128059
11	131408995	131409011	TTTG	snp	G	131409000	AK128059
11	131408995	131409011	TTTG	snp	G	131409000	NTM

11	131408995	131409011	TTTG	snp	G	131409000	NTM
11	131408995	131409011	TTTG	snp	G	131409000	NTM
12	442604	442624	AAAC	snp	T	442619	KDM5A
12	6220533	6220549	ATTC	snp	T	6220544	VWF
12	10046637	10046653	TTTC	snp	G	10046644	KLRF2
12	101672929	101672945	TGAA	snp	T	101672938	UTP20
12	104300975	104300995	GAAG	snp	T	104300987	GNN
12	109608637	109608653	TTTG	snp	C	109608648	ACACB
12	110251527	110251543	GATG	snp	A	110251535	TRPV4
12	111950109	111950129	TTTG	snp	A	111950116	ATXN2
12	112567444	112567460	TTTA	snp	G	112567449	TRAFD1
12	113876812	113876828	ATCC	snp	G	113876820	SDSL
12	120571871	120571887	AAAC	snp	G	120571875	GCN1L1
12	122209945	122209961	TCTT	snp	C	122209953	TMEM120B
12	124005106	124005134	AAAC	snp	C	124005128	RILPL1
12	126453976	126453992	TCTT	snp	C	126453982	LOC400084
12	132614133	132614161	TTTG	snp	T	132614140	EP400NL
13	23754900	23754916	TTGT	snp	C	23754907	SGCG
13	28563283	28563307	TTTG	snp	C	28563298	PRHOXNB
13	50295955	50295975	CAAA	snp	G	50295960	KPNA3
13	71276432	71276460	AAGG	snp	A	71276451	Y_RNA
14	20693540	20693564	TTTG	snp	A	20693555	OR11H6
14	20756341	20756361	AGAT	snp	C	20756356	TTC5
14	20914592	20914608	TGTT	snp	A	20914597	OSGEP
14	23003530	23003550	TTTA	snp	G	23003545	AV8S2A1N1T
14	23003530	23003550	TTTA	snp	G	23003545	hADV36S1
14	23003530	23003550	TTTA	snp	G	23003545	TCRA
14	23003530	23003550	TTTA	snp	G	23003545	TCRA
14	23003530	23003550	TTTA	snp	G	23003545	TCRA
14	23003530	23003550	TTTA	snp	G	23003545	TCRA
14	23003530	23003550	TTTA	snp	G	23003545	TCRA
14	23003530	23003550	TTTA	snp	G	23003545	TCRA
14	23003530	23003550	TTTA	snp	G	23003545	TCRA

14	23003530	23003550	TTTA	snp	G	23003545	TCRA
14	23003530	23003550	TTTA	snp	G	23003545	TCRA
14	23003530	23003550	TTTA	snp	G	23003545	TRA
14	23003530	23003550	TTTA	snp	G	23003545	TRA
14	23003530	23003550	TTTA	snp	G	23003545	TRA@
14	23003530	23003550	TTTA	snp	G	23003545	TRA@
14	23003530	23003550	TTTA	snp	G	23003545	TRA@
14	23003530	23003550	TTTA	snp	G	23003545	TRA@
14	23003530	23003550	TTTA	snp	G	23003545	TRAC
14	23003530	23003550	TTTA	snp	G	23003545	TRAC
14	23003530	23003550	TTTA	snp	G	23003545	TRAC
14	23003530	23003550	TTTA	snp	G	23003545	TRAC
14	23003530	23003550	TTTA	snp	G	23003545	TRAC
14	23003530	23003550	TTTA	snp	G	23003545	TRAC
14	23003530	23003550	TTTA	snp	G	23003545	TRAC
14	23003530	23003550	TTTA	snp	G	23003545	TRAC
14	23003530	23003550	TTTA	snp	G	23003545	TRD
14	23003530	23003550	TTTA	snp	G	23003545	X74394
14	31840298	31840322	CATT	snp	A	31840310	HEATR5A
14	50132540	50132556	TTTA	snp	C	50132548	NEMF
14	50132540	50132556	TTTA	snp	C	50132548	NEMF
14	50132540	50132556	TTTA	snp	C	50132548	POLE2
14	50132540	50132556	TTTA	snp	C	50132548	POLE2
14	60452681	60452697	TTCT	snp	C	60452689	AK128037
14	68757911	68757927	TTTA	snp	C	68757919	RAD51B
14	90437174	90437198	ATTT	snp	A	90437181	TDP1
14	92497437	92497453	TTTA	snp	T	92497448	TRIP11
14	95048993	95049009	TTCA	snp	G	95049000	SERPINA5
14	103575546	103575562	CTCC	snp	T	103575557	EXOC3L4
14	106677140	106677156	TTCC	snp	T	106677151	abParts
15	21198674	21198698	TATT	snp	C	21198684	LOC348120
15	28115993	28116009	AAGC	snp	G	28116000	OCA2
15	42296584	42296600	AGGG	snp	A	42296591	PLA2G4E

15	44150450	44150470	TTTG	snp	T	44150465	WDR76
15	45049394	45049410	CATT	snp	C	45049401	TRIM69
15	45334697	45334725	AAAT	snp	G	45334716	SORD
15	45334697	45334725	AAAT	snp	G	45334720	SORD
15	52311804	52311820	GCGG	snp	C	52311810	MAPK6
15	53900954	53900982	AAAT	snp	G	53900962	WDR72
15	63111043	63111067	TTTA	snp	G	63111060	TLN2
15	68467170	68467186	AAAC	snp	G	68467179	PIAS1
15	75914379	75914399	AAAC	snp	A	75914386	SNUPN
15	101083687	101083707	CTTC	snp	C	101083697	CERS3
15	101841823	101841839	TTGA	snp	C	101841831	AK130759
16	280188	280204	TTAT	snp	C	280194	LUC7L
16	3601450	3601466	TCAT	snp	C	3601458	NLRC3
16	3789069	3789085	AAAC	snp	T	3789076	CREBBP
16	8734685	8734705	GATG	snp	A	8734700	METTL22
16	30390196	30390220	GAGG	snp	A	30390203	MYLPF
16	30390196	30390220	GAGG	snp	A	30390203	SEPT1
16	30390196	30390220	GAGG	snp	A	30390203	SEPT1
16	30390196	30390220	GAGG	snp	A	30390203	SEPT1
16	30390196	30390220	GAGG	snp	A	30390203	SEPT1
16	30390196	30390220	GAGG	snp	A	30390203	ZNF48
16	30390196	30390220	GAGG	snp	A	30390203	ZNF48
16	30390196	30390220	GAGG	snp	A	30390203	ZNF48
16	30390196	30390220	GAGG	snp	A	30390203	ZNF48
16	57064294	57064322	GATG	snp	C	57064312	NLRC5
16	57763258	57763274	TGAG	snp	T	57763268	CCDC135
16	76533440	76533464	TTTA	snp	T	76533459	CNTNAP4
16	83213762	83213778	GTTT	snp	T	83213770	CDH13
16	84650110	84650138	AAAT	snp	C	84650129	COTL1
16	84905149	84905165	TTTG	snp	C	84905154	CRISPLD2
16	88728584	88728600	AAAG	snp	G	88728593	MVD
16	88793808	88793824	TGCG	snp	C	88793812	PIEZO1
17	3380452	3380468	AAAT	snp	A	3380459	ASPA

17	3380452	3380468	AAAT	snp	A	3380459	SPATA22
17	4146357	4146381	TTTG	snp	T	4146376	ANKFY1
17	5998000	5998020	GGAA	snp	T	5998004	WSCD1
17	7244465	7244481	TTTA	snp	G	7244476	ACAP1
17	7298946	7298970	AAAT	snp	C	7298951	PLSCR3
17	27779235	27779263	AAAT	snp	C	27779244	TAOK1
17	29670565	29670581	AAAT	snp	G	29670571	NF1
17	37810420	37810448	TCAT	snp	G	37810438	STARD3
17	38177832	38177848	GGAA	snp	C	38177838	MED24
17	40834060	40834084	GAAA	snp	T	40834072	CCR10
17	40834060	40834084	GAAA	snp	T	40834072	CNTNAP1
17	41295103	41295127	TATT	snp	C	41295121	BRCA1
17	41295103	41295127	TATT	snp	C	41295121	NBR2
17	44059396	44059412	AAAG	snp	A	44059407	MAPT
17	46210425	46210441	AAAC	snp	C	46210433	SKAP1
17	55346194	55346222	CAAA	snp	A	55346210	MSI2
17	56232459	56232475	TTTG	snp	A	56232467	OR4D1
17	60491927	60491943	TGTT	snp	T	60491932	EFCAB3
17	72845201	72845217	GAGG	snp	C	72845209	GRIN2C
17	73031927	73031951	AAAC	snp	T	73031946	KCTD2
17	73031927	73031951	AAAC	snp	T	73031946	TRNA_Arg
17	76485942	76485962	GGAT	snp	G	76485953	DNAH17
17	76866503	76866531	AAAG	snp	C	76866526	TIMP2
18	117490	117506	ATTG	snp	C	117499	ROCK1P1
18	24040047	24040071	CATT	snp	C	24040053	KCTD1
18	32958099	32958119	AAAT	snp	T	32958108	ZNF396
18	53770221	53770237	TGTT	snp	A	53770228	LOC100505474
19	1275916	1275940	CCCT	snp	T	1275929	C19orf24
19	3477478	3477494	GATG	snp	T	3477485	C19orf77
19	4530685	4530701	ATTT	snp	C	4530693	PLIN5
19	6480493	6480509	TCTG	snp	A	6480500	DENND1C
19	6708572	6708588	TCCT	snp	C	6708583	C3
19	8954553	8954569	TATG	snp	G	8954558	MBD3L1

19	10085539	10085555	TTTG	snp	C	10085547	COL5A3
19	12154107	12154131	TTAT	snp	G	12154119	ZNF878
19	12830830	12830846	TATT	snp	G	12830839	TNPO2
19	13225478	13225494	AAAT	snp	A	13225485	TRMT1
19	13398333	13398349	TTTC	snp	C	13398337	CACNA1A
19	16006981	16007001	TCCA	snp	T	16006991	CYP4F2
19	16503852	16503868	TAAA	snp	C	16503858	EPS15L1
19	16854465	16854485	TATT	snp	G	16854478	NWD1
19	16923482	16923510	AAAC	snp	G	16923493	NWD1
19	17757078	17757102	TGTT	snp	G	17757085	UNC13A
19	17784820	17784840	AAAC	snp	G	17784826	UNC13A
19	18184622	18184638	TTTG	snp	C	18184628	IL12RB1
19	19371024	19371040	TATT	snp	G	19371031	HAPLN4
19	21667879	21667895	CTAT	snp	C	21667886	LOC400680
19	34823950	34823970	TTTG	snp	G	34823955	KIAA0355
19	37063620	37063644	TATC	snp	C	37063630	BC039524
19	37063620	37063644	TATC	snp	C	37063630	ZNF529
19	37063620	37063644	TATC	snp	C	37063630	ZNF529
19	38958610	38958630	CATC	snp	G	38958623	RYR1
19	39692788	39692808	TTTG	snp	G	39692796	NCCRP1
19	39692788	39692808	TTTG	snp	G	39692796	SYCN
19	41449446	41449462	ATTG	snp	T	41449454	CYP2A7
19	41449446	41449462	ATTG	snp	T	41449454	CYP2B7P1
19	42127742	42127762	CTCC	snp	T	42127754	CEACAM4
19	44160409	44160429	AAAT	snp	A	44160416	PLAUR
19	45981019	45981043	AAAG	snp	G	45981036	ERCC1
19	45981019	45981043	AAAG	snp	G	45981036	TRNA_SeC
19	46507725	46507741	TTTG	snp	G	46507735	CCDC61
19	49374935	49374955	AATA	snp	C	49374945	PPP1R15A
19	50472613	50472633	GAAT	snp	G	50472619	SIGLEC16
19	51884592	51884608	AAAG	snp	G	51884596	LIM2
19	52129085	52129105	AAAC	snp	T	52129096	SIGLEC5
19	52824228	52824248	TAAA	snp	G	52824240	AK097759

19	52824228	52824248	TAAA	snp	G	52824240	ZNF480
19	54229121	54229137	GATT	snp	G	54229130	MIR516B2
19	55965206	55965226	AAAT	snp	G	55965221	ISOC2
19	59087775	59087791	CTTC	snp	C	59087781	MGC2752
19	59087775	59087791	CTTC	snp	T	59087782	MGC2752
1	3821301	3821321	TGGC	snp	C	3821311	LOC100133612
1	16341726	16341742	TGTC	snp	C	16341732	HSPB7
1	17663620	17663636	TTTA	snp	G	17663629	PADI4
1	27952451	27952471	AAAC	snp	C	27952455	FGR
1	33938839	33938855	TACC	snp	G	33938844	ZSCAN20
1	51939251	51939271	AAAC	snp	A	51939258	EPS15
1	52377776	52377792	TTTC	snp	C	52377786	RAB3B
1	53363886	53363906	TTTA	snp	C	53363894	ECHDC2
1	53526872	53526892	CATT	snp	C	53526878	PODN
1	63085993	63086013	AAAT	snp	A	63086000	DOCK7
1	78099856	78099876	AAAT	snp	A	78099863	ZZZ3
1	92199610	92199634	GAGG	snp	A	92199617	TGFBR3
1	153788202	153788218	TAGA	snp	C	153788210	GATAD2B
1	154173599	154173619	TTTA	snp	C	154173604	C1orf189
1	165566486	165566502	AAAG	snp	T	165566494	TRNA_Pseudo
1	167816642	167816666	TCTT	snp	T	167816655	ADCY10
1	169661385	169661405	ATTT	snp	A	169661390	C1orf112
1	169661385	169661405	ATTT	snp	A	169661390	SELL
1	179326340	179326356	TTTG	snp	C	179326349	SOAT1
1	186318805	186318821	AAAT	snp	A	186318816	MIR548F1
1	186318805	186318821	AAAT	snp	A	186318816	TPR
1	197272237	197272261	TTTA	snp	G	197272247	CRB1
1	207270518	207270534	AAAT	snp	A	207270525	C4BPB
1	207641016	207641032	ATTG	snp	C	207641022	CR2
1	220701165	220701189	ACAA	snp	G	220701184	MARK1
1	224419731	224419751	AAAT	snp	G	224419743	NVL
1	245674886	245674910	TTTG	snp	G	245674895	KIF26B
20	259699	259719	GGAG	snp	C	259709	C20orf96

20	2308269	2308289	ATCT	snp	G	2308273	TGM3
20	44280025	44280041	TTTG	snp	G	44280029	WFDC11
20	49624385	49624401	AAAT	snp	G	49624391	KCNG1
20	57122536	57122552	ATGA	snp	G	57122540	LOC149773
20	62164407	62164423	TTTC	snp	G	62164414	PTK6
20	62609664	62609680	AATG	snp	G	62609669	SAMD10
20	62613003	62613019	CCCG	snp	G	62613007	PRPF6
21	34619535	34619559	TTTC	snp	T	34619554	IFNAR2
21	37587688	37587704	ATGA	snp	C	37587697	DOPEY2
22	18082702	18082718	AAGG	snp	C	18082707	ATP6V1E1
22	18572460	18572484	TTTG	snp	C	18572475	PEX26
22	22736711	22736731	TTTA	snp	A	22736715	abParts
22	42208663	42208679	ATTC	snp	G	42208671	bK250D10.C22.8
22	42208663	42208679	ATTC	snp	G	42208671	CCDC134
22	43466905	43466925	TATG	snp	C	43466915	TTLL1
22	43607925	43607941	GACA	snp	G	43607932	SCUBE1
22	46594151	46594167	AATA	snp	G	46594162	PPARA
2	11880848	11880872	AAAG	snp	C	11880855	LPIN1
2	26612879	26612895	TGTC	snp	T	26612888	EPT1
2	58277019	58277039	TGAA	snp	G	58277030	VRK2
2	59760308	59760328	TATT	snp	C	59760321	Mir_548
2	75425096	75425116	AAAC	snp	T	75425111	TACR1
2	109108220	109108244	ATTT	snp	T	109108236	GCC2
2	110323689	110323705	ATTT	snp	T	110323693	SEPT10
2	113824832	113824852	TGGA	snp	T	113824839	IL1F10
2	128292556	128292576	TTTG	snp	T	128292571	MYO7B
2	167301885	167301901	GGGA	snp	A	167301894	SCN7A
2	191923017	191923037	TTTA	snp	C	191923025	STAT4
2	196675131	196675147	TTAT	snp	G	196675139	DNAH7
2	217025386	217025414	TTAA	snp	T	217025404	XRCC5
2	217025386	217025414	TTAA	snp	T	217025408	XRCC5
2	220032460	220032476	CAAA	snp	G	220032466	NHEJ1
2	220032460	220032476	CAAA	snp	G	220032466	NHEJ1

2	220032460	220032476	CAAA	snp	G	220032466	NHEJ1
2	220032460	220032476	CAAA	snp	G	220032466	SLC23A3
2	220032460	220032476	CAAA	snp	G	220032466	SLC23A3
2	220032460	220032476	CAAA	snp	G	220032466	SLC23A3
2	231558605	231558625	TTTG	snp	T	231558620	LOC151475
2	234968181	234968205	AAAC	snp	G	234968193	SPP2
2	236791624	236791640	TTTA	snp	G	236791631	AGAP1
2	238622268	238622292	AATG	snp	G	238622273	LRRFIP1
2	242371225	242371245	TTTA	snp	T	242371240	FARP2
3	8686194	8686210	AGGA	snp	C	8686201	C3orf32
3	27327020	27327036	AAAC	snp	A	27327031	NEK10
3	52619538	52619558	ATTT	snp	A	52619545	PBRM1
3	124052740	124052756	AAAC	snp	T	124052751	KALRN
3	133468005	133468021	AAAT	snp	G	133468011	TF
3	183696109	183696125	TTTG	snp	C	183696114	ABCC5
3	186290127	186290151	ATTT	snp	T	186290143	DNAJB11
3	195944496	195944512	GAAG	snp	T	195944503	OSTalpha
4	6238402	6238422	TATT	snp	C	6238407	LOC285484
4	28821926	28821942	AAAG	snp	G	28821932	MIR4275
4	40438200	40438220	AAAC	snp	A	40438207	RBM47
4	68383561	68383577	ATAC	snp	C	68383566	CENPC1
4	81188887	81188911	TAGA	snp	A	81188893	FGF5
4	83787256	83787272	AGGG	snp	T	83787266	SEC31A
4	89725403	89725419	AAAT	snp	G	89725413	FAM13A
4	101343954	101343974	TTTC	snp	T	101343969	EMCN
4	105593759	105593775	CTTT	snp	C	105593768	AK094561
4	122748140	122748160	AAAC	snp	T	122748154	BBS7
4	151207961	151207981	TGGT	snp	C	151207968	LRBA
4	151207961	151207981	TGGT	snp	C	151207969	LRBA
4	169140541	169140557	TGAT	snp	C	169140552	DDX60
4	185983994	185984018	TTTG	snp	C	185984013	BC043280
4	190944992	190945008	CTCC	snp	T	190945002	FRG2
4	190944992	190945008	CTCC	snp	T	190945002	LOC100288255

5	21779303	21779319	TATT	snp	C	21779309	BC038535
5	21779303	21779319	TATT	snp	C	21779309	CDH12
5	31193619	31193647	AAAG	snp	A	31193642	CDH6
5	60921669	60921685	GCGG	snp	T	60921674	BC032910
5	60921669	60921685	GCGG	snp	A	60921679	BC032910
5	68424246	68424262	TTTG	snp	G	68424252	SLC30A5
5	78352365	78352381	AATA	snp	C	78352371	DMGDH
5	79408607	79408623	AAAT	snp	A	79408614	SERINC5
5	90606422	90606446	CTTT	snp	A	90606430	AK091866
5	109183334	109183350	TTGT	snp	T	109183340	MAN2A1
5	110713602	110713618	TGAA	snp	G	110713609	CAMK4
5	114481211	114481239	ATTT	snp	C	114481229	TRIM36
5	148384555	148384583	TTTG	snp	C	148384562	SH3TC2
6	2668985	2669001	TTTA	snp	C	2668989	MYLK4
6	12750746	12750762	GGAA	snp	A	12750755	PHACTR1
6	17102574	17102602	AAAC	snp	G	17102595	FLJ23152
6	28776224	28776240	TGTT	snp	C	28776233	TRNA_Phe
6	32311867	32311883	ATTT	snp	A	32311874	C6orf10
6	35756467	35756487	GAAG	snp	A	35756471	C6orf127
6	38748578	38748598	AACA	snp	T	38748592	DNAH8
6	42849205	42849225	AAAT	snp	A	42849212	RPL7L1
6	151131686	151131702	GAAA	snp	G	151131697	PLEKHG1
6	151870025	151870041	TTTG	snp	C	151870032	C6orf97
6	155468429	155468453	TTTA	snp	G	155468436	TIAM2
7	32111087	32111103	TCCC	snp	T	32111093	PDE1C
7	66237904	66237928	AAAT	snp	A	66237911	RABGEF1
7	66578600	66578616	TTTC	snp	T	66578611	MIR4650-1
7	66578600	66578616	TTTC	snp	T	66578611	TYW1
7	74112277	74112305	TTTG	snp	T	74112284	GTF2I
7	97616993	97617013	TTTA	snp	C	97617005	OCM2
7	102116372	102116388	AAAC	snp	A	102116383	POLR2J
7	130060612	130060636	AATA	snp	A	130060622	CEP41
7	148826887	148826903	TTTA	snp	G	148826898	ZNF398

8	2090631	2090655	TCCC	snp	T	2090648	MYOM2
8	22104944	22104964	AAAT	snp	G	22104954	POLR3D
8	38837192	38837216	TTTA	snp	T	38837207	HTRA4
8	39862087	39862107	GATA	snp	T	39862092	IDO2
8	42806209	42806225	TTAT	snp	G	42806217	HOOK3
8	79471149	79471173	GTTT	snp	C	79471162	BC036404
8	79471149	79471173	GTTT	snp	C	79471162	PKIA
8	99163310	99163326	TGTT	snp	T	99163315	POP1
8	103226994	103227018	AAAT	snp	A	103227001	RRM2B
8	125060782	125060798	TACA	snp	G	125060790	AK057332
8	125060782	125060798	TACA	snp	G	125060790	FER1L6
8	125592431	125592447	ATAC	snp	T	125592442	MTSS1
8	131414553	131414573	GACA	snp	T	131414563	ASAP1
8	145617629	145617653	CCCT	snp	C	145617640	ADCK5
8	145617629	145617653	CCCT	snp	C	145617640	CPSF1
9	418269	418285	TTTG	snp	G	418275	DOCK8
9	428960	428980	TTTA	snp	G	428971	DOCK8
9	21031462	21031478	CCGC	snp	A	21031470	PTPLAD2
9	35834933	35834949	TTTC	snp	G	35834941	TMEM8B
9	36355839	36355855	GTTT	snp	C	36355849	RNF38
9	39087486	39087502	TTTG	snp	T	39087497	CNTNAP3
9	77427804	77427824	TGAA	snp	G	77427818	TRPM6
9	95047049	95047069	CAAA	snp	C	95047054	IARS
9	101514373	101514401	ACAA	snp	C	101514396	ANKS6
9	101531421	101531437	AAAG	snp	G	101531425	ANKS6
9	131814092	131814120	TAAT	snp	C	131814097	FAM73B
9	133330404	133330420	CATC	snp	A	133330411	ASS1
X	1741831	1741847	AAAT	snp	A	1741842	ASMT
X	2161519	2161539	TTTA	snp	A	2161523	DHRSX
X	15308146	15308166	AAAT	snp	T	15308158	ASB11
X	32828060	32828084	AAAG	snp	A	32828071	DMD
X	49767231	49767251	GTCT	snp	A	49767240	CLCN5
X	49767231	49767251	GTCT	snp	A	49767240	CLCN5

X	49767231	49767251	GTCT	snp	A	49767240	MIR188
X	49767231	49767251	GTCT	snp	A	49767240	MIR532
X	108724145	108724161	GGAA	snp	T	108724151	GUCY2F
X	138288179	138288207	AAAG	snp	G	138288196	FGF13
X	153538780	153538796	CAAA	snp	C	153538785	TKTL1
X	153679937	153679957	CTTC	snp	C	153679942	FAM50A
10	5029662	5029671	T	ins	C	5029664	AKR1C2
10	5029662	5029671	T	ins	C	5029664	AKR1C3
10	14970264	14970273	A	ins	AAT	14970271	DCLRE1C
10	17495036	17495045	A	ins	AG	17495040	ST8SIA6
10	26593898	26593907	T	ins	G	26593901	GAD2
10	27508245	27508255	A	ins	AC	27508246	ACBD5
10	55942749	55942758	T	ins	G	55942753	PCDH15
10	70157355	70157363	A	ins	AAAATT	70157358	RUFY2
10	70181573	70181582	T	ins	C	70181580	DNA2
10	71992610	71992618	C	ins	CA	71992611	PPA1
10	75083089	75083099	G	ins	T	75083091	TTC18
10	75083089	75083099	G	ins	GC	75083095	TTC18
10	89006360	89006370	T	ins	C	89006362	LOC728190
10	90068198	90068208	A	ins	C	90068205	RNLS
10	98429537	98429547	A	ins	C	98429539	PIK3AP1
10	99019602	99019610	T	ins	G	99019605	ARHGAP19
10	104241136	104241144	G	ins	A	104241137	ACTR1A
10	115347097	115347106	A	ins	AC	115347098	HABP2
10	115347097	115347106	A	ins	AC	115347098	NRAP
10	117855993	117856003	T	ins	C	117855994	GFRA1
10	127397438	127397446	C	ins	T	127397441	FLJ37035
10	127397438	127397446	C	ins	T	127397441	LOC283038
11	1891030	1891038	G	ins	A	1891034	LSP1
11	3861766	3861774	C	ins	A	3861772	RHOG
11	5013421	5013431	T	ins	G	5013422	MMP26
11	8941194	8941203	T	ins	G	8941195	AKIP1
11	8941194	8941203	T	ins	G	8941195	C11orf16

11	10546628	10546638	A	ins	G	10546636	RNF141
11	17035487	17035497	C	ins	A	17035492	PLEKHA7
11	32450244	32450252	G	ins	T	32450249	WT1
11	32852033	32852042	T	ins	G	32852038	PRRG4
11	35228216	35228224	G	ins	CA	35228217	CD44
11	56112867	56112877	T	ins	C	56112872	OR8K1
11	61735381	61735391	C	ins	A	61735389	BC132896
11	61735381	61735391	C	ins	A	61735389	FTH1
11	65662427	65662436	T	ins	CC	65662434	FOSL1
11	72414453	72414463	G	ins	GC	72414456	ARAP1
11	74411661	74411669	T	ins	TA	74411664	CHRDL2
11	90280986	90280994	T	ins	TTC	90280987	HP11113
11	90280986	90280994	T	ins	TC	90280988	HP11113
11	90280986	90280994	T	ins	C	90280989	HP11113
11	93170909	93170918	C	ins	CG	93170913	CCDC67
11	117168337	117168347	A	ins	C	117168338	BACE1
12	1909656	1909666	C	ins	A	1909663	CACNA2D4
12	3918186	3918196	T	ins	TTTTTATAAACACA	3918187	PARP11
12	4870783	4870793	T	ins	G	4870784	GALNT8
12	6717210	6717219	C	ins	A	6717216	CHD4
12	9067053	9067063	G	ins	GT	9067057	PHC1
12	9555620	9555629	G	ins	GGA	9555623	DQ599803
12	15096338	15096346	T	ins	G	15096339	ARHGDIB
12	27522232	27522240	G	ins	T	27522233	ARNTL2
12	28125848	28125857	C	ins	CG	28125849	PTHLH
12	30949712	30949721	C	ins	A	30949716	LOC100287314
12	32760459	32760467	T	ins	G	32760460	FGD4
12	39070797	39070807	A	ins	C	39070803	CPNE8
12	50291547	50291556	C	ins	A	50291548	FAIM2
12	50291547	50291556	C	ins	A	50291549	FAIM2
12	50532188	50532197	A	ins	C	50532191	CERS5
12	51403995	51404005	A	ins	C	51403997	SLC11A2
12	51403995	51404005	A	ins	C	51403997	SLC11A2

12	51403995	51404005	A	ins	C	51403997	U7
12	52696625	52696635	G	ins	GA	52696627	KRT81
12	52696625	52696635	G	ins	GA	52696627	KRT81
12	52696625	52696635	G	ins	GA	52696627	KRT86
12	52696625	52696635	G	ins	GA	52696627	KRT86
12	52696625	52696635	G	ins	A	52696628	KRT81
12	52696625	52696635	G	ins	A	52696628	KRT81
12	52696625	52696635	G	ins	A	52696628	KRT86
12	52696625	52696635	G	ins	A	52696628	KRT86
12	64058129	64058138	A	ins	C	64058131	DPY19L2
12	66232376	66232384	T	ins	G	66232382	HMGA2
12	70328970	70328979	T	ins	G	70328977	C12orf28
12	71016729	71016738	G	ins	A	71016730	PTPRB
12	92821236	92821244	G	ins	A	92821237	CLLU1
12	92821236	92821244	G	ins	A	92821237	CLLU1
12	92821236	92821244	G	ins	A	92821237	CLLU1OS
12	98896608	98896618	C	ins	CA	98896614	LOC643770
12	98896608	98896618	C	ins	CA	98896614	LOC643770
12	98896608	98896618	C	ins	CA	98896614	TRNA_Asp
12	104415852	104415861	A	ins	G	104415854	GLT8D2
12	104496721	104496730	C	ins	T	104496722	HCFC2
12	117657108	117657116	T	ins	G	117657111	NOS1
12	124022055	124022064	A	ins	AC	124022056	MIR3908
12	130930564	130930574	C	ins	T	130930567	RIMBP2
12	132575528	132575536	T	ins	TTTG	132575530	EP400NL
13	19419837	19419846	A	ins	C	19419844	ANKRD20A9P
13	21988035	21988043	A	ins	CTT	21988037	ZDHHC20
13	21988035	21988043	A	ins	CTGT	21988039	ZDHHC20
13	30881149	30881158	T	ins	TC	30881156	KATNAL1
13	32524869	32524879	T	ins	G	32524870	DKFZp666K117
13	32524869	32524879	T	ins	G	32524870	EEF1DP3
13	91545152	91545160	C	ins	A	91545158	LINC00410
13	95747080	95747089	A	ins	C	95747086	ABCC4

13	99128494	99128503	C	ins	CG	99128498	STK24
13	108884457	108884466	A	ins	C	108884464	ABHD13
14	20811075	20811084	G	ins	T	20811076	PARP2
14	20811075	20811084	G	ins	T	20811076	RPPH1
14	22771751	22771759	T	ins	TTTTTC	22771753	av27s1
14	22771751	22771759	T	ins	TTTTTC	22771753	av27s1
14	22771751	22771759	T	ins	TTTTTC	22771753	AV4S1
14	22771751	22771759	T	ins	TTTTTC	22771753	AV4S1
14	22771751	22771759	T	ins	TTTTTC	22771753	hADV29S1
14	22771751	22771759	T	ins	TTTTTC	22771753	hADV29S1
14	22771751	22771759	T	ins	TTTTTC	22771753	hADV36S1
14	22771751	22771759	T	ins	TTTTTC	22771753	hADV36S1
14	22771751	22771759	T	ins	TTTTTC	22771753	hADV38S2
14	22771751	22771759	T	ins	TTTTTC	22771753	hADV38S2
14	22771751	22771759	T	ins	TTTTTC	22771753	T-Cell
14	22771751	22771759	T	ins	TTTTTC	22771753	T-Cell
14	22771751	22771759	T	ins	TTTTTC	22771753	TCRA
14	22771751	22771759	T	ins	TTTTTC	22771753	TCRA
14	22771751	22771759	T	ins	TTTTTC	22771753	TCRA
14	22771751	22771759	T	ins	TTTTTC	22771753	TCRA
14	22771751	22771759	T	ins	TTTTTC	22771753	TCRA
14	22771751	22771759	T	ins	TTTTTC	22771753	TCRA
14	22771751	22771759	T	ins	TTTTTC	22771753	TCRA
14	22771751	22771759	T	ins	TTTTTC	22771753	TCRA
14	22771751	22771759	T	ins	TTTTTC	22771753	TCRA
14	22771751	22771759	T	ins	TTTTTC	22771753	TCRA
14	22771751	22771759	T	ins	TTTTTC	22771753	TCR-alpha
14	22771751	22771759	T	ins	TTTTTC	22771753	TCR-alpha
14	22771751	22771759	T	ins	TTTTTC	22771753	TCR-alpha
14	22771751	22771759	T	ins	TTTTTC	22771753	TCR-alpha
14	22771751	22771759	T	ins	TTTTTC	22771753	TRA
14	22771751	22771759	T	ins	TTTTTC	22771753	TRA
14	22771751	22771759	T	ins	TTTTTC	22771753	TRA

14	22771751	22771759	T	ins	TTTTTC	22771753	TRA
14	22771751	22771759	T	ins	TTTTTC	22771753	TRA
14	22771751	22771759	T	ins	TTTTTC	22771753	TRA
14	22771751	22771759	T	ins	TTTTTC	22771753	TRA@
14	22771751	22771759	T	ins	TTTTTC	22771753	TRA@
14	22771751	22771759	T	ins	TTTTTC	22771753	TRAC
14	22771751	22771759	T	ins	TTTTTC	22771753	TRAC
14	22771751	22771759	T	ins	TTTTTC	22771753	TRAC
14	22771751	22771759	T	ins	TTTTTC	22771753	TRAC
14	22771751	22771759	T	ins	TTTTTC	22771753	TRD
14	22771751	22771759	T	ins	TTTTTC	22771753	TRD
14	24026963	24026971	T	ins	TTC	24026968	AP1G2
14	24026963	24026971	T	ins	TTC	24026968	THTPA
14	24026963	24026971	T	ins	TTC	24026968	THTPA
14	29261304	29261312	A	ins	C	29261306	C14orf23
14	35032940	35032948	T	ins	TC	35032941	SNX6
14	35032940	35032948	T	ins	C	35032942	SNX6
14	48258395	48258403	A	ins	C	48258399	LOC100506433
14	50847520	50847530	T	ins	TG	50847521	CDKL1
14	50847520	50847530	T	ins	G	50847522	CDKL1
14	51311620	51311630	A	ins	C	51311622	SnoU83B
14	51311620	51311630	A	ins	AC	51311623	SnoU83B
14	51311620	51311630	A	ins	C	51311624	SnoU83B
14	55159594	55159603	C	ins	CCCG	55159596	SAMD4A
14	55159594	55159603	C	ins	CCA	55159599	SAMD4A
14	55454950	55454959	T	ins	C	55454955	WDHD1
14	55906332	55906341	C	ins	T	55906336	TBPL2
14	64896048	64896056	T	ins	TG	64896049	MTHFD1
14	67940982	67940992	A	ins	AAC	67940983	TMEM229B
14	70419866	70419875	T	ins	G	70419867	SMOC1
14	73008813	73008821	A	ins	AAAAAAAAC	73008819	RGS6
14	74024571	74024579	T	ins	C	74024575	ACOT1
14	74024571	74024579	T	ins	C	74024575	HEATR4

14	92491853	92491861	C	ins	A	92491858	TRIP11
14	93307108	93307118	T	ins	TCTC	93307109	GOLGA5
14	94547060	94547069	A	ins	AC	94547061	DDX24
14	94547060	94547069	A	ins	AC	94547061	IFI27L1
14	94547060	94547069	A	ins	C	94547062	DDX24
14	94547060	94547069	A	ins	C	94547062	IFI27L1
14	106545788	106545797	A	ins	C	106545794	abParts
15	31065488	31065498	T	ins	G	31065490	LOC100288637
15	32393654	32393662	A	ins	G	32393660	CHRFAM7A
15	32393654	32393662	A	ins	G	32393660	CHRNA7
15	32393654	32393662	A	ins	G	32393660	CHRNA7
15	35812431	35812441	A	ins	C	35812436	ATPBD4
15	40932274	40932282	T	ins	TC	40932276	CASC5
15	41656747	41656756	A	ins	AAAG	41656752	NUSAP1
15	42111745	42111755	G	ins	GGGGC	42111750	MAPKBP1
15	50787683	50787691	G	ins	T	50787688	AX746640
15	50787683	50787691	G	ins	T	50787688	USP8
15	52566867	52566877	C	ins	A	52566868	MYO5C
15	55835150	55835158	A	ins	C	55835156	AK055370
15	59373730	59373738	A	ins	C	59373731	RNF111
15	60786381	60786389	A	ins	C	60786387	BC035094
15	60786381	60786389	A	ins	C	60786387	RORA
15	65688554	65688564	C	ins	T	65688557	IGDCC4
15	67692820	67692828	T	ins	TTTTC	67692823	IQCH
15	72074590	72074599	G	ins	T	72074592	THSD4
15	82558486	82558496	A	ins	C	82558487	FAM154B
15	86029515	86029525	T	ins	C	86029519	AKAP13
16	634159	634169	G	ins	A	634161	PIGQ
16	773313	773321	C	ins	A	773318	CCDC78
16	773313	773321	C	ins	A	773318	FAM173A
16	2720101	2720111	C	ins	CCA	2720104	ERVK13-1
16	15976922	15976930	A	ins	AAG	15976928	FOPNL
16	19713306	19713315	T	ins	C	19713313	C16orf62

16	20826864	20826872	T	ins	TC	20826866	ERI2
16	20826864	20826872	T	ins	TC	20826866	ERI2
16	20826864	20826872	T	ins	TC	20826866	LOC81691
16	20826864	20826872	T	ins	TC	20826866	LOC81691
16	20927404	20927414	T	ins	G	20927405	LYRM1
16	21628753	21628763	A	ins	C	21628757	LOC23117
16	21628753	21628763	A	ins	C	21628757	METTL9
16	24830828	24830838	T	ins	TC	24830829	TNRC6A
16	24830828	24830838	T	ins	C	24830830	TNRC6A
16	48268283	48268293	T	ins	GA	48268285	ABCC11
16	48292428	48292437	T	ins	G	48292429	LONP2
16	48292428	48292437	T	ins	G	48292429	MIR548AE2
16	50347346	50347356	A	ins	C	50347347	ADCY7
16	57071226	57071236	C	ins	T	57071233	NLRC5
16	66807296	66807305	T	ins	C	66807300	CCDC79
16	74497624	74497632	T	ins	TTG	74497626	GLG1
16	74497624	74497632	T	ins	TG	74497627	GLG1
16	74497624	74497632	T	ins	G	74497628	GLG1
16	75202445	75202455	A	ins	G	75202446	ZFP1
16	75299920	75299928	C	ins	T	75299923	BCAR1
17	1964759	1964769	G	ins	T	1964762	AX747853
17	1964759	1964769	G	ins	T	1964762	SMG6
17	5420670	5420680	C	ins	CTCCCCCG	5420672	NLRP1
17	7166926	7166934	T	ins	G	7166931	CLDN7
17	7459290	7459298	T	ins	TTTTG	7459291	TNFSF12
17	7459290	7459298	T	ins	TTTTG	7459291	TNFSF12-TNFSF13
17	7459290	7459298	T	ins	TTTG	7459292	TNFSF12
17	7459290	7459298	T	ins	TTTG	7459292	TNFSF12-TNFSF13
17	7588774	7588782	T	ins	G	7588776	TP53
17	7588774	7588782	T	ins	G	7588776	WRAP53
17	10274592	10274601	A	ins	C	10274594	MYH13
17	11826368	11826378	T	ins	G	11826374	DNAH9
17	16874694	16874703	A	ins	C	16874697	TNFRSF13B

17	17761540	17761550	T	ins	C	17761545	TOM1L2
17	18605672	18605682	T	ins	TTTTC	18605678	TRIM16L
17	20906266	20906274	G	ins	T	20906271	USP22
17	28950663	28950672	C	ins	A	28950665	LRRC37BP1
17	28950663	28950672	C	ins	A	28950665	SH3GL1P2
17	31899321	31899329	C	ins	A	31899324	ACCN1
17	31899321	31899329	C	ins	A	31899324	AK057317
17	33764396	33764404	G	ins	A	33764397	SLFN13
17	37312311	37312320	T	ins	G	37312315	ARL5C
17	38186919	38186929	T	ins	TA	38186920	MED24
17	40264592	40264601	G	ins	GT	40264595	DHX58
17	40264592	40264601	G	ins	GT	40264595	KAT2A
17	40264592	40264601	G	ins	GA	40264598	DHX58
17	40264592	40264601	G	ins	GA	40264598	KAT2A
17	44845139	44845148	T	ins	TTTG	44845144	WNT3
17	45559459	45559467	A	ins	C	45559465	MRPL45P2
17	48542130	48542140	G	ins	T	48542138	ACSF2
17	48542130	48542140	G	ins	T	48542138	ACSF2
17	48542130	48542140	G	ins	T	48542138	ACSF2
17	48542130	48542140	G	ins	T	48542138	CHAD
17	48542130	48542140	G	ins	T	48542138	CHAD
17	48542130	48542140	G	ins	T	48542138	CHAD
17	59116003	59116011	T	ins	TTTG	59116006	BCAS3
17	61779377	61779386	G	ins	A	61779380	STRADA
17	65358802	65358812	A	ins	C	65358810	PSMD12
17	65882484	65882492	T	ins	G	65882490	BPTF
17	73909551	73909560	T	ins	TC	73909558	FBF1
17	74935936	74935945	T	ins	C	74935937	MGAT5B
17	79101066	79101075	C	ins	A	79101069	AATK
18	5956909	5956919	T	ins	G	5956917	L3MBTL4
18	7031986	7031995	A	ins	G	7031987	LAMA1
18	12123030	12123038	T	ins	G	12123034	ANKRD62
18	21124907	21124916	C	ins	CCCT	21124910	NPC1

18	24268642	24268651	T	ins	TTTAA	24268643	LOC728606
18	57365448	57365457	C	ins	CT	57365453	CCBE1
18	61652114	61652124	T	ins	TC	61652122	SERPINB8
18	72124959	72124967	C	ins	CCCCCT	72124960	FAM69C
18	72124959	72124967	C	ins	T	72124965	FAM69C
19	628631	628639	G	ins	GT	628633	POLRMT
19	680001	680010	C	ins	CT	680008	FSTL3
19	709911	709921	G	ins	T	709912	PALM
19	1925909	1925918	C	ins	A	1925913	SCAMP4
19	3295841	3295850	G	ins	T	3295843	CELF5
19	3366957	3366966	C	ins	A	3366964	NFIC
19	3819850	3819859	A	ins	G	3819853	ZFR2
19	3881864	3881874	C	ins	A	3881868	ATCAY
19	8191932	8191942	T	ins	G	8191940	FBN3
19	10676487	10676496	C	ins	CCA	10676490	CDKN2D
19	10676487	10676496	C	ins	CCA	10676490	KRI1
19	11545049	11545059	A	ins	C	11545051	CCDC151
19	12764202	12764210	C	ins	A	12764206	MAN2B1
19	18118930	18118939	C	ins	A	18118933	ARRDC2
19	20003794	20003803	T	ins	TTG	20003800	ZNF253
19	30021342	30021350	C	ins	T	30021344	VSTM2B
19	36169725	36169733	A	ins	AG	36169729	UPK1A
19	36368404	36368414	A	ins	C	36368408	APLP1
19	36673143	36673152	T	ins	C	36673149	ZNF565
19	37488677	37488686	A	ins	G	37488681	AX747376
19	37488677	37488686	A	ins	G	37488681	ZNF568
19	37489607	37489617	A	ins	C	37489615	AX747376
19	37489607	37489617	A	ins	C	37489615	ZNF568
19	41737854	41737862	A	ins	AC	41737855	AXL
19	44426463	44426471	T	ins	G	44426466	ZNF45
19	44906133	44906141	C	ins	CA	44906137	ZFP112
19	44906133	44906141	C	ins	CA	44906137	ZNF285
19	44906133	44906141	C	ins	A	44906138	ZFP112

19	44906133	44906141	C	ins	A	44906138	ZNF285
19	45974754	45974763	C	ins	CCCCG	45974756	ERCC1
19	45974754	45974763	C	ins	CCCCG	45974756	FOSB
19	46173211	46173220	G	ins	GC	46173214	GIPR
19	46322260	46322269	G	ins	A	46322266	SYMPK
19	46707578	46707586	G	ins	T	46707579	DKFZp434J0226
19	48829364	48829373	G	ins	T	48829370	EMP3
19	50837854	50837864	C	ins	A	50837858	NAPSB
19	50837854	50837864	C	ins	A	50837858	NAPSB
19	50837854	50837864	C	ins	A	50837858	NAPSB
19	50837854	50837864	C	ins	A	50837858	NAPSB
19	50837854	50837864	C	ins	A	50837858	NR1H2
19	50837854	50837864	C	ins	A	50837858	NR1H2
19	50837854	50837864	C	ins	A	50837858	NR1H2
19	50837854	50837864	C	ins	A	50837858	NR1H2
19	51326350	51326359	C	ins	A	51326351	KLK1
19	51583439	51583449	T	ins	TA	51583440	KLK14
19	52469900	52469910	T	ins	TG	52469906	BC014606
19	52469900	52469910	T	ins	TG	52469906	ZNF350
19	52693289	52693297	C	ins	A	52693293	PPP2R1A
19	53514420	53514429	T	ins	C	53514425	AK127846
19	55712319	55712327	A	ins	C	55712323	PTPRH
19	57875090	57875099	C	ins	CG	57875096	TRAPPC2
19	57875090	57875099	C	ins	CG	57875096	ZNF547
1	7740869	7740878	G	ins	T	7740872	CAMTA1
1	8029500	8029510	G	ins	A	8029508	PARK7
1	9613288	9613298	T	ins	G	9613296	SLC25A33
1	10857307	10857317	G	ins	GT	10857308	CASZ1
1	10857307	10857317	G	ins	T	10857309	CASZ1
1	16729978	16729986	T	ins	TTTC	16729979	SPATA21
1	19652132	19652141	G	ins	T	19652135	PQLC2
1	19705580	19705589	T	ins	TTTC	19705585	CAPZB
1	24171481	24171491	A	ins	C	24171489	FUCA1

1	45804416	45804426	T	ins	TG	45804421	MUTYH
1	45804416	45804426	T	ins	TG	45804421	TOE1
1	46119365	46119373	T	ins	CC	46119371	GPBP1L1
1	49208092	49208102	A	ins	G	49208094	AGBL4
1	49208092	49208102	A	ins	G	49208094	BEND5
1	55196891	55196900	T	ins	G	55196896	HEATR8-TTC4
1	55196891	55196900	T	ins	G	55196896	TTC4
1	59132546	59132556	A	ins	AT	59132552	MYSM1
1	62253768	62253778	A	ins	G	62253772	INADL
1	67441666	67441674	T	ins	G	67441671	MIER1
1	67792115	67792123	A	ins	AT	67792119	IL12RB2
1	75203791	75203800	A	ins	C	75203797	TYW3
1	78408922	78408932	A	ins	C	78408930	NEXN
1	86045679	86045689	C	ins	A	86045682	CYR61
1	116224112	116224120	T	ins	TTG	116224117	VANGL1
1	116224124	116224132	T	ins	G	116224125	VANGL1
1	149907175	149907184	C	ins	A	149907182	MTMR11
1	152308781	152308789	T	ins	G	152308786	AK056431
1	156401083	156401091	C	ins	CA	156401089	C1orf61
1	157774036	157774046	C	ins	T	157774040	FCRL1
1	167333796	167333805	T	ins	C	167333800	POU2F1
1	167854360	167854368	T	ins	C	167854361	ADCY10
1	170038975	170038984	A	ins	AT	170038981	KIFAP3
1	170933762	170933770	A	ins	TC	170933763	C1orf129
1	170933762	170933770	A	ins	TC	170933765	C1orf129
1	171289974	171289982	A	ins	AAAT	171289978	FMO4
1	171620483	171620491	C	ins	A	171620488	MYOC
1	184587770	184587780	C	ins	A	184587777	C1orf21
1	203771852	203771862	T	ins	TG	203771857	ZC3H11A
1	205886253	205886261	A	ins	G	205886257	SLC26A9
1	221874860	221874869	T	ins	TTTC	221874861	DUSP10
1	227098195	227098203	T	ins	C	227098196	ADCK3
1	227171735	227171745	G	ins	T	227171737	ADCK3

1	229586482	229586490	T	ins	GTTTG	229586484	NUP133
1	234456783	234456793	C	ins	A	234456791	SLC35F3
1	237752192	237752202	A	ins	C	237752196	RYR2
20	3776005	3776013	A	ins	C	3776008	CDC25B
20	5754003	5754012	T	ins	G	5754007	C20orf196
20	17947986	17947996	A	ins	AC	17947991	AK296947
20	17947986	17947996	A	ins	AC	17947991	SNX5
20	23419817	23419826	T	ins	G	23419822	CSTL1
20	25229530	25229538	T	ins	TG	25229536	PYGB
20	34370184	34370194	T	ins	G	34370187	PHF20
20	42844409	42844417	T	ins	TC	42844410	LOC100505783
20	42844409	42844417	T	ins	C	42844411	LOC100505783
20	44524883	44524892	C	ins	CCG	44524888	CTSA
20	55046623	55046632	A	ins	C	55046626	C20orf43
20	61465240	61465249	T	ins	G	61465241	COL9A3
20	61465240	61465249	T	ins	G	61465242	COL9A3
20	61465240	61465249	T	ins	G	61465245	COL9A3
20	61715656	61715665	G	ins	T	61715659	LOC63930
20	62570835	62570845	C	ins	CG	62570841	UCKL1
21	19274501	19274511	T	ins	G	19274504	CHODL
21	32126534	32126542	T	ins	G	32126540	KRTAP21-1
21	33330618	33330627	T	ins	C	33330620	HUNK
21	34670147	34670155	A	ins	C	34670150	IL10RB
21	34724908	34724916	A	ins	C	34724910	IFNAR1
21	43736264	43736274	T	ins	TG	43736267	TFF3
21	43736264	43736274	T	ins	G	43736269	TFF3
21	44882742	44882750	A	ins	C	44882744	LINC00313
21	46573256	46573266	T	ins	C	46573264	ADARB1
22	20118621	20118630	G	ins	T	20118622	ZDHHC8
22	20878896	20878904	T	ins	TTTTG	20878899	MED15
22	21379156	21379164	T	ins	TG	21379162	P2RX6
22	21802824	21802833	T	ins	C	21802831	HIC2
22	23466377	23466387	T	ins	G	23466385	GNAZ

22	23466377	23466387	T	ins	G	23466385	RTDR1
22	24199764	24199774	C	ins	CT	24199765	SLC2A11
22	24940235	24940243	G	ins	T	24940240	C22orf13
22	25323598	25323608	A	ins	C	25323600	SGSM1
22	30751804	30751814	C	ins	CCCA	30751807	CCDC157
22	30751804	30751814	C	ins	CCCA	30751807	SF3A1
22	37622884	37622892	G	ins	T	37622887	RAC2
22	39079147	39079157	G	ins	GGC	39079148	TOMM22
22	41732670	41732678	A	ins	AT	41732676	ZC3H7B
22	43831373	43831383	T	ins	G	43831376	MPPED1
22	44560289	44560298	C	ins	CCG	44560290	PARVB
22	46664985	46664995	C	ins	A	46664991	TTC38
22	50971639	50971648	A	ins	G	50971642	ODF3B
2	3661724	3661733	T	ins	G	3661731	COLEC11
2	9629280	9629288	T	ins	TTG	9629284	ADAM17
2	9629280	9629288	T	ins	TTG	9629284	IAH1
2	9649120	9649129	A	ins	G	9649122	ADAM17
2	24046670	24046678	A	ins	C	24046671	ATAD2B
2	26717524	26717533	G	ins	GT	26717529	OTOF
2	27824507	27824515	T	ins	TTTTG	27824510	ZNF512
2	30864753	30864761	T	ins	TTTTC	30864755	LCLAT1
2	38538263	38538273	A	ins	C	38538266	ATL2
2	55405088	55405096	A	ins	G	55405093	C2orf63
2	61001236	61001246	T	ins	G	61001241	PAPOLG
2	61709732	61709741	T	ins	TG	61709733	XPO1
2	61709732	61709741	T	ins	G	61709734	XPO1
2	61709732	61709741	T	ins	G	61709736	XPO1
2	69650340	69650349	G	ins	GA	69650342	NFU1
2	106014345	106014354	T	ins	G	106014347	FHL2
2	120022379	120022389	C	ins	A	120022380	STEAP3
2	128697870	128697879	A	ins	C	128697872	SAP130
2	130931545	130931554	A	ins	C	130931550	SMPD4
2	139426377	139426387	T	ins	TTG	139426378	NXPH2

2	151324743	151324752	A	ins	G	151324746	RND3
2	153591841	153591849	T	ins	G	153591844	ARL6IP6
2	158979866	158979875	T	ins	C	158979869	UPP2
2	158979866	158979875	T	ins	C	158979870	UPP2
2	160872982	160872990	G	ins	GA	160872985	PLA2R1
2	174081238	174081248	T	ins	C	174081239	MLK7-AS1
2	174081238	174081248	T	ins	C	174081239	MLK7-AS1
2	174081238	174081248	T	ins	C	174081239	ZAK
2	174081238	174081248	T	ins	C	174081239	ZAK
2	175199471	175199481	C	ins	CCA	175199474	SP9
2	179250161	179250169	T	ins	C	179250162	MIR548N
2	179250161	179250169	T	ins	C	179250162	OSBPL6
2	190603665	190603673	T	ins	C	190603666	ANKAR
2	201485102	201485111	T	ins	G	201485105	AOX1
2	209049820	209049830	T	ins	C	209049828	C2orf80
2	212543025	212543034	A	ins	C	212543030	ERBB4
2	213404028	213404037	C	ins	CCCCA	213404031	ERBB4
2	213869915	213869924	G	ins	GGT	213869921	IKZF2
2	216269022	216269030	T	ins	TTTTG	216269027	FN1
2	219871890	219871899	T	ins	TTTC	219871893	CCDC108
2	220117262	220117272	C	ins	A	220117269	TUBA4A
2	220117262	220117272	C	ins	A	220117269	TUBA4B
2	225721011	225721021	T	ins	G	225721013	DOCK10
2	228142841	228142851	T	ins	TA	228142849	AK056332
2	228142841	228142851	T	ins	TA	228142849	BC035052
2	228142841	228142851	T	ins	TA	228142849	COL4A3
2	233996054	233996062	A	ins	C	233996060	INPP5D
2	241388730	241388739	C	ins	CA	241388733	GPC1
2	241388730	241388739	C	ins	CA	241388733	PP14571
2	241388730	241388739	C	ins	T	241388735	GPC1
2	241388730	241388739	C	ins	T	241388735	PP14571
2	242177134	242177142	A	ins	AC	242177140	HDLBP
2	242200454	242200463	A	ins	C	242200456	DKFZp686L08115

2	242200454	242200463	A	ins	C	242200456	HDLBP
3	2280419	2280429	T	ins	G	2280423	CNTN4
3	2280419	2280429	T	ins	C	2280427	CNTN4
3	14509843	14509853	C	ins	CA	14509849	SLC6A6
3	15315755	15315765	A	ins	AAAAG	15315761	SH3BP5
3	23244943	23244951	C	ins	CG	23244947	UBE2E2
3	31616958	31616967	G	ins	T	31616959	STT3B
3	33191005	33191013	G	ins	GA	33191006	SUSD5
3	38751659	38751668	T	ins	C	38751662	SCN10A
3	46244273	46244283	G	ins	T	46244274	CCR1
3	46244273	46244283	G	ins	T	46244274	CCR3
3	47370693	47370701	T	ins	TG	47370698	KLHL18
3	55018614	55018624	T	ins	G	55018618	CACNA2D3
3	56717973	56717982	C	ins	CCCG	56717975	FAM208A
3	56808954	56808964	A	ins	C	56808955	ARHGEF3
3	86119784	86119793	A	ins	C	86119785	CADM2
3	89521598	89521606	T	ins	C	89521601	EPHA3
3	100551609	100551617	A	ins	G	100551611	ABI3BP
3	108747081	108747089	C	ins	A	108747086	MORC1
3	112557336	112557345	A	ins	AC	112557337	CD200R1L
3	121976532	121976540	T	ins	C	121976534	CASR
3	125249828	125249838	T	ins	TTC	125249833	OSBPL11
3	131625046	131625055	T	ins	G	131625052	CPNE4
3	132966485	132966495	A	ins	G	132966486	BC037929
3	132966485	132966495	A	ins	G	132966486	TMEM108
3	149051248	149051256	T	ins	TC	149051254	TM4SF18
3	161222622	161222632	T	ins	TG	161222629	OTOL1
3	168850277	168850285	T	ins	TC	168850280	MECOM
3	168850277	168850285	T	ins	CA	168850281	MECOM
3	176755297	176755305	G	ins	T	176755298	TBL1XR1
3	176914523	176914531	C	ins	T	176914529	TBL1XR1
3	183164881	183164891	T	ins	TTC	183164888	LOC100505687
3	184100958	184100968	G	ins	T	184100960	CHRD

3	185204958	185204967	G	ins	T	185204960	MAP3K13
3	185204958	185204967	G	ins	T	185204964	MAP3K13
3	185215869	185215879	C	ins	CCA	185215875	TMEM41A
3	190993490	190993500	A	ins	C	190993491	UTS2D
4	3177752	3177761	T	ins	GG	3177754	HTT
4	7434412	7434420	G	ins	GT	7434413	PSAPL1
4	7434412	7434420	G	ins	GT	7434413	SORCS2
4	9706756	9706764	G	ins	A	9706760	DQ584669
4	38825709	38825717	C	ins	A	38825712	TLR6
4	40438220	40438229	A	ins	C	40438222	RBM47
4	42557227	42557235	C	ins	CCG	42557232	ATP8A1
4	57887818	57887827	T	ins	G	57887824	POLR2B
4	71385415	71385425	T	ins	TTTTTA	71385416	AMTN
4	81283670	81283679	T	ins	C	81283673	C4orf22
4	82089195	82089203	A	ins	C	82089201	PRKG2
4	82348573	82348581	A	ins	AC	82348579	RASGEF1B
4	84240325	84240335	A	ins	AC	84240326	HPSE
4	85771476	85771485	T	ins	G	85771483	WDFY3
4	103578379	103578389	G	ins	GA	103578383	MANBA
4	106319242	106319251	A	ins	CC	106319246	PPA2
4	106511292	106511300	A	ins	AAAG	106511296	ARHGEF38
4	120375126	120375136	C	ins	A	120375132	BC070391
4	120375126	120375136	C	ins	A	120375132	LOC645513
4	122721708	122721718	T	ins	G	122721712	EXOSC9
4	154318805	154318815	A	ins	G	154318811	MND1
4	156653163	156653171	A	ins	G	156653169	GUCY1A3
4	156838133	156838141	A	ins	C	156838134	TDO2
4	159817993	159818003	T	ins	TA	159818000	C4orf45
4	159817993	159818003	T	ins	TA	159818000	FNIP2
4	166262191	166262201	T	ins	C	166262195	MSMO1
4	170320722	170320730	A	ins	C	170320726	NEK1
4	183601590	183601599	G	ins	T	183601594	ODZ3
4	187344162	187344172	T	ins	TG	187344166	LOC285441

5	1112986	1112996	C	ins	A	1112993	SLC12A7
5	15937655	15937665	C	ins	A	15937662	FBXL7
5	35002881	35002891	G	ins	T	35002884	AGXT2
5	37396287	37396295	T	ins	TC	37396288	WDR70
5	39387801	39387809	T	ins	C	39387806	DAB2
5	40765853	40765863	A	ins	C	40765861	PRKAA1
5	54398880	54398889	A	ins	G	54398881	GZMA
5	60953736	60953745	A	ins	G	60953742	BC043229
5	60953736	60953745	A	ins	G	60953742	C5orf64
5	73177903	73177911	T	ins	G	73177909	RGNEF
5	75998985	75998994	G	ins	T	75998989	IQGAP2
5	82948212	82948221	A	ins	G	82948219	HAPLN1
5	90051086	90051094	T	ins	C	90051087	GPR98
5	102898517	102898527	G	ins	GA	102898520	NUDT12
5	111066236	111066245	G	ins	GT	111066239	LOC100505678
5	111066236	111066245	G	ins	GT	111066239	NREP
5	111066236	111066245	G	ins	GT	111066239	NREP
5	133842267	133842276	C	ins	A	133842268	BC032795
5	136314132	136314142	A	ins	C	136314133	SPOCK1
5	136976422	136976430	C	ins	A	136976423	KLHL3
5	140803317	140803327	G	ins	GC	140803324	PCDHGA1
5	140803317	140803327	G	ins	GC	140803324	PCDHGA10
5	140803317	140803327	G	ins	GC	140803324	PCDHGA11
5	140803317	140803327	G	ins	GC	140803324	PCDHGA11
5	140803317	140803327	G	ins	GC	140803324	PCDHGA2
5	140803317	140803327	G	ins	GC	140803324	PCDHGA3
5	140803317	140803327	G	ins	GC	140803324	PCDHGA4
5	140803317	140803327	G	ins	GC	140803324	PCDHGA5
5	140803317	140803327	G	ins	GC	140803324	PCDHGA6
5	140803317	140803327	G	ins	GC	140803324	PCDHGA7
5	140803317	140803327	G	ins	GC	140803324	PCDHGA8
5	140803317	140803327	G	ins	GC	140803324	PCDHGA9
5	140803317	140803327	G	ins	GC	140803324	PCDHGB1

5	140803317	140803327	G	ins	GC	140803324	PCDHGB2
5	140803317	140803327	G	ins	GC	140803324	PCDHGB3
5	140803317	140803327	G	ins	GC	140803324	PCDHGB4
5	140803317	140803327	G	ins	GC	140803324	PCDHGB5
5	140803317	140803327	G	ins	GC	140803324	PCDHGB6
5	140803317	140803327	G	ins	GC	140803324	PCDHGB7
5	171534119	171534128	A	ins	C	171534123	STK10
5	173416100	173416108	C	ins	CA	173416102	C5orf47
5	177379531	177379540	C	ins	CCAT	177379532	AK126616
5	178584960	178584968	T	ins	G	178584962	ADAMTS2
6	4088272	4088280	C	ins	A	4088277	C6orf146
6	4088272	4088280	C	ins	A	4088277	C6orf146
6	4088272	4088280	C	ins	A	4088277	C6orf201
6	4088272	4088280	C	ins	A	4088277	C6orf201
6	22295430	22295438	T	ins	C	22295432	PRL
6	27774428	27774438	T	ins	TC	27774436	HIST1H2BL
6	28774804	28774814	A	ins	C	28774809	TRNA_Phe
6	30230518	30230526	T	ins	AC	30230520	HLA-L
6	30972958	30972968	T	ins	C	30972960	MUC22
6	31677035	31677044	T	ins	G	31677036	ABHD16A
6	31677035	31677044	T	ins	G	31677036	LY6G6F
6	32373584	32373594	T	ins	G	32373591	BTNL2
6	32525116	32525124	A	ins	C	32525119	HLA-DRB1
6	32525116	32525124	A	ins	C	32525119	HLA-DRB5
6	32525116	32525124	A	ins	C	32525119	HLA-DRB6
6	32605979	32605987	T	ins	TA	32605981	HLA-DQA1
6	32610152	32610162	T	ins	C	32610154	HLA-DQA1
6	32828219	32828227	A	ins	C	32828221	PSMB9
6	32946824	32946833	T	ins	G	32946826	BRD2
6	33741371	33741381	G	ins	GT	33741372	LEMD2
6	38873661	38873669	T	ins	G	38873666	DNAH8
6	38873661	38873669	T	ins	G	38873666	DNAH8
6	38873661	38873669	T	ins	G	38873666	LOC100131047

6	44123188	44123198	T	ins	TGG	44123195	TMEM63B
6	44279296	44279304	G	ins	A	44279301	AARS2
6	44279296	44279304	G	ins	A	44279301	AARS2
6	44279296	44279304	G	ins	A	44279301	AARS2
6	44279296	44279304	G	ins	A	44279301	SPATS1
6	44279296	44279304	G	ins	A	44279301	SPATS1
6	44279296	44279304	G	ins	A	44279301	SPATS1
6	66227234	66227244	A	ins	AC	66227236	EYS
6	88118739	88118747	T	ins	TTATG	88118740	C6orf165
6	117645778	117645786	T	ins	C	117645781	GOPC
6	117645778	117645786	T	ins	C	117645781	ROS1
6	128304353	128304361	A	ins	C	128304357	PTPRK
6	133066058	133066068	A	ins	AAAC	133066062	VNN2
6	133119843	133119853	C	ins	CG	133119848	C6orf192
6	138644772	138644782	A	ins	C	138644773	KIAA1244
6	143660307	143660317	A	ins	G	143660315	AIG1
6	155732616	155732624	T	ins	G	155732622	NOX3
6	166843612	166843622	T	ins	G	166843619	RPS6KA2
7	5938156	5938166	A	ins	C	5938157	CCZ1
7	6780646	6780656	A	ins	G	6780654	PMS2CL
7	6816695	6816703	T	ins	G	6816698	RSPH10B
7	6816695	6816703	T	ins	G	6816698	RSPH10B2
7	16899723	16899732	A	ins	G	16899726	AGR3
7	21913946	21913955	T	ins	G	21913950	DNAH11
7	37261300	37261309	T	ins	TTTTC	37261304	ELMO1
7	43154329	43154338	T	ins	G	43154334	HECW1
7	55758674	55758682	A	ins	G	55758680	FKBP9L
7	57192779	57192788	A	ins	AG	57192784	ZNF479
7	64343592	64343600	C	ins	A	64343594	AK097702
7	64343592	64343600	C	ins	A	64343594	ZNF273
7	66024978	66024987	A	ins	C	66024981	LOC493754
7	66461205	66461213	A	ins	C	66461210	SBDS
7	66461205	66461213	A	ins	C	66461210	TYW1

7	66768668	66768678	T	ins	TTC	66768676	STAG3L4
7	75988727	75988736	G	ins	GT	75988728	YWHAG
7	93520940	93520950	A	ins	G	93520941	GNGT1
7	93520940	93520950	A	ins	G	93520941	TFPI2
7	98922776	98922785	A	ins	AAAGAAAAAG	98922781	ARPC1A
7	99711461	99711471	C	ins	A	99711466	TAF6
7	106847433	106847441	A	ins	AAC	106847437	COG5
7	111508765	111508775	A	ins	AT	111508771	DOCK4
7	134853043	134853053	C	ins	A	134853044	C7orf49
7	135415711	135415720	T	ins	TC	135415718	FAM180A
7	138357386	138357395	T	ins	TTTTG	138357389	SVOPL
7	139026462	139026471	G	ins	T	139026463	C7orf55
7	139026462	139026471	G	ins	T	139026463	LUC7L2
7	139026462	139026471	G	ins	T	139026463	LUC7L2
7	139026462	139026471	G	ins	T	139026463	TRNA
7	139026462	139026471	G	ins	T	139026463	TRNA_Arg
7	139482261	139482271	T	ins	TC	139482269	TBXAS1
7	143806414	143806424	T	ins	G	143806416	OR2A2
8	413963	413971	T	ins	G	413967	FBXO25
8	1650528	1650537	A	ins	C	1650535	DLGAP2
8	1771615	1771624	G	ins	T	1771617	ARHGEF10
8	2794975	2794985	A	ins	AAC	2794976	CSMD1
8	15094761	15094769	C	ins	CCA	15094763	SGCZ
8	35092779	35092788	G	ins	T	35092780	UNC5D
8	74224237	74224246	T	ins	G	74224238	AK128216
8	74224237	74224246	T	ins	G	74224238	RDH10
8	103225971	103225981	A	ins	C	103225977	RRM2B
8	110542701	110542710	G	ins	A	110542708	PKHD1L1
8	117786951	117786960	T	ins	TTTTTG	117786954	UTP23
8	120860224	120860232	T	ins	AA	120860230	DSCC1
8	128699866	128699875	A	ins	C	128699870	BC042052
9	4826073	4826081	A	ins	AAAG	4826077	RCL1
9	6252056	6252065	A	ins	AC	6252057	IL33

9	18794624	18794633	T	ins	TTG	18794629	ADAMTSL1
9	20622064	20622073	G	ins	T	20622068	MLLT3
9	21439837	21439847	T	ins	G	21439845	IFNA1
9	27005350	27005358	C	ins	CCG	27005352	IFT74
9	27005350	27005358	C	ins	CCG	27005352	LRRC19
9	27551081	27551090	A	ins	C	27551085	C9orf72
9	34991612	34991621	C	ins	T	34991615	DNAJB5
9	34991612	34991621	C	ins	T	34991616	DNAJB5
9	34991612	34991621	C	ins	T	34991618	DNAJB5
9	35058763	35058773	A	ins	C	35058766	VCP
9	37885875	37885883	C	ins	A	37885876	DQ590189
9	37885875	37885883	C	ins	A	37885876	MCART1
9	73458045	73458055	A	ins	G	73458049	TRPM3
9	86614103	86614112	A	ins	AT	86614105	RMI1
9	87636617	87636627	T	ins	C	87636618	NTRK2
9	91978182	91978190	C	ins	A	91978184	SEMA4D
9	93637696	93637704	A	ins	AAAAG	93637699	SYK
9	99526455	99526464	T	ins	C	99526458	ZNF510
9	102988032	102988040	A	ins	G	102988035	INVS
9	103278093	103278103	A	ins	C	103278095	C9orf30-TMEFF1
9	103278093	103278103	A	ins	C	103278095	TMEFF1
9	125001503	125001511	T	ins	TG	125001509	RBM18
9	127076072	127076080	G	ins	A	127076076	NEK6
9	128093265	128093275	T	ins	G	128093273	GAPVD1
9	130421576	130421585	T	ins	G	130421580	STXBP1
9	130634621	130634629	G	ins	A	130634622	AK1
9	134460749	134460758	G	ins	A	134460752	RAPGEF1
9	139653123	139653133	T	ins	TTC	139653129	LCN15
9	139653123	139653133	T	ins	TTC	139653129	LCN8
9	139653123	139653133	T	ins	TC	139653130	LCN15
9	139653123	139653133	T	ins	TC	139653130	LCN8
9	141011736	141011746	G	ins	T	141011739	CACNA1B
X	218114	218122	T	ins	G	218115	PLCXD1

X	9717266	9717274	C	ins	T	9717271	GPR143
X	24076639	24076648	T	ins	TCTTTC	24076642	EIF2S3
X	24076639	24076648	T	ins	CTTTCTTTTC	24076643	EIF2S3
X	45707372	45707381	A	ins	C	45707373	AK098783
X	47342912	47342921	C	ins	A	47342919	ZNF41
X	48435396	48435404	T	ins	C	48435400	RBM3
X	53675483	53675492	A	ins	AC	53675485	HUWE1
X	53675483	53675492	A	ins	C	53675486	HUWE1
X	86086804	86086814	A	ins	G	86086808	DACH2
X	100479171	100479179	T	ins	TTTG	100479174	DRP2
X	100534956	100534966	A	ins	C	100534957	TAF7L
X	117750490	117750500	T	ins	G	117750494	DOCK11
X	128875148	128875157	G	ins	GT	128875150	XPNPEP2
X	134031603	134031611	A	ins	AG	134031606	MOSPD1
X	153185235	153185245	T	ins	AA	153185243	ARHGAP4
10	854691	854707	CA	ins	AT	854695	LARP4B
10	11996642	11996660	AT	del	T	11996644	UPF2
10	14940089	14940103	TA	ins	AC	14940097	DCLRE1C
10	14940089	14940103	TA	ins	AC	14940097	SUV39H2
10	25940011	25940021	AG	del	GAGAGAG	25940013	AK123440
10	26534828	26534838	AT	del	A	26534835	GAD2
10	42863998	42864012	AT	ins	AC	42864009	LOC441666
10	51781267	51781281	TG	del	TGT	51781276	FLJ31813
10	72433020	72433038	GT	ins	TA	72433022	ADAMTS14
10	85993089	85993103	AT	del	A	85993100	LRIT1
10	93030859	93030873	TA	ins	ATATATATATAATCTA	93030861	PCGF5
10	95129015	95129033	GT	ins	TA	95129021	MYOF
10	103023054	103023070	TG	ins	GA	103023060	AX747408
10	105669301	105669311	AT	ins	TG	105669303	OBFC1
10	115989562	115989576	TA	ins	T	115989573	TDRD1
10	134725791	134725801	CT	ins	CTC	134725794	TTC40
11	5689541	5689551	AT	ins	TG	5689543	TRIM5
11	7587270	7587288	CA	del	A	7587284	PPFIBP2

11	22360607	22360621	TA	del	TATATGT	22360616	SLC17A6
11	26701941	26701957	AT	ins	AC	26701954	SLC5A12
11	58909399	58909415	AG	ins	AA	58909412	BC028022
11	58909399	58909415	AG	ins	AA	58909412	FAM111A
11	71133923	71133935	AC	del	C	71133925	FLJ42102
11	71133923	71133935	AC	ins	A	71133926	FLJ42102
11	72284647	72284659	CA	ins	ACACACACAG	72284649	AL832797
11	73072965	73072977	CA	del	ACA	73072973	ARHGEF17
11	104872624	104872642	AC	ins	CACACG	104872636	CASP5
11	108170032	108170042	TC	del	CTC	108170038	ATM
11	116715334	116715344	AT	del	TATATAATATATTATAT	116715338	SIK3
11	130183819	130183831	TG	ins	TA	130183822	AX747213
11	130183819	130183831	TG	ins	TA	130183822	ZBTB44
11	133785151	133785163	TC	del	CTC	133785159	IGSF9B
12	3574295	3574313	AT	del	T	3574297	DQ588965
12	3574295	3574313	AT	del	T	3574297	PRMT8
12	20832828	20832838	AT	del	ATATA	20832833	PDE3A
12	31946004	31946020	TC	del	CTC	31946016	H3F3C
12	41323138	41323156	CA	del	C	41323153	CNTN1
12	50572734	50572750	AT	del	A	50572747	LIMA1
12	51124153	51124165	TC	del	TCTCT	51124160	DIP2B
12	53413207	53413221	TA	del	ATATA	53413215	EIF4B
12	54961629	54961647	AT	del	A	54961644	PDE1B
12	65638047	65638065	CA	ins	C	65638062	LEMD3
12	78513832	78513842	TA	ins	A	78513836	NAV3
12	80661171	80661187	TA	del	A	80661183	OTOGL
12	89408663	89408677	GA	del	G	89408674	LOC728084
12	91574163	91574179	AG	ins	AA	91574174	DCN
12	96947348	96947360	TA	del	T	96947355	AX747187
12	98896617	98896633	CA	del	A	98896619	LOC643770
12	98896617	98896633	CA	del	A	98896619	LOC643770
12	98896617	98896633	CA	del	A	98896619	TRNA_Asp
12	112465112	112465130	AT	del	A	112465127	NAA25

12	112924919	112924937	TC	del	CTCTC	112924931	PTPN11
12	117188696	117188714	AT	ins	T	117188710	RNFT2
12	117513776	117513790	TA	ins	T	117513787	TESC
12	122459822	122459834	TG	ins	TGTA	122459829	BCL7A
12	123211475	123211489	AT	ins	AG	123211484	HCAR1
13	42748100	42748118	GT	ins	GC	42748115	DGKH
13	70281008	70281020	CA	ins	CT	70281011	KLHL1
13	99539379	99539397	TA	del	ATA	99539393	DOCK9
13	114503154	114503168	TG	del	T	114503165	FAM70B
13	114779052	114779070	TC	ins	CA	114779067	RASA3
13	114779496	114779508	TC	ins	TA	114779499	RASA3
14	35571225	35571235	TC	del	CTC	35571231	AK128559
14	35571225	35571235	TC	del	CTC	35571231	PPP2R3C
14	65684969	65684979	AG	del	GAG	65684975	BX161428
14	91108265	91108277	TG	ins	GTGTGC	91108271	BC028746
14	91108265	91108277	TG	ins	GTGTGC	91108271	TTC7B
14	105052059	105052069	AG	ins	AT	105052064	C14orf180
15	20646856	20646868	CA	ins	TA	20646858	HERC2P3
15	33823833	33823849	AT	ins	T	33823845	RYR3
15	43109127	43109143	AT	del	T	43109139	TTBK2
15	44159755	44159773	AT	ins	TG	44159761	WDR76
15	56962071	56962085	TA	del	TATAT	56962080	ZNF280D
15	57810124	57810134	TG	del	T	57810131	CGNL1
15	63444675	63444687	TA	ins	T	63444684	RPS27L
15	72049753	72049765	AT	del	A	72049762	THSD4
15	81643675	81643689	TC	del	TCTCT	81643684	TMC3
15	81643675	81643689	TC	del	CTC	81643685	TMC3
15	102029470	102029480	CG	ins	G	102029474	PCSK6
16	612585	612595	CA	ins	AG	612589	C16orf11
16	1557912	1557928	GT	ins	GC	1557921	TELO2
16	11645511	11645521	CA	del	C	11645518	LITAF
16	11985212	11985226	AT	del	A	11985223	GSPT1
16	19460015	19460025	AT	ins	TATAT	19460017	TMC5

16	19504237	19504255	AT	ins	TC	19504251	TMC5
16	20411376	20411394	TA	del	A	20411390	PDILT
16	23312735	23312751	GA	ins	AA	23312743	SCNN1B
16	23312735	23312751	GA	ins	AGAGAAAGAA	23312745	SCNN1B
16	58195466	58195476	AC	ins	ACAG	58195473	BC053935
16	58195466	58195476	AC	ins	ACAG	58195473	CSNK2A2
16	72007914	72007924	AT	del	A	72007917	PKD1L3
16	81059736	81059746	TA	ins	TG	81059743	CENPN
16	84798545	84798561	AT	ins	TG	84798551	USP10
16	89596555	89596573	TG	del	G	89596557	SPG7
17	3374812	3374826	CA	ins	CACACACACCACC	3374817	SPATA22
17	4385168	4385182	CT	del	C	4385179	AX748345
17	4385168	4385182	CT	del	C	4385179	SPNS3
17	19808330	19808344	AT	del	A	19808341	AKAP10
17	33802931	33802943	AT	ins	TT	33802939	SLFN12L
17	33802931	33802943	AT	del	A	33802940	SLFN12L
17	34303676	34303690	TC	del	TCT	34303687	CCL16
17	34418383	34418399	AT	del	A	34418392	CCL3
17	35871088	35871098	AT	del	ATATATA	35871091	DUSP14
17	35972118	35972132	TG	ins	GC	35972122	DDX52
17	39317842	39317860	AT	del	TATAT	39317854	KRTAP4-4
17	39325142	39325154	AC	ins	ACACAT	39325145	KRTAP4-3
17	39325142	39325154	AC	ins	ACAT	39325147	KRTAP4-3
17	39325142	39325154	AC	ins	AT	39325149	KRTAP4-3
17	42991733	42991751	CA	ins	CC	42991740	GFAP
17	48207167	48207177	GT	ins	GTGTGC	48207172	SAMD14
17	49231510	49231520	TC	ins	T	49231517	NME1
17	49231510	49231520	TC	ins	T	49231517	NME1
17	49231510	49231520	TC	ins	T	49231517	NME1-NME2
17	49231510	49231520	TC	ins	T	49231517	NME1-NME2
17	49231510	49231520	TC	ins	T	49231517	NME2
17	49231510	49231520	TC	ins	T	49231517	NME2
17	56654837	56654851	AT	del	A	56654848	TEX14

17	58126333	58126343	AT	del	ATATATA	58126336	HEATR6
17	58126333	58126343	AT	del	ATATA	58126338	HEATR6
17	58126333	58126343	AT	del	ATA	58126340	HEATR6
17	61779366	61779378	TG	ins	GG	61779374	STRADA
17	65906925	65906937	AT	del	T	65906927	BPTF
17	76165337	76165355	TG	del	TGTGTGTGT	76165342	SYNGR2
17	76165337	76165355	TG	del	T	76165346	SYNGR2
17	76165337	76165355	TG	del	TGTGT	76165348	SYNGR2
17	76165337	76165355	TG	ins	G	76165351	SYNGR2
17	76165337	76165355	TG	del	T	76165352	SYNGR2
17	79562547	79562561	AT	ins	T	79562555	NPLOC4
17	79562547	79562561	AT	del	ATA	79562556	NPLOC4
17	79562547	79562561	AT	ins	T	79562557	NPLOC4
18	267019	267037	TA	del	T	267034	THOC1
18	3174076	3174088	AC	ins	AT	3174083	MYOM1
18	3176190	3176200	AC	ins	AT	3176197	MYOM1
18	5245395	5245409	AG	ins	AGAT	5245404	LOC339290
18	19204662	19204680	AT	del	T	19204664	SNRPD1
18	21723508	21723524	AT	del	A	21723521	CABYR
18	47323336	47323352	TA	del	T	47323349	ACAA2
18	51807390	51807404	TG	ins	TA	51807399	POLI
18	64172797	64172807	AT	del	ATATATA	64172800	CDH19
19	926482	926492	GC	del	G	926487	ARID3A
19	2554541	2554557	TA	del	ATA	2554553	GNG7
19	3601365	3601377	AT	del	T	3601367	TBXA2R
19	3699361	3699373	CT	ins	TT	3699367	PIP5K1C
19	6710192	6710202	GA	ins	AA	6710196	C3
19	7943096	7943110	AT	ins	T	7943104	LOC388499
19	7943096	7943110	AT	del	A	7943105	LOC388499
19	7943096	7943110	AT	ins	T	7943106	LOC388499
19	9053608	9053618	AT	del	ATA	9053615	MUC16
19	13343004	13343018	TC	del	CTC	13343014	CACNA1A
19	13371129	13371147	AT	ins	T	13371143	CACNA1A

19	13371129	13371147	AT	del	A	13371144	CACNA1A
19	14045956	14045974	GT	ins	GC	14045959	PODNL1
19	14768542	14768558	AT	ins	T	14768554	EMR3
19	18122743	18122761	TA	del	ATA	18122757	ARRDC2
19	33694230	33694242	TG	ins	GA	33694232	LRP3
19	39869683	39869697	CT	del	CTC	39869692	SAMD4B
19	39869683	39869697	CT	del	C	39869694	SAMD4B
19	41620675	41620693	TC	del	CTC	41620689	CYP2F1
19	45032851	45032869	AC	del	A	45032860	CEACAM20
19	47918816	47918830	TC	del	CTCTC	47918824	MEIS3
19	51320944	51320954	TC	del	T	51320951	MGC45922
19	51982185	51982195	AC	ins	CACACG	51982189	CEACAM18
19	53281264	53281276	CT	del	TCTTT	53281272	ZNF600
19	55397658	55397676	CA	del	C	55397673	FCAR
19	56006583	56006601	AT	ins	TG	56006591	SSC5D
19	56088095	56088109	CT	del	TCTCA	56088105	ZNF579
19	56348973	56348989	AC	ins	C	56348979	NLRP11
19	56348973	56348989	AC	ins	C	56348979	NLRP4
19	56488580	56488594	AT	ins	TG	56488586	NLRP8
1	4001785	4001797	TC	ins	TG	4001792	LOC728716
1	7849200	7849218	AT	ins	TATG	7849202	PER3
1	7849200	7849218	AT	ins	TG	7849204	PER3
1	12027697	12027715	TA	del	TATTT	12027712	PLOD1
1	17431892	17431908	TG	del	G	17431894	PADI2
1	43107917	43107935	AT	del	T	43107919	CCDC30
1	54705780	54705794	GC	del	GCA	54705791	SSBP3
1	62440059	62440077	TC	del	CTC	62440073	INADL
1	62911299	62911317	TG	ins	TGTA	62911302	USP1
1	85562162	85562176	TA	del	TATATATAAAGAT	85562167	WDR63
1	92596076	92596094	AT	del	T	92596078	BTBD8
1	94355461	94355477	AT	del	A	94355474	GCLM
1	94468544	94468554	TC	ins	CA	94468550	ABCA4
1	95631007	95631023	TG	ins	G	95631019	AK090700

1	95631007	95631023	TG	ins	G	95631019	TMEM56
1	95631007	95631023	TG	ins	G	95631019	TMEM56-RWDD3
1	95631007	95631023	TG	del	T	95631020	AK090700
1	95631007	95631023	TG	del	T	95631020	TMEM56
1	95631007	95631023	TG	del	T	95631020	TMEM56-RWDD3
1	100734597	100734607	TA	ins	TG	100734604	RTCD1
1	109773690	109773706	AT	del	A	109773703	SARS
1	113202196	113202208	TC	ins	T	113202205	CAPZA1
1	153607319	153607333	AT	del	A	153607330	CHTOP
1	153607319	153607333	AT	del	A	153607330	CHTOP
1	153607319	153607333	AT	del	A	153607330	S100A13
1	155797473	155797491	CA	del	C	155797488	GON4L
1	156752695	156752705	AT	del	A	156752702	PRCC
1	161279325	161279343	AT	del	A	161279340	MPZ
1	171251672	171251682	AT	del	A	171251679	FMO1
1	173174063	173174081	AC	ins	AT	173174074	TNFSF4
1	179999450	179999460	TA	ins	TG	179999453	CEP350
1	183114853	183114869	TA	ins	AA	183114863	LAMC1
1	183559707	183559719	AG	ins	T	183559711	NCF2
1	183774608	183774618	CT	del	C	183774611	RGL1
1	200816519	200816537	TG	del	G	200816521	CAMSAP2
1	207243362	207243374	AC	ins	A	207243365	PFKFB2
1	222711754	222711764	GA	ins	AGAT	222711760	HHIPL2
1	233431698	233431714	TC	ins	TGTT	233431711	PCNXL2
1	243389360	243389370	AT	ins	AG	243389363	CEP170
1	243389360	243389370	AT	ins	TT	243389364	CEP170
20	23168849	23168859	TG	del	T	23168856	AX747171
20	30602049	30602065	AT	del	ATA	30602060	C20orf160
20	47258362	47258380	AG	del	A	47258375	PREX1
20	49198278	49198288	TC	ins	T	49198283	PTPN1
20	62166759	62166771	CA	ins	CACT	62166764	PTK6
21	15671076	15671094	AT	del	A	15671091	ABCC13
21	19641254	19641266	CT	del	C	19641261	TMPRSS15

21	23468273	23468291	AT	ins	T	23468287	BC039377
21	37519205	37519221	TC	ins	TTTT	37519214	CBR3
21	37519205	37519221	TC	ins	TTTT	37519214	LOC100506428
21	37519205	37519221	TC	del	CTCTC	37519215	CBR3
21	37519205	37519221	TC	del	CTCTC	37519215	LOC100506428
21	37519205	37519221	TC	del	CTC	37519217	CBR3
21	37519205	37519221	TC	del	CTC	37519217	LOC100506428
22	22995284	22995294	GT	ins	GA	22995289	abParts
22	22995284	22995294	GT	ins	GA	22995289	abParts
22	22995284	22995294	GT	ins	GA	22995289	DKFZp667J0810
22	22995284	22995294	GT	ins	GA	22995289	DKFZp667J0810
22	23082678	23082688	GC	ins	GT	23082683	abParts
22	23082678	23082688	GC	ins	GT	23082683	DKFZp667J0810
22	32545637	32545655	AT	del	T	32545639	C22orf42
22	43044845	43044859	AC	del	A	43044856	CYB5R3
2	1157068	1157078	AG	del	G	1157074	SNTG2
2	4678636	4678648	CA	del	ACAAT	4678644	LOC727982
2	9474139	9474151	TC	ins	TCTCTG	9474146	ASAP2
2	9584269	9584287	TA	del	ATA	9584283	CPSF3
2	27889722	27889738	TA	del	ATA	27889734	SLC4A1AP
2	29430942	29430952	TC	del	T	29430949	ALK
2	37898953	37898963	GC	ins	GT	37898956	CDC42EP3
2	42141970	42141980	TG	del	G	42141972	Mir_544
2	47083032	47083044	TA	del	A	47083040	LOC100134259
2	54095619	54095633	AT	del	T	54095629	PSME4
2	61459977	61459993	TA	del	TAT	61459990	USP34
2	65129344	65129358	CT	ins	TCTT	65129350	LOC400958
2	65129344	65129358	CT	ins	TT	65129352	LOC400958
2	78640581	78640597	TA	ins	TT	78640588	BC024248
2	79601635	79601647	AT	del	A	79601644	CTNNA2
2	85867727	85867743	AT	del	A	85867740	USP39
2	86398970	86398986	AT	ins	TT	86398980	IMMT
2	128567627	128567637	AC	ins	ACATATAT	128567634	WDR33

2	132355365	132355379	TG	ins	T	132355376	POTEKP
2	159660637	159660647	AT	del	A	159660644	DAPL1
2	160872996	160873010	GA	ins	GG	160873005	PLA2R1
2	166768165	166768179	AT	ins	T	166768173	TTC21B
2	166768165	166768179	AT	del	A	166768176	TTC21B
2	167302095	167302109	TC	del	TCT	167302106	SCN7A
2	189654185	189654199	AT	ins	AC	189654190	DIRC1
2	190527954	190527966	AT	del	A	190527961	ASNSD1
2	190527954	190527966	AT	del	A	190527963	ASNSD1
2	190607559	190607577	AC	del	CAC	190607573	ANKAR
2	201347489	201347505	TG	del	G	201347491	SPATS2L
2	202071321	202071335	TA	ins	TT	202071326	CASP10
2	207654314	207654328	AC	ins	AA	207654319	FASTKD2
2	220400049	220400065	TG	del	T	220400062	ACCN4
2	226377535	226377553	TC	del	C	226377541	NYAP2
3	21465793	21465809	TA	ins	T	21465806	ZNF385D
3	36934343	36934361	AT	ins	TATC	36934349	TRANK1
3	36934343	36934361	AT	ins	AG	36934350	TRANK1
3	37088088	37088102	TA	ins	T	37088095	MLH1
3	51743846	51743856	AC	ins	CACACCCT	51743850	GRM2
3	58900403	58900419	AT	del	A	58900416	AK090895
3	58900403	58900419	AT	del	A	58900416	C3orf67
3	58900403	58900419	AT	del	A	58900416	C3orf67
3	68780655	68780671	AT	ins	TATG	68780659	FAM19A4
3	74473543	74473561	AT	ins	AC	74473556	CNTN3
3	119264576	119264586	AC	del	A	119264583	CD80
3	129693077	129693093	TC	ins	TCTT	129693088	TRH
3	129693077	129693093	TC	ins	TT	129693090	TRH
3	133906994	133907012	AT	del	ATA	133907007	RYK
3	133906994	133907012	AT	del	A	133907009	RYK
3	140851494	140851512	TA	del	A	140851508	SPSB4
3	150792800	150792810	TA	ins	AC	150792806	CLRN1-AS1
3	158413966	158413980	TA	ins	TT	158413973	RARRES1

3	173774697	173774709	AT	del	A	173774706	7SK
3	173774697	173774709	AT	del	A	173774706	NLGN1
3	179138243	179138259	AT	del	A	179138256	GNB4
3	182584708	182584720	AT	ins	AC	182584713	ATP11B
3	182584708	182584720	AT	del	A	182584717	ATP11B
3	191359025	191359039	TA	del	ATATA	191359033	Y_RNA
4	2657174	2657192	TG	ins	TC	2657187	FAM193A
4	2701173	2701189	CA	del	ACA	2701175	FAM193A
4	5844349	5844363	CA	ins	AA	5844351	CRMP1
4	7778113	7778123	TG	ins	GTGC	7778119	AFAP1
4	7778113	7778123	TG	ins	GTGC	7778119	AFAP1-AS1
4	24982364	24982382	TC	del	CTCTC	24982376	CCDC149
4	37831469	37831481	AT	ins	AC	37831478	PGM2
4	47940331	47940349	AT	del	A	47940346	BC041434
4	47940331	47940349	AT	del	A	47940346	CNGA1
4	56890292	56890302	TA	del	TATAATTAG	56890297	CEP135
4	57343449	57343467	TC	del	CTC	57343463	SRP72
4	71248323	71248337	TG	del	T	71248334	SMR3B
4	77054200	77054218	AT	del	A	77054215	NUP54
4	87870315	87870333	AT	del	A	87870330	AFF1
4	91839789	91839805	AT	ins	AA	91839792	FAM190A
4	100055531	100055541	TA	ins	ATAA	100055533	ADH4
4	100055531	100055541	TA	ins	ATAA	100055533	LOC100507053
4	103500803	103500821	AT	del	T	103500805	NFKB1
4	110606384	110606394	TC	del	CTC	110606390	CCDC109B
4	114822862	114822876	AT	ins	TT	114822866	ARSJ
4	159093344	159093354	AC	del	ACACA	159093349	AK096792
4	159093344	159093354	AC	del	ACACA	159093349	AK096792
4	159093344	159093354	AC	del	ACACA	159093349	AK126266
4	159093344	159093354	AC	del	ACACA	159093349	FAM198B
4	159093344	159093354	AC	del	ACACA	159093349	FAM198B
4	159093344	159093354	AC	del	ACACA	159093349	FAM198B
4	174235079	174235095	AT	ins	T	174235091	GALNT7

4	174235079	174235095	AT	del	A	174235092	GALNT7
4	185617841	185617853	AT	ins	A	185617844	MLF1IP
4	189030305	189030319	AC	ins	CG	189030313	TRIML2
4	189063800	189063814	AG	ins	AA	189063803	TRIML1
5	412818	412830	TC	del	TCTCT	412825	AHRR
5	412818	412830	TC	del	TCT	412827	AHRR
5	13912589	13912601	AC	ins	AT	13912598	DNAH5
5	32228662	32228674	AT	del	ATATATATC	32228665	MTMR12
5	37516814	37516828	AT	del	A	37516825	WDR70
5	58295380	58295394	TA	ins	TATG	58295389	PDE4D
5	61027630	61027640	AG	ins	A	61027635	BC039381
5	64874562	64874572	AT	ins	A	64874567	PPWD1
5	68472353	68472369	TA	del	A	68472365	CCNB1
5	68472353	68472369	TA	ins	T	68472366	CCNB1
5	76371866	76371880	TA	ins	TG	76371873	ZBED3
5	78360550	78360560	AT	del	A	78360557	DMGDH
5	78964887	78964901	TG	ins	TGTA	78964898	PAPD4
5	81613026	81613042	AG	del	A	81613039	ATP6AP1L
5	89947204	89947214	TC	del	CTC	89947210	GPR98
5	94826193	94826209	AT	del	A	94826206	TTC37
5	110448734	110448752	AT	ins	T	110448748	WDR36
5	110448734	110448752	AT	del	A	110448749	WDR36
5	137683541	137683555	CT	ins	TA	137683543	FAM53C
5	147869706	147869716	TA	ins	ATGTATA	147869708	HTR4
5	150837902	150837918	TA	ins	AC	150837912	SLC36A1
5	167992873	167992891	AT	del	A	167992888	PANK3
5	172584913	172584929	TG	ins	T	172584926	BNIP1
6	28611648	28611664	AT	del	ATATATA	28611655	TRNA_Ala
6	29006981	29006997	TA	ins	T	29006994	LOC100129636
6	31669149	31669167	AC	del	A	31669164	ABHD16A
6	31669149	31669167	AC	del	A	31669164	MIR4646
6	32359921	32359931	TA	del	A	32359923	HCG23
6	32359921	32359931	TA	del	ATATA	32359925	HCG23

6	33625592	33625610	TG	del	T	33625599	ITPR3
6	36894335	36894345	AG	del	G	36894341	C6orf89
6	42995643	42995659	AC	ins	AT	42995654	RRP36
6	49422846	49422860	AT	del	T	49422848	MUT
6	88312613	88312623	AT	del	TATATAT	88312615	ORC3
6	89553669	89553681	TA	ins	AG	89553675	RNGTT
6	89809104	89809120	CT	del	T	89809114	SRSF12
6	132014258	132014276	AC	del	A	132014273	ENPP3
6	154678762	154678776	TA	del	ATATATA	154678768	CNKSR3
6	154678762	154678776	TA	del	ATATATA	154678768	IPCEF1
6	154678762	154678776	TA	del	ATATATA	154678768	IPCEF1
6	160390971	160390989	AT	del	A	160390986	IGF2R
6	160677920	160677934	TC	del	CTC	160677930	SLC22A2
6	166822306	166822320	CA	del	A	166822308	RPS6KA2
7	13936315	13936325	AG	ins	GAGAGAGAAAGAAAGAAAG	13936317	AK055368
7	13936315	13936325	AG	ins	GAGAGAGAAAGAAAGAAAG	13936317	ETV1
7	30898268	30898286	AT	del	T	30898270	AQP1
7	30898268	30898286	AT	del	T	30898270	FAM188B
7	31149737	31149755	CT	ins	T	31149751	ADCYAP1R1
7	31149737	31149755	CT	del	C	31149752	ADCYAP1R1
7	37915818	37915834	TA	ins	AG	37915828	TXNDC3
7	47923748	47923764	GT	ins	TC	47923752	PKD1L1
7	73254451	73254463	TG	del	T	73254460	WBSCR27
7	73628370	73628386	TA	ins	AA	73628374	LAT2
7	73804452	73804466	AG	ins	AGAGAA	73804463	CLIP2
7	74232974	74232990	CT	del	TCT	74232986	GTF2IRD2
7	100071377	100071391	TA	ins	T	100071388	TSC22D4
7	101755512	101755522	TC	ins	T	101755519	CUX1
7	124783258	124783270	TC	del	CTC	124783266	AX746567
7	124783258	124783270	TC	del	CTC	124783266	BC142949
7	124783258	124783270	TC	del	CTC	124783266	BX648695
7	128528349	128528361	AC	ins	AT	128528356	KCP
7	128545037	128545053	GT	del	G	128545050	KCP

7	138340385	138340395	TA	del	TATAT	138340390	SVOPL
7	141720386	141720404	AT	ins	TT	141720400	MGAM
7	142621436	142621450	AT	ins	AC	142621445	TRPV5
7	157449536	157449552	CA	del	CAC	157449547	PTPRN2
8	1949199	1949209	CA	del	ACA	1949205	KBTBD11
8	2148364	2148374	TG	ins	TC	2148367	AX747124
8	11994578	11994594	TG	ins	GG	11994582	FAM66D
8	11994578	11994594	TG	ins	GG	11994582	LOC100506990
8	11994578	11994594	TG	ins	GG	11994582	USP17L2
8	15094179	15094189	AT	del	T	15094185	SGCZ
8	22134531	22134547	AT	ins	T	22134543	PIWIL2
8	22134531	22134547	AT	del	A	22134544	PIWIL2
8	27849408	27849420	CA	ins	CG	27849413	SCARA5
8	69022846	69022856	CA	ins	CG	69022851	PREX2
8	69243029	69243043	AC	ins	C	69243039	C8orf34
8	69243029	69243043	AC	ins	C	69243039	LOC286189
8	71037433	71037447	AT	ins	AC	71037442	NCOA2
8	82395812	82395826	TG	del	T	82395823	FABP4
8	110660491	110660503	AT	ins	A	110660500	AX748380
8	110660491	110660503	AT	ins	A	110660500	SYBU
8	110660491	110660503	AT	ins	A	110660500	SYBU
8	118846854	118846864	AC	ins	ACACAT	118846859	EXT1
8	141677549	141677565	TC	del	CTCTC	141677559	PTK2
9	21801809	21801823	GT	ins	GTGTGTGTGTGC	21801812	MTAP
9	103108150	103108168	AT	del	A	103108165	TEX10
9	114376450	114376468	TA	ins	T	114376465	C9orf29
9	117167336	117167350	TC	del	TCTCT	117167345	DFNB31
9	127616792	127616810	AC	del	C	127616794	WDR38
9	131368751	131368767	AT	del	A	131368764	SPTAN1
X	1402366	1402380	CT	del	C	1402373	CRLF2
X	1402366	1402380	CT	del	C	1402373	CRLF2
X	1402366	1402380	CT	del	C	1402373	CSF2RA
X	1402366	1402380	CT	del	C	1402373	CSF2RA

X	2650603	2650615	CT	ins	TT	2650611	CD99
X	8555161	8555175	TG	del	T	8555172	KAL1
X	55511947	55511965	AT	del	T	55511949	USP51
X	118590953	118590965	AG	del	GAGAAAGAAAG	118590961	Y_RNA
X	133694524	133694542	GT	ins	TA	133694528	LOC100506757
10	7451052	7451070	CCA	ins	A	7451056	SFMBT2
10	7608748	7608763	AAG	ins	G	7608752	ITIH5
10	73973950	73973965	AAC	del	AC	73973959	ANAPC16
10	73973950	73973965	AAC	del	AC	73973959	ASCC1
10	95352569	95352587	ATT	ins	T	95352578	RBP4
10	95352569	95352587	ATT	del	A	95352583	RBP4
10	100992956	100992977	CAC	del	C	100992972	HPSE2
10	131335406	131335418	AAG	del	AAGA	131335414	MGMT
11	5877547	5877559	TAT	del	TA	5877552	OR52E8
11	5877547	5877559	TAT	del	TA	5877552	TRIM5
11	5877547	5877559	TAT	del	TT	5877554	OR52E8
11	5877547	5877559	TAT	del	TT	5877554	TRIM5
11	5877547	5877559	TAT	del	TA	5877555	OR52E8
11	5877547	5877559	TAT	del	TA	5877555	TRIM5
11	34219989	34220001	AAC	ins	A	34219997	ABTB2
11	65035810	65035828	TTA	ins	T	65035824	POLA2
11	117280715	117280730	CCT	del	C	117280723	CEP164
12	16409802	16409817	AAT	del	AT	16409805	SLC15A5
12	31354707	31354728	ATT	del	TA	31354720	OVOS2
12	31354707	31354728	ATT	del	A	31354724	OVOS2
12	32482023	32482041	ATT	ins	T	32482035	BICD1
12	32482023	32482041	ATT	del	A	32482037	BICD1
12	47473124	47473148	GTT	del	G	47473141	AMIGO2
12	47473124	47473148	GTT	del	G	47473141	FAM113B
12	93192111	93192129	ATT	ins	T	93192120	EEA1
12	96884473	96884485	AAT	del	AATA	96884481	C12orf55
12	120189487	120189502	ATT	del	TTATT	120189496	CIT
12	120739162	120739183	AAC	del	AC	120739165	SIRT4

12	122357031	122357049	TTA	del	A	122357044	WDR66
12	124299599	124299617	CAA	ins	AAA	124299608	DNAH10
13	77571275	77571287	TTG	del	TTGT	77571283	CLN5
13	77571275	77571287	TTG	del	TTGT	77571283	Mir_633
13	101709792	101709804	TTG	del	TTGT	101709800	NALCN
14	64606119	64606134	ATT	del	A	64606130	SYNE2
14	74003680	74003692	GTT	del	TTGT	74003686	ACOT1
14	74003680	74003692	GTT	del	TTGT	74003686	ACOT1
14	74003680	74003692	GTT	del	TTGT	74003686	HEATR4
14	74003680	74003692	GTT	del	TG	74003687	ACOT1
14	74003680	74003692	GTT	del	TG	74003687	ACOT1
14	74003680	74003692	GTT	del	TG	74003687	HEATR4
14	74003680	74003692	GTT	del	G	74003688	ACOT1
14	74003680	74003692	GTT	del	G	74003688	ACOT1
14	74003680	74003692	GTT	del	G	74003688	HEATR4
14	74450378	74450399	ATT	del	TA	74450391	ENTPD5
14	74450378	74450399	ATT	del	A	74450392	ENTPD5
14	74450378	74450399	ATT	del	TA	74450394	ENTPD5
14	74450378	74450399	ATT	del	A	74450395	ENTPD5
14	88631472	88631484	CAG	del	A	88631475	DQ574857
14	88631472	88631484	CAG	del	A	88631475	DQ577549
15	65243502	65243517	TTC	del	TTCTT	65243513	ANKDD1A
15	96811957	96811975	TCT	del	T	96811970	AK000872
15	96811957	96811975	TCT	del	T	96811970	AK307134
15	96811957	96811975	TCT	ins	C	96811971	AK000872
15	96811957	96811975	TCT	ins	C	96811971	AK307134
16	2770513	2770531	ATT	del	A	2770527	PRSS27
16	8873107	8873125	TGG	del	GGTG	8873119	ABAT
16	24123864	24123876	CAA	ins	AACAACAAT	24123867	PRKCB
16	30930100	30930112	CTT	del	TC	30930107	FBXL19
16	30930100	30930112	CTT	del	TC	30930107	FBXL19-AS1
16	30971061	30971073	TTC	del	TTCT	30971069	SETD1A
16	50347328	50347346	AAC	del	ACAAC	50347340	ADCY7

16	84766037	84766052	CTT	del	TC	84766047	USP10
16	84766037	84766052	CTT	del	C	84766048	USP10
16	84801372	84801384	ATT	del	A	84801380	USP10
17	637413	637431	TTC	del	C	637426	FAM57A
17	1482442	1482463	AAT	del	T	1482446	SLC43A2
17	45940923	45940935	CTT	ins	T	45940929	BC031827
17	47014823	47014841	TAA	ins	T	47014828	SNF8
17	66919330	66919348	TAT	ins	T	66919343	ABCA8
18	9830003	9830024	TAT	ins	TAA	9830011	Metazoa_SRP
18	9830003	9830024	TAT	ins	TAA	9830011	RAB31
18	24916060	24916072	TTC	del	TTCTT	24916068	AK127888
18	48723137	48723152	CCG	del	CGCCGCCG	48723143	MEX3C
19	5668581	5668596	ATT	del	A	5668592	SAFB
19	7569849	7569864	CTT	del	C	7569860	C19orf45
19	7614370	7614382	CTT	del	TC	7614377	PNPLA6
19	8151331	8151352	TTA	del	A	8151347	FBN3
19	8463827	8463845	ATT	del	A	8463841	RAB11B
19	8642947	8642959	AAC	del	C	8642951	MYO1F
19	8642947	8642959	AAC	del	AC	8642953	MYO1F
19	8642947	8642959	AAC	del	C	8642954	MYO1F
19	9647813	9647831	AAT	del	T	9647817	ZNF426
19	40355497	40355515	TTG	del	G	40355510	FCGBP
19	40914708	40914720	TTG	ins	G	40914715	PRX
19	40954498	40954513	AAC	ins	AACAACAACAAT	40954503	BLVRB
19	44454816	44454840	AAT	ins	TC	44454834	ZNF221
19	47909520	47909532	GGA	del	GGAT	47909528	MEIS3
19	50956766	50956781	TTG	del	TG	50956775	MYBPC2
19	54938780	54938798	AAC	del	C	54938793	TTYH1
19	56309354	56309366	TAT	ins	TT	56309358	NLRP11
19	56309354	56309366	TAT	del	A	56309360	NLRP11
1	6529182	6529206	TCC	del	C	6529185	PLEKHG5
1	12197371	12197389	AAT	ins	ATC	12197383	TNFRSF8
1	12319090	12319102	TTC	ins	T	12319098	VPS13D

1	28298103	28298118	CAA	del	AC	28298113	EYA3
1	53154816	53154834	ATT	del	A	53154830	SELRC1
1	75687634	75687646	TCT	ins	T	75687641	SLC44A5
1	75687634	75687646	TCT	del	TC	75687642	SLC44A5
1	77334276	77334300	GCA	ins	CAGCAA	77334291	ST6GALNAC5
1	89352593	89352611	ATT	ins	T	89352605	GTF2B
1	89352593	89352611	ATT	del	A	89352607	GTF2B
1	167598816	167598834	TCC	del	C	167598819	RCSD1
1	183849509	183849521	AAC	del	AC	183849515	RGL1
1	235618437	235618452	TTA	ins	TTG	235618448	B3GALNT2
1	236157987	236158002	CTT	ins	C	236157998	NID1
20	19867237	19867258	CTT	del	C	19867251	RIN2
20	22382255	22382273	TAT	ins	TA	22382266	LOC284788
20	25423232	25423247	TAT	del	TA	25423243	GINS1
20	47692710	47692722	TTG	del	TTGTT	47692718	CSE1L
22	17526292	17526307	TTA	del	T	17526300	CECR7
22	17668980	17668992	AAG	del	AAGAA	17668988	CECR1
22	23914597	23914618	AAC	del	AACAACAACAA	23914608	IGLL1
22	25165766	25165787	AAT	del	AT	25165769	PIWIL3
22	25165766	25165787	AAT	del	T	25165770	PIWIL3
22	25165766	25165787	AAT	del	AT	25165772	PIWIL3
22	26157346	26157364	GGT	del	GT	26157358	MYO18B
22	39795600	39795624	AAC	ins	AC	39795603	TAB1
22	39795600	39795624	AAC	del	A	39795605	TAB1
22	42121115	42121127	ATT	del	A	42121123	bK250D10.C22.8
22	42121115	42121127	ATT	del	A	42121123	MEI1
2	9629286	9629301	TTG	ins	TTC	9629297	ADAM17
2	9629286	9629301	TTG	ins	TTC	9629297	IAH1
2	24105678	24105696	CAA	del	AC	24105691	ATAD2B
2	24105678	24105696	CAA	del	C	24105692	ATAD2B
2	25365630	25365645	AAT	del	AATA	25365641	EFR3B
2	61576979	61577003	AAC	del	AC	61576997	USP34
2	102498304	102498316	TTG	del	GTTGTTGCTTG	102498308	MAP4K4

2	114380292	114380304	AAC	del	AACA	114380300	RPL23AP7
2	152647894	152647915	TTG	ins	GTC	152647904	ARL5A
2	176989013	176989025	CTT	del	C	176989021	HOXD9
2	192921935	192921947	ATT	del	TA	192921942	TMEFF2
2	192921935	192921947	ATT	del	A	192921943	TMEFF2
2	202698422	202698440	TCT	del	TTCT	202698435	CDK15
2	202698422	202698440	TCT	del	TC	202698436	CDK15
2	211341196	211341208	GGC	ins	C	211341203	LANCL1
3	31639319	31639337	ATT	del	A	31639333	STT3B
3	40301474	40301489	AGC	del	GC	40301477	FLJ33065
3	40301474	40301489	AGC	del	GC	40301477	MYRIP
3	40301474	40301489	AGC	del	AG	40301482	FLJ33065
3	40301474	40301489	AGC	del	AG	40301482	MYRIP
3	42596467	42596488	AAC	ins	C	42596477	SEC22C
3	65463367	65463388	ATT	ins	TT	65463382	MAGI1
3	71064315	71064339	CAA	ins	ACC	71064331	FOXP1
3	126181905	126181923	ATT	del	A	126181916	ZXDC
3	126181905	126181923	ATT	del	A	126181919	ZXDC
3	129697384	129697396	TAA	ins	ATAAT	129697388	TRH
3	156182274	156182298	AAT	del	T	156182278	KCNAB1
3	170009485	170009500	AAT	ins	TT	170009492	PRKCI
3	178740419	178740440	AAT	del	T	178740423	ZMAT3
3	183685912	183685930	TTA	ins	ATC	183685916	ABCC5
3	197711350	197711365	GCT	ins	TGCTGT	197711360	LMLN
4	8224403	8224421	TTG	ins	GTC	8224413	SH3TC1
4	39574698	39574719	TTA	del	TTATTATTATT	39574709	AK056558
4	39574698	39574719	TTA	del	TTATTATTATT	39574709	C4orf34
4	39917816	39917828	AAC	del	AC	39917822	PDS5A
4	39917816	39917828	AAC	del	C	39917823	PDS5A
4	41684683	41684695	TTC	ins	T	41684691	LIMCH1
4	47628988	47629012	GTT	del	G	47629008	CORIN
4	57261021	57261033	ATT	del	A	57261029	PPAT
4	90744151	90744172	ATT	del	A	90744168	SNCA

5	13702266	13702278	CAG	del	GCAGTGTAAG	13702273	DNAH5
5	79049410	79049428	AAC	ins	AAA	79049424	CMYA5
5	134180421	134180436	TTG	del	TTGT	134180432	C5orf24
5	139713507	139713522	CTT	del	C	139713518	HBEGF
5	141045619	141045640	AAT	del	T	141045623	ARAP3
5	149599433	149599454	TTA	ins	T	149599444	CAMK2A
5	149633420	149633441	AGC	ins	CAA	149633424	CAMK2A
5	179665596	179665608	TTC	del	TTCT	179665604	MAPK9
6	24418571	24418583	ATT	del	TA	24418578	MRS2
6	24418571	24418583	ATT	del	A	24418579	MRS2
6	27745584	27745608	AAC	ins	A	27745595	TRNA_Met
6	27745584	27745608	AAC	del	ACAACAAC	27745596	TRNA_Met
6	27745584	27745608	AAC	del	C	27745597	TRNA_Met
6	27745584	27745608	AAC	ins	A	27745601	TRNA_Met
6	31111834	31111849	AAC	del	C	31111841	CCHCR1
6	31765848	31765863	ATT	del	A	31765859	LSM2
6	32361455	32361467	TTC	ins	C	32361462	HCG23
6	42630675	42630690	AAC	ins	A	42630686	UBR2
6	44143522	44143537	AAT	ins	AAC	44143533	CAPN11
6	109906329	109906344	CTT	del	TC	109906339	AKD1
6	109906329	109906344	CTT	del	C	109906340	AKD1
6	144069196	144069208	ATT	ins	T	144069202	PHACTR2
6	144069196	144069208	ATT	del	A	144069204	PHACTR2
7	24847115	24847136	AAT	ins	AT	24847127	OSBPL3
7	73821075	73821099	ATT	del	TA	73821079	CLIP2
7	76240186	76240198	ATT	del	TA	76240193	LOC100133091
7	76240186	76240198	ATT	del	TA	76240193	LOC100133091
7	76240186	76240198	ATT	del	TA	76240193	LOC100133091
7	76240186	76240198	ATT	del	TA	76240193	POMZP3
7	76240186	76240198	ATT	del	TA	76240193	POMZP3
7	76240186	76240198	ATT	del	TA	76240193	POMZP3
7	76240186	76240198	ATT	del	A	76240194	LOC100133091
7	76240186	76240198	ATT	del	A	76240194	LOC100133091

7	76240186	76240198	ATT	del	A	76240194	LOC100133091
7	76240186	76240198	ATT	del	A	76240194	POMZP3
7	76240186	76240198	ATT	del	A	76240194	POMZP3
7	76240186	76240198	ATT	del	A	76240194	POMZP3
7	80122918	80122930	TCC	del	C	80122921	CD36
7	80122918	80122930	TCC	del	C	80122921	GNAT3
7	144462339	144462360	AAC	del	AC	144462354	TPK1
7	144462339	144462360	AAC	ins	AA	144462356	TPK1
8	53554520	53554538	AAT	ins	AAC	53554525	RB1CC1
8	59505464	59505476	AAC	del	C	59505471	NSMAF
8	59505464	59505476	AAC	del	C	59505471	TRNA_Glu
8	59505464	59505476	AAC	ins	A	59505472	NSMAF
8	59505464	59505476	AAC	ins	A	59505472	TRNA_Glu
8	125991724	125991736	ATT	del	TA	125991731	ZNF572
8	125991724	125991736	ATT	del	A	125991732	ZNF572
9	18794631	18794643	TTG	del	TG	18794634	ADAMTSL1
9	18794631	18794643	TTG	ins	T	18794639	ADAMTSL1
9	35490113	35490134	CGC	ins	GCCGCT	35490119	RUSC2
9	36190459	36190471	TCT	del	TTCT	36190466	CLTA
9	36190459	36190471	TCT	del	TC	36190467	CLTA
9	94814803	94814818	ATT	del	A	94814814	SPTLC1
9	109859659	109859671	TTA	ins	ATTA	109859666	AK097706
9	114450023	114450035	TTC	del	TTCT	114450031	C9orf84
9	118093809	118093833	ATG	ins	ATGATT	118093820	DEC1
9	130080012	130080033	AAC	del	C	130080016	GARNL3
9	133364619	133364634	ATT	ins	T	133364628	ASS1
9	133364619	133364634	ATT	del	A	133364630	ASS1
X	50054594	50054609	TTG	del	G	50054598	CCNB3
X	123021841	123021853	AAT	del	TAATAAT	123021845	XIAP
X	123021841	123021853	AAT	del	TAAT	123021848	XIAP
X	139867337	139867349	AAT	ins	ATAAT	139867343	AK054921
X	139867337	139867349	AAT	ins	ATAAT	139867343	CDR1
10	26592373	26592397	AGGG	ins	AGGA	26592384	GAD2

10	52498935	52498959	ATTG	ins	TGA	52498944	ASAH2B
10	95855077	95855105	TTTC	ins	TC	95855082	AK098548
10	95855077	95855105	TTTC	ins	TC	95855082	PLCE1
10	98759684	98759704	TAGA	ins	GATAGAT	98759689	SLIT1
10	101969821	101969849	TTAT	ins	T	101969839	CHUK
10	121435264	121435280	TTCC	ins	TCCTTCCCTCCTTCCC	121435272	BAG3
11	49829394	49829410	AAAT	del	A	49829401	LOC440040
11	65151237	65151253	AAAC	del	C	65151243	SLC25A45
11	65151237	65151253	AAAC	ins	A	65151244	SLC25A45
11	66242189	66242213	ATTT	del	A	66242208	PELI3
11	73768999	73769019	TCAA	ins	CAAC	73769003	C2CD3
11	118365863	118365879	TTTA	ins	T	118365874	MLL
11	123677265	123677281	TTTG	del	TG	123677274	OR6M1
11	123677265	123677281	TTTG	del	G	123677275	OR6M1
12	3737165	3737185	TCCC	del	CT	3737175	EFCAB4B
12	8808349	8808369	CAAA	del	AAACA	8808361	MFAP5
12	32785623	32785647	TTTA	del	TA	32785640	FGD4
12	32831183	32831203	TTTA	del	TTTATTTAT	32831190	DNM1L
12	32831183	32831203	TTTA	del	TTTAT	32831194	DNM1L
12	32831326	32831346	TTTG	ins	T	32831341	DNM1L
12	57601080	57601108	TAAA	ins	A	57601096	LRP1
12	66858713	66858737	CTTT	ins	TT	66858721	GRIP1
12	66858713	66858737	CTTT	del	C	66858724	GRIP1
12	69086841	69086865	TTTA	ins	T	69086860	NUP107
12	88175981	88176001	TTTG	ins	TTGC	88175985	MKRN9P
12	92822582	92822602	CTTT	ins	TCTC	92822592	CLLU1
12	92822582	92822602	CTTT	ins	TCTC	92822592	CLLU1
12	92822582	92822602	CTTT	ins	TCTC	92822592	CLLU1OS
12	105468269	105468285	AAAG	del	AA	105468276	ALDH1L2
12	122097422	122097446	TTTC	del	TTTCTT	122097441	MORN3
12	123071339	123071367	TATT	ins	T	123071360	KNTC1
12	123921874	123921898	TTCT	ins	T	123921892	RILPL2
12	124114340	124114360	AAAT	del	AAATA	124114355	EIF2B1

12	132590359	132590379	TTTC	del	TC	132590372	EP400NL
12	132590359	132590379	TTTC	ins	T	132590374	EP400NL
13	36801636	36801652	AAAT	del	AT	36801645	CCDC169
13	36801636	36801652	AAAT	del	AT	36801645	CCDC169-SOHLH2
13	96252828	96252844	AGAT	ins	GAT	96252836	DZIP1
13	103315467	103315483	ATTT	del	TTA	103315476	TPP2
14	23843921	23843941	TCTT	del	TTC	23843935	IL25
14	55405472	55405500	AAAT	del	AAATA	55405495	WDHD1
14	55408969	55408997	AAAT	ins	ATAC	55408982	WDHD1
14	55862071	55862091	TTTG	ins	TTTT	55862082	ATG14
14	55862071	55862091	TTTG	ins	TTTT	55862082	FBXO34
14	57396888	57396908	TTTC	ins	T	57396903	OTX2OS1
14	71443071	71443091	TCTT	del	TTC	71443085	PCNX
14	71443071	71443091	TCTT	del	TC	71443086	PCNX
14	74288374	74288402	TTTC	del	TTCTTTC	74288394	BC038204
14	75178777	75178797	AAAG	del	AG	75178786	KIAA0317
14	75178777	75178797	AAAG	del	AG	75178786	SNORA7
14	78182656	78182676	CAAA	del	C	78182671	SLIRP
14	106136600	106136616	ATTT	del	T	106136604	abParts
14	106136600	106136616	ATTT	del	T	106136604	abParts
14	106136600	106136616	ATTT	del	T	106136604	DKFZp686O16217
14	106136600	106136616	ATTT	del	T	106136604	DKFZp686O16217
14	106136600	106136616	ATTT	del	T	106136604	IGH@
14	106136600	106136616	ATTT	del	T	106136604	IGH@
14	106136600	106136616	ATTT	del	T	106136604	IGHE
14	106136600	106136616	ATTT	del	T	106136604	IGHE
14	106136600	106136616	ATTT	del	T	106136604	IGHE
14	106136600	106136616	ATTT	del	T	106136604	IGHE
14	106136600	106136616	ATTT	del	T	106136604	IGHG1
14	106136600	106136616	ATTT	del	T	106136604	IGHG1
15	34047817	34047837	AAAC	ins	AA	34047828	RYR3
15	35212107	35212123	AAAC	ins	A	35212118	AQR
15	51689531	51689551	CCTC	ins	CCTT	51689542	GLDN

15	63827115	63827143	TTTA	ins	TTAC	63827135	USP3
16	538401	538417	ATTT	del	A	538412	RAB11FIP3
16	2021224	2021240	TTTC	del	TTC	2021228	TBL3
16	2021224	2021240	TTTC	del	TTC	2021228	TCRBV20S1
16	3708675	3708691	AAAC	ins	A	3708686	DNASE1
16	3708675	3708691	AAAC	ins	A	3708686	TRAP1
16	15488735	15488751	CTTT	del	TC	15488745	MPV17L
16	17198508	17198524	AAAC	del	AAC	17198516	XYLT1
16	17198508	17198524	AAAC	del	C	17198518	XYLT1
16	57956317	57956337	TCTT	ins	T	57956330	CNGB1
16	58553712	58553732	ATCA	ins	A	58553726	CNOT1
16	58553712	58553732	ATCA	ins	A	58553726	SETD6
16	74699634	74699654	TTTC	del	C	74699648	RFWD3
16	74699634	74699654	TTTC	ins	T	74699649	RFWD3
16	89380662	89380678	AAAC	del	A	89380673	ANKRD11
16	89617473	89617489	CAAA	del	C	89617484	SPG7
17	4119211	4119227	AAAC	ins	AACT	4119215	ANKFY1
17	5434464	5434484	TCTA	ins	A	5434474	NLRP1
17	9532791	9532815	CTTT	del	C	9532810	WDR16
17	9886098	9886114	AAAG	del	GAAAG	9886108	GAS7
17	12895444	12895468	TTGA	del	TTGAT	12895459	ARHGAP44
17	12895444	12895468	TTGA	del	TTGAT	12895459	ELAC2
17	18153105	18153129	AAAC	del	A	18153124	FLII
17	37413232	37413248	TTTC	del	TTTCT	37413243	FBXL20
17	48560550	48560570	AAAG	ins	A	48560561	RSAD1
17	56649614	56649642	AAAC	ins	A	56649633	TEX14
17	66398384	66398400	TTGT	del	TG	66398388	ARSG
17	72250306	72250326	TTTA	del	A	72250320	TTYH2
17	76866503	76866531	AAAG	del	G	76866513	TIMP2
17	79527706	79527726	AAAC	del	A	79527721	NPLOC4
17	80686291	80686307	TTCT	ins	T	80686301	FN3KRP
18	117490	117506	ATTG	ins	TGAC	117495	ROCK1P1
18	3567040	3567068	TTCA	del	T	3567063	DLGAP1

18	33713680	33713700	TTAT	ins	T	33713694	ELP2
18	43468266	43468282	TTTA	ins	T	43468277	EPG5
18	56400135	56400159	ATTT	del	A	56400154	MALT1
19	544706	544734	TCCC	ins	A	544727	GZMM
19	1969223	1969243	CCAC	ins	T	1969237	CSNK1G2
19	3435807	3435835	TCTT	del	C	3435811	NFIC
19	6189706	6189726	TTTC	del	C	6189720	ACSBG2
19	6189706	6189726	TTTC	del	C	6189720	AK056073
19	13475453	13475469	TTTA	del	TT	13475464	CACNA1A
19	14697687	14697703	TTTC	ins	T	14697698	CLEC17A
19	14704776	14704804	AAAC	del	C	14704798	CLEC17A
19	16872161	16872177	TCTT	ins	TCC	16872168	NWD1
19	17123136	17123160	TTTC	ins	T	17123155	CPAMD8
19	17285746	17285762	AAAT	del	T	17285752	MYO9B
19	17649540	17649556	AAGA	del	AA	17649550	FAM129C
19	17776809	17776825	TCCT	ins	T	17776815	UNC13A
19	19013557	19013577	AAGG	del	AGG	19013565	COPE
19	21739992	21740012	CAAA	ins	AACT	21740001	ZNF429
19	33587838	33587858	TTTC	ins	T	33587853	GPATCH1
19	35541798	35541818	TTTC	del	T	35541813	HPN
19	48347486	48347506	CTTT	del	TTC	48347499	CRX
19	48848702	48848730	ATTT	ins	TTAC	48848707	Mir_324
19	48848702	48848730	ATTT	ins	TTAC	48848707	TMEM143
19	52431303	52431331	ATCT	del	TCT	52431307	ZNF613
19	55601600	55601616	TTTG	del	TTG	55601604	PPP1R12C
19	55601600	55601616	TTTG	del	TG	55601605	PPP1R12C
19	57649480	57649508	AGGG	ins	A	57649495	ZIM3
1	7838921	7838941	TTGT	del	TTG	7838936	VAMP3
1	20666047	20666063	CAAA	del	AAACA	20666055	VWA5B1
1	21881403	21881423	TTTC	del	TTCTTTC	21881415	ALPL
1	35558204	35558220	CTTT	ins	T	35558208	ZMYM1
1	35558204	35558220	CTTT	del	TC	35558210	ZMYM1
1	45140619	45140647	TGGA	ins	G	45140631	C1orf228

1	45140619	45140647	TGGA	ins	G	45140631	TMEM53
1	52254438	52254454	ATAG	ins	AT	52254449	NRD1
1	52254438	52254454	ATAG	ins	AT	52254449	OSBPL9
1	114392196	114392216	TTTA	del	A	114392210	PTPN22
1	115119981	115120001	ATTT	del	A	115119996	BCAS2
1	115119981	115120001	ATTT	del	A	115119996	DENND2C
1	154698005	154698021	CTTC	del	C	154698015	KCNN3
1	165566486	165566502	AAAG	ins	AAGT	165566490	TRNA_Pseudo
1	180021333	180021349	TTCT	del	T	180021343	CEP350
1	201015280	201015304	TATT	del	TTATTTA	201015294	CACNA1S
1	202934752	202934768	CTTT	ins	T	202934760	CYB5R1
1	202934752	202934768	CTTT	del	TC	202934762	CYB5R1
1	202934752	202934768	CTTT	del	C	202934763	CYB5R1
1	233431732	233431748	TTTC	ins	T	233431743	PCNXL2
1	236226779	236226803	AAAT	del	T	236226785	AX747246
1	236226779	236226803	AAAT	del	T	236226785	NID1
1	236226779	236226803	AAAT	del	T	236226789	AX747246
1	236226779	236226803	AAAT	del	T	236226789	NID1
1	236226779	236226803	AAAT	ins	A	236226790	AX747246
1	236226779	236226803	AAAT	ins	A	236226790	NID1
1	236226779	236226803	AAAT	del	T	236226797	AX747246
1	236226779	236226803	AAAT	del	T	236226797	NID1
20	30612921	30612937	CTTT	del	TTTCT	30612929	C20orf160
20	42195538	42195558	GGAG	ins	GGAC	42195553	SGK2
20	43737235	43737255	TTTA	ins	T	43737250	WFDC5
20	45815997	45816013	AAAG	ins	A	45816008	EYA2
20	62607527	62607547	AAAC	ins	AAC	62607539	SAMD10
21	33757316	33757344	AAAG	del	AAG	33757320	URB1
21	45677576	45677604	AAAT	del	AAT	45677580	DNMT3L
21	45677576	45677604	AAAT	del	AT	45677585	DNMT3L
22	22736711	22736731	TTTA	ins	TTTG	22736722	abParts
2	10867680	10867696	CAAA	ins	AAAT	10867688	ATP6V1C2
2	27872115	27872135	AAAC	ins	A	27872130	GPN1

2	27872115	27872135	AAAC	ins	A	27872130	SUPT7L
2	27875016	27875040	AAAT	del	AAA	27875035	GPN1
2	27875016	27875040	AAAT	del	AAA	27875035	SUPT7L
2	33763570	33763594	AAAT	ins	ATAAATAC	33763575	RASGRP3
2	44123337	44123361	TTTC	del	CTTTC	44123355	LRPPRC
2	55860797	55860817	AAAG	del	AA	55860804	PNPT1
2	65072431	65072447	ACAA	del	A	65072436	AK097952
2	109000660	109000688	TTTA	ins	T	109000679	SULT1C4
2	109000660	109000688	TTTA	ins	T	109000683	SULT1C4
2	109108220	109108244	ATTT	ins	T	109108232	GCC2
2	109108220	109108244	ATTT	del	A	109108235	GCC2
2	109108220	109108244	ATTT	del	A	109108239	GCC2
2	120094618	120094646	ATTT	del	A	120094633	C2orf76
2	120094618	120094646	ATTT	del	A	120094637	C2orf76
2	120094618	120094646	ATTT	del	TA	120094640	C2orf76
2	120094618	120094646	ATTT	del	A	120094641	C2orf76
2	128603824	128603840	AAAT	del	AAATAA	128603835	POLR2D
2	182777786	182777806	TATT	ins	T	182777799	SSFA2
2	207609646	207609674	TAAA	del	AT	207609668	MDH1B
2	217025386	217025414	TTAA	ins	T	217025401	XRCC5
2	233407568	233407584	CCTG	ins	GCCTGCCC	233407578	CHRNG
2	242371225	242371245	TTTA	del	A	242371239	FARP2
3	32762314	32762330	CCCT	ins	CCTCCCTT	32762322	CNOT10
3	66464830	66464846	AATA	del	AAT	66464841	LRIG1
3	101083072	101083100	AAAG	ins	AG	101083089	SENP7
3	108189872	108189888	AAAG	ins	AA	108189883	MYH15
3	159727840	159727860	TGCT	ins	T	159727854	AK097161
3	159727840	159727860	TGCT	del	TGC	159727855	AK097161
3	167167269	167167297	ATTT	del	T	167167285	SERPINI2
3	167167269	167167297	ATTT	ins	TG	167167290	SERPINI2
3	167167269	167167297	ATTT	del	A	167167292	SERPINI2
3	170798212	170798232	TTTA	del	A	170798226	TNIK
3	172242139	172242159	CTTT	del	TTT	172242143	TNFSF10

3	197320475	197320491	AAAC	ins	AAAG	197320486	LOC220729
4	1837191	1837211	AAAC	ins	A	1837206	LETM1
4	38029731	38029751	AAAC	ins	A	38029746	TBC1D1
4	43033167	43033195	ATTT	del	TA	43033189	GRXCR1
4	68443353	68443369	TTTC	ins	TT	68443364	STAP1
4	76709304	76709324	TTTA	del	TA	76709317	USO1
4	76709304	76709324	TTTA	ins	T	76709319	USO1
4	77652940	77652960	AAAC	del	C	77652946	SHROOM3
4	120374476	120374492	CTAG	ins	CTAT	120374487	BC070391
4	141545653	141545669	CAAA	del	AAACA	141545661	TBC1D9
4	141545653	141545669	CAAA	del	C	141545664	TBC1D9
4	156628971	156628991	TTTC	ins	T	156628986	GUCY1A3
4	166962437	166962457	TTCT	del	TTC	166962452	TLL1
4	170863318	170863338	TTTC	ins	TTC	170863326	LOC100506085
4	170863318	170863338	TTTC	del	T	170863329	LOC100506085
4	182895719	182895735	CAAA	del	A	182895727	AK056196
5	31193619	31193647	AAAG	ins	A	31193638	CDH6
5	31193619	31193647	AAAG	del	G	31193641	CDH6
5	37479444	37479460	TGGT	del	TGG	37479455	WDR70
5	52388933	52388949	CAAA	del	A	52388941	ITGA2
5	79934285	79934305	TTTA	del	TTA	79934293	DHFR
5	134002266	134002286	AAAG	del	AA	134002281	SEC24A
5	137590712	137590728	AGGA	del	AG	137590723	GFRA3
5	154211004	154211020	TTGT	ins	G	154211013	C5orf4
5	159641799	159641819	AAAG	ins	AAAT	159641810	FABP6
6	8422225	8422253	TGTT	del	G	8422229	SLC35B3
6	24358969	24358985	TTTA	ins	TATA	24358978	DCDC2
6	24358969	24358985	TTTA	ins	TATA	24358978	KAAG1
6	38897465	38897481	AAAG	del	G	38897471	DNAH8
6	38897465	38897481	AAAG	del	G	38897471	LOC100131047
6	56876556	56876572	AAAG	del	AAAGA	56876567	BEND6
6	94478540	94478564	AAAT	del	T	94478546	TSG1
6	136979431	136979451	TTTC	ins	T	136979442	MAP3K5

6	136979431	136979451	TTTC	del	C	136979445	MAP3K5
6	144760622	144760638	TTTC	ins	TT	144760633	UTRN
7	66281798	66281814	TAAA	del	AAATAAATA	66281802	LOC729156
7	82464268	82464296	TTTG	del	G	82464274	PCLO
7	128522809	128522833	AAAC	del	C	128522827	KCP
7	129331178	129331194	TATT	ins	TTA	129331188	NRF1
7	148107157	148107173	AAAG	del	A	148107168	CNTNAP2
7	148311107	148311123	TTTG	ins	T	148311114	C7orf33
8	27621234	27621258	AAAT	ins	A	27621249	CCDC25
8	82437764	82437788	ATAG	ins	GAT	82437778	FABP12
8	82437764	82437788	ATAG	del	A	82437781	FABP12
8	87061979	87061995	GAAA	ins	G	87061986	PSKH2
8	99163310	99163326	TGTT	del	G	99163318	POP1
8	139161379	139161403	AAAT	del	T	139161385	FAM135B
9	34459432	34459448	TTTC	ins	T	34459443	C9orf25
9	34459432	34459448	TTTC	ins	T	34459443	DNAI1
9	75357543	75357563	AAAC	del	C	75357557	TMC1
9	88841666	88841690	TTTG	del	TTG	88841670	C9orf153
9	88841666	88841690	TTTG	del	TTG	88841670	GOLM1
9	114920090	114920106	TCTT	del	C	114920098	MIR3134
9	114920090	114920106	TCTT	del	C	114920098	SUSD1
X	1394445	1394465	TTTA	ins	TTA	1394457	CRLF2
X	1394445	1394465	TTTA	ins	TTA	1394457	CSF2RA
X	1749796	1749812	TATT	ins	T	1749805	ASMT
X	2840923	2840947	AAAG	del	AA	2840942	ARSD
X	31137272	31137288	AAGT	del	AAG	31137283	DMD
X	47968148	47968164	TTTG	del	T	47968159	LOC100509575
X	49046935	49046955	TTTC	del	CTTTC	49046949	SYP
X	64737111	64737131	TTCT	del	TTC	64737126	LAS1L
X	64809730	64809746	TTTC	del	TTTCT	64809741	BC067907
X	70388734	70388754	AGAA	del	AG	70388745	NLGN3
X	75395953	75395969	TTTG	ins	T	75395964	CXorf26
