# Supplementary material for: Microsatellite Interruptions Stabilize Primate Genomes and Exist as Population-Specific Single Nucleotide Polymorphisms within Individual Human Genomes
Source: PLoS Genet. 2014 Jul 17;10(7):e1004498. doi: 10.1371/journal.pgen.1004498 (PMC4102424; doi:10.1371/journal.pgen.1004498)
Supplement: Dataset S3 — American (AMR) population-specific, exonic interrupted microsatellites. (PDF) [file pgen.1004498.s003.pdf]

Dataset S3. American (AMR) population-specific, exonic interrupted microsatellites.

chr	start	end	motif	interruption_event	interruption_nt	interruption_pos	gene
10	329182	329191	G	snp	T	329187	DIP2C
10	4951629	4951638	T	snp	C	4951636	AKR1C1
10	4951629	4951638	T	snp	C	4951636	tAKR
10	10986558	10986567	T	snp	C	10986563	LOC254312
10	11899934	11899943	T	snp	C	11899937	C10orf47
10	11899934	11899943	T	snp	C	11899937	LOC219731
10	11904828	11904836	A	snp	C	11904833	C10orf47
10	11904828	11904836	A	snp	C	11904833	LOC219731
10	14595390	14595400	A	snp	G	14595391	FAM107B
10	15090297	15090305	C	snp	A	15090303	ACBD7
10	15090297	15090305	C	snp	A	15090303	OLAH
10	18826215	18826223	T	snp	C	18826218	CACNB2
10	18826215	18826223	T	snp	C	18826218	U80764
10	24722200	24722209	T	snp	C	24722204	KIAA1217
10	24809616	24809625	A	snp	C	24809617	KIAA1217
10	28341712	28341721	T	snp	C	28341713	MPP7
10	29163826	29163834	G	snp	T	29163827	5S_rRNA
10	29163826	29163834	G	snp	T	29163829	5S_rRNA
10	32097845	32097853	A	snp	G	32097850	ARHGAP12
10	35898086	35898096	A	snp	C	35898091	GJD4
10	37441066	37441075	T	snp	G	37441071	ANKRD30A
10	47396023	47396031	G	snp	T	47396026	FAM35B2
10	51735343	51735352	G	snp	T	51735344	BC035067
10	51735343	51735352	G	snp	T	51735344	TIMM23
10	51735343	51735352	G	snp	T	51735344	TIMM23B
10	60477694	60477704	A	snp	C	60477701	BICC1
10	60477694	60477704	A	snp	C	60477701	LOC728640
10	61714347	61714355	T	snp	G	61714352	C10orf40
10	61788616	61788625	T	snp	G	61788622	ANK3
10	63982248	63982256	A	snp	C	63982253	RTKN2
10	68860119	68860127	A	snp	G	68860122	CTNNA3
10	68860119	68860127	A	snp	G	68860122	LRRTM3
10	69752752	69752760	A	snp	G	69752758	HERC4
10	69756745	69756755	A	snp	G	69756750	HERC4
10	70049307	70049315	A	snp	G	70049309	PBLD
10	70051808	70051816	A	snp	G	70051809	PBLD
10	70247618	70247628	T	snp	C	70247619	SLC25A16
10	70748013	70748021	A	snp	C	70748019	KIAA1279
10	70930184	70930194	A	snp	G	70930187	VPS26A
10	71017441	71017450	G	snp	T	71017443	HKDC1

10	75884745	75884755	T	snp	C	75884751	AP3M1
10	75898977	75898986	A	snp	C	75898978	AP3M1
10	78839028	78839037	A	snp	G	78839033	KCNMA1
10	78843596	78843604	A	snp	C	78843600	KCNMA1
10	78843596	78843604	A	snp	C	78843601	KCNMA1
10	82184768	82184776	T	snp	C	82184769	C10orf58
10	86178182	86178190	T	snp	G	86178185	FAM190B
10	90033586	90033595	A	snp	G	90033592	RNLS
10	90439214	90439223	A	snp	C	90439216	LIPF
10	91399728	91399736	T	snp	C	91399731	PANK1
10	93611984	93611992	A	snp	C	93611990	TNKS2
10	95274525	95274533	T	snp	C	95274527	CEP55
10	95349360	95349370	G	snp	T	95349364	O3FAR1
10	96988733	96988743	T	snp	G	96988738	BC043227
10	96988733	96988743	T	snp	G	96988738	BC043227
10	96988733	96988743	T	snp	G	96988738	C10orf129
10	96988733	96988743	T	snp	G	96988738	C10orf129
10	97425033	97425043	T	snp	G	97425041	TCTN3
10	97442990	97442999	A	snp	G	97442994	TCTN3
10	98127804	98127812	A	snp	C	98127808	TLL2
10	99019602	99019610	T	snp	G	99019606	ARHGAP19
10	101718910	101718919	T	snp	G	101718915	DNMBP
10	101718910	101718919	T	snp	G	101718915	DNMBP-AS1
10	101815106	101815116	C	snp	T	101815113	CPN1
10	102039637	102039645	A	snp	C	102039642	BLOC1S2
10	104250748	104250758	A	snp	C	104250753	ACTR1A
10	104660686	104660694	A	snp	G	104660687	AS3MT
10	114205635	114205643	T	snp	G	114205637	ZDHHC6
10	114710869	114710877	C	snp	T	114710875	TCF7L2
10	115334304	115334312	T	snp	C	115334305	HABP2
10	115355191	115355199	A	snp	G	115355194	NRAP
10	115962358	115962366	T	snp	G	115962360	TDRD1
10	117855993	117856003	T	snp	C	117855995	GFRA1
10	118390022	118390031	T	snp	C	118390024	PNLIPRP2
10	124035405	124035414	A	snp	C	124035410	BTBD16
10	124248088	124248096	A	snp	G	124248090	HTRA1
10	127680207	127680215	T	snp	C	127680211	FANK1
10	128789801	128789810	T	snp	G	128789804	DOCK1
10	128909140	128909148	T	snp	C	128909144	DOCK1
10	134149079	134149087	G	snp	A	134149083	LRRC27
10	135075427	135075435	G	snp	A	135075431	ADAM8
11	441799	441807	C	snp	A	441805	ANO9
11	640341	640350	G	snp	A	640348	DRD4

11	993679	993688	C	snp	A	993683	AP2A2
11	1248192	1248201	G	snp	T	1248196	MUC5B
11	4202734	4202742	T	snp	C	4202735	RRM1
11	4719274	4719282	T	snp	G	4719276	OR51E2
11	8246319	8246327	G	snp	A	8246325	LMO1
11	8941194	8941203	T	snp	G	8941196	AKIP1
11	8941194	8941203	T	snp	G	8941196	C11orf16
11	9537042	9537051	A	snp	C	9537043	DM376719
11	9537042	9537051	A	snp	C	9537043	ZNF143
11	10522976	10522984	G	snp	T	10522977	AMPD3
11	10522976	10522984	G	snp	T	10522982	AMPD3
11	18044643	18044651	A	snp	G	18044647	TPH1
11	18587437	18587446	T	snp	C	18587442	UEVLD
11	18628415	18628425	T	snp	C	18628417	LOC100506540
11	18628415	18628425	T	snp	C	18628417	SPTY2D1
11	20419998	20420007	T	snp	G	20420004	PRMT3
11	27401922	27401930	T	snp	G	27401926	LGR4
11	27719733	27719741	T	snp	C	27719736	BDNF
11	27719733	27719741	T	snp	C	27719736	BDNF-AS1
11	27719733	27719741	T	snp	C	27719738	BDNF
11	27719733	27719741	T	snp	C	27719738	BDNF-AS1
11	30899834	30899842	A	snp	C	30899835	DCDC5
11	33078639	33078649	T	snp	C	33078642	TCP11L1
11	34979843	34979851	G	snp	A	34979849	PDHX
11	43589965	43589975	T	snp	G	43589968	BC031305
11	46624211	46624221	T	snp	G	46624217	HARBI1
11	46883312	46883321	A	snp	C	46883317	LOC100507401
11	46883312	46883321	A	snp	C	46883317	LRP4
11	46883312	46883321	A	snp	C	46883317	LRP4
11	47835717	47835725	A	snp	C	47835720	NUP160
11	57147920	57147929	A	snp	C	57147922	PRG3
11	58701100	58701110	T	snp	G	58701104	GLYATL1
11	58701100	58701110	T	snp	G	58701104	LOC283194
11	60049400	60049410	A	snp	G	60049406	MS4A4A
11	62495237	62495246	A	snp	C	62495238	HNRNPUL2
11	62495237	62495246	A	snp	C	62495238	TTC9C
11	63528434	63528442	G	snp	T	63528439	C11orf95
11	63528434	63528442	G	snp	T	63528439	RTN3
11	64011554	64011564	A	snp	G	64011555	FKBP2
11	64011554	64011564	A	snp	G	64011555	PPP1R14B
11	64814811	64814820	T	snp	G	64814812	NAALADL1
11	65120386	65120396	T	snp	C	65120390	DPF2
11	65730942	65730952	T	snp	G	65730944	SART1

11	66250504	66250514	T	snp	G	66250512	DPP3
11	66495413	66495422	C	snp	T	66495414	SPTBN2
11	68676656	68676665	G	snp	T	68676663	IGHMBP2
11	71203785	71203793	A	snp	C	71203789	NADSYN1
11	72290195	72290203	G	snp	A	72290198	PDE2A
11	74035531	74035539	T	snp	C	74035534	BC048427
11	74057351	74057360	A	snp	C	74057354	PGM2L1
11	76060869	76060877	T	snp	C	76060872	PRKRIR
11	83190417	83190426	G	snp	A	83190418	DLG2
11	89948684	89948693	T	snp	C	89948688	CHORDC1
11	94301877	94301885	T	snp	C	94301880	PIWIL4
11	94319811	94319819	C	snp	A	94319817	PIWIL4
11	95521369	95521378	T	snp	G	95521374	FAM76B
11	103182317	103182327	A	snp	G	103182319	DYNC2H1
11	103817639	103817647	A	snp	G	103817644	PDGFD
11	104756323	104756332	A	snp	C	104756326	CASP12
11	104774008	104774016	A	snp	G	104774013	LOC643733
11	107382225	107382233	T	snp	C	107382229	ALKBH8
11	110207311	110207320	T	snp	C	110207318	AK124179
11	111383110	111383120	C	snp	T	111383114	BC021736
11	111383110	111383120	C	snp	T	111383114	BC021736
11	111383110	111383120	C	snp	T	111383114	BTG4
11	111383110	111383120	C	snp	T	111383114	MIR34B
11	111383110	111383120	C	snp	T	111383114	MIR34C
11	111384418	111384427	T	snp	G	111384422	BC021736
11	111384418	111384427	T	snp	G	111384422	MIR34B
11	111384418	111384427	T	snp	G	111384422	MIR34C
11	111384418	111384427	T	snp	G	111384425	BC021736
11	111384418	111384427	T	snp	G	111384425	MIR34B
11	111384418	111384427	T	snp	G	111384425	MIR34C
11	112095113	112095122	T	snp	G	112095117	BCO2
11	117023481	117023491	A	snp	C	117023486	PAFAH1B2
11	117168337	117168347	A	snp	C	117168339	BACE1
11	118478396	118478406	C	snp	A	118478403	PHLDB1
11	120190137	120190146	T	snp	C	120190140	POU2F3
11	120348592	120348600	A	snp	G	120348596	ARHGEF12
11	124951303	124951312	T	snp	C	124951310	SLC37A2
11	125829501	125829510	A	snp	C	125829504	CDON
11	126165283	126165291	A	snp	G	126165288	TIRAP
11	128992697	128992705	T	snp	C	128992702	ARHGAP32
11	130714222	130714231	T	snp	C	130714225	BC031979
12	2055261	2055270	T	snp	C	2055265	DCP1B
12	3105016	3105024	A	snp	G	3105018	TEAD4

12	4870783	4870793	T	snp	G	4870785	GALNT8
12	6690186	6690194	A	snp	C	6690189	AK096395
12	6690186	6690194	A	snp	C	6690189	AK096395
12	6690186	6690194	A	snp	C	6690189	AK096395
12	6690186	6690194	A	snp	C	6690189	AK096395
12	6690186	6690194	A	snp	C	6690189	CHD4
12	6690186	6690194	A	snp	C	6690189	CHD4
12	6690186	6690194	A	snp	C	6690189	CHD4
12	6690186	6690194	A	snp	C	6690189	CHD4
12	6690186	6690194	A	snp	C	6690189	SCARNA11
12	7044050	7044059	T	snp	G	7044055	ATN1
12	7261607	7261615	G	snp	T	7261608	C1RL
12	7261607	7261615	G	snp	T	7261608	C1RL
12	7261607	7261615	G	snp	T	7261608	C1RL
12	7261607	7261615	G	snp	T	7261608	MATL2963
12	7261607	7261615	G	snp	T	7261608	MATL2963
12	7261607	7261615	G	snp	T	7261608	MATL2963
12	7350641	7350651	T	snp	C	7350649	PEX5
12	7864969	7864977	A	snp	G	7864975	DPPA3
12	8024578	8024587	A	snp	G	8024583	AY455283
12	8024578	8024587	A	snp	G	8024583	SLC2A14
12	8024578	8024587	A	snp	G	8024583	SLC2A14
12	8194273	8194281	T	snp	G	8194275	FOXJ2
12	8285506	8285516	T	snp	G	8285510	CLEC4A
12	8285506	8285516	T	snp	G	8285510	POU5F1P3
12	8976926	8976934	T	snp	C	8976929	A2ML1
12	9094546	9094554	A	snp	G	9094547	M6PR
12	9094546	9094554	A	snp	G	9094547	M6PR
12	9094546	9094554	A	snp	G	9094547	PHC1
12	9228021	9228029	T	snp	G	9228023	A2M
12	9555620	9555629	G	snp	T	9555623	DQ599803
12	10281842	10281852	A	snp	G	10281846	CLEC7A
12	12046811	12046820	A	snp	G	12046812	ETV6
12	18801880	18801888	A	snp	C	18801885	PIK3C2G
12	21391816	21391824	T	snp	C	21391817	SLCO1B1
12	21451025	21451034	T	snp	C	21451027	SLCO1A2
12	25033390	25033400	A	snp	G	25033397	BCAT1
12	25307002	25307011	T	snp	G	25307007	CASC1
12	25385903	25385912	T	snp	C	25385909	KRAS
12	26100419	26100427	A	snp	G	26100425	LOC100506451
12	27131946	27131955	A	snp	C	27131953	TM7SF3
12	27842372	27842382	A	snp	G	27842379	PPFIBP1
12	27842372	27842382	A	snp	G	27842379	PPFIBP1

12	27842372	27842382	A	snp	G	27842379	TRNA_Lys
12	29725489	29725497	T	snp	C	29725493	TMTC1
12	31545378	31545386	A	snp	G	31545380	DENND5B
12	32903144	32903153	T	snp	G	32903147	YARS2
12	40713869	40713877	A	snp	G	40713872	LRRK2
12	44154690	44154698	T	snp	C	44154696	IRAK4
12	48115078	48115087	T	snp	G	48115085	AL831948
12	48115078	48115087	T	snp	G	48115085	ENDOU
12	50229500	50229510	T	snp	G	50229508	BCDIN3D
12	50229500	50229510	T	snp	G	50229508	LOC100286844
12	50280424	50280432	T	snp	C	50280429	FAIM2
12	50291547	50291556	C	snp	A	50291550	FAIM2
12	50506805	50506815	T	snp	G	50506812	C12orf62
12	50576141	50576150	G	snp	T	50576145	LIMA1
12	51632448	51632457	G	snp	T	51632454	DAZAP2
12	51772666	51772676	C	snp	T	51772673	GALNT6
12	52868448	52868458	T	snp	G	52868455	KRT6C
12	53648257	53648267	T	snp	C	53648261	MFSD5
12	53804300	53804310	T	snp	G	53804306	SP1
12	53825863	53825871	T	snp	G	53825869	AMHR2
12	54332264	54332272	C	snp	A	54332270	HOXC13
12	54511887	54511896	A	snp	C	54511889	FLJ12825
12	57318798	57318808	T	snp	G	57318806	SDR9C7
12	57422572	57422580	T	snp	G	57422575	MYO1A
12	57823582	57823590	A	snp	C	57823584	KIAA1002
12	57823582	57823590	A	snp	C	57823584	R3HDM2
12	64058116	64058124	A	snp	C	64058120	DPY19L2
12	66357207	66357215	G	snp	T	66357210	HMGA2
12	70749630	70749639	A	snp	G	70749631	CNOT2
12	72179690	72179699	T	snp	G	72179691	RAB21
12	75891675	75891684	A	snp	G	75891677	GLIPR1
12	75891675	75891684	A	snp	G	75891677	KRR1
12	77457432	77457442	A	snp	G	77457438	E2F7
12	85269882	85269890	A	snp	G	85269885	SLC6A15
12	93196418	93196427	T	snp	G	93196421	EEA1
12	94646220	94646228	T	snp	G	94646221	PLXNC1
12	96412335	96412345	A	snp	C	96412337	LTA4H
12	97311946	97311956	T	snp	G	97311954	NEDD1
12	98389651	98389660	A	snp	C	98389656	MIR4303
12	101015652	101015661	G	snp	A	101015659	GAS2L3
12	102040416	102040425	A	snp	G	102040417	MYBPC1
12	103248607	103248616	A	snp	C	103248611	PAH
12	104286590	104286598	A	snp	G	104286591	GNN

12	104496721	104496730	C	snp	T	104496723	HCFC2
12	105593585	105593594	T	snp	G	105593591	APPL2
12	106458571	106458580	C	snp	A	106458578	NUAK1
12	107415069	107415079	A	snp	G	107415072	CRY1
12	109886818	109886826	A	snp	C	109886821	KCTD10
12	109886818	109886826	A	snp	C	109886821	MYO1H
12	110835012	110835021	A	snp	C	110835014	ANAPC7
12	113742711	113742720	A	snp	C	113742715	SLC24A6
12	114353836	114353844	T	snp	C	114353837	RBM19
12	117205371	117205381	T	snp	G	117205372	RNFT2
12	117205371	117205381	T	snp	G	117205373	RNFT2
12	117902289	117902298	T	snp	C	117902296	KSR2
12	119865785	119865793	A	snp	C	119865791	AF086288
12	119865785	119865793	A	snp	C	119865791	CCDC60
12	120903592	120903600	A	snp	C	120903597	SRSF9
12	121443112	121443120	A	snp	G	121443115	C12orf43
12	121466420	121466428	C	snp	A	121466421	OASL
12	122389160	122389169	A	snp	G	122389166	WDR66
12	122764620	122764628	T	snp	C	122764625	CLIP1
12	123782951	123782959	T	snp	G	123782956	SBNO1
12	124395884	124395892	G	snp	A	124395887	DNAH10
12	124396125	124396133	G	snp	A	124396128	DNAH10
12	124396125	124396133	G	snp	T	124396130	DNAH10
12	124396688	124396696	G	snp	T	124396693	DNAH10
13	19432718	19432726	T	snp	G	19432724	ANKRD20A9P
13	20578403	20578412	T	snp	G	20578407	ZMYM2
13	21636037	21636047	A	snp	G	21636041	LATS2
13	31715453	31715461	A	snp	C	31715454	HSPH1
13	36939380	36939389	A	snp	G	36939384	SPG20
13	36939380	36939389	A	snp	G	36939384	SPG20
13	36939380	36939389	A	snp	G	36939384	SPG20OS
13	36939380	36939389	A	snp	G	36939384	SPG20OS
13	37007654	37007663	T	snp	C	37007658	CCNA1
13	41486861	41486871	A	snp	G	41486865	LOC100616668
13	41486861	41486871	A	snp	G	41486865	SUGT1P3
13	42866790	42866798	A	snp	G	42866794	AKAP11
13	46626455	46626464	G	snp	T	46626460	AK095119
13	46626455	46626464	G	snp	T	46626460	AK095119
13	46626455	46626464	G	snp	T	46626460	AK124928
13	46626455	46626464	G	snp	T	46626460	AK124928
13	46626455	46626464	G	snp	T	46626460	CPB2
13	46626455	46626464	G	snp	T	46626460	ZC3H13
13	46718218	46718228	A	snp	G	46718225	LCP1

13	49763683	49763693	T	snp	G	49763684	FNDC3A
13	50266571	50266581	A	snp	C	50266573	EBPL
13	64320164	64320173	A	snp	C	64320166	LOC647264
13	70371199	70371207	T	snp	G	70371200	KLHL1
13	75910942	75910952	T	snp	G	75910949	TBC1D4
13	76391053	76391061	T	snp	G	76391056	LMO7
13	99047025	99047033	A	snp	G	99047027	FARP1
13	107197114	107197122	A	snp	C	107197118	ARGLU1
13	111091149	111091158	C	snp	A	111091156	COL4A2
13	111858580	111858588	G	snp	A	111858581	ARHGEF7
13	114503068	114503077	G	snp	T	114503069	FAM70B
13	114507420	114507428	C	snp	T	114507425	FAM70B
13	114765431	114765440	G	snp	A	114765438	RASA3
13	114843800	114843808	T	snp	C	114843805	RASA3
13	114898685	114898693	C	snp	T	114898690	RASA3
14	21082318	21082326	C	snp	A	21082324	TRNA_Pro
14	21082318	21082326	C	snp	A	21082324	TRNA_Thr
14	21926844	21926854	A	snp	C	21926846	RAB2B
14	22309293	22309301	T	snp	C	22309294	TCRA
14	22309293	22309301	T	snp	C	22309294	TCRA
14	22309293	22309301	T	snp	C	22309294	TRA
14	22309293	22309301	T	snp	C	22309294	TRA
14	22309293	22309301	T	snp	C	22309294	TRAV12-1
14	22309293	22309301	T	snp	C	22309294	TRAV12-1
14	22888638	22888646	T	snp	G	22888643	AK093552
14	22888638	22888646	T	snp	G	22888643	AK125397
14	22888638	22888646	T	snp	G	22888643	AV4S1
14	22888638	22888646	T	snp	G	22888643	hADV29S1
14	22888638	22888646	T	snp	G	22888643	hADV36S1
14	22888638	22888646	T	snp	G	22888643	hADV38S2
14	22888638	22888646	T	snp	G	22888643	T-Cell
14	22888638	22888646	T	snp	G	22888643	TCRA
14	22888638	22888646	T	snp	G	22888643	TCRA
14	22888638	22888646	T	snp	G	22888643	TCRA
14	22888638	22888646	T	snp	G	22888643	TCRA
14	22888638	22888646	T	snp	G	22888643	TCRA
14	22888638	22888646	T	snp	G	22888643	TCR-alpha
14	22888638	22888646	T	snp	G	22888643	TCR-alpha
14	22888638	22888646	T	snp	G	22888643	TRA
14	22888638	22888646	T	snp	G	22888643	TRA
14	22888638	22888646	T	snp	G	22888643	TRA
14	22888638	22888646	T	snp	G	22888643	TRA@
14	22888638	22888646	T	snp	G	22888643	TRAC

14	22888638	22888646	T	snp	G	22888643	TRAC
14	22888638	22888646	T	snp	G	22888643	TRD
14	22946226	22946235	A	snp	G	22946230	AK093552
14	22946226	22946235	A	snp	G	22946230	AV4S1
14	22946226	22946235	A	snp	G	22946230	hADV29S1
14	22946226	22946235	A	snp	G	22946230	hADV36S1
14	22946226	22946235	A	snp	G	22946230	hADV38S2
14	22946226	22946235	A	snp	G	22946230	T-Cell
14	22946226	22946235	A	snp	G	22946230	TCRA
14	22946226	22946235	A	snp	G	22946230	TCRA
14	22946226	22946235	A	snp	G	22946230	TCRA
14	22946226	22946235	A	snp	G	22946230	TCRA
14	22946226	22946235	A	snp	G	22946230	TCRA
14	22946226	22946235	A	snp	G	22946230	TCRA
14	22946226	22946235	A	snp	G	22946230	TCR-alpha
14	22946226	22946235	A	snp	G	22946230	TCR-alpha
14	22946226	22946235	A	snp	G	22946230	TRA
14	22946226	22946235	A	snp	G	22946230	TRA
14	22946226	22946235	A	snp	G	22946230	TRA
14	22946226	22946235	A	snp	G	22946230	TRA@
14	22946226	22946235	A	snp	G	22946230	TRA@
14	22946226	22946235	A	snp	G	22946230	TRAC
14	22946226	22946235	A	snp	G	22946230	TRAC
14	22946226	22946235	A	snp	G	22946230	TRD
14	22946226	22946235	A	snp	G	22946230	X61074
14	23289191	23289199	C	snp	A	23289193	SLC7A7
14	23377239	23377247	T	snp	C	23377245	RBM23
14	37148384	37148392	T	snp	C	37148385	SLC25A21
14	39533679	39533687	T	snp	G	39533681	SEC23A
14	39789307	39789316	T	snp	C	39789310	CTAGE5
14	39789797	39789806	A	snp	G	39789801	CTAGE5
14	50578438	50578446	A	snp	C	50578440	METTL21D
14	50671131	50671139	A	snp	G	50671132	SOS2
14	52417704	52417714	A	snp	C	52417706	GNG2
14	53112187	53112195	T	snp	G	53112193	ERO1L
14	55203701	55203709	G	snp	T	55203702	SAMD4A
14	58926037	58926046	A	snp	C	58926038	KIAA0586
14	61450503	61450512	T	snp	G	61450505	SLC38A6
14	61857392	61857402	A	snp	C	61857393	PRKCH
14	62598134	62598142	G	snp	T	62598140	FLJ43390
14	71275978	71275987	C	snp	A	71275985	MAP3K9
14	71570897	71570905	T	snp	C	71570903	PCNX
14	73536922	73536931	T	snp	G	73536927	RBM25

14	73945830	73945838	T	snp	G	73945833	AK055876
14	73945830	73945838	T	snp	G	73945833	HEATR4
14	74432423	74432431	A	snp	G	74432429	ENTPD5
14	75229179	75229188	C	snp	A	75229186	YLPM1
14	75763088	75763096	A	snp	G	75763090	LOC731223
14	76087768	76087778	A	snp	G	76087776	FLVCR2
14	78022655	78022664	A	snp	G	78022662	SPTLC2
14	82458072	82458080	A	snp	C	82458077	Mir_633
14	88657946	88657955	T	snp	G	88657951	KCNK10
14	88857704	88857713	T	snp	G	88857705	SPATA7
14	89312670	89312679	A	snp	G	89312674	TTC8
14	89656890	89656900	A	snp	C	89656895	FOXN3
14	92526234	92526243	A	snp	C	92526239	ATXN3
14	93108456	93108466	T	snp	C	93108463	RIN3
14	94373600	94373609	A	snp	G	94373601	FAM181A-AS1
14	94547060	94547069	A	snp	C	94547063	DDX24
14	94547060	94547069	A	snp	C	94547063	IFI27L1
14	96120989	96120999	A	snp	C	96120996	TCL6
14	96991188	96991196	A	snp	G	96991190	PAPOLA
14	100764835	100764843	T	snp	C	100764838	SLC25A29
14	102358731	102358739	G	snp	A	102358734	PPP2R5C
14	102450009	102450019	T	snp	G	102450016	DYNC1H1
14	102844042	102844050	A	snp	G	102844046	TECPR2
14	105173451	105173460	G	snp	A	105173453	INF2
14	106137078	106137088	T	snp	G	106137080	abParts
14	106137078	106137088	T	snp	G	106137080	abParts
14	106137078	106137088	T	snp	G	106137080	DKFZp686O16217
14	106137078	106137088	T	snp	G	106137080	DKFZp686O16217
14	106137078	106137088	T	snp	G	106137080	IGH@
14	106137078	106137088	T	snp	G	106137080	IGH@
14	106137078	106137088	T	snp	G	106137080	IGHE
14	106137078	106137088	T	snp	G	106137080	IGHE
14	106137078	106137088	T	snp	G	106137080	IGHE
14	106137078	106137088	T	snp	G	106137080	IGHE
14	106137078	106137088	T	snp	G	106137080	IGHG1
14	106137078	106137088	T	snp	G	106137080	IGHG1
14	106476029	106476038	T	snp	G	106476030	abParts
14	106479109	106479117	C	snp	A	106479111	abParts
14	106491904	106491913	T	snp	G	106491907	abParts
14	106648981	106648989	T	snp	G	106648986	abParts
14	106709250	106709258	T	snp	G	106709252	abParts
15	23114369	23114377	A	snp	G	23114373	LOC283683
15	29083886	29083894	A	snp	G	29083892	LOC646278

15	31243170	31243179	A	snp	C	31243172	MTMR10
15	32634695	32634704	G	snp	T	32634702	DKFZp434L187
15	33068280	33068289	A	snp	G	33068283	FMN1
15	33877654	33877662	A	snp	G	33877658	RYR3
15	38805182	38805190	A	snp	C	38805184	RASGRP1
15	40270942	40270951	T	snp	G	40270943	EIF2AK4
15	40360674	40360684	A	snp	C	40360680	LOC100131089
15	40493488	40493497	A	snp	G	40493491	BUB1B
15	41589663	41589672	A	snp	C	41589666	OIP5-AS1
15	41798793	41798803	T	snp	C	41798795	LTK
15	42641027	42641035	T	snp	C	42641029	CAPN3
15	42641027	42641035	T	snp	C	42641029	CAPN3
15	42641027	42641035	T	snp	C	42641029	GANC
15	42641027	42641035	T	snp	C	42641029	GANC
15	49799924	49799934	A	snp	C	49799932	C15orf33
15	49913097	49913107	C	snp	A	49913102	C15orf33
15	49913097	49913107	C	snp	A	49913102	DTWD1
15	50555230	50555239	A	snp	G	50555235	HDC
15	50787867	50787875	T	snp	G	50787870	AX746640
15	50787867	50787875	T	snp	G	50787870	USP8
15	50883255	50883264	T	snp	C	50883262	TRPM7
15	50999324	50999333	T	snp	G	50999331	SPPL2A
15	51029657	51029667	T	snp	C	51029659	SPPL2A
15	51569212	51569221	A	snp	G	51569219	CYP19A1
15	51569212	51569221	A	snp	G	51569219	DQ595419
15	52698839	52698849	T	snp	G	52698843	MYO5A
15	59517573	59517581	T	snp	C	59517577	MYO1E
15	59529297	59529306	T	snp	G	59529300	MYO1E
15	59806278	59806286	G	snp	T	59806283	FAM81A
15	59931125	59931133	A	snp	G	59931130	GTF2A2
15	63357848	63357856	T	snp	G	63357851	TPM1
15	65294896	65294905	A	snp	G	65294902	MTFMT
15	65352639	65352649	T	snp	C	65352640	RASL12
15	65477364	65477374	C	snp	T	65477367	CLPX
15	65822172	65822182	A	snp	C	65822174	PTPLAD1
15	65865625	65865634	T	snp	G	65865631	PTPLAD1
15	66044497	66044505	A	snp	G	66044498	DENND4A
15	66776477	66776486	C	snp	A	66776479	MAP2K1
15	72903112	72903121	A	snp	C	72903114	DQ582071
15	73022775	73022783	A	snp	G	73022777	BBS4
15	74288047	74288056	T	snp	G	74288053	PML
15	78474776	78474784	C	snp	A	78474780	ACSBG1
15	78474776	78474784	C	snp	A	78474781	ACSBG1

15	79042733	79042741	T	snp	C	79042735	DQ596823
15	79501864	79501872	T	snp	G	79501866	LOC729911
15	79501864	79501872	T	snp	G	79501866	MIR184
15	83523157	83523166	A	snp	G	83523162	HOMER2
15	85470033	85470042	G	snp	T	85470039	SLC28A1
15	88726243	88726252	C	snp	T	88726244	NTRK3
15	89171975	89171984	A	snp	C	89171976	AEN
15	91325617	91325626	A	snp	C	91325621	BLM
15	99927265	99927274	T	snp	G	99927271	LRRC28
16	1202929	1202938	G	snp	A	1202933	CACNA1H
16	2050937	2050946	A	snp	C	2050940	TCRBV20S1
16	2050937	2050946	A	snp	C	2050940	TCRBV20S1
16	2050937	2050946	A	snp	C	2050940	TCRBV20S1
16	2050937	2050946	A	snp	C	2050940	TCRBV20S1
16	2050937	2050946	A	snp	C	2050940	ZNF598
16	2050937	2050946	A	snp	C	2050940	ZNF598
16	2050937	2050946	A	snp	C	2050940	ZNF598
16	2050937	2050946	A	snp	C	2050940	ZNF598
16	2087452	2087461	G	snp	T	2087459	SLC9A3R2
16	2087452	2087461	G	snp	T	2087459	SLC9A3R2
16	2087452	2087461	G	snp	T	2087459	SLC9A3R2
16	2087452	2087461	G	snp	T	2087459	SLC9A3R2
16	2087452	2087461	G	snp	T	2087459	SLC9A3R2
16	2087452	2087461	G	snp	T	2087459	TCRBV20S1
16	2087452	2087461	G	snp	T	2087459	TCRBV20S1
16	2087452	2087461	G	snp	T	2087459	TCRBV20S1
16	2087452	2087461	G	snp	T	2087459	TCRBV20S1
16	2087452	2087461	G	snp	T	2087459	TCRBV20S1
16	2255804	2255813	G	snp	A	2255806	MLST8
16	3101221	3101229	G	snp	T	3101222	BC045731
16	3101221	3101229	G	snp	T	3101222	MMP25
16	3101221	3101229	G	snp	T	3101222	MMP25
16	3294923	3294932	A	snp	C	3294930	MEFV
16	4740203	4740211	T	snp	G	4740207	MGRN1
16	11055970	11055980	T	snp	C	11055971	CLEC16A
16	11922056	11922064	A	snp	G	11922062	BCAR4
16	14698482	14698492	T	snp	C	14698485	PARN
16	19085867	19085876	A	snp	G	19085872	COQ7
16	19694192	19694200	A	snp	C	19694194	C16orf62
16	19895405	19895413	C	snp	T	19895411	GPRC5B
16	20493482	20493491	A	snp	G	20493485	ACSM2A
16	23654507	23654517	T	snp	G	23654508	DCTN5
16	24874159	24874168	A	snp	C	24874161	SLC5A11

16	24881800	24881810	A	snp	C	24881801	SLC5A11
16	29128985	29128994	A	snp	G	29128989	NPIPL1
16	29128985	29128994	A	snp	G	29128989	RRN3P2
16	29705850	29705858	C	snp	T	29705853	BOLA2
16	29705850	29705858	C	snp	T	29705853	QPRT
16	30510972	30510980	T	snp	G	30510977	ITGAL
16	30773598	30773608	G	snp	T	30773604	C16orf93
16	30773598	30773608	G	snp	T	30773604	RNF40
16	31238688	31238696	T	snp	G	31238689	TRIM72
16	46865342	46865350	C	snp	A	46865348	C16orf87
16	48268283	48268293	T	snp	G	48268286	ABCC11
16	50323103	50323113	T	snp	G	50323109	ADCY7
16	50347346	50347356	A	snp	C	50347348	ADCY7
16	55360200	55360210	G	snp	T	55360207	IRX6
16	55564404	55564414	G	snp	A	55564407	LPCAT2
16	56838974	56838982	T	snp	G	56838977	NUP93
16	57017792	57017801	G	snp	A	57017795	CETP
16	57071226	57071236	C	snp	A	57071227	NLRC5
16	57470882	57470890	A	snp	C	57470883	CIAPIN1
16	58313561	58313569	T	snp	G	58313565	CCDC113
16	58313561	58313569	T	snp	G	58313565	PRSS54
16	58703574	58703584	A	snp	C	58703581	SLC38A7
16	69365121	69365129	G	snp	T	69365122	COG8
16	69365121	69365129	G	snp	T	69365122	PDF
16	70051037	70051046	T	snp	C	70051042	CLEC18A
16	70051037	70051046	T	snp	C	70051042	PDXDC2P
16	70153827	70153837	T	snp	G	70153828	CLEC18A
16	70153827	70153837	T	snp	G	70153828	PDPR
16	74503028	74503036	G	snp	T	74503030	GLG1
16	74655601	74655609	T	snp	G	74655604	RFWD3
16	74908790	74908799	T	snp	G	74908796	WDR59
16	78061562	78061570	T	snp	G	78061565	CLEC3A
16	81929180	81929189	A	snp	G	81929184	PLCG2
16	84213650	84213658	G	snp	A	84213651	DNAAF1
16	84213650	84213658	G	snp	A	84213651	TAF1C
16	87938693	87938702	C	snp	A	87938698	CA5A
16	88781784	88781794	A	snp	C	88781790	CTU2
16	88781784	88781794	A	snp	C	88781790	MIR4722
16	88781784	88781794	A	snp	C	88781790	PIEZO1
16	88781784	88781794	A	snp	C	88781790	PIEZO1
16	88900867	88900875	C	snp	T	88900868	GALNS
16	89518906	89518915	T	snp	C	89518907	AK097694
16	89518906	89518915	T	snp	C	89518907	ANKRD11

16	89849974	89849984	A	snp	C	89849976	FANCA	
17	1495018	1495027	G	snp	A	1495022	SLC43A2	
17	1606876	1606884	T	snp	G	1606882	TLCD2	
17	1609024	1609033	T	snp	G	1609028	TLCD2	
17	1786457	1786466	T	snp	G	1786460	RPA1	
17	2299192	2299200	G	snp	A	2299194	MNT	
17	2888249	2888259	T	snp	C	2888257	RAP1GAP2	
17	3901499	3901508	T	snp	G	3901501	AB062083	
17	3910802	3910810	G	snp	A	3910808	AB062083	
17	3910802	3910810	G	snp	A	3910808	DKFZp761G0818	
17	3910802	3910810	G	snp	A	3910808	ZZEF1	
17	3967357	3967367	G	snp	A	3967358	ZZEF1	
17	4699264	4699274	G	snp	T	4699272	PSMB6	
17	4801279	4801287	C	snp	T	4801285	CHRNE	
17	4801279	4801287	C	snp	T	4801285	MINK1	
17	5346440	5346448	C	snp	T	5346445	DHX33	
17	6603519	6603527	T	snp	C	6603523	SLC13A5	
17	7384038	7384048	A	snp	C	7384045	SLC35G6	
17	7384038	7384048	A	snp	C	7384045	ZBTB4	
17	7788420	7788428	C	snp	A	7788426	CHD3	
17	9923929	9923939	A	snp	C	9923930	GAS7	
17	10446505	10446513	T	snp	G	10446507	AK097500	
17	10446505	10446513	T	snp	G	10446507	AK097500	
17	10446505	10446513	T	snp	G	10446507	AK097500	
17	10446505	10446513	T	snp	G	10446507	AK097500	
17	10446505	10446513	T	snp	G	10446507	AK097500	
17	10446505	10446513	T	snp	G	10446507	MYH2	
17	10446505	10446513	T	snp	G	10446507	MYH2	
17	10446505	10446513	T	snp	G	10446507	MYH2	
17	10446505	10446513	T	snp	G	10446507	MYH2	
17	10446505	10446513	T	snp	G	10446507	MYH2	
17	10532881	10532890	G	snp	A	10532883	MYH3	
17	11829382	11829390	G	snp	T	11829384	DNAH9	
17	13695078	13695086	A	snp	G	13695082	AK123263	
17	18314136	18314144	C	snp	A	18314141	AX748015	
17	20931280	20931288	A	snp	G	20931285	USP22	
17	20946556	20946564	G	snp	A	20946558	USP22	
17	25929141	25929151	T	snp	C	25929148	KSR1	
17	28846159	28846167	C	snp	T	28846161	GOSR1	
17	29111121	29111130	T	snp	G	29111124	CRLF3	
17	29844224	29844232	G	snp	A	29844225	RAB11FIP4	
17	29845846	29845854	T	snp	G	29845851	RAB11FIP4	
17	30679328	30679338	T	snp	G	30679329	ZNF207	

17	34194660	34194670	A	snp	G	34194665	C17orf66
17	35721921	35721931	T	snp	C	35721929	ACACA
17	36000483	36000492	T	snp	C	36000488	DDX52
17	36099967	36099975	T	snp	C	36099969	HNF1B
17	36396345	36396353	C	snp	T	36396350	LOC440434
17	36689850	36689858	G	snp	T	36689851	SRCIN1
17	36893086	36893094	A	snp	C	36893089	PCGF2
17	37212920	37212929	A	snp	G	37212923	LOC100131347
17	37791481	37791490	A	snp	C	37791486	PPP1R1B
17	38031861	38031869	T	snp	G	38031864	ZPBP2
17	38097004	38097012	T	snp	C	38097007	LRRC3C
17	40272592	40272601	C	snp	A	40272593	KAT2A
17	40705710	40705718	G	snp	A	40705714	BC043620
17	40705710	40705718	G	snp	A	40705714	HSD17B1
17	40705710	40705718	G	snp	A	40705714	HSD17B1
17	41231216	41231224	A	snp	C	41231220	BRCA1
17	41247598	41247607	A	snp	C	41247603	BRCA1
17	46474600	46474608	G	snp	T	46474601	SKAP1
17	47295004	47295013	C	snp	A	47295007	ABI3
17	47578434	47578442	T	snp	C	47578437	NGFR
17	48183468	48183476	G	snp	A	48183470	PDK2
17	48260625	48260633	A	snp	G	48260629	COL1A1
17	48676254	48676262	T	snp	G	48676260	CACNA1G
17	56235810	56235820	C	snp	A	56235818	MSX2P1
17	56691029	56691039	T	snp	C	56691033	TEX14
17	58024322	58024331	A	snp	G	58024323	RPS6KB1
17	58349161	58349169	T	snp	C	58349166	USP32
17	60752062	60752071	G	snp	A	60752068	MRC2
17	61959897	61959907	G	snp	A	61959902	GH2
17	62120040	62120049	A	snp	G	62120044	DQ572107
17	62120040	62120049	A	snp	G	62120044	ERN1
17	66597743	66597752	G	snp	T	66597750	FAM20A
17	67252575	67252584	T	snp	C	67252580	ABCA5
17	75138712	75138722	T	snp	C	75138713	SEC14L1
17	76112961	76112971	A	snp	G	76112964	TMC6
17	76573717	76573726	C	snp	A	76573723	DNAH17
17	77768647	77768655	C	snp	A	77768653	CBX8
17	78181911	78181919	C	snp	A	78181917	CARD14
17	79090442	79090451	C	snp	A	79090447	AATK
17	79090442	79090451	C	snp	A	79090447	AATK
17	79090442	79090451	C	snp	A	79090447	BAIAP2
17	79090442	79090451	C	snp	A	79090447	BAIAP2
17	80091256	80091266	A	snp	G	80091264	CCDC57

17	80160333	80160343	A	snp	C	80160336	CCDC57
17	80623102	80623112	A	snp	C	80623105	RAB40B
18	721558	721568	A	snp	C	721562	YES1
18	5420014	5420024	A	snp	G	5420020	EPB41L3
18	6590774	6590783	T	snp	G	6590780	LOC100130480
18	6788457	6788467	T	snp	G	6788460	ARHGAP28
18	8638120	8638129	T	snp	G	8638125	RAB12
18	12493882	12493890	T	snp	G	12493888	SPIRE1
18	12657425	12657433	G	snp	A	12657430	AK095621
18	12657425	12657433	G	snp	A	12657430	SPIRE1
18	12678942	12678951	A	snp	G	12678948	CEP76
18	12678942	12678951	A	snp	G	12678948	PSMG2
18	12784750	12784758	T	snp	G	12784756	PTPN2
18	13099601	13099611	T	snp	G	13099602	CEP192
18	21758185	21758195	A	snp	G	21758187	OSBPL1A
18	23662441	23662449	T	snp	C	23662444	SS18
18	28722636	28722645	T	snp	C	28722643	DSC1
18	29426549	29426557	A	snp	C	29426551	TRAPPC8
18	29692644	29692653	A	snp	G	29692650	RNF138
18	43591268	43591278	A	snp	G	43591272	PSTPIP2
18	43916347	43916356	A	snp	C	43916351	C18orf23
18	43916347	43916356	A	snp	C	43916351	RNF165
18	43916347	43916356	A	snp	C	43916351	RNF165
18	45458514	45458523	T	snp	C	45458518	SMAD2
18	46903929	46903937	A	snp	G	46903934	DYM
18	50490841	50490849	T	snp	C	50490847	DCC
18	53303614	53303622	A	snp	G	53303615	TCF4
18	53443925	53443933	T	snp	G	53443930	AK127787
18	55213052	55213060	A	snp	C	55213057	FECH
18	55272861	55272870	A	snp	G	55272864	NARS
18	56000375	56000383	G	snp	T	56000377	NEDD4L
18	56368000	56368010	T	snp	G	56368003	MALT1
18	56650343	56650352	A	snp	G	56650346	ZNF532
18	56650831	56650839	C	snp	T	56650833	ZNF532
18	57019271	57019279	T	snp	G	57019273	LMAN1
18	74270534	74270542	T	snp	G	74270540	LOC284276
19	291663	291671	G	snp	A	291665	PPAP2C
19	632536	632546	G	snp	T	632544	POLRMT
19	709911	709921	G	snp	T	709912	PALM
19	1118879	1118889	A	snp	C	1118881	SBNO2
19	1396966	1396974	G	snp	A	1396969	AK126693
19	1396966	1396974	G	snp	A	1396969	GAMT
19	1396966	1396974	G	snp	A	1396969	NDUFS7

19	1812200	1812208	G	snp	A	1812203	ATP8B3
19	2084581	2084591	T	snp	C	2084585	MOB3A
19	2512369	2512379	T	snp	G	2512370	GNG7
19	2822214	2822222	T	snp	G	2822218	ZNF554
19	3115783	3115792	G	snp	A	3115789	GNA11
19	3207859	3207867	G	snp	T	3207864	NCLN
19	3744153	3744161	C	snp	T	3744158	TJP3
19	4307360	4307368	T	snp	C	4307363	FSD1
19	4544963	4544971	T	snp	G	4544967	SEMA6B
19	4771799	4771808	A	snp	G	4771805	MIR7-3HG
19	5152120	5152130	G	snp	T	5152124	KDM4B
19	6477250	6477259	G	snp	T	6477255	DENND1C
19	7163592	7163602	A	snp	G	7163598	INSR
19	7614068	7614076	T	snp	C	7614071	PNPLA6
19	8435611	8435619	T	snp	G	8435612	ANGPTL4
19	8435611	8435619	T	snp	C	8435613	ANGPTL4
19	10071663	10071671	C	snp	T	10071668	COL5A3
19	10171487	10171495	A	snp	G	10171490	C3P1
19	10342010	10342018	G	snp	T	10342012	MIR4322
19	10342010	10342018	G	snp	T	10342012	S1PR2
19	10416441	10416449	G	snp	A	10416443	FDX1L
19	10416441	10416449	G	snp	A	10416443	FDX1L
19	10416441	10416449	G	snp	A	10416443	ZGLP1
19	10416441	10416449	G	snp	A	10416443	ZGLP1
19	10691181	10691189	A	snp	C	10691183	AP1M2
19	11274872	11274882	A	snp	C	11274875	KANK2
19	11656786	11656796	A	snp	G	11656791	CNN1
19	12625187	12625196	A	snp	C	12625193	ZNF709
19	12813201	12813210	T	snp	G	12813205	TNPO2
19	14267378	14267386	G	snp	T	14267379	LOC100507373
19	14267378	14267386	G	snp	T	14267379	LOC100507373
19	14267378	14267386	G	snp	T	14267379	LOC100507373
19	14267378	14267386	G	snp	T	14267379	LOC100507373
19	14267378	14267386	G	snp	T	14267379	LPHN1
19	14267378	14267386	G	snp	T	14267379	LPHN1
19	14267378	14267386	G	snp	T	14267379	LPHN1
19	14267378	14267386	G	snp	T	14267379	LPHN1
19	15589163	15589172	A	snp	G	15589164	PGLYRP2
19	15648966	15648976	T	snp	C	15648968	CYP4F22
19	16275653	16275661	C	snp	T	16275654	CIB3
19	16278052	16278060	A	snp	C	16278053	CIB3
19	17355544	17355552	G	snp	A	17355548	NR2F6
19	17572178	17572187	A	snp	G	17572183	NXNL1

19	17694244	17694254	G	snp	T	17694251	GLT25D1
19	17798110	17798118	C	snp	T	17798111	UNC13A
19	18169442	18169451	C	snp	A	18169449	IL12RB1
19	18311106	18311115	A	snp	G	18311110	RAB3A
19	18379172	18379180	G	snp	A	18379178	KIAA1683
19	19294384	19294392	T	snp	C	19294388	MEF2B
19	19294384	19294392	T	snp	C	19294388	MEF2BNB
19	19294384	19294392	T	snp	C	19294388	MEF2BNB-MEF2B
19	19294384	19294392	T	snp	C	19294388	MEF2BNB-MEF2B
19	19842878	19842887	A	snp	C	19842879	ZNF14
19	23316138	23316148	T	snp	G	23316142	ZNF730
19	23556412	23556422	A	snp	C	23556420	ZNF91
19	23992201	23992210	A	snp	G	23992207	RPSA
19	24115133	24115142	T	snp	G	24115137	AK125686
19	24115133	24115142	T	snp	G	24115137	ZNF726
19	33405536	33405545	A	snp	G	33405538	CEP89
19	39370448	39370458	T	snp	G	39370455	SIRT2
19	39690127	39690137	T	snp	C	39690133	NCCRP1
19	39992837	39992847	A	snp	C	39992845	DLL3
19	42342537	42342545	C	snp	T	42342539	LYPD4
19	43519089	43519097	A	snp	C	43519094	PSG11
19	43519089	43519097	A	snp	C	43519094	PSG6
19	44008368	44008377	C	snp	T	44008369	PHLDB3
19	46972011	46972019	T	snp	G	46972017	PNMAL1
19	47543996	47544006	G	snp	T	47543997	NPAS1
19	47543996	47544006	G	snp	T	47543999	NPAS1
19	47921171	47921181	C	snp	A	47921179	MEIS3
19	48248179	48248188	A	snp	G	48248181	GLTSCR2
19	48285805	48285815	T	snp	C	48285808	AX747088
19	48285805	48285815	T	snp	C	48285808	SEPW1
19	48829364	48829373	G	snp	A	48829366	EMP3
19	49168177	49168187	T	snp	C	49168181	NTN5
19	49168177	49168187	T	snp	C	49168181	NTN5
19	49168177	49168187	T	snp	C	49168181	SEC1
19	49168177	49168187	T	snp	C	49168181	SEC1
19	49218105	49218113	G	snp	T	49218110	MAMSTR
19	49337234	49337242	A	snp	G	49337236	HSD17B14
19	49364108	49364117	A	snp	G	49364109	PLEKHA4
19	49957277	49957287	T	snp	G	49957285	ALDH16A1
19	49965125	49965133	G	snp	A	49965130	ALDH16A1
19	50199879	50199888	T	snp	C	50199884	CPT1C
19	50965853	50965862	T	snp	C	50965857	MYBPC2
19	51326350	51326359	C	snp	A	51326357	KLK1

19	52875440	52875449	A	snp	C	52875444	ZNF880
19	54697599	54697607	G	snp	A	54697601	TSEN34
19	55537073	55537082	A	snp	C	55537080	GP6
19	55570038	55570046	T	snp	C	55570043	RDH13
19	56499766	56499776	A	snp	G	56499768	NLRP8
19	57029834	57029842	T	snp	G	57029836	ZNF471
19	57930251	57930261	T	snp	G	57930255	ZNF17
19	58070632	58070641	G	snp	A	58070639	ZNF550
19	58903697	58903707	A	snp	G	58903698	RPS5
1	870311	870319	G	snp	A	870316	SAMD11
1	6164678	6164688	C	snp	A	6164685	CHD5
1	6196074	6196083	A	snp	C	6196080	CHD5
1	6473522	6473531	T	snp	G	6473529	HES2
1	7725774	7725783	A	snp	G	7725775	CAMTA1
1	7805585	7805593	A	snp	G	7805587	CAMTA1
1	7854551	7854559	A	snp	G	7854554	PER3
1	8029500	8029510	G	snp	A	8029508	PARK7
1	8937769	8937779	A	snp	C	8937771	ENO1
1	8937769	8937779	A	snp	C	8937771	ENO1
1	8937769	8937779	A	snp	C	8937771	ENO1-AS1
1	11825542	11825551	T	snp	C	11825543	C1orf167
1	12184229	12184238	T	snp	C	12184230	TNFRSF8
1	12347382	12347390	T	snp	C	12347386	VPS13D
1	12347382	12347390	T	snp	C	12347387	VPS13D
1	12569949	12569958	A	snp	G	12569953	AK095438
1	12569949	12569958	A	snp	G	12569953	VPS13D
1	12857497	12857505	C	snp	T	12857503	PRAMEF1
1	13943890	13943898	T	snp	G	13943896	PDPN
1	15772049	15772058	G	snp	T	15772056	CTRC
1	21904199	21904207	C	snp	T	21904205	ALPL
1	22304227	22304237	T	snp	C	22304228	CELA3B
1	24104825	24104833	C	snp	T	24104828	LOC100506963
1	24104825	24104833	C	snp	T	24104828	PITHD1
1	24405699	24405707	T	snp	C	24405700	MYOM3
1	24829679	24829687	G	snp	T	24829684	RCAN3
1	24829679	24829687	G	snp	T	24829684	RCAN3AS
1	25171669	25171679	A	snp	C	25171672	CLIC4
1	25171669	25171679	A	snp	C	25171672	Z24749
1	26612291	26612299	C	snp	A	26612296	UBXN11
1	31191436	31191446	C	snp	A	31191437	LOC100129196
1	31191436	31191446	C	snp	A	31191437	MATN1
1	34039109	34039118	A	snp	G	34039111	CSMD2
1	35457392	35457401	A	snp	C	35457395	ZMYM6

1	35855052	35855060	A	snp	G	35855054	ZMYM4
1	36563156	36563164	C	snp	A	36563157	COL8A2
1	36703415	36703423	T	snp	G	36703421	THRAP3
1	39350613	39350622	A	snp	C	39350617	RHBDL2
1	39846584	39846592	A	snp	C	39846589	MACF1
1	40138109	40138118	T	snp	C	40138113	NT5C1A
1	40778843	40778853	T	snp	C	40778848	COL9A2
1	42999892	42999901	G	snp	A	42999898	CCDC30
1	43226329	43226337	T	snp	G	43226335	LEPRE1
1	45803411	45803420	A	snp	G	45803412	MUTYH
1	46180465	46180474	T	snp	C	46180469	IPP
1	47281773	47281782	C	snp	A	47281779	CYP4B1
1	47835242	47835252	T	snp	C	47835243	CMPK1
1	49056762	49056771	C	snp	A	49056769	AGBL4
1	61927732	61927741	T	snp	G	61927735	NFIA
1	62380192	62380200	A	snp	G	62380194	INADL
1	62904568	62904576	T	snp	G	62904574	USP1
1	65247390	65247398	T	snp	C	65247393	RAVER2
1	65342502	65342510	A	snp	C	65342504	AK128734
1	65342502	65342510	A	snp	C	65342504	JAK1
1	65614843	65614851	C	snp	T	65614848	AK4
1	70654261	70654270	A	snp	C	70654262	LRRC40
1	71250281	71250290	T	snp	G	71250288	BC041441
1	76388585	76388594	A	snp	C	76388588	ASB17
1	78308227	78308236	T	snp	G	78308233	FAM73A
1	84465067	84465077	C	snp	A	84465072	TTLL7
1	84878636	84878646	C	snp	A	84878641	DNASE2B
1	93791094	93791104	T	snp	C	93791097	LOC100131564
1	95322293	95322302	T	snp	C	95322300	SLC44A3
1	100128778	100128786	A	snp	G	100128782	PALMD
1	100587243	100587251	A	snp	C	100587249	SASS6
1	109398647	109398657	T	snp	G	109398655	AKNAD1
1	113162029	113162039	C	snp	A	113162036	CAPZA1
1	113162029	113162039	C	snp	A	113162036	ST7L
1	113162029	113162039	C	snp	A	113162036	ST7L
1	117660126	117660134	A	snp	G	117660130	TRIM45
1	118549720	118549728	T	snp	G	118549721	SPAG17
1	118693009	118693017	T	snp	G	118693015	SPAG17
1	145327389	145327399	T	snp	C	145327394	NBPF10
1	145327389	145327399	T	snp	C	145327394	NBPF10
1	145327389	145327399	T	snp	C	145327394	NBPF10
1	145327389	145327399	T	snp	C	145327394	NBPF10
1	145327389	145327399	T	snp	C	145327394	NBPF10

1	145327389	145327399	T	snp	C	145327394	NBPF10
1	145327389	145327399	T	snp	C	145327394	NBPF10
1	145327389	145327399	T	snp	C	145327394	NBPF10
1	145327389	145327399	T	snp	C	145327394	NBPF10
1	145327389	145327399	T	snp	C	145327394	NBPF10
1	145327389	145327399	T	snp	C	145327394	NBPF10
1	145327389	145327399	T	snp	C	145327394	NBPF10
1	145327389	145327399	T	snp	C	145327394	NBPF14
1	145327389	145327399	T	snp	C	145327394	NBPF14
1	145327389	145327399	T	snp	C	145327394	NBPF14
1	145327389	145327399	T	snp	C	145327394	NBPF14
1	145327389	145327399	T	snp	C	145327394	NBPF14
1	145327389	145327399	T	snp	C	145327394	NBPF14
1	145327389	145327399	T	snp	C	145327394	NBPF14
1	145327389	145327399	T	snp	C	145327394	NBPF14
1	145327389	145327399	T	snp	C	145327394	NBPF14
1	145327389	145327399	T	snp	C	145327394	NBPF9
1	145327389	145327399	T	snp	C	145327394	NBPF9
1	145327389	145327399	T	snp	C	145327394	NBPF9
1	145327389	145327399	T	snp	C	145327394	NBPF9
1	145327389	145327399	T	snp	C	145327394	NBPF9
1	145327389	145327399	T	snp	C	145327394	NBPF9
1	146739066	146739075	T	snp	C	146739067	CHD1L
1	150918928	150918937	T	snp	G	150918935	SETDB1
1	151816213	151816223	A	snp	G	151816219	LOC100132111
1	154113312	154113320	A	snp	C	154113314	NUP210L
1	155706209	155706218	T	snp	G	155706216	DAP3
1	155706209	155706218	T	snp	G	155706216	YY1AP1
1	160302354	160302364	A	snp	G	160302355	COPA
1	161751220	161751228	A	snp	G	161751225	ATF6
1	164743401	164743411	A	snp	G	164743403	LOC100505795
1	164743401	164743411	A	snp	G	164743403	PBX1
1	165599802	165599810	A	snp	G	165599803	MGST3
1	165623956	165623964	T	snp	C	165623961	MGST3
1	168663765	168663773	T	snp	G	168663771	DPT
1	169483760	169483769	T	snp	G	169483761	F5
1	169890931	169890939	A	snp	G	169890932	KIFAP3
1	170933762	170933770	A	snp	C	170933765	C1orf129
1	176105061	176105070	A	snp	G	176105065	RFWD2
1	178694076	178694085	C	snp	T	178694083	RALGPS2
1	180144134	180144142	A	snp	C	180144135	QSOX1
1	180164131	180164140	G	snp	T	180164133	QSOX1
1	180164131	180164140	G	snp	T	180164135	QSOX1

1	180946380	180946388	T	snp	G	180946381	AK056657
1	180946380	180946388	T	snp	G	180946381	STX6
1	182812212	182812221	A	snp	G	182812216	DHX9
1	184728083	184728093	A	snp	C	184728087	AX747662
1	186925628	186925638	T	snp	G	186925635	PLA2G4A
1	200567340	200567349	T	snp	C	200567343	KIF14
1	202390779	202390789	A	snp	C	202390781	PPP1R12B
1	202573399	202573407	C	snp	T	202573405	SYT2
1	202827393	202827403	A	snp	G	202827397	BC040684
1	202827393	202827403	A	snp	G	202827397	BC040684
1	202827393	202827403	A	snp	G	202827397	BC049825
1	202827393	202827403	A	snp	G	202827397	BC049825
1	202897424	202897432	A	snp	G	202897430	KLHL12
1	204371720	204371729	T	snp	G	204371721	PPP1R15B
1	210856602	210856611	T	snp	G	210856603	KCNH1
1	212617389	212617398	A	snp	C	212617395	NENF
1	212957348	212957357	A	snp	G	212957353	NSL1
1	213057640	213057650	A	snp	G	213057648	FLVCR1
1	215972001	215972009	T	snp	G	215972002	USH2A
1	216495186	216495196	T	snp	G	216495190	USH2A
1	217792147	217792155	A	snp	G	217792148	GPATCH2
1	223168205	223168215	T	snp	C	223168212	DISP1
1	223286557	223286565	T	snp	G	223286560	TLR5
1	223969507	223969516	A	snp	C	223969512	TP53BP2
1	224563198	224563207	T	snp	G	224563202	CNIH4
1	225143091	225143099	T	snp	C	225143096	DNAH14
1	226334599	226334607	T	snp	C	226334604	ACBD3
1	226352490	226352499	T	snp	G	226352497	ACBD3
1	227098195	227098203	T	snp	C	227098196	ADCK3
1	229586482	229586490	T	snp	G	229586485	NUP133
1	233518877	233518886	T	snp	C	233518884	KIAA1804
1	234606022	234606031	A	snp	C	234606024	TARBP1
1	235633220	235633228	T	snp	G	235633223	B3GALNT2
1	237731675	237731684	A	snp	C	237731676	RYR2
1	241265888	241265896	T	snp	C	241265893	RGS7
1	243293174	243293183	A	snp	G	243293175	CEP170
1	244746525	244746533	A	snp	G	244746527	C1orf101
1	246704820	246704830	A	snp	G	246704828	TFB2M
1	248032205	248032213	T	snp	G	248032211	OR2W3
1	248032205	248032213	T	snp	G	248032211	TRIM58
1	248085911	248085919	T	snp	C	248085912	OR2T8
20	2126561	2126569	T	snp	C	2126566	STK35
20	3856672	3856680	T	snp	C	3856677	MAVS

20	17477747	17477755	A	snp	G	17477750	BFSP1
20	17538764	17538773	G	snp	A	17538771	BFSP1
20	18464104	18464113	T	snp	C	18464109	POLR3F
20	18470642	18470651	A	snp	C	18470645	RBBP9
20	18515840	18515849	C	snp	A	18515847	SEC23B
20	20372830	20372839	A	snp	C	20372836	RALGAPA2
20	23808189	23808199	T	snp	G	23808196	CST2
20	25207766	25207774	C	snp	A	25207772	ENTPD6
20	31218829	31218838	A	snp	C	31218835	C20orf203
20	32210236	32210244	T	snp	G	32210237	CBFA2T2
20	34581973	34581982	T	snp	G	34581978	C20orf152
20	37434845	37434854	C	snp	A	37434851	PPP1R16B
20	42263808	42263816	A	snp	C	42263809	IFT52
20	42844409	42844417	T	snp	C	42844412	LOC100505783
20	43037364	43037372	A	snp	C	43037365	HNF4A
20	43037364	43037372	A	snp	C	43037365	MIR3646
20	43159447	43159455	A	snp	G	43159448	PKIG
20	43737274	43737283	T	snp	G	43737277	WFDC5
20	48462381	48462389	T	snp	G	48462385	SLC9A8
20	50776519	50776528	A	snp	C	50776521	ZFP64
20	55046623	55046632	A	snp	C	55046627	C20orf43
20	55802854	55802862	T	snp	G	55802857	BMP7
20	62167484	62167492	C	snp	A	62167489	PTK6
20	62167507	62167516	C	snp	T	62167513	PTK6
20	62378734	62378742	G	snp	A	62378739	SLC2A4RG
20	62378734	62378742	G	snp	A	62378739	ZBTB46
20	62612996	62613006	C	snp	T	62613004	PRPF6
21	10990693	10990702	C	snp	A	10990696	TPTE
21	15537721	15537731	A	snp	G	15537726	LIPI
21	15744386	15744395	T	snp	C	15744388	HSPA13
21	19274501	19274511	T	snp	G	19274505	CHODL
21	27079048	27079057	T	snp	G	27079053	JAM2
21	32126534	32126542	T	snp	G	32126540	KRTAP21-1
21	32597488	32597496	T	snp	G	32597494	TIAM1
21	35741851	35741861	A	snp	G	35741855	KCNE2
21	38130054	38130062	A	snp	G	38130060	HLCS
21	40553530	40553539	A	snp	C	40553534	PSMG1
21	41013390	41013398	A	snp	G	41013393	B3GALT5
21	42748315	42748323	A	snp	C	42748316	MX2
21	42748315	42748323	A	snp	C	42748318	MX2
21	43322301	43322311	A	snp	C	43322303	C2CD2
21	43443106	43443114	A	snp	C	43443112	ZNF295-AS1
21	45092648	45092656	A	snp	G	45092652	RRP1B

21	47406610	47406618	C	snp	A	47406611	COL6A1
21	47832012	47832021	T	snp	C	47832018	PCNT
22	23094195	23094204	A	snp	C	23094200	abParts
22	23094195	23094204	A	snp	C	23094200	DKFZp667J0810
22	23805125	23805134	A	snp	C	23805129	LOC388882
22	23830152	23830161	T	snp	C	23830154	LOC388882
22	24940235	24940243	G	snp	T	24940241	C22orf13
22	25250782	25250790	A	snp	G	25250786	SGSM1
22	25505934	25505943	A	snp	C	25505935	KIAA1671
22	25505934	25505943	A	snp	C	25505935	KIAA1671
22	25505934	25505943	A	snp	C	25505935	LOC100128531
22	25505934	25505943	A	snp	C	25505935	LOC100128531
22	26240231	26240239	A	snp	G	26240233	MYO18B
22	29120395	29120404	A	snp	C	29120399	CHEK2
22	29835545	29835553	G	snp	T	29835551	RFPL1
22	29835545	29835553	G	snp	T	29835551	RFPL1-AS1
22	30426627	30426637	G	snp	T	30426635	MTMR3
22	30892548	30892557	C	snp	A	30892554	SEC14L4
22	30927417	30927427	A	snp	G	30927421	SEC14L6
22	31984821	31984829	A	snp	G	31984826	SFI1
22	32081927	32081935	T	snp	C	32081928	PRR14L
22	36958996	36959006	T	snp	C	36959004	CACNG2
22	37532506	37532515	C	snp	T	37532513	IL2RB
22	38613195	38613205	A	snp	C	38613197	MAFF
22	38934155	38934165	A	snp	C	38934163	DMC1
22	39639850	39639858	G	snp	T	39639852	PDGFB
22	40697582	40697590	T	snp	G	40697588	TNRC6B
22	41210827	41210835	T	snp	G	41210830	MIR4766
22	41210827	41210835	T	snp	G	41210830	SLC25A17
22	44259072	44259081	A	snp	C	44259074	SULT4A1
22	45794719	45794728	A	snp	C	45794722	SMC1B
22	46436325	46436335	T	snp	C	46436331	LOC100271722
22	46439734	46439744	A	snp	C	46439736	LOC100271722
22	46449889	46449897	G	snp	A	46449890	C22orf26
22	46449889	46449897	G	snp	A	46449890	LOC150381
22	46449889	46449897	G	snp	A	46449890	MIRLET7BHG
22	51112354	51112363	G	snp	T	51112358	SHANK3
22	51112354	51112363	G	snp	A	51112360	SHANK3
2	1521712	1521721	C	snp	A	1521718	TPO
2	7080643	7080651	T	snp	C	7080645	RNF144A
2	26067474	26067483	A	snp	G	26067480	ASXL2
2	26607813	26607822	T	snp	C	26607814	EPT1
2	26718821	26718829	C	snp	A	26718827	OTOF

2	27655441	27655450	T	snp	G	27655446	NRBP1
2	28812034	28812044	A	snp	C	28812035	PLB1
2	29134504	29134514	T	snp	C	29134512	WDR43
2	31597403	31597411	A	snp	G	31597409	XDH
2	33050630	33050638	G	snp	A	33050635	LINC00486
2	33162851	33162859	A	snp	G	33162853	LINC00486
2	33162851	33162859	A	snp	G	33162853	LOC100271832
2	36923896	36923905	G	snp	T	36923897	VIT
2	36923896	36923905	G	snp	T	36923899	VIT
2	37663224	37663233	A	snp	G	37663225	U6
2	38202843	38202852	A	snp	C	38202845	FAM82A1
2	38208970	38208980	A	snp	C	38208978	FAM82A1
2	39102669	39102677	C	snp	T	39102670	DHX57
2	39102669	39102677	C	snp	T	39102670	MORN2
2	43965700	43965709	T	snp	C	43965704	PLEKHH2
2	46583272	46583282	C	snp	T	46583280	EPAS1
2	47347883	47347891	T	snp	G	47347888	C2orf61
2	47629890	47629900	T	snp	G	47629897	MSH2
2	48031272	48031282	T	snp	G	48031279	MSH6
2	48916862	48916872	T	snp	C	48916863	LHCGR
2	48916862	48916872	T	snp	C	48916863	STON1-GTF2A1L
2	49004452	49004460	T	snp	C	49004458	STON1-GTF2A1L
2	54571902	54571910	A	snp	C	54571908	C2orf73
2	56598522	56598530	A	snp	C	56598524	CCDC85A
2	61002842	61002852	T	snp	C	61002845	PAPOLG
2	61709732	61709741	T	snp	G	61709735	XPO1
2	64416873	64416883	T	snp	C	64416879	LINC00309
2	85659508	85659516	T	snp	G	85659512	SH2D6
2	85659532	85659542	T	snp	G	85659536	SH2D6
2	85765431	85765440	C	snp	T	85765438	LOC100630918
2	85765431	85765440	C	snp	T	85765438	MAT2A
2	85789197	85789206	A	snp	C	85789198	GGCX
2	86075962	86075970	T	snp	C	86075966	ST3GAL5
2	87114860	87114869	T	snp	G	87114863	LOC100286979
2	87114860	87114869	T	snp	G	87114863	RMND5A
2	88484805	88484814	C	snp	A	88484807	THNSL2
2	101098490	101098498	G	snp	T	101098493	NMS
2	108619310	108619319	A	snp	G	108619315	SLC5A7
2	109098378	109098386	C	snp	A	109098383	GCC2
2	112550822	112550830	T	snp	G	112550824	ANAPC1
2	113531085	113531094	T	snp	C	113531092	IL1A
2	114004988	114004997	A	snp	C	114004993	LOC654433
2	114004988	114004997	A	snp	C	114004993	PAX8

2	114020843	114020851	T	snp	G	114020847	LOC654433
2	114020843	114020851	T	snp	G	114020847	PAX8
2	128048667	128048677	A	snp	C	128048673	ERCC3
2	128239018	128239027	G	snp	A	128239019	IWS1
2	131805979	131805988	G	snp	T	131805983	FAM168B
2	132259417	132259425	A	snp	G	132259420	LOC150776
2	132263229	132263239	T	snp	G	132263237	LOC150776
2	136625594	136625603	A	snp	G	136625601	MCM6
2	153399490	153399498	A	snp	C	153399496	FMNL2
2	157470260	157470270	T	snp	C	157470261	GPD2
2	160373814	160373824	T	snp	C	160373815	BAZ2B
2	160605853	160605862	T	snp	G	160605859	MARCH7
2	165586010	165586019	A	snp	C	165586014	COBLL1
2	169307386	169307394	A	snp	G	169307390	Metazoa_SRP
2	170072509	170072518	A	snp	C	170072514	LRP2
2	172943883	172943893	T	snp	C	172943885	METAP1D
2	173340191	173340199	A	snp	C	173340197	ITGA6
2	173371274	173371282	A	snp	G	173371277	ITGA6
2	175265663	175265671	A	snp	G	175265665	SCRN3
2	176789404	176789413	A	snp	C	176789408	KIAA1715
2	182374176	182374184	T	snp	C	182374178	ITGA4
2	186625767	186625775	A	snp	G	186625769	FSIP2
2	186629183	186629192	T	snp	G	186629190	FSIP2
2	190429174	190429182	T	snp	C	190429180	SLC40A1
2	191843823	191843831	A	snp	G	191843829	STAT1
2	191863407	191863417	A	snp	G	191863408	STAT1
2	192280330	192280340	T	snp	G	192280334	MYO1B
2	197965158	197965167	A	snp	C	197965162	ANKRD44
2	198051196	198051206	T	snp	C	198051204	ANKRD44
2	198355147	198355157	T	snp	G	198355152	HSPD1
2	198413950	198413960	A	snp	G	198413958	HSPE1-MOB4
2	198413950	198413960	A	snp	G	198413958	MOB4
2	200512634	200512643	T	snp	G	200512640	BC035629
2	200710295	200710305	A	snp	C	200710297	FONG
2	202131584	202131592	T	snp	G	202131586	CASP8
2	207610904	207610913	A	snp	C	207610908	MDH1B
2	208592249	208592258	T	snp	G	208592256	CCNYL1
2	208629376	208629385	A	snp	C	208629380	FZD5
2	211158847	211158856	T	snp	C	211158854	MYL1
2	212523105	212523115	A	snp	C	212523113	ERBB4
2	214012403	214012412	A	snp	C	214012404	IKZF2
2	215631927	215631935	T	snp	C	215631931	BARD1
2	216246205	216246214	T	snp	G	216246209	FN1

2	216256768	216256776	A	snp	G	216256774	FN1
2	219271200	219271208	C	snp	T	219271202	CTDSP1
2	220130651	220130660	A	snp	G	220130652	TUBA4B
2	223496862	223496872	A	snp	G	223496869	FARSB
2	224749908	224749917	G	snp	T	224749909	WDFY1
2	226518756	226518765	A	snp	G	226518760	NYAP2
2	228120429	228120439	T	snp	G	228120430	AK056332
2	228120429	228120439	T	snp	G	228120430	AK056332
2	228120429	228120439	T	snp	G	228120430	AK056332
2	228120429	228120439	T	snp	G	228120430	BC035052
2	228120429	228120439	T	snp	G	228120430	BC035052
2	228120429	228120439	T	snp	G	228120430	BC035052
2	228120429	228120439	T	snp	G	228120430	COL4A3
2	228120429	228120439	T	snp	G	228120430	COL4A3
2	228120429	228120439	T	snp	G	228120430	COL4A3
2	228492859	228492868	T	snp	G	228492860	C2orf83
2	228572453	228572461	T	snp	C	228572459	AX746677
2	228572453	228572461	T	snp	C	228572459	SLC19A3
2	231685193	231685202	A	snp	G	231685197	CAB39
2	233640866	233640874	T	snp	G	233640870	GIGYF2
2	233640866	233640874	T	snp	G	233640870	KCNJ13
2	234248610	234248620	T	snp	C	234248618	SAG
2	238402823	238402831	G	snp	A	238402829	MLPH
2	238427730	238427739	T	snp	G	238427733	MLPH
2	239073512	239073521	A	snp	C	239073514	FAM132B
2	239167483	239167491	G	snp	T	239167485	PER2
2	241529075	241529083	G	snp	A	241529078	CAPN10
2	241808307	241808315	C	snp	T	241808313	AGXT
2	242376400	242376408	A	snp	G	242376401	FARP2
3	3885253	3885262	C	snp	A	3885258	LRRN1
3	3885253	3885262	C	snp	A	3885258	SUMF1
3	9031326	9031334	A	snp	C	9031331	SRGAP3
3	10148979	10148989	T	snp	C	10148982	C3orf24
3	12457435	12457444	A	snp	G	12457440	PPARG
3	14485477	14485486	C	snp	A	14485483	SLC6A6
3	15685007	15685017	A	snp	G	15685010	BTD
3	15804554	15804562	T	snp	G	15804557	ANKRD28
3	15804554	15804562	T	snp	G	15804557	BC041363
3	18457302	18457312	G	snp	T	18457303	SATB1
3	32187877	32187885	A	snp	G	32187879	GPD1L
3	33421173	33421183	A	snp	C	33421177	FBXL2
3	33442888	33442896	A	snp	G	33442889	FBXL2
3	33442888	33442896	A	snp	G	33442889	UBP1

3	36888166	36888174	T	snp	G	36888171	TRANK1
3	38527209	38527217	T	snp	C	38527214	ACVR2B
3	38830111	38830120	G	snp	A	38830116	SCN10A
3	46742521	46742531	C	snp	A	46742522	TMIE
3	49508971	49508979	T	snp	C	49508975	DAG1
3	51975569	51975579	C	snp	T	51975576	PARP3
3	51975569	51975579	C	snp	T	51975576	RRP9
3	53219274	53219282	G	snp	T	53219277	PRKCD
3	57400933	57400943	A	snp	G	57400935	DNAH12
3	58518090	58518099	T	snp	C	58518091	ACOX2
3	58630499	58630509	A	snp	G	58630500	FAM3D
3	62459813	62459822	A	snp	C	62459818	CADPS
3	66397442	66397452	A	snp	C	66397446	SLC25A26
3	69590940	69590948	C	snp	A	69590946	FRMD4B
3	75788826	75788835	T	snp	C	75788833	MIR4273
3	75788826	75788835	T	snp	C	75788833	ZNF717
3	97356224	97356233	T	snp	C	97356229	EPHA6
3	98503989	98503999	T	snp	G	98503992	ST3GAL6
3	100013093	100013102	T	snp	G	100013100	TBC1D23
3	100531731	100531740	T	snp	C	100531735	ABI3BP
3	101219388	101219397	A	snp	C	101219391	SENP7
3	108638335	108638343	T	snp	G	108638341	GUCA1C
3	111767190	111767198	T	snp	C	111767193	TMPRSS7
3	111785622	111785632	T	snp	C	111785628	TMPRSS7
3	112191199	112191207	T	snp	G	112191201	BTLA
3	113302488	113302496	A	snp	G	113302492	SIDT1
3	113848189	113848197	A	snp	C	113848192	DRD3
3	119248319	119248328	A	snp	G	119248320	CD80
3	119423024	119423033	T	snp	C	119423031	C3orf15
3	120133850	120133860	A	snp	G	120133856	FSTL1
3	124453017	124453026	T	snp	G	124453021	UMPS
3	124453108	124453118	A	snp	G	124453113	UMPS
3	125043028	125043036	A	snp	G	125043032	ZNF148
3	125651236	125651246	A	snp	C	125651238	ALG1L
3	126181066	126181075	A	snp	C	126181070	ZXDC
3	128723211	128723219	C	snp	A	128723214	CCDC48
3	130369129	130369137	G	snp	T	130369130	COL6A6
3	130369129	130369137	G	snp	T	130369131	COL6A6
3	132194282	132194291	T	snp	G	132194289	DNAJC13
3	133583007	133583016	G	snp	A	133583012	RAB6B
3	137964255	137964264	T	snp	G	137964262	ARMC8
3	141683503	141683512	A	snp	C	141683507	TFDP2
3	141885108	141885117	A	snp	G	141885111	GK5

3	150345688	150345698	T	snp	G	150345689	SELT
3	150402579	150402589	T	snp	C	150402582	FAM194A
3	156259169	156259179	T	snp	C	156259177	SSR3
3	167171055	167171064	T	snp	C	167171056	SERPINI2
3	169578000	169578008	T	snp	C	169578003	LRRC31
3	172064821	172064829	A	snp	C	172064827	FNDC3B
3	172312817	172312827	T	snp	C	172312825	AK127557
3	186015278	186015286	A	snp	G	186015282	DGKG
3	186562896	186562905	G	snp	T	186562897	ADIPOQ
3	194429509	194429517	T	snp	C	194429512	LOC100507391
3	195944418	195944427	A	snp	G	195944425	OSTalpha
3	195965315	195965324	G	snp	A	195965317	AF088041
3	195965315	195965324	G	snp	A	195965317	PCYT1A
4	7801978	7801986	A	snp	G	7801984	AFAP1
4	8021414	8021422	A	snp	G	8021416	ABLIM2
4	15447544	15447554	A	snp	G	15447547	C1QTNF7
4	17183836	17183845	T	snp	G	17183842	BC029598
4	36162828	36162837	A	snp	C	36162832	ARAP2
4	37585225	37585234	T	snp	G	37585232	C4orf19
4	39267998	39268008	A	snp	C	39268004	WDR19
4	39268012	39268020	A	snp	G	39268015	WDR19
4	39864419	39864429	A	snp	C	39864423	PDS5A
4	40128834	40128842	T	snp	C	40128837	N4BP2
4	42415728	42415737	T	snp	C	42415735	AK027252
4	42415728	42415737	T	snp	C	42415735	ATP8A1
4	44713228	44713238	A	snp	G	44713234	GNPDA2
4	47562534	47562542	A	snp	C	47562537	ATP10D
4	48152854	48152863	A	snp	C	48152861	TEC
4	48173067	48173077	A	snp	C	48173071	TEC
4	54243137	54243145	A	snp	G	54243140	FIP1L1
4	54243137	54243145	A	snp	G	54243140	PDGFRA
4	57786908	57786916	A	snp	C	57786914	REST
4	70936637	70936646	A	snp	G	70936641	CSN1S2AP
4	74007866	74007875	A	snp	G	74007869	ANKRD17
4	76282690	76282698	A	snp	G	76282696	LOC441025
4	76581768	76581776	A	snp	C	76581774	G3BP2
4	81106117	81106125	T	snp	C	81106123	PRDM8
4	81124892	81124901	C	snp	A	81124893	PRDM8
4	81124892	81124901	C	snp	A	81124899	PRDM8
4	83801336	83801346	A	snp	G	83801342	SEC31A
4	84349504	84349512	A	snp	G	84349510	HELQ
4	87141502	87141512	A	snp	C	87141510	BC038746
4	87141502	87141512	A	snp	C	87141510	MAPK10

4	88226473	88226482	A	snp	C	88226479	HSD17B13
4	88728178	88728187	A	snp	C	88728180	IBSP
4	88978134	88978143	T	snp	G	88978138	PKD2
4	90167248	90167256	T	snp	C	90167252	GPRIN3
4	95500621	95500630	A	snp	G	95500627	PDLIM5
4	95588562	95588570	T	snp	G	95588564	PDLIM5
4	96012421	96012431	T	snp	G	96012426	BMPR1B
4	100263712	100263720	A	snp	G	100263714	ADH1C
4	100339590	100339598	A	snp	G	100339595	ADH7
4	100459863	100459871	A	snp	C	100459865	C4orf17
4	106291678	106291687	T	snp	C	106291681	PPA2
4	109779967	109779975	A	snp	G	109779969	COL25A1
4	111396985	111396993	A	snp	G	111396986	ENPEP
4	119257717	119257727	T	snp	C	119257718	PRSS12
4	120058057	120058067	T	snp	C	120058065	MYOZ2
4	120414685	120414694	A	snp	C	120414692	LOC645513
4	120414685	120414694	A	snp	C	120414692	PDE5A
4	123662332	123662341	T	snp	G	123662338	BBS12
4	129018470	129018479	T	snp	G	129018474	LARP1B
4	129778071	129778079	T	snp	C	129778075	PHF17
4	142640634	142640642	A	snp	G	142640636	IL15
4	145792599	145792609	A	snp	C	145792601	BC044611
4	151356126	151356134	A	snp	G	151356129	LRBA
4	151771128	151771137	A	snp	C	151771133	LRBA
4	151829655	151829663	T	snp	C	151829658	LRBA
4	152330289	152330297	C	snp	T	152330293	FAM160A1
4	153875839	153875849	A	snp	G	153875845	FHDC1
4	154266314	154266322	C	snp	A	154266318	MND1
4	154515387	154515395	A	snp	G	154515392	KIAA0922
4	156765478	156765486	A	snp	C	156765481	ACCN5
4	156863065	156863074	T	snp	C	156863068	CTSO
4	158281519	158281529	T	snp	G	158281522	GRIA2
4	165031823	165031831	T	snp	G	165031829	MARCH1
4	166262191	166262201	T	snp	C	166262192	MSMO1
4	175838811	175838820	A	snp	G	175838815	ADAM29
4	178283754	178283763	T	snp	C	178283756	NEIL3
4	183810587	183810595	T	snp	C	183810588	DCTD
4	186272015	186272024	A	snp	C	186272020	SNX25
4	187073865	187073873	T	snp	G	187073871	FAM149A
5	345056	345064	G	snp	T	345057	AHRR
5	1112986	1112996	C	snp	A	1112993	SLC12A7
5	1494529	1494539	G	snp	T	1494531	LPCAT1
5	1501535	1501544	C	snp	A	1501541	LPCAT1

5	1627264	1627272	T	snp	G	1627266	LOC728613
5	5321096	5321105	T	snp	C	5321097	ADAMTS16
5	11383882	11383890	A	snp	C	11383883	CTNND2
5	13701161	13701170	A	snp	C	13701166	DNAH5
5	13876600	13876608	T	snp	C	13876604	DNAH5
5	21779150	21779159	T	snp	C	21779153	BC038535
5	21779150	21779159	T	snp	C	21779153	CDH12
5	31407745	31407753	A	snp	G	31407750	DROSHA
5	35036998	35037007	T	snp	C	35037005	AGXT2
5	37479850	37479859	T	snp	G	37479852	WDR70
5	38923696	38923705	T	snp	C	38923697	OSMR
5	52224234	52224243	A	snp	C	52224240	ITGA1
5	64961018	64961027	A	snp	G	64961019	C5orf44
5	64961018	64961027	A	snp	G	64961019	SGTB
5	67097161	67097171	A	snp	C	67097162	BC042046
5	67097161	67097171	A	snp	C	67097164	BC042046
5	70845810	70845818	T	snp	C	70845812	BDP1
5	76758544	76758552	A	snp	G	76758549	WDR41
5	77451499	77451507	A	snp	G	77451505	AP3B1
5	78250337	78250345	T	snp	C	78250338	ARSB
5	79929168	79929177	T	snp	G	79929173	DHFR
5	82806967	82806977	T	snp	C	82806971	VCAN
5	82948212	82948221	A	snp	G	82948215	HAPLN1
5	96314793	96314803	T	snp	G	96314794	LNPEP
5	114516559	114516568	A	snp	G	114516562	TRIM36
5	118466048	118466057	T	snp	G	118466051	DMXL1
5	118508433	118508442	T	snp	C	118508438	DMXL1
5	122165207	122165216	T	snp	G	122165208	SNX2
5	127855318	127855328	T	snp	C	127855319	FBN2
5	133295085	133295093	T	snp	G	133295088	C5orf15
5	134118926	134118935	T	snp	G	134118930	DDX46
5	140177430	140177440	T	snp	G	140177436	PCDHA1
5	140177430	140177440	T	snp	G	140177436	PCDHA2
5	140177430	140177440	T	snp	G	140177436	PCDHA2
5	141365094	141365102	T	snp	G	141365100	RNF14
5	146755559	146755567	T	snp	G	146755564	STK32A
5	149389570	149389579	A	snp	G	149389572	HMGXB3
5	156679025	156679033	A	snp	G	156679028	ITK
5	157099564	157099573	A	snp	C	157099566	C5orf52
5	159842906	159842916	T	snp	C	159842911	SLU7
5	167379580	167379589	T	snp	C	167379587	ODZ2
5	176830622	176830630	G	snp	A	176830626	F12
5	177379531	177379540	C	snp	A	177379535	AK126616

5	179269516	179269526	A	snp	C	179269523	C5orf45
6	2668340	2668350	T	snp	C	2668347	MYLK4
6	2769643	2769652	A	snp	G	2769648	WRNIP1
6	6724987	6724997	T	snp	C	6724994	BC039678
6	8041888	8041897	T	snp	C	8041891	EEF1E1
6	8041888	8041897	T	snp	C	8041891	MUTED
6	8041888	8041897	T	snp	C	8041891	TXNDC5
6	20152618	20152626	A	snp	G	20152622	MBOAT1
6	21743285	21743295	T	snp	C	21743290	LINC00340
6	22190873	22190881	T	snp	G	22190875	LINC00340
6	24701143	24701153	T	snp	C	24701145	ACOT13
6	24701143	24701153	T	snp	C	24701145	C6orf62
6	26856598	26856606	A	snp	C	26856599	GUSBP2
6	27878735	27878745	T	snp	G	27878737	OR2B2
6	31677035	31677044	T	snp	G	31677036	ABHD16A
6	31677035	31677044	T	snp	G	31677036	LY6G6F
6	31690753	31690761	T	snp	G	31690758	C6orf25
6	32604870	32604879	A	snp	G	32604875	HLA-DQA1
6	32605979	32605987	T	snp	G	32605981	HLA-DQA1
6	32610914	32610924	T	snp	C	32610921	HLA-DQA1
6	32630299	32630307	A	snp	C	32630302	HLA-DQB1
6	32630340	32630348	A	snp	C	32630343	HLA-DQB1
6	33219138	33219148	A	snp	C	33219141	HCG25
6	33219138	33219148	A	snp	C	33219141	HCG25
6	33219138	33219148	A	snp	C	33219141	HCG25
6	33219138	33219148	A	snp	C	33219141	VPS52
6	33219138	33219148	A	snp	C	33219141	VPS52
6	33219138	33219148	A	snp	C	33219141	VPS52
6	34204282	34204292	G	snp	A	34204284	HMGA1
6	39854995	39855003	A	snp	C	39854999	AX747174
6	39854995	39855003	A	snp	C	39854999	DAAM2
6	39855016	39855024	A	snp	C	39855018	AX747174
6	39855016	39855024	A	snp	C	39855018	DAAM2
6	41105716	41105725	A	snp	C	41105720	LOC221442
6	41304929	41304938	A	snp	C	41304934	NCR2
6	41563349	41563357	T	snp	G	41563351	FOXP4
6	42109822	42109830	G	snp	T	42109823	C6orf132
6	42985966	42985974	T	snp	C	42985972	KLHDC3
6	43973227	43973237	G	snp	T	43973231	AK024736
6	43973227	43973237	G	snp	T	43973231	C6orf223
6	46702599	46702607	A	snp	G	46702600	PLA2G7
6	58246662	58246671	A	snp	C	58246668	GUSBP4
6	69684540	69684549	A	snp	G	69684541	BAI3

6	70386647	70386657	A	snp	G	70386652	LMBRD1
6	74231127	74231135	G	snp	T	74231131	EEF1A1
6	84666090	84666098	G	snp	T	84666091	CYB5R4
6	88343956	88343966	T	snp	G	88343964	ORC3
6	97346570	97346580	A	snp	C	97346576	NDUFAF4
6	99978984	99978992	C	snp	A	99978986	LOC100130890
6	101163428	101163436	A	snp	C	101163432	ASCC3
6	105594389	105594397	A	snp	C	105594390	C6orf112
6	107017268	107017278	T	snp	G	107017269	AIM1
6	109312269	109312278	A	snp	C	109312275	SESN1
6	111898615	111898623	T	snp	C	111898619	TRAF3IP2
6	111898615	111898623	T	snp	C	111898619	TRAF3IP2-AS1
6	112114243	112114252	A	snp	G	112114248	FYN
6	114283971	114283981	A	snp	C	114283975	HDAC2
6	117084152	117084162	A	snp	G	117084160	FAM162B
6	121460157	121460165	T	snp	C	121460160	C6orf170
6	123819070	123819078	A	snp	C	123819073	TRDN
6	129960364	129960372	A	snp	G	129960367	ARHGAP18
6	131276466	131276475	A	snp	G	131276472	EPB41L2
6	137326801	137326809	T	snp	G	137326803	IL20RA
6	138644772	138644782	A	snp	C	138644774	KIAA1244
6	141005283	141005293	A	snp	C	141005284	MIR4465
6	141939628	141939636	C	snp	T	141939633	AK097143
6	144742823	144742833	A	snp	G	144742829	UTRN
6	146267869	146267877	A	snp	G	146267872	SHPRH
6	149722186	149722195	A	snp	C	149722188	SUMO4
6	149722186	149722195	A	snp	C	149722188	TAB2
6	150209823	150209831	A	snp	G	150209827	LOC100652739
6	150209823	150209831	A	snp	G	150209827	LOC100652739
6	150209823	150209831	A	snp	G	150209827	RAET1E
6	150209823	150209831	A	snp	G	150209827	RAET1E
6	150209823	150209831	A	snp	G	150209827	RAET1E
6	150383206	150383215	T	snp	C	150383212	ULBP3
6	152264525	152264534	A	snp	C	152264528	ESR1
6	159210275	159210284	T	snp	C	159210282	EZR
6	160101530	160101538	T	snp	G	160101531	BC016015
6	160101530	160101538	T	snp	G	160101531	SOD2
6	167413536	167413544	T	snp	C	167413538	CCR6
6	167413536	167413544	T	snp	C	167413538	CCR6
6	167413536	167413544	T	snp	C	167413538	FGFR1OP
6	167413536	167413544	T	snp	C	167413538	FGFR1OP
6	167738157	167738165	T	snp	G	167738158	TTLL2
6	170034791	170034799	A	snp	G	170034793	WDR27

6	170064772	170064782	A	snp	G	170064775	WDR27
7	1203624	1203633	T	snp	G	1203630	AK090593
7	1476518	1476526	C	snp	A	1476519	MICALL2
7	4166764	4166772	T	snp	C	4166767	SDK1
7	4780954	4780964	T	snp	G	4780960	FOXK1
7	7457170	7457180	G	snp	A	7457178	COL28A1
7	7571927	7571936	T	snp	G	7571928	COL28A1
7	8100233	8100241	T	snp	C	8100235	GLCCI1
7	20437587	20437597	T	snp	G	20437590	ITGB8
7	21631676	21631684	A	snp	C	21631682	DNAH11
7	23234974	23234982	T	snp	G	23234980	NUPL2
7	23347389	23347397	A	snp	C	23347393	BC065766
7	23347389	23347397	A	snp	C	23347393	C7orf30
7	23347389	23347397	A	snp	C	23347393	C7orf30
7	26679247	26679255	C	snp	A	26679253	C7orf71
7	29440022	29440030	A	snp	G	29440023	CHN2
7	29551797	29551807	A	snp	C	29551802	BC038570
7	29551797	29551807	A	snp	C	29551802	CHN2
7	29551797	29551807	A	snp	C	29551802	CHN2
7	32662406	32662414	A	snp	C	32662408	AVL9
7	32662406	32662414	A	snp	C	32662408	DPY19L1P1
7	35352491	35352500	A	snp	C	35352496	LOC401324
7	37874424	37874433	T	snp	C	37874428	BC043356
7	38316139	38316149	A	snp	G	38316141	TARP
7	38316139	38316149	A	snp	G	38316141	TCRGC2
7	38316139	38316149	A	snp	G	38316141	TRGC2
7	43157830	43157840	A	snp	G	43157835	AX748020
7	43157830	43157840	A	snp	G	43157835	AX748020
7	43157830	43157840	A	snp	G	43157835	HECW1
7	43157830	43157840	A	snp	G	43157835	HECW1
7	43480196	43480204	A	snp	G	43480198	HECW1
7	48231587	48231596	T	snp	G	48231592	ABCA13
7	48451933	48451941	T	snp	C	48451938	ABCA13
7	50473604	50473612	T	snp	C	50473609	IKZF1
7	55233857	55233867	A	snp	C	55233862	EGFR
7	56086051	56086061	A	snp	C	56086053	PSPH
7	56150284	56150294	A	snp	G	56150288	PHKG1
7	56150284	56150294	A	snp	G	56150288	PHKG1
7	56150284	56150294	A	snp	G	56150288	PHKG1
7	56150284	56150294	A	snp	G	56150288	PHKG1
7	56150284	56150294	A	snp	G	56150288	PSPH
7	56150284	56150294	A	snp	G	56150288	PSPH
7	56150284	56150294	A	snp	G	56150288	PSPH

7	56150284	56150294	A	snp	G	56150288	PSPH
7	56496576	56496586	T	snp	G	56496583	LOC650226
7	56496593	56496601	T	snp	G	56496595	LOC650226
7	57242222	57242230	T	snp	C	57242224	GUSBP10
7	57242222	57242230	T	snp	C	57242224	GUSBP10
7	57242222	57242230	T	snp	C	57242224	MtDNA_ssA
7	57242222	57242230	T	snp	C	57242224	TRNA
7	63983310	63983320	A	snp	G	63983314	ZNF680
7	66461205	66461213	A	snp	C	66461211	SBDS
7	66461205	66461213	A	snp	C	66461211	TYW1
7	70228800	70228810	T	snp	C	70228804	AUTS2
7	73254462	73254471	G	snp	T	73254463	WBSCR27
7	73479395	73479404	C	snp	A	73479401	ELN
7	75141148	75141157	A	snp	C	75141151	PMS2P3
7	75186655	75186664	A	snp	C	75186659	HIP1
7	76032664	76032672	T	snp	C	76032665	SRCRB4D
7	76032664	76032672	T	snp	C	76032665	ZP3
7	76910834	76910842	T	snp	C	76910839	CCDC146
7	77033934	77033942	A	snp	G	77033936	PION
7	81659639	81659647	A	snp	G	81659640	AK055932
7	81659639	81659647	A	snp	G	81659640	CACNA2D1
7	83024566	83024574	A	snp	C	83024569	SEMA3E
7	86574109	86574119	A	snp	C	86574110	KIAA1324L
7	87445746	87445754	G	snp	T	87445747	RUNDC3B
7	87445746	87445754	G	snp	T	87445752	RUNDC3B
7	87761451	87761461	T	snp	G	87761457	ADAM22
7	89866026	89866034	T	snp	C	89866030	STEAP2
7	90192427	90192435	T	snp	C	90192428	CDK14
7	99009010	99009019	T	snp	G	99009013	BUD31
7	99720988	99720997	A	snp	C	99720993	CNPY4
7	101958616	101958625	T	snp	C	101958621	SH2B2
7	102075973	102075981	C	snp	T	102075975	ORAI2
7	103160505	103160513	T	snp	G	103160510	RELN
7	105672823	105672832	T	snp	G	105672825	CDHR3
7	107414306	107414316	T	snp	G	107414313	SLC26A3
7	107577448	107577458	T	snp	C	107577455	LAMB1
7	115894369	115894377	T	snp	C	115894375	BD495725
7	115894369	115894377	T	snp	C	115894375	TES
7	117398600	117398608	T	snp	G	117398603	CTTNBP2
7	124569378	124569387	T	snp	C	124569381	AX746567
7	124569378	124569387	T	snp	C	124569381	BC142949
7	124569378	124569387	T	snp	C	124569381	BX648695
7	124569378	124569387	T	snp	C	124569381	POT1

7	126891389	126891398	T	snp	G	126891391	GRM8
7	128504835	128504845	T	snp	G	128504843	ATP6V1F
7	128504835	128504845	T	snp	G	128504843	ATP6V1F
7	128504835	128504845	T	snp	G	128504843	ATP6V1F
7	128504835	128504845	T	snp	G	128504843	KCP
7	128504835	128504845	T	snp	G	128504843	KCP
7	128504835	128504845	T	snp	G	128504843	KCP
7	129906353	129906361	T	snp	G	129906357	CPA2
7	129906353	129906361	T	snp	G	129906359	CPA2
7	134853043	134853053	C	snp	A	134853045	C7orf49
7	137790647	137790656	A	snp	C	137790649	AKR1D1
7	137790647	137790656	A	snp	C	137790652	AKR1D1
7	138767621	138767630	T	snp	G	138767626	ZC3HAV1
7	139026462	139026471	G	snp	T	139026464	C7orf55
7	139026462	139026471	G	snp	T	139026464	LUC7L2
7	139026462	139026471	G	snp	T	139026464	LUC7L2
7	139026462	139026471	G	snp	T	139026464	TRNA
7	139026462	139026471	G	snp	T	139026464	TRNA_Arg
7	139255028	139255037	G	snp	T	139255030	HIPK2
7	140049338	140049348	G	snp	T	140049341	SLC37A3
7	140101019	140101028	T	snp	C	140101023	AK131347
7	147074228	147074236	C	snp	A	147074234	CNTNAP2
7	147074228	147074236	C	snp	A	147074234	MIR548F4
7	147074228	147074236	C	snp	A	147074234	MIR548I4
7	153755561	153755570	G	snp	T	153755564	AK127966
7	153755561	153755570	G	snp	T	153755564	DPP6
7	154737178	154737187	A	snp	G	154737179	LOC100132707
7	154737178	154737187	A	snp	G	154737179	LOC100132707
7	154737178	154737187	A	snp	G	154737179	PAXIP1
7	154737178	154737187	A	snp	G	154737179	PAXIP1
8	1650528	1650537	A	snp	C	1650535	DLGAP2
8	1952170	1952178	A	snp	G	1952174	KBTBD11
8	2793300	2793308	T	snp	C	2793301	CSMD1
8	6260751	6260759	T	snp	G	6260752	LOC100287015
8	6390158	6390167	T	snp	C	6390161	ANGPT2
8	6390158	6390167	T	snp	C	6390161	MCPH1
8	6692706	6692714	G	snp	T	6692707	LOC100652791
8	6692706	6692714	G	snp	T	6692707	LOC100652791
8	6692706	6692714	G	snp	T	6692707	XKR5
8	7169616	7169626	G	snp	A	7169618	DEFB109P1B
8	7169616	7169626	G	snp	A	7169618	FAM66B
8	8654518	8654526	A	snp	C	8654520	MFHAS1
8	11929620	11929629	T	snp	G	11929624	LOC100133267

8	12176189	12176198	T	snp	G	12176193	LOC100133267
8	12176189	12176198	T	snp	G	12176193	LOC100506990
8	17271631	17271641	T	snp	G	17271633	MTMR7
8	17486422	17486431	T	snp	G	17486424	PDGFRL
8	22134253	22134261	T	snp	C	22134254	PIWIL2
8	22292005	22292014	A	snp	C	22292012	SLC39A14
8	23541373	23541381	T	snp	G	23541374	BC111574
8	23541373	23541381	T	snp	G	23541374	NKX3-1
8	23712167	23712176	T	snp	G	23712173	STC1
8	24770524	24770533	T	snp	G	24770528	AK308605
8	24770524	24770533	T	snp	G	24770528	NEFM
8	25324671	25324681	T	snp	C	25324677	CDCA2
8	25324671	25324681	T	snp	C	25324677	PPP2R2A
8	26264688	26264697	T	snp	G	26264692	BNIP3L
8	28970581	28970591	A	snp	G	28970585	AF086219
8	28970581	28970591	A	snp	G	28970585	KIF13B
8	35092779	35092788	G	snp	T	35092781	UNC5D
8	42402110	42402119	A	snp	G	42402111	C8orf40
8	59170348	59170356	T	snp	C	59170352	BC032030
8	59328609	59328617	T	snp	G	59328612	UBXN2B
8	62412294	62412303	T	snp	C	62412299	ASPH
8	62412294	62412303	T	snp	C	62412299	CLVS1
8	63162633	63162641	T	snp	G	63162635	NKAIN3
8	68985343	68985351	A	snp	C	68985347	PREX2
8	70414915	70414924	T	snp	C	70414918	SULF1
8	71572359	71572368	T	snp	C	71572364	LACTB2
8	71572359	71572368	T	snp	C	71572364	LOC286190
8	71581553	71581561	G	snp	T	71581558	LACTB2
8	71581553	71581561	G	snp	T	71581558	XKR9
8	72932593	72932602	A	snp	G	72932596	LOC100132891
8	72932593	72932602	A	snp	G	72932596	TRPA1
8	76190005	76190014	A	snp	G	76190007	BC062758
8	77595635	77595643	C	snp	A	77595641	LOC100192378
8	77595635	77595643	C	snp	A	77595641	ZFHX4
8	87570198	87570207	A	snp	C	87570203	CPNE3
8	92970148	92970156	A	snp	G	92970152	RUNX1T1
8	95182984	95182994	T	snp	C	95182985	CDH17
8	95777123	95777131	T	snp	C	95777125	DPY19L4
8	100588605	100588614	T	snp	G	100588609	VPS13B
8	101206173	101206183	T	snp	G	101206174	SPAG1
8	104389630	104389638	A	snp	C	104389635	CTHRC1
8	104479421	104479429	T	snp	C	104479422	BX641143
8	110566177	110566185	T	snp	G	110566183	EBAG9

8	113811440	113811449	A	snp	C	113811441	CSMD3
8	118326032	118326041	A	snp	C	118326037	SNORA31
8	131455461	131455470	C	snp	A	131455468	ASAP1
8	131811739	131811748	T	snp	C	131811741	ADCY8
8	133492917	133492926	C	snp	A	133492920	KCNQ3
8	133765016	133765026	A	snp	G	133765024	TMEM71
8	133960424	133960432	C	snp	T	133960429	TG
8	142443021	142443029	G	snp	T	142443022	FLJ43860
8	142490160	142490168	C	snp	T	142490165	FLJ43860
8	143425266	143425274	C	snp	A	143425270	TSNARE1
8	143620141	143620149	C	snp	T	143620145	BAI1
8	143621143	143621152	C	snp	A	143621149	BAI1
8	144406041	144406049	C	snp	A	144406042	TOP1MT
8	144669503	144669513	T	snp	G	144669511	EEF1D
8	145602159	145602167	C	snp	T	145602165	ADCK5
8	146004106	146004114	T	snp	C	146004110	ZNF34
9	2109787	2109795	T	snp	C	2109791	SMARCA2
9	4834291	4834301	T	snp	G	4834298	RCL1
9	14113901	14113911	A	snp	C	14113902	NFIB
9	14119892	14119900	A	snp	G	14119893	NFIB
9	15579739	15579747	T	snp	G	15579743	C9orf93
9	18904697	18904705	A	snp	C	18904698	ADAMTSL1
9	27283246	27283255	T	snp	C	27283248	LINC00032
9	34486442	34486450	A	snp	G	34486447	DNAI1
9	37523381	37523390	A	snp	C	37523382	FBXO10
9	74331368	74331377	A	snp	C	74331375	TMEM2
9	75243644	75243652	A	snp	G	75243649	TMC1
9	78639193	78639201	T	snp	C	78639195	PCSK5
9	82267109	82267119	T	snp	C	82267114	TLE4
9	88692059	88692069	A	snp	C	88692063	GOLM1
9	93640290	93640298	T	snp	C	93640293	SYK
9	94710845	94710854	C	snp	A	94710846	ROR2
9	94710845	94710854	C	snp	A	94710849	ROR2
9	95100597	95100607	A	snp	C	95100598	CENPP
9	100851192	100851200	T	snp	G	100851197	TRIM14
9	101894355	101894363	T	snp	G	101894360	TGFBR1
9	101985597	101985605	A	snp	C	101985601	SEC61B
9	107591112	107591120	T	snp	C	107591115	ABCA1
9	114130329	114130339	A	snp	G	114130333	KIAA0368
9	114178400	114178409	T	snp	G	114178401	KIAA0368
9	115448191	115448199	A	snp	C	115448194	C9orf80
9	115955547	115955555	T	snp	C	115955552	FKBP15
9	115973613	115973621	T	snp	G	115973619	FKBP15

9	116169299	116169307	A	snp	C	116169302	POLE3
9	116818031	116818039	A	snp	C	116818032	ZNF618
9	117880145	117880154	A	snp	G	117880148	TNC
9	125590010	125590019	A	snp	G	125590014	PDCL
9	125608620	125608628	T	snp	C	125608626	AL833455
9	129565990	129565999	A	snp	C	129565994	AX747444
9	129565990	129565999	A	snp	C	129565994	ZBTB43
9	130187860	130187869	T	snp	G	130187865	ZNF79
9	130251034	130251043	T	snp	G	130251036	LRSAM1
9	130700955	130700963	T	snp	C	130700959	DPM2
9	131456518	131456527	T	snp	G	131456519	SET
9	131456518	131456527	T	snp	G	131456519	SET
9	131456518	131456527	T	snp	G	131456519	Y16709
9	131456518	131456527	T	snp	G	131456520	SET
9	131456518	131456527	T	snp	G	131456520	SET
9	131456518	131456527	T	snp	G	131456520	Y16709
9	132576658	132576666	C	snp	A	132576664	TOR1A
9	133541150	133541158	C	snp	A	133541155	PRDM12
9	134006474	134006483	T	snp	G	134006480	NUP214
9	135157440	135157448	A	snp	G	135157442	SETX
9	136659826	136659834	G	snp	A	136659832	VAV2
9	137966925	137966935	C	snp	T	137966930	OLFM1
9	138456317	138456326	T	snp	C	138456322	PAEP
9	140499265	140499273	G	snp	T	140499266	ARRDC1
9	140632645	140632653	A	snp	C	140632650	EHMT1
X	14868798	14868808	A	snp	G	14868805	FANCB
X	19500369	19500379	A	snp	C	19500377	MAP3K15
X	41073729	41073739	A	snp	G	41073733	USP9X
X	47342912	47342921	C	snp	A	47342919	ZNF41
X	48435396	48435404	T	snp	C	48435401	RBM3
X	53675473	53675482	A	snp	C	53675477	HUWE1
X	53675483	53675492	A	snp	C	53675487	HUWE1
X	55246034	55246044	T	snp	G	55246040	PAGE5
X	67263222	67263232	A	snp	G	67263228	OPHN1
X	69642813	69642822	C	snp	A	69642820	GDPD2
X	70788709	70788719	A	snp	C	70788712	BCYRN1
X	70788709	70788719	A	snp	C	70788712	OGT
X	70838048	70838056	C	snp	T	70838053	BCYRN1
X	70838048	70838056	C	snp	T	70838053	BCYRN1
X	70838048	70838056	C	snp	T	70838053	CXCR3
X	70838048	70838056	C	snp	T	70838053	CXCR3
X	74743316	74743325	C	snp	A	74743323	ZDHHC15
X	84534381	84534391	A	snp	C	84534382	POF1B

X	100534956	100534966	A	snp	C	100534958	TAF7L
X	100630493	100630501	G	snp	A	100630499	BTK
X	107315483	107315491	T	snp	G	107315489	VSIG1
X	109439627	109439637	T	snp	G	109439633	AMMECR1
X	117750513	117750523	T	snp	G	117750521	DOCK11
X	119065169	119065179	A	snp	C	119065172	NKAP
X	119248491	119248500	A	snp	G	119248496	AK123976
X	119248491	119248500	A	snp	G	119248496	RHOXF1
X	149826092	149826102	A	snp	G	149826100	MTM1
X	153714027	153714037	G	snp	T	153714029	UBL4A
10	854664	854680	GT	snp	A	854674	LARP4B
10	1206093	1206107	AT	snp	C	1206101	LINC00200
10	5978790	5978800	AG	snp	G	5978794	FBXO18
10	16873849	16873861	TG	snp	G	16873853	CUBN
10	16979256	16979270	TG	snp	C	16979267	CUBN
10	32750680	32750692	AT	snp	G	32750688	CCDC7
10	46245305	46245319	TA	snp	T	46245310	FAM21C
10	49930702	49930712	CA	snp	T	49930708	WDFY4
10	55944220	55944232	TG	snp	A	55944223	PCDH15
10	72135329	72135347	AC	snp	G	72135332	LRRC20
10	72433020	72433038	GT	snp	A	72433024	ADAMTS14
10	73574402	73574418	AC	snp	G	73574404	CDH23
10	75203008	75203026	AG	snp	A	75203023	PPP3CB
10	81449164	81449174	AC	snp	T	81449168	LOC650623
10	87359559	87359577	AT	snp	C	87359566	GRID1
10	87359559	87359577	AT	snp	C	87359566	LOC100507470
10	88718828	88718838	CA	snp	A	88718832	SNCG
10	90500373	90500387	TG	snp	A	90500380	LIPK
10	90674434	90674452	AT	snp	A	90674439	STAMBPL1
10	91179688	91179698	TG	snp	A	91179693	IFIT5
10	97182355	97182365	GA	snp	C	97182361	SORBS1
10	102036079	102036089	CT	snp	C	102036084	BLOC1S2
10	103754146	103754156	AG	snp	A	103754149	C10orf76
10	112679142	112679152	GA	snp	G	112679149	BBIP1
10	112679142	112679152	GA	snp	G	112679149	SHOC2
10	115423329	115423339	CA	snp	G	115423334	NRAP
10	117308872	117308882	TA	snp	G	117308879	ATRNL1
10	135151344	135151356	AC	snp	G	135151348	CALY
10	135151344	135151356	AC	snp	G	135151348	ZNF511
11	320389	320403	CA	snp	T	320393	BC040735
11	320389	320403	CA	snp	T	320393	BC040735
11	320389	320403	CA	snp	T	320393	IFITM3
11	320389	320403	CA	snp	T	320393	IFITM3

11	614364	614374	TG	snp	C	614366	IRF7
11	1781783	1781795	TG	snp	C	1781789	CTSD
11	1781783	1781795	TG	snp	C	1781789	CTSD
11	1781783	1781795	TG	snp	C	1781789	MOB2
11	1781783	1781795	TG	snp	C	1781789	MOB2
11	8647114	8647128	AC	snp	T	8647123	TRIM66
11	10010622	10010634	AC	snp	G	10010628	SBF2
11	15502911	15502921	TG	snp	C	15502913	SnoMBII_202
11	17125164	17125178	TG	snp	A	17125167	PIK3C2A
11	34668638	34668652	CA	snp	C	34668641	EHF
11	34936814	34936824	TC	snp	A	34936819	APIP
11	34936814	34936824	TC	snp	A	34936819	PDHX
11	47361828	47361840	CA	snp	G	47361837	MYBPC3
11	57154521	57154535	GA	snp	C	57154529	PRG2
11	57822447	57822457	TG	snp	C	57822449	OR9Q1
11	58491264	58491276	TA	snp	G	58491267	GLYAT
11	58909399	58909415	AG	snp	A	58909412	BC028022
11	58909399	58909415	AG	snp	A	58909412	FAM111A
11	60292162	60292174	TC	snp	G	60292171	MS4A13
11	62429577	62429591	AT	snp	A	62429580	C11orf48
11	63232757	63232771	AG	snp	G	63232763	HRASLS5
11	66279474	66279490	AT	snp	A	66279477	BBS1
11	70275965	70275977	TG	snp	T	70275974	CTTN
11	74061964	74061982	AG	snp	A	74061979	PGM2L1
11	77376800	77376814	AC	snp	A	77376805	RSF1
11	83166708	83166722	GT	snp	G	83166719	DLG2
11	88910344	88910362	GA	snp	G	88910349	TYR
11	99828623	99828633	TA	snp	G	99828630	CNTN5
11	102987934	102987950	TA	snp	G	102987937	DYNC2H1
11	107926341	107926357	AT	snp	A	107926344	CUL5
11	110481750	110481760	TA	snp	G	110481755	ARHGAP20
11	117887072	117887088	AG	snp	T	117887083	LOC100526771
11	118852639	118852651	TG	snp	A	118852646	FOXR1
11	123066024	123066034	CT	snp	G	123066031	CLMP
11	125480643	125480657	CA	snp	A	125480653	STT3A
11	126327281	126327291	GT	snp	T	126327287	KIRREL3
11	128992824	128992834	AT	snp	C	128992827	ARHGAP32
12	3737212	3737224	CT	snp	C	3737215	EFCAB4B
12	6629357	6629373	AT	snp	A	6629360	NCAPD2
12	7047139	7047149	GC	snp	A	7047142	ATN1
12	7970335	7970349	AT	snp	G	7970342	SLC2A14
12	9021018	9021032	TG	snp	C	9021020	A2ML1
12	9310553	9310569	TC	snp	T	9310564	PZP

12	10168551	10168565	AC	snp	G	10168562	CLEC12B
12	10780601	10780611	TG	snp	T	10780608	STYK1
12	10871664	10871674	AC	snp	G	10871666	CSDA
12	11508875	11508885	TG	snp	C	11508878	PRB1
12	11548851	11548861	TG	snp	C	11548854	PRB2
12	15806751	15806761	TG	snp	A	15806756	EPS8
12	20889651	20889665	AC	snp	T	20889659	SLCO1C1
12	21477227	21477237	TC	snp	T	21477234	SLCO1A2
12	31299114	31299126	TA	snp	A	31299120	OVOS2
12	31299114	31299126	TA	snp	G	31299123	OVOS2
12	40940479	40940491	GT	snp	G	40940486	MUC19
12	75874076	75874090	TC	snp	T	75874087	GLIPR1
12	86272995	86273005	TA	snp	G	86273000	NTS
12	98896617	98896633	CA	snp	C	98896620	LOC643770
12	98896617	98896633	CA	snp	C	98896620	LOC643770
12	98896617	98896633	CA	snp	C	98896620	TRNA_Asp
12	102148288	102148300	GA	snp	A	102148290	GNPTAB
12	104300927	104300943	AG	snp	A	104300930	GNN
12	114385062	114385078	AC	snp	G	114385065	RBM19
12	124978766	124978780	AC	snp	G	124978772	NCOR2
13	23870300	23870310	AT	snp	G	23870302	SGCG
13	23945531	23945541	AT	snp	C	23945534	SACS
13	24241207	24241225	TG	snp	C	24241215	TNFRSF19
13	32798034	32798046	TG	snp	A	32798041	FRY
13	36920501	36920511	CG	snp	T	36920503	SPG20
13	36920501	36920511	CG	snp	T	36920503	SPG20
13	36920501	36920511	CG	snp	T	36920503	SPG20OS
13	36920501	36920511	CG	snp	T	36920503	SPG20OS
13	46155324	46155334	TA	snp	C	46155328	FAM194B
13	49934041	49934051	AT	snp	C	49934044	CAB39L
13	51077089	51077099	TA	snp	G	51077094	BCMS
13	51077089	51077099	TA	snp	G	51077094	BCMS
13	51077089	51077099	TA	snp	G	51077094	DLEU1
13	51077089	51077099	TA	snp	G	51077096	BCMS
13	51077089	51077099	TA	snp	G	51077096	BCMS
13	51077089	51077099	TA	snp	G	51077096	DLEU1
13	52684801	52684819	AC	snp	T	52684816	NEK5
13	76445400	76445418	TC	snp	G	76445412	AX747676
13	79933924	79933942	AT	snp	C	79933931	RBM26
13	88332062	88332072	TA	snp	C	88332066	SLITRK5
13	91150752	91150762	TC	snp	T	91150755	BC038529
13	99055777	99055787	GT	snp	T	99055781	AF339817
13	99055777	99055787	GT	snp	T	99055781	FARP1

13	111566723	111566737	CG	snp	G	111566731	ANKRD10
13	114289142	114289156	TG	snp	C	114289148	TFDP1
13	114757356	114757366	CA	snp	T	114757360	RASA3
14	21791945	21791963	GT	snp	A	21791947	RPGRIP1
14	36075603	36075613	TG	snp	A	36075610	RALGAPA1
14	60074632	60074644	AG	snp	T	60074635	RTN1
14	70988983	70988999	AG	snp	C	70988992	ADAM20
14	74340920	74340930	AT	snp	G	74340926	PTGR2
14	74340920	74340930	AT	snp	G	74340926	ZNF410
14	78325270	78325282	GT	snp	T	78325276	ADCK1
14	88634030	88634040	TG	snp	A	88634033	DQ599616
14	93360097	93360109	CT	snp	G	93360099	AK093301
14	95113997	95114011	TA	snp	C	95114007	SERPINA13
14	100604328	100604338	GA	snp	G	100604333	EVL
14	101378067	101378081	TA	snp	G	101378078	Mir_370
14	102030523	102030533	AG	snp	C	102030526	DIO3
14	102817131	102817145	TC	snp	G	102817134	CINP
14	104095763	104095773	CG	snp	T	104095768	KLC1
14	106913829	106913845	TC	snp	T	106913842	abParts
15	22709777	22709787	TC	snp	C	22709781	abParts
15	22709777	22709787	TC	snp	C	22709781	abParts
15	22709777	22709787	TC	snp	C	22709781	abParts
15	22709777	22709787	TC	snp	C	22709781	abParts
15	22709777	22709787	TC	snp	C	22709781	GOLGA8DP
15	22709777	22709787	TC	snp	C	22709781	GOLGA8DP
15	22709777	22709787	TC	snp	C	22709781	GOLGA8DP
15	22709777	22709787	TC	snp	C	22709781	GOLGA8DP
15	45701904	45701914	AT	snp	T	45701910	SPATA5L1
15	52028909	52028919	AC	snp	G	52028913	LYSMD2
15	54026348	54026364	AC	snp	A	54026357	WDR72
15	56726205	56726215	TA	snp	C	56726211	MNS1
15	56726205	56726215	TA	snp	C	56726211	TEX9
15	57973962	57973972	AG	snp	A	57973965	GCOM1
15	57973962	57973972	AG	snp	A	57973965	GCOM1
15	57973962	57973972	AG	snp	A	57973965	MYZAP
15	57973962	57973972	AG	snp	A	57973965	MYZAP
15	57973962	57973972	AG	snp	A	57973965	MYZAP
15	57973962	57973972	AG	snp	A	57973965	POLR2M
15	63030391	63030401	TC	snp	C	63030397	TLN2
15	75978614	75978630	CA	snp	G	75978619	CSPG4
15	76023634	76023646	AC	snp	G	76023636	DNM1P35
15	76023634	76023646	AC	snp	G	76023636	DNM1P35
15	76023634	76023646	AC	snp	G	76023636	ODF3L1

15	77765055	77765065	GT	snp	A	77765061	HMG20A
15	78450790	78450806	AT	snp	C	78450793	IDH3A
15	79031198	79031208	TA	snp	G	79031203	DQ586415
15	80036498	80036516	GA	snp	A	80036500	TRNA_Cys
15	84236453	84236467	GT	snp	C	84236463	SH3GL3
15	90544227	90544239	AG	snp	A	90544236	ZNF710
15	93443437	93443455	TC	snp	G	93443440	CHD2
16	420899	420909	AT	snp	C	420906	MRPL28
16	420899	420909	AT	snp	C	420906	TMEM8A
16	1114742	1114754	AG	snp	G	1114750	LOC146336
16	1389744	1389756	CA	snp	G	1389750	BAIAP3
16	1390533	1390547	CA	snp	C	1390544	BAIAP3
16	1657004	1657016	AC	snp	T	1657011	IFT140
16	3209542	3209560	TC	snp	T	3209545	TRNA_Pro
16	3529763	3529781	TG	snp	A	3529778	NAA60
16	4828806	4828818	AT	snp	C	4828809	SEPT12
16	8799116	8799132	GA	snp	C	8799120	ABAT
16	8799116	8799132	GA	snp	C	8799120	U7
16	11072519	11072529	GT	snp	C	11072524	CLEC16A
16	11815958	11815970	TC	snp	T	11815963	TXNDC11
16	17200820	17200834	GA	snp	G	17200829	XYLT1
16	18839166	18839176	TA	snp	A	18839170	SMG1
16	18862714	18862730	AC	snp	G	18862726	SMG1
16	20374770	20374782	GT	snp	C	20374779	PDILT
16	20482746	20482756	AC	snp	G	20482750	ACSM2A
16	27899918	27899930	TC	snp	T	27899925	GSG1L
16	48386254	48386266	TA	snp	G	48386263	LONP2
16	48386254	48386266	TA	snp	G	48386263	MIR548AE2
16	56602109	56602127	AG	snp	A	56602124	MT4
16	58429225	58429241	TA	snp	T	58429238	GINS3
16	58622173	58622183	GA	snp	A	58622177	CNOT1
16	68054784	68054798	TA	snp	G	68054787	DDX28
16	68054784	68054798	TA	snp	G	68054787	DUS2L
16	89705359	89705371	CA	snp	G	89705362	DPEP1
16	89980313	89980327	TG	snp	C	89980315	BC160930
17	6356444	6356462	CT	snp	T	6356454	PITPNM3
17	6558634	6558648	GT	snp	A	6558644	MIR4520A
17	6558634	6558648	GT	snp	A	6558644	MIR4520B
17	7644546	7644556	TC	snp	T	7644553	DNAH2
17	8300905	8300917	TC	snp	C	8300913	RNF222
17	8366124	8366136	TG	snp	C	8366128	NDEL1
17	9569130	9569140	AG	snp	G	9569136	USP43
17	9765507	9765517	GA	snp	C	9765513	GLP2R

17	10435819	10435831	AT	snp	C	10435822	AK097500
17	10435819	10435831	AT	snp	C	10435822	AK097500
17	10435819	10435831	AT	snp	C	10435822	AK097500
17	10435819	10435831	AT	snp	C	10435822	MYH2
17	10435819	10435831	AT	snp	C	10435822	MYH2
17	10435819	10435831	AT	snp	C	10435822	MYH2
17	15587339	15587349	CA	snp	G	15587344	TRIM16
17	16841921	16841931	AC	snp	G	16841923	TNFRSF13B
17	28943054	28943066	AT	snp	G	28943063	LRRC37BP1
17	29206419	29206437	TG	snp	A	29206432	ATAD5
17	33761368	33761382	AG	snp	A	33761371	SLFN13
17	34341995	34342011	TA	snp	G	34341998	CCL23
17	36627165	36627175	AT	snp	C	36627168	ARHGAP23
17	36669310	36669320	TA	snp	A	36669312	ARHGAP23
17	38506450	38506464	TG	snp	C	38506456	RARA
17	39136306	39136316	AG	snp	A	39136311	KRT40
17	40553660	40553674	GA	snp	A	40553670	PTRF
17	40557682	40557696	TC	snp	G	40557692	PTRF
17	41225765	41225783	TA	snp	T	41225780	BRCA1
17	41862429	41862443	AG	snp	A	41862432	C17orf105
17	43722505	43722521	TC	snp	G	43722508	C17orf69
17	43722505	43722521	TC	snp	G	43722508	CRHR1
17	45906662	45906680	AG	snp	A	45906665	MRPL10
17	48349383	48349395	AT	snp	G	48349391	TMEM92
17	48704796	48704808	TA	snp	C	48704799	CACNA1G
17	67160182	67160192	AT	snp	C	67160185	ABCA10
17	67214799	67214809	AT	snp	C	67214804	ABCA10
17	75879811	75879825	TC	snp	C	75879815	FLJ45079
17	77705883	77705897	AT	snp	G	77705893	ENPP7
17	78298596	78298606	TG	snp	T	78298599	RNF213
17	78316533	78316543	TA	snp	G	78316536	RNF213
17	80755143	80755153	GT	snp	A	80755147	TBCD
18	157911	157929	TA	snp	A	157921	USP14
18	3253617	3253635	GT	snp	A	3253627	MYL12A
18	7042093	7042105	CA	snp	G	7042098	LAMA1
18	21375681	21375691	AT	snp	T	21375687	LAMA3
18	28917679	28917695	AG	snp	A	28917692	DSG1
18	29782401	29782413	CA	snp	T	29782403	MEP1B
18	32918631	32918641	AG	snp	C	32918638	ZNF24
18	44112355	44112367	GT	snp	A	44112359	LOXHD1
18	45376875	45376885	TA	snp	G	45376880	SMAD2
18	51899680	51899692	AT	snp	A	51899689	C18orf54
18	56415612	56415624	TA	snp	G	56415618	MALT1

18	61583537	61583547	AT	snp	G	61583543	SERPINB10
18	61583537	61583547	AT	snp	G	61583543	SERPINB2
18	76752541	76752551	GC	snp	T	76752543	SALL3
18	76752541	76752551	GC	snp	T	76752544	SALL3
19	1085060	1085070	AG	snp	T	1085066	HMHA1
19	1085060	1085070	AG	snp	T	1085066	HMHA1
19	1085060	1085070	AG	snp	T	1085066	POLR2E
19	1085448	1085458	TC	snp	C	1085454	HMHA1
19	1085448	1085458	TC	snp	C	1085454	POLR2E
19	2645225	2645241	TC	snp	C	2645229	GNG7
19	4655695	4655707	AT	snp	C	4655702	TNFAIP8L1
19	5743678	5743692	CT	snp	G	5743687	TMEM146
19	6429071	6429085	TA	snp	C	6429073	SLC25A41
19	6589104	6589114	CT	snp	G	6589107	CD70
19	6663506	6663516	TG	snp	A	6663511	TNFSF14
19	6710559	6710575	GA	snp	A	6710571	C3
19	8199859	8199875	AT	snp	T	8199871	FBN3
19	8278946	8278960	CA	snp	G	8278955	CERS4
19	11409054	11409064	CA	snp	T	11409060	TSPAN16
19	12428304	12428314	TG	snp	G	12428306	ZNF563
19	13250334	13250348	GT	snp	A	13250339	NACC1
19	14847477	14847487	AT	snp	G	14847482	EMR2
19	17006695	17006711	GC	snp	T	17006699	CPAMD8
19	17056987	17057001	TG	snp	A	17056998	CPAMD8
19	20295140	20295156	TG	snp	A	20295145	ZNF486
19	20808511	20808521	AT	snp	A	20808514	ZNF626
19	21202563	21202573	AT	snp	A	21202566	ZNF430
19	21579139	21579151	TC	snp	T	21579142	ZNF493
19	23591621	23591637	AT	snp	A	23591624	AK022793
19	23591621	23591637	AT	snp	A	23591624	BC043213
19	29881999	29882011	AG	snp	A	29882002	LOC284395
19	33350474	33350484	AT	snp	G	33350478	JA660679
19	33350474	33350484	AT	snp	G	33350478	SLC7A9
19	33610965	33610977	TA	snp	T	33610974	GPATCH1
19	38826182	38826200	AT	snp	A	38826185	CATSPERG
19	41187573	41187587	AT	snp	G	41187583	NUMBL
19	41523250	41523260	TA	snp	G	41523253	CYP2A7
19	41523250	41523260	TA	snp	G	41523253	CYP2B6
19	42127484	42127494	CT	snp	A	42127487	CEACAM4
19	42570566	42570576	AG	snp	C	42570570	GRIK5
19	44841451	44841463	AT	snp	C	44841460	ZFP112
19	45032851	45032869	AC	snp	G	45032866	CEACAM20
19	49206105	49206115	CA	snp	G	49206107	FUT2

19	50733514	50733532	AG	snp	T	50733527	MYH14
19	50764144	50764156	TG	snp	C	50764146	MYH14
19	52223262	52223272	CT	snp	C	52223265	HAS1
19	52870808	52870822	GT	snp	A	52870814	ZNF610
19	53356602	53356612	AT	snp	C	53356609	ZNF468
19	54562173	54562183	AT	snp	C	54562176	VSTM1
19	55287689	55287707	TA	snp	C	55287693	KIR2DL1
19	55287689	55287707	TA	snp	C	55287693	KIR2DL2
19	55287689	55287707	TA	snp	C	55287693	KIR2DL3
19	55287689	55287707	TA	snp	C	55287693	KIR2DL3
19	55287689	55287707	TA	snp	C	55287693	KIR2DS4
19	55902251	55902261	GA	snp	G	55902256	RPL28
19	56006583	56006601	AT	snp	G	56006593	SSC5D
19	56187091	56187101	TC	snp	T	56187096	EPN1
19	58517205	58517217	TC	snp	A	58517212	LOC100128398
19	58546470	58546482	CA	snp	T	58546474	ZSCAN1
1	4002094	4002108	TC	snp	A	4002097	LOC728716
1	4475209	4475219	TC	snp	A	4475213	LOC284661
1	6604572	6604588	AC	snp	G	6604584	NOL9
1	12027697	12027715	TA	snp	G	12027706	PLOD1
1	18435092	18435102	GT	snp	A	18435094	IGSF21
1	22916667	22916677	AG	snp	A	22916670	EPHA8
1	27433853	27433865	AC	snp	T	27433858	SLC9A1
1	28056138	28056148	TC	snp	A	28056145	FAM76A
1	34083627	34083641	AG	snp	C	34083632	CSMD2
1	36180968	36180980	AC	snp	T	36180971	C1orf216
1	38185403	38185419	AC	snp	G	38185408	EPHA10
1	38221000	38221016	CA	snp	G	38221005	EPHA10
1	43031410	43031426	TG	snp	G	43031412	CCDC30
1	46093678	46093694	TG	snp	A	46093691	GPBP1L1
1	48648844	48648854	AT	snp	C	48648849	SKINTL
1	53109550	53109566	AG	snp	C	53109553	FAM159A
1	54509636	54509646	AT	snp	C	54509643	TMEM59
1	55189432	55189442	CA	snp	T	55189434	HEATR8-TTC4
1	55189432	55189442	CA	snp	T	55189434	TTC4
1	62732164	62732174	CA	snp	T	62732169	KANK4
1	62738005	62738015	AC	snp	T	62738012	KANK4
1	67358736	67358748	AG	snp	A	67358739	WDR78
1	67392604	67392616	TA	snp	G	67392613	MIER1
1	67558756	67558766	AT	snp	G	67558762	C1orf141
1	85562141	85562155	TA	snp	G	85562151	WDR63
1	86847116	86847132	AC	snp	G	86847120	ODF2L
1	89293354	89293368	CT	snp	A	89293362	PKN2

1	89293354	89293368	CT	snp	A	89293364	PKN2
1	92735353	92735363	AT	snp	C	92735358	GLMN
1	92788632	92788648	TA	snp	T	92788643	RPAP2
1	94219918	94219928	AG	snp	A	94219925	BCAR3
1	94219918	94219928	AG	snp	A	94219925	MIG7
1	94317477	94317493	GA	snp	G	94317490	AX746627
1	94549026	94549036	AT	snp	G	94549028	ABCA4
1	94696132	94696142	TA	snp	C	94696138	ARHGAP29
1	95293457	95293469	AG	snp	T	95293466	SLC44A3
1	111059812	111059830	GA	snp	A	111059824	KCNA10
1	115089079	115089093	AT	snp	G	115089090	DENND2C
1	116311366	116311382	CA	snp	G	116311379	CASQ2
1	145375100	145375112	TA	snp	C	145375102	AX747132
1	150319751	150319765	AC	snp	G	150319761	PRPF3
1	152850902	152850912	TG	snp	A	152850905	SMCP
1	152958830	152958842	AC	snp	T	152958839	SPRR1A
1	155268950	155268960	TC	snp	T	155268957	PKLR
1	157096246	157096258	AT	snp	G	157096252	ETV3
1	157665675	157665693	TC	snp	C	157665679	FCRL3
1	158747500	158747512	TC	snp	G	158747505	OR6N2
1	160156023	160156033	TC	snp	G	160156026	ATP1A4
1	160605138	160605150	GC	snp	T	160605143	SLAMF1
1	161590688	161590698	TA	snp	C	161590692	TRNA_Asn
1	161761054	161761064	AT	snp	G	161761061	ATF6
1	164558504	164558514	CA	snp	T	164558511	PBX1
1	169338318	169338330	TG	snp	A	169338325	BLZF1
1	175300235	175300245	TC	snp	T	175300238	TNR
1	176830821	176830835	GT	snp	A	176830825	ASTN1
1	177251785	177251795	GA	snp	A	177251791	FAM5B
1	182429363	182429381	TG	snp	A	182429374	RGSL1
1	183912570	183912580	GA	snp	A	183912574	GLT25D2
1	185926575	185926585	TA	snp	G	185926578	HMCN1
1	196747661	196747673	AT	snp	C	196747668	CFHR1
1	196747661	196747673	AT	snp	C	196747668	CFHR3
1	196747661	196747673	AT	snp	C	196747668	CFHR4
1	200310898	200310910	GT	snp	C	200310901	C1orf98
1	204966740	204966752	AC	snp	G	204966748	NFASC
1	211548493	211548503	GT	snp	T	211548499	TRAF5
1	214792669	214792683	TG	snp	T	214792672	CENPF
1	216404575	216404591	AT	snp	G	216404579	USH2A
1	216404575	216404591	AT	snp	G	216404581	USH2A
1	226818407	226818419	AG	snp	G	226818413	ITPKB
1	233482790	233482804	TG	snp	A	233482793	KIAA1804

1	237063497	237063507	AT	snp	G	237063503	MTR
1	246929093	246929103	CT	snp	G	246929099	SCCPDH
20	8866154	8866164	GA	snp	C	8866161	PLCB1
20	15001930	15001940	TG	snp	A	15001937	MACROD2
20	15001930	15001940	TG	snp	A	15001937	U6
20	15967530	15967544	GT	snp	A	15967532	MACROD2
20	19791508	19791522	TG	snp	T	19791511	BC090059
20	20209826	20209836	TC	snp	C	20209832	C20orf26
20	21346724	21346734	TA	snp	G	21346731	XRN2
20	23470913	23470925	TA	snp	A	23470917	CST8
20	29846756	29846770	AC	snp	T	29846761	DEFB115
20	30620662	30620672	TG	snp	C	30620668	C20orf160
20	32000344	32000354	AG	snp	G	32000350	SNTA1
20	37277265	37277275	TC	snp	T	37277270	ARHGAP40
20	46256785	46256799	TC	snp	G	46256796	NCOA3
20	50809374	50809384	CT	snp	T	50809380	ZFP64
20	61524125	61524135	AC	snp	T	61524132	DIDO1
21	17554887	17554903	TC	snp	T	17554900	LINC00478
21	35883738	35883752	TC	snp	A	35883742	KCNE1
21	35883738	35883752	TC	snp	G	35883743	KCNE1
21	42709734	42709748	GT	snp	C	42709736	FAM3B
21	43987477	43987487	AG	snp	G	43987479	SLC37A1
21	46066315	46066325	CA	snp	T	46066318	KRTAP10-11
21	46066315	46066325	CA	snp	T	46066318	TSPEAR
21	46116859	46116869	AC	snp	G	46116863	KRTAP10-12
21	46116859	46116869	AC	snp	G	46116863	TSPEAR
21	46281994	46282008	GT	snp	A	46281997	PTTG1IP
22	17150673	17150689	CT	snp	T	17150675	ANKRD62P1-PARP4P3
22	17150673	17150689	CT	snp	T	17150675	ANKRD62P1-PARP4P3
22	17150673	17150689	CT	snp	T	17150675	TPTEP1
22	17150673	17150689	CT	snp	T	17150675	TPTEP1
22	17663801	17663811	AC	snp	G	17663803	CECR1
22	20656255	20656271	TA	snp	T	20656268	AK129567
22	20656255	20656271	TA	snp	T	20656268	AK302545
22	23024808	23024818	TG	snp	C	23024814	abParts
22	23024808	23024818	TG	snp	C	23024814	abParts
22	23024808	23024818	TG	snp	C	23024814	DKFZp667J0810
22	23024808	23024818	TG	snp	C	23024814	DKFZp667J0810
22	24096939	24096957	CA	snp	G	24096942	VPREB3
22	25587924	25587934	TA	snp	G	25587929	KIAA1671
22	29075763	29075773	GC	snp	T	29075768	TTC28
22	30151315	30151329	GA	snp	G	30151326	ZMAT5
22	30187694	30187710	AC	snp	T	30187701	ASCC2

22	30407378	30407390	TC	snp	G	30407387	MTMR3
22	32545637	32545655	AT	snp	A	32545640	C22orf42
22	37710562	37710572	GA	snp	T	37710568	CYTH4
22	44554942	44554954	GA	snp	C	44554951	PARVB
22	45128756	45128766	CA	snp	T	45128762	ARHGAP8
22	45128756	45128766	CA	snp	T	45128762	ARHGAP8
22	45128756	45128766	CA	snp	T	45128762	PRR5
22	45128756	45128766	CA	snp	T	45128762	PRR5
22	45128756	45128766	CA	snp	T	45128762	PRR5-ARHGAP8
22	45128756	45128766	CA	snp	T	45128762	PRR5-ARHGAP8
22	45920985	45921001	AG	snp	A	45920998	FBLN1
22	47065462	47065476	TC	snp	G	47065473	GRAMD4
22	48938527	48938537	GT	snp	C	48938533	FAM19A5
22	48938527	48938537	GT	snp	C	48938533	LOC284933
22	51010833	51010843	TC	snp	A	51010837	BC048192
22	51010833	51010843	TC	snp	A	51010837	CHKB
22	51010833	51010843	TC	snp	A	51010837	CHKB
22	51010833	51010843	TC	snp	A	51010837	CHKB-CPT1B
22	51010833	51010843	TC	snp	A	51010837	CHKB-CPT1B
22	51010833	51010843	TC	snp	A	51010837	CPT1B
22	51010833	51010843	TC	snp	A	51010837	CPT1B
2	10138727	10138737	AT	snp	C	10138732	GRHL1
2	10919554	10919568	AT	snp	C	10919557	ATP6V1C2
2	11810711	11810723	TG	snp	C	11810715	NTSR2
2	20823851	20823861	CT	snp	T	20823857	HS1BP3
2	20900827	20900837	TG	snp	C	20900831	C2orf43
2	20900827	20900837	TG	snp	A	20900832	C2orf43
2	27527129	27527145	AT	snp	G	27527142	TRIM54
2	33621252	33621266	GT	snp	A	33621262	LTBP1
2	39054699	39054713	AC	snp	T	39054702	DHX57
2	39412502	39412512	AT	snp	G	39412506	CDKL4
2	39412556	39412566	AT	snp	G	39412560	CDKL4
2	54082255	54082269	CT	snp	C	54082260	GPR75
2	54082255	54082269	CT	snp	C	54082260	GPR75-ASB3
2	55407296	55407312	AT	snp	G	55407306	C2orf63
2	55516585	55516597	GA	snp	C	55516587	CCDC88A
2	63169162	63169172	AC	snp	T	63169165	EHBP1
2	66667181	66667199	CG	snp	T	66667185	MEIS1
2	87114260	87114270	TA	snp	G	87114263	LOC100286979
2	87114260	87114270	TA	snp	G	87114263	LOC100286979
2	87114260	87114270	TA	snp	G	87114263	RMND5A
2	87114260	87114270	TA	snp	G	87114263	RMND5A
2	89373790	89373800	CT	snp	G	89373792	abParts

2	95511833	95511847	TA	snp	C	95511843	ANKRD20A8P
2	99775861	99775879	TC	snp	G	99775864	LIPT1
2	99775861	99775879	TC	snp	G	99775864	MRPL30
2	101878878	101878890	TC	snp	T	101878881	C2orf29
2	102809526	102809540	CA	snp	G	102809533	IL1RL2
2	109092543	109092555	TC	snp	G	109092545	GCC2
2	119912803	119912819	AC	snp	G	119912813	C1QL2
2	132266731	132266741	CT	snp	A	132266737	LOC150776
2	150425404	150425422	AT	snp	C	150425419	MMADHC
2	160054062	160054072	AC	snp	T	160054065	TANC1
2	160731224	160731234	AT	snp	C	160731231	LY75
2	160731224	160731234	AT	snp	C	160731231	LY75
2	160731224	160731234	AT	snp	C	160731231	LY75-CD302
2	166768165	166768179	AT	snp	T	166768175	TTC21B
2	172415019	172415029	TA	snp	T	172415024	CYBRD1
2	179192180	179192190	AG	snp	T	179192187	OSBPL6
2	183829231	183829241	TA	snp	C	183829235	NCKAP1
2	187501641	187501651	AT	snp	G	187501647	ITGAV
2	187702356	187702366	TA	snp	G	187702363	ZSWIM2
2	197586077	197586093	AT	snp	C	197586084	CCDC150
2	197862145	197862159	TA	snp	C	197862153	ANKRD44
2	198669102	198669114	CG	snp	A	198669109	PLCL1
2	200800490	200800500	AC	snp	T	200800497	TYW5
2	201347489	201347505	TG	snp	T	201347492	SPATS2L
2	201644768	201644778	AC	snp	A	201644771	AOX2P
2	202213377	202213387	CA	snp	A	202213381	ALS2CR12
2	208443361	208443375	TG	snp	A	208443371	CREB1
2	220502040	220502050	TG	snp	C	220502042	SLC4A3
2	234682051	234682061	CA	snp	A	234682055	UGT1A1
2	234682051	234682061	CA	snp	A	234682055	UGT1A10
2	234682051	234682061	CA	snp	A	234682055	UGT1A3
2	234682051	234682061	CA	snp	A	234682055	UGT1A4
2	234682051	234682061	CA	snp	A	234682055	UGT1A5
2	234682051	234682061	CA	snp	A	234682055	UGT1A6
2	234682051	234682061	CA	snp	A	234682055	UGT1A7
2	234682051	234682061	CA	snp	A	234682055	UGT1A8
2	234682051	234682061	CA	snp	A	234682055	UGT1A9
2	234682051	234682061	CA	snp	A	234682055	UGT1A9
3	8681721	8681731	CA	snp	G	8681723	C3orf32
3	13759382	13759398	CT	snp	G	13759385	LOC285375
3	20054341	20054351	TA	snp	C	20054345	PP2D1
3	20141251	20141267	AG	snp	C	20141264	KAT2B
3	20160263	20160273	AT	snp	T	20160265	KAT2B

3	32030099	32030113	CT	snp	C	32030110	ZNF860
3	32579429	32579439	AT	snp	T	32579431	DYNC1LI1
3	38173808	38173820	TC	snp	C	38173812	ACAA1
3	42601672	42601682	AT	snp	C	42601677	SEC22C
3	42741498	42741516	TG	snp	C	42741511	HHATL
3	45808193	45808207	AG	snp	C	45808204	SLC6A20
3	50306629	50306639	CA	snp	T	50306631	SEMA3B
3	52273418	52273428	GC	snp	A	52273420	BC039681
3	52273418	52273428	GC	snp	A	52273420	TWF2
3	53125466	53125476	GA	snp	A	53125468	RFT1
3	53767939	53767955	TG	snp	A	53767942	CACNA1D
3	65479523	65479533	GA	snp	C	65479529	MAGI1
3	82513668	82513684	TA	snp	A	82513678	BC031255
3	82513740	82513750	TA	snp	A	82513744	BC031255
3	100977407	100977421	CT	snp	T	100977409	IMPG2
3	108116832	108116844	CA	snp	T	108116834	MYH15
3	108288015	108288029	TA	snp	C	108288025	KIAA1524
3	108705692	108705704	AT	snp	C	108705701	MORC1
3	113898519	113898529	TC	snp	G	113898524	DRD3
3	123946955	123946965	TG	snp	A	123946962	KALRN
3	124483081	124483091	TC	snp	G	124483088	ITGB5
3	132077637	132077647	TG	snp	A	132077644	ACPP
3	138007032	138007044	TG	snp	A	138007037	ARMC8
3	138007032	138007044	TG	snp	A	138007037	NME9
3	141688115	141688129	AT	snp	G	141688119	TFDP2
3	142053553	142053563	TA	snp	G	142053560	XRN1
3	142150734	142150744	AT	snp	G	142150736	XRN1
3	145820681	145820693	GT	snp	A	145820683	PLOD2
3	162920228	162920240	CA	snp	G	162920237	BC073807
3	162920228	162920240	CA	snp	G	162920237	LOC647107
3	167251342	167251352	GT	snp	C	167251347	WDR49
3	167507447	167507457	AG	snp	G	167507451	SERPINI1
3	169830167	169830183	TA	snp	C	169830173	PHC3
3	171323297	171323307	AT	snp	C	171323304	PLD1
3	182584708	182584720	AT	snp	C	182584715	ATP11B
4	653361	653371	GT	snp	A	653363	PDE6B
4	1221130	1221140	GT	snp	C	1221135	CTBP1
4	3517738	3517748	GA	snp	C	3517745	LRPAP1
4	6272300	6272310	CT	snp	C	6272305	WFS1
4	8039021	8039031	CA	snp	G	8039028	ABLIM2
4	12249079	12249089	TG	snp	C	12249084	BC042433
4	14473175	14473185	GT	snp	A	14473179	BC070495
4	14473175	14473185	GT	snp	A	14473179	MGC4836

4	15706189	15706199	TG	snp	A	15706192	BST1
4	17632830	17632844	AT	snp	C	17632833	CR936688
4	17632830	17632844	AT	snp	C	17632833	FAM184B
4	28821507	28821517	TC	snp	A	28821511	MIR4275
4	42456678	42456696	GT	snp	A	42456680	ATP8A1
4	48135774	48135786	TA	snp	G	48135782	TXK
4	48169242	48169252	AT	snp	G	48169248	TEC
4	53728367	53728377	CG	snp	A	53728373	RASL11B
4	55163821	55163837	TG	snp	G	55163825	PDGFRA
4	68356609	68356619	AT	snp	G	68356614	CENPC1
4	71256793	71256803	AT	snp	G	71256799	SMR3B
4	73960354	73960364	AC	snp	T	73960359	ANKRD17
4	82025815	82025827	TA	snp	G	82025824	PRKG2
4	83787302	83787314	AG	snp	G	83787306	SEC31A
4	88402966	88402976	AC	snp	G	88402968	SPARCL1
4	89238363	89238373	TG	snp	C	89238368	BC027846
4	91839789	91839805	AT	snp	A	91839792	FAM190A
4	104120033	104120043	TC	snp	T	104120040	CENPE
4	105416876	105416886	TC	snp	C	105416880	AK094561
4	105416876	105416886	TC	snp	C	105416880	CXXC4
4	110611352	110611368	AC	snp	T	110611354	CASP6
4	113349284	113349294	TA	snp	G	113349287	ALPK1
4	123161611	123161621	GA	snp	G	123161618	KIAA1109
4	128814957	128814967	GA	snp	T	128814963	PLK4
4	132649186	132649196	TG	snp	C	132649192	BC131768
4	134062199	134062209	AT	snp	C	134062203	BC040219
4	141075643	141075653	GT	snp	A	141075647	MAML3
4	146081090	146081100	AC	snp	T	146081095	OTUD4
4	165960294	165960304	TA	snp	C	165960296	TRIM60
4	177189807	177189817	AT	snp	G	177189809	ASB5
4	185696726	185696736	GA	snp	A	185696732	ACSL1
4	187154075	187154089	TG	snp	C	187154081	KLKB1
4	187178952	187178962	CA	snp	G	187178959	KLKB1
4	187195788	187195802	CT	snp	C	187195795	F11
5	462731	462745	CA	snp	T	462733	EXOC3
5	640701	640711	CA	snp	G	640704	CEP72
5	7788815	7788825	TG	snp	A	7788822	ADCY2
5	7835065	7835081	AC	snp	T	7835072	C5orf49
5	13862962	13862972	AT	snp	G	13862968	DNAH5
5	17812200	17812210	AT	snp	C	17812203	BC028204
5	17812212	17812222	AT	snp	C	17812215	BC028204
5	17812224	17812234	AT	snp	C	17812227	BC028204
5	17812236	17812246	AT	snp	C	17812239	BC028204

5	17812248	17812258	AT	snp	C	17812251	BC028204
5	17812260	17812270	AT	snp	C	17812263	BC028204
5	17812272	17812282	AT	snp	G	17812274	BC028204
5	17812272	17812282	AT	snp	C	17812275	BC028204
5	17812284	17812294	AT	snp	C	17812287	BC028204
5	17812296	17812306	AT	snp	C	17812299	BC028204
5	17812460	17812472	AT	snp	G	17812464	BC028204
5	40936899	40936911	GA	snp	C	40936907	C7
5	56527830	56527840	TG	snp	A	56527833	GPBP1
5	60998968	60998982	TC	snp	G	60998977	C5orf64
5	67597325	67597335	AT	snp	G	67597329	PIK3R1
5	72200627	72200637	CA	snp	G	72200630	TNPO1
5	72800589	72800599	AT	snp	C	72800596	BTF3
5	75998482	75998492	CT	snp	G	75998487	IQGAP2
5	76371806	76371816	TA	snp	G	76371811	ZBED3
5	76371826	76371836	TA	snp	G	76371829	ZBED3
5	76371826	76371836	TA	snp	G	76371831	ZBED3
5	76371846	76371856	TA	snp	G	76371849	ZBED3
5	85577431	85577443	AT	snp	C	85577436	NBPF22P
5	94289873	94289885	AT	snp	G	94289875	MCTP1
5	94826193	94826209	AT	snp	T	94826205	TTC37
5	96209831	96209841	AG	snp	A	96209838	AK094985
5	98203262	98203278	TG	snp	A	98203271	CHD1
5	101607313	101607325	AT	snp	C	101607316	SLCO4C1
5	109757117	109757133	AT	snp	A	109757120	TMEM232
5	118605129	118605145	TG	snp	A	118605138	TNFAIP8
5	121977815	121977825	TA	snp	C	121977819	BC043373
5	127301428	127301438	TA	snp	G	127301431	FLJ33630
5	128798292	128798310	TG	snp	G	128798306	ADAMTS19
5	133509745	133509755	AG	snp	T	133509751	SKP1
5	140264852	140264862	AT	snp	G	140264854	PCDHA1
5	140264852	140264862	AT	snp	G	140264854	PCDHA10
5	140264852	140264862	AT	snp	G	140264854	PCDHA11
5	140264852	140264862	AT	snp	G	140264854	PCDHA12
5	140264852	140264862	AT	snp	G	140264854	PCDHA13
5	140264852	140264862	AT	snp	G	140264854	PCDHA13
5	140264852	140264862	AT	snp	G	140264854	PCDHA2
5	140264852	140264862	AT	snp	G	140264854	PCDHA3
5	140264852	140264862	AT	snp	G	140264854	PCDHA4
5	140264852	140264862	AT	snp	G	140264854	PCDHA5
5	140264852	140264862	AT	snp	G	140264854	PCDHA6
5	140264852	140264862	AT	snp	G	140264854	PCDHA7
5	140264852	140264862	AT	snp	G	140264854	PCDHA8

5	140264852	140264862	AT	snp	G	140264854	PCDHA9
5	147502947	147502961	TA	snp	G	147502957	SPINK5
5	147506887	147506897	TG	snp	A	147506890	SPINK5
5	147695645	147695657	AC	snp	T	147695650	AK054753
5	147695645	147695657	AC	snp	T	147695650	SPINK7
5	150845256	150845266	AG	snp	A	150845259	SLC36A1
5	167833971	167833987	AG	snp	A	167833974	WWC1
5	168097577	168097589	AC	snp	T	168097582	SLIT3
5	175386580	175386592	TA	snp	G	175386585	THOC3
5	177165017	177165027	GT	snp	C	177165022	FAM153A
6	2663625	2663641	TC	snp	G	2663628	MYLK4
6	4041976	4041990	CA	snp	G	4041987	PRPF4B
6	8652020	8652030	GA	snp	C	8652024	HULC
6	8652020	8652030	GA	snp	C	8652024	LOC100506207
6	10872857	10872867	GT	snp	C	10872862	GCM2
6	10872857	10872867	GT	snp	C	10872862	SYCP2L
6	11139182	11139196	AC	snp	G	11139188	AK129879
6	11139182	11139196	AC	snp	G	11139188	C6orf228
6	11736743	11736753	AC	snp	T	11736746	C6orf105
6	17986440	17986450	AT	snp	G	17986446	KIF13A
6	24358933	24358943	TA	snp	C	24358939	DCDC2
6	24358933	24358943	TA	snp	C	24358939	KAAG1
6	25600229	25600243	TA	snp	G	25600232	LRRC16A
6	32485533	32485543	AG	snp	C	32485538	HLA-DRB5
6	32485533	32485543	AG	snp	C	32485540	HLA-DRB5
6	32974393	32974405	TG	snp	T	32974400	HLA-DOA
6	36285782	36285792	GA	snp	C	36285788	C6orf222
6	37666042	37666052	CT	snp	G	37666047	MDGA1
6	38975723	38975739	CA	snp	C	38975726	DNAH8
6	39048704	39048716	AC	snp	T	39048707	GLP1R
6	43151475	43151487	CT	snp	T	43151483	CUL9
6	46792630	46792640	TA	snp	T	46792637	MEP1A
6	51936843	51936853	TG	snp	C	51936845	PKHD1
6	55407646	55407658	AT	snp	C	55407651	HMGCLL1
6	56881876	56881886	AT	snp	G	56881882	BEND6
6	66052936	66052948	AT	snp	G	66052938	EYS
6	73952900	73952910	TC	snp	G	73952905	KHDC1
6	84799293	84799303	GA	snp	A	84799299	MRAP2
6	105175387	105175397	AC	snp	T	105175390	HACE1
6	105175483	105175493	AC	snp	T	105175486	HACE1
6	105198750	105198760	AT	snp	C	105198753	HACE1
6	111693077	111693087	AG	snp	C	111693082	REV3L
6	122772921	122772939	AT	snp	A	122772924	SERINC1

6	129854347	129854357	GA	snp	C	129854354	BC035400
6	137320597	137320607	TA	snp	G	137320602	IL20RA
6	143083496	143083506	TA	snp	C	143083502	HIVEP2
6	149783539	149783553	CA	snp	T	149783543	ZC3H12D
6	152127701	152127711	TG	snp	G	152127707	ESR1
6	152731556	152731574	AC	snp	A	152731559	SYNE1
6	166822008	166822026	TG	snp	T	166822017	RPS6KA2
7	5518323	5518333	CA	snp	G	5518330	FBXL18
7	6049605	6049623	AT	snp	G	6049619	AIMP2
7	6049605	6049623	AT	snp	G	6049619	PMS2
7	16842375	16842385	AT	snp	C	16842380	AGR2
7	22856799	22856809	TA	snp	C	22856803	TOMM7
7	29729590	29729604	AC	snp	T	29729601	DPY19L2P3
7	31681404	31681414	AC	snp	G	31681407	CCDC129
7	35671888	35671898	AT	snp	T	35671892	HERPUD2
7	36436540	36436552	CA	snp	T	36436546	ANLN
7	39744767	39744779	AC	snp	C	39744775	RALA
7	47866629	47866639	TG	snp	G	47866635	C7orf69
7	47866629	47866639	TG	snp	G	47866635	PKD1L1
7	56125054	56125072	GT	snp	T	56125058	CCT6A
7	56125054	56125072	GT	snp	T	56125058	CCT6A
7	56125054	56125072	GT	snp	T	56125058	CCT6A
7	56125054	56125072	GT	snp	T	56125058	PSPH
7	56125054	56125072	GT	snp	T	56125058	PSPH
7	56125054	56125072	GT	snp	T	56125058	PSPH
7	66479589	66479601	AC	snp	G	66479596	TYW1
7	72510826	72510836	AT	snp	C	72510829	FKBP6
7	72510826	72510836	AT	snp	C	72510829	FKBP6
7	72510826	72510836	AT	snp	C	72510829	FKBP6
7	72510826	72510836	AT	snp	C	72510829	PMS2L2
7	72510826	72510836	AT	snp	C	72510829	PMS2L2
7	72510826	72510836	AT	snp	C	72510829	PMS2L2
7	72510826	72510836	AT	snp	C	72510829	PMS2L2
7	72510854	72510864	AT	snp	C	72510861	FKBP6
7	72510854	72510864	AT	snp	C	72510861	FKBP6
7	72510854	72510864	AT	snp	C	72510861	FKBP6
7	72510854	72510864	AT	snp	C	72510861	PMS2L2
7	72510854	72510864	AT	snp	C	72510861	PMS2L2
7	72510854	72510864	AT	snp	C	72510861	PMS2L2
7	72510854	72510864	AT	snp	C	72510861	PMS2L2
7	73801563	73801579	TC	snp	G	73801567	CLIP2
7	73802657	73802673	CA	snp	A	73802669	CLIP2
7	73974911	73974925	AG	snp	T	73974914	GTF2IRD1

7	74982114	74982124	TA	snp	C	74982118	PMS2L2
7	79829210	79829220	TG	snp	A	79829213	GNAI1
7	86978344	86978354	CT	snp	A	86978346	CROT
7	96649515	96649529	CA	snp	A	96649521	DLX5
7	101259809	101259819	AT	snp	C	101259814	MYL10
7	111366519	111366529	TA	snp	G	111366526	DOCK4
7	111387986	111387998	AT	snp	G	111387990	DOCK4
7	111639401	111639411	AT	snp	C	111639406	DOCK4
7	114765092	114765102	TA	snp	A	114765098	BC022431
7	115893893	115893903	GT	snp	C	115893896	BD495725
7	115893893	115893903	GT	snp	C	115893896	TES
7	122055871	122055881	AC	snp	G	122055873	CADPS2
7	136912990	136913004	TG	snp	C	136913000	PTN
7	137585923	137585933	GT	snp	A	137585927	CREB3L2
7	141431584	141431594	CT	snp	G	141431591	FLJ40852
7	141431584	141431594	CT	snp	G	141431591	WEE2
7	141431632	141431642	CT	snp	G	141431639	FLJ40852
7	141431632	141431642	CT	snp	G	141431639	WEE2
7	142345171	142345185	CT	snp	T	142345181	TCRBV10S1P
7	142345171	142345185	CT	snp	T	142345181	TCRBV10S1P
7	142345171	142345185	CT	snp	T	142345181	TCRBV2S1
7	142345171	142345185	CT	snp	T	142345181	TCRBV2S1
7	142345171	142345185	CT	snp	T	142345181	TCRBV5S1A1T
7	142345171	142345185	CT	snp	T	142345181	TCRBV5S1A1T
7	142345171	142345185	CT	snp	T	142345181	TCRVB
7	142345171	142345185	CT	snp	T	142345181	TCRVB
7	142373903	142373913	CA	snp	G	142373906	MTRNR2L6
7	142373903	142373913	CA	snp	G	142373906	TCRBV19S1P
7	142373903	142373913	CA	snp	G	142373906	TCRBV2S1
7	142373903	142373913	CA	snp	G	142373906	TCRBV5S1A1T
7	142373903	142373913	CA	snp	G	142373906	TCRVB
7	155189224	155189236	CA	snp	G	155189231	BC150495
7	155531072	155531084	CA	snp	G	155531079	RBM33
8	3165467	3165477	TC	snp	A	3165470	CSMD1
8	3224463	3224479	AC	snp	C	3224475	CSMD1
8	7841922	7841940	GT	snp	C	7841926	FAM66E
8	16977994	16978004	TA	snp	G	16977999	EFHA2
8	20040422	20040432	AC	snp	G	20040426	SLC18A1
8	20040422	20040432	AC	snp	A	20040427	SLC18A1
8	20040705	20040721	TG	snp	C	20040711	SLC18A1
8	29605635	29605645	GA	snp	A	29605639	BC015784
8	29605635	29605645	GA	snp	A	29605639	BC082237
8	29605635	29605645	GA	snp	A	29605639	C8orf75

8	37591002	37591016	GT	snp	T	37591012	BC031939
8	53130772	53130782	CT	snp	A	53130778	ST18
8	62467822	62467832	TA	snp	C	62467828	ASPH
8	82395812	82395826	TG	snp	G	82395822	FABP4
8	82597940	82597952	TA	snp	C	82597942	IMPA1
8	87680828	87680838	TG	snp	C	87680832	CNGB3
8	93728091	93728103	AG	snp	A	93728096	AK128161
8	95678307	95678317	TA	snp	C	95678311	ESRP1
8	100222830	100222846	AT	snp	A	100222833	VPS13B
8	120258392	120258404	CA	snp	T	120258396	MAL2
8	120576923	120576941	AT	snp	A	120576926	ENPP2
8	133758698	133758708	AT	snp	C	133758703	TMEM71
8	135847441	135847453	TG	snp	A	135847446	Mir_652
8	139207902	139207916	AC	snp	T	139207909	FAM135B
8	140999515	140999531	TG	snp	C	140999527	TRAPPC9
9	313761	313773	CA	snp	G	313766	DOCK8
9	2524897	2524911	AG	snp	C	2524902	FLJ35024
9	3271553	3271563	TC	snp	T	3271560	RFX3
9	18721912	18721922	TG	snp	T	18721917	ADAMTSL1
9	27331466	27331482	TG	snp	C	27331476	MOB3B
9	33900925	33900941	TC	snp	G	33900930	UBE2R2
9	36608414	36608430	TA	snp	G	36608425	MELK
9	38425030	38425040	AC	snp	G	38425037	IGFBPL1
9	71819652	71819666	GT	snp	T	71819662	TJP2
9	72374929	72374947	GC	snp	A	72374940	PTAR1
9	75355766	75355778	AT	snp	C	75355769	TMC1
9	75369954	75369970	AG	snp	G	75369966	TMC1
9	78784457	78784469	TA	snp	G	78784466	PCSK5
9	80038193	80038205	CA	snp	G	80038195	GNA14
9	94973631	94973647	TC	snp	A	94973643	AK127087
9	94973631	94973647	TC	snp	A	94973643	IARS
9	113697146	113697156	GA	snp	A	113697148	LPAR1
9	113697146	113697156	GA	snp	A	113697148	Y_RNA
9	114125307	114125321	GA	snp	A	114125317	KIAA0368
9	114996229	114996243	AG	snp	C	114996240	MIR3134
9	114996229	114996243	AG	snp	C	114996240	PTBP3
9	117693496	117693506	GT	snp	C	117693499	TNFSF8
9	118165174	118165184	TA	snp	C	118165180	DEC1
9	123164276	123164286	TA	snp	T	123164283	CDK5RAP2
9	125158424	125158440	AT	snp	G	125158430	PTGS1
9	130628518	130628530	GT	snp	A	130628523	AK1
9	131133976	131133986	CT	snp	A	131133982	URM1
9	131598530	131598548	TC	snp	T	131598533	CCBL1

9	138902786	138902798	CA	snp	G	138902789	NACC2
X	217129	217145	TG	snp	A	217136	PLCXD1
X	1425057	1425067	GA	snp	C	1425063	CRLF2
X	1425057	1425067	GA	snp	C	1425063	CRLF2
X	1425057	1425067	GA	snp	C	1425063	CSF2RA
X	1425057	1425067	GA	snp	C	1425063	CSF2RA
X	1762343	1762355	TC	snp	T	1762352	ASMT
X	37961217	37961233	GT	snp	C	37961230	SYTL5
X	55511947	55511965	AT	snp	A	55511950	USP51
X	55757773	55757783	TA	snp	G	55757776	RRAGB
X	69672455	69672465	GA	snp	C	69672461	DLG3
X	70117066	70117076	AT	snp	T	70117072	TEX11
X	85236210	85236224	TA	snp	T	85236213	CHM
X	109932289	109932299	AT	snp	C	109932294	CHRDL1
X	114397900	114397910	CA	snp	T	114397904	LRCH2
X	114796415	114796425	GA	snp	G	114796420	AK127380
X	114796415	114796425	GA	snp	G	114796420	AK127380
X	114796415	114796425	GA	snp	G	114796420	AK127380
X	114796415	114796425	GA	snp	G	114796420	PLS3
X	114796415	114796425	GA	snp	G	114796420	PLS3
X	114796415	114796425	GA	snp	G	114796420	PLS3
X	117580383	117580393	AT	snp	T	117580385	WDR44
X	130433073	130433085	AG	snp	G	130433081	IGSF1
X	135721657	135721675	AT	snp	A	135721660	LOC100128420
X	147002979	147002995	TA	snp	G	147002991	FMR1
X	147002979	147002995	TA	snp	G	147002991	FMR1-AS1
10	14868193	14868217	AGA	snp	T	14868212	CDNF
10	17631267	17631288	ATC	snp	G	17631275	PTPLA
10	26436282	26436294	TGA	snp	T	26436286	MYO3A
10	26994404	26994416	ATT	snp	C	26994411	PDSS1
10	61413014	61413026	AAC	snp	C	61413020	SLC16A9
10	79397498	79397516	GCC	snp	T	79397503	KCNMA1
10	81003214	81003226	CGG	snp	A	81003221	ZMIZ1
10	95352569	95352587	ATT	snp	T	95352581	RBP4
10	96960372	96960387	AGA	snp	G	96960377	C10orf129
10	98393326	98393338	TAT	snp	C	98393331	PIK3AP1
10	98393326	98393338	TAT	snp	G	98393333	PIK3AP1
10	102049297	102049312	AAT	snp	A	102049302	PKD2L1
10	103454383	103454395	GCC	snp	G	103454390	FBXW4
10	105239328	105239343	CAA	snp	G	105239339	CALHM3
10	118459731	118459743	CAT	snp	T	118459734	HSPA12A
10	121302234	121302252	GAG	snp	A	121302243	RGS10
10	124035025	124035037	CAC	snp	G	124035029	BTBD16

10	124321346	124321358	CCT	snp	C	124321354	DMBT1
11	428481	428493	ATG	snp	C	428488	ANO9
11	535289	535313	CCG	snp	T	535305	HRAS
11	1593694	1593706	CGC	snp	T	1593697	DUSP8
11	1593694	1593706	CGC	snp	T	1593697	LOC338651
11	1593694	1593706	CGC	snp	T	1593697	LOC338651
11	1593694	1593706	CGC	snp	T	1593697	MOB2
11	1593694	1593706	CGC	snp	T	1593697	MOB2
11	1593694	1593706	CGC	snp	T	1593697	MOB2
11	4145061	4145073	AAC	snp	G	4145068	RRM1
11	8893185	8893197	GCA	snp	T	8893192	ST5
11	22271032	22271044	AAC	snp	T	22271037	ANO5
11	30432574	30432586	AAC	snp	G	30432578	MPPED2
11	33604955	33604973	TCA	snp	T	33604959	C11orf41
11	36632366	36632387	TGC	snp	A	36632382	C11orf74
11	43419229	43419241	AAT	snp	G	43419233	TTC17
11	46143525	46143543	GCG	snp	T	46143538	PHF21A
11	60228836	60228848	ATT	snp	C	60228843	MS4A1
11	67888779	67888794	GGC	snp	T	67888785	CHKA
11	75062773	75062788	GCC	snp	A	75062782	ARRB1
11	83771364	83771379	AAT	snp	G	83771368	DLG2
11	93063678	93063690	GCC	snp	T	93063685	CCDC67
11	100998099	100998111	GCC	snp	T	100998104	FJ515873
11	100998099	100998111	GCC	snp	T	100998104	PGR
11	103101921	103101933	TGC	snp	T	103101925	DYNC2H1
11	125035042	125035063	CCG	snp	G	125035058	PKNOX2
11	125619928	125619940	AAT	snp	C	125619934	PATE1
11	128641987	128641999	AGG	snp	A	128641994	FLI1
11	134122245	134122257	TCC	snp	T	134122249	THYN1
12	323599	323614	CCT	snp	G	323608	SLC6A12
12	1100455	1100467	GCA	snp	G	1100460	ERC1
12	2038970	2038985	GGA	snp	A	2038977	LOC100271702
12	4553741	4553756	TTC	snp	A	4553746	FGF6
12	4553741	4553756	TTC	snp	A	4553749	FGF6
12	12484644	12484656	GGA	snp	A	12484648	MANSC1
12	15103596	15103608	TCA	snp	G	15103604	ARHGDIB
12	25386049	25386067	ACC	snp	A	25386062	KRAS
12	26593150	26593174	AAC	snp	G	26593163	ITPR2
12	26986193	26986205	GAG	snp	G	26986197	ITPR2
12	53436064	53436076	CTC	snp	T	53436072	EIF4B
12	53436064	53436076	CTC	snp	T	53436072	LOC283335
12	71897912	71897927	TTG	snp	T	71897923	LGR5
12	75824887	75824902	GAG	snp	A	75824892	GLIPR1L2

12	93192111	93192129	ATT	snp	T	93192123	EEA1
12	100166426	100166447	ACA	snp	T	100166434	ANKS1B
12	100440498	100440513	ATA	snp	A	100440508	UHRF1BP1L
12	117014851	117014869	AAC	snp	G	117014864	MAP1LC3B2
12	124299599	124299617	CAA	snp	A	124299608	DNAH10
12	133445264	133445276	CAA	snp	C	133445268	CHFR
13	43137699	43137711	GAA	snp	C	43137703	TNFSF11
13	45151551	45151563	GGA	snp	A	45151558	LOC641467
13	45151551	45151563	GGA	snp	A	45151558	LOC641467
13	45151551	45151563	GGA	snp	A	45151558	TSC22D1
14	24511653	24511671	TTG	snp	C	24511662	DHRS4L1
14	24511653	24511671	TTG	snp	C	24511662	DHRS4L2
14	29235532	29235544	ACC	snp	G	29235536	FOXG1
14	35245742	35245754	TCA	snp	G	35245747	BAZ1A
14	50705219	50705237	TAA	snp	C	50705222	L2HGDH
14	74423786	74423804	TTA	snp	T	74423800	COQ6
14	74423786	74423804	TTA	snp	T	74423800	ENTPD5
14	77579051	77579066	TTG	snp	T	77579056	KIAA1737
14	94126068	94126083	ATC	snp	G	94126077	UNC79
14	101328249	101328264	CTC	snp	T	101328260	MEG3
14	103429046	103429058	GGT	snp	A	103429051	CDC42BPB
14	106993938	106993953	TAC	snp	C	106993944	abParts
15	23086364	23086388	GCC	snp	C	23086382	NIPA1
15	34816952	34816964	ATC	snp	C	34816959	GOLGA8B
15	35530026	35530044	GGA	snp	T	35530029	ANP32AP1
15	40650455	40650479	CCG	snp	A	40650460	DISP2
15	41663385	41663409	GGA	snp	A	41663392	NUSAP1
15	41989922	41989934	AGT	snp	G	41989925	MGA
15	60803736	60803748	GCT	snp	T	60803740	BC035094
15	60803736	60803748	GCT	snp	T	60803740	RORA
15	69388921	69388933	AGG	snp	A	69388925	LINC00277
15	69388921	69388933	AGG	snp	A	69388925	MIR548H4
15	72523678	72523693	GCG	snp	T	72523688	PKM2
15	78203749	78203761	ATC	snp	T	78203757	DQ586415
15	78369948	78369963	CGC	snp	C	78369958	TBC1D2B
15	90768314	90768326	TGC	snp	T	90768319	SEMA4B
16	284550	284562	GAG	snp	C	284555	ITFG3
16	284550	284562	GAG	snp	C	284555	LUC7L
16	2390589	2390601	GGC	snp	T	2390595	ABCA17P
16	2390589	2390601	GGC	snp	T	2390595	ABCA17P
16	2390589	2390601	GGC	snp	T	2390595	ABCA3
16	3111152	3111164	AAT	snp	G	3111158	BC045731
16	3111152	3111164	AAT	snp	G	3111158	MMP25

16	5121685	5121697	CTA	snp	A	5121691	ALG1
16	8901726	8901738	TAA	snp	C	8901731	PMM2
16	15471570	15471585	GGA	snp	A	15471580	NPIP
16	19503767	19503782	ATT	snp	G	19503772	TMC5
16	56459348	56459363	GCC	snp	T	56459353	AMFR
16	57126476	57126497	GCC	snp	T	57126483	CPNE2
16	88780081	88780093	GTG	snp	A	88780089	CTU2
17	260292	260304	GAG	snp	A	260298	C17orf97
17	1482442	1482463	AAT	snp	A	1482447	SLC43A2
17	3444939	3444957	ATG	snp	C	3444951	TRPV3
17	5185671	5185689	GGC	snp	A	5185679	RABEP1
17	7757138	7757150	CGG	snp	A	7757145	KDM6B
17	15583666	15583678	TAA	snp	C	15583674	TRIM16
17	34942586	34942598	AAG	snp	A	34942594	GGNBP2
17	39189355	39189373	TTA	snp	C	39189359	KRTAP1-3
17	39742849	39742867	GCT	snp	A	39742855	JUP
17	39742849	39742867	GCT	snp	A	39742855	KRT14
17	44160044	44160056	CAG	snp	A	44160049	KIAA1267
17	62915287	62915305	GGC	snp	A	62915290	LRRC37A3
17	62915287	62915305	GGC	snp	A	62915293	LRRC37A3
17	67323162	67323174	CGC	snp	T	67323168	ABCA5
17	78181357	78181375	AAC	snp	A	78181371	CARD14
17	79918820	79918838	GGC	snp	A	79918832	NOTUM
18	20953712	20953724	AGG	snp	A	20953719	TMEM241
18	25081558	25081570	AAC	snp	G	25081561	AK127888
18	40038869	40038881	GAT	snp	A	40038874	LOC284260
18	72011110	72011122	TTA	snp	A	72011116	C18orf63
19	520504	520516	AAC	snp	A	520512	TPGS1
19	857094	857109	ATA	snp	C	857104	ELANE
19	2859698	2859716	CCA	snp	G	2859706	ZNF555
19	4211639	4211651	CAA	snp	G	4211644	ANKRD24
19	8001990	8002011	AAC	snp	G	8001998	TIMM44
19	8151331	8151352	TTA	snp	C	8151347	FBN3
19	8151331	8151352	TTA	snp	T	8151348	FBN3
19	9004550	9004571	CAC	snp	A	9004562	MUC16
19	9010296	9010320	ATC	snp	T	9010316	MUC16
19	11307560	11307572	GGT	snp	A	11307563	KANK2
19	11536507	11536531	AAC	snp	T	11536516	CCDC151
19	17721853	17721868	AAC	snp	T	17721858	UNC13A
19	17921697	17921715	AAT	snp	A	17921702	B3GNT3
19	17932851	17932863	TTA	snp	G	17932854	INSL3
19	38011746	38011758	TGG	snp	T	38011751	ZNF793
19	38634012	38634024	TCC	snp	T	38634019	SIPA1L3

19	39957684	39957699	AAT	snp	G	39957687	SUPT5H
19	41173874	41173895	TGC	snp	T	41173879	NUMBL
19	41889414	41889429	TTA	snp	T	41889422	BCKDHA
19	41889414	41889429	TTA	snp	T	41889422	TMEM91
19	44454816	44454840	AAT	snp	C	44454831	ZNF221
19	44454816	44454840	AAT	snp	C	44454834	ZNF221
19	46996180	46996192	CCT	snp	C	46996185	BC132841
19	46996180	46996192	CCT	snp	C	46996185	LOC100506012
19	46996180	46996192	CCT	snp	C	46996185	PNMAL2
19	47657037	47657055	TAT	snp	G	47657044	SAE1
19	48494882	48494897	CCT	snp	T	48494886	BSPH1
19	48494882	48494897	CCT	snp	A	48494890	BSPH1
19	55399308	55399320	AAG	snp	T	55399313	FCAR
19	55693895	55693916	AAC	snp	G	55693907	PTPRH
19	56114231	56114246	GAG	snp	A	56114236	FIZ1
19	56114231	56114246	GAG	snp	A	56114236	ZNF524
1	6529182	6529206	TCC	snp	T	6529187	PLEKHG5
1	11711167	11711179	TAT	snp	G	11711175	FBXO2
1	12572771	12572795	CAA	snp	T	12572780	AK095438
1	12572771	12572795	CAA	snp	T	12572780	VPS13D
1	23107827	23107839	AAG	snp	G	23107831	EPHB2
1	29508342	29508354	GCG	snp	T	29508349	SRSF4
1	31466479	31466494	CAC	snp	G	31466487	PUM1
1	31653725	31653740	GTG	snp	C	31653732	NKAIN1
1	37941983	37941995	GTT	snp	A	37941989	ZC3H12A
1	53930350	53930368	GCC	snp	A	53930359	DMRTB1
1	55063126	55063138	CTC	snp	G	55063129	ACOT11
1	63153860	63153875	GGC	snp	G	63153865	DOCK7
1	68566989	68567010	TAT	snp	C	68566992	LOC100289178
1	68566989	68567010	TAT	snp	C	68566992	WLS
1	79129689	79129701	AAT	snp	G	79129693	IFI44
1	84944985	84944997	AGC	snp	G	84944988	RPF1
1	85593831	85593846	AAG	snp	G	85593840	WDR63
1	89665514	89665526	AAG	snp	A	89665522	GBP4
1	109102744	109102765	GGC	snp	T	109102761	FAM102B
1	112162406	112162418	CGC	snp	T	112162409	RAP1A
1	113120589	113120601	ATC	snp	G	113120592	ST7L
1	146697781	146697793	AGT	snp	C	146697789	FMO5
1	154301252	154301267	GGC	snp	A	154301260	ATP8B2
1	156101066	156101084	AAT	snp	A	156101071	LMNA
1	174245273	174245285	ATT	snp	C	174245277	RABGAP1L
1	186086569	186086584	TTG	snp	A	186086577	HMCN1
1	186086569	186086584	TTG	snp	A	186086577	HMCN1

1	186086569	186086584	TTG	snp	A	186086577	MIR548F1
1	186086569	186086584	TTG	snp	A	186086577	MIR548F1
1	203667527	203667542	CCA	snp	G	203667535	ATP2B4
1	204411140	204411152	CAC	snp	T	204411143	PIK3C2B
1	216693168	216693180	AAG	snp	A	216693173	ESRRG
1	231298894	231298906	CGC	snp	A	231298897	TRIM67
1	237754389	237754413	CTC	snp	C	237754402	RYR2
1	237754433	237754445	CCT	snp	T	237754436	RYR2
20	1115672	1115687	GCC	snp	T	1115680	PSMF1
20	2297211	2297226	AAT	snp	G	2297221	TGM3
20	4765996	4766014	AAC	snp	G	4766009	RASSF2
20	23731647	23731659	CTC	snp	T	23731652	CST1
20	30556073	30556088	GGC	snp	T	30556081	XKR7
20	44182832	44182844	TTG	snp	T	44182840	WFDC8
20	48099617	48099629	TTC	snp	T	48099622	KCNB1
20	49547656	49547668	GGC	snp	T	49547664	ADNP
20	61847465	61847480	GGC	snp	G	61847476	YTHDF1
21	22129886	22129898	TGA	snp	C	22129891	LINC00320
21	32554054	32554075	CTT	snp	G	32554057	TIAM1
21	43167543	43167555	TTA	snp	C	43167549	RIPK4
22	18050630	18050642	CCT	snp	G	18050635	SLC25A18
22	18167324	18167342	GTG	snp	G	18167334	BCL2L13
22	19166259	19166271	GGC	snp	A	19166262	CLTCL1
22	19166259	19166271	GGC	snp	A	19166262	SLC25A1
22	21318545	21318557	GGT	snp	T	21318551	AIFM3
22	21318545	21318557	GGT	snp	T	21318551	BC127858
22	23605416	23605428	TGC	snp	A	23605420	BCR
22	23605416	23605428	TGC	snp	A	23605420	FBXW4P1
22	25567693	25567711	AAT	snp	A	25567698	KIAA1671
2	20490838	20490856	AAT	snp	A	20490843	PUM2
2	27608109	27608121	CTC	snp	T	27608114	PPM1G
2	39005199	39005217	ATT	snp	C	39005204	GEMIN6
2	42274848	42274860	GCC	snp	A	42274851	PKDCC
2	48589159	48589171	ATT	snp	C	48589165	FOXN2
2	71221962	71221980	CCG	snp	C	71221967	TEX261
2	71662817	71662835	AAC	snp	A	71662825	ZNF638
2	79601264	79601276	TCA	snp	T	79601272	CTNNA2
2	89235790	89235802	TTG	snp	C	89235794	abParts
2	106810757	106810772	GCG	snp	T	106810766	UXS1
2	112974131	112974143	AAC	snp	T	112974139	ZC3H8
2	120006720	120006738	TTG	snp	C	120006726	STEAP3
2	160605860	160605881	TTG	snp	T	160605877	MARCH7
2	169727859	169727871	ATA	snp	G	169727862	SPC25

2	174129767	174129782	ACA	snp	T	174129771	MLK7-AS1
2	174129767	174129782	ACA	snp	T	174129771	ZAK
2	176957810	176957825	GCG	snp	A	176957821	HOXD13
2	178129390	178129405	GGC	snp	T	178129398	NFE2L2
2	204305087	204305099	GGT	snp	C	204305092	RAPH1
2	209054064	209054076	CCA	snp	G	209054072	C2orf80
2	217498281	217498293	GCC	snp	T	217498289	IGFBP2
2	225449893	225449917	GGC	snp	G	225449898	CUL3
2	233411049	233411061	TGT	snp	A	233411056	CHRNG
2	242405161	242405173	GAC	snp	T	242405169	FARP2
3	14106326	14106338	CAG	snp	C	14106331	TPRXL
3	16555218	16555233	CCG	snp	T	16555222	RFTN1
3	24379563	24379575	TTG	snp	C	24379566	THRB
3	39229896	39229908	TGC	snp	C	39229899	XIRP1
3	45267303	45267321	CGC	snp	T	45267309	TMEM158
3	46064621	46064633	GCG	snp	G	46064625	XCR1
3	46414019	46414040	ACA	snp	G	46414034	CCR5
3	85962165	85962177	TCA	snp	T	85962169	CADM2
3	133660840	133660852	ATT	snp	C	133660847	SLCO2A1
3	154801359	154801371	AGT	snp	C	154801364	MME
3	171756904	171756919	AAC	snp	G	171756908	FNDC3B
3	178866320	178866335	CGC	snp	T	178866326	BC032034
3	178866320	178866335	CGC	snp	T	178866326	PIK3CA
3	182511419	182511431	GGC	snp	G	182511427	ATP11B
3	190123384	190123396	CCA	snp	T	190123391	CLDN16
4	7716927	7716939	CTC	snp	C	7716931	SORCS2
4	9557645	9557663	GTG	snp	A	9557656	MIR548I2
4	20396582	20396594	TGG	snp	A	20396590	SLIT2
4	20396582	20396594	TGG	snp	A	20396590	SLIT2-IT1
4	38666560	38666572	CCA	snp	C	38666565	FLJ13197
4	38666560	38666572	CCA	snp	C	38666565	FLJ13197
4	38666560	38666572	CCA	snp	C	38666565	KLF3
4	38666560	38666572	CCA	snp	C	38666565	KLF3
4	48014760	48014772	ACA	snp	T	48014764	CNGA1
4	69097705	69097717	AAT	snp	G	69097709	TMPRSS11B
4	76792609	76792621	TTC	snp	C	76792615	PPEF2
4	76957165	76957177	TGC	snp	T	76957170	ART3
4	76957165	76957177	TGC	snp	T	76957170	ART3
4	76957165	76957177	TGC	snp	T	76957170	ART3
4	76957165	76957177	TGC	snp	T	76957170	CXCL11
4	76957165	76957177	TGC	snp	T	76957170	CXCL11
4	76957165	76957177	TGC	snp	T	76957170	CXCL11
4	86937092	86937110	ATT	snp	C	86937095	MAPK10

4	86937092	86937110	ATT	snp	C	86937098	MAPK10
4	126338119	126338131	AAT	snp	G	126338123	FAT4
4	126399435	126399447	TTA	snp	G	126399439	FAT4
4	141677315	141677330	GCG	snp	A	141677324	TBC1D9
4	153457150	153457165	GCG	snp	A	153457161	DKFZP434I0714
4	153457150	153457165	GCG	snp	A	153457161	FBXW7
4	169108193	169108205	AAT	snp	G	169108200	ANXA10
4	185973185	185973209	TTG	snp	C	185973188	BC043280
4	186298674	186298692	AAC	snp	G	186298688	BC128459
4	186298674	186298692	AAC	snp	G	186298688	LRP2BP
4	187071513	187071531	TTG	snp	T	187071518	FAM149A
4	187542975	187542990	AGT	snp	C	187542985	FAT1
5	10244641	10244653	AAC	snp	C	10244648	FAM173B
5	60193330	60193342	TAT	snp	C	60193338	ERCC8
5	76114959	76114971	CGG	snp	T	76114962	F2RL1
5	121798278	121798296	GAT	snp	C	121798286	BC029465
5	121798278	121798296	GAT	snp	C	121798286	SNCAIP
5	127419931	127419952	GCG	snp	A	127419948	FLJ33630
5	127419931	127419952	GCG	snp	A	127419948	SLC12A2
5	156278069	156278081	ATG	snp	A	156278077	PPP1R2P3
5	174951424	174951436	TTG	snp	C	174951430	SFXN1
5	177614360	177614372	GCC	snp	T	177614365	GMCL1P1
5	178772620	178772635	GCC	snp	G	178772630	ADAMTS2
6	3457325	3457349	CTC	snp	T	3457339	AK096549
6	3457325	3457349	CTC	snp	T	3457339	SLC22A23
6	26578017	26578041	GTT	snp	T	26578020	TRNA_Tyr
6	26682080	26682092	TTG	snp	C	26682086	TRNA_Ala
6	29759439	29759451	AAC	snp	T	29759444	HCG4
6	29759439	29759451	AAC	snp	T	29759444	LOC554223
6	31504010	31504022	GGT	snp	A	31504014	DDX39B
6	31504010	31504022	GGT	snp	A	31504014	DDX39B
6	31504010	31504022	GGT	snp	A	31504014	SNORD117
6	31588701	31588716	GGC	snp	T	31588706	PRRC2A
6	32407460	32407475	TTG	snp	C	32407467	HLA-DRA
6	35197026	35197038	AAT	snp	A	35197034	SCUBE3
6	38566699	38566717	AAT	snp	A	38566704	BTBD9
6	53159394	53159409	ATT	snp	C	53159398	ELOVL5
6	70926925	70926946	TGA	snp	C	70926932	COL9A1
6	90366299	90366311	CAA	snp	C	90366306	MDN1
6	91006512	91006536	GCT	snp	C	91006520	BACH2
6	107435941	107435956	GGC	snp	A	107435950	BEND3
6	121526444	121526465	AAG	snp	T	121526458	C6orf170
6	153311966	153311978	AAC	snp	T	153311969	MTRF1L

6	158505845	158505857	AAC	snp	T	158505853	SYNJ2
6	163955711	163955723	ATG	snp	A	163955718	QKI
7	1191681	1191693	TGC	snp	A	1191688	ZFAND2A
7	4944886	4944901	GAG	snp	C	4944889	MMD2
7	5112026	5112044	TGC	snp	T	5112034	LOC389458
7	5112026	5112044	TGC	snp	T	5112034	LOC389458
7	5112026	5112044	TGC	snp	T	5112034	LOC389458
7	5112026	5112044	TGC	snp	T	5112034	RBAK-LOC389458
7	5112026	5112044	TGC	snp	T	5112034	RBAK-LOC389458
7	5112026	5112044	TGC	snp	T	5112034	RBAK-LOC389458
7	12382305	12382323	ATT	snp	G	12382313	VWDE
7	21743366	21743381	AAC	snp	A	21743377	DNAH11
7	32768518	32768533	GGC	snp	A	32768525	AK057321
7	32768518	32768533	GGC	snp	A	32768525	AVL9
7	32768518	32768533	GGC	snp	A	32768525	ZNRF2P1
7	47440708	47440720	CTG	snp	T	47440711	TNS3
7	53834846	53834858	CCA	snp	T	53834849	FLJ45974
7	128423333	128423345	GGA	snp	C	128423340	TRNA
7	128423333	128423345	GGA	snp	C	128423340	TRNA_Pro
7	141888274	141888286	AAC	snp	T	141888282	LOC100124692
7	150864905	150864917	CCG	snp	T	150864912	GBX1
7	155727501	155727513	CTC	snp	T	155727509	Mir_598
7	155727609	155727621	TCC	snp	T	155727617	Mir_598
8	1808558	1808570	CCT	snp	T	1808565	ARHGEF10
8	3000624	3000642	AAC	snp	A	3000638	CSMD1
8	11707580	11707592	TGG	snp	A	11707588	CTSB
8	22861392	22861404	CCA	snp	T	22861396	RHOBTB2
8	25271890	25271902	TCC	snp	T	25271894	DKFZp451J181
8	25271890	25271902	TCC	snp	T	25271894	PPP2R2A
8	38324421	38324433	CCA	snp	A	38324425	FGFR1
8	104153022	104153034	CGC	snp	A	104153027	BAALC
8	104153022	104153034	CGC	snp	A	104153027	BAALC
8	104153022	104153034	CGC	snp	A	104153027	C8orf56
8	104153022	104153034	CGC	snp	A	104153027	C8orf56
8	121824054	121824072	GCC	snp	A	121824062	SNTB1
8	130365020	130365032	CCT	snp	T	130365024	CCDC26
8	140743192	140743204	GGA	snp	A	140743199	TRAPPC9
8	143425132	143425156	GAG	snp	G	143425151	TSNARE1
9	12775885	12775897	AGC	snp	G	12775888	C9orf150
9	27573206	27573230	CGC	snp	T	27573212	C9orf72
9	34016243	34016255	GAG	snp	A	34016248	UBAP2
9	34016279	34016291	GAG	snp	A	34016284	UBAP2
9	35906583	35906598	CCA	snp	C	35906594	HRCT1

9	75315433	75315445	AGA	snp	A	75315437	TMC1
9	89561422	89561434	GCG	snp	A	89561430	GAS1
9	92112892	92112913	GGC	snp	A	92112901	SEMA4D
9	117373813	117373837	GGC	snp	C	117373829	C9orf91
9	118093809	118093833	ATG	snp	T	118093820	DEC1
9	131187328	131187349	TTG	snp	T	131187333	CERCAM
9	134758087	134758099	CTC	snp	T	134758095	MED27
9	140647810	140647822	CAG	snp	T	140647816	EHMT1
X	1715371	1715389	TCC	snp	T	1715384	AKAP17A
X	1715371	1715389	TCC	snp	T	1715384	ASMT
X	18668097	18668109	CCG	snp	A	18668105	CDKL5
X	18668097	18668109	CCG	snp	A	18668105	RS1
X	20134976	20134988	GCC	snp	A	20134979	MAP7D2
X	140993905	140993917	CCT	snp	T	140993911	MAGEC1
X	144901395	144901407	AGA	snp	A	144901399	SLITRK2
10	13901065	13901081	TTCA	snp	T	13901075	FRMD4A
10	14969613	14969633	GAGG	snp	A	14969620	DCLRE1C
10	26592373	26592397	AGGG	snp	A	26592388	GAD2
10	26592373	26592397	AGGG	snp	A	26592392	GAD2
10	55954482	55954502	TCTA	snp	A	55954487	PCDH15
10	57360638	57360662	ATTT	snp	A	57360645	MTRNR2L5
10	57360638	57360662	ATTT	snp	A	57360645	PCDH15
10	59957061	59957089	AAAT	snp	C	59957070	IPMK
10	68934890	68934918	AGGG	snp	A	68934897	CTNNA3
10	71695825	71695845	AGGG	snp	A	71695838	COL13A1
10	81838055	81838075	TCAT	snp	C	81838070	FAM213A
10	81838055	81838075	TCAT	snp	C	81838070	FAM213A
10	81838055	81838075	TCAT	snp	C	81838070	LOC219347
10	97081357	97081373	ATGA	snp	A	97081363	SORBS1
10	97604496	97604516	GATG	snp	C	97604502	ENTPD1
10	97604496	97604516	GATG	snp	C	97604502	ENTPD1
10	97604496	97604516	GATG	snp	C	97604502	ENTPD1
10	97604496	97604516	GATG	snp	C	97604502	LOC728558
10	97604496	97604516	GATG	snp	C	97604502	LOC728558
10	116228028	116228052	TTTG	snp	T	116228047	ABLIM1
10	121435391	121435411	CCTT	snp	C	121435406	BAG3
11	31494405	31494421	TTTA	snp	T	31494416	IMMP1L
11	66242189	66242213	ATTT	snp	T	66242205	PELI3
11	126325704	126325724	AAAC	snp	C	126325716	KIRREL3
12	442604	442624	AAAC	snp	T	442619	KDM5A
12	10046637	10046653	TTTC	snp	G	10046644	KLRF2
12	104300975	104300995	GAAG	snp	T	104300987	GNN
12	109608637	109608653	TTTG	snp	C	109608648	ACACB

12	111950109	111950129	TTTG	snp	A	111950116	ATXN2
12	112567444	112567460	TTTA	snp	G	112567449	TRAFD1
12	122209945	122209961	TCTT	snp	C	122209953	TMEM120B
12	124005106	124005134	AAAC	snp	C	124005128	RILPL1
13	23754900	23754916	TTGT	snp	C	23754907	SGCG
13	28563283	28563307	TTTG	snp	C	28563298	PRHOXNB
13	50295955	50295975	CAAA	snp	G	50295960	KPNA3
13	71276432	71276460	AAGG	snp	A	71276451	Y_RNA
14	20914592	20914608	TGTT	snp	A	20914597	OSGEP
14	23003530	23003550	TTTA	snp	G	23003545	AV8S2A1N1T
14	23003530	23003550	TTTA	snp	G	23003545	hADV36S1
14	23003530	23003550	TTTA	snp	G	23003545	TCRA
14	23003530	23003550	TTTA	snp	G	23003545	TCRA
14	23003530	23003550	TTTA	snp	G	23003545	TCRA
14	23003530	23003550	TTTA	snp	G	23003545	TCRA
14	23003530	23003550	TTTA	snp	G	23003545	TCRA
14	23003530	23003550	TTTA	snp	G	23003545	TCRA
14	23003530	23003550	TTTA	snp	G	23003545	TCRA
14	23003530	23003550	TTTA	snp	G	23003545	TCRA
14	23003530	23003550	TTTA	snp	G	23003545	TCRA
14	23003530	23003550	TTTA	snp	G	23003545	TRA
14	23003530	23003550	TTTA	snp	G	23003545	TRA
14	23003530	23003550	TTTA	snp	G	23003545	TRA@
14	23003530	23003550	TTTA	snp	G	23003545	TRA@
14	23003530	23003550	TTTA	snp	G	23003545	TRA@
14	23003530	23003550	TTTA	snp	G	23003545	TRA@
14	23003530	23003550	TTTA	snp	G	23003545	TRAC
14	23003530	23003550	TTTA	snp	G	23003545	TRAC
14	23003530	23003550	TTTA	snp	G	23003545	TRAC
14	23003530	23003550	TTTA	snp	G	23003545	TRAC
14	23003530	23003550	TTTA	snp	G	23003545	TRAC
14	23003530	23003550	TTTA	snp	G	23003545	TRAC
14	23003530	23003550	TTTA	snp	G	23003545	TRAC
14	23003530	23003550	TTTA	snp	G	23003545	TRAC
14	23003530	23003550	TTTA	snp	G	23003545	TRD
14	23003530	23003550	TTTA	snp	G	23003545	X74394
14	31840298	31840322	CATT	snp	A	31840310	HEATR5A
14	60452681	60452697	TTCT	snp	C	60452689	AK128037
14	90437174	90437198	ATTT	snp	A	90437181	TDP1
14	106677140	106677156	TTCC	snp	T	106677151	abParts
15	34047817	34047837	AAAC	snp	G	34047831	RYR3
15	42296584	42296600	AGGG	snp	A	42296591	PLA2G4E
15	42374601	42374625	TCAT	snp	G	42374610	PLA2G4D

15	44150450	44150470	TTTG	snp	T	44150465	WDR76
15	52311804	52311820	GCGG	snp	C	52311810	MAPK6
15	53900954	53900982	AAAT	snp	G	53900962	WDR72
15	63111043	63111067	TTTA	snp	G	63111060	TLN2
15	63827115	63827143	TTTA	snp	C	63827135	USP3
15	75914379	75914399	AAAC	snp	A	75914386	SNUPN
15	101841823	101841839	TTGA	snp	C	101841831	AK130759
16	280188	280204	TTAT	snp	C	280194	LUC7L
16	3601450	3601466	TCAT	snp	C	3601458	NLRC3
16	21281592	21281608	TTTC	snp	C	21281600	CRYM
16	30390196	30390220	GAGG	snp	A	30390203	MYLPF
16	30390196	30390220	GAGG	snp	A	30390203	SEPT1
16	30390196	30390220	GAGG	snp	A	30390203	SEPT1
16	30390196	30390220	GAGG	snp	A	30390203	SEPT1
16	30390196	30390220	GAGG	snp	A	30390203	SEPT1
16	30390196	30390220	GAGG	snp	A	30390203	ZNF48
16	30390196	30390220	GAGG	snp	A	30390203	ZNF48
16	30390196	30390220	GAGG	snp	A	30390203	ZNF48
16	30390196	30390220	GAGG	snp	A	30390203	ZNF48
16	57763258	57763274	TGAG	snp	T	57763268	CCDC135
16	68861181	68861201	AATA	snp	G	68861194	CDH1
16	76533440	76533464	TTTA	snp	T	76533459	CNTNAP4
16	88793808	88793824	TGCG	snp	C	88793812	PIEZO1
17	4146357	4146381	TTTG	snp	T	4146376	ANKFY1
17	5998000	5998020	GGAA	snp	T	5998004	WSCD1
17	7244465	7244481	TTTA	snp	G	7244476	ACAP1
17	7253619	7253635	CGGG	snp	T	7253623	ACAP1
17	16248079	16248099	ATTT	snp	A	16248086	CENPV
17	27779235	27779263	AAAT	snp	C	27779244	TAOK1
17	38177832	38177848	GGAA	snp	C	38177838	MED24
17	40834060	40834084	GAAA	snp	T	40834072	CCR10
17	40834060	40834084	GAAA	snp	T	40834072	CNTNAP1
17	44059396	44059412	AAAG	snp	A	44059407	MAPT
17	46210425	46210441	AAAC	snp	C	46210433	SKAP1
17	60491927	60491943	TGTT	snp	T	60491932	EFCAB3
17	76485942	76485962	GGAT	snp	G	76485953	DNAH17
17	76866503	76866531	AAAG	snp	C	76866526	TIMP2
18	117490	117506	ATTG	snp	C	117499	ROCK1P1
18	53770221	53770237	TGTT	snp	A	53770228	LOC100505474
19	6480493	6480509	TCTG	snp	A	6480500	DENND1C
19	7051867	7051891	TTTG	snp	C	7051874	MBD3L2
19	8954553	8954569	TATG	snp	G	8954558	MBD3L1
19	10712912	10712932	ATGA	snp	G	10712925	SLC44A2

19	13225478	13225494	AAAT	snp	A	13225485	TRMT1
19	13398333	13398349	TTTC	snp	C	13398337	CACNA1A
19	16503852	16503868	TAAA	snp	C	16503858	EPS15L1
19	16923482	16923510	AAAC	snp	G	16923493	NWD1
19	17784820	17784840	AAAC	snp	G	17784826	UNC13A
19	18184622	18184638	TTTG	snp	C	18184628	IL12RB1
19	34823950	34823970	TTTG	snp	G	34823955	KIAA0355
19	37063620	37063644	TATC	snp	C	37063630	BC039524
19	37063620	37063644	TATC	snp	C	37063630	ZNF529
19	37063620	37063644	TATC	snp	C	37063630	ZNF529
19	37701792	37701808	TTTG	snp	G	37701801	ZNF585B
19	37855729	37855745	ATTT	snp	C	37855733	HKR1
19	41449446	41449462	ATTG	snp	T	41449454	CYP2A7
19	41449446	41449462	ATTG	snp	T	41449454	CYP2B7P1
19	42127742	42127762	CTCC	snp	T	42127754	CEACAM4
19	44160409	44160429	AAAT	snp	A	44160416	PLAUR
19	45981019	45981043	AAAG	snp	G	45981036	ERCC1
19	45981019	45981043	AAAG	snp	G	45981036	TRNA_SeC
19	46507725	46507741	TTTG	snp	G	46507735	CCDC61
19	51884592	51884608	AAAG	snp	G	51884596	LIM2
19	52129085	52129105	AAAC	snp	T	52129096	SIGLEC5
19	52824228	52824248	TAAA	snp	G	52824240	AK097759
19	52824228	52824248	TAAA	snp	G	52824240	ZNF480
19	54229121	54229137	GATT	snp	G	54229130	MIR516B2
19	55965206	55965226	AAAT	snp	G	55965221	ISOC2
19	59087775	59087791	CTTC	snp	C	59087781	MGC2752
19	59087775	59087791	CTTC	snp	T	59087782	MGC2752
1	16341726	16341742	TGTC	snp	C	16341732	HSPB7
1	17663620	17663636	TTTA	snp	G	17663629	PADI4
1	37963177	37963205	TATT	snp	C	37963191	MEAF6
1	52377776	52377792	TTTC	snp	C	52377786	RAB3B
1	53526872	53526892	CATT	snp	C	53526878	PODN
1	63085993	63086013	AAAT	snp	A	63086000	DOCK7
1	78099856	78099876	AAAT	snp	A	78099863	ZZZ3
1	92199610	92199634	GAGG	snp	A	92199617	TGFBR3
1	153788202	153788218	TAGA	snp	C	153788210	GATAD2B
1	167816642	167816666	TCTT	snp	T	167816655	ADCY10
1	169661385	169661405	ATTT	snp	A	169661390	C1orf112
1	169661385	169661405	ATTT	snp	A	169661390	SELL
1	179326340	179326356	TTTG	snp	C	179326349	SOAT1
1	186318805	186318821	AAAT	snp	A	186318816	MIR548F1
1	186318805	186318821	AAAT	snp	A	186318816	TPR
1	197272237	197272261	TTTA	snp	G	197272247	CRB1

1	207641016	207641032	ATTG	snp	C	207641022	CR2
1	220701165	220701189	ACAA	snp	G	220701184	MARK1
1	245674886	245674910	TTTG	snp	G	245674895	KIF26B
20	259699	259719	GGAG	snp	C	259709	C20orf96
20	2308269	2308289	ATCT	snp	G	2308273	TGM3
20	49624385	49624401	AAAT	snp	G	49624391	KCNG1
20	57122536	57122552	ATGA	snp	G	57122540	LOC149773
20	62609664	62609680	AATG	snp	G	62609669	SAMD10
21	34619535	34619559	TTTC	snp	T	34619554	IFNAR2
21	37587688	37587704	ATGA	snp	C	37587697	DOPEY2
22	18082702	18082718	AAGG	snp	C	18082707	ATP6V1E1
22	18572460	18572484	TTTG	snp	C	18572475	PEX26
22	27062920	27062936	GGGA	snp	G	27062927	AK026502
22	27062920	27062936	GGGA	snp	G	27062927	AK124820
22	27062920	27062936	GGGA	snp	G	27062927	MIAT
22	42208663	42208679	ATTC	snp	G	42208671	bK250D10.C22.8
22	42208663	42208679	ATTC	snp	G	42208671	CCDC134
22	43466905	43466925	TATG	snp	C	43466915	TTLL1
22	43607925	43607941	GACA	snp	G	43607932	SCUBE1
2	11880848	11880872	AAAG	snp	C	11880855	LPIN1
2	58277019	58277039	TGAA	snp	G	58277030	VRK2
2	59760308	59760328	TATT	snp	C	59760321	Mir_548
2	75425096	75425116	AAAC	snp	T	75425111	TACR1
2	110323689	110323705	ATTT	snp	T	110323693	SEPT10
2	113824832	113824852	TGGA	snp	T	113824839	IL1F10
2	114508920	114508936	TTTG	snp	C	114508928	SLC35F5
2	128292556	128292576	TTTG	snp	T	128292571	MYO7B
2	166851160	166851188	AAAC	snp	T	166851179	SCN1A
2	191923017	191923037	TTTA	snp	C	191923025	STAT4
2	217025386	217025414	TTAA	snp	T	217025404	XRCC5
2	217025386	217025414	TTAA	snp	T	217025408	XRCC5
2	231558605	231558625	TTTG	snp	T	231558620	LOC151475
2	234968181	234968205	AAAC	snp	G	234968193	SPP2
3	8686194	8686210	AGGA	snp	C	8686201	C3orf32
3	124052740	124052756	AAAC	snp	T	124052751	KALRN
3	133468005	133468021	AAAT	snp	G	133468011	TF
3	183696109	183696125	TTTG	snp	C	183696114	ABCC5
3	186290127	186290151	ATTT	snp	T	186290143	DNAJB11
4	28821926	28821942	AAAG	snp	G	28821932	MIR4275
4	40438200	40438220	AAAC	snp	A	40438207	RBM47
4	68383561	68383577	ATAC	snp	C	68383566	CENPC1
4	83787256	83787272	AGGG	snp	T	83787266	SEC31A
4	101343954	101343974	TTTC	snp	T	101343969	EMCN

4	105593759	105593775	CTTT	snp	C	105593768	AK094561
4	151207961	151207981	TGGT	snp	C	151207968	LRBA
4	151207961	151207981	TGGT	snp	C	151207969	LRBA
4	169140541	169140557	TGAT	snp	C	169140552	DDX60
4	185983994	185984018	TTTG	snp	C	185984013	BC043280
4	190944992	190945008	CTCC	snp	T	190945002	FRG2
4	190944992	190945008	CTCC	snp	T	190945002	LOC100288255
5	31193619	31193647	AAAG	snp	A	31193642	CDH6
5	60921669	60921685	GCGG	snp	A	60921679	BC032910
5	68424246	68424262	TTTG	snp	G	68424252	SLC30A5
5	86543617	86543637	GAAT	snp	A	86543621	BC034940
5	109183334	109183350	TTGT	snp	T	109183340	MAN2A1
5	110713602	110713618	TGAA	snp	G	110713609	CAMK4
5	114481211	114481239	ATTT	snp	C	114481229	TRIM36
5	145494297	145494317	CAAA	snp	A	145494301	LARS
6	2668985	2669001	TTTA	snp	C	2668989	MYLK4
6	17102574	17102602	AAAC	snp	G	17102595	FLJ23152
6	29799797	29799813	GGAG	snp	C	29799802	HLA-G
6	29799797	29799813	GGAG	snp	C	29799802	HLA-G
6	29799797	29799813	GGAG	snp	C	29799802	HLA-H
6	32311867	32311883	ATTT	snp	A	32311874	C6orf10
6	35756467	35756487	GAAG	snp	A	35756471	C6orf127
6	38864354	38864382	AAAC	snp	A	38864377	DNAH8
6	117730378	117730394	AAAT	snp	G	117730386	GOPC
6	117730378	117730394	AAAT	snp	G	117730386	ROS1
6	151131686	151131702	GAAA	snp	G	151131697	PLEKHG1
7	23181228	23181252	ATTC	snp	A	23181235	KLHL7
7	32111087	32111103	TCCC	snp	T	32111093	PDE1C
7	66237904	66237928	AAAT	snp	A	66237911	RABGEF1
7	66578600	66578616	TTTC	snp	T	66578611	MIR4650-1
7	66578600	66578616	TTTC	snp	T	66578611	TYW1
7	102116372	102116388	AAAC	snp	A	102116383	POLR2J
7	130060612	130060636	AATA	snp	A	130060622	CEP41
8	2090631	2090655	TCCC	snp	T	2090648	MYOM2
8	38837192	38837216	TTTA	snp	T	38837207	HTRA4
8	39862087	39862107	GATA	snp	T	39862092	IDO2
8	79471149	79471173	GTTT	snp	C	79471162	BC036404
8	79471149	79471173	GTTT	snp	C	79471162	PKIA
8	103226994	103227018	AAAT	snp	A	103227001	RRM2B
8	125592431	125592447	ATAC	snp	T	125592442	MTSS1
8	131414553	131414573	GACA	snp	T	131414563	ASAP1
8	145617629	145617653	CCCT	snp	C	145617640	ADCK5
8	145617629	145617653	CCCT	snp	C	145617640	CPSF1

9	418269	418285	TTTG	snp	G	418275	DOCK8
9	428960	428980	TTTA	snp	G	428971	DOCK8
9	21031462	21031478	CCGC	snp	A	21031470	PTPLAD2
9	35834933	35834949	TTTC	snp	G	35834941	TMEM8B
9	36355839	36355855	GTTT	snp	C	36355849	RNF38
9	39087486	39087502	TTTG	snp	T	39087497	CNTNAP3
9	77427804	77427824	TGAA	snp	G	77427818	TRPM6
9	132662012	132662032	AAGG	snp	A	132662019	FNBP1
X	1741831	1741847	AAAT	snp	A	1741842	ASMT
X	2161519	2161539	TTTA	snp	A	2161523	DHRSX
X	30714805	30714821	AAAC	snp	A	30714816	GK
X	32828060	32828084	AAAG	snp	A	32828071	DMD
X	153679937	153679957	CTTC	snp	C	153679942	FAM50A
10	24737175	24737183	G	ins	T	24737176	BC141952
10	24737175	24737183	G	ins	T	24737176	KIAA1217
10	26593898	26593907	T	ins	G	26593901	GAD2
10	27508245	27508255	A	ins	AC	27508246	ACBD5
10	70157355	70157363	A	ins	AAAATT	70157358	RUFY2
10	75083089	75083099	G	ins	T	75083091	TTC18
10	78843596	78843604	A	ins	C	78843600	KCNMA1
10	90068198	90068208	A	ins	C	90068205	RNLS
10	99019602	99019610	T	ins	G	99019605	ARHGAP19
10	104241136	104241144	G	ins	A	104241137	ACTR1A
10	105234166	105234174	C	ins	A	105234167	CALHM3
10	115347097	115347106	A	ins	AC	115347098	HABP2
10	115347097	115347106	A	ins	AC	115347098	NRAP
10	117855993	117856003	T	ins	C	117855994	GFRA1
10	123658707	123658717	T	ins	C	123658715	ATE1
10	127397438	127397446	C	ins	A	127397444	FLJ37035
10	127397438	127397446	C	ins	A	127397444	LOC283038
11	1891030	1891038	G	ins	A	1891034	LSP1
11	3861766	3861774	C	ins	A	3861772	RHOG
11	5013421	5013431	T	ins	G	5013422	MMP26
11	8941194	8941203	T	ins	G	8941195	AKIP1
11	8941194	8941203	T	ins	G	8941195	C11orf16
11	10546628	10546638	A	ins	G	10546636	RNF141
11	17035487	17035497	C	ins	A	17035492	PLEKHA7
11	27384242	27384250	A	ins	G	27384246	CCDC34
11	32852033	32852042	T	ins	G	32852038	PRRG4
11	65662427	65662436	T	ins	CC	65662434	FOSL1
11	72414453	72414463	G	ins	GC	72414456	ARAP1
11	74411661	74411669	T	ins	TA	74411664	CHRDL2
11	90280986	90280994	T	ins	TTC	90280987	HP11113

11	90280986	90280994	T	ins	TC	90280988	HP11113
11	90280986	90280994	T	ins	C	90280989	HP11113
11	93170909	93170918	C	ins	CG	93170913	CCDC67
11	117168337	117168347	A	ins	C	117168338	BACE1
12	3918186	3918196	T	ins	TTTTTATAAACACA	3918187	PARP11
12	4870783	4870793	T	ins	G	4870784	GALNT8
12	6717210	6717219	C	ins	A	6717216	CHD4
12	8089305	8089315	T	ins	C	8089313	SLC2A3
12	9067053	9067063	G	ins	GT	9067057	PHC1
12	15096338	15096346	T	ins	G	15096339	ARHGDIB
12	27522232	27522240	G	ins	T	27522233	ARNTL2
12	28125848	28125857	C	ins	CG	28125849	PTHLH
12	30949712	30949721	C	ins	A	30949716	LOC100287314
12	39070797	39070807	A	ins	C	39070803	CPNE8
12	51403995	51404005	A	ins	C	51403997	SLC11A2
12	51403995	51404005	A	ins	C	51403997	SLC11A2
12	51403995	51404005	A	ins	C	51403997	U7
12	52696625	52696635	G	ins	GA	52696627	KRT81
12	52696625	52696635	G	ins	GA	52696627	KRT81
12	52696625	52696635	G	ins	GA	52696627	KRT86
12	52696625	52696635	G	ins	GA	52696627	KRT86
12	52696625	52696635	G	ins	A	52696628	KRT81
12	52696625	52696635	G	ins	A	52696628	KRT81
12	52696625	52696635	G	ins	A	52696628	KRT86
12	52696625	52696635	G	ins	A	52696628	KRT86
12	66232376	66232384	T	ins	G	66232382	HMGA2
12	70328970	70328979	T	ins	G	70328977	C12orf28
12	92821236	92821244	G	ins	A	92821237	CLLU1
12	92821236	92821244	G	ins	A	92821237	CLLU1
12	92821236	92821244	G	ins	A	92821237	CLLU1OS
12	98896608	98896618	C	ins	CA	98896614	LOC643770
12	98896608	98896618	C	ins	CA	98896614	LOC643770
12	98896608	98896618	C	ins	CA	98896614	TRNA_Asp
12	104415852	104415861	A	ins	G	104415854	GLT8D2
12	104496721	104496730	C	ins	T	104496722	HCFC2
12	124022055	124022064	A	ins	AC	124022056	MIR3908
12	130930564	130930574	C	ins	T	130930567	RIMBP2
12	132575528	132575536	T	ins	TTTG	132575530	EP400NL
13	21988035	21988043	A	ins	CTT	21988037	ZDHHC20
13	21988035	21988043	A	ins	CTGT	21988039	ZDHHC20
13	30881149	30881158	T	ins	TC	30881156	KATNAL1
13	32524869	32524879	T	ins	G	32524870	DKFZp666K117
13	32524869	32524879	T	ins	G	32524870	EEF1DP3

13	45491179	45491187	A	ins	C	45491181	TRNA
13	45491179	45491187	A	ins	C	45491181	TRNA_Glu
13	91545152	91545160	C	ins	A	91545158	LINC00410
13	95747080	95747089	A	ins	C	95747086	ABCC4
13	108884457	108884466	A	ins	C	108884464	ABHD13
14	22771751	22771759	T	ins	TTTTTC	22771753	av27s1
14	22771751	22771759	T	ins	TTTTTC	22771753	av27s1
14	22771751	22771759	T	ins	TTTTTC	22771753	AV4S1
14	22771751	22771759	T	ins	TTTTTC	22771753	AV4S1
14	22771751	22771759	T	ins	TTTTTC	22771753	hADV29S1
14	22771751	22771759	T	ins	TTTTTC	22771753	hADV29S1
14	22771751	22771759	T	ins	TTTTTC	22771753	hADV36S1
14	22771751	22771759	T	ins	TTTTTC	22771753	hADV36S1
14	22771751	22771759	T	ins	TTTTTC	22771753	hADV38S2
14	22771751	22771759	T	ins	TTTTTC	22771753	hADV38S2
14	22771751	22771759	T	ins	TTTTTC	22771753	T-Cell
14	22771751	22771759	T	ins	TTTTTC	22771753	T-Cell
14	22771751	22771759	T	ins	TTTTTC	22771753	TCRA
14	22771751	22771759	T	ins	TTTTTC	22771753	TCRA
14	22771751	22771759	T	ins	TTTTTC	22771753	TCRA
14	22771751	22771759	T	ins	TTTTTC	22771753	TCRA
14	22771751	22771759	T	ins	TTTTTC	22771753	TCRA
14	22771751	22771759	T	ins	TTTTTC	22771753	TCRA
14	22771751	22771759	T	ins	TTTTTC	22771753	TCRA
14	22771751	22771759	T	ins	TTTTTC	22771753	TCRA
14	22771751	22771759	T	ins	TTTTTC	22771753	TCRA
14	22771751	22771759	T	ins	TTTTTC	22771753	TCRA
14	22771751	22771759	T	ins	TTTTTC	22771753	TCR-alpha
14	22771751	22771759	T	ins	TTTTTC	22771753	TCR-alpha
14	22771751	22771759	T	ins	TTTTTC	22771753	TCR-alpha
14	22771751	22771759	T	ins	TTTTTC	22771753	TCR-alpha
14	22771751	22771759	T	ins	TTTTTC	22771753	TRA
14	22771751	22771759	T	ins	TTTTTC	22771753	TRA
14	22771751	22771759	T	ins	TTTTTC	22771753	TRA
14	22771751	22771759	T	ins	TTTTTC	22771753	TRA
14	22771751	22771759	T	ins	TTTTTC	22771753	TRA
14	22771751	22771759	T	ins	TTTTTC	22771753	TRA
14	22771751	22771759	T	ins	TTTTTC	22771753	TRA@
14	22771751	22771759	T	ins	TTTTTC	22771753	TRA@
14	22771751	22771759	T	ins	TTTTTC	22771753	TRAC
14	22771751	22771759	T	ins	TTTTTC	22771753	TRAC
14	22771751	22771759	T	ins	TTTTTC	22771753	TRAC
14	22771751	22771759	T	ins	TTTTTC	22771753	TRAC

14	22771751	22771759	T	ins	TTTTTC	22771753	TRD
14	22771751	22771759	T	ins	TTTTTC	22771753	TRD
14	24739363	24739371	G	ins	T	24739364	HP08474
14	24739363	24739371	G	ins	T	24739364	RABGGTA
14	29261304	29261312	A	ins	C	29261306	C14orf23
14	35032940	35032948	T	ins	TC	35032941	SNX6
14	35032940	35032948	T	ins	C	35032942	SNX6
14	50847520	50847530	T	ins	TG	50847521	CDKL1
14	50847520	50847530	T	ins	G	50847522	CDKL1
14	51311620	51311630	A	ins	C	51311622	SnoU83B
14	51311620	51311630	A	ins	AC	51311623	SnoU83B
14	51311620	51311630	A	ins	C	51311624	SnoU83B
14	55159594	55159603	C	ins	CCCG	55159596	SAMD4A
14	55159594	55159603	C	ins	CCA	55159599	SAMD4A
14	55906332	55906341	C	ins	T	55906335	TBPL2
14	67940982	67940992	A	ins	AAC	67940983	TMEM229B
14	69444517	69444526	C	ins	A	69444523	ACTN1
14	70419866	70419875	T	ins	G	70419867	SMOC1
14	73739996	73740004	T	ins	GA	73740002	PAPLN
14	74024571	74024579	T	ins	C	74024575	ACOT1
14	74024571	74024579	T	ins	C	74024575	HEATR4
14	93307108	93307118	T	ins	TCTC	93307109	GOLGA5
14	94547060	94547069	A	ins	AC	94547061	DDX24
14	94547060	94547069	A	ins	AC	94547061	IFI27L1
14	94547060	94547069	A	ins	C	94547062	DDX24
14	94547060	94547069	A	ins	C	94547062	IFI27L1
14	106545788	106545797	A	ins	C	106545794	abParts
15	32393654	32393662	A	ins	G	32393660	CHRFAM7A
15	32393654	32393662	A	ins	G	32393660	CHRNA7
15	32393654	32393662	A	ins	G	32393660	CHRNA7
15	35812431	35812441	A	ins	C	35812436	ATPBD4
15	50863050	50863060	A	ins	C	50863054	TRPM7
15	55835150	55835158	A	ins	C	55835156	AK055370
15	60786381	60786389	A	ins	C	60786387	BC035094
15	60786381	60786389	A	ins	C	60786387	RORA
15	65688554	65688564	C	ins	T	65688557	IGDCC4
15	67692820	67692828	T	ins	TTTTC	67692823	IQCH
15	69744166	69744176	C	ins	A	69744172	RPLP1
15	79231523	79231533	A	ins	G	79231525	CTSH
16	773313	773321	C	ins	A	773318	CCDC78
16	773313	773321	C	ins	A	773318	FAM173A
16	2720101	2720111	C	ins	CCA	2720104	ERVK13-1
16	15976922	15976930	A	ins	AAG	15976928	FOPNL

16	19713306	19713315	T	ins	C	19713313	C16orf62
16	20826864	20826872	T	ins	TC	20826866	ERI2
16	20826864	20826872	T	ins	TC	20826866	ERI2
16	20826864	20826872	T	ins	TC	20826866	LOC81691
16	20826864	20826872	T	ins	TC	20826866	LOC81691
16	20927404	20927414	T	ins	G	20927405	LYRM1
16	23654507	23654517	T	ins	G	23654514	DCTN5
16	24830828	24830838	T	ins	TC	24830829	TNRC6A
16	24830828	24830838	T	ins	C	24830830	TNRC6A
16	48268283	48268293	T	ins	GA	48268285	ABCC11
16	50347346	50347356	A	ins	C	50347347	ADCY7
16	66807296	66807305	T	ins	C	66807300	CCDC79
16	74497624	74497632	T	ins	TTG	74497626	GLG1
16	74497624	74497632	T	ins	TG	74497627	GLG1
16	74497624	74497632	T	ins	G	74497628	GLG1
16	75299920	75299928	C	ins	T	75299923	BCAR1
17	4890939	4890947	C	ins	CG	4890944	CAMTA2
17	4890939	4890947	C	ins	CG	4890944	INCA1
17	7166926	7166934	T	ins	G	7166931	CLDN7
17	7459290	7459298	T	ins	TTTTG	7459291	TNFSF12
17	7459290	7459298	T	ins	TTTTG	7459291	TNFSF12-TNFSF13
17	7459290	7459298	T	ins	TTTG	7459292	TNFSF12
17	7459290	7459298	T	ins	TTTG	7459292	TNFSF12-TNFSF13
17	7588774	7588782	T	ins	G	7588776	TP53
17	7588774	7588782	T	ins	G	7588776	WRAP53
17	8052531	8052541	A	ins	C	8052535	PER1
17	10274592	10274601	A	ins	C	10274594	MYH13
17	11826368	11826378	T	ins	G	11826374	DNAH9
17	16874694	16874703	A	ins	C	16874697	TNFRSF13B
17	18605672	18605682	T	ins	TTTTC	18605678	TRIM16L
17	20906266	20906274	G	ins	T	20906271	USP22
17	31899321	31899329	C	ins	A	31899324	ACCN1
17	31899321	31899329	C	ins	A	31899324	AK057317
17	37312311	37312320	T	ins	G	37312315	ARL5C
17	38186919	38186929	T	ins	TA	38186920	MED24
17	45559459	45559467	A	ins	C	45559465	MRPL45P2
17	48542130	48542140	G	ins	T	48542138	ACSF2
17	48542130	48542140	G	ins	T	48542138	ACSF2
17	48542130	48542140	G	ins	T	48542138	ACSF2
17	48542130	48542140	G	ins	T	48542138	CHAD
17	48542130	48542140	G	ins	T	48542138	CHAD
17	48542130	48542140	G	ins	T	48542138	CHAD
17	59116003	59116011	T	ins	TTTG	59116006	BCAS3

17	61628668	61628676	C	ins	CT	61628671	DCAF7
17	61779377	61779386	G	ins	A	61779380	STRADA
17	65358802	65358812	A	ins	C	65358810	PSMD12
17	74935936	74935945	T	ins	C	74935937	MGAT5B
18	5956909	5956919	T	ins	G	5956917	L3MBTL4
18	12123030	12123038	T	ins	G	12123034	ANKRD62
18	21124907	21124916	C	ins	CCCT	21124910	NPC1
18	24268642	24268651	T	ins	TTTAA	24268643	LOC728606
18	57365448	57365457	C	ins	CT	57365453	CCBE1
18	61652114	61652124	T	ins	TC	61652122	SERPINB8
18	72124959	72124967	C	ins	T	72124965	FAM69C
19	680001	680010	C	ins	CT	680008	FSTL3
19	1925909	1925918	C	ins	A	1925913	SCAMP4
19	3366957	3366966	C	ins	A	3366964	NFIC
19	3819850	3819859	A	ins	G	3819853	ZFR2
19	10676487	10676496	C	ins	CCA	10676490	CDKN2D
19	10676487	10676496	C	ins	CCA	10676490	KRI1
19	12764202	12764210	C	ins	A	12764206	MAN2B1
19	20003794	20003803	T	ins	TTG	20003800	ZNF253
19	30021342	30021350	C	ins	T	30021344	VSTM2B
19	34923465	34923473	T	ins	G	34923471	UBA2
19	36169725	36169733	A	ins	AG	36169729	UPK1A
19	37489607	37489617	A	ins	C	37489615	AX747376
19	37489607	37489617	A	ins	C	37489615	ZNF568
19	41737854	41737862	A	ins	AC	41737855	AXL
19	44426463	44426471	T	ins	G	44426466	ZNF45
19	44906133	44906141	C	ins	CA	44906137	ZFP112
19	44906133	44906141	C	ins	CA	44906137	ZNF285
19	44906133	44906141	C	ins	A	44906138	ZFP112
19	44906133	44906141	C	ins	A	44906138	ZNF285
19	46173211	46173220	G	ins	GC	46173214	GIPR
19	46707578	46707586	G	ins	T	46707579	DKFZp434J0226
19	46972011	46972019	T	ins	G	46972017	PNMAL1
19	48827911	48827921	T	ins	G	48827914	EMP3
19	50787349	50787359	A	ins	C	50787352	MYH14
19	50837854	50837864	C	ins	A	50837858	NAPSB
19	50837854	50837864	C	ins	A	50837858	NAPSB
19	50837854	50837864	C	ins	A	50837858	NAPSB
19	50837854	50837864	C	ins	A	50837858	NAPSB
19	50837854	50837864	C	ins	A	50837858	NR1H2
19	50837854	50837864	C	ins	A	50837858	NR1H2
19	50837854	50837864	C	ins	A	50837858	NR1H2
19	50837854	50837864	C	ins	A	50837858	NR1H2

19	51335568	51335577	T	ins	TTTC	51335573	KLK15
19	52693289	52693297	C	ins	A	52693293	PPP2R1A
19	55712319	55712327	A	ins	C	55712323	PTPRH
19	57875090	57875099	C	ins	CG	57875096	TRAPPC2
19	57875090	57875099	C	ins	CG	57875096	ZNF547
1	7740869	7740878	G	ins	T	7740872	CAMTA1
1	8029500	8029510	G	ins	A	8029508	PARK7
1	16729978	16729986	T	ins	TC	16729980	SPATA21
1	16729978	16729986	T	ins	C	16729981	SPATA21
1	19652132	19652141	G	ins	T	19652135	PQLC2
1	19705580	19705589	T	ins	TTTC	19705585	CAPZB
1	41975890	41975900	T	ins	G	41975891	HIVEP3
1	45804416	45804426	T	ins	TG	45804421	MUTYH
1	45804416	45804426	T	ins	TG	45804421	TOE1
1	46119365	46119373	T	ins	CC	46119371	GPBP1L1
1	49208092	49208102	A	ins	G	49208094	AGBL4
1	49208092	49208102	A	ins	G	49208094	BEND5
1	59132546	59132556	A	ins	AT	59132552	MYSM1
1	62253768	62253778	A	ins	G	62253772	INADL
1	67441666	67441674	T	ins	G	67441671	MIER1
1	116224112	116224120	T	ins	TTG	116224117	VANGL1
1	116224124	116224132	T	ins	G	116224125	VANGL1
1	152308781	152308789	T	ins	G	152308786	AK056431
1	167333796	167333805	T	ins	C	167333800	POU2F1
1	167854360	167854368	T	ins	C	167854361	ADCY10
1	170933762	170933770	A	ins	TC	170933763	C1orf129
1	170933762	170933770	A	ins	TC	170933765	C1orf129
1	171620483	171620491	C	ins	A	171620488	MYOC
1	202722792	202722802	T	ins	G	202722793	KDM5B
1	203771852	203771862	T	ins	TG	203771857	ZC3H11A
1	205886253	205886261	A	ins	G	205886257	SLC26A9
1	227098195	227098203	T	ins	C	227098196	ADCK3
1	227171735	227171745	G	ins	T	227171737	ADCK3
1	229586482	229586490	T	ins	GTTTG	229586484	NUP133
1	234456783	234456793	C	ins	A	234456791	SLC35F3
1	234564092	234564101	A	ins	C	234564094	TARBP1
1	237752192	237752202	A	ins	C	237752196	RYR2
20	3776005	3776013	A	ins	C	3776008	CDC25B
20	17947986	17947996	A	ins	AC	17947991	AK296947
20	17947986	17947996	A	ins	AC	17947991	SNX5
20	23419817	23419826	T	ins	G	23419822	CSTL1
20	42844409	42844417	T	ins	TC	42844410	LOC100505783
20	42844409	42844417	T	ins	C	42844411	LOC100505783

20	42974469	42974477	T	ins	C	42974471	R3HDML
20	61465240	61465249	T	ins	G	61465241	COL9A3
20	61465240	61465249	T	ins	G	61465242	COL9A3
20	61465240	61465249	T	ins	G	61465245	COL9A3
20	61715656	61715665	G	ins	T	61715659	LOC63930
21	19274501	19274511	T	ins	G	19274504	CHODL
21	32126534	32126542	T	ins	G	32126540	KRTAP21-1
21	46573256	46573266	T	ins	C	46573264	ADARB1
22	21379156	21379164	T	ins	TG	21379162	P2RX6
22	23466377	23466387	T	ins	G	23466385	GNAZ
22	23466377	23466387	T	ins	G	23466385	RTDR1
22	24199764	24199774	C	ins	CT	24199765	SLC2A11
22	37622884	37622892	G	ins	T	37622887	RAC2
22	39079147	39079157	G	ins	GGC	39079148	TOMM22
22	44560289	44560298	C	ins	CCG	44560290	PARVB
22	50971639	50971648	A	ins	G	50971642	ODF3B
2	24046670	24046678	A	ins	C	24046671	ATAD2B
2	26717524	26717533	G	ins	GT	26717529	OTOF
2	27824507	27824515	T	ins	TTTTG	27824510	ZNF512
2	29456859	29456868	T	ins	G	29456864	ALK
2	30864753	30864761	T	ins	TTTTC	30864755	LCLAT1
2	55405088	55405096	A	ins	G	55405093	C2orf63
2	61001236	61001246	T	ins	G	61001241	PAPOLG
2	61709732	61709741	T	ins	TG	61709733	XPO1
2	61709732	61709741	T	ins	G	61709734	XPO1
2	106014345	106014354	T	ins	G	106014347	FHL2
2	120022379	120022389	C	ins	A	120022380	STEAP3
2	128697870	128697879	A	ins	C	128697872	SAP130
2	139426377	139426387	T	ins	TTG	139426378	NXPH2
2	151324743	151324752	A	ins	G	151324746	RND3
2	158979866	158979875	T	ins	C	158979869	UPP2
2	158979866	158979875	T	ins	C	158979870	UPP2
2	175199471	175199481	C	ins	CCA	175199474	SP9
2	176789404	176789413	A	ins	C	176789409	KIAA1715
2	201485102	201485111	T	ins	G	201485105	AOX1
2	206861639	206861648	A	ins	C	206861643	INO80D
2	209049820	209049830	T	ins	C	209049828	C2orf80
2	212543025	212543034	A	ins	C	212543030	ERBB4
2	213404028	213404037	C	ins	CCCCA	213404031	ERBB4
2	213869915	213869924	G	ins	GGT	213869921	IKZF2
2	216269022	216269030	T	ins	TTTTG	216269027	FN1
2	228142841	228142851	T	ins	TA	228142849	AK056332
2	228142841	228142851	T	ins	TA	228142849	BC035052

2	228142841	228142851	T	ins	TA	228142849	COL4A3
2	241388730	241388739	C	ins	T	241388735	GPC1
2	241388730	241388739	C	ins	T	241388735	PP14571
2	242177134	242177142	A	ins	AC	242177140	HDLBP
3	33191005	33191013	G	ins	GA	33191006	SUSD5
3	47370693	47370701	T	ins	TG	47370698	KLHL18
3	55018614	55018624	T	ins	G	55018618	CACNA2D3
3	56717973	56717982	C	ins	CCCG	56717975	FAM208A
3	56808954	56808964	A	ins	C	56808955	ARHGEF3
3	86119784	86119793	A	ins	C	86119785	CADM2
3	89521598	89521606	T	ins	C	89521601	EPHA3
3	100551609	100551617	A	ins	G	100551611	ABI3BP
3	108747081	108747089	C	ins	A	108747086	MORC1
3	112557336	112557345	A	ins	AC	112557337	CD200R1L
3	121976532	121976540	T	ins	C	121976534	CASR
3	125249828	125249838	T	ins	TTC	125249833	OSBPL11
3	125249828	125249838	T	ins	TC	125249834	OSBPL11
3	131625046	131625055	T	ins	G	131625052	CPNE4
3	149051248	149051256	T	ins	TC	149051254	TM4SF18
3	161222622	161222632	T	ins	TG	161222629	OTOL1
3	168850277	168850285	T	ins	TC	168850280	MECOM
3	168850277	168850285	T	ins	CA	168850281	MECOM
3	176755297	176755305	G	ins	T	176755298	TBL1XR1
3	176914523	176914531	C	ins	T	176914529	TBL1XR1
3	183164881	183164891	T	ins	TTC	183164888	LOC100505687
3	184100958	184100968	G	ins	T	184100960	CHRD
3	185215869	185215879	C	ins	CCA	185215875	TMEM41A
3	190993490	190993500	A	ins	C	190993491	UTS2D
3	195802754	195802762	G	ins	T	195802756	TFRC
4	9706756	9706764	G	ins	A	9706760	DQ584669
4	38825709	38825717	C	ins	A	38825712	TLR6
4	40438220	40438229	A	ins	C	40438222	RBM47
4	57887818	57887827	T	ins	G	57887824	POLR2B
4	71385415	71385425	T	ins	TTTTTA	71385416	AMTN
4	81283670	81283679	T	ins	C	81283673	C4orf22
4	82089195	82089203	A	ins	C	82089201	PRKG2
4	84240325	84240335	A	ins	AC	84240326	HPSE
4	85771476	85771485	T	ins	G	85771483	WDFY3
4	106319242	106319251	A	ins	CC	106319246	PPA2
4	120375126	120375136	C	ins	A	120375132	BC070391
4	120375126	120375136	C	ins	A	120375132	LOC645513
4	122721708	122721718	T	ins	G	122721712	EXOSC9
4	154318805	154318815	A	ins	G	154318811	MND1

4	156653163	156653171	A	ins	G	156653169	GUCY1A3
4	159817993	159818003	T	ins	TA	159818000	C4orf45
4	159817993	159818003	T	ins	TA	159818000	FNIP2
4	183601590	183601599	G	ins	T	183601594	ODZ3
4	187344162	187344172	T	ins	TG	187344166	LOC285441
5	1112986	1112996	C	ins	A	1112993	SLC12A7
5	15937655	15937665	C	ins	A	15937662	FBXL7
5	35002881	35002891	G	ins	T	35002884	AGXT2
5	39387801	39387809	T	ins	C	39387806	DAB2
5	40765853	40765863	A	ins	C	40765861	PRKAA1
5	54398880	54398889	A	ins	G	54398881	GZMA
5	60953736	60953745	A	ins	G	60953742	BC043229
5	60953736	60953745	A	ins	G	60953742	C5orf64
5	73177903	73177911	T	ins	G	73177909	RGNEF
5	75998985	75998994	G	ins	T	75998989	IQGAP2
5	90051086	90051094	T	ins	C	90051087	GPR98
5	102898517	102898527	G	ins	GA	102898520	NUDT12
5	111066236	111066245	G	ins	GT	111066239	LOC100505678
5	111066236	111066245	G	ins	GT	111066239	NREP
5	111066236	111066245	G	ins	GT	111066239	NREP
5	133842267	133842276	C	ins	A	133842268	BC032795
5	136314132	136314142	A	ins	C	136314133	SPOCK1
5	136976422	136976430	C	ins	A	136976423	KLHL3
5	140803317	140803327	G	ins	GC	140803324	PCDHGA1
5	140803317	140803327	G	ins	GC	140803324	PCDHGA10
5	140803317	140803327	G	ins	GC	140803324	PCDHGA11
5	140803317	140803327	G	ins	GC	140803324	PCDHGA11
5	140803317	140803327	G	ins	GC	140803324	PCDHGA2
5	140803317	140803327	G	ins	GC	140803324	PCDHGA3
5	140803317	140803327	G	ins	GC	140803324	PCDHGA4
5	140803317	140803327	G	ins	GC	140803324	PCDHGA5
5	140803317	140803327	G	ins	GC	140803324	PCDHGA6
5	140803317	140803327	G	ins	GC	140803324	PCDHGA7
5	140803317	140803327	G	ins	GC	140803324	PCDHGA8
5	140803317	140803327	G	ins	GC	140803324	PCDHGA9
5	140803317	140803327	G	ins	GC	140803324	PCDHGB1
5	140803317	140803327	G	ins	GC	140803324	PCDHGB2
5	140803317	140803327	G	ins	GC	140803324	PCDHGB3
5	140803317	140803327	G	ins	GC	140803324	PCDHGB4
5	140803317	140803327	G	ins	GC	140803324	PCDHGB5
5	140803317	140803327	G	ins	GC	140803324	PCDHGB6
5	140803317	140803327	G	ins	GC	140803324	PCDHGB7
5	171534119	171534128	A	ins	C	171534123	STK10

5	173416100	173416108	C	ins	CA	173416102	C5orf47
5	177379531	177379540	C	ins	CCAT	177379532	AK126616
5	178584960	178584968	T	ins	G	178584962	ADAMTS2
6	4088272	4088280	C	ins	A	4088277	C6orf146
6	4088272	4088280	C	ins	A	4088277	C6orf146
6	4088272	4088280	C	ins	A	4088277	C6orf201
6	4088272	4088280	C	ins	A	4088277	C6orf201
6	27774428	27774438	T	ins	TC	27774436	HIST1H2BL
6	28774804	28774814	A	ins	C	28774809	TRNA_Phe
6	30230518	30230526	T	ins	AC	30230520	HLA-L
6	30972958	30972968	T	ins	C	30972960	MUC22
6	31677035	31677044	T	ins	G	31677036	ABHD16A
6	31677035	31677044	T	ins	G	31677036	LY6G6F
6	32373584	32373594	T	ins	G	32373591	BTNL2
6	32525116	32525124	A	ins	C	32525119	HLA-DRB1
6	32525116	32525124	A	ins	C	32525119	HLA-DRB5
6	32525116	32525124	A	ins	C	32525119	HLA-DRB6
6	32605979	32605987	T	ins	TA	32605981	HLA-DQA1
6	32610152	32610162	T	ins	C	32610154	HLA-DQA1
6	32828219	32828227	A	ins	C	32828221	PSMB9
6	33741371	33741381	G	ins	GT	33741372	LEMD2
6	35745086	35745094	G	ins	A	35745089	C6orf126
6	41515515	41515524	C	ins	CA	41515521	FOXP4
6	44123188	44123198	T	ins	TGG	44123195	TMEM63B
6	44279296	44279304	G	ins	A	44279301	AARS2
6	44279296	44279304	G	ins	A	44279301	AARS2
6	44279296	44279304	G	ins	A	44279301	AARS2
6	44279296	44279304	G	ins	A	44279301	SPATS1
6	44279296	44279304	G	ins	A	44279301	SPATS1
6	44279296	44279304	G	ins	A	44279301	SPATS1
6	66227234	66227244	A	ins	AC	66227236	EYS
6	88118739	88118747	T	ins	TTATG	88118740	C6orf165
6	128304353	128304361	A	ins	C	128304357	PTPRK
6	133119843	133119853	C	ins	A	133119847	C6orf192
6	138644772	138644782	A	ins	C	138644773	KIAA1244
7	5938156	5938166	A	ins	C	5938157	CCZ1
7	6780646	6780656	A	ins	G	6780654	PMS2CL
7	11293706	11293715	A	ins	C	11293713	BC040327
7	16899723	16899732	A	ins	G	16899726	AGR3
7	21913946	21913955	T	ins	G	21913950	DNAH11
7	37261300	37261309	T	ins	TTTTC	37261304	ELMO1
7	55758674	55758682	A	ins	G	55758680	FKBP9L
7	64329825	64329834	G	ins	T	64329831	AK097702

7	66024978	66024987	A	ins	C	66024981	LOC493754
7	66461205	66461213	A	ins	C	66461210	SBDS
7	66461205	66461213	A	ins	C	66461210	TYW1
7	66768668	66768678	T	ins	TTC	66768676	STAG3L4
7	93520940	93520950	A	ins	G	93520941	GNGT1
7	93520940	93520950	A	ins	G	93520941	TFPI2
7	98922776	98922785	A	ins	AAAGAAAAAG	98922781	ARPC1A
7	106847433	106847441	A	ins	AAC	106847437	COG5
7	111508765	111508775	A	ins	AT	111508771	DOCK4
7	128312703	128312711	G	ins	A	128312708	FAM71F2
7	134853043	134853053	C	ins	A	134853044	C7orf49
7	135415711	135415720	T	ins	TC	135415718	FAM180A
7	138357386	138357395	T	ins	TTTTG	138357389	SVOPL
7	139026462	139026471	G	ins	T	139026463	C7orf55
7	139026462	139026471	G	ins	T	139026463	LUC7L2
7	139026462	139026471	G	ins	T	139026463	LUC7L2
7	139026462	139026471	G	ins	T	139026463	TRNA
7	139026462	139026471	G	ins	T	139026463	TRNA_Arg
7	139482261	139482271	T	ins	TC	139482269	TBXAS1
7	143806414	143806424	T	ins	G	143806416	OR2A2
8	413963	413971	T	ins	G	413967	FBXO25
8	1650528	1650537	A	ins	C	1650535	DLGAP2
8	1771615	1771624	G	ins	T	1771617	ARHGEF10
8	2794975	2794985	A	ins	AAC	2794976	CSMD1
8	15094761	15094769	C	ins	CCA	15094763	SGCZ
8	35092779	35092788	G	ins	T	35092780	UNC5D
8	74224237	74224246	T	ins	G	74224238	AK128216
8	74224237	74224246	T	ins	G	74224238	RDH10
8	103225971	103225981	A	ins	C	103225977	RRM2B
8	120860224	120860232	T	ins	AA	120860230	DSCC1
9	18794624	18794633	T	ins	TTG	18794629	ADAMTSL1
9	27005350	27005358	C	ins	CCG	27005352	IFT74
9	27005350	27005358	C	ins	CCG	27005352	LRRC19
9	27551081	27551090	A	ins	C	27551085	C9orf72
9	34624216	34624225	G	ins	GGC	34624217	ARID3C
9	34991612	34991621	C	ins	T	34991615	DNAJB5
9	34991612	34991621	C	ins	T	34991616	DNAJB5
9	34991612	34991621	C	ins	T	34991618	DNAJB5
9	35058763	35058773	A	ins	C	35058766	VCP
9	86614103	86614112	A	ins	AT	86614105	RMI1
9	87636617	87636627	T	ins	C	87636618	NTRK2
9	91978182	91978190	C	ins	A	91978184	SEMA4D
9	93637696	93637704	A	ins	AAAAG	93637699	SYK

9	102988032	102988040	A	ins	G	102988035	INVS
9	103278093	103278103	A	ins	C	103278095	C9orf30-TMEFF1
9	103278093	103278103	A	ins	C	103278095	TMEFF1
9	125001503	125001511	T	ins	TG	125001509	RBM18
9	129171760	129171768	G	ins	GC	129171761	FAM125B
9	129171760	129171768	G	ins	GC	129171761	NRON
9	134460749	134460758	G	ins	A	134460752	RAPGEF1
9	139653123	139653133	T	ins	TTC	139653129	LCN15
9	139653123	139653133	T	ins	TTC	139653129	LCN8
9	139653123	139653133	T	ins	TC	139653130	LCN15
9	139653123	139653133	T	ins	TC	139653130	LCN8
9	141011736	141011746	G	ins	T	141011739	CACNA1B
X	218114	218122	T	ins	G	218115	PLCXD1
X	9717266	9717274	C	ins	T	9717271	GPR143
X	24076639	24076648	T	ins	TCTTTC	24076642	EIF2S3
X	24076639	24076648	T	ins	CTTTCTTTTTC	24076643	EIF2S3
X	45707372	45707381	A	ins	C	45707373	AK098783
X	47342912	47342921	C	ins	A	47342919	ZNF41
X	48435396	48435404	T	ins	C	48435400	RBM3
X	53675483	53675492	A	ins	AC	53675485	HUWE1
X	53675483	53675492	A	ins	C	53675486	HUWE1
X	86086804	86086814	A	ins	G	86086808	DACH2
X	100479171	100479179	T	ins	TTTG	100479174	DRP2
X	100534956	100534966	A	ins	C	100534957	TAF7L
X	101576162	101576172	C	ins	CA	101576164	NXF2
X	101576162	101576172	C	ins	CA	101576164	NXF2
X	101576162	101576172	C	ins	CA	101576164	NXF2
X	101576162	101576172	C	ins	CA	101576164	NXF2
X	101576162	101576172	C	ins	CA	101576164	NXF2
X	101576162	101576172	C	ins	CA	101576164	NXF2
X	101576162	101576172	C	ins	CA	101576164	NXF2B
X	101576162	101576172	C	ins	CA	101576164	NXF2B
X	101576162	101576172	C	ins	CA	101576164	NXF2B
X	101576162	101576172	C	ins	CA	101576164	NXF2B
X	101576162	101576172	C	ins	CA	101576164	NXF2B
X	101576162	101576172	C	ins	CA	101576164	NXF2B
X	134031603	134031611	A	ins	AG	134031606	MOSPD1
X	153185235	153185245	T	ins	AA	153185243	ARHGAP4
10	854691	854707	CA	ins	AT	854695	LARP4B
10	11996642	11996660	AT	del	T	11996644	UPF2
10	14940089	14940103	TA	ins	AC	14940097	DCLRE1C
10	14940089	14940103	TA	ins	AC	14940097	SUV39H2
10	25940011	25940021	AG	del	GAGAGAG	25940013	AK123440

10	42863998	42864012	AT	ins	AC	42864009	LOC441666
10	72433020	72433038	GT	ins	TA	72433022	ADAMTS14
10	85993089	85993103	AT	del	A	85993100	LRIT1
10	93030859	93030873	TA	ins	ATATATATATAATCTA	93030861	PCGF5
10	95129015	95129033	GT	ins	TA	95129021	MYOF
10	103023054	103023070	TG	ins	GA	103023060	AX747408
10	105669301	105669311	AT	ins	TG	105669303	OBFC1
10	134725791	134725801	CT	ins	CTC	134725794	TTC40
11	5689541	5689551	AT	ins	TG	5689543	TRIM5
11	22360607	22360621	TA	del	TATATGT	22360616	SLC17A6
11	26701941	26701957	AT	ins	AC	26701954	SLC5A12
11	58909399	58909415	AG	ins	AA	58909412	BC028022
11	58909399	58909415	AG	ins	AA	58909412	FAM111A
11	73072965	73072977	CA	del	ACA	73072973	ARHGEF17
11	116715334	116715344	AT	del	TATATAATATATTATAT	116715338	SIK3
11	118373018	118373032	AT	ins	AN	118373029	MLL
11	133785151	133785163	TC	del	CTC	133785159	IGSF9B
12	3574295	3574313	AT	del	T	3574297	DQ588965
12	3574295	3574313	AT	del	T	3574297	PRMT8
12	20832828	20832838	AT	del	ATATA	20832833	PDE3A
12	21453018	21453032	AG	del	GAGAG	21453026	SLCO1A2
12	26637108	26637118	TA	ins	TG	26637111	ITPR2
12	31946004	31946020	TC	del	CTC	31946016	H3F3C
12	41323138	41323156	CA	del	C	41323153	CNTN1
12	50572734	50572750	AT	del	A	50572747	LIMA1
12	51124153	51124165	TC	del	TCTCT	51124160	DIP2B
12	53413207	53413221	TA	del	ATATA	53413215	EIF4B
12	54961629	54961647	AT	del	A	54961644	PDE1B
12	65638047	65638065	CA	ins	C	65638062	LEMD3
12	78513832	78513842	TA	ins	A	78513836	NAV3
12	98896617	98896633	CA	del	A	98896619	LOC643770
12	98896617	98896633	CA	del	A	98896619	LOC643770
12	98896617	98896633	CA	del	A	98896619	TRNA_Asp
12	112465112	112465130	AT	del	A	112465127	NAA25
12	113321659	113321673	AT	del	A	113321666	RPH3A
12	113321659	113321673	AT	del	T	113321667	RPH3A
12	117188696	117188714	AT	ins	T	117188710	RNFT2
12	122459822	122459834	TG	ins	TGTA	122459829	BCL7A
12	123211475	123211489	AT	ins	AG	123211484	HCAR1
13	31220478	31220492	TG	del	TGT	31220489	USPL1
13	42748100	42748118	GT	ins	GC	42748115	DGKH
13	70281008	70281020	CA	ins	CT	70281011	KLHL1
13	99539379	99539397	TA	del	ATA	99539393	DOCK9

13	114503154	114503168	TG	del	T	114503165	FAM70B
13	114779052	114779070	TC	ins	CA	114779067	RASA3
13	114779496	114779508	TC	ins	TA	114779499	RASA3
14	35571225	35571235	TC	del	CTC	35571231	AK128559
14	35571225	35571235	TC	del	CTC	35571231	PPP2R3C
14	65684969	65684979	AG	del	GAG	65684975	BX161428
14	91108265	91108277	TG	ins	GTGTGC	91108271	BC028746
14	91108265	91108277	TG	ins	GTGTGC	91108271	TTC7B
15	20646856	20646868	CA	ins	TA	20646858	HERC2P3
15	43109127	43109143	AT	del	T	43109139	TTBK2
15	44159755	44159773	AT	ins	TG	44159761	WDR76
15	54841997	54842007	TG	ins	TC	54842002	UNC13C
15	56962071	56962085	TA	del	TATAT	56962080	ZNF280D
15	57810124	57810134	TG	del	T	57810131	CGNL1
15	72049753	72049765	AT	del	A	72049762	THSD4
15	81643675	81643689	TC	del	TCTCT	81643684	TMC3
15	81643675	81643689	TC	del	CTC	81643685	TMC3
15	102029470	102029480	CG	ins	G	102029474	PCSK6
16	612585	612595	CA	ins	AG	612589	C16orf11
16	11645511	11645521	CA	del	C	11645518	LITAF
16	11985212	11985226	AT	del	A	11985223	GSPT1
16	19460015	19460025	AT	ins	TATAT	19460017	TMC5
16	20411376	20411394	TA	del	A	20411390	PDILT
16	58195466	58195476	AC	ins	ACAG	58195473	BC053935
16	58195466	58195476	AC	ins	ACAG	58195473	CSNK2A2
16	72007914	72007924	AT	del	A	72007917	PKD1L3
16	81059736	81059746	TA	ins	TG	81059743	CENPN
16	89596555	89596573	TG	del	G	89596557	SPG7
17	4385168	4385182	CT	del	C	4385179	AX748345
17	4385168	4385182	CT	del	C	4385179	SPNS3
17	16842761	16842771	TC	del	CTC	16842767	TNFRSF13B
17	19808330	19808344	AT	del	A	19808341	AKAP10
17	33802931	33802943	AT	ins	TT	33802939	SLFN12L
17	34303676	34303690	TC	del	TCT	34303687	CCL16
17	34418383	34418399	AT	del	A	34418392	CCL3
17	35871088	35871098	AT	del	ATATATA	35871091	DUSP14
17	42991733	42991751	CA	ins	CC	42991740	GFAP
17	48207167	48207177	GT	ins	GTGTGC	48207172	SAMD14
17	49231510	49231520	TC	ins	T	49231517	NME1
17	49231510	49231520	TC	ins	T	49231517	NME1
17	49231510	49231520	TC	ins	T	49231517	NME1-NME2
17	49231510	49231520	TC	ins	T	49231517	NME1-NME2
17	49231510	49231520	TC	ins	T	49231517	NME2

17	49231510	49231520	TC	ins	T	49231517	NME2
17	56654837	56654851	AT	del	A	56654848	TEX14
17	58126333	58126343	AT	del	ATATATA	58126336	HEATR6
17	58126333	58126343	AT	del	ATATA	58126338	HEATR6
17	58126333	58126343	AT	del	ATA	58126340	HEATR6
17	61779366	61779378	TG	ins	GG	61779374	STRADA
17	65906925	65906937	AT	del	T	65906927	BPTF
17	76165337	76165355	TG	del	TGTGTGTGT	76165342	SYNGR2
17	76165337	76165355	TG	del	T	76165346	SYNGR2
17	76165337	76165355	TG	del	TGTGT	76165348	SYNGR2
17	76165337	76165355	TG	ins	G	76165351	SYNGR2
17	76165337	76165355	TG	del	T	76165352	SYNGR2
17	79562547	79562561	AT	ins	T	79562555	NPLOC4
17	79562547	79562561	AT	del	ATA	79562556	NPLOC4
17	79562547	79562561	AT	ins	T	79562557	NPLOC4
18	267019	267037	TA	del	T	267034	THOC1
18	3174076	3174088	AC	ins	AT	3174083	MYOM1
18	3176190	3176200	AC	ins	AT	3176197	MYOM1
18	5245395	5245409	AG	ins	AGAT	5245404	LOC339290
18	19204662	19204680	AT	del	T	19204664	SNRPD1
18	21723508	21723524	AT	del	A	21723521	CABYR
18	51807390	51807404	TG	ins	TA	51807399	POLI
19	926482	926492	GC	del	G	926487	ARID3A
19	3601365	3601377	AT	del	T	3601367	TBXA2R
19	3699361	3699373	CT	ins	TT	3699367	PIP5K1C
19	6710192	6710202	GA	ins	AA	6710196	C3
19	7943096	7943110	AT	ins	T	7943106	LOC388499
19	9053608	9053618	AT	del	ATA	9053615	MUC16
19	13371129	13371147	AT	ins	T	13371143	CACNA1A
19	13371129	13371147	AT	del	A	13371144	CACNA1A
19	14768542	14768558	AT	ins	T	14768554	EMR3
19	33694230	33694242	TG	ins	GA	33694232	LRP3
19	45032851	45032869	AC	del	A	45032860	CEACAM20
19	45900720	45900732	TC	del	C	45900724	PPP1R13L
19	47918816	47918830	TC	del	CTCTC	47918824	MEIS3
19	51320944	51320954	TC	del	T	51320951	MGC45922
19	51982185	51982195	AC	ins	CACACG	51982189	CEACAM18
19	53281264	53281276	CT	del	TCTTT	53281272	ZNF600
19	55397658	55397676	CA	del	C	55397673	FCAR
19	56348973	56348989	AC	ins	C	56348979	NLRP11
19	56348973	56348989	AC	ins	C	56348979	NLRP4
19	56488580	56488594	AT	ins	TG	56488586	NLRP8
1	7849200	7849218	AT	ins	TATG	7849202	PER3

1	7849200	7849218	AT	ins	TG	7849204	PER3
1	9496942	9496960	TA	del	ATATATA	9496952	5S_rRNA
1	43107917	43107935	AT	del	T	43107919	CCDC30
1	54705780	54705794	GC	del	GCA	54705791	SSBP3
1	62911299	62911317	TG	ins	TGTA	62911302	USP1
1	85562162	85562176	TA	del	TATATATAAAGAT	85562167	WDR63
1	92596076	92596094	AT	del	T	92596078	BTBD8
1	94468544	94468554	TC	ins	CA	94468550	ABCA4
1	95631007	95631023	TG	ins	G	95631019	AK090700
1	95631007	95631023	TG	ins	G	95631019	TMEM56
1	95631007	95631023	TG	ins	G	95631019	TMEM56-RWDD3
1	95631007	95631023	TG	del	T	95631020	AK090700
1	95631007	95631023	TG	del	T	95631020	TMEM56
1	95631007	95631023	TG	del	T	95631020	TMEM56-RWDD3
1	109773690	109773706	AT	del	A	109773703	SARS
1	113202196	113202208	TC	ins	T	113202205	CAPZA1
1	156752695	156752705	AT	del	A	156752702	PRCC
1	161279325	161279343	AT	ins	T	161279339	MPZ
1	161279325	161279343	AT	del	A	161279340	MPZ
1	171251672	171251682	AT	del	A	171251679	FMO1
1	173174063	173174081	AC	ins	AT	173174074	TNFSF4
1	179999450	179999460	TA	ins	TG	179999453	CEP350
1	183114853	183114869	TA	ins	AA	183114863	LAMC1
1	200816519	200816537	TG	del	G	200816521	CAMSAP2
1	207243362	207243374	AC	ins	A	207243365	PFKFB2
1	222711754	222711764	GA	ins	AGAT	222711760	HHIPL2
1	233431698	233431714	TC	ins	TGTT	233431711	PCNXL2
1	243389360	243389370	AT	ins	AG	243389363	CEP170
1	243389360	243389370	AT	ins	TT	243389364	CEP170
20	42817214	42817224	GT	del	G	42817217	JPH2
20	47258362	47258380	AG	del	A	47258375	PREX1
20	62166759	62166771	CA	ins	CACT	62166764	PTK6
21	15671076	15671094	AT	del	A	15671091	ABCC13
21	19641254	19641266	CT	del	C	19641261	TMPRSS15
21	23468273	23468291	AT	ins	T	23468287	BC039377
21	37519205	37519221	TC	ins	TTTT	37519214	CBR3
21	37519205	37519221	TC	ins	TTTT	37519214	LOC100506428
21	37519205	37519221	TC	del	CTCTC	37519215	CBR3
21	37519205	37519221	TC	del	CTCTC	37519215	LOC100506428
21	37519205	37519221	TC	del	CTC	37519217	CBR3
21	37519205	37519221	TC	del	CTC	37519217	LOC100506428
22	23082678	23082688	GC	ins	GT	23082683	abParts
22	23082678	23082688	GC	ins	GT	23082683	DKFZp667J0810

22	24407619	24407631	CG	ins	CA	24407622	CABIN1
22	32545637	32545655	AT	del	T	32545639	C22orf42
22	39438305	39438315	TC	del	TCTCT	39438310	APOBEC3F
22	39438305	39438315	TC	del	TCTCT	39438310	APOBEC3G
22	39438305	39438315	TC	del	CTC	39438311	APOBEC3F
22	39438305	39438315	TC	del	CTC	39438311	APOBEC3G
22	40363000	40363018	CA	del	A	40363012	GRAP2
22	43044845	43044859	AC	del	A	43044856	CYB5R3
2	1157068	1157078	AG	del	G	1157074	SNTG2
2	9584269	9584287	TA	del	ATA	9584283	CPSF3
2	10548388	10548404	TC	del	C	10548398	HPCAL1
2	29430942	29430952	TC	del	T	29430949	ALK
2	37898953	37898963	GC	ins	GT	37898956	CDC42EP3
2	42141970	42141980	TG	del	G	42141972	Mir_544
2	47083032	47083044	TA	del	A	47083040	LOC100134259
2	54095619	54095633	AT	del	T	54095629	PSME4
2	61459977	61459993	TA	del	TAT	61459990	USP34
2	65129344	65129358	CT	ins	TCTT	65129350	LOC400958
2	65129344	65129358	CT	ins	TT	65129352	LOC400958
2	78640581	78640597	TA	ins	TT	78640588	BC024248
2	79601635	79601647	AT	del	A	79601644	CTNNA2
2	85867727	85867743	AT	del	A	85867740	USP39
2	128567627	128567637	AC	ins	ACATATAT	128567634	WDR33
2	159660637	159660647	AT	del	A	159660644	DAPL1
2	166768165	166768179	AT	ins	T	166768173	TTC21B
2	166768165	166768179	AT	del	A	166768176	TTC21B
2	167302095	167302109	TC	del	TCT	167302106	SCN7A
2	189654185	189654199	AT	ins	AC	189654190	DIRC1
2	190527954	190527966	AT	del	A	190527961	ASNSD1
2	190527954	190527966	AT	del	A	190527963	ASNSD1
2	201347489	201347505	TG	del	G	201347491	SPATS2L
2	207654314	207654328	AC	ins	AA	207654319	FASTKD2
2	220400049	220400065	TG	del	T	220400062	ACCN4
3	21465793	21465809	TA	ins	T	21465806	ZNF385D
3	37088088	37088102	TA	ins	T	37088095	MLH1
3	58900403	58900419	AT	del	A	58900416	AK090895
3	58900403	58900419	AT	del	A	58900416	C3orf67
3	58900403	58900419	AT	del	A	58900416	C3orf67
3	68780655	68780671	AT	ins	TATG	68780659	FAM19A4
3	74473543	74473561	AT	ins	AC	74473556	CNTN3
3	129693077	129693093	TC	ins	TCTT	129693088	TRH
3	129693077	129693093	TC	ins	TT	129693090	TRH
3	133906994	133907012	AT	ins	T	133907006	RYK

3	133906994	133907012	AT	del	ATA	133907007	RYK
3	133906994	133907012	AT	del	A	133907009	RYK
3	140851494	140851512	TA	del	A	140851508	SPSB4
3	150792800	150792810	TA	ins	AC	150792806	CLRN1-AS1
3	158413966	158413980	TA	ins	TT	158413973	RARRES1
3	173774697	173774709	AT	del	A	173774706	7SK
3	173774697	173774709	AT	del	A	173774706	NLGN1
3	179138243	179138259	AT	del	A	179138256	GNB4
3	182584708	182584720	AT	ins	AC	182584713	ATP11B
3	191359025	191359039	TA	del	ATATA	191359033	Y_RNA
3	196043922	196043932	GT	ins	G	196043929	TCTEX1D2
3	196043922	196043932	GT	ins	G	196043929	TCTEX1D2
3	196043922	196043932	GT	ins	G	196043929	TM4SF19
3	196043922	196043932	GT	ins	G	196043929	TM4SF19
4	2701173	2701189	CA	del	ACA	2701175	FAM193A
4	37831469	37831481	AT	ins	AC	37831478	PGM2
4	47940331	47940349	AT	del	A	47940346	BC041434
4	47940331	47940349	AT	del	A	47940346	CNGA1
4	57343449	57343467	TC	del	CTC	57343463	SRP72
4	71248323	71248337	TG	del	T	71248334	SMR3B
4	77054200	77054218	AT	del	A	77054215	NUP54
4	82065748	82065764	TA	del	A	82065760	PRKG2
4	87870315	87870333	AT	del	A	87870330	AFF1
4	91839789	91839805	AT	ins	AA	91839792	FAM190A
4	100055531	100055541	TA	ins	ATAA	100055533	ADH4
4	100055531	100055541	TA	ins	ATAA	100055533	LOC100507053
4	103500803	103500821	AT	del	T	103500805	NFKB1
4	110606384	110606394	TC	del	CTC	110606390	CCDC109B
4	114286834	114286846	TC	del	TCTTA	114286843	ANK2
4	114822862	114822876	AT	ins	TT	114822866	ARSJ
4	159093344	159093354	AC	del	ACACA	159093349	AK096792
4	159093344	159093354	AC	del	ACACA	159093349	AK096792
4	159093344	159093354	AC	del	ACACA	159093349	AK126266
4	159093344	159093354	AC	del	ACACA	159093349	FAM198B
4	159093344	159093354	AC	del	ACACA	159093349	FAM198B
4	159093344	159093354	AC	del	ACACA	159093349	FAM198B
4	174235079	174235095	AT	del	A	174235092	GALNT7
4	185617841	185617853	AT	ins	A	185617844	MLF1IP
4	189030305	189030319	AC	ins	CG	189030313	TRIML2
5	412818	412830	TC	del	TCT	412827	AHRR
5	13912589	13912601	AC	ins	AT	13912598	DNAH5
5	37516814	37516828	AT	del	A	37516825	WDR70
5	58295380	58295394	TA	ins	TATG	58295389	PDE4D

5	61027630	61027640	AG	ins	A	61027635	BC039381	
5	64874562	64874572	AT	ins	A	64874567	PPWD1	
5	68472353	68472369	TA	ins	T	68472366	CCNB1	
5	76371866	76371880	TA	ins	TG	76371873	ZBED3	
5	78360550	78360560	AT	del	A	78360557	DMGDH	
5	81550695	81550709	AG	ins	GT	81550703	ATG10	
5	89947204	89947214	TC	del	CTC	89947210	GPR98	
5	94826193	94826209	AT	del	A	94826206	TTC37	
5	110448734	110448752	AT	del	A	110448749	WDR36	
5	137683541	137683555	CT	ins	TA	137683543	FAM53C	
5	150837902	150837918	TA	ins	AC	150837912	SLC36A1	
5	167992873	167992891	AT	del	A	167992888	PANK3	
5	172584913	172584929	TG	ins	T	172584926	BNIP1	
6	28611648	28611664	AT	del	ATATATA	28611655	TRNA_Ala	
6	29006981	29006997	TA	ins	T	29006994	LOC100129636	
6	32359921	32359931	TA	del	A	32359923	HCG23	
6	32359921	32359931	TA	del	ATATA	32359925	HCG23	
6	33625592	33625610	TG	del	T	33625599	ITPR3	
6	35006853	35006869	TG	ins	G	35006865	ANKS1A	
6	36894335	36894345	AG	del	G	36894341	C6orf89	
6	42995643	42995659	AC	ins	AT	42995654	RRP36	
6	43304668	43304686	AT	ins	T	43304678	ZNF318	
6	88312613	88312623	AT	del	TATATAT	88312615	ORC3	
6	89553669	89553681	TA	ins	AG	89553675	RNGTT	
6	89809104	89809120	CT	del	T	89809114	SRSF12	
6	154678762	154678776	TA	del	ATATATA	154678768	CNKSR3	
6	154678762	154678776	TA	del	ATATATA	154678768	IPCEF1	
6	154678762	154678776	TA	del	ATATATA	154678768	IPCEF1	
6	160390971	160390989	AT	del	A	160390986	IGF2R	
6	160677920	160677934	TC	del	CTC	160677930	SLC22A2	
7	13936315	13936325	AG	ins	GAGAGAGAAAGAAAGAAAG		13936317	AK055368
7	13936315	13936325	AG	ins	GAGAGAGAAAGAAAGAAAG		13936317	ETV1
7	30898268	30898286	AT	del	T	30898270	AQP1	
7	30898268	30898286	AT	del	T	30898270	FAM188B	
7	31149737	31149755	CT	ins	T	31149751	ADCYAP1R1	
7	31149737	31149755	CT	del	C	31149752	ADCYAP1R1	
7	66309053	66309063	TC	del	C	66309057	LOC729156	
7	66309053	66309063	TC	del	C	66309059	LOC729156	
7	73254451	73254463	TG	del	TGT	73254458	WBSCR27	
7	73254451	73254463	TG	del	T	73254460	WBSCR27	
7	73804452	73804466	AG	ins	AGAGAA	73804463	CLIP2	
7	74232974	74232990	CT	del	TCT	74232986	GTF2IRD2	
7	101755512	101755522	TC	ins	T	101755519	CUX1	

7	124783258	124783270	TC	del	CTC	124783266	AX746567
7	124783258	124783270	TC	del	CTC	124783266	BC142949
7	124783258	124783270	TC	del	CTC	124783266	BX648695
7	128528349	128528361	AC	ins	AT	128528356	KCP
7	128545037	128545053	GT	del	G	128545050	KCP
7	138340385	138340395	TA	del	TATAT	138340390	SVOPL
7	157449536	157449552	CA	del	CAC	157449547	PTPRN2
8	1949199	1949209	CA	del	ACA	1949205	KBTBD11
8	2148364	2148374	TG	ins	TC	2148367	AX747124
8	15094179	15094189	AT	del	T	15094185	SGCZ
8	20007083	20007099	TC	ins	CC	20007093	SLC18A1
8	22134531	22134547	AT	ins	T	22134543	PIWIL2
8	22134531	22134547	AT	del	A	22134544	PIWIL2
8	27529159	27529169	CA	ins	AT	27529163	SCARA3
8	77595867	77595877	AG	del	A	77595874	LOC100192378
8	77595867	77595877	AG	del	A	77595874	ZFHX4
8	82395812	82395826	TG	ins	G	82395822	FABP4
8	82395812	82395826	TG	del	T	82395823	FABP4
8	118846854	118846864	AC	ins	ACACAT	118846859	EXT1
8	141677549	141677565	TC	del	CTCTC	141677559	PTK2
9	21801809	21801823	GT	ins	GTGTGTGTGTGC	21801812	MTAP
9	114376450	114376468	TA	ins	T	114376465	C9orf29
9	127616792	127616810	AC	del	C	127616794	WDR38
9	131368751	131368767	AT	del	A	131368764	SPTAN1
X	1402366	1402380	CT	del	C	1402373	CRLF2
X	1402366	1402380	CT	del	C	1402373	CRLF2
X	1402366	1402380	CT	del	C	1402373	CSF2RA
X	1402366	1402380	CT	del	C	1402373	CSF2RA
X	2650603	2650615	CT	ins	TT	2650611	CD99
X	8555161	8555175	TG	del	T	8555172	KAL1
X	55511947	55511965	AT	del	T	55511949	USP51
X	118590953	118590965	AG	del	GAGAAAGAAAG	118590961	Y_RNA
X	133694524	133694542	GT	ins	TA	133694528	LOC100506757
10	7608748	7608763	AAG	ins	G	7608752	ITIH5
10	95352569	95352587	ATT	del	A	95352583	RBP4
10	100992956	100992977	CAC	del	C	100992972	HPSE2
10	131335406	131335418	AAG	del	AAGA	131335414	MGMT
11	5877547	5877559	TAT	del	TA	5877552	OR52E8
11	5877547	5877559	TAT	del	TA	5877552	TRIM5
11	5877547	5877559	TAT	del	TT	5877554	OR52E8
11	5877547	5877559	TAT	del	TT	5877554	TRIM5
11	5877547	5877559	TAT	del	TA	5877555	OR52E8
11	5877547	5877559	TAT	del	TA	5877555	TRIM5

11	34219989	34220001	AAC	ins	A	34219997	ABTB2
11	65035810	65035828	TTA	ins	T	65035824	POLA2
11	117280715	117280730	CCT	del	C	117280723	CEP164
12	32482023	32482041	ATT	ins	T	32482035	BICD1
12	32482023	32482041	ATT	del	A	32482037	BICD1
12	47473124	47473148	GTT	del	G	47473141	AMIGO2
12	47473124	47473148	GTT	del	G	47473141	FAM113B
12	54113690	54113702	CTG	del	G	54113697	CALCOCO1
12	93192111	93192129	ATT	ins	T	93192120	EEA1
12	96884473	96884485	AAT	del	AATA	96884481	C12orf55
12	120739162	120739183	AAC	del	AC	120739165	SIRT4
12	124299599	124299617	CAA	ins	AAA	124299608	DNAH10
14	65402047	65402059	ATA	del	AATAA	65402054	CHURC1
14	65402047	65402059	ATA	del	AATAA	65402054	CHURC1-FNTB
14	65545228	65545252	TGT	ins	T	65545247	MAX
14	74003680	74003692	GTT	del	TTGT	74003686	ACOT1
14	74003680	74003692	GTT	del	TTGT	74003686	ACOT1
14	74003680	74003692	GTT	del	TTGT	74003686	HEATR4
14	74003680	74003692	GTT	del	TG	74003687	ACOT1
14	74003680	74003692	GTT	del	TG	74003687	ACOT1
14	74003680	74003692	GTT	del	TG	74003687	HEATR4
14	74003680	74003692	GTT	del	G	74003688	ACOT1
14	74003680	74003692	GTT	del	G	74003688	ACOT1
14	74003680	74003692	GTT	del	G	74003688	HEATR4
14	74450378	74450399	ATT	del	TA	74450391	ENTPD5
14	74450378	74450399	ATT	del	A	74450392	ENTPD5
14	74450378	74450399	ATT	del	TA	74450394	ENTPD5
14	74450378	74450399	ATT	del	A	74450395	ENTPD5
14	88631472	88631484	CAG	del	A	88631475	DQ574857
14	88631472	88631484	CAG	del	A	88631475	DQ577549
15	96811957	96811975	TCT	del	T	96811970	AK000872
15	96811957	96811975	TCT	del	T	96811970	AK307134
15	96811957	96811975	TCT	ins	C	96811971	AK000872
15	96811957	96811975	TCT	ins	C	96811971	AK307134
16	2770513	2770531	ATT	del	A	2770527	PRSS27
16	8873107	8873125	TGG	del	GGTG	8873119	ABAT
16	30971061	30971073	TTC	del	TTCT	30971069	SETD1A
16	84766037	84766052	CTT	del	TC	84766047	USP10
16	84766037	84766052	CTT	del	C	84766048	USP10
16	84801372	84801384	ATT	del	A	84801380	USP10
17	637413	637431	TTC	del	C	637426	FAM57A
17	1482442	1482463	AAT	del	T	1482446	SLC43A2
17	8145342	8145357	ATT	ins	T	8145348	CTC1

17	8145342	8145357	ATT	del	TA	8145349	CTC1
17	8145342	8145357	ATT	del	TA	8145352	CTC1
17	45940923	45940935	CTT	ins	T	45940929	BC031827
17	47014823	47014841	TAA	ins	T	47014828	SNF8
17	49255306	49255321	TAA	del	AT	49255316	MBTD1
17	66919330	66919348	TAT	ins	T	66919343	ABCA8
18	9830003	9830024	TAT	ins	TAA	9830011	Metazoa_SRP
18	9830003	9830024	TAT	ins	TAA	9830011	RAB31
18	24916060	24916072	TTC	del	TTCTT	24916068	AK127888
18	48723137	48723152	CCG	del	CGCCGCCG	48723143	MEX3C
19	520504	520516	AAC	ins	A	520509	TPGS1
19	5668581	5668596	ATT	del	A	5668592	SAFB
19	7614370	7614382	CTT	del	TC	7614377	PNPLA6
19	8151331	8151352	TTA	del	A	8151347	FBN3
19	8463827	8463845	ATT	del	A	8463841	RAB11B
19	10285394	10285412	AAC	ins	A	10285408	DNMT1
19	40355497	40355515	TTG	del	G	40355510	FCGBP
19	40954498	40954513	AAC	ins	AACAACAACAAT	40954503	BLVRB
19	44454816	44454840	AAT	ins	TC	44454834	ZNF221
19	47909520	47909532	GGA	del	GGAT	47909528	MEIS3
19	50956766	50956781	TTG	del	TG	50956775	MYBPC2
19	54938780	54938798	AAC	del	C	54938793	TTYH1
19	56309354	56309366	TAT	ins	TT	56309358	NLRP11
19	56309354	56309366	TAT	del	A	56309360	NLRP11
1	12197371	12197389	AAT	ins	ATC	12197383	TNFRSF8
1	28298103	28298118	CAA	del	AC	28298113	EYA3
1	53154816	53154834	ATT	del	A	53154830	SELRC1
1	75687634	75687646	TCT	ins	T	75687641	SLC44A5
1	75687634	75687646	TCT	del	TC	75687642	SLC44A5
1	89352593	89352611	ATT	del	A	89352607	GTF2B
1	183849509	183849521	AAC	del	AC	183849515	RGL1
1	235618437	235618452	TTA	ins	TTG	235618448	B3GALNT2
1	236157987	236158002	CTT	ins	C	236157998	NID1
20	25423232	25423247	TAT	del	TA	25423243	GINS1
20	47692710	47692722	TTG	del	TTGTT	47692718	CSE1L
22	17668980	17668992	AAG	del	AAGAA	17668988	CECR1
22	23914597	23914618	AAC	del	AACAACAACAA	23914608	IGLL1
22	42121115	42121127	ATT	del	A	42121123	bK250D10.C22.8
22	42121115	42121127	ATT	del	A	42121123	MEI1
2	9629286	9629301	TTG	ins	TTC	9629297	ADAM17
2	9629286	9629301	TTG	ins	TTC	9629297	IAH1
2	24105678	24105696	CAA	del	AC	24105691	ATAD2B
2	24105678	24105696	CAA	del	C	24105692	ATAD2B

2	24888877	24888892	TTA	del	A	24888887	NCOA1
2	25365630	25365645	AAT	del	AATA	25365641	EFR3B
2	55864006	55864030	AAC	del	C	55864013	PNPT1
2	61576979	61577003	AAC	del	AC	61576997	USP34
2	114380292	114380304	AAC	del	AACA	114380300	RPL23AP7
2	160290662	160290686	ATT	del	A	160290682	BAZ2B
2	176989013	176989025	CTT	del	C	176989021	HOXD9
2	192921935	192921947	ATT	del	TA	192921942	TMEFF2
2	192921935	192921947	ATT	del	A	192921943	TMEFF2
2	202698422	202698440	TCT	del	TTCT	202698435	CDK15
2	202698422	202698440	TCT	del	TC	202698436	CDK15
2	211341196	211341208	GGC	ins	C	211341203	LANCL1
3	31639319	31639337	ATT	del	A	31639333	STT3B
3	126181905	126181923	ATT	del	A	126181916	ZXDC
3	126181905	126181923	ATT	del	A	126181919	ZXDC
3	129697384	129697396	TAA	ins	ATAAT	129697388	TRH
3	178740419	178740440	AAT	del	T	178740423	ZMAT3
3	183685912	183685930	TTA	ins	ATC	183685916	ABCC5
3	197711350	197711365	GCT	ins	TGCTGT	197711360	LMLN
4	8224403	8224421	TTG	ins	GTC	8224413	SH3TC1
4	39917816	39917828	AAC	del	AC	39917822	PDS5A
4	39917816	39917828	AAC	del	C	39917823	PDS5A
4	41684683	41684695	TTC	ins	T	41684691	LIMCH1
4	47628988	47629012	GTT	del	G	47629008	CORIN
4	48089443	48089458	TTA	ins	T	48089454	TXK
4	57261021	57261033	ATT	del	A	57261029	PPAT
4	171979888	171979906	AAC	del	AACAACAACAACAA	171979893	LOC100506122
5	134180421	134180436	TTG	del	TTGT	134180432	C5orf24
5	141045619	141045640	AAT	del	T	141045623	ARAP3
5	149599433	149599454	TTA	ins	T	149599444	CAMK2A
5	149633420	149633441	AGC	ins	CAA	149633424	CAMK2A
5	179665596	179665608	TTC	del	TTCT	179665604	MAPK9
6	24418571	24418583	ATT	del	TA	24418578	MRS2
6	24418571	24418583	ATT	del	A	24418579	MRS2
6	24422375	24422387	CAA	del	AC	24422382	MRS2
6	24422375	24422387	CAA	del	C	24422383	MRS2
6	27745584	27745608	AAC	ins	A	27745595	TRNA_Met
6	27745584	27745608	AAC	del	ACAACAAC	27745596	TRNA_Met
6	31765848	31765863	ATT	del	A	31765859	LSM2
6	32361455	32361467	TTC	ins	C	32361462	HCG23
6	42630675	42630690	AAC	ins	A	42630686	UBR2
6	44143522	44143537	AAT	ins	AAC	44143533	CAPN11
6	109906329	109906344	CTT	del	TC	109906339	AKD1

6	109906329	109906344	CTT	del	C	109906340	AKD1
6	116263809	116263821	CAA	del	C	116263817	FRK
6	144069196	144069208	ATT	ins	T	144069202	PHACTR2
6	144069196	144069208	ATT	del	A	144069204	PHACTR2
7	73821075	73821099	ATT	del	TA	73821079	CLIP2
7	76240186	76240198	ATT	del	TA	76240193	LOC100133091
7	76240186	76240198	ATT	del	TA	76240193	LOC100133091
7	76240186	76240198	ATT	del	TA	76240193	LOC100133091
7	76240186	76240198	ATT	del	TA	76240193	POMZP3
7	76240186	76240198	ATT	del	TA	76240193	POMZP3
7	76240186	76240198	ATT	del	TA	76240193	POMZP3
7	76240186	76240198	ATT	del	A	76240194	LOC100133091
7	76240186	76240198	ATT	del	A	76240194	LOC100133091
7	76240186	76240198	ATT	del	A	76240194	LOC100133091
7	76240186	76240198	ATT	del	A	76240194	POMZP3
7	76240186	76240198	ATT	del	A	76240194	POMZP3
7	76240186	76240198	ATT	del	A	76240194	POMZP3
7	129592528	129592543	GCC	del	G	129592539	UBE2H
7	144462339	144462360	AAC	ins	AA	144462356	TPK1
8	39836864	39836876	TTA	del	A	39836871	IDO2
8	59505464	59505476	AAC	del	C	59505471	NSMAF
8	59505464	59505476	AAC	del	C	59505471	TRNA_Glu
8	59505464	59505476	AAC	ins	A	59505472	NSMAF
8	59505464	59505476	AAC	ins	A	59505472	TRNA_Glu
8	125991724	125991736	ATT	del	TA	125991731	ZNF572
8	125991724	125991736	ATT	del	A	125991732	ZNF572
9	18794631	18794643	TTG	del	TG	18794634	ADAMTSL1
9	18794631	18794643	TTG	ins	T	18794639	ADAMTSL1
9	36190459	36190471	TCT	del	TTCT	36190466	CLTA
9	36190459	36190471	TCT	del	TC	36190467	CLTA
9	94814803	94814818	ATT	del	A	94814814	SPTLC1
9	109859659	109859671	TTA	ins	ATTA	109859666	AK097706
9	114450023	114450035	TTC	del	TTCT	114450031	C9orf84
9	118093809	118093833	ATG	ins	ATGATT	118093820	DEC1
9	133364619	133364634	ATT	ins	T	133364628	ASS1
9	133364619	133364634	ATT	del	A	133364630	ASS1
X	123021841	123021853	AAT	del	TAATAAT	123021845	XIAP
X	123021841	123021853	AAT	del	TAAT	123021848	XIAP
X	139867337	139867349	AAT	ins	ATAAT	139867343	AK054921
X	139867337	139867349	AAT	ins	ATAAT	139867343	CDR1
10	26592373	26592397	AGGG	ins	AGGA	26592384	GAD2
10	52498935	52498959	ATTG	ins	TGA	52498944	ASAH2B
10	91341166	91341186	ATTT	del	TTTAT	91341178	PANK1

10	95855077	95855105	TTTC	ins	TC	95855082	AK098548
10	95855077	95855105	TTTC	ins	TC	95855082	PLCE1
10	98759684	98759704	TAGA	ins	GATAGAT	98759689	SLIT1
10	111000308	111000332	AGGA	ins	G	111000320	U6
10	111000308	111000332	AGGA	del	GAAGGAA	111000321	U6
10	121435264	121435280	TTCC	ins	TCCTTCCCTCCTTCCC	121435272	BAG3
11	49829394	49829410	AAAT	del	A	49829401	LOC440040
11	50234633	50234649	GGAA	ins	A	50234642	TRNA_Ala
11	50234633	50234649	GGAA	ins	AG	50234644	TRNA_Ala
11	65151237	65151253	AAAC	del	C	65151243	SLC25A45
11	65151237	65151253	AAAC	ins	A	65151244	SLC25A45
11	66242189	66242213	ATTT	del	A	66242208	PELI3
11	123677265	123677281	TTTG	del	TG	123677274	OR6M1
11	123677265	123677281	TTTG	del	G	123677275	OR6M1
11	128785857	128785877	TGGA	del	G	128785869	KCNJ5
12	3737165	3737185	TCCC	del	CT	3737175	EFCAB4B
12	8808349	8808369	CAAA	del	AAACA	8808361	MFAP5
12	32831183	32831203	TTTA	del	TTTAT	32831194	DNM1L
12	32831326	32831346	TTTG	ins	T	32831341	DNM1L
12	53678812	53678832	AAGA	del	G	53678821	ESPL1
12	57601080	57601108	TAAA	ins	A	57601096	LRP1
12	66858713	66858737	CTTT	ins	TT	66858721	GRIP1
12	66858713	66858737	CTTT	del	C	66858724	GRIP1
12	92822582	92822602	CTTT	ins	TCTC	92822592	CLLU1
12	92822582	92822602	CTTT	ins	TCTC	92822592	CLLU1
12	92822582	92822602	CTTT	ins	TCTC	92822592	CLLU1OS
12	123071339	123071367	TATT	ins	T	123071360	KNTC1
12	123921874	123921898	TTCT	ins	T	123921892	RILPL2
13	36801636	36801652	AAAT	del	AT	36801645	CCDC169
13	36801636	36801652	AAAT	del	AT	36801645	CCDC169-SOHLH2
13	79233447	79233471	AAAG	ins	A	79233462	RNF219
13	96252828	96252844	AGAT	ins	GAT	96252836	DZIP1
13	103315467	103315483	ATTT	del	TTA	103315476	TPP2
14	23843921	23843941	TCTT	del	TTC	23843935	IL25
14	55408969	55408997	AAAT	ins	ATAC	55408982	WDHD1
14	55862071	55862091	TTTG	ins	TTTT	55862082	ATG14
14	55862071	55862091	TTTG	ins	TTTT	55862082	FBXO34
14	57396888	57396908	TTTC	ins	T	57396903	OTX2OS1
14	71443071	71443091	TCTT	del	TTC	71443085	PCNX
14	71443071	71443091	TCTT	del	TC	71443086	PCNX
14	74288374	74288402	TTTC	del	TTCTTTC	74288394	BC038204
14	75178777	75178797	AAAG	del	AG	75178786	KIAA0317
14	75178777	75178797	AAAG	del	AG	75178786	SNORA7

15	34047817	34047837	AAAC	ins	AA	34047828	RYR3
15	51689531	51689551	CCTC	ins	CCTT	51689542	GLDN
15	63674488	63674504	TTTG	del	G	63674494	CA12
15	63827115	63827143	TTTA	ins	TTAC	63827135	USP3
16	15488735	15488751	CTTT	del	TC	15488745	MPV17L
16	21281592	21281608	TTTC	del	CTTTC	21281602	CRYM
16	57993509	57993529	AAAC	del	C	57993515	CNGB1
16	58553712	58553732	ATCA	ins	A	58553726	CNOT1
16	58553712	58553732	ATCA	ins	A	58553726	SETD6
16	74699634	74699654	TTTC	ins	T	74699649	RFWD3
16	89617473	89617489	CAAA	del	C	89617484	SPG7
17	4119211	4119227	AAAC	ins	AACT	4119215	ANKFY1
17	4927833	4927849	GTCT	ins	TC	4927841	KIF1C
17	9532791	9532815	CTTT	del	C	9532810	WDR16
17	12895163	12895187	ACTT	ins	T	12895176	ARHGAP44
17	12895163	12895187	ACTT	ins	T	12895176	ELAC2
17	12895444	12895468	TTGA	del	TTGAT	12895459	ARHGAP44
17	12895444	12895468	TTGA	del	TTGAT	12895459	ELAC2
17	18153105	18153129	AAAC	del	A	18153124	FLII
17	33502079	33502107	AAGA	ins	AG	33502095	UNC45B
17	72250306	72250326	TTTA	del	A	72250320	TTYH2
17	76866503	76866531	AAAG	del	G	76866513	TIMP2
17	79527706	79527726	AAAC	del	A	79527721	NPLOC4
18	117490	117506	ATTG	ins	TGAC	117495	ROCK1P1
18	56400135	56400159	ATTT	del	A	56400154	MALT1
19	544706	544734	TCCC	ins	A	544727	GZMM
19	1969223	1969243	CCAC	ins	T	1969237	CSNK1G2
19	13475453	13475469	TTTA	del	TT	13475464	CACNA1A
19	17123136	17123160	TTTC	ins	T	17123155	CPAMD8
19	17285746	17285762	AAAT	del	T	17285752	MYO9B
19	17649540	17649556	AAGA	del	AA	17649550	FAM129C
19	17776809	17776825	TCCT	ins	T	17776815	UNC13A
19	19013557	19013577	AAGG	del	AGG	19013565	COPE
19	21739992	21740012	CAAA	ins	AACT	21740001	ZNF429
19	33587838	33587858	TTTC	ins	T	33587853	GPATCH1
19	37063620	37063644	TATC	ins	T	37063627	BC039524
19	37063620	37063644	TATC	ins	T	37063627	ZNF529
19	37063620	37063644	TATC	ins	T	37063627	ZNF529
19	48347486	48347506	CTTT	del	TTC	48347499	CRX
19	48848702	48848730	ATTT	ins	TTAC	48848707	Mir_324
19	48848702	48848730	ATTT	ins	TTAC	48848707	TMEM143
19	52431262	52431286	CTAT	ins	A	52431275	ZNF613
19	52431262	52431286	CTAT	del	TCT	52431276	ZNF613

19	52431303	52431331	ATCT	del	TCT	52431307	ZNF613
19	54602339	54602363	TTTG	del	G	54602345	OSCAR
19	55601600	55601616	TTTG	del	TTG	55601604	PPP1R12C
19	55601600	55601616	TTTG	del	TG	55601605	PPP1R12C
1	7838921	7838941	TTGT	del	TTG	7838936	VAMP3
1	21881403	21881423	TTTC	del	TTCTTTC	21881415	ALPL
1	114392196	114392216	TTTA	del	TA	114392205	PTPN22
1	114392196	114392216	TTTA	del	A	114392210	PTPN22
1	115119981	115120001	ATTT	del	A	115119996	BCAS2
1	115119981	115120001	ATTT	del	A	115119996	DENND2C
1	154698005	154698021	CTTC	del	C	154698015	KCNN3
1	165566486	165566502	AAAG	ins	AAGT	165566490	TRNA_Pseudo
1	201015280	201015304	TATT	del	TTATTTA	201015294	CACNA1S
1	202934752	202934768	CTTT	ins	T	202934760	CYB5R1
1	202934752	202934768	CTTT	del	TC	202934762	CYB5R1
1	202934752	202934768	CTTT	del	C	202934763	CYB5R1
1	205865727	205865743	CTTT	del	TTC	205865736	AK055746
1	233431732	233431748	TTTC	ins	T	233431743	PCNXL2
1	236226779	236226803	AAAT	del	T	236226785	AX747246
1	236226779	236226803	AAAT	del	T	236226785	NID1
20	4779475	4779491	AAAC	ins	A	4779486	RASSF2
20	42195538	42195558	GGAG	ins	GGAC	42195553	SGK2
20	43737235	43737255	TTTA	ins	T	43737250	WFDC5
20	62607527	62607547	AAAC	ins	AAC	62607539	SAMD10
21	33757316	33757344	AAAG	del	AAG	33757320	URB1
21	45677576	45677604	AAAT	del	AAT	45677580	DNMT3L
21	45677576	45677604	AAAT	del	AT	45677585	DNMT3L
22	22736711	22736731	TTTA	ins	TTTG	22736722	abParts
2	27872115	27872135	AAAC	ins	A	27872130	GPN1
2	27872115	27872135	AAAC	ins	A	27872130	SUPT7L
2	27875016	27875040	AAAT	del	AAA	27875035	GPN1
2	27875016	27875040	AAAT	del	AAA	27875035	SUPT7L
2	33763570	33763594	AAAT	ins	ATAAATAC	33763575	RASGRP3
2	44123337	44123361	TTTC	del	CTTTC	44123355	LRPPRC
2	109000660	109000688	TTTA	ins	T	109000683	SULT1C4
2	109108220	109108244	ATTT	del	A	109108235	GCC2
2	109108220	109108244	ATTT	del	A	109108239	GCC2
2	120094618	120094646	ATTT	del	A	120094633	C2orf76
2	120094618	120094646	ATTT	del	A	120094637	C2orf76
2	120094618	120094646	ATTT	del	TA	120094640	C2orf76
2	120094618	120094646	ATTT	del	A	120094641	C2orf76
2	207609646	207609674	TAAA	del	AT	207609668	MDH1B
2	217025386	217025414	TTAA	ins	T	217025401	XRCC5

3	32762314	32762330	CCCT	ins	CCTCCCTT	32762322	CNOT10
3	108189872	108189888	AAAG	ins	AA	108189883	MYH15
3	159727840	159727860	TGCT	ins	T	159727854	AK097161
3	159727840	159727860	TGCT	del	TGC	159727855	AK097161
3	172242139	172242159	CTTT	del	TTT	172242143	TNFSF10
4	68443353	68443369	TTTC	ins	TT	68443364	STAP1
4	77652940	77652960	AAAC	del	C	77652946	SHROOM3
4	120374476	120374492	CTAG	ins	CTAT	120374487	BC070391
4	141545653	141545669	CAAA	del	AAACA	141545661	TBC1D9
4	141545653	141545669	CAAA	del	C	141545664	TBC1D9
4	156628971	156628991	TTTC	ins	T	156628986	GUCY1A3
4	166962437	166962457	TTCT	del	TTC	166962452	TLL1
4	170863318	170863338	TTTC	ins	TTC	170863326	LOC100506085
4	170863318	170863338	TTTC	del	T	170863329	LOC100506085
4	182895719	182895735	CAAA	del	A	182895727	AK056196
5	31193619	31193647	AAAG	ins	A	31193638	CDH6
5	31193619	31193647	AAAG	del	G	31193641	CDH6
5	37479444	37479460	TGGT	del	TGG	37479455	WDR70
5	52388933	52388949	CAAA	del	A	52388941	ITGA2
5	79934285	79934305	TTTA	del	TTA	79934293	DHFR
5	134002266	134002286	AAAG	del	AA	134002281	SEC24A
5	137590712	137590728	AGGA	del	AG	137590723	GFRA3
5	154211004	154211020	TTGT	ins	G	154211013	C5orf4
5	159641799	159641819	AAAG	ins	AAAT	159641810	FABP6
6	8422225	8422253	TGTT	del	G	8422229	SLC35B3
6	24358969	24358985	TTTA	ins	TATA	24358978	DCDC2
6	24358969	24358985	TTTA	ins	TATA	24358978	KAAG1
6	94478540	94478564	AAAT	del	T	94478546	TSG1
6	136979431	136979451	TTTC	ins	T	136979442	MAP3K5
6	136979431	136979451	TTTC	del	C	136979445	MAP3K5
7	66281798	66281814	TAAA	del	AAATAAATA	66281802	LOC729156
7	66281798	66281814	TAAA	ins	A	66281806	LOC729156
7	129331178	129331194	TATT	ins	TTA	129331188	NRF1
7	148107157	148107173	AAAG	del	A	148107168	CNTNAP2
7	148311107	148311123	TTTG	ins	T	148311114	C7orf33
8	27621234	27621258	AAAT	ins	A	27621249	CCDC25
8	82437764	82437788	ATAG	ins	GAT	82437778	FABP12
8	99163310	99163326	TGTT	del	G	99163318	POP1
8	139161379	139161403	AAAT	del	T	139161385	FAM135B
9	34459432	34459448	TTTC	ins	T	34459443	C9orf25
9	34459432	34459448	TTTC	ins	T	34459443	DNAI1
9	75357543	75357563	AAAC	del	C	75357557	TMC1
X	31137272	31137288	AAGT	del	AAG	31137283	DMD

X	47968148	47968164	TTTG	del	T	47968159	LOC100509575
X	49046935	49046955	TTTC	del	CTTTC	49046949	SYP
X	64809730	64809746	TTTC	del	TTTCT	64809741	BC067907
