# Supplementary figures and images for: Microsatellite Interruptions Stabilize Primate Genomes and Exist as Population-Specific Single Nucleotide Polymorphisms within Individual Human Genomes
Source: PLoS Genet. 2014 Jul 17;10(7):e1004498. doi: 10.1371/journal.pgen.1004498 (PMC4102424; doi:10.1371/journal.pgen.1004498)

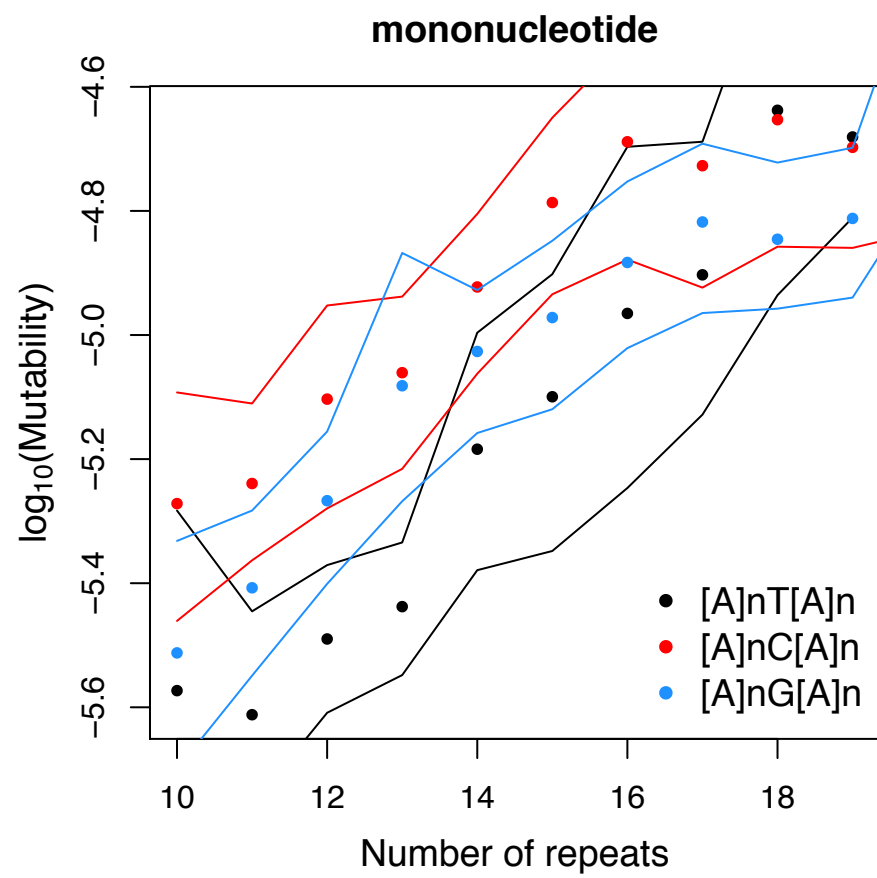

Supplement: Figure S1 — Effect of interruption identity on microsatellite mutability. Mutability of singly-interrupted poly-A microsatellites binned according to their interruption ([A]nT[A]n, [A]nC[A]n and [A]nG[A]n). Number of repeats of a microsatellite was calculated by dividing the total length of the microsatellite, excepting the interrupting nucleotides, by the size of its repeating motif. At each repeat number the lines designate the 2.5th and 97.5th percentiles of empirical distributions that were obtained through resampling. (PDF) [file pgen.1004498.s007.pdf]

**A.**

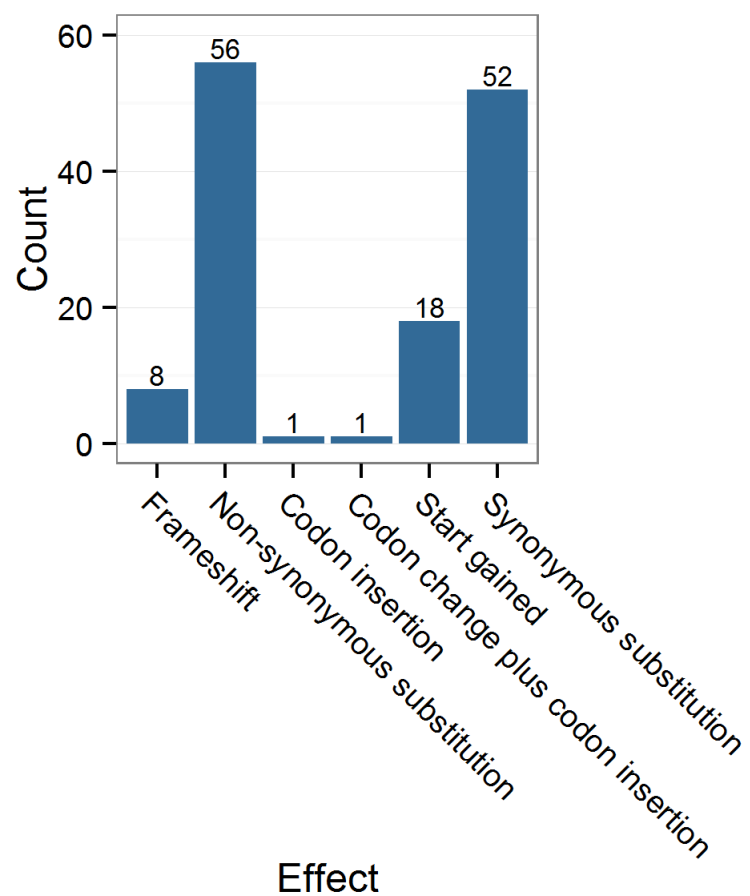

**B.**

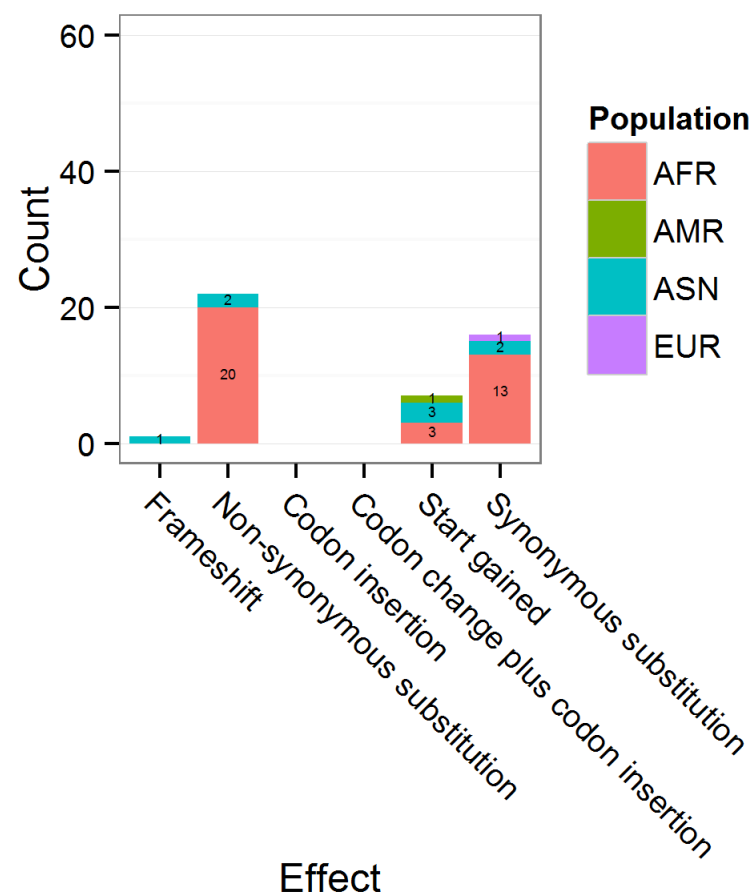

Supplement: Figure S2 — The effect of microsatellite interruptions on protein-coding sequences. (A). Interruptions present in more than one population group. (B). Interruptions present in individual population groups. (PDF) [file pgen.1004498.s008.pdf]

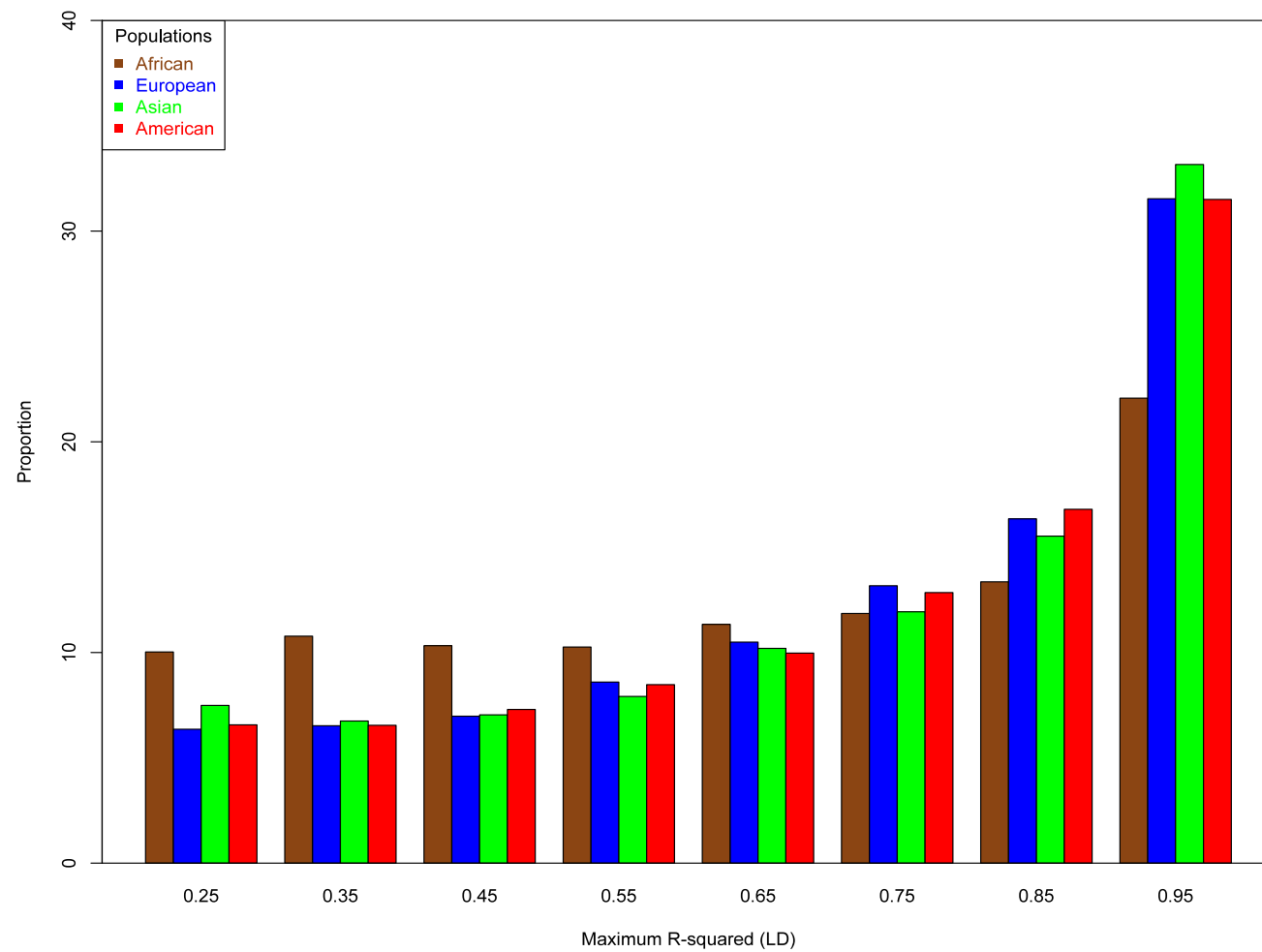

Supplement: Figure S4 — Proportion of iMS alleles at different levels of linkage disequilibrium with neighboring, population-matched SNPs. (PDF) [file pgen.1004498.s010.pdf]

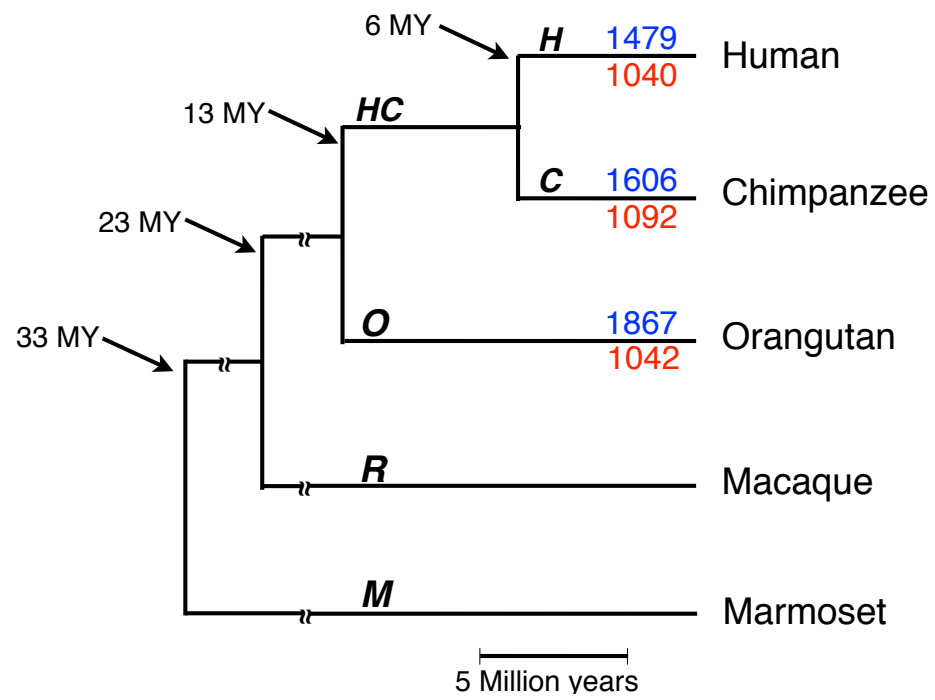

Supplement: Figure S5 — Microsatellite loci are saturated by gain/loss events. Numbers in blue and red indicate the number of interruptions gained and lost in the respective branch of the five-species primate tree. (PDF) [file pgen.1004498.s011.xls]

**A.****AMR interruptions**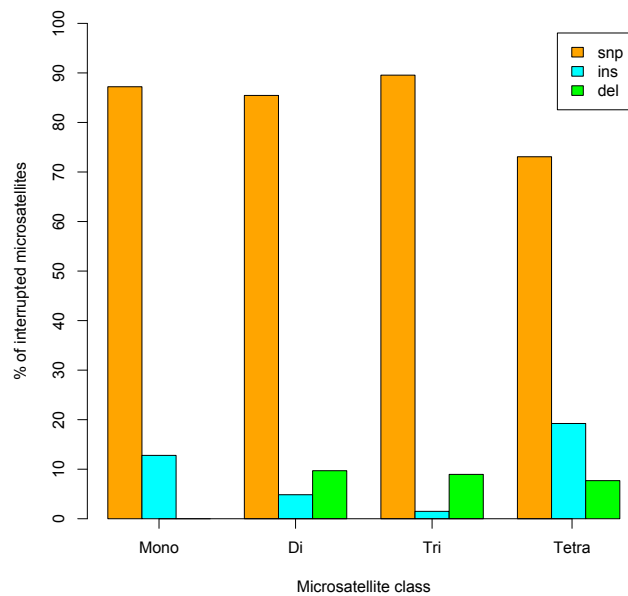**B.****ASN interruptions**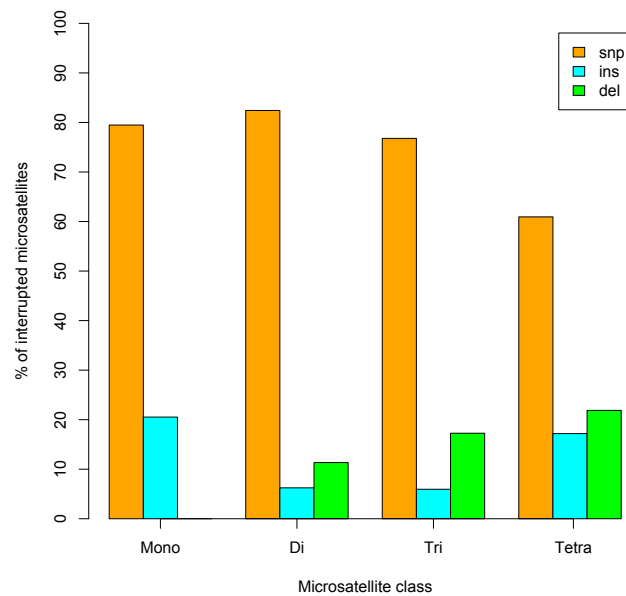**C.****EUR interruptions**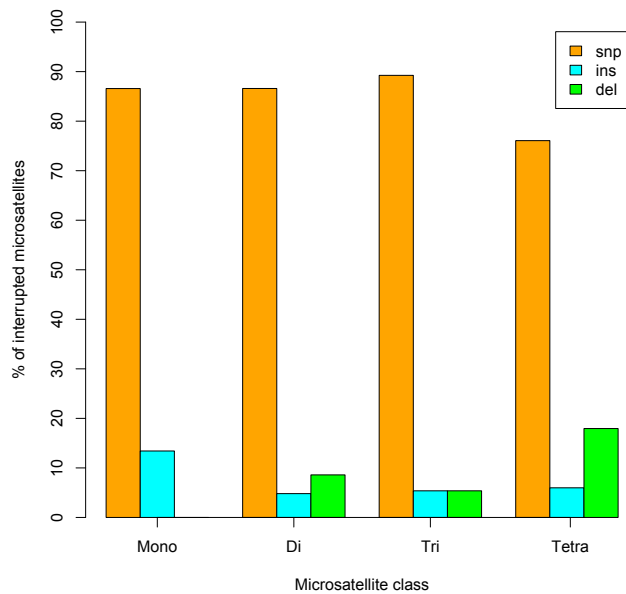

Supplement: Figure S6 — Effect of motif size on population-specific interruptions in 1000 genomes datasets. (A). American population; (B). Asian population; (C). European population. (PDF) [file pgen.1004498.s012.pd]

**A.**

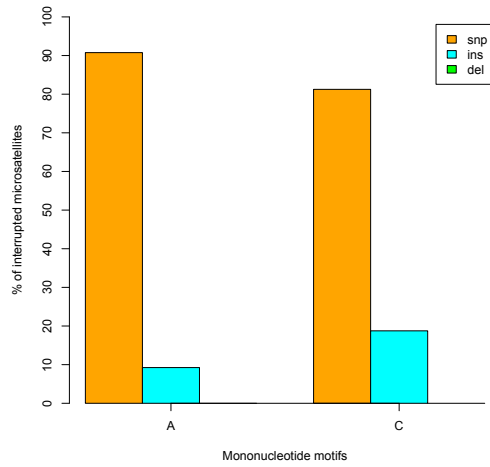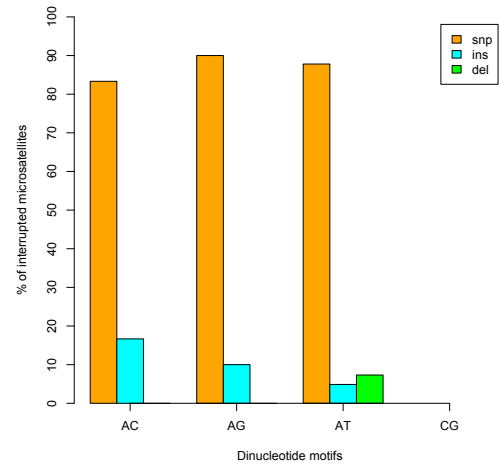

**B.**

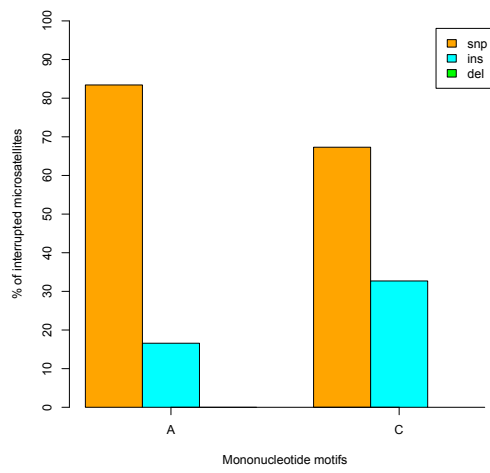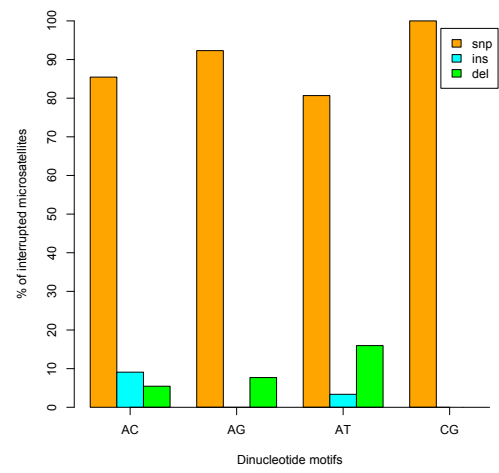

**C.**

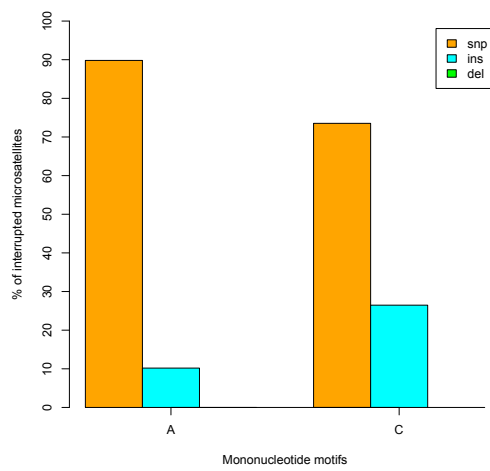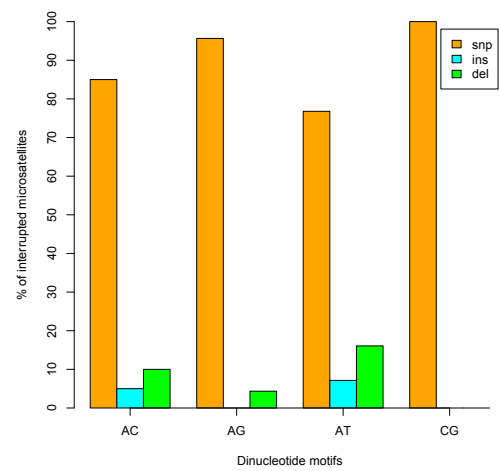

Supplement: Figure S8 — Effect of motif composition on population-specific interruptions in 1000 genomes datasets. (A). American population; (B). Asian population; (C). European population. Individual panels are data for mono- and dinucleotide microsatellites within each population. (PDF) [file pgen.1004498.s014.pdf]
