# Supplementary material for: Microsatellite Interruptions Stabilize Primate Genomes and Exist as Population-Specific Single Nucleotide Polymorphisms within Individual Human Genomes
Source: PLoS Genet. 2014 Jul 17;10(7):e1004498. doi: 10.1371/journal.pgen.1004498 (PMC4102424; doi:10.1371/journal.pgen.1004498)
Supplement: Figure S3 — Proportion of iMS and perfect MS alleles at different levels of heterozygosity. (A). African population; (B). Asian population; (C). European population; (D). American population. The density of iMSs with heterozygosity below 10% is likely an underestimate since our data did not include variants with frequency below 5%. (PDF) [file pgen.1004498.s009.pdf]

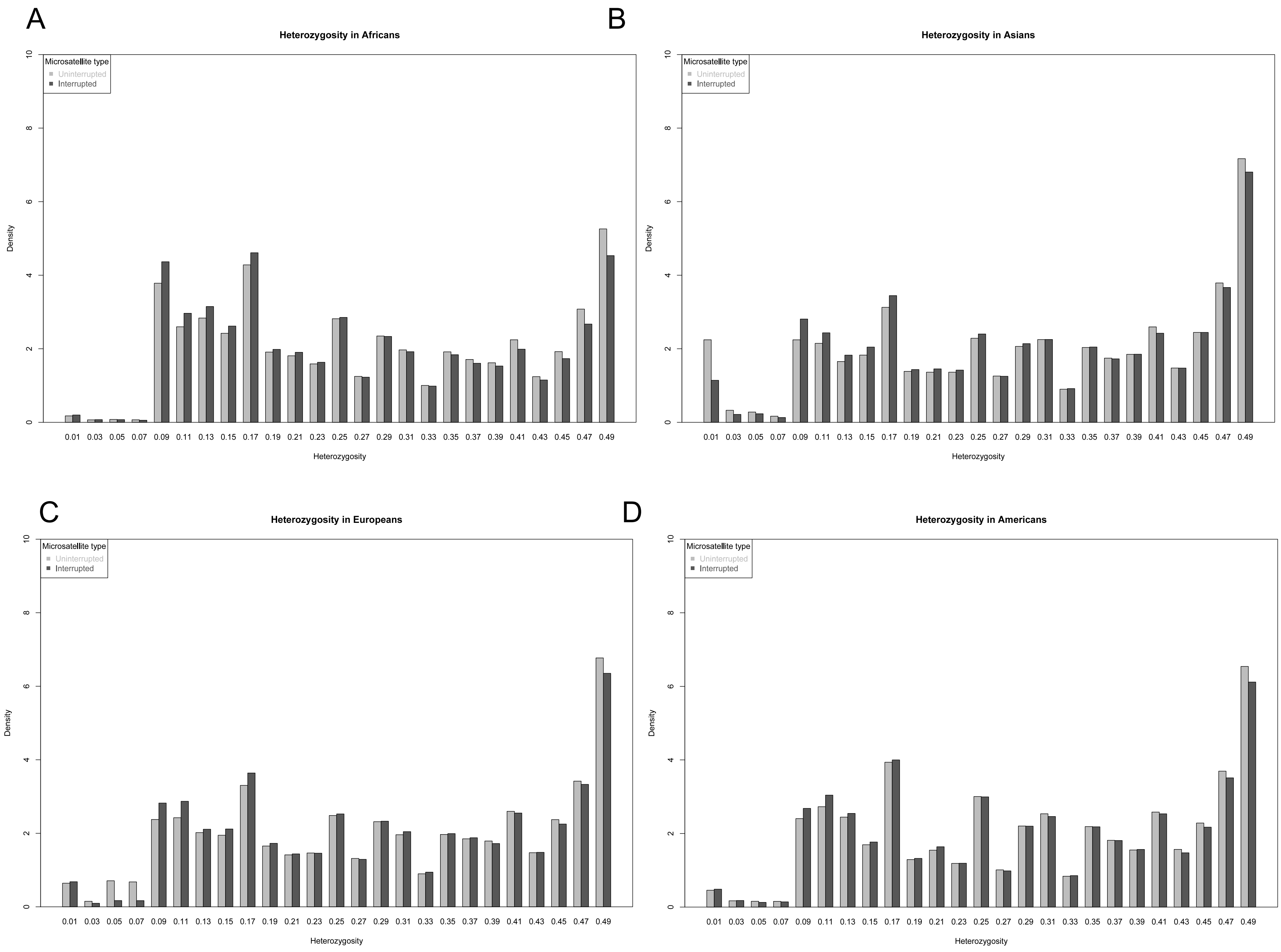

A
Heterozygosity in Africans
B
Heterozygosity in Asians
C
Heterozygosity in Europeans
D
Heterozygosity in Americans
Microsatellite type
Uninterrupted
Interrupted
Density
Heterozygosity
0
2
4
6
8
10
0.01 0.03 0.05 0.07 0.09 0.11 0.13 0.15 0.17 0.19 0.21 0.23 0.25 0.27 0.29 0.31 0.33 0.35 0.37 0.39 0.41 0.43 0.45 0.47 0.49
