# Supplementary material for: Microsatellite Interruptions Stabilize Primate Genomes and Exist as Population-Specific Single Nucleotide Polymorphisms within Individual Human Genomes
Source: PLoS Genet. 2014 Jul 17;10(7):e1004498. doi: 10.1371/journal.pgen.1004498 (PMC4102424; doi:10.1371/journal.pgen.1004498)
Supplement: Figure S7 — Effect of repeat number on population-specific interruptions in 1000 genomes datasets. (A). American population; (B). Asian population; (C). European population. Individual panels are data for mono-, di-, tri-, and tetranucleotide microsatellites within each population. (PDF) [file pgen.1004498.s013.pdf]

A.

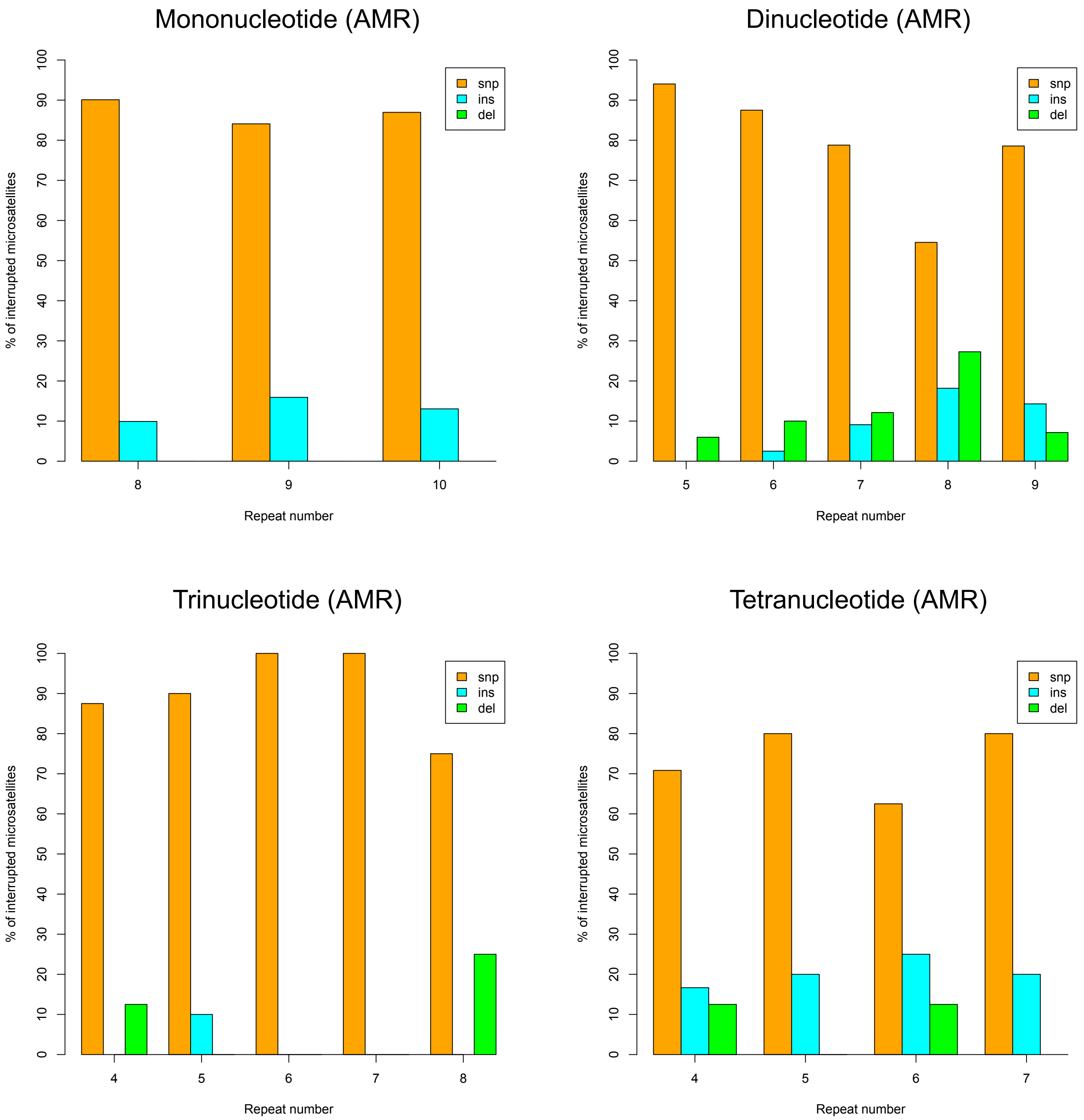

Mononucleotide (AMR)
% of interrupted microsatellites
0
10
20
30
40
50
60
70
80
90
100
snp
ins
del
8
9
10
Repeat number
Dinucleotide (AMR)
% of interrupted microsatellites
snp
ins
del
5
6
7
8
9
Repeat number
Trinucleotide (AMR)
% of interrupted microsatellites
snp
ins
del
4
5
6
7
8
Repeat number
Tetranucleotide (AMR)
% of interrupted microsatellites
snp
ins
del
4
5
6
7
Repeat number

B.

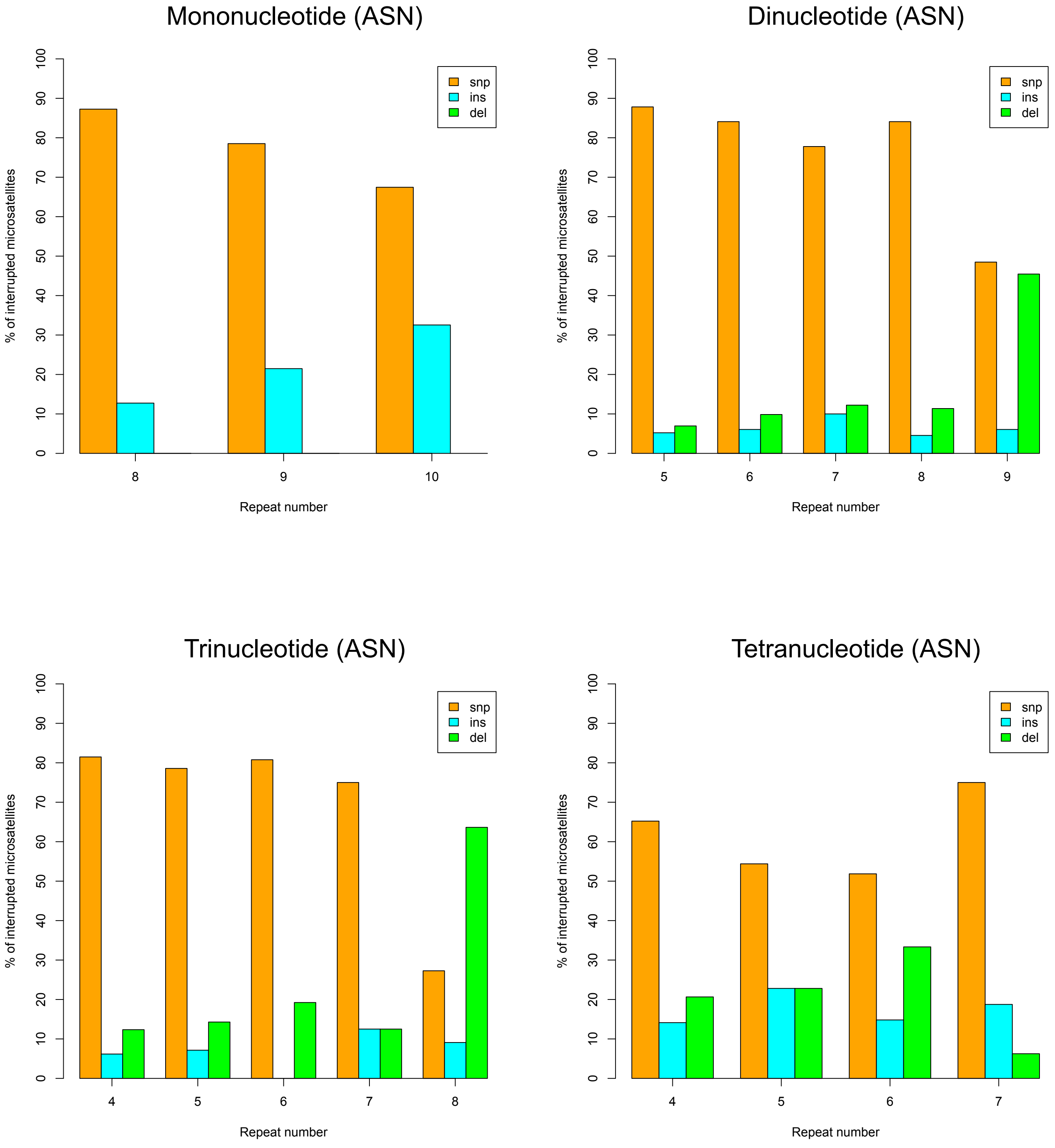
Mononucleotide (ASN)
Dinucleotide (ASN)
Trinucleotide (ASN)
Tetranucleotide (ASN)
% of interrupted microsatellites
Repeat number
snp
ins
del
0
10
20
30
40
50
60
70
80
90
100
4
5
6
7
8
9
10

C.

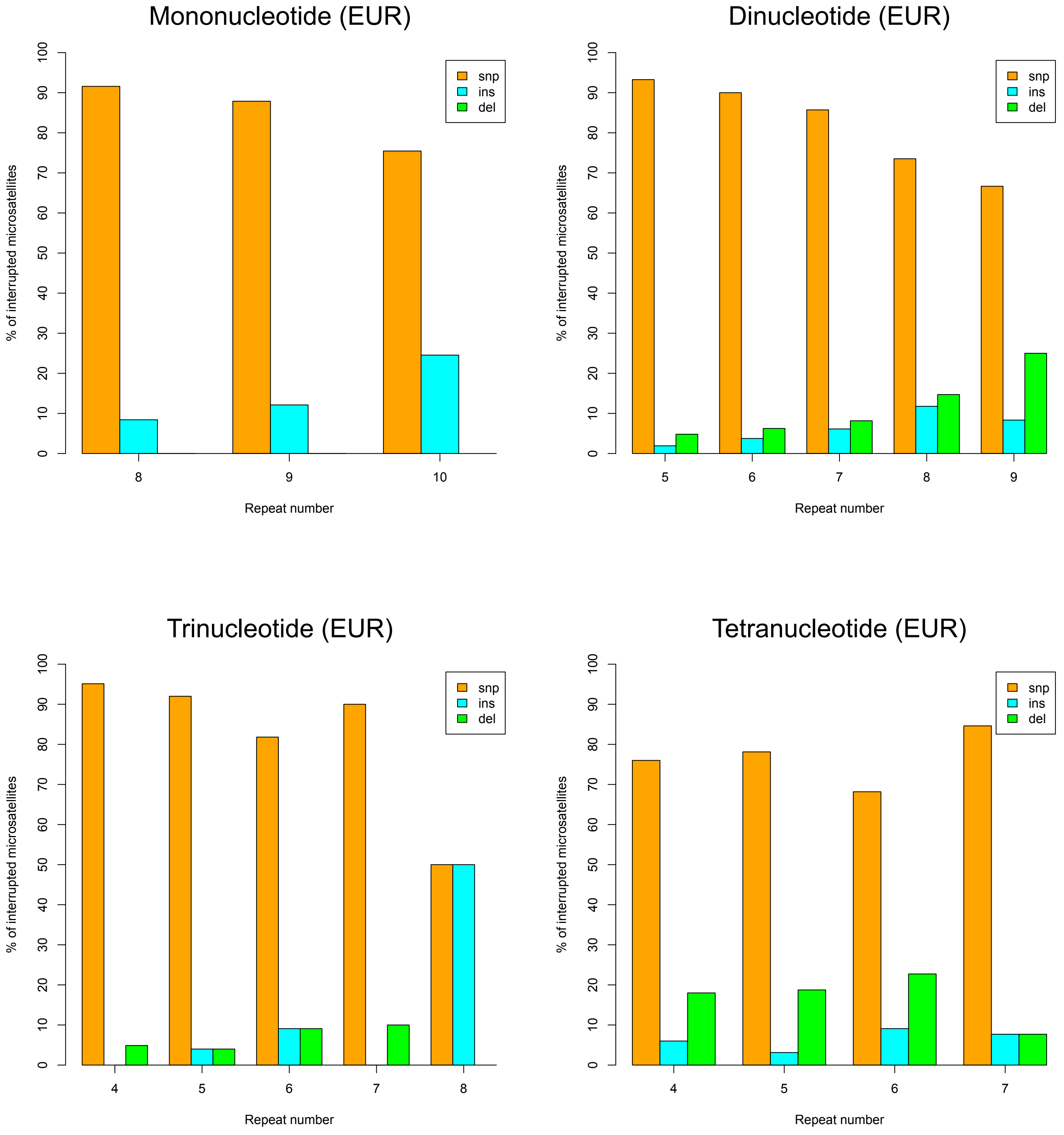

Mononucleotide (EUR)
Dinucleotide (EUR)
Trinucleotide (EUR)
Tetranucleotide (EUR)
% of interrupted microsatellites
Repeat number
0 10 20 30 40 50 60 70 80 90 100
snp
ins
del
8 9 10
5 6 7 8 9
4 5 6 7 8
4 5 6 7
