# Supplementary material for: Microsatellite Interruptions Stabilize Primate Genomes and Exist as Population-Specific Single Nucleotide Polymorphisms within Individual Human Genomes
Source: PLoS Genet. 2014 Jul 17;10(7):e1004498. doi: 10.1371/journal.pgen.1004498 (PMC4102424; doi:10.1371/journal.pgen.1004498)
Supplement: Table S1 — Numbers of perfect (pure) and interrupted orthologous microsatellites in primate genomes. (DOCX) [file pgen.1004498.s017.docx]

**Table S1. Numbers of perfect and interrupted orthologous microsatellites found in primate genomes^a^.**

| Species | Motif size | All pure microsatellites | All microsatellites with any number of interruptions | Orthologous microsatellites with 1 or 2 interruptions for mutability calulations | Orthologous microsatellites with 1 interruption, used for inferring mutational pathways^b^ |
| --- | --- | --- | --- | --- | --- |
| Human | 1 | 89573 | 113676 | 38182 | 34227 |
|  | 2 | 20752 | 39752 | 12778 | 11697 |
|  | 3 | 14592 | 16152 | 6439 | 6748 |
|  | 4 | 42037 | 27701 | 10415 | 13296 |
|  |  |  |  |  |  |
| Chimpanzee | 1 | 89521 | 110238 | 38182 | 33769 |
|  | 2 | 20936 | 39877 | 12778 | 11735 |
|  | 3 | 14731 | 16325 | 6439 | 6693 |
|  | 4 | 42545 | 28142 | 10415 | 13172 |
|  |  |  |  |  |  |
| Orangutan | 1 | 90959 | 114484 | - | 34746 |
|  | 2 | 20635 | 39641 | - | 11612 |
|  | 3 | 14509 | 16165 | - | 6603 |
|  | 4 | 41902 | 27818 | - | 12871 |
|  |  |  |  |  |  |
| Macaque | 1 | 85071 | 102306 | - | - |
|  | 2 | 20436 | 38035 | - | - |
|  | 3 | 14367 | 16003 | - | - |
|  | 4 | 39434 | 26809 | - | - |
|  |  |  |  |  |  |
| Marmoset | 1 | 60370 | 69171 | - | - |
|  | 2 | 15744 | 29763 | - | - |
|  | 3 | 9899 | 10361 | - | - |
|  | 4 | 30027 | 17340 | - | - |

^a^ We implemented a modified version of Sputnik (C. Abajian, http://espressosoftware.com/pages/sputnik.jsp) to identify ‘seeds’ of microsatellites. The modified Sputnik, available on request, identifies mononucleotide microsatellites in addition to microsatellites of larger motif sizes. In order to obtain very small pure repeats the parameters were set to:

(A) EXACT_MATCH_POINTS 1

(B) ERROR_MATCH_POINTS -1000

(C) MATCH_MIN_SCORE 4

(D) MATCH_FAIL_SCORE -1

(E) MAX_RECURSION 5

^b^ Microsatellite loci whose mutational history in the five primate genomes could be deciphered using the principle of maximum parsimony. Macaque and Marmoset genomes were used as out-groups.
