# Supplementary material for: Microsatellite Interruptions Stabilize Primate Genomes and Exist as Population-Specific Single Nucleotide Polymorphisms within Individual Human Genomes
Source: PLoS Genet. 2014 Jul 17;10(7):e1004498. doi: 10.1371/journal.pgen.1004498 (PMC4102424; doi:10.1371/journal.pgen.1004498)
Supplement: Table S2 — Functional effects of exonic iMS SNP/InDel polymorphisms in four populations. (DOCX) [file pgen.1004498.s018.docx]

**Table S2. Functional effects of exonic iMS SNP/Indel polymorphisms in four populations.**

| **Location/Change**  **(reference->sample)** | **Genes / Functional Region^a^** | | |
| --- | --- | --- | --- |
|  | **Coding** | | **Non-coding** |
| **African-Specific** | | | |
| 3:14106103 /  rs144265507: A->T | TPRXL/ S143C |  | |
| 3:14106104/  rs148665086: G->C | TPRXL/ S143T |  | |
| 3:142443450/  rs78715340:T->A | TRPC1/S17T |  | |
| 3:51743034/  rs75461739:T->C | GRM2/L12P | TEX264 /DOWNSTREAM | |
| 5:180030325/  rs115824945:C->T | FLT4/ R1320Q |  | |
| 6:75899300/  rs75535959:T->G | COL12A1/ K209T |  | |
| 8:101076174/  rs73702154:T->G | RGS22/ E262D,E274D,E93D |  | |
| 8:117950738/  rs187578345:G->A | C8orf85/A86T |  | |
| 8:144357345/  rs56871834:C->G | GLI4/P141A | RP13582O9.5.1/DOWNSTREAMZFP41/ INTRON | |
| 10:120789548/  rs79170274:T->A | NANOS1/ S79T | EIF3A/ DOWNSTREAM  RP11-498J9.4.1/ EXON | |
| 11:72535847/  rs77419620:G->C | ATG16L2/ C150S,C319S |  | |
| 12:123466190/  rs190753885:C->T | ARL6IP4/ S182F,S190F,S193F,S201F,S59F,S60F,S67F,S70F | ABCB9 / 5’UTR  OGFOD2/ DOWNSTREAM  PITPNM2/ DOWNSTREAM  RP11-197N18.2.1/ EXON | |
| 12:123466304/  rs74758272:A->G | ARL6IP4/  K105R,K108R,K220R,K228R,K231R,K239R,K97R,K98R | ABCB9 /UPSTREAM  OGFOD2/ DOWNSTREAM  PITPNM2/ DOWNSTREAM  RP11-197N18.2.1/ EXON | |
| 12:49580279/  rs112063173:G->C | TUBA1A/S114C | TUBA1C/UPSTREAM | |
| 16:838637/  rs139737807:G->T | CHTF18/ G182C | RPUSD1/ UPSTREAM | |
| 17:27401061/  rs73986791:C->T | TIAF1/ V53M | MYO18A/ DOWNSTREAM  MYO18A/ EXON  MYO18A/ 3’UTR | |
| 17:65899980/  rs75015132:A->G | BPTF/R797G |  | |
| 19:1009189/  rs74549450:A->G | GRIN3B/ Q907R | C19orf6/ DOWNSTREAM | |
| 19:50832217/  rs185017345:C->A | KCNC3/Q41H | NAPSB/ DOWNSTREAM  NR1H2/ UPSTREAM) | |
| X:92928114/  rs62641622:T->C | NAP1L3/S57G,S64G | FAM133A/ UPSTREAM | |
| 1:156642744/  rs6696672:G->C | NES/L412 |  | |
| 1:156694024/  rs61736207:G->T | ISG20L2/P288 | RRNAD1/ UPSTREAM | |
| 1:3645989/  rs61736049:G->A | TP73/ Q320,Q342,Q391 |  | |
| 2:166868775/  rs36031496:A->G | SCN1A/ Y1213,Y1230,Y1241 |  | |
| 2:69693397/  rs56033551:G->T | AC114772.1/P76 | AAK1/ INTRON | |
| 5:76749694/  rs35356211:C->T | WDR41/ V102,V149,V150,V151,V152,V157 |  | |
| 5:96078051/  rs56299553:T->G | CAST/ start site gained | CTC-506B8.1.1/ INTRON | |
| 6:44219926/  rs35518699:G->A | HSP90AB1/K551 | SLC35B2/ DOWNSTREAM | |
| 11:124413248/  rs61743599:G->A | OR8B12/F101 | RP11-728D14.6.1/ UPSTREAM | |
| 16:88496791/  rs60462217:C->A | ZNF469/ G971 |  | |
| 17:18908070/  rs188674532:G->A | FAM83G/ start site gained | SLC5A10/ INTRON | |
| 19:36211359/  rs76025946:A->G | MLL4.1/E370 | ZBTB32/ DOWNSTREAM | |
| 19:48800725/  rs73585332:G->A | CCDC114/A507 |  | |
| 20:44692170  rs35481630:T->G | NCOA5/R327 | SLC12A5/ DOWNSTREAM | |
| 22:29709359/  rs8142725:G->A | RASL10A/R181 | GAS2L1/DOWNSTREAM | |
| X:134477006/  rs5975504:A->G | ZNF75D/ start site gained | ZNF449/ UPSTREAM | |
| **Asian-Specific** | | | |
| 6:30996492/  rs201042516:CCA->C | MUC22/ frameshift |  | |
| 4:129864275/  rs77885682:C->T | SCLT1/R503K |  | |
| 12:114793297/  rs10850326:T->C | TBX5/E383G |  | |
| 1:94883995/  rs4148058:C->T | ABCD3/ start site gained |  | |
| 2:25384472/  rs28930368:G->A | POMC/S94 | RP11509E16.1.1/  DOWNSTREAM | |
| 7:91509446/  rs76174712:C->T | MTERF/ start site gained | CTB-104F4.2.1/ INTRON | |
| 12:53491531/  rs17123156:G->A | IGFBP6/ L10 |  | |
| 17:36613810/  rs12051675:G->A | ARHGAP23/ start site gained |  | |
| **South American-specific** | | | |
| 15:93443441/  rs182142010:C->G | CHD2/ start site gained |  | |
| **European-specific** | | | |
| 18:67534632/  rs72481819:C->T | CD226/E282 |  | |

^a^ Multiple codons represent alternative transcripts for each gene.
