# Supplementary material for: Microsatellite Interruptions Stabilize Primate Genomes and Exist as Population-Specific Single Nucleotide Polymorphisms within Individual Human Genomes
Source: PLoS Genet. 2014 Jul 17;10(7):e1004498. doi: 10.1371/journal.pgen.1004498 (PMC4102424; doi:10.1371/journal.pgen.1004498)
Supplement: Table S3 — Gene Ontology functions significantly overrepresented (p<0.01) in genes containing African population-specific iMSs. (DOCX) [file pgen.1004498.s019.docx]

**Table S3. Gene Ontology functions significantly overrepresented (p<0.01) in genes containing African population-specific iMSs.**

| **GOBPID** | **Pvalue** | **Odds**  **Ratio** | **Exp.**  **Count** | **Count** | **Size** | **Term** |
| --- | --- | --- | --- | --- | --- | --- |
| GO:0001840 | 0.0003 | 23.1 | 0.38 | 4 | 7 | neuralplate development |
| GO:0010886 | 0.0029 | 17.3 | 0.33 | 3 | 6 | positive regulation of cholesterol storage |
| GO:0042159 | 0.0029 | 17.3 | 0.33 | 3 | 6 | lipoprotein catabolic process |
| GO:0001768 | 0.0030 | Inf | 0.11 | 2 | 2 | establishment of Tcell polarity |
| GO:0021990 | 0.0030 | Inf | 0.11 | 2 | 2 | neural plate formation |
| GO:0030505 | 0.0030 | Inf | 0.11 | 2 | 2 | inorganic diphosphate transport |
| GO:0032456 | 0.0030 | Inf | 0.11 | 2 | 2 | endocytic recycling |
| GO:0033875 | 0.0030 | Inf | 0.11 | 2 | 2 | ribonucleoside bisphosphate metabolic process |
| GO:0034032 | 0.0030 | Inf | 0.11 | 2 | 2 | purine nucleoside bisphosphate metabolic process |
| GO:0042062 | 0.0030 | Inf | 0.11 | 2 | 2 | long-term strengthening of neuromuscular junction |
| GO:0050427 | 0.0030 | Inf | 0.11 | 2 | 2 | 3'-phosphoadenosine 5'-phosphosulfate metabolic process |
| GO:0070997 | 0.0040 | 2.6 | 4.99 | 12 | 91 | neuron death |
| GO:0007018 | 0.0041 | 2.4 | 6.31 | 14 | 115 | microtubule-based movement |
| GO:0048546 | 0.0048 | 5.4 | 1.15 | 5 | 21 | digestive tract morphogenesis |
| GO:0000052 | 0.0049 | 13. | 0.38 | 3 | 7 | citrulline metabolic process |
| GO:0043523 | 0.0050 | 2.7 | 4.50 | 11 | 82 | regulation of neuron apoptosis |
| GO:0001889 | 0.0055 | 3.3 | 2.74 | 8 | 50 | liver development |
| GO:0071044 | 0.0058 | 6.9 | 0.77 | 4 | 14 | histone mRNA catabolic process |
| GO:0014003 | 0.0075 | 6.3 | 0.82 | 4 | 15 | oligodendrocyte development |
| GO:0046849 | 0.0083 | 3.8 | 1.81 | 6 | 33 | bone remodeling |
| GO:0006772 | 0.0087 | 34.6 | 0.16 | 2 | 3 | thiamin metabolic process |
| GO:0018076 | 0.0087 | 34.6 | 0.16 | 2 | 3 | N-terminal peptidyl-lysine acetylation |
| GO:0006805 | 0.0087 | 4.6 | 1.32 | 5 | 24 | xenobiotic metabolic process |
| GO:0051301 | 0.0094 | 1.7 | 18.26 | 29 | 333 | cell division |
| GO:0006725 | 0.0099 | 2.1 | 7.68 | 15 | 140 | cellular aromatic compound metabolic process |
| **GOCCID** |  |  |  |  |  |  |
| GO:0005856 | 0.0004 | 1.5 | 74.47 | 103 | 1370 | cytoskeleton |
| GO:0005622 | 0.0010 | 1.3 | 600.56 | 642 | 11049 | intracellular |
| GO:0016591 | 0.0026 | 3.0 | 4.13 | 11 | 76 | DNA-directed RNA polymerase II, holoenzyme |
| GO:0005777 | 0.0029 | 2.6 | 5.44 | 13 | 100 | peroxisome |
| GO:0005760 | 0.0030 | Inf | 0.11 | 2 | 2 | gamma DNA polymerase complex |
| GO:0005737 | 0.0041 | 1.2 | 402.44 | 441 | 7404 | cytoplasm |
| GO:0030175 | 0.0041 | 4.6 | 1.58 | 6 | 29 | filopodium |
| GO:0005874 | 0.0069 | 1.8 | 14.73 | 25 | 271 | microtubule |
| GO:0034706 | 0.0073 | 6.4 | 0.82 | 4 | 15 | sodium channel complex |
| GO:0043228 | 0.0087 | 1.2 | 139.96 | 166 | 2575 | non-membrane-bounded organelle |
| **GOMFID** |  |  |  |  |  |  |
| GO:0060589 | 0.0001 | 2.0 | 22.87 | 42 | 419 | nucleoside-triphosphatase regulator activity |
| GO:0005083 | 0.0002 | 2.2 | 15.50 | 31 | 284 | small GTPase regulator activity |
| GO:0001882 | 0.0003 | 1.4 | 87.82 | 119 | 1609 | nucleoside binding |
| GO:0030554 | 0.0005 | 1.4 | 85.25 | 115 | 1562 | adenyl nucleotide binding |
| GO:0005085 | 0.0005 | 2.6 | 8.13 | 19 | 149 | guanyl-nucleotide exchange factor activity |
| GO:0019904 | 0.0006 | 2.0 | 17.96 | 33 | 329 | protein domain specific binding |
| GO:0047115 | 0.0006 | 52.2 | 0.22 | 3 | 4 | trans-1,2-dihydrobenzene-1,2-diol dehydrogenase activity |
| GO:0005524 | 0.0007 | 1.4 | 79.90 | 108 | 1464 | ATP binding |
| GO:0005515 | 0.0007 | 1.3 | 418.38 | 463 | 7808 | protein binding |
| GO:0030546 | 0.0009 | 13.9 | 0.49 | 4 | 9 | receptor activator activity |
| GO:0009975 | 0.0012 | 6.2 | 1.26 | 6 | 23 | cyclase activity |
| GO:0016818 | 0.0017 | 1.5 | 41.64 | 61 | 763 | hydrolase activity, acting on acid anhydrides, in phosphorus-containing anhydrides |
| GO:0003777 | 0.0030 | 2.9 | 4.20 | 11 | 77 | microtubule motor activity |
| GO:0004329 | 0.0030 | Inf | 0.11 | 2 | 2 | formate-tetrahydrofolate ligase activity |
| GO:0047026 | 0.0030 | Inf | 0.11 | 2 | 2 | 3-alpha-hydroxysteroid dehydrogenase (A-specific) activity |
| GO:0004725 | 0.0036 | 2.6 | 5.57 | 13 | 102 | protein tyrosine phosphatase activity |
| GO:0017111 | 0.0041 | 1.5 | 39.90 | 57 | 731 | nucleoside-triphosphatase activity |
| GO:0051015 | 0.0047 | 3.4 | 2.67 | 8 | 49 | actin filament binding |
| GO:0000166 | 0.0049 | 1.3 | 121.33 | 148 | 2223 | nucleotide binding |
| GO:0016849 | 0.0058 | 5.1 | 1.20 | 5 | 22 | phosphorus-oxygen lyase activity |
| GO:0032555 | 0.0067 | 1.3 | 99.44 | 123 | 1822 | purine ribonucleotide binding |
| GO:0016888 | 0.0074 | 10.4 | 0.44 | 3 | 8 | endodeoxyribonuclease activity, producing 5'-phosphomonoesters |
| GO:0030375 | 0.0086 | 34.7 | 0.16 | 2 | 3 | thyroid hormone receptor coactivator activity |
| GO:0042975 | 0.0086 | 34.7 | 0.16 | 2 | 3 | peroxisome proliferator activated receptor binding |
