# Supplementary material for: Microsatellite Interruptions Stabilize Primate Genomes and Exist as Population-Specific Single Nucleotide Polymorphisms within Individual Human Genomes
Source: PLoS Genet. 2014 Jul 17;10(7):e1004498. doi: 10.1371/journal.pgen.1004498 (PMC4102424; doi:10.1371/journal.pgen.1004498)
Supplement: Table S4 — Gene Ontology functions significantly overrepresented (p<0.01) in genes containing European population-specific iMSs. (DOCX) [file pgen.1004498.s020.docx]

**Table S4. Gene Ontology functions significantly (p<0.01) overrepresented in genes containing European population-specific iMSs.**

| **GOBPID** | **Pvalue** | **Odds**  **Ratio** | **Exp**  **Count** | **Count** | **Size** | **Term** |
| --- | --- | --- | --- | --- | --- | --- |
| GO:0002250 | 0.0009 | 10.1 | 0.44 | 4 | 113 | adaptive immune response |
| GO:0002028 | 0.0011 | 48.5 | 0.05 | 2 | 13 | regulation of sodium ion transport |
| GO:0006821 | 0.0013 | 15.3 | 0.22 | 3 | 56 | chloride transport |
| GO:0001912 | 0.0024 | 31.4 | 0.07 | 2 | 19 | positive regulation of leukocyte mediated cytotoxicity |
| GO:0006121 | 0.0039 | Inf | 0.00 | 1 | 1 | mitochondrial electron transport, succinate to ubiquinone |
| GO:0010760 | 0.0039 | Inf | 0.00 | 1 | 1 | negative regulation of macrophage chemotaxis |
| GO:0018293 | 0.0039 | Inf | 0.00 | 1 | 1 | protein-FAD linkage |
| GO:0043132 | 0.0039 | Inf | 0.00 | 1 | 1 | NAD transport |
| GO:0045407 | 0.0039 | Inf | 0.00 | 1 | 1 | positive regulation of interleukin-5 biosynthetic process |
| GO:0002824 | 0.0042 | 23.2 | 0.10 | 2 | 25 | positive regulation of adaptive immune response based on somatic recombination of immune receptors |
| GO:0031341 | 0.0045 | 22.2 | 0.10 | 2 | 26 | regulation of cell killing |
| GO:0044243 | 0.0048 | 21.3 | 0.10 | 2 | 27 | multicellular organismal catabolic process |
| GO:0002708 | 0.0056 | 19.7 | 0.11 | 2 | 29 | positive regulation of lymphocyte mediated immunity |
| GO:0045333 | 0.0066 | 8.45 | 0.38 | 3 | 99 | cellular respiration |
| GO:0001845 | 0.0077 | 262.2 | 0.01 | 1 | 2 | phagolysosome assembly |
| GO:0006649 | 0.0077 | 262.2 | 0.01 | 1 | 2 | phospholipid transfer to membrane |
| GO:0014054 | 0.0077 | 262.2 | 0.01 | 1 | 2 | positive regulation of gamma-aminobutyric acid secretion |
| GO:0042637 | 0.0077 | 262.2 | 0.01 | 1 | 2 | catagen |
| GO:0045919 | 0.0077 | 262.2 | 0.01 | 1 | 2 | positive regulation of cytolysis |
| GO:0060369 | 0.0077 | 262.2 | 0.01 | 1 | 2 | positive regulation of Fc receptor mediated stimulatory signaling pathway |
| GO:0048705 | 0.0080 | 7.9 | 0.41 | 3 | 106 | skeletal system morphogenesis |
| GO:0016485 | 0.0082 | 7.8 | 0.41 | 3 | 107 | protein processing |
| GO:0001816 | 0.0084 | 5.4 | 0.80 | 4 | 207 | cytokine production |
| GO:0006885 | 0.0095 | 14.8 | 0.15 | 2 | 38 | regulation of pH |
| GO:0022900 | 0.0097 | 7.3 | 0.44 | 3 | 114 | electron transport chain |
| GO:0032963 | 0.0099 | 14.4 | 0.15 | 2 | 39 | collagen metabolic process |
| **GOCCID** |  |  |  |  |  |  |
| GO:0016281 | 0.0004 | 85.3 | 0.03 | 2 | 9 | eukaryotic translation initiation factor 4F complex |
| GO:0044463 | 0.0018 | 6.2 | 0.88 | 5 | 255 | cell projection part |
| GO:0005749 | 0.0035 | Inf | 0.00 | 1 | 1 | mitochondrial respiratory chain complex II |
| GO:0032590 | 0.0035 | Inf | 0.00 | 1 | 1 | dendrite membrane |
| GO:0045281 | 0.0035 | Inf | 0.00 | 1 | 1 | succinate dehydrogenase complex |
| GO:0000120 | 0.0069 | 293.3 | 0.01 | 1 | 2 | RNA polymerase I transcription factor complex |
| **GOMFID** |  |  |  |  |  |  |
| GO:0005230 | 0.0025 | 12.1 | 0.27 | 3 | 73 | extracellular ligand-gated ion channel activity |
| GO:0004721 | 0.0032 | 7.1 | 0.61 | 4 | 165 | phosphoprotein phosphatase activity |
| GO:0004506 | 0.0037 | Inf | 0.00 | 1 | 1 | squalene monooxygenase activity |
| GO:0004438 | 0.0074 | 274.4 | 0.01 | 1 | 2 | phosphatidylinositol-3-phosphatase activity |
| GO:0004658 | 0.0074 | 274.4 | 0.01 | 1 | 2 | propionyl-CoA carboxylase activity |
| GO:0008177 | 0.0074 | 274.4 | 0.01 | 1 | 2 | succinate dehydrogenase (ubiquinone) activity |
| GO:0015375 | 0.0074 | 274.4 | 0.01 | 1 | 2 | glycine:sodium symporter activity |
