# Supplementary material for: Microsatellite Interruptions Stabilize Primate Genomes and Exist as Population-Specific Single Nucleotide Polymorphisms within Individual Human Genomes
Source: PLoS Genet. 2014 Jul 17;10(7):e1004498. doi: 10.1371/journal.pgen.1004498 (PMC4102424; doi:10.1371/journal.pgen.1004498)
Supplement: Table S5 — Numbers of interruption-causing indels in linkage disequilibrium with SNPs. (DOCX) [file pgen.1004498.s021.docx]

| **Population** | **Numbers of interruption-causing indels** | | | | |
| --- | --- | --- | --- | --- | --- |
|  | **Total** | **R-sq > 0.8** | | **R-sq = 1.0** | |
|  |  | **Exonic** | **Non-exonic** | **Exonic** | **Non-exonic** |
| **Africans** | 13,269 | 54 | 4,647 | 6 | 834 |
| **Europeans** | 9,690 | 64 | 4,576 | 8 | 678 |
| **Asians** | 9,161 | 60 | 4,400 | 11 | 920 |
| **Americans** | 10,418 | 72 | 4,960 | 10 | 990 |

**Table S5. Numbers of interruption-causing indels in linkage disequilibrium with SNPs.**
