# Supplementary material for: Microsatellite Interruptions Stabilize Primate Genomes and Exist as Population-Specific Single Nucleotide Polymorphisms within Individual Human Genomes
Source: PLoS Genet. 2014 Jul 17;10(7):e1004498. doi: 10.1371/journal.pgen.1004498 (PMC4102424; doi:10.1371/journal.pgen.1004498)
Supplement: Table S6 — Phenotype and disease associations of perfect linkage disequilibrium indel-SNP pairs. (DOCX) [file pgen.1004498.s022.docx]

**Table S6. Phenotype and disease associations of perfect linkage disequilibrium indel-SNP pairs.**

| **iMS** | **linked SNP/s** | **Pop** | **gene/s** | **Disease/**  **phenotypes** | **Disease classes** | **PUBMED IDs** |
| --- | --- | --- | --- | --- | --- | --- |
| 1:118411373 | rs116823944  rs118048268  rs138800555  rs75697527 | ASN | SPAG17 | height | Developmental | 18391952 |
| 1:118411373 | rs3738422 | ASN | SPAG17 | adenocarcinoma,  height | Cancer, Developmental | 18391952,  COSMIC |
| 1:58933605 | rs6685601 rs72669882  rs72669887 | ASN | PDE4D | asthma,  neuroticism,  sleepiness | Immune, Neurological,  Psych | 17667963,  17903308,  19426955 |
| 3:40301473 | rs7625538 | AFR | MYRIP | sleep duration | Neurological | 17903308 |
| 4:38825712 | rs5743798  rs73236627  rs73236630 | EUR | TLR1 | Meningeal  meningioma | Cancer | 20406964 |
| 4:38825712 | rs5743818 | EUR | TLR1  TLR6 | Meningeal meningioma,  aspergillosis, prostate cancer | Cancer, Infection | 15812078,  16461792,  20406964 |
| 5:52388947 | rs10051425  rs10051483  rs10058715  rs1042324  rs10471829  rs10471830  rs13357783  rs3212606  rs3212612  rs3212613  rs3212615  rs3212628  rs3212629  rs3212633  rs3212634  rs3212635  rs3212643  rs75427194  rs76529944  rs7700416  rs7737412  rs875657 | AMR | ITGA2 | Glomerulonephritis, IGA, atherosclerosis, coronary; hematology indices, diabetes type 2, stroke, ischemic  thrombocytopenic purpura, immune, deep vein thrombosis; Behcet's Disease, Vascular Disease, bleeding compli-cations, cardio-vascular mortality, diabetic retinopathy, platelet GPIa and GPIIIa, hypertension, intima-media thickness; carotid plaque, limb deficiency anomalies, myocardial infarct, angina, oral cancer, platelet integrin α2 β1 density | Cancer, Cardiovascular,  Developmental,  Hematological,  Immune, Metabolic, Other,  Renal | 10194421,  10688808,  11015342,  11472360,  11568114,  11812069,  11978651,  12412731,  12482840,  12540964,  14556196,  15892865,  15947241,  16513317,  16697311,  16793669,  17036337,  17070428,  17534386,  19420105,  9058714,  9746778,  9950439 |
| 13:  108884464 | rs11841388  rs12853854  rs12854202  rs12855308  rs12855465  rs12855964  rs12856208  rs12856974  rs35286748  rs35950764  rs36041277 | AFR, EUR, ASN, AMR | ABHD13 | Attention deficit hyperactivity disorder and conduct disorder | Psych | 18951430 |
