# Supplementary material for: Microsatellite Interruptions Stabilize Primate Genomes and Exist as Population-Specific Single Nucleotide Polymorphisms within Individual Human Genomes
Source: PLoS Genet. 2014 Jul 17;10(7):e1004498. doi: 10.1371/journal.pgen.1004498 (PMC4102424; doi:10.1371/journal.pgen.1004498)
Supplement: Table S7 — Polymerase error frequencies within perfect and interrupted APC gene model templates. (DOCX) [file pgen.1004498.s023.docx]

**Table S7. Polymerase error frequencies within perfect and interrupted *APC* gene model templates.**

| Template | Polymerase | Observed HSV-tk frequency^a^ x 10^-4^ (±SD) | Pol EF_est_ x 10^-4^ (No. mutational events observed) | | |
| --- | --- | --- | --- | --- | --- |
|  |  |  | Overall^b^ | HSV-tk Coding^c^ | Microsatellite^c^ |
| A8 | None^d^ | 0.71 | n.d. | n.d. | n.d. |
|  | Human pol α | 57 | 55 (77) | 4.3(6) | 51 (71) |
|  | Rat pol β | 410 ± 85 | 400 (82) | 15 (3) | 390 (79) |
|  | Human pol η | 240 ± 31 | 270 (67) | 87 (22) | 180 (45) |
| T8 | None | 1.1 | n.d. | n.d. | n.d. |
|  | Human pol α | 50 | 47 (77) | 1.8 (3) | 45 (74) |
|  | Rat pol β | 430 ± 118 | 380 (83) | 32 (7) | 350 (76) |
|  | Human pol η | 430 ± 144 | 440 (35) | 215 (17) | 230 (18) |
| A3TA4 | None | 0.81 | n.d. | n.d. | n.d. |
|  | Human pol α | 11 ±1 .5 | 5.8(72) | 2.6 (32) | 3.2 (40) |
|  | Rat pol β | 59 ± 19 | 39 (70) | 36 (65) | 2.8 (5) |
|  | Human pol η | 130 ± 12 | 130 (58) | 76 (33) | 58 (25) |
| T3AT4 | None | 0.74 | n.d. | n.d. | n.d. |
|  | Human pol α | 8.1 ± 3.2 | 6.3 (60) | 2.7 (26) | 3.6 (34) |
|  | Rat pol β | 51 ± 21 | 35 (80) | 22 (50) | 13 (30) |
|  | Human pol η | 110 ± 10 | 100 (57) | 71 (40) | 30 (17) |

^a^Observed mutant frequencies are mean of two, or mean ± SD of 3-6 independent reactions.

^b^Overall polymerase error frequency (Pol EF) was calculated using this equation: Pol EF = (Observed MF) – (ssDNA Background MF) – (Outside Target MF). Pol EFs were adjusted for those mutants that had 2 or more mutational events in the target (see Methods). Mutants analyzed for DNA sequence changes were isolated from 2-3 independent polymerase reactions.

^c^Coding and microsatellite PolEF_est_ were calculated by multiplying the proportion of mutational events at either site by the overall PolEF_est_.

^d^Mutant frequency measured by electroporation of the ssDNA used to create DNA substrates.

n.d. not determined.
