# Supplementary material for: Microsatellite Interruptions Stabilize Primate Genomes and Exist as Population-Specific Single Nucleotide Polymorphisms within Individual Human Genomes
Source: PLoS Genet. 2014 Jul 17;10(7):e1004498. doi: 10.1371/journal.pgen.1004498 (PMC4102424; doi:10.1371/journal.pgen.1004498)
Supplement: Table S8 — Specificity of microsatellite interruptions created by DNA polymerases δ, β and κ. (DOCX) [file pgen.1004498.s024.docx]

**Table S8. Specificity of microsatellite interruptions created by DNA polymerases δ, β, and κ**

| Type of Interruption | Microsatellite Allele^a^ | | | | | |
| --- | --- | --- | --- | --- | --- | --- |
|  | **[A]_8_ or [T]_8_** | **[GT]_10_** | **[GT]_13_** | **[GT]_19_** | **[TC]_11_** | **[TC]_14_** |
| **Pol Delta (δ)** |  |  |  |  |  |  |
| Base Deletion | [A]_8_ N/A | [GT]_8_ T GT | n.a. | n.d. | [TC]_3_ T [TC]_7_ | n.a. |
|  | [T]_8_ N/A |  |  |  | [TC]_6_ T [TC]_4_ |  |
| Base Insertion | [A]_8_  n.a. | n.d. | n.a. | n.d. |  | n.a. |
|  | [T]_8_ n.d. |  |  |  |  |  |
| Base Subst. | [A]_8_  n.a. | GC [GT]_9_ | n.a. | [GT]_3_ GA [GT]_15_ | [TC]_6_ AC [TC]_3_^b^ | n.a. |
|  | [T]_8_ n.d. | [GT]_6_ TT [GT]_2_ (2)^b^ |  | [GT]_15_ GA [GT]_3_ | [TC]_7_ AC [TC]_4_^c^ |  |
|  |  |  |  | [GT]_15_ AT [GT]_3_ | [TC]_10_ TA TC^c^ |  |
|  |  |  |  |  | [TC]_11_ TA^c^ |  |
|  |  |  |  |  |  |  |
| **Total** | **[A]_8_ n.a.** | **4** | **n.a.** | **3** | **6** | **n.a.** |
| **Events^f^** | **[T]_8_  0** |  |  |  |  |  |
| **Pol Beta (β)** |  |  |  |  |  |  |
| Base Deletion | [A]_8_ N/A | [GT]_5_ T [GT]_4_ | [GT]_5_ G [GT]_7_ | T [GT]_18_ | C [TC]_10_ | n.d. |
|  | [T]_8_ N/A | [GT]_9_ G (2) | [GT]_10_ T [GT]_2_ | [GT]_9_ G [GT]_9_ | T [TC]_10_ (3) |  |
|  |  |  |  | [GT]_10_ G [GT]_8_ | [TC] C [TC]_9_ |  |
|  |  |  |  | [GT]_13_ G [GT]_5_ | [TC]_3_ C [TC]_7_ |  |
|  |  |  |  |  | [TC]_5_ C [TC]_5_ |  |
| Base Insertion | [A]_8_ n.d. | [GT]_3_ T [GT]_7_ | n.d. | n.d. | [TC]_3_ C [TC]_8_ | [TC]_2_ T [TC]_12_ |
|  | [T]_8_ n.d. |  |  |  |  |  |
| Base Subst. | [A]_8_ n.d. | **GT GC [GT]_6_ GG^b^** | [GT]_5_ GC [GT]_7_ | GT GC [GT]_18_^c^ | GC [TC]_12_^e^ | n.d. |
|  | [T]_8_ n.d. | [GT]_2_ GC [GT]_7_ | [GT]_11_ GG^b^ | [GT]_16_ AT [GT]_3_^c^ | [TC]_2_ TA [TC]_9_^c^ |  |
|  |  | [GT]_4_ TT [GT]_4_^b^ | [GT]_12_ GG (2) | [GT]_16_ GG GT^b^ |  |  |
|  |  | [GT]_6_ GA [GT]_3_ |  | [GT]_17_ GG (2)^b^ |  |  |
|  |  | [GT]_8_ TT (2)^b^ |  | [GT]_18_ GG (2) |  |  |
|  |  | [GT]_8_ GG (3)^b^ |  |  |  |  |
|  |  | [GT]_9_ GA |  |  |  |  |
|  |  |  |  |  |  |  |
| **Total** | **[A]_8_ 0** | **15** | **6** | **11** | **10** | **1** |
| **Events** | **[T]_8_ 0** |  |  |  |  |  |
| **Pol Kappa (κ)** |  |  |  |  |  |  |
| Base Deletion | [A]_8_ N/A | T [GT]_9_ (4) | T [GT]_12_ | T [GT]_18_ | T [TC]_10_ | TC T [TC]_12_ (2) |
|  | [T]_8_ N/A | [GT]_8_ T GT | GT T [GT]_11_ | [GT]_2_ T [GT]_16_ | [TC]_2_ T [TC]_8_ | [TC]_13_ C |
|  |  | [GT]_9_ T | [GT]_6_ T [GT]_6_ | [GT]_4_ T [GT]_14_ | [TC]_3_ T [TC]_7_ |  |
|  |  | [GT]_9_ G | [GT]_8_ G [GT]_4_ | [GT]_11_ T [GT]_7_ |  |  |
|  |  |  |  | [GT]_17_ T GT |  |  |
|  |  |  |  | [GT]_18_ T |  |  |
| Base Insertion | [A]_8_ n.d. | [GT]_2_ T [GT]_8_ | [GT]_3_ GCT [GT]_9_ | [GT]_3_ T [GT]_16_ | TC TGC [TC]_9_ | [TC]_3_ C [TC]_11_ |
|  | [T]_8_ n.d. | [GT]_5_ T [GT]_5_ |  | [GT]_8_ T [GT]_11_ | [TC]_6_ TGC [TC]_4_ | [TC]_7_ T [TC]_7_ |
|  |  | [GT]_8_ T [GT]_2_ (2) |  | [GT]_18_ T GT | [TC]_10_ T [TC] | [TC]_12_ TGC TC |
|  |  | [GT]_9_ T GT |  |  |  |  |
| Base Subst. | [A]_8_ n.d. | [GT]_6_ GA [GT]_3_ | n.d. | [GT]_6_ GG [GT]_12_ | [TC] GC [TC]_9_ | TT [TC]_13_ |
|  | [T]_8_ n.d. | [GT]_7_ TT [GT]_2_ |  | [GT]_11_ CT [GT]_7_ (2) | [TC]_3_ TA [TC]_7_ | [TC]_4_ GC [TC]_9_ |
|  |  | [GT]_9_ CT |  | [GT]_14_ CT [GT]_4_ | [TC]_6_ GC [TC]_3_^b^ | [TC]_4_ GC [TC]_10_^c^ |
|  |  | [GT]_10_ TT^c^ |  |  |  | **[TC]_7_ TT [TC]_4_ GC [TC]_3_^e^** |
|  |  |  |  |  |  | [TC]_13_ GC |
|  |  |  |  |  |  |  |
| Compound | [A]_8_ n.d. |  |  | **[GT]_2_ T [GT]_15_ TT GT** |  |  |
|  | [T]_8_ n.d. |  |  |  |  |  |
|  |  |  |  |  |  |  |
| **Total** | **[A]_8_ 0** | **16** | **5** | **15** | **9** | **12** |
| **Events** | **[T]_8_ 0** |  |  |  |  |  |

^a^Single mutation occurrences, unless otherwise indicated in parentheses. Red, interrupting base(s); **Bold Blue**, new alleles which have 2 or more interruption events. N/A, not applicable for this microsatellite allele. n.a. = not analyzed; n.d. = none detected

^b^A substitution occurred with a 1 unit deletion

^c^A substitution occurred with a 1 unit insertion

^d^A substitution occurred with a 2 unit deletion

^e^A substitution occurred with a 2 unit insertion

^f^Each mutation shown in red was counted as an individual event
