# Supplementary material for: Microsatellite Interruptions Stabilize Primate Genomes and Exist as Population-Specific Single Nucleotide Polymorphisms within Individual Human Genomes
Source: PLoS Genet. 2014 Jul 17;10(7):e1004498. doi: 10.1371/journal.pgen.1004498 (PMC4102424; doi:10.1371/journal.pgen.1004498)
Supplement: Dataset S1 — All interrupted microsatellites in the four 1000 genomes populations, with functional effects. (PDF) [file pgen.1004498.s001.pdf]

Dataset S1. All Interrupted microsatellites in four 1000 genomes populations, with functional effects.

Chr.	motif start	motif end	iMS position	original base	variant base	iMS type	AFR	ASN	AMR	EUR	High	Moderate	Low	Modifier
X	217130	217145	217137	TG	A	snp	1	1	1	1	.	.	.	GTPBP6(DOWNSTREAM);PLCXD1(INTRON);PLCXD1(UTR_3_PRIME)
X	218115	218122	218116	T	G	ins	1	1	1	1	.	.	.	GTPBP6(DOWNSTREAM);PLCXD1(INTRON);PLCXD1(UTR_3_PRIME)
X	283245	283260	283251	AAAG	G	del	1	1	1	1	.	.	.	INTERGENIC(INTERGENIC);PPP2R3B-AS1(DOWNSTREAM)
X	302962	302971	302966	GA	A	snp	0	0	0	1	.	.	.	PPP2R3B(DOWNSTREAM);PPP2R3B(EXON);PPP2R3B(INTRON);PPP2R3B(UPSTREAM)
X	363670	363683	363675	TG	C	snp	1	0	0	0	.	.	.	INTERGENIC(INTERGENIC)
X	391269	391276	391274	G	T	snp	1	0	0	0	.	.	.	INTERGENIC(INTERGENIC)
X	393918	393927	393919	T	G	ins	1	1	1	1	.	.	.	INTERGENIC(INTERGENIC)
X	397446	397459	397456	GA	AC	ins	1	0	0	0	.	.	.	INTERGENIC(INTERGENIC)
X	397446	397459	397456	GA	C	snp	1	0	0	0	.	.	.	INTERGENIC(INTERGENIC)
X	410154	410169	410164	TTAA	T	snp	1	1	1	1	.	.	.	INTERGENIC(INTERGENIC)
X	451383	451398	451390	TTTC	T	ins	1	0	0	0	.	.	.	INTERGENIC(INTERGENIC)
X	451383	451398	451393	TTTC	C	del	1	1	1	0	.	.	.	INTERGENIC(INTERGENIC)
X	458407	458422	458420	AT	A	del	1	1	1	0	.	.	.	INTERGENIC(INTERGENIC)
X	462730	462739	462737	AC	T	snp	1	1	1	1	.	.	.	INTERGENIC(INTERGENIC)
X	463735	463748	463737	AC	CG	ins	1	1	1	1	.	.	.	INTERGENIC(INTERGENIC)
X	467951	467962	467957	AT	G	snp	1	1	1	1	.	.	.	INTERGENIC(INTERGENIC)
X	477242	477253	477247	TC	T	snp	1	1	1	1	.	.	.	INTERGENIC(INTERGENIC)
X	507102	507115	507112	AG	G	del	0	1	1	1	.	.	.	INTERGENIC(INTERGENIC);KRT18P53(UPSTREAM)
X	512042	512061	512055	TCTT	T	del	1	1	1	1	.	.	.	INTERGENIC(INTERGENIC)
X	526815	526830	526821	ATGG	A	snp	0	1	1	1	.	.	.	INTERGENIC(INTERGENIC)
X	539793	539802	539800	AG	T	snp	1	1	1	1	.	.	.	INTERGENIC(INTERGENIC)
X	543461	543478	543476	TC	TCT	del	1	1	1	0	.	.	.	INTERGENIC(INTERGENIC)
X	566054	566063	566060	T	G	snp	1	0	0	1	.	.	.	INTERGENIC(INTERGENIC)
X	570420	570431	570423	AT	A	snp	1	0	0	0	.	.	.	INTERGENIC(INTERGENIC)
X	572819	572826	572820	T	G	snp	1	1	1	1	.	.	.	INTERGENIC(INTERGENIC)
X	582148	582157	582155	A	C	snp	1	0	0	0	.	.	.	INTERGENIC(INTERGENIC);SHOX(UPSTREAM)
X	583059	583072	583062	AG	C	snp	1	1	1	1	.	.	.	INTERGENIC(INTERGENIC);SHOX(UPSTREAM)
X	583370	583383	583373	AG	C	snp	1	1	1	1	.	.	.	INTERGENIC(INTERGENIC);SHOX(UPSTREAM)
X	587278	587289	587283	TC	TT	ins	1	1	1	1	.	.	.	SHOX(INTRON);SHOX(UPSTREAM)
X	589737	589746	589742	TC	A	snp	1	1	1	1	.	.	.	SHOX(INTRON);SHOX(UPSTREAM)
X	603036	603045	603038	G	T	snp	0	1	1	0	.	.	.	SHOX(DOWNSTREAM);SHOX(INTRON)
X	629133	629140	629138	T	C	snp	1	0	0	0	.	.	.	INTERGENIC(INTERGENIC)
X	643097	643112	643104	AG	C	snp	1	1	0	1	.	.	.	INTERGENIC(INTERGENIC)
X	643479	643488	643482	AG	T	snp	1	0	0	0	.	.	.	INTERGENIC(INTERGENIC)
X	643479	643488	643484	AG	C	snp	1	0	0	0	.	.	.	INTERGENIC(INTERGENIC)
X	652660	652668	652667	T	G	ins	1	0	0	0	.	.	.	INTERGENIC(INTERGENIC)
X	676980	676988	676981	T	C	snp	1	0	0	0	.	.	.	INTERGENIC(INTERGENIC)
X	709957	709972	709967	AGAA	AAG	del	1	1	1	1	.	.	.	INTERGENIC(INTERGENIC)
X	721246	721253	721247	A	G	snp	1	1	1	1	.	.	.	INTERGENIC(INTERGENIC)
X	728393	728402	728395	CA	T	snp	1	1	1	1	.	.	.	INTERGENIC(INTERGENIC)
X	741676	741695	741691	AGAT	C	snp	1	1	1	1	.	.	.	INTERGENIC(INTERGENIC)
X	757296	757303	757301	T	TTA	ins	1	0	0	0	.	.	.	INTERGENIC(INTERGENIC)
X	763019	763028	763026	TC	T	snp	1	0	0	1	.	.	.	INTERGENIC(INTERGENIC)
X	769557	769568	769565	TTG	A	snp	1	1	0	1	.	.	.	INTERGENIC(INTERGENIC)
X	778988	778999	778996	ATT	A	snp	1	0	0	1	.	.	.	INTERGENIC(INTERGENIC)
X	803646	803655	803651	CA	C	snp	1	1	0	1	.	.	.	INTERGENIC(INTERGENIC)
X	827730	827738	827737	A	C	snp	1	0	0	1	.	.	.	INTERGENIC(INTERGENIC)
X	843696	843709	843703	AG	C	snp	1	1	1	1	.	.	.	INTERGENIC(INTERGENIC)
X	854282	854305	854294	GATA	ATAA	ins	1	0	1	0	.	.	.	INTERGENIC(INTERGENIC)
X	867128	867145	867132	GA	A	del	0	0	1	0	.	.	.	INTERGENIC(INTERGENIC)
X	884329	884344	884336	ATCT	ATCTATCTATC	del	1	1	1	1	.	.	.	INTERGENIC(INTERGENIC)
X	884329	884344	884340	ATCT	ATCTATC	del	1	1	1	1	.	.	.	INTERGENIC(INTERGENIC)
X	885659	885674	885670	GATA	G	del	0	0	1	0	.	.	.	INTERGENIC(INTERGENIC)
X	893117	893126	893119	AT	G	snp	1	1	0	1	.	.	.	INTERGENIC(INTERGENIC)
X	893353	893362	893355	AT	G	snp	1	0	0	0	.	.	.	INTERGENIC(INTERGENIC)
X	900184	900197	900187	TC	G	snp	0	1	0	1	.	.	.	INTERGENIC(INTERGENIC)
X	902846	902857	902851	AG	A	snp	1	1	1	0	.	.	.	INTERGENIC(INTERGENIC)
X	905840	905855	905847	ATAG	C	snp	1	0	0	0	.	.	.	INTERGENIC(INTERGENIC)
X	925647	925655	925653	T	G	snp	1	1	1	1	.	.	.	INTERGENIC(INTERGENIC)
X	957763	957778	957771	TATG	C	snp	0	1	1	1	.	.	.	INTERGENIC(INTERGENIC);RP11-309M23.1.1(DOWNSTREAM)
X	988557	988564	988563	T	C	snp	1	1	1	1	.	.	.	INTERGENIC(INTERGENIC)
X	1009518	1009533	1009522	GAAA	A	snp	1	1	1	1	.	.	.	INTERGENIC(INTERGENIC)
X	1015900	1015919	1015915	TTTA	TT	del	1	1	1	1	.	.	.	INTERGENIC(INTERGENIC)
X	1018822	1018829	1018823	A	G	snp	1	1	1	1	.	.	.	INTERGENIC(INTERGENIC)

X	1026593	1026600	1026596	T	C	snp	1	0	0	0	.	.	.	INTERGENIC(INTERGENIC)
X	1031233	1031260	1031240	AGGG	A	snp	1	1	1	1	.	.	.	INTERGENIC(INTERGENIC)
X	1206652	1206663	1206659	GA	C	snp	1	0	0	0	.	.	.	INTERGENIC(INTERGENIC)
X	1206652	1206663	1206660	GA	A	snp	1	1	1	1	.	.	.	INTERGENIC(INTERGENIC)
X	1210343	1210352	1210345	GT	C	snp	1	1	1	1	.	.	.	INTERGENIC(INTERGENIC)
X	1219012	1219031	1219026	AAAG	G	snp	1	1	1	1	.	.	.	INTERGENIC(INTERGENIC)
X	1225203	1225218	1225210	ATAG	C	snp	1	0	0	0	.	.	.	INTERGENIC(INTERGENIC)
X	1230371	1230380	1230374	AC	A	snp	1	0	0	0	.	.	.	INTERGENIC(INTERGENIC)
X	1250262	1250289	1250280	GATA	C	snp	1	1	1	1	.	.	.	INTERGENIC(INTERGENIC)
X	1250262	1250289	1250284	GATA	C	snp	1	1	1	1	.	.	.	INTERGENIC(INTERGENIC)
X	1260930	1260939	1260934	GA	C	snp	1	1	0	1	.	.	.	INTERGENIC(INTERGENIC)
X	1261674	1261689	1261682	GT	A	snp	1	1	1	1	.	.	.	INTERGENIC(INTERGENIC)
X	1262170	1262178	1262175	A	G	snp	1	1	1	1	.	.	.	INTERGENIC(INTERGENIC)
X	1315930	1315938	1315936	A	C	snp	1	0	0	0	.	.	.	CRLF2(DOWNSTREAM);CRLF2(INTRON)
X	1337642	1337657	1337652	GATA	C	snp	1	1	1	1	.	.	.	INTERGENIC(INTERGENIC)
X	1350854	1350862	1350859	A	C	snp	1	0	0	0	.	.	.	INTERGENIC(INTERGENIC)
X	1350877	1350886	1350880	A	C	snp	1	0	0	0	.	.	.	INTERGENIC(INTERGENIC)
X	1350877	1350886	1350885	A	C	snp	1	1	1	1	.	.	.	INTERGENIC(INTERGENIC)
X	1363748	1363755	1363754	G	T	snp	1	1	0	1	.	.	.	INTERGENIC(INTERGENIC)
X	1392392	1392401	1392393	T	G	snp	1	1	1	1	.	.	.	CSF2RA(INTRON)
X	1394446	1394465	1394458	TTTA	TTA	ins	1	0	0	0	.	.	.	CSF2RA(INTRON)
X	1399646	1399657	1399651	TTC	T	snp	0	1	0	1	.	.	.	CSF2RA(INTRON);CSF2RA(UPSTREAM)
X	1402367	1402380	1402374	CT	C	del	1	1	1	1	.	.	.	CSF2RA(DOWNSTREAM);CSF2RA(INTRON);CSF2RA(UPSTREAM)
X	1425058	1425067	1425064	GA	C	snp	1	1	1	1	.	.	.	CSF2RA(DOWNSTREAM);CSF2RA(INTRON)
X	1434629	1434644	1434640	ATAG	A	snp	1	0	0	0	.	.	.	INTERGENIC(INTERGENIC)
X	1471625	1471638	1471633	AG	T	snp	1	0	0	0	.	.	.	IL3RA(INTRON)
X	1602537	1602544	1602538	A	G	snp	1	0	0	0	.	.	.	P2RY8(INTRON)
X	1603640	1603660	1603647	AAG	G	del	1	1	1	1	.	.	.	P2RY8(INTRON)
X	1619069	1619077	1619075	T	C	snp	1	1	1	1	.	.	.	P2RY8(INTRON)
X	1628645	1628672	1628666	TCTA	G	snp	1	1	1	1	.	.	.	P2RY8(INTRON)
X	1634121	1634132	1634124	TTA	G	snp	0	0	0	1	.	.	.	P2RY8(INTRON)
X	1643243	1643262	1643258	CATC	CACT	ins	1	0	0	1	.	.	.	P2RY8(INTRON)
X	1681966	1681973	1681969	A	G	snp	1	1	1	1	.	.	.	INTERGENIC(INTERGENIC)
X	1703105	1703119	1703115	TAT	T	ins	1	1	1	1	.	.	.	INTERGENIC(INTERGENIC)
X	1704867	1704882	1704877	TTTG	C	snp	1	1	1	1	.	.	.	INTERGENIC(INTERGENIC)
X	1715372	1715389	1715385	TCC	T	snp	1	1	1	1	.	.	.	AKAP17A(INTRON)
X	1741832	1741847	1741843	AAAT	A	snp	1	1	1	1	.	.	.	ASMT(INTRON);RP13-297E16.3.1(UPSTREAM)
X	1749797	1749812	1749806	TATT	T	ins	1	0	0	0	.	.	.	ASMT(INTRON);RP13-297E16.3.1(INTRON)
X	1762344	1762355	1762353	TC	T	snp	1	1	1	1	.	.	.	ASMT(DOWNSTREAM);INTERGENIC(INTERGENIC)
X	1765530	1765539	1765537	TC	T	snp	0	0	1	0	.	.	.	ASMT(DOWNSTREAM);INTERGENIC(INTERGENIC)
X	1772459	1772468	1772464	T	C	snp	1	1	1	1	.	.	.	INTERGENIC(INTERGENIC)
X	1773524	1773539	1773530	AAAG	G	del	1	1	0	0	.	.	.	INTERGENIC(INTERGENIC)
X	1775323	1775332	1775325	CA	T	snp	0	1	1	1	.	.	.	INTERGENIC(INTERGENIC)
X	1800592	1800603	1800595	CT	C	snp	1	1	1	1	.	.	.	INTERGENIC(INTERGENIC)
X	1822387	1822396	1822394	AT	A	del	1	1	1	1	.	.	.	INTERGENIC(INTERGENIC)
X	1843696	1843711	1843703	ATCC	T	snp	1	0	0	0	.	.	.	INTERGENIC(INTERGENIC)
X	1877502	1877515	1877504	TG	C	snp	1	1	1	1	.	.	.	INTERGENIC(INTERGENIC);RP13-297E16.4.1(DOWNSTREAM)
X	1953641	1953649	1953645	A	G	snp	0	0	1	0	.	.	.	INTERGENIC(INTERGENIC)
X	1972521	1972544	1972525	GGAA	GAAGGAAT	ins	1	1	1	1	.	.	.	. INTERGENIC(INTERGENIC)
X	1982736	1982744	1982742	T	G	snp	1	1	1	1	.	.	.	INTERGENIC(INTERGENIC)
X	2014590	2014599	2014594	AC	G	snp	0	1	1	0	.	.	.	INTERGENIC(INTERGENIC)
X	2036552	2036560	2036559	G	GT	ins	0	0	0	1	.	.	.	INTERGENIC(INTERGENIC)
X	2053820	2053833	2053823	GA	G	snp	1	1	1	1	.	.	.	INTERGENIC(INTERGENIC)
X	2056344	2056364	2056347	AGG	GG	del	1	0	0	0	.	.	.	INTERGENIC(INTERGENIC)
X	2161520	2161539	2161524	TTTA	A	snp	1	1	1	1	.	.	.	DHRSX(INTRON)
X	2200131	2200142	2200135	AGG	A	snp	1	1	1	1	.	.	.	DHRSX(INTRON)
X	2202396	2202411	2202406	TTCA	AA	ins	1	1	1	1	.	.	.	DHRSX(INTRON)
X	2222381	2222400	2222391	GATG	C	snp	1	0	0	0	.	.	.	DHRSX(INTRON)
X	2240723	2240731	2240728	A	C	ins	1	1	0	1	.	.	.	DHRSX(INTRON)
X	2253492	2253509	2253496	GT	TA	ins	1	1	1	1	.	.	.	DHRSX(DOWNSTREAM);DHRSX(INTRON);DHRSX-IT1(INTRON)
X	2264687	2264696	2264691	T	TTTTG	ins	1	1	1	1	.	.	.	DHRSX(INTRON)
X	2272085	2272096	2272091	GGA	A	snp	0	1	1	1	.	.	.	DHRSX(INTRON)
X	2283054	2283073	2283059	TATC	G	snp	1	1	1	1	.	.	.	DHRSX(INTRON)
X	2329794	2329802	2329797	A	C	ins	1	1	1	1	.	.	.	DHRSX(INTRON)
X	2349074	2349081	2349075	A	G	snp	1	0	0	0	.	.	.	DHRSX(INTRON)
X	2388446	2388455	2388454	A	G	snp	1	0	0	0	.	.	.	DHRSX(INTRON)
X	2401922	2401929	2401923	T	C	snp	0	1	1	1	.	.	.	DHRSX(INTRON);RP11-325D5.3.1(DOWNSTREAM);ZBED1(DOWNSTREAM)

X	2412608	2412619	2412613	TTA	G	snp	1	1	1	1	.	.	.	DHRSX(INTRON);ZBED1(INTRON)
X	2426845	2426856	2426850	TC	T	snp	1	0	0	0	.	.	.	INTERGENIC(INTERGENIC)
X	2433585	2433594	2433592	AT	A	del	1	1	0	1	.	.	.	INTERGENIC(INTERGENIC)
X	2453227	2453238	2453235	AAT	AA	del	1	0	1	1	.	.	.	INTERGENIC(INTERGENIC)
X	2453872	2453883	2453877	TTA	G	snp	1	0	1	1	.	.	.	INTERGENIC(INTERGENIC)
X	2503008	2503021	2503014	TC	G	snp	1	0	0	0	.	.	.	INTERGENIC(INTERGENIC)
X	2514072	2514081	2514077	A	C	snp	1	0	1	0	.	.	.	INTERGENIC(INTERGENIC)
X	2519582	2519591	2519589	AG	C	snp	1	0	0	0	.	.	.	INTERGENIC(INTERGENIC)
X	2529279	2529286	2529284	T	G	ins	0	0	1	1	.	.	.	CD99P1(INTRON);LINC00102(DOWNSTREAM)
X	2551956	2551969	2551961	AT	G	snp	1	0	0	0	.	.	.	CD99P1(INTRON)
X	2611180	2611191	2611183	TGG	GGG	ins	1	1	1	0	.	.	.	CD99(DOWNSTREAM);CD99(INTRON)
X	2650604	2650615	2650612	CT	T	snp	1	0	0	0	.	.	.	CD99(INTRON)
X	2650604	2650615	2650612	CT	TT	ins	1	1	1	1	.	.	.	CD99(INTRON)
X	2654915	2654938	2654925	AATA	AAATAAA	del	0	1	0	1	.	.	.	CD99(DOWNSTREAM);CD99(INTRON)
X	2657347	2657356	2657354	TG	T	snp	0	1	0	1	.	.	.	CD99(INTRON)
X	2667391	2667402	2667394	CT	G	snp	1	1	0	1	.	.	.	INTERGENIC(INTERGENIC);XG(UPSTREAM)
X	2668798	2668811	2668804	CA	G	snp	1	1	1	1	.	.	.	INTERGENIC(INTERGENIC);XG(UPSTREAM)
X	2683231	2683238	2683232	A	C	snp	1	0	0	0	.	.	.	XG(INTRON)
X	2699899	2699907	2699906	T	G	snp	1	0	0	0	.	.	.	XG(INTRON)
X	2840924	2840947	2840943	AAAG	AA	del	1	0	0	0	.	.	.	ARSD(INTRON)
X	2919854	2919861	2919855	C	A	snp	1	0	0	0	.	.	.	ARSH(UPSTREAM);INTERGENIC(INTERGENIC)
X	2919854	2919861	2919857	C	A	snp	1	1	0	1	.	.	.	ARSH(UPSTREAM);INTERGENIC(INTERGENIC)
X	2954976	2954987	2954978	AT	T	snp	1	0	0	0	.	.	.	ARSF(UPSTREAM);ARSH(DOWNSTREAM);INTERGENIC(INTERGENIC)
X	3083816	3083824	3083822	A	C	snp	1	1	1	1	.	.	.	INTERGENIC(INTERGENIC)
X	3131244	3131257	3131255	TA	G	snp	1	1	1	1	.	.	.	INTERGENIC(INTERGENIC)
X	3136732	3136739	3136737	A	G	ins	1	1	1	1	.	.	.	INTERGENIC(INTERGENIC)
X	3158503	3158518	3158511	CTTC	T	snp	1	0	0	0	.	.	.	INTERGENIC(INTERGENIC)
X	3172094	3172103	3172096	GA	AGACAA	ins	1	1	1	1	.	.	.	INTERGENIC(INTERGENIC)
X	3172094	3172103	3172098	GA	AC	ins	1	1	1	1	.	.	.	INTERGENIC(INTERGENIC)
X	3224862	3224877	3224873	ATCT	ATCTATC	del	1	1	0	1	.	.	.	INTERGENIC(INTERGENIC);MXRA5(DOWNSTREAM)
X	3224937	3224956	3224950	TATC	G	snp	1	0	0	0	.	.	.	INTERGENIC(INTERGENIC);MXRA5(DOWNSTREAM)
X	3259600	3259611	3259608	TAT	C	snp	1	1	0	0	.	.	.	MXRA5(INTRON)
X	3281987	3281998	3281993	TTG	C	snp	1	1	1	1	.	.	.	ASS1P4(UPSTREAM);INTERGENIC(INTERGENIC)
X	3392910	3392918	3392914	A	AAAAGAAAG	ins	1	1	1	1	.	.	.	INTERGENIC(INTERGENIC)
X	3405665	3405674	3405667	TA	C	snp	1	1	1	1	.	.	.	INTERGENIC(INTERGENIC)
X	3515137	3515144	3515138	T	G	snp	1	1	1	0	.	.	.	INTERGENIC(INTERGENIC)
X	3536109	3536126	3536122	AG	A	snp	1	1	1	1	.	.	.	PRKX(INTRON)
X	3635674	3635689	3635684	AAAC	C	snp	1	0	1	0	.	.	.	INTERGENIC(INTERGENIC);PRKX(UPSTREAM);RP11-558O12.1.1(DOWNSTREAM)
X	3655559	3655566	3655560	A	C	snp	1	1	1	1	.	.	.	INTERGENIC(INTERGENIC)
X	3740982	3740991	3740988	GT	TGT	del	1	1	1	1	.	.	.	RP11-706O15.1.1(DOWNSTREAM);RP11-706O15.1.1(INTRON);RP11-706O15.1.1(UPSTREAM)
X	3869493	3869510	3869508	AT	A	del	0	0	1	0	.	.	.	INTERGENIC(INTERGENIC);RP11-706O15.8.1(UPSTREAM)
X	3881862	3881871	3881865	GA	G	snp	1	1	0	0	.	.	.	INTERGENIC(INTERGENIC)
X	3965805	3965822	3965815	CT	A	snp	1	1	1	0	.	.	.	INTERGENIC(INTERGENIC)
X	3992138	3992149	3992140	AT	G	snp	0	1	1	1	.	.	.	INTERGENIC(INTERGENIC)
X	4002999	4003010	4003005	ATT	T	snp	1	1	1	0	.	.	.	INTERGENIC(INTERGENIC)
X	4008599	4008619	4008609	AAC	G	snp	1	0	1	0	.	.	.	INTERGENIC(INTERGENIC)
X	4039881	4039894	4039892	CT	C	snp	1	0	0	0	.	.	.	INTERGENIC(INTERGENIC)
X	4069628	4069636	4069633	T	G	snp	1	1	0	1	.	.	.	INTERGENIC(INTERGENIC)
X	4088434	4088441	4088438	T	C	snp	1	1	1	1	.	.	.	INTERGENIC(INTERGENIC)
X	4145173	4145190	4145187	AT	G	snp	1	0	0	0	.	.	.	INTERGENIC(INTERGENIC)
X	4186529	4186540	4186538	AT	A	del	1	1	1	1	.	.	.	INTERGENIC(INTERGENIC)
X	4204973	4204981	4204980	C	A	snp	1	1	1	1	.	.	.	INTERGENIC(INTERGENIC)
X	4211782	4211801	4211789	TATC	G	snp	0	0	1	0	.	.	.	INTERGENIC(INTERGENIC)
X	4267975	4267982	4267977	A	G	snp	1	1	1	1	.	.	.	INTERGENIC(INTERGENIC)
X	4270925	4270932	4270927	A	C	snp	1	1	1	0	.	.	.	INTERGENIC(INTERGENIC)
X	4310650	4310661	4310659	AG	C	snp	1	0	0	0	.	.	.	INTERGENIC(INTERGENIC)
X	4325260	4325277	4325274	TTA	T	ins	1	0	0	0	.	.	.	INTERGENIC(INTERGENIC)
X	4361649	4361658	4361652	TA	G	snp	1	1	1	1	.	.	.	INTERGENIC(INTERGENIC)
X	4362125	4362134	4362131	TA	C	snp	1	0	0	0	.	.	.	INTERGENIC(INTERGENIC)
X	4383146	4383155	4383153	AG	A	snp	1	1	1	0	.	.	.	INTERGENIC(INTERGENIC)
X	4410991	4411002	4410999	AG	G	snp	1	0	0	0	.	.	.	INTERGENIC(INTERGENIC)
X	4500708	4500721	4500716	GT	A	snp	1	0	0	0	.	.	.	INTERGENIC(INTERGENIC)
X	4599898	4599913	4599909	GAAG	GAA	del	1	1	1	1	.	.	.	INTERGENIC(INTERGENIC)
X	4631540	4631548	4631543	T	G	snp	1	0	0	0	.	.	.	INTERGENIC(INTERGENIC)
X	4642421	4642429	4642424	C	A	snp	1	0	0	0	.	.	.	INTERGENIC(INTERGENIC)
X	4642586	4642594	4642591	T	G	ins	1	0	0	0	.	.	.	INTERGENIC(INTERGENIC)

X	4656117	4656124	4656119	T	C	snp	1	0	0	0	.	.	.	INTERGENIC(INTERGENIC)
X	4704311	4704319	4704312	T	C	snp	1	1	1	1	.	.	.	INTERGENIC(INTERGENIC)
X	4776714	4776725	4776721	AT	C	snp	1	1	1	1	.	.	.	INTERGENIC(INTERGENIC)
X	4785425	4785436	4785427	AT	G	snp	0	1	0	0	.	.	.	INTERGENIC(INTERGENIC)
X	4879244	4879259	4879255	AAAC	A	del	1	1	0	1	.	.	.	INTERGENIC(INTERGENIC)
X	4898832	4898839	4898834	A	G	snp	1	1	1	1	.	.	.	INTERGENIC(INTERGENIC)
X	4904622	4904639	4904629	TG	T	snp	1	0	1	1	.	.	.	INTERGENIC(INTERGENIC)
X	5045942	5045953	5045951	TC	G	snp	1	1	1	1	.	.	.	INTERGENIC(INTERGENIC)
X	5058111	5058119	5058115	T	TC	ins	1	1	1	1	.	.	.	INTERGENIC(INTERGENIC)
X	5069266	5069273	5069268	T	C	snp	1	1	1	1	.	.	.	INTERGENIC(INTERGENIC)
X	5175521	5175530	5175524	TG	TA	ins	1	0	0	0	.	.	.	INTERGENIC(INTERGENIC)
X	5276979	5276992	5276990	AC	ACTTTCATAT	ins	1	0	0	0	.	.	.	INTERGENIC(INTERGENIC)
X	5328295	5328306	5328304	TA	T	del	1	0	0	0	.	.	.	INTERGENIC(INTERGENIC);SNORA20(DOWNSTREAM)
X	5328374	5328391	5328386	TA	ATATA	del	1	0	0	0	.	.	.	INTERGENIC(INTERGENIC);SNORA20(DOWNSTREAM)
X	5396574	5396594	5396579	AAT	A	snp	1	1	0	1	.	.	.	INTERGENIC(INTERGENIC)
X	5439230	5439243	5439241	TA	G	snp	1	1	1	1	.	.	.	INTERGENIC(INTERGENIC)
X	5449362	5449379	5449376	AAC	A	ins	1	1	1	1	.	.	.	INTERGENIC(INTERGENIC)
X	5466809	5466820	5466817	CTC	T	snp	1	1	1	1	.	.	.	INTERGENIC(INTERGENIC)
X	5534366	5534373	5534369	T	G	snp	1	1	1	1	.	.	.	INTERGENIC(INTERGENIC)
X	5558524	5558541	5558539	AC	A	snp	1	0	0	0	.	.	.	INTERGENIC(INTERGENIC)
X	5593884	5593892	5593885	A	C	snp	1	1	1	1	.	.	.	RP11-733O18.1.1(INTRON)
X	5605729	5605740	5605733	AT	G	snp	1	1	1	1	.	.	.	RP11-733O18.1.1(INTRON)
X	5660954	5660968	5660963	GAA	A	snp	1	1	1	1	.	.	.	INTERGENIC(INTERGENIC)
X	5708289	5708304	5708292	AG	A	snp	1	1	1	1	.	.	.	INTERGENIC(INTERGENIC)
X	5759850	5759859	5759853	TC	G	snp	1	0	0	0	.	.	.	NLGN4X(INTRON)
X	5825976	5825985	5825978	AG	G	snp	1	0	0	0	.	.	.	NLGN4X(INTRON);NLGN4X(UPSTREAM)
X	5916759	5916766	5916765	A	G	snp	1	0	0	0	.	.	.	NLGN4X(INTRON)
X	5942041	5942050	5942043	A	C	snp	0	1	1	1	.	.	.	NLGN4X(INTRON)
X	5985147	5985155	5985149	C	CA	ins	0	1	1	1	.	.	.	NLGN4X(INTRON)
X	6139119	6139142	6139135	TTAT	G	snp	1	1	1	1	.	.	.	NLGN4X(INTRON)
X	6146000	6146009	6146007	G	A	snp	0	0	1	0	.	.	.	NLGN4X(INTRON);NLGN4X(UPSTREAM)
X	6148311	6148319	6148318	A	C	snp	1	1	1	1	.	.	.	INTERGENIC(INTERGENIC);NLGN4X(UPSTREAM)
X	6318999	6319010	6319003	TCT	TT	del	1	0	0	0	.	.	.	INTERGENIC(INTERGENIC)
X	6318999	6319010	6319004	TCT	C	snp	1	1	0	1	.	.	.	INTERGENIC(INTERGENIC)
X	6336170	6336185	6336177	AAAC	A	snp	0	0	1	0	.	.	.	INTERGENIC(INTERGENIC);RP11-459A10.1.1(DOWNSTREAM)
X	6500331	6500346	6500342	ATCT	A	snp	1	1	0	1	.	.	.	INTERGENIC(INTERGENIC)
X	6738014	6738023	6738021	AT	A	del	1	0	1	0	.	.	.	INTERGENIC(INTERGENIC)
X	6812311	6812319	6812312	A	G	snp	1	0	0	0	.	.	.	INTERGENIC(INTERGENIC)
X	6827210	6827219	6827215	AT	C	snp	1	1	1	1	.	.	.	INTERGENIC(INTERGENIC)
X	6973291	6973300	6973298	T	G	snp	1	1	1	1	.	.	.	HDHD1(DOWNSTREAM);HDHD1(INTRON)
X	7001929	7001946	7001941	CAA	A	ins	0	1	1	1	.	.	.	HDHD1(DOWNSTREAM);HDHD1(INTRON)
X	7001929	7001946	7001943	CAA	C	del	1	1	1	1	.	.	.	HDHD1(DOWNSTREAM);HDHD1(INTRON)
X	7064174	7064181	7064178	G	A	snp	1	1	1	1	.	.	.	HDHD1(INTRON)
X	7122561	7122580	7122566	CCTC	G	snp	1	0	0	0	.	.	.	INTERGENIC(INTERGENIC)
X	7217050	7217061	7217058	TA	A	snp	1	1	1	1	.	.	.	STS(INTRON)
X	7227640	7227649	7227644	A	G	ins	1	1	1	1	.	.	.	STS(INTRON)
X	7314533	7314542	7314536	C	CCCA	ins	1	0	0	0	.	.	.	INTERGENIC(INTERGENIC)
X	7348143	7348151	7348147	T	TTTTG	ins	1	0	1	1	.	.	.	INTERGENIC(INTERGENIC)
X	7841493	7841504	7841498	AT	A	del	1	1	1	1	.	.	.	INTERGENIC(INTERGENIC)
X	7841493	7841504	7841502	AT	A	del	1	1	1	1	.	.	.	INTERGENIC(INTERGENIC)
X	8150194	8150203	8150196	TA	C	snp	1	0	0	0	.	.	.	INTERGENIC(INTERGENIC)
X	8261103	8261111	8261104	A	AC	ins	1	1	1	1	.	.	.	INTERGENIC(INTERGENIC)
X	8282353	8282366	8282356	TG	A	snp	1	0	0	0	.	.	.	INTERGENIC(INTERGENIC)
X	8322410	8322417	8322412	T	G	snp	0	1	0	0	.	.	.	INTERGENIC(INTERGENIC)
X	8352675	8352684	8352679	T	C	snp	1	0	0	0	.	.	.	INTERGENIC(INTERGENIC)
X	8476511	8476518	8476517	T	C	snp	1	1	1	1	.	.	.	INTERGENIC(INTERGENIC)
X	8476524	8476531	8476527	T	G	snp	0	1	0	1	.	.	.	INTERGENIC(INTERGENIC)
X	8487183	8487198	8487196	TC	T	ins	1	1	1	1	.	.	.	INTERGENIC(INTERGENIC)
X	8555162	8555175	8555173	TG	T	del	1	1	1	1	.	.	.	KAL1(INTRON)
X	8562793	8562808	8562802	AT	ATATA	del	1	1	0	1	.	.	.	KAL1(INTRON)
X	8562793	8562808	8562803	AT	TATAT	del	1	1	0	1	.	.	.	KAL1(INTRON)
X	8562793	8562808	8562806	AT	A	del	1	1	0	1	.	.	.	KAL1(INTRON)
X	8693623	8693632	8693628	AT	C	snp	1	1	1	1	.	.	.	KAL1(INTRON)
X	8693651	8693662	8693656	AT	C	snp	0	1	0	0	.	.	.	KAL1(INTRON)
X	8693723	8693734	8693728	AT	C	snp	1	1	1	1	.	.	.	KAL1(INTRON)
X	8693753	8693764	8693758	AT	C	snp	1	1	1	1	.	.	.	KAL1(INTRON)
X	8693799	8693808	8693806	AT	C	snp	0	1	0	0	.	.	.	KAL1(INTRON)

X	8707888	8707907	8707895	AAAT	A	snp	1	1	1	1	.	.	.	INTERGENIC(INTERGENIC)
X	8804380	8804387	8804385	A	C	snp	1	0	0	0	.	.	.	INTERGENIC(INTERGENIC)
X	8879215	8879226	8879218	GA	G	snp	1	1	1	1	.	.	.	INTERGENIC(INTERGENIC)
X	8888722	8888733	8888729	AT	C	snp	1	0	0	0	.	.	.	INTERGENIC(INTERGENIC)
X	8941608	8941617	8941613	T	G	snp	1	1	0	1	.	.	.	INTERGENIC(INTERGENIC)
X	8953575	8953598	8953593	ATAG	GATAGAT	del	1	0	0	0	.	.	.	INTERGENIC(INTERGENIC);RP11-66N5.2.1(UPSTREAM)
X	8987871	8987880	8987876	AC	T	snp	1	0	0	0	.	.	.	FAM9B(DOWNSTREAM);INTERGENIC(INTERGENIC)
X	8989324	8989333	8989329	TA	C	snp	1	1	0	0	.	.	.	FAM9B(DOWNSTREAM);INTERGENIC(INTERGENIC)
X	9004243	9004254	9004246	TTG	C	snp	1	0	0	0	.	.	.	FAM9B(INTRON);FAM9B(UPSTREAM)
X	9050315	9050328	9050323	GA	AA	ins	1	1	1	1	.	.	.	FAM9B(INTRON)
X	9099183	9099192	9099190	TC	T	snp	1	0	0	0	.	.	.	5S_rRNA(UPSTREAM);FAM9B(INTRON)
X	9141836	9141845	9141840	A	C	snp	1	0	0	0	.	.	.	INTERGENIC(INTERGENIC)
X	9234464	9234471	9234465	T	G	snp	0	1	0	0	.	.	.	GS1-519E5.1.1(INTRON)
X	9238978	9238993	9238986	GGAA	A	snp	1	0	0	0	.	.	.	GS1-519E5.1.1(INTRON)
X	9282604	9282611	9282607	T	G	ins	1	0	0	0	.	.	.	INTERGENIC(INTERGENIC)
X	9282604	9282611	9282608	T	G	snp	1	1	1	1	.	.	.	INTERGENIC(INTERGENIC)
X	9312235	9312244	9312238	G	T	ins	1	0	0	0	.	.	.	INTERGENIC(INTERGENIC)
X	9312235	9312244	9312239	G	T	snp	1	0	0	0	.	.	.	INTERGENIC(INTERGENIC)
X	9349453	9349468	9349460	AAAG	AAAGAAAGAAA	del	1	0	0	0	.	.	.	INTERGENIC(INTERGENIC)
X	9349453	9349468	9349461	AAAG	T	snp	1	1	1	1	.	.	.	INTERGENIC(INTERGENIC)
X	9355905	9355912	9355910	T	C	snp	0	0	0	1	.	.	.	INTERGENIC(INTERGENIC)
X	9390640	9390648	9390647	T	C	snp	1	1	0	0	.	.	.	INTERGENIC(INTERGENIC)
X	9524230	9524239	9524231	A	AAAC	ins	1	1	1	1	.	.	.	TBL1X(INTRON)
X	9524230	9524239	9524234	A	C	ins	1	1	1	1	.	.	.	TBL1X(INTRON)
X	9561446	9561466	9561449	AAT	AT	del	1	0	0	0	.	.	.	TBL1X(INTRON)
X	9561446	9561466	9561461	AAT	G	snp	1	0	0	0	.	.	.	TBL1X(INTRON)
X	9704503	9704510	9704508	T	G	snp	1	0	0	0	.	.	.	GPR143(DOWNSTREAM);GPR143(INTRON)
X	9717267	9717274	9717272	C	T	ins	1	1	0	0	.	.	.	GPR143(INTRON)
X	9756014	9756029	9756025	TTTC	T	del	0	1	1	1	.	.	.	SHROOM2(INTRON)
X	9797982	9797989	9797985	T	TTTTA	ins	1	1	1	1	.	.	.	SHROOM2(INTRON)
X	9836282	9836297	9836289	TTTA	G	snp	1	0	1	0	.	.	.	HMGN1P33(UPSTREAM);SHROOM2(INTRON)
X	9968060	9968074	9968064	GTT	C	snp	1	0	0	0	.	.	.	INTERGENIC(INTERGENIC)
X	9981689	9981698	9981696	T	G	snp	1	0	0	0	.	.	.	INTERGENIC(INTERGENIC);WWC3(UPSTREAM)
X	10011885	10011898	10011892	AG	C	snp	1	0	0	0	.	.	.	WWC3(INTRON)
X	10017626	10017639	10017632	GT	A	snp	1	0	0	0	.	.	.	WWC3(INTRON)
X	10137541	10137560	10137555	AAAC	C	del	1	0	0	1	.	.	.	CLCN4(INTRON)
X	10137541	10137560	10137556	AAAC	A	snp	1	0	0	1	.	.	.	CLCN4(INTRON)
X	10143954	10143967	10143957	CT	C	snp	1	1	1	1	.	.	.	CLCN4(INTRON)
X	10197427	10197444	10197429	AT	T	del	1	0	0	0	.	.	.	CLCN4(INTRON)
X	10225759	10225776	10225773	AC	T	snp	1	0	0	0	.	.	.	RP6-102.1.1(INTRON)
X	10374070	10374078	10374072	A	G	snp	1	0	0	0	.	.	.	INTERGENIC(INTERGENIC)
X	10429345	10429356	10429352	GT	C	snp	1	0	0	0	.	.	.	MID1(INTRON);MID1(UPSTREAM)
X	10471443	10471456	10471452	GA	G	snp	1	0	0	0	.	.	.	MID1(INTRON)
X	10568342	10568351	10568347	A	G	snp	1	0	0	0	.	.	.	MID1(INTRON)
X	10723379	10723388	10723386	T	G	snp	1	1	0	1	.	.	.	MID1(INTRON)
X	10730504	10730518	10730511	TTG	A	snp	1	0	0	0	.	.	.	MID1(INTRON)
X	10823163	10823171	10823169	T	G	snp	1	0	0	0	.	.	.	MID1(INTRON)
X	11248840	11248849	11248842	AT	G	snp	1	1	0	1	.	.	.	ARHGAP6(INTRON)
X	11503158	11503167	11503159	A	C	ins	1	1	1	0	.	.	.	ARHGAP6(INTRON)
X	11503158	11503167	11503160	A	C	snp	1	1	1	1	.	.	.	ARHGAP6(INTRON)
X	11575569	11575577	11575570	T	G	snp	1	1	1	1	.	.	.	ARHGAP6(INTRON)
X	11740105	11740116	11740109	AAG	T	snp	0	1	1	1	.	.	.	INTERGENIC(INTERGENIC)
X	11881108	11881117	11881115	TC	T	ins	1	1	1	1	.	.	.	INTERGENIC(INTERGENIC)
X	11982273	11982293	11982276	TTG	TG	del	1	0	0	0	.	.	.	INTERGENIC(INTERGENIC)
X	11996610	11996617	11996612	A	G	snp	0	0	0	1	.	.	.	INTERGENIC(INTERGENIC)
X	12057320	12057339	12057335	ATTT	A	del	0	1	1	0	.	.	.	INTERGENIC(INTERGENIC)
X	12104198	12104207	12104204	GT	TA	ins	0	1	1	0	.	.	.	INTERGENIC(INTERGENIC)
X	12200697	12200710	12200700	AT	AA	ins	1	1	0	1	.	.	.	FRMPD4(INTRON)
X	12200697	12200710	12200704	AT	A	snp	0	0	0	1	.	.	.	FRMPD4(INTRON)
X	12249150	12249167	12249153	TA	G	snp	1	0	0	0	.	.	.	FRMPD4(INTRON)
X	12260070	12260090	12260085	GGA	A	snp	1	1	1	1	.	.	.	FRMPD4(INTRON)
X	12276785	12276796	12276792	TTA	C	snp	0	1	0	0	.	.	.	FRMPD4(INTRON)
X	12435017	12435024	12435018	A	C	snp	1	1	1	1	.	.	.	FRMPD4(INTRON)
X	12459561	12459568	12459564	T	G	snp	1	0	0	0	.	.	.	FRMPD4(INTRON)
X	12481801	12481810	12481802	T	G	snp	1	1	1	1	.	.	.	FRMPD4(INTRON)
X	12555563	12555572	12555565	T	C	snp	1	0	0	0	.	.	.	FRMPD4(INTRON)
X	12597772	12597779	12597773	T	G	snp	1	0	0	0	.	.	.	FRMPD4(INTRON)

X	12785889	12785900	12785896	AT	C	snp	1	1	1	1	.	.	.	INTERGENIC(INTERGENIC)
X	12912854	12912865	12912861	TC	TCTCA	del	1	1	1	1	.	.	.	INTERGENIC(INTERGENIC);TLR7(DOWNSTREAM)
X	13242082	13242089	13242084	T	C	snp	0	0	1	0	.	.	.	INTERGENIC(INTERGENIC)
X	13466822	13466845	13466841	AAAC	A	snp	1	1	1	1	.	.	.	INTERGENIC(INTERGENIC)
X	13472323	13472332	13472330	AT	A	ins	1	1	1	1	.	.	.	INTERGENIC(INTERGENIC)
X	13494443	13494454	13494446	TTC	C	snp	1	1	1	1	.	.	.	INTERGENIC(INTERGENIC)
X	13589230	13589250	13589235	CCA	C	snp	1	0	1	1	.	.	.	EGFL6(INTRON);RPL30P15(UPSTREAM)
X	13593554	13593563	13593561	AC	G	snp	1	0	1	0	.	.	.	EGFL6(INTRON);RPL30P15(DOWNSTREAM)
X	13595559	13595568	13595562	TA	G	snp	1	0	0	0	.	.	.	EGFL6(INTRON);RPL30P15(DOWNSTREAM)
X	13662144	13662153	13662147	T	G	snp	1	1	1	1	.	.	.	INTERGENIC(INTERGENIC)
X	13693526	13693533	13693530	A	G	snp	1	0	0	0	.	.	.	INTERGENIC(INTERGENIC)
X	13807956	13807965	13807962	T	C	snp	0	1	0	0	.	.	.	GPM6B(INTRON)
X	13812290	13812305	13812301	TC	T	snp	1	1	1	1	.	.	.	GPM6B(INTRON)
X	13817505	13817522	13817519	TAT	TA	del	1	1	1	1	.	.	.	GPM6B(INTRON)
X	13820672	13820687	13820674	TA	AC	ins	1	1	1	1	.	.	.	GPM6B(INTRON)
X	13822537	13822544	13822538	G	A	snp	0	0	0	1	.	.	.	GPM6B(INTRON)
X	13883679	13883694	13883690	AAAG	A	ins	1	1	1	1	.	.	.	GPM6B(INTRON)
X	13893057	13893065	13893063	C	T	snp	1	0	0	0	.	.	.	GPM6B(INTRON)
X	13893057	13893065	13893064	C	A	ins	0	0	1	0	.	.	.	GPM6B(INTRON)
X	14017199	14017207	14017203	T	C	snp	1	1	0	1	.	.	.	INTERGENIC(INTERGENIC)
X	14063301	14063309	14063306	T	C	snp	1	1	0	1	.	.	.	INTERGENIC(INTERGENIC)
X	14135219	14135227	14135220	G	T	snp	1	0	0	0	.	.	.	INTERGENIC(INTERGENIC)
X	14145738	14145746	14145739	T	C	snp	1	0	0	0	.	.	.	INTERGENIC(INTERGENIC)
X	14268822	14268833	14268827	TA	TG	ins	1	0	1	0	.	.	.	INTERGENIC(INTERGENIC)
X	14415574	14415589	14415587	AT	A	del	1	1	1	1	.	.	.	INTERGENIC(INTERGENIC)
X	14419731	14419740	14419737	AT	G	snp	1	1	0	1	.	.	.	INTERGENIC(INTERGENIC)
X	14420337	14420348	14420339	AT	G	snp	1	0	0	0	.	.	.	INTERGENIC(INTERGENIC)
X	14420337	14420348	14420341	AT	G	snp	1	0	0	0	.	.	.	INTERGENIC(INTERGENIC)
X	14456907	14456914	14456909	T	C	snp	1	0	0	0	.	.	.	INTERGENIC(INTERGENIC)
X	14583067	14583074	14583073	A	C	snp	0	1	1	1	.	.	.	GLRA2(INTRON)
X	14593134	14593147	14593145	AC	T	snp	0	0	0	1	.	.	.	GLRA2(INTRON)
X	14607799	14607814	14607802	AT	A	snp	0	0	0	1	.	.	.	GLRA2(INTRON)
X	14607823	14607832	14607828	TA	TATACACATATATATATG	ins	1	1	1	1	.	.	.	GLRA2(INTRON)
X	14681122	14681133	14681128	TAG	C	snp	1	0	0	0	.	.	.	GLRA2(INTRON)
X	14715593	14715604	14715601	AAT	AATC	del	1	0	0	0	.	.	.	GLRA2(INTRON)
X	14779119	14779128	14779121	A	C	snp	1	1	1	1	.	.	.	AC003658.1(DOWNSTREAM);INTERGENIC(INTERGENIC)
X	14868799	14868808	14868806	A	G	snp	1	1	1	1	.	.	.	FANCB(INTRON)
X	14909686	14909701	14909690	ATTT	T	snp	1	1	1	1	.	.	.	MOSPD2(INTRON)
X	14961030	14961049	14961043	TTAT	C	snp	0	0	1	0	.	.	.	INTERGENIC(INTERGENIC)
X	14995717	14995724	14995720	T	G	snp	1	0	0	0	.	.	.	INTERGENIC(INTERGENIC);RP13-928P6.3.1(DOWNSTREAM)
X	15183136	15183143	15183139	T	C	snp	1	0	0	0	.	.	.	INTERGENIC(INTERGENIC)
X	15246210	15246219	15246212	A	G	snp	1	1	0	1	.	.	.	INTERGENIC(INTERGENIC)
X	15248629	15248638	15248634	T	C	snp	0	1	0	0	.	.	.	ASB9(DOWNSTREAM);INTERGENIC(INTERGENIC)
X	15308147	15308166	15308159	AAAT	T	snp	1	0	0	0	.	.	.	ASB11(INTRON)
X	15319651	15319664	15319662	AG	A	snp	1	1	1	1	.	.	.	ASB11(INTRON)
X	15368888	15368903	15368899	TTTC	TT	del	1	0	0	0	.	.	.	FIGF(INTRON);FIGF(UPSTREAM)
X	15368976	15368995	15368983	TCTT	C	snp	1	1	1	1	.	.	.	FIGF(INTRON);FIGF(UPSTREAM)
X	15386077	15386100	15386096	AAAG	AAAGAA	del	1	1	0	1	.	.	.	FIGF(INTRON)
X	15444212	15444221	15444217	TA	T	snp	1	0	0	0	.	.	.	PIR(DOWNSTREAM);PIR(INTRON)
X	15480590	15480598	15480592	A	AAC	ins	1	0	0	0	.	.	.	BMX(UPSTREAM);PIR(INTRON)
X	15531516	15531523	15531517	T	C	snp	1	0	0	0	.	.	.	BMX(INTRON)
X	15665654	15665662	15665656	G	T	snp	1	1	1	1	.	.	.	GS1-594A7.5.1(UPSTREAM);TMEM27(INTRON)
X	15759612	15759620	15759615	T	G	snp	1	1	1	1	.	.	.	CA5B(INTRON)
X	15961185	15961208	15961201	ATTT	T	ins	1	0	0	0	.	.	.	INTERGENIC(INTERGENIC)
X	15961185	15961208	15961204	ATTT	A	del	1	1	0	0	.	.	.	INTERGENIC(INTERGENIC)
X	16039796	16039805	16039803	T	G	snp	1	1	0	1	.	.	.	INTERGENIC(INTERGENIC)
X	16084312	16084319	16084316	T	G	snp	1	1	0	0	.	.	.	INTERGENIC(INTERGENIC)
X	16113842	16113850	16113849	T	G	snp	1	1	1	1	.	.	.	INTERGENIC(INTERGENIC)
X	16118361	16118372	16118366	AC	AT	ins	1	0	0	0	.	.	.	INTERGENIC(INTERGENIC)
X	16118476	16118487	16118484	CA	T	snp	1	1	0	1	.	.	.	INTERGENIC(INTERGENIC)
X	16119774	16119783	16119778	AG	G	snp	1	1	0	1	.	.	.	INTERGENIC(INTERGENIC)
X	16273847	16273856	16273850	TA	C	snp	1	1	1	1	.	.	.	INTERGENIC(INTERGENIC)
X	16273861	16273870	16273867	TA	G	snp	1	1	1	1	.	.	.	INTERGENIC(INTERGENIC)
X	16300300	16300307	16300302	A	C	ins	1	1	1	1	.	.	.	INTERGENIC(INTERGENIC)
X	16318786	16318801	16318793	TG	T	snp	1	0	0	0	.	.	.	AC078993.1(DOWNSTREAM);INTERGENIC(INTERGENIC)
X	16400448	16400457	16400450	A	C	snp	0	0	1	0	.	.	.	INTERGENIC(INTERGENIC)
X	16400448	16400457	16400453	A	C	snp	1	0	0	0	.	.	.	INTERGENIC(INTERGENIC)

X	16414990	16414997	16414996	T	C	snp	0	0	1	0	.	.	.	INTERGENIC(INTERGENIC)
X	16724985	16724994	16724991	A	C	snp	1	0	0	0	.	.	.	CTPS2(INTRON)
X	16934984	16934993	16934992	T	G	ins	1	1	1	1	.	.	.	INTERGENIC(INTERGENIC);Y_RNA(UPSTREAM)
X	16955649	16955657	16955654	A	C	snp	1	0	0	0	.	.	.	INTERGENIC(INTERGENIC)
X	17063044	17063051	17063045	T	C	snp	1	0	0	0	.	.	.	REPS2(INTRON);SNORA16(UPSTREAM)
X	17293505	17293516	17293509	CAT	G	snp	1	1	1	1	.	.	.	INTERGENIC(INTERGENIC)
X	17620296	17620305	17620302	CA	ACA	del	0	1	0	0	.	.	.	NHS(INTRON)
X	17848608	17848617	17848615	T	G	snp	1	0	0	0	.	.	.	RAI2(INTRON);RP3-389A20.4.1(INTRON)
X	17920065	17920079	17920076	GAA	G	snp	0	0	1	0	.	.	.	INTERGENIC(INTERGENIC)
X	17920095	17920115	17920109	GAA	G	snp	1	0	0	0	.	.	.	INTERGENIC(INTERGENIC)
X	17948795	17948806	17948801	CA	G	snp	1	0	0	0	.	.	.	INTERGENIC(INTERGENIC);RP1-272E8.1.1(UPSTREAM)
X	17976962	17976971	17976970	C	A	snp	1	1	0	0	.	.	.	INTERGENIC(INTERGENIC)
X	18157226	18157237	18157229	TG	C	snp	1	1	1	1	.	.	.	INTERGENIC(INTERGENIC)
X	18200600	18200607	18200603	A	G	snp	1	0	0	0	.	.	.	BEND2(INTRON)
X	18277875	18277882	18277880	A	G	snp	1	0	0	0	.	.	.	SCML2(INTRON)
X	18329519	18329536	18329531	AG	G	del	0	0	1	0	.	.	.	SCML2(INTRON)
X	18607052	18607061	18607054	A	C	snp	1	0	1	0	.	.	.	CDKL5(INTRON)
X	18668098	18668109	18668106	CCG	A	snp	1	1	1	1	.	.	.	CDKL5(INTRON);RS1(INTRON)
X	18698741	18698752	18698748	TTA	G	snp	1	1	1	1	.	.	.	PPEF1(INTRON)
X	18721675	18721690	18721686	TTCA	G	snp	1	1	1	1	.	.	.	PPEF1(INTRON);PPEF1(UPSTREAM)
X	18899539	18899556	18899542	AT	A	snp	1	0	0	0	.	.	.	INTERGENIC(INTERGENIC)
X	18899569	18899580	18899577	AT	G	snp	1	0	0	0	.	.	.	INTERGENIC(INTERGENIC)
X	19014007	19014015	19014011	T	G	snp	1	0	0	0	.	.	.	GPR64(INTRON)
X	19036412	19036420	19036415	T	G	snp	1	0	0	0	.	.	.	GPR64(DOWNSTREAM);GPR64(INTRON)
X	19496038	19496049	19496044	AG	G	snp	1	0	0	0	.	.	.	MAP3K15(INTRON)
X	19500370	19500379	19500378	A	C	snp	1	1	1	1	.	.	.	MAP3K15(EXON);MAP3K15(INTRON)
X	19582688	19582695	19582694	C	A	snp	1	1	0	1	.	.	.	SH3KBP1(INTRON)
X	19671106	19671114	19671111	T	G	snp	1	1	0	1	.	.	.	SH3KBP1(INTRON)
X	19778204	19778221	19778219	AC	AA	ins	1	0	0	0	.	.	.	SH3KBP1(INTRON)
X	19905572	19905586	19905583	GGC	G	snp	0	0	0	1	.	.	.	SH3KBP1(UPSTREAM);SH3KBP1(UTR_5_PRIME)
X	19917740	19917755	19917747	TTTG	T	snp	1	1	0	1	.	.	.	INTERGENIC(INTERGENIC)
X	19941949	19941964	19941959	TTAT	T	ins	1	0	0	0	.	.	.	CXorf23(INTRON)
X	20134977	20134988	20134980	GCC	A	snp	1	1	0	1	.	.	MAP7D2(SYNONYMOUS_CODING):G6	MAP7D2(UPSTREAM);MAP7D2(UTR_5_PRIME)
X	20442844	20442859	20442852	GTTT	T	ins	1	0	0	0	.	.	.	INTERGENIC(INTERGENIC)
X	20468299	20468312	20468310	TC	A	snp	1	0	0	0	.	.	.	7SK(UPSTREAM);INTERGENIC(INTERGENIC)
X	20468329	20468340	20468332	TA	C	snp	1	1	0	1	.	.	.	7SK(UPSTREAM);INTERGENIC(INTERGENIC)
X	20720195	20720202	20720196	T	C	snp	1	1	0	1	.	.	.	INTERGENIC(INTERGENIC)
X	21052932	21052949	21052946	AAT	AA	del	1	0	0	0	.	.	.	INTERGENIC(INTERGENIC)
X	21100843	21100851	21100844	G	T	snp	1	1	1	1	.	.	.	INTERGENIC(INTERGENIC)
X	21274119	21274136	21274131	TTA	C	snp	1	0	0	0	.	.	.	INTERGENIC(INTERGENIC)
X	21370301	21370308	21370304	A	C	snp	1	0	0	0	.	.	.	INTERGENIC(INTERGENIC)
X	21535981	21535988	21535983	T	C	snp	0	0	0	1	.	.	.	CNKSR2(INTRON)
X	21906929	21906944	21906941	TA	ATA	del	0	1	1	1	.	.	.	INTERGENIC(INTERGENIC);MBTPS2(DOWNSTREAM);RP11-147O5.1.1(UPSTREAM)
X	21919292	21919300	21919297	A	G	snp	0	0	1	0	.	.	.	RP11-147O5.1.1(EXON)
X	21944211	21944220	21944216	T	C	snp	0	1	1	0	.	.	.	INTERGENIC(INTERGENIC)
X	21963753	21963762	21963757	C	A	snp	1	0	0	0	.	.	.	SMS(INTRON)
X	21970525	21970540	21970533	TTGT	TGTTTG	del	1	1	1	1	.	.	.	SMS(INTRON)
X	22037202	22037213	22037208	ATA	G	snp	1	1	1	1	.	.	.	INTERGENIC(INTERGENIC)
X	22038279	22038286	22038280	T	C	snp	0	1	1	1	.	.	.	INTERGENIC(INTERGENIC)
X	22048112	22048126	22048122	TTG	C	snp	1	0	0	0	.	.	.	INTERGENIC(INTERGENIC);PHEX(UPSTREAM)
X	22063139	22063148	22063147	T	G	snp	1	0	0	0	.	.	.	PHEX(INTRON)
X	22063148	22063163	22063151	TG	T	snp	1	1	1	1	.	.	.	PHEX(INTRON)
X	22123843	22123854	22123849	ATA	G	snp	1	1	0	0	.	.	.	PHEX(INTRON)
X	22258699	22258708	22258704	A	G	snp	1	0	0	0	.	.	.	PHEX(INTRON)
X	22281191	22281199	22281198	A	C	snp	0	1	0	0	.	.	.	INTERGENIC(INTERGENIC)
X	22393654	22393669	22393665	AG	T	snp	1	1	1	1	.	.	.	INTERGENIC(INTERGENIC)
X	22421678	22421689	22421687	TA	G	snp	0	0	0	1	.	.	.	RP11-40F8.2.1(INTRON)
X	22426357	22426365	22426358	C	T	snp	1	0	0	0	.	.	.	RP11-40F8.2.1(INTRON)
X	22426357	22426365	22426364	C	A	snp	0	0	1	1	.	.	.	RP11-40F8.2.1(INTRON)
X	22449622	22449633	22449625	TGC	C	snp	1	1	1	1	.	.	.	METTL15P3(UPSTREAM);RP11-40F8.2.1(INTRON);RP11-494I9.2.1(INTRON)
X	22518063	22518076	22518065	GA	C	snp	1	0	0	0	.	.	.	RP11-40F8.2.1(INTRON)
X	22562439	22562448	22562447	A	C	snp	1	1	1	1	.	.	.	RP11-40F8.2.1(INTRON)
X	22623827	22623846	22623831	TAAA	C	snp	1	0	0	0	.	.	.	RP11-40F8.2.1(INTRON)
X	22732687	22732706	22732696	AGGA	A	snp	0	1	1	0	.	.	.	GS1-433O24.1.1(INTRON);RP11-40F8.2.1(INTRON)

X	22860925	22860933	22860928	A	C	snp	1	0	0	0	.	.	.	RP11-40F8.2.1(INTRON)
X	22891670	22891678	22891674	A	C	ins	1	0	0	0	.	.	.	RP11-40F8.2.1(INTRON)
X	22916564	22916573	22916568	TA	C	snp	1	0	0	0	.	.	.	RP11-40F8.2.1(INTRON)
X	22917137	22917146	22917139	TA	A	snp	1	1	1	1	.	.	.	RP11-40F8.2.1(INTRON)
X	22917868	22917877	22917875	AT	C	snp	1	1	1	0	.	.	.	RP11-40F8.2.1(INTRON)
X	22957236	22957247	22957239	TA	TATATG	ins	1	1	1	1	.	.	.	RP11-40F8.2.1(INTRON)
X	22966285	22966292	22966290	A	G	snp	1	0	0	0	.	.	.	RP11-40F8.2.1(INTRON)
X	22982241	22982248	22982243	A	G	snp	1	0	0	0	.	.	.	RP11-40F8.2.1(INTRON)
X	23088878	23088885	23088879	A	G	snp	1	0	0	0	.	.	.	FAM3C2(UPSTREAM);INTERGENIC(INTERGENIC)
X	23090294	23090305	23090302	AAC	A	ins	1	0	0	0	.	.	.	FAM3C2(UPSTREAM);INTERGENIC(INTERGENIC)
X	23104297	23104306	23104299	A	C	snp	1	1	1	1	.	.	.	INTERGENIC(INTERGENIC)
X	23122913	23122921	23122919	A	G	snp	1	1	1	1	.	.	.	INTERGENIC(INTERGENIC)
X	23151014	23151028	23151021	ATA	C	snp	1	1	1	1	.	.	.	INTERGENIC(INTERGENIC)
X	23253617	23253626	23253623	TG	C	snp	1	0	0	0	.	.	.	INTERGENIC(INTERGENIC)
X	23256236	23256243	23256238	T	C	snp	1	1	1	1	.	.	.	INTERGENIC(INTERGENIC)
X	23312325	23312336	23312332	AT	C	snp	1	1	1	1	.	.	.	INTERGENIC(INTERGENIC)
X	23317891	23317906	23317902	AAAT	C	snp	1	0	0	0	.	.	.	INTERGENIC(INTERGENIC)
X	23405509	23405517	23405511	T	C	snp	1	0	0	0	.	.	.	PTCHD1(INTRON)
X	23418982	23418990	23418985	T	G	snp	1	1	1	1	.	.	.	PTCHD1(UTR_3_PRIME)
X	23658524	23658531	23658525	A	C	snp	1	1	1	1	.	.	.	INTERGENIC(INTERGENIC)
X	23662792	23662801	23662796	A	C	snp	1	1	1	1	.	.	.	INTERGENIC(INTERGENIC)
X	23687697	23687704	23687703	A	G	snp	1	0	0	0	.	.	.	PRDX4(INTRON)
X	23771945	23771972	23771963	CTTT	TCTTTC	del	1	1	1	1	.	.	.	ACOT9(INTRON)
X	23777853	23777862	23777856	AG	A	snp	1	1	1	1	.	.	.	ACOT9(INTRON)
X	23830552	23830571	23830558	AAAT	G	snp	1	0	1	0	.	.	.	INTERGENIC(INTERGENIC)
X	23830552	23830571	23830558	AAAT	TAAG	ins	1	1	1	0	.	.	.	INTERGENIC(INTERGENIC)
X	23830552	23830571	23830562	AAAT	G	snp	1	1	1	0	.	.	.	INTERGENIC(INTERGENIC)
X	23997229	23997238	23997236	AG	C	snp	1	0	0	0	.	.	.	INTERGENIC(INTERGENIC);KLHL15(DOWNSTREAM)
X	24076640	24076648	24076643	T	TCTTTC	ins	1	1	1	1	.	.	.	EIF2S3(INTRON)
X	24076640	24076648	24076644	T	CTTTCTTTTTC	ins	1	1	1	1	.	.	.	EIF2S3(INTRON)
X	24143745	24143753	24143751	T	G	snp	1	0	0	0	.	.	.	INTERGENIC(INTERGENIC)
X	24151213	24151224	24151220	AAT	G	snp	1	1	1	0	.	.	.	INTERGENIC(INTERGENIC);SNORA68(UPSTREAM)
X	24151878	24151897	24151891	AGAA	A	ins	1	0	0	0	.	.	.	INTERGENIC(INTERGENIC);SNORA68(DOWNSTREAM)
X	24231006	24231014	24231009	T	G	snp	1	0	0	0	.	.	.	ZFX(DOWNSTREAM);ZFX(UTR_3_PRIME)
X	24248967	24248981	24248972	TTG	T	snp	1	1	1	1	.	.	.	INTERGENIC(INTERGENIC)
X	24273417	24273426	24273420	A	C	snp	1	0	0	0	.	.	.	INTERGENIC(INTERGENIC)
X	24401170	24401185	24401181	TTTA	G	snp	0	1	1	1	.	.	.	INTERGENIC(INTERGENIC)
X	24497902	24497909	24497906	A	C	snp	1	0	1	0	.	.	.	PDK3(INTRON)
X	24574578	24574586	24574582	A	G	snp	1	1	1	1	.	.	.	INTERGENIC(INTERGENIC);PCYT1B(DOWNSTREAM)
X	24672202	24672217	24672211	TTTC	TCTTTC	del	1	1	1	1	.	.	.	PCYT1B(INTRON);PCYT1B-AS1(INTRON)
X	24672202	24672217	24672213	TTTC	T	snp	1	1	1	1	.	.	.	PCYT1B(INTRON);PCYT1B-AS1(INTRON)
X	24775198	24775205	24775200	A	G	snp	1	0	0	0	.	.	.	POLA1(INTRON)
X	24894799	24894808	24894803	G	A	snp	1	0	0	0	.	.	.	POLA1(INTRON)
X	24967635	24967644	24967639	T	C	snp	1	0	0	0	.	.	.	POLA1(INTRON)
X	25097931	25097946	25097934	TA	TG	ins	1	1	1	1	.	.	.	INTERGENIC(INTERGENIC)
X	25097931	25097946	25097936	TA	G	snp	1	0	0	0	.	.	.	INTERGENIC(INTERGENIC)
X	25223971	25223980	25223976	TA	G	snp	1	1	1	1	.	.	.	AC112778.1(DOWNSTREAM);INTERGENIC(INTERGENIC)
X	25521895	25521904	25521897	T	G	snp	0	0	0	1	.	.	.	INTERGENIC(INTERGENIC)
X	25582412	25582421	25582419	A	G	snp	1	1	0	0	.	.	.	INTERGENIC(INTERGENIC)
X	25630082	25630097	25630093	TTTC	T	del	1	1	1	1	.	.	.	INTERGENIC(INTERGENIC)
X	25726488	25726496	25726495	T	C	snp	1	0	0	0	.	.	.	INTERGENIC(INTERGENIC)
X	25865317	25865326	25865321	T	G	snp	1	0	0	0	.	.	.	INTERGENIC(INTERGENIC)
X	25899366	25899377	25899375	TA	C	snp	1	1	1	1	.	.	.	RP11-86A5.1.1(INTRON)
X	25924923	25924932	25924925	A	C	snp	1	1	1	1	.	.	.	INTERGENIC(INTERGENIC)
X	26006401	26006412	26006406	TTA	T	snp	1	1	1	1	.	.	.	INTERGENIC(INTERGENIC)
X	26074044	26074052	26074045	A	G	snp	1	1	0	0	.	.	.	INTERGENIC(INTERGENIC)
X	26090414	26090421	26090420	T	C	ins	1	1	1	1	.	.	.	INTERGENIC(INTERGENIC)
X	26106420	26106429	26106421	A	C	ins	1	1	1	1	.	.	.	INTERGENIC(INTERGENIC)
X	26106420	26106429	26106422	A	C	snp	1	1	1	1	.	.	.	INTERGENIC(INTERGENIC)
X	26130927	26130934	26130932	A	C	snp	1	0	0	0	.	.	.	INTERGENIC(INTERGENIC)
X	26225017	26225032	26225027	AT	T	snp	1	1	1	1	.	.	.	INTERGENIC(INTERGENIC)
X	26225017	26225032	26225029	AT	T	snp	1	1	1	1	.	.	.	INTERGENIC(INTERGENIC)
X	26273307	26273316	26273308	T	G	snp	0	1	1	0	.	.	.	INTERGENIC(INTERGENIC)
X	26362833	26362841	26362836	T	C	snp	1	0	0	0	.	.	.	INTERGENIC(INTERGENIC);RP11-617O8.1.1(UPSTREAM)
X	26548817	26548832	26548820	AT	A	snp	1	1	1	1	.	.	.	INTERGENIC(INTERGENIC)
X	26619904	26619919	26619906	AT	G	snp	1	1	1	1	.	.	.	INTERGENIC(INTERGENIC)
X	26660806	26660817	26660813	AT	C	snp	1	1	1	1	.	.	.	INTERGENIC(INTERGENIC)

X	26685085	26685094	26685086	T	G	snp	1	0	0	0	.	.	.	INTERGENIC(INTERGENIC)
X	26705323	26705331	26705328	G	T	snp	0	1	0	1	.	.	.	GS1-541M1.1.1(INTRON)
X	26711415	26711423	26711418	A	G	snp	1	1	1	1	.	.	.	INTERGENIC(INTERGENIC)
X	26849405	26849422	26849418	TTA	A	del	0	1	1	1	.	.	.	INTERGENIC(INTERGENIC)
X	26849405	26849422	26849419	TTA	T	snp	1	1	1	1	.	.	.	INTERGENIC(INTERGENIC)
X	26965433	26965448	26965441	CTTT	T	del	1	0	0	0	.	.	.	INTERGENIC(INTERGENIC)
X	27011938	27011953	27011949	CTTT	C	del	1	1	1	1	.	.	.	INTERGENIC(INTERGENIC)
X	27059064	27059084	27059078	TTA	T	ins	1	1	1	1	.	.	.	INTERGENIC(INTERGENIC)
X	27059064	27059084	27059079	TTA	TA	del	1	1	1	1	.	.	.	INTERGENIC(INTERGENIC)
X	27059064	27059084	27059080	TTA	A	del	1	1	1	1	.	.	.	INTERGENIC(INTERGENIC)
X	27088618	27088627	27088624	AT	G	snp	0	1	0	1	.	.	.	INTERGENIC(INTERGENIC)
X	27152154	27152167	27152156	TA	A	del	1	1	1	1	.	.	.	INTERGENIC(INTERGENIC)
X	27234704	27234711	27234710	A	G	snp	1	0	0	0	.	.	.	RP11-268G12.1.1(INTRON);RP11-268G12.2.1(DOWNSTREAM)
X	27248927	27248936	27248934	T	G	snp	1	0	0	1	.	.	.	RP11-268G12.1.1(INTRON)
X	27406530	27406541	27406533	TTG	C	snp	1	1	1	1	.	.	.	RP11-268G12.1.1(INTRON)
X	27451108	27451117	27451116	A	C	ins	1	1	1	1	.	.	.	INTERGENIC(INTERGENIC);PTP4A1P5(UPSTREAM)
X	27461441	27461448	27461445	A	G	snp	1	1	1	1	.	.	.	INTERGENIC(INTERGENIC)
X	27480404	27480411	27480409	A	C	snp	1	0	0	0	.	.	.	SMEK3P(EXON)
X	27576731	27576742	27576735	TTA	G	snp	1	1	0	1	.	.	.	INTERGENIC(INTERGENIC)
X	27591965	27591982	27591978	TC	TCTCT	del	1	0	0	0	.	.	.	INTERGENIC(INTERGENIC)
X	27591965	27591982	27591979	TC	CTC	del	1	0	0	0	.	.	.	INTERGENIC(INTERGENIC)
X	27624440	27624453	27624451	TA	C	snp	1	0	0	1	.	.	.	INTERGENIC(INTERGENIC)
X	27637519	27637534	27637532	AG	C	snp	1	0	0	0	.	.	.	INTERGENIC(INTERGENIC)
X	27640108	27640125	27640122	CAA	C	del	1	1	0	1	.	.	.	INTERGENIC(INTERGENIC)
X	27640124	27640133	27640126	A	C	snp	1	1	0	1	.	.	.	INTERGENIC(INTERGENIC)
X	27706338	27706345	27706340	T	C	snp	1	1	1	1	.	.	.	INTERGENIC(INTERGENIC)
X	27738477	27738494	27738486	ATT	T	ins	1	1	1	0	.	.	.	RP11-501H19.4.1(INTRON)
X	27738477	27738494	27738488	ATT	A	del	1	1	1	1	.	.	.	RP11-501H19.4.1(INTRON)
X	27738477	27738494	27738489	ATT	T	ins	1	1	1	1	.	.	.	RP11-501H19.4.1(INTRON)
X	27738477	27738494	27738489	ATT	T	snp	1	1	1	1	.	.	.	RP11-501H19.4.1(INTRON)
X	27738477	27738494	27738491	ATT	A	del	1	1	1	1	.	.	.	RP11-501H19.4.1(INTRON)
X	27784412	27784421	27784417	TA	T	ins	0	1	0	1	.	.	.	INTERGENIC(INTERGENIC)
X	27784412	27784421	27784418	TA	AGAG	ins	1	1	1	1	.	.	.	INTERGENIC(INTERGENIC)
X	27792403	27792416	27792411	AG	G	snp	1	0	0	0	.	.	.	INTERGENIC(INTERGENIC)
X	27865079	27865098	27865094	TTTG	T	ins	1	0	0	0	.	.	.	INTERGENIC(INTERGENIC);RP11-12D5.3.1(DOWNSTREAM)
X	27865099	27865106	27865102	T	G	snp	0	0	1	0	.	.	.	INTERGENIC(INTERGENIC);RP11-12D5.3.1(DOWNSTREAM)
X	27970636	27970645	27970641	GA	G	snp	1	1	1	1	.	.	.	INTERGENIC(INTERGENIC)
X	28011352	28011369	28011357	TTA	T	snp	1	0	0	0	.	.	.	INTERGENIC(INTERGENIC)
X	28060892	28060900	28060896	G	GC	ins	0	0	0	1	.	.	.	INTERGENIC(INTERGENIC)
X	28154889	28154900	28154895	TTA	C	snp	1	1	1	0	.	.	.	INTERGENIC(INTERGENIC)
X	28238119	28238132	28238126	AC	T	snp	0	0	1	0	.	.	.	INTERGENIC(INTERGENIC)
X	28241511	28241520	28241512	A	C	snp	1	1	1	1	.	.	.	INTERGENIC(INTERGENIC)
X	28279150	28279161	28279158	AC	G	snp	1	1	0	0	.	.	.	INTERGENIC(INTERGENIC)
X	28304405	28304414	28304406	A	C	snp	1	0	0	0	.	.	.	INTERGENIC(INTERGENIC)
X	28304405	28304414	28304408	A	C	snp	1	0	0	0	.	.	.	INTERGENIC(INTERGENIC)
X	28326334	28326351	28326346	CA	T	snp	1	1	1	1	.	.	.	INTERGENIC(INTERGENIC)
X	28577872	28577883	28577880	GA	AGA	del	1	0	0	0	.	.	.	INTERGENIC(INTERGENIC)
X	28690343	28690352	28690344	A	G	snp	1	1	1	1	.	.	.	IL1RAPL1(INTRON)
X	28741443	28741452	28741447	GT	A	snp	1	1	1	1	.	.	.	IL1RAPL1(INTRON)
X	28756124	28756132	28756129	A	G	snp	1	1	1	1	.	.	.	IL1RAPL1(INTRON)
X	29186997	29187006	29187002	TA	G	snp	1	1	1	1	.	.	.	IL1RAPL1(INTRON)
X	29328735	29328750	29328748	CA	C	del	1	0	0	0	.	.	.	IL1RAPL1(INTRON)
X	29423019	29423034	29423029	TTTG	G	del	1	1	1	1	.	.	.	IL1RAPL1(INTRON)
X	29423478	29423493	29423485	TTTA	T	snp	1	1	0	0	.	.	.	IL1RAPL1(INTRON)
X	29423972	29423980	29423973	T	TG	ins	1	0	0	0	.	.	.	IL1RAPL1(INTRON)
X	29434153	29434160	29434159	A	G	snp	1	0	0	0	.	.	.	IL1RAPL1(INTRON)
X	29538827	29538844	29538838	TTA	G	snp	1	0	0	0	.	.	.	IL1RAPL1(INTRON)
X	29600403	29600418	29600411	AATT	T	snp	1	1	1	1	.	.	.	IL1RAPL1(INTRON)
X	29705913	29705920	29705915	A	G	snp	1	1	1	0	.	.	.	IL1RAPL1(INTRON)
X	30015688	30015705	30015701	AG	C	snp	1	0	0	0	.	.	.	INTERGENIC(INTERGENIC)
X	30038543	30038552	30038548	AT	A	snp	1	0	0	0	.	.	.	CTD-2225F20.1.1(UPSTREAM);INTERGENIC(INTERGENIC)
X	30071219	30071232	30071221	TC	G	snp	1	0	0	0	.	.	.	INTERGENIC(INTERGENIC)
X	30412996	30413005	30413001	T	C	snp	1	0	0	0	.	.	.	INTERGENIC(INTERGENIC)
X	30478336	30478343	30478341	A	C	snp	1	0	0	0	.	.	.	INTERGENIC(INTERGENIC)
X	30564904	30564913	30564909	TC	G	snp	1	0	0	0	.	.	.	INTERGENIC(INTERGENIC)
X	30572336	30572351	30572341	TTCT	G	snp	1	0	0	0	.	.	.	CXorf21(DOWNSTREAM);INTERGENIC(INTERGENIC)

X	30638605	30638620	30638618	CT	G	snp	1	0	0	0	.	.	.	CKS1BP6(DOWNSTREAM);INTERGENIC(INTERGENIC)
X	30667596	30667605	30667602	A	C	ins	1	1	1	1	.	.	.	GK(UPSTREAM);INTERGENIC(INTERGENIC)
X	30674717	30674724	30674723	C	T	snp	0	1	0	1	.	.	.	GK(INTRON)
X	30687819	30687830	30687827	TGG	T	snp	1	0	0	0	.	.	.	GK(INTRON);GK-IT1(UPSTREAM)
X	30714806	30714821	30714817	AAAC	A	snp	0	1	0	1	.	.	.	GK(DOWNSTREAM);GK(INTRON);GK-AS1(DOWNSTREAM);RP11-242C19.2.1(UPSTREAM)
X	30772107	30772115	30772109	C	T	snp	1	0	0	0	.	.	.	INTERGENIC(INTERGENIC)
X	30839517	30839526	30839524	C	T	snp	1	1	1	1	.	.	.	INTERGENIC(INTERGENIC)
X	30882904	30882921	30882919	GT	G	del	1	0	0	0	.	.	.	TAB3(INTRON)
X	30882921	30882930	30882922	T	G	ins	1	0	0	0	.	.	.	TAB3(INTRON)
X	31074392	31074399	31074394	A	G	snp	1	0	0	0	.	.	.	INTERGENIC(INTERGENIC)
X	31137273	31137288	31137284	AAGT	AAG	del	1	1	1	1	.	.	.	DMD(DOWNSTREAM);DMD(INTRON);DMD-AS1(INTRON)
X	31244696	31244707	31244702	TC	C	snp	1	1	1	1	.	.	.	DMD(INTRON)
X	31317474	31317482	31317476	A	C	snp	1	1	0	1	.	.	.	DMD(INTRON);DMD-AS2(INTRON)
X	31361775	31361788	31361784	AG	A	snp	1	1	1	1	.	.	.	DMD(INTRON);U6(UPSTREAM)
X	31362155	31362163	31362161	G	GGA	ins	0	1	0	1	.	.	.	DMD(INTRON);U6(UPSTREAM)
X	31385985	31385996	31385989	TAT	G	snp	1	1	1	1	.	.	.	DMD(INTRON)
X	31478702	31478711	31478709	AT	G	snp	1	1	1	1	.	.	.	DMD(INTRON)
X	31529430	31529453	31529449	TTTA	T	del	0	0	1	0	.	.	.	DMD(INTRON);DMD(UPSTREAM)
X	31531836	31531853	31531841	AGC	T	snp	1	1	1	0	.	.	.	DMD(INTRON)
X	31581171	31581194	31581186	TATT	TA	del	1	0	1	0	.	.	.	DMD(INTRON)
X	31581171	31581194	31581190	TATT	TA	del	1	1	1	1	.	.	.	DMD(INTRON)
X	31754120	31754128	31754121	T	G	snp	1	1	1	1	.	.	.	DMD(INTRON)
X	31852559	31852570	31852562	AT	G	snp	1	1	1	1	.	.	.	DMD(INTRON)
X	31853285	31853298	31853296	TC	G	snp	1	1	1	1	.	.	.	DMD(INTRON)
X	31890638	31890646	31890643	A	G	snp	1	1	1	1	.	.	.	DMD(INTRON)
X	31957781	31957788	31957783	A	G	snp	1	0	0	0	.	.	.	DMD(INTRON)
X	31991413	31991422	31991420	A	G	snp	1	1	1	1	.	.	.	DMD(INTRON)
X	32046933	32046942	32046937	T	C	snp	1	0	1	0	.	.	.	DMD(INTRON)
X	32068353	32068360	32068356	T	C	snp	1	1	1	0	.	.	.	DMD(INTRON)
X	32182912	32182920	32182914	C	A	snp	1	0	0	0	.	.	.	DMD(INTRON)
X	32196505	32196514	32196508	TA	GT	ins	1	0	0	0	.	.	.	DMD(INTRON)
X	32201689	32201706	32201694	AT	AA	ins	0	1	1	1	.	.	.	DMD(INTRON)
X	32201689	32201706	32201703	AT	G	snp	1	1	1	1	.	.	.	DMD(INTRON)
X	32298262	32298279	32298265	TA	TATATATATATATACAG	del	1	1	1	1	.	.	.	DMD(INTRON)
X	32298262	32298279	32298275	TA	TATACAG	del	1	1	1	1	.	.	.	DMD(INTRON)
X	32317483	32317490	32317488	A	C	snp	1	1	1	1	.	.	.	DMD(INTRON)
X	32448135	32448142	32448139	T	G	snp	1	1	1	1	.	.	.	DMD(INTRON)
X	32573057	32573068	32573060	AG	A	snp	1	1	1	1	.	.	.	DMD(INTRON)
X	32740219	32740226	32740223	T	C	snp	1	0	0	0	.	.	.	DMD(INTRON)
X	32828061	32828084	32828072	AAAG	A	snp	1	1	1	1	.	.	.	DMD(INTRON)
X	32845174	32845183	32845182	C	A	snp	1	1	1	1	.	.	.	DMD(INTRON)
X	32899062	32899069	32899064	A	G	snp	1	1	1	1	.	.	.	DMD(INTRON)
X	32905464	32905471	32905468	A	AAAT	ins	1	0	0	0	.	.	.	DMD(INTRON)
X	32954170	32954179	32954177	CA	C	del	1	0	0	0	.	.	.	DMD(INTRON)
X	33016768	33016781	33016777	AC	G	snp	0	1	1	0	.	.	.	DMD(INTRON)
X	33018355	33018362	33018356	T	G	snp	1	1	1	0	.	.	.	DMD(INTRON)
X	33107931	33107939	33107933	A	C	snp	1	1	0	1	.	.	.	DMD(INTRON)
X	33257573	33257580	33257577	A	G	ins	0	1	1	1	.	.	.	DMD(INTRON)
X	33378806	33378814	33378810	T	C	snp	1	1	1	1	.	.	.	INTERGENIC(INTERGENIC)
X	33386014	33386022	33386021	T	C	snp	1	0	0	0	.	.	.	INTERGENIC(INTERGENIC)
X	33482842	33482850	33482844	T	C	ins	1	0	0	0	.	.	.	INTERGENIC(INTERGENIC)
X	33527459	33527474	33527464	AT	C	snp	1	0	0	0	.	.	.	INTERGENIC(INTERGENIC)
X	33536120	33536129	33536123	AT	C	snp	1	0	0	1	.	.	.	INTERGENIC(INTERGENIC)
X	33550006	33550019	33550017	AC	A	snp	1	1	0	1	.	.	.	INTERGENIC(INTERGENIC)
X	33556103	33556112	33556107	GT	A	snp	1	0	0	0	.	.	.	INTERGENIC(INTERGENIC)
X	33600501	33600508	33600505	A	G	ins	1	1	1	1	.	.	.	INTERGENIC(INTERGENIC)
X	33624628	33624637	33624636	G	T	snp	1	0	0	1	.	.	.	AL591378.1(UPSTREAM);INTERGENIC(INTERGENIC)
X	33729128	33729139	33729136	AT	TG	ins	1	0	0	0	.	.	.	INTERGENIC(INTERGENIC)
X	33788133	33788142	33788137	C	CG	ins	1	1	1	1	.	.	.	RP11-305F18.1.1(INTRON)
X	33788133	33788142	33788138	C	CA	ins	1	1	1	1	.	.	.	RP11-305F18.1.1(INTRON)
X	33937285	33937293	33937288	T	C	snp	1	1	1	1	.	.	.	RP11-305F18.1.1(INTRON)
X	33967803	33967812	33967808	A	G	snp	0	0	1	0	.	.	.	INTERGENIC(INTERGENIC)
X	34118858	34118877	34118865	TTTG	T	snp	1	1	1	1	.	.	.	INTERGENIC(INTERGENIC)
X	34160663	34160670	34160668	T	G	snp	1	0	0	0	.	.	.	FTH1P14(UPSTREAM);INTERGENIC(INTERGENIC)
X	34381453	34381464	34381458	AT	C	snp	1	0	0	0	.	.	.	RP11-545D19.1.1(INTRON)
X	34415350	34415357	34415351	A	C	snp	1	1	0	1	.	.	.	RP11-545D19.1.1(INTRON)

X	34489085	34489098	34489088	TA	C	snp	1	1	1	1	.	.	.	INTERGENIC(INTERGENIC)
X	34489085	34489098	34489090	TA	C	snp	1	1	1	1	.	.	.	INTERGENIC(INTERGENIC)
X	34563239	34563247	34563242	T	G	snp	1	1	1	1	.	.	.	INTERGENIC(INTERGENIC)
X	34579576	34579585	34579583	TA	TATT	ins	1	0	0	0	.	.	.	INTERGENIC(INTERGENIC)
X	34608185	34608194	34608189	T	C	snp	1	1	1	1	.	.	.	INTERGENIC(INTERGENIC)
X	34720149	34720158	34720151	GA	A	snp	1	1	1	1	.	.	.	INTERGENIC(INTERGENIC)
X	34759985	34759993	34759989	T	G	snp	1	0	0	0	.	.	.	INTERGENIC(INTERGENIC)
X	34759985	34759993	34759991	T	TTTG	ins	1	1	1	0	.	.	.	INTERGENIC(INTERGENIC)
X	34806458	34806466	34806463	T	TTTC	ins	1	1	0	0	.	.	.	INTERGENIC(INTERGENIC)
X	34809763	34809772	34809769	GA	A	snp	1	0	0	0	.	.	.	INTERGENIC(INTERGENIC)
X	34983506	34983520	34983511	ATT	G	snp	1	1	0	0	.	.	.	INTERGENIC(INTERGENIC)
X	35004680	35004688	35004683	A	G	snp	0	1	0	0	.	.	.	INTERGENIC(INTERGENIC)
X	35124277	35124300	35124285	AAAT	AAT	ins	1	0	0	0	.	.	.	INTERGENIC(INTERGENIC)
X	35211092	35211109	35211104	TTG	G	snp	0	1	1	1	.	.	.	INTERGENIC(INTERGENIC)
X	35604315	35604329	35604323	ATA	ATAAT	del	1	0	0	0	.	.	.	INTERGENIC(INTERGENIC)
X	35665121	35665130	35665125	TA	A	snp	1	1	1	1	.	.	.	INTERGENIC(INTERGENIC)
X	35689371	35689380	35689374	T	C	snp	1	0	0	0	.	.	.	INTERGENIC(INTERGENIC)
X	35850989	35850996	35850992	A	G	snp	1	1	1	1	.	.	.	INTERGENIC(INTERGENIC)
X	35856771	35856779	35856774	T	C	snp	1	1	1	1	.	.	.	INTERGENIC(INTERGENIC)
X	36024023	36024032	36024028	AC	G	snp	1	1	1	1	.	.	.	INTERGENIC(INTERGENIC)
X	36144195	36144215	36144209	ATT	A	del	1	0	0	0	.	.	.	CXorf59(INTRON)
X	36167692	36167701	36167695	AT	C	snp	1	1	1	1	.	.	.	CXorf59(DOWNSTREAM);INTERGENIC(INTERGENIC)
X	36167702	36167729	36167725	TTTA	T	ins	1	0	0	0	.	.	.	CXorf59(DOWNSTREAM);INTERGENIC(INTERGENIC)
X	36173215	36173226	36173221	TA	C	snp	1	0	0	0	.	.	.	INTERGENIC(INTERGENIC)
X	36277813	36277822	36277816	T	C	snp	1	0	0	0	.	.	.	CXorf30(INTRON)
X	36364343	36364351	36364344	T	G	snp	1	1	1	1	.	.	.	CXorf30(INTRON)
X	36519674	36519682	36519680	C	A	snp	0	1	0	0	.	.	.	INTERGENIC(INTERGENIC)
X	36602472	36602481	36602474	TA	C	snp	1	1	0	0	.	.	.	INTERGENIC(INTERGENIC)
X	36654384	36654391	36654386	T	C	snp	1	0	0	1	.	.	.	INTERGENIC(INTERGENIC)
X	36717032	36717049	36717047	TG	C	snp	1	1	0	1	.	.	.	INTERGENIC(INTERGENIC)
X	36717032	36717049	36717047	TG	TC	ins	1	1	0	1	.	.	.	INTERGENIC(INTERGENIC)
X	36852175	36852183	36852179	A	G	snp	1	0	0	0	.	.	.	INTERGENIC(INTERGENIC)
X	36871368	36871376	36871373	T	G	snp	1	1	0	1	.	.	.	INTERGENIC(INTERGENIC)
X	36947701	36947724	36947720	AAAC	A	ins	1	0	1	0	.	.	.	INTERGENIC(INTERGENIC)
X	36947725	36947734	36947727	A	C	ins	1	1	0	1	.	.	.	INTERGENIC(INTERGENIC)
X	37339390	37339401	37339393	AG	A	snp	1	0	0	0	.	.	.	RP5-972B16.2.1(INTRON)
X	37434830	37434845	37434837	AATG	A	snp	1	0	0	0	.	.	.	LANCL3(INTRON);RP5-972B16.2.1(INTRON)
X	37719494	37719509	37719500	ATTC	C	snp	1	0	0	0	.	.	.	RP5-972B16.2.1(INTRON)
X	37719882	37719895	37719885	AT	C	snp	0	1	1	0	.	.	.	RP5-972B16.2.1(INTRON)
X	37721179	37721187	37721185	T	G	snp	1	0	0	0	.	.	.	RP5-972B16.2.1(INTRON)
X	37798533	37798541	37798538	A	G	snp	1	1	1	0	.	.	.	AL121578.2.1(INTRON);RP5-972B16.2.1(INTRON)
X	37961218	37961233	37961231	GT	C	snp	1	1	0	1	.	.	.	RP5-972B16.2.1(INTRON);SYTL5(INTRON)
X	38032147	38032160	38032153	GA	A	snp	1	0	0	0	.	.	.	RP5-972B16.2.1(INTRON);SRPX(INTRON)
X	38254147	38254155	38254152	G	A	ins	1	1	0	1	.	.	.	OTC(INTRON);RP5-972B16.2.1(INTRON);TDGF1P1(DOWNSTREAM)
X	38254147	38254155	38254152	G	A	snp	1	1	0	1	.	.	.	OTC(INTRON);RP5-972B16.2.1(INTRON);TDGF1P1(DOWNSTREAM)
X	38272932	38272940	38272935	T	G	snp	1	1	1	1	.	.	.	OTC(DOWNSTREAM);OTC(INTRON);RP5-972B16.2.1(INTRON)
X	38383235	38383242	38383241	A	G	snp	1	1	1	1	.	.	.	RP5-972B16.2.1(INTRON)
X	38495314	38495321	38495319	T	C	snp	1	0	0	0	.	.	.	RP5-972B16.2.1(INTRON);TSPAN7(INTRON)
X	38660109	38660117	38660113	A	C	snp	1	0	0	0	.	.	.	INTERGENIC(INTERGENIC);MID1IP1(UPSTREAM);MID1IP1-AS1(DOWNSTREAM);RP4-646N3.1.1(DOWNSTREAM)
X	38674303	38674316	38674310	TC	T	snp	1	1	0	1	.	.	.	INTERGENIC(INTERGENIC)
X	38850184	38850195	38850190	TA	ATATA	del	0	1	1	0	.	.	.	INTERGENIC(INTERGENIC)
X	38884249	38884258	38884251	T	G	ins	1	1	1	1	.	.	.	INTERGENIC(INTERGENIC)
X	38893721	38893729	38893724	T	G	snp	1	1	1	1	.	.	.	INTERGENIC(INTERGENIC)
X	38921776	38921785	38921779	TC	T	snp	1	1	1	1	.	.	.	INTERGENIC(INTERGENIC)
X	39049387	39049396	39049389	A	C	snp	1	0	0	0	.	.	.	INTERGENIC(INTERGENIC)
X	39327812	39327823	39327817	TTG	T	snp	1	0	0	0	.	.	.	INTERGENIC(INTERGENIC)
X	39418994	39419007	39418999	AG	AA	ins	1	1	1	1	.	.	.	INTERGENIC(INTERGENIC)
X	39523010	39523018	39523011	T	C	snp	1	0	0	0	.	.	.	INTERGENIC(INTERGENIC)
X	39700528	39700539	39700536	GTG	C	snp	0	1	0	0	.	.	.	MIR1587(INTRON)
X	39730446	39730459	39730448	GA	A	snp	0	0	0	1	.	.	.	INTERGENIC(INTERGENIC)
X	39811531	39811539	39811533	T	C	ins	1	1	0	0	.	.	.	INTERGENIC(INTERGENIC)
X	39901575	39901586	39901577	CA	A	del	1	0	0	0	.	.	.	INTERGENIC(INTERGENIC)
X	39973064	39973079	39973075	TTTC	TT	del	1	0	0	0	.	.	.	BCOR(INTRON)

X	40079539	40079554	40079548	TG	A	snp	1	1	0	1	.	.	.	INTERGENIC(INTERGENIC)
X	40174032	40174041	40174035	A	C	snp	1	1	1	1	.	.	.	INTERGENIC(INTERGENIC)
X	40191045	40191056	40191052	TA	G	snp	1	1	0	1	.	.	.	INTERGENIC(INTERGENIC)
X	40196193	40196208	40196205	AT	TT	ins	1	1	0	1	.	.	.	INTERGENIC(INTERGENIC)
X	40333908	40333915	40333914	G	A	snp	1	0	0	0	.	.	.	INTERGENIC(INTERGENIC)
X	40389694	40389717	40389710	AAGA	AG	del	1	0	0	0	.	.	.	INTERGENIC(INTERGENIC)
X	40524785	40524792	40524788	A	C	snp	1	1	1	1	.	.	.	MED14(INTRON);MED14(UPSTREAM)
X	40599113	40599136	40599118	AAC	A	snp	1	1	1	1	.	.	.	INTERGENIC(INTERGENIC);MED14(UPSTREAM);MED14-AS1(DOWNSTREAM)
X	40599113	40599136	40599122	AAC	G	snp	0	1	0	0	.	.	.	INTERGENIC(INTERGENIC);MED14(UPSTREAM);MED14-AS1(DOWNSTREAM)
X	40641962	40641969	40641965	T	C	ins	0	1	0	1	.	.	.	INTERGENIC(INTERGENIC)
X	40642944	40642959	40642951	ATTC	T	snp	1	0	0	0	.	.	.	INTERGENIC(INTERGENIC)
X	40744828	40744847	40744843	TTTC	T	ins	1	0	0	0	.	.	.	INTERGENIC(INTERGENIC)
X	40766035	40766042	40766041	A	G	snp	1	1	0	0	.	.	.	INTERGENIC(INTERGENIC);RP11-77G22.2.1(UPSTREAM)
X	40813807	40813826	40813819	AAAT	G	snp	1	0	0	0	.	.	.	INTERGENIC(INTERGENIC)
X	40816770	40816789	40816781	AAAC	A	ins	0	1	0	1	.	.	.	INTERGENIC(INTERGENIC)
X	40816770	40816789	40816784	AAAC	C	del	0	1	0	1	.	.	.	INTERGENIC(INTERGENIC)
X	40816770	40816789	40816785	AAAC	A	snp	0	1	0	1	.	.	.	INTERGENIC(INTERGENIC)
X	40843549	40843560	40843555	TA	C	snp	1	0	0	0	.	.	.	INTERGENIC(INTERGENIC)
X	40859836	40859843	40859841	T	C	snp	1	1	0	1	.	.	.	INTERGENIC(INTERGENIC)
X	40924749	40924757	40924755	A	AT	ins	1	1	1	1	.	.	.	INTERGENIC(INTERGENIC)
X	41073730	41073739	41073734	A	G	snp	1	1	1	1	.	.	.	USP9X(DOWNSTREAM);USP9X(INTRON)
X	41079311	41079318	41079316	T	C	snp	1	0	0	0	.	.	.	USP9X(INTRON)
X	41108121	41108144	41108140	GTTT	G	snp	1	0	0	0	.	.	.	INTERGENIC(INTERGENIC)
X	41110990	41110997	41110991	A	G	snp	1	1	1	1	.	.	.	INTERGENIC(INTERGENIC)
X	41276499	41276522	41276503	CAAA	A	snp	1	0	0	0	.	.	.	INTERGENIC(INTERGENIC)
X	41751592	41751599	41751596	T	C	snp	1	0	0	0	.	.	.	CASK(INTRON)
X	41898929	41898936	41898934	T	C	snp	0	1	1	0	.	.	.	INTERGENIC(INTERGENIC)
X	42019007	42019014	42019013	T	G	snp	0	1	1	1	.	.	.	INTERGENIC(INTERGENIC)
X	42298627	42298642	42298630	AG	A	snp	1	0	0	0	.	.	.	RP1-154K9.2.1(INTRON)
X	42298627	42298642	42298631	AG	G	del	1	0	0	0	.	.	.	RP1-154K9.2.1(INTRON)
X	42477073	42477082	42477075	TC	A	snp	1	1	1	1	.	.	.	RP1-154K9.2.1(INTRON)
X	42549723	42549732	42549726	T	G	snp	1	0	0	0	.	.	.	RP1-154K9.2.1(INTRON)
X	42589189	42589216	42589201	GTTT	T	ins	1	0	1	0	.	.	.	INTERGENIC(INTERGENIC)
X	42602134	42602141	42602136	T	C	snp	1	1	1	1	.	.	.	INTERGENIC(INTERGENIC)
X	42605415	42605430	42605417	AT	T	del	1	1	1	1	.	.	.	INTERGENIC(INTERGENIC)
X	42605415	42605430	42605418	AT	A	snp	1	1	1	1	.	.	.	INTERGENIC(INTERGENIC)
X	42735342	42735350	42735343	T	G	snp	1	0	0	0	.	.	.	INTERGENIC(INTERGENIC)
X	42792590	42792597	42792591	T	G	snp	1	1	1	1	.	.	.	INTERGENIC(INTERGENIC)
X	42999701	42999714	42999710	AC	T	snp	1	1	1	1	.	.	.	INTERGENIC(INTERGENIC)
X	43150383	43150400	43150388	TTG	T	snp	0	0	1	0	.	.	.	INTERGENIC(INTERGENIC)
X	43194409	43194417	43194414	T	G	snp	1	0	0	0	.	.	.	INTERGENIC(INTERGENIC)
X	43304443	43304451	43304444	A	G	snp	1	1	1	1	.	.	.	INTERGENIC(INTERGENIC)
X	43638035	43638043	43638038	A	G	snp	1	0	0	0	.	.	.	MAOB(INTRON)
X	43834091	43834098	43834094	A	G	snp	1	1	1	1	.	.	.	INTERGENIC(INTERGENIC);NDP(UPSTREAM)
X	44086163	44086182	44086169	GAAG	T	snp	1	1	1	1	.	.	.	EFHC2(INTRON);EFHC2(UPSTREAM)
X	44156995	44157009	44157005	GAT	T	snp	1	0	0	0	.	.	.	EFHC2(INTRON)
X	44209221	44209238	44209235	TC	CTC	del	1	1	0	1	.	.	.	INTERGENIC(INTERGENIC)
X	44326001	44326018	44326003	AT	T	del	0	0	1	0	.	.	.	RP4-551E13.2.1(INTRON)
X	44326001	44326018	44326004	AT	A	snp	1	0	1	0	.	.	.	RP4-551E13.2.1(INTRON)
X	44326001	44326018	44326006	AT	A	snp	0	0	1	0	.	.	.	RP4-551E13.2.1(INTRON)
X	44408860	44408867	44408863	A	C	ins	1	0	0	0	.	.	.	INTERGENIC(INTERGENIC)
X	44484328	44484335	44484332	T	C	snp	0	0	0	1	.	.	.	INTERGENIC(INTERGENIC)
X	44523089	44523102	44523100	AT	A	del	0	1	1	0	.	.	.	INTERGENIC(INTERGENIC)
X	44541197	44541206	44541200	AG	C	snp	1	0	0	1	.	.	.	INTERGENIC(INTERGENIC)
X	44597974	44597981	44597975	T	TG	ins	1	0	0	0	.	.	.	INTERGENIC(INTERGENIC);RP6-105D16.1.1(UPSTREAM)
X	44652840	44652859	44652852	TTTG	C	snp	1	1	1	1	.	.	.	INTERGENIC(INTERGENIC)
X	44760608	44760623	44760610	AT	TATAT	del	1	0	0	0	.	.	.	KDM6A(INTRON)
X	44952620	44952629	44952626	A	C	snp	1	0	0	0	.	.	.	KDM6A(INTRON)
X	44995885	44995894	44995888	TA	T	snp	1	1	1	1	.	.	.	INTERGENIC(INTERGENIC)
X	44996735	44996746	44996739	TA	C	snp	1	0	0	0	.	.	.	INTERGENIC(INTERGENIC)
X	45032742	45032757	45032749	TTCT	TTC	ins	1	1	1	1	.	.	.	CXorf36(INTRON)
X	45032742	45032757	45032752	TTCT	T	del	1	1	1	1	.	.	.	CXorf36(INTRON)
X	45032759	45032774	45032765	TTTC	C	del	1	1	1	1	.	.	.	CXorf36(INTRON)
X	45042876	45042883	45042878	T	C	snp	1	1	1	1	.	.	.	CXorf36(INTRON);RP11-342D14.1.1(INTRON)
X	45169912	45169921	45169918	T	C	snp	1	1	1	1	.	.	.	RP11-342D14.1.1(INTRON)

X	45388097	45388111	45388100	CAA	A	ins	0	1	1	0	.	.	.	RP11-245M24.1.1(INTRON)
X	45536649	45536656	45536654	A	G	snp	1	0	0	0	.	.	.	INTERGENIC(INTERGENIC)
X	45539921	45539932	45539927	TTG	C	snp	1	0	0	0	.	.	.	INTERGENIC(INTERGENIC)
X	45671870	45671879	45671874	A	C	ins	1	0	0	0	.	.	.	INTERGENIC(INTERGENIC)
X	45671870	45671879	45671875	A	C	snp	1	0	0	0	.	.	.	INTERGENIC(INTERGENIC)
X	45707373	45707381	45707374	A	C	ins	1	1	1	1	.	.	.	RP5-1158E12.3.1(DOWNSTREAM);RP5-1158E12.3.1(EXON)
X	45969297	45969305	45969299	G	A	snp	1	1	1	1	.	.	.	INTERGENIC(INTERGENIC)
X	45986155	45986163	45986160	A	G	snp	1	0	0	0	.	.	.	INTERGENIC(INTERGENIC)
X	46172186	46172200	46172197	TTA	T	ins	1	1	1	1	.	.	.	INTERGENIC(INTERGENIC)
X	46188297	46188306	46188305	T	C	ins	1	1	1	0	.	.	.	INTERGENIC(INTERGENIC);RP1-30G7.2.1(UPSTREAM)
X	46200004	46200012	46200010	A	C	snp	0	0	0	1	.	.	.	INTERGENIC(INTERGENIC)
X	46400205	46400213	46400210	A	G	snp	1	1	1	1	.	.	.	RP1-71L16.2.1(UPSTREAM);ZNF674(INTRON)
X	46597462	46597469	46597466	A	G	snp	1	0	0	0	.	.	.	SLC9A7(INTRON)
X	46634208	46634225	46634210	GT	T	del	1	1	1	1	.	.	.	INTERGENIC(INTERGENIC)
X	46642325	46642339	46642329	TTG	C	snp	1	0	0	0	.	.	.	INTERGENIC(INTERGENIC)
X	46707180	46707189	46707187	TG	A	snp	1	0	0	0	.	.	.	RP2(INTRON)
X	46707202	46707211	46707209	TG	TA	ins	1	0	0	0	.	.	.	RP2(INTRON)
X	47008732	47008740	47008737	T	G	snp	1	0	0	0	.	.	.	NDUFB11(UPSTREAM);RBM10(INTRON)
X	47166745	47166760	47166747	AT	T	del	1	1	0	1	.	.	.	INTERGENIC(INTERGENIC)
X	47166745	47166760	47166748	AT	A	snp	1	1	0	1	.	.	.	INTERGENIC(INTERGENIC)
X	47194902	47194916	47194912	TTA	C	snp	1	1	0	1	.	.	.	INTERGENIC(INTERGENIC)
X	47257909	47257917	47257913	T	C	ins	1	0	0	0	.	.	.	ZNF157(INTRON)
X	47294973	47294984	47294981	TG	G	del	1	0	0	0	.	.	.	INTERGENIC(INTERGENIC);RP13-479F17.2.1(DOWNSTREAM)
X	47294973	47294984	47294982	TG	T	snp	1	0	0	0	.	.	.	INTERGENIC(INTERGENIC);RP13-479F17.2.1(DOWNSTREAM)
X	47294973	47294984	47294982	TG	TTTT	ins	1	0	0	0	.	.	.	INTERGENIC(INTERGENIC);RP13-479F17.2.1(DOWNSTREAM)
X	47342913	47342921	47342920	C	A	ins	1	1	1	1	.	.	.	CXorf24(UPSTREAM);INTERGENIC(INTERGENIC);ZNF41(UPSTREAM)
X	47342913	47342921	47342920	C	A	snp	1	1	1	1	.	.	.	CXorf24(UPSTREAM);INTERGENIC(INTERGENIC);ZNF41(UPSTREAM)
X	47492415	47492426	47492423	TC	CTC	del	1	0	0	0	.	.	.	CFP(UPSTREAM);ELK1(DOWNSTREAM);INTERGENIC(INTERGENIC)
X	47968149	47968164	47968160	TTTG	T	del	1	1	1	0	.	.	.	SSX6(INTRON)
X	48139418	48139441	48139432	TTTG	C	snp	1	0	0	0	.	.	.	RP11-344N17.11.1(INTRON);RP11-344N17.2.1(UPSTREAM);RP11-344N17.3.1(UPSTREAM)
X	48218423	48218443	48218437	ATT	A	del	1	1	0	1	.	.	.	RP11-344N17.9.1(INTRON);SSX3(UPSTREAM)
X	48369586	48369595	48369593	CT	C	snp	1	0	0	0	.	.	.	AF196972.9.1(UPSTREAM);PORCN(INTRON);PORCN(UPSTREAM)
X	48394467	48394476	48394474	G	T	ins	1	1	0	0	.	.	.	INTERGENIC(INTERGENIC);TBC1D25(UPSTREAM)
X	48435397	48435404	48435401	T	C	ins	1	1	1	1	.	.	.	AC115618.1(UPSTREAM);RBM3(DOWNSTREAM);RBM3(INTRON);RP11-1148L6.5.1(DOWNSTREAM)
X	48435397	48435404	48435402	T	C	snp	1	1	1	1	.	.	.	AC115618.1(UPSTREAM);RBM3(DOWNSTREAM);RBM3(INTRON);RP11-1148L6.5.1(DOWNSTREAM)
X	48451876	48451891	48451883	TTTG	TT	ins	1	1	1	1	.	.	.	WDR13(INTRON);WDR13(UPSTREAM)
X	48451876	48451891	48451885	TTTG	TG	del	1	1	1	1	.	.	.	WDR13(INTRON);WDR13(UPSTREAM)
X	48451876	48451891	48451886	TTTG	G	del	1	1	1	1	.	.	.	WDR13(INTRON);WDR13(UPSTREAM)
X	48565501	48565509	48565508	C	A	snp	1	0	0	0	.	.	.	AF196970.3.1(INTRON);SUV39H1(INTRON)
X	48697711	48697720	48697718	T	G	snp	1	1	1	1	.	.	.	INTERGENIC(INTERGENIC);PCSK1N(UPSTREAM)
X	48786667	48786682	48786673	AAAT	G	snp	1	0	0	0	.	.	.	OTUD5(INTRON)
X	48809316	48809325	48809320	A	C	snp	0	0	1	0	.	.	.	OTUD5(INTRON)
X	49038030	49038039	49038036	TA	G	snp	1	0	0	0	.	.	.	PRICKLE3(DOWNSTREAM);PRICKLE3(INTRON)
X	49038046	49038055	49038049	TA	C	snp	1	0	0	0	.	.	.	PRICKLE3(DOWNSTREAM);PRICKLE3(INTRON)
X	49046936	49046955	49046950	TTTC	CTTTC	del	1	1	1	1	.	.	.	PRICKLE3(UPSTREAM);SYP(DOWNSTREAM);SYP(INTRON)
X	49413773	49413782	49413776	T	G	snp	1	0	0	0	.	.	.	INTERGENIC(INTERGENIC)
X	49452830	49452837	49452833	A	C	snp	1	0	0	0	.	.	.	PAGE1(INTRON)
X	49767232	49767251	49767241	GTCT	A	snp	1	0	0	0	.	.	.	CLCN5(INTRON);MIR188(UPSTREAM);MIR532(UPSTREAM)
X	49898591	49898610	49898598	ATTT	A	snp	1	1	0	1	.	.	.	INTERGENIC(INTERGENIC)
X	49909404	49909419	49909416	AT	TT	ins	1	0	1	0	.	.	.	INTERGENIC(INTERGENIC)
X	49959474	49959485	49959483	TG	T	snp	1	0	0	0	.	.	.	AKAP4(INTRON)
X	49970605	49970613	49970612	A	G	snp	0	1	1	1	.	.	.	AKAP4(UPSTREAM);CCNB3(INTRON)
X	50054595	50054609	50054599	TTG	G	del	1	0	0	0	.	.	.	CCNB3(INTRON)
X	50181013	50181021	50181014	A	C	snp	1	1	1	1	.	.	.	DGKK(INTRON)
X	50181013	50181021	50181016	A	C	snp	1	0	0	0	.	.	.	DGKK(INTRON)
X	50306507	50306522	50306520	AT	C	snp	1	1	1	1	.	.	.	INTERGENIC(INTERGENIC)
X	50311688	50311695	50311693	T	G	snp	1	1	1	1	.	.	.	INTERGENIC(INTERGENIC)

X	50538128	50538145	50538143	AT	ATG	del	1	1	1	1	.	.	.	SHROOM4(INTRON)
X	50625134	50625145	50625143	TA	G	snp	1	1	1	1	.	.	.	INTERGENIC(INTERGENIC)
X	50647931	50647940	50647937	T	G	snp	1	1	1	1	.	.	.	H3F3AP5(UPSTREAM);INTERGENIC(INTERGENIC)
X	50865757	50865766	50865763	AT	G	snp	1	0	0	0	.	.	.	RP11-104D21.3.1(INTRON)
X	50980499	50980513	50980505	AAC	G	snp	1	1	1	1	.	.	.	INTERGENIC(INTERGENIC)
X	51145038	51145047	51145041	TA	T	snp	1	1	0	1	.	.	.	CXorf67(UPSTREAM);RP11-348F1.2.1(DOWNSTREAM);RP11-348F1.2.1(INTRON)
X	51394646	51394653	51394650	T	G	ins	1	0	0	0	.	.	.	INTERGENIC(INTERGENIC)
X	51896233	51896247	51896242	GAT	T	snp	0	0	1	0	.	.	.	INTERGENIC(INTERGENIC)
X	51902463	51902470	51902467	A	C	snp	1	1	0	1	.	.	.	INTERGENIC(INTERGENIC)
X	51912828	51912851	51912847	ATCT	ATCTATC	del	1	1	0	1	.	.	.	INTERGENIC(INTERGENIC)
X	52875460	52875469	52875468	A	G	snp	1	1	0	0	.	.	.	INTERGENIC(INTERGENIC)
X	52881110	52881133	52881123	TTTG	C	snp	1	0	0	0	.	.	.	INTERGENIC(INTERGENIC)
X	52911571	52911579	52911577	T	G	ins	1	1	0	0	.	.	.	INTERGENIC(INTERGENIC)
X	53046745	53046758	53046754	AT	A	del	1	0	0	0	.	.	.	INTERGENIC(INTERGENIC)
X	53055227	53055238	53055229	AT	C	snp	0	0	1	1	.	.	.	INTERGENIC(INTERGENIC)
X	53055337	53055348	53055341	AT	G	snp	1	1	1	1	.	.	.	INTERGENIC(INTERGENIC)
X	53055365	53055376	53055369	AT	G	snp	1	1	1	1	.	.	.	INTERGENIC(INTERGENIC)
X	53055393	53055404	53055397	AT	G	snp	1	1	1	1	.	.	.	INTERGENIC(INTERGENIC)
X	53055421	53055432	53055425	AT	G	snp	1	1	1	1	.	.	.	INTERGENIC(INTERGENIC)
X	53210318	53210326	53210323	T	G	snp	1	0	0	0	.	.	.	INTERGENIC(INTERGENIC)
X	53239526	53239537	53239533	CCA	T	snp	1	0	0	0	.	.	.	KDM5C(INTRON);KDM5C-IT1(DOWNSTREAM)
X	53274140	53274155	53274145	CA	C	snp	1	1	1	1	.	.	.	IQSEC2(INTRON)
X	53288027	53288050	53288038	AAAC	A	snp	1	0	0	0	.	.	.	IQSEC2(INTRON)
X	53318352	53318361	53318359	CA	G	snp	1	1	1	1	.	.	.	IQSEC2(INTRON)
X	53371924	53371931	53371925	A	C	snp	1	1	0	0	.	.	.	INTERGENIC(INTERGENIC);RP6-29D12.2.1(DOWNSTREAM);RP6-29D12.3.1(UPSTREAM)
X	53406703	53406712	53406709	CA	A	snp	1	0	0	0	.	.	.	SMC1A(DOWNSTREAM);SMC1A(UTR_3_PRIME)
X	53536736	53536759	53536743	CAAC	T	snp	1	1	0	0	.	.	.	INTERGENIC(INTERGENIC)
X	53643042	53643051	53643045	T	C	snp	0	0	0	1	.	.	.	HUWE1(INTRON)
X	53675474	53675482	53675478	A	C	snp	1	1	1	1	.	.	.	HUWE1(INTRON)
X	53675484	53675492	53675486	A	AC	ins	1	1	1	1	.	.	.	HUWE1(INTRON)
X	53675484	53675492	53675487	A	C	ins	1	1	1	1	.	.	.	HUWE1(INTRON)
X	53675484	53675492	53675488	A	C	snp	1	1	1	1	.	.	.	HUWE1(INTRON)
X	53755791	53755808	53755806	TC	T	snp	0	1	0	0	.	.	.	INTERGENIC(INTERGENIC)
X	54063668	54063675	54063672	T	G	snp	1	0	0	0	.	.	.	PHF8(DOWNSTREAM);PHF8(INTRON)
X	54608305	54608312	54608306	T	G	snp	1	1	1	1	.	.	.	INTERGENIC(INTERGENIC)
X	54686462	54686469	54686466	T	C	snp	0	0	0	1	.	.	.	INTERGENIC(INTERGENIC)
X	54902250	54902258	54902257	T	C	snp	1	0	0	0	.	.	.	INTERGENIC(INTERGENIC)
X	54937195	54937210	54937197	TA	A	snp	1	1	1	1	.	.	.	INTERGENIC(INTERGENIC)
X	54937285	54937296	54937292	TA	G	snp	1	1	1	1	.	.	.	INTERGENIC(INTERGENIC)
X	55097598	55097605	55097601	T	G	snp	1	1	1	0	.	.	.	INTERGENIC(INTERGENIC);PAGE2B(UPSTREAM)
X	55138250	55138257	55138252	G	T	snp	0	0	0	1	.	.	.	INTERGENIC(INTERGENIC)
X	55152922	55152935	55152933	TA	G	snp	1	1	1	1	.	.	.	INTERGENIC(INTERGENIC)
X	55246035	55246044	55246041	T	G	snp	1	1	1	1	.	.	.	INTERGENIC(INTERGENIC);PAGE5(UPSTREAM)
X	55289272	55289281	55289275	AT	C	snp	1	0	0	0	.	.	.	PAGE3(INTRON)
X	55336779	55336790	55336782	TC	G	snp	1	1	1	1	.	.	.	INTERGENIC(INTERGENIC)
X	55511948	55511965	55511950	AT	T	del	1	1	1	1	.	.	.	USP51(UTR_3_PRIME)
X	55511948	55511965	55511951	AT	A	snp	1	1	1	1	.	.	.	USP51(UTR_3_PRIME)
X	55516640	55516655	55516643	GA	G	snp	1	1	1	0	.	.	.	INTERGENIC(INTERGENIC);USP51(UPSTREAM)
X	55638055	55638064	55638061	TA	C	snp	1	1	1	1	.	.	.	INTERGENIC(INTERGENIC)
X	55638296	55638311	55638305	AT	C	snp	1	0	0	0	.	.	.	INTERGENIC(INTERGENIC)
X	55639901	55639910	55639904	AT	C	snp	1	1	1	1	.	.	.	INTERGENIC(INTERGENIC)
X	55641049	55641058	55641052	TA	G	snp	1	1	1	1	.	.	.	INTERGENIC(INTERGENIC)
X	55757774	55757783	55757777	TA	G	snp	0	1	1	0	.	.	.	RRAGB(INTRON);RRAGB(UPSTREAM)
X	55826570	55826578	55826573	A	G	snp	0	1	1	0	.	.	.	INTERGENIC(INTERGENIC)
X	55830464	55830473	55830468	AG	G	snp	1	1	1	1	.	.	.	INTERGENIC(INTERGENIC)
X	55911470	55911481	55911478	TTA	C	snp	1	1	1	1	.	.	.	INTERGENIC(INTERGENIC)
X	55953212	55953220	55953214	T	G	snp	1	1	1	1	.	.	.	RP13-188A5.1.1(INTRON)
X	56037271	56037288	56037282	AAC	T	snp	1	0	0	0	.	.	.	RP13-188A5.1.1(INTRON)
X	56223302	56223309	56223307	A	G	snp	1	0	0	0	.	.	.	INTERGENIC(INTERGENIC)
X	56385390	56385397	56385392	A	AC	ins	1	1	1	1	.	.	.	INTERGENIC(INTERGENIC)
X	56489224	56489232	56489228	T	C	snp	1	1	1	1	.	.	.	INTERGENIC(INTERGENIC)
X	56653874	56653881	56653878	A	C	snp	1	1	1	1	.	.	.	INTERGENIC(INTERGENIC);RP11-431N15.2.1(DOWNSTREAM)
X	56752573	56752584	56752580	TA	T	del	1	1	1	1	.	.	.	INTERGENIC(INTERGENIC);RP11-622K12.1.1(UPSTREAM)
X	56990250	56990261	56990252	CA	A	snp	1	1	0	0	.	.	.	INTERGENIC(INTERGENIC)

X	56990250	56990261	56990257	CA	G	snp	1	0	0	0	.	.	.	INTERGENIC(INTERGENIC)
X	56995116	56995125	56995119	A	G	snp	1	0	0	0	.	.	.	INTERGENIC(INTERGENIC);RP11-445O16.3.1(UPSTREAM)
X	57037738	57037749	57037743	TTA	G	snp	1	1	0	0	.	.	.	INTERGENIC(INTERGENIC)
X	57043529	57043538	57043536	TA	G	snp	1	0	0	0	.	.	.	INTERGENIC(INTERGENIC)
X	57116138	57116147	57116139	T	G	snp	1	1	1	1	.	.	.	INTERGENIC(INTERGENIC)
X	57135652	57135659	57135653	C	A	ins	1	0	0	0	.	.	.	INTERGENIC(INTERGENIC)
X	57242936	57242951	57242941	TA	G	snp	1	0	0	0	.	.	.	INTERGENIC(INTERGENIC)
X	57242936	57242951	57242946	TA	C	snp	1	1	1	1	.	.	.	INTERGENIC(INTERGENIC)
X	57242936	57242951	57242947	TA	G	snp	1	0	0	0	.	.	.	INTERGENIC(INTERGENIC)
X	57242960	57242975	57242970	TA	C	snp	1	1	1	1	.	.	.	INTERGENIC(INTERGENIC)
X	57464645	57464654	57464651	TA	C	snp	1	1	1	1	.	.	.	FAAH2(INTRON)
X	57780984	57780995	57780987	TAA	C	snp	1	1	0	0	.	.	.	INTERGENIC(INTERGENIC)
X	57827967	57827984	57827981	ACA	AC	del	1	1	1	1	.	.	.	INTERGENIC(INTERGENIC)
X	58035644	58035655	58035652	TTG	TTGT	del	1	0	0	0	.	.	.	INTERGENIC(INTERGENIC)
X	58035653	58035672	58035668	TTGT	TTG	del	1	0	0	0	.	.	.	INTERGENIC(INTERGENIC)
X	58372118	58372125	58372123	A	G	snp	1	0	0	0	.	.	.	INTERGENIC(INTERGENIC)
X	61946726	61946733	61946729	A	G	snp	0	1	0	1	.	.	.	INTERGENIC(INTERGENIC)
X	62029065	62029072	62029069	A	C	snp	1	0	0	0	.	.	.	INTERGENIC(INTERGENIC)
X	62602301	62602308	62602306	A	G	snp	1	0	0	0	.	.	.	INTERGENIC(INTERGENIC)
X	62614239	62614246	62614240	G	T	snp	0	1	0	0	.	.	.	INTERGENIC(INTERGENIC)
X	62619367	62619374	62619368	A	C	ins	1	1	0	1	.	.	.	INTERGENIC(INTERGENIC)
X	62664808	62664816	62664815	T	G	snp	0	1	0	0	.	.	.	INTERGENIC(INTERGENIC)
X	63195696	63195705	63195703	A	G	ins	1	0	0	0	.	.	.	INTERGENIC(INTERGENIC)
X	63243165	63243174	63243171	C	A	snp	0	0	1	0	.	.	.	INTERGENIC(INTERGENIC)
X	63243165	63243174	63243173	C	A	snp	1	1	1	1	.	.	.	INTERGENIC(INTERGENIC)
X	63247083	63247092	63247090	T	G	snp	1	1	0	0	.	.	.	INTERGENIC(INTERGENIC)
X	63293644	63293651	63293647	A	G	snp	1	1	0	0	.	.	.	INTERGENIC(INTERGENIC)
X	63315701	63315716	63315706	AAAC	T	snp	1	0	0	0	.	.	.	INTERGENIC(INTERGENIC)
X	63612463	63612478	63612470	TTTG	TTTT	ins	1	0	0	0	.	.	.	MTMR8(INTRON)
X	63616570	63616577	63616572	T	C	snp	1	0	0	0	.	.	.	INTERGENIC(INTERGENIC);MTMR8(UPSTREAM)
X	63710629	63710648	63710643	AGAT	T	ins	1	1	1	1	.	.	.	INTERGENIC(INTERGENIC)
X	63982745	63982753	63982749	T	C	snp	1	0	0	0	.	.	.	INTERGENIC(INTERGENIC)
X	64279100	64279109	64279105	C	A	ins	1	0	0	0	.	.	.	INTERGENIC(INTERGENIC)
X	64349138	64349153	64349142	TA	A	del	1	0	0	0	.	.	.	INTERGENIC(INTERGENIC)
X	64349138	64349153	64349143	TA	TTATATATGGCTCTAT	ins	1	0	0	0	.	.	.	INTERGENIC(INTERGENIC)
X	64504118	64504131	64504129	AC	A	ins	1	0	0	0	.	.	.	INTERGENIC(INTERGENIC)
X	64504990	64505013	64504995	ATT	G	snp	1	0	0	0	.	.	.	INTERGENIC(INTERGENIC)
X	64586051	64586059	64586054	A	G	snp	1	0	0	0	.	.	.	INTERGENIC(INTERGENIC)
X	64670616	64670627	64670621	TA	G	snp	0	0	1	0	.	.	.	INTERGENIC(INTERGENIC)
X	64670670	64670679	64670675	TA	G	snp	1	1	1	1	.	.	.	INTERGENIC(INTERGENIC)
X	64737112	64737131	64737127	TTCT	TTC	del	1	0	0	0	.	.	.	LAS1L(DOWNSTREAM);LAS1L(INTRON)
X	64794103	64794116	64794114	TG	T	snp	1	1	0	0	.	.	.	INTERGENIC(INTERGENIC)
X	64809731	64809746	64809742	TTTC	TTTCT	del	1	1	0	0	.	.	.	RP11-368D24__A.1.1(DOWNSTREAM);RP11-368D24__A.1.1(INTRON)
X	64831025	64831044	64831038	CTTT	A	ins	1	0	0	0	.	.	.	INTERGENIC(INTERGENIC)
X	64831025	64831044	64831040	CTTT	CTTTCTA	del	1	0	0	0	.	.	.	INTERGENIC(INTERGENIC)
X	64970442	64970456	64970447	AAC	T	snp	1	0	0	0	.	.	.	INTERGENIC(INTERGENIC)
X	65112327	65112335	65112334	T	G	snp	1	0	0	0	.	.	.	INTERGENIC(INTERGENIC)
X	65350004	65350027	65350023	CAA	AC	del	1	1	1	1	.	.	.	INTERGENIC(INTERGENIC)
X	65370884	65370893	65370890	AT	G	snp	1	1	0	1	.	.	.	INTERGENIC(INTERGENIC)
X	65406982	65406989	65406987	T	G	snp	1	1	0	1	.	.	.	HEPH(INTRON)
X	65670519	65670532	65670530	TA	TAT	del	1	1	1	1	.	.	.	INTERGENIC(INTERGENIC)
X	65779672	65779685	65779681	TA	G	snp	0	1	1	1	.	.	.	INTERGENIC(INTERGENIC)
X	65779672	65779685	65779683	TA	G	snp	0	0	1	0	.	.	.	INTERGENIC(INTERGENIC)
X	65848207	65848215	65848209	A	C	snp	0	0	0	1	.	.	.	EDA2R(INTRON)
X	66000722	66000731	66000728	T	G	snp	1	1	0	1	.	.	.	INTERGENIC(INTERGENIC)
X	66034135	66034143	66034137	T	G	snp	1	1	1	1	.	.	.	INTERGENIC(INTERGENIC)
X	66300961	66300974	66300971	AG	T	snp	1	0	0	0	.	.	.	INTERGENIC(INTERGENIC)
X	66370145	66370154	66370151	T	C	snp	1	1	1	1	.	.	.	INTERGENIC(INTERGENIC)
X	66538419	66538427	66538421	T	G	snp	0	1	1	0	.	.	.	INTERGENIC(INTERGENIC)
X	66569437	66569444	66569440	A	C	snp	1	1	0	1	.	.	.	INTERGENIC(INTERGENIC)
X	66598260	66598268	66598266	A	C	snp	1	1	0	1	.	.	.	INTERGENIC(INTERGENIC);RP6-22P16.1.1(DOWNSTREAM)
X	66998226	66998245	66998239	TAGA	GAT	ins	1	1	0	1	.	.	.	INTERGENIC(INTERGENIC)
X	67046816	67046824	67046823	A	G	snp	1	1	0	0	.	.	.	INTERGENIC(INTERGENIC)
X	67073001	67073009	67073002	T	C	snp	1	0	0	0	.	.	.	INTERGENIC(INTERGENIC)
X	67073031	67073040	67073033	T	C	snp	1	0	0	0	.	.	.	INTERGENIC(INTERGENIC)
X	67222416	67222425	67222420	A	C	snp	1	0	0	0	.	.	.	INTERGENIC(INTERGENIC)

X	67256552	67256567	67256559	AAAC	G	snp	1	1	1	1	.	.	.	INTERGENIC(INTERGENIC)
X	67263223	67263232	67263229	A	G	snp	1	1	0	1	.	.	.	OPHN1(DOWNSTREAM);OPHN1(UTR_3_PRIME)
X	67348346	67348357	67348351	GA	GAGAGAG	del	1	1	1	1	.	.	.	OPHN1(INTRON);RP11-516A11.3.1(UPSTREAM)
X	67348346	67348357	67348353	GA	GAGAG	del	1	1	1	1	.	.	.	OPHN1(INTRON);RP11-516A11.3.1(UPSTREAM)
X	67357758	67357767	67357765	AC	T	snp	1	0	0	0	.	.	.	OPHN1(INTRON);RP11-516A11.3.1(DOWNSTREAM)
X	67387358	67387373	67387370	TG	G	snp	1	1	0	1	.	.	.	OPHN1(INTRON)
X	67527123	67527132	67527128	A	AT	ins	1	0	0	0	.	.	.	OPHN1(INTRON)
X	67554818	67554841	67554836	AATT	C	snp	0	1	0	0	.	.	.	OPHN1(INTRON)
X	67594991	67594999	67594992	A	C	snp	1	0	0	0	.	.	.	OPHN1(INTRON)
X	67642622	67642631	67642625	AG	A	snp	1	1	1	1	.	.	.	OPHN1(INTRON)
X	67646395	67646412	67646397	TA	A	del	1	0	0	0	.	.	.	OPHN1(INTRON)
X	67676468	67676483	67676477	CA	T	snp	1	1	1	1	.	.	.	AL672138.1(DOWNSTREAM);INTERGENIC(INTERGENIC)
X	67813111	67813120	67813118	TA	G	snp	1	0	0	0	.	.	.	INTERGENIC(INTERGENIC)
X	68113756	68113765	68113758	CT	T	snp	1	1	1	1	.	.	.	INTERGENIC(INTERGENIC)
X	68125232	68125249	68125246	AT	T	snp	1	1	1	1	.	.	.	INTERGENIC(INTERGENIC)
X	68132991	68133014	68132996	GCA	C	snp	1	1	0	1	.	.	.	INTERGENIC(INTERGENIC)
X	68264242	68264249	68264245	T	G	snp	1	0	0	0	.	.	.	INTERGENIC(INTERGENIC)
X	68312487	68312498	68312491	CAC	G	snp	1	0	0	0	.	.	.	INTERGENIC(INTERGENIC)
X	68325375	68325384	68325382	AC	T	snp	1	0	0	0	.	.	.	INTERGENIC(INTERGENIC)
X	68499306	68499314	68499309	G	T	snp	0	0	1	0	.	.	.	INTERGENIC(INTERGENIC)
X	68513883	68513892	68513890	CA	G	snp	1	1	1	1	.	.	.	INTERGENIC(INTERGENIC)
X	68540880	68540888	68540886	C	T	ins	1	1	1	1	.	.	.	INTERGENIC(INTERGENIC)
X	68547903	68547914	68547909	CT	T	snp	0	0	1	0	.	.	.	INTERGENIC(INTERGENIC)
X	68641257	68641266	68641263	CT	T	snp	0	0	1	0	.	.	.	INTERGENIC(INTERGENIC)
X	68761738	68761745	68761740	T	C	snp	0	0	0	1	.	.	.	INTERGENIC(INTERGENIC)
X	68813933	68813942	68813940	A	G	snp	1	1	1	1	.	.	.	INTERGENIC(INTERGENIC)
X	68826721	68826732	68826723	AT	TATATATATTC	del	1	1	1	0	.	.	.	INTERGENIC(INTERGENIC)
X	69119376	69119393	69119389	TG	T	del	1	1	0	1	.	.	.	EDA(INTRON)
X	69119376	69119393	69119391	TG	T	del	1	1	0	1	.	.	.	EDA(INTRON)
X	69126609	69126622	69126617	CA	T	snp	1	0	0	0	.	.	.	EDA(INTRON)
X	69280686	69280694	69280689	A	G	snp	1	0	0	0	.	.	.	INTERGENIC(INTERGENIC);OTUD6A(UPSTREAM)
X	69416365	69416373	69416366	A	G	snp	0	1	1	1	.	.	.	DGAT2L6(INTRON)
X	69467749	69467760	69467756	TC	TCTCT	del	1	1	1	1	.	.	.	INTERGENIC(INTERGENIC)
X	69467749	69467760	69467757	TC	CTC	del	1	1	1	1	.	.	.	INTERGENIC(INTERGENIC)
X	69482679	69482690	69482687	AAT	C	snp	1	0	0	0	.	.	.	INTERGENIC(INTERGENIC);P2RY4(UPSTREAM)
X	69642814	69642822	69642821	C	A	snp	1	1	1	1	.	.	.	GDPD2(UPSTREAM);INTERGENIC(INTERGENIC);KIF4A(DOWNSTREAM)
X	69672456	69672465	69672462	GA	C	snp	1	1	1	1	.	.	.	DLG3(INTRON);DLG3(UPSTREAM);DLG3(UTR_5_PRIME);DLG3-AS1(DOWNSTREAM);U4(DOWNSTREAM)
X	69681891	69681899	69681896	C	T	snp	1	0	0	0	.	.	.	DLG3(INTRON)
X	69751095	69751110	69751103	TGAA	G	snp	1	1	1	0	.	.	.	TEX11(INTRON)
X	69792849	69792868	69792858	AATA	G	snp	1	1	0	1	.	.	.	TEX11(INTRON)
X	69907030	69907043	69907035	AC	T	snp	0	0	0	1	.	.	.	TEX11(INTRON)
X	69944499	69944510	69944505	TAA	C	snp	1	0	0	0	.	.	.	TEX11(INTRON);TEX11(UPSTREAM)
X	69992881	69992888	69992883	A	C	snp	1	0	1	1	.	.	.	TEX11(INTRON)
X	70117067	70117076	70117073	AT	T	snp	1	1	0	1	.	.	.	TEX11(INTRON)
X	70135990	70135999	70135997	A	C	snp	1	1	1	1	.	.	.	INTERGENIC(INTERGENIC)
X	70181101	70181108	70181107	A	G	snp	1	1	1	1	.	.	.	INTERGENIC(INTERGENIC);RPS23P8(UPSTREAM)
X	70224323	70224332	70224331	A	C	snp	1	1	1	0	.	.	.	INTERGENIC(INTERGENIC)
X	70294216	70294223	70294222	A	G	snp	1	1	1	1	.	.	.	INTERGENIC(INTERGENIC)
X	70388735	70388754	70388746	AGAA	AG	del	1	0	0	0	.	.	.	NLGN3(DOWNSTREAM);NLGN3(INTRON)
X	70415210	70415218	70415215	T	G	ins	1	1	1	1	.	.	.	RP5-1091N2.9.1(INTRON)
X	70547360	70547375	70547371	AACA	AAC	del	0	1	0	1	.	.	.	INTERGENIC(INTERGENIC)
X	70619931	70619939	70619936	T	G	snp	1	0	0	0	.	.	.	TAF1(INTRON)
X	70788710	70788719	70788713	A	C	snp	0	1	0	0	.	.	.	OGT(DOWNSTREAM);OGT(INTRON)
X	70837777	70837785	70837784	C	T	snp	1	0	0	0	.	.	.	ACRC(DOWNSTREAM);CXCR3(INTRON)
X	70838049	70838056	70838054	C	T	snp	1	1	1	1	.	.	.	ACRC(DOWNSTREAM);CXCR3(INTRON)
X	71115781	71115800	71115796	AAAG	AA	del	1	1	1	1	.	.	.	INTERGENIC(INTERGENIC)
X	71121623	71121631	71121625	T	C	snp	1	1	1	1	.	.	.	INTERGENIC(INTERGENIC)
X	71200390	71200407	71200397	GT	GTGTGTGTGTGGG	del	1	1	1	1	.	.	.	NHSL2(INTRON)
X	71444597	71444612	71444607	AAAC	G	snp	0	0	0	1	.	.	.	ERCC6L(INTRON);PIN4(INTRON)
X	71558537	71558552	71558546	AT	C	snp	1	1	1	1	.	.	.	HDAC8(INTRON)
X	71646757	71646766	71646762	AG	A	snp	1	0	0	0	.	.	.	HDAC8(INTRON)
X	71648857	71648876	71648871	CTTT	TC	del	1	1	1	1	.	.	.	HDAC8(INTRON)
X	71648857	71648876	71648872	CTTT	C	del	1	1	1	1	.	.	.	HDAC8(INTRON)
X	71711941	71711948	71711944	A	C	snp	1	1	1	0	.	.	.	HDAC8(DOWNSTREAM);HDAC8(INTRON)
X	72035583	72035592	72035587	TA	C	snp	0	1	0	0	.	.	.	DMRTC1B(INTRON)

X	72088511	72088520	72088518	T	G	snp	1	0	0	0	.	.	.	DMRTC1(DOWNSTREAM);INTERGENIC(INTERGENIC)
X	72666210	72666221	72666217	TC	T	snp	1	0	0	0	.	.	.	CDX4(UPSTREAM);INTERGENIC(INTERGENIC)
X	72762614	72762623	72762615	A	C	snp	1	1	1	0	.	.	.	INTERGENIC(INTERGENIC)
X	73268063	73268080	73268065	AT	T	del	0	1	0	1	.	.	.	INTERGENIC(INTERGENIC)
X	73268387	73268395	73268391	A	G	snp	0	1	0	1	.	.	.	INTERGENIC(INTERGENIC)
X	73285222	73285239	73285237	AT	A	del	1	1	1	1	.	.	.	INTERGENIC(INTERGENIC);RP13-33H18.1.1(UPSTREAM);RP13-36G14.3.1(UPSTREAM)
X	73382205	73382216	73382210	GCT	C	snp	1	0	0	1	.	.	.	RP13-204A15.3.1(EXON)
X	73427257	73427266	73427261	G	A	ins	1	1	1	1	.	.	.	INTERGENIC(INTERGENIC);RAB11FIP1P1(DOWNSTREAM)
X	73649140	73649147	73649145	A	G	snp	1	0	0	0	.	.	.	SLC16A2(INTRON)
X	73666456	73666479	73666471	AAAC	A	ins	1	0	1	0	.	.	.	SLC16A2(INTRON)
X	73715026	73715041	73715034	TG	C	snp	1	1	0	1	.	.	.	SLC16A2(INTRON)
X	73726893	73726900	73726897	C	T	snp	1	0	0	0	.	.	.	SLC16A2(INTRON)
X	73820144	73820152	73820148	A	C	snp	0	0	1	0	.	.	.	RLIM(INTRON)
X	73991657	73991674	73991664	TG	C	snp	0	1	0	1	.	.	.	KIAA2022(INTRON)
X	74023407	74023434	74023414	AAAT	A	snp	1	0	0	0	.	.	.	KIAA2022(INTRON)
X	74092810	74092818	74092815	T	G	snp	0	1	0	1	.	.	.	KIAA2022(INTRON)
X	74308711	74308720	74308713	A	C	snp	1	1	0	0	.	.	.	ABCB7(INTRON)
X	74743317	74743325	74743324	C	A	snp	1	1	1	1	.	.	.	ZDHHC15(UPSTREAM);ZDHHC15(UTR_5_PRIME)
X	74863644	74863653	74863648	T	G	snp	1	0	0	0	.	.	.	INTERGENIC(INTERGENIC)
X	74882995	74883002	74882997	T	G	snp	1	0	0	0	.	.	.	INTERGENIC(INTERGENIC)
X	75061995	75062010	75062005	TTGT	T	snp	1	0	0	0	.	.	.	INTERGENIC(INTERGENIC)
X	75361124	75361131	75361126	A	G	snp	1	1	1	1	.	.	.	INTERGENIC(INTERGENIC)
X	75395954	75395969	75395965	TTTG	T	ins	1	0	0	0	.	.	.	CXorf26(INTRON)
X	75429200	75429209	75429202	TA	C	snp	1	0	0	0	.	.	.	INTERGENIC(INTERGENIC)
X	75429226	75429235	75429228	TA	C	snp	1	1	1	1	.	.	.	INTERGENIC(INTERGENIC)
X	75429241	75429250	75429248	AT	G	snp	0	1	0	0	.	.	.	INTERGENIC(INTERGENIC)
X	75500150	75500159	75500155	T	TG	ins	1	1	0	0	.	.	.	INTERGENIC(INTERGENIC)
X	75500150	75500159	75500156	T	TG	ins	1	1	0	0	.	.	.	INTERGENIC(INTERGENIC)
X	75532228	75532237	75532236	T	C	snp	1	1	1	1	.	.	.	INTERGENIC(INTERGENIC)
X	75716450	75716459	75716455	AC	T	snp	1	1	1	1	.	.	.	INTERGENIC(INTERGENIC)
X	75835989	75835996	75835993	A	AC	ins	1	1	1	1	.	.	.	INTERGENIC(INTERGENIC)
X	75835989	75835996	75835994	A	C	ins	1	1	1	1	.	.	.	INTERGENIC(INTERGENIC)
X	75940440	75940448	75940441	T	G	snp	1	1	0	1	.	.	.	INTERGENIC(INTERGENIC)
X	75950817	75950825	75950822	A	G	snp	1	1	1	0	.	.	.	INTERGENIC(INTERGENIC)
X	76043639	76043646	76043645	T	G	snp	1	0	0	0	.	.	.	INTERGENIC(INTERGENIC)
X	76074900	76074911	76074904	TAT	G	snp	1	0	0	0	.	.	.	INTERGENIC(INTERGENIC)
X	76164689	76164697	76164690	T	C	snp	1	1	0	1	.	.	.	INTERGENIC(INTERGENIC)
X	76183170	76183178	76183171	T	C	snp	1	1	0	1	.	.	.	INTERGENIC(INTERGENIC)
X	76256249	76256276	76256259	AGAA	G	snp	1	0	0	0	.	.	.	INTERGENIC(INTERGENIC)
X	76351003	76351012	76351005	TA	ATATATAATATATACATATATATTATATAAC	del	1	1	1	1	.	.	.	INTERGENIC(INTERGENIC)
X	76364843	76364851	76364844	A	C	snp	1	1	1	1	.	.	.	INTERGENIC(INTERGENIC)
X	76459235	76459258	76459253	AAT	G	snp	1	1	1	1	.	.	.	INTERGENIC(INTERGENIC)
X	76529561	76529568	76529567	A	G	snp	1	1	1	1	.	.	.	INTERGENIC(INTERGENIC)
X	76545344	76545351	76545347	G	T	snp	1	1	0	1	.	.	.	INTERGENIC(INTERGENIC)
X	76623932	76623940	76623937	T	C	snp	1	0	0	0	.	.	.	INTERGENIC(INTERGENIC)
X	76786084	76786111	76786106	AAAC	CAAAC	del	1	1	1	1	.	.	.	ATRX(INTRON)
X	77144859	77144874	77144872	AT	A	del	1	1	0	1	.	.	.	MAGT1(INTRON)
X	77180772	77180779	77180775	A	G	snp	1	1	1	0	.	.	.	ATP7A(INTRON)
X	77335690	77335697	77335696	T	C	snp	1	1	1	1	.	.	.	PGK1(INTRON)
X	77450484	77450495	77450488	ATA	C	snp	1	1	1	1	.	.	.	INTERGENIC(INTERGENIC)
X	77900703	77900720	77900718	CA	C	del	1	0	0	0	.	.	.	INTERGENIC(INTERGENIC)
X	77939705	77939716	77939713	AAT	A	ins	1	0	0	0	.	.	.	INTERGENIC(INTERGENIC)
X	77980743	77980756	77980749	AC	G	snp	1	1	1	1	.	.	.	INTERGENIC(INTERGENIC)
X	78035478	78035485	78035483	A	C	snp	1	1	1	1	.	.	.	INTERGENIC(INTERGENIC)
X	78076076	78076089	78076083	AG	A	snp	1	1	1	1	.	.	.	INTERGENIC(INTERGENIC)
X	78182213	78182222	78182216	AT	C	snp	1	1	1	1	.	.	.	INTERGENIC(INTERGENIC)
X	78182237	78182246	78182240	AT	C	snp	1	1	1	1	.	.	.	INTERGENIC(INTERGENIC)
X	78182261	78182270	78182264	AT	C	snp	1	1	1	1	.	.	.	INTERGENIC(INTERGENIC)
X	78325744	78325753	78325749	T	G	snp	1	1	1	1	.	.	.	INTERGENIC(INTERGENIC)
X	78541543	78541554	78541552	AT	A	ins	1	1	1	1	.	.	.	INTERGENIC(INTERGENIC)
X	78541556	78541567	78541562	AT	G	snp	0	0	0	1	.	.	.	INTERGENIC(INTERGENIC)
X	78563669	78563688	78563684	AAAC	A	ins	1	0	0	0	.	.	.	INTERGENIC(INTERGENIC)
X	78574216	78574223	78574220	T	C	snp	1	0	0	0	.	.	.	INTERGENIC(INTERGENIC);RP4-696H22.2.1(UPSTREAM)
X	78609152	78609175	78609172	TAT	G	ins	1	1	1	1	.	.	.	INTERGENIC(INTERGENIC)
X	78744555	78744562	78744559	T	G	snp	1	0	0	0	.	.	.	INTERGENIC(INTERGENIC)
X	78823421	78823430	78823428	AT	A	del	1	1	1	1	.	.	.	INTERGENIC(INTERGENIC)

X	78848064	78848073	78848072	A	C	snp	1	1	1	1	.	.	.	INTERGENIC(INTERGENIC)
X	78860851	78860865	78860861	CAA	AC	del	1	1	1	1	.	.	.	INTERGENIC(INTERGENIC)
X	78860851	78860865	78860862	CAA	C	del	1	1	1	1	.	.	.	INTERGENIC(INTERGENIC)
X	78945399	78945412	78945401	AC	C	del	1	1	1	1	.	.	.	INTERGENIC(INTERGENIC)
X	78945399	78945412	78945402	AC	A	snp	1	1	1	1	.	.	.	INTERGENIC(INTERGENIC)
X	79240703	79240718	79240710	TTTA	C	snp	1	1	1	1	.	.	.	INTERGENIC(INTERGENIC)
X	79324561	79324576	79324569	TTTG	C	snp	1	1	1	1	.	.	.	INTERGENIC(INTERGENIC)
X	79328609	79328618	79328616	T	C	snp	1	0	0	0	.	.	.	INTERGENIC(INTERGENIC)
X	79568511	79568524	79568516	TC	T	snp	1	1	1	1	.	.	.	INTERGENIC(INTERGENIC);RP11-217H19.1.1(UPSTREAM)
X	79772218	79772227	79772223	A	C	snp	1	1	1	1	.	.	.	INTERGENIC(INTERGENIC)
X	79927880	79927895	79927885	AGGA	A	snp	0	0	0	1	.	.	.	BRWD3(DOWNSTREAM);BRWD3(UTR_3_PRIME)
X	80053606	80053629	80053620	TAAA	G	snp	0	0	0	1	.	.	.	BRWD3(INTRON)
X	80115235	80115244	80115240	AT	C	snp	0	0	0	1	.	.	.	ACA64(UPSTREAM);INTERGENIC(INTERGENIC)
X	80117979	80117987	80117980	T	G	snp	1	1	1	1	.	.	.	INTERGENIC(INTERGENIC)
X	80480647	80480655	80480650	T	G	snp	1	0	0	0	.	.	.	SH3BGRL(INTRON)
X	80807573	80807580	80807577	A	C	snp	1	1	1	1	.	.	.	INTERGENIC(INTERGENIC)
X	80952613	80952621	80952616	A	G	snp	1	1	1	1	.	.	.	INTERGENIC(INTERGENIC)
X	81112499	81112506	81112502	A	G	snp	1	1	1	1	.	.	.	INTERGENIC(INTERGENIC)
X	81144521	81144529	81144522	T	C	snp	0	1	0	0	.	.	.	INTERGENIC(INTERGENIC)
X	81205073	81205081	81205080	C	A	snp	1	0	1	0	.	.	.	INTERGENIC(INTERGENIC)
X	81327411	81327419	81327416	A	G	snp	1	0	0	0	.	.	.	INTERGENIC(INTERGENIC)
X	81345374	81345382	81345381	T	C	snp	1	1	1	1	.	.	.	INTERGENIC(INTERGENIC)
X	81372768	81372776	81372775	A	C	snp	1	1	0	1	.	.	.	INTERGENIC(INTERGENIC)
X	81468559	81468568	81468565	TA	C	snp	1	0	0	0	.	.	.	INTERGENIC(INTERGENIC)
X	81746095	81746106	81746099	AT	G	snp	1	0	0	0	.	.	.	INTERGENIC(INTERGENIC)
X	81766019	81766028	81766022	A	G	snp	1	1	0	1	.	.	.	INTERGENIC(INTERGENIC);RPL22P22(UPSTREAM)
X	81799184	81799191	81799190	T	G	snp	1	1	0	1	.	.	.	INTERGENIC(INTERGENIC)
X	81917243	81917254	81917252	AC	A	snp	1	0	0	0	.	.	.	INTERGENIC(INTERGENIC)
X	81977090	81977099	81977096	AG	G	snp	1	0	0	0	.	.	.	INTERGENIC(INTERGENIC)
X	81995968	81995979	81995972	TA	A	snp	1	1	0	1	.	.	.	INTERGENIC(INTERGENIC)
X	82082284	82082291	82082287	A	C	snp	0	0	1	0	.	.	.	INTERGENIC(INTERGENIC)
X	82102455	82102462	82102461	T	G	ins	1	1	1	1	.	.	.	INTERGENIC(INTERGENIC)
X	82226796	82226809	82226799	AT	G	snp	1	1	1	1	.	.	.	INTERGENIC(INTERGENIC)
X	82260244	82260252	82260248	A	G	snp	1	0	0	0	.	.	.	INTERGENIC(INTERGENIC)
X	82341809	82341816	82341814	T	C	snp	1	0	0	0	.	.	.	INTERGENIC(INTERGENIC)
X	82470567	82470582	82470575	TAAA	A	snp	1	0	0	0	.	.	.	INTERGENIC(INTERGENIC)
X	82704867	82704876	82704872	T	C	snp	1	0	0	0	.	.	.	INTERGENIC(INTERGENIC)
X	82767449	82767460	82767457	TC	G	snp	0	0	0	1	.	.	.	INTERGENIC(INTERGENIC);POU3F4(DOWNSTREAM)
X	82807844	82807851	82807848	T	G	snp	1	0	0	0	.	.	.	INTERGENIC(INTERGENIC)
X	82938783	82938792	82938785	T	G	ins	0	1	1	1	.	.	.	INTERGENIC(INTERGENIC)
X	82993904	82993915	82993912	AG	C	snp	1	1	0	1	.	.	.	INTERGENIC(INTERGENIC)
X	83132918	83132933	83132926	AT	T	snp	1	1	1	1	.	.	.	CYLC1(INTRON)
X	83160723	83160730	83160725	T	C	snp	1	0	0	0	.	.	.	CYLC1(INTRON)
X	83201334	83201342	83201339	T	TTTG	ins	1	1	0	0	.	.	.	CYLC1(INTRON)
X	83210624	83210633	83210626	AT	G	snp	1	1	0	1	.	.	.	CYLC1(INTRON)
X	83273393	83273400	83273397	A	C	snp	1	1	1	1	.	.	.	INTERGENIC(INTERGENIC)
X	83446750	83446759	83446756	AT	G	snp	1	1	1	1	.	.	.	INTERGENIC(INTERGENIC);RPS6KA6(UPSTREAM)
X	83446790	83446801	83446798	AT	G	snp	1	1	1	1	.	.	.	INTERGENIC(INTERGENIC);RPS6KA6(UPSTREAM)
X	83596441	83596454	83596449	AG	G	snp	0	0	0	1	.	.	.	HDX(INTRON)
X	83705270	83705277	83705275	A	C	snp	1	1	1	1	.	.	.	HDX(INTRON)
X	83803032	83803039	83803034	T	G	snp	1	1	1	1	.	.	.	INTERGENIC(INTERGENIC)
X	83827742	83827750	83827746	A	G	snp	1	0	0	0	.	.	.	INTERGENIC(INTERGENIC)
X	83878413	83878424	83878416	AT	A	snp	1	0	0	0	.	.	.	INTERGENIC(INTERGENIC)
X	83894467	83894478	83894474	CAT	G	snp	1	1	1	1	.	.	.	INTERGENIC(INTERGENIC)
X	83909591	83909600	83909597	TG	GTGC	ins	1	1	1	1	.	.	.	INTERGENIC(INTERGENIC)
X	84010005	84010013	84010006	A	G	snp	1	0	0	0	.	.	.	SETP4(UPSTREAM);TEX16P(INTRON)
X	84049236	84049253	84049242	CAA	A	snp	1	1	1	1	.	.	.	INTERGENIC(INTERGENIC)
X	84074292	84074303	84074295	ATA	G	snp	0	0	0	1	.	.	.	INTERGENIC(INTERGENIC)
X	84096675	84096684	84096680	A	G	snp	1	0	0	0	.	.	.	INTERGENIC(INTERGENIC)
X	84129940	84129949	84129942	AT	G	snp	1	1	0	1	.	.	.	INTERGENIC(INTERGENIC)
X	84381845	84381852	84381851	C	A	snp	1	1	1	0	.	.	.	INTERGENIC(INTERGENIC)
X	84390556	84390565	84390558	A	G	snp	1	1	1	1	.	.	.	INTERGENIC(INTERGENIC)
X	84450025	84450042	84450037	TA	A	snp	1	1	1	1	.	.	.	INTERGENIC(INTERGENIC)
X	84515612	84515621	84515619	C	T	ins	1	1	1	1	.	.	.	ZNF711(INTRON)
X	84534382	84534391	84534383	A	C	snp	1	1	1	1	.	.	.	POF1B(DOWNSTREAM);POF1B(UTR_3_PRIME)
X	84654523	84654532	84654524	G	T	ins	1	0	0	0	.	.	.	INTERGENIC(INTERGENIC)
X	84671509	84671516	84671515	T	G	snp	1	0	0	0	.	.	.	INTERGENIC(INTERGENIC)

X	84728441	84728450	84728444	AT	A	snp	1	1	1	1	.	.	.	INTERGENIC(INTERGENIC)
X	84790219	84790233	84790223	ATA	C	snp	1	0	0	0	.	.	.	INTERGENIC(INTERGENIC)
X	84956030	84956047	84956043	TAA	G	snp	1	0	0	0	.	.	.	INTERGENIC(INTERGENIC)
X	84975911	84975925	84975921	GAA	G	snp	1	0	1	0	.	.	.	INTERGENIC(INTERGENIC)
X	84995882	84995893	84995891	AC	T	snp	1	1	1	1	.	.	.	INTERGENIC(INTERGENIC)
X	85017709	85017728	85017721	TTTA	G	snp	1	1	1	1	.	.	.	INTERGENIC(INTERGENIC)
X	85236211	85236224	85236214	TA	T	snp	1	1	1	1	.	.	.	CHM(INTRON)
X	85383240	85383249	85383243	T	TA	ins	1	1	0	1	.	.	.	INTERGENIC(INTERGENIC);RP6-145B8.4.1(DOWNSTREAM)
X	85540592	85540599	85540594	T	C	snp	1	0	0	0	.	.	.	DACH2(INTRON)
X	85540937	85540944	85540938	T	G	snp	1	1	1	1	.	.	.	DACH2(INTRON)
X	85559839	85559850	85559847	AGC	A	snp	1	1	1	1	.	.	.	DACH2(INTRON);GEMIN8P3(EXON)
X	85585586	85585597	85585593	AAT	G	snp	1	0	0	0	.	.	.	DACH2(INTRON)
X	85666024	85666041	85666039	TA	G	snp	1	1	0	1	.	.	.	DACH2(INTRON)
X	85888073	85888092	85888085	AAAC	AAC	ins	1	0	0	0	.	.	.	DACH2(INTRON)
X	85943045	85943053	85943050	A	G	snp	1	1	1	1	.	.	.	DACH2(INTRON)
X	85960054	85960069	85960066	AT	C	snp	1	1	1	1	.	.	.	DACH2(INTRON)
X	86031590	86031607	86031598	TTA	G	snp	1	1	1	1	.	.	.	DACH2(INTRON)
X	86081495	86081503	86081500	A	G	snp	1	1	0	1	.	.	.	DACH2(INTRON)
X	86086805	86086814	86086809	A	G	ins	1	1	1	1	.	.	.	DACH2(DOWNSTREAM);DACH2(INTRON);DACH2(UTR_3_PRIME)
X	86108228	86108235	86108234	T	G	snp	1	1	1	1	.	.	.	INTERGENIC(INTERGENIC)
X	86146066	86146074	86146069	T	G	snp	1	0	0	0	.	.	.	INTERGENIC(INTERGENIC);RP3-431C21.1.1(DOWNSTREAM)
X	86279632	86279649	86279647	TC	T	snp	1	1	0	1	.	.	.	INTERGENIC(INTERGENIC)
X	86280126	86280137	86280133	AT	A	snp	1	1	1	1	.	.	.	INTERGENIC(INTERGENIC)
X	86287625	86287644	86287640	TTTA	TTT	ins	1	1	1	1	.	.	.	INTERGENIC(INTERGENIC)
X	86377448	86377459	86377451	TA	G	snp	1	1	1	1	.	.	.	INTERGENIC(INTERGENIC)
X	86489638	86489647	86489645	TA	G	snp	1	1	0	1	.	.	.	INTERGENIC(INTERGENIC)
X	86489696	86489711	86489705	TA	T	ins	1	1	1	1	.	.	.	INTERGENIC(INTERGENIC)
X	86489696	86489711	86489709	TA	T	ins	1	1	1	1	.	.	.	INTERGENIC(INTERGENIC)
X	86631487	86631495	86631493	A	G	snp	1	0	0	0	.	.	.	INTERGENIC(INTERGENIC)
X	86636780	86636787	86636781	T	TA	ins	1	1	0	1	.	.	.	INTERGENIC(INTERGENIC)
X	86695520	86695529	86695527	TC	T	ins	1	1	1	1	.	.	.	INTERGENIC(INTERGENIC)
X	86742133	86742141	86742134	G	T	snp	1	1	1	1	.	.	.	INTERGENIC(INTERGENIC)
X	86792501	86792510	86792508	AT	G	snp	1	1	1	1	.	.	.	KLHL4(INTRON)
X	86894864	86894875	86894870	AAC	AC	del	1	0	0	0	.	.	.	KLHL4(INTRON)
X	86965128	86965137	86965130	C	CA	ins	1	0	0	0	.	.	.	INTERGENIC(INTERGENIC)
X	86965128	86965137	86965131	C	A	ins	1	0	0	0	.	.	.	INTERGENIC(INTERGENIC)
X	86998334	86998342	86998338	T	G	ins	1	0	0	0	.	.	.	INTERGENIC(INTERGENIC)
X	87002464	87002477	87002474	TC	CTC	del	0	0	1	0	.	.	.	INTERGENIC(INTERGENIC)
X	87009446	87009453	87009451	T	G	ins	1	0	0	0	.	.	.	INTERGENIC(INTERGENIC)
X	87013333	87013340	87013335	T	C	ins	1	1	0	1	.	.	.	INTERGENIC(INTERGENIC)
X	87102754	87102761	87102755	A	G	snp	1	0	0	0	.	.	.	INTERGENIC(INTERGENIC)
X	87134405	87134412	87134411	T	TA	ins	1	1	1	1	.	.	.	INTERGENIC(INTERGENIC)
X	87204870	87204877	87204876	A	C	ins	1	1	1	1	.	.	.	INTERGENIC(INTERGENIC)
X	87217598	87217607	87217601	AT	C	snp	1	0	0	0	.	.	.	INTERGENIC(INTERGENIC)
X	87223919	87223928	87223924	TA	TATG	ins	1	0	1	1	.	.	.	INTERGENIC(INTERGENIC)
X	87452834	87452842	87452838	A	G	snp	1	0	1	0	.	.	.	INTERGENIC(INTERGENIC)
X	87491789	87491800	87491797	TGT	G	snp	1	1	1	1	.	.	.	INTERGENIC(INTERGENIC)
X	87517286	87517293	87517288	T	C	snp	1	1	1	1	.	.	.	INTERGENIC(INTERGENIC)
X	87529281	87529295	87529292	AAC	AAT	ins	1	0	0	0	.	.	.	INTERGENIC(INTERGENIC)
X	87563248	87563257	87563256	A	AT	ins	1	0	0	0	.	.	.	INTERGENIC(INTERGENIC)
X	87642943	87642951	87642945	A	G	snp	1	1	0	1	.	.	.	INTERGENIC(INTERGENIC)
X	87669139	87669146	87669141	A	G	snp	1	1	1	1	.	.	.	INTERGENIC(INTERGENIC)
X	87722230	87722238	87722234	A	G	snp	1	1	1	1	.	.	.	INTERGENIC(INTERGENIC)
X	87738845	87738859	87738848	CTT	TTA	ins	1	1	1	1	.	.	.	INTERGENIC(INTERGENIC)
X	87738845	87738859	87738855	CTT	TC	del	1	1	1	1	.	.	.	INTERGENIC(INTERGENIC)
X	87738845	87738859	87738856	CTT	C	del	1	1	1	1	.	.	.	INTERGENIC(INTERGENIC)
X	87755374	87755383	87755382	A	G	snp	1	1	1	1	.	.	.	INTERGENIC(INTERGENIC)
X	87832849	87832856	87832854	A	C	snp	0	1	0	0	.	.	.	INTERGENIC(INTERGENIC)
X	87972560	87972567	87972565	A	G	ins	1	1	0	1	.	.	.	INTERGENIC(INTERGENIC)
X	87990751	87990758	87990754	T	C	snp	0	0	0	1	.	.	.	INTERGENIC(INTERGENIC)
X	88028417	88028426	88028419	TA	C	snp	0	1	0	1	.	.	.	INTERGENIC(INTERGENIC)
X	88222230	88222237	88222233	A	G	snp	1	1	1	1	.	.	.	INTERGENIC(INTERGENIC)
X	88251583	88251591	88251584	A	C	snp	1	0	0	0	.	.	.	INTERGENIC(INTERGENIC)
X	88276407	88276418	88276415	AAT	C	snp	1	0	0	0	.	.	.	INTERGENIC(INTERGENIC)
X	88291521	88291530	88291524	TG	A	snp	1	0	0	0	.	.	.	INTERGENIC(INTERGENIC)
X	88567481	88567492	88567489	TTG	TTGT	ins	1	0	0	0	.	.	.	INTERGENIC(INTERGENIC)

X	88624520	88624535	88624522	AT	T	del	1	0	0	0	.	.	.	INTERGENIC(INTERGENIC);SRIP2(UPSTREAM)
X	88819543	88819552	88819549	A	AG	ins	1	1	1	1	.	.	.	INTERGENIC(INTERGENIC)
X	88819543	88819552	88819550	A	G	ins	1	1	1	1	.	.	.	INTERGENIC(INTERGENIC)
X	88976056	88976075	88976071	TTTG	T	ins	1	0	0	0	.	.	.	INTERGENIC(INTERGENIC)
X	88997413	88997420	88997418	A	C	ins	1	1	1	1	.	.	.	INTERGENIC(INTERGENIC)
X	89022500	89022509	89022507	T	G	snp	1	1	0	1	.	.	.	INTERGENIC(INTERGENIC)
X	89068976	89068984	89068981	A	G	snp	0	1	0	1	.	.	.	INTERGENIC(INTERGENIC)
X	89102766	89102779	89102777	AT	A	del	1	0	1	0	.	.	.	INTERGENIC(INTERGENIC)
X	89199545	89199556	89199548	AC	T	snp	1	0	0	0	.	.	.	INTERGENIC(INTERGENIC)
X	89202770	89202793	89202775	ATA	T	snp	1	0	0	0	.	.	.	INTERGENIC(INTERGENIC)
X	89277614	89277621	89277618	A	G	snp	1	0	0	0	.	.	.	INTERGENIC(INTERGENIC)
X	89348436	89348443	89348441	T	C	snp	1	1	0	0	.	.	.	INTERGENIC(INTERGENIC)
X	89370901	89370908	89370905	A	G	ins	1	1	0	1	.	.	.	INTERGENIC(INTERGENIC);USP12PX(DOWNSTREAM)
X	89471535	89471542	89471539	C	A	snp	1	1	1	1	.	.	.	INTERGENIC(INTERGENIC)
X	89480584	89480597	89480592	TA	A	snp	1	1	0	1	.	.	.	INTERGENIC(INTERGENIC)
X	89506445	89506454	89506446	A	AC	ins	1	1	1	1	.	.	.	INTERGENIC(INTERGENIC)
X	89506445	89506454	89506447	A	C	ins	1	1	1	1	.	.	.	INTERGENIC(INTERGENIC)
X	89709979	89709987	89709982	T	C	snp	1	0	0	0	.	.	.	INTERGENIC(INTERGENIC)
X	89713857	89713866	89713864	TA	G	snp	1	0	0	0	.	.	.	INTERGENIC(INTERGENIC)
X	89739596	89739605	89739599	AG	C	snp	1	1	0	1	.	.	.	INTERGENIC(INTERGENIC)
X	89761512	89761529	89761526	GA	A	snp	1	1	1	1	.	.	.	INTERGENIC(INTERGENIC)
X	89888779	89888790	89888788	AT	G	snp	1	1	1	1	.	.	.	INTERGENIC(INTERGENIC)
X	89969416	89969423	89969420	A	C	snp	1	0	0	0	.	.	.	INTERGENIC(INTERGENIC)
X	90119393	90119400	90119397	A	G	snp	1	1	1	1	.	.	.	INTERGENIC(INTERGENIC)
X	90353804	90353813	90353811	T	G	snp	1	0	0	0	.	.	.	INTERGENIC(INTERGENIC)
X	90575774	90575781	90575778	T	C	snp	1	1	1	1	.	.	.	INTERGENIC(INTERGENIC)
X	90606181	90606200	90606196	AAAT	A	ins	1	0	1	0	.	.	.	INTERGENIC(INTERGENIC)
X	90613058	90613065	90613059	T	G	snp	1	1	1	1	.	.	.	INTERGENIC(INTERGENIC)
X	90618353	90618360	90618354	T	G	snp	0	0	1	0	.	.	.	INTERGENIC(INTERGENIC)
X	90645005	90645028	90645024	AAAT	A	snp	1	0	0	0	.	.	.	INTERGENIC(INTERGENIC)
X	90647718	90647729	90647721	GGA	T	snp	1	0	1	0	.	.	.	INTERGENIC(INTERGENIC)
X	90799207	90799215	90799212	A	C	snp	1	0	0	0	.	.	.	INTERGENIC(INTERGENIC)
X	91014740	91014757	91014742	AT	T	del	1	0	0	0	.	.	.	INTERGENIC(INTERGENIC)
X	91217980	91217989	91217984	A	G	snp	1	0	0	0	.	.	.	PCDH11X(INTRON)
X	91276875	91276882	91276878	A	G	snp	1	1	1	0	.	.	.	PCDH11X(INTRON)
X	91511897	91511904	91511898	T	G	snp	1	0	1	0	.	.	.	PCDH11X(INTRON)
X	91516636	91516644	91516643	A	G	ins	1	1	1	0	.	.	.	PCDH11X(INTRON)
X	91576184	91576192	91576185	T	G	ins	1	1	0	1	.	.	.	PCDH11X(INTRON)
X	92009362	92009377	92009366	TG	C	snp	1	1	1	1	.	.	.	INTERGENIC(INTERGENIC)
X	92009378	92009385	92009379	T	G	snp	1	0	0	0	.	.	.	INTERGENIC(INTERGENIC)
X	92240588	92240596	92240593	A	C	snp	0	0	0	1	.	.	.	INTERGENIC(INTERGENIC)
X	92243662	92243669	92243663	T	C	snp	1	1	1	1	.	.	.	INTERGENIC(INTERGENIC)
X	92248769	92248777	92248773	A	G	snp	1	1	1	1	.	.	.	INTERGENIC(INTERGENIC)
X	92269380	92269394	92269389	ATT	G	snp	1	0	0	0	.	.	.	INTERGENIC(INTERGENIC)
X	92342485	92342494	92342487	TA	C	snp	1	1	1	1	.	.	.	INTERGENIC(INTERGENIC)
X	92372252	92372260	92372257	A	G	snp	1	0	0	0	.	.	.	INTERGENIC(INTERGENIC)
X	92567963	92567972	92567965	G	GT	ins	0	0	1	0	.	.	.	INTERGENIC(INTERGENIC)
X	92611119	92611127	92611121	T	G	ins	1	0	0	0	.	.	.	INTERGENIC(INTERGENIC)
X	92611119	92611127	92611121	T	G	snp	1	0	0	0	.	.	.	INTERGENIC(INTERGENIC)
X	92653082	92653089	92653086	A	G	snp	1	1	1	1	.	.	.	INTERGENIC(INTERGENIC)
X	92714393	92714402	92714396	TC	G	snp	1	1	1	1	.	.	.	INTERGENIC(INTERGENIC)
X	92825986	92825995	92825988	T	C	snp	1	0	1	0	.	.	.	INTERGENIC(INTERGENIC);RPL7P55(UPSTREAM)
X	92890037	92890046	92890041	TG	G	del	1	1	1	1	.	.	.	INTERGENIC(INTERGENIC)
X	92928109	92928126	92928114	GCT	C	snp	1	0	0	0	.	"NAP1L3(NON_SYNONYMOUS_CODING):S57G,S64G"	.	FAM133A(UPSTREAM);NAP1L3(EXON);NAP1L3(INTRON)
X	93004178	93004186	93004181	G	GT	ins	1	1	1	1	.	.	.	INTERGENIC(INTERGENIC)
X	93037975	93037988	93037979	AT	TG	ins	1	1	1	1	.	.	.	INTERGENIC(INTERGENIC)
X	93037975	93037988	93037981	AT	TG	ins	1	1	1	1	.	.	.	INTERGENIC(INTERGENIC)
X	93079196	93079205	93079199	TG	A	snp	1	1	1	1	.	.	.	INTERGENIC(INTERGENIC)
X	93208420	93208427	93208422	T	G	snp	1	1	1	0	.	.	.	INTERGENIC(INTERGENIC)
X	93232628	93232647	93232635	GATA	G	snp	1	1	1	1	.	.	.	INTERGENIC(INTERGENIC)
X	93310097	93310108	93310099	AC	G	snp	0	0	1	1	.	.	.	INTERGENIC(INTERGENIC)
X	93379755	93379762	93379761	T	C	snp	1	0	0	0	.	.	.	INTERGENIC(INTERGENIC)
X	93565347	93565355	93565352	C	A	ins	1	0	0	0	.	.	.	INTERGENIC(INTERGENIC)
X	93823638	93823646	93823640	T	G	ins	1	0	0	0	.	.	.	INTERGENIC(INTERGENIC)
X	93843400	93843407	93843405	A	C	snp	1	0	0	0	.	.	.	INTERGENIC(INTERGENIC)
X	93981833	93981848	93981842	GTTT	TTG	del	1	0	0	0	.	.	.	INTERGENIC(INTERGENIC)

X	93983186	93983193	93983190	A	G	snp	1	1	0	1	.	.	.	INTERGENIC(INTERGENIC)
X	94056570	94056578	94056573	T	C	snp	1	0	1	0	.	.	.	INTERGENIC(INTERGENIC)
X	94209914	94209922	94209918	T	G	snp	1	0	0	0	.	.	.	INTERGENIC(INTERGENIC)
X	94274859	94274870	94274866	AAC	G	snp	1	0	0	0	.	.	.	INTERGENIC(INTERGENIC)
X	94302038	94302045	94302040	A	C	ins	1	1	1	1	.	.	.	INTERGENIC(INTERGENIC)
X	94354220	94354229	94354226	AC	G	snp	1	1	0	0	.	.	.	INTERGENIC(INTERGENIC)
X	94395787	94395796	94395792	TA	G	snp	1	1	1	1	.	.	.	INTERGENIC(INTERGENIC)
X	94489972	94489989	94489987	AT	A	del	0	1	1	1	.	.	.	INTERGENIC(INTERGENIC)
X	94507387	94507404	94507402	TA	C	snp	1	0	0	0	.	.	.	INTERGENIC(INTERGENIC)
X	94549372	94549383	94549376	AT	G	snp	1	0	0	0	.	.	.	INTERGENIC(INTERGENIC)
X	94637804	94637811	94637809	A	AAG	ins	1	1	0	0	.	.	.	INTERGENIC(INTERGENIC)
X	94938264	94938283	94938278	ATAG	GATAGAT	del	1	0	0	0	.	.	.	INTERGENIC(INTERGENIC);RPS7P13(UPSTREAM)
X	94938893	94938900	94938895	G	A	snp	1	0	0	0	.	.	.	INTERGENIC(INTERGENIC);RPS7P13(UPSTREAM)
X	95014280	95014289	95014283	AG	A	snp	1	1	1	1	.	.	.	INTERGENIC(INTERGENIC)
X	95054041	95054050	95054045	GT	A	snp	1	0	0	0	.	.	.	INTERGENIC(INTERGENIC)
X	95075459	95075467	95075460	A	AC	ins	1	1	0	1	.	.	.	INTERGENIC(INTERGENIC)
X	95099388	95099397	95099394	AC	C	snp	1	1	0	1	.	.	.	INTERGENIC(INTERGENIC)
X	95129925	95129942	95129931	CA	T	snp	1	0	0	0	.	.	.	INTERGENIC(INTERGENIC)
X	95169265	95169272	95169270	T	G	snp	1	1	1	1	.	.	.	INTERGENIC(INTERGENIC)
X	95172207	95172215	95172209	A	G	snp	1	0	0	0	.	.	.	INTERGENIC(INTERGENIC)
X	95189966	95189974	95189967	A	C	snp	1	1	1	1	.	.	.	INTERGENIC(INTERGENIC)
X	95202708	95202715	95202712	A	C	ins	1	0	0	0	.	.	.	INTERGENIC(INTERGENIC)
X	95238276	95238283	95238281	T	C	snp	1	1	1	1	.	.	.	INTERGENIC(INTERGENIC)
X	95281873	95281881	95281878	A	G	snp	1	0	0	0	.	.	.	AL137845.1(DOWNSTREAM);INTERGENIC(INTERGENIC)
X	95374638	95374649	95374646	CAA	C	del	1	0	0	0	.	.	.	INTERGENIC(INTERGENIC)
X	95374648	95374656	95374650	A	C	snp	0	1	0	0	.	.	.	INTERGENIC(INTERGENIC)
X	95475652	95475663	95475659	ATA	G	snp	1	0	0	0	.	.	.	INTERGENIC(INTERGENIC)
X	95500739	95500750	95500747	TAT	C	snp	1	0	0	0	.	.	.	INTERGENIC(INTERGENIC)
X	95581364	95581377	95581368	TA	ATATATATATGTTATATATAC	del	1	1	1	1	.	.	.	INTERGENIC(INTERGENIC)
X	95612627	95612636	95612631	AT	TATAT	del	1	0	0	0	.	.	.	INTERGENIC(INTERGENIC)
X	95759193	95759201	95759196	G	A	snp	1	1	1	1	.	.	.	INTERGENIC(INTERGENIC)
X	95838452	95838461	95838455	AT	C	snp	1	1	1	1	.	.	.	INTERGENIC(INTERGENIC)
X	95872361	95872378	95872376	AC	A	snp	1	0	0	0	.	.	.	INTERGENIC(INTERGENIC);RP11-25C15.1.1(UPSTREAM)
X	96006581	96006595	96006590	AAT	G	snp	0	1	0	1	.	.	.	DIAPH2(INTRON)
X	96365982	96365989	96365985	A	C	snp	1	0	0	0	.	.	.	DIAPH2(INTRON)
X	96425868	96425887	96425881	TTAT	A	del	1	1	0	0	.	.	.	DIAPH2(INTRON)
X	96444430	96444438	96444436	C	A	snp	1	0	0	0	.	.	.	DIAPH2(INTRON)
X	96719818	96719829	96719821	AT	C	snp	1	1	1	1	.	.	.	DIAPH2(INTRON);DIAPH2-AS1(INTRON)
X	96848395	96848402	96848400	A	G	snp	1	0	0	0	.	.	.	DIAPH2(INTRON);DIAPH2-AS1(INTRON)
X	96902098	96902107	96902105	T	G	ins	1	0	0	0	.	.	.	DIAPH2-AS1(UPSTREAM);INTERGENIC(INTERGENIC)
X	96928691	96928698	96928692	G	A	snp	1	1	1	1	.	.	.	INTERGENIC(INTERGENIC)
X	96928691	96928698	96928695	G	A	snp	0	1	0	0	.	.	.	INTERGENIC(INTERGENIC)
X	96937391	96937402	96937394	AAT	G	snp	1	1	1	1	.	.	.	INTERGENIC(INTERGENIC)
X	96961270	96961277	96961276	A	C	snp	1	0	0	0	.	.	.	INTERGENIC(INTERGENIC)
X	96990564	96990571	96990567	T	C	snp	1	1	1	1	.	.	.	INTERGENIC(INTERGENIC)
X	97278623	97278632	97278625	TA	G	snp	1	1	1	1	.	.	.	INTERGENIC(INTERGENIC)
X	97278731	97278740	97278733	TA	G	snp	1	1	1	1	.	.	.	INTERGENIC(INTERGENIC)
X	97278759	97278768	97278761	TA	G	snp	1	1	1	1	.	.	.	INTERGENIC(INTERGENIC)
X	97278787	97278796	97278789	TA	G	snp	1	0	0	0	.	.	.	INTERGENIC(INTERGENIC)
X	97429945	97429952	97429951	A	C	snp	1	1	0	1	.	.	.	INTERGENIC(INTERGENIC)
X	97574493	97574501	97574500	T	C	ins	1	1	1	1	.	.	.	INTERGENIC(INTERGENIC)
X	97661458	97661473	97661469	TTTA	C	snp	1	0	0	0	.	.	.	INTERGENIC(INTERGENIC)
X	97668795	97668806	97668798	TA	T	del	1	1	1	1	.	.	.	INTERGENIC(INTERGENIC)
X	97760457	97760470	97760466	AT	G	snp	1	0	0	0	.	.	.	INTERGENIC(INTERGENIC)
X	97791621	97791630	97791623	TA	G	snp	1	1	1	1	.	.	.	INTERGENIC(INTERGENIC)
X	97793383	97793390	97793384	A	G	snp	1	0	0	0	.	.	.	INTERGENIC(INTERGENIC)
X	97846878	97846887	97846882	AG	G	ins	1	1	1	1	.	.	.	INTERGENIC(INTERGENIC)
X	97857089	97857104	97857100	AT	C	snp	1	1	1	1	.	.	.	INTERGENIC(INTERGENIC)
X	97880135	97880150	97880145	TCTT	TTC	del	1	1	1	1	.	.	.	INTERGENIC(INTERGENIC)
X	97909340	97909349	97909343	AT	A	snp	1	1	1	1	.	.	.	INTERGENIC(INTERGENIC)
X	97909340	97909349	97909347	AT	A	snp	1	1	1	1	.	.	.	INTERGENIC(INTERGENIC)
X	97980785	97980792	97980789	T	TTTC	ins	1	1	1	1	.	.	.	INTERGENIC(INTERGENIC)
X	98008782	98008796	98008790	CCA	C	del	1	0	0	0	.	.	.	INTERGENIC(INTERGENIC)
X	98086240	98086251	98086244	AT	T	ins	1	1	1	1	.	.	.	INTERGENIC(INTERGENIC)
X	98087353	98087368	98087361	TTCC	TCCTTCCC	ins	1	0	0	0	.	.	.	INTERGENIC(INTERGENIC)
X	98104261	98104270	98104267	AT	G	snp	1	0	0	0	.	.	.	INTERGENIC(INTERGENIC)
X	98104300	98104309	98104306	AT	G	snp	0	1	1	0	.	.	.	INTERGENIC(INTERGENIC)

X	98104315	98104324	98104321	AT	G	snp	0	1	1	0	.	.	.	INTERGENIC(INTERGENIC)
X	98356887	98356894	98356893	G	GC	ins	1	1	1	1	.	.	.	INTERGENIC(INTERGENIC)
X	98505274	98505281	98505278	T	C	snp	1	0	0	0	.	.	.	INTERGENIC(INTERGENIC)
X	98577355	98577362	98577361	T	G	snp	1	0	0	0	.	.	.	INTERGENIC(INTERGENIC)
X	98682550	98682559	98682557	AT	C	snp	1	0	0	0	.	.	.	INTERGENIC(INTERGENIC)
X	98798804	98798813	98798807	G	T	ins	1	0	0	0	.	.	.	RP3-377O6.1.1(INTRON)
X	98835811	98835818	98835813	T	C	snp	0	0	0	1	.	.	.	RP3-377O6.1.1(INTRON)
X	98933709	98933718	98933715	TG	G	snp	1	0	0	0	.	.	.	RP3-377O6.1.1(INTRON)
X	99269434	99269457	99269441	CATA	C	snp	1	0	0	0	.	.	.	INTERGENIC(INTERGENIC)
X	99281772	99281781	99281779	AT	ATAC	ins	1	1	1	1	.	.	.	INTERGENIC(INTERGENIC)
X	99320487	99320501	99320494	TTA	C	snp	1	0	0	0	.	.	.	INTERGENIC(INTERGENIC)
X	99531624	99531633	99531625	A	C	ins	0	1	1	1	.	.	.	INTERGENIC(INTERGENIC)
X	99709985	99710000	99709991	AAGG	A	snp	1	1	1	1	.	.	.	INTERGENIC(INTERGENIC)
X	99743248	99743257	99743256	C	A	snp	1	1	1	1	.	.	.	INTERGENIC(INTERGENIC)
X	99990923	99990932	99990924	T	TG	ins	0	1	1	1	.	.	.	INTERGENIC(INTERGENIC);SYTL4(UPSTREAM)
X	100018511	100018525	100018517	TTG	C	snp	1	0	0	0	.	.	.	INTERGENIC(INTERGENIC)
X	100026700	100026708	100026701	A	G	snp	1	1	1	1	.	.	.	INTERGENIC(INTERGENIC)
X	100153575	100153586	100153583	AAC	A	ins	1	0	0	0	.	.	.	INTERGENIC(INTERGENIC)
X	100239090	100239099	100239097	T	C	snp	1	0	0	0	.	.	.	ARL13A(INTRON);ARL13A(UPSTREAM)
X	100300630	100300637	100300633	A	C	snp	1	0	0	0	.	.	.	TRMT2B(INTRON);TRMT2B-AS1(DOWNSTREAM)
X	100315176	100315184	100315177	A	C	snp	1	1	1	1	.	.	.	INTERGENIC(INTERGENIC)
X	100479172	100479179	100479175	T	TTTG	ins	1	1	0	1	.	.	.	DRP2(INTRON)
X	100534957	100534966	100534958	A	C	ins	1	1	1	1	.	.	.	TAF7L(INTRON)
X	100534957	100534966	100534959	A	C	snp	1	1	1	1	.	.	.	TAF7L(INTRON)
X	100534957	100534966	100534962	A	C	snp	1	0	0	0	.	.	.	TAF7L(INTRON)
X	100595464	100595473	100595468	TA	C	snp	1	0	0	0	.	.	.	RPL21P132(INTRON)
X	100630494	100630501	100630500	G	A	snp	0	1	1	1	.	.	.	BTK(INTRON)
X	100636024	100636037	100636033	TA	G	snp	1	0	0	0	.	.	.	BTK(INTRON)
X	100764433	100764441	100764439	T	G	snp	1	0	0	0	.	.	.	ARMCX4(INTRON)
X	100826094	100826103	100826096	AT	C	snp	1	1	1	1	.	.	.	INTERGENIC(INTERGENIC)
X	100826094	100826103	100826097	AT	ATATATAATAA	del	1	0	0	0	.	.	.	INTERGENIC(INTERGENIC)
X	100831745	100831754	100831749	T	G	snp	1	0	0	0	.	.	.	INTERGENIC(INTERGENIC)
X	100952911	100952924	100952917	CT	G	snp	1	0	0	0	.	.	.	INTERGENIC(INTERGENIC)
X	101093566	101093574	101093567	G	T	snp	1	0	0	0	.	.	.	NXF5(INTRON)
X	101162846	101162854	101162853	A	G	snp	1	1	1	1	.	.	.	RP1-3E10.2.1(UPSTREAM);ZMAT1(INTRON)
X	101166118	101166126	101166120	A	G	snp	1	1	0	1	.	.	.	RP1-3E10.2.1(UPSTREAM);U6(UPSTREAM);ZMAT1(INTRON)
X	101306129	101306136	101306134	T	G	snp	1	0	0	0	.	.	.	INTERGENIC(INTERGENIC)
X	101576163	101576172	101576165	C	CA	ins	0	1	0	1	.	.	.	NXF2(INTRON);NXF2(UPSTREAM)
X	101870740	101870749	101870746	T	G	snp	1	0	1	1	.	.	.	ARMCX5(INTRON);RP5-1100E15.4.1(EXON)
X	102110617	102110626	102110623	T	G	snp	1	0	0	0	.	.	.	LL0XNC01-157D4.1.1(INTRON)
X	102208474	102208482	102208478	T	G	snp	1	1	0	1	.	.	.	INTERGENIC(INTERGENIC)
X	102211396	102211403	102211400	T	G	snp	1	1	1	1	.	.	.	INTERGENIC(INTERGENIC)
X	102308165	102308173	102308172	T	C	snp	0	1	1	0	.	.	.	INTERGENIC(INTERGENIC)
X	102407598	102407607	102407605	AG	A	snp	1	0	0	0	.	.	.	INTERGENIC(INTERGENIC);Y_RNA(DOWNSTREAM)
X	102408182	102408197	102408195	AG	A	snp	1	1	1	1	.	.	.	INTERGENIC(INTERGENIC);Y_RNA(DOWNSTREAM)
X	102489788	102489797	102489789	A	G	snp	0	1	1	0	.	.	.	INTERGENIC(INTERGENIC)
X	102719440	102719447	102719444	G	A	snp	0	1	1	0	.	.	.	INTERGENIC(INTERGENIC)
X	102789800	102789808	102789807	C	A	snp	1	1	1	1	.	.	.	INTERGENIC(INTERGENIC)
X	102995895	102995904	102995898	A	AAAAAAC	ins	1	1	1	1	.	.	.	INTERGENIC(INTERGENIC)
X	103137401	103137410	103137407	AT	TATACATGATCCAGAAG	del	1	0	0	0	.	.	.	INTERGENIC(INTERGENIC)
X	103247870	103247881	103247872	GA	A	snp	1	0	0	0	.	.	.	INTERGENIC(INTERGENIC)
X	103470462	103470475	103470465	TA	C	snp	1	1	1	0	.	.	.	INTERGENIC(INTERGENIC)
X	103532876	103532883	103532879	T	C	snp	1	0	0	0	.	.	.	INTERGENIC(INTERGENIC)
X	103698146	103698154	103698152	T	G	snp	1	0	0	0	.	.	.	INTERGENIC(INTERGENIC)
X	103708002	103708029	103708025	AAAC	AAACA	del	1	0	0	0	.	.	.	INTERGENIC(INTERGENIC)
X	104195365	104195384	104195380	TTTG	TT	ins	0	1	1	1	.	.	.	IL1RAPL2(INTRON)
X	104336764	104336771	104336770	T	C	snp	1	0	0	0	.	.	.	IL1RAPL2(INTRON)
X	104487981	104487994	104487991	CT	G	snp	1	0	1	0	.	.	.	IL1RAPL2(INTRON)
X	104531219	104531226	104531223	A	G	snp	1	0	0	0	.	.	.	IL1RAPL2(INTRON)
X	104553710	104553733	104553722	AATT	T	snp	1	0	0	0	.	.	.	IL1RAPL2(INTRON)
X	104553710	104553733	104553726	AATT	T	snp	1	0	0	0	.	.	.	IL1RAPL2(INTRON)
X	104718851	104718860	104718857	AC	T	snp	1	0	0	0	.	.	.	IL1RAPL2(INTRON)
X	105030329	105030338	105030334	AT	G	snp	1	1	1	1	.	.	.	INTERGENIC(INTERGENIC)
X	105055611	105055619	105055618	T	G	snp	1	1	0	0	.	.	.	INTERGENIC(INTERGENIC)
X	105240082	105240089	105240087	T	G	snp	1	0	0	0	.	.	.	INTERGENIC(INTERGENIC)
X	105296049	105296057	105296050	A	C	snp	1	0	0	0	.	.	.	INTERGENIC(INTERGENIC)
X	105691167	105691174	105691168	A	G	snp	1	0	0	0	.	.	.	INTERGENIC(INTERGENIC)

X	105823551	105823574	105823557	AAAT	G	snp	1	0	0	0	.	.	.	INTERGENIC(INTERGENIC)	
X	106207108	106207122	106207116	AAC	A	ins	1	1	1	1	.	.	.	AL158821.1(INTRON);MORC4(INTRON)	
X	106207108	106207122	106207119	AAC	A	snp	1	1	1	1	.	.	.	AL158821.1(INTRON);MORC4(INTRON)	
X	106301102	106301109	106301106	A	C	snp	1	1	1	1	.	.	.	INTERGENIC(INTERGENIC)	
X	106380491	106380498	106380493	A	C	snp	0	0	1	0	.	.	.	NUP62CL(INTRON)	
X	106417299	106417310	106417302	CT	C	snp	1	1	1	1	.	.	.	NUP62CL(INTRON)	
X	106417299	106417310	106417308	CT	C	snp	1	1	1	0	.	.	.	NUP62CL(INTRON)	
X	106591046	106591055	106591054	T	G	snp	1	0	0	0	.	.	.	DNAJA1P3(DOWNSTREAM);INTERGENIC(INTERGENIC)	
X	106676598	106676615	106676601	ATT	T	snp	1	1	1	1	.	.	.		
AL035088.1(DOWNSTREAM);INTERGENIC(INTERGENIC);KRT18P49(DOWNSTREAM)															
X	106841057	106841074	106841068	GAG	A	snp	1	1	0	0	.	.	"FRMPD3(SYNONYMOUS_CODING):E634,E686"	.	
X	106865657	106865666	106865659	AG	G	snp	0	1	0	1	.	.	.	INTERGENIC(INTERGENIC)	
X	106953797	106953806	106953798	C	A	snp	1	0	0	0	.	.	.	INTERGENIC(INTERGENIC);TSC22D3(DOWNSTREAM)	
X	107025453	107025464	107025456	AT	A	snp	0	1	1	1	.	.	.	RP5-820B18.3.1(INTRON);TSC22D3(UPSTREAM)	
X	107025517	107025528	107025522	AT	A	snp	1	1	0	0	.	.	.	RP5-820B18.3.1(INTRON);TSC22D3(UPSTREAM)	
X	107076642	107076659	107076657	TA	T	ins	1	1	1	1	.	.	.	MID2(INTRON)	
X	107076642	107076659	107076657	TA	T	snp	1	0	0	1	.	.	.	MID2(INTRON)	
X	107216210	107216225	107216213	AT	A	snp	1	1	1	1	.	.	.	INTERGENIC(INTERGENIC)	
X	107251220	107251227	107251225	T	G	snp	1	1	1	0	.	.	.	INTERGENIC(INTERGENIC)	
X	107315484	107315491	107315490	T	G	snp	1	1	1	1	.	.	.	VSIG1(INTRON);VSIG1(UPSTREAM)	
X	107481326	107481343	107481339	GT	GTG	del	1	0	0	0	.	.	.	COL4A6(INTRON)	
X	107481326	107481343	107481340	GT	T	snp	1	0	0	0	.	.	.	COL4A6(INTRON)	
X	107481326	107481343	107481341	GT	G	del	1	1	1	0	.	.	.	COL4A6(INTRON)	
X	107712386	107712399	107712397	TC	T	snp	1	0	0	0	.	.	.	COL4A5(INTRON)	
X	107775992	107776007	107776000	GAAA	T	snp	1	0	1	0	.	.	.	COL4A5(INTRON)	
X	107795440	107795449	107795446	A	G	snp	1	1	1	1	.	.	.	COL4A5(INTRON)	
X	108002791	108002798	108002795	A	G	snp	1	0	0	0	.	.	.	INTERGENIC(INTERGENIC)	
X	108068903	108068911	108068908	T	TTTG	ins	1	1	1	1	.	.	.	INTERGENIC(INTERGENIC);U6(UPSTREAM)	
X	108085450	108085457	108085454	T	C	snp	1	1	1	1	.	.	.	INTERGENIC(INTERGENIC)	
X	108142102	108142111	108142107	AG	A	snp	1	1	0	0	.	.	.	INTERGENIC(INTERGENIC)	
X	108262562	108262573	108262570	TTA	G	snp	1	0	0	0	.	.	.	INTERGENIC(INTERGENIC)	
X	108327536	108327544	108327543	T	G	snp	1	0	0	0	.	.	.	INTERGENIC(INTERGENIC)	
X	108367686	108367703	108367700	TA	AC	ins	1	1	0	0	.	.	.	INTERGENIC(INTERGENIC)	
X	108398946	108398957	108398955	AG	C	snp	1	0	0	0	.	.	.	INTERGENIC(INTERGENIC)	
X	108417993	108418008	108418003	ATTT	TA	del	1	1	1	1	.	.	.	INTERGENIC(INTERGENIC)	
X	108417993	108418008	108418004	ATTT	A	del	1	1	1	1	.	.	.	INTERGENIC(INTERGENIC)	
X	108580475	108580483	108580482	T	C	snp	1	1	1	1	.	.	.	INTERGENIC(INTERGENIC);RP1-31B8.1.1(UPSTREAM)	
X	108609418	108609427	108609420	TA	C	snp	1	0	0	0	.	.	.	INTERGENIC(INTERGENIC)	
X	108724146	108724161	108724152	GGAA	T	snp	1	0	0	0	.	.	.	GUCY2F(INTRON)	
X	108803935	108803962	108803941	TATC	C	snp	1	0	0	0	.	.	.	INTERGENIC(INTERGENIC)	
X	108997552	108997560	108997555	T	G	snp	1	1	0	1	.	.	.	INTERGENIC(INTERGENIC)	
X	109004548	109004557	109004550	A	C	snp	1	0	0	0	.	.	.	INTERGENIC(INTERGENIC)	
X	109205968	109205981	109205975	AT	A	del	1	0	0	0	.	.	.	INTERGENIC(INTERGENIC)	
X	109205968	109205981	109205978	AT	TAT	del	1	1	1	1	.	.	.	INTERGENIC(INTERGENIC)	
X	109205968	109205981	109205979	AT	A	del	1	1	1	1	.	.	.	INTERGENIC(INTERGENIC)	
X	109260673	109260690	109260688	TC	T	snp	1	1	1	1	.	.	.	TMEM164(INTRON)	
X	109270988	109270995	109270991	A	C	snp	1	0	0	0	.	.	.	TMEM164(INTRON)	
X	109439628	109439637	109439634	T	G	snp	0	1	0	1	.	.	.	AMMECR1(DOWNSTREAM);AMMECR1(UTR_3_PRIME)	
X	109601254	109601261	109601260	A	C	snp	1	0	0	0	.	.	.	AMMECR1(INTRON);RGAG1(UPSTREAM)	
X	109646684	109646695	109646687	AC	G	snp	1	0	0	0	.	.	.	AMMECR1(INTRON);RGAG1(INTRON)	
X	109834341	109834354	109834343	AT	G	snp	1	0	0	0	.	.	.	INTERGENIC(INTERGENIC)	
X	109932290	109932299	109932295	AT	C	snp	1	1	0	1	.	.	.	CHRDL1(INTRON)	
X	110112901	110112918	110112912	CAT	A	snp	1	0	0	0	.	.	.	INTERGENIC(INTERGENIC)	
X	110210870	110210878	110210873	T	G	snp	1	1	1	1	.	.	.	PAK3(INTRON)	
X	110283264	110283272	110283267	A	G	snp	1	1	1	1	.	.	.	PAK3(INTRON)	
X	110321479	110321488	110321484	T	G	snp	1	0	0	0	.	.	.	PAK3(INTRON)	
X	110382586	110382593	110382591	A	G	snp	1	0	0	0	.	.	.	PAK3(INTRON);PAK3(UPSTREAM)	
X	110481194	110481202	110481196	T	C	snp	0	1	0	0	.	.	.	INTERGENIC(INTERGENIC)	
X	110608293	110608313	110608309	TAT	T	snp	1	0	0	0	.	.	.	DCX(INTRON)	
X	110759861	110759876	110759864	TC	A	snp	1	0	0	0	.	.	.	RP6-170F5.2.1(INTRON)	
X	110763287	110763294	110763293	A	G	snp	1	0	0	0	.	.	.	RP6-170F5.2.1(DOWNSTREAM);RP6-170F5.2.1(EXON)	
X	110878885	110878900	110878892	AC	ACACACACAT	ins	1	1	0	0	.	.	.	.	INTERGENIC(INTERGENIC)
X	110903049	110903068	110903059	TCTT	TTCTTTC	del	1	0	0	0	.	.	.	INTERGENIC(INTERGENIC)	
X	110903049	110903068	110903061	TCTT	C	del	1	0	0	0	.	.	.	INTERGENIC(INTERGENIC)	
X	110903049	110903068	110903063	TCTT	TTC	del	1	0	0	0	.	.	.	INTERGENIC(INTERGENIC)	
X	111152352	111152375	111152364	AAAT	T	snp	0	0	0	1	.	.	.	TRPC5(INTRON)	
X	111152352	111152375	111152368	AAAT	T	snp	1	1	1	1	.	.	.	TRPC5(INTRON)	

X	111270496	111270505	111270499	TG	T	snp	0	0	1	0	.	.	.	TRPC5(INTRON)
X	111325621	111325632	111325627	GA	A	snp	1	0	0	0	.	.	.	TRPC5(UTR_5_PRIME)
X	111412313	111412336	111412319	TTTC	C	del	1	1	1	1	.	.	.	INTERGENIC(INTERGENIC)
X	111433119	111433136	111433130	AAC	T	snp	1	1	1	1	.	.	.	INTERGENIC(INTERGENIC)
X	111433508	111433517	111433511	T	C	snp	1	0	0	0	.	.	.	INTERGENIC(INTERGENIC)
X	111501135	111501144	111501141	AT	C	snp	0	1	1	1	.	.	.	INTERGENIC(INTERGENIC)
X	111520062	111520069	111520068	A	G	snp	1	0	0	0	.	.	.	INTERGENIC(INTERGENIC)
X	111524080	111524095	111524090	TTAT	G	snp	0	0	1	0	.	.	.	INTERGENIC(INTERGENIC)
X	111646098	111646105	111646100	A	G	snp	1	1	1	1	.	.	.	INTERGENIC(INTERGENIC)
X	111666340	111666348	111666341	G	T	snp	1	1	1	1	.	.	.	INTERGENIC(INTERGENIC)
X	111683017	111683026	111683020	CT	C	snp	1	1	1	1	.	.	.	INTERGENIC(INTERGENIC)
X	111714825	111714838	111714828	TC	T	snp	1	0	0	0	.	.	.	INTERGENIC(INTERGENIC)
X	111813476	111813487	111813480	TTC	A	snp	1	1	1	1	.	.	.	INTERGENIC(INTERGENIC)
X	111983590	111983605	111983593	GA	G	snp	1	0	0	0	.	.	.	INTERGENIC(INTERGENIC)
X	111983590	111983605	111983602	GA	A	snp	1	1	1	1	.	.	.	INTERGENIC(INTERGENIC)
X	111997313	111997330	111997321	TTG	A	snp	1	0	0	0	.	.	.	INTERGENIC(INTERGENIC)
X	112062449	112062458	112062453	CA	A	snp	1	0	0	0	.	.	.	AMOT(DOWNSTREAM);AMOT(INTRON);AMOT(UPSTREAM)
X	112369258	112369267	112369265	TC	T	snp	1	1	1	0	.	.	.	INTERGENIC(INTERGENIC)
X	112541560	112541573	112541565	TA	C	snp	1	0	0	0	.	.	.	INTERGENIC(INTERGENIC)
X	112636878	112636889	112636883	TAA	C	snp	1	0	0	0	.	.	.	INTERGENIC(INTERGENIC)
X	112722538	112722545	112722540	G	GT	ins	1	0	0	0	.	.	.	INTERGENIC(INTERGENIC)
X	112743827	112743835	112743834	T	G	snp	1	0	0	0	.	.	.	INTERGENIC(INTERGENIC)
X	112773409	112773423	112773417	TTC	TTCTTCT	del	1	1	1	1	.	.	.	INTERGENIC(INTERGENIC)
X	112773409	112773423	112773419	TTC	CTTC	del	1	1	1	1	.	.	.	INTERGENIC(INTERGENIC)
X	112906675	112906682	112906681	A	C	snp	1	1	1	1	.	.	.	INTERGENIC(INTERGENIC)
X	113065252	113065271	113065263	TTTA	C	snp	0	1	0	1	.	.	.	INTERGENIC(INTERGENIC)
X	113621737	113621746	113621740	AT	C	snp	1	1	1	1	.	.	.	CTD-2230M5.1.1(DOWNSTREAM);INTERGENIC(INTERGENIC);SSU72P1(UPSTREAM)
X	113658803	113658816	113658814	CT	C	del	1	0	0	0	.	.	.	INTERGENIC(INTERGENIC)
X	113667527	113667542	113667534	TTCC	T	snp	0	0	0	1	.	.	.	INTERGENIC(INTERGENIC)
X	113693552	113693565	113693563	TA	G	snp	1	0	0	1	.	.	.	INTERGENIC(INTERGENIC)
X	113736014	113736031	113736027	AC	T	snp	1	0	0	0	.	.	.	INTERGENIC(INTERGENIC)
X	113879933	113879946	113879942	GA	G	snp	1	1	0	1	.	.	.	HTR2C(INTRON)
X	113892538	113892545	113892542	T	G	ins	1	1	1	1	.	.	.	HTR2C(INTRON)
X	113974571	113974580	113974573	TA	C	snp	0	0	1	0	.	.	.	HTR2C(INTRON)
X	113987323	113987332	113987330	A	C	snp	1	0	0	0	.	.	.	HTR2C(INTRON)
X	114017280	114017289	114017282	A	G	snp	1	0	0	0	.	.	.	HTR2C(INTRON)
X	114084978	114084985	114084979	A	C	snp	1	1	1	1	.	.	.	HTR2C(INTRON)
X	114228419	114228434	114228431	AT	G	snp	1	1	1	1	.	.	.	INTERGENIC(INTERGENIC)
X	114305528	114305537	114305529	C	A	ins	1	0	0	0	.	.	.	INTERGENIC(INTERGENIC);YAP1P2(DOWNSTREAM)
X	114365814	114365822	114365819	C	T	ins	1	1	1	1	.	.	.	LRCH2(INTRON);SNORA35(UPSTREAM)
X	114397901	114397910	114397905	CA	T	snp	1	1	1	1	.	.	.	LRCH2(INTRON)
X	114506115	114506132	114506122	AG	C	snp	0	0	0	1	.	.	.	INTERGENIC(INTERGENIC)
X	114616365	114616372	114616366	A	C	snp	1	0	1	0	.	.	.	INTERGENIC(INTERGENIC)
X	114725160	114725171	114725164	TGG	A	snp	1	1	0	1	.	.	.	INTERGENIC(INTERGENIC)
X	114768701	114768709	114768705	T	G	snp	1	0	0	0	.	.	.	INTERGENIC(INTERGENIC)
X	114789615	114789638	114789620	TCCC	G	snp	1	1	1	1	.	.	.	INTERGENIC(INTERGENIC)
X	114796416	114796425	114796421	GA	G	snp	1	1	1	1	.	.	.	PLS3(INTRON)
X	114801945	114801953	114801948	A	C	snp	1	0	0	0	.	.	.	PLS3(INTRON)
X	114818179	114818187	114818182	T	C	snp	1	1	0	0	.	.	.	PLS3(INTRON)
X	114819699	114819707	114819704	T	G	snp	1	0	0	0	.	.	.	PLS3(INTRON)
X	114933159	114933168	114933161	AT	G	snp	1	0	0	0	.	.	.	INTERGENIC(INTERGENIC);RP1-241P17.3.1(UPSTREAM)
X	114933185	114933194	114933187	AT	G	snp	1	0	0	0	.	.	.	INTERGENIC(INTERGENIC);RP1-241P17.3.1(UPSTREAM)
X	114933315	114933324	114933317	AT	T	snp	1	0	0	0	.	.	.	INTERGENIC(INTERGENIC);RP1-241P17.3.1(UPSTREAM)
X	114939992	114940001	114939995	TC	T	snp	1	1	1	1	.	.	.	INTERGENIC(INTERGENIC);RP1-241P17.3.1(DOWNSTREAM)
X	114939992	114940001	114939999	TC	T	snp	1	1	1	1	.	.	.	INTERGENIC(INTERGENIC);RP1-241P17.3.1(DOWNSTREAM)
X	114940172	114940185	114940175	TC	T	snp	1	1	0	0	.	.	.	INTERGENIC(INTERGENIC);RP1-241P17.3.1(DOWNSTREAM)
X	114942936	114942953	114942941	AAT	C	snp	1	0	0	0	.	.	.	INTERGENIC(INTERGENIC);RP1-241P17.3.1(DOWNSTREAM)
X	115008900	115008908	115008907	A	G	snp	1	1	1	0	.	.	.	INTERGENIC(INTERGENIC)
X	115027738	115027749	115027745	GAA	AG	del	1	1	1	1	.	.	.	INTERGENIC(INTERGENIC);RP11-761E20.1.1(DOWNSTREAM)
X	115045480	115045487	115045484	T	C	snp	0	1	1	1	.	.	.	RP11-761E20.1.1(INTRON)
X	115083631	115083648	115083642	TG	T	snp	1	1	1	1	.	.	.	RP11-761E20.1.1(INTRON)
X	115154450	115154457	115154452	T	G	snp	0	0	0	1	.	.	.	INTERGENIC(INTERGENIC)
X	115219706	115219714	115219707	T	G	snp	1	0	0	0	.	.	.	INTERGENIC(INTERGENIC)
X	115272634	115272642	115272640	C	A	snp	1	1	1	1	.	.	.	INTERGENIC(INTERGENIC)
X	115391853	115391870	115391861	GA	A	snp	1	1	1	1	.	.	.	INTERGENIC(INTERGENIC)

X	115400900	115400907	115400905	T	C	snp	1	1	1	1	.	.	.	INTERGENIC(INTERGENIC)
X	115414865	115414878	115414872	TA	TC	ins	1	0	0	0	.	.	.	INTERGENIC(INTERGENIC)
X	115415024	115415035	115415033	TA	T	del	1	0	0	0	.	.	.	INTERGENIC(INTERGENIC)
X	115430410	115430419	115430413	T	C	snp	1	1	1	1	.	.	.	INTERGENIC(INTERGENIC)
X	115458455	115458466	115458462	AAT	G	snp	1	0	0	0	.	.	.	INTERGENIC(INTERGENIC)
X	115549779	115549788	115549787	T	C	snp	0	0	1	0	.	.	.	INTERGENIC(INTERGENIC)
X	115565812	115565820	115565817	C	A	snp	1	0	0	0	.	.	.	INTERGENIC(INTERGENIC);SLC6A14(UPSTREAM)
X	115571358	115571366	115571362	A	G	snp	1	0	0	0	.	.	.	SLC6A14(INTRON)
X	115657945	115657960	115657950	TTTC	TC	del	0	1	1	1	.	.	.	INTERGENIC(INTERGENIC)
X	115797963	115797972	115797968	A	G	snp	1	0	0	0	.	.	.	INTERGENIC(INTERGENIC)
X	115867689	115867698	115867693	AG	G	ins	1	0	1	0	.	.	.	INTERGENIC(INTERGENIC)
X	115867689	115867698	115867695	AG	GAG	del	1	1	1	1	.	.	.	INTERGENIC(INTERGENIC)
X	115981787	115981800	115981795	CT	G	snp	1	1	1	1	.	.	.	INTERGENIC(INTERGENIC);U6(DOWNSTREAM)
X	115985397	115985408	115985400	GT	C	snp	1	0	0	0	.	.	.	INTERGENIC(INTERGENIC);U6(UPSTREAM)
X	115985907	115985914	115985911	T	C	snp	1	0	0	0	.	.	.	INTERGENIC(INTERGENIC);U6(UPSTREAM)
X	116110390	116110405	116110397	TAAC	T	snp	1	1	1	1	.	.	.	INTERGENIC(INTERGENIC)
X	116246538	116246545	116246543	A	C	snp	1	0	0	0	.	.	.	INTERGENIC(INTERGENIC)
X	116365710	116365725	116365721	TTTG	T	ins	0	1	1	1	.	.	.	INTERGENIC(INTERGENIC)
X	116365710	116365725	116365721	TTTG	T	snp	1	1	1	1	.	.	.	INTERGENIC(INTERGENIC)
X	116559233	116559242	116559235	AT	G	snp	1	1	1	1	.	.	.	INTERGENIC(INTERGENIC)
X	116628508	116628515	116628512	T	C	snp	1	1	1	1	.	.	.	INTERGENIC(INTERGENIC)
X	116631261	116631270	116631263	TA	A	snp	1	1	1	1	.	.	.	INTERGENIC(INTERGENIC)
X	116673488	116673501	116673495	AG	T	snp	1	0	0	0	.	.	.	INTERGENIC(INTERGENIC)
X	116765845	116765862	116765859	AC	CAC	del	1	1	0	1	.	.	.	INTERGENIC(INTERGENIC)
X	116872613	116872622	116872619	AT	G	snp	1	1	1	1	.	.	.	INTERGENIC(INTERGENIC)
X	117165493	117165502	117165495	TA	C	snp	1	0	0	0	.	.	.	KLHL13(INTRON);KLHL13(UPSTREAM)
X	117215418	117215425	117215422	T	G	snp	1	0	0	0	.	.	.	KLHL13(INTRON)
X	117245862	117245869	117245867	A	G	snp	1	0	0	0	.	.	.	KLHL13(INTRON);RP1-169K13.3.1(DOWNSTREAM)
X	117398521	117398532	117398527	GTA	T	snp	1	0	0	0	.	.	.	INTERGENIC(INTERGENIC)
X	117401211	117401220	117401212	A	C	snp	1	1	1	1	.	.	.	INTERGENIC(INTERGENIC)
X	117457885	117457894	117457888	TA	C	snp	1	1	0	0	.	.	.	INTERGENIC(INTERGENIC)
X	117457922	117457931	117457925	TA	C	snp	1	1	1	1	.	.	.	INTERGENIC(INTERGENIC)
X	117580384	117580393	117580386	AT	T	snp	1	1	1	0	.	.	.	WDR44(INTRON)
X	117597133	117597148	117597143	TTGT	T	ins	1	1	1	1	.	.	.	INTERGENIC(INTERGENIC)
X	117624329	117624344	117624334	TTTC	C	snp	1	0	0	0	.	.	.	INTERGENIC(INTERGENIC)
X	117624345	117624353	117624347	T	C	ins	1	1	1	1	.	.	.	INTERGENIC(INTERGENIC)
X	117750491	117750500	117750495	T	G	ins	1	0	0	0	.	.	.	DOCK11(INTRON)
X	117750514	117750523	117750522	T	G	snp	1	1	1	1	.	.	.	DOCK11(INTRON)
X	117854653	117854662	117854661	T	C	snp	1	0	0	0	.	.	.	INTERGENIC(INTERGENIC)
X	117985377	117985392	117985379	TA	C	snp	1	0	0	0	.	.	.	RP4-562J12.2.1(INTRON)
X	118031826	118031834	118031831	A	G	snp	1	1	1	1	.	.	.	INTERGENIC(INTERGENIC)
X	118039302	118039313	118039307	TCT	C	snp	1	1	1	0	.	.	.	INTERGENIC(INTERGENIC)
X	118078873	118078884	118078880	GGT	A	snp	0	0	1	0	.	.	.	INTERGENIC(INTERGENIC)
X	118149436	118149443	118149439	C	A	snp	1	0	0	0	.	.	.	LONRF3(INTRON)
X	118169962	118169971	118169965	A	C	snp	1	0	0	0	.	.	.	INTERGENIC(INTERGENIC)
X	118228726	118228735	118228728	AG	G	snp	1	0	0	0	.	.	.	KIAA1210(INTRON)
X	118295878	118295887	118295882	T	C	snp	1	1	1	0	.	.	.	INTERGENIC(INTERGENIC)
X	118427766	118427774	118427768	T	G	snp	1	0	0	0	.	.	.	RP5-1139I1.1.1(INTRON)
X	118508257	118508276	118508266	GAAA	AAG	del	1	1	1	1	.	.	.	RP5-1139I1.2.1(INTRON)
X	118514833	118514840	118514839	T	C	snp	1	0	0	0	.	.	.	MRPS17P9(DOWNSTREAM);RP5-1139I1.2.1(INTRON)
X	118577396	118577411	118577409	TC	TCTTT	del	0	0	1	0	.	.	.	SLC25A43(INTRON)
X	118590954	118590965	118590962	AG	GAGAAAGAAAG	del	1	1	0	1	.	.	.	INTERGENIC(INTERGENIC);SLC25A43(DOWNSTREAM);Y_RNA(DOWNSTREAM)
X	118608472	118608479	118608477	T	G	ins	1	1	1	1	.	.	.	INTERGENIC(INTERGENIC);SLC25A5(DOWNSTREAM)
X	118756017	118756025	118756018	A	C	ins	0	0	1	0	.	.	.	SEPT6(INTRON)
X	118802298	118802307	118802300	AG	G	del	1	1	1	1	.	.	.	SEPT6(INTRON)
X	118802298	118802307	118802301	AG	AA	ins	1	1	1	1	.	.	.	SEPT6(INTRON)
X	118845693	118845708	118845701	AG	G	snp	1	1	1	1	.	.	.	INTERGENIC(INTERGENIC)
X	118896406	118896414	118896408	T	C	ins	1	1	1	1	.	.	.	ANKRD58(DOWNSTREAM);INTERGENIC(INTERGENIC)
X	118912197	118912212	118912205	AAAT	AATAAATTAATT	ins	1	1	1	1	.	.	.	INTERGENIC(INTERGENIC)
X	118912197	118912212	118912207	AAAT	T	ins	1	0	0	0	.	.	.	INTERGENIC(INTERGENIC)
X	118997552	118997561	118997559	TC	A	snp	1	0	0	0	.	.	.	INTERGENIC(INTERGENIC)
X	119025379	119025390	119025386	AGC	A	snp	1	0	0	0	.	.	.	AKAP14(UPSTREAM);INTERGENIC(INTERGENIC)
X	119035982	119036005	119035999	CAAA	AAC	del	1	0	1	0	.	.	.	AKAP14(INTRON)
X	119038852	119038866	119038857	AAT	A	snp	1	0	1	0	.	.	.	AKAP14(DOWNSTREAM);AKAP14(INTRON)
X	119050145	119050154	119050148	T	C	snp	0	1	1	1	.	.	.	AKAP14(INTRON);AKAP14(UPSTREAM)
X	119052204	119052213	119052208	T	G	snp	1	1	1	1	.	.	.	

AKAP14(INTRON);AKAP14(UPSTREAM);RP3-327A19.5.1(DOWNSTREAM)

X	119065170	119065179	119065173	A	C	snp	1	1	1	1	.	.	.	NKAP(INTRON)
X	119067904	119067911	119067907	A	C	snp	1	0	0	0	.	.	.	NKAP(INTRON);NKAP(UPSTREAM)
X	119110577	119110586	119110579	TA	C	snp	1	0	0	0	.	.	.	INTERGENIC(INTERGENIC)
X	119248492	119248500	119248497	A	G	snp	1	1	0	0	.	.	.	GS1-421I3.4.1(DOWNSTREAM);RHOXF1(INTRON);RP4-755D9.1.1(INTRON)
X	119302141	119302150	119302146	T	G	snp	1	1	1	1	.	.	.	NKAPP1(INTRON);RHOXF2(DOWNSTREAM)
X	119306473	119306482	119306478	TA	T	snp	0	0	1	0	.	.	.	NKAPP1(INTRON)
X	119430480	119430491	119430483	AT	C	snp	1	1	1	1	.	.	.	FAM70A(INTRON)
X	119733321	119733330	119733326	A	C	snp	1	0	0	0	.	.	.	MCTS1(INTRON);MCTS1(UPSTREAM)
X	119767022	119767029	119767026	A	G	snp	1	0	0	0	.	.	.	C1GALT1C1(UPSTREAM);INTERGENIC(INTERGENIC)
X	119814827	119814835	119814831	T	TG	ins	1	1	1	1	.	.	.	INTERGENIC(INTERGENIC)
X	119814827	119814835	119814832	T	G	ins	1	1	1	1	.	.	.	INTERGENIC(INTERGENIC)
X	119850605	119850612	119850611	T	C	snp	1	0	0	0	.	.	.	INTERGENIC(INTERGENIC)
X	119938960	119938971	119938967	TA	C	snp	1	0	0	0	.	.	.	INTERGENIC(INTERGENIC)
X	119955287	119955296	119955295	T	G	snp	1	1	1	1	.	.	.	INTERGENIC(INTERGENIC)
X	120145101	120145116	120145108	AAAC	AAAG	ins	1	1	1	1	.	.	.	INTERGENIC(INTERGENIC)
X	120380085	120380092	120380091	A	C	snp	1	1	1	1	.	.	.	INTERGENIC(INTERGENIC)
X	120498738	120498747	120498745	T	G	snp	1	1	1	1	.	.	.	INTERGENIC(INTERGENIC)
X	120520078	120520087	120520086	A	C	ins	0	0	1	0	.	.	.	INTERGENIC(INTERGENIC)
X	120589877	120589886	120589878	T	G	snp	1	1	1	1	.	.	.	INTERGENIC(INTERGENIC)
X	120591958	120591967	120591964	AT	G	snp	1	0	0	0	.	.	.	INTERGENIC(INTERGENIC)
X	120610178	120610187	120610181	AT	C	snp	1	0	0	0	.	.	.	INTERGENIC(INTERGENIC)
X	120751540	120751551	120751545	ATT	A	del	1	1	1	1	.	.	.	INTERGENIC(INTERGENIC)
X	120751540	120751551	120751548	ATT	A	del	1	1	1	1	.	.	.	INTERGENIC(INTERGENIC)
X	120904551	120904562	120904560	AT	C	snp	1	1	1	1	.	.	.	INTERGENIC(INTERGENIC)
X	120968391	120968398	120968395	T	C	snp	1	1	1	1	.	.	.	INTERGENIC(INTERGENIC)
X	121017378	121017385	121017382	A	G	snp	1	0	0	0	.	.	.	INTERGENIC(INTERGENIC)
X	121029478	121029487	121029483	GT	C	snp	0	0	1	0	.	.	.	INTERGENIC(INTERGENIC)
X	121065068	121065075	121065069	T	G	snp	1	1	1	0	.	.	.	INTERGENIC(INTERGENIC)
X	121149138	121149147	121149144	AT	G	snp	1	1	1	1	.	.	.	INTERGENIC(INTERGENIC);RP11-417D4.1.1(UPSTREAM)
X	121158520	121158529	121158527	TC	TCT	del	1	1	1	1	.	.	.	INTERGENIC(INTERGENIC)
X	121172181	121172189	121172182	T	C	snp	1	0	0	0	.	.	.	INTERGENIC(INTERGENIC)
X	121172181	121172189	121172186	T	C	snp	1	0	0	0	.	.	.	INTERGENIC(INTERGENIC)
X	121365501	121365518	121365513	AT	G	snp	1	1	1	1	.	.	.	INTERGENIC(INTERGENIC)
X	121365501	121365518	121365515	AT	G	snp	1	1	1	1	.	.	.	INTERGENIC(INTERGENIC)
X	121499851	121499862	121499858	TCT	TTCTT	del	1	0	0	0	.	.	.	INTERGENIC(INTERGENIC)
X	121526332	121526349	121526334	AT	T	del	1	0	0	0	.	.	.	INTERGENIC(INTERGENIC)
X	121526332	121526349	121526335	AT	A	snp	1	0	0	0	.	.	.	INTERGENIC(INTERGENIC)
X	121610832	121610839	121610834	T	C	snp	1	0	1	0	.	.	.	INTERGENIC(INTERGENIC)
X	121649750	121649761	121649753	AT	A	snp	1	0	0	0	.	.	.	INTERGENIC(INTERGENIC)
X	121676882	121676889	121676883	A	C	snp	1	1	1	0	.	.	.	INTERGENIC(INTERGENIC);RPL3P12(DOWNSTREAM)
X	121780038	121780047	121780042	T	C	snp	1	1	1	1	.	.	.	INTERGENIC(INTERGENIC)
X	121800397	121800406	121800400	T	C	snp	0	1	1	1	.	.	.	INTERGENIC(INTERGENIC)
X	121825768	121825776	121825773	A	G	snp	1	1	1	1	.	.	.	INTERGENIC(INTERGENIC)
X	121846323	121846330	121846328	A	G	snp	1	1	1	1	.	.	.	INTERGENIC(INTERGENIC)
X	121847756	121847763	121847759	A	G	snp	1	1	1	1	.	.	.	INTERGENIC(INTERGENIC)
X	121875309	121875320	121875313	TA	G	snp	1	0	0	0	.	.	.	INTERGENIC(INTERGENIC)
X	121905612	121905621	121905614	T	C	snp	1	1	1	0	.	.	.	INTERGENIC(INTERGENIC)
X	121988878	121988891	121988885	AC	A	snp	1	1	0	1	.	.	.	INTERGENIC(INTERGENIC)
X	122003334	122003342	122003337	C	CCA	ins	1	1	0	1	.	.	.	INTERGENIC(INTERGENIC)
X	122037689	122037696	122037692	A	G	snp	1	1	0	1	.	.	.	INTERGENIC(INTERGENIC)
X	122164771	122164786	122164776	CCCT	T	snp	0	1	1	1	.	.	.	INTERGENIC(INTERGENIC)
X	122177011	122177019	122177012	T	C	snp	0	1	1	1	.	.	.	INTERGENIC(INTERGENIC)
X	122202971	122202979	122202977	A	G	snp	0	1	1	1	.	.	.	INTERGENIC(INTERGENIC)
X	122332489	122332497	122332496	A	C	snp	1	1	1	1	.	.	.	GRIA3(INTRON)
X	122579766	122579773	122579772	T	C	snp	1	1	1	1	.	.	.	GRIA3(INTRON)
X	122643079	122643090	122643088	TG	TA	ins	0	0	1	0	.	.	.	INTERGENIC(INTERGENIC)
X	122680553	122680560	122680556	T	C	snp	1	1	1	1	.	.	.	INTERGENIC(INTERGENIC)
X	122846844	122846852	122846850	A	C	snp	1	0	0	0	.	.	.	THOC2(INTRON);THOC2(UPSTREAM)
X	122904269	122904280	122904278	AG	A	snp	1	1	1	1	.	.	.	INTERGENIC(INTERGENIC);MEMO1P4(DOWNSTREAM);RNU7-69P(DOWNSTREAM)
X	123021842	123021853	123021846	AAT	TAATAAT	del	1	1	1	1	.	.	.	AL121601.1(UPSTREAM);XIAP(DOWNSTREAM);XIAP(INTRON)
X	123021842	123021853	123021849	AAT	TAAT	del	1	1	1	1	.	.	.	AL121601.1(UPSTREAM);XIAP(DOWNSTREAM);XIAP(INTRON)
X	123038641	123038648	123038647	T	G	snp	1	1	1	1	.	.	.	XIAP(DOWNSTREAM);XIAP(INTRON)

X	123092106	123092113	123092108	A	G	snp	1	0	0	0	.	.	.	INTERGENIC(INTERGENIC);RP1-315G1.3.1(DOWNSTREAM);STAG2(UPSTREAM)
X	123206724	123206733	123206730	T	C	snp	1	0	1	0	.	.	.	STAG2(INTRON)
X	123220007	123220016	123220012	A	C	ins	0	0	1	0	.	.	.	STAG2(INTRON)
X	123332586	123332594	123332593	A	C	snp	1	1	1	1	.	.	.	SNORA40(DOWNSTREAM);STAG2(INTRON)
X	123333282	123333293	123333285	AG	C	snp	1	0	0	0	.	.	.	SNORA40(DOWNSTREAM);STAG2(INTRON)
X	123432186	123432194	123432192	A	C	snp	1	1	1	1	.	.	.	STAG2(INTRON)
X	123435999	123436014	123436001	TA	ATATATATATATA	del	1	1	1	1	.	.	.	. STAG2(INTRON)
X	123451819	123451828	123451827	A	G	snp	1	1	1	1	.	.	.	STAG2(INTRON)
X	123484836	123484845	123484843	TA	T	ins	1	1	1	1	.	.	.	SH2D1A(INTRON);STAG2(INTRON)
X	123527254	123527262	123527261	T	G	snp	1	1	1	1	.	.	.	ODZ1(INTRON);STAG2(INTRON)
X	123636141	123636148	123636147	C	A	snp	0	1	0	0	.	.	.	ODZ1(INTRON)
X	123728177	123728184	123728181	A	C	snp	1	1	1	1	.	.	.	ODZ1(INTRON)
X	123829653	123829662	123829661	T	G	snp	1	1	1	1	.	.	.	ODZ1(INTRON)
X	124049979	124049986	124049985	A	AC	ins	0	1	0	0	.	.	.	ODZ1(INTRON)
X	124125376	124125387	124125385	AG	T	snp	1	1	1	1	.	.	.	INTERGENIC(INTERGENIC)
X	124162536	124162543	124162541	A	C	snp	1	0	0	0	.	.	.	INTERGENIC(INTERGENIC)
X	124386270	124386281	124386277	AAC	G	snp	1	0	0	0	.	.	.	INTERGENIC(INTERGENIC)
X	124401510	124401521	124401519	AT	A	del	1	1	0	0	.	.	.	INTERGENIC(INTERGENIC)
X	124592262	124592277	124592274	AC	G	snp	1	0	0	0	.	.	.	INTERGENIC(INTERGENIC)
X	124677637	124677656	124677646	AAAT	C	snp	1	1	1	1	.	.	.	INTERGENIC(INTERGENIC)
X	124700774	124700797	124700793	AAAC	A	snp	1	0	0	0	.	.	.	INTERGENIC(INTERGENIC)
X	124769346	124769353	124769347	G	T	snp	1	1	1	1	.	.	.	INTERGENIC(INTERGENIC)
X	124867933	124867940	124867934	T	C	snp	1	1	0	1	.	.	.	INTERGENIC(INTERGENIC)
X	124883320	124883329	124883321	A	G	snp	1	0	0	0	.	.	.	INTERGENIC(INTERGENIC)
X	124943447	124943462	124943457	AT	C	snp	1	1	1	1	.	.	.	INTERGENIC(INTERGENIC)
X	125042468	125042485	125042483	AG	A	snp	1	1	1	1	.	.	.	INTERGENIC(INTERGENIC)
X	125072386	125072394	125072387	A	G	snp	1	0	0	0	.	.	.	INTERGENIC(INTERGENIC)
X	125169331	125169344	125169335	TA	A	snp	1	0	0	0	.	.	.	INTERGENIC(INTERGENIC)
X	125185430	125185441	125185438	TTA	G	snp	1	0	0	0	.	.	.	INTERGENIC(INTERGENIC)
X	125244332	125244341	125244333	A	G	snp	1	0	1	1	.	.	.	RP11-13E5.2.1(INTRON)
X	125257022	125257030	125257029	T	TG	ins	1	1	1	0	.	.	.	INTERGENIC(INTERGENIC)
X	125325378	125325389	125325384	AAT	G	snp	1	0	0	0	.	.	.	INTERGENIC(INTERGENIC)
X	125427353	125427362	125427360	AT	A	snp	1	1	0	1	.	.	.	INTERGENIC(INTERGENIC)
X	125548230	125548239	125548237	A	AT	ins	1	1	1	1	.	.	.	INTERGENIC(INTERGENIC)
X	125599647	125599656	125599655	A	G	snp	1	0	0	0	.	.	.	INTERGENIC(INTERGENIC)
X	125638586	125638595	125638589	T	G	snp	1	0	0	0	.	.	.	INTERGENIC(INTERGENIC)
X	125683149	125683157	125683156	A	G	snp	1	0	0	0	.	.	.	DCAF12L1(DOWNSTREAM);INTERGENIC(INTERGENIC)
X	125765004	125765017	125765011	TG	A	snp	1	1	1	1	.	.	.	INTERGENIC(INTERGENIC)
X	125932457	125932468	125932465	AAC	A	snp	1	1	0	1	.	.	.	INTERGENIC(INTERGENIC)
X	126061973	126061982	126061980	T	G	snp	1	0	0	0	.	.	.	INTERGENIC(INTERGENIC)
X	126079087	126079096	126079093	AT	C	snp	0	1	0	1	.	.	.	INTERGENIC(INTERGENIC)
X	126098316	126098324	126098323	T	C	snp	1	0	0	0	.	.	.	INTERGENIC(INTERGENIC)
X	126195436	126195443	126195440	A	G	snp	1	1	1	1	.	.	.	INTERGENIC(INTERGENIC)
X	126268416	126268424	126268418	T	C	ins	1	1	1	1	.	.	.	INTERGENIC(INTERGENIC)
X	126369864	126369871	126369867	T	C	snp	0	0	0	1	.	.	.	INTERGENIC(INTERGENIC)
X	126611112	126611120	126611118	A	C	snp	1	1	0	1	.	.	.	INTERGENIC(INTERGENIC)
X	126861488	126861497	126861493	TA	T	snp	1	1	0	1	.	.	.	INTERGENIC(INTERGENIC);RP1-107N3.1.1(DOWNSTREAM)
X	126895578	126895587	126895581	AC	A	snp	1	1	0	1	.	.	.	INTERGENIC(INTERGENIC)
X	126903309	126903318	126903314	AC	T	snp	1	1	1	1	.	.	.	INTERGENIC(INTERGENIC)
X	126982144	126982152	126982145	T	C	snp	1	1	0	1	.	.	.	INTERGENIC(INTERGENIC)
X	127042196	127042207	127042205	AT	AC	ins	1	1	0	1	.	.	.	INTERGENIC(INTERGENIC)
X	127133096	127133105	127133103	AG	T	snp	1	1	1	1	.	.	.	INTERGENIC(INTERGENIC)
X	127239989	127239997	127239994	T	C	snp	1	1	0	1	.	.	.	INTERGENIC(INTERGENIC)
X	127278125	127278132	127278127	A	G	snp	1	1	1	1	.	.	.	INTERGENIC(INTERGENIC)
X	127400122	127400135	127400130	AT	G	snp	1	0	0	0	.	.	.	INTERGENIC(INTERGENIC)
X	127671554	127671565	127671557	TGA	G	snp	1	0	0	0	.	.	.	RP1-30E17.2.1(INTRON)
X	127883173	127883184	127883178	AT	C	snp	0	0	1	0	.	.	.	INTERGENIC(INTERGENIC)
X	127890700	127890711	127890706	TA	C	snp	0	0	0	1	.	.	.	INTERGENIC(INTERGENIC)
X	127998257	127998264	127998261	A	C	snp	1	1	1	1	.	.	.	INTERGENIC(INTERGENIC)
X	128113550	128113563	128113560	TA	G	snp	1	0	0	0	.	.	.	INTERGENIC(INTERGENIC)
X	128300519	128300527	128300523	A	G	snp	1	0	0	0	.	.	.	INTERGENIC(INTERGENIC)
X	128518275	128518284	128518279	TA	A	snp	0	0	1	0	.	.	.	INTERGENIC(INTERGENIC)
X	128798444	128798451	128798446	T	C	ins	1	0	1	0	.	.	.	INTERGENIC(INTERGENIC)
X	128833234	128833241	128833238	C	A	snp	0	0	0	1	.	.	.	INTERGENIC(INTERGENIC)
X	128875149	128875157	128875151	G	GT	ins	1	0	1	1	.	.	.	XPNPEP2(INTRON)
X	129002154	129002165	129002160	TGA	C	snp	1	1	1	0	.	.	.	

														INTERGENIC(INTERGENIC);RP4-537K23.4.1(DOWNSTREAM);SALL4P2(UPSTREAM)
X	129272440	129272448	129272441	A	G	snp	1	0	0	0	.	.	.	AIFM1(INTRON);AIFM1(UPSTREAM)
X	129441753	129441760	129441756	A	G	snp	1	0	0	0	.	.	.	INTERGENIC(INTERGENIC);U6(DOWNSTREAM)
X	129465505	129465520	129465512	TTTG	TT	del	1	0	0	0	.	.	.	INTERGENIC(INTERGENIC);RP1-20I3.3.1(DOWNSTREAM)
X	129496247	129496261	129496251	ATT	A	snp	0	1	1	0	.	.	.	SLC25A14(DOWNSTREAM);SLC25A14(INTRON);SLC25A14(UPSTREAM)
X	129636512	129636529	129636525	TC	G	snp	1	0	0	0	.	.	.	RP1-274L7.1.1(INTRON)
X	129649094	129649102	129649095	A	C	snp	1	0	0	0	.	.	.	RP1-274L7.1.1(INTRON)
X	129667067	129667075	129667069	A	C	snp	1	0	0	0	.	.	.	INTERGENIC(INTERGENIC)
X	130264490	130264497	130264496	T	G	snp	1	0	0	0	.	.	.	AL590131.1(DOWNSTREAM);INTERGENIC(INTERGENIC)
X	130433074	130433085	130433082	AG	G	snp	1	1	1	1	.	.	.	IGSF1(INTRON)
X	130492036	130492045	130492041	TA	C	snp	1	0	0	0	.	.	.	IGSF1(INTRON)
X	130492066	130492077	130492071	TA	TC	ins	1	1	1	1	.	.	.	IGSF1(INTRON)
X	130586107	130586116	130586110	GC	T	snp	1	0	0	0	.	.	.	INTERGENIC(INTERGENIC)
X	130600155	130600163	130600158	A	C	snp	1	0	0	0	.	.	.	INTERGENIC(INTERGENIC)
X	130744928	130744937	130744932	TG	C	snp	1	1	0	0	.	.	.	INTERGENIC(INTERGENIC)
X	130761516	130761525	130761524	A	G	snp	1	1	1	1	.	.	.	INTERGENIC(INTERGENIC)
X	130768914	130768922	130768916	A	G	snp	1	0	0	0	.	.	.	INTERGENIC(INTERGENIC)
X	130995844	130995859	130995847	AC	T	snp	1	1	1	1	.	.	.	INTERGENIC(INTERGENIC)
X	131019886	131019893	131019889	T	C	snp	1	1	1	1	.	.	.	INTERGENIC(INTERGENIC)
X	131086113	131086126	131086121	TA	G	snp	1	1	1	1	.	.	.	INTERGENIC(INTERGENIC)
X	131086113	131086126	131086123	TA	G	snp	1	1	1	1	.	.	.	INTERGENIC(INTERGENIC)
X	131138150	131138159	131138157	TA	G	snp	1	1	1	0	.	.	.	INTERGENIC(INTERGENIC)
X	131178752	131178763	131178760	TA	AG	ins	1	1	0	1	.	.	.	MST4.1(INTRON)
X	131182995	131183010	131183006	TTTG	TTTGT	del	1	0	0	1	.	.	.	MST4.1(INTRON)
X	131320929	131320936	131320935	T	C	ins	1	0	0	1	.	.	.	INTERGENIC(INTERGENIC)
X	131370867	131370874	131370870	A	G	snp	1	0	0	0	.	.	.	RP5-842K24.2.1(INTRON)
X	131389534	131389549	131389542	CTTT	TTTCT	del	1	1	1	1	.	.	.	RP5-842K24.2.1(INTRON)
X	131389534	131389549	131389543	CTTT	TTC	del	1	1	1	1	.	.	.	RP5-842K24.2.1(INTRON)
X	131726463	131726472	131726470	T	G	ins	1	1	1	1	.	.	.	INTERGENIC(INTERGENIC)
X	131726463	131726472	131726470	T	G	snp	1	1	1	1	.	.	.	INTERGENIC(INTERGENIC)
X	131813781	131813789	131813782	G	T	snp	1	0	0	0	.	.	.	HS6ST2(INTRON)
X	131899447	131899462	131899454	TG	TA	ins	1	1	1	1	.	.	.	HS6ST2(INTRON)
X	132064144	132064153	132064150	T	C	snp	1	1	1	1	.	.	.	HS6ST2(INTRON)
X	132107937	132107945	132107941	T	C	snp	1	1	1	1	.	.	.	INTERGENIC(INTERGENIC)
X	132217058	132217067	132217060	TA	C	snp	1	0	1	0	.	.	.	USP26(INTRON)
X	132323820	132323827	132323825	T	C	snp	1	0	0	0	.	.	.	INTERGENIC(INTERGENIC)
X	132381494	132381501	132381500	T	G	ins	1	0	0	0	.	.	.	INTERGENIC(INTERGENIC)
X	132435034	132435043	132435036	A	G	snp	0	0	0	1	.	.	.	GPC4(DOWNSTREAM);GPC4(UTR_3_PRIME)
X	132644359	132644378	132644370	TTCC	T	snp	1	1	1	1	.	.	.	INTERGENIC(INTERGENIC)
X	132722745	132722764	132722752	AAAT	A	snp	1	0	0	0	.	.	.	GPC3(INTRON)
X	132989558	132989575	132989567	AT	G	snp	1	0	0	0	.	.	.	GPC3(INTRON)
X	133287135	133287158	133287154	TTTC	TTTCTTT	del	1	0	1	1	.	.	.	INTERGENIC(INTERGENIC)
X	133345554	133345562	133345555	A	G	ins	1	1	1	1	.	.	.	INTERGENIC(INTERGENIC)
X	133383415	133383424	133383423	T	G	snp	1	1	0	1	.	.	.	CCDC160(DOWNSTREAM);INTERGENIC(INTERGENIC)
X	133479413	133479422	133479418	T	G	snp	1	1	1	1	.	.	.	INTERGENIC(INTERGENIC)
X	133578400	133578413	133578403	TA	C	snp	1	1	0	1	.	.	.	INTERGENIC(INTERGENIC)
X	133580254	133580265	133580262	AT	TT	ins	1	0	0	0	.	.	.	INTERGENIC(INTERGENIC)
X	133580254	133580265	133580263	AT	A	del	1	1	1	1	.	.	.	INTERGENIC(INTERGENIC)
X	133612302	133612310	133612303	T	G	snp	1	0	0	0	.	.	.	HPRT1(INTRON)
X	133637092	133637101	133637099	T	G	ins	1	0	0	0	.	.	.	HPRT1(DOWNSTREAM);HPRT1(INTRON)
X	133638921	133638936	133638932	TTTC	T	ins	1	0	0	0	.	.	.	HPRT1(DOWNSTREAM);HPRT1(INTRON)
X	133654568	133654587	133654583	AAAG	AA	ins	1	1	0	0	.	.	.	AC004383.3.1(UPSTREAM);HPRT1(DOWNSTREAM);INTERGENIC(INTERGENIC)
X	133658067	133658090	133658085	AAAT	C	snp	1	1	1	1	.	.	.	AC004383.1(DOWNSTREAM);AC004383.3.1(DOWNSTREAM);HPRT1(DOWNSTREAM);INTERGENIC(INTERGENIC)
X	133662014	133662022	133662020	T	C	snp	1	1	1	1	.	.	.	AC004383.1(UPSTREAM);AC004383.3.1(DOWNSTREAM);INTERGENIC(INTERGENIC)
X	133688371	133688390	133688386	CAAA	C	del	1	1	0	0	.	.	.	AC004383.5.1(INTRON)
X	133694525	133694542	133694529	GT	TA	ins	1	1	1	1	.	.	.	AC004383.5.1(DOWNSTREAM);INTERGENIC(INTERGENIC)
X	133709475	133709483	133709480	A	C	snp	1	1	0	0	.	.	.	PLAC1(INTRON)
X	133724885	133724892	133724890	C	T	snp	1	0	0	0	.	.	.	PLAC1(INTRON)
X	133750688	133750696	133750689	A	C	snp	1	1	0	0	.	.	.	PLAC1(INTRON)
X	133854682	133854689	133854683	A	G	snp	0	0	0	1	.	.	.	PLAC1(INTRON)
X	134019187	134019196	134019190	GC	T	snp	1	1	0	1	.	.	.	INTERGENIC(INTERGENIC);MOSPD1(DOWNSTREAM)
X	134031604	134031611	134031607	A	AG	ins	1	1	1	1	.	.	.	MOSPD1(DOWNSTREAM);MOSPD1(INTRON);Z83826.1(DOWNSTREAM)

X	134072764	134072779	134072770	AAAT	G	snp	1	1	1	1	.	.	.	INTERGENIC(INTERGENIC)
X	134086027	134086034	134086030	G	A	snp	1	1	1	1	.	.	.	RP3-473B4.3.1(INTRON)
X	134120656	134120671	134120660	ATTT	TTTATTTAT	del	1	1	1	1	.	.	.	CXorf69(UPSTREAM);INTERGENIC(INTERGENIC)
X	134120656	134120671	134120663	ATTT	ATTTA	del	1	1	1	1	.	.	.	CXorf69(UPSTREAM);INTERGENIC(INTERGENIC)
X	134476991	134477011	134477006	ACA	G	snp	1	0	0	0	.	.	ZNF75D(START_GAINED)	ZNF449(UPSTREAM);ZNF75D(INTRON)
X	134792853	134792868	134792861	TTTG	C	snp	1	0	0	0	.	.	.	INTERGENIC(INTERGENIC)
X	134839177	134839188	134839184	ATA	AATAA	del	0	1	1	1	.	.	.	INTERGENIC(INTERGENIC)
X	135049992	135050001	135049999	T	G	snp	1	0	0	0	.	.	.	MMGT1(INTRON)
X	135070589	135070608	135070597	TTTC	C	snp	0	0	1	0	.	.	.	SLC9A6(INTRON)
X	135134715	135134724	135134722	T	G	snp	1	1	0	1	.	.	.	INTERGENIC(INTERGENIC)
X	135163732	135163740	135163736	T	C	snp	1	1	1	1	.	.	.	GAPDHP67(DOWNSTREAM);INTERGENIC(INTERGENIC)
X	135163732	135163740	135163737	T	C	snp	1	1	1	1	.	.	.	GAPDHP67(DOWNSTREAM);INTERGENIC(INTERGENIC)
X	135195536	135195543	135195542	A	G	snp	1	1	1	1	.	.	.	INTERGENIC(INTERGENIC)
X	135204773	135204790	135204776	TG	T	snp	1	1	1	1	.	.	.	INTERGENIC(INTERGENIC)
X	135207404	135207417	135207413	GT	C	snp	1	0	0	0	.	.	.	INTERGENIC(INTERGENIC)
X	135215278	135215289	135215282	TTG	C	snp	1	0	0	0	.	.	.	INTERGENIC(INTERGENIC);snoU13(DOWNSTREAM)
X	135265284	135265293	135265287	A	C	snp	1	1	1	1	.	.	.	FHL1(INTRON)
X	135382746	135382755	135382749	T	C	snp	1	0	0	0	.	.	.	GPR112(UPSTREAM);INTERGENIC(INTERGENIC);RP11-535K18.2.1(UPSTREAM)
X	135651183	135651191	135651187	T	C	snp	1	1	1	1	.	.	.	INTERGENIC(INTERGENIC);Y_RNA(DOWNSTREAM)
X	135721658	135721675	135721661	AT	A	snp	1	1	0	0	.	.	.	INTERGENIC(INTERGENIC);RP3-527F8.2.1(UPSTREAM)
X	135809555	135809572	135809570	TC	T	snp	1	1	1	1	.	.	.	ARHGEF6(INTRON)
X	135993722	135993729	135993728	T	C	snp	0	1	1	1	.	.	.	RP11-308D16.4.1(INTRON)
X	136058241	136058250	136058248	A	C	snp	1	1	1	1	.	.	.	RP11-308D16.2.1(INTRON);RP11-308D16.4.1(INTRON)
X	136180830	136180841	136180833	TC	T	snp	1	1	0	0	.	.	.	INTERGENIC(INTERGENIC)
X	136307175	136307184	136307178	TA	G	snp	1	1	1	1	.	.	.	INTERGENIC(INTERGENIC)
X	136367016	136367033	136367018	GA	A	snp	1	0	0	0	.	.	.	INTERGENIC(INTERGENIC)
X	136546074	136546081	136546076	C	CCG	ins	1	0	0	0	.	.	.	INTERGENIC(INTERGENIC)
X	136546074	136546081	136546079	C	T	ins	1	1	1	1	.	.	.	INTERGENIC(INTERGENIC)
X	136568123	136568131	136568124	A	G	snp	1	0	0	0	.	.	.	INTERGENIC(INTERGENIC)
X	136577865	136577874	136577872	TC	TCT	del	1	1	1	1	.	.	.	INTERGENIC(INTERGENIC)
X	136673421	136673430	136673428	TC	T	ins	1	1	1	1	.	.	.	AL035443.1(UPSTREAM);INTERGENIC(INTERGENIC)
X	136691534	136691545	136691542	AAC	T	snp	1	0	0	0	.	.	.	INTERGENIC(INTERGENIC)
X	136707288	136707299	136707297	TG	A	snp	1	0	0	0	.	.	.	INTERGENIC(INTERGENIC)
X	136714875	136714888	136714884	TA	TC	ins	1	1	1	1	.	.	.	INTERGENIC(INTERGENIC)
X	136752294	136752309	136752300	AAGA	A	snp	1	0	0	0	.	.	.	INTERGENIC(INTERGENIC)
X	137125768	137125775	137125774	A	G	snp	1	1	1	1	.	.	.	INTERGENIC(INTERGENIC)
X	137133759	137133770	137133767	AG	G	del	0	1	1	0	.	.	.	INTERGENIC(INTERGENIC)
X	137241485	137241493	137241487	T	G	snp	1	0	0	0	.	.	.	INTERGENIC(INTERGENIC)
X	137264844	137264855	137264852	TTC	T	ins	1	1	0	1	.	.	.	INTERGENIC(INTERGENIC)
X	137265267	137265280	137265276	AG	C	snp	1	1	1	1	.	.	.	INTERGENIC(INTERGENIC)
X	137474116	137474131	137474127	AAAC	A	del	1	1	0	1	.	.	.	INTERGENIC(INTERGENIC)
X	137633983	137633991	137633987	A	C	ins	1	0	0	0	.	.	.	INTERGENIC(INTERGENIC)
X	137753195	137753206	137753202	CCA	ACT	ins	1	1	1	1	.	.	.	FGF13(INTRON);MIR504(UPSTREAM)
X	137959391	137959400	137959397	T	C	snp	1	0	0	0	.	.	.	FGF13(INTRON)
X	138004169	138004176	138004170	A	G	snp	1	0	0	0	.	.	.	FGF13(INTRON)
X	138023945	138023952	138023946	A	G	snp	1	1	1	1	.	.	.	FGF13(INTRON)
X	138025011	138025018	138025015	T	G	snp	1	0	0	0	.	.	.	FGF13(INTRON)
X	138106232	138106240	138106237	T	C	snp	1	0	0	0	.	.	.	FGF13(INTRON)
X	138130190	138130197	138130196	T	C	snp	1	0	0	0	.	.	.	FGF13(INTRON)
X	138285670	138285679	138285675	G	A	snp	1	0	0	0	.	.	.	FGF13(INTRON);FGF13(UTR_5_PRIME)
X	138288180	138288207	138288197	AAAG	G	snp	1	0	0	0	.	.	.	FGF13(INTRON);FGF13(UPSTREAM)
X	138325568	138325575	138325571	A	C	snp	1	0	0	0	.	.	.	INTERGENIC(INTERGENIC)
X	138352436	138352447	138352441	ATA	C	snp	1	0	0	0	.	.	.	INTERGENIC(INTERGENIC)
X	138370296	138370305	138370300	AT	C	snp	1	1	1	1	.	.	.	INTERGENIC(INTERGENIC)
X	138370296	138370305	138370302	AT	C	snp	1	1	1	1	.	.	.	INTERGENIC(INTERGENIC)
X	138476671	138476680	138476676	TG	TA	ins	1	1	1	1	.	.	.	INTERGENIC(INTERGENIC)
X	138595251	138595260	138595253	A	C	snp	1	1	1	1	.	.	.	INTERGENIC(INTERGENIC)
X	138722100	138722109	138722107	TA	TAT	del	1	1	1	1	.	.	.	MCF2(INTRON)
X	138748119	138748128	138748121	TA	G	snp	1	0	0	0	.	.	.	MCF2(INTRON)
X	138904559	138904582	138904578	TTTG	TTTGT	del	1	1	1	0	.	.	.	ATP11C(INTRON)
X	138904583	138904590	138904586	T	G	snp	0	1	0	0	.	.	.	ATP11C(INTRON)
X	138978007	138978014	138978009	T	C	snp	0	0	0	1	.	.	.	ATP11C(INTRON)
X	138991280	138991287	138991281	T	C	snp	0	1	1	0	.	.	.	ATP11C(INTRON);SNORA18(UPSTREAM)
X	139058409	139058426	139058412	CAA	T	snp	1	0	0	0	.	.	.	INTERGENIC(INTERGENIC)
X	139068973	139068993	139068986	CTT	TCTTC	del	1	1	1	1	.	.	.	INTERGENIC(INTERGENIC)
X	139068973	139068993	139068987	CTT	CTTC	del	1	1	1	1	.	.	.	INTERGENIC(INTERGENIC)

X	139068973	139068993	139068989	CTT	TC	del	1	1	1	1	.	.	.	INTERGENIC(INTERGENIC)
X	139068973	139068993	139068990	CTT	C	del	1	1	1	1	.	.	.	INTERGENIC(INTERGENIC)
X	139077493	139077500	139077496	T	TTTTGTTTTG	ins	1	1	1	1	.	.	.	INTERGENIC(INTERGENIC)
X	139127671	139127680	139127676	TG	A	snp	1	1	1	1	.	.	.	INTERGENIC(INTERGENIC)
X	139172505	139172514	139172511	T	G	snp	1	0	0	0	.	.	.	AL589987.1(DOWNSTREAM);INTERGENIC(INTERGENIC);RP11-364B14.3.1(UPSTREAM)
X	139197142	139197150	139197146	T	G	snp	1	0	0	0	.	.	.	INTERGENIC(INTERGENIC)
X	139447701	139447710	139447704	AC	T	snp	1	0	0	0	.	.	.	INTERGENIC(INTERGENIC)
X	139549422	139549429	139549425	A	C	snp	1	1	0	0	.	.	.	INTERGENIC(INTERGENIC);RPS17P17(DOWNSTREAM)
X	139597287	139597310	139597304	TTTA	TATTTA	del	1	1	1	1	.	.	.	INTERGENIC(INTERGENIC)
X	139623981	139623996	139623994	TA	TG	ins	1	1	1	1	.	.	.	INTERGENIC(INTERGENIC)
X	139701497	139701505	139701503	T	G	snp	1	1	0	1	.	.	.	INTERGENIC(INTERGENIC)
X	139710043	139710058	139710049	ATTT	G	snp	1	1	0	1	.	.	.	INTERGENIC(INTERGENIC)
X	139818590	139818601	139818598	TTC	TTCT	del	0	1	1	1	.	.	.	RP1-177G6.2.1(INTRON)
X	139867338	139867349	139867344	AAT	ATAAT	ins	1	1	0	1	.	.	.	CDR1(UPSTREAM);INTERGENIC(INTERGENIC)
X	139895903	139895912	139895909	T	C	snp	1	1	1	1	.	.	.	INTERGENIC(INTERGENIC)
X	140218321	140218329	140218322	A	AC	ins	1	0	0	0	.	.	.	INTERGENIC(INTERGENIC)
X	140289754	140289769	140289759	TA	C	snp	1	0	0	0	.	.	.	INTERGENIC(INTERGENIC)
X	140303708	140303716	140303712	A	C	ins	1	0	0	0	.	.	.	INTERGENIC(INTERGENIC)
X	140303708	140303716	140303712	A	C	snp	1	0	0	0	.	.	.	INTERGENIC(INTERGENIC)
X	140345799	140345807	140345806	T	G	snp	1	1	1	1	.	.	.	INTERGENIC(INTERGENIC)
X	140355351	140355360	140355357	A	G	snp	1	1	1	1	.	.	.	INTERGENIC(INTERGENIC);RBMX2P2(DOWNSTREAM)
X	140359327	140359338	140359335	ATT	A	del	0	0	1	0	.	.	.	INTERGENIC(INTERGENIC);RBMX2P2(UPSTREAM)
X	140843677	140843684	140843680	T	G	snp	1	0	0	0	.	.	.	INTERGENIC(INTERGENIC)
X	140847554	140847571	140847566	AC	TT	ins	1	1	1	0	.	.	.	INTERGENIC(INTERGENIC)
X	140879396	140879405	140879403	TA	G	snp	1	1	1	1	.	.	.	INTERGENIC(INTERGENIC)
X	140946552	140946561	140946555	TA	G	snp	1	0	0	0	.	.	.	MAGEC3(INTRON)
X	140993906	140993917	140993912	CCT	T	snp	1	1	1	1	.	"MAGEC1(NON_SYNONYMOUS_CODING):S241F,S42F,S43F"	.	MAGEC1(INTRON)
X	141035302	141035329	141035324	AATA	A	del	1	0	0	0	.	.	.	INTERGENIC(INTERGENIC)
X	141109501	141109514	141109509	CT	G	snp	0	1	1	0	.	.	.	INTERGENIC(INTERGENIC)
X	141206830	141206847	141206833	AT	C	snp	1	0	0	0	.	.	.	INTERGENIC(INTERGENIC)
X	141219927	141219935	141219934	T	G	snp	1	1	1	1	.	.	.	INTERGENIC(INTERGENIC)
X	141378596	141378607	141378604	AAC	T	snp	1	1	0	1	.	.	.	INTERGENIC(INTERGENIC)
X	141381233	141381241	141381234	G	T	snp	1	1	1	1	.	.	.	INTERGENIC(INTERGENIC)
X	141482708	141482715	141482713	T	C	snp	1	0	0	0	.	.	.	INTERGENIC(INTERGENIC)
X	141518493	141518508	141518499	TCTA	C	snp	1	1	1	1	.	.	.	INTERGENIC(INTERGENIC)
X	141529318	141529333	141529327	TCTT	TT	del	1	1	1	1	.	.	.	INTERGENIC(INTERGENIC)
X	141529728	141529737	141529731	TC	T	snp	1	1	1	1	.	.	.	INTERGENIC(INTERGENIC)
X	141529728	141529737	141529735	TC	T	snp	1	1	1	1	.	.	.	INTERGENIC(INTERGENIC)
X	141530023	141530032	141530030	TC	T	snp	1	0	0	0	.	.	.	INTERGENIC(INTERGENIC)
X	141547848	141547855	141547850	A	G	snp	1	1	0	0	.	.	.	INTERGENIC(INTERGENIC)
X	141657020	141657031	141657023	CT	C	snp	1	1	1	1	.	.	.	INTERGENIC(INTERGENIC)
X	141657120	141657129	141657126	CT	T	snp	1	1	1	1	.	.	.	INTERGENIC(INTERGENIC)
X	141719322	141719333	141719330	TA	G	snp	1	0	0	0	.	.	.	INTERGENIC(INTERGENIC)
X	141720661	141720670	141720665	G	T	snp	1	1	1	1	.	.	.	INTERGENIC(INTERGENIC)
X	141778312	141778327	141778323	AT	C	snp	1	0	0	0	.	.	.	INTERGENIC(INTERGENIC)
X	141791586	141791594	141791591	T	G	snp	1	1	1	1	.	.	.	INTERGENIC(INTERGENIC)
X	141801623	141801631	141801627	A	AG	ins	1	1	1	1	.	.	.	INTERGENIC(INTERGENIC)
X	141861945	141861953	141861946	T	G	snp	1	1	1	1	.	.	.	INTERGENIC(INTERGENIC)
X	141886214	141886222	141886216	A	C	ins	1	0	0	0	.	.	.	INTERGENIC(INTERGENIC)
X	141907699	141907707	141907704	C	A	snp	1	1	0	0	.	.	.	INTERGENIC(INTERGENIC)
X	141976828	141976839	141976832	AT	T	snp	1	1	1	1	.	.	.	INTERGENIC(INTERGENIC)
X	141977400	141977409	141977407	TA	G	snp	1	1	0	0	.	.	.	INTERGENIC(INTERGENIC)
X	141983015	141983023	141983016	T	G	snp	1	0	0	0	.	.	.	INTERGENIC(INTERGENIC)
X	142067209	142067220	142067212	AT	G	snp	1	1	0	1	.	.	.	INTERGENIC(INTERGENIC)
X	142067209	142067220	142067216	AT	ATA	del	1	1	0	1	.	.	.	INTERGENIC(INTERGENIC)
X	142067209	142067220	142067217	AT	T	del	1	1	0	1	.	.	.	INTERGENIC(INTERGENIC)
X	142067209	142067220	142067218	AT	A	del	1	1	0	1	.	.	.	INTERGENIC(INTERGENIC)
X	142137396	142137405	142137401	T	G	snp	1	0	0	0	.	.	.	INTERGENIC(INTERGENIC)
X	142206136	142206149	142206138	TG	A	snp	1	0	0	0	.	.	.	INTERGENIC(INTERGENIC)
X	142299917	142299932	142299926	TTTA	TA	del	1	1	1	1	.	.	.	INTERGENIC(INTERGENIC)
X	142299917	142299932	142299927	TTTA	A	del	1	1	1	1	.	.	.	INTERGENIC(INTERGENIC)
X	142324526	142324535	142324529	TG	A	snp	1	0	0	0	.	.	.	INTERGENIC(INTERGENIC)
X	142429789	142429796	142429791	T	G	snp	1	0	0	0	.	.	.	GS1-256O22.5.1(INTRON);RP1-231L4.2.1(UPSTREAM)
X	142535746	142535754	142535751	A	C	snp	1	0	0	0	.	.	.	AL030997.1(UPSTREAM);GS1-256O22.5.1(INTRON)
X	142554936	142554945	142554943	A	C	snp	1	1	1	1	.	.	.	GS1-256O22.5.1(INTRON)

X	142603940	142603951	142603942	TC	C	snp	1	1	1	1	.	.	.	GS1-256O22.5.1(INTRON);SPANXN3(INTRON)
X	142603940	142603951	142603949	TC	T	snp	1	1	1	1	.	.	.	GS1-256O22.5.1(INTRON);SPANXN3(INTRON)
X	142607261	142607268	142607267	T	C	snp	1	1	0	1	.	.	.	GS1-256O22.5.1(DOWNSTREAM);INTERGENIC(INTERGENIC);SPANXN3(UPSTREAM)
X	142628676	142628685	142628678	A	C	snp	1	1	1	1	.	.	.	GS1-256O22.1.1(INTRON)
X	142689983	142689994	142689990	AC	A	snp	1	1	1	1	.	.	.	INTERGENIC(INTERGENIC)
X	142701683	142701692	142701688	A	G	snp	1	1	1	1	.	.	.	INTERGENIC(INTERGENIC)
X	142802212	142802219	142802217	C	A	ins	1	0	0	0	.	.	.	SPANXN2(INTRON)
X	142872866	142872881	142872872	AGAC	G	snp	1	1	1	1	.	.	.	RP3-526F5.2.1(INTRON)
X	142897496	142897513	142897507	AT	C	snp	1	0	0	0	.	.	.	RP3-526F5.2.1(INTRON)
X	142905334	142905343	142905338	A	C	snp	1	0	0	0	.	.	.	RP3-526F5.2.1(INTRON)
X	143002866	143002880	143002875	GGA	C	snp	1	0	0	0	.	.	.	INTERGENIC(INTERGENIC)
X	143040666	143040674	143040667	T	G	snp	1	0	0	0	.	.	.	INTERGENIC(INTERGENIC)
X	143206593	143206604	143206602	AT	C	snp	1	0	0	0	.	.	.	INTERGENIC(INTERGENIC)
X	143245948	143245965	143245959	AAC	A	ins	1	1	1	1	.	.	.	INTERGENIC(INTERGENIC)
X	143263545	143263560	143263548	TG	A	snp	1	1	1	1	.	.	.	INTERGENIC(INTERGENIC)
X	143347795	143347804	143347796	A	G	ins	1	1	1	1	.	.	.	INTERGENIC(INTERGENIC)
X	143388408	143388417	143388413	AG	C	snp	1	1	0	1	.	.	.	INTERGENIC(INTERGENIC);RP1-169P22.2.1(DOWNSTREAM);RRM2P4(UPSTREAM)
X	143456781	143456792	143456786	AG	A	snp	0	0	1	0	.	.	.	INTERGENIC(INTERGENIC)
X	143497115	143497124	143497121	CA	T	snp	1	0	0	0	.	.	.	INTERGENIC(INTERGENIC)
X	143725278	143725287	143725279	A	G	snp	1	1	1	1	.	.	.	INTERGENIC(INTERGENIC)
X	143794104	143794119	143794107	AG	C	snp	1	0	0	0	.	.	.	INTERGENIC(INTERGENIC)
X	143821787	143821798	143821795	AG	C	snp	1	0	0	0	.	.	.	INTERGENIC(INTERGENIC)
X	143857513	143857527	143857521	GTT	G	del	1	1	0	1	.	.	.	INTERGENIC(INTERGENIC)
X	143857513	143857527	143857523	GTT	TG	del	1	1	1	1	.	.	.	INTERGENIC(INTERGENIC)
X	143920102	143920117	143920108	CCTT	TCCTTC	del	1	0	0	0	.	.	.	INTERGENIC(INTERGENIC);RP11-480M11.1.1(UPSTREAM)
X	144015160	144015174	144015163	TTG	C	snp	1	1	1	1	.	.	.	INTERGENIC(INTERGENIC)
X	144027779	144027788	144027782	T	C	ins	1	1	0	1	.	.	.	INTERGENIC(INTERGENIC)
X	144062465	144062476	144062470	AG	C	snp	1	0	0	0	.	.	.	INTERGENIC(INTERGENIC)
X	144160527	144160542	144160534	ATCT	A	ins	1	1	1	1	.	.	.	INTERGENIC(INTERGENIC)
X	144254850	144254877	144254858	GAAA	AAA	ins	1	1	1	1	.	.	.	GS1-103B18.1.1(UPSTREAM);INTERGENIC(INTERGENIC)
X	144254850	144254877	144254861	GAAA	G	del	1	1	1	1	.	.	.	GS1-103B18.1.1(UPSTREAM);INTERGENIC(INTERGENIC)
X	144281969	144281984	144281979	AT	TATN	ins	1	0	0	0	.	.	.	INTERGENIC(INTERGENIC)
X	144289531	144289538	144289533	A	G	snp	1	1	1	1	.	.	.	INTERGENIC(INTERGENIC)
X	144292368	144292376	144292373	A	C	snp	1	0	0	0	.	.	.	INTERGENIC(INTERGENIC)
X	144368130	144368153	144368137	TAAA	G	snp	0	0	1	0	.	.	.	INTERGENIC(INTERGENIC)
X	144445553	144445561	144445560	T	C	snp	1	1	0	1	.	.	.	INTERGENIC(INTERGENIC)
X	144550053	144550068	144550062	AGGG	A	snp	0	0	0	1	.	.	.	INTERGENIC(INTERGENIC)
X	144583505	144583513	144583511	C	T	snp	1	0	0	0	.	.	.	INTERGENIC(INTERGENIC)
X	144588724	144588735	144588732	TG	C	snp	1	0	0	0	.	.	.	INTERGENIC(INTERGENIC)
X	144676474	144676483	144676482	A	C	snp	1	1	1	1	.	.	.	INTERGENIC(INTERGENIC)
X	144783120	144783133	144783131	AG	A	snp	1	1	1	1	.	.	.	INTERGENIC(INTERGENIC)
X	144853871	144853886	144853881	TTTA	C	ins	1	1	1	1	.	.	.	INTERGENIC(INTERGENIC)
X	144901396	144901407	144901400	AGA	A	snp	1	1	1	1	.	.	.	SLITRK2(INTRON);SLITRK2(UPSTREAM);SLITRK2(UTR_5_PRIME)
X	145030002	145030009	145030003	A	G	snp	1	1	1	1	.	.	.	INTERGENIC(INTERGENIC)
X	145192411	145192418	145192416	T	C	snp	1	0	0	0	.	.	.	INTERGENIC(INTERGENIC)
X	145205415	145205424	145205420	T	G	snp	1	0	0	0	.	.	.	INTERGENIC(INTERGENIC)
X	145316951	145316960	145316958	TA	G	snp	0	0	0	1	.	.	.	INTERGENIC(INTERGENIC)
X	145340974	145340983	145340977	GT	C	snp	1	1	1	1	.	.	.	INTERGENIC(INTERGENIC)
X	145341009	145341024	145341017	TG	C	snp	1	1	1	1	.	.	.	INTERGENIC(INTERGENIC)
X	145341009	145341024	145341018	TG	A	snp	1	1	1	1	.	.	.	INTERGENIC(INTERGENIC)
X	145343050	145343059	145343052	TA	C	snp	1	1	1	1	.	.	.	INTERGENIC(INTERGENIC)
X	145403383	145403391	145403386	T	C	snp	1	0	0	0	.	.	.	INTERGENIC(INTERGENIC)
X	145407390	145407397	145407395	T	C	snp	1	0	0	0	.	.	.	INTERGENIC(INTERGENIC)
X	145429443	145429451	145429444	G	GC	ins	1	0	0	0	.	.	.	INTERGENIC(INTERGENIC)
X	145484651	145484670	145484658	AAAT	G	snp	1	0	0	0	.	.	.	INTERGENIC(INTERGENIC)
X	145499950	145499957	145499953	A	C	snp	0	1	1	1	.	.	.	INTERGENIC(INTERGENIC)
X	145569147	145569158	145569149	TC	G	snp	1	1	1	1	.	.	.	INTERGENIC(INTERGENIC)
X	145577118	145577129	145577122	AAT	T	del	1	1	1	1	.	.	.	INTERGENIC(INTERGENIC)
X	145577118	145577129	145577123	AAT	A	snp	1	1	1	1	.	.	.	INTERGENIC(INTERGENIC)
X	145603896	145603904	145603897	A	C	snp	1	1	1	1	.	.	.	INTERGENIC(INTERGENIC)
X	145603896	145603904	145603899	A	C	snp	1	0	0	0	.	.	.	INTERGENIC(INTERGENIC)
X	145615846	145615855	145615850	A	G	snp	1	0	0	0	.	.	.	INTERGENIC(INTERGENIC);RP11-183K14.1.1(UPSTREAM)
X	145637325	145637332	145637331	A	C	ins	1	1	1	1	.	.	.	ELL2P4(DOWNSTREAM);INTERGENIC(INTERGENIC)
X	145638268	145638275	145638271	T	C	snp	1	0	0	0	.	.	.	ELL2P4(DOWNSTREAM);INTERGENIC(INTERGENIC)

X	145688333	145688342	145688338	AG	C	snp	1	0	0	0	.	.	.	INTERGENIC(INTERGENIC)
X	145694235	145694244	145694239	TG	C	snp	1	1	1	1	.	.	.	INTERGENIC(INTERGENIC)
X	145766161	145766176	145766171	ATTA	C	snp	1	1	1	1	.	.	.	INTERGENIC(INTERGENIC)
X	145821152	145821160	145821153	A	C	snp	1	0	0	0	.	.	.	INTERGENIC(INTERGENIC)
X	145904871	145904880	145904873	GT	A	snp	1	1	1	1	.	.	.	INTERGENIC(INTERGENIC)
X	145904871	145904880	145904877	GT	A	snp	1	1	1	1	.	.	.	INTERGENIC(INTERGENIC)
X	145904909	145904918	145904911	GT	A	snp	1	1	1	1	.	.	.	INTERGENIC(INTERGENIC)
X	145904909	145904918	145904915	GT	A	snp	1	1	1	1	.	.	.	INTERGENIC(INTERGENIC)
X	145904947	145904956	145904949	GT	A	snp	1	1	1	1	.	.	.	INTERGENIC(INTERGENIC)
X	145904947	145904956	145904953	GT	A	snp	1	1	1	1	.	.	.	INTERGENIC(INTERGENIC)
X	145904985	145904994	145904987	GT	A	snp	1	1	1	1	.	.	.	INTERGENIC(INTERGENIC)
X	145904985	145904994	145904991	GT	A	snp	1	1	1	1	.	.	.	INTERGENIC(INTERGENIC)
X	145905023	145905032	145905025	GT	A	snp	1	1	1	1	.	.	.	INTERGENIC(INTERGENIC)
X	145905023	145905032	145905029	GT	A	snp	1	1	1	1	.	.	.	INTERGENIC(INTERGENIC)
X	145905061	145905070	145905063	GT	A	snp	1	1	1	1	.	.	.	INTERGENIC(INTERGENIC)
X	145905061	145905070	145905067	GT	A	snp	1	1	1	1	.	.	.	INTERGENIC(INTERGENIC)
X	145905099	145905108	145905101	GT	A	snp	1	1	1	1	.	.	.	INTERGENIC(INTERGENIC)
X	145905099	145905108	145905105	GT	A	snp	1	1	1	1	.	.	.	INTERGENIC(INTERGENIC)
X	145905385	145905392	145905388	T	G	ins	1	0	0	0	.	.	.	INTERGENIC(INTERGENIC)
X	146021258	146021269	146021266	ATT	G	snp	1	1	1	1	.	.	.	INTERGENIC(INTERGENIC)
X	146036038	146036057	146036053	GTTT	G	del	1	1	0	0	.	.	.	INTERGENIC(INTERGENIC);RP1-73A14.1.1(UPSTREAM)
X	146093339	146093348	146093346	TA	G	snp	1	0	0	0	.	.	.	INTERGENIC(INTERGENIC)
X	146133076	146133083	146133080	A	G	snp	1	1	1	1	.	.	.	INTERGENIC(INTERGENIC)
X	146153341	146153350	146153345	AC	G	snp	1	1	1	1	.	.	.	INTERGENIC(INTERGENIC)
X	146191890	146191897	146191894	A	G	snp	1	0	1	1	.	.	.	INTERGENIC(INTERGENIC)
X	146216646	146216653	146216649	A	G	snp	1	0	0	0	.	.	.	INTERGENIC(INTERGENIC)
X	146314884	146314891	146314890	A	C	snp	1	0	0	0	.	.	.	INTERGENIC(INTERGENIC);MIR506(UPSTREAM);MIR507(UPSTREAM);MIR508(DOWNSTREAM)
X	146320903	146320918	146320906	CT	C	snp	1	1	1	1	.	.	.	INTERGENIC(INTERGENIC);MIR508(UPSTREAM)
X	146320903	146320918	146320912	CT	C	snp	1	0	0	0	.	.	.	INTERGENIC(INTERGENIC);MIR508(UPSTREAM)
X	146324705	146324714	146324711	GA	C	snp	1	0	0	0	.	.	.	INTERGENIC(INTERGENIC)
X	146546862	146546873	146546867	AAT	G	snp	1	1	1	0	.	.	.	INTERGENIC(INTERGENIC)
X	146626112	146626121	146626118	AG	G	snp	0	0	1	0	.	.	.	INTERGENIC(INTERGENIC)
X	146654520	146654527	146654524	A	G	snp	1	1	0	1	.	.	.	INTERGENIC(INTERGENIC)
X	146727456	146727465	146727459	AC	AT	ins	1	0	0	0	.	.	.	INTERGENIC(INTERGENIC)
X	147002980	147002995	147002992	TA	G	snp	1	1	0	1	.	.	.	FMR1(INTRON)
X	147060461	147060470	147060462	G	T	snp	1	1	1	1	.	.	.	FMR1NB(UPSTREAM);INTERGENIC(INTERGENIC)
X	147079368	147079375	147079372	A	G	snp	0	1	1	1	.	.	.	FMR1NB(INTRON)
X	147117269	147117276	147117273	A	G	snp	1	0	0	0	.	.	.	INTERGENIC(INTERGENIC)
X	147129836	147129843	147129840	T	C	snp	1	1	0	1	.	.	.	FTH1P8(UPSTREAM);INTERGENIC(INTERGENIC)
X	147161149	147161156	147161150	A	G	snp	1	0	0	0	.	.	.	INTERGENIC(INTERGENIC)
X	147246770	147246777	147246775	T	C	ins	1	0	0	0	.	.	.	INTERGENIC(INTERGENIC)
X	147246770	147246777	147246775	T	C	snp	1	0	0	0	.	.	.	INTERGENIC(INTERGENIC)
X	147365299	147365306	147365303	T	G	snp	0	1	0	1	.	.	.	INTERGENIC(INTERGENIC)
X	147394211	147394225	147394222	TTC	T	ins	0	1	1	1	.	.	.	INTERGENIC(INTERGENIC)
X	147520756	147520769	147520766	GA	A	snp	1	1	1	1	.	.	.	INTERGENIC(INTERGENIC)
X	147717538	147717553	147717551	AT	A	del	1	0	0	0	.	.	.	AFF2(INTRON)
X	147748190	147748204	147748194	CAA	G	snp	1	0	0	0	.	.	.	AFF2(INTRON)
X	147785627	147785640	147785632	TA	G	snp	1	1	1	1	.	.	.	AFF2(INTRON)
X	147786043	147786056	147786046	TA	G	snp	1	0	0	0	.	.	.	AFF2(INTRON)
X	147793509	147793520	147793512	GTG	A	snp	1	0	0	0	.	.	.	AFF2(INTRON)
X	148045422	148045429	148045428	A	C	snp	1	0	0	0	.	.	.	AFF2(INTRON)
X	148193980	148193988	148193987	A	G	snp	1	1	1	1	.	.	.	INTERGENIC(INTERGENIC)
X	148196413	148196420	148196414	A	C	snp	1	1	1	1	.	.	.	INTERGENIC(INTERGENIC)
X	148196413	148196420	148196416	A	C	snp	1	0	0	0	.	.	.	INTERGENIC(INTERGENIC)
X	148285827	148285850	148285838	TCTT	G	snp	1	0	0	0	.	.	.	INTERGENIC(INTERGENIC)
X	148372658	148372665	148372659	A	C	snp	1	0	0	0	.	.	.	INTERGENIC(INTERGENIC)
X	148552375	148552382	148552377	G	GC	ins	1	0	0	0	.	.	.	INTERGENIC(INTERGENIC)
X	148639312	148639321	148639318	T	G	snp	1	0	0	0	.	.	.	INTERGENIC(INTERGENIC)
X	149127051	149127064	149127062	AT	G	snp	0	0	1	0	.	.	.	RP13-507I23.1.1(INTRON);RP13-507I23.1.1(UPSTREAM)
X	149416175	149416192	149416186	AC	A	ins	0	1	0	0	.	.	.	INTERGENIC(INTERGENIC)
X	149496193	149496202	149496196	CT	C	snp	0	0	1	1	.	.	.	INTERGENIC(INTERGENIC)
X	149505640	149505649	149505645	CA	G	snp	1	0	0	0	.	.	.	INTERGENIC(INTERGENIC)
X	149525596	149525604	149525600	T	C	snp	1	0	0	0	.	.	.	INTERGENIC(INTERGENIC);MAMLD1(UPSTREAM)
X	149541760	149541769	149541767	AC	T	snp	0	0	1	0	.	.	.	MAMLD1(INTRON)
X	149691457	149691476	149691472	CTTT	C	del	1	0	0	0	.	.	.	INTERGENIC(INTERGENIC)
X	149778855	149778870	149778866	AGAC	AGACAG	del	1	1	1	1	.	.	.	MTM1(INTRON)

X	149826093	149826102	149826101	A	G	snp	1	1	1	1	.	.	.	MTM1(INTRON)
X	149980644	149980651	149980645	A	C	snp	1	1	1	1	.	.	.	AF274573.1(DOWNSTREAM);CD99L2(DOWNSTREAM);CD99L2(INTRON)
X	149981688	149981697	149981693	AC	T	snp	1	0	0	0	.	.	.	AF274573.1(DOWNSTREAM);CD99L2(DOWNSTREAM);CD99L2(INTRON)
X	149985684	149985711	149985691	AAAT	A	snp	1	1	1	1	.	.	.	CD99L2(INTRON);RP11-316K19.3.1(UPSTREAM)
X	149985684	149985711	149985699	AAAT	A	snp	1	1	0	1	.	.	.	CD99L2(INTRON);RP11-316K19.3.1(UPSTREAM)
X	149985684	149985711	149985707	AAAT	A	snp	1	1	1	1	.	.	.	CD99L2(INTRON);RP11-316K19.3.1(UPSTREAM)
X	150064876	150064891	150064886	AATT	C	snp	1	1	1	0	.	.	.	CD99L2(INTRON)
X	150166045	150166062	150166059	TTC	T	snp	1	0	0	0	.	.	.	INTERGENIC(INTERGENIC);RPL19P21(DOWNSTREAM)
X	150174493	150174504	150174498	TTA	G	snp	1	1	1	0	.	.	.	INTERGENIC(INTERGENIC)
X	150184491	150184499	150184497	C	A	snp	1	1	1	0	.	.	.	INTERGENIC(INTERGENIC)
X	150220447	150220454	150220451	C	A	ins	1	1	1	1	.	.	.	INTERGENIC(INTERGENIC)
X	150220447	150220454	150220452	C	A	snp	1	1	1	1	.	.	.	INTERGENIC(INTERGENIC)
X	150241819	150241834	150241830	TTTC	TT	del	1	1	1	0	.	.	.	INTERGENIC(INTERGENIC)
X	150427489	150427504	150427496	AAAG	A	snp	1	1	1	1	.	.	.	INTERGENIC(INTERGENIC)
X	150454432	150454447	150454438	TTCT	TTT	del	0	1	1	1	.	.	.	INTERGENIC(INTERGENIC)
X	150454504	150454519	150454514	TCTT	TTC	del	1	1	1	1	.	.	.	INTERGENIC(INTERGENIC)
X	150465022	150465037	150465033	AAAG	A	ins	0	0	1	0	.	.	.	INTERGENIC(INTERGENIC)
X	150487614	150487627	150487624	TC	CTC	del	1	1	1	1	.	.	.	INTERGENIC(INTERGENIC)
X	150551383	150551394	150551391	AAC	G	snp	1	0	0	0	.	.	.	INTERGENIC(INTERGENIC)
X	150672246	150672253	150672250	T	C	snp	1	0	0	0	.	.	.	INTERGENIC(INTERGENIC)
X	150834529	150834544	150834542	TC	T	snp	1	0	0	0	.	.	.	PASD1(INTRON);RP11-45D17.1.1(INTRON)
X	150911398	150911413	150911409	AT	C	snp	1	0	0	0	.	.	.	CNGA2(INTRON)
X	151013156	151013164	151013159	A	C	snp	0	1	0	1	.	.	.	INTERGENIC(INTERGENIC)
X	151047840	151047863	151047855	TTG	C	snp	1	0	1	0	.	.	.	INTERGENIC(INTERGENIC)
X	151074037	151074051	151074048	ATT	C	snp	1	1	1	1	.	.	.	RP11-366F6.2.1(DOWNSTREAM);RP11-366F6.2.1(INTRON)
X	151122555	151122562	151122556	C	A	snp	1	0	0	0	.	.	.	GABRE(DOWNSTREAM);GABRE(EXON);GABRE(UTR_3_PRIME);MIR224(DOWNSTREAM)
X	151160932	151160949	151160946	TG	GTG	del	1	1	1	1	.	.	.	INTERGENIC(INTERGENIC)
X	151274989	151274998	151274995	A	C	snp	1	1	1	1	.	.	.	INTERGENIC(INTERGENIC)
X	151363749	151363756	151363753	A	G	snp	1	1	1	1	.	.	.	GABRA3(INTRON)
X	151383510	151383527	151383523	AT	A	del	1	0	0	0	.	.	.	GABRA3(INTRON)
X	151383510	151383527	151383525	AT	A	del	1	1	1	1	.	.	.	GABRA3(INTRON)
X	151448068	151448075	151448071	T	C	snp	1	0	1	0	.	.	.	GABRA3(INTRON)
X	151483436	151483443	151483438	T	C	snp	1	1	1	1	.	.	.	GABRA3(INTRON)
X	151540639	151540647	151540645	A	C	snp	1	0	0	0	.	.	.	GABRA3(INTRON)
X	151558396	151558404	151558403	A	AG	ins	0	0	1	0	.	.	.	GABRA3(INTRON);GABRA3(UPSTREAM);MIR105-1(DOWNSTREAM);MIR105-2(DOWNSTREAM);MIR767(DOWNSTREAM)
X	151622429	151622436	151622431	A	C	snp	1	1	1	0	.	.	.	GABRA3(UPSTREAM);INTERGENIC(INTERGENIC)
X	151713856	151713867	151713861	CAT	C	snp	0	1	1	1	.	.	.	INTERGENIC(INTERGENIC)
X	151842801	151842809	151842804	A	AAAAAGAAAG	ins	1	0	0	0	.	.	.	INTERGENIC(INTERGENIC)
X	152071967	152071974	152071968	A	G	snp	1	1	1	1	.	.	.	INTERGENIC(INTERGENIC)
X	152195070	152195087	152195077	CT	C	snp	1	1	1	1	.	.	.	INTERGENIC(INTERGENIC);U82671.8.1(UPSTREAM)
X	152356423	152356438	152356427	CATT	T	snp	1	1	1	1	.	.	.	INTERGENIC(INTERGENIC)
X	152677254	152677265	152677262	GCG	A	snp	0	1	1	1	.	.	.	INTERGENIC(INTERGENIC)
X	152681145	152681153	152681149	T	G	snp	1	0	0	0	.	.	.	INTERGENIC(INTERGENIC);ZFP92(UPSTREAM)
X	152779292	152779303	152779296	TG	C	snp	0	0	0	1	.	.	.	ATP2B3(UPSTREAM);BGN(DOWNSTREAM);INTERGENIC(INTERGENIC)
X	152811079	152811090	152811086	AC	T	snp	1	0	1	0	.	.	.	ATP2B3(INTRON)
X	152897681	152897690	152897684	AT	A	snp	0	1	0	0	.	.	.	INTERGENIC(INTERGENIC)
X	153137317	153137324	153137322	C	T	snp	1	0	0	0	.	.	.	L1CAM(DOWNSTREAM);L1CAM(INTRON);L1CAM(UPSTREAM)
X	153155667	153155675	153155674	G	T	snp	1	0	0	0	.	.	.	L1CAM(INTRON);L1CAM(UPSTREAM);U52112.12.1(DOWNSTREAM)
X	153185236	153185245	153185244	T	AA	ins	1	1	1	1	.	.	.	ARHGAP4(DOWNSTREAM);ARHGAP4(INTRON)
X	153240776	153240795	153240784	TATT	G	snp	1	0	0	0	.	.	.	HCFC1(UPSTREAM);TMEM187(INTRON)
X	153301042	153301055	153301051	TC	TCTCT	del	1	0	0	0	.	.	.	MECP2(INTRON);MECP2(UPSTREAM)
X	153538781	153538796	153538786	CAAA	C	snp	1	0	0	0	.	.	.	TKTL1(INTRON)
X	153679938	153679957	153679943	CTTC	C	snp	1	1	1	1	.	.	.	FAM50A(DOWNSTREAM);INTERGENIC(INTERGENIC)
X	153714028	153714037	153714030	G	T	snp	1	1	1	1	.	.	.	SLC10A3(DOWNSTREAM);UBL4A(DOWNSTREAM);UBL4A(INTRON)
X	154002211	154002219	154002215	C	A	snp	0	0	1	0	.	.	.	DKC1(DOWNSTREAM);DKC1(INTRON);DKC1(UPSTREAM);MPP1(DOWNSTREAM);SNORA56(UPSTREAM)
X	154101076	154101084	154101078	A	G	snp	1	1	1	1	.	.	.	F8(INTRON)
X	154192357	154192372	154192359	CA	ACACACACACACAT	ins	0	0	0	1	.	.	.	F8(DOWNSTREAM);F8(INTRON)
X	154823502	154823511	154823507	A	G	snp	1	0	0	0	.	.	.	RP11-954J6.3.1(INTRON);TMLHE(INTRON)
X	155035037	155035060	155035052	AAC	AC	del	1	1	1	1	.	.	.	INTERGENIC(INTERGENIC)

X	155047972	155047979	155047975	G	A	snp	1	1	1	1	.	.	.	INTERGENIC(INTERGENIC)
X	155057076	155057087	155057083	ACT	G	snp	1	1	0	1	.	.	.	AMDP1(DOWNSTREAM);INTERGENIC(INTERGENIC)
X	155142801	155142808	155142803	T	C	snp	1	0	0	1	.	.	.	VAMP7(INTRON)
X	155218290	155218299	155218292	GA	A	snp	1	1	1	1	.	.	.	INTERGENIC(INTERGENIC);TRPC6P(UPSTREAM)
1	870312	870319	870317	G	A	snp	1	1	1	1	.	.	.	SAMD11(INTRON)
1	961365	961373	961366	G	T	snp	0	0	1	0	.	.	.	AGRN(INTRON)
1	961365	961373	961367	G	A	snp	0	0	1	0	.	.	.	AGRN(INTRON)
1	961365	961373	961372	G	A	snp	1	0	0	0	.	.	.	AGRN(INTRON)
1	1015810	1015821	1015817	GGA	A	snp	1	1	1	1	.	.	.	C1orf159(DOWNSTREAM);INTERGENIC(INTERGENIC)
1	1125107	1125121	1125110	TTA	C	snp	0	1	1	1	.	.	.	TTLL10(DOWNSTREAM);TTLL10(INTRON)
1	1193428	1193437	1193432	T	G	snp	1	0	0	0	.	.	.	UBE2J2(DOWNSTREAM);UBE2J2(INTRON);UBE2J2(UPSTREAM)
1	1344533	1344541	1344539	A	G	snp	1	1	0	0	.	.	.	INTERGENIC(INTERGENIC);MRPL20(UPSTREAM)
1	1360474	1360481	1360475	A	G	snp	1	0	0	0	.	.	.	ANKRD65(UPSTREAM);INTERGENIC(INTERGENIC);RP4-758J18.7.1(DOWNSTREAM);RP4-758J18.8.1(DOWNSTREAM);RP4-758J18.9.1(DOWNSTREAM);TMEM88B(UPSTREAM)
1	1547941	1547948	1547942	T	C	ins	1	1	1	1	.	.	.	INTERGENIC(INTERGENIC);MIB2(UPSTREAM)
1	1741515	1741528	1741525	AT	G	snp	0	1	1	1	.	.	.	GNB1(INTRON)
1	1792152	1792160	1792157	A	C	snp	0	1	1	1	.	.	.	GNB1(INTRON)
1	1869904	1869915	1869906	CA	T	snp	1	1	1	1	.	.	.	C1orf222(INTRON)
1	1975552	1975561	1975559	AC	A	del	1	0	0	0	.	.	.	INTERGENIC(INTERGENIC)
1	1997798	1997809	1997801	TC	G	snp	0	0	1	0	.	.	.	PRKCZ(INTRON)
1	2224867	2224875	2224873	C	A	ins	1	0	1	0	.	.	.	SKI(INTRON)
1	2775788	2775807	2775803	AGGA	AGGAG	del	0	1	1	1	.	.	.	INTERGENIC(INTERGENIC)
1	2809519	2809526	2809521	G	A	ins	1	0	0	0	.	.	.	INTERGENIC(INTERGENIC)
1	2886952	2886961	2886954	G	A	ins	1	1	1	1	.	.	.	INTERGENIC(INTERGENIC)
1	2967927	2967942	2967932	CACC	T	snp	1	1	1	0	.	.	.	INTERGENIC(INTERGENIC)
1	3120406	3120413	3120408	G	T	snp	0	1	0	1	.	.	.	PRDM16(INTRON)
1	3247859	3247867	3247865	C	T	snp	1	1	0	1	.	.	.	PRDM16(INTRON)
1	3517594	3517601	3517600	C	T	snp	1	0	0	0	.	.	.	MEGF6(INTRON)
1	3645986	3645997	3645989	GCA	A	snp	1	0	0	0	.	.	"TP73(SYNONYMOUS_CODING):Q320,Q342,Q391"	.
1	3650312	3650319	3650316	C	T	snp	1	0	0	0	.	.	.	RP5-1092A11.3.1(DOWNSTREAM);TP73(DOWNSTREAM);TP73(UTR_3_PRIME);TP73-AS1(DOWNSTREAM)
1	3821302	3821321	3821312	TGGC	C	snp	1	0	0	0	.	.	.	C1orf174(UPSTREAM);RP13-15E13.1.1(INTRON)
1	3998886	3998893	3998891	T	C	snp	1	0	0	0	.	.	.	INTERGENIC(INTERGENIC);RP13-614K11.1.1(UPSTREAM)
1	4001786	4001797	4001793	TC	TG	ins	1	0	1	0	.	.	.	RP13-614K11.1.1(INTRON)
1	4001786	4001797	4001795	TC	G	snp	1	0	1	0	.	.	.	RP13-614K11.1.1(INTRON)
1	4002095	4002108	4002098	TC	A	snp	1	1	1	1	.	.	.	RP13-614K11.1.1(INTRON)
1	4095481	4095498	4095493	AAT	AT	del	1	1	0	1	.	.	.	INTERGENIC(INTERGENIC)
1	4120166	4120175	4120173	A	C	snp	1	1	1	1	.	.	.	INTERGENIC(INTERGENIC)
1	4164638	4164649	4164644	AC	G	snp	1	1	1	1	.	.	.	INTERGENIC(INTERGENIC)
1	4330014	4330028	4330021	AAC	G	snp	1	0	0	0	.	.	.	INTERGENIC(INTERGENIC)
1	4413779	4413790	4413787	TG	C	snp	1	0	0	0	.	.	.	INTERGENIC(INTERGENIC)
1	4475210	4475219	4475214	TC	A	snp	1	1	1	0	.	.	.	RP5-1166F10.1.1(INTRON)
1	4509911	4509918	4509917	A	G	snp	1	0	0	0	.	.	.	INTERGENIC(INTERGENIC)
1	4515504	4515513	4515507	A	C	snp	0	1	0	1	.	.	.	INTERGENIC(INTERGENIC)
1	4603937	4603952	4603942	ATCT	A	snp	1	0	0	0	.	.	.	INTERGENIC(INTERGENIC)
1	4604467	4604476	4604474	GA	G	snp	1	1	1	1	.	.	.	INTERGENIC(INTERGENIC)
1	4701163	4701177	4701168	TCA	T	snp	1	0	1	0	.	.	.	INTERGENIC(INTERGENIC)
1	4728404	4728421	4728407	GT	C	snp	1	0	0	0	.	.	.	AJAP1(INTRON)
1	4993036	4993045	4993039	TG	A	snp	0	1	0	1	.	.	.	INTERGENIC(INTERGENIC)
1	4993843	4993852	4993850	AT	C	snp	1	1	1	1	.	.	.	INTERGENIC(INTERGENIC)
1	5068756	5068767	5068762	GT	T	snp	0	1	0	1	.	.	.	INTERGENIC(INTERGENIC)
1	5129558	5129585	5129574	TTAA	A	snp	1	1	1	1	.	.	.	INTERGENIC(INTERGENIC)
1	5129558	5129585	5129578	TTAA	A	snp	1	1	1	1	.	.	.	INTERGENIC(INTERGENIC)
1	5143801	5143810	5143804	TC	T	snp	1	1	0	1	.	.	.	INTERGENIC(INTERGENIC);RP11-542C10.1.1(DOWNSTREAM)
1	5149059	5149074	5149066	TTTA	T	snp	1	1	1	1	.	.	.	RP11-542C10.1.1(INTRON)
1	5153956	5153965	5153963	TA	G	snp	1	0	0	0	.	.	.	INTERGENIC(INTERGENIC);RP11-542C10.1.1(UPSTREAM)
1	5194655	5194672	5194666	TTG	C	snp	1	0	0	0	.	.	.	INTERGENIC(INTERGENIC)
1	5258977	5258986	5258982	CA	C	snp	1	1	1	1	.	.	.	INTERGENIC(INTERGENIC)
1	5374882	5374889	5374886	C	CCG	ins	1	1	1	1	.	.	.	INTERGENIC(INTERGENIC)
1	5491279	5491287	5491280	G	T	ins	1	0	0	0	.	.	.	INTERGENIC(INTERGENIC)
1	5539178	5539198	5539191	TGC	C	snp	1	0	0	0	.	.	.	INTERGENIC(INTERGENIC)
1	5638215	5638226	5638220	TC	G	snp	1	0	0	0	.	.	.	INTERGENIC(INTERGENIC)
1	5694369	5694380	5694372	TG	T	snp	1	0	0	0	.	.	.	RP11-154H17.1.1(INTRON)
1	5702865	5702873	5702869	A	G	snp	0	1	0	0	.	.	.	RP11-154H17.1.1(INTRON)
1	5713679	5713687	5713682	A	AAAC	ins	1	1	1	1	.	.	.	RP11-154H17.1.1(INTRON)
1	5804942	5804951	5804947	AC	T	snp	1	1	0	1	.	.	.	INTERGENIC(INTERGENIC)
1	5806156	5806163	5806157	G	T	snp	1	0	0	0	.	.	.	INTERGENIC(INTERGENIC)

1	5815913	5815922	5815918	C	A	snp	0	1	0	0	.	.	.	INTERGENIC(INTERGENIC)
1	5815913	5815922	5815919	C	A	snp	1	0	0	0	.	.	.	INTERGENIC(INTERGENIC)
1	5963667	5963675	5963669	A	C	snp	1	1	0	1	.	.	.	NPHP4(INTRON)
1	6032935	6032943	6032938	T	C	snp	1	0	0	0	.	.	.	NPHP4(INTRON)
1	6032935	6032943	6032939	T	C	snp	1	0	0	0	.	.	.	NPHP4(INTRON)
1	6042168	6042177	6042169	T	C	snp	1	0	0	0	.	.	.	NPHP4(INTRON)
1	6044595	6044603	6044602	A	G	snp	1	0	0	0	.	.	.	NPHP4(INTRON)
1	6164679	6164688	6164686	C	A	snp	0	1	0	1	.	.	.	CHD5(DOWNSTREAM);CHD5(INTRON);CHD5(UTR_3_PRIME);KCNAB2(DOWNSTREAM)
1	6196075	6196083	6196081	A	C	snp	1	1	1	1	.	.	.	CHD5(INTRON);CHD5(UPSTREAM)
1	6308924	6308933	6308929	C	A	snp	0	0	1	0	.	.	.	GPR153(UTR_3_PRIME);HES3(DOWNSTREAM)
1	6337051	6337059	6337052	C	A	snp	1	0	1	0	.	.	.	ACOT7(DOWNSTREAM);ACOT7(INTRON)
1	6473523	6473531	6473530	T	G	snp	1	1	1	1	.	.	.	HES2(DOWNSTREAM);HES2(INTRON)
1	6529183	6529206	6529186	TCC	C	del	1	0	0	0	"PLEKHG5(FRAME_SHIFT):-628,-722,-759,-778,-791,-799,-801"	.	.	PLEKHG5(EXON);TNFRSF25(UPSTREAM)
1	6529183	6529206	6529188	TCC	T	snp	1	1	0	1	.	.	"PLEKHG5(SYNONYMOUS_CODING):E627,E721,E758,E777,E790,E798,E800"	PLEKHG5(EXON);TNFRSF25(UPSTREAM)
1	6604573	6604588	6604585	AC	G	snp	1	1	1	1	.	.	.	NOL9(DOWNSTREAM);NOL9(INTRON);RP11-58A11.2.1(UPSTREAM);U6(DOWNSTREAM)
1	6620887	6620894	6620890	A	C	snp	1	1	1	1	.	.	.	TAS1R1(INTRON)
1	6775198	6775205	6775200	C	T	snp	1	0	0	0	.	.	.	INTERGENIC(INTERGENIC)
1	6830345	6830362	6830350	AAT	A	snp	1	1	1	1	.	.	.	INTERGENIC(INTERGENIC)
1	6842975	6842984	6842978	A	C	ins	0	0	1	1	.	.	.	CAMTA1(UPSTREAM);INTERGENIC(INTERGENIC);RP11-312B8.1.1(DOWNSTREAM)
1	6842975	6842984	6842979	A	C	snp	1	1	1	1	.	.	.	CAMTA1(UPSTREAM);INTERGENIC(INTERGENIC);RP11-312B8.1.1(DOWNSTREAM)
1	6853086	6853097	6853091	TA	G	snp	1	1	1	1	.	.	.	CAMTA1(INTRON)
1	6854951	6854958	6854953	T	G	ins	1	0	0	0	.	.	.	CAMTA1(INTRON)
1	6854951	6854958	6854956	T	G	ins	1	1	0	1	.	.	.	CAMTA1(INTRON)
1	6918547	6918556	6918551	G	T	ins	0	1	0	1	.	.	.	CAMTA1(INTRON)
1	6937117	6937128	6937121	CCT	G	snp	1	1	0	1	.	.	.	CAMTA1(INTRON)
1	6987403	6987410	6987405	T	C	snp	1	1	1	1	.	.	.	CAMTA1(INTRON)
1	7094983	7094990	7094986	T	C	snp	1	0	0	0	.	.	.	CAMTA1(INTRON)
1	7107764	7107781	7107777	TC	TCTCT	del	1	0	0	0	.	.	.	CAMTA1(INTRON)
1	7119197	7119208	7119202	GCT	C	snp	1	1	1	1	.	.	.	CAMTA1(INTRON)
1	7124129	7124146	7124133	CTC	C	snp	1	0	0	0	.	.	.	CAMTA1(INTRON)
1	7199061	7199078	7199064	AT	A	snp	1	1	1	1	.	.	.	CAMTA1(INTRON)
1	7199061	7199078	7199066	AT	A	snp	1	1	1	1	.	.	.	CAMTA1(INTRON)
1	7215448	7215456	7215449	G	T	ins	1	1	1	1	.	.	.	CAMTA1(INTRON)
1	7215448	7215456	7215450	G	T	ins	1	0	0	0	.	.	.	CAMTA1(INTRON)
1	7339929	7339937	7339930	A	G	snp	1	1	1	1	.	.	.	CAMTA1(INTRON)
1	7490158	7490169	7490162	CTG	G	snp	1	0	0	0	.	.	.	CAMTA1(INTRON)
1	7559291	7559300	7559297	GT	A	snp	1	1	1	1	.	.	.	CAMTA1(INTRON)
1	7591232	7591251	7591240	GGGA	A	snp	1	1	0	1	.	.	.	CAMTA1(INTRON)
1	7725775	7725783	7725776	A	G	snp	1	1	1	0	.	.	.	CAMTA1(INTRON)
1	7740870	7740878	7740873	G	T	ins	1	1	1	1	.	.	.	CAMTA1(INTRON)
1	7740922	7740942	7740932	CAG	T	snp	0	0	0	1	.	.	.	CAMTA1(INTRON)
1	7805586	7805593	7805588	A	G	snp	1	1	0	1	.	.	.	CAMTA1(INTRON)
1	7838922	7838941	7838937	TTGT	TTG	del	1	1	0	1	.	.	.	VAMP3(DOWNSTREAM);VAMP3(INTRON)
1	7849201	7849218	7849203	AT	TATG	ins	1	1	1	1	.	.	.	PER3(DOWNSTREAM);PER3(INTRON)
1	7849201	7849218	7849205	AT	TG	ins	1	1	1	1	.	.	.	PER3(DOWNSTREAM);PER3(INTRON)
1	7854552	7854559	7854555	A	G	snp	1	1	1	1	.	.	.	PER3(INTRON)
1	7937204	7937213	7937208	A	G	ins	1	1	1	1	.	.	.	INTERGENIC(INTERGENIC)
1	7996479	7996502	7996497	AAAC	G	snp	1	0	0	0	.	.	.	TNFRSF9(INTRON);TNFRSF9(UPSTREAM)
1	8014437	8014445	8014444	G	A	snp	1	0	0	0	.	.	.	PARK7(INTRON)
1	8029501	8029510	8029509	G	A	ins	1	1	1	1	.	.	.	PARK7(INTRON)
1	8029501	8029510	8029509	G	A	snp	1	1	1	1	.	.	.	PARK7(INTRON)
1	8050340	8050351	8050348	CAC	CAT	ins	1	1	1	1	.	.	.	INTERGENIC(INTERGENIC);PARK7(DOWNSTREAM)
1	8055184	8055195	8055193	TC	T	snp	1	0	0	0	.	.	.	INTERGENIC(INTERGENIC)
1	8163928	8163935	8163929	T	G	snp	0	1	0	1	.	.	.	RP11-431K24.1.1(INTRON)
1	8163928	8163935	8163934	T	G	ins	1	1	0	1	.	.	.	RP11-431K24.1.1(INTRON)
1	8250739	8250758	8250746	AAAT	A	snp	1	0	0	0	.	.	.	RP11-431K24.2.1(INTRON)
1	8250739	8250758	8250753	AAAT	C	snp	1	0	0	0	.	.	.	RP11-431K24.2.1(INTRON)
1	8295858	8295881	8295875	AAAT	ATAC	ins	1	0	0	0	.	.	.	INTERGENIC(INTERGENIC)
1	8514888	8514896	8514889	A	C	ins	1	0	0	0	.	.	.	RERE(INTRON)
1	8551677	8551696	8551681	CAAA	A	snp	1	0	0	0	.	.	.	RERE(DOWNSTREAM);RERE(INTRON)
1	8613414	8613423	8613418	CA	A	snp	1	1	1	1	.	.	.	RERE(INTRON)
1	8706598	8706606	8706603	A	C	snp	1	0	0	0	.	.	.	RERE(INTRON)
1	8729239	8729246	8729245	C	T	ins	1	0	0	0	.	.	.	RERE(INTRON)
1	8817971	8817988	8817976	AC	ACACACACACACAT	ins	1	1	1	1	.	.	.	RERE(INTRON);RERE(UPSTREAM)
1	8873003	8873012	8873011	A	C	snp	1	1	1	1	.	.	.	RERE(INTRON)

1	8937770	8937779	8937772	A	C	snp	1	1	1	1	.	.	.	ENO1(INTRON);ENO1(UPSTREAM);ENO1-AS1(UPSTREAM);ENO1-IT1(EXON)
1	8962073	8962082	8962080	AT	A	del	1	1	1	1	.	.	.	INTERGENIC(INTERGENIC)
1	8962822	8962830	8962827	T	C	snp	1	1	1	1	.	.	.	INTERGENIC(INTERGENIC);RP3-477M7.5.1(DOWNSTREAM)
1	9032274	9032281	9032275	T	G	snp	1	0	0	0	.	.	.	CA6(DOWNSTREAM);CA6(INTRON)
1	9120288	9120297	9120295	AC	A	del	1	1	1	1	.	.	.	SLC2A5(INTRON)
1	9120611	9120620	9120618	CA	T	snp	1	0	1	0	.	.	.	SLC2A5(INTRON)
1	9122934	9122957	9122945	AAAT	AAC	ins	1	1	0	0	.	.	.	SLC2A5(INTRON)
1	9122934	9122957	9122953	AAAT	A	snp	1	1	0	0	.	.	.	SLC2A5(INTRON)
1	9123431	9123438	9123436	T	G	snp	1	1	1	1	.	.	.	SLC2A5(INTRON)
1	9176240	9176249	9176244	TA	A	del	1	1	1	1	.	.	.	GPR157(INTRON);GPR157(UPSTREAM)
1	9183854	9183865	9183863	TA	T	snp	1	1	1	1	.	.	.	GPR157(INTRON)
1	9206352	9206375	9206357	CTC	T	snp	1	1	1	1	.	.	.	INTERGENIC(INTERGENIC)
1	9206352	9206375	9206359	CTC	CCTC	del	1	0	0	0	.	.	.	INTERGENIC(INTERGENIC)
1	9206352	9206375	9206363	CTC	T	snp	1	0	0	1	.	.	.	INTERGENIC(INTERGENIC)
1	9206352	9206375	9206366	CTC	T	snp	1	0	0	0	.	.	.	INTERGENIC(INTERGENIC)
1	9247019	9247027	9247020	G	T	snp	1	0	0	0	.	.	.	RP3-510D11.1.1(INTRON);RP3-510D11.2.1(DOWNSTREAM)
1	9307012	9307019	9307017	C	T	snp	1	0	0	0	.	.	.	H6PD(INTRON)
1	9333928	9333937	9333935	AT	A	del	1	1	1	1	.	.	.	H6PD(DOWNSTREAM);INTERGENIC(INTERGENIC);Z98044.1(UPSTREAM)
1	9340317	9340336	9340328	TTCA	G	snp	1	1	1	1	.	.	.	INTERGENIC(INTERGENIC);Z98044.1(DOWNSTREAM)
1	9496943	9496960	9496953	TA	ATATATA	del	0	1	1	0	.	.	.	INTERGENIC(INTERGENIC);RN5S40(DOWNSTREAM)
1	9521770	9521779	9521771	A	G	ins	1	0	0	0	.	.	.	INTERGENIC(INTERGENIC)
1	9521770	9521779	9521772	A	G	snp	1	0	0	0	.	.	.	INTERGENIC(INTERGENIC)
1	9613289	9613298	9613297	T	G	ins	1	0	0	0	.	.	.	SLC25A33(INTRON)
1	9696940	9696947	9696943	T	TTTTC	ins	1	1	1	0	.	.	.	INTERGENIC(INTERGENIC)
1	9698166	9698193	9698184	AAAT	G	snp	1	0	0	0	.	.	.	INTERGENIC(INTERGENIC)
1	9810276	9810287	9810284	AAT	C	snp	1	0	0	0	.	.	.	CLSTN1(INTRON);CLSTN1(UPSTREAM)
1	9836847	9836870	9836864	TCTA	T	del	1	0	0	0	.	.	.	CLSTN1(INTRON)
1	9903544	9903551	9903546	G	T	snp	1	0	0	0	.	.	.	CTNNBIP1(DOWNSTREAM);INTERGENIC(INTERGENIC);RP11-84A14.5.1(UPSTREAM)
1	9903544	9903551	9903548	G	T	snp	1	1	1	1	.	.	.	CTNNBIP1(DOWNSTREAM);INTERGENIC(INTERGENIC);RP11-84A14.5.1(UPSTREAM)
1	9957206	9957221	9957217	AAAC	A	snp	1	0	0	0	.	.	.	CTNNBIP1(INTRON);CTNNBIP1(UPSTREAM);RP11-84A14.2.1(UPSTREAM)
1	9959822	9959845	9959828	AAAG	C	snp	0	0	1	0	.	.	.	CTNNBIP1(INTRON);RP11-84A14.2.1(UPSTREAM)
1	9985658	9985666	9985665	T	G	snp	1	1	0	1	.	.	.	LZIC(DOWNSTREAM);LZIC(INTRON)
1	10011514	10011522	10011520	T	G	snp	1	1	1	1	.	.	.	7SK(DOWNSTREAM);NMNAT1(INTRON);RP11-84A14.4.1(DOWNSTREAM)
1	10038414	10038422	10038418	A	G	snp	0	1	0	0	.	.	.	NMNAT1(INTRON);NMNAT1(UPSTREAM);RP11-807G9.2.1(UPSTREAM)
1	10065182	10065193	10065191	AC	A	snp	1	0	0	0	.	.	.	RBP7(INTRON)
1	10083987	10083996	10083992	T	G	snp	1	0	0	0	.	.	.	INTERGENIC(INTERGENIC)
1	10323337	10323348	10323341	AAT	T	del	1	1	1	1	.	.	.	KIF1B(INTRON)
1	10323337	10323348	10323342	AAT	A	snp	1	1	1	1	.	.	.	KIF1B(INTRON)
1	10385045	10385053	10385047	A	G	snp	1	0	0	0	.	.	.	KIF1B(INTRON)
1	10432868	10432883	10432879	TTTA	G	snp	1	1	1	1	.	.	.	KIF1B(DOWNSTREAM);KIF1B(INTRON)
1	10492695	10492712	10492700	GGA	G	snp	1	0	0	0	.	.	.	APITD1(INTRON);APITD1-CORT(INTRON)
1	10568272	10568281	10568277	T	G	snp	1	1	1	1	.	.	.	PEX14(INTRON)
1	10735850	10735859	10735856	C	A	ins	1	1	1	1	.	.	.	CASZ1(INTRON)
1	10794734	10794745	10794739	AG	A	snp	1	0	0	0	.	.	.	CASZ1(INTRON)
1	10801848	10801861	10801851	TA	TTTT	ins	0	0	1	0	.	.	.	CASZ1(INTRON)
1	10828263	10828290	10828279	GTTT	TT	ins	0	0	1	0	.	.	.	CASZ1(INTRON)
1	10857308	10857317	10857309	G	GT	ins	1	0	1	0	.	.	.	CASZ1(UPSTREAM);INTERGENIC(INTERGENIC)
1	10857308	10857317	10857310	G	T	ins	1	0	0	0	.	.	.	CASZ1(UPSTREAM);INTERGENIC(INTERGENIC)
1	10857308	10857317	10857312	G	A	snp	1	0	0	0	.	.	.	CASZ1(UPSTREAM);INTERGENIC(INTERGENIC)
1	10941664	10941683	10941670	CAAA	C	snp	1	1	1	0	.	.	.	INTERGENIC(INTERGENIC)
1	10992680	10992697	10992695	TA	T	ins	1	1	1	1	.	.	.	INTERGENIC(INTERGENIC)
1	10992680	10992697	10992695	TA	T	snp	1	1	1	1	.	.	.	INTERGENIC(INTERGENIC)
1	11040110	11040125	11040117	AAAG	A	snp	0	0	0	1	.	.	.	C1orf127(INTRON)
1	11325327	11325342	11325330	AT	A	snp	1	1	1	1	.	.	.	INTERGENIC(INTERGENIC);MTOR(UPSTREAM)
1	11327333	11327356	11327337	GTTT	T	snp	1	1	1	0	.	.	.	INTERGENIC(INTERGENIC);MTOR(UPSTREAM)
1	11354361	11354372	11354366	TTA	G	snp	1	0	0	0	.	.	.	UBIAD1(INTRON)
1	11405654	11405662	11405658	T	G	snp	1	1	1	1	.	.	.	INTERGENIC(INTERGENIC)
1	11422475	11422482	11422481	T	G	snp	0	1	0	0	.	.	.	INTERGENIC(INTERGENIC)
1	11431689	11431698	11431693	T	C	snp	1	0	0	0	.	.	.	INTERGENIC(INTERGENIC)
1	11460618	11460629	11460626	TG	C	snp	1	0	0	0	.	.	.	INTERGENIC(INTERGENIC)
1	11464763	11464770	11464766	T	C	snp	1	1	1	1	.	.	.	INTERGENIC(INTERGENIC)
1	11526094	11526103	11526097	C	T	ins	1	0	0	0	.	.	.	INTERGENIC(INTERGENIC)
1	11530363	11530380	11530369	ATA	C	snp	0	1	1	1	.	.	.	INTERGENIC(INTERGENIC)
1	11616755	11616766	11616762	TG	A	snp	1	1	0	1	.	.	.	INTERGENIC(INTERGENIC)
1	11621923	11621934	11621930	GA	C	snp	1	0	0	0	.	.	.	INTERGENIC(INTERGENIC)

1	11657578	11657585	11657579	A	G	snp	1	1	1	1	.	.	.	INTERGENIC(INTERGENIC)
1	11684692	11684701	11684696	AT	G	snp	0	1	1	0	.	.	.	INTERGENIC(INTERGENIC)
1	11703839	11703850	11703843	CAT	G	snp	1	1	1	1	.	.	.	FBXO2(DOWNSTREAM);INTERGENIC(INTERGENIC)
1	11711168	11711179	11711176	TAT	G	snp	1	1	0	0	.	.	.	FBXO2(INTRON);FBXO2(UPSTREAM);FBXO44(UPSTREAM)
1	11755669	11755686	11755671	AT	T	del	1	1	1	1	.	.	.	C1orf187(INTRON);MAD2L2(UPSTREAM)
1	11825543	11825551	11825544	T	C	snp	0	1	1	1	.	.	.	C1orf167(INTRON);C1orf167(UPSTREAM)
1	11833968	11833976	11833974	A	G	ins	1	1	1	1	.	.	.	C1orf167(INTRON);RP11-56N19.5.1(DOWNSTREAM)
1	12003344	12003352	12003349	T	TTTC	ins	1	1	1	1	.	.	.	PLOD1(INTRON)
1	12027698	12027715	12027707	TA	G	snp	1	1	1	1	.	.	.	PLOD1(DOWNSTREAM);PLOD1(INTRON);PLOD1(UPSTREAM)
1	12027698	12027715	12027713	TA	TATTT	del	1	0	1	1	.	.	.	PLOD1(DOWNSTREAM);PLOD1(INTRON);PLOD1(UPSTREAM)
1	12027698	12027715	12027713	TA	T	snp	1	0	0	0	.	.	.	PLOD1(DOWNSTREAM);PLOD1(INTRON);PLOD1(UPSTREAM)
1	12184230	12184238	12184231	T	C	snp	1	1	0	1	.	.	.	TNFRSF8(INTRON);TNFRSF8(UPSTREAM)
1	12197372	12197389	12197384	AAT	ATC	ins	1	1	1	1	.	.	.	TNFRSF8(INTRON)
1	12224297	12224305	12224303	C	A	snp	0	1	0	1	.	.	.	INTERGENIC(INTERGENIC);TNFRSF1B(UPSTREAM)
1	12264848	12264871	12264866	CAC	A	ins	1	0	0	0	.	.	.	TNFRSF1B(INTRON)
1	12264848	12264871	12264868	CAC	A	snp	1	1	0	1	.	.	.	TNFRSF1B(INTRON)
1	12298560	12298568	12298567	T	C	snp	0	1	0	1	.	.	.	VPS13D(INTRON)
1	12319091	12319102	12319099	TTC	T	ins	1	0	0	0	.	.	.	VPS13D(DOWNSTREAM);VPS13D(INTRON)
1	12347383	12347390	12347387	T	C	snp	1	1	1	1	.	.	.	VPS13D(INTRON)
1	12347383	12347390	12347388	T	C	snp	1	1	1	1	.	.	.	VPS13D(INTRON)
1	12569950	12569958	12569954	A	G	snp	0	1	0	0	.	.	.	SNORA59A(DOWNSTREAM);VPS13D(DOWNSTREAM);VPS13D(EXON);VPS13D(UTR_3_PRIME)
1	12572772	12572795	12572781	CAA	T	snp	0	1	0	0	.	.	.	INTERGENIC(INTERGENIC);VPS13D(DOWNSTREAM)
1	12604991	12604999	12604995	T	C	snp	1	1	1	1	.	.	.	INTERGENIC(INTERGENIC)
1	12619999	12620007	12620006	T	G	snp	1	0	0	0	.	.	.	INTERGENIC(INTERGENIC)
1	12620872	12620879	12620873	T	C	ins	1	1	1	1	.	.	.	INTERGENIC(INTERGENIC)
1	12694069	12694076	12694072	C	T	snp	1	0	0	0	.	.	.	INTERGENIC(INTERGENIC)
1	12857498	12857505	12857504	C	T	snp	1	1	1	1	.	.	.	INTERGENIC(INTERGENIC);PRAMEF1(DOWNSTREAM)
1	12874003	12874012	12874006	T	C	snp	0	1	1	1	.	.	.	INTERGENIC(INTERGENIC)
1	13828047	13828066	13828062	TTTG	T	snp	1	1	1	1	.	.	.	LRRC38(INTRON)
1	13852286	13852294	13852287	T	G	ins	1	1	1	1	.	.	.	INTERGENIC(INTERGENIC)
1	13857652	13857661	13857653	A	G	snp	1	0	0	0	.	.	.	INTERGENIC(INTERGENIC)
1	13894367	13894378	13894370	AG	AA	ins	1	0	0	0	.	.	.	INTERGENIC(INTERGENIC)
1	13894652	13894661	13894654	AG	G	snp	1	0	0	0	.	.	.	INTERGENIC(INTERGENIC)
1	13943891	13943898	13943897	T	G	snp	1	1	1	1	.	.	.	PDPN(DOWNSTREAM);PDPN(UTR_3_PRIME)
1	13970025	13970033	13970029	T	C	snp	1	1	1	1	.	.	.	INTERGENIC(INTERGENIC)
1	14039266	14039273	14039269	A	C	snp	1	0	1	0	.	.	.	PRDM2(INTRON)
1	14127185	14127194	14127188	AC	G	snp	1	0	0	0	.	.	.	PRDM2(INTRON)
1	14153145	14153154	14153148	AT	C	snp	0	0	0	1	.	.	.	INTERGENIC(INTERGENIC);PRDM2(DOWNSTREAM)
1	14164720	14164727	14164726	T	C	ins	1	1	1	1	.	.	.	INTERGENIC(INTERGENIC)
1	14267704	14267713	14267706	A	C	snp	1	1	1	1	.	.	.	C1orf196(INTRON)
1	14267704	14267713	14267709	A	C	snp	1	1	1	1	.	.	.	C1orf196(INTRON)
1	14269954	14269961	14269960	A	G	snp	1	1	1	1	.	.	.	C1orf196(INTRON)
1	14272062	14272071	14272069	AT	C	snp	1	1	1	1	.	.	.	C1orf196(INTRON)
1	14282797	14282804	14282802	T	TC	ins	1	1	1	1	.	.	.	C1orf196(INTRON)
1	14282797	14282804	14282803	T	C	ins	1	1	1	1	.	.	.	C1orf196(INTRON)
1	14311405	14311413	14311412	T	C	snp	1	0	0	0	.	.	.	C1orf196(INTRON)
1	14394616	14394624	14394623	C	T	snp	0	1	0	1	.	.	.	C1orf196(INTRON)
1	14456282	14456293	14456286	GA	C	snp	1	1	1	1	.	.	.	C1orf196(INTRON)
1	14767872	14767892	14767877	GCA	G	snp	1	1	1	0	.	.	.	INTERGENIC(INTERGENIC)
1	14797084	14797095	14797093	AG	C	snp	1	0	0	0	.	.	.	INTERGENIC(INTERGENIC)
1	14807921	14807928	14807925	A	AC	ins	1	1	1	1	.	.	.	INTERGENIC(INTERGENIC)
1	15084574	15084597	15084593	TTTG	C	snp	0	0	1	0	.	.	.	KAZN(INTRON)
1	15090883	15090890	15090884	C	A	snp	1	1	1	1	.	.	.	KAZN(INTRON)
1	15160068	15160075	15160070	C	T	snp	1	1	0	1	.	.	.	KAZN(INTRON)
1	15186456	15186467	15186459	GCT	A	snp	0	0	1	0	.	.	.	KAZN(INTRON)
1	15397750	15397758	15397755	T	G	snp	1	0	0	0	.	.	.	KAZN(DOWNSTREAM);KAZN(INTRON)
1	15504072	15504079	15504073	G	T	snp	0	1	1	1	.	.	.	TMEM51(INTRON)
1	15518927	15518946	15518942	TTTC	T	ins	1	1	1	1	.	.	.	RP1-21O18.3.1(INTRON);TMEM51(INTRON)
1	15581068	15581077	15581074	TA	C	snp	1	1	1	1	.	.	.	FHAD1(INTRON)
1	15605435	15605443	15605439	T	C	snp	1	1	1	1	.	.	.	FHAD1(INTRON)
1	15772050	15772058	15772057	G	T	snp	1	1	1	1	.	.	.	CTRC(DOWNSTREAM);CTRC(INTRON)
1	15923730	15923738	15923731	T	G	ins	1	0	0	0	.	.	.	DNAJC16(DOWNSTREAM);RP4-680D5.2.1(INTRON)
1	15950053	15950076	15950058	TTA	C	snp	1	1	0	1	.	.	.	DDI2(INTRON)
1	15950053	15950076	15950061	TTA	C	snp	1	1	0	1	.	.	.	DDI2(INTRON)
1	15950053	15950076	15950064	TTA	C	snp	1	1	0	1	.	.	.	DDI2(INTRON)
1	16040114	16040129	16040124	TTTG	G	del	1	1	0	0	.	.	.	PLEKHM2(INTRON)

1	16040114	16040129	16040125	TTTG	T	snp	1	1	0	0	.	.	.	PLEKHM2(INTRON)
1	16045966	16045979	16045969	AG	T	snp	1	0	0	0	.	.	.	PLEKHM2(INTRON);RP11-288I21.1.1(DOWNSTREAM)
1	16132330	16132338	16132332	A	C	snp	0	0	1	0	.	.	.	INTERGENIC(INTERGENIC);RP11-169K16.7.1(DOWNSTREAM)
1	16205099	16205106	16205102	T	C	snp	1	1	1	1	.	.	.	SPEN(DOWNSTREAM);SPEN(INTRON)
1	16286004	16286027	16286015	GAGG	GAGGGAGGGAGGAAGGGAGGGAGGA	del	1	0	0	0	.	.	.	ZBTB17(INTRON)
1	16290255	16290264	16290262	TA	G	snp	0	1	1	1	.	.	.	ZBTB17(INTRON)
1	16341727	16341742	16341733	TGTC	C	snp	1	1	1	1	.	.	.	CLCNKA(UPSTREAM);HSPB7(DOWNSTREAM);HSPB7(EXON);HSPB7(UTR_3_PRIME)
1	16392402	16392411	16392406	T	C	ins	1	1	0	1	.	.	.	FAM131C(INTRON)
1	16574037	16574051	16574040	TCC	C	del	1	1	1	1	.	.	.	FBXO42(DOWNSTREAM);FBXO42(UTR_3_PRIME)
1	16606350	16606359	16606354	T	C	ins	1	0	0	0	.	.	.	FBXO42(INTRON)
1	16611744	16611753	16611751	A	G	snp	0	0	1	1	.	.	.	FBXO42(INTRON)
1	16715772	16715781	16715775	G	A	snp	1	0	0	0	.	.	.	C1orf144(INTRON);SPATA21(INTRON)
1	16729979	16729986	16729980	T	TTTC	ins	1	0	0	0	.	.	.	SPATA21(INTRON)
1	16729979	16729986	16729981	T	TC	ins	0	1	0	1	.	.	.	SPATA21(INTRON)
1	16729979	16729986	16729982	T	C	ins	0	1	0	1	.	.	.	SPATA21(INTRON)
1	17268504	17268513	17268506	T	G	snp	0	0	0	1	.	.	.	CROCC(DOWNSTREAM);CROCC(INTRON);CROCC(UPSTREAM)
1	17343193	17343204	17343198	AC	T	snp	1	1	0	1	.	.	.	ATP13A2(UPSTREAM);INTERGENIC(INTERGENIC);SDHB(DOWNSTREAM)
1	17375201	17375220	17375208	TATT	G	snp	1	0	0	0	.	.	.	SDHB(INTRON);SDHB(UPSTREAM)
1	17426541	17426549	17426545	G	A	ins	0	0	0	1	.	.	.	PADI2(INTRON)
1	17431893	17431908	17431895	TG	G	del	1	0	0	0	.	.	.	PADI2(INTRON)
1	17472574	17472593	17472589	TTTC	TTT	ins	1	0	1	0	.	.	.	INTERGENIC(INTERGENIC)
1	17518283	17518300	17518292	TTC	C	snp	1	1	0	1	.	.	.	RP11-380J14.1.1(INTRON)
1	17621274	17621288	17621279	AAC	A	del	0	1	1	1	.	.	.	INTERGENIC(INTERGENIC)
1	17663621	17663636	17663630	TTTA	G	snp	1	1	1	1	.	.	.	PADI4(DOWNSTREAM);PADI4(INTRON)
1	17697570	17697578	17697572	A	C	snp	1	1	1	1	.	.	.	INTERGENIC(INTERGENIC);PADI6(UPSTREAM)
1	17703011	17703018	17703017	T	G	ins	1	0	0	0	.	.	.	PADI6(INTRON)
1	17713954	17713963	17713956	G	A	snp	1	0	0	0	.	.	.	PADI6(INTRON)
1	17797510	17797518	17797511	T	C	snp	1	0	0	0	.	.	.	INTERGENIC(INTERGENIC)
1	17806701	17806708	17806705	T	G	snp	1	1	1	1	.	.	.	INTERGENIC(INTERGENIC)
1	17855477	17855488	17855482	GAT	A	snp	1	0	0	0	.	.	.	INTERGENIC(INTERGENIC)
1	17938473	17938500	17938482	TCCA	CAT	ins	1	1	0	1	.	.	.	ARHGEF10L(INTRON);ARHGEF10L(UPSTREAM)
1	18012569	18012576	18012573	T	C	snp	1	1	1	1	.	.	.	ARHGEF10L(INTRON)
1	18054226	18054249	18054230	CTTT	A	snp	1	0	0	0	.	.	.	RP11-473A10.2.1(INTRON)
1	18054226	18054249	18054234	CTTT	TT	del	0	0	1	0	.	.	.	RP11-473A10.2.1(INTRON)
1	18101856	18101870	18101861	GGA	GG	del	1	1	1	1	.	.	.	ACTL8(INTRON)
1	18101856	18101870	18101864	GGA	G	del	1	1	1	1	.	.	.	ACTL8(INTRON)
1	18206264	18206273	18206270	CT	T	snp	1	0	0	0	.	.	.	INTERGENIC(INTERGENIC)
1	18208882	18208890	18208887	T	G	ins	1	1	1	1	.	.	.	INTERGENIC(INTERGENIC)
1	18224061	18224070	18224064	TC	T	snp	1	1	1	1	.	.	.	INTERGENIC(INTERGENIC)
1	18224061	18224070	18224068	TC	T	snp	1	1	1	1	.	.	.	INTERGENIC(INTERGENIC)
1	18435093	18435102	18435095	GT	A	snp	1	1	1	1	.	.	.	IGSF21(INTRON)
1	18448672	18448687	18448683	TTTC	TTTCTT	del	1	1	0	1	.	.	.	IGSF21(INTRON)
1	18519890	18519905	18519894	AT	G	snp	1	0	0	0	.	.	.	IGSF21(INTRON)
1	18526387	18526394	18526393	C	A	snp	1	0	0	0	.	.	.	IGSF21(INTRON)
1	18547553	18547570	18547567	CAC	CAA	ins	1	1	1	1	.	.	.	IGSF21(INTRON)
1	18610397	18610405	18610402	A	C	snp	1	1	1	1	.	.	.	IGSF21(INTRON)
1	18619379	18619388	18619383	CA	T	snp	1	0	0	0	.	.	.	IGSF21(INTRON)
1	18717319	18717330	18717328	TG	T	del	1	1	1	1	.	.	.	INTERGENIC(INTERGENIC);RP11-422P22.1.1(DOWNSTREAM)
1	18837042	18837051	18837047	T	G	snp	0	0	0	1	.	.	.	INTERGENIC(INTERGENIC);RP1-8B22.1.1(UPSTREAM)
1	18929071	18929086	18929081	TGAA	C	snp	1	0	0	0	.	.	.	INTERGENIC(INTERGENIC)
1	18968448	18968457	18968455	C	A	snp	1	0	0	0	.	.	.	PAX7(INTRON)
1	19013379	19013396	19013392	TTA	A	del	1	1	1	1	.	.	.	PAX7(INTRON)
1	19032941	19032954	19032952	TC	TCT	del	1	1	1	1	.	.	.	PAX7(INTRON)
1	19104107	19104115	19104110	C	A	snp	0	1	1	1	.	.	.	INTERGENIC(INTERGENIC)
1	19123564	19123571	19123567	A	G	snp	1	1	1	1	.	.	.	INTERGENIC(INTERGENIC)
1	19124599	19124608	19124602	C	CG	ins	0	1	0	1	.	.	.	INTERGENIC(INTERGENIC)
1	19124599	19124608	19124605	C	CCA	ins	1	1	1	1	.	.	.	INTERGENIC(INTERGENIC)
1	19132006	19132017	19132014	AGG	A	snp	1	1	1	1	.	.	.	INTERGENIC(INTERGENIC)
1	19240303	19240317	19240312	TTG	C	snp	1	0	0	0	.	.	.	IFFO2(INTRON);IFFO2(UPSTREAM)
1	19284095	19284118	19284099	CATT	A	snp	0	0	1	1	.	.	.	IFFO2(UPSTREAM);INTERGENIC(INTERGENIC)
1	19300850	19300858	19300852	A	C	snp	1	1	1	1	.	.	.	INTERGENIC(INTERGENIC)
1	19303147	19303162	19303154	TTTA	T	snp	1	1	1	1	.	.	.	INTERGENIC(INTERGENIC)
1	19335682	19335690	19335685	C	T	ins	1	1	1	1	.	.	.	INTERGENIC(INTERGENIC)

1	19459079	19459087	19459081	A	C	snp	1	0	0	1	.	.	.	UBR4(INTRON);UBR4(UPSTREAM)
1	19567778	19567789	19567785	TTG	A	snp	1	0	0	0	.	.	.	KIAA0090(DOWNSTREAM);KIAA0090(INTRON);KIAA0090(UPSTREAM);RP1-43E13.2.1(DOWNSTREAM)
1	19606486	19606497	19606490	TTC	C	ins	1	1	0	1	.	.	.	AKR7A3(DOWNSTREAM);INTERGENIC(INTERGENIC)
1	19652133	19652141	19652136	G	T	ins	1	1	0	1	.	.	.	PQLC2(INTRON)
1	19662077	19662091	19662085	AAC	T	snp	1	0	0	0	.	.	.	CAPZB(DOWNSTREAM);INTERGENIC(INTERGENIC)
1	19705581	19705589	19705586	T	TTTC	ins	1	1	0	0	.	.	.	CAPZB(INTRON)
1	19707675	19707683	19707678	A	C	snp	1	0	0	0	.	.	.	CAPZB(INTRON)
1	19818592	19818606	19818602	AAC	G	snp	1	0	0	0	.	.	.	INTERGENIC(INTERGENIC)
1	19841922	19841936	19841925	GCT	A	snp	1	1	1	1	.	.	.	INTERGENIC(INTERGENIC);U4(DOWNSTREAM)
1	19874826	19874839	19874832	AT	T	snp	1	0	1	0	.	.	.	INTERGENIC(INTERGENIC)
1	20153899	20153913	20153909	CTT	TC	del	1	1	0	0	.	.	.	INTERGENIC(INTERGENIC)
1	20168084	20168111	20168098	AAAT	G	snp	1	0	0	0	.	.	.	INTERGENIC(INTERGENIC)
1	20254884	20254893	20254887	T	C	snp	1	1	1	1	.	.	.	INTERGENIC(INTERGENIC);PLA2G2E(UPSTREAM)
1	20276293	20276301	20276295	A	G	snp	1	0	0	0	.	.	.	INTERGENIC(INTERGENIC)
1	20287022	20287031	20287029	C	A	snp	0	0	0	1	.	.	.	INTERGENIC(INTERGENIC)
1	20293552	20293563	20293560	TCC	T	snp	1	1	1	1	.	.	.	AL358253.1(UPSTREAM);INTERGENIC(INTERGENIC)
1	20309191	20309198	20309196	T	G	snp	1	1	1	1	.	.	.	INTERGENIC(INTERGENIC);PLA2G2A(UPSTREAM)
1	20398037	20398046	20398043	AG	G	snp	1	1	1	1	.	.	.	PLA2G5(DOWNSTREAM);PLA2G5(INTRON)
1	20631046	20631059	20631050	GT	A	snp	1	1	1	1	.	.	.	RP4-745E8.2.1(INTRON);VWA5B1(INTRON)
1	20666017	20666026	20666023	AG	C	snp	1	0	0	0	.	.	.	VWA5B1(DOWNSTREAM);VWA5B1(INTRON)
1	20666048	20666063	20666056	CAAA	AAACA	del	1	0	0	0	.	.	.	VWA5B1(DOWNSTREAM);VWA5B1(INTRON)
1	20918333	20918341	20918337	T	G	ins	1	1	1	1	.	.	.	CDA(INTRON)
1	20918333	20918341	20918337	T	G	snp	1	0	1	1	.	.	.	CDA(INTRON)
1	21014538	21014547	21014541	T	G	snp	1	0	0	0	.	.	.	KIF17(INTRON);KIF17(UPSTREAM);RP5-930J4.5.1(DOWNSTREAM)
1	21227030	21227039	21227031	A	G	snp	0	0	0	1	.	.	.	EIF4G3(INTRON);EIF4G3(UPSTREAM)
1	21461368	21461377	21461370	T	C	snp	1	1	1	1	.	.	.	EIF4G3(INTRON)
1	21472426	21472435	21472429	T	C	snp	1	1	1	1	.	.	.	EIF4G3(INTRON)
1	21506748	21506761	21506757	TA	G	snp	1	1	1	1	.	.	.	EIF4G3(UPSTREAM);INTERGENIC(INTERGENIC)
1	21645840	21645851	21645846	AAT	C	snp	1	1	0	1	.	.	.	ECE1(INTRON)
1	21673289	21673302	21673298	TG	A	snp	1	1	0	1	.	.	.	ECE1(UPSTREAM);INTERGENIC(INTERGENIC)
1	21683194	21683209	21683205	TTTC	TTTCT	del	0	1	0	1	.	.	.	INTERGENIC(INTERGENIC)
1	21871793	21871801	21871798	T	G	snp	1	1	1	1	.	.	.	ALPL(INTRON)
1	21871793	21871801	21871800	T	G	snp	0	1	1	1	.	.	.	ALPL(INTRON)
1	21881404	21881423	21881416	TTTC	TTCTTTC	del	1	1	0	1	.	.	.	ALPL(INTRON)
1	21904200	21904207	21904206	C	T	snp	1	1	1	1	.	.	.	ALPL(UTR_3_PRIME)
1	21908475	21908488	21908484	AT	AC	ins	1	1	1	1	.	.	.	ALPL(DOWNSTREAM);INTERGENIC(INTERGENIC);RP11-63N8.3.1(UPSTREAM)
1	21916687	21916694	21916688	C	A	snp	0	1	0	1	.	.	.	AL359815.1(DOWNSTREAM);RP11-63N8.3.1(INTRON)
1	22242553	22242564	22242558	AAG	A	ins	1	1	1	1	.	.	.	HSPG2(INTRON)
1	22268117	22268124	22268118	C	T	snp	0	1	0	0	.	.	.	HSPG2(UPSTREAM);INTERGENIC(INTERGENIC)
1	22280693	22280701	22280695	T	C	ins	1	1	1	1	.	.	.	INTERGENIC(INTERGENIC)
1	22303590	22303599	22303593	TG	C	snp	1	0	0	0	.	.	.	CELA3B(INTRON);CELA3B(UPSTREAM)
1	22304228	22304237	22304229	T	C	snp	1	1	1	1	.	.	.	CELA3B(INTRON);CELA3B(UPSTREAM)
1	22311439	22311448	22311440	A	C	snp	1	0	0	0	.	.	.	AL590556.1(UPSTREAM);CELA3B(DOWNSTREAM);CELA3B(INTRON);U6(UPSTREAM)
1	22336811	22336819	22336812	A	C	ins	0	0	0	1	.	.	.	AL590556.2(UPSTREAM);CELA3A(DOWNSTREAM);CELA3A(INTRON);U6(UPSTREAM)
1	22364955	22364970	22364962	TTTA	C	snp	1	0	0	0	.	.	.	INTERGENIC(INTERGENIC)
1	22372680	22372691	22372683	TA	C	snp	1	1	1	1	.	.	.	INTERGENIC(INTERGENIC)
1	22372680	22372691	22372685	TA	C	snp	1	0	0	0	.	.	.	INTERGENIC(INTERGENIC)
1	22554085	22554102	22554088	TC	T	snp	1	1	1	1	.	.	.	INTERGENIC(INTERGENIC)
1	22594082	22594093	22594084	TG	C	snp	0	1	0	1	.	.	.	INTERGENIC(INTERGENIC)
1	22621126	22621145	22621137	AATT	G	snp	1	0	0	1	.	.	.	INTERGENIC(INTERGENIC)
1	22625308	22625319	22625311	AAG	G	snp	1	1	1	1	.	.	.	INTERGENIC(INTERGENIC)
1	22625582	22625590	22625585	T	C	snp	1	0	0	0	.	.	.	INTERGENIC(INTERGENIC)
1	22672356	22672367	22672363	AC	G	snp	0	1	1	1	.	.	.	INTERGENIC(INTERGENIC)
1	22676348	22676365	22676359	CA	G	snp	1	0	0	0	.	.	.	INTERGENIC(INTERGENIC)
1	22686884	22686895	22686887	AG	C	snp	0	0	0	1	.	.	.	INTERGENIC(INTERGENIC)
1	22719479	22719488	22719487	A	C	ins	0	0	1	0	.	.	.	INTERGENIC(INTERGENIC)
1	22737395	22737409	22737400	AAT	C	snp	1	1	1	1	.	.	.	INTERGENIC(INTERGENIC)
1	22739439	22739450	22739448	AG	C	snp	1	1	1	1	.	.	.	INTERGENIC(INTERGENIC)
1	22874234	22874245	22874241	TC	G	snp	1	1	1	1	.	.	.	INTERGENIC(INTERGENIC)
1	22916668	22916677	22916671	AG	A	snp	0	1	1	1	.	.	.	EPHA8(DOWNSTREAM);EPHA8(INTRON)
1	22936456	22936471	22936469	TC	T	snp	1	0	0	0	.	.	.	INTERGENIC(INTERGENIC)
1	22983001	22983008	22983007	A	C	snp	1	1	1	1	.	.	.	C1QB(INTRON)

1	23015920	23015935	23015924	GA	A	snp	0	0	1	0	.	.	.	INTERGENIC(INTERGENIC)
1	23024970	23024979	23024974	TA	C	snp	0	0	1	0	.	.	.	INTERGENIC(INTERGENIC)
1	23107828	23107839	23107832	AAG	G	snp	1	1	1	1	.	.	.	EPHB2(INTRON)
1	23363608	23363615	23363614	A	AT	ins	1	1	0	0	.	.	.	KDM1A(INTRON);RP1-184J9.2.1(INTRON)
1	23372165	23372174	23372173	T	G	snp	1	0	0	0	.	.	.	KDM1A(INTRON);KDM1A(UPSTREAM);RP1-184J9.2.1(INTRON)
1	23508674	23508681	23508676	A	C	snp	1	1	1	1	.	.	.	INTERGENIC(INTERGENIC);LUZP1(UPSTREAM)
1	23562682	23562693	23562689	TAT	T	ins	1	1	0	1	.	.	.	INTERGENIC(INTERGENIC)
1	23580407	23580415	23580408	A	G	snp	1	0	0	0	.	.	.	INTERGENIC(INTERGENIC)
1	23692212	23692235	23692229	AATT	C	snp	1	1	1	1	.	.	.	C1orf213(UPSTREAM);Y_RNA(UPSTREAM);ZNF436(INTRON)
1	23957654	23957662	23957659	A	C	snp	1	1	1	1	.	.	.	MDS2(INTRON)
1	24042809	24042820	24042812	AGG	GG	del	1	0	0	0	.	.	.	INTERGENIC(INTERGENIC)
1	24084737	24084752	24084742	ATTT	TTA	del	1	1	1	1	.	.	.	RP5-886K2.3.1(DOWNSTREAM);TCEB3(INTRON)
1	24104826	24104833	24104829	C	T	snp	1	1	1	1	.	.	.	INTERGENIC(INTERGENIC);PITHD1(UPSTREAM);RP5-886K2.3.1(UPSTREAM)
1	24171482	24171491	24171490	A	C	ins	1	0	0	0	.	.	.	FUCA1(DOWNSTREAM);INTERGENIC(INTERGENIC)
1	24183482	24183491	24183487	A	G	snp	1	0	0	0	.	.	.	FUCA1(INTRON)
1	24215462	24215477	24215473	AAAT	G	snp	1	1	1	1	.	.	.	CNR2(INTRON)
1	24217361	24217372	24217367	TCT	C	del	1	0	0	0	.	.	.	CNR2(INTRON)
1	24217361	24217372	24217369	TCT	TC	del	1	1	1	1	.	.	.	CNR2(INTRON)
1	24226360	24226371	24226367	AG	A	snp	1	1	1	1	.	.	.	CNR2(INTRON);RP11-4M23.2.1(DOWNSTREAM)
1	24334061	24334070	24334065	CA	A	snp	1	1	1	1	.	.	.	INTERGENIC(INTERGENIC);RP11-4M23.7.1(DOWNSTREAM)
1	24334061	24334070	24334067	CA	A	snp	0	1	1	1	.	.	.	INTERGENIC(INTERGENIC);RP11-4M23.7.1(DOWNSTREAM)
1	24405700	24405707	24405701	T	C	snp	1	1	1	1	.	.	.	MYOM3(DOWNSTREAM);MYOM3(INTRON);RP11-293P20.2.1(INTRON);RP11-293P20.3.1(INTRON);RP11-293P20.4.1(INTRON)
1	24519431	24519438	24519437	C	A	snp	1	1	1	1	.	.	.	IL28RA(UPSTREAM);INTERGENIC(INTERGENIC)
1	24532687	24532702	24532693	GAGG	C	snp	1	1	1	1	.	.	.	RP11-10N16.2.1(INTRON)
1	24586771	24586778	24586772	G	GGGGGGC	ins	1	0	0	1	.	.	.	INTERGENIC(INTERGENIC)
1	24586771	24586778	24586773	G	A	snp	1	0	0	0	.	.	.	INTERGENIC(INTERGENIC)
1	24642441	24642458	24642446	TTG	TTC	ins	1	0	0	0	.	.	.	GRHL3(UPSTREAM);RP11-10N16.3.1(INTRON)
1	24802290	24802297	24802296	A	G	ins	1	0	0	0	.	.	.	INTERGENIC(INTERGENIC);NIPAL3(DOWNSTREAM)
1	24829680	24829687	24829685	G	T	snp	1	1	1	1	.	.	.	RCAN3(INTRON);RCAN3(UPSTREAM)
1	24881037	24881044	24881042	C	T	snp	1	1	1	1	.	.	.	C1orf130(UPSTREAM);INTERGENIC(INTERGENIC)
1	24895518	24895526	24895521	T	G	ins	1	1	1	1	.	.	.	C1orf130(INTRON)
1	24895518	24895526	24895522	T	G	snp	1	1	1	1	.	.	.	C1orf130(INTRON)
1	24924170	24924185	24924180	AAAG	G	snp	0	1	1	1	.	.	.	C1orf130(INTRON)
1	24929862	24929870	24929869	A	G	ins	1	0	0	0	.	.	.	C1orf130(INTRON)
1	24938707	24938714	24938713	G	A	snp	1	0	0	0	.	.	.	C1orf130(DOWNSTREAM);INTERGENIC(INTERGENIC)
1	25064759	25064774	25064763	AT	G	snp	1	0	0	0	.	.	.	INTERGENIC(INTERGENIC)
1	25139517	25139540	25139536	CTTT	C	del	1	1	1	1	.	.	.	CLIC4(INTRON)
1	25171670	25171679	25171673	A	C	snp	1	1	1	1	.	.	.	CLIC4(DOWNSTREAM);INTERGENIC(INTERGENIC)
1	25192262	25192269	25192268	A	G	snp	1	0	0	0	.	.	.	INTERGENIC(INTERGENIC)
1	25250852	25250859	25250853	A	C	snp	1	0	0	0	.	.	.	RUNX3(INTRON)
1	25348949	25348956	25348950	G	T	ins	1	1	1	1	.	.	.	INTERGENIC(INTERGENIC)
1	25391263	25391271	25391265	T	G	snp	1	1	1	1	.	.	.	RP4-799D16.1.1(INTRON)
1	25462504	25462513	25462508	C	CA	ins	1	0	0	0	.	.	.	INTERGENIC(INTERGENIC)
1	25466197	25466216	25466206	TTTG	TG	del	1	0	0	0	.	.	.	INTERGENIC(INTERGENIC)
1	25495955	25495970	25495966	AAGG	A	snp	1	1	1	1	.	.	.	INTERGENIC(INTERGENIC)
1	25615688	25615695	25615694	T	G	snp	1	0	0	0	.	.	.	C1orf63(INTRON);RHD(INTRON);SDHDP6(DOWNSTREAM)
1	25743640	25743649	25743644	A	C	ins	1	0	0	0	.	.	.	RHCE(INTRON)
1	25948104	25948111	25948106	T	G	snp	1	0	0	0	.	.	.	MAN1C1(INTRON)
1	25961767	25961774	25961769	G	T	snp	1	0	0	0	.	.	.	MAN1C1(INTRON)
1	25969722	25969745	25969733	TTAA	T	ins	1	1	1	0	.	.	.	MAN1C1(INTRON);RP1-187B23.1.1(DOWNSTREAM)
1	25969722	25969745	25969736	TTAA	T	snp	1	1	1	0	.	.	.	MAN1C1(INTRON);RP1-187B23.1.1(DOWNSTREAM)
1	25969722	25969745	25969737	TTAA	T	ins	1	1	1	0	.	.	.	MAN1C1(INTRON);RP1-187B23.1.1(DOWNSTREAM)
1	25969722	25969745	25969740	TTAA	TTT	ins	1	1	1	0	.	.	.	MAN1C1(INTRON);RP1-187B23.1.1(DOWNSTREAM)
1	25969722	25969745	25969741	TTAA	T	ins	1	1	0	0	.	.	.	MAN1C1(INTRON);RP1-187B23.1.1(DOWNSTREAM)
1	25978440	25978455	25978449	CTGC	GCCG	ins	1	0	0	0	.	.	.	MAN1C1(INTRON);RP1-187B23.1.1(INTRON)
1	26025811	26025828	26025816	AAG	A	snp	0	1	1	0	.	.	.	MAN1C1(INTRON)
1	26066047	26066058	26066054	CAT	G	snp	1	0	0	0	.	.	.	MAN1C1(INTRON)
1	26093751	26093762	26093753	CA	T	snp	1	0	0	0	.	.	.	MAN1C1(INTRON);MAN1C1(UPSTREAM)
1	26326614	26326621	26326618	T	C	snp	1	0	0	0	.	.	.	INTERGENIC(INTERGENIC);PAFAH2(UPSTREAM)
1	26375816	26375825	26375819	CA	G	snp	1	1	1	1	.	.	.	INTERGENIC(INTERGENIC);SLC30A2(UPSTREAM);TRIM63(DOWNSTREAM)
1	26439826	26439834	26439829	A	C	snp	0	0	0	1	.	.	.	PDIK1L(INTRON)
1	26510358	26510365	26510363	C	T	snp	1	0	0	0	.	.	.	CNKSR1(DOWNSTREAM);CNKSR1(INTRON);CNKSR1(UPSTREAM)

1	26612292	26612299	26612297	C	A	snp	1	1	0	1	.	.	.	SH3BGRL3(DOWNSTREAM);UBXN11(DOWNSTREAM);UBXN11(INTRON)
1	26658998	26659015	26659003	TTG	T	snp	1	0	0	0	.	.	.	AIM1L(DOWNSTREAM);AIM1L(INTRON)
1	26733660	26733680	26733665	AAG	A	snp	1	1	1	1	.	.	.	INTERGENIC(INTERGENIC);LIN28A(UPSTREAM)
1	26799164	26799175	26799168	CCG	T	snp	1	0	0	0	.	.	.	DHDDS(DOWNSTREAM);HMGN2(INTRON);HMGN2(UPSTREAM)
1	26829055	26829068	26829058	GT	A	snp	1	1	1	0	.	.	.	INTERGENIC(INTERGENIC)
1	26852283	26852291	26852285	T	C	ins	1	1	1	1	.	.	.	INTERGENIC(INTERGENIC);RPS6KA1(UPSTREAM)
1	26852283	26852291	26852286	T	C	snp	1	1	1	1	.	.	.	INTERGENIC(INTERGENIC);RPS6KA1(UPSTREAM)
1	26905561	26905569	26905567	A	G	snp	0	0	1	1	.	.	.	INTERGENIC(INTERGENIC);RPS6KA1(DOWNSTREAM)
1	27193137	27193144	27193141	T	TA	ins	1	1	0	1	.	.	.	INTERGENIC(INTERGENIC);SFN(DOWNSTREAM)
1	27195899	27195906	27195903	A	C	snp	1	1	1	1	.	.	.	INTERGENIC(INTERGENIC);SFN(DOWNSTREAM)
1	27231629	27231649	27231646	TAT	TA	del	1	1	1	1	.	.	.	GPATCH3(UPSTREAM);NUDC(INTRON)
1	27316116	27316133	27316131	TC	T	snp	0	1	0	0	.	.	.	INTERGENIC(INTERGENIC);OSTCP2(UPSTREAM);TRNP1(UPSTREAM)
1	27382067	27382074	27382071	A	C	snp	1	0	0	0	.	.	.	INTERGENIC(INTERGENIC)
1	27433854	27433865	27433859	AC	T	snp	1	1	1	1	.	.	.	SLC9A1(INTRON);SLC9A1(UPSTREAM)
1	27790302	27790316	27790312	TAT	T	ins	1	1	1	1	.	.	.	RP4-752I6.1.1(DOWNSTREAM);WASF2(INTRON)
1	27865957	27865966	27865963	T	G	snp	1	1	1	1	.	.	.	AHDC1(INTRON)
1	27952452	27952471	27952456	AAAC	C	snp	1	0	0	0	.	.	.	FGR(INTRON);FGR(UPSTREAM)
1	28031448	28031471	28031460	CAAA	A	snp	1	0	0	0	.	.	.	INTERGENIC(INTERGENIC)
1	28056139	28056148	28056146	TC	A	snp	1	1	1	0	.	.	.	FAM76A(INTRON)
1	28105855	28105863	28105857	T	G	ins	1	0	0	0	.	.	.	STX12(INTRON)
1	28115725	28115734	28115727	T	G	snp	0	0	1	0	.	.	.	STX12(INTRON)
1	28150601	28150608	28150606	A	G	snp	1	0	0	0	.	.	.	STX12(UTR_3_PRIME);U6(UPSTREAM)
1	28186502	28186509	28186506	A	C	snp	1	1	0	1	.	.	.	INTERGENIC(INTERGENIC)
1	28251508	28251515	28251514	T	G	snp	0	1	1	1	.	.	.	INTERGENIC(INTERGENIC)
1	28298104	28298118	28298114	CAA	AC	del	1	1	1	1	.	.	.	EYA3(DOWNSTREAM);EYA3(UTR_3_PRIME);XKR8(DOWNSTREAM)
1	28388415	28388423	28388420	T	G	ins	1	1	1	1	.	.	.	EYA3(INTRON);EYA3(UPSTREAM)
1	28459575	28459592	28459590	TC	TCT	del	1	1	1	1	.	.	.	INTERGENIC(INTERGENIC)
1	28589439	28589446	28589442	A	C	snp	1	1	0	1	.	.	.	SESN2(INTRON)
1	28637961	28637978	28637966	AAT	A	snp	1	0	0	0	.	.	.	AL353622.1(DOWNSTREAM);INTERGENIC(INTERGENIC)
1	28692994	28693013	28693005	TTTC	TTTT	ins	1	1	1	1	.	.	.	INTERGENIC(INTERGENIC);PHACTR4(UPSTREAM)
1	28797045	28797052	28797051	C	A	snp	1	0	0	0	.	.	.	PHACTR4(DOWNSTREAM);PHACTR4(INTRON)
1	28884904	28884913	28884910	T	G	snp	1	1	1	1	.	.	.	TRNAU1AP(DOWNSTREAM);TRNAU1AP(INTRON);TRNAU1AP(UPSTREAM)
1	28925792	28925803	28925800	CT	T	snp	1	0	0	0	.	.	.	RAB42(DOWNSTREAM);TAF12(DOWNSTREAM);TAF12(INTRON)
1	29004812	29004835	29004819	TTTG	TT	ins	1	0	0	0	.	.	.	GMEB1(INTRON)
1	29112550	29112557	29112554	T	TTTG	ins	1	0	0	0	.	.	.	INTERGENIC(INTERGENIC)
1	29144227	29144235	29144230	T	G	snp	1	0	0	1	.	.	.	OPRD1(INTRON)
1	29199036	29199045	29199041	TG	C	snp	1	0	0	0	.	.	.	RP1-212P9.2.1(DOWNSTREAM);RP1-212P9.3.1(INTRON)
1	29226793	29226804	29226800	CTT	TC	del	1	0	1	1	.	.	.	EPB41(INTRON)
1	29226793	29226804	29226801	CTT	C	del	1	0	1	1	.	.	.	EPB41(INTRON)
1	29234953	29234961	29234954	T	ATTA	ins	1	1	1	1	.	.	.	EPB41(INTRON)
1	29297670	29297679	29297676	T	C	snp	1	1	1	1	.	.	.	EPB41(INTRON)
1	29508343	29508354	29508350	GCG	T	snp	1	1	1	1	.	.	.	SRSF4(EXON);SRSF4(UTR_5_PRIME)
1	29575671	29575682	29575677	CA	AT	ins	1	1	1	1	.	.	.	AL645859.1(DOWNSTREAM);PTPRU(INTRON)
1	30148227	30148236	30148229	AG	CG	ins	1	1	1	1	.	.	.	INTERGENIC(INTERGENIC)
1	30148227	30148236	30148230	AG	C	snp	1	1	1	1	.	.	.	INTERGENIC(INTERGENIC)
1	30148227	30148236	30148231	AG	G	snp	1	1	1	1	.	.	.	INTERGENIC(INTERGENIC)
1	30191950	30191961	30191955	CCA	C	del	1	0	0	0	.	.	.	INTERGENIC(INTERGENIC)
1	30286816	30286831	30286819	TC	T	snp	0	1	1	1	.	.	.	INTERGENIC(INTERGENIC)
1	30371713	30371732	30371719	ATAA	AATAAATAAATAAAT	ins	1	1	1	1	.	.	.	INTERGENIC(INTERGENIC)
1	30371897	30371904	30371898	G	A	ins	1	1	1	1	.	.	.	INTERGENIC(INTERGENIC)
1	30377371	30377384	30377382	AC	G	snp	0	1	1	1	.	.	.	INTERGENIC(INTERGENIC)
1	30499932	30499939	30499935	A	G	snp	1	1	1	1	.	.	.	RP11-111I12.1.1(INTRON)
1	30528991	30529006	30528996	TCAT	T	snp	1	1	1	1	.	.	.	INTERGENIC(INTERGENIC)
1	30571304	30571315	30571313	GA	G	snp	1	1	1	1	.	.	.	INTERGENIC(INTERGENIC)
1	30582504	30582511	30582510	A	C	snp	1	1	1	1	.	.	.	INTERGENIC(INTERGENIC)
1	30659378	30659401	30659392	CATC	A	snp	0	1	0	1	.	.	.	INTERGENIC(INTERGENIC)
1	30734598	30734607	30734602	AG	C	snp	1	1	1	1	.	.	.	INTERGENIC(INTERGENIC)
1	30744744	30744751	30744747	A	G	snp	1	0	0	0	.	.	.	INTERGENIC(INTERGENIC)
1	30775193	30775204	30775196	ATT	G	snp	1	1	1	1	.	.	.	INTERGENIC(INTERGENIC)
1	30840779	30840788	30840786	AC	T	snp	1	0	0	0	.	.	.	INTERGENIC(INTERGENIC)
1	30866211	30866218	30866216	A	G	snp	1	1	1	1	.	.	.	INTERGENIC(INTERGENIC)
1	30943943	30943951	30943945	A	C	snp	1	0	0	0	.	.	.	INTERGENIC(INTERGENIC)
1	30972424	30972431	30972427	A	C	snp	1	1	1	1	.	.	.	INTERGENIC(INTERGENIC)

1	30997571	30997586	30997574	AG	A	snp	1	0	1	1	.	.	.	INTERGENIC(INTERGENIC)
1	31025776	31025791	31025779	CT	G	snp	1	1	1	1	.	.	.	INTERGENIC(INTERGENIC)
1	31101481	31101490	31101485	G	T	snp	0	0	1	0	.	.	.	INTERGENIC(INTERGENIC)
1	31191437	31191446	31191438	C	A	snp	1	1	1	1	.	.	.	MATN1(INTRON);MATN1(UPSTREAM);RP5-1166H10.2.1(EXON);RP5-1166H10.2.1(UPSTREAM)
1	31285966	31285981	31285977	TTTA	C	snp	0	1	0	1	.	.	.	RP5-1125N11.1.1(INTRON)
1	31295426	31295445	31295440	TTTA	A	del	1	1	1	1	.	.	.	INTERGENIC(INTERGENIC);RP1-65J11.1.1(UPSTREAM)
1	31297232	31297241	31297238	AG	GAG	del	1	0	0	0	.	.	.	RP1-65J11.1.1(INTRON);RP1-65J11.1.1(UPSTREAM)
1	31466480	31466494	31466488	CAC	G	snp	1	1	0	1	.	.	.	PUM1(DOWNSTREAM);PUM1(INTRON)
1	31515155	31515182	31515170	AAAT	AAAC	ins	1	1	1	1	.	.	.	PUM1(INTRON)
1	31564728	31564743	31564739	AAAT	A	ins	1	1	1	1	.	.	.	INTERGENIC(INTERGENIC);SEPW1P(DOWNSTREAM)
1	31605866	31605875	31605874	A	G	snp	1	1	0	1	.	.	.	INTERGENIC(INTERGENIC)
1	31631799	31631826	31631803	TTTG	C	snp	1	1	0	1	.	.	.	INTERGENIC(INTERGENIC)
1	31653726	31653740	31653733	GTG	C	snp	1	1	1	1	.	.	.	NKAIN1(DOWNSTREAM);NKAIN1(INTRON);NKAIN1(UTR_3_PRIME)
1	31668476	31668484	31668481	A	C	snp	0	0	1	0	.	.	.	NKAIN1(INTRON);NKAIN1(UPSTREAM)
1	31686860	31686887	31686883	AATT	A	snp	1	1	1	1	.	.	.	NKAIN1(INTRON)
1	31722239	31722246	31722240	T	G	snp	0	0	1	0	.	.	.	INTERGENIC(INTERGENIC)
1	31746817	31746826	31746822	T	G	snp	0	0	1	0	.	.	.	SNRNP40(INTRON);SNRNP40(UPSTREAM)
1	31773795	31773802	31773800	A	G	snp	0	1	0	0	.	.	.	SNRNP40(UPSTREAM);ZCCHC17(INTRON)
1	31775075	31775083	31775079	A	G	snp	1	1	1	0	.	.	.	ZCCHC17(INTRON)
1	31822064	31822073	31822071	A	G	snp	0	0	1	0	.	.	.	RP11-266K22.2.1(UPSTREAM);ZCCHC17(DOWNSTREAM);ZCCHC17(INTRON)
1	31862186	31862193	31862190	T	TTTGTTA	ins	0	0	1	0	.	.	.	INTERGENIC(INTERGENIC)
1	31923731	31923740	31923734	AT	C	snp	1	1	1	1	.	.	.	INTERGENIC(INTERGENIC)
1	31967004	31967013	31967007	TC	T	snp	0	0	1	0	.	.	.	INTERGENIC(INTERGENIC);RP11-439L8.3.1(UPSTREAM);U6(DOWNSTREAM)
1	32173727	32173735	32173730	T	G	snp	1	0	0	0	.	.	.	COL16A1(UPSTREAM);INTERGENIC(INTERGENIC)
1	32434005	32434012	32434009	T	G	ins	1	0	0	0	.	.	.	INTERGENIC(INTERGENIC)
1	32491518	32491531	32491528	AT	T	snp	1	0	0	0	.	.	.	KHDRBS1(INTRON)
1	32533467	32533474	32533470	A	C	snp	0	1	0	1	.	.	.	RP11-277A4.4.1(INTRON);TMEM39B(UPSTREAM)
1	32556216	32556224	32556222	T	G	snp	1	1	1	1	.	.	.	TMEM39B(INTRON)
1	32572238	32572255	32572251	TC	TCTCT	del	1	1	1	1	.	.	.	INTERGENIC(INTERGENIC);KPNA6(UPSTREAM);TMEM39B(DOWNSTREAM)
1	32725231	32725251	32725235	AAT	T	del	1	1	1	0	.	.	.	LCK(INTRON)
1	32725231	32725251	32725236	AAT	A	ins	1	1	1	0	.	.	.	LCK(INTRON)
1	32725231	32725251	32725236	AAT	A	snp	1	1	1	0	.	.	.	LCK(INTRON)
1	32725231	32725251	32725239	AAT	A	snp	1	0	0	0	.	.	.	LCK(INTRON)
1	33146736	33146747	33146744	TTG	A	snp	1	0	0	0	.	.	.	RBBP4(DOWNSTREAM);SYNC(UTR_3_PRIME)
1	33156839	33156846	33156841	A	C	snp	1	1	1	1	.	.	.	SYNC(DOWNSTREAM);SYNC(INTRON)
1	33380658	33380667	33380662	TC	C	snp	0	0	1	0	.	.	.	INTERGENIC(INTERGENIC)
1	33442964	33442987	33442967	AAG	T	snp	1	1	0	1	.	.	.	INTERGENIC(INTERGENIC);RP4-803A2.1.1(UPSTREAM)
1	33472295	33472304	33472299	T	C	snp	1	0	0	0	.	.	.	AK2(DOWNSTREAM);RP1-117O3.2.1(INTRON)
1	33503442	33503449	33503448	C	T	snp	1	0	0	0	.	.	.	AK2(INTRON);AK2(UPSTREAM)
1	33679479	33679486	33679482	A	C	snp	1	1	1	1	.	.	.	INTERGENIC(INTERGENIC)
1	33857958	33857966	33857960	T	G	snp	1	1	1	1	.	.	.	PHC2(INTRON)
1	33873005	33873013	33873010	T	C	snp	1	1	1	1	.	.	.	PHC2(INTRON)
1	33877052	33877072	33877057	TTG	T	snp	1	1	1	1	.	.	.	PHC2(INTRON)
1	33877052	33877072	33877060	TTG	T	snp	1	1	1	1	.	.	.	PHC2(INTRON)
1	33878548	33878557	33878554	T	G	snp	1	1	1	1	.	.	.	PHC2(INTRON)
1	33897843	33897851	33897845	T	G	ins	1	0	1	0	.	.	.	INTERGENIC(INTERGENIC);PHC2(UPSTREAM)
1	33916608	33916616	33916612	A	G	snp	1	1	0	0	.	.	.	INTERGENIC(INTERGENIC)
1	33938840	33938855	33938845	TACC	G	snp	1	0	1	0	.	.	.	TLR12P(DOWNSTREAM);ZSCAN20(INTRON)
1	34039110	34039118	34039112	A	G	snp	1	1	0	1	.	.	.	CSMD2(INTRON);CSMD2(UPSTREAM)
1	34039110	34039118	34039115	A	C	snp	1	0	0	0	.	.	.	CSMD2(INTRON);CSMD2(UPSTREAM)
1	34040731	34040742	34040737	AGG	G	snp	1	1	1	1	.	.	.	CSMD2(INTRON);CSMD2(UPSTREAM)
1	34083628	34083641	34083633	AG	C	snp	1	1	1	1	.	.	.	CSMD2(INTRON)
1	34117525	34117532	34117526	G	T	snp	1	0	0	0	.	.	.	CSMD2(INTRON)
1	34224916	34224924	34224921	T	TTTC	ins	1	1	1	1	.	.	.	CSMD2(INTRON)
1	34421352	34421366	34421358	CAT	ATCATCACT	ins	1	1	1	1	.	.	.	CSMD2(INTRON)
1	34493512	34493529	34493521	AGG	GGG	ins	1	1	1	1	.	.	.	CSMD2(INTRON)
1	34573874	34573885	34573883	GA	G	del	1	1	1	1	.	.	.	CSMD2(INTRON);RN5S42(UPSTREAM)
1	34617416	34617423	34617421	C	T	ins	1	0	1	0	.	.	.	CSMD2(INTRON)
1	34631521	34631528	34631524	G	A	snp	1	0	0	0	.	.	.	C1orf94(UPSTREAM);CSMD2(UPSTREAM);INTERGENIC(INTERGENIC)
1	34713168	34713183	34713176	AG	G	snp	1	1	1	1	.	.	.	INTERGENIC(INTERGENIC)
1	34874996	34875005	34875002	T	G	ins	1	1	1	1	.	.	.	INTERGENIC(INTERGENIC)

1	34913137	34913144	34913138	A	G	snp	1	0	0	0	.	.	.	INTERGENIC(INTERGENIC)
1	35038904	35038919	35038916	AG	T	snp	1	0	0	0	.	.	.	INTERGENIC(INTERGENIC)
1	35128978	35128989	35128985	GAG	G	snp	1	1	1	1	.	.	.	INTERGENIC(INTERGENIC)
1	35183832	35183841	35183838	A	C	snp	1	1	1	1	.	.	.	C1orf212(INTRON)
1	35266931	35266948	35266939	TG	GA	ins	1	1	1	1	.	.	.	C1orf212(INTRON)
1	35298102	35298111	35298104	CA	T	snp	1	1	0	1	.	.	.	C1orf212(INTRON)
1	35304763	35304774	35304769	GA	C	snp	1	1	1	1	.	.	.	C1orf212(INTRON)
1	35457372	35457379	35457376	A	C	snp	0	0	1	0	.	.	.	ZMYM6(INTRON)
1	35457393	35457401	35457396	A	C	snp	1	1	1	0	.	.	.	ZMYM6(INTRON)
1	35558205	35558220	35558209	CTTT	T	ins	1	0	0	0	.	.	.	ZMYM1(INTRON);ZMYM1(UPSTREAM)
1	35558205	35558220	35558211	CTTT	TC	del	1	0	0	0	.	.	.	ZMYM1(INTRON);ZMYM1(UPSTREAM)
1	35701806	35701815	35701807	T	G	snp	1	0	0	0	.	.	.	INTERGENIC(INTERGENIC)
1	35706301	35706309	35706305	A	C	snp	1	0	0	0	.	.	.	INTERGENIC(INTERGENIC)
1	35747263	35747280	35747271	AT	TAT	del	1	1	1	0	.	.	.	ZMYM4(INTRON)
1	35750726	35750735	35750727	T	C	snp	1	0	0	0	.	.	.	ZMYM4(INTRON)
1	35773120	35773143	35773137	TTA	T	ins	1	0	1	0	.	.	.	SNORA62(UPSTREAM);ZMYM4(INTRON)
1	35773120	35773143	35773140	TTA	T	snp	1	1	1	1	.	.	.	SNORA62(UPSTREAM);ZMYM4(INTRON)
1	35781540	35781551	35781546	ATT	G	snp	1	1	1	1	.	.	.	ZMYM4(INTRON)
1	35855053	35855060	35855055	A	G	snp	1	1	1	0	.	.	.	ZMYM4(INTRON);ZMYM4(UPSTREAM)
1	35904441	35904458	35904455	AAC	T	snp	1	0	0	0	.	.	.	KIAA0319L(DOWNSTREAM);KIAA0319L(EXON);KIAA0319L(INTRON)
1	35992033	35992050	35992036	TA	G	snp	1	1	1	0	.	.	.	KIAA0319L(INTRON)
1	36062984	36062992	36062987	C	A	snp	1	0	0	0	.	.	.	INTERGENIC(INTERGENIC);PSMB2(DOWNSTREAM);RP5-983H21.3.1(DOWNSTREAM);TFAP2E(DOWNSTREAM)
1	36156062	36156073	36156070	TTG	A	snp	1	0	1	0	.	.	.	INTERGENIC(INTERGENIC)
1	36159065	36159082	36159077	AG	C	snp	1	0	0	0	.	.	.	INTERGENIC(INTERGENIC)
1	36168067	36168075	36168074	C	A	snp	1	1	1	1	.	.	.	INTERGENIC(INTERGENIC)
1	36180969	36180980	36180972	AC	T	snp	1	1	1	0	.	.	.	C1orf216(DOWNSTREAM);C1orf216(UTR_3_PRIME);CLSPN(DOWNSTREAM)
1	36247559	36247566	36247563	T	G	snp	1	1	1	0	.	.	.	INTERGENIC(INTERGENIC)
1	36253384	36253393	36253387	AG	A	snp	0	0	1	0	.	.	.	INTERGENIC(INTERGENIC)
1	36563157	36563164	36563158	C	A	snp	1	1	1	1	.	.	.	ADPRHL2(DOWNSTREAM);COL8A2(UTR_3_PRIME)
1	36592164	36592173	36592171	TG	T	snp	1	0	1	0	.	.	.	COL8A2(UPSTREAM);INTERGENIC(INTERGENIC)
1	36592224	36592237	36592226	TG	C	snp	1	1	1	1	.	.	.	COL8A2(UPSTREAM);INTERGENIC(INTERGENIC)
1	36592313	36592322	36592319	GT	A	snp	0	1	0	0	.	.	.	COL8A2(UPSTREAM);INTERGENIC(INTERGENIC)
1	36592652	36592661	36592654	GT	A	snp	1	1	1	1	.	.	.	COL8A2(UPSTREAM);INTERGENIC(INTERGENIC)
1	36619945	36619964	36619959	CCAT	G	snp	1	0	0	0	.	.	.	INTERGENIC(INTERGENIC);MAP7D1(UPSTREAM);TRAPPC3(UPSTREAM)
1	36650996	36651007	36650998	AT	G	snp	1	0	0	0	.	.	.	INTERGENIC(INTERGENIC);MAP7D1(DOWNSTREAM)
1	36703416	36703423	36703422	T	G	snp	1	1	0	1	.	.	.	RP11-238O13.1.1(DOWNSTREAM);THRAP3(INTRON)
1	36991537	36991545	36991538	T	C	ins	1	0	0	1	.	.	.	INTERGENIC(INTERGENIC)
1	36991537	36991545	36991539	T	C	snp	1	1	0	1	.	.	.	INTERGENIC(INTERGENIC)
1	37010248	37010259	37010257	AT	A	snp	0	0	1	0	.	.	.	INTERGENIC(INTERGENIC)
1	37095358	37095365	37095359	T	C	snp	0	1	1	1	.	.	.	FTLP18(UPSTREAM);INTERGENIC(INTERGENIC)
1	37150086	37150095	37150088	T	C	snp	1	1	1	1	.	.	.	INTERGENIC(INTERGENIC)
1	37190594	37190603	37190597	T	C	ins	0	1	0	0	.	.	.	INTERGENIC(INTERGENIC)
1	37559697	37559708	37559700	AC	T	snp	0	0	1	0	.	.	.	INTERGENIC(INTERGENIC)
1	37575837	37575846	37575841	AG	G	snp	1	1	1	1	.	.	.	INTERGENIC(INTERGENIC)
1	37592454	37592469	37592456	GA	A	snp	1	1	0	1	.	.	.	INTERGENIC(INTERGENIC)
1	37722180	37722197	37722189	TC	T	del	1	0	0	0	.	.	.	INTERGENIC(INTERGENIC)
1	37778327	37778336	37778331	CT	G	snp	1	1	1	1	.	.	.	RP5-1180C18.1.1(INTRON)
1	37788786	37788799	37788796	TA	G	snp	1	1	1	1	.	.	.	RP5-1180C18.1.1(INTRON)
1	37788811	37788824	37788822	AT	AG	ins	1	1	1	1	.	.	.	RP5-1180C18.1.1(INTRON)
1	37788827	37788838	37788836	AT	G	snp	1	1	0	1	.	.	.	RP5-1180C18.1.1(INTRON)
1	37823645	37823652	37823650	T	G	ins	0	1	0	0	.	.	.	INTERGENIC(INTERGENIC)
1	37908438	37908453	37908443	TG	TTTC	ins	1	0	0	0	.	.	.	INTERGENIC(INTERGENIC)
1	37941984	37941995	37941990	GTT	A	snp	0	1	0	0	.	.	.	RP11-422J8.1.1(UPSTREAM);ZC3H12A(INTRON);ZC3H12A(UPSTREAM)
1	37963178	37963205	37963192	TATT	C	snp	0	1	1	1	.	.	.	MEAF6(DOWNSTREAM);MEAF6(INTRON)
1	38013338	38013352	38013346	AAC	T	snp	1	1	1	1	.	.	.	FTH1P1(DOWNSTREAM);SNIP1(DOWNSTREAM);SNIP1(INTRON)
1	38185404	38185419	38185409	AC	G	snp	1	1	0	1	.	.	.	EPHA10(DOWNSTREAM);EPHA10(INTRON);EPHA10(UPSTREAM)
1	38221001	38221016	38221006	CA	G	snp	1	1	1	1	.	.	.	EPHA10(DOWNSTREAM);EPHA10(INTRON);EPHA10(UPSTREAM)
1	38241711	38241718	38241715	A	C	snp	1	1	1	1	.	.	.	INTERGENIC(INTERGENIC);RP11-109P14.1.1(UPSTREAM)
1	38534514	38534529	38534523	AG	A	snp	1	1	1	1	.	.	.	RP5-884C9.2.1(INTRON)

1	38643092	38643107	38643096	TTTC	C	snp	1	1	0	1	.	.	.	INTERGENIC(INTERGENIC)
1	38729884	38729893	38729888	AT	T	ins	0	0	1	0	.	.	.	INTERGENIC(INTERGENIC)
1	38736367	38736374	38736368	C	T	snp	1	0	1	0	.	.	.	INTERGENIC(INTERGENIC)
1	38806863	38806882	38806869	TTCT	TTTCTTTCTTTCTT	del	1	1	1	1	.	.	.	INTERGENIC(INTERGENIC)
1	38938508	38938516	38938515	A	C	snp	1	0	0	0	.	.	.	INTERGENIC(INTERGENIC);RP11-329N22.1.1(UPSTREAM)
1	38952350	38952365	38952357	AGGG	A	snp	1	1	1	0	.	.	.	INTERGENIC(INTERGENIC)
1	39249336	39249344	39249339	G	GT	ins	1	1	1	1	.	.	.	INTERGENIC(INTERGENIC)
1	39292764	39292787	39292782	TCAT	G	snp	1	0	0	0	.	.	.	INTERGENIC(INTERGENIC)
1	39350614	39350622	39350618	A	C	snp	0	1	1	1	.	.	.	GJA9(UPSTREAM);MYCBP(UPSTREAM);RHBDL2(DOWNSTREAM);RP5-864K19.4.1(INTRON)
1	39360370	39360378	39360374	T	C	snp	1	1	1	1	.	.	.	RHBDL2(INTRON);RP5-864K19.4.1(INTRON)
1	39415504	39415518	39415513	TTG	C	snp	1	1	1	0	.	.	.	INTERGENIC(INTERGENIC);Y_RNA(DOWNSTREAM)
1	39433530	39433539	39433538	A	C	ins	1	1	0	1	.	.	.	INTERGENIC(INTERGENIC)
1	39446819	39446828	39446820	A	G	snp	1	0	0	0	.	.	.	INTERGENIC(INTERGENIC)
1	39487136	39487143	39487141	T	G	snp	1	0	0	0	.	.	.	INTERGENIC(INTERGENIC);NDUFS5(UPSTREAM)
1	39491560	39491569	39491565	TC	T	snp	1	0	0	0	.	.	.	INTERGENIC(INTERGENIC);NDUFS5(UPSTREAM)
1	39570994	39571008	39570999	GCC	T	snp	1	1	1	1	.	.	.	MACF1(DOWNSTREAM);MACF1(INTRON);MACF1(UPSTREAM)
1	39803450	39803459	39803451	T	G	snp	1	0	0	0	.	.	.	MACF1(INTRON)
1	39846585	39846592	39846590	A	C	snp	0	1	1	1	.	.	.	MACF1(INTRON)
1	39848006	39848017	39848014	TAT	C	snp	1	0	0	0	.	.	.	MACF1(INTRON)
1	39933037	39933044	39933038	A	G	snp	1	0	0	0	.	.	.	MACF1(DOWNSTREAM);MACF1(INTRON);MACF1(UPSTREAM)
1	40002386	40002393	40002392	A	C	snp	1	1	1	1	.	.	.	PPIEL(INTRON);RP11-69E11.4.1(INTRON)
1	40045076	40045083	40045082	T	G	ins	0	1	1	1	.	.	.	INTERGENIC(INTERGENIC);PABPC4(UPSTREAM)
1	40084595	40084604	40084603	T	G	snp	1	1	0	0	.	.	.	INTERGENIC(INTERGENIC)
1	40138110	40138118	40138114	T	C	snp	1	1	1	1	.	.	.	INTERGENIC(INTERGENIC);NT5C1A(UPSTREAM)
1	40180747	40180755	40180750	T	AG	ins	0	1	1	1	.	.	.	PPIE(INTRON);RP1-144F13.4.1(UPSTREAM)
1	40180747	40180755	40180752	T	G	snp	0	1	1	1	.	.	.	PPIE(INTRON);RP1-144F13.4.1(UPSTREAM)
1	40180753	40180776	40180772	TTTG	T	ins	1	1	0	1	.	.	.	PPIE(INTRON);RP1-144F13.4.1(UPSTREAM)
1	40495091	40495102	40495098	CA	C	del	1	1	1	1	.	.	.	INTERGENIC(INTERGENIC)
1	40495091	40495102	40495100	CA	C	del	1	1	1	1	.	.	.	INTERGENIC(INTERGENIC)
1	40533954	40533961	40533958	T	C	snp	1	0	0	0	.	.	.	CAP1(DOWNSTREAM);CAP1(INTRON);CAP1(UPSTREAM);PPT1(DOWNSTREAM)
1	40622025	40622040	40622033	GAAA	C	snp	1	1	0	1	.	.	.	INTERGENIC(INTERGENIC)
1	40631454	40631461	40631460	C	T	snp	1	1	1	1	.	.	.	RLF(INTRON)
1	40666645	40666656	40666653	AC	C	snp	1	0	0	0	.	.	.	RLF(INTRON)
1	40666714	40666721	40666720	C	T	snp	1	0	0	0	.	.	.	RLF(INTRON)
1	40666728	40666741	40666735	CA	C	ins	1	1	1	1	.	.	.	RLF(INTRON)
1	40673058	40673067	40673061	TC	G	snp	1	0	0	0	.	.	.	RLF(DOWNSTREAM);RLF(INTRON)
1	40778844	40778853	40778849	T	C	snp	1	1	1	0	.	.	.	COL9A2(DOWNSTREAM);COL9A2(INTRON)
1	40793442	40793450	40793444	T	G	snp	1	1	1	1	.	.	.	INTERGENIC(INTERGENIC)
1	40795080	40795103	40795083	AAT	AT	del	1	1	1	1	.	.	.	INTERGENIC(INTERGENIC);RP1-228H13.1.1(DOWNSTREAM)
1	40795080	40795103	40795084	AAT	T	del	1	1	1	1	.	.	.	INTERGENIC(INTERGENIC);RP1-228H13.1.1(DOWNSTREAM)
1	40795080	40795103	40795087	AAT	T	del	0	1	1	0	.	.	.	INTERGENIC(INTERGENIC);RP1-228H13.1.1(DOWNSTREAM)
1	40864082	40864089	40864084	A	C	ins	1	1	1	1	.	.	.	SMAP2(INTRON)
1	40906530	40906538	40906534	T	G	snp	0	0	1	0	.	.	.	INTERGENIC(INTERGENIC)
1	40930012	40930023	40930019	ATT	C	snp	1	0	1	0	.	.	.	RP1-228H13.5.1(EXON);ZNF643(DOWNSTREAM)
1	40937117	40937124	40937121	A	G	snp	1	0	0	0	.	.	.	INTERGENIC(INTERGENIC);RP1-228H13.5.1(DOWNSTREAM)
1	41001045	41001053	41001050	T	C	snp	0	0	1	0	.	.	.	ZNF684(INTRON)
1	41022602	41022609	41022604	A	G	snp	1	0	0	0	.	.	.	INTERGENIC(INTERGENIC)
1	41046112	41046119	41046116	A	C	snp	1	0	0	0	.	.	.	INTERGENIC(INTERGENIC)
1	41110881	41110890	41110883	GT	T	snp	1	1	1	1	.	.	.	RIMS3(INTRON)
1	41191798	41191806	41191799	T	G	ins	1	1	1	1	.	.	.	NFYC(INTRON)
1	41358825	41358836	41358830	AAG	A	snp	1	0	0	0	.	.	.	INTERGENIC(INTERGENIC)
1	41358968	41358977	41358970	AG	G	snp	0	1	1	1	.	.	.	INTERGENIC(INTERGENIC)
1	41380150	41380169	41380163	AGAA	A	ins	1	0	0	0	.	.	.	INTERGENIC(INTERGENIC)
1	41392212	41392220	41392215	C	A	ins	1	1	1	1	.	.	.	INTERGENIC(INTERGENIC)
1	41392212	41392220	41392218	C	A	snp	0	1	0	1	.	.	.	INTERGENIC(INTERGENIC)
1	41396550	41396557	41396555	T	G	snp	1	1	1	1	.	.	.	INTERGENIC(INTERGENIC)
1	41416900	41416911	41416908	TA	G	snp	1	1	1	1	.	.	.	INTERGENIC(INTERGENIC)
1	41505880	41505889	41505882	TG	G	snp	0	0	1	0	.	.	.	SCMH1(DOWNSTREAM);SCMH1(INTRON);SCMH1(UPSTREAM)
1	41585111	41585120	41585115	A	C	snp	1	0	0	0	.	.	.	SCMH1(INTRON)
1	41857816	41857827	41857824	TG	G	snp	1	1	0	1	.	.	.	INTERGENIC(INTERGENIC)
1	41878981	41878992	41878988	CT	C	snp	1	1	1	1	.	.	.	INTERGENIC(INTERGENIC)
1	41975891	41975900	41975892	T	G	ins	0	1	0	1	.	.	.	HIVEP3(DOWNSTREAM);HIVEP3(UTR_3_PRIME)
1	42016032	42016039	42016033	T	C	snp	1	1	1	1	.	.	.	HIVEP3(INTRON)
1	42151753	42151760	42151757	T	G	snp	1	0	0	0	.	.	.	HIVEP3(INTRON)
1	42229853	42229860	42229859	A	G	snp	0	0	1	0	.	.	.	HIVEP3(INTRON)

1	42471066	42471075	42471070	TA	C	snp	0	0	1	0	.	.	.	HIVEP3(INTRON)
1	42512224	42512232	42512225	T	TA	ins	1	1	1	1	.	.	.	INTERGENIC(INTERGENIC);RP1-21K4.1.1(DOWNSTREAM)
1	42548840	42548849	42548846	AT	G	snp	1	1	1	1	.	.	.	INTERGENIC(INTERGENIC)
1	42568369	42568377	42568376	A	C	snp	1	0	0	0	.	.	.	INTERGENIC(INTERGENIC)
1	42624436	42624444	42624442	T	G	snp	0	1	1	1	.	.	.	GUCA2A(DOWNSTREAM);GUCA2B(DOWNSTREAM);INTERGENIC(INTERGENIC)
1	42687671	42687682	42687676	GGA	G	snp	1	1	1	1	.	.	.	FOXJ3(INTRON)
1	42687671	42687682	42687678	GGA	A	snp	1	1	1	1	.	.	.	FOXJ3(INTRON)
1	42703051	42703062	42703060	AT	C	snp	1	0	0	0	.	.	.	FOXJ3(INTRON)
1	42999893	42999901	42999899	G	A	snp	1	1	1	1	.	.	.	CCDC30(INTRON);CCDC30(UPSTREAM)
1	43018865	43018874	43018872	T	G	snp	1	1	1	1	.	.	.	CCDC30(INTRON)
1	43031411	43031426	43031413	TG	G	snp	1	1	1	1	.	.	.	CCDC30(INTRON);U6(UPSTREAM)
1	43033622	43033631	43033624	AG	G	del	1	0	0	0	.	.	.	CCDC30(INTRON);RP11-163G10.4.1(UPSTREAM);U6(UPSTREAM)
1	43064565	43064579	43064569	CAA	G	snp	1	0	0	0	.	.	.	CCDC30(DOWNSTREAM);CCDC30(INTRON)
1	43107896	43107911	43107907	AAAC	A	ins	0	0	1	0	.	.	.	CCDC30(INTRON)
1	43107918	43107935	43107920	AT	T	del	1	1	1	1	.	.	.	CCDC30(INTRON)
1	43197502	43197509	43197508	G	A	snp	1	1	1	1	.	.	.	CLDN19(DOWNSTREAM);INTERGENIC(INTERGENIC)
1	43226330	43226337	43226336	T	G	snp	1	1	1	1	.	.	.	LEPRE1(EXON);LEPRE1(INTRON);RP5-994D16.7.1(DOWNSTREAM)
1	43241565	43241579	43241572	AAC	G	snp	0	0	1	0	.	.	.	C1orf50(DOWNSTREAM);C1orf50(INTRON);RP5-994D16.9.1(EXON)
1	43364018	43364032	43364023	TTG	T	ins	1	1	1	1	.	.	.	ATP6V1E1P1(UPSTREAM);INTERGENIC(INTERGENIC)
1	43453175	43453190	43453184	TATT	T	ins	1	1	1	1	.	.	.	INTERGENIC(INTERGENIC);RP5-848E13.3.1(DOWNSTREAM);U6(UPSTREAM)
1	43540829	43540838	43540831	TC	A	snp	1	1	0	0	.	.	.	INTERGENIC(INTERGENIC)
1	43581987	43581998	43581995	TGT	G	snp	1	1	1	0	.	.	.	INTERGENIC(INTERGENIC)
1	43593616	43593635	43593623	TTTC	G	snp	1	0	0	0	.	.	.	INTERGENIC(INTERGENIC)
1	43639845	43639856	43639850	AT	C	snp	1	0	0	0	.	.	.	EBNA1BP2(INTRON);EBNA1BP2(UPSTREAM);WDR65(INTRON)
1	43822377	43822391	43822388	TTA	G	snp	1	0	0	0	.	.	.	CDC20(UPSTREAM);MPL(DOWNSTREAM);RP1-92O14.3.1(INTRON)
1	43845859	43845870	43845863	AGA	T	snp	1	1	1	1	.	.	.	INTERGENIC(INTERGENIC);MED8(DOWNSTREAM);RP1-92O14.6.1(UPSTREAM)
1	43962442	43962457	43962453	TA	T	ins	1	0	0	0	.	.	.	INTERGENIC(INTERGENIC)
1	43962442	43962457	43962454	TA	A	del	1	0	0	0	.	.	.	INTERGENIC(INTERGENIC)
1	43989576	43989584	43989579	A	C	snp	0	0	1	0	.	.	.	INTERGENIC(INTERGENIC);PTPRF(UPSTREAM)
1	44175517	44175524	44175520	A	G	snp	1	0	0	0	.	.	.	KDM4A(DOWNSTREAM);RP11-184I16.2.1(UPSTREAM);RP11-184I16.4.1(INTRON);ST3GAL3(INTRON)
1	44186924	44186933	44186931	A	G	snp	1	0	0	0	.	.	.	RP11-184I16.4.1(INTRON);ST3GAL3(INTRON);U6(DOWNSTREAM)
1	44506415	44506422	44506420	A	C	snp	1	1	1	1	.	.	.	INTERGENIC(INTERGENIC);RP5-1198O20.4.1(UPSTREAM)
1	44565945	44565956	44565952	AG	A	snp	1	1	1	1	.	.	.	RP5-1198O20.4.1(INTRON)
1	44704704	44704712	44704711	C	A	snp	1	0	0	1	.	.	.	ERI3(INTRON);ERI3-IT1(DOWNSTREAM)
1	44827831	44827839	44827837	A	G	snp	1	1	1	1	.	.	.	INTERGENIC(INTERGENIC)
1	44850582	44850602	44850589	TTA	C	snp	0	0	0	1	.	.	.	INTERGENIC(INTERGENIC)
1	44927767	44927775	44927769	T	C	snp	0	0	1	0	.	.	.	RNF220(INTRON)
1	44934128	44934137	44934132	TA	C	snp	1	1	1	1	.	.	.	RNF220(INTRON)
1	45140620	45140647	45140632	TGGA	G	ins	1	0	0	0	.	.	.	C1orf228(INTRON);TMEM53(UPSTREAM)
1	45364029	45364040	45364034	TC	T	snp	1	0	0	0	.	.	.	EIF2B3(INTRON)
1	45387558	45387569	45387567	AT	A	del	1	1	1	1	.	.	.	EIF2B3(DOWNSTREAM);EIF2B3(INTRON)
1	45510477	45510492	45510488	AT	A	snp	1	1	1	1	.	.	.	ZSWIM5(INTRON)
1	45778863	45778872	45778871	A	C	snp	1	0	1	0	.	.	.	INTERGENIC(INTERGENIC)
1	45803412	45803420	45803413	A	G	snp	0	1	0	1	.	.	.	MUTYH(INTRON);MUTYH(UPSTREAM);TOE1(UPSTREAM)
1	45804417	45804426	45804422	T	TG	ins	1	1	1	1	.	.	.	MUTYH(INTRON);MUTYH(UPSTREAM);TOE1(UPSTREAM)
1	46093397	46093406	46093404	A	AC	ins	0	0	1	0	.	.	.	CCDC17(UPSTREAM);GPBP1L1(DOWNSTREAM);GPBP1L1(EXON);GPBP1L1(UTR_3_PRIME)
1	46093679	46093694	46093692	TG	A	snp	1	1	1	1	.	.	.	CCDC17(UPSTREAM);GPBP1L1(DOWNSTREAM);GPBP1L1(EXON);GPBP1L1(UTR_3_PRIME)
1	46119366	46119373	46119372	T	CC	ins	1	1	1	1	.	.	.	GPBP1L1(DOWNSTREAM);GPBP1L1(INTRON);RP11-767N6.2.1(UPSTREAM)
1	46158830	46158839	46158833	AT	C	snp	1	0	0	0	.	.	.	IPP(DOWNSTREAM);RP11-767N6.7.1(DOWNSTREAM);TMEM69(INTRON)
1	46180466	46180474	46180470	T	C	snp	1	1	1	1	.	.	.	IPP(INTRON);IPP(UPSTREAM)
1	46220900	46220911	46220903	GGA	T	snp	0	0	0	1	.	.	.	INTERGENIC(INTERGENIC);IPP(UPSTREAM)
1	46235583	46235590	46235586	A	G	snp	1	1	1	1	.	.	.	INTERGENIC(INTERGENIC)
1	46255759	46255766	46255762	T	C	snp	1	0	0	0	.	.	.	MAST2(INTRON)
1	46278991	46279005	46278997	ATT	G	snp	0	0	0	1	.	.	.	MAST2(INTRON)

1	46349585	46349592	46349586	C	T	ins	1	1	1	1	.	.	.	MAST2(INTRON)
1	46349585	46349592	46349587	C	T	snp	1	1	1	1	.	.	.	MAST2(INTRON)
1	46358869	46358888	46358882	TTAT	C	snp	1	1	1	1	.	.	.	MAST2(INTRON)
1	46360977	46360986	46360978	T	G	snp	0	0	1	0	.	.	.	MAST2(INTRON)
1	46376689	46376700	46376692	TG	T	snp	1	1	1	1	.	.	.	MAST2(INTRON);MAST2(UPSTREAM)
1	46620993	46621004	46620996	CAA	G	snp	0	0	1	0	.	.	.	PIK3R3(INTRON)
1	46698481	46698496	46698490	CTTT	G	snp	1	1	1	1	.	.	.	INTERGENIC(INTERGENIC)
1	46950478	46950495	46950485	AG	C	snp	1	0	0	0	.	.	.	INTERGENIC(INTERGENIC);LINC00505(DOWNSTREAM)
1	46968558	46968565	46968561	A	C	snp	0	0	1	0	.	.	.	DMBX1(UPSTREAM);INTERGENIC(INTERGENIC)
1	47001418	47001425	47001423	A	G	snp	1	1	1	1	.	.	.	AL136373.1(DOWNSTREAM);INTERGENIC(INTERGENIC)
1	47019702	47019717	47019710	CAAA	A	ins	0	0	1	0	.	.	.	INTERGENIC(INTERGENIC);KNCN(UPSTREAM);MKNK1(DOWNSTREAM)
1	47126626	47126635	47126627	T	C	snp	1	1	1	1	.	.	.	ATPAF1(DOWNSTREAM);ATPAF1(INTRON);KIAA0494(INTRON);RP11-8J9.1.1(DOWNSTREAM)
1	47163515	47163526	47163523	TC	CTC	del	0	1	1	0	.	.	.	KIAA0494(INTRON);KIAA0494(UPSTREAM)
1	47265990	47266001	47265999	TC	T	snp	1	1	1	1	.	.	.	CYP4B1(INTRON)
1	47274849	47274857	47274853	A	AAAAC	ins	0	0	1	0	.	.	.	CYP4B1(INTRON);CYP4B1(UPSTREAM)
1	47274849	47274857	47274853	A	C	snp	1	0	0	0	.	.	.	CYP4B1(INTRON);CYP4B1(UPSTREAM)
1	47281774	47281782	47281780	C	A	snp	1	1	1	1	.	.	.	CYP4B1(DOWNSTREAM);CYP4B1(INTRON);CYP4B1(UPSTREAM)
1	47282621	47282632	47282626	GTG	A	snp	1	0	0	0	.	.	.	CYP4B1(DOWNSTREAM);CYP4B1(INTRON);CYP4B1(UPSTREAM)
1	47716491	47716500	47716492	A	G	snp	1	0	0	0	.	.	.	STIL(UTR_3_PRIME)
1	47831858	47831869	47831863	AAC	A	snp	1	1	1	1	.	.	.	CMPK1(INTRON)
1	47835243	47835252	47835244	T	C	snp	1	1	1	1	.	.	.	CMPK1(INTRON)
1	48214668	48214679	48214671	GGA	A	snp	1	0	0	0	.	.	.	INTERGENIC(INTERGENIC)
1	48214668	48214679	48214672	GGA	A	snp	1	0	0	0	.	.	.	INTERGENIC(INTERGENIC)
1	48357528	48357537	48357529	A	G	snp	0	1	1	0	.	.	.	RP11-543D5.3.1(INTRON)
1	48375479	48375498	48375486	CCTT	C	snp	1	1	1	1	.	.	.	RP11-543D5.3.1(INTRON)
1	48375569	48375577	48375572	G	T	snp	1	1	1	1	.	.	.	RP11-543D5.3.1(INTRON)
1	48488684	48488698	48488695	ATT	G	snp	1	0	0	0	.	.	.	INTERGENIC(INTERGENIC)
1	48517735	48517743	48517739	T	C	snp	1	1	1	1	.	.	.	INTERGENIC(INTERGENIC);RP4-683M8.2.1(UPSTREAM)
1	48566097	48566104	48566099	A	C	ins	1	1	0	1	.	.	.	INTERGENIC(INTERGENIC);SKINTL(DOWNSTREAM)
1	48648845	48648854	48648850	AT	C	snp	1	1	1	1	.	.	.	INTERGENIC(INTERGENIC);RP5-1024N4.1.1(UPSTREAM)
1	48749370	48749393	48749389	AAAG	A	ins	1	0	0	0	.	.	.	INTERGENIC(INTERGENIC)
1	48799997	48800006	48799999	A	C	snp	1	1	0	0	.	.	.	SPATA6(INTRON)
1	48799997	48800006	48800005	A	G	snp	0	1	0	1	.	.	.	SPATA6(INTRON)
1	48897071	48897080	48897078	AC	A	snp	1	0	0	0	.	.	.	RP11-329A14.1.1(UPSTREAM);SPATA6(INTRON)
1	49043369	49043378	49043373	AG	C	snp	1	1	0	1	.	.	.	AGBL4(INTRON)
1	49056763	49056771	49056770	C	A	snp	1	1	1	1	.	.	.	AGBL4(INTRON)
1	49135752	49135759	49135756	A	G	snp	1	1	1	1	.	.	.	AGBL4(INTRON)
1	49140206	49140213	49140208	G	A	snp	1	0	0	0	.	.	.	AGBL4(INTRON)
1	49146123	49146130	49146126	A	G	snp	1	0	0	0	.	.	.	AGBL4(INTRON)
1	49208093	49208102	49208095	A	G	ins	1	1	1	1	.	.	.	AGBL4(INTRON);BEND5(INTRON)
1	49244328	49244343	49244331	TG	A	snp	1	1	1	1	.	.	.	AGBL4(INTRON);BEND5(UPSTREAM)
1	49254950	49254967	49254962	AT	T	ins	0	1	1	0	.	.	.	AGBL4(INTRON)
1	49254950	49254967	49254963	AT	ATA	del	1	1	1	1	.	.	.	AGBL4(INTRON)
1	49274249	49274256	49274253	T	C	snp	1	0	0	0	.	.	.	AGBL4(INTRON)
1	49350597	49350606	49350600	AG	A	snp	1	1	1	1	.	.	.	AGBL4(INTRON)
1	49374004	49374012	49374007	A	G	snp	1	1	1	1	.	.	.	AGBL4(INTRON)
1	49616954	49616971	49616957	AT	A	snp	1	1	1	1	.	.	.	AGBL4(INTRON);RP11-141A19.1.1(INTRON);snoU13(UPSTREAM)
1	50045748	50045755	50045749	T	C	snp	0	0	0	1	.	.	.	AGBL4(INTRON)
1	50336230	50336239	50336236	AT	G	snp	1	0	0	0	.	.	.	AGBL4(INTRON);AGBL4(UPSTREAM)
1	50631100	50631113	50631109	AT	G	snp	1	0	0	0	.	.	.	ELAVL4(INTRON)
1	50669365	50669373	50669370	T	C	snp	0	1	1	1	.	.	.	ELAVL4(DOWNSTREAM);ELAVL4(UTR_3_PRIME);RP11-567C20.3.1(DOWNSTREAM)
1	50876431	50876448	50876444	TA	TATG	ins	1	1	0	1	.	.	.	INTERGENIC(INTERGENIC);RP11-183G22.3.1(UPSTREAM)
1	51011273	51011282	51011274	A	G	snp	1	0	0	0	.	.	.	FAF1(INTRON)
1	51060298	51060305	51060301	G	T	snp	0	1	0	0	.	.	.	FAF1(INTRON)
1	51109005	51109014	51109013	T	G	snp	1	0	0	0	.	.	.	FAF1(INTRON)
1	51126805	51126812	51126807	T	C	snp	1	0	0	0	.	.	.	FAF1(INTRON)
1	51288419	51288446	51288435	TTTC	A	snp	1	0	0	0	.	.	.	FAF1(INTRON)
1	51304177	51304186	51304184	TA	G	snp	1	0	0	0	.	.	.	FAF1(INTRON)
1	51314781	51314789	51314782	A	C	snp	1	1	1	1	.	.	.	FAF1(INTRON);MRPS6P2(DOWNSTREAM)
1	51388159	51388168	51388164	AG	AGAGA	del	1	0	1	0	.	.	.	FAF1(INTRON)
1	51460274	51460281	51460280	C	A	snp	1	1	1	1	.	.	.	INTERGENIC(INTERGENIC)

1	51529341	51529356	51529349	CATA	A	snp	1	0	0	0	.	.	.	INTERGENIC(INTERGENIC)
1	51664119	51664126	51664122	A	G	snp	1	0	0	0	.	.	.	RP11-296A18.3.1(INTRON)
1	51747430	51747439	51747438	A	AC	ins	1	0	0	0	.	.	.	INTERGENIC(INTERGENIC)
1	51933630	51933641	51933636	TA	C	snp	1	0	0	0	.	.	.	EPS15(INTRON);EPS15(UPSTREAM);RP11-253A20.1.1(DOWNSTREAM)
1	51939252	51939271	51939259	AAAC	A	snp	1	0	0	0	.	.	.	EPS15(INTRON)
1	52057482	52057489	52057483	A	G	snp	1	0	1	0	.	.	.	CALR4P(INTRON);OSBPL9(INTRON)
1	52254439	52254454	52254450	ATAG	AT	ins	1	0	0	0	.	.	.	NRD1(DOWNSTREAM);OSBPL9(DOWNSTREAM);OSBPL9(UTR_3_PRIME)
1	52254511	52254530	52254526	TATC	T	del	0	0	0	1	.	.	.	NRD1(DOWNSTREAM);OSBPL9(DOWNSTREAM);OSBPL9(UTR_3_PRIME)
1	52308575	52308583	52308577	A	G	snp	0	0	0	1	.	.	.	NRD1(INTRON);NRD1(UPSTREAM)
1	52343184	52343195	52343189	CA	G	snp	0	0	1	0	.	.	.	NRD1(INTRON)
1	52377777	52377792	52377787	TTTC	C	snp	1	1	1	1	.	.	.	RAB3B(UTR_3_PRIME)
1	52430915	52430930	52430920	AAAG	C	snp	1	1	0	1	.	.	.	RAB3B(INTRON)
1	52517045	52517060	52517052	ATTT	A	del	1	1	1	0	.	.	.	BTF3L4(UPSTREAM);RP11-91A18.5.1(INTRON);TXNDC12(INTRON)
1	52517045	52517060	52517053	ATTT	T	snp	1	1	1	0	.	.	.	BTF3L4(UPSTREAM);RP11-91A18.5.1(INTRON);TXNDC12(INTRON)
1	52517045	52517060	52517056	ATTT	A	del	1	1	1	0	.	.	.	BTF3L4(UPSTREAM);RP11-91A18.5.1(INTRON);TXNDC12(INTRON)
1	52630323	52630346	52630334	TCCA	G	snp	1	0	0	0	.	.	.	RP4-800M22.1.1(UPSTREAM);RP4-800M22.2.1(UPSTREAM);ZFYVE9(INTRON)
1	52649870	52649881	52649875	TA	G	snp	0	0	1	0	.	.	.	RP4-800M22.3.1(DOWNSTREAM);ZFYVE9(INTRON)
1	52993857	52993880	52993862	AAT	A	snp	1	1	1	1	.	.	.	ZCCHC11(INTRON);ZCCHC11(UPSTREAM)
1	53008422	53008441	53008436	TTTA	A	del	1	0	0	1	.	.	.	ZCCHC11(INTRON)
1	53008422	53008441	53008437	TTTA	T	snp	1	1	0	1	.	.	.	ZCCHC11(INTRON)
1	53109551	53109566	53109554	AG	C	snp	1	1	1	1	.	.	.	FAM159A(INTRON)
1	53154817	53154834	53154831	ATT	A	del	1	1	0	0	.	.	.	SELRC1(INTRON)
1	53199333	53199352	53199339	ATCA	T	snp	1	1	0	0	.	.	.	ZYG11B(INTRON)
1	53285458	53285466	53285464	A	AT	ins	1	0	0	0	.	.	.	ZYG11B(DOWNSTREAM);ZYG11B(INTRON)
1	53298952	53298961	53298959	AG	C	snp	0	0	1	0	.	.	.	INTERGENIC(INTERGENIC)
1	53363887	53363906	53363895	TTTA	C	snp	1	0	0	0	.	.	.	ECHDC2(DOWNSTREAM);ECHDC2(EXON);ECHDC2(INTRON);ZYG11A(DOWNSTREAM)
1	53455941	53455949	53455942	A	C	ins	1	1	1	0	.	.	.	AL445183.1(DOWNSTREAM);RP11-334A14.2.1(DOWNSTREAM);RP11-334A14.3.1(UPSTREAM);SCP2(DOWNSTREAM);SCP2(INTRON)
1	53490240	53490248	53490242	T	C	ins	1	1	1	1	.	.	.	SCP2(INTRON)
1	53490240	53490248	53490243	T	C	snp	1	1	1	1	.	.	.	SCP2(INTRON)
1	53501395	53501406	53501402	AAC	G	snp	1	1	1	0	.	.	.	SCP2(INTRON)
1	53501917	53501926	53501921	AT	G	snp	1	1	1	0	.	.	.	SCP2(INTRON)
1	53502329	53502338	53502336	AT	A	ins	1	1	1	0	.	.	.	SCP2(INTRON)
1	53503871	53503880	53503874	TA	G	snp	1	0	0	0	.	.	.	SCP2(INTRON)
1	53521004	53521023	53521019	TAAA	T	del	1	1	1	1	.	.	.	INTERGENIC(INTERGENIC);SCP2(DOWNSTREAM)
1	53526873	53526892	53526879	CATT	C	snp	1	1	1	1	.	.	.	INTERGENIC(INTERGENIC);PODN(UPSTREAM)
1	53690489	53690504	53690502	AT	G	snp	1	1	1	1	.	.	.	C1orf123(UPSTREAM);INTERGENIC(INTERGENIC);MAGOH(DOWNSTREAM);RP5-1024G6.7.1(DOWNSTREAM)
1	53806778	53806801	53806783	TGAA	A	snp	0	0	0	1	.	.	.	INTERGENIC(INTERGENIC);RP4-784A16.5.1(DOWNSTREAM)
1	53813093	53813100	53813099	T	G	snp	1	0	0	0	.	.	.	INTERGENIC(INTERGENIC);RP11-117D22.1.1(DOWNSTREAM)
1	53831395	53831406	53831399	CCA	T	snp	1	0	0	0	.	.	.	INTERGENIC(INTERGENIC);RP11-117D22.2.1(DOWNSTREAM)
1	53930351	53930368	53930360	GCC	A	snp	0	1	0	1	.	.	"DMRTB1(SYNONYMOUS_CODING):P114,P267"	DMRTB1(DOWNSTREAM)
1	54004918	54004933	54004924	CA	AG	ins	1	1	1	1	.	.	.	GLIS1(INTRON)
1	54005058	54005067	54005060	CA	T	snp	1	1	1	1	.	.	.	GLIS1(INTRON)
1	54022212	54022223	54022217	GT	C	snp	1	1	1	1	.	.	.	GLIS1(INTRON)
1	54100735	54100744	54100737	T	G	snp	1	1	1	1	.	.	.	GLIS1(INTRON)
1	54113847	54113855	54113854	A	C	snp	1	1	1	1	.	.	.	GLIS1(INTRON)
1	54204857	54204874	54204865	GCC	A	snp	1	1	1	1	.	.	.	GLIS1(UPSTREAM);INTERGENIC(INTERGENIC)
1	54215196	54215213	54215198	GA	A	snp	1	0	0	0	.	.	.	INTERGENIC(INTERGENIC)
1	54456323	54456332	54456325	A	C	snp	0	1	0	1	.	.	.	INTERGENIC(INTERGENIC)
1	54481319	54481326	54481325	T	G	snp	1	1	1	1	.	.	.	AL353898.1(UPSTREAM);LDLRAD1(INTRON)
1	54509637	54509646	54509644	AT	C	snp	1	1	1	1	.	.	TMEM59(START_GAINED)	TMEM59(DOWNSTREAM);TMEM59(INTRON)
1	54585459	54585470	54585461	CA	G	snp	1	0	0	0	.	.	.	INTERGENIC(INTERGENIC)
1	54700030	54700037	54700034	A	AT	ins	1	1	1	1	.	.	.	C1orf191(UPSTREAM);SSBP3(DOWNSTREAM);SSBP3(INTRON)
1	54705781	54705794	54705792	GC	GCA	del	1	1	1	1	.	.	.	

C1orf191(DOWNSTREAM);SSBP3(DOWNSTREAM);SSBP3(INTRON)

1	54841010	54841033	54841026	TGCA	GCA	ins	1	1	1	1	.	.	.	SSBP3(INTRON)
1	54894539	54894546	54894541	A	G	ins	1	1	1	1	.	.	.	INTERGENIC(INTERGENIC)
1	54901016	54901033	54901029	TTA	A	del	1	0	1	0	.	.	.	INTERGENIC(INTERGENIC)
1	54901016	54901033	54901030	TTA	T	snp	1	0	1	0	.	.	.	INTERGENIC(INTERGENIC)
1	54928760	54928767	54928766	G	T	snp	1	1	1	1	.	.	.	INTERGENIC(INTERGENIC)
1	54953361	54953370	54953368	AG	C	snp	0	1	1	1	.	.	.	INTERGENIC(INTERGENIC)
1	55038243	55038251	55038249	G	A	snp	1	0	0	0	.	.	.	ACOT11(INTRON)
1	55063127	55063138	55063130	CTC	G	snp	0	1	1	0	.	.	.	ACOT11(EXON);ACOT11(INTRON);ACOT11(UPSTREAM)
1	55185758	55185767	55185763	AG	A	snp	1	1	1	1	.	.	.	HEATR8(INTRON);TTC4(DOWNSTREAM);TTC4(INTRON)
1	55189433	55189442	55189435	CA	T	snp	1	1	1	1	.	.	.	HEATR8(INTRON);TTC4(INTRON)
1	55196892	55196900	55196897	T	G	ins	1	0	1	0	.	.	.	HEATR8(INTRON);TTC4(INTRON)
1	55284057	55284068	55284064	TC	G	snp	1	0	0	0	.	.	.	C1orf177(INTRON)
1	55339131	55339138	55339133	A	C	snp	0	0	1	0	.	.	.	DHCR24(INTRON)
1	55345699	55345710	55345706	AC	A	snp	1	1	1	1	.	.	.	DHCR24(INTRON);DHCR24(UPSTREAM)
1	55383681	55383688	55383685	T	C	snp	1	0	1	0	.	.	.	INTERGENIC(INTERGENIC)
1	55396910	55396937	55396927	AACA	G	snp	1	1	1	1	.	.	.	INTERGENIC(INTERGENIC)
1	55431285	55431292	55431286	A	G	snp	1	1	1	1	.	.	.	INTERGENIC(INTERGENIC)
1	55466129	55466136	55466134	G	A	snp	1	1	1	0	.	.	.	BSND(INTRON)
1	55971024	55971031	55971030	C	A	snp	0	1	0	0	.	.	.	INTERGENIC(INTERGENIC)
1	56140951	56140959	56140956	T	C	snp	1	0	0	0	.	.	.	RP11-466L17.1.1(INTRON)
1	56313992	56313999	56313997	G	T	ins	1	1	1	1	.	.	.	RP11-90C4.1.1(INTRON)
1	56321864	56321871	56321865	T	C	snp	1	1	1	1	.	.	.	RP11-90C4.1.1(INTRON)
1	56339178	56339187	56339179	T	C	snp	0	1	0	1	.	.	.	INTERGENIC(INTERGENIC)
1	56407530	56407541	56407537	TTA	C	snp	0	1	1	0	.	.	.	RP11-90C4.2.1(INTRON);RP11-90C4.3.1(UPSTREAM);RP11-90C4.4.1(UPSTREAM)
1	56577406	56577415	56577412	A	C	snp	1	1	1	1	.	.	.	INTERGENIC(INTERGENIC)
1	56589450	56589459	56589453	T	C	ins	1	1	1	1	.	.	.	INTERGENIC(INTERGENIC)
1	56618133	56618142	56618138	A	G	snp	0	1	1	1	.	.	.	RP1-158P9.1.1(INTRON)
1	56629172	56629180	56629179	T	C	snp	0	1	1	1	.	.	.	INTERGENIC(INTERGENIC)
1	56765286	56765297	56765289	AC	T	snp	1	1	1	1	.	.	.	INTERGENIC(INTERGENIC)
1	56783356	56783373	56783362	TTA	G	snp	1	1	1	1	.	.	.	INTERGENIC(INTERGENIC)
1	56810594	56810602	56810599	A	C	snp	1	0	0	0	.	.	.	INTERGENIC(INTERGENIC)
1	56859352	56859360	56859357	A	G	ins	1	0	0	0	.	.	.	INTERGENIC(INTERGENIC)
1	56898227	56898236	56898232	A	G	snp	1	0	0	0	.	.	.	INTERGENIC(INTERGENIC)
1	56916913	56916927	56916916	ACC	C	del	1	1	0	1	.	.	.	INTERGENIC(INTERGENIC)
1	56922035	56922042	56922037	T	C	snp	0	1	1	0	.	.	.	INTERGENIC(INTERGENIC)
1	57086843	57086850	57086846	A	C	snp	1	1	1	1	.	.	.	PPAP2B(INTRON);RP11-377K22.2.1(UPSTREAM)
1	57277429	57277456	57277436	CAAA	C	del	1	1	1	1	.	.	.	C1orf168(INTRON)
1	57277429	57277456	57277437	CAAA	A	del	1	1	1	1	.	.	.	C1orf168(INTRON)
1	57277429	57277456	57277440	CAAA	C	del	1	1	1	1	.	.	.	C1orf168(INTRON)
1	57325432	57325440	57325434	A	C	ins	1	1	1	1	.	.	.	C8A(INTRON)
1	57541029	57541038	57541033	TG	C	snp	1	0	0	0	.	.	.	DAB1(INTRON)
1	57618693	57618704	57618700	TAT	G	snp	0	0	1	0	.	.	.	DAB1(INTRON)
1	57621161	57621169	57621166	T	G	snp	1	0	0	0	.	.	.	DAB1(INTRON)
1	57678781	57678788	57678787	A	C	snp	0	1	1	1	.	.	.	DAB1(INTRON)
1	57709149	57709158	57709154	TG	T	del	0	1	0	0	.	.	.	DAB1(INTRON)
1	57746494	57746501	57746498	T	C	snp	1	1	1	1	.	.	.	DAB1(INTRON)
1	57892532	57892547	57892544	AT	T	snp	1	0	0	0	.	.	.	DAB1(INTRON);DAB1(UPSTREAM)
1	57894565	57894572	57894570	T	G	snp	1	0	0	0	.	.	.	DAB1(INTRON);DAB1(UPSTREAM)
1	57916079	57916086	57916085	A	G	snp	0	1	1	1	.	.	.	DAB1(INTRON)
1	57941927	57941935	57941931	A	C	ins	0	0	1	0	.	.	.	DAB1(INTRON)
1	57941927	57941935	57941932	A	C	snp	0	0	1	0	.	.	.	DAB1(INTRON)
1	57988795	57988804	57988801	AT	G	snp	1	1	1	1	.	.	.	DAB1(INTRON)
1	57989597	57989605	57989600	A	C	snp	1	0	0	0	.	.	.	DAB1(INTRON)
1	58001012	58001027	58001022	GAAT	C	snp	1	1	1	1	.	.	.	DAB1(INTRON)
1	58080522	58080541	58080537	TTCT	TTC	del	1	1	1	1	.	.	.	DAB1(INTRON)
1	58117338	58117347	58117340	A	C	ins	0	1	1	1	.	.	.	DAB1(INTRON)
1	58117338	58117347	58117341	A	C	snp	1	1	1	1	.	.	.	DAB1(INTRON)
1	58194266	58194274	58194272	A	C	snp	1	0	0	0	.	.	.	DAB1(INTRON)
1	58202305	58202316	58202308	ATT	T	del	1	0	0	0	.	.	.	DAB1(INTRON)
1	58217507	58217530	58217513	AATA	G	snp	0	1	1	1	.	.	.	DAB1(INTRON)
1	58262212	58262232	58262226	ATA	C	snp	1	0	0	0	.	.	.	DAB1(INTRON)
1	58262212	58262232	58262229	ATA	C	snp	1	0	0	0	.	.	.	DAB1(INTRON)
1	58428562	58428570	58428566	A	C	snp	1	1	0	1	.	.	.	DAB1(INTRON)
1	58446977	58446988	58446985	AGG	C	snp	1	0	1	0	.	.	.	DAB1(INTRON)
1	58449663	58449672	58449669	CA	T	snp	1	1	1	1	.	.	.	DAB1(INTRON)

1	58481679	58481686	58481683	G	T	snp	0	1	0	1	.	.	.	DAB1(INTRON)
1	58648541	58648548	58648546	T	G	snp	0	1	1	1	.	.	.	DAB1(INTRON)
1	58658332	58658343	58658336	TG	G	snp	1	1	1	1	.	.	.	DAB1(INTRON)
1	58933592	58933611	58933606	TTTG	G	del	0	0	1	0	.	.	.	DAB1(INTRON);OMA1(INTRON)
1	59132547	59132556	59132553	A	AT	ins	1	1	1	1	.	.	.	MYSM1(INTRON)
1	59163757	59163774	59163770	TA	TATAT	del	1	1	1	1	.	.	.	MYSM1(INTRON)
1	59216446	59216453	59216447	A	C	snp	0	1	0	1	.	.	.	AL136985.1(DOWNSTREAM);INTERGENIC(INTERGENIC)
1	59234979	59234987	59234986	A	G	snp	1	0	0	0	.	.	.	INTERGENIC(INTERGENIC)
1	59515968	59515977	59515972	A	C	snp	0	1	0	1	.	.	.	INTERGENIC(INTERGENIC);RP11-145M4.1.1(DOWNSTREAM)
1	59632315	59632324	59632317	TG	C	snp	1	1	1	1	.	.	.	RP11-470E16.1.1(INTRON)
1	59642928	59642935	59642933	T	C	snp	1	1	1	1	.	.	.	RP11-470E16.1.1(INTRON)
1	60220053	60220062	60220060	T	G	snp	1	0	0	0	.	.	.	FGGY(INTRON);FGGY(UPSTREAM);RP4-782L23.2.1(DOWNSTREAM)
1	60223712	60223721	60223716	AC	T	snp	1	0	0	0	.	.	.	FGGY(INTRON);RP4-782L23.2.1(INTRON)
1	60230069	60230076	60230074	T	G	snp	1	1	1	1	.	.	.	FGGY(DOWNSTREAM);FGGY(INTRON);RP4-782L23.2.1(INTRON)
1	60236375	60236392	60236386	TTG	C	snp	1	0	0	0	.	.	.	FGGY(DOWNSTREAM);RP4-782L23.1.1(UPSTREAM);RP4-782L23.2.1(INTRON)
1	60261716	60261723	60261722	A	C	snp	1	0	0	0	.	.	.	INTERGENIC(INTERGENIC)
1	60420261	60420268	60420264	G	GA	ins	1	0	0	0	.	.	.	INTERGENIC(INTERGENIC)
1	60469158	60469166	60469159	A	C	snp	1	1	1	1	.	.	.	C1orf87(INTRON)
1	60529960	60529969	60529962	TA	C	snp	0	0	1	0	.	.	.	C1orf87(INTRON)
1	60643582	60643591	60643589	TA	G	snp	1	0	0	0	.	.	.	INTERGENIC(INTERGENIC)
1	60667931	60667939	60667936	A	C	snp	1	1	1	1	.	.	.	INTERGENIC(INTERGENIC)
1	60691841	60691849	60691843	T	C	snp	0	0	0	1	.	.	.	INTERGENIC(INTERGENIC)
1	60709782	60709791	60709786	A	G	ins	1	0	1	1	.	.	.	INTERGENIC(INTERGENIC)
1	60736909	60736920	60736913	GC	A	snp	0	1	0	1	.	.	.	INTERGENIC(INTERGENIC)
1	60762238	60762253	60762251	TA	TT	ins	1	1	0	1	.	.	.	INTERGENIC(INTERGENIC)
1	61019510	61019519	61019512	TA	C	snp	0	0	0	1	.	.	.	RP11-776H12.1.1(INTRON)
1	61059439	61059450	61059442	TG	A	snp	1	0	0	0	.	.	.	RP11-776H12.1.1(INTRON)
1	61103398	61103425	61103421	AGAT	C	snp	1	0	0	0	.	.	.	RP11-776H12.1.1(INTRON)
1	61145141	61145152	61145148	TG	TGT	del	1	1	1	1	.	.	.	RP11-436K8.1.1(INTRON)
1	61329861	61329868	61329865	A	G	snp	1	0	0	1	.	.	.	INTERGENIC(INTERGENIC);NFIA(UPSTREAM)
1	61335721	61335728	61335726	T	C	snp	1	1	1	1	.	.	.	NFIA(INTRON)
1	61499201	61499216	61499213	TA	A	del	1	0	0	0	.	.	.	NFIA(INTRON)
1	61603601	61603618	61603614	TC	G	snp	1	1	1	1	.	.	.	NFIA(DOWNSTREAM);NFIA(INTRON)
1	61719677	61719688	61719685	TGG	T	snp	0	0	1	0	.	.	.	NFIA(INTRON);RP5-833A20.1.1(UPSTREAM)
1	61794887	61794894	61794893	T	G	snp	1	1	1	1	.	.	.	NFIA(INTRON)
1	61927733	61927741	61927736	T	G	snp	1	1	1	1	.	.	.	NFIA(DOWNSTREAM);NFIA(UTR_3_PRIME)
1	61939669	61939678	61939673	CT	G	snp	1	1	1	0	.	.	.	INTERGENIC(INTERGENIC)
1	61941242	61941251	61941249	AC	T	snp	1	1	1	1	.	.	.	INTERGENIC(INTERGENIC)
1	61946471	61946482	61946473	AT	T	del	1	1	1	1	.	.	.	INTERGENIC(INTERGENIC)
1	61946471	61946482	61946474	AT	A	snp	1	1	1	1	.	.	.	INTERGENIC(INTERGENIC)
1	61979029	61979040	61979032	TTG	C	snp	1	0	0	0	.	.	.	INTERGENIC(INTERGENIC)
1	62054752	62054765	62054761	TC	A	snp	0	0	1	0	.	.	.	INTERGENIC(INTERGENIC)
1	62229672	62229680	62229679	T	G	snp	1	0	0	0	.	.	.	INADL(INTRON)
1	62253769	62253778	62253773	A	G	ins	1	1	1	1	.	.	.	INADL(INTRON)
1	62263879	62263886	62263880	T	G	snp	1	0	0	0	.	.	.	INADL(INTRON)
1	62325286	62325294	62325292	T	G	snp	1	0	0	1	.	.	.	INADL(INTRON);INADL(UPSTREAM)
1	62333981	62333990	62333987	CT	G	snp	1	0	0	0	.	.	.	INADL(DOWNSTREAM);INADL(INTRON)
1	62356473	62356480	62356477	T	C	snp	0	1	1	0	.	.	.	INADL(INTRON)
1	62380193	62380200	62380195	A	G	snp	1	1	1	1	.	.	.	INADL(INTRON)
1	62384991	62385000	62384996	T	C	snp	1	0	0	0	.	.	.	INADL(INTRON)
1	62440060	62440077	62440074	TC	CTC	del	1	0	0	0	.	.	.	INADL(INTRON)
1	62449821	62449838	62449832	ATT	A	del	1	0	0	0	.	.	.	INADL(INTRON)
1	62479456	62479463	62479457	T	C	snp	1	0	0	0	.	.	.	INADL(INTRON)
1	62509399	62509406	62509400	G	A	ins	1	1	0	1	.	.	.	HBXIPP1(DOWNSTREAM);INADL(INTRON)
1	62551979	62551990	62551984	TG	C	snp	0	0	1	0	.	.	.	INADL(INTRON)
1	62558399	62558418	62558414	TTTA	T	snp	1	0	0	0	.	.	.	INADL(INTRON)
1	62564050	62564057	62564055	A	G	snp	1	0	0	0	.	.	.	INADL(INTRON)
1	62671046	62671059	62671054	TC	C	snp	0	1	0	1	.	.	.	L1TD1(INTRON);RP5-1155K23.4.1(DOWNSTREAM)
1	62682284	62682292	62682287	T	C	snp	0	0	1	0	.	.	.	INTERGENIC(INTERGENIC);L1TD1(DOWNSTREAM);Y_RNA(DOWNSTREAM)
1	62732165	62732174	62732170	CA	T	snp	1	1	1	1	.	.	.	KANK4(INTRON);KANK4(UPSTREAM)
1	62738006	62738015	62738013	AC	T	snp	1	1	1	1	.	.	KANK4(START_GAINED)	KANK4(INTRON)
1	62790264	62790279	62790275	TTTA	T	ins	0	1	0	0	.	.	.	INTERGENIC(INTERGENIC)
1	62863971	62863986	62863981	CCAC	C	snp	1	0	0	0	.	.	.	INTERGENIC(INTERGENIC)

1	62904569	62904576	62904575	T	G	snp	1	1	1	1	.	.	.	USP1(INTRON)
1	62911300	62911317	62911303	TG	TGTA	ins	1	1	1	1	.	.	.	USP1(DOWNSTREAM);USP1(INTRON)
1	63085994	63086013	63086001	AAAT	A	snp	1	1	1	1	.	.	.	DOCK7(DOWNSTREAM);DOCK7(INTRON)
1	63153861	63153875	63153866	GGC	G	snp	1	1	0	1	.	.	.	DOCK7(INTRON);RP11-230B22.1.1(UPSTREAM)
1	63201530	63201541	63201538	TC	A	snp	1	1	1	1	.	.	.	INTERGENIC(INTERGENIC)
1	63408910	63408919	63408915	CT	G	snp	1	0	0	0	.	.	.	INTERGENIC(INTERGENIC)
1	63412755	63412763	63412761	T	TTTG	ins	1	1	1	1	.	.	.	INTERGENIC(INTERGENIC)
1	63419891	63419898	63419892	G	A	snp	1	0	0	0	.	.	.	INTERGENIC(INTERGENIC)
1	63443038	63443045	63443041	C	A	snp	1	0	0	0	.	.	.	INTERGENIC(INTERGENIC)
1	63462331	63462340	63462332	A	G	snp	1	1	1	1	.	.	.	INTERGENIC(INTERGENIC);RP11-5P4.1.1(DOWNSTREAM)
1	63683869	63683876	63683875	A	C	snp	1	0	0	0	.	.	.	LINC00466(DOWNSTREAM);LINC00466(INTRON)
1	63888104	63888113	63888109	AT	A	snp	1	1	1	1	.	.	.	ALG6(INTRON)
1	63888134	63888143	63888140	AT	G	snp	1	0	0	0	.	.	.	ALG6(INTRON)
1	63958442	63958450	63958446	A	C	snp	1	1	1	1	.	.	.	ITGB3BP(INTRON);ITGB3BP(UPSTREAM)
1	64017378	64017387	64017384	T	G	snp	1	0	0	0	.	.	.	DLEU2L(DOWNSTREAM);EFCAB7(INTRON);EFCAB7(UPSTREAM);ITGB3BP(INTRON)
1	64061518	64061527	64061522	A	G	snp	1	1	1	1	.	.	.	ITGB3BP(UPSTREAM);PGM1(INTRON)
1	64092737	64092750	64092748	CA	G	snp	1	1	1	1	.	.	.	PGM1(INTRON)
1	64196336	64196351	64196347	ATTT	A	del	1	1	1	1	.	.	.	INTERGENIC(INTERGENIC)
1	64222189	64222196	64222195	C	A	ins	1	1	1	1	.	.	.	INTERGENIC(INTERGENIC)
1	64333802	64333819	64333807	TTG	T	snp	1	1	1	1	.	.	.	ROR1(INTRON)
1	64350809	64350823	64350816	ATT	C	snp	1	1	1	1	.	.	.	ROR1(INTRON)
1	64445823	64445838	64445833	AT	G	snp	1	1	1	1	.	.	.	ROR1(INTRON)
1	64480386	64480399	64480393	TA	TATATATATATATATATATG	ins	1	1	1	1	.	.	.	ROR1(INTRON)
1	64518520	64518529	64518525	T	C	snp	1	1	1	1	.	.	.	ROR1(INTRON)
1	64548542	64548551	64548547	TC	G	snp	1	1	1	1	.	.	.	ROR1(INTRON)
1	64560232	64560243	64560235	TG	A	snp	1	0	0	0	.	.	.	ROR1(INTRON);RP11-24J23.2.1(EXON)
1	64704729	64704749	64704739	AAG	G	snp	1	1	0	1	.	.	.	UBE2U(INTRON)
1	64704729	64704749	64704745	AAG	G	snp	1	1	1	1	.	.	.	UBE2U(INTRON)
1	64730634	64730643	64730640	A	AAATT	ins	0	0	1	0	.	.	.	UBE2U(INTRON)
1	64750708	64750717	64750716	T	G	snp	1	1	0	1	.	.	.	INTERGENIC(INTERGENIC)
1	64764595	64764604	64764599	TA	A	ins	1	1	0	1	.	.	.	INTERGENIC(INTERGENIC)
1	64840268	64840277	64840272	T	C	snp	1	1	1	1	.	.	.	INTERGENIC(INTERGENIC)
1	64882717	64882728	64882723	ATA	T	del	1	1	1	1	.	.	.	INTERGENIC(INTERGENIC)
1	65042578	65042593	65042586	GTTT	T	ins	1	1	1	1	.	.	.	CACHD1(INTRON)
1	65091456	65091464	65091462	A	G	snp	1	0	0	0	.	.	.	CACHD1(INTRON)
1	65099458	65099467	65099461	TG	A	snp	1	0	0	0	.	.	.	CACHD1(INTRON)
1	65175759	65175767	65175761	T	G	snp	0	1	1	0	.	.	.	INTERGENIC(INTERGENIC)
1	65247391	65247398	65247394	T	C	snp	1	1	1	1	.	.	.	RAVER2(INTRON)
1	65318894	65318903	65318900	AG	C	snp	1	0	0	0	.	.	.	JAK1(INTRON)
1	65342503	65342510	65342505	A	C	snp	0	1	0	1	.	.	.	JAK1(INTRON)
1	65387832	65387841	65387837	A	G	snp	1	1	1	0	.	.	.	JAK1(INTRON);RP11-182I10.1.1(DOWNSTREAM)
1	65448453	65448461	65448459	T	C	snp	1	1	1	0	.	.	.	RP11-182I10.3.1(INTRON);RP11-182I10.4.1(DOWNSTREAM)
1	65498206	65498213	65498208	A	C	snp	1	1	0	1	.	.	.	INTERGENIC(INTERGENIC)
1	65585492	65585511	65585503	AAAC	T	snp	1	1	1	1	.	.	.	INTERGENIC(INTERGENIC)
1	65614844	65614851	65614849	C	T	snp	1	1	1	0	.	.	.	AK4(INTRON)
1	65615955	65615968	65615964	TC	TCTCT	del	1	1	1	1	.	.	.	AK4(INTRON);RP4-700A9.1.1(UPSTREAM)
1	65636071	65636078	65636072	A	C	snp	1	1	1	1	.	.	.	AK4(INTRON)
1	65706605	65706614	65706611	T	TG	ins	1	1	1	0	.	.	.	INTERGENIC(INTERGENIC)
1	65747290	65747299	65747295	T	G	snp	0	1	1	1	.	.	.	DNAJC6(INTRON);RP5-1044H5.1.1(INTRON)
1	65790051	65790060	65790055	T	G	snp	1	1	1	0	.	.	.	DNAJC6(INTRON)
1	65796143	65796152	65796148	T	C	snp	0	0	0	1	.	.	.	DNAJC6(INTRON)
1	65801888	65801899	65801894	GCA	C	snp	1	1	1	0	.	.	.	DNAJC6(INTRON)
1	65813417	65813428	65813424	CA	G	snp	1	0	0	0	.	.	.	AL355487.1(DOWNSTREAM);DNAJC6(INTRON)
1	65865573	65865580	65865574	T	C	snp	1	1	1	1	.	.	.	DNAJC6(INTRON)
1	66003853	66003862	66003854	T	G	snp	1	1	1	1	.	.	.	LEPR(INTRON)
1	66129953	66129962	66129958	AT	ATATA	del	1	1	1	1	.	.	.	INTERGENIC(INTERGENIC)
1	66225861	66225869	66225862	T	C	snp	0	1	1	1	.	.	.	AL513493.1(DOWNSTREAM);INTERGENIC(INTERGENIC)
1	66365352	66365365	66365355	TA	G	snp	1	0	0	0	.	.	.	PDE4B(INTRON)
1	66430930	66430938	66430934	C	A	snp	1	0	0	0	.	.	.	PDE4B(INTRON)
1	66468747	66468755	66468749	T	G	snp	0	1	1	0	.	.	.	PDE4B(INTRON)
1	66483521	66483536	66483523	AT	T	del	0	1	1	0	.	.	.	PDE4B(INTRON)
1	66618293	66618302	66618300	AT	A	snp	0	1	0	1	.	.	.	PDE4B(INTRON)
1	66866188	66866208	66866204	GAG	G	snp	1	1	1	1	.	.	.	INTERGENIC(INTERGENIC)
1	66886532	66886539	66886536	T	G	snp	1	1	1	1	.	.	.	INTERGENIC(INTERGENIC)
1	66897004	66897013	66897008	T	G	ins	1	1	0	1	.	.	.	INTERGENIC(INTERGENIC)

1	66906226	66906235	66906228	AT	T	snp	1	0	0	0	.	.	.	INTERGENIC(INTERGENIC)
1	66906226	66906235	66906230	AT	T	snp	1	0	0	0	.	.	.	INTERGENIC(INTERGENIC)
1	67062879	67062888	67062885	TC	C	snp	1	1	1	1	.	.	.	SGIP1(INTRON)
1	67095259	67095268	67095264	A	G	snp	1	1	1	1	.	.	.	SGIP1(DOWNSTREAM);SGIP1(INTRON)
1	67132863	67132871	67132870	C	A	snp	1	0	0	0	.	.	.	AL139147.1(INTRON);SGIP1(INTRON);SGIP1(UPSTREAM)
1	67215434	67215441	67215438	T	C	snp	1	1	1	1	.	.	.	INTERGENIC(INTERGENIC);SGIP1(DOWNSTREAM);TCTEX1D1(UPSTREAM)
1	67307045	67307052	67307046	A	G	snp	1	0	0	0	.	.	.	WDR78(INTRON)
1	67334800	67334815	67334802	AT	TG	ins	1	0	0	0	.	.	.	WDR78(DOWNSTREAM);WDR78(INTRON)
1	67358737	67358748	67358740	AG	A	snp	1	1	0	1	.	.	.	WDR78(DOWNSTREAM);WDR78(INTRON)
1	67392605	67392616	67392614	TA	G	snp	1	1	1	1	.	.	.	MIER1(INTRON);MIER1(UPSTREAM);WDR78(UPSTREAM)
1	67441667	67441674	67441672	T	G	ins	1	1	1	1	.	.	.	MIER1(INTRON)
1	67444781	67444790	67444783	T	C	snp	1	1	0	1	.	.	.	MIER1(INTRON)
1	67477333	67477340	67477339	T	G	snp	1	1	1	1	.	.	.	SLC35D1(INTRON)
1	67541432	67541439	67541435	T	G	snp	1	0	0	0	.	.	.	INTERGENIC(INTERGENIC)
1	67558757	67558766	67558763	AT	G	snp	1	1	1	1	.	.	C1orf141(SYNONYMOUS_CODING):Y376	C1orf141(DOWNSTREAM);C1orf141(UTR_3_PRIME)
1	67583111	67583120	67583113	A	G	snp	1	1	1	1	.	.	.	C1orf141(INTRON)
1	67593324	67593335	67593328	GTT	C	snp	1	0	0	0	.	.	.	C1orf141(INTRON);C1orf141(UPSTREAM)
1	67676153	67676162	67676156	T	C	snp	1	0	0	0	.	.	.	IL23R(DOWNSTREAM);IL23R(INTRON)
1	67683955	67683968	67683964	AT	AC	ins	1	1	0	1	.	.	.	IL23R(INTRON)
1	67792116	67792123	67792120	A	AT	ins	1	0	0	0	.	.	.	IL12RB2(INTRON)
1	67819084	67819092	67819087	A	C	snp	1	1	1	1	.	.	.	IL12RB2(INTRON)
1	67825397	67825406	67825399	A	C	snp	1	1	1	1	.	.	.	IL12RB2(INTRON)
1	67912072	67912081	67912079	A	G	snp	1	1	1	1	.	.	.	INTERGENIC(INTERGENIC)
1	67953205	67953224	67953217	AAAC	G	snp	1	1	1	1	.	.	.	INTERGENIC(INTERGENIC)
1	67986459	67986474	67986470	CT	CTC	del	1	1	1	1	.	.	.	INTERGENIC(INTERGENIC)
1	68004632	68004643	68004641	AG	C	snp	1	1	1	1	.	.	.	INTERGENIC(INTERGENIC);U6(UPSTREAM)
1	68044015	68044032	68044030	GT	G	del	1	1	1	1	.	.	.	INTERGENIC(INTERGENIC)
1	68065164	68065172	68065165	A	G	ins	1	1	1	1	.	.	.	INTERGENIC(INTERGENIC)
1	68081885	68081905	68081896	TTG	TTGTTGTTGT	del	1	1	1	1	.	.	.	INTERGENIC(INTERGENIC)
1	68148190	68148205	68148203	AT	ATA	del	1	0	0	0	.	.	.	GADD45A(UPSTREAM);INTERGENIC(INTERGENIC)
1	68175734	68175745	68175743	TA	G	snp	1	1	1	1	.	.	.	GNG12(INTRON)
1	68268511	68268522	68268514	TC	T	snp	0	0	0	1	.	.	.	GNG12(INTRON)
1	68268511	68268522	68268520	TC	T	snp	1	1	1	1	.	.	.	GNG12(INTRON)
1	68279374	68279382	68279381	A	C	ins	1	1	1	1	.	.	.	GNG12(INTRON)
1	68337210	68337221	68337217	CA	T	snp	1	0	0	0	.	.	.	RP11-518D3.1.1(INTRON)
1	68464419	68464433	68464422	TAT	C	snp	1	1	1	1	.	.	.	AL157407.1(UPSTREAM);RP11-518D3.1.1(INTRON)
1	68488369	68488392	68488385	AAAT	T	snp	1	0	0	0	.	.	.	RP11-518D3.1.1(INTRON)
1	68566990	68567010	68566993	TAT	C	snp	1	1	1	1	.	.	.	RP11-518D3.1.1(INTRON);WLS(INTRON)
1	68594689	68594698	68594694	CA	G	snp	1	1	1	1	.	.	.	RP11-518D3.1.1(INTRON);WLS(INTRON)
1	68598747	68598767	68598760	CAG	G	snp	1	0	0	0	.	.	.	RP11-518D3.1.1(INTRON);WLS(INTRON)
1	68598844	68598855	68598846	GT	A	snp	1	0	0	0	.	.	.	RP11-518D3.1.1(INTRON);WLS(INTRON)
1	68658266	68658275	68658267	A	C	snp	1	1	1	1	.	.	.	RP11-518D3.1.1(INTRON);WLS(DOWNSTREAM);WLS(INTRON)
1	68658266	68658275	68658269	A	C	snp	0	1	1	1	.	.	.	RP11-518D3.1.1(INTRON);WLS(DOWNSTREAM);WLS(INTRON)
1	68686787	68686794	68686790	T	C	snp	1	1	1	1	.	.	.	WLS(INTRON)
1	68787901	68787908	68787902	A	C	ins	1	1	1	1	.	.	.	INTERGENIC(INTERGENIC)
1	68900403	68900426	68900411	TGTT	G	del	1	0	0	0	.	.	.	RPE65(INTRON)
1	68900403	68900426	68900412	TGTT	T	snp	1	1	1	1	.	.	.	RPE65(INTRON)
1	69076354	69076362	69076360	T	G	snp	1	1	1	1	.	.	.	INTERGENIC(INTERGENIC)
1	69119327	69119336	69119330	TG	A	snp	1	1	1	1	.	.	.	INTERGENIC(INTERGENIC)
1	69192654	69192662	69192657	T	C	snp	1	1	1	1	.	.	.	INTERGENIC(INTERGENIC)
1	69259250	69259261	69259253	TA	C	snp	1	1	1	1	.	.	.	INTERGENIC(INTERGENIC)
1	69452017	69452026	69452018	A	C	ins	1	1	1	1	.	.	.	INTERGENIC(INTERGENIC)
1	69613149	69613158	69613155	T	TTC	ins	1	1	1	1	.	.	.	RP11-424D14.1.1(INTRON)
1	69942994	69943002	69943001	T	C	snp	1	1	1	1	.	.	.	INTERGENIC(INTERGENIC)
1	69970480	69970499	69970489	TTTG	C	snp	1	1	1	1	.	.	.	INTERGENIC(INTERGENIC)
1	69974302	69974311	69974303	T	G	snp	0	1	0	0	.	.	.	INTERGENIC(INTERGENIC)
1	69984315	69984326	69984321	TA	C	snp	1	0	0	0	.	.	.	INTERGENIC(INTERGENIC)
1	69992355	69992370	69992366	TA	G	snp	1	1	1	1	.	.	.	INTERGENIC(INTERGENIC)
1	70107173	70107182	70107178	AT	C	snp	1	1	0	1	.	.	.	LRRC7(INTRON)
1	70118967	70118974	70118969	T	C	ins	1	1	1	1	.	.	.	LRRC7(INTRON)
1	70167182	70167189	70167183	A	G	snp	0	1	0	1	.	.	.	LRRC7(INTRON)
1	70187822	70187837	70187835	AT	A	del	1	1	1	1	.	.	.	LRRC7(INTRON)
1	70247363	70247370	70247367	A	G	snp	1	1	0	1	.	.	.	LRRC7(INTRON)

1	70261585	70261592	70261588	T	TTGG	ins	1	1	0	1	.	.	.	LRRC7(INTRON)	
1	70261585	70261592	70261589	T	TGG	ins	1	1	0	1	.	.	.	LRRC7(INTRON)	
1	70308898	70308907	70308900	A	C	snp	1	0	0	0	.	.	.	LRRC7(DOWNSTREAM);LRRC7(INTRON)	
1	70361830	70361837	70361833	T	C	snp	1	1	1	1	.	.	.	LRRC7(INTRON)	
1	70381987	70382000	70381993	TA	C	snp	1	0	0	0	.	.	.	LRRC7(INTRON);PIN1P1(UPSTREAM)	
1	70395571	70395582	70395577	CT	T	snp	0	1	1	1	.	.	.	LRRC7(INTRON);LRRC7(UPSTREAM)	
1	70415666	70415673	70415667	T	TA	ins	1	0	0	0	.	.	.	LRRC7(INTRON)	
1	70446814	70446823	70446821	AG	A	ins	0	0	1	1	.	.	.	LRRC7(INTRON)	
1	70654262	70654270	70654263	A	C	snp	0	1	1	1	.	.	.	LRRC40(INTRON)	
1	70841901	70841914	70841906	AC	T	snp	0	1	0	1	.	.	.	HHLA3(INTRON)	
1	70920865	70920872	70920870	T	G	snp	0	0	0	1	.	.	.	INTERGENIC(INTERGENIC)	
1	71005076	71005085	71005078	CT	A	snp	1	1	0	1	.	.	.	INTERGENIC(INTERGENIC)	
1	71070045	71070053	71070050	C	A	snp	1	1	1	1	.	.	.	INTERGENIC(INTERGENIC)	
1	71089752	71089769	71089766	AT	G	snp	1	1	0	0	.	.	.	INTERGENIC(INTERGENIC)	
1	71109583	71109594	71109586	ATC	T	snp	1	1	1	1	.	.	.	INTERGENIC(INTERGENIC)	
1	71211556	71211564	71211562	T	G	snp	0	0	1	0	.	.	.	RP5-952N6.1.1(INTRON)	
1	71250282	71250290	71250289	T	G	snp	1	1	1	1	.	.	.	RP5-952N6.1.1(INTRON)	
1	71279514	71279531	71279527	AG	A	del	1	0	0	0	.	.	.	INTERGENIC(INTERGENIC)	
1	71327937	71327945	71327942	A	G	snp	0	0	1	0	.	.	.	PTGER3(DOWNSTREAM);PTGER3(INTRON)	
1	71354184	71354195	71354186	TG	C	snp	1	0	1	0	.	.	.	PTGER3(INTRON)	
1	71454716	71454723	71454718	C	T	snp	1	0	0	0	.	.	.	PTGER3(INTRON)	
1	71455570	71455581	71455579	AC	A	snp	1	0	0	0	.	.	.	PTGER3(INTRON)	
1	71486161	71486169	71486168	T	C	snp	1	1	1	1	.	.	.	PTGER3(INTRON)	
1	71698466	71698481	71698477	AAAC	A	del	1	1	1	1	.	.	.	ZRANB2-AS2(INTRON)	
1	72141487	72141496	72141492	T	G	ins	1	1	1	1	.	.	.	NEGR1(INTRON)	
1	72157975	72157983	72157977	A	C	snp	0	0	1	0	.	.	.	NEGR1(INTRON)	
1	72312100	72312109	72312105	AC	T	snp	0	0	1	0	.	.	.	NEGR1(INTRON)	
1	72359901	72359915	72359910	ATA	C	snp	1	0	0	0	.	.	.	NEGR1(INTRON)	
1	72411733	72411741	72411740	T	G	ins	1	0	1	0	.	.	.	NEGR1(INTRON)	
1	72411733	72411741	72411740	T	G	snp	1	0	1	0	.	.	.	NEGR1(INTRON)	
1	72456587	72456602	72456598	AT	A	del	1	0	0	0	.	.	.	NEGR1(INTRON)	
1	72456587	72456602	72456600	AT	A	del	1	1	1	1	.	.	.	NEGR1(INTRON)	
1	72492222	72492233	72492230	AAC	AACA	del	1	1	1	1	.	.	.	NEGR1(INTRON)	
1	72492619	72492626	72492623	T	C	snp	1	1	0	1	.	.	.	NEGR1(INTRON)	
1	72557036	72557055	72557040	TCCC	A	snp	1	0	0	0	.	.	.	NEGR1(INTRON)	
1	72558134	72558141	72558138	T	C	snp	1	1	1	1	.	.	.	NEGR1(INTRON)	
1	72582977	72583000	72582989	TTTG	TTG	del	1	0	0	1	.	.	.	NEGR1(INTRON)	
1	72586198	72586205	72586200	T	C	ins	0	1	0	1	.	.	.	NEGR1(INTRON)	
1	72640441	72640448	72640442	A	C	snp	1	1	0	1	.	.	.	NEGR1(INTRON)	
1	72685938	72685953	72685945	TC	T	snp	1	0	0	0	.	.	.	NEGR1(INTRON)	
1	72702114	72702121	72702119	A	G	snp	1	0	0	0	.	.	.	NEGR1(INTRON)	
1	72866572	72866581	72866574	T	G	snp	1	1	1	1	.	.	.	INTERGENIC(INTERGENIC)	
1	72967245	72967264	72967259	TGTT	TTG	del	1	0	0	0	.	.	.	INTERGENIC(INTERGENIC)	
1	72983635	72983644	72983639	TA	AC	ins	1	0	0	0	.	.	.	INTERGENIC(INTERGENIC)	
1	73160614	73160623	73160618	A	G	snp	1	0	0	0	.	.	.	INTERGENIC(INTERGENIC)	
1	73219930	73219937	73219933	A	G	snp	1	0	0	0	.	.	.	RP4-660H19.1.1(INTRON)	
1	73345613	73345628	73345616	AG	A	snp	1	0	0	0	.	.	.	RP4-660H19.1.1(INTRON)	
1	73379308	73379315	73379312	T	C	snp	0	1	1	0	.	.	.	INTERGENIC(INTERGENIC)	
1	73429438	73429447	73429445	TA	TG	ins	1	0	0	0	.	.	.	INTERGENIC(INTERGENIC)	
1	73537022	73537031	73537025	T	C	ins	1	1	1	0	.	.	.	INTERGENIC(INTERGENIC)	
1	73562406	73562419	73562413	TG	TA	ins	1	1	1	0	.	.	.	INTERGENIC(INTERGENIC)	
1	73567870	73567881	73567876	AAC	ACG	ins	1	1	1	0	.	.	.	INTERGENIC(INTERGENIC);KRT8P21(UPSTREAM)	
1	73604464	73604475	73604469	AT	ATATATAC	ins	1	1	1	1	.	.	.	INTERGENIC(INTERGENIC)	
1	73604548	73604561	73604555	TA	G	snp	1	1	1	0	.	.	.	INTERGENIC(INTERGENIC)	
1	73655310	73655321	73655313	AC	A	snp	1	1	1	1	.	.	.	7SK(DOWNSTREAM);INTERGENIC(INTERGENIC)	
1	73676334	73676343	73676338	AT	T	snp	1	0	0	0	.	.	.	INTERGENIC(INTERGENIC)	
1	73730841	73730848	73730846	A	C	snp	1	0	0	0	.	.	.	INTERGENIC(INTERGENIC)	
1	73762953	73762962	73762959	AT	G	snp	1	1	1	1	.	.	.	INTERGENIC(INTERGENIC)	
1	73934320	73934343	73934330	AGAT	G	snp	1	1	1	1	.	.	.	INTERGENIC(INTERGENIC)	
1	74036005	74036013	74036009	A	G	snp	1	1	1	1	.	.	.	INTERGENIC(INTERGENIC)	
1	74084589	74084597	74084595	A	G	snp	1	1	1	1	.	.	.	INTERGENIC(INTERGENIC)	
1	74135297	74135306	74135301	TA	C	snp	1	0	0	1	.	.	.	RP11-275A6.1.1(INTRON)	
1	74184492	74184507	74184496	AGAT	GATAGATAGAT	del	1	1	1	1	.	.	.	INTERGENIC(INTERGENIC);RP11-275A6.2.1(DOWNSTREAM)
1	74184492	74184507	74184500	AGAT	GATAGAT	del	1	1	1	1	.	.	.	INTERGENIC(INTERGENIC);RP11-275A6.2.1(DOWNSTREAM)	
1	74250058	74250065	74250062	A	G	snp	0	1	1	0	.	.	.	INTERGENIC(INTERGENIC)	
1	74262616	74262631	74262629	GT	C	snp	1	1	1	1	.	.	.	INTERGENIC(INTERGENIC)	

1	74286415	74286422	74286416	T	G	snp	1	0	0	0	.	.	.	INTERGENIC(INTERGENIC)
1	74372016	74372031	74372029	TC	T	snp	1	1	1	1	.	.	.	INTERGENIC(INTERGENIC)
1	74372130	74372147	74372133	TC	T	snp	1	1	1	0	.	.	.	INTERGENIC(INTERGENIC)
1	74425051	74425059	74425052	T	G	ins	1	1	1	0	.	.	.	INTERGENIC(INTERGENIC)
1	74512437	74512444	74512443	A	G	snp	1	0	0	0	.	.	.	LRRIQ3(INTRON)
1	74623233	74623241	74623239	T	G	snp	0	1	1	0	.	.	.	LRRIQ3(INTRON);LRRIQ3(UPSTREAM)
1	74695700	74695708	74695704	A	G	snp	1	1	1	1	.	.	.	FPGT(INTRON);FPGT-TNNI3K(INTRON);FPGT-TNNI3K.1(INTRON)
1	74749756	74749776	74749766	TTC	A	snp	1	1	1	1	.	.	.	FPGT-TNNI3K(INTRON);FPGT-TNNI3K.1(INTRON)
1	74768870	74768893	74768888	ATT	T	snp	1	1	1	1	.	.	.	FPGT-TNNI3K(INTRON);FPGT-TNNI3K.1(INTRON)
1	74782367	74782386	74782378	TTTA	T	snp	0	1	1	0	.	.	.	FPGT-TNNI3K(INTRON);FPGT-TNNI3K.1(INTRON)
1	74782367	74782386	74782382	TTTA	T	snp	1	1	1	1	.	.	.	FPGT-TNNI3K(INTRON);FPGT-TNNI3K.1(INTRON)
1	74817556	74817564	74817558	T	C	snp	1	1	1	0	.	.	.	FPGT-TNNI3K(INTRON);FPGT-TNNI3K.1(INTRON);RP11-439H8.4.1(INTRON)
1	75057580	75057587	75057586	T	C	snp	1	1	1	1	.	.	.	C1orf173(INTRON);RP11-17E13.3.1(INTRON);RP4-612J11.1.1(INTRON)
1	75203792	75203800	75203798	A	C	ins	1	0	1	1	.	.	.	CRYZ(UPSTREAM);RP11-17E13.3.1(INTRON);TYW3(INTRON)
1	75248067	75248075	75248072	T	C	snp	0	0	1	0	.	.	.	INTERGENIC(INTERGENIC)
1	75421128	75421136	75421135	A	C	snp	1	1	1	1	.	.	.	INTERGENIC(INTERGENIC)
1	75538121	75538130	75538128	TG	A	snp	1	1	0	1	.	.	.	INTERGENIC(INTERGENIC)
1	75604736	75604745	75604743	A	G	snp	1	0	0	0	.	.	.	LHX8(INTRON)
1	75633908	75633923	75633916	TGTT	A	snp	1	0	0	0	.	.	.	INTERGENIC(INTERGENIC)
1	75687635	75687646	75687642	TCT	T	ins	1	1	0	1	.	.	.	SLC44A5(INTRON)
1	75687635	75687646	75687643	TCT	TC	del	1	1	1	1	.	.	.	SLC44A5(INTRON)
1	75746029	75746038	75746031	AT	C	snp	1	0	1	0	.	.	.	SLC44A5(INTRON)
1	75818124	75818133	75818125	T	G	snp	1	1	1	1	.	.	.	SLC44A5(INTRON)
1	75969120	75969129	75969124	CT	T	snp	1	1	1	1	.	.	.	SLC44A5(INTRON)
1	76044114	76044129	76044120	GATA	G	snp	1	1	0	1	.	.	.	RP4-595K12.1.1(UPSTREAM);SLC44A5(INTRON)
1	76081087	76081100	76081089	CT	A	snp	1	0	0	0	.	.	.	INTERGENIC(INTERGENIC);SLC44A5(UPSTREAM)
1	76089276	76089284	76089281	T	C	snp	1	0	0	0	.	.	.	INTERGENIC(INTERGENIC)
1	76125068	76125083	76125075	GAAA	G	snp	1	1	0	1	.	.	.	INTERGENIC(INTERGENIC)
1	76162429	76162438	76162430	A	C	ins	1	1	1	1	.	.	.	INTERGENIC(INTERGENIC)
1	76350327	76350336	76350332	AC	A	snp	1	0	0	0	.	.	.	MSH4(INTRON)
1	76388586	76388594	76388589	A	C	snp	1	1	1	1	.	.	.	ASB17(INTRON)
1	76428341	76428352	76428347	TA	C	snp	1	1	0	1	.	.	.	INTERGENIC(INTERGENIC)
1	76476243	76476251	76476248	T	G	snp	1	1	1	1	.	.	.	INTERGENIC(INTERGENIC);RP11-550H2.1.1(UPSTREAM)
1	76659283	76659294	76659287	AAT	C	snp	1	1	0	1	.	.	.	ST6GALNAC3(INTRON)
1	76669210	76669217	76669211	T	AA	ins	1	1	1	1	.	.	.	ST6GALNAC3(INTRON)
1	76742938	76742949	76742942	TG	C	snp	1	1	1	0	.	.	.	ST6GALNAC3(INTRON)
1	76903255	76903264	76903258	T	C	snp	1	1	1	1	.	.	.	ST6GALNAC3(INTRON)
1	76908823	76908830	76908826	T	C	snp	1	1	1	1	.	.	.	ST6GALNAC3(INTRON)
1	76981072	76981079	76981073	T	G	snp	1	1	1	1	.	.	.	ST6GALNAC3(INTRON)
1	77105021	77105034	77105023	AC	G	snp	1	0	0	0	.	.	.	INTERGENIC(INTERGENIC);ST6GALNAC3(DOWNSTREAM)
1	77168329	77168338	77168330	A	C	snp	1	0	0	0	.	.	.	INTERGENIC(INTERGENIC);TPI1P1(DOWNSTREAM)
1	77168655	77168664	77168662	GA	G	snp	1	0	0	0	.	.	.	INTERGENIC(INTERGENIC);TPI1P1(DOWNSTREAM)
1	77305418	77305431	77305423	AT	G	snp	1	1	1	1	.	.	.	INTERGENIC(INTERGENIC)
1	77334277	77334300	77334292	GCA	CAGCAA	ins	1	0	0	1	.	ST6GALNAC5(CODON_INSERTION):-43QQ	.	ST6GALNAC5(EXON);ST6GALNAC5(INTRON)
1	77389412	77389419	77389416	A	G	snp	1	1	1	1	.	.	.	ST6GALNAC5(INTRON)
1	77471822	77471829	77471827	T	G	ins	0	0	1	1	.	.	.	ST6GALNAC5(INTRON)
1	77536443	77536460	77536456	AT	A	del	1	0	0	1	.	.	.	RP4-706A16.2.1(INTRON)
1	77536443	77536460	77536457	AT	T	snp	1	1	1	1	.	.	.	RP4-706A16.2.1(INTRON)
1	77536443	77536460	77536458	AT	A	del	1	1	1	1	.	.	.	RP4-706A16.2.1(INTRON)
1	77546085	77546092	77546091	C	A	snp	1	1	1	1	.	.	.	INTERGENIC(INTERGENIC);RP4-706A16.2.1(DOWNSTREAM)
1	77553450	77553461	77553458	AG	G	del	1	0	0	0	.	.	.	INTERGENIC(INTERGENIC);PIGK(DOWNSTREAM)
1	77690135	77690148	77690138	TC	T	del	1	1	1	1	.	.	.	INTERGENIC(INTERGENIC)
1	77857157	77857166	77857162	TG	TGTA	ins	1	1	1	1	.	.	.	AC095030.1(EXON);AK5(INTRON)
1	77863585	77863604	77863589	TAAA	C	snp	0	0	0	1	.	.	.	AK5(INTRON)
1	78099857	78099876	78099864	AAAT	A	snp	1	1	1	1	.	.	.	ZZZ3(INTRON);ZZZ3(UPSTREAM)
1	78153551	78153560	78153552	T	C	snp	1	0	0	0	.	.	.	INTERGENIC(INTERGENIC);ZZZ3(UPSTREAM)
1	78308228	78308236	78308234	T	G	snp	1	1	1	1	.	.	.	FAM73A(INTRON);NSRP1P1(UPSTREAM)
1	78408923	78408932	78408931	A	C	ins	1	0	0	0	.	.	.	FUBP1(DOWNSTREAM);NEXN(DOWNSTREAM);NEXN(UTR_3_PRIME)
1	78443044	78443051	78443047	A	C	snp	0	1	0	1	.	.	.	DNAJB4(UPSTREAM);FUBP1(INTRON);GIPC2(UPSTREAM)
1	78529189	78529198	78529190	A	AC	ins	1	0	0	0	.	.	.	GIPC2(INTRON)
1	78741344	78741351	78741345	C	A	snp	1	1	1	1	.	.	.	RP11-183M13.1.1(INTRON)

1	78741344	78741351	78741346	C	A	ins	1	1	1	1	.	.	.	RP11-183M13.1.1(INTRON)
1	78747760	78747769	78747761	T	C	snp	0	1	1	1	.	.	.	RP11-183M13.1.1(INTRON)
1	78779224	78779231	78779227	A	G	snp	1	0	0	0	.	.	.	PTGFR(INTRON);RP11-183M13.1.1(INTRON);RP11-472F19.1.1(DOWNSTREAM)
1	78939882	78939890	78939887	A	C	snp	1	1	1	1	.	.	.	PTGFR(INTRON)
1	78954868	78954876	78954875	A	G	snp	1	1	1	1	.	.	.	PTGFR(INTRON);PTGFR(UPSTREAM)
1	78976566	78976573	78976570	A	G	snp	1	1	1	1	.	.	.	PTGFR(INTRON)
1	79016996	79017003	79016998	A	C	snp	1	1	0	1	.	.	.	INTERGENIC(INTERGENIC)
1	79036990	79036999	79036992	CT	T	snp	0	1	0	1	.	.	.	INTERGENIC(INTERGENIC)
1	79129690	79129701	79129694	AAT	G	snp	0	1	1	1	.	.	.	IFI44(DOWNSTREAM);IFI44(EXON);IFI44(UTR_3_PRIME);RP4-641G12.3.1(UPSTREAM)
1	79141656	79141671	79141662	GT	TGTA	ins	1	1	1	1	.	.	.	INTERGENIC(INTERGENIC)
1	79208676	79208685	79208678	TA	C	snp	1	1	1	0	.	.	.	INTERGENIC(INTERGENIC)
1	79348074	79348081	79348078	A	C	snp	1	1	1	1	.	.	.	INTERGENIC(INTERGENIC)
1	79372039	79372046	79372042	T	G	snp	0	0	0	1	.	.	.	ELTD1(INTRON)
1	79475384	79475393	79475391	T	G	snp	1	1	1	1	.	.	.	ELTD1(UPSTREAM);INTERGENIC(INTERGENIC)
1	79541128	79541139	79541137	AT	A	del	1	1	1	1	.	.	.	INTERGENIC(INTERGENIC)
1	79607046	79607069	79607060	TTTA	A	del	0	1	1	1	.	.	.	INTERGENIC(INTERGENIC)
1	79719756	79719764	79719757	A	C	snp	1	1	1	1	.	.	.	INTERGENIC(INTERGENIC)
1	79745589	79745612	79745603	CTTT	TC	ins	1	0	0	0	.	.	.	INTERGENIC(INTERGENIC)
1	79862579	79862586	79862583	T	G	snp	1	1	1	1	.	.	.	INTERGENIC(INTERGENIC)
1	80004560	80004571	80004566	GT	A	snp	1	1	1	1	.	.	.	INTERGENIC(INTERGENIC)
1	80112291	80112298	80112295	A	C	snp	1	0	0	0	.	.	.	INTERGENIC(INTERGENIC)
1	80125039	80125047	80125042	T	C	ins	1	0	0	0	.	.	.	INTERGENIC(INTERGENIC)
1	80197789	80197796	80197792	G	T	snp	1	1	1	1	.	.	.	INTERGENIC(INTERGENIC)
1	80197789	80197796	80197795	G	T	snp	0	1	0	1	.	.	.	INTERGENIC(INTERGENIC)
1	80240636	80240645	80240639	AT	A	snp	0	0	1	0	.	.	.	INTERGENIC(INTERGENIC)
1	80249109	80249116	80249110	T	G	snp	0	1	0	1	.	.	.	INTERGENIC(INTERGENIC)
1	80301889	80301896	80301890	T	C	snp	0	0	1	0	.	.	.	INTERGENIC(INTERGENIC)
1	80381753	80381764	80381762	TA	G	snp	1	0	0	0	.	.	.	INTERGENIC(INTERGENIC)
1	80404052	80404071	80404067	TTTA	T	snp	1	1	1	1	.	.	.	INTERGENIC(INTERGENIC)
1	80524920	80524928	80524922	T	C	snp	1	1	1	1	.	.	.	INTERGENIC(INTERGENIC)
1	80591055	80591063	80591058	T	C	snp	1	1	1	1	.	.	.	INTERGENIC(INTERGENIC)
1	80734619	80734627	80734620	T	C	snp	1	0	1	0	.	.	.	INTERGENIC(INTERGENIC)
1	80881298	80881305	80881299	A	G	snp	1	0	0	0	.	.	.	INTERGENIC(INTERGENIC)
1	80902481	80902490	80902488	A	C	ins	1	1	1	0	.	.	.	INTERGENIC(INTERGENIC)
1	80908005	80908016	80908010	CCT	G	snp	1	0	0	0	.	.	.	INTERGENIC(INTERGENIC)
1	80950244	80950251	80950250	A	G	snp	1	0	0	0	.	.	.	INTERGENIC(INTERGENIC)
1	80953830	80953843	80953840	CA	A	snp	1	1	1	1	.	.	.	INTERGENIC(INTERGENIC)
1	81005065	81005072	81005071	T	C	snp	1	0	0	0	.	.	.	RP11-115A15.1.1(INTRON);RP5-887A10.1.1(INTRON)
1	81122396	81122403	81122397	T	C	snp	1	0	1	0	.	.	.	INTERGENIC(INTERGENIC)
1	81177127	81177135	81177128	A	G	snp	1	1	0	1	.	.	.	INTERGENIC(INTERGENIC)
1	81291267	81291276	81291275	T	G	snp	1	1	1	1	.	.	.	INTERGENIC(INTERGENIC)
1	81343369	81343382	81343380	AT	ATC	del	1	0	0	0	.	.	.	INTERGENIC(INTERGENIC)
1	81356611	81356619	81356618	A	AT	ins	1	0	0	0	.	.	.	INTERGENIC(INTERGENIC)
1	81367992	81368007	81367997	TA	TG	ins	1	1	1	1	.	.	.	INTERGENIC(INTERGENIC)
1	81514959	81514972	81514966	TC	T	snp	1	1	1	1	.	.	.	INTERGENIC(INTERGENIC)
1	81535957	81535964	81535960	T	C	snp	1	0	1	0	.	.	.	INTERGENIC(INTERGENIC)
1	81535957	81535964	81535962	T	C	snp	1	1	1	1	.	.	.	INTERGENIC(INTERGENIC)
1	81642881	81642888	81642887	T	C	snp	0	1	0	1	.	.	.	INTERGENIC(INTERGENIC)
1	81827950	81827959	81827957	CT	C	snp	1	1	1	1	.	.	.	LPHN2(INTRON)
1	81846464	81846478	81846472	TGG	C	snp	1	0	0	0	.	.	.	LPHN2(INTRON)
1	81857465	81857472	81857468	T	C	snp	1	1	1	1	.	.	.	LPHN2(INTRON)
1	81906706	81906715	81906713	TC	T	del	1	0	0	0	.	.	.	LPHN2(INTRON)
1	81969064	81969078	81969071	CAG	T	snp	1	1	1	1	.	.	.	LPHN2(INTRON);RP5-837I24.1.1(EXON);RP5-837I24.6.1(UPSTREAM)
1	82033746	82033754	82033752	A	G	snp	1	1	0	1	.	.	.	LPHN2(INTRON)
1	82116076	82116083	82116078	A	G	ins	1	0	0	1	.	.	.	LPHN2(INTRON)
1	82169108	82169128	82169124	TGT	T	snp	0	1	0	1	.	.	.	LPHN2(INTRON)
1	82243850	82243857	82243853	A	G	snp	1	1	1	1	.	.	.	LPHN2(INTRON)
1	82362256	82362265	82362258	AT	G	snp	1	1	0	1	.	.	.	LPHN2(INTRON)
1	82367255	82367268	82367266	AT	A	del	1	1	1	1	.	.	.	LPHN2(INTRON)
1	82464422	82464433	82464427	TA	C	snp	1	0	1	0	.	.	.	INTERGENIC(INTERGENIC)
1	82467044	82467051	82467045	T	C	snp	1	1	1	1	.	.	.	INTERGENIC(INTERGENIC)
1	82574938	82574949	82574940	AT	G	snp	1	1	1	1	.	.	.	INTERGENIC(INTERGENIC)
1	82783298	82783307	82783302	T	G	snp	1	1	1	1	.	.	.	INTERGENIC(INTERGENIC)
1	82850415	82850422	82850421	T	C	snp	1	1	1	1	.	.	.	INTERGENIC(INTERGENIC)

1	82893522	82893529	82893526	A	G	snp	1	1	1	1	.	.	.	INTERGENIC(INTERGENIC)
1	82902797	82902814	82902800	AT	G	snp	1	0	0	0	.	.	.	INTERGENIC(INTERGENIC)
1	82902797	82902814	82902811	AT	C	snp	1	0	0	0	.	.	.	INTERGENIC(INTERGENIC)
1	82959253	82959264	82959261	AT	TC	ins	1	1	1	1	.	.	.	INTERGENIC(INTERGENIC)
1	83084297	83084311	83084303	TTA	A	snp	0	0	1	0	.	.	.	INTERGENIC(INTERGENIC)
1	83143726	83143740	83143736	CAA	G	snp	1	0	0	0	.	.	.	INTERGENIC(INTERGENIC)
1	83149214	83149225	83149219	TTC	A	snp	1	0	0	0	.	.	.	INTERGENIC(INTERGENIC)
1	83239745	83239758	83239749	GA	C	snp	1	1	1	1	.	.	.	INTERGENIC(INTERGENIC)
1	83252696	83252705	83252700	T	G	snp	0	0	1	1	.	.	.	INTERGENIC(INTERGENIC)
1	83252696	83252705	83252703	T	G	snp	1	1	1	1	.	.	.	INTERGENIC(INTERGENIC)
1	83329049	83329057	83329050	T	G	snp	1	0	0	0	.	.	.	INTERGENIC(INTERGENIC)
1	83700350	83700357	83700354	A	G	snp	1	0	1	1	.	.	.	INTERGENIC(INTERGENIC)
1	83804643	83804651	83804648	T	TTTG	ins	1	1	1	1	.	.	.	INTERGENIC(INTERGENIC)
1	83874618	83874627	83874620	AC	C	snp	1	0	0	0	.	.	.	INTERGENIC(INTERGENIC)
1	84067228	84067237	84067232	A	C	snp	1	0	1	0	.	.	.	RP11-475O6.1.1(INTRON)
1	84237445	84237456	84237453	CA	A	snp	1	0	0	0	.	.	.	RP11-475O6.1.1(INTRON)
1	84271402	84271421	84271409	ATAC	T	snp	1	0	0	0	.	.	.	RP11-475O6.1.1(INTRON);RP5-836J3.1.1(DOWNSTREAM)
1	84276829	84276838	84276837	A	G	snp	1	1	1	1	.	.	.	RP11-475O6.1.1(INTRON)
1	84465068	84465077	84465073	C	A	snp	1	1	1	1	.	.	.	INTERGENIC(INTERGENIC);TTLL7(UPSTREAM)
1	84809556	84809563	84809562	A	G	snp	1	0	0	0	.	.	.	RP11-376N17.4.1(UPSTREAM);SAMD13(INTRON);SAMD13(UPSTREAM)
1	84878637	84878646	84878642	C	A	snp	1	1	1	1	.	.	.	DNASE2B(INTRON)
1	84944986	84944997	84944989	AGC	G	snp	1	1	1	1	.	RPF1(NON_SYNONYMOUS_CODING):S9G	.	.
1	85088303	85088316	85088306	TC	G	snp	1	1	1	1	.	.	.	INTERGENIC(INTERGENIC)
1	85088303	85088316	85088312	TC	T	snp	1	1	1	1	.	.	.	INTERGENIC(INTERGENIC)
1	85167552	85167560	85167553	G	T	snp	1	1	1	1	.	.	.	INTERGENIC(INTERGENIC)
1	85222467	85222478	85222473	GA	A	snp	1	0	0	0	.	.	.	INTERGENIC(INTERGENIC)
1	85225530	85225541	85225536	TG	A	snp	1	0	0	0	.	.	.	INTERGENIC(INTERGENIC)
1	85264687	85264696	85264690	AG	A	snp	1	1	1	1	.	.	.	INTERGENIC(INTERGENIC)
1	85322230	85322238	85322235	T	C	ins	0	0	0	1	.	.	.	LPAR3(INTRON)
1	85371069	85371080	85371077	AG	GAGG	ins	0	1	0	1	.	.	.	INTERGENIC(INTERGENIC)
1	85383745	85383758	85383750	TA	C	snp	1	1	1	1	.	.	.	INTERGENIC(INTERGENIC)
1	85410237	85410244	85410242	A	G	snp	1	1	0	1	.	.	.	MCOLN2(INTRON)
1	85455824	85455831	85455825	A	C	snp	1	1	1	1	.	.	.	MCOLN2(INTRON)
1	85471590	85471599	85471598	A	C	snp	1	1	0	1	.	.	.	WDR63(INTRON)
1	85562142	85562155	85562152	TA	G	snp	1	1	0	1	.	.	.	WDR63(INTRON)
1	85562163	85562176	85562168	TA	TATATATAAAGAT	del	1	1	1	1	.	.	.	WDR63(INTRON)
1	85593832	85593846	85593841	AAG	G	snp	1	1	1	1	.	.	.	WDR63(INTRON)
1	85675445	85675459	85675456	TTA	T	ins	0	0	1	0	.	.	.	INTERGENIC(INTERGENIC)
1	85696854	85696861	85696856	T	G	snp	1	0	0	0	.	.	.	INTERGENIC(INTERGENIC)
1	85797107	85797122	85797110	TA	G	snp	1	1	1	1	.	.	.	DDAH1(INTRON);RP11-131L23.1.1(INTRON)
1	85797107	85797122	85797115	TA	C	snp	0	0	1	0	.	.	.	DDAH1(INTRON);RP11-131L23.1.1(INTRON)
1	85930747	85930761	85930750	GGC	C	snp	1	0	0	0	.	.	.	DDAH1(INTRON);DDAH1(UPSTREAM);DDAH1(UTR_5_PRIME)
1	85939170	85939183	85939177	AC	T	snp	1	1	1	1	.	.	.	DDAH1(INTRON)
1	86042638	86042652	86042647	GCC	A	snp	1	1	1	1	.	.	.	CYR61(UPSTREAM);DDAH1(INTRON);DDAH1(UPSTREAM)
1	86045680	86045689	86045683	C	A	ins	1	0	0	0	.	.	.	CYR61(UPSTREAM);DDAH1(UPSTREAM);INTERGENIC(INTERGENIC)
1	86052092	86052099	86052097	C	T	snp	1	0	0	0	.	.	.	CYR61(DOWNSTREAM);INTERGENIC(INTERGENIC)
1	86135754	86135765	86135757	TC	T	snp	1	0	0	0	.	.	.	ZNHIT6(INTRON)
1	86183748	86183757	86183756	A	C	ins	1	1	1	1	.	.	.	INTERGENIC(INTERGENIC)
1	86267627	86267638	86267632	TC	TCTCTCCCTCA	del	1	0	0	0	.	.	.	COL24A1(INTRON)
1	86597055	86597064	86597061	TA	C	snp	0	0	1	0	.	.	.	COL24A1(INTRON)
1	86807741	86807749	86807748	T	G	snp	0	0	1	0	.	.	.	ODF2L(DOWNSTREAM);ODF2L(INTRON)
1	86847117	86847132	86847121	AC	G	snp	1	1	0	0	.	.	.	ODF2L(DOWNSTREAM);ODF2L(INTRON)
1	86945571	86945594	86945582	AAAC	G	snp	1	1	1	1	.	.	.	CLCA1(INTRON)
1	87182125	87182133	87182132	T	G	snp	1	0	0	0	.	.	.	SH3GLB1(INTRON)
1	87227429	87227440	87227431	AG	T	snp	1	1	1	1	.	.	.	INTERGENIC(INTERGENIC)
1	87259915	87259923	87259918	A	C	snp	1	0	0	0	.	.	.	INTERGENIC(INTERGENIC)
1	87330896	87330923	87330915	CAAA	C	del	1	0	0	0	.	.	.	RP4-604K5.1.1(DOWNSTREAM);RP4-604K5.1.1(INTRON)
1	87330896	87330923	87330919	CAAA	C	del	1	0	0	1	.	.	.	RP4-604K5.1.1(DOWNSTREAM);RP4-604K5.1.1(INTRON)
1	87354348	87354357	87354350	AT	G	snp	0	1	0	1	.	.	.	RP4-604K5.1.1(INTRON)
1	87364460	87364467	87364463	T	G	snp	1	0	0	0	.	.	.	RP4-604K5.1.1(INTRON)
1	87365102	87365109	87365107	C	T	snp	1	1	0	1	.	.	.	RP4-604K5.1.1(INTRON)
1	87409402	87409411	87409410	A	G	ins	1	1	0	0	.	.	.	HS2ST1(INTRON);RP4-604K5.2.1(EXON)
1	87482157	87482168	87482160	TC	G	snp	1	1	1	1	.	.	.	HS2ST1(INTRON)
1	87753605	87753612	87753611	T	G	ins	0	0	0	1	.	.	.	INTERGENIC(INTERGENIC)
1	87842991	87842998	87842994	T	C	snp	1	1	1	1	.	.	.	INTERGENIC(INTERGENIC)

1	87847047	87847055	87847053	T	C	snp	1	1	1	1	.	.	.	INTERGENIC(INTERGENIC)
1	87922405	87922412	87922411	T	C	snp	1	1	1	1	.	.	.	INTERGENIC(INTERGENIC);RN5S52(UPSTREAM)
1	87932229	87932236	87932235	G	A	snp	1	1	1	1	.	.	.	INTERGENIC(INTERGENIC)
1	87974054	87974062	87974057	T	G	snp	1	0	0	0	.	.	.	INTERGENIC(INTERGENIC)
1	88036572	88036580	88036573	T	G	snp	0	1	1	1	.	.	.	INTERGENIC(INTERGENIC)
1	88074813	88074820	88074815	T	G	snp	1	0	0	0	.	.	.	INTERGENIC(INTERGENIC)
1	88157348	88157365	88157354	AAT	G	snp	0	0	1	0	.	.	.	INTERGENIC(INTERGENIC)
1	88233284	88233295	88233286	AC	C	del	1	1	1	1	.	.	.	INTERGENIC(INTERGENIC)
1	88233284	88233295	88233287	AC	A	snp	1	1	1	1	.	.	.	INTERGENIC(INTERGENIC)
1	88332846	88332853	88332847	C	CA	ins	1	0	0	0	.	.	.	INTERGENIC(INTERGENIC)
1	88341847	88341854	88341853	T	C	snp	1	1	0	1	.	.	.	INTERGENIC(INTERGENIC)
1	88345319	88345336	88345333	GAT	C	snp	1	0	0	0	.	.	.	INTERGENIC(INTERGENIC)
1	88352133	88352140	88352138	A	C	snp	1	0	0	0	.	.	.	INTERGENIC(INTERGENIC)
1	88416920	88416929	88416925	AG	C	snp	1	0	0	0	.	.	.	INTERGENIC(INTERGENIC)
1	88451110	88451129	88451122	CCTC	T	snp	1	0	0	0	.	.	.	INTERGENIC(INTERGENIC)
1	88644275	88644284	88644277	A	C	snp	1	0	0	0	.	.	.	INTERGENIC(INTERGENIC)
1	88662705	88662722	88662716	AGG	A	snp	1	1	1	1	.	.	.	INTERGENIC(INTERGENIC)
1	88662705	88662722	88662717	AGG	GG	del	0	0	1	0	.	.	.	INTERGENIC(INTERGENIC)
1	88818479	88818488	88818487	A	G	snp	1	1	1	1	.	.	.	INTERGENIC(INTERGENIC)
1	88852369	88852376	88852375	T	TG	ins	1	0	0	0	.	.	.	INTERGENIC(INTERGENIC)
1	88906509	88906520	88906516	AGG	T	snp	0	0	1	0	.	.	.	INTERGENIC(INTERGENIC)
1	88972874	88972881	88972879	A	G	snp	1	1	1	0	.	.	.	INTERGENIC(INTERGENIC)
1	88983149	88983166	88983156	AGG	A	snp	1	1	0	1	.	.	.	INTERGENIC(INTERGENIC)
1	89038801	89038809	89038804	T	G	snp	1	0	0	0	.	.	.	RP11-76N22.2.1(INTRON);RP11-76N22.3.1(UPSTREAM)
1	89293355	89293368	89293363	CT	A	snp	1	1	1	1	.	.	.	PKN2(INTRON);TCEB1P19(UPSTREAM)
1	89293355	89293368	89293365	CT	A	snp	1	1	1	1	.	.	.	PKN2(INTRON);TCEB1P19(UPSTREAM)
1	89301241	89301252	89301243	AT	T	snp	1	0	0	0	.	.	.	PKN2(DOWNSTREAM);PKN2(UTR_3_PRIME)
1	89331524	89331533	89331529	A	C	snp	1	0	1	0	.	.	.	GTF2B(INTRON)
1	89352594	89352611	89352606	ATT	T	ins	1	0	0	0	.	.	.	GTF2B(INTRON)
1	89352594	89352611	89352606	ATT	T	snp	1	0	0	0	.	.	.	GTF2B(INTRON)
1	89352594	89352611	89352608	ATT	A	del	1	1	0	0	.	.	.	GTF2B(INTRON)
1	89372852	89372860	89372856	T	G	snp	1	1	1	1	.	.	.	INTERGENIC(INTERGENIC)
1	89491943	89491950	89491944	G	A	ins	1	0	0	0	.	.	.	GBP3(UPSTREAM);INTERGENIC(INTERGENIC)
1	89606119	89606133	89606128	ATA	G	snp	0	0	1	0	.	.	.	GBP2(INTRON);GBP7(INTRON)
1	89610407	89610414	89610413	T	G	snp	1	0	0	0	.	.	.	GBP2(INTRON);GBP7(INTRON)
1	89665515	89665526	89665523	AAG	A	snp	1	1	1	1	.	.	.	GBP4(UPSTREAM);INTERGENIC(INTERGENIC)
1	89840820	89840827	89840823	A	G	snp	1	0	0	0	.	.	.	GBP6(INTRON)
1	89884221	89884228	89884224	T	C	snp	1	1	1	1	.	.	.	GBP1P1(INTRON)
1	89927097	89927106	89927104	A	C	ins	1	1	1	1	.	.	.	INTERGENIC(INTERGENIC)
1	89942002	89942013	89942009	AC	A	snp	1	0	0	0	.	.	.	INTERGENIC(INTERGENIC)
1	90246899	90246910	90246906	ATA	C	snp	1	1	1	1	.	.	.	LRRC8C(INTRON)
1	90373661	90373681	90373674	TGA	A	snp	1	1	1	1	.	.	.	LRRC8C(INTRON);LRRC8D(INTRON)
1	90499432	90499447	90499438	ATGT	T	snp	1	0	0	0	.	.	.	INTERGENIC(INTERGENIC)
1	90713025	90713032	90713029	T	C	snp	0	1	1	1	.	.	.	INTERGENIC(INTERGENIC)
1	90845811	90845824	90845813	CA	T	snp	1	1	0	1	.	.	.	INTERGENIC(INTERGENIC)
1	90908078	90908089	90908083	AT	C	snp	1	1	1	0	.	.	.	INTERGENIC(INTERGENIC)
1	90929676	90929683	90929682	T	C	snp	0	1	0	1	.	.	.	INTERGENIC(INTERGENIC)
1	90944878	90944887	90944883	AT	AA	ins	1	1	1	1	.	.	.	INTERGENIC(INTERGENIC)
1	90944920	90944935	90944933	AT	GC	ins	1	1	1	1	.	.	.	INTERGENIC(INTERGENIC)
1	90944938	90944951	90944942	AT	T	ins	1	1	0	1	.	.	.	INTERGENIC(INTERGENIC)
1	91037457	91037472	91037468	TG	TGT	del	1	1	1	1	.	.	.	INTERGENIC(INTERGENIC)
1	91037457	91037472	91037469	TG	GTG	del	1	1	1	1	.	.	.	INTERGENIC(INTERGENIC)
1	91092779	91092798	91092790	AAAT	C	snp	1	0	0	0	.	.	.	INTERGENIC(INTERGENIC)
1	91111828	91111836	91111835	A	G	snp	0	1	1	1	.	.	.	INTERGENIC(INTERGENIC)
1	91133201	91133210	91133204	AC	T	snp	1	1	1	1	.	.	.	INTERGENIC(INTERGENIC)
1	91148772	91148779	91148773	A	C	snp	1	1	1	1	.	.	.	INTERGENIC(INTERGENIC)
1	91267796	91267807	91267804	TC	CTC	del	1	1	1	1	.	.	.	INTERGENIC(INTERGENIC)
1	91271842	91271849	91271844	G	A	ins	1	1	1	1	.	.	.	INTERGENIC(INTERGENIC)
1	91271875	91271883	91271881	T	G	snp	1	1	1	1	.	.	.	INTERGENIC(INTERGENIC)
1	91340240	91340247	91340246	T	G	snp	1	1	1	1	.	.	.	INTERGENIC(INTERGENIC)
1	91555090	91555097	91555091	A	C	snp	1	1	0	1	.	.	.	INTERGENIC(INTERGENIC)
1	91606333	91606342	91606340	AT	A	del	1	1	1	1	.	.	.	INTERGENIC(INTERGENIC)
1	91642121	91642138	91642126	TTG	T	snp	1	0	0	0	.	.	.	INTERGENIC(INTERGENIC)
1	91647065	91647076	91647068	GT	C	snp	1	1	1	1	.	.	.	INTERGENIC(INTERGENIC)
1	91648237	91648252	91648250	AT	A	del	1	1	1	1	.	.	.	INTERGENIC(INTERGENIC)
1	91934439	91934448	91934444	TA	G	snp	1	1	0	1	.	.	.	INTERGENIC(INTERGENIC)
1	92072830	92072847	92072843	AT	A	del	0	0	1	0	.	.	.	INTERGENIC(INTERGENIC)

1	92144639	92144650	92144643	TA	C	snp	1	1	1	1	.	.	.	INTERGENIC(INTERGENIC);TGFBR3(DOWNSTREAM)
1	92199611	92199634	92199618	GAGG	A	snp	1	1	1	1	.	.	.	TGFBR3(INTRON)
1	92259763	92259772	92259770	T	G	ins	1	1	1	1	.	.	.	TGFBR3(DOWNSTREAM);TGFBR3(INTRON)
1	92307665	92307680	92307670	AAAG	C	snp	1	0	0	0	.	.	.	TGFBR3(INTRON)
1	92321467	92321476	92321468	T	C	snp	1	1	1	1	.	.	.	TGFBR3(INTRON)
1	92386382	92386389	92386385	A	C	snp	1	0	0	0	.	.	.	INTERGENIC(INTERGENIC)
1	92472756	92472764	92472757	T	C	snp	1	1	0	1	.	.	.	BRDT(INTRON)
1	92481568	92481575	92481570	G	T	snp	1	1	0	1	.	.	.	BRDT(DOWNSTREAM);INTERGENIC(INTERGENIC)
1	92493214	92493222	92493219	T	G	snp	1	1	1	1	.	.	.	EPHX4(UPSTREAM);INTERGENIC(INTERGENIC)
1	92509765	92509772	92509766	A	G	snp	1	1	1	1	.	.	.	EPHX4(DOWNSTREAM);EPHX4(INTRON)
1	92596077	92596094	92596079	AT	T	del	1	1	1	1	.	.	.	BTBD8(INTRON);RP4-621B10.6.1(DOWNSTREAM)
1	92602502	92602509	92602505	T	C	snp	1	0	0	0	.	.	.	BTBD8(INTRON)
1	92690318	92690325	92690321	T	C	snp	1	0	0	0	.	.	.	ACTBP12(DOWNSTREAM);C1orf146(INTRON)
1	92735354	92735363	92735359	AT	C	snp	1	1	1	1	.	.	.	GLMN(INTRON)
1	92788633	92788648	92788644	TA	T	snp	1	1	0	1	.	.	.	RPAP2(INTRON)
1	92925957	92925964	92925962	T	G	snp	0	1	0	1	.	.	.	INTERGENIC(INTERGENIC)
1	93001137	93001145	93001143	A	C	snp	1	1	1	1	.	.	.	EVI5(INTRON)
1	93246395	93246404	93246400	A	C	snp	1	1	0	1	.	.	.	EVI5(INTRON)
1	93254059	93254068	93254062	A	C	ins	1	0	0	0	.	.	.	EVI5(INTRON);EVI5(UPSTREAM)
1	93254059	93254068	93254063	A	C	snp	1	1	0	0	.	.	.	EVI5(INTRON);EVI5(UPSTREAM)
1	93254369	93254376	93254371	A	C	snp	1	0	0	0	.	.	.	EVI5(INTRON);EVI5(UPSTREAM)
1	93254868	93254879	93254875	CTT	TC	del	1	1	0	1	.	.	.	EVI5(INTRON);EVI5(UPSTREAM)
1	93447091	93447099	93447095	T	C	snp	1	0	0	0	.	.	.	AC093577.1(DOWNSTREAM);INTERGENIC(INTERGENIC)
1	93486447	93486454	93486450	T	C	snp	1	0	0	0	.	.	.	INTERGENIC(INTERGENIC)
1	93559648	93559655	93559649	T	C	snp	1	1	1	1	.	.	.	MTF2(INTRON)
1	93571239	93571248	93571240	T	G	snp	1	0	0	0	.	.	.	AC093577.4(UPSTREAM);MTF2(INTRON);MTF2(UPSTREAM)
1	93588808	93588819	93588815	TC	G	snp	1	0	0	0	.	.	.	MTF2(DOWNSTREAM);MTF2(INTRON)
1	93791095	93791104	93791098	T	C	snp	1	1	0	1	.	.	.	RP4-717I23.3.1(INTRON);RP4-717I23.3.1(UPSTREAM)
1	93826601	93826608	93826602	G	T	snp	1	0	0	0	.	.	.	DR1(DOWNSTREAM);DR1(UTR_3_PRIME)
1	94039167	94039190	94039186	TTA	A	del	1	1	0	1	.	.	.	BCAR3(INTRON)
1	94154335	94154358	94154344	TTTG	TG	del	1	1	0	0	.	.	.	BCAR3(INTRON)
1	94219919	94219928	94219926	AG	A	snp	1	1	1	1	.	.	.	BCAR3(INTRON);RP11-488P3.1.1(INTRON)
1	94243099	94243116	94243107	TCC	T	snp	1	1	1	1	.	.	.	BCAR3(INTRON);RP11-488P3.1.1(UPSTREAM)
1	94295713	94295720	94295717	T	C	snp	1	1	1	1	.	.	.	BCAR3(INTRON)
1	94317478	94317493	94317491	GA	G	snp	1	1	1	1	.	.	.	BCAR3(UPSTREAM);INTERGENIC(INTERGENIC);RP4-561L24.3.1(DOWNSTREAM)
1	94355462	94355477	94355475	AT	A	del	1	0	0	0	.	.	.	GCLM(DOWNSTREAM);GCLM(INTRON)
1	94414194	94414203	94414196	T	TA	ins	1	0	0	0	.	.	.	INTERGENIC(INTERGENIC)
1	94468545	94468554	94468551	TC	CA	ins	1	1	1	1	.	.	.	ABCA4(DOWNSTREAM);ABCA4(INTRON)
1	94549027	94549036	94549029	AT	G	snp	1	1	1	1	.	.	.	ABCA4(INTRON)
1	94594846	94594853	94594847	A	G	ins	1	1	1	1	.	.	.	INTERGENIC(INTERGENIC)
1	94594846	94594853	94594848	A	G	snp	1	1	1	1	.	.	.	INTERGENIC(INTERGENIC)
1	94696133	94696142	94696139	TA	C	snp	1	1	1	1	.	.	.	ARHGAP29(INTRON)
1	94734347	94734362	94734351	AT	G	snp	1	1	1	1	.	.	.	ARHGAP29(INTRON)
1	94878630	94878645	94878641	ATTT	A	del	1	1	1	1	.	.	.	INTERGENIC(INTERGENIC)
1	94883978	94883998	94883995	GCC	T	snp	0	0	1	0	.	.	ABCD3(START_GAINED)	ABCD3(UPSTREAM)
1	95115462	95115470	95115467	T	C	snp	1	0	1	0	.	.	.	RP11-86H7.1.1(INTRON);RP11-86H7.3.1(DOWNSTREAM)
1	95293458	95293469	95293467	AG	T	snp	1	1	1	1	.	.	.	SLC44A3(INTRON)
1	95309435	95309442	95309437	G	T	snp	1	1	1	1	.	.	.	RP11-465K1.2.1(INTRON);SLC44A3(INTRON)
1	95322294	95322302	95322301	T	C	snp	1	1	0	1	.	.	.	RP11-465K1.2.1(UPSTREAM);SLC44A3(INTRON)
1	95406214	95406223	95406219	A	G	snp	1	0	0	0	.	.	.	RP4-639F20.1.1(INTRON)
1	95441401	95441408	95441405	A	C	snp	1	1	1	1	.	.	.	INTERGENIC(INTERGENIC)
1	95441414	95441423	95441417	A	C	snp	0	1	1	1	.	.	.	INTERGENIC(INTERGENIC)
1	95450493	95450500	95450495	T	TTTG	ins	1	0	0	0	.	.	.	ALG14(INTRON);RP4-639F20.3.1(INTRON)
1	95467801	95467809	95467803	G	A	snp	1	1	1	1	.	.	.	ALG14(INTRON);RP4-639F20.3.1(INTRON)
1	95601801	95601809	95601808	T	G	snp	1	1	1	1	.	.	.	RP11-57H12.2.1(INTRON);TMEM56(INTRON);TMEM56(UPSTREAM)
1	95631008	95631023	95631020	TG	G	ins	1	1	1	1	.	.	.	RP11-57H12.3.1(INTRON);TMEM56(INTRON)
1	95631008	95631023	95631021	TG	T	del	1	1	1	1	.	.	.	RP11-57H12.3.1(INTRON);TMEM56(INTRON)
1	95690822	95690837	95690828	AAAT	T	del	1	1	1	1	.	.	.	RP11-57H12.3.1(INTRON)
1	95739459	95739466	95739460	A	C	snp	1	1	1	1	.	.	.	INTERGENIC(INTERGENIC)
1	96023947	96023954	96023951	T	C	snp	1	0	0	1	.	.	.	RP11-286B14.1.1(INTRON)
1	96067378	96067385	96067379	T	C	snp	1	1	1	1	.	.	.	RP11-286B14.1.1(INTRON)
1	96251699	96251708	96251707	T	C	snp	1	0	0	0	.	.	.	INTERGENIC(INTERGENIC);RP11-286B14.1.1(DOWNSTREAM)
1	96417769	96417776	96417773	T	G	snp	1	0	0	0	.	.	.	RP11-147C23.1.1(INTRON)
1	96426998	96427021	96427004	CTC	T	snp	1	0	0	0	.	.	.	RP11-147C23.1.1(INTRON)

1	96542817	96542828	96542825	ATC	A	snp	1	1	1	1	.	.	.	INTERGENIC(INTERGENIC)
1	96713914	96713931	96713929	TA	C	snp	1	0	0	0	.	.	.	INTERGENIC(INTERGENIC)
1	96713914	96713931	96713929	TA	TATATATATC	ins	1	1	1	1	.	.	.	INTERGENIC(INTERGENIC)
1	96824392	96824405	96824402	AT	G	snp	1	1	1	1	.	.	.	RP5-898J17.1.1(INTRON)
1	96938247	96938254	96938248	G	A	snp	1	1	1	1	.	.	.	INTERGENIC(INTERGENIC)
1	96952657	96952668	96952664	AGC	C	snp	1	1	1	1	.	.	.	INTERGENIC(INTERGENIC)
1	97026869	97026877	97026871	T	C	snp	0	1	0	1	.	.	.	INTERGENIC(INTERGENIC)
1	97081501	97081509	97081506	T	G	snp	1	0	0	0	.	.	.	INTERGENIC(INTERGENIC)
1	97148733	97148740	97148738	C	A	snp	0	1	1	0	.	.	.	INTERGENIC(INTERGENIC);RP11-122C9.1.1(UPSTREAM)
1	97209870	97209881	97209879	AT	A	del	1	1	1	1	.	.	.	PTBP2(INTRON)
1	97596273	97596288	97596282	TCTT	T	del	0	0	0	1	.	.	.	DPYD(INTRON);DPYD-AS1(INTRON)
1	97605848	97605855	97605851	T	G	snp	1	1	1	1	.	.	.	DPYD(INTRON);DPYD-AS1(INTRON)
1	97757534	97757541	97757537	T	C	snp	1	1	1	1	.	.	.	DPYD(INTRON);DPYD-AS1(INTRON)
1	97866863	97866871	97866868	T	G	snp	1	1	0	1	.	.	.	DPYD(INTRON);DPYD-IT1(INTRON)
1	97892856	97892865	97892858	T	C	snp	1	1	1	1	.	.	.	DPYD(INTRON)
1	98108433	98108444	98108440	ATA	AATAAAATTA	del	1	0	0	0	.	.	.	DPYD(INTRON)
1	98150119	98150127	98150122	A	C	snp	1	0	0	0	.	.	.	DPYD(INTRON)
1	98166770	98166779	98166772	AT	C	snp	1	0	0	0	.	.	.	DPYD(INTRON)
1	98251971	98251978	98251973	T	G	snp	1	0	0	0	.	.	.	DPYD(INTRON)
1	98280581	98280596	98280592	AGGA	AG	del	1	0	0	0	.	.	.	DPYD(INTRON)
1	98377143	98377152	98377149	A	G	snp	0	1	1	1	.	.	.	DPYD(INTRON)
1	98387375	98387384	98387379	C	A	snp	1	0	0	0	.	.	.	DPYD(UPSTREAM);INTERGENIC(INTERGENIC)
1	98403227	98403238	98403233	TTG	TGTTG	del	1	1	1	1	.	.	.	INTERGENIC(INTERGENIC)
1	98597307	98597316	98597309	AG	G	snp	1	1	1	1	.	.	.	INTERGENIC(INTERGENIC)
1	98651914	98651929	98651924	TATC	C	del	0	1	0	1	.	.	.	INTERGENIC(INTERGENIC)
1	98657112	98657121	98657119	T	G	snp	1	1	1	1	.	.	.	INTERGENIC(INTERGENIC)
1	98691782	98691791	98691789	AT	A	snp	1	0	0	0	.	.	.	RP5-1070A16.1.1(INTRON)
1	98728466	98728475	98728468	TG	C	snp	1	0	0	0	.	.	.	RP5-1070A16.1.1(INTRON)
1	98782744	98782753	98782751	AC	AT	ins	1	1	1	1	.	.	.	INTERGENIC(INTERGENIC)
1	98876623	98876631	98876628	T	G	snp	1	1	1	1	.	.	.	INTERGENIC(INTERGENIC)
1	98884768	98884785	98884774	CAA	AAA	ins	0	1	0	1	.	.	.	INTERGENIC(INTERGENIC)
1	98961447	98961458	98961453	AAG	AG	del	1	1	1	1	.	.	.	INTERGENIC(INTERGENIC)
1	98961447	98961458	98961454	AAG	G	del	1	1	1	1	.	.	.	INTERGENIC(INTERGENIC)
1	98975586	98975595	98975593	A	G	snp	1	0	0	0	.	.	.	INTERGENIC(INTERGENIC)
1	99005109	99005123	99005119	AAG	G	ins	1	0	0	0	.	.	.	INTERGENIC(INTERGENIC)
1	99026885	99026893	99026889	A	G	snp	0	1	1	0	.	.	.	INTERGENIC(INTERGENIC)
1	99073491	99073518	99073500	ACAA	A	del	1	1	1	1	.	.	.	INTERGENIC(INTERGENIC)
1	99169282	99169291	99169289	AG	T	snp	1	1	1	1	.	.	.	SNX7(INTRON)
1	99266336	99266343	99266340	T	G	snp	1	1	1	0	.	.	.	INTERGENIC(INTERGENIC)
1	99400506	99400514	99400507	A	C	snp	1	1	0	1	.	.	.	LPPR5.1(INTRON)
1	99492048	99492057	99492050	A	G	snp	1	1	1	1	.	.	.	RP5-896L10.1.1(INTRON)
1	99563891	99563899	99563895	A	C	ins	1	1	1	1	.	.	.	RP5-896L10.1.1(INTRON)
1	99675321	99675328	99675325	A	G	snp	1	0	0	0	.	.	.	INTERGENIC(INTERGENIC)
1	99757484	99757499	99757494	TAGA	AT	del	0	0	0	1	.	.	.	RP4-788L13.1.1(INTRON)
1	99820326	99820334	99820327	A	C	snp	1	0	0	0	.	.	.	INTERGENIC(INTERGENIC)
1	99838480	99838489	99838483	CT	G	snp	1	0	0	0	.	.	.	INTERGENIC(INTERGENIC)
1	99865383	99865394	99865389	TTA	TATTA	del	1	0	1	0	.	.	.	INTERGENIC(INTERGENIC)
1	99894177	99894186	99894181	CT	G	snp	1	0	1	0	.	.	.	INTERGENIC(INTERGENIC)
1	99902021	99902029	99902028	A	C	snp	1	0	0	0	.	.	.	INTERGENIC(INTERGENIC)
1	99955892	99955900	99955894	T	G	snp	0	0	1	0	.	.	.	RP11-413P11.1.1(INTRON)
1	100128779	100128786	100128783	A	G	snp	1	1	1	1	.	.	.	PALMD(INTRON)
1	100208937	100208946	100208940	AT	A	snp	1	1	1	1	.	.	.	FRRS1(DOWNSTREAM);FRRS1(INTRON)
1	100220044	100220061	100220051	AG	C	snp	1	0	0	0	.	.	.	FRRS1(INTRON)
1	100232920	100232935	100232928	TTTG	C	snp	1	0	0	0	.	.	.	FRRS1(UPSTREAM);INTERGENIC(INTERGENIC)
1	100232920	100232935	100232931	TTTG	TTTGT	ins	1	1	1	1	.	.	.	FRRS1(UPSTREAM);INTERGENIC(INTERGENIC)
1	100587244	100587251	100587250	A	C	snp	1	1	1	1	.	.	.	SASS6(INTRON)
1	100617763	100617772	100617766	CT	C	snp	1	0	0	0	.	.	.	CCDC76(DOWNSTREAM);LRRC39(INTRON)
1	100638796	100638805	100638801	A	C	snp	1	0	0	1	.	.	.	LRRC39(INTRON)
1	100734598	100734607	100734605	TA	TG	ins	1	0	0	0	.	.	.	RP11-305E17.6.1(UPSTREAM);RTCD1(DOWNSTREAM);RTCD1(INTRON)
1	100829684	100829693	100829685	A	C	snp	1	0	0	0	.	.	.	CDC14A(INTRON)
1	100859668	100859687	100859683	TTAT	C	snp	1	0	0	0	.	.	.	BCAS2P2(UPSTREAM);CDC14A(INTRON)
1	101032579	101032586	101032581	T	G	snp	1	1	1	1	.	.	.	INTERGENIC(INTERGENIC)
1	101055451	101055468	101055457	AC	C	del	0	1	0	1	.	.	.	INTERGENIC(INTERGENIC);RP11-84012.2.1(UPSTREAM)
1	101055451	101055468	101055459	AC	C	del	0	1	0	1	.	.	.	INTERGENIC(INTERGENIC);RP11-84012.2.1(UPSTREAM)
1	101085869	101085876	101085875	C	A	snp	1	1	1	1	.	.	.	INTERGENIC(INTERGENIC)
1	101136597	101136605	101136601	T	C	snp	1	1	1	1	.	.	.	INTERGENIC(INTERGENIC)

1	101212064	101212091	101212076	AGAA	G	snp	1	0	1	0	.	.	.	INTERGENIC(INTERGENIC)
1	101270760	101270783	101270770	TTTA	C	snp	1	0	0	0	.	.	.	INTERGENIC(INTERGENIC)
1	101284020	101284028	101284027	A	G	snp	1	1	1	1	.	.	.	INTERGENIC(INTERGENIC)
1	101620483	101620494	101620490	TG	A	snp	1	1	1	1	.	.	.	INTERGENIC(INTERGENIC)
1	101630827	101630835	101630828	A	G	snp	1	1	1	1	.	.	.	INTERGENIC(INTERGENIC)
1	101648508	101648535	101648518	TTCT	TT	ins	1	1	1	1	.	.	.	INTERGENIC(INTERGENIC)
1	101649478	101649487	101649483	TC	G	snp	1	0	0	0	.	.	.	INTERGENIC(INTERGENIC)
1	101672502	101672521	101672517	TTTA	T	snp	0	1	1	1	.	.	.	INTERGENIC(INTERGENIC)
1	101673054	101673062	101673058	T	C	snp	1	1	1	1	.	.	.	INTERGENIC(INTERGENIC)
1	101811786	101811793	101811788	A	C	snp	1	1	0	1	.	.	.	RP11-157N3.1.1(INTRON);RP11-157N3.1.1(UPSTREAM)
1	101861741	101861748	101861746	A	G	snp	1	1	1	1	.	.	.	INTERGENIC(INTERGENIC)
1	101880516	101880531	101880524	CAAT	A	del	1	0	1	0	.	.	.	INTERGENIC(INTERGENIC)
1	102167213	102167220	102167216	C	T	snp	1	1	0	0	.	.	.	INTERGENIC(INTERGENIC)
1	102213486	102213494	102213490	A	G	snp	0	1	0	0	.	.	.	INTERGENIC(INTERGENIC)
1	102225505	102225512	102225506	T	G	snp	0	1	1	1	.	.	.	INTERGENIC(INTERGENIC)
1	102357126	102357134	102357129	T	C	snp	1	0	0	0	.	.	.	DNAJA1P5(UPSTREAM);OLFM3(INTRON)
1	102464712	102464719	102464716	T	C	snp	1	1	0	1	.	.	.	INTERGENIC(INTERGENIC);OLFM3(UPSTREAM)
1	102486261	102486270	102486262	T	G	snp	1	0	0	0	.	.	.	INTERGENIC(INTERGENIC)
1	102491700	102491707	102491704	T	C	snp	1	1	1	1	.	.	.	INTERGENIC(INTERGENIC)
1	102559511	102559528	102559525	TA	A	del	1	1	1	1	.	.	.	INTERGENIC(INTERGENIC)
1	102559511	102559528	102559526	TA	T	snp	1	1	1	1	.	.	.	INTERGENIC(INTERGENIC)
1	102645872	102645891	102645885	TTAA	AATTA	del	1	1	1	1	.	.	.	INTERGENIC(INTERGENIC)
1	102711921	102711928	102711925	T	C	snp	1	1	1	1	.	.	.	RP11-202K23.1.1(INTRON)
1	102717326	102717335	102717330	TA	C	snp	1	0	0	0	.	.	.	RP11-202K23.1.1(INTRON)
1	102728283	102728291	102728287	A	G	snp	1	1	1	1	.	.	.	RP11-202K23.1.1(INTRON)
1	102734222	102734229	102734224	T	G	snp	1	1	0	1	.	.	.	RP11-202K23.1.1(INTRON)
1	102740763	102740770	102740768	T	C	snp	1	0	0	0	.	.	.	RP11-202K23.1.1(INTRON)
1	102794141	102794156	102794146	AAAC	C	snp	1	0	0	0	.	.	.	RP11-202K23.1.1(INTRON)
1	102799520	102799533	102799528	AC	T	snp	1	0	0	0	.	.	.	RP11-202K23.1.1(INTRON)
1	102799520	102799533	102799530	AC	G	snp	1	0	0	0	.	.	.	RP11-202K23.1.1(INTRON)
1	102813760	102813768	102813764	A	C	snp	1	0	0	0	.	.	.	RP11-202K23.1.1(INTRON)
1	102814061	102814070	102814066	TA	T	snp	1	0	0	0	.	.	.	RP11-202K23.1.1(INTRON)
1	102858079	102858088	102858085	A	G	snp	1	1	1	1	.	.	.	INTERGENIC(INTERGENIC);RP11-202K23.1.1(UPSTREAM)
1	102895212	102895229	102895220	AAT	C	snp	1	0	0	0	.	.	.	INTERGENIC(INTERGENIC)
1	102918948	102918955	102918950	T	C	snp	1	0	0	0	.	.	.	INTERGENIC(INTERGENIC)
1	102943604	102943627	102943620	TTA	ATTATTA	del	1	1	1	1	.	.	.	INTERGENIC(INTERGENIC)
1	102947361	102947369	102947367	A	C	snp	1	1	1	1	.	.	.	INTERGENIC(INTERGENIC)
1	103044145	103044153	103044146	T	C	snp	1	0	0	0	.	.	.	INTERGENIC(INTERGENIC)
1	103049059	103049070	103049067	TA	C	snp	1	1	1	1	.	.	.	INTERGENIC(INTERGENIC)
1	103049083	103049092	103049089	TA	AC	ins	0	0	1	1	.	.	.	INTERGENIC(INTERGENIC)
1	103082775	103082784	103082780	A	G	snp	1	0	0	1	.	.	.	INTERGENIC(INTERGENIC)
1	103083813	103083822	103083820	T	G	snp	0	1	0	1	.	.	.	INTERGENIC(INTERGENIC)
1	103114167	103114178	103114170	CA	C	snp	1	0	1	1	.	.	.	INTERGENIC(INTERGENIC)
1	103167761	103167768	103167762	A	G	snp	1	0	0	0	.	.	.	INTERGENIC(INTERGENIC)
1	103200470	103200479	103200474	A	G	snp	1	1	1	0	.	.	.	INTERGENIC(INTERGENIC)
1	103208481	103208488	103208485	A	C	snp	1	0	0	0	.	.	.	INTERGENIC(INTERGENIC)
1	103222099	103222107	103222101	A	C	snp	0	0	0	1	.	.	.	INTERGENIC(INTERGENIC)
1	103426034	103426043	103426041	GT	GTG	del	1	1	1	1	.	.	.	COL11A1(DOWNSTREAM);COL11A1(INTRON)
1	103520160	103520168	103520162	A	C	snp	1	0	0	0	.	.	.	COL11A1(INTRON)
1	103531130	103531137	103531131	A	AC	ins	1	0	0	0	.	.	.	COL11A1(INTRON)
1	103542883	103542891	103542888	T	G	snp	1	0	0	0	.	.	.	COL11A1(INTRON)
1	103558538	103558546	103558541	A	C	snp	1	0	0	0	.	.	.	COL11A1(INTRON)
1	103613166	103613180	103613177	TTG	TTGTT	del	1	1	0	1	.	.	.	INTERGENIC(INTERGENIC)
1	103623448	103623461	103623453	AT	G	snp	1	0	0	0	.	.	.	INTERGENIC(INTERGENIC)
1	103743795	103743804	103743801	TC	CTC	del	1	1	1	1	.	.	.	INTERGENIC(INTERGENIC)
1	103743795	103743804	103743802	TC	T	snp	1	1	1	1	.	.	.	INTERGENIC(INTERGENIC)
1	104384854	104384862	104384858	A	G	snp	1	1	1	1	.	.	.	INTERGENIC(INTERGENIC)
1	104393639	104393656	104393654	AT	C	snp	1	0	0	0	.	.	.	INTERGENIC(INTERGENIC)
1	104398188	104398205	104398194	AAT	C	snp	1	0	0	0	.	.	.	INTERGENIC(INTERGENIC)
1	104493517	104493528	104493524	AC	T	snp	1	1	1	1	.	.	.	INTERGENIC(INTERGENIC)
1	104525363	104525372	104525370	GA	G	del	1	1	1	1	.	.	.	INTERGENIC(INTERGENIC)
1	104634019	104634028	104634024	CA	G	snp	1	1	1	1	.	.	.	INTERGENIC(INTERGENIC)
1	104795172	104795179	104795178	T	G	snp	1	1	1	1	.	.	.	INTERGENIC(INTERGENIC)
1	104799659	104799670	104799668	AG	A	del	1	0	0	0	.	.	.	INTERGENIC(INTERGENIC)
1	104830746	104830753	104830750	A	G	snp	1	1	1	1	.	.	.	INTERGENIC(INTERGENIC)
1	104855986	104856001	104855991	TC	G	snp	1	1	1	1	.	.	.	INTERGENIC(INTERGENIC)
1	104886611	104886620	104886613	TC	G	snp	1	1	1	1	.	.	.	INTERGENIC(INTERGENIC)

1	104924107	104924114	104924111	C	T	snp	1	0	0	0	.	.	.	INTERGENIC(INTERGENIC)
1	104945285	104945293	104945287	T	G	snp	1	1	1	1	.	.	.	INTERGENIC(INTERGENIC)
1	104950633	104950640	104950634	C	T	ins	1	1	1	1	.	.	.	INTERGENIC(INTERGENIC)
1	104950633	104950640	104950637	C	A	ins	1	1	1	1	.	.	.	INTERGENIC(INTERGENIC)
1	104960233	104960244	104960236	TC	G	snp	0	1	1	0	.	.	.	INTERGENIC(INTERGENIC)
1	104975137	104975145	104975142	A	C	ins	1	1	1	1	.	.	.	INTERGENIC(INTERGENIC)
1	105000001	105000012	105000006	ATT	A	ins	1	1	1	1	.	.	.	INTERGENIC(INTERGENIC)
1	105014524	105014533	105014527	AT	C	snp	1	0	0	0	.	.	.	INTERGENIC(INTERGENIC)
1	105074499	105074506	105074501	T	C	snp	1	1	1	1	.	.	.	INTERGENIC(INTERGENIC)
1	105097624	105097632	105097631	A	G	snp	1	1	0	1	.	.	.	INTERGENIC(INTERGENIC)
1	105186487	105186496	105186494	AG	T	snp	1	1	0	1	.	.	.	INTERGENIC(INTERGENIC)
1	105208155	105208162	105208161	A	C	snp	1	1	0	0	.	.	.	INTERGENIC(INTERGENIC)
1	105269516	105269525	105269523	TA	G	snp	1	1	0	1	.	.	.	INTERGENIC(INTERGENIC)
1	105494488	105494495	105494489	A	G	snp	1	1	1	1	.	.	.	INTERGENIC(INTERGENIC)
1	105550768	105550776	105550769	T	C	ins	1	1	1	1	.	.	.	INTERGENIC(INTERGENIC)
1	105557842	105557851	105557847	T	G	snp	1	1	1	1	.	.	.	INTERGENIC(INTERGENIC)
1	105572391	105572399	105572392	A	C	snp	1	1	0	1	.	.	.	INTERGENIC(INTERGENIC)
1	105586493	105586501	105586495	A	C	snp	1	1	1	1	.	.	.	INTERGENIC(INTERGENIC)
1	105622189	105622200	105622193	TAA	G	snp	1	1	1	1	.	.	.	INTERGENIC(INTERGENIC)
1	105774626	105774635	105774627	A	C	snp	1	0	0	0	.	.	.	INTERGENIC(INTERGENIC)
1	105808000	105808023	105808004	CAAA	A	snp	1	0	1	0	.	.	.	INTERGENIC(INTERGENIC)
1	105840993	105841002	105840999	A	C	ins	1	1	0	0	.	.	.	INTERGENIC(INTERGENIC)
1	105846355	105846364	105846362	TA	G	snp	1	0	0	0	.	.	.	INTERGENIC(INTERGENIC)
1	105884050	105884067	105884056	AAC	G	snp	1	0	0	0	.	.	.	INTERGENIC(INTERGENIC)
1	105888601	105888609	105888605	T	G	snp	1	0	0	0	.	.	.	INTERGENIC(INTERGENIC)
1	105900560	105900567	105900563	T	G	snp	1	1	1	1	.	.	.	INTERGENIC(INTERGENIC)
1	105921354	105921361	105921359	T	C	snp	1	0	0	0	.	.	.	INTERGENIC(INTERGENIC)
1	106073241	106073249	106073248	T	C	ins	1	1	1	0	.	.	.	INTERGENIC(INTERGENIC)
1	106129110	106129119	106129112	AC	G	snp	1	1	1	1	.	.	.	INTERGENIC(INTERGENIC);RP11-251P6.1.1(DOWNSTREAM)
1	106159341	106159349	106159344	T	C	snp	1	0	0	0	.	.	.	RP11-251P6.1.1(INTRON)
1	106159341	106159349	106159345	T	G	snp	1	1	1	1	.	.	.	RP11-251P6.1.1(INTRON)
1	106164806	106164815	106164808	T	C	ins	1	1	1	1	.	.	.	INTERGENIC(INTERGENIC);RP11-251P6.1.1(UPSTREAM)
1	106164806	106164815	106164809	T	C	snp	1	1	1	1	.	.	.	INTERGENIC(INTERGENIC);RP11-251P6.1.1(UPSTREAM)
1	106205281	106205289	106205288	A	C	ins	1	0	0	0	.	.	.	INTERGENIC(INTERGENIC)
1	106334313	106334320	106334317	A	G	snp	0	0	1	0	.	.	.	INTERGENIC(INTERGENIC)
1	106413948	106413962	106413958	TTG	A	snp	1	0	0	0	.	.	.	INTERGENIC(INTERGENIC)
1	106482200	106482217	106482214	AT	G	snp	1	0	0	0	.	.	.	RP11-24P14.1.1(INTRON)
1	106504026	106504035	106504033	TA	TATTT	del	1	1	1	1	.	.	.	RP11-24P14.1.1(INTRON)
1	106522523	106522534	106522532	TA	G	snp	1	0	0	0	.	.	.	RP11-24P14.1.1(INTRON)
1	106652344	106652351	106652345	A	C	ins	0	1	1	1	.	.	.	INTERGENIC(INTERGENIC)
1	106796081	106796089	106796084	A	C	ins	1	0	1	0	.	.	.	INTERGENIC(INTERGENIC)
1	106864596	106864609	106864602	TA	A	snp	0	1	1	1	.	.	.	INTERGENIC(INTERGENIC)
1	106944868	106944881	106944874	TA	C	snp	1	0	0	0	.	.	.	INTERGENIC(INTERGENIC)
1	106969786	106969797	106969788	TA	AGAC	ins	1	1	0	1	.	.	.	INTERGENIC(INTERGENIC)
1	107085831	107085839	107085837	T	C	snp	1	0	0	0	.	.	.	INTERGENIC(INTERGENIC)
1	107207948	107207971	107207962	TTAT	T	snp	1	1	1	1	.	.	.	INTERGENIC(INTERGENIC)
1	107248936	107248945	107248943	AC	T	snp	1	1	1	1	.	.	.	INTERGENIC(INTERGENIC)
1	107327793	107327806	107327804	GA	G	del	1	0	0	0	.	.	.	INTERGENIC(INTERGENIC)
1	107352792	107352800	107352799	T	G	snp	1	0	0	0	.	.	.	INTERGENIC(INTERGENIC);RP11-233E12.2.1(UPSTREAM)
1	107379332	107379341	107379337	TA	T	ins	1	0	0	0	.	.	.	RP11-233E12.1-001.1(INTRON)
1	107387742	107387757	107387753	GAAG	A	snp	1	0	0	0	.	.	.	INTERGENIC(INTERGENIC)
1	107439015	107439026	107439019	TA	C	snp	1	1	1	1	.	.	.	INTERGENIC(INTERGENIC)
1	107555624	107555639	107555631	AAAT	G	snp	1	1	1	1	.	.	.	INTERGENIC(INTERGENIC)
1	107610377	107610385	107610381	T	C	snp	1	0	0	0	.	.	.	INTERGENIC(INTERGENIC)
1	107631630	107631641	107631638	CT	A	snp	1	1	1	1	.	.	.	INTERGENIC(INTERGENIC)
1	107631737	107631746	107631739	TA	C	snp	1	1	1	1	.	.	.	INTERGENIC(INTERGENIC)
1	107654367	107654386	107654381	TTTA	C	snp	1	1	1	1	.	.	.	INTERGENIC(INTERGENIC)
1	107654367	107654386	107654382	TTTA	TTTT	ins	1	0	0	0	.	.	.	INTERGENIC(INTERGENIC)
1	107682154	107682163	107682160	TA	C	snp	1	0	0	0	.	.	.	INTERGENIC(INTERGENIC);NTNG1(UPSTREAM)
1	107737552	107737559	107737554	A	G	snp	1	1	0	0	.	.	.	NTNG1(INTRON)
1	107853490	107853499	107853493	A	G	snp	1	1	1	1	.	.	.	NTNG1(INTRON)
1	107940598	107940613	107940610	AC	G	snp	0	0	1	0	.	.	.	NTNG1(INTRON)
1	107988034	107988045	107988041	ATT	C	snp	1	1	1	1	.	.	.	AC114491.1(DOWNSTREAM);NTNG1(INTRON)
1	108044586	108044594	108044593	A	G	snp	1	1	1	1	.	.	.	INTERGENIC(INTERGENIC);RP11-270C12.3.1(UPSTREAM)
1	108168642	108168650	108168647	A	G	snp	1	1	0	1	.	.	.	VAV3(INTRON)
1	108191693	108191701	108191696	A	C	snp	1	1	1	1	.	.	.	VAV3(INTRON)
1	108203640	108203649	108203647	AC	A	snp	1	1	1	1	.	.	.	VAV3(INTRON)

1	108365275	108365282	108365277	A	AAAT	ins	0	1	1	1	.	.	.	VAV3(INTRON);VAV3(UPSTREAM)
1	108501251	108501259	108501256	T	C	ins	1	1	1	0	.	.	.	VAV3(INTRON)
1	108538330	108538339	108538336	T	G	snp	1	0	1	0	.	.	.	INTERGENIC(INTERGENIC);RP11-356N1.1.1(DOWNSTREAM)
1	108538330	108538339	108538338	T	G	snp	1	1	1	1	.	.	.	INTERGENIC(INTERGENIC);RP11-356N1.1.1(DOWNSTREAM)
1	108552484	108552497	108552495	CT	C	del	1	1	1	1	.	.	.	INTERGENIC(INTERGENIC)
1	108566349	108566358	108566355	AT	G	snp	1	1	1	1	.	.	.	INTERGENIC(INTERGENIC)
1	108611635	108611654	108611646	AAAC	A	snp	1	1	1	1	.	.	.	INTERGENIC(INTERGENIC);RP11-356N1.2.1(UPSTREAM)
1	108722170	108722179	108722174	TA	C	snp	1	1	1	1	.	.	.	SLC25A24(INTRON)
1	108730550	108730567	108730555	AT	ATAC	ins	1	1	1	1	.	.	.	SLC25A24(INTRON)
1	108730550	108730567	108730557	AT	G	snp	1	1	1	1	.	.	.	SLC25A24(INTRON)
1	108730550	108730567	108730561	AT	G	snp	1	0	1	1	.	.	.	SLC25A24(INTRON)
1	108732493	108732506	108732496	AT	A	snp	1	1	1	1	.	.	.	SLC25A24(INTRON)
1	108737293	108737304	108737302	AC	G	snp	1	1	0	1	.	.	.	SLC25A24(DOWNSTREAM);SLC25A24(INTRON);SLC25A24(UPSTREAM)
1	108753382	108753393	108753387	TG	A	snp	1	1	1	1	.	.	.	INTERGENIC(INTERGENIC)
1	108870151	108870162	108870158	GTG	C	snp	1	1	1	1	.	.	.	RP11-483I13.3.1(INTRON);RP11-483I13.4.1(INTRON)
1	108908541	108908548	108908547	G	A	ins	1	0	0	0	.	.	.	RP11-483I13.4.1(INTRON)
1	108978455	108978463	108978457	C	CCA	ins	1	1	1	1	.	.	.	NBPF6(INTRON);RP11-131J3.1.1(DOWNSTREAM)
1	108978455	108978463	108978458	C	CA	ins	1	1	1	1	.	.	.	NBPF6(INTRON);RP11-131J3.1.1(DOWNSTREAM)
1	109086227	109086236	109086232	AG	T	snp	1	0	0	0	.	.	.	INTERGENIC(INTERGENIC)
1	109102745	109102765	109102762	GGC	T	snp	1	1	1	1	.	.	.	FAM102B(UPSTREAM);FAM102B(UTR_5_PRIME)
1	109136603	109136610	109136609	T	C	snp	1	1	1	1	.	.	.	FAM102B(INTRON)
1	109215390	109215398	109215397	A	G	snp	1	0	0	0	.	.	.	INTERGENIC(INTERGENIC)
1	109241122	109241129	109241128	T	C	snp	1	0	0	0	.	.	.	PRPF38B(DOWNSTREAM);PRPF38B(EXON);PRPF38B(INTRON)
1	109387078	109387089	109387084	AG	G	snp	1	1	1	1	.	.	.	AKNAD1(INTRON)
1	109398648	109398657	109398656	T	G	snp	1	1	0	1	.	.	.	AKNAD1(INTRON);AKNAD1(UPSTREAM);RP11-475E11.5.1(UPSTREAM)
1	109462789	109462796	109462790	A	G	snp	1	1	1	1	.	.	.	AKNAD1(INTRON);GPSM2(INTRON)
1	109592257	109592266	109592261	A	G	snp	1	1	1	1	.	.	.	INTERGENIC(INTERGENIC);RANP5(DOWNSTREAM)
1	109627132	109627141	109627135	A	C	snp	1	0	0	0	.	.	.	INTERGENIC(INTERGENIC)
1	109765004	109765013	109765011	TG	A	snp	0	1	1	1	.	.	.	SARS(INTRON)
1	109773691	109773706	109773704	AT	A	del	1	1	0	1	.	.	.	SARS(INTRON);SARS(UPSTREAM)
1	110067582	110067605	110067589	AAAT	A	snp	1	1	1	1	.	.	.	INTERGENIC(INTERGENIC)
1	110271422	110271430	110271429	T	G	snp	1	1	1	1	.	.	.	RP4-735C1.4.1(INTRON)
1	110341464	110341471	110341466	A	G	ins	1	0	0	0	.	.	.	INTERGENIC(INTERGENIC)
1	110366151	110366162	110366156	CTG	A	snp	1	1	0	1	.	.	.	INTERGENIC(INTERGENIC);RP4-735C1.6.1(UPSTREAM)
1	110374374	110374389	110374381	TTTC	TTTCTTTCTT	del	1	1	1	1	.	.	.	RP4-735C1.6.1(INTRON)
1	110412101	110412120	110412108	TATG	TATGTATGTAT	ins	1	1	1	1	.	.	.	RP4-735C1.6.1(INTRON)
1	110500859	110500867	110500865	C	A	snp	1	1	1	1	.	.	.	INTERGENIC(INTERGENIC)
1	110501826	110501833	110501827	A	G	snp	1	1	1	1	.	.	.	INTERGENIC(INTERGENIC)
1	110516529	110516538	110516530	T	C	snp	1	0	0	0	.	.	.	INTERGENIC(INTERGENIC)
1	110622059	110622070	110622068	GT	G	snp	1	0	0	0	.	.	.	INTERGENIC(INTERGENIC);RP4-773N10.5.1(DOWNSTREAM);RP4-773N10.6.1(UPSTREAM)
1	110642290	110642309	110642303	AGGG	GGAGGGGGCCGGCAGGGAGGGAGGGAA	del	0	1	0	0	.	.	.	RP4-773N10.6.1(INTRON)
1	110642290	110642309	110642304	AGGG	A	snp	1	0	0	0	.	.	.	RP4-773N10.6.1(INTRON)
1	110791076	110791084	110791080	T	G	ins	1	0	0	0	.	.	.	KCNC4(INTRON)
1	110796713	110796722	110796716	G	GT	ins	1	0	0	0	.	.	.	KCNC4(INTRON)
1	110806412	110806421	110806418	A	G	snp	1	1	1	0	.	.	.	KCNC4(INTRON)
1	110806446	110806453	110806448	G	T	snp	1	1	1	1	.	.	.	KCNC4(INTRON)
1	110838355	110838364	110838358	TC	T	snp	1	1	1	1	.	.	.	RP5-1074L1.1.1(INTRON)
1	110858866	110858875	110858871	AT	C	snp	0	0	1	0	.	.	.	RP5-1074L1.1.1(INTRON)
1	111002040	111002048	111002047	T	C	snp	1	1	1	1	.	.	.	INTERGENIC(INTERGENIC);PROK1(DOWNSTREAM)
1	111059813	111059830	111059825	GA	A	snp	1	1	1	1	.	.	.	INTERGENIC(INTERGENIC);KCNA10(DOWNSTREAM)
1	111072789	111072803	111072799	GGT	C	snp	1	0	0	0	.	.	.	INTERGENIC(INTERGENIC)
1	111077626	111077643	111077629	ATC	G	snp	0	1	0	1	.	.	.	INTERGENIC(INTERGENIC)
1	111107355	111107362	111107361	T	G	ins	1	0	0	0	.	.	.	INTERGENIC(INTERGENIC)
1	111253388	111253395	111253392	T	G	snp	1	1	1	1	.	.	.	INTERGENIC(INTERGENIC)
1	111264862	111264869	111264863	G	T	snp	1	0	0	0	.	.	.	INTERGENIC(INTERGENIC)
1	111311855	111311868	111311864	TA	G	snp	0	0	0	1	.	.	.	INTERGENIC(INTERGENIC);Y_RNA(UPSTREAM)
1	111316414	111316425	111316418	AG	G	del	1	1	1	1	.	.	.	INTERGENIC(INTERGENIC)
1	111316456	111316467	111316460	AG	G	ins	1	1	1	1	.	.	.	INTERGENIC(INTERGENIC)
1	111316512	111316523	111316514	AG	G	del	1	1	1	1	.	.	.	INTERGENIC(INTERGENIC)
1	111316512	111316523	111316516	AG	G	del	1	1	1	1	.	.	.	INTERGENIC(INTERGENIC)
1	111331692	111331699	111331694	A	G	snp	0	0	1	0	.	.	.	INTERGENIC(INTERGENIC)
1	111404134	111404143	111404140	AT	G	snp	1	1	1	1	.	.	.	INTERGENIC(INTERGENIC)
1	111422186	111422194	111422188	T	C	snp	0	1	1	1	.	.	.	CD53(INTRON);CD53(UPSTREAM)
1	111462218	111462225	111462221	A	G	snp	1	0	0	0	.	.	.	INTERGENIC(INTERGENIC)

1	111533991	111534008	111533996	TTG	T	snp	1	1	0	1	.	.	.	INTERGENIC(INTERGENIC)
1	111630800	111630807	111630804	T	C	snp	1	1	1	1	.	.	.	INTERGENIC(INTERGENIC)
1	111908230	111908243	111908241	TA	T	snp	1	1	1	1	.	.	.	RP5-1125M8.5.1(INTRON)
1	111972947	111972955	111972948	A	G	snp	1	1	1	1	.	.	.	INTERGENIC(INTERGENIC);OVGP1(UPSTREAM);RP11-552M11.8.1(DOWNSTREAM)
1	112087916	112087923	112087919	T	G	snp	1	0	0	0	.	.	.	ADORA3(INTRON);RAP1A(INTRON)
1	112090631	112090654	112090645	TTTG	A	snp	1	1	1	1	.	.	.	ADORA3(INTRON);RAP1A(INTRON)
1	112123488	112123497	112123491	A	C	ins	1	1	1	1	.	.	.	RAP1A(INTRON)
1	112162407	112162418	112162410	CGC	T	snp	1	1	1	0	.	.	RAP1A(START_GAINED)	RAP1A(INTRON);RAP1A(UPSTREAM)
1	112166216	112166225	112166221	AG	C	snp	1	1	1	1	.	.	.	RAP1A(INTRON);RAP1A(UPSTREAM)
1	112193426	112193435	112193430	T	G	snp	0	1	0	0	.	.	.	RAP1A(INTRON);RP4-773A18.2.1(UPSTREAM);U6(UPSTREAM)
1	112222140	112222148	112222144	T	C	snp	0	1	1	1	.	.	.	FAM212B(DOWNSTREAM);RAP1A(INTRON)
1	112364486	112364497	112364489	GT	C	snp	1	1	1	1	.	.	.	KCND3(INTRON)
1	112389976	112389989	112389980	GT	C	snp	1	1	0	1	.	.	.	KCND3(INTRON)
1	112584191	112584202	112584200	TC	TT	ins	0	0	1	0	.	.	.	INTERGENIC(INTERGENIC)
1	112618474	112618489	112618482	GTGG	A	snp	1	0	0	0	.	.	.	INTERGENIC(INTERGENIC)
1	112621471	112621479	112621472	A	C	ins	1	0	0	0	.	.	.	INTERGENIC(INTERGENIC)
1	112880202	112880210	112880203	T	C	snp	1	0	0	0	.	.	.	RP5-965F6.2.1(INTRON)
1	112925635	112925650	112925637	AT	T	del	1	0	0	0	.	.	.	INTERGENIC(INTERGENIC)
1	112975878	112975893	112975882	GT	T	snp	0	1	0	1	.	.	.	CTTNBP2NL(INTRON)
1	112980140	112980148	112980141	T	G	snp	1	1	1	1	.	.	.	CTTNBP2NL(INTRON)
1	113055743	113055751	113055744	A	AAAAAAAC	ins	1	0	0	0	.	.	.	WNT2B(INTRON);WNT2B(UPSTREAM)
1	113120590	113120601	113120593	ATC	G	snp	1	1	1	1	.	.	.	ST7L(DOWNSTREAM);ST7L(INTRON)
1	113122950	113122957	113122956	T	G	snp	1	1	1	1	.	.	.	ST7L(DOWNSTREAM);ST7L(INTRON)
1	113162030	113162039	113162037	C	A	snp	0	1	1	1	.	.	.	CAPZA1(UPSTREAM);CAPZA1(UTR_5_PRIME);ST7L(EXON);ST7L(INTRON);ST7L(UPSTREAM);ST7L(UTR_5_PRIME)
1	113202197	113202208	113202206	TC	T	ins	1	1	1	1	.	.	.	CAPZA1(EXON);CAPZA1(INTRON)
1	113371009	113371017	113371012	A	G	snp	1	0	0	0	.	.	.	RP11-426L16.8.1(INTRON)
1	113385025	113385034	113385031	T	G	ins	1	1	0	1	.	.	.	RP11-426L16.8.1(INTRON)
1	113537390	113537407	113537403	TTG	G	snp	1	0	0	0	.	.	.	RP11-31F15.1.1(INTRON)
1	113554895	113554906	113554899	GA	A	snp	1	0	0	0	.	.	.	RP11-31F15.2.1(EXON);RP11-389O22.1.1(INTRON)
1	113554895	113554906	113554902	GA	T	snp	1	0	0	0	.	.	.	RP11-31F15.2.1(EXON);RP11-389O22.1.1(INTRON)
1	113713185	113713192	113713190	A	G	ins	1	1	0	1	.	.	.	INTERGENIC(INTERGENIC);RP11-389O22.4.1(DOWNSTREAM)
1	114085388	114085397	114085390	CA	G	snp	1	0	0	0	.	.	.	MAGI3(INTRON)
1	114087706	114087715	114087710	T	G	snp	1	1	1	1	.	.	.	MAGI3(INTRON)
1	114339127	114339138	114339133	AT	C	snp	1	0	0	0	.	.	.	RSBN1(INTRON)
1	114392197	114392216	114392206	TTTA	TA	del	0	1	1	1	.	.	.	PTPN22(INTRON)
1	114392197	114392216	114392211	TTTA	A	del	1	1	1	1	.	.	.	PTPN22(INTRON)
1	114403581	114403592	114403583	GT	A	snp	1	0	0	0	.	.	.	PTPN22(INTRON);RP5-1073O3.5.1(INTRON)
1	114604889	114604904	114604899	ATCT	G	snp	1	1	0	1	.	.	.	INTERGENIC(INTERGENIC)
1	114785878	114785889	114785885	ATG	C	snp	1	1	1	1	.	.	.	RP4-543J13.1.1(INTRON)
1	114786344	114786359	114786354	AATA	A	snp	1	1	1	1	.	.	.	RP4-543J13.1.1(INTRON)
1	114807104	114807113	114807111	AT	ATCTGAA	del	1	1	0	0	.	.	.	INTERGENIC(INTERGENIC);RP4-543J13.1.1(DOWNSTREAM)
1	115089080	115089093	115089091	AT	G	snp	1	1	0	1	.	.	.	INTERGENIC(INTERGENIC)
1	115119982	115120001	115119997	ATTT	A	del	1	1	0	1	.	.	.	BCAS2(INTRON)
1	115277355	115277364	115277362	AT	C	snp	0	0	0	1	.	.	.	CSDE1(DOWNSTREAM);CSDE1(INTRON)
1	115473708	115473717	115473713	A	G	snp	0	1	0	1	.	.	.	SYCP1(INTRON)
1	115677526	115677535	115677533	CA	C	del	1	0	0	0	.	.	.	INTERGENIC(INTERGENIC)
1	115679774	115679783	115679781	TG	A	snp	1	0	0	0	.	.	.	INTERGENIC(INTERGENIC)
1	116107015	116107029	116107025	CGC	A	snp	1	1	1	1	.	.	.	INTERGENIC(INTERGENIC);RP11-710N8.2.1(UPSTREAM)
1	116107015	116107029	116107025	CGC	CCA	ins	1	1	1	1	.	.	.	INTERGENIC(INTERGENIC);RP11-710N8.2.1(UPSTREAM)
1	116168356	116168365	116168358	T	TA	ins	1	1	1	1	.	.	.	INTERGENIC(INTERGENIC);SNORA42(UPSTREAM)
1	116224113	116224120	116224118	T	TTG	ins	1	1	1	1	.	.	.	VANGL1(INTRON)
1	116224125	116224132	116224126	T	G	ins	1	1	1	1	.	.	.	VANGL1(INTRON)
1	116229381	116229392	116229385	AAT	G	snp	1	1	1	1	.	.	.	VANGL1(DOWNSTREAM);VANGL1(INTRON)
1	116230651	116230666	116230657	ATAG	G	snp	1	1	0	1	.	.	.	VANGL1(DOWNSTREAM);VANGL1(INTRON)
1	116265876	116265895	116265888	TATC	ATC	ins	1	1	1	1	.	.	.	CASQ2(DOWNSTREAM);CASQ2(INTRON)
1	116265876	116265895	116265891	TATC	T	del	1	1	1	1	.	.	.	CASQ2(DOWNSTREAM);CASQ2(INTRON)
1	116299576	116299591	116299581	TAAA	G	snp	1	0	0	0	.	.	.	CASQ2(INTRON)
1	116311367	116311382	116311380	CA	G	snp	1	1	1	1	.	.	.	CASQ2(UPSTREAM);CASQ2(UTR_5_PRIME)
1	116351367	116351381	116351378	TTA	T	snp	1	0	0	0	.	.	.	INTERGENIC(INTERGENIC)
1	116402042	116402062	116402048	GTG	A	snp	1	1	1	1	.	.	.	INTERGENIC(INTERGENIC);RP11-485H8.2.1(UPSTREAM)
1	116422551	116422564	116422557	CA	T	snp	1	0	0	0	.	.	.	INTERGENIC(INTERGENIC)
1	116499595	116499602	116499596	C	T	snp	1	0	0	0	.	.	.	INTERGENIC(INTERGENIC)
1	116528825	116528833	116528830	T	G	snp	1	0	0	0	.	.	.	SLC22A15(INTRON)

1	116530162	116530170	116530163	A	C	snp	1	1	1	1	.	.	.	SLC22A15(INTRON)
1	116638673	116638681	116638675	T	C	snp	1	0	0	0	.	.	.	INTERGENIC(INTERGENIC)
1	116662097	116662105	116662099	T	C	snp	1	0	0	0	.	.	.	MAB21L3(INTRON);MAB21L3(UPSTREAM)
1	116816360	116816367	116816361	C	T	snp	1	1	0	1	.	.	.	INTERGENIC(INTERGENIC);U3(UPSTREAM)
1	116851483	116851492	116851484	T	G	snp	1	0	0	0	.	.	.	INTERGENIC(INTERGENIC)
1	116866801	116866810	116866804	TA	C	snp	1	1	1	1	.	.	.	INTERGENIC(INTERGENIC)
1	116910431	116910438	116910434	T	C	snp	1	1	1	1	.	.	.	ATP1A1(UPSTREAM);INTERGENIC(INTERGENIC)
1	116958648	116958657	116958650	G	A	snp	1	1	0	0	.	.	.	ATP1A1OS(INTRON);U6(UPSTREAM)
1	116979595	116979606	116979598	AT	G	snp	1	0	0	0	.	.	.	RP4-655J12.4.1(INTRON);RP4-655J12.5.1(INTRON);RP4-655J12.5.1(UPSTREAM)
1	117213889	117213898	117213896	AG	A	snp	1	0	0	0	.	.	.	IGSF3(UPSTREAM);INTERGENIC(INTERGENIC);MIR320B1(UPSTREAM)
1	117213913	117213930	117213916	AG	A	snp	1	0	0	0	.	.	.	IGSF3(UPSTREAM);INTERGENIC(INTERGENIC);MIR320B1(UPSTREAM)
1	117325563	117325580	117325566	AC	AA	ins	1	0	1	0	.	.	.	INTERGENIC(INTERGENIC)
1	117335833	117335852	117335848	AACA	C	snp	1	0	0	0	.	.	.	INTERGENIC(INTERGENIC)
1	117417523	117417531	117417525	A	C	snp	1	1	1	1	.	.	.	INTERGENIC(INTERGENIC)
1	117541319	117541334	117541325	GATA	A	ins	1	0	0	0	.	.	.	CD101(UPSTREAM);INTERGENIC(INTERGENIC)
1	117660127	117660134	117660131	A	G	snp	1	1	1	1	.	.	.	TRIM45(DOWNSTREAM);TRIM45(INTRON)
1	117694686	117694697	117694694	TTG	A	snp	1	0	0	0	.	.	.	VTCN1(DOWNSTREAM);VTCN1(INTRON)
1	117718378	117718395	117718391	TC	T	ins	1	0	0	0	.	.	.	VTCN1(INTRON);VTCN1(UPSTREAM)
1	117758915	117758922	117758917	A	G	snp	1	1	1	1	.	.	.	INTERGENIC(INTERGENIC)
1	117823893	117823904	117823901	GA	A	snp	1	1	1	1	.	.	.	INTERGENIC(INTERGENIC)
1	117932336	117932345	117932344	T	G	snp	1	1	0	1	.	.	.	MAN1A2(INTRON)
1	118001120	118001133	118001128	TA	G	snp	1	0	0	0	.	.	.	MAN1A2(INTRON)
1	118001120	118001133	118001129	TA	G	snp	1	1	1	1	.	.	.	MAN1A2(INTRON)
1	118001135	118001146	118001138	AT	AG	ins	1	1	1	1	.	.	.	MAN1A2(INTRON)
1	118071065	118071074	118071072	AC	A	snp	1	1	0	1	.	.	.	MAN1A2(DOWNSTREAM);MAN1A2(UTR_3_PRIME)
1	118183242	118183255	118183244	AG	G	snp	0	0	0	1	.	.	.	AL365331.2.1(DOWNSTREAM);INTERGENIC(INTERGENIC)
1	118293345	118293353	118293350	T	C	snp	1	1	1	1	.	.	.	INTERGENIC(INTERGENIC)
1	118293345	118293353	118293352	T	C	snp	1	1	1	1	.	.	.	INTERGENIC(INTERGENIC)
1	118411369	118411377	118411374	A	C	ins	0	0	1	0	.	.	.	GDAP2(DOWNSTREAM);INTERGENIC(INTERGENIC)
1	118549721	118549728	118549722	T	G	snp	1	1	0	0	.	.	.	SPAG17(INTRON)
1	118693010	118693017	118693016	T	G	snp	0	1	1	1	.	.	.	SPAG17(DOWNSTREAM);SPAG17(INTRON)
1	118943876	118943885	118943882	AT	ATATATT	ins	1	1	1	1	.	.	.	INTERGENIC(INTERGENIC)
1	118943936	118943945	118943938	TA	AA	ins	1	1	1	1	.	.	.	INTERGENIC(INTERGENIC)
1	118973021	118973048	118973038	CCTT	T	snp	1	0	0	0	.	.	.	INTERGENIC(INTERGENIC)
1	119006804	119006812	119006810	C	T	snp	0	0	1	0	.	.	.	INTERGENIC(INTERGENIC)
1	119113309	119113317	119113310	T	C	snp	1	1	1	1	.	.	.	INTERGENIC(INTERGENIC)
1	119121023	119121030	119121026	A	C	snp	1	1	1	1	.	.	.	INTERGENIC(INTERGENIC)
1	119125815	119125824	119125818	TA	G	snp	1	1	1	1	.	.	.	INTERGENIC(INTERGENIC)
1	119125815	119125824	119125819	TA	C	snp	1	1	1	1	.	.	.	INTERGENIC(INTERGENIC)
1	119125815	119125824	119125822	TA	G	snp	1	0	0	0	.	.	.	INTERGENIC(INTERGENIC)
1	119159902	119159910	119159904	T	C	snp	1	0	0	0	.	.	.	INTERGENIC(INTERGENIC);PSMC1P12(UPSTREAM)
1	119162211	119162219	119162216	T	G	snp	1	0	0	0	.	.	.	INTERGENIC(INTERGENIC);PSMC1P12(UPSTREAM)
1	119233362	119233370	119233364	T	C	snp	1	0	0	0	.	.	.	INTERGENIC(INTERGENIC)
1	119234498	119234507	119234503	CA	C	snp	1	1	1	1	.	.	.	INTERGENIC(INTERGENIC)
1	119245974	119245989	119245987	AT	A	del	0	0	1	0	.	.	.	INTERGENIC(INTERGENIC)
1	119251808	119251817	119251809	A	G	snp	1	1	1	1	.	.	.	INTERGENIC(INTERGENIC);RP4-630J13.1.1(UPSTREAM)
1	119362472	119362481	119362474	A	C	snp	1	0	0	0	.	.	.	INTERGENIC(INTERGENIC)
1	119516397	119516410	119516405	AC	G	snp	0	1	0	1	.	.	.	TBX15(INTRON)
1	119541259	119541267	119541265	C	A	ins	1	0	1	0	.	.	.	INTERGENIC(INTERGENIC);RP4-712E4.1.1(DOWNSTREAM);RP4-712E4.2.1(DOWNSTREAM)
1	119542179	119542194	119542187	AG	G	snp	1	1	1	1	.	.	.	INTERGENIC(INTERGENIC);RP4-712E4.1.1(DOWNSTREAM);RP4-712E4.2.1(DOWNSTREAM)
1	119615950	119615969	119615956	CCAT	C	snp	1	1	1	1	.	.	.	WARS2(INTRON)
1	119686098	119686105	119686104	A	C	snp	1	1	1	1	.	.	.	RP11-418J17.1.1(DOWNSTREAM);RP11-418J17.1.1(INTRON);RP11-418J17.1.1(UPSTREAM);WARS2(UPSTREAM)
1	119712755	119712762	119712759	T	C	snp	1	1	1	1	.	.	.	RP11-418J17.1.1(INTRON)
1	119773786	119773795	119773793	A	G	snp	0	1	1	1	.	.	.	RP11-418J17.1.1(INTRON)
1	120062340	120062349	120062341	T	TTA	ins	1	1	1	1	.	.	.	HSD3B1(DOWNSTREAM);INTERGENIC(INTERGENIC)
1	120062340	120062349	120062342	T	TA	ins	1	1	1	1	.	.	.	HSD3B1(DOWNSTREAM);INTERGENIC(INTERGENIC)
1	120084651	120084660	120084657	T	TTC	ins	1	0	0	0	.	.	.	HSD3BP3(INTRON)
1	120093421	120093428	120093422	T	C	snp	1	0	0	0	.	.	.	HSD3BP3(DOWNSTREAM);INTERGENIC(INTERGENIC)
1	120158598	120158605	120158599	G	T	snp	1	1	1	1	.	.	.	INTERGENIC(INTERGENIC);ZNF697(DOWNSTREAM)
1	121251788	121251795	121251791	A	C	snp	1	0	0	0	.	.	.	AL592494.4.1(INTRON)
1	143520075	143520082	143520077	T	G	snp	0	0	1	0	.	.	.	BX004987.6.1(DOWNSTREAM);INTERGENIC(INTERGENIC)

1	143529326	143529333	143529330	A	G	ins	1	0	0	0	.	.	.	BX004987.7.1(INTRON);BX004987.7.1(UPSTREAM)
1	144471831	144471839	144471832	T	G	snp	1	1	1	1	.	.	.	AL592284.1(INTRON);AL592284.1.1(INTRON);AL592284.1.1(UPSTREAM)
1	144471831	144471839	144471834	T	G	snp	0	1	0	1	.	.	.	AL592284.1(INTRON);AL592284.1.1(INTRON);AL592284.1.1(UPSTREAM)
1	145048529	145048537	145048535	C	T	ins	0	1	1	1	.	.	.	7SK(DOWNSTREAM);PDE4DIP(INTRON)
1	145327390	145327399	145327395	T	C	snp	0	1	0	0	.	.	.	NBPF10(DOWNSTREAM);NBPF10(INTRON)
1	145375101	145375112	145375103	TA	C	snp	1	1	1	0	.	.	.	NBPF10(DOWNSTREAM);RP11-458D21.1.1(DOWNSTREAM);RP11-458D21.2.1(INTRON)
1	145506522	145506530	145506523	G	T	snp	1	1	0	1	.	.	.	GNRHR2(DOWNSTREAM);LIX1L(DOWNSTREAM);RBM8A(UPSTREAM);RP11-315I20.1.1(DOWNSTREAM);RP11-315I20.1.1(INTRON)
1	146393242	146393253	146393248	TA	C	snp	1	1	0	1	.	.	.	NBPF12(INTRON);PFN1P8(UPSTREAM)
1	146472542	146472555	146472544	TA	C	snp	1	0	0	0	.	.	.	INTERGENIC(INTERGENIC);NBPF12(DOWNSTREAM);RP4-704D21.2.1(DOWNSTREAM)
1	146670442	146670465	146670448	AAAG	GAAAG	del	1	1	1	1	.	.	.	CCT8P1(DOWNSTREAM);FMO5(INTRON)
1	146670442	146670465	146670452	AAAG	G	del	1	1	1	1	.	.	.	CCT8P1(DOWNSTREAM);FMO5(INTRON)
1	146689974	146689981	146689980	T	G	snp	1	0	0	0	.	.	.	FMO5(INTRON)
1	146697782	146697793	146697790	AGT	C	snp	1	1	1	1	.	.	.	FMO5(INTRON);FMO5(UPSTREAM);RP11-337C18.7.1(DOWNSTREAM)
1	146739067	146739075	146739068	T	C	snp	1	1	1	1	.	.	.	CHD1L(INTRON);CHD1L(UPSTREAM)
1	146753472	146753481	146753478	AT	T	snp	0	1	1	1	.	.	.	CHD1L(INTRON)
1	146753472	146753481	146753478	AT	TT	ins	0	1	1	1	.	.	.	CHD1L(INTRON)
1	146791946	146791953	146791952	A	G	snp	1	0	0	0	.	.	.	INTERGENIC(INTERGENIC);RP11-337C18.4.1(DOWNSTREAM)
1	146871078	146871087	146871082	T	C	snp	1	1	1	1	.	.	.	INTERGENIC(INTERGENIC)
1	146955751	146955759	146955754	A	C	snp	1	0	0	0	.	.	.	INTERGENIC(INTERGENIC)
1	147058827	147058844	147058842	TC	A	snp	1	1	1	1	.	.	.	BCL9(INTRON)
1	147278193	147278220	147278200	AAAT	AA	ins	1	0	0	1	.	.	.	INTERGENIC(INTERGENIC)
1	147746458	147746467	147746462	A	G	snp	0	1	0	1	.	.	.	AL592207.3(DOWNSTREAM);RP11-495P10.6.1(INTRON)
1	148352384	148352399	148352393	TG	A	snp	1	1	1	1	.	.	.	INTERGENIC(INTERGENIC);NBPF20(UPSTREAM);PFN1P3(DOWNSTREAM)
1	149083767	149083776	149083773	AT	G	snp	1	1	1	1	.	.	.	INTERGENIC(INTERGENIC);TRNAQ37P(DOWNSTREAM)
1	149159913	149159928	149159921	TGTC	C	snp	1	0	0	0	.	.	.	INTERGENIC(INTERGENIC);TRNAE30P(DOWNSTREAM)
1	149332308	149332315	149332309	T	G	snp	1	1	1	1	.	.	.	FCGR1C(INTRON)
1	149707470	149707487	149707476	TC	C	snp	0	1	1	1	.	.	.	INTERGENIC(INTERGENIC);MIR548U(UPSTREAM)
1	149720967	149720976	149720969	AC	G	snp	0	1	1	1	.	.	.	INTERGENIC(INTERGENIC);RP11-196G18.1.1(DOWNSTREAM)
1	149872772	149872779	149872775	T	G	snp	1	0	0	0	.	.	.	BOLA1(DOWNSTREAM);INTERGENIC(INTERGENIC);SV2A(DOWNSTREAM)
1	149907176	149907184	149907183	C	A	ins	1	0	0	1	.	.	.	MTMR11(INTRON);MTMR11(UPSTREAM)
1	149907176	149907184	149907183	C	A	snp	1	0	0	1	.	.	.	MTMR11(INTRON);MTMR11(UPSTREAM)
1	149954082	149954105	149954098	TAAA	AA	del	1	0	0	0	.	.	.	OTUD7B(INTRON)
1	150025374	150025382	150025377	T	C	snp	1	1	0	1	.	.	.	INTERGENIC(INTERGENIC);RP11-458I7.1.1(UPSTREAM)
1	150221931	150221940	150221934	C	A	snp	1	1	1	1	.	.	.	INTERGENIC(INTERGENIC)
1	150251113	150251124	150251120	AGG	A	snp	1	0	0	0	.	.	.	C1orf51(UPSTREAM);C1orf54(INTRON)
1	150302231	150302238	150302237	T	G	snp	1	0	0	0	.	.	.	PRPF3(EXON);PRPF3(INTRON)
1	150319752	150319765	150319762	AC	G	snp	1	1	1	1	.	.	.	PRPF3(DOWNSTREAM);PRPF3(INTRON);PRPF3(UPSTREAM)
1	150335531	150335542	150335534	CA	G	snp	0	1	0	1	.	.	.	INTERGENIC(INTERGENIC);RPRD2(UPSTREAM)
1	150335531	150335542	150335536	CA	G	snp	0	1	0	1	.	.	.	INTERGENIC(INTERGENIC);RPRD2(UPSTREAM)
1	150346314	150346323	150346316	AG	G	snp	1	0	0	0	.	.	.	RPRD2(INTRON)
1	150360893	150360902	150360895	TA	C	snp	1	0	0	0	.	.	.	RPRD2(INTRON)
1	150383947	150383956	150383951	A	G	snp	1	1	1	1	.	.	.	RPRD2(INTRON)
1	150385473	150385480	150385476	A	G	snp	1	0	0	0	.	.	.	RPRD2(INTRON)
1	150467813	150467828	150467817	AC	G	snp	1	1	0	1	.	.	.	TARS2(DOWNSTREAM);TARS2(INTRON)
1	150697777	150697785	150697781	T	TG	ins	0	0	1	1	.	.	.	CTSS(DOWNSTREAM);HORMAD1(UPSTREAM);INTERGENIC(INTERGENIC)
1	150747411	150747419	150747414	G	A	snp	0	0	1	1	.	.	.	INTERGENIC(INTERGENIC)
1	150766071	150766086	150766082	AT	A	del	0	0	0	1	.	.	.	CTSK(DOWNSTREAM);INTERGENIC(INTERGENIC)
1	150766071	150766086	150766084	AT	A	del	1	1	1	1	.	.	.	CTSK(DOWNSTREAM);INTERGENIC(INTERGENIC)
1	150777705	150777714	150777707	G	A	snp	1	0	0	0	.	.	.	ARNT(DOWNSTREAM);CTSK(INTRON);UBE2D3P3(DOWNSTREAM)
1	150837242	150837269	150837249	AAAT	A	snp	1	1	1	1	.	.	.	ARNT(INTRON)
1	150839681	150839688	150839686	A	C	snp	1	1	1	1	.	.	.	ARNT(INTRON)
1	150839687	150839698	150839695	AAC	A	ins	0	1	0	1	.	.	.	ARNT(INTRON)
1	150918929	150918937	150918936	T	G	snp	1	1	1	1	.	.	.	SETDB1(DOWNSTREAM);SETDB1(INTRON)
1	150949679	150949686	150949682	T	C	snp	1	1	1	1	.	.	.	ANXA9(UPSTREAM);CERS2(UPSTREAM);INTERGENIC(INTERGENIC);RP11-316M1.3.1(DOWNSTREAM)

1	151010862	151010871	151010864	A	G	snp	1	0	0	0	.	.	.	BNIPL(INTRON);BNIPL(UPSTREAM);PRUNE(DOWNSTREAM)
1	151022041	151022048	151022043	T	C	snp	1	0	0	0	.	.	.	BNIPL(DOWNSTREAM);C1orf56(INTRON);CDC42SE1(DOWNSTREAM)
1	151048192	151048199	151048198	C	A	ins	0	1	1	1	.	.	.	GABPB2(INTRON)
1	151228841	151228868	151228847	AAAT	T	del	0	1	1	1	.	.	.	PSMD4(INTRON);PSMD4(UPSTREAM)
1	151369983	151369992	151369984	A	C	ins	1	1	0	1	.	.	.	INTERGENIC(INTERGENIC);PSMB4(UPSTREAM)
1	151492611	151492620	151492614	T	C	snp	1	0	0	0	.	.	.	CGN(DOWNSTREAM);CGN(INTRON)
1	151576167	151576182	151576174	AAAT	AAATAAATA	del	1	1	1	1	.	.	.	INTERGENIC(INTERGENIC)
1	151576167	151576182	151576178	AAAT	AAATA	del	1	1	1	1	.	.	.	INTERGENIC(INTERGENIC)
1	151576472	151576487	151576476	AAAT	G	snp	1	1	1	1	.	.	.	INTERGENIC(INTERGENIC)
1	151602363	151602376	151602373	AT	T	snp	1	1	1	1	.	.	.	SNX27(INTRON);U6(DOWNSTREAM)
1	151614117	151614126	151614123	AT	C	snp	1	1	1	1	.	.	.	SNX27(INTRON)
1	151625702	151625710	151625708	T	G	snp	1	1	1	1	.	.	.	SNX27(INTRON)
1	151816214	151816223	151816220	A	G	snp	1	1	0	1	.	.	.	AL450992.2.1(INTRON);C2CD4D(UPSTREAM);THEM5(DOWNSTREAM);Y_RNA(UPSTREAM)
1	151854507	151854516	151854515	T	C	snp	1	0	0	0	.	.	.	THEM4(INTRON)
1	151859908	151859925	151859913	AAT	A	snp	1	0	0	0	.	.	.	THEM4(INTRON)
1	151936569	151936580	151936578	TA	G	snp	1	1	1	1	.	.	.	INTERGENIC(INTERGENIC)
1	151985016	151985025	151985018	T	C	snp	1	1	1	1	.	.	.	AL591893.1.1(INTRON)
1	152028441	152028456	152028446	TATC	G	snp	1	1	1	1	.	.	.	INTERGENIC(INTERGENIC)
1	152209452	152209469	152209467	AT	A	del	1	1	1	1	.	.	.	RP1-14N1.2.1(INTRON)
1	152240404	152240417	152240413	TA	T	snp	1	1	1	1	.	.	.	RP1-14N1.2.1(INTRON)
1	152240440	152240449	152240445	TA	T	snp	1	1	1	1	.	.	.	RP1-14N1.2.1(INTRON)
1	152240494	152240503	152240499	TA	T	snp	1	1	1	1	.	.	.	RP1-14N1.2.1(INTRON)
1	152263992	152263999	152263995	A	G	snp	0	1	1	1	.	.	.	RP1-14N1.2.1(INTRON)
1	152308782	152308789	152308787	T	G	ins	1	1	1	1	.	.	.	RP1-14N1.2.1(INTRON);RP1-14N1.2.1(UPSTREAM)
1	152343198	152343207	152343201	A	G	snp	1	0	0	0	.	.	.	INTERGENIC(INTERGENIC);RP1-14N1.2.1(DOWNSTREAM);RP1-91G5.3.1(UPSTREAM)
1	152402331	152402346	152402337	CAAA	T	snp	1	1	1	1	.	.	.	RP1-91G5.3.1(INTRON)
1	152424475	152424483	152424479	T	C	snp	0	1	1	1	.	.	.	INTERGENIC(INTERGENIC)
1	152436806	152436813	152436810	C	A	snp	1	1	1	1	.	.	.	INTERGENIC(INTERGENIC)
1	152706809	152706822	152706819	TC	CTC	del	1	1	0	1	.	.	.	INTERGENIC(INTERGENIC);RP1-43O17.1.1(UPSTREAM)
1	152811364	152811387	152811370	TTTA	A	del	1	0	0	0	.	.	.	INTERGENIC(INTERGENIC);LCE6A(UPSTREAM)
1	152850903	152850912	152850906	TG	A	snp	1	1	1	0	.	.	SMCP(START_GAINED)	.
1	152890849	152890862	152890852	AG	A	snp	1	1	0	0	.	.	.	INTERGENIC(INTERGENIC)
1	152923067	152923082	152923072	CATC	T	snp	0	0	0	1	.	.	.	INTERGENIC(INTERGENIC);RP1-13P20.6.1(UPSTREAM)
1	152927271	152927288	152927275	GT	A	snp	1	0	0	0	.	.	.	INTERGENIC(INTERGENIC)
1	152958831	152958842	152958833	AC	G	snp	1	0	0	0	.	.	.	INTERGENIC(INTERGENIC);SPRR1A(DOWNSTREAM)
1	152958831	152958842	152958840	AC	T	snp	1	1	1	1	.	.	.	INTERGENIC(INTERGENIC);SPRR1A(DOWNSTREAM)
1	152966292	152966299	152966293	A	C	snp	1	0	0	0	.	.	.	INTERGENIC(INTERGENIC)
1	152976910	152976921	152976919	AG	A	snp	1	0	0	0	.	.	.	INTERGENIC(INTERGENIC);SPRR3(DOWNSTREAM)
1	153068947	153068958	153068950	AGC	G	snp	1	1	1	1	.	.	.	SPRR2B(INTRON);SPRR2E(INTRON);SPRR2E(UPSTREAM)
1	153137250	153137257	153137253	T	C	snp	0	0	1	0	.	.	.	INTERGENIC(INTERGENIC)
1	153268861	153268868	153268864	C	T	snp	1	1	1	1	.	.	.	INTERGENIC(INTERGENIC);PGLYRP3(DOWNSTREAM)
1	153308198	153308225	153308203	TAAA	AATAAATAAATG	ins	1	1	1	1	.	.	.	PGLYRP4(INTRON);U6(DOWNSTREAM)
1	153308198	153308225	153308207	TAAA	AATAAATG	ins	1	1	1	1	.	.	.	PGLYRP4(INTRON);U6(DOWNSTREAM)
1	153454029	153454038	153454031	T	C	ins	1	0	0	1	.	.	.	INTERGENIC(INTERGENIC)
1	153495764	153495773	153495771	AT	C	snp	1	0	0	0	.	.	.	INTERGENIC(INTERGENIC)
1	153572945	153572972	153572954	AAAT	C	snp	0	1	1	0	.	.	.	INTERGENIC(INTERGENIC)
1	153607320	153607333	153607331	AT	A	del	1	0	0	0	.	.	.	CHTOP(INTRON);CHTOP(UPSTREAM);S100A1(DOWNSTREAM);S100A13(UPSTREAM)
1	153677642	153677649	153677645	T	G	snp	0	1	0	0	.	.	.	AL713889.1(DOWNSTREAM);INTERGENIC(INTERGENIC)
1	153757548	153757565	153757562	TTA	T	ins	1	1	1	1	.	.	.	INTERGENIC(INTERGENIC);SLC27A3(DOWNSTREAM);Y_RNA(DOWNSTREAM)
1	153773145	153773152	153773150	C	T	ins	1	1	1	1	.	.	.	GATAD2B(DOWNSTREAM);RP11-216N14.5.1(EXON)
1	153788203	153788218	153788211	TAGA	C	snp	1	1	1	1	.	.	.	GATAD2B(INTRON)
1	153850653	153850662	153850658	T	C	snp	0	1	1	0	.	.	.	GATAD2B(INTRON)
1	153878577	153878592	153878587	CA	T	snp	1	1	1	1	.	.	.	GATAD2B(INTRON)
1	154106635	154106655	154106639	AAT	T	del	1	1	1	1	.	.	.	NUP210L(INTRON)
1	154106635	154106655	154106640	AAT	A	snp	1	1	1	1	.	.	.	NUP210L(INTRON)
1	154113313	154113320	154113315	A	C	snp	1	1	1	0	.	.	.	NUP210L(INTRON)
1	154173600	154173619	154173605	TTTA	C	snp	1	0	0	0	.	.	.	C1orf189(INTRON)
1	154257716	154257723	154257718	T	C	snp	0	1	1	1	.	.	.	INTERGENIC(INTERGENIC);SNORD59(UPSTREAM)
1	154257878	154257895	154257892	AT	T	snp	1	0	0	0	.	.	.	INTERGENIC(INTERGENIC);SNORD59(UPSTREAM)
1	154288087	154288095	154288089	A	C	ins	1	1	1	1	.	.	.	INTERGENIC(INTERGENIC)
1	154301253	154301267	154301261	GGC	A	snp	1	1	1	1	.	.	.	AQP10(DOWNSTREAM);ATP8B2(INTRON);ATP8B2(UPSTREAM)
1	154342019	154342032	154342022	AT	A	snp	1	1	1	1	.	.	.	INTERGENIC(INTERGENIC)

1	154360830	154360839	154360838	T	C	snp	1	1	1	1	.	.	.	INTERGENIC(INTERGENIC)
1	154365995	154366004	154365998	T	C	snp	0	1	1	1	.	.	.	INTERGENIC(INTERGENIC)
1	154418414	154418423	154418415	T	C	snp	1	1	1	1	.	.	.	IL6R(INTRON)
1	154626043	154626050	154626046	T	G	snp	1	0	0	0	.	.	.	INTERGENIC(INTERGENIC)
1	154698006	154698021	154698016	CTTC	C	del	1	1	1	1	.	.	.	KCNN3(INTRON)
1	154728762	154728776	154728769	AGG	A	snp	0	0	1	0	.	.	.	KCNN3(INTRON)
1	154734434	154734457	154734450	AACA	AC	del	1	1	1	1	.	.	.	KCNN3(INTRON)
1	154738273	154738281	154738277	T	G	snp	1	0	0	0	.	.	.	KCNN3(INTRON)
1	154819681	154819688	154819684	C	T	snp	0	1	0	1	.	.	.	KCNN3(INTRON)
1	154845527	154845538	154845534	AGG	C	snp	1	1	1	1	.	.	.	INTERGENIC(INTERGENIC);KCNN3(UPSTREAM)
1	155085890	155085901	155085898	AAC	A	snp	1	1	1	1	.	.	.	INTERGENIC(INTERGENIC)
1	155119317	155119328	155119325	AAT	A	del	1	1	1	1	.	.	.	HMGN2P18(UPSTREAM);INTERGENIC(INTERGENIC);U7(DOWNSTREAM)
1	155237634	155237642	155237639	C	T	snp	0	0	0	1	.	.	.	CLK2(DOWNSTREAM);CLK2(INTRON)
1	155268951	155268960	155268958	TC	T	snp	1	1	1	1	.	.	.	PKLR(INTRON)
1	155347814	155347823	155347819	A	C	snp	1	0	0	0	.	.	.	ASH1L(INTRON)
1	155509233	155509248	155509242	TA	TG	ins	1	1	1	1	.	.	.	ASH1L(INTRON)
1	155544252	155544265	155544256	TA	C	snp	1	1	1	1	.	.	.	INTERGENIC(INTERGENIC)
1	155546871	155546878	155546875	T	G	snp	0	0	0	1	.	.	.	INTERGENIC(INTERGENIC)
1	155571091	155571099	155571095	T	G	snp	1	0	0	0	.	.	.	DAP3P1(INTRON)
1	155706210	155706218	155706217	T	G	snp	1	1	1	1	.	.	.	DAP3(DOWNSTREAM);DAP3(INTRON);DAP3(UPSTREAM);MSTO1(INTRON);RP11-243J18.3.1(INTRON)
1	155757567	155757586	155757579	AAAT	G	snp	1	0	0	0	.	.	.	GON4L(INTRON);GON4L(UPSTREAM)
1	155779768	155779781	155779774	TA	C	snp	1	1	1	1	.	.	.	GON4L(INTRON);GON4L(UPSTREAM)
1	155797474	155797491	155797489	CA	C	del	1	0	1	0	.	.	.	GON4L(INTRON)
1	155941694	155941705	155941703	AT	C	snp	0	1	0	0	.	.	.	ARHGEF2(INTRON);ARHGEF2(UPSTREAM)
1	155944620	155944629	155944626	GA	A	snp	0	1	1	1	.	.	.	ARHGEF2(INTRON);ARHGEF2(UPSTREAM)
1	156017773	156017781	156017780	A	G	snp	1	0	0	0	.	.	.	UBQLN4(DOWNSTREAM);UBQLN4(INTRON)
1	156094074	156094089	156094087	CT	C	del	1	1	0	1	.	.	.	LMNA(INTRON);LMNA(UPSTREAM)
1	156101067	156101084	156101072	AAT	A	snp	1	1	0	1	.	.	.	LMNA(INTRON);LMNA(UPSTREAM)
1	156102614	156102629	156102627	CT	C	del	1	1	0	1	.	.	.	LMNA(INTRON);LMNA(UPSTREAM)
1	156401084	156401091	156401090	C	CA	ins	1	0	0	0	.	.	.	C1orf61(UPSTREAM);INTERGENIC(INTERGENIC)
1	156494545	156494552	156494547	G	T	snp	1	0	0	0	.	.	.	INTERGENIC(INTERGENIC);IQGAP3(DOWNSTREAM);snoU13(UPSTREAM)
1	156566451	156566462	156566458	GCT	T	snp	1	0	1	0	.	.	.	APOA1BP(DOWNSTREAM);GPATCH4(DOWNSTREAM);GPATCH4(EXON);GPATCH4(INTRON);GPATCH4(UPSTREAM)
1	156642739	156642748	156642744	AG	C	snp	1	0	0	0	.	.	NES(SYNONYMOUS_CODING):L412	.
1	156687939	156687959	156687956	ATT	A	del	1	1	0	0	.	.	.	INTERGENIC(INTERGENIC);ISG20L2(DOWNSTREAM);RP11-66D17.5.1(UPSTREAM)
1	156694023	156694030	156694024	G	T	snp	1	0	0	0	.	.	ISG20L2(SYNONYMOUS_CODING):P288	ISG20L2(DOWNSTREAM);ISG20L2(EXON);RRNAD1(UPSTREAM)
1	156721556	156721567	156721562	CTC	T	snp	1	0	0	0	.	.	.	HDGF(INTRON);HDGF(UPSTREAM);PRCC(INTRON)
1	156735250	156735265	156735260	TTCT	TTTCT	del	1	1	0	1	.	.	.	HDGF(INTRON);PRCC(INTRON);PRCC(UPSTREAM)
1	156746261	156746270	156746269	T	G	snp	1	0	0	0	.	.	.	PRCC(INTRON)
1	156752696	156752705	156752703	AT	A	del	1	1	1	1	.	.	.	PRCC(INTRON);PRCC(UPSTREAM)
1	156826087	156826102	156826095	ATGA	G	snp	1	0	0	0	.	.	.	INSRR(INTRON);NTRK1(INTRON);NTRK1(UPSTREAM)
1	156832872	156832879	156832878	G	A	snp	1	0	0	0	.	.	.	INSRR(UPSTREAM);NTRK1(INTRON)
1	156842025	156842034	156842030	AT	C	snp	1	0	0	0	.	.	.	NTRK1(INTRON);NTRK1(UPSTREAM)
1	156898317	156898331	156898322	TCT	C	snp	1	0	0	0	.	.	.	LRRC71(INTRON);LRRC71(UPSTREAM)
1	157096247	157096258	157096253	AT	G	snp	1	1	1	0	.	.	.	CYCSP52(UPSTREAM);ETV3(INTRON)
1	157115444	157115459	157115447	AT	C	snp	1	0	0	0	.	.	.	INTERGENIC(INTERGENIC)
1	157135122	157135137	157135132	CT	TCTCT	del	1	1	1	1	.	.	.	INTERGENIC(INTERGENIC)
1	157156856	157156873	157156865	GAA	C	snp	1	1	1	1	.	.	.	RP11-71G12.1.1(EXON)
1	157180058	157180066	157180064	A	C	ins	1	1	1	1	.	.	.	INTERGENIC(INTERGENIC)
1	157229842	157229850	157229844	A	G	ins	1	1	1	0	.	.	.	INTERGENIC(INTERGENIC)
1	157278928	157278935	157278933	T	C	ins	1	1	1	0	.	.	.	INTERGENIC(INTERGENIC)
1	157341362	157341373	157341365	TC	T	snp	1	1	1	1	.	.	.	INTERGENIC(INTERGENIC)
1	157356022	157356030	157356025	C	CA	ins	1	0	0	0	.	.	.	INTERGENIC(INTERGENIC)
1	157356022	157356030	157356028	C	A	ins	1	0	0	0	.	.	.	INTERGENIC(INTERGENIC)
1	157360832	157360839	157360834	G	GGC	ins	1	1	1	1	.	.	.	INTERGENIC(INTERGENIC)
1	157360832	157360839	157360837	G	GGC	ins	1	1	1	1	.	.	.	INTERGENIC(INTERGENIC)
1	157407961	157407970	157407963	AT	G	snp	1	0	0	0	.	.	.	INTERGENIC(INTERGENIC)
1	157421394	157421401	157421400	A	G	snp	1	1	1	1	.	.	.	INTERGENIC(INTERGENIC)
1	157585797	157585812	157585804	TTTG	T	snp	0	1	0	1	.	.	.	INTERGENIC(INTERGENIC)
1	157633762	157633770	157633764	A	C	snp	1	0	1	0	.	.	.	INTERGENIC(INTERGENIC)
1	157663517	157663534	157663530	TC	TT	ins	1	1	1	1	.	.	.	FCRL3(DOWNSTREAM);FCRL3(INTRON);FCRL3(UPSTREAM);RP11-367J7.3.1(INTRON)

1	157665676	157665693	157665680	TC	C	snp	1	1	1	1	.	.	.	FCRL3(DOWNSTREAM);FCRL3(INTRON);RP11-367J7.3.1(INTRON)
1	157693399	157693408	157693406	A	AC	ins	1	0	0	0	.	.	.	INTERGENIC(INTERGENIC);VDAC1P9(DOWNSTREAM)
1	157774037	157774046	157774041	C	T	ins	1	0	0	0	.	.	.	FCRL1(DOWNSTREAM);FCRL1(INTRON);FCRL1(UPSTREAM)
1	157835486	157835505	157835499	ATAG	C	snp	1	1	1	1	.	.	.	CD5L(INTRON);RP11-120D12.2.1(DOWNSTREAM)
1	157845772	157845780	157845773	T	G	ins	1	1	1	1	.	.	.	CD5L(INTRON)
1	157857465	157857473	157857466	A	G	snp	1	0	0	0	.	.	.	CD5L(INTRON)
1	157926223	157926232	157926227	A	AC	ins	0	1	1	1	.	.	.	INTERGENIC(INTERGENIC)
1	157934155	157934172	157934165	GA	C	snp	1	0	0	0	.	.	.	INTERGENIC(INTERGENIC)
1	157967671	157967686	157967682	ATTT	A	del	0	0	1	0	.	.	.	KIRREL(INTRON)
1	158004779	158004788	158004784	TG	TT	ins	1	1	1	1	.	.	.	KIRREL(INTRON);KIRREL-IT1(DOWNSTREAM)
1	158077909	158077918	158077910	A	G	snp	1	0	0	0	.	.	.	INTERGENIC(INTERGENIC)
1	158165971	158165990	158165984	CCTT	T	snp	1	1	1	1	.	.	.	INTERGENIC(INTERGENIC);RP11-404O13.4.1(UPSTREAM);RP11-404O13.5.1(DOWNSTREAM)
1	158199257	158199266	158199264	TC	T	snp	1	1	1	1	.	.	.	INTERGENIC(INTERGENIC)
1	158292197	158292204	158292198	T	G	snp	1	0	0	0	.	.	.	INTERGENIC(INTERGENIC)
1	158292422	158292429	158292423	T	C	snp	1	0	0	0	.	.	.	INTERGENIC(INTERGENIC)
1	158426043	158426060	158426045	CA	AT	ins	1	0	0	0	.	.	.	INTERGENIC(INTERGENIC);RP11-144L1.2.1(DOWNSTREAM)
1	158432157	158432172	158432167	TTCC	T	snp	1	1	1	1	.	.	.	INTERGENIC(INTERGENIC);OR10K1(UPSTREAM)
1	158696458	158696473	158696465	TATC	G	snp	1	1	1	1	.	.	.	INTERGENIC(INTERGENIC);OR6K4P(UPSTREAM)
1	158729632	158729641	158729636	TA	C	snp	1	1	1	1	.	.	.	INTERGENIC(INTERGENIC);OR6K6(DOWNSTREAM)
1	158747501	158747512	158747506	TC	G	snp	1	1	1	1	.	.	.	INTERGENIC(INTERGENIC);OR6N2(UPSTREAM)
1	158882874	158882882	158882880	T	C	snp	0	0	0	1	.	.	.	INTERGENIC(INTERGENIC)
1	158922284	158922291	158922287	T	C	snp	1	1	1	1	.	.	.	PYHIN1(INTRON)
1	158941216	158941231	158941219	AT	C	snp	1	1	1	1	.	.	.	PYHIN1(INTRON)
1	158961668	158961691	158961672	CAAA	A	snp	1	0	1	0	.	.	.	INTERGENIC(INTERGENIC)
1	159312841	159312850	159312842	G	T	snp	1	1	1	1	.	.	.	INTERGENIC(INTERGENIC);RP11-550P17.5.1(DOWNSTREAM)
1	159343391	159343400	159343398	A	C	snp	0	1	0	1	.	.	.	RP11-550P17.5.1(INTRON)
1	159389745	159389756	159389752	TC	G	snp	0	1	1	1	.	.	.	RP11-550P17.5.1(INTRON)
1	159552182	159552191	159552190	A	G	snp	1	0	0	0	.	.	.	INTERGENIC(INTERGENIC)
1	159576473	159576484	159576477	CAA	G	snp	1	1	1	1	.	.	.	INTERGENIC(INTERGENIC)
1	159576473	159576484	159576479	CAA	G	snp	1	1	1	1	.	.	.	INTERGENIC(INTERGENIC)
1	159582071	159582094	159582074	CAC	A	snp	1	0	0	0	.	.	.	INTERGENIC(INTERGENIC)
1	159603396	159603403	159603401	T	G	snp	0	1	1	1	.	.	.	INTERGENIC(INTERGENIC)
1	159655720	159655731	159655726	GAA	T	snp	1	1	1	1	.	.	.	INTERGENIC(INTERGENIC)
1	159775909	159775918	159775916	AG	C	snp	1	0	0	0	.	.	.	FCRL6(INTRON)
1	159983022	159983030	159983029	A	G	snp	1	1	1	1	.	.	.	INTERGENIC(INTERGENIC)
1	159985287	159985302	159985294	AG	A	del	1	1	1	1	.	.	.	INTERGENIC(INTERGENIC);RP11-226L15.1.1(UPSTREAM)
1	160020233	160020241	160020237	T	C	snp	0	1	1	1	.	.	.	KCNJ10(INTRON)
1	160156024	160156033	160156027	TC	G	snp	1	1	1	1	.	.	.	ATP1A4(DOWNSTREAM);ATP1A4(INTRON);CASQ1(UPSTREAM)
1	160186890	160186897	160186893	G	A	snp	1	0	0	0	.	.	.	DCAF8(DOWNSTREAM);DCAF8(EXON);DCAF8(INTRON);DCAF8(UTR_3_PRIME);PEA15(DOWNSTREAM);RP11-574F21.3.1(DOWNSTREAM)
1	160302355	160302364	160302356	A	G	snp	1	1	1	1	.	.	.	COPA(DOWNSTREAM);COPA(INTRON)
1	160506169	160506177	160506175	T	TG	ins	1	1	1	1	.	.	.	CD84(DOWNSTREAM);INTERGENIC(INTERGENIC);RP11-528G1.2.1(UPSTREAM)
1	160568956	160568964	160568957	T	C	snp	1	0	0	0	.	.	.	INTERGENIC(INTERGENIC)
1	160605139	160605150	160605144	GC	T	snp	1	1	1	1	.	.	.	SLAMF1(DOWNSTREAM);SLAMF1(INTRON)
1	160764446	160764459	160764449	TC	G	snp	1	1	1	1	.	.	.	INTERGENIC(INTERGENIC);LY9(UPSTREAM)
1	160785130	160785137	160785133	A	C	ins	0	0	1	1	.	.	.	LY9(DOWNSTREAM);LY9(INTRON)
1	160885904	160885912	160885906	G	A	snp	1	1	1	1	.	.	.	INTERGENIC(INTERGENIC)
1	161112659	161112674	161112667	AAAG	C	snp	0	0	1	0	.	.	.	ACA64(UPSTREAM);INTERGENIC(INTERGENIC)
1	161238055	161238064	161238059	A	G	snp	1	1	1	1	.	.	.	PCP4L1(INTRON)
1	161243216	161243231	161243223	AAAC	A	snp	1	0	0	0	.	.	.	PCP4L1(INTRON)
1	161248881	161248888	161248883	T	G	snp	0	0	1	0	.	.	.	PCP4L1(INTRON)
1	161279326	161279343	161279340	AT	T	ins	0	1	1	1	.	.	.	MPZ(INTRON);MPZ(UPSTREAM);SDHC(UPSTREAM)
1	161279326	161279343	161279341	AT	A	del	1	1	1	1	.	.	.	MPZ(INTRON);MPZ(UPSTREAM);SDHC(UPSTREAM)
1	161296353	161296370	161296366	AT	A	del	1	1	1	0	.	.	.	SDHC(INTRON);SDHC(UPSTREAM)
1	161296353	161296370	161296368	AT	A	del	1	1	1	1	.	.	.	SDHC(INTRON);SDHC(UPSTREAM)
1	161375210	161375217	161375213	G	A	snp	1	1	1	1	.	.	.	INTERGENIC(INTERGENIC);RP11-122G18.7.1(UPSTREAM);U6(DOWNSTREAM)
1	161590689	161590698	161590693	TA	C	snp	1	1	1	1	.	.	.	FCGR2B(INTRON);FCGR3A(INTRON);FCGR3B(DOWNSTREAM);RP11-25K21.5.1(UPSTREAM)
1	161640884	161640893	161640886	GA	C	snp	1	0	0	0	.	.	.	FCGR2B(INTRON);RP11-25K21.1.1(DOWNSTREAM)
1	161662010	161662019	161662015	T	G	snp	1	0	0	0	.	.	.	INTERGENIC(INTERGENIC)
1	161690064	161690072	161690065	A	C	snp	0	1	1	1	.	.	.	FCRLB(UPSTREAM);INTERGENIC(INTERGENIC)
1	161706319	161706334	161706325	GA	AC	ins	0	1	1	1	.	.	.	AL359541.1(DOWNSTREAM);INTERGENIC(INTERGENIC)

1	161751221	161751228	161751226	A	G	snp	1	1	1	1	.	.	.	ATF6(INTRON)
1	161761055	161761064	161761062	AT	G	snp	1	1	1	1	.	.	.	ATF6(INTRON)
1	161773631	161773639	161773635	T	C	snp	1	0	0	0	.	.	.	ATF6(INTRON)
1	161862945	161862964	161862953	TTTC	C	snp	1	0	0	0	.	.	.	ATF6(INTRON);RP11-456P18.2.1(DOWNSTREAM)
1	162012520	162012543	162012525	AAT	A	snp	1	1	0	1	.	.	.	INTERGENIC(INTERGENIC);RP11-227F8.2.1(DOWNSTREAM)
1	162097757	162097765	162097764	T	G	snp	1	1	1	1	.	.	.	NOS1AP(INTRON)
1	162198652	162198659	162198657	A	C	snp	0	1	0	0	.	.	.	NOS1AP(INTRON)
1	162329510	162329518	162329514	G	A	snp	1	1	1	1	.	.	.	NOS1AP(INTRON);NOS1AP(UPSTREAM)
1	162431219	162431234	162431226	AAAT	A	snp	1	0	0	0	.	.	.	INTERGENIC(INTERGENIC)
1	162501493	162501501	162501497	A	G	snp	1	0	0	0	.	.	.	INTERGENIC(INTERGENIC);UHMK1(DOWNSTREAM)
1	162697791	162697802	162697794	TA	C	snp	1	1	1	0	.	.	.	DDR2(INTRON)
1	162767064	162767077	162767067	AC	G	snp	1	0	0	0	.	.	.	HSD17B7(DOWNSTREAM);HSD17B7(EXON);HSD17B7(INTRON);HSD17B7(UPSTREAM)
1	162849596	162849604	162849602	T	C	snp	1	1	1	1	.	.	.	INTERGENIC(INTERGENIC)
1	162906258	162906273	162906266	TTTC	TTC	del	0	0	1	0	.	.	.	INTERGENIC(INTERGENIC)
1	162906258	162906273	162906269	TTTC	T	ins	1	1	1	1	.	.	.	INTERGENIC(INTERGENIC)
1	163382899	163382918	163382903	AAAC	AAC	ins	1	1	1	1	.	.	.	INTERGENIC(INTERGENIC)
1	163422314	163422321	163422320	T	G	snp	1	1	1	1	.	.	.	INTERGENIC(INTERGENIC)
1	163434482	163434495	163434487	TC	G	snp	0	1	1	0	.	.	.	INTERGENIC(INTERGENIC);RN5S62(DOWNSTREAM)
1	163500782	163500789	163500784	T	C	snp	1	1	1	1	.	.	.	INTERGENIC(INTERGENIC)
1	163530773	163530788	163530786	TG	A	snp	0	0	0	1	.	.	.	INTERGENIC(INTERGENIC)
1	163540423	163540432	163540429	AC	G	snp	1	1	1	1	.	.	.	INTERGENIC(INTERGENIC)
1	163545353	163545367	163545356	AAT	AT	del	1	1	1	1	.	.	.	INTERGENIC(INTERGENIC)
1	163545353	163545367	163545357	AAT	T	del	1	1	1	1	.	.	.	INTERGENIC(INTERGENIC)
1	163591217	163591226	163591224	A	C	snp	1	0	0	0	.	.	.	INTERGENIC(INTERGENIC)
1	163645848	163645863	163645851	AC	AA	ins	1	0	0	0	.	.	.	INTERGENIC(INTERGENIC)
1	163689516	163689523	163689520	A	G	snp	0	0	1	0	.	.	.	INTERGENIC(INTERGENIC)
1	163734450	163734465	163734458	TA	ATATATATATAG	ins	1	1	1	1	.	.	.	INTERGENIC(INTERGENIC);RP4-640E24.1.1(DOWNSTREAM)
1	163785260	163785269	163785264	T	GTTG	ins	1	1	1	1	.	.	.	INTERGENIC(INTERGENIC)
1	163785260	163785269	163785266	T	G	ins	1	1	1	1	.	.	.	INTERGENIC(INTERGENIC)
1	163881441	163881450	163881445	A	G	snp	1	1	0	1	.	.	.	INTERGENIC(INTERGENIC)
1	163911491	163911498	163911493	A	G	snp	1	0	0	0	.	.	.	INTERGENIC(INTERGENIC)
1	164057173	164057190	164057179	GA	C	snp	1	1	1	1	.	.	.	INTERGENIC(INTERGENIC)
1	164105974	164105983	164105976	A	C	snp	1	1	1	1	.	.	.	INTERGENIC(INTERGENIC)
1	164170082	164170091	164170088	TA	C	snp	1	1	1	1	.	.	.	INTERGENIC(INTERGENIC)
1	164170210	164170219	164170214	TA	C	snp	1	1	1	1	.	.	.	INTERGENIC(INTERGENIC)
1	164245855	164245864	164245861	AG	G	snp	1	1	1	1	.	.	.	INTERGENIC(INTERGENIC)
1	164252014	164252022	164252017	A	G	snp	0	0	1	0	.	.	.	INTERGENIC(INTERGENIC)
1	164309567	164309574	164309571	T	TTTG	ins	0	0	1	0	.	.	.	INTERGENIC(INTERGENIC);RP11-541J2.1.1(UPSTREAM)
1	164375098	164375121	164375103	AGAA	A	snp	1	1	1	1	.	.	.	INTERGENIC(INTERGENIC)
1	164375098	164375121	164375104	AGAA	AAG	del	1	0	0	0	.	.	.	INTERGENIC(INTERGENIC)
1	164375098	164375121	164375108	AGAA	G	snp	1	0	0	0	.	.	.	INTERGENIC(INTERGENIC)
1	164377888	164377895	164377893	T	G	snp	1	1	1	1	.	.	.	INTERGENIC(INTERGENIC)
1	164558505	164558514	164558512	CA	T	snp	1	1	1	1	.	.	.	PBX1(INTRON);PBX1(UPSTREAM)
1	164685986	164685994	164685990	A	C	snp	0	1	1	0	.	.	.	PBX1(INTRON)
1	164735956	164735975	164735971	TTTA	T	ins	0	1	1	1	.	.	.	PBX1(INTRON);RP11-506O24.1.1(DOWNSTREAM)
1	164743402	164743411	164743404	A	G	snp	1	1	1	1	.	.	.	PBX1(INTRON);RP11-506O24.1.1(INTRON)
1	164775537	164775544	164775542	A	C	snp	1	0	0	0	.	.	.	PBX1(INTRON)
1	164845226	164845243	164845229	TA	G	snp	1	1	1	1	.	.	.	PBX1(INTRON)
1	164873854	164873861	164873859	A	G	snp	1	1	1	1	.	.	.	INTERGENIC(INTERGENIC)
1	164959238	164959245	164959244	T	C	ins	1	1	1	1	.	.	.	INTERGENIC(INTERGENIC)
1	164977005	164977014	164977009	CA	T	snp	1	0	0	0	.	.	.	INTERGENIC(INTERGENIC)
1	165001799	165001822	165001817	TTTG	C	snp	1	0	0	0	.	.	.	INTERGENIC(INTERGENIC)
1	165097921	165097936	165097932	TTTC	T	ins	1	0	0	0	.	.	.	INTERGENIC(INTERGENIC)
1	165502859	165502866	165502862	A	G	snp	1	1	1	1	.	.	.	RP11-280O1.2.1(INTRON);RP11-280O1.2.1(UPSTREAM)
1	165566487	165566502	165566491	AAAG	AAGT	ins	1	1	0	0	.	.	.	INTERGENIC(INTERGENIC);RP11-306I1.2.1(UPSTREAM);TRNAK35P(DOWNSTREAM)
1	165566487	165566502	165566495	AAAG	T	snp	1	0	0	0	.	.	.	INTERGENIC(INTERGENIC);RP11-306I1.2.1(UPSTREAM);TRNAK35P(DOWNSTREAM)
1	165576953	165576962	165576956	CA	G	snp	1	1	0	1	.	.	.	RP11-306I1.2.1(INTRON)
1	165599803	165599810	165599804	A	G	snp	1	1	1	1	.	.	.	INTERGENIC(INTERGENIC);MGST3(UPSTREAM)
1	165623957	165623964	165623962	T	C	snp	1	1	1	1	.	.	.	MGST3(DOWNSTREAM);MGST3(INTRON)
1	165657204	165657215	165657206	GA	C	snp	1	1	1	1	.	.	.	ALDH9A1(INTRON)
1	165699401	165699410	165699406	AC	A	snp	1	1	1	1	.	.	.	TMCO1(INTRON)
1	165699401	165699410	165699408	AC	A	snp	1	1	1	1	.	.	.	TMCO1(INTRON)
1	165702833	165702840	165702836	A	G	snp	1	0	0	0	.	.	.	TMCO1(INTRON)

1	165706672	165706681	165706676	AG	G	snp	1	0	0	0	.	.	.	TMCO1(INTRON)
1	165754643	165754651	165754647	T	C	snp	1	1	1	1	.	.	.	INTERGENIC(INTERGENIC)
1	165754643	165754651	165754649	T	C	snp	1	0	0	0	.	.	.	INTERGENIC(INTERGENIC)
1	165832035	165832044	165832038	AG	A	snp	1	0	0	0	.	.	.	UCK2(INTRON)
1	165843061	165843076	165843069	CTCC	T	snp	1	1	1	1	.	.	.	UCK2(INTRON)
1	165850591	165850599	165850596	T	G	snp	1	1	1	1	.	.	.	UCK2(INTRON)
1	165940106	165940114	165940109	T	G	ins	1	0	0	0	.	.	.	INTERGENIC(INTERGENIC)
1	165981681	165981695	165981690	CTG	T	snp	1	1	1	1	.	.	.	INTERGENIC(INTERGENIC)
1	166040758	166040775	166040766	AGG	A	snp	0	0	0	1	.	.	.	FAM78B(INTRON)
1	166079052	166079067	166079061	TA	G	snp	1	1	1	1	.	.	.	FAM78B(INTRON)
1	166287501	166287518	166287512	TA	TATATAT	del	1	1	1	1	.	.	.	INTERGENIC(INTERGENIC)
1	166287501	166287518	166287514	TA	TATAT	del	1	1	1	1	.	.	.	INTERGENIC(INTERGENIC)
1	166412825	166412832	166412828	T	C	ins	1	1	1	1	.	.	.	RP11-479J7.1.1(INTRON)
1	166560119	166560126	166560125	T	G	snp	1	1	1	1	.	.	.	INTERGENIC(INTERGENIC)
1	166599165	166599176	166599172	TG	A	snp	0	0	1	0	.	.	.	FMO9P(DOWNSTREAM);FMO9P(INTRON)
1	166620962	166620970	166620964	T	C	ins	1	1	1	1	.	.	.	INTERGENIC(INTERGENIC)
1	166637501	166637509	166637504	T	C	snp	1	1	1	1	.	.	.	FMO10P(INTRON)
1	166653609	166653618	166653612	A	AG	ins	1	0	0	0	.	.	.	FMO10P(DOWNSTREAM);INTERGENIC(INTERGENIC)
1	166686092	166686115	166686098	GATA	A	del	1	1	1	1	.	.	.	INTERGENIC(INTERGENIC)
1	166710072	166710083	166710074	CA	T	snp	1	0	0	0	.	.	.	INTERGENIC(INTERGENIC)
1	166721284	166721293	166721285	A	C	snp	1	1	1	1	.	.	.	INTERGENIC(INTERGENIC);RP11-54B9.2.1(DOWNSTREAM)
1	166843858	166843867	166843859	A	G	snp	1	1	1	1	.	.	.	TADA1(INTRON)
1	167005807	167005814	167005811	A	C	ins	1	1	0	1	.	.	.	INTERGENIC(INTERGENIC);snoU13(UPSTREAM)
1	167065495	167065503	167065498	A	G	snp	1	0	0	0	.	.	.	DUSP27(INTRON)
1	167085358	167085371	167085367	TC	G	snp	1	0	0	0	.	.	.	DUSP27(INTRON)
1	167333797	167333805	167333801	T	C	ins	1	1	1	1	.	.	.	POU2F1(INTRON)
1	167350316	167350325	167350323	T	G	snp	1	1	1	1	.	.	.	POU2F1(INTRON);POU2F1(UPSTREAM)
1	167362744	167362751	167362745	T	C	snp	1	1	1	1	.	.	.	POU2F1(DOWNSTREAM);POU2F1(INTRON)
1	167379747	167379767	167379764	TTA	T	snp	1	1	1	1	.	.	.	POU2F1(INTRON)
1	167383469	167383477	167383472	T	C	snp	0	1	1	1	.	.	.	POU2F1(DOWNSTREAM);POU2F1(INTRON)
1	167530037	167530060	167530050	AAAG	AG	ins	1	1	1	1	.	.	.	INTERGENIC(INTERGENIC)
1	167549208	167549223	167549211	AG	A	snp	1	1	1	1	.	.	.	INTERGENIC(INTERGENIC)
1	167598817	167598834	167598820	TCC	C	del	1	0	0	0	.	.	.	RCSD1(UPSTREAM);RP3-455J7.4.1(EXON)
1	167627830	167627839	167627838	T	C	ins	0	1	1	1	.	.	.	RCSD1(INTRON)
1	167670554	167670561	167670557	T	C	snp	1	1	1	1	.	.	.	RCSD1(DOWNSTREAM);RCSD1(INTRON)
1	167816643	167816666	167816656	TCTT	T	snp	1	1	0	1	.	.	.	ADCY10(INTRON)
1	167854361	167854368	167854362	T	C	ins	1	1	1	1	.	.	.	ADCY10(INTRON)
1	168086739	168086746	168086743	C	T	snp	1	1	1	1	.	.	.	GPR161(DOWNSTREAM);GPR161(INTRON)
1	168088434	168088447	168088441	AT	ATAC	ins	1	1	1	1	.	.	.	GPR161(INTRON)
1	168095702	168095709	168095706	T	G	snp	1	0	0	0	.	.	.	GPR161(INTRON)
1	168101970	168101981	168101973	TTG	C	snp	1	0	0	0	.	.	.	GPR161(INTRON)
1	168144270	168144279	168144275	A	C	snp	1	0	0	0	.	.	.	INTERGENIC(INTERGENIC);TIPRL(UPSTREAM)
1	168158908	168158917	168158915	T	G	snp	1	1	1	1	.	.	.	TIPRL(INTRON)
1	168246625	168246633	168246629	T	C	snp	1	0	1	0	.	.	.	INTERGENIC(INTERGENIC);TBX19(UPSTREAM)
1	168257513	168257522	168257514	T	G	ins	1	1	1	1	.	.	.	TBX19(INTRON)
1	168268136	168268149	168268139	TC	T	snp	1	0	0	0	.	.	.	TBX19(INTRON)
1	168279899	168279912	168279907	TA	C	snp	1	1	0	1	.	.	.	TBX19(INTRON)
1	168318094	168318108	168318105	ATT	A	del	1	1	1	1	.	.	.	INTERGENIC(INTERGENIC)
1	168398723	168398732	168398729	T	G	snp	1	0	0	0	.	.	.	INTERGENIC(INTERGENIC)
1	168398723	168398732	168398731	T	G	snp	1	1	1	1	.	.	.	INTERGENIC(INTERGENIC)
1	168441311	168441320	168441314	AT	A	snp	1	1	1	1	.	.	.	RP5-968D22.3.1(INTRON)
1	168477903	168477912	168477910	T	G	ins	1	0	0	0	.	.	.	INTERGENIC(INTERGENIC)
1	168555322	168555337	168555327	TC	A	snp	1	0	0	0	.	.	.	INTERGENIC(INTERGENIC);XCL1(DOWNSTREAM)
1	168570945	168570958	168570947	AT	T	del	0	1	0	1	.	.	.	INTERGENIC(INTERGENIC)
1	168663766	168663773	168663772	T	G	snp	1	1	1	1	.	.	.	DPT(DOWNSTREAM);INTERGENIC(INTERGENIC)
1	168735695	168735704	168735702	TA	G	snp	1	0	0	0	.	.	.	RP1-10C16.1.1(INTRON)
1	168824329	168824344	168824338	TTTC	G	snp	1	1	1	1	.	.	.	INTERGENIC(INTERGENIC)
1	168838777	168838790	168838784	AT	C	snp	1	1	1	1	.	.	.	INTERGENIC(INTERGENIC)
1	168882427	168882434	168882428	T	C	snp	1	1	1	1	.	.	.	RP11-375F2.2.1(INTRON)
1	168993641	168993650	168993647	AG	GAG	del	0	1	0	1	.	.	.	RP11-375F2.2.1(INTRON)
1	168998177	168998192	168998180	AG	A	snp	1	1	1	1	.	.	.	RP11-375F2.2.1(INTRON)
1	169001507	169001518	169001510	AT	C	snp	1	1	1	1	.	.	.	RP11-375F2.2.1(INTRON)
1	169001813	169001820	169001814	T	G	snp	1	0	0	0	.	.	.	RP11-375F2.2.1(INTRON)
1	169008524	169008533	169008525	A	C	snp	1	1	1	1	.	.	.	RP11-375F2.2.1(INTRON)
1	169008524	169008533	169008527	A	C	snp	1	0	0	0	.	.	.	RP11-375F2.2.1(INTRON)
1	169183069	169183082	169183072	AT	A	snp	0	1	0	1	.	.	.	NME7(INTRON)
1	169201795	169201804	169201797	G	T	snp	0	1	0	1	.	.	.	NME7(INTRON);NME7(UPSTREAM)

1	169298421	169298430	169298423	A	C	snp	1	1	1	1	.	.	.	NME7(INTRON);NME7(UPSTREAM)
1	169338319	169338330	169338326	TG	A	snp	0	1	1	1	.	.	.	BLZF1(INTRON);NME7(UPSTREAM)
1	169483761	169483769	169483762	T	G	snp	0	1	0	1	.	.	.	F5(INTRON)
1	169606587	169606604	169606590	AC	A	snp	0	1	0	1	.	.	.	INTERGENIC(INTERGENIC)
1	169633085	169633092	169633091	T	C	snp	1	1	0	1	.	.	.	C1orf112(INTRON)
1	169661386	169661405	169661391	ATTT	A	snp	1	1	0	1	.	.	.	C1orf112(INTRON);SELL(INTRON)
1	169704236	169704247	169704243	TA	C	snp	0	1	0	1	.	.	.	C1orf112(INTRON);SELE(UPSTREAM)
1	169890932	169890939	169890933	A	G	snp	1	1	1	1	.	.	.	KIFAP3(INTRON)
1	170038976	170038984	170038982	A	AT	ins	1	0	1	0	.	.	.	KIFAP3(INTRON);KIFAP3(UPSTREAM)
1	170212062	170212075	170212073	TG	T	del	0	1	1	1	.	.	.	INTERGENIC(INTERGENIC);RP11-297H3.3.1(DOWNSTREAM)
1	170314174	170314183	170314178	T	C	snp	1	1	0	1	.	.	.	INTERGENIC(INTERGENIC)
1	170333050	170333061	170333055	TTA	C	snp	1	1	0	1	.	.	.	INTERGENIC(INTERGENIC)
1	170344689	170344697	170344693	T	C	snp	1	1	1	1	.	.	.	INTERGENIC(INTERGENIC)
1	170353000	170353013	170353008	TG	C	snp	1	1	1	1	.	.	.	INTERGENIC(INTERGENIC)
1	170378475	170378490	170378483	TAGA	C	snp	1	0	0	0	.	.	.	INTERGENIC(INTERGENIC)
1	170382957	170382972	170382965	TA	G	snp	1	1	1	1	.	.	.	INTERGENIC(INTERGENIC)
1	170414582	170414591	170414584	AT	G	snp	1	1	0	1	.	.	.	INTERGENIC(INTERGENIC)
1	170440345	170440362	170440359	TCA	G	snp	0	1	1	1	.	.	.	RP11-576I22.2.1(INTRON)
1	170441729	170441736	170441735	T	C	snp	1	0	0	0	.	.	.	RP11-576I22.2.1(INTRON)
1	170460177	170460184	170460179	A	G	snp	1	1	1	1	.	.	.	RP11-576I22.2.1(INTRON)
1	170534649	170534657	170534655	T	G	snp	1	1	1	1	.	.	.	INTERGENIC(INTERGENIC)
1	170553434	170553443	170553442	T	G	snp	1	1	1	1	.	.	.	INTERGENIC(INTERGENIC);RP11-545I10.2.1(DOWNSTREAM)
1	170563080	170563087	170563083	A	C	snp	1	1	1	1	.	.	.	INTERGENIC(INTERGENIC);RP1-79C4.1.1(UPSTREAM)
1	170674260	170674268	170674261	T	C	snp	0	1	0	1	.	.	.	PRRX1(INTRON)
1	170696233	170696242	170696240	TG	T	snp	0	0	0	1	.	.	.	PRRX1(INTRON);PRRX1(UPSTREAM);PRRX1(UTR_3_PRIME)
1	170923003	170923020	170923012	AG	A	snp	1	1	1	1	.	.	.	C1orf129(INTRON)
1	170933763	170933770	170933764	A	TC	ins	1	1	1	1	.	.	.	C1orf129(INTRON)
1	170933763	170933770	170933766	A	C	snp	1	1	1	1	.	.	.	C1orf129(INTRON)
1	170933763	170933770	170933766	A	TC	ins	1	1	1	1	.	.	.	C1orf129(INTRON)
1	170945586	170945594	170945587	T	G	snp	0	1	0	1	.	.	.	C1orf129(INTRON)
1	170981024	170981031	170981025	T	G	snp	0	0	1	0	.	.	.	C1orf129(INTRON)
1	171088187	171088198	171088193	TA	ATATAG	ins	1	1	1	0	.	.	.	FMO3(DOWNSTREAM);INTERGENIC(INTERGENIC)
1	171135068	171135077	171135075	T	C	snp	1	1	1	1	.	.	.	FMO6P(DOWNSTREAM);INTERGENIC(INTERGENIC);RP1-45C12.1.1(DOWNSTREAM)
1	171251673	171251682	171251680	AT	A	del	1	1	0	1	.	.	.	FMO1(DOWNSTREAM);FMO1(EXON);FMO1(INTRON)
1	171289975	171289982	171289979	A	AAAT	ins	1	0	0	0	.	.	.	FMO4(INTRON);FMO4(UPSTREAM)
1	171299709	171299718	171299714	AG	C	snp	1	1	1	1	.	.	.	FMO4(INTRON);FMO4(UPSTREAM)
1	171319360	171319367	171319364	A	G	snp	1	0	0	0	.	.	.	INTERGENIC(INTERGENIC);RP4-798A17.5.1(UPSTREAM)
1	171391268	171391275	171391273	C	A	snp	1	1	1	1	.	.	.	INTERGENIC(INTERGENIC);U6(UPSTREAM)
1	171474687	171474695	171474693	T	G	snp	0	0	0	1	.	.	.	PRRC2C(INTRON);PRRC2C(UPSTREAM)
1	171593749	171593762	171593752	TG	T	snp	1	1	1	1	.	.	.	RP3-454G6.2.1(INTRON)
1	171593763	171593771	171593764	T	G	snp	1	1	1	1	.	.	.	RP3-454G6.2.1(INTRON)
1	171620484	171620491	171620489	C	A	ins	1	1	1	1	.	.	.	MYOC(INTRON)
1	171651623	171651632	171651627	T	G	snp	1	1	1	1	.	.	.	INTERGENIC(INTERGENIC);RP4-560B9.1.1(DOWNSTREAM)
1	171700552	171700566	171700562	ATA	A	ins	1	0	0	0	.	.	.	VAMP4(INTRON)
1	171739425	171739432	171739430	T	G	snp	1	1	1	1	.	.	.	INTERGENIC(INTERGENIC);SCARNA20(DOWNSTREAM)
1	171743703	171743712	171743705	AT	G	snp	1	0	0	0	.	.	.	INTERGENIC(INTERGENIC)
1	171772440	171772453	171772451	GT	G	snp	1	0	0	0	.	.	.	METTL13(INTRON);RP4-612B18.3.1(UPSTREAM)
1	171817838	171817847	171817845	TG	A	snp	1	0	0	0	.	.	.	DNM3(INTRON)
1	171839634	171839642	171839637	A	C	snp	1	0	0	0	.	.	.	DNM3(INTRON)
1	171870085	171870094	171870091	A	C	ins	1	1	0	1	.	.	.	DNM3(INTRON)
1	171977273	171977282	171977276	T	C	ins	1	0	0	0	.	.	.	DNM3(INTRON)
1	172042039	172042046	172042045	A	G	snp	1	1	1	0	.	.	.	DNM3(INTRON)
1	172179113	172179124	172179121	ATA	G	snp	1	1	1	1	.	.	.	DNM3(INTRON)
1	172316335	172316342	172316341	C	T	ins	1	0	0	1	.	.	.	DNM3(INTRON);SNORD112(UPSTREAM)
1	172327397	172327406	172327399	TG	C	snp	1	1	1	1	.	.	.	DNM3(INTRON)
1	172443517	172443526	172443520	A	C	snp	0	1	0	1	.	.	.	INTERGENIC(INTERGENIC)
1	172451576	172451584	172451581	T	G	snp	1	1	1	1	.	.	.	INTERGENIC(INTERGENIC)
1	172492651	172492660	172492658	TG	A	snp	1	0	0	0	.	.	.	INTERGENIC(INTERGENIC)
1	172624231	172624238	172624233	C	T	snp	1	1	1	1	.	.	.	FASLG(UPSTREAM);INTERGENIC(INTERGENIC)
1	172663556	172663565	172663561	AT	C	snp	1	0	0	0	.	.	.	INTERGENIC(INTERGENIC)
1	172663570	172663579	172663575	AT	C	snp	1	0	0	0	.	.	.	INTERGENIC(INTERGENIC)
1	172663768	172663777	172663772	AT	G	snp	1	1	1	1	.	.	.	INTERGENIC(INTERGENIC)
1	172989772	172989783	172989778	CT	T	snp	1	0	0	0	.	.	.	RP1-15D23.2.1(INTRON)
1	173016021	173016044	173016039	TTG	TGTTG	del	1	1	1	1	.	.	.	RP1-15D23.2.1(INTRON);TNFSF18(INTRON)
1	173090546	173090554	173090547	A	C	snp	1	1	1	0	.	.	.	INTERGENIC(INTERGENIC)

1	173146730	173146737	173146732	T	G	snp	1	1	0	1	.	.	.	INTERGENIC(INTERGENIC);RP3-395P12.2.1(UPSTREAM)
1	173174064	173174081	173174075	AC	AT	ins	1	1	0	1	.	.	.	TNFSF4(INTRON)
1	173318164	173318178	173318175	TTA	T	ins	1	0	0	0	.	.	.	INTERGENIC(INTERGENIC);RP11-296O14.1.1(UPSTREAM)
1	173389045	173389054	173389050	G	A	snp	1	1	1	1	.	.	.	RP11-296O14.3.1(INTRON)
1	173392099	173392108	173392103	AT	G	snp	1	1	1	1	.	.	.	RP11-296O14.3.1(INTRON)
1	173712200	173712208	173712204	A	G	snp	1	0	0	0	.	.	.	KLHL20(INTRON);RP11-360D2.2.1(DOWNSTREAM)
1	173867244	173867252	173867251	T	G	snp	1	0	0	0	.	.	.	INTERGENIC(INTERGENIC);ZBTB37(DOWNSTREAM)
1	173968530	173968541	173968532	AG	G	del	1	1	1	1	.	.	.	RC3H1(INTRON)
1	174031018	174031033	174031021	CT	C	snp	1	1	1	1	.	.	.	INTERGENIC(INTERGENIC)
1	174062736	174062743	174062741	G	T	snp	1	0	1	1	.	.	.	INTERGENIC(INTERGENIC);RP11-160H22.4.1(UPSTREAM)
1	174164870	174164889	174164883	TTTC	C	snp	1	1	0	1	.	.	.	RABGAP1L(INTRON);snoU13(DOWNSTREAM)
1	174184606	174184617	174184610	AAT	TAAT	del	1	1	1	1	.	.	.	RABGAP1L(INTRON);RABGAP1L(UPSTREAM)
1	174184606	174184617	174184613	AAT	T	del	1	1	1	1	.	.	.	RABGAP1L(INTRON);RABGAP1L(UPSTREAM)
1	174245274	174245285	174245278	ATT	C	snp	1	1	1	1	.	.	.	RABGAP1L(INTRON)
1	174270005	174270024	174270010	AATG	G	snp	1	0	0	0	.	.	.	RABGAP1L(INTRON)
1	174473102	174473111	174473103	T	C	ins	1	1	1	1	.	.	.	RABGAP1L(INTRON)
1	174594567	174594578	174594575	GT	T	snp	1	1	1	1	.	.	.	RABGAP1L(INTRON)
1	174605510	174605517	174605511	A	C	snp	1	0	0	0	.	.	.	RABGAP1L(INTRON);RABGAP1L(UPSTREAM)
1	174694569	174694580	174694577	TTG	A	snp	1	1	1	1	.	.	.	RABGAP1L(INTRON)
1	174800866	174800874	174800872	A	G	snp	1	1	1	1	.	.	.	RABGAP1L(INTRON)
1	174931334	174931343	174931340	C	A	ins	1	1	1	1	.	.	.	RABGAP1L(DOWNSTREAM);RABGAP1L(INTRON);RABGAP1L(UPSTREAM)
1	175080017	175080025	175080019	T	G	snp	1	1	1	1	.	.	.	TNN(INTRON)
1	175143939	175143954	175143948	AT	C	snp	1	1	1	1	.	.	.	KIAA0040(INTRON)
1	175235706	175235725	175235710	CAAA	AAA	ins	1	1	1	1	.	.	.	INTERGENIC(INTERGENIC)
1	175283989	175283998	175283993	AG	G	ins	1	1	1	1	.	.	.	RP3-518E13.2.1(INTRON)
1	175300236	175300245	175300239	TC	T	snp	1	1	1	1	.	.	.	RP3-518E13.2.1(INTRON);TNR(INTRON)
1	175344613	175344620	175344614	G	T	snp	0	1	1	1	.	.	.	TNR(INTRON)
1	175396868	175396891	175396885	TTCC	C	ins	1	0	0	0	.	.	.	TNR(INTRON)
1	175516809	175516818	175516812	AG	A	snp	1	0	0	0	.	.	.	TNR(INTRON);TNR-IT1(INTRON)
1	175655192	175655207	175655199	CA	G	snp	1	0	0	0	.	.	.	TNR(INTRON)
1	175668377	175668384	175668379	A	G	snp	1	1	1	1	.	.	.	TNR(INTRON)
1	175686987	175687002	175686999	CA	A	snp	0	0	1	1	.	.	.	TNR(INTRON)
1	175900916	175900931	175900923	AAAG	A	snp	1	0	0	0	.	.	.	INTERGENIC(INTERGENIC)
1	175941253	175941273	175941265	AAC	G	snp	1	0	0	0	.	.	.	RFWD2(INTRON);SCARNA3(UPSTREAM)
1	175993544	175993552	175993545	A	G	snp	1	1	1	1	.	.	.	RFWD2(INTRON)
1	176053987	176053996	176053992	T	C	ins	0	0	1	0	.	.	.	RFWD2(DOWNSTREAM);RFWD2(INTRON);RFWD2(UPSTREAM)
1	176064660	176064668	176064661	A	C	snp	1	1	1	1	.	.	.	RFWD2(INTRON)
1	176105062	176105070	176105066	A	G	snp	1	1	1	1	.	.	.	RFWD2(INTRON)
1	176307788	176307796	176307789	T	G	snp	1	0	0	0	.	.	.	INTERGENIC(INTERGENIC)
1	176366857	176366864	176366860	T	C	snp	1	1	1	1	.	.	.	INTERGENIC(INTERGENIC)
1	176388286	176388295	176388293	GT	C	snp	1	0	0	0	.	.	.	INTERGENIC(INTERGENIC)
1	176478996	176479004	176478999	A	C	snp	1	1	1	1	.	.	.	PAPPA2(INTRON);RP4-652L8.1.1(DOWNSTREAM)
1	176551039	176551058	176551054	TTTC	TTTCTTT	del	1	1	0	1	.	.	.	PAPPA2(INTRON)
1	176830822	176830835	176830826	GT	A	snp	0	1	0	1	.	.	.	ASTN1(INTRON);ASTN1(UTR_3_PRIME)
1	177090220	177090233	177090226	AT	TATC	ins	1	1	1	1	.	.	.	ASTN1(INTRON)
1	177119016	177119025	177119022	TA	C	snp	0	1	0	0	.	.	.	ASTN1(INTRON)
1	177119016	177119025	177119023	TA	T	snp	1	0	0	0	.	.	.	ASTN1(INTRON)
1	177146418	177146433	177146429	AAAC	A	del	0	0	1	0	.	.	.	FAM5B(INTRON)
1	177251786	177251795	177251792	GA	A	snp	1	1	1	1	.	.	.	FAM5B(DOWNSTREAM);INTERGENIC(INTERGENIC)
1	177368552	177368563	177368558	TA	A	ins	1	1	1	1	.	.	.	RP1-35C21.2.1(INTRON)
1	177368552	177368563	177368560	TA	ATA	del	1	1	1	1	.	.	.	RP1-35C21.2.1(INTRON)
1	177368552	177368563	177368561	TA	T	del	1	1	1	1	.	.	.	RP1-35C21.2.1(INTRON)
1	177389090	177389097	177389095	T	G	snp	1	1	1	1	.	.	.	RP1-35C21.2.1(INTRON)
1	177538392	177538407	177538395	AC	T	snp	1	1	1	1	.	.	.	RP1-35C21.2.1(INTRON)
1	177574985	177574994	177574992	A	G	snp	1	1	1	1	.	.	.	INTERGENIC(INTERGENIC)
1	177810563	177810570	177810569	T	G	ins	0	0	1	0	.	.	.	INTERGENIC(INTERGENIC)
1	177821755	177821763	177821756	A	C	snp	1	1	1	1	.	.	.	INTERGENIC(INTERGENIC)
1	177821755	177821763	177821758	A	C	snp	1	1	1	1	.	.	.	INTERGENIC(INTERGENIC)
1	177837590	177837599	177837596	TA	C	snp	1	1	1	1	.	.	.	INTERGENIC(INTERGENIC)
1	177949928	177949937	177949930	A	G	snp	1	0	0	0	.	.	.	SEC16B(INTRON);SEC16B.1(INTRON)
1	177987569	177987584	177987576	AAAT	C	snp	1	0	0	0	.	.	.	RP11-568K15.1.1(DOWNSTREAM);RP11-568K15.1.1(INTRON);SEC16B(INTRON);SEC16B.1(INTRON)
1	178049533	178049556	178049544	CTCC	CTCT	ins	1	1	1	1	.	.	.	INTERGENIC(INTERGENIC)
1	178083198	178083206	178083202	T	G	snp	0	1	0	1	.	.	.	RASAL2(INTRON)
1	178130249	178130258	178130253	A	G	ins	1	0	1	0	.	.	.	RASAL2(INTRON)
1	178196086	178196101	178196094	ACAA	T	snp	0	1	1	1	.	.	.	RASAL2(INTRON)

1	178353827	178353836	178353828	A	C	snp	1	1	0	0	.	.	.	RASAL2(INTRON)
1	178353827	178353836	178353830	A	C	snp	1	0	0	0	.	.	.	RASAL2(INTRON)
1	178429887	178429896	178429891	T	C	snp	1	0	0	0	.	.	.	RASAL2(DOWNSTREAM);RASAL2(INTRON)
1	178609757	178609770	178609760	CA	C	snp	1	0	0	0	.	.	.	INTERGENIC(INTERGENIC)
1	178621427	178621436	178621432	CA	T	snp	1	0	0	0	.	.	.	INTERGENIC(INTERGENIC)
1	178633595	178633604	178633602	AC	T	snp	1	1	1	1	.	.	.	INTERGENIC(INTERGENIC)
1	178694077	178694085	178694084	C	T	snp	1	1	1	1	.	.	.	INTERGENIC(INTERGENIC);RALGPS2(UPSTREAM)
1	178728868	178728876	178728873	T	G	snp	0	0	1	0	.	.	.	RALGPS2(INTRON)
1	178819641	178819649	178819644	T	C	snp	1	0	0	0	.	.	.	ANGPTL1(DOWNSTREAM);ANGPTL1(UTR_3_PRIME);RALGPS2(INTRON)
1	178939892	178939901	178939894	CA	T	snp	1	0	0	0	.	.	.	RALGPS2(INTRON)
1	178966795	178966806	178966802	AG	C	snp	1	1	1	1	.	.	.	RALGPS2(INTRON)
1	178966795	178966806	178966803	AG	T	snp	1	1	1	1	.	.	.	RALGPS2(INTRON)
1	179163336	179163343	179163337	T	C	snp	1	0	0	0	.	.	.	ABL2(INTRON);SNORA67(UPSTREAM)
1	179225582	179225591	179225583	G	T	ins	1	1	0	1	.	.	.	COX5BP8(DOWNSTREAM);INTERGENIC(INTERGENIC)
1	179225582	179225591	179225584	G	T	snp	1	1	0	1	.	.	.	COX5BP8(DOWNSTREAM);INTERGENIC(INTERGENIC)
1	179248765	179248772	179248766	T	G	snp	1	1	1	1	.	.	.	INTERGENIC(INTERGENIC)
1	179326341	179326356	179326350	TTTG	C	snp	1	1	1	1	.	.	.	SOAT1(DOWNSTREAM);SOAT1(UTR_3_PRIME)
1	179372279	179372288	179372282	TA	G	snp	1	1	1	1	.	.	.	AXDND1(INTRON)
1	179372279	179372288	179372286	TA	G	snp	1	1	1	1	.	.	.	AXDND1(INTRON)
1	179376353	179376360	179376357	T	C	snp	0	1	1	0	.	.	.	AXDND1(INTRON)
1	179391904	179391912	179391910	T	C	snp	1	1	1	1	.	.	.	AXDND1(INTRON)
1	179394930	179394947	179394945	TG	T	del	1	1	1	1	.	.	.	AXDND1(INTRON)
1	179441038	179441047	179441043	T	C	snp	1	1	1	1	.	.	.	AXDND1(DOWNSTREAM);AXDND1(INTRON)
1	179457878	179457887	179457883	T	C	snp	0	1	1	0	.	.	.	AXDND1(INTRON);AXDND1(UPSTREAM)
1	179518258	179518267	179518260	A	G	snp	1	1	1	1	.	.	.	AXDND1(INTRON);AXDND1(UPSTREAM);NPHS2(DOWNSTREAM);RP11-545A16.1.1(UPSTREAM)
1	179601742	179601750	179601749	T	C	snp	1	1	1	1	.	.	.	TDRD5(INTRON)
1	179904292	179904299	179904297	T	G	snp	0	1	0	1	.	.	.	RP11-533E19.2.1(INTRON)
1	179906693	179906702	179906698	TA	T	snp	0	1	0	1	.	.	.	RP11-533E19.2.1(INTRON)
1	179999451	179999460	179999454	TA	TG	ins	1	1	0	1	.	.	.	CEP350(INTRON)
1	180021334	180021349	180021344	TTCT	T	del	1	0	0	0	.	.	.	CEP350(DOWNSTREAM);CEP350(INTRON)
1	180107947	180107956	180107952	TA	C	snp	1	1	1	1	.	.	.	INTERGENIC(INTERGENIC)
1	180108823	180108838	180108826	TC	AA	ins	1	0	0	0	.	.	.	INTERGENIC(INTERGENIC)
1	180144135	180144142	180144136	A	C	snp	1	1	1	0	.	.	.	QSOX1(INTRON)
1	180164132	180164140	180164134	G	T	snp	1	1	1	1	.	.	.	QSOX1(INTRON)
1	180164132	180164140	180164136	G	T	snp	1	1	1	1	.	.	.	QSOX1(INTRON)
1	180210923	180210930	180210926	T	G	snp	1	1	1	1	.	.	.	LHX4(INTRON)
1	180297980	180297993	180297984	TA	C	snp	0	1	1	0	.	.	.	ACBD6(INTRON)
1	180384256	180384263	180384259	A	C	snp	1	1	1	1	.	.	.	ACBD6(INTRON);ACBD6(UPSTREAM)
1	180412134	180412143	180412139	T	C	ins	1	1	1	1	.	.	.	ACBD6(INTRON)
1	180480502	180480509	180480504	T	C	snp	1	1	1	1	.	.	.	INTERGENIC(INTERGENIC)
1	180483711	180483722	180483719	GAG	GA	del	1	1	1	1	.	.	.	INTERGENIC(INTERGENIC);Y_RNA(DOWNSTREAM)
1	180501502	180501513	180501505	ATT	G	snp	1	1	0	0	.	.	.	INTERGENIC(INTERGENIC)
1	180501657	180501668	180501665	ATT	A	snp	1	0	0	0	.	.	.	INTERGENIC(INTERGENIC)
1	180505147	180505154	180505153	A	C	snp	1	1	1	1	.	.	.	INTERGENIC(INTERGENIC)
1	180553715	180553722	180553721	C	A	snp	1	1	1	1	.	.	.	INTERGENIC(INTERGENIC)
1	180624523	180624536	180624532	TC	TCTCT	del	1	0	0	0	.	.	.	XPR1(INTRON)
1	180625447	180625456	180625452	TA	G	snp	0	0	0	1	.	.	.	XPR1(INTRON)
1	180625492	180625501	180625499	TA	T	snp	0	0	0	1	.	.	.	XPR1(INTRON)
1	180760140	180760154	180760147	AGG	A	snp	1	0	0	0	.	.	.	XPR1(INTRON)
1	180890572	180890581	180890574	GA	C	snp	1	1	1	1	.	.	.	KIAA1614(INTRON)
1	180946381	180946388	180946382	T	G	snp	1	1	1	1	.	.	.	AL162431.1(INTRON);RP11-46A10.5.1(UPSTREAM);STX6(INTRON)
1	181113949	181113964	181113953	TATT	C	snp	1	0	0	0	.	.	.	INTERGENIC(INTERGENIC)
1	181283187	181283196	181283189	G	GC	ins	1	1	1	1	.	.	.	INTERGENIC(INTERGENIC)
1	181283187	181283196	181283193	G	GT	ins	1	1	1	1	.	.	.	INTERGENIC(INTERGENIC)
1	181307220	181307227	181307224	T	C	snp	1	1	1	1	.	.	.	INTERGENIC(INTERGENIC)
1	181404652	181404671	181404662	TTAT	G	snp	1	1	1	1	.	.	.	CACNA1E(INTRON)
1	181439178	181439189	181439187	AG	T	snp	1	0	0	1	.	.	.	CACNA1E(INTRON)
1	181554148	181554156	181554155	C	A	snp	1	0	0	0	.	.	.	CACNA1E(INTRON)
1	181585482	181585489	181585485	T	G	ins	0	1	1	1	.	.	.	CACNA1E(INTRON)
1	181974401	181974416	181974414	AT	A	del	1	1	0	0	.	.	.	INTERGENIC(INTERGENIC)
1	182010687	182010694	182010689	T	C	snp	1	1	1	0	.	.	.	INTERGENIC(INTERGENIC)
1	182093275	182093284	182093281	T	C	snp	1	1	1	1	.	.	.	GS1-122H1.1.1(DOWNSTREAM);INTERGENIC(INTERGENIC)
1	182099157	182099166	182099159	T	G	snp	1	1	1	1	.	.	.	GS1-122H1.1.1(UPSTREAM);GS1-122H1.2.1(INTRON)
1	182100650	182100665	182100661	AAAC	A	del	1	1	1	1	.	.	.	GS1-122H1.1.1(UPSTREAM);GS1-122H1.2.1(INTRON)

1	182100678	182100693	182100689	TAAA	T	del	0	0	1	0	.	.	.	GS1-122H1.1.1(UPSTREAM);GS1-122H1.2.1(INTRON)
1	182127158	182127166	182127160	T	C	snp	1	0	0	0	.	.	.	GS1-122H1.2.1(INTRON)
1	182153737	182153752	182153749	TG	G	snp	0	1	1	1	.	.	.	GS1-122H1.2.1(INTRON);GS1-122H1.3.1(UPSTREAM)
1	182164243	182164270	182164250	ATTC	T	snp	1	1	1	1	.	.	.	GS1-122H1.2.1(INTRON)
1	182250496	182250513	182250510	AT	G	snp	1	1	0	1	.	.	.	GS1-122H1.2.1(INTRON)
1	182264688	182264697	182264689	A	G	snp	1	1	1	1	.	.	.	GS1-122H1.2.1(INTRON)
1	182412143	182412162	182412154	TTTG	A	snp	0	0	0	1	.	.	.	INTERGENIC(INTERGENIC);TEDDM1P1(DOWNSTREAM)
1	182429364	182429381	182429375	TG	A	snp	0	1	1	1	.	.	.	RGSL1(DOWNSTREAM);RGSL1(INTRON)
1	182632194	182632201	182632200	T	C	snp	1	0	0	0	.	.	.	RGS8(DOWNSTREAM);RGS8(INTRON)
1	182685610	182685619	182685614	T	C	snp	1	0	0	0	.	.	.	INTERGENIC(INTERGENIC);RP11-317P15.3.1(DOWNSTREAM)
1	182730011	182730024	182730017	AT	TG	ins	1	0	0	0	.	.	.	INTERGENIC(INTERGENIC)
1	182796747	182796756	182796754	AT	A	del	1	0	0	0	.	.	.	NPL(DOWNSTREAM);NPL(INTRON)
1	182812213	182812221	182812217	A	G	snp	0	1	0	0	.	.	.	DHX9(INTRON)
1	182876698	182876715	182876703	TTA	C	snp	1	0	0	1	.	.	.	SHCBP1L(INTRON)
1	182901604	182901631	182901611	TTAT	C	snp	1	0	0	0	.	.	.	SHCBP1L(INTRON)
1	182924309	182924317	182924310	T	C	snp	1	0	0	0	.	.	.	INTERGENIC(INTERGENIC);KRT18P28(DOWNSTREAM);RP11-505O17.1.1(DOWNSTREAM);SHCBP1L(UPSTREAM)
1	182989602	182989617	182989611	TCTT	TTTCT	del	1	1	1	1	.	.	.	INTERGENIC(INTERGENIC);LAMC1(UPSTREAM)
1	183037370	183037378	183037377	T	G	snp	1	1	1	1	.	.	.	LAMC1(INTRON)
1	183114854	183114869	183114864	TA	AA	ins	1	1	1	1	.	.	.	INTERGENIC(INTERGENIC);LAMC1(DOWNSTREAM);RP11-181K3.4.1(DOWNSTREAM)
1	183150651	183150659	183150658	T	G	snp	1	1	1	1	.	.	.	INTERGENIC(INTERGENIC);LAMC2(UPSTREAM)
1	183286578	183286587	183286581	TG	T	snp	1	1	1	1	.	.	.	NMNAT2(INTRON)
1	183290878	183290887	183290883	A	C	snp	1	0	0	0	.	.	.	NMNAT2(INTRON)
1	183298372	183298383	183298374	TG	G	snp	0	1	0	1	.	.	.	NMNAT2(INTRON)
1	183298386	183298395	183298391	TG	A	snp	1	0	0	0	.	.	.	NMNAT2(INTRON)
1	183310822	183310837	183310824	AG	G	del	1	1	1	1	.	.	.	NMNAT2(INTRON)
1	183317352	183317361	183317360	T	C	snp	1	0	0	0	.	.	.	NMNAT2(INTRON)
1	183409427	183409440	183409437	TA	ATAG	ins	0	0	1	0	.	.	.	INTERGENIC(INTERGENIC)
1	183464286	183464297	183464292	AT	G	snp	1	1	1	1	.	.	.	SMG7(INTRON)
1	183559708	183559719	183559712	AG	T	ins	1	0	0	0	.	.	.	NCF2(INTRON);NCF2(UTR_5_PRIME);SMG7(INTRON)
1	183761304	183761313	183761310	T	G	snp	1	1	0	1	.	.	.	RGL1(INTRON)
1	183774609	183774618	183774612	CT	C	del	1	0	0	0	.	.	.	RGL1(INTRON)
1	183788015	183788022	183788017	A	C	snp	1	1	1	1	.	.	.	RGL1(INTRON)
1	183849510	183849521	183849516	AAC	AC	del	1	1	1	1	.	.	.	RGL1(INTRON)
1	183875613	183875628	183875617	AT	G	snp	1	0	0	0	.	.	.	RGL1(INTRON)
1	183912571	183912580	183912575	GA	A	snp	0	1	0	1	.	.	.	GLT25D2(INTRON);GLT25D2(UPSTREAM)
1	184009827	184009836	184009828	A	AG	ins	1	1	1	1	.	.	.	GLT25D2(UPSTREAM);INTERGENIC(INTERGENIC)
1	184031887	184031895	184031890	T	G	snp	1	0	0	0	.	.	.	TSEN15(INTRON)
1	184033681	184033700	184033696	AAAC	T	snp	1	0	0	0	.	.	.	TSEN15(INTRON)
1	184154054	184154074	184154068	GTT	G	del	1	0	0	0	.	.	.	INTERGENIC(INTERGENIC)
1	184154054	184154074	184154071	GTT	G	del	1	1	1	1	.	.	.	INTERGENIC(INTERGENIC)
1	184154073	184154082	184154075	T	G	snp	1	1	0	1	.	.	.	INTERGENIC(INTERGENIC)
1	184178627	184178644	184178635	TTA	G	snp	1	0	1	0	.	.	.	INTERGENIC(INTERGENIC)
1	184186537	184186544	184186542	A	G	snp	1	1	1	1	.	.	.	INTERGENIC(INTERGENIC)
1	184220369	184220386	184220375	CT	T	del	1	0	0	0	.	.	.	INTERGENIC(INTERGENIC)
1	184220369	184220386	184220377	CT	T	del	1	0	0	0	.	.	.	INTERGENIC(INTERGENIC)
1	184229424	184229431	184229427	A	G	snp	1	1	1	1	.	.	.	INTERGENIC(INTERGENIC)
1	184235053	184235062	184235060	T	G	snp	1	1	1	1	.	.	.	INTERGENIC(INTERGENIC)
1	184290405	184290420	184290416	GA	G	snp	1	1	1	1	.	.	.	INTERGENIC(INTERGENIC)
1	184346765	184346780	184346772	CAAA	C	snp	1	0	0	0	.	.	.	INTERGENIC(INTERGENIC)
1	184424010	184424019	184424014	AT	G	snp	1	0	0	1	.	.	.	C1orf21(INTRON)
1	184426595	184426608	184426604	AT	A	del	1	0	0	0	.	.	.	C1orf21(INTRON)
1	184426595	184426608	184426606	AT	A	del	1	1	0	1	.	.	.	C1orf21(INTRON)
1	184528611	184528634	184528615	TCCT	CCCC	ins	1	0	0	0	.	.	.	C1orf21(INTRON)
1	184587771	184587780	184587778	C	A	ins	1	0	0	1	.	.	.	C1orf21(INTRON)
1	184640426	184640441	184640429	TG	A	snp	1	0	0	0	.	.	.	INTERGENIC(INTERGENIC)
1	184641822	184641833	184641830	TTG	TTGT	del	1	1	1	1	.	.	.	INTERGENIC(INTERGENIC)
1	184710534	184710542	184710541	A	C	snp	1	1	0	1	.	.	.	EDEM3(INTRON);EDEM3(UPSTREAM)
1	184728084	184728093	184728088	A	C	snp	1	1	1	1	.	.	.	EDEM3(UPSTREAM);INTERGENIC(INTERGENIC)
1	184865075	184865082	184865079	T	C	snp	1	1	1	1	.	.	.	FAM129A(INTRON)
1	184882271	184882286	184882273	TA	ATATATATATATATG	ins	1	1	1	1	.	.	.	FAM129A(INTRON)
1	185012941	185012955	185012952	TTG	TTGTT	del	0	0	1	0	.	.	.	INTERGENIC(INTERGENIC);RNF2(UPSTREAM)
1	185080094	185080103	185080101	T	G	snp	1	1	1	1	.	.	.	INTERGENIC(INTERGENIC)
1	185080102	185080113	185080110	TTG	TTGTT	del	0	0	1	0	.	.	.	INTERGENIC(INTERGENIC)
1	185151790	185151799	185151796	CT	G	snp	1	0	0	0	.	.	.	SWT1(INTRON)

1	185339977	185339985	185339980	T	C	snp	1	0	0	0	.	.	.	INTERGENIC(INTERGENIC)
1	185346445	185346453	185346451	A	C	snp	1	1	0	1	.	.	.	INTERGENIC(INTERGENIC)
1	185350467	185350476	185350471	GT	A	snp	0	0	1	0	.	.	.	INTERGENIC(INTERGENIC)
1	185368328	185368343	185368332	AT	G	snp	0	1	0	1	.	.	.	INTERGENIC(INTERGENIC)
1	185564686	185564695	185564692	AG	G	snp	1	0	0	0	.	.	.	INTERGENIC(INTERGENIC)
1	185589182	185589195	185589187	TA	G	snp	1	0	0	0	.	.	.	GS1-204I12.1.1(INTRON)
1	185589182	185589195	185589188	TA	AC	ins	1	0	0	0	.	.	.	GS1-204I12.1.1(INTRON)
1	185589182	185589195	185589189	TA	G	snp	1	0	0	0	.	.	.	GS1-204I12.1.1(INTRON)
1	185633337	185633350	185633344	AC	T	snp	1	0	0	0	.	.	.	INTERGENIC(INTERGENIC)
1	185638796	185638805	185638800	TC	G	snp	1	0	0	0	.	.	.	INTERGENIC(INTERGENIC)
1	185695037	185695046	185695040	TA	G	snp	1	0	0	0	.	.	.	INTERGENIC(INTERGENIC)
1	185781932	185781939	185781938	C	T	ins	1	0	0	0	.	.	.	HMCN1(INTRON)
1	185926576	185926585	185926579	TA	G	snp	1	1	0	0	.	.	.	HMCN1(INTRON)
1	186086570	186086584	186086578	TTG	A	snp	1	1	1	1	.	.	.	HMCN1(INTRON)
1	186117810	186117818	186117813	T	C	snp	1	0	0	0	.	.	.	HMCN1(INTRON)
1	186209226	186209233	186209229	T	G	snp	0	1	1	1	.	.	.	INTERGENIC(INTERGENIC)
1	186260309	186260318	186260311	A	C	snp	1	1	1	1	.	.	.	INTERGENIC(INTERGENIC)
1	186318806	186318821	186318817	AAAT	A	snp	1	1	1	1	.	.	.	TPR(DOWNSTREAM);TPR(INTRON)
1	186508988	186508995	186508991	A	G	snp	1	1	1	1	.	.	.	INTERGENIC(INTERGENIC)
1	186584746	186584763	186584760	AT	TT	ins	0	1	1	1	.	.	.	INTERGENIC(INTERGENIC)
1	186588893	186588908	186588901	ACAA	G	snp	1	1	1	1	.	.	.	INTERGENIC(INTERGENIC)
1	186767876	186767899	186767880	TCTA	C	snp	1	0	0	0	.	.	.	INTERGENIC(INTERGENIC)
1	186863694	186863711	186863702	AG	G	snp	1	0	0	0	.	.	.	PLA2G4A(INTRON)
1	186918447	186918462	186918458	GAAG	A	snp	1	0	0	0	.	.	.	PLA2G4A(INTRON)
1	186925629	186925638	186925633	T	G	snp	1	0	0	0	.	.	.	PLA2G4A(INTRON)
1	186925629	186925638	186925636	T	G	snp	1	1	1	1	.	.	.	PLA2G4A(INTRON)
1	187011558	187011567	187011566	A	G	snp	0	0	0	1	.	.	.	INTERGENIC(INTERGENIC)
1	187045806	187045821	187045812	TTTG	G	snp	1	0	0	0	.	.	.	INTERGENIC(INTERGENIC)
1	187056982	187057005	187056986	TTG	G	del	1	1	0	1	.	.	.	INTERGENIC(INTERGENIC);RP11-108M21.1.1(UPSTREAM)
1	187066829	187066852	187066838	AATT	T	snp	1	0	0	0	.	.	.	RP11-108M21.1.1(INTRON)
1	187066829	187066852	187066848	AATT	A	del	1	1	1	1	.	.	.	RP11-108M21.1.1(INTRON)
1	187103004	187103013	187103011	AC	ACA	del	1	1	0	1	.	.	.	RP11-108M21.1.1(INTRON)
1	187265400	187265417	187265405	TAT	G	snp	0	1	1	1	.	.	.	RP11-108M21.1.1(INTRON)
1	187323671	187323680	187323679	A	G	snp	0	1	1	1	.	.	.	RP11-108M21.1.1(INTRON)
1	187326847	187326858	187326852	GT	C	snp	0	0	1	0	.	.	.	RP11-108M21.1.1(INTRON)
1	187405971	187405980	187405978	T	G	snp	1	0	1	0	.	.	.	INTERGENIC(INTERGENIC)
1	187424193	187424200	187424198	T	C	snp	1	1	1	1	.	.	.	RP11-445P19.1.1(INTRON)
1	187547775	187547783	187547778	T	C	ins	0	1	1	1	.	.	.	INTERGENIC(INTERGENIC)
1	187588928	187588943	187588934	CATT	C	snp	0	0	0	1	.	.	.	INTERGENIC(INTERGENIC)
1	187605110	187605125	187605119	CAAA	AAC	del	1	1	0	1	.	.	.	INTERGENIC(INTERGENIC)
1	187825265	187825276	187825273	AT	TGTG	ins	1	1	1	1	.	.	.	INTERGENIC(INTERGENIC)
1	187850796	187850807	187850800	TG	A	snp	1	0	0	0	.	.	.	INTERGENIC(INTERGENIC)
1	187904687	187904696	187904692	T	C	snp	1	1	1	1	.	.	.	INTERGENIC(INTERGENIC)
1	188065412	188065419	188065418	A	C	snp	1	0	0	0	.	.	.	INTERGENIC(INTERGENIC)
1	188142015	188142024	188142017	A	C	snp	0	1	0	1	.	.	.	INTERGENIC(INTERGENIC)
1	188168272	188168287	188168275	AG	A	snp	1	1	1	1	.	.	.	INTERGENIC(INTERGENIC)
1	188207864	188207881	188207879	AT	AAA	ins	1	1	1	1	.	.	.	INTERGENIC(INTERGENIC);RP11-405P11.1.1(DOWNSTREAM)
1	188228295	188228308	188228303	CT	G	snp	1	1	1	1	.	.	.	INTERGENIC(INTERGENIC)
1	188251779	188251790	188251782	AT	C	snp	1	1	1	1	.	.	.	INTERGENIC(INTERGENIC)
1	188362242	188362251	188362247	TA	G	snp	1	1	1	1	.	.	.	INTERGENIC(INTERGENIC)
1	188461924	188461931	188461928	A	C	snp	1	1	1	1	.	.	.	INTERGENIC(INTERGENIC)
1	188463701	188463724	188463708	AATA	AAT	ins	1	1	1	1	.	.	.	INTERGENIC(INTERGENIC)
1	188494501	188494512	188494510	TA	TAT	del	1	1	0	1	.	.	.	RP11-669M2.1.1(INTRON)
1	188515179	188515187	188515185	T	C	snp	1	0	0	0	.	.	.	INTERGENIC(INTERGENIC)
1	188518824	188518839	188518831	TATT	A	snp	0	0	0	1	.	.	.	INTERGENIC(INTERGENIC)
1	188538019	188538026	188538021	T	G	snp	1	1	1	1	.	.	.	INTERGENIC(INTERGENIC)
1	188540989	188540998	188540990	A	C	ins	1	0	1	0	.	.	.	INTERGENIC(INTERGENIC)
1	188590437	188590445	188590442	T	G	snp	1	0	0	0	.	.	.	INTERGENIC(INTERGENIC)
1	188598253	188598260	188598254	A	C	snp	0	0	0	1	.	.	.	INTERGENIC(INTERGENIC)
1	188626435	188626444	188626438	TA	G	snp	1	1	1	1	.	.	.	INTERGENIC(INTERGENIC)
1	188697799	188697814	188697804	AAAC	C	snp	1	0	0	0	.	.	.	INTERGENIC(INTERGENIC)
1	188838248	188838265	188838258	CT	TT	ins	1	0	1	0	.	.	.	INTERGENIC(INTERGENIC);RP11-316I3.2.1(DOWNSTREAM)
1	188873217	188873226	188873218	A	AG	ins	1	0	1	0	.	.	.	INTERGENIC(INTERGENIC);RP11-373J16.1.1(UPSTREAM)
1	189184699	189184712	189184705	GT	TA	ins	1	1	1	1	.	.	.	INTERGENIC(INTERGENIC)
1	189294417	189294428	189294422	TTA	T	snp	1	0	0	0	.	.	.	INTERGENIC(INTERGENIC)
1	189376126	189376141	189376138	TA	A	del	1	0	0	0	.	.	.	INTERGENIC(INTERGENIC)

1	189376126	189376141	189376139	TA	T	ins	0	1	1	1	.	.	.	INTERGENIC(INTERGENIC)
1	189379741	189379749	189379742	T	G	snp	1	0	0	0	.	.	.	INTERGENIC(INTERGENIC)
1	189397960	189397973	189397971	TA	T	ins	1	1	1	1	.	.	.	INTERGENIC(INTERGENIC)
1	189414459	189414473	189414462	CAC	G	snp	1	0	0	0	.	.	.	INTERGENIC(INTERGENIC)
1	189437869	189437880	189437875	TTA	C	snp	1	0	0	0	.	.	.	INTERGENIC(INTERGENIC)
1	189480872	189480887	189480879	AAAC	G	snp	1	0	0	0	.	.	.	INTERGENIC(INTERGENIC)
1	189502994	189503005	189503003	TG	A	snp	1	0	0	0	.	.	.	INTERGENIC(INTERGENIC)
1	189559218	189559227	189559224	AT	G	snp	1	0	0	0	.	.	.	INTERGENIC(INTERGENIC)
1	189618809	189618829	189618813	TTA	C	snp	1	0	0	0	.	.	.	INTERGENIC(INTERGENIC)
1	189618809	189618829	189618816	TTA	C	snp	1	0	0	0	.	.	.	INTERGENIC(INTERGENIC)
1	189649231	189649239	189649234	T	TG	ins	1	1	1	1	.	.	.	INTERGENIC(INTERGENIC)
1	189649231	189649239	189649235	T	G	ins	1	1	1	1	.	.	.	INTERGENIC(INTERGENIC)
1	189649553	189649562	189649556	T	C	ins	1	1	1	1	.	.	.	INTERGENIC(INTERGENIC)
1	189698251	189698258	189698256	T	G	ins	1	0	0	0	.	.	.	INTERGENIC(INTERGENIC)
1	189717890	189717903	189717893	TA	TATC	ins	1	1	1	1	.	.	.	INTERGENIC(INTERGENIC)
1	189717926	189717937	189717931	TA	C	snp	1	1	1	1	.	.	.	INTERGENIC(INTERGENIC)
1	189717926	189717937	189717932	TA	G	snp	1	1	1	1	.	.	.	INTERGENIC(INTERGENIC)
1	189717987	189717996	189717992	AT	G	snp	1	1	1	1	.	.	.	INTERGENIC(INTERGENIC)
1	189718005	189718014	189718010	AT	G	snp	1	1	1	1	.	.	.	INTERGENIC(INTERGENIC)
1	189742715	189742724	189742722	A	G	snp	1	1	0	0	.	.	.	INTERGENIC(INTERGENIC);RP11-398M15.1.1(UPSTREAM)
1	189783581	189783592	189783584	ATA	T	snp	1	1	1	1	.	.	.	RP11-398M15.1.1(INTRON)
1	189810384	189810391	189810386	T	C	snp	1	1	1	1	.	.	.	INTERGENIC(INTERGENIC)
1	189816223	189816231	189816230	A	C	ins	1	1	1	1	.	.	.	INTERGENIC(INTERGENIC)
1	189820278	189820286	189820285	A	G	snp	1	1	1	1	.	.	.	INTERGENIC(INTERGENIC)
1	189823453	189823468	189823458	TCTT	A	snp	1	1	1	1	.	.	.	INTERGENIC(INTERGENIC)
1	189823453	189823468	189823459	TCTT	G	snp	1	1	1	1	.	.	.	INTERGENIC(INTERGENIC)
1	189847562	189847577	189847572	AT	G	snp	1	0	0	0	.	.	.	INTERGENIC(INTERGENIC)
1	189863742	189863751	189863747	A	G	snp	1	0	0	0	.	.	.	INTERGENIC(INTERGENIC)
1	189900637	189900645	189900642	A	C	snp	1	0	0	0	.	.	.	INTERGENIC(INTERGENIC)
1	189989831	189989840	189989836	TC	A	snp	1	1	1	1	.	.	.	INTERGENIC(INTERGENIC)
1	190029138	190029151	190029146	AG	G	snp	1	0	0	0	.	.	.	INTERGENIC(INTERGENIC)
1	190035408	190035417	190035414	A	TG	ins	1	1	1	1	.	.	.	INTERGENIC(INTERGENIC)
1	190038808	190038821	190038811	CT	G	snp	1	1	1	1	.	.	.	INTERGENIC(INTERGENIC)
1	190048573	190048580	190048574	A	G	snp	1	0	0	0	.	.	.	INTERGENIC(INTERGENIC)
1	190062469	190062482	190062473	AT	C	snp	0	1	1	1	.	.	.	FAM5C(DOWNSTREAM);INTERGENIC(INTERGENIC)
1	190137340	190137357	190137353	TA	TAT	del	1	1	1	1	.	.	.	FAM5C(INTRON)
1	190137340	190137357	190137355	TA	T	del	1	1	1	1	.	.	.	FAM5C(INTRON)
1	190143611	190143620	190143614	AT	C	snp	1	0	1	1	.	.	.	FAM5C(INTRON)
1	190186047	190186054	190186049	T	C	snp	1	0	0	0	.	.	.	FAM5C(DOWNSTREAM);FAM5C(INTRON)
1	190270375	190270388	190270380	AT	C	snp	1	1	1	1	.	.	.	FAM5C(INTRON);RP11-547I7.1.1(INTRON)
1	190298567	190298576	190298573	CA	T	snp	1	0	0	0	.	.	.	FAM5C(INTRON);RP11-547I7.1.1(INTRON)
1	190298576	190298585	190298577	A	C	snp	1	1	1	1	.	.	.	FAM5C(INTRON);RP11-547I7.1.1(INTRON)
1	190306338	190306345	190306342	A	G	snp	1	1	1	1	.	.	.	FAM5C(INTRON);RP11-547I7.1.1(INTRON)
1	190381587	190381595	190381591	T	G	snp	1	1	1	1	.	.	.	FAM5C(INTRON)
1	190444974	190444983	190444979	T	C	snp	0	0	1	0	.	.	.	FAM5C(INTRON);FAM5C(UPSTREAM);RP11-161I10.1.1(UPSTREAM);RP11-547I7.2.1(UPSTREAM)
1	190539859	190539867	190539862	T	C	snp	0	1	1	1	.	.	.	INTERGENIC(INTERGENIC)
1	190574011	190574020	190574013	TA	C	snp	0	1	1	1	.	.	.	INTERGENIC(INTERGENIC)
1	190575004	190575012	190575005	G	T	snp	1	1	1	1	.	.	.	INTERGENIC(INTERGENIC)
1	190677818	190677837	190677828	TTCC	CTTCCTT	del	1	1	1	1	.	.	.	RP11-463J7.2.1(INTRON)
1	190677818	190677837	190677829	TTCC	TTCCTT	del	1	1	1	1	.	.	.	RP11-463J7.2.1(INTRON)
1	190677818	190677837	190677832	TTCC	CTT	del	1	1	1	1	.	.	.	RP11-463J7.2.1(INTRON)
1	190819444	190819457	190819455	AT	A	del	1	1	1	1	.	.	.	INTERGENIC(INTERGENIC)
1	190832403	190832410	190832405	T	C	snp	1	1	1	1	.	.	.	INTERGENIC(INTERGENIC)
1	190940587	190940594	190940592	C	A	snp	1	1	1	1	.	.	.	INTERGENIC(INTERGENIC)
1	190993815	190993822	190993821	A	C	snp	1	1	1	1	.	.	.	INTERGENIC(INTERGENIC)
1	191025626	191025633	191025632	A	C	snp	1	0	0	0	.	.	.	INTERGENIC(INTERGENIC)
1	191138156	191138167	191138165	AC	A	snp	1	1	1	1	.	.	.	INTERGENIC(INTERGENIC)
1	191140173	191140184	191140178	AT	C	snp	1	1	1	1	.	.	.	INTERGENIC(INTERGENIC)
1	191203758	191203773	191203760	TA	C	snp	1	1	1	1	.	.	.	INTERGENIC(INTERGENIC)
1	191203758	191203773	191203769	TA	T	ins	1	1	1	1	.	.	.	INTERGENIC(INTERGENIC)
1	191249343	191249352	191249347	A	C	snp	1	1	1	1	.	.	.	INTERGENIC(INTERGENIC)
1	191295121	191295135	191295131	GTT	TG	del	1	1	1	1	.	.	.	INTERGENIC(INTERGENIC)
1	191384607	191384615	191384610	T	C	snp	1	0	0	0	.	.	.	INTERGENIC(INTERGENIC)
1	191431437	191431452	191431441	TA	C	snp	1	0	0	0	.	.	.	INTERGENIC(INTERGENIC)
1	191508079	191508090	191508082	TA	C	snp	1	1	0	0	.	.	.	INTERGENIC(INTERGENIC)
1	191582893	191582913	191582907	TAA	T	snp	0	0	0	1	.	.	.	INTERGENIC(INTERGENIC)

1	191687725	191687740	191687737	TA	C	snp	1	1	1	1	.	.	.	INTERGENIC(INTERGENIC)
1	191789421	191789430	191789424	TA	G	snp	1	1	1	1	.	.	.	INTERGENIC(INTERGENIC)
1	191801129	191801138	191801133	T	C	snp	1	1	1	1	.	.	.	INTERGENIC(INTERGENIC)
1	191835044	191835052	191835046	A	G	snp	1	1	1	1	.	.	.	RP11-541F9.1.1(INTRON)
1	191842755	191842762	191842761	A	G	snp	1	0	0	0	.	.	.	RP11-541F9.1.1(INTRON);RP11-541F9.2.1(UPSTREAM)
1	191868213	191868220	191868216	A	C	snp	1	1	1	1	.	.	.	RP11-541F9.2.1(INTRON)
1	191926812	191926820	191926818	C	T	snp	0	1	1	1	.	.	.	RP11-541F9.2.1(INTRON)
1	191980753	191980761	191980754	T	C	snp	1	0	0	0	.	.	.	INTERGENIC(INTERGENIC);RP11-541F9.2.1(DOWNSTREAM)
1	191995756	191995764	191995760	T	TTTTG	ins	1	1	1	1	.	.	.	INTERGENIC(INTERGENIC)
1	191995786	191995794	191995790	T	G	snp	1	1	1	1	.	.	.	INTERGENIC(INTERGENIC)
1	192017834	192017851	192017846	AGA	G	snp	1	1	1	1	.	.	.	INTERGENIC(INTERGENIC)
1	192075592	192075601	192075596	AT	G	snp	0	1	1	1	.	.	.	INTERGENIC(INTERGENIC)
1	192403482	192403495	192403493	TG	A	snp	1	1	1	1	.	.	.	INTERGENIC(INTERGENIC)
1	192517792	192517815	192517795	TTG	TGTTGTTGA	ins	1	1	1	1	.	.	.	RP5-1011O1.2.1(INTRON)
1	192575098	192575117	192575105	GAGG	A	snp	1	0	0	0	.	.	.	INTERGENIC(INTERGENIC)
1	192602065	192602073	192602066	A	G	snp	1	1	1	0	.	.	.	INTERGENIC(INTERGENIC);RGS13(UPSTREAM)
1	192621733	192621742	192621736	T	G	snp	1	1	1	1	.	.	.	RGS13(INTRON)
1	192726994	192727003	192727001	CT	C	del	1	1	1	1	.	.	.	INTERGENIC(INTERGENIC)
1	192930091	192930098	192930095	T	G	snp	1	1	0	1	.	.	.	INTERGENIC(INTERGENIC)
1	192930096	192930123	192930103	TTTG	T	snp	1	0	0	0	.	.	.	INTERGENIC(INTERGENIC)
1	192994275	192994282	192994278	A	G	snp	1	0	0	0	.	.	.	UCHL5(INTRON)
1	193068462	193068470	193068466	T	G	snp	0	1	1	1	.	.	.	GLRX2(INTRON)
1	193093085	193093104	193093090	TGAA	C	snp	1	0	0	0	.	.	.	CDC73(INTRON)
1	193216513	193216530	193216516	TC	G	snp	1	0	0	0	.	.	.	CDC73(INTRON)
1	193321984	193321993	193321990	AT	G	snp	1	0	0	0	.	.	.	RP11-452J13.1.1(INTRON)
1	193468167	193468174	193468168	T	G	snp	1	1	0	1	.	.	.	INTERGENIC(INTERGENIC)
1	193480806	193480813	193480807	T	C	snp	1	1	1	1	.	.	.	INTERGENIC(INTERGENIC)
1	193533165	193533174	193533168	TA	C	snp	1	0	0	0	.	.	.	INTERGENIC(INTERGENIC)
1	193597402	193597411	193597404	AT	G	snp	1	1	1	1	.	.	.	INTERGENIC(INTERGENIC)
1	193726869	193726880	193726873	AT	TATATAT	del	1	1	0	1	.	.	.	INTERGENIC(INTERGENIC);RPL23AP22(UPSTREAM)
1	193726869	193726880	193726875	AT	TATAT	del	1	1	1	1	.	.	.	INTERGENIC(INTERGENIC);RPL23AP22(UPSTREAM)
1	193726869	193726880	193726877	AT	TAT	del	1	1	1	1	.	.	.	INTERGENIC(INTERGENIC);RPL23AP22(UPSTREAM)
1	193743266	193743273	193743268	T	G	snp	1	1	1	1	.	.	.	INTERGENIC(INTERGENIC)
1	193851238	193851255	193851252	GT	TGT	del	1	1	1	0	.	.	.	INTERGENIC(INTERGENIC)
1	193851238	193851255	193851253	GT	GTG	del	1	1	1	1	.	.	.	INTERGENIC(INTERGENIC)
1	193923772	193923791	193923782	GATA	C	snp	0	0	1	0	.	.	.	INTERGENIC(INTERGENIC)
1	193997606	193997613	193997607	T	C	ins	1	1	1	1	.	.	.	INTERGENIC(INTERGENIC)
1	194068406	194068415	194068408	AT	T	snp	1	1	1	1	.	.	.	INTERGENIC(INTERGENIC)
1	194068406	194068415	194068409	AT	C	snp	1	1	0	1	.	.	.	INTERGENIC(INTERGENIC)
1	194091818	194091833	194091821	TG	T	del	1	0	0	0	.	.	.	INTERGENIC(INTERGENIC)
1	194091818	194091833	194091821	TG	T	snp	1	1	0	0	.	.	.	INTERGENIC(INTERGENIC)
1	194091818	194091833	194091823	TG	T	snp	1	0	0	0	.	.	.	INTERGENIC(INTERGENIC)
1	194107874	194107883	194107876	AT	G	snp	1	0	0	0	.	.	.	INTERGENIC(INTERGENIC)
1	194187327	194187336	194187334	T	G	ins	1	1	1	1	.	.	.	INTERGENIC(INTERGENIC)
1	194188994	194189003	194189000	TA	A	ins	1	0	0	0	.	.	.	INTERGENIC(INTERGENIC)
1	194230470	194230479	194230471	A	C	snp	1	0	0	0	.	.	.	INTERGENIC(INTERGENIC)
1	194248338	194248345	194248341	A	AG	ins	1	0	0	0	.	.	.	INTERGENIC(INTERGENIC)
1	194248776	194248783	194248779	A	G	snp	1	0	0	0	.	.	.	INTERGENIC(INTERGENIC)
1	194275166	194275177	194275173	GTA	C	snp	1	1	1	1	.	.	.	INTERGENIC(INTERGENIC)
1	194280028	194280036	194280031	A	G	snp	1	1	1	1	.	.	.	INTERGENIC(INTERGENIC)
1	194445130	194445141	194445133	AG	T	snp	1	1	1	1	.	.	.	INTERGENIC(INTERGENIC)
1	194494118	194494135	194494126	GA	C	snp	1	0	0	0	.	.	.	INTERGENIC(INTERGENIC)
1	194526879	194526894	194526884	TA	G	snp	1	1	1	1	.	.	.	INTERGENIC(INTERGENIC)
1	194557455	194557478	194557467	AAAT	T	snp	1	0	0	0	.	.	.	INTERGENIC(INTERGENIC)
1	194557455	194557478	194557471	AAAT	T	snp	1	0	0	0	.	.	.	INTERGENIC(INTERGENIC)
1	194636200	194636207	194636202	T	G	snp	1	0	0	0	.	.	.	INTERGENIC(INTERGENIC)
1	194686779	194686794	194686792	AC	T	snp	0	1	0	1	.	.	.	INTERGENIC(INTERGENIC);RP11-489C13.1.1(UPSTREAM)
1	194702356	194702367	194702360	TA	C	snp	1	1	1	0	.	.	.	INTERGENIC(INTERGENIC)
1	194702427	194702436	194702434	AC	T	snp	1	1	1	1	.	.	.	INTERGENIC(INTERGENIC)
1	194739437	194739444	194739442	A	C	snp	1	0	0	0	.	.	.	INTERGENIC(INTERGENIC)
1	194761929	194761938	194761934	AT	A	ins	1	1	1	1	.	.	.	INTERGENIC(INTERGENIC)
1	194784103	194784114	194784111	AAT	C	snp	1	1	1	0	.	.	.	INTERGENIC(INTERGENIC)
1	194822086	194822095	194822093	TA	G	snp	1	1	1	1	.	.	.	INTERGENIC(INTERGENIC)
1	194942945	194942952	194942946	A	C	snp	1	1	1	1	.	.	.	INTERGENIC(INTERGENIC)
1	194980065	194980074	194980072	A	G	snp	0	1	1	1	.	.	.	INTERGENIC(INTERGENIC)
1	195061835	195061843	195061836	A	C	snp	1	1	1	1	.	.	.	INTERGENIC(INTERGENIC)
1	195086075	195086082	195086081	A	AT	ins	1	1	1	1	.	.	.	INTERGENIC(INTERGENIC)

1	195087455	195087462	195087460	A	C	snp	1	1	1	1	.	.	.	INTERGENIC(INTERGENIC)
1	195142022	195142036	195142025	GAT	T	snp	1	1	1	1	.	.	.	INTERGENIC(INTERGENIC)
1	195403372	195403380	195403378	T	C	snp	0	1	0	1	.	.	.	INTERGENIC(INTERGENIC)
1	195403372	195403380	195403378	T	TTC	ins	1	1	0	1	.	.	.	INTERGENIC(INTERGENIC)
1	195590591	195590608	195590602	TA	G	snp	1	0	0	0	.	.	.	INTERGENIC(INTERGENIC)
1	195627381	195627390	195627383	GT	C	snp	0	1	0	1	.	.	.	INTERGENIC(INTERGENIC)
1	195654726	195654735	195654732	AT	G	snp	1	0	0	0	.	.	.	INTERGENIC(INTERGENIC)
1	195740912	195740920	195740915	A	G	snp	1	0	0	0	.	.	.	INTERGENIC(INTERGENIC)
1	195791631	195791644	195791640	AT	ATATG	del	1	0	0	0	.	.	.	INTERGENIC(INTERGENIC)
1	195791631	195791644	195791641	AT	TATGT	del	1	0	0	0	.	.	.	INTERGENIC(INTERGENIC)
1	195835523	195835532	195835525	A	AG	ins	1	1	1	1	.	.	.	INTERGENIC(INTERGENIC)
1	195835523	195835532	195835526	A	G	ins	1	1	1	1	.	.	.	INTERGENIC(INTERGENIC)
1	195909572	195909583	195909580	CAA	C	del	1	1	1	1	.	.	.	INTERGENIC(INTERGENIC)
1	195979502	195979510	195979505	A	C	snp	0	1	1	1	.	.	.	INTERGENIC(INTERGENIC)
1	196047693	196047702	196047699	AT	G	snp	1	0	0	0	.	.	.	INTERGENIC(INTERGENIC)
1	196106561	196106568	196106565	G	A	snp	1	1	0	1	.	.	.	INTERGENIC(INTERGENIC)
1	196106889	196106898	196106896	T	G	ins	1	1	0	1	.	.	.	INTERGENIC(INTERGENIC)
1	196116661	196116668	196116662	A	C	ins	1	1	1	1	.	.	.	INTERGENIC(INTERGENIC)
1	196116661	196116668	196116663	A	C	snp	1	1	1	1	.	.	.	INTERGENIC(INTERGENIC)
1	196167822	196167830	196167828	A	G	snp	0	0	0	1	.	.	.	INTERGENIC(INTERGENIC)
1	196241564	196241571	196241568	T	C	snp	1	0	0	0	.	.	.	KCNT2(INTRON)
1	196406511	196406520	196406514	TA	T	snp	0	0	0	1	.	.	.	KCNT2(INTRON)
1	196469356	196469373	196469364	GA	A	snp	0	0	0	1	.	.	.	KCNT2(INTRON)
1	196542564	196542572	196542567	T	C	snp	0	1	0	1	.	.	.	KCNT2(INTRON)
1	196561859	196561870	196561864	AT	C	snp	1	1	1	1	.	.	.	KCNT2(INTRON)
1	196609486	196609493	196609491	G	T	snp	1	0	0	0	.	.	.	INTERGENIC(INTERGENIC)
1	196689056	196689063	196689062	T	C	snp	1	1	1	1	.	.	.	CFH(INTRON)
1	196690945	196690952	196690948	T	TG	ins	1	1	1	1	.	.	.	CFH(INTRON)
1	196747662	196747673	196747669	AT	C	snp	1	1	1	1	.	.	.	CFHR3(INTRON)
1	196804129	196804138	196804131	A	AC	ins	1	0	0	0	.	.	.	CFHR1(DOWNSTREAM);CFHR2(INTRON)
1	196804290	196804297	196804291	A	C	snp	1	0	0	0	.	.	.	CFHR1(DOWNSTREAM);CFHR2(INTRON)
1	196995637	196995646	196995638	T	C	snp	1	1	1	1	.	.	.	INTERGENIC(INTERGENIC)
1	197268757	197268766	197268763	A	C	snp	1	1	1	1	.	.	.	CRB1(INTRON)
1	197272238	197272261	197272248	TTTA	G	snp	1	1	1	1	.	.	.	CRB1(INTRON)
1	197448679	197448687	197448685	T	G	snp	1	1	1	1	.	.	.	CRB1(DOWNSTREAM);INTERGENIC(INTERGENIC)
1	197512786	197512793	197512789	A	C	snp	1	1	1	1	.	.	.	DENND1B(DOWNSTREAM);DENND1B(INTRON)
1	197534606	197534615	197534609	T	C	snp	1	1	1	1	.	.	.	DENND1B(INTRON)
1	197575395	197575408	197575398	CA	C	snp	1	0	0	0	.	.	.	DENND1B(EXON);DENND1B(INTRON)
1	197780336	197780344	197780338	A	C	snp	1	1	1	1	.	.	.	INTERGENIC(INTERGENIC)
1	197804342	197804353	197804349	AT	C	snp	1	0	0	0	.	.	.	INTERGENIC(INTERGENIC)
1	197826254	197826261	197826258	G	A	ins	1	0	0	0	.	.	.	INTERGENIC(INTERGENIC)
1	197826254	197826261	197826258	G	A	snp	1	1	1	1	.	.	.	INTERGENIC(INTERGENIC)
1	197880173	197880182	197880175	C	T	ins	0	1	1	1	.	.	.	C1orf53(DOWNSTREAM);INTERGENIC(INTERGENIC);LHX9(UPSTREAM)
1	197928655	197928662	197928658	A	C	ins	1	0	0	0	.	.	.	INTERGENIC(INTERGENIC)
1	198044730	198044753	198044744	TCCC	T	snp	1	0	0	0	.	.	.	INTERGENIC(INTERGENIC)
1	198172965	198172974	198172973	T	G	snp	1	1	1	1	.	.	.	NEK7(INTRON);NEK7(UPSTREAM)
1	198178273	198178282	198178278	TA	TG	ins	1	1	1	1	.	.	.	NEK7(INTRON)
1	198235862	198235871	198235869	TA	G	snp	1	1	1	1	.	.	.	NEK7(DOWNSTREAM);NEK7(INTRON)
1	198379414	198379425	198379422	AGC	A	snp	1	1	1	1	.	.	.	INTERGENIC(INTERGENIC)
1	198430881	198430889	198430885	A	G	snp	1	1	1	0	.	.	.	INTERGENIC(INTERGENIC)
1	198439323	198439332	198439326	A	C	snp	1	1	1	1	.	.	.	INTERGENIC(INTERGENIC)
1	198477320	198477333	198477323	AT	G	snp	1	0	0	1	.	.	.	INTERGENIC(INTERGENIC)
1	198486141	198486154	198486146	TA	C	snp	1	1	1	1	.	.	.	INTERGENIC(INTERGENIC)
1	198486257	198486270	198486267	TA	G	snp	1	1	1	1	.	.	.	INTERGENIC(INTERGENIC)
1	198564641	198564649	198564645	T	G	snp	1	1	1	1	.	.	.	INTERGENIC(INTERGENIC);RP11-553K8.2.1(DOWNSTREAM)
1	198584951	198584965	198584960	TTC	TCTTC	del	0	1	1	1	.	.	.	INTERGENIC(INTERGENIC)
1	198584951	198584965	198584962	TTC	TTCT	del	1	1	1	1	.	.	.	INTERGENIC(INTERGENIC)
1	198618267	198618275	198618270	A	C	snp	0	1	0	1	.	.	.	PTPRC(INTRON)
1	198665297	198665314	198665312	AT	G	snp	1	0	0	0	.	.	.	PTPRC(DOWNSTREAM);PTPRC(INTRON);PTPRC(UPSTREAM)
1	198716147	198716156	198716150	GT	G	snp	1	1	1	1	.	.	.	PTPRC(INTRON)
1	198754471	198754480	198754474	TA	G	snp	1	0	0	0	.	.	.	INTERGENIC(INTERGENIC)
1	199034901	199034908	199034906	A	G	snp	1	1	1	1	.	.	.	RP11-16L9.4.1(INTRON)
1	199043933	199043944	199043939	TA	C	snp	0	0	0	1	.	.	.	RP11-16L9.4.1(INTRON)
1	199065127	199065142	199065133	AG	G	snp	1	0	0	0	.	.	.	INTERGENIC(INTERGENIC)
1	199070158	199070166	199070162	A	G	snp	1	0	0	0	.	.	.	INTERGENIC(INTERGENIC)
1	199105913	199105921	199105918	A	C	snp	1	0	0	0	.	.	.	INTERGENIC(INTERGENIC)

1	199169373	199169380	199169374	T	G	snp	1	0	0	0	.	.	.	AC105941.1(UPSTREAM);RP11-382E9.1.1(INTRON)
1	199396672	199396679	199396676	A	G	snp	1	1	0	1	.	.	.	INTERGENIC(INTERGENIC)
1	199404202	199404213	199404210	AT	T	snp	1	1	0	1	.	.	.	INTERGENIC(INTERGENIC)
1	199419358	199419378	199419361	TGT	GTA	ins	1	1	1	1	.	.	.	INTERGENIC(INTERGENIC)
1	199447708	199447717	199447711	TA	G	snp	1	1	1	1	.	.	.	INTERGENIC(INTERGENIC)
1	199447743	199447752	199447746	TA	G	snp	1	1	1	1	.	.	.	INTERGENIC(INTERGENIC)
1	199531019	199531027	199531025	A	G	snp	1	1	1	1	.	.	.	INTERGENIC(INTERGENIC)
1	199531835	199531850	199531841	GAAG	GGAAGGAAG	del	1	1	1	1	.	.	.	INTERGENIC(INTERGENIC)
1	199570950	199570957	199570953	A	G	snp	1	0	1	0	.	.	.	INTERGENIC(INTERGENIC)
1	199644997	199645006	199645004	AT	C	snp	1	0	0	0	.	.	.	INTERGENIC(INTERGENIC)
1	199724754	199724765	199724763	AT	A	del	1	1	1	1	.	.	.	INTERGENIC(INTERGENIC);RP11-2L13.1.1(DOWNSTREAM)
1	199738384	199738399	199738391	AAAC	G	snp	0	0	0	1	.	.	.	INTERGENIC(INTERGENIC)
1	199858951	199858962	199858960	TG	A	snp	1	1	1	1	.	.	.	INTERGENIC(INTERGENIC);U6(DOWNSTREAM)
1	199872976	199872985	199872983	AT	A	del	1	1	1	1	.	.	.	INTERGENIC(INTERGENIC)
1	199910372	199910389	199910382	GA	T	snp	0	0	0	1	.	.	.	INTERGENIC(INTERGENIC)
1	199956485	199956493	199956487	A	C	snp	1	1	1	1	.	.	.	INTERGENIC(INTERGENIC)
1	200028241	200028258	200028251	AAT	C	snp	1	0	0	1	.	.	.	NR5A2(DOWNSTREAM);NR5A2(INTRON);U6(DOWNSTREAM)
1	200032325	200032333	200032326	T	G	snp	1	0	0	0	.	.	.	NR5A2(INTRON)
1	200052752	200052763	200052761	TA	G	snp	1	1	1	1	.	.	.	NR5A2(INTRON)
1	200086835	200086843	200086839	T	C	snp	1	1	1	1	.	.	.	NR5A2(INTRON)
1	200310899	200310910	200310902	GT	C	snp	1	1	1	1	.	.	.	C1orf98(DOWNSTREAM);INTERGENIC(INTERGENIC)
1	200329187	200329195	200329192	T	C	snp	1	0	0	0	.	.	.	C1orf98(INTRON)
1	200348326	200348335	200348331	AG	A	snp	1	0	0	0	.	.	.	C1orf98(UPSTREAM);INTERGENIC(INTERGENIC)
1	200440495	200440503	200440500	T	G	snp	1	0	0	0	.	.	.	INTERGENIC(INTERGENIC);RP11-469A15.2.1(UPSTREAM)
1	200511515	200511526	200511522	AG	T	snp	0	0	0	1	.	.	.	INTERGENIC(INTERGENIC)
1	200567341	200567349	200567344	T	C	snp	0	1	1	0	.	.	.	KIF14(INTRON)
1	200593411	200593419	200593418	T	G	ins	1	1	1	1	.	.	.	DDX59(UTR_3_PRIME);KIF14(UPSTREAM)
1	200604347	200604366	200604355	TTTA	C	snp	0	0	0	1	.	.	.	DDX59(INTRON)
1	200658170	200658179	200658171	T	G	ins	1	1	1	1	.	.	.	RP11-92G12.3.1(INTRON)
1	200658170	200658179	200658175	T	G	snp	1	1	1	1	.	.	.	RP11-92G12.3.1(INTRON)
1	200681960	200681968	200681967	T	C	snp	1	1	1	1	.	.	.	INTERGENIC(INTERGENIC)
1	200694835	200694844	200694839	A	C	ins	1	1	1	1	.	.	.	INTERGENIC(INTERGENIC)
1	200737778	200737786	200737784	T	G	snp	0	0	1	0	.	.	.	CAMSAP2(INTRON)
1	200753723	200753730	200753725	T	G	snp	0	0	0	1	.	.	.	CAMSAP2(INTRON)
1	200790297	200790311	200790308	CTT	C	del	1	1	1	1	.	.	.	CAMSAP2(INTRON)
1	200816520	200816537	200816522	TG	G	del	1	1	1	1	.	.	.	CAMSAP2(INTRON)
1	200851354	200851369	200851366	TC	CTC	del	1	1	1	1	.	.	.	INTERGENIC(INTERGENIC)
1	200851354	200851369	200851367	TC	TCT	del	1	1	1	1	.	.	.	INTERGENIC(INTERGENIC)
1	200905886	200905893	200905887	A	G	snp	1	0	0	0	.	.	.	C1orf81(INTRON);U6(UPSTREAM)
1	201015281	201015304	201015295	TATT	TTATTTA	del	1	1	1	1	.	.	.	CACNA1S(INTRON);RP11-168O16.2.1(DOWNSTREAM)
1	201049861	201049869	201049867	T	G	snp	0	1	1	1	.	.	.	CACNA1S(INTRON)
1	201408696	201408705	201408698	A	G	snp	0	0	1	0	.	.	.	INTERGENIC(INTERGENIC)
1	201432551	201432571	201432554	TTG	TGC	ins	1	1	0	1	.	.	.	INTERGENIC(INTERGENIC);PHLDA3(DOWNSTREAM);RP11-134G8.8.1(DOWNSTREAM)
1	201820139	201820158	201820149	TTTA	C	snp	1	0	0	0	.	.	.	IPO9(DOWNSTREAM);IPO9(INTRON)
1	201861903	201861911	201861906	G	T	snp	1	0	0	0	.	.	.	INTERGENIC(INTERGENIC);LMOD1(DOWNSTREAM);RP11-307B6.3.1(UPSTREAM);SHISA4(DOWNSTREAM)
1	201893789	201893796	201893793	A	G	snp	1	1	1	0	.	.	.	LMOD1(INTRON)
1	201952891	201952898	201952894	T	G	snp	1	0	0	0	.	.	.	RNPEP(INTRON);RP11-465N4.2.1(DOWNSTREAM)
1	201980846	201980854	201980853	A	G	snp	1	0	0	0	.	.	.	ELF3(EXON);ELF3(INTRON);ELF3(UPSTREAM);RP11-465N4.4.1(UPSTREAM);RP11-510N19.5.1(INTRON)
1	202028480	202028487	202028482	T	G	snp	1	0	0	0	.	.	.	INTERGENIC(INTERGENIC)
1	202062138	202062155	202062150	ATT	T	snp	1	1	1	1	.	.	.	INTERGENIC(INTERGENIC);RP11-510N19.4.1(UPSTREAM)
1	202242580	202242588	202242585	T	C	snp	1	1	1	1	.	.	.	LGR6(INTRON);LGR6(UPSTREAM)
1	202324493	202324500	202324496	A	C	snp	1	0	0	0	.	.	.	PPP1R12B(INTRON)
1	202341960	202341971	202341963	AGT	G	snp	1	0	0	0	.	.	.	PPP1R12B(INTRON);RP11-396D18.1.1(UPSTREAM)
1	202390780	202390789	202390782	A	C	snp	0	1	0	1	.	.	.	PPP1R12B(INTRON)
1	202573400	202573407	202573406	C	T	snp	1	1	1	1	.	.	.	RP11-569A11.1.1(EXON);SYT2(INTRON)
1	202575902	202575913	202575908	GA	C	snp	1	0	0	0	.	.	.	RP11-569A11.1.1(DOWNSTREAM);SYT2(INTRON)
1	202616585	202616592	202616589	C	A	snp	1	1	1	1	.	.	.	SYT2(INTRON);SYT2(UPSTREAM)
1	202722793	202722802	202722794	T	G	ins	0	1	1	1	.	.	.	KDM5B(INTRON);KDM5B(UPSTREAM)
1	202798499	202798506	202798501	A	G	snp	1	1	1	1	.	.	.	INTERGENIC(INTERGENIC);RP11-480I12.4.1(DOWNSTREAM)
1	202803308	202803317	202803312	A	C	ins	0	0	0	1	.	.	.	INTERGENIC(INTERGENIC)
1	202827394	202827403	202827398	A	G	snp	1	1	1	1	.	.	.	RP11-480I12.5.1(INTRON);RP11-480I12.7.1(UPSTREAM)
1	202897425	202897432	202897431	A	G	snp	1	1	1	1	.	.	.	KLHL12(INTRON);KLHL12(UPSTREAM)
1	202934753	202934768	202934761	CTTT	T	ins	1	1	1	1	.	.	.	

CYB5R1(DOWNSTREAM);CYB5R1(INTRON);CYB5R1(UPSTREAM)														
1	202934753	202934768	202934763	CTTT	TC	del	1	1	1	1	.	.	.	
CYB5R1(DOWNSTREAM);CYB5R1(INTRON);CYB5R1(UPSTREAM)														
1	202934753	202934768	202934764	CTTT	C	del	1	1	1	1	.	.	.	
CYB5R1(DOWNSTREAM);CYB5R1(INTRON);CYB5R1(UPSTREAM)														
1	203162395	203162404	203162401	A	G	snp	1	1	1	1	.	.	.	INTERGENIC(INTERGENIC)
1	203288096	203288105	203288098	T	C	snp	1	0	0	0	.	.	.	INTERGENIC(INTERGENIC);U6(DOWNSTREAM)
1	203470662	203470671	203470664	CT	T	snp	0	1	1	0	.	.	.	OPTC(DOWNSTREAM);OPTC(INTRON)
1	203492889	203492904	203492900	AAAC	A	snp	1	1	1	0	.	.	.	INTERGENIC(INTERGENIC)
1	203496231	203496238	203496237	C	A	ins	1	1	1	1	.	.	.	INTERGENIC(INTERGENIC)
1	203520583	203520592	203520591	T	C	snp	1	0	0	0	.	.	.	INTERGENIC(INTERGENIC)
1	203531129	203531140	203531134	GAA	G	snp	1	1	1	1	.	.	.	INTERGENIC(INTERGENIC)
1	203540576	203540591	203540587	TTTC	T	ins	1	1	1	1	.	.	.	INTERGENIC(INTERGENIC)
1	203565063	203565078	203565070	AG	A	del	0	0	1	0	.	.	.	INTERGENIC(INTERGENIC)
1	203606486	203606494	203606493	G	T	snp	1	0	0	0	.	.	.	ATP2B4(INTRON)
1	203667528	203667542	203667536	CCA	G	snp	1	1	1	1	.	.	.	ATP2B4(INTRON)
1	203686483	203686491	203686488	T	G	snp	1	0	0	0	.	.	.	ATP2B4(INTRON)
1	203728426	203728433	203728428	T	C	snp	1	1	1	1	.	.	.	INTERGENIC(INTERGENIC)
1	203771853	203771862	203771858	T	TG	ins	1	1	1	1	.	.	.	
RP11-397P13.6.1(DOWNSTREAM);ZBED6(DOWNSTREAM);ZC3H11A(DOWNSTREAM);ZC3H11A(INTRON);ZC3H11A(UTR_5_PRIME)														
1	203874765	203874776	203874773	TTA	G	snp	0	0	1	0	.	.	.	INTERGENIC(INTERGENIC);RP11-450D21.1.1(UPSTREAM)
1	203928983	203928997	203928994	AAT	AATAA	del	1	0	0	0	.	.	.	CBX1P3(DOWNSTREAM);INTERGENIC(INTERGENIC)
1	203931385	203931412	203931402	AAAC	G	snp	0	0	0	1	.	.	.	INTERGENIC(INTERGENIC)
1	203935492	203935499	203935495	T	G	snp	1	0	0	0	.	.	.	INTERGENIC(INTERGENIC)
1	203961247	203961258	203961254	TCT	TTCT	del	1	0	0	0	.	.	.	INTERGENIC(INTERGENIC)
1	203991535	203991548	203991546	AT	A	del	1	1	1	1	.	.	.	INTERGENIC(INTERGENIC)
1	204022399	204022413	204022408	AAT	G	snp	1	1	1	1	.	.	.	INTERGENIC(INTERGENIC)
1	204023110	204023117	204023113	A	G	snp	1	1	1	1	.	.	.	INTERGENIC(INTERGENIC)
1	204033508	204033523	204033519	TCCC	T	snp	1	0	0	0	.	.	.	INTERGENIC(INTERGENIC)
1	204081659	204081668	204081666	TC	T	snp	1	0	0	0	.	.	.	SOX13(INTRON);SOX13(UPSTREAM)
1	204371721	204371729	204371722	T	G	snp	1	1	1	0	.	.	.	PPP1R15B(DOWNSTREAM);RP11-739N20.2.1(INTRON)
1	204411141	204411152	204411144	CAC	T	snp	0	1	1	1	.	.	.	PIK3C2B(INTRON)
1	204474148	204474157	204474153	A	G	snp	1	0	0	0	.	.	.	INTERGENIC(INTERGENIC)
1	204524989	204524996	204524991	A	G	snp	1	0	0	0	.	.	.	MDM4(DOWNSTREAM);MDM4(INTRON);MDM4(UTR_3_PRIME)
1	204553102	204553111	204553104	A	C	snp	1	1	1	1	.	.	.	INTERGENIC(INTERGENIC)
1	204632060	204632068	204632065	A	G	snp	1	1	1	1	.	.	.	LRRN2(INTRON);RP11-23I7.1.1(UPSTREAM)
1	204702533	204702548	204702535	TC	C	snp	1	0	0	0	.	.	.	INTERGENIC(INTERGENIC)
1	204728537	204728548	204728542	AAC	A	snp	1	1	1	0	.	.	.	INTERGENIC(INTERGENIC)
1	204759909	204759924	204759916	TTCC	T	snp	0	0	0	1	.	.	.	INTERGENIC(INTERGENIC)
1	204827035	204827049	204827046	CTT	C	del	1	1	1	1	.	.	.	NFASC(INTRON)
1	204900211	204900220	204900213	A	G	snp	1	0	0	0	.	.	.	NFASC(INTRON)
1	204966741	204966752	204966749	AC	G	snp	1	1	1	1	.	.	.	NFASC(EXON);NFASC(INTRON)
1	204980336	204980343	204980342	C	CG	ins	1	0	0	1	.	.	.	NFASC(DOWNSTREAM);NFASC(INTRON)
1	205095846	205095854	205095848	G	GA	ins	1	1	1	1	.	.	.	INTERGENIC(INTERGENIC);RBBP5(UPSTREAM)
1	205167322	205167331	205167328	A	G	snp	0	1	1	1	.	.	.	DSTYK(INTRON);RP11-383G10.3.1(UPSTREAM)
1	205288566	205288575	205288571	CA	T	snp	0	1	1	0	.	.	.	NUAK2(INTRON)
1	205369972	205369981	205369973	C	T	ins	1	0	0	0	.	.	.	LEMD1(INTRON)
1	205400855	205400863	205400858	T	C	snp	1	0	0	0	.	.	.	LEMD1(INTRON)
1	205477459	205477468	205477463	AC	T	snp	0	1	0	1	.	.	.	CDK18(INTRON)
1	205508045	205508052	205508047	T	C	snp	0	1	0	0	.	.	.	INTERGENIC(INTERGENIC)
1	205612072	205612085	205612080	TG	G	snp	1	1	1	1	.	.	.	INTERGENIC(INTERGENIC)
1	205666307	205666318	205666310	CAC	T	snp	1	0	0	0	.	.	.	INTERGENIC(INTERGENIC)
1	205684223	205684230	205684228	A	G	snp	1	0	0	0	.	.	.	NUCKS1(DOWNSTREAM);NUCKS1(UTR_3_PRIME)
1	205822565	205822580	205822575	AT	G	snp	1	0	0	0	.	.	.	INTERGENIC(INTERGENIC);PM20D1(UPSTREAM)
1	205865728	205865743	205865737	CTTT	TTC	del	0	1	0	1	.	.	.	INTERGENIC(INTERGENIC)
1	205886254	205886261	205886258	A	G	ins	1	1	0	1	.	.	.	SLC26A9(INTRON)
1	206209016	206209024	206209023	T	G	snp	1	1	1	1	.	.	.	INTERGENIC(INTERGENIC)
1	206215988	206215997	206215989	A	C	ins	1	0	0	0	.	.	.	RP11-38J22.3.1(INTRON)
1	206217164	206217183	206217168	CAAA	A	snp	1	1	0	1	.	.	.	RP11-38J22.3.1(INTRON)
1	206295853	206295870	206295866	AT	A	del	1	0	0	0	.	.	.	RP11-38J22.6.1(DOWNSTREAM);RP11-38J22.6.1(INTRON)
1	206295853	206295870	206295867	AT	T	snp	1	0	0	0	.	.	.	RP11-38J22.6.1(DOWNSTREAM);RP11-38J22.6.1(INTRON)
1	206304780	206304789	206304784	T	C	snp	1	1	0	0	.	.	.	RP11-38J22.6.1(INTRON)
1	206739554	206739563	206739558	CA	T	snp	1	0	0	0	.	.	.	RASSF5(INTRON)
1	206754955	206754964	206754962	AG	A	del	1	1	1	1	.	.	.	
EIF2D(INTRON);EIF2D(UPSTREAM);RASSF5(INTRON);RASSF5(UPSTREAM)														
1	206896619	206896626	206896622	C	A	snp	1	1	1	1	.	.	.	MAPKAPK2(INTRON)
1	206948562	206948570	206948566	T	C	snp	1	1	0	1	.	.	.	IL10(UPSTREAM);INTERGENIC(INTERGENIC)

1	207190486	207190505	207190497	CATC	CATCCATT	ins	1	1	1	1	.	.	.	C1orf116(DOWNSTREAM);INTERGENIC(INTERGENIC)
1	207190486	207190505	207190501	CATC	CATT	ins	1	1	1	1	.	.	.	C1orf116(DOWNSTREAM);INTERGENIC(INTERGENIC)
1	207243363	207243374	207243366	AC	A	ins	1	1	1	1	.	.	.	PFKFB2(INTRON);PFKFB2(UPSTREAM)
1	207270519	207270534	207270526	AAAT	A	snp	1	0	0	0	.	.	.	C4BPB(DOWNSTREAM);C4BPB(INTRON);C4BPB(UPSTREAM)
1	207285447	207285456	207285454	A	G	snp	1	0	0	0	.	.	.	C4BPA(INTRON)
1	207404341	207404349	207404344	T	C	snp	1	1	1	1	.	.	.	C4BPAP2(DOWNSTREAM);INTERGENIC(INTERGENIC)
1	207593560	207593567	207593565	A	G	snp	1	0	1	1	.	.	.	INTERGENIC(INTERGENIC)
1	207625534	207625542	207625541	T	G	snp	0	1	1	1	.	.	.	CR2(UPSTREAM);INTERGENIC(INTERGENIC)
1	207629413	207629422	207629416	AT	C	snp	1	1	1	1	.	.	.	CR2(INTRON)
1	207641017	207641032	207641023	ATTG	C	snp	1	1	1	1	.	.	.	CR2(DOWNSTREAM);CR2(INTRON);CR2(UPSTREAM)
1	207848349	207848357	207848351	T	G	snp	1	0	0	0	.	.	.	CR1L(INTRON);CR1L(UPSTREAM)
1	208006745	208006754	208006749	AT	G	snp	1	1	1	1	.	.	.	INTERGENIC(INTERGENIC)
1	208068160	208068169	208068167	A	G	snp	0	1	1	1	.	.	.	CD34(INTRON);CD34(UPSTREAM)
1	208118672	208118679	208118675	T	C	snp	1	1	1	1	.	.	.	INTERGENIC(INTERGENIC)
1	208317593	208317608	208317601	ATGG	G	snp	0	1	1	1	.	.	.	PLXNA2(INTRON)
1	208376428	208376437	208376434	GT	T	snp	0	1	1	0	.	.	.	PLXNA2(INTRON)
1	208376428	208376437	208376435	GT	G	snp	0	1	1	0	.	.	.	PLXNA2(INTRON)
1	208468842	208468857	208468844	TG	GC	ins	1	1	1	0	.	.	.	INTERGENIC(INTERGENIC)
1	208503202	208503213	208503208	GT	TA	ins	1	0	0	0	.	.	.	INTERGENIC(INTERGENIC)
1	208527712	208527720	208527715	A	C	snp	1	1	0	0	.	.	.	INTERGENIC(INTERGENIC)
1	208552717	208552732	208552728	ACAG	C	snp	1	0	0	0	.	.	.	INTERGENIC(INTERGENIC)
1	208646422	208646429	208646428	C	T	ins	1	1	0	1	.	.	.	INTERGENIC(INTERGENIC)
1	208663916	208663925	208663917	T	G	ins	1	1	1	1	.	.	.	INTERGENIC(INTERGENIC)
1	208679820	208679828	208679826	A	C	ins	1	0	0	0	.	.	.	INTERGENIC(INTERGENIC)
1	208933992	208933999	208933995	T	C	snp	0	1	1	1	.	.	.	INTERGENIC(INTERGENIC)
1	208939842	208939850	208939849	A	TG	ins	1	1	0	0	.	.	.	INTERGENIC(INTERGENIC)
1	209008487	209008496	209008491	CA	T	snp	0	0	1	0	.	.	.	INTERGENIC(INTERGENIC)
1	209119897	209119905	209119898	A	C	snp	1	0	0	0	.	.	.	INTERGENIC(INTERGENIC)
1	209154025	209154032	209154026	T	G	snp	0	1	1	0	.	.	.	INTERGENIC(INTERGENIC)
1	209258732	209258749	209258734	AT	T	del	1	0	0	0	.	.	.	INTERGENIC(INTERGENIC)
1	209261573	209261582	209261575	A	C	snp	1	0	0	0	.	.	.	INTERGENIC(INTERGENIC)
1	209899943	209899952	209899945	A	C	snp	0	1	1	0	.	.	.	HSD11B1(INTRON);RP1-28O10.1.1(UPSTREAM)
1	209945246	209945255	209945250	GT	T	ins	1	1	1	1	.	.	.	TRAF3IP3(INTRON);TRAF3IP3(UPSTREAM)
1	210165481	210165489	210165487	T	G	snp	1	0	0	0	.	.	.	SYT14(INTRON)
1	210203475	210203482	210203477	T	TTTTTG	ins	0	1	1	1	.	.	.	SYT14(INTRON)
1	210256720	210256727	210256723	T	G	snp	1	1	1	1	.	.	.	SYT14(INTRON)
1	210307410	210307429	210307421	TTTA	G	snp	1	0	0	0	.	.	.	SYT14(INTRON)
1	210308030	210308041	210308035	TCC	T	snp	1	0	0	0	.	.	.	SYT14(INTRON)
1	210350139	210350147	210350140	T	C	snp	0	1	1	0	.	.	.	INTERGENIC(INTERGENIC)
1	210394009	210394020	210394014	TG	T	snp	1	0	0	0	.	.	.	INTERGENIC(INTERGENIC)
1	210408392	210408401	210408397	T	C	snp	1	0	0	0	.	.	.	C1orf133(UPSTREAM);SERTAD4(INTRON);SERTAD4(UPSTREAM)
1	210450009	210450028	210450016	AATT	A	snp	1	0	0	0	.	.	.	INTERGENIC(INTERGENIC)
1	210463001	210463010	210463006	A	G	snp	1	1	1	1	.	.	.	INTERGENIC(INTERGENIC)
1	210561170	210561181	210561174	GGT	C	snp	1	0	0	0	.	.	.	HHAT(INTRON)
1	210593994	210594005	210594001	ATA	G	snp	1	1	1	1	.	.	.	HHAT(INTRON)
1	210699090	210699097	210699091	A	G	snp	1	1	0	1	.	.	.	HHAT(INTRON)
1	210715841	210715858	210715851	AT	G	snp	1	1	1	1	.	.	.	HHAT(INTRON)
1	210856603	210856611	210856604	T	G	snp	0	1	0	1	.	.	.	KCNH1(UTR_3_PRIME)
1	210883050	210883065	210883059	AGAA	A	del	0	1	1	0	.	.	.	KCNH1(INTRON)
1	210883065	210883082	210883067	AG	G	del	1	1	1	1	.	.	.	KCNH1(INTRON)
1	211030122	211030135	211030131	TA	TATATT	ins	1	1	1	1	.	.	.	IPO8P1(DOWNSTREAM);KCNH1(INTRON)
1	211134659	211134666	211134663	T	C	snp	1	1	1	1	.	.	.	KCNH1(INTRON)
1	211175369	211175376	211175373	G	A	snp	1	1	1	1	.	.	.	KCNH1(INTRON)
1	211218384	211218401	211218391	AT	G	snp	1	1	1	1	.	.	.	KCNH1(INTRON)
1	211231950	211231957	211231953	A	G	snp	0	1	0	1	.	.	.	KCNH1(INTRON)
1	211237903	211237910	211237908	A	G	snp	1	0	0	0	.	.	.	KCNH1(INTRON)
1	211247642	211247653	211247644	TA	C	snp	1	0	0	0	.	.	.	KCNH1(INTRON)
1	211317167	211317174	211317171	A	G	snp	1	0	0	0	.	.	.	INTERGENIC(INTERGENIC)
1	211352711	211352734	211352720	TTG	A	snp	1	1	1	1	.	.	.	INTERGENIC(INTERGENIC);RP11-543B16.3.1(DOWNSTREAM)
1	211548494	211548503	211548500	GT	T	snp	0	1	1	0	.	.	.	INTERGENIC(INTERGENIC);TRAF5(DOWNSTREAM)
1	211592847	211592856	211592852	A	C	snp	1	0	0	0	.	.	.	LINC00467(INTRON);SNX25P1(DOWNSTREAM)
1	211615082	211615089	211615085	T	G	ins	0	0	0	1	.	.	.	ARPC3P2(UPSTREAM);INTERGENIC(INTERGENIC)
1	211615082	211615089	211615086	T	G	snp	1	0	0	1	.	.	.	ARPC3P2(UPSTREAM);INTERGENIC(INTERGENIC)
1	211820099	211820122	211820106	TTCT	C	snp	1	1	1	1	.	.	.	RP11-354K1.1.1(INTRON);RP11-354K1.2.1(DOWNSTREAM)

1	211859526	211859533	211859529	C	T	snp	0	1	0	0	.	.	.	RP11-122M14.1.1(INTRON);RP11-122M14.2.1(UPSTREAM)
1	211885320	211885327	211885322	T	TTTTTC	ins	1	0	0	0	.	.	.	INTERGENIC(INTERGENIC);RP11-122M14.3.1(DOWNSTREAM)
1	212093156	212093173	212093166	TTA	C	snp	1	1	0	1	.	.	.	INTERGENIC(INTERGENIC)
1	212226072	212226083	212226081	AT	A	del	1	1	1	1	.	.	.	DTL(INTRON);RP11-565J7.3.1(UPSTREAM)
1	212345550	212345561	212345555	GTG	C	snp	1	1	0	1	.	.	.	RP11-15I11.2.1(INTRON)
1	212350852	212350861	212350857	A	G	snp	1	1	1	1	.	.	.	RP11-15I11.2.1(INTRON)
1	212373829	212373838	212373835	A	G	snp	0	1	1	1	.	.	.	INTERGENIC(INTERGENIC)
1	212375382	212375395	212375393	CT	C	del	1	1	1	1	.	.	.	INTERGENIC(INTERGENIC)
1	212384018	212384038	212384033	CTT	TTCTT	del	0	1	0	1	.	.	.	INTERGENIC(INTERGENIC)
1	212426119	212426130	212426127	CA	G	snp	0	1	0	1	.	.	.	INTERGENIC(INTERGENIC)
1	212446659	212446668	212446664	T	G	snp	1	0	0	0	.	.	.	INTERGENIC(INTERGENIC)
1	212482357	212482370	212482360	AT	C	snp	0	0	0	1	.	.	.	PPP2R5A(INTRON);RP11-384C4.3.1(DOWNSTREAM);RP11-384C4.7.1(INTRON)
1	212501672	212501680	212501675	A	G	snp	1	0	0	0	.	.	.	PPP2R5A(INTRON);RP11-384C4.7.1(INTRON)
1	212505147	212505158	212505155	GT	T	snp	1	1	1	1	.	.	.	PPP2R5A(INTRON);RP11-384C4.7.1(UPSTREAM)
1	212545652	212545661	212545655	A	C	snp	1	1	0	0	.	.	.	TMEM206(DOWNSTREAM);TMEM206(INTRON)
1	212613199	212613207	212613205	C	A	snp	0	1	1	0	.	.	.	NENF(DOWNSTREAM);NENF(INTRON)
1	212617390	212617398	212617396	A	C	snp	1	1	1	1	.	.	.	NENF(INTRON)
1	212950063	212950080	212950076	AAC	CAAC	del	1	0	0	1	.	.	.	NSL1(INTRON)
1	212957349	212957357	212957354	A	G	snp	1	1	1	1	.	.	.	NSL1(DOWNSTREAM);NSL1(INTRON)
1	212977390	212977398	212977397	A	G	snp	1	0	0	0	.	.	.	TATDN3(INTRON)
1	212980264	212980275	212980272	AT	T	snp	1	0	0	0	.	.	.	TATDN3(DOWNSTREAM);TATDN3(INTRON)
1	212995094	212995109	212995105	AAAT	C	snp	1	0	0	1	.	.	.	INTERGENIC(INTERGENIC);RP11-348H3.2.1(UPSTREAM)
1	213057641	213057650	213057649	A	G	snp	1	1	1	1	.	.	.	FLVCR1(INTRON)
1	213125698	213125713	213125701	TG	T	snp	1	0	0	0	.	.	.	VASH2(DOWNSTREAM);VASH2(INTRON)
1	213500580	213500607	213500600	AAAG	G	snp	1	0	0	0	.	.	.	INTERGENIC(INTERGENIC)
1	213575128	213575143	213575137	TTTA	G	snp	1	0	0	0	.	.	.	INTERGENIC(INTERGENIC)
1	213655861	213655868	213655864	T	G	snp	0	1	1	1	.	.	.	INTERGENIC(INTERGENIC)
1	213718777	213718794	213718779	AG	G	del	1	1	1	1	.	.	.	RP11-554K11.2.1(INTRON)
1	213757190	213757198	213757196	A	G	snp	0	0	1	1	.	.	.	INTERGENIC(INTERGENIC)
1	213761446	213761457	213761455	TC	G	snp	1	1	1	1	.	.	.	INTERGENIC(INTERGENIC)
1	213859283	213859291	213859290	T	G	snp	1	1	0	1	.	.	.	INTERGENIC(INTERGENIC)
1	213964513	213964522	213964518	A	C	snp	1	1	1	1	.	.	.	RP11-323I1.1.1(INTRON)
1	214037460	214037473	214037469	CA	C	snp	0	0	1	1	.	.	.	RP11-478J18.1.1(INTRON)
1	214105975	214105984	214105978	T	C	ins	0	1	0	1	.	.	.	RP11-478J18.1.1(INTRON)
1	214111243	214111250	214111247	A	G	snp	1	1	1	1	.	.	.	RP11-478J18.1.1(INTRON)
1	214173371	214173384	214173376	TC	TCTG	ins	1	1	1	1	.	.	.	PROX1(DOWNSTREAM);PROX1(INTRON);PROX1-IT1(DOWNSTREAM)
1	214181752	214181769	214181765	AAC	C	del	1	1	1	1	.	.	.	PROX1(INTRON)
1	214265914	214265922	214265921	C	A	snp	1	1	1	1	.	.	.	INTERGENIC(INTERGENIC)
1	214332130	214332141	214332136	AG	GAGAG	del	1	0	0	0	.	.	.	INTERGENIC(INTERGENIC)
1	214343706	214343713	214343707	A	C	snp	0	1	0	1	.	.	.	INTERGENIC(INTERGENIC)
1	214370978	214370998	214370992	TAA	G	snp	1	0	0	0	.	.	.	INTERGENIC(INTERGENIC)
1	214402787	214402796	214402794	AT	C	snp	1	0	0	0	.	.	.	INTERGENIC(INTERGENIC)
1	214403053	214403062	214403059	T	C	snp	1	1	1	1	.	.	.	INTERGENIC(INTERGENIC)
1	214412696	214412703	214412700	A	G	snp	0	1	1	0	.	.	.	INTERGENIC(INTERGENIC)
1	214412696	214412703	214412702	A	C	snp	1	1	1	1	.	.	.	INTERGENIC(INTERGENIC)
1	214414397	214414406	214414402	AT	C	snp	1	0	0	0	.	.	.	INTERGENIC(INTERGENIC)
1	214536180	214536187	214536186	A	G	snp	1	1	0	1	.	.	.	PTPN14(INTRON)
1	214641902	214641909	214641906	A	AAAGG	ins	1	1	1	1	.	.	.	PTPN14(INTRON);PTPN14(UPSTREAM)
1	214715444	214715455	214715447	AC	T	snp	1	1	1	1	.	.	.	PTPN14(INTRON)
1	214749048	214749075	214749055	AAAC	A	snp	1	1	1	1	.	.	.	INTERGENIC(INTERGENIC)
1	214792670	214792683	214792673	TG	T	snp	0	1	0	0	.	.	.	CENPF(DOWNSTREAM);CENPF(INTRON)
1	214858860	214858875	214858873	AT	C	snp	1	1	0	1	.	.	.	INTERGENIC(INTERGENIC)
1	214922480	214922488	214922484	T	C	snp	1	1	1	1	.	.	.	INTERGENIC(INTERGENIC)
1	214925146	214925160	214925149	AAT	G	snp	1	0	0	0	.	.	.	INTERGENIC(INTERGENIC)
1	214971473	214971480	214971474	T	C	snp	1	0	0	0	.	.	.	INTERGENIC(INTERGENIC)
1	215043910	215043921	215043916	GGC	A	snp	1	1	1	1	.	.	.	GAPDHP24(UPSTREAM);INTERGENIC(INTERGENIC)
1	215106704	215106717	215106707	AT	C	snp	1	0	0	0	.	.	.	INTERGENIC(INTERGENIC)
1	215118914	215118923	215118919	T	TTTC	ins	1	1	1	1	.	.	.	INTERGENIC(INTERGENIC);RP11-323K10.1.1(DOWNSTREAM)
1	215160494	215160502	215160498	T	C	snp	1	1	0	1	.	.	.	INTERGENIC(INTERGENIC)
1	215348793	215348802	215348798	T	G	ins	0	1	0	1	.	.	.	KCNK2(INTRON)
1	215403361	215403374	215403364	TA	TC	ins	1	0	0	0	.	.	.	KCNK2(INTRON)
1	215406821	215406832	215406826	AC	G	snp	1	1	1	1	.	.	.	KCNK2(INTRON)
1	215445554	215445561	215445559	T	C	snp	1	1	1	1	.	.	.	INTERGENIC(INTERGENIC)

1	215542118	215542125	215542120	T	C	snp	1	0	0	0	.	.	.	INTERGENIC(INTERGENIC)	
1	215556696	215556703	215556699	A	G	snp	0	1	1	1	.	.	.	INTERGENIC(INTERGENIC)	
1	215561162	215561171	215561164	A	C	snp	1	0	0	0	.	.	.	INTERGENIC(INTERGENIC)	
1	215748145	215748154	215748148	TA	G	snp	1	0	0	0	.	.	.	KCTD3(INTRON)	
1	215748145	215748154	215748152	TA	G	snp	1	0	0	0	.	.	.	KCTD3(INTRON)	
1	215812320	215812328	215812326	A	C	snp	1	0	1	0	.	.	.	USH2A(INTRON)	
1	215921213	215921232	215921228	TTTG	T	snp	1	1	1	1	.	.	.	USH2A(INTRON)	
1	215972002	215972009	215972003	T	G	snp	1	1	1	1	.	.	.	USH2A(INTRON)	
1	216003447	216003454	216003448	T	G	snp	1	0	0	0	.	.	.	USH2A(INTRON)	
1	216009807	216009822	216009811	TA	A	snp	1	0	0	0	.	.	.	USH2A(INTRON)	
1	216009807	216009822	216009820	TA	G	snp	0	0	1	0	.	.	.	USH2A(INTRON)	
1	216035912	216035919	216035913	A	C	snp	1	1	0	1	.	.	.	USH2A(INTRON)	
1	216056774	216056783	216056779	T	C	snp	1	1	0	1	.	.	.	RP5-1111A8.3.1(UPSTREAM);USH2A(INTRON)	
1	216093428	216093435	216093434	A	C	ins	1	1	1	1	.	.	.	USH2A(INTRON)	
1	216097995	216098003	216098000	A	G	snp	1	0	0	0	.	.	.	USH2A(INTRON)	
1	216121634	216121642	216121638	T	C	snp	1	0	0	0	.	.	.	USH2A(INTRON)	
1	216141672	216141679	216141677	T	G	snp	1	0	1	0	.	.	.	USH2A(INTRON)	
1	216147422	216147433	216147431	AC	G	snp	1	0	0	0	.	.	.	USH2A(INTRON)	
1	216160394	216160401	216160398	A	G	snp	1	0	0	0	.	.	.	USH2A(INTRON)	
1	216192140	216192157	216192153	TTA	ATC	ins	1	1	1	1	.	.	.	USH2A(INTRON)	
1	216195372	216195387	216195382	CT	G	snp	1	1	1	1	.	.	.	USH2A(INTRON)	
1	216404576	216404591	216404580	AT	G	snp	0	1	0	1	.	.	.	USH2A(INTRON)	
1	216404576	216404591	216404582	AT	G	snp	1	1	0	1	.	.	.	USH2A(INTRON)	
1	216446825	216446833	216446831	A	G	snp	1	1	1	1	.	.	.	USH2A(INTRON)	
1	216495187	216495196	216495191	T	G	snp	1	1	1	1	.	.	.	USH2A(INTRON)	
1	216621052	216621060	216621057	A	G	snp	1	0	0	0	.	.	.	INTERGENIC(INTERGENIC)	
1	216693169	216693180	216693174	AAG	A	snp	1	1	1	1	.	.	.	ESRRG(INTRON)	
1	216703647	216703674	216703660	TCTA	TATCTATT		ins	1	1	1	1	.	.	.	ESRRG(INTRON)
1	216745909	216745918	216745913	A	G	snp	1	1	1	1	.	.	.	ESRRG(INTRON)	
1	216917029	216917040	216917034	TCA	G	snp	1	1	1	1	.	.	.	ESRRG(INTRON)	
1	216940611	216940626	216940621	AC	G	snp	1	1	1	1	.	.	.	ESRRG(INTRON)	
1	217128717	217128725	217128719	T	C	snp	1	1	1	1	.	.	.	ESRRG(INTRON)	
1	217210184	217210193	217210188	A	AG	ins	0	1	1	1	.	.	.	ESRRG(INTRON)	
1	217258792	217258801	217258798	T	C	snp	1	1	1	1	.	.	.	ESRRG(INTRON)	
1	217318591	217318600	217318595	T	C	snp	1	1	1	1	.	.	.	INTERGENIC(INTERGENIC)	
1	217388500	217388507	217388502	C	CCG	ins	1	1	1	1	.	.	.	INTERGENIC(INTERGENIC)	
1	217389024	217389031	217389029	T	G	snp	1	0	0	0	.	.	.	INTERGENIC(INTERGENIC)	
1	217404287	217404296	217404293	T	G	snp	1	1	1	1	.	.	.	INTERGENIC(INTERGENIC)	
1	217476521	217476528	217476525	T	G	snp	1	0	0	0	.	.	.	INTERGENIC(INTERGENIC)	
1	217500269	217500286	217500281	TAA	C	snp	1	1	1	1	.	.	.	INTERGENIC(INTERGENIC)	
1	217558809	217558816	217558812	A	G	snp	1	1	1	1	.	.	.	INTERGENIC(INTERGENIC)	
1	217582679	217582686	217582680	A	G	snp	1	0	0	0	.	.	.	INTERGENIC(INTERGENIC)	
1	217602810	217602817	217602811	T	G	snp	1	1	1	1	.	.	.	GPATCH2(UTR_3_PRIME)	
1	217751479	217751486	217751484	G	A	snp	1	0	0	0	.	.	.	GPATCH2(INTRON)	
1	217760260	217760267	217760264	A	C	snp	1	1	1	1	.	.	.	GPATCH2(INTRON)	
1	217764594	217764603	217764598	AT	G	snp	1	0	0	0	.	.	.	GPATCH2(INTRON)	
1	217792148	217792155	217792149	A	G	snp	1	1	1	1	.	.	.	GPATCH2(INTRON)	
1	217872057	217872070	217872064	TG	A	snp	1	0	0	0	.	.	.	SPATA17(INTRON)	
1	217886597	217886606	217886598	T	G	snp	1	0	0	0	.	.	.	SPATA17(INTRON)	
1	217900294	217900302	217900301	A	C	snp	1	0	0	0	.	.	.	SPATA17(INTRON)	
1	217949030	217949045	217949042	TA	G	snp	1	1	1	1	.	.	.	SPATA17(DOWNSTREAM);SPATA17(INTRON)	
1	218117518	218117525	218117521	T	C	snp	1	0	0	0	.	.	.	INTERGENIC(INTERGENIC)	
1	218123001	218123010	218123006	AT	C	snp	1	1	1	1	.	.	.	INTERGENIC(INTERGENIC)	
1	218196136	218196143	218196137	T	G	snp	1	1	1	0	.	.	.	INTERGENIC(INTERGENIC)	
1	218261477	218261484	218261483	A	C	snp	1	1	1	1	.	.	.	INTERGENIC(INTERGENIC)	
1	218329943	218329950	218329946	G	GC	ins	1	1	1	0	.	.	.	INTERGENIC(INTERGENIC)	
1	218381164	218381172	218381166	T	TG	ins	1	1	0	1	.	.	.	INTERGENIC(INTERGENIC)	
1	218381164	218381172	218381167	T	G	ins	1	1	0	0	.	.	.	INTERGENIC(INTERGENIC)	
1	218381174	218381183	218381176	T	TG	ins	1	0	0	0	.	.	.	INTERGENIC(INTERGENIC)	
1	218436705	218436719	218436713	AGA	AGAAG	del	0	1	1	0	.	.	.	INTERGENIC(INTERGENIC)	
1	218457166	218457180	218457171	TGG	T	snp	1	0	0	0	.	.	.	INTERGENIC(INTERGENIC);RRP15(UPSTREAM)	
1	218494097	218494106	218494101	A	C	snp	1	0	0	0	.	.	.	RRP15(INTRON)	
1	218521418	218521425	218521422	A	G	snp	1	0	0	0	.	.	.	RP11-224O19.2.1(UPSTREAM);TGFB2(INTRON)	
1	218552116	218552125	218552123	T	TC	ins	1	0	0	0	.	.	.	TGFB2(INTRON)	
1	218813626	218813635	218813634	A	C	ins	0	0	1	0	.	.	.	INTERGENIC(INTERGENIC)	
1	218842194	218842203	218842201	A	C	snp	1	1	1	1	.	.	.	INTERGENIC(INTERGENIC)	
1	218843290	218843298	218843291	T	G	snp	0	1	0	1	.	.	.	INTERGENIC(INTERGENIC)	

1	218953160	218953169	218953166	AC	C	snp	1	1	1	1	.	.	.	INTERGENIC(INTERGENIC)
1	219084119	219084128	219084122	AC	G	snp	0	0	1	0	.	.	.	INTERGENIC(INTERGENIC);RP11-412H9.2.1(DOWNSTREAM)
1	219085468	219085475	219085469	A	G	snp	1	1	1	1	.	.	.	INTERGENIC(INTERGENIC);RP11-412H9.2.1(DOWNSTREAM)
1	219171325	219171332	219171330	A	C	snp	0	0	1	0	.	.	.	INTERGENIC(INTERGENIC)
1	219178347	219178370	219178352	TAGA	T	snp	1	1	1	1	.	.	.	INTERGENIC(INTERGENIC)
1	219289482	219289499	219289487	AAT	A	snp	1	0	0	0	.	.	.	RP11-135J2.4.1(INTRON)
1	219307983	219307992	219307991	A	C	ins	1	0	1	1	.	.	.	RP11-135J2.4.1(INTRON)
1	219377374	219377383	219377377	AG	T	snp	1	1	1	1	.	.	.	LYPLAL1(INTRON);RIMKLBP2(UPSTREAM)
1	219529841	219529855	219529846	CAA	T	snp	1	0	0	0	.	.	.	INTERGENIC(INTERGENIC)
1	219632241	219632250	219632249	A	C	ins	1	0	1	1	.	.	.	INTERGENIC(INTERGENIC)
1	219712381	219712394	219712390	TG	A	snp	1	1	1	1	.	.	.	INTERGENIC(INTERGENIC)
1	219816738	219816746	219816743	A	G	snp	1	1	1	1	.	.	.	INTERGENIC(INTERGENIC)
1	219828855	219828870	219828865	ATAG	GATAGAT	del	1	1	1	1	.	.	.	INTERGENIC(INTERGENIC)
1	219877366	219877377	219877373	TGT	A	snp	1	1	1	1	.	.	.	SLC30A10(INTRON)
1	219908768	219908783	219908775	AAAC	G	snp	0	1	1	1	.	.	.	SLC30A10(INTRON)
1	219916886	219916903	219916890	AAG	G	del	1	1	1	1	.	.	.	SLC30A10(INTRON)
1	219972479	219972488	219972486	GA	G	snp	1	0	0	0	.	.	.	SLC30A10(INTRON)
1	220133832	220133841	220133835	AT	G	snp	1	1	1	1	.	.	.	INTERGENIC(INTERGENIC);SLC30A10(UPSTREAM);U3(UPSTREAM)
1	220134283	220134292	220134285	AT	G	snp	0	0	0	1	.	.	.	INTERGENIC(INTERGENIC);SLC30A10(UPSTREAM);U3(UPSTREAM)
1	220289800	220289807	220289803	T	C	snp	1	1	1	1	.	.	.	IARS2(INTRON);MIR194-1(DOWNSTREAM);MIR215(DOWNSTREAM)
1	220434096	220434110	220434105	GAA	A	snp	1	1	1	1	.	.	.	RAB3GAP2(INTRON)
1	220574657	220574664	220574661	T	C	snp	1	0	0	0	.	.	.	RP11-410C4.5.1(EXON)
1	220620887	220620898	220620893	TA	C	snp	0	1	1	1	.	.	.	INTERGENIC(INTERGENIC);RP11-410C4.1.1(UPSTREAM)
1	220701166	220701189	220701185	ACAA	G	snp	1	1	1	1	.	.	.	INTERGENIC(INTERGENIC);MARK1(UPSTREAM)
1	220760655	220760666	220760658	CT	C	snp	1	1	1	1	.	.	.	MARK1(DOWNSTREAM);MARK1(INTRON)
1	220913400	220913409	220913401	T	G	snp	1	1	1	0	.	.	.	INTERGENIC(INTERGENIC)
1	220932174	220932191	220932183	TG	TA	ins	1	1	1	1	.	.	.	MARC2(DOWNSTREAM);MARC2(INTRON)
1	220946026	220946041	220946028	GT	TA	ins	1	0	0	0	.	.	.	MARC2(INTRON)
1	220946026	220946041	220946030	GT	A	snp	1	0	1	0	.	.	.	MARC2(INTRON)
1	220961354	220961373	220961360	AAAG	G	del	0	0	1	0	.	.	.	MARC1(INTRON);MARC1(UPSTREAM);MARC2(DOWNSTREAM)
1	220961354	220961373	220961364	AAAG	G	del	0	0	1	0	.	.	.	MARC1(INTRON);MARC1(UPSTREAM);MARC2(DOWNSTREAM)
1	221010507	221010522	221010513	AATA	G	snp	1	1	1	1	.	.	.	HLA-AS1(INTRON);RP11-295M18.2.1(DOWNSTREAM)
1	221017713	221017726	221017724	TA	C	snp	1	1	1	1	.	.	.	HLA-AS1(INTRON)
1	221103386	221103394	221103388	A	G	snp	1	1	1	1	.	.	.	INTERGENIC(INTERGENIC)
1	221103577	221103588	221103582	TG	C	snp	0	0	1	0	.	.	.	INTERGENIC(INTERGENIC)
1	221175863	221175880	221175874	TCA	G	snp	1	0	0	0	.	.	.	INTERGENIC(INTERGENIC)
1	221263253	221263261	221263260	T	G	snp	1	1	1	1	.	.	.	INTERGENIC(INTERGENIC)
1	221350605	221350614	221350612	T	G	snp	1	1	1	1	.	.	.	INTERGENIC(INTERGENIC)
1	221356136	221356153	221356141	GAG	A	snp	1	0	0	0	.	.	.	INTERGENIC(INTERGENIC)
1	221356136	221356153	221356142	GAG	T	snp	1	0	0	0	.	.	.	INTERGENIC(INTERGENIC)
1	221496663	221496680	221496674	TG	A	snp	1	1	1	1	.	.	.	INTERGENIC(INTERGENIC)
1	221529881	221529889	221529886	T	G	snp	1	1	1	1	.	.	.	INTERGENIC(INTERGENIC)
1	221529881	221529889	221529888	T	G	snp	1	1	1	1	.	.	.	INTERGENIC(INTERGENIC)
1	221543376	221543383	221543377	T	G	snp	1	1	1	1	.	.	.	INTERGENIC(INTERGENIC)
1	221569195	221569204	221569202	GA	G	snp	1	1	1	1	.	.	.	INTERGENIC(INTERGENIC)
1	221686951	221686959	221686958	T	C	snp	0	1	1	1	.	.	.	INTERGENIC(INTERGENIC);RP11-103C3.1.1(DOWNSTREAM)
1	221700588	221700597	221700591	A	C	ins	1	0	0	0	.	.	.	INTERGENIC(INTERGENIC)
1	221732440	221732448	221732447	A	G	snp	0	0	1	0	.	.	.	INTERGENIC(INTERGENIC)
1	221752871	221752884	221752881	AT	T	ins	1	0	0	0	.	.	.	INTERGENIC(INTERGENIC)
1	221752871	221752884	221752882	AT	A	del	1	1	1	1	.	.	.	INTERGENIC(INTERGENIC)
1	221777359	221777368	221777365	TA	C	snp	1	0	1	0	.	.	.	INTERGENIC(INTERGENIC)
1	221874861	221874869	221874862	T	TTTC	ins	1	0	0	0	.	.	.	DUSP10(DOWNSTREAM);DUSP10(EXON);DUSP10(UTR_3_PRIME)
1	221976738	221976747	221976739	A	G	snp	1	1	1	1	.	.	.	INTERGENIC(INTERGENIC)
1	222014247	222014262	222014257	AAAC	G	snp	1	1	1	1	.	.	.	INTERGENIC(INTERGENIC);RP11-191N8.2.1(UPSTREAM);U6(UPSTREAM)
1	222087997	222088006	222088002	T	G	snp	0	0	1	0	.	.	.	RP11-815M8.1.1(INTRON)
1	222107149	222107156	222107150	A	C	snp	1	1	1	1	.	.	.	RP11-815M8.1.1(INTRON)
1	222153843	222153850	222153848	C	T	snp	1	1	1	1	.	.	.	RP11-400N13.2.1(INTRON);RP11-815M8.1.1(UPSTREAM)
1	222153843	222153850	222153849	C	T	snp	0	0	1	0	.	.	.	RP11-400N13.2.1(INTRON);RP11-815M8.1.1(UPSTREAM)
1	222158016	222158025	222158021	TA	G	snp	0	0	1	0	.	.	.	RP11-400N13.2.1(DOWNSTREAM);RP11-400N13.2.1(EXON)
1	222182430	222182437	222182434	A	AAAG	ins	0	1	0	1	.	.	.	AC092765.1(UPSTREAM);INTERGENIC(INTERGENIC)
1	222265405	222265412	222265407	T	C	snp	0	0	1	0	.	.	.	RP11-400N13.1.1(INTRON)
1	222311483	222311492	222311488	TA	G	snp	1	1	1	1	.	.	.	RP11-400N13.1.1(INTRON)

1	222359134	222359141	222359140	T	C	ins	1	1	1	1	.	.	.	RP11-400N13.1.1(INTRON)
1	222524297	222524305	222524298	A	C	snp	1	0	0	0	.	.	.	AL513363.1(DOWNSTREAM);RP11-400N13.1.1(INTRON)
1	222584849	222584858	222584853	TG	C	snp	1	1	1	1	.	.	.	INTERGENIC(INTERGENIC)
1	222666072	222666081	222666080	A	G	ins	1	1	1	1	.	.	.	INTERGENIC(INTERGENIC)
1	222711755	222711764	222711761	GA	AGAT	ins	1	1	1	1	.	.	.	HHIPL2(DOWNSTREAM);HHIPL2(INTRON)
1	222887925	222887934	222887927	T	G	snp	1	0	1	0	.	.	.	AIDA(UPSTREAM);BROX(DOWNSTREAM);BROX(INTRON)
1	222985037	222985045	222985040	A	C	snp	1	0	0	0	.	.	.	DISP1(UPSTREAM);INTERGENIC(INTERGENIC);RP11-452F19.3.1(UPSTREAM)
1	223023765	223023773	223023769	T	C	snp	1	1	1	1	.	.	.	DISP1(INTRON)
1	223126192	223126211	223126201	AAAC	AC	ins	0	0	1	0	.	.	.	DISP1(INTRON)
1	223150381	223150388	223150382	T	G	snp	1	1	1	1	.	.	.	DISP1(INTRON)
1	223168206	223168215	223168213	T	C	snp	1	1	1	1	.	.	.	DISP1(INTRON)
1	223286558	223286565	223286561	T	G	snp	1	1	1	1	.	.	.	TLR5(INTRON)
1	223297809	223297817	223297811	A	C	snp	1	0	0	0	.	.	.	TLR5(INTRON)
1	223355999	223356007	223356005	A	G	snp	1	1	1	1	.	.	.	RP11-239E10.3.1(INTRON)
1	223379198	223379206	223379202	A	G	ins	1	0	0	0	.	.	.	INTERGENIC(INTERGENIC)
1	223426062	223426071	223426065	T	TG	ins	1	1	1	1	.	.	.	SUSD4(INTRON)
1	223589030	223589044	223589036	AAG	G	snp	0	1	0	1	.	.	.	INTERGENIC(INTERGENIC)
1	223877830	223877843	223877833	AT	A	snp	1	1	1	1	.	.	.	INTERGENIC(INTERGENIC);U6(DOWNSTREAM)
1	223894939	223894954	223894942	TC	TCTG	ins	1	1	1	1	.	.	.	CAPN2(INTRON)
1	223894939	223894954	223894946	TC	G	snp	1	0	0	0	.	.	.	CAPN2(INTRON)
1	223894979	223894990	223894986	TC	G	snp	0	0	1	0	.	.	.	CAPN2(INTRON)
1	223896608	223896616	223896612	A	G	snp	1	1	1	1	.	.	.	CAPN2(INTRON);CAPN2(UPSTREAM)
1	223964807	223964822	223964810	CT	C	snp	1	0	0	0	.	.	.	CAPN2(DOWNSTREAM);INTERGENIC(INTERGENIC);TP53BP2(DOWNSTREAM)
1	223969508	223969516	223969513	A	C	snp	1	1	0	1	.	.	.	TP53BP2(INTRON)
1	224119368	224119377	224119370	TG	C	snp	0	1	0	1	.	.	.	PARP1(INTRON)
1	224125075	224125084	224125082	TA	C	snp	1	1	1	1	.	.	.	PARP1(INTRON)
1	224125238	224125247	224125245	TA	C	snp	1	1	0	1	.	.	.	PARP1(INTRON)
1	224179670	224179679	224179675	C	CG	ins	1	1	1	1	.	.	.	PARP1(INTRON);RP11-504P24.2.1(UPSTREAM);RP11-504P24.3.1(UPSTREAM);RP11-504P24.4.1(UPSTREAM)
1	224290526	224290537	224290535	AG	AA	ins	1	1	1	1	.	.	.	7SK(DOWNSTREAM);PARP1(INTRON)
1	224360133	224360148	224360141	GAAG	A	snp	1	1	1	1	.	.	.	DEGS1(UPSTREAM);PARP1(INTRON);RP11-365O16.1.1(DOWNSTREAM)
1	224417003	224417026	224417010	AAAT	A	snp	1	1	1	1	.	.	.	NVL(DOWNSTREAM);NVL(INTRON);PARP1(INTRON);RP11-365O16.6.1(DOWNSTREAM)
1	224419732	224419751	224419744	AAAT	G	snp	1	0	0	0	.	.	.	NVL(DOWNSTREAM);NVL(INTRON);PARP1(INTRON);RP11-365O16.6.1(DOWNSTREAM)
1	224563199	224563207	224563203	T	G	snp	1	1	0	0	.	.	.	CNIH4(DOWNSTREAM);CNIH4(INTRON);CNIH4(UPSTREAM);PARP1(INTRON)
1	224631358	224631367	224631364	TA	ATAG	ins	1	1	0	1	.	.	.	CNIH3(INTRON);PARP1(INTRON)
1	224631358	224631367	224631364	TA	G	snp	0	0	0	1	.	.	.	CNIH3(INTRON);PARP1(INTRON)
1	224631401	224631414	224631406	AT	A	del	0	0	0	1	.	.	.	CNIH3(INTRON);PARP1(INTRON)
1	224631401	224631414	224631408	AT	A	del	0	0	0	1	.	.	.	CNIH3(INTRON);PARP1(INTRON)
1	224631401	224631414	224631409	AT	TTT	ins	1	1	0	1	.	.	.	CNIH3(INTRON);PARP1(INTRON)
1	224631401	224631414	224631411	AT	T	snp	1	1	0	1	.	.	.	CNIH3(INTRON);PARP1(INTRON)
1	224631401	224631414	224631411	AT	TTT	ins	1	1	0	1	.	.	.	CNIH3(INTRON);PARP1(INTRON)
1	224631401	224631414	224631412	AT	A	del	1	1	0	1	.	.	.	CNIH3(INTRON);PARP1(INTRON)
1	224676160	224676168	224676167	T	G	ins	1	1	0	0	.	.	.	CNIH3(INTRON);PARP1(INTRON)
1	224698312	224698321	224698316	A	C	ins	1	1	1	1	.	.	.	CNIH3(INTRON);CNIH3(UPSTREAM);PARP1(INTRON)
1	224725647	224725661	224725658	ATT	A	del	1	1	1	1	.	.	.	CNIH3(DOWNSTREAM);CNIH3(INTRON);PARP1(INTRON)
1	225143092	225143099	225143097	T	C	snp	0	1	1	0	.	.	.	DNAH14(DOWNSTREAM);DNAH14(INTRON);PARP1(INTRON)
1	225222755	225222762	225222761	T	G	ins	1	1	1	1	.	.	.	DNAH14(INTRON);PARP1(INTRON)
1	225261336	225261353	225261350	TTG	A	snp	1	0	0	0	.	.	.	DNAH14(INTRON);PARP1(INTRON)
1	225319896	225319905	225319898	T	G	snp	1	0	1	0	.	.	.	DNAH14(INTRON);PARP1(INTRON)
1	225383701	225383728	225383705	ATTT	G	snp	1	1	1	1	.	.	.	DNAH14(DOWNSTREAM);DNAH14(INTRON);PARP1(INTRON)
1	225561181	225561196	225561187	AGGG	A	snp	1	1	1	1	.	.	.	DNAH14(INTRON);PARP1(INTRON)
1	225581517	225581526	225581523	T	G	ins	1	1	1	1	.	.	.	DNAH14(INTRON);PARP1(INTRON)
1	225644953	225644962	225644959	TA	C	snp	1	1	1	1	.	.	.	PARP1(INTRON);RP11-496N12.6.1(INTRON)
1	225677294	225677302	225677301	A	G	snp	1	0	0	0	.	.	.	ENAH(DOWNSTREAM);PARP1(INTRON)
1	225702605	225702614	225702610	GA	G	snp	0	0	1	0	.	.	.	ENAH(DOWNSTREAM);ENAH(EXON);ENAH(INTRON);ENAH(UPSTREAM);PARP1(INTRON)
1	225853431	225853450	225853445	TTAT	T	snp	1	0	0	0	.	.	.	PARP1(INTRON)
1	225867153	225867168	225867166	CT	C	del	1	1	1	1	.	.	.	PARP1(INTRON)
1	225940633	225940640	225940637	T	G	snp	1	0	0	0	.	.	.	PARP1(INTRON);RP11-145A3.1.1(DOWNSTREAM)
1	225992540	225992551	225992549	TA	TAT	del	1	1	1	1	.	.	.	PARP1(INTRON);RP11-145A3.4.1(DOWNSTREAM)
1	226188548	226188555	226188551	A	G	snp	0	1	0	1	.	.	.	C1orf55(UPSTREAM);PARP1(INTRON)

1	226213550	226213558	226213556	T	C	snp	1	0	1	0	.	.	.	PARP1(INTRON)
1	226236021	226236035	226236032	TTG	TTGTT	del	1	0	1	0	.	.	.	PARP1(INTRON)
1	226334600	226334607	226334605	T	C	snp	0	1	0	1	.	.	.	ACBD3(INTRON);PARP1(INTRON);RP11-275I14.4.1(UPSTREAM)
1	226352491	226352499	226352498	T	G	snp	1	1	1	1	.	ACBD3(NON_SYNONYMOUS_CODING):E187D	.	ACBD3(UPSTREAM);PARP1(INTRON)
1	226444254	226444271	226444261	AT	G	snp	1	0	0	0	.	.	.	LIN9(INTRON);PARP1(INTRON)
1	226500826	226500835	226500831	AT	C	snp	1	1	1	1	.	.	.	LIN9(UPSTREAM);PARP1(INTRON);YBX1P9(DOWNSTREAM);snoU13(DOWNSTREAM)
1	226637597	226637604	226637600	A	G	snp	1	1	1	1	.	.	.	7SK(DOWNSTREAM);INTERGENIC(INTERGENIC)
1	226696755	226696774	226696760	TTTC	C	snp	1	0	1	1	.	.	.	INTERGENIC(INTERGENIC)
1	226697206	226697213	226697210	T	C	snp	1	1	1	1	.	.	.	INTERGENIC(INTERGENIC)
1	226735154	226735165	226735157	GT	A	snp	0	0	1	0	.	.	.	C1orf95(UPSTREAM);INTERGENIC(INTERGENIC)
1	226818408	226818419	226818414	AG	G	snp	1	1	1	1	.	.	.	INTERGENIC(INTERGENIC);ITPKB(DOWNSTREAM)
1	226857569	226857578	226857573	T	G	snp	0	1	1	0	.	.	.	ITPKB(INTRON);ITPKB-AS1(INTRON);ITPKB-IT1(INTRON)
1	226984302	226984309	226984305	A	AC	ins	1	1	1	0	.	.	.	INTERGENIC(INTERGENIC)
1	227047579	227047590	227047587	ATT	A	del	1	1	1	1	.	.	.	INTERGENIC(INTERGENIC)
1	227098196	227098203	227098197	T	C	ins	1	1	1	1	.	.	.	ADCK3(INTRON)
1	227098196	227098203	227098197	T	C	snp	1	1	1	1	.	.	.	ADCK3(INTRON)
1	227135502	227135519	227135504	AT	T	del	1	1	1	1	.	.	.	ADCK3(INTRON)
1	227171736	227171745	227171738	G	T	ins	1	1	1	1	.	.	.	ADCK3(EXON);ADCK3(INTRON)
1	227318555	227318563	227318560	A	C	snp	0	1	1	1	.	.	.	CDC42BPA(INTRON)
1	227372466	227372474	227372471	A	G	snp	1	1	1	1	.	.	.	CDC42BPA(INTRON);RP11-1B20.1.1(DOWNSTREAM)
1	227395069	227395078	227395074	T	G	snp	1	0	0	0	.	.	.	CDC42BPA(INTRON)
1	227399794	227399801	227399797	T	G	snp	1	1	1	1	.	.	.	CDC42BPA(DOWNSTREAM);CDC42BPA(INTRON)
1	227517485	227517492	227517487	T	G	snp	1	1	1	1	.	.	.	INTERGENIC(INTERGENIC)
1	227562759	227562776	227562773	TC	C	del	0	0	0	1	.	.	.	INTERGENIC(INTERGENIC)
1	227593964	227593987	227593981	AAC	A	ins	1	0	1	0	.	.	.	CTD-2090I13.1.1(INTRON);CTD-2090I13.1.1(UPSTREAM);CTD-2090I13.2.1(DOWNSTREAM)
1	227610119	227610126	227610121	C	T	snp	1	0	0	0	.	.	.	CTD-2090I13.1.1(INTRON)
1	227770486	227770499	227770497	TG	A	snp	1	0	0	0	.	.	.	ZNF678(INTRON)
1	227909587	227909596	227909593	GT	T	snp	0	0	1	0	.	.	.	INTERGENIC(INTERGENIC)
1	227922141	227922149	227922142	A	C	snp	1	0	0	0	.	.	.	JMJD4(INTRON);JMJD4(UPSTREAM);SNAP47(DOWNSTREAM);SNAP47(INTRON);SNAP47(UPSTREAM)
1	228590239	228590250	228590246	CTC	C	snp	1	0	0	0	.	.	.	RP11-245P10.4.1(UPSTREAM);TRIM11(INTRON);TRIM11(UPSTREAM)
1	228656051	228656060	228656056	T	G	ins	1	1	1	0	.	.	.	HIST3H2BA(UPSTREAM);INTERGENIC(INTERGENIC)
1	228720272	228720279	228720273	A	C	snp	1	1	1	1	.	.	.	INTERGENIC(INTERGENIC)
1	228742941	228742952	228742947	TGG	C	snp	1	1	1	1	.	.	.	DUSP5P(UPSTREAM);INTERGENIC(INTERGENIC);RN5S1(DOWNSTREAM);RN5S19(UPSTREAM)
1	228775824	228775839	228775828	AC	CACG	ins	1	1	1	1	.	.	.	DUSP5P(INTRON);RN5S13(UPSTREAM);RN5S14(UPSTREAM);RN5S15(DOWNSTREAM);RN5S16(DOWNSTREAM)
1	228842966	228842975	228842970	AT	G	snp	1	0	0	0	.	.	.	INTERGENIC(INTERGENIC)
1	228855515	228855522	228855517	A	C	snp	1	0	0	0	.	.	.	INTERGENIC(INTERGENIC)
1	228868573	228868581	228868574	A	C	snp	1	1	1	1	.	.	.	INTERGENIC(INTERGENIC);RHOU(UPSTREAM)
1	228900812	228900825	228900823	TC	TCT	del	1	1	1	1	.	.	.	INTERGENIC(INTERGENIC)
1	229070749	229070758	229070754	C	A	ins	1	0	0	0	.	.	.	INTERGENIC(INTERGENIC)
1	229097256	229097267	229097265	TA	G	snp	1	0	0	0	.	.	.	INTERGENIC(INTERGENIC)
1	229097330	229097337	229097333	T	G	snp	1	0	0	0	.	.	.	INTERGENIC(INTERGENIC)
1	229122973	229122981	229122980	C	A	snp	1	1	1	1	.	.	.	INTERGENIC(INTERGENIC)
1	229141064	229141075	229141071	GT	GC	ins	1	0	0	0	.	.	.	INTERGENIC(INTERGENIC)
1	229156993	229157010	229157006	TCT	G	snp	1	0	0	0	.	.	.	INTERGENIC(INTERGENIC)
1	229196223	229196236	229196226	TG	TGTGTA	ins	1	1	1	1	.	.	.	INTERGENIC(INTERGENIC)
1	229213817	229213825	229213824	T	C	snp	1	1	1	1	.	.	.	INTERGENIC(INTERGENIC)
1	229297468	229297483	229297477	AT	C	snp	1	1	1	1	.	.	.	INTERGENIC(INTERGENIC)
1	229347353	229347380	229347367	ATAC	T	snp	0	1	1	0	.	.	.	INTERGENIC(INTERGENIC)
1	229354167	229354178	229354170	TA	TG	ins	1	1	1	1	.	.	.	INTERGENIC(INTERGENIC)
1	229354713	229354724	229354717	TC	C	snp	1	0	0	0	.	.	.	INTERGENIC(INTERGENIC);RP5-1061H20.5.1(DOWNSTREAM)
1	229437652	229437661	229437659	TG	A	snp	1	0	0	0	.	.	.	RAB4A(INTRON);SPHAR(UPSTREAM)
1	229469816	229469831	229469825	TTTG	G	snp	1	0	0	0	.	.	.	C1orf96(INTRON)
1	229586483	229586490	229586485	T	GTTTG	ins	1	1	1	1	.	.	.	NUP133(EXON);NUP133(INTRON)
1	229586483	229586490	229586486	T	G	snp	1	1	1	1	.	.	.	NUP133(EXON);NUP133(INTRON)
1	229627525	229627534	229627530	A	C	snp	1	0	0	0	.	.	.	NUP133(INTRON)
1	229655718	229655726	229655720	T	C	snp	1	1	1	1	.	.	.	ABCB10(INTRON);ABCB10(UPSTREAM)
1	229705464	229705487	229705471	TTAT	A	snp	1	0	0	0	.	.	.	HMGN2P19(UPSTREAM);INTERGENIC(INTERGENIC)
1	229719384	229719403	229719399	TTTA	G	snp	1	0	0	0	.	.	.	INTERGENIC(INTERGENIC)

1	229735796	229735803	229735802	T	C	snp	1	0	0	0	.	.	.	TAF5L(INTRON);TAF5L(UTR_3_PRIME)
1	229847444	229847459	229847455	AAAC	A	del	1	1	0	1	.	.	.	INTERGENIC(INTERGENIC)
1	230186701	230186710	230186707	TA	C	snp	1	1	1	1	.	.	.	BX323860.1(UPSTREAM);INTERGENIC(INTERGENIC)
1	230205009	230205017	230205012	T	G	ins	1	1	1	0	.	.	.	GALNT2(INTRON)
1	230205009	230205017	230205013	T	G	snp	1	1	1	1	.	.	.	GALNT2(INTRON)
1	230246800	230246809	230246806	TG	C	snp	1	1	1	1	.	.	.	GALNT2(INTRON)
1	230426577	230426584	230426580	A	G	ins	0	1	0	1	.	.	.	INTERGENIC(INTERGENIC)
1	230624220	230624235	230624231	CATC	T	snp	1	0	0	0	.	.	.	INTERGENIC(INTERGENIC)
1	230702171	230702180	230702173	TA	G	snp	0	1	1	1	.	.	.	INTERGENIC(INTERGENIC)
1	230762419	230762439	230762432	TAT	T	ins	1	0	0	0	.	.	.	INTERGENIC(INTERGENIC)
1	230762419	230762439	230762435	TAT	T	ins	1	1	1	1	.	.	.	INTERGENIC(INTERGENIC)
1	231019774	231019781	231019780	A	C	ins	0	0	1	0	.	.	.	INTERGENIC(INTERGENIC)
1	231298895	231298906	231298898	CGC	A	snp	0	1	1	1	.	.	TRIM67(SYNONYMOUS_CODING):A61	TRIM67(INTRON)
1	231378493	231378512	231378504	TTTG	A	snp	1	1	1	1	.	.	.	C1orf131(UPSTREAM);GNPAT(INTRON)
1	231425975	231425982	231425980	A	C	snp	1	0	1	0	.	.	.	INTERGENIC(INTERGENIC)
1	231437281	231437288	231437287	C	A	snp	1	1	1	1	.	.	.	INTERGENIC(INTERGENIC)
1	231674951	231674958	231674953	T	C	snp	1	0	0	0	.	.	.	TSNAX(INTRON)
1	231695710	231695733	231695727	ATT	A	del	1	1	1	0	.	.	.	TSNAX(INTRON)
1	231695710	231695733	231695728	ATT	T	snp	0	1	1	0	.	.	.	TSNAX(INTRON)
1	231695710	231695733	231695730	ATT	A	del	1	1	1	1	.	.	.	TSNAX(INTRON)
1	231801054	231801062	231801061	T	G	snp	1	0	0	0	.	.	.	DISC1(INTRON)
1	231807043	231807052	231807047	T	C	snp	0	0	1	0	.	.	.	DISC1(INTRON);RNU5A-5P(DOWNSTREAM)
1	231869538	231869549	231869546	GTG	A	snp	0	0	1	0	.	.	.	DISC1(INTRON)
1	232038347	232038356	232038355	A	C	snp	1	0	0	0	.	.	.	DISC1(INTRON)
1	232058874	232058882	232058875	A	C	snp	1	0	0	0	.	.	.	DISC1(INTRON);DISC1-IT1(UPSTREAM)
1	232167036	232167051	232167045	AGGA	GAA	del	1	1	1	1	.	.	.	DISC1(INTRON)
1	232167036	232167051	232167047	AGGA	AGG	del	1	1	1	1	.	.	.	DISC1(INTRON)
1	232172768	232172777	232172774	GA	C	snp	1	0	0	0	.	.	.	DISC1(UTR_3_PRIME)
1	232224637	232224654	232224639	AT	T	del	1	0	0	0	.	.	.	INTERGENIC(INTERGENIC)
1	232266483	232266490	232266485	G	T	snp	0	1	1	1	.	.	.	INTERGENIC(INTERGENIC)
1	232266483	232266490	232266486	G	T	snp	1	1	1	1	.	.	.	INTERGENIC(INTERGENIC)
1	232277103	232277110	232277104	A	G	snp	1	1	1	1	.	.	.	INTERGENIC(INTERGENIC)
1	232316600	232316608	232316602	A	C	snp	1	1	1	1	.	.	.	RP5-865N13.1.1(UPSTREAM);RP5-865N13.2.1(INTRON)
1	232387565	232387573	232387566	A	G	snp	1	0	0	0	.	.	.	INTERGENIC(INTERGENIC)
1	232686362	232686373	232686370	AGC	T	snp	1	0	0	0	.	.	.	SIPA1L2(INTRON)
1	232805933	232805942	232805937	T	C	snp	1	0	0	0	.	.	.	INTERGENIC(INTERGENIC)
1	232903979	232904006	232903995	CAAA	T	snp	0	1	0	1	.	.	.	INTERGENIC(INTERGENIC)
1	232955070	232955078	232955073	T	G	snp	1	0	0	0	.	.	.	INTERGENIC(INTERGENIC)
1	232962588	232962601	232962592	GT	A	snp	0	0	1	0	.	.	.	INTERGENIC(INTERGENIC)
1	232982872	232982879	232982873	A	C	snp	1	1	1	1	.	.	.	INTERGENIC(INTERGENIC)
1	233043977	233043986	233043985	T	G	snp	1	0	0	0	.	.	.	INTERGENIC(INTERGENIC)
1	233051095	233051108	233051100	TG	T	snp	1	0	0	0	.	.	.	INTERGENIC(INTERGENIC);RP4-659I19.1.1(UPSTREAM)
1	233072072	233072089	233072086	TC	CTCC	ins	1	1	1	1	.	.	.	INTERGENIC(INTERGENIC)
1	233158351	233158358	233158353	G	GGA	ins	1	1	1	1	.	.	.	PCNXL2(DOWNSTREAM);PCNXL2(INTRON)
1	233158351	233158358	233158353	G	T	snp	1	1	1	1	.	.	.	PCNXL2(DOWNSTREAM);PCNXL2(INTRON)
1	233209352	233209369	233209357	TTA	C	snp	1	1	1	1	.	.	.	PCNXL2(INTRON)
1	233215197	233215208	233215200	GGA	A	snp	1	0	0	0	.	.	.	PCNXL2(INTRON)
1	233227524	233227533	233227530	T	C	snp	1	1	1	1	.	.	.	PCNXL2(DOWNSTREAM);PCNXL2(INTRON)
1	233301927	233301950	233301939	AAAT	T	snp	1	0	0	0	.	.	.	PCNXL2(INTRON)
1	233301927	233301950	233301943	AAAT	T	snp	1	0	1	0	.	.	.	PCNXL2(INTRON)
1	233301927	233301950	233301945	AAAT	T	ins	1	1	1	1	.	.	.	PCNXL2(INTRON)
1	233353499	233353512	233353508	TA	G	snp	1	0	0	0	.	.	.	PCNXL2(INTRON)
1	233416724	233416735	233416732	ATC	ATT	ins	1	1	1	1	.	.	.	PCNXL2(INTRON)
1	233431699	233431714	233431710	TC	G	snp	0	0	0	1	.	.	.	INTERGENIC(INTERGENIC);PCNXL2(UPSTREAM);RP5-862P8.3.1(UPSTREAM)
1	233431699	233431714	233431712	TC	TGTT	ins	1	1	1	1	.	.	.	INTERGENIC(INTERGENIC);PCNXL2(UPSTREAM);RP5-862P8.3.1(UPSTREAM)
1	233431699	233431714	233431712	TC	T	snp	0	0	0	1	.	.	.	INTERGENIC(INTERGENIC);PCNXL2(UPSTREAM);RP5-862P8.3.1(UPSTREAM)
1	233431733	233431748	233431744	TTTC	T	ins	1	1	1	1	.	.	.	INTERGENIC(INTERGENIC);PCNXL2(UPSTREAM);RP5-862P8.3.1(UPSTREAM)
1	233439392	233439400	233439394	T	C	ins	1	0	0	0	.	.	.	INTERGENIC(INTERGENIC)
1	233445290	233445310	233445294	AAT	T	del	1	1	1	1	.	.	.	INTERGENIC(INTERGENIC)
1	233475217	233475234	233475230	CTT	TC	del	1	1	1	1	.	.	.	RP5-862P8.2.1(INTRON)
1	233482791	233482804	233482794	TG	A	snp	0	1	1	1	.	.	.	RP5-862P8.2.1(INTRON)
1	233518878	233518886	233518885	T	C	snp	1	1	1	1	.	.	.	RP5-862P8.2.1(UTR_3_PRIME)
1	233523316	233523330	233523327	AAC	A	snp	1	1	1	1	.	.	.	INTERGENIC(INTERGENIC);RP5-862P8.2.1(DOWNSTREAM)

1	233528364	233528375	233528372	TTC	T	snp	0	1	1	0	.	.	. INTERGENIC(INTERGENIC)
1	233546999	233547008	233547001	G	GGT	ins	0	1	1	1	.	.	. INTERGENIC(INTERGENIC)
1	233613412	233613421	233613415	T	C	snp	1	1	1	1	.	.	. INTERGENIC(INTERGENIC)
1	233634592	233634600	233634596	T	G	snp	1	1	1	1	.	.	. INTERGENIC(INTERGENIC)
1	233784217	233784226	233784222	T	G	snp	1	1	1	1	.	.	. KCNK1(INTRON);KCNK1(UPSTREAM)
1	233895283	233895294	233895290	AGG	A	snp	0	0	0	1	.	.	. INTERGENIC(INTERGENIC)
1	233914492	233914501	233914495	TA	G	snp	0	0	1	0	.	.	. INTERGENIC(INTERGENIC)
1	233916106	233916117	233916114	TTG	TTGT	del	1	1	1	1	.	.	. INTERGENIC(INTERGENIC)
1	233954866	233954873	233954869	T	G	snp	1	1	1	1	.	.	. INTERGENIC(INTERGENIC)
1	233967655	233967666	233967660	TC	G	snp	1	1	1	1	.	.	. INTERGENIC(INTERGENIC);RP11-301G21.1.1(DOWNSTREAM)
1	234054525	234054542	234054540	TC	T	snp	1	1	1	1	.	.	. SLC35F3(INTRON)
1	234054783	234054792	234054790	TA	G	snp	1	0	0	0	.	.	. SLC35F3(INTRON)
1	234104807	234104816	234104811	G	A	snp	0	1	1	1	.	.	. SLC35F3(INTRON)
1	234141254	234141268	234141264	GCT	T	snp	1	1	1	0	.	.	. SLC35F3(INTRON)
1	234244219	234244228	234244224	AT	C	snp	0	1	0	0	.	.	. SLC35F3(INTRON)
1	234244370	234244379	234244373	AT	C	snp	1	0	0	1	.	.	. SLC35F3(INTRON)
1	234244404	234244413	234244410	TA	A	del	1	1	1	1	.	.	. SLC35F3(INTRON)
1	234252252	234252271	234252267	TTTA	T	snp	0	1	1	1	.	.	. SLC35F3(INTRON)
1	234342245	234342253	234342252	G	T	snp	1	0	0	0	.	.	. SLC35F3(INTRON)
1	234365082	234365089	234365086	T	G	snp	1	1	1	1	.	.	. SLC35F3(INTRON)
1	234430649	234430656	234430650	G	A	snp	1	1	1	1	.	.	. SLC35F3(INTRON)
1	234437899	234437916	234437913	GTT	G	del	1	1	1	1	.	.	. SLC35F3(INTRON)
1	234456784	234456793	234456792	C	A	ins	1	1	0	1	.	.	. SLC35F3(INTRON)
1	234512874	234512881	234512876	T	G	snp	1	1	1	1	.	.	. C1orf31(INTRON);RP5-827C21.4.1(UPSTREAM)
1	234512874	234512881	234512879	T	G	snp	1	1	1	1	.	.	. C1orf31(INTRON);RP5-827C21.4.1(UPSTREAM)
1	234564093	234564101	234564095	A	C	ins	0	1	0	0	.	.	. TARBP1(INTRON)
1	234606023	234606031	234606025	A	C	snp	1	1	0	1	.	.	. TARBP1(INTRON)
1	234616691	234616700	234616699	A	C	ins	1	1	1	0	.	.	. INTERGENIC(INTERGENIC);TARBP1(UPSTREAM)
1	234650544	234650567	234650550	TTG	C	snp	1	1	1	1	.	.	. INTERGENIC(INTERGENIC)
1	234650544	234650567	234650564	TTG	A	snp	1	0	0	0	.	.	. INTERGENIC(INTERGENIC)
1	234669686	234669695	234669693	TG	A	snp	1	0	0	0	.	.	. INTERGENIC(INTERGENIC);RP5-855F14.1.1(UPSTREAM)
1	234674952	234674960	234674954	T	G	snp	0	0	1	0	.	.	. INTERGENIC(INTERGENIC)
1	234785254	234785281	234785271	GATA	G	snp	0	1	0	1	.	.	. RP4-781K5.4.1(INTRON)
1	234785254	234785281	234785275	GATA	G	snp	1	1	0	1	.	.	. RP4-781K5.4.1(INTRON)
1	234816050	234816059	234816057	TG	T	del	1	0	0	1	.	.	. RP4-781K5.4.1(INTRON);RP4-781K5.6.1(INTRON)
1	234852084	234852099	234852095	AAAG	T	snp	0	0	0	1	.	.	. RP4-781K5.7.1(DOWNSTREAM);RP4-781K5.7.1(INTRON)
1	234894528	234894536	234894532	G	A	ins	1	1	1	1	.	.	. INTERGENIC(INTERGENIC)
1	234958841	234958850	234958846	A	C	snp	1	1	1	1	.	.	. INTERGENIC(INTERGENIC)
1	235169066	235169085	235169080	ATTA	G	snp	0	0	0	1	.	.	. INTERGENIC(INTERGENIC)
1	235433017	235433026	235433019	AC	G	snp	1	0	0	0	.	.	. ARID4B(INTRON)
1	235440552	235440561	235440556	CT	G	snp	1	1	1	1	.	.	. ARID4B(INTRON)
1	235576113	235576130	235576127	ATT	A	del	1	0	0	0	.	.	. TBCE(INTRON)
1	235618438	235618452	235618449	TTA	TTG	ins	1	1	0	1	.	.	. B3GALNT2(INTRON)
1	235633221	235633228	235633224	T	G	snp	1	1	1	1	.	.	. B3GALNT2(INTRON);B3GALNT2(UPSTREAM)
1	235669416	235669425	235669418	A	C	ins	1	1	1	1	.	.	. B3GALNT2(UPSTREAM);INTERGENIC(INTERGENIC)
1	235697468	235697483	235697473	CT	C	snp	1	0	0	0	.	.	. AL672238.4(UPSTREAM);INTERGENIC(INTERGENIC);MTND5P19(DOWNSTREAM);MTND6P14(UPSTREAM)
1	235750613	235750624	235750616	GT	G	snp	1	1	1	1	.	.	. GNG4(INTRON)
1	235790512	235790527	235790520	TTTG	C	snp	1	0	1	0	.	.	. GNG4(INTRON)
1	235791497	235791517	235791500	ACA	T	snp	1	0	0	0	.	.	. GNG4(INTRON)
1	235791717	235791725	235791723	G	A	ins	1	0	0	0	.	.	. GNG4(INTRON)
1	235798254	235798261	235798257	A	C	ins	1	1	1	1	.	.	. GNG4(INTRON)
1	235985934	235985945	235985942	AAT	C	snp	1	0	0	0	.	.	. LYST(INTRON);LYST-IT1(DOWNSTREAM)
1	236079337	236079354	236079350	AGA	A	del	1	1	1	1	.	.	. INTERGENIC(INTERGENIC);U6(DOWNSTREAM)
1	236098374	236098387	236098384	TG	G	ins	1	0	0	0	.	.	. INTERGENIC(INTERGENIC)
1	236098387	236098394	236098392	G	A	ins	0	0	1	1	.	.	. INTERGENIC(INTERGENIC)
1	236102528	236102535	236102532	A	C	snp	1	1	1	1	.	.	. INTERGENIC(INTERGENIC);RP5-940F7.2.1(DOWNSTREAM)
1	236118777	236118788	236118783	AT	TG	ins	1	0	0	0	.	.	. INTERGENIC(INTERGENIC);RP4-580N22.2.1(DOWNSTREAM)
1	236118777	236118788	236118785	AT	G	snp	1	0	0	0	.	.	. INTERGENIC(INTERGENIC);RP4-580N22.2.1(DOWNSTREAM)
1	236157988	236158002	236157999	CTT	C	ins	1	1	1	1	.	.	. NID1(INTRON)
1	236226780	236226803	236226786	AAAT	T	del	1	1	0	1	.	.	. NID1(INTRON);Y_RNA(DOWNSTREAM)
1	236226780	236226803	236226790	AAAT	T	del	1	0	0	0	.	.	. NID1(INTRON);Y_RNA(DOWNSTREAM)
1	236226780	236226803	236226791	AAAT	A	ins	1	0	0	0	.	.	. NID1(INTRON);Y_RNA(DOWNSTREAM)
1	236226780	236226803	236226798	AAAT	T	del	1	0	0	0	.	.	. NID1(INTRON);Y_RNA(DOWNSTREAM)
1	236236478	236236497	236236485	AAAG	A	ins	1	0	0	0	.	.	. INTERGENIC(INTERGENIC)
1	236408456	236408471	236408467	ATTT	A	snp	1	1	1	0	.	.	. ERO1LB(INTRON)

1	236409465	236409474	236409468	T	G	snp	1	1	1	1	.	.	.	ERO1LB(INTRON)
1	236469046	236469053	236469047	A	G	snp	0	1	1	1	.	.	.	INTERGENIC(INTERGENIC);snoU13(DOWNSTREAM)
1	236518791	236518802	236518796	CTT	G	snp	1	0	0	0	.	.	.	EDARADD(INTRON)
1	236539202	236539211	236539205	A	G	snp	1	1	1	1	.	.	.	EDARADD(INTRON)
1	236576714	236576723	236576717	TC	T	snp	1	0	0	0	.	.	.	EDARADD(INTRON)
1	236598246	236598255	236598250	TG	C	snp	1	0	0	0	.	.	.	EDARADD(INTRON)
1	236611131	236611140	236611138	T	TC	ins	1	1	0	1	.	.	.	EDARADD(INTRON)
1	236615802	236615815	236615804	CT	T	del	1	1	1	1	.	.	.	EDARADD(INTRON)
1	236639082	236639091	236639089	AC	A	ins	1	0	0	0	.	.	.	EDARADD(INTRON)
1	236656679	236656688	236656685	TA	C	snp	1	1	1	1	.	.	.	INTERGENIC(INTERGENIC)
1	236663788	236663802	236663798	AAG	C	snp	1	0	0	0	.	.	.	INTERGENIC(INTERGENIC)
1	236668865	236668873	236668872	A	C	snp	1	1	1	1	.	.	.	INTERGENIC(INTERGENIC)
1	236707832	236707841	236707839	T	G	snp	0	0	1	0	.	.	.	HEATR1(DOWNSTREAM);LGALS8(DOWNSTREAM);LGALS8(INTRON);RP11-385F5.4.1(INTRON)
1	236764978	236764987	236764979	A	AC	ins	1	1	1	1	.	.	.	HEATR1(DOWNSTREAM);HEATR1(INTRON);HEATR1(UPSTREAM)
1	236782420	236782437	236782433	AT	A	del	0	0	0	1	.	.	.	INTERGENIC(INTERGENIC)
1	236782420	236782437	236782435	AT	A	del	1	1	1	1	.	.	.	INTERGENIC(INTERGENIC)
1	236796317	236796336	236796321	TTCT	C	snp	1	1	1	1	.	.	.	INTERGENIC(INTERGENIC)
1	236842903	236842926	236842919	TTTC	TTC	del	1	1	1	1	.	.	.	INTERGENIC(INTERGENIC)
1	236842903	236842926	236842920	TTTC	TC	del	1	1	1	1	.	.	.	INTERGENIC(INTERGENIC)
1	236842903	236842926	236842922	TTTC	T	ins	0	0	1	0	.	.	.	INTERGENIC(INTERGENIC)
1	236864551	236864559	236864558	T	C	snp	1	0	0	0	.	.	.	ACTN2(INTRON)
1	236886492	236886503	236886495	CTT	T	snp	1	1	1	1	.	.	.	ACTN2(INTRON)
1	236961259	236961267	236961263	A	C	snp	0	0	1	0	.	.	.	MTR(INTRON)
1	237063498	237063507	237063504	AT	G	snp	1	1	1	1	.	.	.	MTR(DOWNSTREAM);MTR(EXON);MTR(UTR_3_PRIME)
1	237127084	237127095	237127089	CT	C	snp	0	1	1	1	.	.	.	INTERGENIC(INTERGENIC)
1	237157967	237157976	237157971	CA	T	snp	0	1	1	1	.	.	.	INTERGENIC(INTERGENIC)
1	237177512	237177520	237177517	A	G	snp	1	0	0	0	.	.	.	INTERGENIC(INTERGENIC)
1	237214517	237214524	237214523	C	A	snp	0	1	0	1	.	.	.	RYR2(INTRON)
1	237222267	237222275	237222270	T	G	snp	1	0	0	0	.	.	.	RYR2(INTRON)
1	237228759	237228766	237228763	T	C	ins	1	1	1	1	.	.	.	RYR2(INTRON)
1	237273031	237273045	237273035	AAT	T	del	1	1	1	1	.	.	.	RYR2(INTRON)
1	237278537	237278545	237278541	C	A	snp	1	0	0	1	.	.	.	RYR2(INTRON)
1	237339549	237339564	237339554	AT	A	snp	1	1	1	1	.	.	.	RYR2(INTRON)
1	237394607	237394626	237394622	TTTC	TTTCT	del	1	1	1	0	.	.	.	RYR2(INTRON)
1	237582602	237582611	237582610	A	C	snp	0	0	0	1	.	.	.	RYR2(INTRON)
1	237588483	237588490	237588487	A	C	snp	1	1	1	1	.	.	.	RYR2(INTRON)
1	237636720	237636733	237636727	TC	T	snp	1	1	1	1	.	.	.	RYR2(INTRON)
1	237636720	237636733	237636731	TC	T	snp	1	1	1	1	.	.	.	RYR2(INTRON)
1	237731676	237731684	237731677	A	C	snp	1	1	1	1	.	.	.	RYR2(INTRON)
1	237752193	237752202	237752197	A	C	ins	1	1	1	1	.	.	.	RYR2(INTRON)
1	237754390	237754413	237754403	CTC	C	snp	1	1	1	1	.	.	.	RYR2(INTRON)
1	237754434	237754445	237754437	CCT	T	snp	1	1	1	1	.	.	.	RYR2(INTRON)
1	237832810	237832817	237832811	C	T	ins	1	1	1	1	.	.	.	RYR2(INTRON)
1	237860644	237860655	237860650	TA	C	snp	1	0	0	0	.	.	.	RYR2(INTRON)
1	237866905	237866918	237866912	AT	A	snp	1	1	1	1	.	.	.	RYR2(INTRON)
1	237907956	237907967	237907962	CAA	A	ins	1	0	0	0	.	.	.	RYR2(INTRON)
1	238128060	238128069	238128064	T	C	ins	1	1	0	1	.	.	.	RYR2(INTRON)
1	238140357	238140364	238140358	A	C	snp	1	0	0	0	.	.	.	INTERGENIC(INTERGENIC)
1	238200417	238200428	238200422	TTA	T	snp	1	1	1	1	.	.	.	INTERGENIC(INTERGENIC)
1	238326061	238326068	238326067	T	G	snp	1	1	1	1	.	.	.	INTERGENIC(INTERGENIC)
1	238329023	238329038	238329027	ATTT	C	snp	1	1	1	1	.	.	.	INTERGENIC(INTERGENIC)
1	238457267	238457275	238457274	C	T	snp	0	0	1	0	.	.	.	INTERGENIC(INTERGENIC)
1	238506958	238506965	238506963	T	G	snp	1	1	1	1	.	.	.	INTERGENIC(INTERGENIC)
1	238541235	238541244	238541238	A	C	snp	1	1	1	1	.	.	.	INTERGENIC(INTERGENIC)
1	238683543	238683550	238683549	T	C	snp	1	1	0	1	.	.	.	RP11-177F15.1.1(INTRON)
1	238722569	238722580	238722576	GT	G	snp	1	1	1	1	.	.	.	INTERGENIC(INTERGENIC)
1	238795576	238795589	238795585	TA	TAT	del	1	0	0	0	.	.	.	INTERGENIC(INTERGENIC)
1	238895171	238895178	238895175	A	G	snp	1	0	0	0	.	.	.	INTERGENIC(INTERGENIC)
1	238951043	238951052	238951050	AC	T	snp	0	1	0	1	.	.	.	INTERGENIC(INTERGENIC)
1	238984282	238984297	238984290	ATAC	G	snp	0	1	1	1	.	.	.	INTERGENIC(INTERGENIC)
1	239014180	239014191	239014183	ACA	C	snp	1	1	0	1	.	.	.	INTERGENIC(INTERGENIC)
1	239056752	239056759	239056758	T	C	snp	1	1	1	1	.	.	.	INTERGENIC(INTERGENIC)
1	239073497	239073506	239073501	A	G	snp	1	1	1	1	.	.	.	INTERGENIC(INTERGENIC)
1	239167099	239167108	239167104	AC	A	snp	1	0	0	0	.	.	.	INTERGENIC(INTERGENIC)
1	239332106	239332117	239332114	CA	T	snp	0	1	1	1	.	.	.	INTERGENIC(INTERGENIC)

1	239370250	239370267	239370256	TTA	C	snp	1	0	0	0	.	.	.	INTERGENIC(INTERGENIC)
1	239382413	239382422	239382418	T	G	snp	1	0	0	0	.	.	.	INTERGENIC(INTERGENIC)
1	239507592	239507615	239507605	AAAT	T	snp	1	0	0	0	.	.	.	INTERGENIC(INTERGENIC)
1	239507592	239507615	239507609	AAAT	T	snp	1	0	0	0	.	.	.	INTERGENIC(INTERGENIC)
1	239571093	239571100	239571098	A	C	snp	1	0	0	0	.	.	.	CHRM3(INTRON);CHRM3(UPSTREAM)
1	239573668	239573677	239573670	TA	G	snp	0	1	1	1	.	.	.	CHRM3(INTRON)
1	239631200	239631208	239631205	C	T	ins	1	1	1	1	.	.	.	CHRM3(INTRON)
1	239693189	239693206	239693194	TTA	G	snp	1	1	1	1	.	.	.	CHRM3(INTRON)
1	239694099	239694107	239694101	A	C	snp	1	1	1	1	.	.	.	CHRM3(INTRON)
1	239717753	239717770	239717764	TG	C	snp	1	1	1	1	.	.	.	CHRM3(INTRON)
1	239770381	239770388	239770384	T	C	snp	0	1	1	1	.	.	.	CHRM3(DOWNSTREAM);CHRM3(INTRON)
1	239797677	239797704	239797691	GAAA	C	snp	1	1	1	1	.	.	.	CHRM3(INTRON)
1	239797730	239797737	239797736	A	C	ins	1	1	1	1	.	.	.	CHRM3(INTRON)
1	239834072	239834079	239834073	C	CG	ins	1	1	1	1	.	.	.	CHRM3(INTRON)
1	239927233	239927241	239927235	A	C	snp	1	0	0	0	.	.	.	CHRM3(INTRON)
1	239980998	239981011	239981003	AC	G	snp	1	1	1	1	.	.	.	CHRM3(INTRON)
1	240003101	240003108	240003103	G	T	snp	1	0	0	0	.	.	.	CHRM3(INTRON)
1	240022696	240022703	240022701	T	G	snp	1	1	1	1	.	.	.	CHRM3(INTRON)
1	240105545	240105552	240105548	T	G	ins	1	1	1	1	.	.	.	INTERGENIC(INTERGENIC)
1	240215339	240215354	240215350	TTTG	T	ins	1	0	0	1	.	.	.	FMN2(INTRON)
1	240224927	240224942	240224934	AAAC	T	snp	1	1	0	1	.	.	.	FMN2(INTRON)
1	240226415	240226424	240226420	T	C	ins	1	0	0	0	.	.	.	FMN2(INTRON)
1	240233133	240233148	240233138	AT	ATATATA	ins	1	0	0	0	.	.	.	FMN2(INTRON)
1	240246455	240246466	240246461	TA	C	snp	1	1	1	1	.	.	.	FMN2(INTRON)
1	240246470	240246481	240246476	TA	C	snp	1	0	0	1	.	.	.	FMN2(INTRON)
1	240262616	240262624	240262619	T	G	ins	1	0	0	0	.	.	.	FMN2(INTRON)
1	240262616	240262624	240262620	T	G	snp	1	1	1	0	.	.	.	FMN2(INTRON)
1	240481715	240481724	240481720	T	C	snp	0	1	1	1	.	.	.	FMN2(INTRON)
1	240564105	240564118	240564110	CA	C	snp	1	1	0	0	.	.	.	FMN2(INTRON);RP11-177F11.1.1(EXON)
1	240590495	240590504	240590499	T	G	snp	1	0	0	0	.	.	.	FMN2(INTRON)
1	240659248	240659257	240659252	T	G	ins	1	1	1	1	.	.	.	FMN2(DOWNSTREAM);GREM2(INTRON)
1	240788932	240788947	240788937	AG	AA	ins	1	0	0	0	.	.	.	INTERGENIC(INTERGENIC)
1	240789333	240789342	240789339	A	G	snp	1	0	0	0	.	.	.	INTERGENIC(INTERGENIC)
1	240804651	240804665	240804662	AAT	G	snp	1	0	0	0	.	.	.	INTERGENIC(INTERGENIC);RP11-467I20.3.1(UPSTREAM)
1	240817091	240817099	240817095	A	C	snp	1	1	0	1	.	.	.	INTERGENIC(INTERGENIC);RNU5F-8P(DOWNSTREAM);RP11-467I20.5.1(UPSTREAM)
1	240817116	240817124	240817120	A	C	snp	1	0	0	0	.	.	.	INTERGENIC(INTERGENIC);RNU5F-8P(DOWNSTREAM);RP11-467I20.5.1(UPSTREAM)
1	240849563	240849572	240849570	AT	A	ins	0	1	0	1	.	.	.	INTERGENIC(INTERGENIC)
1	241025054	241025061	241025057	T	C	snp	1	0	0	0	.	.	.	RGS7(INTRON)
1	241170209	241170223	241170216	TTC	C	snp	1	0	0	0	.	.	.	RGS7(INTRON)
1	241204102	241204121	241204113	AAAC	A	snp	1	1	0	1	.	.	.	RGS7(INTRON)
1	241236716	241236724	241236721	A	G	snp	1	0	0	0	.	.	.	RGS7(INTRON)
1	241265889	241265896	241265894	T	C	snp	1	1	0	1	.	.	.	RGS7(INTRON)
1	241332021	241332036	241332034	TC	T	del	1	1	1	0	.	.	.	RGS7(INTRON)
1	241362396	241362403	241362400	A	C	snp	1	1	0	1	.	.	.	RGS7(INTRON)
1	241370649	241370660	241370652	AT	C	snp	1	1	0	1	.	.	.	RGS7(INTRON)
1	241385253	241385262	241385255	AC	G	snp	0	1	1	1	.	.	.	RGS7(INTRON)
1	241398983	241398992	241398990	TC	T	snp	1	1	1	1	.	.	.	RGS7(INTRON)
1	241401825	241401834	241401831	CT	TCT	del	0	1	1	1	.	.	.	RGS7(INTRON)
1	241410053	241410061	241410059	T	C	snp	1	1	1	1	.	.	.	RGS7(INTRON)
1	241448160	241448168	241448165	T	G	snp	1	1	1	1	.	.	.	RGS7(INTRON)
1	241487634	241487641	241487640	A	C	ins	1	1	1	1	.	.	.	RGS7(INTRON)
1	241525164	241525171	241525167	T	G	snp	1	0	0	0	.	.	.	INTERGENIC(INTERGENIC);RGS7(UPSTREAM)
1	241603940	241603955	241603944	GGGA	C	snp	1	0	0	0	.	.	.	INTERGENIC(INTERGENIC)
1	241670728	241670739	241670735	AC	G	snp	1	0	0	0	.	.	.	FH(DOWNSTREAM);FH(INTRON)
1	241720194	241720217	241720209	AAT	ATC	ins	1	0	0	0	.	.	.	KMO(DOWNSTREAM);KMO(INTRON)
1	241727863	241727872	241727869	AT	G	snp	0	0	1	0	.	.	.	KMO(DOWNSTREAM);KMO(INTRON)
1	241752260	241752271	241752265	AC	AT	ins	0	0	1	0	.	.	.	KMO(DOWNSTREAM);KMO(INTRON);OPN3(DOWNSTREAM)
1	241806576	241806593	241806590	CT	G	snp	1	1	1	1	.	.	.	OPN3(INTRON);OPN3(UPSTREAM)
1	241853771	241853780	241853779	A	C	snp	1	0	0	0	.	.	.	WDR64(INTRON)
1	241864581	241864596	241864588	CA	G	snp	0	1	0	1	.	.	.	WDR64(INTRON)
1	241876808	241876823	241876812	AAAG	AAT	ins	1	1	0	1	.	.	.	WDR64(INTRON)
1	241892978	241892985	241892984	T	C	snp	1	1	1	1	.	.	.	WDR64(INTRON)
1	241895604	241895623	241895609	TATC	TCTATCTATCTAT	del	1	1	1	1	.	.	.	WDR64(INTRON)
1	241896121	241896130	241896129	A	C	snp	1	1	1	1	.	.	.	WDR64(INTRON)
1	241936768	241936783	241936773	TTGT	C	snp	0	0	1	0	.	.	.	WDR64(INTRON)

1	241995108	241995121	241995114	CA	T	snp	1	1	1	1	.	.	.	INTERGENIC(INTERGENIC);RP11-553N16.1.1(UPSTREAM)
1	242028355	242028364	242028356	G	T	snp	1	1	1	1	.	.	.	EXO1(DOWNSTREAM);EXO1(INTRON)
1	242028355	242028364	242028358	G	T	snp	1	1	1	1	.	.	.	EXO1(DOWNSTREAM);EXO1(INTRON)
1	242028355	242028364	242028360	G	T	snp	1	1	1	1	.	.	.	EXO1(DOWNSTREAM);EXO1(INTRON)
1	242028480	242028497	242028492	TA	A	del	1	1	1	1	.	.	.	EXO1(DOWNSTREAM);EXO1(INTRON)
1	242028480	242028497	242028494	TA	A	del	1	1	1	1	.	.	.	EXO1(DOWNSTREAM);EXO1(INTRON)
1	242039809	242039820	242039815	AGG	G	snp	1	0	0	0	.	.	.	EXO1(INTRON)
1	242056145	242056153	242056150	A	C	ins	1	1	1	1	.	.	.	EXO1(DOWNSTREAM);EXO1(INTRON)
1	242068456	242068465	242068462	AG	G	snp	1	1	1	1	.	.	.	INTERGENIC(INTERGENIC)
1	242068456	242068465	242068463	AG	C	snp	1	1	1	1	.	.	.	INTERGENIC(INTERGENIC)
1	242069244	242069257	242069255	AC	T	snp	1	1	1	1	.	.	.	INTERGENIC(INTERGENIC)
1	242085142	242085150	242085148	A	C	snp	1	1	1	1	.	.	.	INTERGENIC(INTERGENIC)
1	242101223	242101231	242101224	T	TG	ins	1	0	0	0	.	.	.	INTERGENIC(INTERGENIC)
1	242101223	242101231	242101225	T	G	ins	1	0	0	0	.	.	.	INTERGENIC(INTERGENIC)
1	242101223	242101231	242101227	T	TTTTG	ins	1	1	1	1	.	.	.	INTERGENIC(INTERGENIC)
1	242191835	242191854	242191844	GTTT	C	snp	1	0	0	0	.	.	.	INTERGENIC(INTERGENIC);U6(UPSTREAM)
1	242230467	242230476	242230471	T	G	snp	1	0	0	0	.	.	.	INTERGENIC(INTERGENIC)
1	242253557	242253565	242253559	A	C	snp	1	0	0	0	.	.	.	PLD5(INTRON)
1	242273379	242273393	242273384	TAT	A	snp	1	0	0	0	.	.	.	PLD5(INTRON)
1	242344960	242344968	242344964	T	TG	ins	1	1	1	1	.	.	.	PLD5(INTRON)
1	242420211	242420234	242420218	ATCT	A	ins	1	0	0	0	.	.	.	PLD5(INTRON)
1	242480881	242480889	242480885	T	C	snp	1	1	1	1	.	.	.	PLD5(INTRON)
1	242488798	242488813	242488801	AG	A	snp	1	1	1	1	.	.	.	PLD5(INTRON)
1	242561072	242561086	242561083	TTC	TTCT	del	1	1	1	1	.	.	.	PLD5(INTRON)
1	242623412	242623421	242623414	AC	G	snp	1	0	0	0	.	.	.	PLD5(INTRON)
1	242661277	242661284	242661281	T	G	snp	1	1	1	1	.	.	.	PLD5(INTRON)
1	242792663	242792671	242792668	T	G	snp	1	1	1	1	.	.	.	INTERGENIC(INTERGENIC)
1	242800383	242800390	242800385	A	G	snp	1	0	0	0	.	.	.	INTERGENIC(INTERGENIC)
1	242906209	242906216	242906212	A	G	snp	1	0	0	0	.	.	.	INTERGENIC(INTERGENIC)
1	242972487	242972498	242972492	TG	T	snp	1	0	0	0	.	.	.	INTERGENIC(INTERGENIC)
1	242972838	242972857	242972853	TTTG	T	snp	1	1	1	1	.	.	.	INTERGENIC(INTERGENIC)
1	243002289	243002297	243002292	C	T	ins	0	0	1	0	.	.	.	INTERGENIC(INTERGENIC)
1	243002289	243002297	243002296	C	A	snp	1	1	1	1	.	.	.	INTERGENIC(INTERGENIC)
1	243051462	243051477	243051470	TATT	C	snp	0	1	1	1	.	.	.	INTERGENIC(INTERGENIC)
1	243102057	243102065	243102062	A	C	snp	0	1	1	0	.	.	.	INTERGENIC(INTERGENIC)
1	243104450	243104461	243104453	AG	C	snp	1	1	1	0	.	.	.	INTERGENIC(INTERGENIC)
1	243120306	243120314	243120310	A	C	snp	1	1	1	1	.	.	.	INTERGENIC(INTERGENIC)
1	243293175	243293183	243293176	A	G	snp	1	1	1	0	.	.	.	CEP170(INTRON);CEP170(UPSTREAM)
1	243389361	243389370	243389364	AT	AG	ins	1	1	1	1	.	.	.	CEP170(INTRON)
1	243389361	243389370	243389365	AT	TT	ins	1	1	1	1	.	.	.	CEP170(INTRON)
1	243891559	243891572	243891570	AC	T	snp	1	1	1	1	.	.	.	AKT3(INTRON);RP11-370K11.1.1(INTRON)
1	243936510	243936527	243936512	AT	T	del	1	0	0	0	.	.	.	AKT3(INTRON)
1	244036146	244036160	244036157	CAA	C	del	1	1	1	1	.	.	.	INTERGENIC(INTERGENIC)
1	244041916	244041923	244041920	T	G	snp	0	1	0	0	.	.	.	INTERGENIC(INTERGENIC)
1	244044532	244044547	244044536	ATAC	G	snp	1	0	0	0	.	.	.	INTERGENIC(INTERGENIC)
1	244075724	244075739	244075731	GAAG	A	snp	1	0	0	0	.	.	.	INTERGENIC(INTERGENIC);RP11-278H7.1.1(UPSTREAM)
1	244113106	244113115	244113110	T	G	snp	0	0	1	0	.	.	.	RP11-278H7.1.1(INTRON)
1	244272787	244272795	244272793	T	G	ins	1	0	0	0	.	.	.	INTERGENIC(INTERGENIC);RP11-278H7.3.1(DOWNSTREAM)
1	244314189	244314202	244314197	AT	G	snp	0	1	0	0	.	.	.	INTERGENIC(INTERGENIC)
1	244318718	244318733	244318727	GGAG	C	snp	0	1	1	1	.	.	.	INTERGENIC(INTERGENIC)
1	244460005	244460013	244460012	A	C	snp	1	0	0	0	.	.	.	INTERGENIC(INTERGENIC)
1	244518687	244518710	244518706	ATTG	C	snp	1	1	1	1	.	.	.	C1orf100(INTRON)
1	244523031	244523046	244523041	AATT	G	snp	1	0	0	0	.	.	.	C1orf100(INTRON)
1	244526310	244526319	244526317	T	G	ins	1	0	0	0	.	.	.	C1orf100(INTRON)
1	244694824	244694833	244694825	T	TA	ins	1	1	0	1	.	.	.	C1orf101(INTRON)
1	244698416	244698431	244698429	TC	G	snp	1	1	1	1	.	.	.	C1orf101(INTRON)
1	244712724	244712737	244712732	TA	AC	ins	1	1	1	1	.	.	.	C1orf101(INTRON)
1	244746526	244746533	244746528	A	G	snp	1	1	1	1	.	.	.	C1orf101(INTRON)
1	244836958	244836965	244836962	T	G	snp	0	1	0	1	.	.	.	PPPDE1(INTRON)
1	244975119	244975134	244975130	AAAG	C	snp	1	0	0	0	.	.	.	INTERGENIC(INTERGENIC)
1	245085888	245085897	245085894	CT	G	snp	1	0	0	0	.	.	.	INTERGENIC(INTERGENIC)
1	245092248	245092256	245092255	T	C	snp	1	0	0	0	.	.	.	INTERGENIC(INTERGENIC)
1	245112979	245112986	245112984	A	G	snp	0	1	0	1	.	.	.	INTERGENIC(INTERGENIC)
1	245119175	245119184	245119179	A	G	snp	1	1	1	1	.	.	.	INTERGENIC(INTERGENIC);U6(DOWNSTREAM)
1	245130108	245130123	245130111	TA	C	snp	1	1	1	1	.	.	.	EFCAB2(UPSTREAM);INTERGENIC(INTERGENIC)
1	245142165	245142173	245142166	T	C	snp	1	0	0	0	.	.	.	EFCAB2(INTRON)
1	245147431	245147440	245147432	G	T	snp	1	0	0	0	.	.	.	EFCAB2(INTRON)

1	245211380	245211397	245211389	GAG	C	snp	1	1	0	1	.	.	.	EFCAB2(INTRON);U6(DOWNSTREAM)
1	245228158	245228165	245228159	T	C	snp	1	1	1	1	.	.	.	EFCAB2(INTRON)
1	245386850	245386857	245386855	T	G	snp	0	1	1	1	.	.	.	KIF26B(INTRON);RP11-62I21.1.1(INTRON)
1	245408944	245408951	245408946	A	C	snp	0	0	1	0	.	.	.	KIF26B(INTRON)
1	245450789	245450800	245450795	TC	C	del	1	1	1	1	.	.	.	KIF26B(INTRON);RP11-62I21.2.1(UPSTREAM)
1	245450789	245450800	245450797	TC	CTC	del	1	1	1	1	.	.	.	KIF26B(INTRON);RP11-62I21.2.1(UPSTREAM)
1	245451354	245451361	245451358	T	C	snp	1	1	1	1	.	.	.	KIF26B(INTRON);RP11-62I21.2.1(UPSTREAM)
1	245483951	245483958	245483956	T	G	snp	1	1	1	1	.	.	.	KIF26B(INTRON)
1	245509402	245509410	245509409	T	G	ins	1	0	0	0	.	.	.	KIF26B(INTRON)
1	245509402	245509410	245509409	T	G	snp	1	0	0	0	.	.	.	KIF26B(INTRON)
1	245580473	245580481	245580477	T	C	snp	1	1	1	1	.	.	.	KIF26B(INTRON)
1	245590912	245590927	245590920	AAAC	T	snp	1	1	1	1	.	.	.	KIF26B(INTRON)
1	245619287	245619294	245619288	A	G	snp	1	1	1	1	.	.	.	KIF26B(INTRON)
1	245674887	245674910	245674896	TTTG	G	snp	1	1	1	1	.	.	.	KIF26B(INTRON)
1	245825009	245825018	245825015	AC	CAC	del	1	1	1	1	.	.	.	KIF26B(INTRON)
1	245827746	245827755	245827748	T	TA	ins	1	1	1	1	.	.	.	KIF26B(INTRON)
1	245854043	245854052	245854047	G	T	snp	1	1	1	1	.	.	.	KIF26B(DOWNSTREAM);KIF26B(INTRON)
1	245914822	245914833	245914828	GA	AA	ins	1	1	1	1	.	.	.	SMYD3(INTRON)
1	245949516	245949524	245949517	G	GGT	ins	1	1	1	1	.	.	.	SMYD3(INTRON)
1	245970654	245970663	245970655	A	G	snp	0	0	1	0	.	.	.	SMYD3(INTRON)
1	246017896	246017911	246017898	TC	C	del	1	0	0	0	.	.	.	SMYD3(DOWNSTREAM);SMYD3(INTRON)
1	246030993	246031002	246030997	CA	T	snp	1	1	1	1	.	.	.	SMYD3(INTRON)
1	246038009	246038017	246038016	A	G	snp	1	1	1	1	.	.	.	SMYD3(INTRON)
1	246113128	246113135	246113130	C	T	snp	1	1	1	1	.	.	.	SMYD3(INTRON);SMYD3(UPSTREAM)
1	246122427	246122446	246122442	AAAC	A	del	0	1	1	1	.	.	.	SMYD3(INTRON);SMYD3(UPSTREAM)
1	246212588	246212595	246212592	T	G	snp	1	0	0	0	.	.	.	SMYD3(DOWNSTREAM);SMYD3(INTRON)
1	246214419	246214427	246214422	T	C	snp	1	1	1	1	.	.	.	SMYD3(DOWNSTREAM);SMYD3(INTRON)
1	246249942	246249951	246249949	AG	A	snp	1	0	0	0	.	.	.	SMYD3(INTRON)
1	246253815	246253824	246253820	TC	T	ins	1	1	0	1	.	.	.	SMYD3(INTRON)
1	246301313	246301320	246301315	T	G	snp	1	1	1	1	.	.	.	SMYD3(INTRON)
1	246343665	246343674	246343670	TA	C	snp	1	0	0	1	.	.	.	RP11-36N20.2.1(DOWNSTREAM);SMYD3(INTRON)
1	246389487	246389498	246389491	AGA	A	snp	1	1	1	1	.	.	.	SMYD3(INTRON)
1	246403245	246403253	246403251	T	C	snp	1	1	1	1	.	.	.	SMYD3(INTRON)
1	246432836	246432844	246432841	T	C	ins	1	0	1	0	.	.	.	SMYD3(INTRON)
1	246491918	246491926	246491925	T	C	snp	1	1	1	1	.	.	.	SMYD3(INTRON)
1	246547348	246547355	246547354	A	G	snp	0	1	0	0	.	.	.	SMYD3(INTRON)
1	246605998	246606009	246606003	AT	C	snp	1	0	0	0	.	.	.	SMYD3(INTRON)
1	246644922	246644937	246644927	ATAC	C	snp	1	1	1	1	.	.	.	SMYD3(INTRON)
1	246644922	246644937	246644929	ATAC	T	snp	1	1	1	1	.	.	.	SMYD3(INTRON)
1	246644922	246644937	246644931	ATAC	C	snp	1	1	1	1	.	.	.	SMYD3(INTRON)
1	246668732	246668749	246668742	AGC	A	snp	1	1	0	1	.	.	.	RP11-690C23.4.1(DOWNSTREAM);SMYD3(INTRON)
1	246704821	246704830	246704829	A	G	snp	1	1	0	1	.	.	.	TFB2M(INTRON)
1	246710269	246710284	246710274	AC	ACAT	ins	1	1	1	1	.	.	.	TFB2M(DOWNSTREAM);TFB2M(INTRON)
1	246883764	246883772	246883770	C	A	ins	1	0	0	1	.	.	.	INTERGENIC(INTERGENIC);SCCPDH(UPSTREAM)
1	246883764	246883772	246883771	C	T	ins	1	1	1	1	.	.	.	INTERGENIC(INTERGENIC);SCCPDH(UPSTREAM)
1	246900921	246900932	246900930	TC	T	snp	0	1	1	1	.	.	.	SCCPDH(INTRON)
1	246929094	246929103	246929100	CT	G	snp	1	1	1	0	.	.	.	SCCPDH(INTRON)
1	246973875	246973886	246973877	AT	T	del	1	1	1	1	.	.	.	RP11-439E19.5.1(INTRON);SNORD112(DOWNSTREAM)
1	247010609	247010618	247010611	A	C	ins	1	0	1	0	.	.	.	AHCTF1(DOWNSTREAM);AHCTF1(INTRON)
1	247044687	247044694	247044692	A	C	snp	1	1	1	1	.	.	.	AHCTF1(INTRON);AHCTF1(UPSTREAM)
1	247100217	247100226	247100219	A	C	snp	1	0	0	0	.	.	.	AHCTF1(UPSTREAM);INTERGENIC(INTERGENIC)
1	247137448	247137463	247137453	AAAC	AC	del	1	1	1	1	.	.	.	ZNF670(INTRON);ZNF695(INTRON)
1	247137448	247137463	247137454	AAAC	C	del	1	1	1	1	.	.	.	ZNF670(INTRON);ZNF695(INTRON)
1	247137464	247137472	247137467	A	C	snp	1	1	1	1	.	.	.	ZNF670(INTRON);ZNF695(INTRON)
1	247139304	247139313	247139306	AG	G	snp	1	0	0	0	.	.	.	ZNF670(INTRON);ZNF695(INTRON)
1	247168995	247169002	247168998	A	C	snp	1	0	0	0	.	.	.	ZNF670(INTRON);ZNF695(INTRON)
1	247173546	247173555	247173550	T	C	snp	1	0	0	0	.	.	.	RP11-551G24.3.1(UPSTREAM);ZNF670(INTRON);ZNF695(UPSTREAM)
1	247196843	247196852	247196850	A	G	snp	1	0	0	0	.	.	.	ZNF670(DOWNSTREAM);ZNF670(INTRON)
1	247214572	247214581	247214573	A	C	ins	1	1	0	1	.	.	.	ZNF670(INTRON)
1	247216948	247216963	247216959	AAAC	A	ins	1	0	0	0	.	.	.	ZNF670(INTRON)
1	247232746	247232754	247232747	A	G	snp	0	0	1	0	.	.	.	ZNF670(INTRON)
1	247340496	247340503	247340501	T	G	ins	1	1	1	1	.	.	.	INTERGENIC(INTERGENIC);RP11-488L18.6.1(DOWNSTREAM)
1	247356828	247356835	247356834	A	G	snp	1	0	0	0	.	.	.	RP11-488L18.4.1(INTRON)
1	247374429	247374438	247374433	AT	TG	ins	1	0	0	0	.	.	.	RP11-488L18.4.1(UPSTREAM);RP11-488L18.8.1(INTRON)
1	247377386	247377399	247377391	AT	C	snp	1	1	1	1	.	.	.	RP11-488L18.4.1(UPSTREAM);RP11-488L18.8.1(INTRON)

1	247413472	247413479	247413473	T	G	snp	1	1	1	1	.	.	.	INTERGENIC(INTERGENIC)
1	247480873	247480882	247480879	C	T	snp	1	0	0	0	.	.	.	ZNF496(INTRON);ZNF496(UPSTREAM)
1	247561276	247561285	247561279	AC	A	snp	1	0	0	0	.	.	.	INTERGENIC(INTERGENIC)
1	247574301	247574312	247574307	ATT	T	ins	1	0	0	0	.	.	.	INTERGENIC(INTERGENIC)
1	247574301	247574312	247574309	ATT	A	del	1	0	0	0	.	.	.	INTERGENIC(INTERGENIC)
1	247622795	247622802	247622800	T	C	snp	1	1	1	1	.	.	.	INTERGENIC(INTERGENIC)
1	247693341	247693358	247693351	GT	A	snp	1	0	0	0	.	.	.	C1orf150(INTRON);OR2C3(DOWNSTREAM);RP11-978I15.9.1(UPSTREAM)
1	247713955	247713963	247713957	A	C	ins	1	0	0	1	.	.	.	C1orf150(DOWNSTREAM);C1orf150(INTRON)
1	247752621	247752629	247752627	A	C	snp	1	0	0	0	.	.	.	OR2G2(DOWNSTREAM);RP11-978I15.10.1(INTRON)
1	247817992	247818000	247817998	T	G	snp	0	1	0	1	.	.	.	RP11-634B7.4.1(INTRON)
1	247849464	247849479	247849473	TC	TT	ins	1	1	1	1	.	.	.	RP11-634B7.4.1(INTRON)
1	247905570	247905581	247905578	AC	C	snp	1	0	0	0	.	.	.	OR14K1(DOWNSTREAM);RP11-634B7.4.1(INTRON)
1	247905673	247905684	247905675	CA	T	snp	0	1	0	1	.	.	.	OR14K1(DOWNSTREAM);RP11-634B7.4.1(INTRON)
1	247972191	247972202	247972195	TTA	C	snp	1	0	0	0	.	.	.	INTERGENIC(INTERGENIC)
1	248032206	248032213	248032212	T	G	snp	1	1	1	1	.	.	.	OR2W3(INTRON);TRIM58(INTRON)
1	248032615	248032623	248032618	G	T	snp	1	1	1	1	.	.	.	OR2W3(INTRON);TRIM58(INTRON)
1	248085912	248085919	248085913	T	C	snp	1	1	1	1	.	.	.	INTERGENIC(INTERGENIC);OR2T8(DOWNSTREAM)
1	248114677	248114688	248114684	AAT	T	snp	1	1	1	1	.	.	.	OR2L13(INTRON);OR2L8(DOWNSTREAM)
1	248118248	248118263	248118260	AT	T	ins	1	1	1	1	.	.	.	OR2L13(INTRON)
1	248118248	248118263	248118261	AT	A	del	1	1	1	1	.	.	.	OR2L13(INTRON)
1	248119014	248119021	248119020	T	G	snp	1	1	1	1	.	.	.	OR2L13(INTRON)
1	248183057	248183071	248183062	CTT	C	snp	1	0	0	0	.	.	.	OR2L13(INTRON);OR2L5(UPSTREAM)
1	248212216	248212225	248212222	TC	C	del	1	0	0	0	.	.	.	OR2L13(INTRON);Y_RNA(DOWNSTREAM)
1	248212216	248212225	248212223	TC	T	ins	1	0	0	0	.	.	.	OR2L13(INTRON);Y_RNA(DOWNSTREAM)
1	248218704	248218713	248218705	T	C	snp	1	1	1	1	.	.	.	OR2L13(INTRON)
1	248449740	248449747	248449742	T	C	snp	1	0	0	0	.	.	.	INTERGENIC(INTERGENIC)
1	248506025	248506033	248506028	T	G	snp	1	1	1	1	.	.	.	INTERGENIC(INTERGENIC)
1	248516676	248516691	248516688	TA	A	del	1	1	1	1	.	.	.	INTERGENIC(INTERGENIC);OR14C36(DOWNSTREAM)
1	248545825	248545840	248545835	CTTT	TC	del	1	1	1	1	.	.	.	INTERGENIC(INTERGENIC)
1	248642151	248642158	248642155	T	C	snp	1	1	1	1	.	.	.	INTERGENIC(INTERGENIC);OR2T3(DOWNSTREAM)
1	248649158	248649167	248649163	CT	A	snp	1	1	1	1	.	.	.	INTERGENIC(INTERGENIC);OR2T5(UPSTREAM);RP11-407H12.8.1(UPSTREAM)
1	248689782	248689789	248689787	A	C	snp	1	1	1	1	.	.	.	INTERGENIC(INTERGENIC);OR2G6(DOWNSTREAM)
1	248732553	248732560	248732556	A	G	snp	1	0	0	0	.	.	.	INTERGENIC(INTERGENIC);OR2T34(DOWNSTREAM)
1	248836427	248836444	248836430	AT	A	snp	1	1	1	1	.	.	.	INTERGENIC(INTERGENIC)
1	248871448	248871471	248871464	AAC	C	del	1	1	1	1	.	.	.	INTERGENIC(INTERGENIC)
1	248871448	248871471	248871468	AAC	A	snp	1	1	1	1	.	.	.	INTERGENIC(INTERGENIC)
1	248879104	248879113	248879106	TA	C	snp	0	0	1	0	.	.	.	INTERGENIC(INTERGENIC);XX-CR54.3.1(DOWNSTREAM)
1	248906457	248906465	248906462	A	C	snp	0	1	1	1	.	.	.	INTERGENIC(INTERGENIC)
1	249205319	249205328	249205323	T	C	snp	1	0	1	0	.	.	.	PGBD2(INTRON);U6(DOWNSTREAM)
2	136331	136338	136336	A	G	snp	1	1	1	1	.	.	.	INTERGENIC(INTERGENIC)
2	151657	151672	151666	TA	C	snp	1	1	1	1	.	.	.	INTERGENIC(INTERGENIC)
2	172884	172892	172886	A	C	snp	1	1	1	1	.	.	.	INTERGENIC(INTERGENIC)
2	237822	237831	237826	A	C	snp	1	0	0	0	.	.	.	SH3YL1(DOWNSTREAM);SH3YL1(INTRON);SH3YL1(UPSTREAM)
2	434729	434744	434738	TCTT	T	del	1	1	1	1	.	.	.	INTERGENIC(INTERGENIC)
2	434966	434981	434971	TCCT	CTTCCTTCCTT	del	0	1	0	1	.	.	.	INTERGENIC(INTERGENIC)
2	590178	590187	590181	AC	T	snp	0	1	1	1	.	.	.	INTERGENIC(INTERGENIC)
2	724999	725006	725003	T	C	snp	1	1	1	1	.	.	.	AC116609.1.1(DOWNSTREAM);AC116609.1.1(EXON)
2	730299	730306	730305	A	C	snp	1	0	0	0	.	.	.	AC116609.1.1(INTRON)
2	733001	733008	733007	G	A	snp	1	1	1	1	.	.	.	AC116609.1.1(UPSTREAM);INTERGENIC(INTERGENIC)
2	733018	733025	733024	G	A	snp	1	1	1	1	.	.	.	AC116609.1.1(UPSTREAM);INTERGENIC(INTERGENIC)
2	765641	765650	765647	TG	C	snp	1	0	0	0	.	.	.	INTERGENIC(INTERGENIC)
2	854317	854334	854321	TG	C	snp	1	1	1	1	.	.	.	AC113607.1.1(INTRON)
2	856816	856823	856817	C	T	snp	1	1	0	1	.	.	.	AC113607.1.1(INTRON)
2	859462	859471	859467	C	A	snp	1	0	0	0	.	.	.	AC113607.1.1(INTRON)
2	893474	893482	893478	G	A	snp	1	0	0	0	.	.	.	AC113607.2.1(DOWNSTREAM);INTERGENIC(INTERGENIC)
2	935604	935621	935618	CT	A	snp	1	1	0	1	.	.	.	INTERGENIC(INTERGENIC)
2	935671	935684	935676	TA	C	snp	1	1	0	1	.	.	.	INTERGENIC(INTERGENIC)
2	935671	935684	935678	TA	C	snp	1	0	0	0	.	.	.	INTERGENIC(INTERGENIC)
2	962897	962904	962902	T	C	snp	1	1	1	1	.	.	.	SNTG2(INTRON)
2	1019486	1019495	1019488	TG	C	snp	1	1	0	1	.	.	.	SNTG2(INTRON)
2	1119721	1119728	1119723	T	C	ins	1	1	1	1	.	.	.	SNTG2(INTRON)
2	1139572	1139580	1139574	C	A	snp	0	1	1	1	.	.	.	SNTG2(INTRON)
2	1150752	1150761	1150756	CA	T	snp	1	1	1	1	.	.	.	AC114808.2.1(DOWNSTREAM);SNTG2(INTRON)
2	1151038	1151047	1151044	CA	T	snp	1	1	1	1	.	.	.	AC114808.2.1(DOWNSTREAM);SNTG2(INTRON)
2	1157069	1157078	1157075	AG	G	del	1	1	1	1	.	.	.	AC114808.2.1(UPSTREAM);SNTG2(INTRON);SNTG2(UPSTREAM);SNTG2(UTR_3_PRIME)

2	1192428	1192435	1192434	A	C	snp	1	1	1	1	.	.	.	SNTG2(INTRON)
2	1246013	1246020	1246019	T	C	snp	1	1	1	1	.	.	.	SNTG2(INTRON)
2	1367751	1367759	1367754	T	TTG	ins	1	1	1	1	.	.	.	SNTG2(INTRON)
2	1367751	1367759	1367756	T	G	ins	1	1	1	1	.	.	.	SNTG2(INTRON)
2	1393932	1393939	1393934	C	T	ins	1	1	1	1	.	.	.	TPO(INTRON)
2	1422769	1422778	1422774	AT	C	snp	0	0	1	0	.	.	.	TPO(INTRON)
2	1450758	1450771	1450768	GT	A	snp	1	0	1	1	.	.	.	TPO(INTRON)
2	1451487	1451496	1451492	GT	C	snp	1	1	1	1	.	.	.	TPO(INTRON)
2	1521713	1521721	1521719	C	A	snp	1	1	1	1	.	.	.	TPO(DOWNSTREAM);TPO(INTRON)
2	1540414	1540423	1540419	C	CA	ins	1	1	1	1	.	.	.	TPO(INTRON)
2	1551887	1551896	1551894	CA	CAC	del	1	0	0	0	.	.	.	AC144450.1.1(INTRON);TPO(DOWNSTREAM)
2	1589304	1589317	1589313	AT	C	snp	1	0	0	0	.	.	.	AC144450.1.1(INTRON)
2	1628111	1628118	1628115	T	G	snp	1	0	0	0	.	.	.	AC144450.1.1(UPSTREAM);AC144450.2.1(INTRON)
2	1632437	1632444	1632440	T	TTTTG	ins	1	0	0	0	.	.	.	AC144450.2.1(UPSTREAM);INTERGENIC(INTERGENIC);PXDN(DOWNSTREAM)
2	1660917	1660924	1660922	G	T	snp	1	0	0	0	.	.	.	PXDN(INTRON);PXDN(UPSTREAM)
2	1706993	1707000	1706999	G	A	snp	1	1	0	1	.	.	.	PXDN(INTRON)
2	1723649	1723658	1723655	AG	G	snp	1	1	1	1	.	.	.	PXDN(INTRON)
2	1723702	1723713	1723710	GA	AGA	del	0	0	1	0	.	.	.	PXDN(INTRON)
2	1745501	1745509	1745504	T	C	snp	1	1	1	1	.	.	.	PXDN(INTRON)
2	1870360	1870369	1870365	AG	C	snp	1	1	1	1	.	.	.	MYT1L(INTRON)
2	1883385	1883394	1883389	TG	C	snp	1	1	1	1	.	.	.	MYT1L(INTRON)
2	2041577	2041586	2041584	A	C	ins	1	1	0	1	.	.	.	MYT1L(INTRON)
2	2201262	2201271	2201267	CA	T	snp	1	0	0	0	.	.	.	MYT1L(INTRON)
2	2266163	2266171	2266165	A	C	snp	1	0	0	0	.	.	.	MYT1L(INTRON)
2	2324264	2324272	2324271	A	C	snp	0	0	1	0	.	.	.	AC009232.2.1(INTRON);MYT1L(INTRON)
2	2337139	2337146	2337140	A	G	snp	0	1	1	1	.	.	.	INTERGENIC(INTERGENIC);MYT1L(UPSTREAM)
2	2397350	2397369	2397357	AAAT	A	snp	1	1	1	1	.	.	.	INTERGENIC(INTERGENIC)
2	2422910	2422929	2422921	TTTG	A	snp	1	0	0	0	.	.	.	INTERGENIC(INTERGENIC)
2	2422910	2422929	2422925	TTTG	A	snp	1	0	0	0	.	.	.	INTERGENIC(INTERGENIC)
2	2425929	2425938	2425933	A	C	snp	1	0	0	0	.	.	.	INTERGENIC(INTERGENIC)
2	2480639	2480659	2480656	TTC	TTCT	del	1	1	1	1	.	.	.	INTERGENIC(INTERGENIC)
2	2483822	2483829	2483827	T	G	ins	0	1	1	1	.	.	.	INTERGENIC(INTERGENIC)
2	2564715	2564722	2564720	T	C	snp	1	1	1	1	.	.	.	INTERGENIC(INTERGENIC)
2	2616839	2616853	2616850	ATT	A	del	1	1	1	1	.	.	.	INTERGENIC(INTERGENIC)
2	2640080	2640087	2640081	T	G	snp	1	1	1	1	.	.	.	INTERGENIC(INTERGENIC)
2	2753888	2753897	2753891	GT	C	snp	1	1	1	1	.	.	.	INTERGENIC(INTERGENIC)
2	2788736	2788744	2788737	G	T	snp	1	1	1	1	.	.	.	INTERGENIC(INTERGENIC)
2	2788736	2788744	2788741	G	A	snp	0	0	0	1	.	.	.	INTERGENIC(INTERGENIC)
2	2790664	2790683	2790679	AAAT	G	snp	1	1	1	1	.	.	.	INTERGENIC(INTERGENIC)
2	2795559	2795567	2795561	G	T	ins	0	0	0	1	.	.	.	INTERGENIC(INTERGENIC)
2	2795559	2795567	2795565	G	T	ins	1	1	1	1	.	.	.	INTERGENIC(INTERGENIC)
2	3004202	3004210	3004209	T	C	snp	0	1	1	1	.	.	.	AC019118.2.1(INTRON)
2	3231545	3231556	3231551	GCT	C	snp	1	1	1	1	.	.	.	TSSC1(INTRON)
2	3250639	3250666	3250650	GGAG	A	snp	1	1	1	1	.	.	.	TSSC1(INTRON)
2	3349056	3349065	3349061	A	G	snp	0	1	1	1	.	.	.	TSSC1(INTRON)
2	3379175	3379182	3379176	A	G	snp	1	1	1	1	.	.	.	TRAPPC12(UPSTREAM);TSSC1(INTRON)
2	3487427	3487434	3487433	C	T	snp	0	1	0	0	.	.	.	TRAPPC12(DOWNSTREAM);TRAPPC12(INTRON);TRAPPC12-AS1(UPSTREAM)
2	3661725	3661733	3661732	T	G	ins	1	0	0	0	.	.	.	COLEC11(DOWNSTREAM);COLEC11(INTRON);TMSB4XP2(DOWNSTREAM)
2	3662717	3662736	3662721	TTTA	TTA	del	1	1	1	1	.	.	.	COLEC11(DOWNSTREAM);COLEC11(INTRON);TMSB4XP2(DOWNSTREAM)
2	3662717	3662736	3662723	TTTA	A	del	1	1	1	1	.	.	.	COLEC11(DOWNSTREAM);COLEC11(INTRON);TMSB4XP2(DOWNSTREAM)
2	3971143	3971151	3971145	G	T	snp	1	1	0	1	.	.	.	INTERGENIC(INTERGENIC)
2	3971143	3971151	3971148	G	T	ins	0	0	1	0	.	.	.	INTERGENIC(INTERGENIC)
2	4003042	4003053	4003051	AT	ATG	del	1	1	0	0	.	.	.	AC107070.1.1(DOWNSTREAM);INTERGENIC(INTERGENIC)
2	4034176	4034183	4034182	A	G	ins	1	0	0	0	.	.	.	INTERGENIC(INTERGENIC)
2	4134856	4134865	4134860	CA	A	snp	1	0	0	0	.	.	.	INTERGENIC(INTERGENIC)
2	4150810	4150817	4150813	T	C	snp	1	1	0	1	.	.	.	INTERGENIC(INTERGENIC)
2	4159592	4159615	4159611	TTTA	TTT	ins	1	1	0	1	.	.	.	INTERGENIC(INTERGENIC)
2	4205395	4205403	4205398	G	A	snp	1	1	0	1	.	.	.	INTERGENIC(INTERGENIC)
2	4221585	4221596	4221591	AAC	G	snp	1	0	0	0	.	.	.	INTERGENIC(INTERGENIC)
2	4280089	4280097	4280096	A	C	snp	1	0	0	0	.	.	.	AC068292.1(DOWNSTREAM);INTERGENIC(INTERGENIC)
2	4402181	4402188	4402182	A	C	snp	1	1	1	1	.	.	.	INTERGENIC(INTERGENIC)
2	4459664	4459679	4459674	TG	G	del	1	1	1	1	.	.	.	INTERGENIC(INTERGENIC)
2	4517888	4517899	4517893	GAG	A	snp	1	0	0	0	.	.	.	INTERGENIC(INTERGENIC)
2	4519632	4519643	4519640	TCT	C	snp	1	0	0	0	.	.	.	INTERGENIC(INTERGENIC)
2	4648809	4648818	4648814	TG	C	snp	0	1	1	1	.	.	.	INTERGENIC(INTERGENIC)
2	4648859	4648870	4648861	AT	G	snp	1	1	1	1	.	.	.	INTERGENIC(INTERGENIC)
2	4678637	4678648	4678645	CA	ACAAT	del	1	0	0	0	.	.	.	AC022311.1.1(INTRON)

2	4834197	4834204	4834199	T	C	snp	1	1	1	1	.	.	.	INTERGENIC(INTERGENIC)
2	4874679	4874702	4874691	AAAC	C	snp	1	0	0	0	.	.	.	INTERGENIC(INTERGENIC);SNORA31(UPSTREAM)
2	4874679	4874702	4874698	AAAC	A	ins	0	1	0	0	.	.	.	INTERGENIC(INTERGENIC);SNORA31(UPSTREAM)
2	4969506	4969517	4969512	TA	AA	ins	1	0	0	0	.	.	.	INTERGENIC(INTERGENIC)
2	5122798	5122806	5122799	A	G	snp	1	1	1	1	.	.	.	INTERGENIC(INTERGENIC)
2	5175855	5175870	5175863	AC	C	snp	1	1	1	1	.	.	.	INTERGENIC(INTERGENIC)
2	5186044	5186055	5186050	TG	C	snp	1	0	0	0	.	.	.	INTERGENIC(INTERGENIC)
2	5186058	5186067	5186065	TG	T	snp	1	0	0	0	.	.	.	INTERGENIC(INTERGENIC)
2	5195591	5195598	5195593	A	G	snp	1	0	0	0	.	.	.	INTERGENIC(INTERGENIC)
2	5302054	5302068	5302061	TAA	G	snp	1	0	0	0	.	.	.	INTERGENIC(INTERGENIC)
2	5425611	5425620	5425619	A	C	snp	1	1	1	1	.	.	.	INTERGENIC(INTERGENIC)
2	5507079	5507092	5507090	TC	TCT	del	1	1	0	1	.	.	.	INTERGENIC(INTERGENIC)
2	5540112	5540125	5540119	AT	C	snp	1	1	1	1	.	.	.	INTERGENIC(INTERGENIC)
2	5543323	5543338	5543328	TC	T	snp	1	0	0	0	.	.	.	INTERGENIC(INTERGENIC)
2	5569640	5569649	5569646	T	G	snp	1	0	0	0	.	.	.	INTERGENIC(INTERGENIC)
2	5582046	5582054	5582053	A	AG	ins	1	1	1	1	.	.	.	INTERGENIC(INTERGENIC)
2	5619085	5619092	5619091	T	C	snp	0	0	0	1	.	.	.	INTERGENIC(INTERGENIC)
2	5704253	5704260	5704254	A	G	snp	1	1	1	1	.	.	.	INTERGENIC(INTERGENIC)
2	5731842	5731849	5731843	G	T	snp	1	1	1	1	.	.	.	INTERGENIC(INTERGENIC)
2	5810407	5810415	5810412	A	G	snp	1	0	0	0	.	.	.	AC107057.2.1(INTRON);AC108025.2.1(INTRON)
2	5843328	5843337	5843330	AC	G	snp	1	0	0	0	.	.	.	AC010729.1.1(INTRON);SOX11(DOWNSTREAM)
2	5875945	5875954	5875951	T	G	ins	1	0	0	0	.	.	.	INTERGENIC(INTERGENIC)
2	5883929	5883946	5883932	AG	A	snp	1	1	1	1	.	.	.	INTERGENIC(INTERGENIC)
2	6006128	6006135	6006129	T	G	snp	1	1	0	1	.	.	.	INTERGENIC(INTERGENIC)
2	6151829	6151836	6151832	A	C	snp	1	0	0	0	.	.	.	INTERGENIC(INTERGENIC)
2	6157174	6157185	6157176	CT	T	snp	1	1	1	1	.	.	.	INTERGENIC(INTERGENIC)
2	6194155	6194163	6194161	T	G	snp	1	1	0	1	.	.	.	INTERGENIC(INTERGENIC)
2	6263917	6263928	6263923	CT	T	ins	0	0	1	0	.	.	.	INTERGENIC(INTERGENIC)
2	6270039	6270048	6270044	A	G	snp	1	0	1	0	.	.	.	INTERGENIC(INTERGENIC)
2	6360644	6360657	6360649	AG	A	snp	1	0	0	0	.	.	.	INTERGENIC(INTERGENIC)
2	6368076	6368091	6368089	TA	T	del	1	0	0	0	.	.	.	INTERGENIC(INTERGENIC)
2	6394068	6394077	6394075	TC	G	snp	1	1	1	1	.	.	.	INTERGENIC(INTERGENIC)
2	6396824	6396844	6396837	CCA	A	snp	1	1	1	1	.	.	.	INTERGENIC(INTERGENIC)
2	6396824	6396844	6396839	CCA	A	snp	1	1	1	1	.	.	.	INTERGENIC(INTERGENIC)
2	6403290	6403305	6403301	TTTC	TT	ins	1	1	1	1	.	.	.	INTERGENIC(INTERGENIC)
2	6416488	6416496	6416489	T	C	snp	1	1	1	1	.	.	.	INTERGENIC(INTERGENIC)
2	6419242	6419249	6419246	T	G	snp	1	1	1	1	.	.	.	INTERGENIC(INTERGENIC)
2	6505278	6505289	6505280	GT	A	snp	1	1	1	1	.	.	.	AC105253.1.1(DOWNSTREAM);INTERGENIC(INTERGENIC)
2	6515917	6515940	6515929	ATT	T	ins	1	0	0	0	.	.	.	AC105253.1.1(UPSTREAM);INTERGENIC(INTERGENIC)
2	6515917	6515940	6515931	ATT	A	del	1	1	1	1	.	.	.	AC105253.1.1(UPSTREAM);INTERGENIC(INTERGENIC)
2	6515917	6515940	6515932	ATT	T	snp	1	1	0	1	.	.	.	AC105253.1.1(UPSTREAM);INTERGENIC(INTERGENIC)
2	6515917	6515940	6515934	ATT	A	del	1	1	1	1	.	.	.	AC105253.1.1(UPSTREAM);INTERGENIC(INTERGENIC)
2	6515917	6515940	6515935	ATT	T	snp	1	1	1	1	.	.	.	AC105253.1.1(UPSTREAM);INTERGENIC(INTERGENIC)
2	6515917	6515940	6515937	ATT	A	del	1	1	1	1	.	.	.	AC105253.1.1(UPSTREAM);INTERGENIC(INTERGENIC)
2	6548547	6548562	6548558	TCTT	TC	del	1	1	1	1	.	.	.	INTERGENIC(INTERGENIC)
2	6557193	6557206	6557202	AT	AG	ins	1	1	1	1	.	.	.	INTERGENIC(INTERGENIC)
2	6583523	6583531	6583524	A	C	snp	0	0	0	1	.	.	.	INTERGENIC(INTERGENIC)
2	6656187	6656198	6656190	TG	TA	ins	1	1	1	1	.	.	.	INTERGENIC(INTERGENIC)
2	6684933	6684942	6684939	AG	G	snp	1	0	0	0	.	.	.	INTERGENIC(INTERGENIC)
2	6884538	6884547	6884543	AT	A	snp	1	1	1	1	.	.	.	LINC00487(INTRON)
2	6889845	6889864	6889860	AAAT	G	snp	1	1	1	1	.	.	.	LINC00487(INTRON)
2	6899856	6899865	6899858	TG	G	snp	0	1	0	1	.	.	.	LINC00487(INTRON)
2	6986094	6986101	6986097	T	G	snp	0	1	0	1	.	.	.	CMPK2(DOWNSTREAM);CMPK2(INTRON)
2	7048206	7048223	7048217	AC	G	snp	1	1	1	1	.	.	.	AC017076.4.1(DOWNSTREAM);INTERGENIC(INTERGENIC)
2	7080644	7080651	7080646	T	C	snp	1	1	1	1	.	.	.	RNF144A(INTRON)
2	7265330	7265339	7265338	T	TA	ins	1	1	1	1	.	.	.	INTERGENIC(INTERGENIC)
2	7278570	7278581	7278575	ATA	G	snp	0	1	0	1	.	.	.	7SK(DOWNSTREAM);INTERGENIC(INTERGENIC)
2	7386188	7386197	7386192	TG	C	snp	0	1	0	1	.	.	.	INTERGENIC(INTERGENIC)
2	7446538	7446552	7446548	TTG	G	snp	1	0	0	0	.	.	.	INTERGENIC(INTERGENIC)
2	7461831	7461839	7461836	T	G	snp	1	0	0	0	.	.	.	AC010904.1.1(DOWNSTREAM);INTERGENIC(INTERGENIC)
2	7578968	7578995	7578978	AATA	C	snp	1	0	0	0	.	.	.	AC013460.1.1(INTRON)
2	7733868	7733883	7733870	CT	G	snp	1	1	1	1	.	.	.	INTERGENIC(INTERGENIC)
2	7733975	7733984	7733979	AT	C	snp	1	0	1	1	.	.	.	INTERGENIC(INTERGENIC)
2	7734003	7734012	7734007	AT	C	snp	1	1	0	1	.	.	.	INTERGENIC(INTERGENIC)
2	7734031	7734040	7734035	AT	C	snp	1	0	1	1	.	.	.	INTERGENIC(INTERGENIC)
2	7750514	7750523	7750518	GA	C	snp	0	1	0	0	.	.	.	INTERGENIC(INTERGENIC)
2	7803923	7803938	7803936	AG	A	snp	1	0	0	0	.	.	.	INTERGENIC(INTERGENIC)

2	7862695	7862702	7862696	T	G	snp	1	0	0	0	.	.	.	AC092580.4.1(UPSTREAM);INTERGENIC(INTERGENIC)
2	7881098	7881115	7881105	GT	C	snp	1	1	1	1	.	.	.	AC092580.1.1(UPSTREAM);INTERGENIC(INTERGENIC)
2	7881686	7881693	7881692	T	G	snp	1	1	1	1	.	.	.	AC092580.1.1(UPSTREAM);INTERGENIC(INTERGENIC)
2	7970992	7970999	7970996	T	C	snp	1	0	0	0	.	.	.	INTERGENIC(INTERGENIC)
2	8038985	8039004	8038994	AACA	G	snp	1	1	1	1	.	.	.	AC007463.2.1(INTRON);AC007463.2.1(UPSTREAM)
2	8055353	8055360	8055359	A	G	snp	1	0	0	0	.	.	.	INTERGENIC(INTERGENIC)
2	8126788	8126801	8126791	TC	T	snp	1	1	1	0	.	.	.	AC007464.1.1(INTRON)
2	8209548	8209555	8209553	A	G	snp	1	0	0	0	.	.	.	AC007464.1.1(INTRON);LINC00299(INTRON)
2	8276544	8276551	8276549	A	C	snp	1	0	0	0	.	.	.	AC007464.1.1(INTRON);LINC00299(INTRON);U91324.1.1(UPSTREAM)
2	8431556	8431571	8431568	AC	T	snp	1	0	0	0	.	.	.	LINC00299(INTRON)
2	8448416	8448423	8448419	G	T	snp	1	1	1	1	.	.	.	LINC00299(DOWNSTREAM);LINC00299(INTRON)
2	8448416	8448423	8448422	G	A	snp	1	1	1	1	.	.	.	LINC00299(DOWNSTREAM);LINC00299(INTRON)
2	8500700	8500707	8500704	T	C	snp	1	0	0	0	.	.	.	LINC00299(INTRON)
2	8729692	8729703	8729695	AG	T	snp	0	1	0	0	.	.	.	INTERGENIC(INTERGENIC)
2	8845872	8845879	8845875	A	G	snp	1	1	1	0	.	.	.	INTERGENIC(INTERGENIC)
2	9203271	9203286	9203273	GA	A	snp	1	0	0	0	.	.	.	INTERGENIC(INTERGENIC)
2	9255668	9255685	9255682	TC	CTC	del	0	0	0	1	.	.	.	RP11-734K21.3.1(INTRON)
2	9260099	9260108	9260104	A	G	snp	1	1	0	1	.	.	.	INTERGENIC(INTERGENIC);RP11-734K21.3.1(DOWNSTREAM)
2	9426577	9426590	9426585	AT	G	snp	0	0	1	0	.	.	.	ASAP2(INTRON)
2	9474140	9474151	9474147	TC	TCTCTG	ins	1	0	0	0	.	.	.	ASAP2(INTRON)
2	9478477	9478492	9478485	TC	C	del	1	0	0	0	.	.	.	ASAP2(INTRON)
2	9550879	9550886	9550880	T	C	snp	1	1	0	1	.	.	.	ITGB1BP1(DOWNSTREAM);ITGB1BP1(INTRON);ITGB1BP1(UPSTREAM)
2	9555536	9555544	9555537	A	C	snp	1	0	0	0	.	.	.	ITGB1BP1(INTRON);ITGB1BP1(UPSTREAM)
2	9584270	9584287	9584284	TA	ATA	del	1	1	0	0	.	.	.	CPSF3(INTRON)
2	9629281	9629288	9629285	T	TTG	ins	1	0	0	0	.	.	.	ADAM17(UTR_3_PRIME);IAH1(DOWNSTREAM);IAH1(INTRON);IAH1(UTR_3_PRIME)
2	9629287	9629301	9629298	TTG	TTC	ins	1	1	0	1	.	.	.	ADAM17(UTR_3_PRIME);IAH1(DOWNSTREAM);IAH1(INTRON);IAH1(UTR_3_PRIME)
2	9649121	9649129	9649123	A	G	ins	1	0	0	0	.	.	.	ADAM17(INTRON);RP11-400L8.2.1(INTRON)
2	9705276	9705287	9705282	TTC	C	snp	1	1	1	0	.	.	.	INTERGENIC(INTERGENIC)
2	9740835	9740843	9740836	A	G	snp	1	0	0	0	.	.	.	YWHAQ(INTRON)
2	9792308	9792331	9792312	TCCA	C	snp	1	1	1	1	.	.	.	INTERGENIC(INTERGENIC);RP11-521D12.1.1(DOWNSTREAM)
2	9880364	9880379	9880369	ACCC	T	snp	1	1	1	1	.	.	.	INTERGENIC(INTERGENIC);U4(DOWNSTREAM)
2	9880364	9880379	9880370	ACCC	A	snp	0	1	1	1	.	.	.	INTERGENIC(INTERGENIC);U4(DOWNSTREAM)
2	9918368	9918377	9918372	CA	T	snp	1	0	0	0	.	.	.	INTERGENIC(INTERGENIC)
2	10017637	10017648	10017642	CT	C	snp	0	1	0	1	.	.	.	TAF1B(INTRON)
2	10040668	10040682	10040672	AAT	T	snp	1	1	1	1	.	.	.	TAF1B(INTRON)
2	10042021	10042030	10042023	A	C	snp	1	1	1	1	.	.	.	TAF1B(INTRON)
2	10088692	10088699	10088693	G	GGTGGT	ins	1	1	1	1	.	.	.	GRHL1(INTRON);GRHL1(UPSTREAM)
2	10097114	10097131	10097128	TTC	T	ins	1	1	1	1	.	.	.	GRHL1(INTRON);GRHL1(UPSTREAM)
2	10138695	10138704	10138702	TA	G	snp	0	0	0	1	.	.	.	AC010969.1.1(DOWNSTREAM);GRHL1(INTRON)
2	10138728	10138737	10138733	AT	C	snp	1	1	1	1	.	.	.	AC010969.1.1(DOWNSTREAM);GRHL1(INTRON)
2	10204838	10204853	10204849	TTTA	T	snp	1	0	0	0	.	.	.	CYS1(INTRON)
2	10227361	10227372	10227368	AC	G	snp	1	1	1	1	.	.	.	INTERGENIC(INTERGENIC);SNORA26(DOWNSTREAM)
2	10309099	10309114	10309106	TATT	G	snp	1	0	0	0	.	.	.	C2orf48(INTRON)
2	10321864	10321881	10321877	CT	CTC	del	1	1	1	1	.	.	.	C2orf48(INTRON)
2	10322493	10322501	10322496	A	C	snp	1	1	0	0	.	.	.	C2orf48(INTRON)
2	10474822	10474829	10474825	T	TC	ins	1	0	0	0	.	.	.	HPCAL1(INTRON)
2	10474822	10474829	10474826	T	C	ins	1	0	0	0	.	.	.	HPCAL1(INTRON)
2	10548389	10548404	10548399	TC	C	del	0	1	0	1	.	.	.	HPCAL1(INTRON)
2	10558391	10558410	10558405	ATAA	AAT	ins	1	1	1	1	.	.	.	HPCAL1(INTRON)
2	10658826	10658835	10658830	CA	A	snp	1	1	0	1	.	.	.	INTERGENIC(INTERGENIC)
2	10727639	10727646	10727640	C	A	snp	1	0	0	0	.	.	.	AC092687.5.1(UPSTREAM);NOL10(INTRON)
2	10767801	10767808	10767805	A	C	snp	1	0	0	0	.	.	.	NOL10(INTRON)
2	10841809	10841816	10841814	T	C	snp	1	1	1	1	.	.	.	INTERGENIC(INTERGENIC)
2	10867681	10867696	10867689	CAAA	AAAT	ins	1	0	0	0	.	.	.	ATP6V1C2(INTRON)
2	10892190	10892199	10892197	A	C	snp	1	1	1	1	.	.	.	ATP6V1C2(INTRON)
2	10892198	10892209	10892206	AAC	A	snp	1	0	0	0	.	.	.	ATP6V1C2(INTRON)
2	10919555	10919568	10919558	AT	C	snp	1	1	0	1	.	.	.	ATP6V1C2(INTRON);PDIA6(DOWNSTREAM)
2	10946755	10946774	10946762	AGAA	C	snp	0	0	0	1	.	.	.	PDIA6(INTRON)
2	10980729	10980738	10980732	C	CA	ins	1	1	1	0	.	.	.	INTERGENIC(INTERGENIC);PDIA6(UPSTREAM)
2	10980729	10980738	10980733	C	A	ins	1	0	0	0	.	.	.	INTERGENIC(INTERGENIC);PDIA6(UPSTREAM)
2	10996265	10996273	10996270	G	T	snp	1	1	1	1	.	.	.	AC092687.4.1(DOWNSTREAM);INTERGENIC(INTERGENIC)
2	11116095	11116103	11116101	A	C	snp	0	1	0	1	.	.	.	INTERGENIC(INTERGENIC)
2	11116641	11116652	11116644	AAT	G	snp	0	1	0	1	.	.	.	INTERGENIC(INTERGENIC)
2	11159288	11159296	11159290	T	G	snp	1	0	0	0	.	.	.	INTERGENIC(INTERGENIC)
2	11187420	11187437	11187422	AT	T	del	1	0	1	1	.	.	.	INTERGENIC(INTERGENIC)
2	11188829	11188842	11188834	TG	A	snp	0	1	0	1	.	.	.	INTERGENIC(INTERGENIC)
2	11188891	11188900	11188893	GT	A	snp	1	1	1	1	.	.	.	INTERGENIC(INTERGENIC)

2	11213634	11213645	11213643	CA	G	snp	1	1	1	1	.	.	.	INTERGENIC(INTERGENIC)
2	11227581	11227598	11227590	TTG	C	snp	0	0	0	1	.	.	.	INTERGENIC(INTERGENIC)
2	11253174	11253189	11253185	AAGG	A	snp	1	1	1	1	.	.	.	AC062028.1.1(INTRON)
2	11265651	11265658	11265654	T	C	snp	1	0	0	0	.	.	.	AC062028.1.1(INTRON)
2	11442449	11442458	11442451	T	G	snp	1	1	1	1	.	.	.	ROCK2(INTRON)
2	11469227	11469235	11469228	A	C	ins	0	1	1	1	.	.	.	ROCK2(INTRON)
2	11519311	11519320	11519315	GA	A	snp	0	0	0	1	.	.	.	INTERGENIC(INTERGENIC)
2	11520817	11520826	11520819	AT	G	snp	1	0	0	0	.	.	.	INTERGENIC(INTERGENIC)
2	11521538	11521547	11521539	T	G	snp	0	1	1	1	.	.	.	INTERGENIC(INTERGENIC)
2	11542753	11542767	11542762	CAC	A	del	1	1	1	1	.	.	.	AC099344.4.1(DOWNSTREAM);AC099344.4.1(EXON)
2	11576240	11576263	11576246	AATA	A	del	1	0	0	0	.	.	.	INTERGENIC(INTERGENIC)
2	11582986	11582995	11582987	G	A	snp	1	0	0	0	.	.	.	E2F6(DOWNSTREAM);INTERGENIC(INTERGENIC)
2	11626433	11626440	11626439	A	C	snp	1	0	0	0	.	.	.	INTERGENIC(INTERGENIC)
2	11678886	11678901	11678892	CTTC	C	ins	1	1	0	1	.	.	.	GREB1(INTRON);GREB1(UPSTREAM)
2	11678886	11678901	11678893	CTTC	CTT	del	1	1	1	1	.	.	.	GREB1(INTRON);GREB1(UPSTREAM)
2	11685689	11685696	11685694	A	C	snp	1	1	1	1	.	.	.	GREB1(INTRON)
2	11711165	11711178	11711167	AT	G	snp	0	1	0	0	.	.	.	GREB1(INTRON)
2	11810712	11810723	11810716	TG	C	snp	1	1	1	1	.	.	.	AC110754.3.1(UPSTREAM);INTERGENIC(INTERGENIC);NTSR2(UPSTREAM)
2	11824854	11824873	11824869	TCCC	G	snp	1	0	0	0	.	.	.	AC106875.1.1(DOWNSTREAM);LPIN1(INTRON)
2	11853720	11853728	11853722	T	C	snp	1	0	1	0	.	.	.	LPIN1(INTRON)
2	11880849	11880872	11880856	AAAG	C	snp	1	1	0	0	.	.	.	AC012456.4.1(DOWNSTREAM);LPIN1(INTRON)
2	11929690	11929699	11929697	GT	C	snp	1	1	1	1	.	.	.	LPIN1(DOWNSTREAM);LPIN1(INTRON)
2	12020543	12020554	12020552	CT	C	del	1	1	0	1	.	.	.	AC096559.1.1(INTRON)
2	12261101	12261110	12261103	GA	C	snp	1	1	1	1	.	.	.	AC096559.1.1(INTRON)
2	12302648	12302659	12302655	AC	T	snp	1	1	1	1	.	.	.	AC096559.1.1(INTRON)
2	12374319	12374328	12374323	TG	G	snp	0	0	0	1	.	.	.	AC096559.1.1(INTRON)
2	12385412	12385423	12385418	CTT	TTCT	del	1	1	1	1	.	.	.	AC096559.1.1(INTRON)
2	12385412	12385423	12385419	CTT	TC	del	1	1	1	1	.	.	.	AC096559.1.1(INTRON)
2	12385412	12385423	12385420	CTT	C	del	1	1	1	1	.	.	.	AC096559.1.1(INTRON)
2	12606175	12606186	12606181	AC	T	snp	1	1	1	1	.	.	.	AC096559.1.1(INTRON)
2	12661858	12661881	12661869	TTAT	TTA	ins	1	1	1	1	.	.	.	AC096559.1.1(INTRON)
2	12783330	12783339	12783333	T	G	snp	1	0	0	0	.	.	.	INTERGENIC(INTERGENIC)
2	12918386	12918394	12918393	A	G	snp	1	0	1	1	.	.	.	INTERGENIC(INTERGENIC)
2	13145794	13145811	13145807	CA	CAC	del	1	1	1	1	.	.	.	AC064875.2.1(INTRON)
2	13154949	13154962	13154956	TG	A	snp	1	1	1	0	.	.	.	INTERGENIC(INTERGENIC)
2	13169761	13169770	13169768	TC	A	snp	1	1	1	1	.	.	.	INTERGENIC(INTERGENIC)
2	13209419	13209426	13209425	A	G	snp	1	1	1	1	.	.	.	INTERGENIC(INTERGENIC)
2	13245472	13245483	13245475	AT	AC	ins	0	1	1	1	.	.	.	INTERGENIC(INTERGENIC)
2	13365225	13365234	13365231	AT	G	snp	1	1	1	1	.	.	.	INTERGENIC(INTERGENIC)
2	13381132	13381139	13381137	C	T	snp	1	1	1	1	.	.	.	INTERGENIC(INTERGENIC)
2	13459819	13459826	13459823	T	C	snp	1	0	0	0	.	.	.	INTERGENIC(INTERGENIC)
2	13532083	13532092	13532088	TC	T	snp	1	1	1	1	.	.	.	INTERGENIC(INTERGENIC)
2	13539983	13539992	13539984	A	G	snp	1	1	1	1	.	.	.	INTERGENIC(INTERGENIC)
2	13611617	13611628	13611622	AT	C	snp	1	0	0	0	.	.	.	INTERGENIC(INTERGENIC)
2	13613633	13613640	13613639	T	G	snp	1	1	1	1	.	.	.	INTERGENIC(INTERGENIC)
2	13669486	13669495	13669487	T	C	ins	1	0	0	0	.	.	.	INTERGENIC(INTERGENIC)
2	13727065	13727076	13727073	AC	G	snp	1	0	0	0	.	.	.	AC092635.1.1(INTRON)
2	13756592	13756600	13756597	A	G	ins	1	0	1	0	.	.	.	INTERGENIC(INTERGENIC)
2	13756592	13756600	13756599	A	G	ins	1	1	1	1	.	.	.	INTERGENIC(INTERGENIC)
2	13789481	13789492	13789490	GA	T	snp	1	0	1	0	.	.	.	INTERGENIC(INTERGENIC)
2	13869373	13869382	13869379	A	G	ins	1	1	1	1	.	.	.	AC016730.1.1(INTRON);LINC00276(INTRON)
2	13890984	13890993	13890988	TA	C	snp	1	1	1	0	.	.	.	AC016730.1.1(INTRON);LINC00276(INTRON)
2	13959017	13959026	13959024	TA	T	snp	1	1	1	1	.	.	.	LINC00276(INTRON)
2	13959037	13959048	13959040	TA	T	snp	1	1	1	1	.	.	.	LINC00276(INTRON)
2	13959097	13959106	13959102	TA	T	snp	1	1	1	1	.	.	.	LINC00276(INTRON)
2	13959673	13959690	13959683	AT	T	snp	1	1	1	0	.	.	.	LINC00276(INTRON)
2	13968691	13968704	13968702	TA	T	snp	0	0	1	0	.	.	.	LINC00276(INTRON)
2	13990490	13990497	13990496	A	G	snp	1	1	1	1	.	.	.	LINC00276(INTRON)
2	13991603	13991612	13991608	AG	C	snp	1	1	1	1	.	.	.	LINC00276(INTRON)
2	14009170	14009179	14009177	TG	C	snp	1	0	0	0	.	.	.	LINC00276(INTRON)
2	14017527	14017535	14017531	T	C	ins	0	1	1	1	.	.	.	LINC00276(INTRON)
2	14040886	14040899	14040897	TG	A	snp	1	1	1	1	.	.	.	LINC00276(INTRON)
2	14125668	14125677	14125672	A	C	snp	0	1	0	0	.	.	.	LINC00276(INTRON)
2	14143185	14143198	14143195	TA	ACAC	ins	1	1	0	1	.	.	.	LINC00276(INTRON)
2	14157092	14157107	14157104	GA	C	snp	0	0	1	0	.	.	.	LINC00276(INTRON)
2	14192093	14192112	14192099	AAAT	T	ins	0	0	1	0	.	.	.	LINC00276(INTRON)

2	14214604	14214618	14214614	TTG	C	snp	1	0	1	0	.	.	.	LINC00276(INTRON)
2	14265644	14265652	14265649	A	C	snp	1	0	0	0	.	.	.	LINC00276(INTRON)
2	14274563	14274578	14274570	AGGG	C	snp	1	1	1	1	.	.	.	LINC00276(INTRON)
2	14295235	14295250	14295239	GA	C	snp	0	1	1	1	.	.	.	LINC00276(INTRON)
2	14308312	14308321	14308317	CA	T	snp	1	1	1	1	.	.	.	LINC00276(INTRON)
2	14386753	14386760	14386758	T	C	snp	1	1	1	1	.	.	.	LINC00276(INTRON)
2	14392647	14392662	14392649	AC	G	snp	1	0	0	0	.	.	.	LINC00276(INTRON)
2	14495037	14495045	14495040	A	C	snp	1	1	1	1	.	.	.	LINC00276(INTRON)
2	14505061	14505074	14505063	CT	G	snp	1	0	0	0	.	.	.	LINC00276(INTRON)
2	14518020	14518031	14518028	CT	TCT	del	1	0	0	0	.	.	.	LINC00276(INTRON)
2	14603225	14603236	14603234	AC	A	snp	1	0	0	0	.	.	.	INTERGENIC(INTERGENIC)
2	14622810	14622829	14622815	AAAT	T	snp	1	0	0	0	.	.	.	INTERGENIC(INTERGENIC)
2	14819165	14819172	14819168	A	G	snp	1	0	0	0	.	.	.	INTERGENIC(INTERGENIC)
2	14900631	14900646	14900634	TA	TATATATATAATATATATATACCAAAG	ins	1	1	1	1	.	.	.	INTERGENIC(INTERGENIC)
2	14965771	14965779	14965773	A	G	snp	0	0	0	1	.	.	.	INTERGENIC(INTERGENIC)
2	15007086	15007097	15007094	ATA	G	snp	1	1	1	1	.	.	.	INTERGENIC(INTERGENIC)
2	15030979	15030987	15030980	A	C	ins	1	1	0	1	.	.	.	AC068286.1.1(INTRON)
2	15030979	15030987	15030983	A	C	ins	1	1	0	1	.	.	.	AC068286.1.1(INTRON)
2	15088856	15088864	15088858	A	C	ins	1	0	0	0	.	.	.	INTERGENIC(INTERGENIC)
2	15141377	15141388	15141380	AG	T	snp	1	1	0	1	.	.	.	INTERGENIC(INTERGENIC)
2	15141377	15141388	15141382	AG	T	snp	1	0	0	0	.	.	.	INTERGENIC(INTERGENIC)
2	15141470	15141479	15141476	TA	C	snp	1	1	0	1	.	.	.	INTERGENIC(INTERGENIC)
2	15273445	15273454	15273449	G	T	snp	1	1	1	1	.	.	.	INTERGENIC(INTERGENIC)
2	15343975	15343986	15343982	TG	C	snp	0	1	0	1	.	.	.	NBAS(INTRON)
2	15689856	15689871	15689866	AAAT	G	snp	1	0	0	0	.	.	.	NBAS(INTRON)
2	15779684	15779693	15779689	T	C	snp	0	1	1	1	.	.	.	INTERGENIC(INTERGENIC)
2	15810008	15810016	15810012	T	G	snp	1	0	0	0	.	.	.	AC008278.3.1(INTRON)
2	15865863	15865872	15865869	AG	G	snp	1	0	0	0	.	.	.	INTERGENIC(INTERGENIC)
2	16001533	16001540	16001536	A	G	snp	1	0	0	0	.	.	.	INTERGENIC(INTERGENIC);RNU5E-7P(UPSTREAM)
2	16066701	16066715	16066705	TAT	G	snp	0	0	0	1	.	.	.	AC010145.3.1(INTRON);MYCNOS(INTRON)
2	16073007	16073014	16073011	A	G	snp	0	0	1	0	.	.	.	AC010145.3.1(INTRON);MYCNOS(DOWNSTREAM);MYCNOS(INTRON)
2	16073012	16073031	16073024	AAAG	C	snp	1	1	1	1	.	.	.	AC010145.3.1(INTRON);MYCNOS(DOWNSTREAM);MYCNOS(INTRON)
2	16115193	16115202	16115200	AG	T	snp	0	1	1	1	.	.	.	INTERGENIC(INTERGENIC)
2	16134189	16134204	16134196	TC	T	snp	1	1	1	1	.	.	.	AC010145.4.1(DOWNSTREAM);INTERGENIC(INTERGENIC)
2	16157122	16157136	16157126	TAT	G	snp	1	0	0	0	.	.	.	AC010145.4.1(INTRON)
2	16158303	16158318	16158315	CT	T	snp	1	1	1	0	.	.	.	AC010145.4.1(INTRON)
2	16169563	16169571	16169569	T	G	snp	1	1	1	1	.	.	.	AC010145.4.1(INTRON)
2	16182363	16182370	16182365	G	A	snp	1	0	0	0	.	.	.	AC010145.4.1(INTRON)
2	16523989	16524004	16523991	AT	T	del	1	1	1	1	.	.	.	INTERGENIC(INTERGENIC)
2	16539535	16539544	16539537	TA	C	snp	1	1	1	1	.	.	.	AC010880.1.1(INTRON)
2	16721016	16721025	16721021	AC	T	snp	1	0	1	0	.	.	.	INTERGENIC(INTERGENIC)
2	16724799	16724808	16724806	TA	G	snp	1	1	1	1	.	.	.	INTERGENIC(INTERGENIC)
2	16750919	16750934	16750923	GTCT	A	snp	1	1	0	1	.	.	.	FAM49A(INTRON)
2	16787651	16787660	16787656	TC	TCT	del	1	1	1	1	.	.	.	FAM49A(INTRON)
2	16824260	16824271	16824267	TTA	G	snp	1	0	0	0	.	.	.	FAM49A(INTRON)
2	16844171	16844179	16844177	T	C	snp	1	0	0	0	.	.	.	FAM49A(INTRON)
2	16858595	16858612	16858598	TA	G	snp	1	1	1	1	.	.	.	INTERGENIC(INTERGENIC)
2	16858595	16858612	16858600	TA	G	snp	1	0	1	0	.	.	.	INTERGENIC(INTERGENIC)
2	16902671	16902686	16902681	TCAT	G	snp	1	0	1	0	.	.	.	INTERGENIC(INTERGENIC)
2	16916971	16916984	16916975	GT	C	snp	1	0	0	0	.	.	.	AC008069.1.1(INTRON)
2	17064477	17064488	17064480	CAA	T	snp	1	1	1	1	.	.	.	INTERGENIC(INTERGENIC)
2	17064477	17064488	17064483	CAA	A	snp	1	1	1	1	.	.	.	INTERGENIC(INTERGENIC)
2	17320088	17320096	17320090	A	C	snp	0	1	0	1	.	.	.	INTERGENIC(INTERGENIC)
2	17387340	17387348	17387344	T	G	snp	1	1	1	1	.	.	.	INTERGENIC(INTERGENIC)
2	17444273	17444284	17444281	AT	T	snp	1	0	0	0	.	.	.	INTERGENIC(INTERGENIC)
2	17510527	17510535	17510529	A	G	snp	1	1	0	1	.	.	.	INTERGENIC(INTERGENIC)
2	17566103	17566117	17566109	TAT	G	snp	0	1	1	0	.	.	.	INTERGENIC(INTERGENIC);PSMC1P10(UPSTREAM)
2	17672475	17672484	17672483	A	C	snp	1	0	0	0	.	.	.	INTERGENIC(INTERGENIC)
2	17713315	17713322	17713316	G	A	snp	0	0	1	0	.	.	.	INTERGENIC(INTERGENIC)
2	17801149	17801156	17801155	C	T	snp	1	1	1	1	.	.	.	VSNL1(INTRON)
2	17964336	17964344	17964339	T	G	snp	1	0	0	0	.	.	.	GEN1(DOWNSTREAM);GEN1(UTR_3_PRIME);SMC6(INTRON)
2	18013326	18013335	18013329	AC	T	snp	1	1	0	1	.	.	.	INTERGENIC(INTERGENIC)
2	18031488	18031511	18031493	TTAT	A	snp	1	0	0	0	.	.	.	INTERGENIC(INTERGENIC)
2	18073552	18073561	18073558	T	C	snp	1	1	0	1	.	.	.	KCNS3(INTRON)
2	18097626	18097637	18097632	AGG	G	ins	1	0	0	0	.	.	.	KCNS3(INTRON)

2	18145802	18145815	18145808	GT	A	snp	1	1	1	1	.	.	.	KCNS3(INTRON)
2	18475441	18475456	18475446	TGTA	A	snp	1	1	0	0	.	.	.	KCNS3(INTRON)
2	18576099	18576106	18576101	A	C	ins	1	1	1	1	.	.	.	INTERGENIC(INTERGENIC)
2	18631760	18631767	18631765	T	G	snp	0	1	1	1	.	.	.	INTERGENIC(INTERGENIC)
2	18680176	18680185	18680181	T	C	snp	1	1	0	1	.	.	.	INTERGENIC(INTERGENIC)
2	18702433	18702441	18702440	C	CT	ins	1	1	1	1	.	.	.	INTERGENIC(INTERGENIC)
2	18801114	18801121	18801117	T	C	snp	1	0	0	0	.	.	.	INTERGENIC(INTERGENIC)
2	18913479	18913488	18913485	CA	T	snp	0	0	1	0	.	.	.	INTERGENIC(INTERGENIC)
2	18962271	18962278	18962275	T	G	snp	1	1	0	1	.	.	.	AC106053.1.1(UPSTREAM);INTERGENIC(INTERGENIC)
2	18978308	18978317	18978316	T	C	snp	1	1	1	1	.	.	.	INTERGENIC(INTERGENIC)
2	19013617	19013624	19013622	C	T	ins	1	0	0	0	.	.	.	INTERGENIC(INTERGENIC)
2	19138509	19138518	19138514	A	G	snp	1	1	1	1	.	.	.	INTERGENIC(INTERGENIC)
2	19256431	19256438	19256435	T	G	snp	1	0	0	0	.	.	.	AC092594.1.1(INTRON)
2	19613723	19613746	19613730	AAT	T	snp	1	0	0	0	.	.	.	INTERGENIC(INTERGENIC)
2	19623095	19623118	19623109	TTTG	C	snp	1	0	0	0	.	.	.	INTERGENIC(INTERGENIC)
2	19713869	19713884	19713880	TTTG	A	snp	1	1	1	1	.	.	.	INTERGENIC(INTERGENIC)
2	19768349	19768366	19768351	CA	T	snp	1	1	1	1	.	.	.	INTERGENIC(INTERGENIC)
2	19770775	19770789	19770786	CTT	C	del	1	1	1	1	.	.	.	INTERGENIC(INTERGENIC)
2	19773073	19773081	19773077	A	G	snp	1	0	0	0	.	.	.	INTERGENIC(INTERGENIC)
2	19804206	19804213	19804210	T	C	snp	1	0	0	1	.	.	.	INTERGENIC(INTERGENIC)
2	19805071	19805080	19805072	T	G	ins	1	1	1	1	.	.	.	INTERGENIC(INTERGENIC)
2	19805071	19805080	19805073	T	G	snp	1	1	1	1	.	.	.	INTERGENIC(INTERGENIC)
2	19822501	19822508	19822503	A	C	snp	1	1	1	1	.	.	.	INTERGENIC(INTERGENIC)
2	19836423	19836431	19836427	A	C	snp	0	1	0	1	.	.	.	INTERGENIC(INTERGENIC)
2	19912791	19912802	19912794	CT	A	snp	1	1	0	1	.	.	.	AC019055.1.1(INTRON)
2	19983699	19983710	19983706	CA	T	snp	1	0	0	0	.	.	.	INTERGENIC(INTERGENIC)
2	20014693	20014700	20014695	A	G	snp	1	1	1	1	.	.	.	INTERGENIC(INTERGENIC)
2	20055213	20055220	20055215	T	C	snp	1	1	1	1	.	.	.	INTERGENIC(INTERGENIC)
2	20128205	20128214	20128211	TG	G	snp	1	1	1	1	.	.	.	WDR35(INTRON)
2	20128205	20128214	20128212	TG	T	snp	1	1	1	1	.	.	.	WDR35(INTRON)
2	20187651	20187662	20187657	AT	C	snp	1	1	1	1	.	.	.	AC079145.4.1(UPSTREAM);MATN3(DOWNSTREAM);WDR35(INTRON)
2	20228594	20228602	20228601	A	G	snp	1	0	1	0	.	.	.	INTERGENIC(INTERGENIC);LAPTM4A(DOWNSTREAM)
2	20246517	20246526	20246522	AT	A	snp	1	0	0	0	.	.	.	LAPTM4A(INTRON)
2	20246541	20246550	20246546	AT	ATATA	del	1	0	0	0	.	.	.	LAPTM4A(INTRON)
2	20311992	20312000	20311996	T	G	snp	1	1	1	1	.	.	.	INTERGENIC(INTERGENIC)
2	20420125	20420138	20420130	CA	G	snp	1	1	1	1	.	.	.	SDC1(INTRON)
2	20429318	20429337	20429325	AAGG	A	snp	0	1	0	1	.	.	.	INTERGENIC(INTERGENIC);SDC1(UPSTREAM)
2	20490839	20490856	20490844	AAT	A	snp	0	1	0	1	.	.	.	PUM2(INTRON)
2	20542726	20542735	20542732	AT	T	snp	1	1	1	1	.	.	.	PUM2(INTRON)
2	20644385	20644392	20644387	A	C	snp	1	1	0	1	.	.	.	INTERGENIC(INTERGENIC);RHOB(UPSTREAM)
2	20795175	20795186	20795182	TCC	T	snp	1	0	0	0	.	.	.	HS1BP3(INTRON);HS1BP3-IT1(UPSTREAM)
2	20823852	20823861	20823858	CT	T	snp	1	1	1	1	.	.	.	HS1BP3(INTRON)
2	20900828	20900837	20900832	TG	C	snp	1	1	1	0	.	.	.	C2orf43(DOWNSTREAM);C2orf43(INTRON)
2	20900828	20900837	20900833	TG	A	snp	0	1	1	1	.	.	.	C2orf43(DOWNSTREAM);C2orf43(INTRON)
2	21038595	21038602	21038600	A	G	snp	1	0	0	0	.	.	.	INTERGENIC(INTERGENIC)
2	21132399	21132410	21132407	AG	G	snp	1	0	0	0	.	.	.	INTERGENIC(INTERGENIC)
2	21203193	21203206	21203198	TA	G	snp	1	0	0	0	.	.	.	INTERGENIC(INTERGENIC)
2	21254725	21254736	21254730	AAC	T	snp	1	0	0	0	.	.	.	APOB(INTRON)
2	21268807	21268820	21268818	AT	A	del	1	1	1	1	.	.	.	APOB(UPSTREAM);INTERGENIC(INTERGENIC)
2	21313380	21313397	21313384	TA	ATATATATATATAC	ins	1	1	1	1	.	.	.	INTERGENIC(INTERGENIC)
2	21419636	21419651	21419649	GA	G	del	1	1	0	1	.	.	.	INTERGENIC(INTERGENIC)
2	21697710	21697719	21697714	TG	C	snp	1	1	1	1	.	.	.	AC011752.1.1(INTRON);AC067959.1.1(INTRON)
2	21855219	21855227	21855226	T	G	snp	1	0	1	0	.	.	.	AC009411.2.1(INTRON);AC067959.1.1(INTRON)
2	22055936	22055953	22055946	AT	G	snp	1	0	0	0	.	.	.	AC067959.1.1(INTRON)
2	22091402	22091409	22091407	C	A	snp	1	0	0	0	.	.	.	AC067959.1.1(INTRON)
2	22111151	22111158	22111153	A	G	ins	1	0	0	0	.	.	.	AC067959.1.1(INTRON)
2	22139671	22139685	22139681	ATA	C	snp	1	0	0	0	.	.	.	AC067959.1.1(INTRON)
2	22232428	22232437	22232432	A	G	snp	1	0	0	0	.	.	.	AC068490.2.1(INTRON)
2	22236856	22236865	22236862	GA	C	snp	1	0	0	0	.	.	.	AC068490.2.1(INTRON)
2	22260136	22260143	22260142	A	G	ins	1	1	1	1	.	.	.	AC068490.2.1(INTRON)
2	22260136	22260143	22260142	A	G	snp	1	1	1	1	.	.	.	AC068490.2.1(INTRON)
2	22290121	22290138	22290136	AT	A	del	1	1	0	0	.	.	.	AC068490.2.1(INTRON)
2	22322106	22322115	22322108	TA	C	snp	1	0	0	0	.	.	.	AC068490.2.1(INTRON)
2	22322453	22322462	22322459	AT	T	del	1	0	0	0	.	.	.	AC068490.2.1(INTRON)
2	22400704	22400713	22400708	A	C	snp	0	1	1	0	.	.	.	AC068490.2.1(INTRON)
2	22435807	22435815	22435808	T	C	ins	1	0	0	0	.	.	.	AC068490.2.1(INTRON)

2	22469625	22469632	22469631	T	C	snp	1	0	0	0	.	.	.	AC068490.2.1(INTRON)
2	22482337	22482350	22482348	AT	ATGTGAG	del	1	1	1	1	.	.	.	AC068490.2.1(INTRON)
2	22482356	22482369	22482366	AT	C	snp	1	1	0	0	.	.	.	AC068490.2.1(INTRON)
2	22545282	22545301	22545297	CAAA	C	del	0	1	1	1	.	.	.	AC068490.2.1(INTRON)
2	22823557	22823564	22823562	T	C	snp	1	0	0	0	.	.	.	INTERGENIC(INTERGENIC)
2	22916432	22916445	22916434	AG	G	snp	1	0	0	0	.	.	.	INTERGENIC(INTERGENIC)
2	22962406	22962413	22962407	A	C	snp	1	1	1	0	.	.	.	INTERGENIC(INTERGENIC)
2	22986997	22987006	22987002	TA	G	snp	1	1	1	0	.	.	.	INTERGENIC(INTERGENIC)
2	23118479	23118492	23118482	AG	A	snp	0	1	1	1	.	.	.	INTERGENIC(INTERGENIC)
2	23118479	23118492	23118483	AG	G	ins	0	1	1	1	.	.	.	INTERGENIC(INTERGENIC)
2	23118479	23118492	23118484	AG	AGA	del	0	1	1	1	.	.	.	INTERGENIC(INTERGENIC)
2	23118479	23118492	23118487	AG	G	ins	0	1	0	1	.	.	.	INTERGENIC(INTERGENIC)
2	23193983	23193992	23193986	G	A	snp	0	0	1	0	.	.	.	INTERGENIC(INTERGENIC)
2	23249618	23249626	23249625	A	C	snp	0	1	1	1	.	.	.	AC016768.1.1(DOWNSTREAM);AC016768.1.1(INTRON)
2	23369468	23369477	23369473	AC	T	snp	1	1	1	1	.	.	.	AC016768.1.1(INTRON)
2	23394594	23394601	23394598	C	A	snp	1	1	1	1	.	.	.	AC016768.1.1(INTRON)
2	23788098	23788106	23788104	T	C	snp	0	0	0	1	.	.	.	KLHL29(INTRON)
2	23803773	23803784	23803781	AGG	A	snp	1	1	1	1	.	.	.	KLHL29(INTRON)
2	23893870	23893879	23893876	C	A	snp	1	1	1	1	.	.	.	KLHL29(INTRON)
2	24046671	24046678	24046672	A	C	ins	1	1	1	1	.	.	.	ATAD2B(INTRON)
2	24105679	24105696	24105692	CAA	AC	del	1	1	0	1	.	.	.	ATAD2B(INTRON)
2	24105679	24105696	24105693	CAA	C	del	1	1	0	1	.	.	.	ATAD2B(INTRON)
2	24158255	24158272	24158268	CAA	AC	del	1	1	0	1	.	.	.	UBXN2A(INTRON)
2	24158255	24158272	24158269	CAA	C	del	1	1	0	1	.	.	.	UBXN2A(INTRON)
2	24184716	24184725	24184724	T	C	snp	1	1	0	1	.	.	.	UBXN2A(INTRON)
2	24280244	24280252	24280251	T	G	ins	1	1	1	1	.	.	.	FKBP1B(INTRON);MFSD2B(INTRON)
2	24563614	24563621	24563620	A	C	snp	0	0	1	0	.	.	.	ITSN2(INTRON)
2	24597514	24597529	24597525	TTTA	G	snp	1	1	1	1	.	.	.	INTERGENIC(INTERGENIC)
2	24640213	24640228	24640225	AT	T	ins	1	1	1	1	.	.	.	INTERGENIC(INTERGENIC)
2	24655372	24655387	24655383	TTTC	T	ins	1	0	0	0	.	.	.	INTERGENIC(INTERGENIC)
2	24691828	24691848	24691842	TTG	T	snp	1	1	1	1	.	.	.	INTERGENIC(INTERGENIC)
2	24692006	24692023	24692010	TGA	A	snp	1	1	1	1	.	.	.	INTERGENIC(INTERGENIC)
2	24722970	24722985	24722977	TTTC	A	snp	1	1	0	1	.	.	.	NCOA1(INTRON)
2	24761724	24761731	24761730	A	G	snp	0	0	1	0	.	.	.	NCOA1(INTRON)
2	24865188	24865205	24865203	AT	A	del	1	1	1	1	.	.	.	NCOA1(INTRON)
2	24888878	24888892	24888888	TTA	A	del	0	1	1	1	.	.	.	NCOA1(INTRON)
2	25127390	25127398	25127394	A	G	snp	1	1	1	1	.	.	.	ADCY3(INTRON)
2	25146682	25146689	25146684	C	A	snp	1	1	0	1	.	.	.	ADCY3(UPSTREAM);INTERGENIC(INTERGENIC)
2	25233517	25233524	25233521	T	G	snp	1	0	0	0	.	.	.	DNAJC27-AS1(INTRON);Y_RNA(UPSTREAM)
2	25233517	25233524	25233523	T	G	snp	1	1	1	1	.	.	.	DNAJC27-AS1(INTRON);Y_RNA(UPSTREAM)
2	25233733	25233748	25233746	GT	G	del	1	1	1	1	.	.	.	DNAJC27-AS1(INTRON);Y_RNA(UPSTREAM)
2	25234364	25234379	25234377	AG	AGAA	ins	1	1	1	1	.	.	.	DNAJC27-AS1(INTRON);Y_RNA(UPSTREAM)
2	25248814	25248823	25248819	CA	G	snp	1	1	1	1	.	.	.	DNAJC27-AS1(INTRON)
2	25276341	25276350	25276344	CA	G	snp	0	1	1	1	.	.	.	AC013267.1(UPSTREAM);EFR3B(INTRON)
2	25365631	25365645	25365642	AAT	AATA	del	1	1	1	1	.	.	.	EFR3B(INTRON)
2	25384469	25384480	25384472	GCT	A	snp	0	0	1	0	.	.	POMC(SYNONYMOUS_CODING):S94	RP11-509E16.1.1(DOWNSTREAM)
2	25390152	25390159	25390153	A	C	snp	1	1	0	0	.	.	.	POMC(INTRON)
2	25692300	25692319	25692305	ATTC	C	snp	1	0	0	0	.	.	.	DTNB(INTRON)
2	25863424	25863435	25863432	CT	A	snp	1	1	1	1	.	.	.	DTNB(INTRON);DTNB(UPSTREAM)
2	25941404	25941413	25941412	T	G	snp	1	0	0	0	.	.	.	AC010150.1(UPSTREAM);INTERGENIC(INTERGENIC)
2	26067475	26067483	26067481	A	G	snp	1	1	1	1	.	.	.	ASXL2(INTRON)
2	26127470	26127487	26127485	TA	T	ins	1	0	0	0	.	.	.	INTERGENIC(INTERGENIC)
2	26139930	26139945	26139932	AT	T	del	1	1	0	1	.	.	.	INTERGENIC(INTERGENIC)
2	26147436	26147444	26147438	T	G	snp	1	0	0	0	.	.	.	INTERGENIC(INTERGENIC);KIF3C(DOWNSTREAM)
2	26523004	26523019	26523017	AT	A	ins	1	0	0	0	.	.	.	INTERGENIC(INTERGENIC)
2	26578596	26578611	26578609	TC	T	del	1	1	1	1	.	.	.	EPT1(INTRON)
2	26600735	26600744	26600742	T	G	snp	1	0	0	0	.	.	.	EPT1(DOWNSTREAM);EPT1(INTRON)
2	26607814	26607822	26607815	T	C	snp	0	1	1	1	.	.	.	EPT1(INTRON)
2	26612880	26612895	26612889	TGTC	T	snp	1	0	0	0	.	.	.	EPT1(DOWNSTREAM);EPT1(UTR_3_PRIME)
2	26627521	26627529	26627523	T	G	snp	1	1	1	1	.	.	.	CCDC164(INTRON)
2	26705516	26705529	26705527	TG	T	snp	0	0	0	1	.	.	.	OTOF(INTRON);OTOF(UPSTREAM)
2	26717525	26717533	26717530	G	GT	ins	1	1	0	1	.	.	.	OTOF(INTRON)
2	26718822	26718829	26718828	C	A	snp	1	1	1	1	.	.	.	OTOF(INTRON)
2	26784891	26784900	26784894	CA	G	snp	1	0	0	0	.	.	.	C2orf70(UPSTREAM);INTERGENIC(INTERGENIC);OTOF(UPSTREAM)
2	26885571	26885598	26885576	AAAT	AT	del	1	1	1	1	.	.	.	INTERGENIC(INTERGENIC)

2	26885571	26885598	26885578	AAAT	A	ins	1	1	1	1	.	.	.	INTERGENIC(INTERGENIC)
2	27007087	27007095	27007088	T	C	snp	1	0	0	0	.	.	.	C2orf18(DOWNSTREAM);CENPA(INTRON);CENPA(UPSTREAM)
2	27026687	27026696	27026691	TA	C	snp	1	0	0	0	.	.	.	CENPA(DOWNSTREAM);INTERGENIC(INTERGENIC)
2	27053639	27053647	27053644	A	G	snp	1	1	1	1	.	.	.	CDKN2AIPNLP2(UPSTREAM);INTERGENIC(INTERGENIC)
2	27073850	27073859	27073853	AT	C	snp	1	1	1	1	.	.	.	DPYSL5(INTRON)
2	27077663	27077670	27077667	T	C	snp	1	0	0	0	.	.	.	DPYSL5(INTRON)
2	27185312	27185321	27185317	A	C	snp	1	1	1	1	.	.	.	AC013472.4.1(INTRON)
2	27229074	27229083	27229079	A	C	snp	1	1	1	1	.	.	.	AC013472.3.1(INTRON);AC013472.4.1(INTRON);MAPRE3(INTRON)
2	27330119	27330126	27330121	T	G	ins	1	0	0	0	.	.	.	CGREF1(INTRON);CGREF1(UPSTREAM)
2	27339407	27339416	27339408	A	G	snp	1	0	0	0	.	.	.	CGREF1(INTRON)
2	27527130	27527145	27527143	AT	G	snp	1	1	1	1	.	.	.	TRIM54(INTRON);TRIM54(UPSTREAM);UCN(DOWNSTREAM)
2	27608110	27608121	27608115	CTC	T	snp	1	1	1	1	.	.	.	PPM1G(DOWNSTREAM);PPM1G(EXON);PPM1G(INTRON);PPM1G(UPSTREAM);ZNF513(UPSTREAM)
2	27648659	27648667	27648661	A	C	snp	1	0	0	0	.	.	.	INTERGENIC(INTERGENIC);NRBP1(UPSTREAM)
2	27655442	27655450	27655447	T	G	snp	1	1	1	1	.	.	.	NRBP1(INTRON);NRBP1(UPSTREAM)
2	27752688	27752696	27752692	A	G	snp	1	1	1	1	.	.	.	INTERGENIC(INTERGENIC)
2	27774695	27774704	27774702	T	G	snp	1	1	1	1	.	.	.	AC109829.1.1(INTRON)
2	27824508	27824515	27824511	T	TTTTG	ins	1	1	1	1	.	.	.	RP11-158I13.2.1(INTRON);ZNF512(DOWNSTREAM);ZNF512(INTRON)
2	27872116	27872135	27872131	AAAC	A	ins	1	1	1	1	.	.	.	GPN1(DOWNSTREAM);GPN1(INTRON);SUPT7L(DOWNSTREAM)
2	27875017	27875040	27875036	AAAT	AAA	del	1	1	1	1	.	.	.	GPN1(DOWNSTREAM);SUPT7L(DOWNSTREAM);SUPT7L(UTR_3_PRIME)
2	27889723	27889738	27889735	TA	ATA	del	1	0	0	0	.	.	.	SLC4A1AP(INTRON);SUPT7L(UPSTREAM)
2	28036535	28036546	28036537	TA	G	snp	1	0	0	0	.	.	.	MRPL33(INTRON);RBKS(INTRON)
2	28096699	28096708	28096707	A	C	ins	1	1	1	1	.	.	.	MRPL33(INTRON);RBKS(INTRON)
2	28096699	28096708	28096707	A	C	snp	1	1	1	1	.	.	.	MRPL33(INTRON);RBKS(INTRON)
2	28288386	28288397	28288392	CA	T	snp	1	1	1	1	.	.	.	BRE(INTRON)
2	28373752	28373759	28373753	A	C	snp	1	1	1	1	.	.	.	BRE(INTRON)
2	28471935	28471950	28471942	TTTG	A	snp	1	1	0	1	.	.	.	BRE(INTRON)
2	28585655	28585663	28585662	T	C	snp	1	1	1	1	.	.	.	INTERGENIC(INTERGENIC)
2	28674158	28674173	28674162	AATG	T	snp	0	1	1	0	.	.	.	AC104695.4.1(UPSTREAM);INTERGENIC(INTERGENIC)
2	28685353	28685364	28685357	TTG	G	snp	0	1	1	1	.	.	.	PLB1(INTRON);SNRPGP7(UPSTREAM)
2	28812035	28812044	28812036	A	C	snp	0	1	0	1	.	.	.	PLB1(INTRON)
2	29134505	29134514	29134513	T	C	snp	1	1	1	1	.	.	.	WDR43(INTRON)
2	29229289	29229298	29229295	TG	GA	ins	1	1	1	1	.	.	.	FAM179A(INTRON)
2	29243330	29243339	29243335	TG	T	snp	1	1	1	1	.	.	.	FAM179A(INTRON)
2	29279252	29279260	29279259	T	C	ins	1	1	1	1	.	.	.	FAM179A(DOWNSTREAM);INTERGENIC(INTERGENIC)
2	29281517	29281524	29281520	A	G	snp	1	0	0	0	.	.	.	C2orf71(DOWNSTREAM);INTERGENIC(INTERGENIC)
2	29308818	29308829	29308823	TA	TATATAATT	del	1	1	1	1	.	.	.	. INTERGENIC(INTERGENIC)
2	29320628	29320639	29320634	CCA	T	snp	1	0	0	0	.	.	.	AC105398.3.1(UPSTREAM);CLIP4(UTR_5_PRIME)
2	29326558	29326567	29326566	T	TA	ins	1	0	1	0	.	.	.	CLIP4(INTRON)
2	29430943	29430952	29430950	TC	T	del	1	1	1	1	.	.	.	ALK(INTRON)
2	29431780	29431789	29431782	A	G	snp	1	0	0	0	.	.	.	ALK(INTRON)
2	29439978	29439987	29439979	G	T	snp	1	1	1	1	.	.	.	ALK(DOWNSTREAM);ALK(INTRON)
2	29456860	29456868	29456865	T	G	ins	0	1	0	1	.	.	.	ALK(INTRON)
2	29487874	29487883	29487881	T	G	ins	0	1	0	1	.	.	.	ALK(INTRON)
2	29501846	29501855	29501849	G	A	ins	1	1	1	1	.	.	.	ALK(INTRON)
2	29874235	29874246	29874242	AC	T	snp	1	0	0	0	.	.	.	ALK(INTRON)
2	29876933	29876944	29876940	AAT	C	snp	1	1	1	1	.	.	.	ALK(INTRON)
2	29914848	29914855	29914852	A	G	snp	1	1	1	1	.	.	.	ALK(INTRON)
2	29967432	29967439	29967436	T	C	snp	1	0	0	0	.	.	.	ALK(INTRON)
2	30037535	30037543	30037542	A	G	snp	1	1	1	1	.	.	.	ALK(INTRON)
2	30406031	30406040	30406035	A	C	snp	1	1	0	1	.	.	.	INTERGENIC(INTERGENIC);SNORA64(UPSTREAM)
2	30493583	30493597	30493586	CAA	T	snp	1	0	0	0	.	.	.	LBH(INTRON)
2	30532168	30532183	30532174	TTTA	G	snp	1	0	0	0	.	.	.	LBH(INTRON)
2	30534968	30534979	30534975	CTT	TC	del	1	0	0	0	.	.	.	LBH(INTRON)
2	30708329	30708336	30708333	T	G	snp	1	1	1	1	.	.	.	LCLAT1(INTRON)
2	30864754	30864761	30864756	T	TTTTC	ins	1	1	1	1	.	.	.	LCLAT1(DOWNSTREAM);LCLAT1(UTR_3_PRIME)
2	30897989	30898008	30897996	TTTG	A	snp	0	1	0	1	.	.	.	INTERGENIC(INTERGENIC)
2	31204274	31204285	31204281	CTG	C	snp	0	1	1	0	.	.	.	GALNT14(INTRON)
2	31299479	31299496	31299494	AT	A	del	1	1	1	1	.	.	.	GALNT14(INTRON)
2	31363783	31363800	31363794	AAC	A	ins	1	0	0	0	.	.	.	GALNT14(INTRON);GALNT14(UPSTREAM)
2	31363783	31363800	31363797	AAC	A	snp	1	0	0	0	.	.	.	GALNT14(INTRON);GALNT14(UPSTREAM)
2	31365628	31365637	31365634	C	A	snp	1	1	1	1	.	.	.	GALNT14(INTRON);GALNT14(UPSTREAM)
2	31475323	31475343	31475338	TTG	C	snp	1	1	1	1	.	.	.	EHD3(INTRON)
2	31480310	31480325	31480314	AGGG	G	del	1	0	0	0	.	.	.	EHD3(INTRON)

2	31480310	31480325	31480318	AGGG	G	del	1	0	0	0	.	.	.	EHD3(INTRON)
2	31495387	31495404	31495401	GT	C	snp	1	0	0	0	.	.	.	EHD3(DOWNSTREAM);INTERGENIC(INTERGENIC)
2	31528732	31528743	31528736	TA	C	snp	1	1	1	1	.	.	.	INTERGENIC(INTERGENIC)
2	31557740	31557748	31557743	T	C	snp	1	0	0	0	.	.	.	XDH(UTR_3_PRIME)
2	31597404	31597411	31597410	A	G	snp	1	1	1	1	.	.	.	XDH(INTRON)
2	31604837	31604846	31604844	GT	C	snp	1	0	0	0	.	.	.	XDH(DOWNSTREAM);XDH(INTRON)
2	31638587	31638600	31638595	AC	G	snp	1	0	1	0	.	.	.	INTERGENIC(INTERGENIC);XDH(UPSTREAM)
2	31727508	31727519	31727513	ATA	C	snp	1	0	0	0	.	.	.	INTERGENIC(INTERGENIC)
2	31871251	31871262	31871256	CA	G	snp	1	1	1	1	.	.	.	INTERGENIC(INTERGENIC)
2	31912160	31912171	31912164	TG	C	snp	1	1	1	1	.	.	.	INTERGENIC(INTERGENIC)
2	32034700	32034723	32034707	AAAT	A	snp	0	0	1	0	.	.	.	AL121652.2(DOWNSTREAM);INTERGENIC(INTERGENIC)
2	32292127	32292134	32292130	T	C	snp	1	0	0	0	.	.	.	SPAST(INTRON)
2	32335641	32335650	32335645	TA	G	snp	1	0	0	0	.	.	.	SPAST(INTRON)
2	32338192	32338206	32338203	ATT	A	del	1	1	1	1	.	.	.	AL121658.1(UPSTREAM);SPAST(INTRON)
2	32456644	32456653	32456648	G	GT	ins	1	1	0	1	.	.	.	NLRC4(INTRON)
2	32456644	32456653	32456649	G	GT	ins	1	1	0	1	.	.	.	NLRC4(INTRON)
2	32505190	32505205	32505203	AT	A	del	1	0	0	0	.	.	.	YIPF4(INTRON)
2	32588389	32588404	32588400	CAAA	C	del	1	0	0	0	.	.	.	BIRC6(INTRON)
2	32787122	32787133	32787126	ATT	C	snp	0	0	0	1	.	.	.	BIRC6(INTRON)
2	32808802	32808809	32808804	T	C	snp	1	1	1	1	.	.	.	BIRC6(INTRON)
2	32877448	32877463	32877455	ATAA	G	snp	1	1	1	1	.	.	.	TTC27(INTRON)
2	32937615	32937623	32937622	T	C	snp	1	0	0	0	.	.	.	TTC27(INTRON)
2	33015338	33015347	33015344	T	G	snp	1	0	0	0	.	.	.	TTC27(INTRON)
2	33015338	33015347	33015346	T	G	snp	1	1	1	1	.	.	.	TTC27(INTRON)
2	33023781	33023804	33023785	AAT	T	del	1	1	1	1	.	.	.	TTC27(INTRON)
2	33050631	33050638	33050636	G	A	snp	1	1	1	1	.	.	.	LINC00486(INTRON);TTC27(DOWNSTREAM)
2	33072187	33072200	33072197	AT	TC	ins	0	0	0	1	.	.	.	LINC00486(INTRON)
2	33098583	33098590	33098586	A	C	snp	1	0	0	0	.	.	.	LINC00486(INTRON)
2	33162852	33162859	33162854	A	G	snp	1	1	1	1	.	.	.	INTERGENIC(INTERGENIC)
2	33165135	33165150	33165146	ATAA	T	snp	1	1	0	1	.	.	.	INTERGENIC(INTERGENIC)
2	33263904	33263912	33263907	T	G	snp	1	1	0	1	.	.	.	LTBP1(INTRON)
2	33270031	33270039	33270032	T	G	snp	1	0	0	0	.	.	.	LTBP1(INTRON)
2	33273010	33273019	33273016	T	C	snp	1	1	1	1	.	.	.	LTBP1(INTRON)
2	33276493	33276500	33276499	A	C	snp	1	1	0	1	.	.	.	AL133244.1.1(DOWNSTREAM);LTBP1(INTRON)
2	33343286	33343293	33343287	A	G	snp	1	1	1	1	.	.	.	LTBP1(INTRON)
2	33346871	33346879	33346877	T	G	ins	1	1	0	1	.	.	.	LTBP1(INTRON)
2	33354190	33354197	33354194	A	G	snp	1	1	1	1	.	.	.	LTBP1(INTRON)
2	33415407	33415418	33415413	GT	TA	ins	1	0	0	0	.	.	.	LTBP1(DOWNSTREAM);LTBP1(INTRON)
2	33419050	33419065	33419055	TTTA	G	snp	1	1	1	1	.	.	.	LTBP1(INTRON)
2	33505583	33505594	33505589	AC	G	snp	1	0	0	0	.	.	.	LTBP1(INTRON)
2	33621253	33621266	33621263	GT	A	snp	1	1	1	1	.	.	.	LTBP1(INTRON)
2	33730181	33730192	33730186	TCC	T	snp	1	0	0	0	.	.	.	RASGRP3(INTRON)
2	33762391	33762398	33762395	C	A	ins	1	1	1	1	.	.	.	AC020594.1(DOWNSTREAM);RASGRP3(INTRON);RASGRP3(UPSTREAM)
2	33763571	33763594	33763576	AAAT	ATAAATAC	ins	1	1	1	1	.	.	.	AC020594.1(DOWNSTREAM);RASGRP3(INTRON);RASGRP3(UPSTREAM)
2	33786617	33786624	33786619	A	G	snp	1	0	0	0	.	.	.	AC020594.5.1(INTRON);RASGRP3(INTRON)
2	33826491	33826498	33826496	A	G	snp	0	1	0	0	.	.	.	ATP6V0E1P3(UPSTREAM);FAM98A(UPSTREAM);INTERGENIC(INTERGENIC)
2	33831765	33831774	33831770	TC	T	snp	1	1	1	1	.	.	.	ATP6V0E1P3(DOWNSTREAM);INTERGENIC(INTERGENIC)
2	33839767	33839782	33839776	TCTG	TGTT	ins	1	1	1	1	.	.	.	INTERGENIC(INTERGENIC)
2	33849949	33849957	33849956	T	C	ins	1	0	0	0	.	.	.	INTERGENIC(INTERGENIC)
2	33856311	33856318	33856317	T	G	snp	0	0	0	1	.	.	.	INTERGENIC(INTERGENIC)
2	34007694	34007703	34007699	AG	A	snp	1	0	0	0	.	.	.	AC009499.1.1(INTRON)
2	34007694	34007703	34007700	AG	C	snp	1	0	0	0	.	.	.	AC009499.1.1(INTRON)
2	34019375	34019382	34019376	A	G	snp	1	1	1	1	.	.	.	AC009499.1.1(INTRON)
2	34031354	34031365	34031358	TCT	A	snp	0	0	0	1	.	.	.	AC009499.1.1(INTRON)
2	34049334	34049345	34049342	CT	T	snp	0	1	1	1	.	.	.	AC009499.1.1(INTRON)
2	34050765	34050774	34050772	T	TA	ins	0	1	0	1	.	.	.	AC009499.1.1(INTRON)
2	34062575	34062583	34062576	A	C	snp	1	0	0	0	.	.	.	AC009499.1.1(INTRON);AC069303.1.1(DOWNSTREAM)
2	34097974	34097982	34097979	A	C	snp	1	0	0	0	.	.	.	AC009499.1.1(INTRON)
2	34131462	34131469	34131463	T	C	snp	1	1	1	1	.	.	.	AC009499.1.1(INTRON)
2	34148919	34148928	34148924	A	C	ins	1	0	0	0	.	.	.	AC009499.1.1(INTRON)
2	34242202	34242215	34242212	AT	C	snp	1	1	1	1	.	.	.	AC009499.1.1(INTRON)
2	34248621	34248629	34248627	A	C	snp	1	1	1	1	.	.	.	AC009499.1.1(INTRON)
2	34337125	34337133	34337126	A	G	snp	1	0	0	0	.	.	.	AC009499.1.1(INTRON)
2	34452903	34452918	34452914	TTCA	G	snp	1	0	0	0	.	.	.	AC009499.1.1(INTRON)

2	34454500	34454511	34454506	CAG	A	snp	1	1	1	1	.	.	.	AC009499.1.1(INTRON)
2	34519801	34519810	34519806	T	G	snp	1	0	0	0	.	.	.	AC009499.1.1(INTRON)
2	34522688	34522695	34522689	T	C	snp	1	1	1	1	.	.	.	AC009499.1.1(EXON)
2	34554629	34554638	34554631	A	G	snp	0	1	0	1	.	.	.	INTERGENIC(INTERGENIC)
2	34642169	34642177	34642175	A	G	snp	1	1	1	1	.	.	.	AC073218.1.1(INTRON)
2	34646499	34646522	34646517	TTCT	A	snp	1	0	0	0	.	.	.	AC073218.1.1(INTRON)
2	34665599	34665606	34665604	A	G	snp	1	1	1	1	.	.	.	AC073218.1.1(INTRON)
2	34704872	34704895	34704888	TTTC	TTCTTTC	del	1	1	1	1	.	.	.	AC073218.1.1(INTRON)
2	34704872	34704895	34704889	TTTC	TCTTTC	del	1	1	1	1	.	.	.	AC073218.1.1(INTRON)
2	34770264	34770272	34770266	G	T	ins	1	0	0	0	.	.	.	AC073218.1.1(INTRON)
2	34813724	34813733	34813730	T	C	snp	1	0	0	0	.	.	.	AC073218.1.1(INTRON)
2	34828346	34828354	34828349	A	G	snp	1	0	0	0	.	.	.	AC073218.1.1(INTRON)
2	34960703	34960710	34960706	A	C	snp	1	1	1	1	.	.	.	AC073218.2.1(INTRON);AC073218.3.1(INTRON)
2	34968579	34968590	34968585	ATA	G	snp	0	0	1	0	.	.	.	INTERGENIC(INTERGENIC)
2	34978389	34978396	34978390	A	C	ins	1	0	0	1	.	.	.	INTERGENIC(INTERGENIC)
2	34978389	34978396	34978390	A	C	snp	1	1	1	1	.	.	.	INTERGENIC(INTERGENIC)
2	34978389	34978396	34978391	A	C	ins	1	0	0	1	.	.	.	INTERGENIC(INTERGENIC)
2	34978389	34978396	34978392	A	C	snp	1	0	0	1	.	.	.	INTERGENIC(INTERGENIC)
2	35022146	35022161	35022150	AC	C	del	1	0	0	0	.	.	.	AC012593.1.1(UPSTREAM);INTERGENIC(INTERGENIC)
2	35022146	35022161	35022152	AC	C	del	1	0	0	0	.	.	.	AC012593.1.1(UPSTREAM);INTERGENIC(INTERGENIC)
2	35040929	35040938	35040936	TA	G	snp	0	1	1	1	.	.	.	AC012593.1.1(INTRON)
2	35041138	35041151	35041141	AC	T	snp	1	1	1	1	.	.	.	AC012593.1.1(INTRON)
2	35041510	35041525	35041512	AC	C	snp	1	1	0	1	.	.	.	AC012593.1.1(INTRON)
2	35089156	35089165	35089163	AC	A	snp	1	0	0	0	.	.	.	AC012593.1.1(INTRON)
2	35100103	35100114	35100107	TAT	G	snp	0	0	0	1	.	.	.	AC012593.1.1(INTRON)
2	35172360	35172367	35172366	A	C	snp	1	0	0	0	.	.	.	AC012593.1.1(INTRON)
2	35186912	35186923	35186918	TTG	C	snp	1	1	1	0	.	.	.	AC012593.1.1(INTRON)
2	35308743	35308750	35308749	A	AGAT	ins	1	1	1	1	.	.	.	AC012593.1.1(INTRON)
2	35397502	35397511	35397504	AT	G	snp	0	0	0	1	.	.	.	INTERGENIC(INTERGENIC)
2	35397502	35397511	35397509	AT	C	snp	1	0	0	0	.	.	.	INTERGENIC(INTERGENIC)
2	35399967	35399976	35399968	A	AC	ins	1	0	0	0	.	.	.	INTERGENIC(INTERGENIC)
2	35476837	35476844	35476842	T	C	snp	1	1	1	1	.	.	.	INTERGENIC(INTERGENIC)
2	35513694	35513701	35513700	T	C	snp	1	0	0	0	.	.	.	INTERGENIC(INTERGENIC)
2	35517788	35517795	35517792	T	G	snp	1	1	1	1	.	.	.	INTERGENIC(INTERGENIC)
2	35521741	35521748	35521745	A	C	snp	1	1	1	1	.	.	.	INTERGENIC(INTERGENIC)
2	35532214	35532222	35532215	A	C	snp	1	1	1	1	.	.	.	INTERGENIC(INTERGENIC)
2	35532214	35532222	35532217	A	C	snp	1	1	0	1	.	.	.	INTERGENIC(INTERGENIC)
2	35639969	35639984	35639972	TA	G	snp	1	1	1	1	.	.	.	INTERGENIC(INTERGENIC)
2	35639969	35639984	35639974	TA	G	snp	1	1	1	1	.	.	.	INTERGENIC(INTERGENIC)
2	35643907	35643924	35643913	AT	T	ins	0	1	0	0	.	.	.	INTERGENIC(INTERGENIC)
2	35647992	35648005	35648003	AT	ATAG	ins	1	1	1	1	.	.	.	INTERGENIC(INTERGENIC)
2	35761466	35761475	35761471	AT	C	snp	0	1	0	1	.	.	.	INTERGENIC(INTERGENIC)
2	35762628	35762635	35762630	A	G	snp	1	0	0	0	.	.	.	INTERGENIC(INTERGENIC)
2	35863772	35863783	35863778	TGT	C	snp	1	0	0	0	.	.	.	INTERGENIC(INTERGENIC)
2	35914043	35914051	35914048	T	G	snp	1	0	0	0	.	.	.	INTERGENIC(INTERGENIC)
2	36005160	36005173	36005171	TG	T	snp	1	1	1	1	.	.	.	INTERGENIC(INTERGENIC)
2	36046121	36046130	36046124	AT	AA	ins	1	0	0	0	.	.	.	INTERGENIC(INTERGENIC)
2	36046306	36046315	36046311	AC	G	snp	1	1	1	1	.	.	.	INTERGENIC(INTERGENIC)
2	36061573	36061586	36061584	AC	A	snp	1	1	1	1	.	.	.	INTERGENIC(INTERGENIC)
2	36174034	36174041	36174035	A	C	snp	1	1	1	1	.	.	.	INTERGENIC(INTERGENIC)
2	36182161	36182170	36182166	AC	T	snp	1	1	1	1	.	.	.	INTERGENIC(INTERGENIC)
2	36182161	36182170	36182167	AC	G	snp	1	1	1	1	.	.	.	INTERGENIC(INTERGENIC)
2	36182197	36182206	36182202	AC	T	snp	1	1	1	1	.	.	.	INTERGENIC(INTERGENIC)
2	36182197	36182206	36182203	AC	G	snp	1	1	1	1	.	.	.	INTERGENIC(INTERGENIC)
2	36226103	36226114	36226112	TG	A	snp	1	1	1	1	.	.	.	INTERGENIC(INTERGENIC)
2	36293805	36293819	36293814	AAT	ATAAT	del	1	0	0	0	.	.	.	INTERGENIC(INTERGENIC)
2	36326840	36326847	36326845	T	C	snp	1	0	0	0	.	.	.	INTERGENIC(INTERGENIC)
2	36411905	36411914	36411910	TA	G	snp	1	1	0	1	.	.	.	INTERGENIC(INTERGENIC)
2	36436298	36436307	36436303	T	G	snp	0	1	0	0	.	.	.	INTERGENIC(INTERGENIC)
2	36492683	36492692	36492690	CA	C	del	1	1	1	1	.	.	.	INTERGENIC(INTERGENIC)
2	36502617	36502628	36502626	GT	G	snp	1	0	0	0	.	.	.	INTERGENIC(INTERGENIC)
2	36556278	36556293	36556286	ATGA	G	snp	1	0	0	0	.	.	.	INTERGENIC(INTERGENIC)
2	36594095	36594104	36594100	TG	C	snp	1	0	0	0	.	.	.	CRIM1(INTRON);CRIM1(UPSTREAM)
2	36634373	36634382	36634379	CA	T	snp	1	0	1	0	.	.	.	CRIM1(INTRON)
2	36677027	36677035	36677030	A	C	ins	1	1	1	1	.	.	.	CRIM1(INTRON)
2	36687247	36687256	36687249	A	G	snp	1	0	0	0	.	.	.	CRIM1(INTRON)
2	36708037	36708045	36708041	A	G	snp	1	1	1	1	.	.	.	CRIM1(INTRON)

2	36869769	36869792	36869772	AAC	G	snp	1	0	0	0	.	.	.	FEZ2(INTRON)
2	36923897	36923905	36923898	G	T	snp	1	1	1	1	.	.	.	VIT(UPSTREAM);VIT(UTR_5_PRIME)
2	36923897	36923905	36923900	G	T	snp	1	1	1	1	.	.	.	VIT(UPSTREAM);VIT(UTR_5_PRIME)
2	36931034	36931049	36931041	TTTG	C	snp	1	1	1	1	.	.	.	VIT(INTRON)
2	36958774	36958781	36958780	A	G	snp	1	1	1	1	.	.	.	VIT(INTRON)
2	37064355	37064362	37064359	T	C	snp	1	0	0	0	.	.	.	INTERGENIC(INTERGENIC)
2	37136142	37136150	37136145	A	AAC	ins	1	1	1	1	.	.	.	STRN(INTRON)
2	37144312	37144321	37144316	T	C	snp	0	0	1	0	.	.	.	STRN(INTRON)
2	37148228	37148239	37148233	AAT	A	snp	1	1	0	1	.	.	.	STRN(INTRON)
2	37264289	37264302	37264294	AT	G	snp	1	0	1	0	.	.	.	HEATR5B(DOWNSTREAM);HEATR5B(INTRON)
2	37264289	37264302	37264297	AT	G	snp	1	0	0	0	.	.	.	HEATR5B(DOWNSTREAM);HEATR5B(INTRON)
2	37329775	37329784	37329781	CA	T	snp	0	0	0	1	.	.	.	CCDC75(DOWNSTREAM);EIF2AK2(DOWNSTREAM);EIF2AK2(UTR_3_PRIME)
2	37519655	37519662	37519656	T	G	snp	1	0	0	0	.	.	.	PRKD3(DOWNSTREAM);PRKD3(INTRON);PRKD3(UPSTREAM)
2	37527460	37527469	37527466	A	G	snp	0	0	0	1	.	.	.	PRKD3(INTRON)
2	37555006	37555025	37555021	TTTA	G	snp	0	1	0	1	.	.	.	AC007391.2.1(DOWNSTREAM);INTERGENIC(INTERGENIC);PRKD3(UPSTREAM);U6(DOWNSTREAM)
2	37556860	37556871	37556864	AAC	G	snp	1	1	0	1	.	.	.	AC007391.2.1(DOWNSTREAM);INTERGENIC(INTERGENIC);PRKD3(UPSTREAM);U6(DOWNSTREAM)
2	37607571	37607578	37607576	T	C	snp	0	0	1	0	.	.	.	INTERGENIC(INTERGENIC)
2	37663225	37663233	37663226	A	G	snp	0	1	0	0	.	.	.	INTERGENIC(INTERGENIC);U6(UPSTREAM)
2	37668607	37668614	37668611	T	G	snp	1	0	0	0	.	.	.	INTERGENIC(INTERGENIC)
2	37819395	37819412	37819399	TA	AAAG	ins	1	1	1	1	.	.	.	INTERGENIC(INTERGENIC)
2	37865753	37865762	37865755	T	C	ins	1	0	0	0	.	.	.	AC006369.2.1(INTRON);CDC42EP3(DOWNSTREAM)
2	37865753	37865762	37865755	T	C	snp	1	0	0	0	.	.	.	AC006369.2.1(INTRON);CDC42EP3(DOWNSTREAM)
2	37898954	37898963	37898957	GC	GT	ins	1	1	1	1	.	.	.	CDC42EP3(INTRON);CDC42EP3(UTR_5_PRIME)
2	37919550	37919565	37919557	AAAT	A	snp	1	0	0	0	.	.	.	CDC42EP3(INTRON);CDC42EP3(UPSTREAM)
2	37948474	37948481	37948476	T	C	ins	1	1	0	1	.	.	.	CDC42EP3(INTRON)
2	37948474	37948481	37948480	T	C	ins	1	0	0	0	.	.	.	CDC42EP3(INTRON)
2	38007302	38007311	38007309	AG	C	snp	1	1	0	1	.	.	.	INTERGENIC(INTERGENIC)
2	38097628	38097635	38097631	T	C	snp	1	1	1	1	.	.	.	LINC00211(INTRON)
2	38202844	38202852	38202846	A	C	snp	0	1	0	1	.	.	.	AC016689.1.1(INTRON);FAM82A1(DOWNSTREAM);FAM82A1(INTRON)
2	38208971	38208980	38208979	A	C	snp	1	1	1	1	.	.	.	AC016689.1.1(INTRON);FAM82A1(INTRON)
2	38214223	38214232	38214226	A	G	snp	1	1	1	1	.	.	.	AC016689.1.1(INTRON);FAM82A1(INTRON)
2	38242723	38242730	38242727	A	G	snp	1	0	0	0	.	.	.	AC016689.1.1(INTRON);FAM82A1(INTRON)
2	38289913	38289922	38289916	C	A	snp	1	1	0	1	.	.	.	CYP1B1(DOWNSTREAM);FAM82A1(INTRON)
2	38316024	38316037	38316031	CT	C	snp	1	0	0	0	.	.	.	CYP1B1(INTRON);CYP1B1-AS1(INTRON)
2	38317299	38317314	38317304	ATAG	G	snp	1	1	0	1	.	.	.	CYP1B1(INTRON);CYP1B1-AS1(INTRON)
2	38327354	38327361	38327356	T	C	snp	1	1	1	1	.	.	.	CYP1B1(INTRON);CYP1B1-AS1(INTRON)
2	38395417	38395426	38395423	CA	A	snp	1	0	0	0	.	.	.	CYP1B1-AS1(INTRON)
2	38456899	38456916	38456904	AAT	A	del	1	0	0	0	.	.	.	AC009229.4.1(UPSTREAM);AC009229.5.1(INTRON)
2	38480371	38480378	38480377	T	C	snp	0	0	1	0	.	.	.	INTERGENIC(INTERGENIC)
2	38481983	38481990	38481987	T	G	snp	1	0	0	0	.	.	.	INTERGENIC(INTERGENIC)
2	38486567	38486574	38486570	T	C	snp	1	1	1	1	.	.	.	INTERGENIC(INTERGENIC)
2	38538264	38538273	38538267	A	C	ins	1	0	0	0	.	.	.	ATL2(DOWNSTREAM);ATL2(INTRON)
2	38547444	38547451	38547450	A	C	snp	1	0	0	0	.	.	.	ATL2(INTRON)
2	38553030	38553045	38553040	TTTG	GTTTGTTTAAAAAAAAAAA	del	1	1	1	1	.	.	.	ATL2(INTRON)
2	38561496	38561507	38561504	TAA	C	snp	0	0	1	0	.	.	.	ATL2(INTRON)
2	38594590	38594598	38594593	A	G	snp	1	1	1	0	.	.	.	ATL2(INTRON)
2	38651159	38651168	38651163	AC	G	snp	1	0	0	0	.	.	.	AC016995.3.1(INTRON)
2	38679878	38679886	38679884	C	T	snp	1	1	1	1	.	.	.	AC016995.3.1(INTRON)
2	38691174	38691182	38691181	A	C	snp	1	0	0	0	.	.	.	AC016995.3.1(INTRON);AC016995.3.1(UPSTREAM)
2	38731749	38731764	38731760	AAAG	AAAGA	del	0	1	0	1	.	.	.	AC016995.3.1(INTRON)
2	38745157	38745172	38745165	TATT	A	snp	1	0	0	0	.	.	.	AC016995.3.1(UPSTREAM);INTERGENIC(INTERGENIC)
2	38772158	38772181	38772162	TTG	G	del	1	0	1	0	.	.	.	INTERGENIC(INTERGENIC)
2	38772158	38772181	38772168	TTG	G	del	1	0	1	0	.	.	.	INTERGENIC(INTERGENIC)
2	38814500	38814511	38814507	TAT	TTAT	del	1	0	0	0	.	.	.	HNRPLL(DOWNSTREAM);HNRPLL(INTRON);HNRPLL(UPSTREAM)
2	38883889	38883903	38883898	AAC	AC	del	1	1	1	1	.	.	.	INTERGENIC(INTERGENIC)
2	38984492	38984503	38984500	TTA	TTAT	del	0	1	0	1	.	.	.	GEMIN6(INTRON)
2	39005200	39005217	39005205	ATT	C	snp	1	1	1	1	.	.	.	GEMIN6(INTRON);GEMIN6(UPSTREAM)
2	39009118	39009127	39009123	TG	T	snp	0	0	1	0	.	.	.	GEMIN6(DOWNSTREAM);GEMIN6(UTR_3_PRIME)
2	39054700	39054713	39054703	AC	T	snp	1	1	1	1	.	.	.	DHX57(INTRON)
2	39102670	39102677	39102671	C	T	snp	1	1	1	1	.	.	.	DHX57(INTRON);MORN2(UPSTREAM)
2	39104409	39104432	39104425	AAAT	T	snp	1	1	1	1	.	.	.	DHX57(UPSTREAM);MORN2(INTRON)
2	39412503	39412512	39412507	AT	G	snp	1	1	1	1	.	.	.	CDKL4(INTRON)

2	39412557	39412566	39412561	AT	G	snp	1	1	1	1	.	.	.	CDKL4(INTRON)
2	39445754	39445769	39445760	GGCA	A	snp	1	0	0	0	.	.	.	CDKL4(INTRON)
2	39522442	39522451	39522448	AC	C	snp	1	1	1	1	.	.	.	MAP4K3(INTRON);MAP4K3(UPSTREAM)
2	39581815	39581824	39581818	A	C	snp	1	0	0	0	.	.	.	MAP4K3(DOWNSTREAM);MAP4K3(INTRON)
2	39689278	39689289	39689282	AAT	T	del	1	1	1	1	.	.	.	AC007246.3.1(INTRON)
2	39689278	39689289	39689283	AAT	A	snp	1	1	1	1	.	.	.	AC007246.3.1(INTRON)
2	39700421	39700430	39700429	A	C	snp	0	0	1	0	.	.	.	AC007246.3.1(INTRON)
2	39716687	39716695	39716690	T	C	snp	1	0	0	0	.	.	.	AC007246.3.1(INTRON);AC007388.1(UPSTREAM)
2	39730566	39730575	39730572	T	G	snp	1	1	1	0	.	.	.	AC007246.3.1(INTRON)
2	39889057	39889074	39889066	TG	TA	ins	1	0	0	0	.	.	.	AC007246.3.1(INTRON);TMEM178(UPSTREAM)
2	39965743	39965752	39965749	AT	T	ins	1	0	0	0	.	.	.	THUMPD2(INTRON)
2	39967114	39967128	39967122	TAG	T	ins	1	0	0	0	.	.	.	THUMPD2(INTRON)
2	40016337	40016345	40016341	A	C	snp	1	1	1	1	.	.	.	INTERGENIC(INTERGENIC)
2	40019671	40019679	40019672	A	G	snp	0	0	0	1	.	.	.	INTERGENIC(INTERGENIC)
2	40108315	40108322	40108321	A	C	snp	1	0	0	0	.	.	.	INTERGENIC(INTERGENIC)
2	40125269	40125276	40125273	T	G	snp	1	0	0	0	.	.	.	INTERGENIC(INTERGENIC)
2	40177519	40177528	40177523	AC	G	snp	0	1	0	1	.	.	.	AC007254.3.1(INTRON)
2	40242939	40242958	40242946	AAAT	A	snp	1	0	0	0	.	.	.	AC007254.3.1(INTRON)
2	40242939	40242958	40242954	AAAT	C	snp	1	0	0	0	.	.	.	AC007254.3.1(INTRON)
2	40411160	40411167	40411166	A	C	snp	1	1	1	1	.	.	.	AC007254.3.1(INTRON);SLC8A1(INTRON)
2	40427314	40427331	40427326	AT	TATATTT	del	1	1	1	1	.	.	.	AC007254.3.1(INTRON);SLC8A1(INTRON)
2	40520190	40520201	40520193	TTG	C	snp	1	0	0	0	.	.	.	SLC8A1(INTRON)
2	40591650	40591658	40591656	T	G	ins	1	1	0	1	.	.	.	SLC8A1(INTRON)
2	40648209	40648223	40648218	CAC	A	snp	1	0	0	0	.	.	.	SLC8A1(INTRON)
2	40708267	40708278	40708276	GT	C	snp	0	1	1	1	.	.	.	SLC8A1(INTRON)
2	40782948	40782962	40782953	GGT	C	snp	0	1	1	0	.	.	.	SLC8A1(INTRON)
2	40832092	40832106	40832095	GAA	A	snp	1	0	0	0	.	.	.	SLC8A1(INTRON)
2	40874981	40874988	40874987	T	G	snp	1	1	1	1	.	.	.	INTERGENIC(INTERGENIC)
2	40948560	40948575	40948564	GAAA	AAAGAAAGAA	del	1	1	1	1	.	.	.	INTERGENIC(INTERGENIC)
2	40948560	40948575	40948568	GAAA	AAAGAA	del	1	1	1	1	.	.	.	INTERGENIC(INTERGENIC)
2	40974655	40974666	40974661	CT	A	snp	1	1	0	1	.	.	.	AC007317.1.1(INTRON)
2	41024110	41024118	41024112	T	G	snp	0	1	0	1	.	.	.	INTERGENIC(INTERGENIC)
2	41045655	41045670	41045662	AAGG	A	snp	1	1	0	1	.	.	.	INTERGENIC(INTERGENIC)
2	41142085	41142094	41142087	A	C	snp	1	1	1	1	.	.	.	INTERGENIC(INTERGENIC)
2	41153540	41153547	41153544	A	C	snp	0	0	1	0	.	.	.	INTERGENIC(INTERGENIC)
2	41184141	41184148	41184144	A	G	snp	1	0	0	0	.	.	.	INTERGENIC(INTERGENIC)
2	41193306	41193319	41193310	GA	C	snp	0	1	1	0	.	.	.	INTERGENIC(INTERGENIC)
2	41193306	41193319	41193311	GA	T	snp	1	0	0	0	.	.	.	INTERGENIC(INTERGENIC)
2	41197702	41197709	41197708	T	G	snp	1	1	1	1	.	.	.	INTERGENIC(INTERGENIC)
2	41219025	41219034	41219028	AT	C	snp	1	1	1	1	.	.	.	INTERGENIC(INTERGENIC)
2	41254971	41254984	41254974	AT	C	snp	0	1	0	1	.	.	.	INTERGENIC(INTERGENIC)
2	41297884	41297893	41297891	T	G	snp	1	1	1	1	.	.	.	INTERGENIC(INTERGENIC)
2	41345761	41345778	41345774	AG	C	snp	1	1	0	0	.	.	.	INTERGENIC(INTERGENIC)
2	41443850	41443863	41443853	TC	T	snp	1	0	0	0	.	.	.	INTERGENIC(INTERGENIC)
2	41480242	41480250	41480246	T	TTTTCTTTTC	ins	1	1	1	1	.	.	.	INTERGENIC(INTERGENIC)
2	41502433	41502444	41502441	ATA	G	snp	1	1	1	1	.	.	.	INTERGENIC(INTERGENIC)
2	41651565	41651572	41651569	T	G	ins	1	1	1	1	.	.	.	INTERGENIC(INTERGENIC)
2	41651565	41651572	41651570	T	G	ins	1	0	0	0	.	.	.	INTERGENIC(INTERGENIC)
2	41651578	41651585	41651581	T	G	snp	1	0	0	0	.	.	.	INTERGENIC(INTERGENIC)
2	41714635	41714644	41714641	AT	G	snp	1	0	0	0	.	.	.	INTERGENIC(INTERGENIC)
2	41759755	41759764	41759760	CA	G	snp	1	1	1	1	.	.	.	INTERGENIC(INTERGENIC)
2	41827865	41827874	41827868	A	G	snp	1	0	0	0	.	.	.	AC010739.1(DOWNSTREAM);INTERGENIC(INTERGENIC)
2	41958313	41958322	41958316	TG	T	snp	1	1	1	1	.	.	.	INTERGENIC(INTERGENIC);snoZ247(UPSTREAM)
2	42034068	42034076	42034069	A	G	snp	1	1	1	1	.	.	.	INTERGENIC(INTERGENIC)
2	42113482	42113493	42113489	CT	A	snp	1	1	1	1	.	.	.	AC104654.1.1(INTRON)
2	42141971	42141980	42141973	TG	G	del	1	1	0	1	.	.	.	INTERGENIC(INTERGENIC)
2	42203990	42203999	42203995	AT	A	del	1	0	0	0	.	.	.	INTERGENIC(INTERGENIC)
2	42203990	42203999	42203997	AT	A	del	1	1	1	0	.	.	.	INTERGENIC(INTERGENIC)
2	42240388	42240397	42240392	TA	C	snp	1	0	0	0	.	.	.	AC013480.2.1(DOWNSTREAM);INTERGENIC(INTERGENIC)
2	42274849	42274860	42274852	GCC	A	snp	1	1	1	1	.	.	.	INTERGENIC(INTERGENIC);PKDCC(UPSTREAM)
2	42300244	42300255	42300250	CA	AA	ins	1	1	1	1	.	.	.	INTERGENIC(INTERGENIC)
2	42315672	42315681	42315678	A	G	snp	1	0	0	0	.	.	.	INTERGENIC(INTERGENIC)
2	42337452	42337461	42337454	GA	A	snp	1	1	1	1	.	.	.	INTERGENIC(INTERGENIC)
2	42377242	42377249	42377243	A	C	snp	1	1	0	1	.	.	.	AC083949.1.1(INTRON)
2	42437080	42437099	42437091	AAAC	T	snp	1	0	0	0	.	.	.	EML4(INTRON)
2	42442210	42442217	42442212	T	C	snp	1	1	1	1	.	.	.	EML4(INTRON)
2	42685943	42685952	42685951	A	G	ins	1	0	1	0	.	.	.	AC025750.6.1(DOWNSTREAM);KCNG3(INTRON)

2	42687342	42687349	42687343	A	G	snp	1	0	0	0	.	.	.	AC025750.6.1(DOWNSTREAM);KCNG3(INTRON)
2	42700488	42700497	42700494	T	G	snp	1	1	1	1	.	.	.	AC025750.5.1(DOWNSTREAM);KCNG3(INTRON)
2	42705024	42705035	42705033	AT	A	del	1	1	1	1	.	.	.	KCNG3(INTRON)
2	42733756	42733767	42733764	TTA	TTAT	del	1	1	1	1	.	.	.	MTA3(INTRON)
2	42739157	42739164	42739163	A	C	snp	1	1	1	1	.	.	.	MTA3(INTRON)
2	42751435	42751446	42751443	ATT	A	del	0	1	0	1	.	.	.	MTA3(INTRON)
2	42827338	42827345	42827341	A	C	snp	1	1	1	1	.	.	.	MTA3(INTRON)
2	42947333	42947340	42947335	T	C	snp	1	1	1	1	.	.	.	MTA3(INTRON)
2	42969266	42969285	42969270	CTTT	T	snp	1	1	1	1	.	.	.	MTA3(INTRON)
2	43048619	43048626	43048620	T	C	snp	1	1	1	1	.	.	.	AC098824.6.1(DOWNSTREAM);INTERGENIC(INTERGENIC)
2	43193569	43193577	43193571	G	GTT	ins	1	1	1	1	.	.	.	INTERGENIC(INTERGENIC)
2	43211660	43211667	43211663	C	T	snp	1	1	1	0	.	.	.	INTERGENIC(INTERGENIC)
2	43223616	43223627	43223622	AT	G	snp	1	1	1	1	.	.	.	AC016735.1.1(DOWNSTREAM);INTERGENIC(INTERGENIC)
2	43227269	43227280	43227273	TG	C	snp	1	1	1	0	.	.	.	AC016735.1.1(DOWNSTREAM);INTERGENIC(INTERGENIC)
2	43239242	43239251	43239248	AT	G	snp	1	0	0	0	.	.	.	INTERGENIC(INTERGENIC)
2	43257130	43257153	43257149	AAAC	AA	del	1	0	0	0	.	.	.	AC016735.2.1(DOWNSTREAM);AC016735.2.1(INTRON)
2	43317354	43317363	43317358	CT	G	snp	1	1	0	1	.	.	.	INTERGENIC(INTERGENIC);U6(DOWNSTREAM)
2	43369624	43369631	43369625	A	G	snp	1	0	0	0	.	.	.	INTERGENIC(INTERGENIC)
2	43380682	43380689	43380684	C	T	ins	1	0	0	0	.	.	.	INTERGENIC(INTERGENIC)
2	43396131	43396140	43396134	T	C	ins	1	1	1	1	.	.	.	THADA(INTRON)
2	43419879	43419887	43419886	A	G	snp	1	1	1	1	.	.	.	THADA(INTRON)
2	43475201	43475209	43475203	A	C	ins	1	1	0	1	.	.	.	THADA(INTRON)
2	43717647	43717654	43717650	T	C	snp	1	1	1	1	.	.	.	THADA(INTRON);THADA(UPSTREAM)
2	43748075	43748090	43748079	GGAG	A	snp	1	1	0	1	.	.	.	THADA(INTRON)
2	43878285	43878308	43878303	TTCA	T	snp	1	1	0	1	.	.	.	PLEKHH2(INTRON)
2	43904971	43904979	43904973	C	T	snp	1	1	0	1	.	.	.	AC011242.5.1(DOWNSTREAM);AC011242.6.1(UPSTREAM);PLEKHH2(INTRON)
2	43965701	43965709	43965705	T	C	snp	1	1	1	1	.	.	.	PLEKHH2(INTRON);PLEKHH2(UPSTREAM)
2	43979052	43979065	43979055	TC	T	snp	1	0	0	0	.	.	.	PLEKHH2(INTRON)
2	44060824	44060839	44060832	TTTA	C	snp	1	1	1	1	.	.	.	ABCG5(INTRON)
2	44092792	44092801	44092798	CT	A	snp	1	0	0	0	.	.	.	ABCG8(INTRON)
2	44123338	44123361	44123356	TTTC	CTTTC	del	1	1	1	1	.	.	.	LRPPRC(DOWNSTREAM);LRPPRC(INTRON);U6(DOWNSTREAM)
2	44161455	44161464	44161457	AT	G	snp	1	0	0	0	.	.	.	LRPPRC(INTRON)
2	44193877	44193885	44193881	A	G	snp	1	0	0	0	.	.	.	LRPPRC(INTRON);LRPPRC(UPSTREAM)
2	44338910	44338921	44338919	TA	T	del	1	1	0	1	.	.	.	INTERGENIC(INTERGENIC)
2	44352120	44352127	44352121	T	G	snp	1	1	1	1	.	.	.	INTERGENIC(INTERGENIC)
2	44373098	44373111	44373109	AT	A	del	1	0	0	0	.	.	.	INTERGENIC(INTERGENIC)
2	44540541	44540550	44540548	TG	A	snp	1	0	0	0	.	.	.	PREPL(DOWNSTREAM);SLC3A1(DOWNSTREAM);SLC3A1(INTRON)
2	44580730	44580749	44580737	AAAT	A	snp	1	1	1	1	.	.	.	PREPL(INTRON)
2	44647705	44647718	44647716	TA	T	snp	0	0	0	1	.	.	.	CAMKMT(INTRON)
2	44764698	44764707	44764704	T	C	snp	1	0	0	0	.	.	.	CAMKMT(INTRON)
2	44824867	44824886	44824873	TTTG	A	snp	1	0	0	0	.	.	.	CAMKMT(INTRON)
2	44858616	44858623	44858618	T	G	snp	1	0	0	0	.	.	.	CAMKMT(INTRON)
2	44891953	44891961	44891958	A	C	snp	1	0	0	0	.	.	.	CAMKMT(INTRON)
2	44988932	44988941	44988937	GT	G	snp	0	0	0	1	.	.	.	CAMKMT(INTRON)
2	45071198	45071206	45071204	G	A	snp	1	1	1	1	.	.	.	INTERGENIC(INTERGENIC)
2	45073509	45073517	45073513	A	C	snp	1	1	1	1	.	.	.	INTERGENIC(INTERGENIC)
2	45084183	45084191	45084188	A	G	snp	1	0	1	0	.	.	.	INTERGENIC(INTERGENIC)
2	45125507	45125530	45125526	AAAC	A	snp	1	0	0	0	.	.	.	INTERGENIC(INTERGENIC)
2	45196038	45196053	45196049	AATA	AATATTT	del	1	0	1	0	.	.	.	AC012354.6.1(DOWNSTREAM);INTERGENIC(INTERGENIC)
2	45242779	45242788	45242785	CA	T	snp	1	1	1	1	.	.	.	AC093702.1.1(UPSTREAM);INTERGENIC(INTERGENIC)
2	45328774	45328781	45328780	T	C	snp	1	1	1	1	.	.	.	INTERGENIC(INTERGENIC)
2	45560456	45560465	45560463	AC	T	snp	1	1	1	1	.	.	.	INTERGENIC(INTERGENIC)
2	45620529	45620540	45620535	TTG	C	snp	1	0	0	0	.	.	.	SRBD1(INTRON)
2	45641887	45641896	45641894	AC	AT	ins	1	1	1	0	.	.	.	SRBD1(DOWNSTREAM);SRBD1(INTRON)
2	45719577	45719591	45719587	TGT	C	snp	1	1	0	1	.	.	.	SRBD1(INTRON)
2	45767462	45767471	45767467	AG	C	snp	1	0	0	0	.	.	.	SRBD1(INTRON)
2	45935652	45935661	45935656	CT	G	snp	1	1	1	1	.	.	.	PRKCE(INTRON)
2	45959705	45959716	45959710	AGT	C	snp	1	0	0	0	.	.	.	PRKCE(INTRON)
2	45977528	45977537	45977533	TG	A	snp	1	0	0	0	.	.	.	PRKCE(INTRON)
2	46004076	46004085	46004084	T	C	snp	1	1	1	1	.	.	.	PRKCE(INTRON)
2	46036709	46036717	46036716	T	C	snp	1	1	1	1	.	.	.	PRKCE(INTRON)
2	46096836	46096845	46096841	TC	T	ins	0	1	1	1	.	.	.	PRKCE(INTRON)
2	46160780	46160788	46160787	T	G	snp	0	0	1	0	.	.	.	PRKCE(INTRON)
2	46431652	46431659	46431658	T	C	snp	0	1	0	1	.	.	.	INTERGENIC(INTERGENIC)
2	46438191	46438200	46438199	T	G	ins	1	1	1	1	.	.	.	INTERGENIC(INTERGENIC)

2	46466187	46466195	46466188	T	C	snp	1	0	0	0	.	.	.	INTERGENIC(INTERGENIC)
2	46471228	46471236	46471231	A	C	snp	1	1	1	1	.	.	.	INTERGENIC(INTERGENIC)
2	46481068	46481076	46481069	G	T	snp	1	0	0	0	.	.	.	AC016696.1.1(DOWNSTREAM);INTERGENIC(INTERGENIC)
2	46481068	46481076	46481070	G	T	snp	1	1	1	0	.	.	.	AC016696.1.1(DOWNSTREAM);INTERGENIC(INTERGENIC)
2	46491479	46491487	46491485	A	G	snp	0	1	0	1	.	.	.	INTERGENIC(INTERGENIC)
2	46583273	46583282	46583281	C	T	snp	1	1	1	1	.	.	.	EPAS1(EXON);EPAS1(INTRON)
2	46657042	46657051	46657050	A	C	snp	1	0	0	0	.	.	.	AC018682.6.1(INTRON);AC018682.6.1(UPSTREAM)
2	46736657	46736680	46736676	AAAC	A	ins	1	0	0	0	.	.	.	ATP6V1E2(DOWNSTREAM);ATP6V1E2(INTRON)
2	46847876	46847887	46847883	TA	T	snp	1	0	0	0	.	.	.	CRIPT(INTRON);PIGF(UPSTREAM)
2	47022542	47022550	47022543	A	C	snp	1	1	1	1	.	.	.	INTERGENIC(INTERGENIC)
2	47083033	47083044	47083041	TA	A	del	1	1	1	1	.	.	.	AC016722.2.1(EXON);AC016722.2.1(INTRON);AC016722.3.1(UPSTREAM)
2	47097253	47097267	47097257	TAT	G	snp	1	1	1	1	.	.	.	INTERGENIC(INTERGENIC)
2	47106968	47106987	47106979	TGGA	G	snp	1	0	0	0	.	.	.	INTERGENIC(INTERGENIC)
2	47140144	47140155	47140152	GT	A	snp	1	1	1	1	.	.	.	AC016722.4.1(DOWNSTREAM);MCFD2(INTRON);MCFD2(UPSTREAM);TTC7A(UPSTREAM)
2	47302451	47302460	47302456	TA	G	snp	1	0	0	0	.	.	.	AC073283.7.1(DOWNSTREAM);CALM2(INTRON);TTC7A(DOWNSTREAM);TTC7A(EXON);TTC7A(UTR_3_PRIME)
2	47307631	47307646	47307640	TCTT	T	del	1	1	1	1	.	.	.	CALM2(INTRON);TTC7A(DOWNSTREAM)
2	47345055	47345070	47345068	AT	A	del	1	1	1	1	.	.	.	C2orf61(INTRON);CALM2(INTRON)
2	47347884	47347891	47347889	T	G	snp	1	1	1	1	.	.	.	C2orf61(INTRON);CALM2(INTRON)
2	47471938	47471949	47471946	TTG	A	snp	1	0	0	0	.	.	.	AC073283.4.1(INTRON)
2	47473176	47473185	47473179	AG	A	snp	1	0	0	0	.	.	.	AC073283.4.1(INTRON)
2	47496327	47496350	47496338	AATA	AAT	del	1	1	1	1	.	.	.	AC073283.4.1(INTRON)
2	47505758	47505773	47505769	TTCC	T	snp	1	0	0	0	.	.	.	AC073283.4.1(INTRON)
2	47521919	47521930	47521925	AT	G	snp	0	1	1	1	.	.	.	AC073283.4.1(INTRON)
2	47524190	47524198	47524192	A	G	snp	1	1	1	1	.	.	.	AC073283.4.1(INTRON)
2	47594368	47594377	47594374	T	G	snp	0	0	0	1	.	.	.	EPCAM(INTRON);EPCAM(UPSTREAM)
2	47622466	47622479	47622474	CA	A	snp	1	0	0	0	.	.	.	INTERGENIC(INTERGENIC)
2	47629891	47629900	47629898	T	G	snp	1	1	1	1	.	.	.	INTERGENIC(INTERGENIC);MSH2(UPSTREAM)
2	47647775	47647783	47647779	T	G	snp	1	0	0	0	.	.	.	MSH2(INTRON)
2	47662227	47662235	47662233	T	G	snp	1	1	1	1	.	.	.	MSH2(INTRON)
2	47662227	47662235	47662234	T	GA	ins	1	1	1	1	.	.	.	MSH2(INTRON)
2	47679353	47679360	47679356	T	C	ins	1	1	0	0	.	.	.	MSH2(INTRON)
2	47713847	47713855	47713849	T	C	snp	1	0	0	0	.	.	.	AC138655.6.1(EXON);MSH2(DOWNSTREAM);MSH2(INTRON)
2	47734250	47734265	47734259	CT	C	snp	1	1	1	1	.	.	.	MSH2(INTRON)
2	47936802	47936815	47936807	TC	T	ins	1	1	0	1	.	.	.	AC006509.4.1(DOWNSTREAM);AC006509.6.1(INTRON)
2	48031273	48031282	48031280	T	G	snp	1	1	0	1	.	.	.	FBXO11(DOWNSTREAM);FBXO11(INTRON);MSH6(DOWNSTREAM);MSH6(INTRON)
2	48120306	48120325	48120317	TAAA	TAAATAAATAAC	ins	1	1	0	1	.	.	.	FBXO11(INTRON);FBXO11(UPSTREAM)
2	48128146	48128155	48128153	AT	C	snp	1	0	0	0	.	.	.	AC079807.2.1(UPSTREAM);FBXO11(INTRON)
2	48161428	48161443	48161438	AAAT	T	del	1	0	0	1	.	.	.	INTERGENIC(INTERGENIC)
2	48161428	48161443	48161439	AAAT	A	snp	1	0	0	0	.	.	.	INTERGENIC(INTERGENIC)
2	48290703	48290714	48290706	AG	C	snp	1	0	0	0	.	.	.	AC079807.4.1(INTRON)
2	48296482	48296501	48296490	TTTG	C	snp	1	1	1	1	.	.	.	AC079807.4.1(INTRON)
2	48489055	48489070	48489062	TTTG	T	snp	1	0	0	0	.	.	.	INTERGENIC(INTERGENIC)
2	48577095	48577106	48577103	AGA	G	snp	1	1	1	1	.	.	.	FOXN2(INTRON)
2	48589160	48589171	48589166	ATT	C	snp	1	1	1	1	.	.	.	FOXN2(INTRON)
2	48675118	48675127	48675122	A	G	snp	1	0	0	0	.	.	.	PPP1R21(INTRON)
2	48696628	48696643	48696641	AT	AGAG	ins	1	1	1	1	.	.	.	PPP1R21(INTRON)
2	48731794	48731802	48731797	A	C	snp	1	0	0	0	.	.	.	PPP1R21(INTRON);PPP1R21(UPSTREAM);U6(UPSTREAM)
2	48765003	48765011	48765004	A	G	snp	0	1	0	1	.	.	.	STON1(INTRON);STON1-GTF2A1L(INTRON)
2	48834954	48834967	48834960	GA	A	snp	1	0	0	0	.	.	.	STON1-GTF2A1L(INTRON)
2	48887233	48887242	48887234	T	G	ins	1	1	1	1	.	.	.	GTF2A1L(INTRON);LHCGR(INTRON);STON1-GTF2A1L(INTRON)
2	48908695	48908706	48908703	AAT	G	snp	1	1	1	1	.	.	.	GTF2A1L(DOWNSTREAM);GTF2A1L(INTRON);LHCGR(INTRON);STON1-GTF2A1L(DOWNSTREAM);STON1-GTF2A1L(INTRON)
2	48916863	48916872	48916864	T	C	snp	0	1	1	1	.	.	.	GTF2A1L(INTRON);LHCGR(INTRON);STON1-GTF2A1L(INTRON)
2	49004453	49004460	49004459	T	C	snp	1	1	1	1	.	.	.	INTERGENIC(INTERGENIC);STON1-GTF2A1L(DOWNSTREAM);TCEB2P3(UPSTREAM)
2	49009105	49009113	49009112	T	G	snp	1	1	0	1	.	.	.	INTERGENIC(INTERGENIC);TCEB2P3(DOWNSTREAM)
2	49042899	49042912	49042903	TA	C	snp	0	1	0	0	.	.	.	INTERGENIC(INTERGENIC)
2	49096649	49096662	49096659	TG	C	snp	1	1	1	1	.	.	.	INTERGENIC(INTERGENIC)
2	49187209	49187226	49187211	GT	A	snp	1	1	1	1	.	.	.	FSHR(DOWNSTREAM);INTERGENIC(INTERGENIC)
2	49208529	49208542	49208532	TG	A	snp	1	1	1	1	.	.	.	FSHR(DOWNSTREAM);FSHR(INTRON)
2	49240238	49240249	49240247	CT	C	snp	1	0	0	0	.	.	.	FSHR(INTRON)

2	49240625	49240638	49240628	AT	A	snp	1	0	0	1	.	.	.	FSHR(INTRON)
2	49240641	49240654	49240652	AT	A	snp	1	1	1	1	.	.	.	FSHR(INTRON)
2	49354878	49354889	49354881	TTC	TC	del	1	1	1	1	.	.	.	FSHR(INTRON)
2	49454433	49454452	49454444	AAAC	A	del	1	1	1	1	.	.	.	INTERGENIC(INTERGENIC)
2	49454433	49454452	49454446	AAAC	AC	del	1	1	1	1	.	.	.	INTERGENIC(INTERGENIC)
2	49485133	49485150	49485147	AT	TG	ins	1	0	0	0	.	.	.	INTERGENIC(INTERGENIC)
2	49499173	49499188	49499180	CCTT	C	snp	1	1	1	1	.	.	.	INTERGENIC(INTERGENIC)
2	49499396	49499407	49499404	AAC	T	snp	1	0	1	1	.	.	.	INTERGENIC(INTERGENIC)
2	49537382	49537391	49537384	G	T	snp	1	1	1	1	.	.	.	INTERGENIC(INTERGENIC)
2	49606471	49606482	49606478	GAG	G	del	1	1	1	1	.	.	.	INTERGENIC(INTERGENIC)
2	49805468	49805476	49805470	A	G	snp	0	0	0	1	.	.	.	INTERGENIC(INTERGENIC)
2	49909668	49909677	49909670	A	C	snp	1	1	1	1	.	.	.	INTERGENIC(INTERGENIC)
2	49946292	49946299	49946295	A	G	snp	1	1	1	1	.	.	.	INTERGENIC(INTERGENIC)
2	49982415	49982422	49982417	T	G	snp	1	0	0	0	.	.	.	INTERGENIC(INTERGENIC)
2	50057309	50057316	50057312	T	G	snp	1	1	1	1	.	.	.	INTERGENIC(INTERGENIC)
2	50121750	50121758	50121751	T	G	ins	1	1	0	1	.	.	.	INTERGENIC(INTERGENIC)
2	50134581	50134590	50134585	TG	G	snp	1	0	0	0	.	.	.	INTERGENIC(INTERGENIC)
2	50209615	50209622	50209621	A	C	ins	1	1	1	1	.	.	.	NRXN1(INTRON)
2	50241234	50241243	50241238	T	G	snp	1	1	1	1	.	.	.	NRXN1(INTRON)
2	50244356	50244367	50244362	ATT	G	snp	1	0	0	0	.	.	.	NRXN1(INTRON)
2	50380533	50380542	50380540	T	G	ins	1	0	0	0	.	.	.	NRXN1(INTRON)
2	50383158	50383167	50383160	TA	A	snp	0	1	1	1	.	.	.	NRXN1(INTRON)
2	50386213	50386236	50386231	TTG	C	snp	1	0	0	0	.	.	.	NRXN1(INTRON)
2	50393138	50393147	50393141	GT	G	snp	1	0	0	0	.	.	.	NRXN1(INTRON)
2	50393464	50393479	50393467	TG	C	snp	1	1	1	1	.	.	.	NRXN1(INTRON)
2	50456468	50456481	50456470	TG	G	del	0	1	0	1	.	.	.	NRXN1(INTRON)
2	50505368	50505379	50505371	TA	TATATATATTATATATATGTAT	ins	1	1	1	1	.	.	.	NRXN1(INTRON)
2	50505380	50505393	50505390	AT	G	snp	1	1	1	1	.	.	.	NRXN1(INTRON)
2	50541655	50541663	50541657	A	G	snp	0	1	0	0	.	.	.	NRXN1(INTRON)
2	50651471	50651485	50651480	GGA	A	snp	1	0	0	0	.	.	.	NRXN1(INTRON)
2	50745687	50745695	50745692	A	C	snp	1	1	1	1	.	.	.	NRXN1(INTRON)
2	50834134	50834145	50834137	GGT	A	snp	1	1	1	1	.	.	.	NRXN1(INTRON)
2	50841779	50841796	50841782	AT	A	snp	0	1	1	1	.	.	.	NRXN1(INTRON)
2	50954584	50954597	50954595	AC	AT	ins	1	1	1	1	.	.	.	NRXN1(INTRON)
2	50974046	50974054	50974053	T	C	snp	0	1	1	1	.	.	.	NRXN1(INTRON)
2	50999646	50999655	50999647	A	G	snp	1	0	0	0	.	.	.	NRXN1(INTRON)
2	51103365	51103374	51103370	T	C	snp	0	1	1	1	.	.	.	NRXN1(INTRON)
2	51183299	51183310	51183305	GA	C	snp	1	1	0	1	.	.	.	NRXN1(INTRON)
2	51280248	51280257	51280251	TA	G	snp	1	1	1	1	.	.	.	AC007682.1.1(INTRON)
2	51402585	51402598	51402589	TA	C	snp	1	0	0	0	.	.	.	AC007682.1.1(INTRON)
2	51434442	51434457	51434452	TTTA	C	snp	1	1	1	1	.	.	.	AC007680.2.1(INTRON);AC007682.1.1(INTRON)
2	51506354	51506361	51506355	A	C	snp	1	0	0	0	.	.	.	AC007682.1.1(INTRON)
2	51512151	51512160	51512152	A	G	snp	1	1	0	1	.	.	.	AC007682.1.1(INTRON)
2	51699604	51699615	51699608	TTG	C	snp	1	1	1	1	.	.	.	AC007682.1.1(INTRON)
2	51775856	51775865	51775858	AT	G	snp	1	1	1	1	.	.	.	AC007682.1.1(INTRON)
2	51776858	51776867	51776864	AT	G	snp	1	0	0	0	.	.	.	AC007682.1.1(INTRON)
2	51900048	51900055	51900053	C	T	snp	1	1	1	1	.	.	.	AC007682.1.1(INTRON)
2	51953884	51953891	51953886	A	G	snp	0	0	1	0	.	.	.	AC007682.1.1(INTRON)
2	51984878	51984892	51984889	TTG	C	snp	1	0	0	0	.	.	.	AC007682.1.1(INTRON)
2	52003379	52003388	52003384	AG	A	snp	0	1	1	1	.	.	.	AC007682.1.1(INTRON);AC064868.1.1(UPSTREAM)
2	52111888	52111895	52111894	T	C	snp	1	0	0	0	.	.	.	AC007682.1.1(INTRON)
2	52159048	52159071	52159065	TTTC	TC	del	1	1	1	1	.	.	.	AC007682.1.1(INTRON);AC097463.1.1(UPSTREAM)
2	52163145	52163156	52163147	TA	C	snp	1	1	1	0	.	.	.	AC007682.1.1(INTRON)
2	52194217	52194228	52194219	TA	C	snp	1	0	0	0	.	.	.	AC007682.1.1(INTRON)
2	52194217	52194228	52194226	TA	T	ins	1	0	0	0	.	.	.	AC007682.1.1(INTRON)
2	52294646	52294655	52294653	AC	G	snp	1	1	1	1	.	.	.	AC007682.1.1(INTRON);ZNF863P(UPSTREAM)
2	52306420	52306435	52306422	AC	T	snp	1	1	1	1	.	.	.	AC007682.1.1(INTRON);ZNF863P(DOWNSTREAM)
2	52306420	52306435	52306431	AC	T	snp	1	1	1	1	.	.	.	AC007682.1.1(INTRON);ZNF863P(DOWNSTREAM)
2	52381153	52381164	52381155	TA	C	snp	1	1	1	1	.	.	.	AC007682.1.1(INTRON)
2	52473527	52473534	52473531	A	G	snp	1	0	0	0	.	.	.	AC007682.1.1(INTRON)
2	52567767	52567782	52567780	AT	A	del	0	1	1	1	.	.	.	AC007682.1.1(INTRON)
2	52598750	52598758	52598752	T	C	snp	1	1	0	1	.	.	.	AC007682.1.1(INTRON);AC087073.1.1(UPSTREAM)
2	52709261	52709268	52709262	A	G	snp	1	0	0	0	.	.	.	INTERGENIC(INTERGENIC)
2	52714243	52714266	52714246	GTT	TT	del	1	1	0	1	.	.	.	INTERGENIC(INTERGENIC)
2	52782719	52782726	52782724	T	C	snp	1	1	0	1	.	.	.	INTERGENIC(INTERGENIC)
2	53251057	53251065	53251060	T	C	snp	1	1	0	1	.	.	.	INTERGENIC(INTERGENIC)
2	53298295	53298304	53298297	A	G	ins	1	1	0	1	.	.	.	INTERGENIC(INTERGENIC)

2	53381749	53381762	53381756	AT	A	snp	1	0	0	1	.	.	.	INTERGENIC(INTERGENIC)
2	53463831	53463839	53463835	A	C	snp	1	1	1	1	.	.	.	INTERGENIC(INTERGENIC)
2	53533359	53533366	53533361	A	G	snp	1	1	1	1	.	.	.	INTERGENIC(INTERGENIC)
2	53605528	53605539	53605535	TTC	C	del	1	0	0	0	.	.	.	INTERGENIC(INTERGENIC)
2	53673176	53673184	53673182	A	G	snp	1	1	1	1	.	.	.	INTERGENIC(INTERGENIC)
2	53731699	53731708	53731702	A	C	snp	1	1	1	1	.	.	.	INTERGENIC(INTERGENIC)
2	53734144	53734152	53734146	T	C	snp	1	1	1	1	.	.	.	INTERGENIC(INTERGENIC)
2	53844092	53844111	53844101	TATT	G	snp	1	0	0	0	.	.	.	ASB3(INTRAGENIC)
2	53856046	53856053	53856049	T	G	snp	1	1	1	1	.	.	.	ASB3(INTRAGENIC)
2	53939907	53939922	53939917	GC	A	snp	1	1	1	1	.	.	.	ASB3(DOWNSTREAM);ASB3(INTRON)
2	54048936	54048943	54048939	T	C	snp	1	1	1	1	.	.	.	AC007883.1(DOWNSTREAM);ASB3(INTRON);ERLEC1(DOWNSTREAM)
2	54051482	54051489	54051486	T	G	ins	1	1	1	1	.	.	.	AC007883.1(UPSTREAM);ASB3(INTRON)
2	54051501	54051508	54051502	T	G	snp	0	0	0	1	.	.	.	AC007883.1(UPSTREAM);ASB3(INTRON)
2	54051501	54051508	54051502	T	TG	ins	1	1	1	1	.	.	.	AC007883.1(UPSTREAM);ASB3(INTRON)
2	54078806	54078821	54078812	TTTC	G	snp	0	0	1	1	.	.	.	ASB3(INTRON);GPR75(DOWNSTREAM)
2	54082256	54082269	54082261	CT	C	snp	1	1	0	1	.	.	.	ASB3(INTRON);GPR75(INTRON)
2	54095620	54095633	54095630	AT	T	del	1	1	1	1	.	.	.	PSME4(INTRON)
2	54111358	54111365	54111360	T	C	snp	0	0	1	0	.	.	.	PSME4(DOWNSTREAM);PSME4(INTRON);PSME4(UPSTREAM)
2	54161104	54161112	54161109	A	C	snp	0	0	0	1	.	.	.	PSME4(DOWNSTREAM);PSME4(INTRON);PSME4(UPSTREAM)
2	54217923	54217932	54217928	T	G	snp	1	0	0	0	.	.	.	AC008280.5.1(INTRON)
2	54235030	54235039	54235036	A	C	snp	1	1	1	1	.	.	.	AC008280.5.1(INTRON)
2	54265553	54265562	54265560	AT	C	snp	1	0	0	1	.	.	.	AC008280.5.1(INTRON)
2	54303855	54303866	54303861	GA	C	snp	1	0	0	0	.	.	.	AC008280.3.1(DOWNSTREAM);AC008280.5.1(INTRON)
2	54325008	54325023	54325021	TC	T	del	1	1	1	1	.	.	.	INTERGENIC(INTERGENIC)
2	54347306	54347315	54347313	TC	T	snp	0	1	0	1	.	.	.	ACYP2(INTRON)
2	54428236	54428245	54428243	TC	TCT	del	1	1	1	1	.	.	.	ACYP2(INTRON)
2	54428818	54428835	54428821	TC	T	snp	1	1	0	0	.	.	.	ACYP2(INTRON)
2	54436001	54436014	54436006	TG	A	snp	1	1	1	1	.	.	.	ACYP2(INTRON)
2	54437869	54437877	54437873	T	G	ins	1	0	0	0	.	.	.	ACYP2(INTRON)
2	54556903	54556916	54556913	AC	C	snp	1	0	0	0	.	.	.	C2orf73(UPSTREAM);INTERGENIC(INTERGENIC)
2	54571903	54571910	54571909	A	C	snp	1	1	0	1	.	.	.	C2orf73(INTRON)
2	54627638	54627645	54627639	A	G	snp	1	1	1	1	.	.	.	INTERGENIC(INTERGENIC)
2	54725820	54725827	54725824	T	C	snp	1	1	1	1	.	.	.	SPTBN1(INTRON)
2	54778966	54778974	54778970	T	C	snp	1	0	0	0	.	.	.	SPTBN1(INTRON)
2	54789017	54789026	54789025	A	AC	ins	1	1	1	1	.	.	.	SPTBN1(INTRON)
2	54790280	54790289	54790283	TA	G	snp	1	0	0	0	.	.	.	SPTBN1(INTRON)
2	54797109	54797118	54797113	C	A	snp	1	1	1	1	.	.	.	SPTBN1(INTRON)
2	54805593	54805613	54805607	GCT	C	snp	1	1	1	1	.	.	.	SPTBN1(INTRON)
2	54895850	54895857	54895851	G	T	snp	1	0	0	0	.	.	.	SPTBN1(EXON);SPTBN1(UTR_3_PRIME)
2	54912693	54912702	54912700	TC	T	snp	1	0	0	0	.	.	.	INTERGENIC(INTERGENIC)
2	55184128	55184139	55184133	TAC	T	snp	1	0	0	0	.	.	.	EML6(INTRON)
2	55388995	55389012	55389005	CTT	C	snp	1	1	0	1	.	.	.	INTERGENIC(INTERGENIC)
2	55388995	55389012	55389008	CTT	TC	del	1	0	1	0	.	.	.	INTERGENIC(INTERGENIC)
2	55405089	55405096	55405094	A	G	ins	1	1	1	1	.	.	.	C2orf63(INTRON)
2	55407297	55407312	55407307	AT	G	snp	1	1	0	1	.	.	.	C2orf63(INTRON)
2	55483021	55483029	55483028	T	GG	ins	1	0	0	0	.	.	.	MTIF2(INTRON);MTIF2(UPSTREAM)
2	55516586	55516597	55516588	GA	C	snp	1	1	1	1	.	.	.	CCDC88A(DOWNSTREAM);CCDC88A(INTRON);CCDC88A(UTR_3_PRIME);PRORSD1P(DOWNSTREAM);Y_RNA(DOWNSTREAM)
2	55575104	55575113	55575107	T	G	ins	1	0	0	0	.	.	.	CCDC88A(INTRON)
2	55587869	55587877	55587876	T	C	snp	1	1	1	1	.	.	.	CCDC88A(INTRON)
2	55611656	55611667	55611659	TA	TATATGTG		ins	1	1	1	1	.	.	. CCDC88A(INTRON)
2	55860798	55860817	55860805	AAAG	AA	del	1	0	0	1	.	.	.	INTERGENIC(INTERGENIC);PNPT1(DOWNSTREAM)
2	55864007	55864030	55864014	AAC	C	del	0	1	0	0	.	.	.	PNPT1(INTRON)
2	55926491	55926500	55926498	AG	A	snp	1	1	1	1	.	.	.	INTERGENIC(INTERGENIC)
2	56051407	56051416	56051414	T	G	ins	1	1	0	0	.	.	.	INTERGENIC(INTERGENIC)
2	56051407	56051416	56051414	T	G	snp	1	1	1	1	.	.	.	INTERGENIC(INTERGENIC)
2	56248087	56248096	56248095	A	G	snp	1	0	0	0	.	.	.	AC011306.2.1(INTRON)
2	56331371	56331386	56331375	AAGG	G	snp	1	1	1	1	.	.	.	INTERGENIC(INTERGENIC)
2	56355696	56355704	56355703	T	G	snp	1	0	0	0	.	.	.	INTERGENIC(INTERGENIC)
2	56385139	56385148	56385145	TA	C	snp	1	1	1	1	.	.	.	INTERGENIC(INTERGENIC)
2	56577289	56577298	56577297	T	C	snp	1	1	1	1	.	.	.	CCDC85A(INTRON)
2	56598523	56598530	56598525	A	C	snp	1	1	1	1	.	.	.	CCDC85A(INTRON)
2	56607765	56607784	56607772	AAAT	A	snp	0	1	0	1	.	.	.	CCDC85A(INTRON)
2	56624942	56624950	56624947	T	C	snp	1	1	1	1	.	.	.	INTERGENIC(INTERGENIC)
2	56721300	56721308	56721305	T	G	snp	1	1	1	0	.	.	.	INTERGENIC(INTERGENIC)
2	56721300	56721308	56721307	T	G	snp	1	1	1	1	.	.	.	INTERGENIC(INTERGENIC)

2	56767564	56767571	56767565	T	GA	ins	1	1	1	1	.	.	.	INTERGENIC(INTERGENIC)
2	56767564	56767571	56767566	T	G	snp	1	1	1	1	.	.	.	INTERGENIC(INTERGENIC)
2	56767564	56767571	56767567	T	GA	ins	1	1	1	1	.	.	.	INTERGENIC(INTERGENIC)
2	56844630	56844643	56844639	AT	A	snp	1	1	1	1	.	.	.	INTERGENIC(INTERGENIC)
2	56844645	56844654	56844648	TA	G	snp	1	0	0	0	.	.	.	INTERGENIC(INTERGENIC)
2	56858182	56858197	56858192	TCTT	TTC	del	1	1	1	1	.	.	.	INTERGENIC(INTERGENIC)
2	56858182	56858197	56858193	TCTT	TC	del	1	1	1	1	.	.	.	INTERGENIC(INTERGENIC)
2	56970179	56970186	56970182	A	C	snp	1	0	0	0	.	.	.	INTERGENIC(INTERGENIC)
2	56990401	56990410	56990404	T	G	snp	1	0	0	0	.	.	.	INTERGENIC(INTERGENIC)
2	57036590	57036597	57036592	T	C	snp	1	1	1	0	.	.	.	INTERGENIC(INTERGENIC)
2	57196288	57196297	57196290	GA	A	snp	1	0	1	0	.	.	.	INTERGENIC(INTERGENIC)
2	57243631	57243638	57243633	T	G	snp	1	1	1	1	.	.	.	INTERGENIC(INTERGENIC);snoU13(UPSTREAM)
2	57261628	57261637	57261631	T	G	snp	1	1	1	1	.	.	.	INTERGENIC(INTERGENIC)
2	57306377	57306390	57306388	TC	G	snp	1	1	1	1	.	.	.	INTERGENIC(INTERGENIC)
2	57388610	57388617	57388614	T	C	snp	1	0	1	0	.	.	.	INTERGENIC(INTERGENIC)
2	57416137	57416148	57416142	AC	T	snp	1	1	1	1	.	.	.	INTERGENIC(INTERGENIC)
2	57455900	57455909	57455905	TA	TATAT	del	1	1	1	1	.	.	.	INTERGENIC(INTERGENIC)
2	57484547	57484558	57484553	GAT	A	snp	0	1	1	1	.	.	.	INTERGENIC(INTERGENIC)
2	57487064	57487075	57487067	AT	G	snp	1	0	0	0	.	.	.	INTERGENIC(INTERGENIC)
2	57520177	57520185	57520184	T	G	ins	1	1	1	1	.	.	.	INTERGENIC(INTERGENIC)
2	57564269	57564276	57564274	A	G	snp	1	1	1	1	.	.	.	INTERGENIC(INTERGENIC)
2	57682250	57682257	57682251	A	G	snp	1	1	1	1	.	.	.	INTERGENIC(INTERGENIC)
2	57682701	57682710	57682708	TA	T	snp	1	1	1	1	.	.	.	INTERGENIC(INTERGENIC)
2	57682787	57682796	57682794	TA	T	snp	1	1	1	1	.	.	.	INTERGENIC(INTERGENIC)
2	57711365	57711372	57711369	T	G	snp	0	1	0	1	.	.	.	INTERGENIC(INTERGENIC)
2	57711365	57711372	57711371	T	G	snp	1	1	1	1	.	.	.	INTERGENIC(INTERGENIC)
2	57748412	57748423	57748418	TTG	C	snp	1	0	0	1	.	.	.	INTERGENIC(INTERGENIC)
2	57793011	57793018	57793015	T	G	snp	1	0	0	0	.	.	.	INTERGENIC(INTERGENIC)
2	57842022	57842030	57842023	A	C	snp	1	1	1	1	.	.	.	INTERGENIC(INTERGENIC)
2	57866351	57866358	57866356	A	G	snp	0	0	1	0	.	.	.	INTERGENIC(INTERGENIC)
2	58059051	58059060	58059057	TA	C	snp	1	0	0	0	.	.	.	INTERGENIC(INTERGENIC)
2	58074450	58074458	58074455	A	C	snp	1	0	0	0	.	.	.	INTERGENIC(INTERGENIC)
2	58136612	58136621	58136613	T	G	snp	1	1	1	1	.	.	.	VRK2(INTRON)
2	58160799	58160808	58160803	T	C	ins	1	0	1	0	.	.	.	VRK2(INTRON)
2	58171218	58171226	58171220	A	C	snp	1	1	1	1	.	.	.	VRK2(INTRON)
2	58277020	58277039	58277031	TGAA	G	snp	1	1	1	1	.	.	.	VRK2(INTRON)
2	58335654	58335662	58335657	T	C	snp	1	0	0	0	.	.	.	VRK2(INTRON)
2	58404952	58404959	58404956	A	G	snp	1	1	0	1	.	.	.	FANCL(INTRON)
2	58492223	58492246	58492235	AAAT	T	snp	1	1	1	1	.	.	.	INTERGENIC(INTERGENIC)
2	58492223	58492246	58492239	AAAT	T	snp	1	1	1	1	.	.	.	INTERGENIC(INTERGENIC)
2	58572870	58572878	58572874	A	C	snp	1	1	1	1	.	.	.	INTERGENIC(INTERGENIC)
2	58619763	58619772	58619764	T	G	snp	1	1	1	1	.	.	.	INTERGENIC(INTERGENIC)
2	58688842	58688855	58688845	CT	G	snp	1	1	1	1	.	.	.	AC007092.1.1(INTRON);AC007238.1.1(EXON)
2	59120067	59120090	59120084	ATT	A	del	1	1	1	1	.	.	.	AC007092.1.1(INTRON)
2	59120067	59120090	59120086	ATT	TA	del	1	1	1	1	.	.	.	AC007092.1.1(INTRON)
2	59120067	59120090	59120087	ATT	A	del	1	1	1	1	.	.	.	AC007092.1.1(INTRON)
2	59421866	59421879	59421876	TG	C	snp	1	1	1	1	.	.	.	INTERGENIC(INTERGENIC)
2	59648408	59648416	59648411	A	G	snp	1	1	1	1	.	.	.	AC007131.2.1(INTRON)
2	59739937	59739952	59739943	AAAC	C	del	1	1	1	1	.	.	.	AC007131.2.1(INTRON)
2	59739937	59739952	59739944	AAAC	A	ins	1	1	0	1	.	.	.	AC007131.2.1(INTRON)
2	59739937	59739952	59739947	AAAC	C	del	1	1	1	1	.	.	.	AC007131.2.1(INTRON)
2	59739937	59739952	59739948	AAAC	A	ins	1	1	1	1	.	.	.	AC007131.2.1(INTRON)
2	59760309	59760328	59760322	TATT	C	snp	1	1	1	1	.	.	.	AC007131.2.1(INTRON);AC007179.1(DOWNSTREAM)
2	59769264	59769278	59769273	GAG	T	snp	1	1	1	1	.	.	.	AC007131.2.1(INTRON)
2	59878431	59878438	59878436	A	C	snp	1	1	1	0	.	.	.	AC007131.2.1(INTRON);U6(DOWNSTREAM)
2	59905359	59905366	59905361	T	C	snp	1	0	0	0	.	.	.	AC007131.2.1(INTRON)
2	59940201	59940210	59940206	A	G	snp	1	0	0	0	.	.	.	AC007131.2.1(INTRON)
2	59963084	59963107	59963097	TATC	G	snp	0	0	0	1	.	.	.	AC007131.2.1(UPSTREAM);INTERGENIC(INTERGENIC)
2	59978982	59978989	59978985	A	G	snp	1	1	1	1	.	.	.	INTERGENIC(INTERGENIC)
2	60117108	60117119	60117115	GGA	A	snp	1	1	0	0	.	.	.	INTERGENIC(INTERGENIC)
2	60163598	60163611	60163600	GT	T	ins	0	1	1	1	.	.	.	INTERGENIC(INTERGENIC)
2	60384093	60384112	60384101	TTTA	C	snp	1	1	1	1	.	.	.	INTERGENIC(INTERGENIC)
2	60452274	60452283	60452278	TG	A	snp	1	1	1	1	.	.	.	INTERGENIC(INTERGENIC)
2	60522301	60522312	60522304	GA	G	snp	0	0	1	0	.	.	.	INTERGENIC(INTERGENIC)
2	60533908	60533917	60533913	AG	A	snp	1	1	1	1	.	.	.	INTERGENIC(INTERGENIC)
2	60681180	60681197	60681187	AG	A	snp	0	1	0	1	.	.	.	BCL11A(DOWNSTREAM);BCL11A(INTRON)
2	60895111	60895122	60895116	TTA	T	ins	1	0	0	0	.	.	.	INTERGENIC(INTERGENIC)

2	60902427	60902441	60902430	GCT	A	snp	1	1	1	1	.	.	.	INTERGENIC(INTERGENIC)
2	61001237	61001246	61001242	T	G	ins	1	1	0	1	.	.	.	PAPOLG(INTRON)
2	61002843	61002852	61002846	T	C	snp	1	1	1	1	.	.	.	PAPOLG(INTRON);PAPOLG(UPSTREAM)
2	61286377	61286385	61286382	T	C	snp	1	0	0	0	.	.	.	INTERGENIC(INTERGENIC)
2	61307872	61307880	61307874	A	C	snp	1	0	0	0	.	.	.	KIAA1841(INTRON)
2	61349915	61349922	61349916	T	C	snp	0	0	1	1	.	.	.	KIAA1841(DOWNSTREAM);KIAA1841(INTRON);KIAA1841(UTR_3_PRIME)
2	61388387	61388398	61388390	TTG	C	snp	1	1	1	1	.	.	.	C2orf74(INTRON);C2orf74(UPSTREAM);KIAA1841(INTRON);KIAA1841(UPSTREAM)
2	61395664	61395691	61395668	TATT	A	del	1	1	1	1	.	.	.	C2orf74(DOWNSTREAM);INTERGENIC(INTERGENIC);KIAA1841(DOWNSTREAM)
2	61395664	61395691	61395669	TATT	T	snp	1	1	1	1	.	.	.	C2orf74(DOWNSTREAM);INTERGENIC(INTERGENIC);KIAA1841(DOWNSTREAM)
2	61395664	61395691	61395673	TATT	T	snp	1	1	1	1	.	.	.	C2orf74(DOWNSTREAM);INTERGENIC(INTERGENIC);KIAA1841(DOWNSTREAM)
2	61427185	61427194	61427187	TG	A	snp	0	0	1	1	.	.	.	USP34(DOWNSTREAM);USP34(INTRON)
2	61459978	61459993	61459991	TA	TAT	del	1	1	1	1	.	.	.	USP34(INTRON)
2	61489252	61489267	61489265	AT	G	snp	1	1	1	1	.	.	.	USP34(DOWNSTREAM);USP34(INTRON)
2	61576980	61577003	61576998	AAC	AC	del	1	1	1	1	.	.	.	USP34(INTRON);USP34(UPSTREAM)
2	61709733	61709741	61709734	T	TG	ins	1	1	0	1	.	.	.	XPO1(DOWNSTREAM);XPO1(INTRON)
2	61709733	61709741	61709735	T	G	ins	1	1	0	1	.	.	.	XPO1(DOWNSTREAM);XPO1(INTRON)
2	61709733	61709741	61709736	T	G	snp	1	1	0	1	.	.	.	XPO1(DOWNSTREAM);XPO1(INTRON)
2	61709733	61709741	61709737	T	G	ins	1	0	0	0	.	.	.	XPO1(DOWNSTREAM);XPO1(INTRON)
2	61721952	61721965	61721962	TA	G	snp	1	0	0	0	.	.	.	XPO1(DOWNSTREAM);XPO1(INTRON);XPO1(UPSTREAM)
2	61793331	61793344	61793336	TA	G	snp	1	0	0	0	.	.	.	INTERGENIC(INTERGENIC)
2	61808763	61808770	61808768	T	C	snp	0	1	1	1	.	.	.	INTERGENIC(INTERGENIC)
2	61854278	61854286	61854281	T	C	snp	1	0	0	0	.	.	.	INTERGENIC(INTERGENIC)
2	61880399	61880418	61880411	ATTT	TTTAT	del	1	1	1	1	.	.	.	INTERGENIC(INTERGENIC)
2	61948124	61948131	61948127	A	G	snp	0	0	1	0	.	.	.	INTERGENIC(INTERGENIC)
2	61965580	61965591	61965583	GAT	A	snp	1	1	1	1	.	.	.	INTERGENIC(INTERGENIC)
2	61967425	61967436	61967430	AT	C	snp	0	1	1	1	.	.	.	INTERGENIC(INTERGENIC)
2	61974998	61975009	61975005	CTT	TC	del	1	1	0	1	.	.	.	INTERGENIC(INTERGENIC)
2	62022921	62022929	62022925	T	G	snp	1	0	0	0	.	.	.	INTERGENIC(INTERGENIC)
2	62059375	62059386	62059383	CA	A	ins	1	1	1	1	.	.	.	FAM161A(DOWNSTREAM);FAM161A(INTRON)
2	62059375	62059386	62059384	CA	C	del	1	1	1	1	.	.	.	FAM161A(DOWNSTREAM);FAM161A(INTRON)
2	62059396	62059413	62059401	CAA	C	snp	0	1	1	0	.	.	.	FAM161A(DOWNSTREAM);FAM161A(INTRON)
2	62075998	62076007	62076005	TA	T	snp	1	1	1	1	.	.	.	FAM161A(INTRON)
2	62076162	62076171	62076169	TA	T	snp	1	1	1	1	.	.	.	FAM161A(INTRON)
2	62190305	62190320	62190308	AT	A	snp	1	1	1	1	.	.	.	COMMD1(INTRON)
2	62342968	62342995	62342989	TTTC	TCTTTC	del	1	1	1	1	.	.	.	AC018462.2.1(INTRON);COMMD1(INTRON)
2	62351436	62351459	62351443	TAAA	G	snp	1	0	0	0	.	.	.	AC018462.2.1(INTRON);COMMD1(INTRON)
2	62372822	62372829	62372824	T	G	snp	1	0	0	0	.	.	.	AC018462.2.1(INTRON);AC018462.3.1(UPSTREAM);COMMD1(INTRON)
2	62448649	62448656	62448655	A	G	snp	1	0	0	0	.	.	.	B3GNT2(INTRON)
2	62473352	62473361	62473353	A	C	ins	1	1	1	1	.	.	.	INTERGENIC(INTERGENIC)
2	62482598	62482607	62482600	CA	T	snp	1	0	0	0	.	.	.	INTERGENIC(INTERGENIC)
2	62487825	62487836	62487832	AG	A	snp	0	1	1	1	.	.	.	AC093401.1(DOWNSTREAM);INTERGENIC(INTERGENIC);snoU13(UPSTREAM)
2	62491667	62491674	62491670	C	A	snp	0	1	1	1	.	.	.	AC093401.1(UPSTREAM);INTERGENIC(INTERGENIC);snoU13(UPSTREAM)
2	62596272	62596281	62596280	A	G	snp	1	0	0	0	.	.	.	INTERGENIC(INTERGENIC)
2	62679700	62679707	62679706	T	C	ins	1	1	1	0	.	.	.	INTERGENIC(INTERGENIC)
2	62752273	62752284	62752277	TTG	C	snp	1	1	1	1	.	.	.	INTERGENIC(INTERGENIC)
2	62793186	62793195	62793191	TA	G	snp	0	1	1	1	.	.	.	INTERGENIC(INTERGENIC);RSL24D1P2(UPSTREAM)
2	62804320	62804329	62804327	T	G	snp	1	1	1	1	.	.	.	INTERGENIC(INTERGENIC)
2	62926133	62926142	62926138	TG	A	snp	1	1	1	1	.	.	.	EHBP1(INTRON)
2	63127805	63127814	63127808	GT	G	snp	1	0	0	0	.	.	.	EHBP1(INTRON)
2	63169163	63169172	63169166	AC	T	snp	0	1	0	1	.	.	.	EHBP1(INTRON);EHBP1(UPSTREAM);RP11-443F16.1.1(DOWNSTREAM)
2	63297241	63297252	63297247	TA	C	snp	1	1	0	1	.	.	.	INTERGENIC(INTERGENIC)
2	63518337	63518344	63518343	A	C	ins	1	1	1	1	.	.	.	WDPCP(INTRON)
2	63522573	63522580	63522578	A	G	snp	1	1	1	1	.	.	.	WDPCP(INTRON)
2	63556030	63556037	63556036	T	G	snp	1	1	1	1	.	.	.	WDPCP(INTRON)
2	63610846	63610865	63610853	TCAA	G	snp	1	0	0	0	.	.	.	WDPCP(INTRON);WDPCP(UPSTREAM)
2	63692480	63692487	63692482	A	G	snp	1	0	0	0	.	.	.	WDPCP(INTRON)
2	64037046	64037061	64037053	AAAT	A	snp	1	1	1	1	.	.	.	WDPCP(INTRON)
2	64049988	64050001	64049995	AT	C	snp	1	1	0	1	.	.	.	WDPCP(INTRON)

2	64298655	64298663	64298660	T	G	snp	1	0	0	0	.	.	.	INTERGENIC(INTERGENIC)
2	64416874	64416883	64416880	T	C	snp	1	1	1	1	.	.	.	AC074289.1.1(INTRON);LINC00309(INTRON)
2	64505083	64505096	64505086	TC	T	snp	1	1	1	1	.	.	.	INTERGENIC(INTERGENIC)
2	64505083	64505096	64505090	TC	T	snp	1	1	1	1	.	.	.	INTERGENIC(INTERGENIC)
2	64505083	64505096	64505094	TC	T	snp	1	1	1	1	.	.	.	INTERGENIC(INTERGENIC)
2	64746575	64746602	64746581	AAAC	C	del	1	1	1	1	.	.	.	AFTPH(UPSTREAM);INTERGENIC(INTERGENIC);RP11-568N6.1.1(DOWNSTREAM)
2	64746575	64746602	64746582	AAAC	A	snp	1	1	1	1	.	.	.	AFTPH(UPSTREAM);INTERGENIC(INTERGENIC);RP11-568N6.1.1(DOWNSTREAM)
2	64784149	64784163	64784160	CAA	G	snp	1	0	0	0	.	.	.	AFTPH(INTRON)
2	64873283	64873291	64873285	A	C	ins	1	1	1	1	.	.	.	AC007365.3.1(INTRON);SERTAD2(INTRON)
2	64904528	64904547	64904543	AAAC	A	del	1	0	0	0	.	.	.	SERTAD2(INTRON)
2	64968174	64968193	64968189	ATTT	A	del	1	1	1	1	.	.	.	SERTAD2(INTRON)
2	64985014	64985028	64985024	CAA	AC	del	1	1	1	1	.	.	.	INTERGENIC(INTERGENIC)
2	64985014	64985028	64985025	CAA	C	del	1	1	1	1	.	.	.	INTERGENIC(INTERGENIC)
2	65072432	65072447	65072437	ACAA	A	del	1	0	0	0	.	.	.	AC007880.1.1(DOWNSTREAM);INTERGENIC(INTERGENIC)
2	65129345	65129358	65129351	CT	TCTT	ins	1	1	1	1	.	.	.	AC007386.1(DOWNSTREAM);AC007386.2.1(EXON)
2	65129345	65129358	65129353	CT	TT	ins	1	1	1	1	.	.	.	AC007386.1(DOWNSTREAM);AC007386.2.1(EXON)
2	65218467	65218475	65218468	A	C	ins	1	1	1	1	.	.	.	SLC1A4(INTRON);U6(UPSTREAM)
2	65272887	65272894	65272889	C	T	snp	1	0	0	0	.	.	.	AC007386.4.1(INTRON)
2	65305842	65305849	65305848	T	G	snp	1	0	0	0	.	.	.	CEP68(DOWNSTREAM);CEP68(INTRON);RAB1A(INTRON)
2	65354876	65354885	65354877	T	C	snp	1	0	0	0	.	.	.	RAB1A(INTRON)
2	65369448	65369457	65369451	CA	C	snp	1	0	0	0	.	.	.	INTERGENIC(INTERGENIC)
2	65402013	65402021	65402019	A	C	snp	1	1	1	1	.	.	.	INTERGENIC(INTERGENIC)
2	65412841	65412860	65412855	TTCT	T	del	1	1	1	1	.	.	.	INTERGENIC(INTERGENIC)
2	65516138	65516147	65516144	T	G	snp	1	1	1	1	.	.	.	INTERGENIC(INTERGENIC)
2	65711586	65711593	65711587	A	G	snp	1	0	0	0	.	.	.	AC074391.1.1(DOWNSTREAM);AC074391.1.1(INTRON)
2	65737991	65738014	65738002	TTTC	G	snp	0	1	0	1	.	.	.	AC007389.2.1(DOWNSTREAM);AC074391.1.1(INTRON)
2	65757354	65757362	65757357	A	C	snp	1	1	1	1	.	.	.	AC074391.1.1(INTRON)
2	65815122	65815133	65815125	CTG	T	snp	1	1	1	1	.	.	.	AC007389.3.1(DOWNSTREAM);AC074391.1.1(INTRON)
2	65902660	65902671	65902667	CTT	TCTTCTGG	del	1	1	0	1	.	.	.	AC074391.1.1(INTRON)
2	65930956	65930967	65930963	AAC	G	snp	1	1	1	1	.	.	.	AC074391.1.1(INTRAGENIC)
2	65961191	65961199	65961192	T	G	ins	1	0	0	0	.	.	.	AC074391.1.1(INTRON)
2	65967696	65967713	65967709	AT	A	del	1	0	1	0	.	.	.	AC074391.1.1(INTRON)
2	65967696	65967713	65967710	AT	T	snp	1	0	1	0	.	.	.	AC074391.1.1(INTRON)
2	66053890	66053899	66053897	CA	G	snp	0	0	1	0	.	.	.	AC074391.1.1(INTRAGENIC)
2	66102987	66102996	66102991	T	C	snp	0	0	0	1	.	.	.	AC074391.1.1(INTRAGENIC)
2	66142990	66143003	66143000	TG	C	snp	1	1	1	1	.	.	.	INTERGENIC(INTERGENIC)
2	66242130	66242137	66242132	G	A	ins	1	0	1	0	.	.	.	INTERGENIC(INTERGENIC)
2	66243055	66243062	66243060	A	C	snp	1	0	0	0	.	.	.	INTERGENIC(INTERGENIC)
2	66254129	66254136	66254133	A	C	snp	1	0	0	0	.	.	.	INTERGENIC(INTERGENIC)
2	66281889	66281897	66281893	T	G	snp	0	0	1	0	.	.	.	INTERGENIC(INTERGENIC)
2	66361922	66361930	66361926	A	G	snp	1	1	1	1	.	.	.	INTERGENIC(INTERGENIC)
2	66374686	66374694	66374689	A	G	snp	1	0	0	0	.	.	.	INTERGENIC(INTERGENIC)
2	66422388	66422395	66422390	A	G	snp	1	0	0	0	.	.	.	INTERGENIC(INTERGENIC)
2	66641285	66641294	66641292	T	C	snp	1	0	0	0	.	.	.	INTERGENIC(INTERGENIC)
2	66667182	66667199	66667186	CG	T	snp	1	1	1	1	.	.	.	AC092669.1.1(INTRON);AC092669.4.1(INTRON);MEIS1(DOWNSTREAM);MEIS1(INTRON);MEIS1(UPSTREAM)
2	66792414	66792429	66792425	CT	CTC	del	1	1	1	1	.	.	.	MEIS1(INTRON)
2	66850130	66850139	66850138	T	TG	ins	1	1	0	1	.	.	.	AC007392.3.1(INTRON)
2	66869936	66869943	66869941	C	A	snp	1	1	0	1	.	.	.	AC007392.3.1(INTRON)
2	66897882	66897891	66897888	A	C	snp	1	0	0	0	.	.	.	AC007392.3.1(INTRON)
2	66912934	66912949	66912947	AT	A	del	1	1	1	1	.	.	.	AC007392.3.1(INTRON);AC007392.3.1(UPSTREAM)
2	66989961	66989972	66989968	AT	C	snp	0	1	0	1	.	.	.	INTERGENIC(INTERGENIC)
2	67000840	67000847	67000843	T	C	snp	1	0	0	1	.	.	.	INTERGENIC(INTERGENIC)
2	67002388	67002397	67002391	TA	G	snp	1	1	1	1	.	.	.	INTERGENIC(INTERGENIC)
2	67019050	67019063	67019061	TC	T	snp	1	0	0	0	.	.	.	INTERGENIC(INTERGENIC)
2	67070585	67070604	67070593	TGAA	C	snp	1	0	0	0	.	.	.	INTERGENIC(INTERGENIC)
2	67082856	67082865	67082861	TG	T	snp	1	0	0	0	.	.	.	INTERGENIC(INTERGENIC)
2	67416674	67416697	67416692	AAC	AC	del	1	1	1	1	.	.	.	AC023115.3.1(INTRON);AC078941.1.1(INTRON)
2	67416702	67416711	67416704	A	C	ins	1	1	1	1	.	.	.	AC023115.3.1(INTRON);AC078941.1.1(INTRON)
2	67438729	67438736	67438731	A	C	snp	1	1	1	1	.	.	.	AC023115.1.1(UPSTREAM);AC023115.3.1(INTRON);AC078941.1.1(INTRON)
2	67532990	67533005	67532999	TA	T	snp	1	1	1	1	.	.	.	INTERGENIC(INTERGENIC)
2	67698662	67698670	67698665	T	G	ins	0	1	0	1	.	.	.	INTERGENIC(INTERGENIC)
2	67741693	67741701	67741695	A	C	snp	1	1	1	1	.	.	.	INTERGENIC(INTERGENIC)
2	67760443	67760462	67760451	GATG	ATGGATGGATGC	ins	1	1	1	1	.	.	.	INTERGENIC(INTERGENIC)

2	67826598	67826607	67826601	TC	G	snp	1	0	0	0	.	.	.	AC010987.5.1(INTRON)
2	67868477	67868485	67868480	A	C	snp	1	1	1	1	.	.	.	AC010987.5.1(INTRON)
2	67899836	67899847	67899841	AC	A	snp	1	1	1	1	.	.	.	AC010987.5.1(INTRON)
2	67915926	67915941	67915939	TC	T	del	1	1	1	1	.	.	.	AC010987.5.1(UPSTREAM);INTERGENIC(INTERGENIC)
2	67917195	67917206	67917202	TG	A	snp	1	1	1	1	.	.	.	INTERGENIC(INTERGENIC)
2	67994519	67994528	67994523	CA	G	snp	1	1	0	0	.	.	.	INTERGENIC(INTERGENIC)
2	67994519	67994528	67994524	CA	C	del	1	1	0	0	.	.	.	INTERGENIC(INTERGENIC)
2	68009816	68009823	68009817	T	G	snp	1	1	1	1	.	.	.	INTERGENIC(INTERGENIC)
2	68035137	68035148	68035140	CTT	T	del	1	1	1	1	.	.	.	AC010987.6.1(INTRON)
2	68045406	68045415	68045409	A	AG	ins	1	0	0	0	.	.	.	AC010987.6.1(INTRON)
2	68173044	68173057	68173052	AT	TTT	ins	1	1	1	1	.	.	.	INTERGENIC(INTERGENIC)
2	68173044	68173057	68173053	AT	ATA	del	1	1	1	1	.	.	.	INTERGENIC(INTERGENIC)
2	68173044	68173057	68173054	AT	TTTT	ins	1	1	1	1	.	.	.	INTERGENIC(INTERGENIC)
2	68230009	68230016	68230013	A	C	snp	1	0	0	0	.	.	.	INTERGENIC(INTERGENIC)
2	68242623	68242632	68242630	AC	T	snp	1	1	1	1	.	.	.	INTERGENIC(INTERGENIC)
2	68494211	68494219	68494214	A	C	ins	0	1	1	1	.	.	.	INTERGENIC(INTERGENIC)
2	68494211	68494219	68494215	A	C	snp	1	1	1	1	.	.	.	INTERGENIC(INTERGENIC)
2	68517690	68517698	68517697	A	C	snp	1	0	0	0	.	.	.	CNRIP1(DOWNSTREAM);CNRIP1(INTRON)
2	68535358	68535365	68535361	A	C	ins	1	0	0	0	.	.	.	CNRIP1(INTRON)
2	68606305	68606314	68606310	AG	A	snp	1	0	0	0	.	.	.	PLEK(INTRON)
2	68677112	68677123	68677114	AT	T	snp	1	1	1	1	.	.	.	AC127383.1.1(UPSTREAM);INTERGENIC(INTERGENIC)
2	68677148	68677157	68677152	TA	C	snp	1	1	0	1	.	.	.	AC127383.1.1(UPSTREAM);INTERGENIC(INTERGENIC)
2	68677214	68677223	68677218	TA	C	snp	1	1	1	1	.	.	.	AC127383.1.1(UPSTREAM);INTERGENIC(INTERGENIC)
2	68828222	68828239	68828226	AT	G	snp	0	1	0	1	.	.	.	APLF(INTRON)
2	68828222	68828239	68828235	AT	C	snp	1	0	0	1	.	.	.	APLF(INTRON)
2	68828222	68828239	68828236	AT	G	snp	1	0	0	0	.	.	.	APLF(INTRON)
2	68828222	68828239	68828236	AT	T	del	1	1	0	1	.	.	.	APLF(INTRON)
2	69025233	69025240	69025238	A	G	snp	1	0	0	0	.	.	.	ARHGAP25(INTRON)
2	69090962	69090969	69090966	A	G	snp	1	0	0	0	.	.	.	BMP10(DOWNSTREAM);INTERGENIC(INTERGENIC)
2	69203443	69203451	69203446	T	G	snp	1	0	0	0	.	.	.	GKN1(INTRON)
2	69224855	69224866	69224862	AC	A	snp	1	1	1	1	.	.	.	INTERGENIC(INTERGENIC)
2	69250627	69250641	69250632	TGG	A	snp	1	1	1	1	.	.	.	ANTXR1(INTRON)
2	69256174	69256183	69256182	C	A	snp	1	0	0	0	.	.	.	ANTXR1(INTRON)
2	69328387	69328394	69328389	A	G	snp	1	1	1	1	.	.	.	ANTXR1(INTRON)
2	69354890	69354899	69354896	AG	C	snp	1	1	1	1	.	.	.	ANTXR1(INTRON)
2	69373509	69373516	69373512	T	G	snp	1	0	0	0	.	.	.	ANTXR1(INTRON);ANTXR1(UTR_3_PRIME)
2	69510927	69510934	69510932	A	G	snp	1	0	0	0	.	.	.	INTERGENIC(INTERGENIC)
2	69540839	69540846	69540843	T	C	snp	0	0	0	1	.	.	.	INTERGENIC(INTERGENIC)
2	69640431	69640439	69640436	A	G	snp	0	0	1	0	.	.	.	NFU1(INTRON)
2	69650341	69650349	69650343	G	GA	ins	1	0	0	0	.	.	.	NFU1(INTRON)
2	69654201	69654216	69654207	AAAG	G	del	1	0	0	0	.	.	.	NFU1(INTRON)
2	69693392	69693399	69693397	G	T	snp	1	0	0	0	.	.	AC114772.1(SYNONYMOUS_CODING):P76	AAK1(INTRON)
2	69853625	69853642	69853639	ATT	A	del	1	1	1	1	.	.	.	AAK1(INTRON)
2	69861304	69861313	69861309	AT	A	del	0	1	1	1	.	.	.	AAK1(INTRON)
2	70150213	70150222	70150215	A	C	snp	1	1	1	1	.	.	.	MXD1(INTRON)
2	70187728	70187735	70187734	C	A	snp	0	0	1	0	.	.	.	ASPRV1(UTR_3_PRIME);PCBP1-AS1(DOWNSTREAM);snoU13(DOWNSTREAM)
2	70449281	70449290	70449284	A	C	snp	1	0	0	0	.	.	.	C2orf42(INTRON);TIA1(DOWNSTREAM);TIA1(INTRON);TIA1(UPSTREAM)
2	70462705	70462714	70462707	TG	C	snp	0	0	1	1	.	.	.	C2orf42(INTRON);TIA1(INTRON)
2	70565727	70565744	70565729	AT	TAT	del	1	1	1	1	.	.	.	INTERGENIC(INTERGENIC)
2	70730701	70730715	70730712	ATA	AT	del	1	1	1	1	.	.	.	TGFA(INTRON)
2	70818962	70818971	70818964	T	G	snp	1	1	0	0	.	.	.	INTERGENIC(INTERGENIC)
2	70839802	70839810	70839805	C	T	ins	1	0	0	0	.	.	.	ADD2(INTRON)
2	70839802	70839810	70839809	C	CT	ins	0	1	0	0	.	.	.	ADD2(INTRON)
2	71051166	71051189	71051172	AAAG	G	del	1	1	1	1	.	.	.	CLEC4F(UPSTREAM);INTERGENIC(INTERGENIC)
2	71051166	71051189	71051176	AAAG	G	del	1	1	1	1	.	.	.	CLEC4F(UPSTREAM);INTERGENIC(INTERGENIC)
2	71061923	71061931	71061924	A	C	snp	1	0	0	0	.	.	.	CD207(INTRON)
2	71097331	71097350	71097345	TTAA	T	snp	1	0	0	0	.	.	.	INTERGENIC(INTERGENIC)
2	71221963	71221980	71221968	CCG	C	snp	1	1	0	1	.	.	.	AC007040.11.1(UPSTREAM);AC007040.6.1(INTRON);TEX261(EXON);TEX261(UPSTREAM);TEX261(UTR_5_PRIME)
2	71338078	71338085	71338084	A	C	snp	1	0	0	0	.	.	.	MCEE(INTRON)
2	71355192	71355201	71355198	AT	G	snp	1	0	0	0	.	.	.	MCEE(INTRON);MCEE(UPSTREAM);MPHOSPH10(UPSTREAM)
2	71444259	71444270	71444268	TA	C	snp	1	1	1	1	.	.	.	PAIP2B(INTRON)
2	71540801	71540810	71540806	TC	G	snp	1	1	0	1	.	.	.	ZNF638(INTRON)
2	71573801	71573810	71573808	TG	A	snp	1	1	1	1	.	.	.	ZNF638(INTRON)
2	71578683	71578690	71578684	T	G	snp	1	1	0	1	.	.	.	ZNF638(DOWNSTREAM);ZNF638(INTRON)

2	71637500	71637509	71637505	T	G	ins	1	0	0	0	.	.	.	ZNF638(INTRON)
2	71637500	71637509	71637506	T	G	ins	1	1	0	0	.	.	.	ZNF638(INTRON)
2	71638571	71638578	71638572	T	TTTA	ins	1	1	1	1	.	.	.	ZNF638(INTRON)
2	71662818	71662835	71662826	AAC	A	snp	1	1	1	1	.	.	.	INTERGENIC(INTERGENIC);ZNF638(DOWNSTREAM)
2	71675910	71675917	71675916	G	T	snp	1	1	1	1	.	.	.	DYSF(UPSTREAM);INTERGENIC(INTERGENIC)
2	71722384	71722399	71722389	TGAA	T	snp	1	0	0	0	.	.	.	DYSF(INTRON)
2	71962682	71962691	71962685	AT	A	snp	1	1	1	1	.	.	.	INTERGENIC(INTERGENIC)
2	72039707	72039722	72039714	CATT	G	snp	1	1	1	1	.	.	.	INTERGENIC(INTERGENIC)
2	72053190	72053198	72053196	T	G	snp	0	1	1	1	.	.	.	INTERGENIC(INTERGENIC)
2	72196121	72196128	72196124	G	A	snp	1	0	0	0	.	.	.	INTERGENIC(INTERGENIC)
2	72289900	72289911	72289902	AT	G	snp	0	1	0	1	.	.	.	INTERGENIC(INTERGENIC)
2	72573201	72573210	72573205	T	C	snp	1	1	0	1	.	.	.	EXOC6B(INTRON)
2	72637961	72637969	72637965	A	G	snp	1	1	0	1	.	.	.	EXOC6B(INTRON)
2	72662844	72662852	72662851	T	G	ins	1	1	0	1	.	.	.	EXOC6B(INTRON)
2	72682949	72682958	72682956	AG	C	snp	1	1	0	1	.	.	.	EXOC6B(INTRON)
2	72789312	72789319	72789317	A	G	snp	1	1	1	1	.	.	.	EXOC6B(INTRON)
2	72809294	72809305	72809297	AT	C	snp	1	0	0	0	.	.	.	EXOC6B(INTRON)
2	72822414	72822423	72822419	AT	AG	ins	1	1	1	1	.	.	.	EXOC6B(INTRON)
2	72822414	72822423	72822421	AT	G	snp	1	1	1	1	.	.	.	EXOC6B(INTRON)
2	72822425	72822436	72822429	TA	G	snp	0	0	1	0	.	.	.	EXOC6B(INTRON)
2	72833752	72833767	72833760	CTTT	T	ins	1	1	1	1	.	.	.	EXOC6B(INTRON)
2	72833752	72833767	72833763	CTTT	C	del	1	1	1	1	.	.	.	EXOC6B(INTRON)
2	72835885	72835892	72835886	A	G	snp	1	0	0	0	.	.	.	EXOC6B(INTRON)
2	73024965	73024976	73024968	TAA	AA	del	1	0	0	0	.	.	.	EXOC6B(INTRON)
2	73346008	73346023	73346019	TAAA	G	snp	1	1	1	1	.	.	.	RAB11FIP5(INTRON)
2	73458555	73458562	73458560	T	C	snp	1	1	1	1	.	.	.	CCT7(UPSTREAM);PRADC1(INTRON);SMYD5(DOWNSTREAM)
2	73501854	73501862	73501859	T	G	ins	1	0	0	0	.	.	.	FBXO41(INTRON);FBXO41(UPSTREAM)
2	73537730	73537757	73537738	TATC	ATCTATCTATC	ins	1	1	1	1	.	.	.	INTERGENIC(INTERGENIC)
2	73584774	73584791	73584785	AT	A	del	1	1	1	1	.	.	.	INTERGENIC(INTERGENIC)
2	73621495	73621506	73621500	TTG	A	snp	0	0	1	0	.	.	.	ALMS1(INTRON)
2	73637516	73637523	73637519	A	G	snp	1	1	0	1	.	.	.	ALMS1(INTRON)
2	73749049	73749057	73749054	T	G	snp	1	1	0	1	.	.	.	ALMS1(INTRON)
2	73806490	73806499	73806495	T	C	snp	1	1	0	0	.	.	.	ALMS1(INTRON)
2	73854470	73854477	73854473	A	G	snp	1	0	0	0	.	.	.	INTERGENIC(INTERGENIC)
2	73861094	73861101	73861095	T	G	snp	1	0	0	0	.	.	.	INTERGENIC(INTERGENIC)
2	73913159	73913172	73913169	CT	G	snp	1	0	0	0	.	.	.	ALMS1P(DOWNSTREAM);INTERGENIC(INTERGENIC)
2	73966039	73966056	73966042	AT	A	snp	1	0	0	1	.	.	.	INTERGENIC(INTERGENIC);TPRKB(UPSTREAM)
2	74037728	74037737	74037732	AT	T	snp	0	0	0	1	.	.	.	C2orf78(INTRON)
2	74152746	74152755	74152754	T	G	ins	1	1	1	1	.	.	.	DGUOK(UPSTREAM);INTERGENIC(INTERGENIC)
2	74205884	74205893	74205889	C	T	snp	1	0	0	0	.	.	.	DGUOK-AS1(INTRON)
2	74221742	74221765	74221758	TTTC	TTCTTTC	del	1	1	1	1	.	.	.	INTERGENIC(INTERGENIC);RP11-711M9.1.1(DOWNSTREAM)
2	74355310	74355319	74355311	T	C	ins	1	1	1	1	.	.	.	INTERGENIC(INTERGENIC);RP11-287D1.1.1(DOWNSTREAM)
2	74355310	74355319	74355312	T	C	snp	1	1	1	1	.	.	.	INTERGENIC(INTERGENIC);RP11-287D1.1.1(DOWNSTREAM)
2	74545262	74545270	74545265	A	G	snp	1	0	0	0	.	.	.	SLC4A5(INTRON);SLC4A5(UPSTREAM);U6(UPSTREAM)
2	74632392	74632412	74632395	AAT	AT	del	1	0	0	0	.	.	.	INTERGENIC(INTERGENIC)
2	74632392	74632412	74632397	AAT	A	ins	1	0	0	0	.	.	.	INTERGENIC(INTERGENIC)
2	74672676	74672687	74672685	TA	TT	ins	1	1	1	1	.	.	.	INTERGENIC(INTERGENIC);RTKN(UPSTREAM)
2	74705187	74705198	74705191	AT	G	snp	1	0	0	0	.	.	.	CCDC142(DOWNSTREAM);CCDC142(INTRON);CCDC142(UPSTREAM);MRPL53(UPSTREAM)
2	74942712	74942719	74942714	C	A	snp	0	1	1	0	.	.	.	INTERGENIC(INTERGENIC)
2	74988147	74988156	74988155	A	G	snp	0	0	1	0	.	.	.	INTERGENIC(INTERGENIC)
2	75391092	75391101	75391100	A	G	snp	1	1	1	1	.	.	.	TACR1(INTRON)
2	75425097	75425116	75425112	AAAC	T	snp	1	1	1	1	.	.	.	TACR1(INTRON)
2	75501069	75501077	75501076	T	G	snp	1	0	0	0	.	.	.	INTERGENIC(INTERGENIC)
2	75538425	75538436	75538433	GT	T	snp	0	0	0	1	.	.	.	INTERGENIC(INTERGENIC)
2	75550029	75550038	75550033	A	C	snp	1	1	1	1	.	.	.	INTERGENIC(INTERGENIC)
2	75550655	75550664	75550659	AC	G	snp	1	1	1	1	.	.	.	INTERGENIC(INTERGENIC)
2	75569318	75569329	75569325	TG	A	snp	0	1	0	1	.	.	.	INTERGENIC(INTERGENIC)
2	75674775	75674784	75674776	C	A	snp	1	1	1	1	.	.	.	INTERGENIC(INTERGENIC)
2	75674775	75674784	75674782	C	A	snp	0	1	1	1	.	.	.	INTERGENIC(INTERGENIC)
2	75685203	75685214	75685208	AAG	A	snp	1	1	0	1	.	.	.	GAPDHP57(UPSTREAM);INTERGENIC(INTERGENIC)
2	75758661	75758674	75758669	TA	C	snp	0	0	1	0	.	.	.	FAM176A(INTRON)
2	75811203	75811211	75811210	A	C	snp	1	0	0	0	.	.	.	INTERGENIC(INTERGENIC)
2	75817699	75817712	75817705	TA	A	snp	1	0	0	0	.	.	.	INTERGENIC(INTERGENIC)
2	75867925	75867932	75867929	T	C	snp	1	1	1	1	.	.	.	INTERGENIC(INTERGENIC)
2	76041545	76041552	76041547	T	C	snp	1	1	1	1	.	.	.	INTERGENIC(INTERGENIC)
2	76239404	76239413	76239406	TA	C	snp	1	1	1	1	.	.	.	INTERGENIC(INTERGENIC)

2	76291642	76291649	76291645	T	C	snp	1	1	1	1	.	.	.	INTERGENIC(INTERGENIC)
2	76300863	76300870	76300869	T	G	ins	0	0	1	0	.	.	.	INTERGENIC(INTERGENIC)
2	76356615	76356634	76356625	TTTG	A	snp	1	0	0	0	.	.	.	INTERGENIC(INTERGENIC)
2	76366900	76366907	76366901	A	C	ins	1	1	1	1	.	.	.	INTERGENIC(INTERGENIC)
2	76457216	76457225	76457217	T	G	snp	0	0	1	0	.	.	.	INTERGENIC(INTERGENIC)
2	76475097	76475104	76475098	A	C	snp	1	0	0	0	.	.	.	INTERGENIC(INTERGENIC)
2	76477087	76477106	76477098	TAAA	T	snp	1	1	1	1	.	.	.	INTERGENIC(INTERGENIC)
2	76647819	76647838	76647834	AAAC	A	del	0	1	1	1	.	.	.	INTERGENIC(INTERGENIC)
2	76695799	76695813	76695809	AAC	G	snp	1	1	1	1	.	.	.	INTERGENIC(INTERGENIC)
2	76912279	76912288	76912285	A	G	snp	1	1	1	1	.	.	.	INTERGENIC(INTERGENIC)
2	76990534	76990543	76990538	CT	G	snp	1	0	0	0	.	.	.	LRRTM4(INTRON)
2	77010134	77010147	77010142	AT	G	snp	1	0	0	0	.	.	.	LRRTM4(INTRON)
2	77022719	77022728	77022722	AC	T	snp	1	0	1	0	.	.	.	LRRTM4(INTRON)
2	77049926	77049941	77049930	TTAT	C	snp	1	1	1	1	.	.	.	LRRTM4(INTRON)
2	77058332	77058343	77058341	TG	A	snp	1	1	1	1	.	.	.	LRRTM4(INTRON)
2	77201282	77201293	77201291	AT	AC	ins	1	1	1	1	.	.	.	LRRTM4(INTRON)
2	77206689	77206702	77206699	TA	G	snp	1	0	0	0	.	.	.	LRRTM4(INTRON)
2	77246117	77246124	77246118	A	G	snp	1	0	0	0	.	.	.	LRRTM4(INTRON)
2	77256198	77256209	77256201	AT	C	snp	0	1	1	1	.	.	.	LRRTM4(INTRON)
2	77262387	77262410	77262394	AAAC	A	snp	1	1	1	0	.	.	.	LRRTM4(INTRON)
2	77288505	77288512	77288507	A	C	snp	1	0	0	0	.	.	.	LRRTM4(INTRON)
2	77300882	77300895	77300891	TC	A	snp	1	0	0	0	.	.	.	LRRTM4(INTRON)
2	77300882	77300895	77300893	TC	A	snp	1	1	1	1	.	.	.	LRRTM4(INTRON)
2	77326579	77326590	77326582	GT	C	snp	1	1	1	1	.	.	.	LRRTM4(INTRON)
2	77330791	77330800	77330793	AT	C	snp	1	0	0	0	.	.	.	LRRTM4(INTRON)
2	77333915	77333929	77333922	ATA	C	snp	1	1	1	1	.	.	.	LRRTM4(INTRON)
2	77377281	77377289	77377286	A	G	snp	1	1	1	1	.	.	.	LRRTM4(INTRON)
2	77392360	77392369	77392367	AC	G	snp	1	0	0	0	.	.	.	LRRTM4(INTRON)
2	77397967	77397982	77397970	TA	G	snp	1	0	0	0	.	.	.	LRRTM4(INTRON)
2	77472663	77472674	77472666	AAG	AGAAGAAG	del	1	1	1	1	.	.	.	LRRTM4(INTRON)
2	77472663	77472674	77472669	AAG	AGAAG	del	1	1	1	1	.	.	.	LRRTM4(INTRON)
2	77472750	77472758	77472757	T	TG	ins	1	1	1	1	.	.	.	LRRTM4(INTRON)
2	77488306	77488315	77488312	A	G	snp	1	1	1	1	.	.	.	LRRTM4(INTRON)
2	77489632	77489641	77489633	T	G	snp	1	0	0	0	.	.	.	LRRTM4(INTRON)
2	77491453	77491466	77491455	AG	GG	ins	1	0	0	0	.	.	.	LRRTM4(INTRON)
2	77530147	77530156	77530153	AT	TG	ins	1	1	1	0	.	.	.	LRRTM4(INTRON)
2	77552378	77552385	77552381	A	C	snp	0	0	0	1	.	.	.	LRRTM4(INTRON)
2	77579876	77579884	77579882	T	G	snp	1	1	1	1	.	.	.	LRRTM4(INTRON)
2	77684227	77684236	77684232	T	C	snp	1	0	0	0	.	.	.	LRRTM4(INTRON)
2	77825981	77825988	77825986	A	G	snp	0	1	1	1	.	.	.	INTERGENIC(INTERGENIC)
2	77943875	77943890	77943882	ATAG	A	snp	1	1	1	1	.	.	.	INTERGENIC(INTERGENIC)
2	78019278	78019286	78019280	T	C	snp	1	1	1	1	.	.	.	AC105399.2.1(INTRON);AC105399.2.1(UPSTREAM)
2	78065525	78065532	78065529	T	G	snp	1	0	0	0	.	.	.	INTERGENIC(INTERGENIC)
2	78085959	78085972	78085968	TG	T	snp	1	1	0	0	.	.	.	INTERGENIC(INTERGENIC)
2	78102196	78102205	78102204	A	G	snp	1	0	0	0	.	.	.	INTERGENIC(INTERGENIC)
2	78158817	78158824	78158820	A	G	ins	1	1	1	1	.	.	.	INTERGENIC(INTERGENIC)
2	78190304	78190321	78190318	TG	C	snp	1	1	1	1	.	.	.	INTERGENIC(INTERGENIC)
2	78202859	78202867	78202860	A	C	snp	1	1	1	1	.	.	.	INTERGENIC(INTERGENIC)
2	78206229	78206238	78206235	A	C	snp	1	1	1	1	.	.	.	INTERGENIC(INTERGENIC)
2	78263711	78263720	78263717	CA	A	snp	1	1	1	1	.	.	.	INTERGENIC(INTERGENIC)
2	78312604	78312621	78312618	GA	A	del	1	0	0	0	.	.	.	AC012494.1.1(UPSTREAM);INTERGENIC(INTERGENIC)
2	78315637	78315650	78315648	AC	AT	ins	1	1	1	1	.	.	.	AC012494.1.1(UPSTREAM);INTERGENIC(INTERGENIC)
2	78328991	78329002	78328999	AAG	A	snp	1	1	1	1	.	.	.	AC012494.1.1(INTRON)
2	78348175	78348184	78348180	TC	G	snp	1	0	0	0	.	.	.	AC012494.1.1(INTRON)
2	78420355	78420364	78420363	A	G	snp	1	0	0	0	.	.	.	INTERGENIC(INTERGENIC)
2	78439922	78439930	78439926	A	C	snp	1	1	1	1	.	.	.	INTERGENIC(INTERGENIC)
2	78462935	78462944	78462942	TA	C	snp	0	1	1	1	.	.	.	INTERGENIC(INTERGENIC)
2	78528552	78528560	78528553	T	G	ins	0	0	1	1	.	.	.	INTERGENIC(INTERGENIC)
2	78556159	78556166	78556165	T	G	snp	1	1	1	1	.	.	.	INTERGENIC(INTERGENIC)
2	78624631	78624638	78624633	A	C	ins	1	1	1	1	.	.	.	INTERGENIC(INTERGENIC)
2	78640582	78640597	78640589	TA	TT	ins	1	1	0	0	.	.	.	AC064872.1.1(UPSTREAM);INTERGENIC(INTERGENIC)
2	78681322	78681329	78681328	T	C	snp	0	0	0	1	.	.	.	INTERGENIC(INTERGENIC)
2	78682670	78682678	78682674	A	G	snp	1	1	1	1	.	.	.	INTERGENIC(INTERGENIC)
2	78739156	78739163	78739157	A	C	snp	1	1	1	1	.	.	.	INTERGENIC(INTERGENIC)
2	78745238	78745258	78745248	TTG	C	snp	1	1	1	1	.	.	.	INTERGENIC(INTERGENIC)
2	78918960	78918971	78918966	CT	TG	ins	1	1	1	1	.	.	.	INTERGENIC(INTERGENIC)
2	79258139	79258146	79258144	T	G	snp	1	1	1	1	.	.	.	INTERGENIC(INTERGENIC);REG3G(DOWNSTREAM)

2	79346520	79346528	79346526	A	G	snp	1	1	1	1	.	.	.	INTERGENIC(INTERGENIC);REG1A(UPSTREAM)
2	79521314	79521329	79521327	AG	A	ins	1	0	0	0	.	.	.	CTNNA2(INTRON)
2	79532500	79532517	79532502	AT	TATATG	ins	1	1	1	1	.	.	.	CTNNA2(INTRON)
2	79535006	79535013	79535011	T	C	snp	1	1	0	1	.	.	.	CTNNA2(INTRON);CTNNA2(UPSTREAM)
2	79571997	79572006	79572000	TA	G	snp	1	0	0	0	.	.	.	CTNNA2(DOWNSTREAM);CTNNA2(INTRON)
2	79601265	79601276	79601273	TCA	T	snp	1	1	1	1	.	.	.	CTNNA2(INTRON)
2	79601636	79601647	79601645	AT	A	del	1	1	0	1	.	.	.	CTNNA2(INTRON)
2	79736058	79736069	79736063	AT	C	snp	1	0	1	0	.	.	.	CTNNA2(INTRON);CTNNA2(UPSTREAM)
2	79774163	79774174	79774167	GT	A	snp	0	1	0	1	.	.	.	CTNNA2(INTRON)
2	79866697	79866705	79866700	T	G	snp	0	0	1	0	.	.	.	CTNNA2(INTRON);U6(UPSTREAM)
2	79890762	79890770	79890768	T	G	snp	1	1	0	1	.	.	.	CTNNA2(INTRON)
2	79890861	79890870	79890866	TG	A	snp	1	1	1	1	.	.	.	CTNNA2(INTRON)
2	79904629	79904640	79904633	AC	G	snp	1	1	1	1	.	.	.	CTNNA2(INTRON)
2	79925181	79925196	79925192	TTTA	G	snp	1	1	1	1	.	.	.	AC096753.1.1(INTRON);CTNNA2(INTRON)
2	79927696	79927710	79927703	CAA	C	snp	1	1	0	1	.	.	.	AC096753.1.1(INTRON);CTNNA2(INTRON)
2	80035919	80035928	80035922	T	C	ins	1	1	1	1	.	.	.	CTNNA2(INTRON)
2	80053305	80053312	80053308	A	AT	ins	1	0	0	0	.	.	.	CTNNA2(INTRON)
2	80054162	80054170	80054166	T	G	snp	0	1	1	0	.	.	.	CTNNA2(INTRON)
2	80077450	80077465	80077455	TCCC	G	snp	1	1	1	1	.	.	.	CTNNA2(INTRON)
2	80103949	80103958	80103953	T	G	snp	1	0	0	1	.	.	.	CTNNA2(INTRON)
2	80103949	80103958	80103956	T	G	snp	1	1	1	1	.	.	.	CTNNA2(INTRON)
2	80144187	80144210	80144193	TTAT	T	snp	1	1	1	1	.	.	.	CTNNA2(INTRON)
2	80144187	80144210	80144202	TTAT	TTA	ins	1	1	1	1	.	.	.	CTNNA2(INTRON)
2	80144187	80144210	80144205	TTAT	T	del	1	1	1	1	.	.	.	CTNNA2(INTRON)
2	80216772	80216789	80216784	AAC	G	snp	1	1	1	1	.	.	.	CTNNA2(INTRON)
2	80270220	80270237	80270222	TC	C	del	1	1	1	1	.	.	.	CTNNA2(INTRON)
2	80270220	80270237	80270223	TC	T	snp	1	1	1	1	.	.	.	CTNNA2(INTRON)
2	80270220	80270237	80270227	TC	T	snp	1	1	1	1	.	.	.	CTNNA2(INTRON)
2	80288789	80288800	80288793	AT	G	snp	1	0	0	0	.	.	.	CTNNA2(INTRON)
2	80358178	80358197	80358191	TTTG	C	snp	0	0	0	1	.	.	.	CTNNA2(INTRON)
2	80435473	80435488	80435481	GATA	C	snp	1	1	1	1	.	.	.	CTNNA2(INTRON)
2	80546433	80546441	80546439	T	G	snp	1	1	1	1	.	.	.	CTNNA2(INTRON)
2	80584366	80584375	80584372	T	G	ins	1	0	0	0	.	.	.	CTNNA2(INTRON)
2	80689742	80689757	80689748	TTCT	A	snp	0	1	0	0	.	.	.	CTNNA2(INTRON)
2	80716053	80716066	80716055	CA	T	snp	0	0	1	0	.	.	.	CTNNA2(INTRON)
2	80717406	80717415	80717413	C	CG	ins	1	1	1	1	.	.	.	CTNNA2(INTRON)
2	80730297	80730306	80730301	A	G	snp	1	1	1	1	.	.	.	CTNNA2(INTRON)
2	80746556	80746563	80746561	A	G	snp	1	0	0	0	.	.	.	CTNNA2(INTRON)
2	80804813	80804827	80804823	TTG	GTC	ins	1	1	1	0	.	.	.	AC008067.2.1(INTRON);CTNNA2(INTRON)
2	80820473	80820488	80820477	TTTG	A	snp	1	0	0	0	.	.	.	AC008067.2.1(INTRON);CTNNA2(INTRON)
2	80864320	80864331	80864323	ATA	G	snp	1	1	1	1	.	.	.	CTNNA2(INTRON)
2	80962419	80962428	80962425	T	C	ins	1	1	1	1	.	.	.	AC084193.1.1(INTRON)
2	81087401	81087410	81087406	AC	A	snp	1	0	0	0	.	.	.	AC084193.1.1(INTRON)
2	81113049	81113056	81113052	T	C	ins	1	1	1	1	.	.	.	INTERGENIC(INTERGENIC)
2	81154780	81154791	81154783	TC	A	snp	1	1	1	1	.	.	.	INTERGENIC(INTERGENIC)
2	81162636	81162645	81162641	TA	C	snp	1	1	1	1	.	.	.	INTERGENIC(INTERGENIC)
2	81182125	81182134	81182131	T	G	snp	1	1	1	1	.	.	.	INTERGENIC(INTERGENIC)
2	81298938	81298945	81298944	A	C	snp	1	0	0	0	.	.	.	INTERGENIC(INTERGENIC)
2	81423355	81423362	81423356	G	A	snp	1	1	1	1	.	.	.	AC105342.1.1(EXON)
2	81508648	81508655	81508653	T	C	snp	0	1	0	1	.	.	.	INTERGENIC(INTERGENIC)
2	81553634	81553641	81553639	T	C	snp	0	1	0	1	.	.	.	INTERGENIC(INTERGENIC)
2	81599766	81599777	81599770	TA	G	snp	1	1	0	0	.	.	.	INTERGENIC(INTERGENIC)
2	81617466	81617475	81617472	CA	ACAT	ins	1	1	1	1	.	.	.	INTERGENIC(INTERGENIC)
2	81623970	81623977	81623974	A	C	snp	1	1	1	1	.	.	.	INTERGENIC(INTERGENIC)
2	81635214	81635222	81635220	A	G	ins	1	1	1	1	.	.	.	INTERGENIC(INTERGENIC)
2	81657538	81657553	81657550	AT	T	snp	1	1	1	1	.	.	.	INTERGENIC(INTERGENIC)
2	81697417	81697428	81697423	AT	C	snp	1	1	1	1	.	.	.	AC012075.2.1(UPSTREAM);INTERGENIC(INTERGENIC)
2	81751607	81751622	81751617	TCTT	TTC	del	1	1	1	1	.	.	.	INTERGENIC(INTERGENIC)
2	81755878	81755889	81755883	AT	G	snp	1	0	0	0	.	.	.	INTERGENIC(INTERGENIC)
2	81852855	81852864	81852857	A	C	snp	1	1	1	1	.	.	.	INTERGENIC(INTERGENIC)
2	81874403	81874411	81874404	T	G	ins	0	0	1	0	.	.	.	INTERGENIC(INTERGENIC)
2	82018693	82018701	82018694	T	G	ins	1	1	1	1	.	.	.	INTERGENIC(INTERGENIC)
2	82057472	82057487	82057483	AAAC	AA	ins	1	0	0	0	.	.	.	INTERGENIC(INTERGENIC)
2	82195349	82195357	82195355	A	C	ins	1	1	1	1	.	.	.	AC079896.1(DOWNSTREAM);INTERGENIC(INTERGENIC)
2	82239415	82239424	82239420	AT	C	snp	1	1	0	1	.	.	.	INTERGENIC(INTERGENIC)
2	82268207	82268220	82268212	TA	G	snp	0	1	0	1	.	.	.	INTERGENIC(INTERGENIC)
2	82362278	82362293	82362286	CTAT	GA	ins	0	1	0	1	.	.	.	INTERGENIC(INTERGENIC)

2	82375262	82375277	82375275	TG	A	snp	1	0	0	0	.	.	.	INTERGENIC(INTERGENIC)
2	82432555	82432564	82432558	A	G	snp	1	0	0	0	.	.	.	INTERGENIC(INTERGENIC)
2	82432555	82432564	82432561	A	G	snp	1	1	1	1	.	.	.	INTERGENIC(INTERGENIC)
2	82435892	82435900	82435897	T	G	snp	1	1	1	1	.	.	.	INTERGENIC(INTERGENIC)
2	82523520	82523533	82523529	AT	G	snp	1	0	0	0	.	.	.	INTERGENIC(INTERGENIC)
2	82547058	82547069	82547067	AC	TAT	ins	1	1	0	1	.	.	.	INTERGENIC(INTERGENIC)
2	82556849	82556857	82556853	T	G	snp	1	0	0	0	.	.	.	INTERGENIC(INTERGENIC)
2	82602002	82602025	82602014	AAT	ATAATAATAAT	del	1	0	0	0	.	.	.	INTERGENIC(INTERGENIC)
2	82686173	82686181	82686177	T	C	snp	1	1	0	1	.	.	.	INTERGENIC(INTERGENIC)
2	82828314	82828321	82828320	C	T	snp	0	1	0	1	.	.	.	INTERGENIC(INTERGENIC)
2	82959137	82959146	82959142	AG	C	snp	1	0	0	0	.	.	.	INTERGENIC(INTERGENIC)
2	83037124	83037133	83037125	A	G	snp	1	1	0	1	.	.	.	INTERGENIC(INTERGENIC);MTND4P25(UPSTREAM)
2	83211917	83211926	83211924	T	C	snp	1	1	1	1	.	.	.	INTERGENIC(INTERGENIC)
2	83250794	83250801	83250797	A	G	snp	1	0	0	0	.	.	.	INTERGENIC(INTERGENIC)
2	83321972	83321991	83321980	AAAT	T	snp	0	1	0	0	.	.	.	INTERGENIC(INTERGENIC)
2	83384538	83384545	83384540	T	G	ins	1	0	0	0	.	.	.	INTERGENIC(INTERGENIC)
2	83384538	83384545	83384541	T	G	snp	1	0	0	0	.	.	.	INTERGENIC(INTERGENIC)
2	83510222	83510229	83510226	T	G	snp	1	1	1	1	.	.	.	INTERGENIC(INTERGENIC)
2	83515917	83515925	83515924	C	A	snp	1	1	1	1	.	.	.	INTERGENIC(INTERGENIC)
2	83515925	83515934	83515929	CA	T	snp	1	0	0	0	.	.	.	INTERGENIC(INTERGENIC)
2	83527557	83527568	83527562	GT	GTGTGTG	del	1	1	1	1	.	.	.	INTERGENIC(INTERGENIC)
2	83565911	83565928	83565925	AAT	A	snp	1	1	0	1	.	.	.	INTERGENIC(INTERGENIC)
2	83589896	83589911	83589903	TAAA	G	snp	0	1	1	1	.	.	.	INTERGENIC(INTERGENIC)
2	83649044	83649071	83649065	CAAA	AAC	del	1	0	1	0	.	.	.	INTERGENIC(INTERGENIC)
2	83660981	83660988	83660987	T	C	snp	0	1	0	0	.	.	.	INTERGENIC(INTERGENIC)
2	83738286	83738306	83738303	TGC	TGA	ins	1	0	0	0	.	.	.	INTERGENIC(INTERGENIC)
2	83738598	83738613	83738608	TAGA	C	snp	1	0	1	0	.	.	.	INTERGENIC(INTERGENIC)
2	83787416	83787423	83787422	T	G	snp	1	1	1	1	.	.	.	INTERGENIC(INTERGENIC)
2	83788471	83788482	83788475	ATT	C	snp	0	1	0	1	.	.	.	INTERGENIC(INTERGENIC)
2	83943735	83943750	83943747	TC	CTC	del	1	1	1	1	.	.	.	INTERGENIC(INTERGENIC)
2	83963216	83963225	83963220	T	C	snp	1	1	1	1	.	.	.	INTERGENIC(INTERGENIC)
2	84013965	84013976	84013968	CTA	A	snp	1	0	0	0	.	.	.	INTERGENIC(INTERGENIC)
2	84136461	84136472	84136465	TG	A	snp	0	1	1	1	.	.	.	INTERGENIC(INTERGENIC)
2	84216275	84216283	84216280	A	C	snp	1	0	0	0	.	.	.	INTERGENIC(INTERGENIC)
2	84362123	84362134	84362132	AG	T	snp	1	1	1	1	.	.	.	INTERGENIC(INTERGENIC)
2	84424826	84424835	84424829	AT	C	snp	1	1	0	1	.	.	.	INTERGENIC(INTERGENIC)
2	84531339	84531352	84531344	AC	T	snp	1	0	0	0	.	.	.	INTERGENIC(INTERGENIC)
2	84587084	84587092	84587090	A	C	snp	1	0	0	0	.	.	.	INTERGENIC(INTERGENIC)
2	84655045	84655053	84655052	T	C	snp	1	1	1	1	.	.	.	SUCLG1(INTRON);SUCLG1(UPSTREAM)
2	84681746	84681754	84681747	A	C	snp	1	0	0	0	.	.	.	SUCLG1(INTRON)
2	84681746	84681754	84681753	A	C	snp	1	0	1	0	.	.	.	SUCLG1(INTRON)
2	85018333	85018342	85018341	A	C	ins	1	0	0	0	.	.	.	DNAH6(INTRON)
2	85107120	85107127	85107121	C	T	snp	1	0	0	0	.	.	.	C2orf89(INTRON);RPL12P18(DOWNSTREAM)
2	85119545	85119554	85119551	TA	A	snp	1	1	0	1	.	.	.	C2orf89(INTRON)
2	85171010	85171033	85171014	AAAT	C	snp	1	1	1	1	.	.	.	INTERGENIC(INTERGENIC)
2	85318883	85318898	85318885	AT	T	del	1	1	1	1	.	.	.	INTERGENIC(INTERGENIC)
2	85318883	85318898	85318886	AT	A	snp	1	1	1	1	.	.	.	INTERGENIC(INTERGENIC)
2	85430867	85430875	85430869	A	G	snp	1	0	0	0	.	.	.	TCF7L1(INTRON);U6(UPSTREAM)
2	85445763	85445771	85445766	G	GGC	ins	1	0	0	0	.	.	.	TCF7L1(INTRON)
2	85649289	85649298	85649292	T	C	snp	1	0	0	0	.	.	.	CAPG(UPSTREAM);SH2D6(INTRON)
2	85659509	85659516	85659513	T	G	snp	1	1	1	1	.	.	.	SH2D6(DOWNSTREAM);SH2D6(INTRON);SH2D6(UPSTREAM);Y_RNA(UPSTREAM)
2	85659533	85659542	85659537	T	G	snp	1	1	1	1	.	.	.	SH2D6(DOWNSTREAM);SH2D6(INTRON);SH2D6(UPSTREAM);Y_RNA(UPSTREAM)
2	85696270	85696281	85696279	TG	T	del	0	1	0	1	.	.	.	INTERGENIC(INTERGENIC)
2	85736674	85736685	85736676	AG	G	del	1	0	0	0	.	.	.	INTERGENIC(INTERGENIC)
2	85765432	85765440	85765439	C	T	snp	0	1	0	1	.	.	.	INTERGENIC(INTERGENIC);MAT2A(UPSTREAM)
2	85789198	85789206	85789199	A	C	snp	0	1	0	0	.	.	.	GGCX(UPSTREAM);VAMP8(INTRON)
2	85801067	85801084	85801079	ATT	T	ins	1	1	1	1	.	.	.	VAMP8(INTRON);VAMP8(UPSTREAM)
2	85801067	85801084	85801081	ATT	A	del	1	1	1	1	.	.	.	VAMP8(INTRON);VAMP8(UPSTREAM)
2	85867728	85867743	85867741	AT	A	del	1	1	1	1	.	.	.	USP39(DOWNSTREAM);USP39(INTRON);USP39(UPSTREAM)
2	85878717	85878730	85878728	GT	GTG	del	0	1	0	1	.	.	.	INTERGENIC(INTERGENIC);USP39(DOWNSTREAM)
2	86075963	86075970	86075967	T	C	snp	1	1	1	1	.	.	.	ST3GAL5(DOWNSTREAM);ST3GAL5(EXON);ST3GAL5(INTRON)
2	86138952	86138959	86138958	T	G	snp	1	0	0	0	.	.	.	INTERGENIC(INTERGENIC)
2	86182601	86182615	86182606	CTC	T	snp	1	0	0	0	.	.	.	INTERGENIC(INTERGENIC)
2	86189954	86189967	86189963	TC	T	snp	0	0	1	0	.	.	.	INTERGENIC(INTERGENIC)
2	86398971	86398986	86398981	AT	TT	ins	1	0	0	0	.	.	.	IMMT(DOWNSTREAM);IMMT(INTRON);IMMT(UPSTREAM)

2	86464642	86464656	86464653	TGT	C	snp	1	0	0	0	.	.	.	REEP1(INTRON)
2	86493823	86493832	86493828	CA	G	snp	1	0	0	0	.	.	.	REEP1(INTRON)
2	86522162	86522170	86522166	A	G	snp	1	0	0	0	.	.	.	REEP1(INTRON)
2	86628601	86628614	86628604	TC	TG	ins	1	1	1	1	.	.	.	INTERGENIC(INTERGENIC)
2	86637141	86637168	86637159	CAAA	AC	del	1	0	1	0	.	.	.	INTERGENIC(INTERGENIC)
2	86637141	86637168	86637163	CAAA	AC	del	1	0	1	0	.	.	.	INTERGENIC(INTERGENIC)
2	86666455	86666462	86666459	A	G	snp	1	1	1	1	.	.	.	INTERGENIC(INTERGENIC);KDM3A(UPSTREAM)
2	86755161	86755170	86755167	T	G	snp	0	1	1	0	.	.	.	CHMP3(INTRON);RNF103-CHMP3(INTRON)
2	86807828	86807855	86807851	ATCT	AG	ins	1	1	1	1	.	.	.	AC015971.2.1(INTRON);CHMP3(INTRON);RNF103-CHMP3(INTRON)
2	86915642	86915649	86915648	T	C	snp	1	0	0	0	.	.	.	CHMP3(INTRON)
2	87111276	87111284	87111283	T	C	snp	1	0	0	0	.	.	.	AC111200.10.1(INTRON);AC111200.2.1(DOWNSTREAM)
2	87111877	87111884	87111882	T	G	snp	1	1	1	1	.	.	.	AC111200.10.1(INTRON);AC111200.2.1(DOWNSTREAM)
2	87114261	87114270	87114264	TA	G	snp	1	1	1	1	.	.	.	AC111200.10.1(INTRON);AC111200.2.1(UPSTREAM)
2	87114861	87114869	87114864	T	G	snp	1	1	1	1	.	.	.	AC111200.10.1(INTRON);AC111200.2.1(UPSTREAM)
2	87132307	87132318	87132314	TA	G	snp	1	1	1	1	.	.	.	AC111200.10.1(INTRON);RGPD1(UPSTREAM)
2	87170397	87170406	87170402	C	T	snp	0	0	0	1	.	.	.	RGPD1(INTRON)
2	87319116	87319131	87319123	TTCC	T	snp	1	1	1	1	.	.	.	INTERGENIC(INTERGENIC)
2	87520093	87520102	87520098	T	G	snp	0	1	0	0	.	.	.	INTERGENIC(INTERGENIC)
2	87786013	87786024	87786016	CA	G	snp	1	1	0	0	.	.	.	LINC00152(INTRON)
2	87841500	87841509	87841505	G	A	snp	1	1	1	1	.	.	.	LINC00152(INTRON)
2	87898680	87898689	87898682	T	G	snp	0	1	0	0	.	.	.	LINC00152(INTRON)
2	88144148	88144159	88144153	TA	G	snp	0	1	0	0	.	.	.	RGPD2(INTRON)
2	88215180	88215191	88215185	TA	G	snp	1	0	0	0	.	.	.	RGPD2(INTRON)
2	88258561	88258576	88258573	AT	T	snp	1	0	1	0	.	.	.	RGPD2(INTRON)
2	88258561	88258576	88258574	AT	A	del	1	0	0	0	.	.	.	RGPD2(INTRON)
2	88329359	88329374	88329372	AT	A	del	1	1	1	1	.	.	.	KRCC1(INTRON)
2	88484806	88484814	88484808	C	A	snp	0	1	1	1	.	.	.	THNSL2(DOWNSTREAM);THNSL2(INTRON)
2	88496177	88496194	88496184	CA	G	snp	1	1	1	1	.	.	.	INTERGENIC(INTERGENIC)
2	88504646	88504659	88504648	TG	G	del	0	1	0	1	.	.	.	INTERGENIC(INTERGENIC)
2	88647191	88647206	88647198	TC	G	snp	1	1	0	0	.	.	.	INTERGENIC(INTERGENIC)
2	88728730	88728757	88728736	TTTG	A	snp	1	0	0	0	.	.	.	AC012671.2.1(DOWNSTREAM);INTERGENIC(INTERGENIC)
2	88728730	88728757	88728748	TTTG	A	snp	1	0	0	0	.	.	.	AC012671.2.1(DOWNSTREAM);INTERGENIC(INTERGENIC)
2	88734547	88734560	88734557	CT	T	snp	1	1	1	1	.	.	.	INTERGENIC(INTERGENIC)
2	88755884	88755898	88755889	ATT	C	snp	1	1	1	1	.	.	.	AC012671.3.1(UPSTREAM);INTERGENIC(INTERGENIC)
2	88777723	88777738	88777730	TTCC	T	snp	1	1	1	0	.	.	.	INTERGENIC(INTERGENIC)
2	88996007	88996018	88996016	TG	T	del	1	0	0	0	.	.	.	RPIA(INTRON)
2	89001001	89001008	89001005	A	G	snp	1	1	1	1	.	.	.	RPIA(INTRON)
2	89023835	89023842	89023838	T	G	snp	1	0	0	0	.	.	.	RPIA(INTRON);RPIA(UPSTREAM)
2	89235791	89235802	89235795	TTG	C	snp	1	1	1	1	.	.	.	IGKV2-4(UPSTREAM);INTERGENIC(INTERGENIC)
2	89267987	89267994	89267990	T	C	snp	1	1	1	1	.	.	.	IGKV1-6(UPSTREAM);INTERGENIC(INTERGENIC)
2	89314850	89314857	89314853	A	C	snp	1	1	1	1	.	.	.	IGKV1-9(UPSTREAM);IGKV2-10(DOWNSTREAM);INTERGENIC(INTERGENIC)
2	89326077	89326084	89326083	T	C	snp	1	0	0	0	.	.	.	IGKV3-11(DOWNSTREAM);INTERGENIC(INTERGENIC)
2	89373791	89373800	89373793	CT	G	snp	1	1	1	1	.	.	.	IGKV2-14(DOWNSTREAM);INTERGENIC(INTERGENIC)
2	89528524	89528533	89528529	AC	G	snp	0	1	0	0	.	.	.	INTERGENIC(INTERGENIC)
2	89885975	89885986	89885980	TAT	C	snp	1	0	0	1	.	.	.	IGKV2D-40(UPSTREAM);INTERGENIC(INTERGENIC)
2	89956374	89956383	89956379	TA	G	snp	0	0	0	1	.	.	.	IGKV1D-33(DOWNSTREAM);INTERGENIC(INTERGENIC)
2	89956712	89956719	89956714	G	A	snp	0	0	0	1	.	.	.	IGKV1D-33(DOWNSTREAM);INTERGENIC(INTERGENIC)
2	90093694	90093702	90093697	A	G	snp	1	1	1	1	.	.	.	IGKV2D-18(DOWNSTREAM);INTERGENIC(INTERGENIC)
2	90200261	90200269	90200263	A	C	ins	1	1	0	1	.	.	.	IGKV1D-12(DOWNSTREAM);INTERGENIC(INTERGENIC)
2	90450612	90450620	90450613	C	T	snp	1	1	1	1	.	.	.	INTERGENIC(INTERGENIC)
2	91635490	91635498	91635492	C	T	ins	1	1	1	1	.	.	.	AC018696.1.1(UPSTREAM);INTERGENIC(INTERGENIC)
2	91689254	91689262	91689259	A	C	snp	1	1	1	1	.	.	.	AC018696.1(DOWNSTREAM);AC018696.3.1(UPSTREAM);INTERGENIC(INTERGENIC)
2	92148920	92148928	92148925	T	G	snp	0	0	0	1	.	.	.	CHEK2P3(INTRON)
2	95341849	95341862	95341860	GT	C	snp	1	0	0	0	.	.	.	INTERGENIC(INTERGENIC)
2	95507343	95507358	95507347	AAAT	G	snp	1	1	0	1	.	.	.	ANKRD20A8P(INTRON);U6(DOWNSTREAM)
2	95507343	95507358	95507349	AAAT	T	del	1	1	0	1	.	.	.	ANKRD20A8P(INTRON);U6(DOWNSTREAM)
2	95511834	95511847	95511844	TA	C	snp	1	1	0	1	.	.	.	ANKRD20A8P(INTRON);U6(DOWNSTREAM)
2	95736731	95736740	95736736	TA	G	snp	1	1	1	1	.	.	.	INTERGENIC(INTERGENIC)
2	95875817	95875826	95875819	AT	TATATATG	ins	0	0	1	0	.	.	.	AC092835.2.1(INTRON);AC092835.2.1(UPSTREAM)
2	95976924	95976933	95976928	CA	T	snp	1	0	0	0	.	.	.	KCNIP3(EXON);KCNIP3(INTRON)
2	95991957	95991966	95991962	AC	T	snp	1	1	1	1	.	.	.	KCNIP3(INTRON)
2	96085114	96085125	96085122	AG	G	del	0	1	0	1	.	.	.	FAHD2A(DOWNSTREAM);INTERGENIC(INTERGENIC)
2	96409745	96409758	96409755	AG	GAG	del	1	0	0	0	.	.	.	INTERGENIC(INTERGENIC)

2	96409745	96409758	96409756	AG	AGA	del	1	1	1	1	.	.	.	INTERGENIC(INTERGENIC)
2	96669128	96669136	96669134	A	C	snp	1	1	1	0	.	.	.	AC013272.1(DOWNSTREAM);INTERGENIC(INTERGENIC)
2	96767576	96767583	96767577	G	T	snp	1	0	0	0	.	.	.	INTERGENIC(INTERGENIC)
2	96896540	96896547	96896544	T	TTTG	ins	1	1	1	1	.	.	.	AC012307.3.1(INTRON)
2	97114604	97114623	97114619	AAAT	AAATA	del	1	1	1	0	.	.	.	INTERGENIC(INTERGENIC)
2	97139870	97139885	97139873	TA	G	snp	1	1	1	1	.	.	.	INTERGENIC(INTERGENIC)
2	97411192	97411203	97411200	AAC	G	snp	1	0	0	0	.	.	.	INTERGENIC(INTERGENIC)
2	97431515	97431534	97431530	TTTC	TTTCT	del	1	1	1	1	.	.	.	CNNM4(INTRON)
2	97538069	97538096	97538091	AAAC	CAAAC	del	1	1	1	1	.	.	.	FAM178B(DOWNSTREAM);INTERGENIC(INTERGENIC);SEMA4C(UPSTREAM)
2	97660527	97660544	97660530	TG	C	snp	1	1	1	1	.	.	.	FAM178B(INTRON)
2	97693965	97693974	97693966	A	G	snp	1	1	1	1	.	.	.	AC018892.4.1(INTRON)
2	97700868	97700877	97700870	CA	A	snp	1	1	1	1	.	.	.	AC018892.3.1(DOWNSTREAM);AC018892.4.1(UPSTREAM);AC018892.8.1(DOWNSTREAM);INTERGENIC(INTERGENIC)
2	97975857	97975864	97975860	T	C	snp	1	1	1	1	.	.	.	INTERGENIC(INTERGENIC)
2	98178789	98178796	98178792	T	C	snp	1	0	0	0	.	.	.	ANKRD36B(INTRON)
2	98336017	98336026	98336025	G	T	snp	0	0	0	1	.	.	.	ZAP70(INTRON);ZAP70(UPSTREAM)
2	98466169	98466180	98466177	TC	A	snp	1	0	0	0	.	.	.	TMEM131(INTRON)
2	98466169	98466180	98466178	TC	A	snp	1	0	0	0	.	.	.	TMEM131(INTRON)
2	98554819	98554826	98554824	A	C	snp	1	1	1	1	.	.	.	TMEM131(INTRON)
2	98592336	98592355	98592348	CAAA	A	snp	1	0	0	0	.	.	.	TMEM131(INTRON)
2	98624545	98624552	98624551	G	A	snp	1	1	0	1	.	.	.	INTERGENIC(INTERGENIC)
2	98887115	98887130	98887120	TGTT	T	snp	0	0	0	1	.	.	.	VWA3B(INTRON)
2	99009058	99009066	99009062	A	G	snp	1	0	0	0	.	.	.	CNGA3(DOWNSTREAM);CNGA3(INTRON)
2	99084698	99084717	99084708	ATTG	G	snp	1	1	1	1	.	.	.	INPP4A(INTRON)
2	99139830	99139837	99139832	T	G	snp	1	1	1	1	.	.	.	INPP4A(DOWNSTREAM);INPP4A(INTRON)
2	99228432	99228439	99228433	C	A	snp	1	0	0	0	.	.	.	COA5(UPSTREAM);UNC50(INTRON);UNC50(UPSTREAM)
2	99336040	99336047	99336044	A	G	snp	1	1	1	1	.	.	.	MGAT4A(INTRON)
2	99350353	99350362	99350358	A	G	snp	1	1	1	1	.	.	.	INTERGENIC(INTERGENIC);MGAT4A(UPSTREAM)
2	99363782	99363790	99363784	A	C	snp	1	0	0	0	.	.	.	INTERGENIC(INTERGENIC)
2	99443370	99443378	99443371	G	T	snp	0	0	0	1	.	.	.	C2orf55(DOWNSTREAM);C2orf55(INTRON);C2orf55(UPSTREAM)
2	99505618	99505633	99505622	GAAG	A	snp	1	1	1	0	.	.	.	C2orf55(INTRON)
2	99505618	99505633	99505629	GAAG	A	snp	0	1	1	0	.	.	.	C2orf55(INTRON)
2	99590705	99590714	99590713	C	A	snp	1	1	1	1	.	.	.	INTERGENIC(INTERGENIC)
2	99593387	99593396	99593394	TA	T	ins	1	1	1	1	.	.	.	INTERGENIC(INTERGENIC)
2	99607971	99607980	99607977	AT	G	snp	1	1	1	1	.	.	.	INTERGENIC(INTERGENIC)
2	99759769	99759777	99759774	T	G	ins	0	0	1	0	.	.	.	C2orf15(INTRON);TSGA10(DOWNSTREAM);TSGA10(INTRON);TSGA10(UPSTREAM)
2	99775862	99775879	99775865	TC	G	snp	1	1	1	1	.	.	.	C2orf15(INTRON);LIPT1(DOWNSTREAM);LIPT1(INTRON);MITD1(DOWNSTREAM);MRPL30(INTRON);TSGA10(UPSTREAM)
2	99777171	99777180	99777177	CT	T	snp	1	0	0	0	.	.	.	C2orf15(INTRON);LIPT1(DOWNSTREAM);LIPT1(INTRON);MITD1(DOWNSTREAM);MRPL30(INTRON)
2	99875750	99875758	99875752	A	C	snp	1	1	1	1	.	.	.	C2orf15(INTRON);LYG2(UPSTREAM)
2	100100847	100100856	100100848	A	C	snp	1	1	0	1	.	.	.	REV1(INTRON)
2	100228015	100228022	100228020	T	C	snp	0	0	1	0	.	.	.	AFF3(INTRON)
2	100456063	100456070	100456067	T	C	snp	0	1	1	0	.	.	.	AFF3(INTRON)
2	100501036	100501045	100501037	T	C	snp	1	1	1	1	.	.	.	AFF3(INTRON)
2	100544479	100544488	100544482	CT	G	snp	1	0	0	1	.	.	.	AFF3(INTRON)
2	100590152	100590160	100590154	T	C	snp	1	0	0	1	.	.	.	AFF3(INTRON)
2	100619778	100619787	100619785	GT	C	snp	0	1	1	1	.	.	.	AFF3(DOWNSTREAM);AFF3(INTRON)
2	100749763	100749771	100749769	A	G	snp	1	0	0	0	.	.	.	AFF3(INTRON)
2	100854259	100854268	100854261	A	AC	ins	1	1	1	1	.	.	.	AC104782.3.1(INTRON);AC104782.3.1(UPSTREAM)
2	100922537	100922548	100922540	TG	T	snp	0	1	0	1	.	.	.	LONRF2(INTRON)
2	100997468	100997485	100997471	CAC	T	snp	1	0	0	0	.	.	.	INTERGENIC(INTERGENIC)
2	101098491	101098498	101098494	G	T	snp	1	1	1	1	.	.	.	NMS(INTRON)
2	101109435	101109446	101109439	AAG	G	snp	1	0	1	0	.	.	.	INTERGENIC(INTERGENIC)
2	101119680	101119688	101119686	A	C	ins	1	1	1	1	.	.	.	INTERGENIC(INTERGENIC)
2	101155651	101155666	101155653	AT	TATG	ins	1	1	1	1	.	.	.	INTERGENIC(INTERGENIC)
2	101232994	101233005	101233000	TC	A	snp	1	0	0	0	.	.	.	INTERGENIC(INTERGENIC)
2	101238526	101238543	101238531	TTC	T	snp	1	1	1	1	.	.	.	INTERGENIC(INTERGENIC)
2	101249778	101249787	101249786	T	G	ins	1	0	0	0	.	.	.	INTERGENIC(INTERGENIC)
2	101432780	101432795	101432789	TTAA	A	ins	1	1	1	1	.	.	.	INTERGENIC(INTERGENIC);NPAS2(UPSTREAM)
2	101432780	101432795	101432790	TTAA	ATT	del	1	1	1	1	.	.	.	INTERGENIC(INTERGENIC);NPAS2(UPSTREAM)
2	101448530	101448543	101448538	TG	C	snp	0	0	1	0	.	.	.	AC092168.2.1(INTRON);NPAS2(INTRON)
2	101528716	101528727	101528720	TTA	A	ins	1	1	1	1	.	.	.	NPAS2(INTRON)
2	101641546	101641555	101641548	GT	A	snp	0	0	1	0	.	.	.	

TBC1D8(DOWNSTREAM);TBC1D8(INTRON);TBC1D8(UPSTREAM)

2	101689723	101689730	101689726	T	G	snp	1	1	1	1	.	.	.	TBC1D8(INTRON)
2	101711856	101711864	101711858	C	T	snp	1	0	0	0	.	.	.	TBC1D8(INTRON)
2	101821205	101821212	101821210	C	T	snp	0	1	0	0	.	.	.	TBC1D8(INTRON)
2	101878879	101878890	101878882	TC	T	snp	0	1	0	1	.	.	.	C2orf29(INTRON);C2orf29(UPSTREAM)
2	102164744	102164764	102164757	TAT	T	ins	0	1	0	0	.	.	.	INTERGENIC(INTERGENIC)
2	102164744	102164764	102164760	TAT	T	snp	1	1	1	1	.	.	.	INTERGENIC(INTERGENIC)
2	102185686	102185695	102185688	GA	T	snp	1	0	0	0	.	.	.	INTERGENIC(INTERGENIC)
2	102211714	102211731	102211722	AAC	T	snp	1	1	1	1	.	.	.	INTERGENIC(INTERGENIC)
2	102221627	102221638	102221636	CA	C	del	1	0	1	0	.	.	.	INTERGENIC(INTERGENIC)
2	102224854	102224865	102224857	TC	T	snp	1	1	1	1	.	.	.	INTERGENIC(INTERGENIC)
2	102227618	102227625	102227623	A	C	snp	1	1	1	1	.	.	.	INTERGENIC(INTERGENIC)
2	102253089	102253096	102253091	T	C	snp	0	1	1	1	.	.	.	INTERGENIC(INTERGENIC)
2	102299908	102299915	102299914	A	C	snp	1	0	0	0	.	.	.	INTERGENIC(INTERGENIC)
2	102425261	102425278	102425270	TG	TA	ins	1	0	0	0	.	.	.	MAP4K4(INTRON)
2	102426760	102426767	102426762	T	G	snp	1	0	0	0	.	.	.	MAP4K4(INTRON)
2	102498305	102498316	102498309	TTG	GTTGTTGCTTG	del	1	0	0	0	.	.	.	MAP4K4(INTRON)
2	102597604	102597613	102597608	G	T	snp	1	0	0	0	.	.	.	INTERGENIC(INTERGENIC)
2	102629633	102629641	102629640	A	G	ins	1	1	1	1	.	.	.	IL1R2(DOWNSTREAM);IL1R2(INTRON)
2	102629633	102629641	102629640	A	G	snp	1	1	1	1	.	.	.	IL1R2(DOWNSTREAM);IL1R2(INTRON)
2	102710482	102710501	102710496	AACC	T	snp	1	0	0	0	.	.	.	IL1R1(INTRON)
2	102748108	102748116	102748115	A	AC	ins	1	1	1	1	.	.	.	IL1R1(INTRON)
2	102798231	102798246	102798243	TG	G	ins	0	1	1	1	.	.	.	AC007271.3.1(INTRON);IL1R1(DOWNSTREAM)
2	102798231	102798246	102798244	TG	T	del	1	1	1	1	.	.	.	AC007271.3.1(INTRON);IL1R1(DOWNSTREAM)
2	102798246	102798254	102798247	G	T	snp	1	1	1	0	.	.	.	AC007271.3.1(INTRON);IL1R1(DOWNSTREAM)
2	102809527	102809540	102809534	CA	G	snp	1	1	1	1	.	.	.	IL1RL2(INTRON)
2	102837688	102837699	102837691	TC	T	snp	1	1	1	1	.	.	.	IL1RL2(INTRON)
2	102845336	102845343	102845337	T	C	ins	1	0	0	0	.	.	.	IL1RL2(INTRON)
2	102916958	102916966	102916965	T	C	snp	1	1	0	1	.	.	.	INTERGENIC(INTERGENIC)
2	102941815	102941826	102941818	TCA	C	snp	1	1	1	1	.	.	.	IL18R1(INTRON);IL1RL1(INTRON)
2	102970241	102970249	102970243	T	G	snp	1	1	1	1	.	.	.	IL18R1(INTRON);IL18R1(UPSTREAM);IL1RL1(DOWNSTREAM)
2	103035369	103035376	103035375	T	C	snp	1	0	0	1	.	.	.	IL18RAP(UPSTREAM);IL18RAP(UTR_5_PRIME)
2	103073127	103073136	103073133	TA	AA	ins	1	1	1	1	.	.	.	IL18RAP(DOWNSTREAM);INTERGENIC(INTERGENIC)
2	103074807	103074816	103074813	A	C	snp	1	1	1	1	.	.	.	INTERGENIC(INTERGENIC)
2	103218359	103218367	103218360	T	C	snp	1	1	1	1	.	.	.	INTERGENIC(INTERGENIC)
2	103243787	103243794	103243790	A	G	snp	1	0	1	0	.	.	.	SLC9A2(INTRON)
2	103295830	103295843	103295841	TA	T	del	1	1	1	1	.	.	.	SLC9A2(INTRON);SLC9A2(UPSTREAM)
2	103369654	103369662	103369659	C	CG	ins	1	1	1	1	.	.	.	TMEM182(INTRON)
2	103439342	103439353	103439350	AAG	AAGAAAGAAGA	del	1	0	0	0	.	.	.	TMEM182(INTRON)
2	103479667	103479675	103479673	A	G	snp	1	1	0	1	.	.	.	INTERGENIC(INTERGENIC)
2	103503246	103503261	103503253	TTTC	T	ins	0	1	1	1	.	.	.	AC007251.2.1(INTRON)
2	103830962	103830979	103830973	AC	AA	ins	1	0	0	0	.	.	.	INTERGENIC(INTERGENIC)
2	103970756	103970764	103970758	G	T	ins	1	1	0	0	.	.	.	INTERGENIC(INTERGENIC)
2	103975953	103975964	103975960	ATA	C	snp	1	0	0	0	.	.	.	INTERGENIC(INTERGENIC)
2	103977496	103977505	103977499	GT	C	snp	1	1	1	1	.	.	.	INTERGENIC(INTERGENIC)
2	103977496	103977505	103977503	GT	C	snp	1	0	0	0	.	.	.	INTERGENIC(INTERGENIC)
2	103980307	103980316	103980311	T	C	snp	1	0	0	0	.	.	.	INTERGENIC(INTERGENIC)
2	103983792	103983799	103983795	T	G	snp	1	0	0	0	.	.	.	INTERGENIC(INTERGENIC)
2	104008615	104008626	104008622	AT	C	snp	1	0	0	0	.	.	.	INTERGENIC(INTERGENIC)
2	104009021	104009030	104009028	AT	A	snp	1	1	1	1	.	.	.	INTERGENIC(INTERGENIC)
2	104009691	104009698	104009696	T	C	snp	1	0	0	0	.	.	.	INTERGENIC(INTERGENIC)
2	104022121	104022132	104022126	AAC	A	snp	1	0	0	0	.	.	.	INTERGENIC(INTERGENIC)
2	104043011	104043020	104043016	TA	G	snp	1	0	0	0	.	.	.	INTERGENIC(INTERGENIC)
2	104050915	104050930	104050926	TTTA	T	ins	1	1	1	0	.	.	.	INTERGENIC(INTERGENIC)
2	104123609	104123618	104123611	CT	G	snp	1	1	0	1	.	.	.	INTERGENIC(INTERGENIC)
2	104190317	104190325	104190318	T	G	snp	1	1	1	0	.	.	.	INTERGENIC(INTERGENIC)
2	104197415	104197422	104197421	C	A	snp	0	1	1	0	.	.	.	INTERGENIC(INTERGENIC)
2	104208148	104208155	104208153	A	G	snp	1	1	1	1	.	.	.	INTERGENIC(INTERGENIC)
2	104257487	104257494	104257491	G	A	snp	1	0	0	0	.	.	.	INTERGENIC(INTERGENIC)
2	104270718	104270729	104270723	AAT	AA	del	1	0	0	0	.	.	.	INTERGENIC(INTERGENIC)
2	104320146	104320157	104320154	TA	AG	ins	0	0	1	0	.	.	.	INTERGENIC(INTERGENIC)
2	104321382	104321397	104321388	AT	G	snp	0	0	1	0	.	.	.	INTERGENIC(INTERGENIC)
2	104321523	104321532	104321529	TA	C	snp	1	1	1	1	.	.	.	INTERGENIC(INTERGENIC)
2	104330096	104330105	104330099	T	G	ins	1	1	1	1	.	.	.	INTERGENIC(INTERGENIC)
2	104346305	104346313	104346306	A	C	ins	1	0	1	0	.	.	.	INTERGENIC(INTERGENIC)
2	104373985	104373994	104373990	CA	G	snp	0	1	0	0	.	.	.	INTERGENIC(INTERGENIC)

2	104450992	104451015	104451009	TTGT	C	snp	1	1	1	1	.	.	.	INTERGENIC(INTERGENIC)
2	104522269	104522282	104522279	TA	G	snp	1	1	1	1	.	.	.	RP11-76I14.1.1(INTRON)
2	104522269	104522282	104522280	TA	G	snp	0	1	0	0	.	.	.	RP11-76I14.1.1(INTRON)
2	104540677	104540686	104540682	TA	T	snp	1	0	0	0	.	.	.	RP11-76I14.1.1(INTRON)
2	104728790	104728799	104728795	GT	A	snp	1	1	1	1	.	.	.	INTERGENIC(INTERGENIC)
2	104818493	104818502	104818497	T	C	snp	1	1	1	1	.	.	.	INTERGENIC(INTERGENIC)
2	104915980	104915989	104915982	A	G	snp	1	0	0	0	.	.	.	INTERGENIC(INTERGENIC)
2	105146235	105146242	105146237	A	G	snp	1	0	0	0	.	.	.	INTERGENIC(INTERGENIC);U3(UPSTREAM)
2	105213047	105213054	105213051	A	G	snp	1	0	0	0	.	.	.	INTERGENIC(INTERGENIC)
2	105252459	105252467	105252464	T	G	ins	1	0	0	0	.	.	.	INTERGENIC(INTERGENIC)
2	105255299	105255307	105255306	A	G	snp	1	1	0	1	.	.	.	INTERGENIC(INTERGENIC)
2	105266888	105266899	105266894	AG	G	snp	1	1	0	1	.	.	.	INTERGENIC(INTERGENIC)
2	105397603	105397612	105397609	A	C	ins	1	1	1	1	.	.	.	INTERGENIC(INTERGENIC)
2	105490225	105490234	105490228	AC	T	snp	1	0	0	0	.	.	.	AC018730.3.1(UPSTREAM);INTERGENIC(INTERGENIC)
2	105580196	105580203	105580197	T	C	snp	1	1	1	1	.	.	.	AC010884.1.1(INTRON)
2	105620059	105620070	105620061	TA	C	snp	1	1	1	1	.	.	.	AC010884.1.1(INTRON)
2	105637363	105637370	105637366	T	C	ins	1	1	1	1	.	.	.	AC010884.1.1(INTRON)
2	105678594	105678609	105678606	AC	C	snp	1	0	0	0	.	.	.	MRPS9(INTRON)
2	105757621	105757629	105757626	A	C	snp	0	1	1	1	.	.	.	AC104655.3.1(UPSTREAM);INTERGENIC(INTERGENIC)
2	105787510	105787525	105787515	CA	G	snp	1	0	0	0	.	.	.	INTERGENIC(INTERGENIC)
2	105823115	105823124	105823122	T	C	snp	1	1	1	1	.	.	.	INTERGENIC(INTERGENIC)
2	105936402	105936415	105936412	AC	G	snp	1	1	1	1	.	.	.	AC012360.4.1(UPSTREAM);TGFBRAP1(INTRON)
2	105986673	105986682	105986674	A	C	snp	1	1	1	1	.	.	.	AC108058.1.1(UPSTREAM);FHL2(INTRON)
2	106014346	106014354	106014348	T	G	ins	1	1	1	1	.	.	.	FHL2(INTRON);FHL2(UPSTREAM)
2	106049625	106049639	106049634	TTC	TCTTC	del	1	1	1	0	.	.	.	FHL2(INTRON)
2	106049625	106049639	106049636	TTC	TTCT	del	1	1	1	1	.	.	.	FHL2(INTRON)
2	106056287	106056294	106056288	G	T	snp	1	0	0	0	.	.	.	FHL2(UPSTREAM);INTERGENIC(INTERGENIC)
2	106069589	106069598	106069594	A	G	snp	0	1	1	0	.	.	.	INTERGENIC(INTERGENIC)
2	106087140	106087149	106087141	T	G	snp	1	0	0	0	.	.	.	INTERGENIC(INTERGENIC)
2	106096746	106096755	106096748	CA	T	snp	0	1	0	1	.	.	.	INTERGENIC(INTERGENIC)
2	106105790	106105803	106105800	GA	AGA	del	0	1	0	1	.	.	.	INTERGENIC(INTERGENIC)
2	106106581	106106590	106106584	TC	G	snp	1	1	1	1	.	.	.	INTERGENIC(INTERGENIC)
2	106272742	106272751	106272744	TC	C	snp	1	1	0	1	.	.	.	INTERGENIC(INTERGENIC)
2	106272942	106272951	106272944	TC	C	snp	1	1	0	1	.	.	.	INTERGENIC(INTERGENIC)
2	106284226	106284234	106284229	T	G	snp	0	0	1	0	.	.	.	INTERGENIC(INTERGENIC)
2	106318216	106318224	106318223	A	AC	ins	1	1	1	1	.	.	.	INTERGENIC(INTERGENIC)
2	106424271	106424286	106424279	CTCC	T	snp	1	1	1	1	.	.	.	NCK2(INTRON)
2	106566512	106566539	106566526	AAAG	GAAAG	del	1	1	1	1	.	.	.	INTERGENIC(INTERGENIC)
2	106566512	106566539	106566530	AAAG	G	del	1	1	1	1	.	.	.	INTERGENIC(INTERGENIC)
2	106566573	106566584	106566581	AG	G	snp	0	0	1	0	.	.	.	INTERGENIC(INTERGENIC)
2	106568452	106568461	106568457	GA	G	snp	1	1	1	1	.	.	.	INTERGENIC(INTERGENIC)
2	106601441	106601452	106601448	TC	G	snp	0	0	1	0	.	.	.	INTERGENIC(INTERGENIC)
2	106614997	106615006	106615004	CT	G	snp	0	0	0	1	.	.	.	INTERGENIC(INTERGENIC)
2	106629563	106629572	106629568	TG	A	snp	1	1	0	1	.	.	.	INTERGENIC(INTERGENIC)
2	106666544	106666555	106666552	TA	A	snp	1	1	1	1	.	.	.	INTERGENIC(INTERGENIC)
2	106687833	106687842	106687838	A	G	snp	0	0	1	0	.	.	.	C2orf40(INTRON);C2orf40(UPSTREAM)
2	106692185	106692194	106692193	A	G	snp	1	0	0	0	.	.	.	C2orf40(DOWNSTREAM);C2orf40(INTRON)
2	106810758	106810772	106810767	GCG	T	snp	1	1	1	1	.	.	.	UXS1(UPSTREAM);UXS1(UTR_5_PRIME)
2	106861006	106861013	106861009	A	G	snp	1	1	1	1	.	.	.	AC092106.2.1(UPSTREAM);INTERGENIC(INTERGENIC)
2	106941761	106941780	106941767	TTCT	TTTG	ins	1	0	0	0	.	.	.	INTERGENIC(INTERGENIC)
2	106969286	106969295	106969290	T	G	snp	1	0	0	0	.	.	.	INTERGENIC(INTERGENIC)
2	106992519	106992528	106992527	T	C	snp	1	1	1	1	.	.	.	AC114755.7.1(INTRON)
2	106992528	106992541	106992533	TC	G	snp	1	1	1	1	.	.	.	AC114755.7.1(INTRON)
2	107137517	107137526	107137524	AG	T	snp	1	1	1	1	.	.	.	AC108868.5.1(DOWNSTREAM);INTERGENIC(INTERGENIC)
2	107141112	107141123	107141119	TGC	C	snp	1	1	1	1	.	.	.	AC108868.5.1(INTRON)
2	107214934	107214947	107214936	TA	G	snp	1	0	0	0	.	.	.	INTERGENIC(INTERGENIC)
2	107262758	107262771	107262760	GT	C	snp	1	1	0	1	.	.	.	INTERGENIC(INTERGENIC)
2	107340934	107340943	107340937	TG	A	snp	1	1	1	1	.	.	.	INTERGENIC(INTERGENIC)
2	107369093	107369102	107369097	CT	T	snp	0	1	1	1	.	.	.	INTERGENIC(INTERGENIC)
2	107382266	107382279	107382274	GA	AC	ins	1	1	0	1	.	.	.	INTERGENIC(INTERGENIC)
2	107388956	107388967	107388959	GT	C	snp	1	1	1	1	.	.	.	INTERGENIC(INTERGENIC)
2	107388956	107388967	107388960	GT	A	snp	1	1	1	1	.	.	.	INTERGENIC(INTERGENIC)
2	107398251	107398258	107398255	T	C	snp	1	1	1	1	.	.	.	INTERGENIC(INTERGENIC)
2	107439964	107439981	107439972	GA	C	snp	1	1	1	1	.	.	.	ST6GAL2(INTRON);ST6GAL2-IT1(INTRON)
2	107513146	107513154	107513148	G	T	snp	0	0	0	1	.	.	.	INTERGENIC(INTERGENIC)
2	107542545	107542554	107542549	AT	T	snp	1	0	0	0	.	.	.	INTERGENIC(INTERGENIC)
2	107760425	107760433	107760428	T	C	snp	1	1	1	1	.	.	.	INTERGENIC(INTERGENIC)

2	107906454	107906463	107906455	A	G	snp	1	0	0	0	.	.	.	AC006227.1.1(INTRON)
2	107993754	107993765	107993762	AG	G	snp	1	0	0	0	.	.	.	AC096669.1.1(INTRON);AC096669.1.1(UPSTREAM)
2	107994645	107994656	107994652	GA	GAG	del	1	1	1	1	.	.	.	AC096669.1.1(INTRON);AC096669.1.1(UPSTREAM)
2	108036204	108036212	108036205	A	C	snp	1	0	0	0	.	.	.	AC096669.2.1(INTRON)
2	108053188	108053195	108053189	A	C	snp	1	1	1	1	.	.	.	AC096669.2.1(INTRON)
2	108062228	108062239	108062233	AT	A	del	0	0	1	0	.	.	.	AC096669.2.1(INTRON)
2	108087819	108087830	108087822	TA	C	snp	0	0	0	1	.	.	.	AC096669.2.1(INTRON)
2	108137123	108137130	108137129	T	C	snp	1	1	1	1	.	.	.	AC096669.2.1(INTRON)
2	108148461	108148478	108148464	AC	A	snp	1	0	0	0	.	.	.	AC096669.2.1(INTRON);AC096669.3.1(INTRON)
2	108191261	108191280	108191270	TGTT	A	snp	1	0	1	1	.	.	.	INTERGENIC(INTERGENIC)
2	108234597	108234605	108234599	A	G	snp	1	0	0	0	.	.	.	INTERGENIC(INTERGENIC)
2	108256570	108256583	108256577	TC	G	snp	1	0	0	0	.	.	.	INTERGENIC(INTERGENIC)
2	108341620	108341635	108341630	GT	C	snp	1	1	0	0	.	.	.	INTERGENIC(INTERGENIC)
2	108347062	108347069	108347063	T	C	snp	1	0	0	0	.	.	.	INTERGENIC(INTERGENIC)
2	108360825	108360836	108360834	AT	A	snp	1	1	1	1	.	.	.	INTERGENIC(INTERGENIC)
2	108364679	108364686	108364682	T	G	ins	1	1	1	1	.	.	.	INTERGENIC(INTERGENIC)
2	108364679	108364686	108364682	T	G	snp	1	1	1	1	.	.	.	INTERGENIC(INTERGENIC)
2	108364837	108364844	108364839	T	C	snp	1	0	0	0	.	.	.	INTERGENIC(INTERGENIC)
2	108619311	108619319	108619316	A	G	snp	1	1	1	1	.	.	.	SLC5A7(INTRON)
2	108771495	108771504	108771498	A	C	snp	1	0	0	0	.	.	.	INTERGENIC(INTERGENIC)
2	108795714	108795731	108795721	ATG	C	snp	1	0	0	0	.	.	.	AC019100.3.1(EXON)
2	108945272	108945281	108945274	TG	C	snp	1	0	0	0	.	.	.	SULT1C2P1(INTRON)
2	109000661	109000688	109000680	TTTA	T	ins	1	0	0	0	.	.	.	SULT1C4(DOWNSTREAM);SULT1C4(INTRON)
2	109000661	109000688	109000684	TTTA	T	ins	1	1	1	1	.	.	.	SULT1C4(DOWNSTREAM);SULT1C4(INTRON)
2	109092544	109092555	109092546	TC	G	snp	1	1	1	1	.	.	.	GCC2(DOWNSTREAM);GCC2(INTRON)
2	109098379	109098386	109098384	C	A	snp	1	1	1	1	.	.	.	GCC2(INTRON);GCC2(UPSTREAM)
2	109108221	109108244	109108233	ATTT	T	ins	1	0	0	0	.	.	.	GCC2(DOWNSTREAM);GCC2(INTRON);GCC2(UPSTREAM)
2	109108221	109108244	109108236	ATTT	A	del	1	1	0	1	.	.	.	GCC2(DOWNSTREAM);GCC2(INTRON);GCC2(UPSTREAM)
2	109108221	109108244	109108237	ATTT	T	snp	1	0	0	1	.	.	.	GCC2(DOWNSTREAM);GCC2(INTRON);GCC2(UPSTREAM)
2	109108221	109108244	109108240	ATTT	A	del	1	1	1	1	.	.	.	GCC2(DOWNSTREAM);GCC2(INTRON);GCC2(UPSTREAM)
2	109119624	109119633	109119631	A	G	ins	1	1	1	1	.	.	.	AC012487.2.1(DOWNSTREAM);GCC2(INTRON)
2	109142019	109142028	109142021	AG	G	snp	1	1	1	1	.	.	.	AC012487.2.1(INTRON)
2	109250317	109250330	109250321	GT	A	snp	1	0	0	0	.	.	.	LIMS1(INTRON)
2	109317345	109317360	109317355	AT	TATAT	del	1	1	0	1	.	.	.	INTERGENIC(INTERGENIC)
2	109333364	109333381	109333374	TAT	C	snp	1	1	1	1	.	.	.	AC010095.7.1(UPSTREAM);INTERGENIC(INTERGENIC);RANBP2(UPSTREAM)
2	109395632	109395639	109395634	T	G	snp	1	0	0	0	.	.	.	RANBP2(INTRON)
2	109574891	109574898	109574897	A	C	snp	1	0	0	0	.	.	.	EDAR(INTRON)
2	109595893	109595902	109595898	TC	T	snp	1	1	1	1	.	.	.	EDAR(INTRON)
2	109633858	109633867	109633865	GA	C	snp	0	0	1	0	.	.	.	INTERGENIC(INTERGENIC)
2	109786773	109786784	109786781	CT	T	del	0	1	1	0	.	.	.	SH3RF3(INTRON)
2	109981508	109981531	109981526	AAAC	G	snp	1	0	0	0	.	.	.	SH3RF3(INTRON)
2	110091421	110091428	110091425	T	G	snp	0	0	0	1	.	.	.	SH3RF3(INTRON)
2	110278203	110278210	110278207	A	G	snp	1	0	0	0	.	.	.	INTERGENIC(INTERGENIC)
2	110282331	110282346	110282340	ATAA	A	snp	1	0	0	0	.	.	.	INTERGENIC(INTERGENIC)
2	110323690	110323705	110323694	ATTT	T	snp	1	1	1	1	.	.	.	SEPT10(DOWNSTREAM);SEPT10(INTRON)
2	110347314	110347321	110347318	A	C	snp	1	1	1	0	.	.	.	AC011753.5.1(DOWNSTREAM);SEPT10(INTRON)
2	110429581	110429595	110429588	CTC	C	del	1	0	0	0	.	.	.	AC011753.7.1(DOWNSTREAM);INTERGENIC(INTERGENIC)
2	110495053	110495062	110495056	T	G	snp	0	1	1	0	.	.	.	AC109815.2.1(DOWNSTREAM);RPL22P11(EXON)
2	110944424	110944443	110944430	AAAG	G	snp	1	1	1	1	.	.	.	AC092664.1.1(DOWNSTREAM);NPHP1(INTRON)
2	111417959	111417968	111417964	A	C	snp	1	0	0	0	.	.	.	BUB1(DOWNSTREAM);BUB1(INTRON)
2	111471334	111471345	111471340	CA	T	snp	1	0	0	0	.	.	.	AC114776.3.1(INTRON)
2	111779575	111779586	111779582	AG	A	snp	1	1	1	1	.	.	.	ACOXL(DOWNSTREAM);ACOXL(INTRON)
2	111834482	111834491	111834487	TC	G	snp	0	0	1	0	.	.	.	ACOXL(INTRON)
2	111840764	111840771	111840765	A	C	snp	1	1	1	1	.	.	.	ACOXL(INTRON)
2	112062927	112062946	112062940	ATTT	C	snp	0	0	1	0	.	.	.	AC068491.1.1(INTRON)
2	112197264	112197271	112197268	A	C	snp	1	0	0	0	.	.	.	AC068491.1.1(INTRON)
2	112390572	112390587	112390574	GT	C	snp	0	1	1	0	.	.	.	INTERGENIC(INTERGENIC)
2	112416566	112416580	112416577	AAC	A	ins	1	0	0	0	.	.	.	INTERGENIC(INTERGENIC)
2	112448913	112448928	112448924	GGAT	C	snp	1	1	1	1	.	.	.	INTERGENIC(INTERGENIC)
2	112500563	112500582	112500575	AAAG	C	snp	0	1	1	1	.	.	.	AC093166.3.1(INTRON)
2	112500707	112500722	112500715	AGAA	G	ins	1	0	0	0	.	.	.	AC093166.3.1(INTRON)
2	112550823	112550830	112550825	T	G	snp	1	1	0	1	.	.	.	ANAPC1(INTRON)
2	112567880	112567888	112567881	G	A	ins	1	0	0	0	.	.	.	ANAPC1(INTRON)
2	112567880	112567888	112567882	G	T	snp	1	0	0	0	.	.	.	ANAPC1(INTRON)
2	112567880	112567888	112567884	G	A	ins	1	1	1	1	.	.	.	ANAPC1(INTRON)
2	112682853	112682870	112682868	AT	A	del	1	1	1	1	.	.	.	AC104651.1(UPSTREAM);MERTK(INTRON)

2	112769336	112769343	112769339	A	G	snp	1	1	1	1	.	.	.	MERTK(INTRON)
2	112808267	112808286	112808278	TTTG	A	snp	1	1	1	1	.	.	.	INTERGENIC(INTERGENIC);TMEM87B(UPSTREAM)
2	112956217	112956244	112956223	AAAG	G	del	0	1	0	1	.	.	.	INTERGENIC(INTERGENIC)
2	112974132	112974143	112974140	AAC	T	snp	0	1	1	0	.	.	.	ZC3H8(INTRON)
2	113366636	113366651	113366640	TTTA	TTA	del	1	1	1	1	.	.	.	AC012442.5.1(INTRON)
2	113473912	113473919	113473916	T	G	snp	1	0	0	1	.	.	.	INTERGENIC(INTERGENIC)
2	113531086	113531094	113531093	T	C	snp	1	1	1	1	.	.	.	IL1A(DOWNSTREAM);INTERGENIC(INTERGENIC)
2	113824833	113824852	113824840	TGGA	T	snp	1	1	1	1	.	.	.	AC016724.8.1(DOWNSTREAM);IL1F10(UPSTREAM);IL36RN(DOWNSTREAM);INTERGENIC(INTERGENIC)
2	113848883	113848892	113848889	T	G	ins	1	1	1	1	.	.	.	INTERGENIC(INTERGENIC);U6(DOWNSTREAM)
2	113893297	113893311	113893308	TTA	G	snp	0	1	1	1	.	.	.	IL1RN(DOWNSTREAM);INTERGENIC(INTERGENIC)
2	113905945	113905958	113905954	AG	C	snp	1	1	1	1	.	.	.	INTERGENIC(INTERGENIC)
2	113978281	113978290	113978288	GT	A	snp	1	0	0	0	.	.	.	AC016683.6.1(INTRON);PAX8(DOWNSTREAM);PAX8(INTRON);PAX8(UPSTREAM)
2	114004989	114004997	114004994	A	C	snp	1	1	1	1	.	.	.	AC016683.6.1(INTRON);AC016683.6.1(UPSTREAM);PAX8(INTRON)
2	114013024	114013033	114013029	A	C	snp	1	0	0	0	.	.	.	AC016683.6.1(EXON);AC016683.6.1(INTRON);PAX8(INTRON)
2	114020844	114020851	114020848	T	G	snp	1	1	1	1	.	.	.	AC016683.6.1(INTRON);PAX8(INTRON)
2	114099795	114099804	114099799	CT	T	snp	1	1	1	1	.	.	.	INTERGENIC(INTERGENIC)
2	114100027	114100035	114100028	T	C	snp	0	1	1	1	.	.	.	INTERGENIC(INTERGENIC)
2	114176416	114176431	114176424	CAAA	T	snp	1	1	1	1	.	.	.	INTERGENIC(INTERGENIC)
2	114380293	114380304	114380301	AAC	AACA	del	1	1	1	1	.	.	.	RABL2A(UPSTREAM);RPL23AP7(INTRON)
2	114424970	114424979	114424974	TA	G	snp	1	1	1	1	.	.	.	AC017074.1.1(UPSTREAM);AL078621.13.1(DOWNSTREAM);INTERGENIC(INTERGENIC)
2	114508921	114508936	114508929	TTTG	C	snp	0	1	0	1	.	.	.	SLC35F5(INTRON)
2	114891911	114891918	114891917	T	G	snp	1	1	1	1	.	.	.	INTERGENIC(INTERGENIC)
2	114906564	114906581	114906568	TAA	CT	ins	1	1	1	1	.	.	.	INTERGENIC(INTERGENIC)
2	115130095	115130104	115130097	AT	T	snp	1	1	1	1	.	.	.	INTERGENIC(INTERGENIC)
2	115130095	115130104	115130102	AT	C	snp	1	1	1	1	.	.	.	INTERGENIC(INTERGENIC)
2	115130118	115130127	115130124	TA	A	snp	1	1	1	1	.	.	.	INTERGENIC(INTERGENIC)
2	115238605	115238613	115238611	A	G	snp	1	0	0	0	.	.	.	DPP10(INTRON)
2	115334225	115334244	115334234	CAAA	G	snp	0	0	0	1	.	.	.	DPP10(INTRON)
2	115334451	115334460	115334457	T	G	ins	1	0	0	0	.	.	.	DPP10(INTRON)
2	115468659	115468667	115468663	A	C	snp	1	0	0	0	.	.	.	DPP10(INTRON)
2	115514973	115514984	115514976	GA	G	snp	1	0	0	0	.	.	.	DPP10(INTRON)
2	115680269	115680276	115680274	T	C	snp	1	0	0	0	.	.	.	DPP10(INTRON)
2	115696850	115696857	115696852	G	T	snp	1	0	0	0	.	.	.	AC068542.1(UPSTREAM);DPP10(INTRON)
2	115709831	115709840	115709834	AT	C	snp	1	1	1	1	.	.	.	DPP10(INTRON)
2	115722725	115722732	115722731	T	C	snp	1	1	1	1	.	.	.	DPP10(INTRON)
2	115824814	115824821	115824818	T	G	snp	1	1	1	1	.	.	.	DPP10(DOWNSTREAM);DPP10(INTRON)
2	115919056	115919065	115919061	C	A	snp	1	0	1	0	.	.	.	AC066593.1.1(UPSTREAM);DPP10(INTRON);DPP10(UPSTREAM)
2	115919056	115919065	115919062	C	A	snp	1	0	0	0	.	.	.	AC066593.1.1(UPSTREAM);DPP10(INTRON);DPP10(UPSTREAM)
2	115925016	115925031	115925027	AAAG	A	snp	1	0	0	0	.	.	.	DPP10(INTRON)
2	116477826	116477835	116477830	TG	G	snp	1	0	0	0	.	.	.	DPP10(INTRON)
2	116487377	116487390	116487380	AG	AGAGAGAGAGA	del	1	1	1	1	.	.	.	DPP10(INTRON)
2	116570037	116570060	116570048	AAAC	G	snp	0	1	0	1	.	.	.	DPP10(INTRON);DPP10(UPSTREAM)
2	116579074	116579085	116579076	AG	G	snp	1	1	1	1	.	.	.	DPP10(INTRON)
2	116797409	116797432	116797425	TGC	CT	del	0	0	1	0	.	.	.	INTERGENIC(INTERGENIC)
2	116835803	116835820	116835812	AC	A	snp	1	1	1	1	.	.	.	INTERGENIC(INTERGENIC)
2	116844734	116844742	116844735	A	AC	ins	1	0	0	0	.	.	.	INTERGENIC(INTERGENIC)
2	116851062	116851069	116851067	T	C	snp	0	1	0	1	.	.	.	INTERGENIC(INTERGENIC)
2	116877271	116877280	116877277	T	C	snp	1	0	0	0	.	.	.	INTERGENIC(INTERGENIC)
2	116891939	116891950	116891945	CCT	T	snp	1	0	0	0	.	.	.	INTERGENIC(INTERGENIC)
2	116970924	116970931	116970928	A	G	snp	1	0	0	0	.	.	.	INTERGENIC(INTERGENIC)
2	116978702	116978709	116978704	A	G	snp	1	0	0	0	.	.	.	INTERGENIC(INTERGENIC)
2	117017297	117017306	117017298	A	AC	ins	0	1	1	1	.	.	.	INTERGENIC(INTERGENIC)
2	117031926	117031940	117031929	AAC	G	snp	1	1	1	1	.	.	.	INTERGENIC(INTERGENIC)
2	117123226	117123233	117123229	A	G	snp	0	1	1	1	.	.	.	INTERGENIC(INTERGENIC)
2	117127589	117127597	117127591	T	C	snp	1	0	0	0	.	.	.	INTERGENIC(INTERGENIC)
2	117243444	117243451	117243449	A	G	ins	1	1	1	1	.	.	.	INTERGENIC(INTERGENIC)
2	117353324	117353331	117353329	T	G	snp	1	1	1	1	.	.	.	INTERGENIC(INTERGENIC)
2	117358759	117358766	117358765	T	G	snp	1	1	1	1	.	.	.	INTERGENIC(INTERGENIC)
2	117377746	117377757	117377750	AT	G	snp	0	1	0	0	.	.	.	INTERGENIC(INTERGENIC)
2	117472543	117472550	117472546	A	G	snp	1	0	0	0	.	.	.	INTERGENIC(INTERGENIC)

2	117513226	117513233	117513232	T	C	snp	1	0	0	0	.	.	.	AC062016.2.1(DOWNSTREAM);INTERGENIC(INTERGENIC)
2	117530383	117530390	117530385	A	C	ins	1	1	1	1	.	.	.	INTERGENIC(INTERGENIC)
2	117573788	117573795	117573792	T	G	snp	1	0	0	0	.	.	.	AC062016.3.1(UPSTREAM);INTERGENIC(INTERGENIC)
2	117588261	117588272	117588266	AT	G	ins	1	1	1	1	.	.	.	INTERGENIC(INTERGENIC)
2	117592241	117592250	117592244	AT	ATATATTCTTC	del	1	1	1	1	.	.	.	INTERGENIC(INTERGENIC)
2	117592303	117592314	117592308	AT	C	snp	1	0	0	0	.	.	.	INTERGENIC(INTERGENIC)
2	117598994	117599001	117598998	A	G	snp	1	1	1	1	.	.	.	INTERGENIC(INTERGENIC)
2	117627810	117627818	117627815	T	G	snp	0	0	1	0	.	.	.	INTERGENIC(INTERGENIC)
2	117657689	117657700	117657692	AT	C	snp	1	1	1	1	.	.	.	INTERGENIC(INTERGENIC)
2	117690798	117690807	117690803	A	G	snp	1	0	0	0	.	.	.	INTERGENIC(INTERGENIC)
2	117690798	117690807	117690804	A	G	snp	1	1	1	1	.	.	.	INTERGENIC(INTERGENIC)
2	117715283	117715290	117715289	A	C	snp	1	0	0	0	.	.	.	INTERGENIC(INTERGENIC)
2	117799703	117799714	117799707	TA	C	snp	1	0	0	0	.	.	.	INTERGENIC(INTERGENIC)
2	117799767	117799776	117799774	TA	G	snp	1	0	0	0	.	.	.	INTERGENIC(INTERGENIC)
2	117847847	117847855	117847848	A	C	snp	1	0	0	0	.	.	.	INTERGENIC(INTERGENIC)
2	117998935	117998943	117998940	A	G	snp	1	0	0	0	.	.	.	INTERGENIC(INTERGENIC)
2	118126212	118126221	118126217	A	G	ins	1	0	0	0	.	.	.	INTERGENIC(INTERGENIC)
2	118169364	118169373	118169367	TC	G	snp	1	0	0	0	.	.	.	INTERGENIC(INTERGENIC)
2	118266462	118266473	118266470	GT	TGTA	ins	1	1	1	1	.	.	.	INTERGENIC(INTERGENIC)
2	118314004	118314012	118314007	A	G	snp	1	1	1	1	.	.	.	INTERGENIC(INTERGENIC)
2	118386854	118386863	118386861	A	AG	ins	1	0	0	0	.	.	.	INTERGENIC(INTERGENIC)
2	118444973	118444982	118444976	T	G	snp	1	1	1	1	.	.	.	INTERGENIC(INTERGENIC)
2	118512921	118512934	118512924	CT	C	snp	0	0	0	1	.	.	.	AC009312.1.1(DOWNSTREAM);INTERGENIC(INTERGENIC)
2	118535132	118535141	118535137	A	G	snp	1	1	1	1	.	.	.	AC009312.1.1(INTRON)
2	118726375	118726384	118726381	A	C	snp	0	1	1	1	.	.	.	CCDC93(INTRON)
2	118979844	118979859	118979853	AG	A	snp	1	0	0	0	.	.	.	INTERGENIC(INTERGENIC)
2	119008372	119008381	119008376	G	GC	ins	0	1	0	1	.	.	.	INTERGENIC(INTERGENIC)
2	119066804	119066811	119066809	C	A	snp	1	1	1	1	.	.	.	INTERGENIC(INTERGENIC)
2	119129070	119129077	119129071	A	G	snp	1	1	1	1	.	.	.	INTERGENIC(INTERGENIC)
2	119154109	119154124	119154120	TTTG	TTTGT	del	0	0	0	1	.	.	.	INTERGENIC(INTERGENIC)
2	119259656	119259671	119259665	AAGA	G	ins	1	1	1	1	.	.	.	INTERGENIC(INTERGENIC)
2	119330393	119330407	119330404	CTC	CTCCTCCTT	ins	1	1	0	0	.	.	.	INTERGENIC(INTERGENIC)
2	119534327	119534334	119534331	A	G	snp	1	1	1	1	.	.	.	INTERGENIC(INTERGENIC)
2	119549498	119549506	119549499	A	G	snp	1	0	0	0	.	.	.	INTERGENIC(INTERGENIC)
2	119646341	119646350	119646345	AT	G	snp	1	0	0	0	.	.	.	INTERGENIC(INTERGENIC)
2	119649712	119649719	119649715	T	G	ins	1	1	1	1	.	.	.	INTERGENIC(INTERGENIC)
2	119649712	119649719	119649716	T	G	snp	1	1	1	1	.	.	.	INTERGENIC(INTERGENIC)
2	119816524	119816539	119816535	TTTC	T	ins	1	0	0	0	.	.	.	INTERGENIC(INTERGENIC)
2	119825785	119825794	119825788	TG	T	snp	1	1	1	1	.	.	.	INTERGENIC(INTERGENIC)
2	119912804	119912819	119912814	AC	G	snp	1	1	1	1	.	.	.	C1QL2(DOWNSTREAM);INTERGENIC(INTERGENIC)
2	119950913	119950922	119950918	A	G	snp	1	1	1	1	.	.	.	INTERGENIC(INTERGENIC)
2	119953236	119953245	119953243	AG	A	snp	0	1	0	1	.	.	.	INTERGENIC(INTERGENIC)
2	119953427	119953442	119953432	AGGA	A	snp	1	1	1	1	.	.	.	INTERGENIC(INTERGENIC)
2	119953451	119953466	119953460	AGGA	A	snp	0	1	0	0	.	.	.	INTERGENIC(INTERGENIC)
2	120006721	120006738	120006727	TTG	C	snp	0	1	0	1	.	.	.	STEAP3(INTRON);STEAP3-AS1(UPSTREAM)
2	120019947	120019956	120019950	TA	T	snp	1	0	0	0	.	.	.	STEAP3(DOWNSTREAM);STEAP3(INTRON)
2	120022380	120022389	120022381	C	A	ins	1	1	1	1	.	.	.	STEAP3(DOWNSTREAM);STEAP3(UTR_3_PRIME)
2	120094619	120094646	120094634	ATTT	A	del	1	1	1	1	.	.	.	C2orf76(DOWNSTREAM);C2orf76(INTRON)
2	120094619	120094646	120094638	ATTT	A	del	1	1	1	1	.	.	.	C2orf76(DOWNSTREAM);C2orf76(INTRON)
2	120094619	120094646	120094641	ATTT	TA	del	1	1	1	1	.	.	.	C2orf76(DOWNSTREAM);C2orf76(INTRON)
2	120094619	120094646	120094642	ATTT	A	del	1	1	1	1	.	.	.	C2orf76(DOWNSTREAM);C2orf76(INTRON)
2	120121602	120121617	120121615	AG	C	snp	1	1	1	1	.	.	.	C2orf76(INTRON);DBI(UPSTREAM)
2	120144771	120144778	120144772	T	TTGA	ins	1	1	1	1	.	.	.	INTERGENIC(INTERGENIC)
2	120171246	120171253	120171250	T	G	snp	1	0	0	0	.	.	.	INTERGENIC(INTERGENIC)
2	120277439	120277447	120277443	A	C	snp	1	1	1	1	.	.	.	SCTR(INTRON)
2	120442828	120442841	120442833	AC	G	snp	1	0	0	0	.	.	.	TMEM177(DOWNSTREAM);TMEM177(INTRON)
2	120442828	120442841	120442838	AC	G	snp	1	0	0	0	.	.	.	TMEM177(DOWNSTREAM);TMEM177(INTRON)
2	120630771	120630778	120630773	T	G	snp	1	0	0	0	.	.	.	AC092455.1(DOWNSTREAM);PTPN4(INTRON);PTPN4(UPSTREAM)
2	121342748	121342756	121342752	A	G	snp	0	0	1	0	.	.	.	INTERGENIC(INTERGENIC)
2	121483760	121483769	121483764	GA	C	snp	1	1	1	1	.	.	.	INTERGENIC(INTERGENIC)
2	121506520	121506539	121506534	CCAT	G	snp	0	1	1	1	.	.	.	GLI2(INTRON)
2	121652186	121652194	121652190	G	T	snp	1	0	0	0	.	.	.	GLI2(INTRON)
2	121732418	121732427	121732425	AC	T	snp	1	0	0	0	.	.	.	GLI2(DOWNSTREAM);GLI2(INTRON)
2	121864710	121864727	121864713	AT	A	snp	1	1	1	1	.	.	.	INTERGENIC(INTERGENIC)
2	121891674	121891683	121891680	GT	A	snp	1	1	1	1	.	.	.	INTERGENIC(INTERGENIC)
2	121949831	121949838	121949835	A	G	snp	1	1	1	1	.	.	.	AC079988.1(DOWNSTREAM);INTERGENIC(INTERGENIC)

2	122072172	122072195	122072190	TTGT	T	snp	1	0	0	0	.	.	.	AC013399.3.1(DOWNSTREAM);INTERGENIC(INTERGENIC)
2	122081672	122081691	122081687	TTCT	C	snp	0	1	1	0	.	.	.	INTERGENIC(INTERGENIC)
2	122184935	122184952	122184949	CAA	C	del	0	0	1	0	.	.	.	CLASP1(INTRON)
2	122380647	122380658	122380649	TA	C	snp	1	0	0	0	.	.	.	CLASP1(INTRON)
2	122434488	122434496	122434489	A	G	snp	0	1	1	0	.	.	.	AC018737.1.1(INTRON)
2	122476952	122476959	122476957	A	C	snp	1	1	1	0	.	.	.	AC018737.1.1(INTRON)
2	122587946	122587954	122587949	T	C	snp	1	1	0	1	.	.	.	INTERGENIC(INTERGENIC)
2	122631223	122631230	122631224	C	A	snp	1	1	1	1	.	.	.	INTERGENIC(INTERGENIC)
2	122681077	122681084	122681082	T	C	snp	1	1	1	1	.	.	.	INTERGENIC(INTERGENIC)
2	122755315	122755326	122755323	CT	T	snp	1	1	1	1	.	.	.	INTERGENIC(INTERGENIC)
2	122817635	122817644	122817639	AT	G	snp	1	0	0	0	.	.	.	INTERGENIC(INTERGENIC)
2	122817666	122817675	122817670	AT	G	snp	1	0	0	0	.	.	.	INTERGENIC(INTERGENIC)
2	122836770	122836779	122836775	A	C	snp	1	0	0	0	.	.	.	INTERGENIC(INTERGENIC)
2	122898465	122898476	122898473	TCT	TC	del	1	1	0	1	.	.	.	INTERGENIC(INTERGENIC)
2	122969374	122969388	122969379	CTT	C	snp	1	0	0	0	.	.	.	INTERGENIC(INTERGENIC)
2	123051667	123051680	123051677	GA	T	snp	1	1	1	1	.	.	.	INTERGENIC(INTERGENIC)
2	123087285	123087304	123087292	AAAC	A	snp	1	1	1	1	.	.	.	INTERGENIC(INTERGENIC)
2	123192835	123192858	123192850	GTTT	G	snp	1	1	1	1	.	.	.	INTERGENIC(INTERGENIC)
2	123243607	123243616	123243612	A	C	snp	1	1	1	1	.	.	.	INTERGENIC(INTERGENIC)
2	123271686	123271694	123271691	A	G	snp	1	0	0	0	.	.	.	INTERGENIC(INTERGENIC)
2	123378461	123378470	123378465	T	C	snp	1	0	0	0	.	.	.	INTERGENIC(INTERGENIC)
2	123381001	123381009	123381003	T	C	snp	1	0	0	0	.	.	.	INTERGENIC(INTERGENIC)
2	123444910	123444921	123444913	TA	G	snp	1	1	1	1	.	.	.	INTERGENIC(INTERGENIC)
2	123539227	123539244	123539239	TA	ATA	del	0	0	1	1	.	.	.	INTERGENIC(INTERGENIC)
2	123609944	123609953	123609947	CT	G	snp	1	1	1	1	.	.	.	INTERGENIC(INTERGENIC)
2	123623614	123623621	123623616	A	G	snp	1	1	1	1	.	.	.	INTERGENIC(INTERGENIC)
2	123738753	123738761	123738759	T	TTTG	ins	1	1	1	1	.	.	.	INTERGENIC(INTERGENIC)
2	123769294	123769301	123769295	A	C	ins	1	0	1	0	.	.	.	INTERGENIC(INTERGENIC)
2	123771303	123771310	123771306	A	G	snp	1	0	0	0	.	.	.	INTERGENIC(INTERGENIC)
2	123840266	123840277	123840270	CCA	A	snp	1	1	1	1	.	.	.	INTERGENIC(INTERGENIC)
2	123842608	123842616	123842612	A	G	snp	1	1	1	1	.	.	.	INTERGENIC(INTERGENIC)
2	123842935	123842958	123842948	CAAA	AACG	ins	1	1	1	1	.	.	.	INTERGENIC(INTERGENIC)
2	123867877	123867886	123867884	AC	G	snp	1	0	0	0	.	.	.	INTERGENIC(INTERGENIC)
2	123874022	123874029	123874028	T	G	snp	0	0	1	0	.	.	.	INTERGENIC(INTERGENIC)
2	123879709	123879720	123879713	TA	C	snp	1	0	0	0	.	.	.	INTERGENIC(INTERGENIC)
2	123879862	123879873	123879871	AC	T	snp	1	0	0	0	.	.	.	INTERGENIC(INTERGENIC)
2	123931270	123931277	123931274	A	G	snp	0	1	0	1	.	.	.	INTERGENIC(INTERGENIC)
2	123947603	123947610	123947607	A	C	snp	1	1	1	1	.	.	.	INTERGENIC(INTERGENIC)
2	123947624	123947633	123947626	A	C	ins	1	0	0	0	.	.	.	INTERGENIC(INTERGENIC)
2	123947624	123947633	123947627	A	C	snp	1	1	1	1	.	.	.	INTERGENIC(INTERGENIC)
2	123983504	123983521	123983518	CA	A	snp	1	1	1	1	.	.	.	INTERGENIC(INTERGENIC)
2	124005453	124005462	124005457	AT	G	snp	1	1	1	1	.	.	.	INTERGENIC(INTERGENIC)
2	124066619	124066626	124066621	A	C	snp	1	1	0	1	.	.	.	INTERGENIC(INTERGENIC)
2	124103275	124103284	124103281	TA	G	snp	1	1	1	1	.	.	.	INTERGENIC(INTERGENIC)
2	124114389	124114398	124114394	AT	C	snp	1	0	0	0	.	.	.	INTERGENIC(INTERGENIC)
2	124159739	124159752	124159742	TA	C	snp	1	1	1	1	.	.	.	INTERGENIC(INTERGENIC)
2	124159739	124159752	124159745	TA	A	snp	1	1	1	1	.	.	.	INTERGENIC(INTERGENIC)
2	124165930	124165945	124165932	AT	T	del	1	1	1	1	.	.	.	INTERGENIC(INTERGENIC)
2	124171696	124171711	124171706	AGAA	AAG	del	1	0	0	0	.	.	.	INTERGENIC(INTERGENIC)
2	124187021	124187030	124187023	TA	C	snp	1	1	0	1	.	.	.	INTERGENIC(INTERGENIC)
2	124386077	124386085	124386082	T	C	ins	1	1	1	1	.	.	.	INTERGENIC(INTERGENIC)
2	124514184	124514193	124514188	AT	G	snp	1	1	1	1	.	.	.	INTERGENIC(INTERGENIC)
2	124518572	124518579	124518575	T	G	snp	0	1	0	1	.	.	.	AC092646.2.1(UPSTREAM);INTERGENIC(INTERGENIC)
2	124526798	124526813	124526802	AAAT	C	snp	1	1	0	1	.	.	.	INTERGENIC(INTERGENIC)
2	124566133	124566140	124566138	C	T	ins	1	1	1	1	.	.	.	INTERGENIC(INTERGENIC)
2	124682405	124682412	124682410	T	C	ins	1	1	1	1	.	.	.	INTERGENIC(INTERGENIC)
2	124753507	124753515	124753513	T	G	snp	1	1	0	1	.	.	.	INTERGENIC(INTERGENIC)
2	124890888	124890896	124890892	A	C	snp	1	1	1	1	.	.	.	CNTNAP5(INTRON)
2	124895152	124895159	124895153	A	C	snp	1	1	1	1	.	.	.	CNTNAP5(DOWNSTREAM);CNTNAP5(INTRON)
2	124895152	124895159	124895158	A	C	snp	1	0	0	0	.	.	.	CNTNAP5(DOWNSTREAM);CNTNAP5(INTRON)
2	124927815	124927828	124927824	AT	AC	ins	0	1	1	1	.	.	.	CNTNAP5(INTRON)
2	124939033	124939042	124939036	T	G	ins	1	1	0	1	.	.	.	CNTNAP5(INTRON)
2	125037748	125037757	125037753	TA	G	snp	1	0	0	0	.	.	.	CNTNAP5(INTRON)
2	125183632	125183640	125183636	A	C	snp	1	1	1	1	.	.	.	CNTNAP5(INTRON)
2	125350683	125350706	125350693	TATT	G	snp	1	1	1	1	.	.	.	CNTNAP5(INTRON)
2	125440979	125440986	125440980	T	C	snp	1	1	0	1	.	.	.	AC019159.1(UPSTREAM);CNTNAP5(INTRON);MTND5P22(UPSTREAM)

2	125569284	125569291	125569285	G	T	ins	1	1	1	1	.	.	.	CNTNAP5(INTRON)
2	125575011	125575019	125575012	A	C	snp	1	0	0	0	.	.	.	CNTNAP5(INTRON)
2	125578114	125578121	125578116	G	GT	ins	1	0	0	0	.	.	.	CNTNAP5(INTRON)
2	125719973	125719980	125719978	T	G	snp	0	0	1	0	.	.	.	INTERGENIC(INTERGENIC)
2	125792025	125792033	125792027	T	G	snp	1	0	0	0	.	.	.	INTERGENIC(INTERGENIC)
2	126016313	126016322	126016317	T	G	snp	1	1	0	1	.	.	.	INTERGENIC(INTERGENIC)
2	126027141	126027152	126027148	TCC	T	snp	1	0	0	0	.	.	.	INTERGENIC(INTERGENIC)
2	126142733	126142740	126142735	A	C	snp	0	1	0	1	.	.	.	INTERGENIC(INTERGENIC)
2	126167599	126167612	126167607	AC	T	snp	0	0	0	1	.	.	.	INTERGENIC(INTERGENIC)
2	126198112	126198127	126198123	TTTC	T	snp	1	1	1	1	.	.	.	INTERGENIC(INTERGENIC)
2	126214233	126214244	126214238	AT	C	snp	1	1	0	1	.	.	.	INTERGENIC(INTERGENIC)
2	126214233	126214244	126214242	AT	A	del	1	1	1	1	.	.	.	INTERGENIC(INTERGENIC)
2	126266023	126266032	126266026	AT	C	snp	1	0	0	0	.	.	.	INTERGENIC(INTERGENIC)
2	126301989	126301998	126301994	TG	A	snp	1	1	1	1	.	.	.	INTERGENIC(INTERGENIC)
2	126541097	126541104	126541099	A	G	snp	1	0	0	0	.	.	.	INTERGENIC(INTERGENIC)
2	126576058	126576065	126576061	G	A	snp	1	1	1	1	.	.	.	AC097499.2.1(UPSTREAM);INTERGENIC(INTERGENIC)
2	126631750	126631758	126631751	A	C	ins	1	0	0	1	.	.	.	INTERGENIC(INTERGENIC)
2	126652495	126652506	126652498	AT	C	snp	1	0	0	0	.	.	.	INTERGENIC(INTERGENIC)
2	126652635	126652646	126652639	AT	G	snp	1	1	1	1	.	.	.	INTERGENIC(INTERGENIC)
2	126652791	126652800	126652797	AT	C	snp	1	1	1	0	.	.	.	INTERGENIC(INTERGENIC)
2	126689158	126689167	126689164	T	G	snp	0	1	0	1	.	.	.	INTERGENIC(INTERGENIC)
2	126720573	126720581	126720576	T	G	snp	1	1	0	1	.	.	.	INTERGENIC(INTERGENIC)
2	126826078	126826087	126826082	CA	T	snp	1	1	1	1	.	.	.	INTERGENIC(INTERGENIC)
2	126863794	126863805	126863802	TG	C	snp	0	1	1	0	.	.	.	AC114813.1.1(DOWNSTREAM);INTERGENIC(INTERGENIC)
2	126972619	126972628	126972627	A	C	snp	1	1	1	1	.	.	.	INTERGENIC(INTERGENIC)
2	127034372	127034381	127034376	TA	A	snp	1	0	0	0	.	.	.	INTERGENIC(INTERGENIC)
2	127083387	127083395	127083389	T	G	snp	1	1	0	1	.	.	.	AC023347.1.1(INTRON)
2	127234776	127234784	127234779	A	G	snp	1	1	1	1	.	.	.	INTERGENIC(INTERGENIC)
2	127318447	127318474	127318458	ATTT	C	snp	1	1	1	1	.	.	.	INTERGENIC(INTERGENIC);YWHAZP2(UPSTREAM)
2	127326009	127326024	127326022	AT	A	del	1	1	0	1	.	.	.	INTERGENIC(INTERGENIC)
2	127494707	127494726	127494718	GATA	GATAGATAGAT		del	1	1	0	1	.	.	. INTERGENIC(INTERGENIC)
2	127494839	127494854	127494844	GATA	T	ins	1	0	0	0	.	.	.	INTERGENIC(INTERGENIC)
2	127572607	127572615	127572613	T	C	snp	1	1	1	1	.	.	.	INTERGENIC(INTERGENIC)
2	127575038	127575055	127575050	CA	ACACAT	ins	1	0	0	0	.	.	.	INTERGENIC(INTERGENIC)
2	127575038	127575055	127575050	CA	T	snp	1	0	0	0	.	.	.	INTERGENIC(INTERGENIC)
2	127772130	127772137	127772136	C	A	snp	1	1	0	1	.	.	.	INTERGENIC(INTERGENIC);U7(UPSTREAM)
2	127835761	127835768	127835767	C	T	snp	1	0	0	0	.	.	.	BIN1(INTRON)
2	127881153	127881160	127881156	T	TTTTG	ins	1	1	1	1	.	.	.	INTERGENIC(INTERGENIC)
2	127905536	127905545	127905542	T	C	snp	1	1	1	1	.	.	.	INTERGENIC(INTERGENIC)
2	127961971	127961980	127961978	AG	A	snp	0	0	1	0	.	.	.	CYP27C1(INTRON)
2	128001903	128001912	128001905	A	G	snp	1	1	1	1	.	.	.	AC110926.4.1(UPSTREAM);INTERGENIC(INTERGENIC)
2	128048668	128048677	128048674	A	C	snp	1	1	1	1	.	.	.	AC110926.1(DOWNSTREAM);ERCC3(DOWNSTREAM);ERCC3(INTRON);ERCC3(UPSTREAM)
2	128132976	128132995	128132982	TTAT	T	ins	0	0	1	0	.	.	.	MAP3K2(INTRON)
2	128132976	128132995	128132984	TTAT	TA	del	0	1	1	1	.	.	.	MAP3K2(INTRON)
2	128132976	128132995	128132989	TTAT	A	del	1	1	1	1	.	.	.	MAP3K2(INTRON)
2	128239019	128239027	128239020	G	A	snp	1	1	1	1	.	.	.	AC010976.2.1(INTRON);IWS1(INTRON)
2	128292557	128292576	128292572	TTTG	T	snp	1	1	1	1	.	.	.	INTERGENIC(INTERGENIC);MYO7B(UPSTREAM)
2	128312939	128312953	128312942	TTG	A	snp	0	1	1	1	.	.	.	MYO7B(INTRON);MYO7B(UPSTREAM)
2	128567628	128567637	128567635	AC	ACATATAT		ins	1	1	1	1	.	.	. WDR33(INTRON)
2	128595537	128595556	128595551	TCTT	TTC	del	1	1	0	1	.	.	.	INTERGENIC(INTERGENIC)
2	128603825	128603840	128603836	AAAT	AAATAA	del	1	0	0	0	.	.	.	AC006011.4.1(DOWNSTREAM);INTERGENIC(INTERGENIC);POLR2D(DOWNSTREAM);U6(DOWNSTREAM)
2	128697871	128697879	128697873	A	C	ins	1	1	1	1	.	.	.	INTERGENIC(INTERGENIC);SAP130(DOWNSTREAM)
2	128723367	128723378	128723369	AG	G	snp	1	0	0	0	.	.	.	SAP130(INTRON)
2	129003089	129003100	129003092	TA	C	snp	1	0	0	0	.	.	.	HS6ST1(INTRON)
2	129198548	129198555	129198553	C	CG	ins	1	1	0	1	.	.	.	INTERGENIC(INTERGENIC);RN5S103(DOWNSTREAM)
2	129201409	129201416	129201411	G	T	snp	1	1	1	1	.	.	.	INTERGENIC(INTERGENIC);RN5S103(DOWNSTREAM)
2	129201409	129201416	129201412	G	T	snp	1	1	1	1	.	.	.	INTERGENIC(INTERGENIC);RN5S103(DOWNSTREAM)
2	129302290	129302297	129302296	G	A	snp	1	0	0	0	.	.	.	INTERGENIC(INTERGENIC)
2	129444327	129444342	129444336	TTTC	G	snp	1	0	0	0	.	.	.	INTERGENIC(INTERGENIC)
2	129467884	129467895	129467890	AGC	G	snp	1	1	1	1	.	.	.	INTERGENIC(INTERGENIC)
2	129475312	129475320	129475314	A	C	ins	0	1	0	1	.	.	.	INTERGENIC(INTERGENIC)
2	129483878	129483889	129483885	AG	A	snp	1	1	1	1	.	.	.	INTERGENIC(INTERGENIC)
2	129512787	129512794	129512792	A	G	snp	1	1	1	1	.	.	.	INTERGENIC(INTERGENIC)
2	129583004	129583015	129583012	TTA	G	snp	0	1	1	1	.	.	.	INTERGENIC(INTERGENIC)
2	129597758	129597778	129597765	AAC	G	ins	1	0	0	0	.	.	.	INTERGENIC(INTERGENIC)

2	129616223	129616230	129616229	T	G	snp	1	1	1	1	.	.	.	INTERGENIC(INTERGENIC)
2	129713347	129713356	129713353	T	G	snp	1	1	1	1	.	.	.	INTERGENIC(INTERGENIC)
2	129859154	129859163	129859156	T	G	snp	1	1	1	1	.	.	.	INTERGENIC(INTERGENIC)
2	129859154	129859163	129859162	T	G	snp	1	1	1	1	.	.	.	INTERGENIC(INTERGENIC)
2	129872360	129872367	129872365	A	G	snp	1	0	0	0	.	.	.	INTERGENIC(INTERGENIC)
2	129873497	129873504	129873502	A	C	snp	0	0	1	0	.	.	.	INTERGENIC(INTERGENIC)
2	129908941	129908949	129908948	T	G	snp	1	1	1	1	.	.	.	INTERGENIC(INTERGENIC)
2	129915236	129915247	129915241	AAT	A	snp	1	0	0	0	.	.	.	INTERGENIC(INTERGENIC)
2	129915248	129915255	129915253	A	C	snp	1	1	1	1	.	.	.	INTERGENIC(INTERGENIC)
2	129924224	129924231	129924229	A	G	snp	1	0	0	0	.	.	.	INTERGENIC(INTERGENIC)
2	129932487	129932495	129932489	A	G	snp	1	0	0	0	.	.	.	INTERGENIC(INTERGENIC)
2	130039764	130039773	130039771	T	G	snp	1	0	0	0	.	.	.	INTERGENIC(INTERGENIC)
2	130118704	130118723	130118709	TTTG	TG	del	1	1	1	1	.	.	.	INTERGENIC(INTERGENIC)
2	130118704	130118723	130118710	TTTG	G	del	1	1	1	1	.	.	.	INTERGENIC(INTERGENIC)
2	130146241	130146249	130146245	A	G	snp	1	1	1	1	.	.	.	INTERGENIC(INTERGENIC)
2	130167554	130167567	130167565	AT	A	del	0	0	0	1	.	.	.	INTERGENIC(INTERGENIC)
2	130322619	130322632	130322622	AT	A	snp	1	0	0	0	.	.	.	INTERGENIC(INTERGENIC)
2	130375216	130375230	130375225	CTC	A	snp	1	1	1	1	.	.	.	INTERGENIC(INTERGENIC)
2	130547022	130547031	130547023	T	G	snp	1	0	0	0	.	.	.	INTERGENIC(INTERGENIC)
2	130686592	130686603	130686597	AG	A	snp	1	0	0	0	.	.	.	AC079776.2.1(INTRON);AC079776.2.1(UPSTREAM);AC079776.3.1(INTRON)
2	130931546	130931554	130931551	A	C	ins	1	0	0	0	.	.	.	SMPD4(INTRON);SMPD4(UPSTREAM)
2	131070476	131070490	131070482	GTG	A	snp	1	1	1	1	.	.	.	INTERGENIC(INTERGENIC)
2	131075878	131075893	131075888	CTTT	TC	del	1	1	1	1	.	.	.	INTERGENIC(INTERGENIC)
2	131075878	131075893	131075889	CTTT	C	del	1	1	1	1	.	.	.	INTERGENIC(INTERGENIC)
2	131107132	131107143	131107138	CAA	A	ins	1	0	0	0	.	.	.	IMP4(DOWNSTREAM);INTERGENIC(INTERGENIC)
2	131134054	131134063	131134060	AG	G	snp	1	0	0	0	.	.	.	INTERGENIC(INTERGENIC);PTPN18(DOWNSTREAM)
2	131264835	131264844	131264840	A	C	snp	1	1	1	1	.	.	.	POTEI(INTRON)
2	131361297	131361306	131361305	A	G	snp	1	0	0	0	.	.	.	CFC1(UPSTREAM);INTERGENIC(INTERGENIC)
2	131503625	131503642	131503640	AT	A	del	1	0	0	0	.	.	.	AC140481.8.1(DOWNSTREAM);AC140481.8.1(INTRON)
2	131578698	131578706	131578705	T	TG	ins	1	1	1	1	.	.	.	INTERGENIC(INTERGENIC)
2	131643931	131643938	131643937	A	G	ins	1	0	0	0	.	.	.	ARHGEF4(INTRON)
2	131677651	131677659	131677658	T	G	snp	1	0	0	0	.	.	.	ARHGEF4(INTRON)
2	131742318	131742329	131742327	AC	A	snp	1	1	1	1	.	.	.	ARHGEF4(INTRON)
2	131743522	131743533	131743524	TG	C	snp	1	1	1	1	.	.	.	ARHGEF4(INTRON)
2	131805980	131805988	131805984	G	T	snp	1	1	1	1	.	.	.	ARHGEF4(DOWNSTREAM);FAM168B(DOWNSTREAM);FAM168B(INTRON);FAM168B(UTR_3_PRIME)
2	131991177	131991186	131991179	T	G	ins	1	1	1	1	.	.	.	AC131180.1(INTRON);PLEKHB2(INTRON);POTEE(INTRON);U6(DOWNSTREAM)
2	132026361	132026370	132026364	A	C	ins	1	0	0	0	.	.	.	AC131180.1(DOWNSTREAM);PLEKHB2(INTRON);POTEE(DOWNSTREAM)
2	132177802	132177811	132177809	A	AT	ins	1	1	1	0	.	.	.	GNAQP1(DOWNSTREAM);INTERGENIC(INTERGENIC)
2	132181046	132181057	132181055	AG	C	snp	1	1	1	1	.	.	.	GNAQP1(DOWNSTREAM);INTERGENIC(INTERGENIC)
2	132217908	132217915	132217914	T	TTC	ins	0	1	1	0	.	.	.	INTERGENIC(INTERGENIC);RHOQP2(UPSTREAM)
2	132222988	132222996	132222995	A	C	snp	0	1	0	0	.	.	.	INTERGENIC(INTERGENIC);MZT2A(DOWNSTREAM);RHOQP2(DOWNSTREAM)
2	132259418	132259425	132259421	A	G	snp	1	1	1	1	.	.	.	AC093838.4.1(INTRON)
2	132263230	132263239	132263238	T	G	snp	1	1	1	1	.	.	.	AC093838.4.1(INTRON)
2	132266732	132266741	132266738	CT	A	snp	1	1	1	1	.	.	.	AC093838.4.1(INTRON)
2	132342463	132342470	132342469	A	G	snp	1	0	0	0	.	.	.	INTERGENIC(INTERGENIC)
2	132355366	132355379	132355377	TG	T	ins	1	0	0	0	.	.	.	POTEKP(INTRON);U6(UPSTREAM)
2	132378602	132378610	132378608	C	T	snp	1	0	0	0	.	.	.	POTEKP(INTRON)
2	132530820	132530831	132530828	CA	T	snp	0	1	1	1	.	.	.	AC103564.9.1(UPSTREAM);INTERGENIC(INTERGENIC)
2	132588887	132588896	132588888	G	T	snp	1	1	1	1	.	.	.	AC103564.6.1(DOWNSTREAM);AC103564.7.1(INTRON)
2	132588887	132588896	132588895	G	A	snp	1	1	1	1	.	.	.	AC103564.6.1(DOWNSTREAM);AC103564.7.1(INTRON)
2	132616550	132616561	132616555	TA	C	snp	1	1	1	1	.	.	.	INTERGENIC(INTERGENIC)
2	132616572	132616583	132616577	TA	C	snp	1	1	1	1	.	.	.	INTERGENIC(INTERGENIC)
2	132616572	132616583	132616581	TA	C	snp	0	1	1	1	.	.	.	INTERGENIC(INTERGENIC)
2	132641010	132641019	132641016	A	G	snp	1	1	1	1	.	.	.	INTERGENIC(INTERGENIC)
2	132743405	132743416	132743410	TG	A	snp	1	1	1	1	.	.	.	RP1-156L9.1.1(INTRON)
2	132766713	132766722	132766720	A	C	snp	1	1	1	1	.	.	.	INTERGENIC(INTERGENIC)
2	132947459	132947467	132947464	A	C	snp	1	1	0	0	.	.	.	ANKRD30BL(INTRON)
2	132960009	132960017	132960012	T	G	snp	1	0	0	0	.	.	.	ANKRD30BL(INTRON)
2	133037889	133037900	133037893	CAC	G	snp	1	1	1	1	.	.	.	AC097532.1(DOWNSTREAM);INTERGENIC(INTERGENIC)
2	133165453	133165461	133165454	A	C	snp	1	1	1	1	.	.	.	INTERGENIC(INTERGENIC);U6(UPSTREAM)
2	133250239	133250248	133250241	AT	C	snp	1	0	0	0	.	.	.	GPR39(INTRON);YBX1P7(UPSTREAM)
2	133251110	133251123	133251117	AT	G	snp	1	0	0	0	.	.	.	GPR39(INTRON);YBX1P7(UPSTREAM)

2	133347911	133347920	133347918	AC	T	snp	1	1	1	1	.	.	.	GPR39(INTRON)
2	133371751	133371760	133371755	T	TG	ins	1	0	0	0	.	.	.	GPR39(INTRON)
2	133456430	133456439	133456433	A	C	snp	1	0	0	0	.	.	.	NCKAP5(INTRON)
2	133577809	133577818	133577814	T	G	snp	1	0	0	0	.	.	.	NCKAP5(INTRON)
2	133593985	133593994	133593991	T	G	snp	0	1	1	0	.	.	.	NCKAP5(INTRON)
2	133631059	133631068	133631064	TG	T	snp	0	1	1	0	.	.	.	NCKAP5(INTRON)
2	133661911	133661926	133661921	AAAT	T	snp	1	0	0	0	.	.	.	NCKAP5(INTRON)
2	133678261	133678274	133678269	TA	A	snp	1	1	1	1	.	.	.	AC016909.1.1(INTRON);NCKAP5(INTRON)
2	133802957	133802968	133802960	TA	C	snp	1	0	0	0	.	.	.	NCKAP5(INTRON)
2	133802957	133802968	133802964	TA	C	snp	1	0	0	0	.	.	.	NCKAP5(INTRON)
2	133930213	133930222	133930220	T	G	snp	0	1	1	1	.	.	.	NCKAP5(INTRON)
2	133962708	133962716	133962709	A	C	snp	0	1	1	1	.	.	.	NCKAP5(INTRON)
2	134095693	134095702	134095700	T	G	snp	0	1	0	0	.	.	.	NCKAP5(INTRON);U6(DOWNSTREAM)
2	134096417	134096424	134096418	A	C	snp	1	0	0	0	.	.	.	NCKAP5(INTRON);U6(DOWNSTREAM)
2	134119585	134119594	134119591	A	G	snp	1	0	0	0	.	.	.	NCKAP5(INTRON)
2	134232372	134232381	134232379	T	G	snp	0	1	0	0	.	.	.	NCKAP5(INTRON)
2	134357909	134357918	134357914	GA	GAG	del	1	1	1	1	.	.	.	7SK(DOWNSTREAM);INTERGENIC(INTERGENIC)
2	134357964	134357971	134357967	A	G	snp	1	1	1	1	.	.	.	7SK(DOWNSTREAM);INTERGENIC(INTERGENIC)
2	134363711	134363718	134363717	A	C	ins	1	1	1	1	.	.	.	INTERGENIC(INTERGENIC)
2	134363711	134363718	134363717	A	C	snp	1	1	1	1	.	.	.	INTERGENIC(INTERGENIC)
2	134381503	134381512	134381508	TA	G	snp	1	0	0	0	.	.	.	INTERGENIC(INTERGENIC)
2	134413828	134413843	134413835	AAAC	G	snp	1	1	0	1	.	.	.	INTERGENIC(INTERGENIC)
2	134481675	134481683	134481679	A	G	snp	1	0	0	0	.	.	.	INTERGENIC(INTERGENIC)
2	134606552	134606561	134606555	AG	AC	ins	1	0	0	0	.	.	.	INTERGENIC(INTERGENIC)
2	134659424	134659435	134659431	ACA	AACA	del	1	1	0	1	.	.	.	INTERGENIC(INTERGENIC)
2	134667500	134667517	134667510	TA	A	ins	1	0	0	0	.	.	.	INTERGENIC(INTERGENIC)
2	134713095	134713104	134713097	A	C	ins	1	0	0	0	.	.	.	INTERGENIC(INTERGENIC)
2	134713095	134713104	134713098	A	C	snp	1	0	0	0	.	.	.	INTERGENIC(INTERGENIC)
2	134878140	134878147	134878142	C	A	snp	1	1	1	1	.	.	.	MGAT5(INTRON)
2	134934716	134934733	134934722	GT	A	snp	0	1	0	1	.	.	.	MGAT5(INTRON)
2	134994113	134994120	134994119	C	A	snp	1	1	0	1	.	.	.	MGAT5(INTRON)
2	135004947	135004956	135004955	A	C	snp	1	0	0	0	.	.	.	MGAT5(INTRON)
2	135018199	135018207	135018206	T	G	snp	1	1	1	1	.	.	.	MGAT5(INTRON)
2	135077741	135077752	135077748	TCC	T	snp	0	1	1	0	.	.	.	AC016906.1.1(UPSTREAM);MGAT5(INTRON)
2	135151905	135151912	135151909	A	C	snp	0	0	1	0	.	.	.	MGAT5(INTRON)
2	135217647	135217655	135217648	C	CG	ins	1	1	1	1	.	.	.	AC013718.1(UPSTREAM);TMEM163(INTRON);TMEM163(UPSTREAM)
2	135219734	135219745	135219739	TC	G	snp	1	1	1	0	.	.	.	TMEM163(INTRON);TMEM163(UPSTREAM)
2	135262023	135262031	135262027	A	AC	ins	1	1	1	1	.	.	.	TMEM163(INTRON)
2	135262023	135262031	135262028	A	C	ins	1	1	1	1	.	.	.	TMEM163(INTRON)
2	135268725	135268733	135268728	G	GGGA	ins	1	0	1	0	.	.	.	TMEM163(INTRON)
2	135293309	135293316	135293312	T	G	snp	0	1	0	0	.	.	.	TMEM163(INTRON)
2	135408074	135408081	135408078	T	G	snp	1	0	0	0	.	.	.	TMEM163(INTRON)
2	135408079	135408102	135408094	TTTG	T	ins	1	1	1	1	.	.	.	TMEM163(INTRON)
2	135408079	135408102	135408095	TTTG	TTG	del	1	1	1	1	.	.	.	TMEM163(INTRON)
2	135408079	135408102	135408097	TTTG	G	del	1	1	1	1	.	.	.	TMEM163(INTRON)
2	135426351	135426358	135426352	A	C	snp	1	0	0	0	.	.	.	TMEM163(INTRON)
2	135625400	135625415	135625408	TA	C	snp	1	0	0	0	.	.	.	AC016725.4.1(INTRON);ACMSD(INTRON)
2	135643804	135643818	135643813	ATT	T	ins	0	0	1	0	.	.	.	AC016725.4.1(INTRON);ACMSD(INTRON)
2	135653091	135653100	135653094	T	G	snp	0	1	1	0	.	.	.	AC016725.4.1(INTRON);ACMSD(INTRON)
2	135764407	135764418	135764416	AG	A	snp	1	0	0	0	.	.	.	YSK4(INTRON)
2	135790154	135790163	135790156	T	G	ins	1	1	1	1	.	.	.	YSK4(INTRON)
2	135895390	135895399	135895394	T	G	snp	1	0	0	0	.	.	.	RAB3GAP1(INTRON);SNORA40(DOWNSTREAM);ZRANB3(INTRON)
2	135946641	135946664	135946660	AAAT	C	snp	1	0	0	0	.	.	.	ZRANB3(INTRON)
2	135980336	135980347	135980342	TA	C	snp	1	0	0	0	.	.	.	ZRANB3(DOWNSTREAM);ZRANB3(INTRON)
2	136074554	136074571	136074561	AT	A	snp	1	0	0	0	.	.	.	ZRANB3(INTRON)
2	136337166	136337174	136337169	G	GGT	ins	1	0	0	0	.	.	.	R3HDM1(INTRON)
2	136337166	136337174	136337170	G	GT	ins	1	0	0	0	.	.	.	R3HDM1(INTRON)
2	136340814	136340822	136340817	T	C	snp	1	0	0	0	.	.	.	R3HDM1(INTRON);R3HDM1(UPSTREAM)
2	136619193	136619200	136619195	T	G	snp	0	0	1	0	.	.	.	MCM6(INTRON)
2	136625595	136625603	136625602	A	G	snp	1	1	1	1	.	.	.	MCM6(INTRON);MCM6(UPSTREAM)
2	136711915	136711924	136711921	AG	C	snp	1	0	0	0	.	.	.	DARS(INTRON)
2	136785472	136785483	136785475	AAT	G	snp	1	0	0	0	.	.	.	INTERGENIC(INTERGENIC)
2	136976766	136976775	136976767	A	G	snp	1	1	1	1	.	.	.	INTERGENIC(INTERGENIC)
2	136987599	136987607	136987605	A	G	snp	1	1	1	1	.	.	.	INTERGENIC(INTERGENIC)
2	136987618	136987626	136987622	A	G	snp	0	1	0	0	.	.	.	INTERGENIC(INTERGENIC)

2	137060135	137060150	137060145	TTTG	G	snp	1	0	0	0	.	.	.	INTERGENIC(INTERGENIC)
2	137094979	137094994	137094986	TC	T	snp	1	0	0	0	.	.	.	INTERGENIC(INTERGENIC)
2	137110675	137110692	137110677	AT	T	del	1	1	1	0	.	.	.	INTERGENIC(INTERGENIC)
2	137150763	137150774	137150771	ATT	A	del	1	0	0	0	.	.	.	7SK(DOWNSTREAM);INTERGENIC(INTERGENIC)
2	137334165	137334173	137334171	A	G	snp	1	1	1	1	.	.	.	INTERGENIC(INTERGENIC)
2	137479245	137479260	137479252	TTTA	T	del	1	1	1	1	.	.	.	INTERGENIC(INTERGENIC)
2	137514550	137514570	137514565	ATT	TTG	ins	1	0	0	0	.	.	.	INTERGENIC(INTERGENIC)
2	137519057	137519074	137519070	TA	T	del	1	1	1	1	.	.	.	INTERGENIC(INTERGENIC);THSD7B(UPSTREAM)
2	137524555	137524566	137524563	TG	G	del	1	1	1	1	.	.	.	THSD7B(INTRON)
2	137542561	137542572	137542570	TC	G	snp	1	1	1	1	.	.	.	THSD7B(INTRON)
2	137597028	137597039	137597032	TAA	G	snp	1	1	1	1	.	.	.	THSD7B(INTRON)
2	137648088	137648111	137648105	TTC	T	snp	1	1	1	1	.	.	.	THSD7B(INTRON)
2	137648088	137648111	137648108	TTC	T	snp	1	1	1	1	.	.	.	THSD7B(INTRON)
2	137682665	137682674	137682672	CA	G	snp	1	1	1	1	.	.	.	THSD7B(INTRON)
2	137740929	137740940	137740936	AC	G	snp	0	0	1	0	.	.	.	THSD7B(INTRON)
2	137792957	137792964	137792960	T	C	snp	0	0	1	0	.	.	.	THSD7B(INTRON)
2	137822521	137822530	137822528	T	G	snp	1	1	1	1	.	.	.	THSD7B(INTRON)
2	137896921	137896928	137896926	A	C	snp	1	0	0	0	.	.	.	THSD7B(INTRON)
2	137900672	137900679	137900678	A	C	snp	1	1	1	0	.	.	.	THSD7B(INTRON)
2	137916510	137916517	137916515	A	G	snp	1	1	1	1	.	.	.	THSD7B(INTRON)
2	138051067	138051078	138051076	TC	T	snp	0	1	1	1	.	.	.	THSD7B(INTRON)
2	138126721	138126738	138126735	AT	TT	ins	1	0	0	0	.	.	.	THSD7B(INTRON)
2	138146797	138146808	138146806	AT	G	snp	1	1	1	1	.	.	.	THSD7B(INTRON)
2	138283969	138283992	138283987	TTG	TG	del	1	0	0	0	.	.	.	THSD7B(INTRON)
2	138287542	138287549	138287546	T	G	snp	1	1	1	1	.	.	.	THSD7B(INTRON)
2	138337109	138337122	138337111	CA	AT	ins	1	1	1	1	.	.	.	THSD7B(INTRON)
2	138349493	138349500	138349496	T	C	snp	1	0	0	0	.	.	.	THSD7B(INTRON)
2	138368559	138368576	138368562	ATA	G	snp	1	0	0	0	.	.	.	THSD7B(INTRON)
2	138476998	138477007	138477000	A	C	snp	1	1	1	1	.	.	.	INTERGENIC(INTERGENIC);Y_RNA(DOWNSTREAM)
2	138507089	138507100	138507097	ATA	AT	del	1	1	1	1	.	.	.	INTERGENIC(INTERGENIC)
2	138537629	138537638	138537633	GT	A	snp	1	1	1	1	.	.	.	INTERGENIC(INTERGENIC)
2	138688771	138688786	138688776	GAAG	AGGAAGGAAGGG	ins	1	1	1	1	.	.	.	AC020601.1.1(UPSTREAM);INTERGENIC(INTERGENIC)
2	138688771	138688786	138688782	GAAG	A	snp	1	1	1	1	.	.	.	AC020601.1.1(UPSTREAM);INTERGENIC(INTERGENIC)
2	138760800	138760817	138760810	ATA	A	ins	1	0	0	0	.	.	.	HNMT(DOWNSTREAM);HNMT(INTRON)
2	138760800	138760817	138760812	ATA	T	del	1	0	0	0	.	.	.	HNMT(DOWNSTREAM);HNMT(INTRON)
2	138760800	138760817	138760813	ATA	A	ins	1	0	0	0	.	.	.	HNMT(DOWNSTREAM);HNMT(INTRON)
2	138942599	138942610	138942607	GA	C	snp	1	1	1	1	.	.	.	INTERGENIC(INTERGENIC)
2	139293908	139293917	139293914	C	A	snp	1	1	1	1	.	.	.	SPOPL(INTRON)
2	139426378	139426387	139426379	T	TTG	ins	1	1	0	1	.	.	.	INTERGENIC(INTERGENIC);NXPH2(DOWNSTREAM)
2	139560010	139560023	139560018	AG	G	snp	1	0	0	0	.	.	.	INTERGENIC(INTERGENIC)
2	139603067	139603087	139603083	CTT	TC	del	1	1	1	1	.	.	.	INTERGENIC(INTERGENIC)
2	139603067	139603087	139603084	CTT	C	del	1	1	1	1	.	.	.	INTERGENIC(INTERGENIC)
2	139815692	139815699	139815696	A	G	snp	1	0	0	0	.	.	.	INTERGENIC(INTERGENIC)
2	139894248	139894257	139894252	T	C	snp	1	1	1	1	.	.	.	INTERGENIC(INTERGENIC)
2	139949187	139949196	139949193	T	G	snp	1	1	1	1	.	.	.	INTERGENIC(INTERGENIC)
2	140014632	140014641	140014634	AT	G	snp	1	0	0	0	.	.	.	INTERGENIC(INTERGENIC)
2	140014632	140014641	140014639	AT	A	snp	0	1	1	1	.	.	.	INTERGENIC(INTERGENIC)
2	140035670	140035683	140035675	TG	A	snp	1	0	0	0	.	.	.	INTERGENIC(INTERGENIC)
2	140097393	140097404	140097397	GA	A	snp	0	1	1	1	.	.	.	INTERGENIC(INTERGENIC)
2	140115142	140115153	140115151	AT	C	snp	1	1	1	1	.	.	.	INTERGENIC(INTERGENIC)
2	140124276	140124284	140124280	A	G	snp	1	1	1	1	.	.	.	AC062021.1.1(INTRON)
2	140133601	140133621	140133610	GTG	T	snp	0	1	0	1	.	.	.	AC062021.1.1(INTRON)
2	140149292	140149305	140149298	TA	ATATATA	del	1	1	1	1	.	.	.	INTERGENIC(INTERGENIC)
2	140152448	140152455	140152449	A	G	snp	1	1	1	1	.	.	.	INTERGENIC(INTERGENIC)
2	140152448	140152455	140152453	A	C	snp	1	1	1	1	.	.	.	INTERGENIC(INTERGENIC)
2	140169285	140169294	140169289	T	C	snp	0	0	0	1	.	.	.	INTERGENIC(INTERGENIC)
2	140171019	140171028	140171022	T	C	ins	1	0	0	0	.	.	.	INTERGENIC(INTERGENIC)
2	140265449	140265456	140265454	A	G	snp	0	1	1	1	.	.	.	INTERGENIC(INTERGENIC);SNORA72(UPSTREAM)
2	140374922	140374933	140374930	ATT	A	del	1	1	1	1	.	.	.	INTERGENIC(INTERGENIC)
2	140483258	140483265	140483260	T	G	snp	1	0	0	0	.	.	.	INTERGENIC(INTERGENIC)
2	140515125	140515132	140515131	T	G	snp	1	1	1	1	.	.	.	INTERGENIC(INTERGENIC)
2	140674840	140674855	140674852	AC	CTC	ins	1	1	0	1	.	.	.	INTERGENIC(INTERGENIC)
2	140700424	140700433	140700428	CT	T	snp	0	1	0	1	.	.	.	INTERGENIC(INTERGENIC)
2	140755921	140755930	140755926	TC	T	snp	1	1	1	1	.	.	.	INTERGENIC(INTERGENIC)
2	140755921	140755930	140755928	TC	T	snp	0	0	1	0	.	.	.	INTERGENIC(INTERGENIC)
2	140781911	140781920	140781916	AC	T	snp	1	0	1	0	.	.	.	INTERGENIC(INTERGENIC)

2	140790576	140790584	140790583	T	TA	ins	0	0	1	0	.	.	.	INTERGENIC(INTERGENIC)
2	140875527	140875534	140875531	A	G	snp	1	1	1	1	.	.	.	AC073928.2.1(INTRON)
2	140900521	140900528	140900525	A	G	snp	1	1	1	1	.	.	.	INTERGENIC(INTERGENIC)
2	140941962	140941971	140941968	GT	A	snp	1	1	1	1	.	.	.	INTERGENIC(INTERGENIC)
2	141097648	141097657	141097650	T	C	snp	1	0	0	0	.	.	.	LRP1B(INTRON)
2	141100574	141100581	141100580	T	G	ins	1	0	0	0	.	.	.	LRP1B(INTRON)
2	141290499	141290516	141290511	AT	C	snp	1	1	1	1	.	.	.	LRP1B(INTRON)
2	141368598	141368609	141368605	TTA	C	snp	1	0	0	0	.	.	.	LRP1B(INTRON)
2	141417712	141417720	141417715	A	C	ins	1	1	0	1	.	.	.	LRP1B(INTRON)
2	141489749	141489758	141489756	T	C	snp	0	1	1	0	.	.	.	LRP1B(INTRON)
2	141506001	141506010	141506008	TA	T	ins	1	1	0	1	.	.	.	LRP1B(INTRON)
2	141545789	141545806	141545792	CAA	T	snp	1	1	1	1	.	.	.	LRP1B(INTRON)
2	141553623	141553642	141553627	TATC	A	snp	1	1	1	0	.	.	.	LRP1B(INTRON)
2	141620030	141620041	141620038	ATT	G	snp	0	1	1	0	.	.	.	LRP1B(INTRON)
2	141848738	141848753	141848741	AG	T	snp	1	0	0	0	.	.	.	LRP1B(INTRON)
2	141861906	141861913	141861910	A	G	snp	1	1	1	1	.	.	.	LRP1B(INTRON)
2	141884119	141884126	141884120	T	C	snp	1	0	0	0	.	.	.	LRP1B(INTRON)
2	141892491	141892504	141892496	TA	G	snp	0	1	0	1	.	.	.	LRP1B(INTRON)
2	142065835	142065846	142065843	AAC	A	snp	1	0	0	0	.	.	.	LRP1B(INTRON)
2	142068168	142068181	142068173	TC	G	snp	1	1	1	1	.	.	.	LRP1B(INTRON)
2	142073349	142073357	142073353	T	G	snp	1	1	1	1	.	.	.	LRP1B(INTRON)
2	142221392	142221401	142221394	TA	G	snp	1	1	1	1	.	.	.	LRP1B(INTRON)
2	142231790	142231799	142231795	A	C	ins	1	1	1	1	.	.	.	LRP1B(INTRON)
2	142267134	142267157	142267147	CAAA	AAC	del	1	0	0	0	.	.	.	LRP1B(INTRON)
2	142267134	142267157	142267149	CAAA	C	del	1	0	0	0	.	.	.	LRP1B(INTRON)
2	142278297	142278304	142278298	A	C	snp	1	1	1	1	.	.	.	LRP1B(INTRON)
2	142282335	142282346	142282341	AG	G	snp	0	1	0	1	.	.	.	LRP1B(INTRON)
2	142378520	142378529	142378528	A	G	ins	1	1	1	1	.	.	.	LRP1B(INTRON)
2	142396563	142396580	142396566	AT	A	snp	1	1	1	1	.	.	.	LRP1B(INTRON)
2	142399550	142399573	142399564	AAAC	C	del	0	1	1	0	.	.	.	LRP1B(INTRON)
2	142470238	142470245	142470239	T	C	snp	1	1	1	1	.	.	.	LRP1B(INTRON)
2	142622454	142622481	142622477	AAAC	G	snp	1	1	1	1	.	.	.	LRP1B(INTRON)
2	142636381	142636389	142636383	T	C	snp	1	1	1	1	.	.	.	LRP1B(INTRON)
2	142706968	142706977	142706975	AT	C	snp	1	1	1	1	.	.	.	LRP1B(INTRON)
2	142723341	142723360	142723349	AATA	ATAC	ins	1	0	0	0	.	.	.	LRP1B(INTRON)
2	142763468	142763477	142763470	A	G	ins	0	0	1	0	.	.	.	LRP1B(INTRON)
2	142896475	142896492	142896484	AT	C	snp	1	1	1	1	.	.	.	AC078882.1.1(UPSTREAM);INTERGENIC(INTERGENIC)
2	143010168	143010182	143010173	AGC	A	snp	1	0	0	0	.	.	.	INTERGENIC(INTERGENIC)
2	143031824	143031831	143031829	T	C	ins	1	0	0	0	.	.	.	INTERGENIC(INTERGENIC)
2	143034450	143034457	143034456	G	T	snp	1	0	0	0	.	.	.	INTERGENIC(INTERGENIC)
2	143047073	143047088	143047084	AAAC	AAACA	del	1	0	0	0	.	.	.	INTERGENIC(INTERGENIC)
2	143169382	143169391	143169387	AT	C	snp	1	0	0	0	.	.	.	INTERGENIC(INTERGENIC)
2	143409842	143409859	143409853	TG	A	snp	1	0	0	0	.	.	.	INTERGENIC(INTERGENIC)
2	143450636	143450643	143450639	T	G	snp	1	0	0	0	.	.	.	INTERGENIC(INTERGENIC)
2	143450733	143450741	143450737	T	C	snp	1	0	0	0	.	.	.	INTERGENIC(INTERGENIC)
2	143462939	143462948	143462946	TG	A	snp	1	1	0	1	.	.	.	INTERGENIC(INTERGENIC)
2	143616007	143616016	143616015	A	G	snp	1	1	1	1	.	.	.	AC013444.1.1(DOWNSTREAM);INTERGENIC(INTERGENIC)
2	143760855	143760862	143760857	G	A	snp	1	0	0	0	.	.	.	AC013444.1(UPSTREAM);KYNU(INTRON)
2	143770822	143770835	143770830	TC	A	snp	1	1	0	1	.	.	.	KYNU(INTRON)
2	143829060	143829068	143829067	G	A	snp	1	0	0	0	.	.	.	INTERGENIC(INTERGENIC)
2	143853963	143853972	143853970	AT	C	snp	1	1	1	1	.	.	.	AC013437.1(DOWNSTREAM);AC013437.2(DOWNSTREAM);AC013437.2.1(UPSTREAM);AC013437.3(DOWNSTREAM);AC013437.4(DOWNSTREAM);AC013437.4.1(DOWNSTREAM);AC013437.5(DOWNSTREAM);AC013437.6(UPSTREAM);AC013437.6.1(DOWNSTREAM);AC013437.7.1(DOWNSTREAM);AC013437.8(DOWNSTREAM);AC013437.9(DOWNSTREAM);ARHGAP15(INTRON);MTND3P9(DOWNSTREAM);MTND4P22(DOWNSTREAM);MTND5P24(EXON);MTND6P11(DOWNSTREAM)
2	144049862	144049869	144049865	T	C	snp	1	0	0	0	.	.	.	AC096558.1.1(DOWNSTREAM);ARHGAP15(INTRON);RP11-570L15.2.1(DOWNSTREAM)
2	144218013	144218024	144218021	AT	G	snp	1	1	0	1	.	.	.	AC096558.1.1(INTRON);ARHGAP15(INTRON);RP11-570L15.2.1(INTRON)
2	144227722	144227729	144227728	A	C	ins	1	1	1	1	.	.	.	AC096558.1.1(INTRON);ARHGAP15(INTRON);RP11-570L15.2.1(INTRON)
2	144228825	144228834	144228832	AT	A	snp	0	0	1	0	.	.	.	AC096558.1.1(INTRON);ARHGAP15(INTRON);RP11-570L15.2.1(INTRON)
2	144257417	144257428	144257423	ATT	G	snp	1	1	1	1	.	.	.	ARHGAP15(INTRON);RP11-570L15.2.1(INTRON)
2	144430706	144430713	144430711	A	G	snp	0	1	1	1	.	.	.	ARHGAP15(INTRON);RP11-434H14.1.1(DOWNSTREAM)
2	144506977	144506986	144506983	TC	C	snp	1	0	0	0	.	.	.	ARHGAP15(INTRON)
2	144584543	144584552	144584550	TC	G	snp	1	0	0	0	.	.	.	INTERGENIC(INTERGENIC)
2	144607374	144607383	144607382	T	G	ins	1	0	0	0	.	.	.	INTERGENIC(INTERGENIC)

2	144607374	144607383	144607382	T	G	snp	1	0	0	0	.	.	.	INTERGENIC(INTERGENIC)
2	144627655	144627666	144627663	AAC	T	snp	1	1	1	1	.	.	.	INTERGENIC(INTERGENIC)
2	144890729	144890738	144890736	AT	C	snp	1	1	1	1	.	.	.	GTDC1(INTRON)
2	144940469	144940482	144940480	TA	T	del	1	0	0	0	.	.	.	GTDC1(INTRON);GTDC1(UPSTREAM)
2	145203004	145203013	145203011	TC	G	snp	1	0	0	0	.	.	.	ZEB2(INTRON)
2	145203029	145203040	145203034	CT	A	snp	1	0	0	0	.	.	.	ZEB2(INTRON)
2	145203029	145203040	145203035	CT	T	snp	1	0	0	0	.	.	.	ZEB2(INTRON)
2	145222133	145222140	145222136	T	C	snp	1	0	0	0	.	.	.	AC009951.1(UPSTREAM);ZEB2(INTRON)
2	145255424	145255437	145255430	AT	T	ins	1	1	1	1	.	.	.	ZEB2(INTRON)
2	145309358	145309367	145309362	CT	G	snp	0	0	0	1	.	.	.	INTERGENIC(INTERGENIC)
2	145817926	145817935	145817933	A	C	snp	1	1	0	1	.	.	.	AC074093.1.1(INTRON)
2	145841450	145841459	145841453	TA	TATATATGT	del	0	1	0	1	.	.	.	AC074093.1.1(INTRON)
2	146140778	146140793	146140782	TA	A	snp	1	0	0	0	.	.	.	INTERGENIC(INTERGENIC)
2	146140816	146140825	146140821	AT	A	snp	1	1	1	1	.	.	.	INTERGENIC(INTERGENIC)
2	146140849	146140858	146140854	AT	A	snp	1	1	1	1	.	.	.	INTERGENIC(INTERGENIC)
2	146171803	146171817	146171813	AGA	A	snp	1	1	1	1	.	.	.	INTERGENIC(INTERGENIC)
2	146268619	146268626	146268620	T	C	ins	1	0	0	0	.	.	.	INTERGENIC(INTERGENIC)
2	146357927	146357936	146357930	AT	ATATATA	del	1	1	1	0	.	.	.	AC079163.1.1(DOWNSTREAM);INTERGENIC(INTERGENIC)
2	146373810	146373818	146373817	A	G	snp	1	1	1	1	.	.	.	INTERGENIC(INTERGENIC)
2	146398293	146398313	146398308	ATT	T	snp	1	1	1	1	.	.	.	INTERGENIC(INTERGENIC)
2	146553539	146553546	146553543	A	G	snp	1	0	0	0	.	.	.	INTERGENIC(INTERGENIC)
2	146608024	146608033	146608028	A	C	snp	0	1	1	0	.	.	.	INTERGENIC(INTERGENIC)
2	146648812	146648825	146648814	TA	AC	ins	1	1	1	0	.	.	.	INTERGENIC(INTERGENIC)
2	146648868	146648877	146648873	TA	G	snp	1	0	0	0	.	.	.	INTERGENIC(INTERGENIC)
2	146648880	146648889	146648885	TA	G	snp	1	1	1	1	.	.	.	INTERGENIC(INTERGENIC)
2	146648928	146648937	146648933	TA	G	snp	1	0	0	0	.	.	.	INTERGENIC(INTERGENIC)
2	146682357	146682372	146682368	ATTT	A	del	1	1	1	1	.	.	.	AC062031.1.1(INTRON)
2	146747295	146747302	146747301	T	C	snp	1	1	1	1	.	.	.	INTERGENIC(INTERGENIC)
2	146803502	146803511	146803506	AT	TATG	ins	1	1	1	1	.	.	.	INTERGENIC(INTERGENIC)
2	146803502	146803511	146803508	AT	TG	ins	1	1	1	1	.	.	.	INTERGENIC(INTERGENIC)
2	146999112	146999119	146999114	T	G	snp	1	0	0	0	.	.	.	INTERGENIC(INTERGENIC)
2	147012570	147012585	147012576	GATA	C	snp	1	0	0	0	.	.	.	INTERGENIC(INTERGENIC)
2	147110519	147110534	147110523	AC	CACT	ins	1	1	1	1	.	.	.	INTERGENIC(INTERGENIC)
2	147145959	147145974	147145968	ATTT	TTA	del	1	1	1	1	.	.	.	INTERGENIC(INTERGENIC)
2	147145959	147145974	147145969	ATTT	TA	del	1	1	1	1	.	.	.	INTERGENIC(INTERGENIC)
2	147182182	147182189	147182184	A	G	snp	1	0	0	0	.	.	.	INTERGENIC(INTERGENIC)
2	147601564	147601571	147601570	A	G	snp	1	1	1	1	.	.	.	AC103881.1.1(INTRON)
2	147621311	147621318	147621315	A	G	snp	0	1	1	1	.	.	.	INTERGENIC(INTERGENIC)
2	147669049	147669064	147669058	GAAG	AGG	del	0	1	1	1	.	.	.	INTERGENIC(INTERGENIC)
2	147672481	147672498	147672487	GA	C	snp	0	1	0	1	.	.	.	INTERGENIC(INTERGENIC)
2	147760693	147760708	147760706	AT	A	del	1	0	0	0	.	.	.	INTERGENIC(INTERGENIC)
2	148112753	148112768	148112756	AT	C	snp	1	0	0	0	.	.	.	INTERGENIC(INTERGENIC)
2	148127679	148127686	148127683	A	G	snp	1	0	0	0	.	.	.	INTERGENIC(INTERGENIC)
2	148142929	148142940	148142936	TA	G	snp	1	0	0	0	.	.	.	INTERGENIC(INTERGENIC)
2	148433206	148433215	148433207	A	TC	ins	1	0	0	0	.	.	.	INTERGENIC(INTERGENIC)
2	148503622	148503629	148503623	A	G	snp	0	1	0	1	.	.	.	INTERGENIC(INTERGENIC)
2	148553478	148553493	148553487	TG	T	snp	0	1	0	1	.	.	.	INTERGENIC(INTERGENIC)
2	148825777	148825784	148825780	A	G	snp	1	1	1	1	.	.	.	MBD5(INTRON);MBD5(UPSTREAM)
2	148925351	148925360	148925358	T	G	snp	1	1	1	1	.	.	.	MBD5(INTRON)
2	148980226	148980239	148980230	TG	C	snp	1	1	1	1	.	.	.	MBD5(INTRON)
2	148996566	148996583	148996571	AT	C	snp	1	1	1	1	.	.	.	MBD5(INTRON)
2	149257825	149257834	149257828	TG	A	snp	1	0	0	0	.	.	.	MBD5(INTRON)
2	149392954	149392963	149392961	C	CA	ins	0	0	1	0	.	.	.	INTERGENIC(INTERGENIC)
2	149563261	149563284	149563270	CCT	T	snp	1	0	0	0	.	.	.	INTERGENIC(INTERGENIC)
2	149573509	149573516	149573514	C	A	snp	1	0	0	0	.	.	.	INTERGENIC(INTERGENIC)
2	149591548	149591555	149591551	T	C	snp	1	0	0	0	.	.	.	INTERGENIC(INTERGENIC)
2	149591664	149591673	149591667	TC	T	snp	1	1	1	1	.	.	.	INTERGENIC(INTERGENIC)
2	149611549	149611558	149611552	AT	A	snp	0	0	0	1	.	.	.	INTERGENIC(INTERGENIC)
2	149677668	149677677	149677671	T	C	snp	1	1	1	1	.	.	.	KIF5C(INTRON)
2	149817811	149817818	149817817	G	T	snp	1	0	0	0	.	.	.	KIF5C(INTRON)
2	149903897	149903906	149903900	A	C	snp	1	0	0	0	.	.	.	LYPD6B(INTRON)
2	149926454	149926467	149926462	GT	T	snp	1	1	1	1	.	.	.	AC073271.1.1(UPSTREAM);LYPD6B(INTRON)
2	149948705	149948712	149948711	G	T	snp	1	1	1	1	.	.	.	LYPD6B(INTRON)
2	150244681	150244694	150244690	TC	TCTCT	del	1	1	1	1	.	.	.	LYPD6(INTRON)
2	150272112	150272121	150272119	GA	G	snp	1	0	0	0	.	.	.	LYPD6(INTRON)
2	150425405	150425422	150425420	AT	C	snp	1	1	1	1	.	.	.	INTERGENIC(INTERGENIC);MMADHC(DOWNSTREAM)
2	150473185	150473194	150473193	T	C	snp	1	0	0	0	.	.	.	AC144449.1.1(INTRON)

2	150531534	150531549	150531537	TA	C	snp	1	1	1	0	.	.	.	AC144449.1.1(INTRON)
2	150585119	150585130	150585122	TAA	A	snp	1	1	1	1	.	.	.	AC144449.1.1(INTRON)
2	150753868	150753881	150753871	TA	C	snp	1	1	1	1	.	.	.	INTERGENIC(INTERGENIC)
2	150753868	150753881	150753875	TA	C	snp	0	0	1	0	.	.	.	INTERGENIC(INTERGENIC)
2	150878728	150878739	150878734	GT	C	snp	1	1	1	1	.	.	.	INTERGENIC(INTERGENIC)
2	150879415	150879428	150879418	TG	T	snp	0	0	0	1	.	.	.	INTERGENIC(INTERGENIC)
2	150881088	150881097	150881093	AT	A	snp	1	1	1	1	.	.	.	INTERGENIC(INTERGENIC)
2	150928991	150928998	150928992	A	C	snp	1	0	0	0	.	.	.	INTERGENIC(INTERGENIC)
2	151030440	151030447	151030444	A	G	snp	1	1	1	1	.	.	.	AC016682.1.1(INTRON)
2	151116325	151116332	151116331	T	C	snp	1	0	0	0	.	.	.	AC016682.1.1(INTRON);AC113610.1.1(UPSTREAM)
2	151146423	151146432	151146430	TA	G	snp	1	1	1	1	.	.	.	AC016682.1.1(INTRON)
2	151222753	151222780	151222769	GATA	ATAC	ins	1	1	1	1	.	.	.	INTERGENIC(INTERGENIC);TRNAE38P(DOWNSTREAM)
2	151270720	151270735	151270731	AAGG	A	snp	1	1	0	1	.	.	.	INTERGENIC(INTERGENIC)
2	151324744	151324752	151324747	A	G	ins	1	1	1	1	.	.	.	RND3(DOWNSTREAM);RND3(UTR_3_PRIME)
2	151352856	151352863	151352861	A	C	snp	1	1	1	1	.	.	.	RND3(INTRON)
2	151375999	151376010	151376002	CA	G	snp	1	0	0	0	.	.	.	RND3(INTRON)
2	151481486	151481494	151481490	T	G	snp	1	0	0	0	.	.	.	AC104777.1.1(UPSTREAM);AC104777.4.1(INTRON);AC104777.4.1(UPSTREAM)
2	151489757	151489764	151489760	T	G	ins	1	0	0	0	.	.	.	AC104777.4.1(INTRON)
2	151644706	151644714	151644711	T	G	snp	1	1	1	1	.	.	.	INTERGENIC(INTERGENIC)
2	151772214	151772225	151772223	AT	G	snp	0	1	1	1	.	.	.	INTERGENIC(INTERGENIC)
2	151805930	151805939	151805933	A	AC	ins	1	1	1	1	.	.	.	INTERGENIC(INTERGENIC)
2	151805930	151805939	151805934	A	C	ins	1	1	1	1	.	.	.	INTERGENIC(INTERGENIC)
2	151960871	151960888	151960874	TC	G	snp	1	1	1	1	.	.	.	INTERGENIC(INTERGENIC)
2	151988971	151988984	151988982	AG	A	snp	1	1	1	1	.	.	.	AC018731.3.1(INTRON)
2	152116153	152116160	152116157	A	G	snp	0	0	1	0	.	.	.	RBM43(DOWNSTREAM);RBM43(INTRON)
2	152491240	152491257	152491245	TTA	C	snp	1	0	0	0	.	.	.	NEB(INTRON)
2	152615427	152615442	152615438	TCCA	C	snp	0	0	1	0	.	.	.	INTERGENIC(INTERGENIC)
2	152647895	152647915	152647905	TTG	GTC	ins	1	0	1	0	.	.	.	ARL5A(INTRON)
2	152675974	152675983	152675981	T	C	snp	1	1	1	1	.	.	.	ARL5A(INTRON)
2	152776815	152776832	152776827	TTC	TCTTC	del	1	0	1	1	.	.	.	CACNB4(INTRON)
2	152781064	152781075	152781070	TA	C	snp	1	0	0	0	.	.	.	CACNB4(INTRON)
2	152906128	152906137	152906133	TC	T	snp	1	0	0	0	.	.	.	CACNB4(INTRON)
2	152998096	152998115	152998106	TTTG	C	snp	0	0	0	1	.	.	.	STAM2(DOWNSTREAM);STAM2(INTRON)
2	153024678	153024687	153024685	T	G	snp	1	0	0	0	.	.	.	STAM2(INTRON)
2	153029908	153029921	153029918	AT	T	ins	1	0	0	0	.	.	.	STAM2(INTRON)
2	153274342	153274353	153274349	TTA	A	snp	1	1	1	1	.	.	.	FMNL2(INTRON)
2	153303040	153303048	153303041	A	G	snp	1	1	1	1	.	.	.	FMNL2(INTRON)
2	153335623	153335631	153335629	T	G	snp	1	0	0	0	.	.	.	FMNL2(INTRON)
2	153371271	153371284	153371282	TC	G	snp	1	1	1	1	.	.	.	FMNL2(INTRON)
2	153397937	153397944	153397942	T	C	snp	1	1	1	1	.	.	.	FMNL2(INTRON)
2	153399491	153399498	153399497	A	C	snp	1	1	1	1	.	.	.	FMNL2(INTRON)
2	153441333	153441340	153441336	T	C	snp	1	1	1	1	.	.	.	FMNL2(INTRON)
2	153446531	153446540	153446535	AT	G	snp	0	1	1	1	.	.	.	FMNL2(INTRON)
2	153546796	153546809	153546799	AT	C	snp	1	0	0	0	.	.	.	PRPF40A(DOWNSTREAM);PRPF40A(INTRON)
2	153591842	153591849	153591845	T	G	ins	1	0	0	0	.	.	.	ARL6IP6(INTRON)
2	153704675	153704683	153704677	T	G	snp	1	1	1	1	.	.	.	AC009969.1.1(DOWNSTREAM);INTERGENIC(INTERGENIC)
2	153710699	153710708	153710703	TG	C	snp	1	1	1	1	.	.	.	AC009969.1.1(UPSTREAM);INTERGENIC(INTERGENIC)
2	153710699	153710708	153710704	TG	A	snp	1	1	1	1	.	.	.	AC009969.1.1(UPSTREAM);INTERGENIC(INTERGENIC)
2	153717072	153717095	153717083	CAA	G	snp	0	1	1	1	.	.	.	INTERGENIC(INTERGENIC)
2	153778240	153778248	153778241	A	C	ins	1	1	1	1	.	.	.	INTERGENIC(INTERGENIC)
2	153818032	153818041	153818040	T	C	snp	1	1	1	1	.	.	.	INTERGENIC(INTERGENIC)
2	153822550	153822557	153822552	T	G	snp	1	1	1	1	.	.	.	INTERGENIC(INTERGENIC)
2	153870792	153870800	153870795	A	AAAAAT	ins	1	1	1	1	.	.	.	INTERGENIC(INTERGENIC)
2	154067649	154067658	154067656	T	G	ins	0	0	1	0	.	.	.	AC079150.2.1(UPSTREAM);INTERGENIC(INTERGENIC)
2	154129019	154129026	154129022	A	G	snp	1	1	1	1	.	.	.	INTERGENIC(INTERGENIC)
2	154179853	154179861	154179854	T	G	ins	0	0	1	0	.	.	.	INTERGENIC(INTERGENIC)
2	154265179	154265188	154265184	T	G	snp	1	1	1	1	.	.	.	INTERGENIC(INTERGENIC)
2	154265813	154265824	154265821	TA	C	snp	1	1	0	0	.	.	.	INTERGENIC(INTERGENIC)
2	154267389	154267406	154267392	AG	T	snp	1	0	0	0	.	.	.	INTERGENIC(INTERGENIC)
2	154391026	154391041	154391031	TCTT	T	ins	1	1	1	1	.	.	.	AC012501.2.1(INTRON)
2	154391026	154391041	154391034	TCTT	C	del	1	1	1	1	.	.	.	AC012501.2.1(INTRON)
2	154391026	154391041	154391035	TCTT	T	snp	1	1	1	1	.	.	.	AC012501.2.1(INTRON)
2	154393691	154393700	154393697	GA	C	snp	1	0	0	0	.	.	.	AC012501.2.1(INTRON)
2	154398859	154398872	154398863	AC	CACACAC	del	1	1	1	1	.	.	.	AC012501.2.1(INTRON)
2	154464231	154464238	154464234	T	C	snp	1	1	1	1	.	.	.	INTERGENIC(INTERGENIC)
2	154605383	154605391	154605388	A	AAAG	ins	0	1	0	0	.	.	.	INTERGENIC(INTERGENIC)

2	154607878	154607887	154607882	T	G	snp	1	0	1	0	.	.	.	INTERGENIC(INTERGENIC)
2	154708221	154708232	154708226	AAC	G	snp	1	1	0	1	.	.	.	INTERGENIC(INTERGENIC)
2	154715069	154715080	154715078	GA	C	snp	1	0	0	0	.	.	.	INTERGENIC(INTERGENIC)
2	154738691	154738698	154738692	G	T	snp	1	1	1	1	.	.	.	GALNT13(INTRON)
2	154849837	154849848	154849840	AGT	GT	del	0	1	1	1	.	.	.	GALNT13(INTRON)
2	154871091	154871099	154871097	T	G	ins	1	1	1	1	.	.	.	GALNT13(INTRON)
2	154963528	154963535	154963531	T	G	snp	0	1	1	1	.	.	.	GALNT13(INTRON)
2	154976581	154976596	154976592	TTTG	T	snp	1	0	1	0	.	.	.	GALNT13(INTRON)
2	155228279	155228290	155228282	TG	T	ins	1	1	1	1	.	.	.	GALNT13(INTRON)
2	155297401	155297408	155297402	T	G	snp	0	1	1	1	.	.	.	AC009227.2.1(INTRON);GALNT13(DOWNSTREAM);GALNT13(INTRON);GALNT13(UPSTREAM)
2	155354989	155354996	155354991	T	C	snp	1	1	1	1	.	.	.	INTERGENIC(INTERGENIC)
2	155505484	155505493	155505485	T	G	snp	1	1	1	1	.	.	.	INTERGENIC(INTERGENIC)
2	155542864	155542873	155542868	CT	T	del	1	1	1	1	.	.	.	INTERGENIC(INTERGENIC)
2	155617239	155617246	155617244	T	C	snp	1	1	1	1	.	.	.	KCNJ3(INTRON)
2	155636034	155636043	155636037	TA	T	snp	1	1	1	1	.	.	.	KCNJ3(INTRON)
2	155775257	155775265	155775263	T	C	snp	1	0	0	0	.	.	.	INTERGENIC(INTERGENIC)
2	155833708	155833725	155833723	AT	A	del	1	1	1	1	.	.	.	INTERGENIC(INTERGENIC)
2	155934063	155934072	155934070	T	G	snp	1	1	1	1	.	.	.	INTERGENIC(INTERGENIC)
2	156026132	156026141	156026135	T	C	snp	1	1	1	1	.	.	.	INTERGENIC(INTERGENIC)
2	156034739	156034746	156034745	T	C	snp	1	0	0	0	.	.	.	INTERGENIC(INTERGENIC)
2	156157481	156157490	156157483	TA	C	snp	1	0	0	1	.	.	.	INTERGENIC(INTERGENIC)
2	156338183	156338190	156338188	T	G	snp	0	0	0	1	.	.	.	INTERGENIC(INTERGENIC)
2	156366358	156366366	156366363	A	C	snp	0	0	0	1	.	.	.	INTERGENIC(INTERGENIC)
2	156388823	156388836	156388826	AT	G	ins	1	1	1	1	.	.	.	INTERGENIC(INTERGENIC)
2	156527831	156527850	156527846	AAAG	T	snp	1	1	0	0	.	.	.	INTERGENIC(INTERGENIC)
2	156581785	156581793	156581786	T	G	snp	1	1	0	1	.	.	.	INTERGENIC(INTERGENIC)
2	156590450	156590469	156590457	AGAA	AGAG	ins	0	1	0	1	.	.	.	INTERGENIC(INTERGENIC)
2	156594208	156594217	156594212	TA	C	snp	0	1	0	1	.	.	.	INTERGENIC(INTERGENIC)
2	156677118	156677127	156677120	TA	C	snp	1	1	1	1	.	.	.	INTERGENIC(INTERGENIC)
2	156801893	156801904	156801896	TA	C	snp	1	1	0	1	.	.	.	INTERGENIC(INTERGENIC)
2	156801893	156801904	156801898	TA	C	snp	1	0	0	0	.	.	.	INTERGENIC(INTERGENIC)
2	156843848	156843859	156843856	TTC	TT	del	1	1	1	1	.	.	.	INTERGENIC(INTERGENIC)
2	157026355	157026362	157026357	T	C	ins	1	1	0	1	.	.	.	INTERGENIC(INTERGENIC)
2	157064045	157064056	157064051	AG	C	snp	1	1	1	1	.	.	.	INTERGENIC(INTERGENIC)
2	157340956	157340963	157340961	A	G	snp	1	1	1	1	.	.	.	GPD2(INTRON)
2	157359375	157359382	157359378	T	G	ins	1	1	1	1	.	.	.	GPD2(DOWNSTREAM);GPD2(INTRON)
2	157470261	157470270	157470262	T	C	snp	1	1	1	1	.	.	.	GPD2(DOWNSTREAM);INTERGENIC(INTERGENIC)
2	157477851	157477858	157477855	T	C	snp	1	0	0	0	.	.	.	INTERGENIC(INTERGENIC)
2	157513692	157513705	157513694	GT	T	snp	0	0	0	1	.	.	.	INTERGENIC(INTERGENIC)
2	157576002	157576010	157576004	A	C	snp	1	0	0	0	.	.	.	INTERGENIC(INTERGENIC)
2	157597358	157597365	157597364	T	C	snp	1	0	0	0	.	.	.	INTERGENIC(INTERGENIC)
2	157627200	157627223	157627219	AAAC	A	snp	1	0	1	0	.	.	.	INTERGENIC(INTERGENIC)
2	157627328	157627335	157627334	T	G	snp	1	1	1	1	.	.	.	INTERGENIC(INTERGENIC)
2	157639095	157639110	157639098	TA	TC	ins	1	0	0	0	.	.	.	AC021849.1.1(UPSTREAM);INTERGENIC(INTERGENIC)
2	157639095	157639110	157639100	TA	C	snp	1	0	0	0	.	.	.	AC021849.1.1(UPSTREAM);INTERGENIC(INTERGENIC)
2	157699510	157699521	157699518	AAT	C	snp	1	0	0	0	.	.	.	INTERGENIC(INTERGENIC)
2	157711292	157711305	157711298	AC	T	snp	1	0	0	0	.	.	.	INTERGENIC(INTERGENIC)
2	157919660	157919669	157919662	G	T	snp	1	1	1	1	.	.	.	INTERGENIC(INTERGENIC)
2	157948250	157948259	157948256	A	C	snp	1	1	1	1	.	.	.	INTERGENIC(INTERGENIC)
2	157953754	157953771	157953768	TA	A	del	1	1	0	1	.	.	.	INTERGENIC(INTERGENIC)
2	158059962	158059977	158059966	TTTA	TTA	del	0	0	1	0	.	.	.	INTERGENIC(INTERGENIC)
2	158075904	158075911	158075906	T	C	snp	1	1	1	1	.	.	.	INTERGENIC(INTERGENIC)
2	158148146	158148154	158148150	C	CCT	ins	1	0	0	0	.	.	.	GALNT5(INTRON)
2	158194561	158194572	158194569	ATA	C	snp	0	0	1	0	.	.	.	INTERGENIC(INTERGENIC)
2	158410794	158410809	158410796	AG	G	snp	1	0	0	0	.	.	.	ACVR1C(INTRON)
2	158503588	158503599	158503591	AC	A	snp	1	0	0	0	.	.	.	INTERGENIC(INTERGENIC)
2	158668300	158668320	158668317	CAA	C	del	1	0	0	0	.	.	.	ACVR1(INTRON);ACVR1(UPSTREAM)
2	158740976	158740991	158740979	AG	A	snp	1	1	0	1	.	.	.	INTERGENIC(INTERGENIC)
2	158756346	158756355	158756354	T	C	snp	1	0	0	0	.	.	.	INTERGENIC(INTERGENIC)
2	158771043	158771066	158771048	AAGG	G	del	1	0	0	0	.	.	.	INTERGENIC(INTERGENIC);U6(DOWNSTREAM)
2	158822713	158822720	158822718	C	A	snp	1	1	1	1	.	.	.	AC005539.2.1(UPSTREAM);INTERGENIC(INTERGENIC)
2	158856346	158856355	158856352	CA	T	snp	1	1	1	1	.	.	.	UPP2(INTRON)
2	158879743	158879751	158879745	G	T	snp	1	0	0	0	.	.	.	UPP2(INTRON)
2	158951079	158951086	158951083	A	G	snp	1	1	1	1	.	.	.	UPP2(INTRON)
2	158979867	158979875	158979870	T	C	ins	1	1	0	1	.	.	.	UPP2(INTRON);UPP2(UPSTREAM);UPP2-IT1(UPSTREAM)
2	158979867	158979875	158979871	T	C	ins	1	1	0	1	.	.	.	UPP2(INTRON);UPP2(UPSTREAM);UPP2-IT1(UPSTREAM)

2	158986286	158986295	158986289	T	G	snp	0	0	1	0	.	.	.	UPP2(INTRON);UPP2-IT1(INTRON)
2	159436357	159436368	159436361	AT	G	snp	0	1	1	1	.	.	.	PKP4(INTRON)
2	159436386	159436395	159436392	TA	G	snp	1	1	1	1	.	.	.	PKP4(INTRON)
2	159509759	159509766	159509760	T	G	ins	1	1	1	1	.	.	.	PKP4(INTRON)
2	159570516	159570523	159570518	T	C	ins	1	1	1	1	.	.	.	AC005042.4.1(INTRON);AC005042.5.1(INTRON)
2	159660638	159660647	159660645	AT	A	del	1	1	1	1	.	.	.	DAPL1(INTRON)
2	159689516	159689529	159689527	AT	A	del	1	1	0	1	.	.	.	DAPL1(INTRON)
2	159692769	159692778	159692774	A	G	snp	1	0	0	0	.	.	.	DAPL1(INTRON)
2	159732867	159732875	159732868	T	TTG	ins	1	1	0	1	.	.	.	AC064843.3.1(DOWNSTREAM);INTERGENIC(INTERGENIC)
2	159732867	159732875	159732871	T	TTTTTG	ins	1	1	1	1	.	.	.	AC064843.3.1(DOWNSTREAM);INTERGENIC(INTERGENIC)
2	159772183	159772198	159772193	AC	G	snp	1	1	1	1	.	.	.	INTERGENIC(INTERGENIC)
2	159951287	159951296	159951294	G	T	ins	1	1	1	1	.	.	.	TANC1(INTRON)
2	159951287	159951296	159951294	G	T	snp	1	1	1	1	.	.	.	TANC1(INTRON)
2	160038557	160038564	160038563	A	C	snp	1	0	0	0	.	.	.	TANC1(INTRON)
2	160048535	160048544	160048538	A	G	snp	1	0	1	0	.	.	.	TANC1(INTRON)
2	160054063	160054072	160054066	AC	T	snp	0	1	0	1	.	.	.	TANC1(INTRON);TANC1(UPSTREAM)
2	160072419	160072433	160072430	ATG	AT	del	1	1	1	1	.	.	.	TANC1(INTRON)
2	160118121	160118130	160118126	AC	G	snp	1	0	1	0	.	.	.	WDSUB1(INTRON)
2	160197752	160197767	160197759	AAAC	A	del	1	1	1	1	.	.	.	BAZ2B(INTRON)
2	160290663	160290686	160290683	ATT	A	del	0	1	1	1	.	.	.	BAZ2B(DOWNSTREAM);BAZ2B(EXON);BAZ2B(INTRON)
2	160366704	160366711	160366706	T	G	snp	1	1	1	1	.	.	.	BAZ2B(INTRON)
2	160367154	160367161	160367155	A	G	snp	1	1	1	1	.	.	.	BAZ2B(INTRON)
2	160373815	160373824	160373816	T	C	snp	0	1	0	1	.	.	.	BAZ2B(INTRON);BAZ2B(UPSTREAM)
2	160387478	160387497	160387482	AAAC	C	snp	0	1	0	1	.	.	.	BAZ2B(INTRON)
2	160430847	160430862	160430858	AAAC	A	ins	1	1	1	1	.	.	.	BAZ2B(INTRON)
2	160488400	160488411	160488409	TA	T	ins	1	0	0	0	.	.	.	INTERGENIC(INTERGENIC)
2	160526489	160526496	160526493	T	C	snp	1	0	0	0	.	.	.	AC009961.3.1(DOWNSTREAM);INTERGENIC(INTERGENIC)
2	160539480	160539489	160539486	T	G	snp	0	1	1	1	.	.	.	AC009961.3.1(INTRON)
2	160548668	160548677	160548675	T	C	snp	1	0	0	0	.	.	.	AC009961.2.1(UPSTREAM);AC009961.3.1(INTRON)
2	160571870	160571877	160571876	G	A	snp	1	0	0	0	.	.	.	AC009961.3.1(UPSTREAM);MARCH7(INTRON)
2	160588313	160588320	160588319	A	C	snp	1	1	1	1	.	.	.	MARCH7(INTRON);MARCH7(UPSTREAM)
2	160589183	160589194	160589185	CA	A	snp	1	1	1	1	.	.	.	MARCH7(INTRON);MARCH7(UPSTREAM)
2	160605854	160605862	160605860	T	G	snp	1	1	1	1	.	.	.	MARCH7(DOWNSTREAM);MARCH7(INTRON);MARCH7(UPSTREAM)
2	160605861	160605881	160605878	TTG	T	snp	1	1	1	1	.	.	.	MARCH7(DOWNSTREAM);MARCH7(INTRON);MARCH7(UPSTREAM)
2	160731225	160731234	160731232	AT	C	snp	1	1	0	1	.	.	.	LY75(INTRON);LY75-CD302(INTRON)
2	160781241	160781250	160781243	G	T	ins	1	0	0	0	.	.	.	INTERGENIC(INTERGENIC)
2	160872983	160872990	160872986	G	GA	ins	1	0	0	0	.	.	.	PLA2R1(INTRON)
2	160872997	160873010	160873006	GA	GG	ins	1	0	1	0	.	.	.	PLA2R1(INTRON)
2	160904504	160904519	160904513	TTGT	C	snp	0	1	1	1	.	.	.	PLA2R1(INTRON)
2	161114204	161114223	161114219	AAAC	A	ins	1	0	0	0	.	.	.	ITGB6(DOWNSTREAM);ITGB6(INTRON);ITGB6(UPSTREAM)
2	161178002	161178009	161178007	T	C	snp	0	0	0	1	.	.	.	RBMS1(INTRON);RBMS1(UPSTREAM)
2	161240709	161240728	161240718	AAAT	C	snp	0	1	0	1	.	.	.	RBMS1(INTRON)
2	161333870	161333878	161333872	A	C	snp	1	1	1	1	.	.	.	RBMS1(INTRON)
2	161366690	161366701	161366696	AGG	C	snp	1	1	1	1	.	.	.	INTERGENIC(INTERGENIC)
2	161449691	161449718	161449712	TTAT	A	del	0	0	1	1	.	.	.	INTERGENIC(INTERGENIC)
2	161471249	161471260	161471257	TG	G	snp	1	1	1	1	.	.	.	INTERGENIC(INTERGENIC)
2	161479869	161479878	161479872	T	C	ins	0	0	1	1	.	.	.	INTERGENIC(INTERGENIC)
2	161535470	161535478	161535477	T	TG	ins	1	1	1	1	.	.	.	INTERGENIC(INTERGENIC)
2	161600616	161600627	161600619	GTT	TT	del	1	0	0	0	.	.	.	INTERGENIC(INTERGENIC)
2	161703880	161703888	161703886	A	C	snp	1	1	1	1	.	.	.	INTERGENIC(INTERGENIC)
2	161969020	161969035	161969031	AAAT	C	snp	0	1	1	1	.	.	.	AC009313.1.1(INTRON)
2	162037951	162037959	162037953	A	C	snp	1	1	1	0	.	.	.	TANK(DOWNSTREAM);TANK(INTRON)
2	162095001	162095012	162095009	AAT	AATA	del	1	0	0	0	.	.	.	AC009299.2.1(INTRON);TANK(DOWNSTREAM)
2	162198374	162198381	162198380	A	AT	ins	1	1	0	1	.	.	.	AC009299.5.1(UPSTREAM);PSMD14(INTRON)
2	162249447	162249455	162249448	T	C	snp	1	1	1	1	.	.	.	PSMD14(INTRON)
2	162299705	162299714	162299712	T	G	snp	1	1	1	1	.	.	.	SLC4A10(INTRON)
2	162374311	162374324	162374319	AT	G	snp	0	1	1	1	.	.	.	SLC4A10(INTRON)
2	162398187	162398198	162398195	ATA	T	snp	1	0	0	0	.	.	.	SLC4A10(INTRON)
2	162450367	162450376	162450369	T	C	snp	1	1	1	1	.	.	.	SLC4A10(INTRON)
2	162583425	162583440	162583433	CAAA	G	snp	1	0	0	0	.	.	.	SLC4A10(INTRON)
2	162598930	162598949	162598945	TTTC	T	ins	1	0	0	0	.	.	.	SLC4A10(INTRON)
2	162742853	162742860	162742855	A	G	snp	1	1	0	1	.	.	.	SLC4A10(INTRON)
2	162917410	162917425	162917414	CCTC	A	snp	0	0	1	0	.	.	.	DPP4(INTRON)
2	162990686	162990693	162990692	A	G	snp	1	1	1	1	.	.	.	INTERGENIC(INTERGENIC)
2	163116727	163116746	163116734	CTAG	T	snp	1	0	0	0	.	.	.	INTERGENIC(INTERGENIC)

2	163278996	163279003	163279002	T	C	snp	0	0	1	0	.	.	.	KCNH7(DOWNSTREAM);KCNH7(INTRON)
2	163398759	163398776	163398766	TTA	G	snp	1	0	0	0	.	.	.	KCNH7(INTRON)
2	163398759	163398776	163398770	TTA	G	snp	1	0	1	0	.	.	.	KCNH7(INTRON)
2	163578753	163578764	163578758	AAG	C	snp	1	1	1	1	.	.	.	KCNH7(INTRON)
2	163579739	163579746	163579744	A	C	snp	0	0	1	0	.	.	.	KCNH7(INTRON)
2	163604970	163604978	163604977	T	G	snp	1	1	1	1	.	.	.	KCNH7(INTRON)
2	163618723	163618730	163618726	T	G	snp	1	0	0	0	.	.	.	KCNH7(INTRON)
2	163679749	163679757	163679753	A	G	ins	1	1	1	1	.	.	.	KCNH7(INTRON)
2	163790807	163790818	163790811	TC	C	snp	1	1	1	1	.	.	.	INTERGENIC(INTERGENIC)
2	163790828	163790839	163790837	CT	C	snp	0	0	1	0	.	.	.	INTERGENIC(INTERGENIC)
2	163819041	163819049	163819044	T	G	ins	1	1	1	1	.	.	.	INTERGENIC(INTERGENIC)
2	163871644	163871657	163871647	TG	T	snp	0	1	1	0	.	.	.	INTERGENIC(INTERGENIC)
2	163900102	163900113	163900110	TA	C	snp	0	1	1	0	.	.	.	INTERGENIC(INTERGENIC)
2	163900235	163900242	163900238	A	G	snp	1	0	0	0	.	.	.	INTERGENIC(INTERGENIC)
2	163948122	163948130	163948126	T	G	ins	1	0	0	0	.	.	.	INTERGENIC(INTERGENIC)
2	164022906	164022921	164022909	TG	A	snp	0	0	1	0	.	.	.	INTERGENIC(INTERGENIC)
2	164076307	164076318	164076315	CA	A	ins	0	0	1	0	.	.	.	INTERGENIC(INTERGENIC)
2	164076307	164076318	164076316	CA	C	del	0	1	1	0	.	.	.	INTERGENIC(INTERGENIC)
2	164076318	164076326	164076322	A	G	snp	1	0	0	0	.	.	.	INTERGENIC(INTERGENIC)
2	164112113	164112124	164112117	TG	C	snp	1	0	0	0	.	.	.	INTERGENIC(INTERGENIC)
2	164126497	164126511	164126508	AAC	A	snp	1	1	0	1	.	.	.	INTERGENIC(INTERGENIC)
2	164243769	164243778	164243776	CA	G	snp	1	1	1	1	.	.	.	INTERGENIC(INTERGENIC)
2	164257338	164257347	164257342	TC	G	snp	0	0	1	0	.	.	.	INTERGENIC(INTERGENIC)
2	164296429	164296438	164296430	T	C	ins	0	0	1	0	.	.	.	INTERGENIC(INTERGENIC)
2	164297200	164297208	164297207	G	A	ins	1	0	1	0	.	.	.	INTERGENIC(INTERGENIC)
2	164366142	164366151	164366149	AC	T	snp	1	0	0	0	.	.	.	INTERGENIC(INTERGENIC)
2	164376642	164376649	164376646	T	C	snp	1	1	1	1	.	.	.	INTERGENIC(INTERGENIC)
2	164424344	164424363	164424357	AAAG	AG	ins	1	1	1	1	.	.	.	INTERGENIC(INTERGENIC)
2	164581319	164581328	164581321	AT	T	snp	1	0	1	0	.	.	.	FIGN(INTRON)
2	164611134	164611145	164611137	ACA	G	snp	1	1	1	1	.	.	.	AC092684.1.1(INTRON)
2	165034336	165034345	165034340	AG	GAGAG	del	1	0	0	0	.	.	.	AC092684.1.1(INTRON)
2	165043821	165043830	165043823	TG	C	snp	0	1	0	1	.	.	.	AC092684.1.1(INTRON)
2	165105766	165105779	165105777	TA	T	snp	1	1	0	1	.	.	.	AC092684.1.1(INTRON)
2	165112998	165113006	165113004	A	C	snp	1	1	1	1	.	.	.	AC092684.1.1(INTRON)
2	165168148	165168157	165168149	T	C	snp	1	1	1	1	.	.	.	AC092684.1.1(INTRON)
2	165226799	165226806	165226803	A	G	snp	1	1	0	0	.	.	.	INTERGENIC(INTERGENIC)
2	165265121	165265136	165265127	AATA	A	snp	1	1	1	1	.	.	.	INTERGENIC(INTERGENIC)
2	165281637	165281645	165281639	T	C	snp	1	0	0	0	.	.	.	INTERGENIC(INTERGENIC)
2	165438913	165438932	165438928	TTAT	TTA	del	1	1	1	1	.	.	.	GRB14(INTRON)
2	165448634	165448643	165448639	T	G	snp	0	0	1	0	.	.	.	GRB14(INTRON)
2	165460182	165460193	165460186	AT	T	del	1	1	1	1	.	.	.	GRB14(INTRON)
2	165496091	165496111	165496105	TTA	T	ins	1	1	0	1	.	.	.	INTERGENIC(INTERGENIC)
2	165496091	165496111	165496107	TTA	A	del	1	1	0	1	.	.	.	INTERGENIC(INTERGENIC)
2	165496091	165496111	165496108	TTA	T	snp	1	1	0	1	.	.	.	INTERGENIC(INTERGENIC)
2	165586011	165586019	165586015	A	C	snp	1	1	1	1	.	.	.	COBLL1(DOWNSTREAM);COBLL1(INTRON);COBLL1(UPSTREAM)
2	165626300	165626307	165626304	C	A	snp	1	1	1	1	.	.	.	COBLL1(INTRON)
2	165834696	165834711	165834706	AATA	AAATA	del	1	0	0	0	.	.	.	INTERGENIC(INTERGENIC)
2	165883762	165883769	165883767	A	G	snp	1	1	1	1	.	.	.	AC013463.2.1(INTRON)
2	165978090	165978097	165978091	T	C	snp	1	1	1	1	.	.	.	SCN3A(DOWNSTREAM);SCN3A(INTRON)
2	166009436	166009447	166009442	AT	T	snp	1	1	1	1	.	.	.	SCN3A(INTRON)
2	166047097	166047108	166047101	TA	G	snp	1	1	1	1	.	.	.	SCN3A(INTRON)
2	166101002	166101013	166101005	TAA	C	snp	1	1	0	1	.	.	.	SCN2A(INTRON)
2	166106379	166106386	166106383	A	G	snp	1	0	0	0	.	.	.	SCN2A(INTRON)
2	166139417	166139426	166139419	GA	C	snp	0	0	1	0	.	.	.	MAPRE1P3(UPSTREAM);SCN2A(INTRON)
2	166242991	166242998	166242997	A	G	snp	1	0	0	0	.	.	.	SCN2A(INTRON)
2	166295835	166295843	166295842	A	G	snp	0	0	1	0	.	.	.	INTERGENIC(INTERGENIC)
2	166318572	166318579	166318574	T	C	snp	1	1	1	1	.	.	.	INTERGENIC(INTERGENIC)
2	166334531	166334540	166334537	AG	G	snp	1	1	1	1	.	.	.	CSRNP3(INTRON)
2	166377201	166377220	166377210	TATC	G	snp	1	0	0	0	.	.	.	CSRNP3(INTRON)
2	166377281	166377292	166377288	TA	TATAT	del	1	1	1	1	.	.	.	CSRNP3(INTRON)
2	166702903	166702912	166702906	TA	G	snp	0	1	1	0	.	.	.	AC009495.4.1(UPSTREAM);INTERGENIC(INTERGENIC)
2	166768166	166768179	166768174	AT	T	ins	1	1	0	1	.	.	.	TTC21B(INTRON);TTC21B(UPSTREAM)
2	166768166	166768179	166768176	AT	T	snp	1	1	1	1	.	.	.	TTC21B(INTRON);TTC21B(UPSTREAM)
2	166768166	166768179	166768177	AT	A	del	1	1	1	1	.	.	.	TTC21B(INTRON);TTC21B(UPSTREAM)
2	166783731	166783746	166783733	TA	C	snp	1	1	1	1	.	.	.	TTC21B(DOWNSTREAM);TTC21B(INTRON)
2	166783781	166783794	166783783	TA	C	snp	1	1	1	1	.	.	.	TTC21B(DOWNSTREAM);TTC21B(INTRON)

2	166783868	166783885	166783873	TA	T	del	1	1	1	1	.	.	.	TTC21B(DOWNSTREAM);TTC21B(INTRON)
2	166851161	166851188	166851180	AAAC	T	snp	0	1	1	0	.	.	.	SCN1A(DOWNSTREAM);SCN1A(INTRON)
2	166868773	166868782	166868775	AT	G	snp	1	0	0	0	.	.	"SCN1A(SYNONYMOUS_CODING):Y1213,Y1230,Y1241"	.
2	167007109	167007117	167007115	A	G	snp	0	1	1	0	.	.	.	AC010127.3.1(INTRON)
2	167032646	167032653	167032651	A	C	snp	0	1	1	0	.	.	.	7SK(DOWNSTREAM);AC010127.3.1(INTRON)
2	167058140	167058149	167058145	TA	G	snp	0	1	0	1	.	.	.	AC010127.3.1(INTRON);SCN9A(INTRON)
2	167205637	167205664	167205659	AAAC	C	del	1	0	0	0	.	.	.	SCN9A(INTRON)
2	167301886	167301901	167301895	GGGA	A	snp	1	0	0	0	.	.	.	SCN7A(INTRON)
2	167302096	167302109	167302107	TC	TCT	del	1	1	0	1	.	.	.	SCN7A(INTRON)
2	167409927	167409938	167409933	TTG	C	snp	1	1	1	1	.	.	.	INTERGENIC(INTERGENIC)
2	167420725	167420736	167420727	TG	C	snp	0	0	1	0	.	.	.	INTERGENIC(INTERGENIC)
2	167423325	167423334	167423327	A	C	snp	1	0	0	0	.	.	.	INTERGENIC(INTERGENIC)
2	167445737	167445746	167445741	AT	G	snp	1	0	0	0	.	.	.	INTERGENIC(INTERGENIC)
2	167594610	167594619	167594614	T	C	ins	1	1	1	1	.	.	.	INTERGENIC(INTERGENIC)
2	167671319	167671326	167671324	A	C	snp	0	1	0	1	.	.	.	INTERGENIC(INTERGENIC)
2	167681240	167681251	167681243	AT	A	snp	1	0	0	0	.	.	.	INTERGENIC(INTERGENIC)
2	167956692	167956700	167956693	A	C	snp	1	0	0	0	.	.	.	XIRP2(INTRON)
2	167969118	167969127	167969125	TC	A	snp	1	1	1	1	.	.	.	XIRP2(INTRON)
2	167988503	167988512	167988507	CT	G	snp	1	0	0	0	.	.	.	XIRP2(INTRON);XIRP2-AS1(INTRON)
2	167990802	167990811	167990806	TA	A	snp	1	1	1	1	.	.	.	XIRP2(INTRON);XIRP2-AS1(INTRON)
2	168080929	168080938	168080934	T	G	snp	1	0	0	0	.	.	.	XIRP2(INTRON)
2	168188591	168188600	168188595	CT	G	snp	1	1	1	1	.	.	.	AC074363.1.1(INTRON)
2	168203256	168203265	168203258	G	T	ins	1	1	1	1	.	.	.	AC074363.1.1(INTRON)
2	168235912	168235923	168235918	TAT	C	snp	1	0	0	0	.	.	.	AC074363.1.1(INTRON)
2	168374547	168374554	168374549	A	C	snp	1	1	1	1	.	.	.	AC074363.1.1(INTRON)
2	168381781	168381788	168381784	T	C	snp	0	1	1	0	.	.	.	AC074363.1.1(INTRON)
2	168423007	168423024	168423011	ATA	C	snp	1	0	1	0	.	.	.	INTERGENIC(INTERGENIC)
2	168423062	168423070	168423063	A	G	snp	1	0	0	0	.	.	.	INTERGENIC(INTERGENIC)
2	168439847	168439854	168439849	A	G	snp	1	0	0	0	.	.	.	INTERGENIC(INTERGENIC)
2	168506685	168506692	168506686	T	G	snp	1	1	1	1	.	.	.	INTERGENIC(INTERGENIC)
2	168542171	168542180	168542174	AG	A	snp	0	1	0	1	.	.	.	INTERGENIC(INTERGENIC)
2	168542171	168542180	168542178	AG	C	snp	0	1	1	1	.	.	.	INTERGENIC(INTERGENIC)
2	168669507	168669515	168669511	C	A	ins	1	1	1	1	.	.	.	AC016723.4.1(DOWNSTREAM);INTERGENIC(INTERGENIC)
2	168669507	168669515	168669514	C	A	ins	1	1	1	1	.	.	.	AC016723.4.1(DOWNSTREAM);INTERGENIC(INTERGENIC)
2	168716001	168716010	168716005	CA	T	snp	0	0	0	1	.	.	.	AC016723.4.1(INTRON);B3GALT1(INTRON)
2	168875797	168875808	168875803	GA	C	snp	1	1	1	0	.	.	.	STK39(INTRON)
2	168922551	168922558	168922553	A	C	snp	1	0	0	0	.	.	.	STK39(INTRON)
2	169012072	169012079	169012073	C	T	ins	1	1	1	1	.	.	.	STK39(INTRON)
2	169012072	169012079	169012074	C	A	snp	1	0	1	0	.	.	.	STK39(INTRON)
2	169070767	169070776	169070768	A	C	ins	1	0	1	1	.	.	.	STK39(INTRON)
2	169188380	169188387	169188382	A	G	snp	1	1	1	1	.	.	.	INTERGENIC(INTERGENIC)
2	169284335	169284342	169284340	A	G	snp	1	1	1	1	.	.	.	INTERGENIC(INTERGENIC)
2	169307387	169307394	169307391	A	G	snp	0	1	1	1	.	.	.	AC019086.2(UPSTREAM);CERS6(UPSTREAM);INTERGENIC(INTERGENIC)
2	169532122	169532139	169532129	ACA	G	snp	0	0	0	1	.	.	.	CERS6(INTRON)
2	169599658	169599673	169599670	AT	G	snp	1	1	0	0	.	.	.	CERS6(INTRON)
2	169604349	169604366	169604362	AC	T	snp	1	0	0	0	.	.	.	CERS6(INTRON)
2	169617249	169617276	169617271	TTTA	ATTTA	del	0	1	1	1	.	.	.	CERS6(INTRON)
2	169727860	169727871	169727863	ATA	G	snp	1	1	0	1	.	.	.	SPC25(INTRON);SPC25(UTR_3_PRIME)
2	169728495	169728503	169728497	T	G	ins	0	0	0	1	.	.	.	SPC25(INTRON)
2	169745274	169745285	169745278	TA	C	snp	1	0	0	0	.	.	.	SPC25(INTRON)
2	169806733	169806742	169806738	TG	A	snp	1	1	1	1	.	.	.	ABCB11(INTRON)
2	169853320	169853329	169853323	AT	A	snp	1	0	0	0	.	.	.	ABCB11(INTRON)
2	169894797	169894810	169894801	CA	T	snp	1	0	1	0	.	.	.	INTERGENIC(INTERGENIC)
2	169902305	169902313	169902310	A	G	snp	1	0	1	0	.	.	.	INTERGENIC(INTERGENIC)
2	169902916	169902925	169902917	A	C	snp	1	1	1	1	.	.	.	INTERGENIC(INTERGENIC)
2	170072510	170072518	170072515	A	C	snp	1	1	1	1	.	.	.	LRP2(INTRON)
2	170162680	170162691	170162682	AT	G	snp	1	1	1	1	.	.	.	LRP2(INTRON)
2	170240678	170240687	170240683	A	G	snp	1	0	0	0	.	.	.	INTERGENIC(INTERGENIC)
2	170294989	170294998	170294994	A	C	snp	1	0	0	0	.	.	.	INTERGENIC(INTERGENIC)
2	170324622	170324631	170324625	TA	T	snp	1	1	1	1	.	.	.	INTERGENIC(INTERGENIC)
2	170391982	170391997	170391989	TTTA	G	snp	1	0	0	0	.	.	.	FASTKD1(DOWNSTREAM);FASTKD1(INTRON);FASTKD1(UPSTREAM)
2	170509601	170509612	170509608	TA	C	snp	0	0	0	1	.	.	.	C2orf77(INTRON);PPIG(DOWNSTREAM)
2	170517099	170517114	170517102	TA	G	snp	0	0	1	0	.	.	.	AC016772.4.1(INTRON);C2orf77(DOWNSTREAM);C2orf77(INTRON)
2	170517099	170517114	170517112	TA	TAT	del	1	1	1	1	.	.	.	

														AC016772.4.1(INTRON);C2orf77(DOWNSTREAM);C2orf77(INTRON)
2	170611380	170611391	170611388	GCT	C	snp	1	1	1	1	.	.	.	KLHL23(DOWNSTREAM);KLHL23(INTRON)
2	170633709	170633724	170633721	AT	T	snp	0	1	1	1	.	.	.	INTERGENIC(INTERGENIC);KLHL23(DOWNSTREAM)
2	170671388	170671397	170671396	T	C	snp	0	0	0	1	.	.	.	METTL5(INTRON);METTL5(UPSTREAM);SSB(DOWNSTREAM);U3(UPSTREAM)
2	170791564	170791575	170791570	ACA	G	snp	1	1	1	1	.	.	.	UBR3(INTRON)
2	170823776	170823784	170823779	C	T	snp	1	1	1	1	.	.	.	UBR3(INTRON)
2	170823776	170823784	170823783	C	A	snp	1	0	0	0	.	.	.	UBR3(INTRON)
2	170856664	170856673	170856668	A	C	snp	1	0	0	0	.	.	.	UBR3(INTRON);UBR3(UPSTREAM)
2	170892421	170892430	170892428	G	GA	ins	0	0	1	0	.	.	.	UBR3(INTRON)
2	170892421	170892430	170892429	G	A	ins	1	0	0	0	.	.	.	UBR3(INTRON)
2	170893979	170893990	170893984	CCT	A	snp	1	1	1	1	.	.	.	UBR3(INTRON)
2	170945070	170945077	170945072	G	T	snp	1	0	1	1	.	.	.	INTERGENIC(INTERGENIC);UBR3(DOWNSTREAM)
2	171017472	171017480	171017478	T	C	snp	1	0	1	0	.	.	.	INTERGENIC(INTERGENIC)
2	171086516	171086523	171086520	A	G	snp	1	1	1	1	.	.	.	MYO3B(INTRON)
2	171136821	171136829	171136824	T	G	snp	1	1	1	1	.	.	.	MYO3B(INTRON)
2	171150489	171150496	171150494	T	TG	ins	1	1	1	1	.	.	.	MYO3B(INTRON)
2	171204739	171204762	171204750	TGT	C	snp	1	1	1	1	.	.	.	AC012594.1.1(INTRON);AC068280.1.1(UPSTREAM);MYO3B(INTRON)
2	171456632	171456643	171456639	TTG	C	snp	1	1	0	1	.	.	.	HMGB1P4(DOWNSTREAM);MYO3B(INTRON)
2	171520631	171520650	171520637	TGTT	C	snp	1	1	1	1	.	.	.	AC007277.3.1(INTRON)
2	171528290	171528305	171528301	AT	A	snp	0	1	0	0	.	.	.	AC007277.3.1(INTRON)
2	171544636	171544649	171544639	TG	T	snp	0	1	1	1	.	.	.	AC007277.3.1(INTRON)
2	171591700	171591717	171591712	AT	G	snp	1	1	1	1	.	.	.	AC007405.4.1(INTRON)
2	171645442	171645453	171645450	TTC	TTCT	del	1	1	1	1	.	.	.	AC007405.7.1(INTRON)
2	171692993	171693002	171692995	TA	C	snp	1	0	0	0	.	.	.	GAD1(INTRON)
2	171783467	171783484	171783482	AG	A	snp	1	1	1	1	.	.	.	GORASP2(UPSTREAM);INTERGENIC(INTERGENIC)
2	171833054	171833069	171833065	ATAC	T	snp	1	1	1	1	.	.	.	INTERGENIC(INTERGENIC)
2	171964568	171964577	171964575	A	C	snp	1	1	1	1	.	.	.	TLK1(INTRON)
2	172074807	172074816	172074809	A	C	snp	1	1	1	1	.	.	.	TLK1(INTRON)
2	172161663	172161672	172161664	A	AC	ins	1	1	1	1	.	.	.	INTERGENIC(INTERGENIC)
2	172393379	172393388	172393381	T	G	snp	1	1	1	1	.	.	.	CYBRD1(INTRON)
2	172406617	172406628	172406622	TTG	A	snp	1	0	0	0	.	.	.	CYBRD1(INTRON)
2	172408457	172408465	172408460	T	C	snp	1	1	1	1	.	.	.	CYBRD1(DOWNSTREAM);CYBRD1(INTRON)
2	172415020	172415029	172415025	TA	T	snp	0	1	0	1	.	.	.	CYBRD1(DOWNSTREAM);INTERGENIC(INTERGENIC)
2	172447118	172447131	172447123	CT	C	snp	0	0	1	0	.	.	.	AC064826.2.1(DOWNSTREAM);INTERGENIC(INTERGENIC)
2	172474314	172474325	172474321	ATA	AATA	del	1	0	0	0	.	.	.	INTERGENIC(INTERGENIC)
2	172753804	172753813	172753812	T	C	ins	0	1	0	0	.	.	.	SLC25A12(INTRON);SLC25A12(UPSTREAM)
2	172754681	172754696	172754689	TTTC	C	snp	1	0	0	0	.	.	.	SLC25A12(INTRON);SLC25A12(UPSTREAM)
2	172893082	172893091	172893089	AG	A	del	0	0	0	1	.	.	.	METAP1D(INTRON)
2	172894982	172895001	172894993	TTTA	T	ins	0	0	0	1	.	.	.	METAP1D(INTRON)
2	172894982	172895001	172894997	TTTA	T	snp	1	1	1	1	.	.	.	METAP1D(INTRON)
2	172900051	172900060	172900056	T	TTG	ins	1	0	0	0	.	.	.	METAP1D(INTRON)
2	172909622	172909641	172909628	AAAT	T	del	0	0	0	1	.	.	.	METAP1D(INTRON)
2	172943884	172943893	172943886	T	C	snp	0	1	0	1	.	.	.	METAP1D(INTRON)
2	172962311	172962320	172962314	A	C	snp	1	1	1	1	.	.	.	DLX2(DOWNSTREAM);INTERGENIC(INTERGENIC)
2	173034730	173034738	173034736	A	C	snp	1	1	1	1	.	.	.	INTERGENIC(INTERGENIC)
2	173138568	173138577	173138572	AT	G	snp	0	0	1	0	.	.	.	INTERGENIC(INTERGENIC)
2	173152047	173152062	173152055	ATAA	G	snp	1	0	0	0	.	.	.	INTERGENIC(INTERGENIC)
2	173175545	173175554	173175553	A	C	ins	1	0	0	0	.	.	.	AC104088.1.1(UPSTREAM);INTERGENIC(INTERGENIC)
2	173192134	173192157	173192152	AAT	G	snp	1	1	0	1	.	.	.	AC104088.1.1(DOWNSTREAM);INTERGENIC(INTERGENIC)
2	173197242	173197257	173197253	AC	GCACGCATGCAT	ins	1	1	1	1	.	.	.	INTERGENIC(INTERGENIC)
2	173197861	173197870	173197867	AC	C	snp	1	1	1	1	.	.	.	INTERGENIC(INTERGENIC)
2	173250983	173250990	173250988	A	C	snp	1	0	0	1	.	.	.	INTERGENIC(INTERGENIC)
2	173340192	173340199	173340198	A	C	snp	0	1	1	1	.	.	.	ITGA6(DOWNSTREAM);ITGA6(INTRON)
2	173371275	173371282	173371278	A	G	snp	0	1	1	0	.	.	.	AC093818.1.1(DOWNSTREAM);AC093818.1.1(INTRON);ITGA6(DOWNSTREAM)
2	173446632	173446639	173446638	T	G	snp	1	1	0	1	.	.	.	PDK1(INTRON)
2	173501644	173501651	173501647	A	C	snp	1	1	1	1	.	.	.	INTERGENIC(INTERGENIC)
2	173505731	173505746	173505742	TTTC	TT	del	1	1	1	1	.	.	.	INTERGENIC(INTERGENIC)
2	173511234	173511242	173511235	A	G	snp	1	0	0	0	.	.	.	INTERGENIC(INTERGENIC)
2	173533669	173533678	173533673	AG	G	snp	0	1	1	0	.	.	.	INTERGENIC(INTERGENIC)
2	173542898	173542905	173542899	A	G	snp	1	1	1	1	.	.	.	AC018712.2.1(UPSTREAM);AC018712.3.1(INTRON)
2	173573775	173573782	173573776	T	C	snp	0	1	1	1	.	.	.	AC018712.3.1(INTRON)
2	173842927	173842934	173842932	C	T	snp	1	0	0	0	.	.	.	RAPGEF4(INTRON)
2	174081239	174081248	174081240	T	C	ins	1	0	0	0	.	.	.	AC013461.1.1(DOWNSTREAM);AC013461.1.1(INTRON);MLK7-AS1(INTRON)

2	174129768	174129782	174129772	ACA	T	snp	1	1	1	1	.	.	.	AC013461.1.1(INTRON);MLK7-AS1(INTRON)
2	174227478	174227486	174227480	T	G	snp	1	0	0	0	.	.	.	CDCA7(INTRON)
2	174263557	174263580	174263564	AAAT	A	snp	1	1	1	1	.	.	.	INTERGENIC(INTERGENIC)
2	174263557	174263580	174263568	AAAT	A	snp	1	1	1	1	.	.	.	INTERGENIC(INTERGENIC)
2	174308590	174308597	174308594	T	G	snp	1	1	1	1	.	.	.	INTERGENIC(INTERGENIC)
2	174355574	174355597	174355592	CAA	AACAA	del	0	0	1	0	.	.	.	AC073465.3.1(UPSTREAM);INTERGENIC(INTERGENIC)
2	174432503	174432514	174432506	CTC	A	snp	1	1	1	1	.	.	.	INTERGENIC(INTERGENIC)
2	174432503	174432514	174432508	CTC	A	snp	1	0	0	0	.	.	.	INTERGENIC(INTERGENIC)
2	174455828	174455839	174455831	TA	G	snp	1	1	1	1	.	.	.	INTERGENIC(INTERGENIC)
2	174530965	174530972	174530968	T	C	snp	1	0	0	0	.	.	.	INTERGENIC(INTERGENIC)
2	174596054	174596063	174596056	TG	A	snp	1	0	0	0	.	.	.	INTERGENIC(INTERGENIC)
2	174600951	174600974	174600957	AAAC	G	snp	1	0	0	0	.	.	.	INTERGENIC(INTERGENIC)
2	174713799	174713810	174713807	AG	G	snp	1	1	0	0	.	.	.	INTERGENIC(INTERGENIC)
2	174747899	174747910	174747907	GA	T	snp	1	1	1	1	.	.	.	AC106900.6.1(INTRON)
2	174807638	174807647	174807645	TA	G	snp	1	1	0	0	.	.	.	SP3(INTRON)
2	174839417	174839424	174839420	T	C	snp	1	1	0	1	.	.	.	INTERGENIC(INTERGENIC)
2	175071117	175071125	175071120	A	C	snp	1	1	1	1	.	.	.	OLA1(INTRON)
2	175118365	175118380	175118375	AAAC	G	snp	1	1	1	1	.	.	.	INTERGENIC(INTERGENIC);OLA1(UPSTREAM)
2	175199472	175199481	175199475	C	CCA	ins	1	1	0	1	.	.	.	AC018470.4.1(DOWNSTREAM);INTERGENIC(INTERGENIC);SP9(UPSTREAM)
2	175265664	175265671	175265666	A	G	snp	1	1	1	1	.	.	.	SCRN3(DOWNSTREAM);SCRN3(INTRON)
2	175354563	175354572	175354571	A	G	snp	1	0	0	0	.	.	.	AC010894.3.1(DOWNSTREAM);GPR155(UPSTREAM);INTERGENIC(INTERGENIC)
2	175457752	175457759	175457757	T	C	snp	1	0	0	0	.	.	.	AC018890.6.1(INTRON);WIPF1(INTRON)
2	175470165	175470184	175470176	CAAA	G	snp	1	0	0	0	.	.	.	AC018890.6.1(INTRON);WIPF1(INTRON)
2	175571088	175571096	175571091	C	T	ins	1	1	1	1	.	.	.	AC018890.6.1(INTRON)
2	175671163	175671170	175671164	A	G	snp	1	0	0	0	.	.	.	CHN1(DOWNSTREAM);CHN1(INTRON);CHN1(UPSTREAM)
2	175704685	175704693	175704686	A	C	snp	0	1	0	1	.	.	.	CHN1(INTRON)
2	175772387	175772396	175772391	A	G	snp	0	1	0	1	.	.	.	CHN1(INTRON)
2	175819824	175819833	175819831	TA	C	snp	1	0	0	0	.	.	.	CHN1(INTRON)
2	175819913	175819928	175819917	TA	AG	ins	1	1	1	1	.	.	.	CHN1(INTRON)
2	175819913	175819928	175819924	TA	TATAATTGATATATATATATATC	del	1	1	1	1	.	.	.	CHN1(INTRON)
2	175880885	175880894	175880889	TA	C	snp	1	1	1	1	.	.	.	INTERGENIC(INTERGENIC)
2	175971265	175971274	175971266	A	C	ins	1	1	1	1	.	.	.	ATF2(INTRON)
2	175971265	175971274	175971267	A	C	snp	1	1	1	1	.	.	.	ATF2(INTRON)
2	175999835	175999846	175999839	GGA	A	snp	0	1	0	0	.	.	.	ATF2(INTRON)
2	176302636	176302655	176302651	CTTT	C	del	0	1	0	1	.	.	.	AC096649.3.1(INTRON)
2	176323883	176323892	176323891	A	C	snp	1	1	0	1	.	.	.	AC096649.3.1(DOWNSTREAM);INTERGENIC(INTERGENIC)
2	176449874	176449887	176449877	TA	C	snp	1	0	1	0	.	.	.	INTERGENIC(INTERGENIC)
2	176584716	176584725	176584721	AC	T	snp	1	1	1	1	.	.	.	INTERGENIC(INTERGENIC)
2	176604155	176604170	176604159	TA	C	snp	1	0	0	0	.	.	.	INTERGENIC(INTERGENIC)
2	176761234	176761245	176761240	TA	G	snp	1	0	0	0	.	.	.	AC016751.3.1(UPSTREAM);INTERGENIC(INTERGENIC)
2	176789405	176789413	176789409	A	C	snp	1	1	1	1	.	.	.	KIAA1715(DOWNSTREAM);KIAA1715(UTR_3_PRIME)
2	176789405	176789413	176789410	A	C	ins	0	1	1	1	.	.	.	KIAA1715(DOWNSTREAM);KIAA1715(UTR_3_PRIME)
2	176840741	176840749	176840746	A	G	snp	1	1	0	0	.	.	.	KIAA1715(INTRON)
2	176957811	176957825	176957822	GCG	A	snp	1	1	1	1	.	.	HOXD13(SYNONYMOUS_CODING):A68	.
2	176989014	176989025	176989022	CTT	C	del	1	1	1	1	.	.	.	AC009336.1(DOWNSTREAM);AC009336.21.1(INTRON);HOXD10(DOWNSTREAM);HOXD9(INTRON);HOXD9(UTR_3_PRIME)
2	177030983	177030992	177030986	AT	C	snp	1	1	1	1	.	.	.	HOXD3(INTRON)
2	177128741	177128750	177128745	T	G	snp	1	0	1	1	.	.	.	INTERGENIC(INTERGENIC)
2	177225329	177225338	177225335	T	G	ins	1	1	1	1	.	.	.	INTERGENIC(INTERGENIC)
2	177391010	177391029	177391022	ATTT	T	ins	1	1	1	1	.	.	.	AC017048.2.1(INTRON)
2	177391010	177391029	177391025	ATTT	A	del	1	1	1	1	.	.	.	AC017048.2.1(INTRON)
2	177416863	177416876	177416872	AT	C	snp	1	1	0	1	.	.	.	INTERGENIC(INTERGENIC)
2	177416863	177416876	177416874	AT	C	snp	1	1	1	1	.	.	.	INTERGENIC(INTERGENIC)
2	177464575	177464582	177464576	A	G	snp	1	1	1	1	.	.	.	INTERGENIC(INTERGENIC)
2	177521658	177521681	177521664	AGG	C	snp	0	1	0	0	.	.	.	AC017048.4.1(DOWNSTREAM);INTERGENIC(INTERGENIC)
2	177598458	177598465	177598464	T	G	snp	1	1	0	1	.	.	.	AC092162.1.1(INTRON)
2	177654972	177654985	177654979	TC	T	snp	1	1	0	1	.	.	.	AC092162.1.1(INTRON)
2	177834320	177834327	177834323	T	G	ins	1	1	0	1	.	.	.	INTERGENIC(INTERGENIC)
2	177834320	177834327	177834324	T	G	snp	1	1	0	1	.	.	.	INTERGENIC(INTERGENIC)
2	177899402	177899411	177899404	C	CCCG	ins	1	1	1	1	.	.	.	AC079305.11.1(INTRON)
2	178043476	178043487	178043481	AT	ATATATAAAGG	del	1	1	1	1	.	.	.	AC079305.5.1(DOWNSTREAM);AC079305.8.1(INTRON)
2	178043476	178043487	178043485	AT	ATAAAGG	del	1	1	1	1	.	.	.	AC079305.5.1(DOWNSTREAM);AC079305.8.1(INTRON)
2	178129391	178129405	178129399	GGC	T	snp	1	1	1	0	.	.	.	AC079305.10.1(EXON);NFE2L2(INTRON);NFE2L2(UPSTREAM);NFE2L2(UTR_5_PRIME)

2	178155704	178155723	178155719	TTTC	TTTCT	del	1	1	0	1	.	.	.	AC074286.1.1(INTRON);NFE2L2(INTRON)
2	178161629	178161637	178161630	T	C	ins	0	1	1	1	.	.	.	AC074286.1.1(INTRON);NFE2L2(INTRON)
2	178162369	178162376	178162375	T	G	snp	1	0	0	0	.	.	.	AC074286.1.1(INTRON);NFE2L2(INTRON)
2	178403474	178403483	178403475	T	G	snp	1	0	0	0	.	.	.	AGPS(DOWNSTREAM);AGPS(UTR_3_PRIME)
2	178431533	178431541	178431537	T	C	snp	1	1	1	1	.	.	.	INTERGENIC(INTERGENIC)
2	178473140	178473149	178473141	A	C	ins	1	1	1	1	.	.	.	AC073834.3.1(INTRON)
2	178497021	178497032	178497026	TGA	G	snp	1	1	1	1	.	.	.	PDE11A(INTRON)
2	178524826	178524839	178524836	TC	C	del	1	1	1	1	.	.	.	PDE11A(INTRON)
2	178530216	178530227	178530221	AAT	A	ins	1	1	1	1	.	.	.	PDE11A(DOWNSTREAM);PDE11A(INTRON)
2	178530216	178530227	178530222	AAT	AT	del	1	1	1	1	.	.	.	PDE11A(DOWNSTREAM);PDE11A(INTRON)
2	178530216	178530227	178530223	AAT	T	del	1	1	1	1	.	.	.	PDE11A(DOWNSTREAM);PDE11A(INTRON)
2	178530216	178530227	178530224	AAT	A	ins	1	1	1	1	.	.	.	PDE11A(DOWNSTREAM);PDE11A(INTRON)
2	178584607	178584615	178584609	A	C	snp	1	0	0	0	.	.	.	AC012499.1.1(INTRON);PDE11A(INTRON)
2	178765485	178765493	178765492	A	C	snp	1	1	1	1	.	.	.	PDE11A(INTRON)
2	178801109	178801117	178801115	A	C	snp	1	1	1	1	.	.	.	PDE11A(INTRON)
2	178871278	178871285	178871280	A	G	snp	0	0	0	1	.	.	.	AC011998.1.1(INTRON);PDE11A(INTRON)
2	179035709	179035717	179035710	T	C	ins	1	1	1	1	.	.	.	INTERGENIC(INTERGENIC)
2	179051549	179051568	179051560	AAAC	T	snp	1	0	0	0	.	.	.	INTERGENIC(INTERGENIC)
2	179192181	179192190	179192188	AG	T	snp	1	1	0	1	.	.	.	OSBPL6(INTRON)
2	179250162	179250169	179250163	T	C	ins	1	0	1	0	.	.	.	OSBPL6(DOWNSTREAM);OSBPL6(INTRON)
2	179367848	179367857	179367855	TA	G	snp	0	0	1	0	.	.	.	PLEKHA3(INTRON)
2	179846429	179846437	179846432	A	G	snp	1	1	1	1	.	.	.	CCDC141(INTRON)
2	179889118	179889133	179889123	ATTC	C	snp	1	0	0	0	.	.	.	ACA59(UPSTREAM);CCDC141(INTRON)
2	179909699	179909707	179909701	T	C	ins	1	0	1	1	.	.	.	CCDC141(INTRON)
2	179938497	179938512	179938502	GTTG	C	snp	1	1	0	0	.	.	.	INTERGENIC(INTERGENIC)
2	179960484	179960492	179960489	T	G	snp	1	0	0	0	.	.	.	INTERGENIC(INTERGENIC)
2	180098518	180098533	180098528	CATG	C	snp	1	1	1	1	.	.	.	SESTD1(INTRON)
2	180117963	180117972	180117966	A	C	snp	1	1	1	1	.	.	.	SESTD1(INTRON)
2	180131506	180131515	180131509	AT	G	snp	0	1	0	1	.	.	.	INTERGENIC(INTERGENIC);SESTD1(UPSTREAM)
2	180139065	180139080	180139072	TTCA	T	snp	1	0	0	0	.	.	.	AC093911.1.1(INTRON)
2	180266126	180266133	180266132	T	C	snp	1	1	1	1	.	.	.	AC068042.1.1(UPSTREAM);INTERGENIC(INTERGENIC)
2	180338391	180338398	180338392	C	T	snp	1	1	1	1	.	.	.	ZNF385B(INTRON)
2	180376969	180376983	180376975	TAA	G	snp	1	1	1	1	.	.	.	ZNF385B(INTRON)
2	180402470	180402493	180402478	AAAC	AC	ins	1	1	0	1	.	.	.	ZNF385B(INTRON);ZNF385B(UPSTREAM)
2	180402470	180402493	180402481	AAAC	A	del	1	1	0	1	.	.	.	ZNF385B(INTRON);ZNF385B(UPSTREAM)
2	180402470	180402493	180402484	AAAC	C	del	1	1	0	1	.	.	.	ZNF385B(INTRON);ZNF385B(UPSTREAM)
2	180405206	180405223	180405220	AAC	A	ins	1	1	0	1	.	.	.	ZNF385B(DOWNSTREAM);ZNF385B(INTRON);ZNF385B(UPSTREAM)
2	180424370	180424377	180424371	T	C	ins	1	1	0	1	.	.	.	ZNF385B(INTRON)
2	180535222	180535230	180535223	A	AC	ins	1	1	1	1	.	.	.	ZNF385B(INTRON)
2	180590176	180590185	180590183	AT	A	snp	1	0	0	0	.	.	.	ZNF385B(INTRON)
2	180892182	180892191	180892187	AC	A	snp	1	0	0	0	.	.	.	INTERGENIC(INTERGENIC)
2	180914460	180914469	180914463	AC	A	snp	1	1	1	1	.	.	.	INTERGENIC(INTERGENIC)
2	181029421	181029444	181029430	TAT	C	snp	1	1	1	1	.	.	.	INTERGENIC(INTERGENIC)
2	181029421	181029444	181029433	TAT	C	snp	0	1	0	1	.	.	.	INTERGENIC(INTERGENIC)
2	181075013	181075022	181075020	CT	C	del	1	1	1	1	.	.	.	INTERGENIC(INTERGENIC)
2	181091457	181091466	181091463	TC	CTC	del	1	1	1	1	.	.	.	INTERGENIC(INTERGENIC)
2	181124244	181124251	181124250	A	C	ins	1	0	0	0	.	.	.	INTERGENIC(INTERGENIC)
2	181133904	181133915	181133912	TAT	C	snp	1	1	1	1	.	.	.	INTERGENIC(INTERGENIC)
2	181137246	181137253	181137247	T	G	snp	1	0	1	0	.	.	.	INTERGENIC(INTERGENIC)
2	181215891	181215898	181215893	T	G	snp	0	1	0	0	.	.	.	INTERGENIC(INTERGENIC)
2	181238622	181238631	181238630	T	C	snp	1	1	1	1	.	.	.	INTERGENIC(INTERGENIC)
2	181352801	181352808	181352806	T	G	snp	1	1	0	1	.	.	.	INTERGENIC(INTERGENIC)
2	181401787	181401795	181401792	A	G	snp	1	1	0	1	.	.	.	INTERGENIC(INTERGENIC)
2	181417004	181417011	181417009	T	C	snp	1	1	1	1	.	.	.	INTERGENIC(INTERGENIC)
2	181419701	181419712	181419708	ATT	TA	del	0	1	1	1	.	.	.	INTERGENIC(INTERGENIC)
2	181419701	181419712	181419709	ATT	A	del	0	1	1	1	.	.	.	INTERGENIC(INTERGENIC)
2	181426985	181426993	181426992	A	C	snp	1	1	1	1	.	.	.	INTERGENIC(INTERGENIC)
2	181431354	181431367	181431357	AG	A	snp	1	0	0	0	.	.	.	INTERGENIC(INTERGENIC)
2	181455442	181455450	181455443	T	G	snp	1	1	1	1	.	.	.	AC009478.1.1(INTRON)
2	181456975	181456984	181456978	TC	T	snp	1	1	1	1	.	.	.	AC009478.1.1(INTRON)
2	181532730	181532743	181532734	GT	A	ins	1	0	0	1	.	.	.	AC009478.1.1(INTRON)
2	181666839	181666846	181666841	A	G	snp	0	0	1	0	.	.	.	INTERGENIC(INTERGENIC)
2	181677800	181677820	181677803	AAT	G	snp	0	1	1	1	.	.	.	INTERGENIC(INTERGENIC)
2	181807877	181807888	181807885	AAT	A	snp	1	1	1	1	.	.	.	AC104076.2.1(UPSTREAM);INTERGENIC(INTERGENIC)
2	181835064	181835073	181835066	CA	T	snp	1	0	0	0	.	.	.	UBE2E3(INTRON)
2	181894894	181894911	181894898	TGG	A	snp	1	1	1	1	.	.	.	UBE2E3(INTRON)

2	181953410	181953419	181953412	AT	G	snp	1	1	1	1	.	.	.	AC068196.1.1(INTRON)
2	181977150	181977169	181977157	CAAA	T	snp	0	1	0	0	.	.	.	AC104820.2.1(INTRON)
2	182150411	182150420	182150419	A	G	snp	1	1	1	1	.	.	.	AC104820.2.1(INTRON)
2	182156334	182156341	182156337	T	C	snp	1	0	0	0	.	.	.	AC104820.2.1(INTRON)
2	182157837	182157846	182157839	A	G	snp	1	1	1	1	.	.	.	AC104820.2.1(INTRON)
2	182247921	182247928	182247926	A	G	snp	1	0	0	0	.	.	.	AC104820.2.1(INTRON)
2	182249873	182249881	182249877	T	TTTTC	ins	1	1	1	1	.	.	.	AC104820.2.1(INTRON)
2	182316463	182316472	182316464	A	C	snp	1	1	1	1	.	.	.	INTERGENIC(INTERGENIC)
2	182374177	182374184	182374179	T	C	snp	1	1	1	1	.	.	.	ITGA4(INTRON)
2	182777787	182777806	182777796	TATT	T	ins	0	0	1	0	.	.	.	SSFA2(INTRON);SSFA2(UPSTREAM)
2	182777787	182777806	182777799	TATT	A	del	0	0	1	0	.	.	.	SSFA2(INTRON);SSFA2(UPSTREAM)
2	182777787	182777806	182777800	TATT	T	ins	1	0	1	0	.	.	.	SSFA2(INTRON);SSFA2(UPSTREAM)
2	182867658	182867667	182867662	C	CT	ins	1	0	0	0	.	.	.	PPP1R1C(INTRON)
2	182902770	182902779	182902775	TA	G	snp	1	0	0	0	.	.	.	PPP1R1C(INTRON)
2	182956202	182956211	182956209	A	AT	ins	1	0	0	0	.	.	.	PPP1R1C(INTRON)
2	182959862	182959869	182959868	A	C	snp	1	1	1	1	.	.	.	PPP1R1C(INTRON)
2	182995928	182995941	182995930	GT	TT	ins	1	1	1	1	.	.	.	PPP1R1C(DOWNSTREAM);PPP1R1C(UTR_3_PRIME)
2	183134140	183134151	183134148	AGG	A	snp	1	0	0	0	.	.	.	PDE1A(INTRON)
2	183492157	183492168	183492159	AT	TATC	ins	1	1	1	1	.	.	.	INTERGENIC(INTERGENIC)
2	183496012	183496021	183496019	T	TAA	ins	1	1	1	1	.	.	.	INTERGENIC(INTERGENIC)
2	183543897	183543920	183543917	ATT	A	del	1	1	1	1	.	.	.	INTERGENIC(INTERGENIC)
2	183679606	183679615	183679610	CT	G	snp	1	1	1	1	.	.	.	INTERGENIC(INTERGENIC)
2	183685219	183685226	183685222	T	G	ins	1	0	0	0	.	.	.	INTERGENIC(INTERGENIC)
2	183716801	183716808	183716807	T	C	snp	1	1	1	1	.	.	.	FRZB(INTRON)
2	183741529	183741538	183741533	A	G	ins	1	1	1	1	.	.	.	INTERGENIC(INTERGENIC);U6(DOWNSTREAM)
2	183829232	183829241	183829236	TA	C	snp	1	1	1	0	.	.	.	NCKAP1(INTRON)
2	183852610	183852617	183852615	G	A	snp	1	0	0	0	.	.	.	NCKAP1(INTRON)
2	183872663	183872671	183872665	T	C	snp	0	1	0	1	.	.	.	NCKAP1(INTRON)
2	184074984	184074991	184074989	T	C	snp	1	1	0	1	.	.	.	INTERGENIC(INTERGENIC)
2	184150919	184150928	184150924	AT	A	ins	1	1	1	1	.	.	.	INTERGENIC(INTERGENIC)
2	184151014	184151027	184151018	TA	A	snp	1	1	1	1	.	.	.	INTERGENIC(INTERGENIC)
2	184151014	184151027	184151022	TA	A	snp	0	1	0	1	.	.	.	INTERGENIC(INTERGENIC)
2	184209879	184209892	184209889	GT	C	snp	0	0	0	1	.	.	.	INTERGENIC(INTERGENIC)
2	184239248	184239256	184239252	G	T	snp	1	0	0	0	.	.	.	INTERGENIC(INTERGENIC)
2	184259716	184259724	184259720	G	T	snp	0	0	1	0	.	.	.	INTERGENIC(INTERGENIC)
2	184259716	184259724	184259723	G	A	snp	0	1	0	0	.	.	.	INTERGENIC(INTERGENIC)
2	184295105	184295114	184295109	T	C	snp	1	1	1	1	.	.	.	INTERGENIC(INTERGENIC)
2	184299559	184299570	184299563	AAT	G	snp	1	0	0	0	.	.	.	INTERGENIC(INTERGENIC)
2	184329246	184329255	184329251	T	G	snp	1	1	1	1	.	.	.	INTERGENIC(INTERGENIC)
2	184516580	184516591	184516582	TC	C	snp	1	0	0	0	.	.	.	INTERGENIC(INTERGENIC)
2	184557948	184557957	184557953	A	G	ins	1	1	0	0	.	.	.	INTERGENIC(INTERGENIC)
2	184585805	184585812	184585807	A	G	snp	1	1	1	1	.	.	.	INTERGENIC(INTERGENIC)
2	184722560	184722575	184722571	AGAT	C	snp	1	0	1	0	.	.	.	INTERGENIC(INTERGENIC)
2	184783777	184783784	184783778	T	C	snp	1	0	0	0	.	.	.	AC093639.1.1(INTRON)
2	184788968	184788975	184788970	A	G	snp	0	1	0	1	.	.	.	AC093639.1.1(INTRON)
2	184865277	184865286	184865283	A	C	ins	1	1	1	1	.	.	.	INTERGENIC(INTERGENIC)
2	185040165	185040172	185040170	A	C	snp	1	0	0	0	.	.	.	INTERGENIC(INTERGENIC)
2	185084123	185084130	185084125	T	G	ins	1	1	1	1	.	.	.	INTERGENIC(INTERGENIC)
2	185111514	185111522	185111521	A	C	snp	1	0	0	0	.	.	.	INTERGENIC(INTERGENIC)
2	185117184	185117192	185117189	A	G	snp	1	1	1	1	.	.	.	INTERGENIC(INTERGENIC)
2	185189697	185189706	185189698	T	C	ins	1	1	1	1	.	.	.	INTERGENIC(INTERGENIC)
2	185189697	185189706	185189699	T	C	snp	1	1	1	1	.	.	.	INTERGENIC(INTERGENIC)
2	185219540	185219547	185219543	A	G	snp	0	0	1	0	.	.	.	INTERGENIC(INTERGENIC)
2	185534492	185534507	185534495	CA	CACACT	ins	1	1	1	1	.	.	.	ZNF804A(INTRON)
2	185630333	185630342	185630340	C	CG	ins	1	0	0	0	.	.	.	ZNF804A(INTRON)
2	185673389	185673397	185673390	A	C	snp	0	1	0	1	.	.	.	ZNF804A(INTRON)
2	185683935	185683943	185683942	A	C	snp	1	1	0	1	.	.	.	ZNF804A(INTRON)
2	185708157	185708166	185708163	T	G	snp	1	0	0	0	.	.	.	ZNF804A(INTRON)
2	185708157	185708166	185708165	T	G	snp	1	1	1	1	.	.	.	ZNF804A(INTRON)
2	185776339	185776347	185776345	T	C	snp	1	1	1	1	.	.	.	ZNF804A(INTRON)
2	185784848	185784863	185784855	AACA	G	snp	1	0	0	0	.	.	.	ZNF804A(INTRON)
2	185823876	185823885	185823879	TA	G	snp	1	1	1	1	.	.	.	INTERGENIC(INTERGENIC)
2	185850329	185850340	185850338	TG	T	del	1	1	0	1	.	.	.	INTERGENIC(INTERGENIC)
2	185925995	185926006	185925999	ACT	A	snp	1	0	0	0	.	.	.	INTERGENIC(INTERGENIC)
2	186017003	186017012	186017005	T	C	snp	1	1	1	1	.	.	.	INTERGENIC(INTERGENIC)
2	186135146	186135153	186135147	T	G	snp	1	1	1	1	.	.	.	INTERGENIC(INTERGENIC)
2	186142368	186142381	186142373	AG	A	snp	0	0	1	0	.	.	.	INTERGENIC(INTERGENIC)

2	186143035	186143052	186143046	TA	TATATAT	del	1	1	1	1	.	.	.	INTERGENIC(INTERGENIC)
2	186143035	186143052	186143047	TA	ATATA	del	1	1	1	1	.	.	.	INTERGENIC(INTERGENIC)
2	186230429	186230438	186230434	A	C	snp	1	1	1	1	.	.	.	INTERGENIC(INTERGENIC)
2	186281537	186281545	186281538	T	G	ins	1	1	1	1	.	.	.	INTERGENIC(INTERGENIC)
2	186392042	186392051	186392046	TG	C	snp	1	0	0	0	.	.	.	INTERGENIC(INTERGENIC)
2	186515912	186515921	186515916	AT	G	snp	1	0	0	0	.	.	.	INTERGENIC(INTERGENIC)
2	186564864	186564881	186564878	GT	C	snp	1	1	0	1	.	.	.	INTERGENIC(INTERGENIC)
2	186625768	186625775	186625770	A	G	snp	1	1	1	1	.	.	"FSIP2(SYNONYMOUS_CODING):K378,K467"	FSIP2(UTR_3_PRIME)
2	186629184	186629192	186629191	T	G	snp	1	1	1	1	.	.	.	FSIP2(INTRON)
2	186641030	186641041	186641036	ATA	T	snp	1	0	0	0	.	.	.	FSIP2(INTRON)
2	186652815	186652824	186652818	TA	G	snp	0	0	1	0	.	.	.	AC008174.3.1(EXON);AC008174.3.1(INTRON);FSIP2(INTRON)
2	186768488	186768497	186768492	TA	ATATA	del	1	0	0	0	.	.	.	INTERGENIC(INTERGENIC)
2	186868528	186868551	186868542	GAAA	AG	ins	1	1	0	0	.	.	.	INTERGENIC(INTERGENIC)
2	186985503	186985511	186985507	T	G	ins	1	0	0	0	.	.	.	INTERGENIC(INTERGENIC)
2	186990284	186990292	186990285	G	T	snp	1	1	1	1	.	.	.	INTERGENIC(INTERGENIC)
2	187047473	187047481	187047480	C	A	ins	0	0	1	0	.	.	.	INTERGENIC(INTERGENIC)
2	187102402	187102413	187102405	AT	C	snp	1	1	1	1	.	.	.	INTERGENIC(INTERGENIC)
2	187106372	187106387	187106379	AAAT	C	snp	1	1	1	1	.	.	.	INTERGENIC(INTERGENIC)
2	187501642	187501651	187501648	AT	G	snp	1	1	0	1	.	.	.	AC017101.10.1(DOWNSTREAM);ITGAV(INTRON)
2	187549353	187549360	187549354	A	G	snp	1	1	1	1	.	.	.	AC017101.10.1(INTRON);ITGAV(DOWNSTREAM)
2	187676899	187676918	187676910	GTTT	G	del	1	1	1	1	.	.	.	INTERGENIC(INTERGENIC)
2	187676899	187676918	187676911	GTTT	T	snp	1	1	1	1	.	.	.	INTERGENIC(INTERGENIC)
2	187676899	187676918	187676914	GTTT	G	del	1	1	1	1	.	.	.	INTERGENIC(INTERGENIC)
2	187692281	187692289	187692285	T	G	snp	1	0	0	0	.	.	.	INTERGENIC(INTERGENIC);ZSWIM2(DOWNSTREAM)
2	187702357	187702366	187702364	TA	G	snp	1	1	0	1	.	.	.	ZSWIM2(INTRON)
2	187743192	187743199	187743196	T	G	snp	1	1	1	1	.	.	.	INTERGENIC(INTERGENIC)
2	187743192	187743199	187743198	T	G	snp	1	1	1	1	.	.	.	INTERGENIC(INTERGENIC)
2	187784373	187784390	187784386	TTC	CTTC	del	0	1	1	1	.	.	.	INTERGENIC(INTERGENIC)
2	187895484	187895499	187895493	ACAA	A	ins	1	0	0	0	.	.	.	AC007319.1.1(INTRON)
2	187900978	187900986	187900985	A	G	snp	1	1	1	1	.	.	.	AC007319.1.1(INTRON)
2	187963101	187963108	187963102	T	G	snp	0	1	1	1	.	.	.	AC007319.1.1(INTRON)
2	187972674	187972685	187972680	TA	C	snp	1	0	0	0	.	.	.	AC007319.1.1(INTRON)
2	187979370	187979377	187979373	T	G	snp	1	0	0	0	.	.	.	AC007319.1.1(INTRON)
2	188007264	188007279	188007266	AT	G	snp	0	1	0	1	.	.	.	AC007319.1.1(INTRON)
2	188007264	188007279	188007267	AT	C	snp	1	1	1	1	.	.	.	AC007319.1.1(INTRON)
2	188023884	188023893	188023887	TC	G	snp	1	1	1	1	.	.	.	AC007319.1.1(INTRON)
2	188048921	188048936	188048929	AT	G	snp	0	1	1	1	.	.	.	AC007319.1.1(INTRON)
2	188171333	188171342	188171337	AG	G	snp	1	1	1	1	.	.	.	AC007319.1.1(INTRON)
2	188175997	188176014	188176012	AT	A	del	1	1	1	1	.	.	.	AC007319.1.1(INTRON)
2	188199737	188199746	188199741	A	G	ins	1	1	1	1	.	.	.	AC007319.1.1(INTRON)
2	188288337	188288346	188288343	TG	G	snp	1	0	0	0	.	.	.	AC007319.1.1(INTRON);CALCRL(DOWNSTREAM);CALCRL(INTRON)
2	188388309	188388316	188388311	T	C	snp	1	1	1	1	.	.	.	AC007319.1.1(INTRON);TFPI(INTRON);TFPI(UPSTREAM)
2	188437349	188437357	188437356	T	C	snp	1	0	0	0	.	.	.	INTERGENIC(INTERGENIC)
2	188499928	188499937	188499929	A	G	ins	1	1	0	1	.	.	.	INTERGENIC(INTERGENIC)
2	188610888	188610897	188610896	A	G	snp	1	0	0	0	.	.	.	AC068718.1.1(INTRON)
2	188657632	188657640	188657635	A	C	snp	1	1	1	1	.	.	.	AC068718.1.1(INTRON)
2	188665863	188665870	188665869	A	C	snp	1	0	0	0	.	.	.	AC068718.1.1(INTRON)
2	188780478	188780487	188780483	T	G	snp	0	1	0	0	.	.	.	AC068718.1.1(INTRON)
2	188865151	188865171	188865164	AAG	G	del	1	1	1	1	.	.	.	AC068718.1.1(INTRON)
2	188975165	188975184	188975172	AAAC	A	snp	1	1	0	1	.	.	.	AC068718.1.1(INTRON)
2	188975165	188975184	188975180	AAAC	AAACAAA	del	1	0	0	0	.	.	.	AC068718.1.1(INTRON)
2	189479538	189479545	189479544	T	G	snp	0	1	0	1	.	.	.	AC079613.1.1(INTRON)
2	189525936	189525943	189525939	T	G	snp	1	1	1	1	.	.	.	AC079613.1.1(INTRON)
2	189583534	189583541	189583540	T	G	snp	1	1	1	1	.	.	.	AC079613.1.1(INTRON)
2	189654186	189654199	189654191	AT	AC	ins	1	1	0	1	.	.	.	AC079613.1.1(INTRON);DIRC1(INTRON)
2	189707714	189707731	189707724	AAC	C	del	1	0	0	0	.	.	.	AC079613.1.1(UPSTREAM);INTERGENIC(INTERGENIC)
2	189711896	189711923	189711916	ATTT	T	ins	1	0	0	0	.	.	.	INTERGENIC(INTERGENIC)
2	189798090	189798101	189798096	TA	C	snp	1	1	1	1	.	.	.	INTERGENIC(INTERGENIC)
2	189798090	189798101	189798099	TA	T	snp	1	1	0	1	.	.	.	INTERGENIC(INTERGENIC)
2	189830238	189830246	189830239	A	G	snp	1	0	0	0	.	.	.	INTERGENIC(INTERGENIC)
2	190018840	190018849	190018844	CA	T	snp	1	1	1	1	.	.	.	COL5A2(INTRON)
2	190082230	190082247	190082234	TG	C	snp	0	1	1	1	.	.	.	INTERGENIC(INTERGENIC)
2	190094143	190094152	190094149	CA	T	snp	1	1	1	1	.	.	.	INTERGENIC(INTERGENIC)
2	190175919	190175927	190175923	T	C	snp	1	1	0	0	.	.	.	INTERGENIC(INTERGENIC);KRT18P19(DOWNSTREAM)

2	190284595	190284612	190284609	GAG	GA	del	1	0	0	0	.	.	.	INTERGENIC(INTERGENIC)
2	190429175	190429182	190429181	T	C	snp	1	1	1	1	.	.	.	SLC40A1(DOWNSTREAM);SLC40A1(INTRON)
2	190527955	190527966	190527962	AT	A	del	1	1	0	0	.	.	.	ASNSD1(INTRON)
2	190527955	190527966	190527964	AT	A	del	1	1	1	1	.	.	.	ASNSD1(INTRON)
2	190599700	190599717	190599711	AT	C	snp	1	1	1	1	.	.	.	ANKAR(INTRON);ANKAR(UPSTREAM)
2	190603666	190603673	190603667	T	C	ins	1	0	1	0	.	.	.	ANKAR(INTRON)
2	190607560	190607577	190607574	AC	CAC	del	1	0	0	0	.	.	.	ANKAR(INTRON);OSGEPL1(DOWNSTREAM)
2	190790060	190790071	190790068	AAT	G	snp	1	1	1	1	.	.	.	AC019178.1.1(DOWNSTREAM);AC019178.2.1(DOWNSTREAM);C2orf88(INTRON)
2	190820895	190820902	190820898	T	C	snp	1	0	0	0	.	.	.	C2orf88(INTRON)
2	190863475	190863498	190863493	TTCT	TTTCT	del	1	0	0	0	.	.	.	C2orf88(INTRON)
2	190903605	190903632	190903626	TCTT	T	del	1	1	1	1	.	.	.	C2orf88(INTRON)
2	191010598	191010613	191010609	TA	T	del	1	1	1	1	.	.	.	C2orf88(INTRON);C2orf88(UPSTREAM)
2	191010598	191010613	191010611	TA	T	del	1	1	1	1	.	.	.	C2orf88(INTRON);C2orf88(UPSTREAM)
2	191025065	191025074	191025069	T	G	snp	1	1	1	1	.	.	.	C2orf88(INTRON)
2	191093276	191093284	191093283	A	G	ins	1	1	1	1	.	.	.	HIBCH(INTRON)
2	191243077	191243104	191243081	TTTG	TTG	del	1	1	1	1	.	.	.	INTERGENIC(INTERGENIC)
2	191305821	191305828	191305824	T	C	snp	1	1	1	1	.	.	.	MFSD6(DOWNSTREAM);MFSD6(INTRON);MFSD6(UPSTREAM)
2	191331295	191331312	191331304	GAT	C	snp	1	1	1	1	.	.	.	MFSD6(INTRON);MFSD6(UPSTREAM)
2	191501005	191501012	191501007	A	C	snp	1	0	0	0	.	.	.	INTERGENIC(INTERGENIC)
2	191506931	191506940	191506938	T	G	snp	1	1	1	1	.	.	.	INTERGENIC(INTERGENIC);NAB1(UPSTREAM)
2	191530870	191530879	191530877	T	C	snp	1	0	0	0	.	.	.	NAB1(INTRON)
2	191633315	191633330	191633325	CTTT	TC	del	1	1	1	1	.	.	.	INTERGENIC(INTERGENIC)
2	191633315	191633330	191633326	CTTT	C	del	1	1	1	1	.	.	.	INTERGENIC(INTERGENIC)
2	191669499	191669512	191669502	AC	T	snp	0	0	1	0	.	.	.	INTERGENIC(INTERGENIC)
2	191669927	191669938	191669930	AG	A	snp	1	1	1	1	.	.	.	INTERGENIC(INTERGENIC)
2	191701203	191701212	191701210	AT	C	snp	1	1	1	1	.	.	.	INTERGENIC(INTERGENIC)
2	191701224	191701233	191701230	AT	G	snp	1	0	0	0	.	.	.	INTERGENIC(INTERGENIC)
2	191756715	191756722	191756718	A	G	snp	1	0	0	0	.	.	.	GLS(INTRON);GLS(UPSTREAM)
2	191825766	191825773	191825769	T	C	snp	1	1	1	1	.	.	.	GLS(INTRON)
2	191843824	191843831	191843830	A	G	snp	1	1	1	1	.	.	.	STAT1(INTRON)
2	191863408	191863417	191863409	A	G	snp	1	1	0	1	.	.	.	STAT1(DOWNSTREAM);STAT1(INTRON)
2	191923018	191923037	191923026	TTTA	C	snp	1	1	1	1	.	.	.	STAT4(INTRON)
2	192280331	192280340	192280335	T	G	snp	0	1	1	0	.	.	.	MYO1B(INTRON)
2	192290991	192291000	192290998	TG	T	snp	1	0	0	0	.	.	.	INTERGENIC(INTERGENIC);MYO1B(DOWNSTREAM)
2	192317819	192317828	192317824	AT	A	snp	1	1	0	1	.	.	.	INTERGENIC(INTERGENIC)
2	192436541	192436558	192436556	TA	T	ins	1	0	0	0	.	.	.	INTERGENIC(INTERGENIC)
2	192496565	192496572	192496566	A	G	snp	1	0	0	0	.	.	.	INTERGENIC(INTERGENIC)
2	192499273	192499286	192499284	TG	A	snp	1	0	0	0	.	.	.	INTERGENIC(INTERGENIC)
2	192537931	192537948	192537934	AT	A	snp	1	0	1	0	.	.	.	INTERGENIC(INTERGENIC);OBFC2A(UPSTREAM)
2	192660309	192660318	192660313	TG	A	snp	1	1	1	1	.	.	.	AC098872.3.1(INTRON)
2	192801245	192801254	192801251	AC	G	snp	1	0	0	0	.	.	.	AC098617.1.1(INTRON);AC098617.2.1(INTRON)
2	192838988	192838995	192838994	T	G	snp	1	1	0	1	.	.	.	AC098617.1.1(INTRON);AC098617.2.1(INTRON);TMEFF2(INTRON)
2	192921936	192921947	192921943	ATT	TA	del	1	1	1	1	.	.	.	TMEFF2(INTRON)
2	192921936	192921947	192921944	ATT	A	del	1	1	1	1	.	.	.	TMEFF2(INTRON)
2	193070039	193070048	193070041	T	G	snp	0	1	1	0	.	.	.	INTERGENIC(INTERGENIC)
2	193128907	193128916	193128913	T	G	snp	1	1	1	1	.	.	.	INTERGENIC(INTERGENIC)
2	193147848	193147863	193147851	TA	G	snp	1	1	0	1	.	.	.	INTERGENIC(INTERGENIC)
2	193175138	193175145	193175140	T	AAAA	ins	1	1	1	1	.	.	.	INTERGENIC(INTERGENIC)
2	193396939	193396946	193396943	A	C	snp	1	1	1	1	.	.	.	INTERGENIC(INTERGENIC)
2	193582072	193582079	193582077	T	G	snp	1	1	1	1	.	.	.	INTERGENIC(INTERGENIC)
2	193738840	193738849	193738847	AT	C	snp	0	0	1	1	.	.	.	INTERGENIC(INTERGENIC)
2	193797516	193797531	193797525	TTTA	G	snp	0	0	0	1	.	.	.	INTERGENIC(INTERGENIC)
2	193921294	193921305	193921299	TG	T	ins	1	1	1	1	.	.	.	INTERGENIC(INTERGENIC)
2	194155798	194155807	194155803	A	C	snp	1	1	1	1	.	.	.	INTERGENIC(INTERGENIC)
2	194163085	194163092	194163087	T	G	snp	1	0	0	0	.	.	.	INTERGENIC(INTERGENIC)
2	194440225	194440232	194440231	A	G	snp	0	0	1	0	.	.	.	INTERGENIC(INTERGENIC)
2	194493742	194493749	194493743	A	C	snp	1	1	1	1	.	.	.	INTERGENIC(INTERGENIC)
2	194733903	194733912	194733907	TC	C	snp	1	1	1	1	.	.	.	INTERGENIC(INTERGENIC)
2	194862833	194862848	194862837	TGTA	C	snp	1	1	1	1	.	.	.	INTERGENIC(INTERGENIC)
2	194876825	194876832	194876830	A	C	snp	1	1	1	1	.	.	.	INTERGENIC(INTERGENIC)
2	194895513	194895520	194895518	T	C	snp	1	1	1	1	.	.	.	AC068135.1.1(DOWNSTREAM);INTERGENIC(INTERGENIC)
2	194940392	194940400	194940395	T	C	snp	1	1	1	1	.	.	.	INTERGENIC(INTERGENIC)
2	194962577	194962585	194962580	T	C	snp	1	0	0	0	.	.	.	INTERGENIC(INTERGENIC)
2	194987069	194987092	194987086	CAAA	C	snp	1	0	0	0	.	.	.	INTERGENIC(INTERGENIC)
2	194987090	194987099	194987093	A	C	snp	0	0	1	0	.	.	.	INTERGENIC(INTERGENIC)

2	195042900	195042909	195042906	CT	T	snp	1	0	0	0	.	.	.	INTERGENIC(INTERGENIC)
2	195075993	195076000	195075997	T	G	snp	1	1	1	1	.	.	.	INTERGENIC(INTERGENIC)
2	195149991	195149998	195149993	T	C	snp	1	0	0	0	.	.	.	INTERGENIC(INTERGENIC)
2	195197037	195197048	195197040	AG	T	snp	1	1	1	1	.	.	.	INTERGENIC(INTERGENIC)
2	195259809	195259818	195259811	TA	C	snp	1	0	0	0	.	.	.	AC018799.1.1(DOWNSTREAM);AC018799.1.1(INTRON)
2	195331666	195331673	195331672	T	C	snp	1	0	1	1	.	.	.	INTERGENIC(INTERGENIC)
2	195362280	195362289	195362283	AT	C	snp	0	1	1	1	.	.	.	INTERGENIC(INTERGENIC)
2	195384567	195384582	195384579	TA	A	del	0	1	0	1	.	.	.	INTERGENIC(INTERGENIC)
2	195384567	195384582	195384580	TA	T	snp	1	1	1	1	.	.	.	INTERGENIC(INTERGENIC)
2	195498163	195498176	195498173	AT	T	del	1	0	0	0	.	.	.	INTERGENIC(INTERGENIC)
2	195568002	195568011	195568007	AT	C	snp	1	1	1	1	.	.	.	INTERGENIC(INTERGENIC)
2	195568028	195568037	195568033	AT	C	snp	1	0	1	1	.	.	.	INTERGENIC(INTERGENIC)
2	195568041	195568050	195568043	AT	G	snp	1	0	0	1	.	.	.	INTERGENIC(INTERGENIC)
2	195568041	195568050	195568046	AT	C	snp	1	1	1	1	.	.	.	INTERGENIC(INTERGENIC)
2	195568054	195568065	195568056	AT	G	snp	1	1	1	1	.	.	.	INTERGENIC(INTERGENIC)
2	195568069	195568078	195568074	AT	C	snp	1	1	1	1	.	.	.	INTERGENIC(INTERGENIC)
2	195568155	195568164	195568159	AT	G	snp	1	1	0	1	.	.	.	INTERGENIC(INTERGENIC)
2	195568168	195568179	195568172	AT	G	snp	1	1	1	1	.	.	.	INTERGENIC(INTERGENIC)
2	195568183	195568192	195568187	AT	G	snp	1	1	1	1	.	.	.	INTERGENIC(INTERGENIC)
2	195568211	195568220	195568215	AT	G	snp	1	1	1	1	.	.	.	INTERGENIC(INTERGENIC)
2	195568491	195568500	195568496	AT	C	snp	1	1	1	1	.	.	.	INTERGENIC(INTERGENIC)
2	195568504	195568515	195568509	AT	C	snp	1	1	1	1	.	.	.	INTERGENIC(INTERGENIC)
2	195568519	195568528	195568524	AT	C	snp	1	1	1	1	.	.	.	INTERGENIC(INTERGENIC)
2	195568556	195568567	195568561	AT	C	snp	0	1	0	1	.	.	.	INTERGENIC(INTERGENIC)
2	195568580	195568591	195568587	AT	C	snp	1	1	1	1	.	.	.	INTERGENIC(INTERGENIC)
2	195568664	195568677	195568673	AT	C	snp	1	1	1	1	.	.	.	INTERGENIC(INTERGENIC)
2	195568681	195568690	195568686	AT	C	snp	1	1	1	1	.	.	.	INTERGENIC(INTERGENIC)
2	195568694	195568709	195568705	AT	C	snp	1	1	1	1	.	.	.	INTERGENIC(INTERGENIC)
2	195591610	195591619	195591617	AT	A	snp	1	0	0	0	.	.	.	AC006196.1.1(UPSTREAM);INTERGENIC(INTERGENIC)
2	195650111	195650118	195650116	A	C	snp	1	1	1	1	.	.	.	INTERGENIC(INTERGENIC)
2	195679182	195679189	195679184	T	C	snp	1	1	1	1	.	.	.	INTERGENIC(INTERGENIC)
2	195761438	195761451	195761441	CT	A	snp	1	0	0	0	.	.	.	INTERGENIC(INTERGENIC)
2	195787379	195787388	195787383	TA	C	snp	1	0	0	0	.	.	.	INTERGENIC(INTERGENIC)
2	195817664	195817681	195817671	ACA	T	snp	1	0	0	0	.	.	.	INTERGENIC(INTERGENIC)
2	195817681	195817690	195817682	A	C	ins	1	1	1	1	.	.	.	INTERGENIC(INTERGENIC)
2	195841991	195842010	195842005	TGAT	T	del	1	0	0	0	.	.	.	INTERGENIC(INTERGENIC)
2	195851653	195851667	195851664	CAA	C	del	1	0	1	1	.	.	.	INTERGENIC(INTERGENIC)
2	195851666	195851674	195851668	A	C	snp	1	0	0	0	.	.	.	INTERGENIC(INTERGENIC)
2	195887706	195887715	195887713	TA	TGT	ins	1	1	1	1	.	.	.	AC010983.1.1(INTRON)
2	195956763	195956778	195956766	AT	C	snp	1	0	0	0	.	.	.	INTERGENIC(INTERGENIC)
2	196053071	196053078	196053075	T	C	snp	1	1	1	1	.	.	.	INTERGENIC(INTERGENIC)
2	196062096	196062105	196062100	A	G	snp	1	0	0	0	.	.	.	INTERGENIC(INTERGENIC)
2	196125486	196125494	196125491	A	C	snp	0	1	1	1	.	.	.	INTERGENIC(INTERGENIC)
2	196156967	196156976	196156969	AT	C	snp	0	1	1	0	.	.	.	INTERGENIC(INTERGENIC)
2	196216993	196217006	196216997	CA	A	snp	0	1	0	0	.	.	.	INTERGENIC(INTERGENIC)
2	196271498	196271505	196271503	A	G	snp	1	1	1	0	.	.	.	INTERGENIC(INTERGENIC)
2	196341493	196341508	196341502	TTAT	C	snp	0	1	1	0	.	.	.	AC064834.1.1(INTRON)
2	196428005	196428012	196428006	C	T	snp	0	1	1	1	.	.	.	INTERGENIC(INTERGENIC)
2	196428005	196428012	196428011	C	T	snp	1	1	1	0	.	.	.	INTERGENIC(INTERGENIC)
2	196489941	196489949	196489943	A	G	ins	0	0	1	0	.	.	.	SLC39A10(INTRON)
2	196501099	196501106	196501104	T	C	snp	1	1	1	1	.	.	.	SLC39A10(INTRON)
2	196663435	196663443	196663437	T	G	snp	1	0	0	0	.	.	.	DNAH7(INTRON);DNAH7(UPSTREAM)
2	196675132	196675147	196675140	TTAT	G	snp	1	0	0	0	.	.	.	DNAH7(INTRON)
2	196693318	196693335	196693333	AT	A	del	1	1	0	1	.	.	.	DNAH7(INTRON)
2	196821416	196821423	196821417	T	C	snp	1	0	0	0	.	.	.	DNAH7(INTRON)
2	196870032	196870047	196870040	AT	G	snp	1	0	0	0	.	.	.	DNAH7(INTRON)
2	196923890	196923899	196923895	T	C	snp	1	0	0	0	.	.	.	AC114760.1.1(DOWNSTREAM);DNAH7(INTRON)
2	197076570	197076578	197076574	T	G	snp	0	0	1	0	.	.	.	HECW2(INTRON);snoU13(DOWNSTREAM)
2	197103160	197103183	197103178	TTTC	C	del	1	0	0	0	.	.	.	HECW2(DOWNSTREAM);HECW2(INTRON)
2	197293965	197293972	197293971	G	T	ins	1	0	0	0	.	.	.	HECW2(INTRON)
2	197367383	197367398	197367391	TTCC	G	snp	1	0	0	0	.	.	.	HECW2(INTRON)
2	197398192	197398201	197398199	TG	T	del	1	0	0	0	.	.	.	HECW2(INTRON)
2	197409754	197409767	197409758	TG	C	snp	1	0	0	0	.	.	.	HECW2(INTRON)
2	197438582	197438596	197438592	CAA	AC	del	1	0	0	0	.	.	.	HECW2(INTRON)
2	197438595	197438604	197438597	A	C	snp	1	1	0	1	.	.	.	HECW2(INTRON)
2	197438595	197438604	197438600	A	C	snp	0	0	0	1	.	.	.	HECW2(INTRON)
2	197453384	197453397	197453393	AC	T	snp	1	1	1	1	.	.	.	HECW2(INTRON)

2	197453384	197453397	197453395	AC	T	snp	1	0	0	0	.	.	.	HECW2(INTRON)
2	197562024	197562031	197562026	T	C	ins	1	0	0	0	.	.	.	CCDC150(DOWNSTREAM);CCDC150(INTRON);CCDC150(UPSTREAM)
2	197586078	197586093	197586085	AT	C	snp	1	1	1	1	.	.	.	CCDC150(DOWNSTREAM);CCDC150(INTRON)
2	197862146	197862159	197862154	TA	C	snp	1	1	1	1	.	.	.	ANKRD44(INTRON);ANKRD44(UPSTREAM)
2	197921008	197921017	197921009	A	C	snp	1	0	0	0	.	.	.	ANKRD44(INTRON)
2	197965159	197965167	197965163	A	C	snp	0	1	1	1	.	.	.	ANKRD44(INTRON)
2	198004373	198004382	198004376	AT	AG	ins	1	0	0	0	.	.	.	ANKRD44(INTRON)
2	198005943	198005962	198005948	ATAC	C	snp	0	0	1	0	.	.	.	ANKRD44(INTRON)
2	198051197	198051206	198051205	T	C	snp	1	1	1	1	.	.	.	ANKRD44(INTRON)
2	198170332	198170347	198170342	AAAG	G	snp	1	1	1	1	.	.	.	ANKRD44(INTRON);ANKRD44-IT1(UPSTREAM)
2	198200211	198200220	198200213	T	G	snp	1	1	1	1	.	.	.	INTERGENIC(INTERGENIC)
2	198305447	198305455	198305449	A	C	ins	1	1	1	1	.	.	.	INTERGENIC(INTERGENIC)
2	198340995	198341004	198341001	A	G	snp	1	1	1	1	.	.	.	COQ10B(DOWNSTREAM);INTERGENIC(INTERGENIC)
2	198355148	198355157	198355153	T	G	snp	1	1	1	1	.	.	.	HSPD1(DOWNSTREAM);HSPD1(EXON);HSPD1(INTRON)
2	198413951	198413960	198413959	A	G	snp	0	1	0	1	.	.	.	MOB4(INTRON)
2	198466957	198466966	198466965	T	G	snp	1	0	0	0	.	.	.	RFTN2(INTRON)
2	198669103	198669114	198669110	CG	A	snp	1	1	1	1	.	.	.	INTERGENIC(INTERGENIC);PLCL1(UPSTREAM)
2	198746246	198746263	198746259	TG	A	snp	1	1	1	1	.	.	.	PLCL1(INTRON)
2	198958783	198958792	198958789	TC	A	snp	1	0	0	0	.	.	.	PLCL1(INTRON)
2	198961387	198961394	198961389	T	G	snp	1	1	1	1	.	.	.	PLCL1(INTRON)
2	199080893	199080900	199080894	A	C	snp	1	0	0	0	.	.	.	PLCL1(INTRAGENIC)
2	199187371	199187378	199187376	A	G	snp	1	1	1	1	.	.	.	AC005235.1.1(INTRON);PLCL1(INTRAGENIC)
2	199207411	199207428	199207426	AT	A	del	1	1	1	1	.	.	.	AC005235.1.1(INTRON);PLCL1(INTRAGENIC);PLCL1(UPSTREAM)
2	199222784	199222791	199222790	T	G	snp	1	0	0	0	.	.	.	AC005235.1.1(INTRON);PLCL1(INTRON)
2	199227020	199227029	199227024	GT	T	snp	1	0	0	0	.	.	.	AC005235.1.1(INTRON);PLCL1(INTRON)
2	199284347	199284358	199284356	TA	G	snp	1	1	1	1	.	.	.	PLCL1(INTRON)
2	199342044	199342061	199342056	GT	A	snp	1	1	1	1	.	.	.	PLCL1(INTRON)
2	199435603	199435614	199435611	TTA	G	snp	1	0	0	0	.	.	.	AC019330.1(UPSTREAM);AC019330.1.1(INTRON);PLCL1(INTRON)
2	199615274	199615288	199615283	TTG	C	snp	1	0	0	0	.	.	.	AC019330.1.1(INTRON)
2	199713729	199713738	199713734	TA	G	snp	1	1	0	0	.	.	.	INTERGENIC(INTERGENIC)
2	199741579	199741586	199741584	T	G	ins	1	1	1	0	.	.	.	INTERGENIC(INTERGENIC)
2	199775310	199775327	199775321	AT	G	snp	1	1	1	1	.	.	.	AC018717.1.1(INTRON)
2	199803753	199803762	199803758	AT	C	snp	1	1	1	1	.	.	.	AC018717.1.1(INTRON)
2	199871650	199871658	199871656	T	C	snp	1	1	1	0	.	.	.	AC018717.1.1(INTRON)
2	199872121	199872148	199872141	TTTA	TTATTTA	del	0	1	0	1	.	.	.	AC018717.1.1(INTRON)
2	199903420	199903431	199903423	AG	C	snp	1	0	0	0	.	.	.	AC018717.1.1(INTRON)
2	200143081	200143096	200143085	AAAC	G	snp	1	0	0	0	.	.	.	SATB2(INTRON)
2	200159453	200159460	200159457	A	C	snp	1	0	0	0	.	.	.	SATB2(INTRON)
2	200229939	200229948	200229946	T	G	snp	1	0	0	0	.	.	.	SATB2(DOWNSTREAM);SATB2(INTRON)
2	200301148	200301156	200301152	A	C	snp	1	0	0	0	.	.	.	SATB2(INTRON)
2	200450385	200450393	200450386	A	G	snp	1	1	1	1	.	.	.	INTERGENIC(INTERGENIC)
2	200467364	200467372	200467367	A	C	snp	1	1	1	1	.	.	.	INTERGENIC(INTERGENIC)
2	200484659	200484682	200484663	TTTC	TTCA	ins	1	1	1	1	.	.	.	AC093590.1.1(INTRON);AC093590.1.1(UPSTREAM)
2	200484683	200484690	200484686	T	C	snp	1	1	1	1	.	.	.	AC093590.1.1(INTRON);AC093590.1.1(UPSTREAM)
2	200491177	200491192	200491187	AAGG	A	snp	1	1	1	1	.	.	.	AC093590.1.1(INTRON)
2	200512635	200512643	200512641	T	G	snp	1	1	1	1	.	.	.	AC093590.1.1(INTRON)
2	200564185	200564196	200564192	AAG	G	snp	0	1	0	1	.	.	.	INTERGENIC(INTERGENIC)
2	200710296	200710305	200710298	A	C	snp	0	1	1	0	.	.	.	AC073043.2.1(INTRON)
2	200800491	200800500	200800498	AC	T	snp	0	1	0	0	.	.	.	C2orf69(INTRON);TYW5(INTRON)
2	201006242	201006251	201006247	AT	C	snp	0	1	1	1	.	.	.	INTERGENIC(INTERGENIC)
2	201158232	201158241	201158237	AT	C	snp	1	1	0	1	.	.	.	INTERGENIC(INTERGENIC)
2	201347490	201347505	201347492	TG	G	del	1	1	1	1	.	.	.	INTERGENIC(INTERGENIC);SPATS2L(DOWNSTREAM)
2	201347490	201347505	201347493	TG	T	snp	1	1	1	1	.	.	.	INTERGENIC(INTERGENIC);SPATS2L(DOWNSTREAM)
2	201395383	201395398	201395394	AGGT	A	snp	1	1	0	1	.	.	.	SGOL2(INTRON);SGOL2(UPSTREAM)
2	201485103	201485111	201485106	T	G	ins	1	1	1	1	.	.	.	AOX1(INTRON)
2	201556513	201556530	201556520	ATC	C	snp	1	1	0	1	.	.	.	AC007163.4.1(INTRON);AC007163.4.1(UPSTREAM);AOX2P(UPSTREAM)
2	201638788	201638802	201638791	AAC	AC	del	0	0	0	1	.	.	.	AOX2P(INTRON);U1(UPSTREAM)
2	201644769	201644778	201644772	AC	A	snp	0	1	1	1	.	.	.	AC007163.6.1(DOWNSTREAM);AOX2P(INTRON);U1(DOWNSTREAM)
2	201657732	201657741	201657734	AC	C	snp	1	0	0	0	.	.	.	AC007163.6.1(INTRON);AOX2P(DOWNSTREAM);AOX2P(INTRON)
2	201934324	201934339	201934337	AC	T	snp	0	1	1	0	.	.	.	AC005037.4.1(DOWNSTREAM);FAM126B(INTRON);NDUFB3(UPSTREAM)

2	202071322	202071335	202071327	TA	TT	ins	1	0	0	0	.	.	.	CASP10(DOWNSTREAM);CASP10(INTRON)
2	202131585	202131592	202131587	T	G	snp	1	1	1	1	.	.	.	CASP8(INTRON)
2	202213378	202213387	202213382	CA	A	snp	1	1	1	1	.	.	.	ALS2CR12(DOWNSTREAM);ALS2CR12(INTRON);ALS2CR12(UPSTREAM)
2	202281129	202281137	202281136	A	G	snp	1	1	1	1	.	.	.	AC007256.5.1(UPSTREAM);STRADB(INTRON);TRAK2(DOWNSTREAM);TRAK2(INTRON)
2	202377840	202377847	202377843	A	G	snp	1	0	0	0	.	.	.	ALS2CR11(INTRON);ALS2CR11(UPSTREAM)
2	202435141	202435152	202435145	ATG	C	snp	1	1	1	1	.	.	.	ALS2CR11(INTRON)
2	202686404	202686411	202686408	T	G	snp	0	1	0	0	.	.	.	AC007242.3.1(UPSTREAM);CDK15(INTRON)
2	202698423	202698440	202698436	TCT	TTCT	del	1	1	1	1	.	.	.	CDK15(INTRON)
2	202698423	202698440	202698437	TCT	TC	del	1	1	1	1	.	.	.	CDK15(INTRON)
2	202763033	202763040	202763039	A	G	snp	1	1	1	0	.	.	.	CDK15(DOWNSTREAM);INTERGENIC(INTERGENIC)
2	202927047	202927056	202927048	T	C	snp	1	1	1	1	.	.	.	INTERGENIC(INTERGENIC)
2	202997480	202997489	202997487	TA	G	snp	1	1	1	1	.	.	.	AC079354.1.1(INTRON);AC079354.1.1(UPSTREAM)
2	203033163	203033171	203033168	A	G	snp	1	0	0	0	.	.	.	AC079354.1.1(INTRON)
2	203131933	203131942	203131937	AT	G	snp	0	1	0	1	.	.	.	NOP58(INTRON)
2	203180282	203180295	203180284	TA	A	del	1	1	1	1	.	.	.	INTERGENIC(INTERGENIC)
2	203206246	203206263	203206250	AAT	T	del	1	1	0	0	.	.	.	AC064836.1(DOWNSTREAM);AC064836.3.1(UPSTREAM);INTERGENIC(INTERGENIC)
2	203206246	203206263	203206251	AAT	A	snp	1	0	0	0	.	.	.	AC064836.1(DOWNSTREAM);AC064836.3.1(UPSTREAM);INTERGENIC(INTERGENIC)
2	203238792	203238800	203238793	A	C	ins	1	0	0	0	.	.	.	BMPR2(UPSTREAM);INTERGENIC(INTERGENIC)
2	203258185	203258194	203258192	AT	C	snp	0	1	1	1	.	.	.	BMPR2(INTRON)
2	203321225	203321240	203321236	TTTC	T	ins	1	1	1	1	.	.	.	BMPR2(INTRON)
2	203408875	203408883	203408879	A	C	snp	1	0	0	0	.	.	.	BMPR2(INTRON)
2	203487593	203487602	203487597	T	C	snp	1	0	0	0	.	.	.	AC009960.3.1(UPSTREAM);AC009960.5(DOWNSTREAM);AC009960.6.1(DOWNSTREAM);INTERGENIC(INTERGENIC);MTND4P30(DOWNSTREAM)
2	203492919	203492946	203492933	TTTG	G	del	1	0	0	0	.	.	.	INTERGENIC(INTERGENIC)
2	203513242	203513251	203513250	C	A	ins	1	0	0	0	.	.	.	FAM117B(INTRON)
2	203530328	203530342	203530332	TTG	C	snp	1	1	1	1	.	.	.	FAM117B(INTRON)
2	203531219	203531227	203531223	T	C	snp	1	0	0	0	.	.	.	FAM117B(INTRON)
2	203711456	203711479	203711473	TCAT	ATT	del	1	1	1	1	.	.	.	ICA1L(INTRON);KRT8P15(UPSTREAM)
2	203792047	203792056	203792049	T	C	snp	1	0	0	0	.	.	.	AC010900.2.1(DOWNSTREAM);ALS2CR8(INTRON);WDR12(INTRON)
2	203820977	203820996	203820984	TATT	G	snp	0	0	1	0	.	.	.	ALS2CR8(DOWNSTREAM);ALS2CR8(INTRON);WDR12(INTRON)
2	203870943	203870952	203870946	A	C	snp	1	1	1	1	.	.	.	WDR12(INTRON)
2	203941202	203941217	203941208	AAAC	C	del	1	1	1	1	.	.	.	AC023271.2.1(UPSTREAM);NBEAL1(INTRON)
2	203941202	203941217	203941213	AAAC	A	ins	1	1	1	1	.	.	.	AC023271.2.1(UPSTREAM);NBEAL1(INTRON)
2	204028978	204028989	204028986	AT	C	snp	1	1	1	1	.	.	.	NBEAL1(INTRON);NBEAL1(UPSTREAM)
2	204207563	204207572	204207570	AC	A	ins	1	0	0	0	.	.	.	ABI2(INTRON)
2	204305088	204305099	204305093	GGT	C	snp	1	1	1	1	.	.	"RAPH1(SYNONYMOUS_CODING):P940,P992"	ABI2(DOWNSTREAM);RAPH1(DOWNSTREAM);RAPH1(INTRON)
2	204346732	204346741	204346740	A	C	snp	1	0	0	1	.	.	.	RAPH1(INTRON)
2	204347526	204347533	204347529	A	C	snp	1	1	1	0	.	.	.	RAPH1(INTRON)
2	204371466	204371479	204371468	TA	G	snp	0	1	1	1	.	.	.	RAPH1(INTRON)
2	204371466	204371479	204371469	TA	C	snp	1	1	1	1	.	.	.	RAPH1(INTRON)
2	204371466	204371479	204371477	TA	C	snp	1	1	1	0	.	.	.	RAPH1(INTRON)
2	204371482	204371491	204371484	TA	G	snp	0	0	0	1	.	.	.	RAPH1(INTRON)
2	204371518	204371527	204371520	TA	G	snp	1	0	0	0	.	.	.	RAPH1(INTRON)
2	204455238	204455246	204455241	T	C	snp	1	0	1	0	.	.	.	INTERGENIC(INTERGENIC)
2	204456783	204456790	204456787	T	C	snp	1	0	0	0	.	.	.	INTERGENIC(INTERGENIC)
2	204499213	204499227	204499224	AAT	AATA	del	1	1	1	1	.	.	.	AC125238.2.1(DOWNSTREAM);INTERGENIC(INTERGENIC)
2	204576924	204576931	204576925	A	C	ins	1	1	1	1	.	.	.	CD28(INTRON)
2	204615785	204615812	204615802	TTAT	A	ins	1	1	1	0	.	.	.	INTERGENIC(INTERGENIC)
2	204653889	204653898	204653892	TA	T	snp	1	1	0	1	.	.	.	INTERGENIC(INTERGENIC)
2	204664451	204664460	204664454	AC	T	snp	1	1	1	1	.	.	.	INTERGENIC(INTERGENIC)
2	204912538	204912551	204912546	TC	CTCTC	del	0	1	1	0	.	.	.	INTERGENIC(INTERGENIC)
2	205028706	205028721	205028711	TA	TG	ins	1	1	1	1	.	.	.	INTERGENIC(INTERGENIC)
2	205089985	205089994	205089987	T	G	snp	1	1	1	1	.	.	.	AC009498.1.1(DOWNSTREAM);INTERGENIC(INTERGENIC)
2	205089985	205089994	205089988	T	C	snp	1	0	0	0	.	.	.	AC009498.1.1(DOWNSTREAM);INTERGENIC(INTERGENIC)
2	205094576	205094589	205094587	AC	T	snp	1	1	1	1	.	.	.	AC009498.1.1(INTRON)
2	205342065	205342080	205342072	AAAG	A	snp	1	1	1	1	.	.	.	AC016903.1.1(UPSTREAM);AC016903.2.1(INTRON)
2	205351412	205351423	205351419	TGG	T	snp	1	1	1	1	.	.	.	AC016903.2.1(INTRON)
2	205638111	205638122	205638120	AT	A	del	1	1	1	1	.	.	.	PARD3B(INTRON)
2	205763474	205763485	205763480	CT	T	snp	1	1	1	1	.	.	.	PARD3B(INTRON)
2	205899827	205899835	205899834	C	A	snp	1	0	0	0	.	.	.	PARD3B(INTRON)
2	206195918	206195927	206195920	AC	G	snp	0	1	0	1	.	.	.	PARD3B(INTRON)

2	206213594	206213608	206213598	ATA	A	del	1	1	0	1	.	.	.	PARD3B(INTRON)
2	206245977	206245986	206245982	TA	T	snp	1	1	1	1	.	.	.	PARD3B(INTRON)
2	206381474	206381489	206381478	CTTT	A	snp	1	0	1	0	.	.	.	PARD3B(INTRON)
2	206386277	206386284	206386278	T	GA	ins	0	1	0	0	.	.	.	PARD3B(INTRON)
2	206446020	206446035	206446027	ATAG	T	snp	1	0	0	1	.	.	.	PARD3B(INTRON)
2	206446020	206446035	206446031	ATAG	C	snp	0	1	1	1	.	.	.	PARD3B(INTRON)
2	206454013	206454036	206454018	TCAA	T	snp	1	1	1	1	.	.	.	PARD3B(INTRON)
2	206597598	206597605	206597601	T	C	snp	1	0	0	0	.	.	.	NRP2(INTRON)
2	206599124	206599132	206599127	C	A	ins	1	1	0	1	.	.	.	NRP2(INTRON)
2	206599124	206599132	206599130	C	A	snp	1	0	0	0	.	.	.	NRP2(INTRON)
2	206602239	206602250	206602242	TG	A	snp	1	1	1	1	.	.	.	NRP2(INTRON);NRP2(UPSTREAM)
2	206616070	206616077	206616074	T	C	snp	1	0	0	0	.	.	.	NRP2(INTRON);NRP2(UPSTREAM)
2	206685162	206685177	206685173	AT	ATA	del	1	0	0	0	.	.	.	INTERGENIC(INTERGENIC)
2	206777520	206777528	206777522	C	CG	ins	1	1	1	1	.	.	.	INTERGENIC(INTERGENIC)
2	206777520	206777528	206777524	C	CA	ins	1	1	1	1	.	.	.	INTERGENIC(INTERGENIC)
2	206777520	206777528	206777525	C	CA	ins	1	1	1	1	.	.	.	INTERGENIC(INTERGENIC)
2	206777532	206777540	206777534	A	C	snp	1	0	0	0	.	.	.	INTERGENIC(INTERGENIC)
2	206861640	206861648	206861644	A	C	ins	0	1	0	1	.	.	.	AC007679.3.1(INTRON);INO80D(UTR_3_PRIME)
2	207115551	207115559	207115554	T	TG	ins	1	0	0	0	.	.	.	INTERGENIC(INTERGENIC)
2	207115551	207115559	207115555	T	G	ins	1	0	0	0	.	.	.	INTERGENIC(INTERGENIC)
2	207122452	207122460	207122456	T	G	snp	1	1	1	1	.	.	.	7SK(DOWNSTREAM);HMGN1P6(DOWNSTREAM);INTERGENIC(INTERGENIC)
2	207132842	207132851	207132845	T	TTTTTTC	ins	1	1	1	1	.	.	.	INTERGENIC(INTERGENIC)
2	207134265	207134288	207134284	TTTC	T	ins	1	1	1	1	.	.	.	INTERGENIC(INTERGENIC)
2	207163238	207163245	207163240	T	G	snp	1	0	0	0	.	.	.	ZDBF2(INTRON)
2	207187736	207187744	207187737	A	AAAC	ins	1	1	1	1	.	.	.	INTERGENIC(INTERGENIC)
2	207270950	207270958	207270954	C	A	snp	1	1	1	1	.	.	.	AC017081.2.1(DOWNSTREAM);INTERGENIC(INTERGENIC)
2	207281779	207281787	207281782	T	G	snp	1	0	0	0	.	.	.	AC017081.3.1(DOWNSTREAM);INTERGENIC(INTERGENIC)
2	207329306	207329320	207329315	AAT	C	snp	1	0	0	0	.	.	.	ADAM23(INTRON)
2	207609647	207609674	207609669	TAAA	AT	del	1	1	1	1	.	.	.	MDH1B(INTRON)
2	207610905	207610913	207610909	A	C	snp	1	1	1	1	.	.	.	MDH1B(INTRON)
2	207623506	207623515	207623507	A	C	snp	1	1	1	1	.	.	.	MDH1B(INTRON)
2	207654315	207654328	207654320	AC	AA	ins	1	1	1	0	.	.	.	FASTKD2(EXON);FASTKD2(INTRON)
2	207679023	207679031	207679030	T	G	snp	1	1	0	1	.	.	.	INTERGENIC(INTERGENIC)
2	207762764	207762771	207762768	T	C	snp	1	0	0	0	.	.	.	INTERGENIC(INTERGENIC)
2	207860169	207860188	207860182	TCTT	TT	del	1	1	1	1	.	.	.	INTERGENIC(INTERGENIC)
2	207860169	207860188	207860184	TCTT	TC	del	1	1	1	1	.	.	.	INTERGENIC(INTERGENIC)
2	207863017	207863025	207863024	A	G	snp	1	0	0	0	.	.	.	INTERGENIC(INTERGENIC)
2	207911098	207911118	207911103	AGG	A	snp	1	1	1	1	.	.	.	INTERGENIC(INTERGENIC)
2	207946713	207946726	207946722	TG	T	snp	1	0	0	0	.	.	.	KLF7(INTRON)
2	208207888	208207901	208207895	TG	A	snp	1	1	1	1	.	.	.	AC007879.5.1(INTRON)
2	208443362	208443375	208443372	TG	A	snp	1	1	0	1	.	.	.	CREB1(DOWNSTREAM);CREB1(INTRON);METTL21A(DOWNSTREAM)
2	208450709	208450718	208450713	GA	A	snp	1	0	0	0	.	.	.	CREB1(INTRON);METTL21A(INTRON)
2	208568482	208568489	208568488	A	G	snp	1	1	1	1	.	.	.	INTERGENIC(INTERGENIC)
2	208592250	208592258	208592257	T	G	snp	1	1	1	1	.	.	.	CCNYL1(INTRON)
2	208614220	208614228	208614226	A	C	snp	1	1	1	1	.	.	.	CCNYL1(INTRON);CCNYL1(UPSTREAM)
2	208629377	208629385	208629381	A	C	snp	1	1	1	1	.	.	.	CCNYL1(DOWNSTREAM);FZD5(UTR_3_PRIME)
2	208745531	208745539	208745534	A	C	snp	1	0	0	0	.	.	.	PLEKHM3(INTRON)
2	208758398	208758409	208758407	AG	C	snp	1	1	1	1	.	.	.	PLEKHM3(INTRON)
2	208792731	208792738	208792736	A	C	snp	1	1	1	1	.	.	.	PLEKHM3(INTRON)
2	208821039	208821047	208821040	A	C	snp	1	1	1	1	.	.	.	PLEKHM3(INTRON)
2	208868773	208868782	208868780	T	C	snp	1	1	1	1	.	.	.	PLEKHM3(INTRON)
2	209013168	209013179	209013177	AT	A	del	1	1	1	1	.	.	.	CRYGB(UPSTREAM);INTERGENIC(INTERGENIC)
2	209019104	209019115	209019109	CAC	G	snp	1	0	0	0	.	.	.	INTERGENIC(INTERGENIC)
2	209049821	209049830	209049829	T	C	ins	1	1	0	1	.	.	.	C2orf80(INTRON);C2orf80(UPSTREAM)
2	209054065	209054076	209054073	CCA	G	snp	1	1	1	1	.	.	.	C2orf80(INTRON);C2orf80(UPSTREAM)
2	209107475	209107482	209107480	T	C	snp	1	0	0	0	.	.	.	IDH1(DOWNSTREAM);IDH1(INTRON);IDH1(UPSTREAM)
2	209230084	209230091	209230087	T	C	snp	1	1	1	1	.	.	.	AC012362.3.1(UPSTREAM);PTH2R(INTRON)
2	209423155	209423178	209423170	TTTC	T	ins	1	1	1	1	.	.	.	PTH2R(INTRAGENIC)
2	209423155	209423178	209423173	TTTC	C	del	1	1	1	1	.	.	.	PTH2R(INTRAGENIC)
2	209423155	209423178	209423174	TTTC	T	snp	1	1	1	1	.	.	.	PTH2R(INTRAGENIC)
2	209533774	209533783	209533777	A	C	snp	1	0	0	0	.	.	.	PTH2R(INTRAGENIC)
2	209541977	209541986	209541981	T	G	ins	1	0	0	0	.	.	.	PTH2R(INTRAGENIC)
2	209586259	209586266	209586262	T	C	snp	1	0	0	0	.	.	.	PTH2R(INTRAGENIC)
2	209734383	209734394	209734390	TG	T	snp	1	1	1	1	.	.	.	INTERGENIC(INTERGENIC)
2	209737744	209737752	209737748	A	C	snp	0	0	1	0	.	.	.	INTERGENIC(INTERGENIC)

2	209792200	209792209	209792207	CT	C	snp	1	1	1	1	.	.	.	INTERGENIC(INTERGENIC)
2	209858044	209858059	209858047	AC	AT	ins	1	0	1	0	.	.	.	INTERGENIC(INTERGENIC)
2	209882523	209882534	209882527	AT	G	snp	1	1	1	1	.	.	.	INTERGENIC(INTERGENIC)
2	209957199	209957212	209957209	CT	G	snp	1	1	1	1	.	.	.	INTERGENIC(INTERGENIC)
2	209991620	209991631	209991626	TTA	G	snp	1	1	1	1	.	.	.	INTERGENIC(INTERGENIC)
2	210045914	210045921	210045917	T	G	snp	1	1	1	1	.	.	.	MEAF6P1(INTRON);PKP4P1(DOWNSTREAM)
2	210071382	210071389	210071388	A	G	snp	1	1	1	1	.	.	.	INTERGENIC(INTERGENIC)
2	210114891	210114899	210114896	A	G	snp	1	1	1	1	.	.	.	INTERGENIC(INTERGENIC)
2	210210717	210210725	210210718	A	C	snp	1	1	1	1	.	.	.	INTERGENIC(INTERGENIC)
2	210372978	210372985	210372984	T	C	snp	1	0	0	0	.	.	.	MAP2(INTRON)
2	210513905	210513913	210513909	A	C	snp	1	1	1	1	.	.	.	MAP2(INTRON);MAP2(UPSTREAM)
2	210768006	210768017	210768008	TG	C	snp	1	0	0	0	.	.	.	UNC80(INTRON)
2	210816022	210816035	210816024	TC	C	del	1	1	0	0	.	.	.	UNC80(INTRON);UNC80(UPSTREAM)
2	210972244	210972259	210972247	AT	A	snp	1	1	1	1	.	.	.	C2orf67(INTRON);C2orf67(UPSTREAM)
2	211158848	211158856	211158855	T	C	snp	1	1	0	1	.	.	.	MYL1(INTRON)
2	211248094	211248101	211248098	A	G	snp	1	1	1	1	.	.	.	AC007970.1.1(INTRON)
2	211341197	211341208	211341204	GGC	C	ins	1	1	1	1	.	.	.	CPS1(UPSTREAM);LANCL1(INTRON);LANCL1(UTR_5_PRIME)
2	211350807	211350816	211350813	TA	G	snp	1	1	1	1	.	.	.	CPS1(INTRON)
2	211377588	211377599	211377597	AT	G	snp	1	1	1	1	.	.	.	CPS1(INTRON)
2	211377844	211377855	211377847	AT	C	snp	1	1	1	1	.	.	.	CPS1(INTRON)
2	211383442	211383450	211383444	T	C	snp	1	1	1	1	.	.	.	CPS1(INTRON)
2	211462813	211462822	211462814	T	C	ins	1	1	1	1	.	.	.	CPS1(INTRON)
2	211588177	211588186	211588178	T	C	snp	1	1	1	1	.	.	.	INTERGENIC(INTERGENIC)
2	211629326	211629335	211629329	TG	A	snp	1	0	0	0	.	.	.	INTERGENIC(INTERGENIC)
2	211842593	211842602	211842598	AT	A	snp	1	0	0	0	.	.	.	INTERGENIC(INTERGENIC)
2	211859406	211859417	211859410	TAT	C	snp	1	0	0	0	.	.	.	INTERGENIC(INTERGENIC)
2	211886590	211886597	211886595	T	G	snp	1	1	1	0	.	.	.	INTERGENIC(INTERGENIC)
2	211973097	211973106	211973105	A	TT	ins	1	1	1	1	.	.	.	INTERGENIC(INTERGENIC)
2	212000302	212000311	212000308	TA	C	snp	1	1	1	1	.	.	.	INTERGENIC(INTERGENIC)
2	212013833	212013844	212013839	TA	ATATA	del	1	1	1	1	.	.	.	INTERGENIC(INTERGENIC)
2	212030531	212030550	212030540	AGGG	T	snp	1	1	1	1	.	.	.	INTERGENIC(INTERGENIC)
2	212053032	212053047	212053035	AG	A	snp	1	0	0	0	.	.	.	INTERGENIC(INTERGENIC)
2	212182750	212182759	212182756	AT	G	snp	0	1	0	0	.	.	.	INTERGENIC(INTERGENIC)
2	212316912	212316920	212316914	T	C	ins	1	0	0	0	.	.	.	ERBB4(INTRON)
2	212326640	212326648	212326642	A	C	snp	1	1	1	1	.	.	.	ERBB4(INTRON)
2	212371183	212371190	212371188	T	C	snp	0	0	0	1	.	.	.	ERBB4(INTRON)
2	212523106	212523115	212523114	A	C	snp	1	1	1	1	.	.	.	ERBB4(INTRON)
2	212543026	212543034	212543031	A	C	ins	1	1	0	1	.	.	.	ERBB4(INTRON)
2	212583556	212583570	212583563	AAC	G	snp	1	1	1	1	.	.	.	ERBB4(INTRON)
2	212617533	212617542	212617539	CA	A	ins	1	1	1	1	.	.	.	ERBB4(INTRON)
2	212617533	212617542	212617540	CA	C	del	1	1	1	1	.	.	.	ERBB4(INTRON)
2	212637315	212637324	212637319	CT	G	snp	1	0	0	0	.	.	.	AC105921.6(DOWNSTREAM);AC105921.8(DOWNSTREAM);ERBB4(INTRON);MTND2P23(UPSTREAM)
2	212717967	212717975	212717969	T	G	snp	0	1	0	1	.	.	.	ERBB4(INTRON)
2	212786133	212786140	212786138	A	G	snp	0	1	0	1	.	.	.	ERBB4(INTRON)
2	212941250	212941261	212941258	GA	C	snp	0	1	1	1	.	.	.	ERBB4(INTRON)
2	212956427	212956434	212956433	A	G	snp	1	1	0	1	.	.	.	ERBB4(INTRON)
2	212966076	212966091	212966079	AT	ATATATATATATAC	ins	1	1	1	1	.	.	.	ERBB4(INTRON)
2	212980059	212980066	212980064	T	C	snp	1	0	0	0	.	.	.	ERBB4(INTRON)
2	212998122	212998131	212998128	T	G	ins	1	0	0	0	.	.	.	ERBB4(INTRON)
2	213088138	213088153	213088151	AT	A	del	1	1	1	1	.	.	.	ERBB4(INTRON)
2	213105396	213105403	213105401	A	G	snp	1	1	1	1	.	.	.	ERBB4(INTRON)
2	213120323	213120334	213120328	GT	C	snp	1	0	0	0	.	.	.	ERBB4(INTRON)
2	213145742	213145749	213145745	T	C	snp	1	0	0	0	.	.	.	ERBB4(INTRON)
2	213179862	213179869	213179868	T	G	snp	1	1	1	1	.	.	.	ERBB4(INTRON)
2	213238665	213238674	213238668	AT	A	snp	1	1	0	1	.	.	.	ERBB4(INTRON)
2	213239341	213239356	213239348	AC	T	snp	1	1	0	1	.	.	.	ERBB4(INTRON)
2	213311303	213311314	213311311	AT	C	snp	0	1	0	0	.	.	.	ERBB4(INTRON)
2	213362841	213362858	213362852	TG	T	del	1	0	0	0	.	.	.	ERBB4(INTRON)
2	213362841	213362858	213362854	TG	T	del	1	0	0	0	.	.	.	ERBB4(INTRON)
2	213362841	213362858	213362855	TG	G	snp	1	0	0	0	.	.	.	ERBB4(INTRON)
2	213369225	213369234	213369228	A	G	snp	1	1	0	1	.	.	.	ERBB4(INTRON)
2	213401857	213401877	213401874	GGC	G	snp	1	1	0	1	.	.	.	ERBB4(INTRON)
2	213404029	213404037	213404032	C	CCCCA	ins	1	1	0	1	.	.	.	ERBB4(UPSTREAM);INTERGENIC(INTERGENIC)
2	213505213	213505228	213505226	AT	A	del	1	1	0	0	.	.	.	AC093865.1.1(INTRON)
2	213508182	213508199	213508196	TTA	G	snp	1	0	0	0	.	.	.	AC093865.1.1(INTRON)
2	213567359	213567374	213567370	AAAT	A	ins	1	0	0	0	.	.	.	AC093865.1.1(INTRON)

2	213605196	213605205	213605199	AT	C	snp	1	0	0	1	.	.	.	AC093865.1.1(INTRON)
2	213632272	213632295	213632291	TTTA	T	ins	1	1	1	1	.	.	.	AC093865.1(UPSTREAM);AC093865.1.1(INTRON)
2	213768946	213768969	213768951	AAT	A	snp	1	1	1	1	.	.	.	AC093865.1.1(INTRON)
2	213812036	213812047	213812043	AT	C	snp	0	0	1	0	.	.	.	AC093865.1.1(INTRON)
2	213829568	213829585	213829571	AT	A	snp	1	1	1	1	.	.	.	AC093865.1.1(INTRON)
2	213859249	213859256	213859254	A	G	snp	1	0	0	0	.	.	.	AC093865.1.1(INTRON)
2	213869916	213869924	213869919	G	A	snp	1	0	0	0	.	.	.	AC093865.1.1(INTRON);IKZF2(DOWNSTREAM);IKZF2(UTR_3_PRIME)
2	213869916	213869924	213869922	G	GGT	ins	1	1	1	1	.	.	.	AC093865.1.1(INTRON);IKZF2(DOWNSTREAM);IKZF2(UTR_3_PRIME)
2	213906064	213906071	213906066	G	T	ins	0	1	0	1	.	.	.	IKZF2(INTRON)
2	213906064	213906071	213906069	G	T	ins	0	1	0	1	.	.	.	IKZF2(INTRON)
2	213940651	213940662	213940657	CAC	T	snp	1	0	0	0	.	.	.	IKZF2(INTRON)
2	213944220	213944231	213944227	AG	A	snp	1	0	0	0	.	.	.	IKZF2(INTRON)
2	213986828	213986837	213986831	AG	A	snp	1	0	0	0	.	.	.	IKZF2(INTRON)
2	214012404	214012412	214012405	A	C	snp	1	1	1	1	.	IKZF2(NON_SYNONYMOUS_CODING):L62V	.	IKZF2(INTRON)
2	214012404	214012412	214012407	A	C	snp	1	0	0	0	.	IKZF2(NON_SYNONYMOUS_CODING):F61C	.	IKZF2(INTRON)
2	214082955	214082964	214082959	CA	T	snp	1	0	0	0	.	.	.	INTERGENIC(INTERGENIC)
2	214157410	214157418	214157416	A	AAAC	ins	1	1	0	1	.	.	.	SPAG16(INTRON);SPAG16(UPSTREAM)
2	214173151	214173158	214173156	T	C	snp	1	1	0	1	.	.	.	SPAG16(INTRON)
2	214201893	214201904	214201900	AAC	C	snp	1	1	1	1	.	.	.	SPAG16(INTRON)
2	214271817	214271826	214271822	TG	C	snp	0	1	0	1	.	.	.	SPAG16(INTRON)
2	214326627	214326636	214326631	TG	C	snp	1	0	0	0	.	.	.	SPAG16(INTRON)
2	214333205	214333214	214333211	AT	C	snp	1	1	0	1	.	.	.	SPAG16(INTRON)
2	214423197	214423206	214423205	T	TG	ins	1	0	0	0	.	.	.	SPAG16(INTRON)
2	214601019	214601027	214601022	T	C	snp	1	1	1	1	.	.	.	SPAG16(INTRON)
2	214725198	214725211	214725200	TA	AG	ins	1	1	1	1	.	.	.	SPAG16(INTRON)
2	214725198	214725211	214725205	TA	TG	ins	0	0	0	1	.	.	.	SPAG16(INTRON)
2	214735792	214735801	214735797	AC	T	snp	1	1	1	1	.	.	.	SPAG16(INTRON)
2	214845200	214845209	214845202	TA	C	snp	1	1	1	1	.	.	.	SPAG16(INTRON)
2	214898409	214898417	214898413	T	G	snp	1	1	0	1	.	.	.	SPAG16(INTRON)
2	214918028	214918036	214918031	A	G	snp	1	1	1	1	.	.	.	SPAG16(INTRON)
2	214920457	214920464	214920463	A	C	snp	1	0	0	0	.	.	.	SPAG16(INTRON)
2	215034954	215034963	215034959	AC	T	snp	0	0	0	1	.	.	.	SPAG16(INTRON)
2	215098799	215098806	215098803	C	T	ins	0	1	1	1	.	.	.	SPAG16(INTRON)
2	215098799	215098806	215098804	C	T	ins	0	1	1	1	.	.	.	SPAG16(INTRON)
2	215347373	215347388	215347386	AT	A	del	1	0	0	0	.	.	.	AC107218.3.1(INTRON);VWC2L(INTRON)
2	215470338	215470347	215470345	T	G	snp	1	0	0	0	.	.	.	AC107218.3.1(INTRON)
2	215527423	215527430	215527428	A	C	snp	1	1	1	1	.	.	.	AC107218.3.1(INTRON)
2	215558726	215558733	215558731	T	C	snp	1	1	1	1	.	.	.	ENSAP3(UPSTREAM);INTERGENIC(INTERGENIC)
2	215631928	215631935	215631932	T	C	snp	1	1	1	1	.	.	.	BARD1(INTRON)
2	215774706	215774713	215774709	A	C	snp	1	1	1	1	.	.	.	INTERGENIC(INTERGENIC)
2	215789994	215790003	215790001	T	G	snp	0	1	0	1	.	.	.	INTERGENIC(INTERGENIC)
2	215931789	215931796	215931791	A	G	snp	0	1	1	1	.	.	.	ABCA12(INTRON)
2	215964722	215964739	215964736	TC	CTC	del	1	1	0	1	.	.	.	ABCA12(INTRON)
2	216005840	216005855	216005844	TTTG	C	snp	1	0	0	0	.	.	.	ABCA12(UPSTREAM);INTERGENIC(INTERGENIC)
2	216027446	216027461	216027452	TTTA	G	snp	1	0	0	0	.	.	.	INTERGENIC(INTERGENIC)
2	216036676	216036685	216036681	AC	G	snp	0	1	0	1	.	.	.	INTERGENIC(INTERGENIC)
2	216066902	216066911	216066905	T	G	snp	1	0	0	0	.	.	.	INTERGENIC(INTERGENIC)
2	216101272	216101287	216101283	AAAT	AAATA	del	0	1	1	1	.	.	.	INTERGENIC(INTERGENIC)
2	216136994	216137005	216137002	TC	CTC	del	1	1	1	1	.	.	.	AC073284.4.1(UPSTREAM);INTERGENIC(INTERGENIC)
2	216162434	216162443	216162439	T	C	snp	1	0	0	0	.	.	.	INTERGENIC(INTERGENIC)
2	216202408	216202417	216202415	T	G	snp	1	1	1	1	.	.	.	ATIC(DOWNSTREAM);ATIC(INTRON);ATIC(UPSTREAM)
2	216246206	216246214	216246210	T	G	snp	1	1	1	1	.	.	.	FN1(DOWNSTREAM);FN1(EXON);FN1(INTRON);FN1(UPSTREAM)
2	216256769	216256776	216256775	A	G	snp	1	1	1	1	.	.	.	FN1(EXON);FN1(INTRON)
2	216269023	216269030	216269028	T	TTTTG	ins	1	1	1	1	.	.	.	FN1(DOWNSTREAM);FN1(INTRON)
2	216371451	216371460	216371454	AG	A	ins	1	0	0	0	.	.	.	INTERGENIC(INTERGENIC)
2	216411145	216411152	216411146	T	G	snp	1	1	1	1	.	.	.	AC012668.1.1(UPSTREAM);AC012668.2.1(INTRON)
2	216422780	216422787	216422783	T	G	ins	1	0	0	0	.	.	.	AC012668.2.1(INTRON)
2	216422780	216422787	216422784	T	G	ins	1	0	0	0	.	.	.	AC012668.2.1(INTRON)
2	216422780	216422787	216422786	T	G	snp	1	1	0	0	.	.	.	AC012668.2.1(INTRON)
2	216530938	216530946	216530939	T	C	snp	0	1	0	0	.	.	.	AC012668.2.1(INTRON);AC122136.1.1(INTRON)
2	216534159	216534172	216534169	GA	C	snp	1	0	0	0	.	.	.	AC012668.2.1(INTRON);AC122136.1.1(INTRON)
2	216625679	216625686	216625680	G	T	snp	0	1	1	0	.	.	.	AC122136.1.1(INTRON)
2	216704900	216704911	216704907	AC	T	snp	0	1	1	1	.	.	.	AC122136.1.1(INTRON)
2	216763330	216763337	216763335	T	C	snp	1	1	1	1	.	.	.	INTERGENIC(INTERGENIC)

2	216783539	216783562	216783554	AAAG	AA	del	1	1	1	1	.	.	.	INTERGENIC(INTERGENIC)
2	216891077	216891100	216891092	TTTA	TTTAT	del	1	1	1	1	.	.	.	MREG(INTRON);PECR(INTRON)
2	216895729	216895740	216895736	CT	G	snp	1	1	1	1	.	.	.	MREG(INTRON);PECR(INTRON)
2	216911000	216911011	216911008	TA	AC	ins	1	1	1	1	.	.	.	PECR(DOWNSTREAM);PECR(INTRON)
2	217025387	217025414	217025402	TTAA	T	ins	1	1	0	1	.	.	.	AC012513.3.1(UPSTREAM);XRCC5(INTRON)
2	217025387	217025414	217025405	TTAA	T	snp	1	1	1	1	.	.	.	AC012513.3.1(UPSTREAM);XRCC5(INTRON)
2	217025387	217025414	217025409	TTAA	T	snp	1	1	1	1	.	.	.	AC012513.3.1(UPSTREAM);XRCC5(INTRON)
2	217048231	217048245	217048236	AAT	G	snp	0	1	0	1	.	.	.	XRCC5(INTRON)
2	217146999	217147008	217147002	TG	C	snp	1	1	1	1	.	.	.	MARCH4(INTRON)
2	217165041	217165050	217165044	AT	AC	ins	1	0	0	0	.	.	.	AC069155.1.1(UPSTREAM);MARCH4(INTRON)
2	217165074	217165091	217165081	TA	TATGTG	ins	1	0	0	0	.	.	.	AC069155.1.1(UPSTREAM);MARCH4(INTRON)
2	217232039	217232053	217232046	CCT	T	snp	0	1	1	1	.	.	.	MARCH4(INTRON)
2	217415687	217415694	217415689	A	G	snp	1	0	0	0	.	.	.	RPL37A(INTRON)
2	217451678	217451686	217451680	T	C	ins	1	0	0	0	.	.	.	AC073321.3.1(DOWNSTREAM);INTERGENIC(INTERGENIC)
2	217459885	217459893	217459888	A	G	snp	0	0	1	0	.	.	.	AC073321.3.1(INTRON)
2	217498282	217498293	217498290	GCC	T	snp	1	1	1	1	.	IGFBP2(NON_SYNONYMOUS_CODING):P15L	.	IGFBP2(EXON);IGFBP2(INTRON)
2	217518802	217518809	217518805	A	G	snp	1	0	0	0	.	.	.	IGFBP2(INTRON)
2	217568494	217568503	217568497	CT	C	snp	1	1	1	1	.	.	.	AC007563.5.1(INTRON)
2	217666003	217666010	217666009	T	TC	ins	1	1	1	1	.	.	.	AC007563.3.1(INTRON);AC007563.5.1(INTRON);FABP5P14(DOWNSTREAM)
2	217772857	217772864	217772859	A	C	snp	1	0	0	0	.	.	.	AC007563.5.1(INTRON)
2	217800394	217800409	217800406	TC	C	snp	1	0	1	0	.	.	.	AC007563.5.1(INTRON)
2	218210166	218210181	218210176	GATA	G	snp	1	1	1	1	.	.	.	DIRC3(INTRON)
2	218239804	218239819	218239814	GGAG	T	snp	1	1	1	1	.	.	.	DIRC3(INTRON)
2	218260068	218260077	218260074	T	G	snp	1	0	0	0	.	.	.	DIRC3(INTRON)
2	218326647	218326658	218326654	AG	A	snp	1	0	0	0	.	.	.	DIRC3(INTRON)
2	218390845	218390856	218390853	AG	GAGACAGAG	del	1	1	1	1	.	.	.	DIRC3(INTRON)
2	218407271	218407288	218407280	AG	A	snp	1	0	0	0	.	.	.	DIRC3(INTRON)
2	218469702	218469710	218469708	T	G	snp	0	0	0	1	.	.	.	DIRC3(INTRON)
2	218487165	218487174	218487170	GA	T	snp	1	0	0	0	.	.	.	DIRC3(INTRON)
2	218716868	218716876	218716875	A	C	snp	1	1	1	1	.	.	.	TNS1(INTRON)
2	218787662	218787670	218787663	T	C	snp	1	0	0	0	.	.	.	TNS1(INTRON);TNS1(UPSTREAM)
2	218843804	218843811	218843805	G	A	snp	1	0	0	0	.	.	.	AC010136.2.1(INTRON);TNS1(INTRON);TNS1(UPSTREAM)
2	218869092	218869100	218869097	C	A	snp	1	0	1	0	.	.	.	INTERGENIC(INTERGENIC);TNS1(UPSTREAM)
2	219179718	219179733	219179729	TTTC	TTTCTT	ins	1	1	1	1	.	.	.	PNKD(INTRON);PNKD(UPSTREAM)
2	219189681	219189689	219189688	A	AG	ins	1	0	0	0	.	.	.	AC021016.8.1(DOWNSTREAM);PNKD(INTRON);snoU13(UPSTREAM)
2	219203685	219203692	219203691	T	G	snp	1	0	0	0	.	.	.	AC021016.8.1(INTRON);PNKD(INTRON);PNKD(UPSTREAM)
2	219271201	219271208	219271203	C	T	snp	1	1	1	1	.	.	.	CTDSP1(DOWNSTREAM);INTERGENIC(INTERGENIC);MIR26B(DOWNSTREAM)
2	219279665	219279674	219279671	T	G	snp	1	0	0	0	.	.	.	INTERGENIC(INTERGENIC);VIL1(UPSTREAM)
2	219279665	219279674	219279673	T	G	snp	1	1	1	1	.	.	.	INTERGENIC(INTERGENIC);VIL1(UPSTREAM)
2	219312464	219312473	219312469	T	C	snp	1	1	1	1	.	.	.	USP37(DOWNSTREAM);VIL1(INTRON)
2	219322794	219322803	219322798	A	G	snp	1	0	0	0	.	.	.	USP37(INTRON);VIL1(DOWNSTREAM)
2	219330668	219330679	219330674	TTA	C	snp	1	0	0	0	.	.	.	USP37(DOWNSTREAM);USP37(INTRON)
2	219332081	219332089	219332082	T	C	snp	1	0	0	0	.	.	.	USP37(INTRON)
2	219332081	219332089	219332085	T	C	snp	1	0	0	1	.	.	.	USP37(INTRON)
2	219372991	219372999	219372998	T	C	ins	1	0	0	0	.	.	.	USP37(DOWNSTREAM);USP37(INTRON)
2	219424720	219424729	219424723	A	C	snp	1	0	1	0	.	.	.	USP37(INTRON)
2	219639019	219639032	219639024	AG	A	snp	1	1	1	1	.	.	.	INTERGENIC(INTERGENIC)
2	219644618	219644627	219644625	T	G	snp	1	1	1	1	.	.	.	CYP27A1(UPSTREAM);INTERGENIC(INTERGENIC)
2	219843935	219843954	219843950	TTTA	T	ins	1	0	0	0	.	.	.	AC097468.5.1(DOWNSTREAM);AC097468.5.1(EXON);FEV(DOWNSTREAM)
2	219869553	219869562	219869556	G	T	snp	1	0	0	0	.	.	.	AC097468.4.1(INTRON);CCDC108(INTRON);MIR375(UPSTREAM)
2	219871891	219871899	219871894	T	TTTC	ins	1	0	0	0	.	.	.	AC097468.4.1(INTRON);CCDC108(INTRON)
2	219945273	219945290	219945276	AT	A	snp	1	1	0	1	.	.	.	NHEJ1(INTRON);SLC23A3(INTRON)
2	220032461	220032476	220032467	CAAA	G	snp	1	0	0	0	.	.	.	CNPPD1(DOWNSTREAM);SLC23A3(DOWNSTREAM);SLC23A3(INTRON)
2	220117263	220117272	220117270	C	A	ins	1	0	0	0	.	.	.	STK16(DOWNSTREAM);TUBA4A(INTRON);TUBA4B(UPSTREAM)
2	220130652	220130660	220130653	A	G	snp	1	1	1	1	.	.	.	TUBA4A(INTRON);TUBA4B(INTRON);TUBA4B(UPSTREAM)
2	220182093	220182106	220182099	TG	C	snp	0	1	0	1	.	.	.	INTERGENIC(INTERGENIC)
2	220222915	220222924	220222923	C	A	snp	1	0	0	0	.	.	.	AC053503.2.1(DOWNSTREAM);INTERGENIC(INTERGENIC)
2	220231909	220231918	220231912	A	C	snp	1	1	1	1	.	.	.	INTERGENIC(INTERGENIC)
2	220376115	220376124	220376120	C	A	snp	1	0	0	0	.	.	.	AC053503.11.1(INTRON);ACCN4(UPSTREAM);DNPEP(INTRON);GMPPA(DOWNSTREAM)

2	220376115	220376124	220376122	C	T	snp	0	0	1	0	.	.	.	AC053503.11.1(INTRON);ACCN4(UPSTREAM);DNPEP(INTRON);GMPPA(DOWNSTREAM)
2	220393253	220393273	220393258	TTG	T	snp	1	0	0	0	.	.	.	ACCN4(INTRON);DNPEP(INTRON)
2	220400050	220400065	220400063	TG	T	del	1	1	1	1	.	.	.	ACCN4(DOWNSTREAM);ACCN4(INTRON);CHPF(DOWNSTREAM);DNPEP(INTRON)
2	220502041	220502050	220502043	TG	C	snp	1	1	1	1	.	.	.	DNPEP(INTRON);SLC4A3(DOWNSTREAM);SLC4A3(INTRON)
2	220603787	220603798	220603793	GT	C	snp	1	0	0	0	.	.	.	AC009502.3.1(DOWNSTREAM);INTERGENIC(INTERGENIC)
2	220603799	220603806	220603800	G	T	snp	1	1	1	1	.	.	.	AC009502.3.1(DOWNSTREAM);INTERGENIC(INTERGENIC)
2	220670072	220670081	220670075	AT	C	snp	1	1	1	1	.	.	.	INTERGENIC(INTERGENIC)
2	220684502	220684510	220684509	T	G	ins	1	1	1	1	.	.	.	INTERGENIC(INTERGENIC)
2	220704005	220704020	220704013	TTCT	C	snp	1	0	0	0	.	.	.	INTERGENIC(INTERGENIC)
2	220767471	220767478	220767472	G	T	snp	1	1	1	1	.	.	.	AC008281.1.1(DOWNSTREAM);INTERGENIC(INTERGENIC)
2	220802571	220802594	220802589	TAA	C	snp	0	1	0	0	.	.	.	INTERGENIC(INTERGENIC)
2	220820107	220820122	220820112	TCTT	TTTCTTTCT	del	1	1	1	1	.	.	.	INTERGENIC(INTERGENIC)
2	220839306	220839315	220839310	T	C	snp	1	1	1	1	.	.	.	INTERGENIC(INTERGENIC)
2	220912283	220912294	220912292	TG	A	snp	0	1	1	1	.	.	.	AC009310.1.1(DOWNSTREAM);INTERGENIC(INTERGENIC)
2	221028089	221028098	221028093	TA	G	snp	1	0	0	0	.	.	.	AC114765.1.1(INTRON);AC114765.2.1(INTRON)
2	221174924	221174931	221174927	T	C	snp	1	1	1	1	.	.	.	AC114765.1.1(INTRON)
2	221271915	221271934	221271922	TTTG	C	snp	1	0	0	0	.	.	.	AC114765.1.1(INTRON)
2	221296193	221296201	221296200	T	C	snp	1	1	1	1	.	.	.	AC114765.1.1(INTRON)
2	221310127	221310134	221310132	T	C	snp	1	0	0	0	.	.	.	AC114765.1.1(INTRON)
2	221344082	221344105	221344094	AAAT	T	snp	1	0	0	0	.	.	.	AC019051.1(UPSTREAM);AC067956.1.1(INTRON)
2	221344082	221344105	221344098	AAAT	T	snp	1	1	0	0	.	.	.	AC019051.1(UPSTREAM);AC067956.1.1(INTRON)
2	221346252	221346260	221346255	A	C	snp	1	1	1	1	.	.	.	AC019051.1(UPSTREAM);AC067956.1.1(INTRON)
2	221421954	221421961	221421960	A	C	snp	1	0	0	0	.	.	.	AC067956.1.1(INTRON)
2	221614542	221614553	221614549	CAA	T	snp	1	1	1	1	.	.	.	INTERGENIC(INTERGENIC)
2	221692468	221692475	221692470	T	C	snp	1	1	1	1	.	.	.	INTERGENIC(INTERGENIC)
2	221808808	221808815	221808813	A	G	snp	1	0	0	0	.	.	.	INTERGENIC(INTERGENIC)
2	221826836	221826851	221826842	CCTG	A	snp	1	1	0	1	.	.	.	INTERGENIC(INTERGENIC)
2	221910193	221910216	221910208	ATTA	G	snp	1	0	0	0	.	.	.	INTERGENIC(INTERGENIC)
2	222046330	222046343	222046341	TA	T	snp	1	1	1	1	.	.	.	INTERGENIC(INTERGENIC)
2	222083997	222084020	222084002	AATT	TT	del	1	1	1	1	.	.	.	INTERGENIC(INTERGENIC)
2	222119324	222119333	222119330	GA	C	snp	1	0	0	0	.	.	.	INTERGENIC(INTERGENIC)
2	222211781	222211790	222211786	A	C	snp	1	1	0	1	.	.	.	INTERGENIC(INTERGENIC)
2	222342941	222342950	222342943	A	C	snp	1	0	0	0	.	.	.	EPHA4(DOWNSTREAM);EPHA4(INTRON)
2	222402718	222402726	222402724	A	C	snp	1	1	1	1	.	.	.	AC079834.2.1(DOWNSTREAM);EPHA4(INTRON)
2	222431575	222431595	222431580	AGA	G	snp	1	0	1	0	.	.	.	EPHA4(INTRON)
2	222431575	222431595	222431586	AGA	G	snp	1	1	1	1	.	.	.	EPHA4(INTRON)
2	222431575	222431595	222431592	AGA	G	snp	1	1	1	1	.	.	.	EPHA4(INTRON)
2	222512395	222512408	222512406	CA	C	del	1	0	0	0	.	.	.	INTERGENIC(INTERGENIC)
2	222553843	222553852	222553849	T	G	snp	1	0	0	0	.	.	.	INTERGENIC(INTERGENIC)
2	222657056	222657067	222657058	CT	TA	ins	1	0	0	0	.	.	.	INTERGENIC(INTERGENIC)
2	222661776	222661791	222661780	CAAA	T	snp	1	0	0	0	.	.	.	INTERGENIC(INTERGENIC)
2	222713527	222713535	222713533	G	A	snp	1	1	1	1	.	.	.	INTERGENIC(INTERGENIC)
2	222740394	222740403	222740397	T	G	ins	1	0	0	0	.	.	.	INTERGENIC(INTERGENIC)
2	222883874	222883883	222883878	G	GC	ins	0	0	1	0	.	.	.	INTERGENIC(INTERGENIC)
2	222908015	222908026	222908022	AAT	G	snp	0	0	1	0	.	.	.	AC009302.4.1(DOWNSTREAM);INTERGENIC(INTERGENIC)
2	223034759	223034782	223034770	TTTG	TTTT	ins	1	0	0	0	.	.	.	INTERGENIC(INTERGENIC)
2	223040756	223040765	223040764	A	C	snp	1	1	1	1	.	.	.	INTERGENIC(INTERGENIC)
2	223250708	223250725	223250723	AT	A	del	1	1	1	1	.	.	.	INTERGENIC(INTERGENIC)
2	223305072	223305080	223305076	T	G	ins	1	0	1	1	.	.	.	SGPP2(INTRON)
2	223452772	223452780	223452774	A	C	snp	1	1	1	1	.	.	.	FARSB(INTRON)
2	223496863	223496872	223496870	A	G	snp	1	1	1	1	.	.	.	FARSB(INTRON)
2	223566302	223566317	223566307	AAAG	AGAAAGAAAAAG	ins	1	1	1	1	.	.	.	AC016712.1.1(UPSTREAM);MOGAT1(INTRON);snoU13(UPSTREAM)
2	223657588	223657602	223657592	CCT	G	snp	1	1	0	1	.	.	.	INTERGENIC(INTERGENIC)
2	223694210	223694233	223694229	GTTT	G	snp	0	1	0	1	.	.	.	INTERGENIC(INTERGENIC)
2	223906823	223906836	223906833	CT	T	snp	1	1	1	1	.	.	.	INTERGENIC(INTERGENIC)
2	223975393	223975402	223975395	T	TTTTTTTGTTGTTG	ins	1	1	1	1	.	.	.	KCNE4(INTRON)
2	223986868	223986875	223986872	T	C	snp	0	1	0	1	.	.	.	KCNE4(INTRON)
2	224000455	224000466	224000460	AAT	AATAATAA	del	1	1	1	1	.	.	.	KCNE4(INTRON)
2	224145162	224145177	224145174	AT	T	snp	1	0	0	0	.	.	.	INTERGENIC(INTERGENIC)
2	224219196	224219205	224219199	A	C	snp	1	0	0	0	.	.	.	INTERGENIC(INTERGENIC)
2	224429089	224429104	224429100	AAAC	AAACA	del	1	0	0	0	.	.	.	INTERGENIC(INTERGENIC)
2	224429105	224429114	224429108	A	C	snp	1	0	0	1	.	.	.	INTERGENIC(INTERGENIC)
2	224474510	224474530	224474513	AAT	C	ins	1	0	0	0	.	.	.	INTERGENIC(INTERGENIC)
2	224490771	224490788	224490783	AAG	C	snp	1	0	1	0	.	.	.	INTERGENIC(INTERGENIC)

2	224574589	224574597	224574591	T	C	snp	1	0	0	0	.	.	.	AC012512.1.1(DOWNSTREAM);INTERGENIC(INTERGENIC)
2	224653497	224653514	224653505	AAG	A	snp	1	1	1	1	.	.	.	AP1S3(INTRON)
2	224653923	224653932	224653930	AC	T	snp	1	0	0	0	.	.	.	AP1S3(INTRON)
2	224655477	224655488	224655480	TG	T	snp	1	0	0	0	.	.	.	AP1S3(INTRON)
2	224693004	224693013	224693007	CT	CA	ins	1	1	1	1	.	.	.	AP1S3(INTRON)
2	224693004	224693013	224693009	CT	A	snp	1	1	1	1	.	.	.	AP1S3(INTRON)
2	224711237	224711248	224711245	TTG	C	snp	1	0	0	0	.	.	.	INTERGENIC(INTERGENIC)
2	224729370	224729378	224729373	T	C	snp	1	1	1	1	.	.	.	WDFY1(INTRON)
2	224749909	224749917	224749910	G	T	snp	1	1	1	1	.	.	.	WDFY1(INTRON)
2	224798499	224798507	224798504	C	T	snp	1	0	0	0	.	.	.	WDFY1(INTRON)
2	224798499	224798507	224798505	C	A	snp	0	1	1	1	.	.	.	WDFY1(INTRON)
2	224830545	224830553	224830552	T	C	snp	1	0	0	0	.	.	.	AC073641.2.1(EXON);MRPL44(INTRON)
2	224832411	224832418	224832414	T	G	snp	1	0	0	0	.	.	.	AC073641.2.1(EXON);MRPL44(UTR_3_PRIME)
2	224995827	224995836	224995834	CA	T	snp	1	1	1	1	.	.	.	INTERGENIC(INTERGENIC)
2	224998044	224998051	224998047	A	G	snp	1	1	1	1	.	.	.	INTERGENIC(INTERGENIC)
2	225042432	225042445	225042437	TA	G	snp	1	1	1	1	.	.	.	INTERGENIC(INTERGENIC)
2	225153726	225153737	225153732	TAT	C	snp	0	1	0	1	.	.	.	INTERGENIC(INTERGENIC)
2	225188149	225188160	225188154	AC	T	snp	0	0	0	1	.	.	.	INTERGENIC(INTERGENIC)
2	225226992	225227006	225226997	AGA	AG	del	0	1	1	0	.	.	.	INTERGENIC(INTERGENIC)
2	225227093	225227116	225227101	GAAA	A	del	1	0	0	0	.	.	.	INTERGENIC(INTERGENIC)
2	225227093	225227116	225227109	GAAA	A	ins	1	0	0	0	.	.	.	INTERGENIC(INTERGENIC)
2	225283400	225283413	225283404	AT	G	snp	1	0	0	0	.	.	.	INTERGENIC(INTERGENIC)
2	225449894	225449917	225449899	GGC	G	snp	1	1	1	1	.	.	.	CUL3(UTR_5_PRIME)
2	225492051	225492059	225492057	T	G	snp	1	0	0	0	.	.	.	INTERGENIC(INTERGENIC)
2	225494380	225494399	225494384	TTTG	TTGTTTG	del	0	1	1	1	.	.	.	INTERGENIC(INTERGENIC)
2	225494380	225494399	225494394	TTTG	G	del	0	1	1	0	.	.	.	INTERGENIC(INTERGENIC)
2	225571607	225571624	225571621	TA	AC	ins	1	1	0	1	.	.	.	INTERGENIC(INTERGENIC)
2	225608291	225608299	225608295	T	C	snp	1	0	1	0	.	.	.	INTERGENIC(INTERGENIC)
2	225620868	225620879	225620877	TA	TATG	ins	1	1	1	1	.	.	.	INTERGENIC(INTERGENIC)
2	225721012	225721021	225721014	T	G	ins	1	0	0	0	.	.	.	DOCK10(DOWNSTREAM);DOCK10(INTRON)
2	225783052	225783063	225783060	GT	TC	ins	1	1	1	1	.	.	.	DOCK10(INTRON)
2	225863958	225863967	225863963	TC	TCTCT	del	1	0	0	0	.	.	.	DOCK10(INTRON)
2	225914640	225914651	225914646	TTG	C	snp	1	1	1	1	.	.	.	INTERGENIC(INTERGENIC)
2	225950436	225950445	225950439	TG	A	snp	1	1	1	1	.	.	.	INTERGENIC(INTERGENIC)
2	226024768	226024781	226024770	CA	A	snp	0	1	0	1	.	.	.	INTERGENIC(INTERGENIC)
2	226046548	226046557	226046552	AT	G	snp	1	1	1	1	.	.	.	INTERGENIC(INTERGENIC)
2	226078014	226078025	226078022	AAC	A	ins	1	0	0	0	.	.	.	INTERGENIC(INTERGENIC)
2	226370776	226370785	226370777	A	G	snp	1	1	1	1	.	.	.	NYAP2(INTRON)
2	226370776	226370785	226370779	A	G	snp	1	1	1	1	.	.	.	NYAP2(INTRON)
2	226377536	226377553	226377542	TC	C	del	1	0	0	0	.	.	.	NYAP2(INTRON)
2	226448775	226448784	226448778	A	C	snp	1	0	0	0	.	.	.	NYAP2(INTRON)
2	226503780	226503789	226503788	A	G	ins	1	0	1	1	.	.	.	NYAP2(INTRON)
2	226518757	226518765	226518761	A	G	snp	1	1	0	1	.	.	.	INTERGENIC(INTERGENIC);NYAP2(DOWNSTREAM)
2	226536706	226536715	226536709	T	G	snp	1	1	1	1	.	.	.	INTERGENIC(INTERGENIC)
2	226692506	226692515	226692507	T	C	snp	1	1	1	1	.	.	.	INTERGENIC(INTERGENIC)
2	226959438	226959446	226959439	A	G	ins	1	0	0	0	.	.	.	INTERGENIC(INTERGENIC)
2	226971789	226971797	226971793	T	C	snp	1	1	1	1	.	.	.	INTERGENIC(INTERGENIC)
2	227013796	227013816	227013812	AAC	C	del	0	1	1	1	.	.	.	INTERGENIC(INTERGENIC)
2	227127671	227127679	227127672	A	C	snp	1	1	1	1	.	.	.	INTERGENIC(INTERGENIC)
2	227177531	227177548	227177546	AC	A	snp	1	1	1	1	.	.	.	INTERGENIC(INTERGENIC)
2	227215908	227215919	227215912	CCT	T	snp	1	1	1	1	.	.	.	INTERGENIC(INTERGENIC)
2	227334251	227334264	227334254	AT	C	snp	1	0	0	0	.	.	.	INTERGENIC(INTERGENIC)
2	227335365	227335378	227335368	AT	C	snp	1	1	0	1	.	.	.	INTERGENIC(INTERGENIC)
2	227335481	227335492	227335484	AT	C	snp	1	1	1	1	.	.	.	INTERGENIC(INTERGENIC)
2	227335481	227335492	227335489	AT	G	snp	1	1	0	0	.	.	.	INTERGENIC(INTERGENIC)
2	227335541	227335550	227335547	AT	G	snp	1	0	0	0	.	.	.	INTERGENIC(INTERGENIC)
2	227335703	227335712	227335706	AT	C	snp	1	1	0	1	.	.	.	INTERGENIC(INTERGENIC)
2	227335783	227335792	227335786	AT	C	snp	1	1	1	1	.	.	.	INTERGENIC(INTERGENIC)
2	227379153	227379168	227379164	AT	A	del	1	0	0	0	.	.	.	INTERGENIC(INTERGENIC)
2	227547467	227547475	227547470	A	G	ins	1	1	1	0	.	.	.	INTERGENIC(INTERGENIC)
2	227547467	227547475	227547471	A	G	snp	1	1	1	1	.	.	.	INTERGENIC(INTERGENIC)
2	227669915	227669934	227669920	TTTG	C	snp	1	0	0	0	.	.	.	INTERGENIC(INTERGENIC);RP11-395N3.1.1(DOWNSTREAM)
2	227800753	227800760	227800757	T	G	snp	1	0	0	0	.	.	.	RHBDD1(INTRON)
2	227924352	227924360	227924354	A	C	snp	1	0	0	0	.	.	.	COL4A4(INTRON)
2	228039215	228039238	228039230	ATT	TTG	ins	1	0	0	0	.	.	.	COL4A3(INTRON)
2	228043428	228043437	228043434	T	C	snp	1	0	0	0	.	.	.	COL4A3(INTRON)
2	228089951	228089958	228089955	T	C	snp	1	1	1	1	.	.	.	AC097662.2.1(INTRON);COL4A3(INTRON)

2	228120430	228120439	228120431	T	G	snp	1	1	1	1	.	.	.	AC097662.2.1(INTRON);COL4A3(INTRON)
2	228142842	228142851	228142850	T	TA	ins	1	1	1	1	.	.	.	AC097662.2.1(INTRON);COL4A3(INTRON)
2	228256324	228256332	228256328	A	G	snp	1	1	1	1	.	.	.	INTERGENIC(INTERGENIC)
2	228263773	228263784	228263781	GA	AGA	del	1	1	1	1	.	.	.	INTERGENIC(INTERGENIC)
2	228286513	228286524	228286518	CCT	G	snp	1	1	1	1	.	.	.	INTERGENIC(INTERGENIC)
2	228447044	228447053	228447050	CT	T	snp	1	0	0	1	.	.	.	INTERGENIC(INTERGENIC)
2	228491236	228491244	228491241	T	C	snp	1	0	0	0	.	.	.	C2orf83(INTRON)
2	228492860	228492868	228492861	T	G	snp	1	1	1	1	.	.	.	AC064853.3.1(DOWNSTREAM);C2orf83(INTRON)
2	228510332	228510339	228510336	T	C	snp	0	0	1	0	.	.	.	AC064853.3.1(INTRON)
2	228527081	228527088	228527083	G	T	snp	1	1	0	1	.	.	.	INTERGENIC(INTERGENIC)
2	228528008	228528016	228528014	A	C	snp	1	0	0	0	.	.	.	INTERGENIC(INTERGENIC)
2	228548060	228548071	228548063	TTA	A	snp	1	0	0	0	.	.	.	AC064853.4.1(DOWNSTREAM);INTERGENIC(INTERGENIC);SLC19A3(DOWNSTREAM)
2	228572454	228572461	228572460	T	C	snp	1	1	1	1	.	.	.	SLC19A3(INTRON);SLC19A3(UPSTREAM)
2	228636828	228636841	228636839	TC	G	snp	1	1	1	0	.	.	.	INTERGENIC(INTERGENIC)
2	228669243	228669252	228669249	AC	T	snp	1	0	0	0	.	.	.	AC073065.3.1(EXON)
2	228722746	228722753	228722747	G	T	snp	0	1	1	1	.	.	.	INTERGENIC(INTERGENIC)
2	228835378	228835389	228835384	AG	G	snp	1	0	0	0	.	.	.	INTERGENIC(INTERGENIC)
2	228994452	228994463	228994458	AT	G	snp	1	0	0	0	.	.	.	SPHKAP(INTRON)
2	229118423	229118437	229118430	TAT	G	snp	1	1	1	1	.	.	.	INTERGENIC(INTERGENIC)
2	229133679	229133686	229133683	T	C	snp	1	1	1	1	.	.	.	INTERGENIC(INTERGENIC)
2	229176727	229176742	229176737	GA	T	snp	1	1	0	1	.	.	.	INTERGENIC(INTERGENIC)
2	229212931	229212946	229212934	CT	C	snp	1	1	1	1	.	.	.	AC074019.2.1(DOWNSTREAM);INTERGENIC(INTERGENIC)
2	229351933	229351942	229351938	TA	G	snp	1	1	1	0	.	.	.	AC009410.1.1(INTRON)
2	229452739	229452750	229452747	TTC	TTCT	del	1	1	1	1	.	.	.	AC009410.1.1(INTRON);SNORD112(UPSTREAM)
2	229458547	229458564	229458558	AC	AT	ins	1	0	0	0	.	.	.	AC009410.1.1(INTRON)
2	229504347	229504355	229504348	A	G	snp	1	1	1	1	.	.	.	INTERGENIC(INTERGENIC)
2	229629195	229629206	229629201	AAG	G	snp	0	0	1	0	.	.	.	INTERGENIC(INTERGENIC)
2	229758313	229758322	229758321	T	TA	ins	1	1	0	1	.	.	.	PID1(INTRON)
2	229758359	229758368	229758360	A	C	snp	1	1	1	1	.	.	.	PID1(INTRON)
2	229845846	229845853	229845851	T	G	snp	1	0	0	0	.	.	.	PID1(INTRON)
2	229884761	229884768	229884765	A	C	snp	1	1	1	1	.	.	.	PID1(DOWNSTREAM);PID1(INTRON)
2	229918813	229918821	229918816	A	G	ins	1	1	1	1	.	.	.	PID1(INTRON)
2	229919420	229919435	229919430	GT	TGTGT	del	1	1	1	1	.	.	.	PID1(INTRON)
2	229921329	229921344	229921340	AT	G	snp	1	0	0	1	.	.	.	PID1(INTRON)
2	229926698	229926705	229926702	T	C	snp	1	0	0	0	.	.	.	PID1(INTRON)
2	230049650	230049659	230049653	AT	A	snp	1	0	0	0	.	.	.	AC006368.1(UPSTREAM);PID1(INTRON)
2	230052211	230052218	230052213	C	T	snp	1	1	1	1	.	.	.	PID1(INTRON)
2	230190891	230190900	230190893	A	C	snp	1	0	0	0	.	.	.	INTERGENIC(INTERGENIC)
2	230205966	230205977	230205975	AC	A	snp	1	1	0	1	.	.	.	INTERGENIC(INTERGENIC)
2	230212607	230212616	230212614	T	G	snp	1	1	0	1	.	.	.	INTERGENIC(INTERGENIC)
2	230257270	230257281	230257278	GT	A	snp	1	0	0	0	.	.	.	DNER(INTRON)
2	230258791	230258804	230258797	CA	G	snp	1	0	0	0	.	.	.	DNER(INTRON)
2	230310482	230310495	230310493	TC	T	del	1	1	1	1	.	.	.	DNER(INTRON)
2	230406037	230406048	230406045	AGA	G	ins	0	1	1	1	.	.	.	DNER(INTRON)
2	230464154	230464161	230464160	T	C	snp	1	1	1	1	.	.	.	DNER(INTRON)
2	230498971	230498994	230498986	TTCC	T	snp	1	1	1	1	.	.	.	DNER(INTRON)
2	230594526	230594533	230594529	G	T	snp	1	0	0	0	.	.	.	INTERGENIC(INTERGENIC)
2	230917171	230917178	230917177	A	G	snp	1	1	1	1	.	.	.	SLC16A14(INTRON)
2	230925395	230925402	230925401	T	TTG	ins	1	1	1	1	.	.	.	RNY4P19(UPSTREAM);SLC16A14(INTRON)
2	230961673	230961681	230961679	T	C	ins	1	1	1	1	.	.	.	INTERGENIC(INTERGENIC)
2	231070314	231070323	231070322	A	G	snp	1	1	1	1	.	.	.	AC009950.1.1(INTRON);SP110(INTRON)
2	231286220	231286237	231286231	AT	AC	ins	1	1	0	1	.	.	.	SP100(INTRON)
2	231421159	231421182	231421163	TAAA	AAATAAAC	ins	1	1	1	1	.	.	.	AC010149.4.1(INTRON)
2	231498091	231498102	231498099	TTA	G	snp	1	1	0	1	.	.	.	INTERGENIC(INTERGENIC)
2	231514086	231514109	231514096	TTTC	C	ins	1	0	0	0	.	.	.	INTERGENIC(INTERGENIC)
2	231515005	231515013	231515008	A	AG	ins	1	0	0	0	.	.	.	INTERGENIC(INTERGENIC)
2	231542875	231542889	231542878	ACA	T	snp	1	0	0	0	.	.	.	INTERGENIC(INTERGENIC)
2	231558606	231558625	231558621	TTTG	T	snp	1	1	1	1	.	.	.	AC098823.3.1(INTRON)
2	231685194	231685202	231685198	A	G	snp	1	1	1	1	.	.	.	CAB39(DOWNSTREAM);CAB39(UTR_3_PRIME)
2	232033866	232033874	232033873	T	G	snp	1	0	0	0	.	.	.	PSMD1(DOWNSTREAM);PSMD1(INTRON);PSMD1(UPSTREAM)
2	232049465	232049478	232049471	AT	TATATAT	del	1	1	1	1	.	.	.	INTERGENIC(INTERGENIC)
2	232216335	232216344	232216337	AT	G	snp	1	1	0	0	.	.	.	ARMC9(INTRON)
2	232402902	232402917	232402909	TTTG	T	ins	1	1	1	1	.	.	.	INTERGENIC(INTERGENIC)
2	232437835	232437842	232437838	A	G	snp	1	1	1	1	.	.	.	INTERGENIC(INTERGENIC)
2	232472930	232472950	232472935	AAT	A	snp	1	0	0	0	.	.	.	INTERGENIC(INTERGENIC)
2	232504580	232504588	232504587	C	A	snp	0	1	1	1	.	.	.	INTERGENIC(INTERGENIC)

2	232538478	232538495	232538492	AT	T	snp	1	1	1	1	.	.	.	INTERGENIC(INTERGENIC)
2	232538478	232538495	232538493	AT	A	del	1	1	1	1	.	.	.	INTERGENIC(INTERGENIC)
2	232541811	232541834	232541831	TTA	G	snp	1	0	0	0	.	.	.	INTERGENIC(INTERGENIC)
2	232659467	232659484	232659481	TTG	A	snp	1	0	0	0	.	.	.	COPS7B(DOWNSTREAM);COPS7B(INTRON);COPS7B(UPSTREAM);RP11-690I21.1.1(UPSTREAM)
2	232670124	232670141	232670138	TA	A	del	1	0	0	0	.	.	.	COPS7B(INTRON);RP11-690I21.2.1(UPSTREAM);RP11-690I21.3.1(DOWNSTREAM)
2	232747891	232747898	232747892	G	T	snp	1	1	1	1	.	.	.	INTERGENIC(INTERGENIC)
2	232845419	232845428	232845420	A	G	snp	1	1	0	1	.	.	.	AC105461.1.1(INTRON);DIS3L2(INTRON)
2	233031054	233031063	233031057	GC	T	snp	0	1	1	1	.	.	.	DIS3L2(DOWNSTREAM);DIS3L2(INTRON)
2	233041480	233041503	233041498	TTAA	T	snp	1	1	1	1	.	.	.	DIS3L2(INTRON);MIR562(DOWNSTREAM)
2	233259966	233259973	233259967	A	C	snp	1	0	0	0	.	.	.	INTERGENIC(INTERGENIC)
2	233407569	233407584	233407579	CCTG	GCCTGCCC	ins	1	0	0	0	.	.	.	CHRNG(DOWNSTREAM);CHRNG(INTRON)
2	233411050	233411061	233411057	TGT	A	snp	1	1	1	1	.	.	.	CHRNG(DOWNSTREAM);CHRNG(UTR_3_PRIME);EIF4E2(UPSTREAM);TIGD1(DOWNSTREAM)
2	233461770	233461778	233461777	A	C	snp	1	0	1	0	.	.	.	INTERGENIC(INTERGENIC)
2	233470172	233470187	233470184	AG	G	snp	1	0	0	0	.	.	.	EFHD1(UPSTREAM);INTERGENIC(INTERGENIC)
2	233582788	233582795	233582793	T	G	snp	1	1	1	1	.	.	.	GIGYF2(INTRON)
2	233585296	233585323	233585319	TTTG	T	snp	1	1	1	1	.	.	.	GIGYF2(INTRON)
2	233640867	233640874	233640871	T	G	snp	0	1	1	1	.	.	.	GIGYF2(DOWNSTREAM);GIGYF2(INTRON);GIGYF2(UPSTREAM);KCNJ13(INTRON);KCNJ13(UPSTREAM)
2	233649790	233649801	233649796	CTT	TTCT	del	1	1	1	1	.	.	.	GIGYF2(INTRON);GIGYF2(UPSTREAM);U6(DOWNSTREAM)
2	233649790	233649801	233649797	CTT	TC	del	1	1	1	1	.	.	.	GIGYF2(INTRON);GIGYF2(UPSTREAM);U6(DOWNSTREAM)
2	233649790	233649801	233649798	CTT	C	del	1	1	1	1	.	.	.	GIGYF2(INTRON);GIGYF2(UPSTREAM);U6(DOWNSTREAM)
2	233673655	233673666	233673658	AT	C	snp	1	0	0	0	.	.	.	GIGYF2(INTRON);GIGYF2(UPSTREAM)
2	233688065	233688080	233688074	CTTT	TTC	del	1	1	1	1	.	.	.	GIGYF2(DOWNSTREAM);GIGYF2(INTRON)
2	233728795	233728803	233728801	C	A	snp	1	1	1	1	.	.	.	C2orf82(INTRON);C2orf82(UPSTREAM);GIGYF2(DOWNSTREAM)
2	233787801	233787818	233787811	AAC	G	snp	0	1	0	0	.	.	.	NGEF(INTRON)
2	233818074	233818085	233818081	AG	AGAGA	del	1	1	1	1	.	.	.	NGEF(INTRON)
2	233836986	233836993	233836988	A	C	snp	0	1	0	1	.	.	.	NGEF(INTRON)
2	233857747	233857756	233857751	AT	T	snp	1	1	1	1	.	.	.	NGEF(INTRON)
2	233865304	233865311	233865309	A	C	snp	1	0	0	0	.	.	.	NGEF(INTRON)
2	233940310	233940327	233940317	TG	A	snp	1	0	0	0	.	.	.	INPP5D(INTRON);INPP5D(UPSTREAM)
2	233967403	233967410	233967405	A	G	snp	1	0	0	0	.	.	.	INPP5D(INTRON)
2	233996055	233996062	233996061	A	C	ins	1	0	0	0	.	.	.	INPP5D(DOWNSTREAM);INPP5D(INTRON)
2	234118220	234118229	234118224	C	A	snp	1	1	0	1	.	.	.	ATG16L1(UPSTREAM);INPP5D(DOWNSTREAM);INTERGENIC(INTERGENIC)
2	234150166	234150180	234150170	AAG	G	del	1	1	1	1	.	.	.	ATG16L1(INTRON)
2	234212487	234212496	234212494	T	TGAGACAGGGTCTCGTTCTG	ins	1	1	0	1	.	.	.	INTERGENIC(INTERGENIC);SAG(UPSTREAM)
2	234248611	234248620	234248619	T	C	snp	0	1	0	0	.	.	.	SAG(INTRON);SAG(UPSTREAM)
2	234305805	234305812	234305811	G	T	snp	1	0	0	0	.	.	.	AC019221.4.1(DOWNSTREAM);DGKD(INTRON)
2	234480753	234480761	234480760	T	C	snp	0	1	0	0	.	.	.	INTERGENIC(INTERGENIC)
2	234607857	234607865	234607863	A	G	snp	1	0	0	0	.	.	.	AC114812.8.1(INTRON);UGT1A10(INTRON);UGT1A6(INTRON);UGT1A7(INTRON);UGT1A8(INTRON);UGT1A9(INTRON)
2	234617405	234617413	234617407	C	T	snp	1	1	1	1	.	.	.	AC114812.8.1(UPSTREAM);UGT1A10(INTRON);UGT1A5(UPSTREAM);UGT1A6(INTRON);UGT1A7(INTRON);UGT1A8(INTRON);UGT1A9(INTRON)
2	234682052	234682061	234682056	CA	A	snp	0	1	1	1	.	.	.	HEATR7B1(UPSTREAM);INTERGENIC(INTERGENIC);UGT1A1(DOWNSTREAM);UGT1A10(DOWNSTREAM);UGT1A3(DOWNSTREAM);UGT1A4(DOWNSTREAM);UGT1A5(DOWNSTREAM);UGT1A6(DOWNSTREAM);UGT1A7(DOWNSTREAM);UGT1A8(DOWNSTREAM);UGT1A9(DOWNSTREAM)
2	234804322	234804329	234804326	T	G	snp	1	1	1	1	.	.	.	INTERGENIC(INTERGENIC)
2	234824897	234824906	234824902	TC	G	snp	1	0	0	0	.	.	.	INTERGENIC(INTERGENIC);TRPM8(UPSTREAM)
2	234830285	234830292	234830287	T	TTTTTC	ins	0	0	1	0	.	.	.	TRPM8(INTRON)
2	234831680	234831687	234831685	T	G	snp	1	1	1	1	.	.	.	TRPM8(INTRON)
2	234836554	234836567	234836557	TC	T	snp	1	1	1	1	.	.	.	TRPM8(INTRON);TRPM8(UPSTREAM)
2	234836554	234836567	234836561	TC	T	snp	1	1	1	1	.	.	.	TRPM8(INTRON);TRPM8(UPSTREAM)
2	234836554	234836567	234836565	TC	T	snp	1	1	1	0	.	.	.	TRPM8(INTRON);TRPM8(UPSTREAM)
2	234866152	234866159	234866153	A	C	snp	1	1	1	1	.	.	.	AC005538.5.1(UPSTREAM);TRPM8(INTRON)
2	234873229	234873236	234873231	A	C	snp	1	0	0	0	.	.	.	TRPM8(DOWNSTREAM);TRPM8(INTRON);TRPM8(UPSTREAM)
2	234911439	234911446	234911444	T	C	snp	1	0	0	0	.	.	.	TRPM8(INTRON)
2	234948897	234948906	234948900	TG	T	snp	1	1	0	0	.	.	.	INTERGENIC(INTERGENIC)
2	234968182	234968205	234968194	AAAC	G	snp	1	1	0	0	.	.	.	SPP2(DOWNSTREAM);SPP2(INTRON)
2	235021803	235021811	235021808	T	G	snp	0	1	1	1	.	.	.	AC006037.2.1(DOWNSTREAM);INTERGENIC(INTERGENIC)
2	235034815	235034826	235034823	AAG	AAGA	del	1	1	1	1	.	.	.	INTERGENIC(INTERGENIC)
2	235133214	235133229	235133227	AT	A	del	1	1	1	0	.	.	.	INTERGENIC(INTERGENIC);RP11-309M7.1.1(DOWNSTREAM)
2	235165064	235165073	235165066	GT	T	snp	1	1	0	1	.	.	.	INTERGENIC(INTERGENIC)

2	235204529	235204537	235204535	T	C	snp	1	0	0	0	.	.	.	INTERGENIC(INTERGENIC)	
2	235206306	235206315	235206309	TC	T	snp	1	1	1	1	.	.	.	INTERGENIC(INTERGENIC)	
2	235239002	235239015	235239010	TG	C	snp	0	1	0	0	.	.	.	AC097713.5.1(DOWNSTREAM);INTERGENIC(INTERGENIC)	
2	235291718	235291727	235291725	A	G	snp	1	1	0	1	.	.	.	INTERGENIC(INTERGENIC)	
2	235437109	235437116	235437115	T	C	snp	1	0	0	0	.	.	.	INTERGENIC(INTERGENIC)	
2	235438774	235438783	235438778	TG	G	snp	0	0	1	0	.	.	.	INTERGENIC(INTERGENIC)	
2	235470363	235470372	235470366	AG	C	snp	1	0	1	0	.	.	.	INTERGENIC(INTERGENIC)	
2	235513409	235513423	235513420	CTC	G	snp	0	1	0	1	.	.	.	INTERGENIC(INTERGENIC)	
2	235582733	235582742	235582737	CT	G	snp	0	1	0	0	.	.	.	INTERGENIC(INTERGENIC)	
2	235614294	235614311	235614305	AT	A	del	1	0	0	0	.	.	.	INTERGENIC(INTERGENIC)	
2	235614294	235614311	235614309	AT	A	del	1	1	1	1	.	.	.	INTERGENIC(INTERGENIC)	
2	235654609	235654616	235654613	A	C	snp	1	1	1	1	.	.	.	INTERGENIC(INTERGENIC)	
2	235668513	235668530	235668521	GT	T	snp	1	0	0	0	.	.	.	INTERGENIC(INTERGENIC)	
2	235690717	235690728	235690725	AAT	AATAA	del	1	1	0	1	.	.	.	INTERGENIC(INTERGENIC)	
2	235705458	235705465	235705461	A	G	snp	1	1	1	1	.	.	.	INTERGENIC(INTERGENIC)	
2	235712266	235712281	235712275	AGAA	AAAGA	del	1	1	1	1	.	.	.	INTERGENIC(INTERGENIC)	
2	235783894	235783909	235783904	TCTT	C	snp	1	0	0	0	.	.	.	AC010148.1.1(INTRON)	
2	235946883	235946892	235946888	TA	C	snp	1	0	0	0	.	.	.	SH3BP4(INTRON);SH3BP4(UPSTREAM)	
2	235958770	235958777	235958774	T	C	snp	1	1	1	1	.	.	.	SH3BP4(INTRON)	
2	235984775	235984783	235984782	T	C	snp	1	1	1	1	.	.	.	INTERGENIC(INTERGENIC)	
2	235987906	235987915	235987908	GT	TGTGTGTGA		del	1	1	1	1	.	.	.	INTERGENIC(INTERGENIC)
2	235999023	235999037	235999033	AAT	TAC	ins	1	0	0	0	.	.	.	AC114814.4.1(UPSTREAM);INTERGENIC(INTERGENIC)	
2	236006542	236006550	236006544	T	G	snp	1	0	0	0	.	.	.	INTERGENIC(INTERGENIC)	
2	236017785	236017794	236017792	CA	CG	ins	0	0	1	0	.	.	.	INTERGENIC(INTERGENIC)	
2	236032698	236032705	236032703	A	G	snp	1	0	0	0	.	.	.	INTERGENIC(INTERGENIC)	
2	236156741	236156754	236156751	CG	T	snp	1	1	1	1	.	.	.	INTERGENIC(INTERGENIC)	
2	236156760	236156769	236156765	GT	C	snp	1	0	0	0	.	.	.	INTERGENIC(INTERGENIC)	
2	236166135	236166144	236166140	A	G	snp	1	0	0	0	.	.	.	INTERGENIC(INTERGENIC)	
2	236210558	236210569	236210562	CTC	C	ins	1	0	0	0	.	.	.	INTERGENIC(INTERGENIC)	
2	236252893	236252902	236252898	AC	T	snp	1	0	0	0	.	.	.	INTERGENIC(INTERGENIC)	
2	236290419	236290438	236290424	GGTT	A	snp	1	1	1	1	.	.	.	INTERGENIC(INTERGENIC)	
2	236294738	236294746	236294739	T	C	snp	1	1	1	1	.	.	.	INTERGENIC(INTERGENIC)	
2	236331198	236331207	236331202	AC	G	snp	1	0	0	0	.	.	.	INTERGENIC(INTERGENIC)	
2	236341083	236341092	236341088	A	G	snp	1	0	0	0	.	.	.	INTERGENIC(INTERGENIC)	
2	236342991	236343000	236342993	T	C	ins	0	1	1	1	.	.	.	INTERGENIC(INTERGENIC)	
2	236342991	236343000	236342994	T	C	snp	0	1	1	1	.	.	.	INTERGENIC(INTERGENIC)	
2	236349654	236349681	236349662	TGAT	GATTGATTGATA		ins	1	1	1	1	.	.	.	INTERGENIC(INTERGENIC)
2	236373198	236373207	236373202	T	C	ins	1	0	0	0	.	.	.	INTERGENIC(INTERGENIC)	
2	236396713	236396740	236396736	TCTT	G	snp	0	0	0	1	.	.	.	INTERGENIC(INTERGENIC)	
2	236609446	236609463	236609449	TC	G	snp	1	0	0	0	.	.	.	AGAP1(INTRON)	
2	236660832	236660839	236660837	T	C	snp	1	0	0	0	.	.	.	AGAP1(INTRON)	
2	236791625	236791640	236791632	TTTA	G	snp	1	0	1	0	.	.	.	AGAP1(INTRON);TMSB10P1(UPSTREAM)	
2	236828354	236828369	236828361	AC	G	snp	0	1	1	1	.	.	.	AGAP1(INTRON)	
2	236966196	236966203	236966198	C	CA	ins	1	0	0	0	.	.	.	AGAP1(INTRON)	
2	236966196	236966203	236966200	C	A	snp	1	0	0	0	.	.	.	AGAP1(INTRON)	
2	236971287	236971306	236971298	TTTG	TTTGTTTGTT		del	1	0	0	0	.	.	.	AGAP1(INTRON)
2	236988250	236988259	236988254	AT	G	snp	1	1	1	1	.	.	.	AGAP1(INTRON)	
2	237129868	237129891	237129887	CAA	G	snp	1	0	0	0	.	.	.	ASB18(INTRON)	
2	237171341	237171348	237171342	C	A	snp	1	0	0	0	.	.	.	ASB18(INTRON)	
2	237265603	237265612	237265605	AT	C	snp	1	1	0	0	.	.	.	IQCA1(INTRON)	
2	237267866	237267877	237267872	TA	C	snp	1	1	0	1	.	.	.	IQCA1(INTRON)	
2	237424505	237424520	237424512	TTCT	TTC	ins	1	0	1	0	.	.	.	INTERGENIC(INTERGENIC)	
2	237501886	237501897	237501894	TG	C	snp	1	1	0	1	.	.	.	INTERGENIC(INTERGENIC)	
2	237534226	237534239	237534237	TG	T	del	1	0	0	0	.	.	.	INTERGENIC(INTERGENIC)	
2	237534239	237534247	237534240	G	T	snp	1	0	0	0	.	.	.	INTERGENIC(INTERGENIC)	
2	237592063	237592072	237592065	CT	G	snp	1	1	1	1	.	.	.	INTERGENIC(INTERGENIC)	
2	237598200	237598217	237598214	TCT	TC	del	1	1	1	1	.	.	.	INTERGENIC(INTERGENIC)	
2	237617680	237617689	237617686	CA	A	snp	1	1	0	1	.	.	.	INTERGENIC(INTERGENIC)	
2	237620538	237620547	237620543	TC	T	snp	1	0	0	1	.	.	.	INTERGENIC(INTERGENIC)	
2	237630879	237630894	237630884	AATA	C	snp	1	1	0	1	.	.	.	INTERGENIC(INTERGENIC)	
2	237705785	237705792	237705788	A	C	snp	1	1	1	1	.	.	.	INTERGENIC(INTERGENIC)	
2	237787924	237787933	237787928	A	C	snp	1	0	0	0	.	.	.	INTERGENIC(INTERGENIC)	
2	237832949	237832960	237832954	TC	G	snp	1	1	0	1	.	.	.	INTERGENIC(INTERGENIC)	
2	237863144	237863151	237863149	T	C	snp	1	1	1	1	.	.	.	INTERGENIC(INTERGENIC)	
2	238009516	238009527	238009519	AT	C	snp	1	0	0	0	.	.	.	COPS8(DOWNSTREAM);INTERGENIC(INTERGENIC)	
2	238037823	238037832	238037827	TG	C	snp	1	1	0	1	.	.	.	INTERGENIC(INTERGENIC)	
2	238146530	238146538	238146532	A	C	snp	1	1	1	1	.	.	.	INTERGENIC(INTERGENIC)	

2	238317944	238317955	238317947	TTA	C	snp	1	0	0	0	.	.	.	COL6A3(INTRON)	
2	238402824	238402831	238402830	G	A	snp	0	1	1	1	.	.	.	MLPH(INTRON)	
2	238405359	238405366	238405360	G	A	snp	1	0	0	0	.	.	.	MLPH(INTRON)	
2	238427731	238427739	238427734	T	G	snp	1	1	1	1	.	.	.	AC112721.1(DOWNSTREAM);AC112721.2(DOWNSTREAM);AC112721.3(DOWNSTREAM);MLPH(DOWNSTREAM);MLPH(INTRON);MLPH(UPSTREAM)	
2	238465725	238465738	238465730	CT	C	snp	1	0	0	0	.	.	.	INTERGENIC(INTERGENIC);MLPH(DOWNSTREAM)	
2	238465827	238465838	238465833	CA	T	snp	1	0	0	0	.	.	.	INTERGENIC(INTERGENIC);MLPH(DOWNSTREAM)	
2	238479394	238479403	238479401	AG	A	snp	0	0	0	1	.	.	.	INTERGENIC(INTERGENIC);PRLH(DOWNSTREAM);RAB17(DOWNSTREAM)	
2	238622269	238622292	238622274	AATG	G	snp	1	0	0	0	.	.	.	LRRFIP1(INTRON)	
2	238706051	238706074	238706068	GAAT	C	snp	1	1	0	1	.	.	.	LRRFIP1(INTRON);RBM44(UPSTREAM)	
2	238823230	238823241	238823234	GT	C	snp	1	1	1	1	.	.	.	INTERGENIC(INTERGENIC);RAMP1(DOWNSTREAM)	
2	238870875	238870888	238870879	CA	T	snp	1	1	1	1	.	.	.	INTERGENIC(INTERGENIC);RP11-526L8.1.1(UPSTREAM);UBE2F(UPSTREAM)	
2	239073513	239073521	239073515	A	C	snp	1	1	1	1	.	.	.	AC016757.1(INTRON);FAM132B(DOWNSTREAM);FAM132B(INTRON)	
2	239167484	239167491	239167486	G	T	snp	0	1	1	1	.	.	.	AC096574.4.1(DOWNSTREAM);PER2(DOWNSTREAM);PER2(INTRON)	
2	239283140	239283147	239283146	T	G	snp	0	1	1	1	.	.	.	TRAF3IP1(INTRON)	
2	239304325	239304334	239304327	A	G	snp	1	1	1	1	.	.	.	TRAF3IP1(INTRON);TRAF3IP1(UPSTREAM)	
2	239564497	239564508	239564503	GAG	A	snp	1	1	1	1	.	.	.	INTERGENIC(INTERGENIC)	
2	239622166	239622177	239622173	CA	CAC	del	1	0	0	0	.	.	.	INTERGENIC(INTERGENIC)	
2	239743956	239743976	239743969	TCC	T	snp	1	0	0	0	.	.	.	INTERGENIC(INTERGENIC)	
2	239751513	239751528	239751517	AT	TATATATATCT	del	1	1	1	1	.	.	.	.	INTERGENIC(INTERGENIC)
2	239751513	239751528	239751519	AT	TATATATCT	del	1	1	0	1	.	.	.	.	INTERGENIC(INTERGENIC)
2	239833621	239833630	239833628	CA	G	snp	1	1	0	0	.	.	.	INTERGENIC(INTERGENIC)	
2	240034945	240034960	240034956	CA	C	del	0	1	0	0	.	.	.	HDAC4(INTRON)	
2	240042099	240042110	240042101	AC	G	snp	0	0	1	0	.	.	.	HDAC4(INTRON)	
2	240127360	240127371	240127368	GAA	C	snp	1	0	0	0	.	.	.	HDAC4(INTRON)	
2	240210522	240210531	240210525	CA	G	snp	1	1	0	1	.	.	.	HDAC4(INTRON)	
2	240287277	240287290	240287284	AC	AG	ins	1	1	1	1	.	.	.	HDAC4(INTRON)	
2	240295155	240295169	240295166	TTG	TTGT	del	1	1	1	1	.	.	.	HDAC4(INTRON)	
2	240485199	240485207	240485205	T	G	snp	1	1	1	1	.	.	.	INTERGENIC(INTERGENIC)	
2	240593793	240593807	240593802	TGA	C	snp	1	1	1	1	.	.	.	INTERGENIC(INTERGENIC)	
2	240634827	240634840	240634833	GT	A	snp	1	1	1	1	.	.	.	INTERGENIC(INTERGENIC)	
2	240660191	240660200	240660193	AC	C	snp	1	0	0	0	.	.	.	INTERGENIC(INTERGENIC);RP11-315F22.1.1(DOWNSTREAM)	
2	240676989	240677004	240676997	TGGA	C	snp	1	0	0	0	.	.	.	INTERGENIC(INTERGENIC)	
2	240771992	240771999	240771997	A	C	snp	0	1	1	1	.	.	.	INTERGENIC(INTERGENIC)	
2	240812231	240812246	240812242	TTTG	T	del	1	1	1	1	.	.	.	INTERGENIC(INTERGENIC)	
2	240902986	240902995	240902988	GA	C	snp	1	1	1	1	.	.	.	NDUFA10(INTRON)	
2	241086570	241086579	241086575	TG	TC	ins	1	1	0	0	.	.	.	INTERGENIC(INTERGENIC);OTOS(UPSTREAM)	
2	241086570	241086579	241086577	TG	T	del	1	0	0	0	.	.	.	INTERGENIC(INTERGENIC);OTOS(UPSTREAM)	
2	241216108	241216117	241216111	AG	A	snp	1	0	0	0	.	.	.	INTERGENIC(INTERGENIC)	
2	241253777	241253800	241253793	AAAT	AAT	del	1	1	1	1	.	.	.	INTERGENIC(INTERGENIC)	
2	241388731	241388739	241388734	C	CA	ins	1	0	0	0	.	.	.	AC110619.1(DOWNSTREAM);AC110619.2.1(DOWNSTREAM);AC110619.2.1(EXON);GPC1(INTRON)	
2	241388731	241388739	241388736	C	T	ins	1	1	0	0	.	.	.	AC110619.1(DOWNSTREAM);AC110619.2.1(DOWNSTREAM);AC110619.2.1(EXON);GPC1(INTRON)	
2	241483909	241483916	241483910	G	A	snp	0	1	0	1	.	.	.	ANKMY1(INTRON)	
2	241529076	241529083	241529079	G	A	snp	0	1	1	1	.	.	.	CAPN10(EXON);CAPN10(INTRON);CAPN10(UPSTREAM);RP11-27M15.1.1(UPSTREAM)	
2	241563427	241563434	241563429	G	T	snp	1	1	1	0	.	.	.	GPR35(INTRON);GPR35(UPSTREAM)	
2	241595452	241595459	241595456	T	G	snp	1	0	0	0	.	.	.	INTERGENIC(INTERGENIC)	
2	241647805	241647814	241647809	T	C	ins	1	1	1	1	.	.	.	INTERGENIC(INTERGENIC)	
2	241732443	241732450	241732446	G	T	snp	1	0	0	0	.	.	.	KIF1A(INTRON)	
2	241808308	241808315	241808314	C	T	snp	1	1	0	1	.	AGXT(NON_SYNONYMOUS_CODING):P11L	.	AGXT(EXON);AGXT(UPSTREAM)	
2	241850548	241850555	241850554	A	C	snp	1	1	1	1	.	.	.	INTERGENIC(INTERGENIC)	
2	241913943	241913950	241913947	T	C	snp	1	1	1	1	.	.	.	AC104809.3.1(INTRON)	
2	242177135	242177142	242177141	A	AC	ins	1	1	1	1	.	.	.	HDLBP(DOWNSTREAM);HDLBP(INTRON);HDLBP(UPSTREAM)	
2	242184226	242184233	242184231	T	C	snp	1	0	0	0	.	.	.	HDLBP(DOWNSTREAM);HDLBP(INTRON);HDLBP(UPSTREAM)	
2	242200455	242200463	242200457	A	C	ins	1	0	1	1	.	.	.	HDLBP(DOWNSTREAM);HDLBP(INTRON);HDLBP(UPSTREAM)	
2	242218481	242218488	242218487	A	C	ins	1	0	0	0	.	.	.	HDLBP(INTRON)	
2	242332432	242332439	242332438	T	C	snp	1	1	1	1	.	.	.	FARP2(INTRON)	
2	242371226	242371245	242371240	TTTA	A	del	1	0	0	0	.	.	.	FARP2(INTRON);FARP2(UPSTREAM)	
2	242371226	242371245	242371241	TTTA	T	snp	1	0	0	0	.	.	.	FARP2(INTRON);FARP2(UPSTREAM)	

2	242376401	242376408	242376402	A	G	snp	0	1	0	0	.	.	.	FARP2(DOWNSTREAM);FARP2(INTRON)
2	242405162	242405173	242405170	GAC	T	snp	0	1	1	0	.	.	.	FARP2(DOWNSTREAM);FARP2(INTRON);FARP2(UTR_3_PRIME)
2	242443156	242443164	242443157	A	C	snp	0	1	1	1	.	.	.	STK25(INTRON);STK25(UPSTREAM)
2	242451417	242451426	242451421	CA	G	snp	1	1	1	1	.	.	.	INTERGENIC(INTERGENIC);STK25(UPSTREAM)
2	242485336	242485345	242485341	CA	G	snp	1	1	1	1	.	.	.	BOK-AS1(INTRON)
2	242583005	242583014	242583011	AT	G	snp	1	1	1	0	.	.	.	ATG4B(INTRON)
2	242634434	242634442	242634436	A	G	snp	0	1	1	0	.	.	.	AC133528.2.1(DOWNSTREAM);INTERGENIC(INTERGENIC)
2	242747248	242747263	242747253	CCTC	T	snp	1	0	0	0	.	.	.	AC114730.3.1(DOWNSTREAM);GAL3ST2(DOWNSTREAM);INTERGENIC(INTERGENIC);NEU4(UPSTREAM)
2	242766706	242766723	242766716	CTT	TCTTC	del	1	1	0	1	.	.	.	INTERGENIC(INTERGENIC)
2	242787568	242787577	242787574	AC	C	snp	0	1	1	0	.	.	.	AC114730.2.1(DOWNSTREAM);INTERGENIC(INTERGENIC);PDCD1(DOWNSTREAM)
2	242806382	242806391	242806385	GT	C	snp	0	1	0	1	.	.	.	INTERGENIC(INTERGENIC)
2	242838115	242838123	242838116	G	T	ins	1	1	0	1	.	.	.	AC131097.3.1(INTRON);AC131097.4.1(INTRON)
2	242838115	242838123	242838122	G	T	ins	1	1	0	1	.	.	.	AC131097.3.1(INTRON);AC131097.4.1(INTRON)
2	242838869	242838878	242838874	GT	G	snp	1	1	1	1	.	.	.	AC131097.3.1(INTRON);AC131097.4.1(INTRON)
2	242842221	242842232	242842229	AAC	A	snp	1	0	0	0	.	.	.	AC131097.3.1(INTRON);AC131097.4.1(INTRON);AC131097.4.1(UPSTREAM)
2	242861889	242861896	242861891	A	C	snp	0	1	0	1	.	.	.	AC131097.3.1(INTRON)
2	242960139	242960162	242960154	AAAG	A	snp	1	1	1	1	.	.	.	AC131097.3.1(INTRON)
2	242965087	242965094	242965090	G	T	snp	0	1	1	0	.	.	.	AC093642.4.1(DOWNSTREAM);AC131097.3.1(INTRON)
3	154947	154956	154949	CT	A	snp	1	0	0	0	.	.	.	INTERGENIC(INTERGENIC)
3	174460	174469	174463	GT	C	snp	1	1	1	1	.	.	.	INTERGENIC(INTERGENIC)
3	184359	184366	184365	T	G	snp	1	1	1	1	.	.	.	INTERGENIC(INTERGENIC)
3	189254	189263	189256	TG	C	snp	0	0	0	1	.	.	.	INTERGENIC(INTERGENIC)
3	243196	243205	243202	TG	G	snp	1	1	0	1	.	.	.	CHL1(INTRON);CHL1-AS2(UPSTREAM)
3	367139	367146	367143	A	G	snp	1	0	0	0	.	.	.	CHL1(INTRON)
3	453351	453362	453354	AAC	C	snp	0	1	1	0	.	.	.	CHL1(DOWNSTREAM);INTERGENIC(INTERGENIC)
3	514228	514237	514229	T	G	ins	1	0	0	0	.	.	.	INTERGENIC(INTERGENIC)
3	514228	514237	514230	T	G	snp	1	0	0	0	.	.	.	INTERGENIC(INTERGENIC)
3	572377	572384	572381	A	C	snp	1	0	0	0	.	.	.	INTERGENIC(INTERGENIC)
3	742820	742847	742833	TTTG	C	snp	1	1	1	1	.	.	.	AC090044.1.1(INTRON)
3	838615	838624	838622	TA	G	snp	1	0	0	0	.	.	.	AC090044.1.1(INTRON)
3	925618	925631	925627	GT	G	snp	0	1	0	1	.	.	.	INTERGENIC(INTERGENIC)
3	979385	979392	979386	T	C	snp	1	0	0	0	.	.	.	INTERGENIC(INTERGENIC)
3	1069569	1069578	1069571	CT	T	snp	1	1	1	1	.	.	.	INTERGENIC(INTERGENIC)
3	1186267	1186278	1186271	AAT	T	del	1	0	0	0	.	.	.	CNTN6(INTRON)
3	1225432	1225440	1225435	A	G	snp	0	1	0	1	.	.	.	CNTN6(INTRON)
3	1243080	1243087	1243081	T	C	snp	1	1	0	1	.	.	.	CNTN6(INTRON)
3	1258654	1258668	1258665	GAT	C	snp	1	0	0	0	.	.	.	CNTN6(INTRON)
3	1297110	1297133	1297125	AAAG	AAAT	ins	1	0	0	0	.	.	.	CNTN6(INTRON)
3	1312577	1312592	1312582	TTTG	G	snp	1	0	0	0	.	.	.	CNTN6(INTRON)
3	1366388	1366395	1366393	T	C	snp	1	1	1	1	.	.	.	CNTN6(INTRON)
3	1391082	1391090	1391083	T	G	snp	1	0	0	0	.	.	.	CNTN6(INTRON)
3	1425349	1425357	1425354	A	C	snp	1	0	0	0	.	.	.	CNTN6(INTRON)
3	1486305	1486319	1486313	AAC	AAT	ins	1	1	1	1	.	.	.	INTERGENIC(INTERGENIC)
3	1491024	1491031	1491030	T	G	snp	1	1	0	1	.	.	.	INTERGENIC(INTERGENIC)
3	1493181	1493196	1493185	AAGA	AG	del	1	1	0	1	.	.	.	INTERGENIC(INTERGENIC)
3	1536982	1536989	1536987	A	C	snp	0	1	0	1	.	.	.	INTERGENIC(INTERGENIC)
3	1619668	1619685	1619683	CA	C	del	1	1	1	0	.	.	.	INTERGENIC(INTERGENIC)
3	1688317	1688332	1688320	TG	A	ins	0	1	1	1	.	.	.	INTERGENIC(INTERGENIC)
3	1725657	1725665	1725664	A	G	snp	1	1	1	1	.	.	.	INTERGENIC(INTERGENIC)
3	1790006	1790014	1790012	A	C	ins	1	1	1	1	.	.	.	INTERGENIC(INTERGENIC)
3	1831352	1831359	1831355	T	C	snp	1	0	0	0	.	.	.	INTERGENIC(INTERGENIC)
3	1885891	1885898	1885895	A	G	snp	1	0	0	0	.	.	.	INTERGENIC(INTERGENIC)
3	1901468	1901475	1901469	T	C	ins	1	1	1	1	.	.	.	INTERGENIC(INTERGENIC)
3	1968737	1968748	1968743	TA	A	snp	0	0	1	0	.	.	.	INTERGENIC(INTERGENIC)
3	2062128	2062137	2062130	GT	A	snp	0	0	0	1	.	.	.	INTERGENIC(INTERGENIC)
3	2079334	2079343	2079342	T	G	snp	0	1	1	1	.	.	.	INTERGENIC(INTERGENIC)
3	2095541	2095548	2095542	T	G	snp	1	1	1	1	.	.	.	INTERGENIC(INTERGENIC)
3	2191982	2191989	2191987	A	G	snp	1	0	0	0	.	.	.	CNTN4(INTRON)
3	2205916	2205925	2205923	A	C	snp	1	1	1	1	.	.	.	CNTN4(INTRON)
3	2267818	2267826	2267820	T	G	snp	1	1	1	1	.	.	.	CNTN4(INTRON)
3	2269248	2269257	2269251	TC	T	snp	0	1	1	1	.	.	.	CNTN4(INTRON)
3	2280420	2280429	2280424	T	G	ins	1	0	0	0	.	.	.	CNTN4(INTRON);CNTN4(UPSTREAM)
3	2280420	2280429	2280428	T	C	ins	1	0	0	0	.	.	.	CNTN4(INTRON);CNTN4(UPSTREAM)

3	2327921	2327928	2327926	C	A	ins	1	1	0	1	.	.	.	CNTN4(INTRON)
3	2377908	2377916	2377910	G	T	snp	1	1	1	1	.	.	.	CNTN4(INTRON)
3	2420951	2420959	2420955	T	C	snp	1	0	0	0	.	.	.	CNTN4(INTRON)
3	2447811	2447818	2447813	A	G	snp	1	1	1	1	.	.	.	CNTN4(INTRON)
3	2522718	2522745	2522739	TTTC	C	snp	1	1	0	1	.	.	.	CNTN4(INTRON)
3	2528639	2528646	2528643	A	C	snp	1	0	0	0	.	.	.	CNTN4(INTRON)
3	2558628	2558636	2558631	A	G	snp	1	0	0	0	.	.	.	CNTN4(INTRON)
3	2739401	2739409	2739402	A	G	snp	0	1	0	1	.	.	.	CNTN4(INTRON)
3	2747143	2747154	2747148	AT	G	snp	1	0	0	0	.	.	.	CNTN4(INTRON)
3	2747143	2747154	2747150	AT	G	snp	1	1	1	1	.	.	.	CNTN4(INTRON)
3	2747314	2747323	2747321	AT	C	snp	1	0	1	0	.	.	.	CNTN4(INTRON)
3	2820930	2820953	2820934	TTTG	TTG	del	1	1	1	1	.	.	.	CNTN4(INTRON)
3	2846422	2846430	2846424	T	AG	ins	0	0	1	1	.	.	.	CNTN4(INTRON)
3	2882063	2882074	2882069	CTC	G	snp	1	1	1	1	.	.	.	CNTN4(INTRON)
3	2902663	2902671	2902668	T	C	snp	1	1	1	1	.	.	.	CNTN4(INTRON)
3	2930733	2930748	2930737	AAGG	AGG	del	1	0	0	0	.	.	.	CNTN4(DOWNSTREAM);CNTN4(INTRON);CNTN4(UPSTREAM)
3	2985297	2985314	2985309	TA	ATATA	del	1	1	0	1	.	.	.	CNTN4(INTRON)
3	3011201	3011224	3011206	ATT	G	snp	1	0	0	0	.	.	.	CNTN4(INTRON)
3	3011201	3011224	3011215	ATT	G	snp	1	1	0	0	.	.	.	CNTN4(INTRON)
3	3040333	3040342	3040336	A	G	snp	0	1	1	1	.	.	.	CNTN4(INTRON)
3	3105119	3105128	3105125	T	G	ins	1	1	1	1	.	.	.	CNTN4-AS1(UPSTREAM);INTERGENIC(INTERGENIC)
3	3122694	3122701	3122695	T	G	snp	1	1	1	1	.	.	.	IL5RA(INTRON)
3	3128355	3128363	3128356	A	G	ins	1	1	0	0	.	.	.	IL5RA(DOWNSTREAM);IL5RA(INTRON)
3	3148241	3148250	3148245	A	C	snp	1	1	1	1	.	.	.	IL5RA(DOWNSTREAM);IL5RA(INTRON);IL5RA(UPSTREAM);SNORA43(DOWNSTREAM)
3	3153609	3153624	3153615	AAAC	G	snp	1	1	1	1	.	.	.	IL5RA(INTRON);IL5RA(UPSTREAM)
3	3167324	3167335	3167329	AG	C	snp	0	0	0	1	.	.	.	IL5RA(INTRON);TRNT1(UPSTREAM)
3	3205512	3205521	3205515	A	C	snp	1	1	1	1	.	.	.	CRBN(INTRON)
3	3296585	3296608	3296592	ATCT	ATC	ins	0	0	1	0	.	.	.	AC026188.1.1(INTRON)
3	3310615	3310622	3310616	T	C	snp	1	0	0	0	.	.	.	AC026188.1.1(INTRON)
3	3366683	3366696	3366692	TC	TCTCT	del	1	0	0	0	.	.	.	AC026188.1.1(INTRON)
3	3366683	3366696	3366693	TC	CTC	del	1	0	0	0	.	.	.	AC026188.1.1(INTRON)
3	3388753	3388762	3388760	AC	G	snp	1	0	0	0	.	.	.	AC026188.1.1(INTRON)
3	3447890	3447897	3447895	A	C	ins	1	1	1	1	.	.	.	AC026188.1.1(INTRON)
3	3469617	3469636	3469621	TTAA	G	snp	1	1	1	1	.	.	.	AC026188.1.1(INTRON)
3	3504684	3504691	3504690	A	G	snp	1	0	0	0	.	.	.	AC026188.1.1(INTRON)
3	3528565	3528574	3528567	TG	C	snp	1	1	1	1	.	.	.	AC026188.1.1(INTRON);AC026188.1.1(UPSTREAM)
3	3556310	3556319	3556317	AT	C	snp	0	0	1	0	.	.	.	AC026188.1.1(INTRON)
3	3658812	3658821	3658817	CT	G	snp	1	0	0	0	.	.	.	AC026188.1.1(INTRON)
3	3740321	3740328	3740325	A	C	snp	1	0	0	0	.	.	.	INTERGENIC(INTERGENIC);SUMF1(DOWNSTREAM)
3	3767062	3767070	3767068	A	C	snp	0	0	0	1	.	.	.	SUMF1(INTRON)
3	3771138	3771151	3771148	AC	G	snp	1	1	1	1	.	.	.	SUMF1(INTRON)
3	3773209	3773218	3773216	CA	C	snp	0	1	1	1	.	.	.	SUMF1(INTRON)
3	3781435	3781444	3781442	CA	C	snp	1	1	1	1	.	.	.	SUMF1(INTRON)
3	3866184	3866193	3866190	A	G	snp	1	0	0	0	.	.	.	LRRN1(INTRON);SUMF1(DOWNSTREAM);SUMF1(INTRON)
3	3885254	3885262	3885259	C	A	snp	1	1	1	1	.	.	.	LRRN1(INTRON);SUMF1(INTRON)
3	3901420	3901429	3901427	A	C	snp	1	1	1	1	.	.	.	SUMF1(INTRON)
3	3914593	3914601	3914595	A	C	snp	0	0	1	0	.	.	.	SUMF1(INTRON)
3	3995121	3995141	3995125	AAC	C	del	0	0	0	1	.	.	.	SUMF1(INTRON)
3	4073108	4073117	4073114	AT	TATG	ins	1	0	0	0	.	.	.	SUMF1(INTRON)
3	4294917	4294924	4294921	A	C	snp	1	1	1	1	.	.	.	SUMF1(INTRON)
3	4300950	4300969	4300957	AAAT	A	snp	1	1	1	1	.	.	.	SUMF1(INTRON)
3	4310561	4310574	4310567	TG	C	snp	1	0	0	0	.	.	.	SUMF1(INTRON)
3	4322179	4322188	4322184	A	C	snp	1	1	1	1	.	.	.	SUMF1(INTRON)
3	4346657	4346664	4346660	G	A	snp	0	0	0	1	.	.	.	SETMAR(INTRON);SUMF1(INTRON)
3	4484107	4484116	4484114	T	C	snp	1	1	1	1	.	.	.	SUMF1(INTRON)
3	4498779	4498786	4498782	T	G	snp	0	1	0	1	.	.	.	SUMF1(INTRON)
3	4585732	4585740	4585736	T	G	snp	1	0	0	0	.	.	.	ITPR1(INTRON)
3	4795733	4795742	4795739	G	T	ins	1	0	0	0	.	.	.	EGOT(UPSTREAM);ITPR1(INTRON)
3	4900389	4900398	4900395	A	AT	ins	1	0	0	0	.	.	.	AC018816.3.1(INTRON);AC018816.3.1(UPSTREAM)
3	4901342	4901359	4901354	CAA	T	snp	1	1	1	1	.	.	.	AC018816.3.1(INTRON);AC018816.3.1(UPSTREAM)
3	4911187	4911195	4911192	T	C	snp	1	0	0	0	.	.	.	AC018816.3.1(INTRON)
3	4964268	4964276	4964272	A	TT	ins	1	0	0	0	.	.	.	AC018816.4.1(INTRON)
3	4969010	4969018	4969012	A	C	ins	1	0	1	1	.	.	.	AC018816.4.1(INTRON)
3	4969010	4969018	4969013	A	C	snp	1	1	1	1	.	.	.	AC018816.4.1(INTRON)
3	5002601	5002608	5002604	T	C	snp	1	0	0	0	.	.	.	AC018816.4.1(INTRON)
3	5076674	5076681	5076675	A	G	snp	1	1	1	1	.	.	.	INTERGENIC(INTERGENIC)
3	5152530	5152539	5152537	T	G	ins	1	0	1	0	.	.	.	INTERGENIC(INTERGENIC)

3	5267037	5267044	5267043	C	AG	ins	1	1	1	1	.	.	.	INTERGENIC(INTERGENIC)
3	5267527	5267550	5267546	TTA	ATTA	del	1	1	1	1	.	.	.	INTERGENIC(INTERGENIC)
3	5300759	5300768	5300763	T	G	ins	1	0	0	0	.	.	.	AC026202.5.1(UPSTREAM);INTERGENIC(INTERGENIC)
3	5304581	5304590	5304582	T	C	ins	1	1	1	1	.	.	.	INTERGENIC(INTERGENIC)
3	5386396	5386413	5386399	TG	T	snp	1	1	1	1	.	.	.	INTERGENIC(INTERGENIC)
3	5386396	5386413	5386409	TG	A	snp	1	1	1	1	.	.	.	INTERGENIC(INTERGENIC)
3	5387219	5387226	5387220	A	C	snp	1	0	0	0	.	.	.	INTERGENIC(INTERGENIC)
3	5415469	5415483	5415478	TTA	C	snp	1	0	0	0	.	.	.	INTERGENIC(INTERGENIC)
3	5506405	5506414	5506410	TG	T	snp	1	0	0	0	.	.	.	INTERGENIC(INTERGENIC)
3	5573340	5573347	5573342	T	G	snp	1	1	1	1	.	.	.	INTERGENIC(INTERGENIC)
3	5783566	5783573	5783571	A	G	ins	1	1	1	1	.	.	.	INTERGENIC(INTERGENIC)
3	5819189	5819196	5819193	T	C	ins	0	0	1	0	.	.	.	INTERGENIC(INTERGENIC)
3	5906716	5906729	5906724	AT	G	snp	0	0	1	0	.	.	.	INTERGENIC(INTERGENIC)
3	5908699	5908714	5908708	TGAA	C	snp	0	0	1	0	.	.	.	INTERGENIC(INTERGENIC)
3	5915469	5915480	5915473	AT	T	ins	1	1	1	1	.	.	.	INTERGENIC(INTERGENIC)
3	5925031	5925038	5925036	A	C	snp	0	1	1	1	.	.	.	INTERGENIC(INTERGENIC)
3	5935080	5935095	5935087	ATAA	C	snp	1	1	1	1	.	.	.	INTERGENIC(INTERGENIC)
3	6109055	6109064	6109059	TC	C	snp	1	1	0	1	.	.	.	AC027119.1.1(INTRON)
3	6109055	6109064	6109062	TC	T	snp	0	1	0	1	.	.	.	AC027119.1.1(INTRON)
3	6139409	6139416	6139410	A	G	snp	1	1	1	1	.	.	.	AC027119.1.1(INTRON)
3	6162155	6162163	6162158	T	C	snp	1	1	1	1	.	.	.	AC027119.1.1(INTRON)
3	6188351	6188365	6188362	TTA	G	snp	1	0	0	0	.	.	.	AC026167.1.1(UPSTREAM);INTERGENIC(INTERGENIC)
3	6214790	6214798	6214795	T	C	snp	1	1	1	1	.	.	.	AC026167.1.1(INTRON)
3	6220121	6220130	6220127	TA	C	snp	0	1	1	1	.	.	.	AC026167.1.1(INTRON)
3	6228975	6228986	6228983	TTA	T	ins	1	0	0	0	.	.	.	AC026167.1.1(INTRON)
3	6370062	6370075	6370065	AT	A	snp	1	1	1	1	.	.	.	AC026167.1.1(INTRON)
3	6448029	6448044	6448038	AG	C	snp	1	0	0	0	.	.	.	INTERGENIC(INTERGENIC)
3	6621962	6621973	6621969	TA	G	snp	1	0	0	0	.	.	.	AC069277.2.1(INTRON)
3	6638691	6638698	6638692	T	G	snp	1	0	0	0	.	.	.	AC069277.2.1(INTRON)
3	6642078	6642101	6642090	AGGA	G	snp	1	1	1	1	.	.	.	AC069277.2.1(INTRON)
3	6736881	6736908	6736900	TTTC	G	ins	1	0	0	0	.	.	.	AC024162.2.1(DOWNSTREAM);AC069277.2.1(DOWNSTREAM);AC069277.2.1(INTRON);GRM7-AS3(INTRON)
3	6791723	6791731	6791724	A	C	snp	0	0	1	0	.	.	.	GRM7-AS3(INTRON)
3	6900519	6900528	6900524	TC	T	del	0	1	0	1	.	.	.	GRM7(INTRON);GRM7(UPSTREAM)
3	6911678	6911687	6911682	CT	G	snp	0	1	1	1	.	.	.	GRM7(INTRON)
3	6924235	6924244	6924236	A	C	snp	0	1	1	0	.	.	.	GRM7(INTRON)
3	7010776	7010785	7010784	T	C	ins	1	1	0	0	.	.	.	GRM7(INTRON)
3	7145529	7145544	7145537	CT	TCTT	ins	1	1	1	1	.	.	.	GRM7(INTRON)
3	7145529	7145544	7145541	CT	T	snp	1	1	1	1	.	.	.	GRM7(INTRON)
3	7232386	7232397	7232395	AT	C	snp	1	0	0	0	.	.	.	GRM7(INTRON)
3	7280422	7280429	7280425	T	TTTTC	ins	1	0	0	0	.	.	.	GRM7(INTRON)
3	7301824	7301833	7301826	T	C	snp	1	0	0	0	.	.	.	GRM7(INTRON)
3	7364121	7364129	7364127	T	G	snp	1	0	0	0	.	.	.	GRM7(INTRON)
3	7367858	7367867	7367866	A	G	snp	1	1	1	1	.	.	.	GRM7(INTRON)
3	7416163	7416180	7416169	ATT	G	snp	1	1	1	1	.	.	.	GRM7(INTRON)
3	7530546	7530573	7530557	TTAT	C	snp	0	0	0	1	.	.	.	GRM7(INTRON)
3	7535864	7535878	7535867	ATT	G	snp	1	1	1	1	.	.	.	GRM7(INTRON)
3	7608491	7608500	7608493	A	G	snp	1	1	1	1	.	.	.	GRM7(INTRON)
3	7708968	7708985	7708970	AG	G	del	1	1	0	1	.	.	.	GRM7(INTRON)
3	7807102	7807117	7807110	CAAA	AAACA	del	1	0	0	0	.	.	.	INTERGENIC(INTERGENIC)
3	7807847	7807858	7807855	AGG	A	snp	1	1	1	1	.	.	.	INTERGENIC(INTERGENIC)
3	7820474	7820489	7820487	AT	C	snp	1	1	1	1	.	.	.	INTERGENIC(INTERGENIC)
3	7968754	7968767	7968763	GA	G	snp	1	1	1	1	.	.	.	INTERGENIC(INTERGENIC)
3	7970326	7970335	7970329	AG	A	snp	1	1	1	1	.	.	.	INTERGENIC(INTERGENIC)
3	7970326	7970335	7970333	AG	A	snp	1	1	1	1	.	.	.	INTERGENIC(INTERGENIC)
3	7975867	7975875	7975873	T	C	snp	1	1	1	1	.	.	.	INTERGENIC(INTERGENIC)
3	8052006	8052017	8052010	TA	ATATAC	ins	1	1	1	1	.	.	.	AC087859.1.1(INTRON)
3	8111934	8111941	8111937	C	A	snp	1	1	1	1	.	.	.	AC087859.1.1(INTRON)
3	8135532	8135543	8135538	TA	G	snp	1	1	0	1	.	.	.	AC018832.1.1(INTRON);AC087859.1.1(INTRON)
3	8157933	8157950	8157937	CT	G	snp	1	1	1	1	.	.	.	AC018832.1.1(DOWNSTREAM);AC087859.1.1(INTRON)
3	8233375	8233386	8233381	CTT	T	snp	1	1	1	1	.	.	.	AC087859.1.1(DOWNSTREAM);AC087859.1.1(INTRON)
3	8316363	8316370	8316368	G	T	snp	1	0	0	0	.	.	.	AC087859.1.1(INTRON)
3	8418871	8418880	8418877	A	C	snp	1	1	1	1	.	.	.	AC087859.1.1(INTRON)
3	8437885	8437893	8437886	A	C	snp	0	0	1	0	.	.	.	AC087859.1.1(INTRON)
3	8681722	8681731	8681724	CA	G	snp	1	1	1	0	.	.	.	C3orf32(EXON);C3orf32(INTRON);C3orf32(UPSTREAM);C3orf32(UTR_3_PRIME);C3orf32(UTR_5_PRIME)
3	8686195	8686210	8686202	AGGA	C	snp	1	1	0	1	.	.	.	C3orf32(INTRON);C3orf32(UPSTREAM)

3	8722848	8722855	8722852	G	T	snp	1	0	0	0	.	.	.	C3orf32(INTRON);C3orf32(UPSTREAM)
3	8779725	8779742	8779733	TC	CC	ins	1	1	1	1	.	.	.	C3orf32(INTRON);CAV3(INTRON)
3	8814812	8814825	8814823	TA	T	del	1	1	1	1	.	.	.	CAV3(INTRON);OXTR(UPSTREAM)
3	8814838	8814849	8814843	TA	C	snp	1	1	1	1	.	.	.	CAV3(INTRON);OXTR(UPSTREAM)
3	8814863	8814874	8814868	TA	C	snp	1	1	1	1	.	.	.	CAV3(INTRON);OXTR(UPSTREAM)
3	8814888	8814899	8814893	TA	C	snp	1	1	1	1	.	.	.	CAV3(INTRON);OXTR(UPSTREAM)
3	8814947	8814958	8814952	TA	C	snp	1	1	1	1	.	.	.	CAV3(INTRON);OXTR(UPSTREAM)
3	8880971	8880985	8880974	TGA	A	snp	1	1	1	1	.	.	.	CAV3(INTRON)
3	8925035	8925046	8925041	TTG	C	snp	1	1	1	1	.	.	.	RAD18(INTRON)
3	8937322	8937331	8937328	T	G	snp	1	1	1	1	.	.	.	RAD18(INTRON)
3	8969934	8969941	8969938	A	G	snp	1	1	1	1	.	.	.	RAD18(INTRON);SNORA43(UPSTREAM)
3	9031327	9031334	9031332	A	C	snp	1	1	1	1	.	.	.	SRGAP3(DOWNSTREAM);SRGAP3(INTRON)
3	9111631	9111639	9111636	A	G	snp	0	0	0	1	.	.	.	SRGAP3(INTRON);SRGAP3(UPSTREAM)
3	9179965	9179972	9179971	C	A	snp	1	1	1	1	.	.	.	SRGAP3(INTRON)
3	9263825	9263834	9263829	GA	C	snp	1	1	1	1	.	.	.	SRGAP3(INTRON);SRGAP3-AS3(DOWNSTREAM)
3	9336235	9336243	9336236	A	AC	ins	1	1	1	1	.	.	.	RP11-380O24.1.1(INTRON);SRGAP3(INTRON)
3	9336235	9336243	9336237	A	C	ins	1	1	1	1	.	.	.	RP11-380O24.1.1(INTRON);SRGAP3(INTRON)
3	9550931	9550944	9550934	CT	C	snp	1	1	1	1	.	.	.	LHFPL4(INTRON)
3	9568426	9568437	9568434	AC	C	ins	0	0	0	1	.	.	.	LHFPL4(INTRON)
3	9583976	9583983	9583982	A	G	snp	0	0	1	0	.	.	.	LHFPL4(INTRON)
3	9587401	9587416	9587412	AAAT	C	snp	1	1	1	1	.	.	.	LHFPL4(DOWNSTREAM);LHFPL4(INTRON)
3	9599156	9599165	9599159	AG	A	snp	1	1	1	1	.	.	.	INTERGENIC(INTERGENIC);LHFPL4(UPSTREAM)
3	9602075	9602086	9602078	AT	A	snp	0	1	0	1	.	.	.	INTERGENIC(INTERGENIC)
3	9619606	9619615	9619610	T	TA	ins	1	1	1	1	.	.	.	INTERGENIC(INTERGENIC)
3	9675850	9675857	9675852	T	C	snp	1	0	0	0	.	.	.	INTERGENIC(INTERGENIC)
3	9678756	9678775	9678766	TTTA	G	snp	1	0	0	0	.	.	.	INTERGENIC(INTERGENIC)
3	9691543	9691554	9691548	GA	G	snp	0	0	0	1	.	.	.	MTMR14(INTRON)
3	9763845	9763853	9763848	C	T	snp	1	0	0	0	.	.	.	CPNE9(INTRON)
3	9806302	9806311	9806308	T	G	ins	1	0	0	0	.	.	.	CAMK1(INTRON);CAMK1(UPSTREAM);OGG1(INTRON)
3	9816134	9816141	9816138	T	G	snp	1	1	1	1	.	.	.	CAMK1(UPSTREAM);INTERGENIC(INTERGENIC)
3	9903626	9903633	9903632	C	T	snp	1	0	0	0	.	.	.	CIDEC(DOWNSTREAM);INTERGENIC(INTERGENIC)
3	10032503	10032526	10032517	TTTA	A	del	1	0	0	0	.	.	.	AC034193.5.1(INTRON);TMEM111(INTRON);TMEM111(UPSTREAM)
3	10032503	10032526	10032520	TTTA	TA	del	1	1	1	1	.	.	.	AC034193.5.1(INTRON);TMEM111(INTRON);TMEM111(UPSTREAM)
3	10096038	10096051	10096045	TA	G	snp	1	1	1	1	.	.	.	AC034193.7.1(DOWNSTREAM);FANCD2(DOWNSTREAM);FANCD2(INTRON)
3	10096063	10096072	10096070	AT	C	snp	1	0	0	0	.	.	.	AC034193.7.1(DOWNSTREAM);FANCD2(DOWNSTREAM);FANCD2(INTRON)
3	10126148	10126157	10126153	T	G	snp	1	1	1	1	.	.	.	C3orf24(INTRON);FANCD2(INTRON)
3	10148980	10148989	10148983	T	C	snp	1	1	0	1	.	.	.	C3orf24(INTRON);C3orf24(UPSTREAM)
3	10173162	10173169	10173166	T	C	snp	0	0	1	0	.	.	.	BRK1(DOWNSTREAM);INTERGENIC(INTERGENIC);snoU13(UPSTREAM)
3	10578104	10578118	10578114	TCT	T	snp	1	0	0	0	.	.	.	ATP2B2(INTRON);ATP2B2(UPSTREAM)
3	10686794	10686809	10686803	AG	C	snp	1	1	1	1	.	.	.	ATP2B2(INTRON)
3	10929134	10929141	10929140	T	C	snp	0	0	0	1	.	.	.	SLC6A11(INTRON)
3	11084938	11084946	11084942	A	C	snp	1	1	1	0	.	.	.	INTERGENIC(INTERGENIC);SLC6A1(DOWNSTREAM)
3	11221443	11221452	11221445	GT	TT	ins	1	1	0	1	.	.	.	HRH1(INTRON)
3	11232037	11232052	11232048	AT	ATA	del	1	1	1	1	.	.	.	HRH1(INTRON)
3	11232037	11232052	11232049	AT	T	snp	1	1	1	1	.	.	.	HRH1(INTRON)
3	11232037	11232052	11232050	AT	A	del	1	1	1	1	.	.	.	HRH1(INTRON)
3	11252084	11252099	11252095	AAAT	C	snp	1	1	1	1	.	.	.	HRH1(INTRON)
3	11270163	11270170	11270164	T	C	snp	1	1	1	1	.	.	.	HRH1(INTRON)
3	11277645	11277652	11277648	A	G	snp	1	0	0	0	.	.	.	AC083855.4.1(UPSTREAM);HRH1(INTRON)
3	11364569	11364578	11364572	A	G	snp	1	0	0	0	.	.	.	ATG7(INTRON)
3	11433433	11433442	11433441	G	T	ins	1	0	1	0	.	.	.	ATG7(INTRON)
3	11490850	11490861	11490856	CAA	A	snp	1	1	1	1	.	.	.	ATG7(DOWNSTREAM);ATG7(INTRON)
3	11633638	11633645	11633642	A	G	snp	1	1	1	1	.	.	.	VGLL4(INTRON)
3	11654409	11654420	11654412	GAG	A	snp	1	1	1	1	.	.	.	VGLL4(INTRON);VGLL4(UPSTREAM)
3	11714723	11714734	11714728	AT	C	snp	1	1	1	1	.	.	.	VGLL4(INTRON)
3	11770976	11770999	11770995	TTTC	T	snp	1	1	1	1	.	.	.	AC090939.1.1(UPSTREAM);AC090939.2.1(DOWNSTREAM);INTERGENIC(INTERGENIC)
3	11781567	11781574	11781569	A	AC	ins	1	1	1	1	.	.	.	INTERGENIC(INTERGENIC)
3	11788067	11788074	11788070	A	G	snp	1	0	0	0	.	.	.	INTERGENIC(INTERGENIC)
3	11827733	11827756	11827748	ATTT	A	ins	1	0	0	0	.	.	.	INTERGENIC(INTERGENIC);TAMM41(DOWNSTREAM)
3	11860265	11860273	11860268	G	A	snp	1	1	1	1	.	.	.	TAMM41(INTRON);TAMM41(UPSTREAM)
3	11985147	11985156	11985154	TA	T	snp	1	1	1	1	.	.	.	INTERGENIC(INTERGENIC)

3	12004261	12004268	12004267	A	AG	ins	1	0	1	0	.	.	.	INTERGENIC(INTERGENIC)
3	12008056	12008063	12008059	A	G	snp	1	1	1	0	.	.	.	INTERGENIC(INTERGENIC)
3	12232974	12232989	12232984	GT	A	snp	1	0	0	0	.	.	.	INTERGENIC(INTERGENIC);SYN2(DOWNSTREAM)
3	12457436	12457444	12457441	A	G	snp	0	1	1	1	.	.	.	PPARG(INTRON)
3	12527450	12527461	12527457	AT	C	snp	1	1	0	0	.	.	.	TSEN2(INTRON);TSEN2(UPSTREAM)
3	12654920	12654927	12654921	C	A	snp	1	1	0	1	.	.	.	RAF1(INTRON);RAF1(UPSTREAM)
3	12699356	12699365	12699361	A	C	snp	1	1	0	1	.	.	.	RAF1(INTRON)
3	12708979	12708998	12708990	AATA	AATAAAT	ins	1	1	0	1	.	.	.	INTERGENIC(INTERGENIC);RAF1(UPSTREAM)
3	12737067	12737078	12737074	GTT	TG	del	1	1	1	1	.	.	.	INTERGENIC(INTERGENIC)
3	12739710	12739719	12739715	A	G	ins	1	0	1	0	.	.	.	INTERGENIC(INTERGENIC)
3	12944790	12944799	12944792	CA	A	snp	1	0	0	0	.	.	.	IQSEC1(INTRON)
3	12987072	12987079	12987076	A	G	snp	1	0	0	0	.	.	.	IQSEC1(INTRON)
3	13319749	13319757	13319755	C	A	snp	1	0	0	0	.	.	.	INTERGENIC(INTERGENIC)
3	13469458	13469485	13469473	TAAA	G	snp	1	1	1	1	.	.	.	INTERGENIC(INTERGENIC)
3	13471119	13471127	13471125	T	G	ins	1	0	0	1	.	.	.	INTERGENIC(INTERGENIC)
3	13494014	13494031	13494028	AAC	A	ins	1	0	0	0	.	.	.	INTERGENIC(INTERGENIC)
3	13516805	13516813	13516806	T	G	snp	1	1	0	1	.	.	.	HDAC11(UPSTREAM);HDAC11-AS1(DOWNSTREAM);INTERGENIC(INTERGENIC)
3	13564335	13564344	13564342	T	C	snp	1	1	1	1	.	.	.	INTERGENIC(INTERGENIC)
3	13611479	13611490	13611487	TG	C	snp	1	0	0	0	.	.	.	FBLN2(INTRON)
3	13644399	13644414	13644410	TTTC	T	ins	1	1	1	0	.	.	.	FBLN2(INTRON)
3	13688537	13688545	13688540	G	GGA	ins	1	1	1	1	.	.	.	AC093611.1.1(UPSTREAM);INTERGENIC(INTERGENIC)
3	13759383	13759398	13759386	CT	G	snp	1	1	1	1	.	.	.	AC093611.1.1(INTRON)
3	13789290	13789307	13789294	CA	AG	ins	1	1	1	0	.	.	.	AC093611.1.1(DOWNSTREAM);INTERGENIC(INTERGENIC)
3	13848072	13848083	13848077	TC	G	snp	1	0	0	0	.	.	.	INTERGENIC(INTERGENIC)
3	13904862	13904877	13904873	TGAA	G	snp	0	1	0	0	.	.	.	WNT7A(DOWNSTREAM);WNT7A(INTRON)
3	13910308	13910321	13910319	AG	AA	ins	1	1	1	1	.	.	.	WNT7A(INTRON)
3	13951417	13951425	13951421	A	G	snp	1	0	0	0	.	.	.	INTERGENIC(INTERGENIC)
3	13953837	13953844	13953841	A	G	snp	1	0	0	0	.	.	.	INTERGENIC(INTERGENIC)
3	14057087	14057095	14057091	T	C	snp	1	1	0	1	.	.	.	TPRXL(INTRON);TPRXL(UPSTREAM)
3	14106093	14106110	14106103	CAG	T	snp	1	0	0	0	.	TPRXL(NON_SYNONYMOUS_CODING):S143C	.	TPRXL(DOWNSTREAM);TPRXL(INTRON)
3	14106093	14106110	14106104	CAG	C	snp	1	0	0	0	.	TPRXL(NON_SYNONYMOUS_CODING):S143T	.	TPRXL(DOWNSTREAM);TPRXL(INTRON)
3	14106327	14106338	14106332	CAG	C	snp	1	1	1	1	.	TPRXL(NON_SYNONYMOUS_CODING):S219T	.	TPRXL(DOWNSTREAM);TPRXL(INTRON)
3	14123419	14123436	14123434	CA	G	snp	1	0	0	0	.	.	.	TPRXL(INTRON)
3	14226872	14226880	14226875	A	C	ins	1	0	0	0	.	.	.	LSM3(INTRON)
3	14226872	14226880	14226876	A	C	snp	1	0	0	0	.	.	.	LSM3(INTRON)
3	14372593	14372602	14372599	CA	T	snp	1	1	1	1	.	.	.	INTERGENIC(INTERGENIC)
3	14414514	14414529	14414521	TC	G	snp	1	1	0	1	.	.	.	INTERGENIC(INTERGENIC)
3	14485478	14485486	14485480	C	T	snp	1	0	0	0	.	.	.	SLC6A6(EXON);SLC6A6(INTRON)
3	14485478	14485486	14485484	C	A	snp	1	1	1	1	.	.	.	SLC6A6(EXON);SLC6A6(INTRON)
3	14509844	14509853	14509850	C	CA	ins	1	0	0	0	.	.	.	SLC6A6(INTRON)
3	14557144	14557159	14557149	TATT	G	snp	1	0	1	0	.	.	.	GRIP2(INTRON);GRIP2(UPSTREAM)
3	14594126	14594141	14594138	TC	CTC	del	0	1	0	1	.	.	.	INTERGENIC(INTERGENIC)
3	14604521	14604530	14604523	A	G	ins	1	1	1	1	.	.	.	INTERGENIC(INTERGENIC)
3	14604521	14604530	14604524	A	G	snp	1	1	1	1	.	.	.	INTERGENIC(INTERGENIC)
3	14833965	14833978	14833968	AT	A	snp	1	1	1	1	.	.	.	INTERGENIC(INTERGENIC)
3	14850587	14850598	14850595	CCA	T	snp	1	1	1	1	.	.	.	RP11-95M5.1.1(INTRON)
3	14971324	14971339	14971329	AATG	G	snp	1	0	0	0	.	.	.	FGD5(INTRON);FGD5(UPSTREAM);FGD5-AS1(INTRON)
3	14978366	14978374	14978368	T	C	ins	1	0	0	0	.	.	.	FGD5(DOWNSTREAM);FGD5-AS1(INTRON)
3	14993005	14993013	14993011	T	C	snp	1	0	0	0	.	.	.	FGD5-AS1(UPSTREAM);NR2C2(INTRON)
3	15013637	15013644	15013639	A	G	snp	1	0	0	0	.	.	.	NR2C2(INTRON)
3	15019469	15019486	15019472	AAG	AG	del	1	0	0	1	.	.	.	NR2C2(INTRON)
3	15113899	15113906	15113900	A	G	snp	1	0	0	0	.	.	.	ZFYVE20(DOWNSTREAM);ZFYVE20(UTR_3_PRIME)
3	15221877	15221885	15221882	T	C	snp	1	0	0	0	.	.	.	COL6A4P1(INTRON)
3	15248806	15248815	15248812	A	G	snp	1	0	0	0	.	.	.	AC087590.1(UPSTREAM);CAPN7(INTRON);COL6A4P1(UPSTREAM)
3	15282164	15282172	15282165	A	G	snp	1	0	0	0	.	.	.	CAPN7(INTRON);Y_RNA(UPSTREAM)
3	15315756	15315765	15315762	A	AAAAG	ins	1	0	0	0	.	.	.	SH3BP5(INTRON)
3	15315756	15315765	15315762	A	G	snp	1	0	0	0	.	.	.	SH3BP5(INTRON)
3	15392664	15392672	15392669	A	C	snp	1	0	0	0	.	.	.	INTERGENIC(INTERGENIC)
3	15447503	15447512	15447508	TG	T	snp	1	0	0	0	.	.	.	METTL6(INTRON)
3	15605482	15605490	15605484	A	C	snp	1	0	0	0	.	.	.	HACL1(INTRON)
3	15655136	15655143	15655141	T	C	snp	1	0	0	0	.	.	.	BTD(INTRON)
3	15655619	15655634	15655625	TCTT	TTCTTTC	del	1	0	0	0	.	.	.	BTD(INTRON)

3	15655619	15655634	15655630	TCTT	TC	del	1	1	1	1	.	.	.	BTD(INTRON)
3	15684129	15684136	15684135	T	G	snp	1	0	0	0	.	.	.	BTD(DOWNSTREAM);BTD(INTRON)
3	15685008	15685017	15685011	A	G	snp	0	1	1	0	.	.	.	BTD(DOWNSTREAM);BTD(INTRON)
3	15742336	15742344	15742337	A	G	snp	1	0	0	0	.	.	.	ANKRD28(INTRON);ANKRD28(UPSTREAM)
3	15746836	15746847	15746843	TG	C	snp	1	0	0	0	.	.	.	ANKRD28(DOWNSTREAM);ANKRD28(INTRON)
3	15778813	15778822	15778819	GT	T	snp	1	0	0	0	.	.	.	ANKRD28(INTRON)
3	15804555	15804562	15804558	T	G	snp	1	1	1	1	.	.	.	ANKRD28(INTRON)
3	15883090	15883097	15883094	A	G	snp	1	1	1	1	.	.	.	ANKRD28(INTRON)
3	15919333	15919341	15919336	A	C	snp	1	1	1	1	.	.	.	AC090945.1.1(UPSTREAM);INTERGENIC(INTERGENIC);MIR563(DOWNSTREAM)
3	15943042	15943050	15943049	T	G	snp	1	0	0	0	.	.	.	INTERGENIC(INTERGENIC)
3	16038301	16038310	16038302	A	G	snp	1	0	0	0	.	.	.	INTERGENIC(INTERGENIC)
3	16065090	16065097	16065092	T	TTG	ins	1	1	1	1	.	.	.	INTERGENIC(INTERGENIC)
3	16065090	16065097	16065093	T	TG	ins	1	1	1	1	.	.	.	INTERGENIC(INTERGENIC)
3	16065090	16065097	16065094	T	G	ins	1	1	1	1	.	.	.	INTERGENIC(INTERGENIC)
3	16138850	16138861	16138855	TC	T	snp	1	0	0	0	.	.	.	INTERGENIC(INTERGENIC)
3	16208113	16208128	16208116	AC	G	snp	1	0	0	0	.	.	.	INTERGENIC(INTERGENIC)
3	16366197	16366208	16366201	TA	A	snp	1	1	1	1	.	.	.	OXNAD1(INTRON);RFTN1(INTRON)
3	16406165	16406180	16406174	AAAT	C	snp	0	1	1	1	.	.	.	RFTN1(INTRON)
3	16458902	16458911	16458905	CA	G	snp	1	1	1	1	.	.	.	RFTN1(INTRON)
3	16482195	16482204	16482197	G	T	snp	0	0	0	1	.	.	.	RFTN1(INTRON)
3	16533257	16533276	16533272	AAAC	A	ins	0	0	1	0	.	.	.	RFTN1(INTRON)
3	16546945	16546958	16546950	TG	T	snp	0	0	0	1	.	.	.	RFTN1(INTRON)
3	16555219	16555233	16555223	CCG	T	snp	1	1	1	1	.	.	.	RFTN1(INTRON);RFTN1(UPSTREAM)
3	16619214	16619225	16619221	CCA	ACT	ins	1	1	1	1	.	.	.	INTERGENIC(INTERGENIC)
3	16678737	16678746	16678739	T	C	ins	1	1	1	1	.	.	.	DAZL(INTRON)
3	16715727	16715736	16715731	A	C	ins	1	0	0	0	.	.	.	DAZL(UPSTREAM);INTERGENIC(INTERGENIC)
3	16767198	16767205	16767202	A	G	snp	1	1	0	1	.	.	.	INTERGENIC(INTERGENIC)
3	16792457	16792465	16792459	T	C	snp	1	0	0	0	.	.	.	INTERGENIC(INTERGENIC)
3	16856772	16856786	16856778	TGG	G	snp	1	1	1	1	.	.	.	PLCL2(INTRON)
3	17030510	17030521	17030514	TC	CTC	del	1	1	1	1	.	.	.	PLCL2(INTRON)
3	17063992	17063999	17063993	A	C	snp	1	0	0	0	.	.	.	PLCL2(INTRON)
3	17081614	17081621	17081619	T	C	snp	1	1	1	1	.	.	.	PLCL2(INTRON);PLCL2-AS1(DOWNSTREAM)
3	17161306	17161317	17161314	CTC	CT	del	1	1	1	1	.	.	.	INTERGENIC(INTERGENIC)
3	17171836	17171844	17171837	G	A	snp	1	1	1	1	.	.	.	INTERGENIC(INTERGENIC)
3	17171836	17171844	17171838	G	T	snp	1	0	0	0	.	.	.	INTERGENIC(INTERGENIC)
3	17393422	17393430	17393426	T	C	ins	1	0	0	0	.	.	.	TBC1D5(INTRON)
3	17417166	17417175	17417172	A	C	snp	1	0	0	0	.	.	.	TBC1D5(INTRON);TBC1D5(UPSTREAM)
3	17438644	17438652	17438647	T	G	snp	1	0	0	0	.	.	.	TBC1D5(INTRON)
3	17538566	17538577	17538570	TC	C	snp	1	0	0	0	.	.	.	TBC1D5(INTRON)
3	17571126	17571143	17571140	ATT	A	del	1	0	0	0	.	.	.	TBC1D5(INTRON)
3	17803971	17803984	17803974	TC	T	snp	1	1	1	1	.	.	.	TBC1D5(INTRON)
3	17930440	17930451	17930444	AT	G	snp	1	1	1	1	.	.	.	TBC1D5(INTRON)
3	17941362	17941369	17941365	A	C	snp	0	0	0	1	.	.	.	TBC1D5(INTRON)
3	17945764	17945771	17945770	T	C	snp	1	1	1	1	.	.	.	TBC1D5(INTRON)
3	17945771	17945786	17945778	TC	G	snp	1	1	1	1	.	.	.	TBC1D5(INTRON)
3	17997085	17997100	17997094	CCTT	TT	del	0	1	0	1	.	.	.	TBC1D5(INTRON)
3	18027299	18027314	18027308	CT	C	snp	1	0	0	0	.	.	.	AC132807.1.1(UPSTREAM);TBC1D5(INTRON)
3	18071526	18071541	18071536	TGCA	T	snp	0	0	1	1	.	.	.	TBC1D5(INTRON)
3	18106682	18106699	18106687	TTG	T	snp	1	0	0	0	.	.	.	TBC1D5(INTRON)
3	18149438	18149451	18149444	AT	G	snp	0	0	1	0	.	.	.	TBC1D5(INTRON)
3	18432611	18432619	18432613	A	G	snp	1	1	0	1	.	.	.	SATB1(DOWNSTREAM);SATB1(INTRON);TBC1D5(INTRON)
3	18457303	18457312	18457304	G	T	ins	0	0	1	0	.	.	.	SATB1(DOWNSTREAM);SATB1(INTRON);SATB1(UPSTREAM);TBC1D5(INTRON)
3	18457303	18457312	18457304	G	T	snp	1	1	1	1	.	.	.	SATB1(DOWNSTREAM);SATB1(INTRON);SATB1(UPSTREAM);TBC1D5(INTRON)
3	18466369	18466380	18466376	TGT	T	snp	1	0	0	0	.	.	.	SATB1(INTRON);SATB1(UPSTREAM);SATB1(UTR_5_PRIME);TBC1D5(INTRON)
3	18672792	18672811	18672804	CAAA	A	ins	1	0	0	0	.	.	.	INTERGENIC(INTERGENIC)
3	18700399	18700408	18700402	AT	ATATATATAC	ins	1	1	1	1	.	.	.	INTERGENIC(INTERGENIC)
3	18790283	18790292	18790284	A	C	ins	1	1	0	1	.	.	.	AC105750.1.1(DOWNSTREAM);AC105750.1.1(INTRON)
3	18822777	18822784	18822782	C	A	ins	1	0	0	0	.	.	.	AC105750.1.1(INTRON)
3	18841577	18841586	18841584	TG	A	snp	0	0	1	1	.	.	.	AC105750.1.1(INTRON)
3	18845858	18845867	18845860	TC	C	snp	0	1	1	1	.	.	.	AC105750.1.1(INTRON)
3	18868102	18868111	18868108	AT	C	snp	1	0	1	1	.	.	.	AC105750.1.1(INTRON)
3	18904135	18904150	18904140	TTTC	TCTTTCTTTC	del	1	1	1	1	.	.	.	AC105750.1.1(INTRON)
3	19215131	19215142	19215138	CA	G	snp	1	0	0	0	.	.	.	KCNH8(INTRON)

3	19230448	19230457	19230451	T	G	snp	0	1	1	1	.	.	.	KCNH8(INTRON)
3	19239975	19239982	19239980	A	G	snp	1	0	0	0	.	.	.	KCNH8(INTRON)
3	19253066	19253073	19253068	T	G	snp	1	0	0	0	.	.	.	KCNH8(INTRON)
3	19354793	19354801	19354797	A	G	snp	1	0	0	0	.	.	.	KCNH8(INTRON)
3	19424167	19424181	19424177	TAA	ATG	ins	1	1	0	1	.	.	.	KCNH8(INTRON)
3	19546459	19546474	19546462	AT	C	snp	1	0	0	0	.	.	.	KCNH8(INTRON)
3	19665307	19665327	19665315	TTA	G	snp	0	0	1	0	.	.	.	INTERGENIC(INTERGENIC)
3	19812607	19812616	19812609	CT	G	snp	1	1	1	1	.	.	.	INTERGENIC(INTERGENIC)
3	19870475	19870488	19870483	TA	A	ins	1	0	0	0	.	.	.	INTERGENIC(INTERGENIC)
3	19870497	19870510	19870502	TA	G	snp	1	0	0	1	.	.	.	INTERGENIC(INTERGENIC)
3	19875653	19875668	19875658	ATTT	C	snp	1	0	0	0	.	.	.	INTERGENIC(INTERGENIC)
3	19936482	19936491	19936485	AT	A	snp	1	0	0	0	.	.	.	EFHB(DOWNSTREAM);EFHB(INTRON)
3	19969449	19969458	19969453	TA	A	snp	1	1	0	1	.	.	.	EFHB(INTRON)
3	20000937	20000950	20000944	TA	G	snp	1	0	0	0	.	.	.	RAB5A(INTRON)
3	20054342	20054351	20054346	TA	C	snp	0	1	0	1	.	.	.	AC097635.4.1(UPSTREAM);AC097635.5.1(DOWNSTREAM);INTERGENIC(INTERGENIC);PP2D1(UPSTREAM)
3	20072269	20072277	20072273	A	G	snp	1	0	0	0	.	.	.	INTERGENIC(INTERGENIC)
3	20092714	20092721	20092720	A	G	snp	0	1	0	1	.	.	.	KAT2B(INTRON)
3	20119674	20119697	20119685	AAAT	C	snp	1	0	0	0	.	.	.	KAT2B(INTRON)
3	20119674	20119697	20119692	AAAT	T	snp	1	1	1	1	.	.	.	KAT2B(INTRON)
3	20141252	20141267	20141265	AG	C	snp	1	1	0	1	.	.	.	KAT2B(INTRON)
3	20160264	20160273	20160266	AT	T	snp	1	1	0	1	.	.	.	KAT2B(INTRON)
3	20180384	20180399	20180391	TTTG	T	snp	1	1	1	1	.	.	.	KAT2B(INTRON)
3	20184378	20184395	20184382	TG	C	snp	1	1	1	1	.	.	.	KAT2B(INTRON)
3	20255120	20255131	20255123	CT	C	snp	1	0	0	0	.	.	.	INTERGENIC(INTERGENIC)
3	20285742	20285759	20285748	AAC	C	snp	0	0	1	0	.	.	.	INTERGENIC(INTERGENIC)
3	20296230	20296238	20296234	A	G	snp	1	1	1	1	.	.	.	INTERGENIC(INTERGENIC)
3	20481532	20481540	20481534	A	G	snp	0	1	0	0	.	.	.	INTERGENIC(INTERGENIC)
3	20542368	20542377	20542375	T	G	snp	1	1	1	0	.	.	.	INTERGENIC(INTERGENIC)
3	20636034	20636043	20636041	T	G	snp	1	0	0	0	.	.	.	INTERGENIC(INTERGENIC)
3	20693690	20693697	20693692	T	C	snp	1	1	0	0	.	.	.	INTERGENIC(INTERGENIC)
3	20852537	20852544	20852538	A	C	snp	1	0	0	0	.	.	.	INTERGENIC(INTERGENIC)
3	20897823	20897842	20897835	CCTT	A	snp	1	1	1	1	.	.	.	INTERGENIC(INTERGENIC)
3	20897856	20897865	20897862	CT	G	snp	1	1	1	1	.	.	.	INTERGENIC(INTERGENIC)
3	20915394	20915413	20915398	TTTA	G	snp	1	0	0	0	.	.	.	INTERGENIC(INTERGENIC)
3	20941153	20941172	20941166	AGAA	A	snp	1	0	0	0	.	.	.	INTERGENIC(INTERGENIC)
3	20941153	20941172	20941167	AGAA	T	snp	1	0	0	0	.	.	.	INTERGENIC(INTERGENIC)
3	21002691	21002700	21002698	T	G	snp	1	1	1	1	.	.	.	INTERGENIC(INTERGENIC)
3	21077422	21077437	21077427	TATT	G	snp	1	1	1	1	.	.	.	INTERGENIC(INTERGENIC)
3	21112076	21112084	21112082	C	A	ins	1	1	1	1	.	.	.	INTERGENIC(INTERGENIC)
3	21170055	21170064	21170058	AC	A	ins	1	1	1	1	.	.	.	INTERGENIC(INTERGENIC)
3	21184913	21184922	21184919	AT	G	snp	1	1	1	1	.	.	.	INTERGENIC(INTERGENIC)
3	21184961	21184970	21184967	AT	G	snp	1	1	1	1	.	.	.	INTERGENIC(INTERGENIC)
3	21185453	21185464	21185457	TA	A	snp	1	1	1	1	.	.	.	INTERGENIC(INTERGENIC)
3	21267126	21267135	21267129	TA	G	snp	0	1	1	1	.	.	.	INTERGENIC(INTERGENIC)
3	21301780	21301787	21301784	T	C	snp	0	1	1	0	.	.	.	INTERGENIC(INTERGENIC)
3	21353258	21353266	21353262	T	G	snp	1	0	0	0	.	.	.	INTERGENIC(INTERGENIC)
3	21402895	21402906	21402901	AAT	AT	del	0	1	1	1	.	.	.	INTERGENIC(INTERGENIC)
3	21465794	21465809	21465807	TA	T	ins	1	1	1	1	.	.	.	ZNF385D(INTRON)
3	21515460	21515471	21515464	TA	G	snp	0	1	1	1	.	.	.	ZNF385D(INTRON)
3	21538021	21538030	21538024	AT	ATATATC	del	1	1	1	1	.	.	.	ZNF385D(INTRON)
3	21538021	21538030	21538026	AT	ATATC	del	1	1	1	1	.	.	.	ZNF385D(INTRON)
3	21570554	21570561	21570560	T	G	snp	1	1	1	1	.	.	.	ZNF385D(INTRON)
3	21662479	21662488	21662485	TG	C	snp	1	1	1	1	.	.	.	ZNF385D(INTRON)
3	21677026	21677043	21677035	TTG	G	snp	0	1	1	1	.	.	.	ZNF385D(INTRON)
3	21731690	21731699	21731697	TG	T	snp	1	1	1	1	.	.	.	ZNF385D(INTRON)
3	21740135	21740142	21740139	A	C	snp	1	1	1	1	.	.	.	ZNF385D(INTRON)
3	21807136	21807145	21807138	A	C	snp	1	1	1	1	.	.	.	ZNF385D(INTRON)
3	21826864	21826871	21826870	T	G	snp	1	1	1	1	.	.	.	ZNF385D(INTRON)
3	21863294	21863301	21863295	T	G	snp	0	1	1	1	.	.	.	ZNF385D(INTRON)
3	21889671	21889678	21889675	T	C	snp	1	0	0	0	.	.	.	ZNF385D(INTRON)
3	21898928	21898939	21898931	TTG	C	snp	1	1	1	1	.	.	.	ZNF385D(INTRON)
3	21984811	21984820	21984815	TA	C	snp	1	0	0	0	.	.	.	ZNF385D(INTRON);ZNF385D-AS2(INTRON)
3	22002216	22002225	22002219	AT	A	snp	1	1	1	1	.	.	.	ZNF385D(INTRON);ZNF385D-AS2(INTRON)
3	22012100	22012117	22012103	AG	T	snp	1	1	1	1	.	.	.	ZNF385D(INTRON);ZNF385D-AS2(INTRON)
3	22071959	22071970	22071962	GA	G	snp	1	1	1	1	.	.	.	ZNF385D(INTRON)
3	22071959	22071970	22071966	GA	G	snp	1	1	1	1	.	.	.	ZNF385D(INTRON)

3	22121434	22121449	22121445	ATAG	C	snp	1	1	1	1	.	.	.	ZNF385D(INTRON)
3	22272857	22272866	22272858	A	G	snp	1	0	1	0	.	.	.	ZNF385D(INTRON)
3	22321131	22321144	22321136	TA	TTC	ins	1	1	1	1	.	.	.	ZNF385D(INTRON)
3	22321131	22321144	22321137	TA	ATA	del	1	1	1	1	.	.	.	ZNF385D(INTRON)
3	22427686	22427693	22427687	A	C	snp	1	1	1	1	.	.	.	HMGB1P5(DOWNSTREAM);INTERGENIC(INTERGENIC)
3	22428736	22428756	22428747	ACC	T	snp	1	0	1	1	.	.	.	HMGB1P5(DOWNSTREAM);INTERGENIC(INTERGENIC)
3	22491944	22491961	22491954	CA	T	snp	1	0	0	0	.	.	.	INTERGENIC(INTERGENIC)
3	22492376	22492384	22492378	A	C	snp	1	0	0	0	.	.	.	INTERGENIC(INTERGENIC)
3	22515358	22515375	22515366	AAG	A	ins	1	1	1	1	.	.	.	INTERGENIC(INTERGENIC)
3	22515358	22515375	22515367	AAG	AGAAG	del	1	1	1	1	.	.	.	INTERGENIC(INTERGENIC)
3	22515358	22515375	22515369	AAG	A	ins	1	1	1	1	.	.	.	INTERGENIC(INTERGENIC)
3	22579372	22579380	22579375	T	G	snp	1	0	0	0	.	.	.	INTERGENIC(INTERGENIC)
3	22589693	22589700	22589696	A	G	snp	0	0	1	0	.	.	.	INTERGENIC(INTERGENIC)
3	22621567	22621574	22621568	A	C	snp	1	1	1	1	.	.	.	INTERGENIC(INTERGENIC)
3	22656278	22656287	22656281	A	G	snp	1	0	0	0	.	.	.	INTERGENIC(INTERGENIC)
3	22670221	22670230	22670228	T	G	ins	1	0	0	0	.	.	.	INTERGENIC(INTERGENIC)
3	22705451	22705460	22705456	CA	G	snp	0	1	1	0	.	.	.	INTERGENIC(INTERGENIC)
3	22716220	22716229	22716224	CT	G	snp	1	1	1	1	.	.	.	AC092421.1.1(UPSTREAM);INTERGENIC(INTERGENIC)
3	22853873	22853884	22853881	TTG	TTGT	del	1	0	0	0	.	.	.	INTERGENIC(INTERGENIC)
3	22884107	22884114	22884110	T	C	snp	1	0	0	0	.	.	.	INTERGENIC(INTERGENIC)
3	22928815	22928822	22928819	A	C	snp	1	1	1	1	.	.	.	INTERGENIC(INTERGENIC)
3	23007424	23007432	23007426	A	C	snp	1	1	1	1	.	.	.	INTERGENIC(INTERGENIC)
3	23067790	23067801	23067794	TA	C	snp	1	1	1	1	.	.	.	INTERGENIC(INTERGENIC)
3	23102502	23102513	23102509	AT	C	snp	1	0	0	0	.	.	.	INTERGENIC(INTERGENIC)
3	23205871	23205878	23205872	A	G	ins	1	1	1	1	.	.	.	INTERGENIC(INTERGENIC)
3	23218854	23218865	23218858	AAT	G	snp	0	1	1	0	.	.	.	INTERGENIC(INTERGENIC)
3	23244944	23244951	23244948	C	CG	ins	1	0	1	0	.	.	.	AC135966.1.1(UPSTREAM);UBE2E2(INTRON);UBE2E2(UTR_5_PRIME)
3	23296394	23296402	23296397	T	C	snp	0	0	0	1	.	.	.	UBE2E2(INTRON)
3	23314863	23314872	23314864	A	C	snp	0	1	1	0	.	.	.	UBE2E2(DOWNSTREAM);UBE2E2(INTRON);Y_RNA(UPSTREAM)
3	23353670	23353677	23353671	A	G	snp	1	1	1	1	.	.	.	UBE2E2(INTRON)
3	23373643	23373650	23373646	T	C	snp	1	1	1	1	.	.	.	UBE2E2(INTRON)
3	23445632	23445645	23445638	CA	A	snp	1	1	1	1	.	.	.	AC092038.1(UPSTREAM);UBE2E2(INTRON)
3	23454683	23454694	23454686	ATA	G	snp	1	1	1	1	.	.	.	UBE2E2(INTRON)
3	23512305	23512314	23512312	AT	A	del	1	1	1	1	.	.	.	UBE2E2(INTRON)
3	23520371	23520388	23520386	AT	A	del	1	1	1	1	.	.	.	UBE2E2(INTRON)
3	23642819	23642827	23642823	T	C	snp	1	0	0	0	.	.	.	INTERGENIC(INTERGENIC)
3	23671762	23671777	23671765	AG	A	snp	1	0	0	0	.	.	.	INTERGENIC(INTERGENIC)
3	23675250	23675258	23675252	A	G	snp	1	0	0	0	.	.	.	INTERGENIC(INTERGENIC)
3	23769862	23769871	23769865	A	C	snp	1	0	0	0	.	.	.	INTERGENIC(INTERGENIC)
3	23780284	23780303	23780294	AAAC	C	del	1	0	0	0	.	.	.	INTERGENIC(INTERGENIC)
3	23780284	23780303	23780298	AAAC	C	del	1	0	0	0	.	.	.	INTERGENIC(INTERGENIC)
3	23951346	23951354	23951351	T	TTTGG	ins	0	0	1	0	.	.	.	NKIRAS1(INTRON)
3	24012251	24012265	24012262	TTG	TTGT	del	1	0	0	0	.	.	.	NR1D2(DOWNSTREAM);NR1D2(INTRON)
3	24039292	24039301	24039297	A	C	snp	1	1	0	1	.	.	.	INTERGENIC(INTERGENIC)
3	24058811	24058820	24058817	CT	G	snp	0	1	0	1	.	.	.	INTERGENIC(INTERGENIC)
3	24259759	24259767	24259761	A	G	snp	1	0	1	0	.	.	.	THRB(INTRON)
3	24265376	24265385	24265383	TG	T	del	1	0	0	1	.	.	.	THRB(INTRON)
3	24341261	24341280	24341276	TTTA	T	del	1	1	1	1	.	.	.	THRB(INTRON)
3	24379564	24379575	24379567	TTG	C	snp	1	1	1	1	.	.	.	THRB(INTRON)
3	24416626	24416635	24416629	AG	C	snp	1	0	0	0	.	.	.	THRB(INTRON)
3	24564663	24564671	24564664	G	T	snp	0	0	1	0	.	.	.	INTERGENIC(INTERGENIC)
3	24568271	24568280	24568275	AT	TATG	ins	1	0	0	0	.	.	.	INTERGENIC(INTERGENIC)
3	24574135	24574143	24574142	T	C	snp	1	1	1	1	.	.	.	INTERGENIC(INTERGENIC)
3	24578772	24578787	24578782	TTTA	C	snp	1	0	0	0	.	.	.	INTERGENIC(INTERGENIC)
3	24613940	24613951	24613946	AC	G	snp	1	1	1	1	.	.	.	INTERGENIC(INTERGENIC)
3	24649111	24649118	24649114	T	G	snp	1	0	0	0	.	.	.	INTERGENIC(INTERGENIC)
3	24669339	24669347	24669346	A	G	snp	0	0	0	1	.	.	.	INTERGENIC(INTERGENIC)
3	24822853	24822860	24822859	T	G	snp	0	0	1	0	.	.	.	AC133680.1.1(INTRON)
3	24954880	24954893	24954891	TC	TCT	del	0	1	1	1	.	.	.	AC133680.1.1(INTRON)
3	25019702	25019710	25019708	T	G	ins	1	0	0	0	.	.	.	AC133680.1.1(INTRON)
3	25119222	25119233	25119231	TA	G	snp	0	1	0	1	.	.	.	AC133680.1.1(INTRON)
3	25187987	25188002	25187994	TTTG	T	snp	1	1	1	1	.	.	.	AC133680.1.1(INTRON)
3	25234223	25234232	25234228	AC	T	snp	0	1	1	1	.	.	.	RARB(INTRON)
3	25276138	25276145	25276139	T	C	ins	1	0	1	0	.	.	.	RARB(INTRON)
3	25305856	25305864	25305858	T	C	snp	1	0	0	0	.	.	.	RARB(INTRON)
3	25341247	25341254	25341248	A	AAT	ins	1	0	0	0	.	.	.	RARB(INTRON)

3	25341252	25341271	25341265	AAAT	ATAG	ins	1	0	0	0	.	.	.	RARB(INTRON)
3	25391234	25391241	25391240	T	G	snp	1	0	0	0	.	.	.	RARB(INTRON);RN5S126(DOWNSTREAM)
3	25393703	25393712	25393708	TC	T	snp	1	0	0	0	.	.	.	RARB(INTRON);RN5S126(DOWNSTREAM)
3	25466171	25466178	25466176	G	A	snp	0	1	0	1	.	.	.	RARB(INTRON);RARB(UPSTREAM)
3	25516893	25516901	25516894	G	T	snp	0	0	0	1	.	.	.	RARB(INTRON)
3	25706341	25706358	25706350	GCC	A	snp	1	0	0	0	.	.	.	TOP2B(INTRON);TOP2B(UPSTREAM);TOP2B(UTR_5_PRIME)
3	25979091	25979098	25979093	A	G	snp	1	1	0	1	.	.	.	INTERGENIC(INTERGENIC)
3	26086227	26086246	26086233	TATT	C	snp	1	0	0	0	.	.	.	INTERGENIC(INTERGENIC)
3	26133835	26133850	26133845	TA	G	snp	1	1	1	1	.	.	.	INTERGENIC(INTERGENIC)
3	26133835	26133850	26133847	TA	G	snp	1	1	1	1	.	.	.	INTERGENIC(INTERGENIC)
3	26215322	26215331	26215327	AT	C	snp	1	0	0	0	.	.	.	INTERGENIC(INTERGENIC)
3	26298333	26298341	26298338	T	G	snp	1	1	0	0	.	.	.	INTERGENIC(INTERGENIC)
3	26298333	26298341	26298340	T	G	snp	1	1	0	0	.	.	.	INTERGENIC(INTERGENIC)
3	26306669	26306678	26306675	AC	G	snp	1	0	0	0	.	.	.	INTERGENIC(INTERGENIC)
3	26329539	26329562	26329546	AAAT	A	ins	0	1	0	0	.	.	.	INTERGENIC(INTERGENIC)
3	26456631	26456640	26456635	T	C	snp	1	1	1	1	.	.	.	INTERGENIC(INTERGENIC)
3	26469813	26469820	26469815	A	G	snp	1	1	1	1	.	.	.	INTERGENIC(INTERGENIC)
3	26604449	26604460	26604457	ATT	A	del	1	1	1	1	.	.	.	INTERGENIC(INTERGENIC)
3	26605886	26605893	26605892	A	C	snp	1	1	1	1	.	.	.	INTERGENIC(INTERGENIC)
3	26678464	26678475	26678467	TC	T	snp	1	1	1	0	.	.	.	LRRC3B(INTRON)
3	26678464	26678475	26678469	TC	T	snp	1	1	1	1	.	.	.	LRRC3B(INTRON)
3	26678490	26678505	26678500	TCTT	C	snp	1	0	1	0	.	.	.	LRRC3B(INTRON)
3	26755664	26755672	26755665	A	C	ins	1	1	1	1	.	.	.	AC114877.3.1(DOWNSTREAM);INTERGENIC(INTERGENIC);LRRC3B(DOWNSTREAM)
3	26844503	26844514	26844505	GT	T	snp	1	0	0	0	.	.	.	INTERGENIC(INTERGENIC)
3	26944981	26944989	26944983	A	C	snp	1	1	1	1	.	.	.	INTERGENIC(INTERGENIC)
3	26994023	26994032	26994028	T	G	snp	1	0	0	0	.	.	.	INTERGENIC(INTERGENIC)
3	27052675	27052694	27052686	TTTA	C	snp	0	1	1	1	.	.	.	INTERGENIC(INTERGENIC)
3	27267845	27267852	27267849	A	G	snp	1	0	0	0	.	.	.	NEK10(INTRON)
3	27327021	27327036	27327032	AAAC	A	snp	1	0	1	1	.	.	.	NEK10(INTRON);NEK10(UPSTREAM)
3	27365096	27365104	27365100	A	G	snp	1	0	0	0	.	.	.	NEK10(INTRON)
3	27401500	27401509	27401505	AC	G	snp	1	1	0	1	.	.	.	AC099535.1(DOWNSTREAM);NEK10(INTRON)
3	27507455	27507474	27507461	AAAG	G	del	1	1	1	1	.	.	.	AC099535.3.1(DOWNSTREAM);SLC4A7(INTRON)
3	27583081	27583098	27583087	CA	T	snp	1	0	0	0	.	.	.	INTERGENIC(INTERGENIC)
3	27597083	27597100	27597086	AAT	AT	del	1	1	1	1	.	.	.	INTERGENIC(INTERGENIC)
3	27597083	27597100	27597087	AAT	T	del	1	1	1	1	.	.	.	INTERGENIC(INTERGENIC)
3	27597083	27597100	27597090	AAT	T	del	0	0	1	0	.	.	.	INTERGENIC(INTERGENIC)
3	27628085	27628094	27628093	T	AG	ins	1	1	1	1	.	.	.	INTERGENIC(INTERGENIC)
3	27634820	27634827	27634823	T	C	snp	1	1	1	1	.	.	.	INTERGENIC(INTERGENIC)
3	27741985	27741992	27741991	T	C	snp	1	0	0	0	.	.	.	AC098614.3.1(DOWNSTREAM);INTERGENIC(INTERGENIC)
3	27780621	27780640	27780636	TCTT	TC	del	1	1	0	1	.	.	.	INTERGENIC(INTERGENIC)
3	27895272	27895280	27895277	A	C	snp	1	1	0	1	.	.	.	AC098973.2.1(INTRON)
3	27925407	27925416	27925410	AT	C	snp	1	0	0	0	.	.	.	AC098973.2.1(INTRON)
3	27933595	27933615	27933603	TCC	T	snp	1	0	0	0	.	.	.	AC098973.2.1(DOWNSTREAM);INTERGENIC(INTERGENIC)
3	27966812	27966821	27966816	T	C	snp	1	1	1	1	.	.	.	INTERGENIC(INTERGENIC)
3	28118986	28118997	28118994	ATA	G	snp	1	0	0	0	.	.	.	INTERGENIC(INTERGENIC)
3	28151699	28151708	28151703	T	G	snp	1	1	1	1	.	.	.	INTERGENIC(INTERGENIC)
3	28233644	28233652	28233649	A	G	snp	1	0	0	0	.	.	.	INTERGENIC(INTERGENIC)
3	28321241	28321249	28321247	A	G	snp	1	1	1	1	.	.	.	CMC1(INTRON)
3	28425048	28425056	28425051	T	C	snp	1	1	1	1	.	.	.	ZCWPW2(INTRON)
3	28446161	28446170	28446168	AT	C	snp	1	1	0	1	.	.	.	ZCWPW2(INTRON)
3	28464259	28464274	28464264	TG	A	snp	1	0	0	0	.	.	.	ZCWPW2(INTRON)
3	28464275	28464283	28464276	T	G	snp	1	1	1	1	.	.	.	ZCWPW2(INTRON)
3	28639525	28639538	28639535	GT	A	snp	1	0	0	0	.	.	.	AC109586.1.1(INTRON)
3	28680902	28680911	28680907	TA	G	snp	1	0	0	0	.	.	.	AC109586.1.1(INTRON)
3	28683494	28683503	28683497	TA	G	snp	1	0	0	0	.	.	.	AC109586.1.1(INTRON)
3	28699157	28699174	28699159	TC	C	del	1	1	1	1	.	.	.	AC109586.1.1(INTRON)
3	28760577	28760586	28760584	GT	C	snp	1	0	0	0	.	.	.	AC109586.1.1(INTRON)
3	28839086	28839103	28839089	TC	T	snp	1	1	1	1	.	.	.	INTERGENIC(INTERGENIC)
3	28839086	28839103	28839093	TC	T	snp	1	1	1	1	.	.	.	INTERGENIC(INTERGENIC)
3	28839086	28839103	28839101	TC	T	snp	1	1	1	0	.	.	.	INTERGENIC(INTERGENIC)
3	28856681	28856689	28856688	A	C	snp	1	0	1	0	.	.	.	INTERGENIC(INTERGENIC)
3	28857339	28857354	28857342	AC	T	snp	1	1	1	1	.	.	.	INTERGENIC(INTERGENIC)
3	28857339	28857354	28857346	AC	A	snp	1	1	1	1	.	.	.	INTERGENIC(INTERGENIC)
3	28889078	28889091	28889081	CT	C	snp	1	0	1	0	.	.	.	INTERGENIC(INTERGENIC)
3	28893340	28893353	28893344	AT	TC	ins	1	0	0	0	.	.	.	INTERGENIC(INTERGENIC)
3	28906214	28906234	28906231	AAC	A	snp	1	1	1	0	.	.	.	INTERGENIC(INTERGENIC)

3	29044281	29044289	29044288	T	G	snp	1	1	1	1	.	.	.	INTERGENIC(INTERGENIC)
3	29048836	29048845	29048842	TA	C	snp	1	1	1	1	.	.	.	AC097361.1(UPSTREAM);INTERGENIC(INTERGENIC)
3	29131667	29131676	29131675	A	C	ins	1	0	0	0	.	.	.	INTERGENIC(INTERGENIC);MESTP4(DOWNSTREAM)
3	29148965	29148982	29148970	TC	A	snp	0	0	0	1	.	.	.	INTERGENIC(INTERGENIC)
3	29162821	29162834	29162827	GA	A	snp	0	0	1	0	.	.	.	INTERGENIC(INTERGENIC)
3	29265950	29265958	29265952	T	C	snp	1	1	1	1	.	.	.	INTERGENIC(INTERGENIC)
3	29272319	29272328	29272326	TC	G	snp	1	1	1	1	.	.	.	INTERGENIC(INTERGENIC)
3	29317835	29317846	29317838	TA	G	snp	0	1	0	0	.	.	.	RBMS3(UPSTREAM);RBMS3-AS3(INTRON)
3	29350398	29350405	29350401	T	C	snp	0	0	1	0	.	.	.	RBMS3(INTRON)
3	29380184	29380195	29380188	CTC	C	del	1	1	1	1	.	.	.	RBMS3(INTRON)
3	29380184	29380195	29380189	CTC	CTCCT	del	1	1	1	1	.	.	.	RBMS3(INTRON)
3	29395975	29395988	29395979	GT	A	snp	1	1	1	1	.	.	.	RBMS3(INTRON)
3	29398537	29398546	29398540	TA	G	snp	0	1	0	1	.	.	.	RBMS3(INTRON)
3	29434922	29434935	29434929	CA	G	snp	1	1	1	1	.	.	.	RBMS3(INTRON);RPS12P5(DOWNSTREAM)
3	29462052	29462060	29462055	T	G	ins	1	1	1	1	.	.	.	RBMS3(INTRON)
3	29471425	29471452	29471447	TTTA	A	del	0	1	1	0	.	.	.	RBMS3(INTRON);RBMS3(UPSTREAM)
3	29556119	29556128	29556125	AT	G	snp	0	1	1	1	.	.	.	RBMS3(INTRON)
3	29575268	29575287	29575280	TAAA	AAA	del	1	0	0	0	.	.	.	RBMS3(INTRON)
3	29722826	29722834	29722829	T	G	snp	1	0	0	0	.	.	.	RBMS3(INTRON)
3	29802427	29802435	29802430	T	G	snp	1	1	1	1	.	.	.	RBMS3(INTRON);RBMS3(UPSTREAM)
3	29906539	29906554	29906545	GGAA	C	snp	1	1	1	1	.	.	.	RBMS3(INTRON)
3	29940517	29940524	29940521	A	G	snp	1	0	0	0	.	.	.	RBMS3(INTRON)
3	30004043	30004058	30004056	AT	C	snp	1	0	0	0	.	.	.	RBMS3(INTRON)
3	30011939	30011946	30011942	T	C	snp	1	1	1	1	.	.	.	RBMS3(INTRON)
3	30019938	30019946	30019942	A	G	snp	1	0	0	0	.	.	.	RBMS3(INTRON)
3	30020650	30020673	30020653	AAC	G	snp	1	1	1	1	.	.	.	RBMS3(INTRON)
3	30020650	30020673	30020666	AAC	G	snp	1	1	1	1	.	.	.	RBMS3(INTRON)
3	30041395	30041406	30041402	ATA	AATAA	del	0	0	1	0	.	.	.	RBMS3(INTRON)
3	30052614	30052629	30052617	AT	G	snp	1	0	0	0	.	.	.	INTERGENIC(INTERGENIC);RBMS3(DOWNSTREAM)
3	30061173	30061181	30061177	T	G	snp	1	0	0	0	.	.	.	INTERGENIC(INTERGENIC)
3	30076280	30076295	30076283	AG	A	snp	1	1	1	1	.	.	.	INTERGENIC(INTERGENIC)
3	30118987	30119004	30118993	AC	G	snp	1	1	1	1	.	.	.	INTERGENIC(INTERGENIC)
3	30120698	30120713	30120704	CCTC	C	del	1	1	1	1	.	.	.	INTERGENIC(INTERGENIC)
3	30239040	30239049	30239044	A	G	snp	1	1	1	1	.	.	.	INTERGENIC(INTERGENIC)
3	30278055	30278062	30278058	A	C	snp	0	1	0	1	.	.	.	INTERGENIC(INTERGENIC)
3	30294941	30294949	30294942	A	G	snp	1	1	1	1	.	.	.	INTERGENIC(INTERGENIC)
3	30330126	30330135	30330130	GT	A	snp	0	1	1	1	.	.	.	INTERGENIC(INTERGENIC)
3	30331509	30331523	30331519	CTT	TC	del	1	0	0	0	.	.	.	INTERGENIC(INTERGENIC)
3	30394875	30394882	30394877	T	C	snp	1	1	0	0	.	.	.	INTERGENIC(INTERGENIC)
3	30539366	30539373	30539372	T	C	snp	1	0	0	0	.	.	.	INTERGENIC(INTERGENIC)
3	30540676	30540683	30540679	A	C	snp	0	0	1	0	.	.	.	INTERGENIC(INTERGENIC)
3	30651818	30651826	30651824	T	G	ins	1	1	1	1	.	.	.	TGFBR2(INTRON);TGFBR2(UPSTREAM)
3	30695224	30695231	30695229	A	G	snp	0	1	0	0	.	.	.	TGFBR2(INTRON)
3	30839163	30839172	30839167	T	G	ins	0	1	0	1	.	.	.	GADL1(DOWNSTREAM);GADL1(INTRON)
3	30904570	30904583	30904578	CT	A	snp	1	1	1	1	.	.	.	GADL1(INTRON)
3	30977268	30977285	30977273	TTG	T	ins	1	0	0	0	.	.	.	INTERGENIC(INTERGENIC)
3	31033317	31033332	31033319	CT	G	snp	1	1	1	1	.	.	.	INTERGENIC(INTERGENIC)
3	31096611	31096622	31096619	AT	T	snp	1	0	0	0	.	.	.	INTERGENIC(INTERGENIC)
3	31168335	31168346	31168343	AC	T	ins	1	1	1	1	.	.	.	INTERGENIC(INTERGENIC)
3	31275089	31275106	31275093	CAA	G	snp	1	1	1	0	.	.	.	INTERGENIC(INTERGENIC);RN5S127(DOWNSTREAM)
3	31404440	31404454	31404444	CCT	T	snp	1	0	0	0	.	.	.	INTERGENIC(INTERGENIC)
3	31413275	31413282	31413280	T	C	snp	1	0	0	0	.	.	.	INTERGENIC(INTERGENIC)
3	31434540	31434567	31434559	GAAT	G	del	1	1	1	0	.	.	.	AC099540.1(UPSTREAM);INTERGENIC(INTERGENIC)
3	31523540	31523547	31523545	C	CT	ins	1	1	0	1	.	.	.	INTERGENIC(INTERGENIC)
3	31523540	31523547	31523546	C	T	ins	1	1	0	1	.	.	.	INTERGENIC(INTERGENIC)
3	31601820	31601829	31601823	TG	A	snp	1	1	1	1	.	.	.	STT3B(INTRON)
3	31603343	31603354	31603347	TG	C	snp	0	0	1	1	.	.	.	STT3B(INTRON)
3	31616959	31616967	31616960	G	T	ins	1	0	0	0	.	.	.	STT3B(INTRON)
3	31639320	31639337	31639334	ATT	A	del	1	1	1	1	.	.	.	STT3B(INTRON)
3	31692070	31692079	31692075	A	C	snp	1	1	1	1	.	.	.	INTERGENIC(INTERGENIC)
3	31868800	31868809	31868805	A	G	snp	1	1	1	1	.	.	.	OSBPL10(INTRON)
3	31903977	31903985	31903980	T	G	snp	1	0	1	0	.	.	.	OSBPL10(INTRON)
3	32030100	32030113	32030111	CT	C	snp	1	1	1	1	.	.	.	OSBPL10(INTRON);ZNF860(INTRON)
3	32107516	32107529	32107519	AG	A	snp	1	1	1	1	.	.	.	AC094019.2.1(INTRON);OSBPL10(INTRON)
3	32129025	32129033	32129027	A	C	snp	1	1	1	1	.	.	.	INTERGENIC(INTERGENIC)
3	32139281	32139288	32139287	A	C	snp	1	1	1	1	.	.	.	INTERGENIC(INTERGENIC)
3	32157104	32157115	32157107	GGA	A	snp	1	1	1	1	.	.	.	GPD1L(INTRON)

3	32163040	32163049	32163043	TA	G	snp	1	0	0	0	.	.	.	GPD1L(INTRON)
3	32163139	32163148	32163144	AT	A	snp	1	1	1	1	.	.	.	GPD1L(INTRON)
3	32163244	32163255	32163253	TA	G	snp	1	1	0	1	.	.	.	GPD1L(INTRON)
3	32187878	32187885	32187880	A	G	snp	1	1	1	1	.	.	.	AC094019.4.1(DOWNSTREAM);GPD1L(INTRON)
3	32270360	32270368	32270362	A	C	ins	1	1	1	1	.	.	.	INTERGENIC(INTERGENIC)
3	32331606	32331613	32331609	A	G	snp	0	1	0	0	.	.	.	CMTM8(INTRON)
3	32356603	32356620	32356617	TTC	T	ins	1	1	1	1	.	.	.	CMTM8(INTRON)
3	32360550	32360567	32360553	AT	C	snp	1	0	0	0	.	.	.	CMTM8(INTRON)
3	32360550	32360567	32360563	AT	ATA	del	1	1	1	1	.	.	.	CMTM8(INTRON)
3	32402779	32402787	32402781	T	G	ins	1	1	1	1	.	.	.	CMTM8(INTRON)
3	32408489	32408496	32408493	T	C	snp	1	0	1	0	.	.	.	CMTM8(INTRON)
3	32428861	32428872	32428865	TC	G	snp	1	1	1	1	.	.	.	CMTM7(UPSTREAM);INTERGENIC(INTERGENIC)
3	32456688	32456708	32456705	AAT	A	snp	1	0	0	0	.	.	.	CMTM7(INTRON)
3	32459307	32459316	32459313	T	G	ins	1	1	1	1	.	.	.	CMTM7(INTRON)
3	32512512	32512529	32512517	GAA	G	snp	1	1	1	1	.	.	.	INTERGENIC(INTERGENIC)
3	32512512	32512529	32512523	GAA	G	snp	1	1	1	1	.	.	.	INTERGENIC(INTERGENIC)
3	32579430	32579439	32579432	AT	T	snp	1	1	1	1	.	.	.	DYNC1LI1(DOWNSTREAM);DYNC1LI1(INTRON);DYNC1LI1(UPSTREAM)
3	32736080	32736095	32736090	TTTG	C	snp	0	0	1	0	.	.	.	AC138972.1(UPSTREAM);CNOT10(INTRON);CNOT10(UPSTREAM)
3	32762315	32762330	32762323	CCCT	CCTCCCTT	ins	1	1	1	1	.	.	.	CNOT10(DOWNSTREAM);CNOT10(INTRON)
3	32785349	32785356	32785355	T	G	snp	1	1	1	1	.	.	.	CNOT10(INTRON)
3	32826224	32826231	32826228	T	G	snp	0	0	1	0	.	.	.	AC139452.2.1(UPSTREAM);INTERGENIC(INTERGENIC)
3	32848478	32848493	32848489	TCTT	TC	del	1	1	1	1	.	.	.	INTERGENIC(INTERGENIC)
3	32859515	32859532	32859520	CTC	T	snp	0	0	1	0	.	.	.	TRIM71(UTR_5_PRIME)
3	32891758	32891766	32891765	T	G	snp	0	1	0	1	.	.	.	TRIM71(INTRON)
3	32954517	32954524	32954521	A	G	snp	1	1	1	1	.	.	.	INTERGENIC(INTERGENIC)
3	32980905	32980918	32980907	AC	CACACACACAC	del	1	0	1	0	.	.	.	INTERGENIC(INTERGENIC)
3	33020242	33020257	33020253	TTTC	T	ins	1	1	1	0	.	.	.	INTERGENIC(INTERGENIC)
3	33032468	33032488	33032482	AAT	C	snp	1	1	1	1	.	.	.	INTERGENIC(INTERGENIC)
3	33191006	33191013	33191007	G	GA	ins	1	1	1	1	.	.	.	INTERGENIC(INTERGENIC);SUSD5(DOWNSTREAM)
3	33208678	33208691	33208681	AT	A	snp	0	1	0	1	.	.	.	SUSD5(INTRON)
3	33219928	33219937	33219932	T	TG	ins	1	1	1	1	.	.	.	SUSD5(INTRON)
3	33221855	33221863	33221861	A	AT	ins	1	1	1	1	.	.	.	SUSD5(INTRON)
3	33421174	33421183	33421178	A	C	snp	1	1	1	1	.	.	.	FBXL2(DOWNSTREAM);FBXL2(INTRON);FBXL2(UPSTREAM)
3	33442889	33442896	33442890	A	G	snp	1	1	1	1	.	.	.	FBXL2(INTRON);UBP1(INTRON);UBP1(UPSTREAM)
3	33556314	33556331	33556329	AC	A	del	1	0	0	0	.	.	.	CLASP2(INTRON)
3	33566161	33566172	33566169	AAT	AATC	del	1	0	0	0	.	.	.	CLASP2(INTRON)
3	33917714	33917722	33917716	T	G	snp	0	0	0	1	.	.	.	INTERGENIC(INTERGENIC)
3	33918752	33918763	33918755	CTT	T	snp	1	0	0	0	.	.	.	INTERGENIC(INTERGENIC)
3	33918752	33918763	33918760	CTT	C	snp	1	1	0	1	.	.	.	INTERGENIC(INTERGENIC)
3	34146628	34146636	34146629	G	A	ins	1	1	1	1	.	.	.	INTERGENIC(INTERGENIC)
3	34197812	34197820	34197815	T	C	snp	1	0	0	0	.	.	.	AC018359.1.1(UPSTREAM);INTERGENIC(INTERGENIC)
3	34209722	34209729	34209724	T	C	snp	1	1	0	1	.	.	.	AC018359.1.1(INTRON)
3	34236197	34236208	34236205	CAT	C	snp	1	1	1	1	.	.	.	AC018359.1.1(INTRON)
3	34292279	34292294	34292291	TA	A	snp	1	1	1	1	.	.	.	AC018359.1.1(INTRON);AC123023.1.1(INTRON)
3	34322477	34322494	34322487	AC	CC	ins	1	0	0	1	.	.	.	AC018359.1.1(INTRON)
3	34322477	34322494	34322487	AC	C	snp	1	0	0	0	.	.	.	AC018359.1.1(INTRON)
3	34339059	34339068	34339064	CT	G	snp	0	1	0	0	.	.	.	AC018359.1.1(INTRON)
3	34663330	34663338	34663334	T	G	snp	1	1	1	1	.	.	.	INTERGENIC(INTERGENIC)
3	34719633	34719646	34719644	TA	T	del	1	1	1	1	.	.	.	INTERGENIC(INTERGENIC)
3	34762105	34762114	34762109	T	G	snp	1	0	0	0	.	.	.	INTERGENIC(INTERGENIC)
3	34779668	34779676	34779669	A	C	snp	0	1	0	1	.	.	.	INTERGENIC(INTERGENIC)
3	34875809	34875820	34875814	AAT	A	snp	0	1	1	1	.	.	.	INTERGENIC(INTERGENIC)
3	34902200	34902217	34902205	TC	G	snp	0	1	0	1	.	.	.	INTERGENIC(INTERGENIC)
3	35105672	35105681	35105673	A	AG	ins	1	1	1	1	.	.	.	INTERGENIC(INTERGENIC)
3	35114556	35114567	35114562	TTA	A	snp	1	1	1	1	.	.	.	INTERGENIC(INTERGENIC)
3	35282660	35282667	35282665	A	G	snp	0	1	1	0	.	.	.	INTERGENIC(INTERGENIC)
3	35309486	35309499	35309491	TA	G	snp	1	0	0	0	.	.	.	INTERGENIC(INTERGENIC)
3	35330831	35330854	35330842	AAAC	A	snp	1	1	1	1	.	.	.	INTERGENIC(INTERGENIC)
3	35346146	35346155	35346152	A	C	ins	1	1	1	1	.	.	.	INTERGENIC(INTERGENIC)
3	35383980	35383995	35383986	GAGG	A	snp	1	0	0	0	.	.	.	INTERGENIC(INTERGENIC)
3	35526716	35526724	35526721	A	G	snp	1	1	1	1	.	.	.	INTERGENIC(INTERGENIC)
3	35550301	35550318	35550316	TA	T	snp	1	1	1	1	.	.	.	INTERGENIC(INTERGENIC)
3	35550301	35550318	35550316	TA	TT	ins	1	1	1	1	.	.	.	INTERGENIC(INTERGENIC)
3	35581153	35581162	35581157	CA	T	snp	1	0	0	0	.	.	.	INTERGENIC(INTERGENIC)
3	35581368	35581377	35581370	TA	C	snp	1	1	1	1	.	.	.	INTERGENIC(INTERGENIC)

3	35768077	35768085	35768081	T	C	snp	1	1	1	1	.	.	.	ARPP21(INTRON)
3	35872306	35872315	35872310	AT	G	snp	0	1	0	0	.	.	.	INTERGENIC(INTERGENIC)
3	35912604	35912627	35912616	CAG	T	snp	0	0	0	1	.	.	.	AC104308.2.1(UPSTREAM);INTERGENIC(INTERGENIC)
3	35992971	35992985	35992975	ATT	G	snp	0	0	0	1	.	.	.	INTERGENIC(INTERGENIC)
3	35992971	35992985	35992976	ATT	A	snp	1	0	0	0	.	.	.	INTERGENIC(INTERGENIC)
3	36153830	36153837	36153834	A	G	snp	1	1	1	1	.	.	.	INTERGENIC(INTERGENIC)
3	36206833	36206842	36206835	CA	G	snp	0	0	1	0	.	.	.	AC105901.1.1(DOWNSTREAM);INTERGENIC(INTERGENIC)
3	36211105	36211114	36211110	TA	G	snp	1	1	1	1	.	.	.	AC105901.1.1(DOWNSTREAM);INTERGENIC(INTERGENIC)
3	36296260	36296268	36296265	A	C	snp	1	1	1	1	.	.	.	INTERGENIC(INTERGENIC)
3	36341818	36341827	36341821	AC	T	snp	1	1	1	1	.	.	.	INTERGENIC(INTERGENIC)
3	36479265	36479276	36479269	ATA	C	snp	0	1	1	1	.	.	.	STAC(INTRON)
3	36695583	36695590	36695587	A	C	snp	1	1	1	1	.	.	.	INTERGENIC(INTERGENIC)
3	36770727	36770740	36770738	TG	T	del	1	1	1	1	.	.	.	DCLK3(INTRON)
3	36788930	36788941	36788936	TA	C	snp	0	1	1	1	.	.	.	INTERGENIC(INTERGENIC)
3	36864517	36864525	36864523	T	G	snp	1	0	0	0	.	.	.	INTERGENIC(INTERGENIC);TRANK1(DOWNSTREAM)
3	36888167	36888174	36888172	T	G	snp	1	1	1	1	.	.	.	TRANK1(INTRON)
3	36907425	36907432	36907431	A	AG	ins	0	1	1	1	.	.	.	TRANK1(INTRON)
3	36934344	36934361	36934350	AT	TATC	ins	1	0	0	0	.	.	.	TRANK1(INTRON)
3	36934344	36934361	36934351	AT	AG	ins	1	0	0	0	.	.	.	TRANK1(INTRON)
3	36934344	36934361	36934353	AT	G	snp	1	0	0	0	.	.	.	TRANK1(INTRON)
3	36934344	36934361	36934354	AT	C	snp	1	0	0	0	.	.	.	TRANK1(INTRON)
3	36990352	36990360	36990356	T	G	ins	1	1	1	1	.	.	.	INTERGENIC(INTERGENIC);TRANK1(UPSTREAM)
3	36996006	36996020	36996014	AAC	A	ins	1	0	0	0	.	.	.	INTERGENIC(INTERGENIC)
3	37088089	37088102	37088096	TA	T	ins	1	1	1	1	.	.	.	MLH1(INTRON)
3	37167505	37167522	37167519	TTG	TTGT	del	1	0	0	0	.	.	.	LRRFIP2(DOWNSTREAM);LRRFIP2(INTRON)
3	37280825	37280832	37280830	C	A	snp	1	0	0	0	.	.	.	AC097359.1(DOWNSTREAM);GOLGA4(UPSTREAM);INTERGENIC(INTERGENIC)
3	37280825	37280832	37280831	C	T	ins	0	1	0	1	.	.	.	AC097359.1(DOWNSTREAM);GOLGA4(UPSTREAM);INTERGENIC(INTERGENIC)
3	37296646	37296666	37296662	ATT	C	snp	1	1	1	1	.	.	.	GOLGA4(INTRON)
3	37352210	37352227	37352225	GT	G	del	1	1	0	0	.	.	.	GOLGA4(INTRON)
3	37483900	37483907	37483902	A	C	snp	1	1	0	1	.	.	.	INTERGENIC(INTERGENIC)
3	37643769	37643778	37643773	A	C	snp	1	1	1	1	.	.	.	ITGA9(INTRON)
3	37647376	37647384	37647380	T	C	snp	1	0	0	0	.	.	.	ITGA9(INTRON)
3	37711133	37711141	37711134	A	G	snp	0	1	1	1	.	.	.	ITGA9(INTRON)
3	37751892	37751900	37751897	C	A	ins	1	0	0	0	.	.	.	ITGA9(INTRON)
3	37813899	37813908	37813903	A	G	snp	1	0	0	0	.	.	.	AC093415.2.1(INTRON);ITGA9(INTRON)
3	37825132	37825146	37825138	ATT	G	snp	1	1	1	1	.	.	.	AC093415.2.1(INTRON);ITGA9(INTRON)
3	37876662	37876670	37876669	G	T	snp	1	0	0	0	.	.	.	AC093415.2.1(INTRON)
3	38034922	38034930	38034928	A	G	snp	1	0	0	0	.	.	.	VILL(EXON);VILL(INTRON);VILL(UPSTREAM)
3	38173809	38173820	38173813	TC	C	snp	1	1	0	1	.	.	.	ACAA1(DOWNSTREAM);ACAA1(EXON);ACAA1(INTRON)
3	38259031	38259038	38259034	T	G	snp	1	1	1	1	.	.	.	OXSR1(INTRON)
3	38378043	38378054	38378050	TCT	TTCT	del	1	1	1	1	.	.	.	INTERGENIC(INTERGENIC)
3	38378043	38378054	38378051	TCT	TC	del	1	1	1	1	.	.	.	INTERGENIC(INTERGENIC)
3	38395556	38395571	38395562	TGTT	C	snp	1	1	1	1	.	.	.	XYLB(INTRON)
3	38464559	38464573	38464565	TTA	C	snp	1	1	1	1	.	.	.	INTERGENIC(INTERGENIC);XYLB(DOWNSTREAM)
3	38527210	38527217	38527215	T	C	snp	1	1	1	1	.	.	.	ACVR2B(DOWNSTREAM);INTERGENIC(INTERGENIC)
3	38691859	38691868	38691861	AC	G	snp	1	0	0	0	.	.	.	INTERGENIC(INTERGENIC);SCN5A(UPSTREAM)
3	38751660	38751668	38751663	T	C	ins	1	0	0	0	.	.	.	SCN10A(INTRON)
3	38775824	38775833	38775829	A	C	snp	0	0	1	0	.	.	.	SCN10A(INTRON)
3	38779261	38779270	38779264	CT	C	snp	1	1	1	0	.	.	.	SCN10A(INTRON)
3	38830112	38830120	38830117	G	A	snp	1	1	1	1	.	.	.	SCN10A(INTRON)
3	38894244	38894251	38894245	A	G	snp	1	0	0	0	.	.	.	SCN11A(INTRON)
3	38981549	38981566	38981562	AT	G	snp	1	0	0	0	.	.	.	SCN11A(INTRON)
3	38994540	38994548	38994546	T	G	snp	1	0	0	1	.	.	.	INTERGENIC(INTERGENIC);SCN11A(UPSTREAM)
3	39010619	39010646	39010625	AGGC	A	snp	1	1	1	1	.	.	.	INTERGENIC(INTERGENIC)
3	39050917	39050926	39050920	AT	C	snp	1	0	1	0	.	.	.	INTERGENIC(INTERGENIC)
3	39059205	39059213	39059207	T	C	snp	1	0	0	0	.	.	.	INTERGENIC(INTERGENIC)
3	39074000	39074014	39074005	AAC	T	snp	1	0	0	0	.	.	.	AC123903.1(DOWNSTREAM);AC123903.2(DOWNSTREAM);INTERGENIC(INTERGENIC)
3	39101930	39101941	39101934	CA	AT	ins	1	1	1	1	.	.	.	WDR48(INTRON)
3	39119881	39119889	39119886	T	G	snp	1	0	0	0	.	.	.	WDR48(DOWNSTREAM);WDR48(INTRON)
3	39168035	39168050	39168047	CA	T	snp	1	0	0	0	.	.	.	TTC21A(INTRON);TTC21A(UPSTREAM)
3	39168035	39168050	39168048	CA	G	snp	1	0	0	0	.	.	.	TTC21A(INTRON);TTC21A(UPSTREAM)
3	39229897	39229908	39229900	TGC	C	snp	1	1	1	1	.	XIRP1(NON_SYNONYMOUS_CODING):Q346R	.	XIRP1(INTRON)
3	39366534	39366543	39366540	T	G	snp	1	1	1	1	.	.	.	CCR8(UPSTREAM);INTERGENIC(INTERGENIC)
3	39381236	39381243	39381238	A	G	snp	1	1	1	1	.	.	.	

AC104850.1(UPSTREAM);HNRNPA1P21(DOWNSTREAM);INTERGENIC(INTERGENIC);RP11-241K18.2.1(UPSTREAM)

3	39442108	39442116	39442110	T	G	snp	1	0	0	0	.	.	.	INTERGENIC(INTERGENIC);SLC25A38(DOWNSTREAM)
3	39459414	39459427	39459418	CT	T	snp	1	1	1	1	.	.	.	INTERGENIC(INTERGENIC)
3	39469686	39469706	39469700	ATA	C	snp	1	1	1	1	.	.	.	INTERGENIC(INTERGENIC)
3	39537998	39538007	39538006	T	C	snp	1	1	0	1	.	.	.	MOBP(INTRON)
3	39810353	39810366	39810356	AC	A	snp	1	1	0	1	.	.	.	INTERGENIC(INTERGENIC)
3	39820434	39820441	39820438	T	TG	ins	1	1	0	1	.	.	.	INTERGENIC(INTERGENIC)
3	39912018	39912026	39912024	T	G	snp	1	1	1	1	.	.	.	MYRIP(INTRON)
3	39912018	39912026	39912025	T	G	snp	1	1	1	1	.	.	.	MYRIP(INTRON)
3	39951374	39951382	39951375	T	TTTG	ins	1	1	1	1	.	.	.	MYRIP(INTRON)
3	39951374	39951382	39951376	T	TTG	ins	1	1	1	1	.	.	.	MYRIP(INTRON)
3	39951374	39951382	39951378	T	G	ins	1	1	1	1	.	.	.	MYRIP(INTRON)
3	40004885	40004900	40004890	CATC	T	snp	1	1	1	1	.	.	.	MYRIP(INTRON)
3	40061206	40061214	40061207	C	T	snp	1	0	0	0	.	.	.	MYRIP(INTRON)
3	40268879	40268896	40268892	CT	C	snp	1	0	0	0	.	.	.	MYRIP(INTRON)
3	40301475	40301489	40301478	AGC	GC	del	1	0	0	0	.	.	.	MYRIP(DOWNSTREAM);MYRIP(UTR_3_PRIME)
3	40301475	40301489	40301483	AGC	AG	del	1	0	0	0	.	.	.	MYRIP(DOWNSTREAM);MYRIP(UTR_3_PRIME)
3	40369872	40369881	40369879	C	A	ins	0	0	0	1	.	.	.	ENTPD3-AS1(INTRON)
3	40377396	40377403	40377399	T	G	snp	1	1	1	1	.	.	.	ENTPD3-AS1(INTRON)
3	40418972	40418989	40418987	GA	G	snp	1	1	1	1	.	.	.	ENTPD3-AS1(INTRON)
3	40419048	40419063	40419050	GA	T	snp	1	0	0	0	.	.	.	ENTPD3-AS1(INTRON)
3	40419048	40419063	40419051	GA	G	snp	1	0	0	0	.	.	.	ENTPD3-AS1(INTRON)
3	40428602	40428609	40428603	G	T	snp	1	0	0	0	.	.	.	ENTPD3(UPSTREAM);ENTPD3-AS1(DOWNSTREAM);ENTPD3-AS1(INTRON)
3	40439833	40439842	40439836	TC	T	snp	1	1	1	1	.	.	.	ENTPD3(INTRON);ENTPD3-AS1(INTRON)
3	40497389	40497398	40497392	A	G	snp	1	0	0	0	.	.	.	ENTPD3-AS1(UPSTREAM);INTERGENIC(INTERGENIC);RPL14(UPSTREAM)
3	40632919	40632930	40632922	TA	G	snp	1	0	0	0	.	.	.	INTERGENIC(INTERGENIC);RP11-528N21.2.1(DOWNSTREAM);RP11-528N21.3.1(UPSTREAM)
3	40653837	40653846	40653845	T	C	ins	1	0	1	0	.	.	.	INTERGENIC(INTERGENIC)
3	40960924	40960933	40960926	AG	G	snp	1	0	0	0	.	.	.	INTERGENIC(INTERGENIC)
3	41120302	41120311	41120307	TC	G	snp	1	1	1	1	.	.	.	INTERGENIC(INTERGENIC)
3	41331365	41331378	41331376	TG	A	snp	1	1	1	1	.	.	.	ULK4(INTRON)
3	41523450	41523457	41523455	T	G	snp	1	1	1	1	.	.	.	ULK4(INTRON)
3	41578684	41578693	41578691	T	TG	ins	1	0	0	0	.	.	.	ULK4(INTRON)
3	41610992	41610999	41610997	A	G	snp	1	1	1	1	.	.	.	ULK4(INTRON)
3	41635546	41635555	41635551	AG	A	snp	0	0	1	0	.	.	.	ULK4(INTRON)
3	41688590	41688597	41688593	T	C	snp	1	0	0	0	.	.	.	AC099537.1(UPSTREAM);ULK4(INTRON)
3	41702986	41703001	41702990	AGAT	GATAGATAGAT	del	1	1	1	0	.	.	.	. ULK4(INTRON)
3	41702986	41703001	41702994	AGAT	GATAGAT	del	1	1	1	0	.	.	.	ULK4(INTRON)
3	41702986	41703001	41702997	AGAT	AGAG	ins	1	0	0	0	.	.	.	ULK4(INTRON)
3	41744337	41744345	41744340	T	G	snp	1	0	0	0	.	.	.	ULK4(INTRON)
3	41792465	41792476	41792472	AG	C	snp	1	0	0	0	.	.	.	ULK4(INTRON)
3	41797497	41797506	41797504	AG	C	snp	1	1	0	1	.	.	.	ULK4(INTRON)
3	41834681	41834690	41834682	A	C	ins	1	1	0	1	.	.	.	ULK4(INTRON)
3	41850681	41850689	41850685	A	C	snp	1	1	0	1	.	.	.	ULK4(INTRON)
3	42001020	42001031	42001027	AAT	TAAT	del	1	1	0	1	.	.	.	RP11-626A1.2.1(DOWNSTREAM);ULK4(INTRON);ULK4(UPSTREAM)
3	42049623	42049632	42049629	T	G	snp	1	1	0	1	.	.	.	INTERGENIC(INTERGENIC)
3	42085108	42085117	42085110	G	A	snp	1	0	0	0	.	.	.	TRAK1(INTRON)
3	42214647	42214655	42214650	A	C	snp	1	0	0	0	.	.	.	TRAK1(INTRON)
3	42310435	42310448	42310441	TC	C	snp	1	1	1	1	.	.	.	CCK(UPSTREAM);INTERGENIC(INTERGENIC)
3	42366213	42366221	42366217	T	C	snp	0	1	1	0	.	.	.	INTERGENIC(INTERGENIC);SALL4P6(DOWNSTREAM)
3	42368497	42368505	42368500	T	C	snp	1	1	1	1	.	.	.	INTERGENIC(INTERGENIC);SALL4P6(DOWNSTREAM)
3	42415048	42415057	42415052	TG	C	snp	1	0	0	0	.	.	.	INTERGENIC(INTERGENIC)
3	42427592	42427600	42427597	T	C	snp	1	0	0	0	.	.	.	INTERGENIC(INTERGENIC)
3	42472743	42472750	42472745	A	G	snp	1	1	1	1	.	.	.	AC092047.1(UPSTREAM);INTERGENIC(INTERGENIC)
3	42483194	42483205	42483197	AAC	G	snp	1	0	0	0	.	.	.	INTERGENIC(INTERGENIC)
3	42494918	42494927	42494921	T	C	ins	1	1	1	1	.	.	.	INTERGENIC(INTERGENIC)
3	42509403	42509412	42509407	CT	G	snp	1	0	0	0	.	.	.	INTERGENIC(INTERGENIC);RP11-219I21.1.1(UPSTREAM)
3	42533055	42533070	42533062	TTTG	TTTGTTTGTTT	del	0	1	1	1	.	.	.	. VIPR1(INTRON)
3	42596468	42596488	42596478	AAC	C	ins	1	0	0	0	.	.	.	SEC22C(DOWNSTREAM);SEC22C(INTRON)
3	42596468	42596488	42596485	AAC	A	snp	1	0	0	0	.	.	.	SEC22C(DOWNSTREAM);SEC22C(INTRON)
3	42601673	42601682	42601678	AT	C	snp	1	1	1	1	.	.	.	SEC22C(DOWNSTREAM);SEC22C(INTRON)
3	42637495	42637502	42637497	T	C	snp	1	0	1	0	.	.	.	NKTR(UPSTREAM);SEC22C(INTRON);SS18L2(DOWNSTREAM)
3	42645872	42645881	42645878	T	C	snp	1	0	0	0	.	.	.	NKTR(DOWNSTREAM);NKTR(INTRON);RP4-613B23.1.1(INTRON);SEC22C(UPSTREAM)

3	42740778	42740789	42740781	TAA	A	snp	1	0	0	0	.	.	.	CCDC13(INTRON);HHATL(INTRON);HHATL-AS1(UPSTREAM)
3	42741499	42741516	42741512	TG	C	snp	1	1	1	1	.	.	.	CCDC13(INTRON);HHATL(INTRON);HHATL-AS1(UPSTREAM)
3	42757113	42757121	42757120	A	C	snp	1	0	0	0	.	.	.	CCDC13(DOWNSTREAM);CCDC13(INTRON);CCDC13(UPSTREAM)
3	42815403	42815422	42815417	TCTA	A	ins	0	0	1	1	.	.	.	CCDC13(UPSTREAM);HIGD1A(INTRON);RP11-70C1.1.1(DOWNSTREAM)
3	42869567	42869584	42869582	TA	T	ins	1	0	0	0	.	.	.	CCBP2(INTRON);KRBOX1(INTRON);RP11-70C1.3.1(INTRON)
3	43040493	43040502	43040496	GT	C	snp	0	0	1	0	.	.	.	FAM198A(INTRON);KRBOX1(INTRON)
3	43049472	43049491	43049484	TTGC	C	snp	0	1	1	0	.	.	.	FAM198A(INTRON);KRBOX1(INTRON)
3	43156583	43156592	43156590	TC	T	del	0	1	1	1	.	.	.	INTERGENIC(INTERGENIC)
3	43161071	43161094	43161089	TTTA	A	del	1	1	1	1	.	.	.	INTERGENIC(INTERGENIC)
3	43192577	43192585	43192583	A	C	snp	1	1	1	1	.	.	.	INTERGENIC(INTERGENIC)
3	43219119	43219128	43219125	C	CCT	ins	1	1	1	1	.	.	.	INTERGENIC(INTERGENIC)
3	43265245	43265253	43265247	A	C	snp	0	1	0	1	.	.	.	INTERGENIC(INTERGENIC)
3	43274325	43274334	43274329	TA	C	snp	1	1	1	1	.	.	.	AC104434.2(DOWNSTREAM);AC104434.3(UPSTREAM);INTERGENIC(INTERGENIC)
3	43289611	43289626	43289618	AAAG	A	snp	1	1	1	1	.	.	.	INTERGENIC(INTERGENIC)
3	43337908	43337925	43337911	AT	ATATATATATAC	ins	1	1	1	1	.	.	.	SNRK(INTRON)
3	43337908	43337925	43337922	AT	G	snp	1	0	0	0	.	.	.	SNRK(INTRON)
3	43337927	43337938	43337930	TA	G	snp	1	1	0	0	.	.	.	SNRK(INTRON)
3	43502487	43502496	43502488	A	G	snp	0	0	0	1	.	.	.	ANO10(INTRON)
3	43586875	43586884	43586882	T	C	snp	1	1	1	1	.	.	.	ANO10(INTRON)
3	43782850	43782861	43782857	CA	G	snp	0	1	0	0	.	.	.	INTERGENIC(INTERGENIC)
3	43850773	43850788	43850775	AT	TG	ins	1	1	1	1	.	.	.	INTERGENIC(INTERGENIC)
3	43850773	43850788	43850778	AT	AC	ins	1	1	1	1	.	.	.	INTERGENIC(INTERGENIC)
3	44050471	44050488	44050485	TCC	T	snp	1	0	0	0	.	.	.	INTERGENIC(INTERGENIC)
3	44080076	44080083	44080077	T	C	snp	1	1	0	1	.	.	.	INTERGENIC(INTERGENIC)
3	44205103	44205116	44205105	GT	T	snp	0	1	0	0	.	.	.	INTERGENIC(INTERGENIC)
3	44279655	44279662	44279656	T	G	snp	1	1	0	1	.	.	.	C3orf77(UPSTREAM);INTERGENIC(INTERGENIC)
3	44302468	44302476	44302472	T	C	snp	1	1	1	1	.	.	.	C3orf77(INTRON)
3	44473811	44473820	44473816	AG	C	snp	1	0	0	0	.	.	.	INTERGENIC(INTERGENIC)
3	44617453	44617460	44617455	T	G	snp	1	0	0	0	.	.	.	RP11-944L7.2.1(DOWNSTREAM);RP11-944L7.4.1(INTRON);ZNF167(DOWNSTREAM);ZNF167(INTRON);ZNF660(UPSTREAM)
3	44747555	44747564	44747559	T	C	snp	1	0	0	0	.	.	.	INTERGENIC(INTERGENIC);RP11-348P10.1.1(DOWNSTREAM)
3	45017688	45017696	45017690	G	T	snp	0	0	0	1	.	.	.	EXOSC7(INTRON);EXOSC7(UPSTREAM);ZDHHC3(UPSTREAM)
3	45069429	45069438	45069431	A	G	snp	1	1	1	1	.	.	.	CLEC3B(INTRON);CLEC3B(UPSTREAM);EXOSC7(INTRON)
3	45087873	45087882	45087876	T	C	snp	1	1	1	1	.	.	.	INTERGENIC(INTERGENIC)
3	45150250	45150261	45150254	TA	C	snp	1	1	0	1	.	.	.	CDCP1(DOWNSTREAM);CDCP1(INTRON)
3	45150286	45150297	45150290	TA	C	snp	1	0	0	1	.	.	.	CDCP1(DOWNSTREAM);CDCP1(INTRON)
3	45150429	45150446	45150443	AT	T	snp	1	1	0	1	.	.	.	CDCP1(DOWNSTREAM);CDCP1(INTRON)
3	45210684	45210692	45210691	A	C	snp	0	1	1	1	.	.	.	INTERGENIC(INTERGENIC)
3	45229932	45229941	45229933	T	G	snp	1	0	0	0	.	.	.	INTERGENIC(INTERGENIC)
3	45252278	45252285	45252284	G	T	snp	1	0	0	1	.	.	.	INTERGENIC(INTERGENIC)
3	45267304	45267321	45267310	CGC	T	snp	1	1	1	1	.	.	TMEM158(SYNONYMOUS_CODING):A70	AC010170.1(EXON)
3	45325737	45325756	45325752	TTTG	T	ins	0	1	1	1	.	.	.	INTERGENIC(INTERGENIC)
3	45373441	45373450	45373444	AT	A	snp	1	0	0	0	.	.	.	INTERGENIC(INTERGENIC)
3	45383225	45383240	45383229	TGAA	C	snp	1	1	1	1	.	.	.	INTERGENIC(INTERGENIC)
3	45450097	45450112	45450105	TC	A	snp	1	0	1	0	.	.	.	LARS2(INTRON)
3	45495352	45495361	45495358	A	G	snp	1	1	1	1	.	.	.	LARS2(INTRON)
3	45525141	45525149	45525145	A	G	snp	0	0	0	1	.	.	.	LARS2(INTRON);LARS2-AS1(DOWNSTREAM)
3	45619094	45619109	45619107	AG	AA	ins	1	1	1	1	.	.	.	LIMD1(INTRON)
3	45672204	45672211	45672209	G	T	snp	0	0	0	1	.	.	.	LIMD1(INTRON)
3	45672204	45672211	45672210	G	A	snp	1	1	1	1	.	.	.	LIMD1(INTRON)
3	45691252	45691261	45691256	TA	C	snp	1	0	0	0	.	.	.	LIMD1(INTRON)
3	45691264	45691273	45691269	TA	G	snp	1	0	0	0	.	.	.	LIMD1(INTRON)
3	45718598	45718609	45718602	TGC	A	snp	1	0	0	0	.	.	.	LIMD1(DOWNSTREAM);LIMD1(UTR_3_PRIME);RP11-697K23.1.1(DOWNSTREAM)
3	45808194	45808207	45808205	AG	C	snp	0	1	1	1	.	.	.	SLC6A20(DOWNSTREAM);SLC6A20(INTRON);SLC6A20(UPSTREAM)
3	46064622	46064633	46064626	GCG	G	snp	0	1	1	1	.	.	.	RP11-91E8.1.1(UPSTREAM);XCR1(INTRON)
3	46086599	46086606	46086602	A	C	snp	1	1	1	1	.	.	.	INTERGENIC(INTERGENIC)
3	46096041	46096048	46096043	G	A	snp	0	1	1	1	.	.	.	INTERGENIC(INTERGENIC)
3	46106611	46106619	46106614	A	G	snp	1	1	1	1	.	.	.	INTERGENIC(INTERGENIC)
3	46147203	46147212	46147209	GT	A	snp	1	0	0	0	.	.	.	INTERGENIC(INTERGENIC)
3	46244274	46244283	46244275	G	T	ins	1	0	0	0	.	.	.	CCR1(UTR_3_PRIME);CCR3(INTRON)

3	46356042	46356049	46356048	T	C	snp	0	0	0	1	.	.	.	INTERGENIC(INTERGENIC);RP13-546I2.1.1(UPSTREAM)
3	46414020	46414040	46414035	ACA	G	snp	1	1	1	1	.	.	.	CCR5(INTRON);CCR5(UPSTREAM);RP11-24F11.2.1(INTRON)
3	46552298	46552309	46552301	GTA	C	snp	0	1	1	0	.	.	.	INTERGENIC(INTERGENIC);LRRC2(DOWNSTREAM)
3	46576909	46576918	46576916	AT	C	snp	0	1	1	1	.	.	.	LRRC2(INTRON)
3	46636297	46636306	46636298	A	G	snp	1	1	1	1	.	.	.	TDGF1(INTRON)
3	46649701	46649714	46649711	AC	G	snp	0	1	1	0	.	.	.	TDGF1(INTRON);TDGF1(UPSTREAM)
3	46742522	46742531	46742523	C	A	snp	1	1	1	1	.	.	.	INTERGENIC(INTERGENIC);TMIE(UPSTREAM)
3	46787687	46787694	46787689	T	G	snp	1	1	0	0	.	.	.	PRSS45(INTRON);PRSS45(UPSTREAM);PRSS50(INTRON)
3	46916218	46916226	46916225	C	A	snp	1	1	1	1	.	.	.	MYL3(INTRON);PTH1R(UPSTREAM)
3	46954597	46954616	46954612	CAAA	C	del	1	1	1	1	.	.	.	INTERGENIC(INTERGENIC)
3	47069047	47069058	47069054	ATA	C	snp	1	1	1	1	.	.	.	SETD2(INTRON)
3	47138237	47138245	47138244	A	C	snp	0	1	1	0	.	.	.	SETD2(INTRON)
3	47252401	47252409	47252408	A	G	snp	1	1	1	1	.	.	.	RP11-447D11.2.1(INTRON)
3	47370694	47370701	47370699	T	TG	ins	1	1	1	1	.	.	.	KLHL18(INTRON)
3	47604271	47604280	47604272	G	T	snp	1	0	0	0	.	.	.	AC099778.1(UPSTREAM);CSPG5(INTRON)
3	47637019	47637034	47637030	AAAT	AAATA	del	1	1	1	1	.	.	.	SMARCC1(INTRON)
3	47669536	47669549	47669547	TA	T	snp	1	1	1	1	.	.	.	SMARCC1(INTRON)
3	47708224	47708232	47708228	T	G	snp	1	0	0	0	.	.	.	RP11-395P16.1.1(DOWNSTREAM);SMARCC1(DOWNSTREAM);SMARCC1(INTRON)
3	47829251	47829274	47829268	AAT	G	snp	1	0	1	0	.	.	.	INTERGENIC(INTERGENIC)
3	47829251	47829274	47829271	AAT	G	snp	1	0	0	0	.	.	.	INTERGENIC(INTERGENIC)
3	47971950	47971965	47971961	AAAC	A	ins	1	1	0	0	.	.	.	MAP4(INTRON);MAP4(UPSTREAM);Y_RNA(DOWNSTREAM)
3	47995167	47995174	47995168	A	C	snp	1	1	1	1	.	.	.	MAP4(INTRON)
3	47998961	47998969	47998966	A	G	snp	1	1	1	1	.	.	.	MAP4(INTRON);RP11-88B8.2.1(DOWNSTREAM)
3	48086450	48086459	48086454	T	G	snp	0	1	0	0	.	.	.	MAP4(INTRON)
3	48503077	48503085	48503083	T	G	snp	1	0	0	0	.	.	.	ATRIP(DOWNSTREAM);ATRIP(INTRON);TREX1(UPSTREAM)
3	48535336	48535349	48535340	AG	G	snp	1	0	0	0	.	.	.	SHISA5(DOWNSTREAM);SHISA5(INTRON)
3	49106517	49106540	49106536	TTA	A	del	1	1	1	1	.	.	.	QRICH1(INTRON)
3	49106517	49106540	49106537	TTA	T	ins	0	1	1	1	.	.	.	QRICH1(INTRON)
3	49113084	49113091	49113086	A	C	ins	0	1	1	1	.	.	.	QRICH1(DOWNSTREAM);QRICH1(INTRON)
3	49120039	49120047	49120040	A	G	snp	0	1	1	1	.	.	.	AC135506.1(UPSTREAM);QRICH1(INTRON)
3	49319464	49319471	49319469	A	C	snp	1	0	0	0	.	.	.	C3orf62(UPSTREAM);USP4(DOWNSTREAM);USP4(INTRON);USP4(UPSTREAM)
3	49379359	49379366	49379364	A	C	snp	1	0	0	0	.	.	.	INTERGENIC(INTERGENIC);USP4(UPSTREAM)
3	49448580	49448589	49448583	T	C	snp	1	0	0	0	.	.	.	RHOA(DOWNSTREAM);RHOA(INTRON);TCTA(UPSTREAM)
3	49508972	49508979	49508976	T	C	snp	1	1	0	1	.	.	.	DAG1(INTRON);RN5S130(UPSTREAM)
3	49746564	49746573	49746570	T	C	snp	0	0	0	1	.	.	.	RNF123(INTRON);RNF123(UPSTREAM)
3	49809748	49809757	49809752	TA	C	snp	0	1	0	1	.	.	.	IP6K1(INTRON);RP13-1056D16.2.1(UPSTREAM)
3	50029377	50029396	50029386	TTTA	G	snp	1	0	0	1	.	.	.	RBM6(INTRON)
3	50041740	50041747	50041743	C	T	ins	1	1	1	1	.	.	.	RBM6(INTRON)
3	50125090	50125105	50125100	TTTA	A	del	1	0	0	0	.	.	.	RBM5(UPSTREAM);RBM6(INTRON)
3	50238146	50238157	50238155	AT	A	del	1	0	0	0	.	.	.	GNAT1(DOWNSTREAM);INTERGENIC(INTERGENIC);SLC38A3(UPSTREAM)
3	50306630	50306639	50306632	CA	T	snp	1	1	0	0	.	.	.	SEMA3B(EXON);SEMA3B(UPSTREAM);SEMA3B(UTR_5_PRIME);U73167.7.1(UPSTREAM)
3	50627332	50627343	50627339	AAT	G	snp	0	1	1	0	.	.	.	HEMK1(DOWNSTREAM);INTERGENIC(INTERGENIC)
3	50692829	50692844	50692835	CT	T	snp	1	1	1	0	.	.	.	INTERGENIC(INTERGENIC)
3	50776497	50776505	50776498	G	T	snp	0	1	1	1	.	.	.	DOCK3(INTRON)
3	50825243	50825252	50825246	CT	A	snp	1	1	1	1	.	.	.	DOCK3(INTRON);ZNF652P1(EXON)
3	50832461	50832470	50832462	T	C	snp	1	0	0	0	.	.	.	DOCK3(INTRON)
3	50866498	50866521	50866507	ATTT	C	snp	1	1	1	0	.	.	.	DOCK3(INTRON)
3	51170601	51170612	51170607	TTA	C	snp	1	0	0	0	.	.	.	DOCK3(INTRON)
3	51259074	51259082	51259079	A	C	snp	1	1	1	1	.	.	.	DOCK3(INTRON)
3	51559874	51559885	51559880	TTG	C	snp	0	1	0	0	.	.	.	INTERGENIC(INTERGENIC)
3	51562742	51562749	51562745	A	C	snp	0	0	0	1	.	.	.	INTERGENIC(INTERGENIC)
3	51743027	51743038	51743034	CTG	C	snp	1	0	0	0	.	GRM2(NON_SYNONYMOUS_CODING):L12P	.	GRM2(EXON);GRM2(INTRON);GRM2(UPSTREAM);TEX264(DOWNSTREAM)
3	51743847	51743856	51743851	AC	CACACCT	ins	1	0	0	0	.	.	.	GRM2(DOWNSTREAM);GRM2(INTRON);GRM2(UPSTREAM)
3	51975570	51975579	51975577	C	T	snp	1	1	1	1	.	.	.	PARP3(UPSTREAM);RRP9(INTRON)
3	52131252	52131261	52131256	GT	C	snp	1	0	0	0	.	.	.	POC1A(INTRON)
3	52207880	52207887	52207886	T	G	snp	1	1	1	1	.	.	.	INTERGENIC(INTERGENIC)
3	52208047	52208056	52208054	T	G	snp	1	0	0	0	.	.	.	INTERGENIC(INTERGENIC)
3	52273419	52273428	52273421	GC	A	snp	1	1	1	1	.	.	.	RP5-1157M23.2.1(EXON);TWF2(UPSTREAM)
3	52619539	52619558	52619546	ATTT	A	snp	1	0	0	0	.	.	.	PBRM1(DOWNSTREAM);PBRM1(EXON);PBRM1(INTRON)
3	52759548	52759557	52759550	A	C	ins	1	0	0	0	.	.	.	NEK4(INTRON)

3	52891768	52891779	52891774	TAT	C	snp	1	0	0	0	.	.	.	TMEM110(INTRON);TMEM110-MUSTN1(INTRON)
3	53074817	53074826	53074824	T	G	snp	1	1	1	1	.	.	.	SFMBT1(DOWNSTREAM);SFMBT1(INTRON)
3	53099706	53099715	53099713	TC	T	snp	1	1	0	0	.	.	.	INTERGENIC(INTERGENIC);RP11-5O17.2.1(UPSTREAM)
3	53125467	53125476	53125469	GA	A	snp	1	1	1	1	.	.	.	RFT1(DOWNSTREAM);RFT1(UTR_3_PRIME)
3	53219275	53219282	53219278	G	T	snp	1	1	1	1	.	.	.	PRKCD(DOWNSTREAM);PRKCD(INTRON)
3	53231291	53231298	53231295	C	T	snp	1	1	1	1	.	.	.	INTERGENIC(INTERGENIC);PRKCD(DOWNSTREAM)
3	53282982	53282997	53282990	TTTA	C	snp	1	1	1	1	.	.	.	TKT(INTRON)
3	53339376	53339384	53339378	T	C	ins	1	1	1	1	.	.	.	DCP1A(INTRON)
3	53596682	53596689	53596683	C	T	ins	1	1	1	1	.	.	.	CACNA1D(INTRON)
3	53596682	53596689	53596688	C	A	snp	1	0	0	0	.	.	.	CACNA1D(INTRON)
3	53666777	53666788	53666784	AG	AGAGATGGAGA	del	1	0	0	0	.	.	.	CACNA1D(INTRON)
3	53703993	53704000	53703995	T	C	snp	0	0	1	0	.	.	.	CACNA1D(INTRON);CACNA1D(UPSTREAM)
3	53767940	53767955	53767943	TG	A	snp	0	1	1	0	.	.	.	CACNA1D(INTRON)
3	53789349	53789358	53789354	TG	T	snp	0	1	1	1	.	.	.	CACNA1D(INTRON)
3	53936637	53936646	53936640	AT	C	snp	1	0	0	0	.	.	.	INTERGENIC(INTERGENIC)
3	54176620	54176640	54176631	TGA	G	snp	1	1	1	1	.	.	.	CACNA2D3(INTRON)
3	54352900	54352908	54352903	A	C	snp	1	1	1	1	.	.	.	CACNA2D3(INTRON)
3	54371105	54371128	54371124	AGAC	G	snp	1	1	1	1	.	.	.	CACNA2D3(INTRON)
3	54425519	54425546	54425539	CTTT	T	ins	1	1	1	1	.	.	.	CACNA2D3(INTRON)
3	54447436	54447445	54447443	TA	TATC	ins	0	1	1	1	.	.	.	CACNA2D3(INTRON)
3	54605471	54605478	54605477	G	A	snp	1	0	0	0	.	.	.	CACNA2D3(INTRON)
3	54637289	54637300	54637292	CCT	T	snp	0	0	0	1	.	.	.	CACNA2D3(INTRON)
3	54743379	54743392	54743390	CT	C	del	1	1	1	1	.	.	.	CACNA2D3(INTRON)
3	54745309	54745318	54745313	CA	T	snp	1	0	0	0	.	.	.	CACNA2D3(INTRON)
3	54770022	54770033	54770029	AT	C	snp	1	1	0	1	.	.	.	CACNA2D3(INTRON)
3	54770052	54770061	54770058	AT	G	snp	1	0	0	0	.	.	.	CACNA2D3(INTRON)
3	55010286	55010297	55010291	TG	A	snp	1	0	0	0	.	.	.	CACNA2D3(INTRON)
3	55018615	55018624	55018619	T	G	ins	1	1	1	1	.	.	.	CACNA2D3(INTRON);CACNA2D3(UPSTREAM)
3	55089589	55089596	55089590	T	G	snp	1	1	1	1	.	.	.	CACNA2D3(INTRON)
3	55135620	55135637	55135635	CA	C	del	1	1	1	1	.	.	.	INTERGENIC(INTERGENIC)
3	55194490	55194499	55194492	C	A	ins	1	1	1	1	.	.	.	INTERGENIC(INTERGENIC)
3	55369253	55369261	55369256	C	A	snp	1	1	0	0	.	.	.	INTERGENIC(INTERGENIC);RP11-875H7.1.1(DOWNSTREAM)
3	55380919	55380936	55380933	CAA	C	del	1	1	1	1	.	.	.	RP11-875H7.1.1(INTRON)
3	55759057	55759068	55759066	AG	C	snp	1	0	1	0	.	.	.	ERC2(INTRON)
3	55811105	55811114	55811112	A	AT	ins	1	1	1	1	.	.	.	ERC2(INTRON)
3	55833391	55833404	55833393	TA	G	snp	1	1	1	1	.	.	.	ERC2(INTRON)
3	55839697	55839708	55839702	ACA	ACG	ins	1	1	1	1	.	.	.	ERC2(INTRON)
3	55861328	55861347	55861340	AGAA	GAAG	ins	1	1	1	1	.	.	.	ERC2(INTRON)
3	55898199	55898208	55898204	AT	A	snp	1	0	0	0	.	.	.	ERC2(INTRON)
3	55946238	55946247	55946240	GA	A	snp	1	0	0	0	.	.	.	ERC2(INTRON)
3	56005652	56005661	56005655	GA	G	snp	1	1	1	1	.	.	.	ERC2(INTRON)
3	56066914	56066927	56066917	AG	A	snp	1	1	1	1	.	.	.	ERC2(INTRON)
3	56066914	56066927	56066921	AG	A	snp	0	1	1	1	.	.	.	ERC2(INTRON)
3	56066914	56066927	56066925	AG	A	snp	0	1	1	1	.	.	.	ERC2(INTRON)
3	56145346	56145361	56145358	TC	C	snp	1	1	1	1	.	.	.	ERC2(INTRON)
3	56168594	56168603	56168597	T	G	snp	0	0	1	0	.	.	.	ERC2(INTRON)
3	56195671	56195684	56195682	TG	A	snp	1	1	1	1	.	.	.	ERC2(INTRON)
3	56450717	56450726	56450722	TC	T	snp	1	0	0	0	.	.	.	ERC2(INTRON)
3	56450717	56450726	56450723	TC	C	snp	1	1	1	1	.	.	.	ERC2(INTRON)
3	56461289	56461308	56461293	TTGG	TGG	del	1	0	0	0	.	.	.	ERC2(INTRON)
3	56655212	56655221	56655214	A	C	snp	1	0	0	0	.	.	.	CCDC66(DOWNSTREAM);CCDC66(INTRON);FAM208A(DOWNSTREAM);FAM208A(EXON);FAM208A(UTR_3_PRIME)
3	56690622	56690630	56690628	C	A	snp	1	1	1	1	.	.	.	FAM208A(DOWNSTREAM);FAM208A(INTRON)
3	56717974	56717982	56717976	C	CCCG	ins	1	1	1	1	.	.	.	FAM208A(UPSTREAM);INTERGENIC(INTERGENIC)
3	56734704	56734723	56734710	TCCT	T	snp	0	0	1	0	.	.	.	INTERGENIC(INTERGENIC)
3	56808955	56808964	56808956	A	C	ins	1	1	1	1	.	.	.	ARHGEF3(INTRON)
3	56848694	56848702	56848697	T	C	ins	0	1	1	0	.	.	.	ARHGEF3(INTRON)
3	56929482	56929501	56929495	TTAT	A	del	1	1	1	1	.	.	.	ARHGEF3(INTRON)
3	56929482	56929501	56929497	TTAT	TTA	del	1	1	1	1	.	.	.	ARHGEF3(INTRON)
3	56947201	56947208	56947202	A	C	ins	1	1	1	1	.	.	.	ARHGEF3(INTRON)
3	57019967	57019978	57019973	GCT	A	snp	0	1	0	1	.	.	.	ARHGEF3(INTRON)
3	57036491	57036504	57036500	TA	TATATGT	del	1	1	1	1	.	.	.	ARHGEF3(INTRON)
3	57039000	57039017	57039003	AT	A	snp	0	1	0	1	.	.	.	ARHGEF3(INTRON)
3	57161270	57161279	57161273	AT	A	snp	1	1	1	1	.	.	.	IL17RD(INTRON)
3	57161394	57161405	57161403	AT	A	snp	1	1	1	1	.	.	.	IL17RD(INTRON)
3	57161424	57161441	57161427	AT	A	snp	1	1	1	1	.	.	.	IL17RD(INTRON)
3	57161424	57161441	57161436	AT	TATAT	del	1	1	1	1	.	.	.	IL17RD(INTRON)

3	57161424	57161441	57161439	AT	A	del	1	1	1	1	.	.	.	IL17RD(INTRON)
3	57223488	57223499	57223495	TA	TATATG	ins	1	1	1	1	.	.	.	INTERGENIC(INTERGENIC)
3	57372039	57372050	57372046	CT	C	snp	1	1	0	1	.	.	.	DNAH12(INTRON)
3	57400934	57400943	57400936	A	G	snp	1	1	1	1	.	.	.	DNAH12(INTRON)
3	57568472	57568479	57568478	A	TT	ins	1	1	1	1	.	.	.	ARF4(DOWNSTREAM);ARF4(INTRON)
3	57770755	57770768	57770760	TA	G	snp	0	1	1	1	.	.	.	SLMAP(INTRON)
3	57791747	57791756	57791749	T	G	snp	1	0	0	0	.	.	.	SLMAP(INTRON)
3	57808915	57808923	57808916	T	G	snp	1	0	0	0	.	.	.	SLMAP(INTRON)
3	57839836	57839845	57839842	AT	G	snp	1	1	1	1	.	.	.	SLMAP(INTRON)
3	58056372	58056387	58056383	TC	TCTCTTT	del	1	1	1	1	.	.	.	FLNB(INTRON)
3	58361071	58361078	58361074	A	G	snp	1	0	0	0	.	.	.	PXK(INTRON)
3	58442033	58442044	58442036	CCT	T	snp	1	0	0	0	.	.	.	INTERGENIC(INTERGENIC)
3	58470767	58470776	58470772	AC	A	snp	1	0	0	0	.	.	.	INTERGENIC(INTERGENIC)
3	58518091	58518099	58518092	T	C	snp	1	1	1	1	.	.	.	ACOX2(DOWNSTREAM);ACOX2(INTRON);ACOX2(UPSTREAM)
3	58620098	58620107	58620105	AG	A	snp	1	0	0	0	.	.	.	FAM3D(DOWNSTREAM);FAM3D(INTRON);RP11-475O23.3.1(EXON)
3	58630500	58630509	58630501	A	G	snp	1	1	1	1	.	.	.	FAM3D(INTRON)
3	58725140	58725147	58725145	T	C	snp	0	1	0	0	.	.	.	C3orf67(DOWNSTREAM);C3orf67(INTRON)
3	58900404	58900419	58900417	AT	A	del	1	1	0	1	.	.	.	C3orf67(INTRON);RP11-147N17.1.1(INTRON)
3	58903013	58903028	58903026	AC	T	snp	1	0	0	0	.	.	.	C3orf67(INTRON);RP11-147N17.1.1(INTRON)
3	58968334	58968341	58968335	A	G	snp	1	0	0	0	.	.	.	C3orf67(INTRON);RP11-147N17.1.1(INTRON)
3	58994582	58994593	58994589	ATA	C	snp	1	0	0	0	.	.	.	C3orf67(INTRON);RP11-147N17.1.1(INTRON)
3	59097294	59097302	59097295	A	G	snp	1	1	0	0	.	.	.	CTD-2185K10.1.1(INTRON)
3	59105495	59105504	59105502	A	C	snp	1	1	0	0	.	.	.	CTD-2185K10.1.1(DOWNSTREAM);INTERGENIC(INTERGENIC)
3	59187036	59187053	59187050	TA	A	del	1	1	0	1	.	.	.	INTERGENIC(INTERGENIC)
3	59187036	59187053	59187051	TA	T	ins	1	0	1	1	.	.	.	INTERGENIC(INTERGENIC)
3	59187036	59187053	59187051	TA	T	snp	1	1	0	1	.	.	.	INTERGENIC(INTERGENIC)
3	59201240	59201255	59201252	AT	G	snp	1	0	0	0	.	.	.	INTERGENIC(INTERGENIC)
3	59212746	59212759	59212749	TG	A	snp	1	0	0	0	.	.	.	INTERGENIC(INTERGENIC)
3	59282267	59282276	59282271	A	G	snp	0	1	1	0	.	.	.	INTERGENIC(INTERGENIC)
3	59361204	59361212	59361210	T	C	snp	1	1	1	1	.	.	.	INTERGENIC(INTERGENIC);RP11-285B24.1.1(DOWNSTREAM)
3	59364664	59364671	59364670	T	C	snp	1	0	0	0	.	.	.	INTERGENIC(INTERGENIC);RP11-285B24.1.1(DOWNSTREAM)
3	59392310	59392325	59392318	AAAT	C	snp	1	0	0	0	.	.	.	INTERGENIC(INTERGENIC)
3	59394860	59394880	59394873	AAC	G	snp	1	0	0	0	.	.	.	INTERGENIC(INTERGENIC)
3	59476466	59476481	59476475	CTTT	TTC	del	1	1	1	1	.	.	.	RP11-719N22.2.1(INTRON)
3	59476466	59476481	59476476	CTTT	TC	del	1	1	1	1	.	.	.	RP11-719N22.2.1(INTRON)
3	59570566	59570573	59570569	A	G	snp	1	1	1	1	.	.	.	INTERGENIC(INTERGENIC)
3	59644665	59644684	59644680	TTTG	A	snp	1	1	1	1	.	.	.	INTERGENIC(INTERGENIC)
3	59840530	59840539	59840535	AG	A	del	1	1	1	1	.	.	.	FHIT(INTRON)
3	59866750	59866758	59866751	A	G	snp	1	0	0	0	.	.	.	FHIT(INTRON)
3	59948834	59948842	59948841	A	C	snp	1	1	1	1	.	.	.	FHIT(INTRON)
3	59970121	59970128	59970125	G	A	ins	1	0	0	0	.	.	.	FHIT(INTRON)
3	60094869	60094880	60094877	GA	C	snp	0	1	1	1	.	.	.	FHIT(INTRON)
3	60113493	60113502	60113496	AT	C	snp	1	0	0	0	.	.	.	FHIT(INTRON)
3	60144797	60144804	60144803	T	C	snp	1	0	0	0	.	.	.	FHIT(INTRON)
3	60191878	60191901	60191888	AAT	T	ins	1	0	0	0	.	.	.	FHIT(INTRON)
3	60191878	60191901	60191890	AAT	T	snp	1	1	1	1	.	.	.	FHIT(INTRON)
3	60235865	60235872	60235868	A	G	snp	1	1	1	1	.	.	.	FHIT(INTRON)
3	60236501	60236508	60236506	A	G	snp	1	0	0	0	.	.	.	FHIT(INTRON)
3	60263533	60263544	60263537	TG	A	snp	1	0	0	0	.	.	.	FHIT(INTRON)
3	60327733	60327740	60327737	A	G	snp	1	0	0	0	.	.	.	FHIT(INTRON)
3	60349966	60349975	60349971	TG	C	snp	1	1	1	1	.	.	.	FHIT(INTRON)
3	60358515	60358523	60358517	T	C	snp	1	1	1	1	.	.	.	FHIT(INTRON)
3	60506982	60506993	60506990	TAT	TA	del	1	1	1	1	.	.	.	FHIT(INTRON)
3	60568359	60568367	60568366	A	C	snp	1	0	0	0	.	.	.	FHIT(INTRON)
3	60600543	60600552	60600550	T	G	ins	1	1	1	1	.	.	.	FHIT(INTRON);RP11-641C17.4.1(UPSTREAM)
3	60675734	60675745	60675740	TTG	C	snp	1	1	1	1	.	.	.	FHIT(INTRON);RP11-641C17.2.1(DOWNSTREAM)
3	60800577	60800585	60800582	T	G	ins	1	1	1	1	.	.	.	FHIT(INTRON)
3	60846953	60846962	60846954	T	C	snp	1	0	0	0	.	.	.	FHIT(INTRON);U3(UPSTREAM)
3	60866411	60866434	60866427	AAC	T	snp	1	1	0	1	.	.	.	FHIT(INTRON)
3	60936149	60936156	60936155	T	G	ins	1	0	0	0	.	.	.	FHIT(INTRON)
3	60994572	60994587	60994583	TG	C	snp	1	1	1	1	.	.	.	FHIT(INTRON)
3	61063159	61063167	61063162	A	C	snp	1	1	0	1	.	.	.	FHIT(INTRON)
3	61264889	61264906	61264892	CA	CACACT	ins	1	1	1	1	.	.	.	INTERGENIC(INTERGENIC)

3	61306946	61306954	61306949	T	C	snp	1	0	0	0	.	.	.	INTERGENIC(INTERGENIC)
3	61432004	61432013	61432006	GA	C	snp	1	0	0	0	.	.	.	INTERGENIC(INTERGENIC)
3	61450938	61450945	61450940	A	G	snp	1	1	1	1	.	.	.	INTERGENIC(INTERGENIC)
3	61451653	61451672	61451668	TTTC	T	ins	1	0	0	0	.	.	.	INTERGENIC(INTERGENIC)
3	61535176	61535184	61535178	T	C	snp	1	1	1	1	.	.	.	INTERGENIC(INTERGENIC)
3	61578304	61578323	61578318	TTTC	C	del	1	1	0	1	.	.	.	PTPRG(INTRON)
3	61596457	61596466	61596464	CT	C	snp	1	0	0	0	.	.	.	PTPRG(INTRON)
3	61658487	61658496	61658492	GA	G	snp	1	0	0	0	.	.	.	PTPRG(INTRON)
3	61802649	61802658	61802653	TA	A	snp	1	1	1	1	.	.	.	PTPRG(INTRON)
3	61874002	61874017	61874013	TTTG	T	snp	1	1	0	0	.	.	.	PTPRG(INTRON)
3	62055375	62055390	62055382	CA	G	snp	1	1	1	1	.	.	.	PTPRG(INTRON)
3	62056623	62056631	62056626	A	G	snp	1	1	1	1	.	.	.	PTPRG(INTRON)
3	62061651	62061668	62061664	ATG	C	snp	0	1	0	0	.	.	.	PTPRG(INTRON)
3	62373826	62373841	62373832	TTCT	T	snp	0	1	0	0	.	.	.	INTERGENIC(INTERGENIC)
3	62403524	62403532	62403526	A	C	ins	1	1	1	1	.	.	.	CADPS(INTRON)
3	62455045	62455054	62455052	TA	C	snp	1	0	0	0	.	.	.	CADPS(DOWNSTREAM);CADPS(INTRON);CADPS(UPSTREAM)
3	62455196	62455205	62455200	T	C	snp	1	0	0	0	.	.	.	CADPS(DOWNSTREAM);CADPS(INTRON);CADPS(UPSTREAM)
3	62459814	62459822	62459819	A	C	snp	0	1	0	1	.	.	.	CADPS(DOWNSTREAM);CADPS(EXON);CADPS(INTRON);CADPS(UPSTREAM)
3	62463077	62463088	62463080	TGA	C	snp	0	0	0	1	.	.	.	CADPS(DOWNSTREAM);CADPS(INTRON)
3	62473738	62473747	62473743	TG	T	snp	0	1	0	1	.	.	.	CADPS(INTRON)
3	62480484	62480492	62480489	T	G	snp	1	0	0	0	.	.	.	CADPS(INTRON);CADPS(UPSTREAM)
3	62494980	62494999	62494986	AAAG	G	del	1	1	1	1	.	.	.	CADPS(INTRON)
3	62579833	62579848	62579843	TTTA	A	del	1	1	1	1	.	.	.	CADPS(INTRON)
3	62579833	62579848	62579844	TTTA	T	ins	1	1	1	1	.	.	.	CADPS(INTRON)
3	62713213	62713220	62713217	A	G	snp	1	1	1	1	.	.	.	CADPS(INTRON)
3	62785469	62785483	62785475	ACC	G	snp	1	1	1	1	.	.	.	AC012519.1(UPSTREAM);CADPS(INTRON)
3	62785973	62785993	62785990	CAT	C	snp	1	1	1	1	.	.	.	AC012519.1(UPSTREAM);CADPS(INTRON)
3	62826264	62826273	62826266	GT	A	snp	1	0	0	0	.	.	.	CADPS(INTRON)
3	62884814	62884825	62884823	AG	A	ins	1	1	1	0	.	.	.	INTERGENIC(INTERGENIC)
3	62938254	62938265	62938259	TTG	C	snp	1	0	0	0	.	.	.	RP11-129K20.2.1(INTRON)
3	62958106	62958115	62958113	TG	TGTA	ins	1	0	0	0	.	.	.	RP11-129K20.2.1(INTRON)
3	63010905	63010914	63010913	T	C	snp	0	0	1	0	.	.	.	RP11-129K20.2.1(INTRON)
3	63018819	63018826	63018822	T	C	snp	1	0	0	0	.	.	.	RP11-129K20.2.1(INTRON)
3	63047914	63047925	63047920	CA	A	snp	1	0	1	0	.	.	.	RP11-129K20.2.1(INTRON)
3	63071363	63071380	63071372	TTG	C	snp	1	1	1	1	.	.	.	RP11-129K20.2.1(INTRON)
3	63141542	63141561	63141552	AAAT	T	snp	1	0	1	0	.	.	.	INTERGENIC(INTERGENIC)
3	63141542	63141561	63141557	AAAT	A	snp	1	1	1	0	.	.	.	INTERGENIC(INTERGENIC)
3	63155761	63155770	63155766	A	G	snp	1	1	1	1	.	.	.	INTERGENIC(INTERGENIC)
3	63176613	63176622	63176618	T	G	snp	1	1	1	1	.	.	.	INTERGENIC(INTERGENIC)
3	63299381	63299392	63299389	AT	G	snp	1	1	1	1	.	.	.	SYNPR(INTRON)
3	63299409	63299420	63299417	AT	G	snp	1	0	0	0	.	.	.	SYNPR(INTRON)
3	63417486	63417493	63417491	A	C	snp	1	1	1	1	.	.	.	SYNPR(INTRON);SYNPR-AS1(INTRON)
3	63434343	63434351	63434350	T	C	snp	1	1	0	1	.	.	.	SYNPR(INTRON);SYNPR-AS1(INTRON)
3	63486740	63486748	63486745	A	G	snp	1	1	1	1	.	.	.	SYNPR(INTRON);SYNPR-AS1(INTRON)
3	63509401	63509416	63509408	TTTG	A	snp	1	0	0	0	.	.	.	SYNPR(INTRON);SYNPR-AS1(INTRON)
3	63572363	63572376	63572373	TA	C	snp	1	0	0	0	.	.	.	SYNPR(INTRON)
3	63605962	63605981	63605975	TTTC	TCTTTC	del	1	1	1	0	.	.	.	INTERGENIC(INTERGENIC);SYNPR(DOWNSTREAM)
3	63629606	63629613	63629611	T	C	snp	1	1	1	1	.	.	.	INTERGENIC(INTERGENIC)
3	63742423	63742434	63742427	TA	C	snp	1	1	1	1	.	.	.	AC136289.1.1(INTRON)
3	63926229	63926240	63926236	AAT	G	snp	0	1	1	1	.	.	.	ATXN7(INTRON)
3	64055495	64055503	64055501	T	G	snp	1	1	0	1	.	.	.	RP11-129B22.1.1(INTRON);U3(DOWNSTREAM)
3	64092434	64092445	64092441	GT	A	snp	1	0	0	0	.	.	.	PRICKLE2(INTRON);PRICKLE2-AS2(DOWNSTREAM);RP11-129B22.1.1(DOWNSTREAM)
3	64262694	64262701	64262700	T	G	snp	1	1	1	0	.	.	.	PRICKLE2(INTRON)
3	64283271	64283282	64283278	AC	T	snp	1	1	1	1	.	.	.	PRICKLE2(INTRON)
3	64300951	64300960	64300958	AT	A	snp	1	1	1	1	.	.	.	PRICKLE2(INTRON)
3	64312630	64312641	64312635	TG	C	snp	1	1	1	1	.	.	.	PRICKLE2(INTRON)
3	64347192	64347201	64347194	A	C	ins	0	1	1	0	.	.	.	PRICKLE2(INTRON)
3	64347192	64347201	64347195	A	C	snp	0	1	1	1	.	.	.	PRICKLE2(INTRON)
3	64403635	64403642	64403638	T	G	snp	0	1	1	1	.	.	.	PRICKLE2(INTRON)
3	64691374	64691389	64691379	AC	T	snp	1	1	1	1	.	.	.	ADAMTS9-AS2(INTRON)
3	64719573	64719596	64719588	TTTC	T	ins	1	1	0	1	.	.	.	ADAMTS9-AS2(INTRON)
3	64719573	64719596	64719591	TTTC	C	del	1	1	0	1	.	.	.	ADAMTS9-AS2(INTRON)
3	64722625	64722632	64722628	A	G	snp	1	1	0	1	.	.	.	ADAMTS9-AS2(INTRON)
3	64762569	64762577	64762570	T	C	ins	1	1	1	1	.	.	.	ADAMTS9-AS2(INTRON)
3	64796627	64796635	64796631	T	G	ins	1	0	0	0	.	.	.	ADAMTS9-AS2(INTRON)

3	64798076	64798083	64798078	T	G	snp	1	1	1	1	.	.	.	ADAMTS9-AS2(INTRON)
3	64800621	64800628	64800623	T	G	snp	1	1	1	1	.	.	.	ADAMTS9-AS2(INTRON)
3	64830177	64830185	64830184	T	G	snp	1	0	0	0	.	.	.	ADAMTS9-AS2(INTRON)
3	64956203	64956210	64956208	T	C	snp	1	1	1	1	.	.	.	ADAMTS9-AS2(INTRON)
3	64970415	64970430	64970425	TCAC	C	del	1	0	0	0	.	.	.	ADAMTS9-AS2(INTRON)
3	65134984	65134995	65134987	TA	C	snp	1	1	1	1	.	.	.	INTERGENIC(INTERGENIC)
3	65147860	65147869	65147864	T	C	snp	1	0	1	0	.	.	.	INTERGENIC(INTERGENIC)
3	65223745	65223756	65223753	TG	C	snp	1	1	1	1	.	.	.	INTERGENIC(INTERGENIC)
3	65238418	65238425	65238423	T	G	snp	1	0	0	0	.	.	.	INTERGENIC(INTERGENIC)
3	65463368	65463388	65463383	ATT	TT	ins	1	0	0	0	.	.	.	MAGI1(INTRON)
3	65479524	65479533	65479530	GA	C	snp	1	1	1	1	.	.	.	MAGI1(INTRON)
3	65501866	65501873	65501867	T	C	snp	1	0	0	0	.	.	.	MAGI1(INTRON)
3	65516834	65516843	65516835	A	C	snp	1	1	1	1	.	.	.	MAGI1(INTRON)
3	65529431	65529439	65529435	G	A	snp	0	1	1	1	.	.	.	MAGI1(INTRON)
3	65850980	65850988	65850983	A	G	snp	0	0	0	1	.	.	.	MAGI1(INTRON)
3	65876099	65876110	65876107	CA	T	snp	1	0	0	0	.	.	.	MAGI1(INTRON);MAGI1-AS1(UPSTREAM);MAGI1-IT1(INTRON)
3	65897212	65897219	65897218	T	C	snp	0	1	0	1	.	.	.	MAGI1(INTRON);MAGI1(UPSTREAM);MAGI1-AS1(INTRON);MAGI1-IT1(INTRON)
3	65954238	65954247	65954243	GT	C	snp	1	0	0	0	.	.	.	MAGI1(INTRON)
3	65964764	65964773	65964767	C	CT	ins	1	0	0	0	.	.	.	MAGI1(INTRON)
3	66005294	66005305	66005299	AAT	C	snp	0	1	1	1	.	.	.	MAGI1(INTRON)
3	66058496	66058503	66058502	T	G	snp	1	1	1	1	.	.	.	INTERGENIC(INTERGENIC)
3	66085311	66085325	66085315	AAC	G	snp	1	1	1	1	.	.	.	INTERGENIC(INTERGENIC)
3	66150120	66150129	66150123	T	G	snp	1	0	0	0	.	.	.	SLC25A26(INTRON)
3	66329419	66329427	66329426	T	G	snp	1	1	1	1	.	.	.	SLC25A26(INTRON)
3	66360752	66360759	66360756	T	G	snp	1	0	0	0	.	.	.	SLC25A26(INTRON)
3	66382353	66382368	66382362	TTTG	G	snp	1	0	0	0	.	.	.	SLC25A26(INTRON)
3	66397443	66397452	66397447	A	C	snp	1	1	1	1	.	.	.	SLC25A26(INTRON)
3	66422359	66422374	66422369	AAAT	G	snp	1	1	1	1	.	.	.	SLC25A26(DOWNSTREAM);SLC25A26(INTRON)
3	66464831	66464846	66464842	AATA	AAT	del	1	0	0	0	.	.	.	LRIG1(DOWNSTREAM);LRIG1(INTRON);LRIG1(UPSTREAM)
3	66478901	66478910	66478907	A	C	snp	1	1	1	1	.	.	.	LRIG1(INTRON)
3	66480615	66480624	66480616	A	C	ins	1	1	1	1	.	.	.	LRIG1(INTRON)
3	66480615	66480624	66480617	A	C	snp	1	1	1	1	.	.	.	LRIG1(INTRON)
3	66495390	66495399	66495397	TA	G	snp	0	1	0	1	.	.	.	LRIG1(INTRON)
3	66542858	66542865	66542863	A	G	snp	0	1	1	1	.	.	.	LRIG1(INTRON)
3	66544260	66544273	66544263	AG	A	snp	1	1	1	1	.	.	.	LRIG1(INTRON)
3	66587585	66587592	66587586	T	G	snp	1	0	0	0	.	.	.	INTERGENIC(INTERGENIC)
3	66602251	66602278	66602270	AATA	AAT	del	1	0	0	0	.	.	.	INTERGENIC(INTERGENIC)
3	66638437	66638445	66638440	A	G	snp	1	1	1	1	.	.	.	INTERGENIC(INTERGENIC)
3	66675553	66675568	66675556	AT	A	snp	1	0	1	0	.	.	.	INTERGENIC(INTERGENIC)
3	66684286	66684299	66684291	AG	A	snp	1	0	0	0	.	.	.	INTERGENIC(INTERGENIC);RP11-814M22.1.1(UPSTREAM)
3	66699223	66699232	66699228	AG	A	snp	1	0	0	0	.	.	.	INTERGENIC(INTERGENIC)
3	66734453	66734476	66734464	TTC	T	snp	1	1	1	1	.	.	.	INTERGENIC(INTERGENIC)
3	66743699	66743706	66743701	A	C	snp	1	1	1	1	.	.	.	INTERGENIC(INTERGENIC)
3	66769431	66769439	66769432	G	T	snp	0	1	1	1	.	.	.	INTERGENIC(INTERGENIC)
3	66924517	66924528	66924522	AT	C	snp	1	1	0	1	.	.	.	INTERGENIC(INTERGENIC)
3	66924650	66924659	66924655	AT	C	snp	1	1	1	1	.	.	.	INTERGENIC(INTERGENIC)
3	66924710	66924719	66924715	AT	C	snp	1	1	1	1	.	.	.	INTERGENIC(INTERGENIC)
3	66925458	66925467	66925460	TA	A	snp	1	1	1	1	.	.	.	INTERGENIC(INTERGENIC)
3	67046204	67046212	67046208	A	C	snp	1	0	0	0	.	.	.	INTERGENIC(INTERGENIC);KBTBD8(UPSTREAM)
3	67106683	67106690	67106688	T	C	snp	0	0	1	1	.	.	.	INTERGENIC(INTERGENIC)
3	67163603	67163611	67163607	G	T	snp	1	1	1	1	.	.	.	INTERGENIC(INTERGENIC)
3	67183992	67184000	67183993	T	C	snp	1	1	0	0	.	.	.	INTERGENIC(INTERGENIC)
3	67352518	67352525	67352521	C	T	snp	1	1	1	1	.	.	.	RP11-85I21.1.1(INTRON);RP11-85I21.1.1(UPSTREAM)
3	67352999	67353008	67353002	AC	T	snp	0	1	0	0	.	.	.	RP11-85I21.1.1(INTRON);RP11-85I21.1.1(UPSTREAM)
3	67387345	67387353	67387346	A	C	ins	1	1	1	1	.	.	.	INTERGENIC(INTERGENIC)
3	67524684	67524693	67524690	A	G	snp	1	0	0	0	.	.	.	SUCLG2(INTRON)
3	67527730	67527737	67527731	T	C	snp	1	0	0	0	.	.	.	SUCLG2(INTRON)
3	67592374	67592385	67592379	CCT	A	snp	0	0	1	0	.	.	.	SUCLG2(INTRON)
3	67609859	67609868	67609861	GA	A	del	1	0	0	0	.	.	.	SUCLG2(INTRON)
3	67680593	67680602	67680600	A	G	snp	0	0	1	1	.	.	.	SUCLG2(INTRON)
3	67745706	67745714	67745713	T	G	snp	1	0	0	0	.	.	.	RP11-81N13.1.1(INTRON)
3	67777262	67777269	67777268	A	C	snp	1	1	1	1	.	.	.	RP11-81N13.1.1(INTRON)
3	67852570	67852578	67852572	T	G	snp	1	0	0	0	.	.	.	RP11-81N13.1.1(INTRON)
3	68109220	68109229	68109228	A	C	ins	1	0	0	0	.	.	.	FAM19A1(INTRON)
3	68168723	68168732	68168729	T	C	ins	1	1	1	1	.	.	.	FAM19A1(INTRON)

3	68264003	68264010	68264009	T	C	snp	1	0	0	0	.	.	.	FAM19A1(INTRON)
3	68459408	68459416	68459412	T	C	snp	0	0	1	0	.	.	.	FAM19A1(INTRON)
3	68476805	68476812	68476807	C	T	snp	1	1	1	1	.	.	.	FAM19A1(INTRON)
3	68481203	68481217	68481207	TAT	T	snp	0	0	1	0	.	.	.	FAM19A1(INTRON)
3	68608280	68608291	68608285	AG	A	snp	0	0	1	0	.	.	.	INTERGENIC(INTERGENIC)
3	68623659	68623668	68623662	A	C	snp	1	0	0	0	.	.	.	INTERGENIC(INTERGENIC)
3	68674836	68674844	68674837	G	T	ins	1	0	1	0	.	.	.	INTERGENIC(INTERGENIC)
3	68674836	68674844	68674842	G	GC	ins	1	1	0	1	.	.	.	INTERGENIC(INTERGENIC)
3	68683716	68683727	68683724	CT	T	snp	1	0	0	0	.	.	.	AC096922.1(UPSTREAM);INTERGENIC(INTERGENIC);PSMC1P1(UPSTREAM);RP11-436J20.1.1(UPSTREAM)
3	68687811	68687825	68687816	TTA	T	snp	1	1	1	1	.	.	.	AC096922.1(DOWNSTREAM);INTERGENIC(INTERGENIC);PSMC1P1(DOWNSTREAM);RP11-436J20.1.1(UPSTREAM)
3	68765631	68765640	68765638	TC	G	snp	1	1	1	1	.	.	.	INTERGENIC(INTERGENIC)
3	68780656	68780671	68780660	AT	TATG	ins	1	1	1	1	.	.	.	FAM19A4(DOWNSTREAM);INTERGENIC(INTERGENIC)
3	68890489	68890497	68890495	A	G	snp	1	1	1	1	.	.	.	FAM19A4(INTRON)
3	69022304	69022315	69022306	GA	C	snp	1	1	1	1	.	.	.	C3orf64(DOWNSTREAM);INTERGENIC(INTERGENIC)
3	69236720	69236731	69236725	AT	C	snp	1	0	0	0	.	.	.	FRMD4B(INTRON)
3	69448060	69448071	69448065	AT	C	snp	1	0	0	0	.	.	.	FRMD4B(INTRON)
3	69542855	69542864	69542857	TC	A	snp	1	1	1	1	.	.	.	FRMD4B(INTRON)
3	69590941	69590948	69590947	C	A	snp	1	1	1	1	.	.	.	FRMD4B(INTRON);FRMD4B(UPSTREAM)
3	69609745	69609752	69609751	T	G	snp	1	0	0	0	.	.	.	INTERGENIC(INTERGENIC);RP11-62G11.2.1(DOWNSTREAM)
3	69695660	69695669	69695667	GT	C	snp	1	0	0	0	.	.	.	INTERGENIC(INTERGENIC)
3	69771982	69771993	69771990	GA	A	snp	1	1	1	1	.	.	.	INTERGENIC(INTERGENIC)
3	69772102	69772113	69772104	GA	A	snp	1	1	1	1	.	.	.	INTERGENIC(INTERGENIC)
3	69795657	69795664	69795658	T	TG	ins	1	1	1	1	.	.	.	MITF(INTRON)
3	69795657	69795664	69795659	T	G	ins	1	1	1	1	.	.	.	MITF(INTRON)
3	69795657	69795664	69795660	T	G	snp	1	1	1	1	.	.	.	MITF(INTRON)
3	70094156	70094167	70094159	TA	T	del	1	1	0	1	.	.	.	INTERGENIC(INTERGENIC)
3	70165667	70165675	70165668	T	G	snp	1	1	1	1	.	.	.	INTERGENIC(INTERGENIC)
3	70209505	70209524	70209510	CCTC	A	snp	0	1	0	1	.	.	.	INTERGENIC(INTERGENIC)
3	70220767	70220775	70220771	G	A	snp	1	1	1	1	.	.	.	INTERGENIC(INTERGENIC)
3	70249862	70249871	70249865	TC	G	snp	1	1	1	1	.	.	.	RP11-231I13.2.1(INTRON)
3	70412597	70412604	70412598	A	C	snp	0	0	1	0	.	.	.	INTERGENIC(INTERGENIC)
3	70437120	70437127	70437124	T	G	ins	1	1	1	1	.	.	.	INTERGENIC(INTERGENIC)
3	70489325	70489333	70489326	G	T	snp	1	1	0	1	.	.	.	INTERGENIC(INTERGENIC)
3	70581500	70581511	70581505	TG	A	snp	1	0	0	0	.	.	.	INTERGENIC(INTERGENIC)
3	70585174	70585189	70585180	CCTT	C	snp	1	1	1	0	.	.	.	INTERGENIC(INTERGENIC)
3	70658767	70658774	70658772	A	G	snp	1	1	1	1	.	.	.	INTERGENIC(INTERGENIC)
3	70680356	70680369	70680359	AG	A	snp	1	1	1	1	.	.	.	INTERGENIC(INTERGENIC)
3	70680356	70680369	70680367	AG	AA	ins	1	1	1	1	.	.	.	INTERGENIC(INTERGENIC)
3	70680462	70680473	70680467	AG	A	snp	1	1	1	1	.	.	.	INTERGENIC(INTERGENIC)
3	70685048	70685055	70685054	A	C	snp	1	0	0	0	.	.	.	INTERGENIC(INTERGENIC)
3	70829320	70829328	70829323	A	G	snp	1	0	1	0	.	.	.	INTERGENIC(INTERGENIC)
3	70965042	70965049	70965044	T	G	snp	1	1	1	1	.	.	.	INTERGENIC(INTERGENIC)
3	70996718	70996726	70996719	C	A	snp	1	0	0	0	.	.	.	INTERGENIC(INTERGENIC)
3	71064316	71064339	71064332	CAA	ACC	ins	1	0	0	0	.	.	.	FOXP1(DOWNSTREAM);FOXP1(INTRON)
3	71185971	71185980	71185972	A	C	ins	1	0	0	0	.	.	.	FOXP1(INTRON)
3	71203248	71203256	71203250	T	C	snp	1	0	0	0	.	.	.	FOXP1(INTRON)
3	71231204	71231212	71231205	A	AAG	ins	1	1	1	1	.	.	.	FOXP1(INTRON)
3	71278969	71278983	71278974	AAC	T	snp	1	0	0	0	.	.	.	FOXP1(INTRON)
3	71454868	71454891	71454885	TATT	T	snp	1	1	1	1	.	.	.	FOXP1(INTRON)
3	71484403	71484422	71484409	GAGG	C	snp	1	1	0	1	.	.	.	FOXP1(INTRON)
3	71498557	71498568	71498561	TG	C	snp	1	1	0	1	.	.	.	FOXP1(INTRON)
3	71641335	71641346	71641341	GCA	A	snp	1	0	0	0	.	.	.	INTERGENIC(INTERGENIC)
3	71679659	71679668	71679664	T	G	snp	1	1	1	1	.	.	.	INTERGENIC(INTERGENIC)
3	71763471	71763486	71763480	GAAA	AAG	ins	1	0	0	0	.	.	.	EIF4E3(INTRON)
3	71810201	71810209	71810206	T	G	snp	1	1	1	1	.	.	.	GPR27(DOWNSTREAM);INTERGENIC(INTERGENIC)
3	71819727	71819740	71819734	TA	T	snp	1	0	0	0	.	.	.	INTERGENIC(INTERGENIC);PROK2(DOWNSTREAM)
3	71855379	71855386	71855380	A	G	snp	1	1	1	1	.	.	.	INTERGENIC(INTERGENIC)
3	71907531	71907544	71907534	AC	A	snp	1	0	0	1	.	.	.	INTERGENIC(INTERGENIC)
3	71947842	71947851	71947848	T	G	snp	1	1	0	1	.	.	.	INTERGENIC(INTERGENIC)
3	71959615	71959632	71959630	AG	A	snp	1	1	1	1	.	.	.	INTERGENIC(INTERGENIC)
3	71976482	71976491	71976489	T	C	snp	1	1	1	1	.	.	.	INTERGENIC(INTERGENIC)
3	72095643	72095654	72095647	ACA	T	snp	1	1	1	1	.	.	.	RP11-398A8.3.1(INTRON)
3	72101938	72101945	72101942	A	C	snp	1	1	1	1	.	.	.	RP11-398A8.3.1(INTRON)
3	72114072	72114087	72114079	ATCT	G	snp	1	0	0	1	.	.	.	RP11-398A8.3.1(INTRON)
3	72119831	72119848	72119845	TTA	TTG	ins	1	1	0	1	.	.	.	RP11-398A8.3.1(INTRON)

3	72131055	72131063	72131056	A	AC	ins	1	1	1	1	.	.	.	RP11-398A8.1.1(UPSTREAM);RP11-398A8.2.1(UPSTREAM);RP11-398A8.3.1(INTRON)
3	72204032	72204043	72204040	CT	T	snp	1	1	1	1	.	.	.	RP11-398A8.3.1(INTRON);RP11-433A10.1.1(INTRON)
3	72408700	72408715	72408711	AAAC	A	snp	1	0	0	0	.	.	.	INTERGENIC(INTERGENIC)
3	72435313	72435320	72435318	T	C	snp	1	0	0	0	.	.	.	INTERGENIC(INTERGENIC)
3	72518854	72518862	72518859	A	C	snp	1	0	0	0	.	.	.	INTERGENIC(INTERGENIC)
3	72544091	72544098	72544096	A	G	snp	1	0	1	0	.	.	.	INTERGENIC(INTERGENIC)
3	72582882	72582893	72582889	GT	C	snp	1	1	1	1	.	.	.	RP11-654C22.2.1(INTRON)
3	72690556	72690563	72690562	C	A	snp	1	0	0	0	.	.	.	INTERGENIC(INTERGENIC)
3	72784850	72784863	72784861	AG	AGGA	ins	1	1	1	1	.	.	.	INTERGENIC(INTERGENIC)
3	72840873	72840882	72840877	T	G	snp	1	1	0	1	.	.	.	SHQ1(INTRON)
3	72913122	72913129	72913126	T	G	snp	1	0	0	0	.	.	.	INTERGENIC(INTERGENIC);SHQ1(UPSTREAM)
3	72947891	72947899	72947895	T	C	snp	1	1	0	1	.	.	.	GXYLT2(INTRON)
3	72964385	72964392	72964391	T	C	snp	1	0	0	0	.	.	.	GXYLT2(INTRON)
3	72988367	72988376	72988370	TA	G	snp	1	1	1	1	.	.	.	GXYLT2(INTRON)
3	73052859	73052867	73052862	T	G	ins	1	1	1	1	.	.	.	PPP4R2(INTRON)
3	73093372	73093392	73093386	TTG	T	snp	1	0	0	0	.	.	.	PPP4R2(INTRON)
3	73099605	73099616	73099608	TA	T	snp	1	1	1	1	.	.	.	PPP4R2(INTRON)
3	73099605	73099616	73099610	TA	G	snp	1	1	1	1	.	.	.	PPP4R2(INTRON)
3	73138060	73138073	73138070	AT	T	ins	1	1	1	1	.	.	.	INTERGENIC(INTERGENIC);U6(DOWNSTREAM)
3	73138060	73138073	73138071	AT	A	del	1	1	1	1	.	.	.	INTERGENIC(INTERGENIC);U6(DOWNSTREAM)
3	73234463	73234470	73234466	T	G	snp	1	1	1	1	.	.	.	CCDC75P1(UPSTREAM);INTERGENIC(INTERGENIC)
3	73457740	73457749	73457741	G	A	ins	1	1	1	1	.	.	.	PDZRN3(INTRON);PDZRN3(UPSTREAM)
3	73457740	73457749	73457742	G	A	snp	1	1	1	1	.	.	.	PDZRN3(INTRON);PDZRN3(UPSTREAM)
3	73463585	73463593	73463589	T	G	snp	1	1	1	1	.	.	.	PDZRN3(INTRON)
3	73466002	73466011	73466008	T	G	snp	1	0	0	0	.	.	.	PDZRN3(INTRON)
3	73572605	73572616	73572607	TA	AC	ins	1	1	1	1	.	.	.	PDZRN3(INTRON)
3	73612336	73612345	73612343	T	G	snp	1	1	1	1	.	.	.	PDZRN3(INTRON);PDZRN3(UPSTREAM)
3	73753304	73753327	73753323	AAAC	A	del	1	1	1	1	.	.	.	INTERGENIC(INTERGENIC)
3	73920381	73920395	73920387	TTG	A	snp	1	0	1	0	.	.	.	RP11-20B7.1.1(INTRON)
3	73975511	73975518	73975515	A	G	snp	1	1	1	1	.	.	.	INTERGENIC(INTERGENIC)
3	73997777	73997788	73997784	AT	C	snp	1	0	0	0	.	.	.	INTERGENIC(INTERGENIC)
3	74072182	74072191	74072185	TC	G	snp	0	1	0	1	.	.	.	INTERGENIC(INTERGENIC)
3	74094666	74094677	74094671	TC	G	snp	1	0	0	0	.	.	.	INTERGENIC(INTERGENIC)
3	74113579	74113594	74113587	GA	A	snp	1	0	0	0	.	.	.	INTERGENIC(INTERGENIC)
3	74174386	74174393	74174390	T	C	snp	0	1	0	0	.	.	.	INTERGENIC(INTERGENIC)
3	74324845	74324854	74324849	A	C	snp	1	1	1	1	.	.	.	CNTN3(INTRON)
3	74348882	74348893	74348888	CAG	T	snp	1	0	0	0	.	.	.	CNTN3(INTRON)
3	74358254	74358269	74358264	TTAT	T	snp	1	0	0	0	.	.	.	CNTN3(INTRON)
3	74373357	74373368	74373364	TCT	TTCT	del	0	1	1	1	.	.	.	CNTN3(INTRON)
3	74407748	74407771	74407755	ATTT	G	snp	1	0	0	0	.	.	.	CNTN3(INTRON)
3	74441532	74441547	74441537	TCCC	T	snp	1	1	1	1	.	.	.	CNTN3(INTRON)
3	74473544	74473561	74473557	AT	AC	ins	1	1	1	1	.	.	.	CNTN3(INTRON)
3	74698667	74698684	74698679	TC	G	snp	1	1	1	1	.	.	.	INTERGENIC(INTERGENIC)
3	74750185	74750194	74750190	T	TC	ins	1	1	1	1	.	.	.	INTERGENIC(INTERGENIC)
3	74769171	74769178	74769177	T	C	snp	1	1	0	1	.	.	.	INTERGENIC(INTERGENIC)
3	74921963	74921971	74921967	T	C	snp	1	0	0	0	.	.	.	INTERGENIC(INTERGENIC)
3	74961193	74961200	74961196	C	T	snp	1	1	1	1	.	.	.	INTERGENIC(INTERGENIC)
3	75103870	75103879	75103874	CA	A	ins	1	1	1	1	.	.	.	INTERGENIC(INTERGENIC)
3	75105794	75105809	75105803	AGAA	A	del	1	0	0	0	.	.	.	INTERGENIC(INTERGENIC)
3	75309452	75309465	75309457	TG	A	snp	1	0	0	0	.	.	.	INTERGENIC(INTERGENIC)
3	75357839	75357847	75357840	G	T	snp	1	1	0	1	.	.	.	INTERGENIC(INTERGENIC)
3	75357839	75357847	75357842	G	A	ins	1	1	1	0	.	.	.	INTERGENIC(INTERGENIC)
3	75435190	75435205	75435202	AG	G	snp	0	0	1	0	.	.	.	RP11-803B1.2.1(INTRON)
3	75455616	75455629	75455625	TG	A	snp	1	1	1	1	.	.	.	INTERGENIC(INTERGENIC)
3	75567885	75567892	75567891	C	A	ins	1	1	1	0	.	.	.	CTD-2026G6.2.1(UPSTREAM);INTERGENIC(INTERGENIC)
3	75574584	75574601	75574588	CA	A	snp	1	1	0	1	.	.	.	CTD-2026G6.2.1(DOWNSTREAM);INTERGENIC(INTERGENIC)
3	75596631	75596638	75596632	A	G	snp	1	0	0	0	.	.	.	AC133041.1(UPSTREAM);CTD-2026G6.1.1(UPSTREAM);INTERGENIC(INTERGENIC)
3	75788827	75788835	75788834	T	C	snp	1	1	1	1	.	.	.	ZNF717(INTRON)
3	76072808	76072817	76072810	TA	C	snp	1	0	0	0	.	.	.	ROBO2(INTRON)
3	76128618	76128632	76128629	TTA	G	snp	1	1	1	1	.	.	.	ROBO2(INTRON)
3	76161353	76161360	76161359	T	C	snp	0	1	1	0	.	.	.	ROBO2(INTRON)
3	76190212	76190227	76190215	AT	C	snp	1	1	1	0	.	.	.	ROBO2(INTRON)
3	76321848	76321857	76321851	TA	G	snp	1	1	0	0	.	.	.	ROBO2(INTRON)
3	76322069	76322078	76322074	AT	A	snp	1	0	1	0	.	.	.	ROBO2(INTRON)
3	76353956	76353964	76353958	T	C	snp	1	0	0	0	.	.	.	ROBO2(INTRON)

3	76383299	76383310	76383306	AT	G	snp	1	1	1	1	.	.	.	ROBO2(INTRON)
3	76404196	76404211	76404199	TG	TA	ins	1	0	0	0	.	.	.	ROBO2(INTRON)
3	76664551	76664562	76664559	TAA	C	snp	1	1	1	1	.	.	.	ROBO2(INTRON)
3	76742061	76742070	76742067	GT	C	snp	1	0	0	0	.	.	.	ROBO2(INTRON)
3	76742416	76742425	76742423	TC	TA	ins	1	0	0	0	.	.	.	ROBO2(INTRON)
3	76782146	76782154	76782148	G	T	snp	1	1	1	1	.	.	.	ROBO2(INTRON)
3	76791936	76791945	76791942	CA	A	snp	1	1	1	1	.	.	.	ROBO2(INTRON)
3	76793505	76793519	76793515	CTT	TC	del	1	1	1	1	.	.	.	ROBO2(INTRON)
3	76829340	76829349	76829346	T	G	snp	0	1	1	0	.	.	.	ROBO2(INTRON)
3	76829359	76829368	76829361	T	G	ins	1	1	1	1	.	.	.	ROBO2(INTRON)
3	76829359	76829368	76829362	T	G	snp	1	1	1	1	.	.	.	ROBO2(INTRON)
3	76840603	76840616	76840606	TC	TCTTTCTCTG	ins	1	0	0	0	.	.	.	ROBO2(INTRON)
3	76878630	76878638	76878635	A	C	snp	1	0	0	0	.	.	.	ROBO2(INTRON)
3	76883323	76883340	76883326	TC	T	snp	1	1	1	1	.	.	.	ROBO2(INTRON)
3	76883323	76883340	76883330	TC	T	snp	0	0	1	1	.	.	.	ROBO2(INTRON)
3	76883323	76883340	76883334	TC	T	snp	0	0	1	1	.	.	.	ROBO2(INTRON)
3	76883323	76883340	76883338	TC	T	snp	0	0	1	0	.	.	.	ROBO2(INTRON)
3	76889773	76889784	76889776	TA	G	snp	1	0	1	0	.	.	.	ROBO2(INTRON)
3	76917318	76917325	76917321	T	C	snp	1	1	1	1	.	.	.	ROBO2(INTRON)
3	77000755	77000762	77000757	T	C	snp	1	0	0	0	.	.	.	ROBO2(INTRON)
3	77070351	77070358	77070355	A	G	snp	1	0	0	0	.	.	.	ROBO2(INTRON)
3	77070356	77070371	77070364	AAAG	G	snp	1	1	1	1	.	.	.	ROBO2(INTRON)
3	77099744	77099751	77099750	T	C	snp	1	0	0	0	.	.	.	ROBO2(INTRON)
3	77157569	77157576	77157571	C	A	snp	1	0	0	0	.	.	.	ROBO2(INTRON)
3	77172111	77172119	77172117	T	C	snp	1	0	0	0	.	.	.	ROBO2(INTRON)
3	77175849	77175864	77175862	AT	A	del	1	0	0	0	.	.	.	ROBO2(INTRON)
3	77260201	77260212	77260207	TG	C	snp	1	1	1	1	.	.	.	ROBO2(INTRON)
3	77276287	77276294	77276289	T	C	snp	1	0	0	0	.	.	.	ROBO2(INTRON)
3	77318268	77318276	77318275	T	C	ins	1	1	1	1	.	.	.	ROBO2(INTRON)
3	77337906	77337913	77337910	A	AAT	ins	1	0	0	0	.	.	.	ROBO2(INTRON)
3	77399236	77399245	77399238	AC	G	snp	1	1	1	1	.	.	.	ROBO2(INTRON)
3	77500868	77500875	77500874	T	G	snp	1	1	1	1	.	.	.	ROBO2(INTRON)
3	77618609	77618617	77618614	T	G	snp	1	0	0	0	.	.	.	ROBO2(INTRON)
3	77662104	77662112	77662105	A	G	snp	0	0	0	1	.	.	.	ROBO2(INTRON)
3	77676438	77676446	77676441	T	C	snp	1	0	0	0	.	.	.	ROBO2(INTRON);ROBO2(UPSTREAM)
3	77803480	77803489	77803485	TA	C	snp	1	0	0	0	.	.	.	INTERGENIC(INTERGENIC)
3	77970515	77970524	77970518	CT	C	snp	1	1	1	1	.	.	.	INTERGENIC(INTERGENIC)
3	78011938	78011945	78011943	T	C	snp	1	0	0	0	.	.	.	INTERGENIC(INTERGENIC)
3	78051870	78051890	78051882	TTG	C	snp	1	1	1	1	.	.	.	INTERGENIC(INTERGENIC)
3	78399655	78399663	78399659	T	G	snp	1	1	0	0	.	.	.	INTERGENIC(INTERGENIC)
3	78642324	78642343	78642339	TTTG	T	del	1	1	1	1	.	.	.	INTERGENIC(INTERGENIC);ROBO1(DOWNSTREAM)
3	78800446	78800469	78800466	TAA	G	snp	1	0	0	0	.	.	.	ROBO1(INTRON)
3	79001122	79001131	79001128	TA	A	snp	1	0	0	0	.	.	.	ROBO1(INTRON)
3	79020892	79020899	79020897	T	G	snp	1	1	0	1	.	.	.	ROBO1(INTRON)
3	79263866	79263877	79263869	TA	T	snp	1	0	0	0	.	.	.	ROBO1(INTRON)
3	79263866	79263877	79263871	TA	T	snp	1	0	0	0	.	.	.	ROBO1(INTRON)
3	79263962	79263977	79263975	AT	A	del	1	1	1	0	.	.	.	ROBO1(INTRON)
3	79404338	79404347	79404341	A	C	snp	1	1	0	1	.	.	.	ROBO1(INTRON)
3	79433161	79433168	79433163	T	G	snp	1	0	0	0	.	.	.	ROBO1(INTRON)
3	79635121	79635128	79635127	A	C	ins	1	1	1	1	.	.	.	AC117479.1(UPSTREAM);ROBO1(INTRON)
3	79662535	79662550	79662544	AGAA	T	snp	1	1	1	1	.	.	.	ROBO1(INTRON)
3	79680350	79680357	79680352	A	C	snp	1	1	1	1	.	.	.	ROBO1(INTRON)
3	79689476	79689485	79689478	CT	TCTCTCTTGCCTGCCACCAT	ins	1	1	1	1	.	.	.	ROBO1(INTRON)
3	79712450	79712457	79712452	A	C	snp	0	1	1	1	.	.	.	ROBO1(INTRON)
3	79727444	79727459	79727452	TA	A	snp	1	0	0	0	.	.	.	ROBO1(INTRON)
3	79768329	79768338	79768334	A	C	snp	1	1	1	1	.	.	.	ROBO1(INTRON)
3	79838889	79838897	79838893	T	C	snp	1	1	1	1	.	.	.	INTERGENIC(INTERGENIC)
3	79867688	79867695	79867694	T	G	snp	1	1	1	1	.	.	.	INTERGENIC(INTERGENIC)
3	79899125	79899136	79899134	TA	T	snp	1	1	1	1	.	.	.	INTERGENIC(INTERGENIC)
3	79940787	79940800	79940790	AC	T	snp	1	1	1	1	.	.	.	INTERGENIC(INTERGENIC)
3	79986570	79986577	79986571	T	G	snp	0	1	0	0	.	.	.	INTERGENIC(INTERGENIC)
3	79999634	79999643	79999638	TG	C	snp	1	0	0	0	.	.	.	INTERGENIC(INTERGENIC)
3	80062012	80062021	80062016	AT	G	snp	1	1	1	1	.	.	.	INTERGENIC(INTERGENIC)
3	80150859	80150886	80150873	TTAT	T	del	1	1	1	1	.	.	.	INTERGENIC(INTERGENIC)
3	80427547	80427554	80427549	A	C	snp	1	0	0	0	.	.	.	INTERGENIC(INTERGENIC)
3	80514829	80514840	80514833	TG	C	snp	1	1	1	1	.	.	.	INTERGENIC(INTERGENIC)
3	80651417	80651424	80651423	A	C	snp	1	1	1	1	.	.	.	INTERGENIC(INTERGENIC)

3	80656785	80656796	80656794	TG	A	snp	1	1	1	1	.	.	.	INTERGENIC(INTERGENIC)
3	80657510	80657525	80657521	AGGA	G	snp	1	0	0	0	.	.	.	INTERGENIC(INTERGENIC)
3	80701590	80701597	80701593	T	C	snp	1	1	1	1	.	.	.	INTERGENIC(INTERGENIC)
3	80705315	80705338	80705325	CTAA	C	snp	0	1	1	0	.	.	.	INTERGENIC(INTERGENIC)
3	80959442	80959450	80959447	A	G	snp	1	1	1	1	.	.	.	INTERGENIC(INTERGENIC)
3	81020502	81020510	81020504	A	C	snp	1	1	1	1	.	.	.	INTERGENIC(INTERGENIC)
3	81087177	81087184	81087181	T	C	snp	1	1	1	1	.	.	.	RP11-6B4.1.1(INTRON)
3	81158838	81158845	81158843	C	CG	ins	1	1	1	1	.	.	.	INTERGENIC(INTERGENIC)
3	81212017	81212036	81212030	CATA	G	snp	1	1	1	1	.	.	.	INTERGENIC(INTERGENIC)
3	81276629	81276636	81276633	T	C	snp	1	1	1	1	.	.	.	INTERGENIC(INTERGENIC)
3	81279195	81279203	81279198	T	C	ins	1	0	1	0	.	.	.	INTERGENIC(INTERGENIC)
3	81314815	81314832	81314819	AAC	C	del	0	0	1	1	.	.	.	RP11-520D19.2.1(INTRON)
3	81541861	81541884	81541868	TTCC	T	ins	1	1	1	1	.	.	.	GBE1(INTRON)
3	81707262	81707269	81707264	T	C	snp	1	1	1	1	.	.	.	GBE1(INTRON)
3	81713168	81713176	81713171	A	C	snp	0	0	0	1	.	.	.	GBE1(INTRON)
3	81734555	81734570	81734560	TGTT	A	snp	0	1	1	0	.	.	.	GBE1(INTRON)
3	81770356	81770383	81770363	AAAT	A	snp	1	1	1	1	.	.	.	GBE1(INTRON)
3	81822025	81822032	81822030	A	G	snp	1	0	0	0	.	.	.	INTERGENIC(INTERGENIC)
3	81921525	81921533	81921526	T	G	snp	1	1	1	1	.	.	.	RP11-359D24.1.1(INTRON)
3	81968593	81968612	81968601	ATCT	TCTG	ins	1	1	1	1	.	.	.	RP11-359D24.1.1(INTRON)
3	81980706	81980723	81980715	AC	T	snp	1	0	0	0	.	.	.	INTERGENIC(INTERGENIC)
3	82054864	82054871	82054868	T	G	snp	1	0	0	0	.	.	.	RP11-260O18.1.1(INTRON)
3	82094619	82094628	82094624	CA	G	snp	1	1	1	0	.	.	.	RP11-260O18.1.1(INTRON)
3	82345154	82345165	82345158	ATT	TA	del	1	0	1	0	.	.	.	RP11-260O18.1.1(INTRON)
3	82345154	82345165	82345161	ATT	TA	del	1	1	1	1	.	.	.	RP11-260O18.1.1(INTRON)
3	82471583	82471596	82471585	TA	C	snp	0	1	0	0	.	.	.	RP11-260O18.1.1(INTRON)
3	82513669	82513684	82513679	TA	A	snp	1	1	1	1	.	.	.	INTERGENIC(INTERGENIC);RP11-260O18.1.1(DOWNSTREAM)
3	82513741	82513750	82513745	TA	A	snp	1	1	1	1	.	.	.	INTERGENIC(INTERGENIC);RP11-260O18.1.1(DOWNSTREAM)
3	82513859	82513876	82513869	TA	A	snp	1	1	1	1	.	.	.	INTERGENIC(INTERGENIC);RP11-260O18.1.1(DOWNSTREAM)
3	82513902	82513917	82513912	AT	T	snp	1	1	1	1	.	.	.	INTERGENIC(INTERGENIC);RP11-260O18.1.1(DOWNSTREAM)
3	82514227	82514236	82514230	TA	G	snp	1	1	1	1	.	.	.	INTERGENIC(INTERGENIC);RP11-260O18.1.1(DOWNSTREAM)
3	82527439	82527448	82527440	A	G	snp	1	0	0	0	.	.	.	INTERGENIC(INTERGENIC)
3	82560873	82560882	82560880	AG	C	snp	1	1	1	1	.	.	.	INTERGENIC(INTERGENIC)
3	82645910	82645927	82645914	AAT	T	del	1	1	0	0	.	.	.	INTERGENIC(INTERGENIC)
3	82645910	82645927	82645915	AAT	A	snp	1	1	0	0	.	.	.	INTERGENIC(INTERGENIC)
3	82661892	82661899	82661893	T	C	snp	1	0	0	0	.	.	.	INTERGENIC(INTERGENIC)
3	82668531	82668539	82668536	T	C	snp	1	1	1	1	.	.	.	INTERGENIC(INTERGENIC)
3	82794486	82794495	82794492	A	C	snp	1	0	0	0	.	.	.	INTERGENIC(INTERGENIC)
3	82905860	82905867	82905862	A	G	snp	1	0	0	0	.	.	.	INTERGENIC(INTERGENIC)
3	82965599	82965610	82965604	TA	G	snp	1	1	1	1	.	.	.	INTERGENIC(INTERGENIC)
3	83121270	83121277	83121275	A	G	snp	1	1	1	1	.	.	.	INTERGENIC(INTERGENIC)
3	83125158	83125165	83125164	A	C	snp	1	1	1	1	.	.	.	INTERGENIC(INTERGENIC)
3	83125817	83125825	83125824	T	G	snp	1	0	0	0	.	.	.	INTERGENIC(INTERGENIC)
3	83133577	83133585	83133580	T	C	ins	0	0	1	0	.	.	.	INTERGENIC(INTERGENIC)
3	83263724	83263737	83263726	CA	T	snp	0	1	1	1	.	.	.	INTERGENIC(INTERGENIC)
3	83307363	83307372	83307370	AT	C	snp	1	1	1	1	.	.	.	INTERGENIC(INTERGENIC)
3	83482772	83482783	83482774	TA	C	snp	1	1	1	1	.	.	.	INTERGENIC(INTERGENIC)
3	83519258	83519273	83519271	AG	A	snp	1	1	1	1	.	.	.	INTERGENIC(INTERGENIC)
3	83542189	83542197	83542196	A	G	snp	1	0	0	0	.	.	.	INTERGENIC(INTERGENIC)
3	83544563	83544574	83544568	AT	C	snp	1	0	0	0	.	.	.	INTERGENIC(INTERGENIC)
3	83582171	83582198	83582176	TTTG	TG	del	1	0	0	0	.	.	.	INTERGENIC(INTERGENIC)
3	83582171	83582198	83582178	TTTG	TT	ins	1	0	0	0	.	.	.	INTERGENIC(INTERGENIC)
3	83629800	83629811	83629808	AT	T	del	1	0	0	0	.	.	.	INTERGENIC(INTERGENIC)
3	83652859	83652867	83652866	T	C	snp	1	1	1	1	.	.	.	INTERGENIC(INTERGENIC)
3	83749046	83749069	83749057	TAT	C	snp	0	1	0	1	.	.	.	INTERGENIC(INTERGENIC)
3	83867047	83867055	83867053	C	A	ins	1	1	1	1	.	.	.	INTERGENIC(INTERGENIC)
3	83877680	83877691	83877684	ATT	G	snp	1	1	1	1	.	.	.	INTERGENIC(INTERGENIC)
3	83982986	83982995	83982991	A	G	snp	0	1	1	1	.	.	.	INTERGENIC(INTERGENIC);SRRM1P2(UPSTREAM)
3	84026798	84026805	84026803	T	C	snp	1	1	0	1	.	.	.	INTERGENIC(INTERGENIC)
3	84067250	84067270	84067262	ATT	G	snp	1	0	0	0	.	.	.	INTERGENIC(INTERGENIC)
3	84068177	84068186	84068182	AT	C	snp	1	0	0	0	.	.	.	INTERGENIC(INTERGENIC)
3	84068975	84068984	84068978	TC	T	snp	1	1	1	1	.	.	.	INTERGENIC(INTERGENIC)

3	84161989	84161996	84161993	A	C	snp	1	0	0	0	.	.	.	INTERGENIC(INTERGENIC)
3	84211587	84211594	84211593	T	C	snp	1	1	0	1	.	.	.	INTERGENIC(INTERGENIC)
3	84223103	84223118	84223116	TG	C	snp	1	1	1	1	.	.	.	INTERGENIC(INTERGENIC)
3	84297225	84297239	84297229	AAT	T	del	1	1	1	1	.	.	.	INTERGENIC(INTERGENIC)
3	84332972	84332981	84332975	AT	A	ins	1	0	0	0	.	.	.	INTERGENIC(INTERGENIC)
3	84387025	84387032	84387030	T	C	snp	1	1	1	1	.	.	.	INTERGENIC(INTERGENIC)
3	84424652	84424659	84424653	T	C	snp	1	1	1	1	.	.	.	INTERGENIC(INTERGENIC)
3	84547801	84547810	84547803	GA	A	snp	1	0	0	0	.	.	.	INTERGENIC(INTERGENIC)
3	84566530	84566539	84566535	AT	C	snp	1	1	1	1	.	.	.	INTERGENIC(INTERGENIC)
3	84589069	84589082	84589072	TC	G	snp	1	1	1	1	.	.	.	INTERGENIC(INTERGENIC)
3	84631489	84631496	84631490	A	G	snp	1	1	1	1	.	.	.	INTERGENIC(INTERGENIC)
3	84658201	84658218	84658216	AT	A	del	1	1	1	1	.	.	.	INTERGENIC(INTERGENIC)
3	84667239	84667256	84667254	CA	C	del	1	0	1	0	.	.	.	INTERGENIC(INTERGENIC)
3	84698405	84698413	84698407	A	C	snp	1	1	0	1	.	.	.	RP11-735B13.1.1(INTRON)
3	84706444	84706453	84706446	T	C	ins	1	1	1	1	.	.	.	RP11-735B13.1.1(INTRON)
3	84714393	84714402	84714395	TA	C	snp	1	1	0	1	.	.	.	RP11-735B13.1.1(INTRON)
3	84728022	84728035	84728025	CA	G	snp	1	0	0	0	.	.	.	RP11-735B13.1.1(INTRON)
3	84875980	84875988	84875984	T	G	snp	1	1	1	1	.	.	.	RP11-735B13.1.1(INTRON)
3	84916594	84916609	84916603	AATT	T	snp	0	1	0	1	.	.	.	RP11-735B13.1.1(DOWNSTREAM);RP11-735B13.1.1(INTRON)
3	84920065	84920076	84920071	TC	C	snp	1	1	0	1	.	.	.	RP11-735B13.1.1(INTRON);RP11-735B13.1.1(UPSTREAM)
3	84922326	84922345	84922338	CTAT	T	del	0	1	0	1	.	.	.	RP11-735B13.1.1(INTRON);RP11-735B13.1.1(UPSTREAM)
3	84946602	84946611	84946607	T	G	ins	1	1	1	1	.	.	.	INTERGENIC(INTERGENIC);RP11-735B13.2.1(DOWNSTREAM)
3	84958021	84958035	84958031	GAA	G	snp	1	1	1	1	.	.	.	INTERGENIC(INTERGENIC)
3	85003607	85003614	85003612	T	G	snp	1	0	1	0	.	.	.	CADM2(UPSTREAM);INTERGENIC(INTERGENIC)
3	85049435	85049444	85049439	T	C	snp	1	0	0	0	.	.	.	CADM2(INTRON)
3	85123107	85123118	85123114	TAT	G	snp	1	0	0	0	.	.	.	CADM2(INTRON)
3	85324915	85324924	85324922	TA	G	snp	1	0	0	0	.	.	.	CADM2(INTRON)
3	85360515	85360535	85360532	TTA	T	ins	1	1	0	0	.	.	.	CADM2(INTRON)
3	85462312	85462335	85462316	AAT	T	del	1	1	1	1	.	.	.	CADM2(INTRON)
3	85481011	85481022	85481015	AT	G	snp	1	1	1	1	.	.	.	CADM2(INTRON)
3	85509430	85509438	85509431	A	G	ins	1	0	0	0	.	.	.	CADM2(INTRON)
3	85672015	85672022	85672018	T	G	snp	1	1	1	1	.	.	.	CADM2(INTRON)
3	85677827	85677840	85677837	AT	G	snp	1	1	1	1	.	.	.	CADM2(INTRON)
3	85678550	85678563	85678554	CA	G	snp	1	0	0	0	.	.	.	CADM2(INTRON)
3	85847259	85847278	85847263	GTTT	T	snp	1	0	0	0	.	.	.	CADM2(INTRON);CADM2-AS2(DOWNSTREAM)
3	85882928	85882939	85882936	ATC	T	snp	1	0	0	0	.	.	.	CADM2(INTRON)
3	85893039	85893048	85893045	TG	G	ins	1	1	1	1	.	.	.	CADM2(INTRON)
3	85893039	85893048	85893046	TG	T	del	1	1	1	1	.	.	.	CADM2(INTRON)
3	85948088	85948105	85948091	TA	G	snp	1	1	0	1	.	.	.	CADM2(INTRON)
3	85962166	85962177	85962170	TCA	T	snp	0	1	0	1	.	.	.	CADM2(INTRON)
3	86057276	86057284	86057280	T	G	snp	1	1	1	1	.	.	.	CADM2(INTRON);CADM2-AS1(INTRON);PRKRIRP2(UPSTREAM)
3	86111137	86111146	86111141	G	T	ins	1	1	1	1	.	.	.	CADM2(INTRON)
3	86119785	86119793	86119786	A	C	ins	1	1	1	0	.	.	.	CADM2(DOWNSTREAM);CADM2(UTR_3_PRIME)
3	86136778	86136789	86136781	CA	T	snp	0	1	0	1	.	.	.	INTERGENIC(INTERGENIC)
3	86136805	86136818	86136810	CA	T	snp	0	0	1	0	.	.	.	INTERGENIC(INTERGENIC)
3	86185001	86185009	86185002	T	C	snp	1	1	1	1	.	.	.	INTERGENIC(INTERGENIC)
3	86185868	86185877	86185874	GA	C	snp	1	0	0	0	.	.	.	INTERGENIC(INTERGENIC)
3	86217817	86217826	86217822	AG	C	snp	1	0	0	0	.	.	.	INTERGENIC(INTERGENIC)
3	86238351	86238360	86238353	AT	G	snp	1	1	1	1	.	.	.	INTERGENIC(INTERGENIC)
3	86245491	86245499	86245493	T	C	snp	0	0	1	0	.	.	.	INTERGENIC(INTERGENIC)
3	86251630	86251641	86251633	GTG	T	snp	1	1	1	1	.	.	.	INTERGENIC(INTERGENIC)
3	86353264	86353279	86353270	TTAT	G	snp	1	0	1	0	.	.	.	INTERGENIC(INTERGENIC)
3	86357097	86357104	86357099	T	C	snp	1	0	0	0	.	.	.	INTERGENIC(INTERGENIC)
3	86375498	86375506	86375503	A	G	snp	1	0	1	0	.	.	.	INTERGENIC(INTERGENIC)
3	86401157	86401165	86401158	T	TC	ins	1	0	1	0	.	.	.	INTERGENIC(INTERGENIC)
3	86401157	86401165	86401159	T	C	ins	1	0	1	0	.	.	.	INTERGENIC(INTERGENIC)
3	86434215	86434224	86434222	AT	A	del	1	0	0	1	.	.	.	INTERGENIC(INTERGENIC)
3	86545160	86545169	86545162	CA	A	snp	1	0	0	0	.	.	.	RP11-331K15.1.1(EXON)
3	86747554	86747562	86747558	G	GT	ins	0	1	0	1	.	.	.	INTERGENIC(INTERGENIC)
3	86846468	86846476	86846471	T	C	ins	1	1	1	0	.	.	.	INTERGENIC(INTERGENIC)
3	86875914	86875922	86875919	A	C	snp	1	1	1	0	.	.	.	INTERGENIC(INTERGENIC)
3	86880210	86880223	86880221	TA	T	snp	1	0	0	0	.	.	.	INTERGENIC(INTERGENIC)
3	86908742	86908751	86908747	AT	C	snp	0	0	1	0	.	.	.	INTERGENIC(INTERGENIC)
3	86909912	86909921	86909916	AT	G	snp	1	1	1	1	.	.	.	INTERGENIC(INTERGENIC)

3	86997102	86997111	86997106	T	C	snp	1	0	0	0	.	.	.	VGLL3(INTRON)
3	87204680	87204687	87204685	A	C	snp	0	1	1	0	.	.	.	INTERGENIC(INTERGENIC)
3	87210545	87210554	87210550	AC	ACAG	ins	1	1	1	1	.	.	.	INTERGENIC(INTERGENIC)
3	87233261	87233269	87233263	T	C	snp	1	0	0	0	.	.	.	INTERGENIC(INTERGENIC)
3	87461275	87461290	87461288	AT	A	del	1	1	1	1	.	.	.	INTERGENIC(INTERGENIC)
3	87521688	87521697	87521693	TA	T	snp	1	1	1	1	.	.	.	INTERGENIC(INTERGENIC)
3	87547029	87547038	87547034	CA	G	snp	1	1	1	1	.	.	.	INTERGENIC(INTERGENIC)
3	87552190	87552203	87552192	TG	A	snp	1	0	0	0	.	.	.	INTERGENIC(INTERGENIC)
3	87759549	87759556	87759551	A	G	snp	1	0	0	0	.	.	.	INTERGENIC(INTERGENIC)
3	87814943	87814950	87814945	A	G	snp	1	1	1	1	.	.	.	RP11-451B8.1.1(INTRON)
3	87817310	87817317	87817313	T	C	snp	1	0	0	0	.	.	.	RP11-451B8.1.1(INTRON)
3	87929781	87929790	87929788	TG	TATT	ins	1	0	0	0	.	.	.	INTERGENIC(INTERGENIC)
3	88038703	88038710	88038706	T	G	snp	0	0	1	0	.	.	.	HTR1F(UPSTREAM);INTERGENIC(INTERGENIC)
3	88169225	88169233	88169227	A	C	ins	1	1	1	1	.	.	.	CGGBP1(INTRON);RP11-159G9.5.1(INTRON)
3	88298204	88298212	88298205	T	G	snp	1	1	1	1	.	.	.	INTERGENIC(INTERGENIC)
3	88429351	88429358	88429354	A	G	snp	1	0	0	0	.	.	.	INTERGENIC(INTERGENIC)
3	88462760	88462769	88462763	TG	A	snp	0	1	0	0	.	.	.	INTERGENIC(INTERGENIC)
3	88478887	88478894	88478892	T	G	snp	1	1	1	1	.	.	.	INTERGENIC(INTERGENIC);Y_RNA(UPSTREAM)
3	88495265	88495272	88495268	T	C	snp	1	0	0	0	.	.	.	INTERGENIC(INTERGENIC)
3	88513459	88513470	88513466	ATA	AATA	del	1	1	1	1	.	.	.	INTERGENIC(INTERGENIC)
3	88562812	88562823	88562819	ATA	C	snp	0	1	0	1	.	.	.	INTERGENIC(INTERGENIC)
3	88570094	88570103	88570097	AT	C	snp	1	1	1	1	.	.	.	INTERGENIC(INTERGENIC)
3	88618201	88618210	88618209	A	C	snp	1	0	0	0	.	.	.	INTERGENIC(INTERGENIC)
3	88626874	88626883	88626877	TA	T	snp	1	1	1	1	.	.	.	INTERGENIC(INTERGENIC)
3	88645099	88645107	88645106	A	G	snp	0	1	0	0	.	.	.	INTERGENIC(INTERGENIC)
3	88679483	88679491	88679489	C	A	ins	1	0	0	0	.	.	.	INTERGENIC(INTERGENIC)
3	88679483	88679491	88679490	C	A	snp	1	1	1	1	.	.	.	INTERGENIC(INTERGENIC)
3	88752254	88752268	88752258	TCC	A	snp	0	1	1	1	.	.	.	INTERGENIC(INTERGENIC)
3	88820416	88820425	88820423	A	C	snp	1	1	1	1	.	.	.	INTERGENIC(INTERGENIC)
3	88927180	88927191	88927188	TC	A	snp	1	0	0	0	.	.	.	INTERGENIC(INTERGENIC)
3	89252501	89252510	89252505	A	C	snp	1	1	1	1	.	.	.	EPHA3(INTRON)
3	89408824	89408837	89408833	TG	A	snp	0	0	1	0	.	.	.	EPHA3(INTRON)
3	89521599	89521606	89521602	T	C	ins	1	1	1	1	.	.	.	EPHA3(INTRON)
3	89612923	89612943	89612938	CAA	A	snp	1	1	1	1	.	.	.	INTERGENIC(INTERGENIC)
3	89612923	89612943	89612940	CAA	C	del	1	1	1	1	.	.	.	INTERGENIC(INTERGENIC)
3	89757398	89757417	89757413	AAAC	A	ins	1	0	0	0	.	.	.	INTERGENIC(INTERGENIC)
3	89758187	89758198	89758196	AT	G	snp	1	0	0	0	.	.	.	INTERGENIC(INTERGENIC)
3	89766082	89766090	89766083	T	C	snp	1	0	0	0	.	.	.	INTERGENIC(INTERGENIC)
3	89825421	89825438	89825433	TCT	C	snp	1	1	1	1	.	.	.	INTERGENIC(INTERGENIC)
3	93612365	93612372	93612369	T	C	snp	1	0	0	0	.	.	.	PROS1(INTRON)
3	93964083	93964094	93964091	GTT	C	snp	1	1	1	1	.	.	.	INTERGENIC(INTERGENIC)
3	93972619	93972628	93972620	A	C	ins	1	0	0	0	.	.	.	INTERGENIC(INTERGENIC)
3	94127950	94127957	94127953	T	G	snp	1	0	0	0	.	.	.	INTERGENIC(INTERGENIC)
3	94203187	94203196	94203195	T	C	snp	1	0	0	0	.	.	.	INTERGENIC(INTERGENIC)
3	94236032	94236047	94236041	CAAA	AAC	del	1	1	1	1	.	.	.	INTERGENIC(INTERGENIC)
3	94246316	94246327	94246319	TTG	C	snp	1	1	0	1	.	.	.	INTERGENIC(INTERGENIC)
3	94273485	94273494	94273489	TA	ATATA	del	1	1	1	1	.	.	.	INTERGENIC(INTERGENIC)
3	94459680	94459689	94459687	TG	T	snp	1	0	0	0	.	.	.	INTERGENIC(INTERGENIC)
3	94472072	94472080	94472079	T	G	ins	1	1	1	1	.	.	.	INTERGENIC(INTERGENIC)
3	94478169	94478180	94478176	TAA	G	snp	1	1	1	1	.	.	.	INTERGENIC(INTERGENIC)
3	94581584	94581598	94581590	TAT	C	snp	1	1	1	1	.	.	.	INTERGENIC(INTERGENIC)
3	94598958	94598969	94598966	ATT	C	snp	1	0	0	0	.	.	.	INTERGENIC(INTERGENIC)
3	94811431	94811440	94811433	AG	G	snp	1	0	0	0	.	.	.	RP11-118N24.3.1(INTRON)
3	94826266	94826273	94826270	A	G	snp	1	1	1	1	.	.	.	RP11-118N24.3.1(INTRON)
3	94918828	94918839	94918834	TTA	A	snp	1	1	1	1	.	.	.	INTERGENIC(INTERGENIC)
3	95035905	95035914	95035912	GA	T	snp	1	1	1	1	.	.	.	INTERGENIC(INTERGENIC)
3	95208045	95208056	95208049	AAT	G	snp	1	0	0	0	.	.	.	INTERGENIC(INTERGENIC)
3	95212201	95212212	95212210	AG	C	snp	1	0	0	0	.	.	.	INTERGENIC(INTERGENIC)
3	95227577	95227596	95227581	AAAT	C	snp	1	0	0	0	.	.	.	INTERGENIC(INTERGENIC)
3	95252780	95252797	95252786	TTG	C	snp	0	0	1	0	.	.	.	INTERGENIC(INTERGENIC)
3	95371346	95371353	95371348	T	C	snp	0	0	1	0	.	.	.	INTERGENIC(INTERGENIC);RP11-219E24.1.1(DOWNSTREAM)
3	95376152	95376166	95376156	TTA	C	snp	0	1	1	1	.	.	.	RP11-219E24.1.1(INTRON);RP11-219E24.1.1(UPSTREAM)
3	95397858	95397867	95397861	AG	A	snp	1	1	1	1	.	.	.	RP11-219E24.1.1(INTRON)
3	95448240	95448251	95448245	AT	AG	ins	1	0	0	0	.	.	.	INTERGENIC(INTERGENIC)
3	95478044	95478051	95478045	G	T	snp	1	0	0	0	.	.	.	INTERGENIC(INTERGENIC)
3	95503160	95503171	95503165	AT	C	snp	1	1	1	1	.	.	.	INTERGENIC(INTERGENIC)

3	95503160	95503171	95503166	AT	G	snp	1	1	1	1	.	.	.	INTERGENIC(INTERGENIC)
3	95503185	95503196	95503190	AT	C	snp	1	1	1	1	.	.	.	INTERGENIC(INTERGENIC)
3	95503185	95503196	95503191	AT	G	snp	1	1	1	1	.	.	.	INTERGENIC(INTERGENIC)
3	95632812	95632820	95632815	T	C	snp	1	0	0	0	.	.	.	INTERGENIC(INTERGENIC)
3	95676433	95676440	95676436	A	G	snp	1	1	1	1	.	.	.	INTERGENIC(INTERGENIC)
3	95676518	95676529	95676525	ATA	C	snp	1	1	1	1	.	.	.	INTERGENIC(INTERGENIC)
3	95691001	95691010	95691005	TA	C	snp	1	1	1	1	.	.	.	INTERGENIC(INTERGENIC)
3	95691001	95691010	95691007	TA	C	snp	1	1	1	1	.	.	.	INTERGENIC(INTERGENIC)
3	95696814	95696821	95696816	G	A	snp	1	0	0	0	.	.	.	INTERGENIC(INTERGENIC)
3	95842936	95842951	95842947	AAAC	A	del	1	0	0	0	.	.	.	INTERGENIC(INTERGENIC)
3	95958473	95958484	95958482	TA	G	snp	1	1	1	1	.	.	.	INTERGENIC(INTERGENIC)
3	96198354	96198362	96198359	A	G	snp	1	0	0	0	.	.	.	INTERGENIC(INTERGENIC)
3	96232417	96232426	96232419	A	C	snp	1	0	0	0	.	.	.	INTERGENIC(INTERGENIC)
3	96232417	96232426	96232422	A	C	snp	1	0	0	0	.	.	.	INTERGENIC(INTERGENIC)
3	96300657	96300672	96300663	AAAT	G	snp	1	0	0	0	.	.	.	INTERGENIC(INTERGENIC)
3	96305070	96305081	96305075	CA	T	snp	1	1	1	1	.	.	.	INTERGENIC(INTERGENIC)
3	96330020	96330039	96330035	GTTT	G	del	1	1	1	1	.	.	.	INTERGENIC(INTERGENIC);U6(UPSTREAM)
3	96460017	96460032	96460025	TA	ATAC	ins	1	1	1	0	.	.	.	INTERGENIC(INTERGENIC)
3	96729499	96729518	96729505	TTTA	C	snp	1	1	0	1	.	.	.	EPHA6(DOWNSTREAM);EPHA6(INTRON)
3	96777132	96777141	96777139	T	G	snp	1	0	0	0	.	.	.	EPHA6(INTRON)
3	96777132	96777141	96777139	T	TTG	ins	1	0	0	0	.	.	.	EPHA6(INTRON)
3	96796237	96796252	96796241	TA	C	snp	1	0	0	0	.	.	.	EPHA6(INTRON)
3	96877121	96877130	96877126	AC	T	snp	1	0	0	0	.	.	.	EPHA6(INTRON)
3	96917719	96917728	96917724	TA	G	snp	1	0	0	0	.	.	.	EPHA6(INTRON)
3	97212049	97212062	97212060	TG	A	snp	1	0	0	0	.	.	.	EPHA6(INTRON)
3	97215650	97215659	97215657	CA	C	del	1	1	0	0	.	.	.	EPHA6(INTRON)
3	97215659	97215668	97215660	A	C	snp	0	1	1	0	.	.	.	EPHA6(INTRON)
3	97341800	97341808	97341806	T	G	ins	1	0	1	0	.	.	.	EPHA6(INTRON)
3	97356225	97356233	97356230	T	C	snp	0	1	0	1	.	.	.	EPHA6(INTRON)
3	97390128	97390139	97390131	CT	A	snp	1	0	0	0	.	.	.	EPHA6(INTRON)
3	97623545	97623553	97623552	T	G	snp	0	0	1	0	.	.	.	CRYBG3(INTRON)
3	97642418	97642426	97642422	A	G	snp	1	1	1	1	.	.	.	CRYBG3(INTRON);CRYBG3(UPSTREAM)
3	97700208	97700221	97700219	AT	A	del	0	1	1	1	.	.	.	INTERGENIC(INTERGENIC)
3	97793238	97793249	97793246	AT	T	snp	1	1	1	0	.	.	.	INTERGENIC(INTERGENIC)
3	97800985	97800992	97800987	A	G	snp	1	1	1	1	.	.	.	INTERGENIC(INTERGENIC)
3	97908791	97908798	97908796	T	TG	ins	1	1	1	1	.	.	.	INTERGENIC(INTERGENIC)
3	97925901	97925909	97925905	T	G	snp	1	0	0	0	.	.	.	INTERGENIC(INTERGENIC);OR5H3P(UPSTREAM)
3	97950094	97950101	97950096	A	AAAAAAAC	ins	1	0	0	0	.	.	.	INTERGENIC(INTERGENIC);RP11-325B23.2.1(UPSTREAM)
3	97967334	97967341	97967336	T	C	snp	1	1	1	1	.	.	.	RP11-325B23.2.1(INTRON)
3	97970581	97970590	97970584	TC	G	snp	0	1	1	1	.	.	.	RP11-325B23.2.1(INTRON)
3	98155124	98155133	98155126	TA	A	del	1	1	1	1	.	.	.	RP11-325B23.2.1(INTRON);UBFD1P1(DOWNSTREAM)
3	98163906	98163925	98163916	AAAC	C	del	1	0	0	0	.	.	.	RP11-325B23.2.1(INTRON)
3	98163926	98163933	98163929	A	G	snp	1	0	0	0	.	.	.	RP11-325B23.2.1(INTRON)
3	98354186	98354193	98354191	C	A	snp	1	1	1	1	.	.	.	INTERGENIC(INTERGENIC)
3	98383352	98383360	98383359	T	TC	ins	1	1	0	0	.	.	.	INTERGENIC(INTERGENIC);RP11-569H14.1.1(DOWNSTREAM)
3	98386354	98386362	98386359	A	C	snp	1	1	0	0	.	.	.	INTERGENIC(INTERGENIC)
3	98395213	98395220	98395217	T	C	snp	1	1	0	0	.	.	.	INTERGENIC(INTERGENIC)
3	98462746	98462754	98462751	C	A	snp	1	0	0	0	.	.	.	ST3GAL6(INTRON)
3	98503990	98503999	98503993	T	G	snp	1	1	1	1	.	.	.	RP11-319J24.3.1(DOWNSTREAM);ST3GAL6(DOWNSTREAM);ST3GAL6(INTRON)
3	98515112	98515120	98515116	A	C	snp	1	0	0	0	.	.	.	DCBLD2(DOWNSTREAM);DCBLD2(UTR_3_PRIME);ST3GAL6(DOWNSTREAM);ST3GAL6(INTRON)
3	98601264	98601273	98601270	T	C	snp	1	0	0	1	.	.	.	DCBLD2(INTRON)
3	98713475	98713484	98713480	TC	T	snp	1	1	1	1	.	.	.	CTD-2021J15.1.1(INTRON)
3	98777241	98777260	98777256	TCCT	A	snp	1	0	0	0	.	.	.	INTERGENIC(INTERGENIC)
3	98777241	98777260	98777256	TCCT	TCCA	ins	1	0	0	0	.	.	.	INTERGENIC(INTERGENIC)
3	98777339	98777348	98777340	T	G	ins	0	0	0	1	.	.	.	INTERGENIC(INTERGENIC)
3	98953577	98953584	98953583	T	G	snp	1	1	1	1	.	.	.	INTERGENIC(INTERGENIC)
3	98954631	98954642	98954640	TG	T	snp	1	0	0	0	.	.	.	INTERGENIC(INTERGENIC)
3	98979342	98979351	98979345	AT	C	snp	1	1	1	1	.	.	.	INTERGENIC(INTERGENIC)
3	99047294	99047305	99047299	TA	TG	ins	1	1	1	1	.	.	.	INTERGENIC(INTERGENIC);RP11-121C1.1.1(UPSTREAM)
3	99101601	99101610	99101608	A	C	snp	1	0	1	0	.	.	.	INTERGENIC(INTERGENIC)
3	99171246	99171253	99171248	A	C	snp	1	1	1	1	.	.	.	INTERGENIC(INTERGENIC)
3	99332641	99332650	99332649	C	A	snp	1	0	1	1	.	.	.	RP11-201E8.1.1(INTRON)
3	99574226	99574235	99574232	AT	G	snp	0	1	0	1	.	.	.	C3orf26(INTRON);FILIP1L(INTRON);FILIP1L(UPSTREAM)

3	99706081	99706090	99706082	A	G	snp	0	0	1	0	.	.	.	C3orf26(INTRON);FILIP1L(INTRON)
3	99754119	99754133	99754122	TAT	G	snp	1	0	0	0	.	.	.	C3orf26(INTRON);FILIP1L(INTRON)
3	99919667	99919675	99919668	A	C	snp	0	1	0	1	.	.	.	INTERGENIC(INTERGENIC)
3	100013094	100013102	100013101	T	G	snp	1	1	1	1	.	.	.	TBC1D23(DOWNSTREAM);TBC1D23(INTRON)
3	100389408	100389415	100389411	T	C	snp	1	1	1	1	.	.	.	GPR128(INTRON)
3	100516761	100516768	100516767	T	C	snp	1	1	1	1	.	.	.	ABI3BP(INTRON)
3	100531732	100531740	100531736	T	C	snp	0	1	1	1	.	.	.	ABI3BP(DOWNSTREAM);ABI3BP(INTRON);ABI3BP(UPSTREAM)
3	100551610	100551617	100551612	A	G	ins	1	1	1	1	.	.	.	ABI3BP(INTRON)
3	100567070	100567077	100567076	A	C	ins	0	0	0	1	.	.	.	ABI3BP(INTRON);ABI3BP(UPSTREAM)
3	100628414	100628423	100628418	A	G	snp	1	1	1	1	.	.	.	ABI3BP(INTRON);RP11-301G23.1.1(DOWNSTREAM)
3	100650461	100650469	100650462	A	C	snp	1	1	1	1	.	.	.	ABI3BP(INTRON);ABI3BP(UPSTREAM)
3	100869146	100869155	100869149	A	C	ins	0	0	1	0	.	.	.	INTERGENIC(INTERGENIC)
3	100977408	100977421	100977410	CT	T	snp	1	1	1	1	.	.	.	IMPG2(INTRON)
3	101083073	101083100	101083090	AAAG	AG	ins	1	0	0	0	.	.	.	SENP7(INTRON)
3	101164536	101164545	101164542	AT	G	snp	1	1	0	1	.	.	.	SENP7(INTRON)
3	101219389	101219397	101219392	A	C	snp	1	1	1	1	.	.	.	SENP7(INTRON);snoU13(UPSTREAM)
3	101230656	101230665	101230663	T	G	snp	1	1	1	1	.	.	.	SENP7(INTRON)
3	101325580	101325587	101325586	T	G	snp	1	1	1	1	.	.	.	INTERGENIC(INTERGENIC)
3	101345959	101345966	101345965	A	G	snp	1	1	1	1	.	.	.	INTERGENIC(INTERGENIC);Y_RNA(DOWNSTREAM)
3	101346214	101346223	101346219	A	G	snp	0	1	0	1	.	.	.	INTERGENIC(INTERGENIC);Y_RNA(DOWNSTREAM)
3	101425702	101425710	101425703	A	G	snp	1	1	1	1	.	.	.	INTERGENIC(INTERGENIC)
3	101475600	101475607	101475604	A	G	snp	1	0	0	0	.	.	.	CEP97(DOWNSTREAM);CEP97(INTRON)
3	101641719	101641727	101641723	T	G	snp	1	0	0	0	.	.	.	INTERGENIC(INTERGENIC)
3	101733383	101733390	101733389	T	C	snp	1	1	1	1	.	.	.	INTERGENIC(INTERGENIC)
3	101757901	101757928	101757922	TTCC	C	snp	1	1	1	1	.	.	.	INTERGENIC(INTERGENIC)
3	101767063	101767082	101767075	ATTT	G	snp	1	1	1	1	.	.	.	INTERGENIC(INTERGENIC)
3	101839411	101839420	101839415	T	G	ins	1	1	1	1	.	.	.	ZPLD1(INTRON)
3	101921241	101921248	101921246	T	C	snp	1	0	0	0	.	.	.	ZPLD1(INTRON)
3	101924272	101924285	101924277	TC	G	snp	1	0	0	0	.	.	.	ZPLD1(INTRON)
3	102006477	102006484	102006481	T	G	snp	1	1	1	1	.	.	.	ZPLD1(INTRON)
3	102223574	102223585	102223579	AAC	T	snp	1	0	0	0	.	.	.	INTERGENIC(INTERGENIC)
3	102293825	102293833	102293826	T	C	snp	1	0	0	0	.	.	.	INTERGENIC(INTERGENIC)
3	102416639	102416654	102416650	AAAT	A	snp	1	1	1	1	.	.	.	INTERGENIC(INTERGENIC)
3	102430749	102430763	102430758	GTG	T	snp	1	0	0	0	.	.	.	INTERGENIC(INTERGENIC)
3	102462395	102462406	102462402	AAT	G	snp	1	0	0	0	.	.	.	INTERGENIC(INTERGENIC)
3	102576781	102576798	102576786	CTC	A	snp	1	1	1	1	.	.	.	INTERGENIC(INTERGENIC)
3	102576781	102576798	102576787	CTC	T	snp	1	1	1	1	.	.	.	INTERGENIC(INTERGENIC)
3	102642987	102642995	102642988	T	G	ins	1	0	0	0	.	.	.	INTERGENIC(INTERGENIC)
3	102672004	102672013	102672008	TA	C	snp	1	0	0	0	.	.	.	INTERGENIC(INTERGENIC)
3	102709584	102709593	102709590	AT	G	snp	1	1	1	1	.	.	.	INTERGENIC(INTERGENIC)
3	102732658	102732675	102732669	AC	AG	ins	1	1	1	1	.	.	.	INTERGENIC(INTERGENIC)
3	102773786	102773795	102773790	T	C	snp	1	0	0	0	.	.	.	INTERGENIC(INTERGENIC)
3	102813048	102813059	102813054	TA	AG	ins	1	1	1	1	.	.	.	INTERGENIC(INTERGENIC)
3	102826165	102826176	102826173	TTG	TTGT	del	1	1	0	0	.	.	.	INTERGENIC(INTERGENIC)
3	102999119	102999133	102999122	TGT	G	snp	0	0	0	1	.	.	.	INTERGENIC(INTERGENIC)
3	103131868	103131875	103131871	T	G	snp	1	0	0	0	.	.	.	INTERGENIC(INTERGENIC)
3	103177712	103177719	103177715	T	C	snp	1	0	0	0	.	.	.	INTERGENIC(INTERGENIC)
3	103196378	103196387	103196383	A	C	snp	1	1	1	1	.	.	.	INTERGENIC(INTERGENIC)
3	103347012	103347035	103347017	TTTG	C	snp	1	1	1	1	.	.	.	INTERGENIC(INTERGENIC);RP11-628J14.2.1(UPSTREAM)
3	103420637	103420660	103420647	ATCT	T	del	0	0	1	0	.	.	.	INTERGENIC(INTERGENIC)
3	103580092	103580099	103580098	T	G	snp	1	1	1	1	.	.	.	INTERGENIC(INTERGENIC)
3	103661021	103661038	103661034	TC	TG	ins	1	1	1	1	.	.	.	INTERGENIC(INTERGENIC)
3	103661021	103661038	103661036	TC	G	snp	1	1	1	1	.	.	.	INTERGENIC(INTERGENIC)
3	103754550	103754559	103754555	CA	T	snp	1	0	0	0	.	.	.	INTERGENIC(INTERGENIC)
3	103804917	103804924	103804920	T	C	ins	1	0	0	0	.	.	.	INTERGENIC(INTERGENIC)
3	103824433	103824442	103824436	TA	C	snp	1	0	0	1	.	.	.	INTERGENIC(INTERGENIC)
3	103873056	103873065	103873061	T	G	ins	1	1	1	1	.	.	.	INTERGENIC(INTERGENIC)
3	103900987	103900996	103900994	AT	C	snp	0	1	0	0	.	.	.	INTERGENIC(INTERGENIC)
3	103928237	103928244	103928242	T	C	snp	1	1	1	1	.	.	.	INTERGENIC(INTERGENIC)
3	103932787	103932795	103932791	A	G	snp	0	1	1	1	.	.	.	INTERGENIC(INTERGENIC)
3	103964315	103964324	103964322	AG	T	snp	1	0	0	0	.	.	.	INTERGENIC(INTERGENIC)
3	103979028	103979039	103979035	AT	C	snp	1	1	1	1	.	.	.	INTERGENIC(INTERGENIC)
3	104160827	104160838	104160833	AGC	G	snp	1	0	0	1	.	.	.	INTERGENIC(INTERGENIC)
3	104222636	104222645	104222642	CA	T	snp	1	0	0	0	.	.	.	INTERGENIC(INTERGENIC);RP11-281P11.1.1(DOWNSTREAM)
3	104239522	104239529	104239527	A	C	snp	1	1	1	1	.	.	.	INTERGENIC(INTERGENIC)

3	104342391	104342399	104342398	G	A	snp	1	1	0	1	.	.	.	INTERGENIC(INTERGENIC)
3	104364921	104364928	104364927	T	G	snp	1	0	0	0	.	.	.	INTERGENIC(INTERGENIC)
3	104423019	104423027	104423026	A	AC	ins	1	1	1	1	.	.	.	INTERGENIC(INTERGENIC)
3	104434197	104434204	104434198	A	G	snp	1	1	1	1	.	.	.	INTERGENIC(INTERGENIC)
3	104507975	104507990	104507986	AGAA	G	snp	1	0	0	0	.	.	.	INTERGENIC(INTERGENIC)
3	104519835	104519842	104519838	T	G	snp	1	0	0	0	.	.	.	INTERGENIC(INTERGENIC)
3	104561863	104561872	104561871	T	C	snp	1	1	1	1	.	.	.	INTERGENIC(INTERGENIC)
3	104576845	104576852	104576848	A	G	snp	1	0	0	0	.	.	.	INTERGENIC(INTERGENIC)
3	104576845	104576852	104576849	A	G	snp	1	0	0	0	.	.	.	INTERGENIC(INTERGENIC)
3	104584118	104584126	104584119	T	G	snp	1	1	0	1	.	.	.	INTERGENIC(INTERGENIC)
3	104584118	104584126	104584122	T	C	snp	1	0	0	0	.	.	.	INTERGENIC(INTERGENIC)
3	104669754	104669777	104669766	TAAA	C	snp	1	1	1	1	.	.	.	INTERGENIC(INTERGENIC)
3	104682834	104682849	104682838	AT	G	snp	1	0	0	0	.	.	.	INTERGENIC(INTERGENIC)
3	104770390	104770399	104770396	T	C	snp	0	1	0	1	.	.	.	INTERGENIC(INTERGENIC)
3	104810442	104810450	104810448	A	G	snp	1	1	1	1	.	.	.	INTERGENIC(INTERGENIC)
3	104823530	104823541	104823538	ATT	G	snp	1	0	0	0	.	.	.	INTERGENIC(INTERGENIC)
3	104849851	104849860	104849856	T	G	snp	1	1	0	1	.	.	.	INTERGENIC(INTERGENIC)
3	104855953	104855961	104855958	A	C	snp	1	0	0	0	.	.	.	INTERGENIC(INTERGENIC)
3	104940370	104940378	104940373	A	AC	ins	0	1	1	1	.	.	.	INTERGENIC(INTERGENIC)
3	104991927	104991942	104991938	TG	T	snp	0	1	1	1	.	.	.	INTERGENIC(INTERGENIC)
3	105071125	105071136	105071129	TA	C	snp	1	1	1	1	.	.	.	INTERGENIC(INTERGENIC)
3	105154644	105154652	105154647	T	G	snp	1	0	0	0	.	.	.	ALCAM(INTRON);ALCAM(UPSTREAM)
3	105305455	105305469	105305464	GTT	TTGT	del	1	0	0	0	.	.	.	INTERGENIC(INTERGENIC)
3	105356690	105356697	105356691	T	C	snp	1	0	0	0	.	.	.	INTERGENIC(INTERGENIC)
3	105375130	105375138	105375132	A	G	snp	1	1	1	1	.	.	.	CBLB(DOWNSTREAM);CBLB(UTR_3_PRIME)
3	105559819	105559826	105559821	T	C	snp	1	1	1	1	.	.	.	CBLB(INTRON)
3	105627467	105627475	105627474	T	G	snp	1	1	1	1	.	.	.	INTERGENIC(INTERGENIC)
3	105691765	105691782	105691780	AT	A	del	1	1	1	1	.	.	.	INTERGENIC(INTERGENIC)
3	105733865	105733876	105733868	TG	A	snp	1	1	1	0	.	.	.	INTERGENIC(INTERGENIC)
3	105737820	105737837	105737835	TC	T	snp	0	1	0	1	.	.	.	INTERGENIC(INTERGENIC)
3	105741298	105741307	105741300	T	G	snp	1	0	0	0	.	.	.	INTERGENIC(INTERGENIC)
3	105757115	105757123	105757118	T	G	snp	1	1	1	0	.	.	.	INTERGENIC(INTERGENIC)
3	105886633	105886642	105886635	A	C	ins	0	0	1	0	.	.	.	INTERGENIC(INTERGENIC)
3	105886633	105886642	105886636	A	C	snp	0	0	1	0	.	.	.	INTERGENIC(INTERGENIC)
3	105939720	105939729	105939727	TG	TGTC	ins	0	1	1	1	.	.	.	INTERGENIC(INTERGENIC)
3	105947567	105947576	105947572	TA	G	snp	1	1	1	1	.	.	.	INTERGENIC(INTERGENIC)
3	105959933	105959941	105959940	A	G	snp	1	0	0	0	.	.	.	INTERGENIC(INTERGENIC)
3	105970742	105970757	105970752	AAGA	T	snp	1	1	1	1	.	.	.	INTERGENIC(INTERGENIC)
3	105972292	105972299	105972298	A	G	snp	0	1	1	0	.	.	.	INTERGENIC(INTERGENIC)
3	106020240	106020253	106020251	TC	T	del	0	1	0	1	.	.	.	INTERGENIC(INTERGENIC)
3	106102027	106102036	106102028	T	TTG	ins	1	1	1	1	.	.	.	INTERGENIC(INTERGENIC)
3	106102027	106102036	106102030	T	G	ins	1	1	1	1	.	.	.	INTERGENIC(INTERGENIC)
3	106174274	106174282	106174275	A	C	snp	1	1	1	1	.	.	.	INTERGENIC(INTERGENIC)
3	106232848	106232855	106232849	A	C	snp	1	1	1	1	.	.	.	INTERGENIC(INTERGENIC);Y_RNA(UPSTREAM)
3	106262168	106262177	106262171	A	C	snp	1	0	0	0	.	.	.	INTERGENIC(INTERGENIC)
3	106299575	106299602	106299592	TATT	TTT	ins	1	0	1	0	.	.	.	INTERGENIC(INTERGENIC)
3	106299575	106299602	106299595	TATT	A	del	1	0	1	0	.	.	.	INTERGENIC(INTERGENIC)
3	106351311	106351322	106351314	TC	T	snp	0	0	1	0	.	.	.	INTERGENIC(INTERGENIC)
3	106352695	106352708	106352697	AT	G	snp	0	1	1	1	.	.	.	INTERGENIC(INTERGENIC)
3	106384001	106384009	106384006	T	C	snp	1	1	1	1	.	.	.	INTERGENIC(INTERGENIC)
3	106898428	106898448	106898443	TAA	C	snp	1	0	0	0	.	.	.	RP11-280F2.2.1(INTRON)
3	107062573	107062582	107062578	A	G	snp	0	0	0	1	.	.	.	INTERGENIC(INTERGENIC)
3	107074190	107074198	107074197	T	G	snp	1	1	1	1	.	.	.	INTERGENIC(INTERGENIC)
3	107105231	107105238	107105237	A	C	snp	1	1	1	1	.	.	.	INTERGENIC(INTERGENIC)
3	107123125	107123134	107123127	GT	T	ins	1	0	0	0	.	.	.	INTERGENIC(INTERGENIC)
3	107125143	107125154	107125151	AT	G	snp	1	1	1	1	.	.	.	INTERGENIC(INTERGENIC)
3	107152359	107152367	107152361	C	A	ins	1	1	1	1	.	.	.	INTERGENIC(INTERGENIC)
3	107152359	107152367	107152363	C	A	snp	1	1	0	1	.	.	.	INTERGENIC(INTERGENIC)
3	107235701	107235712	107235710	AT	C	snp	1	1	1	1	.	.	.	INTERGENIC(INTERGENIC)
3	107382649	107382656	107382655	T	C	snp	0	1	1	1	.	.	.	BBX(INTRON)
3	107501678	107501686	107501680	T	G	snp	0	1	0	1	.	.	.	BBX(INTRON)
3	107681569	107681578	107681573	TG	C	snp	1	0	0	0	.	.	.	INTERGENIC(INTERGENIC)
3	107703733	107703742	107703736	T	G	ins	1	0	1	0	.	.	.	INTERGENIC(INTERGENIC)
3	107703997	107704008	107704000	TA	TATG	ins	1	1	1	1	.	.	.	INTERGENIC(INTERGENIC)
3	107703997	107704008	107704002	TA	TG	ins	1	1	1	1	.	.	.	INTERGENIC(INTERGENIC)
3	107780735	107780742	107780738	T	C	snp	1	1	1	1	.	.	.	CD47(INTRON)
3	107856255	107856264	107856258	G	A	snp	1	1	1	1	.	.	.	INTERGENIC(INTERGENIC)

3	107972006	107972015	107972007	T	G	ins	1	1	1	1	.	.	.	INTERGENIC(INTERGENIC)
3	108116833	108116844	108116835	CA	T	snp	1	1	1	1	.	.	.	MYH15(INTRON)
3	108189873	108189888	108189884	AAAG	AA	ins	1	1	1	1	.	.	.	MYH15(INTRON)
3	108192529	108192540	108192532	ATT	G	snp	1	1	0	1	.	.	.	MYH15(INTRON)
3	108250624	108250651	108250646	TAAA	ATAC	ins	1	1	0	1	.	.	.	INTERGENIC(INTERGENIC);MYH15(UPSTREAM)
3	108288016	108288029	108288026	TA	C	snp	1	1	1	1	.	.	.	KIAA1524(INTRON)
3	108359797	108359806	108359799	T	C	snp	0	0	1	0	.	.	.	DZIP3(INTRON)
3	108587983	108588003	108588000	AAC	AACA	del	1	0	0	0	.	.	.	INTERGENIC(INTERGENIC)
3	108629507	108629514	108629509	T	C	snp	1	0	0	0	.	.	.	GUCA1C(INTRON)
3	108638336	108638343	108638342	T	G	snp	1	1	1	1	.	.	.	GUCA1C(INTRON)
3	108696735	108696742	108696736	T	G	snp	1	1	0	1	.	.	.	MORC1(INTRON)
3	108698024	108698031	108698028	A	C	snp	1	0	0	0	.	.	.	MORC1(INTRON)
3	108705693	108705704	108705702	AT	C	snp	0	1	0	1	.	.	.	MORC1(INTRON)
3	108747082	108747089	108747087	C	A	ins	1	1	0	1	.	.	.	MORC1(INTRON)
3	108910198	108910207	108910201	TG	T	snp	1	0	0	0	.	.	.	INTERGENIC(INTERGENIC)
3	108963148	108963157	108963150	TA	C	snp	0	0	0	1	.	.	.	INTERGENIC(INTERGENIC);RP11-59E19.3.1(DOWNSTREAM)
3	109120433	109120441	109120439	G	A	ins	1	0	1	0	.	.	.	INTERGENIC(INTERGENIC);RP11-702L6.1.1(DOWNSTREAM)
3	109152579	109152592	109152589	TG	G	snp	1	0	0	0	.	.	.	RP11-702L6.4.1(INTRON)
3	109251859	109251870	109251866	CCA	T	snp	1	0	0	0	.	.	.	INTERGENIC(INTERGENIC)
3	109647737	109647746	109647743	GA	A	snp	1	1	1	1	.	.	.	INTERGENIC(INTERGENIC)
3	109744950	109744959	109744955	TG	C	snp	1	1	1	1	.	.	.	INTERGENIC(INTERGENIC)
3	109914905	109914922	109914909	CA	T	snp	1	0	1	0	.	.	.	INTERGENIC(INTERGENIC)
3	109944836	109944844	109944840	A	G	snp	1	1	0	1	.	.	.	INTERGENIC(INTERGENIC)
3	110111875	110111882	110111881	C	A	ins	1	1	1	1	.	.	.	INTERGENIC(INTERGENIC)
3	110111875	110111882	110111881	C	A	snp	1	1	1	1	.	.	.	INTERGENIC(INTERGENIC)
3	110128554	110128562	110128558	T	G	snp	1	1	1	1	.	.	.	INTERGENIC(INTERGENIC)
3	110220498	110220515	110220510	TA	C	snp	1	1	1	1	.	.	.	INTERGENIC(INTERGENIC)
3	110497803	110497811	110497805	A	G	snp	1	1	1	1	.	.	.	INTERGENIC(INTERGENIC)
3	110548033	110548048	110548039	AT	G	snp	0	1	1	0	.	.	.	INTERGENIC(INTERGENIC)
3	110674035	110674043	110674040	T	TTTC	ins	0	1	1	0	.	.	.	PVRL3-AS1(INTRON)
3	110674053	110674060	110674055	T	C	ins	1	1	1	1	.	.	.	PVRL3-AS1(INTRON)
3	110676220	110676235	110676229	AC	T	snp	1	0	0	0	.	.	.	PVRL3-AS1(INTRON)
3	110839524	110839533	110839526	TC	C	snp	1	1	1	0	.	.	.	PVRL3(DOWNSTREAM);PVRL3(INTRON)
3	111020974	111020983	111020981	TC	A	snp	1	1	1	1	.	.	.	RP11-615J4.1.1(INTRON)
3	111040069	111040082	111040071	TG	G	snp	1	1	1	1	.	.	.	RP11-615J4.1.1(INTRON)
3	111138316	111138327	111138319	GTG	T	snp	0	1	1	1	.	.	.	RP11-615J4.1.1(INTRON)
3	111142591	111142602	111142598	AT	C	snp	1	1	1	1	.	.	.	RP11-615J4.1.1(INTRON)
3	111149404	111149413	111149405	T	C	snp	1	0	0	0	.	.	.	RP11-615J4.1.1(INTRON)
3	111201061	111201068	111201063	G	A	snp	1	0	0	0	.	.	.	ATP6V0CP2(DOWNSTREAM);RP11-615J4.1.1(INTRON);RP11-615J4.3.1(INTRON)
3	111220261	111220270	111220268	A	C	snp	1	0	0	0	.	.	.	RP11-615J4.1.1(INTRON);RP11-615J4.3.1(UPSTREAM)
3	111321980	111321987	111321982	A	G	snp	0	1	1	0	.	.	.	CD96(INTRON)
3	111447261	111447270	111447266	TG	A	snp	1	1	1	1	.	.	.	PHLDB2(UPSTREAM);PLCXD2(INTRON);PLCXD2(UPSTREAM)
3	111471096	111471113	111471099	TG	TC	ins	1	1	1	1	.	.	.	PHLDB2(INTRON);PLCXD2(INTRON)
3	111499119	111499142	111499135	AAG	G	snp	0	0	1	1	.	.	.	PHLDB2(INTRON);PLCXD2(DOWNSTREAM);PLCXD2(INTRON)
3	111507061	111507070	111507065	T	C	snp	1	1	1	1	.	.	.	PHLDB2(INTRON);PLCXD2(INTRON)
3	111552459	111552468	111552461	TA	C	snp	1	1	1	1	.	.	.	PHLDB2(INTRON);PLCXD2(INTRON)
3	111552589	111552598	111552593	TA	C	snp	1	1	1	1	.	.	.	PHLDB2(INTRON);PLCXD2(INTRON)
3	111590509	111590516	111590511	A	C	ins	1	1	1	1	.	.	.	PHLDB2(INTRON)
3	111637040	111637049	111637045	TG	A	snp	1	0	0	0	.	.	.	PHLDB2(DOWNSTREAM);PHLDB2(INTRON)
3	111743033	111743040	111743036	T	C	ins	1	0	0	0	.	.	.	INTERGENIC(INTERGENIC)
3	111767191	111767198	111767194	T	C	snp	1	1	1	1	.	.	.	TMPRSS7(DOWNSTREAM);TMPRSS7(INTRON)
3	111785623	111785632	111785629	T	C	snp	1	1	1	1	.	.	.	TMPRSS7(INTRON)
3	111802517	111802524	111802518	A	C	snp	0	0	1	0	.	.	.	C3orf52(UPSTREAM);INTERGENIC(INTERGENIC);TMPRSS7(DOWNSTREAM)
3	111807509	111807532	111807525	TCTA	C	del	1	1	0	0	.	.	.	C3orf52(INTRON)
3	112079174	112079181	112079175	A	AAAT	ins	1	1	1	1	.	.	.	CD200(INTRON)
3	112133478	112133489	112133482	GT	T	snp	0	1	1	0	.	.	.	INTERGENIC(INTERGENIC)
3	112191200	112191207	112191202	T	G	snp	1	1	1	1	.	.	.	BTLA(INTRON);BTLA(UPSTREAM)
3	112259608	112259617	112259609	A	G	snp	1	1	1	0	.	.	.	ATG3(DOWNSTREAM);ATG3(INTRON);ATG3(UPSTREAM)
3	112334635	112334646	112334641	CCA	A	snp	0	0	0	1	.	.	.	CCDC80(INTRON)
3	112369173	112369184	112369177	AT	G	snp	1	1	1	0	.	.	.	CCDC80(UPSTREAM);INTERGENIC(INTERGENIC)
3	112466392	112466401	112466399	G	T	snp	0	0	1	1	.	.	.	RP11-572C15.5.1(INTRON)
3	112523339	112523362	112523349	CTT	C	snp	1	1	1	1	.	.	.	RP11-180K7.1.1(DOWNSTREAM);RP11-180K7.1.1(INTRON)
3	112557316	112557323	112557320	A	C	snp	0	0	1	0	.	.	.	CD200R1L(INTRON)
3	112557337	112557345	112557338	A	AC	ins	1	1	0	1	.	.	.	CD200R1L(INTRON)
3	112609263	112609272	112609270	T	C	snp	1	1	1	1	.	.	.	INTERGENIC(INTERGENIC)

3	112639496	112639507	112639503	ATA	C	snp	0	0	1	0	.	.	.	CD200R1(DOWNSTREAM);INTERGENIC(INTERGENIC)
3	112664927	112664936	112664928	A	G	snp	1	1	1	1	.	.	.	CD200R1(INTRON)
3	112684612	112684621	112684618	A	G	snp	1	0	0	0	.	.	.	CD200R1(INTRON)
3	112700060	112700068	112700062	T	G	snp	1	0	1	0	.	.	.	INTERGENIC(INTERGENIC)
3	112741853	112741880	112741872	TTTG	C	snp	0	0	1	0	.	.	.	C3orf17(UPSTREAM);RP11-572M11.4.1(INTRON)
3	112840292	112840301	112840297	T	C	ins	0	0	1	0	.	.	.	RP11-572M11.4.1(INTRON)
3	112966716	112966725	112966721	CT	C	snp	1	1	1	1	.	.	.	BOC(INTRON)
3	113069232	113069241	113069237	AG	C	snp	1	1	1	1	.	.	.	WDR52(INTRON)
3	113284380	113284388	113284385	A	C	snp	1	0	0	0	.	.	.	SIDT1(INTRON)
3	113287474	113287481	113287478	A	G	snp	1	0	0	0	.	.	.	SIDT1(DOWNSTREAM);SIDT1(INTRON)
3	113290792	113290799	113290793	A	C	snp	1	1	1	1	.	.	.	SIDT1(DOWNSTREAM);SIDT1(INTRON)
3	113302489	113302496	113302493	A	G	snp	1	1	1	1	.	.	.	SIDT1(DOWNSTREAM);SIDT1(INTRON);SIDT1(UPSTREAM)
3	113319180	113319189	113319185	A	G	snp	1	1	1	1	.	.	.	SIDT1(INTRON);SIDT1(UPSTREAM)
3	113433901	113433910	113433903	T	G	snp	1	1	1	1	.	.	.	INTERGENIC(INTERGENIC);NAA50(DOWNSTREAM)
3	113550887	113550894	113550892	T	C	ins	1	0	0	0	.	.	.	GRAMD1C(INTRON)
3	113848190	113848197	113848193	A	C	snp	1	1	1	1	.	.	.	DRD3(INTRON)
3	113898520	113898529	113898525	TC	G	snp	1	1	0	1	.	.	.	DRD3(INTRON);DRD3(UPSTREAM)
3	113934209	113934216	113934212	T	G	snp	0	0	0	1	.	.	.	RP11-553L6.2.1(INTRON)
3	114047075	114047083	114047080	T	G	snp	0	1	1	1	.	.	.	INTERGENIC(INTERGENIC)
3	114126759	114126766	114126761	G	T	snp	1	0	0	0	.	.	.	ZBTB20(INTRON)
3	114175061	114175078	114175064	GA	TG	ins	1	1	1	0	.	.	.	RP11-197K3.1.1(INTRON);ZBTB20(INTRON);ZBTB20(UPSTREAM)
3	114180144	114180153	114180149	C	T	snp	1	1	1	1	.	.	.	RP11-197K3.1.1(INTRON);ZBTB20(INTRON)
3	114339148	114339156	114339152	T	C	snp	0	1	0	1	.	.	.	ZBTB20(INTRON)
3	114387835	114387846	114387840	AAC	G	snp	1	0	0	0	.	.	.	ZBTB20(INTRON)
3	114483084	114483099	114483097	CT	C	del	1	0	0	0	.	.	.	ZBTB20(INTRON);ZBTB20(UPSTREAM)
3	114722258	114722267	114722264	T	G	snp	1	1	1	1	.	.	.	ZBTB20(INTRON)
3	114853331	114853340	114853336	T	C	snp	1	0	0	0	.	.	.	ZBTB20(INTRON)
3	114961224	114961237	114961235	TA	TG	ins	1	1	1	1	.	.	.	INTERGENIC(INTERGENIC)
3	115047967	115047982	115047974	TCTT	C	ins	1	0	0	0	.	.	.	INTERGENIC(INTERGENIC)
3	115064968	115064987	115064975	TCTT	G	snp	1	0	0	0	.	.	.	INTERGENIC(INTERGENIC)
3	115229846	115229853	115229847	A	C	ins	1	1	1	1	.	.	.	INTERGENIC(INTERGENIC)
3	115289077	115289086	115289084	AC	ACA	del	1	1	1	0	.	.	.	INTERGENIC(INTERGENIC)
3	115378466	115378475	115378472	AG	GAG	del	0	1	1	1	.	.	.	GAP43(INTRON);RP11-326J18.1.1(INTRON)
3	115417164	115417175	115417173	AT	C	snp	1	0	0	0	.	.	.	GAP43(INTRON)
3	115430342	115430359	115430357	AC	A	del	1	1	1	1	.	.	.	GAP43(INTRON)
3	115462950	115462961	115462958	TTC	T	ins	1	1	0	0	.	.	.	INTERGENIC(INTERGENIC)
3	115660407	115660418	115660410	CA	C	snp	1	1	1	1	.	.	.	LSAMP(INTRON)
3	115852099	115852108	115852106	T	G	snp	1	1	1	1	.	.	.	LSAMP(INTRON)
3	115872546	115872554	115872548	A	G	snp	1	1	1	1	.	.	.	LSAMP(INTRON)
3	115879298	115879307	115879300	GA	C	snp	1	1	1	1	.	.	.	LSAMP(INTRON)
3	115992632	115992641	115992638	AT	TATC	ins	1	1	1	1	.	.	.	LSAMP(INTRON)
3	116049396	116049404	116049402	C	T	snp	0	0	0	1	.	.	.	LSAMP(INTRON)
3	116116234	116116243	116116242	C	A	ins	1	1	1	1	.	.	.	LSAMP(INTRON)
3	116119240	116119253	116119244	TC	C	snp	1	1	1	1	.	.	.	LSAMP(INTRON)
3	116133290	116133305	116133294	TATT	C	snp	1	0	0	0	.	.	.	LSAMP(INTRON)
3	116135641	116135658	116135655	CTT	C	del	0	1	0	1	.	.	.	LSAMP(INTRON)
3	116238333	116238344	116238337	ATG	C	snp	1	1	1	1	.	.	.	LSAMP(INTRON)
3	116314391	116314408	116314404	AAC	C	del	1	1	1	1	.	.	.	LSAMP(INTRON)
3	116378401	116378410	116378404	AT	C	snp	1	0	0	0	.	.	.	LSAMP(INTRON)
3	116423877	116423888	116423883	TTA	G	snp	1	1	1	1	.	.	.	LSAMP(INTRON);LSAMP-AS3(UPSTREAM)
3	116448576	116448589	116448585	AC	G	snp	1	0	0	0	.	.	.	LSAMP(INTRON)
3	116452753	116452761	116452754	T	C	snp	1	1	1	1	.	.	.	LSAMP(INTRON)
3	116582724	116582743	116582736	AAAC	AC	ins	1	0	0	0	.	.	.	LSAMP(INTRON)
3	116753419	116753428	116753425	TG	G	ins	1	1	1	1	.	.	.	LSAMP(INTRON)
3	116761447	116761455	116761451	A	G	snp	1	0	0	0	.	.	.	LSAMP(INTRON)
3	116971855	116971864	116971857	CT	T	snp	1	0	0	0	.	.	.	LSAMP(INTRON)
3	117003905	117003916	117003908	TTG	TGTTG	del	1	1	1	1	.	.	.	LSAMP(INTRON)
3	117003905	117003916	117003909	TTG	G	del	1	1	1	1	.	.	.	LSAMP(INTRON)
3	117003905	117003916	117003911	TTG	TG	del	1	1	1	1	.	.	.	LSAMP(INTRON)
3	117127913	117127922	117127917	CT	T	snp	1	0	0	0	.	.	.	LSAMP(INTRON)
3	117166368	117166375	117166372	A	G	snp	1	0	0	0	.	.	.	LSAMP(INTRON)
3	117198688	117198697	117198694	TA	C	snp	0	1	1	1	.	.	.	LSAMP(INTRON)
3	117292181	117292190	117292184	A	G	snp	1	0	0	0	.	.	.	LSAMP(INTRON)
3	117301693	117301700	117301697	A	G	snp	1	1	1	1	.	.	.	LSAMP(INTRON)
3	117310931	117310939	117310938	T	C	snp	1	1	1	1	.	.	.	LSAMP(INTRON)
3	117312210	117312219	117312215	TC	G	snp	1	1	1	1	.	.	.	LSAMP(INTRON)

3	117485149	117485166	117485154	AAC	A	snp	1	1	1	1	.	.	.	LSAMP(INTRON);RP11-384F7.2.1(INTRON)
3	117544867	117544880	117544873	GT	A	snp	0	1	0	0	.	.	.	LSAMP(INTRON);RP11-384F7.2.1(INTRON)
3	117783527	117783535	117783532	T	C	snp	1	1	1	0	.	.	.	RP11-384F7.1.1(INTRON)
3	117791865	117791876	117791868	TA	TATATATAT	del	1	1	1	1	.	.	.	RP11-384F7.1.1(INTRON)
3	117791865	117791876	117791870	TA	TATATAT	del	1	1	1	1	.	.	.	RP11-384F7.1.1(INTRON)
3	117791902	117791913	117791910	AT	G	snp	0	0	0	1	.	.	.	RP11-384F7.1.1(INTRON)
3	118001199	118001213	118001204	CTC	T	snp	1	1	1	1	.	.	.	RP11-384F7.1.1(INTRON)
3	118015032	118015039	118015037	T	G	snp	1	0	1	0	.	.	.	RP11-384F7.1.1(INTRON)
3	118038946	118038963	118038950	GTT	C	snp	1	1	1	1	.	.	.	RP11-384F7.1.1(INTRON)
3	118053040	118053049	118053046	TC	C	snp	1	1	1	1	.	.	.	RP11-384F7.1.1(INTRON)
3	118130421	118130432	118130426	TG	A	snp	1	1	0	1	.	.	.	RP11-384F7.1.1(INTRON)
3	118293176	118293191	118293178	AT	T	del	1	1	1	1	.	.	.	INTERGENIC(INTERGENIC)
3	118324213	118324222	118324220	T	G	snp	0	1	1	0	.	.	.	INTERGENIC(INTERGENIC)
3	118367415	118367424	118367416	A	C	snp	0	0	1	0	.	.	.	INTERGENIC(INTERGENIC)
3	118367872	118367889	118367877	TA	T	del	1	0	0	0	.	.	.	INTERGENIC(INTERGENIC)
3	118423386	118423393	118423392	A	C	snp	1	1	1	1	.	.	.	INTERGENIC(INTERGENIC)
3	118527333	118527348	118527336	AT	A	snp	1	1	0	1	.	.	.	INTERGENIC(INTERGENIC)
3	118579301	118579309	118579302	T	G	ins	1	1	1	1	.	.	.	INTERGENIC(INTERGENIC)
3	118579895	118579902	118579896	T	C	snp	0	1	0	1	.	.	.	INTERGENIC(INTERGENIC)
3	118640875	118640884	118640879	A	G	snp	0	1	1	0	.	.	.	IGSF11(DOWNSTREAM);IGSF11(INTRON)
3	118654084	118654093	118654091	AT	C	snp	1	1	1	1	.	.	.	IGSF11(INTRON);IGSF11(UPSTREAM)
3	118716374	118716382	118716377	C	A	snp	1	1	1	1	.	.	.	IGSF11(INTRON)
3	118856059	118856068	118856066	A	G	snp	1	1	1	1	.	.	.	IGSF11(INTRON)
3	119248320	119248328	119248321	A	G	snp	1	1	1	1	.	.	.	AC073352.1(EXON);CD80(INTRON);TIMMDC1(DOWNSTREAM)
3	119264577	119264586	119264584	AC	A	del	1	0	0	0	.	.	.	CD80(INTRON)
3	119289911	119289919	119289916	A	C	snp	1	1	1	1	.	.	.	INTERGENIC(INTERGENIC)
3	119340935	119340955	119340950	AAG	G	snp	1	1	1	1	.	.	.	PLA1A(DOWNSTREAM);PLA1A(INTRON)
3	119423025	119423033	119423032	T	C	snp	1	1	1	1	.	.	.	C3orf15(INTRON);C3orf15(UPSTREAM)
3	119446957	119446968	119446963	AAT	G	snp	1	0	0	0	.	.	.	C3orf15(DOWNSTREAM);C3orf15(INTRON)
3	119453835	119453843	119453840	T	G	snp	0	1	1	1	.	.	.	C3orf15(INTRON)
3	119614143	119614152	119614145	AT	G	snp	0	1	1	1	.	.	.	GSK3B(INTRON)
3	119655185	119655202	119655200	AT	A	del	1	1	1	1	.	.	.	GSK3B(INTRON)
3	119689654	119689661	119689657	A	C	snp	1	1	0	1	.	.	.	GSK3B(INTRON)
3	119726309	119726317	119726311	A	C	snp	1	0	0	0	.	.	.	GSK3B(INTRON)
3	119921950	119921959	119921953	AC	T	snp	1	0	1	1	.	.	.	GPR156(INTRON)
3	119930853	119930862	119930855	T	C	snp	1	1	1	1	.	.	.	GPR156(INTRON);Y_RNA(DOWNSTREAM)
3	120019714	120019723	120019717	AC	G	snp	1	0	0	0	.	.	.	INTERGENIC(INTERGENIC)
3	120133851	120133860	120133857	A	G	snp	1	1	1	1	.	.	.	FSTL1(INTRON);FSTL1(UPSTREAM)
3	120226807	120226815	120226809	A	AC	ins	0	1	0	1	.	.	.	INTERGENIC(INTERGENIC);RP11-359H3.1.1(UPSTREAM)
3	120226807	120226815	120226810	A	C	ins	1	1	0	1	.	.	.	INTERGENIC(INTERGENIC);RP11-359H3.1.1(UPSTREAM)
3	120253168	120253176	120253170	T	C	snp	1	1	1	1	.	.	.	INTERGENIC(INTERGENIC)
3	120340908	120340917	120340910	CT	T	snp	1	0	0	0	.	.	.	INTERGENIC(INTERGENIC)
3	120340937	120340946	120340941	CT	T	snp	1	0	0	0	.	.	.	INTERGENIC(INTERGENIC)
3	120373937	120373948	120373942	AAC	T	snp	0	1	0	1	.	.	.	HGD(INTRON);HGD(UPSTREAM)
3	120661431	120661439	120661438	T	C	snp	1	1	1	1	.	.	.	STXBP5L(INTRON)
3	120690720	120690731	120690722	CA	A	snp	1	0	0	0	.	.	.	STXBP5L(INTRON)
3	120699844	120699853	120699851	T	C	snp	0	0	1	0	.	.	.	STXBP5L(INTRON)
3	120801518	120801527	120801524	GT	A	snp	0	1	0	1	.	.	.	STXBP5L(INTRON)
3	120814043	120814052	120814047	CT	G	snp	1	1	1	1	.	.	.	RP11-678L1.1.1(UPSTREAM);STXBP5L(INTRON)
3	120906448	120906457	120906456	A	G	ins	1	0	1	0	.	.	.	STXBP5L(INTRON)
3	120932518	120932532	120932529	TTG	T	del	1	1	1	1	.	.	.	STXBP5L(INTRON)
3	121011878	121011886	121011883	A	C	snp	1	0	0	0	.	.	.	STXBP5L(INTRON)
3	121063795	121063804	121063800	TA	G	snp	0	1	0	1	.	.	.	STXBP5L(INTRON)
3	121171320	121171327	121171322	T	C	snp	0	1	0	1	.	.	.	POLQ(INTRON)
3	121270183	121270191	121270187	A	G	snp	1	0	0	0	.	.	.	INTERGENIC(INTERGENIC);POLQ(UPSTREAM)
3	121287940	121287948	121287941	A	C	snp	1	1	0	1	.	.	.	ARGFX(UPSTREAM);INTERGENIC(INTERGENIC)
3	121393084	121393093	121393089	T	G	snp	1	1	1	1	.	.	.	GOLGB1(INTRON)
3	121543079	121543088	121543084	T	G	snp	1	1	1	1	.	.	.	IQCB1(DOWNSTREAM);IQCB1(INTRON)
3	121567726	121567734	121567733	C	A	ins	1	1	1	1	.	.	.	EAF2(INTRON)
3	121616525	121616533	121616528	A	G	snp	1	0	0	0	.	.	.	SLC15A2(INTRON)
3	121628833	121628841	121628839	T	C	ins	1	1	1	1	.	.	.	SLC15A2(INTRON);SLC15A2(UPSTREAM)
3	121671878	121671887	121671880	T	G	snp	1	1	1	1	.	.	.	INTERGENIC(INTERGENIC)
3	121789968	121789982	121789974	ATT	G	snp	0	0	0	1	.	.	.	CD86(INTRON)
3	121912037	121912046	121912042	T	G	ins	1	1	0	1	.	.	.	CASR(INTRON)
3	121971293	121971300	121971296	A	G	ins	0	0	1	0	.	.	.	CASR(INTRON);CASR(UPSTREAM)
3	121976533	121976540	121976535	T	C	ins	1	1	1	1	.	.	.	CASR(DOWNSTREAM);CASR(INTRON)
3	122017599	122017608	122017603	AC	CACACATACATGG	del	1	1	1	1	.	.	.	INTERGENIC(INTERGENIC)

3	122073455	122073466	122073460	TTA	T	snp	1	0	0	0	.	.	.	CCDC58(DOWNSTREAM);INTERGENIC(INTERGENIC)
3	122202201	122202208	122202204	T	G	ins	0	0	0	1	.	.	.	KPNA1(INTRON)
3	122505972	122505979	122505978	A	C	snp	1	0	0	0	.	.	.	HSPBAP1(INTRON)
3	122587181	122587188	122587185	A	C	snp	1	0	0	0	.	.	.	DIRC2(INTRON)
3	122601821	122601844	122601832	TTTG	A	snp	0	1	0	0	.	.	.	DIRC2(DOWNSTREAM);INTERGENIC(INTERGENIC)
3	122696002	122696016	122696011	GAG	A	snp	1	1	0	1	.	.	.	SEMA5B(INTRON);SEMA5B(UPSTREAM)
3	122702318	122702325	122702322	A	G	snp	1	0	0	0	.	.	.	SEMA5B(INTRON)
3	122716366	122716374	122716372	C	T	snp	0	1	1	1	.	.	.	SEMA5B(INTRON);SEMA5B(UPSTREAM)
3	122722682	122722705	122722701	AATA	T	snp	1	0	0	0	.	.	.	SEMA5B(INTRON)
3	122798995	122799004	122799002	GT	G	snp	1	1	1	1	.	.	.	PDIA5(INTRON)
3	122843200	122843211	122843204	GT	A	snp	1	0	0	0	.	.	.	PDIA5(INTRON)
3	122952502	122952510	122952504	A	C	ins	1	0	0	0	.	.	.	SEC22A(INTRON)
3	123115311	123115326	123115316	AAAG	C	snp	0	1	1	0	.	.	.	ADCY5(INTRON)
3	123115325	123115334	123115328	AG	C	snp	1	0	0	0	.	.	.	ADCY5(INTRON)
3	123115380	123115399	123115391	AAAG	A	ins	1	0	0	0	.	.	.	ADCY5(INTRON)
3	123129163	123129174	123129170	CCT	T	snp	1	1	0	1	.	.	.	ADCY5(INTRON)
3	123312221	123312230	123312228	T	G	snp	1	1	1	1	.	.	.	MYLK-AS1(INTRON)
3	123352677	123352690	123352684	TG	C	snp	1	0	0	0	.	.	.	MYLK(INTRON);MYLK-AS1(DOWNSTREAM);MYLK-AS1(INTRON)
3	123360541	123360560	123360555	CTTC	C	ins	1	0	0	0	.	.	.	MYLK(INTRON);MYLK-AS1(INTRON)
3	123373192	123373200	123373194	T	G	snp	1	1	1	0	.	.	.	MYLK(DOWNSTREAM);MYLK(INTRON)
3	123482413	123482421	123482419	T	C	snp	1	0	0	0	.	.	.	MYLK(INTRON)
3	123556385	123556394	123556392	TA	G	snp	1	1	1	1	.	.	.	MYLK(INTRON)
3	123716606	123716621	123716618	CA	A	ins	1	1	1	0	.	.	.	INTERGENIC(INTERGENIC)
3	123787626	123787635	123787628	A	G	snp	1	0	0	0	.	.	.	INTERGENIC(INTERGENIC)
3	123822820	123822828	123822821	A	G	snp	1	0	0	0	.	.	.	KALRN(INTRON)
3	123828551	123828560	123828558	AT	C	snp	1	1	1	1	.	.	.	KALRN(INTRON)
3	123828611	123828620	123828616	AT	C	snp	1	1	1	1	.	.	.	KALRN(INTRON)
3	123828611	123828620	123828618	AT	C	snp	1	1	1	1	.	.	.	KALRN(INTRON)
3	123828731	123828740	123828738	AT	C	snp	1	1	1	1	.	.	.	KALRN(INTRON)
3	123828761	123828770	123828768	AT	C	snp	1	1	1	1	.	.	.	KALRN(INTRON)
3	123946956	123946965	123946963	TG	A	snp	0	1	1	0	.	.	.	KALRN(INTRON)
3	123973788	123973799	123973794	TA	AA	ins	1	1	1	1	.	.	.	KALRN(INTRON)
3	123993608	123993617	123993610	T	G	snp	0	1	1	0	.	.	.	KALRN(INTRON)
3	124012480	124012488	124012485	A	G	snp	1	1	1	1	.	.	.	KALRN(INTRON)
3	124046442	124046451	124046449	AC	T	snp	1	1	1	1	.	.	.	KALRN(DOWNSTREAM);KALRN(INTRON)
3	124052741	124052756	124052752	AAAC	T	snp	1	1	0	1	.	.	.	KALRN(INTRON);KALRN(UPSTREAM)
3	124117164	124117175	124117166	TG	A	snp	1	0	0	0	.	.	.	KALRN(DOWNSTREAM);KALRN(INTRON)
3	124187191	124187198	124187193	G	A	snp	1	0	0	0	.	.	.	KALRN(INTRON)
3	124187191	124187198	124187197	G	T	snp	1	1	1	1	.	.	.	KALRN(INTRON)
3	124197762	124197771	124197765	AC	T	snp	0	1	1	0	.	.	.	KALRN(INTRON)
3	124299795	124299818	124299799	AAT	TAATAG	ins	1	1	1	1	.	.	.	KALRN(INTRON);KALRN(UPSTREAM)
3	124310037	124310046	124310042	TC	G	snp	1	1	1	0	.	.	.	KALRN(INTRON)
3	124329482	124329489	124329483	G	A	ins	1	1	1	1	.	.	.	KALRN(INTRON)
3	124447114	124447128	124447125	TTG	TTGT	del	1	1	1	1	.	.	.	INTERGENIC(INTERGENIC);RP11-71H17.7.1(DOWNSTREAM);UMPS(UPSTREAM)
3	124453018	124453026	124453022	T	G	snp	0	1	1	1	.	.	.	RP11-71H17.1.1(UPSTREAM);UMPS(INTRON);UMPS(UPSTREAM)
3	124453109	124453118	124453114	A	G	snp	1	1	1	1	.	.	.	RP11-71H17.1.1(UPSTREAM);UMPS(INTRON);UMPS(UPSTREAM)
3	124483082	124483091	124483089	TC	G	snp	1	1	1	1	.	.	.	ITGB5(DOWNSTREAM);ITGB5(INTRON)
3	124513654	124513667	124513657	AG	AA	ins	1	0	0	0	.	.	.	ITGB5(DOWNSTREAM);ITGB5(INTRON)
3	124541741	124541752	124541748	GAG	C	snp	1	0	0	0	.	.	.	ITGB5(INTRON)
3	124590702	124590721	124590715	AATA	G	snp	0	0	1	0	.	.	.	ITGB5(INTRON)
3	124641388	124641395	124641391	T	C	snp	1	0	0	0	.	.	.	MUC13(INTRON)
3	124806628	124806635	124806634	T	G	snp	1	0	0	0	.	.	.	SLC12A8(DOWNSTREAM);SLC12A8(INTRON)
3	124871462	124871471	124871467	GA	G	snp	1	1	1	1	.	.	.	SLC12A8(INTRON);SLC12A8(UPSTREAM)
3	125043029	125043036	125043033	A	G	snp	0	1	1	1	.	.	.	ZNF148(INTRON)
3	125098986	125098994	125098989	A	G	snp	0	1	0	1	.	.	.	INTERGENIC(INTERGENIC);ZNF148(UPSTREAM)
3	125112174	125112182	125112177	T	G	ins	1	0	0	0	.	.	.	INTERGENIC(INTERGENIC);U6(DOWNSTREAM)
3	125174848	125174867	125174856	AAAC	G	snp	0	1	0	1	.	.	.	SNX4(INTRON)
3	125186408	125186419	125186416	TTA	T	ins	1	0	0	0	.	.	.	SNX4(DOWNSTREAM);SNX4(INTRON)
3	125234201	125234218	125234203	AT	T	del	1	0	0	0	.	.	.	SNX4(INTRON);Y_RNA(UPSTREAM)
3	125249829	125249838	125249834	T	TTC	ins	1	1	1	1	.	.	.	OSBPL11(INTRON);Y_RNA(UPSTREAM)
3	125249829	125249838	125249835	T	TC	ins	0	1	1	1	.	.	.	OSBPL11(INTRON);Y_RNA(UPSTREAM)
3	125261520	125261527	125261525	T	C	snp	0	1	1	1	.	.	.	OSBPL11(INTRON)
3	125264463	125264476	125264467	TG	G	snp	0	1	0	1	.	.	.	OSBPL11(INTRON)

3	125344289	125344304	125344299	TCTT	TTC	del	1	1	1	1	.	.	.	INTERGENIC(INTERGENIC)
3	125347880	125347888	125347882	T	G	snp	1	1	0	1	.	.	.	INTERGENIC(INTERGENIC)
3	125384809	125384823	125384816	CTT	C	snp	1	0	0	0	.	.	.	INTERGENIC(INTERGENIC)
3	125493620	125493628	125493621	A	G	snp	1	1	1	1	.	.	.	INTERGENIC(INTERGENIC)
3	125545055	125545064	125545060	A	C	ins	1	1	1	1	.	.	.	INTERGENIC(INTERGENIC)
3	125555434	125555442	125555435	A	C	ins	1	1	0	1	.	.	.	AC092902.1(UPSTREAM);INTERGENIC(INTERGENIC)
3	125555434	125555442	125555436	A	C	snp	1	1	1	1	.	.	.	AC092902.1(UPSTREAM);INTERGENIC(INTERGENIC)
3	125591962	125591970	125591968	T	G	snp	1	0	0	0	.	.	.	RP11-379B18.4.1(INTRON);RP11-379B18.5.1(INTRON)
3	125651237	125651246	125651239	A	C	snp	1	1	0	1	.	.	.	ALG1L(INTRON);RP11-666A20.1.1(DOWNSTREAM)
3	125671340	125671347	125671342	A	C	snp	1	1	1	1	.	.	.	INTERGENIC(INTERGENIC)
3	125763888	125763896	125763893	A	G	snp	1	1	1	1	.	.	.	SLC41A3(INTRON)
3	125782793	125782801	125782798	C	CT	ins	1	1	1	1	.	.	.	SLC41A3(DOWNSTREAM);SLC41A3(INTRON)
3	125793524	125793533	125793526	T	G	snp	1	1	1	1	.	.	.	SLC41A3(INTRON);SLC41A3(UPSTREAM)
3	126017264	126017287	126017272	ATT	ATC	ins	1	1	0	1	.	.	.	INTERGENIC(INTERGENIC);RP11-71E19.2.1(UPSTREAM)
3	126020782	126020791	126020789	T	C	ins	1	0	0	0	.	.	.	INTERGENIC(INTERGENIC)
3	126020782	126020791	126020790	T	C	ins	1	1	1	1	.	.	.	INTERGENIC(INTERGENIC)
3	126181067	126181075	126181071	A	C	snp	1	1	1	1	.	.	.	ZXDC(INTRON)
3	126181906	126181923	126181917	ATT	A	del	1	1	1	1	.	.	.	ZXDC(INTRON)
3	126181906	126181923	126181920	ATT	A	del	1	1	1	1	.	.	.	ZXDC(INTRON)
3	126204430	126204445	126204441	ATTT	A	del	1	1	1	1	.	.	.	UROC1(INTRON)
3	126258282	126258291	126258285	AC	A	snp	0	1	1	1	.	.	.	C3orf22(INTRON);CHST13(INTRON)
3	126619851	126619858	126619856	C	A	snp	1	1	1	0	.	.	.	CHCHD6(INTRON)
3	126801233	126801241	126801238	T	C	snp	1	0	0	0	.	.	.	INTERGENIC(INTERGENIC)
3	126873793	126873802	126873800	GT	G	del	0	1	1	0	.	.	.	INTERGENIC(INTERGENIC)
3	127044033	127044042	127044035	A	C	snp	0	0	0	1	.	.	.	RP11-88I21.2.1(INTRON)
3	127258920	127258929	127258928	G	T	ins	1	0	0	0	.	.	.	INTERGENIC(INTERGENIC);RP11-59J16.1.1(UPSTREAM)
3	127473202	127473211	127473204	A	G	ins	1	1	0	1	.	.	.	MGLL(INTRON)
3	127596828	127596841	127596837	AT	C	snp	1	1	0	1	.	.	.	INTERGENIC(INTERGENIC)
3	127755387	127755395	127755392	T	C	snp	1	0	0	0	.	.	.	INTERGENIC(INTERGENIC)
3	127814589	127814597	127814594	T	C	snp	1	0	0	0	.	.	.	RUVBL1(INTRON)
3	127825302	127825311	127825305	C	T	ins	1	1	0	1	.	.	.	RUVBL1(INTRON)
3	127825302	127825311	127825306	C	T	snp	1	1	0	1	.	.	.	RUVBL1(INTRON)
3	127869431	127869440	127869438	AT	C	snp	1	0	0	0	.	.	.	EEFSEC(UPSTREAM);RUVBL1(INTRON)
3	127961171	127961186	127961178	AAAT	A	snp	1	1	1	1	.	.	.	EEFSEC(INTRON)
3	128075391	128075400	128075398	GT	G	snp	1	1	1	1	.	.	.	EEFSEC(INTRON)
3	128118434	128118441	128118439	C	A	snp	1	1	0	0	.	.	.	EEFSEC(INTRON)
3	128263028	128263039	128263036	AT	C	snp	1	0	0	0	.	.	.	INTERGENIC(INTERGENIC)
3	128274644	128274651	128274645	G	A	snp	1	1	0	1	.	.	.	INTERGENIC(INTERGENIC)
3	128392045	128392062	128392050	TA	TATC	ins	1	1	1	1	.	.	.	AC079945.1(DOWNSTREAM);POU5F1P6(DOWNSTREAM);RPN1(INTRON)
3	128428506	128428513	128428510	C	T	snp	1	1	1	1	.	.	.	INTERGENIC(INTERGENIC);SNORA24(DOWNSTREAM)
3	128645941	128645948	128645947	A	G	snp	0	0	0	1	.	.	.	KIAA1257(INTRON);RP11-723O4.6.1(INTRON)
3	128709956	128709967	128709963	ACA	A	snp	1	0	0	0	.	.	.	KIAA1257(INTRON)
3	128723212	128723219	128723215	C	A	snp	1	1	1	1	.	.	.	CCDC48(INTRON);KIAA1257(UPSTREAM);RP11-723O4.8.1(UPSTREAM)
3	128734263	128734270	128734265	G	A	snp	1	0	0	0	.	.	.	CCDC48(INTRON)
3	128774476	128774491	128774487	TTTC	T	ins	1	0	1	0	.	.	.	INTERGENIC(INTERGENIC)
3	128857124	128857133	128857129	A	C	snp	0	0	0	1	.	.	.	ISY1(INTRON);ISY1-RAB43(INTRON)
3	129135262	129135289	129135285	AAAC	T	snp	1	1	1	0	.	.	.	C3orf25(INTRON)
3	129213060	129213069	129213063	T	G	snp	1	1	1	0	.	.	.	IFT122(INTRON);IFT122(UPSTREAM)
3	129378991	129379002	129378999	TTG	TTGT	del	1	0	0	0	.	.	.	TMCC1(INTRON);TMCC1(UPSTREAM)
3	129647906	129647915	129647910	A	G	ins	1	1	1	1	.	.	.	INTERGENIC(INTERGENIC)
3	129693078	129693093	129693089	TC	TCTT	ins	1	1	1	1	.	.	.	INTERGENIC(INTERGENIC);RP11-93K22.6.1(UPSTREAM);TRH(UPSTREAM)
3	129693078	129693093	129693091	TC	TT	ins	1	1	1	1	.	.	.	INTERGENIC(INTERGENIC);RP11-93K22.6.1(UPSTREAM);TRH(UPSTREAM)
3	129697385	129697396	129697389	TAA	ATAAT	ins	1	1	1	1	.	.	.	INTERGENIC(INTERGENIC);TRH(DOWNSTREAM)
3	129716945	129716960	129716952	TTTC	T	snp	1	0	0	0	.	.	.	INTERGENIC(INTERGENIC);RP11-93K22.1.1(UPSTREAM)
3	129717457	129717470	129717468	CA	C	del	1	1	1	1	.	.	.	RP11-93K22.1.1(EXON)
3	129768929	129768940	129768934	AAG	A	snp	1	1	1	1	.	.	.	INTERGENIC(INTERGENIC)
3	129785908	129785917	129785912	TC	C	snp	1	1	1	1	.	.	.	INTERGENIC(INTERGENIC)
3	129786260	129786268	129786265	A	C	snp	1	1	1	1	.	.	.	INTERGENIC(INTERGENIC)
3	129788445	129788453	129788448	T	G	ins	1	1	1	1	.	.	.	INTERGENIC(INTERGENIC)
3	129788445	129788453	129788448	T	G	snp	1	1	1	1	.	.	.	INTERGENIC(INTERGENIC)
3	129832757	129832766	129832765	T	C	snp	1	0	0	0	.	.	.	FAM86HP(UPSTREAM);RP11-77P16.4.1(INTRON);RP11-93K22.11.1(DOWNSTREAM)
3	129846310	129846317	129846313	T	C	snp	1	1	1	1	.	.	.	INTERGENIC(INTERGENIC)

3	130013287	130013294	130013289	T	G	snp	1	0	0	0	.	.	.	INTERGENIC(INTERGENIC);RP11-77P16.1.1(DOWNSTREAM)
3	130061575	130061584	130061576	A	G	snp	0	1	0	1	.	.	.	COL6A5(UPSTREAM);INTERGENIC(INTERGENIC)
3	130369130	130369137	130369131	G	T	snp	1	1	1	1	.	.	.	COL6A6(INTRON)
3	130369130	130369137	130369132	G	T	snp	1	1	1	1	.	.	.	COL6A6(INTRON)
3	130494801	130494808	130494803	A	C	snp	0	0	1	0	.	.	.	INTERGENIC(INTERGENIC)
3	130503976	130503983	130503978	T	G	snp	1	0	1	0	.	.	.	INTERGENIC(INTERGENIC)
3	130559893	130559916	130559904	ATT	A	del	1	0	0	0	.	.	.	INTERGENIC(INTERGENIC)
3	130559893	130559916	130559913	ATT	A	del	1	1	1	1	.	.	.	INTERGENIC(INTERGENIC)
3	130638059	130638074	130638070	TTTC	T	ins	1	1	1	1	.	.	.	ATP2C1(INTRON);RP11-39E3.3.1(INTRON);RP11-39E3.4.1(UPSTREAM);Y_RNA(UPSTREAM)
3	130849644	130849653	130849646	AT	G	snp	1	0	0	0	.	.	.	NEK11(INTRON)
3	130950044	130950051	130950045	T	TTTTTTC	ins	1	1	1	0	.	.	.	NEK11(DOWNSTREAM);NEK11(INTRON)
3	131023851	131023860	131023857	TG	G	snp	1	0	0	0	.	.	.	NEK11(INTRON)
3	131049771	131049780	131049772	G	T	snp	0	1	0	1	.	.	.	NEK11(INTRON);RP11-933H2.4.1(INTRON);RP11-933H2.4.1(UPSTREAM)
3	131114404	131114419	131114414	AT	G	snp	1	1	1	1	.	.	.	INTERGENIC(INTERGENIC)
3	131114404	131114419	131114415	AT	C	snp	1	1	1	1	.	.	.	INTERGENIC(INTERGENIC)
3	131114404	131114419	131114417	AT	C	snp	1	1	1	1	.	.	.	INTERGENIC(INTERGENIC)
3	131114422	131114433	131114428	AT	G	snp	1	0	1	0	.	.	.	INTERGENIC(INTERGENIC)
3	131114458	131114473	131114461	AT	C	snp	1	1	1	1	.	.	.	INTERGENIC(INTERGENIC)
3	131138496	131138505	131138503	TC	T	snp	1	0	0	0	.	.	.	AC107027.1(UPSTREAM);INTERGENIC(INTERGENIC)
3	131289152	131289175	131289169	TTTA	TA	del	1	1	0	0	.	.	.	CPNE4(INTRON)
3	131312546	131312569	131312550	GTTT	T	snp	1	1	1	1	.	.	.	CPNE4(INTRON)
3	131436840	131436849	131436844	TC	C	snp	1	1	1	1	.	.	.	CPNE4(INTRON)
3	131520200	131520211	131520209	AT	A	del	1	0	0	0	.	.	.	CPNE4(INTRON)
3	131545898	131545906	131545902	A	C	snp	1	0	0	0	.	.	.	CPNE4(INTRON)
3	131574559	131574570	131574561	TG	C	snp	0	1	1	1	.	.	.	CPNE4(INTRON)
3	131591040	131591053	131591043	AG	C	snp	0	1	1	1	.	.	.	CPNE4(INTRON)
3	131597960	131597971	131597962	GT	A	snp	1	1	1	1	.	.	.	CPNE4(INTRON)
3	131598959	131598972	131598964	TA	G	snp	1	1	1	1	.	.	.	CPNE4(INTRON)
3	131606415	131606426	131606423	TTC	A	snp	1	1	1	1	.	.	.	CPNE4(INTRON)
3	131614407	131614414	131614408	T	TAA	ins	1	1	1	1	.	.	.	CPNE4(INTRON)
3	131614407	131614414	131614409	T	AA	ins	1	1	1	1	.	.	.	CPNE4(INTRON)
3	131622319	131622326	131622322	A	C	snp	1	1	1	1	.	.	.	CPNE4(INTRON)
3	131625047	131625055	131625053	T	G	ins	1	1	1	1	.	.	.	CPNE4(INTRON)
3	131681266	131681277	131681271	CAC	T	snp	0	1	1	1	.	.	.	CPNE4(INTRON)
3	131724238	131724247	131724242	AT	G	snp	1	1	1	1	.	.	.	CPNE4(INTRON)
3	131733283	131733294	131733286	GAG	A	snp	1	1	1	1	.	.	.	CPNE4(INTRON)
3	131775315	131775323	131775316	T	G	snp	0	1	1	1	.	.	.	CPNE4(INTRON)
3	131783271	131783278	131783273	A	G	snp	1	0	0	0	.	.	.	CPNE4(INTRON)
3	131844102	131844119	131844106	TCC	T	snp	1	1	1	1	.	.	.	CPNE4(INTRON)
3	131946904	131946911	131946909	A	G	snp	1	0	0	0	.	.	.	CPNE4(INTRON)
3	131953147	131953160	131953156	TG	A	snp	1	0	0	0	.	.	.	CPNE4(INTRON)
3	131982095	131982104	131982101	CA	A	snp	1	1	1	1	.	.	.	CPNE4(INTRON)
3	132020877	132020886	132020881	TC	C	snp	1	0	0	0	.	.	.	INTERGENIC(INTERGENIC)
3	132077638	132077647	132077645	TG	A	snp	0	1	0	1	.	.	.	ACPP(DOWNSTREAM);ACPP(INTRON)
3	132194283	132194291	132194290	T	G	snp	0	1	1	1	.	.	.	DNAJC13(INTRON);DNAJC13(UPSTREAM)
3	132251559	132251567	132251565	A	C	ins	1	1	1	0	.	.	.	DNAJC13(INTRON)
3	132551939	132551946	132551940	T	G	snp	1	1	1	1	.	.	.	NPHP3-AS1(INTRON)
3	132618973	132618988	132618975	TA	A	ins	0	1	1	1	.	.	.	INTERGENIC(INTERGENIC)
3	132625583	132625592	132625588	AT	C	snp	0	1	1	1	.	.	.	INTERGENIC(INTERGENIC)
3	132636893	132636901	132636899	A	G	snp	1	0	0	0	.	.	.	INTERGENIC(INTERGENIC)
3	132638236	132638243	132638242	A	G	snp	1	1	1	1	.	.	.	INTERGENIC(INTERGENIC)
3	132708373	132708380	132708376	A	G	snp	0	1	1	0	.	.	.	INTERGENIC(INTERGENIC)
3	132759035	132759050	132759046	TTTG	T	ins	1	1	1	0	.	.	.	RP11-469L4.1.1(UPSTREAM);TMEM108(INTRON);TMEM108(UPSTREAM)
3	132813078	132813085	132813084	C	A	snp	1	1	1	1	.	.	.	TMEM108(INTRON)
3	132966486	132966495	132966487	A	G	ins	1	0	0	0	.	.	.	RP11-402L6.1.1(EXON);RP11-402L6.1.1(INTRON);TMEM108(INTRON)
3	133158773	133158788	133158776	AG	C	snp	0	0	0	1	.	.	.	BFSP2(INTRON);RP11-91K8.1.1(INTRON)
3	133252445	133252454	133252450	T	C	snp	1	1	1	1	.	.	.	INTERGENIC(INTERGENIC)
3	133409830	133409839	133409835	AC	T	snp	1	1	1	1	.	.	.	TFP1(INTRON)
3	133468006	133468021	133468012	AAAT	G	snp	1	1	1	1	.	.	.	TF(INTRON);TFP1(INTRON)
3	133501717	133501724	133501718	A	C	snp	1	1	1	1	.	.	.	INTERGENIC(INTERGENIC);SRPRB(UPSTREAM);TF(DOWNSTREAM)
3	133583008	133583016	133583013	G	A	snp	0	1	0	1	.	.	.	RAB6B(INTRON)
3	133595748	133595757	133595749	A	C	snp	1	1	1	1	.	.	.	RAB6B(INTRON)

3	133598668	133598679	133598675	AGC	C	snp	1	1	1	1	.	.	.	RAB6B(INTRON)
3	133660841	133660852	133660848	ATT	C	snp	1	1	0	1	.	.	.	SLCO2A1(DOWNSTREAM);SLCO2A1(INTRON)
3	133681293	133681301	133681299	A	G	ins	0	0	1	0	.	.	.	SLCO2A1(INTRON)
3	133853694	133853707	133853696	TC	C	snp	1	0	0	0	.	.	.	RYK(INTRON)
3	133903980	133903995	133903987	AATT	A	del	1	1	1	1	.	.	.	RYK(INTRON);RYK(UPSTREAM)
3	133903980	133903995	133903988	AATT	AT	del	1	1	1	1	.	.	.	RYK(INTRON);RYK(UPSTREAM)
3	133906995	133907012	133907007	AT	T	ins	0	1	0	1	.	.	.	RYK(INTRON);RYK(UPSTREAM)
3	133906995	133907012	133907008	AT	ATA	del	1	1	1	1	.	.	.	RYK(INTRON);RYK(UPSTREAM)
3	133906995	133907012	133907010	AT	A	del	1	1	1	1	.	.	.	RYK(INTRON);RYK(UPSTREAM)
3	134023960	134023969	134023966	TG	C	snp	1	1	1	1	.	.	.	INTERGENIC(INTERGENIC)
3	134034933	134034952	134034940	ATTC	T	snp	1	1	1	1	.	.	.	RP11-200A1.1.1(INTRON)
3	134105279	134105288	134105281	TA	A	ins	0	1	1	1	.	.	.	HMGN1P9(DOWNSTREAM);INTERGENIC(INTERGENIC)
3	134153609	134153616	134153614	T	TTG	ins	1	1	1	1	.	.	.	HMGB3P13(UPSTREAM);INTERGENIC(INTERGENIC)
3	134186493	134186502	134186496	A	C	snp	1	1	1	1	.	.	.	INTERGENIC(INTERGENIC)
3	134187831	134187840	134187838	AG	A	snp	1	0	0	0	.	.	.	INTERGENIC(INTERGENIC)
3	134380650	134380658	134380654	C	T	snp	1	1	0	1	.	.	.	EPHB1(INTRON)
3	134464911	134464918	134464916	C	T	snp	0	0	0	1	.	.	.	EPHB1(INTRON)
3	134526409	134526418	134526410	A	G	snp	1	0	1	0	.	.	.	EPHB1(INTRON)
3	134830531	134830539	134830532	T	C	snp	1	1	1	1	.	.	.	EPHB1(INTRON)
3	134866444	134866453	134866451	AC	G	snp	1	1	1	1	.	.	.	EPHB1(INTRON)
3	134973518	134973525	134973524	T	G	snp	0	1	0	1	.	.	.	EPHB1(INTRON)
3	134983463	134983471	134983468	A	G	snp	1	0	0	0	.	.	.	EPHB1(DOWNSTREAM);INTERGENIC(INTERGENIC)
3	135003628	135003637	135003636	A	G	ins	1	0	0	0	.	.	.	INTERGENIC(INTERGENIC)
3	135110989	135110996	135110991	A	G	snp	1	1	1	1	.	.	.	RP11-657O9.1.1(INTRON)
3	135112355	135112370	135112360	GATT	C	snp	1	0	0	0	.	.	.	RP11-657O9.1.1(INTRON)
3	135142517	135142526	135142519	A	G	ins	1	0	1	1	.	.	.	RP11-657O9.1.1(INTRON)
3	135320185	135320193	135320189	A	C	snp	1	0	0	0	.	.	.	INTERGENIC(INTERGENIC)
3	135516398	135516406	135516405	G	T	snp	1	0	0	0	.	.	.	INTERGENIC(INTERGENIC);U8(DOWNSTREAM)
3	135594631	135594638	135594635	A	G	snp	1	0	0	0	.	.	.	INTERGENIC(INTERGENIC)
3	135611906	135611921	135611917	GAAG	A	snp	1	0	0	0	.	.	.	INTERGENIC(INTERGENIC)
3	135671418	135671437	135671433	AAGG	AAGT	ins	1	1	0	1	.	.	.	INTERGENIC(INTERGENIC)
3	135949732	135949741	135949737	T	G	snp	1	1	1	1	.	.	.	INTERGENIC(INTERGENIC)
3	136273943	136273952	136273947	AT	G	snp	1	0	0	0	.	.	.	STAG1(INTRON)
3	136275413	136275422	136275419	T	G	snp	1	0	0	0	.	.	.	STAG1(INTRON)
3	136429903	136429920	136429906	AT	A	snp	1	1	0	1	.	.	.	STAG1(INTRON)
3	136597974	136597982	136597975	A	C	ins	1	0	1	0	.	.	.	NCK1(INTRON)
3	136613722	136613731	136613725	T	G	ins	1	1	1	1	.	.	.	NCK1(INTRON);RP11-85F14.1.1(DOWNSTREAM)
3	136772088	136772103	136772099	TTAA	G	snp	1	1	1	1	.	.	.	INTERGENIC(INTERGENIC)
3	136846822	136846830	136846823	G	A	snp	1	1	1	0	.	.	.	INTERGENIC(INTERGENIC)
3	136849965	136849980	136849972	TTTA	G	snp	1	1	1	1	.	.	.	INTERGENIC(INTERGENIC)
3	136873903	136873912	136873907	T	G	snp	1	1	1	1	.	.	.	INTERGENIC(INTERGENIC)
3	137030179	137030186	137030183	T	C	snp	1	1	1	1	.	.	.	INTERGENIC(INTERGENIC)
3	137032220	137032227	137032221	T	C	snp	1	1	1	1	.	.	.	INTERGENIC(INTERGENIC)
3	137037135	137037142	137037136	T	C	ins	1	1	1	1	.	.	.	INTERGENIC(INTERGENIC)
3	137286644	137286651	137286648	T	C	snp	1	1	1	1	.	.	.	INTERGENIC(INTERGENIC)
3	137615024	137615033	137615025	A	C	snp	1	1	1	1	.	.	.	INTERGENIC(INTERGENIC)
3	137628507	137628515	137628514	A	G	snp	1	1	0	1	.	.	.	INTERGENIC(INTERGENIC)
3	137756487	137756498	137756491	TCT	T	ins	1	1	1	1	.	.	.	CLDN18(DOWNSTREAM);INTERGENIC(INTERGENIC)
3	137874708	137874717	137874715	T	C	ins	1	0	0	0	.	.	.	INTERGENIC(INTERGENIC)
3	137964256	137964264	137964263	T	G	snp	0	1	0	0	.	.	.	ARMC8(EXON);ARMC8(INTRON);ARMC8(UTR_3_PRIME)
3	138007033	138007044	138007038	TG	A	snp	1	1	0	1	.	.	.	ARMC8(INTRON);ARMC8(UPSTREAM);NME9(INTRON)
3	138144854	138144861	138144855	A	C	snp	1	0	1	0	.	.	.	INTERGENIC(INTERGENIC)
3	138402351	138402359	138402354	A	G	snp	1	0	0	0	.	.	.	PIK3CB(DOWNSTREAM);PIK3CB(INTRON)
3	138449406	138449417	138449410	AT	G	snp	1	0	0	0	.	.	.	PIK3CB(INTRON)
3	138604293	138604300	138604297	T	C	ins	1	1	1	1	.	.	.	ATP5G1P3(DOWNSTREAM);INTERGENIC(INTERGENIC)
3	138704309	138704318	138704317	A	C	snp	1	0	0	0	.	.	.	INTERGENIC(INTERGENIC)
3	138835173	138835182	138835177	AT	G	snp	0	1	0	0	.	.	.	BPESC1(INTRON);MRPS22(INTRON)
3	138859319	138859334	138859321	GT	A	snp	1	1	1	1	.	.	.	MRPS22(INTRON)
3	138979501	138979520	138979508	AGGG	A	snp	1	1	1	1	.	.	.	MRPS22(INTRON)
3	138979501	138979520	138979511	AGGG	A	snp	1	1	0	0	.	.	.	MRPS22(INTRON)
3	139024746	139024757	139024750	CT	A	snp	1	0	0	0	.	.	.	MRPS22(INTRON)
3	139470740	139470748	139470745	A	C	snp	0	1	0	1	.	.	.	INTERGENIC(INTERGENIC)
3	139471690	139471701	139471695	AAC	T	snp	0	1	0	1	.	.	.	INTERGENIC(INTERGENIC)
3	139480511	139480524	139480522	TA	T	del	1	1	0	1	.	.	.	INTERGENIC(INTERGENIC)
3	139521583	139521594	139521590	TA	T	del	1	1	1	1	.	.	.	INTERGENIC(INTERGENIC)
3	139561573	139561581	139561579	A	C	snp	0	0	0	1	.	.	.	RP11-166D18.1.1(INTRON);RP11-166D18.2.1(DOWNSTREAM)

3	139612240	139612247	139612246	C	A	snp	1	1	1	1	.	.	.	INTERGENIC(INTERGENIC)
3	139620431	139620444	139620442	AG	AC	ins	1	1	1	1	.	.	.	INTERGENIC(INTERGENIC)
3	139620431	139620444	139620442	AG	C	snp	1	1	1	1	.	.	.	INTERGENIC(INTERGENIC)
3	139689161	139689168	139689163	G	T	snp	1	1	1	1	.	.	.	CLSTN2(INTRON)
3	140057407	140057414	140057408	C	T	ins	1	0	0	0	.	.	.	CLSTN2(INTRON)
3	140223054	140223069	140223059	ATGA	C	snp	0	0	1	0	.	.	.	CLSTN2(INTRON);RP11-13L2.2.1(DOWNSTREAM)
3	140245486	140245495	140245488	A	C	snp	1	1	0	0	.	.	.	CLSTN2(INTRON)
3	140279192	140279201	140279198	CA	T	snp	1	1	1	1	.	.	.	CLSTN2(DOWNSTREAM);CLSTN2(INTRON)
3	140434503	140434512	140434507	TA	C	snp	1	0	0	0	.	.	.	INTERGENIC(INTERGENIC)
3	140509991	140509998	140509995	A	G	snp	1	1	1	1	.	.	.	INTERGENIC(INTERGENIC)
3	140598512	140598521	140598514	CT	G	snp	1	1	1	0	.	.	.	INTERGENIC(INTERGENIC)
3	140774850	140774859	140774853	GA	T	snp	1	1	1	1	.	.	.	SPSB4(INTRON)
3	140851495	140851512	140851509	TA	A	del	1	1	0	0	.	.	.	SPSB4(INTRON)
3	140870586	140870597	140870590	CA	T	snp	1	1	1	0	.	.	.	INTERGENIC(INTERGENIC);SPSB4(DOWNSTREAM)
3	140903087	140903110	140903103	GTTT	TTTA	ins	1	1	1	0	.	.	.	INTERGENIC(INTERGENIC)
3	141004367	141004375	141004369	A	C	snp	1	1	1	1	.	.	.	ACPL2(INTRON);ACPL2(UPSTREAM);RP11-438D8.2.1(INTRON)
3	141008784	141008795	141008787	ATC	G	snp	1	1	1	1	.	.	.	ACPL2(DOWNSTREAM);ACPL2(INTRON);ACPL2(UPSTREAM);RP11-438D8.2.1(INTRON)
3	141110020	141110027	141110025	A	G	snp	0	0	1	0	.	.	.	ZBTB38(DOWNSTREAM);ZBTB38(INTRON)
3	141128517	141128528	141128524	TGC	A	snp	1	0	0	0	.	.	.	ZBTB38(DOWNSTREAM);ZBTB38(INTRON)
3	141138830	141138837	141138833	A	G	snp	1	1	1	1	.	.	.	ZBTB38(INTRON)
3	141155346	141155353	141155352	T	G	snp	1	0	0	0	.	.	.	ZBTB38(INTRON)
3	141253215	141253222	141253216	G	T	snp	1	1	1	1	.	.	.	RASA2(INTRON)
3	141281497	141281505	141281498	A	C	snp	1	0	0	0	.	.	.	RASA2(INTRON)
3	141356882	141356890	141356883	A	G	snp	0	1	1	1	.	.	.	INTERGENIC(INTERGENIC)
3	141577794	141577813	141577806	AAAG	G	snp	1	0	0	0	.	.	.	INTERGENIC(INTERGENIC)
3	141591277	141591288	141591283	TAA	C	snp	0	0	0	1	.	.	.	ATP1B3(UPSTREAM);INTERGENIC(INTERGENIC)
3	141608920	141608927	141608926	T	G	snp	0	1	1	1	.	.	.	ATP1B3(INTRON)
3	141683504	141683512	141683508	A	C	snp	0	1	1	1	.	.	.	TFDP2(INTRON)
3	141688075	141688088	141688085	AT	G	snp	1	0	0	0	.	.	.	TFDP2(DOWNSTREAM);TFDP2(INTRON)
3	141688116	141688129	141688120	AT	G	snp	0	1	1	1	.	.	.	TFDP2(DOWNSTREAM);TFDP2(INTRON)
3	141695932	141695949	141695935	AT	G	snp	0	1	1	1	.	.	.	TFDP2(DOWNSTREAM);TFDP2(INTRON)
3	141695932	141695949	141695939	AT	G	snp	0	1	1	1	.	.	.	TFDP2(DOWNSTREAM);TFDP2(INTRON)
3	141885109	141885117	141885112	A	G	snp	0	1	1	1	.	.	.	GK5(DOWNSTREAM);GK5(INTRON)
3	141968357	141968364	141968359	T	G	snp	1	1	1	1	.	.	.	INTERGENIC(INTERGENIC)
3	142015057	142015068	142015064	ATA	A	ins	1	0	0	0	.	.	.	INTERGENIC(INTERGENIC)
3	142019458	142019467	142019465	A	C	snp	0	0	0	1	.	.	.	INTERGENIC(INTERGENIC)
3	142041518	142041533	142041529	CAAA	G	ins	1	0	0	0	.	.	.	XRN1(INTRON)
3	142053554	142053563	142053561	TA	G	snp	1	1	1	1	.	.	.	XRN1(INTRON);XRN1(UPSTREAM)
3	142150735	142150744	142150737	AT	G	snp	1	1	1	1	.	.	.	XRN1(DOWNSTREAM);XRN1(INTRON);XRN1(UPSTREAM)
3	142193770	142193778	142193775	T	C	snp	1	0	0	0	.	.	.	ATR(INTRON);RP11-383G6.3.1(UPSTREAM)
3	142320076	142320085	142320081	TA	G	snp	0	1	1	1	.	.	.	PLS1(INTRON)
3	142436785	142436808	142436804	AAC	C	del	1	1	1	1	.	.	.	INTERGENIC(INTERGENIC);PLS1(DOWNSTREAM)
3	142443442	142443453	142443450	CCT	A	snp	1	0	0	0	.	TRPC1(NON_SYNONYMOUS_CODING):S17T	.	TRPC1(UTR_5_PRIME)
3	142454618	142454635	142454622	GT	A	snp	0	0	1	1	.	.	.	TRPC1(INTRON)
3	142466185	142466193	142466188	A	G	snp	1	0	0	0	.	.	.	TRPC1(INTRON)
3	142477896	142477903	142477900	T	C	snp	1	1	1	1	.	.	.	TRPC1(INTRON)
3	142540749	142540757	142540755	T	G	snp	1	0	1	0	.	.	.	PCOLCE2(INTRON)
3	142552019	142552028	142552024	T	C	snp	1	1	0	1	.	.	.	PCOLCE2(INTRON);PCOLCE2(UPSTREAM)
3	142577578	142577595	142577592	TG	C	snp	1	1	0	1	.	.	.	PCOLCE2(INTRON)
3	142596768	142596783	142596775	AATG	T	snp	1	0	0	1	.	.	.	PCOLCE2(INTRON)
3	142676411	142676426	142676419	GTTT	TTTGT	del	1	0	0	0	.	.	.	PAQR9(DOWNSTREAM);PAQR9(INTRON);RP11-372E1.7.1(UPSTREAM)
3	142688840	142688851	142688843	TA	G	snp	1	1	0	0	.	.	.	RP11-372E1.6.1(DOWNSTREAM);RP11-372E1.6.1(INTRON)
3	142863555	142863563	142863559	C	CT	ins	1	1	1	1	.	.	.	INTERGENIC(INTERGENIC)
3	142863555	142863563	142863560	C	T	ins	1	1	1	1	.	.	.	INTERGENIC(INTERGENIC)
3	142863555	142863563	142863561	C	T	snp	1	1	1	1	.	.	.	INTERGENIC(INTERGENIC)
3	142880026	142880043	142880029	TA	G	snp	1	1	0	1	.	.	.	AC018450.1(UPSTREAM);INTERGENIC(INTERGENIC)
3	142880026	142880043	142880041	TA	T	ins	1	1	0	1	.	.	.	AC018450.1(UPSTREAM);INTERGENIC(INTERGENIC)
3	142880026	142880043	142880041	TA	T	snp	0	1	1	1	.	.	.	AC018450.1(UPSTREAM);INTERGENIC(INTERGENIC)
3	142896799	142896808	142896803	CA	A	del	1	0	0	0	.	.	.	PBX2P1(DOWNSTREAM);PBX2P1(EXON)
3	143064862	143064871	143064866	T	G	snp	1	1	1	1	.	.	.	SLC9A9(INTRON);SLC9A9-AS1(INTRON)
3	143107355	143107364	143107361	AT	C	snp	1	1	1	1	.	.	.	SLC9A9(INTRON)
3	143112988	143112995	143112989	A	C	snp	0	1	1	1	.	.	.	SLC9A9(INTRON)
3	143157266	143157279	143157268	GT	T	snp	0	1	1	1	.	.	.	SLC9A9(INTRON)

3	143279572	143279581	143279579	A	AAG	ins	1	1	1	1	.	.	.	SLC9A9(INTRON)
3	143294355	143294362	143294356	T	C	snp	1	1	1	0	.	.	.	SLC9A9(INTRON)
3	143334890	143334897	143334891	T	G	snp	1	1	1	1	.	.	.	RP11-501M7.1.1(UPSTREAM);SLC9A9(INTRON)
3	143353497	143353510	143353506	CA	G	snp	1	1	1	1	.	.	.	SLC9A9(INTRON)
3	143377428	143377435	143377429	T	G	snp	1	0	0	0	.	.	.	SLC9A9(INTRON)
3	143395233	143395248	143395242	AAAC	G	snp	1	0	0	0	.	.	.	SLC9A9(INTRON)
3	143439082	143439093	143439089	TA	G	snp	1	1	1	1	.	.	.	SLC9A9(INTRON)
3	143439082	143439093	143439091	TA	T	ins	0	1	1	1	.	.	.	SLC9A9(INTRON)
3	143485416	143485423	143485422	T	C	ins	1	0	0	0	.	.	.	SLC9A9(INTRON)
3	143534711	143534730	143534726	AAAC	A	snp	1	1	1	1	.	.	.	SLC9A9(INTRON);SLC9A9(UPSTREAM)
3	143697445	143697456	143697448	TA	C	snp	1	1	1	1	.	.	.	C3orf58(INTRON)
3	143701753	143701760	143701755	A	G	snp	1	1	1	1	.	.	.	C3orf58(INTRON)
3	143713828	143713843	143713835	ATTT	C	snp	1	1	1	1	.	.	.	C3orf58(DOWNSTREAM);C3orf58(INTRON)
3	143758982	143758993	143758990	ACA	C	snp	1	0	0	0	.	.	.	C3orf58(INTRON)
3	143779059	143779074	143779070	TTTG	T	snp	1	1	0	0	.	.	.	INTERGENIC(INTERGENIC)
3	143871819	143871830	143871826	TG	TT	ins	1	0	1	0	.	.	.	INTERGENIC(INTERGENIC)
3	144096055	144096062	144096058	A	G	snp	1	1	0	1	.	.	.	INTERGENIC(INTERGENIC)
3	144112505	144112516	144112513	CA	T	snp	1	1	1	1	.	.	.	INTERGENIC(INTERGENIC)
3	144204362	144204373	144204370	TTA	G	snp	1	0	0	0	.	.	.	INTERGENIC(INTERGENIC)
3	144212046	144212054	144212050	A	G	snp	1	1	0	1	.	.	.	INTERGENIC(INTERGENIC)
3	144323627	144323635	144323628	T	G	snp	1	1	0	1	.	.	.	INTERGENIC(INTERGENIC)
3	144328300	144328309	144328307	TG	T	snp	1	1	0	1	.	.	.	INTERGENIC(INTERGENIC)
3	144342393	144342408	144342403	TATT	C	snp	1	0	0	0	.	.	.	INTERGENIC(INTERGENIC)
3	144359162	144359173	144359168	TG	G	snp	1	1	1	1	.	.	.	INTERGENIC(INTERGENIC)
3	144382102	144382109	144382107	T	C	snp	0	0	1	0	.	.	.	INTERGENIC(INTERGENIC)
3	144393651	144393660	144393658	AT	C	snp	1	0	1	0	.	.	.	INTERGENIC(INTERGENIC)
3	144521749	144521760	144521752	TAT	C	snp	1	0	1	0	.	.	.	INTERGENIC(INTERGENIC)
3	144556704	144556713	144556709	T	G	snp	1	0	0	0	.	.	.	INTERGENIC(INTERGENIC)
3	144575709	144575716	144575712	T	C	snp	1	1	0	0	.	.	.	INTERGENIC(INTERGENIC)
3	144593532	144593541	144593536	TG	A	snp	0	1	0	1	.	.	.	INTERGENIC(INTERGENIC)
3	144617140	144617149	144617142	T	C	snp	1	0	0	0	.	.	.	INTERGENIC(INTERGENIC)
3	144617140	144617149	144617144	T	G	snp	0	0	0	1	.	.	.	INTERGENIC(INTERGENIC)
3	144897569	144897588	144897574	AGGG	A	snp	1	1	1	1	.	.	.	INTERGENIC(INTERGENIC)
3	144991022	144991037	144991029	AT	ATATG	ins	1	1	1	1	.	.	.	INTERGENIC(INTERGENIC)
3	144991022	144991037	144991033	AT	G	ins	1	1	1	1	.	.	.	INTERGENIC(INTERGENIC)
3	144991065	144991076	144991073	AT	G	snp	1	0	0	0	.	.	.	INTERGENIC(INTERGENIC)
3	145003678	145003689	145003686	ATT	A	del	1	0	0	0	.	.	.	INTERGENIC(INTERGENIC)
3	145093915	145093930	145093920	AG	C	snp	1	0	0	0	.	.	.	INTERGENIC(INTERGENIC)
3	145216512	145216521	145216518	TA	A	snp	1	0	0	0	.	.	.	INTERGENIC(INTERGENIC)
3	145253882	145253889	145253885	A	G	snp	1	1	1	1	.	.	.	INTERGENIC(INTERGENIC)
3	145267762	145267770	145267764	T	G	snp	1	1	1	1	.	.	.	INTERGENIC(INTERGENIC)
3	145305393	145305401	145305394	A	C	snp	1	1	1	1	.	.	.	INTERGENIC(INTERGENIC)
3	145415689	145415697	145415694	A	C	snp	1	0	0	0	.	.	.	INTERGENIC(INTERGENIC)
3	145437847	145437862	145437852	TA	G	snp	1	0	0	0	.	.	.	INTERGENIC(INTERGENIC)
3	145439052	145439065	145439058	TA	AA	ins	1	1	1	1	.	.	.	INTERGENIC(INTERGENIC)
3	145440756	145440765	145440759	TG	TA	ins	1	0	0	0	.	.	.	INTERGENIC(INTERGENIC)
3	145470106	145470119	145470113	AG	C	snp	1	1	1	1	.	.	.	INTERGENIC(INTERGENIC)
3	145539053	145539064	145539056	TTA	A	snp	1	1	1	1	.	.	.	INTERGENIC(INTERGENIC);RP11-88H10.1.1(DOWNSTREAM)
3	145543375	145543383	145543378	T	C	snp	1	0	0	0	.	.	.	INTERGENIC(INTERGENIC);RP11-88H10.1.1(UPSTREAM)
3	145619189	145619198	145619195	AC	G	snp	1	1	1	1	.	.	.	AC055758.1(DOWNSTREAM);INTERGENIC(INTERGENIC)
3	145652302	145652309	145652305	C	A	ins	1	0	0	0	.	.	.	INTERGENIC(INTERGENIC)
3	145693497	145693504	145693502	C	A	snp	0	0	1	1	.	.	.	INTERGENIC(INTERGENIC)
3	145730527	145730536	145730530	TA	G	snp	1	0	0	0	.	.	.	INTERGENIC(INTERGENIC)
3	145753271	145753279	145753275	A	G	snp	1	1	1	1	.	.	.	INTERGENIC(INTERGENIC)
3	145815591	145815611	145815596	AAT	A	snp	1	1	1	1	.	.	.	PLOD2(DOWNSTREAM);PLOD2(INTRON);RP11-274H2.2.1(INTRON)
3	145820682	145820693	145820684	GT	A	snp	1	1	1	1	.	.	.	PLOD2(INTRON);RP11-274H2.2.1(INTRON)
3	145888875	145888886	145888883	TC	C	snp	1	1	1	1	.	.	.	INTERGENIC(INTERGENIC)
3	145957806	145957825	145957817	AAAT	G	snp	1	0	0	0	.	.	.	PLSCR4(INTRON)
3	145989875	145989884	145989881	TG	G	snp	1	0	0	0	.	.	.	INTERGENIC(INTERGENIC)
3	146030808	146030816	146030815	T	G	snp	1	1	1	1	.	.	.	INTERGENIC(INTERGENIC)
3	146112063	146112071	146112065	T	TC	ins	0	1	0	0	.	.	.	PLSCR2(INTRON);RP11-758I14.3.1(DOWNSTREAM);RP11-758I14.3.1(INTRON)
3	146112063	146112071	146112066	T	C	ins	0	1	0	0	.	.	.	PLSCR2(INTRON);RP11-758I14.3.1(DOWNSTREAM);RP11-758I14.3.1(INTRON)
3	146120257	146120266	146120264	T	G	snp	0	1	0	0	.	.	.	PLSCR2(INTRON);RP11-758I14.3.1(INTRON)
3	146344930	146344937	146344932	C	T	ins	1	1	1	1	.	.	.	INTERGENIC(INTERGENIC)

3	146344930	146344937	146344934	C	T	ins	1	1	1	1	.	.	.	INTERGENIC(INTERGENIC)
3	146353419	146353430	146353425	TA	AC	ins	1	1	0	1	.	.	.	INTERGENIC(INTERGENIC)
3	146353419	146353430	146353425	TA	C	snp	0	0	0	1	.	.	.	INTERGENIC(INTERGENIC)
3	146377870	146377877	146377876	A	G	snp	0	0	0	1	.	.	.	INTERGENIC(INTERGENIC)
3	146442887	146442900	146442892	AT	C	snp	1	0	0	0	.	.	.	INTERGENIC(INTERGENIC)
3	146450506	146450513	146450507	T	TTTTTTC	ins	0	1	0	1	.	.	.	INTERGENIC(INTERGENIC)
3	146457233	146457241	146457239	T	C	snp	0	1	0	1	.	.	.	INTERGENIC(INTERGENIC)
3	146508619	146508626	146508620	A	C	snp	1	0	0	0	.	.	.	INTERGENIC(INTERGENIC)
3	146518321	146518330	146518324	TA	C	snp	1	1	1	1	.	.	.	INTERGENIC(INTERGENIC)
3	146520524	146520532	146520526	A	C	snp	1	1	1	1	.	.	.	INTERGENIC(INTERGENIC)
3	146579237	146579254	146579248	TGA	G	snp	0	1	1	1	.	.	.	INTERGENIC(INTERGENIC)
3	146632667	146632676	146632673	CA	T	snp	0	1	0	1	.	.	.	RP11-649A16.1.1(INTRON)
3	146655171	146655180	146655174	AT	G	snp	1	0	0	0	.	.	.	RP11-649A16.1.1(INTRON)
3	146765616	146765625	146765620	TA	C	snp	0	1	0	1	.	.	.	RP11-649A16.1.1(INTRON)
3	146787500	146787511	146787503	TTA	C	snp	1	0	0	0	.	.	.	RP11-649A16.1.1(INTRON)
3	146824131	146824140	146824133	T	G	ins	1	1	1	1	.	.	.	RP11-649A16.1.1(INTRON)
3	146854115	146854122	146854117	A	G	snp	1	0	0	0	.	.	.	RP11-649A16.1.1(INTRON)
3	146854894	146854902	146854896	T	G	snp	1	0	0	0	.	.	.	RP11-649A16.1.1(INTRON)
3	146942250	146942269	146942264	TCTT	TTC	del	0	1	0	1	.	.	.	RP11-649A16.1.1(INTRON)
3	146942268	146942276	146942271	T	C	snp	0	0	1	0	.	.	.	RP11-649A16.1.1(INTRON)
3	146943452	146943460	146943453	T	G	snp	0	1	0	1	.	.	.	RP11-649A16.1.1(INTRON)
3	146943590	146943605	146943593	TG	T	snp	1	1	1	1	.	.	.	RP11-649A16.1.1(INTRON)
3	146944735	146944746	146944737	GA	C	snp	1	1	0	1	.	.	.	RP11-649A16.1.1(INTRON)
3	147235170	147235179	147235176	TA	ATAC	ins	0	0	0	1	.	.	.	INTERGENIC(INTERGENIC)
3	147251907	147251914	147251908	T	G	snp	0	1	0	1	.	.	.	INTERGENIC(INTERGENIC)
3	147276536	147276543	147276540	A	G	ins	1	1	1	1	.	.	.	INTERGENIC(INTERGENIC)
3	147368778	147368787	147368782	AC	G	snp	1	1	1	1	.	.	.	INTERGENIC(INTERGENIC)
3	147415628	147415641	147415633	TC	TCTA	ins	0	1	0	1	.	.	.	INTERGENIC(INTERGENIC)
3	147614655	147614662	147614656	T	C	snp	1	0	0	0	.	.	.	INTERGENIC(INTERGENIC)
3	147728655	147728663	147728660	A	G	snp	1	0	0	0	.	.	.	INTERGENIC(INTERGENIC);RP11-71N10.1.1(DOWNSTREAM)
3	147904540	147904548	147904544	A	C	snp	0	0	1	0	.	.	.	RP11-501O2.3.1(INTRON);RP11-501O2.3.1(UPSTREAM)
3	147922990	147922998	147922991	T	C	snp	1	0	0	0	.	.	.	RP11-501O2.3.1(INTRON);RP11-501O2.4.1(INTRON)
3	148121188	148121197	148121192	T	C	snp	1	0	0	0	.	.	.	INTERGENIC(INTERGENIC);RP11-501O2.5.1(DOWNSTREAM)
3	148263203	148263218	148263216	TG	A	snp	1	1	1	1	.	.	.	INTERGENIC(INTERGENIC)
3	148390642	148390649	148390647	T	C	snp	1	1	1	1	.	.	.	INTERGENIC(INTERGENIC)
3	148574595	148574602	148574597	T	G	snp	0	0	1	0	.	.	.	CPB1(EXON);CPB1(INTRON);CPB1(UPSTREAM);RP11-680B3.2.1(INTRON)
3	148581722	148581729	148581728	A	G	snp	1	0	0	0	.	.	.	CPA3(UPSTREAM);CPB1(DOWNSTREAM);RP11-680B3.2.1(INTRON)
3	148689274	148689283	148689276	A	G	snp	0	1	0	0	.	.	.	INTERGENIC(INTERGENIC)
3	148701962	148701971	148701965	T	C	ins	1	0	0	0	.	.	.	INTERGENIC(INTERGENIC);UBQLN4P1(DOWNSTREAM)
3	148806496	148806509	148806498	CA	T	snp	1	1	1	1	.	.	.	HLTF(UPSTREAM);HLTF-AS1(INTRON)
3	148828434	148828442	148828439	A	C	snp	1	1	1	1	.	.	.	INTERGENIC(INTERGENIC)
3	149012505	149012514	149012512	TC	G	snp	0	1	0	1	.	.	.	RP11-206M11.4.1(UPSTREAM);RP11-206M11.7.1(INTRON)
3	149051249	149051256	149051255	T	TC	ins	1	1	1	1	.	.	.	RP11-206M11.7.1(INTRON);TM4SF18(INTRON);TM4SF18(UPSTREAM);TM4SF18(UTR_5_PRIME)
3	149067190	149067201	149067192	AC	G	snp	1	0	0	0	.	.	.	INTERGENIC(INTERGENIC)
3	149081675	149081684	149081677	A	C	snp	1	1	1	1	.	.	.	INTERGENIC(INTERGENIC)
3	149108375	149108382	149108378	A	G	snp	0	1	1	1	.	.	.	INTERGENIC(INTERGENIC);RP11-278L15.2.1(UPSTREAM);TM4SF1-AS1(DOWNSTREAM)
3	149233662	149233670	149233665	T	TTG	ins	1	1	1	1	.	.	.	INTERGENIC(INTERGENIC);WWTR1(DOWNSTREAM)
3	149233662	149233670	149233667	T	G	ins	1	1	1	1	.	.	.	INTERGENIC(INTERGENIC);WWTR1(DOWNSTREAM)
3	149233662	149233670	149233668	T	G	snp	1	1	1	1	.	.	.	INTERGENIC(INTERGENIC);WWTR1(DOWNSTREAM)
3	149292885	149292894	149292886	A	C	snp	1	1	1	1	.	.	.	WWTR1(INTRON)
3	149321338	149321345	149321343	C	T	snp	1	0	0	0	.	.	.	WWTR1(INTRON)
3	149353286	149353300	149353290	AGA	T	snp	1	1	1	1	.	.	.	WWTR1(INTRON)
3	149538434	149538441	149538439	A	C	snp	1	0	0	0	.	.	.	ANKUB1(INTRON);RNF13(INTRON)
3	149774641	149774655	149774652	TAT	TATTAC	ins	1	0	0	0	.	.	.	RP11-167H9.4.1(INTRON)
3	149799340	149799351	149799349	CA	C	snp	1	1	1	0	.	.	.	RP11-167H9.3.1(UPSTREAM);RP11-167H9.4.1(INTRON);snoU13(UPSTREAM)
3	149964362	149964373	149964367	GAA	C	snp	1	0	0	0	.	.	.	INTERGENIC(INTERGENIC)
3	150045401	150045409	150045404	T	G	snp	0	1	0	1	.	.	.	INTERGENIC(INTERGENIC)
3	150058395	150058403	150058398	T	C	snp	1	0	0	0	.	.	.	INTERGENIC(INTERGENIC)
3	150062586	150062594	150062592	T	G	snp	1	1	1	1	.	.	.	INTERGENIC(INTERGENIC)
3	150066228	150066236	150066233	T	G	snp	1	1	1	1	.	.	.	INTERGENIC(INTERGENIC)
3	150066234	150066253	150066238	TTTG	G	ins	0	0	0	1	.	.	.	INTERGENIC(INTERGENIC)
3	150102386	150102393	150102390	A	G	snp	1	1	1	1	.	.	.	INTERGENIC(INTERGENIC)

3	150105695	150105702	150105701	T	C	snp	0	1	1	1	.	.	.	INTERGENIC(INTERGENIC)
3	150345689	150345698	150345690	T	G	snp	1	1	1	1	.	.	.	RP11-392O18.1.1(DOWNSTREAM);RP11-392O18.1.1(EXON);RP11-392O18.1.1(UTR_3_PRIME)
3	150402580	150402589	150402583	T	C	snp	1	1	1	1	.	.	.	FAM194A(DOWNSTREAM);FAM194A(INTRON);FAM194A(UPSTREAM)
3	150436186	150436197	150436194	ATT	A	del	1	1	1	1	.	.	.	RP11-103G8.1.1(DOWNSTREAM);RP11-103G8.2.1(INTRON)
3	150543659	150543667	150543664	C	A	ins	1	1	0	1	.	.	.	INTERGENIC(INTERGENIC)
3	150639159	150639174	150639165	CA	AA	ins	1	1	1	1	.	.	.	AC020636.1(DOWNSTREAM);CLRN1(DOWNSTREAM);CLRN1-AS1(INTRON);RP11-166N6.2.1(INTRON)
3	150696139	150696146	150696143	T	G	snp	1	1	1	1	.	.	.	CLRN1-AS1(INTRON);RP11-166N6.2.1(INTRON)
3	150696752	150696761	150696758	AT	G	snp	1	1	1	1	.	.	.	CLRN1-AS1(INTRON);RP11-166N6.2.1(INTRON)
3	150760888	150760899	150760894	TAT	G	snp	1	0	0	0	.	.	.	CLRN1-AS1(INTRON);RP11-166N6.2.1(DOWNSTREAM)
3	150792801	150792810	150792807	TA	AC	ins	1	1	1	1	.	.	.	CLRN1-AS1(INTRON)
3	150814444	150814451	150814445	G	T	snp	0	1	1	1	.	.	.	MED12L(INTRON)
3	151062094	151062102	151062101	A	AC	ins	1	1	1	1	.	.	.	MED12L(INTRON);P2RY12(INTRON);P2RY12(UPSTREAM)
3	151125654	151125661	151125658	T	C	snp	0	1	1	0	.	.	.	MED12L(INTRON)
3	151203035	151203044	151203036	A	G	snp	1	1	1	1	.	.	.	INTERGENIC(INTERGENIC)
3	151211641	151211656	151211647	AAAC	C	del	1	1	1	1	.	.	.	INTERGENIC(INTERGENIC)
3	151211641	151211656	151211650	AAAC	ACAAAC	del	1	1	1	1	.	.	.	INTERGENIC(INTERGENIC)
3	151230947	151230954	151230952	A	G	snp	0	0	1	0	.	.	.	INTERGENIC(INTERGENIC)
3	151271762	151271771	151271763	A	C	ins	1	1	1	1	.	.	.	INTERGENIC(INTERGENIC)
3	151278491	151278498	151278496	C	A	ins	1	1	1	1	.	.	.	INTERGENIC(INTERGENIC)
3	151368086	151368097	151368090	TC	G	snp	1	1	1	1	.	.	.	RP11-454C18.1.1(INTRON)
3	151430215	151430223	151430218	A	G	snp	1	0	0	0	.	.	.	INTERGENIC(INTERGENIC)
3	151436147	151436154	151436149	A	G	snp	1	1	1	1	.	.	.	INTERGENIC(INTERGENIC)
3	151467273	151467281	151467274	T	C	snp	1	1	1	1	.	.	.	AADACL2(INTRON);RP11-454C18.2.1(DOWNSTREAM)
3	151478933	151478946	151478939	AT	TT	ins	1	1	1	1	.	.	.	AADACL2(DOWNSTREAM);AADACL2(UTR_3_PRIME);RP11-454C18.2.1(INTRON);RP11-64D22.1.1(EXON)
3	151549566	151549575	151549573	A	G	snp	1	1	1	1	.	.	.	AADAC(DOWNSTREAM);RP11-454C18.2.1(INTRON)
3	151564825	151564832	151564831	T	CA	ins	1	0	0	0	.	.	.	RP11-454C18.2.1(INTRON)
3	151567869	151567877	151567875	T	TG	ins	1	1	1	1	.	.	.	RP11-454C18.2.1(INTRON)
3	151567876	151567890	151567884	TTG	T	ins	1	1	1	1	.	.	.	RP11-454C18.2.1(INTRON)
3	151567876	151567890	151567887	TTG	TT	ins	1	1	1	1	.	.	.	RP11-454C18.2.1(INTRON)
3	151615207	151615214	151615208	A	G	snp	0	1	0	1	.	.	.	RP11-454C18.2.1(INTRON)
3	151622165	151622172	151622168	T	G	snp	1	1	1	1	.	.	.	RP11-454C18.2.1(INTRON)
3	151723768	151723783	151723779	AAAG	AAAGAA	del	1	1	1	1	.	.	.	INTERGENIC(INTERGENIC)
3	151997384	151997391	151997387	A	AC	ins	1	1	1	1	.	.	.	MBNL1(INTRON)
3	151997384	151997391	151997388	A	C	ins	1	1	1	1	.	.	.	MBNL1(INTRON)
3	152011746	152011759	152011752	AT	G	snp	0	0	1	0	.	.	.	MBNL1(INTRON)
3	152047463	152047474	152047470	TA	T	ins	1	1	1	1	.	.	.	MBNL1(INTRON)
3	152196937	152196945	152196940	T	C	snp	0	1	0	1	.	.	.	RP11-362A9.3.1(INTRON)
3	152203248	152203259	152203250	TA	G	snp	1	1	0	1	.	.	.	RP11-362A9.3.1(INTRON)
3	152232811	152232826	152232822	AAAG	A	snp	1	1	1	1	.	.	.	INTERGENIC(INTERGENIC)
3	152362655	152362666	152362664	AT	G	snp	1	0	0	0	.	.	.	INTERGENIC(INTERGENIC);RP11-788A4.1.1(UPSTREAM)
3	152385439	152385452	152385444	TC	T	snp	1	1	1	0	.	.	.	INTERGENIC(INTERGENIC)
3	152412109	152412118	152412115	AT	G	snp	1	1	1	1	.	.	.	INTERGENIC(INTERGENIC)
3	152422292	152422301	152422298	A	C	snp	1	1	1	1	.	.	.	INTERGENIC(INTERGENIC)
3	152453884	152453893	152453892	A	AG	ins	1	0	0	0	.	.	.	INTERGENIC(INTERGENIC)
3	152483471	152483480	152483476	AC	T	snp	1	0	0	0	.	.	.	INTERGENIC(INTERGENIC)
3	152484761	152484770	152484762	A	G	snp	1	0	0	0	.	.	.	INTERGENIC(INTERGENIC)
3	152491056	152491065	152491064	T	G	snp	0	1	1	1	.	.	.	INTERGENIC(INTERGENIC)
3	152513768	152513776	152513774	A	G	snp	0	1	1	1	.	.	.	ATP5LP5(DOWNSTREAM);INTERGENIC(INTERGENIC)
3	152534051	152534070	152534062	TTTG	T	snp	1	1	1	1	.	.	.	INTERGENIC(INTERGENIC)
3	152534051	152534070	152534066	TTTG	T	ins	1	1	1	1	.	.	.	INTERGENIC(INTERGENIC)
3	152683318	152683327	152683326	A	G	ins	0	0	1	0	.	.	.	INTERGENIC(INTERGENIC)
3	152691202	152691217	152691213	TCAC	T	snp	1	1	1	1	.	.	.	INTERGENIC(INTERGENIC)
3	152707922	152707931	152707929	AT	C	snp	1	1	1	1	.	.	.	INTERGENIC(INTERGENIC)
3	152768594	152768602	152768599	A	C	snp	1	0	0	0	.	.	.	INTERGENIC(INTERGENIC)
3	152779823	152779832	152779825	T	C	snp	0	1	0	0	.	.	.	INTERGENIC(INTERGENIC)
3	152795259	152795266	152795262	A	C	snp	0	0	1	0	.	.	.	INTERGENIC(INTERGENIC)
3	152820367	152820375	152820370	A	C	ins	0	0	0	1	.	.	.	INTERGENIC(INTERGENIC)
3	152893561	152893569	152893568	T	C	snp	1	0	0	0	.	.	.	INTERGENIC(INTERGENIC)
3	152942912	152942920	152942913	A	C	snp	1	0	0	0	.	.	.	INTERGENIC(INTERGENIC)
3	152961684	152961703	152961692	TTTC	C	snp	1	1	1	1	.	.	.	INTERGENIC(INTERGENIC)
3	152985055	152985064	152985063	T	G	snp	1	1	1	1	.	.	.	INTERGENIC(INTERGENIC)
3	152986750	152986758	152986756	C	A	ins	1	1	1	1	.	.	.	INTERGENIC(INTERGENIC)
3	153062775	153062792	153062780	TTA	T	snp	1	0	0	0	.	.	.	INTERGENIC(INTERGENIC)

3	153116153	153116164	153116162	TA	TAT	del	1	1	1	1	.	.	.	RP11-23D24.2.1(INTRON)
3	153165502	153165519	153165514	AT	G	snp	1	0	0	0	.	.	.	RP11-23D24.2.1(INTRON);RP11-292E2.4.1(DOWNSTREAM)
3	153205161	153205184	153205171	TTTG	A	snp	1	1	1	0	.	.	.	C3orf79(INTRON);RP11-23D24.2.1(INTRON)
3	153207000	153207008	153207001	A	G	snp	1	1	1	1	.	.	.	C3orf79(INTRON);RP11-23D24.2.1(INTRON)
3	153207000	153207008	153207007	A	C	snp	1	0	1	0	.	.	.	C3orf79(INTRON);RP11-23D24.2.1(INTRON)
3	153218178	153218187	153218182	T	C	snp	1	0	1	0	.	.	.	C3orf79(INTRON);RP11-23D24.2.1(INTRON)
3	153270681	153270688	153270683	T	G	snp	1	1	1	1	.	.	.	RP11-23D24.2.1(INTRON)
3	153373341	153373350	153373346	AC	A	snp	0	1	1	1	.	.	.	AC069509.1(DOWNSTREAM);AC069509.2(UPSTREAM);RP11-23D24.2.1(INTRON)
3	153401743	153401752	153401750	AT	C	snp	1	1	0	1	.	.	.	RP11-23D24.2.1(INTRON)
3	153401876	153401887	153401880	TAA	G	snp	1	0	0	0	.	.	.	RP11-23D24.2.1(INTRON)
3	153556017	153556024	153556023	A	G	ins	1	1	1	1	.	.	.	RP11-23D24.2.1(INTRON)
3	153569562	153569571	153569566	A	G	snp	1	1	1	1	.	.	.	RP11-23D24.2.1(INTRON)
3	153678011	153678026	153678016	AG	A	snp	1	0	0	0	.	.	.	RP11-23D24.2.1(INTRON)
3	153801176	153801184	153801181	A	C	ins	1	1	1	1	.	.	.	ARHGEF26-AS1(INTRON)
3	153862750	153862759	153862757	TC	T	snp	1	1	1	1	.	.	.	ARHGEF26(INTRON)
3	153968174	153968181	153968176	G	A	snp	1	1	1	1	.	.	.	ARHGEF26(INTRON)
3	153983878	153983885	153983879	A	G	snp	1	0	0	0	.	.	.	INTERGENIC(INTERGENIC)
3	154045189	154045197	154045191	A	G	snp	1	1	1	1	.	.	.	DHX36(UPSTREAM);INTERGENIC(INTERGENIC);snoU13(UPSTREAM)
3	154295003	154295011	154295010	A	AC	ins	1	0	0	0	.	.	.	INTERGENIC(INTERGENIC)
3	154297099	154297110	154297108	AG	AA	ins	1	1	0	1	.	.	.	INTERGENIC(INTERGENIC)
3	154297123	154297130	154297126	A	G	snp	1	1	0	1	.	.	.	INTERGENIC(INTERGENIC)
3	154345941	154345950	154345944	A	C	ins	1	1	0	1	.	.	.	INTERGENIC(INTERGENIC)
3	154346955	154346963	154346962	C	A	ins	1	0	0	0	.	.	.	INTERGENIC(INTERGENIC)
3	154346955	154346963	154346962	C	A	snp	1	0	0	0	.	.	.	INTERGENIC(INTERGENIC)
3	154351953	154351961	154351957	T	C	snp	1	0	0	0	.	.	.	INTERGENIC(INTERGENIC)
3	154404642	154404651	154404648	TA	C	snp	1	0	0	0	.	.	.	INTERGENIC(INTERGENIC)
3	154410032	154410052	154410048	CAA	AC	del	1	1	0	1	.	.	.	INTERGENIC(INTERGENIC)
3	154526338	154526347	154526342	T	TC	ins	1	0	1	0	.	.	.	INTERGENIC(INTERGENIC)
3	154526539	154526548	154526544	AG	A	del	1	0	0	0	.	.	.	INTERGENIC(INTERGENIC)
3	154560803	154560810	154560804	T	TA	ins	1	0	1	0	.	.	.	RP11-439C8.1.1(INTRON)
3	154570103	154570110	154570106	C	CCA	ins	1	1	0	1	.	.	.	RP11-439C8.1.1(INTRON)
3	154600425	154600434	154600432	CA	G	snp	1	0	0	0	.	.	.	INTERGENIC(INTERGENIC)
3	154640431	154640439	154640436	A	C	snp	1	0	0	0	.	.	.	INTERGENIC(INTERGENIC)
3	154643236	154643245	154643239	AG	T	snp	1	0	0	0	.	.	.	INTERGENIC(INTERGENIC)
3	154756560	154756567	154756563	T	C	snp	1	0	0	0	.	.	.	MME(INTRON)
3	154801360	154801371	154801365	AGT	C	snp	1	1	1	1	.	.	.	MME(INTRON);MME(UPSTREAM);MME(UTR_5_PRIME)
3	154844333	154844341	154844339	T	G	snp	1	1	1	1	.	.	.	MME(INTRON)
3	154891783	154891794	154891788	TG	C	snp	0	0	1	0	.	.	.	MME(INTRON);MME-AS1(INTRON)
3	154899708	154899717	154899711	A	G	snp	0	0	1	0	.	.	.	MME(DOWNSTREAM);MME(UTR_3_PRIME);MME-AS1(INTRON)
3	154940416	154940423	154940420	T	G	snp	0	0	1	0	.	.	.	INTERGENIC(INTERGENIC)
3	154985650	154985658	154985651	A	C	snp	1	1	1	1	.	.	.	INTERGENIC(INTERGENIC)
3	155090610	155090625	155090616	TCAT	T	snp	1	1	1	1	.	.	.	INTERGENIC(INTERGENIC);PLCH1(DOWNSTREAM);RP11-639F1.1.1(DOWNSTREAM)
3	155318774	155318782	155318778	T	C	snp	1	1	1	1	.	.	.	PLCH1(INTRON)
3	155320941	155320950	155320942	A	C	ins	1	1	1	1	.	.	.	PLCH1(INTRON)
3	155380085	155380092	155380086	A	C	snp	1	1	1	1	.	.	.	PLCH1(INTRON);RPL6P7(DOWNSTREAM)
3	155583280	155583289	155583281	A	G	snp	1	0	0	0	.	.	.	INTERGENIC(INTERGENIC)
3	155666516	155666525	155666521	A	G	snp	1	1	0	1	.	.	.	INTERGENIC(INTERGENIC)
3	155706837	155706844	155706838	A	C	snp	0	1	1	1	.	.	.	INTERGENIC(INTERGENIC);SETP14(DOWNSTREAM)
3	155829918	155829926	155829925	T	G	snp	1	1	1	1	.	.	.	KCNAB1(INTRON);RP11-294L13.1.1(INTRON)
3	156019001	156019010	156019005	T	G	snp	1	0	0	0	.	.	.	KCNAB1(INTRON)
3	156115407	156115415	156115411	T	C	snp	1	0	0	0	.	.	.	KCNAB1(INTRON)
3	156182275	156182298	156182279	AAT	T	del	1	0	1	0	.	.	.	KCNAB1(DOWNSTREAM);KCNAB1(INTRON)
3	156187020	156187039	156187033	TCTT	T	ins	1	0	0	0	.	.	.	KCNAB1(INTRON)
3	156210221	156210228	156210222	T	C	snp	1	0	1	0	.	.	.	KCNAB1(INTRON)
3	156231001	156231010	156231008	A	C	snp	1	0	0	0	.	.	.	KCNAB1(INTRON)
3	156259170	156259179	156259178	T	C	snp	1	1	1	1	.	.	.	KCNAB1(DOWNSTREAM);SSR3(DOWNSTREAM);SSR3(UTR_3_PRIME)
3	156301041	156301058	156301050	TTG	G	snp	1	0	0	0	.	.	.	INTERGENIC(INTERGENIC)
3	156302715	156302732	156302724	AC	A	snp	0	1	1	1	.	.	.	INTERGENIC(INTERGENIC)
3	156315596	156315603	156315598	T	G	snp	1	0	0	0	.	.	.	INTERGENIC(INTERGENIC)
3	156319218	156319226	156319224	T	G	snp	1	0	1	0	.	.	.	INTERGENIC(INTERGENIC)
3	156384791	156384800	156384797	AT	T	snp	1	1	1	1	.	.	.	INTERGENIC(INTERGENIC);TIPARP-AS1(DOWNSTREAM)
3	156438513	156438522	156438518	A	C	snp	0	0	1	0	.	.	.	INTERGENIC(INTERGENIC)
3	156486641	156486648	156486645	A	C	snp	1	0	0	0	.	.	.	RP11-392A22.2.1(INTRON)

3	156504509	156504528	156504519	TTTA	C	snp	0	0	1	0	.	.	.	RP11-392A22.2.1(INTRON)
3	156576057	156576070	156576067	AG	C	snp	1	1	0	1	.	.	.	LEKR1(INTRON);RP11-6F2.7.1(INTRON)
3	156595566	156595585	156595578	TTTA	C	snp	1	0	0	0	.	.	.	LEKR1(INTRON);RP11-6F2.7.1(INTRON)
3	156595566	156595585	156595579	TTTA	G	snp	1	0	0	0	.	.	.	LEKR1(INTRON);RP11-6F2.7.1(INTRON)
3	156600964	156600972	156600967	A	G	snp	1	0	0	0	.	.	.	LEKR1(INTRON);RP11-6F2.7.1(INTRON)
3	156724993	156725004	156725001	AAT	A	ins	1	0	0	0	.	.	.	LEKR1(INTRON)
3	156789964	156789979	156789969	AGGA	A	snp	1	0	0	0	.	.	.	INTERGENIC(INTERGENIC)
3	156902142	156902150	156902146	T	G	snp	1	1	1	1	.	.	.	RP11-550I24.2.1(INTRON)
3	156934439	156934462	156934458	TTTA	T	del	1	0	1	0	.	.	.	RP11-550I24.2.1(INTRON)
3	156934460	156934483	156934463	TTA	TA	del	1	0	0	0	.	.	.	RP11-550I24.2.1(INTRON)
3	156934460	156934483	156934477	TTA	TT	ins	1	1	1	1	.	.	.	RP11-550I24.2.1(INTRON)
3	156934460	156934483	156934478	TTA	TA	del	1	1	1	1	.	.	.	RP11-550I24.2.1(INTRON)
3	156934460	156934483	156934479	TTA	A	del	1	1	1	1	.	.	.	RP11-550I24.2.1(INTRON)
3	156952052	156952060	156952057	T	C	snp	1	0	0	0	.	.	.	RP11-550I24.2.1(INTRON)
3	156953869	156953884	156953882	AT	A	del	1	1	1	1	.	.	.	RP11-550I24.2.1(INTRON)
3	156990107	156990120	156990117	CT	G	snp	1	1	1	1	.	.	.	RP11-550I24.2.1(INTRON);VEPH1(INTRON)
3	157070482	157070493	157070488	CAA	A	snp	1	1	0	1	.	.	.	RP11-550I24.2.1(INTRON);VEPH1(INTRON)
3	157206280	157206299	157206295	CTTT	C	del	1	1	1	1	.	.	.	VEPH1(DOWNSTREAM);VEPH1(INTRON)
3	157227984	157227992	157227991	A	C	snp	1	0	0	0	.	.	.	VEPH1(INTRON)
3	157266367	157266382	157266374	TTTA	T	snp	0	0	1	0	.	.	.	C3orf55(INTRON)
3	157340461	157340472	157340470	TG	C	snp	1	0	0	0	.	.	.	C3orf55(INTRON)
3	157434101	157434108	157434106	T	G	ins	1	1	0	0	.	.	.	INTERGENIC(INTERGENIC)
3	157439548	157439555	157439550	T	G	snp	1	1	1	1	.	.	.	INTERGENIC(INTERGENIC)
3	157440277	157440284	157440283	T	C	snp	1	1	0	0	.	.	.	INTERGENIC(INTERGENIC)
3	157485939	157485946	157485940	T	G	ins	0	0	0	1	.	.	.	INTERGENIC(INTERGENIC)
3	157500724	157500733	157500731	TA	T	ins	1	1	1	1	.	.	.	INTERGENIC(INTERGENIC)
3	157539565	157539580	157539569	ATGA	G	snp	1	0	0	0	.	.	.	INTERGENIC(INTERGENIC)
3	157636959	157636967	157636963	A	G	snp	1	0	0	0	.	.	.	INTERGENIC(INTERGENIC)
3	157671964	157671975	157671970	CA	T	snp	1	1	0	1	.	.	.	INTERGENIC(INTERGENIC)
3	157685116	157685124	157685117	T	G	snp	1	1	1	1	.	.	.	INTERGENIC(INTERGENIC)
3	157777684	157777697	157777690	AC	G	snp	1	0	0	0	.	.	.	RP11-290K4.2.1(INTRON)
3	157836956	157836965	157836961	T	G	snp	1	1	1	1	.	.	.	RSRC1(INTRON)
3	157947134	157947143	157947137	TA	G	snp	1	0	0	0	.	.	.	RSRC1(INTRON)
3	157947134	157947143	157947139	TA	G	snp	1	0	0	0	.	.	.	RSRC1(INTRON)
3	157947204	157947219	157947215	TGTA	TG	del	1	1	1	1	.	.	.	RSRC1(INTRON)
3	157976128	157976137	157976135	T	G	snp	1	0	0	0	.	.	.	RSRC1(INTRON)
3	158132621	158132628	158132626	A	G	snp	1	0	0	0	.	.	.	RSRC1(INTRON)
3	158154201	158154214	158154206	AT	A	snp	1	1	1	1	.	.	.	RSRC1(INTRON)
3	158175554	158175563	158175561	AT	C	snp	1	0	0	0	.	.	.	RSRC1(INTRON)
3	158269148	158269155	158269154	A	C	snp	1	1	1	1	.	.	.	RP11-538P18.2.1(DOWNSTREAM);RP11-538P18.2.1(INTRON)
3	158270297	158270304	158270299	T	C	snp	1	1	1	1	.	.	.	RP11-538P18.2.1(DOWNSTREAM);RP11-538P18.2.1(INTRON)
3	158413967	158413980	158413974	TA	TT	ins	1	1	1	1	.	.	.	GFM1(DOWNSTREAM);INTERGENIC(INTERGENIC);RARRES1(DOWNSTREAM)
3	158561403	158561410	158561406	A	G	snp	1	1	1	1	.	.	.	GPR79(UPSTREAM);INTERGENIC(INTERGENIC)
3	158627733	158627744	158627735	TG	C	snp	1	0	0	0	.	.	.	INTERGENIC(INTERGENIC)
3	158639190	158639197	158639193	T	C	snp	1	1	1	1	.	.	.	INTERGENIC(INTERGENIC)
3	158802703	158802712	158802707	T	C	ins	0	0	1	0	.	.	.	IQCJ(INTRON);IQCJ-SCHIP1(INTRON)
3	158915703	158915711	158915707	T	G	snp	0	1	1	1	.	.	.	IQCJ(INTRON);IQCJ-SCHIP1(INTRON)
3	159033341	159033350	159033347	TA	C	snp	1	0	0	0	.	.	.	IQCJ-SCHIP1(INTRON)
3	159136854	159136862	159136857	A	C	snp	1	1	1	1	.	.	.	IQCJ-SCHIP1(INTRON)
3	159136854	159136862	159136858	A	CG	ins	1	1	1	1	.	.	.	IQCJ-SCHIP1(INTRON)
3	159136854	159136862	159136858	A	G	snp	1	1	1	1	.	.	.	IQCJ-SCHIP1(INTRON)
3	159343970	159343997	159343984	TAGA	T	snp	1	0	1	0	.	.	.	IQCJ-SCHIP1(INTRON)
3	159343970	159343997	159343988	TAGA	T	snp	1	0	1	0	.	.	.	IQCJ-SCHIP1(INTRON)
3	159430636	159430645	159430639	AG	A	snp	1	0	1	0	.	.	.	IQCJ-SCHIP1(INTRON)
3	159589149	159589156	159589154	G	A	snp	0	1	0	1	.	.	.	IQCJ-SCHIP1(INTRON);SCHIP1(INTRON)
3	159680235	159680242	159680238	A	G	snp	1	1	1	1	.	.	.	CTD-2049J23.2.1(INTRON)
3	159686530	159686553	159686537	TTAT	A	snp	1	0	0	0	.	.	.	CTD-2049J23.2.1(INTRON)
3	159727841	159727860	159727855	TGCT	T	ins	1	1	1	1	.	.	.	CTD-2049J23.2.1(INTRON)
3	159727841	159727860	159727856	TGCT	TGC	del	1	1	1	1	.	.	.	CTD-2049J23.2.1(INTRON)
3	159897410	159897418	159897411	A	C	snp	1	0	1	0	.	.	.	CTD-2049J23.2.1(INTRON)
3	159957474	159957501	159957494	TTTA	G	snp	1	1	1	1	.	.	.	RP11-432B6.3.1(INTRON)
3	159961519	159961526	159961524	A	AAG	ins	1	0	0	0	.	.	.	RP11-432B6.3.1(INTRON)
3	160039174	160039185	160039176	TC	G	snp	1	0	0	0	.	.	.	IFT80(INTRON);IFT80(UPSTREAM);RP11-432B6.3.1(INTRON)

3	160144020	160144029	160144022	A	G	snp	0	0	0	1	.	.	.	RP11-432B6.3.1(INTRON);SMC4(DOWNSTREAM);SMC4(INTRON);SMC4(UPSTREAM)
3	160220152	160220161	160220158	T	C	snp	1	0	0	0	.	.	.	KPNA4(DOWNSTREAM);KPNA4(INTRON)
3	160289274	160289288	160289284	TTA	C	snp	1	1	1	1	.	.	.	INTERGENIC(INTERGENIC);KRT8P12(DOWNSTREAM)
3	160402681	160402689	160402687	C	T	ins	1	0	1	0	.	.	.	INTERGENIC(INTERGENIC)
3	160482249	160482264	160482262	CT	C	del	1	1	1	1	.	.	.	PPM1L(INTRON)
3	160515974	160515989	160515979	AAAC	AC	del	1	1	1	0	.	.	.	PPM1L(INTRON)
3	160561184	160561193	160561192	T	G	snp	1	1	0	1	.	.	.	PPM1L(INTRON)
3	160622559	160622572	160622570	AG	C	snp	1	1	1	1	.	.	.	PPM1L(INTRON)
3	160828864	160828871	160828865	A	C	snp	1	1	1	1	.	.	.	NMD3(INTRON)
3	160870692	160870699	160870695	A	G	snp	0	1	1	0	.	.	.	NMD3(INTRON)
3	161158751	161158758	161158752	T	G	snp	1	1	1	1	.	.	.	RP11-3P17.4.1(INTRON)
3	161188590	161188599	161188595	AT	C	snp	1	0	0	0	.	.	.	AC112491.1(UPSTREAM);INTERGENIC(INTERGENIC)
3	161222623	161222632	161222630	T	TG	ins	1	1	1	1	.	.	.	INTERGENIC(INTERGENIC);OTOL1(DOWNSTREAM)
3	161432437	161432454	161432451	TC	A	snp	1	1	0	0	.	.	.	INTERGENIC(INTERGENIC)
3	161582954	161582963	161582961	TC	T	snp	1	0	0	0	.	.	.	INTERGENIC(INTERGENIC)
3	161587162	161587171	161587166	T	C	snp	1	1	0	1	.	.	.	INTERGENIC(INTERGENIC)
3	161587809	161587818	161587815	CA	A	ins	1	0	0	0	.	.	.	INTERGENIC(INTERGENIC)
3	161621438	161621446	161621441	T	G	snp	1	1	1	1	.	.	.	INTERGENIC(INTERGENIC)
3	161646748	161646757	161646754	GT	A	snp	1	1	1	1	.	.	.	INTERGENIC(INTERGENIC)
3	161792702	161792717	161792714	TC	CTC	del	0	0	0	1	.	.	.	INTERGENIC(INTERGENIC)
3	161809755	161809772	161809760	TAT	C	snp	1	1	1	1	.	.	.	INTERGENIC(INTERGENIC)
3	161832131	161832142	161832134	TA	G	snp	1	1	1	1	.	.	.	INTERGENIC(INTERGENIC)
3	161856967	161856978	161856975	AT	T	ins	1	1	1	1	.	.	.	INTERGENIC(INTERGENIC)
3	161856967	161856978	161856976	AT	A	del	1	1	1	1	.	.	.	INTERGENIC(INTERGENIC)
3	161885482	161885489	161885483	T	C	snp	1	1	1	1	.	.	.	INTERGENIC(INTERGENIC)
3	161999500	161999507	161999506	T	G	snp	1	1	1	1	.	.	.	INTERGENIC(INTERGENIC)
3	162027484	162027493	162027488	AT	TATAT	del	1	1	1	1	.	.	.	INTERGENIC(INTERGENIC)
3	162040435	162040442	162040436	T	G	snp	1	1	1	1	.	.	.	INTERGENIC(INTERGENIC)
3	162157209	162157220	162157212	GGT	T	snp	1	1	1	1	.	.	.	INTERGENIC(INTERGENIC)
3	162163260	162163275	162163271	TTTA	TT	del	1	1	0	1	.	.	.	INTERGENIC(INTERGENIC)
3	162182228	162182235	162182234	T	C	snp	1	1	1	1	.	.	.	INTERGENIC(INTERGENIC)
3	162188729	162188738	162188734	T	C	snp	1	1	1	1	.	.	.	INTERGENIC(INTERGENIC)
3	162273198	162273205	162273203	T	G	snp	1	1	1	1	.	.	.	INTERGENIC(INTERGENIC)
3	162306813	162306821	162306815	T	C	ins	1	1	1	1	.	.	.	INTERGENIC(INTERGENIC)
3	162345783	162345794	162345789	TAA	C	snp	1	0	0	0	.	.	.	INTERGENIC(INTERGENIC)
3	162346125	162346139	162346129	AAC	C	del	0	1	0	1	.	.	.	INTERGENIC(INTERGENIC)
3	162367586	162367601	162367597	TTTG	T	del	1	1	1	1	.	.	.	INTERGENIC(INTERGENIC)
3	162368595	162368603	162368602	A	C	snp	1	1	1	1	.	.	.	INTERGENIC(INTERGENIC)
3	162412176	162412184	162412181	A	G	ins	1	1	1	1	.	.	.	INTERGENIC(INTERGENIC)
3	162465469	162465477	162465473	T	C	ins	1	0	1	0	.	.	.	INTERGENIC(INTERGENIC)
3	162471630	162471645	162471642	AT	TATATATT	ins	1	1	1	1	.	.	.	INTERGENIC(INTERGENIC)
3	162490953	162490960	162490957	A	C	snp	1	1	1	1	.	.	.	INTERGENIC(INTERGENIC)
3	162505504	162505515	162505511	TG	A	snp	1	1	1	1	.	.	.	INTERGENIC(INTERGENIC)
3	162600091	162600100	162600094	AC	T	snp	1	0	0	0	.	.	.	INTERGENIC(INTERGENIC)
3	162610815	162610824	162610820	T	TTTC	ins	1	1	1	1	.	.	.	INTERGENIC(INTERGENIC)
3	162610815	162610824	162610821	T	TTC	ins	1	1	1	1	.	.	.	INTERGENIC(INTERGENIC)
3	162610815	162610824	162610822	T	TC	ins	1	1	1	1	.	.	.	INTERGENIC(INTERGENIC)
3	162688453	162688462	162688460	T	C	snp	1	1	1	1	.	.	.	INTERGENIC(INTERGENIC)
3	162739764	162739775	162739768	TG	C	snp	1	1	1	1	.	.	.	INTERGENIC(INTERGENIC);RP11-10O22.1.1(UPSTREAM)
3	162920229	162920240	162920238	CA	G	snp	1	1	1	0	.	.	.	RP11-10O22.1.1(INTRON);RP11-10O22.2.1(INTRON)
3	163066558	163066566	163066560	A	C	snp	1	0	0	0	.	.	.	RP11-10O22.2.1(INTRON)
3	163138828	163138839	163138832	CT	TT	ins	1	1	0	1	.	.	.	INTERGENIC(INTERGENIC)
3	163139785	163139798	163139788	TG	A	snp	1	0	0	1	.	.	.	INTERGENIC(INTERGENIC)
3	163144492	163144503	163144495	AG	A	snp	0	1	0	1	.	.	.	INTERGENIC(INTERGENIC)
3	163151743	163151750	163151748	A	C	snp	1	1	1	1	.	.	.	INTERGENIC(INTERGENIC)
3	163165808	163165816	163165814	A	G	snp	0	1	1	1	.	.	.	INTERGENIC(INTERGENIC)
3	163241991	163242006	163242002	AT	C	snp	0	0	1	0	.	.	.	INTERGENIC(INTERGENIC)
3	163534354	163534362	163534358	A	C	snp	1	1	1	1	.	.	.	INTERGENIC(INTERGENIC)
3	163960814	163960827	163960821	TC	T	snp	0	0	1	0	.	.	.	INTERGENIC(INTERGENIC)
3	163985920	163985937	163985923	AT	A	snp	0	0	1	0	.	.	.	INTERGENIC(INTERGENIC)
3	164018640	164018648	164018645	A	G	snp	1	0	0	0	.	.	.	INTERGENIC(INTERGENIC)
3	164068213	164068222	164068218	CA	G	snp	1	1	1	1	.	.	.	INTERGENIC(INTERGENIC)
3	164091168	164091176	164091173	T	C	snp	1	1	1	1	.	.	.	INTERGENIC(INTERGENIC)
3	164121707	164121714	164121708	A	G	snp	1	1	1	1	.	.	.	INTERGENIC(INTERGENIC)
3	164225257	164225266	164225259	AT	G	snp	1	1	0	1	.	.	.	INTERGENIC(INTERGENIC)
3	164229583	164229598	164229595	AT	T	snp	1	1	1	1	.	.	.	INTERGENIC(INTERGENIC)

3	164254760	164254773	164254771	AT	G	snp	1	1	1	1	.	.	.	INTERGENIC(INTERGENIC)
3	164306510	164306523	164306516	AT	T	ins	1	1	1	1	.	.	.	INTERGENIC(INTERGENIC)
3	164389306	164389315	164389310	CT	A	snp	1	1	1	1	.	.	.	RP11-71H9.2.1(INTRON)
3	164390488	164390496	164390491	T	G	ins	1	0	1	0	.	.	.	RP11-71H9.2.1(INTRON)
3	164391538	164391545	164391542	T	C	snp	1	1	1	1	.	.	.	RP11-71H9.2.1(INTRON)
3	164500139	164500146	164500145	T	G	snp	1	0	0	0	.	.	.	RP11-71H9.1.1(INTRON)
3	164503272	164503280	164503277	A	C	ins	1	0	1	1	.	.	.	RP11-71H9.1.1(INTRON)
3	164522388	164522403	164522395	AT	ATATAC	ins	1	0	0	0	.	.	.	RP11-71H9.1.1(INTRON)
3	164522426	164522435	164522429	AT	C	snp	1	0	0	0	.	.	.	RP11-71H9.1.1(INTRON)
3	164652701	164652708	164652703	A	C	snp	1	0	0	0	.	.	.	INTERGENIC(INTERGENIC)
3	164671100	164671107	164671104	A	G	snp	0	1	0	1	.	.	.	INTERGENIC(INTERGENIC)
3	164684146	164684155	164684148	T	G	snp	1	0	0	0	.	.	.	INTERGENIC(INTERGENIC)
3	164706877	164706896	164706889	AATA	C	snp	1	1	1	1	.	.	.	SI(INTRON)
3	164747421	164747434	164747429	TA	A	snp	0	1	0	0	.	.	.	SI(INTRON)
3	164790202	164790216	164790213	AAC	A	ins	1	0	0	0	.	.	.	SI(DOWNSTREAM);SI(INTRON)
3	164813695	164813704	164813702	GA	G	ins	1	1	1	1	.	.	.	INTERGENIC(INTERGENIC)
3	164816469	164816476	164816470	T	C	snp	1	0	0	0	.	.	.	INTERGENIC(INTERGENIC)
3	164898203	164898214	164898207	AAT	T	snp	1	1	1	1	.	.	.	INTERGENIC(INTERGENIC)
3	164931007	164931015	164931010	A	C	snp	1	0	0	0	.	.	.	RP11-85M11.2.1(INTRON)
3	165018797	165018805	165018801	T	C	snp	1	0	0	0	.	.	.	RP11-85M11.2.1(INTRON)
3	165033830	165033844	165033835	TTA	T	ins	1	0	0	0	.	.	.	RP11-85M11.2.1(INTRON)
3	165131965	165131974	165131968	A	G	snp	1	0	0	0	.	.	.	RP11-85M11.2.1(INTRON)
3	165186405	165186412	165186409	T	C	snp	1	0	0	0	.	.	.	RP11-85M11.2.1(INTRON)
3	165364405	165364416	165364412	AT	C	snp	1	1	1	1	.	.	.	RP11-85M11.2.1(INTRON)
3	165412666	165412673	165412668	T	G	snp	1	0	0	0	.	.	.	INTERGENIC(INTERGENIC)
3	165421903	165421918	165421913	TATT	G	snp	0	1	0	1	.	.	.	INTERGENIC(INTERGENIC)
3	165450866	165450874	165450872	A	G	snp	1	1	1	1	.	.	.	INTERGENIC(INTERGENIC)
3	165474785	165474794	165474787	T	TA	ins	1	1	1	1	.	.	.	INTERGENIC(INTERGENIC)
3	165512551	165512562	165512556	TG	A	snp	1	1	0	0	.	.	.	BCHE(INTRON)
3	165578933	165578950	165578936	TC	T	snp	1	1	1	1	.	.	.	INTERGENIC(INTERGENIC)
3	165579170	165579183	165579181	TC	T	snp	1	1	1	1	.	.	.	INTERGENIC(INTERGENIC)
3	165603424	165603431	165603426	A	C	ins	1	0	0	0	.	.	.	INTERGENIC(INTERGENIC)
3	165633352	165633363	165633361	TA	TAAAG	del	1	1	1	1	.	.	.	INTERGENIC(INTERGENIC)
3	165663225	165663233	165663227	A	C	ins	1	1	0	1	.	.	.	INTERGENIC(INTERGENIC)
3	165663225	165663233	165663227	A	C	snp	1	0	0	0	.	.	.	INTERGENIC(INTERGENIC)
3	165663225	165663233	165663228	A	C	snp	1	1	0	1	.	.	.	INTERGENIC(INTERGENIC)
3	165727608	165727628	165727624	ATT	TA	del	1	1	1	1	.	.	.	INTERGENIC(INTERGENIC)
3	165728278	165728287	165728281	T	TG	ins	1	1	1	1	.	.	.	INTERGENIC(INTERGENIC)
3	165728278	165728287	165728282	T	G	ins	1	1	1	1	.	.	.	INTERGENIC(INTERGENIC)
3	165747369	165747377	165747374	C	T	ins	1	1	1	1	.	.	.	INTERGENIC(INTERGENIC)
3	165761048	165761056	165761055	T	G	snp	1	1	1	1	.	.	.	INTERGENIC(INTERGENIC)
3	165767974	165767985	165767977	TA	G	snp	1	1	1	1	.	.	.	INTERGENIC(INTERGENIC)
3	165799728	165799735	165799730	T	G	ins	1	1	1	1	.	.	.	INTERGENIC(INTERGENIC)
3	165811419	165811427	165811423	A	G	ins	1	1	1	1	.	.	.	INTERGENIC(INTERGENIC)
3	165834584	165834591	165834586	T	G	snp	0	1	0	1	.	.	.	INTERGENIC(INTERGENIC)
3	165895356	165895365	165895360	A	C	ins	1	0	0	0	.	.	.	INTERGENIC(INTERGENIC);RP11-12N13.2.1(DOWNSTREAM)
3	165962210	165962221	165962219	TA	G	snp	1	1	1	1	.	.	.	INTERGENIC(INTERGENIC)
3	165973221	165973228	165973224	T	C	snp	1	0	0	0	.	.	.	INTERGENIC(INTERGENIC)
3	165984116	165984123	165984118	T	C	snp	1	1	1	1	.	.	.	INTERGENIC(INTERGENIC)
3	166002885	166002893	166002887	A	G	snp	1	1	1	1	.	.	.	INTERGENIC(INTERGENIC)
3	166030883	166030892	166030885	TG	C	snp	1	0	0	0	.	.	.	INTERGENIC(INTERGENIC)
3	166079430	166079443	166079441	CA	G	snp	1	1	1	1	.	.	.	INTERGENIC(INTERGENIC)
3	166087862	166087873	166087866	CCT	T	snp	1	0	1	1	.	.	.	INTERGENIC(INTERGENIC)
3	166141282	166141293	166141286	ACA	A	del	1	0	1	1	.	.	.	INTERGENIC(INTERGENIC)
3	166167400	166167411	166167404	TA	C	snp	1	0	0	0	.	.	.	INTERGENIC(INTERGENIC)
3	166176340	166176348	166176347	T	C	snp	1	1	1	1	.	.	.	INTERGENIC(INTERGENIC)
3	166256943	166256950	166256947	T	G	snp	1	0	0	0	.	.	.	INTERGENIC(INTERGENIC)
3	166285134	166285145	166285142	TG	C	snp	1	1	0	1	.	.	.	INTERGENIC(INTERGENIC);RP11-450H5.1.1(DOWNSTREAM)
3	166355395	166355403	166355397	T	C	snp	1	0	0	0	.	.	.	INTERGENIC(INTERGENIC)
3	166362361	166362370	166362365	TC	G	snp	1	0	0	1	.	.	.	INTERGENIC(INTERGENIC)
3	166383227	166383236	166383233	TA	C	snp	1	1	1	1	.	.	.	7SK(DOWNSTREAM);INTERGENIC(INTERGENIC)
3	166442965	166442972	166442971	A	G	snp	1	1	1	1	.	.	.	INTERGENIC(INTERGENIC);RP11-450H5.3.1(DOWNSTREAM)
3	166566963	166566974	166566970	TGT	A	snp	0	1	0	1	.	.	.	INTERGENIC(INTERGENIC);RP11-12K11.2.1(UPSTREAM)
3	166574314	166574321	166574317	A	C	snp	1	0	0	0	.	.	.	INTERGENIC(INTERGENIC)
3	166597067	166597076	166597071	T	G	snp	1	0	0	0	.	.	.	INTERGENIC(INTERGENIC)
3	166597067	166597076	166597074	T	G	ins	1	1	1	1	.	.	.	INTERGENIC(INTERGENIC)
3	166597067	166597076	166597074	T	G	snp	1	1	1	1	.	.	.	INTERGENIC(INTERGENIC)

3	166624310	166624317	166624314	T	C	snp	1	0	0	0	.	.	.	INTERGENIC(INTERGENIC)
3	166696273	166696281	166696279	T	G	snp	1	1	1	1	.	.	.	INTERGENIC(INTERGENIC)
3	166746898	166746905	166746902	T	G	snp	1	1	1	1	.	.	.	AC092965.1(DOWNSTREAM);INTERGENIC(INTERGENIC)
3	167066157	167066164	167066160	A	G	snp	1	1	1	1	.	.	.	ZBBX(DOWNSTREAM);ZBBX(INTRON)
3	167072283	167072291	167072288	A	C	snp	1	1	1	1	.	.	.	ZBBX(INTRON)
3	167167270	167167297	167167286	ATTT	T	del	1	0	0	0	.	.	.	SERPINI2(INTRON);SERPINI2(UPSTREAM)
3	167167270	167167297	167167291	ATTT	TG	ins	1	0	0	0	.	.	.	SERPINI2(INTRON);SERPINI2(UPSTREAM)
3	167167270	167167297	167167293	ATTT	A	del	1	0	0	0	.	.	.	SERPINI2(INTRON);SERPINI2(UPSTREAM)
3	167171056	167171064	167171057	T	C	snp	0	1	1	1	.	.	.	SERPINI2(INTRON)
3	167251343	167251352	167251348	GT	C	snp	1	1	0	0	.	.	.	WDR49(INTRON)
3	167263274	167263283	167263276	AT	TATATAT	del	1	0	0	1	.	.	.	WDR49(INTRON)
3	167362989	167363008	167363001	ATTT	T	snp	1	1	1	1	.	.	.	WDR49(INTRON)
3	167507448	167507457	167507452	AG	G	snp	1	1	1	1	.	.	.	SERPINI1(INTRON);SERPINI1(UPSTREAM)
3	167586500	167586517	167586510	AG	G	snp	1	0	0	0	.	.	.	RP11-298O21.2.1(INTRON);RP11-298O21.3.1(INTRON);RP11-298O21.6.1(INTRON);RP11-298O21.7.1(DOWNSTREAM)
3	167600171	167600180	167600176	TC	G	snp	1	1	1	1	.	.	.	RP11-298O21.2.1(INTRON)
3	167620027	167620034	167620028	A	C	snp	1	0	0	1	.	.	.	RP11-298O21.5.1(INTRON);RP11-298O21.5.1(UPSTREAM)
3	167656249	167656258	167656254	TA	T	snp	1	1	1	1	.	.	.	INTERGENIC(INTERGENIC)
3	167656303	167656320	167656312	TA	T	snp	1	1	0	1	.	.	.	INTERGENIC(INTERGENIC)
3	167665817	167665824	167665823	A	C	snp	1	0	0	0	.	.	.	INTERGENIC(INTERGENIC)
3	167703627	167703636	167703635	A	G	snp	1	1	1	0	.	.	.	INTERGENIC(INTERGENIC)
3	167732372	167732380	167732379	A	G	snp	1	1	1	1	.	.	.	GOLIM4(INTRON)
3	167820444	167820453	167820446	T	G	snp	1	0	0	0	.	.	.	INTERGENIC(INTERGENIC)
3	167838899	167838906	167838903	A	G	snp	0	0	0	1	.	.	.	INTERGENIC(INTERGENIC)
3	168121197	168121205	168121201	T	C	snp	1	0	0	0	.	.	.	EGFEM1P(INTRON)
3	168224662	168224681	168224669	CTCC	CT	ins	1	0	0	0	.	.	.	EGFEM1P(INTRON)
3	168306752	168306767	168306755	AT	C	snp	1	1	1	1	.	.	.	EGFEM1P(INTRON)
3	168340277	168340290	168340279	GT	T	snp	0	1	1	1	.	.	.	EGFEM1P(INTRON)
3	168399154	168399163	168399159	CT	C	snp	1	0	0	0	.	.	.	EGFEM1P(INTRON)
3	168407942	168407949	168407948	T	G	snp	1	1	1	0	.	.	.	EGFEM1P(INTRON)
3	168570106	168570125	168570121	TTCT	TTC	del	1	1	1	1	.	.	.	INTERGENIC(INTERGENIC)
3	168587707	168587721	168587712	AAT	A	snp	1	0	0	0	.	.	.	INTERGENIC(INTERGENIC)
3	168606677	168606686	168606681	T	G	ins	1	0	0	0	.	.	.	INTERGENIC(INTERGENIC)
3	168616269	168616277	168616275	A	C	snp	1	1	1	0	.	.	.	INTERGENIC(INTERGENIC);RP11-368I23.2.1(UPSTREAM)
3	168616700	168616707	168616705	T	G	ins	1	0	1	0	.	.	.	INTERGENIC(INTERGENIC);RP11-368I23.2.1(UPSTREAM)
3	168685573	168685590	168685588	AT	A	del	1	1	1	1	.	.	.	INTERGENIC(INTERGENIC)
3	168746509	168746518	168746516	A	G	ins	1	1	1	1	.	.	.	INTERGENIC(INTERGENIC)
3	168746664	168746675	168746668	ATT	C	snp	1	1	1	1	.	.	.	INTERGENIC(INTERGENIC)
3	168795474	168795481	168795475	T	G	snp	1	1	1	1	.	.	.	INTERGENIC(INTERGENIC)
3	168799152	168799179	168799156	AAAT	AAT	del	1	1	1	1	.	.	.	INTERGENIC(INTERGENIC);MECOM(DOWNSTREAM)
3	168799152	168799179	168799158	AAAT	TAAAT	del	1	1	1	1	.	.	.	INTERGENIC(INTERGENIC);MECOM(DOWNSTREAM)
3	168850278	168850285	168850281	T	TC	ins	1	1	1	1	.	.	.	MECOM(INTRON);MECOM(UPSTREAM)
3	168850278	168850285	168850282	T	CA	ins	1	1	1	1	.	.	.	MECOM(INTRON);MECOM(UPSTREAM)
3	168865504	168865521	168865510	GT	A	snp	1	0	0	0	.	.	.	MECOM(DOWNSTREAM);MECOM(INTRON);MECOM(UPSTREAM);MECOM(UTR_5_PRIME)
3	168915108	168915131	168915127	AAAT	AAATA	del	1	1	1	1	.	.	.	MECOM(INTRON)
3	169008287	169008294	169008292	A	G	snp	1	1	1	0	.	.	.	MECOM(INTRON)
3	169168464	169168473	169168467	AG	T	snp	0	0	1	0	.	.	.	MECOM(INTRON);RP11-641D5.2.1(INTRON)
3	169199954	169199961	169199959	A	C	snp	1	0	1	0	.	.	.	MECOM(INTRON);RP11-641D5.1.1(DOWNSTREAM)
3	169262077	169262091	169262087	CAT	G	snp	1	0	0	0	.	.	.	MECOM(INTRON)
3	169265979	169265987	169265980	A	C	snp	1	1	1	1	.	.	.	MECOM(INTRON)
3	169331903	169331912	169331911	T	C	ins	1	1	1	1	.	.	.	MECOM(INTRON);RP11-3K16.2.1(INTRON)
3	169379094	169379102	169379101	C	A	snp	1	1	0	1	.	.	.	MECOM(INTRON)
3	169390749	169390757	169390754	A	G	snp	1	0	0	1	.	.	.	INTERGENIC(INTERGENIC)
3	169425401	169425408	169425402	A	G	snp	1	1	1	1	.	.	.	INTERGENIC(INTERGENIC);RP11-3K16.1.1(UPSTREAM)
3	169468106	169468126	169468123	TTG	T	snp	0	0	1	0	.	.	.	INTERGENIC(INTERGENIC)
3	169578001	169578008	169578004	T	C	snp	1	1	0	1	.	.	.	LRRC31(INTRON)
3	169584832	169584847	169584842	TTTG	G	del	1	1	0	1	.	.	.	LRRC31(INTRON)
3	169611314	169611325	169611317	TG	T	snp	1	1	1	1	.	.	.	INTERGENIC(INTERGENIC)
3	169611314	169611325	169611319	TG	T	snp	1	1	1	1	.	.	.	INTERGENIC(INTERGENIC)
3	169611314	169611325	169611321	TG	T	snp	1	1	1	1	.	.	.	INTERGENIC(INTERGENIC)
3	169640286	169640297	169640293	TTG	C	snp	1	0	0	0	.	.	.	SAMD7(INTRON)
3	169749218	169749227	169749221	AC	T	snp	1	1	1	1	.	.	.	INTERGENIC(INTERGENIC)
3	169830168	169830183	169830174	TA	C	snp	1	1	1	1	.	.	.	PHC3(INTRON)
3	169839326	169839333	169839328	T	C	snp	1	0	0	0	.	.	.	PHC3(INTRON)
3	170009486	170009500	170009493	AAT	TT	ins	1	0	0	0	.	.	.	PRKCI(EXON);PRKCI(INTRON);PRKCI(UPSTREAM)
3	170103589	170103596	170103592	T	C	snp	1	1	1	1	.	.	.	SKIL(DOWNSTREAM);SKIL(INTRON)

3	170103912	170103935	170103931	TTTG	T	snp	1	0	0	0	.	.	.	SKIL(DOWNSTREAM);SKIL(INTRON)
3	170110901	170110909	170110903	T	G	snp	1	0	0	0	.	.	.	SKIL(DOWNSTREAM);SKIL(UTR_3_PRIME)
3	170169551	170169562	170169554	AG	C	snp	1	0	0	0	.	.	.	CLDN11(INTRON)
3	170228187	170228210	170228192	AAAT	AT	del	1	1	1	1	.	.	.	CLDN11(INTRON);SLC7A14(INTRON)
3	170228187	170228210	170228193	AAAT	T	del	1	1	1	1	.	.	.	CLDN11(INTRON);SLC7A14(INTRON)
3	170228187	170228210	170228194	AAAT	A	ins	1	1	1	1	.	.	.	CLDN11(INTRON);SLC7A14(INTRON)
3	170228187	170228210	170228196	AAAT	AT	del	1	1	1	1	.	.	.	CLDN11(INTRON);SLC7A14(INTRON)
3	170228187	170228210	170228197	AAAT	T	del	1	1	1	1	.	.	.	CLDN11(INTRON);SLC7A14(INTRON)
3	170233419	170233426	170233420	G	T	snp	1	1	0	1	.	.	.	CLDN11(INTRON);SLC7A14(INTRON)
3	170233419	170233426	170233425	G	A	snp	1	0	0	0	.	.	.	CLDN11(INTRON);SLC7A14(INTRON)
3	170272964	170272977	170272967	AT	A	snp	1	1	0	1	.	.	.	CLDN11(INTRON);SLC7A14(INTRON)
3	170281193	170281208	170281202	TGTT	T	ins	0	1	1	1	.	.	.	CLDN11(INTRON);SLC7A14(INTRON)
3	170321493	170321500	170321494	A	C	snp	0	1	1	1	.	.	.	CLDN11(INTRON)
3	170341349	170341357	170341352	A	C	snp	1	1	1	1	.	.	.	CLDN11(INTRON)
3	170380161	170380176	170380169	CTTT	T	ins	1	1	1	1	.	.	.	CLDN11(INTRON);RP11-373E16.3.1(DOWNSTREAM)
3	170380161	170380176	170380172	CTTT	C	del	1	1	1	1	.	.	.	CLDN11(INTRON);RP11-373E16.3.1(DOWNSTREAM)
3	170400127	170400136	170400129	GA	T	snp	1	0	0	0	.	.	.	CLDN11(INTRON)
3	170413403	170413410	170413409	A	C	ins	1	1	1	1	.	.	.	CLDN11(INTRON)
3	170429505	170429512	170429507	T	C	snp	1	0	0	0	.	.	.	CLDN11(DOWNSTREAM);CLDN11(INTRON)
3	170436673	170436681	170436680	T	C	ins	1	1	1	1	.	.	.	CLDN11(INTRON)
3	170503388	170503399	170503397	AG	A	snp	0	1	1	0	.	.	.	CLDN11(INTRON)
3	170516042	170516051	170516045	A	G	snp	0	1	1	1	.	.	.	CLDN11(INTRON)
3	170543645	170543652	170543646	A	C	snp	1	1	0	0	.	.	.	CLDN11(INTRON)
3	170613633	170613642	170613638	AT	G	snp	1	0	1	0	.	.	.	EIF5A2(INTRON)
3	170687268	170687276	170687273	T	C	snp	1	1	0	1	.	.	.	INTERGENIC(INTERGENIC)
3	170777809	170777816	170777810	T	C	snp	1	1	0	1	.	.	.	INTERGENIC(INTERGENIC);TNIK(DOWNSTREAM)
3	170777809	170777816	170777813	T	C	snp	1	1	0	1	.	.	.	INTERGENIC(INTERGENIC);TNIK(DOWNSTREAM)
3	170798213	170798232	170798227	TTTA	A	del	1	0	0	0	.	.	.	TNIK(INTRON);TNIK(UPSTREAM)
3	170852701	170852709	170852703	A	G	snp	1	0	0	0	.	.	.	TNIK(INTRON)
3	170926142	170926150	170926147	A	G	snp	1	0	0	0	.	.	.	TNIK(INTRON)
3	170949087	170949101	170949098	TTG	TTGTT	del	1	0	0	0	.	.	.	TNIK(INTRON)
3	170961828	170961835	170961833	T	G	snp	0	0	1	0	.	.	.	TNIK(INTRON)
3	170995290	170995299	170995291	A	G	snp	1	0	1	0	.	.	.	TNIK(INTRON)
3	171028923	171028936	171028928	AG	C	snp	1	1	1	1	.	.	.	TNIK(INTRON)
3	171042889	171042912	171042896	AAAT	A	snp	1	0	0	0	.	.	.	TNIK(INTRON)
3	171219957	171219980	171219973	TTTC	GTA	ins	1	1	1	1	.	.	.	INTERGENIC(INTERGENIC);U6(DOWNSTREAM)
3	171240965	171240974	171240972	AC	G	snp	1	0	0	0	.	.	.	INTERGENIC(INTERGENIC)
3	171275687	171275701	171275696	AAG	C	snp	1	1	1	1	.	.	.	INTERGENIC(INTERGENIC)
3	171323298	171323307	171323305	AT	C	snp	1	1	1	1	.	.	.	PLD1(INTRON);PLD1(UPSTREAM)
3	171580070	171580079	171580073	CA	G	snp	1	1	1	1	.	.	.	TMEM212(DOWNSTREAM);TMEM212(INTRAGENIC)
3	171631104	171631111	171631108	C	A	ins	1	1	1	0	.	.	.	TMEM212(INTRON)
3	171684514	171684522	171684515	A	G	snp	1	1	1	1	.	.	.	INTERGENIC(INTERGENIC)
3	171687416	171687423	171687420	C	T	snp	1	1	1	1	.	.	.	INTERGENIC(INTERGENIC)
3	171756905	171756919	171756909	AAC	G	snp	1	1	1	1	.	.	.	FNDC3B(UPSTREAM);INTERGENIC(INTERGENIC)
3	171898244	171898253	171898246	TG	C	snp	1	1	0	1	.	.	.	FNDC3B(INTRON)
3	171910899	171910918	171910907	CCTC	T	snp	1	0	0	0	.	.	.	FNDC3B(INTRON)
3	171998990	171999001	171998998	TTA	TTAT	del	1	1	1	1	.	.	.	FNDC3B(INTRON)
3	171999709	171999717	171999716	A	G	snp	1	1	1	1	.	.	.	FNDC3B(INTRON)
3	172064822	172064829	172064828	A	C	snp	1	1	1	1	.	.	.	FNDC3B(INTRON)
3	172073307	172073315	172073311	G	T	snp	1	0	0	0	.	.	.	FNDC3B(DOWNSTREAM);FNDC3B(INTRON)
3	172080402	172080411	172080403	T	G	snp	1	0	0	0	.	.	.	FNDC3B(INTRON)
3	172104539	172104548	172104543	A	G	snp	1	0	0	0	.	.	.	FNDC3B(INTRON)
3	172141120	172141137	172141131	TTG	T	del	1	1	1	1	.	.	.	BZW1P1(DOWNSTREAM);INTERGENIC(INTERGENIC)
3	172184532	172184552	172184544	AAT	G	snp	0	1	0	1	.	.	.	INTERGENIC(INTERGENIC)
3	172204944	172204952	172204950	T	TTG	ins	1	1	1	1	.	.	.	INTERGENIC(INTERGENIC)
3	172218793	172218804	172218800	ATA	AATA	del	1	1	1	1	.	.	.	INTERGENIC(INTERGENIC);TNFSF10(DOWNSTREAM)
3	172242140	172242159	172242144	CTTT	TTT	del	1	1	1	1	.	.	.	AC007919.18.1(DOWNSTREAM);INTERGENIC(INTERGENIC);TNFSF10(UPSTREAM)
3	172312818	172312827	172312826	T	C	snp	1	1	1	1	.	.	.	RP11-408H1.3.1(INTRON);RP11-408H1.3.1(UPSTREAM)
3	172430190	172430199	172430191	T	C	snp	1	1	1	1	.	.	.	INTERGENIC(INTERGENIC);NCEH1(UPSTREAM)
3	172453951	172453959	172453958	T	G	snp	1	1	1	1	.	.	.	INTERGENIC(INTERGENIC)
3	172453959	172453970	172453962	TG	T	snp	1	0	0	0	.	.	.	INTERGENIC(INTERGENIC)
3	172477399	172477407	172477402	T	G	snp	1	0	0	0	.	.	.	ECT2(DOWNSTREAM);ECT2(INTRON)
3	172577334	172577351	172577343	TAA	G	snp	0	1	0	1	.	.	.	INTERGENIC(INTERGENIC)
3	172678674	172678683	172678677	TC	T	snp	1	1	1	1	.	.	.	SPATA16(INTRON)
3	172704464	172704471	172704468	G	A	snp	1	1	1	1	.	.	.	SPATA16(INTRON)
3	172717186	172717209	172717204	TTTC	C	del	0	0	1	1	.	.	.	SPATA16(INTRON)

3	172763665	172763680	172763672	AATG	A	snp	1	0	0	0	.	.	.	SPATA16(INTRON)
3	172772534	172772542	172772540	T	C	snp	1	0	0	0	.	.	.	SPATA16(INTRON)
3	172833678	172833687	172833686	T	C	ins	0	1	1	0	.	.	.	SPATA16(INTRON)
3	172866705	172866720	172866709	AG	GG	ins	1	1	1	1	.	.	.	INTERGENIC(INTERGENIC);RP11-324C10.1.1(UPSTREAM)
3	172872921	172872936	172872927	TTCC	T	snp	1	0	0	0	.	.	.	RP11-324C10.1.1(INTRON)
3	172962606	172962614	172962608	C	A	snp	0	0	0	1	.	.	.	RP11-324C10.1.1(INTRON)
3	172984197	172984208	172984203	GT	T	snp	1	0	0	0	.	.	.	RP11-324C10.1.1(INTRON)
3	172984788	172984796	172984790	T	C	snp	1	0	0	0	.	.	.	RP11-324C10.1.1(INTRON)
3	172992471	172992494	172992478	AAAC	A	snp	1	1	1	1	.	.	.	RP11-324C10.1.1(INTRON)
3	173093273	173093290	173093286	TC	T	snp	1	0	0	0	.	.	.	INTERGENIC(INTERGENIC)
3	173257169	173257178	173257176	TA	T	del	1	0	0	0	.	.	.	NLGN1(INTRON)
3	173262829	173262846	173262832	TG	T	snp	1	0	0	0	.	.	.	NLGN1(INTRON)
3	173335995	173336002	173335999	T	C	ins	1	1	1	0	.	.	.	NLGN1(INTRON)
3	173393633	173393642	173393636	TC	G	snp	0	0	0	1	.	.	.	NLGN1(INTRON)
3	173462621	173462630	173462622	T	TA	ins	1	0	0	0	.	.	.	NLGN1(INTRON)
3	173464978	173464987	173464982	AT	G	snp	1	1	1	1	.	.	.	NLGN1(INTRON)
3	173471788	173471796	173471795	T	C	snp	1	0	0	0	.	.	.	NLGN1(INTRON)
3	173774698	173774709	173774707	AT	A	del	1	1	1	1	.	.	.	7SK(UPSTREAM);NLGN1(INTRON)
3	173813324	173813339	173813327	TA	TC	ins	1	1	1	1	.	.	.	NLGN1(INTRON)
3	173858993	173859000	173858995	T	C	snp	1	1	1	0	.	.	.	NLGN1(INTRON)
3	173956151	173956160	173956158	T	G	ins	1	0	1	0	.	.	.	NLGN1(INTRON)
3	174184133	174184144	174184141	TTC	TT	del	1	1	1	0	.	.	.	NAALADL2(INTRON)
3	174193859	174193870	174193866	TGT	A	snp	1	1	1	1	.	.	.	NAALADL2(INTRON)
3	174227793	174227801	174227797	T	G	snp	1	1	1	0	.	.	.	NAALADL2(INTRON)
3	174303453	174303462	174303457	T	C	ins	1	1	1	1	.	.	.	NAALADL2(INTRON)
3	174436707	174436714	174436712	A	C	snp	1	0	0	0	.	.	.	NAALADL2(INTRON)
3	174474740	174474747	174474742	A	C	ins	0	0	0	1	.	.	.	NAALADL2(INTRON)
3	174539950	174539959	174539954	AC	CG	ins	1	1	0	1	.	.	.	NAALADL2(INTRON)
3	174546888	174546911	174546904	TTG	C	snp	1	0	0	0	.	.	.	NAALADL2(INTRON)
3	174590512	174590521	174590519	AC	T	snp	0	0	1	0	.	.	.	NAALADL2(INTRON)
3	174757640	174757647	174757642	T	C	snp	1	1	1	1	.	.	.	NAALADL2(INTRON)
3	174767891	174767902	174767897	TG	C	snp	1	1	1	1	.	.	.	NAALADL2(INTRON)
3	174767891	174767902	174767898	TG	A	snp	1	1	1	1	.	.	.	NAALADL2(INTRON)
3	174846716	174846724	174846721	T	G	snp	1	0	0	0	.	.	.	NAALADL2(INTRON)
3	174924067	174924076	174924073	TG	GTG	del	1	1	1	1	.	.	.	NAALADL2(INTRON)
3	174975564	174975571	174975570	A	G	snp	1	1	1	1	.	.	.	NAALADL2(DOWNSTREAM);NAALADL2(INTRON);NAALADL2-AS2(INTRON)
3	174995915	174995935	174995927	AAT	G	snp	1	0	0	0	.	.	.	NAALADL2(INTRON)
3	174998902	174998909	174998908	A	AT	ins	1	0	0	0	.	.	.	NAALADL2(INTRON)
3	175024459	175024478	175024464	ACAA	T	snp	1	0	0	0	.	.	.	NAALADL2(INTRON)
3	175102540	175102555	175102545	TTTA	C	snp	1	0	0	0	.	.	.	NAALADL2(INTRON)
3	175235566	175235575	175235571	GA	G	snp	1	0	0	0	.	.	.	NAALADL2(INTRON)
3	175309630	175309639	175309635	TA	G	snp	1	0	0	0	.	.	.	NAALADL2(INTRON)
3	175325687	175325700	175325689	AC	CG	ins	1	1	0	1	.	.	.	NAALADL2(INTRON);U4(UPSTREAM)
3	175392217	175392225	175392221	T	C	ins	1	0	0	0	.	.	.	NAALADL2(INTRON);RP11-744O11.2.1(DOWNSTREAM)
3	175464154	175464163	175464160	T	C	snp	1	0	0	0	.	.	.	NAALADL2(INTRON)
3	175476580	175476588	175476583	T	C	snp	1	1	0	1	.	.	.	NAALADL2(INTRON)
3	175574475	175574483	175574481	T	G	snp	1	0	0	0	.	.	.	INTERGENIC(INTERGENIC)
3	175595837	175595848	175595842	TC	G	snp	1	0	0	0	.	.	.	INTERGENIC(INTERGENIC)
3	175609668	175609676	175609671	A	C	snp	1	1	1	1	.	.	.	INTERGENIC(INTERGENIC)
3	175657485	175657496	175657494	AT	A	del	1	0	0	0	.	.	.	RP11-809F4.3.1(EXON)
3	175669531	175669539	175669533	A	C	snp	1	1	1	1	.	.	.	INTERGENIC(INTERGENIC)
3	175677100	175677109	175677102	AT	C	snp	1	1	1	1	.	.	.	INTERGENIC(INTERGENIC)
3	175771619	175771636	175771629	TA	AC	ins	1	1	1	1	.	.	.	INTERGENIC(INTERGENIC)
3	175856941	175856949	175856942	A	G	snp	1	1	1	1	.	.	.	INTERGENIC(INTERGENIC)
3	175919942	175919949	175919946	T	C	snp	1	0	0	0	.	.	.	INTERGENIC(INTERGENIC)
3	175982408	175982415	175982413	T	TG	ins	1	1	1	1	.	.	.	INTERGENIC(INTERGENIC)
3	176028278	176028286	176028281	T	G	snp	1	1	1	1	.	.	.	INTERGENIC(INTERGENIC)
3	176200254	176200262	176200259	T	G	snp	1	0	0	0	.	.	.	INTERGENIC(INTERGENIC)
3	176270587	176270598	176270589	AG	G	snp	1	0	0	0	.	.	.	INTERGENIC(INTERGENIC)
3	176345378	176345387	176345383	AG	T	snp	0	1	1	0	.	.	.	RP11-71G7.1.1(INTRON)
3	176469531	176469544	176469534	AG	A	snp	1	1	1	1	.	.	.	RP11-644C3.1.1(INTRON)
3	176469639	176469654	176469649	GAAA	AG	del	1	0	0	0	.	.	.	RP11-644C3.1.1(INTRON)
3	176478602	176478611	176478609	AT	A	ins	1	1	0	1	.	.	.	RN5S147(UPSTREAM);RP11-644C3.1.1(INTRON)
3	176479884	176479891	176479885	A	G	snp	1	1	0	0	.	.	.	RN5S147(UPSTREAM);RP11-644C3.1.1(INTRON)
3	176483882	176483890	176483883	A	C	snp	1	1	0	0	.	.	.	RN5S147(DOWNSTREAM);RP11-644C3.1.1(INTRON)
3	176486710	176486725	176486723	AG	A	snp	1	1	1	1	.	.	.	RP11-644C3.1.1(INTRON)

3	176526687	176526706	176526701	TTAT	T	ins	0	1	1	0	.	.	.	RP11-644C3.1.1(INTRON)
3	176561809	176561823	176561815	GAA	A	snp	1	1	0	1	.	.	.	RP11-644C3.1.1(INTRON)
3	176563896	176563904	176563902	A	G	snp	1	1	0	1	.	.	.	RP11-644C3.1.1(INTRON)
3	176647957	176647968	176647960	CA	CAC	del	1	1	1	1	.	.	.	INTERGENIC(INTERGENIC)
3	176755298	176755305	176755299	G	T	ins	1	1	1	0	.	.	.	TBL1XR1(INTRON)
3	176914524	176914531	176914530	C	T	ins	1	1	1	1	.	.	.	TBL1XR1(INTRON);TBL1XR1(UPSTREAM)
3	176945706	176945717	176945714	TTG	C	ins	1	1	1	0	.	.	.	INTERGENIC(INTERGENIC)
3	176964108	176964131	176964122	AGAA	AAGAAAG	del	1	1	1	0	.	.	.	INTERGENIC(INTERGENIC)
3	176964108	176964131	176964124	AGAA	G	ins	1	1	1	0	.	.	.	INTERGENIC(INTERGENIC)
3	176966087	176966096	176966091	C	A	snp	1	1	1	0	.	.	.	INTERGENIC(INTERGENIC)
3	176982600	176982609	176982603	T	C	snp	1	0	0	0	.	.	.	INTERGENIC(INTERGENIC)
3	176996537	176996548	176996546	TA	CG	ins	1	1	1	1	.	.	.	INTERGENIC(INTERGENIC)
3	177007213	177007220	177007214	A	C	snp	1	1	1	1	.	.	.	INTERGENIC(INTERGENIC)
3	177112579	177112598	177112583	TCCC	A	snp	1	1	1	1	.	.	.	INTERGENIC(INTERGENIC)
3	177171476	177171489	177171479	CT	C	snp	1	1	1	1	.	.	.	RP11-335L9.1.1(INTRON)
3	177171476	177171489	177171480	CT	T	snp	1	1	1	1	.	.	.	RP11-335L9.1.1(INTRON)
3	177270084	177270093	177270086	T	G	snp	1	1	1	1	.	.	.	RP11-335L9.1.1(INTRON)
3	177270084	177270093	177270089	T	C	snp	1	1	0	1	.	.	.	RP11-335L9.1.1(INTRON)
3	177341531	177341540	177341538	TG	A	snp	1	1	1	1	.	.	.	RP11-114M1.2.1(INTRON);RP11-335L9.1.1(INTRON);SNORA18(DOWNSTREAM)
3	177363018	177363031	177363028	AC	CT	ins	1	1	1	1	.	.	.	RP11-335L9.1.1(INTRON)
3	177422949	177422957	177422954	T	C	snp	0	1	0	1	.	.	.	RP11-335L9.1.1(INTRON)
3	177435114	177435125	177435119	TCT	C	snp	1	1	1	1	.	.	.	RP11-335L9.1.1(INTRON)
3	177442893	177442900	177442896	A	G	snp	0	0	0	1	.	.	.	RP11-335L9.1.1(INTRON)
3	177549151	177549160	177549158	GT	C	snp	0	1	0	1	.	.	.	RP11-91K9.1.1(INTRON)
3	177576056	177576063	177576062	T	C	snp	1	0	0	0	.	.	.	RP11-91K9.1.1(INTRON)
3	177597765	177597782	177597770	AAT	A	snp	1	1	1	1	.	.	.	RP11-91K9.1.1(INTRON)
3	177671825	177671834	177671832	T	GA	ins	1	1	0	1	.	.	.	INTERGENIC(INTERGENIC)
3	177685611	177685619	177685618	T	C	snp	1	1	0	1	.	.	.	INTERGENIC(INTERGENIC)
3	177736644	177736659	177736655	TC	TCTCT	del	1	0	0	0	.	.	.	INTERGENIC(INTERGENIC)
3	177829886	177829897	177829889	ATA	C	snp	0	0	1	1	.	.	.	INTERGENIC(INTERGENIC)
3	177894546	177894555	177894548	AT	T	del	1	1	0	1	.	.	.	INTERGENIC(INTERGENIC)
3	178043730	178043738	178043733	T	C	snp	1	0	0	0	.	.	.	KCNMB2(INTRON);RP11-33A14.1.1(INTRON)
3	178124356	178124370	178124367	AAC	A	ins	1	1	1	1	.	.	.	KCNMB2(INTRON)
3	178138766	178138774	178138767	T	C	snp	1	0	0	0	.	.	.	KCNMB2(INTRON);KCNMB2-IT1(INTRON)
3	178257558	178257567	178257562	GT	A	snp	1	1	1	1	.	.	.	KCNMB2(INTRON);RP11-385J1.2.1(INTRON)
3	178335522	178335529	178335523	A	C	snp	1	1	1	1	.	.	.	KCNMB2(INTRON);RP11-385J1.2.1(INTRON)
3	178357848	178357857	178357854	A	AG	ins	1	1	1	1	.	.	.	KCNMB2(INTRON);RP11-385J1.2.1(INTRON)
3	178357848	178357857	178357855	A	G	ins	1	1	1	1	.	.	.	KCNMB2(INTRON);RP11-385J1.2.1(INTRON)
3	178471370	178471377	178471375	A	G	snp	0	1	1	1	.	.	.	KCNMB2(INTRON);RP11-385J1.2.1(INTRON)
3	178475928	178475945	178475940	AT	G	snp	1	1	1	0	.	.	.	KCNMB2(INTRON);RP11-385J1.2.1(INTRON)
3	178476282	178476299	178476297	AT	AC	ins	1	1	1	1	.	.	.	KCNMB2(INTRON);RP11-385J1.2.1(INTRON)
3	178477105	178477122	178477119	AT	C	snp	0	1	0	1	.	.	.	KCNMB2(INTRON);RP11-385J1.2.1(INTRON)
3	178477425	178477440	178477434	AT	C	snp	0	0	0	1	.	.	.	KCNMB2(INTRON);RP11-385J1.2.1(INTRON)
3	178477571	178477584	178477573	TA	C	snp	1	1	0	0	.	.	.	KCNMB2(INTRON);RP11-385J1.2.1(INTRON)
3	178477631	178477646	178477634	TA	C	snp	1	0	0	1	.	.	.	KCNMB2(INTRON);RP11-385J1.2.1(INTRON)
3	178477663	178477678	178477666	TA	C	snp	1	1	0	1	.	.	.	KCNMB2(INTRON);RP11-385J1.2.1(INTRON)
3	178477977	178477992	178477987	TA	G	snp	1	1	1	1	.	.	.	KCNMB2(INTRON);RP11-385J1.2.1(INTRON)
3	178605703	178605712	178605708	A	G	snp	1	0	0	0	.	.	.	INTERGENIC(INTERGENIC)
3	178618317	178618326	178618324	AC	T	snp	1	1	1	0	.	.	.	INTERGENIC(INTERGENIC)
3	178641894	178641901	178641899	C	T	snp	1	0	0	0	.	.	.	INTERGENIC(INTERGENIC)
3	178699609	178699618	178699612	T	C	snp	1	1	1	1	.	.	.	INTERGENIC(INTERGENIC)
3	178718294	178718305	178718297	TA	T	snp	1	1	1	1	.	.	.	INTERGENIC(INTERGENIC)
3	178740420	178740440	178740424	AAT	T	del	1	1	1	1	.	.	.	ZMAT3(DOWNSTREAM);ZMAT3(UTR_3_PRIME)
3	178787902	178787910	178787903	G	A	ins	1	1	1	1	.	.	.	ZMAT3(INTRON)
3	178817792	178817801	178817797	AC	AT	ins	1	0	0	0	.	.	.	INTERGENIC(INTERGENIC);RP11-360P21.2.1(DOWNSTREAM);Y_RNA(DOWNSTREAM)
3	178866321	178866335	178866327	CGC	T	snp	1	1	1	1	.	.	PIK3CA(START_GAINED)	PIK3CA(INTRON);PIK3CA(UPSTREAM);RP11-360P21.2.1(UPSTREAM)
3	178908096	178908103	178908098	C	A	ins	1	1	1	1	.	.	.	PIK3CA(INTRON)
3	178979118	178979126	178979122	C	A	snp	0	0	0	1	.	.	.	KCNMB3(INTRON);KCNMB3(UPSTREAM);LRRFIP1P1(UPSTREAM)
3	178994327	178994335	178994331	T	G	snp	1	0	0	0	.	.	.	INTERGENIC(INTERGENIC)
3	179063929	179063940	179063935	CA	T	snp	1	0	0	0	.	.	.	INTERGENIC(INTERGENIC);MFN1(UPSTREAM);RP11-255C15.3.1(DOWNSTREAM)
3	179088811	179088818	179088815	T	C	snp	1	1	0	0	.	.	.	MFN1(DOWNSTREAM);MFN1(INTRON)
3	179138244	179138259	179138257	AT	A	del	1	1	1	1	.	.	.	GNB4(INTRON)

3	179140275	179140282	179140278	A	C	ins	1	0	0	0	.	.	.	GNB4(INTRON)
3	179302576	179302584	179302578	A	G	snp	1	1	0	0	.	.	.	ACTL6A(DOWNSTREAM);ACTL6A(INTRON);ACTL6A(UPSTREAM);MRPL47(DOWNSTREAM)
3	179397997	179398010	179398000	AG	A	snp	1	1	1	1	.	.	.	USP13(INTRON)
3	179584482	179584490	179584483	T	G	snp	1	0	0	0	.	.	.	PEX5L(INTRON)
3	179661789	179661797	179661796	C	A	ins	1	0	0	0	.	.	.	PEX5L(INTRON)
3	179661789	179661797	179661796	C	A	snp	1	0	0	0	.	.	.	PEX5L(INTRON)
3	179733887	179733907	179733902	CAC	ACT	ins	1	0	0	1	.	.	.	PEX5L(INTRON)
3	179764194	179764207	179764203	TC	TCTCT	del	1	1	1	1	.	.	.	INTERGENIC(INTERGENIC)
3	179814299	179814308	179814301	TA	C	snp	1	0	0	0	.	.	.	INTERGENIC(INTERGENIC)
3	179863240	179863247	179863243	T	G	ins	1	1	1	0	.	.	.	INTERGENIC(INTERGENIC)
3	180058668	180058677	180058671	TA	G	snp	1	1	0	1	.	.	.	INTERGENIC(INTERGENIC)
3	180099464	180099475	180099472	CA	A	ins	0	1	0	1	.	.	.	INTERGENIC(INTERGENIC)
3	180099464	180099475	180099473	CA	C	del	1	1	0	1	.	.	.	INTERGENIC(INTERGENIC)
3	180125749	180125756	180125750	A	C	snp	1	1	0	1	.	.	.	INTERGENIC(INTERGENIC)
3	180276569	180276582	180276572	AT	A	snp	0	0	1	0	.	.	.	INTERGENIC(INTERGENIC)
3	180316674	180316681	180316679	A	G	snp	1	0	0	0	.	.	.	RP11-496B10.3.1(INTRON);TTC14(UPSTREAM)
3	180446599	180446606	180446605	T	G	snp	1	0	0	0	.	.	.	CCDC39(INTRON)
3	180472852	180472859	180472855	A	G	snp	1	1	1	1	.	.	.	CCDC39(INTRON)
3	180475818	180475829	180475821	AG	A	snp	1	1	1	1	.	.	.	CCDC39(INTRON)
3	180563082	180563091	180563084	A	C	snp	1	1	1	1	.	.	.	CCDC39(INTRON)
3	180976175	180976192	180976179	AG	GC	ins	1	1	1	0	.	.	.	SOX2-OT(INTRON)
3	180976175	180976192	180976181	AG	C	snp	1	1	1	0	.	.	.	SOX2-OT(INTRON)
3	180976193	180976200	180976194	A	G	snp	1	1	1	0	.	.	.	SOX2-OT(INTRON)
3	181063245	181063256	181063252	TAT	TTAT	del	1	1	1	1	.	.	.	SOX2-OT(INTRON)
3	181163761	181163768	181163763	T	C	snp	0	0	1	0	.	.	.	RP11-275H4.1.1(UPSTREAM);SOX2-OT(INTRON)
3	181371816	181371825	181371823	AT	C	snp	1	1	1	1	.	.	.	SOX2-OT(INTRON)
3	181383888	181383895	181383890	T	TTTTTG	ins	1	0	0	0	.	.	.	SOX2-OT(INTRON)
3	181460738	181460749	181460746	TA	C	snp	1	1	1	0	.	.	.	SOX2-OT(DOWNSTREAM);SOX2-OT(INTRON)
3	181475230	181475243	181475240	AC	G	snp	1	0	0	0	.	.	.	SOX2-OT(INTRON)
3	181567154	181567168	181567165	AAC	A	ins	1	0	0	0	.	.	.	INTERGENIC(INTERGENIC);RP11-252O18.2.1(UPSTREAM)
3	181657635	181657644	181657636	T	G	ins	1	0	0	0	.	.	.	INTERGENIC(INTERGENIC)
3	181834891	181834900	181834893	GT	A	snp	1	0	0	0	.	.	.	INTERGENIC(INTERGENIC);RP11-416O18.2.1(UPSTREAM)
3	181899343	181899350	181899346	A	C	snp	1	1	1	1	.	.	.	INTERGENIC(INTERGENIC)
3	182258397	182258406	182258400	A	C	snp	1	0	0	0	.	.	.	INTERGENIC(INTERGENIC)
3	182289864	182289875	182289870	CCA	T	snp	1	0	0	0	.	.	.	INTERGENIC(INTERGENIC)
3	182318172	182318180	182318174	A	C	snp	1	1	1	1	.	.	.	INTERGENIC(INTERGENIC)
3	182341377	182341386	182341382	AT	C	snp	1	1	1	1	.	.	.	INTERGENIC(INTERGENIC)
3	182356802	182356819	182356811	TC	A	snp	1	0	0	0	.	.	.	INTERGENIC(INTERGENIC)
3	182489296	182489304	182489300	A	C	ins	1	1	1	1	.	.	.	INTERGENIC(INTERGENIC)
3	182490335	182490343	182490341	T	G	snp	0	0	1	0	.	.	.	INTERGENIC(INTERGENIC)
3	182511420	182511431	182511428	GGC	G	snp	1	1	1	1	.	.	.	ATP11B(UTR_5_PRIME);RP11-225N10.1.1(UPSTREAM)
3	182518663	182518670	182518667	C	A	snp	1	0	0	0	.	.	.	ATP11B(INTRON)
3	182584709	182584720	182584714	AT	AC	ins	1	1	1	1	.	.	.	ATP11B(INTRON)
3	182584709	182584720	182584716	AT	C	snp	1	1	1	1	.	.	.	ATP11B(INTRON)
3	182584709	182584720	182584718	AT	A	del	1	0	0	0	.	.	.	ATP11B(INTRON)
3	182629060	182629068	182629064	C	CCCG	ins	1	1	1	1	.	.	.	ATP11B(INTRON)
3	182647595	182647602	182647597	A	C	snp	1	1	1	1	.	.	.	INTERGENIC(INTERGENIC)
3	182719477	182719485	182719484	T	C	snp	1	0	1	0	.	.	.	INTERGENIC(INTERGENIC)
3	182748127	182748134	182748128	A	C	snp	1	0	0	0	.	.	.	MCCC1(INTRON)
3	182795812	182795821	182795815	TA	G	snp	1	0	1	0	.	.	.	MCCC1(INTRON)
3	182820414	182820421	182820417	A	G	snp	1	0	0	0	.	.	.	MCCC1(INTRON);MCCC1(UPSTREAM)
3	182824663	182824674	182824669	AC	T	snp	1	0	0	0	.	.	.	MCCC1(INTRON)
3	182875533	182875540	182875536	A	G	ins	1	0	0	0	.	.	.	LAMP3(DOWNSTREAM);LAMP3(INTRON)
3	182904926	182904934	182904927	A	C	snp	1	0	0	0	.	.	.	MCF2L2(INTRON)
3	182995982	182995999	182995984	AT	T	del	1	0	0	0	.	.	.	B3GNT5(DOWNSTREAM);B3GNT5(INTRON);MCF2L2(INTRON)
3	183070311	183070319	183070315	A	G	snp	1	1	1	1	.	.	.	MCF2L2(INTRON)
3	183164882	183164891	183164889	T	TTC	ins	1	1	1	1	.	.	.	INTERGENIC(INTERGENIC);RP11-393B14.1.1(UPSTREAM);SNORA63(UPSTREAM)
3	183189616	183189623	183189620	T	C	snp	1	1	1	1	.	.	.	INTERGENIC(INTERGENIC)
3	183241491	183241498	183241497	T	C	ins	1	1	1	1	.	.	.	KLHL6(INTRON)
3	183252722	183252739	183252736	AT	T	ins	1	1	1	1	.	.	.	KLHL6(INTRON)
3	183252722	183252739	183252737	AT	A	del	1	1	1	1	.	.	.	KLHL6(INTRON)
3	183268213	183268226	183268223	AC	G	snp	1	0	0	0	.	.	.	KLHL6(INTRON);KLHL6-AS1(INTRON)
3	183320779	183320788	183320786	CT	C	del	1	1	1	1	.	.	.	INTERGENIC(INTERGENIC)
3	183591348	183591363	183591357	AATT	G	snp	1	0	0	0	.	.	.	PARL(INTRON)
3	183605004	183605015	183605006	CT	T	snp	1	0	0	0	.	.	.	

														PARL(UPSTREAM);RP11-315J22.5.1(INTRON);RP11-315J22.5.1(UPSTREAM)
3	183685913	183685930	183685917	TTA	ATC	ins	1	1	1	1	.	.	.	ABCC5(INTRON)
3	183696110	183696125	183696115	TTTG	C	snp	1	1	1	1	.	.	.	ABCC5(INTRON)
3	183769680	183769687	183769683	T	C	snp	1	1	1	1	.	.	.	HTR3C(UPSTREAM);INTERGENIC(INTERGENIC)
3	183991206	183991233	183991218	AAAC	G	snp	1	0	0	0	.	.	.	ECE2(INTRON);ECE2(UPSTREAM);EIF2B5(INTRON)
3	184100959	184100968	184100961	G	T	ins	1	1	1	1	.	.	.	CHRD(DOWNSTREAM);CHRD(EXON);CHRD(INTRON);CHRD(UPSTREAM);EIF2B5(INTRON)
3	184125525	184125532	184125531	C	T	snp	1	0	0	0	.	.	.	EIF2B5(INTRON);RP11-433C9.2.1(INTRON)
3	184132440	184132449	184132447	GT	C	snp	1	1	1	1	.	.	.	EIF2B5(INTRON);RP11-433C9.2.1(INTRON)
3	184158004	184158011	184158005	A	C	snp	1	1	1	1	.	.	.	EIF2B5(INTRON);RP11-433C9.2.1(DOWNSTREAM)
3	184215210	184215225	184215220	CCTC	C	ins	1	0	1	1	.	.	.	EIF2B5(INTRON)
3	184215210	184215225	184215221	CCTC	CCT	del	1	1	1	1	.	.	.	EIF2B5(INTRON)
3	184260397	184260410	184260407	GA	C	snp	1	1	1	1	.	.	.	EIF2B5(INTRON);EIF2B5-AS1(DOWNSTREAM)
3	184262334	184262348	184262345	CCT	C	snp	1	1	0	1	.	.	.	EIF2B5(INTRON);EIF2B5-AS1(DOWNSTREAM)
3	184262426	184262437	184262432	CCT	A	snp	1	1	0	1	.	.	.	EIF2B5(INTRON);EIF2B5-AS1(DOWNSTREAM)
3	184264489	184264503	184264500	TTG	TTGT	del	1	1	1	1	.	.	.	EIF2B5(INTRON);EIF2B5-AS1(DOWNSTREAM);EIF2B5-AS1(EXON)
3	184283815	184283822	184283817	G	T	snp	1	1	0	0	.	.	.	EIF2B5(INTRON);EPHB3(INTRON)
3	184354166	184354174	184354167	A	C	snp	1	0	0	0	.	.	.	EIF2B5(INTRON)
3	184490646	184490661	184490650	AAGG	AGG	del	1	1	0	1	.	.	.	RP11-329B9.1.1(INTRON)
3	184809685	184809696	184809694	AC	T	snp	1	1	1	1	.	.	.	C3orf70(INTRON)
3	184874041	184874049	184874042	A	G	snp	1	1	0	1	.	.	.	C3orf70(UPSTREAM);INTERGENIC(INTERGENIC)
3	185172155	185172164	185172160	AT	C	snp	0	0	1	0	.	.	.	MAP3K13(DOWNSTREAM);MAP3K13(INTRON)
3	185172252	185172261	185172255	AT	C	snp	0	1	1	0	.	.	.	MAP3K13(DOWNSTREAM);MAP3K13(INTRON)
3	185172600	185172609	185172607	AT	C	snp	0	0	1	0	.	.	.	MAP3K13(DOWNSTREAM);MAP3K13(INTRON)
3	185173075	185173084	185173080	AT	C	snp	1	1	1	1	.	.	.	MAP3K13(DOWNSTREAM);MAP3K13(INTRON)
3	185173575	185173584	185173580	AT	C	snp	1	0	0	0	.	.	.	MAP3K13(DOWNSTREAM);MAP3K13(INTRON)
3	185204959	185204967	185204961	G	T	ins	1	0	1	0	.	.	.	MAP3K13(DOWNSTREAM);MAP3K13(UTR_3_PRIME);TMEM41A(DOWNSTREAM);TMEM41A(INTRON)
3	185204959	185204967	185204965	G	T	ins	1	0	0	0	.	.	.	MAP3K13(DOWNSTREAM);MAP3K13(UTR_3_PRIME);TMEM41A(DOWNSTREAM);TMEM41A(INTRON)
3	185215870	185215879	185215876	C	CCA	ins	1	1	1	1	.	.	.	TMEM41A(INTRON)
3	185262128	185262136	185262132	T	G	snp	0	1	1	0	.	.	.	LIPH(INTRON)
3	185354942	185354956	185354952	AAC	CAAC	del	1	1	1	1	.	.	.	INTERGENIC(INTERGENIC);SENP2(DOWNSTREAM)
3	185388350	185388373	185388363	ATTT	C	snp	0	0	0	1	.	.	.	IGF2BP2(DOWNSTREAM);IGF2BP2(INTRON)
3	185503454	185503465	185503459	AAT	A	snp	1	1	1	1	.	.	.	IGF2BP2(INTRON)
3	185702265	185702274	185702267	CT	A	snp	1	1	1	1	.	.	.	INTERGENIC(INTERGENIC);RP11-443P15.2.1(DOWNSTREAM)
3	185752434	185752443	185752438	A	C	snp	1	0	0	0	.	.	.	INTERGENIC(INTERGENIC)
3	185787333	185787340	185787334	A	G	snp	1	1	0	1	.	.	.	ETV5(INTRON);ETV5(UPSTREAM)
3	185844435	185844446	185844444	GT	C	snp	1	1	1	1	.	.	.	DGKG(INTRON)
3	186015279	186015286	186015283	A	G	snp	1	1	1	1	.	.	.	DGKG(INTRON)
3	186290128	186290151	186290144	ATTT	T	snp	1	1	0	0	.	.	.	DNAJB11(INTRON);TBCCD1(UPSTREAM)
3	186340632	186340647	186340642	TG	G	ins	1	1	0	0	.	.	.	AHSG(DOWNSTREAM);INTERGENIC(INTERGENIC)
3	186427839	186427846	186427840	A	C	snp	1	1	1	1	.	.	.	INTERGENIC(INTERGENIC);RP11-795A2.3.1(DOWNSTREAM)
3	186548632	186548645	186548634	TG	C	snp	1	1	1	1	.	.	.	INTERGENIC(INTERGENIC);RP11-573D15.1.1(DOWNSTREAM)
3	186562897	186562905	186562898	G	T	snp	1	1	1	1	.	.	.	ADIPOQ(INTRON)
3	186579687	186579694	186579692	T	G	snp	1	0	0	0	.	.	.	ADIPOQ(DOWNSTREAM);INTERGENIC(INTERGENIC)
3	186604330	186604339	186604335	AG	A	del	1	0	0	0	.	.	.	INTERGENIC(INTERGENIC)
3	186604330	186604339	186604336	AG	G	snp	0	1	1	1	.	.	.	INTERGENIC(INTERGENIC)
3	186620692	186620703	186620695	TC	G	snp	1	1	1	1	.	.	.	INTERGENIC(INTERGENIC);RP11-42D20.2.1(DOWNSTREAM)
3	186652550	186652569	186652557	TATT	G	snp	1	0	0	0	.	.	.	AC007690.1(DOWNSTREAM);ST6GAL1(INTRON)
3	186692515	186692524	186692521	G	T	snp	0	1	1	1	.	.	.	ST6GAL1(INTRON);ST6GAL1(UPSTREAM)
3	186704969	186704988	186704984	AGGG	A	ins	1	1	1	1	.	.	.	ST6GAL1(INTRON)
3	186712737	186712746	186712738	A	AC	ins	1	1	1	1	.	.	.	ST6GAL1(INTRON);ST6GAL1(UPSTREAM)
3	186810229	186810244	186810236	TTTG	A	snp	1	0	0	0	.	.	.	INTERGENIC(INTERGENIC)
3	186818133	186818142	186818137	A	C	snp	1	0	0	0	.	.	.	INTERGENIC(INTERGENIC)
3	186874831	186874839	186874835	T	G	snp	1	1	1	1	.	.	.	RPL39L(INTRON)
3	187259938	187259961	187259951	AAAT	T	snp	1	0	1	0	.	.	.	INTERGENIC(INTERGENIC)
3	187281300	187281317	187281315	AC	T	snp	1	0	0	0	.	.	.	INTERGENIC(INTERGENIC)
3	187297651	187297662	187297654	TG	A	snp	1	1	1	1	.	.	.	INTERGENIC(INTERGENIC)
3	187353454	187353463	187353461	T	G	snp	1	1	1	1	.	.	.	INTERGENIC(INTERGENIC)
3	187376840	187376851	187376846	TA	A	snp	1	1	1	1	.	.	.	INTERGENIC(INTERGENIC)
3	187383339	187383348	187383344	GA	G	snp	1	1	1	1	.	.	.	INTERGENIC(INTERGENIC);SST(DOWNSTREAM)
3	187507812	187507831	187507825	AAGG	C	snp	1	0	0	0	.	.	.	INTERGENIC(INTERGENIC)
3	187553402	187553409	187553408	T	G	ins	1	0	0	0	.	.	.	INTERGENIC(INTERGENIC)

3	187563779	187563794	187563783	CCTT	CTT	del	1	0	0	0	.	.	.	INTERGENIC(INTERGENIC)
3	187592423	187592432	187592427	A	G	snp	1	0	0	0	.	.	.	INTERGENIC(INTERGENIC)
3	187615372	187615380	187615376	C	T	snp	1	1	0	1	.	.	.	INTERGENIC(INTERGENIC)
3	187628006	187628014	187628007	T	G	snp	1	1	0	1	.	.	.	INTERGENIC(INTERGENIC)
3	187891290	187891297	187891293	A	C	snp	1	1	1	1	.	.	.	LPP(INTRON)
3	187953872	187953879	187953874	A	G	snp	1	0	0	0	.	.	.	LPP(INTRON);LPP(UPSTREAM)
3	187962504	187962513	187962509	CT	C	ins	1	0	0	0	.	.	.	LPP(INTRON)
3	187967673	187967690	187967687	AT	TAT	del	1	1	1	1	.	.	.	LPP(INTRON)
3	187967673	187967690	187967688	AT	A	del	1	1	1	1	.	.	.	LPP(INTRON)
3	187968473	187968486	187968483	CT	T	snp	1	0	0	0	.	.	.	LPP(INTRON)
3	187968908	187968923	187968919	TCTT	C	snp	1	0	0	0	.	.	.	LPP(INTRON)
3	187987424	187987432	187987431	T	TTA	ins	1	1	1	1	.	.	.	LPP(INTRON)
3	188006582	188006597	188006591	TC	A	snp	0	0	1	0	.	.	.	LPP(INTRON)
3	188031811	188031818	188031813	T	C	snp	1	1	1	1	.	.	.	LPP(INTRON)
3	188078311	188078319	188078316	T	C	snp	1	1	1	1	.	.	.	LPP(INTRON)
3	188180020	188180040	188180024	ATT	C	snp	1	1	1	1	.	.	.	LPP(INTRON)
3	188191203	188191212	188191208	AG	C	snp	1	0	0	0	.	.	.	LPP(INTRON)
3	188209373	188209384	188209378	AGC	G	snp	1	1	1	1	.	.	.	LPP(INTRON)
3	188281507	188281515	188281514	T	G	ins	0	1	1	1	.	.	.	LPP(INTRON);LPP(UPSTREAM);LPP-AS1(INTRON)
3	188295636	188295647	188295639	TC	TG	ins	1	1	1	1	.	.	.	LPP(INTRON)
3	188383233	188383250	188383247	CTT	C	del	1	1	1	1	.	.	.	LPP(INTRON)
3	188420114	188420128	188420125	TTC	TTCT	del	0	1	0	1	.	.	.	LPP(INTRON)
3	188451788	188451799	188451791	TG	A	snp	0	0	0	1	.	.	.	LPP(INTRON)
3	188513478	188513495	188513486	TCA	G	snp	1	1	1	1	.	.	.	LPP(INTRON)
3	188531892	188531901	188531897	CA	G	snp	1	0	0	0	.	.	.	LPP(INTRON)
3	188538084	188538099	188538096	TG	C	snp	1	1	1	1	.	.	.	LPP(INTRON)
3	188610826	188610839	188610830	TA	C	snp	0	1	0	1	.	.	.	INTERGENIC(INTERGENIC);LPP(DOWNSTREAM)
3	188625504	188625513	188625509	AG	C	snp	1	0	0	0	.	.	.	INTERGENIC(INTERGENIC)
3	188630542	188630549	188630544	G	A	ins	1	1	1	1	.	.	.	INTERGENIC(INTERGENIC)
3	188691986	188691993	188691989	A	C	ins	1	1	1	1	.	.	.	TPRG1(INTRON);TPRG1(UPSTREAM)
3	188794713	188794730	188794717	AT	TG	ins	1	0	0	0	.	.	.	TPRG1(INTRON)
3	188807689	188807696	188807695	T	G	snp	1	0	0	0	.	.	.	TPRG1(INTRON)
3	188823843	188823860	188823848	CAA	G	snp	1	1	1	1	.	.	.	TPRG1(INTRON)
3	188828907	188828914	188828909	A	C	ins	1	1	1	0	.	.	.	TPRG1(INTRON)
3	188831703	188831714	188831710	ATA	AATAA	del	1	1	1	1	.	.	.	TPRG1(INTRON)
3	188966292	188966301	188966298	TA	C	snp	1	0	0	0	.	.	.	TPRG1(INTRON)
3	188966292	188966301	188966299	TA	G	snp	0	1	1	1	.	.	.	TPRG1(INTRON)
3	189019360	189019367	189019361	T	G	snp	1	0	0	1	.	.	.	TPRG1(INTRON)
3	189062650	189062657	189062655	T	G	ins	1	1	1	1	.	.	.	INTERGENIC(INTERGENIC)
3	189062650	189062657	189062655	T	G	snp	1	1	1	1	.	.	.	INTERGENIC(INTERGENIC)
3	189078939	189078948	189078942	A	C	snp	1	1	1	1	.	.	.	INTERGENIC(INTERGENIC)
3	189099346	189099365	189099353	TTTG	T	ins	1	1	0	0	.	.	.	INTERGENIC(INTERGENIC)
3	189099346	189099365	189099357	TTTG	T	snp	1	1	1	1	.	.	.	INTERGENIC(INTERGENIC)
3	189099346	189099365	189099361	TTTG	T	snp	1	1	1	1	.	.	.	INTERGENIC(INTERGENIC)
3	189102482	189102491	189102485	T	G	snp	1	1	1	1	.	.	.	INTERGENIC(INTERGENIC)
3	189102482	189102491	189102488	T	G	snp	0	0	0	1	.	.	.	INTERGENIC(INTERGENIC)
3	189126846	189126855	189126847	T	G	ins	1	1	1	1	.	.	.	INTERGENIC(INTERGENIC)
3	189240487	189240494	189240489	A	C	snp	0	1	0	1	.	.	.	INTERGENIC(INTERGENIC)
3	189280845	189280853	189280846	A	G	snp	1	1	1	1	.	.	.	INTERGENIC(INTERGENIC)
3	189565162	189565179	189565167	ATC	A	snp	1	1	1	1	.	.	.	TP63(INTRON)
3	189619674	189619691	189619688	AT	T	snp	1	0	0	0	.	.	.	INTERGENIC(INTERGENIC);TP63(DOWNSTREAM)
3	189619674	189619691	189619689	AT	A	del	1	1	1	1	.	.	.	INTERGENIC(INTERGENIC);TP63(DOWNSTREAM)
3	189633957	189633964	189633963	A	G	snp	1	0	0	0	.	.	.	INTERGENIC(INTERGENIC)
3	189635501	189635520	189635516	AAAT	A	ins	1	0	0	0	.	.	.	INTERGENIC(INTERGENIC)
3	189635501	189635520	189635516	AAAT	C	snp	0	1	1	1	.	.	.	INTERGENIC(INTERGENIC)
3	189667073	189667096	189667091	TCCT	T	snp	1	0	0	0	.	.	.	INTERGENIC(INTERGENIC)
3	189683834	189683853	189683847	AAAG	AGAAAG	del	1	0	0	0	.	.	.	LEPREL1(INTRON);LEPREL1(UPSTREAM);MTAPP2(DOWNSTREAM)
3	189697058	189697067	189697059	A	C	snp	1	0	0	0	.	.	.	LEPREL1(INTRON)
3	189753287	189753295	189753291	T	C	snp	1	1	1	1	.	.	.	LEPREL1(INTRON)
3	189901925	189901936	189901932	TGT	A	snp	1	1	0	1	.	.	.	INTERGENIC(INTERGENIC)
3	190021684	190021691	190021686	A	C	ins	1	1	1	1	.	.	.	CLDN1(DOWNSTREAM);INTERGENIC(INTERGENIC)
3	190053900	190053908	190053907	A	G	snp	1	1	1	0	.	.	.	CLDN16(INTRON)
3	190076456	190076463	190076461	A	AAC	ins	0	1	0	0	.	.	.	CLDN16(INTRON)
3	190123385	190123396	190123392	CCA	T	snp	1	1	1	1	.	.	.	CLDN16(DOWNSTREAM);CLDN16(INTRON)
3	190164297	190164305	190164304	T	G	ins	1	1	1	1	.	.	.	TMEM207(INTRON)
3	190219171	190219178	190219174	T	G	snp	1	0	0	0	.	.	.	INTERGENIC(INTERGENIC)

3	190258021	190258030	190258027	CT	G	snp	1	0	0	0	.	.	.	IL1RAP(INTRON)
3	190280161	190280170	190280165	T	G	snp	1	1	1	1	.	.	.	IL1RAP(INTRON);IL1RAP(UPSTREAM)
3	190397011	190397018	190397014	T	G	snp	0	0	1	0	.	.	.	INTERGENIC(INTERGENIC)
3	190519727	190519735	190519729	A	C	ins	1	1	1	1	.	.	.	INTERGENIC(INTERGENIC)
3	190519727	190519735	190519730	A	C	snp	1	1	1	1	.	.	.	INTERGENIC(INTERGENIC)
3	190524125	190524133	190524126	T	C	snp	1	1	1	1	.	.	.	INTERGENIC(INTERGENIC)
3	190530826	190530835	190530830	TA	C	snp	1	1	1	1	.	.	.	INTERGENIC(INTERGENIC)
3	190742583	190742592	190742590	AG	T	snp	0	0	1	0	.	.	.	INTERGENIC(INTERGENIC)
3	190748594	190748601	190748599	T	G	ins	0	0	1	0	.	.	.	INTERGENIC(INTERGENIC)
3	190812602	190812609	190812608	T	C	snp	1	1	1	1	.	.	.	INTERGENIC(INTERGENIC)
3	190923233	190923248	190923236	AT	A	snp	1	1	1	0	.	.	.	OSTN(INTRON)
3	190993491	190993500	190993492	A	C	ins	1	1	1	1	.	.	.	UTS2D(DOWNSTREAM);UTS2D(INTRON)
3	191014836	191014844	191014839	A	C	snp	1	1	1	1	.	.	.	UTS2D(INTRON)
3	191029219	191029230	191029225	TC	C	snp	1	0	0	0	.	.	.	UTS2D(INTRON)
3	191067756	191067764	191067763	C	T	ins	1	0	1	0	.	.	.	CCDC50(INTRON)
3	191131684	191131692	191131687	A	C	snp	1	0	0	0	.	.	.	INTERGENIC(INTERGENIC)
3	191180871	191180882	191180879	TC	C	snp	1	1	0	1	.	.	.	INTERGENIC(INTERGENIC);PYDC2(DOWNSTREAM)
3	191227244	191227252	191227246	T	C	snp	0	1	0	1	.	.	.	INTERGENIC(INTERGENIC)
3	191253286	191253295	191253293	T	G	snp	1	1	1	1	.	.	.	INTERGENIC(INTERGENIC)
3	191289735	191289742	191289739	A	G	snp	1	0	0	0	.	.	.	RP11-197K6.1.1(INTRON)
3	191302119	191302130	191302126	TTA	C	snp	1	0	0	0	.	.	.	RP11-197K6.1.1(INTRON)
3	191331246	191331254	191331251	A	G	snp	1	0	0	0	.	.	.	INTERGENIC(INTERGENIC)
3	191345142	191345153	191345147	CT	A	snp	0	0	1	0	.	.	.	INTERGENIC(INTERGENIC)
3	191359026	191359039	191359034	TA	ATATA	del	1	1	1	1	.	.	.	INTERGENIC(INTERGENIC);Y_RNA(DOWNSTREAM)
3	191660492	191660519	191660514	TTTA	A	del	1	1	1	0	.	.	.	INTERGENIC(INTERGENIC)
3	191660492	191660519	191660515	TTTA	T	snp	1	1	1	0	.	.	.	INTERGENIC(INTERGENIC)
3	191681845	191681859	191681856	TTG	A	snp	0	1	1	1	.	.	.	INTERGENIC(INTERGENIC)
3	191738147	191738154	191738150	A	G	ins	1	1	1	1	.	.	.	INTERGENIC(INTERGENIC)
3	191738147	191738154	191738151	A	G	snp	1	1	1	1	.	.	.	INTERGENIC(INTERGENIC)
3	191747750	191747761	191747759	GA	GT	ins	1	1	1	1	.	.	.	RP11-655G22.1.1(EXON);RP11-655G22.1.1(INTRON);RP11-655G22.1.1(UPSTREAM)
3	191788597	191788605	191788604	A	C	snp	1	1	0	1	.	.	.	INTERGENIC(INTERGENIC)
3	191879229	191879242	191879237	AC	G	snp	1	1	1	1	.	.	.	FGF12(INTRON)
3	192051432	192051439	192051436	A	G	snp	1	0	0	0	.	.	.	FGF12(INTRON)
3	192171227	192171234	192171233	C	A	snp	1	1	0	0	.	.	.	FGF12(INTRON)
3	192178451	192178464	192178456	AC	T	snp	1	0	0	0	.	.	.	FGF12(INTRON)
3	192244331	192244340	192244336	AC	T	snp	1	0	0	0	.	.	.	FGF12(INTRON)
3	192378774	192378783	192378778	A	AT	ins	1	1	1	1	.	.	.	FGF12(INTRON)
3	192542630	192542637	192542632	T	G	snp	1	1	1	1	.	.	.	MB21D2(INTRON)
3	192599280	192599289	192599283	AC	A	snp	1	0	0	0	.	.	.	MB21D2(INTRON)
3	192697998	192698007	192698000	A	C	snp	1	1	1	1	.	.	.	INTERGENIC(INTERGENIC)
3	192723502	192723509	192723506	T	G	snp	1	0	0	0	.	.	.	INTERGENIC(INTERGENIC)
3	192756773	192756780	192756779	A	G	snp	0	1	0	0	.	.	.	INTERGENIC(INTERGENIC)
3	192806718	192806725	192806724	C	A	ins	1	0	1	0	.	.	.	INTERGENIC(INTERGENIC)
3	192809330	192809337	192809332	A	C	snp	1	0	1	0	.	.	.	INTERGENIC(INTERGENIC)
3	192810677	192810691	192810680	AAT	G	snp	1	1	1	1	.	.	.	INTERGENIC(INTERGENIC)
3	192812327	192812342	192812340	CT	TG	ins	0	1	1	1	.	.	.	INTERGENIC(INTERGENIC)
3	192895602	192895609	192895603	A	C	snp	0	1	1	0	.	.	.	INTERGENIC(INTERGENIC);RP11-143P4.2.1(UPSTREAM)
3	192904553	192904561	192904554	T	G	snp	0	0	1	0	.	.	.	INTERGENIC(INTERGENIC)
3	193075752	193075771	193075762	ATTC	C	del	1	1	1	1	.	.	.	ATP13A5(DOWNSTREAM);ATP13A5(INTRON)
3	193107820	193107829	193107822	A	C	snp	1	1	1	1	.	.	.	INTERGENIC(INTERGENIC)
3	193192502	193192511	193192509	AG	A	snp	1	1	1	1	.	.	.	ATP13A4(INTRON)
3	193352072	193352081	193352073	A	AAC	ins	1	1	1	1	.	.	.	OPA1(INTRON);OPA1(UPSTREAM)
3	193509497	193509512	193509503	GT	A	snp	1	0	0	0	.	.	.	INTERGENIC(INTERGENIC)
3	193535633	193535640	193535638	T	G	ins	1	1	0	0	.	.	.	INTERGENIC(INTERGENIC)
3	193547073	193547084	193547081	TTC	TT	del	1	1	1	1	.	.	.	INTERGENIC(INTERGENIC)
3	193731565	193731574	193731569	CA	A	snp	1	1	1	1	.	.	.	RP11-407B7.1.1(DOWNSTREAM);RP11-407B7.1.1(INTRON)
3	193738173	193738192	193738187	TTGT	TTT	ins	1	1	1	1	.	.	.	RP11-407B7.1.1(INTRON)
3	193834223	193834230	193834224	A	C	snp	1	1	1	1	.	.	.	INTERGENIC(INTERGENIC)
3	193870144	193870155	193870153	TC	T	snp	1	0	1	0	.	.	.	INTERGENIC(INTERGENIC)
3	193886056	193886063	193886057	T	G	snp	1	1	0	0	.	.	.	AC080129.1(DOWNSTREAM);INTERGENIC(INTERGENIC)
3	193928595	193928603	193928599	T	G	snp	1	1	1	1	.	.	.	RP11-513G11.4.1(INTRON);RP11-513G11.4.1(UPSTREAM)
3	194024548	194024556	194024553	A	C	snp	1	0	0	0	.	.	.	RP11-513G11.1.1(INTRON);RP11-513G11.1.1(UPSTREAM)
3	194041350	194041358	194041351	C	A	snp	1	1	1	1	.	.	.	INTERGENIC(INTERGENIC)
3	194138772	194138787	194138782	GAAG	G	snp	1	0	0	0	.	.	.	ATP13A3(INTRON);ATP13A3(UPSTREAM)
3	194268444	194268461	194268457	TG	T	snp	1	1	1	1	.	.	.	INTERGENIC(INTERGENIC)
3	194277800	194277809	194277804	T	C	snp	1	0	0	0	.	.	.	INTERGENIC(INTERGENIC)

3	194321485	194321492	194321490	A	G	snp	1	1	1	1	.	.	.	TMEM44(INTRON)
3	194364258	194364267	194364266	A	G	snp	1	1	1	1	.	.	.	AC046143.3.1(INTRON);LSG1(INTRON)
3	194429510	194429517	194429513	T	C	snp	1	1	1	1	.	.	.	AC090505.6.1(EXON);AC090505.6.1(UPSTREAM);AC106706.1.1(DOWNSTREAM)
3	194468121	194468129	194468124	T	C	snp	1	0	0	0	.	.	.	AC090505.6.1(INTRON)
3	194576792	194576801	194576797	CA	T	snp	1	0	0	0	.	.	.	INTERGENIC(INTERGENIC)
3	194759188	194759199	194759193	TTG	T	snp	1	1	1	1	.	.	.	INTERGENIC(INTERGENIC)
3	194828364	194828384	194828375	GGA	G	snp	1	1	1	1	.	.	.	XXYLT1(INTRON)
3	194864601	194864618	194864615	AT	T	snp	1	0	0	0	.	.	.	AC011325.1.1(UPSTREAM);AC106705.1(DOWNSTREAM);XXYLT1(INTRON)
3	194890582	194890599	194890595	AT	ATA	del	1	1	1	1	.	.	.	XXYLT1(INTRON)
3	194890582	194890599	194890597	AT	A	del	1	1	1	1	.	.	.	XXYLT1(INTRON)
3	194953055	194953063	194953061	A	C	ins	1	1	1	0	.	.	.	XXYLT1(INTRON)
3	195080195	195080210	195080205	TTTG	G	snp	0	0	0	1	.	.	.	ACAP2(INTRON)
3	195109755	195109764	195109760	A	C	snp	1	1	1	1	.	.	.	ACAP2(INTRON)
3	195160362	195160369	195160365	T	C	snp	1	0	0	0	.	.	.	ACAP2(DOWNSTREAM);ACAP2(INTRON)
3	195179268	195179277	195179276	A	G	snp	1	0	0	0	.	.	.	AC091633.1(UPSTREAM);INTERGENIC(INTERGENIC)
3	195327407	195327416	195327412	GA	G	snp	1	1	0	1	.	.	.	INTERGENIC(INTERGENIC)
3	195364091	195364098	195364093	G	T	ins	1	0	0	0	.	.	.	MUC20(INTRON)
3	195387478	195387487	195387483	T	C	snp	1	0	0	0	.	.	.	AC069513.4.1(UPSTREAM);MUC20(INTRON);SDHAP2(INTRON)
3	195657677	195657696	195657687	TTAA	T	snp	1	0	0	0	.	.	.	INTERGENIC(INTERGENIC);U2(UPSTREAM)
3	195721250	195721261	195721254	GAG	T	snp	1	1	1	1	.	.	.	AC024937.4.1(UPSTREAM);INTERGENIC(INTERGENIC);SDHAP1(UPSTREAM)
3	195783273	195783282	195783280	T	G	snp	1	1	1	1	.	.	.	TFRC(DOWNSTREAM);TFRC(INTRON);TFRC(UPSTREAM)
3	195802755	195802762	195802757	G	T	ins	0	1	0	1	.	.	.	TFRC(INTRON);U7(DOWNSTREAM)
3	195811278	195811293	195811289	AAAG	AAAGA	del	1	1	1	1	.	.	.	INTERGENIC(INTERGENIC);TFRC(UPSTREAM)
3	195822803	195822818	195822813	TTAT	G	snp	0	1	0	1	.	.	.	INTERGENIC(INTERGENIC)
3	195854983	195854990	195854988	C	T	ins	1	1	1	1	.	.	.	INTERGENIC(INTERGENIC)
3	195860284	195860299	195860297	CT	C	del	1	1	0	1	.	.	.	INTERGENIC(INTERGENIC)
3	195879712	195879721	195879718	T	C	ins	1	0	0	0	.	.	.	AC139666.1.1(INTRON)
3	195896636	195896653	195896640	AAT	T	del	1	0	0	0	.	.	.	INTERGENIC(INTERGENIC)
3	195941852	195941859	195941853	C	A	snp	1	0	0	0	.	.	.	AC069257.9.1(INTRON);AC069257.9.1(UPSTREAM);PCYT1A(INTRON);ZDHHC19(UPSTREAM)
3	195944419	195944427	195944426	A	G	snp	0	1	0	0	.	.	.	AC069257.9.1(INTRON);PCYT1A(INTRON)
3	195944497	195944512	195944504	GAAG	T	snp	1	0	0	0	.	.	.	AC069257.9.1(INTRON);PCYT1A(INTRON)
3	195948558	195948577	195948572	TTTG	G	snp	0	0	1	0	.	.	.	AC069257.9.1(DOWNSTREAM);AC069257.9.1(INTRON);AC069257.9.1(UPSTREAM);PCYT1A(INTRON)
3	195965316	195965324	195965318	G	A	snp	1	1	1	1	.	.	.	AC069257.9.1(DOWNSTREAM);AC069257.9.1(INTRON);PCYT1A(DOWNSTREAM);PCYT1A(INTRON);PCYT1A(UTR_3_PRIME)
3	196043677	196043692	196043685	GT	A	snp	0	0	1	0	.	.	.	TCTEX1D2(INTRON);TM4SF19(DOWNSTREAM);TM4SF19(INTRON);TM4SF19-AS1(UPSTREAM)
3	196043923	196043932	196043930	GT	G	ins	0	1	0	1	.	.	.	TCTEX1D2(INTRON);TM4SF19(DOWNSTREAM);TM4SF19(INTRON);TM4SF19-AS1(UPSTREAM)
3	196256591	196256614	196256598	AATT	A	snp	1	1	1	1	.	.	.	AC092933.4.1(UPSTREAM);INTERGENIC(INTERGENIC)
3	196318557	196318565	196318560	A	G	snp	0	1	0	0	.	.	.	FBXO45(DOWNSTREAM);INTERGENIC(INTERGENIC)
3	196367927	196367938	196367930	TG	A	snp	1	1	1	1	.	.	.	LRRC33(INTRON);PIGX(INTRON)
3	196367927	196367938	196367936	TG	A	snp	1	1	1	1	.	.	.	LRRC33(INTRON);PIGX(INTRON)
3	196423024	196423031	196423026	T	C	snp	1	0	0	0	.	.	.	PIGX(INTRON)
3	196491496	196491511	196491502	AAAT	T	del	1	1	1	1	.	.	.	PAK2(INTRON)
3	196500903	196500911	196500909	A	C	snp	1	1	1	1	.	.	.	PAK2(INTRON)
3	196550415	196550442	196550438	CAAA	C	del	1	1	0	1	.	.	.	PAK2(INTRON)
3	196553347	196553355	196553351	T	G	snp	1	0	0	0	.	.	.	PAK2(INTRON)
3	196623326	196623345	196623341	TTGA	T	ins	1	1	1	1	.	.	.	SENP5(INTRON);SENP5(UPSTREAM)
3	196768330	196768353	196768345	ATT	T	snp	0	1	1	1	.	.	.	DLG1(DOWNSTREAM);INTERGENIC(INTERGENIC)
3	196768330	196768353	196768348	ATT	T	snp	0	1	1	1	.	.	.	DLG1(DOWNSTREAM);INTERGENIC(INTERGENIC)
3	196883557	196883564	196883562	A	G	snp	0	1	0	1	.	.	.	DLG1(INTRON)
3	196981769	196981777	196981771	A	G	snp	1	1	1	1	.	.	.	DLG1(INTRON)
3	197245185	197245193	197245188	C	A	ins	1	0	0	0	.	.	.	BDH1(INTRON);BDH1(UPSTREAM)
3	197275737	197275745	197275740	A	C	ins	0	0	0	1	.	.	.	BDH1(INTRON)
3	197316876	197316891	197316888	AT	TAT	del	1	1	1	1	.	.	.	AC024560.3.1(INTRON)
3	197320476	197320491	197320487	AAAC	AAAG	ins	1	0	0	0	.	.	.	AC024560.3.1(INTRON)
3	197324208	197324215	197324210	T	G	snp	1	0	0	0	.	.	.	AC024560.3.1(INTRON)
3	197491624	197491633	197491629	A	G	snp	1	0	0	0	.	.	.	FYTTD1(INTRON);FYTTD1(UPSTREAM)
3	197624012	197624021	197624020	A	C	snp	1	1	1	1	.	.	.	IQCG(INTRON);U6(DOWNSTREAM)
3	197711351	197711365	197711361	GCT	TGCTGT	ins	1	1	1	1	.	.	.	LMLN(DOWNSTREAM);LMLN(INTRON);LMLN(UPSTREAM)
3	197800766	197800775	197800767	T	C	ins	1	1	1	1	.	.	.	ANKRD18DP(INTRON);U6(UPSTREAM)

3	197837964	197837973	197837967	T	G	snp	1	1	1	1	.	.	.	AC073135.3.1(DOWNSTREAM);AC073135.3.1(INTRON)
4	128478	128487	128484	T	G	snp	0	1	0	1	.	.	.	AC118278.1(UPSTREAM);Z95704.3(UPSTREAM);ZNF718(DOWNSTREAM);ZNF718(INTRON)
4	224439	224448	224446	T	C	snp	1	0	0	0	.	.	.	ZNF876P(INTRON)
4	281428	281441	281438	AT	T	ins	0	1	0	0	.	.	.	ZNF732(INTRON)
4	461006	461015	461011	A	C	ins	0	1	1	1	.	.	.	ABCA11P(INTRON);AC092574.2(DOWNSTREAM);ZNF721(INTRON)
4	467312	467319	467317	T	C	snp	1	0	0	0	.	.	.	ABCA11P(INTRON);ZNF721(INTRON)
4	485978	485987	485981	A	C	snp	1	1	0	0	.	.	.	ZNF721(INTRON)
4	485978	485987	485986	A	C	snp	1	0	0	0	.	.	.	ZNF721(INTRON)
4	532607	532614	532608	G	T	snp	1	0	0	0	.	.	.	PIGG(DOWNSTREAM);PIGG(INTRON)
4	565557	565572	565564	AAAC	A	ins	1	1	1	1	.	.	.	INTERGENIC(INTERGENIC)
4	565573	565582	565575	A	AC	ins	1	0	0	0	.	.	.	INTERGENIC(INTERGENIC)
4	566174	566183	566177	CA	G	snp	1	1	1	1	.	.	.	INTERGENIC(INTERGENIC)
4	653362	653371	653364	GT	A	snp	1	1	0	1	.	.	.	PDE6B(DOWNSTREAM);PDE6B(INTRON);PDE6B(UPSTREAM);RP11-1191J2.2.1(UPSTREAM)
4	718586	718599	718590	CA	T	snp	1	1	1	1	.	.	.	PCGF3(INTRON);PCGF3(UPSTREAM);RP11-1191J2.4.1(UPSTREAM)
4	721337	721344	721340	T	G	snp	0	0	0	1	.	.	.	PCGF3(INTRON);PCGF3(UPSTREAM);RP11-1191J2.4.1(UPSTREAM)
4	758637	758644	758638	C	T	snp	0	0	0	1	.	.	.	AC139887.4.1(INTRON);PCGF3(DOWNSTREAM);PCGF3(INTRON);RP11-440L14.1.1(DOWNSTREAM);RP11-440L14.1.1(INTRON)
4	763999	764010	764008	CA	G	snp	1	0	0	0	.	.	.	PCGF3(DOWNSTREAM);PCGF3(UTR_3_PRIME);RP11-440L14.1.1(INTRON)
4	802028	802047	802037	ATAA	AAATAAATAA	del	1	1	0	1	.	.	.	CPLX1(INTRON);CPLX1(UPSTREAM)
4	867936	867945	867941	TG	T	ins	0	0	1	0	.	.	.	GAK(INTRON);GAK(UPSTREAM)
4	912472	912479	912473	A	C	snp	1	1	1	1	.	.	.	GAK(INTRON);GAK(UPSTREAM)
4	1096362	1096371	1096367	A	G	snp	1	1	1	1	.	.	.	RNF212(INTRON);RNF212(UPSTREAM);RP11-20I20.1.1(UPSTREAM)
4	1143405	1143412	1143408	T	C	snp	0	1	1	0	.	.	.	AC092535.3.1(UPSTREAM);TMED11P(INTRON)
4	1190900	1190909	1190904	TC	C	snp	1	0	0	0	.	.	.	AC092535.1(INTRON);SPON2(DOWNSTREAM);SPON2(EXON);SPON2(INTRON);SPON2(UPSTREAM)
4	1221131	1221140	1221136	GT	C	snp	1	1	1	1	.	.	.	CTBP1(INTRON)
4	1247948	1247956	1247955	T	C	snp	1	1	1	1	.	.	.	C4orf42(DOWNSTREAM);CTBP1(UPSTREAM);RP11-20I20.4.1(EXON)
4	1278197	1278216	1278206	AATA	G	snp	1	1	1	1	.	.	.	INTERGENIC(INTERGENIC)
4	1293174	1293185	1293177	GCT	C	snp	1	0	0	0	.	.	.	MAEA(INTRON)
4	1537243	1537254	1537252	GT	C	snp	1	1	1	1	.	.	.	INTERGENIC(INTERGENIC)
4	1551034	1551041	1551040	G	A	snp	1	1	1	1	.	.	.	INTERGENIC(INTERGENIC)
4	1586987	1587004	1586998	CT	CTCTC	del	1	1	1	1	.	.	.	INTERGENIC(INTERGENIC)
4	1638612	1638620	1638613	G	GC	ins	0	1	0	1	.	.	.	FAM53A(DOWNSTREAM);INTERGENIC(INTERGENIC)
4	1652530	1652539	1652534	T	G	snp	1	1	1	1	.	.	.	FAM53A(DOWNSTREAM);FAM53A(INTRON)
4	1837192	1837211	1837207	AAAC	A	ins	1	0	0	0	.	.	.	LETM1(INTRON);LETM1(UPSTREAM)
4	1908368	1908381	1908377	TC	G	snp	1	0	0	0	.	.	.	WHSC1(INTRON)
4	1999824	1999841	1999836	CAA	AAT	ins	1	1	1	1	.	.	.	WHSC2(INTRON)
4	2185426	2185434	2185429	T	C	snp	1	1	1	1	.	.	.	POLN(INTRON)
4	2224124	2224133	2224127	A	C	snp	1	0	0	0	.	.	.	POLN(INTRON)
4	2285305	2285332	2285327	CCAT	G	snp	1	0	0	0	.	.	.	ZFYVE28(INTRON)
4	2310328	2310337	2310330	AG	G	snp	1	0	0	0	.	.	.	RP11-478C1.7.1(INTRON);ZFYVE28(INTRON)
4	2376373	2376384	2376377	AGG	A	snp	1	1	1	1	.	.	.	ZFYVE28(INTRON)
4	2406785	2406794	2406792	AT	C	snp	1	0	0	0	.	.	.	ZFYVE28(INTRON)
4	2490540	2490555	2490551	TTTA	T	snp	1	1	1	1	.	.	.	RNF4(INTRON);RNF4(UPSTREAM)
4	2521329	2521349	2521344	TAT	A	del	1	1	1	1	.	.	.	INTERGENIC(INTERGENIC);RNF4(DOWNSTREAM)
4	2527881	2527888	2527883	A	C	snp	1	1	1	1	.	.	.	INTERGENIC(INTERGENIC)
4	2582869	2582878	2582871	A	C	snp	1	0	0	0	.	.	.	INTERGENIC(INTERGENIC)
4	2657175	2657192	2657188	TG	C	snp	1	0	0	0	.	.	.	FAM193A(INTRON);FAM193A(UPSTREAM)
4	2657175	2657192	2657188	TG	TC	ins	1	0	0	0	.	.	.	FAM193A(INTRON);FAM193A(UPSTREAM)
4	2701174	2701189	2701176	CA	ACA	del	1	1	1	1	.	.	.	FAM193A(INTRON)
4	2726936	2726945	2726943	TA	G	snp	1	0	0	0	.	.	.	FAM193A(INTRON)
4	2782195	2782206	2782199	AAT	T	del	1	1	1	1	.	.	.	INTERGENIC(INTERGENIC)
4	2865444	2865452	2865448	T	G	ins	1	1	0	1	.	.	.	ADD1(INTRON);ADD1(UPSTREAM)
4	2990908	2990916	2990913	A	G	snp	1	0	0	0	.	.	.	GRK4(INTRON)
4	3034951	3034966	3034958	AAAG	C	snp	1	1	1	1	.	.	.	GRK4(INTRON)
4	3068998	3069007	3069002	GA	A	snp	1	0	0	0	.	.	.	HTT-AS1(INTRON)
4	3102937	3102944	3102940	T	G	snp	1	0	0	0	.	.	.	HTT(INTRON)
4	3110794	3110809	3110796	AT	T	del	1	1	1	1	.	.	.	HTT(INTRON)
4	3111473	3111487	3111476	GGA	A	snp	1	0	0	0	.	.	.	HTT(INTRON)
4	3129691	3129698	3129696	A	C	snp	1	0	0	0	.	.	.	HTT(DOWNSTREAM);HTT(INTRON);HTT(UPSTREAM)
4	3150525	3150534	3150528	A	G	snp	1	0	0	0	.	.	.	HTT(INTRON)
4	3177753	3177761	3177755	T	GG	ins	1	0	0	0	.	.	.	HTT(INTRON);HTT(UPSTREAM)
4	3380085	3380093	3380088	T	C	snp	0	1	1	1	.	.	.	RGS12(EXON);RGS12(INTRON)
4	3485053	3485060	3485054	G	T	snp	1	1	1	1	.	.	.	DOK7(INTRON);DOK7(UPSTREAM)
4	3517739	3517748	3517746	GA	C	snp	0	1	0	0	.	.	.	LRPAP1(DOWNSTREAM);LRPAP1(INTRON);LRPAP1(UTR_3_PRIME)
4	3687680	3687687	3687686	C	A	snp	1	1	1	1	.	.	.	INTERGENIC(INTERGENIC)
4	3716088	3716097	3716090	TG	A	snp	1	1	1	1	.	.	.	INTERGENIC(INTERGENIC)

4	3728081	3728090	3728085	GA	C	snp	0	0	0	1	.	.	.	INTERGENIC(INTERGENIC)
4	3728175	3728184	3728180	GA	GG	ins	1	1	1	1	.	.	.	INTERGENIC(INTERGENIC)
4	3912811	3912824	3912821	AC	T	snp	0	0	1	0	.	.	.	AC226119.4.1(DOWNSTREAM);INTERGENIC(INTERGENIC);RP11-718M20.5.1(DOWNSTREAM)
4	3991590	3991599	3991596	T	C	snp	1	0	0	0	.	.	.	INTERGENIC(INTERGENIC)
4	3992139	3992147	3992141	T	G	snp	0	1	0	0	.	.	.	INTERGENIC(INTERGENIC)
4	4037259	4037273	4037270	TTC	T	snp	1	0	0	0	.	.	.	AC116562.2(UPSTREAM);INTERGENIC(INTERGENIC)
4	4091432	4091455	4091451	ATA	A	snp	1	1	1	1	.	.	.	INTERGENIC(INTERGENIC);RP11-489M13.3.1(DOWNSTREAM)
4	4137623	4137630	4137624	G	A	snp	1	0	0	0	.	.	.	INTERGENIC(INTERGENIC)
4	4180732	4180751	4180747	TTTG	T	snp	0	1	1	1	.	.	.	INTERGENIC(INTERGENIC);RP11-324I10.1.1(UPSTREAM)
4	4265541	4265564	4265554	CTTT	C	snp	1	1	0	1	.	.	.	INTERGENIC(INTERGENIC);LYAR(DOWNSTREAM)
4	4265541	4265564	4265560	CTTT	C	ins	1	1	0	1	.	.	.	INTERGENIC(INTERGENIC);LYAR(DOWNSTREAM)
4	4267708	4267719	4267714	CTT	T	snp	1	1	1	1	.	.	.	INTERGENIC(INTERGENIC);LYAR(DOWNSTREAM)
4	4306760	4306769	4306762	CT	G	snp	1	0	1	0	.	.	.	ZBTB49(DOWNSTREAM);ZBTB49(INTRON)
4	4342563	4342570	4342565	A	C	snp	1	1	1	1	.	.	.	INTERGENIC(INTERGENIC)
4	4485589	4485598	4485593	T	C	snp	1	0	0	0	.	.	.	STX18(INTRON);STX18-IT1(UPSTREAM)
4	4510129	4510137	4510136	T	G	snp	0	0	1	0	.	.	.	RP11-323F5.1.1(DOWNSTREAM);STX18(INTRON)
4	4591101	4591112	4591108	TCT	TTCT	del	1	1	1	1	.	.	.	RP11-323F5.2.1(INTRON)
4	4591101	4591112	4591109	TCT	TC	del	1	1	1	1	.	.	.	RP11-323F5.2.1(INTRON)
4	4599973	4599982	4599981	T	C	ins	0	0	1	0	.	.	.	RP11-323F5.2.1(INTRON)
4	4675521	4675538	4675526	TC	T	snp	1	1	1	1	.	.	.	INTERGENIC(INTERGENIC)
4	4684991	4685008	4685003	ATT	C	ins	1	1	1	1	.	.	.	INTERGENIC(INTERGENIC)
4	4684991	4685008	4685005	ATT	A	del	1	1	1	1	.	.	.	INTERGENIC(INTERGENIC)
4	4885341	4885356	4885350	AAAC	AC	del	1	1	0	1	.	.	.	INTERGENIC(INTERGENIC)
4	4934312	4934321	4934319	TC	G	snp	0	1	0	1	.	.	.	INTERGENIC(INTERGENIC)
4	4944546	4944573	4944565	TGAA	G	snp	1	1	1	0	.	.	.	INTERGENIC(INTERGENIC)
4	4962216	4962235	4962224	ATTT	G	snp	1	0	0	0	.	.	.	INTERGENIC(INTERGENIC)
4	4967671	4967680	4967675	TA	ATATA	del	1	1	1	0	.	.	.	INTERGENIC(INTERGENIC)
4	5010863	5010870	5010866	T	G	snp	1	1	0	1	.	.	.	INTERGENIC(INTERGENIC)
4	5019929	5019946	5019932	AC	G	snp	1	1	1	1	.	.	.	CYTL1(INTRON)
4	5102591	5102610	5102596	TCCT	T	snp	1	1	1	1	.	.	.	STK32B(INTRON)
4	5186423	5186437	5186427	AAG	G	del	1	1	1	1	.	.	.	STK32B(INTRON)
4	5189352	5189359	5189354	G	A	snp	1	1	1	1	.	.	.	STK32B(INTRON)
4	5304074	5304085	5304078	TAT	C	snp	1	1	1	0	.	.	.	STK32B(INTRON)
4	5318029	5318038	5318033	AT	TATATTG	del	1	0	1	0	.	.	.	STK32B(INTRON)
4	5319250	5319259	5319252	TA	C	snp	1	0	1	1	.	.	.	STK32B(INTRON)
4	5368819	5368826	5368822	A	G	snp	1	0	1	0	.	.	.	STK32B(INTRON)
4	5376507	5376514	5376508	G	T	snp	1	1	1	1	.	.	.	STK32B(INTRON)
4	5377237	5377244	5377242	T	C	snp	1	1	1	1	.	.	.	STK32B(INTRON)
4	5391455	5391462	5391459	C	CCCG	ins	1	0	0	0	.	.	.	STK32B(INTRON)
4	5431890	5431899	5431896	AT	G	snp	0	0	1	0	.	.	.	STK32B(INTRON);STK32B(UPSTREAM);Y_RNA(UPSTREAM)
4	5498781	5498789	5498782	T	G	snp	1	1	1	0	.	.	.	STK32B(INTRON)
4	5522555	5522562	5522557	T	G	ins	1	0	0	0	.	.	.	C4orf6(UPSTREAM);INTERGENIC(INTERGENIC)
4	5838190	5838199	5838192	CA	T	snp	1	0	0	0	.	.	.	CRMP1(INTRON)
4	5844350	5844363	5844352	CA	AA	ins	1	0	0	0	.	.	.	CRMP1(INTRON)
4	5916099	5916106	5916105	T	C	snp	1	0	0	0	.	.	.	INTERGENIC(INTERGENIC)
4	5938755	5938764	5938760	A	C	snp	1	1	1	1	.	.	.	INTERGENIC(INTERGENIC)
4	5940971	5940979	5940976	A	G	snp	1	1	0	1	.	.	.	INTERGENIC(INTERGENIC)
4	5962580	5962591	5962587	ATA	A	snp	1	1	1	1	.	.	.	C4orf50(INTRON)
4	5994758	5994765	5994764	C	A	snp	1	1	0	1	.	.	.	C4orf50(UPSTREAM);INTERGENIC(INTERGENIC)
4	6100252	6100267	6100260	AG	G	snp	1	1	0	1	.	.	.	JAKMIP1(INTRON)
4	6121298	6121306	6121299	A	C	ins	1	1	1	1	.	.	.	JAKMIP1(INTRON)
4	6127294	6127307	6127299	AC	G	snp	1	0	0	1	.	.	.	JAKMIP1(INTRON)
4	6134752	6134759	6134756	T	C	snp	1	0	0	0	.	.	.	JAKMIP1(INTRON)
4	6211526	6211549	6211545	AAAC	A	ins	1	0	0	0	.	.	.	RP11-586D19.1.1(INTRON);RP11-586D19.2.1(DOWNSTREAM)
4	6238403	6238422	6238408	TATT	C	snp	1	0	0	0	.	.	.	RP11-586D19.1.1(INTRON)
4	6272301	6272310	6272306	CT	C	snp	1	1	1	1	.	.	.	WFS1(INTRON)
4	6399577	6399592	6399585	AATA	T	snp	1	1	1	0	.	.	.	PPP2R2C(INTRON)
4	6409292	6409299	6409293	T	C	snp	1	0	0	0	.	.	.	PPP2R2C(INTRON)
4	6457716	6457725	6457720	G	T	snp	1	1	1	1	.	.	.	PPP2R2C(INTRON)
4	6457716	6457725	6457721	G	A	ins	1	1	1	1	.	.	.	PPP2R2C(INTRON)
4	6466645	6466660	6466655	AGGG	A	snp	1	0	0	0	.	.	.	PPP2R2C(INTRON)
4	6513191	6513202	6513195	GGT	T	snp	1	0	0	0	.	.	.	PPP2R2C(INTRON)
4	6535863	6535872	6535865	AC	C	del	1	0	0	0	.	.	.	PPP2R2C(INTRON)
4	6539874	6539882	6539875	A	G	snp	1	1	1	1	.	.	.	PPP2R2C(INTRON)
4	6701674	6701688	6701685	TTG	TTGTTTT	del	1	1	1	1	.	.	.	INTERGENIC(INTERGENIC);S100P(DOWNSTREAM)
4	6729206	6729217	6729209	GCT	A	snp	1	0	0	0	.	.	.	INTERGENIC(INTERGENIC)

4	6857275	6857286	6857278	AT	C	snp	1	1	1	1	.	.	.	KIAA0232(INTRON);KIAA0232(UPSTREAM)	
4	6901916	6901923	6901919	A	G	snp	1	1	1	1	.	.	.	INTERGENIC(INTERGENIC)	
4	6921348	6921363	6921358	TATT	C	snp	1	0	0	0	.	.	.	TBC1D14(INTRON)	
4	6954558	6954566	6954564	T	G	snp	1	1	0	1	.	.	.	TBC1D14(INTRON);TBC1D14(UPSTREAM)	
4	7103407	7103416	7103414	GT	G	snp	1	1	1	1	.	.	.	RP11-367J11.3.1(INTRON);RP11-367J11.4.1(EXON)	
4	7108431	7108440	7108433	A	G	snp	1	1	1	1	.	.	.	INTERGENIC(INTERGENIC);RP11-367J11.3.1(UPSTREAM);RP11-367J11.4.1(UPSTREAM)	
4	7123943	7123958	7123952	CCCT	T	snp	1	1	1	1	.	.	.	INTERGENIC(INTERGENIC)	
4	7192491	7192502	7192493	AT	G	snp	1	1	1	1	.	.	.	INTERGENIC(INTERGENIC);SORCS2(UPSTREAM)	
4	7240018	7240025	7240019	G	A	ins	1	1	1	1	.	.	.	SORCS2(INTRON)	
4	7240018	7240025	7240020	G	A	snp	1	1	1	1	.	.	.	SORCS2(INTRON)	
4	7286749	7286764	7286762	AT	A	del	1	1	0	1	.	.	.	SORCS2(INTRON)	
4	7329531	7329538	7329537	C	A	snp	1	1	1	1	.	.	.	SORCS2(INTRON)	
4	7374030	7374038	7374035	T	G	snp	1	0	0	0	.	.	.	SORCS2(INTRON)	
4	7400787	7400795	7400793	T	G	snp	1	1	1	1	.	.	.	SORCS2(INTRON)	
4	7434413	7434420	7434414	G	GT	ins	1	0	0	0	.	.	.	PSAPL1(UTR_3_PRIME);SORCS2(INTRON)	
4	7434413	7434420	7434415	G	T	snp	1	0	0	0	.	.	.	PSAPL1(UTR_3_PRIME);SORCS2(INTRON)	
4	7450533	7450552	7450539	TCCC	T	snp	1	1	1	1	.	.	.	SORCS2(INTRON)	
4	7450574	7450589	7450581	CCTC	T	snp	1	1	1	1	.	.	.	SORCS2(INTRON)	
4	7450909	7450928	7450924	CCTC	T	snp	1	1	1	1	.	.	.	SORCS2(INTRON)	
4	7458207	7458215	7458208	G	T	snp	1	0	0	0	.	.	.	SORCS2(INTRON)	
4	7470920	7470931	7470926	TGG	C	snp	1	0	0	0	.	.	.	SORCS2(INTRON)	
4	7472119	7472134	7472127	TCCA	G	snp	0	1	0	1	.	.	.	SORCS2(INTRON)	
4	7490707	7490722	7490717	TTCA	G	snp	1	1	1	1	.	.	.	SORCS2(INTRON)	
4	7591123	7591138	7591134	TTTG	T	ins	1	1	1	1	.	.	.	SORCS2(INTRON)	
4	7716928	7716939	7716932	CTC	C	snp	1	1	1	1	.	"SORCS2(NON_SYNONYMOUS_CODING):S544P,S716P"	.	.	
4	7778114	7778123	7778120	TG	GTGC	ins	1	0	0	0	.	.	.	AFAP1(INTRON)	
4	7801979	7801986	7801985	A	G	snp	1	1	1	1	.	.	.	AFAP1(INTRON);AFAP1(UPSTREAM)	
4	7819363	7819370	7819369	G	A	snp	1	1	1	1	.	.	.	AFAP1(INTRON)	
4	7847139	7847159	7847154	ATT	TTATTACT	del	1	1	0	1	.	.	.	.	AFAP1(INTRON)
4	8021415	8021422	8021417	A	G	snp	1	1	1	1	.	.	.	ABLIM2(DOWNSTREAM);ABLIM2(INTRON)	
4	8039022	8039031	8039029	CA	G	snp	1	1	1	1	.	.	.	ABLIM2(INTRON)	
4	8078464	8078471	8078469	G	A	snp	1	0	0	0	.	.	.	ABLIM2(INTRON);ABLIM2(UPSTREAM)	
4	8092997	8093006	8093001	TA	C	snp	1	1	1	1	.	.	.	ABLIM2(INTRON)	
4	8096736	8096749	8096745	TC	TCT	del	1	1	1	0	.	.	.	ABLIM2(INTRON)	
4	8135714	8135721	8135719	G	T	snp	1	1	1	1	.	.	.	ABLIM2(INTRON)	
4	8147779	8147786	8147784	T	C	snp	1	1	0	1	.	.	.	ABLIM2(INTRON)	
4	8224404	8224421	8224414	TTG	GTC	ins	1	1	1	1	.	.	.	SH3TC1(DOWNSTREAM);SH3TC1(INTRON);SH3TC1(UPSTREAM)	
4	8499650	8499657	8499652	G	T	snp	0	1	1	1	.	.	.	METTL19(DOWNSTREAM);RP11-689P11.2.1(INTRON)	
4	8570849	8570860	8570852	TA	TATATATG	ins	1	0	0	0	.	.	.	.	GPR78(INTRON)
4	8636999	8637022	8637014	AAAT	C	snp	1	1	1	1	.	.	.	INTERGENIC(INTERGENIC)	
4	8726020	8726031	8726023	AG	C	snp	1	0	1	0	.	.	.	INTERGENIC(INTERGENIC)	
4	8766137	8766144	8766140	C	T	snp	1	1	1	1	.	.	.	INTERGENIC(INTERGENIC)	
4	9028392	9028406	9028402	GTT	C	snp	0	1	0	0	.	.	.	AC073648.2(UPSTREAM);INTERGENIC(INTERGENIC);RP11-747H12.5.1(UPSTREAM)	
4	9040415	9040428	9040424	TA	TG	ins	1	0	0	0	.	.	.	INTERGENIC(INTERGENIC)	
4	9045492	9045499	9045496	A	G	snp	0	1	1	1	.	.	.	INTERGENIC(INTERGENIC)	
4	9110284	9110295	9110287	CCT	T	snp	1	0	0	0	.	.	.	RP11-1286E23.1.1(INTRON)	
4	9115240	9115249	9115246	TC	C	snp	1	0	0	0	.	.	.	RP11-1286E23.1.1(INTRON)	
4	9122809	9122832	9122827	AAAC	C	del	1	0	0	0	.	.	.	RP11-1286E23.1.1(INTRON)	
4	9136964	9136975	9136967	TC	A	snp	1	0	0	0	.	.	.	RP11-1286E23.1.1(INTRON)	
4	9138280	9138287	9138282	A	G	snp	1	1	1	1	.	.	.	RP11-1286E23.1.1(INTRON)	
4	9192236	9192253	9192247	ACA	C	snp	1	1	1	1	.	.	.	INTERGENIC(INTERGENIC)	
4	9409869	9409877	9409876	A	C	snp	1	0	0	0	.	.	.	INTERGENIC(INTERGENIC);RN5S153(DOWNSTREAM);RP11-1396O13.14.1(DOWNSTREAM)	
4	9440767	9440776	9440772	T	C	snp	1	0	0	0	.	.	.	INTERGENIC(INTERGENIC)	
4	9442447	9442458	9442450	AG	A	snp	1	0	0	0	.	.	.	DEFB131(UPSTREAM);INTERGENIC(INTERGENIC)	
4	9550847	9550855	9550849	A	C	ins	0	1	1	1	.	.	.	INTERGENIC(INTERGENIC);RP11-1396O13.1.1(DOWNSTREAM)	
4	9557646	9557663	9557657	GTG	A	snp	0	1	0	0	.	.	.	INTERGENIC(INTERGENIC);MIR548I2(DOWNSTREAM);RP11-1396O13.1.1(UPSTREAM)	
4	9634140	9634163	9634145	AAAC	AC	del	1	0	0	0	.	.	.	INTERGENIC(INTERGENIC)	
4	9652578	9652593	9652583	TTTA	A	snp	1	1	1	1	.	.	.	INTERGENIC(INTERGENIC)	
4	9674056	9674067	9674061	TA	TATATAT	ins	1	1	1	1	.	.	.	INTERGENIC(INTERGENIC);RP11-180A12.4.1(UPSTREAM)	
4	9706757	9706764	9706761	G	A	ins	1	1	1	0	.	.	.	RP11-180A12.1.1(INTRON);RP11-180A12.3.1(DOWNSTREAM)	
4	9723644	9723653	9723645	A	G	snp	1	0	0	0	.	.	.	INTERGENIC(INTERGENIC)	
4	9723668	9723675	9723672	A	G	snp	1	1	1	1	.	.	.	INTERGENIC(INTERGENIC)	
4	9780431	9780439	9780436	T	C	snp	1	0	0	0	.	.	.	DRD5(UPSTREAM);SLC2A9(DOWNSTREAM);SLC2A9(INTRON)	
4	9801317	9801326	9801325	C	A	ins	1	0	0	0	.	.	.	SLC2A9(INTRON)	
4	9829795	9829808	9829799	TC	C	snp	1	1	1	1	.	.	.	SLC2A9(INTRON);SLC2A9(UPSTREAM)	
4	10055903	10055912	10055905	TG	A	snp	1	1	1	1	.	.	.	SLC2A9(INTRON)	

4	10090633	10090640	10090638	C	A	snp	0	0	1	0	.	.	.	WDR1(INTRON)
4	10241989	10241998	10241991	G	T	snp	1	1	1	1	.	.	.	INTERGENIC(INTERGENIC)
4	10292443	10292458	10292455	GT	TGT	del	1	1	1	1	.	.	.	AC006499.1.1(DOWNSTREAM);AC006499.2.1(DOWNSTREAM);INTERGENIC(INTERGENIC)
4	10389326	10389334	10389327	A	G	snp	1	0	0	0	.	.	.	INTERGENIC(INTERGENIC)
4	10401773	10401782	10401781	T	G	ins	1	1	1	1	.	.	.	INTERGENIC(INTERGENIC)
4	10477484	10477493	10477485	T	C	snp	1	1	1	1	.	.	.	INTERGENIC(INTERGENIC);RP11-136I13.2.1(DOWNSTREAM)
4	10776942	10776961	10776955	TATT	TTTATTTTTATTTTT	del	1	1	1	1	.	.	.	INTERGENIC(INTERGENIC)
4	10991140	10991149	10991142	TG	C	snp	0	0	1	0	.	.	.	INTERGENIC(INTERGENIC)
4	11081704	11081711	11081705	A	C	snp	1	1	1	1	.	.	.	INTERGENIC(INTERGENIC)
4	11084674	11084682	11084681	A	C	snp	1	1	1	1	.	.	.	INTERGENIC(INTERGENIC)
4	11087030	11087045	11087041	TTTC	A	snp	0	0	0	1	.	.	.	INTERGENIC(INTERGENIC)
4	11104789	11104798	11104791	A	C	snp	1	1	1	1	.	.	.	INTERGENIC(INTERGENIC)
4	11108566	11108573	11108568	T	C	snp	1	0	0	0	.	.	.	INTERGENIC(INTERGENIC)
4	11139367	11139376	11139368	A	C	snp	1	1	0	1	.	.	.	INTERGENIC(INTERGENIC)
4	11172781	11172790	11172788	T	C	snp	1	1	0	1	.	.	.	INTERGENIC(INTERGENIC)
4	11271077	11271092	11271088	TCTA	G	snp	1	0	0	0	.	.	.	INTERGENIC(INTERGENIC)
4	11287536	11287551	11287544	ATAG	C	snp	1	1	1	1	.	.	.	INTERGENIC(INTERGENIC)
4	11445043	11445058	11445054	TTGT	C	snp	1	1	1	1	.	.	.	INTERGENIC(INTERGENIC)
4	11446696	11446705	11446702	TA	A	ins	1	1	1	1	.	.	.	INTERGENIC(INTERGENIC)
4	11446717	11446726	11446723	AT	TATTATT	del	1	1	1	1	.	.	.	INTERGENIC(INTERGENIC)
4	11454765	11454778	11454768	TA	T	ins	0	0	1	0	.	.	.	INTERGENIC(INTERGENIC)
4	11468721	11468730	11468728	AT	AC	ins	1	1	1	1	.	.	.	INTERGENIC(INTERGENIC);RP11-281P23.1.1(UPSTREAM)
4	11469658	11469667	11469660	A	G	snp	1	0	0	0	.	.	.	INTERGENIC(INTERGENIC);RP11-281P23.1.1(UPSTREAM)
4	11470175	11470190	11470178	TA	G	snp	1	1	1	1	.	.	.	INTERGENIC(INTERGENIC);RP11-281P23.1.1(UPSTREAM)
4	11541664	11541672	11541667	A	C	snp	1	0	0	0	.	.	.	INTERGENIC(INTERGENIC)
4	11614606	11614614	11614607	T	C	snp	1	1	1	1	.	.	.	INTERGENIC(INTERGENIC)
4	11744247	11744256	11744251	TG	C	snp	1	1	1	1	.	.	.	RP11-281P23.2.1(INTRON);RP11-74M11.2.1(INTRON)
4	11826776	11826783	11826780	T	C	snp	1	1	0	1	.	.	.	INTERGENIC(INTERGENIC)
4	11882411	11882424	11882417	TG	C	snp	1	0	0	0	.	.	.	INTERGENIC(INTERGENIC)
4	11887955	11887972	11887964	AT	G	snp	1	0	0	0	.	.	.	INTERGENIC(INTERGENIC)
4	11967789	11967796	11967793	T	C	snp	1	1	1	1	.	.	.	INTERGENIC(INTERGENIC)
4	11997789	11997797	11997793	A	C	ins	1	1	1	1	.	.	.	INTERGENIC(INTERGENIC)
4	11997789	11997797	11997794	A	C	snp	1	1	1	1	.	.	.	INTERGENIC(INTERGENIC)
4	12010772	12010781	12010778	A	AT	ins	1	1	1	1	.	.	.	INTERGENIC(INTERGENIC)
4	12020457	12020466	12020461	GA	C	snp	1	0	0	0	.	.	.	INTERGENIC(INTERGENIC)
4	12081225	12081234	12081229	CT	G	snp	1	0	0	0	.	.	.	INTERGENIC(INTERGENIC)
4	12154207	12154214	12154213	T	C	snp	1	1	1	1	.	.	.	INTERGENIC(INTERGENIC)
4	12171950	12171965	12171956	TA	C	snp	1	0	0	0	.	.	.	INTERGENIC(INTERGENIC)
4	12197329	12197340	12197335	AT	G	snp	1	0	1	0	.	.	.	INTERGENIC(INTERGENIC)
4	12249080	12249089	12249085	TG	C	snp	1	1	1	1	.	.	.	RP11-168E17.1.1(INTRON)
4	12254809	12254817	12254815	A	G	snp	1	1	1	1	.	.	.	INTERGENIC(INTERGENIC);RP11-168E17.1.1(DOWNSTREAM)
4	12269272	12269281	12269279	AT	C	snp	1	1	1	1	.	.	.	INTERGENIC(INTERGENIC)
4	12294675	12294684	12294682	TG	T	snp	1	1	1	1	.	.	.	INTERGENIC(INTERGENIC)
4	12294922	12294933	12294925	TTG	C	snp	1	0	0	0	.	.	.	INTERGENIC(INTERGENIC)
4	12387859	12387868	12387862	AT	C	snp	1	0	0	0	.	.	.	INTERGENIC(INTERGENIC)
4	12387883	12387892	12387889	AT	T	snp	1	0	0	0	.	.	.	INTERGENIC(INTERGENIC)
4	12387921	12387930	12387923	TA	C	snp	1	0	0	0	.	.	.	INTERGENIC(INTERGENIC)
4	12398872	12398885	12398882	TA	A	del	1	0	0	0	.	.	.	INTERGENIC(INTERGENIC)
4	12430424	12430431	12430425	T	C	snp	0	1	0	0	.	.	.	INTERGENIC(INTERGENIC)
4	12650285	12650294	12650290	TA	G	snp	1	1	1	1	.	.	.	INTERGENIC(INTERGENIC)
4	12667858	12667867	12667863	AT	C	snp	1	1	1	1	.	.	.	INTERGENIC(INTERGENIC)
4	12667890	12667899	12667895	AT	C	snp	1	1	0	1	.	.	.	INTERGENIC(INTERGENIC)
4	12668923	12668932	12668930	TA	G	snp	1	1	1	1	.	.	.	INTERGENIC(INTERGENIC)
4	12668933	12668942	12668939	AT	C	snp	1	1	1	1	.	.	.	INTERGENIC(INTERGENIC)
4	12669090	12669097	12669094	T	G	ins	1	1	1	1	.	.	.	INTERGENIC(INTERGENIC)
4	12714291	12714306	12714301	AACA	A	snp	1	0	0	0	.	.	.	INTERGENIC(INTERGENIC)
4	12715296	12715305	12715303	AT	C	snp	1	0	0	0	.	.	.	INTERGENIC(INTERGENIC)
4	12794862	12794869	12794866	A	G	snp	1	1	1	1	.	.	.	AC096750.1(DOWNSTREAM);INTERGENIC(INTERGENIC)
4	12794862	12794869	12794867	A	C	snp	1	1	1	1	.	.	.	AC096750.1(DOWNSTREAM);INTERGENIC(INTERGENIC)
4	12921747	12921755	12921751	T	G	ins	1	1	1	1	.	.	.	INTERGENIC(INTERGENIC)
4	12993587	12993595	12993594	A	G	snp	1	1	1	1	.	.	.	INTERGENIC(INTERGENIC)
4	13003785	13003794	13003787	AC	G	snp	1	1	1	1	.	.	.	INTERGENIC(INTERGENIC)
4	13048219	13048226	13048222	A	G	snp	1	0	0	0	.	.	.	INTERGENIC(INTERGENIC);U6(UPSTREAM)
4	13121718	13121725	13121722	T	C	snp	1	1	1	1	.	.	.	INTERGENIC(INTERGENIC)

4	13132300	13132307	13132306	A	C	snp	0	0	0	1	.	.	.	INTERGENIC(INTERGENIC)
4	13139352	13139363	13139357	TG	C	snp	1	1	1	1	.	.	.	INTERGENIC(INTERGENIC)
4	13140206	13140215	13140211	TG	A	snp	1	0	0	0	.	.	.	INTERGENIC(INTERGENIC)
4	13155477	13155485	13155483	A	AC	ins	1	1	1	1	.	.	.	INTERGENIC(INTERGENIC)
4	13197744	13197755	13197751	AC	T	snp	0	1	0	1	.	.	.	INTERGENIC(INTERGENIC)
4	13306035	13306044	13306038	AG	A	snp	1	0	0	0	.	.	.	INTERGENIC(INTERGENIC)
4	13692997	13693005	13693000	T	C	ins	1	1	1	1	.	.	.	RP11-341G5.1.1(INTRON)
4	13736734	13736743	13736737	AG	C	snp	1	1	1	1	.	.	.	RP11-341G5.1.1(INTRON)
4	13894295	13894310	13894308	AG	A	snp	1	1	1	1	.	.	.	RP11-341G5.1.1(INTRON)
4	14068944	14068952	14068950	C	A	ins	1	1	1	1	.	.	.	INTERGENIC(INTERGENIC)
4	14068944	14068952	14068950	C	A	snp	1	1	1	1	.	.	.	INTERGENIC(INTERGENIC)
4	14068944	14068952	14068951	C	T	ins	1	1	1	1	.	.	.	INTERGENIC(INTERGENIC)
4	14075728	14075755	14075738	TTAT	G	snp	1	0	0	0	.	.	.	INTERGENIC(INTERGENIC)
4	14106746	14106755	14106750	AT	C	snp	1	1	1	1	.	.	.	INTERGENIC(INTERGENIC)
4	14123772	14123781	14123775	AG	AA	ins	1	1	1	1	.	.	.	RP11-341G5.3.1(INTRON);RP11-669M16.3.1(UPSTREAM)
4	14265865	14265873	14265869	T	C	snp	1	1	1	1	.	.	.	INTERGENIC(INTERGENIC)
4	14331364	14331377	14331370	GA	T	snp	1	1	1	1	.	.	.	INTERGENIC(INTERGENIC)
4	14388448	14388455	14388453	T	TC	ins	1	1	1	1	.	.	.	AC006296.1.1(INTRON);AC006296.2.1(UPSTREAM)
4	14388448	14388455	14388454	T	C	ins	1	1	1	1	.	.	.	AC006296.1.1(INTRON);AC006296.2.1(UPSTREAM)
4	14469841	14469850	14469847	GT	A	snp	0	1	1	1	.	.	.	INTERGENIC(INTERGENIC)
4	14473176	14473185	14473180	GT	A	snp	1	1	1	1	.	.	.	INTERGENIC(INTERGENIC);LINC00504(DOWNSTREAM)
4	14591296	14591303	14591297	T	G	snp	1	1	1	1	.	.	.	LINC00504(INTRON)
4	14733223	14733232	14733228	AG	C	snp	0	1	0	0	.	.	.	LINC00504(INTRON)
4	14802521	14802536	14802532	CAAA	G	snp	1	0	0	0	.	.	.	LINC00504(INTRON)
4	14832858	14832869	14832866	ATG	C	snp	1	1	1	1	.	.	.	LINC00504(INTRON)
4	14916356	14916365	14916362	TA	G	snp	1	0	0	0	.	.	.	AC006296.3.1(INTRON)
4	14923338	14923347	14923344	GT	C	snp	0	1	0	1	.	.	.	AC006296.3.1(INTRON)
4	14979386	14979395	14979392	AT	G	snp	1	0	0	0	.	.	.	AC006296.3.1(INTRON)
4	15050016	15050024	15050018	T	G	snp	0	1	1	1	.	.	.	CPEB2(INTRON);RP11-665G4.1.1(INTRON)
4	15054632	15054647	15054643	TTTA	T	ins	0	0	1	0	.	.	.	CPEB2(INTRON);CPEB2(UPSTREAM);RP11-665G4.1.1(INTRON)
4	15078250	15078259	15078255	A	G	snp	1	1	1	0	.	.	.	RP11-665G4.1.1(INTRON)
4	15185950	15185964	15185955	AAT	A	snp	1	1	1	1	.	.	.	RP11-665G4.1.1(INTRON)
4	15214468	15214479	15214475	AT	C	snp	1	1	1	1	.	.	.	RP11-665G4.1.1(INTRON)
4	15447545	15447554	15447548	A	G	snp	1	1	1	0	.	.	.	C1QTNF7(DOWNSTREAM);C1QTNF7(UTR_3_PRIME)
4	15706190	15706199	15706193	TG	A	snp	1	1	1	1	.	.	.	BST1(INTRON);FAM200B(INTRON)
4	15724870	15724878	15724877	T	C	snp	1	0	0	0	.	.	.	BST1(INTRON)
4	15727514	15727521	15727516	T	C	snp	0	0	1	0	.	.	.	BST1(INTRON)
4	15865895	15865903	15865901	A	G	snp	1	0	0	0	.	.	.	INTERGENIC(INTERGENIC);RP11-442P12.1.1(DOWNSTREAM)
4	16006456	16006483	16006464	CTTC	TT	ins	1	0	1	0	.	.	.	PROM1(INTRON);U6(DOWNSTREAM)
4	16006529	16006552	16006534	CCTT	TT	del	1	1	1	1	.	.	.	PROM1(INTRON);U6(DOWNSTREAM)
4	16006529	16006552	16006539	CCTT	C	ins	0	1	0	1	.	.	.	PROM1(INTRON);U6(DOWNSTREAM)
4	16051341	16051354	16051347	TA	C	snp	1	0	0	0	.	.	.	PROM1(INTRON)
4	16082668	16082677	16082673	AT	C	snp	1	0	0	0	.	.	.	PROM1(INTRON);PROM1(UPSTREAM)
4	16216756	16216764	16216763	A	C	snp	1	0	0	0	.	.	.	TAPT1(INTRON);TAPT1(UPSTREAM)
4	16242924	16242935	16242930	AT	T	ins	1	1	1	1	.	.	.	INTERGENIC(INTERGENIC)
4	16257402	16257413	16257406	GA	C	snp	1	0	0	0	.	.	.	AC006427.1.1(DOWNSTREAM);INTERGENIC(INTERGENIC)
4	16413017	16413024	16413018	A	C	snp	0	0	1	0	.	.	.	RP11-446J8.1.1(INTRON)
4	16448476	16448485	16448483	T	G	snp	0	1	0	1	.	.	.	RP11-446J8.1.1(INTRON)
4	16520108	16520117	16520110	T	G	ins	0	0	1	0	.	.	.	LDB2(INTRON)
4	16574830	16574839	16574835	AT	C	snp	0	1	1	1	.	.	.	LDB2(INTRON)
4	16757175	16757190	16757183	AG	C	snp	0	0	1	0	.	.	.	LDB2(INTRON)
4	16786632	16786640	16786635	T	C	ins	1	1	1	1	.	.	.	LDB2(INTRON)
4	16789690	16789697	16789695	C	T	snp	1	1	1	1	.	.	.	LDB2(INTRON)
4	16825845	16825860	16825849	TG	G	snp	1	1	1	1	.	.	.	LDB2(INTRON)
4	16897168	16897185	16897177	TG	T	snp	1	1	1	1	.	.	.	LDB2(INTRON)
4	16928544	16928563	16928558	TTCC	T	snp	0	0	1	0	.	.	.	INTERGENIC(INTERGENIC)
4	16960323	16960331	16960330	T	C	snp	1	1	1	1	.	.	.	INTERGENIC(INTERGENIC)
4	17049095	17049118	17049099	AAAT	T	ins	1	1	0	1	.	.	.	RP11-141E13.1.1(INTRON)
4	17183837	17183845	17183843	T	G	snp	1	1	1	1	.	.	.	RP11-576E20.1.1(INTRON)
4	17304561	17304572	17304565	CA	T	snp	1	1	1	1	.	.	.	INTERGENIC(INTERGENIC)
4	17319640	17319648	17319646	A	G	ins	1	1	1	1	.	.	.	INTERGENIC(INTERGENIC);SNORA75(DOWNSTREAM)
4	17326227	17326234	17326230	C	CCA	ins	0	1	1	1	.	.	.	INTERGENIC(INTERGENIC);SNORA75(UPSTREAM)
4	17326227	17326234	17326233	C	CCG	ins	0	1	1	1	.	.	.	INTERGENIC(INTERGENIC);SNORA75(UPSTREAM)
4	17439091	17439098	17439096	G	A	snp	0	1	1	1	.	.	.	INTERGENIC(INTERGENIC)
4	17439091	17439098	17439096	G	T	ins	1	1	1	1	.	.	.	INTERGENIC(INTERGENIC)

4	17439091	17439098	17439097	G	A	snp	1	0	0	0	.	.	.	INTERGENIC(INTERGENIC)
4	17449788	17449796	17449794	T	G	snp	0	1	0	0	.	.	.	INTERGENIC(INTERGENIC)
4	17474537	17474544	17474539	A	C	snp	1	1	1	0	.	.	.	QDPR(INTRON)
4	17548873	17548892	17548884	AAAC	T	snp	1	1	1	1	.	.	.	AC006160.4.1(DOWNSTREAM);INTERGENIC(INTERGENIC)
4	17595593	17595602	17595598	TG	A	snp	1	0	0	0	.	.	.	AC006160.5.1(INTRON);LAP3(INTRON)
4	17632831	17632844	17632834	AT	C	snp	1	1	1	1	.	.	.	AC006160.8.1(EXON);FAM184B(UTR_3_PRIME)
4	17762109	17762124	17762120	AAAC	AAACAAA	del	0	0	1	0	.	.	.	FAM184B(INTRON)
4	17918832	17918840	17918836	T	G	snp	1	1	1	1	.	.	.	LCORL(INTRON);RP11-414P19.1.1(DOWNSTREAM)
4	18023887	18023894	18023891	T	G	snp	1	0	0	0	.	.	.	INTERGENIC(INTERGENIC);LCORL(UPSTREAM)
4	18146027	18146042	18146039	AT	TAT	del	1	1	1	1	.	.	.	INTERGENIC(INTERGENIC)
4	18146027	18146042	18146040	AT	A	del	1	1	1	1	.	.	.	INTERGENIC(INTERGENIC)
4	18154149	18154160	18154152	AC	A	snp	1	1	0	1	.	.	.	INTERGENIC(INTERGENIC)
4	18220908	18220921	18220919	AT	A	del	1	1	1	1	.	.	.	INTERGENIC(INTERGENIC)
4	18363014	18363033	18363018	TTAA	G	snp	1	1	1	1	.	.	.	INTERGENIC(INTERGENIC)
4	18372620	18372627	18372622	A	C	snp	1	1	1	1	.	.	.	INTERGENIC(INTERGENIC)
4	18585458	18585465	18585462	T	TG	ins	1	1	0	0	.	.	.	INTERGENIC(INTERGENIC)
4	18697332	18697347	18697341	AT	ATAC	ins	1	0	1	0	.	.	.	INTERGENIC(INTERGENIC)
4	18697332	18697347	18697344	AT	TT	ins	1	0	0	0	.	.	.	INTERGENIC(INTERGENIC)
4	18697332	18697347	18697345	AT	A	del	1	1	0	0	.	.	.	INTERGENIC(INTERGENIC)
4	18783089	18783108	18783096	TTCA	G	snp	1	1	1	1	.	.	.	INTERGENIC(INTERGENIC)
4	18786667	18786675	18786672	T	C	snp	1	1	1	1	.	.	.	INTERGENIC(INTERGENIC)
4	18813306	18813313	18813310	A	G	snp	0	0	1	0	.	.	.	INTERGENIC(INTERGENIC)
4	18818988	18818999	18818996	AC	C	snp	1	1	1	1	.	.	.	INTERGENIC(INTERGENIC)
4	19042294	19042309	19042303	AAAT	G	snp	1	0	0	0	.	.	.	INTERGENIC(INTERGENIC)
4	19074612	19074623	19074621	CA	C	del	1	1	1	1	.	.	.	INTERGENIC(INTERGENIC)
4	19152187	19152195	19152194	T	C	snp	0	1	0	1	.	.	.	INTERGENIC(INTERGENIC)
4	19245147	19245160	19245157	AG	C	snp	0	0	0	1	.	.	.	RP11-3J1.1.1(INTRON)
4	19394208	19394215	19394213	T	G	snp	1	1	0	1	.	.	.	RP11-3J1.1.1(INTRON)
4	19499379	19499387	19499382	A	C	snp	1	0	0	0	.	.	.	RP11-608O21.1.1(INTRON)
4	19539207	19539224	19539222	TA	TC	ins	1	1	0	1	.	.	.	RP11-608O21.1.1(INTRON)
4	19543058	19543066	19543063	A	G	snp	0	1	0	1	.	.	.	RP11-608O21.1.1(INTRON)
4	19620517	19620525	19620521	A	G	snp	1	1	0	1	.	.	.	RP11-608O21.1.1(INTRON)
4	19748949	19748962	19748959	GA	C	snp	1	1	0	1	.	.	.	RP11-608O21.1.1(INTRON);RP11-94H6.1.1(INTRON)
4	19828545	19828554	19828550	T	G	snp	1	1	1	1	.	.	.	RP11-608O21.1.1(INTRON)
4	20069627	20069634	20069633	T	C	snp	1	1	1	1	.	.	.	INTERGENIC(INTERGENIC)
4	20080992	20081003	20080994	GA	C	snp	1	0	1	1	.	.	.	INTERGENIC(INTERGENIC)
4	20135918	20135929	20135921	GAA	A	snp	1	1	0	1	.	.	.	INTERGENIC(INTERGENIC)
4	20157273	20157290	20157288	TC	T	snp	0	1	1	1	.	.	.	INTERGENIC(INTERGENIC)
4	20164901	20164918	20164914	AC	T	snp	1	1	1	1	.	.	.	INTERGENIC(INTERGENIC)
4	20244469	20244477	20244476	T	C	snp	1	0	0	0	.	.	.	INTERGENIC(INTERGENIC)
4	20327914	20327925	20327921	TTG	A	snp	1	0	1	0	.	.	.	SLIT2(INTRON)
4	20392403	20392412	20392410	AT	AC	ins	1	1	1	1	.	.	.	SLIT2(INTRON);SLIT2-IT1(UPSTREAM)
4	20396583	20396594	20396591	TGG	A	snp	1	1	1	1	.	.	.	SLIT2(INTRON);SLIT2-IT1(DOWNSTREAM)
4	20503594	20503603	20503595	T	CA	ins	1	1	1	1	.	.	.	SLIT2(INTRON)
4	20503594	20503603	20503596	T	C	snp	1	1	1	1	.	.	.	SLIT2(INTRON)
4	20505112	20505121	20505116	AT	G	snp	1	1	1	1	.	.	.	SLIT2(INTRON)
4	20510786	20510795	20510791	AT	C	snp	1	1	0	0	.	.	.	SLIT2(INTRON)
4	20622638	20622653	20622647	AAAC	G	snp	0	1	1	1	.	.	.	INTERGENIC(INTERGENIC);SLIT2(DOWNSTREAM)
4	20694723	20694730	20694725	T	C	snp	1	1	1	1	.	.	.	INTERGENIC(INTERGENIC);PACRGL(UPSTREAM)
4	20805312	20805325	20805317	GA	G	snp	0	0	1	1	.	.	.	KCNIP4(INTRON)
4	20848191	20848198	20848197	A	C	snp	1	1	1	1	.	.	.	KCNIP4(DOWNSTREAM);KCNIP4(INTRON)
4	20865695	20865710	20865699	GTAT	T	snp	1	0	0	0	.	.	.	KCNIP4(INTRON)
4	20869637	20869648	20869645	ATA	AT	del	1	1	1	1	.	.	.	KCNIP4(INTRON)
4	20961223	20961232	20961225	CT	T	snp	1	0	0	0	.	.	.	KCNIP4(INTRON)
4	21007199	21007207	21007200	T	C	snp	1	1	0	1	.	.	.	KCNIP4(INTRON)
4	21017021	21017030	21017023	TA	C	snp	1	0	0	0	.	.	.	KCNIP4(INTRON)
4	21064142	21064151	21064145	TG	T	snp	1	1	0	1	.	.	.	KCNIP4(INTRON)
4	21088552	21088567	21088559	TTCT	C	snp	1	1	1	1	.	.	.	KCNIP4(INTRON)
4	21216055	21216062	21216060	A	G	snp	1	0	0	0	.	.	.	KCNIP4(INTRON)
4	21225413	21225426	21225419	GT	T	snp	0	1	1	1	.	.	.	KCNIP4(INTRON)
4	21287309	21287317	21287313	A	C	snp	1	0	0	0	.	.	.	KCNIP4(INTRON)
4	21293559	21293567	21293561	A	G	ins	1	0	0	0	.	.	.	KCNIP4(INTRON)
4	21377357	21377364	21377360	T	C	snp	1	1	0	0	.	.	.	KCNIP4(INTRON)
4	21505213	21505221	21505217	T	C	snp	1	1	1	1	.	.	.	KCNIP4(INTRON)
4	21542820	21542835	21542829	AG	AC	ins	1	0	0	0	.	.	.	KCNIP4(INTRON)
4	21542820	21542835	21542829	AG	C	snp	1	0	0	0	.	.	.	KCNIP4(INTRON)
4	21617189	21617197	21617192	A	C	ins	1	1	1	1	.	.	.	KCNIP4(INTRON);RP11-556G22.3.1(DOWNSTREAM)

4	21645481	21645495	21645492	TGA	T	del	1	1	1	1	.	.	.	KCNIP4(INTRON)
4	21696557	21696572	21696563	AAAT	T	del	1	1	1	1	.	.	.	KCNIP4(INTRON);RP11-556G22.2.1(UPSTREAM)
4	21696557	21696572	21696568	AAAT	AAA	ins	1	1	0	1	.	.	.	KCNIP4(INTRON);RP11-556G22.2.1(UPSTREAM)
4	21696594	21696601	21696600	A	C	snp	1	0	0	0	.	.	.	KCNIP4(INTRON);RP11-556G22.2.1(UPSTREAM)
4	21702183	21702191	21702185	T	G	snp	1	1	1	1	.	.	.	KCNIP4(INTRON);KCNIP4(UPSTREAM);RP11-556G22.2.1(INTRON)
4	21720087	21720095	21720091	T	C	snp	1	1	1	1	.	.	.	KCNIP4(INTRON);RP11-556G22.2.1(INTRON)
4	21760555	21760572	21760560	AAT	A	snp	1	0	0	0	.	.	.	KCNIP4(INTRON)
4	21769933	21769942	21769939	CT	G	snp	1	1	1	1	.	.	.	KCNIP4(INTRON)
4	21783667	21783676	21783672	GA	GAG	del	1	1	1	1	.	.	.	KCNIP4(INTRON)
4	21787215	21787238	21787234	AATA	G	snp	1	0	1	0	.	.	.	KCNIP4(INTRON)
4	21800009	21800018	21800014	CA	C	del	1	0	0	0	.	.	.	KCNIP4(INTRON)
4	21800009	21800018	21800016	CA	C	del	1	0	0	0	.	.	.	KCNIP4(INTRON)
4	21832296	21832315	21832308	CAAA	G	snp	1	1	1	1	.	.	.	KCNIP4(INTRON)
4	21837217	21837225	21837223	T	G	ins	1	1	1	1	.	.	.	KCNIP4(INTRON)
4	21894789	21894800	21894796	TG	C	snp	0	1	1	1	.	.	.	KCNIP4(INTRON)
4	21997490	21997507	21997497	AC	G	snp	1	0	0	0	.	.	.	RP11-17E2.2.1(INTRON)
4	22122123	22122130	22122126	T	G	snp	1	0	0	0	.	.	.	RP11-17E2.2.1(INTRON);U6(DOWNSTREAM)
4	22227817	22227825	22227823	T	C	snp	1	1	1	1	.	.	.	RP11-17E2.2.1(INTRON)
4	22274010	22274017	22274013	A	C	snp	1	0	1	0	.	.	.	RP11-17E2.2.1(INTRON)
4	22319344	22319353	22319351	A	C	ins	1	0	0	0	.	.	.	RP11-17E2.2.1(INTRON)
4	22361843	22361852	22361850	AC	T	snp	0	0	1	0	.	.	.	GPR125(INTRON)
4	22367422	22367431	22367425	AT	C	snp	1	1	0	1	.	.	.	GPR125(INTRON)
4	22367422	22367431	22367428	AT	G	snp	1	1	1	1	.	.	.	GPR125(INTRON)
4	22368284	22368291	22368288	A	G	snp	1	1	1	0	.	.	.	GPR125(INTRON)
4	22386048	22386057	22386052	TA	C	snp	1	0	0	0	.	.	.	GPR125(DOWNSTREAM);GPR125(INTRON)
4	22413583	22413591	22413585	A	C	snp	1	0	0	0	.	.	.	GPR125(DOWNSTREAM);GPR125(INTRON)
4	22419947	22419956	22419951	GA	A	snp	1	0	0	0	.	.	.	GPR125(DOWNSTREAM);GPR125(INTRON);GPR125(UPSTREAM)
4	22432911	22432920	22432917	TA	G	snp	0	1	0	1	.	.	.	GPR125(INTRON)
4	22452526	22452535	22452529	TG	A	snp	1	1	1	1	.	.	.	GPR125(INTRON)
4	22458064	22458073	22458069	CT	C	snp	1	0	0	0	.	.	.	GPR125(INTRON);GPR125(UPSTREAM)
4	22470419	22470434	22470424	GAAG	G	snp	1	1	0	1	.	.	.	GPR125(DOWNSTREAM);GPR125(INTRON)
4	22480648	22480656	22480652	A	C	snp	1	1	1	1	.	.	.	GPR125(INTRON);GPR125(UPSTREAM)
4	22535800	22535809	22535802	A	C	snp	1	1	1	1	.	.	.	INTERGENIC(INTERGENIC)
4	22549956	22549963	22549959	T	C	snp	1	1	1	1	.	.	.	INTERGENIC(INTERGENIC)
4	22599338	22599347	22599343	TG	TGTC	ins	1	1	1	0	.	.	.	INTERGENIC(INTERGENIC);RP11-10G12.1.1(DOWNSTREAM)
4	22605052	22605060	22605059	A	C	snp	1	1	1	1	.	.	.	INTERGENIC(INTERGENIC);RP11-10G12.2-001.1(UPSTREAM)
4	22676818	22676827	22676822	TC	C	snp	1	1	1	1	.	.	.	INTERGENIC(INTERGENIC)
4	22711781	22711796	22711786	TGTT	A	snp	1	1	1	1	.	.	.	GBA3(INTRON);GBA3(UPSTREAM)
4	22780841	22780850	22780847	C	A	ins	1	0	0	1	.	.	.	GBA3(INTRON)
4	22805786	22805797	22805792	AT	G	snp	1	1	1	1	.	.	.	GBA3(INTRON)
4	22912292	22912299	22912294	G	T	snp	1	1	1	1	.	.	.	INTERGENIC(INTERGENIC)
4	22928544	22928552	22928551	A	G	snp	1	0	0	0	.	.	.	INTERGENIC(INTERGENIC)
4	22977281	22977290	22977289	T	C	snp	1	1	1	1	.	.	.	INTERGENIC(INTERGENIC)
4	22992699	22992708	22992702	A	G	snp	1	0	0	0	.	.	.	INTERGENIC(INTERGENIC)
4	23133541	23133550	23133543	TG	C	snp	1	0	0	0	.	.	.	INTERGENIC(INTERGENIC)
4	23200960	23200969	23200963	TA	T	snp	1	1	1	1	.	.	.	INTERGENIC(INTERGENIC)
4	23226465	23226476	23226472	AC	T	snp	1	0	0	0	.	.	.	INTERGENIC(INTERGENIC)
4	23226526	23226535	23226533	TC	A	snp	1	0	0	0	.	.	.	INTERGENIC(INTERGENIC)
4	23298013	23298020	23298019	G	T	snp	1	0	0	0	.	.	.	INTERGENIC(INTERGENIC)
4	23303960	23303971	23303969	TG	T	del	0	1	0	1	.	.	.	INTERGENIC(INTERGENIC)
4	23328402	23328425	23328409	CATC	T	snp	0	1	1	1	.	.	.	INTERGENIC(INTERGENIC)
4	23340310	23340318	23340314	A	G	snp	1	0	0	0	.	.	.	INTERGENIC(INTERGENIC)
4	23374464	23374481	23374479	AT	A	del	1	0	0	0	.	.	.	INTERGENIC(INTERGENIC)
4	23398366	23398373	23398372	T	C	snp	1	1	1	1	.	.	.	INTERGENIC(INTERGENIC)
4	23445908	23445917	23445913	AC	AT	ins	1	1	1	1	.	.	.	INTERGENIC(INTERGENIC)
4	23462441	23462449	23462448	A	G	snp	1	0	0	0	.	.	.	INTERGENIC(INTERGENIC)
4	23472802	23472811	23472808	T	G	ins	0	1	1	0	.	.	.	INTERGENIC(INTERGENIC)
4	23472802	23472811	23472808	T	G	snp	0	1	1	0	.	.	.	INTERGENIC(INTERGENIC)
4	23508356	23508365	23508363	T	C	snp	1	1	1	1	.	.	.	INTERGENIC(INTERGENIC)
4	23562135	23562144	23562139	CT	T	snp	1	1	1	1	.	.	.	INTERGENIC(INTERGENIC);RP11-380P13.1.1(UPSTREAM)
4	23567177	23567196	23567191	AAAT	G	snp	1	0	0	0	.	.	.	RP11-380P13.1.1(INTRON)
4	23771012	23771035	23771028	CATC	T	snp	1	0	1	0	.	.	.	PPARGC1A(INTRON);RP11-380P13.1.1(DOWNSTREAM)
4	23823942	23823949	23823946	T	G	snp	1	1	1	1	.	.	.	PPARGC1A(INTRON)
4	23858984	23858995	23858993	GT	GTG	del	1	1	1	1	.	.	.	PPARGC1A(INTRON)

4	23914771	23914782	23914774	CT	A	snp	0	1	1	1	.	.	.	INTERGENIC(INTERGENIC)
4	23940851	23940859	23940852	C	A	snp	1	1	1	1	.	.	.	INTERGENIC(INTERGENIC)
4	23943699	23943708	23943700	A	G	snp	0	1	1	1	.	.	.	INTERGENIC(INTERGENIC)
4	24062159	24062168	24062161	A	C	snp	1	1	1	1	.	.	.	INTERGENIC(INTERGENIC)
4	24120529	24120537	24120534	A	C	snp	1	0	0	0	.	.	.	INTERGENIC(INTERGENIC)
4	24227153	24227162	24227161	A	C	ins	1	1	1	1	.	.	.	INTERGENIC(INTERGENIC)
4	24227162	24227175	24227165	AC	A	snp	1	1	1	1	.	.	.	INTERGENIC(INTERGENIC)
4	24230289	24230296	24230290	A	C	snp	0	0	1	0	.	.	.	INTERGENIC(INTERGENIC)
4	24306875	24306884	24306880	AT	G	snp	1	0	0	0	.	.	.	INTERGENIC(INTERGENIC)
4	24329875	24329884	24329883	C	A	snp	1	0	1	0	.	.	.	INTERGENIC(INTERGENIC)
4	24335551	24335565	24335558	CAA	ACAAC	del	1	1	1	1	.	.	.	INTERGENIC(INTERGENIC)
4	24342307	24342314	24342309	T	G	snp	0	0	1	0	.	.	.	INTERGENIC(INTERGENIC)
4	24346225	24346234	24346228	T	G	snp	1	1	1	1	.	.	.	INTERGENIC(INTERGENIC)
4	24406907	24406916	24406911	T	C	snp	1	1	1	1	.	.	.	INTERGENIC(INTERGENIC)
4	24480714	24480737	24480729	AAG	G	snp	1	1	1	1	.	.	.	INTERGENIC(INTERGENIC)
4	24518402	24518417	24518410	CAAA	AA	ins	1	0	0	1	.	.	.	DHX15(DOWNSTREAM);INTERGENIC(INTERGENIC);MIR573(DOWNSTREAM)
4	24570786	24570795	24570793	TA	G	snp	0	1	1	1	.	.	.	DHX15(INTRON)
4	24629427	24629442	24629440	GT	GC	ins	1	0	1	0	.	.	.	INTERGENIC(INTERGENIC)
4	24645961	24645972	24645965	CT	G	snp	0	0	1	0	.	.	.	INTERGENIC(INTERGENIC)
4	24670192	24670211	24670196	TTAT	G	snp	1	0	0	0	.	.	.	RP11-496D24.2.1(INTRON)
4	24698129	24698140	24698136	TA	TATATG	ins	1	0	0	0	.	.	.	INTERGENIC(INTERGENIC)
4	24803665	24803672	24803671	T	C	snp	1	0	0	0	.	.	.	CCDC149(DOWNSTREAM);INTERGENIC(INTERGENIC);SOD3(DOWNSTREAM)
4	24859651	24859660	24859659	A	G	snp	1	1	1	1	.	.	.	CCDC149(INTRON);CCDC149(UPSTREAM)
4	24972894	24972905	24972902	TGT	G	snp	1	0	0	0	.	.	.	CCDC149(INTRON)
4	24982365	24982382	24982377	TC	CTCTC	del	1	0	0	0	.	.	.	CCDC149(UPSTREAM);INTERGENIC(INTERGENIC)
4	25079095	25079122	25079114	TTTG	TTTGTTTGTT	del	1	1	1	1	.	.	.	INTERGENIC(INTERGENIC)
4	25154684	25154691	25154688	T	C	snp	1	0	0	0	.	.	.	SEPSECS(INTRON)
4	25156235	25156243	25156241	T	C	snp	0	0	1	0	.	.	.	SEPSECS(DOWNSTREAM);SEPSECS(INTRON)
4	25193901	25193924	25193919	TCT	A	snp	0	0	0	1	.	.	.	PI4K2B(INTRON)
4	25220724	25220731	25220728	A	C	snp	0	1	1	0	.	.	.	PI4K2B(INTRON)
4	25297265	25297272	25297271	T	G	ins	0	0	1	0	.	.	.	INTERGENIC(INTERGENIC)
4	25300084	25300091	25300090	T	C	ins	1	1	0	1	.	.	.	INTERGENIC(INTERGENIC)
4	25320412	25320419	25320418	T	G	ins	1	1	1	1	.	.	.	ZCCHC4(INTRON)
4	25329000	25329023	25329004	CTCC	A	snp	1	0	0	0	.	.	.	ZCCHC4(INTRON)
4	25329000	25329023	25329006	CTCC	T	snp	1	1	1	1	.	.	.	ZCCHC4(INTRON)
4	25405995	25406003	25405998	T	C	snp	1	1	1	0	.	.	.	ANAPC4(INTRON);ANAPC4(UPSTREAM)
4	25453519	25453528	25453522	AG	A	snp	1	1	1	1	.	.	.	INTERGENIC(INTERGENIC)
4	25459448	25459456	25459454	T	G	snp	1	1	0	1	.	.	.	INTERGENIC(INTERGENIC)
4	25470770	25470789	25470778	TTTA	C	snp	0	0	1	0	.	.	.	INTERGENIC(INTERGENIC);RP11-206P5.1.1(UPSTREAM)
4	25483052	25483067	25483065	AG	C	snp	1	0	1	0	.	.	.	INTERGENIC(INTERGENIC)
4	25502019	25502026	25502022	A	C	snp	1	0	0	0	.	.	.	INTERGENIC(INTERGENIC);RP11-206P5.2.1(UPSTREAM)
4	25634723	25634730	25634726	C	T	snp	1	1	0	1	.	.	.	INTERGENIC(INTERGENIC)
4	25696734	25696742	25696735	A	AAAG	ins	1	0	0	0	.	.	.	INTERGENIC(INTERGENIC)
4	25715083	25715097	25715092	ATG	G	snp	1	1	0	1	.	.	.	AC092436.3(DOWNSTREAM);AC092436.4(DOWNSTREAM);INTERGENIC(INTERGENIC);MTND4P9(DOWNSTREAM)
4	25742414	25742421	25742419	T	C	snp	1	1	1	1	.	.	.	INTERGENIC(INTERGENIC)
4	25773191	25773199	25773197	A	C	snp	1	1	1	1	.	.	.	RP11-302F12.10.1(INTRON);SEL1L3(INTRON);SEL1L3(UPSTREAM)
4	25824278	25824292	25824283	AGG	A	snp	1	0	0	0	.	.	.	SEL1L3(INTRON)
4	25886921	25886928	25886922	G	T	ins	1	0	0	0	.	.	.	C4orf52(INTRON)
4	25887196	25887205	25887202	TA	A	ins	1	0	0	0	.	.	.	C4orf52(INTRON)
4	25887196	25887205	25887203	TA	T	del	1	1	1	1	.	.	.	C4orf52(INTRON)
4	25887925	25887934	25887932	TG	A	snp	0	1	0	1	.	.	.	C4orf52(INTRON)
4	25962688	25962703	25962698	TA	AC	ins	1	1	1	1	.	.	.	INTERGENIC(INTERGENIC)
4	25962688	25962703	25962698	TA	C	snp	1	1	1	1	.	.	.	INTERGENIC(INTERGENIC)
4	26025265	26025272	26025270	T	C	snp	1	1	1	1	.	.	.	INTERGENIC(INTERGENIC)
4	26085476	26085487	26085480	AT	G	snp	1	1	0	1	.	.	.	INTERGENIC(INTERGENIC)
4	26091196	26091205	26091198	AT	G	snp	1	1	0	1	.	.	.	INTERGENIC(INTERGENIC)
4	26185824	26185831	26185830	A	C	snp	1	0	0	0	.	.	.	RBPJ(INTRON)
4	26283746	26283759	26283757	CT	C	del	1	1	1	1	.	.	.	RBPJ(INTRON)
4	26294420	26294433	26294427	AT	C	snp	1	0	0	0	.	.	.	RBPJ(INTRON)
4	26423410	26423417	26423413	T	C	snp	1	0	0	0	.	.	.	RBPJ(DOWNSTREAM);RBPJ(INTRON)
4	26449236	26449247	26449245	TC	G	snp	1	1	1	1	.	.	.	INTERGENIC(INTERGENIC)
4	26450536	26450544	26450538	A	G	snp	1	1	1	1	.	.	.	INTERGENIC(INTERGENIC)
4	26450719	26450727	26450726	A	C	ins	1	1	1	1	.	.	.	INTERGENIC(INTERGENIC)

4	26575873	26575881	26575878	A	G	ins	1	0	0	0	.	.	.	INTERGENIC(INTERGENIC);TBC1D19(UPSTREAM)	
4	26578234	26578241	26578240	G	A	snp	1	0	0	0	.	.	.	TBC1D19(UPSTREAM);TBC1D19(UTR_5_PRIME)	
4	26578905	26578922	26578920	GT	C	snp	1	0	0	0	.	.	.	TBC1D19(INTRON)	
4	26826306	26826315	26826309	T	C	snp	1	1	1	1	.	.	.	INTERGENIC(INTERGENIC)	
4	26909703	26909710	26909704	A	G	snp	1	0	0	0	.	.	.	STIM2(INTRON)	
4	26912854	26912877	26912869	AAAT	A	ins	0	0	0	1	.	.	.	STIM2(INTRON)	
4	27043445	27043456	27043449	ACA	G	snp	1	1	1	1	.	.	.	INTERGENIC(INTERGENIC)	
4	27061823	27061832	27061829	A	G	ins	1	0	1	1	.	.	.	INTERGENIC(INTERGENIC)	
4	27154032	27154041	27154038	T	C	snp	1	1	1	1	.	.	.	INTERGENIC(INTERGENIC)	
4	27227174	27227183	27227182	C	T	snp	1	0	0	0	.	.	.	RP11-415C15.2.1(INTRON);Y_RNA(UPSTREAM)	
4	27444566	27444574	27444570	T	C	snp	1	1	0	0	.	.	.	INTERGENIC(INTERGENIC)	
4	27522492	27522507	27522497	AAAG	AG	del	1	1	1	1	.	.	.	INTERGENIC(INTERGENIC)	
4	27535628	27535639	27535631	CAA	A	snp	1	1	1	1	.	.	.	INTERGENIC(INTERGENIC)	
4	27649025	27649038	27649035	AT	C	snp	1	1	1	1	.	.	.	INTERGENIC(INTERGENIC)	
4	27699956	27699967	27699964	TTA	G	snp	1	1	1	1	.	.	.	INTERGENIC(INTERGENIC)	
4	27726901	27726908	27726905	A	C	snp	1	0	0	0	.	.	.	INTERGENIC(INTERGENIC)	
4	27778614	27778625	27778620	TC	C	snp	1	1	1	1	.	.	.	INTERGENIC(INTERGENIC)	
4	27813934	27813942	27813941	A	G	snp	1	1	1	1	.	.	.	INTERGENIC(INTERGENIC)	
4	27903381	27903390	27903383	A	C	snp	1	1	1	1	.	.	.	INTERGENIC(INTERGENIC)	
4	27959687	27959696	27959689	A	C	ins	0	1	1	1	.	.	.	INTERGENIC(INTERGENIC)	
4	28021953	28021966	28021962	AT	A	snp	1	1	1	1	.	.	.	INTERGENIC(INTERGENIC)	
4	28034363	28034380	28034376	AAT	T	snp	1	0	0	0	.	.	.	INTERGENIC(INTERGENIC)	
4	28096148	28096157	28096153	TA	T	del	1	1	1	1	.	.	.	INTERGENIC(INTERGENIC)	
4	28156617	28156624	28156622	A	C	snp	1	0	0	0	.	.	.	INTERGENIC(INTERGENIC)	
4	28221079	28221086	28221080	A	G	snp	0	1	1	1	.	.	.	INTERGENIC(INTERGENIC)	
4	28245593	28245608	28245598	AAAT	C	snp	1	1	1	1	.	.	.	INTERGENIC(INTERGENIC)	
4	28258162	28258176	28258167	CTC	T	snp	1	0	0	0	.	.	.	INTERGENIC(INTERGENIC)	
4	28342422	28342431	28342428	AT	TGTG	ins	1	1	1	1	.	.	.	INTERGENIC(INTERGENIC)	
4	28469180	28469193	28469189	CA	C	snp	1	0	0	0	.	.	.	RP11-123O22.1.1(INTRON)	
4	28520768	28520775	28520773	A	C	snp	0	1	0	1	.	.	.	RP11-123O22.1.1(INTRON)	
4	28528225	28528234	28528227	T	C	snp	1	0	0	0	.	.	.	RP11-123O22.1.1(INTRON)	
4	28577143	28577160	28577157	TCA	G	snp	0	1	1	1	.	.	.	RP11-123O22.1.1(INTRON)	
4	28629638	28629653	28629644	ATAA	AATAAATAAATAG		del	1	1	1	0	.	.	.	INTERGENIC(INTERGENIC)
4	28629638	28629653	28629648	ATAA	AATAAATAG		del	1	1	1	0	.	.	.	INTERGENIC(INTERGENIC)
4	28778278	28778289	28778280	AG	T	snp	1	0	0	0	.	.	.	INTERGENIC(INTERGENIC)	
4	28804764	28804779	28804769	AGTG	A	snp	1	1	1	1	.	.	.	INTERGENIC(INTERGENIC)	
4	28821508	28821517	28821512	TC	A	snp	0	1	0	0	.	.	.	INTERGENIC(INTERGENIC);MESTP3(DOWNSTREAM)	
4	28821927	28821942	28821933	AAAG	G	snp	1	1	1	1	.	.	.	INTERGENIC(INTERGENIC);MESTP3(DOWNSTREAM)	
4	28870362	28870369	28870368	A	G	ins	1	1	1	1	.	.	.	INTERGENIC(INTERGENIC)	
4	28955517	28955534	28955529	TC	CTCTC	del	1	1	1	1	.	.	.	INTERGENIC(INTERGENIC)	
4	28961213	28961222	28961218	AT	C	snp	1	1	0	1	.	.	.	INTERGENIC(INTERGENIC)	
4	29067577	29067586	29067585	T	C	snp	1	0	0	0	.	.	.	INTERGENIC(INTERGENIC)	
4	29110158	29110167	29110163	TG	C	snp	1	0	0	0	.	.	.	INTERGENIC(INTERGENIC)	
4	29115070	29115085	29115075	TG	C	snp	1	1	1	1	.	.	.	INTERGENIC(INTERGENIC);RP11-472K22.1.1(UPSTREAM)	
4	29135010	29135018	29135014	T	G	snp	1	1	1	1	.	.	.	RP11-472K22.1.1(INTRON)	
4	29170123	29170132	29170129	AT	G	snp	1	1	0	0	.	.	.	RP11-472K22.1.1(INTRON)	
4	29180754	29180762	29180757	T	C	snp	1	0	0	0	.	.	.	RP11-472K22.1.1(INTRON)	
4	29188622	29188631	29188629	A	C	snp	1	1	1	1	.	.	.	RP11-472K22.1.1(INTRON)	
4	29263417	29263426	29263424	AC	A	ins	1	0	0	0	.	.	.	INTERGENIC(INTERGENIC)	
4	29269984	29269992	29269988	A	G	snp	1	0	0	0	.	.	.	INTERGENIC(INTERGENIC)	
4	29383869	29383878	29383873	TG	C	snp	1	1	1	1	.	.	.	INTERGENIC(INTERGENIC)	
4	29406742	29406751	29406744	GA	AGAGAGA	del	1	1	0	1	.	.	.	INTERGENIC(INTERGENIC);RP11-68D16.1.1(UPSTREAM)	
4	29427673	29427682	29427680	C	T	snp	1	1	1	1	.	.	.	INTERGENIC(INTERGENIC)	
4	29469873	29469882	29469880	AG	A	snp	1	0	0	0	.	.	.	INTERGENIC(INTERGENIC);RP11-68D16.2.1(UPSTREAM)	
4	29711798	29711817	29711803	GAAA	AAG	ins	1	1	1	1	.	.	.	INTERGENIC(INTERGENIC)	
4	29715263	29715276	29715273	TA	C	snp	0	1	1	1	.	.	.	INTERGENIC(INTERGENIC)	
4	29836549	29836560	29836552	TTA	C	snp	1	0	0	0	.	.	.	INTERGENIC(INTERGENIC)	
4	29851959	29851970	29851963	TC	C	snp	1	1	1	1	.	.	.	INTERGENIC(INTERGENIC)	
4	29870780	29870789	29870784	A	C	ins	1	0	0	0	.	.	.	INTERGENIC(INTERGENIC)	
4	29870780	29870789	29870785	A	C	snp	1	0	0	0	.	.	.	INTERGENIC(INTERGENIC)	
4	29938277	29938286	29938283	A	G	snp	1	0	0	0	.	.	.	INTERGENIC(INTERGENIC)	
4	29940547	29940562	29940553	TTTA	C	snp	1	0	0	0	.	.	.	INTERGENIC(INTERGENIC)	
4	29994161	29994184	29994180	GATA	GAT	ins	1	1	1	1	.	.	.	INTERGENIC(INTERGENIC)	
4	30041133	30041141	30041137	T	G	snp	0	0	1	0	.	.	.	INTERGENIC(INTERGENIC)	
4	30075443	30075454	30075445	AT	G	snp	1	1	0	1	.	.	.	INTERGENIC(INTERGENIC)	
4	30075456	30075467	30075461	TA	G	snp	1	1	0	1	.	.	.	INTERGENIC(INTERGENIC)	
4	30076291	30076299	30076293	T	C	snp	1	1	0	1	.	.	.	INTERGENIC(INTERGENIC)	

4	30187899	30187906	30187901	A	G	snp	1	0	0	0	.	.	.	INTERGENIC(INTERGENIC)
4	30249813	30249820	30249814	T	C	ins	1	1	1	1	.	.	.	INTERGENIC(INTERGENIC)
4	30317738	30317745	30317743	A	G	snp	1	1	1	1	.	.	.	INTERGENIC(INTERGENIC)
4	30369334	30369343	30369336	AT	C	snp	0	1	1	0	.	.	.	INTERGENIC(INTERGENIC)
4	30375713	30375720	30375715	T	G	snp	1	1	1	0	.	.	.	INTERGENIC(INTERGENIC)
4	30473534	30473548	30473537	TTA	G	snp	0	1	1	1	.	.	.	INTERGENIC(INTERGENIC)
4	30481011	30481028	30481018	AG	A	snp	0	0	1	0	.	.	.	INTERGENIC(INTERGENIC)
4	30490999	30491006	30491003	T	G	snp	1	1	1	1	.	.	.	INTERGENIC(INTERGENIC)
4	30511075	30511084	30511081	CT	T	snp	0	0	0	1	.	.	.	INTERGENIC(INTERGENIC)
4	30522178	30522187	30522179	A	G	snp	0	0	0	1	.	.	.	INTERGENIC(INTERGENIC)
4	30735692	30735700	30735699	A	C	ins	1	0	0	0	.	.	.	PCDH7(DOWNSTREAM);PCDH7(INTRON)
4	30740772	30740781	30740779	TA	C	snp	1	0	0	0	.	.	.	PCDH7(INTRON)
4	30768271	30768288	30768284	AT	ATATG	del	0	0	1	0	.	.	.	PCDH7(INTRON)
4	30777011	30777020	30777013	GT	A	snp	1	0	0	0	.	.	.	PCDH7(INTRON);RP11-619J20.1.1(DOWNSTREAM)
4	30876501	30876512	30876509	TTA	C	snp	0	1	0	1	.	.	.	PCDH7(INTRON)
4	30928237	30928244	30928243	T	G	snp	1	1	1	1	.	.	.	PCDH7(INTRON)
4	30965845	30965872	30965860	TTTA	T	ins	1	1	1	1	.	.	.	PCDH7(INTRON)
4	30965845	30965872	30965861	TTTA	TTA	del	1	1	1	1	.	.	.	PCDH7(INTRON)
4	30965845	30965872	30965866	TTTA	TA	del	1	1	1	1	.	.	.	PCDH7(INTRON)
4	30965845	30965872	30965867	TTTA	A	del	1	1	1	1	.	.	.	PCDH7(INTRON)
4	31034267	31034280	31034278	AG	AGGAAGGA		ins	1	1	1	1	.	.	. PCDH7(INTRON)
4	31167821	31167830	31167827	CA	T	snp	1	0	0	0	.	.	.	INTERGENIC(INTERGENIC);RP11-617I14.1.1(UPSTREAM)
4	31244264	31244273	31244268	A	C	snp	1	0	0	0	.	.	.	INTERGENIC(INTERGENIC)
4	31275276	31275299	31275295	TTTC	TTTCT	del	1	1	1	1	.	.	.	INTERGENIC(INTERGENIC)
4	31298764	31298772	31298771	T	G	ins	0	0	1	0	.	.	.	INTERGENIC(INTERGENIC)
4	31298764	31298772	31298771	T	G	snp	1	1	1	1	.	.	.	INTERGENIC(INTERGENIC)
4	31304986	31304994	31304992	C	A	snp	0	0	0	1	.	.	.	INTERGENIC(INTERGENIC)
4	31328765	31328772	31328768	T	C	snp	0	1	1	1	.	.	.	INTERGENIC(INTERGENIC)
4	31328765	31328772	31328771	T	G	snp	1	1	1	1	.	.	.	INTERGENIC(INTERGENIC)
4	31335988	31335999	31335993	AG	A	snp	1	1	1	1	.	.	.	INTERGENIC(INTERGENIC)
4	31335988	31335999	31335996	AG	G	snp	1	1	1	1	.	.	.	INTERGENIC(INTERGENIC)
4	31336408	31336417	31336412	T	G	snp	1	1	1	1	.	.	.	INTERGENIC(INTERGENIC)
4	31367097	31367104	31367102	C	CG	ins	0	1	1	0	.	.	.	INTERGENIC(INTERGENIC)
4	31509448	31509461	31509451	TA	TATATG	ins	1	1	1	1	.	.	.	RP11-665I14.1.1(INTRON)
4	31592202	31592209	31592206	T	C	snp	1	1	1	1	.	.	.	INTERGENIC(INTERGENIC)
4	31615429	31615442	31615431	AT	T	del	1	0	0	0	.	.	.	INTERGENIC(INTERGENIC)
4	31677935	31677949	31677946	AAC	A	ins	1	1	1	1	.	.	.	INTERGENIC(INTERGENIC)
4	31794590	31794597	31794594	A	G	snp	0	0	1	0	.	.	.	INTERGENIC(INTERGENIC)
4	31820564	31820573	31820570	AT	G	snp	1	0	0	0	.	.	.	INTERGENIC(INTERGENIC)
4	31869831	31869838	31869832	A	C	snp	1	0	0	0	.	.	.	INTERGENIC(INTERGENIC)
4	31925871	31925880	31925874	TC	T	snp	1	1	1	1	.	.	.	INTERGENIC(INTERGENIC)
4	32006451	32006468	32006462	CAA	G	snp	1	0	0	0	.	.	.	RP11-734I18.1.1(INTRON)
4	32033427	32033442	32033434	TA	G	snp	0	1	0	1	.	.	.	RP11-734I18.1.1(INTRON)
4	32033444	32033457	32033454	AT	G	snp	1	1	1	1	.	.	.	RP11-734I18.1.1(INTRON)
4	32090851	32090860	32090853	CT	G	snp	1	0	0	0	.	.	.	RP11-734I18.1.1(INTRON)
4	32122227	32122235	32122230	T	G	snp	1	0	0	0	.	.	.	RP11-734I18.1.1(INTRON)
4	32135294	32135307	32135305	TA	T	ins	1	1	1	1	.	.	.	RP11-734I18.1.1(INTRON)
4	32149348	32149355	32149354	G	A	snp	1	1	1	1	.	.	.	RP11-734I18.1.1(INTRON)
4	32230881	32230894	32230886	AC	G	snp	1	1	1	1	.	.	.	INTERGENIC(INTERGENIC)
4	32251586	32251594	32251588	T	C	ins	1	1	1	1	.	.	.	INTERGENIC(INTERGENIC)
4	32297796	32297805	32297802	TA	C	snp	1	1	1	1	.	.	.	INTERGENIC(INTERGENIC)
4	32314562	32314579	32314577	AC	A	del	0	1	1	1	.	.	.	INTERGENIC(INTERGENIC)
4	32340025	32340034	32340032	TA	T	del	0	1	0	0	.	.	.	INTERGENIC(INTERGENIC)
4	32341248	32341259	32341255	TAA	G	snp	1	1	1	1	.	.	.	INTERGENIC(INTERGENIC)
4	32393000	32393009	32393008	A	C	ins	1	1	1	0	.	.	.	INTERGENIC(INTERGENIC)
4	32400878	32400893	32400890	TA	AC	ins	1	1	1	1	.	.	.	INTERGENIC(INTERGENIC)
4	32401018	32401029	32401026	TA	C	snp	1	1	1	1	.	.	.	INTERGENIC(INTERGENIC)
4	32418802	32418809	32418803	T	G	snp	0	0	1	0	.	.	.	INTERGENIC(INTERGENIC)
4	32460315	32460324	32460323	A	G	snp	0	1	1	0	.	.	.	INTERGENIC(INTERGENIC)
4	32460684	32460701	32460698	ATT	A	del	1	1	1	0	.	.	.	INTERGENIC(INTERGENIC)
4	32487900	32487908	32487906	A	C	snp	1	1	1	1	.	.	.	INTERGENIC(INTERGENIC)
4	32511332	32511340	32511333	A	C	snp	0	1	0	1	.	.	.	INTERGENIC(INTERGENIC)
4	32512514	32512523	32512518	AT	G	snp	1	1	1	1	.	.	.	INTERGENIC(INTERGENIC)
4	32555493	32555502	32555499	T	G	snp	1	0	0	0	.	.	.	INTERGENIC(INTERGENIC)
4	32570731	32570738	32570732	T	C	snp	1	1	1	1	.	.	.	INTERGENIC(INTERGENIC)
4	32650273	32650288	32650284	TTTG	TTTGTT	del	1	1	1	1	.	.	.	INTERGENIC(INTERGENIC)
4	32666654	32666663	32666659	AT	C	snp	1	0	0	0	.	.	.	INTERGENIC(INTERGENIC)

4	32713619	32713628	32713620	T	G	snp	1	1	0	1	.	.	.	INTERGENIC(INTERGENIC)
4	32735528	32735537	32735530	AT	T	del	1	1	1	1	.	.	.	INTERGENIC(INTERGENIC)
4	32735528	32735537	32735531	AT	A	snp	1	1	0	1	.	.	.	INTERGENIC(INTERGENIC)
4	32740570	32740579	32740573	T	G	snp	1	1	0	1	.	.	.	INTERGENIC(INTERGENIC)
4	32771850	32771858	32771853	A	G	snp	1	1	1	1	.	.	.	INTERGENIC(INTERGENIC)
4	32833819	32833830	32833827	AT	G	snp	0	0	0	1	.	.	.	INTERGENIC(INTERGENIC)
4	32833920	32833929	32833924	AT	T	snp	1	0	1	0	.	.	.	INTERGENIC(INTERGENIC)
4	32868578	32868585	32868581	A	C	snp	1	0	0	0	.	.	.	INTERGENIC(INTERGENIC)
4	32987406	32987413	32987408	G	T	snp	1	1	0	1	.	.	.	INTERGENIC(INTERGENIC)
4	32987406	32987413	32987410	G	T	snp	1	0	1	0	.	.	.	INTERGENIC(INTERGENIC)
4	33028883	33028894	33028892	AT	C	snp	0	0	0	1	.	.	.	INTERGENIC(INTERGENIC)
4	33044170	33044177	33044174	A	G	snp	1	1	1	1	.	.	.	INTERGENIC(INTERGENIC)
4	33135474	33135482	33135479	T	C	snp	1	0	0	0	.	.	.	INTERGENIC(INTERGENIC)
4	33142637	33142646	33142644	CA	T	snp	1	0	0	0	.	.	.	INTERGENIC(INTERGENIC)
4	33233492	33233500	33233494	T	C	snp	1	0	0	0	.	.	.	INTERGENIC(INTERGENIC)
4	33299230	33299239	33299232	GT	A	snp	0	1	1	0	.	.	.	INTERGENIC(INTERGENIC)
4	33446169	33446176	33446175	A	C	ins	1	0	0	0	.	.	.	INTERGENIC(INTERGENIC)
4	33551772	33551785	33551779	AC	T	snp	1	0	0	0	.	.	.	INTERGENIC(INTERGENIC)
4	33630601	33630616	33630607	ATTC	C	snp	1	0	0	0	.	.	.	INTERGENIC(INTERGENIC)
4	33630601	33630616	33630608	ATTC	T	snp	0	1	0	1	.	.	.	INTERGENIC(INTERGENIC)
4	33641543	33641550	33641544	A	AC	ins	0	1	1	1	.	.	.	INTERGENIC(INTERGENIC)
4	33641543	33641550	33641545	A	C	ins	0	1	0	1	.	.	.	INTERGENIC(INTERGENIC)
4	33641543	33641550	33641546	A	C	snp	1	0	0	0	.	.	.	INTERGENIC(INTERGENIC)
4	33654619	33654638	33654630	AATA	G	snp	1	1	1	1	.	.	.	INTERGENIC(INTERGENIC)
4	33731806	33731821	33731818	AT	G	snp	1	1	1	1	.	.	.	INTERGENIC(INTERGENIC)
4	33780172	33780183	33780178	CTA	T	snp	1	0	0	0	.	.	.	RP11-79E3.3.1(INTRON)
4	33921620	33921629	33921627	T	G	snp	1	1	1	1	.	.	.	RP11-79E3.2.1(INTRON);RP11-79E3.3.1(INTRON)
4	34151972	34151995	34151979	AAAT	A	snp	1	1	1	1	.	.	.	RP11-548L20.1.1(INTRON)
4	34211608	34211615	34211614	T	C	snp	1	0	0	0	.	.	.	RP11-548L20.1.1(INTRON)
4	34220706	34220717	34220709	TA	G	snp	1	0	0	0	.	.	.	RP11-548L20.1.1(INTRON)
4	34312125	34312132	34312129	T	C	snp	1	1	1	1	.	.	.	INTERGENIC(INTERGENIC)
4	34355318	34355333	34355325	ATTT	A	del	1	1	0	1	.	.	.	INTERGENIC(INTERGENIC)
4	34396543	34396552	34396545	TA	C	snp	1	0	0	0	.	.	.	INTERGENIC(INTERGENIC)
4	34422014	34422023	34422017	G	A	ins	1	0	0	0	.	.	.	INTERGENIC(INTERGENIC)
4	34497594	34497601	34497598	A	AT	ins	1	1	1	0	.	.	.	INTERGENIC(INTERGENIC)
4	34528889	34528898	34528894	T	G	snp	1	0	0	1	.	.	.	INTERGENIC(INTERGENIC)
4	34682665	34682679	34682676	TTC	TT	del	1	1	1	1	.	.	.	INTERGENIC(INTERGENIC)
4	34685786	34685795	34685790	GA	A	snp	0	1	1	1	.	.	.	INTERGENIC(INTERGENIC)
4	34704996	34705004	34705001	A	C	snp	0	1	1	1	.	.	.	INTERGENIC(INTERGENIC)
4	34754331	34754340	34754338	A	C	snp	1	1	1	1	.	.	.	INTERGENIC(INTERGENIC)
4	34758546	34758555	34758550	AC	T	snp	1	1	1	1	.	.	.	INTERGENIC(INTERGENIC)
4	34909319	34909326	34909322	A	G	snp	0	0	1	0	.	.	.	INTERGENIC(INTERGENIC)
4	34932051	34932060	34932056	AG	C	snp	1	0	0	0	.	.	.	INTERGENIC(INTERGENIC)
4	34943830	34943838	34943832	T	G	ins	1	1	1	1	.	.	.	INTERGENIC(INTERGENIC)
4	34948185	34948196	34948190	TA	G	snp	1	0	0	0	.	.	.	INTERGENIC(INTERGENIC)
4	34977395	34977404	34977399	T	G	ins	0	0	1	0	.	.	.	INTERGENIC(INTERGENIC)
4	34984011	34984026	34984020	TAGA	GAT	ins	1	1	1	1	.	.	.	INTERGENIC(INTERGENIC)
4	34991040	34991049	34991042	AT	G	snp	0	0	1	0	.	.	.	INTERGENIC(INTERGENIC)
4	35015402	35015411	35015405	AT	C	snp	1	1	0	1	.	.	.	INTERGENIC(INTERGENIC)
4	35078499	35078507	35078505	T	TC	ins	1	1	1	1	.	.	.	INTERGENIC(INTERGENIC)
4	35078499	35078507	35078506	T	C	ins	1	1	1	1	.	.	.	INTERGENIC(INTERGENIC)
4	35092410	35092418	35092414	A	G	snp	1	1	1	1	.	.	.	INTERGENIC(INTERGENIC)
4	35120559	35120568	35120562	TA	T	snp	1	0	1	0	.	.	.	INTERGENIC(INTERGENIC)
4	35128971	35128978	35128973	A	G	snp	1	1	1	1	.	.	.	INTERGENIC(INTERGENIC)
4	35163143	35163150	35163149	T	C	snp	1	1	1	1	.	.	.	INTERGENIC(INTERGENIC)
4	35202426	35202433	35202427	T	C	snp	1	1	1	1	.	.	.	INTERGENIC(INTERGENIC)
4	35271429	35271444	35271433	TTTC	TTC	del	1	0	0	0	.	.	.	INTERGENIC(INTERGENIC)
4	35295029	35295036	35295032	A	C	snp	0	0	0	1	.	.	.	INTERGENIC(INTERGENIC)
4	35322727	35322736	35322732	AT	ATA	del	0	0	1	0	.	.	.	INTERGENIC(INTERGENIC)
4	35347167	35347184	35347181	AT	TCTC	ins	1	0	0	0	.	.	.	INTERGENIC(INTERGENIC)
4	35347962	35347973	35347968	AAC	T	snp	1	1	1	1	.	.	.	INTERGENIC(INTERGENIC)
4	35358702	35358709	35358704	T	G	snp	1	1	1	1	.	.	.	INTERGENIC(INTERGENIC)
4	35370299	35370308	35370303	T	G	snp	1	0	0	0	.	.	.	INTERGENIC(INTERGENIC)
4	35370299	35370308	35370306	T	G	snp	1	1	1	1	.	.	.	INTERGENIC(INTERGENIC)
4	35392207	35392216	35392210	AT	C	snp	1	0	0	0	.	.	.	INTERGENIC(INTERGENIC)
4	35471707	35471714	35471711	A	G	snp	1	1	1	1	.	.	.	INTERGENIC(INTERGENIC)
4	35471707	35471714	35471713	A	G	snp	1	0	0	0	.	.	.	INTERGENIC(INTERGENIC)

4	35507954	35507965	35507959	TC	T	snp	1	1	1	1	.	.	.	INTERGENIC(INTERGENIC)
4	35521221	35521229	35521228	T	C	snp	1	1	1	1	.	.	.	INTERGENIC(INTERGENIC)
4	35528111	35528126	35528124	TG	A	snp	1	1	1	1	.	.	.	INTERGENIC(INTERGENIC)
4	35700704	35700713	35700707	AG	T	snp	1	0	0	0	.	.	.	INTERGENIC(INTERGENIC)
4	35704180	35704207	35704203	AGAT	C	snp	1	0	0	0	.	.	.	INTERGENIC(INTERGENIC)
4	35728162	35728170	35728164	A	G	snp	1	0	0	1	.	.	.	INTERGENIC(INTERGENIC)
4	35936110	35936119	35936114	A	G	ins	0	0	1	0	.	.	.	INTERGENIC(INTERGENIC)
4	36157962	36157979	36157977	AG	A	snp	1	1	1	1	.	.	.	ARAP2(DOWNSTREAM);ARAP2(INTRON)
4	36162829	36162837	36162833	A	C	snp	1	1	1	1	.	.	.	ARAP2(INTRON);ARAP2(UPSTREAM)
4	36165154	36165161	36165160	A	G	snp	1	1	1	1	.	.	.	ARAP2(INTRON);ARAP2(UPSTREAM)
4	36263091	36263102	36263093	TA	C	snp	0	0	0	1	.	.	.	RP11-431M7.3.1(INTRON)
4	36356728	36356745	36356743	TC	TCT	del	1	1	1	1	.	.	.	RP11-431M7.2.1(INTRON)
4	36394352	36394361	36394359	CA	C	snp	1	1	0	0	.	.	.	INTERGENIC(INTERGENIC);RP11-431M7.2.1(UPSTREAM)
4	36401003	36401017	36401013	TGG	A	snp	1	0	0	0	.	.	.	INTERGENIC(INTERGENIC)
4	36408415	36408428	36408417	TC	C	del	1	1	1	0	.	.	.	INTERGENIC(INTERGENIC)
4	36505581	36505588	36505585	A	G	snp	1	1	1	1	.	.	.	RP11-698E18.1.1(UPSTREAM);RP11-722M1.1.1(INTRON)
4	36508708	36508716	36508710	T	G	snp	1	1	0	0	.	.	.	RP11-698E18.1.1(EXON);RP11-722M1.1.1(INTRON)
4	36512829	36512837	36512833	T	C	snp	1	1	1	1	.	.	.	RP11-698E18.1.1(DOWNSTREAM);RP11-722M1.1.1(INTRON)
4	36513546	36513557	36513554	ATA	T	snp	1	1	1	1	.	.	.	RP11-698E18.1.1(DOWNSTREAM);RP11-722M1.1.1(INTRON)
4	36604070	36604078	36604073	A	C	snp	1	1	1	1	.	.	.	RP11-722M1.1.1(INTRON)
4	36715990	36715999	36715993	AT	A	snp	1	0	0	0	.	.	.	INTERGENIC(INTERGENIC)
4	36722137	36722152	36722149	AT	C	snp	1	0	0	0	.	.	.	INTERGENIC(INTERGENIC)
4	36791498	36791509	36791505	ACA	T	snp	0	1	1	0	.	.	.	INTERGENIC(INTERGENIC)
4	36862346	36862361	36862355	AC	T	snp	1	1	1	1	.	.	.	INTERGENIC(INTERGENIC)
4	36869035	36869058	36869041	ATGA	A	snp	1	1	1	1	.	.	.	INTERGENIC(INTERGENIC)
4	36909203	36909212	36909206	AG	A	snp	1	0	0	0	.	.	.	INTERGENIC(INTERGENIC)
4	37030694	37030701	37030696	A	C	snp	1	1	1	1	.	.	.	INTERGENIC(INTERGENIC)
4	37036205	37036213	37036207	T	C	snp	1	1	0	1	.	.	.	INTERGENIC(INTERGENIC)
4	37095472	37095480	37095476	T	TG	ins	1	1	1	1	.	.	.	RP11-103J17.1.1(INTRON)
4	37095495	37095509	37095504	TTG	C	snp	1	0	0	0	.	.	.	RP11-103J17.1.1(INTRON)
4	37101868	37101888	37101880	ATT	C	snp	0	1	1	1	.	.	.	RP11-103J17.1.1(INTRON)
4	37129488	37129496	37129494	A	C	snp	0	0	0	1	.	.	.	INTERGENIC(INTERGENIC);RP11-103J17.1.1(DOWNSTREAM)
4	37161258	37161269	37161267	TA	T	del	1	0	0	0	.	.	.	INTERGENIC(INTERGENIC)
4	37165556	37165563	37165561	A	G	snp	1	1	1	1	.	.	.	INTERGENIC(INTERGENIC)
4	37173839	37173858	37173848	AAAC	G	snp	1	1	1	1	.	.	.	INTERGENIC(INTERGENIC)
4	37193415	37193424	37193421	AT	G	snp	1	1	1	1	.	.	.	INTERGENIC(INTERGENIC)
4	37390266	37390275	37390272	AT	G	snp	1	0	0	0	.	.	.	KIAA1239(INTRON)
4	37415743	37415750	37415748	A	C	snp	1	1	1	1	.	.	.	KIAA1239(INTRON)
4	37455628	37455651	37455641	CCG	T	snp	1	0	0	1	.	.	C4orf19(START_GAINED)	KIAA1239(DOWNSTREAM)
4	37518893	37518902	37518898	AT	A	ins	1	0	0	0	.	.	.	C4orf19(INTRON)
4	37528487	37528495	37528493	A	G	snp	0	1	0	1	.	.	.	C4orf19(INTRON)
4	37547684	37547695	37547692	GAT	C	snp	1	1	1	1	.	.	.	C4orf19(INTRON)
4	37549636	37549644	37549643	A	G	ins	1	1	1	1	.	.	.	C4orf19(INTRON)
4	37553672	37553679	37553677	A	C	snp	0	1	0	1	.	.	.	C4orf19(INTRON)
4	37568684	37568692	37568685	A	C	snp	1	1	1	0	.	.	.	C4orf19(INTRON)
4	37585226	37585234	37585233	T	G	snp	1	1	1	0	.	.	.	C4orf19(INTRON);C4orf19(UPSTREAM);RP11-36B15.1.1(DOWNSTREAM)
4	37605426	37605433	37605431	T	G	snp	1	0	0	0	.	.	.	C4orf19(INTRON);RELL1(INTRON)
4	37795521	37795530	37795524	AG	A	snp	1	1	1	0	.	.	.	INTERGENIC(INTERGENIC)
4	37812662	37812671	37812666	T	C	snp	0	0	1	0	.	.	.	INTERGENIC(INTERGENIC)
4	37831470	37831481	37831479	AT	AC	ins	1	1	1	1	.	.	.	PGM2(INTRON)
4	37843821	37843830	37843825	TA	A	ins	1	1	1	0	.	.	.	PGM2(INTRON);PGM2(UPSTREAM)
4	37843821	37843830	37843826	TA	T	del	1	1	1	0	.	.	.	PGM2(INTRON);PGM2(UPSTREAM)
4	37864571	37864578	37864572	T	G	snp	1	0	0	0	.	.	.	INTERGENIC(INTERGENIC);PGM2(DOWNSTREAM)
4	37921736	37921750	37921744	AAC	T	snp	0	0	0	1	.	.	.	TBC1D1(INTRON)
4	37927384	37927391	37927387	A	G	snp	1	1	1	1	.	.	.	TBC1D1(INTRON)
4	38029732	38029751	38029747	AAAC	A	ins	1	0	0	0	.	.	.	TBC1D1(INTRON);TBC1D1(UPSTREAM)
4	38068499	38068508	38068500	T	G	snp	0	0	0	1	.	.	.	TBC1D1(INTRON)
4	38155428	38155436	38155431	T	C	snp	1	1	0	1	.	.	.	INTERGENIC(INTERGENIC)
4	38163603	38163626	38163619	ATCT	G	snp	1	0	0	0	.	.	.	INTERGENIC(INTERGENIC)
4	38178500	38178519	38178515	AAAT	AAATAA	del	1	1	1	1	.	.	.	INTERGENIC(INTERGENIC)
4	38208744	38208751	38208748	A	C	snp	0	1	0	1	.	.	.	INTERGENIC(INTERGENIC)
4	38245385	38245394	38245392	TG	A	snp	1	1	1	0	.	.	.	INTERGENIC(INTERGENIC)
4	38258860	38258871	38258865	AT	C	snp	1	1	1	1	.	.	.	

AC098680.3(DOWNSTREAM);AC098680.4(DOWNSTREAM);INTERGENIC(INTERGENIC)

4	38268734	38268751	38268745	AT	A	del	0	0	0	1	.	.	.	AC098680.2(DOWNSTREAM);INTERGENIC(INTERGENIC)
4	38327119	38327126	38327123	G	A	ins	1	0	0	0	.	.	.	INTERGENIC(INTERGENIC)
4	38441413	38441424	38441421	ATA	AT	del	1	1	1	1	.	.	.	RP11-83C7.2.1(INTRON)
4	38453319	38453326	38453324	A	G	snp	0	1	1	0	.	.	.	RP11-83C7.2.1(INTRON)
4	38484240	38484248	38484247	A	C	snp	0	1	1	0	.	.	.	RP11-83C7.2.1(INTRON)
4	38495145	38495152	38495148	A	G	snp	0	1	1	0	.	.	.	RP11-83C7.2.1(INTRON)
4	38508427	38508434	38508429	A	G	snp	1	1	1	1	.	.	.	RP11-213G21.1.1(UPSTREAM);RP11-83C7.2.1(INTRON)
4	38586762	38586769	38586763	A	C	snp	1	0	0	0	.	.	.	INTERGENIC(INTERGENIC)
4	38603267	38603274	38603272	A	C	snp	1	1	1	1	.	.	.	INTERGENIC(INTERGENIC)
4	38663354	38663363	38663360	T	TG	ins	0	1	1	1	.	.	.	AC021860.1(INTRON);KLF3(UPSTREAM);RP11-617D20.1.1(INTRON)
4	38664129	38664137	38664131	T	C	snp	1	1	0	1	.	.	.	AC021860.1(INTRON);KLF3(UPSTREAM);RP11-617D20.1.1(INTRON)
4	38666561	38666572	38666566	CCA	C	snp	1	1	1	1	.	.	.	AC021860.1(UPSTREAM);KLF3(INTRON);RP11-617D20.1.1(UPSTREAM)
4	38742947	38742954	38742953	T	C	snp	1	1	1	1	.	.	.	INTERGENIC(INTERGENIC)
4	38750298	38750307	38750304	TC	C	snp	0	1	1	1	.	.	.	INTERGENIC(INTERGENIC)
4	38772309	38772322	38772317	AT	TATATATATATATC	ins	1	1	1	1	.	.	.	. INTERGENIC(INTERGENIC);TLR10(DOWNSTREAM)
4	38772309	38772322	38772319	AT	TATCTCC	del	1	1	1	1	.	.	.	INTERGENIC(INTERGENIC);TLR10(DOWNSTREAM)
4	38772699	38772710	38772706	AT	ATATAG	ins	1	0	1	0	.	.	.	INTERGENIC(INTERGENIC);TLR10(DOWNSTREAM)
4	38787595	38787604	38787601	CA	T	snp	1	1	1	1	.	.	.	INTERGENIC(INTERGENIC);TLR1(DOWNSTREAM);TLR10(UPSTREAM)
4	38790629	38790648	38790643	AAAT	G	snp	1	0	0	0	.	.	.	INTERGENIC(INTERGENIC);TLR1(DOWNSTREAM)
4	38825710	38825717	38825713	C	A	ins	1	1	0	1	.	.	.	TLR1(INTRON);TLR6(DOWNSTREAM);TLR6(UTR_3_PRIME)
4	38846521	38846530	38846524	A	C	snp	1	1	1	1	.	.	.	TLR1(INTRON);TLR6(INTRON)
4	38900647	38900662	38900659	TA	AG	ins	1	1	1	1	.	.	.	FAM114A1(INTRON)
4	38994369	38994382	38994380	TA	T	snp	1	0	0	1	.	.	.	TMEM156(DOWNSTREAM);TMEM156(INTRON)
4	39021314	39021323	39021320	TA	C	snp	1	1	1	1	.	.	.	TMEM156(INTRON)
4	39027993	39028002	39027996	A	AGC	ins	1	1	1	1	.	.	.	TMEM156(DOWNSTREAM);TMEM156(INTRON)
4	39028183	39028192	39028190	AT	C	snp	0	1	1	1	.	.	.	TMEM156(DOWNSTREAM);TMEM156(INTRON)
4	39036649	39036656	39036654	A	G	snp	1	1	1	1	.	.	.	INTERGENIC(INTERGENIC);TMEM156(UPSTREAM)
4	39155566	39155574	39155573	A	AAC	ins	1	1	1	1	.	.	.	INTERGENIC(INTERGENIC)
4	39223487	39223498	39223495	AGT	C	snp	1	0	0	0	.	.	.	WDR19(DOWNSTREAM);WDR19(INTRON)
4	39267999	39268008	39268005	A	C	snp	1	1	1	1	.	.	.	WDR19(INTRON);WDR19(UPSTREAM)
4	39268013	39268020	39268016	A	G	snp	1	1	1	1	.	.	.	WDR19(INTRON);WDR19(UPSTREAM)
4	39389210	39389229	39389225	AAAT	AAATA	del	1	1	1	1	.	.	.	INTERGENIC(INTERGENIC)
4	39410569	39410576	39410575	A	C	ins	1	1	1	1	.	.	.	KLB(INTRON)
4	39574699	39574719	39574710	TTA	TTATTATTATT	del	1	0	0	0	.	.	.	. C4orf34(INTRON);UGDH-AS1(INTRON)
4	39706670	39706678	39706674	T	G	snp	1	1	1	1	.	.	.	UBE2K(INTRON)
4	39755649	39755657	39755655	A	C	snp	0	1	0	0	.	.	.	UBE2K(INTRON)
4	39768941	39768960	39768956	TTTG	T	ins	0	0	1	0	.	.	.	UBE2K(INTRON);ZBTB12B(DOWNSTREAM)
4	39800332	39800352	39800349	TAT	TA	del	1	1	1	1	.	.	.	INTERGENIC(INTERGENIC)
4	39846829	39846837	39846831	A	G	snp	0	0	1	0	.	.	.	PDS5A(INTRON)
4	39864420	39864429	39864424	A	C	snp	0	1	0	1	.	.	.	PDS5A(DOWNSTREAM);PDS5A(INTRON)
4	39917817	39917828	39917823	AAC	AC	del	1	1	1	1	.	.	.	PDS5A(INTRON);PDS5A(UPSTREAM)
4	39917817	39917828	39917824	AAC	C	del	1	1	1	1	.	.	.	PDS5A(INTRON);PDS5A(UPSTREAM)
4	39966565	39966582	39966570	AAT	A	snp	1	0	1	0	.	.	.	PDS5A(INTRON)
4	39988597	39988605	39988599	A	C	snp	1	1	1	1	.	.	.	INTERGENIC(INTERGENIC)
4	40003061	40003070	40003069	T	TA	ins	1	1	0	1	.	.	.	INTERGENIC(INTERGENIC)
4	40005111	40005118	40005113	A	C	ins	1	1	1	1	.	.	.	INTERGENIC(INTERGENIC)
4	40061005	40061024	40061009	TATG	G	snp	1	0	0	0	.	.	.	N4BP2(INTRON);RP11-333E13.4.1(UPSTREAM)
4	40095208	40095223	40095218	TTAT	T	del	0	1	0	1	.	.	.	N4BP2(INTRON)
4	40128835	40128842	40128838	T	C	snp	0	1	0	1	.	.	.	N4BP2(INTRON)
4	40129279	40129292	40129281	AT	G	snp	1	0	0	0	.	.	.	N4BP2(INTRON)
4	40284431	40284439	40284434	T	C	snp	1	1	1	1	.	.	.	INTERGENIC(INTERGENIC)
4	40361134	40361153	40361147	TATT	G	snp	1	0	0	0	.	.	.	CHRNA9(DOWNSTREAM);INTERGENIC(INTERGENIC)
4	40368167	40368186	40368171	AAAG	AAG	del	1	1	1	1	.	.	.	INTERGENIC(INTERGENIC)
4	40438201	40438220	40438208	AAAC	A	snp	1	1	1	1	.	.	.	RBM47(DOWNSTREAM);RBM47(INTRON)
4	40438221	40438229	40438223	A	C	ins	1	1	1	1	.	.	.	RBM47(DOWNSTREAM);RBM47(INTRON)
4	40487011	40487019	40487016	T	G	snp	0	0	1	0	.	.	.	RBM47(INTRON)
4	40538720	40538735	40538733	GT	G	del	1	1	0	0	.	.	.	RBM47(INTRON)
4	40563562	40563571	40563564	T	TC	ins	1	1	1	1	.	.	.	RBM47(INTRON)
4	40563562	40563571	40563565	T	C	ins	1	1	1	1	.	.	.	RBM47(INTRON)
4	40563562	40563571	40563566	T	C	snp	1	1	1	1	.	.	.	RBM47(INTRON)
4	40568094	40568103	40568096	A	C	snp	1	1	1	1	.	.	.	RBM47(INTRON)
4	40575801	40575814	40575812	AG	A	snp	1	1	1	1	.	.	.	RBM47(INTRON)

4	40610120	40610129	40610128	A	G	snp	1	0	1	1	.	.	.	RBM47(INTRON)
4	40617148	40617159	40617153	AAT	A	snp	1	0	1	1	.	.	.	RBM47(INTRON)
4	40673462	40673469	40673463	T	G	snp	1	0	0	0	.	.	.	INTERGENIC(INTERGENIC)
4	40691031	40691039	40691036	T	C	snp	1	1	1	1	.	.	.	INTERGENIC(INTERGENIC)
4	40852794	40852801	40852795	A	C	snp	1	1	1	1	.	.	.	APBB2(INTRON)
4	40872727	40872740	40872730	TG	A	snp	0	1	1	1	.	.	.	APBB2(INTRON);snoU13(UPSTREAM)
4	40950108	40950116	40950113	A	G	snp	1	1	1	1	.	.	.	APBB2(INTRON)
4	41040231	41040250	41040246	ATAA	G	snp	1	0	0	0	.	.	.	APBB2(INTRON)
4	41119298	41119312	41119309	TTA	T	ins	0	1	1	0	.	.	.	APBB2(INTRON);U6(DOWNSTREAM)
4	41359591	41359599	41359598	A	C	ins	1	0	0	0	.	.	.	INTERGENIC(INTERGENIC);LIMCH1(UPSTREAM)
4	41371692	41371699	41371698	T	G	snp	1	1	1	1	.	.	.	LIMCH1(INTRON)
4	41470369	41470384	41470373	CCTC	T	snp	1	1	0	1	.	.	.	LIMCH1(INTRON)
4	41509868	41509879	41509874	TGG	G	snp	1	0	1	0	.	.	.	LIMCH1(INTRON)
4	41548878	41548887	41548884	GA	T	snp	1	1	0	1	.	.	.	LIMCH1(INTRON)
4	41642829	41642836	41642833	A	G	snp	1	1	1	1	.	.	.	LIMCH1(INTRON)
4	41680747	41680762	41680755	GT	A	snp	1	0	0	0	.	.	.	LIMCH1(INTRON)
4	41684684	41684695	41684692	TTC	T	ins	1	1	0	1	.	.	.	LIMCH1(INTRON)
4	41715365	41715376	41715368	AT	C	snp	1	1	0	1	.	.	.	INTERGENIC(INTERGENIC)
4	41793385	41793400	41793396	AAAC	A	snp	0	1	0	1	.	.	.	RP11-227F19.1.1(INTRON)
4	41810769	41810778	41810770	A	C	snp	1	1	1	1	.	.	.	RP11-227F19.1.1(INTRON)
4	41935345	41935354	41935346	A	G	snp	1	0	0	1	.	.	.	INTERGENIC(INTERGENIC);TMEM33(UPSTREAM)
4	42047783	42047790	42047784	A	C	snp	1	1	1	1	.	.	.	SLC30A9(INTRON)
4	42178777	42178794	42178791	AAC	A	snp	1	1	0	1	.	.	.	INTERGENIC(INTERGENIC)
4	42205961	42205978	42205973	TA	A	del	1	0	0	0	.	.	.	INTERGENIC(INTERGENIC)
4	42205961	42205978	42205975	TA	A	del	1	1	1	1	.	.	.	INTERGENIC(INTERGENIC)
4	42205961	42205978	42205976	TA	T	snp	1	1	1	1	.	.	.	INTERGENIC(INTERGENIC)
4	42205992	42206007	42206002	TA	G	snp	1	0	0	0	.	.	.	INTERGENIC(INTERGENIC)
4	42209362	42209371	42209369	TG	A	snp	1	1	1	1	.	.	.	INTERGENIC(INTERGENIC)
4	42221609	42221618	42221615	A	G	snp	1	1	1	1	.	.	.	INTERGENIC(INTERGENIC)
4	42258762	42258770	42258767	T	G	snp	1	1	1	1	.	.	.	INTERGENIC(INTERGENIC)
4	42259443	42259451	42259450	A	G	snp	1	1	1	1	.	.	.	INTERGENIC(INTERGENIC)
4	42262029	42262043	42262040	TTG	T	ins	1	1	1	1	.	.	.	INTERGENIC(INTERGENIC)
4	42386513	42386520	42386519	T	C	snp	1	1	0	1	.	.	.	RP11-63A11.1.1(INTRON)
4	42415729	42415737	42415736	T	C	snp	1	1	0	1	.	.	.	ATP8A1(INTRON)
4	42456679	42456696	42456681	GT	A	snp	1	1	1	1	.	.	.	ATP8A1(DOWNSTREAM);ATP8A1(INTRON)
4	42480581	42480590	42480582	A	C	snp	1	1	1	1	.	.	.	ATP8A1(INTRON)
4	42480581	42480590	42480584	A	C	snp	1	1	0	0	.	.	.	ATP8A1(INTRON)
4	42557228	42557235	42557233	C	CCG	ins	1	0	0	0	.	.	.	ATP8A1(INTRON)
4	42604014	42604023	42604017	T	G	ins	1	1	1	1	.	.	.	ATP8A1(INTRON)
4	42684087	42684094	42684091	T	C	snp	1	1	1	0	.	.	.	INTERGENIC(INTERGENIC)
4	42795970	42795983	42795972	AC	C	snp	0	1	1	1	.	.	.	INTERGENIC(INTERGENIC)
4	42852420	42852434	42852429	TTG	C	snp	1	1	1	1	.	.	.	INTERGENIC(INTERGENIC)
4	42951227	42951235	42951228	A	C	snp	1	1	1	0	.	.	.	GRXCR1(INTRON)
4	43005316	43005327	43005320	ATA	A	snp	1	0	0	0	.	.	.	GRXCR1(INTRON)
4	43033168	43033195	43033190	ATTT	TA	del	1	0	1	0	.	.	.	GRXCR1(DOWNSTREAM);INTERGENIC(INTERGENIC)
4	43066786	43066795	43066788	A	G	snp	1	0	0	0	.	.	.	INTERGENIC(INTERGENIC)
4	43104630	43104639	43104638	A	C	snp	1	0	1	0	.	.	.	INTERGENIC(INTERGENIC)
4	43257998	43258005	43258000	G	GT	ins	1	0	0	0	.	.	.	INTERGENIC(INTERGENIC)
4	43330372	43330387	43330385	TA	T	del	1	1	1	1	.	.	.	INTERGENIC(INTERGENIC)
4	43386948	43386957	43386955	TC	G	snp	0	0	1	0	.	.	.	INTERGENIC(INTERGENIC)
4	43560828	43560839	43560834	AT	C	snp	1	1	1	0	.	.	.	INTERGENIC(INTERGENIC)
4	43603478	43603489	43603486	ATT	A	del	1	0	1	0	.	.	.	AC114774.1(UPSTREAM);INTERGENIC(INTERGENIC)
4	43675968	43675975	43675971	T	G	snp	1	0	0	0	.	.	.	INTERGENIC(INTERGENIC)
4	43717962	43717969	43717964	T	G	snp	1	1	1	1	.	.	.	INTERGENIC(INTERGENIC)
4	43733078	43733086	43733083	A	G	snp	1	0	0	0	.	.	.	INTERGENIC(INTERGENIC)
4	43803165	43803172	43803167	A	G	snp	0	1	0	1	.	.	.	INTERGENIC(INTERGENIC)
4	43885619	43885626	43885622	T	G	snp	1	1	1	0	.	.	.	INTERGENIC(INTERGENIC)
4	43912826	43912835	43912827	A	C	ins	1	0	0	0	.	.	.	INTERGENIC(INTERGENIC)
4	44024920	44024943	44024934	CTC	A	snp	0	1	1	1	.	.	.	INTERGENIC(INTERGENIC);RP11-328N19.1.1(DOWNSTREAM)
4	44190030	44190039	44190031	A	C	snp	1	0	0	0	.	.	.	KCTD8(INTRON)
4	44218911	44218920	44218915	T	C	snp	0	0	1	0	.	.	.	KCTD8(INTRON)
4	44298244	44298251	44298245	T	C	snp	1	1	1	1	.	.	.	KCTD8(INTRON)
4	44479814	44479821	44479815	T	G	snp	0	1	1	1	.	.	.	INTERGENIC(INTERGENIC)
4	44523556	44523563	44523562	T	C	snp	1	1	1	1	.	.	.	INTERGENIC(INTERGENIC)
4	44593794	44593809	44593800	TC	C	snp	1	1	1	1	.	.	.	INTERGENIC(INTERGENIC)
4	44593794	44593809	44593801	TC	T	snp	1	0	0	0	.	.	.	INTERGENIC(INTERGENIC)

4	44674829	44674838	44674832	TG	T	snp	1	0	0	0	.	.	.	YIPF7(INTRON)
4	44713229	44713238	44713235	A	G	snp	1	1	1	1	.	.	.	GNPDA2(INTRON)
4	44732056	44732064	44732063	T	G	snp	1	1	1	0	.	.	.	GNPDA2(UPSTREAM);INTERGENIC(INTERGENIC)
4	44798079	44798086	44798080	T	C	snp	1	1	1	1	.	.	.	INTERGENIC(INTERGENIC)
4	44932656	44932671	44932666	AT	G	snp	0	0	1	0	.	.	.	INTERGENIC(INTERGENIC)
4	45028160	45028171	45028162	AT	C	snp	1	1	1	1	.	.	.	RP11-362I1.1.1(INTRON)
4	45028160	45028171	45028169	AT	G	snp	0	1	0	0	.	.	.	RP11-362I1.1.1(INTRON)
4	45098662	45098669	45098666	T	G	snp	0	1	1	1	.	.	.	INTERGENIC(INTERGENIC)
4	45113934	45113942	45113940	T	C	snp	1	1	1	1	.	.	.	INTERGENIC(INTERGENIC)
4	45150444	45150455	45150446	AT	G	snp	1	0	0	0	.	.	.	INTERGENIC(INTERGENIC)
4	45198420	45198431	45198425	AT	C	snp	1	1	1	1	.	.	.	INTERGENIC(INTERGENIC)
4	45214254	45214267	45214256	TA	A	snp	1	0	0	0	.	.	.	INTERGENIC(INTERGENIC)
4	45215898	45215907	45215905	TG	A	snp	1	0	0	0	.	.	.	INTERGENIC(INTERGENIC)
4	45310898	45310909	45310901	TTG	C	snp	1	0	0	0	.	.	.	INTERGENIC(INTERGENIC)
4	45315782	45315789	45315787	A	G	snp	1	0	0	0	.	.	.	INTERGENIC(INTERGENIC)
4	45402906	45402921	45402911	AATT	G	snp	1	1	1	1	.	.	.	INTERGENIC(INTERGENIC)
4	45580565	45580572	45580571	A	C	ins	1	1	1	1	.	.	.	INTERGENIC(INTERGENIC)
4	45678796	45678807	45678802	AC	T	snp	1	0	0	0	.	.	.	INTERGENIC(INTERGENIC)
4	45867799	45867806	45867805	T	G	snp	1	0	1	0	.	.	.	INTERGENIC(INTERGENIC)
4	45876685	45876694	45876692	AT	AC	ins	1	1	1	1	.	.	.	INTERGENIC(INTERGENIC)
4	46154241	46154248	46154246	A	C	snp	1	0	0	0	.	.	.	INTERGENIC(INTERGENIC)
4	46267403	46267412	46267410	AT	AC	ins	1	1	1	1	.	.	.	GABRA2(INTRON)
4	46291897	46291924	46291912	TTTA	T	del	1	1	1	1	.	.	.	GABRA2(INTRON)
4	46372927	46372938	46372930	AC	G	snp	1	1	0	1	.	.	.	GABRA2(INTRON)
4	46461174	46461182	46461181	A	C	snp	0	1	1	1	.	.	.	GABRA2(INTRON)
4	46822852	46822869	46822855	AT	A	snp	1	1	1	1	.	.	.	COX7B2(INTRON)
4	46822852	46822869	46822867	AT	G	snp	1	1	1	1	.	.	.	COX7B2(INTRON)
4	46880501	46880518	46880509	TA	C	snp	1	1	1	1	.	.	.	COX7B2(INTRON);COX7B2(UPSTREAM)
4	46934011	46934018	46934014	T	G	snp	1	1	1	1	.	.	.	GABRA4(INTRON)
4	47037659	47037670	47037664	AC	T	snp	0	1	0	1	.	.	.	GABRB1(DOWNSTREAM);GABRB1(INTRON)
4	47039546	47039554	47039552	T	G	snp	1	0	0	0	.	.	.	GABRB1(DOWNSTREAM);GABRB1(INTRON)
4	47215549	47215558	47215553	TC	C	snp	1	1	1	1	.	.	.	GABRB1(INTRON)
4	47319470	47319477	47319474	A	C	snp	1	1	0	1	.	.	.	GABRB1(INTRON)
4	47385508	47385517	47385516	A	C	snp	0	1	0	1	.	.	.	GABRB1(INTRON)
4	47476390	47476399	47476393	A	C	ins	1	0	0	0	.	.	.	INTERGENIC(INTERGENIC)
4	47482835	47482842	47482838	T	C	snp	1	1	1	1	.	.	.	ATP10D(UPSTREAM);INTERGENIC(INTERGENIC)
4	47484439	47484450	47484447	TCT	TCTTCC	ins	1	1	1	1	.	.	.	ATP10D(UPSTREAM);INTERGENIC(INTERGENIC)
4	47562535	47562542	47562538	A	C	snp	0	1	0	1	.	.	.	AC092597.3.1(UPSTREAM);ATP10D(INTRON)
4	47628989	47629012	47629009	GTT	G	del	1	1	1	1	.	.	.	CORIN(INTRON)
4	47741049	47741056	47741050	A	C	snp	1	1	1	1	.	.	.	CORIN(INTRON)
4	47759885	47759894	47759887	AT	TATTATG	ins	0	1	0	1	.	.	.	CORIN(INTRON)
4	47759885	47759894	47759890	AT	A	del	0	1	0	1	.	.	.	CORIN(INTRON)
4	47807367	47807374	47807369	T	G	snp	1	0	0	0	.	.	.	CORIN(INTRON)
4	47817329	47817337	47817331	T	C	ins	1	1	1	1	.	.	.	7SK(UPSTREAM);CORIN(INTRON)
4	47940332	47940349	47940347	AT	A	del	1	1	1	1	.	.	.	CNGA1(DOWNSTREAM);CNGA1(INTRON);NIPAL1(INTRON)
4	47962733	47962741	47962739	A	C	ins	1	1	1	1	.	.	.	CNGA1(INTRON);NIPAL1(DOWNSTREAM);NIPAL1(INTRON)
4	47987785	47987792	47987787	A	G	snp	1	1	0	1	.	.	.	CNGA1(INTRON);CNGA1(UPSTREAM);NIPAL1(INTRON)
4	48014761	48014772	48014765	ACA	T	snp	0	1	1	1	.	.	.	CNGA1(INTRON);NIPAL1(INTRON);NIPAL1(UPSTREAM)
4	48043603	48043610	48043609	A	G	snp	1	1	1	1	.	.	.	INTERGENIC(INTERGENIC);NIPAL1(DOWNSTREAM)
4	48089444	48089458	48089455	TTA	T	ins	0	1	0	1	.	.	.	TXK(DOWNSTREAM);TXK(INTRON)
4	48106577	48106588	48106579	AT	T	snp	1	0	0	0	.	.	.	TXK(INTRON);U6(DOWNSTREAM)
4	48135775	48135786	48135783	TA	G	snp	1	1	1	1	.	.	.	TEC(DOWNSTREAM);TXK(INTRON)
4	48152855	48152863	48152862	A	C	snp	0	1	1	0	.	.	.	TEC(INTRON);TEC(UPSTREAM);Y_RNA(UPSTREAM)
4	48169243	48169252	48169249	AT	G	snp	1	1	1	1	.	.	.	TEC(INTRON)
4	48173068	48173077	48173072	A	C	snp	1	1	1	1	.	.	.	TEC(INTRON)
4	48184351	48184358	48184357	T	G	snp	1	1	1	1	.	.	.	TEC(INTRON)
4	48227718	48227726	48227719	T	C	snp	1	1	1	1	.	.	.	TEC(INTRON)
4	48337802	48337811	48337805	T	C	snp	0	1	1	1	.	.	.	INTERGENIC(INTERGENIC)
4	48339659	48339674	48339672	AC	A	del	1	1	1	1	.	.	.	INTERGENIC(INTERGENIC);SLAIN2(UPSTREAM)
4	48649690	48649697	48649693	A	C	snp	1	1	1	1	.	.	.	FRYL(INTRON)
4	48655825	48655848	48655845	AGC	A	snp	0	0	1	0	.	.	.	FRYL(EXON);FRYL(INTRON)
4	48669112	48669119	48669113	T	C	snp	0	1	0	1	.	.	.	FRYL(INTRON)
4	48719293	48719301	48719300	A	C	snp	1	0	0	0	.	.	.	FRYL(INTRON)
4	48794927	48794947	48794942	TTC	A	snp	1	1	1	1	.	.	.	INTERGENIC(INTERGENIC)
4	48885163	48885176	48885166	TA	C	snp	1	0	1	0	.	.	.	INTERGENIC(INTERGENIC);OCIAD2(DOWNSTREAM)
4	48885163	48885176	48885169	TA	C	snp	1	0	0	0	.	.	.	INTERGENIC(INTERGENIC);OCIAD2(DOWNSTREAM)
4	49311282	49311293	49311291	CA	C	snp	0	0	1	0	.	.	.	INTERGENIC(INTERGENIC)

4	49501125	49501132	49501128	A	G	snp	1	1	1	1	.	.	.	ANKRD20A17P(DOWNSTREAM);INTERGENIC(INTERGENIC)
4	49613027	49613036	49613032	A	C	snp	1	1	1	1	.	.	.	INTERGENIC(INTERGENIC)
4	49624378	49624385	49624379	A	G	snp	1	1	1	1	.	.	.	INTERGENIC(INTERGENIC)
4	53078051	53078060	53078052	A	C	ins	1	1	0	1	.	.	.	INTERGENIC(INTERGENIC)
4	53100104	53100113	53100111	TG	C	snp	1	1	1	1	.	.	.	INTERGENIC(INTERGENIC)
4	53150561	53150569	53150566	T	TG	ins	1	1	1	1	.	.	.	INTERGENIC(INTERGENIC)
4	53199606	53199623	53199621	GT	C	snp	1	1	1	1	.	.	.	INTERGENIC(INTERGENIC)
4	53285700	53285707	53285702	T	C	snp	1	0	0	0	.	.	.	INTERGENIC(INTERGENIC)
4	53343843	53343850	53343848	A	G	snp	1	1	1	1	.	.	.	INTERGENIC(INTERGENIC)
4	53344267	53344278	53344271	GA	A	snp	1	0	0	0	.	.	.	INTERGENIC(INTERGENIC)
4	53352211	53352220	53352213	T	C	snp	1	1	1	1	.	.	.	INTERGENIC(INTERGENIC)
4	53363886	53363895	53363893	AC	T	snp	1	0	0	0	.	.	.	INTERGENIC(INTERGENIC);U6(DOWNSTREAM)
4	53374507	53374514	53374509	A	G	snp	1	0	0	0	.	.	.	INTERGENIC(INTERGENIC);snoU13(UPSTREAM)
4	53557487	53557494	53557489	A	C	snp	0	1	0	1	.	.	.	RP11-177B4.2.1(EXON)
4	53618931	53618940	53618934	A	C	snp	1	1	0	0	.	.	.	ERVMER34-1(UPSTREAM);INTERGENIC(INTERGENIC);SNORA26(DOWNSTREAM)
4	53653422	53653442	53653427	ATT	A	snp	1	1	1	1	.	.	.	INTERGENIC(INTERGENIC);RP11-752D24.3.1(UPSTREAM);Y_RNA(DOWNSTREAM)
4	53660681	53660692	53660684	AT	C	snp	1	0	0	0	.	.	.	RP11-752D24.3.1(INTRON)
4	53661676	53661690	53661680	GAA	G	snp	1	0	0	0	.	.	.	RP11-752D24.3.1(INTRON)
4	53728368	53728377	53728374	CG	A	snp	1	1	1	1	.	.	.	INTERGENIC(INTERGENIC);RASL11B(UPSTREAM)
4	53790890	53790903	53790892	AC	G	snp	1	0	0	0	.	.	.	SCFD2(INTRON)
4	53878509	53878520	53878518	TC	TCTT	ins	1	0	0	0	.	.	.	SCFD2(INTRON)
4	53968578	53968585	53968580	A	G	snp	1	1	1	1	.	.	.	SCFD2(INTRON)
4	54064837	54064850	54064842	TG	TGTGTGTGCA	ins	1	1	1	1	.	.	.	SCFD2(INTRON)
4	54121352	54121360	54121358	T	C	snp	1	0	0	0	.	.	.	SCFD2(INTRON)
4	54191345	54191358	54191356	TG	A	snp	1	1	1	1	.	.	.	SCFD2(INTRON)
4	54222602	54222613	54222605	AG	T	snp	1	0	0	0	.	.	.	SCFD2(INTRON)
4	54239824	54239835	54239827	CCT	T	snp	0	1	0	0	.	.	.	FIP1L1(UPSTREAM);INTERGENIC(INTERGENIC)
4	54243138	54243145	54243141	A	G	snp	1	1	1	1	.	.	.	FIP1L1(UPSTREAM);INTERGENIC(INTERGENIC)
4	54456243	54456251	54456246	C	A	ins	1	1	1	1	.	.	.	FIP1L1(INTRON);LNX1(INTRON);LNX1-AS2(UPSTREAM)
4	54456243	54456251	54456247	C	A	snp	1	1	1	1	.	.	.	FIP1L1(INTRON);LNX1(INTRON);LNX1-AS2(UPSTREAM)
4	54496261	54496268	54496263	G	T	snp	1	1	1	1	.	.	.	FIP1L1(INTRON);LNX1(INTRON)
4	54682335	54682344	54682339	A	C	snp	1	1	0	1	.	.	.	FIP1L1(INTRON)
4	54689706	54689714	54689707	T	G	snp	1	1	1	1	.	.	.	FIP1L1(INTRON)
4	54696029	54696044	54696042	AT	A	del	1	0	0	0	.	.	.	FIP1L1(INTRON)
4	54733473	54733482	54733480	AG	A	snp	1	0	0	0	.	.	.	FIP1L1(INTRON)
4	54836835	54836844	54836836	G	GC	ins	1	0	0	0	.	.	.	FIP1L1(INTRON)
4	54918292	54918300	54918293	T	C	ins	1	1	1	1	.	.	.	CHIC2(INTRON);FIP1L1(INTRON)
4	54946282	54946293	54946291	TA	G	snp	1	0	0	0	.	.	.	FIP1L1(INTRON);RP11-571I18.2.1(DOWNSTREAM)
4	55040270	55040277	55040271	T	TTTTTTC	ins	1	1	1	1	.	.	.	FIP1L1(INTRON)
4	55071340	55071351	55071349	CA	C	del	1	0	0	0	.	.	.	FIP1L1(INTRON)
4	55083048	55083059	55083056	TC	C	snp	1	1	1	1	.	.	.	FIP1L1(INTRON);RP11-601I15.1.1(UPSTREAM)
4	55163822	55163837	55163826	TG	G	snp	1	1	1	1	.	.	.	FIP1L1(DOWNSTREAM);PDGFRA(UTR_3_PRIME)
4	55343715	55343726	55343724	TC	TG	ins	1	1	1	1	.	.	.	INTERGENIC(INTERGENIC)
4	55556214	55556222	55556221	T	TG	ins	1	0	0	0	.	.	.	KIT(INTRON)
4	55615302	55615311	55615308	TA	ATAC	ins	1	1	1	1	.	.	.	INTERGENIC(INTERGENIC)
4	55652395	55652404	55652398	TA	G	snp	1	1	0	1	.	.	.	INTERGENIC(INTERGENIC)
4	55707076	55707089	55707078	CA	A	snp	1	1	1	1	.	.	.	RP11-273B19.1.1(UPSTREAM);RP11-273B19.2.1(INTRON)
4	55707076	55707089	55707081	CA	C	snp	1	1	1	1	.	.	.	RP11-273B19.1.1(UPSTREAM);RP11-273B19.2.1(INTRON)
4	55850981	55850988	55850984	T	G	snp	1	1	1	1	.	.	.	INTERGENIC(INTERGENIC)
4	55891314	55891321	55891315	A	G	snp	1	1	1	1	.	.	.	AC111194.1(UPSTREAM);INTERGENIC(INTERGENIC)
4	55942370	55942377	55942374	C	A	snp	0	0	1	0	.	.	.	KDR(DOWNSTREAM);RP11-530I17.1.1(INTRON)
4	55969947	55969956	55969949	A	C	snp	1	0	0	0	.	.	.	KDR(DOWNSTREAM);KDR(INTRON)
4	56045476	56045483	56045478	T	G	snp	1	0	0	0	.	.	.	INTERGENIC(INTERGENIC)
4	56048319	56048330	56048328	CA	T	snp	1	0	0	0	.	.	.	INTERGENIC(INTERGENIC)
4	56141809	56141822	56141820	AC	A	snp	1	1	1	1	.	.	.	AC108209.1(DOWNSTREAM);INTERGENIC(INTERGENIC)
4	56206959	56206968	56206966	AG	C	snp	1	0	0	0	.	.	.	INTERGENIC(INTERGENIC)
4	56259151	56259166	56259160	AAAT	C	snp	1	0	0	0	.	.	.	INTERGENIC(INTERGENIC);TMEM165(UPSTREAM)
4	56266329	56266338	56266334	TA	G	snp	1	1	1	1	.	.	.	TMEM165(DOWNSTREAM);TMEM165(INTRON);TMEM165(UPSTREAM)
4	56274668	56274677	56274673	TA	G	snp	1	0	0	0	.	.	.	AC069200.1(DOWNSTREAM);TMEM165(INTRON);Y_RNA(UPSTREAM)
4	56286274	56286287	56286277	AT	A	snp	1	1	1	1	.	.	.	TMEM165(DOWNSTREAM);TMEM165(INTRON);TMEM165(UPSTREAM)
4	56286295	56286314	56286302	TATT	A	snp	1	1	1	1	.	.	.	TMEM165(DOWNSTREAM);TMEM165(INTRON);TMEM165(UPSTREAM)

4	56286295	56286314	56286304	TATT	TTTATTTAT	del	1	1	1	1	.	.	. TMEM165(DOWNSTREAM);TMEM165(INTRON);TMEM165(UPSTREAM)
4	56286295	56286314	56286306	TATT	A	snp	1	1	1	1	.	.	. TMEM165(DOWNSTREAM);TMEM165(INTRON);TMEM165(UPSTREAM)
4	56328464	56328471	56328468	T	C	snp	1	1	1	1	.	.	. CLOCK(DOWNSTREAM);CLOCK(INTRON)
4	56366360	56366369	56366364	A	C	snp	1	0	0	0	.	.	. CLOCK(INTRON);Y_RNA(DOWNSTREAM)
4	56387386	56387393	56387392	T	G	snp	1	1	1	1	.	.	. CLOCK(INTRON)
4	56390412	56390419	56390413	A	C	snp	1	1	1	1	.	.	. CLOCK(INTRON)
4	56420699	56420708	56420701	TG	C	snp	1	0	0	0	.	.	. INTERGENIC(INTERGENIC);PDCL2(DOWNSTREAM)
4	56617294	56617302	56617299	T	G	snp	1	1	0	1	.	.	. INTERGENIC(INTERGENIC)
4	56714071	56714079	56714072	A	G	snp	0	1	1	0	.	.	. INTERGENIC(INTERGENIC)
4	56890293	56890302	56890298	TA	TATAATTAG	del	1	0	0	0	.	.	. CEP135(INTRON)
4	56922765	56922774	56922771	A	AC	ins	1	0	0	0	.	.	. INTERGENIC(INTERGENIC)
4	57090315	57090323	57090317	T	C	snp	1	0	0	0	.	.	. KIAA1211(INTRON)
4	57223799	57223808	57223805	TA	C	snp	1	0	0	0	.	.	. AASDH(INTRON);RPL7AP31(DOWNSTREAM)
4	57255068	57255076	57255073	T	C	snp	1	1	1	1	.	.	. AASDH(UPSTREAM);INTERGENIC(INTERGENIC);PPAT(DOWNSTREAM)
4	57261022	57261033	57261030	ATT	A	del	1	1	1	1	.	.	. PPAT(EXON);PPAT(UTR_3_PRIME)
4	57343450	57343467	57343464	TC	CTC	del	1	1	1	1	.	.	. SRP72(DOWNSTREAM);SRP72(INTRON)
4	57375368	57375375	57375369	T	TTC	ins	1	1	1	1	.	.	. ARL9(INTRON)
4	57375368	57375375	57375370	T	TC	ins	1	1	1	1	.	.	. ARL9(INTRON)
4	57375368	57375375	57375371	T	C	ins	1	1	1	1	.	.	. ARL9(INTRON)
4	57446817	57446825	57446824	A	C	snp	1	0	0	0	.	.	. RP11-630A13.2.1(INTRON)
4	57453075	57453082	57453081	A	C	ins	1	0	1	0	.	.	. RP11-630A13.2.1(INTRON)
4	57462448	57462455	57462449	A	C	ins	1	0	0	0	.	.	. GLDCP.1(DOWNSTREAM);RP11-630A13.2.1(INTRON)
4	57505372	57505379	57505376	T	C	snp	1	1	1	0	.	.	. INTERGENIC(INTERGENIC)
4	57528624	57528647	57528641	TTTC	TC	del	1	1	1	1	.	.	. HOPX(INTRON);HOPX(UPSTREAM)
4	57649363	57649383	57649380	AAT	A	del	1	0	1	0	.	.	. INTERGENIC(INTERGENIC)
4	57699418	57699435	57699429	TTG	T	snp	1	1	1	1	.	.	. INTERGENIC(INTERGENIC)
4	57786909	57786916	57786915	A	C	snp	0	1	0	1	.	.	. REST(INTRON)
4	57887819	57887827	57887825	T	G	ins	1	1	1	1	.	.	. POLR2B(INTRON);POLR2B(UPSTREAM)
4	57923173	57923181	57923177	T	C	snp	1	0	0	0	.	.	. IGFBP7(INTRON);RP11-738E22.2.1(INTRON)
4	57979325	57979333	57979326	T	TCA	ins	0	1	1	0	.	.	. IGFBP7(UPSTREAM);RP11-12A1.1.1(INTRON)
4	58090723	58090730	58090725	G	A	snp	1	1	0	1	.	.	. INTERGENIC(INTERGENIC)
4	58112900	58112908	58112903	A	C	snp	1	1	0	1	.	.	. INTERGENIC(INTERGENIC)
4	58123438	58123446	58123442	T	C	snp	1	0	0	0	.	.	. INTERGENIC(INTERGENIC)
4	58150069	58150078	58150073	CT	T	snp	1	1	1	0	.	.	. INTERGENIC(INTERGENIC)
4	58174147	58174174	58174153	CCAT	G	snp	1	0	0	0	.	.	. INTERGENIC(INTERGENIC)
4	58236689	58236696	58236691	C	T	snp	1	0	0	0	.	.	. INTERGENIC(INTERGENIC)
4	58329028	58329037	58329031	AG	C	snp	1	1	1	1	.	.	. RP11-319E12.1.1(INTRON)
4	58348858	58348869	58348862	TA	C	snp	1	0	0	0	.	.	. INTERGENIC(INTERGENIC)
4	58537639	58537650	58537642	TA	C	snp	1	1	1	1	.	.	. INTERGENIC(INTERGENIC)
4	58570994	58571011	58571002	TTA	T	snp	1	1	1	1	.	.	. INTERGENIC(INTERGENIC)
4	58607976	58607983	58607977	T	C	snp	1	0	0	0	.	.	. INTERGENIC(INTERGENIC)
4	58656374	58656401	58656389	TTAT	C	snp	0	0	1	0	.	.	. INTERGENIC(INTERGENIC)
4	58719475	58719486	58719480	TTA	G	snp	1	1	1	1	.	.	. INTERGENIC(INTERGENIC)
4	58744170	58744179	58744172	T	C	snp	1	1	1	1	.	.	. INTERGENIC(INTERGENIC)
4	58765616	58765624	58765619	T	C	snp	1	1	1	1	.	.	. INTERGENIC(INTERGENIC)
4	58778624	58778635	58778629	GT	C	snp	1	0	0	0	.	.	. INTERGENIC(INTERGENIC)
4	58932203	58932223	58932219	AAC	G	snp	1	0	0	0	.	.	. INTERGENIC(INTERGENIC)
4	58946367	58946384	58946372	TG	TA	ins	1	1	1	1	.	.	. INTERGENIC(INTERGENIC)
4	59052414	59052425	59052417	AG	A	snp	1	0	0	0	.	.	. INTERGENIC(INTERGENIC)
4	59060583	59060592	59060585	GT	TA	ins	0	1	0	1	.	.	. INTERGENIC(INTERGENIC)
4	59118605	59118614	59118607	A	C	snp	1	1	1	1	.	.	. INTERGENIC(INTERGENIC)
4	59143595	59143609	59143606	TAT	TA	del	1	0	0	0	.	.	. INTERGENIC(INTERGENIC)
4	59145426	59145437	59145433	TA	C	snp	0	1	1	1	.	.	. INTERGENIC(INTERGENIC)
4	59155976	59155987	59155983	TTA	A	del	0	1	1	1	.	.	. INTERGENIC(INTERGENIC)
4	59235696	59235704	59235697	T	G	snp	1	0	0	0	.	.	. INTERGENIC(INTERGENIC)
4	59262775	59262784	59262779	A	G	ins	1	0	0	0	.	.	. INTERGENIC(INTERGENIC)
4	59308937	59308948	59308939	TG	GTGA	ins	1	1	0	1	.	.	. INTERGENIC(INTERGENIC)
4	59308937	59308948	59308941	TG	GA	ins	1	1	0	1	.	.	. INTERGENIC(INTERGENIC)
4	59349916	59349924	59349920	A	C	snp	1	0	0	0	.	.	. INTERGENIC(INTERGENIC)
4	59405016	59405036	59405019	TTG	C	snp	1	1	0	1	.	.	. INTERGENIC(INTERGENIC);RP11-577G20.1.1(UPSTREAM)
4	59449465	59449476	59449474	AT	A	del	1	1	1	1	.	.	. INTERGENIC(INTERGENIC)
4	59463617	59463624	59463622	A	G	snp	1	1	1	1	.	.	. INTERGENIC(INTERGENIC)
4	59501549	59501572	59501558	TCTT	T	ins	0	1	1	0	.	.	. INTERGENIC(INTERGENIC)
4	59606437	59606446	59606441	TG	G	snp	1	1	1	1	.	.	. INTERGENIC(INTERGENIC)

4	59628270	59628279	59628277	AT	C	snp	1	1	1	1	.	.	.	INTERGENIC(INTERGENIC)
4	59628388	59628397	59628395	AT	C	snp	1	1	1	1	.	.	.	INTERGENIC(INTERGENIC)
4	59714018	59714029	59714022	TA	A	snp	1	1	1	1	.	.	.	RP11-506N2.1.1(DOWNSTREAM);RP11-506N2.1.1(INTRON)
4	59809714	59809722	59809717	T	G	snp	1	1	1	1	.	.	.	RP11-506N2.1.1(INTRON)
4	59809757	59809764	59809762	A	G	ins	1	1	1	1	.	.	.	RP11-506N2.1.1(INTRON)
4	59812180	59812189	59812183	T	C	snp	1	1	1	1	.	.	.	RP11-506N2.1.1(INTRON)
4	59837031	59837038	59837032	A	G	snp	0	1	1	0	.	.	.	RP11-506N2.1.1(INTRON)
4	59906626	59906634	59906632	A	G	snp	1	1	1	1	.	.	.	RP11-340A13.2.1(INTRON)
4	59913411	59913418	59913417	A	C	snp	1	1	1	1	.	.	.	RP11-340A13.2.1(DOWNSTREAM);RP11-340A13.3.1(INTRON)
4	60031790	60031805	60031801	AAAC	A	ins	1	1	1	1	.	.	.	RP11-622J8.1.1(INTRON)
4	60057058	60057065	60057063	A	G	snp	1	1	1	1	.	.	.	INTERGENIC(INTERGENIC)
4	60086193	60086208	60086201	AAAT	G	snp	1	0	0	0	.	.	.	INTERGENIC(INTERGENIC)
4	60128408	60128417	60128415	A	G	ins	1	1	1	1	.	.	.	INTERGENIC(INTERGENIC)
4	60128408	60128417	60128415	A	G	snp	1	1	1	1	.	.	.	INTERGENIC(INTERGENIC)
4	60144175	60144182	60144180	A	G	snp	1	1	1	1	.	.	.	INTERGENIC(INTERGENIC)
4	60217106	60217123	60217119	TG	A	snp	1	0	0	0	.	.	.	INTERGENIC(INTERGENIC)
4	60345749	60345760	60345754	AC	A	snp	1	0	0	0	.	.	.	INTERGENIC(INTERGENIC)
4	60394244	60394251	60394248	T	C	snp	1	1	1	1	.	.	.	INTERGENIC(INTERGENIC)
4	60405866	60405873	60405872	T	TCTTTTA	ins	1	0	0	0	.	.	.	INTERGENIC(INTERGENIC)
4	60488605	60488612	60488609	A	C	snp	1	0	0	0	.	.	.	RP11-725D20.1.1(INTRON)
4	60594781	60594792	60594783	AT	TATATATAT	del	1	0	1	0	.	.	.	INTERGENIC(INTERGENIC)
4	60617650	60617657	60617652	T	G	snp	1	1	1	1	.	.	.	INTERGENIC(INTERGENIC)
4	60817953	60817964	60817960	TG	A	snp	1	0	0	0	.	.	.	INTERGENIC(INTERGENIC)
4	60987633	60987644	60987637	TA	C	snp	1	1	1	0	.	.	.	INTERGENIC(INTERGENIC)
4	60987754	60987765	60987758	TA	C	snp	1	1	1	0	.	.	.	INTERGENIC(INTERGENIC)
4	60992155	60992164	60992161	AT	G	snp	0	1	1	0	.	.	.	INTERGENIC(INTERGENIC)
4	61006351	61006359	61006355	T	C	snp	0	0	1	0	.	.	.	INTERGENIC(INTERGENIC)
4	61014038	61014053	61014044	TATG	C	snp	0	1	1	0	.	.	.	INTERGENIC(INTERGENIC)
4	61040915	61040932	61040923	TA	A	snp	0	0	1	0	.	.	.	INTERGENIC(INTERGENIC)
4	61303401	61303410	61303403	A	G	snp	1	1	1	1	.	.	.	INTERGENIC(INTERGENIC)
4	61361630	61361649	61361634	AAAC	AAC	del	0	1	1	1	.	.	.	INTERGENIC(INTERGENIC)
4	61361630	61361649	61361637	AAAC	A	ins	0	1	1	1	.	.	.	INTERGENIC(INTERGENIC)
4	61366821	61366828	61366823	T	C	snp	1	1	1	1	.	.	.	INTERGENIC(INTERGENIC)
4	61376305	61376312	61376307	A	G	ins	0	1	1	1	.	.	.	INTERGENIC(INTERGENIC)
4	61461077	61461088	61461086	TA	G	snp	1	0	0	0	.	.	.	INTERGENIC(INTERGENIC)
4	61559816	61559824	61559819	T	G	snp	1	0	0	0	.	.	.	INTERGENIC(INTERGENIC)
4	61583398	61583413	61583411	AC	T	snp	1	1	1	1	.	.	.	INTERGENIC(INTERGENIC)
4	61603967	61603982	61603973	ATAG	G	snp	1	1	0	1	.	.	.	INTERGENIC(INTERGENIC)
4	61626366	61626374	61626369	C	CCA	ins	1	1	1	1	.	.	.	RP11-593F5.2.1(INTRON)
4	61730841	61730854	61730847	CT	T	snp	0	0	1	0	.	.	.	INTERGENIC(INTERGENIC)
4	61740853	61740862	61740856	AT	G	snp	1	0	1	1	.	.	.	INTERGENIC(INTERGENIC)
4	61886895	61886903	61886896	A	C	snp	1	1	1	1	.	.	.	INTERGENIC(INTERGENIC)
4	62003705	62003716	62003713	TTG	T	snp	1	1	1	1	.	.	.	INTERGENIC(INTERGENIC)
4	62065772	62065789	62065787	GA	G	snp	1	0	0	0	.	.	.	INTERGENIC(INTERGENIC);LPHN3(UPSTREAM)
4	62195411	62195418	62195412	A	C	snp	1	1	1	1	.	.	.	LPHN3(INTRON)
4	62322094	62322107	62322097	TA	TC	ins	1	1	0	1	.	.	.	LPHN3(INTRON)
4	62322094	62322107	62322099	TA	C	snp	1	1	0	1	.	.	.	LPHN3(INTRON)
4	62322196	62322205	62322200	TA	G	snp	0	1	0	0	.	.	.	LPHN3(INTRON)
4	62349458	62349473	62349465	AAAT	A	del	1	0	0	0	.	.	.	LPHN3(INTRON)
4	62425535	62425543	62425536	T	G	snp	1	0	0	0	.	.	.	LPHN3(INTRON)
4	62442939	62442947	62442943	G	A	snp	1	0	0	0	.	.	.	LPHN3(INTRON)
4	62482547	62482555	62482551	T	C	snp	1	0	0	0	.	.	.	LPHN3(INTRON)
4	62564173	62564196	62564193	AAC	A	snp	1	0	0	0	.	.	.	LPHN3(INTRON)
4	62641602	62641629	62641625	TTTA	T	snp	0	1	0	1	.	.	.	LPHN3(INTRON);RP11-621K7.1.1(UPSTREAM)
4	62713636	62713645	62713640	TA	C	snp	1	1	1	1	.	.	.	LPHN3(INTRON)
4	62722305	62722322	62722317	TC	CTCTC	del	1	1	1	1	.	.	.	LPHN3(INTRON)
4	62917743	62917752	62917745	GT	A	snp	1	0	0	0	.	.	.	LPHN3(INTRON)
4	63137269	63137282	63137276	AT	A	snp	1	1	1	1	.	.	.	INTERGENIC(INTERGENIC)
4	63217331	63217340	63217337	T	G	snp	1	0	0	0	.	.	.	INTERGENIC(INTERGENIC)
4	63229496	63229503	63229498	T	G	snp	1	1	0	1	.	.	.	INTERGENIC(INTERGENIC)
4	63335169	63335178	63335173	A	C	snp	1	0	0	0	.	.	.	INTERGENIC(INTERGENIC)
4	63367172	63367179	63367173	A	G	snp	0	0	0	1	.	.	.	INTERGENIC(INTERGENIC)
4	63453446	63453459	63453452	TA	G	snp	1	1	1	1	.	.	.	INTERGENIC(INTERGENIC)
4	63459036	63459047	63459042	GA	A	snp	1	1	1	1	.	.	.	INTERGENIC(INTERGENIC)
4	63462723	63462732	63462726	AT	C	snp	1	0	0	0	.	.	.	INTERGENIC(INTERGENIC)
4	63476751	63476758	63476756	A	C	snp	1	0	1	0	.	.	.	INTERGENIC(INTERGENIC)

4	63553852	63553861	63553854	AT	G	snp	1	1	1	1	.	.	.	INTERGENIC(INTERGENIC)
4	63553864	63553873	63553866	AT	G	snp	1	1	1	1	.	.	.	INTERGENIC(INTERGENIC)
4	63553908	63553919	63553917	AT	C	snp	0	1	1	1	.	.	.	INTERGENIC(INTERGENIC)
4	63553972	63553981	63553977	AT	G	snp	1	1	1	1	.	.	.	INTERGENIC(INTERGENIC)
4	63554010	63554019	63554012	AT	G	snp	1	1	1	1	.	.	.	INTERGENIC(INTERGENIC)
4	63554036	63554045	63554038	AT	G	snp	1	1	1	1	.	.	.	INTERGENIC(INTERGENIC)
4	63554596	63554605	63554600	AT	G	snp	0	0	1	0	.	.	.	INTERGENIC(INTERGENIC)
4	63573089	63573098	63573095	AT	G	snp	1	0	1	0	.	.	.	INTERGENIC(INTERGENIC)
4	63578190	63578203	63578196	GA	A	snp	1	1	1	1	.	.	.	INTERGENIC(INTERGENIC)
4	63660711	63660720	63660714	T	TTTC	ins	1	1	1	1	.	.	.	INTERGENIC(INTERGENIC)
4	63696025	63696042	63696033	CT	T	snp	1	1	1	1	.	.	.	INTERGENIC(INTERGENIC)
4	63745123	63745130	63745129	A	AT	ins	1	0	0	0	.	.	.	INTERGENIC(INTERGENIC)
4	63817558	63817569	63817564	TAA	AATA	del	1	1	1	1	.	.	.	INTERGENIC(INTERGENIC)
4	63839118	63839125	63839120	A	C	snp	1	0	0	0	.	.	.	INTERGENIC(INTERGENIC)
4	63895604	63895613	63895610	GT	TT	ins	1	0	0	0	.	.	.	INTERGENIC(INTERGENIC)
4	63895604	63895613	63895611	GT	G	del	1	0	0	0	.	.	.	INTERGENIC(INTERGENIC)
4	63968758	63968769	63968765	ATA	AATAA	del	1	0	0	0	.	.	.	INTERGENIC(INTERGENIC)
4	64066835	64066844	64066839	TA	C	snp	1	0	0	0	.	.	.	INTERGENIC(INTERGENIC)
4	64067190	64067205	64067197	TA	G	snp	1	0	0	0	.	.	.	INTERGENIC(INTERGENIC)
4	64067777	64067786	64067780	TA	C	snp	1	1	1	1	.	.	.	INTERGENIC(INTERGENIC)
4	64067884	64067893	64067887	TA	C	snp	0	1	0	1	.	.	.	INTERGENIC(INTERGENIC)
4	64068475	64068486	64068481	TA	C	snp	1	0	0	0	.	.	.	INTERGENIC(INTERGENIC)
4	64068541	64068552	64068549	TA	C	snp	1	0	0	0	.	.	.	INTERGENIC(INTERGENIC)
4	64068607	64068616	64068613	TA	C	snp	1	0	0	0	.	.	.	INTERGENIC(INTERGENIC)
4	64068638	64068647	64068644	TA	C	snp	1	1	1	1	.	.	.	INTERGENIC(INTERGENIC)
4	64078176	64078184	64078180	T	C	snp	1	1	1	1	.	.	.	INTERGENIC(INTERGENIC)
4	64078997	64079008	64079006	TA	TAT	del	1	1	1	1	.	.	.	INTERGENIC(INTERGENIC)
4	64222383	64222394	64222386	AT	C	snp	1	0	0	0	.	.	.	INTERGENIC(INTERGENIC)
4	64224751	64224762	64224758	AC	T	snp	0	1	1	1	.	.	.	INTERGENIC(INTERGENIC)
4	64249321	64249328	64249322	A	C	snp	1	1	1	1	.	.	.	INTERGENIC(INTERGENIC)
4	64314287	64314295	64314294	T	G	ins	1	1	1	1	.	.	.	INTERGENIC(INTERGENIC)
4	64314287	64314295	64314294	T	G	snp	1	1	1	1	.	.	.	INTERGENIC(INTERGENIC)
4	64332376	64332385	64332378	TA	C	snp	1	0	0	0	.	.	.	INTERGENIC(INTERGENIC);RP11-12K22.1.1(DOWNSTREAM)
4	64341848	64341855	64341851	A	C	snp	1	0	0	0	.	.	.	RP11-12K22.1.1(INTRON)
4	64376750	64376759	64376753	AT	G	snp	1	0	0	0	.	.	.	INTERGENIC(INTERGENIC)
4	64379803	64379820	64379818	AT	A	del	1	1	1	1	.	.	.	INTERGENIC(INTERGENIC)
4	64478330	64478337	64478334	T	C	snp	1	1	1	1	.	.	.	INTERGENIC(INTERGENIC)
4	64634958	64634966	64634960	A	C	snp	1	0	0	0	.	.	.	INTERGENIC(INTERGENIC)
4	64641424	64641437	64641427	TA	G	snp	1	0	0	0	.	.	.	INTERGENIC(INTERGENIC)
4	64700265	64700276	64700271	TA	AG	ins	1	1	1	1	.	.	.	INTERGENIC(INTERGENIC)
4	64705808	64705816	64705814	T	C	snp	1	1	1	1	.	.	.	INTERGENIC(INTERGENIC)
4	64722172	64722189	64722177	ATT	A	snp	1	0	0	0	.	.	.	INTERGENIC(INTERGENIC)
4	64770286	64770293	64770292	T	C	snp	1	0	0	0	.	.	.	INTERGENIC(INTERGENIC)
4	64780152	64780159	64780153	T	G	ins	1	1	1	1	.	.	.	INTERGENIC(INTERGENIC)
4	64912032	64912039	64912038	A	G	snp	1	1	1	1	.	.	.	INTERGENIC(INTERGENIC)
4	64914313	64914324	64914322	TA	G	snp	1	1	1	1	.	.	.	INTERGENIC(INTERGENIC)
4	64918821	64918832	64918826	AT	C	snp	0	1	1	0	.	.	.	INTERGENIC(INTERGENIC)
4	64919888	64919895	64919891	A	G	ins	1	1	1	1	.	.	.	INTERGENIC(INTERGENIC)
4	65085863	65085871	65085864	T	G	snp	1	1	1	1	.	.	.	INTERGENIC(INTERGENIC)
4	65159701	65159716	65159714	AT	A	del	1	1	1	1	.	.	.	TECRL(DOWNSTREAM);TECRL(INTRON)
4	65262476	65262483	65262481	T	C	snp	1	1	1	1	.	.	.	TECRL(INTRON);U6(DOWNSTREAM)
4	65361472	65361485	65361476	TA	C	snp	1	1	1	1	.	.	.	INTERGENIC(INTERGENIC)
4	65381933	65381940	65381938	T	C	snp	1	1	1	1	.	.	.	INTERGENIC(INTERGENIC)
4	65470469	65470477	65470473	T	G	snp	1	1	1	1	.	.	.	AC097648.1(DOWNSTREAM);AC097648.2(UPSTREAM);AC097648.4(UPSTREAM);INTERGENIC(INTERGENIC);MTND5P13(DOWNSTREAM);MTND6P16(UPSTREAM);RP11-63H19.1.1(UPSTREAM);RP11-63H19.2.1(DOWNSTREAM)
4	65520306	65520321	65520318	AC	C	del	0	1	1	0	.	.	.	INTERGENIC(INTERGENIC)
4	65566506	65566523	65566519	TG	A	snp	1	0	0	0	.	.	.	INTERGENIC(INTERGENIC)
4	65599630	65599647	65599644	AT	T	ins	1	1	1	1	.	.	.	INTERGENIC(INTERGENIC)
4	65599630	65599647	65599645	AT	A	del	1	1	1	1	.	.	.	INTERGENIC(INTERGENIC)
4	65659587	65659595	65659591	A	G	snp	1	0	0	0	.	.	.	INTERGENIC(INTERGENIC)
4	65702064	65702073	65702068	CT	T	del	1	0	0	0	.	.	.	INTERGENIC(INTERGENIC)
4	65720598	65720605	65720604	T	C	snp	1	0	0	0	.	.	.	INTERGENIC(INTERGENIC)
4	65818703	65818712	65818709	A	G	ins	1	1	1	1	.	.	.	RP11-707A18.1.1(INTRON)
4	65829193	65829204	65829198	AT	A	snp	1	1	1	1	.	.	.	RP11-707A18.1.1(INTRON)
4	65931839	65931847	65931842	T	C	ins	1	1	0	1	.	.	.	INTERGENIC(INTERGENIC)
4	65991272	65991279	65991274	A	C	snp	1	0	0	0	.	.	.	INTERGENIC(INTERGENIC)
4	66040568	66040577	66040569	T	C	snp	1	1	1	1	.	.	.	INTERGENIC(INTERGENIC)

4	66117692	66117719	66117703	AAAT	AA	ins	1	0	1	0	.	.	.	INTERGENIC(INTERGENIC)
4	66150253	66150262	66150260	AG	C	snp	1	1	0	1	.	.	.	INTERGENIC(INTERGENIC)
4	66191750	66191759	66191754	AT	G	snp	1	1	1	1	.	.	.	EPHA5(DOWNSTREAM);EPHA5(INTRON)
4	66223539	66223548	66223544	GT	A	snp	1	0	0	0	.	.	.	EPHA5(INTRON)
4	66273649	66273657	66273655	T	G	ins	1	0	0	0	.	.	.	EPHA5(INTRON)
4	66275065	66275088	66275069	AAAT	G	snp	1	0	0	0	.	.	.	EPHA5(INTRON)
4	66319999	66320010	66320005	TAA	A	ins	1	1	1	1	.	.	.	EPHA5(INTRON)
4	66320010	66320030	66320014	AAT	G	snp	1	0	0	0	.	.	.	EPHA5(INTRON)
4	66334213	66334226	66334215	AC	G	snp	0	1	1	1	.	.	.	EPHA5(INTRON)
4	66370084	66370099	66370087	AT	A	snp	1	1	1	1	.	.	.	EPHA5(INTRON)
4	66370084	66370099	66370097	AT	AG	ins	1	1	1	1	.	.	.	EPHA5(INTRON)
4	66376704	66376715	66376706	TC	C	snp	1	1	1	1	.	.	.	EPHA5(INTRON)
4	66507892	66507900	66507899	A	G	snp	0	1	1	1	.	.	.	EPHA5(INTRON)
4	66532697	66532708	66532701	CA	A	snp	1	0	0	0	.	.	.	EPHA5(INTRON);RP11-807H7.1.1(UPSTREAM)
4	66657973	66657982	66657977	CT	T	snp	0	0	1	1	.	.	.	INTERGENIC(INTERGENIC)
4	66727386	66727395	66727393	AC	T	snp	1	1	1	1	.	.	.	INTERGENIC(INTERGENIC);RP11-51P8.1.1(DOWNSTREAM)
4	66791044	66791057	66791050	CA	T	snp	0	0	0	1	.	.	.	INTERGENIC(INTERGENIC)
4	66801806	66801813	66801808	T	TC	ins	1	1	1	1	.	.	.	INTERGENIC(INTERGENIC)
4	66801806	66801813	66801809	T	C	ins	1	1	1	1	.	.	.	INTERGENIC(INTERGENIC)
4	66852488	66852496	66852495	A	C	ins	1	0	0	0	.	.	.	INTERGENIC(INTERGENIC)
4	66918846	66918854	66918850	A	G	snp	0	1	0	0	.	.	.	RP11-25H12.1.1(INTRON)
4	67053348	67053359	67053357	TG	A	snp	1	0	0	0	.	.	.	INTERGENIC(INTERGENIC)
4	67117449	67117460	67117452	TAA	C	snp	1	0	0	0	.	.	.	INTERGENIC(INTERGENIC)
4	67215358	67215365	67215359	A	C	snp	1	0	0	0	.	.	.	INTERGENIC(INTERGENIC)
4	67340222	67340233	67340228	GTT	T	ins	1	1	0	1	.	.	.	INTERGENIC(INTERGENIC)
4	67340222	67340233	67340230	GTT	G	del	1	1	0	1	.	.	.	INTERGENIC(INTERGENIC)
4	67591060	67591073	67591065	TA	C	snp	1	1	1	1	.	.	.	INTERGENIC(INTERGENIC)
4	67618983	67618990	67618987	A	C	snp	1	0	0	0	.	.	.	INTERGENIC(INTERGENIC)
4	67627286	67627297	67627291	AAC	T	snp	1	0	0	0	.	.	.	INTERGENIC(INTERGENIC)
4	67699121	67699130	67699123	TC	A	snp	1	1	1	1	.	.	.	INTERGENIC(INTERGENIC)
4	67699121	67699130	67699124	TC	AA	ins	1	1	1	1	.	.	.	INTERGENIC(INTERGENIC)
4	67699121	67699130	67699124	TC	A	snp	1	1	1	1	.	.	.	INTERGENIC(INTERGENIC)
4	67731335	67731350	67731344	TGTC	TC	ins	1	1	1	1	.	.	.	INTERGENIC(INTERGENIC)
4	68016599	68016610	68016606	CT	CTC	del	0	0	1	0	.	.	.	INTERGENIC(INTERGENIC)
4	68017626	68017635	68017631	GC	G	ins	1	1	1	1	.	.	.	INTERGENIC(INTERGENIC)
4	68236389	68236404	68236402	CT	C	snp	1	1	1	1	.	.	.	INTERGENIC(INTERGENIC)
4	68258257	68258268	68258264	TTG	A	snp	1	1	1	1	.	.	.	INTERGENIC(INTERGENIC)
4	68356610	68356619	68356615	AT	G	snp	1	1	0	0	.	.	.	CENPC1(DOWNSTREAM);CENPC1(INTRON)
4	68383562	68383577	68383567	ATAC	C	snp	1	1	1	1	.	.	.	CENPC1(INTRON);CENPC1(UPSTREAM)
4	68443354	68443369	68443365	TTTC	TT	ins	1	1	1	1	.	.	.	STAP1(INTRON)
4	68454104	68454111	68454109	T	G	snp	1	1	1	1	.	.	.	STAP1(INTRON)
4	68689543	68689551	68689550	T	C	snp	1	1	1	1	.	.	.	RP11-453E17.1.1(INTRON);TMPRSS11D(DOWNSTREAM);TMPRSS11D(INTRON)
4	68710079	68710088	68710080	T	G	snp	1	1	1	1	.	.	.	RP11-453E17.1.1(INTRON);TMPRSS11D(INTRON)
4	68929899	68929906	68929902	T	C	snp	1	0	0	0	.	.	.	RP11-35D5.1.1(UPSTREAM);RP11-453E17.1.1(INTRON);TMPRSS11F(INTRON)
4	69044999	69045006	69045004	T	G	snp	1	1	1	1	.	.	.	FTLP10(UPSTREAM);INTERGENIC(INTERGENIC);RP11-453E17.3.1(DOWNSTREAM)
4	69097706	69097717	69097710	AAT	G	snp	1	1	0	1	.	.	.	RP11-453E17.3.1(UPSTREAM);TMPRSS11B(EXON);TMPRSS11B(INTRON);TMPRSS11B(UPSTREAM)
4	69108034	69108043	69108041	TA	G	snp	0	0	1	0	.	.	.	TMPRSS11B(INTRON)
4	69120724	69120732	69120726	A	C	snp	1	0	0	0	.	.	.	INTERGENIC(INTERGENIC)
4	69132155	69132162	69132157	T	G	ins	1	0	0	0	.	.	.	INTERGENIC(INTERGENIC)
4	69132155	69132162	69132158	T	G	snp	1	0	0	0	.	.	.	INTERGENIC(INTERGENIC)
4	69146147	69146155	69146154	A	C	snp	0	1	0	0	.	.	.	INTERGENIC(INTERGENIC)
4	69305530	69305545	69305537	TTTA	TTTG	ins	1	1	1	1	.	.	.	INTERGENIC(INTERGENIC)
4	69507379	69507386	69507382	T	G	snp	1	1	0	0	.	.	.	INTERGENIC(INTERGENIC);UGT2B15(DOWNSTREAM)
4	69592049	69592060	69592057	TAA	T	snp	1	1	1	1	.	.	.	INTERGENIC(INTERGENIC)
4	69640814	69640829	69640823	AGAA	AAAT	ins	1	1	1	0	.	.	.	INTERGENIC(INTERGENIC)
4	69644880	69644893	69644889	TG	TGT	del	1	1	1	1	.	.	.	INTERGENIC(INTERGENIC)
4	69644903	69644916	69644905	GT	T	del	1	0	0	0	.	.	.	INTERGENIC(INTERGENIC)
4	69648288	69648296	69648290	T	C	ins	1	1	1	0	.	.	.	CTD-2005D20.1.1(UPSTREAM);INTERGENIC(INTERGENIC)
4	69702944	69702951	69702947	T	G	snp	0	0	1	0	.	.	.	INTERGENIC(INTERGENIC);RP11-468N14.3.1(UPSTREAM)
4	69718016	69718023	69718019	T	G	snp	1	1	1	1	.	.	.	INTERGENIC(INTERGENIC)
4	69721318	69721331	69721325	CA	G	snp	1	0	0	0	.	.	.	INTERGENIC(INTERGENIC)
4	69732254	69732261	69732256	T	G	snp	1	0	0	0	.	.	.	INTERGENIC(INTERGENIC);RP11-468N14.4.1(UPSTREAM)
4	69744146	69744155	69744150	T	C	snp	1	1	1	1	.	.	.	RP11-468N14.5.1(INTRON)

4	69759348	69759357	69759351	TC	T	snp	0	1	0	1	.	.	.	INTERGENIC(INTERGENIC)
4	69861960	69861969	69861962	AT	G	snp	1	1	1	1	.	.	.	INTERGENIC(INTERGENIC);RP11-468N14.11.1(DOWNSTREAM)
4	69990707	69990716	69990713	GA	A	snp	0	1	1	1	.	.	.	INTERGENIC(INTERGENIC);RP13-644M16.1.1(UPSTREAM)
4	69999334	69999343	69999338	AT	G	snp	1	1	1	1	.	.	.	RP13-644M16.3.1(INTRON)
4	70001381	70001389	70001384	A	C	ins	1	1	1	1	.	.	.	INTERGENIC(INTERGENIC);RP13-644M16.3.1(DOWNSTREAM)
4	70056311	70056319	70056315	T	C	ins	1	1	1	1	.	.	.	RP11-704M14.1.1(INTRON)
4	70083522	70083529	70083528	A	C	snp	1	1	1	1	.	.	.	INTERGENIC(INTERGENIC);RP11-704M14.1.1(DOWNSTREAM);RP11-704M14.2.1(UPSTREAM);UGT2B11(UPSTREAM)
4	70104855	70104864	70104860	A	C	ins	1	1	1	1	.	.	.	INTERGENIC(INTERGENIC);RP11-618I10.1.1(UPSTREAM)
4	70128222	70128231	70128226	GA	A	snp	0	1	1	0	.	.	.	INTERGENIC(INTERGENIC)
4	70264012	70264019	70264015	T	C	snp	1	0	0	0	.	.	.	RP11-790I12.1.1(INTRON)
4	70276907	70276916	70276910	AG	T	snp	1	1	1	1	.	.	.	RP11-790I12.2.1(INTRON);RP11-790I12.6.1(DOWNSTREAM)
4	70338871	70338879	70338872	T	G	snp	1	0	0	0	.	.	.	INTERGENIC(INTERGENIC)
4	70346055	70346062	70346057	T	G	snp	1	0	0	0	.	.	.	UGT2B4(DOWNSTREAM);UGT2B4(EXON);UGT2B4(UTR_3_PRIME)
4	70370802	70370809	70370804	A	G	snp	1	1	1	1	.	.	.	UGT2B4(INTRON)
4	70422749	70422756	70422755	T	G	snp	1	1	0	1	.	.	.	INTERGENIC(INTERGENIC)
4	70434469	70434492	70434472	GTT	T	snp	1	1	0	1	.	.	.	INTERGENIC(INTERGENIC);RP11-401E5.2.1(DOWNSTREAM)
4	70434469	70434492	70434473	GTT	TG	del	1	1	0	1	.	.	.	INTERGENIC(INTERGENIC);RP11-401E5.2.1(DOWNSTREAM)
4	70436844	70436851	70436850	T	G	snp	1	1	1	1	.	.	.	INTERGENIC(INTERGENIC);RP11-401E5.2.1(DOWNSTREAM)
4	70452171	70452180	70452175	A	C	snp	1	0	0	0	.	.	.	INTERGENIC(INTERGENIC);RP11-401E5.2.1(UPSTREAM);UGT2A1(DOWNSTREAM)
4	70637857	70637866	70637865	T	C	ins	1	1	0	1	.	.	.	SULT1B1(INTRON)
4	70799756	70799764	70799759	T	C	snp	1	0	0	0	.	.	.	CSN1S1(INTRON)
4	70844293	70844302	70844298	AG	A	snp	1	0	0	0	.	.	.	INTERGENIC(INTERGENIC)
4	70878207	70878215	70878213	T	G	snp	1	0	0	0	.	.	.	INTERGENIC(INTERGENIC)
4	70889934	70889942	70889938	A	G	snp	1	0	0	0	.	.	.	HTN3(UPSTREAM);INTERGENIC(INTERGENIC)
4	70919455	70919475	70919466	TCC	T	snp	1	0	0	0	.	.	.	HTN1(INTRON)
4	70936638	70936646	70936642	A	G	snp	1	1	1	1	.	.	.	CSN1S2AP(INTRON);CSN1S2AP(UPSTREAM)
4	70991200	70991208	70991203	A	G	snp	1	1	1	1	.	.	.	INTERGENIC(INTERGENIC)
4	71089653	71089668	71089657	TG	C	snp	0	1	1	1	.	.	.	FDCSP(UPSTREAM);INTERGENIC(INTERGENIC)
4	71212014	71212023	71212022	T	C	snp	1	1	1	1	.	.	.	INTERGENIC(INTERGENIC)
4	71248324	71248337	71248335	TG	T	del	1	1	1	1	.	.	.	SMR3B(INTRON);SMR3B(UPSTREAM)
4	71251669	71251677	71251671	T	C	snp	1	1	1	1	.	.	.	SMR3B(DOWNSTREAM);SMR3B(INTRON)
4	71253991	71254000	71253998	T	TA	ins	0	1	1	1	.	.	.	SMR3B(DOWNSTREAM);SMR3B(INTRON)
4	71256794	71256803	71256800	AT	G	snp	1	1	1	1	.	.	.	INTERGENIC(INTERGENIC);SMR3B(DOWNSTREAM)
4	71358086	71358093	71358091	T	G	snp	1	1	1	1	.	.	.	INTERGENIC(INTERGENIC)
4	71361162	71361171	71361165	T	G	ins	1	1	0	1	.	.	.	INTERGENIC(INTERGENIC)
4	71385416	71385425	71385417	T	TTTTTA	ins	1	1	1	1	.	.	.	AMTN(INTRON)
4	71403103	71403112	71403107	A	G	snp	0	1	1	1	.	.	.	AMTN(DOWNSTREAM);INTERGENIC(INTERGENIC)
4	71422310	71422319	71422312	T	G	ins	1	1	1	1	.	.	.	INTERGENIC(INTERGENIC)
4	71480433	71480448	71480438	AATT	G	snp	1	0	0	0	.	.	.	INTERGENIC(INTERGENIC)
4	71485883	71485894	71485891	AT	T	snp	1	1	0	0	.	.	.	INTERGENIC(INTERGENIC)
4	71553075	71553094	71553086	TTTA	T	snp	1	0	0	0	.	.	.	ENAM(DOWNSTREAM);INTERGENIC(INTERGENIC);UTP3(UPSTREAM)
4	71571507	71571521	71571516	CGC	T	snp	1	0	0	0	.	.	.	RUFY3(DOWNSTREAM);RUFY3(INTRON);U6(DOWNSTREAM);U6(UPSTREAM)
4	71637715	71637726	71637723	GAG	T	snp	0	1	1	0	.	.	.	RUFY3(DOWNSTREAM);RUFY3(INTRON)
4	71772100	71772107	71772104	A	G	snp	0	0	1	0	.	.	.	MOB1B(INTRON)
4	71798124	71798131	71798125	T	G	snp	1	0	0	0	.	.	.	MOB1B(INTRON)
4	71798124	71798131	71798126	T	C	snp	1	1	1	1	.	.	.	MOB1B(INTRON)
4	71833444	71833451	71833447	A	G	snp	1	0	0	0	.	.	.	MOB1B(INTRON)
4	71966063	71966072	71966071	A	C	snp	0	1	1	1	.	.	.	INTERGENIC(INTERGENIC)
4	71972669	71972676	71972673	A	G	snp	1	0	0	0	.	.	.	INTERGENIC(INTERGENIC)
4	71981093	71981116	71981110	ACAA	A	snp	1	1	1	1	.	.	.	INTERGENIC(INTERGENIC)
4	72259402	72259409	72259408	A	G	snp	1	1	1	0	.	.	.	SLC4A4(INTRON)
4	72367377	72367392	72367384	TTTG	A	snp	1	1	1	1	.	.	.	SLC4A4(INTRON)
4	72452825	72452840	72452827	GT	C	snp	1	0	0	0	.	.	.	INTERGENIC(INTERGENIC)
4	72474963	72474971	72474970	A	C	ins	1	1	1	1	.	.	.	INTERGENIC(INTERGENIC)
4	72505374	72505381	72505375	A	G	snp	1	1	0	1	.	.	.	INTERGENIC(INTERGENIC)
4	72645280	72645291	72645286	GA	A	snp	1	1	1	0	.	.	.	GC(INTRON)
4	72680862	72680869	72680868	T	G	snp	1	1	1	0	.	.	.	INTERGENIC(INTERGENIC)
4	72711362	72711381	72711369	ATTT	A	snp	1	0	0	0	.	.	.	INTERGENIC(INTERGENIC)
4	72756501	72756510	72756508	AT	C	snp	1	1	1	1	.	.	.	INTERGENIC(INTERGENIC)

4	72824833	72824848	72824837	AT	T	ins	1	1	1	1	.	.	.	INTERGENIC(INTERGENIC)
4	72839242	72839255	72839247	AT	C	snp	1	1	1	1	.	.	.	INTERGENIC(INTERGENIC)
4	72840319	72840326	72840321	T	C	snp	1	1	1	1	.	.	.	INTERGENIC(INTERGENIC)
4	72850887	72850895	72850890	T	C	snp	1	1	1	1	.	.	.	INTERGENIC(INTERGENIC)
4	72875035	72875052	72875048	TA	TAT	del	1	0	0	0	.	.	.	INTERGENIC(INTERGENIC)
4	72881880	72881887	72881883	T	C	snp	1	1	1	1	.	.	.	INTERGENIC(INTERGENIC)
4	72938129	72938136	72938132	A	G	snp	1	1	1	1	.	.	.	NPFFR2(INTRON)
4	72945856	72945873	72945868	TTG	TG	del	1	1	1	1	.	.	.	NPFFR2(INTRON)
4	72975465	72975476	72975468	ACA	G	snp	1	0	0	0	.	.	.	NPFFR2(INTRON)
4	73087945	73087952	73087948	T	G	snp	0	0	1	0	.	.	.	INTERGENIC(INTERGENIC)
4	73182410	73182433	73182414	AAT	C	snp	1	0	0	0	.	.	.	ADAMTS3(INTRON);ADAMTS3(UPSTREAM)
4	73226104	73226111	73226107	A	G	snp	1	0	0	0	.	.	.	ADAMTS3(INTRON)
4	73319621	73319636	73319624	AT	A	snp	1	1	1	1	.	.	.	ADAMTS3(INTRON)
4	73321502	73321515	73321504	TA	C	snp	1	0	0	0	.	.	.	ADAMTS3(INTRON)
4	73431084	73431091	73431088	A	G	snp	1	0	0	0	.	.	.	ADAMTS3(INTRON)
4	73513916	73513925	73513924	T	C	snp	1	1	1	1	.	.	.	INTERGENIC(INTERGENIC)
4	73771665	73771682	73771673	TTA	G	snp	1	1	1	1	.	.	.	INTERGENIC(INTERGENIC)
4	73846288	73846297	73846294	T	G	snp	1	0	0	0	.	.	.	INTERGENIC(INTERGENIC)
4	73960355	73960364	73960360	AC	T	snp	0	1	1	1	.	.	.	ANKRD17(DOWNSTREAM);ANKRD17(INTRON);HMGA1P2(UPSTREAM)
4	74007867	74007875	74007870	A	G	snp	1	1	1	1	.	.	.	ANKRD17(INTRON)
4	74164481	74164488	74164483	A	C	snp	1	0	0	0	.	.	.	RP11-692D12.1.1(INTRON)
4	74194399	74194406	74194403	T	C	snp	1	1	1	1	.	.	.	INTERGENIC(INTERGENIC)
4	74207130	74207137	74207135	T	G	snp	1	0	0	0	.	.	.	INTERGENIC(INTERGENIC);RP11-580P21.1.1(UPSTREAM)
4	74326847	74326862	74326855	TTCT	TC	del	1	1	1	1	.	.	.	AFP(DOWNSTREAM);INTERGENIC(INTERGENIC)
4	74356132	74356147	74356137	TTTG	TG	del	1	1	1	1	.	.	.	AFM(INTRON);AFM(UPSTREAM)
4	74356132	74356147	74356138	TTTG	G	del	1	1	1	1	.	.	.	AFM(INTRON);AFM(UPSTREAM)
4	74388171	74388179	74388173	T	G	snp	1	1	1	1	.	.	.	RP11-622A1.2.1(INTRON)
4	74409308	74409317	74409315	TA	T	del	1	1	1	1	.	.	.	INTERGENIC(INTERGENIC);RP11-622A1.1.1(UPSTREAM)
4	74422489	74422500	74422494	ATA	C	snp	1	0	0	0	.	.	.	INTERGENIC(INTERGENIC)
4	74509341	74509352	74509344	AT	C	snp	0	1	1	1	.	.	.	INTERGENIC(INTERGENIC)
4	74654422	74654430	74654429	T	C	snp	1	0	0	0	.	.	.	INTERGENIC(INTERGENIC)
4	74674379	74674386	74674380	A	G	snp	1	0	0	0	.	.	.	INTERGENIC(INTERGENIC)
4	75262092	75262101	75262094	TA	C	snp	1	0	0	0	.	.	.	INTERGENIC(INTERGENIC)
4	75381125	75381134	75381130	A	AG	ins	1	1	1	0	.	.	.	INTERGENIC(INTERGENIC)
4	75381125	75381134	75381131	A	G	ins	1	1	1	0	.	.	.	INTERGENIC(INTERGENIC)
4	75381125	75381134	75381132	A	G	snp	1	1	1	0	.	.	.	INTERGENIC(INTERGENIC)
4	75495106	75495117	75495109	AAT	T	snp	1	1	0	1	.	.	.	AC142293.3.1(INTRON);AREGB(DOWNSTREAM)
4	75514755	75514770	75514764	AGAA	A	del	0	1	0	1	.	.	.	AC142293.3.1(UPSTREAM);INTERGENIC(INTERGENIC)
4	75518297	75518306	75518301	AG	T	snp	1	1	1	1	.	.	.	AC142293.3.1(UPSTREAM);INTERGENIC(INTERGENIC)
4	75580761	75580770	75580764	AC	T	snp	1	0	0	0	.	.	.	INTERGENIC(INTERGENIC)
4	75598690	75598699	75598696	A	G	snp	1	1	0	1	.	.	.	INTERGENIC(INTERGENIC)
4	75825769	75825788	75825775	TTTG	C	snp	1	1	1	1	.	.	.	INTERGENIC(INTERGENIC)
4	75899096	75899109	75899107	AG	A	snp	1	0	0	0	.	.	.	PARM1(INTRON);RP11-44F21.3.1(UPSTREAM)
4	75904358	75904366	75904359	A	C	snp	1	0	0	0	.	.	.	PARM1(INTRON)
4	75940217	75940224	75940218	T	C	snp	1	1	0	1	.	.	.	PARM1(INTRON);RP11-44F21.2.1(INTRON)
4	76112476	76112487	76112484	TA	AC	ins	0	1	0	1	.	.	.	INTERGENIC(INTERGENIC)
4	76282691	76282698	76282697	A	G	snp	1	1	1	1	.	.	.	RP11-542G1.1.1(INTRON);RP11-542G1.2.1(INTRON);RP11-542G1.3.1(INTRON);RP11-567N4.2.1(DOWNSTREAM)
4	76296588	76296605	76296597	CA	CG	ins	1	1	1	1	.	.	.	RP11-567N4.2.1(INTRON)
4	76332428	76332437	76332429	A	G	ins	1	1	1	1	.	.	.	RP11-567N4.2.1(INTRON);RP11-567N4.3.1(INTRON)
4	76376272	76376280	76376276	A	C	snp	1	1	1	1	.	.	.	INTERGENIC(INTERGENIC)
4	76399788	76399796	76399794	A	C	ins	1	1	1	1	.	.	.	INTERGENIC(INTERGENIC);RCHY1(DOWNSTREAM)
4	76399788	76399796	76399795	A	C	snp	1	1	1	1	.	.	.	INTERGENIC(INTERGENIC);RCHY1(DOWNSTREAM)
4	76581769	76581776	76581775	A	C	snp	1	1	1	1	.	.	.	G3BP2(DOWNSTREAM);G3BP2(INTRON)
4	76617048	76617059	76617056	AC	G	snp	1	1	0	1	.	.	.	G3BP2(INTRON)
4	76709305	76709324	76709318	TTTA	TA	del	1	0	0	0	.	.	.	USO1(INTRON)
4	76709305	76709324	76709320	TTTA	T	ins	1	0	0	0	.	.	.	USO1(INTRON)
4	76748475	76748484	76748477	G	T	snp	1	0	0	0	.	.	.	INTERGENIC(INTERGENIC);RP11-556N4.1.1(UPSTREAM);U2(DOWNSTREAM)
4	76792610	76792621	76792616	TTC	C	snp	1	1	1	1	.	.	.	PPEF2(INTRON)
4	76819029	76819038	76819033	AC	G	snp	1	1	0	1	.	.	.	PPEF2(INTRON)
4	76848949	76848957	76848954	A	G	snp	1	1	1	1	.	.	.	NAAA(DOWNSTREAM);NAAA(INTRON)
4	76867822	76867830	76867825	A	G	snp	1	0	0	0	.	.	.	SDAD1(DOWNSTREAM);SDAD1(INTRON)
4	76957166	76957177	76957171	TGC	T	snp	1	1	1	1	.	.	.	ART3(INTRON);CXCL11(UTR_5_PRIME);RP11-630D6.1.1(DOWNSTREAM)
4	76973038	76973051	76973047	TC	T	ins	1	1	1	1	.	.	.	ART3(INTRON)

4	76973038	76973051	76973048	TC	CTC	del	1	1	1	1	.	.	.	ART3(INTRON)
4	77022907	77022916	77022911	A	G	snp	1	0	1	0	.	.	.	ART3(DOWNSTREAM);ART3(INTRON)
4	77054201	77054218	77054216	AT	A	del	1	1	1	1	.	.	.	NUP54(DOWNSTREAM);NUP54(INTRON);NUP54(UPSTREAM)
4	77093158	77093169	77093167	TA	TAC	del	1	0	0	0	.	.	.	SCARB2(INTRON)
4	77112879	77112890	77112884	TC	T	snp	0	1	1	1	.	.	.	SCARB2(INTRON)
4	77342831	77342838	77342835	C	T	snp	1	0	0	0	.	.	.	CCDC158(INTRON)
4	77342831	77342838	77342836	C	A	snp	1	0	0	0	.	.	.	CCDC158(INTRON)
4	77432339	77432362	77432342	AAT	AT	del	1	1	1	1	.	.	.	RP11-688H10.1.1(UPSTREAM);SHROOM3(INTRON)
4	77432339	77432362	77432347	AAT	A	snp	1	1	1	1	.	.	.	RP11-688H10.1.1(UPSTREAM);SHROOM3(INTRON)
4	77439263	77439272	77439268	TG	T	snp	1	1	1	1	.	.	.	SHROOM3(INTRON)
4	77466247	77466256	77466254	T	C	snp	1	1	1	1	.	.	.	SHROOM3(INTRON)
4	77490142	77490149	77490144	A	C	snp	1	1	1	1	.	.	.	SHROOM3(INTRON)
4	77533924	77533943	77533939	TCAA	G	snp	0	1	1	1	.	.	.	SHROOM3(INTRON)
4	77592101	77592116	77592108	AAAT	A	snp	1	0	0	0	.	.	.	SHROOM3(INTRON)
4	77652941	77652960	77652947	AAAC	C	del	1	1	1	1	.	.	.	SHROOM3(INTRON)
4	77677559	77677570	77677568	TC	G	snp	1	0	0	0	.	.	.	RP11-359D14.2.1(DOWNSTREAM);RP11-359D14.3.1(DOWNSTREAM);SHROOM3(DOWNSTREAM);SHROOM3(INTRON)
4	77904107	77904116	77904112	T	G	snp	1	1	1	1	.	.	.	SEPT11(INTRON);SEPT11(UPSTREAM)
4	77982813	77982824	77982820	TTG	C	snp	1	0	0	0	.	.	.	CCNI(DOWNSTREAM);CCNI(INTRON)
4	77982813	77982824	77982821	TTG	T	snp	1	0	0	0	.	.	.	CCNI(DOWNSTREAM);CCNI(INTRON)
4	78016349	78016356	78016353	T	C	snp	1	1	1	1	.	.	.	INTERGENIC(INTERGENIC)
4	78071879	78071886	78071885	C	A	snp	1	1	1	1	.	.	.	INTERGENIC(INTERGENIC);U6(UPSTREAM)
4	78100069	78100088	78100084	TTTC	T	ins	1	1	1	1	.	.	.	CCNG2(INTRON)
4	78100089	78100097	78100093	T	C	ins	0	1	1	1	.	.	.	CCNG2(INTRON)
4	78454096	78454104	78454103	T	C	ins	1	0	0	0	.	.	.	CXCL13(INTRON)
4	78530455	78530464	78530460	T	G	snp	1	0	0	0	.	.	.	CXCL13(INTRON)
4	78626078	78626085	78626082	T	G	snp	1	1	1	1	.	.	.	INTERGENIC(INTERGENIC);SNORD75(UPSTREAM)
4	78768227	78768234	78768230	A	G	snp	1	1	1	1	.	.	.	INTERGENIC(INTERGENIC)
4	78821505	78821514	78821509	GA	T	snp	1	0	0	0	.	.	.	MRPL1(INTRON)
4	78886654	78886663	78886657	TA	TG	ins	1	0	0	0	.	.	.	HMGB1P44(DOWNSTREAM);INTERGENIC(INTERGENIC);MRPL1(DOWNSTREAM)
4	78966272	78966287	78966285	TG	T	del	1	1	1	1	.	.	.	INTERGENIC(INTERGENIC)
4	78977669	78977678	78977671	AC	G	snp	1	1	0	1	.	.	.	FRAS1(UPSTREAM);INTERGENIC(INTERGENIC)
4	78980267	78980278	78980272	GGC	T	snp	1	1	1	1	.	.	.	FRAS1(INTRON)
4	79021920	79021931	79021923	TA	G	snp	1	0	1	0	.	.	.	FRAS1(INTRON)
4	79048789	79048816	79048808	ACAA	AC	del	1	1	1	1	.	.	.	FRAS1(INTRON)
4	79048789	79048816	79048810	ACAA	A	del	1	1	1	1	.	.	.	FRAS1(INTRON)
4	79061101	79061109	79061103	T	C	snp	1	0	0	0	.	.	.	FRAS1(INTRON)
4	79157442	79157465	79157461	ATTT	A	del	0	0	1	0	.	.	.	FRAS1(INTRON)
4	79164343	79164354	79164346	TA	C	snp	1	1	1	1	.	.	.	FRAS1(INTRON)
4	79164343	79164354	79164348	TA	C	snp	1	1	1	1	.	.	.	FRAS1(INTRON)
4	79211911	79211926	79211917	TTTG	C	snp	1	1	1	1	.	.	.	FRAS1(DOWNSTREAM);FRAS1(INTRON)
4	79268929	79268942	79268940	GT	G	del	1	1	1	1	.	.	.	FRAS1(INTRON)
4	79312189	79312196	79312191	T	G	snp	1	0	0	0	.	.	.	FRAS1(INTRON)
4	79415164	79415172	79415167	T	G	snp	1	0	0	0	.	.	.	FRAS1(INTRON)
4	79441561	79441568	79441565	A	C	snp	1	0	0	0	.	.	.	FRAS1(INTRON)
4	79535228	79535235	79535234	T	G	snp	1	1	1	1	.	.	.	ANXA3(DOWNSTREAM);INTERGENIC(INTERGENIC)
4	79544709	79544720	79544717	TA	G	snp	1	0	0	0	.	.	.	INTERGENIC(INTERGENIC)
4	79608788	79608796	79608790	A	G	snp	1	0	0	0	.	.	.	INTERGENIC(INTERGENIC);RP11-792D21.2.1(DOWNSTREAM)
4	79755379	79755393	79755389	ATC	C	snp	1	0	0	0	.	.	.	BMP2K(INTRON)
4	79896322	79896330	79896329	T	C	ins	1	1	1	1	.	.	.	RP11-438E5.1.1(INTRON)
4	79896449	79896458	79896455	T	G	snp	1	0	0	0	.	.	.	RP11-438E5.1.1(INTRON)
4	79922564	79922575	79922567	TTG	C	ins	1	1	1	1	.	.	.	RP11-438E5.1.1(INTRON)
4	80018474	80018494	80018477	TTA	TA	del	1	1	0	1	.	.	.	RP11-438E5.1.1(INTRON)
4	80047862	80047870	80047869	T	G	snp	0	0	1	0	.	.	.	RP11-438E5.1.1(INTRON)
4	80135951	80135959	80135955	A	C	snp	1	0	0	0	.	.	.	RP11-438E5.1.1(INTRON)
4	80274047	80274056	80274052	AG	AGAGACAGAC	ins	1	0	0	0	.	.	.	INTERGENIC(INTERGENIC)
4	80321831	80321842	80321839	AG	C	snp	1	0	0	0	.	.	.	INTERGENIC(INTERGENIC)
4	80540145	80540156	80540147	AC	G	snp	0	0	1	0	.	.	.	INTERGENIC(INTERGENIC)
4	80619170	80619183	80619172	GT	C	snp	0	1	0	1	.	.	.	INTERGENIC(INTERGENIC);RP11-452C8.1.1(DOWNSTREAM)
4	80643333	80643342	80643337	AT	G	snp	1	1	1	1	.	.	.	INTERGENIC(INTERGENIC)
4	80686869	80686884	80686879	TAAA	C	snp	1	1	1	1	.	.	.	INTERGENIC(INTERGENIC)
4	80701101	80701108	80701105	A	AAAGAAAGAAGG	ins	1	1	1	1	.	.	.	INTERGENIC(INTERGENIC)
4	80779824	80779831	80779827	T	C	snp	0	1	1	1	.	.	.	RP11-610O8.1.1(INTRON)
4	80935724	80935735	80935733	AT	A	snp	1	1	0	1	.	.	.	ANTXR2(INTRON)
4	80970611	80970620	80970614	AT	C	snp	1	0	0	0	.	.	.	ANTXR2(DOWNSTREAM);ANTXR2(INTRON)

4	81074664	81074675	81074671	TA	G	snp	0	1	0	1	.	.	.	INTERGENIC(INTERGENIC)
4	81106118	81106125	81106124	T	C	snp	1	1	1	1	.	.	.	PRDM8(INTRON);PRDM8(UPSTREAM);RP11-377G16.2.1(INTRON)
4	81124893	81124901	81124894	C	A	snp	0	1	0	1	.	.	.	PRDM8(DOWNSTREAM);PRDM8(UTR_3_PRIME)
4	81124893	81124901	81124900	C	A	snp	1	1	1	1	.	.	.	PRDM8(DOWNSTREAM);PRDM8(UTR_3_PRIME)
4	81144043	81144052	81144048	T	G	snp	1	0	0	0	.	.	.	INTERGENIC(INTERGENIC)
4	81150040	81150051	81150045	TG	A	snp	0	1	0	1	.	.	.	INTERGENIC(INTERGENIC)
4	81181465	81181472	81181468	A	G	snp	1	1	0	1	.	.	.	INTERGENIC(INTERGENIC)
4	81188888	81188911	81188894	TAGA	A	snp	1	0	0	0	.	.	.	FGF5(INTRON);FGF5(UPSTREAM)
4	81247411	81247422	81247419	GT	A	snp	1	1	1	1	.	.	.	FGF5(INTRON)
4	81283671	81283679	81283674	T	C	ins	1	1	1	0	.	.	.	C4orf22(INTRON)
4	81384043	81384050	81384048	A	C	snp	0	0	1	0	.	.	.	C4orf22(INTRON)
4	81398086	81398097	81398093	TG	A	snp	0	0	1	0	.	.	.	C4orf22(INTRON)
4	81525489	81525497	81525496	A	C	snp	1	1	1	1	.	.	.	C4orf22(INTRON)
4	81590286	81590305	81590301	TCTT	TC	del	1	1	1	1	.	.	.	C4orf22(INTRON)
4	81605830	81605837	81605835	T	G	snp	1	0	0	0	.	.	.	C4orf22(INTRON)
4	81644064	81644072	81644066	A	AC	ins	1	1	0	1	.	.	.	C4orf22(INTRON)
4	81678858	81678881	81678876	TTTG	G	ins	1	0	0	0	.	.	.	C4orf22(INTRON)
4	81708390	81708401	81708396	AT	T	snp	1	1	0	1	.	.	.	C4orf22(INTRON)
4	81720492	81720501	81720494	TA	G	snp	1	1	1	1	.	.	.	C4orf22(INTRON)
4	81721941	81721952	81721947	AT	TG	ins	1	0	0	0	.	.	.	C4orf22(INTRON)
4	81764223	81764234	81764231	TA	C	snp	1	0	0	0	.	.	.	C4orf22(INTRON)
4	81785573	81785582	81785577	TA	C	snp	1	0	0	0	.	.	.	C4orf22(INTRON)
4	81785585	81785602	81785595	TA	C	snp	1	0	0	0	.	.	.	C4orf22(INTRON)
4	81803215	81803223	81803217	A	G	snp	1	0	0	0	.	.	.	C4orf22(INTRON)
4	81821907	81821924	81821915	AAT	C	snp	1	0	0	0	.	.	.	C4orf22(INTRON)
4	81890124	81890137	81890134	CA	A	snp	0	1	0	0	.	.	.	INTERGENIC(INTERGENIC)
4	82022871	82022879	82022876	A	G	snp	1	1	1	0	.	.	.	PRKG2(INTRON)
4	82025816	82025827	82025825	TA	G	snp	1	1	1	1	.	.	.	PRKG2(INTRON)
4	82065749	82065764	82065761	TA	A	del	0	1	0	1	.	.	.	PRKG2(INTRON)
4	82089196	82089203	82089202	A	C	ins	1	1	1	1	.	.	.	PRKG2(INTRON);RP11-100N20.1.1(INTRON)
4	82110536	82110544	82110537	A	C	snp	1	1	1	1	.	.	.	PRKG2(INTRON);RP11-100N20.1.1(INTRON)
4	82124234	82124243	82124238	TA	C	snp	1	1	1	1	.	.	.	PRKG2(INTRON)
4	82172577	82172586	82172581	CT	G	snp	1	0	0	0	.	.	.	INTERGENIC(INTERGENIC)
4	82184142	82184151	82184148	AG	G	snp	1	0	0	0	.	.	.	INTERGENIC(INTERGENIC)
4	82348574	82348581	82348580	A	AC	ins	1	0	0	0	.	.	.	RASGEF1B(DOWNSTREAM);RASGEF1B(UTR_3_PRIME)
4	82367118	82367127	82367125	AG	C	snp	1	0	0	0	.	.	.	RASGEF1B(DOWNSTREAM);RASGEF1B(INTRON)
4	82409792	82409807	82409805	TC	TT	ins	1	1	1	1	.	.	.	RP11-689K5.3.1(INTRON)
4	82436627	82436635	82436634	T	G	snp	1	0	0	0	.	.	.	RP11-689K5.3.1(INTRON)
4	82445487	82445495	82445490	T	C	snp	1	0	1	1	.	.	.	RP11-689K5.3.1(INTRON)
4	82812065	82812080	82812075	AATA	C	snp	1	1	1	1	.	.	.	RP11-689K5.3.1(INTRON)
4	82859156	82859164	82859162	A	G	snp	1	0	0	0	.	.	.	RP11-689K5.3.1(INTRON)
4	82860248	82860255	82860252	A	G	snp	1	1	1	1	.	.	.	RP11-689K5.3.1(INTRON)
4	82863561	82863572	82863569	AG	T	snp	1	0	0	0	.	.	.	RP11-689K5.3.1(INTRON)
4	82964503	82964522	82964509	AGCC	A	snp	1	1	1	1	.	.	.	RP11-689K5.3.1(INTRON)
4	83052227	83052238	83052235	AT	G	snp	0	1	1	1	.	.	.	INTERGENIC(INTERGENIC);RP11-263F19.1.1(DOWNSTREAM)
4	83069525	83069532	83069529	A	AAAG	ins	1	0	0	0	.	.	.	INTERGENIC(INTERGENIC)
4	83124762	83124770	83124764	T	TG	ins	1	1	1	1	.	.	.	INTERGENIC(INTERGENIC)
4	83139038	83139051	83139046	AC	G	snp	1	0	0	0	.	.	.	INTERGENIC(INTERGENIC)
4	83151887	83151896	83151890	AT	C	snp	1	0	1	0	.	.	.	INTERGENIC(INTERGENIC)
4	83153828	83153835	83153833	A	G	snp	1	0	0	0	.	.	.	INTERGENIC(INTERGENIC)
4	83181106	83181113	83181107	T	G	snp	0	1	0	1	.	.	.	INTERGENIC(INTERGENIC)
4	83200405	83200412	83200409	A	AAAC	ins	1	0	0	0	.	.	.	INTERGENIC(INTERGENIC);RP11-429B11.1.1(DOWNSTREAM)
4	83240102	83240119	83240113	AAG	AA	del	1	1	0	1	.	.	.	INTERGENIC(INTERGENIC)
4	83240102	83240119	83240116	AAG	A	del	1	1	0	1	.	.	.	INTERGENIC(INTERGENIC)
4	83251777	83251785	83251780	A	G	snp	1	1	0	1	.	.	.	INTERGENIC(INTERGENIC)
4	83252269	83252282	83252277	TA	C	ins	1	0	0	0	.	.	.	INTERGENIC(INTERGENIC)
4	83440564	83440587	83440582	TTTC	A	snp	1	1	1	1	.	.	.	TMEM150C(INTRON)
4	83449720	83449729	83449723	CT	C	snp	0	0	0	1	.	.	.	TMEM150C(INTRON)
4	83588423	83588440	83588437	AC	G	snp	1	1	1	1	.	.	.	SCD5(INTRON)
4	83651417	83651426	83651419	TA	C	snp	1	1	1	1	.	.	.	SCD5(INTRON)
4	83663007	83663016	83663012	G	T	ins	1	0	1	0	.	.	.	SCD5(INTRON)
4	83787257	83787272	83787267	AGGG	T	snp	1	1	1	1	.	.	.	SEC31A(INTRON);SEC31A(UPSTREAM)
4	83787303	83787314	83787307	AG	G	snp	1	1	1	1	.	.	.	SEC31A(INTRON);SEC31A(UPSTREAM)
4	83801337	83801346	83801343	A	G	snp	1	1	1	1	.	.	.	SEC31A(DOWNSTREAM);SEC31A(INTRON)

4	83982196	83982216	83982201	AAT	A	snp	1	0	0	0	.	.	.	COPS4(DOWNSTREAM);COPS4(INTRON);COPS4(UPSTREAM)
4	84003234	84003242	84003241	G	T	snp	0	1	1	0	.	.	.	INTERGENIC(INTERGENIC)
4	84100881	84100900	84100894	TATC	TC	del	1	1	1	1	.	.	.	INTERGENIC(INTERGENIC)
4	84132193	84132216	84132207	ATTT	C	snp	1	1	1	1	.	.	.	INTERGENIC(INTERGENIC)
4	84240326	84240335	84240327	A	AC	ins	1	1	1	1	.	.	.	HPSE(INTRON)
4	84349505	84349512	84349511	A	G	snp	1	1	1	1	.	.	.	HELQ(INTRON);HELQ(UPSTREAM)
4	84366729	84366740	84366737	TAT	C	snp	1	0	0	0	.	.	.	HELQ(INTRON)
4	84395137	84395145	84395139	A	C	snp	0	0	0	1	.	.	.	FAM175A(DOWNSTREAM);FAM175A(INTRON);FAM175A(UPSTREAM);MRPS18C(DOWNSTREAM);RP11-722P15.2.1(UPSTREAM)
4	84415248	84415255	84415251	A	G	snp	1	1	1	1	.	.	.	FAM175A(INTRON)
4	84442318	84442327	84442321	TC	T	snp	1	1	1	1	.	.	.	FAM175A(INTRON)
4	84483379	84483388	84483385	AT	G	snp	1	0	0	0	.	.	.	AGPAT9(INTRON)
4	84558955	84558964	84558958	T	C	snp	1	1	1	1	.	.	.	INTERGENIC(INTERGENIC);Y_RNA(UPSTREAM)
4	84775653	84775667	84775664	ACA	C	snp	1	0	0	0	.	.	.	RP11-8L2.1.1(INTRON)
4	84805992	84806001	84805993	A	G	snp	1	1	1	1	.	.	.	RP11-8L2.1.1(INTRON)
4	84846212	84846227	84846220	AAAT	T	snp	1	0	0	0	.	.	.	RP11-8L2.1.1(INTRON)
4	84970965	84970974	84970969	GT	A	snp	1	1	1	1	.	.	.	RP11-8L2.1.1(INTRON)
4	85038008	85038016	85038012	T	C	ins	1	0	0	0	.	.	.	RP11-8L2.1.1(INTRON)
4	85043350	85043365	85043353	AT	C	snp	1	0	0	0	.	.	.	RP11-8L2.1.1(INTRON)
4	85372869	85372882	85372878	TG	A	snp	1	1	1	1	.	.	.	INTERGENIC(INTERGENIC)
4	85393226	85393234	85393233	T	C	snp	1	0	0	0	.	.	.	INTERGENIC(INTERGENIC)
4	85720479	85720492	85720489	AT	T	ins	0	0	1	0	.	.	.	WDFY3(DOWNSTREAM);WDFY3(INTRON);WDFY3-AS1(UPSTREAM)
4	85720479	85720492	85720490	AT	A	del	1	1	1	1	.	.	.	WDFY3(DOWNSTREAM);WDFY3(INTRON);WDFY3-AS1(UPSTREAM)
4	85771477	85771485	85771484	T	G	ins	1	1	1	1	.	.	.	WDFY3(INTRON)
4	85835598	85835606	85835602	A	C	snp	1	1	1	1	.	.	.	WDFY3(INTRON)
4	85853400	85853409	85853402	A	C	snp	1	0	0	0	.	.	.	WDFY3(INTRON)
4	85917021	85917032	85917029	ATC	G	snp	1	0	0	0	.	.	.	WDFY3-AS2(INTRON)
4	86023524	86023531	86023528	T	G	snp	1	1	1	1	.	.	.	7SK(DOWNSTREAM);INTERGENIC(INTERGENIC)
4	86128671	86128684	86128682	AC	T	snp	1	1	1	1	.	.	.	INTERGENIC(INTERGENIC)
4	86262013	86262022	86262020	T	C	snp	1	1	1	0	.	.	.	INTERGENIC(INTERGENIC)
4	86285744	86285752	86285751	T	C	snp	1	1	1	1	.	.	.	INTERGENIC(INTERGENIC)
4	86377600	86377609	86377603	TA	C	snp	1	0	1	1	.	.	.	INTERGENIC(INTERGENIC)
4	86502894	86502901	86502897	T	C	snp	1	1	1	1	.	.	.	ARHGAP24(INTRON)
4	86542767	86542775	86542769	T	C	snp	0	1	0	1	.	.	.	ARHGAP24(INTRON)
4	86590176	86590189	86590181	TG	T	snp	0	1	1	1	.	.	.	ARHGAP24(INTRON)
4	86604271	86604280	86604278	G	T	snp	1	0	0	0	.	.	.	ARHGAP24(INTRON)
4	86668862	86668881	86668876	AAAC	G	snp	1	0	0	0	.	.	.	ARHGAP24(INTRON)
4	86937093	86937110	86937096	ATT	C	snp	1	1	1	1	.	.	.	MAPK10(DOWNSTREAM);MAPK10(UTR_3_PRIME);RP13-514E23.1.1(UPSTREAM)
4	86937093	86937110	86937099	ATT	C	snp	1	1	0	1	.	.	.	MAPK10(DOWNSTREAM);MAPK10(UTR_3_PRIME);RP13-514E23.1.1(UPSTREAM)
4	87141503	87141512	87141511	A	C	snp	1	1	1	1	.	.	.	MAPK10(INTRON);RP11-778J15.1.1(DOWNSTREAM)
4	87237528	87237537	87237533	AG	C	snp	1	0	0	0	.	.	.	MAPK10(INTRON)
4	87259262	87259271	87259268	CA	T	snp	1	1	1	1	.	.	.	7SK(UPSTREAM);MAPK10(INTRON)
4	87549093	87549101	87549096	T	C	snp	1	1	1	1	.	.	.	PTPN13(INTRON)
4	87659237	87659246	87659244	T	C	snp	0	1	0	1	.	.	.	PTPN13(INTRON)
4	87711691	87711708	87711693	CA	T	snp	1	1	0	1	.	.	.	PTPN13(INTRON)
4	87823609	87823616	87823612	A	G	snp	0	1	0	1	.	.	.	C4orf36(INTRON)
4	87869210	87869218	87869217	T	G	snp	0	0	0	1	.	.	.	AFF1(INTRON);RP11-397E7.3.1(DOWNSTREAM)
4	87870316	87870333	87870331	AT	A	del	1	1	1	1	.	.	.	AFF1(INTRON);RP11-397E7.3.1(DOWNSTREAM)
4	87894216	87894225	87894219	TG	T	snp	0	0	0	1	.	.	.	AFF1(INTRON)
4	87926960	87926968	87926967	A	AT	ins	1	0	0	0	.	.	.	AFF1(INTRON);AFF1(UPSTREAM)
4	87941037	87941045	87941042	G	GA	ins	1	1	1	1	.	.	.	AFF1(INTRON)
4	88147928	88147937	88147931	TA	T	snp	1	1	1	1	.	.	.	RP11-476C8.3.1(INTRON)
4	88159665	88159674	88159671	CA	T	snp	0	1	0	1	.	.	.	RP11-476C8.3.1(INTRON)
4	88218142	88218161	88218148	AAAT	T	del	1	1	0	1	.	.	.	INTERGENIC(INTERGENIC)
4	88218142	88218161	88218149	AAAT	A	snp	1	1	0	1	.	.	.	INTERGENIC(INTERGENIC)
4	88226474	88226482	88226480	A	C	snp	1	1	1	1	.	.	.	HSD17B13(INTRON)
4	88255631	88255642	88255639	TTC	TTCT	del	1	0	0	0	.	.	.	HSD17B11(DOWNSTREAM);RP11-529H2.2.1(INTRON)
4	88299950	88299963	88299960	TA	A	snp	1	0	0	0	.	.	.	HSD17B11(INTRON);HSD17B11(UPSTREAM)
4	88299984	88299995	88299993	AT	A	snp	1	1	1	1	.	.	.	HSD17B11(INTRON);HSD17B11(UPSTREAM)
4	88300034	88300045	88300043	AT	A	snp	1	1	1	1	.	.	.	HSD17B11(INTRON);HSD17B11(UPSTREAM)
4	88300058	88300069	88300064	AT	G	snp	1	1	1	1	.	.	.	HSD17B11(INTRON);HSD17B11(UPSTREAM)
4	88333459	88333466	88333461	C	A	snp	1	1	1	1	.	.	.	RP11-710E1.1.1(DOWNSTREAM);Y_RNA(EXON)
4	88402967	88402976	88402969	AC	G	snp	1	1	1	1	.	.	.	SPARCL1(INTRON)

4	88464307	88464322	88464316	TC	T	snp	1	1	1	1	.	.	.	INTERGENIC(INTERGENIC)	
4	88501149	88501157	88501154	A	G	ins	0	0	1	0	.	.	.	RP11-742B18.1.1(INTRON)	
4	88511444	88511471	88511460	GAAA	AA	ins	1	1	1	1	.	.	.	RP11-742B18.1.1(INTRON)	
4	88511444	88511471	88511464	GAAA	A	ins	1	1	1	1	.	.	.	RP11-742B18.1.1(INTRON)	
4	88511444	88511471	88511467	GAAA	G	del	1	1	1	1	.	.	.	RP11-742B18.1.1(INTRON)	
4	88566303	88566312	88566310	AT	C	snp	1	0	0	0	.	.	.	RP11-742B18.1.1(INTRON)	
4	88728179	88728187	88728181	A	C	snp	1	1	1	1	.	.	.	IBSP(INTRON)	
4	88776952	88776969	88776960	CAC	T	snp	1	1	0	1	.	.	.	INTERGENIC(INTERGENIC)	
4	88777110	88777117	88777116	G	A	snp	1	1	0	1	.	.	.	INTERGENIC(INTERGENIC)	
4	88789031	88789040	88789034	TA	G	snp	1	1	1	1	.	.	.	INTERGENIC(INTERGENIC)	
4	88824445	88824454	88824447	T	G	ins	1	1	1	1	.	.	.	INTERGENIC(INTERGENIC)	
4	88824445	88824454	88824450	T	G	snp	1	0	0	0	.	.	.	INTERGENIC(INTERGENIC)	
4	88978135	88978143	88978139	T	G	snp	0	1	1	1	.	.	.	PKD2(INTRON)	
4	89025817	89025826	89025818	A	G	ins	1	0	0	0	.	.	.	ABCG2(INTRON)	
4	89053657	89053664	89053658	A	C	snp	1	0	1	1	.	.	.	ABCG2(INTRON)	
4	89112239	89112247	89112240	A	C	snp	0	1	0	1	.	.	.	ABCG2(INTRON)	
4	89238364	89238373	89238369	TG	C	snp	1	1	1	1	.	.	.	RP11-10L7.1.1(INTRON)	
4	89261406	89261421	89261410	AAAT	G	snp	1	1	0	1	.	.	.	INTERGENIC(INTERGENIC)	
4	89262723	89262732	89262730	AC	A	snp	1	0	0	0	.	.	.	INTERGENIC(INTERGENIC)	
4	89295158	89295165	89295163	T	C	snp	1	1	1	1	.	.	.	HERC6(UPSTREAM);INTERGENIC(INTERGENIC)	
4	89640617	89640631	89640628	CTC	CTCCTTCCCTT	ins	1	1	1	1	.	.	.	.	FAM13A-AS1(INTRON);FAM13A-AS1(UPSTREAM)
4	89640640	89640654	89640651	CTC	CT	del	1	1	1	1	.	.	.	FAM13A-AS1(INTRON);FAM13A-AS1(UPSTREAM)	
4	89725404	89725419	89725414	AAAT	G	snp	1	0	0	0	.	.	.	FAM13A(INTRON)	
4	89746819	89746826	89746821	A	G	snp	1	1	1	1	.	.	.	FAM13A(INTRON);FAM13A(UPSTREAM)	
4	89791648	89791655	89791654	G	T	snp	0	1	0	1	.	.	.	FAM13A(INTRON)	
4	89805355	89805362	89805356	A	C	ins	0	0	1	0	.	.	.	FAM13A(INTRON)	
4	89808673	89808688	89808681	CTTT	T	ins	1	1	1	1	.	.	.	FAM13A(INTRON)	
4	89808673	89808688	89808683	CTTT	TC	del	1	1	1	1	.	.	.	FAM13A(INTRON)	
4	89808673	89808688	89808684	CTTT	C	del	1	1	1	1	.	.	.	FAM13A(INTRON)	
4	89850638	89850645	89850640	T	C	ins	1	0	0	0	.	.	.	FAM13A(INTRON)	
4	89916244	89916253	89916251	CA	G	snp	1	1	1	1	.	.	.	FAM13A(INTRON)	
4	89948500	89948507	89948501	T	G	snp	0	0	0	1	.	.	.	FAM13A(INTRON)	
4	90018264	90018275	90018272	AT	G	snp	1	1	1	1	.	.	.	FAM13A(INTRON)	
4	90078267	90078276	90078273	TA	C	snp	1	1	1	1	.	.	.	INTERGENIC(INTERGENIC)	
4	90089019	90089030	90089028	TA	G	snp	1	0	0	0	.	.	.	INTERGENIC(INTERGENIC)	
4	90167249	90167256	90167253	T	C	snp	1	1	1	1	.	.	.	GPRIN3(UTR_3_PRIME)	
4	90186884	90186899	90186892	CCTT	T	snp	1	0	0	0	.	.	.	GPRIN3(INTRON)	
4	90202209	90202216	90202214	G	T	ins	1	0	0	0	.	.	.	GPRIN3(INTRON)	
4	90295627	90295635	90295628	A	AAG	ins	1	1	1	0	.	.	.	INTERGENIC(INTERGENIC)	
4	90513674	90513682	90513676	T	G	snp	0	1	1	1	.	.	.	RP11-115D19.1.1(INTRON)	
4	90717436	90717451	90717438	AT	T	del	1	0	0	0	.	.	.	SNCA(INTRON)	
4	90744152	90744172	90744169	ATT	A	del	1	0	0	1	.	.	.	SNCA(INTRON)	
4	90780804	90780815	90780812	TA	A	snp	1	1	1	0	.	.	.	INTERGENIC(INTERGENIC)	
4	90837525	90837540	90837531	AAAC	C	del	1	1	1	1	.	.	.	MMRN1(INTRON)	
4	90837525	90837540	90837532	AAAC	A	snp	1	1	1	1	.	.	.	MMRN1(INTRON)	
4	90841976	90841983	90841979	T	G	snp	1	0	0	0	.	.	.	MMRN1(INTRON)	
4	90919239	90919248	90919246	A	C	snp	0	1	0	0	.	.	.	INTERGENIC(INTERGENIC)	
4	90975817	90975828	90975820	AAT	G	snp	0	1	0	0	.	.	.	INTERGENIC(INTERGENIC)	
4	91017520	91017529	91017525	TA	G	snp	1	0	0	0	.	.	.	INTERGENIC(INTERGENIC)	
4	91194158	91194173	91194165	AAAC	A	ins	1	0	0	0	.	.	.	FAM190A(INTRON)	
4	91194158	91194173	91194167	AAAC	AC	del	1	0	0	0	.	.	.	FAM190A(INTRON)	
4	91194158	91194173	91194169	AAAC	A	ins	1	0	0	0	.	.	.	FAM190A(INTRON)	
4	91194174	91194182	91194178	A	C	snp	1	0	0	0	.	.	.	FAM190A(INTRON)	
4	91217156	91217164	91217162	A	C	snp	1	0	0	0	.	.	.	FAM190A(INTRON)	
4	91275652	91275661	91275654	AC	G	snp	1	1	1	1	.	.	.	FAM190A(INTRON)	
4	91293912	91293921	91293917	A	G	snp	0	1	0	0	.	.	.	7SK(DOWNSTREAM);FAM190A(INTRON)	
4	91386870	91386879	91386873	TG	A	snp	1	0	0	0	.	.	.	FAM190A(INTRON)	
4	91451088	91451103	91451100	AT	T	ins	1	0	0	0	.	.	.	FAM190A(INTRON)	
4	91451088	91451103	91451101	AT	A	del	1	1	1	1	.	.	.	FAM190A(INTRON)	
4	91453155	91453168	91453166	TA	TT	ins	1	0	0	0	.	.	.	FAM190A(INTRON)	
4	91609469	91609478	91609473	TG	C	snp	1	0	0	0	.	.	.	FAM190A(INTRON)	
4	91628690	91628707	91628693	AT	A	snp	1	1	1	1	.	.	.	FAM190A(INTRON)	
4	91668788	91668797	91668791	T	C	snp	1	1	1	1	.	.	.	FAM190A(INTRON)	
4	91724824	91724835	91724830	TG	C	snp	1	1	1	1	.	.	.	FAM190A(INTRON)	
4	91744382	91744395	91744390	AC	T	snp	1	0	0	0	.	.	.	FAM190A(INTRON)	
4	91789715	91789724	91789722	TG	A	snp	1	0	0	0	.	.	.	FAM190A(INTRON)	
4	91839790	91839805	91839793	AT	AA	ins	1	1	1	1	.	.	.	FAM190A(INTRON)	

4	91839790	91839805	91839793	AT	A	snp	1	1	1	1	.	.	.	FAM190A(INTRON)
4	91863110	91863117	91863114	T	G	snp	0	1	1	0	.	.	.	FAM190A(INTRON)
4	91927409	91927417	91927412	T	G	snp	1	0	0	1	.	.	.	FAM190A(INTRON)
4	91975854	91975862	91975861	T	G	snp	1	1	1	1	.	.	.	FAM190A(INTRON)
4	91980462	91980473	91980465	TA	TATATATACATATAGTGTATATATATGTG	ins	1	1	1	1	.	.	.	FAM190A(INTRON)
4	92081907	92081914	92081909	T	G	snp	1	0	1	0	.	.	.	FAM190A(INTRON)
4	92209300	92209313	92209310	TA	C	snp	1	0	0	0	.	.	.	FAM190A(INTRON)
4	92334104	92334113	92334105	A	AC	ins	1	1	1	1	.	.	.	FAM190A(INTRON)
4	92334104	92334113	92334106	A	C	ins	1	1	1	1	.	.	.	FAM190A(INTRON)
4	92380502	92380513	92380509	AT	C	snp	1	1	0	1	.	.	.	FAM190A(INTRON)
4	92417875	92417888	92417884	AT	A	snp	1	1	1	1	.	.	.	FAM190A(INTRON)
4	92418017	92418026	92418023	AT	G	snp	1	1	1	1	.	.	.	FAM190A(INTRON)
4	92429144	92429161	92429148	TA	G	snp	1	0	0	0	.	.	.	FAM190A(INTRON)
4	92535446	92535454	92535447	A	C	ins	1	0	0	0	.	.	.	INTERGENIC(INTERGENIC)
4	92584491	92584502	92584494	GCA	A	snp	1	0	0	0	.	.	.	INTERGENIC(INTERGENIC)
4	92603418	92603429	92603422	CTA	C	snp	1	1	1	1	.	.	.	INTERGENIC(INTERGENIC)
4	92653785	92653799	92653796	TTC	T	del	1	0	0	0	.	.	.	INTERGENIC(INTERGENIC)
4	92687335	92687344	92687338	AT	AC	ins	1	1	1	1	.	.	.	INTERGENIC(INTERGENIC)
4	92815262	92815270	92815267	A	C	ins	1	1	1	1	.	.	.	RP11-354O24.1.1(INTRON)
4	92850624	92850632	92850630	C	A	ins	1	1	1	1	.	.	.	INTERGENIC(INTERGENIC)
4	92867381	92867388	92867382	T	C	ins	1	0	0	0	.	.	.	INTERGENIC(INTERGENIC)
4	92905957	92905974	92905965	TA	A	ins	1	1	1	1	.	.	.	INTERGENIC(INTERGENIC)
4	92905957	92905974	92905966	TA	T	del	1	1	1	1	.	.	.	INTERGENIC(INTERGENIC)
4	92905957	92905974	92905972	TA	GG	ins	1	1	1	1	.	.	.	INTERGENIC(INTERGENIC)
4	92920134	92920147	92920144	TA	C	snp	1	0	0	0	.	.	.	INTERGENIC(INTERGENIC)
4	92920178	92920187	92920184	TA	C	snp	1	1	1	1	.	.	.	INTERGENIC(INTERGENIC)
4	92920217	92920226	92920223	AT	G	snp	1	1	0	0	.	.	.	INTERGENIC(INTERGENIC)
4	93071516	93071524	93071522	A	G	snp	1	0	0	0	.	.	.	INTERGENIC(INTERGENIC)
4	93092923	93092931	93092927	A	G	snp	1	0	1	0	.	.	.	INTERGENIC(INTERGENIC)
4	93173814	93173821	93173817	T	G	snp	1	0	0	0	.	.	.	INTERGENIC(INTERGENIC)
4	93313692	93313701	93313696	AT	G	snp	0	1	1	1	.	.	.	GRID2(INTRON)
4	93373993	93374004	93374001	AT	T	snp	0	1	0	1	.	.	.	GRID2(INTRON)
4	93452061	93452068	93452066	G	A	snp	0	1	1	0	.	.	.	GRID2(INTRON)
4	93499271	93499285	93499276	TTA	C	snp	1	1	1	1	.	.	.	GRID2(INTRON)
4	93556011	93556028	93556024	AG	AGAA	ins	1	1	1	1	.	.	.	GRID2(INTRON)
4	93585616	93585627	93585623	AT	C	snp	0	0	1	0	.	.	.	GRID2(INTRON)
4	93625918	93625937	93625932	CCCT	T	snp	0	1	1	1	.	.	.	AC022317.1(DOWNSTREAM);GRID2(INTRON);MTND1P19(DOWNSTREAM)
4	93651050	93651061	93651057	AAC	G	snp	0	1	1	1	.	.	.	GRID2(INTRON)
4	93761618	93761626	93761619	T	C	snp	1	1	1	1	.	.	.	GRID2(INTRON);RP11-428L21.1.1(UPSTREAM)
4	93817903	93817912	93817905	TG	G	snp	1	0	0	0	.	.	.	GRID2(INTRON)
4	93990160	93990169	93990164	AG	G	snp	0	1	0	0	.	.	.	GRID2(INTRON)
4	94084495	94084504	94084503	T	G	snp	1	1	1	1	.	.	.	GRID2(INTRON)
4	94133210	94133219	94133218	A	C	snp	0	1	0	1	.	.	.	GRID2(INTRON)
4	94154465	94154488	94154482	ATT	A	del	1	1	1	0	.	.	.	GRID2(INTRON)
4	94154465	94154488	94154485	ATT	A	del	1	1	1	1	.	.	.	GRID2(INTRON)
4	94163861	94163870	94163865	GA	A	snp	1	0	0	0	.	.	.	GRID2(DOWNSTREAM);GRID2(INTRON)
4	94169238	94169255	94169253	AC	A	del	1	0	0	0	.	.	.	GRID2(INTRON)
4	94181550	94181558	94181556	A	G	ins	1	1	1	1	.	.	.	GRID2(INTRON)
4	94191789	94191797	94191793	T	G	snp	1	0	1	0	.	.	.	GRID2(INTRON)
4	94196583	94196600	94196595	AT	G	snp	1	1	0	1	.	.	.	GRID2(INTRON)
4	94252278	94252287	94252285	C	A	snp	1	1	1	1	.	.	.	GRID2(INTRON)
4	94517729	94517738	94517730	A	C	snp	1	0	0	0	.	.	.	GRID2(INTRON)
4	94613070	94613081	94613077	GT	C	snp	1	0	0	0	.	.	.	GRID2(INTRON)
4	94633159	94633173	94633169	TTG	G	del	1	0	0	0	.	.	.	GRID2(INTRON)
4	94634580	94634607	94634600	ATTT	TTTG	ins	1	0	0	0	.	.	.	GRID2(INTRON)
4	94696056	94696063	94696057	A	C	snp	1	0	0	0	.	.	.	GRID2(DOWNSTREAM);INTERGENIC(INTERGENIC)
4	94732714	94732721	94732716	T	C	snp	1	0	0	0	.	.	.	INTERGENIC(INTERGENIC)
4	94764187	94764202	94764198	TG	T	ins	1	1	1	1	.	.	.	INTERGENIC(INTERGENIC)
4	94764187	94764202	94764199	TG	G	del	1	1	1	1	.	.	.	INTERGENIC(INTERGENIC)
4	94787751	94787758	94787752	T	C	ins	1	1	1	1	.	.	.	INTERGENIC(INTERGENIC)
4	94833867	94833876	94833870	TA	T	snp	1	1	1	1	.	.	.	INTERGENIC(INTERGENIC)
4	94969746	94969755	94969749	AT	C	snp	1	0	0	0	.	.	.	INTERGENIC(INTERGENIC)
4	94992966	94992974	94992969	A	G	snp	1	1	1	1	.	.	.	INTERGENIC(INTERGENIC)
4	94993382	94993390	94993387	A	C	snp	1	1	1	1	.	.	.	INTERGENIC(INTERGENIC)
4	95051164	95051171	95051165	A	AAG	ins	1	0	0	0	.	.	.	RP11-363G15.2.1(INTRON)
4	95057964	95057979	95057973	AAGG	G	snp	1	1	1	1	.	.	.	RP11-363G15.2.1(INTRON)

4	95283085	95283092	95283088	A	G	snp	1	1	1	1	.	.	.	INTERGENIC(INTERGENIC)
4	95347388	95347396	95347389	A	G	snp	1	0	0	0	.	.	.	INTERGENIC(INTERGENIC)
4	95500622	95500630	95500628	A	G	snp	1	1	1	1	.	.	.	PDLIM5(DOWNSTREAM);PDLIM5(INTRON);PDLIM5(UPSTREAM)
4	95516216	95516227	95516221	GA	G	snp	1	0	0	0	.	.	.	PDLIM5(INTRON)
4	95588563	95588570	95588565	T	G	snp	1	1	1	1	.	.	.	PDLIM5(DOWNSTREAM);PDLIM5(UTR_3_PRIME)
4	95631888	95631896	95631889	A	C	ins	1	1	1	1	.	.	.	INTERGENIC(INTERGENIC)
4	95631888	95631896	95631891	A	C	ins	1	1	1	1	.	.	.	INTERGENIC(INTERGENIC)
4	95635039	95635048	95635043	AT	G	snp	1	0	0	0	.	.	.	INTERGENIC(INTERGENIC)
4	95750118	95750127	95750123	GT	A	snp	0	1	1	1	.	.	.	BMPR1B(INTRON)
4	95865791	95865802	95865796	ATG	T	snp	1	1	0	1	.	.	.	BMPR1B(INTRON)
4	95883194	95883209	95883200	TTCT	TTTCTTTCT	del	1	0	0	0	.	.	.	BMPR1B(INTRON)
4	96012422	96012431	96012427	T	G	snp	1	1	1	1	.	.	.	BMPR1B(INTRON);BMPR1B(UPSTREAM)
4	96016184	96016195	96016187	TA	G	snp	1	0	0	0	.	.	.	BMPR1B(INTRON)
4	96191038	96191045	96191041	T	C	snp	1	0	0	0	.	.	.	UNC5C(INTRON)
4	96277370	96277379	96277375	A	C	snp	1	1	0	1	.	.	.	UNC5C(INTRON)
4	96367115	96367122	96367118	A	C	ins	1	1	1	1	.	.	.	UNC5C(INTRON)
4	96367115	96367122	96367121	A	C	ins	1	1	1	1	.	.	.	UNC5C(INTRON)
4	96367142	96367149	96367147	A	C	snp	1	1	1	1	.	.	.	UNC5C(INTRON)
4	96473308	96473323	96473319	TG	A	snp	1	0	0	0	.	.	.	INTERGENIC(INTERGENIC);UNC5C(UPSTREAM)
4	96527738	96527747	96527746	T	TA	ins	1	1	1	1	.	.	.	INTERGENIC(INTERGENIC)
4	96580900	96580911	96580908	CA	A	snp	0	1	0	1	.	.	.	INTERGENIC(INTERGENIC)
4	96684887	96684896	96684892	AG	T	snp	1	0	0	0	.	.	.	INTERGENIC(INTERGENIC)
4	96705041	96705048	96705043	A	G	snp	1	0	0	0	.	.	.	INTERGENIC(INTERGENIC)
4	96706090	96706099	96706096	T	G	ins	1	0	0	0	.	.	.	INTERGENIC(INTERGENIC)
4	96758274	96758285	96758280	TA	G	snp	1	1	1	1	.	.	.	INTERGENIC(INTERGENIC);PDHA2(UPSTREAM)
4	96924795	96924806	96924799	AAC	G	snp	1	1	1	1	.	.	.	INTERGENIC(INTERGENIC)
4	97004019	97004026	97004024	A	C	snp	1	1	0	1	.	.	.	INTERGENIC(INTERGENIC)
4	97029299	97029306	97029304	A	G	snp	1	1	0	1	.	.	.	INTERGENIC(INTERGENIC)
4	97208584	97208591	97208588	G	A	snp	1	1	1	1	.	.	.	INTERGENIC(INTERGENIC)
4	97396010	97396021	97396013	AT	C	snp	1	1	0	1	.	.	.	RP11-145G20.1.1(INTRON)
4	97433578	97433585	97433584	A	C	ins	1	1	0	1	.	.	.	RP11-145G20.1.1(INTRON)
4	97470141	97470150	97470144	T	C	snp	1	0	0	0	.	.	.	RP11-145G20.1.1(INTRON)
4	97515070	97515085	97515079	TC	TCT	del	1	1	0	1	.	.	.	RP11-145G20.1.1(INTRON)
4	97515070	97515085	97515082	TC	C	snp	1	1	0	1	.	.	.	RP11-145G20.1.1(INTRON)
4	97573415	97573422	97573419	T	G	snp	1	1	1	1	.	.	.	RP11-145G20.1.1(INTRON)
4	97684009	97684016	97684013	T	G	snp	1	1	0	1	.	.	.	RP11-145G20.1.1(INTRON)
4	97710708	97710723	97710714	TCCC	G	snp	1	0	0	0	.	.	.	RP11-145G20.1.1(INTRON)
4	97720967	97720976	97720974	TA	G	snp	1	1	0	1	.	.	.	RP11-145G20.1.1(INTRON)
4	97794821	97794840	97794828	AAAC	A	snp	1	1	1	1	.	.	.	INTERGENIC(INTERGENIC)
4	97838824	97838841	97838836	AC	C	del	1	1	0	1	.	.	.	INTERGENIC(INTERGENIC)
4	97923017	97923026	97923018	A	G	snp	1	1	1	1	.	.	.	INTERGENIC(INTERGENIC)
4	97923017	97923026	97923020	A	C	snp	1	1	1	1	.	.	.	INTERGENIC(INTERGENIC)
4	97963729	97963736	97963735	T	G	snp	1	1	1	1	.	.	.	INTERGENIC(INTERGENIC)
4	98030092	98030103	98030099	TC	TCTCA	del	1	0	0	0	.	.	.	INTERGENIC(INTERGENIC)
4	98038282	98038299	98038294	TA	ATATA	del	1	0	0	0	.	.	.	INTERGENIC(INTERGENIC);RP11-431J17.1.1(UPSTREAM)
4	98038282	98038299	98038296	TA	A	del	1	0	0	0	.	.	.	INTERGENIC(INTERGENIC);RP11-431J17.1.1(UPSTREAM)
4	98061610	98061625	98061623	AG	C	snp	1	0	0	0	.	.	.	INTERGENIC(INTERGENIC)
4	98142797	98142804	98142800	T	C	snp	1	0	0	0	.	.	.	C4orf37(INTRON)
4	98172595	98172610	98172599	TA	C	snp	1	0	0	0	.	.	.	C4orf37(INTRON)
4	98208565	98208574	98208573	A	C	snp	1	1	1	1	.	.	.	C4orf37(INTRON)
4	98210888	98210897	98210891	AG	T	snp	1	1	1	1	.	.	.	C4orf37(INTRON)
4	98225112	98225119	98225118	A	G	snp	1	1	1	1	.	.	.	C4orf37(INTRON)
4	98552201	98552212	98552204	AC	T	snp	1	0	0	0	.	.	.	C4orf37(INTRON);RP11-681L8.1.1(INTRON)
4	98588136	98588150	98588142	CAC	T	snp	1	1	1	1	.	.	.	C4orf37(INTRON)
4	98608074	98608094	98608081	ATT	G	snp	1	1	1	1	.	.	.	C4orf37(INTRON)
4	98608074	98608094	98608090	ATT	TA	del	1	1	1	1	.	.	.	C4orf37(INTRON)
4	98658811	98658819	98658817	A	G	snp	1	1	1	1	.	.	.	C4orf37(INTRON)
4	98771608	98771616	98771612	T	G	snp	1	0	0	0	.	.	.	C4orf37(INTRON)
4	98940021	98940030	98940028	T	C	snp	1	1	1	1	.	.	.	C4orf37(INTRON)
4	98959562	98959570	98959564	T	G	snp	1	1	1	1	.	.	.	C4orf37(INTRON)
4	98977260	98977268	98977263	C	T	snp	1	1	1	1	.	.	.	C4orf37(INTRON)
4	99011692	99011700	99011693	T	C	snp	1	1	1	1	.	.	.	C4orf37(INTRON)
4	99042872	99042879	99042878	T	G	snp	1	1	1	1	.	.	.	C4orf37(INTRON)
4	99066861	99066869	99066863	T	G	snp	1	0	0	0	.	.	.	C4orf37(UPSTREAM);INTERGENIC(INTERGENIC)
4	99118829	99118846	99118842	AT	A	del	1	1	0	0	.	.	.	INTERGENIC(INTERGENIC)
4	99118829	99118846	99118843	AT	T	snp	1	0	0	0	.	.	.	INTERGENIC(INTERGENIC)

4	99118829	99118846	99118844	AT	A	del	1	1	1	1	.	.	.	INTERGENIC(INTERGENIC)
4	99329242	99329255	99329253	TA	T	ins	1	0	0	0	.	.	.	RAP1GDS1(DOWNSTREAM);RAP1GDS1(INTRON);RP11-810D13.1.1(UPSTREAM)
4	99378618	99378627	99378620	A	C	snp	1	0	0	0	.	.	.	INTERGENIC(INTERGENIC)
4	99378629	99378637	99378636	A	C	snp	1	0	0	0	.	.	.	INTERGENIC(INTERGENIC)
4	99470454	99470462	99470457	T	TGG	ins	1	1	1	1	.	.	.	TSPAN5(INTRON)
4	99470454	99470462	99470458	T	GG	ins	1	1	1	1	.	.	.	TSPAN5(INTRON)
4	99481895	99481904	99481900	TA	G	snp	1	1	1	1	.	.	.	TSPAN5(INTRON);TSPAN5(UPSTREAM)
4	99690130	99690141	99690134	TAA	G	snp	1	1	1	1	.	.	.	INTERGENIC(INTERGENIC)
4	99694650	99694673	99694670	GAA	G	snp	1	0	0	0	.	.	.	INTERGENIC(INTERGENIC)
4	100055532	100055541	100055534	TA	ATAA	ins	1	1	0	1	.	.	.	ADH4(DOWNSTREAM);ADH4(INTRON);RP11-696N14.1.1(INTRON)
4	100263713	100263720	100263715	A	G	snp	1	1	1	1	.	.	.	ADH1C(DOWNSTREAM);ADH1C(INTRON)
4	100339591	100339598	100339596	A	G	snp	1	1	1	1	.	.	.	ADH7(INTRON);ADH7(UPSTREAM)
4	100459864	100459871	100459866	A	C	snp	1	1	1	1	.	.	.	C4orf17(INTRON)
4	100601798	100601806	100601801	A	G	snp	1	0	0	0	.	.	.	INTERGENIC(INTERGENIC)
4	100673889	100673900	100673897	GA	C	snp	1	0	1	0	.	.	.	INTERGENIC(INTERGENIC)
4	100752406	100752415	100752410	TG	C	snp	1	1	1	1	.	.	.	DAPP1(INTRON)
4	100760506	100760517	100760514	AT	C	snp	1	0	0	0	.	.	.	DAPP1(INTRON)
4	101035981	101035990	101035982	T	C	ins	0	0	1	0	.	.	.	RP11-15B17.1.1(INTRON)
4	101044223	101044234	101044229	CAG	A	del	1	1	1	1	.	.	.	RP11-15B17.1.1(INTRON)
4	101189703	101189710	101189706	T	G	snp	1	0	0	0	.	.	.	INTERGENIC(INTERGENIC)
4	101203299	101203318	101203314	TTAT	TTA	del	1	1	1	1	.	.	.	INTERGENIC(INTERGENIC)
4	101234555	101234563	101234562	T	G	snp	0	1	0	1	.	.	.	INTERGENIC(INTERGENIC)
4	101304676	101304684	101304681	A	AC	ins	1	1	1	1	.	.	.	INTERGENIC(INTERGENIC)
4	101304978	101304989	101304983	AT	C	snp	1	0	0	0	.	.	.	INTERGENIC(INTERGENIC)
4	101343955	101343974	101343970	TTTC	T	snp	1	1	1	1	.	.	.	EMCN(INTRON)
4	101348599	101348610	101348608	TC	T	ins	1	1	1	0	.	.	.	EMCN(INTRON)
4	101403454	101403469	101403461	GT	G	snp	1	1	0	1	.	.	.	EMCN(DOWNSTREAM);EMCN(INTRON)
4	101437157	101437172	101437161	GTTT	T	ins	1	1	1	1	.	.	.	EMCN(INTRON)
4	101447418	101447431	101447427	AT	A	del	1	1	1	1	.	.	.	EMCN(INTRON)
4	101488824	101488833	101488825	A	C	snp	1	0	0	0	.	.	.	EMCN(INTRON)
4	101528450	101528461	101528456	TTG	TG	del	1	1	1	1	.	.	.	EMCN(INTRON)
4	101563600	101563609	101563603	AT	C	snp	1	1	0	1	.	.	.	EMCN(INTRON)
4	101566910	101566919	101566913	AT	C	snp	1	1	1	1	.	.	.	EMCN(INTRON)
4	101814931	101814954	101814935	AAAG	AAG	del	1	1	1	1	.	.	.	INTERGENIC(INTERGENIC)
4	101814931	101814954	101814936	AAAG	AG	del	1	1	1	1	.	.	.	INTERGENIC(INTERGENIC)
4	101854181	101854198	101854188	AC	T	snp	1	1	0	0	.	.	.	INTERGENIC(INTERGENIC)
4	101861433	101861442	101861436	AT	G	snp	1	1	0	0	.	.	.	INTERGENIC(INTERGENIC)
4	101939825	101939832	101939830	A	G	snp	1	0	1	0	.	.	.	INTERGENIC(INTERGENIC);PPP3CA(DOWNSTREAM)
4	101950531	101950538	101950536	A	C	snp	1	0	1	0	.	.	.	PPP3CA(INTRON)
4	102077835	102077854	102077846	AAAG	A	del	1	1	1	1	.	.	.	PPP3CA(INTRON)
4	102148277	102148300	102148285	ACAT	CATG	ins	1	1	1	1	.	.	.	PPP3CA(INTRON)
4	102305240	102305249	102305242	A	C	ins	1	0	0	0	.	.	.	INTERGENIC(INTERGENIC)
4	102307289	102307298	102307291	T	TA	ins	1	1	1	1	.	.	.	INTERGENIC(INTERGENIC)
4	102609748	102609757	102609749	T	G	ins	1	0	0	0	.	.	.	BANK1(INTRON)
4	102623947	102623954	102623952	A	G	snp	1	0	0	0	.	.	.	BANK1(INTRON)
4	102672325	102672340	102672330	TTAT	C	snp	1	1	0	1	.	.	.	BANK1(INTRON)
4	102727504	102727512	102727507	A	AC	ins	1	1	1	1	.	.	.	BANK1(INTRON)
4	102727504	102727512	102727508	A	C	ins	1	1	1	1	.	.	.	BANK1(INTRON)
4	102742810	102742829	102742822	TTTG	C	snp	0	0	1	0	.	.	.	BANK1(INTRON)
4	102833198	102833209	102833204	AC	G	snp	0	0	1	0	.	.	.	BANK1(INTRON)
4	102865232	102865243	102865235	AG	C	snp	1	0	0	0	.	.	.	BANK1(INTRON)
4	102908436	102908445	102908441	TG	T	snp	1	1	1	1	.	.	.	BANK1(INTRON);RP11-498M5.2.1(INTRON)
4	102925214	102925222	102925221	T	G	snp	0	1	1	1	.	.	.	BANK1(INTRON);RP11-498M5.2.1(INTRON)
4	102933991	102934000	102933998	A	C	snp	1	0	0	0	.	.	.	BANK1(INTRON);RP11-498M5.2.1(INTRON)
4	103027821	103027830	103027826	AC	A	snp	1	1	1	1	.	.	.	INTERGENIC(INTERGENIC)
4	103027821	103027830	103027827	AC	C	del	1	1	1	1	.	.	.	INTERGENIC(INTERGENIC)
4	103027821	103027830	103027828	AC	A	snp	1	1	1	1	.	.	.	INTERGENIC(INTERGENIC)
4	103052093	103052108	103052099	AAAT	T	snp	0	0	1	0	.	.	.	INTERGENIC(INTERGENIC)
4	103072433	103072444	103072435	GA	C	snp	1	1	1	1	.	.	.	INTERGENIC(INTERGENIC)
4	103076939	103076948	103076941	A	C	ins	0	1	1	1	.	.	.	INTERGENIC(INTERGENIC)
4	103092420	103092429	103092425	TA	G	snp	1	1	1	1	.	.	.	INTERGENIC(INTERGENIC)
4	103094177	103094204	103094192	AAAC	T	ins	1	1	0	1	.	.	.	INTERGENIC(INTERGENIC)
4	103095295	103095303	103095300	T	C	snp	1	0	0	0	.	.	.	INTERGENIC(INTERGENIC)
4	103110218	103110231	103110223	TG	T	del	1	1	1	1	.	.	.	INTERGENIC(INTERGENIC)
4	103357423	103357431	103357425	A	G	snp	1	1	0	1	.	.	.	RP11-499E18.1.1(INTRON);RP11-499E18.1.1(UPSTREAM)

4	103486836	103486851	103486844	CTCC	T	snp	1	0	0	0	.	.	.	AF213884.1(DOWNSTREAM);NFKB1(INTRON)
4	103500804	103500821	103500806	AT	T	del	1	1	1	1	.	.	.	NFKB1(DOWNSTREAM);NFKB1(INTRON)
4	103553958	103553965	103553960	T	C	snp	1	0	0	1	.	.	.	MANBA(DOWNSTREAM);MANBA(INTRON)
4	103578380	103578389	103578384	G	GA	ins	1	0	0	0	.	.	.	MANBA(INTRON)
4	103658648	103658657	103658651	T	G	snp	1	1	1	1	.	.	.	MANBA(INTRON);RP11-10L12.1.1(DOWNSTREAM)
4	103732836	103732845	103732838	A	C	snp	1	0	0	0	.	.	.	UBE2D3(EXON);UBE2D3(INTRON);UBE2D3(UPSTREAM)
4	103898316	103898324	103898318	A	G	snp	1	0	0	0	.	.	.	SLC9B1(INTRON)
4	104023486	104023503	104023499	AT	ATA	del	1	1	1	1	.	.	.	BDH2(UPSTREAM);CENPE(DOWNSTREAM);INTERGENIC(INTERGENIC)
4	104034108	104034117	104034110	TA	C	snp	1	1	0	1	.	.	.	CENPE(DOWNSTREAM);CENPE(INTRON)
4	104120034	104120043	104120041	TC	T	snp	1	1	1	1	.	.	.	CENPE(UPSTREAM);INTERGENIC(INTERGENIC)
4	104127417	104127425	104127421	T	G	snp	1	1	1	1	.	.	.	INTERGENIC(INTERGENIC)
4	104233959	104233966	104233961	G	A	snp	1	0	0	0	.	.	.	RP11-328K4.1.1(INTRON)
4	104358600	104358611	104358604	TA	ATATATAAA	ins	1	1	1	1	.	.	.	RP11-328K4.1.1(INTRON)
4	104358622	104358633	104358628	TA	G	snp	1	0	0	0	.	.	.	RP11-328K4.1.1(INTRON)
4	104440119	104440126	104440120	T	C	snp	1	1	1	1	.	.	.	INTERGENIC(INTERGENIC)
4	104448663	104448671	104448666	A	C	snp	1	0	0	0	.	.	.	INTERGENIC(INTERGENIC)
4	104551051	104551059	104551053	A	C	snp	1	1	1	1	.	.	.	TACR3(INTRON)
4	104551051	104551059	104551055	A	C	ins	0	0	0	1	.	.	.	TACR3(INTRON)
4	104571946	104571955	104571948	AT	G	snp	1	1	1	0	.	.	.	TACR3(INTRON)
4	104601626	104601643	104601641	AT	C	snp	1	1	1	1	.	.	.	TACR3(INTRON)
4	104715026	104715037	104715030	TA	C	snp	1	0	0	0	.	.	.	INTERGENIC(INTERGENIC)
4	104716721	104716730	104716728	TA	T	del	1	1	1	1	.	.	.	INTERGENIC(INTERGENIC)
4	104744782	104744790	104744788	T	C	snp	1	0	0	0	.	.	.	INTERGENIC(INTERGENIC)
4	104883228	104883236	104883235	T	G	snp	1	1	1	1	.	.	.	RP11-703G6.1.1(INTRON)
4	104911132	104911140	104911139	T	G	ins	1	1	1	1	.	.	.	RP11-703G6.1.1(INTRON)
4	104955187	104955194	104955190	T	C	snp	1	0	0	0	.	.	.	RP11-703G6.1.1(INTRON)
4	105006381	105006395	105006391	GAA	G	snp	1	0	0	0	.	.	.	INTERGENIC(INTERGENIC)
4	105190264	105190273	105190268	AT	G	snp	1	0	0	0	.	.	.	RP11-729M20.1.1(INTRON)
4	105416877	105416886	105416881	TC	C	snp	1	1	0	1	.	.	.	AC004053.1.1(INTRON);AC093628.1(DOWNSTREAM);CXXC4(UPSTREAM)
4	105465756	105465764	105465759	T	G	snp	1	1	1	1	.	.	.	AC004053.1.1(INTRON)
4	105466080	105466088	105466081	T	TTG	ins	1	1	1	1	.	.	.	AC004053.1.1(INTRON)
4	105485311	105485326	105485315	CTTT	A	snp	1	1	1	1	.	.	.	AC004053.1.1(INTRON);AC004063.1.1(INTRON)
4	105593760	105593775	105593769	CTTT	C	snp	1	1	0	1	.	.	.	AC004053.1.1(INTRON);RP11-556I14.2.1(INTRON)
4	105782891	105782900	105782897	A	G	snp	1	0	0	0	.	.	.	RP11-556I14.2.1(INTRON)
4	105943618	105943641	105943633	TTTG	A	snp	1	0	0	0	.	.	.	RP11-556I14.1.1(INTRON)
4	106138358	106138367	106138362	A	C	snp	1	1	0	1	.	.	.	TET2(INTRON)
4	106291679	106291687	106291682	T	C	snp	0	1	1	1	.	.	.	PPA2(INTRON)
4	106319243	106319251	106319247	A	CC	ins	1	1	1	1	.	.	.	PPA2(DOWNSTREAM);PPA2(INTRON)
4	106466246	106466253	106466247	A	G	snp	1	1	0	1	.	.	.	AC004066.3.1(INTRON)
4	106494915	106494923	106494920	A	C	snp	1	0	0	0	.	.	.	ARHGEF38(INTRON);ARHGEF38-IT1(DOWNSTREAM)
4	106498710	106498733	106498718	TTG	A	snp	1	1	1	1	.	.	.	ARHGEF38(INTRON)
4	106511293	106511300	106511297	A	AAAG	ins	1	0	0	0	.	.	.	ARHGEF38(INTRON)
4	106511312	106511327	106511315	AG	A	snp	1	0	0	0	.	.	.	ARHGEF38(INTRON)
4	106568434	106568443	106568437	TC	G	snp	1	0	0	0	.	.	.	ARHGEF38(DOWNSTREAM);ARHGEF38(INTRON)
4	106578750	106578765	106578754	AT	G	snp	1	1	0	1	.	.	.	ARHGEF38(INTRON)
4	106586158	106586169	106586162	TGT	T	snp	1	0	0	0	.	.	.	ARHGEF38(INTRON)
4	106876976	106876983	106876977	T	G	snp	1	1	0	1	.	.	.	NPNT(INTRON);NPNT(UPSTREAM)
4	106907181	106907189	106907185	T	C	snp	1	1	1	1	.	.	.	INTERGENIC(INTERGENIC)
4	107074986	107074995	107074988	T	C	snp	0	1	1	0	.	.	.	TBCK(INTRON)
4	107145473	107145482	107145481	T	G	ins	1	0	1	0	.	.	.	TBCK(INTRON)
4	107146617	107146624	107146621	T	G	snp	0	0	0	1	.	.	.	TBCK(INTRON)
4	107219777	107219784	107219779	A	C	snp	1	0	0	0	.	.	.	TBCK(INTRON);TBCK(UPSTREAM)
4	107222332	107222341	107222338	TA	ATA	del	0	1	1	1	.	.	.	TBCK(INTRON);TBCK(UPSTREAM)
4	107429943	107429960	107429957	TA	ATA	del	1	1	1	1	.	.	.	INTERGENIC(INTERGENIC)
4	107575868	107575877	107575870	AT	G	snp	0	1	0	1	.	.	.	INTERGENIC(INTERGENIC)
4	107904119	107904134	107904129	AAGA	C	snp	1	1	0	1	.	.	.	DKK2(INTRON)
4	107974519	107974530	107974527	ACA	T	snp	1	0	0	0	.	.	.	DKK2(INTRON)
4	107976357	107976364	107976363	T	G	snp	1	1	1	1	.	.	.	DKK2(INTRON)
4	107988400	107988409	107988403	TC	T	snp	0	1	0	1	.	.	.	DKK2(INTRON)
4	108022922	108022930	108022929	A	G	snp	1	1	1	1	.	.	.	DKK2(INTRON)
4	108092340	108092351	108092343	ATG	G	snp	1	1	1	0	.	.	.	DKK2(INTRON)
4	108125274	108125281	108125277	C	T	snp	1	1	1	1	.	.	.	DKK2(INTRON);RAC1P5(UPSTREAM)
4	108157159	108157186	108157173	CAAA	ACAC	ins	1	0	0	0	.	.	.	DKK2(INTRON)
4	108291250	108291273	108291269	TTTA	T	snp	1	1	0	1	.	.	.	INTERGENIC(INTERGENIC)
4	108309276	108309284	108309283	C	A	snp	1	1	1	1	.	.	.	INTERGENIC(INTERGENIC)

4	108379145	108379154	108379147	TC	CC	ins	1	1	1	1	.	.	.	INTERGENIC(INTERGENIC)
4	108379145	108379154	108379149	TC	C	snp	1	1	1	1	.	.	.	INTERGENIC(INTERGENIC)
4	108443171	108443186	108443179	GA	T	snp	1	1	1	1	.	.	.	INTERGENIC(INTERGENIC)
4	108443171	108443186	108443180	GA	C	snp	1	1	1	1	.	.	.	INTERGENIC(INTERGENIC)
4	108460561	108460570	108460567	A	C	snp	1	1	1	1	.	.	.	INTERGENIC(INTERGENIC)
4	108477557	108477566	108477560	TA	G	snp	1	1	1	1	.	.	.	INTERGENIC(INTERGENIC)
4	108477557	108477566	108477562	TA	G	snp	1	1	1	0	.	.	.	INTERGENIC(INTERGENIC)
4	108477590	108477599	108477595	TA	G	snp	1	1	1	1	.	.	.	INTERGENIC(INTERGENIC)
4	108502646	108502654	108502650	A	C	snp	1	1	1	1	.	.	.	INTERGENIC(INTERGENIC)
4	108603452	108603461	108603457	T	G	snp	1	0	0	0	.	.	.	PAPSS1(DOWNSTREAM);PAPSS1(INTRON)
4	108865996	108866007	108865998	AT	G	snp	0	0	1	0	.	.	.	CYP2U1(INTRON);RP11-286E11.1.1(INTRON);RP11-286E11.2.1(UPSTREAM)
4	109025653	109025664	109025659	ATA	T	del	0	0	1	0	.	.	.	LEF1(INTRON)
4	109437017	109437028	109437021	AG	T	snp	1	1	1	1	.	.	.	INTERGENIC(INTERGENIC)
4	109446518	109446541	109446533	CAA	A	snp	1	0	0	0	.	.	.	INTERGENIC(INTERGENIC)
4	109523891	109523906	109523895	TTCT	C	snp	1	0	0	0	.	.	.	RP11-462C24.1.1(EXON);RP11-462C24.1.1(INTRON)
4	109557523	109557534	109557532	AC	A	del	1	1	1	1	.	.	.	INTERGENIC(INTERGENIC)
4	109593650	109593663	109593657	AT	C	snp	1	1	1	1	.	.	.	INTERGENIC(INTERGENIC);OSTC(DOWNSTREAM)
4	109641033	109641042	109641037	AT	T	del	1	0	0	0	.	.	.	INTERGENIC(INTERGENIC)
4	109677994	109678002	109677996	A	C	snp	1	0	0	0	.	.	.	AGXT2L1(INTRON);AGXT2L1(UPSTREAM)
4	109750603	109750618	109750607	TG	C	snp	1	0	0	0	.	.	.	COL25A1(DOWNSTREAM);COL25A1(INTRON)
4	109768515	109768522	109768519	T	C	snp	1	1	1	1	.	.	.	COL25A1(INTRON)
4	109776535	109776543	109776541	T	G	ins	1	1	1	1	.	.	.	COL25A1(INTRON)
4	109779968	109779975	109779970	A	G	snp	1	1	1	1	.	.	.	COL25A1(INTRON)
4	109834234	109834242	109834237	T	GC	ins	1	1	1	1	.	.	.	COL25A1(INTRON)
4	109834234	109834242	109834238	T	G	snp	1	1	1	1	.	.	.	COL25A1(INTRON)
4	109834234	109834242	109834239	T	C	snp	1	1	1	1	.	.	.	COL25A1(INTRON)
4	109856677	109856686	109856683	AT	C	snp	0	1	0	1	.	.	.	COL25A1(INTRON)
4	109879306	109879314	109879312	A	G	snp	1	1	1	1	.	.	.	COL25A1(INTRON)
4	109887851	109887858	109887856	A	C	snp	1	1	1	1	.	.	.	COL25A1(INTRON)
4	109965287	109965296	109965293	AT	G	snp	1	1	1	1	.	.	.	COL25A1(INTRON)
4	110012670	110012683	110012673	TG	C	snp	1	1	1	1	.	.	.	COL25A1(INTRON)
4	110071099	110071114	110071108	TG	T	snp	1	0	1	0	.	.	.	COL25A1(INTRON)
4	110274510	110274521	110274519	CA	C	del	1	0	0	0	.	.	.	RP11-264J9.1.1(DOWNSTREAM);RP11-264J9.1.1(INTRON)
4	110527115	110527142	110527134	TTTG	T	snp	1	0	0	0	.	.	.	CCDC109B(INTRON)
4	110606385	110606394	110606391	TC	CTC	del	1	1	0	0	.	.	.	CASP6(DOWNSTREAM);CCDC109B(DOWNSTREAM);CCDC109B(EXON);CCDC109B(INTRON)
4	110611353	110611368	110611355	AC	T	snp	1	1	1	1	.	.	.	CASP6(DOWNSTREAM);CASP6(INTRON);CCDC109B(DOWNSTREAM)
4	110699832	110699840	110699839	A	G	snp	1	1	1	1	.	.	.	CFI(INTRON)
4	110803057	110803072	110803070	TA	T	del	1	0	0	1	.	.	.	INTERGENIC(INTERGENIC);KRT19P3(UPSTREAM)
4	110806038	110806053	110806051	TA	TT	ins	1	0	0	0	.	.	.	INTERGENIC(INTERGENIC);KRT19P3(UPSTREAM)
4	110857163	110857170	110857167	T	C	snp	1	1	1	1	.	.	.	EGF(INTRON);EGF(UPSTREAM)
4	111141108	111141117	111141112	T	C	snp	1	1	0	1	.	.	.	INTERGENIC(INTERGENIC)
4	111183412	111183419	111183416	T	C	snp	1	1	1	1	.	.	.	INTERGENIC(INTERGENIC);RP11-433B3.1.1(DOWNSTREAM)
4	111189227	111189236	111189234	A	G	snp	1	0	0	0	.	.	.	INTERGENIC(INTERGENIC);RP11-433B3.1.1(UPSTREAM)
4	111368821	111368830	111368823	T	G	snp	1	1	1	1	.	.	.	ENPEP(INTRON)
4	111396986	111396993	111396987	A	G	snp	0	1	0	0	.	.	.	ENPEP(INTRON);ENPEP(UPSTREAM)
4	111575870	111575878	111575872	T	G	snp	1	1	1	1	.	.	.	INTERGENIC(INTERGENIC)
4	111697449	111697456	111697451	T	G	snp	1	1	1	1	.	.	.	INTERGENIC(INTERGENIC)
4	111804899	111804910	111804907	AT	T	snp	1	1	1	1	.	.	.	INTERGENIC(INTERGENIC)
4	111804916	111804927	111804924	AT	T	snp	1	1	1	1	.	.	.	INTERGENIC(INTERGENIC)
4	111822272	111822286	111822277	TGG	A	snp	0	0	1	0	.	.	.	INTERGENIC(INTERGENIC)
4	111867074	111867089	111867082	TTTA	C	snp	0	1	1	1	.	.	.	AC004062.1.1(UPSTREAM);INTERGENIC(INTERGENIC)
4	111941047	111941054	111941049	T	C	snp	1	1	1	1	.	.	.	INTERGENIC(INTERGENIC)
4	111956984	111956991	111956987	T	G	snp	1	0	0	0	.	.	.	INTERGENIC(INTERGENIC)
4	112029442	112029451	112029447	GA	G	snp	1	1	1	1	.	.	.	INTERGENIC(INTERGENIC)
4	112044803	112044814	112044810	GA	C	snp	1	0	0	0	.	.	.	INTERGENIC(INTERGENIC)
4	112044848	112044865	112044859	AG	T	snp	1	1	1	1	.	.	.	INTERGENIC(INTERGENIC)
4	112068154	112068162	112068159	A	C	snp	1	1	1	1	.	.	.	INTERGENIC(INTERGENIC)
4	112139364	112139384	112139369	TAA	C	snp	1	0	0	0	.	.	.	INTERGENIC(INTERGENIC)
4	112139364	112139384	112139375	TAA	C	snp	1	1	0	1	.	.	.	INTERGENIC(INTERGENIC)
4	112268997	112269004	112269003	T	C	snp	1	0	0	0	.	.	.	INTERGENIC(INTERGENIC)
4	112347970	112347977	112347974	A	G	snp	1	1	1	1	.	.	.	INTERGENIC(INTERGENIC)
4	112423618	112423641	112423631	AAAT	G	snp	1	1	1	1	.	.	.	INTERGENIC(INTERGENIC)
4	112524978	112524987	112524985	A	C	snp	1	1	1	1	.	.	.	INTERGENIC(INTERGENIC)
4	112583719	112583733	112583727	AGG	A	snp	1	0	0	0	.	.	.	INTERGENIC(INTERGENIC)

4	112653648	112653657	112653651	AT	ATATATC	del	1	1	1	0	.	.	.	INTERGENIC(INTERGENIC)	
4	112653648	112653657	112653653	AT	ATATC	del	1	1	1	0	.	.	.	INTERGENIC(INTERGENIC)	
4	112676768	112676779	112676775	AAG	G	snp	1	1	1	0	.	.	.	INTERGENIC(INTERGENIC)	
4	112764356	112764363	112764361	C	A	snp	1	1	1	1	.	.	.	RP11-269F21.1.1(DOWNSTREAM);RP11-269F21.3.1(INTRON)	
4	112824686	112824697	112824694	TA	C	snp	1	1	1	1	.	.	.	RP11-269F21.3.1(INTRON)	
4	112865497	112865504	112865498	T	G	snp	0	1	1	0	.	.	.	RP11-269F21.3.1(INTRON)	
4	112959744	112959758	112959750	ATT	G	snp	1	1	1	1	.	.	.	RP11-269F21.3.1(INTRON)	
4	113025643	113025658	113025645	AC	G	snp	0	1	1	0	.	.	.	INTERGENIC(INTERGENIC)	
4	113035900	113035909	113035908	A	G	ins	1	1	1	1	.	.	.	INTERGENIC(INTERGENIC);RP11-477G18.1.1(DOWNSTREAM)	
4	113163609	113163617	113163613	A	G	ins	1	1	1	0	.	.	.	AP1AR(INTRON)	
4	113258080	113258089	113258082	T	G	snp	1	1	1	1	.	.	.	ALPK1(INTRON)	
4	113270708	113270716	113270714	C	CCG	ins	1	1	1	1	.	.	.	ALPK1(INTRON)	
4	113337558	113337565	113337560	A	G	snp	1	1	0	1	.	.	.	ALPK1(DOWNSTREAM);ALPK1(INTRON)	
4	113346112	113346127	113346121	AAAC	ACAAACAG		ins	0	0	0	1	.	.	.	ALPK1(INTRON);ALPK1(UPSTREAM)
4	113349285	113349294	113349288	TA	G	snp	1	1	1	1	.	.	.	ALPK1(DOWNSTREAM);ALPK1(INTRON)	
4	113458358	113458373	113458365	AAAT	A	snp	1	1	1	1	.	.	.	C4orf21(DOWNSTREAM);RP11-402J6.1.1(INTRON)	
4	113477224	113477233	113477230	A	G	snp	0	1	0	1	.	.	.	C4orf21(INTRON)	
4	113518680	113518694	113518684	TAA	C	snp	1	0	0	0	.	.	.	C4orf21(INTRON)	
4	113605179	113605188	113605186	TA	G	snp	1	0	0	1	.	.	.	INTERGENIC(INTERGENIC);OSTCP4(UPSTREAM)	
4	113605265	113605276	113605269	TA	A	ins	1	1	1	1	.	.	.	INTERGENIC(INTERGENIC);OSTCP4(UPSTREAM)	
4	113692276	113692284	113692279	T	G	snp	1	1	1	1	.	.	.	INTERGENIC(INTERGENIC)	
4	113692276	113692284	113692282	T	C	snp	1	0	0	0	.	.	.	INTERGENIC(INTERGENIC)	
4	113712620	113712637	113712632	CTT	TTCTT	del	1	1	1	1	.	.	.	INTERGENIC(INTERGENIC);RP11-119H12.2.1(UPSTREAM)	
4	113712620	113712637	113712633	CTT	TC	del	1	1	1	1	.	.	.	INTERGENIC(INTERGENIC);RP11-119H12.2.1(UPSTREAM)	
4	113712620	113712637	113712634	CTT	C	del	1	1	1	1	.	.	.	INTERGENIC(INTERGENIC);RP11-119H12.2.1(UPSTREAM)	
4	113725946	113725957	113725950	ACA	A	snp	1	1	1	1	.	.	.	INTERGENIC(INTERGENIC)	
4	113750328	113750347	113750341	TGAA	A	snp	0	1	0	1	.	.	.	ANK2(INTRON);RP11-119H12.4.1(DOWNSTREAM)	
4	113751762	113751776	113751773	ATT	A	del	1	1	1	1	.	.	.	ANK2(INTRON);RP11-119H12.4.1(DOWNSTREAM)	
4	113814379	113814390	113814384	TAT	C	snp	1	0	0	0	.	.	.	ANK2(INTRON)	
4	113836918	113836937	113836924	AAAT	T	del	1	1	1	0	.	.	.	ANK2(INTRON)	
4	113836918	113836937	113836925	AAAT	A	snp	1	1	1	0	.	.	.	ANK2(INTRON)	
4	113857938	113857947	113857944	T	G	snp	1	1	1	1	.	.	.	ANK2(INTRON)	
4	113887613	113887626	113887621	GT	A	snp	1	1	0	1	.	.	.	ANK2(INTRON)	
4	113907228	113907241	113907232	AT	G	snp	1	1	1	1	.	.	.	ANK2(INTRON);RP11-650J17.1.1(INTRON)	
4	113910381	113910392	113910387	TAA	A	snp	1	0	1	0	.	.	.	ANK2(INTRON);RP11-650J17.1.1(INTRON)	
4	114097623	114097631	114097630	T	G	snp	1	1	1	1	.	.	.	ANK2(INTRON)	
4	114286835	114286846	114286844	TC	TCTTA	del	0	1	0	1	.	.	.	ANK2(DOWNSTREAM);ANK2(INTRON)	
4	114312015	114312022	114312016	G	T	snp	1	0	0	0	.	.	.	ANK2(DOWNSTREAM);INTERGENIC(INTERGENIC)	
4	114312931	114312940	114312932	T	G	ins	0	0	0	1	.	.	.	ANK2(DOWNSTREAM);INTERGENIC(INTERGENIC)	
4	114318550	114318557	114318551	T	G	snp	1	0	0	0	.	.	.	INTERGENIC(INTERGENIC)	
4	114365134	114365147	114365144	TC	C	ins	1	1	1	1	.	.	.	INTERGENIC(INTERGENIC)	
4	114428048	114428056	114428055	C	A	snp	0	1	1	1	.	.	.	CAMK2D(INTRON)	
4	114488305	114488312	114488308	T	TTTTC	ins	0	1	1	0	.	.	.	CAMK2D(INTRON)	
4	114672007	114672014	114672008	A	C	snp	1	1	1	1	.	.	.	CAMK2D(DOWNSTREAM);CAMK2D(INTRON)	
4	114785042	114785050	114785049	A	C	snp	1	1	0	1	.	.	.	INTERGENIC(INTERGENIC)	
4	114822863	114822876	114822867	AT	TT	ins	1	1	1	1	.	.	.	ARSJ(DOWNSTREAM);ARSJ(UTR_3_PRIME)	
4	114825651	114825658	114825652	T	C	snp	0	0	1	1	.	.	.	ARSJ(INTRON);ARSJ(UPSTREAM)	
4	114845179	114845190	114845188	AT	AA	ins	1	1	1	1	.	.	.	ARSJ(INTRON)	
4	114981769	114981776	114981773	T	G	snp	1	1	1	1	.	.	.	INTERGENIC(INTERGENIC)	
4	114981769	114981776	114981773	T	TTTG	ins	1	1	1	1	.	.	.	INTERGENIC(INTERGENIC)	
4	114991182	114991191	114991188	AT	G	snp	1	1	1	1	.	.	.	INTERGENIC(INTERGENIC)	
4	115014799	115014806	115014804	T	G	ins	1	0	0	0	.	.	.	INTERGENIC(INTERGENIC)	
4	115043054	115043077	115043065	TTTC	T	snp	1	0	0	0	.	.	.	INTERGENIC(INTERGENIC)	
4	115074126	115074133	115074129	C	CT	ins	1	0	0	0	.	.	.	INTERGENIC(INTERGENIC)	
4	115134958	115134967	115134961	A	C	snp	1	1	1	1	.	.	.	INTERGENIC(INTERGENIC)	
4	115156930	115156937	115156932	G	A	ins	1	1	1	1	.	.	.	INTERGENIC(INTERGENIC)	
4	115160472	115160481	115160476	T	C	snp	1	1	1	1	.	.	.	INTERGENIC(INTERGENIC)	
4	115170524	115170533	115170531	C	CT	ins	1	1	1	1	.	.	.	INTERGENIC(INTERGENIC)	
4	115215936	115215945	115215940	GA	C	snp	1	1	1	1	.	.	.	INTERGENIC(INTERGENIC)	
4	115229029	115229036	115229032	A	C	ins	0	1	1	1	.	.	.	INTERGENIC(INTERGENIC)	
4	115229029	115229036	115229032	A	C	snp	1	1	1	1	.	.	.	INTERGENIC(INTERGENIC)	
4	115282865	115282873	115282866	T	G	snp	1	0	0	0	.	.	.	INTERGENIC(INTERGENIC)	
4	115375287	115375295	115375294	A	G	snp	1	1	1	1	.	.	.	INTERGENIC(INTERGENIC)	
4	115638571	115638578	115638574	T	C	snp	1	0	0	0	.	.	.	INTERGENIC(INTERGENIC);RP11-201O10.1.1(UPSTREAM)	
4	115712379	115712386	115712383	A	C	snp	1	0	0	0	.	.	.	INTERGENIC(INTERGENIC)	

4	115714456	115714465	115714461	A	C	snp	1	0	0	0	.	.	.	INTERGENIC(INTERGENIC)
4	115942456	115942465	115942459	AT	C	snp	0	0	1	0	.	.	.	NDST4(INTRON)
4	115942527	115942536	115942531	AT	G	snp	1	0	0	0	.	.	.	NDST4(INTRON)
4	116001848	116001865	116001863	GT	A	ins	1	1	1	1	.	.	.	NDST4(INTRON)
4	116030191	116030206	116030202	GGAA	T	snp	0	0	0	1	.	.	.	NDST4(INTRON)
4	116194300	116194309	116194305	TC	G	snp	1	0	0	0	.	.	.	INTERGENIC(INTERGENIC)
4	116331785	116331796	116331788	AG	AC	ins	0	1	1	1	.	.	.	INTERGENIC(INTERGENIC)
4	116587405	116587428	116587416	TCCC	T	snp	1	1	0	1	.	.	.	INTERGENIC(INTERGENIC)
4	116604048	116604055	116604053	T	C	snp	1	1	0	1	.	.	.	INTERGENIC(INTERGENIC)
4	116688979	116688988	116688984	C	A	snp	1	1	0	1	.	.	.	INTERGENIC(INTERGENIC);PGAM4P2(UPSTREAM)
4	116688979	116688988	116688985	C	T	ins	1	0	0	0	.	.	.	INTERGENIC(INTERGENIC);PGAM4P2(UPSTREAM)
4	116751546	116751554	116751548	T	C	snp	1	0	0	0	.	.	.	INTERGENIC(INTERGENIC)
4	116751815	116751824	116751821	A	C	snp	1	1	1	1	.	.	.	INTERGENIC(INTERGENIC)
4	116785720	116785729	116785722	AT	G	snp	1	1	1	1	.	.	.	INTERGENIC(INTERGENIC)
4	116785885	116785894	116785891	TA	C	snp	1	1	1	1	.	.	.	INTERGENIC(INTERGENIC)
4	116800221	116800230	116800226	CA	CACAAG	ins	1	1	1	1	.	.	.	INTERGENIC(INTERGENIC)
4	116800221	116800230	116800228	CA	CAAG	ins	1	1	1	1	.	.	.	INTERGENIC(INTERGENIC)
4	116854612	116854621	116854619	CA	G	snp	1	0	0	0	.	.	.	RP11-65903.1.1(INTRON)
4	116865340	116865348	116865341	T	G	ins	1	0	0	0	.	.	.	RP11-65903.1.1(INTRON)
4	116870053	116870060	116870057	T	G	snp	1	1	1	1	.	.	.	RP11-65903.1.1(INTRON)
4	116904698	116904705	116904704	A	C	ins	1	1	1	1	.	.	.	RP11-65903.1.1(INTRON)
4	116925266	116925274	116925270	A	C	snp	1	1	1	1	.	.	.	RP11-65903.1.1(INTRON)
4	116987532	116987541	116987539	TG	T	snp	1	1	1	1	.	.	.	RP11-65903.1.1(INTRON)
4	117035314	117035331	117035323	AT	AC	ins	1	0	0	0	.	.	.	RP11-65903.1.1(INTRON)
4	117044833	117044842	117044838	AT	C	snp	0	1	1	1	.	.	.	RP11-65903.1.1(INTRON)
4	117073623	117073638	117073633	TTTG	G	snp	1	1	1	1	.	.	.	RP11-65903.1.1(INTRON)
4	117093347	117093354	117093351	T	C	snp	1	0	0	0	.	.	.	RP11-65903.1.1(INTRON)
4	117094870	117094879	117094871	T	TGA	ins	1	1	1	1	.	.	.	RP11-65903.1.1(INTRON)
4	117127971	117127980	117127972	T	G	ins	1	1	0	0	.	.	.	RP11-65903.1.1(INTRON)
4	117127971	117127980	117127973	T	G	snp	1	1	1	1	.	.	.	RP11-65903.1.1(INTRON)
4	117134202	117134210	117134206	A	C	snp	1	0	0	0	.	.	.	RP11-65903.1.1(INTRON)
4	117212080	117212087	117212084	T	C	snp	1	1	1	1	.	.	.	RP11-65903.1.1(INTRON)
4	117419623	117419630	117419624	T	G	snp	0	0	1	0	.	.	.	RP11-55L3.1.1(INTRON)
4	117430050	117430057	117430051	A	G	snp	1	1	1	1	.	.	.	RP11-55L3.1.1(INTRON)
4	117536739	117536762	117536743	GGAA	A	snp	1	1	0	1	.	.	.	INTERGENIC(INTERGENIC)
4	117546912	117546919	117546915	T	C	snp	1	0	0	0	.	.	.	INTERGENIC(INTERGENIC)
4	117582535	117582543	117582538	A	G	snp	1	0	0	0	.	.	.	INTERGENIC(INTERGENIC)
4	117583399	117583408	117583406	A	C	snp	1	1	1	1	.	.	.	INTERGENIC(INTERGENIC)
4	117651014	117651022	117651019	C	CCG	ins	1	0	0	0	.	.	.	INTERGENIC(INTERGENIC)
4	117662949	117662956	117662951	A	G	snp	1	1	1	1	.	.	.	INTERGENIC(INTERGENIC)
4	117820582	117820599	117820585	AT	ATATAG	ins	1	1	0	1	.	.	.	INTERGENIC(INTERGENIC)
4	117820582	117820599	117820590	AT	TT	ins	1	0	0	0	.	.	.	INTERGENIC(INTERGENIC)
4	117823475	117823482	117823479	A	G	snp	1	1	1	1	.	.	.	INTERGENIC(INTERGENIC)
4	117845250	117845259	117845257	TA	G	snp	0	1	0	1	.	.	.	INTERGENIC(INTERGENIC)
4	117901339	117901350	117901346	AG	AGAGA	del	1	1	1	1	.	.	.	INTERGENIC(INTERGENIC)
4	117901339	117901350	117901347	AG	GAG	del	1	1	1	1	.	.	.	INTERGENIC(INTERGENIC)
4	117927145	117927156	117927149	TA	AC	ins	1	0	0	0	.	.	.	INTERGENIC(INTERGENIC)
4	117940601	117940612	117940605	AT	G	snp	1	0	0	0	.	.	.	INTERGENIC(INTERGENIC)
4	117947314	117947323	117947320	AG	GAG	del	1	1	1	1	.	.	.	INTERGENIC(INTERGENIC)
4	117983265	117983273	117983266	A	C	snp	1	1	1	1	.	.	.	INTERGENIC(INTERGENIC)
4	118001606	118001613	118001607	T	G	snp	1	1	1	1	.	.	.	INTERGENIC(INTERGENIC);TRAM1L1(DOWNSTREAM)
4	118136398	118136407	118136405	AT	ATG	del	1	0	0	0	.	.	.	INTERGENIC(INTERGENIC)
4	118151799	118151807	118151802	A	C	snp	1	0	0	0	.	.	.	INTERGENIC(INTERGENIC)
4	118159053	118159061	118159056	T	G	snp	1	1	1	1	.	.	.	INTERGENIC(INTERGENIC)
4	118208561	118208568	118208563	A	G	snp	0	0	0	1	.	.	.	INTERGENIC(INTERGENIC)
4	118293683	118293696	118293690	AT	C	snp	1	1	0	1	.	.	.	AC113617.1.1(EXON);AC113617.1.1(INTRON)
4	118393142	118393151	118393149	TG	A	snp	1	1	1	1	.	.	.	AC092661.1.1(INTRON)
4	118397454	118397463	118397455	A	C	snp	1	1	1	1	.	.	.	AC092661.1.1(INTRON)
4	118465519	118465530	118465527	CAA	C	del	1	0	0	0	.	.	.	AC092661.1.1(DOWNSTREAM);AC092661.1.1(INTRON)
4	118562092	118562106	118562103	CAA	C	del	1	1	1	1	.	.	.	AC092661.1.1(INTRON)
4	118648135	118648143	118648140	T	G	snp	1	1	1	1	.	.	.	INTERGENIC(INTERGENIC)
4	118751986	118751994	118751993	A	G	snp	0	1	1	1	.	.	.	AC108056.1.1(UPSTREAM);INTERGENIC(INTERGENIC)
4	118774571	118774585	118774580	CAA	G	snp	1	0	0	0	.	.	.	AC108056.1.1(INTRON)
4	118849134	118849145	118849138	ATA	AATAATAAATA	del	1	0	0	0	.	.	.	INTERGENIC(INTERGENIC)
4	118932578	118932585	118932584	T	C	snp	1	0	0	0	.	.	.	INTERGENIC(INTERGENIC)
4	119013205	119013232	119013223	TTAT	G	snp	1	1	0	0	.	.	.	NDST3(INTRON)
4	119156201	119156220	119156211	AAGG	A	snp	1	1	1	1	.	.	.	NDST3(INTRON)

4	119181150	119181167	119181163	AT	C	snp	1	1	1	1	.	.	.	INTERGENIC(INTERGENIC);NDST3(DOWNSTREAM)
4	119208286	119208299	119208296	AC	G	snp	1	0	0	0	.	.	.	PRSS12(INTRON)
4	119257718	119257727	119257719	T	C	snp	0	1	1	1	.	.	.	PRSS12(INTRON)
4	119297418	119297438	119297434	CAA	AC	del	1	1	1	1	.	.	.	INTERGENIC(INTERGENIC)
4	119337545	119337552	119337546	A	C	snp	0	0	0	1	.	.	.	INTERGENIC(INTERGENIC)
4	119441052	119441059	119441053	A	C	snp	1	1	1	1	.	.	.	CEP170P1(INTRON)
4	119576409	119576418	119576415	TG	G	del	0	1	1	0	.	.	.	INTERGENIC(INTERGENIC)
4	119728648	119728656	119728652	A	C	snp	0	0	0	1	.	.	.	SEC24D(INTRON)
4	119761068	119761075	119761069	A	C	snp	0	0	0	1	.	.	.	INTERGENIC(INTERGENIC);SEC24D(UPSTREAM)
4	120018328	120018337	120018333	TG	TT	ins	1	1	1	1	.	.	.	INTERGENIC(INTERGENIC)
4	120023018	120023025	120023023	A	G	snp	1	1	0	1	.	.	.	INTERGENIC(INTERGENIC)
4	120058058	120058067	120058066	T	C	snp	1	1	1	1	.	.	.	MYOZ2(INTRON)
4	120362730	120362738	120362735	A	G	snp	1	1	1	1	.	.	.	RP11-33B1.2.1(INTRON)
4	120374477	120374492	120374488	CTAG	CTAT	ins	1	1	1	1	.	.	.	INTERGENIC(INTERGENIC);RP11-33B1.1.1(UPSTREAM);RP11-33B1.2.1(UPSTREAM);RP11-33B1.3.1(UPSTREAM)
4	120375127	120375136	120375133	C	A	ins	1	1	1	1	.	.	.	INTERGENIC(INTERGENIC);RP11-33B1.1.1(UPSTREAM);RP11-33B1.2.1(UPSTREAM);RP11-33B1.3.1(UPSTREAM)
4	120414686	120414694	120414693	A	C	snp	1	1	1	1	.	.	.	PDE5A(DOWNSTREAM);RP11-33B1.1.1(DOWNSTREAM);RP11-33B1.1.1(INTRON);RP11-33B1.1.1(UPSTREAM)
4	120423687	120423696	120423691	A	G	snp	0	0	1	0	.	.	.	PDE5A(EXON);PDE5A(INTRON);RP11-33B1.1.1(DOWNSTREAM);RP11-33B1.1.1(INTRON)
4	120453411	120453422	120453416	TAT	C	snp	1	1	1	1	.	.	.	PDE5A(INTRON);PDE5A(UPSTREAM);RP11-33B1.1.1(INTRON)
4	120596097	120596110	120596108	CT	C	del	0	1	0	1	.	.	.	INTERGENIC(INTERGENIC)
4	120605438	120605447	120605444	T	G	snp	1	1	0	1	.	.	.	INTERGENIC(INTERGENIC)
4	120621018	120621026	120621019	T	G	ins	1	0	0	0	.	.	.	INTERGENIC(INTERGENIC)
4	120622221	120622234	120622231	AT	C	snp	1	1	0	0	.	.	.	INTERGENIC(INTERGENIC)
4	120646455	120646462	120646459	A	G	snp	1	0	0	0	.	.	.	INTERGENIC(INTERGENIC)
4	120679740	120679748	120679746	A	C	snp	1	1	1	1	.	.	.	INTERGENIC(INTERGENIC)
4	120737445	120737452	120737450	T	C	snp	1	0	0	0	.	.	.	INTERGENIC(INTERGENIC)
4	120833078	120833089	120833085	TC	TCTCT	del	1	0	0	0	.	.	.	INTERGENIC(INTERGENIC)
4	120858720	120858729	120858728	T	G	snp	1	1	0	1	.	.	.	INTERGENIC(INTERGENIC);RP11-700N1.1.1(UPSTREAM)
4	120907492	120907501	120907497	TA	G	snp	1	0	0	0	.	.	.	INTERGENIC(INTERGENIC)
4	120907492	120907501	120907499	TA	G	snp	1	1	1	1	.	.	.	INTERGENIC(INTERGENIC)
4	120963280	120963287	120963281	C	CG	ins	1	0	0	0	.	.	.	INTERGENIC(INTERGENIC);RP11-96A1.5.1(UPSTREAM)
4	121121537	121121544	121121540	A	G	snp	1	1	1	1	.	.	.	RP11-679C8.2.1(INTRON)
4	121123421	121123436	121123434	AT	A	del	1	1	1	1	.	.	.	RP11-679C8.2.1(INTRON)
4	121178249	121178258	121178253	TA	A	snp	1	0	0	0	.	.	.	RP11-679C8.2.1(INTRON)
4	121178249	121178258	121178256	TA	G	snp	0	0	0	1	.	.	.	RP11-679C8.2.1(INTRON)
4	121257890	121257899	121257895	TA	G	snp	1	0	0	0	.	.	.	RP11-679C8.2.1(INTRON)
4	121288937	121288948	121288942	GAA	G	snp	1	1	0	1	.	.	.	RP11-679C8.2.1(INTRON)
4	121296576	121296583	121296582	T	C	snp	1	1	1	1	.	.	.	RP11-679C8.2.1(INTRON)
4	121331623	121331632	121331624	A	C	ins	1	0	0	0	.	.	.	RP11-679C8.2.1(INTRON)
4	121416109	121416120	121416116	ATA	C	snp	1	1	0	1	.	.	.	INTERGENIC(INTERGENIC)
4	121421500	121421508	121421501	T	TTC	ins	1	1	1	1	.	.	.	INTERGENIC(INTERGENIC)
4	121538030	121538039	121538033	TC	T	snp	0	1	0	0	.	.	.	INTERGENIC(INTERGENIC)
4	121564321	121564330	121564324	T	C	snp	0	1	1	1	.	.	.	RP11-501E14.1.1(INTRON)
4	121622304	121622313	121622307	A	C	snp	0	1	1	1	.	.	.	PRDM5(INTRON)
4	121655411	121655420	121655414	T	G	snp	0	1	1	1	.	.	.	PRDM5(INTRON)
4	121696044	121696061	121696057	TA	G	snp	1	1	1	1	.	.	.	PRDM5(INTRON)
4	121696044	121696061	121696059	TA	G	snp	1	1	1	1	.	.	.	PRDM5(INTRON)
4	121730562	121730569	121730568	A	G	snp	1	1	1	1	.	.	.	PRDM5(INTRON);U6(UPSTREAM)
4	121765777	121765790	121765788	TC	T	snp	0	1	1	1	.	.	.	PRDM5(INTRON)
4	121862862	121862889	121862881	AAAC	A	del	1	0	0	0	.	.	.	INTERGENIC(INTERGENIC)
4	121862893	121862902	121862894	A	C	ins	0	1	1	1	.	.	.	INTERGENIC(INTERGENIC)
4	121872941	121872950	121872947	TC	C	snp	1	0	0	0	.	.	.	INTERGENIC(INTERGENIC)
4	121919656	121919663	121919662	T	G	snp	1	1	1	1	.	.	.	INTERGENIC(INTERGENIC)
4	121936113	121936122	121936116	AC	T	snp	1	1	1	1	.	.	.	INTERGENIC(INTERGENIC)
4	121945069	121945084	121945080	TTTA	T	ins	1	1	1	1	.	.	.	INTERGENIC(INTERGENIC)
4	121984606	121984623	121984611	AC	A	snp	1	0	0	0	.	.	.	7SK(UPSTREAM);NDNF(INTRON)
4	122046104	122046115	122046107	AG	A	snp	1	1	1	1	.	.	.	INTERGENIC(INTERGENIC)
4	122052334	122052347	122052341	TA	G	snp	1	0	0	0	.	.	.	INTERGENIC(INTERGENIC);TNIP3(DOWNSTREAM)
4	122112245	122112259	122112254	TTG	C	snp	1	0	0	0	.	.	.	TNIP3(INTRON);U6(DOWNSTREAM)
4	122140354	122140381	122140374	ACAA	C	del	1	0	0	0	.	.	.	TNIP3(INTRON);TNIP3(UPSTREAM)
4	122141644	122141652	122141648	T	G	snp	1	1	1	1	.	.	.	TNIP3(INTRON);TNIP3(UPSTREAM)
4	122162013	122162021	122162018	T	C	snp	0	1	1	1	.	.	.	INTERGENIC(INTERGENIC)
4	122168555	122168562	122168556	A	C	snp	1	0	0	0	.	.	.	INTERGENIC(INTERGENIC)

4	122192027	122192034	122192030	T	G	ins	1	1	1	1	.	.	.	INTERGENIC(INTERGENIC)
4	122245096	122245109	122245105	AC	A	del	1	0	0	0	.	.	.	INTERGENIC(INTERGENIC)
4	122264811	122264818	122264815	T	C	snp	1	1	1	1	.	.	.	QRFPR(INTRON)
4	122392205	122392212	122392208	A	C	snp	1	1	1	1	.	.	.	INTERGENIC(INTERGENIC)
4	122423840	122423859	122423847	CCTC	T	snp	1	1	1	1	.	.	.	INTERGENIC(INTERGENIC)
4	122423872	122423887	122423881	CCTC	T	snp	1	1	1	1	.	.	.	INTERGENIC(INTERGENIC)
4	122586385	122586396	122586392	AG	C	snp	1	1	1	1	.	.	.	ANXA5(DOWNSTREAM);INTERGENIC(INTERGENIC)
4	122586810	122586833	122586829	TCCC	G	snp	0	0	0	1	.	.	.	ANXA5(DOWNSTREAM);INTERGENIC(INTERGENIC)
4	122708621	122708640	122708632	TCAT	A	snp	1	1	0	1	.	.	.	INTERGENIC(INTERGENIC)
4	122721709	122721718	122721713	T	G	ins	1	1	1	1	.	.	.	EXOSC9(UPSTREAM);INTERGENIC(INTERGENIC)
4	122721709	122721718	122721714	T	G	snp	1	0	0	0	.	.	.	EXOSC9(UPSTREAM);INTERGENIC(INTERGENIC)
4	122748141	122748160	122748155	AAAC	T	snp	1	0	0	0	.	.	.	BBS7(DOWNSTREAM);BBS7(INTRON);CCNA2(UPSTREAM)
4	122892688	122892701	122892699	CA	G	snp	0	0	1	0	.	.	.	INTERGENIC(INTERGENIC)
4	122898151	122898160	122898154	T	G	snp	1	1	1	1	.	.	.	INTERGENIC(INTERGENIC)
4	122991449	122991460	122991455	TA	C	snp	1	1	1	1	.	.	.	INTERGENIC(INTERGENIC)
4	123000630	123000639	123000634	T	C	snp	1	1	0	1	.	.	.	AC097533.1.1(UPSTREAM);INTERGENIC(INTERGENIC)
4	123044644	123044667	123044659	TTTC	TTTCTTTCT	del	1	1	1	1	.	.	.	INTERGENIC(INTERGENIC)
4	123136990	123136997	123136992	C	CCG	ins	1	0	0	0	.	.	.	KIAA1109(INTRON);KIAA1109(UPSTREAM)
4	123161612	123161621	123161619	GA	G	snp	1	1	1	1	.	.	.	KIAA1109(DOWNSTREAM);KIAA1109(INTRON)
4	123364113	123364120	123364114	T	G	snp	1	0	0	0	.	.	.	INTERGENIC(INTERGENIC)
4	123406109	123406116	123406114	T	C	snp	1	0	0	0	.	.	.	INTERGENIC(INTERGENIC)
4	123420209	123420220	123420216	ATA	AATAA	del	1	1	0	1	.	.	.	INTERGENIC(INTERGENIC)
4	123424763	123424776	123424770	TC	A	snp	1	0	0	0	.	.	.	INTERGENIC(INTERGENIC)
4	123509813	123509826	123509817	CA	A	snp	1	0	0	0	.	.	.	INTERGENIC(INTERGENIC)
4	123627360	123627369	123627364	TC	G	snp	1	1	0	0	.	.	.	INTERGENIC(INTERGENIC)
4	123635339	123635348	123635346	TC	T	snp	1	0	0	0	.	.	.	INTERGENIC(INTERGENIC)
4	123662333	123662341	123662339	T	G	snp	1	1	1	1	.	.	.	BBS12(INTRON)
4	123670560	123670569	123670566	TA	C	snp	1	0	0	0	.	.	.	BBS12(DOWNSTREAM);INTERGENIC(INTERGENIC)
4	123700071	123700080	123700075	T	C	ins	1	0	0	0	.	.	.	INTERGENIC(INTERGENIC)
4	123707232	123707241	123707237	A	G	snp	1	1	1	0	.	.	.	INTERGENIC(INTERGENIC)
4	123715832	123715840	123715835	T	C	snp	1	0	0	0	.	.	.	INTERGENIC(INTERGENIC)
4	123741655	123741663	123741660	T	C	snp	1	1	0	1	.	.	.	INTERGENIC(INTERGENIC);RP11-170N16.1.1(UPSTREAM)
4	123822606	123822615	123822608	TG	G	snp	1	0	0	0	.	.	.	FGF2(DOWNSTREAM);NUDT6(INTRON)
4	123940142	123940150	123940146	A	G	snp	1	0	0	0	.	.	.	SPATA5(INTRON)
4	123963490	123963505	123963502	AG	G	snp	1	0	0	0	.	.	.	SPATA5(INTRON)
4	123968248	123968255	123968251	T	C	snp	1	1	1	1	.	.	.	SPATA5(INTRON)
4	123998114	123998131	123998127	TG	T	del	1	0	0	0	.	.	.	SPATA5(INTRON)
4	124051329	124051340	124051338	AT	A	del	1	1	1	1	.	.	.	SPATA5(INTRON)
4	124078719	124078727	124078726	A	G	snp	1	1	1	1	.	.	.	SPATA5(INTRON)
4	124079529	124079536	124079533	T	G	snp	1	1	1	1	.	.	.	SPATA5(INTRON)
4	124088422	124088431	124088425	TA	G	snp	1	0	0	0	.	.	.	SPATA5(INTRON)
4	124150235	124150242	124150240	A	G	snp	1	1	0	0	.	.	.	SPATA5(INTRON)
4	124169229	124169238	124169230	A	C	snp	1	0	0	0	.	.	.	SPATA5(INTRON)
4	124244242	124244251	124244245	AT	C	snp	1	0	0	0	.	.	.	INTERGENIC(INTERGENIC)
4	124244294	124244303	124244297	AT	C	snp	1	0	0	0	.	.	.	INTERGENIC(INTERGENIC)
4	124335581	124335588	124335586	A	C	snp	1	1	1	1	.	.	.	INTERGENIC(INTERGENIC)
4	124335966	124335983	124335971	AAC	T	snp	0	0	1	0	.	.	.	INTERGENIC(INTERGENIC)
4	124359607	124359615	124359609	A	G	snp	1	1	1	1	.	.	.	INTERGENIC(INTERGENIC)
4	124441922	124441930	124441927	A	G	snp	1	0	0	0	.	.	.	RP11-381N20.1.1(INTRON);RP11-381N20.2.1(INTRON)
4	124533091	124533099	124533096	T	C	snp	1	1	1	1	.	.	.	INTERGENIC(INTERGENIC)
4	124606925	124606933	124606927	A	G	snp	1	1	1	1	.	.	.	RP11-93L9.1.1(INTRON)
4	124685489	124685500	124685491	AT	G	snp	1	0	0	0	.	.	.	RP11-93L9.1.1(INTRON)
4	124685509	124685522	124685518	AC	T	snp	1	1	1	1	.	.	.	RP11-93L9.1.1(INTRON)
4	124753518	124753525	124753519	G	A	snp	1	0	1	0	.	.	.	RP11-93L9.1.1(INTRON)
4	124756463	124756470	124756468	A	C	snp	1	0	0	0	.	.	.	RP11-93L9.1.1(INTRON)
4	124832355	124832364	124832359	A	G	snp	1	0	0	0	.	.	.	RP11-93L9.1.1(INTRON)
4	124860185	124860196	124860189	AT	G	snp	1	1	1	1	.	.	.	INTERGENIC(INTERGENIC)
4	124868106	124868115	124868111	GA	T	snp	0	0	1	1	.	.	.	INTERGENIC(INTERGENIC)
4	124903752	124903765	124903763	AT	A	del	1	1	1	1	.	.	.	INTERGENIC(INTERGENIC)
4	124931029	124931036	124931030	T	G	snp	0	1	1	1	.	.	.	INTERGENIC(INTERGENIC)
4	125060893	125060902	125060900	TA	G	snp	0	0	1	0	.	.	.	INTERGENIC(INTERGENIC)
4	125097025	125097036	125097033	TA	C	snp	0	0	1	0	.	.	.	CTD-2325B11.1.1(INTRON)
4	125195216	125195224	125195217	T	G	snp	1	0	0	0	.	.	.	INTERGENIC(INTERGENIC)
4	125215457	125215464	125215463	A	G	snp	1	0	0	0	.	.	.	INTERGENIC(INTERGENIC)
4	125389440	125389448	125389441	A	G	snp	0	0	1	0	.	.	.	INTERGENIC(INTERGENIC);RP11-93I21.1.1(UPSTREAM)
4	125438157	125438164	125438159	T	G	snp	1	1	1	1	.	.	.	RP11-93I21.3.1(INTRON)
4	125457659	125457670	125457662	TTA	C	snp	1	1	1	1	.	.	.	RP11-93I21.3.1(INTRON)

4	125509423	125509431	125509424	A	C	ins	1	1	1	1	.	.	.	INTERGENIC(INTERGENIC)
4	125572960	125572968	125572963	A	G	snp	1	0	0	0	.	.	.	INTERGENIC(INTERGENIC)
4	125833888	125833895	125833889	T	G	snp	1	0	0	0	.	.	.	INTERGENIC(INTERGENIC)
4	125889627	125889638	125889631	TAA	G	snp	1	0	0	0	.	.	.	INTERGENIC(INTERGENIC)
4	125908065	125908080	125908074	TC	AA	ins	0	1	1	1	.	.	.	INTERGENIC(INTERGENIC)
4	125908065	125908080	125908075	TC	A	snp	0	1	1	1	.	.	.	INTERGENIC(INTERGENIC)
4	125908065	125908080	125908076	TC	A	snp	0	1	1	1	.	.	.	INTERGENIC(INTERGENIC)
4	126119093	126119101	126119096	A	C	snp	1	1	1	1	.	.	.	INTERGENIC(INTERGENIC)
4	126184782	126184790	126184788	A	C	snp	1	0	0	0	.	.	.	INTERGENIC(INTERGENIC)
4	126206485	126206498	126206494	TC	G	snp	0	0	1	0	.	.	.	INTERGENIC(INTERGENIC)
4	126208313	126208322	126208320	AC	A	snp	0	0	0	1	.	.	.	INTERGENIC(INTERGENIC)
4	126235872	126235879	126235877	A	G	ins	1	1	0	1	.	.	.	FAT4(UPSTREAM);INTERGENIC(INTERGENIC)
4	126261949	126261960	126261956	AGT	A	snp	0	1	1	1	.	.	.	FAT4(INTRON)
4	126266604	126266613	126266612	A	C	ins	0	1	1	1	.	.	.	FAT4(INTRON)
4	126289642	126289655	126289649	AT	C	snp	0	1	1	1	.	.	.	FAT4(INTRON)
4	126324936	126324943	126324940	T	C	snp	1	0	0	0	.	.	.	FAT4(INTRON)
4	126338120	126338131	126338124	AAT	G	snp	1	1	1	1	.	.	.	FAT4(INTRON)
4	126399436	126399447	126399440	TTA	G	snp	1	1	1	1	.	.	.	FAT4(INTRON)
4	126426150	126426161	126426156	TG	G	snp	1	1	1	1	.	.	.	INTERGENIC(INTERGENIC)
4	126459061	126459069	126459068	A	G	snp	0	1	1	1	.	.	.	INTERGENIC(INTERGENIC)
4	126477034	126477041	126477037	A	G	snp	1	1	1	1	.	.	.	INTERGENIC(INTERGENIC)
4	126480730	126480749	126480744	TCCT	TT	ins	1	0	1	0	.	.	.	INTERGENIC(INTERGENIC)
4	126612146	126612155	126612148	CT	T	snp	0	0	1	1	.	.	.	RP11-399F2.2.1(INTRON)
4	126615103	126615112	126615110	A	G	snp	1	1	1	1	.	.	.	RP11-399F2.2.1(INTRON)
4	126624390	126624405	126624403	GA	G	snp	1	0	0	0	.	.	.	RP11-399F2.2.1(INTRON)
4	126635381	126635398	126635386	CA	CACACG	ins	1	0	0	0	.	.	.	RP11-399F2.2.1(INTRON)
4	126680662	126680669	126680668	T	G	snp	1	1	1	1	.	.	.	INTERGENIC(INTERGENIC)
4	126772836	126772843	126772841	T	G	snp	1	0	0	0	.	.	.	INTERGENIC(INTERGENIC)
4	126898410	126898421	126898413	TA	G	snp	0	0	0	1	.	.	.	INTERGENIC(INTERGENIC)
4	126933761	126933774	126933768	AT	C	snp	1	0	0	0	.	.	.	INTERGENIC(INTERGENIC)
4	126963886	126963905	126963901	TTTA	C	snp	0	1	1	1	.	.	.	INTERGENIC(INTERGENIC)
4	127022883	127022894	127022888	TGG	A	snp	1	0	0	0	.	.	.	INTERGENIC(INTERGENIC);RP11-318I4.2.1(DOWNSTREAM)
4	127032931	127032938	127032937	T	G	snp	1	0	0	0	.	.	.	INTERGENIC(INTERGENIC);RP11-318I4.2.1(UPSTREAM)
4	127052336	127052345	127052338	TG	C	snp	1	0	0	0	.	.	.	INTERGENIC(INTERGENIC)
4	127066844	127066853	127066845	G	T	snp	1	0	1	0	.	.	.	INTERGENIC(INTERGENIC)
4	127083775	127083782	127083779	A	TG	ins	1	1	1	1	.	.	.	INTERGENIC(INTERGENIC)
4	127168476	127168484	127168477	A	G	snp	1	1	1	1	.	.	.	INTERGENIC(INTERGENIC)
4	127193213	127193228	127193224	ATTT	A	del	1	1	1	1	.	.	.	INTERGENIC(INTERGENIC)
4	127259201	127259212	127259206	TTA	G	snp	1	1	1	1	.	.	.	INTERGENIC(INTERGENIC)
4	127270883	127270906	127270891	CAAA	T	snp	1	1	1	1	.	.	.	INTERGENIC(INTERGENIC)
4	127381087	127381094	127381090	T	C	snp	0	0	0	1	.	.	.	INTERGENIC(INTERGENIC)
4	127482462	127482473	127482469	AT	C	snp	1	0	0	0	.	.	.	INTERGENIC(INTERGENIC);RP11-123G5.1.1(DOWNSTREAM);RP11-662G20.1.1(UPSTREAM)
4	127492304	127492311	127492309	T	C	snp	0	0	0	1	.	.	.	RP11-123G5.1.1(INTRON)
4	127499565	127499572	127499568	T	C	snp	1	1	1	1	.	.	.	RP11-123G5.1.1(INTRON)
4	127644221	127644229	127644224	A	C	snp	0	0	1	0	.	.	.	RP11-123G5.1.1(INTRON)
4	127652999	127653007	127653000	A	G	ins	0	1	0	1	.	.	.	RP11-123G5.1.1(INTRON)
4	127676064	127676081	127676067	AG	AA	ins	1	0	0	0	.	.	.	RP11-123G5.1.1(INTRON)
4	127730966	127730980	127730971	TTG	A	snp	1	1	1	1	.	.	.	RP11-123G5.1.1(INTRON)
4	127782017	127782028	127782022	TTA	C	snp	1	0	0	0	.	.	.	RP11-123G5.1.1(INTRON)
4	127871611	127871618	127871615	T	C	snp	1	1	1	1	.	.	.	RP11-123G5.1.1(INTRON)
4	127881963	127881972	127881966	AG	AA	ins	1	1	1	1	.	.	.	INTERGENIC(INTERGENIC)
4	127924171	127924178	127924172	A	C	snp	1	1	1	1	.	.	.	INTERGENIC(INTERGENIC)
4	128019239	128019254	128019250	TC	TT	ins	1	0	0	0	.	.	.	RP11-125O18.1.1(INTRON)
4	128021687	128021698	128021696	TG	TGTC	ins	1	1	1	0	.	.	.	RP11-125O18.1.1(INTRON)
4	128033592	128033603	128033601	TA	T	snp	1	1	1	0	.	.	.	RP11-125O18.1.1(INTRON)
4	128264527	128264538	128264530	CT	G	snp	1	0	0	0	.	.	.	RP11-125O18.1.1(INTRON)
4	128521643	128521650	128521645	A	G	snp	0	1	1	1	.	.	.	INTERGENIC(INTERGENIC)
4	128532120	128532135	128532131	TA	T	snp	0	1	1	0	.	.	.	INTERGENIC(INTERGENIC)
4	128532120	128532135	128532133	TA	T	snp	1	1	1	0	.	.	.	INTERGENIC(INTERGENIC)
4	128563169	128563182	128563178	AT	A	del	1	0	0	0	.	.	.	INTU(INTRON)
4	128563169	128563182	128563180	AT	A	del	1	0	0	0	.	.	.	INTU(INTRON)
4	128597755	128597766	128597757	AG	G	del	1	1	1	1	.	.	.	INTU(DOWNSTREAM);INTU(INTRON)
4	128657024	128657051	128657040	TTTA	TTA	del	1	1	1	1	.	.	.	SLC25A31(INTRON)
4	128657024	128657051	128657041	TTTA	TA	del	1	1	1	1	.	.	.	SLC25A31(INTRON)
4	128657024	128657051	128657044	TTTA	TTA	del	1	1	1	1	.	.	.	SLC25A31(INTRON)
4	128657024	128657051	128657045	TTTA	TA	del	1	1	1	1	.	.	.	SLC25A31(INTRON)

4	128814958	128814967	128814964	GA	T	snp	1	1	1	1	.	"PLK4(NON_SYNONYMOUS_CODING):E752D,E769D,E76D,E789D,E798D,E830D"	.	PLK4(DOWNSTREAM);PLK4(EXON);U6(DOWNSTREAM)
4	128922033	128922042	128922038	TG	A	snp	1	0	0	0	.	.	.	C4orf29(INTRON);C4orf29(UPSTREAM)
4	128963371	128963380	128963372	A	C	ins	1	0	1	0	.	.	.	C4orf29(DOWNSTREAM);INTERGENIC(INTERGENIC)
4	128963371	128963380	128963373	A	C	snp	1	1	1	0	.	.	.	C4orf29(DOWNSTREAM);INTERGENIC(INTERGENIC)
4	128986076	128986090	128986079	TAA	C	snp	1	0	0	0	.	.	.	LARP1B(INTRON);RP11-214O14.1.1(DOWNSTREAM)
4	129018471	129018479	129018475	T	G	snp	0	1	1	1	.	.	.	LARP1B(INTRON)
4	129026222	129026231	129026226	T	G	snp	1	1	1	1	.	.	.	LARP1B(INTRON)
4	129124474	129124497	129124479	CTCC	C	snp	1	1	1	1	.	.	.	LARP1B(INTRON)
4	129159641	129159656	129159652	TA	T	ins	0	0	0	1	.	.	.	INTERGENIC(INTERGENIC)
4	129159641	129159656	129159653	TA	A	del	1	1	1	1	.	.	.	INTERGENIC(INTERGENIC)
4	129174366	129174377	129174374	CAC	G	snp	1	0	1	0	.	.	.	INTERGENIC(INTERGENIC)
4	129294031	129294050	129294045	TTTC	C	del	1	1	0	1	.	.	.	RP11-420A23.1.1(INTRON)
4	129466367	129466380	129466370	CA	G	snp	1	0	0	0	.	.	.	INTERGENIC(INTERGENIC)
4	129524498	129524515	129524513	TG	A	snp	1	1	1	1	.	.	.	INTERGENIC(INTERGENIC);RP11-184M15.2.1(UPSTREAM)
4	129538567	129538586	129538573	AAAT	T	del	0	1	1	1	.	.	.	INTERGENIC(INTERGENIC)
4	129538567	129538586	129538582	AAAT	A	snp	1	1	1	1	.	.	.	INTERGENIC(INTERGENIC)
4	129569225	129569239	129569235	ACA	A	snp	1	0	0	0	.	.	.	INTERGENIC(INTERGENIC)
4	129613245	129613265	129613255	TTG	C	snp	1	1	0	1	.	.	.	INTERGENIC(INTERGENIC)
4	129615824	129615833	129615831	TA	G	snp	1	0	0	0	.	.	.	INTERGENIC(INTERGENIC)
4	129660133	129660142	129660137	TA	G	snp	1	1	1	1	.	.	.	INTERGENIC(INTERGENIC)
4	129667846	129667853	129667847	A	C	snp	1	0	0	0	.	.	.	INTERGENIC(INTERGENIC)
4	129697128	129697137	129697133	AG	A	snp	1	0	0	0	.	.	.	INTERGENIC(INTERGENIC)
4	129706378	129706401	129706383	ATTT	TAA	ins	1	0	0	0	.	.	.	INTERGENIC(INTERGENIC)
4	129778072	129778079	129778076	T	C	snp	1	1	1	1	.	.	.	PHF17(DOWNSTREAM);PHF17(INTRON)
4	129780351	129780360	129780358	GT	GTGA	ins	1	0	0	0	.	.	.	PHF17(INTRON)
4	129864270	129864279	129864275	TC	T	snp	0	0	1	0	.	SCLT1(NON_SYNONYMOUS_CODING):R503K	.	SCLT1(EXON);SCLT1(INTRON)
4	129904046	129904061	129904050	TTTG	C	snp	1	1	1	1	.	.	.	SCLT1(INTRON)
4	130151851	130151858	130151856	A	G	snp	0	0	0	1	.	.	.	INTERGENIC(INTERGENIC)
4	130170633	130170644	130170639	TG	C	snp	1	1	1	1	.	.	.	INTERGENIC(INTERGENIC)
4	130248813	130248820	130248817	A	G	snp	1	1	1	1	.	.	.	INTERGENIC(INTERGENIC)
4	130272644	130272658	130272655	GCT	GCTACTGCTGCTGCC	ins	1	1	1	1	.	.	.	INTERGENIC(INTERGENIC)
4	130399377	130399391	130399385	TTG	A	snp	1	0	0	0	.	.	.	INTERGENIC(INTERGENIC)
4	130541182	130541189	130541184	T	C	snp	0	1	0	0	.	.	.	INTERGENIC(INTERGENIC)
4	130572799	130572808	130572807	T	G	snp	1	0	0	0	.	.	.	INTERGENIC(INTERGENIC)
4	130677079	130677098	130677087	GTTT	T	del	1	1	1	1	.	.	.	RP11-519M16.1.1(INTRON)
4	130689133	130689156	130689144	ATT	ATC	ins	1	1	1	1	.	.	.	RP11-422J15.1.1(UPSTREAM);RP11-519M16.1.1(INTRON)
4	130753062	130753071	130753065	G	GT	ins	0	1	1	0	.	.	.	RP11-422J15.1.1(INTRON)
4	130997651	130997668	130997665	GT	T	snp	0	0	1	0	.	.	.	INTERGENIC(INTERGENIC)
4	131000546	131000557	131000548	GT	A	snp	1	1	0	0	.	.	.	INTERGENIC(INTERGENIC)
4	131030632	131030640	131030638	T	G	snp	1	0	0	1	.	.	.	INTERGENIC(INTERGENIC)
4	131043939	131043953	131043944	CAA	C	del	1	1	1	1	.	.	.	INTERGENIC(INTERGENIC)
4	131043939	131043953	131043947	CAA	C	del	1	1	1	1	.	.	.	INTERGENIC(INTERGENIC)
4	131043939	131043953	131043950	CAA	C	del	1	1	1	1	.	.	.	INTERGENIC(INTERGENIC)
4	131118519	131118526	131118521	A	G	snp	0	0	0	1	.	.	.	INTERGENIC(INTERGENIC)
4	131142121	131142128	131142127	A	C	ins	0	1	1	0	.	.	.	INTERGENIC(INTERGENIC)
4	131190701	131190708	131190703	T	TATTG	ins	1	1	1	1	.	.	.	INTERGENIC(INTERGENIC)
4	131203237	131203248	131203241	AC	C	del	1	0	0	0	.	.	.	INTERGENIC(INTERGENIC)
4	131203237	131203248	131203243	AC	C	del	1	0	0	0	.	.	.	INTERGENIC(INTERGENIC)
4	131233178	131233187	131233180	T	G	snp	1	0	0	0	.	.	.	INTERGENIC(INTERGENIC)
4	131248140	131248147	131248146	T	C	snp	1	1	1	0	.	.	.	INTERGENIC(INTERGENIC)
4	131396500	131396515	131396510	GT	A	snp	0	1	1	0	.	.	.	INTERGENIC(INTERGENIC)
4	131400129	131400146	131400141	AT	G	snp	1	0	0	0	.	.	.	INTERGENIC(INTERGENIC)
4	131442314	131442341	131442331	TATT	T	del	1	1	1	1	.	.	.	INTERGENIC(INTERGENIC)
4	131442314	131442341	131442335	TATT	T	del	1	0	0	1	.	.	.	INTERGENIC(INTERGENIC)
4	131458045	131458054	131458049	TG	C	snp	1	0	0	0	.	.	.	INTERGENIC(INTERGENIC)
4	131468469	131468476	131468470	A	C	ins	1	1	1	1	.	.	.	INTERGENIC(INTERGENIC)
4	131468469	131468476	131468471	A	C	ins	1	1	1	1	.	.	.	INTERGENIC(INTERGENIC)
4	131473526	131473534	131473531	A	C	snp	1	1	1	1	.	.	.	INTERGENIC(INTERGENIC)
4	131491371	131491378	131491377	A	C	snp	1	1	1	1	.	.	.	INTERGENIC(INTERGENIC)
4	131561317	131561334	131561330	TG	T	snp	1	1	1	1	.	.	.	INTERGENIC(INTERGENIC)
4	131610791	131610798	131610797	T	G	snp	1	0	0	0	.	.	.	INTERGENIC(INTERGENIC)
4	131700627	131700635	131700628	T	G	snp	1	0	0	0	.	.	.	INTERGENIC(INTERGENIC)
4	131710173	131710182	131710174	A	G	snp	1	0	0	0	.	.	.	INTERGENIC(INTERGENIC)
4	131716036	131716045	131716043	TA	GT	ins	0	1	1	1	.	.	.	INTERGENIC(INTERGENIC)
4	132026751	132026760	132026756	TA	G	snp	1	1	1	1	.	.	.	INTERGENIC(INTERGENIC)

4	132066702	132066717	132066709	AGAT	C	snp	1	1	1	1	.	.	.	INTERGENIC(INTERGENIC)
4	132187235	132187262	132187242	TAAT	A	snp	1	0	0	0	.	.	.	INTERGENIC(INTERGENIC)
4	132299606	132299613	132299611	T	C	snp	1	1	1	1	.	.	.	INTERGENIC(INTERGENIC);RP11-314N14.1.1(UPSTREAM)
4	132347209	132347218	132347211	A	G	ins	1	0	0	0	.	.	.	RP11-314N14.1.1(INTRON)
4	132379688	132379697	132379691	TC	A	snp	1	0	0	1	.	.	.	RP11-314N14.1.1(INTRON)
4	132410194	132410203	132410198	T	C	snp	0	1	0	1	.	.	.	RP11-314N14.1.1(INTRON)
4	132418790	132418798	132418795	A	C	snp	0	1	1	1	.	.	.	RP11-314N14.1.1(INTRON)
4	132426168	132426181	132426175	TA	G	snp	0	1	1	1	.	.	.	RP11-314N14.1.1(INTRON)
4	132517333	132517356	132517340	CCTT	C	snp	1	1	1	1	.	.	.	INTERGENIC(INTERGENIC)
4	132548493	132548500	132548499	T	G	snp	1	0	0	0	.	.	.	INTERGENIC(INTERGENIC)
4	132579093	132579106	132579101	AT	G	snp	1	0	0	0	.	.	.	INTERGENIC(INTERGENIC)
4	132649187	132649196	132649193	TG	C	snp	1	1	1	0	.	.	.	INTERGENIC(INTERGENIC)
4	132684010	132684025	132684013	TC	G	snp	0	0	0	1	.	.	.	INTERGENIC(INTERGENIC)
4	132730332	132730340	132730338	T	G	snp	1	1	1	1	.	.	.	INTERGENIC(INTERGENIC)
4	132734891	132734899	132734893	T	C	snp	1	1	1	1	.	.	.	INTERGENIC(INTERGENIC)
4	132772639	132772648	132772643	T	C	snp	0	0	0	1	.	.	.	INTERGENIC(INTERGENIC)
4	132792025	132792032	132792029	T	G	snp	1	0	0	0	.	.	.	INTERGENIC(INTERGENIC)
4	132883920	132883929	132883928	A	AGT	ins	0	1	0	1	.	.	.	RP11-745L13.2.1(INTRON)
4	132917344	132917351	132917348	A	C	snp	1	0	0	0	.	.	.	INTERGENIC(INTERGENIC)
4	132950048	132950063	132950055	CA	G	snp	1	0	0	0	.	.	.	RP11-1E22.1.1(UPSTREAM);RP11-789C2.1.1(INTRON)
4	133086324	133086339	133086330	TTGT	T	ins	1	1	0	1	.	.	.	INTERGENIC(INTERGENIC)
4	133086324	133086339	133086333	TTGT	TTGCCAG	ins	1	1	0	1	.	.	.	INTERGENIC(INTERGENIC)
4	133100363	133100377	133100370	AAT	C	snp	1	0	0	0	.	.	.	INTERGENIC(INTERGENIC)
4	133107069	133107083	133107077	AGC	T	snp	1	0	0	0	.	.	.	INTERGENIC(INTERGENIC)
4	133115047	133115054	133115053	T	C	snp	1	1	0	1	.	.	.	INTERGENIC(INTERGENIC)
4	133192601	133192610	133192608	GT	A	snp	1	0	0	0	.	.	.	INTERGENIC(INTERGENIC)
4	133209340	133209348	133209341	A	C	ins	1	1	1	1	.	.	.	INTERGENIC(INTERGENIC)
4	133312938	133312945	133312942	A	G	ins	1	1	1	0	.	.	.	INTERGENIC(INTERGENIC)
4	133425473	133425482	133425476	AT	A	snp	1	0	1	0	.	.	.	INTERGENIC(INTERGENIC)
4	133532464	133532473	133532469	TG	A	snp	1	0	0	0	.	.	.	RP11-149A7.2.1(INTRON)
4	133545663	133545677	133545671	CTC	G	snp	1	1	1	1	.	.	.	RP11-149A7.2.1(INTRON)
4	133584585	133584594	133584591	AT	C	snp	1	1	1	1	.	.	.	RP11-149A7.2.1(INTRON)
4	133641004	133641011	133641007	T	C	snp	1	0	0	0	.	.	.	INTERGENIC(INTERGENIC)
4	133649984	133649999	133649994	AT	T	ins	1	0	0	1	.	.	.	INTERGENIC(INTERGENIC)
4	133649984	133649999	133649996	AT	T	snp	1	1	1	1	.	.	.	INTERGENIC(INTERGENIC)
4	133649984	133649999	133649997	AT	A	del	1	1	1	1	.	.	.	INTERGENIC(INTERGENIC)
4	133700110	133700119	133700112	TA	C	snp	1	1	1	1	.	.	.	INTERGENIC(INTERGENIC)
4	133762975	133762988	133762978	CT	C	snp	1	0	1	0	.	.	.	RP11-404I7.2.1(INTRON)
4	133768173	133768180	133768174	A	G	snp	1	0	0	0	.	.	.	RP11-404I7.2.1(INTRON)
4	133790192	133790199	133790197	A	G	ins	1	1	1	1	.	.	.	RP11-404I7.2.1(INTRON)
4	133857061	133857070	133857063	CA	T	snp	1	1	1	0	.	.	.	RP11-404I7.2.1(INTRON)
4	133916564	133916573	133916570	CA	T	snp	1	0	0	0	.	.	.	INTERGENIC(INTERGENIC)
4	133933832	133933839	133933833	A	C	ins	1	1	1	1	.	.	.	INTERGENIC(INTERGENIC)
4	134012183	134012190	134012187	A	C	snp	1	1	0	1	.	.	.	RP11-9G1.3.1(DOWNSTREAM);RP11-9G1.3.1(INTRON)
4	134037835	134037846	134037842	CAA	AC	del	1	1	1	1	.	.	.	RP11-9G1.3.1(INTRON)
4	134062200	134062209	134062204	AT	C	snp	1	1	0	1	.	.	.	RP11-9G1.3.1(INTRON)
4	134201515	134201528	134201526	GT	G	del	1	1	1	1	.	.	.	INTERGENIC(INTERGENIC)
4	134248930	134248943	134248934	TA	C	snp	1	0	0	0	.	.	.	INTERGENIC(INTERGENIC)
4	134417300	134417307	134417302	T	C	snp	1	0	0	0	.	.	.	INTERGENIC(INTERGENIC)
4	134446149	134446157	134446154	A	C	snp	0	0	1	0	.	.	.	INTERGENIC(INTERGENIC)
4	134494957	134494964	134494963	T	C	snp	1	0	0	0	.	.	.	INTERGENIC(INTERGENIC);RP11-285A15.1.1(DOWNSTREAM)
4	134529875	134529884	134529881	AT	G	snp	1	0	0	0	.	.	.	INTERGENIC(INTERGENIC)
4	134661111	134661120	134661112	T	G	snp	1	0	0	0	.	.	.	INTERGENIC(INTERGENIC)
4	134695811	134695819	134695817	A	G	snp	1	1	1	1	.	.	.	INTERGENIC(INTERGENIC)
4	134707036	134707047	134707041	AG	A	snp	1	1	1	1	.	.	.	AC133605.1(UPSTREAM);INTERGENIC(INTERGENIC)
4	134761514	134761523	134761518	CT	T	snp	1	1	1	1	.	.	.	INTERGENIC(INTERGENIC)
4	135109254	135109265	135109257	TG	T	snp	1	1	0	1	.	.	.	INTERGENIC(INTERGENIC)
4	135307475	135307484	135307482	AT	C	snp	1	1	1	1	.	.	.	INTERGENIC(INTERGENIC)
4	135332578	135332587	135332579	T	C	ins	1	1	1	1	.	.	.	INTERGENIC(INTERGENIC)
4	135332578	135332587	135332580	T	C	snp	1	1	1	1	.	.	.	INTERGENIC(INTERGENIC)
4	135373046	135373053	135373049	C	T	snp	1	1	0	1	.	.	.	RP11-400D2.3.1(INTRON)
4	135479124	135479137	135479135	TA	G	snp	1	1	0	1	.	.	.	INTERGENIC(INTERGENIC)
4	135485114	135485125	135485122	AAT	A	ins	1	1	0	1	.	.	.	INTERGENIC(INTERGENIC)
4	135518778	135518785	135518782	A	G	snp	1	0	0	0	.	.	.	INTERGENIC(INTERGENIC)
4	135588210	135588217	135588211	T	C	snp	1	1	0	1	.	.	.	INTERGENIC(INTERGENIC)
4	135665259	135665266	135665263	A	G	snp	1	1	1	1	.	.	.	INTERGENIC(INTERGENIC)

4	135717186	135717195	135717189	A	C	snp	1	0	0	0	.	.	.	CTD-2012I17.1.1(INTRON)
4	135773703	135773712	135773705	AC	C	snp	1	0	0	0	.	.	.	INTERGENIC(INTERGENIC)
4	135784241	135784252	135784244	TTA	C	snp	1	0	0	0	.	.	.	INTERGENIC(INTERGENIC)
4	135884451	135884459	135884452	T	C	snp	0	0	0	1	.	.	.	INTERGENIC(INTERGENIC)
4	136113448	136113465	136113461	ATT	C	snp	1	1	1	1	.	.	.	INTERGENIC(INTERGENIC)
4	136164824	136164843	136164833	TTTC	TC	del	1	0	0	0	.	.	.	INTERGENIC(INTERGENIC)
4	136238010	136238019	136238013	AG	A	snp	1	0	0	0	.	.	.	INTERGENIC(INTERGENIC);U6(UPSTREAM)
4	136356023	136356032	136356030	TA	G	snp	1	1	1	1	.	.	.	INTERGENIC(INTERGENIC)
4	136572115	136572124	136572121	TA	A	snp	1	1	1	1	.	.	.	INTERGENIC(INTERGENIC)
4	136688444	136688451	136688447	A	G	snp	1	1	1	1	.	.	.	INTERGENIC(INTERGENIC)
4	136695084	136695092	136695088	T	G	snp	1	1	1	1	.	.	.	INTERGENIC(INTERGENIC)
4	136809733	136809740	136809739	A	AG	ins	1	1	1	1	.	.	.	RP11-782E2.1.1(INTRON);RP11-782E2.1.1(UPSTREAM)
4	136889352	136889367	136889359	AAAT	C	snp	1	0	0	0	.	.	.	INTERGENIC(INTERGENIC)
4	136945307	136945315	136945313	A	AT	ins	1	0	0	0	.	.	.	INTERGENIC(INTERGENIC)
4	136992830	136992837	136992836	A	G	snp	1	0	0	0	.	.	.	INTERGENIC(INTERGENIC)
4	137053957	137053966	137053959	T	G	snp	1	1	1	1	.	.	.	RP11-775H9.1.1(INTRON);RP11-775H9.2.1(INTRON)
4	137199545	137199564	137199560	TTTG	TTTGTT	del	1	0	0	0	.	.	.	RP11-775H9.2.1(INTRON)
4	137209938	137209946	137209942	T	G	snp	1	1	0	0	.	.	.	RP11-775H9.2.1(INTRON)
4	137242651	137242678	137242661	AATA	A	del	1	0	0	0	.	.	.	RP11-775H9.2.1(INTRON)
4	137295712	137295721	137295718	T	G	snp	1	0	0	0	.	.	.	RP11-775H9.2.1(INTRON)
4	137372268	137372283	137372271	AG	T	snp	1	1	1	1	.	.	.	INTERGENIC(INTERGENIC)
4	137400718	137400727	137400725	TC	T	snp	1	1	1	1	.	.	.	INTERGENIC(INTERGENIC)
4	137479015	137479022	137479018	C	T	snp	1	1	1	1	.	.	.	INTERGENIC(INTERGENIC)
4	137488147	137488156	137488149	TG	A	snp	1	1	1	1	.	.	.	INTERGENIC(INTERGENIC)
4	137526974	137526983	137526976	CA	T	snp	1	1	1	1	.	.	.	INTERGENIC(INTERGENIC)
4	137558882	137558891	137558883	A	AC	ins	1	0	0	0	.	.	.	INTERGENIC(INTERGENIC)
4	137558882	137558891	137558886	A	AC	ins	1	0	0	0	.	.	.	INTERGENIC(INTERGENIC)
4	137671958	137671969	137671964	TAA	A	snp	0	0	0	1	.	.	.	INTERGENIC(INTERGENIC)
4	137846397	137846420	137846412	AAAT	A	snp	1	1	1	1	.	.	.	RP11-138I17.1.1(INTRON);RP11-138I17.1.1(UPSTREAM)
4	137854036	137854045	137854042	TA	C	snp	1	0	0	0	.	.	.	RP11-138I17.1.1(INTRON)
4	137864675	137864682	137864681	A	C	snp	0	1	1	1	.	.	.	RP11-138I17.1.1(INTRON)
4	138085858	138085873	138085867	TC	T	snp	1	0	0	0	.	.	.	RP11-138I17.1.1(INTRON)
4	138086060	138086069	138086063	AT	A	snp	1	1	1	0	.	.	.	RP11-138I17.1.1(INTRON)
4	138090393	138090402	138090396	TA	G	snp	1	0	1	1	.	.	.	RP11-138I17.1.1(INTRON)
4	138103452	138103467	138103455	AG	C	snp	1	0	0	0	.	.	.	RP11-138I17.1.1(INTRON)
4	138149235	138149246	138149243	CT	A	snp	1	1	1	1	.	.	.	AC060835.1(UPSTREAM);INTERGENIC(INTERGENIC)
4	138154925	138154934	138154926	T	G	snp	0	0	1	0	.	.	.	INTERGENIC(INTERGENIC)
4	138231348	138231357	138231354	GA	C	snp	0	0	1	0	.	.	.	INTERGENIC(INTERGENIC)
4	138281718	138281727	138281720	CA	G	snp	1	0	0	0	.	.	.	INTERGENIC(INTERGENIC)
4	138292941	138292950	138292942	A	C	snp	1	1	1	1	.	.	.	INTERGENIC(INTERGENIC)
4	138333990	138334001	138333998	CA	ACAT	ins	1	1	1	1	.	.	.	INTERGENIC(INTERGENIC)
4	138488206	138488225	138488217	ATCT	G	snp	1	1	1	1	.	.	.	RP11-714L20.1.1(INTRON)
4	138522766	138522786	138522775	AAC	G	snp	0	1	1	1	.	.	.	RP11-714L20.1.1(EXON)
4	138607045	138607054	138607052	AC	ACA	del	1	1	1	0	.	.	.	INTERGENIC(INTERGENIC);RP13-884E18.4.1(DOWNSTREAM)
4	138647493	138647506	138647497	AT	C	snp	1	1	1	1	.	.	.	RP13-884E18.4.1(INTRON)
4	138647493	138647506	138647504	AT	A	snp	1	1	1	1	.	.	.	RP13-884E18.4.1(INTRON)
4	138653507	138653516	138653514	TC	T	snp	1	0	0	0	.	.	.	RP13-884E18.4.1(INTRON)
4	138698627	138698636	138698629	TG	A	snp	1	1	1	1	.	.	.	INTERGENIC(INTERGENIC)
4	138708493	138708500	138708499	A	C	snp	1	1	1	1	.	.	.	INTERGENIC(INTERGENIC)
4	138712895	138712902	138712900	A	G	snp	1	1	1	1	.	.	.	INTERGENIC(INTERGENIC)
4	138730266	138730275	138730267	A	C	snp	1	1	1	1	.	.	.	RP11-793B23.1.1(INTRON)
4	138866063	138866072	138866066	AC	T	snp	1	0	0	0	.	.	.	INTERGENIC(INTERGENIC)
4	138949557	138949564	138949560	T	G	snp	1	0	0	1	.	.	.	RP11-8P17.1.1(DOWNSTREAM);RP11-8P17.1.1(INTRON)
4	139027190	139027201	139027193	AT	C	snp	1	0	0	0	.	.	.	RP11-725C19.1.1(INTRON);RP11-8P17.1.1(INTRON)
4	139102739	139102747	139102745	A	G	snp	1	0	0	1	.	.	.	RP11-725C19.1.1(DOWNSTREAM);SLC7A11(INTRON)
4	139223869	139223880	139223878	AT	G	snp	1	1	1	1	.	.	.	LINC00498(INTRON)
4	139377404	139377413	139377409	A	G	snp	0	0	1	1	.	.	.	INTERGENIC(INTERGENIC)
4	139409323	139409332	139409326	A	G	snp	0	1	0	1	.	.	.	INTERGENIC(INTERGENIC)
4	139410467	139410476	139410471	AT	G	snp	1	1	1	1	.	.	.	INTERGENIC(INTERGENIC)
4	139496851	139496860	139496853	A	G	ins	0	0	1	0	.	.	.	INTERGENIC(INTERGENIC)
4	139501824	139501832	139501825	T	C	snp	1	1	1	1	.	.	.	INTERGENIC(INTERGENIC)
4	139557617	139557625	139557619	T	C	snp	1	1	1	1	.	.	.	INTERGENIC(INTERGENIC)
4	139640551	139640560	139640556	A	AC	ins	1	1	1	1	.	.	.	INTERGENIC(INTERGENIC)
4	139640551	139640560	139640557	A	C	ins	1	1	1	1	.	.	.	INTERGENIC(INTERGENIC)
4	139706941	139706949	139706942	A	AC	ins	1	0	1	0	.	.	.	RP11-98O2.1.1(INTRON)
4	139706941	139706949	139706943	A	C	ins	1	0	1	0	.	.	.	RP11-98O2.1.1(INTRON)

4	139713810	139713827	139713816	ATG	T	snp	1	1	1	1	.	.	.	RP11-98O2.1.1(INTRON)
4	139731803	139731818	139731813	AAAT	G	snp	1	0	0	0	.	.	.	AC093766.1(DOWNSTREAM);INTERGENIC(INTERGENIC)
4	139802280	139802291	139802284	TGG	T	snp	1	0	0	0	.	.	.	RP11-371F15.3.1(INTRON)
4	139863238	139863249	139863243	TTC	T	ins	1	1	1	1	.	.	.	RP11-371F15.3.1(INTRON);RP11-371F15.3.1(UPSTREAM)
4	139955731	139955745	139955740	GTG	T	snp	1	1	1	1	.	.	.	CCRN4L(INTRON);ELF2(INTRON)
4	140125240	140125247	140125245	A	G	snp	1	0	0	0	.	.	.	INTERGENIC(INTERGENIC);Y_RNA(DOWNSTREAM)
4	140219753	140219760	140219754	A	C	snp	1	1	1	1	.	.	.	NAA15(UPSTREAM);NDUFC1(INTRON);NDUFC1(UPSTREAM)
4	140227752	140227761	140227757	T	G	snp	1	1	1	1	.	.	.	NAA15(INTRON);NDUFC1(UPSTREAM)
4	140341227	140341234	140341233	A	G	snp	0	0	0	1	.	.	.	INTERGENIC(INTERGENIC);NAA15(DOWNSTREAM)
4	140384853	140384868	140384863	TGTC	A	snp	1	1	1	1	.	.	.	RAB33B(INTRON)
4	140481379	140481388	140481381	G	GC	ins	1	1	0	1	.	.	.	INTERGENIC(INTERGENIC);SETD7(UPSTREAM)
4	140481379	140481388	140481384	G	GC	ins	1	1	0	1	.	.	.	INTERGENIC(INTERGENIC);SETD7(UPSTREAM)
4	140500757	140500772	140500766	ATTT	C	snp	1	0	1	1	.	.	.	INTERGENIC(INTERGENIC)
4	140532167	140532184	140532181	TC	CTC	del	1	1	1	1	.	.	.	INTERGENIC(INTERGENIC)
4	140589015	140589024	140589016	A	G	snp	1	1	1	1	.	.	.	MGST2(INTRON)
4	140786640	140786649	140786645	T	TTTG	ins	1	1	1	1	.	.	.	MAML3(INTRON)
4	140786640	140786649	140786648	T	G	ins	1	1	1	1	.	.	.	MAML3(INTRON)
4	140896604	140896615	140896613	CA	T	snp	1	0	0	0	.	.	.	MAML3(INTRON)
4	141012218	141012229	141012226	AAC	AACA	del	1	1	1	1	.	.	.	MAML3(INTRON)
4	141069102	141069111	141069109	GA	G	snp	1	0	0	0	.	.	.	MAML3(INTRON)
4	141075644	141075653	141075648	GT	A	snp	1	1	1	1	.	.	.	INTERGENIC(INTERGENIC);MAML3(UPSTREAM)
4	141081968	141081985	141081975	TA	TG	ins	1	0	0	0	.	.	.	INTERGENIC(INTERGENIC)
4	141081968	141081985	141081981	TA	TATAT	del	1	1	1	1	.	.	.	INTERGENIC(INTERGENIC)
4	141152688	141152697	141152689	C	CCA	ins	1	0	0	0	.	.	.	INTERGENIC(INTERGENIC)
4	141254475	141254484	141254480	TG	A	snp	1	1	1	1	.	.	.	RP11-425I13.3.1(INTRON);SCOC(INTRON)
4	141526407	141526427	141526416	TTG	C	snp	1	1	0	1	.	.	.	INTERGENIC(INTERGENIC);RP11-5K16.3.1(UPSTREAM)
4	141545654	141545669	141545662	CAAA	AAACA	del	1	1	0	1	.	.	.	TBC1D9(INTRON)
4	141545654	141545669	141545665	CAAA	C	del	1	1	0	1	.	.	.	TBC1D9(INTRON)
4	141595831	141595846	141595844	AT	A	del	1	1	0	0	.	.	.	TBC1D9(INTRON)
4	141664043	141664063	141664056	AGG	A	snp	1	0	0	0	.	.	.	TBC1D9(INTRON)
4	141677316	141677330	141677325	GCG	A	snp	0	1	0	1	.	.	.	INTERGENIC(INTERGENIC);TBC1D9(UPSTREAM)
4	141680443	141680466	141680449	ATTC	C	snp	1	1	1	1	.	.	.	INTERGENIC(INTERGENIC);TBC1D9(UPSTREAM)
4	141722864	141722873	141722871	T	C	snp	1	0	0	0	.	.	.	INTERGENIC(INTERGENIC)
4	141732207	141732224	141732215	CT	T	snp	1	0	0	0	.	.	.	INTERGENIC(INTERGENIC)
4	141775389	141775397	141775393	T	C	snp	1	0	0	0	.	.	.	INTERGENIC(INTERGENIC)
4	141801794	141801802	141801799	T	G	ins	1	1	1	1	.	.	.	RNF150(INTRON)
4	141856211	141856222	141856218	TA	T	snp	1	1	1	1	.	.	.	RNF150(INTRON)
4	141965938	141965953	141965949	CA	CAC	del	1	0	1	0	.	.	.	RNF150(INTRON)
4	142011966	142011974	142011970	T	G	snp	1	1	1	1	.	.	.	RNF150(INTRON)
4	142099399	142099416	142099404	TG	A	snp	1	0	0	0	.	.	.	RNF150(INTRON)
4	142219298	142219311	142219309	TC	T	snp	1	0	0	0	.	.	.	INTERGENIC(INTERGENIC);snoU13(UPSTREAM)
4	142275475	142275484	142275478	AC	T	snp	1	1	1	1	.	.	.	INTERGENIC(INTERGENIC)
4	142458952	142458961	142458959	A	C	ins	1	1	1	1	.	.	.	INTERGENIC(INTERGENIC)
4	142515630	142515639	142515632	AC	C	snp	0	1	1	1	.	.	.	INTERGENIC(INTERGENIC)
4	142518083	142518090	142518084	A	G	snp	0	0	0	1	.	.	.	INTERGENIC(INTERGENIC)
4	142530220	142530233	142530231	GA	G	del	1	0	0	0	.	.	.	INTERGENIC(INTERGENIC)
4	142599536	142599544	142599537	T	C	snp	1	0	0	0	.	.	.	IL15(INTRON)
4	142640635	142640642	142640637	A	G	snp	1	1	1	1	.	.	.	IL15(INTRON);IL15(UPSTREAM)
4	142660796	142660813	142660810	AAC	A	ins	1	1	1	1	.	.	.	INTERGENIC(INTERGENIC)
4	142673266	142673274	142673270	T	C	snp	1	0	0	1	.	.	.	INTERGENIC(INTERGENIC)
4	142798491	142798498	142798494	T	C	ins	1	1	1	1	.	.	.	INTERGENIC(INTERGENIC)
4	142933303	142933318	142933308	TTTG	C	snp	1	1	1	1	.	.	.	INTERGENIC(INTERGENIC)
4	142999112	142999121	142999113	A	C	ins	1	1	1	1	.	.	.	INPP4B(DOWNSTREAM);INPP4B(INTRON)
4	143019516	143019525	143019518	A	C	snp	1	1	1	1	.	.	.	INPP4B(INTRON)
4	143043165	143043172	143043169	A	C	snp	1	0	0	0	.	.	.	INPP4B(INTRON)
4	143074123	143074131	143074127	A	C	snp	1	0	0	0	.	.	.	INPP4B(INTRON)
4	143146671	143146684	143146676	AT	AC	ins	1	0	0	0	.	.	.	INPP4B(INTRON)
4	143169472	143169489	143169487	TC	TCT	del	1	0	0	0	.	.	.	INPP4B(INTRON)
4	143416667	143416682	143416676	AT	G	snp	1	1	1	1	.	.	.	INPP4B(INTRON)
4	143614068	143614087	143614083	ATAG	C	snp	1	1	0	1	.	.	.	INPP4B(INTRON)
4	143641885	143641900	143641898	AT	C	snp	1	0	0	0	.	.	.	INPP4B(INTRON)
4	143724337	143724356	143724343	AAAG	G	del	1	1	1	1	.	.	.	INPP4B(INTRON)
4	143727902	143727913	143727910	AAG	A	ins	1	0	0	0	.	.	.	INPP4B(INTRON)
4	143762198	143762205	143762200	A	C	snp	1	0	0	0	.	.	.	INPP4B(INTRON)
4	143773115	143773122	143773121	A	C	snp	1	1	1	1	.	.	.	INPP4B(UPSTREAM);INTERGENIC(INTERGENIC)
4	143779555	143779564	143779562	AC	T	snp	1	0	0	0	.	.	.	INTERGENIC(INTERGENIC)
4	143835757	143835772	143835768	TTTA	T	ins	1	0	0	0	.	.	.	INTERGENIC(INTERGENIC)

4	143902327	143902334	143902333	T	G	snp	1	1	1	1	.	.	.	RP11-284M14.1.1(INTRON)
4	144107342	144107351	144107344	CA	T	snp	1	0	0	0	.	.	.	RP11-284M14.1.1(UPSTREAM);USP38(INTRON)
4	144295022	144295039	144295025	AT	A	snp	1	1	0	1	.	.	.	GAB1(INTRON);GAB1(UPSTREAM)
4	144601268	144601275	144601271	A	G	snp	1	1	1	0	.	.	.	FREM3(INTRON)
4	144656968	144656975	144656970	T	C	snp	1	1	1	1	.	.	.	RP13-578N3.3.1(INTRON)
4	144683762	144683769	144683768	T	G	snp	1	1	1	1	.	.	.	RP11-780M14.1.1(UPSTREAM);RP13-578N3.3.1(INTRON)
4	144700210	144700219	144700216	TA	A	ins	1	1	1	1	.	.	.	RP13-578N3.3.1(INTRON)
4	144711545	144711558	144711553	TC	C	snp	1	1	1	1	.	.	.	RP13-578N3.3.1(INTRON)
4	144713481	144713494	144713492	TA	T	del	1	1	1	1	.	.	.	RP13-578N3.3.1(INTRON)
4	144713481	144713494	144713492	TA	T	snp	1	0	0	0	.	.	.	RP13-578N3.3.1(INTRON)
4	144763683	144763694	144763689	ATT	G	snp	1	1	1	1	.	.	.	RP13-578N3.3.1(INTRON)
4	144786298	144786305	144786301	T	TC	ins	1	1	1	1	.	.	.	INTERGENIC(INTERGENIC);RP13-578N3.3.1(DOWNSTREAM)
4	144953361	144953376	144953372	GT	C	snp	1	1	1	0	.	.	.	GYPB(INTRON);RP11-673E1.4.1(INTRON)
4	145068773	145068787	145068779	ACA	C	snp	1	1	1	1	.	.	.	INTERGENIC(INTERGENIC)
4	145076473	145076488	145076485	TC	CTC	del	1	1	1	1	.	.	.	INTERGENIC(INTERGENIC)
4	145176955	145176966	145176959	TTA	A	snp	0	1	1	1	.	.	.	INTERGENIC(INTERGENIC)
4	145560325	145560334	145560332	AG	A	snp	1	1	1	1	.	.	.	INTERGENIC(INTERGENIC);RP11-291L15.2.1(DOWNSTREAM)
4	145711610	145711621	145711612	TA	ATATATATA	del	1	1	1	1	.	.	.	INTERGENIC(INTERGENIC)
4	145711610	145711621	145711614	TA	ATATATA	del	1	1	1	1	.	.	.	INTERGENIC(INTERGENIC)
4	145711610	145711621	145711616	TA	ATATA	del	1	1	1	1	.	.	.	INTERGENIC(INTERGENIC)
4	145792600	145792609	145792602	A	C	snp	1	1	1	1	.	.	.	RP13-539F13.3.1(INTRON)
4	145863753	145863760	145863759	T	C	snp	1	1	1	1	.	.	.	INTERGENIC(INTERGENIC)
4	146081091	146081100	146081096	AC	T	snp	1	1	1	1	.	.	.	OTUD4(INTRON)
4	146242507	146242522	146242514	AT	A	snp	1	1	1	0	.	.	.	INTERGENIC(INTERGENIC)
4	146311664	146311683	146311676	GAAG	C	snp	1	0	0	0	.	.	.	INTERGENIC(INTERGENIC)
4	146398301	146398324	146398318	TATT	T	snp	1	0	0	0	.	.	.	INTERGENIC(INTERGENIC);SMAD1(UPSTREAM)
4	146410619	146410634	146410630	AAAT	AAATA	del	1	0	1	0	.	.	.	SMAD1(INTRON)
4	146494641	146494656	146494650	TTAT	C	snp	1	0	0	0	.	.	.	INTERGENIC(INTERGENIC)
4	146586688	146586696	146586689	T	G	snp	1	0	0	0	.	.	.	INTERGENIC(INTERGENIC)
4	146783674	146783689	146783679	AT	C	snp	1	0	1	0	.	.	.	ZNF827(INTRON)
4	146876987	146877002	146876998	AG	C	snp	1	1	1	1	.	.	.	INTERGENIC(INTERGENIC);Y_RNA(DOWNSTREAM)
4	146913798	146913809	146913802	TTG	C	snp	1	0	0	0	.	.	.	INTERGENIC(INTERGENIC)
4	146917977	146917984	146917980	T	G	snp	1	0	0	0	.	.	.	INTERGENIC(INTERGENIC)
4	147271160	147271169	147271167	AT	C	snp	1	0	0	0	.	.	.	SLC10A7(INTRON)
4	147309913	147309924	147309921	AAC	AA	ins	1	1	1	1	.	.	.	SLC10A7(INTRON)
4	147345822	147345831	147345826	A	C	snp	1	0	0	0	.	.	.	SLC10A7(INTRON)
4	147549057	147549064	147549059	A	G	snp	1	0	0	0	.	.	.	INTERGENIC(INTERGENIC)
4	147578066	147578077	147578070	TA	ATAC	ins	1	0	0	0	.	.	.	INTERGENIC(INTERGENIC)
4	147578066	147578077	147578074	TA	C	snp	1	0	0	1	.	.	.	INTERGENIC(INTERGENIC)
4	147691272	147691281	147691274	A	G	snp	1	0	0	0	.	.	.	TTC29(INTRON)
4	147954357	147954366	147954364	AG	C	snp	1	1	1	1	.	.	.	INTERGENIC(INTERGENIC)
4	147966305	147966318	147966314	AT	C	snp	0	0	1	0	.	.	.	INTERGENIC(INTERGENIC)
4	147974104	147974113	147974109	AT	C	snp	1	1	1	1	.	.	.	INTERGENIC(INTERGENIC)
4	148016554	148016563	148016559	TA	C	snp	1	0	0	0	.	.	.	INTERGENIC(INTERGENIC)
4	148092038	148092045	148092039	A	G	snp	1	0	1	1	.	.	.	INTERGENIC(INTERGENIC)
4	148188558	148188565	148188559	A	G	snp	0	1	1	1	.	.	.	INTERGENIC(INTERGENIC)
4	148334284	148334292	148334288	T	C	snp	1	0	0	0	.	.	.	INTERGENIC(INTERGENIC)
4	148444621	148444632	148444626	TTG	T	ins	1	1	1	1	.	.	.	EDNRA(DOWNSTREAM);EDNRA(INTRON)
4	148496166	148496173	148496168	T	G	snp	1	1	1	1	.	.	.	RP11-752L20.5.1(INTRON)
4	148519633	148519642	148519640	GT	C	snp	1	1	1	1	.	.	.	INTERGENIC(INTERGENIC);RP11-752L20.5.1(DOWNSTREAM)
4	148616984	148616991	148616985	C	A	snp	1	0	0	0	.	.	.	INTERGENIC(INTERGENIC)
4	148687717	148687726	148687720	GT	G	snp	1	1	1	1	.	.	.	ARHGAP10(INTRON)
4	148780727	148780734	148780729	T	G	snp	1	1	1	1	.	.	.	ARHGAP10(DOWNSTREAM);ARHGAP10(INTRON);ARHGAP10(UPSTREAM)
4	148814586	148814595	148814593	AT	C	snp	1	0	0	0	.	.	.	ARHGAP10(INTRON)
4	148820480	148820495	148820483	GT	C	snp	1	0	0	0	.	.	.	ARHGAP10(INTRON)
4	148821971	148821994	148821982	CAA	G	snp	1	1	1	1	.	.	.	ARHGAP10(INTRON)
4	148970972	148970979	148970975	T	G	snp	1	0	0	0	.	.	.	ARHGAP10(INTRON)
4	149010806	149010814	149010808	T	G	snp	1	0	0	0	.	.	.	NR3C2(INTRON)
4	149055777	149055794	149055787	TC	CTCTCTC	del	1	0	0	0	.	.	.	NR3C2(INTRON)
4	149057257	149057264	149057262	A	C	snp	1	1	1	1	.	.	.	NR3C2(INTRON)
4	149065355	149065363	149065359	T	C	snp	1	1	1	1	.	.	.	NR3C2(DOWNSTREAM);NR3C2(INTRON);RP11-76G10.1.1(UPSTREAM)
4	149068095	149068103	149068096	A	G	snp	1	0	0	0	.	.	.	NR3C2(INTRON);RP11-76G10.1.1(INTRON)
4	149199730	149199739	149199735	CA	C	snp	1	0	0	0	.	.	.	NR3C2(INTRON)

4	149247553	149247572	149247568	AAAT	AAATA	del	1	0	0	0	.	.	.	NR3C2(INTRON)
4	149291526	149291535	149291532	A	C	snp	1	1	1	1	.	.	.	NR3C2(INTRON);NR3C2(UPSTREAM)
4	149414630	149414639	149414633	TA	G	snp	1	1	1	1	.	.	.	INTERGENIC(INTERGENIC)
4	149414812	149414821	149414815	TA	G	snp	1	1	1	1	.	.	.	INTERGENIC(INTERGENIC)
4	149477509	149477522	149477515	GT	C	snp	1	1	1	1	.	.	.	INTERGENIC(INTERGENIC);RN5S166(UPSTREAM)
4	149544286	149544301	149544298	AT	C	snp	0	0	1	0	.	.	.	INTERGENIC(INTERGENIC)
4	149581839	149581850	149581843	TA	C	snp	1	1	1	1	.	.	.	INTERGENIC(INTERGENIC)
4	149595398	149595412	149595408	TTA	C	snp	1	1	0	1	.	.	.	INTERGENIC(INTERGENIC)
4	149632367	149632374	149632373	G	A	ins	1	1	1	1	.	.	.	INTERGENIC(INTERGENIC)
4	149677133	149677140	149677139	C	A	ins	0	1	0	1	.	.	.	INTERGENIC(INTERGENIC)
4	149704903	149704912	149704907	T	C	snp	1	0	0	0	.	.	.	INTERGENIC(INTERGENIC)
4	149728842	149728869	149728857	GAAG	T	snp	1	0	0	0	.	.	.	INTERGENIC(INTERGENIC)
4	149855676	149855684	149855678	A	C	snp	1	1	1	1	.	.	.	INTERGENIC(INTERGENIC)
4	150111020	150111027	150111025	A	C	snp	1	0	0	0	.	.	.	RP11-24I21.1.1(INTRON)
4	150185285	150185293	150185286	A	C	snp	1	1	1	1	.	.	.	RP11-24I21.1.1(INTRON)
4	150255100	150255109	150255105	AC	T	snp	1	1	1	1	.	.	.	INTERGENIC(INTERGENIC)
4	150289909	150289922	150289920	TA	G	snp	0	0	0	1	.	.	.	RP11-526A4.1.1(INTRON)
4	150328316	150328323	150328320	C	T	snp	1	0	0	0	.	.	.	RP11-526A4.1.1(INTRON)
4	150386379	150386386	150386384	T	C	snp	1	1	1	1	.	.	.	RP11-526A4.1.1(INTRON)
4	150386715	150386726	150386723	CAC	T	snp	1	1	1	1	.	.	.	RP11-526A4.1.1(INTRON)
4	150678409	150678417	150678416	A	C	snp	1	1	1	1	.	.	.	RP11-526A4.1.1(INTRON)
4	150788862	150788873	150788870	TA	C	snp	1	0	0	0	.	.	.	RP11-526A4.1.1(INTRON)
4	150789082	150789097	150789092	AT	TC	ins	0	0	1	0	.	.	.	RP11-526A4.1.1(INTRON)
4	150789793	150789802	150789800	AT	AAC	ins	1	0	1	0	.	.	.	RP11-526A4.1.1(INTRON)
4	150850598	150850606	150850602	A	G	snp	1	1	1	1	.	.	.	INTERGENIC(INTERGENIC);U6(UPSTREAM)
4	150908312	150908321	150908317	TG	A	snp	1	0	0	0	.	.	.	INTERGENIC(INTERGENIC)
4	150916295	150916310	150916298	AG	AC	ins	1	1	1	1	.	.	.	INTERGENIC(INTERGENIC)
4	150992509	150992518	150992512	AT	C	snp	0	1	0	1	.	.	.	INTERGENIC(INTERGENIC)
4	150996289	150996302	150996293	GT	TA	ins	1	1	1	1	.	.	.	DCLK2(UPSTREAM);INTERGENIC(INTERGENIC)
4	151002845	151002854	151002847	T	G	snp	1	1	1	1	.	.	.	DCLK2(INTRON)
4	151078692	151078701	151078699	TC	T	snp	1	1	1	1	.	.	.	DCLK2(INTRON)
4	151091498	151091506	151091500	T	C	snp	0	0	1	0	.	.	.	DCLK2(INTRON)
4	151105733	151105753	151105748	TTC	TC	del	1	0	0	0	.	.	.	DCLK2(INTRON)
4	151207962	151207981	151207969	TGGT	C	snp	1	1	1	1	.	.	.	LRBA(INTRON)
4	151207962	151207981	151207970	TGGT	C	snp	1	1	1	1	.	.	.	LRBA(INTRON)
4	151310506	151310533	151310522	TAAA	C	snp	0	1	1	1	.	.	.	LRBA(INTRON)
4	151331201	151331208	151331207	A	C	snp	1	1	1	1	.	.	.	LRBA(INTRON)
4	151356127	151356134	151356130	A	G	snp	1	1	1	1	.	.	.	LRBA(INTRON)
4	151658211	151658218	151658214	T	G	snp	1	1	1	1	.	.	.	LRBA(INTRON)
4	151770175	151770186	151770179	TA	C	snp	1	0	0	0	.	.	.	LRBA(INTRON);LRBA(UPSTREAM)
4	151771129	151771137	151771134	A	C	snp	1	1	1	1	.	.	.	LRBA(INTRON);LRBA(UPSTREAM)
4	151798252	151798260	151798253	A	C	snp	0	1	1	0	.	.	.	LRBA(INTRON)
4	151829656	151829663	151829659	T	C	snp	1	1	1	1	.	.	.	LRBA(INTRON)
4	152016840	152016847	152016844	A	G	snp	0	0	0	1	.	.	.	INTERGENIC(INTERGENIC);RPS3A(UPSTREAM)
4	152166470	152166479	152166472	T	G	ins	1	1	1	1	.	.	.	INTERGENIC(INTERGENIC)
4	152167369	152167378	152167376	TA	C	snp	1	1	1	1	.	.	.	INTERGENIC(INTERGENIC)
4	152196978	152197001	152196993	TAT	ATTATTA	del	1	1	1	1	.	.	.	INTERGENIC(INTERGENIC);PRSS48(UPSTREAM);RP11-731D1.3.1(DOWNSTREAM)
4	152330290	152330297	152330294	C	T	snp	1	1	1	1	.	.	.	FAM160A1(UPSTREAM);INTERGENIC(INTERGENIC);RP11-610P16.1.1(UPSTREAM)
4	152394467	152394474	152394472	T	C	snp	1	1	1	1	.	.	.	FAM160A1(INTRON)
4	152796020	152796027	152796025	T	G	snp	1	1	1	1	.	.	.	INTERGENIC(INTERGENIC)
4	152822098	152822105	152822099	A	C	snp	1	0	0	0	.	.	.	INTERGENIC(INTERGENIC);RP11-73G16.3.1(DOWNSTREAM)
4	152823466	152823474	152823467	T	TA	ins	1	1	1	1	.	.	.	INTERGENIC(INTERGENIC);RP11-73G16.3.1(DOWNSTREAM)
4	152835319	152835326	152835320	T	G	snp	1	1	1	1	.	.	.	RP11-73G16.3.1(INTRON)
4	152885277	152885285	152885279	T	G	snp	1	1	1	1	.	.	.	RP11-73G16.1.1(INTRON)
4	153010689	153010700	153010697	GGT	G	snp	1	0	0	0	.	.	.	INTERGENIC(INTERGENIC)
4	153032936	153032945	153032939	AT	A	snp	1	1	1	1	.	.	.	INTERGENIC(INTERGENIC)
4	153097891	153097899	153097893	T	TCC	ins	1	1	1	1	.	.	.	RP11-18H21.2.1(INTRON)
4	153099202	153099213	153099210	AGG	A	snp	1	0	0	0	.	.	.	RP11-18H21.2.1(INTRON)
4	153112442	153112461	153112451	ATTT	A	snp	1	0	0	0	.	.	.	INTERGENIC(INTERGENIC)
4	153353805	153353814	153353806	A	C	snp	1	0	0	0	.	.	.	FBXW7(INTRON)
4	153457151	153457165	153457162	GCG	A	snp	1	1	1	0	.	.	.	FBXW7(UPSTREAM);INTERGENIC(INTERGENIC)
4	153521192	153521200	153521194	G	T	snp	1	0	0	0	.	.	.	INTERGENIC(INTERGENIC)
4	153581529	153581536	153581530	C	T	snp	0	0	1	1	.	.	.	TMEM154(INTRON)
4	153620174	153620181	153620178	T	C	snp	0	1	1	0	.	.	.	INTERGENIC(INTERGENIC)
4	153790589	153790604	153790600	TTTC	T	ins	1	1	1	1	.	.	.	ARFIP1(INTRON)

4	153834825	153834844	153834837	ATTT	T	ins	1	0	0	0	.	.	.	ARFIP1(DOWNSTREAM);ARFIP1(INTRON)
4	153834825	153834844	153834840	ATTT	A	del	1	1	1	0	.	.	.	ARFIP1(DOWNSTREAM);ARFIP1(INTRON)
4	153875840	153875849	153875846	A	G	snp	1	1	1	1	.	.	.	FHDC1(INTRON)
4	154001458	154001472	154001469	AGC	T	snp	1	1	1	1	.	.	.	RP11-285C1.2.1(INTRON)
4	154013114	154013123	154013118	A	G	snp	1	0	0	0	.	.	.	INTERGENIC(INTERGENIC);RP11-285C1.2.1(DOWNSTREAM)
4	154057129	154057140	154057132	AG	A	snp	1	0	0	0	.	.	.	INTERGENIC(INTERGENIC)
4	154117416	154117424	154117420	C	CA	ins	1	0	0	0	.	.	.	TRIM2(INTRON)
4	154117416	154117424	154117421	C	A	ins	1	0	0	0	.	.	.	TRIM2(INTRON)
4	154233047	154233074	154233069	TTTA	A	del	1	0	1	0	.	.	.	ANXA2P1(UPSTREAM);TRIM2(DOWNSTREAM);TRIM2(INTRON)
4	154266315	154266322	154266319	C	A	snp	1	1	1	1	.	.	.	MND1(INTRON);MND1(UPSTREAM)
4	154269808	154269816	154269815	T	G	snp	1	1	1	1	.	.	.	MND1(INTRON);MND1(UPSTREAM)
4	154318806	154318815	154318812	A	G	ins	1	1	0	0	.	.	.	MND1(DOWNSTREAM);MND1(INTRON)
4	154342475	154342484	154342479	A	C	snp	1	1	0	0	.	.	.	INTERGENIC(INTERGENIC)
4	154407306	154407314	154407307	A	G	snp	0	0	1	0	.	.	.	KIAA0922(INTRON)
4	154468295	154468303	154468297	A	G	snp	1	1	1	1	.	.	.	KIAA0922(INTRON)
4	154515388	154515395	154515393	A	G	snp	1	1	1	1	.	.	.	KIAA0922(INTRON)
4	154565400	154565409	154565405	A	G	snp	1	0	0	0	.	.	.	INTERGENIC(INTERGENIC)
4	154674695	154674712	154674710	TC	TCT	del	1	1	1	1	.	.	.	RNF175(INTRON);RNF175(UPSTREAM)
4	154743752	154743763	154743758	GCA	A	snp	0	0	0	1	.	.	.	INTERGENIC(INTERGENIC)
4	154846648	154846657	154846653	TA	G	snp	1	1	1	1	.	.	.	INTERGENIC(INTERGENIC)
4	154990455	154990464	154990463	A	G	snp	1	0	0	0	.	.	.	INTERGENIC(INTERGENIC)
4	155134994	155135003	155135002	A	G	snp	0	0	0	1	.	.	.	INTERGENIC(INTERGENIC)
4	155331346	155331353	155331350	A	G	snp	1	1	1	1	.	.	.	DCHS2(INTRON)
4	155331346	155331353	155331351	A	G	snp	1	1	1	1	.	.	.	DCHS2(INTRON)
4	155440092	155440099	155440093	A	C	ins	1	0	0	0	.	.	.	INTERGENIC(INTERGENIC);RP11-158C21.2.1(UPSTREAM)
4	155686540	155686551	155686549	AT	G	snp	1	1	1	1	.	.	.	RP11-21G20.3.1(INTRON)
4	155787676	155787684	155787677	A	C	snp	1	1	1	1	.	.	.	INTERGENIC(INTERGENIC)
4	155861725	155861732	155861728	A	G	snp	1	0	0	0	.	.	.	INTERGENIC(INTERGENIC)
4	155919418	155919425	155919419	T	G	snp	1	0	0	0	.	.	.	INTERGENIC(INTERGENIC)
4	155971302	155971321	155971313	AAAG	A	del	1	0	0	0	.	.	.	INTERGENIC(INTERGENIC)
4	156004345	156004359	156004352	TAA	G	snp	1	1	1	1	.	.	.	INTERGENIC(INTERGENIC)
4	156004345	156004359	156004354	TAA	C	snp	1	0	0	0	.	.	.	INTERGENIC(INTERGENIC)
4	156089094	156089102	156089097	A	G	snp	1	0	0	0	.	.	.	INTERGENIC(INTERGENIC)
4	156200606	156200615	156200613	AT	C	snp	1	1	1	1	.	.	.	INTERGENIC(INTERGENIC)
4	156290515	156290524	156290517	A	C	snp	1	0	0	0	.	.	.	MAP9(DOWNSTREAM);MAP9(INTRON)
4	156299144	156299153	156299151	TA	TAT	del	1	1	1	1	.	.	.	INTERGENIC(INTERGENIC);MAP9(UPSTREAM);RP11-27G13.3.1(DOWNSTREAM)
4	156322697	156322706	156322698	A	C	ins	0	0	1	0	.	.	.	INTERGENIC(INTERGENIC)
4	156360530	156360539	156360537	AG	T	snp	1	1	0	0	.	.	.	INTERGENIC(INTERGENIC)
4	156392412	156392421	156392416	T	G	snp	1	1	1	1	.	.	.	INTERGENIC(INTERGENIC)
4	156434369	156434377	156434370	T	G	snp	1	1	1	1	.	.	.	INTERGENIC(INTERGENIC);RP13-487K5.1.1(UPSTREAM)
4	156558265	156558288	156558272	TTTG	A	snp	1	0	0	0	.	.	.	INTERGENIC(INTERGENIC)
4	156594806	156594817	156594808	TG	G	snp	1	1	0	1	.	.	.	GUCY1A3(INTRON)
4	156600285	156600292	156600288	T	G	snp	1	1	1	1	.	.	.	GUCY1A3(INTRON)
4	156628972	156628991	156628987	TTTC	T	ins	1	1	1	1	.	.	.	GUCY1A3(INTRON)
4	156653164	156653171	156653170	A	G	ins	1	1	1	1	.	.	.	GUCY1A3(DOWNSTREAM);GUCY1A3(UTR_3_PRIME);RP11-588K22.2.1(UPSTREAM)
4	156741491	156741502	156741500	TC	G	snp	1	0	0	0	.	.	.	INTERGENIC(INTERGENIC)
4	156765479	156765486	156765482	A	C	snp	1	1	1	1	.	.	.	ACCN5(INTRON)
4	156838134	156838141	156838135	A	C	ins	1	0	0	0	.	.	.	TDO2(INTRON)
4	156838134	156838141	156838136	A	C	snp	1	0	0	0	.	.	.	TDO2(INTRON)
4	156863066	156863074	156863069	T	C	snp	0	1	0	0	.	.	.	CTSO(INTRON)
4	156869218	156869225	156869223	T	G	ins	1	1	1	1	.	.	.	CTSO(INTRON)
4	156878390	156878409	156878397	TTTG	T	snp	1	1	0	1	.	.	.	CTSO(UPSTREAM);INTERGENIC(INTERGENIC)
4	156928316	156928323	156928321	A	C	snp	1	1	1	1	.	.	.	FTH1P21(DOWNSTREAM);INTERGENIC(INTERGENIC)
4	156994260	156994269	156994264	AT	G	snp	1	1	1	1	.	.	.	INTERGENIC(INTERGENIC)
4	156996819	156996827	156996821	T	G	ins	1	1	1	1	.	.	.	INTERGENIC(INTERGENIC)
4	157056440	157056447	157056441	A	C	snp	1	1	1	1	.	.	.	INTERGENIC(INTERGENIC)
4	157176033	157176044	157176042	AC	A	snp	1	0	0	0	.	.	.	INTERGENIC(INTERGENIC)
4	157268283	157268294	157268285	CT	G	snp	0	0	1	0	.	.	.	INTERGENIC(INTERGENIC)
4	157285774	157285785	157285778	AAT	G	snp	1	0	0	0	.	.	.	INTERGENIC(INTERGENIC)
4	157401869	157401876	157401870	A	C	ins	1	1	1	1	.	.	.	INTERGENIC(INTERGENIC)
4	157401869	157401876	157401875	A	C	snp	1	1	1	1	.	.	.	INTERGENIC(INTERGENIC)
4	157453404	157453413	157453407	TA	G	snp	1	1	1	1	.	.	.	INTERGENIC(INTERGENIC)
4	157616493	157616502	157616498	CA	C	snp	0	0	0	1	.	.	.	INTERGENIC(INTERGENIC);RP11-171N4.4.1(DOWNSTREAM)
4	157812070	157812081	157812078	TG	C	snp	0	1	1	0	.	.	.	PDGFC(INTRON)
4	157923930	157923947	157923939	AAT	ATAAT	ins	1	1	1	1	.	.	.	INTERGENIC(INTERGENIC)

4	157974172	157974180	157974175	C	A	ins	0	0	1	1	.	.	.	INTERGENIC(INTERGENIC)
4	157990010	157990017	157990013	A	G	snp	1	0	0	1	.	.	.	INTERGENIC(INTERGENIC);Y_RNA(DOWNSTREAM)
4	158041531	158041542	158041538	ATA	AATAA	del	0	0	1	0	.	.	.	GLRB(INTRON)
4	158177386	158177397	158177388	AT	T	ins	1	1	0	1	.	.	.	GRIA2(INTRON)
4	158281520	158281529	158281523	T	G	snp	1	1	1	1	.	.	.	GRIA2(INTRON);GRIA2(UPSTREAM)
4	158427504	158427511	158427508	A	G	snp	1	1	1	1	.	.	.	INTERGENIC(INTERGENIC)
4	158433214	158433223	158433218	TG	A	snp	1	1	1	1	.	.	.	INTERGENIC(INTERGENIC)
4	158456952	158456963	158456957	CT	G	snp	1	1	1	1	.	.	.	INTERGENIC(INTERGENIC)
4	158457648	158457657	158457649	T	G	snp	1	1	0	1	.	.	.	INTERGENIC(INTERGENIC)
4	158649649	158649656	158649650	C	T	snp	1	1	0	1	.	.	.	INTERGENIC(INTERGENIC)
4	158675273	158675288	158675283	GATA	C	snp	0	1	0	1	.	.	.	INTERGENIC(INTERGENIC)
4	158685708	158685717	158685715	TC	G	snp	1	0	0	0	.	.	.	INTERGENIC(INTERGENIC);Y_RNA(DOWNSTREAM)
4	158687312	158687319	158687318	T	G	snp	1	1	1	1	.	.	.	INTERGENIC(INTERGENIC);Y_RNA(DOWNSTREAM)
4	158863674	158863682	158863675	A	G	snp	1	1	1	1	.	.	.	INTERGENIC(INTERGENIC)
4	158993466	158993475	158993467	A	C	snp	1	1	1	1	.	.	.	INTERGENIC(INTERGENIC)
4	159027154	159027167	159027164	AT	C	snp	1	1	1	1	.	.	.	INTERGENIC(INTERGENIC)
4	159093345	159093354	159093350	AC	ACACA	del	1	1	0	1	.	.	.	FAM198B(INTRON);RP11-597D13.9.1(INTRON);RP11-597D13.9.1(UPSTREAM)
4	159357937	159357946	159357938	A	AG	ins	1	1	1	1	.	.	.	RXFP1(INTRON)
4	159403557	159403564	159403559	T	G	snp	1	1	1	1	.	.	.	RXFP1(INTRON)
4	159584584	159584593	159584588	AG	C	snp	1	0	0	0	.	.	.	C4orf46(DOWNSTREAM);INTERGENIC(INTERGENIC)
4	159697943	159697958	159697948	TGAA	A	snp	1	1	1	1	.	.	.	FNIP2(INTRON)
4	159804264	159804273	159804265	A	C	snp	1	1	1	1	.	.	.	FNIP2(INTRON)
4	159817994	159818003	159818001	T	TA	ins	1	1	1	1	.	.	.	C4orf45(INTRON);FNIP2(INTRON)
4	159914183	159914196	159914186	TG	C	snp	1	1	1	1	.	.	.	C4orf45(INTRON)
4	159974091	159974108	159974105	TAT	TA	del	1	1	1	1	.	.	.	INTERGENIC(INTERGENIC)
4	160045308	160045319	160045313	TTG	A	snp	1	0	0	0	.	.	.	RAPGEF2(INTRON)
4	160062735	160062746	160062743	TA	C	snp	1	0	0	0	.	.	.	RAPGEF2(INTRON)
4	160149868	160149877	160149875	AC	T	snp	1	1	1	1	.	.	.	RAPGEF2(INTRON);RAPGEF2(UPSTREAM)
4	160337978	160337985	160337979	A	C	snp	1	0	0	0	.	.	.	INTERGENIC(INTERGENIC)
4	160351247	160351256	160351251	TG	C	snp	0	1	0	1	.	.	.	INTERGENIC(INTERGENIC)
4	160547484	160547491	160547485	T	G	snp	1	1	1	1	.	.	.	INTERGENIC(INTERGENIC)
4	160653239	160653247	160653245	T	G	snp	1	0	0	0	.	.	.	RP11-6C14.1.1(INTRON)
4	160739140	160739147	160739141	A	G	snp	1	0	0	0	.	.	.	INTERGENIC(INTERGENIC)
4	160740540	160740548	160740547	T	C	snp	1	0	0	0	.	.	.	INTERGENIC(INTERGENIC)
4	160854657	160854664	160854658	T	G	snp	1	0	0	0	.	.	.	INTERGENIC(INTERGENIC)
4	160898897	160898908	160898905	AG	G	snp	1	1	1	1	.	.	.	INTERGENIC(INTERGENIC)
4	160948007	160948016	160948013	TC	A	snp	1	0	0	0	.	.	.	INTERGENIC(INTERGENIC)
4	160953964	160953975	160953971	AAT	G	snp	1	1	0	0	.	.	.	INTERGENIC(INTERGENIC)
4	161050512	161050521	161050518	CA	A	ins	0	1	1	1	.	.	.	INTERGENIC(INTERGENIC)
4	161102154	161102162	161102160	A	C	snp	0	1	0	0	.	.	.	INTERGENIC(INTERGENIC)
4	161246667	161246676	161246674	GA	C	snp	1	0	0	0	.	.	.	INTERGENIC(INTERGENIC)
4	161256145	161256154	161256148	TA	C	snp	1	1	1	1	.	.	.	INTERGENIC(INTERGENIC)
4	161271704	161271715	161271708	TA	ATATAC	ins	1	1	1	1	.	.	.	INTERGENIC(INTERGENIC)
4	161287950	161287957	161287956	A	AT	ins	1	1	1	1	.	.	.	INTERGENIC(INTERGENIC)
4	161318561	161318578	161318570	TA	T	snp	1	1	1	1	.	.	.	INTERGENIC(INTERGENIC)
4	161431953	161431964	161431960	TA	T	snp	1	1	1	1	.	.	.	INTERGENIC(INTERGENIC)
4	161436489	161436498	161436490	T	G	snp	1	1	1	1	.	.	.	INTERGENIC(INTERGENIC)
4	161447161	161447176	161447170	TCTT	TTTATT	ins	1	0	0	0	.	.	.	INTERGENIC(INTERGENIC);RP11-502M1.1.1(UPSTREAM)
4	161450004	161450011	161450009	A	G	snp	0	0	0	1	.	.	.	INTERGENIC(INTERGENIC)
4	161494474	161494481	161494475	T	C	snp	1	1	1	1	.	.	.	RP11-502M1.2.1(INTRON)
4	161494493	161494501	161494497	T	G	snp	1	1	1	1	.	.	.	RP11-502M1.2.1(INTRON)
4	161652899	161652914	161652907	TTCC	C	snp	1	0	1	0	.	.	.	INTERGENIC(INTERGENIC)
4	161654808	161654823	161654812	TA	C	snp	1	0	0	0	.	.	.	INTERGENIC(INTERGENIC)
4	161732729	161732737	161732734	A	G	snp	0	1	0	1	.	.	.	INTERGENIC(INTERGENIC)
4	161775696	161775707	161775698	TA	C	snp	1	1	1	1	.	.	.	INTERGENIC(INTERGENIC)
4	161787090	161787104	161787093	CAT	A	snp	1	1	1	1	.	.	.	INTERGENIC(INTERGENIC)
4	161788785	161788793	161788790	T	G	snp	1	1	1	1	.	.	.	INTERGENIC(INTERGENIC)
4	161812383	161812391	161812387	T	C	snp	1	1	1	1	.	.	.	INTERGENIC(INTERGENIC)
4	161857457	161857464	161857461	T	C	snp	1	1	1	1	.	.	.	INTERGENIC(INTERGENIC)
4	161897000	161897007	161897004	A	C	snp	0	1	1	1	.	.	.	INTERGENIC(INTERGENIC)
4	161900714	161900722	161900715	T	G	snp	1	0	0	0	.	.	.	INTERGENIC(INTERGENIC)
4	161900714	161900722	161900717	T	C	snp	0	1	1	1	.	.	.	INTERGENIC(INTERGENIC)
4	161960212	161960220	161960217	A	G	snp	1	0	0	0	.	.	.	INTERGENIC(INTERGENIC)
4	161987591	161987602	161987598	CAA	G	snp	1	1	1	1	.	.	.	INTERGENIC(INTERGENIC)
4	161994531	161994540	161994537	A	C	ins	1	1	1	1	.	.	.	INTERGENIC(INTERGENIC)
4	162118962	162118973	162118970	TG	GC	ins	0	1	0	1	.	.	.	INTERGENIC(INTERGENIC)

4	162157929	162157936	162157932	A	G	snp	1	0	0	0	.	.	.	INTERGENIC(INTERGENIC)
4	162213525	162213540	162213529	AAAT	G	snp	0	1	1	1	.	.	.	INTERGENIC(INTERGENIC)
4	162238850	162238859	162238851	T	G	ins	1	1	1	1	.	.	.	INTERGENIC(INTERGENIC)
4	162324456	162324469	162324459	TG	T	snp	1	0	0	0	.	.	.	FSTL5(INTRON)
4	162336844	162336857	162336851	AG	C	snp	0	1	1	1	.	.	.	FSTL5(INTRON)
4	162351357	162351365	162351361	A	G	snp	1	0	0	0	.	.	.	FSTL5(INTRON)
4	162368457	162368468	162368462	AT	C	snp	1	1	1	1	.	.	.	FSTL5(INTRON)
4	162450110	162450124	162450121	TCA	G	snp	1	1	1	1	.	.	.	FSTL5(INTRON)
4	162598427	162598438	162598431	GA	C	snp	1	0	0	0	.	.	.	FSTL5(INTRON)
4	162645049	162645056	162645050	A	C	snp	1	0	0	0	.	.	.	FSTL5(INTRON)
4	162672380	162672388	162672385	A	G	snp	0	0	1	1	.	.	.	FSTL5(INTRON)
4	162750250	162750258	162750257	A	C	snp	0	0	0	1	.	.	.	FSTL5(INTRON)
4	162786203	162786212	162786208	A	C	snp	1	1	1	1	.	.	.	FSTL5(INTRON)
4	162855769	162855776	162855771	T	G	snp	1	1	1	1	.	.	.	FSTL5(INTRON)
4	162882677	162882692	162882679	AT	T	del	1	0	1	0	.	.	.	FSTL5(INTRON)
4	162908611	162908620	162908618	TA	G	snp	0	0	0	1	.	.	.	FSTL5(INTRON)
4	162923947	162923956	162923952	T	G	ins	1	1	1	1	.	.	.	FSTL5(INTRON)
4	163135130	163135149	163135137	TTTC	A	snp	1	0	0	0	.	.	.	INTERGENIC(INTERGENIC)
4	163265226	163265234	163265232	T	G	snp	1	1	1	1	.	.	.	INTERGENIC(INTERGENIC)
4	163268254	163268263	163268260	T	C	snp	1	1	1	1	.	.	.	INTERGENIC(INTERGENIC)
4	163279016	163279027	163279025	TC	T	snp	1	1	1	1	.	.	.	INTERGENIC(INTERGENIC)
4	163328189	163328197	163328191	A	C	snp	1	0	0	0	.	.	.	INTERGENIC(INTERGENIC)
4	163332346	163332357	163332350	GTT	C	snp	1	1	1	1	.	.	.	INTERGENIC(INTERGENIC)
4	163346646	163346655	163346654	T	C	ins	1	1	1	1	.	.	.	AC114770.1(UPSTREAM);INTERGENIC(INTERGENIC)
4	163369849	163369858	163369854	AT	C	snp	1	0	0	0	.	.	.	INTERGENIC(INTERGENIC)
4	163374526	163374535	163374531	AT	A	snp	1	0	0	0	.	.	.	INTERGENIC(INTERGENIC)
4	163528726	163528735	163528730	A	C	ins	1	1	1	1	.	.	.	INTERGENIC(INTERGENIC)
4	163641612	163641623	163641620	CAA	C	del	1	1	1	1	.	.	.	INTERGENIC(INTERGENIC)
4	163687820	163687827	163687826	T	C	snp	0	1	0	0	.	.	.	INTERGENIC(INTERGENIC)
4	163706744	163706761	163706748	AAG	G	del	1	1	1	1	.	.	.	INTERGENIC(INTERGENIC)
4	163706744	163706761	163706749	AAG	A	snp	1	1	1	1	.	.	.	INTERGENIC(INTERGENIC)
4	163791497	163791504	163791500	T	C	snp	1	1	1	1	.	.	.	INTERGENIC(INTERGENIC)
4	163917875	163917886	163917881	AT	G	snp	1	0	1	0	.	.	.	INTERGENIC(INTERGENIC)
4	163918068	163918083	163918078	AT	T	snp	1	0	1	0	.	.	.	INTERGENIC(INTERGENIC)
4	164019266	164019279	164019277	TA	T	snp	1	0	0	0	.	.	.	INTERGENIC(INTERGENIC)
4	164054970	164054983	164054973	TG	C	snp	1	0	0	0	.	.	.	NAF1(DOWNSTREAM);NAF1(INTRON);NAF1(UPSTREAM)
4	164100344	164100351	164100346	C	A	snp	0	1	0	0	.	.	.	INTERGENIC(INTERGENIC)
4	164137394	164137409	164137404	GAAG	C	snp	1	0	0	0	.	.	.	INTERGENIC(INTERGENIC)
4	164300552	164300561	164300559	TG	A	snp	1	1	1	1	.	.	.	INTERGENIC(INTERGENIC);RPL35AP12(UPSTREAM)
4	164399715	164399726	164399719	ACC	A	snp	0	0	1	0	.	.	.	INTERGENIC(INTERGENIC);TKTL2(UPSTREAM)
4	164459492	164459505	164459497	CA	G	snp	1	0	0	0	.	.	.	MARCH1(INTRON)
4	164498161	164498168	164498167	T	G	snp	1	1	1	1	.	.	.	MARCH1(INTRON)
4	164521369	164521378	164521372	AC	T	snp	1	1	1	1	.	.	.	MARCH1(INTRON)
4	164521369	164521378	164521374	AC	T	snp	1	1	1	1	.	.	.	MARCH1(INTRON)
4	164580860	164580869	164580867	A	C	snp	1	1	1	1	.	.	.	MARCH1(INTRON)
4	164654380	164654393	164654390	TA	C	snp	1	1	1	1	.	.	.	MARCH1(INTRON)
4	164697007	164697016	164697014	AG	A	snp	0	0	1	0	.	.	.	MARCH1(INTRON)
4	164794196	164794205	164794202	A	C	snp	1	1	1	1	.	.	.	MARCH1(INTRON)
4	164794219	164794227	164794222	A	C	snp	1	1	1	1	.	.	.	MARCH1(INTRON)
4	164794219	164794227	164794224	A	C	snp	1	1	1	1	.	.	.	MARCH1(INTRON)
4	164794241	164794248	164794243	A	C	ins	1	1	1	1	.	.	.	MARCH1(INTRON)
4	164804371	164804382	164804376	AT	C	snp	1	0	1	0	.	.	.	MARCH1(INTRON)
4	164815843	164815852	164815850	AT	C	snp	1	1	1	1	.	.	.	MARCH1(INTRON)
4	164816038	164816047	164816045	AT	C	snp	1	1	1	1	.	.	.	MARCH1(INTRON)
4	164842532	164842539	164842533	A	G	snp	1	1	1	1	.	.	.	MARCH1(INTRON)
4	165031824	165031831	165031830	T	G	snp	1	1	1	1	.	.	.	MARCH1(INTRON)
4	165068991	165068998	165068995	A	AAT	ins	1	1	1	1	.	.	.	MARCH1(INTRON)
4	165220465	165220472	165220468	A	C	snp	1	1	1	0	.	.	.	MARCH1(INTRON)
4	165321998	165322005	165322001	A	G	snp	0	1	1	0	.	.	.	INTERGENIC(INTERGENIC)
4	165324505	165324512	165324506	T	C	snp	1	0	0	0	.	.	.	INTERGENIC(INTERGENIC)
4	165359363	165359372	165359368	TC	TCTCT	del	1	0	0	0	.	.	.	INTERGENIC(INTERGENIC)
4	165392470	165392478	165392477	T	C	snp	1	1	1	0	.	.	.	INTERGENIC(INTERGENIC)
4	165420439	165420448	165420444	TA	G	snp	0	1	0	1	.	.	.	INTERGENIC(INTERGENIC)
4	165450705	165450719	165450712	TTG	A	snp	0	0	0	1	.	.	.	INTERGENIC(INTERGENIC)
4	165562975	165562984	165562979	A	C	snp	1	0	0	0	.	.	.	INTERGENIC(INTERGENIC)
4	165580153	165580164	165580156	TC	T	snp	0	0	1	0	.	.	.	INTERGENIC(INTERGENIC)
4	165677732	165677743	165677735	AAT	AT	del	1	1	1	1	.	.	.	RP11-294O2.2.1(INTRON)

4	165677732	165677743	165677736	AAT	T	del	1	1	1	1	.	.	.	RP11-294O2.2.1(INTRON)
4	165711456	165711463	165711458	A	G	snp	1	1	0	1	.	.	.	RP11-294O2.2.1(INTRON);U6(DOWNSTREAM)
4	165729075	165729082	165729077	T	G	ins	0	0	0	1	.	.	.	INTERGENIC(INTERGENIC);RP11-294O2.2.1(DOWNSTREAM)
4	165765212	165765219	165765216	A	C	ins	1	0	0	0	.	.	.	INTERGENIC(INTERGENIC)
4	165867829	165867844	165867835	TTTA	C	snp	1	1	1	1	.	.	.	INTERGENIC(INTERGENIC);NACA3P(DOWNSTREAM);RP11-366M4.8.1(UPSTREAM)
4	165923064	165923075	165923068	ATG	G	snp	1	0	0	0	.	.	.	INTERGENIC(INTERGENIC)
4	165929852	165929871	165929857	TTTA	TATG	ins	1	1	1	1	.	.	.	INTERGENIC(INTERGENIC);RP11-366M4.1.1(DOWNSTREAM);RP11-366M4.13.1(UPSTREAM);RP11-366M4.14.1(UPSTREAM)
4	165960295	165960304	165960297	TA	C	snp	0	1	0	0	.	.	.	TRIM60(INTRON);TRIM60(UPSTREAM)
4	165970542	165970553	165970547	TA	G	snp	1	1	1	1	.	.	.	INTERGENIC(INTERGENIC);RP11-366M4.17.1(DOWNSTREAM)
4	166004484	166004491	166004490	A	C	snp	0	1	0	0	.	.	.	TMEM192(DOWNSTREAM);TMEM192(INTRON)
4	166038189	166038197	166038191	A	C	snp	1	1	1	1	.	.	.	TMEM192(INTRON);TMEM192(UPSTREAM)
4	166071073	166071086	166071078	TA	G	snp	0	1	0	1	.	.	.	TMEM192(INTRON)
4	166080235	166080242	166080237	T	C	snp	1	1	1	1	.	.	.	TMEM192(INTRON)
4	166161138	166161149	166161145	TA	T	del	1	1	1	1	.	.	.	KLHL2(DOWNSTREAM);KLHL2(INTRON);KLHL2(UPSTREAM)
4	166166031	166166038	166166032	T	G	snp	1	1	1	1	.	.	.	KLHL2(INTRON)
4	166166374	166166381	166166376	A	C	snp	1	0	1	0	.	.	.	KLHL2(INTRON)
4	166220100	166220107	166220103	C	A	snp	1	0	1	0	.	.	.	KLHL2(INTRON)
4	166262192	166262201	166262193	T	C	snp	1	1	0	1	.	.	.	MSMO1(DOWNSTREAM);MSMO1(INTRON)
4	166262192	166262201	166262196	T	C	ins	1	0	0	1	.	.	.	MSMO1(DOWNSTREAM);MSMO1(INTRON)
4	166294517	166294526	166294524	AT	A	del	1	1	1	1	.	.	.	CPE(INTRON)
4	166313343	166313354	166313345	TA	C	snp	1	0	0	0	.	.	.	CPE(INTRON)
4	166324476	166324485	166324481	A	G	snp	1	1	1	1	.	.	.	CPE(INTRON);HADHAP1(DOWNSTREAM);hsa-mir-1979(UPSTREAM)
4	166346490	166346497	166346496	A	C	ins	1	1	1	1	.	.	.	CPE(INTRON)
4	166361607	166361615	166361614	C	A	snp	1	1	1	1	.	.	.	CPE(INTRON)
4	166419901	166419918	166419908	GT	A	snp	1	0	0	0	.	.	.	CPE(DOWNSTREAM);INTERGENIC(INTERGENIC)
4	166447010	166447023	166447015	AG	C	snp	1	0	0	0	.	.	.	INTERGENIC(INTERGENIC)
4	166478402	166478417	166478405	GA	G	snp	1	0	1	0	.	.	.	INTERGENIC(INTERGENIC)
4	166495758	166495767	166495761	A	C	snp	1	1	1	1	.	.	.	INTERGENIC(INTERGENIC)
4	166496308	166496321	166496310	AT	T	snp	1	1	1	1	.	.	.	INTERGENIC(INTERGENIC)
4	166536449	166536464	166536460	TTTA	G	snp	1	1	1	1	.	.	.	INTERGENIC(INTERGENIC)
4	166578289	166578297	166578294	T	G	snp	1	1	0	1	.	.	.	INTERGENIC(INTERGENIC)
4	166679172	166679180	166679175	A	G	snp	1	1	1	1	.	.	.	RP11-340B18.1.1(INTRON)
4	166778073	166778088	166778075	TG	C	snp	1	0	0	0	.	.	.	INTERGENIC(INTERGENIC)
4	166780102	166780109	166780107	A	C	snp	1	0	1	0	.	.	.	INTERGENIC(INTERGENIC)
4	166806752	166806761	166806755	A	C	snp	1	1	1	1	.	.	.	TLL1(INTRON)
4	166857344	166857359	166857355	AT	A	del	1	0	0	0	.	.	.	TLL1(INTRON)
4	166857344	166857359	166857356	AT	T	snp	1	0	0	0	.	.	.	TLL1(INTRON)
4	166857344	166857359	166857357	AT	A	del	1	0	1	0	.	.	.	TLL1(INTRON)
4	166962438	166962457	166962453	TTCT	TTC	del	1	1	1	1	.	.	.	TLL1(INTRON)
4	166974118	166974133	166974121	TA	G	snp	1	1	0	0	.	.	.	RN5S170(DOWNSTREAM);TLL1(INTRON)
4	167161099	167161108	167161101	G	T	ins	1	0	0	0	.	.	.	INTERGENIC(INTERGENIC)
4	167183600	167183608	167183607	T	G	snp	1	1	1	1	.	.	.	INTERGENIC(INTERGENIC)
4	167267688	167267705	167267698	TA	C	snp	1	1	1	1	.	.	.	INTERGENIC(INTERGENIC)
4	167267688	167267705	167267700	TA	C	snp	0	0	1	0	.	.	.	INTERGENIC(INTERGENIC)
4	167280073	167280086	167280082	TC	G	snp	1	0	0	0	.	.	.	INTERGENIC(INTERGENIC)
4	167280073	167280086	167280083	TC	CTC	del	1	0	1	1	.	.	.	INTERGENIC(INTERGENIC)
4	167322513	167322521	167322514	T	C	snp	1	0	1	1	.	.	.	RP11-217C7.1.1(INTRON)
4	167437522	167437545	167437528	AAAG	G	del	1	1	1	1	.	.	.	RP11-217C7.1.1(INTRON)
4	167437522	167437545	167437529	AAAG	A	snp	1	1	1	1	.	.	.	RP11-217C7.1.1(INTRON)
4	167508257	167508266	167508264	AT	C	snp	0	1	1	0	.	.	.	INTERGENIC(INTERGENIC)
4	167539834	167539843	167539836	A	G	snp	1	1	1	1	.	.	.	INTERGENIC(INTERGENIC)
4	167551959	167551978	167551965	ATAG	GATAGATAGATAGAT	ins	1	1	1	1	.	.	.	INTERGENIC(INTERGENIC)
4	167563117	167563130	167563120	TG	A	snp	1	0	0	0	.	.	.	INTERGENIC(INTERGENIC)
4	167570061	167570069	167570065	T	G	snp	1	1	1	0	.	.	.	INTERGENIC(INTERGENIC)
4	167586377	167586386	167586382	AT	C	snp	1	0	0	0	.	.	.	INTERGENIC(INTERGENIC)
4	167587316	167587325	167587319	AT	G	snp	1	0	1	0	.	.	.	INTERGENIC(INTERGENIC)
4	167588508	167588519	167588516	AT	G	snp	0	1	1	0	.	.	.	INTERGENIC(INTERGENIC)
4	167732468	167732477	167732474	T	C	snp	0	0	1	0	.	.	.	SPOCK3(INTRON)
4	167773449	167773466	167773457	AAT	G	snp	1	1	1	1	.	.	.	SPOCK3(INTRON)
4	167781954	167781967	167781963	AT	C	snp	1	1	1	1	.	.	.	SPOCK3(INTRON)
4	167886623	167886630	167886629	T	C	snp	1	1	1	1	.	.	.	SPOCK3(INTRON)
4	167955484	167955493	167955488	A	C	snp	0	1	0	0	.	.	.	SPOCK3(INTRON)
4	168117669	168117678	168117672	T	C	snp	0	0	0	1	.	.	.	SPOCK3(INTRON)

4	168126493	168126502	168126495	TA	A	snp	0	1	1	1	.	.	.	SPOCK3(INTRON)
4	168263819	168263832	168263830	AG	T	snp	1	1	1	1	.	.	.	INTERGENIC(INTERGENIC)
4	168284973	168284980	168284977	T	G	snp	1	0	0	0	.	.	.	INTERGENIC(INTERGENIC)
4	168372611	168372620	168372615	AT	C	snp	1	1	1	1	.	.	.	INTERGENIC(INTERGENIC)
4	168372639	168372648	168372643	AT	C	snp	1	1	1	1	.	.	.	INTERGENIC(INTERGENIC)
4	168372667	168372676	168372671	AT	C	snp	1	1	1	1	.	.	.	INTERGENIC(INTERGENIC)
4	168372695	168372704	168372699	AT	C	snp	1	1	1	1	.	.	.	INTERGENIC(INTERGENIC)
4	168372723	168372732	168372727	AT	C	snp	1	1	1	0	.	.	.	INTERGENIC(INTERGENIC)
4	168372837	168372846	168372841	AT	C	snp	1	1	1	1	.	.	.	INTERGENIC(INTERGENIC)
4	168374856	168374875	168374867	TTTC	T	del	0	0	1	0	.	.	.	INTERGENIC(INTERGENIC)
4	168374856	168374875	168374870	TTTC	C	del	1	0	1	0	.	.	.	INTERGENIC(INTERGENIC)
4	168382052	168382061	168382055	TA	C	snp	1	0	0	0	.	.	.	INTERGENIC(INTERGENIC)
4	168407383	168407392	168407386	T	G	snp	1	1	1	1	.	.	.	INTERGENIC(INTERGENIC)
4	168461297	168461306	168461304	G	T	snp	0	0	1	0	.	.	.	INTERGENIC(INTERGENIC)
4	168529245	168529252	168529251	T	C	snp	1	0	0	0	.	.	.	INTERGENIC(INTERGENIC)
4	168542972	168542979	168542977	T	C	snp	1	0	0	0	.	.	.	INTERGENIC(INTERGENIC)
4	168585898	168585907	168585905	GT	C	snp	1	1	0	1	.	.	.	7SK(DOWNSTREAM);INTERGENIC(INTERGENIC)
4	168586778	168586786	168586784	A	G	snp	0	1	1	0	.	.	.	7SK(DOWNSTREAM);INTERGENIC(INTERGENIC)
4	168664999	168665008	168665004	TA	G	snp	1	1	1	1	.	.	.	INTERGENIC(INTERGENIC)
4	168677847	168677854	168677848	A	C	snp	1	0	0	0	.	.	.	INTERGENIC(INTERGENIC)
4	168765653	168765667	168765657	TTA	G	snp	1	1	1	1	.	.	.	INTERGENIC(INTERGENIC)
4	168920233	168920242	168920237	AT	G	snp	0	1	0	1	.	.	.	RP11-310I9.1.1(INTRON)
4	169108194	169108205	169108201	AAT	G	snp	1	1	1	1	.	.	.	ANXA10(INTRON)
4	169118251	169118260	169118252	A	C	snp	1	1	1	1	.	.	.	INTERGENIC(INTERGENIC)
4	169140542	169140557	169140553	TGAT	C	snp	1	1	1	1	.	.	.	DDX60(INTRON)
4	169271970	169271990	169271983	AAC	C	del	0	1	1	1	.	.	.	INTERGENIC(INTERGENIC)
4	169421691	169421699	169421698	T	C	snp	1	1	1	1	.	.	.	DDX60L(INTRON);PALLD(INTRON)
4	169546654	169546669	169546661	AT	G	snp	1	1	1	1	.	.	.	PALLD(INTRON)
4	169555103	169555114	169555106	AAT	G	snp	0	1	1	1	.	.	.	PALLD(INTRON)
4	169586724	169586731	169586730	A	C	snp	1	1	0	1	.	.	.	PALLD(INTRON)
4	169656123	169656134	169656130	TAG	G	snp	1	1	1	1	.	.	.	PALLD(INTRON)
4	169661218	169661225	169661224	A	C	snp	1	1	1	1	.	.	.	PALLD(INTRON)
4	169673978	169673989	169673981	TAT	G	ins	1	0	0	0	.	.	.	PALLD(INTRON);RPL9P16(UPSTREAM)
4	169692228	169692237	169692229	A	AAC	ins	1	1	1	1	.	.	.	PALLD(INTRON)
4	169730470	169730493	169730483	CTT	TCTTCTTC		del	1	1	1	1	.	.	. PALLD(INTRON)
4	169731979	169731987	169731985	A	G	snp	1	1	1	1	.	.	.	PALLD(INTRON)
4	169734562	169734579	169734573	AAC	A	snp	1	0	0	0	.	.	.	PALLD(INTRON)
4	169744830	169744838	169744837	A	C	snp	0	1	1	1	.	.	.	PALLD(INTRON)
4	169970416	169970425	169970419	T	C	snp	1	0	1	0	.	.	.	INTERGENIC(INTERGENIC)
4	169975636	169975645	169975638	CT	TCTG	ins	1	0	0	0	.	.	.	INTERGENIC(INTERGENIC)
4	169986159	169986168	169986163	A	C	snp	1	0	0	0	.	.	.	INTERGENIC(INTERGENIC)
4	170046353	170046361	170046357	A	C	snp	1	0	0	1	.	.	.	SH3RF1(INTRON)
4	170069872	170069879	170069876	T	G	snp	1	0	0	0	.	.	.	SH3RF1(INTRON)
4	170113971	170113980	170113976	AT	C	snp	1	1	0	1	.	.	.	SH3RF1(INTRON)
4	170165565	170165574	170165573	T	G	ins	1	0	1	1	.	.	.	SH3RF1(INTRON)
4	170203142	170203151	170203147	A	C	snp	1	0	0	0	.	.	.	INTERGENIC(INTERGENIC)
4	170320723	170320730	170320727	A	C	ins	1	0	0	0	.	.	.	NEK1(INTRON)
4	170662485	170662494	170662489	T	G	snp	1	0	0	0	.	.	.	C4orf27(INTRON)
4	170680233	170680240	170680236	T	G	ins	1	0	0	0	.	.	.	C4orf27(UPSTREAM);INTERGENIC(INTERGENIC)
4	170684956	170684965	170684959	AG	A	snp	1	0	0	0	.	.	.	INTERGENIC(INTERGENIC)
4	170758561	170758584	170758565	CAAA	G	snp	1	1	1	1	.	.	.	INTERGENIC(INTERGENIC);RP11-205M3.1.1(DOWNSTREAM)
4	170819870	170819879	170819877	T	G	snp	1	1	1	1	.	.	.	INTERGENIC(INTERGENIC)
4	170821348	170821365	170821356	AAT	AAC	ins	1	1	1	1	.	.	.	INTERGENIC(INTERGENIC)
4	170848253	170848266	170848263	AG	C	snp	1	1	1	0	.	.	.	RP11-205M3.3.1(INTRON)
4	170863319	170863338	170863327	TTTC	TTC	ins	1	1	1	1	.	.	.	RP11-205M3.3.1(INTRON);RP11-6E9.5.1(UPSTREAM)
4	170863319	170863338	170863330	TTTC	T	del	1	1	1	1	.	.	.	RP11-205M3.3.1(INTRON);RP11-6E9.5.1(UPSTREAM)
4	170881985	170881992	170881986	G	T	snp	1	1	1	1	.	.	.	RP11-205M3.3.1(INTRON);RP11-6E9.5.1(INTRON)
4	170881985	170881992	170881988	G	T	snp	1	1	1	1	.	.	.	RP11-205M3.3.1(INTRON);RP11-6E9.5.1(INTRON)
4	170885804	170885812	170885805	T	G	snp	0	1	0	1	.	.	.	RP11-205M3.3.1(INTRON);RP11-6E9.5.1(DOWNSTREAM)
4	170899038	170899046	170899039	A	AC	ins	1	1	1	1	.	.	.	INTERGENIC(INTERGENIC);RP11-205M3.3.1(UPSTREAM);RP11-6E9.4.1(UPSTREAM)
4	171109593	171109604	171109601	GT	T	snp	0	0	1	0	.	.	.	INTERGENIC(INTERGENIC)
4	171140955	171140962	171140958	A	C	snp	1	0	0	0	.	.	.	INTERGENIC(INTERGENIC)
4	171399969	171399978	171399976	AT	A	snp	1	1	1	1	.	.	.	INTERGENIC(INTERGENIC);RP11-796L2.1.1(DOWNSTREAM)
4	171401438	171401449	171401443	TG	A	snp	1	0	0	0	.	.	.	INTERGENIC(INTERGENIC);RP11-796L2.1.1(DOWNSTREAM)
4	171453517	171453524	171453520	T	G	snp	1	0	0	0	.	.	.	INTERGENIC(INTERGENIC)
4	171475204	171475211	171475206	A	C	snp	1	1	1	1	.	.	.	INTERGENIC(INTERGENIC)

4	171507609	171507618	171507614	A	G	snp	1	0	0	0	.	.	.	HSP90AA6P(INTRON)
4	171598552	171598561	171598555	T	C	snp	1	1	1	1	.	.	.	INTERGENIC(INTERGENIC)
4	171604910	171604917	171604914	A	G	snp	0	0	1	0	.	.	.	INTERGENIC(INTERGENIC)
4	171634053	171634064	171634056	ACA	G	snp	1	1	1	1	.	.	.	INTERGENIC(INTERGENIC)
4	171682422	171682439	171682436	TAC	A	snp	0	1	0	0	.	.	.	INTERGENIC(INTERGENIC)
4	171751817	171751826	171751823	AT	G	snp	1	1	1	0	.	.	.	INTERGENIC(INTERGENIC)
4	171826982	171826995	171826987	AT	C	snp	1	0	0	0	.	.	.	INTERGENIC(INTERGENIC)
4	171938261	171938270	171938263	TA	C	snp	1	1	1	0	.	.	.	INTERGENIC(INTERGENIC)
4	171943591	171943598	171943595	G	A	snp	1	1	1	1	.	.	.	INTERGENIC(INTERGENIC)
4	171943787	171943794	171943791	A	C	snp	1	1	1	1	.	.	.	INTERGENIC(INTERGENIC)
4	171979889	171979906	171979894	AAC	AACAACAACAACAA	del	0	1	1	1	.	.	.	RP11-344G13.1.1(EXON)
4	172031643	172031652	172031650	AC	T	snp	1	0	0	0	.	.	.	INTERGENIC(INTERGENIC);RP11-344G13.2.1(UPSTREAM)
4	172097323	172097330	172097324	T	G	snp	0	1	0	1	.	.	.	INTERGENIC(INTERGENIC)
4	172121738	172121747	172121741	AT	C	snp	1	0	0	0	.	.	.	INTERGENIC(INTERGENIC)
4	172141555	172141564	172141558	AT	G	snp	1	1	1	1	.	.	.	INTERGENIC(INTERGENIC)
4	172154238	172154253	172154245	AAAT	A	snp	1	1	1	1	.	.	.	INTERGENIC(INTERGENIC)
4	172160784	172160792	172160790	A	C	snp	1	1	1	1	.	.	.	INTERGENIC(INTERGENIC)
4	172176530	172176537	172176536	A	G	snp	1	0	0	0	.	.	.	INTERGENIC(INTERGENIC)
4	172176715	172176724	172176723	A	G	snp	1	0	0	0	.	.	.	INTERGENIC(INTERGENIC)
4	172189718	172189745	172189738	CTTT	T	ins	1	0	0	0	.	.	.	INTERGENIC(INTERGENIC)
4	172225012	172225029	172225019	TA	TG	ins	1	0	0	0	.	.	.	INTERGENIC(INTERGENIC)
4	172225012	172225029	172225021	TA	G	snp	1	0	0	0	.	.	.	INTERGENIC(INTERGENIC)
4	172287259	172287276	172287272	TA	G	snp	1	1	0	0	.	.	.	INTERGENIC(INTERGENIC)
4	172306103	172306114	172306109	CTC	T	snp	1	1	1	1	.	.	.	INTERGENIC(INTERGENIC)
4	172323222	172323229	172323228	A	C	snp	1	1	1	1	.	.	.	INTERGENIC(INTERGENIC)
4	172358159	172358166	172358164	A	G	snp	1	1	1	1	.	.	.	INTERGENIC(INTERGENIC)
4	172487695	172487702	172487699	T	C	ins	1	0	0	0	.	.	.	INTERGENIC(INTERGENIC);RP11-97E7.1.1(UPSTREAM)
4	172538209	172538216	172538213	T	C	snp	1	1	0	1	.	.	.	RP11-97E7.2.1(INTRON)
4	172703337	172703345	172703343	T	G	snp	1	0	0	0	.	.	.	INTERGENIC(INTERGENIC)
4	172830296	172830315	172830306	AATA	G	snp	1	0	0	0	.	.	.	GALNTL6(INTRON)
4	172830296	172830315	172830310	AATA	G	snp	1	0	0	0	.	.	.	GALNTL6(INTRON)
4	172836156	172836165	172836158	TA	A	del	1	0	0	0	.	.	.	GALNTL6(INTRON)
4	172851831	172851838	172851837	A	C	snp	1	1	1	1	.	.	.	GALNTL6(INTRON)
4	172877150	172877165	172877162	TG	GG	ins	1	1	1	1	.	.	.	GALNTL6(INTRON)
4	172889974	172889983	172889982	T	G	snp	1	0	0	0	.	.	.	GALNTL6(INTRON)
4	172889993	172890002	172889997	G	T	snp	1	1	1	1	.	.	.	GALNTL6(INTRON)
4	172993392	172993401	172993398	T	C	ins	1	0	0	0	.	.	.	GALNTL6(INTRON)
4	173017690	173017699	173017698	A	AC	ins	1	1	1	1	.	.	.	GALNTL6(INTRON)
4	173062317	173062326	173062324	AG	A	snp	1	0	0	0	.	.	.	GALNTL6(INTRON)
4	173127957	173127964	173127958	T	TG	ins	1	1	1	1	.	.	.	GALNTL6(INTRON)
4	173127957	173127964	173127959	T	G	ins	1	1	1	1	.	.	.	GALNTL6(INTRON)
4	173182388	173182395	173182391	T	G	snp	1	0	0	0	.	.	.	GALNTL6(INTRON);GALNTL6(UPSTREAM)
4	173193096	173193111	173193109	AT	A	del	1	1	0	1	.	.	.	GALNTL6(INTRON)
4	173193111	173193119	173193114	T	C	snp	1	0	0	0	.	.	.	GALNTL6(INTRON)
4	173212960	173212967	173212965	A	G	snp	1	1	1	1	.	.	.	GALNTL6(INTRON)
4	173216885	173216896	173216889	CA	T	snp	1	1	1	1	.	.	.	GALNTL6(INTRON)
4	173227801	173227812	173227808	TAT	C	snp	1	1	1	1	.	.	.	GALNTL6(INTRON)
4	173270982	173270997	173270994	AT	TT	ins	1	1	1	1	.	.	.	GALNTL6(INTRON)
4	173320123	173320130	173320124	A	C	snp	1	0	0	0	.	.	.	GALNTL6(INTRON)
4	173399308	173399317	173399314	A	C	snp	1	1	1	1	.	.	.	GALNTL6(INTRON)
4	173527782	173527791	173527787	TA	G	snp	1	1	1	1	.	.	.	GALNTL6(INTRON)
4	173527828	173527837	173527832	TA	C	snp	1	0	1	0	.	.	.	GALNTL6(INTRON)
4	173538594	173538613	173538605	TTTA	T	snp	1	0	0	0	.	.	.	GALNTL6(INTRON)
4	173626365	173626374	173626370	T	TTG	ins	1	1	1	1	.	.	.	GALNTL6(INTRON)
4	173626365	173626374	173626371	T	TG	ins	1	0	0	0	.	.	.	GALNTL6(INTRON)
4	173626365	173626374	173626372	T	TTG	ins	1	1	1	1	.	.	.	GALNTL6(INTRON)
4	173693004	173693013	173693008	T	G	snp	1	0	1	0	.	.	.	GALNTL6(INTRON)
4	173792740	173792747	173792741	G	GA	ins	0	1	0	1	.	.	.	GALNTL6(INTRON)
4	173792740	173792747	173792743	G	A	snp	0	1	0	1	.	.	.	GALNTL6(INTRON)
4	173816536	173816553	173816548	TA	ATA	del	1	0	0	0	.	.	.	GALNTL6(INTRON)
4	173861818	173861838	173861830	TTA	TATTA	del	1	1	1	1	.	.	.	GALNTL6(INTRON)
4	173861818	173861838	173861831	TTA	A	del	1	1	1	1	.	.	.	GALNTL6(INTRON)
4	173861818	173861838	173861833	TTA	TATTA	del	1	1	1	1	.	.	.	GALNTL6(INTRON)
4	173866672	173866687	173866681	TTTG	A	snp	1	0	0	0	.	.	.	GALNTL6(INTRON)
4	173972764	173972775	173972770	AT	G	snp	0	0	1	0	.	.	.	INTERGENIC(INTERGENIC)
4	173991115	173991122	173991119	T	C	snp	1	1	1	1	.	.	.	INTERGENIC(INTERGENIC)
4	174078850	174078863	174078855	TA	TATATATATGTGGTG	del	1	1	0	1	.	.	.	RP11-10K16.1.1(INTRON)

4	174087951	174087958	174087952	A	C	snp	1	0	0	0	.	.	.	GALNT7(UPSTREAM);RP11-10K16.1.1(INTRON)
4	174205873	174205882	174205880	A	C	snp	1	1	1	0	.	.	.	GALNT7(INTRON)
4	174207164	174207171	174207169	A	C	snp	1	0	0	0	.	.	.	GALNT7(INTRON)
4	174235080	174235095	174235092	AT	T	ins	1	0	0	0	.	.	.	GALNT7(INTRON);GALNT7(UPSTREAM)
4	174235080	174235095	174235092	AT	T	snp	0	0	1	0	.	.	.	GALNT7(INTRON);GALNT7(UPSTREAM)
4	174235080	174235095	174235093	AT	A	del	1	1	1	0	.	.	.	GALNT7(INTRON);GALNT7(UPSTREAM)
4	174284272	174284280	174284278	A	G	snp	1	0	0	0	.	.	.	INTERGENIC(INTERGENIC)
4	174302749	174302756	174302755	T	G	snp	1	0	0	0	.	.	.	INTERGENIC(INTERGENIC);SAP30(DOWNSTREAM)
4	174344810	174344818	174344816	T	AAA	ins	1	1	1	1	.	.	.	INTERGENIC(INTERGENIC);RP11-489G11.1.1(UPSTREAM)
4	174347469	174347476	174347475	A	C	snp	1	1	1	1	.	.	.	INTERGENIC(INTERGENIC)
4	174356836	174356843	174356838	T	G	snp	1	1	1	1	.	.	.	INTERGENIC(INTERGENIC)
4	174401500	174401507	174401505	T	C	ins	0	0	0	1	.	.	.	INTERGENIC(INTERGENIC)
4	174401500	174401507	174401506	T	C	snp	0	0	0	1	.	.	.	INTERGENIC(INTERGENIC)
4	174473541	174473552	174473546	GAG	A	snp	1	1	1	1	.	.	.	RP11-471J12.1.1(INTRON)
4	174480794	174480803	174480795	A	C	ins	1	0	0	0	.	.	.	RP11-471J12.1.1(INTRON)
4	174541552	174541560	174541554	T	C	snp	1	0	0	0	.	.	.	INTERGENIC(INTERGENIC);MORF4(UPSTREAM)
4	174721166	174721177	174721174	ATT	A	del	0	1	1	0	.	.	.	INTERGENIC(INTERGENIC)
4	174773106	174773119	174773113	AG	A	snp	1	1	1	0	.	.	.	INTERGENIC(INTERGENIC)
4	174782831	174782838	174782834	A	G	snp	1	1	1	1	.	.	.	INTERGENIC(INTERGENIC)
4	174884102	174884113	174884109	TTA	ATC	ins	1	1	1	1	.	.	.	RP11-161D15.3.1(INTRON)
4	174895297	174895316	174895309	TATT	A	del	1	1	1	1	.	.	.	RP11-161D15.3.1(INTRON)
4	174895297	174895316	174895312	TATT	TA	del	1	1	1	1	.	.	.	RP11-161D15.3.1(INTRON)
4	174917204	174917217	174917215	TC	T	snp	1	0	0	0	.	.	.	INTERGENIC(INTERGENIC)
4	174958226	174958239	174958236	TC	CTC	del	1	0	0	0	.	.	.	INTERGENIC(INTERGENIC)
4	175047280	175047287	175047281	A	G	snp	0	1	0	1	.	.	.	RP11-148L24.1.1(INTRON)
4	175054538	175054553	175054541	AT	A	snp	1	1	1	1	.	.	.	RP11-148L24.1.1(INTRON);RP11-248N22.1.1(DOWNSTREAM)
4	175077738	175077747	175077740	TA	C	snp	0	1	1	0	.	.	.	RP11-148L24.1.1(INTRON);RP11-248N22.1.1(UPSTREAM)
4	175138384	175138391	175138390	C	CG	ins	1	0	1	0	.	.	.	RP11-148L24.1.1(INTRON)
4	175187400	175187417	175187404	AAT	G	snp	1	1	0	1	.	.	.	FBXO8(INTRON)
4	175198184	175198191	175198186	T	G	snp	0	0	1	0	.	.	.	FBXO8(INTRON)
4	175201473	175201481	175201477	A	C	snp	1	1	0	1	.	.	.	CEP44(UPSTREAM);FBXO8(INTRON)
4	175248900	175248909	175248906	GT	A	snp	1	0	0	0	.	.	.	CEP44(INTRON)
4	175250819	175250828	175250827	T	C	snp	1	1	0	1	.	.	.	CEP44(INTRON)
4	175378234	175378242	175378238	T	C	snp	1	1	0	1	.	.	.	INTERGENIC(INTERGENIC)
4	175380184	175380195	175380187	TG	A	snp	1	1	1	1	.	.	.	INTERGENIC(INTERGENIC)
4	175582315	175582330	175582319	CA	A	snp	1	0	0	0	.	.	.	GLRA3(INTRON)
4	175838812	175838820	175838816	A	G	snp	1	1	1	1	.	.	.	ADAM29(INTRON);ADAM29(UPSTREAM)
4	175844208	175844219	175844215	ATT	TA	del	1	1	1	1	.	.	.	ADAM29(INTRON);RP13-577H12.3.1(DOWNSTREAM)
4	175844208	175844219	175844216	ATT	A	del	1	1	1	1	.	.	.	ADAM29(INTRON);RP13-577H12.3.1(DOWNSTREAM)
4	175877663	175877674	175877671	GA	AGA	del	1	1	1	1	.	.	.	ADAM29(INTRON)
4	175935877	175935904	175935894	AACA	C	del	0	1	1	1	.	.	.	INTERGENIC(INTERGENIC);RP11-391J13.1.1(UPSTREAM)
4	176028911	176028928	176028921	ATT	G	snp	0	1	0	1	.	.	.	INTERGENIC(INTERGENIC)
4	176034925	176034934	176034931	A	AG	ins	1	0	0	0	.	.	.	INTERGENIC(INTERGENIC)
4	176053962	176053971	176053965	T	C	snp	1	1	1	1	.	.	.	INTERGENIC(INTERGENIC)
4	176165990	176165999	176165996	TA	C	snp	1	0	0	0	.	.	.	INTERGENIC(INTERGENIC)
4	176180809	176180824	176180819	TTTA	A	del	1	1	1	1	.	.	.	INTERGENIC(INTERGENIC)
4	176186855	176186866	176186858	AT	AG	ins	1	1	1	1	.	.	.	INTERGENIC(INTERGENIC)
4	176186855	176186866	176186860	AT	AG	ins	1	1	1	1	.	.	.	INTERGENIC(INTERGENIC)
4	176200508	176200523	176200521	CA	C	del	0	1	1	0	.	.	.	INTERGENIC(INTERGENIC)
4	176230213	176230236	176230218	CTT	CTC	ins	1	0	0	0	.	.	.	INTERGENIC(INTERGENIC)
4	176230213	176230236	176230230	CTT	C	snp	1	0	0	0	.	.	.	INTERGENIC(INTERGENIC)
4	176230419	176230434	176230424	GT	G	snp	1	0	0	0	.	.	.	INTERGENIC(INTERGENIC)
4	176289496	176289507	176289500	CCT	T	snp	0	0	1	0	.	.	.	RP11-287F9.2.1(INTRON)
4	176355914	176355923	176355916	TA	A	snp	1	0	0	1	.	.	.	INTERGENIC(INTERGENIC)
4	176355914	176355923	176355918	TA	A	snp	1	1	1	1	.	.	.	INTERGENIC(INTERGENIC)
4	176356321	176356330	176356328	AT	A	snp	1	1	1	1	.	.	.	INTERGENIC(INTERGENIC)
4	176445270	176445287	176445276	CAA	A	snp	1	0	0	0	.	.	.	INTERGENIC(INTERGENIC)
4	176547195	176547212	176547199	AG	GG	ins	1	0	0	0	.	.	.	INTERGENIC(INTERGENIC)
4	176547195	176547212	176547201	AG	G	snp	1	0	0	0	.	.	.	INTERGENIC(INTERGENIC)
4	176559037	176559052	176559041	TA	G	snp	0	1	1	0	.	.	.	GPM6A(DOWNSTREAM);GPM6A(INTRON)
4	176577323	176577331	176577324	A	G	snp	0	0	0	1	.	.	.	GPM6A(INTRON);GPM6A(UPSTREAM)
4	176614407	176614418	176614416	AT	C	snp	0	0	0	1	.	.	.	GPM6A(INTRON)
4	176676350	176676361	176676358	AAT	C	snp	1	1	1	1	.	.	.	GPM6A(INTRON)
4	176738005	176738012	176738010	T	C	snp	1	0	0	0	.	.	.	GPM6A(INTRON);GPM6A(UPSTREAM);RP11-806K15.1.1(DOWNSTREAM)
4	176872128	176872137	176872136	T	C	snp	1	1	1	1	.	.	.	GPM6A(INTRON)

4	176912199	176912214	176912204	TCTT	TT	del	1	1	1	1	.	.	.	GPM6A(INTRON)
4	176934201	176934210	176934208	AT	A	del	0	1	1	1	.	.	.	MARK2P4(EXON)
4	177040246	177040255	177040251	TC	G	snp	1	0	0	0	.	.	.	WDR17(INTRON)
4	177054310	177054319	177054318	A	C	snp	1	1	1	1	.	.	.	WDR17(INTRON)
4	177139403	177139414	177139405	AT	G	snp	1	1	0	1	.	.	.	ASB5(DOWNSTREAM);ASB5(INTRON)
4	177139430	177139441	177139432	AT	G	snp	0	0	1	1	.	.	.	ASB5(DOWNSTREAM);ASB5(INTRON)
4	177140610	177140619	177140615	AT	A	snp	1	1	0	1	.	.	.	ASB5(DOWNSTREAM);ASB5(INTRON)
4	177189808	177189817	177189810	AT	G	snp	1	1	1	1	.	.	.	ASB5(DOWNSTREAM);ASB5(INTRON)
4	177215572	177215580	177215578	G	A	ins	1	0	0	0	.	.	.	INTERGENIC(INTERGENIC)
4	177427544	177427552	177427550	A	G	snp	1	1	1	1	.	.	.	INTERGENIC(INTERGENIC)
4	177427662	177427681	177427677	TTTG	T	ins	1	1	1	1	.	.	.	INTERGENIC(INTERGENIC)
4	177808518	177808531	177808525	TC	TT	ins	1	0	0	0	.	.	.	INTERGENIC(INTERGENIC)
4	177808518	177808531	177808529	TC	T	snp	1	0	0	0	.	.	.	INTERGENIC(INTERGENIC)
4	177814721	177814728	177814723	A	G	snp	1	0	0	0	.	.	.	INTERGENIC(INTERGENIC)
4	177846090	177846099	177846097	TA	G	snp	1	0	0	0	.	.	.	INTERGENIC(INTERGENIC)
4	177872919	177872926	177872921	C	T	snp	1	0	0	0	.	.	.	INTERGENIC(INTERGENIC)
4	177894748	177894763	177894751	CA	C	snp	1	0	1	1	.	.	.	INTERGENIC(INTERGENIC)
4	178067042	178067050	178067043	T	C	snp	0	1	1	0	.	.	.	INTERGENIC(INTERGENIC)
4	178070661	178070668	178070663	A	G	snp	1	0	0	0	.	.	.	INTERGENIC(INTERGENIC)
4	178088180	178088189	178088186	AC	G	snp	0	1	1	0	.	.	.	INTERGENIC(INTERGENIC)
4	178142705	178142724	178142713	GTTT	C	snp	1	0	0	0	.	.	.	INTERGENIC(INTERGENIC)
4	178164868	178164876	178164871	A	AC	ins	1	1	1	1	.	.	.	RP11-487E13.1.1(INTRON)
4	178283755	178283763	178283757	T	C	snp	1	1	0	1	.	.	.	NEIL3(UTR_3_PRIME)
4	178391904	178391919	178391909	TTTG	TG	del	1	1	1	1	.	.	.	RP11-130F10.1.1(INTRON)
4	178391904	178391919	178391911	TTTG	T	ins	1	1	1	1	.	.	.	RP11-130F10.1.1(INTRON)
4	178391904	178391919	178391912	TTTG	TTG	del	1	1	1	1	.	.	.	RP11-130F10.1.1(INTRON)
4	178442458	178442467	178442465	TA	G	snp	1	1	1	0	.	.	.	RP11-130F10.1.1(INTRON)
4	178470898	178470905	178470904	A	C	snp	1	0	0	0	.	.	.	RP11-130F10.1.1(INTRON)
4	178480316	178480323	178480320	A	G	snp	1	1	1	1	.	.	.	RP11-130F10.1.1(INTRON)
4	178582046	178582057	178582054	CA	T	snp	1	0	0	0	.	.	.	RP11-130F10.1.1(INTRON)
4	178642267	178642276	178642273	AT	G	snp	0	0	1	0	.	.	.	INTERGENIC(INTERGENIC)
4	178657739	178657752	178657747	TA	C	snp	1	1	1	1	.	.	.	RP11-162G9.1.1(INTRON);RP11-389E17.1.1(INTRON)
4	178657800	178657809	178657805	TA	G	snp	1	1	1	1	.	.	.	RP11-162G9.1.1(INTRON);RP11-389E17.1.1(INTRON)
4	178665735	178665742	178665739	A	C	snp	1	1	1	1	.	.	.	RP11-162G9.1.1(INTRON);RP11-389E17.1.1(INTRON)
4	178680856	178680865	178680862	T	C	snp	0	1	1	1	.	.	.	RP11-162G9.1.1(INTRON);RP11-389E17.1.1(INTRON)
4	178916382	178916393	178916391	TA	C	snp	1	1	1	1	.	.	.	INTERGENIC(INTERGENIC);RP11-389E17.1.1(DOWNSTREAM)
4	178934152	178934159	178934153	T	C	snp	1	0	0	0	.	.	.	INTERGENIC(INTERGENIC)
4	178952042	178952051	178952045	TA	CG	ins	1	0	0	0	.	.	.	INTERGENIC(INTERGENIC)
4	178980635	178980643	178980637	T	G	snp	0	1	0	1	.	.	.	INTERGENIC(INTERGENIC)
4	179096427	179096434	179096428	A	C	snp	1	0	0	0	.	.	.	INTERGENIC(INTERGENIC)
4	179169117	179169125	179169118	A	G	snp	1	0	0	0	.	.	.	INTERGENIC(INTERGENIC)
4	179204444	179204457	179204455	AT	A	del	1	1	1	1	.	.	.	INTERGENIC(INTERGENIC);RP11-84H6.1.1(UPSTREAM)
4	179278369	179278378	179278376	AC	G	snp	0	0	1	0	.	.	.	INTERGENIC(INTERGENIC)
4	179298377	179298384	179298379	A	C	ins	1	0	0	0	.	.	.	INTERGENIC(INTERGENIC)
4	179366795	179366802	179366796	A	C	snp	0	1	1	1	.	.	.	INTERGENIC(INTERGENIC)
4	179403251	179403258	179403257	A	G	snp	0	0	1	0	.	.	.	INTERGENIC(INTERGENIC)
4	179471181	179471192	179471189	GA	A	ins	1	1	1	1	.	.	.	INTERGENIC(INTERGENIC)
4	179509452	179509459	179509457	A	G	snp	1	1	1	0	.	.	.	INTERGENIC(INTERGENIC)
4	179515189	179515212	179515202	AATA	C	snp	1	1	1	1	.	.	.	INTERGENIC(INTERGENIC)
4	179517349	179517357	179517351	A	C	ins	1	1	1	1	.	.	.	INTERGENIC(INTERGENIC)
4	179519220	179519227	179519225	A	C	snp	0	0	1	1	.	.	.	INTERGENIC(INTERGENIC)
4	179552921	179552932	179552927	TA	C	snp	1	1	0	1	.	.	.	INTERGENIC(INTERGENIC)
4	179555381	179555389	179555384	A	G	snp	1	1	0	1	.	.	.	INTERGENIC(INTERGENIC)
4	179569845	179569854	179569849	A	C	snp	1	1	1	1	.	.	.	INTERGENIC(INTERGENIC)
4	179638404	179638413	179638411	AT	C	snp	1	1	1	0	.	.	.	INTERGENIC(INTERGENIC)
4	179864935	179864948	179864944	AT	ATGG	ins	1	0	0	0	.	.	.	INTERGENIC(INTERGENIC)
4	179864935	179864948	179864946	AT	GG	ins	1	0	0	0	.	.	.	INTERGENIC(INTERGENIC)
4	179982823	179982831	179982824	A	C	ins	1	1	1	1	.	.	.	INTERGENIC(INTERGENIC)
4	179982823	179982831	179982825	A	C	snp	1	1	1	1	.	.	.	INTERGENIC(INTERGENIC)
4	179995564	179995572	179995571	A	C	snp	1	1	1	1	.	.	.	INTERGENIC(INTERGENIC);RP11-296L20.1.1(DOWNSTREAM)
4	180009797	180009808	180009800	AT	C	snp	1	0	0	0	.	.	.	INTERGENIC(INTERGENIC)
4	180030081	180030088	180030082	T	C	snp	0	1	1	0	.	.	.	INTERGENIC(INTERGENIC)
4	180085556	180085565	180085557	A	C	snp	1	1	1	1	.	.	.	INTERGENIC(INTERGENIC)
4	180093474	180093481	180093477	A	G	snp	0	0	1	0	.	.	.	INTERGENIC(INTERGENIC)
4	180097117	180097126	180097122	T	C	ins	1	0	1	0	.	.	.	INTERGENIC(INTERGENIC)

4	180098185	180098194	180098191	A	G	snp	0	0	1	0	.	.	.	INTERGENIC(INTERGENIC)
4	180163330	180163338	180163335	A	C	snp	1	1	1	1	.	.	.	INTERGENIC(INTERGENIC)
4	180188978	180188986	180188984	T	G	snp	1	0	0	0	.	.	.	INTERGENIC(INTERGENIC)
4	180199049	180199056	180199055	T	C	snp	1	0	0	0	.	.	.	INTERGENIC(INTERGENIC)
4	180251477	180251492	180251485	ATTT	T	del	1	1	1	1	.	.	.	INTERGENIC(INTERGENIC)
4	180251477	180251492	180251487	ATTT	TA	del	1	1	1	1	.	.	.	INTERGENIC(INTERGENIC)
4	180343232	180343247	180343239	CT	C	snp	0	1	0	1	.	.	.	RP11-404J23.1.1(INTRON)
4	180343269	180343282	180343273	CT	G	snp	0	1	0	1	.	.	.	RP11-404J23.1.1(INTRON)
4	180372818	180372832	180372823	AAT	G	snp	1	1	1	1	.	.	.	RP11-404J23.1.1(INTRON)
4	180414343	180414354	180414351	CAA	G	snp	1	0	0	0	.	.	.	INTERGENIC(INTERGENIC)
4	180415297	180415306	180415300	CA	T	snp	0	1	1	1	.	.	.	INTERGENIC(INTERGENIC)
4	180516401	180516408	180516403	A	G	snp	0	1	1	0	.	.	.	INTERGENIC(INTERGENIC)
4	180607531	180607542	180607536	TA	TG	ins	1	1	1	1	.	.	.	INTERGENIC(INTERGENIC)
4	180727162	180727169	180727164	A	G	snp	0	0	0	1	.	.	.	INTERGENIC(INTERGENIC)
4	180826400	180826411	180826402	AT	C	snp	1	1	1	1	.	.	.	INTERGENIC(INTERGENIC)
4	180852137	180852144	180852142	A	C	snp	1	0	0	0	.	.	.	INTERGENIC(INTERGENIC)
4	180928735	180928744	180928741	CA	A	snp	0	1	0	1	.	.	.	INTERGENIC(INTERGENIC)
4	180935898	180935906	180935905	A	G	snp	1	1	1	0	.	.	.	INTERGENIC(INTERGENIC)
4	180966991	180966998	180966996	A	G	snp	0	1	0	1	.	.	.	INTERGENIC(INTERGENIC)
4	180980137	180980148	180980142	CG	A	snp	0	0	1	0	.	.	.	INTERGENIC(INTERGENIC)
4	181026729	181026746	181026740	TGT	G	snp	1	0	0	0	.	.	.	INTERGENIC(INTERGENIC)
4	181035007	181035014	181035009	A	G	snp	1	1	1	1	.	.	.	INTERGENIC(INTERGENIC)
4	181097956	181097964	181097961	T	C	snp	0	0	1	0	.	.	.	INTERGENIC(INTERGENIC)
4	181182359	181182368	181182365	TA	C	snp	1	1	1	0	.	.	.	INTERGENIC(INTERGENIC)
4	181217204	181217219	181217214	AC	C	snp	1	1	1	1	.	.	.	INTERGENIC(INTERGENIC)
4	181255529	181255537	181255535	T	C	snp	1	1	1	1	.	.	.	INTERGENIC(INTERGENIC)
4	181287134	181287143	181287138	TA	C	snp	1	0	0	0	.	.	.	INTERGENIC(INTERGENIC)
4	181300315	181300330	181300319	CCTT	T	snp	1	1	0	1	.	.	.	INTERGENIC(INTERGENIC)
4	181311384	181311393	181311386	TA	C	snp	1	0	0	0	.	.	.	INTERGENIC(INTERGENIC)
4	181311384	181311393	181311391	TA	G	snp	0	1	0	1	.	.	.	INTERGENIC(INTERGENIC)
4	181311406	181311415	181311408	TA	C	snp	1	1	1	1	.	.	.	INTERGENIC(INTERGENIC)
4	181312012	181312021	181312019	TA	G	snp	1	1	0	1	.	.	.	INTERGENIC(INTERGENIC)
4	181325054	181325063	181325060	AT	T	del	1	0	0	0	.	.	.	INTERGENIC(INTERGENIC)
4	181379518	181379525	181379524	T	G	snp	1	1	1	1	.	.	.	INTERGENIC(INTERGENIC)
4	181438498	181438505	181438500	G	A	ins	1	1	1	1	.	.	.	INTERGENIC(INTERGENIC)
4	181497835	181497844	181497843	A	C	snp	1	0	0	0	.	.	.	INTERGENIC(INTERGENIC);RP11-751A18.1.1(UPSTREAM)
4	181526470	181526477	181526472	A	C	snp	0	0	1	0	.	.	.	INTERGENIC(INTERGENIC)
4	181572293	181572304	181572300	CCT	TCG	ins	0	0	1	0	.	.	.	INTERGENIC(INTERGENIC)
4	181580183	181580198	181580192	TCTT	T	ins	1	1	1	1	.	.	.	INTERGENIC(INTERGENIC)
4	181608954	181608963	181608957	TA	T	snp	1	1	1	1	.	.	.	INTERGENIC(INTERGENIC)
4	181608970	181608979	181608976	TA	C	snp	1	1	1	1	.	.	.	INTERGENIC(INTERGENIC)
4	181621023	181621030	181621025	A	G	ins	1	1	1	1	.	.	.	INTERGENIC(INTERGENIC)
4	181694150	181694159	181694153	GA	G	snp	0	1	0	1	.	.	.	INTERGENIC(INTERGENIC)
4	181733221	181733228	181733222	A	G	snp	1	0	0	0	.	.	.	INTERGENIC(INTERGENIC)
4	181763608	181763616	181763612	T	C	snp	1	1	1	1	.	.	.	INTERGENIC(INTERGENIC)
4	181812332	181812351	181812347	AAAC	A	ins	1	0	0	0	.	.	.	INTERGENIC(INTERGENIC)
4	181822464	181822475	181822468	CCA	T	snp	1	1	1	1	.	.	.	INTERGENIC(INTERGENIC)
4	181828674	181828681	181828675	T	G	snp	1	0	0	0	.	.	.	INTERGENIC(INTERGENIC)
4	181828674	181828681	181828680	T	G	snp	1	1	1	1	.	.	.	INTERGENIC(INTERGENIC)
4	181937087	181937096	181937090	TA	TATATAT	del	1	0	0	0	.	.	.	INTERGENIC(INTERGENIC)
4	181937087	181937096	181937092	TA	TATAT	del	1	0	0	0	.	.	.	INTERGENIC(INTERGENIC)
4	181952583	181952592	181952586	AC	T	snp	1	1	1	1	.	.	.	INTERGENIC(INTERGENIC)
4	181981248	181981259	181981251	ATA	G	snp	0	1	1	1	.	.	.	INTERGENIC(INTERGENIC);LINC00290(DOWNSTREAM)
4	182001426	182001435	182001428	TA	G	snp	1	1	0	1	.	.	.	LINC00290(INTRON)
4	182140874	182140882	182140875	T	TG	ins	1	1	1	1	.	.	.	INTERGENIC(INTERGENIC)
4	182140874	182140882	182140876	T	G	ins	1	1	1	1	.	.	.	INTERGENIC(INTERGENIC)
4	182159916	182159933	182159928	CT	A	snp	1	1	1	1	.	.	.	INTERGENIC(INTERGENIC);RP11-665C14.1.1(UPSTREAM)
4	182273265	182273276	182273273	ATT	A	del	1	0	1	0	.	.	.	INTERGENIC(INTERGENIC)
4	182293827	182293835	182293834	A	G	ins	1	1	1	1	.	.	.	INTERGENIC(INTERGENIC)
4	182409055	182409062	182409058	A	C	snp	0	1	1	0	.	.	.	INTERGENIC(INTERGENIC)
4	182439192	182439199	182439194	A	G	snp	0	1	0	1	.	.	.	INTERGENIC(INTERGENIC);RP11-433O3.1.1(UPSTREAM)
4	182443088	182443097	182443096	A	C	snp	1	1	1	1	.	.	.	INTERGENIC(INTERGENIC);RP11-433O3.1.1(UPSTREAM)
4	182467881	182467895	182467884	AAG	AG	del	1	1	1	1	.	.	.	INTERGENIC(INTERGENIC)
4	182703232	182703241	182703239	T	G	snp	0	1	0	1	.	.	.	INTERGENIC(INTERGENIC)
4	182811581	182811588	182811583	A	G	snp	0	0	0	1	.	.	.	AC108142.1.1(INTRON)
4	182819903	182819912	182819908	TG	A	snp	1	1	1	1	.	.	.	AC108142.1.1(INTRON)
4	182862864	182862875	182862871	TTG	C	snp	1	1	1	1	.	.	.	AC108142.1.1(INTRON)

4	182895720	182895735	182895728	CAAA	A	del	1	1	1	1	.	.	.	AC108142.1.1(DOWNSTREAM);AC108142.1.1(INTRON)
4	183082767	183082778	183082772	TA	G	snp	1	1	1	1	.	.	.	ODZ3(INTRON);RP11-402C9.1.1(INTRON)
4	183098755	183098768	183098766	AT	A	del	0	0	1	0	.	.	.	ODZ3(INTRON);RP11-402C9.1.1(INTRON)
4	183144866	183144875	183144868	GT	A	snp	0	1	0	1	.	.	.	ODZ3(INTRON)
4	183207011	183207019	183207016	A	G	snp	1	0	0	0	.	.	.	ODZ3(INTRON)
4	183345617	183345626	183345625	T	C	snp	1	0	0	0	.	.	.	ODZ3(INTRON)
4	183370363	183370383	183370380	GGC	T	snp	1	1	1	1	.	.	.	ODZ3(INTRON)
4	183484142	183484150	183484147	T	G	snp	1	1	1	1	.	.	.	ODZ3(INTRON)
4	183536223	183536232	183536231	T	C	snp	1	1	1	1	.	.	.	ODZ3(INTRON)
4	183551891	183551906	183551902	TTTA	T	ins	0	1	1	1	.	.	.	7SK(DOWNSTREAM);ODZ3(DOWNSTREAM);ODZ3(INTRON)
4	183601591	183601599	183601592	G	A	ins	0	0	0	1	.	.	.	ODZ3(EXON);ODZ3(INTRON)
4	183601591	183601599	183601595	G	T	ins	1	1	0	1	.	.	.	ODZ3(EXON);ODZ3(INTRON)
4	183787232	183787240	183787239	T	C	snp	1	0	0	0	.	.	.	INTERGENIC(INTERGENIC)
4	183810588	183810595	183810589	T	C	snp	1	1	1	1	.	.	.	DCTD(DOWNSTREAM);INTERGENIC(INTERGENIC)
4	183824599	183824608	183824601	GT	C	snp	1	1	1	1	.	.	.	DCTD(INTRON)
4	183891951	183891974	183891958	AGGA	C	snp	1	0	0	0	.	.	.	INTERGENIC(INTERGENIC)
4	183955207	183955222	183955220	TA	TATTT	del	1	1	1	1	.	.	.	FAM92A3(UPSTREAM);INTERGENIC(INTERGENIC);RP11-335L23.2.1(UPSTREAM)
4	183999513	183999530	183999516	CA	CG	ins	1	0	0	0	.	.	.	INTERGENIC(INTERGENIC)
4	184056698	184056713	184056701	TA	TG	ins	1	0	0	0	.	.	.	WWC2(INTRON)
4	184259438	184259453	184259446	CTTT	G	snp	0	1	1	0	.	.	.	INTERGENIC(INTERGENIC)
4	184344901	184344909	184344906	T	C	snp	0	0	0	1	.	.	.	INTERGENIC(INTERGENIC);RP11-692E14.1.1(UPSTREAM)
4	184525814	184525821	184525815	A	G	snp	1	1	1	1	.	.	.	INTERGENIC(INTERGENIC)
4	184528068	184528077	184528073	GT	G	snp	1	0	0	0	.	.	.	INTERGENIC(INTERGENIC)
4	184552805	184552813	184552812	T	G	snp	0	1	0	1	.	.	.	INTERGENIC(INTERGENIC)
4	184578405	184578413	184578409	A	C	snp	1	1	1	1	.	.	.	RWDD4(INTRON);RWDD4(UPSTREAM);TRAPPC11(UPSTREAM);U6(UPSTREAM)
4	184619258	184619266	184619260	T	C	snp	1	0	0	0	.	.	.	TRAPPC11(INTRON);TRAPPC11(UPSTREAM)
4	184704379	184704386	184704383	T	C	snp	1	0	0	0	.	.	.	INTERGENIC(INTERGENIC)
4	184704379	184704386	184704385	T	C	snp	1	1	1	1	.	.	.	INTERGENIC(INTERGENIC)
4	184792997	184793006	184793000	A	G	snp	1	1	1	1	.	.	.	STOX2(INTRON)
4	184967828	184967836	184967829	T	C	snp	1	0	0	0	.	.	.	INTERGENIC(INTERGENIC)
4	185049206	185049213	185049210	A	C	snp	1	1	1	1	.	.	.	ENPP6(INTRON)
4	185051878	185051889	185051887	AG	T	snp	1	1	1	1	.	.	.	ENPP6(INTRON)
4	185078561	185078580	185078568	TCTT	TCTTTCTC		ins	1	1	1	1	.	.	. ENPP6(INTRON)
4	185082822	185082833	185082831	TA	G	snp	1	1	1	1	.	.	.	ENPP6(INTRON)
4	185113046	185113053	185113051	A	G	snp	1	1	1	1	.	.	.	ENPP6(INTRON)
4	185137291	185137300	185137293	TG	C	snp	1	0	0	0	.	.	.	ENPP6(DOWNSTREAM);ENPP6(INTRON)
4	185162280	185162287	185162285	T	G	snp	1	1	1	1	.	.	.	INTERGENIC(INTERGENIC)
4	185189017	185189025	185189019	G	T	snp	0	1	0	1	.	.	.	AC079080.1(UPSTREAM);INTERGENIC(INTERGENIC)
4	185317570	185317581	185317574	AT	G	snp	0	0	0	1	.	.	.	IRF2(INTRON)
4	185317570	185317581	185317579	AT	A	del	1	0	0	0	.	.	.	IRF2(INTRON)
4	185384802	185384813	185384811	AT	A	del	1	1	1	1	.	.	.	IRF2(INTRON)
4	185495355	185495363	185495359	T	G	snp	1	1	0	1	.	.	.	INTERGENIC(INTERGENIC)
4	185617842	185617853	185617845	AT	A	ins	1	1	0	1	.	.	.	CCDC111(DOWNSTREAM);MLF1IP(DOWNSTREAM);MLF1IP(INTRON)
4	185620486	185620509	185620500	AAAT	TAAG	ins	1	1	0	1	.	.	.	CCDC111(DOWNSTREAM);MLF1IP(DOWNSTREAM);MLF1IP(INTRON);MLF1IP(UPSTREAM)
4	185643870	185643878	185643872	A	AC	ins	1	1	0	1	.	.	.	MLF1IP(INTRON)
4	185643870	185643878	185643873	A	C	ins	1	1	0	1	.	.	.	MLF1IP(INTRON)
4	185696727	185696736	185696733	GA	A	snp	1	1	0	1	.	.	.	ACSL1(INTRON)
4	185730950	185730957	185730954	A	C	snp	1	1	1	1	.	.	.	ACSL1(INTRON);ACSL1(UPSTREAM);RP11-701P16.2.1(UPSTREAM)
4	185741762	185741770	185741763	T	C	snp	1	0	0	0	.	.	.	ACSL1(INTRON);RP11-701P16.2.1(INTRON)
4	185749286	185749306	185749292	AAC	T	snp	1	1	1	1	.	.	.	ACSL1(UPSTREAM);INTERGENIC(INTERGENIC)
4	185953455	185953462	185953457	T	G	snp	1	0	0	0	.	.	.	INTERGENIC(INTERGENIC)
4	185973186	185973209	185973189	TTG	C	snp	1	1	0	0	.	.	.	RP11-386B13.3.1(INTRON)
4	185983995	185984018	185984014	TTTG	C	snp	1	1	1	1	.	.	.	RP11-386B13.1.1(UPSTREAM);RP11-386B13.3.1(DOWNSTREAM);RP11-386B13.3.1(INTRON)
4	186122322	186122330	186122323	A	C	snp	1	1	0	1	.	.	.	KIAA1430(INTRON);SNX25(UPSTREAM)
4	186158893	186158902	186158895	T	C	snp	1	1	1	1	.	.	.	SNX25(INTRON)
4	186272016	186272024	186272021	A	C	snp	1	1	1	1	.	.	.	SNX25(INTRON);SNX25(UPSTREAM)
4	186298675	186298692	186298689	AAC	G	snp	1	1	1	1	.	.	.	LRP2BP(INTRON);LRP2BP(UTR_5_PRIME);RP11-714G18.1.1(INTRON)
4	186401488	186401507	186401501	AAAG	C	snp	1	1	1	1	.	.	.	INTERGENIC(INTERGENIC)
4	186457755	186457764	186457759	CA	T	snp	1	1	0	1	.	.	.	INTERGENIC(INTERGENIC);PDLIM3(UPSTREAM)
4	186588842	186588859	186588852	AT	T	ins	1	1	1	1	.	.	.	RP11-626E13.1.1(INTRON);SORBS2(INTRON)

4	186677808	186677816	186677811	T	C	snp	1	1	1	1	.	.	.	SORBS2(DOWNSTREAM);SORBS2(INTRON)
4	186686195	186686203	186686199	T	C	snp	1	1	1	1	.	.	.	SORBS2(INTRON);SORBS2(UPSTREAM)
4	186849700	186849711	186849706	CTT	T	snp	1	1	1	1	.	.	.	SORBS2(INTRON)
4	186905657	186905666	186905662	T	G	snp	1	1	1	1	.	.	.	INTERGENIC(INTERGENIC)
4	186985605	186985613	186985611	A	G	snp	1	0	0	0	.	.	.	INTERGENIC(INTERGENIC);TLR3(UPSTREAM)
4	187013402	187013409	187013406	A	C	snp	1	1	1	1	.	.	.	INTERGENIC(INTERGENIC)
4	187027458	187027469	187027466	AC	G	snp	1	1	1	1	.	.	.	FAM149A(INTRON)
4	187071514	187071531	187071519	TTG	T	snp	1	1	1	1	.	.	.	FAM149A(DOWNSTREAM);FAM149A(INTRON)
4	187073866	187073873	187073872	T	G	snp	1	1	1	1	.	.	.	FAM149A(DOWNSTREAM);FAM149A(INTRON)
4	187081529	187081538	187081535	AC	CAC	del	1	0	0	0	.	.	.	FAM149A(DOWNSTREAM);FAM149A(INTRON);RP11-173M11.2.1(DOWNSTREAM)
4	187101508	187101515	187101514	T	C	snp	1	0	0	0	.	.	.	INTERGENIC(INTERGENIC)
4	187130933	187130940	187130939	G	T	snp	1	0	0	0	.	.	.	CYP4V2(INTRON);KLKB1(INTRAGENIC)
4	187154076	187154089	187154082	TG	C	snp	0	1	1	1	.	.	.	KLKB1(INTRON)
4	187166957	187166965	187166962	T	G	ins	1	1	1	1	.	.	.	KLKB1(INTRON)
4	187178953	187178962	187178960	CA	G	snp	0	1	1	1	.	.	.	KLKB1(INTRON)
4	187195789	187195802	187195796	CT	C	snp	1	1	0	0	.	.	.	F11(DOWNSTREAM);F11(INTRON);F11(UTR_3_PRIME)
4	187280370	187280381	187280379	AT	ACAC	ins	1	0	1	0	.	.	.	RP11-215A19.1.1(INTRON)
4	187300384	187300397	187300388	AC	C	ins	1	0	0	1	.	.	.	RP11-215A19.1.1(INTRON)
4	187313240	187313263	187313257	GCA	C	snp	1	1	1	1	.	.	.	RP11-215A19.1.1(INTRON)
4	187314537	187314544	187314543	A	C	snp	1	1	1	1	.	.	.	RP11-215A19.1.1(INTRON)
4	187326735	187326742	187326738	A	G	snp	1	1	1	1	.	.	.	RP11-215A19.1.1(INTRON)
4	187344163	187344172	187344167	T	TG	ins	1	1	1	1	.	.	.	RP11-215A19.1.1(DOWNSTREAM);RP11-215A19.1.1(INTRON)
4	187367952	187367963	187367961	TC	T	snp	1	1	1	1	.	.	.	RP11-215A19.1.1(INTRON)
4	187416674	187416682	187416680	C	A	ins	1	1	0	1	.	.	.	RP11-215A19.1.1(INTRON)
4	187416674	187416682	187416681	C	A	snp	1	1	1	1	.	.	.	RP11-215A19.1.1(INTRON)
4	187529533	187529540	187529538	T	G	snp	1	0	0	0	.	.	.	FAT1(DOWNSTREAM);FAT1(INTRON)
4	187542976	187542990	187542986	AGT	C	snp	1	1	1	1	.	.	.	FAT1(INTRON)
4	187608552	187608560	187608559	A	AGAAC	ins	1	1	0	1	.	.	.	FAT1(INTRON)
4	187613459	187613478	187613465	TTAT	T	snp	1	0	0	0	.	.	.	FAT1(INTRON)
4	187649095	187649104	187649098	A	C	ins	1	1	1	1	.	.	.	FAT1(UPSTREAM);INTERGENIC(INTERGENIC)
4	187738032	187738045	187738035	TA	C	snp	0	1	0	1	.	.	.	INTERGENIC(INTERGENIC)
4	187772782	187772791	187772789	TA	G	snp	1	1	1	1	.	.	.	INTERGENIC(INTERGENIC)
4	187798529	187798538	187798533	TG	C	snp	1	1	1	1	.	.	.	INTERGENIC(INTERGENIC)
4	187856872	187856880	187856876	T	G	snp	1	1	1	1	.	.	.	INTERGENIC(INTERGENIC)
4	187923261	187923268	187923265	T	TTTG	ins	0	1	1	1	.	.	.	RP11-217E13.1.1(INTRON)
4	187930902	187930911	187930909	AC	T	snp	1	1	1	0	.	.	.	RP11-217E13.1.1(INTRON)
4	187935074	187935087	187935083	AG	AA	ins	1	0	0	0	.	.	.	RP11-217E13.1.1(INTRON)
4	187951977	187951985	187951984	T	G	snp	1	1	0	0	.	.	.	INTERGENIC(INTERGENIC)
4	187957492	187957505	187957502	AG	G	del	1	1	1	1	.	.	.	INTERGENIC(INTERGENIC)
4	187989271	187989294	187989287	TTTC	TTCTTTC	del	1	1	1	1	.	.	.	INTERGENIC(INTERGENIC)
4	187992515	187992534	187992522	TTCT	C	snp	1	1	1	1	.	.	.	INTERGENIC(INTERGENIC)
4	187992655	187992670	187992663	CCTT	T	snp	1	0	0	1	.	.	.	INTERGENIC(INTERGENIC)
4	188115280	188115288	188115282	T	G	snp	1	1	1	1	.	.	.	INTERGENIC(INTERGENIC)
4	188284317	188284326	188284324	CA	G	snp	1	1	1	1	.	.	.	INTERGENIC(INTERGENIC)
4	188380020	188380035	188380028	ATTT	TTTAT	del	1	1	1	1	.	.	.	INTERGENIC(INTERGENIC)
4	188397446	188397455	188397453	CA	G	snp	1	0	0	0	.	.	.	INTERGENIC(INTERGENIC)
4	188463506	188463517	188463511	ACA	G	snp	1	0	0	0	.	.	.	RP11-565A3.2.1(INTRON)
4	188477280	188477287	188477282	T	C	snp	1	1	1	1	.	.	.	RP11-565A3.2.1(INTRON);RP11-565A3.2.1(UPSTREAM)
4	188557983	188558006	188557995	CTTT	A	snp	1	0	0	0	.	.	.	RP11-565A3.1.1(INTRON);RP11-565A3.2.1(INTRON)
4	188608305	188608319	188608313	AAC	T	snp	1	0	0	0	.	.	.	INTERGENIC(INTERGENIC)
4	188677983	188678006	188677993	TTCT	A	snp	1	1	1	1	.	.	.	INTERGENIC(INTERGENIC)
4	188840520	188840531	188840525	GAG	A	snp	1	0	0	0	.	.	.	INTERGENIC(INTERGENIC)
4	188948154	188948162	188948156	A	G	snp	1	0	0	0	.	.	.	INTERGENIC(INTERGENIC)
4	188995054	188995063	188995062	A	G	ins	0	1	0	1	.	.	.	INTERGENIC(INTERGENIC)
4	189030306	189030319	189030311	AC	T	snp	1	0	0	0	.	.	.	RP11-713C19.1.1(DOWNSTREAM);TRIML2(INTRON);TRIML2(UPSTREAM)
4	189030306	189030319	189030314	AC	CG	ins	1	1	1	1	.	.	.	RP11-713C19.1.1(DOWNSTREAM);TRIML2(INTRON);TRIML2(UPSTREAM)
4	189063801	189063814	189063804	AG	AA	ins	1	0	0	0	.	.	.	RP11-366H4.3.1(INTRON);TRIML1(INTRON);TRIML1(UPSTREAM)
4	189063801	189063814	189063806	AG	A	snp	1	0	0	0	.	.	.	RP11-366H4.3.1(INTRON);TRIML1(INTRON);TRIML1(UPSTREAM)
4	189091406	189091414	189091412	G	T	snp	1	0	0	0	.	.	.	INTERGENIC(INTERGENIC)
4	189104768	189104785	189104781	TG	TA	ins	1	1	0	1	.	.	.	INTERGENIC(INTERGENIC)
4	189134043	189134054	189134047	TTG	C	snp	1	1	1	0	.	.	.	INTERGENIC(INTERGENIC)

4	189137892	189137901	189137894	TC	C	snp	1	1	1	1	.	.	.	INTERGENIC(INTERGENIC)
4	189150274	189150283	189150282	T	C	ins	1	1	1	1	.	.	.	INTERGENIC(INTERGENIC)
4	189157270	189157287	189157284	CAA	C	del	1	1	1	1	.	.	.	INTERGENIC(INTERGENIC)
4	189192101	189192115	189192106	CAG	C	snp	1	1	1	1	.	.	.	INTERGENIC(INTERGENIC)
4	189215310	189215321	189215318	GA	A	snp	1	0	1	0	.	.	.	INTERGENIC(INTERGENIC)
4	189276051	189276058	189276054	T	C	snp	1	1	1	1	.	.	.	INTERGENIC(INTERGENIC);RP11-219G10.1.1(UPSTREAM)
4	189323050	189323059	189323058	A	G	snp	1	1	1	1	.	.	.	RP11-219G10.3.1(INTRON)
4	189323413	189323420	189323419	T	G	snp	0	1	1	0	.	.	.	RP11-219G10.3.1(INTRON)
4	189324745	189324753	189324750	T	G	ins	1	0	1	1	.	.	.	RP11-219G10.3.1(INTRON)
4	189335628	189335645	189335637	AC	A	snp	1	1	1	1	.	.	.	RP11-219G10.3.1(INTRON)
4	189353930	189353941	189353933	GGT	A	snp	0	0	0	1	.	.	.	RP11-219G10.3.1(INTRON)
4	189390792	189390811	189390803	AAAC	A	snp	1	1	1	1	.	.	.	RP11-219G10.3.1(INTRON)
4	189409200	189409209	189409204	AC	G	snp	0	0	1	1	.	.	.	RP11-219G10.3.1(DOWNSTREAM);RP11-219G10.3.1(INTRON)
4	189471135	189471146	189471137	TG	C	snp	1	0	0	0	.	.	.	RP11-219G10.3.1(INTRON)
4	189479424	189479433	189479427	T	C	snp	1	0	0	0	.	.	.	RP11-219G10.3.1(INTRON)
4	189485553	189485562	189485560	TG	A	snp	1	0	0	0	.	.	.	RP11-219G10.3.1(INTRON)
4	189508297	189508306	189508300	T	C	snp	1	1	1	1	.	.	.	RP11-219G10.3.1(INTRON)
4	189544222	189544231	189544226	AT	TAT	del	0	0	1	0	.	.	.	RP11-219G10.3.1(INTRON)
4	189651772	189651781	189651777	T	G	ins	1	1	1	0	.	.	.	INTERGENIC(INTERGENIC)
4	189685539	189685552	189685549	TA	C	snp	1	1	1	1	.	.	.	RP11-756P10.4.1(INTRON)
4	189732630	189732639	189732634	AT	TATATAATAATAT	del	1	1	1	1	.	.	.	INTERGENIC(INTERGENIC)
4	189776253	189776268	189776265	TC	CTC	del	0	1	0	1	.	.	.	INTERGENIC(INTERGENIC)
4	189849201	189849210	189849203	CA	T	snp	1	1	1	1	.	.	.	INTERGENIC(INTERGENIC)
4	189892330	189892337	189892333	A	G	snp	1	1	1	1	.	.	.	INTERGENIC(INTERGENIC)
4	189918360	189918369	189918367	T	TG	ins	0	1	0	1	.	.	.	RP11-818C3.1.1(INTRON)
4	190071179	190071188	190071181	GT	T	snp	1	0	0	0	.	.	.	INTERGENIC(INTERGENIC)
4	190127090	190127097	190127093	A	AT	ins	1	0	0	0	.	.	.	INTERGENIC(INTERGENIC);RP11-706F1.1.1(DOWNSTREAM)
4	190185524	190185539	190185534	GATT	C	snp	0	0	1	0	.	.	.	INTERGENIC(INTERGENIC)
4	190238068	190238076	190238073	A	G	snp	1	0	0	0	.	.	.	INTERGENIC(INTERGENIC)
4	190248562	190248570	190248563	G	T	ins	1	0	0	0	.	.	.	INTERGENIC(INTERGENIC)
4	190360003	190360011	190360007	T	C	ins	1	1	1	1	.	.	.	INTERGENIC(INTERGENIC)
4	190457746	190457755	190457753	G	A	snp	1	1	0	1	.	.	.	INTERGENIC(INTERGENIC)
4	190513571	190513588	190513585	TA	C	snp	1	1	1	1	.	.	.	INTERGENIC(INTERGENIC)
4	190691585	190691594	190691586	T	C	snp	0	0	1	0	.	.	.	INTERGENIC(INTERGENIC)
4	190756817	190756828	190756822	AC	A	snp	1	1	1	1	.	.	.	AF146191.4.1(INTRON)
4	190806515	190806522	190806521	A	G	ins	1	1	1	1	.	.	.	AF146191.4.1(INTRON);RP11-463J17.1.1(DOWNSTREAM)
4	190832084	190832095	190832089	TTC	T	snp	1	1	1	0	.	.	.	AF146191.4.1(INTRON)
4	190879290	190879301	190879293	TC	A	snp	1	0	0	0	.	.	.	FRG1(DOWNSTREAM);FRG1(INTRON);FRG1(UPSTREAM)
4	190937359	190937370	190937364	TC	T	snp	1	1	1	1	.	.	.	INTERGENIC(INTERGENIC);RN5S174(DOWNSTREAM);RN5S175(UPSTREAM)
4	190944993	190945008	190945003	CTCC	T	snp	1	1	1	1	.	.	.	DUX4L9(UPSTREAM);FRG2(DOWNSTREAM);INTERGENIC(INTERGENIC)
5	14775	14784	14782	TA	G	snp	0	0	1	0	.	.	.	INTERGENIC(INTERGENIC)
5	148116	148124	148117	A	G	snp	1	1	0	1	.	.	.	PLEKHG4B(INTRON);Y_RNA(UPSTREAM)
5	196784	196792	196788	C	T	ins	1	1	0	1	.	.	.	CTD-2083E4.7.1(DOWNSTREAM);INTERGENIC(INTERGENIC);LRRC14B(DOWNSTREAM)
5	228999	229008	229005	A	G	snp	0	0	0	1	.	.	.	SDHA(DOWNSTREAM);SDHA(INTRON);SDHA(UPSTREAM)
5	324137	324150	324140	TC	T	snp	1	0	0	0	.	.	.	AHRR(INTRON);PDCD6(INTRON)
5	345057	345064	345058	G	T	snp	1	1	1	1	.	.	.	AHRR(INTRON);PDCD6(INTRON)
5	412819	412830	412826	TC	TCTCT	del	1	0	0	0	.	.	.	AHRR(INTRON)
5	412819	412830	412828	TC	TCT	del	1	1	0	1	.	.	.	AHRR(INTRON)
5	462732	462745	462734	CA	T	snp	1	1	1	1	.	.	.	CTD-2228K2.5.1(DOWNSTREAM);EXOC3(DOWNSTREAM);EXOC3(INTRON);EXOC3(UPSTREAM)
5	492775	492783	492779	G	A	ins	0	0	0	1	.	.	.	SLC9A3(INTRON)
5	640702	640711	640704	CA	T	snp	1	0	0	0	.	.	CEP72(SYNONYMOUS_CODING):H508	CEP72(UPSTREAM);CEP72(UTR_3_PRIME)
5	640702	640711	640705	CA	G	snp	1	1	1	1	.	CEP72(NON_SYNONYMOUS_CODING):T509A	.	CEP72(UPSTREAM);CEP72(UTR_3_PRIME)
5	940706	940714	940713	T	C	snp	1	1	0	1	.	.	.	INTERGENIC(INTERGENIC)
5	1003731	1003738	1003732	G	T	snp	1	0	0	0	.	.	.	AC116351.2(UPSTREAM);INTERGENIC(INTERGENIC)
5	1102350	1102359	1102352	GT	C	snp	1	1	1	1	.	.	.	SLC12A7(INTRON)
5	1112987	1112996	1112994	C	A	ins	1	1	1	1	.	.	.	INTERGENIC(INTERGENIC);SLC12A7(UPSTREAM)
5	1112987	1112996	1112994	C	A	snp	1	1	1	1	.	.	.	INTERGENIC(INTERGENIC);SLC12A7(UPSTREAM)
5	1193022	1193029	1193023	G	T	snp	1	1	1	1	.	.	.	INTERGENIC(INTERGENIC)
5	1198937	1198948	1198946	TC	G	snp	1	1	1	1	.	.	.	INTERGENIC(INTERGENIC);SLC6A19(UPSTREAM)
5	1206355	1206370	1206365	TC	C	snp	1	1	1	1	.	.	.	SLC6A19(INTRON)
5	1287624	1287641	1287626	AT	T	del	1	1	1	1	.	.	.	TERT(INTRON);TERT(UPSTREAM)
5	1297377	1297384	1297379	G	A	snp	0	1	0	0	.	.	.	INTERGENIC(INTERGENIC);TERT(UPSTREAM)
5	1308861	1308868	1308865	T	C	snp	1	0	0	0	.	.	.	INTERGENIC(INTERGENIC)

5	1349698	1349705	1349700	T	G	snp	1	0	0	0	.	.	.	CLPTM1L(UPSTREAM);INTERGENIC(INTERGENIC)
5	1443971	1443985	1443976	TTG	T	snp	1	0	0	0	.	.	.	SLC6A3(INTRON);SLC6A3(UPSTREAM)
5	1465729	1465738	1465732	CA	G	snp	0	0	0	1	.	.	.	LPCAT1(INTRON)
5	1494530	1494539	1494532	G	T	snp	1	1	0	1	.	.	.	LPCAT1(INTRON)
5	1501536	1501544	1501542	C	A	snp	1	1	1	1	.	.	.	LPCAT1(INTRON)
5	1542831	1542848	1542843	AT	TATAT	del	1	1	1	1	.	.	.	CTD-2245E15.3.1(DOWNSTREAM);INTERGENIC(INTERGENIC)
5	1556905	1556914	1556907	G	GGT	ins	1	0	0	0	.	.	.	INTERGENIC(INTERGENIC)
5	1556905	1556914	1556913	G	A	snp	1	1	1	1	.	.	.	INTERGENIC(INTERGENIC)
5	1627265	1627272	1627267	T	G	snp	1	1	1	1	.	.	.	RP11-43F13.1.1(DOWNSTREAM);RP11-43F13.1.1(INTRON)
5	1649912	1649927	1649925	TC	TT	ins	1	1	1	1	.	.	.	INTERGENIC(INTERGENIC)
5	1649940	1649953	1649942	TC	C	snp	0	0	0	1	.	.	.	INTERGENIC(INTERGENIC)
5	1653417	1653425	1653418	T	C	snp	1	1	1	1	.	.	.	INTERGENIC(INTERGENIC)
5	1667674	1667681	1667676	G	T	snp	1	1	1	1	.	.	.	INTERGENIC(INTERGENIC)
5	1684367	1684378	1684376	AG	A	snp	1	1	0	0	.	.	.	INTERGENIC(INTERGENIC)
5	1694603	1694618	1694610	TTTA	C	snp	1	1	1	1	.	.	.	INTERGENIC(INTERGENIC)
5	1960874	1960887	1960877	CA	G	snp	1	1	1	1	.	.	.	INTERGENIC(INTERGENIC);RP11-259O2.1.1(DOWNSTREAM);RP11-259O2.2.1(DOWNSTREAM)
5	2024194	2024201	2024199	T	C	snp	1	1	1	1	.	.	.	INTERGENIC(INTERGENIC)
5	2029721	2029736	2029731	TA	A	del	1	0	0	0	.	.	.	INTERGENIC(INTERGENIC)
5	2029721	2029736	2029733	TA	A	del	1	1	1	1	.	.	.	INTERGENIC(INTERGENIC)
5	2029721	2029736	2029734	TA	T	snp	1	0	0	0	.	.	.	INTERGENIC(INTERGENIC)
5	2054672	2054679	2054673	A	AC	ins	0	0	1	0	.	.	.	INTERGENIC(INTERGENIC)
5	2083668	2083691	2083677	AAT	AT	del	0	1	1	1	.	.	.	INTERGENIC(INTERGENIC)
5	2083668	2083691	2083685	AAT	AATAATAAATA	del	1	1	1	1	.	.	.	INTERGENIC(INTERGENIC)
5	2227200	2227209	2227205	TA	G	snp	1	1	1	1	.	.	.	INTERGENIC(INTERGENIC)
5	2246395	2246404	2246399	AT	G	snp	1	1	0	1	.	.	.	INTERGENIC(INTERGENIC)
5	2325548	2325559	2325552	GA	C	snp	0	0	1	0	.	.	.	INTERGENIC(INTERGENIC)
5	2352259	2352282	2352273	AATA	A	del	1	1	1	1	.	.	.	INTERGENIC(INTERGENIC)
5	2394390	2394398	2394391	A	G	snp	1	1	1	1	.	.	.	INTERGENIC(INTERGENIC)
5	2404601	2404608	2404605	A	AAAG	ins	1	0	0	0	.	.	.	INTERGENIC(INTERGENIC)
5	2404601	2404608	2404605	A	G	snp	1	1	1	1	.	.	.	INTERGENIC(INTERGENIC)
5	2407420	2407435	2407427	TATT	G	snp	1	0	1	0	.	.	.	INTERGENIC(INTERGENIC)
5	2492062	2492073	2492068	AGG	C	snp	1	1	1	1	.	.	.	INTERGENIC(INTERGENIC)
5	2533340	2533351	2533347	GAG	T	ins	1	1	1	1	.	.	.	INTERGENIC(INTERGENIC)
5	2540518	2540533	2540522	ATCC	TCCG	ins	1	1	1	1	.	.	.	INTERGENIC(INTERGENIC)
5	2581368	2581377	2581375	TC	T	del	1	1	1	1	.	.	.	INTERGENIC(INTERGENIC)
5	2601229	2601244	2601241	AT	T	del	1	1	1	1	.	.	.	INTERGENIC(INTERGENIC)
5	2630200	2630209	2630201	A	G	snp	1	1	0	1	.	.	.	INTERGENIC(INTERGENIC)
5	2742015	2742022	2742017	A	C	snp	1	1	1	1	.	.	.	INTERGENIC(INTERGENIC);IRX2(DOWNSTREAM);RP11-129I19.2.1(UPSTREAM)
5	2828091	2828106	2828098	GGAA	T	snp	1	1	1	1	.	.	.	INTERGENIC(INTERGENIC);RP11-468D11.1.1(DOWNSTREAM)
5	2855491	2855508	2855497	TTG	C	snp	1	0	0	0	.	.	.	INTERGENIC(INTERGENIC)
5	3106727	3106734	3106733	C	T	ins	1	1	1	1	.	.	.	INTERGENIC(INTERGENIC)
5	3257607	3257618	3257614	ATA	AATAA	del	0	1	1	0	.	.	.	INTERGENIC(INTERGENIC)
5	3290238	3290246	3290245	A	C	snp	1	0	0	0	.	.	.	INTERGENIC(INTERGENIC)
5	3506050	3506058	3506056	T	G	snp	1	1	1	1	.	.	.	RP11-121L11.1.1(INTRON);RP11-121L11.2.1(UPSTREAM)
5	3511877	3511886	3511881	GT	A	snp	1	0	0	0	.	.	.	RP11-121L11.1.1(INTRON)
5	3529416	3529425	3529423	T	G	snp	1	0	0	0	.	.	.	RP11-121L11.1.1(INTRON)
5	3549331	3549339	3549333	A	C	snp	1	1	1	1	.	.	.	INTERGENIC(INTERGENIC)
5	3581089	3581098	3581094	C	T	snp	1	1	1	1	.	.	.	INTERGENIC(INTERGENIC)
5	3624009	3624016	3624015	A	G	snp	1	0	0	0	.	.	.	INTERGENIC(INTERGENIC)
5	3634800	3634809	3634807	A	C	snp	1	1	1	1	.	.	.	INTERGENIC(INTERGENIC)
5	3653352	3653367	3653362	AGAA	AAG	del	1	1	1	1	.	.	.	INTERGENIC(INTERGENIC)
5	3653366	3653381	3653370	AAGA	G	snp	0	1	1	0	.	.	.	INTERGENIC(INTERGENIC)
5	3653725	3653740	3653732	AGAA	G	snp	1	0	1	0	.	.	.	INTERGENIC(INTERGENIC)
5	3701086	3701097	3701091	CTC	A	snp	1	0	0	0	.	.	.	INTERGENIC(INTERGENIC)
5	3702040	3702048	3702043	T	G	snp	1	1	1	1	.	.	.	INTERGENIC(INTERGENIC)
5	3832099	3832106	3832100	A	G	snp	1	0	0	0	.	.	.	INTERGENIC(INTERGENIC)
5	3911187	3911196	3911191	A	G	snp	0	1	0	1	.	.	.	INTERGENIC(INTERGENIC)
5	3944557	3944568	3944563	CTT	TTCT	del	1	1	0	1	.	.	.	INTERGENIC(INTERGENIC)
5	3944557	3944568	3944564	CTT	TC	del	1	1	0	1	.	.	.	INTERGENIC(INTERGENIC)
5	3994755	3994764	3994756	A	G	snp	1	0	0	0	.	.	.	INTERGENIC(INTERGENIC)
5	4211342	4211355	4211345	TG	A	snp	1	0	0	0	.	.	.	INTERGENIC(INTERGENIC)
5	4285642	4285651	4285646	TA	C	snp	1	1	1	1	.	.	.	INTERGENIC(INTERGENIC)
5	4285793	4285808	4285798	TA	G	snp	1	1	1	0	.	.	.	INTERGENIC(INTERGENIC)
5	4289489	4289498	4289490	A	C	snp	1	1	1	1	.	.	.	INTERGENIC(INTERGENIC)
5	4289489	4289498	4289492	A	C	snp	1	0	0	0	.	.	.	INTERGENIC(INTERGENIC)
5	4330799	4330818	4330809	TTTA	C	snp	0	1	1	1	.	.	.	INTERGENIC(INTERGENIC)

5	4391213	4391220	4391218	T	G	ins	1	1	1	1	.	.	.	INTERGENIC(INTERGENIC)	
5	4429362	4429371	4429368	A	G	snp	1	0	0	0	.	.	.	INTERGENIC(INTERGENIC)	
5	4473270	4473277	4473274	T	C	snp	1	0	0	0	.	.	.	RP11-445O3.2.1(INTRON)	
5	4522064	4522072	4522066	A	G	snp	1	1	1	1	.	.	.	RP11-445O3.2.1(INTRON)	
5	4562257	4562265	4562261	A	C	snp	1	0	0	0	.	.	.	RP11-445O3.2.1(INTRON)	
5	4579174	4579183	4579180	TA	C	ins	1	1	1	0	.	.	.	RP11-445O3.2.1(INTRON)	
5	4667808	4667822	4667813	GTG	C	snp	1	1	0	1	.	.	.	RP11-445O3.2.1(INTRON)	
5	4696088	4696097	4696093	AG	C	snp	1	0	0	0	.	.	.	RP11-445O3.2.1(INTRON)	
5	4753400	4753415	4753404	GT	A	snp	1	1	1	1	.	.	.	RP11-445O3.2.1(INTRON)	
5	4832213	4832228	4832222	GAGG	G	del	0	0	0	1	.	.	.	CTD-2161F6.1.1(INTRON);RP11-445O3.2.1(INTRON)	
5	5047503	5047511	5047507	T	G	snp	1	1	0	0	.	.	.	CTD-2247C11.3.1(INTRON);CTD-2247C11.4.1(EXON);CTD-2247C11.4.1(INTRON)	
5	5125698	5125706	5125705	A	C	ins	1	1	0	1	.	.	.	7SK(UPSTREAM);INTERGENIC(INTERGENIC)	
5	5129115	5129126	5129118	CT	C	snp	1	1	1	1	.	.	.	7SK(DOWNSTREAM);CTD-2297D10.2.1(DOWNSTREAM);INTERGENIC(INTERGENIC)	
5	5144239	5144248	5144241	A	AC	ins	1	0	1	0	.	.	.	ADAMTS16(INTRON);CTD-2297D10.1.1(INTRON);CTD-2297D10.2.1(UPSTREAM)	
5	5162846	5162857	5162855	AG	C	snp	1	1	1	1	.	.	.	ADAMTS16(INTRON);CTD-2297D10.1.1(INTRON)	
5	5215811	5215826	5215819	AT	TATATATGTGG		del	1	0	0	0	.	.	.	ADAMTS16(INTRON)
5	5215811	5215826	5215821	AT	TATATGTGG		del	1	0	0	0	.	.	.	ADAMTS16(INTRON)
5	5289318	5289325	5289321	A	G	snp	0	1	1	1	.	.	.	ADAMTS16(INTRON)	
5	5321097	5321105	5321098	T	C	snp	1	1	1	1	.	.	.	ADAMTS16(DOWNSTREAM);INTERGENIC(INTERGENIC)	
5	5723897	5723904	5723899	T	G	snp	1	1	1	0	.	.	.	INTERGENIC(INTERGENIC)	
5	5754902	5754910	5754905	A	G	snp	1	1	1	1	.	.	.	INTERGENIC(INTERGENIC)	
5	5774944	5774955	5774947	CCT	CTCCTT	ins	1	1	0	1	.	.	.	INTERGENIC(INTERGENIC)	
5	5816110	5816121	5816117	AT	C	snp	1	0	0	0	.	.	.	INTERGENIC(INTERGENIC)	
5	5816314	5816323	5816319	AT	C	snp	1	1	1	1	.	.	.	INTERGENIC(INTERGENIC)	
5	5816480	5816489	5816482	AT	G	snp	1	0	0	0	.	.	.	INTERGENIC(INTERGENIC)	
5	5876275	5876284	5876279	CT	T	snp	1	1	1	1	.	.	.	INTERGENIC(INTERGENIC)	
5	5876336	5876351	5876342	TC	C	snp	0	1	1	1	.	.	.	INTERGENIC(INTERGENIC)	
5	5912129	5912136	5912132	A	G	snp	1	0	0	0	.	.	.	INTERGENIC(INTERGENIC)	
5	5912129	5912136	5912133	A	C	snp	1	0	0	0	.	.	.	INTERGENIC(INTERGENIC)	
5	6156101	6156116	6156107	GA	C	snp	1	1	1	1	.	.	.	INTERGENIC(INTERGENIC)	
5	6201566	6201593	6201577	AATG	T	snp	1	1	1	1	.	.	.	INTERGENIC(INTERGENIC)	
5	6291550	6291558	6291557	A	C	snp	1	1	1	1	.	.	.	HMGB3P3(DOWNSTREAM);INTERGENIC(INTERGENIC)	
5	6302994	6303001	6302999	C	CG	ins	1	1	1	1	.	.	.	INTERGENIC(INTERGENIC)	
5	6406142	6406156	6406147	TTG	T	snp	1	1	1	0	.	.	.	INTERGENIC(INTERGENIC)	
5	6445042	6445050	6445043	T	C	ins	0	0	1	0	.	.	.	INTERGENIC(INTERGENIC);UBE2QL1(UPSTREAM)	
5	6521856	6521867	6521861	AAG	C	snp	1	1	1	1	.	.	.	INTERGENIC(INTERGENIC)	
5	6531564	6531578	6531572	CTC	T	snp	1	1	1	1	.	.	.	INTERGENIC(INTERGENIC)	
5	6580803	6580818	6580812	TA	TG	ins	1	1	1	1	.	.	.	CTD-2195M18.1.1(UPSTREAM);INTERGENIC(INTERGENIC)	
5	6654541	6654550	6654547	T	G	snp	1	0	0	0	.	.	.	SRD5A1(DOWNSTREAM);SRD5A1(INTRON)	
5	6725877	6725886	6725883	CT	A	snp	1	1	0	0	.	.	.	PAPD7(INTRON)	
5	6790379	6790390	6790388	TG	T	del	1	1	1	1	.	.	.	INTERGENIC(INTERGENIC)	
5	6841344	6841353	6841349	AT	G	snp	1	1	1	1	.	.	.	INTERGENIC(INTERGENIC)	
5	6976535	6976544	6976538	CT	C	snp	1	0	0	0	.	.	.	RP11-122F24.1.1(INTRON)	
5	7031747	7031760	7031758	AC	A	del	1	1	0	1	.	.	.	RP11-122F24.1.1(INTRON)	
5	7057702	7057710	7057707	A	G	snp	1	0	0	0	.	.	.	RP11-122F24.1.1(INTRON)	
5	7203128	7203143	7203141	TC	A	snp	1	0	0	0	.	.	.	INTERGENIC(INTERGENIC)	
5	7214012	7214019	7214016	T	G	snp	0	1	0	1	.	.	.	INTERGENIC(INTERGENIC)	
5	7215291	7215302	7215299	ATC	AT	del	0	1	0	0	.	.	.	INTERGENIC(INTERGENIC)	
5	7219791	7219802	7219797	AT	G	snp	1	1	1	1	.	.	.	INTERGENIC(INTERGENIC)	
5	7290616	7290625	7290618	TG	G	snp	1	0	0	0	.	.	.	INTERGENIC(INTERGENIC);RP11-404K5.1.1(UPSTREAM);RP11-404K5.3.1(UPSTREAM)	
5	7532396	7532407	7532399	AG	A	snp	1	0	0	0	.	.	.	ADCY2(INTRON)	
5	7575399	7575407	7575405	T	C	snp	1	1	1	1	.	.	.	ADCY2(INTRON)	
5	7587091	7587106	7587102	ATAG	C	snp	0	1	0	1	.	.	.	ADCY2(INTRON)	
5	7677414	7677422	7677417	A	C	snp	1	1	0	1	.	.	.	ADCY2(INTRON)	
5	7683977	7683988	7683983	CTT	A	snp	1	1	1	1	.	.	.	ADCY2(INTRON);ADCY2(UPSTREAM)	
5	7763160	7763167	7763162	T	G	ins	1	1	1	1	.	.	.	ADCY2(INTRON)	
5	7788816	7788825	7788823	TG	A	snp	1	1	1	1	.	.	.	ADCY2(INTRON);ADCY2(UPSTREAM)	
5	7801011	7801020	7801016	AG	A	snp	1	0	0	0	.	.	.	ADCY2(EXON);ADCY2(INTRON)	
5	7835066	7835081	7835073	AC	T	snp	1	1	1	1	.	.	.	ADCY2(DOWNSTREAM);C5orf49(INTRON)	
5	7981708	7981717	7981716	A	C	ins	1	1	1	1	.	.	.	INTERGENIC(INTERGENIC);U1(DOWNSTREAM)	
5	7984628	7984651	7984632	TTTG	TTG	del	1	1	1	1	.	.	.	INTERGENIC(INTERGENIC);U1(DOWNSTREAM)	
5	8020097	8020105	8020098	T	G	snp	1	1	1	1	.	.	.	INTERGENIC(INTERGENIC)	
5	8045490	8045497	8045493	A	G	snp	0	1	1	1	.	.	.	INTERGENIC(INTERGENIC)	
5	8052006	8052033	8052013	TCAT	TCAC	ins	1	0	1	0	.	.	.	INTERGENIC(INTERGENIC)	
5	8058065	8058074	8058067	CA	T	snp	0	1	1	0	.	.	.	INTERGENIC(INTERGENIC)	
5	8126289	8126298	8126291	AT	T	del	1	1	1	1	.	.	.	INTERGENIC(INTERGENIC)	
5	8135118	8135125	8135123	A	G	snp	1	0	0	0	.	.	.	INTERGENIC(INTERGENIC)	

5	8275209	8275216	8275211	A	G	snp	1	1	1	1	.	.	.	INTERGENIC(INTERGENIC)
5	8306346	8306353	8306347	G	A	snp	1	1	1	1	.	.	.	INTERGENIC(INTERGENIC)
5	8367972	8367979	8367977	A	C	snp	1	0	0	0	.	.	.	RP11-480D4.1.1(INTRON)
5	8368749	8368756	8368750	A	G	snp	1	0	0	0	.	.	.	RP11-480D4.1.1(INTRON)
5	8500198	8500205	8500201	A	C	snp	1	0	0	0	.	.	.	INTERGENIC(INTERGENIC)
5	8549520	8549527	8549521	T	C	snp	0	0	1	0	.	.	.	INTERGENIC(INTERGENIC)
5	8597253	8597262	8597256	TG	A	snp	1	0	0	0	.	.	.	INTERGENIC(INTERGENIC)
5	8616558	8616567	8616566	T	G	snp	1	1	1	1	.	.	.	AC091953.1(DOWNSTREAM);AC091953.3(DOWNSTREAM);INTERGENIC(INTERGENIC);MTND6P2(DOWNSTREAM);RP11-417J1.3.1(UPSTREAM)
5	8649900	8649908	8649906	T	C	snp	1	1	0	1	.	.	.	INTERGENIC(INTERGENIC)
5	8649900	8649908	8649907	T	C	ins	1	0	0	0	.	.	.	INTERGENIC(INTERGENIC)
5	8652700	8652709	8652704	G	T	ins	1	0	0	0	.	.	.	INTERGENIC(INTERGENIC)
5	8672401	8672412	8672408	AT	A	snp	1	1	1	1	.	.	.	INTERGENIC(INTERGENIC)
5	8690187	8690206	8690198	AAAC	A	snp	1	0	0	0	.	.	.	INTERGENIC(INTERGENIC)
5	8690207	8690214	8690209	A	C	ins	1	1	1	1	.	.	.	INTERGENIC(INTERGENIC)
5	8727362	8727373	8727371	TC	T	snp	1	1	1	1	.	.	.	INTERGENIC(INTERGENIC)
5	8788752	8788761	8788758	TC	G	snp	1	0	0	0	.	.	.	INTERGENIC(INTERGENIC);RP11-315A16.1.1(UPSTREAM)
5	8788789	8788804	8788798	CT	C	snp	1	0	0	0	.	.	.	INTERGENIC(INTERGENIC);RP11-315A16.1.1(UPSTREAM)
5	8801745	8801760	8801756	AAAG	AA	del	1	1	1	1	.	.	.	INTERGENIC(INTERGENIC)
5	8931375	8931383	8931378	T	C	snp	1	1	0	1	.	.	.	INTERGENIC(INTERGENIC)
5	8983297	8983316	8983301	AAAG	AAG	del	1	1	1	1	.	.	.	INTERGENIC(INTERGENIC)
5	8983297	8983316	8983302	AAAG	AG	del	1	1	1	1	.	.	.	INTERGENIC(INTERGENIC)
5	8987567	8987576	8987572	TA	G	snp	1	1	1	1	.	.	.	INTERGENIC(INTERGENIC)
5	9047016	9047035	9047029	TCAC	G	snp	1	1	1	1	.	.	.	CTD-2215L10.1.1(DOWNSTREAM);SEMA5A(INTRON)
5	9114571	9114578	9114576	T	C	snp	1	1	1	1	.	.	.	SEMA5A(INTRON)
5	9114571	9114578	9114577	T	G	snp	1	1	1	1	.	.	.	SEMA5A(INTRON)
5	9185572	9185581	9185575	TG	A	snp	1	1	1	1	.	.	.	SEMA5A(INTRON)
5	9347668	9347677	9347676	T	C	snp	0	1	0	1	.	.	.	SEMA5A(INTRON)
5	9493395	9493410	9493403	ATCT	TC	del	1	1	1	1	.	.	.	SEMA5A(INTRON)
5	9496986	9496995	9496993	CA	G	snp	1	1	1	1	.	.	.	SEMA5A(INTRON)
5	9513168	9513179	9513175	AT	A	ins	0	1	0	1	.	.	.	CTD-2201E9.1.1(INTRON);SEMA5A(INTRON)
5	9574623	9574634	9574629	TTG	C	snp	0	0	1	0	.	.	.	INTERGENIC(INTERGENIC)
5	9580146	9580155	9580152	TA	C	snp	1	0	0	0	.	.	.	INTERGENIC(INTERGENIC)
5	9671753	9671760	9671757	A	C	snp	1	1	1	1	.	.	.	CTD-2143L24.1.1(INTRON)
5	9701253	9701264	9701257	TG	C	snp	1	1	1	1	.	.	.	CTD-2143L24.1.1(INTRON)
5	9756715	9756722	9756720	T	G	snp	1	0	0	0	.	.	.	CTD-2143L24.1.1(INTRON);RN5S177(UPSTREAM)
5	9830684	9830699	9830693	GGTA	A	snp	1	1	1	0	.	.	.	CTD-2143L24.1.1(INTRON)
5	9971289	9971296	9971294	T	C	snp	1	1	1	1	.	.	.	INTERGENIC(INTERGENIC)
5	10024526	10024533	10024528	A	G	snp	1	0	0	0	.	.	.	INTERGENIC(INTERGENIC)
5	10087611	10087620	10087614	AC	A	snp	1	1	1	1	.	.	.	INTERGENIC(INTERGENIC)
5	10106411	10106420	10106418	AT	ATAC	ins	1	1	1	1	.	.	.	INTERGENIC(INTERGENIC)
5	10118603	10118622	10118613	TTTC	C	ins	1	1	1	1	.	.	.	INTERGENIC(INTERGENIC)
5	10205918	10205941	10205928	TTA	ATC	ins	1	1	1	0	.	.	.	AC034229.1(DOWNSTREAM);INTERGENIC(INTERGENIC)
5	10244642	10244653	10244649	AAC	C	snp	1	1	1	1	.	.	.	CTD-2256P15.1.1(UPSTREAM);FAM173B(INTRON)
5	10306483	10306494	10306488	GAG	A	snp	0	0	0	1	.	.	.	CMBL(DOWNSTREAM);CMBL(INTRON)
5	10347510	10347519	10347516	AT	T	ins	1	0	0	0	.	.	.	INTERGENIC(INTERGENIC)
5	10393178	10393186	10393182	T	G	snp	1	0	0	0	.	.	.	MARCH6(DOWNSTREAM);MARCH6(INTRON)
5	10427740	10427747	10427742	A	G	snp	0	1	0	0	.	.	.	MARCH6(DOWNSTREAM);MARCH6(INTRON)
5	10545249	10545256	10545251	A	C	snp	0	1	1	1	.	.	.	AC112200.1(DOWNSTREAM);INTERGENIC(INTERGENIC)
5	10641321	10641338	10641335	TCT	TC	del	1	1	0	1	.	.	.	ANKRD33B(INTRON)
5	10661761	10661770	10661766	TG	A	snp	1	1	1	1	.	.	.	INTERGENIC(INTERGENIC);RP11-215G15.4.1(DOWNSTREAM)
5	10735285	10735292	10735289	A	G	ins	1	0	1	0	.	.	.	DAP(INTRON)
5	10830899	10830912	10830907	AT	TC	ins	1	1	0	1	.	.	.	INTERGENIC(INTERGENIC)
5	10863099	10863107	10863106	T	G	snp	0	1	1	0	.	.	.	INTERGENIC(INTERGENIC)
5	10909822	10909831	10909825	A	C	snp	1	1	1	1	.	.	.	INTERGENIC(INTERGENIC)
5	10983407	10983415	10983414	T	C	snp	1	0	0	0	.	.	.	CTNND2(INTRON)
5	11006161	11006168	11006166	A	C	snp	1	1	0	1	.	.	.	CTNND2(INTRON)
5	11059973	11059982	11059977	TA	C	snp	1	0	0	0	.	.	.	CTNND2(INTRON)
5	11088619	11088627	11088620	T	C	snp	0	1	0	1	.	.	.	CTNND2(INTRON)
5	11128843	11128852	11128848	AT	AAA	ins	1	1	1	1	.	.	.	CTNND2(INTRON)
5	11130372	11130379	11130374	C	T	ins	0	0	1	0	.	.	.	CTNND2(INTRON)
5	11248453	11248468	11248456	AT	C	snp	1	0	0	0	.	.	.	CTNND2(INTRON)
5	11277255	11277263	11277256	T	C	snp	1	1	1	1	.	.	.	CTNND2(INTRON)
5	11286165	11286174	11286173	A	G	snp	0	1	1	1	.	.	.	CTNND2(INTRON)
5	11319032	11319046	11319043	ATA	T	ins	1	1	1	1	.	.	.	CTNND2(INTRON)
5	11383883	11383890	11383884	A	C	snp	1	1	1	1	.	.	.	CTNND2(DOWNSTREAM);CTNND2(INTRON)

5	11433366	11433374	11433367	A	C	snp	1	1	1	0	.	.	.	CTNND2(INTRON)
5	11443516	11443523	11443517	G	T	snp	1	1	1	1	.	.	.	CTNND2(INTRON)
5	11530522	11530531	11530526	TG	TA	ins	1	1	1	1	.	.	.	CTNND2(INTRON)
5	11530522	11530531	11530527	TG	T	snp	1	1	1	1	.	.	.	CTNND2(INTRON)
5	11530522	11530531	11530528	TG	A	snp	1	1	1	1	.	.	.	CTNND2(INTRON)
5	11546721	11546728	11546725	A	G	snp	1	1	1	1	.	.	.	CTNND2(INTRON)
5	11600421	11600428	11600425	A	C	snp	1	1	1	1	.	.	.	CTNND2(INTRON)
5	11817084	11817095	11817088	GA	C	snp	1	1	1	1	.	.	.	CTNND2(INTRON)
5	11817190	11817201	11817195	GAG	A	snp	1	1	1	1	.	.	.	CTNND2(INTRON)
5	11817227	11817238	11817232	GAG	A	snp	1	1	1	1	.	.	.	CTNND2(INTRON)
5	11817344	11817361	11817359	GA	G	snp	1	0	1	1	.	.	.	CTNND2(INTRON)
5	11879454	11879469	11879464	TA	AC	ins	1	1	1	0	.	.	.	CTNND2(INTRON)
5	11931664	11931671	11931666	C	T	snp	0	0	1	0	.	.	.	INTERGENIC(INTERGENIC)
5	12026086	12026094	12026089	A	C	snp	1	1	1	1	.	.	.	INTERGENIC(INTERGENIC)
5	12059316	12059329	12059322	TA	C	snp	1	0	0	0	.	.	.	INTERGENIC(INTERGENIC)
5	12124322	12124329	12124324	T	C	ins	1	1	1	1	.	.	.	INTERGENIC(INTERGENIC)
5	12426875	12426884	12426877	TA	A	snp	1	1	1	1	.	.	.	INTERGENIC(INTERGENIC)
5	12426927	12426936	12426929	TA	A	snp	1	1	1	1	.	.	.	INTERGENIC(INTERGENIC)
5	12467158	12467175	12467165	AG	A	snp	1	0	0	0	.	.	.	INTERGENIC(INTERGENIC)
5	12681035	12681046	12681043	AT	TAT	del	1	1	1	1	.	.	.	RP11-308B16.1.1(INTRON)
5	12722418	12722429	12722426	AAT	AATA	del	0	1	0	0	.	.	.	RP11-308B16.1.1(INTRON)
5	12801645	12801660	12801653	TC	C	snp	1	1	1	1	.	.	.	RP11-308B16.1.1(INTRON)
5	12801660	12801669	12801664	C	A	ins	1	1	1	1	.	.	.	RP11-308B16.1.1(INTRON)
5	12809700	12809713	12809710	AT	T	snp	1	0	1	0	.	.	.	INTERGENIC(INTERGENIC)
5	12809700	12809713	12809711	AT	A	del	1	1	1	1	.	.	.	INTERGENIC(INTERGENIC)
5	12939199	12939207	12939200	T	C	snp	0	0	1	0	.	.	.	RP11-419C19.2.1(INTRON)
5	13115890	13115897	13115893	T	C	snp	1	1	1	1	.	.	.	INTERGENIC(INTERGENIC)
5	13119504	13119513	13119508	A	G	snp	1	1	1	1	.	.	.	INTERGENIC(INTERGENIC)
5	13122528	13122537	13122531	TA	G	snp	1	1	1	1	.	.	.	INTERGENIC(INTERGENIC)
5	13310154	13310165	13310161	TG	C	snp	0	0	1	0	.	.	.	INTERGENIC(INTERGENIC)
5	13349513	13349522	13349515	GA	C	snp	0	0	1	0	.	.	.	INTERGENIC(INTERGENIC)
5	13370347	13370355	13370351	A	C	ins	1	1	1	1	.	.	.	INTERGENIC(INTERGENIC)
5	13423175	13423182	13423180	T	C	snp	1	1	1	1	.	.	.	INTERGENIC(INTERGENIC)
5	13437679	13437688	13437683	A	G	ins	1	0	0	0	.	.	.	INTERGENIC(INTERGENIC)
5	13478467	13478474	13478473	C	T	ins	0	0	1	0	.	.	.	INTERGENIC(INTERGENIC)
5	13527767	13527780	13527770	CA	C	snp	1	0	0	0	.	.	.	INTERGENIC(INTERGENIC)
5	13701162	13701170	13701167	A	C	snp	1	1	1	1	.	.	.	DNAH5(INTRON)
5	13702267	13702278	13702274	CAG	GCAGTGTAAG	del	1	0	0	0	.	.	.	DNAH5(INTRON)
5	13847878	13847891	13847889	TG	C	snp	1	0	0	0	.	.	.	DNAH5(INTRON)
5	13862963	13862972	13862969	AT	G	snp	1	1	1	1	.	.	.	CTB-51A17.1.1(INTRON);DNAH5(INTRON)
5	13876601	13876608	13876605	T	C	snp	1	1	1	1	.	.	.	CTB-51A17.1.1(INTRON);DNAH5(INTRON)
5	13905037	13905052	13905044	GAAA	G	snp	1	0	0	0	.	.	.	DNAH5(INTRON)
5	13912590	13912601	13912599	AC	AT	ins	1	1	1	1	.	.	.	DNAH5(INTRON)
5	13962570	13962577	13962573	A	G	snp	0	0	1	0	.	.	.	INTERGENIC(INTERGENIC)
5	13962570	13962577	13962574	A	G	snp	0	0	1	0	.	.	.	INTERGENIC(INTERGENIC)
5	13991687	13991698	13991692	GAG	GAGGAGGGGGAA	ins	1	0	1	0	.	.	.	INTERGENIC(INTERGENIC)
5	14090041	14090056	14090048	AAAC	A	del	1	1	1	1	.	.	.	INTERGENIC(INTERGENIC)
5	14107194	14107201	14107195	T	TC	ins	1	0	0	0	.	.	.	INTERGENIC(INTERGENIC)
5	14157673	14157688	14157683	TC	G	snp	1	0	0	0	.	.	.	TRIO(INTRON)
5	14181282	14181291	14181285	A	G	ins	0	0	1	0	.	.	.	TRIO(INTRON);TRIO(UPSTREAM)
5	14280135	14280142	14280139	T	G	snp	1	0	0	0	.	.	.	TRIO(INTRON)
5	14441111	14441126	14441118	GC	GG	ins	1	1	0	1	.	.	.	TRIO(EXON);TRIO(INTRON)
5	14568621	14568640	14568634	CTTT	TTC	del	1	0	0	0	.	.	.	INTERGENIC(INTERGENIC)
5	14568621	14568640	14568635	CTTT	TC	del	1	0	0	0	.	.	.	INTERGENIC(INTERGENIC)
5	14568621	14568640	14568636	CTTT	C	del	1	0	0	0	.	.	.	INTERGENIC(INTERGENIC)
5	14596258	14596265	14596260	T	C	snp	1	0	0	0	.	.	.	FAM105A(INTRON);FAM105A(UPSTREAM)
5	14660028	14660035	14660033	T	G	snp	1	0	0	0	.	.	.	CTD-2165H16.4.1(DOWNSTREAM);FAM105B(UPSTREAM);INTERGENIC(INTERGENIC)
5	14661902	14661910	14661908	T	G	snp	1	1	0	1	.	.	.	CTD-2165H16.4.1(DOWNSTREAM);FAM105B(UPSTREAM);INTERGENIC(INTERGENIC)
5	14845458	14845475	14845467	TA	TATATATATTTTTTT	del	1	1	1	1	.	.	.	ANKH(INTRON)
5	14919466	14919481	14919470	ATCT	G	snp	1	0	0	0	.	.	.	INTERGENIC(INTERGENIC)
5	14919466	14919481	14919472	ATCT	T	snp	0	0	1	0	.	.	.	INTERGENIC(INTERGENIC)
5	14969669	14969684	14969682	AT	A	del	1	1	1	1	.	.	.	INTERGENIC(INTERGENIC)
5	14979919	14979930	14979923	AAC	G	snp	1	0	0	0	.	.	.	INTERGENIC(INTERGENIC)
5	15026076	15026089	15026083	AC	T	snp	1	0	0	0	.	.	.	INTERGENIC(INTERGENIC)
5	15093305	15093313	15093308	A	C	snp	1	0	0	0	.	.	.	INTERGENIC(INTERGENIC)

5	15152643	15152652	15152650	T	C	snp	1	0	1	0	.	.	.	INTERGENIC(INTERGENIC)
5	15229046	15229053	15229048	T	TTC	ins	1	1	1	1	.	.	.	RP1-137K24.1.1(INTRON)
5	15309650	15309663	15309655	AG	A	snp	1	0	0	0	.	.	.	INTERGENIC(INTERGENIC)
5	15380955	15380962	15380960	C	A	snp	1	0	0	0	.	.	.	INTERGENIC(INTERGENIC);MARK2P5(UPSTREAM)
5	15720921	15720932	15720928	TAT	T	del	1	1	1	1	.	.	.	FBXL7(INTRON)
5	15789316	15789324	15789322	T	G	snp	1	1	1	1	.	.	.	FBXL7(INTRON)
5	15937656	15937665	15937663	C	A	ins	1	1	1	1	.	.	.	FBXL7(DOWNSTREAM);FBXL7(UTR_3_PRIME);MIR887(DOWNSTREAM)
5	16006682	16006705	16006694	ATT	T	ins	1	0	1	0	.	.	.	INTERGENIC(INTERGENIC)
5	16006682	16006705	16006702	ATT	A	del	1	1	1	1	.	.	.	INTERGENIC(INTERGENIC)
5	16038836	16038845	16038838	AG	G	snp	1	1	1	1	.	.	.	INTERGENIC(INTERGENIC)
5	16108756	16108765	16108761	GA	T	snp	1	0	0	0	.	.	.	MARCH11(INTRON)
5	16369625	16369633	16369628	A	C	ins	1	1	1	1	.	.	.	INTERGENIC(INTERGENIC);RP1-167G20.1.1(DOWNSTREAM)
5	16389823	16389846	16389830	AATT	A	snp	1	0	0	0	.	.	.	RP1-167G20.1.1(INTRON)
5	16402651	16402660	16402658	AC	ACA	del	1	0	0	0	.	.	.	RP1-167G20.1.1(INTRON)
5	16515010	16515021	16515019	AT	AC	ins	1	1	1	1	.	.	.	FAM134B(INTRON)
5	16585083	16585092	16585084	T	G	snp	0	0	0	1	.	.	.	FAM134B(INTRON)
5	16615343	16615350	16615345	A	G	snp	1	1	1	1	.	.	.	CTC-461F20.1.1(UPSTREAM);FAM134B(DOWNSTREAM);FAM134B(INTRON);RP11-260E18.1.1(UPSTREAM)
5	16776403	16776412	16776411	T	C	snp	1	1	1	1	.	.	.	MYO10(DOWNSTREAM);MYO10(INTRON)
5	16811273	16811281	16811278	G	A	snp	1	1	1	1	.	.	.	MYO10(DOWNSTREAM);MYO10(INTRON)
5	16811999	16812013	16812004	CAC	G	snp	1	1	1	1	.	.	.	MYO10(DOWNSTREAM);MYO10(INTRON)
5	16835879	16835886	16835880	A	C	snp	1	1	1	1	.	.	.	MYO10(INTRON)
5	16837425	16837433	16837429	A	C	snp	1	0	0	0	.	.	.	MYO10(INTRON)
5	16865921	16865944	16865937	ATA	C	snp	1	0	0	0	.	.	.	MYO10(INTRON)
5	16871010	16871017	16871011	A	C	snp	1	0	0	0	.	.	.	MYO10(INTRON)
5	16871010	16871017	16871013	A	C	snp	1	0	0	0	.	.	.	MYO10(INTRON)
5	16920735	16920744	16920737	CA	T	snp	1	0	0	0	.	.	.	MYO10(INTRON);MYO10(UPSTREAM)
5	16968087	16968095	16968089	T	C	snp	0	1	1	1	.	.	.	INTERGENIC(INTERGENIC)
5	16974856	16974869	16974867	CT	C	del	1	1	1	1	.	.	.	INTERGENIC(INTERGENIC)
5	16981060	16981071	16981066	AAC	AC	del	1	1	1	1	.	.	.	INTERGENIC(INTERGENIC)
5	17009644	17009653	17009651	TA	C	snp	0	0	0	1	.	.	.	INTERGENIC(INTERGENIC)
5	17026164	17026171	17026169	A	C	snp	1	0	0	0	.	.	.	INTERGENIC(INTERGENIC)
5	17040043	17040057	17040054	CTC	T	snp	1	0	0	0	.	.	.	INTERGENIC(INTERGENIC)
5	17052145	17052158	17052156	TA	TG	ins	1	1	1	1	.	.	.	INTERGENIC(INTERGENIC)
5	17063780	17063787	17063781	A	G	snp	0	1	1	1	.	.	.	INTERGENIC(INTERGENIC)
5	17171285	17171293	17171286	T	G	snp	1	0	0	0	.	.	.	AC091878.1.1(INTRON)
5	17201324	17201333	17201330	T	C	snp	1	0	1	0	.	.	.	AC091878.1.1(INTRON);CTD-3065A13.1.1(UPSTREAM)
5	17599789	17599798	17599796	TA	G	snp	1	1	1	1	.	.	.	INTERGENIC(INTERGENIC);RP11-432M8.5.1(UPSTREAM);RP11-432M8.6.1(DOWNSTREAM);RP11-432M8.7.1(UPSTREAM)
5	17778281	17778288	17778283	T	TTTTTTG	ins	1	1	1	1	.	.	.	RP11-454P21.1.1(INTRON)
5	17812201	17812210	17812204	AT	C	snp	1	1	1	1	.	.	.	RP11-454P21.1.1(INTRON)
5	17812213	17812222	17812216	AT	C	snp	1	1	1	1	.	.	.	RP11-454P21.1.1(INTRON)
5	17812225	17812234	17812228	AT	C	snp	1	1	1	1	.	.	.	RP11-454P21.1.1(INTRON)
5	17812237	17812246	17812240	AT	C	snp	1	1	1	1	.	.	.	RP11-454P21.1.1(INTRON)
5	17812249	17812258	17812252	AT	C	snp	1	1	1	1	.	.	.	RP11-454P21.1.1(INTRON)
5	17812261	17812270	17812264	AT	C	snp	1	1	1	1	.	.	.	RP11-454P21.1.1(INTRON)
5	17812273	17812282	17812275	AT	G	snp	1	1	0	1	.	.	.	RP11-454P21.1.1(INTRON)
5	17812273	17812282	17812276	AT	C	snp	1	1	1	1	.	.	.	RP11-454P21.1.1(INTRON)
5	17812285	17812294	17812288	AT	C	snp	1	1	1	1	.	.	.	RP11-454P21.1.1(INTRON)
5	17812297	17812306	17812300	AT	C	snp	1	1	1	1	.	.	.	RP11-454P21.1.1(INTRON)
5	17812461	17812472	17812465	AT	G	snp	1	1	1	1	.	.	.	RP11-454P21.1.1(INTRON)
5	17835112	17835121	17835115	TA	C	snp	1	0	0	1	.	.	.	RP11-454P21.1.1(INTRON)
5	17871908	17871916	17871913	A	G	snp	1	1	0	1	.	.	.	RP11-454P21.1.1(INTRON)
5	18005516	18005523	18005518	T	C	snp	1	1	1	1	.	.	.	INTERGENIC(INTERGENIC)
5	18060979	18060988	18060982	T	C	snp	1	0	0	0	.	.	.	INTERGENIC(INTERGENIC)
5	18076432	18076449	18076444	TC	CTCTC	del	1	0	0	0	.	.	.	INTERGENIC(INTERGENIC)
5	18166176	18166185	18166179	T	C	snp	1	1	1	1	.	.	.	INTERGENIC(INTERGENIC)
5	18241487	18241495	18241493	T	C	snp	1	1	0	1	.	.	.	INTERGENIC(INTERGENIC)
5	18264263	18264272	18264268	A	C	snp	1	1	1	1	.	.	.	INTERGENIC(INTERGENIC)
5	18336196	18336205	18336199	AT	C	snp	1	0	0	0	.	.	.	INTERGENIC(INTERGENIC)
5	18380749	18380762	18380756	CA	G	snp	1	0	0	0	.	.	.	INTERGENIC(INTERGENIC)
5	18386997	18387004	18387000	T	G	snp	0	1	1	0	.	.	.	INTERGENIC(INTERGENIC)
5	18419267	18419276	18419271	TG	C	snp	1	1	1	1	.	.	.	INTERGENIC(INTERGENIC)
5	18430909	18430922	18430912	AC	AA	ins	1	1	1	1	.	.	.	INTERGENIC(INTERGENIC)
5	18550415	18550434	18550419	TTTC	C	snp	1	1	1	1	.	.	.	AC113389.1(DOWNSTREAM);INTERGENIC(INTERGENIC)
5	18556462	18556477	18556465	TC	T	snp	0	0	0	1	.	.	.	INTERGENIC(INTERGENIC)

5	18599664	18599675	18599669	AT	C	snp	1	1	1	1	.	.	.	INTERGENIC(INTERGENIC)
5	18652278	18652287	18652284	CT	T	snp	1	0	0	0	.	.	.	INTERGENIC(INTERGENIC)
5	18678700	18678709	18678701	T	C	snp	1	1	1	1	.	.	.	INTERGENIC(INTERGENIC)
5	18905793	18905800	18905794	G	T	snp	1	1	1	1	.	.	.	INTERGENIC(INTERGENIC);RP11-81B23.1.1(UPSTREAM)
5	18952538	18952545	18952540	A	G	snp	1	1	1	1	.	.	.	INTERGENIC(INTERGENIC)
5	18968597	18968610	18968604	TG	T	snp	1	1	1	1	.	.	.	RP11-124N3.3.1(INTRON)
5	19157309	19157320	19157318	TA	G	snp	1	1	1	1	.	.	.	INTERGENIC(INTERGENIC)
5	19157337	19157352	19157344	AT	AC	ins	1	1	1	1	.	.	.	INTERGENIC(INTERGENIC)
5	19157337	19157352	19157346	AT	C	snp	1	0	0	0	.	.	.	INTERGENIC(INTERGENIC)
5	19348611	19348625	19348619	AAC	AAT	ins	1	0	1	0	.	.	.	INTERGENIC(INTERGENIC)
5	19368125	19368132	19368127	A	G	snp	1	0	0	0	.	.	.	INTERGENIC(INTERGENIC)
5	19421709	19421716	19421715	A	C	snp	1	1	1	1	.	.	.	INTERGENIC(INTERGENIC)
5	19465092	19465101	19465100	A	C	snp	1	1	1	1	.	.	.	INTERGENIC(INTERGENIC)
5	19482224	19482235	19482232	TTA	T	ins	1	0	0	0	.	.	.	CDH18(INTRON);CDH18(UPSTREAM)
5	19495849	19495864	19495857	AAAG	AAG	del	1	0	0	0	.	.	.	CDH18(INTRON)
5	19602570	19602587	19602577	AC	A	snp	0	1	0	1	.	.	.	CDH18(INTRON)
5	19635733	19635752	19635740	AAAC	T	snp	1	0	0	0	.	.	.	CDH18(INTRON)
5	19702237	19702252	19702247	TTCT	TT	del	1	0	0	0	.	.	.	CDH18(INTRON)
5	19709704	19709719	19709709	GAAG	C	snp	1	1	1	1	.	.	.	CDH18(INTRON)
5	19772616	19772623	19772620	A	G	snp	1	1	1	1	.	.	.	CDH18(INTRON)
5	19941898	19941905	19941899	A	G	snp	1	0	1	0	.	.	.	CDH18(INTRON)
5	20045688	20045703	20045691	AT	C	snp	0	0	1	0	.	.	.	CDH18(INTRON)
5	20090661	20090676	20090668	AAAC	T	snp	1	1	1	1	.	.	.	CDH18(INTRON)
5	20241989	20242000	20241997	AT	TATG	ins	1	1	1	1	.	.	.	CDH18(INTRON)
5	20242002	20242017	20242014	TA	A	snp	1	1	1	1	.	.	.	CDH18(INTRON)
5	20296340	20296348	20296345	T	G	snp	1	0	0	0	.	.	.	CDH18(INTRON)
5	20300402	20300411	20300408	TG	GC	ins	1	1	0	1	.	.	.	CDH18(INTRON);RP11-420O16.2.1(DOWNSTREAM)
5	20384937	20384952	20384947	TTAT	G	snp	1	1	0	1	.	.	.	CDH18(INTRON)
5	20424519	20424528	20424526	T	G	snp	1	1	1	1	.	.	.	CDH18(INTRON)
5	20433367	20433380	20433370	TG	A	snp	1	1	1	1	.	.	.	CDH18(INTRON)
5	20486696	20486703	20486699	T	G	snp	0	1	0	1	.	.	.	CDH18(INTRON)
5	20501679	20501690	20501686	AT	C	snp	1	1	1	1	.	.	.	CDH18(INTRON)
5	20505207	20505215	20505211	A	C	ins	0	1	1	1	.	.	.	CDH18(INTRON)
5	20511593	20511602	20511599	AT	G	snp	1	1	1	1	.	.	.	CDH18(INTRON)
5	20567747	20567754	20567752	T	C	snp	1	1	1	1	.	.	.	CDH18(INTRON)
5	20678582	20678591	20678586	TA	C	snp	1	1	1	1	.	.	.	RP11-774D14.1.1(INTRON)
5	20877440	20877448	20877443	A	C	ins	1	1	1	1	.	.	.	RP11-774D14.1.1(INTRON)
5	20926124	20926132	20926130	A	C	snp	1	0	0	0	.	.	.	RP11-774D14.1.1(INTRON)
5	20931584	20931595	20931590	ATA	G	snp	1	0	0	0	.	.	.	RP11-774D14.1.1(INTRON)
5	20959083	20959102	20959090	TTTA	G	snp	1	0	0	0	.	.	.	INTERGENIC(INTERGENIC)
5	20978204	20978213	20978211	AT	C	snp	1	1	1	1	.	.	.	INTERGENIC(INTERGENIC)
5	21035582	21035591	21035586	A	G	ins	1	0	0	0	.	.	.	INTERGENIC(INTERGENIC)
5	21048417	21048426	21048419	TA	G	snp	1	1	1	1	.	.	.	INTERGENIC(INTERGENIC)
5	21138879	21138888	21138887	T	C	ins	0	0	0	1	.	.	.	INTERGENIC(INTERGENIC)
5	21198652	21198659	21198658	A	C	snp	1	1	1	1	.	.	.	INTERGENIC(INTERGENIC);RP11-811J10.1.1(DOWNSTREAM)
5	21233083	21233092	21233086	A	AC	ins	0	0	1	0	.	.	.	INTERGENIC(INTERGENIC)
5	21233083	21233092	21233088	A	C	snp	1	0	0	0	.	.	.	INTERGENIC(INTERGENIC)
5	21298055	21298066	21298062	ATT	C	snp	1	0	0	0	.	.	.	INTERGENIC(INTERGENIC)
5	21374495	21374506	21374500	TA	C	snp	1	1	1	1	.	.	.	RP11-823P9.1.1(INTRON)
5	21418492	21418505	21418500	TA	C	snp	1	1	1	0	.	.	.	RP11-823P9.1.1(INTRON)
5	21438066	21438079	21438075	TA	G	snp	1	0	0	0	.	.	.	RP11-823P9.1.1(INTRON)
5	21475327	21475334	21475333	T	G	snp	1	0	0	0	.	.	.	RP11-823P9.1.1(DOWNSTREAM);RP11-823P9.1.1(INTRON)
5	21624495	21624506	21624503	AT	G	snp	1	0	0	0	.	.	.	RP11-804N13.1.1(INTRON)
5	21668571	21668582	21668576	GT	G	snp	1	1	1	0	.	.	.	RP11-804N13.1.1(INTRON)
5	21711120	21711129	21711127	AT	C	snp	1	1	1	1	.	.	.	RP11-804N13.1.1(INTRON)
5	21711156	21711165	21711163	AT	C	snp	1	1	1	1	.	.	.	RP11-804N13.1.1(INTRON)
5	21711396	21711405	21711403	AT	C	snp	1	1	0	1	.	.	.	RP11-804N13.1.1(INTRON)
5	21711563	21711572	21711565	TA	CA	ins	1	1	1	1	.	.	.	RP11-804N13.1.1(INTRON)
5	21726281	21726294	21726289	TA	C	snp	1	0	0	0	.	.	.	RP11-804N13.1.1(INTRON)
5	21779151	21779159	21779154	T	C	snp	0	1	1	1	.	.	.	CDH12(INTRON);RP11-804N13.1.1(INTRON)
5	21779304	21779319	21779310	TATT	C	snp	1	0	0	0	.	.	.	CDH12(INTRON);RP11-804N13.1.1(INTRON)
5	21825279	21825286	21825283	A	G	snp	1	1	1	1	.	.	.	CDH12(INTRON)
5	21831177	21831185	21831178	A	AC	ins	0	1	1	1	.	.	.	CDH12(INTRON)
5	22019429	22019436	22019431	T	G	snp	1	1	1	1	.	.	.	CDH12(INTRON)
5	22058819	22058834	22058830	AAAG	C	snp	1	0	0	0	.	.	.	CDH12(INTRON)
5	22080802	22080810	22080803	T	C	snp	0	1	1	1	.	.	.	CDH12(INTRON)

5	22165623	22165640	22165626	AC	AT	ins	1	1	1	1	.	.	.	CDH12(INTRON)
5	22229470	22229479	22229473	A	G	ins	1	1	1	1	.	.	.	CDH12(INTRON)
5	22232973	22232982	22232979	TA	A	del	1	0	0	0	.	.	.	CDH12(INTRON)
5	22233415	22233426	22233422	AT	C	snp	1	0	0	1	.	.	.	CDH12(INTRON)
5	22244641	22244664	22244660	AAG	C	snp	1	1	1	1	.	.	.	CDH12(INTRON)
5	22244937	22244946	22244944	AG	A	snp	1	1	1	1	.	.	.	CDH12(INTRON)
5	22257910	22257917	22257916	T	G	snp	1	1	1	1	.	.	.	CDH12(INTRON)
5	22302346	22302355	22302351	AC	T	snp	0	1	0	0	.	.	.	CDH12(INTRON)
5	22563186	22563197	22563192	TA	C	snp	1	1	1	1	.	.	.	CDH12(INTRON)
5	22596555	22596562	22596561	A	G	snp	1	1	1	1	.	.	.	CDH12(INTRON)
5	22654147	22654162	22654155	CCTT	T	snp	1	0	1	0	.	.	.	CDH12(INTRON)
5	22654745	22654759	22654749	TTG	G	del	1	1	1	1	.	.	.	CDH12(INTRON)
5	22670308	22670317	22670313	AG	C	snp	1	1	1	1	.	.	.	CDH12(INTRON)
5	22694040	22694055	22694046	TTAT	G	snp	1	0	0	0	.	.	.	CDH12(INTRON)
5	22694222	22694229	22694224	T	G	snp	1	0	0	0	.	.	.	CDH12(INTRON)
5	22849478	22849485	22849484	T	C	snp	1	1	1	1	.	.	.	CDH12(INTRON)
5	22993028	22993036	22993035	A	AG	ins	0	0	1	0	.	.	.	INTERGENIC(INTERGENIC)
5	22995464	22995475	22995466	TC	C	snp	1	0	0	0	.	.	.	INTERGENIC(INTERGENIC)
5	23147545	23147552	23147546	G	T	snp	1	0	0	0	.	.	.	INTERGENIC(INTERGENIC)
5	23154026	23154033	23154031	T	G	snp	0	1	1	0	.	.	.	INTERGENIC(INTERGENIC)
5	23189295	23189304	23189296	A	C	snp	1	1	1	1	.	.	.	INTERGENIC(INTERGENIC)
5	23196625	23196634	23196631	AT	G	snp	1	1	1	1	.	.	.	INTERGENIC(INTERGENIC)
5	23235771	23235779	23235772	A	C	snp	1	1	1	0	.	.	.	INTERGENIC(INTERGENIC)
5	23244280	23244289	23244285	T	G	snp	0	1	1	0	.	.	.	INTERGENIC(INTERGENIC)
5	23244280	23244289	23244288	T	TGTA	ins	1	1	1	1	.	.	.	INTERGENIC(INTERGENIC)
5	23272159	23272170	23272168	TC	G	snp	1	1	1	1	.	.	.	INTERGENIC(INTERGENIC)
5	23292457	23292477	23292468	TTG	T	ins	1	1	1	1	.	.	.	INTERGENIC(INTERGENIC)
5	23292457	23292477	23292471	TTG	T	snp	1	1	1	1	.	.	.	INTERGENIC(INTERGENIC)
5	23292457	23292477	23292474	TTG	T	snp	1	1	1	1	.	.	.	INTERGENIC(INTERGENIC)
5	23305428	23305435	23305433	C	CG	ins	0	1	0	1	.	.	.	CTD-2272G21.2.1(UPSTREAM);INTERGENIC(INTERGENIC)
5	23504382	23504389	23504387	A	G	snp	1	1	1	1	.	.	.	INTERGENIC(INTERGENIC);PRDM9(UPSTREAM)
5	23572123	23572142	23572136	AAAT	G	snp	1	1	1	1	.	.	.	INTERGENIC(INTERGENIC)
5	23574159	23574168	23574164	TG	A	snp	1	1	1	1	.	.	.	INTERGENIC(INTERGENIC)
5	23633199	23633216	23633212	GT	GTGC	ins	1	0	0	0	.	.	.	INTERGENIC(INTERGENIC)
5	23637688	23637697	23637693	TA	G	snp	1	1	1	1	.	.	.	INTERGENIC(INTERGENIC)
5	23757101	23757108	23757105	T	C	snp	1	1	1	1	.	.	.	INTERGENIC(INTERGENIC)
5	23872412	23872420	23872414	A	C	snp	1	0	0	0	.	.	.	INTERGENIC(INTERGENIC)
5	23998212	23998223	23998221	TA	TG	ins	1	1	1	1	.	.	.	C5orf17(INTRON);Y_RNA(UPSTREAM)
5	24010190	24010197	24010196	A	G	snp	1	1	1	1	.	.	.	C5orf17(INTRON)
5	24127061	24127070	24127068	T	G	snp	1	1	1	1	.	.	.	C5orf17(INTRON)
5	24130917	24130925	24130918	A	C	snp	1	1	1	1	.	.	.	C5orf17(INTRON)
5	24145637	24145652	24145649	GA	A	snp	1	1	1	1	.	.	.	C5orf17(INTRON)
5	24173890	24173899	24173896	AT	G	snp	1	0	0	0	.	.	.	C5orf17(INTRON);CTD-2015B23.2.1(DOWNSTREAM)
5	24209621	24209644	24209638	TTTG	A	snp	1	0	0	0	.	.	.	INTERGENIC(INTERGENIC)
5	24210178	24210187	24210182	TA	C	snp	1	0	0	0	.	.	.	INTERGENIC(INTERGENIC)
5	24217739	24217748	24217742	TG	A	snp	1	0	0	0	.	.	.	INTERGENIC(INTERGENIC)
5	24251406	24251413	24251409	T	G	snp	1	0	0	0	.	.	.	INTERGENIC(INTERGENIC)
5	24251406	24251413	24251412	T	G	snp	1	1	1	1	.	.	.	INTERGENIC(INTERGENIC)
5	24317188	24317195	24317190	T	G	snp	1	1	1	1	.	.	.	INTERGENIC(INTERGENIC)
5	24436646	24436655	24436651	T	G	snp	1	1	0	1	.	.	.	INTERGENIC(INTERGENIC)
5	24443592	24443605	24443603	GT	G	snp	0	0	1	0	.	.	.	INTERGENIC(INTERGENIC)
5	24465578	24465589	24465583	AAG	A	snp	1	1	1	1	.	.	.	INTERGENIC(INTERGENIC)
5	24465590	24465598	24465595	A	C	snp	1	1	1	1	.	.	.	INTERGENIC(INTERGENIC)
5	24505514	24505523	24505520	CA	T	snp	1	0	0	0	.	.	.	CDH10(INTRON)
5	24520833	24520841	24520839	T	G	snp	1	1	1	1	.	.	.	CDH10(INTRON)
5	24522264	24522279	24522269	AAAC	AC	del	1	1	1	1	.	.	.	CDH10(INTRON)
5	24522264	24522279	24522270	AAAC	C	del	1	1	1	1	.	.	.	CDH10(INTRON)
5	24600715	24600732	24600718	GT	C	snp	1	0	0	0	.	.	.	CDH10(INTRON);RP11-116O11.1.1(INTRON)
5	24627022	24627031	24627028	TA	C	snp	1	0	0	0	.	.	.	CDH10(INTRON)
5	24712818	24712827	24712823	AT	C	snp	1	0	0	0	.	.	.	INTERGENIC(INTERGENIC)
5	24813024	24813031	24813030	T	G	snp	1	1	1	1	.	.	.	INTERGENIC(INTERGENIC);SNORD29(DOWNSTREAM)
5	24920933	24920941	24920938	A	C	snp	1	1	1	1	.	.	.	INTERGENIC(INTERGENIC)
5	25265415	25265426	25265422	GC	T	snp	1	0	0	0	.	.	.	RP11-184E9.1.1(INTRON);RP11-192H6.2.1(INTRON)
5	25364859	25364866	25364862	A	C	snp	1	1	1	1	.	.	.	INTERGENIC(INTERGENIC)
5	25459378	25459391	25459388	AC	CG	ins	1	1	1	1	.	.	.	INTERGENIC(INTERGENIC)
5	25514791	25514800	25514797	TA	AA	ins	1	1	1	1	.	.	.	INTERGENIC(INTERGENIC)
5	25695744	25695753	25695750	TC	G	snp	0	0	0	1	.	.	.	INTERGENIC(INTERGENIC)

5	25701017	25701025	25701018	T	C	ins	1	1	1	1	.	.	.	INTERGENIC(INTERGENIC);U6(DOWNSTREAM)
5	25782087	25782094	25782093	T	G	snp	1	1	1	1	.	.	.	INTERGENIC(INTERGENIC)
5	25789261	25789268	25789266	A	AAG	ins	1	0	0	0	.	.	.	INTERGENIC(INTERGENIC)
5	25790394	25790403	25790400	A	G	snp	1	0	0	0	.	.	.	INTERGENIC(INTERGENIC)
5	25817925	25817942	25817938	ACA	A	snp	0	1	1	1	.	.	.	INTERGENIC(INTERGENIC)
5	25870899	25870922	25870905	TTTG	C	snp	1	0	0	0	.	.	.	INTERGENIC(INTERGENIC)
5	25871738	25871749	25871741	AT	A	snp	1	1	0	1	.	.	.	INTERGENIC(INTERGENIC)
5	25903954	25903968	25903963	TAT	A	snp	1	1	0	1	.	.	.	INTERGENIC(INTERGENIC)
5	25944056	25944069	25944063	AG	C	snp	1	1	1	1	.	.	.	INTERGENIC(INTERGENIC)
5	25985274	25985289	25985286	CA	A	del	1	0	0	0	.	.	.	INTERGENIC(INTERGENIC)
5	25985312	25985321	25985318	AT	G	snp	1	1	1	1	.	.	.	INTERGENIC(INTERGENIC)
5	26092515	26092534	26092530	TTTG	T	snp	1	0	0	0	.	.	.	INTERGENIC(INTERGENIC)
5	26201422	26201437	26201433	AT	A	del	1	1	1	1	.	.	.	INTERGENIC(INTERGENIC)
5	26213602	26213610	26213607	A	C	snp	1	0	0	0	.	.	.	INTERGENIC(INTERGENIC)
5	26222077	26222085	26222079	A	C	snp	1	1	1	1	.	.	.	INTERGENIC(INTERGENIC)
5	26242627	26242634	26242632	A	C	snp	1	1	1	0	.	.	.	INTERGENIC(INTERGENIC)
5	26356424	26356432	26356427	T	C	snp	1	0	0	0	.	.	.	INTERGENIC(INTERGENIC)
5	26398612	26398623	26398621	TA	G	snp	1	1	1	1	.	.	.	RP11-351N6.1.1(INTRON)
5	26455313	26455322	26455320	TG	C	snp	1	1	0	1	.	.	.	INTERGENIC(INTERGENIC)
5	26455326	26455337	26455331	AT	C	snp	0	1	0	1	.	.	.	INTERGENIC(INTERGENIC)
5	26519651	26519662	26519658	AGG	A	snp	1	1	1	0	.	.	.	INTERGENIC(INTERGENIC)
5	26535376	26535383	26535378	T	G	ins	1	1	0	1	.	.	.	INTERGENIC(INTERGENIC)
5	26565176	26565191	26565179	AT	A	snp	1	1	1	1	.	.	.	INTERGENIC(INTERGENIC)
5	26569669	26569676	26569670	G	T	snp	1	0	0	1	.	.	.	INTERGENIC(INTERGENIC)
5	26600379	26600388	26600383	AT	G	snp	1	1	1	1	.	.	.	INTERGENIC(INTERGENIC)
5	26638909	26638916	26638915	T	G	snp	1	0	0	0	.	.	.	INTERGENIC(INTERGENIC)
5	26701540	26701549	26701541	T	C	snp	1	0	0	0	.	.	.	INTERGENIC(INTERGENIC)
5	26857026	26857037	26857029	TC	T	snp	1	0	1	0	.	.	.	INTERGENIC(INTERGENIC)
5	26893780	26893791	26893788	AT	TATTT	del	1	1	1	1	.	.	.	CDH9(INTRON);CDH9(UPSTREAM)
5	26924639	26924648	26924641	TA	C	snp	1	1	1	1	.	.	.	CDH9(INTRON)
5	26927026	26927034	26927027	C	CCCT	ins	1	1	1	1	.	.	.	CDH9(INTRON)
5	26927026	26927034	26927028	C	CCT	ins	1	1	1	1	.	.	.	CDH9(INTRON)
5	26971874	26971889	26971887	AC	A	del	0	0	0	1	.	.	.	CDH9(INTRON)
5	26982806	26982813	26982807	T	G	snp	1	1	1	1	.	.	.	CDH9(INTRON)
5	26994676	26994684	26994680	T	G	snp	1	0	0	0	.	.	.	CDH9(INTRON)
5	27155287	27155304	27155290	AT	C	snp	1	0	0	0	.	.	.	INTERGENIC(INTERGENIC)
5	27221749	27221756	27221751	T	C	snp	0	0	0	1	.	.	.	INTERGENIC(INTERGENIC)
5	27352715	27352722	27352716	T	G	snp	1	0	0	0	.	.	.	INTERGENIC(INTERGENIC)
5	27355051	27355062	27355057	AAT	T	snp	1	0	0	0	.	.	.	INTERGENIC(INTERGENIC)
5	27377739	27377754	27377750	AGAT	A	snp	1	0	0	0	.	.	.	INTERGENIC(INTERGENIC)
5	27423724	27423732	27423731	A	C	snp	0	1	1	1	.	.	.	CTD-3007L5.1.1(INTRON)
5	27437217	27437226	27437219	TA	G	snp	1	0	0	0	.	.	.	CTD-3007L5.1.1(UPSTREAM);INTERGENIC(INTERGENIC)
5	27535234	27535247	27535245	TG	T	del	1	1	1	0	.	.	.	INTERGENIC(INTERGENIC)
5	27547446	27547454	27547450	T	G	snp	1	0	0	0	.	.	.	INTERGENIC(INTERGENIC)
5	27565842	27565853	27565847	ATG	ATA	ins	1	1	1	1	.	.	.	INTERGENIC(INTERGENIC)
5	27568751	27568768	27568754	AT	A	snp	1	1	1	1	.	.	.	INTERGENIC(INTERGENIC)
5	27594032	27594039	27594038	A	C	ins	1	1	1	1	.	.	.	INTERGENIC(INTERGENIC)
5	27640425	27640433	27640428	T	G	snp	1	1	0	1	.	.	.	INTERGENIC(INTERGENIC)
5	27769046	27769055	27769048	A	C	snp	1	1	1	1	.	.	.	INTERGENIC(INTERGENIC)
5	27918305	27918314	27918307	A	C	ins	1	0	0	0	.	.	.	INTERGENIC(INTERGENIC)
5	27969260	27969277	27969273	AT	ATA	del	1	1	1	1	.	.	.	INTERGENIC(INTERGENIC)
5	27970522	27970533	27970529	ATA	C	snp	1	1	1	1	.	.	.	INTERGENIC(INTERGENIC)
5	27999192	27999200	27999194	T	C	ins	1	0	0	0	.	.	.	INTERGENIC(INTERGENIC)
5	28086810	28086817	28086816	A	G	snp	0	1	0	1	.	.	.	INTERGENIC(INTERGENIC)
5	28177303	28177312	28177310	T	G	snp	1	1	1	1	.	.	.	INTERGENIC(INTERGENIC)
5	28219158	28219167	28219160	A	C	snp	1	1	1	1	.	.	.	INTERGENIC(INTERGENIC);RP11-560A7.1.1(UPSTREAM)
5	28237947	28237962	28237953	TTTC	C	snp	1	1	1	1	.	.	.	INTERGENIC(INTERGENIC)
5	28277840	28277855	28277846	TCTT	TTCTTTC	del	1	0	0	0	.	.	.	INTERGENIC(INTERGENIC)
5	28277840	28277855	28277850	TCTT	TTC	del	1	0	0	0	.	.	.	INTERGENIC(INTERGENIC)
5	28285436	28285443	28285440	T	C	snp	1	1	1	1	.	.	.	CTD-2061E9.1.1(DOWNSTREAM);INTERGENIC(INTERGENIC)
5	28343487	28343506	28343500	TTTC	TCTG	ins	1	0	0	0	.	.	.	INTERGENIC(INTERGENIC)
5	28364145	28364159	28364151	CAA	T	snp	1	0	0	0	.	.	.	INTERGENIC(INTERGENIC)
5	28429655	28429664	28429661	T	G	snp	1	0	0	0	.	.	.	INTERGENIC(INTERGENIC)
5	28450979	28450988	28450982	TA	C	snp	0	0	1	0	.	.	.	INTERGENIC(INTERGENIC)
5	28457440	28457449	28457441	T	TC	ins	0	0	1	0	.	.	.	INTERGENIC(INTERGENIC)
5	28464288	28464295	28464291	T	G	snp	1	1	1	1	.	.	.	INTERGENIC(INTERGENIC)
5	28517726	28517733	28517728	A	G	ins	1	1	0	1	.	.	.	INTERGENIC(INTERGENIC)

5	28574049	28574057	28574052	A	C	snp	1	0	0	0	.	.	.	INTERGENIC(INTERGENIC)
5	28596319	28596342	28596337	TTTA	A	del	1	0	0	0	.	.	.	INTERGENIC(INTERGENIC)
5	28596319	28596342	28596338	TTTA	T	snp	1	0	0	0	.	.	.	INTERGENIC(INTERGENIC)
5	28806115	28806128	28806119	AC	T	snp	0	1	1	0	.	.	.	CTD-2134P3.1.1(INTRON)
5	28931063	28931070	28931065	A	G	snp	1	0	0	0	.	.	.	CTD-2269E23.2.1(DOWNSTREAM);INTERGENIC(INTERGENIC);LSP1P3(DOWNSTREAM)
5	28933536	28933545	28933542	AT	T	del	1	1	1	1	.	.	.	INTERGENIC(INTERGENIC)
5	29008162	29008173	29008167	TG	A	snp	1	0	0	0	.	.	.	INTERGENIC(INTERGENIC);RP11-42L13.1.1(DOWNSTREAM)
5	29023959	29023966	29023961	T	C	snp	1	1	1	1	.	.	.	INTERGENIC(INTERGENIC)
5	29026175	29026184	29026181	TA	C	snp	1	1	1	1	.	.	.	INTERGENIC(INTERGENIC)
5	29071205	29071224	29071220	AAAT	AAATAAAATAG	del	1	0	0	0	.	.	.	INTERGENIC(INTERGENIC);SNORA18(DOWNSTREAM)
5	29087870	29087878	29087874	T	G	snp	0	1	0	0	.	.	.	INTERGENIC(INTERGENIC)
5	29128301	29128310	29128309	A	G	ins	1	0	0	0	.	.	.	INTERGENIC(INTERGENIC)
5	29232954	29232969	29232957	TC	A	snp	1	0	0	0	.	.	.	INTERGENIC(INTERGENIC)
5	29250750	29250759	29250758	T	C	ins	1	0	0	0	.	.	.	INTERGENIC(INTERGENIC)
5	29258118	29258133	29258121	AC	T	snp	1	1	1	1	.	.	.	INTERGENIC(INTERGENIC)
5	29268806	29268815	29268812	AT	T	snp	0	0	0	1	.	.	.	INTERGENIC(INTERGENIC)
5	29320009	29320024	29320019	ATCT	G	snp	1	1	1	1	.	.	.	INTERGENIC(INTERGENIC)
5	29320799	29320812	29320810	AT	A	del	1	0	0	1	.	.	.	INTERGENIC(INTERGENIC)
5	29445746	29445753	29445747	T	G	snp	0	1	1	0	.	.	.	INTERGENIC(INTERGENIC)
5	29445746	29445753	29445750	T	C	snp	1	1	1	1	.	.	.	INTERGENIC(INTERGENIC)
5	29529465	29529480	29529472	AAAC	T	snp	1	1	1	1	.	.	.	INTERGENIC(INTERGENIC)
5	29559130	29559138	29559135	A	C	snp	1	1	1	1	.	.	.	INTERGENIC(INTERGENIC)
5	29720904	29720911	29720908	T	C	snp	0	1	0	1	.	.	.	INTERGENIC(INTERGENIC)
5	29727594	29727601	29727599	T	G	ins	1	0	1	0	.	.	.	INTERGENIC(INTERGENIC)
5	29773711	29773730	29773726	AAAT	A	snp	1	1	1	1	.	.	.	INTERGENIC(INTERGENIC)
5	29786681	29786692	29786690	TC	TCT	del	1	1	1	1	.	.	.	INTERGENIC(INTERGENIC)
5	29893309	29893320	29893311	AG	G	snp	1	1	0	1	.	.	.	INTERGENIC(INTERGENIC)
5	29927492	29927501	29927493	A	C	snp	0	1	0	1	.	.	.	INTERGENIC(INTERGENIC)
5	29936749	29936762	29936752	AT	A	snp	1	0	0	0	.	.	.	INTERGENIC(INTERGENIC)
5	30010757	30010772	30010768	AAAG	T	snp	0	0	1	0	.	.	.	INTERGENIC(INTERGENIC)
5	30100560	30100573	30100563	GA	T	snp	1	1	1	1	.	.	.	INTERGENIC(INTERGENIC)
5	30230096	30230111	30230102	AAGA	A	ins	1	0	0	0	.	.	.	INTERGENIC(INTERGENIC)
5	30234948	30234975	30234969	TTGT	G	del	1	0	0	0	.	.	.	INTERGENIC(INTERGENIC)
5	30263133	30263160	30263152	AAAC	A	ins	1	1	1	1	.	.	.	INTERGENIC(INTERGENIC)
5	30399104	30399113	30399108	A	C	snp	1	0	0	0	.	.	.	INTERGENIC(INTERGENIC)
5	30406637	30406652	30406646	CATA	C	snp	1	1	1	1	.	.	.	INTERGENIC(INTERGENIC)
5	30442831	30442839	30442836	A	G	snp	1	1	1	1	.	.	.	INTERGENIC(INTERGENIC)
5	30570106	30570113	30570109	A	C	snp	1	0	0	0	.	.	.	INTERGENIC(INTERGENIC)
5	30583308	30583325	30583310	TA	ATA	del	1	1	1	1	.	.	.	INTERGENIC(INTERGENIC)
5	30670763	30670772	30670767	AT	G	snp	1	0	0	0	.	.	.	INTERGENIC(INTERGENIC)
5	30683926	30683945	30683931	AAAT	G	snp	1	0	0	0	.	.	.	INTERGENIC(INTERGENIC)
5	30742695	30742702	30742699	T	C	snp	1	0	0	0	.	.	.	INTERGENIC(INTERGENIC)
5	30783245	30783264	30783259	AAAT	TAAAT	del	1	1	1	0	.	.	.	INTERGENIC(INTERGENIC)
5	30788586	30788595	30788588	AC	T	snp	1	1	1	1	.	.	.	INTERGENIC(INTERGENIC)
5	30880263	30880274	30880271	TG	A	snp	0	0	0	1	.	.	.	INTERGENIC(INTERGENIC)
5	31081675	31081683	31081681	A	G	snp	1	0	0	0	.	.	.	INTERGENIC(INTERGENIC)
5	31097116	31097124	31097119	T	C	ins	1	1	1	1	.	.	.	RP11-152K4.2.1(INTRON)
5	31116872	31116881	31116877	TA	G	snp	1	0	1	0	.	.	.	RP11-152K4.2.1(INTRON)
5	31154693	31154702	31154698	C	A	snp	0	1	1	1	.	.	.	RP11-152K4.2.1(INTRON)
5	31174872	31174880	31174878	T	G	snp	1	1	0	1	.	.	.	RP11-152K4.2.1(INTRON)
5	31193620	31193647	31193639	AAAG	A	ins	1	1	1	1	.	.	.	CDH6(UPSTREAM);RP11-152K4.2.1(INTRON)
5	31193620	31193647	31193642	AAAG	G	del	1	1	1	1	.	.	.	CDH6(UPSTREAM);RP11-152K4.2.1(INTRON)
5	31193620	31193647	31193643	AAAG	A	snp	1	1	1	1	.	.	.	CDH6(UPSTREAM);RP11-152K4.2.1(INTRON)
5	31233186	31233195	31233191	AC	T	snp	1	0	0	0	.	.	.	CDH6(INTRON);RP11-152K4.2.1(INTRON)
5	31242809	31242817	31242811	G	GT	ins	1	1	1	1	.	.	.	CDH6(INTRON);RP11-152K4.2.1(INTRON)
5	31283102	31283125	31283108	GGAT	T	snp	1	0	0	0	.	.	.	CDH6(INTRON)
5	31340786	31340793	31340791	T	C	snp	1	1	1	1	.	.	.	INTERGENIC(INTERGENIC)
5	31354233	31354244	31354240	CTC	C	del	1	1	1	1	.	.	.	INTERGENIC(INTERGENIC)
5	31359755	31359764	31359762	AT	C	snp	1	1	1	1	.	.	.	INTERGENIC(INTERGENIC)
5	31407746	31407753	31407751	A	G	snp	0	1	1	1	.	.	.	DROSHA(DOWNSTREAM);DROSHA(INTRON);DROSHA(UPSTREAM)
5	31544063	31544070	31544067	A	C	snp	1	0	0	0	.	.	.	C5orf22(INTRON);C5orf22(UPSTREAM)
5	31578527	31578544	31578540	GAA	G	snp	1	1	1	1	.	.	.	INTERGENIC(INTERGENIC)
5	31583019	31583028	31583022	T	C	snp	1	0	0	0	.	.	.	INTERGENIC(INTERGENIC)
5	31697990	31698007	31698004	TTA	TTATCATTTT	del	1	1	1	1	.	.	.	PDZD2(INTRON)

5	31699889	31699898	31699891	T	G	ins	1	1	1	1	.	.	.	PDZD2(INTRON)
5	31734414	31734428	31734418	TTG	A	snp	1	1	1	1	.	.	.	PDZD2(INTRON);RP11-5N11.3.1(UPSTREAM)
5	31968765	31968780	31968772	AAAC	T	snp	1	0	0	0	.	.	.	PDZD2(INTRON)
5	32003571	32003578	32003577	C	A	snp	1	1	1	1	.	.	.	PDZD2(INTRON)
5	32018799	32018808	32018804	TC	G	snp	1	0	0	0	.	.	.	PDZD2(INTRON)
5	32116162	32116176	32116168	CCA	T	snp	1	0	0	0	.	.	.	CTD-2152M20.2.1(INTRON)
5	32152528	32152535	32152533	C	T	snp	0	1	1	0	.	.	.	GOLPH3(INTRON)
5	32163770	32163783	32163781	AT	A	del	1	1	1	1	.	.	.	GOLPH3(INTRON)
5	32210523	32210530	32210524	G	A	ins	1	0	1	0	.	.	.	INTERGENIC(INTERGENIC)
5	32210523	32210530	32210529	G	T	snp	1	0	0	0	.	.	.	INTERGENIC(INTERGENIC)
5	32228663	32228674	32228666	AT	ATATATATC	del	1	0	0	0	.	.	.	MTMR12(DOWNSTREAM);MTMR12(UTR_3_PRIME)
5	32254950	32254957	32254953	A	G	snp	1	0	0	0	.	.	.	MTMR12(INTRON)
5	32345887	32345896	32345890	TA	C	snp	1	1	0	1	.	.	.	INTERGENIC(INTERGENIC)
5	32413298	32413305	32413301	A	C	ins	1	1	1	1	.	.	.	ZFR(INTRON)
5	32498556	32498570	32498562	AAC	T	snp	1	1	1	1	.	.	.	INTERGENIC(INTERGENIC)
5	32536898	32536906	32536903	C	T	snp	1	0	0	0	.	.	.	SUB1(INTRON)
5	32719884	32719895	32719891	TGT	T	ins	1	1	0	1	.	.	.	NPR3(INTRON)
5	32719884	32719895	32719892	TGT	TG	del	1	1	1	1	.	.	.	NPR3(INTRON)
5	32753668	32753677	32753672	T	C	snp	1	1	1	1	.	.	.	NPR3(INTRON)
5	32809589	32809597	32809595	T	TTG	ins	1	1	1	1	.	.	.	INTERGENIC(INTERGENIC)
5	32849942	32849957	32849955	AC	AT	ins	1	0	0	0	.	.	.	INTERGENIC(INTERGENIC)
5	32905101	32905108	32905104	T	C	snp	1	1	1	1	.	.	.	INTERGENIC(INTERGENIC)
5	32981132	32981143	32981135	AT	C	snp	0	0	1	0	.	.	.	CTD-2066L21.3.1(INTRON)
5	33038693	33038700	33038696	A	G	snp	0	0	1	0	.	.	.	CTD-2066L21.3.1(INTRON)
5	33203937	33203944	33203939	T	C	snp	1	0	0	0	.	.	.	CTD-2066L21.3.1(INTRON)
5	33247239	33247246	33247241	A	G	snp	1	1	0	1	.	.	.	CTD-2066L21.3.1(INTRON);RP11-113I22.1.1(INTRON)
5	33252556	33252571	33252559	CA	C	snp	1	1	1	1	.	.	.	CTD-2066L21.3.1(INTRON);RP11-113I22.1.1(INTRON)
5	33336641	33336649	33336644	T	C	snp	1	1	1	1	.	.	.	INTERGENIC(INTERGENIC)
5	33371589	33371600	33371595	TGA	G	snp	1	1	0	1	.	.	.	INTERGENIC(INTERGENIC)
5	33490512	33490520	33490519	T	C	snp	1	0	0	0	.	.	.	INTERGENIC(INTERGENIC)
5	33612532	33612541	33612535	TG	A	snp	1	0	0	0	.	.	.	ADAMTS12(INTRON)
5	33647835	33647850	33647843	CAAA	AAACAA	del	1	1	1	1	.	.	.	ADAMTS12(INTRON)
5	33682029	33682036	33682032	A	G	snp	1	1	1	0	.	.	.	ADAMTS12(INTRON)
5	33738413	33738422	33738419	T	G	ins	0	0	1	0	.	.	.	ADAMTS12(INTRON)
5	33914626	33914634	33914633	T	TC	ins	1	0	0	0	.	.	.	INTERGENIC(INTERGENIC)
5	33968314	33968325	33968322	CTT	G	snp	1	1	1	1	.	.	.	RP11-1084J3.1.1(UPSTREAM);SLC45A2(INTRON)
5	33969381	33969390	33969389	A	G	snp	1	1	1	1	.	.	.	RP11-1084J3.1.1(UPSTREAM);SLC45A2(INTRON)
5	34108144	34108159	34108151	AAAG	C	snp	1	1	1	1	.	.	.	C1QTNF3(INTRON)
5	34176525	34176534	34176533	A	AGAC	ins	1	1	1	1	.	.	.	RP11-1023L17.1.1(DOWNSTREAM);RP11-1023L17.1.1(INTRON);RP11-1023L17.1.1(UPSTREAM)
5	34213824	34213833	34213830	TA	C	snp	1	0	0	0	.	.	.	RP11-1023L17.1.1(INTRON)
5	34319617	34319634	34319632	AC	T	snp	0	1	1	0	.	.	.	INTERGENIC(INTERGENIC)
5	34372053	34372068	34372056	GA	G	snp	0	1	0	0	.	.	.	INTERGENIC(INTERGENIC);RP11-1325J9.1.1(UPSTREAM)
5	34372776	34372783	34372777	A	G	snp	0	1	0	0	.	.	.	INTERGENIC(INTERGENIC);RP11-1325J9.1.1(UPSTREAM)
5	34392832	34392840	34392833	T	G	snp	1	1	1	1	.	.	.	INTERGENIC(INTERGENIC)
5	34428851	34428871	34428854	TTA	TATTATTATAATTATTATTATTAC	ins	1	1	1	1	.	.	.	INTERGENIC(INTERGENIC)
5	34428851	34428871	34428855	TTA	ATTATTATA	ins	1	1	1	1	.	.	.	INTERGENIC(INTERGENIC)
5	34441736	34441751	34441748	TA	A	del	1	0	0	0	.	.	.	INTERGENIC(INTERGENIC)
5	34452116	34452125	34452120	CA	G	snp	1	1	0	1	.	.	.	INTERGENIC(INTERGENIC)
5	34549696	34549711	34549704	TTTA	C	snp	1	1	0	1	.	.	.	INTERGENIC(INTERGENIC)
5	34606808	34606825	34606821	CT	CTC	del	0	1	0	1	.	.	.	INTERGENIC(INTERGENIC)
5	34753459	34753466	34753462	T	C	snp	1	1	1	1	.	.	.	RAI14(INTRON);RAI14(UPSTREAM)
5	34777977	34777984	34777983	G	T	snp	1	0	0	0	.	.	.	RAI14(INTRON)
5	34939833	34939842	34939834	T	G	snp	1	0	0	0	.	.	.	DNAJC21(INTRON)
5	35002882	35002891	35002885	G	T	ins	1	1	1	1	.	.	.	AGXT2(INTRON)
5	35036999	35037007	35037006	T	C	snp	1	1	1	1	.	.	.	AC010368.1(DOWNSTREAM);AGXT2(INTRON)
5	35229234	35229245	35229238	AT	G	snp	1	0	0	0	.	.	.	PRLR(INTRON)
5	35272559	35272570	35272567	TA	C	snp	0	1	0	1	.	.	.	INTERGENIC(INTERGENIC)
5	35290842	35290855	35290845	TA	TATATATATAGTT	del	0	0	0	1	.	.	.	INTERGENIC(INTERGENIC)
5	35416485	35416492	35416491	A	C	snp	1	0	0	0	.	.	.	INTERGENIC(INTERGENIC)
5	35464176	35464184	35464183	T	C	snp	1	1	1	1	.	.	.	INTERGENIC(INTERGENIC)
5	35496791	35496800	35496793	TA	C	snp	1	1	0	1	.	.	.	INTERGENIC(INTERGENIC)
5	35595972	35595981	35595975	T	C	snp	0	0	0	1	.	.	.	INTERGENIC(INTERGENIC)
5	35664866	35664883	35664875	GA	G	snp	1	1	1	1	.	.	.	SPEF2(INTRON)
5	35674478	35674495	35674483	TTA	T	snp	1	1	1	1	.	.	.	CTD-2113L7.1.1(DOWNSTREAM);SPEF2(DOWNSTREAM);SPEF2(INTRON);SPEF2(UPSTREAM)
5	35687570	35687577	35687573	T	G	snp	1	0	0	0	.	.	.	CTD-2113L7.1.1(INTRON);SPEF2(INTRON)

5	35710660	35710667	35710666	A	G	snp	1	0	0	0	.	.	.	CTD-2113L7.1.1(INTRON);SPEF2(DOWNSTREAM);SPEF2(EXON);SPEF2(INTRON);SPEF2(UTR_3_PRIME)
5	35794497	35794505	35794504	T	G	snp	1	1	1	1	.	.	.	CTD-2113L7.1.1(INTRON);SPEF2(INTRON)
5	35834732	35834739	35834736	T	C	snp	1	1	0	1	.	.	.	INTERGENIC(INTERGENIC)
5	35913061	35913069	35913067	A	C	snp	0	1	0	1	.	.	.	CAPSL(INTRON)
5	35983696	35983704	35983698	A	G	snp	0	1	1	1	.	.	.	UGT3A1(INTRON)
5	36200230	36200237	36200234	A	G	snp	0	0	1	0	.	.	.	NADKD1(INTRON)
5	36243518	36243526	36243520	A	G	snp	1	0	0	0	.	.	.	INTERGENIC(INTERGENIC);NADKD1(UPSTREAM)
5	36244258	36244273	36244271	TC	G	snp	1	1	1	0	.	.	.	INTERGENIC(INTERGENIC);NADKD1(UPSTREAM);RANBP3L(DOWNSTREAM)
5	36316563	36316577	36316573	ATT	TA	del	1	1	1	1	.	.	.	INTERGENIC(INTERGENIC)
5	36345601	36345610	36345606	TC	G	snp	1	1	1	1	.	.	.	INTERGENIC(INTERGENIC)
5	36492837	36492844	36492841	T	G	snp	1	0	0	0	.	.	.	INTERGENIC(INTERGENIC)
5	36523735	36523744	36523738	TG	A	snp	0	0	0	1	.	.	.	INTERGENIC(INTERGENIC)
5	36734940	36734951	36734946	ATT	G	snp	1	1	1	1	.	.	.	INTERGENIC(INTERGENIC)
5	36916606	36916613	36916610	T	G	snp	1	0	0	0	.	.	.	NIPBL(INTRON)
5	37150627	37150644	37150636	CA	G	snp	1	0	0	0	.	.	.	C5orf42(DOWNSTREAM);C5orf42(INTRON)
5	37150627	37150644	37150638	CA	G	snp	1	0	0	0	.	.	.	C5orf42(DOWNSTREAM);C5orf42(INTRON)
5	37184002	37184010	37184006	A	G	snp	1	0	0	0	.	.	.	C5orf42(INTRON)
5	37244794	37244803	37244795	A	C	snp	1	0	0	0	.	.	.	AC025449.1(DOWNSTREAM);C5orf42(INTRON)
5	37345536	37345547	37345540	GGA	A	snp	1	0	0	0	.	.	.	NUP155(INTRON)
5	37385905	37385918	37385916	AT	A	del	1	0	0	0	.	.	.	WDR70(DOWNSTREAM);WDR70(INTRON)
5	37396288	37396295	37396289	T	TC	ins	1	0	0	0	.	.	.	WDR70(INTRON)
5	37399379	37399390	37399388	CA	C	del	1	0	0	0	.	.	.	WDR70(INTRON)
5	37408865	37408876	37408869	TG	C	snp	1	0	0	0	.	.	.	WDR70(INTRON)
5	37427053	37427060	37427055	T	G	snp	1	0	0	0	.	.	.	WDR70(INTRON)
5	37454186	37454194	37454193	T	C	snp	1	1	1	1	.	.	.	WDR70(INTRON)
5	37462397	37462416	37462407	TTAA	T	snp	1	0	0	0	.	.	.	WDR70(INTRON)
5	37479445	37479460	37479456	TGGT	TGG	del	1	1	1	1	.	.	.	WDR70(INTRON);WDR70(UPSTREAM)
5	37479851	37479859	37479853	T	G	snp	1	1	1	1	.	.	.	WDR70(INTRON)
5	37481433	37481440	37481438	C	A	ins	1	1	1	1	.	.	.	WDR70(INTRON)
5	37481433	37481440	37481438	C	A	snp	1	1	1	1	.	.	.	WDR70(INTRON)
5	37516815	37516828	37516826	AT	A	del	1	1	1	1	.	.	.	WDR70(INTRON)
5	37524622	37524629	37524628	A	G	snp	1	1	1	1	.	.	.	WDR70(INTRON)
5	37545644	37545653	37545649	T	G	snp	0	1	0	0	.	.	.	WDR70(INTRON)
5	37593264	37593271	37593265	T	C	snp	1	0	0	0	.	.	.	WDR70(INTRON)
5	37686425	37686434	37686427	A	G	snp	1	0	0	0	.	.	.	WDR70(INTRON)
5	37742547	37742558	37742552	TTG	A	snp	1	1	1	1	.	.	.	WDR70(INTRON)
5	37783356	37783371	37783364	AT	G	snp	1	1	0	1	.	.	.	INTERGENIC(INTERGENIC)
5	37857413	37857422	37857419	T	G	snp	1	1	1	1	.	.	.	GDNF-AS1(INTRON)
5	37858000	37858009	37858007	AG	A	snp	1	0	0	0	.	.	.	GDNF-AS1(INTRON)
5	37892766	37892775	37892772	A	G	snp	1	0	0	0	.	.	.	INTERGENIC(INTERGENIC)
5	37943935	37943942	37943938	A	C	snp	1	1	1	1	.	.	.	CTD-2130F23.2.1(UPSTREAM);CTD-2194L12.3.1(INTRON)
5	38004751	38004768	38004755	AAT	G	snp	1	1	1	1	.	.	.	INTERGENIC(INTERGENIC)
5	38035256	38035265	38035260	TG	A	snp	1	1	0	0	.	.	.	CTD-2116N24.1.1(INTRON)
5	38062536	38062553	38062550	TCT	TC	del	1	1	1	1	.	.	.	CTD-2116N24.1.1(INTRON)
5	38064691	38064700	38064692	T	G	snp	1	1	1	1	.	.	.	CTD-2116N24.1.1(INTRON)
5	38091867	38091882	38091871	ATTA	G	snp	0	0	1	0	.	.	.	CTD-2116N24.1.1(INTRON)
5	38158030	38158037	38158036	A	G	snp	1	0	1	0	.	.	.	CTD-2116N24.1.1(INTRON);CTD-2207A17.1.1(DOWNSTREAM)
5	38205137	38205146	38205143	T	C	snp	1	0	0	0	.	.	.	INTERGENIC(INTERGENIC)
5	38323754	38323761	38323759	T	C	snp	1	1	0	1	.	.	.	EGFLAM(INTRON)
5	38480233	38480240	38480238	A	C	snp	0	0	1	0	.	.	.	LIFR(DOWNSTREAM);LIFR(UTR_3_PRIME)
5	38483484	38483491	38483489	T	C	snp	1	0	0	0	.	.	.	LIFR(DOWNSTREAM);LIFR(INTRON)
5	38500304	38500313	38500310	TA	C	snp	1	0	0	0	.	.	.	LIFR(INTRON)
5	38514315	38514322	38514316	A	G	snp	1	1	1	1	.	.	.	LIFR(INTRON)
5	38549900	38549908	38549901	A	C	snp	0	0	1	0	.	.	.	LIFR(INTRON)
5	38625693	38625702	38625697	AT	G	snp	1	1	1	1	.	.	.	LIFR-AS1(INTRON)
5	38660687	38660700	38660698	CT	CTC	del	1	1	0	0	.	.	.	LIFR-AS1(INTRON)
5	38699818	38699826	38699821	T	G	snp	1	0	0	0	.	.	.	INTERGENIC(INTERGENIC)
5	38699824	38699843	38699830	TTTG	G	snp	1	0	1	0	.	.	.	INTERGENIC(INTERGENIC)
5	38750545	38750554	38750549	CT	G	snp	0	1	1	1	.	.	.	CTD-2127H9.1.1(INTRON)
5	38759793	38759801	38759800	A	G	snp	1	1	1	1	.	.	.	CTD-2127H9.1.1(INTRON);RP11-122C5.2.1(DOWNSTREAM)
5	38796139	38796146	38796141	A	G	snp	1	1	0	1	.	.	.	CTD-2127H9.1.1(INTRON);RP11-122C5.3.1(DOWNSTREAM)
5	38843527	38843550	38843540	TAAA	T	snp	0	0	1	0	.	.	.	CTD-2127H9.1.1(EXON);CTD-2127H9.1.1(INTRON);OSMR(UPSTREAM)
5	38843527	38843550	38843544	TAAA	T	snp	1	0	1	0	.	.	.	

CTD-2127H9.1.1(EXON);CTD-2127H9.1.1(INTRON);OSMR(UPSTREAM)														
5	38923697	38923705	38923698	T	C	snp	1	1	1	1	.	.	.	OSMR(DOWNSTREAM);OSMR(INTRON)
5	39111194	39111208	39111202	TTG	A	snp	0	0	1	0	.	.	.	FYB(INTRON)
5	39186273	39186282	39186275	AC	T	snp	1	0	0	0	.	.	.	FYB(INTRON)
5	39232626	39232649	39232644	TTTA	A	del	1	0	0	0	.	.	.	FYB(INTRON)
5	39275813	39275832	39275818	TCCT	T	snp	1	1	1	1	.	.	.	FYB(UPSTREAM);INTERGENIC(INTERGENIC)
5	39387802	39387809	39387807	T	C	ins	1	1	1	1	.	.	.	
C9(INTRON);DAB2(DOWNSTREAM);DAB2(EXON);DAB2(INTRON)														
5	39478602	39478619	39478613	TA	TC	ins	1	1	1	0	.	.	.	INTERGENIC(INTERGENIC)
5	39530694	39530701	39530695	T	G	snp	1	1	0	1	.	.	.	INTERGENIC(INTERGENIC)
5	39658290	39658298	39658295	A	G	snp	1	1	1	1	.	.	.	INTERGENIC(INTERGENIC)
5	39732666	39732674	39732672	T	G	snp	1	0	0	0	.	.	.	INTERGENIC(INTERGENIC)
5	39732673	39732684	39732681	TTG	T	ins	1	1	1	1	.	.	.	INTERGENIC(INTERGENIC)
5	39780353	39780376	39780360	TTTG	T	snp	0	1	0	1	.	.	.	INTERGENIC(INTERGENIC)
5	39885060	39885079	39885067	TTTG	T	snp	1	1	0	1	.	.	.	
CTD-2127F10.1.1(DOWNSTREAM);INTERGENIC(INTERGENIC)														
5	39947762	39947771	39947765	TC	G	snp	1	0	0	1	.	.	.	INTERGENIC(INTERGENIC)
5	39988116	39988135	39988123	TTTC	A	snp	0	1	0	1	.	.	.	INTERGENIC(INTERGENIC)
5	40025646	40025655	40025648	A	G	ins	1	1	1	1	.	.	.	INTERGENIC(INTERGENIC)
5	40025646	40025655	40025649	A	G	snp	1	1	1	1	.	.	.	INTERGENIC(INTERGENIC)
5	40042769	40042784	40042780	AAAC	A	del	1	1	1	1	.	.	.	INTERGENIC(INTERGENIC)
5	40087132	40087140	40087133	T	G	snp	0	0	1	0	.	.	.	INTERGENIC(INTERGENIC)
5	40147121	40147130	40147124	AT	C	snp	1	1	1	1	.	.	.	INTERGENIC(INTERGENIC)
5	40168211	40168220	40168214	AT	A	del	1	1	1	1	.	.	.	INTERGENIC(INTERGENIC)
5	40197572	40197579	40197574	T	G	snp	1	1	1	1	.	.	.	INTERGENIC(INTERGENIC)
5	40198445	40198454	40198448	TA	C	snp	1	1	1	1	.	.	.	INTERGENIC(INTERGENIC)
5	40425808	40425817	40425810	TG	C	snp	1	1	1	1	.	.	.	INTERGENIC(INTERGENIC)
5	40449002	40449009	40449005	T	C	snp	1	1	1	1	.	.	.	INTERGENIC(INTERGENIC)
5	40765854	40765863	40765862	A	C	ins	1	1	1	1	.	.	.	
PRKAA1(DOWNSTREAM);PRKAA1(INTRON);PRKAA1(UPSTREAM)														
5	40780564	40780573	40780569	AT	C	snp	1	1	1	1	.	.	.	PRKAA1(INTRON)
5	40890030	40890045	40890033	TC	G	snp	1	1	1	1	.	.	.	INTERGENIC(INTERGENIC)
5	40918482	40918496	40918486	ACA	A	snp	1	0	0	0	.	.	.	C7(INTRON)
5	40936900	40936911	40936908	GA	C	snp	1	1	1	1	.	.	.	C7(INTRON)
5	41102987	41102994	41102988	T	G	snp	1	1	1	1	.	.	.	INTERGENIC(INTERGENIC)
5	41105240	41105249	41105244	AT	G	snp	1	0	0	0	.	.	.	INTERGENIC(INTERGENIC)
5	41135590	41135598	41135594	T	C	snp	1	1	1	1	.	.	.	INTERGENIC(INTERGENIC)
5	41247988	41247995	41247993	A	G	snp	1	0	0	0	.	.	.	C6(INTRON)
5	41260371	41260384	41260377	CA	A	snp	1	0	0	0	.	.	.	C6(INTRON)
5	41285321	41285335	41285326	ACA	C	snp	0	1	0	1	.	.	.	INTERGENIC(INTERGENIC)
5	41338562	41338573	41338567	CA	C	snp	0	1	0	1	.	.	.	PLCXD3(INTRON)
5	41392035	41392046	41392038	GGT	T	snp	1	1	0	1	.	.	.	PLCXD3(INTRON)
5	41402249	41402256	41402253	T	G	snp	1	0	0	0	.	.	.	PLCXD3(INTRON)
5	41422091	41422106	41422104	AT	A	del	1	1	1	1	.	.	.	PLCXD3(INTRON)
5	41555153	41555161	41555158	A	G	snp	1	1	1	1	.	.	.	INTERGENIC(INTERGENIC)
5	41999524	41999533	41999530	AT	G	snp	1	0	0	0	.	.	.	INTERGENIC(INTERGENIC)
5	41999648	41999663	41999657	TA	G	snp	1	1	0	1	.	.	.	INTERGENIC(INTERGENIC)
5	42004838	42004845	42004844	A	C	snp	0	0	1	0	.	.	.	INTERGENIC(INTERGENIC)
5	42016466	42016475	42016471	T	C	snp	1	1	1	1	.	.	.	INTERGENIC(INTERGENIC)
5	42133834	42133845	42133837	AG	A	snp	1	1	1	1	.	.	.	INTERGENIC(INTERGENIC)
5	42220426	42220434	42220433	A	C	snp	1	1	1	1	.	.	.	INTERGENIC(INTERGENIC)
5	42259243	42259250	42259246	T	G	snp	1	1	1	0	.	.	.	INTERGENIC(INTERGENIC)
5	42398087	42398098	42398090	AC	G	snp	1	1	0	1	.	.	.	INTERGENIC(INTERGENIC)
5	42419332	42419349	42419343	AT	G	snp	1	0	0	0	.	.	.	GHR(UPSTREAM);INTERGENIC(INTERGENIC)
5	42433459	42433467	42433460	T	G	snp	1	1	0	1	.	.	.	GHR(INTRON)
5	42676385	42676394	42676393	A	G	snp	1	1	0	1	.	.	.	GHR(INTRON)
5	42821617	42821624	42821621	A	C	snp	0	0	1	0	.	.	.	SEPP1(INTRON)
5	42829825	42829840	42829832	TTTG	A	snp	0	0	1	0	.	.	.	SEPP1(INTRON);SEPP1(UPSTREAM)
5	42895173	42895196	42895178	AAT	AAC	ins	0	0	1	0	.	.	.	
CTD-2325A15.2.1(DOWNSTREAM);CTD-2325A15.3.1(UPSTREAM);INTERGENIC(INTERGENIC)														
5	42949064	42949071	42949066	T	G	snp	1	0	0	0	.	.	.	INTERGENIC(INTERGENIC)
5	42976147	42976155	42976148	A	G	snp	1	0	0	0	.	.	.	
CTD-2201E18.1.1(DOWNSTREAM);INTERGENIC(INTERGENIC)														
5	42994249	42994263	42994260	TTG	TTGT	del	0	0	1	0	.	.	.	INTERGENIC(INTERGENIC)
5	43099666	43099685	43099680	AAAC	C	del	1	1	1	0	.	.	.	ZNF131(DOWNSTREAM);ZNF131(INTRON)
5	43099666	43099685	43099681	AAAC	A	snp	1	1	1	1	.	.	.	ZNF131(DOWNSTREAM);ZNF131(INTRON)
5	43147512	43147531	43147520	TTTA	C	snp	0	1	0	1	.	.	.	ZNF131(INTRON)

5	43413982	43413991	43413985	T	G	snp	1	1	0	0	.	.	.	CCL28(UPSTREAM);INTERGENIC(INTERGENIC)
5	43524415	43524423	43524421	T	C	snp	0	0	1	0	.	.	.	PAIP1(DOWNSTREAM);RP11-159F24.5.1(INTRON);RP11-159F24.6.1(DOWNSTREAM)
5	43665309	43665324	43665320	TTTA	T	del	1	1	1	1	.	.	.	CTD-2210P15.1.1(DOWNSTREAM);NNT(INTRON)
5	43731224	43731241	43731238	TCT	TCC	ins	1	0	1	1	.	.	.	INTERGENIC(INTERGENIC)
5	43759706	43759714	43759709	T	G	snp	1	1	1	1	.	.	.	INTERGENIC(INTERGENIC)
5	43901644	43901653	43901650	T	C	snp	1	0	0	0	.	.	.	INTERGENIC(INTERGENIC)
5	43951829	43951836	43951835	A	G	snp	0	1	1	0	.	.	.	INTERGENIC(INTERGENIC)
5	43975317	43975326	43975323	A	C	snp	1	1	1	1	.	.	.	INTERGENIC(INTERGENIC)
5	43985149	43985156	43985155	T	G	snp	1	1	0	0	.	.	.	INTERGENIC(INTERGENIC)
5	43997190	43997199	43997197	AG	A	snp	0	1	0	0	.	.	.	INTERGENIC(INTERGENIC)
5	44102751	44102759	44102754	T	C	snp	1	0	0	0	.	.	.	INTERGENIC(INTERGENIC)
5	44115400	44115409	44115401	T	G	snp	1	1	1	1	.	.	.	INTERGENIC(INTERGENIC)
5	44134388	44134399	44134396	TA	C	snp	1	1	1	1	.	.	.	INTERGENIC(INTERGENIC)
5	44280205	44280212	44280209	T	G	ins	1	1	0	0	.	.	.	INTERGENIC(INTERGENIC)
5	44300359	44300368	44300362	T	C	ins	1	1	1	1	.	.	.	FGF10(DOWNSTREAM);INTERGENIC(INTERGENIC)
5	44441173	44441180	44441175	T	C	snp	1	1	1	1	.	.	.	INTERGENIC(INTERGENIC)
5	44673349	44673356	44673354	A	C	snp	1	1	1	1	.	.	.	INTERGENIC(INTERGENIC)
5	44782289	44782297	44782295	T	C	snp	1	1	1	1	.	.	.	RP11-53O19.1.1(INTRON)
5	44929217	44929232	44929230	TA	TAATATATATATT	del	1	1	1	1	.	.	.	INTERGENIC(INTERGENIC)
5	44955695	44955702	44955696	A	G	snp	1	0	0	0	.	.	.	INTERGENIC(INTERGENIC)
5	45298087	45298095	45298093	A	C	ins	1	1	0	0	.	.	.	HCN1(INTRON)
5	45372135	45372144	45372139	TA	C	snp	1	1	1	1	.	.	.	HCN1(INTRON)
5	45686215	45686226	45686224	AT	A	del	1	1	1	1	.	.	.	HCN1(INTRON)
5	45733119	45733146	45733141	TTTA	A	del	0	1	0	1	.	.	.	INTERGENIC(INTERGENIC)
5	45808259	45808268	45808262	A	G	snp	1	0	0	0	.	.	.	INTERGENIC(INTERGENIC)
5	45870691	45870698	45870695	A	G	snp	1	0	0	0	.	.	.	INTERGENIC(INTERGENIC)
5	45879405	45879414	45879409	T	G	snp	1	1	1	1	.	.	.	INTERGENIC(INTERGENIC)
5	45977564	45977575	45977572	ATT	A	del	1	1	1	1	.	.	.	INTERGENIC(INTERGENIC)
5	46005460	46005469	46005468	T	G	snp	1	0	0	0	.	.	.	INTERGENIC(INTERGENIC)
5	46045899	46045906	46045900	T	C	snp	1	1	1	1	.	.	.	INTERGENIC(INTERGENIC)
5	46102754	46102763	46102760	GA	A	snp	1	1	1	1	.	.	.	INTERGENIC(INTERGENIC)
5	46156466	46156477	46156474	TA	C	snp	0	1	0	1	.	.	.	INTERGENIC(INTERGENIC)
5	46239017	46239024	46239020	A	G	snp	1	1	1	1	.	.	.	INTERGENIC(INTERGENIC)
5	49646846	49646855	49646852	TG	G	snp	1	1	1	1	.	.	.	INTERGENIC(INTERGENIC)
5	49650436	49650447	49650444	ATT	A	del	1	1	1	1	.	.	.	INTERGENIC(INTERGENIC)
5	49758749	49758757	49758750	A	C	snp	1	1	1	1	.	.	.	INTERGENIC(INTERGENIC)
5	49831760	49831768	49831765	C	T	ins	0	0	0	1	.	.	.	INTERGENIC(INTERGENIC)
5	49908345	49908352	49908350	T	G	snp	1	1	1	1	.	.	.	INTERGENIC(INTERGENIC)
5	49924077	49924088	49924084	AG	C	snp	1	1	1	1	.	.	.	INTERGENIC(INTERGENIC);RP11-269M20.1.1(UPSTREAM)
5	50098628	50098635	50098630	A	G	snp	1	1	0	1	.	.	.	PARP8(INTRON)
5	50203322	50203330	50203328	A	G	ins	1	1	1	1	.	.	.	INTERGENIC(INTERGENIC)
5	50280608	50280615	50280610	A	G	snp	0	1	1	1	.	.	.	INTERGENIC(INTERGENIC)
5	50303561	50303569	50303565	A	C	snp	1	1	1	1	.	.	.	INTERGENIC(INTERGENIC)
5	50486518	50486529	50486522	AC	T	snp	1	1	1	1	.	.	.	INTERGENIC(INTERGENIC)
5	50607396	50607405	50607399	A	G	snp	1	0	0	1	.	.	.	INTERGENIC(INTERGENIC)
5	50804789	50804798	50804791	TA	ATATATAC	ins	1	1	1	1	.	.	.	INTERGENIC(INTERGENIC)
5	50893513	50893532	50893520	TTTG	T	del	1	0	0	0	.	.	.	INTERGENIC(INTERGENIC)
5	50948559	50948570	50948568	TG	T	snp	1	0	0	0	.	.	.	INTERGENIC(INTERGENIC)
5	50962972	50962989	50962976	AAC	G	snp	0	0	0	1	.	.	.	INTERGENIC(INTERGENIC)
5	51032925	51032933	51032927	T	C	snp	1	0	0	0	.	.	.	INTERGENIC(INTERGENIC)
5	51049335	51049346	51049340	AC	T	snp	0	0	0	1	.	.	.	INTERGENIC(INTERGENIC)
5	51088853	51088860	51088854	A	C	snp	1	1	1	1	.	.	.	INTERGENIC(INTERGENIC)
5	51115292	51115315	51115298	AAT	G	snp	1	0	0	0	.	.	.	INTERGENIC(INTERGENIC)
5	51139163	51139171	51139165	T	TTG	ins	1	0	0	0	.	.	.	INTERGENIC(INTERGENIC)
5	51139163	51139171	51139167	T	G	ins	1	0	0	0	.	.	.	INTERGENIC(INTERGENIC)
5	51139163	51139171	51139168	T	G	snp	1	0	1	0	.	.	.	INTERGENIC(INTERGENIC)
5	51139169	51139184	51139177	TTTG	C	snp	0	1	1	1	.	.	.	INTERGENIC(INTERGENIC)
5	51189396	51189410	51189407	TTA	C	snp	1	1	0	1	.	.	.	INTERGENIC(INTERGENIC)
5	51193597	51193604	51193600	A	G	snp	1	1	1	1	.	.	.	INTERGENIC(INTERGENIC)
5	51201468	51201476	51201472	T	C	snp	1	1	1	1	.	.	.	INTERGENIC(INTERGENIC)
5	51336963	51336972	51336967	AT	G	snp	1	0	0	0	.	.	.	CTD-2203A3.1.1(INTRON)
5	51563868	51563887	51563883	TTTG	A	snp	1	1	0	1	.	.	.	INTERGENIC(INTERGENIC)
5	51572155	51572166	51572161	TTA	A	snp	1	1	1	1	.	.	.	INTERGENIC(INTERGENIC)
5	51654661	51654670	51654663	CT	A	snp	1	1	1	1	.	.	.	INTERGENIC(INTERGENIC)
5	51669381	51669390	51669383	AT	G	snp	1	1	1	1	.	.	.	INTERGENIC(INTERGENIC)
5	51724037	51724052	51724046	AAAT	C	snp	1	0	0	0	.	.	.	INTERGENIC(INTERGENIC)

5	51901903	51901912	51901907	T	C	snp	1	1	1	0	.	.	.	INTERGENIC(INTERGENIC)
5	51976340	51976359	51976347	TTTG	C	snp	1	1	0	1	.	.	.	CTD-2288O8.1.1(INTRON)
5	52014829	52014836	52014834	A	C	snp	1	1	1	1	.	.	.	CTD-2288O8.1.1(INTRON)
5	52061431	52061438	52061433	T	G	snp	1	1	1	1	.	.	.	CTD-2288O8.1.1(INTRON)
5	52114071	52114079	52114075	A	C	snp	0	0	0	1	.	.	.	ITGA1(INTRON)
5	52207405	52207414	52207409	TA	C	snp	1	0	0	0	.	.	.	ITGA1(INTRON)
5	52207434	52207443	52207438	TA	C	snp	1	1	1	1	.	.	.	ITGA1(INTRON)
5	52207606	52207615	52207610	TA	C	snp	1	1	1	1	.	.	.	ITGA1(INTRON)
5	52224235	52224243	52224241	A	C	snp	1	1	1	1	.	.	.	CTD-2175A23.1.1(DOWNSTREAM);ITGA1(INTRON);ITGA1(UPSTREAM)
5	52280170	52280178	52280172	T	C	snp	1	0	0	0	.	.	.	CTD-2175A23.1.1(INTRON);ITGA2(UPSTREAM)
5	52303335	52303346	52303344	GA	G	del	0	1	1	1	.	.	.	ITGA2(INTRON)
5	52329421	52329444	52329437	AATT	T	snp	1	0	0	0	.	.	.	ITGA2(INTRON)
5	52388934	52388949	52388942	CAAA	A	del	1	1	1	1	.	.	.	ITGA2(DOWNSTREAM);ITGA2(UTR_3_PRIME);MOCS2(DOWNSTREAM)
5	52432903	52432916	52432907	GT	C	snp	1	1	1	1	.	.	.	INTERGENIC(INTERGENIC)
5	52439588	52439595	52439593	T	G	snp	1	1	1	1	.	.	.	INTERGENIC(INTERGENIC)
5	52547007	52547030	52547010	TTG	TG	del	1	1	1	1	.	.	.	INTERGENIC(INTERGENIC)
5	52578071	52578078	52578073	T	C	snp	1	0	1	0	.	.	.	INTERGENIC(INTERGENIC)
5	52583640	52583649	52583641	T	G	snp	1	0	1	0	.	.	.	INTERGENIC(INTERGENIC)
5	52813370	52813378	52813375	A	G	snp	1	1	1	1	.	.	.	INTERGENIC(INTERGENIC)
5	52843132	52843147	52843135	AT	A	snp	1	1	0	1	.	.	.	INTERGENIC(INTERGENIC)
5	53143451	53143458	53143457	A	G	snp	1	1	1	1	.	.	.	INTERGENIC(INTERGENIC)
5	53144218	53144227	53144224	TA	C	snp	1	1	1	1	.	.	.	INTERGENIC(INTERGENIC)
5	53300606	53300617	53300612	TG	G	snp	1	1	1	1	.	.	.	ARL15(INTRON)
5	53345428	53345445	53345433	TTG	T	snp	1	0	0	0	.	.	.	AC016601.1(UPSTREAM);ARL15(INTRON)
5	53349056	53349070	53349067	AAC	AACA	del	1	0	0	0	.	.	.	AC016601.1(UPSTREAM);ARL15(INTRON)
5	53353819	53353846	53353840	TTTA	A	ins	1	0	0	0	.	.	.	ARL15(INTRON)
5	53654171	53654179	53654172	A	G	snp	0	0	0	1	.	.	.	RP11-461C13.1.1(INTRON)
5	53668037	53668045	53668041	T	G	ins	1	0	0	0	.	.	.	RP11-461C13.1.1(INTRON)
5	53711571	53711586	53711576	AT	C	snp	1	0	0	0	.	.	.	INTERGENIC(INTERGENIC);RP11-461C13.1.1(DOWNSTREAM)
5	53715244	53715255	53715250	TA	C	snp	1	1	1	1	.	.	.	INTERGENIC(INTERGENIC);RP11-461C13.1.1(DOWNSTREAM)
5	53811001	53811016	53811005	GGGA	A	snp	0	0	1	0	.	.	.	INTERGENIC(INTERGENIC);SNX18(UPSTREAM)
5	53833013	53833026	53833021	AG	T	snp	0	1	1	1	.	.	.	SNX18(INTRON)
5	53873689	53873700	53873692	TGG	C	snp	1	1	1	1	.	.	.	INTERGENIC(INTERGENIC)
5	54005617	54005624	54005622	T	C	snp	1	1	1	1	.	.	.	INTERGENIC(INTERGENIC)
5	54083556	54083564	54083557	T	G	snp	1	1	0	1	.	.	.	INTERGENIC(INTERGENIC)
5	54140858	54140881	54140864	AAAC	C	del	1	1	0	1	.	.	.	AC112198.1.1(INTRON)
5	54268026	54268035	54268032	TA	C	snp	1	1	0	1	.	.	.	INTERGENIC(INTERGENIC)
5	54289929	54289943	54289937	CAA	G	snp	1	0	0	0	.	.	.	INTERGENIC(INTERGENIC)
5	54314171	54314178	54314174	A	C	snp	1	1	1	1	.	.	.	CTD-2313F11.1.1(DOWNSTREAM);INTERGENIC(INTERGENIC)
5	54352890	54352897	54352893	T	C	snp	1	0	0	0	.	.	.	CTD-2313F11.2.1(DOWNSTREAM);INTERGENIC(INTERGENIC)
5	54398881	54398889	54398882	A	G	ins	1	1	1	1	.	.	.	GZMA(INTRON)
5	54489968	54489983	54489980	AG	GAG	del	1	1	1	1	.	.	.	INTERGENIC(INTERGENIC)
5	54501846	54501863	54501851	GAA	G	snp	1	1	1	1	.	.	.	INTERGENIC(INTERGENIC)
5	54768485	54768496	54768491	AG	G	del	1	1	0	1	.	.	.	PPAP2A(INTRON);PPAP2A(UPSTREAM)
5	54768485	54768496	54768493	AG	G	del	1	1	0	1	.	.	.	PPAP2A(INTRON);PPAP2A(UPSTREAM)
5	54777816	54777825	54777823	AT	A	snp	1	1	1	1	.	.	.	PPAP2A(INTRON)
5	54810301	54810308	54810306	A	G	snp	1	1	1	1	.	.	.	PPAP2A(INTRON)
5	54876778	54876785	54876779	T	G	ins	1	1	1	0	.	.	.	INTERGENIC(INTERGENIC)
5	54881945	54881960	54881954	AC	A	snp	1	0	0	0	.	.	.	INTERGENIC(INTERGENIC)
5	54956627	54956634	54956632	G	T	snp	1	1	1	1	.	.	.	SLC38A9(DOWNSTREAM);SLC38A9(INTRON);SLC38A9(UPSTREAM)
5	54973967	54973975	54973971	T	G	ins	1	0	0	0	.	.	.	SLC38A9(INTRON)
5	55331234	55331241	55331235	C	A	snp	1	1	1	1	.	.	.	INTERGENIC(INTERGENIC)
5	55332551	55332566	55332561	AT	TATAT	del	1	0	0	0	.	.	.	INTERGENIC(INTERGENIC)
5	55332551	55332566	55332562	AT	ATA	del	1	0	0	0	.	.	.	INTERGENIC(INTERGENIC)
5	55332551	55332566	55332563	AT	TATTT	del	1	1	0	0	.	.	.	INTERGENIC(INTERGENIC)
5	55334061	55334088	55334083	TTTA	A	del	1	0	0	0	.	.	.	INTERGENIC(INTERGENIC)
5	55341086	55341095	55341093	T	C	snp	1	0	0	0	.	.	.	INTERGENIC(INTERGENIC)
5	55345003	55345010	55345009	T	C	snp	0	1	1	1	.	.	.	INTERGENIC(INTERGENIC)
5	55372200	55372209	55372205	A	AAAAGAAAAG	ins	1	0	0	0	.	.	.	INTERGENIC(INTERGENIC)
5	55372200	55372209	55372205	A	G	snp	1	0	0	0	.	.	.	INTERGENIC(INTERGENIC)

5	55485831	55485838	55485832	A	G	snp	1	0	0	0	.	.	.	ANKRD55(INTRON);RP11-365H8.2.1(UPSTREAM)
5	55561284	55561307	55561288	TTCA	TCA	del	1	0	0	0	.	.	.	INTERGENIC(INTERGENIC)
5	55575748	55575756	55575755	C	A	ins	0	0	1	0	.	.	.	INTERGENIC(INTERGENIC);PSMC1P4(UPSTREAM)
5	55609305	55609314	55609308	AT	C	snp	1	1	1	1	.	.	.	INTERGENIC(INTERGENIC);RP11-155L15.1.1(DOWNSTREAM)
5	55754724	55754739	55754731	TTCC	T	snp	0	1	1	1	.	.	.	CTC-236F12.4.1(INTRON)
5	55765861	55765870	55765866	A	C	snp	1	1	1	1	.	.	.	CTC-236F12.4.1(INTRON)
5	55940869	55940878	55940877	A	C	snp	1	1	1	1	.	.	.	INTERGENIC(INTERGENIC)
5	55952999	55953007	55953005	C	A	snp	1	0	0	0	.	.	.	INTERGENIC(INTERGENIC)
5	55993236	55993244	55993242	C	T	snp	1	1	1	1	.	.	.	INTERGENIC(INTERGENIC)
5	55993806	55993817	55993813	AT	C	snp	1	1	1	1	.	.	.	INTERGENIC(INTERGENIC)
5	56011226	56011233	56011232	T	G	snp	1	0	0	0	.	.	.	INTERGENIC(INTERGENIC)
5	56012630	56012639	56012635	A	C	snp	1	1	1	1	.	.	.	INTERGENIC(INTERGENIC)
5	56148734	56148745	56148741	AC	A	snp	1	1	1	1	.	.	.	AC008937.2.1(INTRON);MAP3K1(INTRON);snoU13(UPSTREAM)
5	56199131	56199148	56199142	AC	T	snp	1	0	0	0	.	.	.	AC008937.3.1(INTRON)
5	56320669	56320680	56320674	ATT	C	snp	1	1	1	1	.	.	.	INTERGENIC(INTERGENIC)
5	56326984	56326995	56326987	TC	T	snp	1	1	1	1	.	.	.	INTERGENIC(INTERGENIC)
5	56327055	56327070	56327065	TTTC	G	snp	1	1	1	1	.	.	.	INTERGENIC(INTERGENIC)
5	56389891	56389918	56389897	TTCT	T	snp	0	1	0	1	.	.	.	INTERGENIC(INTERGENIC)
5	56400388	56400395	56400390	T	G	snp	1	1	1	1	.	.	.	INTERGENIC(INTERGENIC)
5	56428130	56428145	56428134	GTTT	A	snp	1	0	1	0	.	.	.	INTERGENIC(INTERGENIC)
5	56428947	56428956	56428955	T	G	snp	1	1	1	1	.	.	.	INTERGENIC(INTERGENIC)
5	56428957	56428966	56428963	GA	C	snp	1	0	0	0	.	.	.	INTERGENIC(INTERGENIC)
5	56454241	56454249	56454242	A	C	snp	0	0	0	1	.	.	.	INTERGENIC(INTERGENIC)
5	56519607	56519616	56519610	TA	G	snp	1	0	0	0	.	.	.	GPBP1(INTRON)
5	56519607	56519616	56519612	TA	G	snp	1	1	1	1	.	.	.	GPBP1(INTRON)
5	56527831	56527840	56527834	TG	A	snp	1	1	1	1	.	.	.	GPBP1(INTRON)
5	56534389	56534412	56534395	AAAT	T	del	0	0	1	0	.	.	.	GPBP1(DOWNSTREAM);GPBP1(INTRON)
5	56534389	56534412	56534396	AAAT	A	snp	0	0	1	0	.	.	.	GPBP1(DOWNSTREAM);GPBP1(INTRON)
5	56638116	56638125	56638123	T	C	snp	0	1	0	1	.	.	.	INTERGENIC(INTERGENIC)
5	56899104	56899119	56899112	GGGT	T	snp	1	1	1	1	.	.	.	CTD-2023N9.2.1(INTRON)
5	56913575	56913583	56913582	C	CA	ins	1	1	1	1	.	.	.	CTD-2023N9.2.1(DOWNSTREAM);CTD-2023N9.3.1(DOWNSTREAM);INTERGENIC(INTERGENIC)
5	56940135	56940142	56940140	T	TTTG	ins	0	0	1	0	.	.	.	INTERGENIC(INTERGENIC)
5	56957192	56957200	56957199	A	C	ins	1	0	0	0	.	.	.	INTERGENIC(INTERGENIC)
5	56966089	56966106	56966102	TG	T	snp	0	1	0	1	.	.	.	INTERGENIC(INTERGENIC)
5	57009917	57009932	57009923	TCTA	ATCTATCTATC	del	1	0	0	0	.	.	.	INTERGENIC(INTERGENIC)
5	57009917	57009932	57009927	TCTA	ATCTATC	del	1	0	0	0	.	.	.	INTERGENIC(INTERGENIC)
5	57053003	57053012	57053006	T	C	ins	1	1	1	1	.	.	.	INTERGENIC(INTERGENIC)
5	57130471	57130480	57130477	A	C	snp	1	0	0	0	.	.	.	INTERGENIC(INTERGENIC)
5	57155358	57155365	57155361	T	TTTTG	ins	1	0	0	0	.	.	.	INTERGENIC(INTERGENIC)
5	57185043	57185052	57185045	TG	A	snp	1	0	0	0	.	.	.	INTERGENIC(INTERGENIC);RP11-478P10.1.1(DOWNSTREAM)
5	57227998	57228007	57228005	A	G	snp	1	0	0	0	.	.	.	INTERGENIC(INTERGENIC)
5	57269251	57269259	57269256	A	G	snp	1	0	0	0	.	.	.	INTERGENIC(INTERGENIC)
5	57302815	57302822	57302819	A	G	snp	1	0	0	0	.	.	.	INTERGENIC(INTERGENIC)
5	57408866	57408873	57408867	A	C	snp	0	0	1	1	.	.	.	CTD-2037L6.2.1(INTRON)
5	57589978	57589989	57589985	ATA	A	ins	1	0	0	0	.	.	.	INTERGENIC(INTERGENIC)
5	57639628	57639635	57639630	A	C	snp	1	1	1	1	.	.	.	INTERGENIC(INTERGENIC)
5	57649049	57649064	57649052	AT	AC	ins	0	0	0	1	.	.	.	INTERGENIC(INTERGENIC)
5	57768733	57768752	57768743	TTTA	A	del	1	1	0	1	.	.	.	INTERGENIC(INTERGENIC)
5	57768733	57768752	57768747	TTTA	A	del	1	1	0	1	.	.	.	INTERGENIC(INTERGENIC)
5	57768733	57768752	57768748	TTTA	T	snp	1	1	0	1	.	.	.	INTERGENIC(INTERGENIC)
5	57837112	57837123	57837120	TGA	G	snp	1	1	1	1	.	.	.	CTD-2117L12.1.1(DOWNSTREAM);INTERGENIC(INTERGENIC)
5	57916615	57916622	57916619	T	C	snp	1	0	0	0	.	.	.	RAB3C(DOWNSTREAM);RAB3C(INTRON)
5	57951425	57951432	57951429	T	C	snp	0	1	1	1	.	.	.	RAB3C(INTRON)
5	57955042	57955055	57955048	TA	C	snp	1	1	1	1	.	.	.	RAB3C(INTRON)
5	58011284	58011298	58011287	GAA	AA	del	1	0	0	0	.	.	.	RAB3C(INTRON)
5	58011284	58011298	58011290	GAA	A	del	1	0	0	0	.	.	.	RAB3C(INTRON)
5	58011284	58011298	58011291	GAA	G	snp	1	1	1	1	.	.	.	RAB3C(INTRON)
5	58088642	58088651	58088649	AC	T	snp	0	1	1	1	.	.	.	RAB3C(INTRON);RP11-479O16.1.1(INTRON)
5	58090095	58090104	58090103	T	TC	ins	1	1	1	1	.	.	.	RAB3C(INTRON);RP11-479O16.1.1(INTRON)
5	58093186	58093195	58093192	TA	ATA	del	1	1	1	1	.	.	.	RAB3C(DOWNSTREAM);RAB3C(INTRON);RP11-479O16.1.1(INTRON)
5	58161410	58161421	58161414	AGA	AAGAAGAAAAA	del	1	0	0	0	.	.	.	

CTD-2176I21.2.1(UPSTREAM);INTERGENIC(INTERGENIC)

5	58245930	58245941	58245938	CTG	T	snp	1	1	1	1	.	.	.	INTERGENIC(INTERGENIC)
5	58295381	58295394	58295390	TA	TATG	ins	1	1	1	1	.	.	.	PDE4D(INTRON);PDE4D(UTR_5_PRIME)
5	58339040	58339049	58339046	A	G	snp	1	1	1	1	.	.	.	PDE4D(INTRON);PDE4D(UPSTREAM);RP11-266N13.2.1(INTRON)
5	58362859	58362868	58362864	AG	C	snp	1	1	1	1	.	.	.	PDE4D(INTRON);RP11-266N13.2.1(DOWNSTREAM)
5	58373289	58373298	58373296	T	C	snp	1	0	1	0	.	.	.	PDE4D(INTRON)
5	58449601	58449609	58449603	T	G	snp	1	1	0	1	.	.	.	PDE4D(DOWNSTREAM);PDE4D(INTRON)
5	58467582	58467595	58467592	CT	A	snp	1	1	1	1	.	.	.	PDE4D(DOWNSTREAM);PDE4D(INTRON)
5	58467929	58467938	58467932	TA	T	snp	1	1	1	1	.	.	.	PDE4D(DOWNSTREAM);PDE4D(INTRON)
5	58467951	58467960	58467954	TA	T	snp	1	1	1	1	.	.	.	PDE4D(DOWNSTREAM);PDE4D(INTRON)
5	58503683	58503692	58503685	T	C	snp	0	1	1	1	.	.	.	PDE4D(INTRON)
5	58524254	58524265	58524262	AAC	A	ins	0	1	1	1	.	.	.	PDE4D(INTRON)
5	58595640	58595648	58595644	A	G	snp	0	1	0	1	.	.	.	PDE4D(INTRON)
5	58601180	58601195	58601191	ATTT	A	del	0	0	0	1	.	.	.	PDE4D(INTRON)
5	58682518	58682541	58682525	AGGG	A	snp	1	1	1	1	.	.	.	PDE4D(INTRON)
5	58822616	58822623	58822619	T	C	snp	1	0	0	0	.	.	.	CTD-2085F10.1.1(UPSTREAM);PDE4D(INTRON)
5	58964541	58964548	58964546	A	AAG	ins	1	1	1	1	.	.	.	PDE4D(INTRON)
5	59058619	59058628	59058622	TA	G	snp	1	1	0	1	.	.	.	PDE4D(INTRON)
5	59070498	59070507	59070501	T	C	snp	0	0	0	1	.	.	.	PDE4D(INTRON)
5	59113569	59113577	59113576	C	A	snp	1	1	0	1	.	.	.	PDE4D(INTRON)
5	59507318	59507327	59507321	AT	C	snp	1	0	0	0	.	.	.	PDE4D(INTRON)
5	59531472	59531481	59531477	A	C	snp	0	1	0	1	.	.	.	PDE4D(INTRON)
5	59542921	59542928	59542924	T	C	snp	1	1	1	1	.	.	.	PDE4D(INTRON)
5	59662921	59662929	59662927	T	G	snp	0	1	1	1	.	.	.	PDE4D(INTRON)
5	59666587	59666604	59666601	TG	C	snp	1	1	1	0	.	.	.	PDE4D(INTRON)
5	59851133	59851144	59851141	AC	G	snp	1	0	0	0	.	.	.	INTERGENIC(INTERGENIC)
5	59930503	59930530	59930510	AAAT	A	snp	1	0	0	0	.	.	.	CTD-2003D5.1.1(DOWNSTREAM);DEPDC1B(INTRON)
5	59963731	59963740	59963738	TA	G	snp	1	1	1	1	.	.	.	AC109133.1(DOWNSTREAM);DEPDC1B(INTRON)
5	60126419	60126427	60126424	T	C	snp	1	1	1	1	.	.	.	ELOVL7(INTRON)
5	60162680	60162689	60162683	T	G	snp	1	1	0	1	.	.	.	INTERGENIC(INTERGENIC)
5	60193331	60193342	60193338	TAT	G	snp	0	0	0	1	.	.	.	ERCC8(DOWNSTREAM);ERCC8(INTRON);GNL3LP1(UPSTREAM)
5	60193331	60193342	60193339	TAT	C	snp	1	1	1	1	.	.	.	ERCC8(DOWNSTREAM);ERCC8(INTRON);GNL3LP1(UPSTREAM)
5	60302794	60302801	60302799	T	C	snp	0	1	1	1	.	.	.	NDUFAF2(INTRON)
5	60338370	60338379	60338374	TG	C	snp	1	0	0	0	.	.	.	NDUFAF2(INTRON)
5	60499201	60499209	60499205	T	G	ins	0	1	1	1	.	.	.	CTC-436P18.1.1(INTRON);CTC-436P18.3.1(INTRON)
5	60506269	60506277	60506274	A	G	snp	1	1	1	1	.	.	.	CTC-436P18.1.1(INTRON);CTC-436P18.3.1(INTRON)
5	60534362	60534370	60534366	A	G	snp	1	1	1	1	.	.	.	CTC-436P18.3.1(INTRON)
5	60565293	60565301	60565297	A	G	snp	1	1	1	1	.	.	.	CTC-436P18.3.1(UPSTREAM);INTERGENIC(INTERGENIC)
5	60577864	60577873	60577869	AT	G	snp	1	1	1	1	.	.	.	INTERGENIC(INTERGENIC)
5	60578165	60578174	60578169	AT	T	snp	1	1	1	1	.	.	.	INTERGENIC(INTERGENIC)
5	60578285	60578294	60578289	AT	T	snp	1	1	1	1	.	.	.	INTERGENIC(INTERGENIC)
5	60578317	60578326	60578321	AT	T	snp	1	1	1	1	.	.	.	INTERGENIC(INTERGENIC)
5	60578349	60578358	60578353	AT	C	snp	1	1	1	1	.	.	.	INTERGENIC(INTERGENIC)
5	60578349	60578358	60578356	AT	C	snp	0	1	0	1	.	.	.	INTERGENIC(INTERGENIC)
5	60578519	60578528	60578526	AT	C	snp	1	1	0	1	.	.	.	INTERGENIC(INTERGENIC)
5	60578603	60578612	60578606	AT	A	snp	1	1	1	1	.	.	.	INTERGENIC(INTERGENIC)
5	60578923	60578932	60578927	AT	T	snp	0	1	1	1	.	.	.	INTERGENIC(INTERGENIC)
5	60630599	60630607	60630604	T	G	snp	1	0	0	0	.	.	.	ZSWIM6(INTRON)
5	60921670	60921685	60921675	GCGG	T	snp	1	0	0	0	.	.	.	INTERGENIC(INTERGENIC)
5	60921670	60921685	60921680	GCGG	A	snp	1	1	1	1	.	.	.	INTERGENIC(INTERGENIC)
5	60953737	60953745	60953743	A	G	ins	1	1	1	1	.	.	.	C5orf64(INTRON);RP11-2O17.2.1(DOWNSTREAM)
5	60998969	60998982	60998978	TC	G	snp	0	1	1	1	.	.	.	C5orf64(INTRON);RP11-2O17.2.1(UPSTREAM)
5	61005901	61005909	61005903	A	C	snp	1	0	0	0	.	.	.	C5orf64(DOWNSTREAM);C5orf64(INTRON)
5	61027631	61027640	61027636	AG	A	ins	1	1	0	1	.	.	.	C5orf64(DOWNSTREAM);C5orf64(INTRON);CTD-2170G1.2.1(DOWNSTREAM)
5	61034165	61034174	61034166	G	GGGA	ins	1	1	1	1	.	.	.	C5orf64(INTRON);CTD-2170G1.2.1(UPSTREAM)
5	61216414	61216421	61216415	C	T	snp	1	1	1	1	.	.	.	INTERGENIC(INTERGENIC)
5	61345633	61345647	61345644	AAC	A	ins	1	0	0	0	.	.	.	INTERGENIC(INTERGENIC)
5	61384949	61384956	61384950	A	C	ins	1	0	0	0	.	.	.	INTERGENIC(INTERGENIC)
5	61518869	61518884	61518882	AT	A	del	1	1	0	1	.	.	.	INTERGENIC(INTERGENIC)
5	61619871	61619886	61619878	AAAT	AAATA	del	1	0	0	0	.	.	.	KIF2A(INTRON)
5	61689741	61689748	61689743	T	C	snp	1	0	0	0	.	.	.	DIMT1(INTRON);DIMT1(UPSTREAM);KIF2A(INTRON)
5	61758641	61758650	61758649	T	C	snp	0	1	0	0	.	.	.	IPO11(INTRON);IPO11(UPSTREAM);KIF2A(INTRON)
5	61804332	61804346	61804337	TTC	T	snp	1	0	0	0	.	.	.	IPO11(INTRON);KIF2A(INTRON)
5	61829025	61829040	61829036	TTCT	TTC	del	1	1	1	1	.	.	.	IPO11(INTRON);KIF2A(INTRON)
5	62078539	62078548	62078543	TG	C	snp	1	1	1	1	.	.	.	INTERGENIC(INTERGENIC)

5	62236929	62236936	62236934	T	C	snp	0	0	1	0	.	.	.	INTERGENIC(INTERGENIC)
5	62288338	62288345	62288342	T	G	snp	1	1	1	1	.	.	.	INTERGENIC(INTERGENIC)
5	62348624	62348633	62348628	GA	A	snp	1	1	1	1	.	.	.	INTERGENIC(INTERGENIC)
5	62436859	62436866	62436863	T	G	snp	1	0	0	0	.	.	.	INTERGENIC(INTERGENIC)
5	62553402	62553410	62553406	T	C	snp	1	1	1	1	.	.	.	INTERGENIC(INTERGENIC)
5	62589401	62589410	62589404	A	G	snp	1	0	0	0	.	.	.	INTERGENIC(INTERGENIC)
5	62606947	62606954	62606953	T	TC	ins	1	0	0	0	.	.	.	INTERGENIC(INTERGENIC)
5	62619005	62619014	62619011	AT	G	snp	1	0	0	0	.	.	.	INTERGENIC(INTERGENIC)
5	62699219	62699226	62699225	A	C	snp	1	0	0	0	.	.	.	INTERGENIC(INTERGENIC)
5	62732989	62732997	62732990	A	G	snp	0	0	0	1	.	.	.	INTERGENIC(INTERGENIC)
5	62747811	62747834	62747817	ATCT	T	del	0	1	1	1	.	.	.	INTERGENIC(INTERGENIC)
5	62777460	62777477	62777468	TTG	T	snp	1	1	1	1	.	.	.	INTERGENIC(INTERGENIC)
5	62850524	62850535	62850526	AT	G	snp	1	0	0	0	.	.	.	INTERGENIC(INTERGENIC)
5	62883743	62883757	62883747	ATA	AAC	ins	1	1	1	1	.	.	.	INTERGENIC(INTERGENIC)
5	62886632	62886641	62886637	A	C	snp	0	1	0	1	.	.	.	INTERGENIC(INTERGENIC)
5	62899215	62899224	62899216	T	G	snp	1	1	0	0	.	.	.	INTERGENIC(INTERGENIC)
5	63079864	63079873	63079869	A	G	snp	1	1	1	1	.	.	.	INTERGENIC(INTERGENIC)
5	63173240	63173249	63173242	AT	G	snp	1	1	1	1	.	.	.	INTERGENIC(INTERGENIC)
5	63585695	63585702	63585697	T	C	snp	1	1	1	1	.	.	.	RNF180(INTRON)
5	63632832	63632841	63632836	T	G	snp	1	0	0	1	.	.	.	RNF180(INTRON)
5	63720727	63720736	63720731	GA	T	snp	1	0	1	0	.	.	.	AC016561.1.1(INTRON)
5	63738590	63738598	63738595	A	G	snp	1	0	0	0	.	.	.	AC016561.1.1(INTRON)
5	63985694	63985703	63985700	T	C	snp	1	0	0	0	.	.	.	FAM159B(UPSTREAM);INTERGENIC(INTERGENIC)
5	64088364	64088371	64088366	T	C	snp	1	1	1	1	.	.	.	CWC27(DOWNSTREAM);CWC27(INTRON)
5	64092652	64092667	64092656	CTTC	G	snp	0	0	1	0	.	.	.	CWC27(INTRON);CWC27(UPSTREAM)
5	64373620	64373629	64373621	C	T	ins	0	1	0	1	.	.	.	INTERGENIC(INTERGENIC)
5	64452205	64452219	64452214	ACC	G	snp	0	1	1	1	.	.	.	ADAMTS6(INTRON)
5	64551086	64551094	64551087	T	C	snp	1	1	1	1	.	.	.	ADAMTS6(DOWNSTREAM);ADAMTS6(INTRON);CTD-2194F4.1.1(UPSTREAM)
5	64558466	64558477	64558472	GAA	A	snp	1	0	0	0	.	.	.	ADAMTS6(INTRON)
5	64722359	64722366	64722363	A	C	snp	1	1	0	1	.	.	.	ADAMTS6(INTRON)
5	64728350	64728365	64728352	CA	AA	ins	1	0	0	0	.	.	.	ADAMTS6(INTRON)
5	64789742	64789750	64789749	A	G	snp	1	0	1	0	.	.	.	INTERGENIC(INTERGENIC)
5	64832459	64832470	64832462	AT	A	snp	1	1	1	1	.	.	.	CENPK(INTRON)
5	64855973	64855982	64855976	T	G	snp	0	1	1	1	.	.	.	CENPK(DOWNSTREAM);CENPK(INTRON);CENPK(UPSTREAM);PPWD1(UPSTREAM)
5	64869286	64869303	64869297	AT	A	del	1	0	0	0	.	.	.	PPWD1(DOWNSTREAM);PPWD1(INTRON);PPWD1(UPSTREAM)
5	64869286	64869303	64869298	AT	T	ins	1	0	0	0	.	.	.	PPWD1(DOWNSTREAM);PPWD1(INTRON);PPWD1(UPSTREAM)
5	64869286	64869303	64869301	AT	A	del	1	1	0	0	.	.	.	PPWD1(DOWNSTREAM);PPWD1(INTRON);PPWD1(UPSTREAM)
5	64874563	64874572	64874568	AT	A	ins	1	1	1	1	.	.	.	PPWD1(DOWNSTREAM);PPWD1(INTRON);PPWD1(UPSTREAM)
5	64947474	64947481	64947479	G	T	snp	1	0	0	0	.	.	.	C5orf44(INTRON);C5orf44(UPSTREAM)
5	64961019	64961027	64961020	A	G	snp	1	1	1	1	.	.	.	C5orf44(DOWNSTREAM);C5orf44(EXON);C5orf44(UTR_3_PRIME);SGTB(DOWNSTREAM)
5	65116545	65116553	65116546	T	C	snp	0	0	0	1	.	.	.	NLN(INTRON)
5	65177492	65177501	65177496	TA	C	snp	1	1	0	0	.	.	.	INTERGENIC(INTERGENIC)
5	65189941	65189956	65189948	TTAT	C	snp	0	0	1	0	.	.	.	INTERGENIC(INTERGENIC)
5	65203053	65203076	65203067	GTTT	C	snp	1	0	0	0	.	.	.	INTERGENIC(INTERGENIC)
5	65277480	65277489	65277481	A	C	ins	1	0	0	0	.	.	.	ERBB2IP(INTRON)
5	65400664	65400679	65400670	AAAG	G	snp	1	0	0	0	.	.	.	INTERGENIC(INTERGENIC)
5	65404937	65404946	65404942	AC	G	snp	1	0	0	0	.	.	.	INTERGENIC(INTERGENIC)
5	65411701	65411716	65411710	AG	T	snp	1	1	1	1	.	.	.	INTERGENIC(INTERGENIC)
5	65417798	65417806	65417803	T	C	snp	1	1	1	1	.	.	.	INTERGENIC(INTERGENIC)
5	65466025	65466033	65466027	T	C	ins	0	0	1	0	.	.	.	SREK1(INTRON);SREK1(UPSTREAM)
5	65740109	65740120	65740115	TTA	C	snp	1	1	1	1	.	.	.	RP11-305P14.1.1(INTRON)
5	65818115	65818126	65818120	AT	A	snp	1	1	0	1	.	.	.	RP11-305P14.1.1(INTRON)
5	65867586	65867597	65867588	AC	G	snp	1	1	1	1	.	.	.	RP11-305P14.1.1(EXON);RP11-5P22.1.1(UPSTREAM)
[truncated: 3,625,049 more chars]
