# Supplementary material for: Microsatellite Interruptions Stabilize Primate Genomes and Exist as Population-Specific Single Nucleotide Polymorphisms within Individual Human Genomes
Source: PLoS Genet. 2014 Jul 17;10(7):e1004498. doi: 10.1371/journal.pgen.1004498 (PMC4102424; doi:10.1371/journal.pgen.1004498)
Supplement: Figure S9 — Unique mutational signatures of polymerase interruption errors within [GT] dinucleotide microsatellites. (A). GT10 template; (B). GT13 template; (C). GT19 template. DNA synthesis proceeds from right to left. The middle line of sequence is the in-frame wild-type HSV-tk gene and subscripts indicate each dinucleotide unit of the microsatellite. Indels are shown above the sequence and base substitutions are shown below. Each symbol represents one mutational event: (▵) one base deletion; (♦) two base deletion; (▴) one base insertion with identity of the inserted base above the symbol. Symbols with subscripts indicate that the interruption event occurred with a 1 dinucleotide unit deletion (−1); 2 dinucleotide unit deletion (−2); 1 dinucleotide unit insertion (+1); or 2 dinucleotide unit insertion (+2). Interruption events for pol δ are gray, pol β are blue, pol κ are red, and pol η are black. (PDF) [file pgen.1004498.s015.pdf]

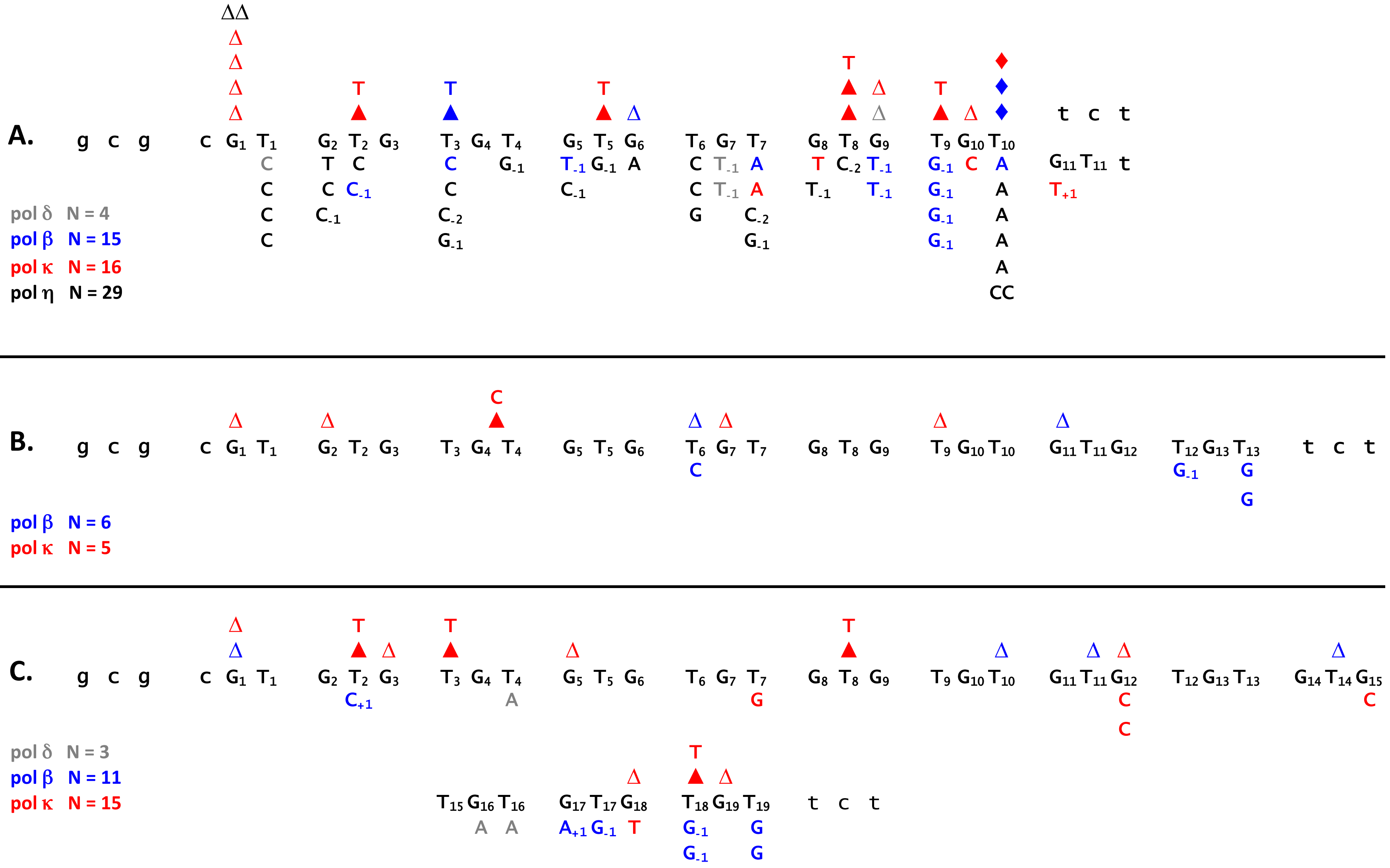
A.
pol δ N = 4
pol β N = 15
pol κ N = 16
pol η N = 29
B.
pol β N = 6
pol κ N = 5
C.
pol δ N = 3
pol β N = 11
pol κ N = 15
