# Supplementary material for: Microsatellite Interruptions Stabilize Primate Genomes and Exist as Population-Specific Single Nucleotide Polymorphisms within Individual Human Genomes
Source: PLoS Genet. 2014 Jul 17;10(7):e1004498. doi: 10.1371/journal.pgen.1004498 (PMC4102424; doi:10.1371/journal.pgen.1004498)
Supplement: Figure S10 — Unique mutational signatures of polymerase interruption errors within [TC] dinucleotide microsatellites. (A). TC11 template; (B). TC14 template. Symbols and subscripts are the same as that indicated in Figure S9. (PDF) [file pgen.1004498.s016.pdf]

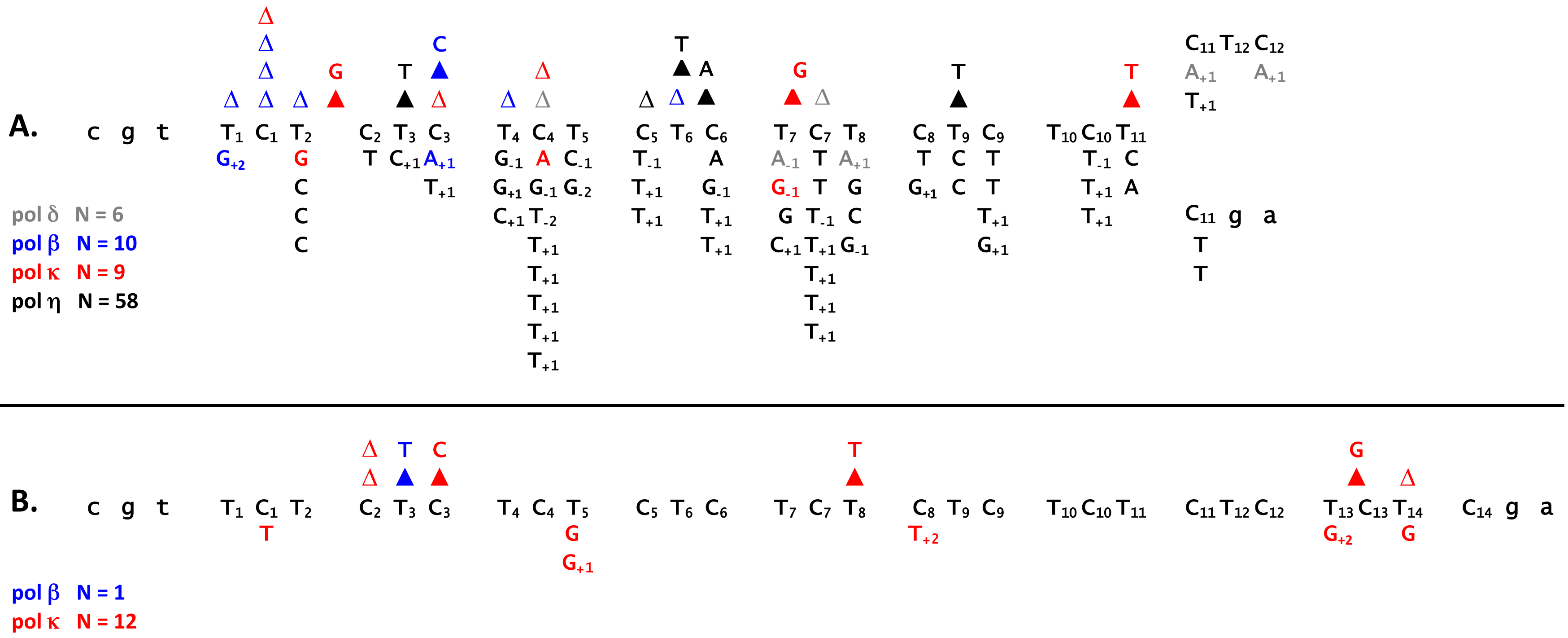
A.
c g t T1 C1 T2 C2 T3 C3 T4 C4 T5 C5 T6 C6 T7 C7 T8 C8 T9 C9 T10 C10 T11 C11 T12 C12
C11 g a
pol δ N = 6
pol β N = 10
pol κ N = 9
pol η N = 58
B.
c g t T1 C1 T2 C2 T3 C3 T4 C4 T5 C5 T6 C6 T7 C7 T8 C8 T9 C9 T10 C10 T11 C11 T12 C12 T13 C13 T14 C14 g a
pol β N = 1
pol κ N = 12
